# Genomic Analysis Enlightens Agaricales Lifestyle Evolution and Increasing Peroxidase Diversity

**DOI:** 10.1093/molbev/msaa301

**Published:** 2020-11-19

**Authors:** Francisco J Ruiz-Dueñas, José M Barrasa, Marisol Sánchez-García, Susana Camarero, Shingo Miyauchi, Ana Serrano, Dolores Linde, Rashid Babiker, Elodie Drula, Iván Ayuso-Fernández, Remedios Pacheco, Guillermo Padilla, Patricia Ferreira, Jorge Barriuso, Harald Kellner, Raúl Castanera, Manuel Alfaro, Lucía Ramírez, Antonio G Pisabarro, Robert Riley, Alan Kuo, William Andreopoulos, Kurt LaButti, Jasmyn Pangilinan, Andrew Tritt, Anna Lipzen, Guifen He, Mi Yan, Vivian Ng, Igor V Grigoriev, Daniel Cullen, Francis Martin, Marie-Noëlle Rosso, Bernard Henrissat, David Hibbett, Angel T Martínez

**Affiliations:** 1 Centro de Investigaciones Biológicas Margarita Salas (CIB), CSIC, Madrid, Spain; 2 Life Sciences Department, Alcalá University, Alcalá de Henares, Spain; 3 Biology Department, Clark University, Worcester, MA, USA; 4 INRAE, Laboratory of Excellence ARBRE, Champenoux, France; 5 Architecture et Fonction des Macromolécules Biologiques, CNRS/Aix-Marseille University, Marseille, France; 6 Biochemistry and Molecular and Cellular Biology Department and BIFI, Zaragoza University, Zaragoza, Spain; 7 International Institute Zittau, Technische Universität Dresden, Zittau, Germany; 8 Institute for Multidisciplinary Research in Applied Biology, IMAB-UPNA, Pamplona, Spain; 9 US Department of Energy (DOE) Joint Genome Institute (JGI), Lawrence Berkeley National Lab, Berkeley, CA, USA; 10 Department of Plant and Microbial Biology, University of California, Berkeley, Berkeley, CA, USA; 11 Forest Products Laboratory, US Department of Agriculture, Madison, WI, USA; 12 INRAE, Biodiversité et Biotechnologie Fongiques, Aix-Marseille University, Marseille, France; 13 Department of Biological Sciences, King Abdulaziz University, Jeddah, Saudi Arabia

**Keywords:** Agaricales, lifestyle evolution, lignocellulose decay, plant cell-wall degrading enzymes, ligninolytic peroxidases, ancestral-sequence reconstruction

## Abstract

As actors of global carbon cycle, Agaricomycetes (Basidiomycota) have developed complex enzymatic machineries that allow them to decompose all plant polymers, including lignin. Among them, saprotrophic Agaricales are characterized by an unparalleled diversity of habitats and lifestyles. Comparative analysis of 52 Agaricomycetes genomes (14 of them sequenced de novo) reveals that Agaricales possess a large diversity of hydrolytic and oxidative enzymes for lignocellulose decay. Based on the gene families with the predicted highest evolutionary rates—namely cellulose-binding CBM1, glycoside hydrolase GH43, lytic polysaccharide monooxygenase AA9, class-II peroxidases, glucose–methanol–choline oxidase/dehydrogenases, laccases, and unspecific peroxygenases—we reconstructed the lifestyles of the ancestors that led to the extant lignocellulose-decomposing Agaricomycetes. The changes in the enzymatic toolkit of ancestral Agaricales are correlated with the evolution of their ability to grow not only on wood but also on leaf litter and decayed wood, with grass-litter decomposers as the most recent eco-physiological group. In this context, the above families were analyzed in detail in connection with lifestyle diversity. Peroxidases appear as a central component of the enzymatic toolkit of saprotrophic Agaricomycetes, consistent with their essential role in lignin degradation and high evolutionary rates. This includes not only expansions/losses in peroxidase genes common to other basidiomycetes but also the widespread presence in Agaricales (and Russulales) of new peroxidases types not found in wood-rotting Polyporales, and other Agaricomycetes orders. Therefore, we analyzed the peroxidase evolution in Agaricomycetes by ancestral-sequence reconstruction revealing several major evolutionary pathways and mapped the appearance of the different enzyme types in a time-calibrated species tree.

## Introduction

Large amounts of organic carbon reside in soil (∼700 Gt in the upper 30 cm) (Batjes 2014) and plant biomass (∼455 Gt, with ∼300 Gt in cell-wall polymers) (Bar-On et al. 2018). Saprotrophic fungi colonizing soil and dead plant biomass play a crucial role in the global carbon cycle. Among them, basidiomycetes of the class Agaricomycetes include most wood-colonizing, soil-inhabiting, and litter-decomposing species in forests and grasslands (Lundell et al. 2014). Cellulose, hemicelluloses, and lignin are the main cell-wall components in vascular plants. Lignin protects cellulose and hemicelluloses, being highly recalcitrant toward degradation due to its aromatic nature and heterogeneous structure (Ralph et al. 2004). Therefore, lignin breakdown represents a critical step for both carbon cycling in terrestrial ecosystems and biomass utilization in lignocellulose biorefineries (Martínez et al. 2009). Wood-colonizing Agaricomycetes have developed two main strategies that allow them to overcome the recalcitrance of the lignin polymer. Typical white-rot fungi can depolymerize (and mineralize) lignin, providing access to cellulose and hemicelluloses, whereas brown-rot fungi are able to degrade wood polysaccharides with only a partial modification of lignin (Martínez et al. 2005, 2011), together with several Agaricomycetes causing undefined weak wood decay (Riley et al. 2014; Floudas et al. 2015). On the other hand, although soft rot is typically caused by fungi of the phylum Ascomycota, and their asexual states, characterized by forming cavity chains inside the secondary wall of wood (Daniel and Nilsson 1998), soft-rot-like decay has been reported for some white-rot Agaricomycetes forming the above characteristic cavities (Daniel et al. 1992; Martínez et al. 2005; Schwarze 2007; Sahu et al. 2020).

Comparative genomics has contributed to our understanding of white-rot and brown-rot decay mechanisms through evolutionary analyses of the enzyme families involved, most of them classified in the CAZy database (www.cazy.org; last accessed November 17, 2020) (Lombard et al. 2014). White rot has been related with the appearance of genes encoding ligninolytic class-II peroxidases (POD, CAZy AA2) in the Late Carboniferous, and subsequent duplication events (Floudas et al. 2012; Ruiz-Dueñas et al. 2013). The evolution of POD enzymes, with changes in their catalytic and other relevant properties, would parallel the evolution of lignin complexity in plants, as recently proposed by resurrection of ancestral Polyporales enzymes (Ayuso-Fernández et al. 2019). Unlike white-rot, brown-rot fungi have repeatedly appeared from white-rot ancestors through a process of contraction of genes involved in plant cell-wall degradation with loss of ligninolytic PODs. Among Agaricomycetes, those of the order Polyporales include most wood rotters and, after sequencing the first white-rot (Martinez et al. 2004) and brown-rot (Martinez et al. 2009) fungal genomes, more than seventy species of this order have been characterized at multiomic level (see Vanden Wymelenberg et al. 2009; Eastwood et al. 2011; Fernández-Fueyo et al. 2012; Floudas et al. 2012; Binder et al. 2013; Ruiz-Dueñas et al. 2013; Miyauchi et al. 2018, 2020; Oghenekaro et al. 2020, among others). In contrast to the state of knowledge on Polyporales, less is known about lignocellulose-degradation mechanisms in Agaricales, the largest order of Agaricomycetes (with ∼13,000 described species), despite their broader variety of substrates, from sound wood and decayed wood to soil. Nevertheless, some omic analyses have anticipated differences in the gene presence and expression in Agaricales with different ecologies (Morin et al. 2012; Bao et al. 2013; Sahu et al. 2020). Such differences would represent an adaptation of the so-called plant cell-wall degrading enzymes (PCWDE) (Kohler et al. 2015)—a repertoire of hydrolases, oxidoreductases, and other enzymes—to the variety of substrates that these fungi colonize in nature.

Hydrolytic enzymes, including glycoside hydrolases and carbohydrate esterases together with polysaccharide lyases, are responsible for: 1) polysaccharide depolymerization, 2) cleavage of bonds between hemicellulose polymers and between hemicelluloses and lignin, and 3) cutin/suberin depolymerization. Likewise, oxidoreductases are involved in both lignin and polysaccharide degradation. The latter includes the more recently discovered copper-containing lytic polysaccharide monooxygenases (LPMO, CAZy AA9-AA11 and AA13-AA16) acting on crystalline cellulose and xylan (Quinlan et al. 2011; Couturier et al. 2018; Filiatrault-Chastel et al. 2019). On the other hand, heme-containing PODs, and eventually heme-thiolate peroxidases/peroxygenases (HTP) and heme dye-decolorizing peroxidases (DyP), together with laccases of the multicopper-oxidase (MCO, CAZy AA1) superfamily, are responsible for lignin degradation acting synergistically with auxiliary oxidoreductases (Martínez et al. 2018). These auxiliary enzymes include the glucose–methanol–choline oxidase/dehydrogenase (GMC, CAZy AA3) superfamily and the copper-radical oxidase (CRO, CAZy AA5) family that provide the H_2_O_2_ required by peroxidases and Fenton-type reactions.

With the aim of shedding light on the evolution of lignocellulose-decaying lifestyles in Agaricales, we analyze here 52 fungal genomes of this and related orders (including 14 new sequenced genomes) focusing on the evolution of the PCWDE repertoires. We aim to correlate different saprotrophic lifestyles (such as white-rot and brown-rot wood decay, forest-litter and grass-litter degradation, and decayed-wood degradation) with different enzymatic machineries. This information would enable us to predict the ecologies of ancestors in the different Agaricomycetes orders. Relevant enzymes with a high evolutionary rate will be examined, and the ancestry of ligninolytic PODs analyzed in detail, given their key contribution to lignocellulose decay including new enzyme types.

## Results and Discussion

### Sequenced Genomes

**Fig. 1.**
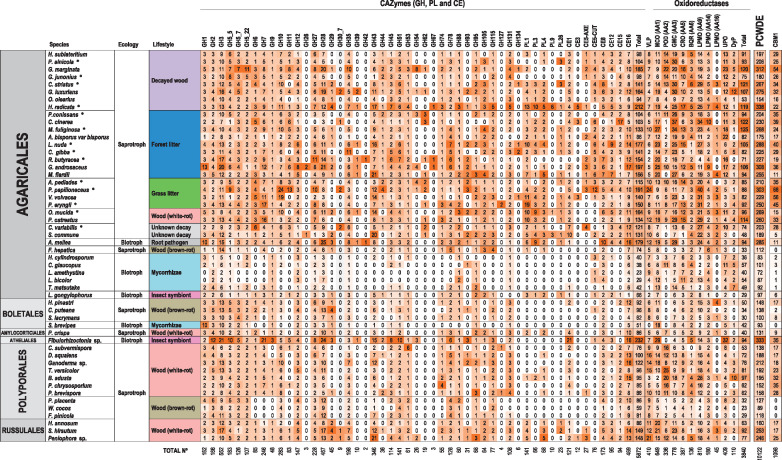
Distribution of classical CAZymes, oxidoreductases, CBM1, and versatile lipases (VLP) in the sequenced genomes of 52 Agaricomycetes species. The CAZyme genes identified correspond to 38 glycoside hydrolase (GH), five polysaccharide-lyase (PL), and seven carbohydrate-esterase (CE) families. MCO (AA1), POD (AA2), GMC (AA3), CRO (AA5), benzoquinone reductase (BQR, AA6), LPMO (AA9, AA14 and AA16), unspecific peroxygenases (UPO), and DyP oxidoreductase genes were identified. In each column, the color goes from orange, for the species with the highest number of genes, to white when an enzyme is absent. PCWDE does not include CBM1. Asterisks indicate the 14 species sequenced in the present study.

The interest for sequencing saprotrophic Agaricales is relatively recent compared with the interest on mycorrhizal Agaricales (Martin et al. 2008) and saprotrophic Polyporales genomes (Floudas et al. 2012), the exception being *Coprinopsis cinerea* as a model for morphogenetic studies (Stajich et al. 2010) and *Agaricus bisporus* as a model edible mushroom (Morin et al. 2012). Therefore, a number of Agaricales genomes have been sequenced in the frame of two JGI projects, and the results are analyzed here, together with previously available genomes from additional Agaricales and a selection of Agaricomycetes, up to a total of 52 species (fig. 1). Information on fungal strains, genome sequencing, assembly, and annotation is provided in supplementary sections I1–I3 (including tables S1–S4), Supplementary Material online.

High-quality-annotated genome assemblies were obtained for the 14 de novo sequenced species, with BUSCO completeness scores (ranging from 92.9% to 98.9%, supplementary table S3, Supplementary Material online) similar to those of other fungal genomes included in this study (Miyauchi et al. 2020). These 14 species cover the saprotrophic lifestyles in Agaricales according to the type of lignocellulosic substrate they preferentially, although not exclusively, degrade in nature. Among them, *Clitocybe gibba*, *Lepista nuda*, *Macrolepiota fuliginosa*, *Pholiota conissans*, and *Rhodocollybia butyracea* are forest-litter decomposers. *Pleurotus eryngii*, *Agrocybe pediades*, and *Panaeolus papilionaceus* are grassland-litter decomposers (the latter species as a late dung colonizer). *Crepidotus variabilis* grows on forest wood debris producing an uncharacterized degradation, whereas *Hymenopellis radicata*, *Pholiota alnicola*, *Cyathus striatus*, and *Gymnopilus junonius* grow on wood at advanced stages of decomposition (decayed-wood degraders). Finally, *Oudemansiella mucida* produces white-rot decay of wood, but soft-rot-like decay has also been reported (Daniel et al. 1992; Sahu et al. 2020). The 38 additional Agaricomycetes include 19 Agaricales and 19 Polyporales, Boletales, Russulales, Amylocorticiales, and Atheliales species, with saprotrophic and biotrophic lifestyles. Comparison of the species from the orders analyzed revealed higher lifestyle diversity in Agaricales (33 species with nine lifestyles in fig. 1) than in Polyporales (ten species with only white-rot and brown-rot lifestyles), with very different diversity-index values (calculated with the same algorithm used below for estimation of enzyme diversity), in agreement with the characteristic wood habitat of the latter fungi reported in literature (whereas no conclusive comparison with Boletales and Russulales could be established at this stage).

The 33 Agaricales genomes range in size from ∼28 to 175 Mb (supplementary figs. S1, left, and S2*A*, Supplementary Material online) due to the high content of transposable elements in some species (representing 66% and 63% of the *Leucoagaricus gongylophorus* and *Tricholoma matsutake* genomes, respectively) (supplementary fig. S7*B*, Supplementary Material online). Analysis of these repetitive sequences (supplementary section I8, including figs. S6–S9 and table S6; and data sets S1 and S2, Supplementary Material online) revealed that they had evolved independently of phylogenetic and lifestyle constraints. Noteworthy differences are also observed in the content of predicted genes in the Agaricales (between 5,000 and 29,000 genes, supplementary figs. S1, right, and S2*B*, Supplementary Material online). Despite these differences, phylogenetic analysis of variance (ANOVA) revealed that gene content and genome size are not significantly correlated to the broader ecology (lifestyles) in this order. Likewise, it was found that the differences do not respond to wide phylogenetic patterns, since they are not significant among the different orders analyzed.

### Phylogenetic Analysis and Molecular Dating

**Fig. 2.**
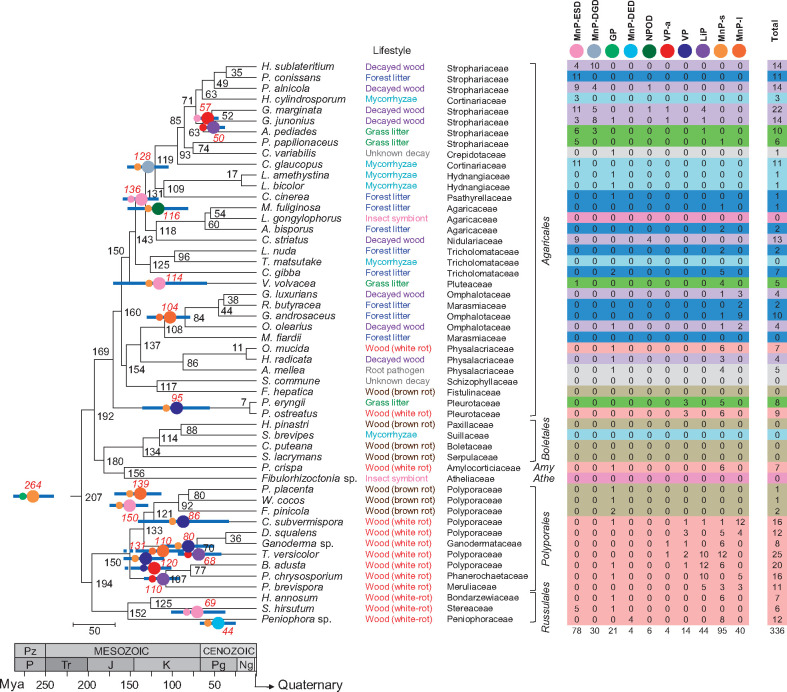
Evolution of Agaricomycetes with different peroxidase sets. (Left) ML organismal phylogeny inferred (with most nodes having >95% support) from the genomes of 52 Agaricales, Boletales, Amylocorticiales (*Amy*), Atheliales (*Athe*), Polyporales, and Russulales species. Mean ages of the ancestral species (Ma) are indicated (black numbers) adjacent to the nodes. Based on figure 5, the tree also shows: 1) the appearance of the different class-II peroxidase (POD) types (large circles colored using the POD color code in the right); 2) their estimated age (Ma), indicated by a red number; 3) the age 95% hpd-interval, indicated by blue bars; and 4) the ancestor from which they derived, indicated by a small circle to their left. (Right) POD types identified including lignin peroxidases (LiP), versatile peroxidases (VP), atypical VPs (VP-a), short (MnP-s), long (MnP-l) and new (MnP-ESD, MnP-DGD, and MnP-DED) manganese peroxidases, new PODs (NPOD), and generic peroxidases (GP), the latter not involved in lignin degradation (the colored background of the different rows correspond to the different lifestyles).

The evolutionary relationships between the 52 sequenced species were shown in a maximum-likelihood (ML) phylogenetic tree (fig. 2, left). The super-matrix used consisted of 25,699 sites from concatenated alignments of single-copy orthologous genes. The tree was time-calibrated using a penalized likelihood algorithm, with secondary calibration using dates for the origin of fungal clades provided by Varga et al. (2019). The resulting dated phylogeny, which recovered the six Agaricomycetes orders as monophyletic, is in good agreement with two recent megaphylogenies that include more than 1,000 (Krah et al. 2018) and 5,000 (Varga et al. 2019) Agaricomycetes, although our divergence dates are somewhat higher. Thus, the appearance of the most recent common ancestor (MRCA) of the Agaricomycetidae (including Agaricales, Boletales, Amylocorticiales, and Atheliales) was dated 192 million years ago (Ma), to the early Jurassic. Those of Agaricales (169 Ma), Polyporales (150 Ma), and Russulales (152 Ma) were also placed within this geological period, and the MRCA of Boletales (134 Ma) in the Early Cretaceous. The subsequent diversification and speciation has been reported to be especially accelerated in Agaricales (Varga et al. 2019).

Our time-calibrated organismal phylogeny shows the evolution of the main lignocellulose-degrading Agaricomycetes groups in the last 200 My. This class also includes biotrophic species derived from an ancestral white-rot saprotroph, as it would be the case of the mycorrhizal fungi and the root pathogen *Armillaria* species included in the present study (Floudas et al. 2012; Kohler et al. 2015; Nagy, Riley, Tritt, et al. 2016; Sipos et al. 2017; Sahu et al. 2020). The evolution maintained wood degradation (white rot and brown rot) widely represented in Polyporales, Russulales, Amylocorticiales, and Boletales. Moreover, new lifestyle evolution was common in Agaricales, with high diversity of lignocellulose-decaying lifestyles even at family level (fig. 2). Thus, in the Pleurotaceae family, *P. eryngii* and *Pleurotus ostreatus*, which in our phylogeny separated only 7 Ma, exhibit very different lignocellulose-decaying lifestyles (grassland litter and wood decay, respectively). Likewise, grass-litter (*A. pediades* and *P. papilionaceus*), forest-litter (*P. conissans*), and decayed-wood (*Hypholoma sublateritium*, *P. alnicola*, *Galerina marginata*, and *G. junonius*) degraders are found in the Strophariaceae family, which arose 85 Ma; and decayed-wood (*Hymenopellis radicata*) and wood (*O. mucida*) decomposers, together with root pathogen *Armillaria mellea*, are identified in the Physalacriaceae family, which also arose in the upper Cretaceous (86 Ma). In the latter family, white-rot decay by *O. mucida*, *A. mellea*, and other *Armillaria* species (the latter in the saprotrophic phase of their lifecycle) has been reported to include some soft-rot characteristics (Daniel et al. 1992; Schwarze 2007; Sahu et al. 2020). These observations taken together indicate not only a wide diversity but also a convergent evolution to different lignocellulose-decaying lifestyles in different lineages of the Agaricales phylogeny.

### Enzyme Repertoires and Lignocellulose-Decay Lifestyles

The genomes of saprotrophic Agaricomycetes encode a vast repertoire of carbohydrate- and lignin-degrading enzymes. A total of 18,645 classical CAZymes, 3,977 oxidoreductases, and 3,536 carbohydrate binding modules (CBMs) were identified in the 52 genomes analyzed (supplementary data set S3, Supplementary Material online). Among them, we focused on the PCWDE repertoire, and the cellulose-binding CBM1, directly or indirectly participating in the decay of plant biomass (Chen et al. 2013; Rytioja et al. 2014; Martínez et al. 2018) shown in figure 1. The white-rot, grass-litter, forest-litter, and decayed-wood Agaricales and Russulales analyzed showed the highest PCWDE numbers (up to >250 genes per genome), whereas similar values are not present in the Polyporales and Boletales genomes analyzed nor in the genomes of Agaricales with other lifestyles (supplementary fig. S10, Supplementary Material online). A first quantitative analysis of these enzymes revealed a larger PCWDE (lignocellulose-degrading CAZymes and oxidoreductases included) variability in Agaricales compared with the other orders analyzed (with diversity-index values Agaricales > Russulales > Polyporales > Boletales) (these differences are already shown in the gene-frequency distributions of supplementary fig. S12, Supplementary Material online).

Previous studies indicated that the white-rot machinery is the result of a complex evolutionary process with increased PCWDE gene numbers (Nagy, Riley, Bergmann, et al. 2016), whereas brown-rot, soft-rot, and ectomycorrhizal Agaricomycetes have reduced this gene complement (Floudas et al. 2012; Nagy, Riley, Tritt, et al. 2016), the latter with the simultaneous evolution of an ectomycorrhizal symbiosis toolkit (Kohler et al. 2015). Therefore, evolutionary changes in the PCWDE type and number are expected to contribute to the diversity of ecologies and lignocellulose-degradation lifestyles found in Agaricales. To visualize this diversity and the underlying relationships, we performed phylogenetic principal-component analysis (pPCA) using the gene-copy numbers of the 62 PCWDE families identified in figure 1, and the organismal tree of figure 2 (left) (for this analysis, the CE5 family was not split, in contrast to fig. 1, only laccases from the MCO superfamily were used, and CBM1 was included).

**Fig. 3.**
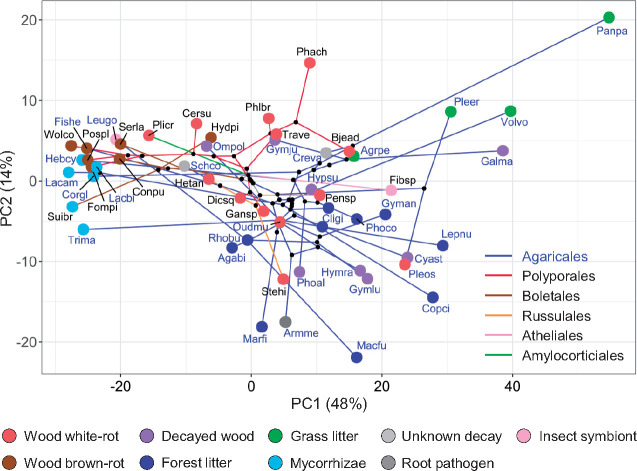
pPCA representing changes of fungal lifestyle through evolutionary time in 52 species from six Agaricomycetes orders. The multivariate phylomorphospace locates the species according to their enzymatic machineries (shown in fig. 1) and phylogenetic relationships (from fig. 2, left dated tree). In the PC1 versus PC2 plot presented, the species are shown as circles colored according to lifestyles, with branch colors of the phylogenetic tree projected into the morphospace indicating the order to which they belong. An interactive 3D plot is also available (as supplementary file S2, Supplementary Material online) and similar results (supplementary fig. S4, Supplementary Material online) were obtained using the seven gene families with higher evolutionary rates identified in figure 4. Species abbreviations: Agabi, *Agaricus bisporus*; Agrpe, *Agrocybe pediades*; Armme, *Armillaria mellea*; Bjead, *Bjerkandera adusta*; Cersu, *Ceriporiopsis subvermispora*; Cligi, *Clitocybe gibba*; Conpu, *Coniophora puteana*; Copci, *Coprinopsis cinerea*; Corgl, *Cortinarius glaucopus*; Creva, *Crepidotus variabilis*; Cyast, *Cyathus striatus*; Dicsq, *Dichomitus squalens*; Fibsp, *Fibulorhizoctonia* sp.; Fishe, *Fistulina hepatica*; Fompi, *Fomitopsis pinicola*; Galma, *Galerina marginata*; Gansp, *Ganoderma* sp.; Gyman, *Gymnopus androsaceus*; Gymlu, *Gymnopus luxurians*; Gymju, *Gymnopilus junonius*; Hebcy, *Hebeloma cylindrosporum*; Hetan, *Heterobasidion annosum*; Hydpi, *Hydnomerulius pinastri*; Hymra, *Hymenopellis radicata*; Hypsu, *Hypholoma sublateritium*; Lacam, *Laccaria amethystina*; Lacbi, *Laccaria bicolor*; Lepnu, *Lepista nuda*; Leugo, *Leucoagaricus gongylophorus*; Macfu, *Macrolepiota fuliginosa*; Marfi, *Marasmius fiardii*; Ompol, *Omphalotus olearius*; Oudmu, *Oudemansiella mucida*; Panpa, *Panaeolus papilionaceus*; Pensp, *Peniophora* sp.; Phach, *Phanerochaete chrysosporium*; Phlbr, *Phlebia brevispora*; Phoal, *Pholiota alnicola*; Phoco, *Pholiota conissans*; Pleer, *Pleurotus eryngii*; Pleos, *Pleurotus ostreatus*; Plicr, *Plicaturopsis crispa*; Pospl, *Postia placenta*; Rhobu, *Rhodocollybia butyracea*; Schco, *Schizophyllum commune*; Serla, *Serpula lacrymans*; Stehi, *Stereum hirsutum*; Suibr, *Suillus brevipes*; Trave, *Trametes versicolor*; Trima, *Tricholoma matsutake*; Volvo, *Volvariella volvacea*; Wolco, *Wolfiporia cocos*.

The resulting figure 3 shows the species phylogenetic relationships and distribution over the 2D pPCA plot, with the first two components explaining 62% of the data variability. The extant species of different lifestyles (colored circles) occupy the final positions of a tree with branch colors identifying the Agaricomycetes orders, and black circles corresponding to ancestral species. An interactive 3D plot, explaining 70% of the variance, is available as supplementary file S2, Supplementary Material online. On PC1, a variety of glycoside hydrolases (GH10 and GH11 xylanases included) and CBM1 have the highest positive loading factors, together with AA9 LPMO (supplementary fig. S3, Supplementary Material online). Concerning PC2, GH127 arabinofuranosidase and PL1 pectin-lyase have high positive factors, and several oxidoreductases (GMC and laccase included) high negative factors. Agaricales (blue branches) appear widely distributed given their above-mentioned eco-physiological diversity. A first distribution of Agaricomycetes lifestyles is evidenced along PC1, with ectomycorrhizal and brown-rot fungi in the negative part, decayed-wood, and forest-litter degraders in the central part, and grass-litter decomposers, including the coprophilous *P. papilionaceus*, in the positive part. White-rot fungi begin to differentiate from decayed-wood and forest-litter species on PC2, and differences with grass-litter fungi are better observed.

Furthermore, the 51 components explaining the whole data variability were used in a permutational analysis of variance (PERMANOVA) (supplementary table S5, Supplementary Material online). The five main lignocellulose-decaying lifestyles analyzed (white rot, brown rot, decayed wood, grass litter, and forest litter) plus mycorrhizae were determined to be collectively different according to the composition of their enzymatic sets, and all but one (forest-litter vs. decayed-wood decomposers) pairwise comparisons were found to be significantly (*P *<* *0.05) distinct. These observations reveal that fungi sharing lifestyle are related in their lignocellulose-degrading machinery, independently of their phylogenetic origin. The above results suggest that convergent evolution of the enzymatic sets toward a diversity of lignocellulose-decay lifestyles, and not only toward white rot and brown rot, has been produced in nature.

### Evolution of Saprotrophic Lifestyles (Computer-Assisted Gene-Family Evolution and Pattern Recognition Analyses)

**Fig. 4.**
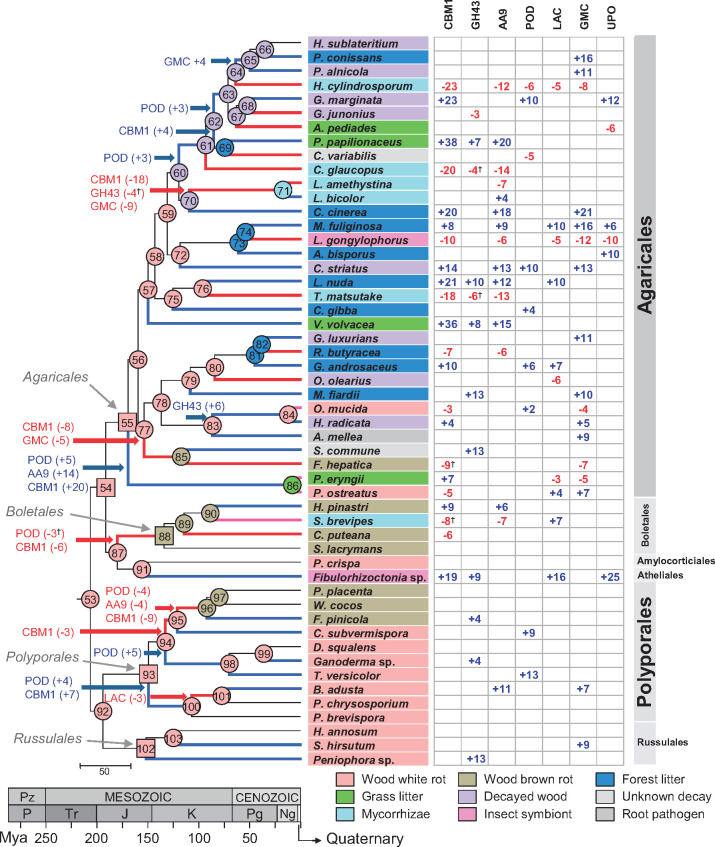
CAFE analysis revealing gene families with higher evolutionary rates in the phylogeny of the 52 species analyzed, and ancestral lifestyle predictions (as color codes at the tree nodes). Changes in gene-copy numbers estimated with CAFE in the seven families with significantly higher evolutionary rates (family-wide *P *<* *0.01)—that is, POD, laccase (LAC), AA9 LPMO, GMC, UPO, GH43, and CBM1—in the different ancestral nodes (left) and in the last evolutionary step leading to the extant species (right) are shown. Blue and red branches indicate significant expansion and contraction of enzyme numbers (Viterbi *P *<* *0.05) in blue and red font, respectively (magenta branches indicate that both expansions and contractions occurred). ^†^Complete loss of a gene type. The ancestral lifestyles reconstruction, carried out with a trained wknn algorithm (Samworth 2012), was based on gene numbers for the above seven families predicted by CAFE at the 53–103 nodes of the species tree. The nodes of the MRCAs of the Agaricomycetidae (node 54), Agaricales (node 55), Boletales (node 88), Polyporales (node 93), and Russulales (node 102) are shown as squares, whereas the rest of nodes are shown as circles (the predicted lifestyles are indicated by color codes).

Computer-assisted gene-family evolution (CAFE) analyses were performed with a 2-fold objective: 1) to determine enzyme diversification in the organismal phylogeny; and 2) to predict the overall content of the enzyme families in the ancestors of the extant species. As inputs, we used the time-calibrated fungal phylogeny (fig. 2, left) and the gene-copy numbers for the different PCWDE families (fig. 1). Twenty-four additional enzyme families participating in amino-acid metabolism (included in supplementary data set S4, Supplementary Material online), which are not expected to be involved in lignocellulose decay, were used as a background gene set. Surprisingly, only five oxidoreductase types (POD, laccase, AA9 LPMO, GMC, and unspecific peroxygenase [UPO]), one glycoside hydrolase (GH43), and CBM1 showed evolutionary rates significantly higher (family-wide *P *<* *0.01) than the average *λ* value for the whole enzyme set. The significant changes in gene-copy numbers in the different branches of the organismal tree (Viterbi *P *<* *0.05), and in the final step leading to the extant species, are shown in figure 4 (left and right, respectively).

The above suggests less abundant and more linear changes in the numbers of hydrolases (GH43 excluded), esterases, and lyases throughout Agaricomycetes evolution, in contrast with more abrupt contraction and expansion changes in oxidoreductases involved in cellulose and lignin degradation (five enzyme types). These conclusions are in agreement with recent results of An et al. (2019) on the evolution of mushroom lignocellulose-decay mechanisms. The expansion of ligninolytic PODs characterizing the evolution of white-rot Polyporales reported by Floudas et al. (2012), and here corroborated, is also observed in the evolution of saprotrophic Agaricales (fig. 4). This expansion of key enzymes in lignin degradation would have significantly contributed to the ecological diversity of this fungal order together with other changes in the enzyme sets, as described below.

As previously shown by PERMANOVA, white-rot, brown-rot, decayed-wood, forest-litter, grass-litter, and mycorrhizal lifestyles have distinct PCWDE repertoires. Based on these results, we hypothesized that it should be possible to assign the ancestors of the extant species to one of the six lifestyles using their enzymatic sets predicted by CAFE. To do this, we firstly trained the weighted k-nearest-neighbor (wknn) algorithm (with three tunable parameters) (Samworth 2012) using the copy numbers of the 62 families analyzed in the extant species as features. The accuracy of lifestyle prediction (i.e., the success rate when applied to the current species with known lifestyles) increased from 66% to 77% if the seven faster-evolving families (i.e., POD, laccase, AA9 LPMO, GMC, UPO, GH43, and CBM1) were used as input instead of the whole 62 families (supplementary fig. S5, Supplementary Material online). Therefore, the latter predictions provide us a useful tool for the prediction of ancestral lifestyles (supplementary section I7, Supplementary Material online) and confirmed the relevance of the above enzymes (and CBM) in lignocellulose-decay types.

By applying the trained algorithm to the selected enzyme sets reconstructed by CAFE (included in supplementary data set S4, Supplementary Material online), we were able to predict the lifestyles of the Agaricomycetes ancestors at the main nodes of the dated organismal tree. In this way, white-rot decay appeared as the ancestral lifestyle in our phylogeny (node 53 in fig. 4). This was also inferred for the MRCA of Agaricales (node 55), Polyporales (node 93), and Russulales (node 102), whereas the MRCA of Boletales appeared as a brown-rot fungus (node 88). Interestingly, the predicted ancestral lifestyles showed higher diversity within the phylogeny of Agaricales than of the other fungal orders (with decayed-wood and forest-litter as transition types between white-rot and other lifestyles, and grass-litter decomposers as the most recent lignocellulose-degrading group). Finally, our analysis showed multiple origins for the different saprotrophic lifestyles in the Agaricales (from white-rot ancestors), and also for mycorrhizae in the Agaricales and Boletales (from white-rot and brown-rot ancestors). Although limited by its current accuracy, the results obtained provide the first computational prediction on lifestyle evolution in saprotrophic Agaricales, and more generally in Agaricomycetes.

### Enzyme Families Contributing to Lifestyle Diversity in Saprotrophic Agaricales

The plant cell-wall decay machineries of the 52 fungal species under study were exhaustively analyzed (supplementary sections I9–I14, Supplementary Material online). However, given their relevance in the lignocellulose degradation process and contribution to the diversity of lignocellulose-decaying lifestyles, this section mainly focuses on the enzyme families, and CBM, with the highest evolutionary rates, previously identified by CAFE.

A variety of classical CAZymes acting on cell-wall polysaccharides were identified (supplementary section I9, including figs. S10–S15 and table S7, Supplementary Material online). The diversity of these enzymes in the genomes analyzed is higher in Agaricales than in Russulales, Polyporales, and Boletales, as shown by diversity-index values of 4.39, 4.29, 4.03, and 3.92, respectively (these differences are already visible in the gene-frequency histograms of supplementary fig. S11, Supplementary Material online). Very recently, diversity in number and types of enzymes has been described by Floudas et al. (2020) in a more reduced set of litter-decomposing Agaricales, and correlated to their ability to decompose crystalline cellulose investigated by Raman spectroscopy. Related to this, the AA9 LPMO family, initiating crystalline cellulose degradation, presents large expansions in some terminal lineages of saprotrophic Agaricales (fig. 4). These expansions have led to grass-litter decomposers as the saprotrophic group with the highest gene-copy numbers of this LPMO family (*P *<* *0.05 for pairwise comparisons, binomial exact test). Similarly, grass-litter Agaricales have a larger average number of PCWDE genes appended to CBM1 (42 vs. 22, 18, 7, 0, and 1 in white-rot Polyporales and Russulales, brown-rot Boletales and Polyporales, and mycorrhizal Agaricales, respectively) (*P *<* *0.05 for pairwise comparisons, binomial exact test) (supplementary table S7, Supplementary Material online). This agrees with the differences observed between the two *Pleurotus* species analyzed, with the grassland-inhabiting *P. eryngii* having more CBM-appended genes than the wood-rotter *Pleurotus ostreatus*. The latter finding could be related to an improved action of CBM-appended catalytic domains preventing enzyme leaching in a loose environment, such as leaf litter, compared with compact wood.

The high evolutionary rates of POD, GMC, laccase, and UPO gene families, paralleling the ecological diversification in Agaricales, support the relevance of the oxidative enzymatic machinery in the evolution of saprotrophic lifestyles in this order. Among these enzymes, laccases are well-known phenoloxidases, but other members of the MCO superfamily also have laccase-like activity. Every genome analyzed presents MCO genes, whose phylogenetic analysis (supplementary fig. S16, Supplementary Material online) revealed clusters of different MCO families (supplementary section I10, including figs. S16–S23 and table S8, Supplementary Material online). The average MCO number per genome was lower (40% decrease) in those species having at least one gene of the lignin peroxidase (LiP) family, discussed below, than in those lacking LiP (*P *=* *2.51 × 10^−5^, binomial exact test) (figs. 1 and 2) where MCOs appear overrepresented in the PCWDE set (*P *=* *2.67 × 10^−5^, Fisher’s exact test). These results suggest that MCOs can be an alternative to ligninolytic PODs in certain fungi and lifestyles. Laccases sensu stricto represent over 70% of all MCO genes (supplementary table S8, Supplementary Material online). Forest-litter Agaricales species have the highest numbers per genome (after *Fibulorhizoctonia* sp.) (*P *<* *0.05, binomial exact test). Among over 70 atypical MCO sequences identified, 28 have been classified as novel laccases containing an arginine residue instead of the conserved aspartate involved in concerted electron and proton transfer (Pardo et al. 2016). These laccases, are only found in Agaricales and Russulales, being particularly abundant in some forest-litter species. Two of them had already been characterized from *Pleurotus* species (Muñoz et al. 1997; Giardina et al. 2007). Another 29 sequences from Agaricales and Russulales also segregated from the rest, and have been classified as novel MCO lacking three of the conserved Cu-coordinating histidines (supplementary fig. S20*A*, Supplementary Material online). All nine genomes with this arrangement also encode the novel laccase described above, with the two enzyme families showing similar evolutionary events along the organismal phylogeny, which finally led to their loss in Polyporales and Boletales (supplementary figs. S19 and S21, Supplementary Material online).

UPO enzymes from the HTP superfamily have also been related to lignin degradation after showing that *Cyclocybe aegerita* UPO is able to demethylate nonphenolic lignin models, and degrade the released phenolic counterparts (Kinne et al. 2011). This enzyme is representative of the long-UPO subfamily, whereas the short-UPO subfamily includes enzymes from basidiomycetes and ascomycetes (Hofrichter et al. 2020). Among the 409 putative UPO genes identified in the 52 genomes analyzed (supplementary table S14, Supplementary Material online), short-UPOs are widely distributed and do not correlate with any lifestyle, whereas long-UPOs are nearly exclusive of Agaricales, and more abundant (*P *<* *0.05, binomial exact test) and overrepresented (*P *<* *0.05, Fisher’s exact test) in forest-litter and decayed-wood species compared with white-rot and brown-rot decomposers. Expression of UPOs, together with PODs discussed below, has been observed in leaf litter across forest ecosystems (Kellner et al. 2014). Despite UPO activity on nonphenolic lignin model dimers, this enzyme has very low activity on polymeric lignin (Kinne et al. 2009), probably because it lacks the long-range electron transfer route found in PODs (Martínez et al. 2018). Therefore, this enzyme most probably contributes to the transformation of lignin-derived compounds and soil organic matter by forest-litter and decayed-wood species, where we have seen it is more abundant.

Hydrogen peroxide plays a central role in lignocellulose degradation by Agaricomycetes, as the oxidizing substrate of peroxidases in white-rot decay (Martínez et al. 2009), and the precursor of hydroxyl radical in brown-rot decay (Baldrian and Valaskova 2008). The analysis of the enzymes of the GMC superfamily and CRO family responsible for H_2_O_2_ production (Kersten and Cullen 2014; Ferreira et al. 2015) in the 52 fungal species is presented in supplementary section I11 (including figs. S24–S30 and tables S9–S13), Supplementary Material online. GMC genes were found in all the genomes analyzed (supplementary table S9, Supplementary Material online). Their average numbers are higher in Agaricales (and Russulales) (*P *<* *0.05 for pairwise comparisons, binomial exact test) (supplementary table S11, Supplementary Material online), and within this order in the forest-litter, decayed-wood, and white-rot degraders (and in *A. mellea*), mainly due to the high content of aryl-alcohol oxidases (AAO, in CAZy AA3_2) (*P *<* *0.05 for pairwise comparisons, binomial exact test) (supplementary table S12, Supplementary Material online). Representing over 60% of the identified GMC genes, phylogenetic analysis revealed four AAO groups (supplementary fig. S25*A*, Supplementary Material online). AAO was absent from Boletales (and two Atheliales/Amylocorticiales). The distribution within the AAO groups also depends on the lifestyle, with most of the enzymes from grassland organisms grouped in the AAO-I subcluster, together with the well-known *P. eryngii* enzyme (Carro et al. 2016) (supplementary fig. S25*B*, Supplementary Material online).

Among the seven PCWDE and CBM families with the highest evolutionary rate, we paid special attention to ligninolytic PODs (supplementary section I12, including figs. S31 and S32, Supplementary Material online) in the superfamily of peroxidase-catalases (Zámocký et al. 2015) due to their key role in the overall process of lignocellulose degradation by white-rot fungi (Martínez et al. 2018). In contrast, they are absent from brown-rot fungi that only have, if any, nonligninolytic generic peroxidases (GP) (Ruiz-Dueñas et al. 2013). They are also absent from basidiomycetes characterized by their weak wood decay capabilities (Riley et al. 2014; Floudas et al. 2015). POD degradation of lignin, described as an enzymatic combustion (Kirk and Farrell 1987), facilitates the access of enzymes acting on the carbohydrate fraction of the plant cell wall (Martínez et al. 2009). Heme-containing DyPs are not discussed here because the corresponding genomic analyses (supplementary tables S15–S17 and figs. S33–S35, Supplementary Material online) do not show relevant relationships with the Agaricales lifestyles analyzed.

**Fig. 5.**
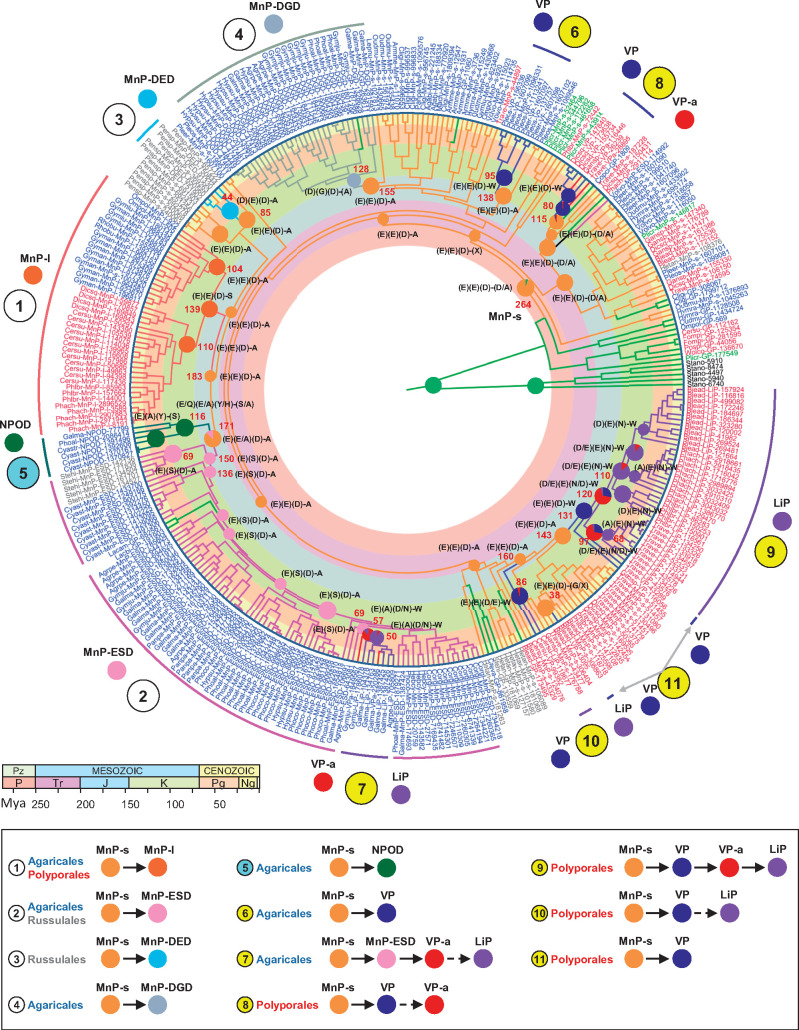
Dated ML phylogenetic tree of the 336 POD sequences identified, with indication of relevant enzyme residues at the tree internal nodes (deduced by sequence reconstruction with PAML) suggesting eleven evolutionary pathways. Mean ages of selected ancestors (in Ma) are indicated in red numbers adjacent to the nodes (95% hpd-intervals are shown in fig. 2, left). Five GP sequences of *Stagonospora nodorum* (Pleosporales, black labels) were used for rooting the tree. The 11 evolutionary pathways leading from ancestral MnP-s to extant POD types are indicated with white (those resulting in MnP subfamilies differing in the three Mn^2+^-binding residues, fig. 6*E*–*G*) or yellow (those giving rise to LiP/VP with a surface tryptophan) circles (the residues at these four positions are indicated adjacent to the nodes). A total of 95 MnP-s enzymes remained as such among the extant enzymes analyzed. The evolutionary pathway-5 (light blue circle) leads to PODs with alternative residues involved in enzyme activation by H_2_O_2_ and a putative catalytic tyrosine (fig. 6*H*). The color of the nodes refers to the POD type to which the reconstructed ancestor belongs (two color sectors indicate reconstruction of two different ancestral types with different probabilities), and the color of the branches indicates the type of the next ancestor (or current enzyme) in the tree: light green, GP; light orange, MnP-s; dark orange, MnP-l; dark blue, VP; red, VPa; purple, LiP; pink, MnP-ESD; gray, MnP-DGD; light blue, MnP-DED; dark green, NPOD. The font color of the enzymes at the tips of the branches denotes the order of the fungi they belong to: blue, Agaricales; green, Amylocorticiales; red, Polyporales; gray, Russulales; black, Pleosporales (Ascomycota). The color code of geological times, included as concentric circles, is explained in the geological timescale below the tree. The abbreviated enzyme names include the species abbreviation (as in fig. 3) followed by the (sub)family name, and the protein identification number at the corresponding JGI genomes.

A comprehensive study—including gene annotation, amino-acid sequence inspection, phylogenetic analysis, and examination of structural models—allowed classifying the 336 POD sequences identified in 42 of the 52 genomes into already-known and new families and subfamilies. The result revealed a surprisingly high diversity of POD enzymes in Agaricales, not seen before in other lignocellulose-degrading fungi. Among them, different manganese peroxidases (MnP), a family characterized by its ability to oxidize phenolic lignin and lignin compounds via Mn^3+^ chelates (Gold et al. 2000), were recognized representing over 82% of the total 336 POD genes (fig. 2, right). To better understand the above POD diversity, the phylogenetic tree was analyzed and enzyme evolution was compared with that described in Polyporales (Ayuso-Fernández et al. 2018). For that, the POD phylogeny was time-calibrated, the amino-acid sequences of ancestors at the different nodes were reconstructed with PAML (Yang 2007), the evolutionary pathways to the extant POD enzymes were determined (fig. 5), and the appearance of the first and other relevant representatives of each POD type was dated in the species tree (fig. 2, left) to correlate the evolution of enzymes and fungi, as discussed in the next section.

### Ligninolytic Peroxidases Revisited

#### Classical Manganese-Oxidizing Peroxidases (in Different Agaricomycetes)

**Fig. 6.**
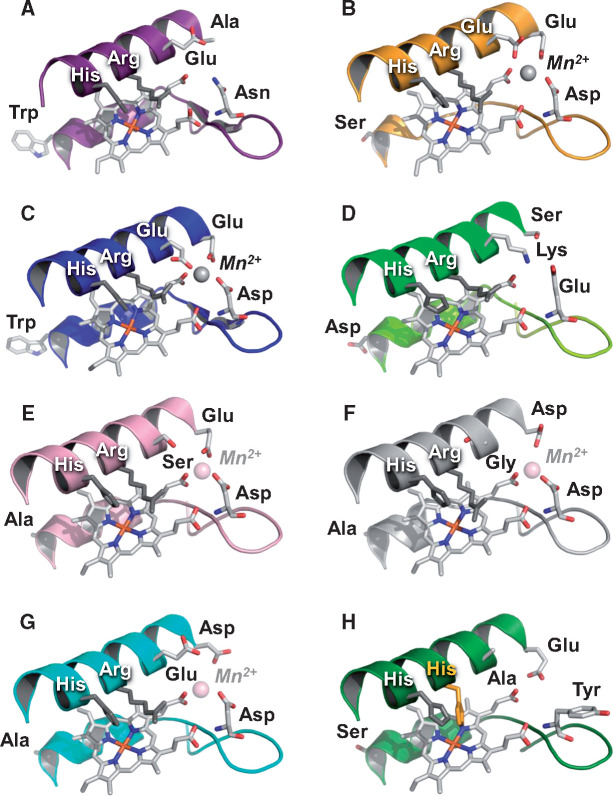
Axial view of the heme region in classical (*A*–*D*) and alternative (*E*–*H*) POD families identified in the 52 fungal genomes. (*A*) LiP of *Phanerochaete chrysosporium* (PDB 1LLP) with catalytic tryptophan exposed to the solvent. (*B*) MnP-l of *P. chrysosporium* (PDB 1MnP) with Mn^2+^-oxidation site formed by Glu/Glu/Asp residues (MnP-s presents the same Mn^2+^-oxidation site). (*C*) VP of *Pleurotus eryngii* (PDB 3FJW) combining the exposed tryptophan of LiP and the Mn^2+^-oxidation site of MnP. (*D*) GP of *Coprinopsis cinerea* (PDB 1ARP) lacking both the Mn^2+^-oxidation site (a lysine residue prevents cation binding) and the exposed tryptophan. (*E*–*G*) Homology models of the MnP-ESD (from *Cyathus striatus*, JGI ID 1429066), MnP-DGD (from *Agrocybe pediades*, JGI ID 699589), and MnP-DED (from *Peniophora* sp., JGI ID 91330) enzymes, characterized by alternative Mn^2+^-oxidation sites (formed by Glu/Ser/Asp, Asp/Gly/Asp, and Asp/Glu/Asp residues, respectively). (*H*) Homology model of the NPOD enzyme (from *C. striatus* JGI ID 1391496) where a Glu/Ala/Tyr triad occupies the position of the Mn^2+^-oxidation site in MnP and VP, with the tyrosine residue of this triad at the same position of the catalytic tyrosine in the *Trametopsis cervina* LiP (Miki et al. 2011), and a His/His couple substituting the conserved His/Arg involved in activation by H_2_O_2_ in other POD enzymes.

MnP enzymes belonging to the long (MnP-l) and short (MnP-s) subfamilies were identified in many of the lignocellulose-degrading Agaricales species (fig. 2, right) with >2 genes per genome in members of the Omphalotaceae, Physalacriaceae, Pleurotaceae, Pluteaceae, and Tricholomataceae families. All these enzymes have a Mn^2+^-oxidation site formed by two glutamates and one aspartate (fig. 6*B*) but differ in the C-terminal tail length (Fernández-Fueyo et al. 2014). Enzymes similar to classical MnP-l, originally described in Polyporales, were found in forest-litter and decayed-wood Agaricales (such as *Omphalotus olearius*, *Gymnopus luxurians*, *Gymnopus androsaceus*, and *R. butyracea*). These enzymes diverged from a MnP-l common ancestor dated ∼139 Ma (mean age, with 95% hpd-interval = 114–167 Ma) (evolutionary pathway-1 in fig. 5). Then, despite following independent evolutionary pathways, it seems that the Agaricales MnP-l has maintained the specificity for Mn^2+^ oxidation (to Mn^3+^), as reported in *R. butyracea* (Valaskova et al. 2007). In contrast, MnP-s enzymes, whose evolutionary origin is commented below, catalyze both Mn^3+^-mediated and Mn^3+^-independent oxidative reactions (Fernández-Fueyo et al. 2014). Moreover, in many Agaricales and some Russulales, these enzymes coexist or have been replaced by those described as atypical MnPs, presenting only two acidic residues at the Mn^2+^-oxidation site.

#### Alternative Manganese-Oxidizing Peroxidases (in Agaricales and Russulales)

The above “atypical MnP” enzymes were until recently only occasionally identified (Lundell et al. 2014; Kohler et al. 2015). However, we found here that they represent the only ligninolytic peroxidases in lignocellulose-decaying *C. striatus* (Nidulariaceae), *P. alnicola*, *P. conissans*, and *Hypholoma sublateritium* (Strophariaceae), as well as in ectomycorrhizal *Hebeloma cylindrosporum* and *Cortinarius glaucopus* (Cortinariaceae) (fig. 2, right). According to these findings, the latter enzymes can no longer be considered “atypical.” Moreover, they form well-defined clusters in the POD tree (fig. 5), and herein have been classified as two novel MnP subfamilies (globally representing 33% of all the POD sequences identified) differing in the Mn^2+^-binding residues: 1) MnP-ESD (Glu/Ser/Asp Mn^2+^-oxidation site, fig. 6*E*) with 78 genes identified (fig. 2, right); and 2) MnP-DGD (Asp/Gly/Asp Mn^2+^-oxidation site, fig. 6*F*) with 30 genes. The Mn^3+^-mediated activity of the novel subfamilies is supported by results reported for the *Agrocybe praecox* MnP (Hilden et al. 2014) herein classified as MnP-ESD. Following the same criterion, a third novel minor MnP type is reported with a different Mn^2+^-oxidation site. Formed by Asp/Glu/Asp residues (MnP-DED in fig. 6*G*), it was identified in four enzymes of *Peniophora* sp. (fig. 2, right) clustering separately from MnP-ESD and MnP-DGD.

In the search for the origin of the above alternative MnP enzymes, ancestral-sequence reconstruction predicted that different MnP-s enzymes were the ancestors of the MnP-ESD, MnP-DGD, and MnP-DED subfamilies. MnP-ESD in Agaricales and Russulales emerged from a MnP-ESD common ancestor dated ∼150 (128–173) Ma (evolutionary pathway-2 in fig. 5). Moreover, an ancestral MnP-s (∼183 Ma) was predicted to be the common ancestor of both MnP-ESD and MnP-l (therefore also representing the beginning of the evolutionary pathway-1). The above results suggest that the MnP-ESD subfamily was present in the MRCA of Russulales and Agaricomycetidae (Agaricales, Boletales, Amylocorticiales, and Atheliales included) which has been reconstructed as a wood white-rot fungus (fig. 4). Although MnP-ESD enzymes can be currently found in wood-degrading *Stereum hirsutum* (Russulales), where they have been related to ligninolytic activity (Presley et al. 2018), this MnP subfamily is characteristic of Agaricales with a variety of lignocellulose-degrading (grass-litter, forest-litter, and decayed-wood) lifestyles (fig. 2, right). Moreover, MnP-ESD enzymes are the only ligninolytic PODs identified in ectomycorrhizal *Hebeloma cylindrosporum* and *Cortinarius glaucopus* (Cortinariaceae), where they have been related to the reworking of recalcitrant (partially lignin-derived) organic matter in forest soils (Bodeker et al. 2014). Their absence in other mycorrhizal Agaricales (such as *Laccaria amethystina*, *Laccaria bicolor*, and *Tricholoma matsutak*e) suggests a different reduction of the PCWDE complement (Kohler et al. 2015) in the independently evolved mycorrhizal lineages these species belong to. Similarly, their absence in all Polyporales indicates reduction events and loss in the MRCA of this order. On the other hand, the six atypical MnP enzymes identified in *Auricularia* (Floudas et al. 2012) can now be classified as MnP-ESD, suggesting an independent evolutionary lineage leading to the extant MnP-ESD enzymes in the early-diverging Auriculariales.

In contrast, MnP-DGD and MnP-DED present single evolutionary origins ∼128 (104–152) Ma and ∼44 (24–66) Ma, respectively (evolutionary pathway-4 and pathway-3, respectively, in fig. 5). According to our analysis, the MnP-DED subfamily arose within the Russulales, and the MnP-DGD subfamily appeared in an ancestral Agaricales species (fig. 2, left), most probably in the transition between white-rot and decayed-wood lifestyles (ancestors at the nodes 59 and 60 in fig. 4). Subsequent expansions resulted in the number of MnP-DGD (up to ten genes per genome) in grass-litter and decayed-wood decomposers of the family Strophariaceae (fig. 2, right). Although the oxidative capabilities of MnP-DED and MnP-DGD have yet to be demonstrated, results from site-directed mutagenesis of the similar Mn^2+^-oxidation site of versatile peroxidase (VP) (Ruiz-Dueñas et al. 2007) suggest that both enzymes should be able to generate Mn^3+^ oxidizer, although maybe less efficiently than typical MnP enzymes.

#### Evolution to “Ligninases” Par Excellence

The evolution of PODs in Agaricales has, however, gone beyond MnP. Sequence reconstructions of the progeny of ancestral MnP-s predict the appearance of a surface tryptophan, conferring the ability to abstract electrons from nonphenolic lignin and transfer them to the cofactor via a long-range electron transfer pathway as “true ligninases” (Sáez-Jiménez et al. 2016). This change led to a first VP (evolutionary pathway-6 in fig. 5) that evolved to the extant *Pleurotus* VPs (fig. 6*C*). These enzymes are characterized by combining the catalytic properties of MnP, associated with the Mn^2+^-oxidation site, and LiP, associated with the surface tryptophan (Ruiz-Dueñas et al. 2007, 2009). In an independent evolutionary pathway, the surface catalytic tryptophan appeared ∼57 (44–71) Ma (fig. 2, left) in an atypical VP (VP-a) of a decayed-wood decomposer (most probably the ancestor at node 67 in fig. 4). This VP-a was an intermediate step (before loss of the Mn^2+^-oxidation site) in MnP evolution toward the LiP enzymes identified in three decayed-wood and grass-litter (*G. marginata*, *A. pediades*, and *G. junonius*) Strophariaceae species (evolutionary pathway-7 in fig. 5). The pathway is characterized by including an ancestral MnP-ESD, unlike the pathway leading to *Pleurotus* VPs and the four pathways leading to Polyporales VPs and LiPs (fig. 6*A*), two of them (pathway-8 and pathway-9 in fig. 5) experimentally demonstrated by enzyme resurrection (Ayuso-Fernández et al. 2018). Finally, six members of a second LiP subfamily that, instead of the tryptophan described above, has a surface tyrosine at the same position of the catalytic tyrosine in the *Trametopsis cervina* (Polyporales) LiP (supplementary fig. S31*A*, Supplementary Material online) (Miki et al. 2013) have also been identified in three decayed-wood Agaricales (*P. alnicola*, *G. Marginata*, and *C. striatus*) (fig. 2, right). However, they were cataloged as a new POD (NPOD) type due to the residues putatively involved in activation by H_2_O_2_ (fig. 6*H*), which differ from those conserved in all POD enzymes characterized to date.

#### Overview of POD Evolution

The above analysis of POD evolution brings to light the large number of changes that occurred in these enzymes along the evolutionary time, leading to different families and subfamilies. MnP-s would be the ancestral type since, according to figure 5, it appeared ∼264 (240–286) Ma, followed by MnP-ESD being ∼150 (128–173) Ma with a probable second origin by convergent evolution in the Auriculariales. From these ancestral enzymes the other POD types arose inside the different lineages able to degrade lignocellulose. Thus, MnP-DGD and NPOD appeared in Agaricales, MnP-DED arose in Russulales, and VP and LiP appeared several times in Agaricales and Polyporales by convergent evolution from different POD ancestors. According to our predictions, most POD subfamilies appeared in white-rot ancestors, with the exception of MnP-DGD that probably appeared in the transition between white-rot and decayed-wood lifestyles, and VP-a from Agaricales in a decayed-wood decomposer. The different POD families would be the result of exploration of new mechanisms to modify lignin (Ayuso-Fernández et al. 2017). The changes in the catalytic residues that have allowed us to establish the different evolutionary pathways are just the tip of the iceberg of the many other changes that would have occurred in their molecular architecture throughout evolution (e.g., affecting substrate affinity and oxidation rate, stability, or redox potential). Some of these changes, identified in Polyporales by ancestral POD resurrection, were related to lignin evolution in plants (Ayuso-Fernández et al. 2019). A similar evolution would be expected in Agaricales, which most likely would have led to an evolutionary adaptation of their enzymes to the different lignocellulosic substrates on which these fungi grow in nature, although this has yet to be demonstrated experimentally.

## Conclusions

The genomic analysis herein presented has revealed that, compared with other orders of Agaricomycetes, the Agaricales possess a larger diversity of PCWDEs, in agreement with their wider contribution to global carbon cycle in both forest and grassland ecosystems. This enzyme diversity has resulted in distinct PCWDE repertoires responsible for the different lignocellulose-decaying lifestyles characterizing this order. Based on the reconstruction of ancestral enzyme inventories, a wood white-rot species was predicted as the MRCA of the Agaricales, Polyporales, Russulales, Boletales, Amylocorticiales, and Atheliales orders. This ancestral lifestyle was maintained in the MRCAs of Agaricales, Polyporales, and Russulales, whereas it changed to brown rot in the ancestor of Boletales. In the latter three orders, the lignocellulolytic systems evolved around a dominant saprotrophic nutritional strategy primarily focused on wood. By contrast, we predict that changes in the composition of the lignocellulolytic system during the last 170 My led Agaricales ancestral species to shift their growth preferences toward a wider diversity of lignocellulosic substrates. Our results indicate that these shifts of the enzyme sets converged several times in different saprotrophic (grass litter, forest litter, and decayed wood) and also biotrophic (e.g., mycorrhizal) lifestyles in the phylogeny of this order.

In these evolutionary processes, expansion/contraction and diversification of key enzymes involved in lignocellulose degradation are produced. Thus, a significant expansion of AA9 LPMOs acting on crystalline cellulose and the increase in CBM1-appended PCWDEs are outstanding evolutionary events in grass-litter decomposers. Similarly, other oxidoreductases both directly involved in lignin degradation (such as POD, GMC, and laccase-type enzymes) and/or contributing to the catabolism of lignin-derived compounds (such as some of the above enzymes plus UPO) markedly evolved in Agaricales. This is evidenced by the increased abundances of laccases sensu stricto and long-UPO enzymes characterizing the forest-litter decomposers (the latter enzymes being also abundant in decayed-wood species) and by the widespread increase of AAO in forest-litter, decayed-wood, and white-rot Agaricales. Finally, POD enzymes, characterized by their key role in lignin degradation, underwent an evolutionary process resulting in the surprisingly high diversity of families and subfamilies identified in the extant Agaricales. Different evolutionary pathways were identified for ligninolytic peroxidases, leading from ancestral short MnP to long MnP, LiP, VP, and several new POD types. The latter include two alternative MnP subfamilies widespread in Agaricales, where they represent over 50% of all the POD genes. One of these subfamilies, MnP-ESD, appeared before the diversification of Russulales and Agaricomycetidae, remaining only in Agaricales (and Russulales) with very different lignocellulose-decaying lifestyles, whereas MnP-DGD arose directly in an ancestral species of the order Agaricales and it is specific to grass-litter and decayed-wood decomposers. All of the above indicates that the lignocellulolytic machinery of leaf-litter decomposers would have differentiated from that of wood-degrading fungi in key enzymes acting on the polysaccharide and lignin fractions of the plant cell wall, resulting in: 1) diversification of ligninolytic peroxidases; 2) increased amount of CBM1-appended PCWDEs, and AA9 LPMO enzymes (in grassland decomposers); and 3) enrichment in laccases sensu stricto and long-UPOs (in forest-litter decomposers).

In short, a detailed analysis of Agaricomycetes lifestyles is herein presented based on comparative genomic data. This analysis unveils how Agaricales have developed complex enzymatic machineries to decompose the different types of lignocellulosic biomass constituting their carbon and energy source. Although it remains to be fully understood how the elements of these machineries engage to be efficient in nature, with this study we have contributed to expand our knowledge of the functional diversity of the Agaricales, one of the most diverse orders of fungi existing in nature.

## Materials and Methods

### Genome Sequencing, Assembly, and Annotation

New Agaricales genomes were sequenced in the JGI projects “Study of the lignocellulolytic machinery in saprobic wood and leaf litter degrading Agaricales” and “Metatranscriptomics of forest soil ecosystems.” Detailed information on the fungal strains, culture conditions, DNA and RNA extraction, and genome sequencing, assembly, and annotation are provided in supplementary sections I1–I3, Supplementary Material online.

Briefly, genomes were sequenced using Illumina and PacBio technologies, assembled with Velvet (Zerbino and Birney 2008), AllPathsLG version R49403 (Gnerre et al. 2011), or Falcon (https://github.com/PacificBiosciences/FALCON; last accessed November 17, 2020), improved with finisherSC (Lam et al. 2015), polished with Quiver (https://github.com/PacificBiosciences/GenomicConsensus; last accessed November 17, 2020), and annotated with the JGI Annotation pipeline. RNA-seq transcriptomic data were used to improve the annotation of the fungal genomes. BUSCO version 4.1.1 assessment tool (Seppey et al. 2019) was used in both genome and protein modes to determine the completeness scores of the new genomic assemblies and annotations with the specific data set agaricales_odb10 (3870 BUSCO groups) downloaded from https://busco-data.ezlab.org/v4/data/lineages; last accessed November 17, 2020. Phylogenetic ANOVA using the function phylANOVA (Garland et al. 1993) implemented in the R phytools package (Core Team 2020) was employed to test for differences in genome size and gene content among fungal orders and among fungal lifestyles.

The 14 de novo sequenced, 35 published (Martin et al. 2008; Martinez et al. 2009; Ohm et al. 2010; Stajich et al. 2010; Eastwood et al. 2011; Fernández-Fueyo et al. 2012; Floudas et al. 2012; Morin et al. 2012; Wawrzyn et al. 2012; Aylward et al. 2013; Bao et al. 2013; Binder et al. 2013; Collins et al. 2013; Ohm et al. 2014; Riley et al. 2014; Branco et al. 2015; Floudas et al. 2015; Kohler et al. 2015; Nagy, Riley, Tritt, et al. 2016; Barbi et al. 2020) and three pending publication genomes analyzed (up to a total of 52) cover 28 families within Agaricales, Polyporales, Russulales, Boletales, Atheliales, and Amylocorticiales. All are available at the JGI MycoCosm (https://mycocosm.jgi.doe.gov; last accessed November 17, 2020).

### Identification of PCWDE and Other Genes in Genomes

Classical CAZymes and LPMO genes were annotated by the CAZy pipeline (Lombard et al. 2014). POD, MCO, GMC, CRO, DyP, and UPO oxidoreductases were identified in the automatically annotated genomes by BLASTing amino-acid sequences of representative enzymes as queries. The identified oxidoreductases were assigned to different families and subfamilies based on analysis of their amino-acid sequences, active sites in structural models generated at the SWISS-MODEL server (Waterhouse et al. 2018), and phylogenetic relationships. Bonferroni-corrected one-tailed exact binomial tests and one-tailed Fisher’s exact tests, implemented in R, were performed to determine the differences in PCWDE families copy number and enrichment between biological groups, respectively. Identification of repetitive sequences was performed by the RECON (Bao and Eddy 2002) and RepeatScout (Price et al. 2005) programs. Gene-family diversity in the fungal groups (e.g., orders) whose genomes were sequenced was estimated using the following algorithm (Brillouin 1962):

$$\mathrm{diversity}=\frac{1}{N}\left(\log_{2}\;N!-\overset{i=s}{\underset{i=1}{\Sigma }}\log_{2}\;n_{i}!\right),$$

where *N* is the total gene number (of PCWDEs, CAZymes, etc.), and *n_i_* are the average numbers of genes of each of the *s* enzyme families.

### Species Tree and Molecular Dating

We reconstructed an organismal phylogeny with orthologous genes identified in the 52 genomes using FastOrtho with the parameters set to 50% identity, 50% coverage, and inflation 3.0 (Wattam et al. 2014). Clusters with single-copy genes were identified and aligned with MAFFT 7.221 (Katoh and Standley 2013), single-gene alignments were concatenated, and ambiguous regions (containing gaps and poorly aligned) were removed with Gblocks 0.91b (Talavera and Castresana 2007). A ML tree was reconstructed with RAxML v. 8 (Stamatakis 2014) using the PROTGAMMAWAG model of sequence evolution and 1,000 bootstrap replicates.

The species phylogeny was time-calibrated using the penalized likelihood algorithm as implemented in r8s 1.8.1 (Sanderson 2003) with the POWELL optimization algorithm. A secondary calibration was performed using the ranges of dates for the origin of Agaricales (160–182 Ma), Boletales (133–153 Ma), and Agaricomycetidae (174–192 Ma) from Varga et al. (2019). A cross-validation analysis was performed in order to identify the appropriate smoothing parameter.

### Phylogenetic PCA

pPCA was performed using the function phyl.pca (Revell 2009) from the R phytools package (Core Team 2020). As input, we used the time-calibrated species tree and the matrix of 62 PCWDE families and CBM1 (included in supplementary data set S4, Supplementary Material online). Then, the phylomorphospace function (phytools) was used to show the species phylogenetic relationships and ancestral node position over the pPCA plot. The significance of the differences observed in the phylomorphospace was tested by PERMANOVA as implemented in the Adonis function of the R package vegan (Oksanen et al. 2019) using the first 51 principal components (explaining the whole data variability) to reduce redundancy. Pairwise tests were performed applying the False Discovery Rate (FDR) correction to the *P* values (Benjamini and Hochberg 1995). An alpha of 0.05 was used as the cutoff for significance.

### Gene-Family Evolution Analysis

The CAFE ver. 4.1 program (De Bie et al. 2006; Han et al. 2013) was used to analyze gene-family expansions and contractions in the 62 enzyme families and CBM1 previously described for pPCA. The time-calibrated species tree and the gene-family numbers, including those of 24 additional families participating in amino-acid metabolism (present in the 52 fungal genomes) as a background gene set (supplementary data set S4, rows 1–53, Supplementary Material online), were used as inputs. The gene-copy numbers of all the above enzymes (and CBM1) at ancestral nodes reconstructed with CAFE are presented in rows 54–104 of supplementary data set S4, Supplementary Material online. The ML value of the birth and death parameter (*λ*) describing the probability that any gene will be gained or lost, over a time interval, was estimated for the whole tree. Gene families with a family-wide *P* value < 0.01 were considered to have a significantly higher rate of evolution. Then, a Viterbi *P* value < 0.05 was used to determine the branches in the species tree in which these fast-evolving families underwent significant contractions or expansions.

### Ancestral Lifestyle Reconstruction

The above CAFE estimations of gene-copy numbers at each node of the organismal phylogeny were used to infer the ancestral lifestyles, using either the whole 62 PCWDE families annotated in the genomes or the seven faster-evolving enzymes and CBM (i.e., POD, laccase, AA9 LPMO, GMC, UPO, GH43, and CBM1) identified by the program. This was done using the machine-learning wknn algorithm (Samworth 2012) as implemented in the kknn R package, which was trained using the known lifestyles and identified enzyme repertoires of the extant species analyzed. Model training consisted of tuning up different parameters, including 1) number of neighbor (*k*) values from 1 to 25 (higher *k* values were not used to avoid bias of the results toward the most abundant lifestyles); 2) distance function (we tested several *d* values and it was found that precision decreases when *d* increases, so we finally used Manhattan distance, *d* = 1); and 3) Kernel function (i.e., rectangular, triangular, epanechnikov, gaussian, rank, and optima) used to translate distances into weights. Combining these parameters, 300 predictive models were evaluated (supplementary fig. S5, Supplementary Material online) using the leave-one-out cross-validation test to optimize the results in terms of accuracy. Finally, the model with the highest accuracy was selected for classification of the ancestral node lifestyles.

### POD Phylogenetic and Molecular Clock Analyses

A total of 336 POD amino-acid sequences were aligned with MUSCLE, as implemented in MEGA X (Kumar et al. 2018). The GP sequences of *Stagonospora nodorum*, available at JGI (http://genome.jgi.doe.gov/Stano1/Stano1.home.html; last accessed November 17, 2020), were included in the alignment for tree rooting. The ML phylogeny was reconstructed with RAxML ver. 8 through the CIPRES Science Gateway ver. 3.3 (Miller et al. 2015) using 1,000 bootstrap replicates, under the WAG model of evolution and gamma-distributed rate of heterogeneity, with empirical amino-acid frequencies and invariant sites (WAG + I + G + F) as suggested by ProtTest (Darriba et al. 2011) (supplementary fig. S32, Supplementary Material online).

The peroxidase phylogeny was time-calibrated with BEAST v. 2.2.1 (Bouckaert et al. 2014) using an uncorrelated lognormal relaxed molecular clock with a birth-death prior and a WAG substitution model. The topology was fixed using the POD phylogenetic tree from the RAxML analysis described above. A secondary calibration was performed using the dates of the split of Dikarya, the origin of Basidiomycota, and the origin of Pezizomycotina (Floudas et al. 2012). Four independent chains were run for 50 million generations each and sampling every 5,000 generations. Chain convergence was assessed using Tracer 1.7.1 (Rambaut et al. 2018). Fifty percent of the samples were discarded as burn-in and a maximum clade credibility tree with mean ages was obtained with TreeAnnotator 2.2.1.

### Reconstruction of Ancestral POD Enzymes

PAML 4.7 (Yang 2007) was used to obtain the most probable sequence at each node of the POD phylogeny, as reported for reconstruction of Polyporales ancestral POD enzymes (Ayuso-Fernández et al. 2019). We used the WAG model of evolution, and the previously obtained MUSCLE alignment and ML phylogeny as inputs for the software. Then, as with extant POD sequences, relevant information was obtained on 1) proximal histidine, 2) distal residues responsible for activation by H_2_O_2_, and 3) Mn^2+^-oxidation site and surface tryptophan (or tyrosine) residue involved in oxidation of lignin. This information enabled us to classify the reconstructed ancestors in the different POD types illustrated in figure 6.

Additional information on gene identification, ancestral lifestyle and sequence predictions, species and POD phylogenetic analyses, and tree time-calibration are provided in Supplementary Material online.

## Supplementary Material

Supplementary data are available at *Molecular Biology and Evolution* online.

## Supplementary Material

msaa301_Supplementary_Data
