# Supplementary material for: Genomic Analysis Enlightens Agaricales Lifestyle Evolution and Increasing Peroxidase Diversity
Source: Mol Biol Evol. 2020 Nov 19;38(4):1428–46. doi: 10.1093/molbev/msaa301 (PMC8480192; doi:10.1093/molbev/msaa301)
Supplement: msaa301_Supplementary_Data [file msaa301_Supplementary_Data.zip › RuizDuenas-SM file S1-revised .pdf]

SUPPLEMENTARY MATERIAL – File S1

Genomic Analysis Enlightens Agaricales Lifestyle Evolution and Increasing Peroxidase Diversity

Francisco J. Ruiz-Dueñas,[*,1] José M. Barrasa,[*,2] Marisol Sánchez-García,[3,#] Susana Camarero,[1] Shingo Miyauchi,[4, ‡] Ana Serrano,[1] Dolores Linde,[1] Rashid Babiker,[1] Elodie Drula,[5] Iván Ayuso-Fernández,[1,¶] Remedios Pacheco,[1] Guillermo Padilla,[1] Patricia Ferreira,[6] Jorge Barriuso,[1] Harald Kellner,[7] Raúl Castanera,[8,§] Manuel Alfaro,[8] Lucía Ramirez,[8] Antonio G. Pisabarro,[8] Robert Riley,[9] Alan Kuo,[9] William Andreopoulos,[9] Kurt LaButti,[9] Jasmyn Pangilinan,[9] Andrew Tritt,[9] Anna Lipzen,[9] Guifen He,[9] Mi Yan,[9] Vivian Ng,[9] Igor V. Grigoriev,[9,10] Daniel Cullen,[11] Francis Martin,[4] Marie-Noëlle Rosso,[12] Bernard Henrissat,[5,13] David Hibbett,[3] Angel T. Martínez[*,1]

[1]Centro de Investigaciones Biológicas Margarita Salas (CIB), CSIC, Madrid, Spain
[2]Life Sciences Department, Alcalá University, Alcalá de Henares, Spain
[3]Clark University, Worcester, MA, USA
[4]INRAE, Laboratory of Excellence ARBRE, Champenoux, France
[5]Architecture et Fonction des Macromolécules Biologiques, CNRS/Aix-Marseille University, France
[6]Biochemistry and Molecular and Cellular Biology Department and BIFI, Zaragoza University, Spain
[7]Technische Universität Dresden, International Institute Zittau, Zittau, Germany
[8]Institue for Multidisciplinary Research in Applied Biology, IMAB-UPNA, Pamplona, Spain
[9]US Department of Energy (DOE) Joint Genome Institute (JGI), Lawrence Berkeley National Laboratory, Berkeley, CA, USA
[10]Department of Plant and Microbial Biology, University of California, Berkeley, CA, USA
[11]US Department of Agriculture Forest Products Laboratory, Madison, WI, USA
[12]INRAE, Aix-Marseille University, Biodiversité et Biotechnologie Fongiques, Marseille, France
[13]Department of Biological Sciences, King Abdulaziz University, Jeddah, Saudi Arabia

*Corresponding authors: fjruiz@cib.csic.es (FJR-D), josembarrasa.61@gmail.com (JMB) and atmartinez@cib.csic.es (ATM)
[#]Current address: Uppsala Biocentre, Swedish University of Agricultural Sciences, Uppsala, Sweden
[‡]Current address: Max Planck Institute for Plant Breeding Research, Köln, Germany
[¶]Current address: Norwegian University of Life Sciences (NMBU), Ås, Norway
[§]Current address: Centre for Research in Agricultural Genomics, CSIC-IRTA-UAB-UB, Barcelona, Spain

Table of Contents

I. Supplementary Methods, Results and Discussion (*page 6*)

1. Fungal strains and culture conditions (*page 6*)

2. DNA and RNA extraction (*page 6*)
   - Table S1 Agaricomycetes genomes analyzed (*page 7*)

3. Genome sequencing, assembly and annotation (*page 8*)
   - Table S2 Summary statistics of genome assemblies (*page 9*)
   - Table S3 Summary statistics of annotated genomes (*page 10*)
   - Table S4 Links to the fungal genomes analyzed in this study available at the DOE JGI (*page 11*)

- Fig. S1 Bar chart showing the genome sizes (megabase pairs, Mb) and gene contents in 52 fungal species belonging to six Agaricomycetes orders (Agaricales, Boletales, Amylocorticiales, Atheliales, Polyporales and Russulales) (*page 12*)
- Fig. S2 Box plots showing the genome size (A) and gene content (B) variation among Agaricales, Polyporales, Boletales and Russulales orders based on the 52 Agaricomycetes species analyzed (*page 13*)

4. Phylogenetic analysis of the Agaricomycetes species and molecular dating (*page13*)

5. Phylogenetic principal-component analysis (pPCA) and phylomorphospace (*page 14*)
   - Fig. S3 pPCA of the 52 species analyzed according to their PCWDE repertoires, including lifestyle information. A) PC1 *vs* PC2 plot showing the distribution of species according to the composition of their enzymatic machineries, with the species as circles colored according to their lifestyles. B) Loading vectors indicating the direction and strength of the most significant enzyme families contributing to the distribution of the species in the 2D pPCA plot (*page 14*)
   - Table S5 Results from PERMANOVA (Overall Adonis p-value = 1.00e−04) showing the probability of two groups of fungi with different lifestyles occupying the same phylomorphospace region based on data obtained from the 51 principal components (which explain 100% of data variability) (*page 15*)

6. Gene family evolution (*page 15*)
   - Fig. S4 PCA of the 52 species analyzed based on the gene families with the fastest evolution rates, including lifestyle information. A) PC1 *vs* PC2 plot showing the distribution of species according to the gene copy numbers of the 7 faster-evolving PCWDE families (i.e. POD, laccase, AA9 LPMO, GMC, UPO, GH43 and CBM1) identified by CAFE analysis, with the species as circles colored according to their lifestyles. B) Loading vectors indicating the direction and strength of the enzyme families contributing to the distribution of the species in the 2D PCA plot (*page 16*)

7. Ancestral lifestyle reconstruction (*page 16*)
   - Fig. S5 wknn algorithm optimization (using LOOCV) for ancestral lifestyle reconstruction of the species at the nodes of the phylogenetic tree of Fig. 4, based on the reconstructed gene copy numbers of: A) 62 PCWDE families; and B) 7 faster-evolving gene families (POD, laccase, AA9 LPMO, GMC, UPO, GH43 and CBM1) (*page 17*)

8. Transposable elements (*page 18*)
   8.1 Annotation and classification of transposable elements in genome assemblies (*page 18*)
   8.2 Estimation of LTR-retrotransposon insertion age (*page 18*)
   8.3 Distribution and characteristics of transposable elements in Agaricomycetes (*page 18*)
   - Fig. S6 TE in genomes. A) Density plot showing the total TE content in the 52 Agaricomycetes (each blue dot represents a single species). B) Number of identified families per TE order (*page 19*)
   - Table S6 Main features of Agaricomycetes TE family consensuses (*page 19*)

   8.4 TE dynamics in the context of phylogeny and lifestyle (*page 19*)
   - Fig. S7 TE in genomes. A) PCA plot at the order level with each dot representing a species. B) Box plot representing TE content in families with 3 or more species sampled (*page 20*)
   - Fig. S8 Box plot showing TE content of Agaricomycetes species grouped by lifestyle (*page 20*)
   - Fig. S9 Density plot showing the distribution of insertion times of full-length LTR-retrotransposons (*page20*)

9. Polysaccharide decay machinery (CAZymes) (*page 21*)
   9.1 Cellulose depolymerization (*page 21*)
   - Fig. S10 Box plots showing the distribution of CAZymes and oxidoreductases contributing to plant cell-wall degradation, and of the total PCWDE numbers among Agaricales, Polyporales, Boletales and Russulales orders, based on the 52 Agaricomycetes species analyzed (*page22*)

- Fig. S11 Histograms showing the average numbers of genes of the different CAZy families (Fig. 1) per sequenced genome of Russulales, Polyporales, Boletales and Agaricales species (from top to bottom) (*page23*)
- Fig. S12 Histograms showing the average numbers of genes of the different PCWDE families and CBM1 (Fig. 1) per sequenced genome of Russulales, Polyporales, Boletales and Agaricales species (from top to bottom) (*page24*)
- Fig. S13 ML phylogenetic tree (RAxML v.8.1.12), constructed with 1000 bootstrap replications, of 85 enzymes of the glycoside hydrolase family 6 (GH6) identified in 42 of the 52 Agaricomycetes genomes analyzed (*page 25*)
- Table S7 A) Content of catalytic domains (GH, glycoside hydrolases; PL, polysaccharide lyases; CE, carbohydrate esterases; AA, oxidoreductases, classified as Auxiliary Activities in CAZy database (http://www.cazy.org); and EXPN, expansin-like proteins appended to carbohydrate binding modules of families CBM1, CBM6, CBM8, CBM35, CBM42, CBM63 or CMB67, able to bind different polymers of the plant cell wall; and B) content of CBM1 modules non-appended (first column) and appended to catalytic domains, identified in the genomes analyzed (*page 26*)

9.2 Hemicelluloses depolymerization (*page 26*)
9.3 Pectin depolymerization (*page 27*)
9.4 Cuticle degradation (*page 28*)
9.5 Feruloyl esterases (*page 28*)

- Fig. S14 ML phylogenetic tree (RAXML, v.8.1.12), constructed with 1000 bootstrap replications, for the CE5 enzymes identified in the 52 Agaricomycetes genomes included in the present study (*page 29*)
- Fig. S15 ML phylogenetic tree, constructed with 1000 bootstrap replications, of 121 enzymes of the carbohydrate esterase family 1 (CE1) identified in 39 of the 52 fungal genomes analyzed (*page 30*)

10. Multicopper oxidases (MCO) (*page 31*)

- Fig. S16 ML phylogenetic tree of the 649 MCO sequences identified in the 52 Agaricomycetes genomes analyzed, formed by distant ascorbate oxidases (AO), ferroxidases (FOX) and hybrid laccase-ferroxidases (LAC-FOX), main laccase (LAC) cluster, and three groups of atypical MCO catalogued here as novel laccases (NLAC), novel laccase-ferroxidases (NLAC-FOX) and novel MCO (NMCO) (*page 31*)

10.1 Laccases *sensu-stricto* (LAC) (*page 31*)

- Table S8 MCO in the 52 Agaricomycetes genomes analyzed, classified as laccases sensu-stricto (LAC), novel laccases (NLAC), novel MCO (NMCO), novel laccase-ferroxidases (NLAC-FOX), laccase-ferroxidases (LAC-FOX), ferroxidases (FOX) and ascorbate oxidases (AO) (*page 32*)

10.2 Novel laccases with Arg206 (NLAC) (*page 33*)

- Fig. S17 Evolution of laccase *sensu-stricto* gene copy numbers by CAFE analysis (*page 34*)
- Fig. S18 Sequence logos for the amino-acid residues delimiting the substrate binding pocket in: A) Polyporales laccases *sensu stricto* (LAC); B) Laccases with Arg206 (NLAC); and C) Novel MCO (NMCO) (*page 35*)
- Fig. S19 Changes in gene copy numbers of NLAC family estimated with CAFE (*page 35*)

10.3 Novel MCO (NMCO) (*page 36*)

- Fig. S20 A) Catalytic site of NMCO from *M. fuliginosa* ID# 778682; and B) Sequence logo for L1 and L2 motifs in the NMCO enzymes found in the Agaricomycetes (*page 36*)

10.4 Novel laccase-ferroxidases (NLAC-FOX) (*page 36*)
10.5 Ferroxidases (FOX) (*page 36*)

- Fig. S21 Evolution of NMCO gene copy numbers by CAFE analysis (*page 37*)
- Fig. S22 Evolution of NLAC-FOX gene copy numbers by CAFE analysis (*page 38*)

10.6 Laccase-ferroxidases (LAC-FOX) (*page 38*)

- Fig. S23 Evolution of FOX gene copy numbers by CAFE analysis (*page 39*)

10.7 Ascorbate oxidases (AO) (*page 39*)

11. H_2O_2-producing enzymes (*page 40*)

11.1 Glucose-methanol-choline (GMC) oxidoreductase superfamily (*page 40*)

11.1.1 GMC gene families in Agaricomycetes (*page 40*)

- Table S9 GMC oxidoreductases in the 52 Agaricomycetes genomes analyzed, classified as AAO, CDH, MOX I, MOX II, P2O and PDH (*page 41*)
- Table S10 Average gene numbers of the different GMC families in the genomes of the 52 Agaricomycetes species analyzed, characterized by presenting different lifestyles (*page 42*)
- Table S11 Average gene numbers of the different GMC families per genome of Agaricales, Boletales, Polyporales and Russulales species (*page 42*)
- Table S12 Average gene numbers of the different GMC families in the genomes of the 33 Agaricales species with different lifestyles (*page 42*)
- Fig. S24 ML phylogenetic tree of the 778 GMC sequences identified in the 52 Agaricomycetes genomes analyzed (*page 43*)
- Fig. S25 Phylogenetic relationships of the 778 GMC sequences colored by order (A) and lifestyle (B) (*page 44*)

11.1.2 Structural features of the different GMC families (*page 44*)

- Fig. S26 Sequence logo of conserved: A) ADP-binding motif; B) signature 1, Prosite PS00623; C) signature 2, Prosite PS00624; and D) catalytic residues, in 761 GMC sequences (P2O excluded) identified in the 52 genomes analyzed (*page 45*)
- Fig. S27 Sequence logo of the flavinylation sequence in P2O (A) and PDH (B) proteins (*page 45*)
- Fig. S28 Structural models of *V. volvacea* MOX from: A) group MOX I (JGI ID# 121657); and B) group MOX II (JGI ID# 120949) (*page 46*)
- Fig. S29 Structural models of representative enzymes from the different AAO clusters: A) *P. eryngii* AAO from AAO I (JGI ID# 1382984); B) *B. adusta* AAO from AAO II (JGI ID# 171002); C) *R. butyracea* AAO from AAO III (JGI ID# 1235264); and D) *A. bisporus* AAO from AAO IV (JGI ID# 121909) (*page 46*)

11.2 Glyoxal oxidases (GLX) and related copper-radical oxidases (CRO) (*page 47*)

11.2.1 Search for copper-radical oxidases (*page 47*)

11.2.2 Analysis of copper-radical enzymes identified in the 52 genomes analyzed (*page 47*)

- Table S13 CRO in the 52 Agaricomycetes genomes analyzed, classified as GLX and CRO1 through CRO6 (*page 48*)
- Fig. S30 Phylogenetic trees, based on Kimura distances, of 387 sequences of copper radical oxidases (all together and CRO1, CRO2, WSC-containing CRO3–CRO5, and CRO6 separately) identified in the 52 Agaricomycetes genomes analyzed (*page 49*)

12. Class-II peroxidases (POD) (*page 52*)

12.1 POD types in 52 Agaricomycetes genomes (*page 52*)

- Fig. S31 Lateral view of the heme region in: A) the crystal structure of LiP from *T. cervina* (PDB 3Q3U); and B) the homology model of NPOD (JGI ID# 1391496) from *C. striatus* (*page 53*)

12.2 Phylogenetic and molecular clock analysis of ligninolytic peroxidases (*page 54*)

- Fig. S32 ML phylogenetic tree (RAxML v.8.1.12), constructed with 1000 bootstrap replications, of 336 peroxidases identified in 42 of the 52 Agaricomycetes genomes analyzed (*page 55*)

12.3 Reconstruction of ancestral ligninolytic peroxidases (*page 56*)

13. Unspecific peroxygenases (UPO) (*page 56*)

- Table S14 Unspecific peroxygenases (UPO) in the 52 Agaricomycetes genomes analyzed, classified as short-UPO and long-UPO families (*page 57*)

14. Dye-decolorizing peroxidases (DyP) (*page 58*)

14.1 DyP general characteristics (*page 58*)

14.2 Identification of new DyP sequences and determination of their evolutionary relationships (*page 58*)

- Fig. S33 ML phylogram of 218 DyP sequences identified in 64 Agaricomycotina genomes adapted from Fernández-Fueyo et al. (2015) (*page 59*)

14.3 DyP enzymes in the 52 fungal species analyzed (*page 59*)

- Fig. S34 ML phylogram of 110 DyP sequences identified in 39 of the 52 genomes analyzed (*page 60*)
- Table S15 DyP enzymes in the Agaricomycetes genomes analyzed, with indication of their lifestyle, classified in the evolutionary clusters type I, III, IV and V-VI previously described by Fernández-Fueyo et al. (2015) (*page 61*)
- Table S16 DyP average distribution in the six orders the 52 fungal species analyzed belong to (*page 61*)
- Table S17 DyP distribution by lifestyles in Agaricales (*page 62*)
- Fig. S35 Phylogram of DyP enzymes from Agaricomycetes (*page 63*)

II. Supplementary references (*page 65*)

III. Dataset and file S2 legends (*page 74*)

IV. List of additional supplementary files (*page 74*)

I. SUPPLEMENTARY METHODS, RESULTS AND DISCUSSION

1. Fungal strains and culture conditions

Genomic DNA (and total RNA) samples supplied to the U.S. Department of Energy Joint Genome Institute (DOE JGI, http://www.jgi.doe.gov) for sequencing were obtained from pure cultures of fourteen species of saprotrophic Agaricales covering a total of eight families (Strophariaceae, Tricholomataceae Crepidotaceae Nidulariaceae, Physalacriaceae, Agaricaceae, Pleurotaceae and Marasmiaceae) (**Table S1**) which are available at six culture collections:

- AH= University of Alcalá Herbarium Culture Collection, Alcalá de Henares, Spain
- ATCC= American Type Culture Collection, Manassas, Virginia, USA
- CBS= Westerdijk Fungal Biodiversity Institute CBS-KNAW Collection, Utrecht, The Netherlands
- CIRM-BRFM= *Centre International Ressources Microbiennes-Champignons Filamenteux* Culture Collection, INRAE, Marseille, France
- GIGM= Grupo de Investigación en Genética y Microbiología, IMAB, UPNA, Pamplona, Spain.
- IJFM= Centro *de Investigaciones Biológicas Margarita Salas* (CIB) Fungal Culture Collection, CSIC, Madrid, Spain

These species include representatives of five lifestyles (decayed-wood, wood white-rot, forest-litter, grass-litter and unknown-decay degraders; see **Fig. S1**), and pure cultures of some of them were obtained from fruiting bodies (basidiomata) specifically collected for this study. Thus, *Agrocybe pediades* was collected from grassland leaf litter in Collado del Hornillo, Cantalojas, Guadalajara (Spain); *Clitocybe gibba* was collected from oak (*Quercus ilex*) leaf litter in Fresno de Cantespino, Segovia (Spain); *Crepidotus variabilis* was collected from beech (*Fagus sylvatica*) fallen twigs at Bremgarten wald, Berne (Switzerland); *Cyathus striatus* was collected from beech (*F. sylvatica*) dead wood in Belagua, Navarra (Spain); *Gymnopilus junonius* was collected from pine (*Pinus radiata*) dead wood in Pared Vieja, La Palma, Canary Islands (Spain); *Hymenopellis radicata* was collected from beech (*F. sylvatica*) buried stumps in Burguete, Navarra (Spain); *Macrolepiota fuliginosa* was collected from beech (*F. sylvatica*) leaf litter in Aspurz (Navascués), Navarra (Spain); *Pholiota alnicola* was collected from birch (*Betula* sp.) dead wood in Cantalojas, Guadalajara (Spain); and *Rhodocollybia butyracea* was collected from chestnut (*Castanea sativa*) leaf litter in Salamanca, Spain. Information about the location where the other strains sequenced in this study were isolated is not available in the fungal culture collections where they are deposited.

Genomic DNA was isolated from 7 days-old mycelium grown on malt extract-glucose-peptone (MEGP) medium (composition per liter: 20 g malt extract, 20 g glucose, 1 g bactopeptone) at 28ºC and 150 rpm. *Oudemansiella mucida*, *C. variabilis* and *M. fuliginosa* were the exception as: i) the two former species were grown at 21ºC; ii) *C. variabilis* was grown in modified Melin-Norkrans medium (Marx 1969); and iii) *M. fuliginosa* was grown in sucrose-malt extract-yeast extract (SMY) medium (composition per liter: 10 g sucrose, 10 g malt extract, 4 g yeast extract) at 25ºC. Total RNA used for RNA-seq library construction by the JGI was a mixture of equal amounts of total RNA isolated from mycelium grown in two culture media under four different experimental conditions: i) Higley's medium (Highley 1973) containing 0.5% glucose, at 28ºC and 150 rpm for 7 days; ii) MEGP medium, at 28ºC and 150 rpm for 7 days; iii) MEGP medium, at 28ºC and 150 rpm for 10 days; and iv) MEGP medium, at 28ºC and 150 rpm for 7 days, followed by further 10 days incubation under static conditions at 4ºC. *M. fuliginosa* was the exception as total RNA was isolated from mycelium grown on SMY medium at 25ºC and 150 rpm for 7 days.

2. DNA and RNA extraction

The DNA was extracted with phenol–chloroform–isoamyl alcohol and treated with ribonuclease (González et al. 1992). Then it was cleaned up with Genomic-tip 100/G (Qiagen) according to manufacturer’s instructions. The identity of all fungal strains was previously confirmed by sequencing the ribosomal ITS1-5.8S-ITS2 region and comparison with nucleotide databases using BLAST (http://blast.ncbi.nlm.nih.gov/Blast.cgi). Concerning total RNA, it was extracted using the RNeasy plant mini Kit (Qiagen), subsequently treated with Turbo DNase (Ambion) and finally extracted with phenol–chloroform–isoamyl alcohol.

Table S1 Agaricomycetes genomes analyzed: Abbreviations, names and taxonomic position. The abbreviated names of the species sequenced for this study are followed by a parenthesis with the culture collection reference (see Fungal strains and culture conditions section).

Agabi	*Agaricus bisporus*	Agaricaceae (Agaricales)
Agrpe (AH 40210)	*Agrocybe pediades*	Strophariaceae (Agaricales)
Armme	*Armillaria mellea*	Physalacriaceae (Agaricales)
Bjead	*Bjerkandera adusta*	Polyporaceae (Polyporales)
Cersu	*Ceriporiopsis subvermispora*	Polyporaceae (Polyporales)
Cligi (IJFM A808)	*Clitocybe gibba*	Tricholomataceae (Agaricales)
Conpu	*Coniophora puteana*	Boletaceae (Boletales)
Copci	*Coprinopsis cinerea*	Psathyrellaceae (Agaricales)
Corgl	*Cortinarius glaucopus*	Cortinariaceae (Agaricales)
Creva (CBS 506.95)	*Crepidotus variabilis*	Crepidotaceae (Agaricales)
Cyast (AH 40144)	*Cyathus striatus*	Nidulariaceae (Agaricales)
Dicsq	*Dichomitus squalens*	Polyporaceae (Polyporales)
Fibsp	*Fibulorhizoctonia* sp	Atheliaceae (Atheliales)
Fishe	*Fistulina hepatica*	Fistulinaceae (Agaricales)
Fompi	*Fomitopsis pinicola*	Polyporaceae (Polyporales)
Galma	*Galerina marginata*	Strophariaceae (Agaricales)
Gansp	*Ganoderma* sp	Ganodermataceae (Polyporales)
Gyman	*Gymnopus androsaceus*	Omphalotaceae (Agaricales)
Gymlu	*Gymnopus luxurians*	Omphalotaceae (Agaricales)
Gymju (AH 44721)	*Gymnopilus junonius*	Strophariaceae (Agaricales)
Hebcy	*Hebeloma cylindrosporum*	Cortinariaceae (Agaricales)
Hetan	*Heterobasidion annosum*	Bondarzewiaceae (Russulales)
Hydpi	*Hydnomerulius pinastri*	Paxillaceae (Boletales)
Hymra (IJFM 160)	*Hymenopellis radicata*	Physalacriaceae (Agaricales)
Hypsu	*Hypholoma sublateritium*	Strophariaceae (Agaricales)
Lacam	*Laccaria amethystina*	Hydnangiaceae (Agaricales)
Lacbi	*Laccaria bicolor*	Hydnangiaceae (Agaricales)
Lepnu (CBS 247.69)	*Lepista nuda*	Tricholomataceae (Agaricales)
Leugo	*Leucoagaricus gongylophorus*	Agaricaceae (Agaricales)
Macfu (GIGM MF-IS2)	*Macrolepiota fuliginosa*	Agaricaceae (Agaricales)
Marfi	*Marasmius fiardii*	Marasmiaceae (Agaricales)
Ompol	*Omphalotus olearius*	Omphalotaceae (Agaricales)
Oudmu (CBS 558.79)	*Oudemansiella mucida*	Physalacriaceae (Agaricales)
Panpa (CIRM-BRFM 715)	*Panaeolus papilionaceus*	Strophariaceae (Agaricales)
Pensp	*Peniophora* sp	Peniophoraceae (Russulales)
Phach	*Phanerochaete chrysosporium*	Phanerochaetaceae (Polyporales)
Phlbr	*Phlebia brevispora*	Meruliaceae (Polyporales)
Phoal (AH 47727)	*Pholiota alnicola*	Strophariaceae (Agaricales)
Phoco (CIRM-BRFM 674)	*Pholiota conissans*	Strophariaceae (Agaricales)
Pleer (ATCC 90797)	*Pleurotus eryngii*	Pleurotaceae(Agaricales)
Pleos	*Pleurotus ostreatus*	Pleurotaceae(Agaricales)
Plicr	*Plicaturopsis crispa*	Amylocorticiaceae (Amylocorticiales)
Pospl	*Postia placenta*	Polyporaceae (Polyporales)
Rhobu (AH 40177)	*Rhodocollybia butyracea*	Marasmiaceae (Agaricales)
Schco	*Schizophyllum commune*	Schizophyllaceae (Agaricales)
Serla	*Serpula lacrymans*	Serpulaceae (Boletales)
Stehi	*Stereum hirsutum*	Stereaceae (Russulales)
Suibr	*Suillus brevipes*	Suillaceae (Boletales)
Trave	*Trametes versicolor*	Polyporaceae (Polyporales)
Trima	*Tricholoma matsutake*	Tricholomataceae (Agaricales)
Volvo	*Volvariella volvacea*	Pluteaceae (Agaricales)
Wolco	*Wolfiporia cocos*	Polyporaceae (Polyporales)

3. Genome sequencing, assembly and annotation

The *A. pediades* AH40210, *Pholiota conissans* CIRM-BRFM 674, *M. fuliginosa* GIGM MF-IS2, *Pleurotus eryngii* IJFM A732 (=ATCC 90797), *Lepista nuda* CBS 247.69 and *C. variabilis* CBS 506.95 genomes were sequenced using the Illumina platform and assembled with Velvet and AllPathsLG version R49403 (Gnerre et al. 2011) (*A. pediades, P. conissans* and *M. fuliginosa*) or only with AllPathsLG (*P. eryngii*, *L. nuda* and *C. variabilis*). *Gymnopilus junonius* AH44721 and *C. striatus* AH40144 were sequenced using both Illumina and PacBio technology, assembled with Falcon (https://github.com/PacificBiosciences/FALCON) (the *C. striatus* genome assembly was improved with finisherSC (Lam et al. 2015)) and polished with Quiver (https://github.com/PacificBiosciences/GenomicConsensus). The remaining six fungal genomes (*C. gibba* IJFM A808, *P. alnicola* AH47727, *Panaeolus papilionaceus* CIRM-BRFM 715, *O. mucida* CBS 558.79, *R. butyracea* AH 40177 and *H. radicata* IJFM A160) were sequenced using PacBio and assembled with Falcon. The *R. butyracea* and *H. radicata* genome assemblies were improved with finisherSC and, together to the *O. mucida* genome assembly, polished with Quiver. Mitochondria were assembled separately with Discovar version 52488 (*A. pediades*), AllPathsLG (*P. conissans*, *P. eryngii*, *L. nuda* and *C. variabilis*), Celera version 8.3 (*C. gibba*, *P. alnicola*, *G. junonius*, *O. mucida*, *R. butyracea* and *H. radicata*) or Velvet (*C. striatus*). Finally, the assembled DNA was annotated with the JGI Annotation pipeline.

RNA-seq transcriptomic data were used to assess the completeness of the final assembly and improve the annotation of the fungal genomes. First, stranded RNAseq libraries were created and quantified by qPCR. Then, sequencing was performed using the Illumina HiSeq 2000 and Illumina HiSeq 2500 platforms. Raw fastq file reads were filtered and trimmed using the JGI QC pipeline resulting in the filtered fastq file. Using BBDuk (https://sourceforge.net/projects/bbmap/), raw reads were evaluated for artifact sequence by kmer matching (kmer=25), allowing 1 mismatch and detected artifact was trimmed from the 3' end of the reads. RNA spike-in reads, PhiX reads and reads containing any Ns were removed. Quality trimming was performed using the phred trimming method set at Q6. Following trimming, reads under the length threshold were removed (minimum length 25 bases or 1/3 of the original read length - whichever is longer). Filtered fastq files were used as input for de novo assembly of RNA contigs. In the case of the transcriptomes of *C. gibba*, *P. papilionaceus*, *O. mucida* and *P. alnicola*, the reads were assembled into consensus sequences using Trinity (ver. 2.1.1) (Grabherr et al. 2011). Trinity was run with the normalize_reads (In-silico normalization routine) and jaccard_clip (Minimizing fusion transcripts derived from gene dense genomes) options. For the rest of fungal species, the reads were assembled into consensus sequences using Rnnotator (v. 3.4.0) (Martin et al. 2010). Rnnotator was also used for Assembly and Post-processing of contigs. Assembly was completed with Velvet (Zerbino and Birney 2008). Eight runs of velveth (v. 1.2.07) were performed in parallel, once for each hash length for the De Bruijn graph. Minimum contig length was set at 100. The read depth minimum was set to 3 reads. Redundant contigs were removed using Vmatch (v.2.2.4) and contigs with significant overlap were further assembled using Minimus2 with a minimum overlap of 40. Contig post-processing included splitting misassembled contigs, contig extension and polishing using the strand information of the reads. Single base errors were corrected by aligning the reads back to each contig with BWA to generate a consensus nucleotide sequence. Post-processed contigs were clustered into loci and putative transcript precursors were identified. In creation of the contigs, all the reads were used not just those that uniquely mapped. This enabled the generation of isoforms as the reads forked off of each other. Individual reads were then aligned uniquely to all the isoforms and "v1" was always the most highly expressed transcript.

BUSCO version 4.1.1 assessment tool (Seppey et al. 2019) was used in both genome and protein modes to determine the completeness scores of the new genomic assemblies and annotations with the specific data set agaricales_odb10 (3870 BUSCO groups) downloaded from https://busco-data.ezlab.org/v4/data/lineages/.

A summary of statistics of genome assembly and annotation data is shown in **Tables S2** and **S3**. The assembled genome sequences are publicly available on JGI MycoCosm Portal (https://mycocosm.jgi.doe.gov) (Grigoriev et al. 2014). The accession links, both to the fourteen genomes sequenced *de novo* and to the additional thirty-five published and three pending publication genomes analyzed in this study are available in **Table S4**.

Table S2 Summary statistics of genome assemblies. BUSCO scores indicate the completeness of the genome assemblies

Species name, culture collection reference, and genome version at JGI	Assembly size (Mbp)	Read coverage depth	Nº of contigs	Nº of scaffolds	Nº of scaffolds >= 2Kbp	Scaffold N50	Scaffold L50 (Mbp)	Nº of gaps	Scaffold length in gaps	Three largest scaffolds (Mbp)	BUSCO (%)
Agrocybe pediades AH 40210 v1.0	45.06	151.6x	2369	1384	1091	107	0.10	985	2.0%	0.70, 0.58, 0.51	98.0
Clitocybe gibba IJFM A808 v1.0	65.97	128.02x	1258	1258	1228	82	0.18	0	0.0%	2.65, 1.84, 1.83	90.4
Crepidotus variabilis CBS 506.95 v1.0	38.58	111.2x	1097	435	359	31	0.32	662	2.5%	1.51, 1.50, 1.08	96.3
Cyathus striatus AH 40144 v1.0	91.18	-	1562	1562	1555	90	0.20	0	0.0%	2.13, 1.39, 1.37	91.6
Gymnopilus junonius AH 44721 v1.0	59.46	101.97x	1174	1174	1132	95	0.15	0	0.0%	1.66, 1.37, 0.82	94.5
Hymenopellis radicata IJFM A160 v1.0	78.59	-	1968	1968	1877	195	0.10	0	0.0%	1.28, 0.93, 0.74	91.8
Lepista nuda CBS 247.69 v1.0	43.49	93.4x	2234	822	603	49	0.27	1412	8.0%	1.46, 0.85, 0.84	97.1
Macrolepiota fuliginosa GIGM MF-IS2 v1.0	46.40	165.4x	4852	3478	2184	199	0.05	1374	1.6%	0.63, 0.54, 0.45	97.7
Oudemansiella mucida CBS 558.79 v1.0	61.73	131x	1386	1386	1338	107	0.12	0	0.0%	2.15, 1.35, 0.84	91.8
Panaeolus papilionaceus CIRM-BRFM 715 v1.0	50.89	79.85x	79	79	77	11	1.82	0	0.0%	3.44, 2.95, 2.66	97.6
Pholiota alnicola AH 47727 v1.0	75.01	128.9x	976	976	960	93	0.21	0	0.0%	1.58, 1.42, 1.37	94.6
Pholiota conissans CIRM-BRFM 674 v1.0	43.96	141.2x	1558	1310	1065	114	0.10	248	0.5%	0.79, 0.53, 0.44	98.4
Pleurotus eryngii ATCC 90797 v1.0	44.61	96.7x	1684	609	487	54	0.24	1075	3.0%	1.22, 1.02, 0.83	98.1
Rhodocollybia butyracea AH 40177 v1.0	96.28	-	1482	1482	1474	150	0.16	0	0.0%	1.54, 1.17, 1.10	93.1

Table S3 Summary statistics of annotated genomes. BUSCO scores indicate the completeness of the annotated genome assemblies

Species, culture collection reference, and genome version at JGI	Number of ESTs	% Mapped to genome	Average gene length (bp)	Average transcript length (bp)	Average exon length (bp)	Average intron length (bp)	Average protein length (aa)	Average number of exons per gene	Nº of gene models	BUSCO (%)
Agrocybe pediades AH 40210 v1.0	43077	92.8%	1692	1381	249	70	401	5.54	17281	98.3
Clitocybe gibba IJFM A808 v1.0	109055	90.4%	1568	1239	205	67	359	6.04	19049	93.4
Crepidotus variabilis CBS 506.95 v1.0	41955	99.2%	1748	1457	258	65	416	5.66	14573	98.3
Cyathus striatus AH 40144 v1.0	59103	93.4%	1461	1143	208	74	334	5.49	23513	94.8
Gymnopilus junonius AH 44721 v1.0	73806	96.0%	1650	1317	226	71	382	5.83	16444	94.4
Hymenopellis radicata IJFM A160 v1.0	55564	87.9%	1454	1179	223	66	345	5.29	27481	92.9
Lepista nuda CBS 247.69 v1.0	63470	95.7%	1700	1391	227	62	396	6.13	14880	97.9
Macrolepiota fuliginosa GIGM MF-IS2 v1.0	51170	92.9%	1576	1271	236	72	376	5.39	15801	96.9
Oudemansiella mucida CBS 558.79 v1.0	78574	93.7%	1573	1299	229	61	378	5.67	18562	93.4
Panaeolus papilionaceus CIRM-BRFM 715 v1.0	104443	98.3%	1700	1380	253	74	401	5.46	17466	97.0
Pholiota alnicola AH 47727 v1.0	89455	96.0%	1662	1325	229	72	389	5.79	18795	95.3
Pholiota conissans CIRM-BRFM 674 v1.0	46281	97.6%	1689	1386	250	69	399	5.53	16589	98.8
Pleurotus eryngii ATCC 90797 v1.0	48628	97.8%	1621	1318	236	68	379	5.59	15960	97.4
Rhodocollybia butyracea AH 40177 v1.0	50467	92.9%	1548	1231	219	71	365	5.62	22870	94.4

Table S4 Links to the fungal genomes analyzed in this study available at the DOE JGI.

Species sequenced for this study

Agrocybe pediades: http://genome.jgi.doe.gov/Agrped1/Agrped1.home.html
Clitocybe gibba: http://genome.jgi.doe.gov/Cligib1/Cligib1.home.html
Crepidotus variabilis: http://genome.jgi.doe.gov/Crevar1/Crevar1.home.html
Cyathus striatus: http://genome.jgi.doe.gov/Cyastr2/Cyastr2.home.html
Gymnopilus junonius: http://genome.jgi.doe.gov/Gymjun1/Gymjun1.home.html
Hymenopellis radicata: http://genome.jgi.doe.gov/Hymrad1/Hymrad1.home.html
Lepista nuda: http://genome.jgi.doe.gov/Lepnud1/Lepnud1.home.html
Macrolepiota fuliginosa: http://genome.jgi.doe.gov/Macfu1/Macfu1.home.html
Oudemansiella mucida: http://genome.jgi.doe.gov/Oudmuc1/Oudmuc1.home.html
Panaeolus papilionaceus: http://genome.jgi.doe.gov/Panpap1/Panpap1.home.html
Pholiota alnicola: http://genome.jgi.doe.gov/Phoaln1/Phoaln1.home.html
Pholiota conissans: http://genome.jgi.doe.gov/Phocon1/Phocon1.home.html
Pleurotus eryngii: http://genome.jgi.doe.gov/Pleery1/Pleery1.home.html
Rhodocollybia butyracea: http://genome.jgi.doe.gov/Rhobut1_1/Rhobut1_1.home.html

Species previously sequenced by the JGI that were also used in this study

Agaricus bisporus: http://genome.jgi.doe.gov/Agabi_varbisH97_2/Agabi_varbisH97_2.home.html
Armillaria mellea: http://genome.jgi.doe.gov/Armme1_1/Armme1_1.home.html
Bjerkandera adusta: http://genome.jgi.doe.gov/Bjead1_1/Bjead1_1.home.html
Ceriporiopsis subvermispora: http://genome.jgi.doe.gov/Cersu1/Cersu1.home.html
Coniophora puteana: http://genome.jgi.doe.gov/Conpu1/Conpu1.home.html
Coprinopsis cinerea: http://genome.jgi.doe.gov/Copci1/Copci1.home.html
Cortinarius glaucopus: http://genome.jgi.doe.gov/Corgl3/Corgl3.home.html
Dichomitus squalens LYAD-421 SS1 v1.0:http://genome.jgi.doe.gov/Dicsq1/Dicsq1.home.html
Fibulorhizoctonia sp.: http://genome.jgi.doe.gov/Fibsp1/Fibsp1.home.html
Fistulina hepatica: http://genome.jgi.doe.gov/Fishe1/Fishe1.home.html
Fomitopsis pinicola: http://genome.jgi.doe.gov/Fompi3/Fompi3.home.html
Galerina marginata: http://genome.jgi.doe.gov/Galma1/Galma1.home.html
Ganoderma sp: http://genome.jgi.doe.gov/Gansp1/Gansp1.home.html
Gymnopus androsaceus: http://genome.jgi.doe.gov/Gyman1/Gyman1.home.html
Gymnopus luxurians: http://genome.jgi.doe.gov/Gymlu1/Gymlu1.home.html
Hebeloma cylindrosporum: http://genome.jgi.doe.gov/Hebcy2/Hebcy2.home.html
Heterobasidion annosum: http://genome.jgi.doe.gov/Hetan2/Hetan2.home.html
Hydnomerulius pinastri: http://genome.jgi.doe.gov/Hydpi2/Hydpi2.home.html
Hypholoma sublateritium: http://genome.jgi.doe.gov/Hypsu1/Hypsu1.home.html
Laccaria amethystina: http://genome.jgi.doe.gov/Lacam2/Lacam2.home.html
Laccaria bicolor: http://genome.jgi.doe.gov/Lacbi2/Lacbi2.home.html
Leucoagaricus gongylophorus: http://genome.jgi.doe.gov/Leugo1_1/Leugo1_1.home.html
Marasmius fiardii: http://genome.jgi.doe.gov/Marfi1/Marfi1.home.html
Omphalotus olearius: http://genome.jgi.doe.gov/Ompol1/Ompol1.home.html
Peniophora sp: http://genome.jgi.doe.gov/Ricme1/Ricme1.download.html
Phanerochaete chrysosporium: http://genome.jgi.doe.gov/Phchr2/Phchr2.home.html
Phlebia brevispora: http://genome.jgi.doe.gov/Phlbr1/Phlbr1.home.html
Pleurotus ostreatus: http://genome.jgi.doe.gov/PleosPC15_2/PleosPC15_2.home.html
Plicaturopsis crispa: http://genome.jgi.doe.gov/Plicr1/Plicr1.home.html
Postia placenta MAD 698-R V1.0: http://genome.jgi.doe.gov/Pospl1/Pospl1.home.html
Schizophyllum commune: http://genome.jgi.doe.gov/Schco3/Schco3.home.html
Serpula lacrymans S7.9 v2.0: http://genome.jgi.doe.gov/SerlaS7_9_2/SerlaS7_9_2.home.html
Stagonospora nodorum SN15*:* https://genome.jgi.doe.gov/Stano1/Stano1.home.html
Stereum hirsutum: http://genome.jgi.doe.gov/Stehi1/Stehi1.home.html
Suillus brevipes: http://genome.jgi.doe.gov/Suibr2/Suibr2.home.html
Trametes versicolor: http://genome.jgi.doe.gov/Trave1/Trave1.home.html
Tricholoma matsutake: http://genome.jgi.doe.gov/Trima3/Trima3.home.html
Volvariella volvacea: http://genome.jgi.doe.gov/Volvo1/Volvo1.home.html
Wolfiporia cocos: http://genome.jgi.doe.gov/Wolco1/Wolco1.home.html

The genome size and gene content in the 52 fungal species of the six Agaricomycetes orders used in this study (i.e. Agaricales, Boletales, Amylocorticiales, Atheliales, Polyporales and Russulales) are shown graphically in **Figs. S1 and S2**.

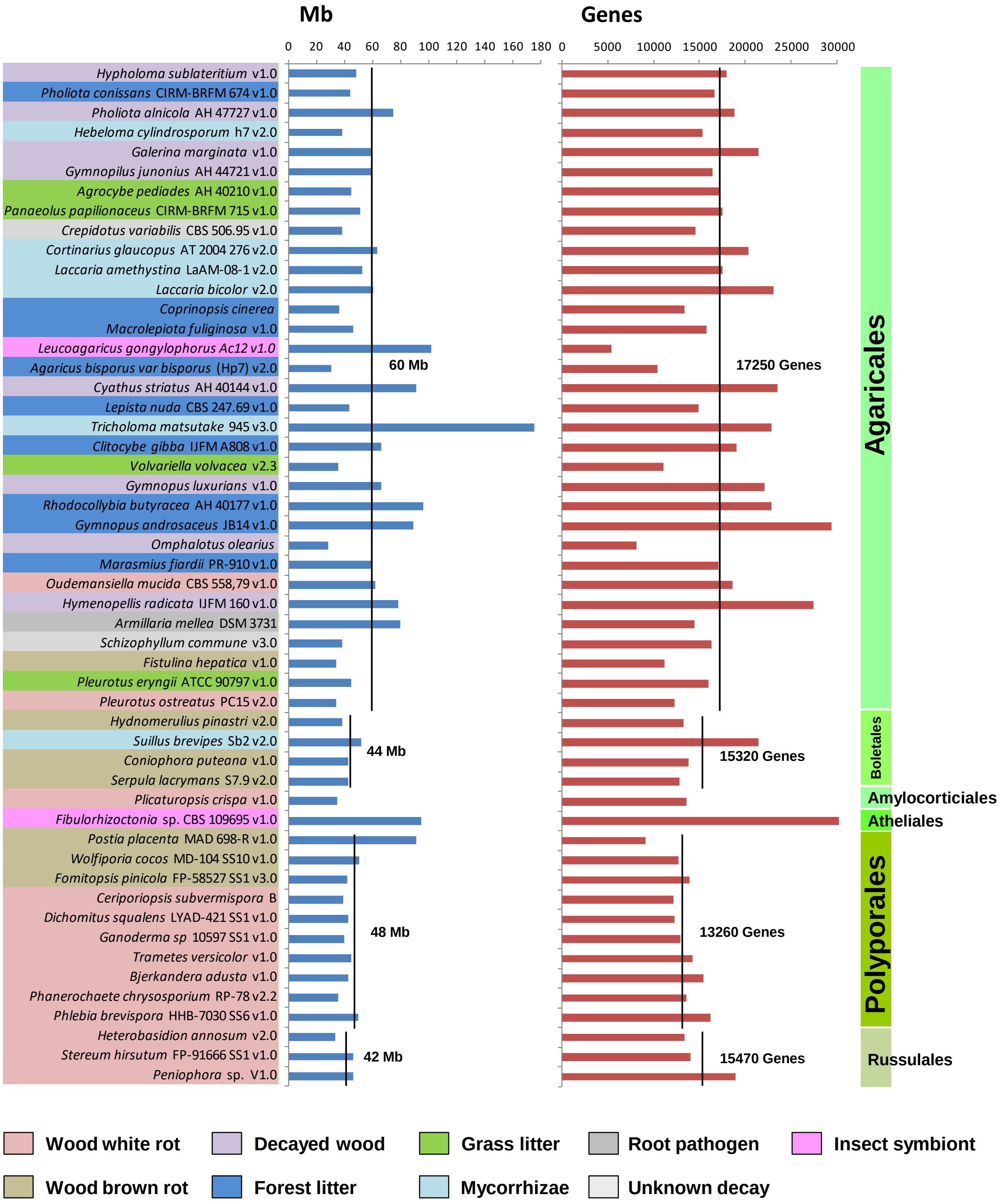

Fig. S1 Bar chart showing the genome sizes (megabase pairs, Mb) and gene contents in 52 fungal species belonging to six Agaricomycetes orders (Agaricales, Boletales, Amylocorticiales, Atheliales, Polyporales and Russulales). The color background of the fungal species corresponds to their lifestyles according to the color code indicated below. Black vertical lines indicate the average Mb and gene numbers in the fungal orders analyzed.

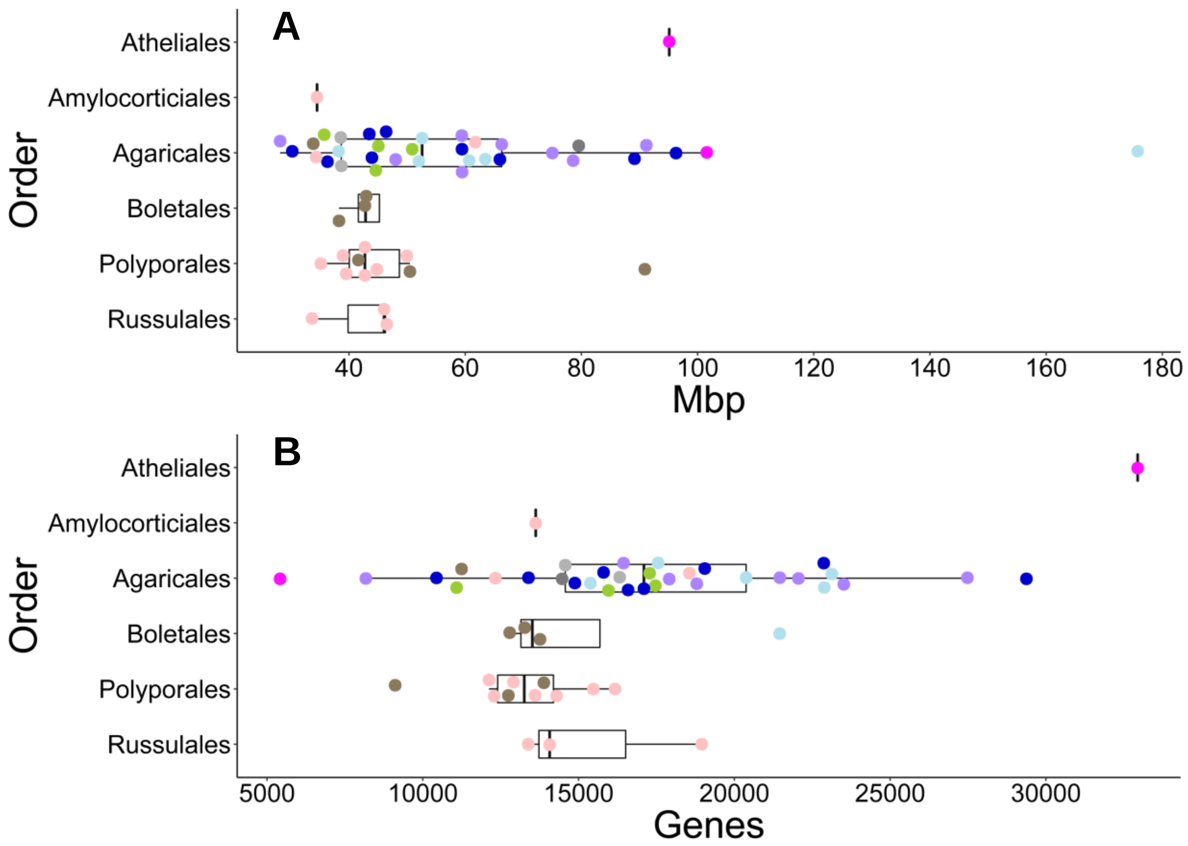

Fig. S2 Box plots showing the genome size (**A**) and gene content (**B**) variation among Agaricales, Polyporales, Boletales and Russulales orders based on the 52 Agaricomycetes species analyzed (only one species of the order Amylocorticiales and one species of the order Atheliales have been included in this study) with lifestyle information included using the **Fig. S1** color code.

4. Phylogenetic analysis of the Agaricomycetes species and molecular dating

An organismal phylogeny was constructed with the 52 genomes of Agaricomycetes selected for this study. Protein sequences were downloaded from MycoCosm. Orthologous genes among the fungi were identified using FastOrtho with the parameters set to 50% identity, 50% coverage, inflation 3.0 (Wattam et al. 2014). Clusters with single copy genes were identified and aligned with MAFFT 7.221 (Katoh and Standley 2013), single-gene alignments were concatenated, and ambiguous regions (containing gaps and poorly aligned) were eliminated with Gblocks 0.91b (Talavera and Castresana 2007). A maximum likelihood (ML) tree was constructed with RAxML (v.8.2.10) (Stamatakis 2014) standard algorithm, the PROTGAMMAWAG model of sequence evolution and 1,000 bootstrap replicates.

The resulting phylogeny was used in the molecular dating analyses. The species phylogeny was time-calibrated using the penalized likelihood algorithm as implemented in r8s 1.8.1 (Sanderson 2003) with the POWELL optimization algorithm. A secondary calibration was performed using the ranges of dates for the origin of the orders Agaricales (min age 160 - max age 182) and Boletales (min age 133 - max age 153), and the subclass Agaricomycetidae (min age 174 - max age 192) including Agaricales, Boletales, Amylocorticiales and Atheliales, obtained from a megaphylogeny generated for 5,284 species by Varga et al. (2019). A cross-validation analysis was performed in order to identify the appropriate smoothing parameter. The resulting dated species tree is shown in **Fig. 2** *left*.

5. *Phylogenetic principal-component analysis (pPCA) and phylomorphospace*

pPCA was performed using the function phyl.pca (Revell 2009) from the R phytools package (www.phytools.org) (Core Team 2020). In this analysis we used, as input, the time-calibrated RAxML tree of the 52 fungal species under study (**Fig. 2** *left*) and a matrix of the following 62 representative enzyme families (included in **Dataset S4**) identified as directly or indirectly involved in plant cell-wall degradation, which are shown in **Fig. 1** of the main manuscript:

i) 50 families encoding carbohydrate-active enzymes (classical CAZymes), including 38 glycoside hydrolases (GH), 5 polysaccharide lyases (PL), and 7 carbohydrate esterases (CE), with the CE5 family containing both acetyl xylan esterases (AXE) and cutinases (CUT).

ii) 10 families encoding oxidoreductases, including laccases [grouping laccases *sensu stricto*, LAC; and novel laccases, NLAC, of the multicopper oxidase (MCO, CAZy AA1) superfamily]; class-II peroxidases (POD, CAZy AA2), glucose-methanol-choline oxidases/dehydrogenases (GMC, CAZy AA3), copper-radical oxidases (CRO, CAZy AA5), lytic polysaccharide monooxygenases (LPMO of CAZy AA9, AA14 and AA16 families), benzoquinone reductases (BQR, CAZy AA6), unspecific peroxygenases (UPO), and dye-decolorizing peroxidases (DyP)

iii) 1 family encoding *Candida rugosa*-like versatile lipases (versatile lipases, VLP)

iv) 1 family encoding carbohydrate binding modules (CAZy CBM1).

The resulting pPCA plot and the loading vectors indicating the contribution of the most significant enzyme families are shown in **Fig. S3.**

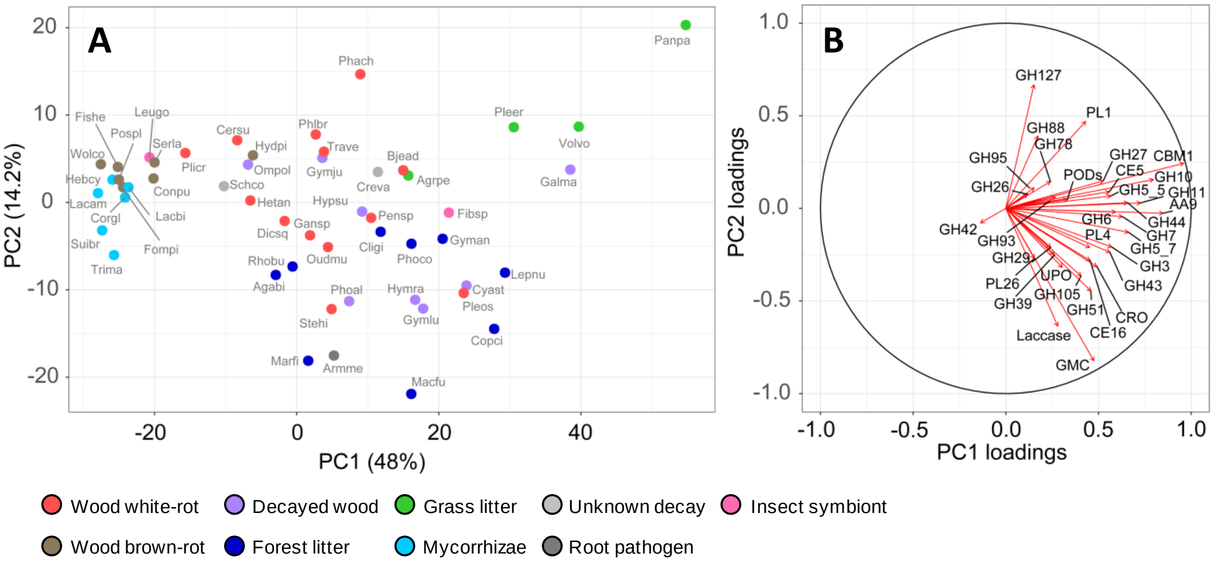

Fig. S3 pPCA of the 52 species analyzed according to their PCWDE repertoires, including lifestyle information. **A**) PC1 *vs* PC2 plot showing the distribution of species according to the composition of their enzymatic machineries, with the species as circles colored according to their lifestyles. **B**) Loading vectors indicating the direction and strength of the most significant enzyme families contributing to the distribution of the species in the 2D pPCA plot.

Then, the function phylomorphospace (phytools) was used to show the phylogenetic relationships of the fungal species over the pPCA plot (**Fig. 3** of the main manuscript). This function, based on Sidlauskas (2008), creates a projection of the phylogenetic tree into a morphospace in such a way that we can visualize how the extant species of the phylogeny diverge and converge from ancestral nodes along evolution. The significance of the differences in the phylomorphospace was tested using PERMANOVA, implemented in the Adonis function of the R package vegan (Oksanen et al. 2019). To reduce redundancy in the input data, the first 51 components explaining the whole data variability were used in this analysis, instead of the 62 initial variables (family numbers). Pairwise tests were

performed using the FDR correction to the p-values (Benjamini and Hochberg 1995), implemented in the pairwise Adonis package (Martinez Arbizu 2020). An alpha of 0.05 was used as the cutoff for significance. The results are shown in **Table S5**. Unknown-decay, root-pathogen and insect-symbiont species were removed from this statistical analysis because their lifestyles have no enough representatives to be statistically compared.

Table S5 Results from PERMANOVA (overall Adonis *p*-value = 1.00e−04) showing the probability of two groups of fungi with different lifestyles occupying the same phylomorphospace region based on data obtained from the 51 principal components (which explain 100% of data variability). Values in gray show significantly different colocalization in the phylomorphospace ($p < 0.05$). Only lifestyles with three or more representative species were included in this analysis. $p < 0.025$, dark gray; $0.025 < p < 0.05$, light gray; $p > 0.05$ white.

	Wood white rot	Decayed wood	Forest litter	Grass litter	Wood brown rot
Decayed wood	0.0169	-	-	-	-
Forest litter	0.0025	0.2960	-	-	-
Grass litter	0.0032	0.0105	0.0066	-	-
Wood brown rot	0.0028	0.0025	0.0024	0.0052	-
Mycorrhizae	0.0023	0.0026	0.0024	0.0072	0.028

6. Gene family evolution

The CAFE program (v4.1) (De Bie et al. 2006; Han et al. 2013) was used to analyze gene family expansions and contractions in the 62 enzyme families previously described for the pPCA and phylomorphospace generation. The time-calibrated RAxML phylogenetic tree of the 52 fungal species under analysis, obtained as described above, and the gene family sizes including those of 24 enzyme families participating in the amino-acid metabolism as a background gene set (a list of these enzymes is found at the end of this section-6) (**Dataset S4**, rows 1-53) were used as input for the program. The ML value of the birth and death parameter describing the probability that any gene will be gained or lost was estimated for the whole tree, resulting in $\lambda = 0.0055$. The gene copy numbers of the enzyme families involved in lignocellulose degradation and CBM1 at ancestral nodes reconstructed with CAFE are presented in **Dataset S4** (rows 54 to 104 in this dataset, corresponding to nodes 53 to 103 of the tree in **Fig. 4**). Seven gene families (POD, laccase, AA9 LPMO, GMC, UPO, GH43 and CBM1) with a family-wide *p*-value <0.01 were considered to have significantly greater rate of evolution. Then a Viterbi *p*-value <0.05 was established to be statistically significant to determine the branches in the species tree in which these seven families underwent significant shifts in λ (contractions or expansions). Significant branch-specific expansions and contractions for all seven non-randomly evolving gene families are shown in **Fig. 4** of the main manuscript.

Given the expected key role in lignocellulose degradation of the seven families with a higher evolutionary rate, we evaluated how they could contribute to the distribution of the fungal species in a PCA as the one previously carried out with the whole 62 PCWDE families. We performed this analysis using, as input, the time-calibrated RAxML tree of the 52 fungal species (**Fig. 2** *left*) and a matrix of the seven enzyme families plus CBM1. The resulting plot and the loading vectors indicating the contribution of the enzymes are shown in **Fig. S4**. Interestingly, the distribution of the species in this PCA was similar to that shown in **Fig. S3** and the first two principal components explained a higher percentage of the variance (78% *vs* 62%).

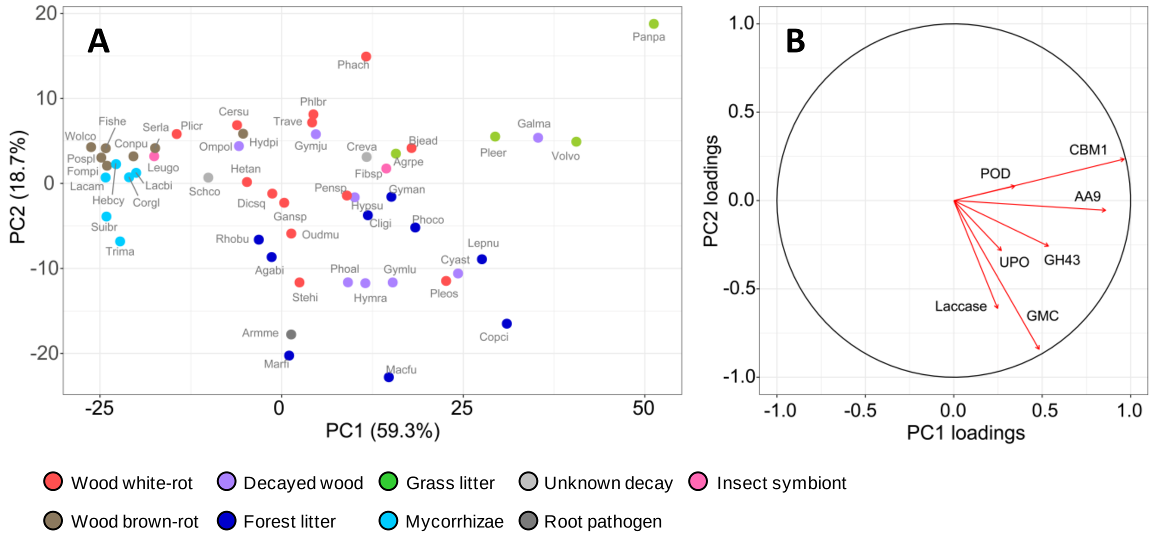

Fig. S4 PCA of the 52 species analyzed based on the gene families with the fastest evolution rates, including lifestyle information. **A)** PC1 *vs* PC2 plot showing the distribution of species according to the gene copy numbers of the 7 faster-evolving PCWDE families (i.e. POD, laccase, AA9 LPMO, GMC, UPO, GH43 and CBM1) identified by CAFE analysis, with the species as circles colored according to their lifestyles. **B)** Loading vectors indicating the direction and strength of the enzyme families contributing to the distribution of the species in the 2D PCA plot.

<u>Amino-acid metabolism enzymes used in CAFE analyses as a background gene set</u>: EC 1.4.1.2, glutamate dehydrogenase; EC 1.8.1.4, dihydrolipoyl dehydrogenase; EC 2.1.3.3, ornithine carbamoyl-transferase; EC 2.3.3.13, 2-isopropylmalate synthase; EC 2.3.3.14, homocitrate synthase; EC 2.5.1.47, cysteine synthase; EC 2.5.1.48, cystathionine gamma-synthase; EC 2.5.1.6, methionine adenosyltransferase; EC 2.6.1.1, aspartate transaminase; EC 2.6.1.2, alanine transaminase; EC 3.4.11.5, prolyl aminopeptidase; EC 3.5.1.1, asparaginase; EC 3.5.3.1, arginase; EC 4.1.1.15, glutamate decarboxilase; EC 4.1.1.17, ornithine decarboxylase; EC4.1.1.28, aromatic-L-amino-acid decarboxylase; EC 4.2.1.20, tryptophan synthase; EC 4.2.1.33, 3-isopropylmalate dehydratase; EC 4.2.3.1, threonine synthase; EC 4.3.1.19, threonine ammonia-lyase; EC 4.3.2.1, argininosuccinate lyase; EC 6.3.1.2, glutamate-ammonia ligase; EC 6.3.5.4, asparagine synthase; and EC 6.4.1.3, propionyl-CoA carboxylase.

7. Ancestral lifestyle reconstruction

The lifestyles of the ancestral fungal species at the nodes of the time-calibrated phylogenetic tree of **Fig. 4** were predicted based on the gene copy numbers reconstructed with CAFE (see **section 6**) encoding: i) the 62 plant cell-wall degrading enzyme (PCWDE) families involved in plant cell-wall degradation (**Fig 1** of the main manuscript); or ii) the seven faster-evolving families (i.e. POD, laccase, AA9 LPMO, GMC, UPO, GH43 and CBM1), all of them included in **Dataset S4** (after manual revision of the predicted values) using the Weighted k-Nearest-Neighbor (wknn) (implemented in the kknn R package).

This method is an extended version of knn algorithm, where the distances of the nearest neighbors are taken into account, so that close neighbors have more influence (Hechenbichler and Schliep 2004; Samworth 2012). This machine learning algorithm has three parameters that can be tuned to improve the classification performance:

i) k = number of neighbors used for classification (k values from 1 to 25 were tested; higher k values were not used to avoid bias of the results towards the most abundant lifestyle among the extant species used in our study).

ii) d value in the Minkowski distance formula (included below), which determines the distance function (we tested several d values and it was found that precision decreases when d increases, so we finally used Manhattan distance, d=1).

iii) Kernel function used to translate distances into weights (kernel transformations are maximal when distance is zero and get smaller with growing absolute value of distance; the following kernel functions were tested: rectangular, triangular, epanechnikov, gaussian, rank and optimal).

$$d(x_i, x_j) = \left(\sum_{s=1}^{p} \left| x_{is} - x_{js} \right|^d \right)^{\frac{1}{d}}$$

Combining these parameters, 150 predictive models were evaluated using the gene copy numbers of the 62 PCWDE families (**Fig S5A**), and the same number of models were evaluated using the gene copy numbers of the seven faster-evolving families (**Fig S5B**), in both cases applying Leave-one-out cross-validation (LOOCV) of the extant species.

The prediction accuracy of the best model achieved with the 62 PCWDE families (wknn conditions: k= 4, d= 1 and kernel= "gaussian"), was significantly increased (from 66% to 77%) when the seven faster-evolving gene families were used as input to the wknn algorithm (conditions: k=8, d =1 and kernel= "epanechnikov"). Given that baseline classifications such as the base rate (accuracy of predicting the most-frequent class; also known as zeroR) and the random rate (accuracy of generating predictions uniformly at random) are way under 50% (27.7% and 16.7%, respectively) a 77% accuracy (1-misclassification) is an acceptable improvement of these naive classifiers. Therefore, the model with the highest accuracy was selected for classification of the ancestral nodes lifestyle and the results are shown in **Fig. 4** of the main manuscript.

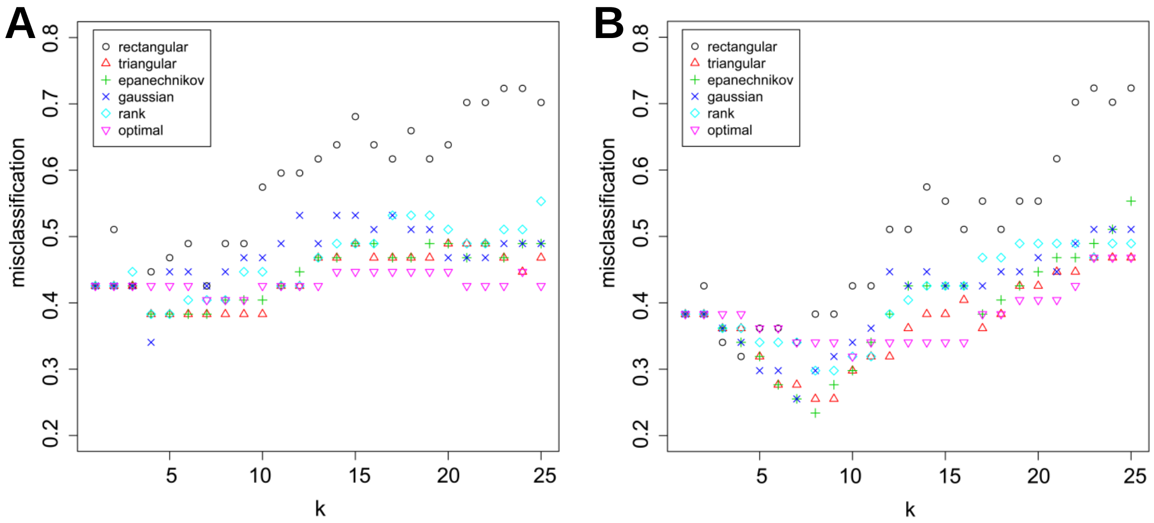

Fig. S5 wknn algorithm optimization (using LOOCV) for ancestral lifestyle reconstruction of the species at the nodes of the phylogenetic tree of **Fig. 4**, based on the reconstructed gene copy numbers of: **A**) 62 PCWDE families; and **B**) 7 faster-evolving gene families (POD, laccase, AA9 LPMO, GMC, UPO, GH43 and CBM1). Y-axis represents misclassification (1-accuracy), x-axis shows the number of neighbors (k) used for classification; and the inset includes the kernel functions tested (using Manhattan distance, d=1).

8. Transposable elements

Transposable elements (TE) are the most important component of the repetitive fraction of eukaryotic genomes. They have a strong mutagenic potential, inherent to their ability to move from one locus to another. In addition to the harmful effects that they can produce in their hosts, TE constitute a source of genomic innovation and play an essential role in heterochromatin maintenance (Lippman et al. 2004). Previous studies carried out in fungi have shown that the genomic fraction occupied by these elements is highly variable, with symbiotic species and broad-range plant pathogens usually carrying higher TE content, and yeasts being practically free of TE, as reviewed by Castanera et al (2017). Despite the repetitive content of many fungal sequenced genomes has been previously estimated, the diversity in the tools and pipelines used hinders the comparative analysis of TE dynamics without annotation bias. Herein, we present a comprehensive, order-wide TE quantification in Agaricales, along with a comparative analysis with other Agaricomycetes species in the Boletales, Atheliales, Amylocorticiales, Russulales and Polyporales orders.

8.1 Annotation and classification of transposable elements in genome assemblies

De novo identification of repetitive sequences in each genome assembly was performed by the RECON (Bao and Eddy 2002) and RepeatScout (Price et al. 2005) programs, of the RepeatModeler pipeline (http://www.repeatmasker.org/RepeatModeler). Structure-based long-terminal repeat (LTR)-retrotransposon predictions were carried out using LTRharvest (Ellinghaus et al. 2008), retaining only the elements showing significant similarity (BLASTX cutoff e-value = 10^{-5}) to any Repbase entry (Jurka 2000). The latter elements were further clustered at 80% similarity with USEARCH (Edgar 2010) to obtain LTR-retrotransposon consensuses. Redundancy between RepeatModeler and LTR-retrotransposon consensuses was eliminated by obtaining centroids at 90% similarity. Classification of TE consensuses into the categories proposed by Wicker et al. (2007) was performed using the PASTEC classifier from the REPET package (Hoede et al. 2014). This process yielded 52 species-specific libraries, which were complemented with a set of 107 full-length consensuses belonging to the most representative Class II TE superfamilies to improve the detection of low-copy DNA transposons. The resulting libraries were used as input for RepeatMasker to annotate and quantify TE content in each genome assembly.

8.2 Estimation of LTR-retrotransposon insertion age

Left and right long terminal repeats of LTR-retrotransposons were extracted and aligned with MUSCLE (Edgar 2004b). Alignments were trimmed using trimal (Capella-Gutiérrez et al. 2009) and used to calculate Kimura 2P distance. Insertion age was calculated following the approach described by SanMiguel et al. (1998) and the fungal substitution rate of 1.05×10^{-9} nucleotides per site per year. Only genomes assembled without gaps were used to avoid introducing bias in the estimation due to the difficulty of assembling highly similar (young) copies.

8.3 Distribution and characteristics of transposable elements in Agaricomycetes

The annotation of 52 Agaricomycetes yielded an impressive variability in TE content, with species ranging from 0.52 % to 65.89 % of their genome occupied by TE (*Omphalotus olearius* and *Leucoagaricus gongylophorus*, respectively (**Dataset S1**). Despite this diversity, the majority of species analyzed carry low to intermediate amounts of TE (mean = 12.61%, median = 8.81%; **Fig. S6**) and only nine species displayed more than 20% TE coverage. We identified a total of 7,401 Class I and Class II TE families (**Dataset S2**, excluding chimeric and unclassified elements) that were confidently assigned to seven out of the nine orders proposed by Wicker et al. (2007).

Class I transposons are the most abundant TE in Agaricomycetes in terms of number of families and genome coverage. Our results reinforce the fact that LTR-retrotransposons in the Gypsy and Copia superfamilies are the most relevant repeated elements in fungal genomes. In fact, in addition to be the most abundant, they display the highest number of families, the highest average length (**Table S6**) and their expansions are responsible of the exceptional repeat content of species such *L. gongylophorus* (65.89%), *Tricholoma matsutake* (62.82%), *Serpula lacrymans* (32.86%) or *Cortinarius glaucopus* (32.17%). DIRS (*Dictyostelium* Intermediate Repeat Sequences) and LINEs (Long Interspersed

Nuclear Elements) are also relatively abundant, but far less than LTRs. Regarding Class II elements, cut-and-paste transposons with Terminal Inverted Repeats (TIRs) and Helitrons are present in most Agaricomycetes, although in low copy number. More specifically, TIRs represent 0-5% of the total genome size whereas Helitrons never reach more than 1 % (**Dataset S1**). Interestingly, up to 78% of the TIR families are non-autonomous MITE (Miniature Inverted-repeat Transposable Elements), elements of short length that do not encode transposases but are able to cross-mobilize using that of other autonomous elements (Jiang et al. 2004). Class I transposons are longer than class II, and both classes have an average GC content of about 50%, which suggests that Agaricomycetes lack the RIP mechanism (Repeat Induced Point mutation) described in Ascomycetes, which inactivates repeats by point mutations.

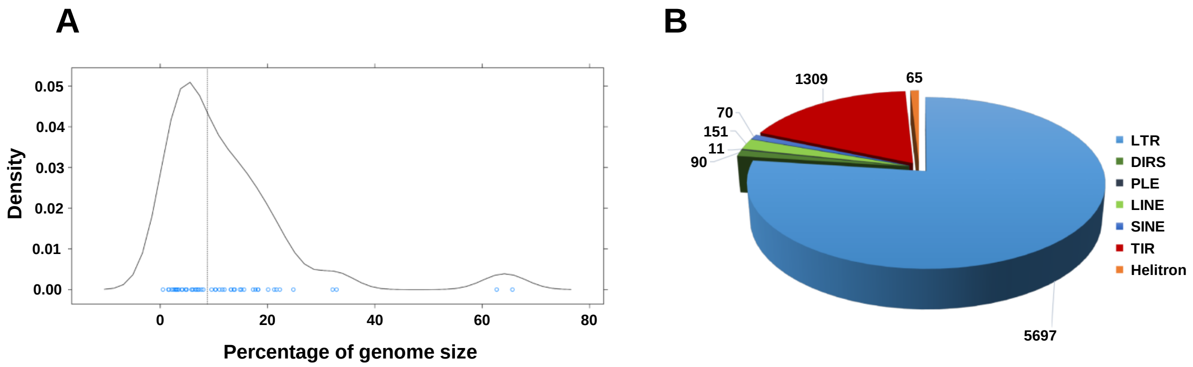

Fig. S6 TE in genomes. **A**) Density plot showing the total TE content in the 52 Agaricomycetes (each blue dot represents a single species). **B**) Number of identified families per TE order.

Table S6 Main features of Agaricomycetes TE family consensuses.

Classification	Mean length	GC content	Class	Number of families
LTR *	6,042	47.6	I	5,697
LTR/Gypsy	6,657	47.2	I	3,493
LTR/Copia	4,970	48.2	I	2,007
DIRS	4,046	53.3	I	90
PLE	1,840	49.8	I	11
LINE	2,815	53.5	I	151
SINE	319	47.9	I	70
TIR	1,747	49.5	II	285
TIR/MITE	448	47.0	II	1,024
Helitron	2,978	45.8	II	65

* Includes Gypsy, Copia, ERV, TRIM and LARD LTR-retrotransposons

8.4 TE dynamics in the context of phylogeny and lifestyle

The diversity found within Agaricomycetes was also observed at the order and even family level, as there is no apparent relationship between TE content and phylogenetic proximity (**Fig. S7**). Examples of this phenomenon are found in the Tricholomataceae family, with *T. matsutake* displaying up to 62.82% of genome TE coverage and *L. nuda* and *C. gibba* carrying 7.66% and 13.82%, respectively. A similar case was found in Cortinariaceae, represented by the ectomycorrhizal species *C. glaucopus* (32.17%) and *Hebeloma cylindrosporum* (2.79%). Fungal symbionts often display highly repetitive genomes (Raffaele and Kamoun 2012). In this sense, we found that on average mycorrhizal species had higher TE content than those with other lifestyles represented by three or more species (**Fig. S8**). Nevertheless, there was a great variability and the Cortinariaceae example indicates that this is not a clear cut rule in Agaricomycetes.

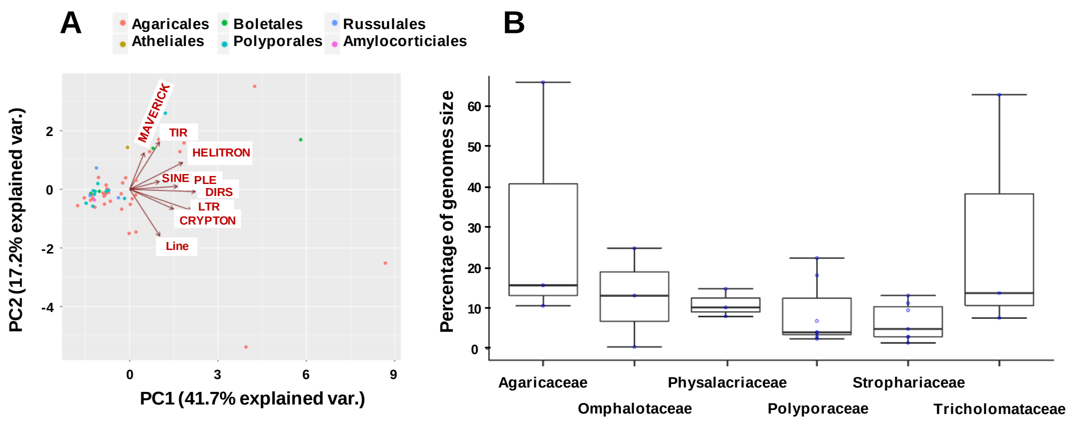

Fig. S7 TE in genomes. **A**) PCA plot at the order level with each dot representing a species. **B**) Box plot representing TE content in families with 3 or more species sampled.

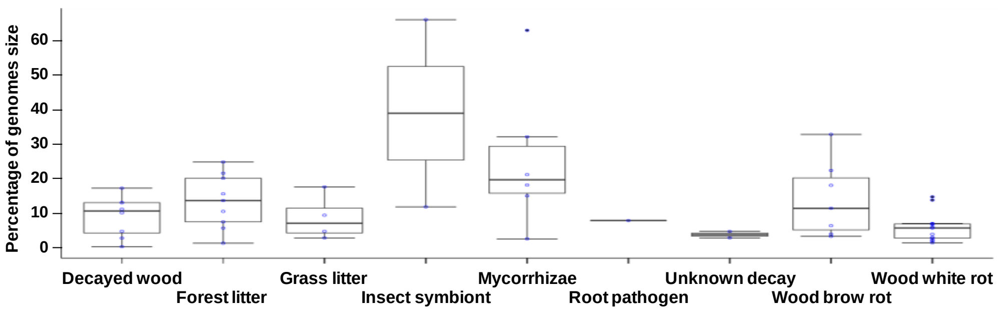

Fig. S8 Box plot showing TE content of Agaricomycetes species grouped by lifestyle.

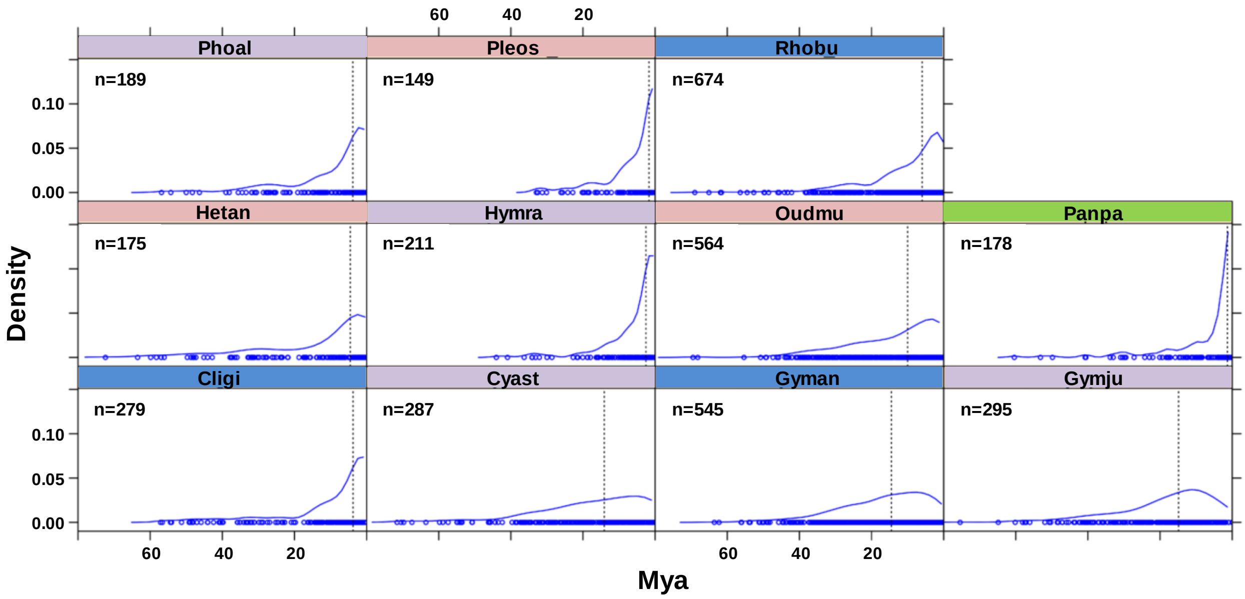

Fig. S9 Density plot showing the distribution of insertion times of full-length LTR-retrotransposons The background color on which the name of the species appears refers to the fungal lifestyle: *purple*, decayed-wood degradation; *dark blue*, forest-litter decay; green, grass-litter decay; and *red*, wood white-rot. Mya = million years ago, n = number of elements included, dotted line = median.

In summary, our data suggest that TE in Agaricomycetes evolve quite independently of phylogenetic and lifestyle constraints. A plausible explanation for this is that most detectable TE expansions might have occurred recently, after the split of closely related species. We tested this hypothesis by estimating the insertion age of LTR-retrotransposons, the most important TE in Agaricomycetes (**Fig. S9**). The results support this hypothesis, as most of the insertions clocked 0 to 20 million years ago (Mya) and the highest number of elements were young copies (0-5 Myr). Interestingly, we found important differences in the amount of time that LTRs survive in the genome, which approximately ranged from 40 to 75 Myr. This suggests that genomic factors, probably related to the defense machinery, modulate the long-term TE dynamics in basidiomycete fungi.

9. Polysaccharide decay machinery (CAZymes)

The numbers of genes encoding plant cell wall-degrading carbohydrate-active enzymes, CAZymes (Lombard et al. 2014) automatically annotated by the JGI Annotation pipeline, and different oxidoreductases, automatically (or generally) manually annotated as indicated below are shown in **Fig. 1** of the main manuscript, together with the total PCWDE numbers (resulting from the sum of the above two groups of enzymes). Box plots showing the number of these enzymes in the 52 fungal species of the orders Agaricales, Boletales, Amylocorticiales, Atheliales, Polyporales and Russulales used in this study are presented in **Fig. S10**, together with the lifestyle of each of them. The higher CAZy, and total PCWDE, gene diversity in Agaricales, compared with the other fungal orders analyzed, are illustrated in the frequency histograms shown in **Figs. S11** and **S12**, respectively.

9.1 Cellulose depolymerization

Saprotrophic Agaricales encode a set of enzymes for cellulose depolymerization consisting of AA9 lytic polysaccharide monooxygenases (LPMO) and glycoside hydrolases from families GH5, GH45, GH6, GH7, GH12, GH1 and GH3 (**Fig. 1,** main manuscript), the members of which comprise endoglucanases (β-1,4-glucanases, EC 3.2.1.4), exoglucanases (cellobiohydrolases, EC 3.2.1.91) and β-1,4-glucosidases (EC 3.2.1.21). Grass-litter decomposers are the saprotrophic group with the highest gene-copy numbers of the AA9 LPMO family acting on crystalline cellulose ($P < 0.05$ for pairwise comparisons, binomial exact test), where they are overrepresented in the PCWDE set compared with mycorrhizae, insect symbionts, and white-rot, brown-rot, decayed-wood and forest-litter decomposers ($P < 0.05$ for pairwise comparisons, Fisher's exact test). This suggests an increased oxidative activity of grass-litter decomposers on cellulose by AA9 LPMO activity.

On the other hand, only the brown-rot degrader *Fistulina hepatica*, which has been suggested to be in an ongoing transition process toward this lifestyle (Floudas et al. 2015), lacks representatives of the GH6 family among the saprotrophic Agaricales analyzed. GH6 and GH7 enzymes, mainly including cellobiohydrolases acting on the non-reducing and reducing end of the cellulose chains, respectively (Chanzy and Henrissat 1985; Vrsanska and Biely 1992), are well represented in most lignocellulose degrading Agaricales. GH6 enzymes are more abundant in some of the Agaricales species analyzed, including white-rot wood and grass-litter degraders, a few forest-litter and decayed-wood decomposers, and the unknown decay fungus *C. variabilis* (containing 3-6 members of this family). By contrast, most of the lignocellulose degraders analyzed from the orders Polyporales, Boletales and Russulales, and even the Atheliales species *Fibulorhizoctonia* sp., which stands out for the high amount of cellulolytic enzymes, only encode one GH6 enzyme in their genomes. This suggests different cellulolytic capabilities in Agaricales with a higher content of GH6 enzymes considering their synergistic action with GH7 enzymes acting on both ends of the cellulose polymer and that, in general, up-regulation of GH6 and GH7 expression has been observed in white-rot fungi growing on lignocellulosic materials (Morin et al. 2012; Fernández-Fueyo et al. 2012a; Barbi et al. 2020).

Two GH6 types concerning cellulose binding are found in Agaricales. These fungi posses CBM1-less and CBM1-containing GH6 enzymes whereas CBM1-less GH6 proteins are rare in the other Agaricomycetes orders. With the aim of finding an evolutionary explanation, we constructed a phylogenetic tree for the enzymes of this family (**Fig. S13**). The basal group of the tree is formed by

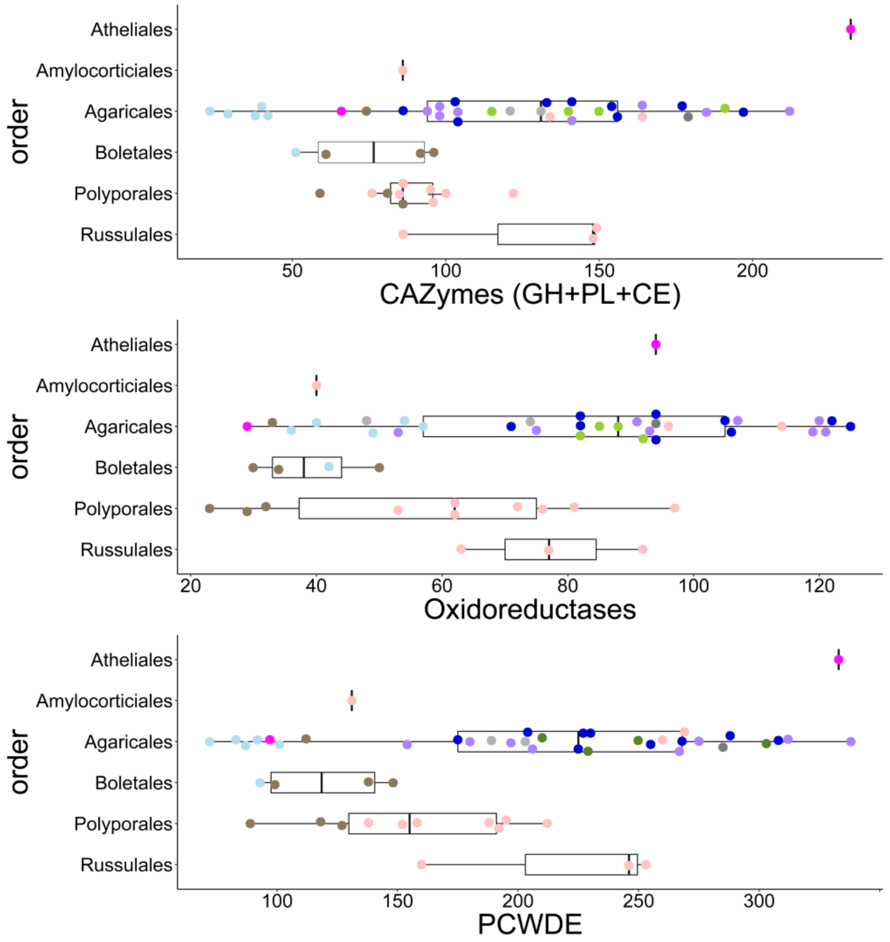

Fig. S10 Box plots showing the distribution of CAZymes and oxidoreductases contributing to plant cell-wall degradation, and of the total PCWDE numbers (data from **Fig. 1** of main manuscript) among Agaricales, Polyporales, Boletales and Russulales orders, based on the 52 Agaricomycetes species analyzed (only one species of the order Amylocorticiales and one species of the order Atheliales have been included in this study). The species are shown as circles colored according to lifestyles as indicated in **Fig. S1**.

CBM1-less GH6 proteins, supporting that GH6 enzymes lacking this CBM, which is characterized by its ability to bind to crystalline cellulose (Boraston et al. 2004), are the ancestors of this GH family in the Agaricomycetes under study. Then, the evolution seems to have followed two paths. On one hand, CBM1-less GH6 proteins give rise to GH6 enzymes lacking CBM1 modules in the extant Agaricales. On the other hand, an ancestral GH6 enzyme linked a CBM1 module and then evolved leading to the CBM1-containing enzymes of this family identified in Agaricales, Polyporales, Russulales, Amylocorticiales, Atheliales and Boletales (most of them with only one CBM1-containing GH6 enzyme). Interestingly the only three CBM1-less GH6 proteins identified outside the order Agaricales (two in *Coniophora puteana* and one in *Plicaturopsis crispa*) appear clustered together with CBM1-containing GH6 enzymes, suggesting they derived later from these enzymes by losing the CBM1 module. The evolutionary process here described for the GH6 family would have provided the extant Agaricales with CBM1-less and CBM1-containing enzymes able to depolymerize cellulose in a wider variety of situations. CBM1 modules increase the efficiency of the enzymes at low concentration of substrates, whereas their absence when not required avoids the unproductive enzyme adsorption on lignin, which reduces the amount of effective enzyme acting on cellulose (Varnai et al. 2014).

On the other hand, the grassland litter decomposers *P. papilionaceus*, *Volvariella volvacea*, and *P. eryngii*, belonging to three phylogenetically distant families (Strophariaceae, Pluteaceae and Pleurotaceae), and the decayed-wood degrader *Galerina marginata* (Strophariaceae) have the highest number of CBM1-containing PCWDE (50, 50, 39 and 43, respectively) and, in general, the largest

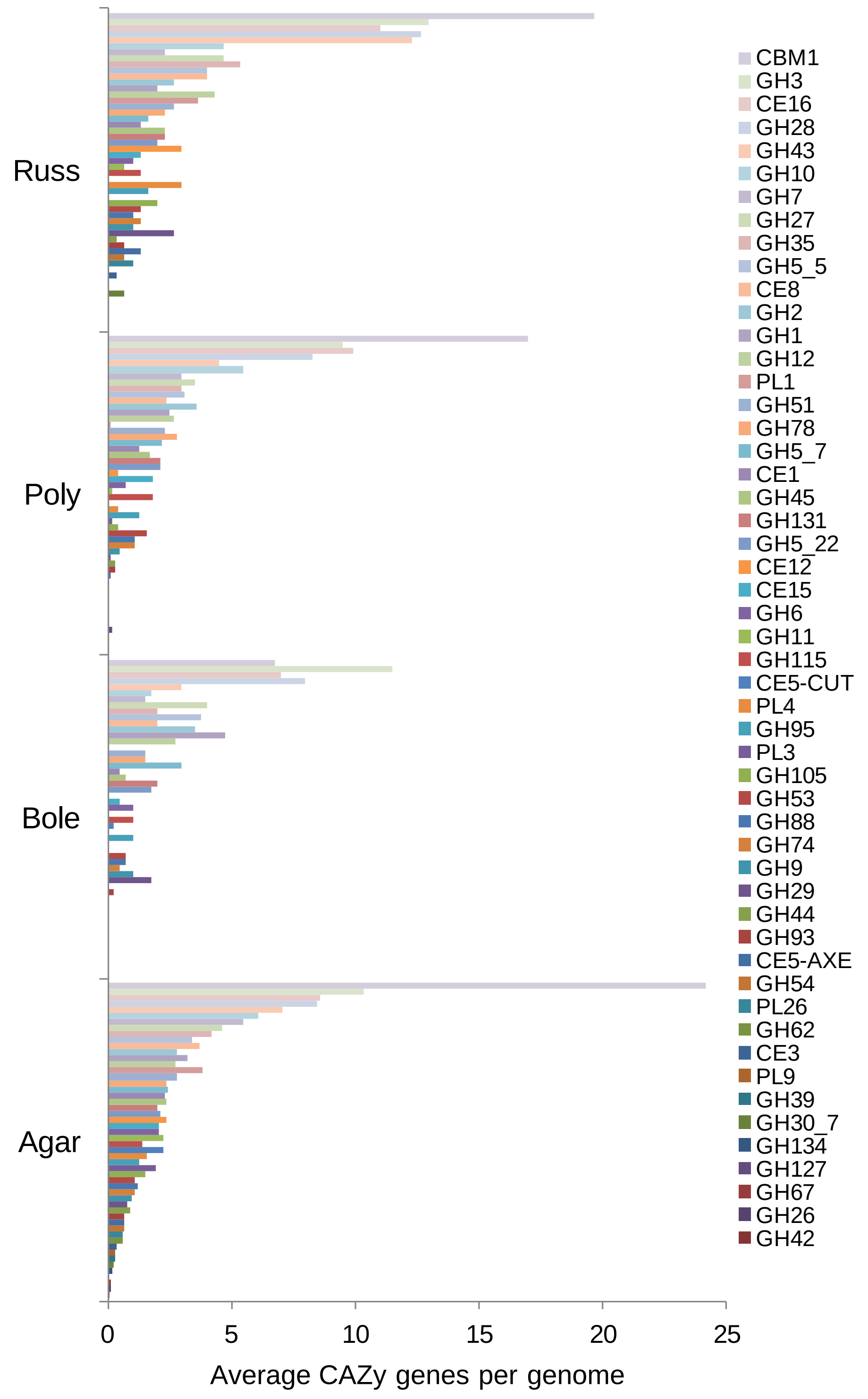

Fig. S11. Histograms showing the average numbers of genes of the different CAZy families (**Fig. 1**) per sequenced genome of Russulales, Polyporales, Boletales and Agaricales species (from top to bottom).

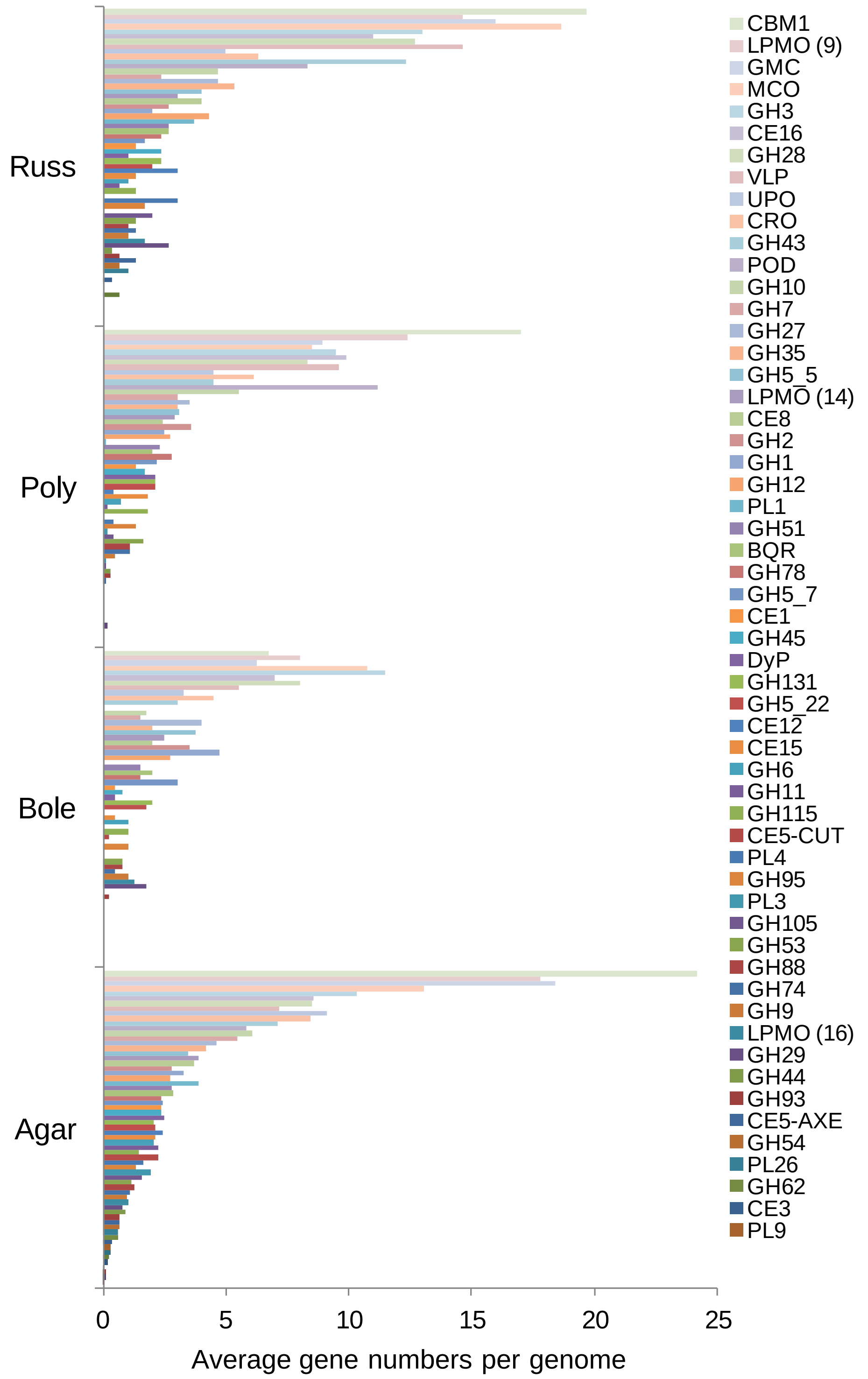

Fig. S12. Histograms showing the average numbers of genes of the different PCWDE families and CBM1 (**Fig. 1**) per sequenced genome of Russulales, Polyporales, Boletales and Agaricales species (from top to bottom).

amount of catalytic domains (55, 51, 41 and 53, respectively) appended to carbohydrate binding modules (CBM1, CBM6, CBM8, CBM35, CBM42, CBM63 or CMB67) able to bind different polymers of the plant cell wall, compared to other saprobic and biotrophic Agaricomycetes (23, 21, 1, 9 and 2 on average in white-rot wood Polyporales and Russulales, brown-rot Polyporales and

Boletales, and mycorrhizal Agaricales, respectively) (**Table S7**). The abundance of CBM-containing PCWDE seems to be a differentiating feature of the grass-litter decay lifestyle, as evidenced by the differences observed between the two *Pleurotus* species analyzed. Thus, the grassland-inhabiting *P. eryngii* has 41 CBM-appended enzymes –including five AA9-LPMOs and members of 12 glycoside hydrolase (GH5_5, GH5_7, GH6, GH7, GH10, GH11, GH27, GH43, GH45, GH62, GH74 and GH131), three carbohydrate esterase (CE1, CE15 and CE16), and two polysaccharide lyase (PL1_9 and PL3) (sub)families active on the plant cell wall– whereas the white-rot wood decomposer *Pleurotus ostreatus* presents a more reduced set of 30 CBM-appended enzymes made up of five AA9-LPMOs, and 25 more enzymes of nine glycoside hydrolase (GH5_5, GH5_7, GH6, GH7, GH10, GH11, GH43, GH62 and GH74), and one carbohydrate esterase (CE1) (sub)families active on the plant cell wall, besides having no appended pectin-active polysaccharide lyases (**Table S7**). The biological significance of these findings in lifestyle adaptation could be related to the fact that CBM1-appended catalytic domains would be a better option to reduce the leaching of enzymes in a loose environment, such as leaf litter, compared with wood.

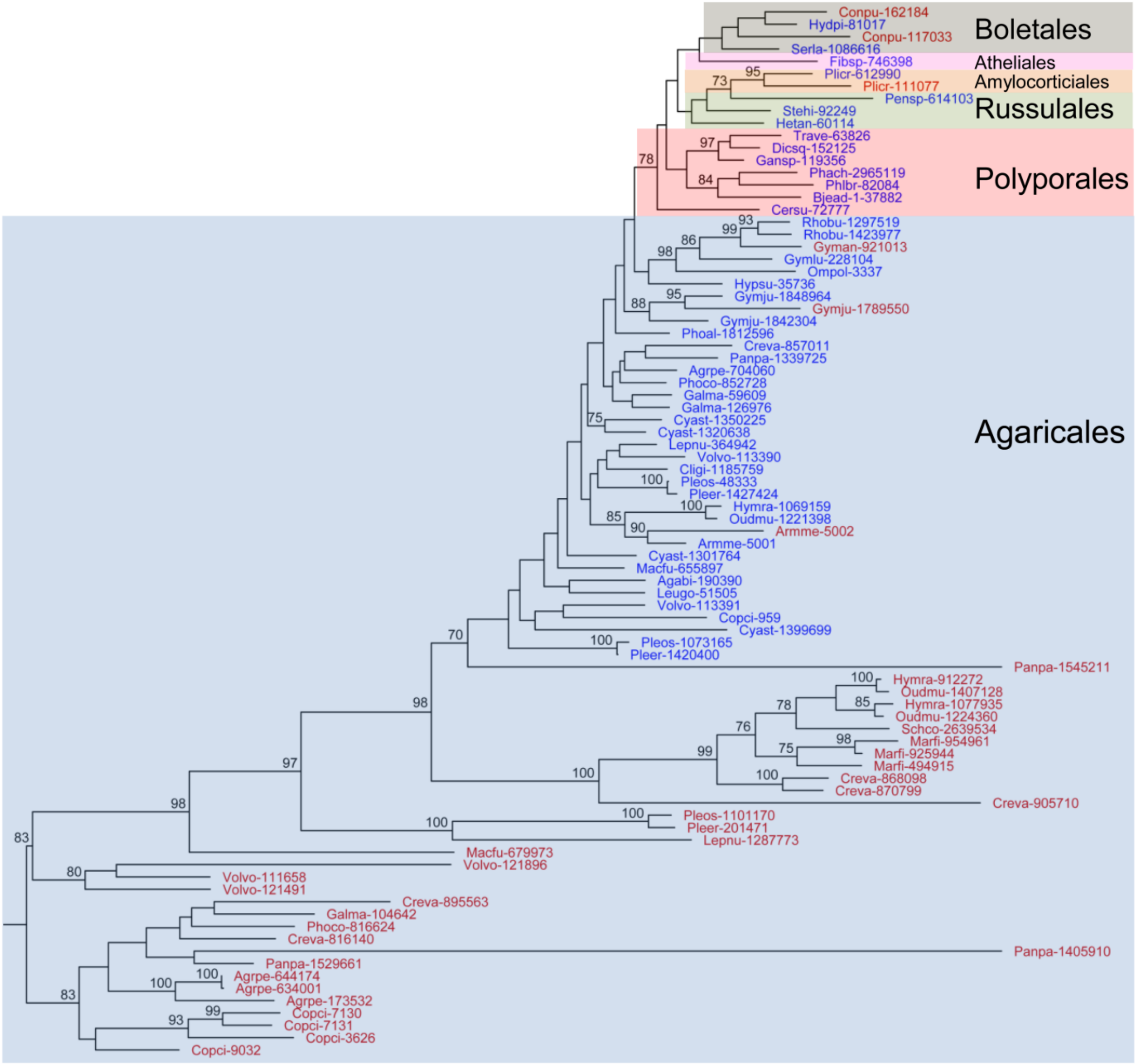

Fig. S13 ML phylogenetic tree (RAxML v.8.1.12), constructed with 1000 bootstrap replications, of 85 enzymes of the glycoside hydrolase family 6 (GH6) identified in 42 of the 52 Agaricomycetes genomes analyzed. The tree was constructed using the evolutionary model WAG+I+G+F suggested by ProtTest (Abascal et al. 2005). The font color code indicates CBM1-less (red font) and CBM1-containing (blue font) GH6 enzymes, and the colored background identifies the different orders. Bootstrap values ≥70% are indicated on the nodes. All sequences are available through the links to JGI fungal genomes included in Supplementary **Table S4**.

Table S7 CBM-containing CAZymes and oxidoreductases. **A**) Content of catalytic domains –GH, glycoside hydrolases; PL, polysaccharide lyases; CE, carbohydrate esterases; AA, oxidoreductases, classified as Auxiliary Activities in CAZy database (http://www.cazy.org); and EXPN, expansin-like proteins (Saloheimo et al. 2002)– appended to carbohydrate binding modules (of families CBM1, CBM6, CBM8, CBM35, CBM42, CBM63 or CMB67) able to bind different polymers of the plant cell wall. **B**) Total content of CBM1 modules non-appended (first column) and appended to catalytic domains, in the genomes analyzed.

				A: CBM1								CBM8	CBM1/42	CBM1	CBM1/35	CBM1			CBM1/6/35	CBM1			CBM67	CBM1											CBM1/63	CBM-CD		B: CBM1		Catalytic domains (CD) appended to CBM1									CBM1-CD	
	species	Ecology	Lifestyle	GH5_5	GH45	GH6	GH7	GH12	GH3	GH131	GH74	GH44	GH54	GH95	GH27	GH10	GH11	GH30_7	GH43	GH62	GH5_7	GH28	GH78	PL1	PL3	CE16	CE5	CE3	CE1	CE15	AA9	AA8-AA12	AA3-2	AA8	EXPN	Total	Mean	Total	Mean	GH	AA9	CE	PL1_9	PL3_2	AA8	AA3_2	AA8-AA12	EXPN	Total	Mean
AGARICALES	*H. sublateritium*	Saprotroph	Decayed wood	4	0	1	2	0	1	1	1	0	0	0	1	3	1	0	1	0	1	0	0	0	0	1	2	0	2	0	2	0	0	0	0	24	28	29	30	15	2	5	-	-	-	-	-	-	22	25
	P. alnicola			3	1	1	1	0	1	0	1	0	0	0	1	2	1	0	1	0	2	0	2	0	0	2	2	0	1	0	4	0	0	0	0	26		25		13	4	5	-	-	-	-	-	-	22	
	G. marginata			5	0	2	5	1	1	1	2	0	8	0	2	5	4	0	1	1	3	2	1	0	0	1	2	0	2	1	3	0	0	0	0	53		54		34	3	6	-	-	-	-	-	-	43	
	G. junonius			5	0	2	1	0	1	1	1	0	0	0	2	3	1	0	1	0	1	0	0	0	0	1	1	0	1	1	1	0	0	0	0	24		26		17	1	4	-	-	-	-	-	-	22	
	C. striatus			4	0	4	0	0	1	1	1	0	0	0	2	3	0	0	1	0	3	0	0	0	1	1	0	0	0	0	5	0	0	0	0	27		34		18	5	1	-	1	-	-	-	-	25	
	G. luxurians			3	0	1	4	0	1	1	0	0	0	0	2	4	1	2	2	0	4	0	0	0	0	1	0	0	2	0	4	0	0	0	0	32		32		22	4	3	-	-	-	-	-	-	29	
	O. olearius			2	0	1	2	0	0	1	1	0	0	0	1	3	0	0	1	0	0	0	0	0	0	0	0	0	2	0	3	0	0	0	0	17		16		10	3	2	-	-	-	-	-	-	15	
	H. radicata			2	0	1	2	0	0	1	1	0	0	0	2	5	0	0	2	0	1	0	0	0	0	0	2	1	3	0	1	0	0	0	0	24		22		14	1	6	-	-	-	-	-	-	21	
	P.conissans		Forest litter	3	0	1	3	0	1	1	1	0	3	0	1	3	1	0	2	3	1	0	0	0	0	1	0	0	2	1	5	0	0	0	0	33	25	35	28	20	5	4	-	-	-	-	-	-	29	22
	C. cinerea			1	0	1	0	0	0	1	0	0	0	0	0	2	1	0	1	1	1	0	0	0	0	0	0	1	4	3	5	3	0	0	0	25		39		8	5	9	-	-	-	-	3	-	25	
	M. fuliginosa			3	0	1	4	0	0	1	1	0	0	0	1	4	0	0	2	0	1	0	0	0	0	1	0	0	2	2	2	0	0	0	0	25		24		15	2	5	-	-	-	-	-	-	22	
	A. bisporus var bisporus			3	0	1	1	0	0	0	1	0	0	0	1	1	1	0		0	1	0	0	0	0	1	1	0	1	1	2	0	0	0	0	16		17		9	2	4	-	-	-	-	-	-	15	
	L. nuda			1	0	1	3	0	0	1	1	0	0	0	3	3	0	0	2	0	2	0	1	0	1	3		0	1	2	3	0	0	0	0	28		40		15	3	6	-	1	-	-	-	-	25	
	C. gibba			3	0	1	4	0	0	1	1	0	0	0	2	3	0	0	1	0	1	0	0	0	1	2		0	3	0	2	0	0	0	0	25		29		15	2	5	-	1	-	-	-	-	23	
	R. butyracea			1	0	2	4	0	0	0	1	0	0	0	1	2	1	0	3	1	1	0	0	0	0	3	2	0	0	0	0	0	0	0	1	23		19		13	-	5	-	-	-	-	-	1	19	
	G. androsaceus			3	0	0	9	0	1	1	1	0	0	0	2	5	2	0	1		2	0	0	0	0	2	3	0	1	0	2	0	0	0	0	35		36		25	2	6	-	-	-	-	-	-	33	
	M. fiardii			0	0	0	0	0	0	1	1	1	0	0	1	2	0	0	2		1	0	0	0	0	2	1	0	1	0	1	0	0	0	0	14		11		5	1	4	-	-	-	-	-	-	10	
	A. pediades		Grass litter	3	0	1	5	0	0	1	1	0	1	0	4	4	2	0	1	1	1	0	1	0	0	1	1	0	2	1	5	0	0	0	0	36	46	35	51	19	5	5	-	-	-	-	-	-	29	42
	P. papilionaceus			5	0	1	2	0	0	1	1	0	1	0	4	10	0	2	5	1	2	0	0	0	0	3	2	0	4	2	5	2	2	0	0	55		68		30	5	11	-	-	-	2	2	-	50	
	V. volvacea			1	0	2	7	0	1	1	1	0	0	1	0	16	0	0	4		1	0	0	0	2	1	0	2	4	1	6	0	0	0	0	51		56		34	6	8	-	2	-	-	-	-	50	
	P. eryngii			3	2	2	10	0	0	1	2	0	0	0	1	3	1	0	1	1	2	0	0	1	2	2	0	0	1	1	5	0	0	0	0	41		45		27	5	4	1	2	-	-	-	-	39	
	O. mucida		Wood (white rot)	2	0	1	0	0	0	1	0	0	0	0	1	5	0	0	1	0	1	0	0	0	0	0	1	0	2	0	1	0	0	0	0	16	23	15	24	10	1	3	-	-	-	-	-	-	14	22
	P. ostreatus			4	0	2	9	0	0	0	2	0	0	0	0	1	1	0	1	1	3	0	0	0	0	0	0	0	1	0	5	0	0	0	0	30		33		23	5	1	-	-	-	-	-	-	29	
	C. variabilis		Unknown decay	1	0	1	1	0	0	1	0	0	0	0	0	2	0	0	1	1	2	0	1	0	0	2	0	0	4	1	3	1	0	0	0	22	14	28	17	9	3	7	-	-	-	-	1	-	20	12
	S. commune		Unknown decay	0	0	0	0	0	0	0	0	0	0	0	0	1	0	0	2	0	0	0	0	0	0	0	0	0	2	0	0	0	0	0	1	6		5		1	-	2	-	-	-	-	-	-	3	
	A. mellea	Biotroph	Root pathogen	0	0	1	0	0	1	1	1	0	0	0	1	2	1	0	0	0	1	0	0	0	0	0	0	0	0	0	1	0	0	0	0	10	10	11	11	8	1	-	-	-	-	-	-	-	9	9
	F. hepatica	Saprotroph	Wood (brown rot)	0	0	0	0	0	0	0	0	0	0	0	1	0	0	0	1	0	0	0	0	0	0	0	0	0	0	0	0	0	0	0	1	3	3	0	0	-	-	-	-	-	-	-	-	-	0	0
	H. cylindrosporum	Biotroph	Mycorrhizae	1	0	0	0	0	0	0	0	0	0	0	0	1	0	0	0	0	0	0	0	0	0	0	0	0	0	0	0	0	0	0	0	2	2	2	2	2	-	-	-	-	-	-	-	-	2	1
	C. glaucopus			0	0	0	0	0	0	0	0	0	0	0	0	2	0	0	0	0	0	0	0	0	0	0	0	0	0	0	0	0	0	0	0	2		3		2	-	-	-	-	-	-	-	-	2	
	L. amethystina			1	0	0	0	0	0	0	0	0	0	0	0	0	0	0	0	0	0	0	0	0	0	0	0	0	0	0	0	0	0	0	0	1		1		1	-	-	-	-	-	-	-	-	1	
	L. bicolor			1	0	0	0	0	0	0	0	0	0	0	0	0	0	0	0	0	0	0	0	0	0	0	0	0	0	0	0	0	0	0	0	1		1		1	-	-	-	-	-	-	-	-	1	
	T. matsutake			1	0	0	0	0	0	0	0	0	0	0	1	0	0	0	0	0	0	0	0	0	0	0	0	0	0	0	0	0	0	0	0	2		1		1	-	-	-	-	-	-	-	-	1	
	L. gongylophorus	Biotroph	Insect symbiont	1	0	1	0	0	0	0	1	1	0	0	0	0	0	0	1	0	0	0	0	0	0	0	0	0	1	1	0	0	0	0	0	7	7	6	6	3	-	2	-	-	-	-	-	-	5	5
BOLETALES	*H. pinastri*	Saprotroph	Wood (brown rot)	3	0	1	1			1	1	0	0	0	1	1	0	0	1	0	1	0	0	0	0	0	0	0	1	0	3	0	0	1	0	16	9	17	9	9	3	1	-	-	1	-	-	-	14	7
	C. puteana			0	0	0	0	0	0	0	0	0	0	0	0	1	0	0	0	0	0	0	0	0	0	0	0	0	0	0	0	0	0	1	0	2		2		1	-	-	-	-	1	-	-	-	2	
	S. lacrymans			2	0	1	0	0	0	1	0	0	0	0	1	0	0	0	1	0	1	0	0	0	0	0	0	0	0	0	0	0	0	1	0	8		8		5	-	-	-	-	1	-	-	-	6	
	S. brevipes	Biotroph	Mycorrhizae	0	0	0	0	0	0	0	0	0	0	0	0	0	0	0	0	0	0	0	0	0	0	0	0	0	0	0	0	0	0	0	0	0	0	0	0	-	-	-	-	-	-	-	-	-	0	0
AMYLOCORTICIALES	*P. crispa*	Saprotroph	Wood (white rot)	1	0	1	0	0	0	1	0	0	0	0	1	1	0	0	1	0	1	0	0	0	0	1	0	0	0	1	0	0	0	0	0	9	9	9	9	6	-	2	-	-	-	-	-	-	8	8
ATHELIALES	*Fibulorhizoctonia* sp.	Biotroph	Insect symbiont	4	4	1	7	0	0	1	1	2	0	0	0	1	1	0	3	0	3	0	0	0	0	2	0	0	3	0	2	0	0	0	0	35	35	35	35	25	2	5	-	-	-	-	-	-	32	32
POLYPORALES	*C. subvermispora*	Saprotroph	Wood (white rot)	2	0	1	2	0	0	0	1	0	0	0	0	3	1	0	0	0	1	0	0	0	0	1	0	0	1	0	3	0	0	1	0	17	23	17	24	11	3	2	-	-	1	-	-	-	17	22
	D. squalens			3	0	1	0	0	1	0	1	0	0	0	0	2	0	0	1	0	1	0	0	0	0	1	0	0	0	1	3	0	0	1	0	16		17		9	3	2	-	-	1	-	-	-	15	
	Ganoderma sp.			3	0	1	0	0	1	1	1	0	0	0	1	3	0	0	1	0	1	0	0	0	0	1	0	0	1	1	1	0	0	1	0	18		18		12	1	3	-	-	1	-	-	-	17	
	T. versicolor			3	0	1	0	0	1	1	1	0	0	0	1	3	0	0	1	0	1	0	0	0	0	1	0	0	3		3	0	0	1	0	21		23		12	3	4	-	-	1	-	-	-	20	
	B. adusta			4	0	1	4	0	1	1	1	0	0	0	1	2	0	0	1	0	1	0	0	0	0	4	0	0	1	1	7	0	0	1	0	31		32		16	7	6	-	-	1	-	-	-	30	
	P. chrysosporium			2	0	1	5	0	1	1	1	0	0	0	1	4	1	0	1	0	2	1	0	0	0	1	0	0	2	1	6	0	0	1	0	32		35		20	6	4	-	-	1	-	-	-	31	
	P. brevispora			4	1	1	4	0	1	1	0	0	0	0	0	4	0	0	1	0	1	0	0	0	0		1	0	1	1	4	0	0	1	2	28		28		17	4	3	-	-	1	-	-	-	25	
	P. placenta		Wood (brown rot)	0	0	0	0	0	0	0	0	0	0	0	0	0	0	0	1	0	0	0	0	0	0		0	0	0	0	0	0	0	0	0	1	1	0	0	-	-	-	-	-	-	-	-	-	0	0
	W. cocos			0	0	0	0	0	0	0	0	0	0	0	0	0	0	0	1	0	0	0	0	0	0	0	0	0	0	0	0	0	0	0	0	1		0		-	-	-	-	-	-	-	-	-	0	
	F. pinicola			0	0	0	0	0	0	0	0	0	0	0	0	0	0	0	1	0	0	0	0	0	0	0	0	0	0	0	0	0	0	0	0	1		0		-	-	-	-	-	-	-	-	-	0	
RUSSULALES	*H. annosum*		Wood (white rot)	3	0	1	0	0	1	1	1	0	0	0	2	1	0	0	1	0	1	0	0	0	0	1	0	0	1	0	3	0	0	1	0	18	21	17	20	10	3	2	-	-	1	-	-	-	16	18
	S. hirsutum			3	0	1	0	0	1	0	1	0	0	0	0	3	1	0	2	0	1	0	0	0	0	0	0	0	1	1	2	0	0	1	0	18		17		11	2	2	-	-	1	-	-	-	16	
	Peniophora sp.			3	0	1	2	0	0	1	1	0	1	0	3	2	0	1	2	0	2	0	0	0	0	0	1	0	1	1	5	0	0	0	0	27		25		15	5	3	-	-	-	-	-	-	23	

9.2 Hemicellulose depolymerization

Unlike cellulose, the depolymerization of hemicelluloses requires a great diversity of hydrolases given the variety and heterogeneity of the polymers included in this group of polysaccharides. Genes encoding enzymes from families GH12 and GH74 where putative xyloglucan β-1,4-endoglucanases (EC 3.2.1.151) responsible for xyloglucan backbone depolymerization are included, and genes from families GH5_7 and GH2 containing putative endo-β-1,4-mannases (EC 3.2.1.78) and β-mannosidases (EC 3.2.1.25), respectively, able to act on the mannan backbone, are found in all saprotrophic fungi analyzed (including Agaricales) with only a few exceptions (**Fig. 1**). These exceptions correspond to the brown-rot fungi *Postia placenta*, *Wolfiporia cocos* and *Fomitopsis pinicola* (order Polyporales) and *C. puteana* (order Boletales), which suffered contraction of genes encoding lignocellulolytic enzymes through the evolution (Floudas et al. 2012) and lack GH74 enzymes.

The minimal differences observed in the composition of the enzyme complex involved in xyloglucan and mannan backbone degradation in saprotrophic Agaricomycetes (brown-rot fungi excluded) contrast with the differences noted in the enzymatic machinery responsible for the depolymerization of xylan, the most abundant hemicellulose in plant cell walls (Fry 1988). The major endoxylanase

families GH10 and GH11 (EC 3.2.1.8) (Biely et al. 1997) have been identified in most saprotrophic Agaricales genomes analyzed as the only ones able to act on xylan **(Fig. 1)**. Of these, GH10 endoxylanases, able to cleave the xylan backbone at the non-reducing site of substituted xylose residues, are also present in the saprotrophic fungi from the orders Boletales, Amylocorticiales, Atheliales, Polyporales and Russulales, independently of their lifestyle. Endoxylanases work synergistically with β-1,4-xylosidases which catalyze both the removal of D-xylose residues from the non-reducing termini and xylobiose hydrolysis. GH3 and GH43 families containing these enzymes have been also identified in a good number in these fungi.

Unlike GH10 endoxylanases, widely distributed in different orders, the GH11 endoxylanases identified are almost exclusive of saprotrophic Agaricales, with a very low presence in other saprotrophic fungi (**Fig. 1**). GH11 xylanases are characterized by their high substrate specificity (Paës et al. 2012). They are smaller in size and can better penetrate the plant cell-wall network compared with GH10 xylanases. GH11 enzymes do not tolerate a high degree of substitutions and only cleave unsubstituted regions of the xylan backbone. To be efficient, they have to work synergistically with debranching enzymes able to remove side chains linked to the xylose backbone. Interestingly, a higher number and variety of debranching enzymes can be found in Agaricales compared with Polyporales and Boletales ($P < 0.05$ for pairwise comparisons, binomial exact test). Thus, whereas putative α-L-arabinofuranosidases (EC 3.2.1.55), α-glucuronidases (EC 3.2.1.131) and acetyl xylan esterases (AXE) (EC 3.1.1.72) from families GH51, GH115 and CE1, respectively, are widely distributed in fungi from the orders Agaricales, Boletales, Amylocorticiales, Atheliales, Polyporales, and Russulales (**Fig. 1**), GH62 α-L-arabinofuranosidases only appear in saprotrophic Agaricales and those of family GH54 are almost limited to this order (they are also present in the Atheliales species *Fibulorhizoctonia* sp and the Russulales species *Peniophora* sp), GH67 α-glucuronidases are only found in three saprotrophic Agaricales (*H. radicata*, *R. butyracea* and *Gymnopus androsaceus*), and CE3 acetyl xylan esterases have been identified in the Agaricales *H. radicata*, *Coprinopsis cinerea* and *V. volvacea* (also in the Russulales species *Peniophora* sp).

9.3 Pectin depolymerization

The enzymatic machinery involved in pectin depolymerization was also analyzed. Among the enzymes specifically acting on this polymer, those of family GH28, which includes exo- (EC 3.2.1.67) and endo-polygalacturonases (EC 3.2.1.15), exo- (EC 3.2.1.-) and endo-rhamnogalacturonases (EC 3.2.1.171) and xylogalacturan hydrolases (EC 3.2.1.-), GH78 α-rhamnosidases (EC 3.2.1.40), GH53 endo-β-1,4-galactanase (EC 3.2.1.89), GH88 unsaturated β-glucuronyl hydrolase (EC 3.2.1.-) and CE8 pectin methylesterases (EC 3.1.1.11) are distributed in most genomes analyzed regardless of the fungal lifestyle and order. Some mycorrhizal fungi lack GH78, GH88 and GH53 enzymes, whereas the Amylocorticiales species *P. crispa* lacks GH53 enzymes, and *C. cinerea* and *V. volvacea* do not contain members of family GH78. On the other hand, GH93 exoarabinases (EC 3.2.1.-) are also found in some species of the different lifestyles analyzed. Pectin, consisting of a family of galacturonic acid-rich polysaccharides, is mainly found in the primary plant cell wall and middle lamella, with a very different composition and percentage of this polymer depending on the plant type and growth stage, being relatively abundant in nonwoody plant tissues and minority in lignified tissues (Sakai et al. 1993; Caffall and Mohnen 2009). In consequence, pectinolytic enzymes were expected to be especially well represented in those fungi growing on non-woody plant biomass in nature.

This is apparently so in saprotrophic Agaricales, although not only in leaf-litter decomposers but also in wood degraders (and to a lesser extent in wood white-rot Russulales). They seem to have further pectinolytic capabilities as most of them possess an additional complement of enzymes active on pectin (barely represented in a few fungi of the other orders) made up of: i) polysaccharide lyases, including PL1 and PL3 pectin and pectate lyases (EC 4.2.2.10 and EC 4.2.2.2) and PL4 rhamnogalacturonan lyases (EC 4.2.2.23) able to cleave glycosidic bonds via β-elimination; ii) GH105 unsaturated rhamnogalacturonyl hydrolases (EC 3.2.1.172); and iii) CE12 pectin acetylesterases (EC 3.1.1.-) and rhamnogalacturonan acetylesterases (EC 3.1.1.-) capable of removing the acetyl residues of the galacturonan and rhamnogalacturonan backbone (**Fig. 1**). In consequence, we can expect that

Agaricales will be in general better pectin degraders compared, for example, with wood white-rot Polyporales.

9.4 Cuticle degradation

Members of the recently renamed as versatile lipase family (Barriuso et al. 2016) were found in 51 of the 52 fungal genomes (**Fig. 1**). These enzymes have been related to the initial degradation of epicuticular waxes and cuticle, which includes a mixture of lipids (Juniper and Jeffree 1983). Degradation of these external layers makes the plant polysaccharides more accessible to the CAZymes described above. For this purpose, most saprotrophic Agaricales analyzed –excluding white-rot fungi, the brown-rot *F. hepatica*, and two (*P. conissans* and *P. eryngii*) of the thirteen grassland/forest leaf-litter fungi– also contain an extra set of enzymes of the family CE5, which contrasts with their scarce presence, if any, in other lignocellulose degraders (**Fig. 1**). A phylogenetic analysis of these enzymes was performed using, as references, characterized cutinases and AXE forming this carbohydrate esterase family (phylogenetic tree in **Fig. S14)**. Two clearly differentiated clusters containing AXE and cutinases were identified in the tree. The only enzymes of the CE5 family identified in wood white-rot fungi (*Peniophora* sp., *Stereum hirsutum* and *Phlebia brevispora*) were grouped with AXE whereas putative cutinases appear represented in most leaf-litter and decayed-wood Agaricales.

Cutinases contribute to degrade the cutin polymer made by hydroxyfatty acids and other components. Cutin, the main lipid plant polymer (Heredia 2003), is a major component of the cuticle that acts as a protective barrier of almost all the aerial surfaces in higher plants, including leaves, fruits and nonwoody stems. The presence of CE5 cutinases in both decayed-wood and leaf-litter Agaricales (and other species without a white-rot lifestyle) suggests that these fungi would initiate the degradation of leaves and other plant remains in soil by acting on cutin. Suberinases, potentially-overlapping with cutinases, have also recently been reported in Agaricales genomes (Almasi et al. 2019). As a result, the access of enzymes capable of degrading the inner pectin layer would be favored facilitating in this way the subsequent access of enzymes capable of degrading other polymers of the plant cell wall. Interestingly, not only white-rot and brown-rot wood Agaricales lack cutinases. These enzymes are also absent in most of the analyzed white-rot and brown-rot wood Boletales, Amylocorticiales, Polyporales and Russulales where they are not so critical for these fungi growing on lignified wood.

9.5 Feruloyl esterases

CE1 feruloyl esterases (FAE; EC 3.1.1.73) also play a key role in lignocellulose degradation by removing the ester bonds between *p*-hydroxycinnamic acids and hemicelluloses (arabinoxylans and certain pectins). As a result, polysaccharide-polysaccharide (connected by diferulic acids) and polysaccharide-lignin crosslinks are broken providing hydrolase and lyase access to cellulose and hemicelluloses (Wong 2006; Dilokpimol et al. 2016). An extensive phylogenetic analysis of the 121 CE1 enzymes identified in 39 of the 52 fungal genomes was performed to discriminate between putative AXE and FAE (**Fig. S15**), both included in this carbohydrate esterase family (the only CAZy family containing FAE). Fungal CE1 enzymes described as AXE and FAE in the CAZy database, and those recently characterized as functional FAE by Dilokpimol et al. (2018) were used as references to classify the 121 enzymes in one of the above two CE1 families.

Most of the identified CE1 enzymes clustered together with AXE and they can be classified as such (their role as debranching enzymes has been described above). Some of these xylanases may also have FAE activity as recently demonstrated for Galma-144217 and Cersu-68569, from *G. marginata* and *Ceriporiopsis subvermispora*, respectively, which are able to use *p*NP-ferulate as substrate, although very low or no activity has been observed on different hydroxycinnamic methyl esters (Dilokpimol et al. 2018). The analysis of the phylogenetic tree revealed that only a minority (38 CE1 enzymes) correspond to putative FAE, belonging to subfamilies 5 and 6 (SF5 and SF6) (Dilokpimol et al. 2016), which appear distributed only in a few Agaricales species, the Amylocorticiales species *P. crispa* and the Atheliales species *Fibulorhizoctonia* sp. SF5 contains Type A and D FAE (Crepin et al. 2004). The broad FAE substrate specificity on methyl esters of different methoxylated and non-methoxylated hydroxycinnamic acids (including ferulic and *p*-coumaric acids, which are the most abundant in the lignocellulosic matrix), as well as the ability of some of them to release diferulic acid from plant cell

walls (Dilokpimol et al. 2016) suggest that putative FAE may have an important role in lignocellulose degradation by some decayed-wood, forest-litter, grass-litter, white-rot and unknown-decay Agaricales compared with both brown-rot fungi, including Agaricales, Boletales and Polyporales, and white-rot wood Polyporales and Russulales species, which lack SF5 CE1 FAE.

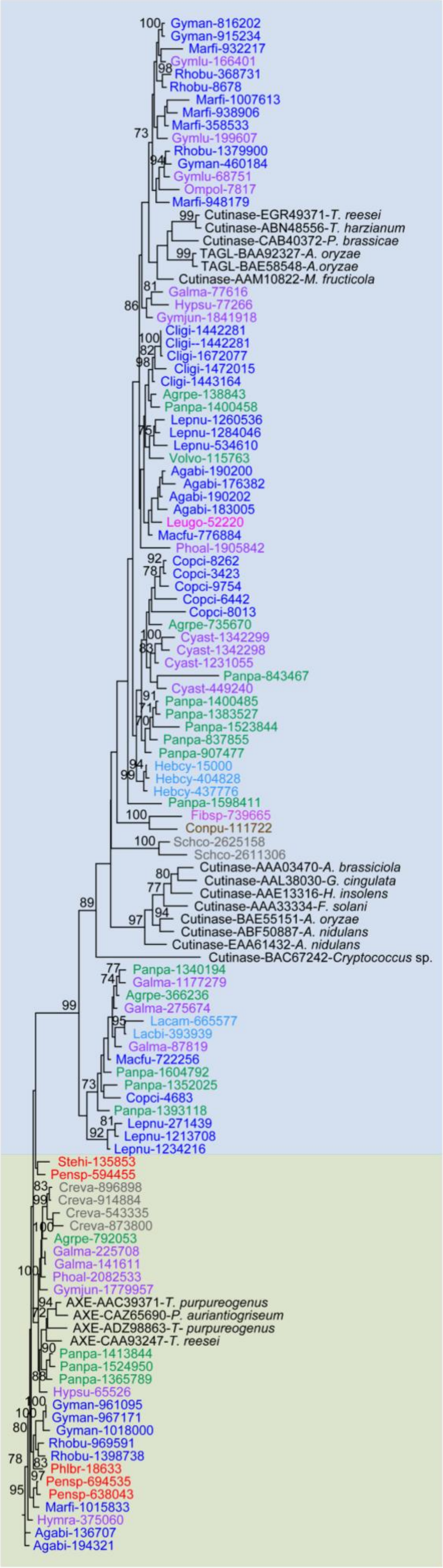

Fig. S14 ML phylogenetic tree (RAXML, v.8.1.12), constructed with 1000 bootstrap replications, for the CE5 enzymes identified in the 52 Agaricomycetes genomes included in the present study (only bootstrap values ≥70% are shown). 18 CE5 enzymes previously characterized, with cutinase (14 enzymes) and AXE (4 enzymes) activity, available at CAZy database were included as references to predict the putative activity of the CE5 enzymes identified in the genomes. Putative cutinases on cyan background and putative AXE on green background. The color code of the enzymes corresponds to the lifestyle of the fungal species where they were found (as indicated in **Fig. S1**).

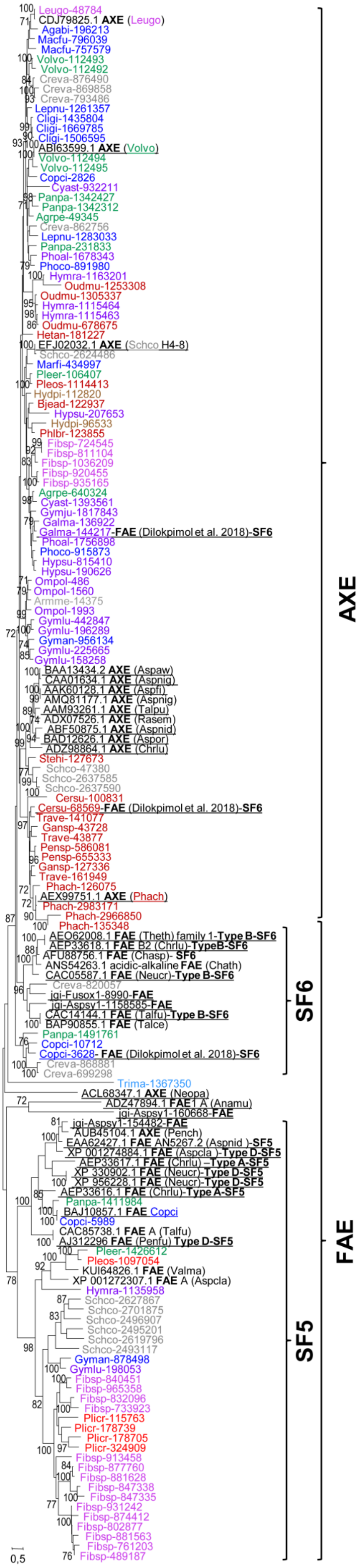

Fig. S15 ML phylogenetic tree, constructed with 1000 bootstrap replications, of 121 enzymes of the carbohydrate esterase family 1 (CE1) identified in 39 of the 52 fungal genomes analyzed (bootstrap values ≥70 are indicated). The color code of the enzymes corresponds to the lifestyle of the fungal species they belong to (see **Fig. S1**). CE1 amino-acid sequences of this family previously classified (shown in bold) and characterized (shown in bold and underlined) as AXE or FAE (of type A, B and D, belonging to subfamilies SF5 and SF6) available in the CAZy database or described by Dilokpimol et al. (2016; 2018), have been included as internal references in the tree to assign the putative function of the CE1 enzymes identified in this study.

The abbreviated names of the fungal species to which the reference enzymes belong are: Anamu, *Anaeromyces mucronatus*; Aspaw, *Aspergillus awamori*; Aspcl, *Aspergillus clavatus;* Aspfi, *Aspergillus ficuum*; Aspnid, *Aspergillus nidulans*; Aspnig, *Aspergillus niger*; Aspor, *Aspergillus oryzae*; Aspsy, *Aspergillus sydowii*; Chasp; *Chaetomium* sp.; Chrlu, *Chrysosporium lucknowense*; Fusox, *Fusarium oxysporum*; Neopa, *Neocallimastix patriciarum*; Nercr, *Neurospora crassa*; Pench, *Penicillium chrysogenum;* Rasem, *Rasamsonia emersonii*; Talce, *Talaromyces cellulolyticus*; Talfu, *Talaromyces funiculosus*; Talpu, *Talaromyces purpureogenus*; Theth, *Thermothelomyces thermophila*; Valma, *Valsa mali*.

10. Multicopper oxidases (MCO)

The MCO genes present in the 52 Agaricomycetes genomes were identified by SEARCHing by keyword using "multicopper oxidase" or “laccase” in the JGI Genome Portal Search application. Both together yielded a total of 649 MCO genes. Then, a multiple sequence alignment of the encoded proteins was performed by MUSCLE as implemented in MEGA X (Kumar et al. 2018) with the aim of both facilitating the identification of conserved motifs and residues that characterize the members of this protein superfamily and performing a phylogenetic analysis. A ML tree was constructed by MEGA X (**Fig. S16**) using the WAG evolutionary model with gamma-distributed rate variation and the amino-acid frequencies of the dataset as suggested by ProtTest (Abascal et al. 2005). ML clade support was estimated by bootstrapping (100 replicates). Finally, molecular homology models of distinctive proteins were automatically built up with Swiss-model Server (Waterhouse et al. 2018) to better determine the different MCO types.

It was observed that every species presents MCO genes, but the number of MCO per species varies greatly (between 2 and 29 genes). In general, MCO sequences assemble in different clusters according to their theoretical activity as laccases *sensu stricto* (LAC), ferroxidases (FOX), laccase-ferroxidases (LAC-FOX) and ascorbate oxidases (AO) (**Fig. S16**). Besides, atypical laccase-like enzymes related to laccases *sensu stricto* grouped together in three novel and well defined clusters named as: i) novel laccases (NLAC); ii) novel MCO (NMCO); and iii) laccases with potential ferroxidase activity (NLAC-FOX). Every species holds different types of MCO in its genome (**Table S8**), with the exception of *V. volvacea*, whose MCO genes (6) exclusively correspond to laccases *sensu stricto*.

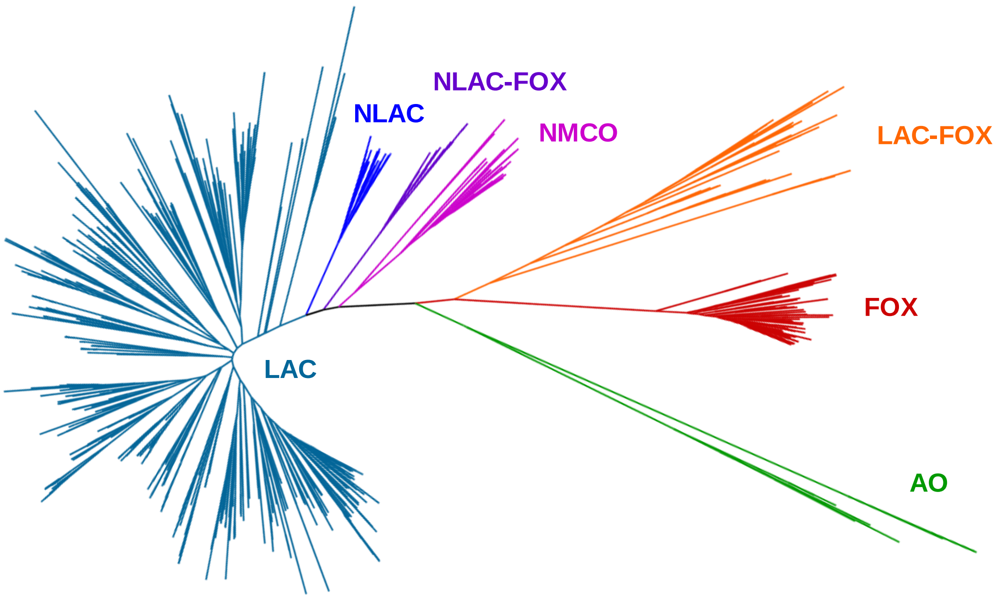

Fig. S16 ML phylogenetic tree of the 649 MCO sequences identified in the 52 Agaricomycetes genomes analyzed, formed by distant ascorbate oxidases (AO), ferroxidases (FOX) and hybrid laccase-ferroxidases (LAC-FOX), main laccase (LAC) cluster, and three groups of atypical MCO catalogued here as novel laccases (NLAC), novel laccase-ferroxidases (NLAC-FOX) and novel MCO (NMCO).

10.1 Laccases sensu stricto (LAC)

The largest cluster of MCO enzymes corresponds to laccases *sensu stricto* (465 sequences, **Table S8**) with multiple enzymes of a same species grouping together in the ML phylogenetic tree of **Fig. S16**. In general, the highest number of laccases are found in forest-litter degrading species compared with decayed-wood decomposers, white-rot wood Polyporales, and brown-rot Polyporales and Boletales (*P* <0.05 for pairwise comparisons, binomial exact test). For instance, *M. fuliginosa*, *R. butyracea*, *L. nuda* or *G. androsaceus* hold between 17-22 laccase genes. This trend has been related to the more complex and heterogeneous substrates that forest-litter degrading fungi living in soil have to face up.

Table S8 MCO in the 52 Agaricomycetes genomes analyzed, classified as laccases *sensu stricto* (LAC), novel laccases (NLAC), novel MCO (NMCO), novel laccase-ferroxidases (NLAC-FOX), laccase-ferroxidases (LAC-FOX), ferroxidases (FOX) and ascorbate oxidases (AO). Each family of enzymes is colored according to their abundance in the fungal genomes (in each column the color goes from dark orange, for the species with the highest number of enzymes of a MCO family, to white when the family is absent).

	species	Ecology	Lifestyle	Multicopper oxidases (MCO)							
				LAC	NLAC	NMCO	NLAC-FOX	LAC-FOX	FOX	AO	Total
AGARICALES	*H. sublateritium*	Saprotroph	Decayed wood	9	0	0	0	0	2	0	11
	P. alnicola			8	0	0	1	0	1	0	10
	G. marginata			7	0	0	1	0	1	0	9
	G. junonius			5	0	0	0	0	1	0	6
	C. striatus			9	1	1	0	0	3	0	14
	G. luxurians			9	2	5	0	1	2	0	19
	O. olearius			5	0	0	0	1	2	0	8
	H. radicata			8	1	0	4	1	4	1	19
	P.conissans		Forest litter	8	0	0	2	0	1	0	11
	C. cinerea			15	0	0	2	0	0	0	17
	M. fuliginosa			17	4	5	0	0	1	0	27
	A. bisporus var bisporus			9	2	0	0	0	1	0	12
	L. nuda			20	2	0	0	0	1	0	23
	C. gibba			3	3	7	0	0	1	0	14
	R. butyracea			17	0	0	0	1	2	0	20
	G. androsaceus			22	0	0	0	1	2	0	25
	M. fiardii			14	2	2	0	0	2	0	20
	A. pediades		Grass litter	11	0	0	1	0	1	0	13
	P. papilionaceus			6	0	0	2	0	1	0	9
	V. volvacea			6	0	0	0	0	0	0	6
	P. eryngii			9	1	0	0	0	1	0	11
	O. mucida		Wood (white rot)	7	2	1	2	1	2	1	16
	P. ostreatus			16	1	0	0	0	2	0	19
	C. variabilis		Unknown decay	6	0	0	0	0	1	0	7
	S. commune			2	0	0	0	1	0	3	6
	A. mellea	Biotroph	Root pathogen	11	2	3	1	0	2	0	19
	F. hepatica	Saprotroph	Wood (brown rot)	5	0	0	0	1	1	1	8
	H. cylindrosporum	Biotroph	Mycorrhizae	2	0	0	0	0	1	0	3
	C. glaucopus			5	0	0	0	0	3	0	8
	L. amethystina			6	0	0	0	0	2	0	8
	L. bicolor			9	0	0	0	0	3	0	12
	T. matsutake			12	0	0	0	0	1	0	13
	L. gongylophorus	Biotroph	Insect symbiont	5	1	0	0	0	1	0	7
BOLETALES	*H. pinastri*	Saprotroph	Wood (brown rot)	9	0	0	0	1	1	0	11
	C. puteana			6	0	0	0	1	1	0	8
	S. lacrymans			4	0	0	0	1	1	0	6
	S. brevipes	Biotroph	Mycorrhizae	16	0	0	0	1	1	0	18
AMYLOCORTICIALES	*P. crispa*	Saprotroph	Wood (white rot)	5	0	0	0	0	1	0	6
ATHELIALES	*Fibulorhizoctonia* sp.	Biotroph	Insect symbiont	26	0	0	0	1	2	0	29
POLYPORALES	*C. subvermispora*	Saprotroph	Wood (white rot)	7	0	0	0	1	1	0	9
	D. squalens			12	0	0	0	1	1	0	14
	Ganoderma sp.			16	0	0	0	1	1	0	18
	T. versicolor			7	0	0	0	1	2	0	10
	B. adusta			0	0	0	0	1	1	0	2
	P. chrysosporium			0	0	0	0	4	1	0	5
	P. brevispora			7	0	0	0	1	2	0	10
	P. placenta		Wood (brown rot)	2	0	0	0	1	2	0	5
	W. cocos			3	0	0	0	1	1	0	5
	F. pinicola			5	0	0	0	1	1	0	7
RUSSULALES	*H. annosum*		Wood (white rot)	12	2	2	0	2	1	1	20
	S. hirsutum			12	1	3	0	2	2	1	21
	Peniophora sp.			13	1	0	0	1	0	0	15
			**Total**	465	28	29	16	31	72	8	649

Wood white-rot fungi also stand out by the important number of laccases in many species like *S. hirsutum*, *Heterobasidion annosum*, *Dichomitus squalens*, *P. ostreatus* or *Ganoderma* sp. The exception are *Phanerochaete chrysosporium* and *Bjerkandera adusta* lacking laccase genes. Interestingly, the number of these enzymes tends to be higher in those wood white-rot fungal genomes with no LiP genes (see **Fig. 2**, main manuscript, containing POD information). Finally, *Fibulorhizoctonia sp.*, a fungus related to species associated with wood-feeding termites, shows outstanding collection of laccase genes (up to 26), but no POD, in its genome. This suggests that presence of these enzymes could aid termites' wood colonization.

Laccases have been classified as high, medium and low redox potential enzymes. The highest redox potential described elsewhere (E^0 T1≈ 780 mV) is found in some Polyporales laccases such as those from *Pycnoporus cinnabarinus* and PM1 basidiomycete. These high redox-potential laccases (HRPLs) hold a phenylalanine residue in the position of the putative 4th axial ligand of the catalytic T1 copper (T1 Cu). Of the total number of laccases, 31% showed a phenylalanine residue in this position, whereas 62 % laccases showed a leucine (including NLAC) that is also typical for fungal laccases with somehow lower redox potential (E^0 T1≤ 700 mV). Surprisingly, 7% of the laccases found showed a methionine as 4th axial ligand of the T1 Cu, a typical feature of low redox-potential laccases (LRPLs) from bacteria and plants (E^0 T1≤ 500 mV). HRPLs are more abundant in the wood white-rot fungi analyzed, although two forest-litter degraders (*R. butyracea* and *G. androsaceus*) and one mycorrhizal fungus (*Suillus brevipes*) stand out for the high number of HRPL genes (11-13). On the other hand, all wood brown-rot fungi have HRPLs except for *F. hepatica*. Several laccases belonging to Agaricales, Boletales or Russulales do not conserve the acidic residue in position 206, using *P. cinnabarinus* laccase (PcL) numbering (Camarero et al. 2012). Seventy-three of these sequences, holding an amino acid different from aspartatre in position 206, gathered indiscriminately with other laccases within the laccase *sensu stricto* cluster in the **Fig. S16** tree, whereas other laccases with arginine in this position formed a separate cluster named as NLAC.

Finally, analysis of the evolution of the laccase *sensu stricto* family using CAFE v4.1 (De Bie et al. 2006; Han et al. 2013) revealed a common ancestor for the 52 fungal species of this study with 8 laccase genes from which the current genes would diverge after speciation (**Fig. S17**). Duplication events rendered up to more than 20 laccases in some species, whereas in others, deletions gave to a reduced number of laccase genes or even to a total lack of them (the latter only in *P. chrysosporium* and *B. adusta*).

10.2 Novel laccases with Arg206 (NLAC)

Up to 28 laccase sequences from different species grouped in a cluster separate from the rest of laccases *sensu stricto* (**Fig. S16**). All of them hold an arginine residue instead of the conserved Asp206 residue (PcL numbering as described above). The acidic residue in this position is involved in the concerted electron-proton transfer during oxidation of phenols by fungal laccases (Galli et al. 2011; Pardo et al. 2016). These NLAC genes were only found in some Agaricales and Russulales species. A slightly higher number of NLAC is found in two forest-litter degrading species (*M. fuliginosa* and *C. gibba*), and they are also present in other leaf-litter, wood white-rot and decayed-wood species. However, we could not demonstrate significant differences in the number of NLAC between the different eco-physiological groups. These enzymes have not been found in Polyporales. As aforementioned, the rest of laccases that hold amino-acid residues different from aspartic acid (or arginine) in the position of the Asp206 residue in PcL, group randomly with other laccases from the same or related species. On the contrary, all NLAC establish a well defined cluster with 1-2 proteins from many different species gathering together.

Both *P. eryngii* and *P. ostreatus* hold one NLAC each. The one found in *P. eryngii* genome (Pleery1-ID# 1521536) is the same enzyme characterized by Muñoz et al. (1997) (as revealed by the Nt sequence: ATKKLDFHIRN). Production of NLAC by *P. ostreatus* (POXA3=Lacc2) (Castanera et al. 2012) and *P. eryngii* (Pleery1-ID# 1521536, unpublished data from secretome of *P. eryngii* grown on wheat-straw) are induced in the presence of lignocellulose. Similarly, production of NLAC enzymes by *M. fuliginosa* is also induced in the presence of lignocellulose (unpublished data).

NLAC from *P. ostreatus* (POXA3) forms an heterodimer with an uncharacterized small subunit supposed to enhance secretion and stability of POXA3 laccase (Giardina et al. 2007). A similar effect could be inferred in *P. eryngii* laccase, since two isoenzymes with different Mw were obtained (Muñoz 1995). Interestingly, all Agaricales genomes having NLAC have at least one gene encoding a small-subunit-like protein. Finally, the amino-acid residues delimiting the substrate-binding pocket in NLAC differ considerably from Polyporales laccases (**Fig. S18**) .

CAFE analysis using the 28 orthologous genes of NLAC family (**Fig. S19**) revealed that these enzymes were lost through evolution in wood brown- and white-rot Polyporales and Boletales, as well as in many Agaricales species (including mycorrhizal, grass-litter and other species). Only several Agaricales and Russulales species maintained NLAC genes, in some cases as a result of some duplication event that led, for example, to 4 and 3 genes in *M. fuliginosa* and *C. gibba* respectively.

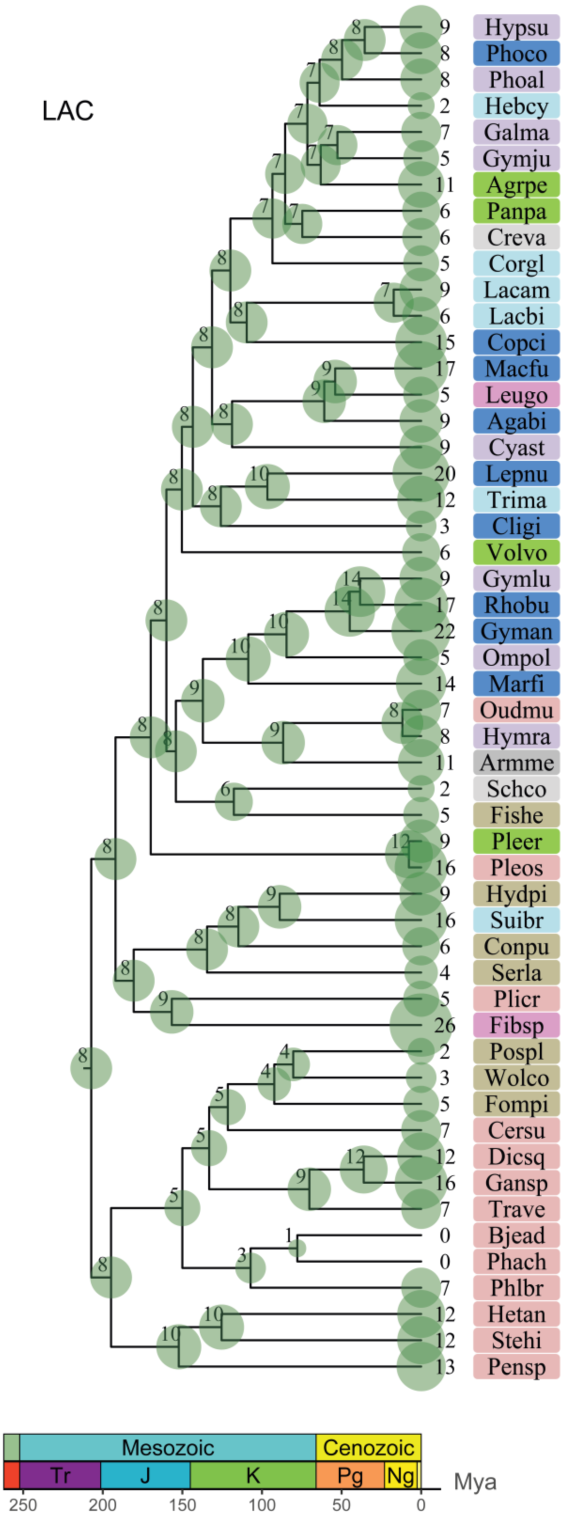

Fig. S17 Evolution of laccase *sensu stricto* gene copy numbers by CAFE analysis.

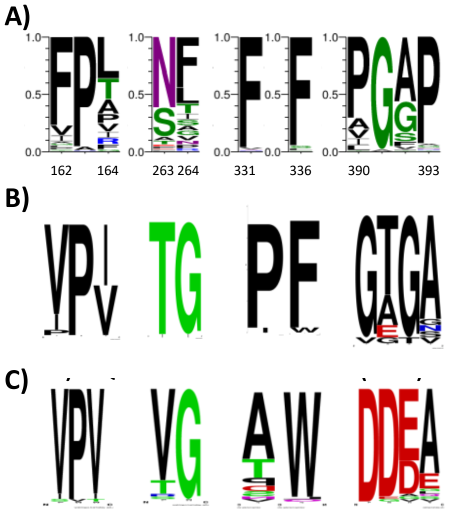

Fig. S18 Sequence logos for the amino-acid residues delimiting the substrate binding pocket in: **A)** Polyporales laccases *sensu stricto* (LAC); **B)** Laccases with Arg206 (NLAC); and **C)** Novel MCO (NMCO) (see description below).

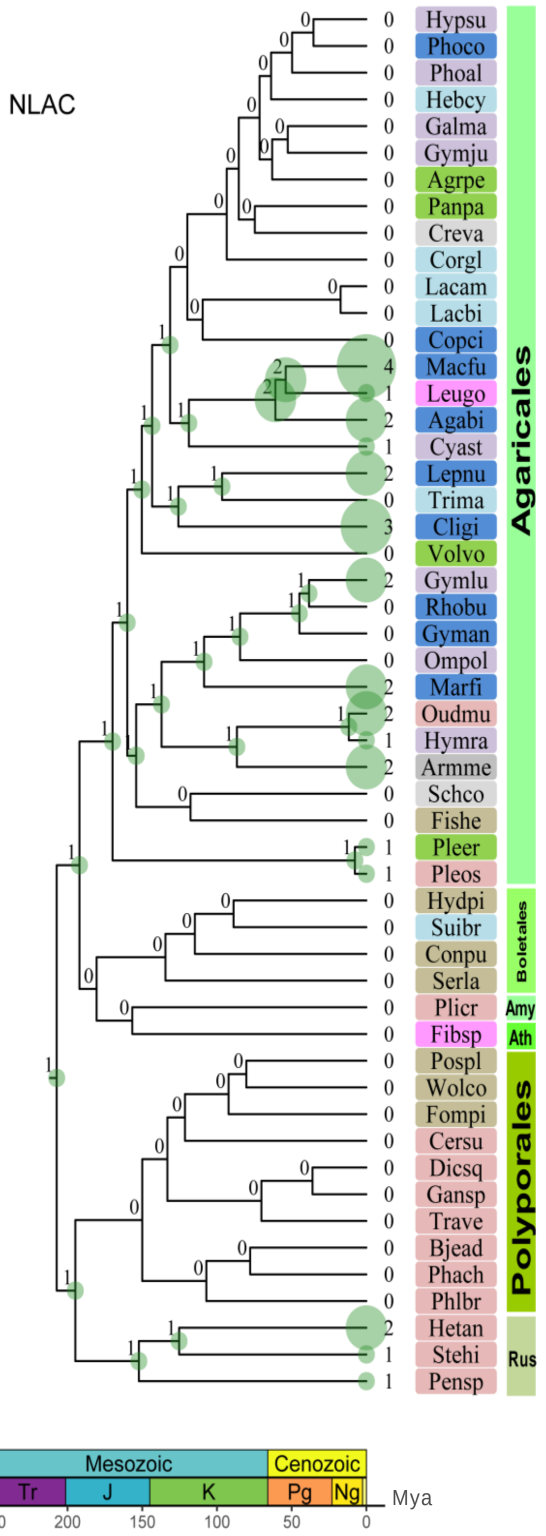

Fig. S19 Changes in gene copy numbers of NLAC family estimated with CAFE.

10.3 Novel MCO (NMCO)

Up to 29 atypical MCO enzymes (**Table S8**) segregate from the rest in a separate cluster (**Fig. S16**). These enzymes (named as NMCO) were only found in a few Agaricales and Russulales species of forest-litter, decayed-wood and white-rot decomposers (and in the root-pathogen *Armillaria mellea*), with highest prevalence in forest-litter species like *C. gibba* and *M. fuliginosa*. The latter species over-expressed all its NMCO in the presence of lignocellulose (alike NLAC) (unpublished data).

NMCO are characterized by the absence of three of the ten histidine residues coordinating the catalytic coppers in all MCO (**Fig. S20A**). In particular, the two histidines in L1 motif (coordinating the T2 and T3 copper ions) and one histidine in L2 motif (coordinating one of the two T3 copper ions) are replaced by Asn/Asp, Asp and Gln/Glu (**Fig. S20B**). In addition, and alike NLAC, many NMCO show arginine instead of aspartic acid in position 206 (PcL numbering). Besides, the substrate binding site is notably more polar (acidic) than the binding pockets of laccases *sensu stricto* and NLAC (**Fig. S18**).

Every genome with NMCO (9 species) also posses NLAC proteins. Interestingly these two families have experienced a similar evolution, as observed when their CAFE analysis are compared. Thus, expansions/contractions of the NLAC (**Fig. S19**) and NMCO (**Fig. S21**) families in the phylogeny of the 52 genomes showed: i) a probable common ancestor for Agaricales and Russulales; ii) their evolution in these two fungal orders; and iii) their loss in orders such as Polyporales and Boletales.

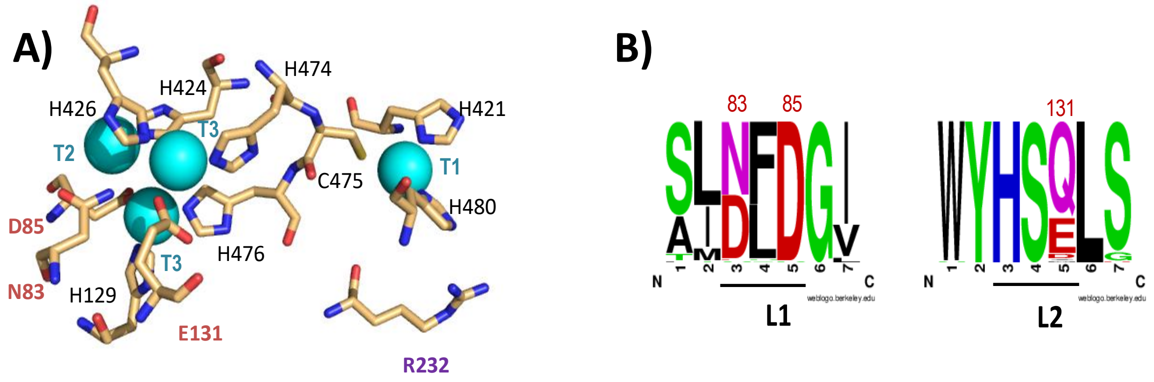

Fig. S20 A) Catalytic site of NMCO from *M. fuliginosa* ID# 778682 (the three residues replacing the typical His ligands of T2 and T3 coppers are depicted in red font)**;** and **B)** Sequence logo for L1 and L2 motifs in the NMCO enzymes found in the Agaricomycetes.

10.4 Novel laccase-ferroxidases (NLAC-FOX)

A few Agaricales species harbor some atypical laccase sequences that group separately, all with one or two of the amino-acid residues needed for Fe^{2+} binding. As this suggest a possible hybrid laccase-ferroxidase activity, we named this MCO group as NLAC-FOX. They have a leucine residue in the position of the 4th axial ligand of T1 Cu, as many fungal laccases. Unlike typical LAC-FOX, NLAC-FOX do not diverge from a common ancestor with FOX. In addition, NLAC-FOX genes (16 sequences) most probably come from a common ancestor within the Agaricales given their absence in other fungal orders. In consequence, their evolution can only be predicted within this order (see **Fig. S22**) (CAFE analysis maintains a representative of this MCO family in the most ancestral nodes of the species phylogenetic tree, although this fact is highly unlikely for this enzyme family).

10.5 Ferroxidases (FOX)

Fet3-like ferroxidases efficiently oxidize Fe^{2+} to Fe^{3+} due to the presence of an iron-binding site constituted by three acid residues: Glu185, Asp283 and Asp409 (*Saccharomyces cerevisiae* Fet3p numbering). Glu185 and Asp409 are part of the electrical wire that connects Fe^{2+} to T1 copper through their H-bonds to the two T1 Cu His ligands. Both acid residues together with Asp283 assist in productive Fe^{2+} binding and in lowering the reduction potential of the bound Fe^{2+} for efficient electron transfer.

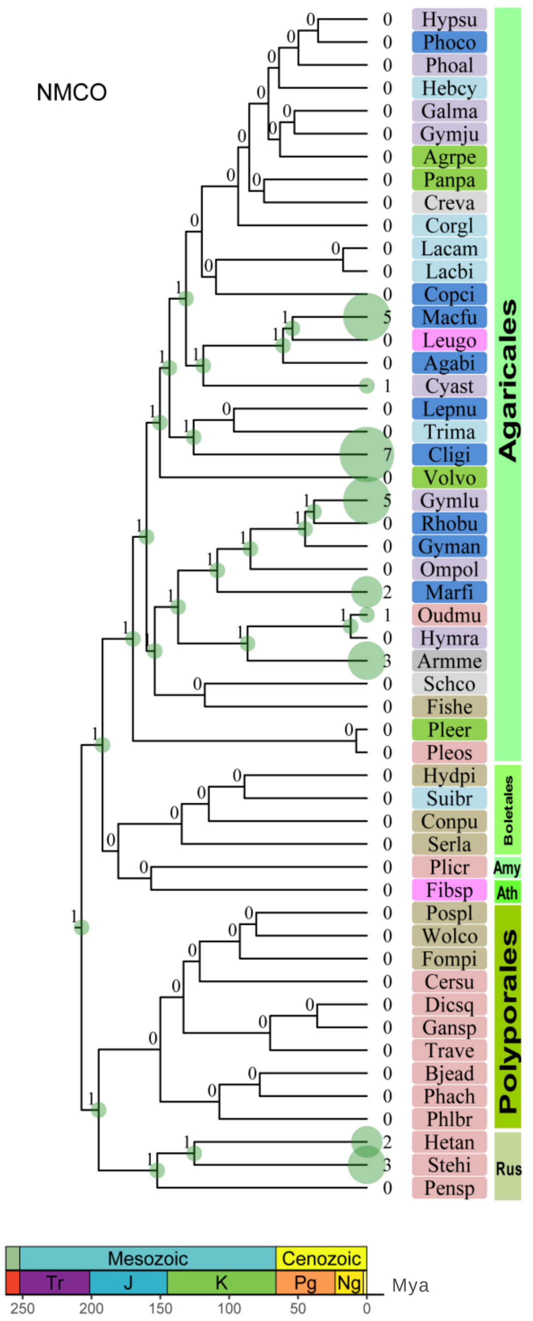

Fig. S21 Evolution of NMCO gene copy numbers by CAFE analysis.

At least one ferroxidase gene is present in almost every species of the 52 fungal genomes studied, except for *V. volvacea*, *C. cinerea*, *Schizophyllum commune* and *Peniophora* sp., which have none. Interestingly, *H. radicata* (decayed-wood degrader) from the Physalacriaceae family (Agaricales) shows an outstanding number of enzymes with supposedly ferroxidase activity including four Fet3p-like enzymes (FOX) and five putative hybrid laccase-ferroxidase enzymes (see previous and next sections). The wood white-rot *O. mucida* from the same family harbors also two FOX and three putative hybrid laccase-ferroxidase enzymes. According to CAFE, Agaricales, Boletales, Russulales and Polyporales ferroxidase genes evolved from a common unique ancestor (**Fig. S23**). Some duplications events occurred recently, resulting in existing species with 2-4 ferroxidases, and others were produced before, as observed in the clade giving rise to species of the Marasmiaceae, Physalacriaceae and Omphalotaceae families (highlighted with a red asterisk in the figure).

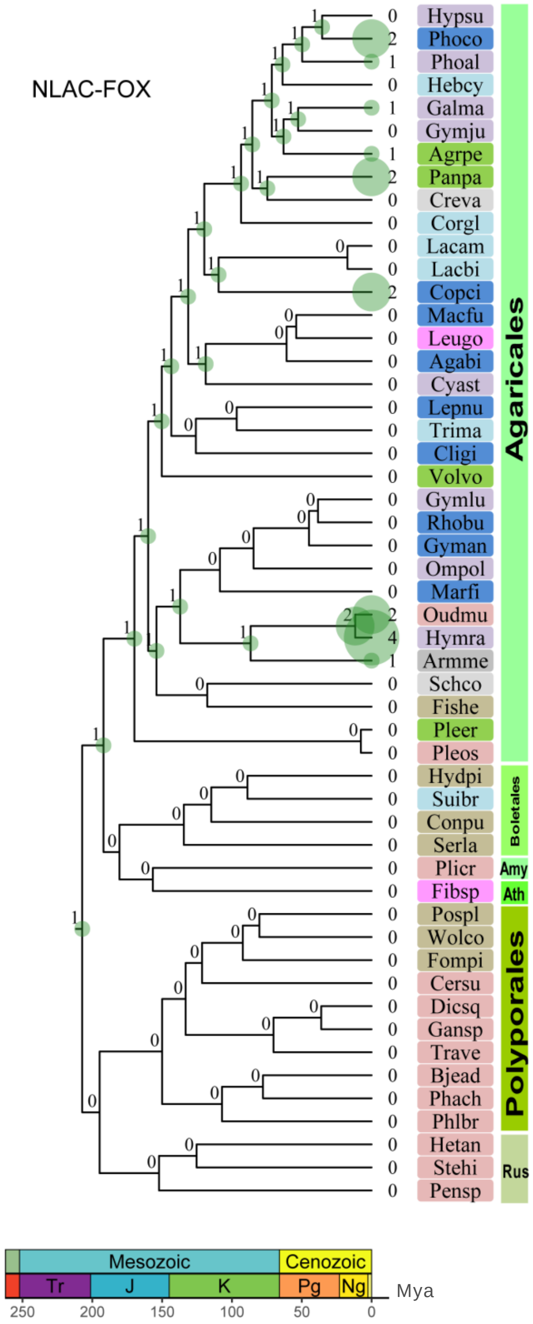

Fig. S22 Evolution of NLAC-FOX gene copy numbers by CAFE analysis.

10.6 Laccase-ferroxidases (LAC-FOX)

MCO with laccase plus ferroxidase activities have been described in Polyporales species such as *P. chrysosporium* and *Phanerochaete flavido-alba* (Larrondo et al. 2003; Rodríguez-Rincón et al. 2010). *Phanerochaete chrysosporium* harbors four MCO with hybrid molecular/structural characteristics, of which MCO1, holding 2 acidic residues equivalent to Glu185 and Asp409 in Fet3p, has been proved to show efficient ferroxidase activity close similar to that of Fet3p, while oxidizes typical laccase substrates like aromatic amines, ABTS and phenols (Larrondo et al. 2003). We named it as LAC-FOX due to its hybrid oxidation abilities and, consequently, all proteins with similar structural features in the same tree cluster (up to 31 proteins) were named as LAC-FOX too.

Of the 52 genomes studied, LAC-FOX were mainly found in wood-rotting fungi. One LAC-FOX is a common feature for every brown-rot species studied independently whether they belong to the

Polyporales, Agaricales or Boletales order. At least one LAC-FOX gene was also found in every white-rot species, except for *P. ostreatus* and *P. crispa* that hold none. Looking to the phylogeny, LAC-FOX diverged from the same node as FOX (**Fig. S16**). All Polyporales LAC-FOX hold two of the three acidic residues of FOX, leucine in the position of 4th axial ligand of the T1 site, phenylalanine replacing Asp206, and one disulfide bond.

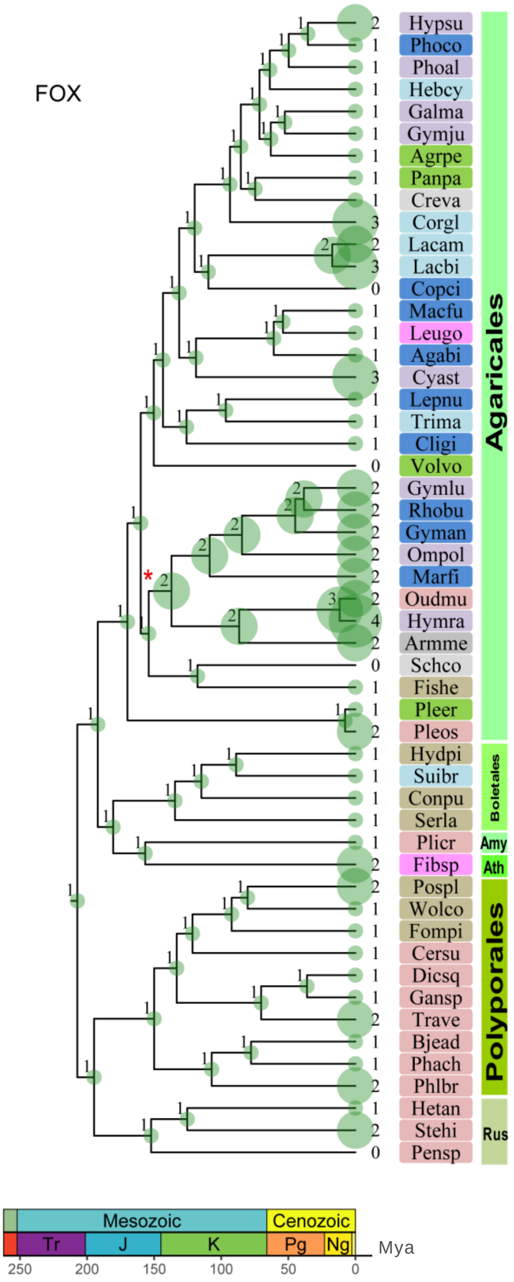

Fig. S23 Evolution of FOX gene copy numbers by CAFE analysis.

10.7 Ascorbate oxidases (AO)

Out of the 52 fungal genomes studied, only a few Agaricales and Russulales species harbor ascorbate oxidases (one per genome). These enzymes, which have been described as involved in overcoming host defense, have a methionine in the position of the 4th axial ligand of T1 copper, leucine instead Asp206, and no disulfide bonds.

11. H_2O_2-producing enzymes

Hydrogen peroxide plays a central role in lignocellulose degradation by Agaricomycetes. It is the substrate required by ligninolytic POD in white-rot decay and also the precursor of hydroxyl radical, which acts as a diffusible oxidizing agent in brown-rot degradation (Xu and Goodell 2001; Cohen et al. 2002). The enzymes that have been involved in H_2O_2 production belong to the GMC and CRO superfamilies (Kersten and Cullen 2014; Ferreira et al. 2015). Genes encoding enzymes of these two superfamilies were identified in the 52 Agaricomycetes genomes under study, classified into different families and analyzed as described below.

11.1 Glucose-methanol-choline (GMC) oxidoreductase superfamily

The screening for each of the GMC families was performed by querying in the filtered model protein database of each of the genomes available at MycoCosm (mycocosm.jgi.doe.gov) with the following reference sequences (GenBank):

i) Aryl-alcohol oxidase (AAO) from *P. eryngii* (AAC72747)

ii) Methanol oxidases (MOX) from *Gloeophyllum trabeum, Pichia methanolica* and *Candida boidinii* (ABI14440, AF141329 and Q00922, respectively)

iii) Cellobiose dehydrogenases (CDH) from *P. cinnabarinus, Trametes versicolor, C. subvermispora, C. puteana* and *P. chrysosporium* (AAC32197, AAC50004, ACF60617, BAD32781 and CAA61359, respectively)

iv) Pyranose-2-oxidases (P2O) from *Peniophora* sp., *P. chrysosporium, T. versicolor, G. trabeum and Lyophyllum shimeji* (AAO13382, AAS93628, ACJ54278, BAA11119 and BAD12079, respectively)

v) Pyranose dehydrogenases (PDH) from *Leucoagaricus meleagris*, *Agaricus xanthodermus* and *Agaricus bisporus* (AAW82997, AAW92123 and AAW92124, respectively).

Scoring matrix BLOSUM62 and cut-off E-values of e^{-100} were used as parameters for the Blastp search. The gene sequences obtained were manually revised, including the position of introns and N- and C-termini of the translated proteins, and annotated (Ballance 1986; Ruiz-Dueñas et al. 2011). SignalP 4.0 (www.cbs.dtu.dk/services/SignalP) (Petersen et al. 2011), TargetP 1.1 www.cbs.dtu.dk/services/TargetP) (Emanuelsson et al. 2000) and TMHMM 2.0 (www.cbs.dtu.dk/services/TMHMM) servers were used to identify the absence/presence of signal peptides and transmembrane domains, and WoLF PSORT (www.genscript.com/wolf-psort.html) (Horton et al. 2007) server was used to predict the putative subcellular location of the mature protein sequences.

Multiple alignments of the different families with MUSCLE (Edgar 2004b) were used to identify conserved motifs (ADP binding domain, Prosite PS00623 and PS00624 sequences) as well as the conserved histidine and histidine/asparagine residues at the active site. The sequences that lacked any of these GMC conserved motifs or the catalytic residues were discarded. Finally, structural homology models of the protein sequences were generated using the Swiss-Model server (https://swissmodel.expasy.org/), which selects the most adequate template for each amino-acid sequence (Waterhouse et al. 2018). The sequences were tested using ProtTest (Abascal et al. 2005) to determine the best evolutionary model. The maximum ML phylogenetic tree was constructed by MEGA X (Kumar et al. 2018) (with 100-iteration bootstrap) under the Whelan and Goldman (2001) model of evolution using gamma-distributed rate of heterogeneity (gamma shape with 4 rates of categories = 1.48) with empirical amino-acid frequencies and invariant sites (proportion of invariant sites = 0.001) (WAG+F+I+G).

11.1.1 GMC gene families in Agaricomycetes

A total of 778 GMC —487 AAO, 208 MOX, 45 CDH, 17 P2O and 21 PDH— proteins were identified in the 52 Agaricomycetes genomes analyzed (**Table S9**).

In general, considering the 52 fungal species analyzed, the number of peroxide-producing GMC enzymes is higher in forest-litter and decayed-wood degraders and the root pathogen *A. mellea* ($P <$ 0.05, binomial exact test) (**Table S10**). When the analysis was performed at order level, we observed

that the average number of GMC enzymes is higher in Agaricales (and Russulales) ($P < 0.05$, binomial exact test) (**Table S11**), and within this order in the forest-litter, decayed-wood and white-rot degraders (and also in *A. mellea*, as mentioned above), mainly due to the high content of AAO enzymes ($P < 0.05$ for pairwise comparisons, binomial exact test) (**Table S12**) capable to produce H_2O_2 being involved in oxidation of a wide range of substrates. Only in the case of fungi with brown-rot lifestyle, the average number of MOX exceeds the number of AAO, being consistent with the different way of lignocellulose decay described for white-rot and brown-rot fungi (Carro et al. 2016). While the fungal genomes contain a large number of genes encoding AAO or MOX proteins, in general P2O or CDH activities are assigned to only one gene (two maximum) so no multigenicity is observed for these two proteins.

Table S9 GMC oxidoreductases in the 52 Agaricomycetes genomes analyzed, classified as AAO, CDH, MOX I, MOX II, P2O and PDH. Each enzyme family is colored according to their abundance in the fungal genomes (in each column the color goes from dark orange, for the species with the highest number of enzymes of a GMC family, to white when the family is absent).

				GMC						GMC	
	species	Ecology	Lifestyle	AAO	MOX I	MOX II	CDH	P2O	PDH	TOTAL	average number
AGARICALES	*H. sublateritium*	Saprotroph	Decayed wood	12	3	1	1	0	2	19	21.8
	P. alnicola			22	6	1	0	1	0	30	
	G. marginata			15	3	1	1	0	0	20	
	G. junonius			8	1	1	1	0	0	11	
	C. striatus			23	5	0	2	0	0	30	
	G. luxurians			20	8	1	1	0	0	30	
	O. olearius			4	3	1	1	0	0	9	
	H. radicata			12	11	1	1	0	0	25	
	P.conissans		Forest litter	20	4	1	1	0	0	26	24.7
	C. cinerea			31	2	2	2	0	0	37	
	M. fuliginosa			16	1	1	3	0	13	34	
	A. bisporus var bisporus			9	3	1	1	0	5	19	
	L. nuda			20	1	1	2	1	0	25	
	C. gibba			21	1	1	0	0	0	23	
	R. butyracea			13	1	1	0	1	0	16	
	G. androsaceus			10	4	1	0	0	0	15	
	M. fiardii			22	2	1	1	1	0	27	
	A. pediades		Grass litter	11	3	1	1	0	0	16	16.8
	P. papilionaceus			1	8	1	1	0	0	11	
	V. volvacea			17	4	1	1	0	0	23	
	P. eryngii			12	3	1	1	0	0	17	
	O. mucida		Wood (white rot)	11	2	1	1	1	0	16	22.5
	P. ostreatus			24	3	1	1	0	0	29	
	C. variabilis		Unknown decay	9	1	1	2	0	0	13	10.0
	S. commune			1	3	1	1	1	0	7	
	A. mellea	Biotroph	Root pathogen	20	6	1	1	1	0	29	
	F. hepatica	Saprotroph	Wood (brown rot)	1	1	1	0	0	0	3	
	H. cylindrosporum	Biotroph	Mycorrhizae	6	1	0	0	0	0	7	8.2
	C. glaucopus			3	4	0	0	1	0	8	
	L. amethystina			4	2	0	0	1	0	7	
	L. bicolor			3	0	2	0	0	0	5	
	T. matsutake			8	3	1	0	2	0	14	
	L. gongylophorus	Biotroph	Insect symbiont	4	1	0	0	0	1	6	
BOLETALES	*H. pinastri*	Saprotroph	Wood (brown rot)	0	3	1	1	1	0	6	5.7
	C. puteana			0	2	1	1	0	0	4	
	S. lacrymans			0	4	1	2	0	0	7	
	S. brevipes	Biotroph	Mycorrhizae	1	6	1	0	0	0	8	
AMYLOCORTICIALES	*P. crispa*	Saprotroph	Wood (white rot)	0	2	2	1	0	0	5	
ATHELIALES	*Fibulorhizoctonia* sp.	Biotroph	Insect symbiont	0	1	0	2	1	0	4	
POLYPORALES	*C. subvermispora*	Saprotroph	Wood (white rot)	4	0	1	1	0	0	6	10.7
	D. squalens			8	3	1	1	0	0	13	
	Ganoderma sp.			7	3	1	1	0	0	12	
	T. versicolor			3	3	1	1	1	0	9	
	B. adusta			11	4	1	1	1	0	18	
	P. chrysosporium			2	2	1	1	1	0	7	
	P. brevispora			2	5	1	1	1	0	10	
	P. placenta		Wood (brown rot)	2	2	1	0	0	0	5	4.7
	W. cocos			0	3	1	0	0	0	4	
	F. pinicola			1	3	1	0	0	0	5	
RUSSULALES	*H. annosum*		Wood (white rot)	7	2	1	1	0	0	11	16.0
	S. hirsutum			15	6	1	1	0	0	23	
	Peniophora sp.			11	1	1	1	0	0	14	
				487	159	49	45	17	21	778	

Table S10 Average gene numbers of the different GMC families in the genomes of the 52 Agaricomycetes species analyzed characterized by presenting different lifestyles. In each row, the color goes from dark orange, for the family with the highest number, to white when the family is absent.

	Average number of GMC genes per genome					
**Lifestyle**	AAO	MOX	CDH	P2O	PDH	Total GMCs
Decayed wood	14.5	5.9	1.0	0.1	0.3	21.8
Forest litter	18	3.2	1.1	0.3	2.0	24.6
Grass litter	10.3	5.5	1.0	0.0	0.0	16.8
Wood (white rot)	8.1	3.8	1.0	0.4	0.0	13.3
Unknown decay	5	3.0	1.5	0.5	0.0	10.0
Root pathogen	20.0	7.0	1.0	1.0	0.0	29.0
Wood (brown rot)	0.6	3.6	0.6	0.1	0.0	4.9
Mycorrhizae	4.2	3.3	0.0	0.7	0.0	8.2
Insect symbiont	2.0	1.0	1.0	0.5	0.5	5.0

Table S11 Average gene numbers of the different GMC families per genome of Agaricales, Boletales, Polyporales and Russulales species. In each row, the color goes from dark orange, for the family with the highest number, to white when the family is absent.

	Average number of GMC genes per genome					
**Lifestyle**	AAO	MOX	CDH	P2O	PDH	Total GMCs
Agaricales	12.5	4.1	0.8	0.3	0.6	18.3
Boletales	0.3	4.8	1.0	0.3	0.0	6.3
Polyporales	4	3.8	0.7	0.4	0.0	8.9
Russulales	11	4.0	1.0	0.0	0.0	16.0

Table S12 Average gene numbers of the different GMC families in the genomes of the 33 Agaricales species with different lifestyles. In each row, the color goes from dark orange, for the family with the highest number, to white when the family is absent.

	Average number of GMCs per genome					
**Lifestyle**	AAO	MOX	CDH	P2O	PDH	Total GMCs
Decayed wood	14.5	5.9	1.0	0.1	0.3	21.8
Forest litter	18	3.2	1.1	0.3	2.0	24.6
Grass litter	10.3	5.5	1.0	0.0	0.0	16.8
Wood (white rot)	17.5	3.5	1.0	0.5	0.0	22.5
Unknown decay	5	3.0	1.5	0.5	0.0	10.0
Root pathogen	20.0	7.0	1.0	1.0	0.0	29.0
Wood (brown rot)	1	2.0	0.0	0.0	0.0	3.0
Mycorrhizae	4.8	2.6	0.0	0.8	0.0	8.2
Insect symbiont	4.0	1.0	0.0	0.0	1.0	6.0

The phylogram obtained for the 778 GMC sequences found in the 52 genomes shows that all the enzymes are distributed in clusters according to the enzyme group that they belong to (**Fig. S24**).

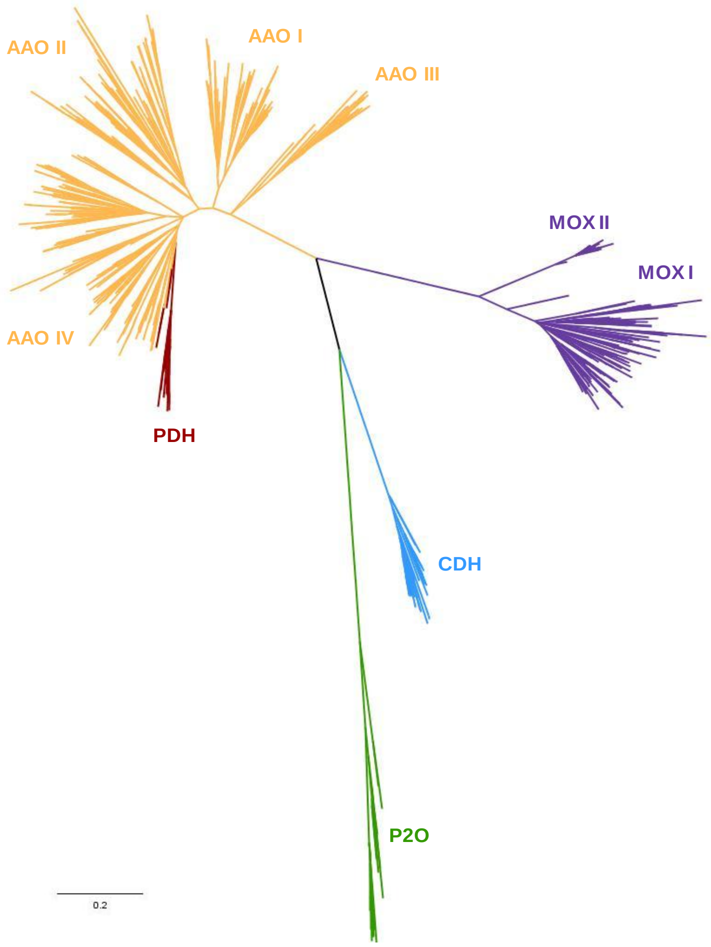

Fig. S24 ML phylogenetic tree of the 778 GMC sequences identified in the 52 Agaricomycetes genomes analyzed. Well-separated clusters corresponding to pyranose 2-oxidase (P2O), cellobiose-dehydrogenase (CDH), methanol-oxidase (MOX) and aryl-alcohol oxidase (AAO) families, the latter including 4 subclusters, were identified (pyranose dehydrogenase, PDH, is included in one of the AAO groups).

CDH and P2O families appear unrelated with the rest of GMC families. MOX are distributed in: i) MOX I, the most abundant; and ii) MOX II, with one representative sequence in each genome (two in the case of *C. cinerea*, *Laccaria bicolor* and *P. crispa* and no sequences in *C. striatus*, *H. cylindrosporum*, *C. glaucopus*, *Laccaria amethystina*, *L. gongylophorus* and *Fibulorhizoctonia* sp). Finally, PDH enzymes are the closest phylogenetic neighbors of AAO, which are distributed in four groups. AAO I and AAO II contain the well-characterized enzymes from *P. eryngii* (JGI ID# 1382984) and *B. adusta* (JGI ID# 171002), respectively (Ferreira et al. 2005; Ruiz-Dueñas et al. 2006; Romero et al. 2009). AAO IV groups together with PDH (only present in Agaricales, **Fig. S25A**). Although the enzymes belonging to this group showed higher score and lower E-values when blasting with AAO template, a dehydrogenase activity, as previously described in *P. cinnabarinus* enzymes secreted during plant biomass degradation (Mathieu et al. 2016), cannot be discarded for the AAO IV enzymes.

AAO enzymes are absent in the Atheliales, Amylocorticiales and Boletales included in this study (with the exception of *S. brevipes* that contains one representative of this GMC family). The members of this family identified in Russulales and Polyporales are mainly distributed in the AAO II and AAO

III groups, while AAO enzymes from groups I and IV belong to Agaricales (a few representatives of the AAO IV group are also found in Russulales) (**Fig. S25A**). As shown in **Fig. S25B**, AAO II and AAO III contain mainly enzymes from wood white-rot organisms, while forest-litter and decayed-wood organisms group their AAO enzymes in cluster IV, together with PDH enzymes (mainly found in forest-litter species). Finally, most AAO from grass-litter species grouped in AAO I, together with *P. eryngii* AAO. CDH can be found in all lifestyles, except in mycorrhizae and wood brown-rot, and P2O are mainly from Agaricales and Polyporales, with wood white-rot and mycorrhizal lifestyle.

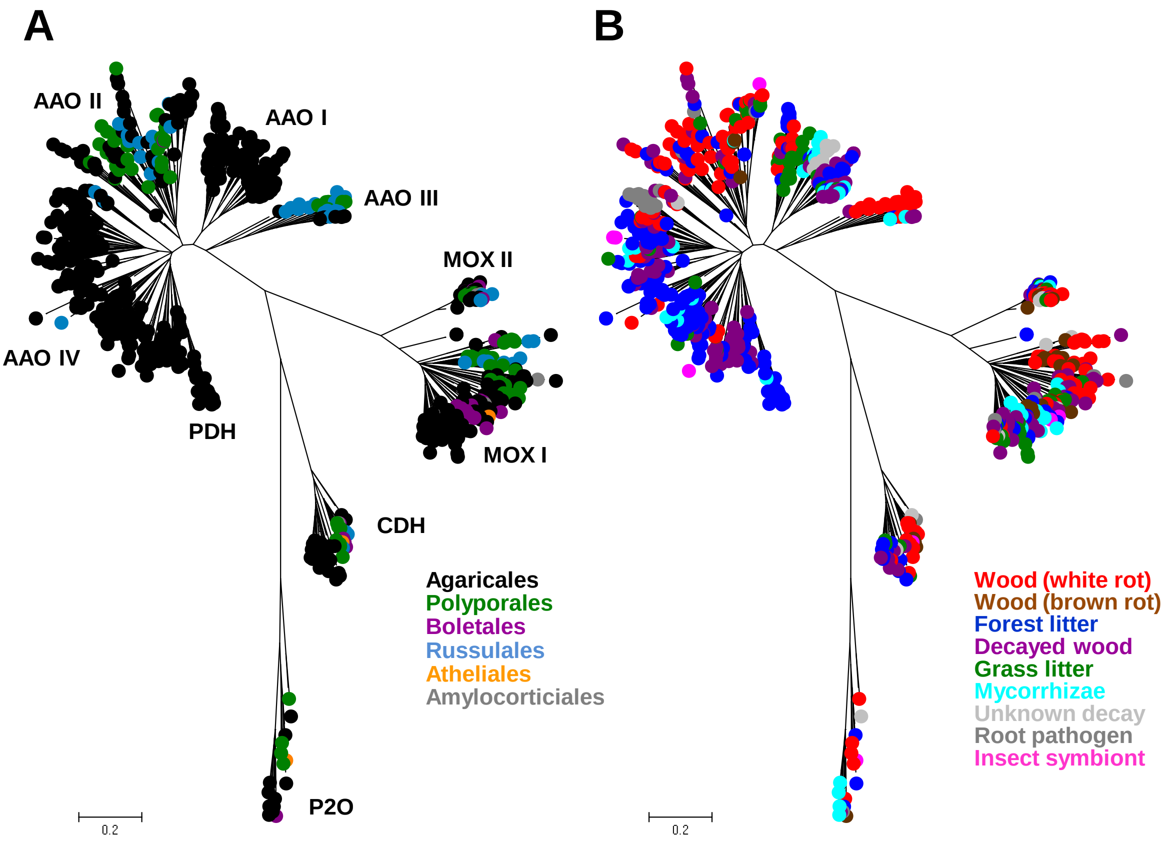

Fig. S25 Phylogenetic relationships of the 778 GMC sequences colored by order (**A**) and lifestyle (**B**).

AAO, CDH and PDH are extracellular proteins with signal peptides of around 20-25 amino-acids. For MOX and P2O no signal peptide has been recognized and a cytosolic sub-cellular localization is predicted. However, some studies suggest the secretion of these enzymes during wood decay (Volc et al. 1996; Daniel et al. 2007) so secretion might be produced through a different mechanism.

11.1.2 Structural features of the different GMC families

All GMC sequences share the conserved regions described for this superfamily, ADP-binding domain and signatures 1 and 2 (Prosite PS00623 and PS00624, respectively) with the only exception of P2O that lacks signature 1) and the two catalytic residues that are facing the *re*-face of the isoalloxazine ring of the flavinic cofactor (**Fig. S26**). The first histidine is strictly conserved in the superfamily. However, the second conserved catalytic residue is also a histidine in AAO but is replaced by an asparagine in MOX, CDH and P2O (**Fig. S26D**).

While the FAD binding domain is highly conserved in the GMC superfamily, the substrate binding (located at the C-terminal), although exhibits a conserved structure, has divergent sequences to accommodate and transform different substrates: aromatic benzylic alcohols for AAO, methanol for MOX, cellobiose for CDH and different sugars for P2O and PDH, using molecular oxygen in the case of oxidases or other electron acceptors in the case of dehydrogenases.

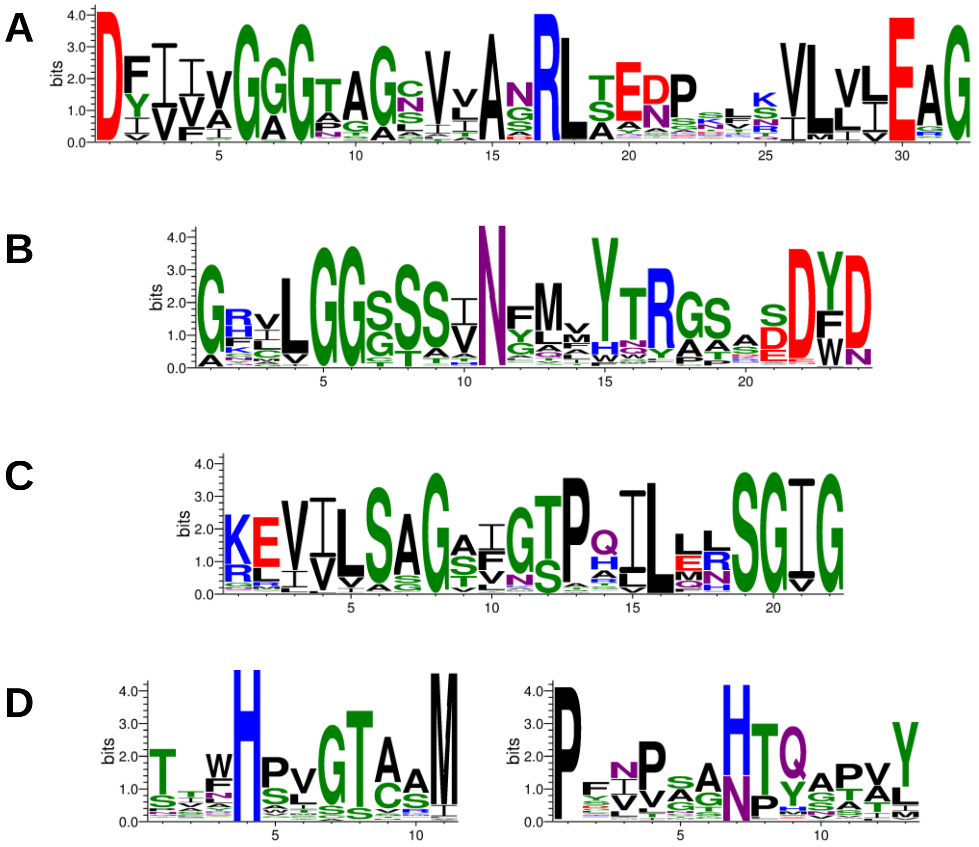

Fig. S26. Sequence logo of conserved: **A**) ADP-binding motif; **B**) signature 1, Prosite PS00623; **C**) signature 2, Prosite PS00624; and **D**) catalytic residues, in 761 GMC sequences (P2O excluded) identified in the 52 genomes analyzed. Created at WebLogo 3.1 server (Crooks et al. 2004).

The overall folding is similar among the different GMC families as previously described (Ferreira et al. 2015). However, some specific features are observed in the different types of enzymes. Unlike the rest of GMC families, P2O and PDH have the flavinic cofactor covalently bound, but with a different organization of the covalent FAD-binding structure. While in P2O, flavinylation takes place at the histidine N^3 atom in the peptide GGM(S/A)THW (Halada et al. 2003) through a 8α-(N^3-histidyl)-FAD linkage, in PDH a bicovalent flavinylation in the conserved sequence GGC(T/S)SHN, through a [6-*S*-cysteinyl-8α-(N^1 -histidyl)-FAD linkage has been described (Kujawa et al. 2007) (**Fig. S27**).

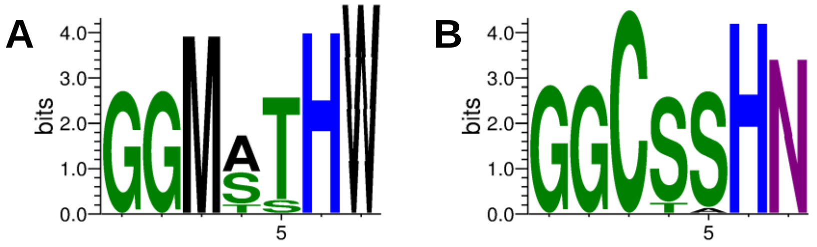

Fig. S27 Sequence logo of the flavinylation sequence in P2O (**A**) and PDH (**B**) proteins. Logo created with WebLogo 3.1 server (Crooks et al. 2004).

CDH has a multi domain organization with a FAD binding dehydrogenase domain (known as CBQ) connected to a cytochrome domain (CytCDH, with a heme group) through a peptide linker rich in serine and threonine residues (Zámocký et al. 2004).

Concerning enzyme structure, MOX II enzymes have an insertion of around 25 amino acids and a longer C-terminal (~20 extra amino-acids) compared to MOX I enzymes (**Fig. S28**). These elements

have been suggested to be involved in the secretion of the enzyme to the extracellular medium (Daniel et al. 2007). All AAO enzymes were modeled with the crystal structures of AAO from *P. eryngii* free (PDB 3FIM) or in complex with *p*-anisic acid (PDB 5OC1) as templates, generating structural models with a secondary structure similar between them (**Fig. S29**). No different patterns in residues near the catalytic ones have been found in the four AAO groups. Regarding the quaternary organization of these enzymes, dehydrogenases (CDH and PDH) and oxidase AAO exist as monomers. However, the active form for MOX and P2O is an octamer and a tetramer, respectively.

Fig. S28 Structural models of *V. volvacea* MOX from: **A)** group MOX I (JGI ID# 121657); and **B)** group MOX II (JGI ID# 120949). The extra structural elements present in MOX II are colored in red.

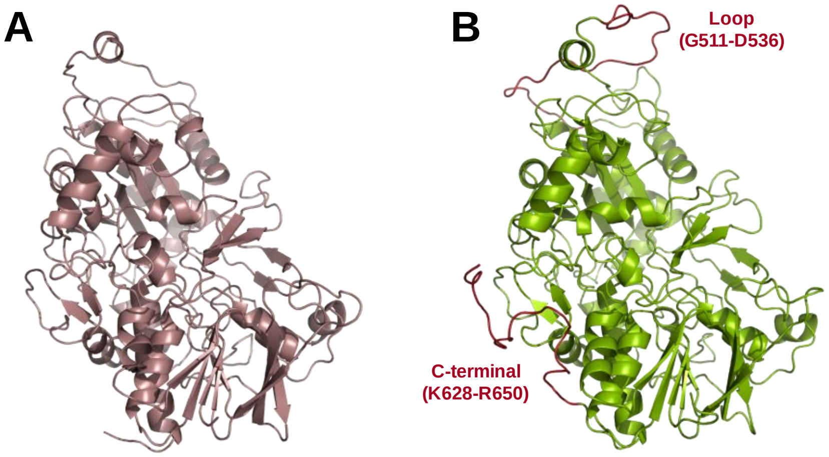

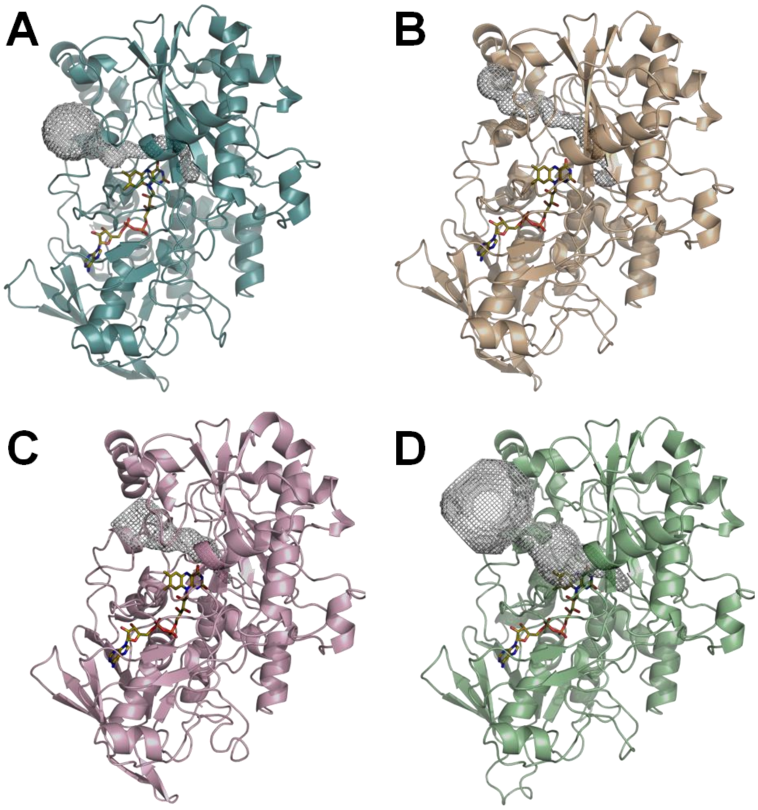

Fig. S29 Structural models of representative enzymes from the different AAO clusters: **A**) *P. eryngii* AAO from AAO I (JGI ID# 1382984); **B**) *B. adusta* AAO from AAO II (JGI ID# 171002); **C**) *R. butyracea* AAO from AAO III (JGI ID# 1235264); and **D**) *A. bisporus* AAO from AAO IV (JGI ID# 121909).

11.2 Glyoxal oxidases (GLX) and related copper-radical oxidases (CRO)

First discovered in *P. chrysosporium* cultures, glyoxal oxidase (GLX) converts simple aldehydes to carboxylic acids with the concomitant reduction of O_2 to H_2O_2. A broad range of substrates, including lignocellulose-derived glyoxal and methylglyoxal, are utilized, and the enzyme is thought to be physiologically connected to ligninolysis through its activation by peroxidases (Kersten and Kirk 1987; Kersten 1990). The active site of GLX is similar to the well characterized CRO of *Dactylium dendroides*, galactose oxidase that shares the reaction mechanism involving a copper cofactor and a catalytic protein radical (Kersten and Cullen 1993; Whittaker et al. 1996; Whittaker et al. 1999). Catalysis by CRO enzymes involves two one-electron acceptors, and alignments have identified the essential ligands in mature GLX as Cys70, Tyr135, Tyr377, His378 and His471 (Kersten and Cullen 1993; Whittaker et al. 1996; Whittaker 2005). Based on overall sequence similarity and conservation of these residues, seven CRO-encoding genes (*cro1, cro2, cro3, cro4, cro5, cro6* and *glx)* have been identified in the *P. chrysosporium* genome (Vanden Wymelenberg et al. 2006).

Previous analysis of polypore genomes has revealed GLX- and related CRO-encoding genes in numerous taxa, especially ligninolytic white-rot fungi. The function of these closely related genes is uncertain, and in the case of *P. chrysosporium* GLX and CRO2, substantial differences in substrate preference were observed (Vanden Wymelenberg et al. 2006). Two *P. cinnabarinus* GLX enzymes also exhibit differences in substrate specificity (Daou et al. 2016). A homolog of *P. chrysosporium cro2*, the *Ustilago maydis glo1* gene, appears to be involved in pathogenicity and filamentous growth (Leuthner et al. 2005). *P. chrysosporium cro3*, *cro4*, and *cro5* feature a repeated N-terminal domain of unknown function, described as a wall stress component (WSC). These WSC-containing genes are common in nearly all Agaricomycetes (**Table S13**).

11.2.1 Search for copper-radical oxidases

In our study, potential copper radical oxidases were identified by BLASTP searches of 52 genomes. Initial MUSCLE alignments (Edgar 2004a; Edgar 2004b) eliminated those protein models lacking catalytic residues (Cys70, Tyr135, Tyr377, His378) and/or containing extended gaps or insertions. The 387 remaining models were aligned with ClustalW (Thompson et al. 1994) (gap penalty 10; gap length penalty 0.20; Protein weight matrix, Gonnet series). Distances were calculated using the Kimura distance formula. Bootstrap values were computed for 100 trials.

11.2.2 Analysis of copper-radical enzymes identified in the 52 genomes analyzed

All 52 Agaricomycetes analyzed feature CRO-encoding genes in their genomes although they differ in the type and amount of these enzymes (**Table S13**). Significant genetic multiplicity has been observed. Most species contain paralogs of different CRO families (e.g. 9 and 5 *glx* paralogs in *L. nuda* and *Gymnopus luxurians*, respectively). Although the high content of CROs is not a general rule among the Agaricales, it is in this order where we find species with the highest number of these enzymes. Thus, six of the fourteen new sequenced Agaricales genomes are among those with more gene copies of this oxidoreductase superfamily (*H. radicata*, *L. nuda, A. pediades, P. eryngii*, *M. fuliginosa* and *O. mucida* with 17, 15, 14, 13, 13 and 12 gene copies, respectively) together with other species of the same order (such as *G. marginata*, *P. ostreatus* or *G. androsaceus* with 16, 15 and 12 copies, respectively). In contrast, *Ganoderma* sp. and *T. versicolor* with 9 gene copies each, *S. hirsutum* with 8 copies; and, *C. puteana* with 6 copies, are the species having the highest amount of *cro* genes among the Polyporales, Russulales and Boletales genomes analyzed, respectively. On the other hand, Clustal analysis of the amino-acid sequences showed remarkable sequence conservation within orders. Thus, Agaricales-derived sequences were generally assigned to one (*cro3*, *cro4* and *cro5*) or two clades (*glx*, *cro6*) **Fig. S30**, suggesting unique characteristics still to be determined for the enzymes of this order.

Table S13 CRO in the 52 Agaricomycetes genomes analyzed, classified as GLX and CRO1 through CRO6. Each family of enzymes is colored according to their abundance in the fungal genomes (in each column the color goes from dark orange, for the species with the highest number of enzymes of a CRO family, to white when the family is absent).

	species	Ecology	Lifestyle	CRO GLX	CRO CRO1	CRO CRO2	CRO CRO3-5	CRO CRO6	CRO TOTAL	CRO average number
AGARICALES	*H. sublateritium*	Saprotroph	Decayed wood	2	2	3	1	1	9	10
	P. alnicola			0	2	2	1	1	6	
	G. marginata			4	2	4	2	4	16	
	G. junonius			2	2	4	1	1	10	
	C. striatus			2	1	2	1	1	7	
	G. luxurians			5	1	2	1	1	10	
	O. olearius			1	1	2	1	2	7	
	H. radicata			4	2	2	1	8	17	
	P.conissans		Forest litter	2	2	3	1	1	9	9
	C. cinerea			0	2	2	1	1	6	
	M. fuliginosa			7	2	2	1	1	13	
	A. bisporus var bisporus			3	2	2	1	1	9	
	L. nuda			9	1	2	2	1	15	
	C. gibba			0	1	2	1	1	5	
	R. butyracea			2	0	3	1	1	7	
	G. androsaceus			1	0	2	1	8	12	
	M. fiardii			0	0	3	1	2	6	
	A. pediades		Grass litter	3	2	7	1	1	14	9
	P. papilionaceus			2	2	1	1	1	7	
	V. volvacea			0	1	0	1	1	3	
	P. eryngii			3	1	2	2	5	13	
	O. mucida		Wood (white rot)	5	1	2	0	4	12	14
	P. ostreatus			3	1	2	3	6	15	
	C. variabilis		Unknown decay	0	1	3	1	1	6	4
	S. commune			0	0	0	0	1	1	
	A. mellea	Biotroph	Root pathogen	0	1	1	1	0	3	
	F. hepatica	Saprotroph	Wood (brown rot)	0	0	1	1	1	3	
	H. cylindrosporum	Biotroph	Mycorrhizae	0	2	3	0	1	6	7
	C. glaucopus			1	2	1	1	1	6	
	L. amethystina			0	2	3	2	0	7	
	L. bicolor			0	7	2	2	0	11	
	T. matsutake			0	1	2	1	1	5	
	L. gongylophorus	Biotroph	Insect symbiont	0	0	2	1	0	3	
BOLETALES	*H. pinastri*	Saprotroph	Wood (brown rot)	0	1	2	1	1	5	5
	C. puteana			0	1	4	0	1	6	
	S. lacrymans			0	0	1	1	1	3	
	S. brevipes	Biotroph	Mycorrhizae	0	1	1	1	1	4	
AMYLOCORTICIALES	*P. crispa*	Saprotroph	Wood (white rot)	0	1	3	0	1	5	
ATHELIALES	*Fibulorhizoctonia* sp.	Biotroph	Insect symbiont	0	1	4	0	0	5	
POLYPORALES	*C. subvermispora*	Saprotroph	Wood (white rot)	0	1	1	1	0	3	7
	D. squalens			5	1	0	1	1	8	
	Ganoderma sp.			5	1	1	1	1	9	
	T. versicolor			5	1	1	1	1	9	
	B. adusta			1	1	1	3	1	7	
	P. chrysosporium			1	1	1	3	1	7	
	P. brevispora			1	1	2	3	1	8	
	P. placenta		Wood (brown rot)	0	0	1	0	1	2	3
	W. cocos			0	1	1	1	1	4	
	F. pinicola			0	1	1	1	1	4	
RUSSULALES	*H. annosum*		Wood (white rot)	0	1	2	1	1	5	6
	S. hirsutum			3	0	3	1	1	8	
	Peniophora sp.			1	0	4	1	0	6	
				83	62	108	58	76	387	

Concerning GLX in particular, not all genomes analyzed have members of this CRO family (**Table S13**) which, as described above, have been more clearly related to ligninolysis. Thus, GLX enzymes are absent in all the analyzed species lacking ligninolytic POD (*C. cinerea*, *Marasmius fiardii*, *C. variabilis*, *S. commune*, *L. amethystina*, *L. bicolor*, *T. matsutake*, *S. brevipes*, *L. gongylophorus*, *Fibulorhizoctonia* sp., *F. hepatica*, *Hydnomerulius pinastri*, *C. puteana*, *S. lacrymans*, *P. placenta*, *W. cocos* and *F. pinicola*) (see **Table S13** and **Fig. 2**-*right* of POD proteins in the main manuscript) irrespective of taxonomic grouping, including brown-rot species and the two Agaricales species included in this study with a weak (unknown) degradation pattern, such as *S. commune* and *C. variabilis*. In the absence of GLX, brown-rot species could express other CRO enzymes and MOX (from the GMC oxidoreductase superfamily), according to the evidence obtained for *P. placenta* and *G. trabeum* growing on aspen, birch and pine wood (Daniel et al. 2007; Martinez et al. 2009), supporting in this way the Fenton chemistry through the generation of extracellular H_2O_2.

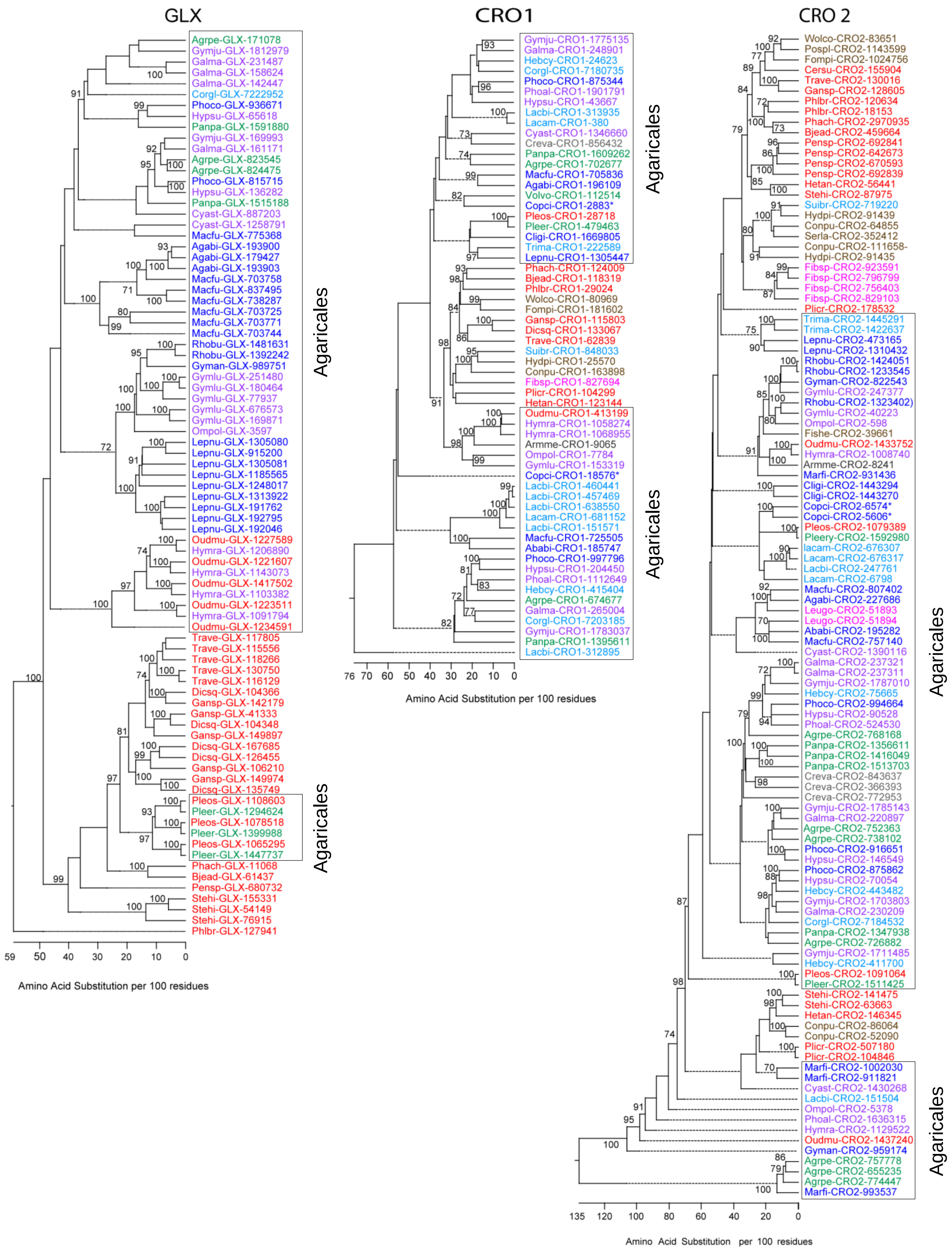

Fig. S30 Phylogenetic trees, based on Kimura distances, of 387 sequences of copper radical oxidases (all together and CRO1, CRO2, WSC-containing CRO3–CRO5, and CRO6 separately) identified in the 52 Agaricomycetes genomes analyzed. Bootstrap values were computed for 100 trials. All sequences are available through the links to the fungal genomes included in **Table S4**. The color code of the enzymes denotes the lifestyle of the fungal species they belong to (*purple*, decayed-wood degradation; *dark blue*, forest-litter decay; *green*, grass-litter decay; *pink*, insect symbiont; *pale blue*, mycorrhizal; *dark gray*, root pathogen; *light gray*, wood unknown decay; *brown*, wood brown-rot; *red*, wood white-rot).

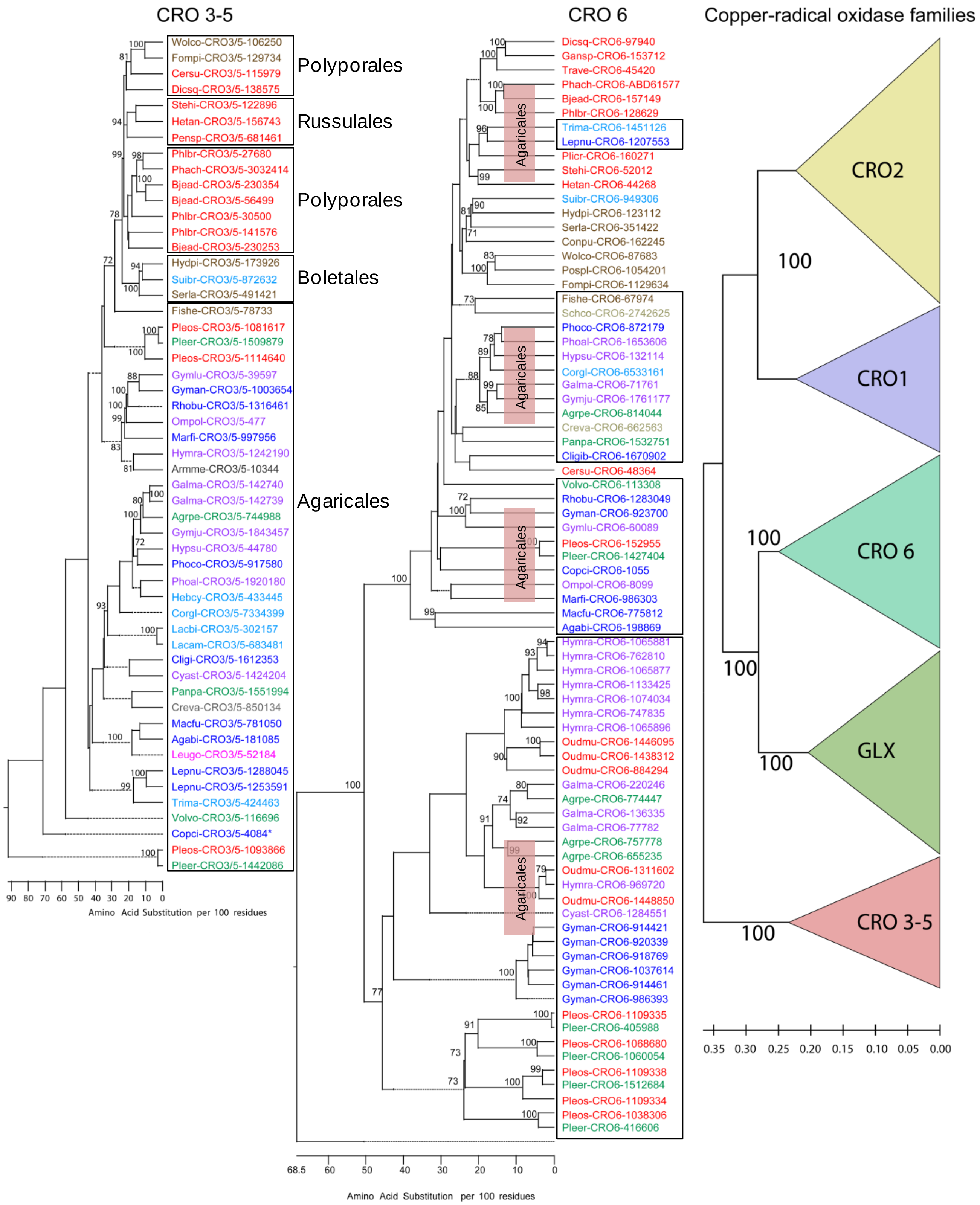

Fig. S30 (continued).

Similarly, *glx* genes are not observed in the genome sequences of the Agaricales and Boletales mycorrhizal species analyzed except in *C. glaucopus*, which has a high POD content (11 MnP-ESD isoenzymes) (**Fig. 2-***right*, main manuscript). GLX could contribute to the ligninolytic capabilities of this fungus that also contributes to decomposition of complex soil organic matter (Bodeker et al. 2014). On the other hand, GLX enzymes are absent in some Agaricales species (*P. alnicola*, *H. cylindrosporum*, *C. gibba*, *V. volvacea* and *A. mellea*), and also in the Atheliales species *P. crispa* and in the selective lignin degrading polypore *C. subvermispora*, which feature genes of ligninolytic POD in their genomes. In the case of *C. subvermispora*, as in others, perhaps the absence of GLX is compensated by *cro1, cro2* or *cro3-5*. Thus, transcript levels have been associated with culture conditions and it has been observed that *C. subvermispora cro2* and *cro5* are upregulated in media containing ground wood and microcrystalline cellulose, respectively (Fernández-Fueyo et al. 2012a). Similarly, *H. cylindrosporum* expresses GMC and CRO enzymes, presumably generating H_2O_2 that could be used by ligninolytic POD (herein described as MnP-ESD) which are upregulated when grown on soil organic matter as substrate (Shah et al. 2016).

According to our genomic evidence and the expression results published by different authors, GLX, other CRO families (CRO1 through CRO6) and AAO described above, would be available for plant cell wall degradation by those species where these enzymes have been identified, supplying H_2O_2 to ligninolytic POD as described for *P. chrysosporium* and different *Pleurotus* species (Kirk and Farrell 1987; Camarero et al. 1997; Vanden Wymelenberg et al. 2006). In summary, although experimental evidence does not definitively demonstrate the role of many of these enzymes, their expression by different fungi suggests that members of different CRO and GMC oxidoreductase families could produce the H_2O_2 necessary for white-rot, brown-rot and even for soil humus degradation. In the absence of expression experiments of the enzymes identified in the Agaricales genomes analyzed, we can speculate that the large number and diversity of CRO and GMC enzymes identified in quite a few fungi of this order could contribute to providing H_2O_2 for degradation of different substrates, under different growth conditions, etc., although this has still to be demonstrated.

12. Class-II peroxidases (POD)

POD have been described as key enzymes in lignin degradation. In fact all the typical lignin-degrading basidiomycetes include genes of at least one of the generally-known as ligninolytic peroxidase families in their genomes (Martínez et al. 2018).

12.1 POD types in 52 Agaricomycetes genomes

A screening of the 52 automatically-annotated Agaricomycetes genomes was performed by BLASTing the amino-acid sequences of five selected POD representatives of different families and subfamilies –i.e. generic peroxidase (GP), JGI ID# 1809, from *C. cinerea*; short manganese peroxidase (short MnP, MnP-s), JGI ID# 1099081, from *P. ostreatus* PC15 v2.0; long manganese peroxidase (long MnP, MnP-l), JGI ID# 8191, and lignin peroxidase (LiP), JGI ID# 2989894, from *P. chrysosporium* RP-78 v2.2; and versatile peroxidase (VP), JGI ID# 137757, from *P. ostreatus* PC9 v1.0– against the filtered model protein databases of these fungi available at MycoCosm (mycocosm.jgi.doe.gov). The BlastP search parameters were: scoring matrix BLOSUM 62, gapped alignment allowed, and cut-off E-value 0.1. The putative POD identified were functionally annotated based on: i) multiple alignment of their amino-acid sequences; and ii) identification of characteristic catalytic sites and/or structurally-relevant residues in the 3D-models generated using the automated protein structure homology-modeling server SWISS-MODEL (Waterhouse et al. 2018).

336 POD were identified, and a table including the number of enzymes of the different POD (sub)families ,and a phylogenetic tree, time-calibrated as described below, are shown in **Figs. 2**-*right* and **5** of the main manuscript, respectively. Most of them (218 enzymes) were classified according to their structural-functional properties within the well-known families characterizing Polyporales (Ruiz-Dueñas et al. 2013) and some Agaricales (Ruiz-Dueñas et al. 1999; Fernández-Fueyo et al. 2014b) as follows:

i) LiP (EC 1.11.1.14) proteins (44 gene models identified) were defined as including a catalytic tryptophan exposed to the solvent, homologous to Trp171 in *P. chrysosporium* LiP-H8 (encoded by LiPA, JGI ID# 2989894) (Doyle et al. 1998) and Trp164 of *P. eryngii* VPL (Pérez-Boada et al. 2005) (**Fig. 6A** and **C**, main manuscript).

ii) MnP (EC 1.11.1.13) proteins, present in all sequenced white-rot Polyporales species, which include short (MnP-s) and long (MnP-l) isoenzymes (95 and 40 gene models identified, respectively) differing in the length of their C-terminal tail affecting catalytic properties and pH stability (Fernández-Fueyo et al. 2014a), were defined as harboring a Mn(II)-oxidation site near the internal propionate of heme formed by three acidic residues homologous to *P. chrysosporium* MnP1 Glu35, Glu39 and Asp179 (Sundaramoorthy et al. 2005) and *P. eryngii* VPL Glu36, Glu40 and Asp175 (Ruiz-Dueñas et al. 2007) (**Fig. 6B** and **C**, main manuscript).

iii) versatile peroxidase (VP; EC 1.11.1.16) proteins (14 gene models identified) were defined by the simultaneous presence of the catalytic tryptophan and the Mn(II)-oxidation site of LiP and MnP, respectively (Ruiz-Dueñas et al. 2009) (**Fig. 6C**, min manuscript).

iv) atypical VP proteins (VP-a) (4 gene models identified) were defined as such due to the presence of a catalytic tryptophan in their molecular structure (like in LiP and VP) but only having two of the three acidic residues characterizing the Mn(II)-oxidation site typical of VP.

In addition to the above ligninolytic peroxidase families, non-ligninolytic generic peroxidase (GP; EC 1.11.1.7) proteins (21 gene models identified) were defined by the simultaneous absence of the two catalytic sites mentioned above (**Fig. 6D**, main manuscript). GP are low redox-potential peroxidases with catalytic properties similar to those of the well-known *C. cinerea* peroxidase (Morita et al. 1988) and *C. subvermispora* GP (Fernández-Fueyo et al. 2012b) (i.e. they are able to oxidize phenolic substrates at the main heme access channel).

112 of the remaining 118 POD sequences, some of them formerly catalogued as atypical MnP isoenzymes (Floudas et al. 2012), were here for the first time classified into three new MnP subfamilies with a broad presence in genomes of fungi of the orders Agaricales (*Hypholoma sublateritium*, *P. alnicola*, *G. marginata*, *G. junonius*, *C. striatus*, *P. conissans*, *A. pediades*, *P.*

papilionaceus, *V. volvacea*, *H. cylindrosporum* and *C. glaucopus*) and Russulales (*S. hirsutum* and *Peniophora* sp.). These new MnP families, hereinafter named MnP-ESD, MnP-DGD and MnP-DED (78, 30 and 4 gene models identified, respectively) are characterized by possessing a Mn(II)-oxidation site formed by Glu/Ser/Asp, Asp/Gly/Asp and Asp/Glu/Asp, respectively (**Fig. 6E, F** and **G**, respectively, of the main manuscript), instead of Glu/Glu/Asp as described above for typical short and long MnP isoenzymes.

Finally, six POD identified in three decayed-wood Agaricales species (*P. alnicola*, *G. marginata* and *C. striatus*) were classified as novel POD (NPOD). In these enzymes, a Ser residue occupies the position of the catalytic tryptophan in LiP, and the Glu/Ala/Tyr triad is located at the manganese oxidation site of MnP (**Fig. 6H**, main manuscript). These NPOD could represent the first member in Agaricales of a new LiP subfamily presenting a tyrosine (included in the Glu/Ala/Tyr triad) at the same position of the catalytic tyrosine reported in a new type of LiP found in *Trametopsis cervina* (order Polyporales) (Miki et al. 2013) (**Fig. S31**). However, they were classified as NPOD due to the presence of two histidines above the heme plane putatively participating in enzyme activation by hydrogen peroxide instead of the arginine and histidine residues characterizing all the native POD described to date (**Fig. S31**). NPOD have been considered functional POD based on previous evidences demonstrating that is possible to enhance the peroxidase activity of myoglobin by construction of two distal histidines mimicking the role of the His-Arg pair (Lei-Bin et al. 2016).

With the sole exception of GP, all the other POD exhibit structural features of ligninolytic enzymes. The ability of LiP, MnP, and VP to oxidize lignin compounds directly or acting in the presence of mediators has been documented (Wariishi et al. 1991; Bao et al. 1994; Johjima et al. 1999; Martínez et al. 2005; Hammel and Cullen 2008; Sáez-Jiménez et al. 2015b). LiP is able to oxidize the major nonphenolic moiety of lignin. These enzymes exhibit the highest redox-potential of the three POD families (Ayuso-Fernández et al. 2019a) and are the most efficient lignin-degrading peroxidases existing in nature (Ayuso-Fernández et al. 2019b). Until recently, LiP had been only identified in white-rot wood Polyporales species. The screening of the fungal genomes here presented reveals that LiP genes are also found in Agaricales (**Fig. 2** *right*, main manuscript), and not only in the decayed-wood degrader *G. marginata* as previously reported by Koheler et al. (2015), but also in other species such as *G. junonius* (also preferentially growing on decayed wood) and *A. pediades* (a grass-litter degrader). Given the different evolutionary origin of LiP from Agaricales and Polyporales (**Fig. 5**, main manuscript), it is logical to think that they will present differences in their catalytic properties and/or stability, although this still needs to be confirmed.

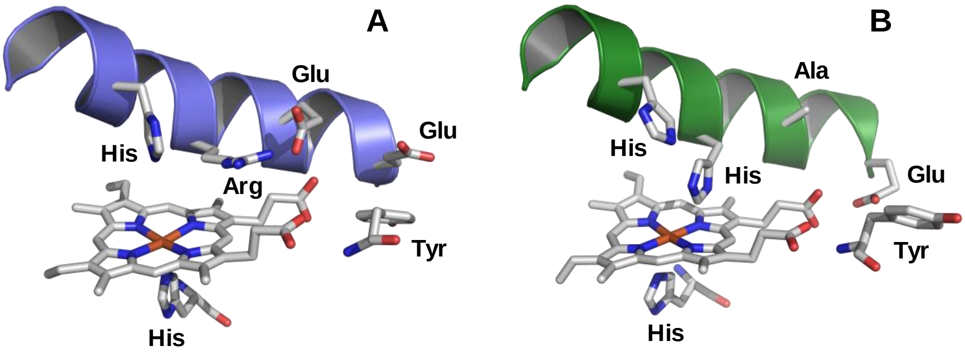

Fig. S31 Lateral view of the heme region in: **A**) the crystal structure of LiP from *T. cervina* (PDB 3Q3U); and **B**) the homology model of NPOD (JGI ID# 1391496) from *C. striatus*. The catalytic tyrosine (and surrounding residues) responsible for the oxidation of non-phenolic aromatic compounds in the *T. cervina* LiP and the homologous tyrosine (and surrounding residues) in NPOD are shown, together with the proximal histidine acting as the fifth ligand of the heme iron (located below the heme plane in both enzymes) and the Arg/His residues involved in enzyme activation by H_2O_2 in LiP (and the other POD described to date) whose position is occupied by His/His residues in NPOD.

Unlike LiP, typical MnP appear as a family of ligninolytic peroxidases widely distributed not only in wood-colonizing white-rot Agaricales, Polyporales, Amylocorticiales and Russulales, but also in decayed-wood, forest-litter and grass-litter Agaricales (**Fig. 2** *right*, main manuscript). MnP is also identified in the root pathogen *Armillaria mellea*, which includes a saprotrophic phase in its life cycle. Members of the short MnP subfamily (Floudas et al. 2012) exhibiting a broad substrate specificity due to their ability to oxidize both Mn(II) and low redox potential substrates in Mn-independent reactions (Fernández-Fueyo et al. 2014b) occur in a large number of species. By contrast, long MnP only oxidizing Mn(II) to Mn(III)) is absent from the grass-litter Agaricales and the white-rot wood Agaricales, Amylocorticiales and Russulales (and the root pathogen) species here analyzed.

Concerning VP, these enzymes are well represented in white-rot wood Polyporales. Out of this order they only appear in two Agaricales species, *P. eryngii* and *P. ostreatus*, differing in their preference for wood and grass-litter, respectively. Like VP, atypical VP is only found in white-rot wood Polyporales (*Ganoderma* sp. and *Trametes versicolor*) and in two Agaricales species also containing LiP (*G. marginata* and *G. junonius*).

Although less is still known about the so far called atypical MnP, this enzyme type has been reported to oxidize Mn(II). Thus, Hilden et al. (2014) purified and characterized one of these POD (now classified as MnP-ESD by us in agreement with the residues forming its Mn(II)-oxidation site) from the litter-decomposing fungus *Agrocybe praecox*, demonstrating its high affinity for this cation (K_m in the same order of magnitude as typical long MnP). In the same way, the MnP activity observed in cultures of *S. hirsutum* growing on lignocellulosic materials was correlated with the secretion of atypical MnP (MnP-ESD, as defined in the new classification). Interestingly, a comparative analysis suggested that this atypical MnP may be more effective than typical MnP enzymes from Polyporales (Presley et al. 2018).

12.2 Phylogenetic and molecular clock analysis of ligninolytic peroxidases

The amino-acid sequences of the 336 POD identified in the 52 Agaricomycetes genomes analyzed were aligned using MUSCLE as implemented in MEGA X (Kumar et al. 2018). The non-ligninolytic GP sequences of the ascomycete *Stagonospora nodorum*, available at JGI Mycocosm (http://genome.jgi.doe.gov/Stano1/Stano1.home.html), were included in the alignment and used for subsequent tree rooting. The sequence alignment was tested using ProtTest (Darriba et al. 2011) to determine the evolutionary model that best fits the data for ML analysis among 60 empirical models of evolution. The ML phylogeny was then constructed with RAxML (v.8.2.10) (Stamatakis 2014) through the CIPRES Science Gateway v.3.3 (Miller et al. 2015) using 1,000 bootstrap replicates, under the Whelan and Goldman (2001) model of evolution and gamma-distributed rate of heterogeneity (gamma shape with 4 rates of categories = 1.147) with empirical amino-acid frequencies and invariant sites (proportion of invariant sites = 0.007) (WAG+I+G+F) (**Fig. S32**).

The peroxidase phylogeny was time-calibrated with BEAST v. 2.2.1 (Bouckaert et al. 2014) using an uncorrelated lognormal relaxed molecular clock with a birth-death prior and a WAG substitution model. The topology was fixed using the POD phylogenetic tree from the RAxML analysis described above and shown in **Fig. S32**. A secondary calibration was performed using three calibration points from Floudas et al. (2012) based on their species phylogeny that estimated the split of Dikarya as 662 (520-831) Mya, the origin of Basidiomycota as 521 (403-665) Mya and the origin of Pezizomycotina as 344 (248-455) Mya. Four independent chains were run for 50 million generations each and sampling every 5000 generations. Chain convergence was assessed using Tracer 1.7.1 (Rambaut et al. 2018). Fifty percent of the samples were discarded as burn-in and a maximum clade credibility (MCC) tree with mean ages was obtained with TreeAnnotatior 2.2.1 (**Fig. 5**, main manuscript).

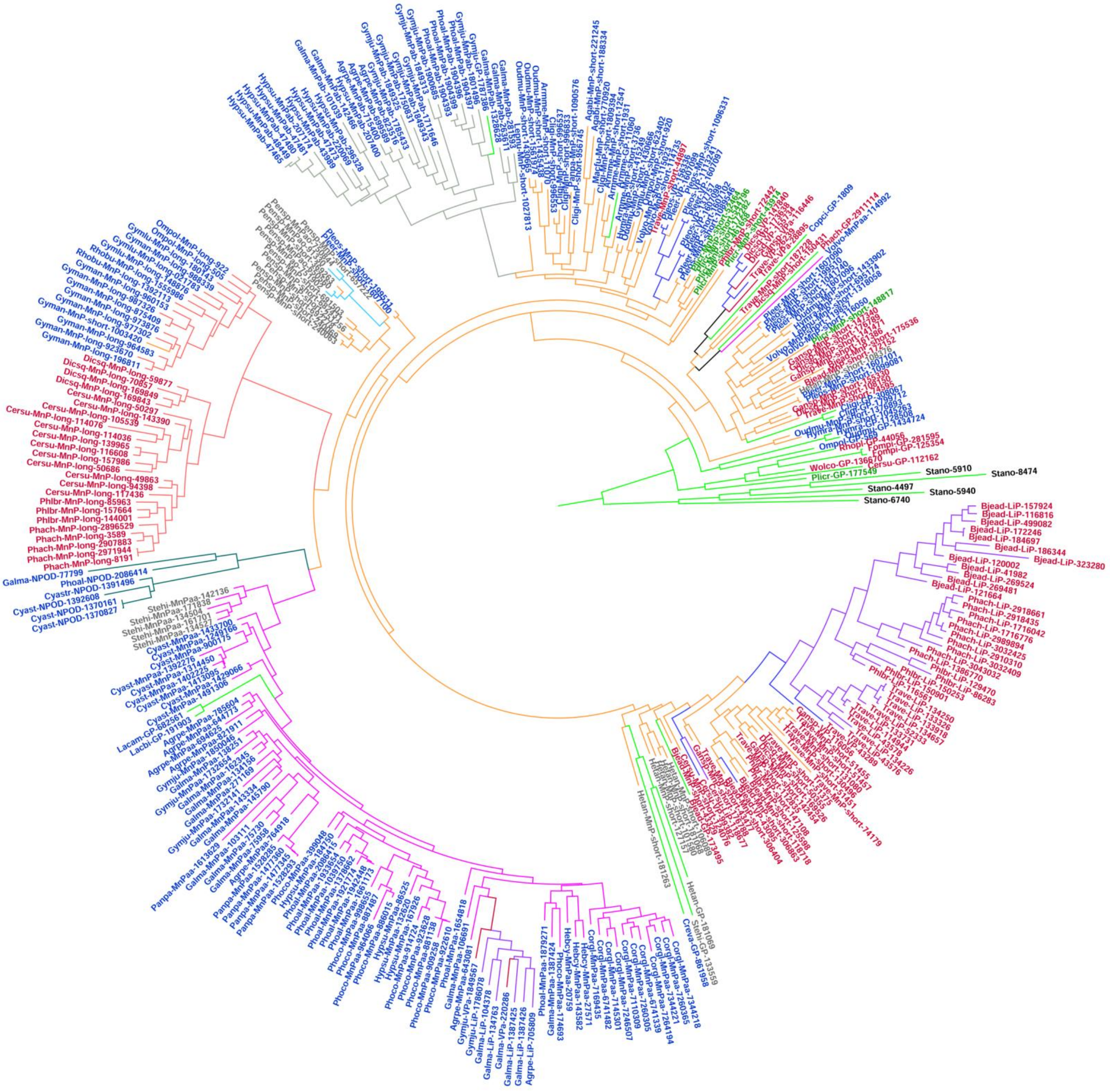

Fig. S32 ML phylogenetic tree (RAxML v.8.1.12), constructed with 1000 bootstrap replications, of 336 peroxidases identified in 42 of the 52 Agaricomycetes genomes analyzed. Five GP enzymes of the ascomycete *S. nodorum* were used as outgroup for rooting the tree. All sequences are available through the links to the JGI genomes included in **Table S4**. The tree was constructed using the evolutionary model WAG+I+G+F suggested by ProtTest (Abascal et al. 2005). The color of the branches indicates the (sub)family of the ancestor deduced by reconstructing sequences with PAML 4.7 (Yang 2007) as described in **section 12.3** (or current enzyme) to which they give rise in the next node of the tree (these ancestors were: *light green*, GP; *light orange*, MnP-s; *dark orange*, MnP-l; *dark blue*, VP; *red*, VP-a; *purple*, LiP; *pink*, MnP-ESD; *gray*, MnP-DGD; *light blue*, MnP-DED; and *dark green*, NPOD). The font color of the enzymes at the tips of the branches denotes the order of the fungi they belong to: *blue*, Agaricales; *green*, Amylocorticiales; *red*, Polyporales; gray, Russulales; and *black*, Pleosporales (Ascomycota).

12.3 Reconstruction of ancestral ligninolytic peroxidases

Ancestral sequence reconstruction was used to study the evolution of ligninolytic POD. PAML 4.7 package (Yang 2007) was used to obtain the most probable sequence at each node of the POD phylogeny and the posterior amino-acid probability per site in each ancestor. We used the WAG model of evolution, and the previously obtained ML phylogeny and the MUSCLE alignment (see section 12.2) as inputs for the software. Both joint and marginal reconstructions were performed and the most probable sequences of the marginal reconstruction were selected. In this study, we focused our analysis on: i) the proximal histidine (located below the heme plane); ii) the amino-acid residues responsible for the enzyme activation by hydrogen peroxide; and iii) the catalytic sites of the reconstructed ancestral enzymes (Mn-oxidation site, and tryptophan or tyrosine residue exposed to the solvent responsible for direct oxidation of the lignin polymer). The predicted function for the reconstructed enzymes is shown in the nodes of **Fig. 5** (main manuscript) (it is also indicated with the color code of the branches in the phylogenetic tree of **Fig. S32**).

13. Unspecific peroxygenases (UPO)

UPO enzymes from the heme-thiolate peroxidase (HTP) superfamily have been related to biodegradation of lignin and lignin-derived compounds by Agaricomycetes (Hofrichter et al. 2010). UPO are characterized by their monooxygenase activity that, in addition to oxygenate a variety of aromatic and aliphatic substrates (introducing hydroxy, oxo, carboxy and epoxy functionalities) present "etherase" activity after ether hydroxylation resulting in bond cleavage. Using these catalytic capabilities, the *Cyclocybe aegerita* (syn.: *Agrocybe aegerita*) UPO, representative of the long-UPO family, has been described to be able to cleave nonphenolic lignin model compounds via initial O-dealkylation (monooxygenase activity) and subsequent oxidation of the introduced phenolic moieties (peroxidase activity) (Kinne et al. 2011). Moreover, this second reaction could be catalyzed by other oxidoreductases such as peroxidases or laccases, the latter secreted coinciding with the production of these UPO (Ullrich et al. 2004; Gröbe et al. 2011).

Identifying a correlation between the presence of HTP (UPO-type) enzymes and a specific fungal lifestyle has been elusive as these enzymes have been found in white-rot and brown-rot fungi, and also in other basidiomycetes with different nutritional strategies (Floudas et al. 2012; Ruiz-Dueñas et al. 2013). In our study, UPO genes were identified in all fungal genomes analyzed (**Table S14**). For that, the amino-acid sequences of the only three HTP enzymes available as crystal structures (i.e. *Caldariomyces* (*Leptoxyphium*) *fumago* PDB-1CPO, *C. aegerita* PDB-2YOR and *Marasmius rotula* PDB-5FUJ) were BLASTed against the filtered model protein databases of the 52 fungal species under analysis, available at MycoCosm (mycocosm.jgi.doe.gov) (cut-off E-value 1.0E-5). However, whereas enzymes of the short-UPO family are widely distributed in the 52 species and their presence do not correlate with any specific lifestyle ($P > 0.05$, binomial exact test), enzymes of the long-UPO family seem to be exclusive of Agaricales (with only three exceptions, corresponding to the white-rot fungi *C. subvermispora* -Polyporales- and *S. hirsutum* -Russulales-, which only contain two and one gene of this family respectively; and *Fibulorhizoctonia* sp., with 18 enzymes).

Moreover, long-UPO enzymes are especially abundant in forest-litter and decayed-wood species ($P < 0.05$, binomial exact test) (**Table S14**). Interestingly those species of these two lifestyles with no long-UPO genes in their genomes (the leaf-litter degraders *R. butyracea* and *M. fiardii*, and the decayed-wood decomposer *G. luxurians*) seem to compensate the lack of these enzymes by increasing the number of genes encoding short-UPO enzymes. Therefore, the ligninolytic capabilities of short UPO, characterized by a wider heme access-channel (Hofrichter et al. 2020) that would favor the access of lignin products to the heme cofactor, are still to be investigated.

Table S14 Unspecific peroxygenases (UPO) in the 52 Agaricomycetes genomes analyzed, classified as short-UPO and long-UPO families. Each family is colored according to their abundance in the fungal genomes (in each column the color goes from dark orange, for the species with the highest number of enzymes of a MCO family, to white when the family is absent).

				UPO			
	species	Ecology	Lifestyle	short-UPO	long-UPO	TOTAL	Average number
AGARICALES	***H. sublateritium***	**Saprotroph**	**Decayed wood**	4	9	13	**12**
	P. alnicola			4	7	11	
	G. marginata			3	20	23	
	G. junonius			2	10	12	
	C. striatus			2	10	12	
	G. luxurians			12	0	12	
	O. olearius			2	2	4	
	H. radicata			7	5	12	
	P.conissans		**Forest litter**	3	8	11	**13**
	C. cinerea			4	7	11	
	M. fuliginosa			4	14	18	
	A. bisporus var bisporus			2	20	22	
	L. nuda			6	0	6	
	C. gibba			6	0	6	
	R. butyracea			16	0	16	
	G. androsaceus			7	10	17	
	M. fiardii			12	0	12	
	A. pediades		**Grass litter**	2	1	3	**4**
	P. papilionaceus			3	2	5	
	V. volvacea			3	0	3	
	P. eryngii			3	0	3	
	O. mucida		**Wood (white-rot)**	9	2	11	**8**
	P. ostreatus			4	0	4	
	C. variabilis		**Unknown decay**	3	7	10	**7**
	S. commune		**Unknown decay**	3	0	3	
	A. mellea	**Biotroph**	**Root pathogen**	3	0	3	**3**
	F. hepatica	**Saprotroph**	**Wood (brown-rot)**	3	0	3	**3**
	H. cylindrosporum	**Biotroph**	**Mycorrhizae**	5	2	7	**7**
	C. glaucopus			5	6	11	
	L. amethystina			6	1	7	
	L. bicolor			3	1	4	
	T. matsutake			3	1	4	
	L. gongylophorus	**Biotroph**	**Insect symbiont**	1	1	2	**2**
BOLETALES	***H. pinastri***	**Saprotroph**	**Wood (brown-rot)**	3	0	3	**3**
	C. puteana			2	0	2	
	S. lacrymans			3	0	3	
	S. brevipes	**Biotroph**	**Mycorrhizae**	5	0	5	**5**
AMYLOCORTICIALES	***P. crispa***	**Saprotroph**	**Wood (white-rot)**	3	0	3	**3**
ATHELIALES	***Fibulorhizoctonia* sp.**	**Biotroph**	**Insect symbiont**	14	18	32	**32**
POLYPORALES	***C. subvermispora***	**Saprotroph**	**Wood (white-rot)**	6	2	8	**4**
	D. squalens			4	0	4	
	***Ganoderma* sp.**			4	0	4	
	T. versicolor			3	0	3	
	B. adusta			4	0	4	
	P. chrysosporium			5	0	5	
	P. brevispora			2	0	2	
	P. placenta		**Wood (brown-rot)**	8	0	8	**5**
	W. cocos			4	0	4	
	F. pinicola			3	0	3	
RUSSULALES	***H. annosum***		**Wood (white-rot)**	4	0	4	**5**
	S. hirsutum			8	1	9	
	***Peniophora* sp.**			2	0	2	

14. Dye-decolorizing peroxidases (DyP)

14.1 DyP general characteristics

DyP are heme-proteins belonging to the peroxidase-chlorite dismutase superfamily (CDE) (Goblirsch et al. 2011). They are not exclusive of fungi and also appear distributed in bacteria and archaea genomes. Four DyP types (A-D) have been defined on the basis of their amino-acid sequence, with types A-C corresponding to prokaryotic enzymes and fungal DyP proteins preferentially included in type D (http://peroxibase.toulouse.inra.fr). Their evolutionary origin is different from that of the peroxidase-catalase superfamily, where ligninolytic POD are included (Zámocký et al. 2015). Fungal DyP enzymes show no sequence or structural homology with ligninolytic POD. However, DyP display similarities with these enzymes in the essential amino-acids located in the heme cavity. Thus, both types of peroxidases contain a histidine below the heme plane (proximal side) acting as the fifth ligand of the heme iron, whose orientation and distance to the metal determine the differences in the redox potential of ligninolytic POD (Ayuso-Fernández et al. 2019a). An arginine residue at the opposite side of the heme (distal side) contributes to the enzyme activation by H_2O_2 together with an aspartic acid in DyP, this being a histidine in POD (Martínez 2002; Linde et al. 2015b).

Despite their differences in tertiary structure, origin and distribution among organisms (Zámocký et al. 2015), DyP functionality is similar to that found in fungal POD enzymes. So, both show similar heme reactivity properties and form surface protein radicals via a long range electron transfer (LRET). There are members of these two types of peroxidases able to oxidize phenolic compounds such as 2,6-dimethoxyphenol, guaiacol and other substituted phenols, as well as several synthetic dyes such as azo or anthraquinone dyes in the absence of Mn^{2+}. Oxidation of Mn^{2+} to Mn^{3+}, a reaction characteristic of ligninolytic MnP and VP (Ruiz-Dueñas et al. 2009) has also been reported for some DyP enzymes, although the amino-acid composition and location of the Mn-binding site at the molecular structure is very different (Fernández-Fueyo et al. 2018). Oxidation of bulky substrates unable to directly interact with the buried heme cofactor occurs using the same mechanism of LiP and VP, which consists in subtracting electrons by a surface-exposed tryptophan or tyrosine radical and transferring them to the heme cofactor via LRET pathways (Linde et al. 2015a). Interestingly, despite all these similarities, DyP enzymes characterized to date do not efficiently degrade non-phenolic aromatic lignin model compounds, thus differing from ligninolytic POD (Linde et al. 2014; Linde et al. 2015b).

A phylogenetic analysis of 218 fungal DyP sequences performed by Fernández-Fueyo et al. (2015) reveled the existence of seven fungal DyP evolutionary clusters (**Fig. S33**). Cluster I contains most of the Agaricales DyP sequences, and most of the characterized DyP enzymes from basidiomycetes, such as those from *P. ostreatus* (Pleos-DyP1) (Fernández-Fueyo et al. 2015), *Auricularia auricula-judae* (Liers et al. 2010; Liers et al. 2013), *B. adusta* (Kim and Shoda 1999), *Irpex lacteus* (Salvachúa et al. 2013), *Mycetinis scorodonius* (Mycsc-DyP1and Mycsc-DyP2) (Scheibner et al. 2008; Zelena et al. 2009), *Termitomyces albuminosus* (Johjima et al. 2003) and *Exidia glandulosa* (Liers et al. 2013). TvDyP1 from *Trametes versicolor* (Amara et al. 2018) and Pleos-DyP4 from *P. ostreatus*, the latter able to oxidizes Mn^{2+} to Mn^{3+}, belong to a large subgroup of Agaricales DyP enzymes in the cluster III, predominantly constituted by sequences from Agaricales and Polyporales.

14.2 Identification of new DyP sequences and determination of their evolutionary relationships

In this study, DyP sequences were found by searching for "DyP" and "dye decolorizing peroxidase" keywords in 52 sequenced Agaricomycetes genomes available at the JGI-DOE Mycocosm portal (mycocosm.jgi.doe.gov). A multiple sequence alignment by MUSCLE using UPGMB as clustering method enabled detection and curation of erroneously processed introns as well as the identification of conserved amino-acid residues (distal arginine and aspartic acid, and proximal histidine). To establish the evolutionary relationships among the enzymes identified, a ML phylogram was constructed by MEGA X (Kumar et al. 2018) under the Whelan and Goldman model of evolution using gamma-distributed rate of heterogeneity (gamma shape with 4 rates of categories = 1.14) with empirical amino-acid frequencies and invariant sites (proportion of invariant sites = 0.018) (WAG+I+G+F) as suggested by ProtTest (Abascal et al. 2005).

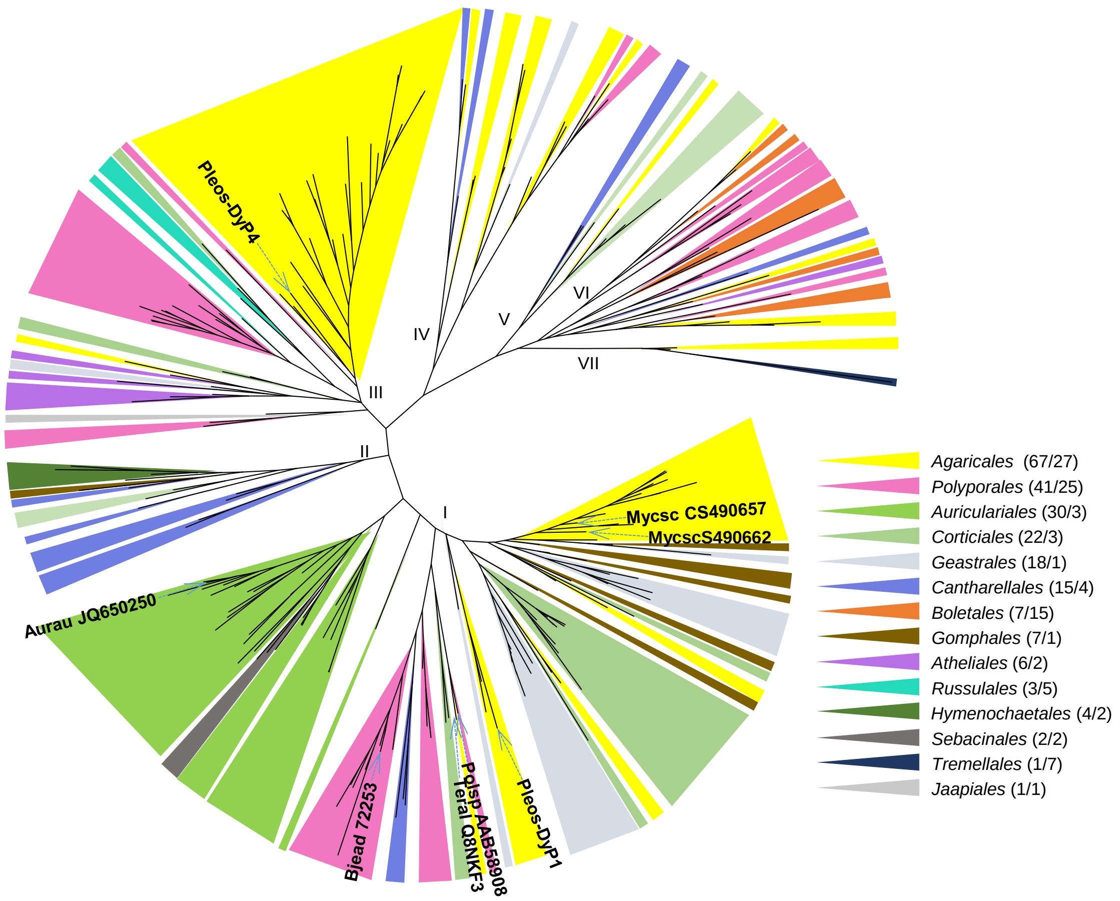

Fig. S33 ML phylogram of 218 DyP sequences identified in 64 Agaricomycotina genomes adapted from Fernández-Fueyo et al. (2015). The position of different DyP enzymes biochemically characterized is shown, including enzymes from *P. ostreatus* (Pleos-DyP1 and Pleos-DyP4, JGI ID# 62271 and 1069077, respectively), *M. scorodonius* (Mycsc-DyP1, CS490662; and Mycsc-DyP2, CS490657) and *T. albuminosus* (Teral-DyP, Q8NKF3) (three Agaricales species); unidentified Polyporaceae species corresponding to *I. lacteus* (Polsp-DyP, AAB58908), *T. versicolor* (Trave 48870) and *B. adusta* (Bjead-DyP, BAA77283; corresponding to JGI *B. adusta* genome protein ID# 72253) (three Polyporales species); and the Auriculariales species *A. auricula-judae* (Aurau-DyP, JQ650250) (GenBank accession numbers are indicated) and *E. glandulosa* (Exigl 767058). Subclusters from species of the orders Agaricales and Polyporales are widespread through the tree, with *P. ostreatus* DyP1 and DyP4 in clusters I and III, respectively. Colored triangles on the phylogram show the position of DyP sequences from 14 fungal orders (including five of the six orders to which the 52 fungal species of this study belong: Agaricales, Polyporales, Boletales, Atheliales and Russulales) with the total gene numbers followed by the number of genomes indicated in the legend for each order.

14.3 DyP enzymes in the 52 fungal species analyzed

A total of 110 DyP sequences were found (**Table S15**). Of these, 82 were identified in 28 of the 33 Agaricales genomes analyzed (86 DyP sequences had been previously automatically annotated as such by the JGI, but four of them were discarded after determining that they lack the proximal histidine necessary for iron heme coordination). A look at the Agaricales genomes containing DyP sequences allowed us to confirm that these enzymes are widely distributed in species of this order with different lifestyles related to lignocellulose degradation (decayed-wood, grass-litter, forest-litter and wood white-rot decomposers and the unknown-decay degrader *C. variabilis*), as well as in mycorrhizal species and root pathogens. Concerning the remaining 28 DyP sequences, 21 were found in 6 of the 10

wood-rotting Polyporales genomes (including white-rot and brown-rot species), 2 genes were identified in 2 of the 4 Boletales genomes (corresponding to species with mycorrhizal and brown-rot lifestyles), 3 genes were localized in 2 of the 3 wood white-rot Russulales genomes and other 2 genes in the only genome analyzed of an Atheliales species (*Fibulorhizoctonia* sp., here studied as a fungus related to species associated with wood-feeding termites). The phylogram obtained to determine the relationships among these enzymes revealed four different evolutionary groups (**Fig. S34**), corresponding to clusters I, III, IV and V-VI previously described.

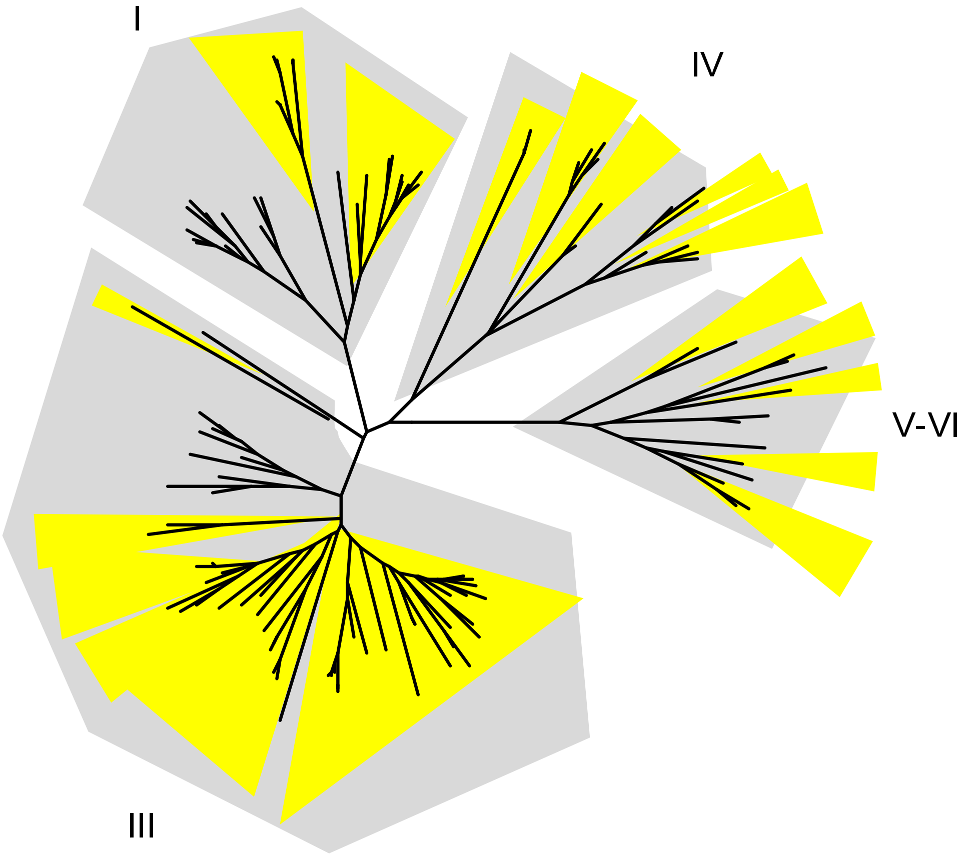

Fig. S34 ML phylogram of 110 DyP sequences identified in 39 of the 52 genomes analyzed (see **Table S15**). Agaricales DyP sequences are highlighted in yellow.

On average, Agaricales show a number slightly higher of DyP sequences per genome (2.5) compared with other orders (**Tables S15** and **S16),** although this is not statistically significant ($P > 0.05$ for pairwise comparisons, binomial exact test) as a very heterogeneous amount of these enzymes is actually detected in the species analyzed. Thus, the decayed-wood decomposer *G. luxurians* has 12 DyP sequences, while other species do not contain any sequence (e.g. the forest-litter degraders *A. bisporus* and *R. butyracea*).

Table S15 DyP enzymes in the Agaricomycetes genomes analyzed, with indication of their lifestyles, classified in the evolutionary clusters type I, III, IV and V-VI previously described by Fernández-Fueyo et al. (2015).

	species	Ecology	Lifestyle	DyP				
				Type I	Type III	Type IV	Type V-VI	TOTAL
AGARICALES	*H. sublateritium*	Saprotroph	Decayed wood	0	2	0	0	2
AGARICALES	*P. alnicola*	Saprotroph	Decayed wood	0	3	0	0	3
AGARICALES	*G. marginata*	Saprotroph	Decayed wood	0	4	0	1	5
AGARICALES	*G. junonius*	Saprotroph	Decayed wood	0	1	0	1	2
AGARICALES	*C. striatus*	Saprotroph	Decayed wood	0	2	0	0	2
AGARICALES	*G. luxurians*	Saprotroph	Decayed wood	7	0	2	3	12
AGARICALES	*O. olearius*	Saprotroph	Decayed wood	1	0	0	0	1
AGARICALES	*H. radicata*	Saprotroph	Decayed wood	0	1	0	0	1
AGARICALES	*P.conissans*	Saprotroph	Forest litter	0	2	0	0	2
AGARICALES	*C. cinerea*	Saprotroph	Forest litter	0	1	1	1	3
AGARICALES	*M. fuliginosa*	Saprotroph	Forest litter	0	1	0	0	1
AGARICALES	*A. bisporus var bisporus*	Saprotroph	Forest litter	0	0	0	0	0
AGARICALES	*L. nuda*	Saprotroph	Forest litter	0	1	1	0	2
AGARICALES	*C. gibba*	Saprotroph	Forest litter	0	3	2	0	5
AGARICALES	*R. butyracea*	Saprotroph	Forest litter	0	0	0	0	0
AGARICALES	*G. androsaceus*	Saprotroph	Forest litter	0	0	2	0	2
AGARICALES	*M. fiardii*	Saprotroph	Forest litter	1	0	0	0	1
AGARICALES	*A. pediades*	Saprotroph	Grass litter	0	2	0	0	2
AGARICALES	*P. papilionaceus*	Saprotroph	Grass litter	0	1	0	0	1
AGARICALES	*V. volvacea*	Saprotroph	Grass litter	0	2	1	0	3
AGARICALES	*P. eryngii*	Saprotroph	Grass litter	3	1	0	0	4
AGARICALES	*O. mucida*	Saprotroph	Wood (white rot)	0	1	0	1	2
AGARICALES	*P. ostreatus*	Saprotroph	Wood (white rot)	3	1	0	0	4
AGARICALES	*C. variabilis*	Saprotroph	Unknown decay	0	2	0	0	2
AGARICALES	*S. commune*	Saprotroph	Unknown decay	0	0	0	0	0
AGARICALES	*A. mellea*	Biotroph	Root pathogen	1	1	0	0	2
AGARICALES	*F. hepatica*	Saprotroph	Wood (brown rot)	0	0	0	0	0
AGARICALES	*H. cylindrosporum*	Biotroph	Mycorrhizae	0	2	0	0	2
AGARICALES	*C. glaucopus*	Biotroph	Mycorrhizae	0	2	3	0	5
AGARICALES	*L. amethystina*	Biotroph	Mycorrhizae	0	1	0	1	2
AGARICALES	*L. bicolor*	Biotroph	Mycorrhizae	0	1	0	1	2
AGARICALES	*T. matsutake*	Biotroph	Mycorrhizae	0	6	0	1	7
AGARICALES	*L. gongylophorus*	Biotroph	Insect symbiont	0	0	0	0	0
BOLETALES	*H. pinastri*	Saprotroph	Wood (brown rot)	0	0	0	1	1
BOLETALES	*C. puteana*	Saprotroph	Wood (brown rot)	0	0	0	0	0
BOLETALES	*S. lacrymans*	Saprotroph	Wood (brown rot)	0	0	0	0	0
BOLETALES	*S. brevipes*	Biotroph	Mycorrhizae	0	0	0	1	1
AMYLOCORTICIALES	*P. crispa*	Saprotroph	Wood (white rot)	0	0	0	0	0
ATHELIALES	*Fibulorhizoctonia* sp.	Biotroph	Insect symbiont	0	1	0	1	2
POLYPORALES	*C. subvermispora*	Saprotroph	Wood (white rot)	0	0	0	0	0
POLYPORALES	*D. squalens*	Saprotroph	Wood (white rot)	0	1	0	0	1
POLYPORALES	*Ganoderma* sp.	Saprotroph	Wood (white rot)	0	3	0	0	3
POLYPORALES	*T. versicolor*	Saprotroph	Wood (white rot)	0	2	0	0	2
POLYPORALES	*B. adusta*	Saprotroph	Wood (white rot)	8	0	2	0	10
POLYPORALES	*P. chrysosporium*	Saprotroph	Wood (white rot)	0	0	0	0	0
POLYPORALES	*P. brevispora*	Saprotroph	Wood (white rot)	3	0	0	0	3
POLYPORALES	*P. placenta*	Saprotroph	Wood (brown rot)	0	0	0	2	2
POLYPORALES	*W. cocos*	Saprotroph	Wood (brown rot)	0	0	0	0	0
POLYPORALES	*F. pinicola*	Saprotroph	Wood (brown rot)	0	0	0	0	0
RUSSULALES	*H. annosum*	Saprotroph	Wood (white rot)	0	1	0	0	1
RUSSULALES	*S. hirsutum*	Saprotroph	Wood (white rot)	0	2	0	0	2
RUSSULALES	*Peniophora* sp.	Saprotroph	Wood (white rot)	0	0	0	0	0
				27	54	14	15	110

Table S16 DyP average distribution in the six orders the 52 fungal species analyzed belong to

	Number of species	DyP total number	Cluster I	Cluster III	Cluster IV	Cluster V-VI	DyP per genome
Agaricales	33	82	16	44	12	10	2.5
Boletales	4	2	0	0	0	2	0.5
Amylocorticiales	1	0	0	0	0	0	0
Atheliales	1	2	0	1	0	1	2.0
Polyporales	10	21	11	6	2	2	2.1
Russulales	3	3	0	3	0	0	1.0

Focusing on Agaricales, a higher average number of DyP sequences per genome is observed in mycorrhizal fungi (3.6), and decayed-wood (3.5), wood white-rot (3) and grass-litter (2.5) decomposers (**Table S17**). However, as described above for the fungal orders, these differences are also not statistically significant ($P > 0.05$ for pairwise comparisons, binomial exact test) . Species with these lifestyles show at least one DyP sequence and, in general, belong to cluster III, which is also the cluster with a higher number of DyP enzymes detected in Agaricales.

Table S17 DyP distribution by lifestyles in Agaricales

	DyP	Cluster I	Cluster III	Cluster IV	Cluster V-VI	DyP per genome
Decayed wood	28	8	13	2	5	3.50
Forest-litter	16	1	8	6	1	1.78
Grass-litter	10	3	6	1	0	2.50
Wood (white-rot)	6	3	2	0	1	3.00
Unknown decay	2	0	2	0	0	1.00
Root pathogen	2	1	1	0	0	2.00
Wood (Brown-rot)	0	0	0	0	0	0.00
Mycorrhizae	18	0	12	3	3	3.60
Insect symbiont	0	0	0	0	0	0.00

Concerning the amino-acid residues coordinating the heme iron (proximal histidine) and involved in enzyme activation by H_2O_2 (distal arginine and aspartic acid) (Sugano et al. 2007), they are well conserved among the analyzed DyP enzymes, with the exception of seven sequences grouped in cluster IV which display glycine instead of the above aspartic acid (**Fig. S35)**. This fact suggests that another residue, probably a different aspartic acid also located in the distal heme cavity, could accept the proton from H_2O_2 during the enzyme activation. These differences in the type of residue and/or its positioning could be responsible for a different activation rate by H_2O_2 in these DyP enzymes, ultimately affecting the overall rate of the catalytic cycle and even contributing to their oxidative stability. In fact, the idea of modifying the residues located at the heme distal side has been already assayed as an strategy to slow down the enzymatic activation rate and in this way improve the stability of a ligninolytic POD towards H_2O_2 (Sáez-Jiménez et al. 2015a).

On the other hand, it is worthy to mention that the Pleos-DyP4 (*P. ostreatus* DyP4, ID# 1069077), classified in Cluster III, oxidizes Mn^{2+} to Mn^{3+} (Fernández-Fueyo et al. 2015). The manganese oxidation site of this enzyme has been recently characterized (Fernández-Fueyo et al. 2018). Four acidic residues (Asp215, Glu345, Asp352 and Asp354) participate in Mn^{2+} binding, with the glutamate also involved in the initial electron transfer to a key tyrosine (Tyr339) as confirmed by the >50-fold decreased k_{cat} in the E345A variant and the complete loss of activity when Tyr339 was removed. Some residues forming this manganese oxidation site are relatively well conserved among DyP sequences of cluster III, which contain glutamic and aspartic acids at positions 345 and 354, respectively (**Fig. S35)**. However, only *P. eryngii* DyP-1429886 maintains the tyrosine absolutely necessary to make possible the functionality of this Mn^{2+} oxidation site**,** suggesting that manganese oxidation is not a common characteristic of fungal DyP enzymes. In fact, other DyPs characterized from Cluster III has no activity towards Mn^{2+}, such as TvDyP1 (*T. versicolor* ID# 48870) (Amara et al. 2018), or very low activity, such as the *Coriolopsis trogii* DyP (AUW34346) (Kolwek et al. 2018).

Finally, we can observe that the residue equivalent to the catalytic tryptophan responsible for oxidation of substrates at protein surface, is also well conserved excluding the DyP sequences of cluster V-VI and some sequences of cluster IV and III. Interestingly, the only three DyP enzymes identified in two of the 7 brown-rot species analyzed (Pospl-105831, Pospl-99677 and Hydpi 122607 from *P. placenta* and *H. pinastri*) are grouped in cluster V-VI and lack the tryptophan residue equivalent to the characterized catalytic Trp-377 of *A. auricula-judae* DyP exposed to the solvent. Accordingly, if DyP had a potential role in lignin degradation, this would be ruled out in brown-rot fungi since their enzymes do not have neither a manganese oxidation site nor a tryptophan exposed to

the solvent for the lignin polymer oxidation. In summary, all the above suggests a wide diversity of DyP enzymes, not only in Agaricales, with catalytic properties that still have to be determined, as well as their putative role in lignocellulose degradation or in other biological processes.

Fig. S35 (*next page*) Phylogram of DyP enzymes from Agaricomycetes. Up to a total 110 DyP sequences were obtained from 39 of the 52 fungal genomes analyzed. The phylogram prepared with MEGA X (Kumar et al. 2018) includes three main clusters (I, III and IV) and a small group of sequences that can be classified as members of the evolutionary clusters V and VI previously obtained by Fernández-Fueyo et al. (Fernández-Fueyo et al. 2015) and shown in **Fig. S33.** Numbers on branches represent bootstrap values supporting that branch; only values ≥70% are presented. The JGI references are provided, together with indication of: i) residues equivalent to those forming the Mn^{2+} oxidation site in Pleos-DyP4 (Asp215, Glu345, Asp352, Asp354 and Tyr339); ii) the residue occupying the equivalent position of the surface catalytic tryptophan in Pleos-Dyp4; and iii) key residues of the heme environment, including a His acting as the fifth ligand of the heme iron and Arg and Asp residues participating in the enzyme activation by H_2O_2. *P. eryngii* DyP-1429886, which is the only enzyme containing the five amino-acid residues responsible for Mn^{2+} oxidation in Pleos-DyP4, is highlighted with an asterisk. The color code of the DyP sequences correspond to the lifestyle of the fugal species they belong to, as shown in **Fig. S1**.

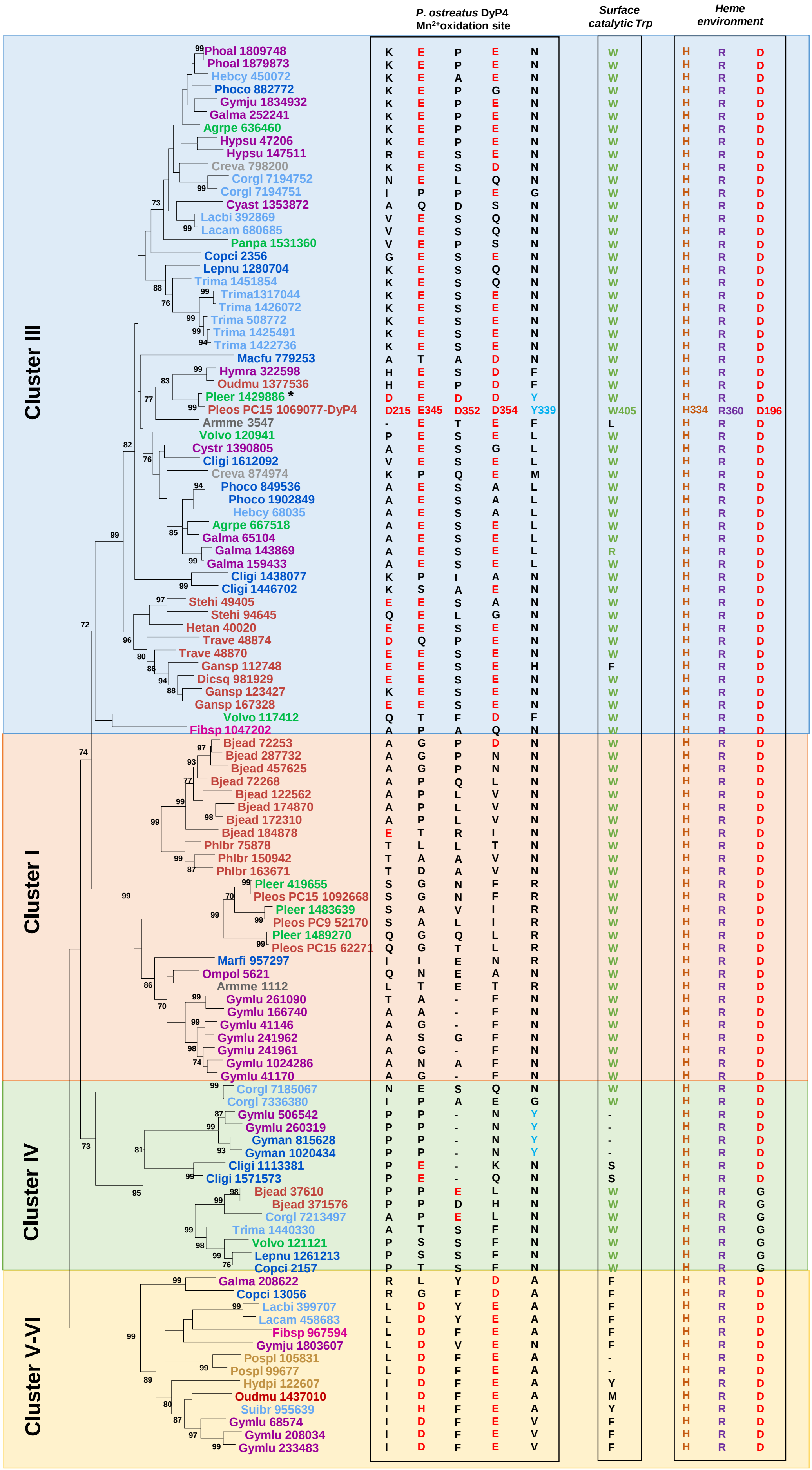

P. ostreatus DyP4
Mn²⁺oxidation site
Surface catalytic Trp
Heme environment
Cluster III
Cluster I
Cluster IV
Cluster V-VI
D215 E345 D352 D354 Y339
W405
H334 R360 D196
Phoal 1809748
Phoal 1879873
Hebcy 450072
Phoco 882772
Gymju 1834932
Galma 252241
Agrpe 636460
Hypsu 47206
Hypsu 147511
Creva 798200
Corgl 7194752
Corgl 7194751
Cyast 1353872
Lacbi 392869
Lacam 680685
Panpa 1531360
Copci 2356
Lepnu 1280704
Trima 1451854
Trima1317044
Trima 1426072
Trima 508772
Trima 1425491
Trima 1422736
Macfu 779253
Hymra 322598
Oudmu 1377536
Pleer 1429886 *
Pleos PC15 1069077-DyP4
Armme 3547
Volvo 120941
Cystr 1390805
Cligi 1612092
Creva 874974
Phoco 849536
Phoco 1902849
Hebcy 68035
Agrpe 667518
Galma 65104
Galma 143869
Galma 159433
Cligi 1438077
Cligi 1446702
Stehi 49405
Stehi 94645
Hetan 40020
Trave 48874
Trave 48870
Gansp 112748
Dicsq 981929
Gansp 123427
Gansp 167328
Volvo 117412
Fibsp 1047202
Bjead 72253
Bjead 287732
Bjead 457625
Bjead 72268
Bjead 122562
Bjead 174870
Bjead 172310
Bjead 184878
Phlbr 75878
Phlbr 150942
Phlbr 163671
Pleer 419655
Pleos PC15 1092668
Pleer 1483639
Pleos PC9 52170
Pleer 1489270
Pleos PC15 62271
Marfi 957297
Ompol 5621
Armme 1112
Gymlu 261090
Gymlu 166740
Gymlu 41146
Gymlu 241962
Gymlu 241961
Gymlu 1024286
Gymlu 41170
Corgl 7185067
Corgl 7336380
Gymlu 506542
Gymlu 260319
Gyman 815628
Gyman 1020434
Cligi 1113381
Cligi 1571573
Bjead 37610
Bjead 371576
Corgl 7213497
Trima 1440330
Volvo 121121
Lepnu 1261213
Copci 2157
Galma 208622
Copci 13056
Lacbi 399707
Lacam 458683
Fibsp 967594
Gymju 1803607
Pospl 105831
Pospl 99677
Hydpi 122607
Oudmu 1437010
Suibr 955639
Gymlu 68574
Gymlu 208034
Gymlu 233483

II. SUPPLEMENTARY REFERENCES

Abascal F, Zardoya R, and Posada D. 2005. ProtTest: selection of best-fit models of protein evolution. *Bioinformatics* 21:2104-2105.

Almasi E, Sahu N, Krizsan K, Balint B, Kovacs GM, Kiss B, Cseklye J, Drula E, Henrissat B, Nagy I et al. 2019. Comparative genomics reveals unique wood-decay strategies and fruiting body development in the Schizophyllaceae. *New Phytol.* 224:902-915.

Amara S, Perrot T, Navarro D, Deroy A, Benkhelfallah A, Chalak A, Daou M, Chevret D, Faulds CB, Berrin JG et al. 2018. Enzyme activities of two recombinant heme-including peroxidases TvDyP1 and TvVP2 identified from the secretome of *Trametes versicolor*. *Appl. Environ. Microbiol.* online doi: 10.1128/AEM.02826-17.

Ayuso-Fernández I, De Lacey AL, Cañada FJ, Ruiz-Dueñas FJ, and Martínez AT. 2019a. Increase of redox potential during the evolution of enzymes degrading recalcitrant lignin. *Chemistry* 25:2708-2712.

Ayuso-Fernández I, Rencoret J, Gutiérrez A, Ruiz-Dueñas FJ, and Martínez AT. 2019b. Peroxidase evolution in white-rot fungi follows wood lignin evolution in plants. *Proc. Natl. Acad. Sci. U. S A* 116:17900-17905.

Ballance DJ. 1986. Sequences important for gene expression in filamentous fungi. *Yeast* 2:229-236.

Bao WL, Fukushima Y, Jensen KA, Moen MA, and Hammel KE. 1994. Oxidative degradation of non-phenolic lignin during lipid peroxidation by fungal manganese peroxidase. *FEBS Lett.* 354:297-300.

Bao Z and Eddy SR. 2002. Automated de novo identification of repeat sequence families in sequenced genomes. *Genome Res.* 12:1269-1276.

Barbi F, Kohler A, Barry K, Baskaran P, Daum C, Fauchery L, Ihrmark K, Kuo A, LaButti K, Lipzen A et al. 2020. Fungal ecological strategies reflected in gene transcription - a case study of two litter decomposers. *Environ. Microbiol.* 22:1089-1103.

Barriuso J, Vaquero ME, Prieto A, and Martínez MJ. 2016. Structural traits and catalytic versatility of the lipases from the *Candida rugosa*-like family: A review. *Biotechnol. Adv.* 34:874-885.

Benjamini Y and Hochberg Y. 1995. Controlling the false discovery rate: a practical and powerful approach to multiple testing. *J. R. Statist. Soc. B* 57:289-300.

Biely P, Vrsanska M, Tenkanen M, and Kluepfel D. 1997. Endo-beta-1,4-xylanase families: differences in catalytic properties. *J. Biotechnol.* 57:151-166.

Bodeker ITM, Clemmensen KE, de Boer W, Martin F, Olson A, and Lindahl BD. 2014. Ectomycorrhizal *Cortinarius* species participate in enzymatic oxidation of humus in northern forest ecosystems. *New Phytol.* 203:245-256.

Boraston AB, Bolam DN, Gilbert HJ, and Davies GJ. 2004. Carbohydrate-binding modules: fine-tuning polysaccharide recognition. *Biochem. J.* 382:769-781.

Bouckaert R, Heled J, Kühnert D, Vaughan T, Wu C-H, Xie D, Suchard MA, Rambaut A, and Drummond AJ. 2014. A software platform for Bayesian evolutionary analysis. *PLoS Comput. Biol.* 10:1-6.

Caffall KH and Mohnen D. 2009. The structure, function, and biosynthesis of plant cell wall pectic polysaccharides. *Carbohydr. Res.* 344:1879-1900.

Camarero,S, Martínez MJ, and Martínez AT. 1997. Lignin-degrading enzymes produced by *Pleurotus* species during solid-state fermentation of wheat straw, p. 335-345. In: Roussos S, Lonsane BK, Raimbault M, and Viniegra-González G, editors. Advances in solid state fermentation. Kluwer Acad. Publ., Dordrecht.

Camarero S, Pardo I, Cañas AI, Molina P, Record E, Martínez AT, Martínez MJ, and Alcalde M. 2012. Engineering platforms for directed evolution of laccase from *Pycnoporus cinnabarinus*. *Appl. Environ. Microbiol.* 78:1370-1384.

Capella-Gutiérrez S, Silla-Martínez JM, and Gabaldón T. 2009. trimAl: a tool for automated alignment trimming in large-scale phylogenetic analyses. *Bioinformatics* 25:1972-1973.

Carro,J, Serrano A, Ferreira P, and Martínez AT. 2016. Fungal aryl-alcohol oxidase in lignocellulose degradation and bioconversion, p. 301-322. In: Gupta VK and Tuohy MG, editors. Microbial Enzymes in Bioconversions of Biomass. Springer, Berlin, Germany.

Castanera R, Borgognone A, Pisabarro AG, and Ramírez L. 2017. Biology, dynamics, and applications of transposable elements in basidiomycete fungi. *Appl. Microbiol. Biotechnol.* 101:1337-1350.

Castanera R, Pérez G, Omarini A, Alfaro M, Pisabarro AG, Faraco V, Amore A, and Ramírez L. 2012. Transcriptional and enzymatic profiling of *Pleurotus ostreatus* laccase genes in submerged and solid-state fermentation cultures. *Appl. Environ. Microbiol.* 78:4037-4045.

Chanzy H and Henrissat B. 1985. Undirectional degradation of *Valonia* cellulose microcrystals subjected to cellulase action. *FEBS Lett.* 184:285-288.

Cohen R, Jensen KA, Houtman CJ, and Hammel KE. 2002. Significant levels of extracellular reactive oxygen species produced by brown rot basidiomycetes on cellulose. *FEBS Lett.* 531:483-488.

Core Team. 2020. R: A language and environment for statistical computin. R Foundation for Statistical Computing, Vienna, Austria (https://www.R-project.org).

Crepin VF, Faulds CB, and Connerton IF. 2004. Functional classification of the microbial feruloyl esterases. *Appl. Microbiol. Biotechnol.* 63:647-652.

Crooks GE, Hon G, Chandonia JM, and Brenner SE. 2004. WebLogo: A sequence logo generator. *Genome Res* 14:1188-1190.

Daniel G, Volc J, Filonova L, Plihal O, Kubátová E, and Halada P. 2007. Characteristics of *Gloeophyllum trabeum* alcohol oxidase, an extracellular source of H2O2 in brown rot decay of wood. *Appl. Environ. Microbiol.* 73:6241-6253.

Daou M, Piumi F, Cullen D, Record E, and Faulds CB. 2016. Heterologous production and characterization of two glyoxal oxidases from *Pycnoporus cinnabarinus*. *Appl. Environ. Microbiol.* 82:4867-4875.

Darriba D, Taboada GL, Doallo R, and Posada D. 2011. ProtTest 3: fast selection of best-fit models of protein evolution. *Bioinformatics* 27:1164-1165.

De Bie T, Cristianini N, Demuth JP, and Hahn MW. 2006. CAFE: a computational tool for the study of gene family evolution. *Bioinformatics* 22:1269-1271.

Dilokpimol A, Makela MR, Varriale S, Zhou M, Cerullo G, Gidijala L, Hinkka H, Bras JLA, Jutten P, Piechot A et al. 2018. Fungal feruloyl esterases: Functional validation of genome mining based enzyme discovery including uncharacterized subfamilies. *Nat. Biotechnol.* 41:9-14.

Dilokpimol A, Mäkelä MR, Aguilar-Pontes MV, Benoit-Gelber I, Hildén KS, and de Vries RP. 2016. Diversity of fungal feruloyl esterases: updated phylogenetic classification, properties, and industrial applications. *Biotechnol. Biofuels* 9:231.

Doyle WA, Blodig W, Veitch NC, Piontek K, and Smith AT. 1998. Two substrate interaction sites in lignin peroxidase revealed by site-directed mutagenesis. *Biochemistry* 37:15097-15105.

Edgar RC. 2004a. MUSCLE: a multiple sequence alignment method with reduced time and space complexity. *BMC Bioinformatics* 5:113.

Edgar RC. 2004b. MUSCLE: multiple sequence alignment with high accuracy and high throughput. *Nucleic Acids Res.* 32:1792-1797.

Edgar RC. 2010. Search and clustering orders of magnitude faster than BLAST. *Bioinformatics* 26:2460-2461.

Ellinghaus D, Kurtz S, and Willhoeft U. 2008. LTRharvest, an efficient and flexible software for de novo detection of LTR retrotransposons. *BMC Bioinformatics* 9:18.

Emanuelsson O, Nielsen H, Brunak S, and von Heijne G. 2000. Predicting subcellular localization of proteins based on their N-terminal amino acid sequence. *J. Mol. Biol.* 300:1005-1016.

Fernández-Fueyo E, Acebes S, Ruiz-Dueñas FJ, Martínez MJ, Romero A, Medrano FJ, Guallar V, and Martínez AT. 2014a. Structural implications of the C-terminal tail in the catalytic and stability properties of manganese peroxidases from ligninolytic fungi. *Acta Crystallogr. D. Biol. Crystallogr.* 70:3253-3265.

Fernández-Fueyo E, Davó-Siguero I, Almendral D, Linde D, Baratto MC, Pogni R, Romero A, Guallar V, and Martínez AT. 2018. Description of a non-canonical Mn(II)-oxidation site in peroxidases. *ACS Catal.* 8:8386-8395.

Fernández-Fueyo E, Linde D, Almendral D, López-Lucendo MF, Ruiz-Dueñas FJ, and Martínez AT. 2015. Description of the first fungal dye-decolorizing peroxidase oxidizing manganese(II). *Appl. Microbiol. Biotechnol.* 99:8927-8942.

Fernández-Fueyo E, Ruiz-Dueñas FJ, Ferreira P, Floudas D, Hibbett DS, Canessa P, Larrondo L, James TY, Seelenfreund D, Lobos S et al. 2012a. Comparative genomics of *Ceriporiopsis subvermispora* and *Phanerochaete chrysosporium* provide insight into selective ligninolysis. *Proc. Natl. Acad. Sci. USA* 109:5458-5463.
Fernández-Fueyo E, Ruiz-Dueñas FJ, Martínez MJ, Romero A, Hammel KE, Medrano FJ, and Martínez AT. 2014b. Ligninolytic peroxidase genes in the oyster mushroom genome: Heterologous expression, molecular structure, catalytic and stability properties and lignin-degrading ability. *Biotechnol. Biofuels* 7:2.
Fernández-Fueyo E, Ruiz-Dueñas FJ, Miki Y, Martínez MJ, Hammel KE, and Martínez AT. 2012b. Lignin-degrading peroxidases from genome of selective ligninolytic fungus *Ceriporiopsis subvermispora*. *J. Biol. Chem.* 287:16903-16916.
Ferreira P, Carro J, Serrano A, and Martínez AT. 2015. A survey of genes encoding H2O2-producing GMC oxidoreductases in 10 Polyporales genomes. *Mycologia* 107:1105-1119.
Ferreira P, Medina M, Guillén F, Martínez MJ, van Berkel WJH, and Martínez AT. 2005. Spectral and catalytic properties of aryl-alcohol oxidase, a fungal flavoenzyme acting on polyunsaturated alcohols. *Biochem. J.* 389:731-738.
Floudas D, Binder M, Riley R, Barry K, Blanchette RA, Henrissat B, Martínez AT, Otillar R, Spatafora JW, Yadav JS et al. 2012. The Paleozoic origin of enzymatic lignin decomposition reconstructed from 31 fungal genomes. *Science* 336:1715-1719.
Floudas D, Held BW, Riley R, Nagy LG, Koehler G, Ransdell AS, Younus H, Chow J, Chiniqui J, Lipzen A et al. 2015. Evolution of novel wood decay mechanisms in Agaricales revealed by the genome sequences of *Fistulina hepatica* and *Cylindrobasidium torrendii*. *Fungal Genet. Biol.* 76:78-92.
Fry SC. 1988. The growing plant cell wall: chemical and metabolic analysis. Longman Group Limited, Harlow, UK.
Galli C, Gentili P, Jolivalt C, Madzak C, and Vadala R. 2011. How is the reactivity of laccase affected by single-point mutations? Engineering laccase for improved activity towards sterically demanding substrates. *Appl. Microbiol. Biotechnol.* 91:123-131.
Giardina P, Autore F, Faraco V, Festa G, Palmieri G, Piscitelli A, and Sannia G. 2007. Structural characterization of heterodimeric laccases from *Pleurotus ostreatus*. *Appl. Microbiol. Biotechnol.* 75:1293-1300.
Gnerre S, Maccallum I, Przybylski D, Ribeiro FJ, Burton JN, Walker BJ, Sharpe T, Hall G, Shea TP, Sykes S et al. 2011. High-quality draft assemblies of mammalian genomes from massively parallel sequence data. *Proc. Natl. Acad. Sci. U. S A* 108:1513-1518.
Goblirsch B, Kurker RC, Streit BR, Wilmot CM, and Dubois JL. 2011. Chlorite dismutases, DyPs, and EfeB: 3 microbial heme enzyme families comprise the CDE structural superfamily. *J. Mol. Biol.* 408:379-398.
González R, Ramón D, and Pérez-González JA. 1992. Cloning, sequencing analysis and yeast expression of the egl1 gene from *Trichoderma longibrachiatum*. *Appl. Microbiol. Biotechnol.* 38:370-378.
Grabherr MG, Haas BJ, Yassour M, Levin JZ, Thompson DA, Amit I, Adiconis X, Fan L, Raychowdhury R, Zeng Q et al. 2011. Full-length transcriptome assembly from RNA-Seq data without a reference genome. *Nat. Biotechnol.* 29:644-652.
Grigoriev IV, Nikitin R, Haridas S, Kuo A, Ohm R, Otillar R, Riley R, Salamov A, Zhao X, Korzeniewski F et al. 2014. MycoCosm portal: gearing up for 1000 fungal genomes. *Nucleic Acids Res.* 42:D699-D704.
Gröbe G, Ullrich M, Pecyna M, Kapturska D, Friedrich S, Hofrichter M, and Scheibner K. 2011. High-yield production of aromatic peroxygenase by the agaric fungus *Marasmius rotula*. *AMB Express* 1:31-42.
Halada P, Leitner C, Sedmera P, Haltrich D, and Volc J. 2003. Identification of the covalent flavin adenine dinucleotide-binding region in pyranose 2-oxidase from *Trametes multicolor*. *Anal. Biochem.* 314:235-242.
Hammel KE and Cullen D. 2008. Role of fungal peroxidases in biological ligninolysis. *Curr. Opin. Plant Biol.* 11:349-355.

Han MV, Thomas GWC, Lugo-Martinez J, and Hahn MW. 2013. Estimating gene gain and loss rates in the presence of error in genome assembly and annotation using CAFE 3. *Mol. Biol. Evol.* 30:1987-1997.

Hechenbichler K and Schliep K. 2004. Weighted k-nearest neighbor techniques and ordinal classification. Discussion paper 399, SFB 386. *Ludwig-Maximilians Univ. Munich* doi.org/10.5282/ubm/epub.1769.

Heredia A. 2003. Biophysical and biochemical characteristics of cutin, a plant barrier biopolymer. *Biochim. Biophys. Acta* 1620:1-7.

Highley TL. 1973. Influence of carbon source on cellulase activity of white-rot and brown-rot fungi. *Wood Fiber* 5:50-58.

Hilden K, Makela MR, Steffen KT, Hofrichter M, Hatakka A, Archer DB, and Lundell TK. 2014. Biochemical and molecular characterization of an atypical manganese peroxidase of the litter-decomposing fungus *Agrocybe praecox*. *Fungal Genet. Biol.* 72:131-136.

Hoede C, Arnoux S, Moisset M, Chaumier T, Inizan O, Jamilloux V, and Quesneville H. 2014. PASTEC: an automatic transposable element classification tool. *PLoS ONE* 9:e91929.

Hofrichter,M, Kellner H, Herzog R, Karich A, Liers C, Scheibner K, Kimani VW, and Ullrich R. 2020. Fungal peroxygenases: A phylogenetically old superfamily of heme enzymes with promiscuity for oxygen transfer reactions, p. 369-404. In: Nevalainen H, editor. Grand challenges in fungal biotechnology. Springer, Switzerland AG.

Hofrichter M, Ullrich R, Pecyna MJ, Liers C, and Lundell T. 2010. New and classic families of secreted fungal heme peroxidases. *Appl. Microbiol. Biotechnol.* 87:871-897.

Horton P, Park KJ, Obayashi T, Fujita N, Harada H, Adams-Collier CJ, and Nakai K. 2007. WoLF PSORT: protein localization predictor. *Nucleic Acids Res.* 35:W585-W587.

Jiang N, Feschotte C, Zhang X, and Wessler SR. 2004. Using rice to understand the origin and amplification of miniature inverted repeat transposable elements (MITEs). *Curr. Opin. Plant Biol.* 7:115-119.

Johjima T, Itoh H, Kabuto M, Tokimura F, Nakagawa T, Wariishi H, and Tanaka H. 1999. Direct interaction of lignin and lignin peroxidase from *Phanerochaete chrysosporium*. *Proc. Natl. Acad. Sci. USA* 96:1989-1994.

Johjima T, Ohkuma M, and Kudo T. 2003. Isolation and cDNA cloning of novel hydrogen peroxide-dependent phenol oxidase from the basidiomycete *Termitomyces albuminosus*. *Appl. Microbiol. Biotechnol.* 61:220-225.

Juniper BE and Jeffree CE. 1983. Plant surfaces. London : Edward Arnold, 1983.

Jurka J. 2000. Repbase update: a database and an electronic journal of repetitive elements. *Trends Genet.* 16:418-420.

Katoh K and Standley DM. 2013. MAFFT multiple sequence alignment software version 7: improvements in performance and usability. *Mol. Biol. Evol.* 30:772-780.

Kersten P and Cullen D. 2014. Copper radical oxidases and related extracellular oxidoreductases of wood-decay Agaricomycetes. *Fungal Genet. Biol.* 72:124-130.

Kersten PJ. 1990. Glyoxal oxidase of *Phanerochaete chrysosporium*: Its characterization and activation by lignin peroxidase. *Proc. Natl. Acad. Sci. USA* 87:2936-2940.

Kersten PJ and Cullen D. 1993. Cloning and characterization of a cDNA encoding glyoxal oxidase, a H_2O_2-producing enzyme from the lignin-degrading basidiomycete *Phanerochaete chrysosporium*. *Proc. Natl. Acad. Sci. USA* 90:7411-7413.

Kersten PJ and Kirk TK. 1987. Involvement of a new enzyme, glyoxal oxidase, in extracellular H_2O_2 production by *Phanerochaete chrysosporium*. *J. Bacteriol.* 169:2195-2201.

Kim SJ and Shoda M. 1999. Purification and characterization of a novel peroxidase from *Geotrichum candidum* Dec 1 involved in decolorization of dyes. *Appl. Environ. Microbiol.* 65:1029-1035.

Kinne M, Poraj-Kobielska M, Ullrich R, Nousiainen P, Sipila J, Scheibner K, Hammel KE, and Hofrichter M. 2011. Oxidative cleavage of non-phenolic β-*O*-4 lignin model dimers by an extracellular aromatic peroxygenase. *Holzforschung* 65:673-679.

Kirk TK and Farrell RL. 1987. Enzymatic "combustion": The microbial degradation of lignin. *Annu. Rev. Microbiol.* 41:465-505.

Kohler A, Kuo A, Nagy LG, Morin E, Barry KW, Buscot F, Canback B, Choi C, Cichocki N, Clum A et al. 2015. Convergent losses of decay mechanisms and rapid turnover of symbiosis genes in mycorrhizal mutualists. *Nat. Genet.* 47:410-415.

Kolwek J, Behrens C, Linke D, Krings U, and Berger RG. 2018. Cell-free one-pot conversion of (+)-valencene to (+)-nootkatone by a unique dye-decolorizing peroxidase combined with a laccase from *Funalia trogii*. *J. Ind. Microbiol. Biotechnol.* 45:89-101.

Kujawa M, Volc J, Halada P, Sedmera P, Divne C, Sygmund C, Leitner C, Peterbauer C, and Haltrich D. 2007. Properties of pyranose dehydrogenase purified from the litter-degrading fungus *Agaricus xanthoderma*. *FEBS J.* 274:879-894.

Kumar S, Stecher G, Li M, Knyaz C, and Tamura K. 2018. MEGA X: Molecular evolutionary genetics analysis across computing platforms. *Mol. Biol. Evol.* 35:1547-1549.

Lam KK, LaButti K, Khalak A, and Tse D. 2015. FinisherSC: a repeat-aware tool for upgrading de novo assembly using long reads. *Bioinformatics* 31:3207-3209.

Larrondo LF, Salas L, Melo F, Vicuña R, and Cullen D. 2003. A novel extracellular multicopper oxidase from *Phanerochaete chrysosporium* with ferroxidase activity. *Appl. Environ. Microbiol.* 69:6257-6263.

Lei-Bin W, Ke-Jie D, Chang-Ming N, Shu-Qin G, Ge-Bo W, Xiangshi T, and Ying-Wu L. 2016. Peroxidase activity enhancement of myoglobin by two cooperative distal histidines and a channel to the heme pocket. *Journal of Molecular Catalysis B: Enzymatic* 134:367-371.

Leuthner B, Aichinger C, Oehmen E, Koopmann E, Muller O, Muller P, Kahmann R, Bolker M, and Schreier PH. 2005. A H2O2-producing glyoxal oxidase is required for filamentous growth and phatogenicity in *Ustilago maydis*. *Mol. Genet. Genomics* 272:639-650.

Liers C, Bobeth C, Pecyna M, Ullrich R, and Hofrichter M. 2010. DyP-like peroxidases of the jelly fungus *Auricularia auricula-judae* oxidize nonphenolic lignin model compounds and high-redox potential dyes. *Appl. Microbiol. Biotechnol.* 85:1869-1879.

Liers C, Pecyna MJ, Kellner H, Worrich A, Zorn H, Steffen KT, Hofrichter M, and Ullrich R. 2013. Substrate oxidation by dye-decolorizing peroxidases (DyPs) from wood- and litter-degrading agaricomycetes compared to other fungal and plant heme-peroxidases. *Appl. Microbiol. Biotechnol.* 87:5839-5849.

Linde D, Coscolín C, Liers C, Hofrichter M, Martínez AT, and Ruiz-Dueñas FJ. 2014. Heterologous expression and physicochemical characterization of a fungal dye-decolorizing peroxidase from *Auricularia auricula-judae*. *Protein Express. Purif.* 103:28-37.

Linde D, Pogni R, Cañellas M, Lucas F, Guallar V, Baratto MC, Sinicropi A, Sáez-Jiménez V, Coscolín C, Romero A et al. 2015a. Catalytic surface radical in dye-decolorizing peroxidase: A computational, spectroscopic and directed mutagenesis study. *Biochem. J.* 466:253-262.

Linde D, Ruiz-Dueñas FJ, Fernández-Fueyo E, Guallar V, Hammel KE, Pogni R, and Martínez AT. 2015b. Basidiomycete DyPs: Genomic diversity, structural-functional aspects, reaction mechanism and environmental significance. *Arch. Biochem. Biophys.* 574:66-74.

Lippman Z, Gendrel AV, Black M, Vaughn MW, Dedhia N, McCombie WR, Lavine K, Mittal V, May B, Kasschau KD et al. 2004. Role of transposable elements in heterochromatin and epigenetic control. *Nature* 430:471-476.

Lombard V, Ramulu HG, Drula E, Coutinho PM, and Henrissat B. 2014. The carbohydrate-active enzymes database (CAZy) in 2013. *Nucleic Acids Res.* 42:D490-D495.

Martin J, Bruno VM, Fang Z, Meng X, Blow M, Zhang T, Sherlock G, Snyder M, and Wang Z. 2010. Rnnotator: an automated de novo transcriptome assembly pipeline from stranded RNA-Seq reads. *BMC Genomics* 11:663.

Martinez Arbizu P. 2020. pairwiseAdonis: Pairwise multilevel comparison using adonis. R Package version 0.4. *URL* https://github.com/pmartinezarbizu/pairwiseAdonis.

Martínez AT. 2002. Molecular biology and structure-function of lignin-degrading heme peroxidases. *Enzyme Microb. Technol.* 30:425-444.

Martínez,AT, Camarero S, Ruiz-Dueñas FJ, and Martínez MJ. 2018. Biological lignin degradation, p. 199-225. In: Beckham GT, editor. Lignin valorization: Emerging approaches. Royal Society of Chemistry, London.

Martínez AT, Speranza M, Ruiz-Dueñas FJ, Ferreira P, Camarero S, Guillén F, Martínez MJ, Gutiérrez A, and del Río JC. 2005. Biodegradation of lignocellulosics: Microbiological, chemical and enzymatic aspects of fungal attack to lignin. *Int. Microbiol.* 8:195-204.
Martinez D, Challacombe J, Morgenstern I, Hibbett DS, Schmoll M, Kubicek CP, Ferreira P, Ruiz-Dueñas FJ, Martínez AT, Kersten P et al. 2009. Genome, transcriptome, and secretome analysis of wood decay fungus *Postia placenta* supports unique mechanisms of lignocellulose conversion. *Proc. Natl. Acad. Sci. USA* 106:1954-1959.
Marx DH. 1969. The influence of ectotrophic mycorrhizal fungi on the resistance of pine roots to pathogenic infections. I. Antagonism of mycorrhizal fungi to root pathogenic fungi and soil bacteria. *Phytopathology* 59:153-163.
Mathieu Y, Piumi F, Valli R, Aramburu JC, Ferreira P, Faulds CB, and Record E. 2016. Activities of secreted aryl alcohol quinone oxidoreductases from *Pycnoporus cinnabarinus* provide insights into fungal degradation of plant biomass. *Appl. Environ. Microbiol.* 82:2411-2423.
Miki Y, Pogni R, Acebes S, Lucas F, Fernández-Fueyo E, Baratto MC, Fernández MI, de los Ríos V, Ruiz-Dueñas FJ, Sinicropi A et al. 2013. Formation of a tyrosine adduct involved in lignin degradation by *Trametopsis cervina* lignin peroxidase: A novel peroxidase activation mechanism. *Biochem. J.* 452:575-584.
Miller MA, Schwartz T, Pickett BE, He S, Klem EB, Scheuermann RH, Passarotti M, Kaufman S, and O'Leary MA. 2015. A RESTful API for Access to Phylogenetic Tools via the CIPRES Science Gateway. *Evol. Bioinform. Online.* 11:43-48.
Morin E, Kohler A, Baker AR, Foulongne-Oriol M, Lombard V, Nagy LG, Ohm RA, Patyshakuliyeva A, Brun A, Aerts AL et al. 2012. Genome sequence of the button mushroom *Agaricus bisporus* reveals mechanisms governing adaptation to a humic-rich ecological niche. *Proc. Natl. Acad. Sci. USA* 109:17501-17506.
Morita Y, Yamashita H, Mikami B, Iwamoto H, Aibara S, Terada M, and Minami J. 1988. Purification, crystallization, and characterization of peroxidase from *Coprinus cinereus*. *J. Biochem. (Tokyo)* 103:693-699.
Muñoz C. 1995. Caracterización y purificación de las lacasas de *Pleurotus eryngii*. Aplicaciones biotecnológicas en relación con la degradación de la lignina. Thesis, Universidad Alcalá de Henares, Madrid.
Muñoz C, Guillén F, Martínez AT, and Martínez MJ. 1997. Laccase isoenzymes of *Pleurotus eryngii:* Characterization, catalytic properties and participation in activation of molecular oxygen and Mn^{2+} oxidation. *Appl. Environ. Microbiol.* 63:2166-2174.
Oksanen J, Blanchet FG, Friendly M, Kindt R, Legendre P, McGlinn D, Minchin PR, O'Hara RB, Simpson GL, Solymos P et al. 2019. vegan: Community ecology package. R package v.2.5-6. *URL* https://CRAN.R-project.org/package=vegan.
Paës G, Berrin JG, and Beaugrand J. 2012. GH11 xylanases: Structure/function/properties relationships and applications. *Biotechnol. Adv.* 30:564-592.
Pardo I, Santiago G, Gentili P, Lucas F, Monza E, Medrano FJ, Galli C, Martínez AT, Guallar V, and Camarero S. 2016. Re-designing the substrate binding pocket of laccase for enhanced oxidation of sinapic acid. *Catal. Sci. Technol.* 6:3900-3910.
Pérez-Boada M, Ruiz-Dueñas FJ, Pogni R, Basosi R, Choinowski T, Martínez MJ, Piontek K, and Martínez AT. 2005. Versatile peroxidase oxidation of high redox potential aromatic compounds: Site-directed mutagenesis, spectroscopic and crystallographic investigations of three long-range electron transfer pathways. *J. Mol. Biol.* 354:385-402.
Petersen TN, Brunak S, von Heijne G, and Nielsen H. 2011. SignalP 4.0: discriminating signal peptides from transmembrane regions. *Nature Methods* 8:785-786.
Presley GN, Panisko E, Purvine SO, and Schilling JS. 2018. Comparing the temporal process of wood metabolism among white and brown rot fungi by coupling secretomics with enzyme activities. *Appl. Environ. Microbiol.* 18:e00159-18.
Price AL, Jones NC, and Pevzner PA. 2005. *De novo* identification of repeat families in large genomes. *Bioinformatics* 21 Suppl 1:i351-i358.
Raffaele S and Kamoun S. 2012. Genome evolution in filamentous plant pathogens: why bigger can be better. *Nat. Rev. Microbiol.* 10:417-430.

Rambaut A, Drummond AJ, Xie D, Baele G, and Suchard MA. 2018. Posterior summarization in Bayesian phylogenetics using Tracer 1.7. *Syst. Biol.* 67:901-904.

Revell LJ. 2009. Size-correction and principal components for interspecific comparative studies. *Evolution* 63:3258-3268.

Rodríguez-Rincón F, Suarez A, Lucas M, Larrondo LF, de la Rubia T, Polaina J, and Martínez J. 2010. Molecular and structural modeling of the *Phanerochaete flavido-alba* extracellular laccase reveals its ferroxidase structure. *Arch. Microbiol.* 192:883-892.

Romero E, Ferreira P, Martínez AT, and Martínez MJ. 2009. New oxidase from *Bjerkandera* arthroconidial anamorph that oxidizes both phenolic and nonphenolic benzyl alcohols. *Biochim. Biophys. Acta* 1794:689-697.

Ruiz-Dueñas FJ, Fernández E, Martínez MJ, and Martínez AT. 2011. *Pleurotus ostreatus* heme peroxidases: An *in silico* analysis from the genome sequence to the enzyme molecular structure. *C. R. Biol.* 334:795-805.

Ruiz-Dueñas FJ, Ferreira P, Martínez MJ, and Martínez AT. 2006. *In vitro* activation, purification, and characterization of *Escherichia coli* expressed aryl-alcohol oxidase, a unique H2O2-producing enzyme. *Protein Express. Purif.* 45:191-199.

Ruiz-Dueñas FJ, Lundell T, Floudas D, Nagy LG, Barrasa JM, Hibbett DS, and Martínez AT. 2013. Lignin-degrading peroxidases in Polyporales: An evolutionary survey based on ten sequenced genomes. *Mycologia* 105:1428-1444.

Ruiz-Dueñas FJ, Martínez MJ, and Martínez AT. 1999. Molecular characterization of a novel peroxidase isolated from the ligninolytic fungus *Pleurotus eryngii*. *Mol. Microbiol.* 31:223-236.

Ruiz-Dueñas FJ, Morales M, García E, Miki Y, Martínez MJ, and Martínez AT. 2009. Substrate oxidation sites in versatile peroxidase and other basidiomycete peroxidases. *J. Exp. Bot.* 60:441-452.

Ruiz-Dueñas FJ, Morales M, Pérez-Boada M, Choinowski T, Martínez MJ, Piontek K, and Martínez AT. 2007. Manganese oxidation site in *Pleurotus eryngii* versatile peroxidase: A site-directed mutagenesis, kinetic and crystallographic study. *Biochemistry* 46:66-77.

Sáez-Jiménez V, Acebes S, Guallar V, Martínez AT, and Ruiz-Dueñas FJ. 2015a. Improving the oxidative stability of a high redox potential fungal peroxidase by rational design. *PLoS ONE*10(4):e0124750. doi:10.1371/journal.pone.0124750.

Sáez-Jiménez V, Baratto MC, Pogni R, Rencoret J, Gutiérrez A, Santos JI, Martínez AT, and Ruiz-Dueñas FJ. 2015b. Demonstration of lignin-to-peroxidase direct electron transfer: A transient-state kinetics, directed mutagenesis, EPR and NMR study. *J. Biol. Chem.* 290:23201-23213.

Sakai T, Sakamoto T, Hallaert J, and Vandamme EJ. 1993. Pectin, pectinase and protopectinase: production, properties, and applications. *Adv. Appl. Microbiol.* 39:213-294.

Saloheimo M, Paloheimo M, Hakola S, Pere J, Swanson B, Nyyssonen E, Bhatia A, Ward M, and Penttilä M. 2002. Swollenin, a *Trichoderma reesei* protein with sequence similarity to the plant expansins, exhibits disruption activity on cellulosic materials. *Eur. J. Biochem.* 269:4202-4211.

Salvachúa D, Prieto A, Martínez AT, and Martínez MJ. 2013. Characterization of a novel dye-decolorizing peroxidase (DyP)-type enzyme from *Irpex lacteus* and its application in enzymatic hydrolysis of wheat straw. *Appl. Environ. Microbiol.* 79:4316-4324.

Samworth RJ. 2012. Optimal weighted nearest neighbour classifiers. *Ann. Statist.* 40:2733-2763.

Sanderson MJ. 2003. r8s: inferring absolute rates of molecular evolution and divergence times in the absence of a molecular clock. *Bioinformatics* 19:301-302.

SanMiguel P, Gaut BS, Tikhonov A, Nakajima Y, and Bennetzen JL. 1998. The paleontology of intergene retrotransposons of maize. *Nature Genetics* 20:43-45.

Scheibner M, Hulsdau B, Zelena K, Nimtz M, de Boer L, Berger RG, and Zorn H. 2008. Novel peroxidases of *Marasmius scorodonius* degrade β-carotene. *Appl. Microbiol. Biotechnol.* 77:1241-1250.

Seppey M, Manni M, and Zdobnov EM. 2019. BUSCO: Assessing Genome Assembly and Annotation Completeness. *Methods Mol. Biol.* 1962:227-245.

Shah F, Nicolas C, Bentzer J, Ellstrom M, Smits M, Rineau F, Canback B, Floudas D, Carleer R, Lackner G et al. 2016. Ectomycorrhizal fungi decompose soil organic matter using oxidative mechanisms adapted from saprotrophic ancestors. *New Phytol.* 209:1705-1719.

Sidlauskas B. 2008. Continuous and arrested morphological diversification in sister clades of characiform fishes: a phylomorphospace approach. *Evolution* 62:3135-3156.
Stamatakis A. 2014. RAxML version 8: a tool for phylogenetic analysis and post-analysis of large phylogenies. *Bioinformatics* 30:1312-1313.
Sugano Y, Muramatsu R, Ichiyanagi A, Sato T, and Shoda M. 2007. DyP, a unique dye-decolorizing peroxidase, represents a novel heme peroxidase family. Asp171 replaces the distal histidine of classical peroxidases. *J. Biol. Chem.* 282:36652-36658.
Sundaramoorthy M, Youngs HL, Gold MH, and Poulos TL. 2005. High-resolution crystal structure of manganese peroxidase: substrate and inhibitor complexes. *Biochemistry* 44:6463-6470.
Talavera G and Castresana J. 2007. Improvement of phylogenies after removing divergent and ambiguously aligned blocks from protein sequence alignments. *Syst. Biol.* 56:564-577.
Thompson JD, Higgins DG, and Gibson TJ. 1994. CLUSTAL W: improving the sensitivity of progressive multiple sequence alignment through sequence weighting, position-specific gap penalties and weight matrix choice. *Nucleic Acids Res.* 22:4673-4680.
Ullrich R, Nuske J, Scheibner K, Spantzel J, and Hofrichter M. 2004. Novel haloperoxidase from the agaric basidiomycete *Agrocybe aegerita* oxidizes aryl alcohols and aldehydes. *Appl. Environ. Microbiol.* 70:4575-4581.
Vanden Wymelenberg A, Sabat G, Mozuch M, Kersten PJ, Cullen D, and Blanchette RA. 2006. Structure, organization, and transcriptional regulation of a family of copper radical oxidase genes in the lignin-degrading basidiomycete *Phanerochaete chrysosporium*. *Appl. Environ. Microbiol.* 72:4871-4877.
Varga T, Krizsán K, Földi C, Dima B, Sánchez-García M, Sánchez-Ramírez S, Szöllosi GJ, Szarkándi JG, Papp V, Albert L et al. 2019. Megaphylogeny resolves global patterns of mushroom evolution. *Nat. Ecol. Evol.* 3:668-678.
Varnai A, Makela MR, Djajadi DT, Rahikainen J, Hatakka A, and Viikari L. 2014. Carbohydrate-binding modules of fungal cellulases: Occurrence in nature, function, and relevance in industrial biomass conversion. *Advances in Applied Microbiology, Vol 88* 88:103-165.
Volc J, Kubátová E, Daniel G, and Prikrylova V. 1996. Only C-2 specific glucose oxidase activity is expressed in ligninolytic cultures of the white rot fungus *Phanerochaete chrysosporium*. *Arch. Microbiol.* 165:421-424.
Vrsanska M and Biely P. 1992. The cellobiohydrolase I from *Trichoderma reesei* QM 9414: action on cello-oligosaccharides. *Carbohydr. Res.* 227:19-27.
Wariishi H, Valli K, and Gold MH. 1991. *In vitro* depolymerization of lignin by manganese peroxidase of *Phanerochaete chrysosporium*. *Biochem. Biophys. Res. Commun.* 176:269-275.
Waterhouse A, Bertoni M, Bienert S, Studer G, Tauriello G, Gumienny R, Heer FT, de Beer TAP, Rempfer C, Bordoli L et al. 2018. SWISS-MODEL: homology modelling of protein structures and complexes. *Nucleic Acids Res.* 46:W296-W303.
Wattam AR, Abraham D, Dalay O, Disz TL, Driscoll T, Gabbard JL, Gillespie JJ, Gough R, Hix D, Kenyon R et al. 2014. PATRIC, the bacterial bioinformatics database and analysis resource. *Nucleic Acids Res.* 42:D581-D591.
Whelan S and Goldman N. 2001. A general empirical model of protein evolution derived from multiple protein families using a maximum-likelihood approach. *Mol. Biol. Evol.* 18:691-699.
Whittaker JW. 2005. The radical chemistry of galactose oxidase. *Arch. Biochem. Biophys.* 433:227-239.
Whittaker MM, Kersten PJ, Cullen D, and Whittaker JW. 1999. Identification of catalytic residues in glyoxal oxidase by targeted mutagenesis. *J. Biol. Chem.* 274:36226-36232.
Whittaker MM, Kersten PJ, Nakamura N, Sanders-Loehr J, Schweizer ES, and Whittaker JW. 1996. Glyoxal oxidase from *Phanerochaete chrysosporium* is a new radical-copper oxidase. *J. Biol. Chem.* 271:681-687.
Wicker T, Sabot F, Hua-Van A, Bennetzen JL, Capy P, Chalhoub B, Flavell A, Leroy P, Morgante M, Panaud O et al. 2007. A unified classification system for eukaryotic transposable elements. *Nat. Rev. Genet.* 8:973-982.
Wong DWS. 2006. Feruloyl esterase - A key enzyme in biomass degradation. *Appl. Biochem. Biotechnol.* 133:87-112.

Xu G and Goodell B. 2001. Mechanisms of wood degradation by brown-rot fungi: chelator-mediated cellulose degradation and binding of iron by cellulose. *J. Biotechnol.* 87:43-57.
Yang ZH. 2007. PAML 4: Phylogenetic analysis by maximum likelihood. *Mol. Biol. Evol.* 24:1586-1591.
Zámocký M, Hallberg M, Ludwig R, Divne C, and Haltrich D. 2004. Ancestral gene fusion in cellobiose dehydrogenases reflects a specific evolution of GMC oxidoreductases in fungi. *Gene* 338:1-14.
Zámocký M, Hofbauer S, Schaffner I, Gasselhuber B, Nicolussi A, Soudi M, Pirker KF, Furtmüller PG, and Obinger C. 2015. Independent evolution of four heme peroxidase superfamilies. *Arch. Biochem. Biophys.* 574:108-119.
Zelena K, Zorn H, Nimtz M, and Berger RG. 2009. Heterologous expression of the *msp2* gene from *Marasmius scorodonius*. *Arch. Microbiol.* 191:397-402.
Zerbino DR and Birney E. 2008. Velvet: algorithms for de novo short read assembly using de Bruijn graphs. *Genome Res.* 18:821-829.

III. DATASET AND FILE S2 LEGENDS

Dataset S1. Classification and abundance of repeats in 52 Agaricomycetes species.

Dataset S2. Consensus sequences of TE families in 52 Agaricomycetes species.

Dataset S3. Repertoire of glycoside hydrolase (GH), polysaccharide lyase (PL), carbohydrate esterase (CE), and glycosyl transferase (GT) CAZymes; oxidoreductases classified as auxiliary activities (AA) in CAZy database (www.cazy.org); *Candida rugosa*-like versatile lipases (VLP); unspecific peroxygenases (UPO); dye-decolorizing peroxidases (DyP); and carbohydrate binding modules (CBM) in the genomes of 52 Agaricomycetes from different orders and with different ecologies and lifestyles.

Dataset S4. Gene copy numbers of both the 62 enzyme families identified as directly or indirectly involved in plant cell-wall degradation and 24 enzyme families participating in the amino-acid metabolism (which are not expected to be involved in lignocellulose-decay patterns) identified in the 52 Agaricomycetes species (nodes 1 to 52), and reconstructed at the nodes of the time-calibrated phylogenetic tree (nodes 53 to 103) of **Fig. 4**: i) 50 families of carbohydrate-active enzymes (CAZymes) including 38 glycoside hydrolases (GH), 5 polysaccharide lyases (PL), and 7 carbohydrate esterases (CE); ii) 10 families of oxidoreductases including laccases *sensu stricto* (LAC) plus novel laccases (NLAC), of the multicopper oxidase (MCO, AA1) superfamily, class-II peroxidases (POD, AA2), glucose-methanol-choline oxidoreductases (GMC, AA3), copper-radical oxidases (CRO, AA5), lytic polysaccharide monooxygenases (LPMO, AA9, AA14 and AA16), benzoquinone reductases (BQR, AA6), unspecific peroxygenases (UPO), and dye-decolorizing peroxidases (DyP); iii) 1 family of *Candida rugosa*-like versatile lipases (VLP); iv) 1 family of carbohydrate binding modules (CBM1); and v) 24 families involved in the amino-acid metabolism including glutamate dehydrogenases (EC 1.4.1.2), dihydrolipoyl dehydrogenases (EC 1.8.1.4), ornithine carbamoyltransferases (EC 2.1.3.3), 2-isopropylmalate synthases (EC 2.3.3.13), homocitrate synthases (EC 2.3.3.14), cysteine synthases (EC 2.5.1.47), cystathionine gamma-synthases (EC 2.5.1.48), methionine adenosyltransferases (EC 2.5.1.6), aspartate transaminases (EC 2.6.1.1), alanine transaminases (EC 2.6.1.2), prolyl aminopeptidases (EC 3.4.11.5), asparaginases (EC 3.5.1.1), arginases (EC 3.5.3.1), glutamate decarboxilases (EC 4.1.1.15), ornithine decarboxylases (EC 4.1.1.17), aromatic-L-amino-acid decarboxylases (EC4.1.1.28), tryptophan synthases (EC 4.2.1.20), 3-isopropylmalate dehydratases (EC 4.2.1.33), threonine synthases (EC 4.2.3.1), threonine ammonia-lyases (EC 4.3.1.19), argininosuccinate lyases (EC 4.3.2.1), glutamate-ammonia ligases (EC 6.3.1.2), asparagine synthases (EC 6.3.5.4), and propionyl-CoA carboxylases (EC 6.4.1.3).

File S2. Interactive phylogenetic 3D pPCA representing changes of fungal lifestyle through evolutionary time in the orders Agaricales, Polyporales, Russulales, Boletales, Amylocorticiales and Atheliales. The multivariate phylomorphospace shows the distribution of species according to the composition of their enzymatic machineries responsible for plant cell-wall degradation (described in **Fig. 1**) and phylogenetic relationships (based on the time-calibrated tree of **Fig. 2** *left*). The species are shown as spheres using the color code assigned to each lifestyle in **Fig. 1**, with branch colors indicating the order to which they belong (blue, Agaricales; red, Polyporales; orange, Russulales; brown, Boletales; green, Amylocorticiales; and pink, Atheliales).

IV. LIST OF ADDITIONAL SUPPLEMENTARY FILES

Supplementary **file S1** (this document), **file S2**, and **Datasets S1** to **S4** are available at *Molecular Biology and Evolution* online (http://www.mbe.oxfordjournals.org).
