# Supplementary material for: Genomic Analysis Enlightens Agaricales Lifestyle Evolution and Increasing Peroxidase Diversity
Source: Mol Biol Evol. 2020 Nov 19;38(4):1428–46. doi: 10.1093/molbev/msaa301 (PMC8480192; doi:10.1093/molbev/msaa301)
Supplement: msaa301_Supplementary_Data [file Dataset S2.pdf › Dataset S2.pdf]

>Agabi_varbisH97_2_Cluster11#LTR/Gypsy
TCGGAGCCATTGCTTAAATATTTCATGTGGACACGAAACCTAGTCGGAGGGTATTGTTATGATCTCCGCAGTA
TGAGAACTGTTTCTCTTTCATGTTACCTGTTTTATCTTTTCTCTCTCATGATCAATCTTCTATTCTCATGATT
GATCATCCTCCTTCCTAGTTTGTACTTTCTTTCTCAGTATTACTCATCTACCTCGGTCATGGTTACTCAGCCC
GTTCTATAGTTAGTTACCTCAGGTAGATAGTCTTAGTATAAGTAAGCGTAGATACTCTCTGTATTTTCCTATG
CTTTACCAACTTGCTTTTCCCCTTACTCGCCCTCAAGTCTTTGCCTTTGCTGCAACCTGAGCTTCGCCTTAAG
GTCGTAACAGAAAAGATATAACAGGTAACATGAAGGATCAATCAAGGGAACAATAAGAATGATCATGAGAAGG
AAAAGACAAACAAGGTAACGTCAAAGAGAAAGCATAGATAGAAATAGAATGACGTATCCTGATGATAGGAATA
GAATGGATAAGGTCCGCATCAGATAAGGATTAGATAAGATCAGATAAGATTTAGAATACTCGGATTTGTTCCA
CAATCATAACAGAATCTGGTTGTCAAGTAAACACCTCAGGACTTTGTTTGTCCCAAAGTTGTGTGTCATGGAG
GTGGGATAATTGTTAAGACTATTACTGGAAAGGGATCTCTAAGCCGGAAAAGACTTGGCAAGTAAACAGCTTT
GGCCTTAGCGACACCAGAACGGTGTGCTATGCCTTCCAACTAAGTCAGTAGCCGATTTGAAGATAGAGCTGAG
CGACCTGCAGAATGCAGAATCATTTGAGGGTGAGAGAGAGATCAAGCTGAGGATAGCGAGGAACAATTGAAGT
TGATTTTTAGACTGAAGTACCGTACAGAATATCTACAGCTTAAATGCATGAAGACTGAATAGAATATGAAAAC
GGAAACATGAAAGATGATAGCACAACAATTGCGCAATGGTTCAGGATGAATAACAGAAAGGAGTCTTTACCTT
AAGGGATTCTGATGGTACAGTGGAAAGGAGGCTTTGAAGGGTAAAAGTCTTGCGATGGAAATAACAACCCATG
TTGTGTTGGTCGTAAAGTGACCATGGGAGCACAACCACAATACCAAAGGTACTGCAGCATTCCATCGGTCAAC
TTGACCAGTAAGTCAGAATTGGATTGCCGGGATTGATGCAAACAGGATGAGTTGGAATGGAATATCCGGGAAG
AATCAGAAACAATGCCGAGAACAGGATTGGTGGAGTTAGCATGTTCCAGATATAACAATAATCCACCCATTTG
ATATTGTAGTCGGGACCTGACTGTGGTATGAAAGGGGAGCTGGGATGCTCCTGGTTATATGCTGCTATGGCAT
CACAATACATGTATGGGGTTGCCAATACAACTGCAAAGTAGCGAGCGAAAGTCACTTGTTGATATCTATCCCG
GGGAGCAAGCGCCCGCAGGAGACTGTATCACAACCTCTATATTTAAATAATACACATGTATTATATAAACATT
ATGCAAATGTTAGCGTAAATATAATTTACATAGTACATATGTAATATGTTTTACATATTTAAACTTTATGCAA
ACAAAAGTCTTAAATTGAGCTTTATTGCATTTTTAATTTACATATGTTTTAAATAGTACACATGTATTATCAA
AGTGATATTTACGGTAAAGTTTGCATAAAGTTTGCATAATACATGTGTACTATTCAAATATAAAGGTTGTGTA
TCTGAAAAGAGACCTATGGTGAACACTGTAGGCATGGTTCATGACAATACTGTGGATCTGGTTGTTAGATCCT
GGATGAGCATGCAGTGTGATCCACTGCCCAACATATTCTATCTCCAGTATCCACTTAGTTTTTGGTGGAGGAA
CACTTGTTCCTCCTGACGGAGCATATTTGTTGCATCTGGTCAGGACGCTGGAGTAATCACAGGGGACAAAATG
TTCATCCCAGGCTCGAGAGTTATCAGGGACATTAGGGGCATATTTCTTGTCATTGAATTGAAGGTCAACAGTG
GCCTGCATTGCCTTTTCCCTAAAAGTGGGCATGACACGCCCAGATATTCCACAGCCTAAAAACTGACAAAAGA
GATGATAAGCTTCTACATGCGTGGACTCCTGTGCATAAACATTATGGATCATCCTCACTAGCTGCTCAACATC
CTATAGTGACATGGGTATCTCCATCAGTGCCTTCTTGTAGAGAGGATTCTTTTTATTGGGCCTCCAAGGGTCT
CTTCCCTTAGTGAGCTTGTCAGCATTGCATGAGGCTACAGTGGATTGTCCCACCACGACTATGTTGGTGAAGG
GGCTGTGATACAGCTGCAGGTGTAGCATTCCTCTTAGCACATTGTTACGTGCGAAACTCTTGTTGACCTTAGC
CATTCCCCAGAAGTTGGGGATTTGCATCAGAAAAATGTAACAAGCCTTATTCATGCCTTATAGGCCTTAGGCA
ATTGCATAAGGCATAAATAAGGCAATTTTGCCCAAGGCAGAGTCTAATCTTAGGTTTGACTTAGGAAAGCCTT
AATATATAGACATAAGTATACAGTATTTCATAAGTTATTTTATAAGAATGCTTAAGGCAATATTGTCATTAAG
ACTCTCCAGATCATCTTGAGTTTATGCATAGGTGTGGTATAACAAAATCACATTCAAATATATTATATATAAT
ATAGTAATATACAATCAAAATTCTGCGGAAAATTTTCCTATTTAGGAAACCAAAAAAGCGGACACTAGGAACA
GACATAGCATCAGCAAAATATCCAAGTCTTATATGGACTTTTTTAGTCTTATTCATGTCATACCTTATTTCAA
ACTTATTTTGGGAGCACTTGAAGGCAGAGTTGATAGCTAAGTTAAAAAAAAGGTGATAAGTTGCAATAATAAC
ACACAAAGCAAAAAAGTCTTGGGAAATTGGCACTACAACAGCTATTTGCTAGTCGAGATTGTTTCGCTAAACT
ATAAATTGTAGCGGCGCTTATGTGCCAGCCCCCGACTCTGTTAATGTTAAACAGTTTTATAAAGATTAGATTG
TATTAAAAGATCAGAACTACTAAAATATATTCATGGATCAGGTGTAATCAAAAGAAAAAAGAAATAGAAAGGA
ATGAAATTATTCAGGACTATTTAACGGATGTCTTTCGCTTGGTTACTCCTTCACTCTTGCTCTTGACCACCTT
TTTCCTCTTCTTGGCTCCTGATTTAGACAATCTTTTCCTTTTCGCCATTTGATCAGAGGTATAACGAATCAGC
TTGGCTGAAGGTCTGGGATTTTTGTTACATCGGCATCCACATTTTTGATTGCACTTTGTCAGTTTTAAGGGTG
GCAGAAGTGATCACTGCAGAGACGAATATGTGGAAGCTATATGGGCCAAGAGTGGTTTTGTGAAATTCATCTT
TGACGTCTAACTCCATCAAAGTTATACTCTTCCCAAAGTTTCAGCAAACAAAAGACAAAAATAAAGGAAAATC
ATACCTCAATCACAACTGGCCAGGATATATTTGGCTGTGCAACAATGGGCAAATAGACCTTATAGGAGTCTTT
CAAACTTCTTCCTCCCTTCAACTTACTCAACCAATATTGGATAGCATATATGTTGAGGATTTTATACCTGCTT
TGAACTCTCTTCACAACCTTTATATTTCAATAATACACATGTATTATGCAAATATTATGCAAACATTACCATA
ATTTTAATTTGCATAGTACATGTATATTATCTAAAACATATCTAAACCTTATGCAAACAAAACCTTTGAAATG
AACATTCTTGAATTATTTTTTTACATATGTTTTACATAATATATGTGTACTATGTAAATTACATTTACGGTAA
TGTTTACATAATGTTTGTATAATATATGTGTATTATTGAAATATAGAGGTTGTGCTTCTTGAAAGAAATATCT
TCCATCGGATTTGATAACAACACAGAATATGCACCTGTCAACCCAGAAACTAGGTTCAAAGAAACAAAAATGT
TATAAAGGTTTTAGGGTTGGAAGCAGTAATACTCACCACCATATCCATTCTCGTCAATCACCGCATTTTTGTC
TCCAAGATTATACAATTTCAACATATCGAGAAATGCTTGCATTGAATTGAAACGTGCTCGACAATAGCAAGAG

ACAGTGCTGAGGAAGTTAGTATGAGCAGCAACATTAAATCGGAATGGTGTTCCACTGGCAGTAGGTAGATGCT
TCCCATTTGGTACTATTCCACCATTTTCTATCCTTAGGTGTTCCTCCTGATACAAGACCAAATTGGTACAAAA
TCAATTCCATAAGTCGGGCGAGTTGCATATATCAAAGATAGGAATGAGGGGAAATATACAAGCATTTGCGAGC
ATATCATTCGTCTCTTGAGCACCATAGCCATTAAAAATGTCTTGCCGTTCCTTAACAATGTTCCAGACTGGTC
GACAACCACGAGCTGTACAAATGGTGTGTTGAAAGTGTTCGAGTCTCTCGAGAAATTGGTCAAGTTCAGAATC
AATATAAGGACCATGGGACGAGCTCCAAAGAAAAGGCCAAATCTTAAAACAAGTAAATAAATGTACCAAATTA
CAATAATTAATCAACTTACCTCACTATCAGGTTCAAGATCTGATTCCCAGAGGATATGACGTAGAGCCATGAC
ATGAAATGATATCATGACATTGTGATCACAGATCAGAGTCCAGTCAGGACATTCAAAAGCAGCCTGGATGGAC
AATATCCCTTCCCTAAGTGGAGAGCCCATCCAGTAATTAGGTGGAATCTTATATCCGAAACTTTTCTCACCTC
GTTTGATGGGAGGTTGAGATATAATGTCGTGATATGCTTCCATAGAAATCCAGAAGCAATGTCCTTCCAAGCT
GAACCCAACATGAAATAAAAACTATATGAATGAAAATTGAACTAACAGTTACAAAAGGAAATAAGAAACTTAC
AGTACCTGATTAATGACAATCATAAGCTCTTTTCCCCTTGATAAAATACAAGGGAGGCCATGAATATTGAGAC
TATTAATGATCTCAGCCGCCGGTTTACCAGACAACGCTGAATAGGTTTTCTAAAAAGATATGAATCAATAATA
TCATATAATTAGTCTCAGGGAAAGAAAGGGACATACTTTCAATTCATTAACTGGTTTATTTTTCAAGAAGAAT
AGATTCACAGGTTTTTTGGTCAAGCTGCAACTGGAACAAGCAGACAGATTGATGGATGTCCTGAATGATGGTG
AAATACAGTATAAGATGTCGGAAAAGGCTCAAAATAATGATCATTGAGCTGGCAGTGGTCTAAAGTGACAGTT
TGACATTGGCCTTCTATAGACGCACAACATATCGGATGGAAATGCCATGTGCCAGCACCTATGGAATGTGAGA
GTAAGGTTAGGGTTATAGTTGAATATCATTGACTTACCAGTAATATGGATTTTGTCAAGCATGTGAGTGATCT
CACGACGGAAAAATGACAATACCAGCAATTCTGGCATTCTTAAGGTACTGACAGTGGATGGAAGGTTGAAAAA
AAGGGACACAACAGTTGAAAAAAAAGAACTAAGCGCTAAACGTGCAGGGTCACTACGGCTTCATCGGAGATTT
GTCCGGTTACCCGAATTTCCTCTCCTTATCCCCATGTTTGGCTTTCCCTTACTACGGATTACCTACATCAGCA
AAAAGTCGCACAACTTATGCAAACTTATGAAAAAGTTGCCTTATGCTTGCCTTATAAAGACTAACTTAAGGCA
CATAAGTCTCCAAAATAGCATAAGTTCATAATTTTTCATAAGCCAGACAAGAAGTTTAGACATAAGTCACACA
TAGGGCCCACCAGGGGTAACCCTAGGGTAGATCACAGGCTACCCTTACCCTTACCCCAGATTACCCTACCCTT
CACCAAGGGTAGAGGGTTACCCTTGAAAATTGCAAGGGTTACCCTTAAATCAGGTCATGTGATGAAAGGGTAC
ACTTGTACCATCAGATGATATAGGTTACACATCAGTTCTTGCCTAATTCACACAAATTCACACTAAACTAATA
TTGAAAGTGAATGATTTCCCAGAGCTCGGGCACAGCTACCTACCCCTACCCTTAAATTTGACAGGGTAACCCT
TCAAAACAGAGGGGGTAGAGGCAAGGGTAGCCAATAGCCTACCCTTACCCTCACCCCTGGTCCTACCCTCAAT
GATGCCCTAGGGTTGAACCTACCCTTGGTGGGCCCTAGTCACACATTAGTTCATAAGTTTATCATAAATTCAT
AAGTTGGATACAAAGAAAGCATAAGCACAGGTCATTTCATAAGTCAGACCAAAGGTTAAATCACAAGTCAGAC
AAAATTTCATAAGTTTATTATGAATTCATAAGGCTACTGACAAGTCAAACAGTATCTCATAAGTGAAACATAG
GCTCATAAGTCAGATAACAAGGCAAGAATCATAGCATTATAAGAACATTATGCTGTATGTCTTGTTCTACTAC
TCTTAAAATTCCGGAAAAATCACGACAAATCTTAGAATATTAAAAAAACATAAGTCTATATGTATGTTTTTTG
TCGAAAACATACCCATTATGGAGATATCAAGATTCCATCATGGGGTGGGGGGTTATTGTCAACTACAGATAGA
CTCGTCATAGAGCCTGCTCTACTAACCGGAAAATTCTGGAAAAATCATGACAAATCATAGAATATTAAAAGAA
TATAAGGTTATATGTATGCTTTTGTCGGAAAACATACCAGTTATGGAGATACAGAATAACATAACAAGAATGC
CATGTTTTCTACTGGTTACTGTGCCGATTCTGACGATTGAAGAGTGTAAGTATATGCTTTCAAGGTCCAGGAA
TTTGAATCTGATTTTAGATTTATCATCCGATCAATATTTGAGGAGCTACAGCGCAATCTTGTGTTTATTATAT
GTTAAATGTGCAATATCTCAATAGCCTTTCATCTAACAAGAAATCTAAGAACATATTCGGACTCCCCGACCTC
GAAACTATAGGAATACACTTTTAGATCATTAAAATCCATGCAGAAATGACCCGAAAATATCTCCTACCTATGA
TTTGAGGAACTTTGACTCGAAAATCCCAAGTCCAGCAGTCATGTGACATCATGTGACATGTTGAACTTCTGTT
TCGAGCACAACGTTGATCCTTCCTCATCACCACCATCATTCCTCCCCTCCCCTCCCTCCCCTTCCCTCCTCGT
TGCTCTCCTAGGCTTATATTCAAGTGAGTAACTTCCCTTCCCTTCCCTTAGCTCTCTCCCAGCTAATTCTAAG
CTTGTTTAGGACACTTTTTTGCCTATTTTGGACGCTTTTTTGGGGCTATTTTGGGAGGGTTTTGAGAGGAGAT
GTGAGGATGATATGTGGATGAGTTTTGGGGGGATCTCTAGTCTTTGGTGAGTGCTTTTTGACTCTTCATCTAC
TCATACTCATTTGTTACTTTTAGAATCACATTTCTCATCATTTTCCCCCCATTTTCCATTTCTACTCCCCTGG
CTGCTGTCATATCCCTCTTGTCCGCTTTTTTCAGGGTCTATTTCCTCTTTAGGAAGTCTGTATTATACATGAT
GTATTGTATTTACTATATGAATATAATTCTTTGTTCCTTATGCATGATGATGTTCCTTATGATTGACCTTATA
CTCTCTTATTACTTCTTACTGTCTTCAGTACTTACTTATGCAACTTAAGATAATTCCTCATGACTTATGATTT
ACTTAAGTATTGCATAAGTCAGAACCTCAGACGGTGACATGCCTTATGCAAAAATGACTTATACTGACTTATG
ATTTTTATAAGTTTGCATAAGTCAAATTTAAGTCACTTTACCTTTTCCTGATGTGCCGAAGGAGATAAAGTTT
GGTTAGAAACGACGAACATTCATTCCAATCGTCCGACTTGGAAACTAGACCACAAACGATATGGACCTTTCGA
GATCTTGAAACAAATCGGCGATCGATCTTACAAACTGAAGTTACCTGATACTTGGGCAATACACGACGTCTTC
CACACATCACTCCTAACGAAAGTCCGAAACCCGGAGTTTGACAGTCAGAAGAAACCCACTCCACCTCCACCTG
ACATTATCAATGAAGAAGAAGAATATGAAGTTGAAGAAATCCGAGGACACCGATGAAAAGGCCGAGGAATACA
ATTTTTAGTTCACTGGAAAAGTTATGGAAACGAAGACGACACATGGTTACCACGCTCTGCTCTAACAAATTCC
GCGGACATCCTAAAAGAGTATCATGAAAGACATCCCTCCCTATAAAAGGATATCTGAAACTTATAACCGGTAT
AGCATCGTCTATTGGCATCTGAAGAAGATCATCATGAGCAACCAACAAACCACAACCACCCGCACTGCCAATT

TCGGAGATTCTCTCCAACGACTCCTAACCATTCACAATGACATCACCGACCTCTTACTATTCCAGTCCAACGT
ACAACTTCCTCTTTTCATGAATCCTCCACCACATATCGAAATATCAGATGAAGAAAAAAGTCAATTAGAGGAG
CAACTCGAGAAGGAGAAGAGGTTAATGAAGCTGGTAGAAAATCTGTTGAGGATGGCAAAAGCGAACAACAGAG
AAGCAAAAGAAGCATTGTCACCTGGATCCACAACATGGACTACAAACAAAGATGGATGGGAGTAACAAGATCA
TATCTGGAATCTAGATCCCAAGAAAGGGGATAATTTATGTATAAAAACCCAACTCCTGTTCTCATTTTTATCA
TCATCTTTTGCCTCTTTATCCTCGAAACTCACAACAAACGTTCACCATGTCTAACATCAACACCCCTATCCCT
ATCTCCACCAACTTCCTCGACACTGACTTCCACACTGGATATTGCGAATCTATATCATCGATCAAAAATAGAC
GTTGAGCCTTGCTCATGTCCATCCAATCCCTAGCAAATATCCGCCAATTTGCCAAACATATTTCCTCCACCGT
CATTAGTGCTACATCTACTGCCATCAAAGTCAACCCGTACATCCAAAACCTGATCAGAAATTCTGCTGAAGCT
ATGGGATATATGACCGTCATCATGGAGACACTCCAAGCCGGAACTCAATGGGACATTGAATATTCTTCAACCA
AAGTCTGCGTTACTCTAGCCAAGGCGCTCAAACAATTGCCAGACGTAGGAAATCAAAATGAGATCGACGCAGC
CCACTCCATCGCTAACGCTTACTTATCCCTTCCATACCCGGCTCCTACCCGCAACAACTCTCCTGAACCCTTA
CCTATTCATCCTCAACCACCGAGTTCCAACAACAAAGATATCAACAACAATGCTCCCAAGCATCCCAATACAA
AAACCCCCATTGACGACATTGACGAAGAAGTCCATCGCCTCACTAATTTTTTGAAATCTGCTCCACCTTTCCT
CATTCCTGACGAATTCCTTCCACCAACCAATTATACTACCTTCGTTGACAAAGGAGAAGAAAACCTGTCTTAC
TGGAACGACATACTCAAAAGATTGTTCAAAAACAATCAACAAGAGGGAAAATACAGAGGATACTCAGTTGGAT
ACGCTGAACTAGGTGTTCCGAACGCTCCTCACTTCTTGGGTATTGATTACAACGGTAAATTCATAATCGCCAA
CACCAAGAAAGCTCTCTTCGGAATAATTGCTTAAATGTTTCATGTGGACACGAAACCTAATCGGAGGGTATTG
TTATGATCTCCACAGTATGAGAACTGTTTCTCTTTCATGTTACCTGTTTTATCTTTTCACTCTCACGATCAAT
CTTCCTTTCTCATGATTAATCATCTTCCTTCCTAGTTTGTACTTTCCTTCTCATTATTACTCATCTACCTCGG
TCATGGTTACTCAGCCCGTTCTATAGTTAGTTACCTCGGGTAGATAGTCTTAGTATAAGTAAGCGTAGATACT
CTCTGTATTTCCCTACGCTTTACCAACTTGCTTTTCCCCTTACTCGCCCTCAAGTCTTTGCCTTTGCTGCAAC
CTGAGCATTCGCTTTAAGGTCGTAACA
>Agabi_varbisH97_2_Cluster12#LTR/Gypsy
TGTTAGGGCGACTGTGACTGAGATGTCACAGGTCACACCTGAACTTAAGAACTTAAGAGTAAAGTGACAGAAG
TTGTATTAGGCGTGAACCTAAGAGAACTCGACGGAGTCTTCTACAAGTTTCTACCTACTTTATATACCTAGAC
TACGGCGCACAGACCAACTACCCTAGGTAACTATTTATATACTGACATGAGAAAAGAAGCACGTATGAGAAAG
TAAACTAATTATACATTTGACATGCGAAAAGAAGAGTGGAGTAAGAAGCAAGTAAACGAAAATAGTTTTGATC
ATAAAAACGTCAACAACACCAAACAAACCATAAAAGTGTCCACAAGTACAAACAACTCCTAAACCGGTAATCC
ACTCACACAAACCAATACCTACCAAAGTAATTCCCCAACATCGCATCACATGGGCATAGTACGAAGAATCCGG
CTATTGCCCAGTGTCGTGGTCCCCAGTATCCCCATCTCTGTATTACCCACGCCTTCTGTTCCTCATAGTCCCA
CATCGCTAAGCATACTCTCCCACACCGCCTGCACCGATGATCCGATAGGTTCCATCCTACCGGCCTGTCTTCT
CTTCCTATACCCGCCGCTCTCCTTCGCCATTCTACTTGTTCCGCATGATTCGGAGGTAAAAAAGATAAGATAA
GATCGATAAGCTCAATTGGTAAATCTGTAGCTTGCGCCATTTTAGCAGTATGCCGATAGTTTAGCCTCCGTCG
AACTGTAGCAGCAGAGGGAAATTATTATGTAATAAAATATGTGACAGATGTGACAGCGTGACGCCGTGACATT
CTGGCAAGGTTCTAACAGTACCCCTTCCTTAAAGTCGGCTCCTCCAGAGACGAACCTTCAAGACCTCCAAGTG
CGCCAATTTCGTTCCAACTTCTTTAGATATTCGGCGTCAAACTGTTTGTCATCCCATCCGAATAATGCTTTGG
CTGTGAACCGATTAGGTAGTTCCCTTTCTTGGAAACCTGTGTCATGTAACACGTGATGTATCCTATTGAGTTC
CCCATAGAGTTGGTGGAATTCAGTAATCAGTTCTTTGGCGTTAGCCAAGTTTTCTTCAGGTTCCCATGAATCC
GCTTCTGCCGTATATCCTTTCCATCGAACTAAGTACCGCTTCTTTCCGCGGAACCATTTGGCGTTGAGGATTT
TCTCAACTTCCCACTCTTGTTGTCCTTCTATTTCCACTGCCGGTGCCGGTTTACTCCCCTGTCCTTCTACTTG
TTCTCGGTAAAGTACTATTCAGCTGACGTTAACCACCGGGTGGATCCGAATAGATCGGGGAAGATCTAGCTCT
ACTGCATTGGGAGAAATGACCTTTTTGATGGTGTATGGTCCGATGAATTTCTCCGTAAGTTTCCGAGTAGGTC
GTCCCTGGATTTGAAGGTCCCGGGTAGCCAATAGAACTTTATCTCCTACTTTGTATTTTACCGCTTCTCCTCT
ACTTCTATCCGCATATCTCCTCATGTCATCTTGTGCCTTTTTTAGGGCCGCTTGAGTTTCCTCGTGTTGAAGT
TTCATGTGGTCCGCAAAGTCTGCTGCTGCCGGGTGATTACCTTGTCGTCGAAGATCAAAGCCAGTTCTCGGGT
GTCTTCCCACGTTGATCATAAATAGAGTTTCCTTTGTTGATGATTGTTCTTTATTGTTATAGGAAAACTCTGC
CGTTGCTAACCAATCAGGCCAATCAGTTTGTTTATGATCAACAAATGATCGGAGATATTGTTCTAGTTCTTGA
TTGGTTCGTTCAGTTTGGCCATCAGTTTGTGGGTGGAACGCCGTCGATAGTCTAGTGTCGATTCCCAACATTT
TATTCAACTCCTTTGTGAAATCTGCAGCAAACTGCGGTCCCCTATCTGAGATCACCGTTTCTGGTAACCCATG
CAACTTCCATACATGATCCCTGAACAGTTTGGCTAGTCCTAGCGCTGATGTCGTTTCCGTAGTTGTGATAAAA
TGAGCCATTTTACTGAATCGATCGCACACCACTAAAATTGAGTCCATTCCCTGAGATTCTGGTAATTTTACTA
TGAAGTCGACAGATACATCGGCCCATGGTTTTGTGGGAACAGGGTTTGGCATCAATTTCCCTGCCGGTGGTGT
TGGTCGATTCTTGTTTCGTTGGCATTGATCACATCCCTCTACGTACTTCTGGACTTGCTTAGTAACGCCTGGC
CACCAATAATTTTGCGTTACCATTTCTACCGTCTTCCATTGTCCTCCGTGTCCGGCAATGGGAGTGTCATGAT
GTAGTCGGATGATTTTCGTCCTCAGAGTTTCGTCCTTTGGCACATAGACTTTCCCTTCTTTCAACACTAATCC
TTCTTCTAACTTCCACTCGTCTCCCCTCAATGATTTCACCTTTGCTCGCTTCATCTCTTCTACCGCTTTTATC
ACATCATCTTCCTTTGCTTCTGATTTTCTGATTTCTTCTAATATTCCTTCTTCCTCGGCATTGATTAACATAT

GACCTTCTTCCATTGCTCGGATGGCGAAATATTCAGGTTTCAACAAGGTTTGATCCTTGTTATCGTCCTTTGT
CCCTTGTTCAAAATCAGGTCTTCTGCTCAACGTGTCCGCCTTCCCCATTGTCTTTCCCGGACGGAATTTTAAC
TCGAAATTGAATCTAGATAAGTATAGAGCCCATCTGGCTTGTCGTCGATTTAGTTTCTGGGCTTTTGTGAAGT
ATTCTAGATTACGATGATCCGTCCAAATTTCGAATTTATGTTTCGCCCCTTCCAGGAAGTGTCTCCATTCTTC
TAAACACTGCATAATCCCTAACATCTCCCTATCGTGAATGTCGTAGTTTCGTTCTGTCTCTGTCATAGATTTG
GAGATGTAGGCTACCGGCCTCCACTTTCCGTCTTCACATTTCATTGATAGTTCTCCGCCTGTGGCATAGGCCG
ATGCGTCAGATTCCACCTTTAGTTCTTTATCCAACTCTGGTGCTACTAGAATTGGATGTGACGTGAATTTTGC
TTTTAATGTTCCAAAGGCTTCCTCTTCTTCATCTCCCCAACTCCATTTCTTGTCCTTTTTGGTTAATGTGTGT
AATGGTTTTGCAATACTGGCGAACTTTTCAATGAACCGTCAATAGTAATTTGCTAGTCCCAAAAATTTTTGTA
CATCCTTCACGCATTTTGGAGTCGGCCATTCTACTACGTCTTTCACTTTCCTTTCCTCCATCTTGATTCCGTT
TCTCCCTATTATACATCCCAAAAATCCCATCTCGCCTTGTTTCCATTCACATTTCTCTGGCTTTGCAAATAAG
TCGTTGTCCATCAGTCTCTGCAACACCTCTTCCACTACTTTGTCATGTTCTTCTTCATTGTCTGTTCCTATGT
ATCAAGATATCGTCAATAAATACAGCTACTTTCCCAGTATTTACTAAGTCCCTAAAGAGTTCGTTCATCATAG
TCTGAAAAGTAGCAGGCAAATTTGTAAGTCCAAAAAACATAACGGTTGGTTCGAAAGCACCCATAAAAGTAGT
AAAGGCTGCCTTCCACTCATCTCCTTCTTTTATTCTAATGTTATTGTAACCCCAACGTAAATCTATCTTAGAA
AATAATCGTTTACTGCCCATAGTGTCTATAAGTTCAGAAATTAAAGGTAAAGGGTAATTGTTTTTAATTGTCC
AATCGTTCAAAAATCGATAGTCCTGTACCATCCGCTTCTTTCCATCCTTTTTTGGCACAAAAAACACCGGTGA
GGTTTGGGGGGATTTTGAAGGTCTGATATATCCCTTTCGGAGTTGGTCCTCGATGAATGCTTGGACTTCCTCC
TTTTCGTTTGGTGATAAGGGATATATCTTTCCTTTCTTGGGTTTGAAATCCTCTTTTAATTCGATAGCATGAT
CCCATGGTTTCCTAGTAGGCATTCTCTCTGATTCCTTCTGTTTGAATACCTTAAGGAACTTGTGGAACCGTCT
GGGTACAAGTTGTTCCACTGAGTCTGTGCTTCGAATTTCTTCTTCTTCCGGCATTTCCTCTGTCATTCCCCTC
ACCCACGTTTCCAGTTGTCCTTCTTCCTCTCTACTCCTATTTTTCCTTTGGGCGCTTATTCGTTTAGGACGTA
CCTGTCCAGCCCCCGCTTGTCCCGCGGTAGCTGTCTTGCTGTGATCTTCTTCTAGACGTTCTGCCTTATCCCC
ATTTAATCGGTCAGCCACGTCACCGGGTGATTGCGCCCACCCCTCAGCTTTCTCTTTGGGTTGTTGCGCCAAC
CCCTCAGCTCTACCATTAGCACGCCTGTTATGTGCCAATTCCCTAGCGAATGCTGCTTCAACTCCTAGTGCGA
TCCCTTCGATGATGTTCCTGGGAGTGGTGAATCTCCCTACTGCCTTGTTCACTCGTTCGTATCTTTCTTCCTC
ATCTCTTATTTCTTCCTGCTCCTTTTCATATGCTTCTATCTTGGCCATAGTTGGCCCTTCCTCCTCGTCATCA
CTTCCTTCGTATGGCATTCCAAACTCGTCTAAGGCAATTTCCTCTCTTGCTGGGCATTTGTGTTTGCTCAACA
AGTGTTCTGATGTTTGGGTTCCACATTCTTCGCATCTATACTGCAACACCGTTCCCCAGTTTGATTTCTTTGG
GAAGGAAACTAATTTCCAGCCTGCATATTGCTTGTGTGACATGTCAGGCTTTTCCCTTTTTTCTTCTTCTCGC
GCTTCTCTCATCAACTCCCTTGTTGATTTCTTGGGTTCCCCCACCCTTCATATTTCCTCCTGTTTTTCCTTTG
CTTTTCCTTTGTCTTTGTACCCACACCCATCTGGGCACCTGGTCATCTTTACCTCTCCTTTCTCCCAGTCGAT
TTCAGGGTTATGCATCTGTAGCCAAGGCATTCCTAATATGACTTTGTTTTTTCCGAGCCCGCATATTTCAAAG
TGCACTCTTTCTTTATGCCCCTTGTAGTATATGTTTGCAGCCACTCGATGTGTTACTGCTCTAGATGCGTTGA
TTGTTCCGTCAACGTTTCTGACCGGAATAGGTCTTTCTAAGGGTTCTAGCTTAAACCCTTCTTCTTCCGCAAA
TGCCTTATCCATGAACATTCCTGTCGCCCCGCTATCTAACAACGCCTGTACTCCAACTCCATCATGTGTATCC
AACTTCTCCACTCCCACGTTCAGCTTAATTTCCCTTAAAGGTCGGATTGTCCTTACGAGGAAACTCTCCTCTC
GTACAGTGTTACAAACATTGATTTCGTTACATAGTACATTGAATTTGTTGTAGGAAGTCAAACGAGGAGACTT
TGCTTCCTTTACTCTTTTAAATTCTTCTGCTCTTCCTCGAACATCCTCTTCCAATATTCCCGCTGTTCATCCA
CATCTAAAGTTCGCACATCTCGAGTTGGAGCCTTGCTCCAGCAATTCTTGGCCATATGCCCCAACTTTCCACA
CTTGTAACACTTGATAGGACCTCGACTGGGTCCTGCTGCGGCGTCGACATCCATCGCGTTGGGATCTCGACGT
TGAGGCTGAGGCCTCACGAAAGTGTTGTTGGAGGTGTGGGTGCGTAGTTGGGGCTTAGCTTGCCCCCGACGAT
AATATTCTTCCTCTGCCTTAGCTTGTCTCCACTGCCTATCTAATTTCATGCTCCTGTCGTACCAACTCCTGAT
GGTGGTTGGTGGATTTTCTGACTCCATCAACGCCTTTCTAATTCTTCCTGATAAACTTCTCTTGAATTCTTCG
ATGAGGGCTCTTCCCTCGTATCCGCTATCTCGTGCTATCCTCTTGAATTCCTGTACATGTTCTTCCGCGCTCT
TGCTTCCTTGCTCGATTAACCTCAACTTCCCTACTGCCGTACTCTCTTCGTCTGCATCTCCGAAATTGTTCCG
AAGACTATCAAAAAGCTCCTCCGTGCTGGCTGGAGCTTCTTGTTCGCCGTTTTCAATCATTTCCATGATGTTC
TCTTTCCAAGACTCCGCTGCTCCTCCTTGTACGTATGATAGTATCCATGTGATTTGATCCCCTTGTAGTCTGT
CTACCATTTTTAAACTCAAGTAAATTTTGCAAGCTGTTATGAAGCCACTCAGCTCCTTCCCATCTCCTGAGAA
TATTTTTGGTCTTGCCACATCAATTTGTGGCGGTACTACTTGGATGATGGGGGTTTCTGCGTCGTCCCCCGGT
GGACCTTGTGGACCTGGCTGTTGGTTGTTCTGCAGCTGGGCGAGTTGCTGCTGCAGTTGGTTGATGAGAGCTT
GTTGCGCGTTTACTTGGTCCATGATTTGTTGGGCAGTAGACATGTTTGGCTCGTGGTATTCGGGGGGAGGTAC
AGTAGTTGATATAGGGGTAGGGGGGATATGGAAGGTTCTCGTTGTTCTAGTTCTGATTCCTCGGCATCAGGAA
AAGGTAAAGTGACTTAAATTTGACTTATGCAAACTTATAAAAATCATAAGTCAGTATAAGTCATTTTTGCATA
AGGCATGTCACCGTCTGAGGTTCTGACTTATGCAATACTTAAGTAAATCATAAGTGCATAAGTCACAATATGG
TCATAAGATTCAAGATTATCATAAGTCATGAGGATTTATCTTAAGTTGCATAAGTAAGTACTTAAGACAGTAA
GAAGATGTAAGAGAGTATAAGCTCAATCATAAGGAACATCATAATGCATAAGGAGCAAAGAATTATATTCATA
TAGTAAATACAATACATCATGTATAATACAGACTTCCTAAAGAGGAAATAGACCCTGAAAAAAGCGGACAAGA

GGGATATGACAGCAGCCAGGGGAGTAGAATTGGAAAATGGGGGAAAATGATGAGAAATGAGATTCTAAAAGT
AACAAATGAGTATGAGAAGATGAAGAGTCAAAAAGCACTCACCAAAGACTAGAAATCCCCCCAAGTATCTCCT
CAAACATCCCCCCAAGTATCCCCTCAAATAAGCCTCCAAAATCCCCCCAAAAAAGCGTCCAAAATAGGCAAAA
AAGCGTCCTAAACCAGCTTAGAATCAGCTAGGGGAGAGCTAGGAAGGTGAAGGGGATGTTACTTACCATATTA
CAGGCCTGGAGGGCAAGAAAGGGGGAGGGGGAGGATGGGGAGGGGAAGAATGATGATGGTGATGAGGAAGGAT
CAACGTTGTGCTCGAAACAGAAGTTCAACATGTCACATGATGTCACATGACTGCTGGACTTGGGATTTTCGAG
TCAAAGTTCCTCAAATCATAGGTAGGAGATATTTTCGGGTCATTTCTGCATGGATTTTAATGATCTAAAAGTG
TATTCCTATAGTTTCGAGGTCGGGGAGTCCGAATATGTTCTTAGATTTCTTGTTAGATGAAAGGCTATTGAGA
TATTGCACATTTAACATATAATAAACACAAGATTGCGCTGTAGCTCCTCAAATATTGATCGGATGATAAATCT
AAAATCAGATTCAAATTCCTGGACCTTGAAAGCATATACTTACACTCTTCAATCGTCAGAATCGGCACAGTAA
CCAGTAGAAAACATGGCATTCTTGTTATGTTATTCTGTATCTCCATAACTGGTATGTTTTCCGACAAAAGCAT
ACATATAACCTTATATTCTTTTAATATTCTATGATTTGTCATGATTTTTCCAGAATTTTCCGGTTAGTAGAGC
AGGCTCTACGACGAGTCTGTCTGCAGTTGAGAATGACCCCCCCACCCCATGATGGAATCTTGATATCTCCATA
ATGGGTATGTTTTCTGACAAAAACATACATATAGACTTATGTTTTTTAATATTTTAAGATTTGTCGTGATTT
TTCCGGAATTTTATGAACAGTAGAACAAGACATACAGCATAATGTTCTTATAATGCTGTGATGCTTGCCTTGT
TATCTGACTTATTCACTTATGAGAACCTGTTTGACTTATGAAACTTTGTCTGACTTGTGGTTTAACCTTTGGT
CTGACTTATGAAATGTCCTGTGCTTATGCTTTCTTTGTATCCAACTTATGAATTTATGGTAAACTTATGAACT
AATTTGTGACTTATGTGTAACTTCTTGTCTGGCTTATGAAAAATTATGAACTTATGCTATTTTGGAGACTTAT
GTGCCTTAAGTTGGTCTTTATAAGGCAAGCATAAGGCAACTTTTTCATAAGTTTGCATAAGTTGTGTGACTTT
TTGCTGATGGGATTGGGTTTCCGAGGCGTGTTCTTCTTCTTCCTCGCTTTCAGAGGAAGGTACTCGGAATGTT
CCGGGCGGTGTTCGGTGAGAAATTTTGAAGAAGGTGAGGGAGGTCTTTTCCTGTCACCTGATGCGTATCCACC
TATCCAATGAAGCCAATAGCCTGCTTCACTTGGATGTGTACACGAAAAGAAAAAAGAAAGATTTTGTATTTGA
GAAGTGCCGCACTGGCGAAGGGAACGACTACTGCCCTAGATAAAGCTATGTACGAAGACGGATCTTCACGCTA
ATCTAGACACGCACTTAATGTGGCAATATCACTCCTCTGCGTGTACCACTCGACAATGCAAGCGCAGCACGAG
GTTTATACAAGAAGTTACAAATGGAGTAATACAGGGGTAGAAGATAACACCTCACGTAGTGAACGTCTCGTGT
TCAACTAGGGCACGTGACACGCCCTAATCTCAACGGGGTACGTTGTGACTGTACCCGATCACCCCTTAAGCCT
GGGGACCGGCTATGAGTGTGGTGGCTGCGACGGCGCGCAGAGAGTAGAGGGCGAATAGAGAGTAAGAGGATAG
GGAGGGCACTGTGCTGTGCGATCCCTGGGTGTGGTCGAGTTCTACTGTTAGGGCGACTGTGACTGAGATGTCA
CAGGTCACACCTGAACTTAAGAACTTAAGAGTAAAGTGACAGAAGTTGTATTAGGCGTGAACCTAAGAGAACT
CGACGGAGTCTTCTACAAGTTTCTACCTACTTTATATACCTAGACTACGGCGCACAGACCAACTACCCTAGGT
AACTATTTATATACTGACATGAGAAAAGAAGCACGTATGAGAAAGTAAACTAATTATACATTTGACATGCGAA
AAGAAGAGTGGAGTAAGAAGCAAGTAAACGAAAATAGTTTTGATCATAAAAACGTCAACAACACCAAACAAAC
CATAAAAGTGTCCACAAGTACAAACAACTCCTAAACCGGTAATCCACTCACACAAACCAATACCTACCAAAGT
AATTCCCCAACATCGCATCACATGGGCATAGTACGAAGAATCCGGCTATTGCCCAGTGTCGTGGTCCCCAGTA
TCCCCATCTCTGTATTACCCACGCCTTCTGTTCCTCATAGTCCCACATCGCTAAGCATACTCTCCCACACCGC
CTGCACCGATGATCCGATAGGTTCCATCCTACCGGCCTGTCTTCTCTTCCTATACCCGCCGCTCTCCTTCGCC
ATTCTACTTGTTCCGCATGATTCGGAGGTAAAAAAGATAAGATAAGATCGATAAGCTCAATTGGTAAATCTGT
AGCTTGCGCCATTTTAGCAGTATGCCGATAGTTTAGCCTCCGTCGAACTGTAGCAGCAGAGGGAAATTATTAT
GTAATAAAATATGTGACAGATGTGACAGCGTGACGCCGTGACATTCTGGCAAGGTTCTAACA
>Agabi_varbisH97_2_rnd-1_family-8#LINE/I
GATGANGAGGAAGTNGCAGAGAGCAGCGACGAAGAAAACGACGACGCAAAGGCCGCCGCCGTACCCAGCCAGA
CTGATTCCAACATCTGGAATCAGTTCTCGACCCCCCCAGCGGAACTCATGACGACTCCGCCCCTCCCATCCCC
CCTCGATGCGTCCCAGTCGACGTACGGGTTCCCCACCCCCTCCCCAGCGCTCCTGGACCACCCCGCGTTGAAG
GACGCGCGTACACCTCCCCGNACCCACGAGGTCTTGCCCGATTTGGACAAGACCCCNACCACGCCCCGGGTAA
TGAAAATTACCGAGGCCGAAGCTTACGCGCTCAAAGTCCGAGCGGCAAACGACNCGGACAGCCATATCATGAC
AGGCGTCAGCATGTCATCAAATCAGTTCGAAGAGAACGTCAACAAGTCGAAGGAGATAGTTGGCGAGATNGTA
GATGAAGAGATTCGGAGCTCGTCGCCCGACCTTCCGTCCGAGCCGTGGACGTTTNCTGANGACCAAGTCGCGG
GCTTCAGAGAAAACTTAAACGGGATCCTCAACAGCATGGCCCGTATCACACCCAGGATGGCGACCAACCCGGA
CGCCATCGCCANNGAACTTGCCATATTCGTGGGCAGGATTATCAACTCNGAGTGGGCCGAAGTCAANGTTGGA
CATGNGTCCATCGCGTCAGCCATCAGAGAAGCGGCAACGTACCATACCCCAGTCGATATTCCCCCTCCCCCGC
CGGTGAAGAACGTCAAGTTCGCGCCGACCCCCATCCCCACCCCAGCAAGCGCACCGACTCCTATGGAAGTCGA
TGCGTCCCCACCGAGGATCCCGGCCGCCGCCAAAGGNAAGAAAAAGGCCTCCCCTCTTCCCCCNACTCCGCAA
CATCCGGTCACCACCTCGCCGGTGATCGCGTCNCCCATCAAGATCAGCGCGGTTCCCAAACCGCTNGTCCAAC
CNTCAAAGACTGGCAAACAAGGTAAGCCAGTCACNTCNTATATGCCCATTAAGCTGTCGGTGGTTACCAAGCC
GACAGCGACTAACGAGACGCTAGCGGGAGTCACGCCCCGTATGCCCGAGTCCGGTTCCAGCCGGCAAGCTAAG
GGCAGAGACGCTAGCGGGAGTAACGCCCCGTCCGCCAGTGCAGTTCCTGCTGCCGGCAGGAGTCGAGACGCTA
GCGGGAGTAACGCCCCGTCGAACCCGCCCAAGAGCGGTAAAGGTGCCCCAGGTAAGTCAGGGGCATCTACCAC
AGCAGAACACGGAACTCGTTCCGGTCCTCCCCCCGTCCCCATGCACTCCAAACCGAACTCTCNATCCAGGAGC

GCNGAGATGAAGAACAACGCGCGTGCGTTCGCTGCACGTGTCGTCCCCAAATCCTATGCCCAGGCGGCTAAAC
AGTCTGCGGCGCCTACCGCCCCCGCTCCCATTGTCATGGAAGAAACCATNCAATGGGCAAAAGCATTGAGAGA
ACAGTGTCCTGAGATGACGATGCAGGACGCTTTGAAAGCGGTAGCTCCGGTGGTCAACACGCCACCGAGCTCG
AACGAAGTCATTGTGCCAAAGTCGAAGAAGGCGCTCGCGGCTCAAAAAGCCGCCCAAGGAATTACGGGTGGAC
TCAATCGGAAGTCCGCTCAGTTCGCNTTCTCCCACATCGTTCAACCGGAACGCTTCGGNGACATGGGGAAAGT
AGTCGATGCCATGAACAGGTATCTGGTTTTCCACAAATCGCAGATGAGGGTCCAAAATGGCCGCTTATTTAAA
AATGTTGTCCACTTTTATCTTAACTTAGTACCGTCCAAACAGGGGTTCATGTTGATAAGAGAGTCGTTATACA
AAGCGTTGGAGGTAGAGCTCCCTGAAGGGGACAGTGACCTTCCCAATGCTCCGCAGTCTGTAGCCCATTTGAT
TCTGAAGGGGTTCGATTATTATACCAGCCAGTACAACAAACGCCCGGAGGATATCCTTACGGGTGATCAAGTG
ATTGAAGCTATGGGATCTGTNGATCAATTCCGTGGCTTGGAATGTGTCAGAAGGCCAGCCGTTGTCAGATCTC
GAGGATCAAGGGACATGGCGGTCGCGTTTGTAGATGTTTGGGATTCGAAGACTGGGTCGCGTACGAAGGACTT
AGTCAATAAAGTATATCATATTCGAGGAAAGCTTATTAAAGTAGAATACGCTAGACAGAGAGAATTCGTTCCA
CAGTGTCAGAAGTGTTGGAAATGGAACCACGGAACATCCCGGTGTCGCCTCAGCCATCAGCTGTGCGCCCGAT
GCGGTCAACCCCACATGACCAAGAACCACACCNTCTTCGCTACATGTTGCGGAGCGGCAAGGAAGAAGGAGGA
TTGGACAGGAGAGTGCAAGCACGAGATAAAGTGCATCAATTGCAAAGGCAATCACACTGCCGACTCCACCAAG
TGCACGTACAAGCGGCACCAGAACAATATTTCGTGGCACGATCAAAGGCGCGCTACGGATATGGCNATCGAAA
GGGAGAAGCGCGACAACAGGCGCCGAACCATCCACCAAGCTCGTCAACTCGACGACGAGCAAATTATCGAAAT
CCCCTCTTCCTCTGAATGAATTGTACAACAGAAAGAGACATTAATACTATTAGCAGACCTAAACAATCTGCAC
CACGGTCGTTACAATACTTAACTATTCTATCATTCAATGCGCGTAAGAGTTGGGAAACCACCTCTATCGTATT
GGACAAATGTGCCGGATATGTGGACATCATCATGTTCCAGGAACCGGGGTGGAAGGGTGTGAGGAGGCAACCA
TCTACCAAGAACAAGGAGGGCGACGTGGCGTGCGGCCCTCCTTTACACAACTCGTGGCGTCCTTTCACGGAAG
CTTTCGACAACAGAGATGAATCCAGAGCAGCCAGGCGGGTGCTCACNTACATCAACAGACGACTGGACGTTTT
CAAACCACAACTCCGGTCAGACATCCTCAAGCATCGTGACGCATCATTAGTCACTTTGCACATTCCTCAACCT
CGTAGGGGCATACCTCACGAGTTCAACATCCTCAATGTCTACAATGATGGGGAAACACATGCGGCGGTGCATG
AACTTAGACGGATCACNGAANCTTTGCCACGGATATCGTTGTGCGCAGGAGACTTCAACATCCAAGATCGTAC
ATGGGACGAAGGAGCGGTCAATCAAATCAGACCCTCTTCTCCCTTCATGAGGCTTCAGGAACTGTTCACAGAC
CTGGAAATTCAATACGTGCACCCCGTTAACAGAGGAACACCAACGAGGATACCAGACGATGCNGANTCGCGAG
CGTCCGTCATNGACCTGGTCGCAGCATCAGCGGCCTTGATCAACCAAGAAGGATTCCACTACAAAGTGAAGGT
GGATGACAGAGAGAGGTGGGGGTCGGATCACCGGCCTCTTCAGATNGAGGTGCCGATCTCGGGGAGTGAGCCG
GAGATGAGGTGTAAGAGGAAGATNGAAGCGTGGTCGGAGGAGGAGGACGACTACGTGACACAGATATGCGGAG
ACTTAGGCAGAATGATAGAGAACGTTTCTGAANCAGCGGAGGATTTGGAAGCGACTATGGGAAGAGTGTCTAC
TGCGTTCGAAAGAGCGTGGGAGGCGTATTCAGAGGAGAGGAAACCAAGCCGTCACGGGAAGAAGTGGTGGAAT
GAGGANTGCAAGAAGGCGTACCAGGAGATGGGAGAGAATGGNGGGCCTAGGAATAGAGAAATGCGTAATAAGA
TGAGGAAGACTTTGCGTGTAGCGAGGCGNCAATACTTTGATAAACAGATTCATAGTATGGCAAGTGACAGGAA
GAGACCATGGGACTTGATGCCGTGGACTAGAGAGAGGAAGATGCCGGCGGTGGAGGCGATCCTAGATAGTGAG
GGAAATTCGTGCAACAATGAGGAGAAGTTGTTCGAAACCCTTCACAACACGTACAATGCGGCGGATAATAGAG
AGGTGGATATGAGCAGCATGTATAGAGAGTTAGAAGAGTTTGAGGAGAGGGAATGGGTGAAGTTCTCTGTTCA
AGAATTTCACGACGCGCTCAAGAATTGTGCCAAGAACACGGCACCTGGGCCAGACCACGTCTCTTGGAGATTG
TGGAAGCGATTTGCGACAGATGACACAGTCTGCCAATTCGTCACGAAGATAGCCAATGCCTGTTTTGATACNG
GGTATTGGCCTCAACACTTCAAACAGTCCATTTCGGTGATCATTCCGAAACCGGGCAAACCGTCATACGACAA
GGCCAAATCGTTCAGGCCTATCGTCTTACTCAATACGATGGGGAAACTCATCGAGAAAATGATCGCGCGGAGG
CTTCAGTTCGAGAGTATTGAAGCCGGCGTCATTCATCCTTGTCAAACNGGAGGTATACTCCAAAGATCGGTCG
AGGATGCAGCAGTCGCACTGACACATCATGTTCGTACGGCGTGGGCGAAGAAAAAGGTGACTTCAGTTTTGGC
TTTCGATGTAGCACAATTCTTTCCTTCTCTAAATCATAACGTTCTTTCNAAAATTTTCAAACATTTTGGATTT
TCGGCTAGTGTAGTTTCTTTTTTCTCTGATTATTTAGTAGGAAGAAAAACGCAATATTCCATAGACAATATCA
CTTCTCCTCTATTCGATAGTAATGTAGGAGTTGGACAAGGTAGCGCACTGTCGCCTATACTCTCTGCTTTGTA
TATAGCGCCGTGTTTTCATTTACTGGACAAATGGGTCAAAGACGAGGAGGACCGTATTCATTTTCCTTTAATT
TCTTTTCTTTCTTTTGTAGACGATGGACTTCTTGTTGCTACGGGGGATTGTAGAATGGACACTCACGATACTC
TAAAAGAGGCGTATGCCCAAATGACTTCCATTTTCAAGGACTTCGGACCTGGTTATGGAACACACAAAGACGG
AATTATTCAATTTTGCGGACTCCGCCCGTAACAAAAACTCGCCAAACCCCAGCATCAATTTGGGTGTGGCCCC
CTTCACCCGCGAATCTCCATGCGTCCCCAAAGATGTGTGGAGATATCTCGGAATCATGTTTGATCGCAATTTG
ACCTTTCGCGATCACGTCAAATTCTATTCCACCAAGTCGGTTTCTGCAGTTCGTTGCATGAAGTCGCTTGGAA
ACTCCAACCGAGGATTGACGCCTAAACAAAAGCGCCTCCTCTACATTTCGTGCATTCAACCAATAGCGACNTT
CGGACTACGCTGTTGGTACAAACCGGGAACGAGAGCATTCAAAACCAACATCAAGATGCTCCGTCTCACNCAC
AACCAAGGGGCGCGCTGGATTACAGGCGCGTTCAGAACTTCTCCTATGGGAGCCATGTCGGCCGTGGCTGGTC
TCATGCCTCTCCACCTCCAACTCAAGAAGCTGTTTGAGAGATCGGTCATCCGCATCAACACGCTTCACCCGCA
CCATCCCGTTCTTTCACTATTAGAGCGGCGAAAAGCCAAAGGATCTACCCTACATCCCAACGCCTTGGAACGC
AAGTCCAAGACGATCCGCAAAGTCATCAAGTCTCCGCTNGACTCGGTGATCGACAACCCTTCCTCAGAGATNT

TCGACCCCCTCTCTGAACATATCAAACCTGGACATCGGCTTGTGGACCGACATCCAGACAAAGTCAAATTCGT
TCTCATGCCAAAGAATCGCAAAGACAGAGACGAATACCACAGCAAACTGAACCTTGCTCCCGTAACGGAACCG
AACGCCTTGCATCTGTTCACAGCAGGCGTCAAACACAAGTTCGATCCGAGCGTGCCCGTGCCGCATCGAACTC
ACTCGCATATCGCGCTTGTCACAGTCTTCAAAGGACAAGCCATCACATCTAAAACGAGGGCCGCCGGAAGGCG
GGTCAACGATGAAACAATTGTCGGATGGTCTACATTTCTGGCATTAATCAAAGCGCACAAGAAGCTTCAAAAG
GGATGGCCTATTTCCAAGGTCGTCCTTTACACTACGTCCCAGTTGATCGTCAAGAATCTGATGTCAACNAACT
CCAAACCGCTCGGATCACAGCTTTCCATTGCTGTATCCTTAGCCTTCGACGCGATCGCCACAGACTTCCCAGA
GGTCGATATTGAAGTGCGAGGCTTTAACAAATCGTGGGCCACGGCTCCGGGTACCTATACAGAGGACTCGGGA
ATGTTACAGCCGTTGGCTCAAAGAGCATACTATTCAGCGGAGACCGCAAGGACGTTTACAGAGCGCTCTCTTT
CATTCCCTCAATCTGCCTCNTATCATTACACGAGGCAGAAAACAACGCGAGACGCCATTTACCTATGGCAAAC
CGGTTTCCAACAAGGAGTCTGCACGCCGAGCGAGCCGCCACCACACATGCCGTCTTCGGATGGTATAGGAACG
CGCTTNGGACTTTTAGTCCGCTCGCAAGGATNCAACGTGCCGCCACCACACATGCTCTCTTCGGGNAGTATAG
GAACGCGCTCAGGACTCGGGAACGCATCAAGCGAGCCGCCACCACACATGCCGTCTTCGGATGGTATAGGAAC
GCGCTCAGGACCTTCGGTCCGCTCGCAGGTNCAAAGTTCATCATATCATATCAAGTCTTGTTTCAAAGGTAAC
AGCTGGTATGACATTACTGACAAGGAAGGCAACGAAGTACTTCCAAGTCACCTTCAAGGCGGTCCATGGTTCC
AAACNTATCAAGGAGTCAAGGCTGCCAACAATTCGCCACTCTTCGCTCGTCTTATGCGAGTCATGTGCAATCA
CGCACCGATAGGGGAATACAGAGTGAGGTTCTTCCCAAACGAACCTTACAAATCGTGCCAATGCGATCTCAAG
TCACCGGAGACGCGGCAACATCTACTAATGGTCTGTCCTCTGTTCGTGAGGCGGATCAATCATTACGACTTGG
ACGGNATCACTACGATACGAGGCCTTATCCTCTTCCTCCAAGACAATCCCCAAGCGTTTAGTTTCGAACCACC
GGAACTTCCGCGACATGCAGACGAAAGCTGGCCGGCGTATAGGAGAGAGTTGGAGTTGGCTAGAGACGAAGAG
AACAAAAGGAGGAAGGAGAAGAGGCTTCCTCCACTAGNCGGACCGATCTTCTTGCACGAGTCGATCGAGTTCG
TAAGGCATTGGAACGTGTGGGAGATAGATGACGGATGGGGAGATATCTTCTGCGCGACGATGAGAGAGATCAA
GCCTGTGTCGCAGGCGGTGTTGAAAAGAGGTAGAAGANGGATGATTTTCTTTTATCTCGTGTATGAACGTTTC
TGTTTCTTTGTTTACTTAGCTGTACTTGTATCCAGATTCGAACAGTCTCTTCTTTCTTTTTCTTTTTCTCTTN
TAGCTTCGACCTCGTCAGCTGGATACGTCTCGCGTAGAGTAGTTAAGTACAGGTGATGNAAAGTACGGGGAAC
GCGTCATGGCACCCTTTAAGTAGTTTTGAAATAAACTATAATGATTACAAAAAAAAAAAAAAAAAAAAAAA
>Agabi_varbisH97_2_rnd-1_family-25#LTR/Gypsy
CCCGAGCCCGAAGGATTTTGGGGGGAATAGAGAGCAGTTTGCAGAATGGTGGAGGAGCATGACACTTTTCCTC
AAATACAACAAGGTCACGGACACCGACCAGAAGATCATCGCTACCATCGTACGATTGAAAGGACCNATCCCCA
GCTGTTTTGCCGAAGTATGGACGGAGAAACTCGCCGGCAGCATCACATACACATGGGATANTTTGAAGAAGA
AATCAAGACTTCATTCGGAAAAGGAAACGAGAAGGATATAGCAGAGGAAAGGATCGAATCCCTCAAACAAGGA
AACAGGAACACCATGGACTTCCTAGTCGAATTCACAGCTCTCATGTACAAAGCCAAAATCGACGACCAACACG
CCATATTCCTCCTTAGACGTCACACCCGTCACGATATTATCAAGACGATTTTGGGATACCCAAGGAAAGACAT
TCCGGAGNCGAGAGAAGACTGGAATAACGCAATTCTACAAGTTGGGTCAGGACTAGAGACGATGAAAAGGATT
GATCAGAGGACCGAAACTGGAATCACTTACGGAGGAAGCGGACAACCGATGGAGATAGGATGGAAGAAATTCG
AATGGGACAAGGATGGGAAACCCAAATGTCACAAATGCGGAAAGTTCGGACACATCGGGAAGGACTGCGAAGA
GAAGAAGAAGAATTTTGGAGAGAAGAAGAGCTTCAAGTGTTTCAGTTGTGGGAAATTNGGACACATTGCGAAG
AATTGCCGAAGCAACAGGAGTGGAAGGATTAGAGCGATGAACGAGGATTCTGACGATGAAGACGACTCCAAGA
AGGAGCAGGGTTTTTCCGATGGTTCCGAGTAGGCACGACCTCGCGAACCAATGTACCTAGACAAATGAACAAT
CGATACGTTGTCCCTCAAAAACGTCTAGGAGTGCAAGAACTGGAAGTAAAACCCCTCCTCACTACAACAAACG
GAAAGAAACTCAAAGTCTCAGCCATGGTCGACTCGGGATGCACACACACATGTATCGACGAAGGATTAGTAAA
GAAGAAGAAGATCCCGACGAAGAAATTGGAACGGCCGATCACATGCAGGAACTCAGATGGAACGATAGCAGGA
AAGAAGGATATCACCAAATTCGCGAAGATAGATCTAAACATCAACGGTCATAACGAGCAACTGGACGCGGTCG
TCACTCCATTACAATCATCCGACCTCTTTCTAGGTCATGATTGGTTAACGAACCACAACCCCGAGATCAATTG
GAAACAAGGAATGATCAAATTCAACCGGTGCCCCACATCATGTTCTTTTCCCCATACCGACATCTCTTTCGAA
CCACGTATACGACGATTACAGTCAAACGAAGACACTGAGGAAAAAGAACCGGATCCAACGAACCCTGAGGACT
TACCAGCATACATGAAACCCTTCGCCCATCTTTTCAACAAAAAGAACTTCGACAAACTNCCTGAACGAACGGA
ATGGGATCACGAAATTAACTTCACGGAAAACGCACCAACGGAGATATCATCAAAGGTTTATAGCATGACACCA
CTGGAGAGAGAGAAGAACTGGACAAATTCTTGGATGAAAACCTGGCTACCAATCGAATCCGACCATCGAAATCNC
CCCACGCNGCACCATGTTTTTTCGTTCCNAAGAAAGACGGTTCACTAAGCGGTTATGGTACAGGACTATNGGN
ANCTGAACGAAATCGGACGATCAAGGACAAGACGCCACTACCCCTCACCNNTGAAGTATTAGATCTGAACTCA
NAGACGCCAAGGTTTTCAACGATTATTTCAGGACAGATTTACGCTGGGGNTATAACAACGTTAGGATACGAGA
AGGAGACGAGTGGAAGGCAGCGTTCTTNACNACTTTCGCGGGTGCNTTTGAACCNACNGTCATGTTCTTTGGA
ATGACNAATTCGCCCGCCACCTTCTCACGTATGATGAGCGACACTTTTTCGGGAANTGGTACAANACGGANNN
GTAGCCGTACTACATGGACGATNTCNTNATTCCAGNAACGGACGATGAAGAATTGGAGCACGAACTATACGT
TNTCTCAGAAGTCGCGGAGAAACACAATTTATCATTCAAACCGGACGAAATGCGAATNGAAACGCANCGACAA
CGGTATTNGGGACGTGTTATTGGGAATGGAAAGGCAATCAAGATGGAGGAAGGGAAGGTNAAAGNCGTAGNAG
ANTGGCCGGCCCCTAAAACGCGTCAAANANGTCGAAAGNTTCTTGGGNTTNGCAAACTNCTATCGACGGTTCA

TCGAAAANTTCGCCANCATCGCGCAACCACTANACGCGCTGAAGTCTAAGAAGGGCGAGAAATGGNGTTGGGG
NGATGAAGAAGAAGAAGCTTTCGAACAAATCAAGCAAGCTATCGCCAGCGAA
>Agabi_varbisH97_2_Cluster42#LTR/Copia
GTTGCGAAAGCAGCGCACAATCTACGCTTAGTGGTAAACCGACTTACATTTGGAGATAGCTTGATAGTAGAGC
GAAGCGGTGTTTGAGAGTAAATGTAGCTGGTTAGGCTAAGAGTTGAGAGCGATTGGGAACTTAAAATTATCGG
TAGACTACAGTGCTCTTATATACTACAACGATATAGGGTTTAGGGTATAGCTAAAAGAACTCATGAAATCATA
GTGCCCTGCGGGCGTAGTCTACGAGAGGATAATGCATGGAGGCACAGTAATAATGTGTATAATGCATGTATTT
GAAACAGGAGTCATTCATACGTCATACCGAGTGTAAGACTCAAGGTATTCATTTCAACACTCCTCCTTGAATG
AACTCATTGTTGCTTTAAGTTAAGCATAGAGCGGAAAAGAGAGAATTTCACTTTTTCTAGGTTTTTAGTTAAA
ACGTCAGCAGGATTCTTTGATCCGTCAATCCAATCAAGATTGATTTGCTTCTGTTTTAAGAGCTCTCGGATAT
AATGGAAGCGAATGTCAATGTGCTTCGATCGTTTCTCCTGAACTTCAGAAGTTGACCAAAATAGACTGCCCAT
ATTGTCTCCATAGAGCATTGGAGTTTGTATCTCGAAACCAATCTCGGTCAAGAGTTGGGAGACCCAAACTAAT
TGTCGCGAACAATCAGAGATAGCCATATATTCGGCTTCCGTCGATGAGAGTGCAACAGTCTTTTGTTTACGAC
TCAGCCAGGAAATAGTTCCATTAGCAATTGTAACAAAGTTGCCGGTAATAGATTTCCGGTCTTCTGGATCAGA
AGCCCAATCCAAATCGGAATAGGCTATGACACCTTGATTCGAGTTATCATCAAACTCAAGAACCATATTTTTA
GTGCCATTAAGGTACCGAAGAAGATGCATGCCGGCAGTATAGTGCTCTTTATTTGGAGTCATCATGTGCTGTG
ATAATTTTACAACGACAAATGCAATATCCGGTCGTGATCCGATCATGAGGTACATCAATGATCCGACCAACTG
TTGGTACTTCGTTCTGAATCCATTATTTGGTACTTTATCGGAAGTTTTGAACACGTAACCTTTCGGTAACGGT
GTTTCTTCAGATCCGGATTTAATACCGAATCGATCAAGGACCTTTTGGAGGTACTTCTGCTGATGCAAGAAGA
TTTTCTTGTTCTGACGATCTTGAGATATTTGCATACCGAGAAATTCTGTCATAACACCTTGATCACGACATTC
CCATTTGGTCGAAAACTTTAGCTTTAACTCTTTGAGGAGCTGGGAGTTTTTCTTTCCCATGAAAGCTACATCA
TCAACATAGACAAGCGCAACTTCAAGTTCACGCGTCTTTAGATGACGGTAATAATATACGCCTGCATCGGATT
TGCATCGGACAAAACCGATCTCGGTCATGCTGGCAGTTAATTGTTTCCACCAACTTAACCCGGCTTGCTTAAG
GCCGTACAATGCTCGGCGTAATCTCCAGACCTTATTTTCTTTGCCGGTGGCTGTGAATCCTTCGGGTTGTGTC
ATATAGATTTCCTCATCCAGTTTGCCATATAAATAGGCAGTTTTTACGTCAACACTAAACATATCCCAATCTT
CAAGGGCAGCGATGCCTAGAAGAAGACGGGCAGTTTCATAGCGGACTACTGGAGAGAAGAGCTCGTTATAGTC
GATTCCTTCGATTTGAGAGAATCCTTTTGCGACTAATCGAGCGCAAAGGCGGCCATCGCTTTTCTCATTAAAG
ACCCATCTATTTTTAATAGTTCTTCGACCTTTCGGGAGATCTACTAATTCATAAACACCACGTTTCTGTAAGG
CTTCTAACTCCTCCAAACAAGCATCAATCCATTTCTTCCGCATTGCTGCAGGTAGTTGCGTTAAGTCCTTGAA
TTGGACTGGCGTTTGGATCTGATTTGCACGTTCAAAGAGAAAGTTATTTAACCTTACTCCTCCTTCTCTGATT
AGAACTTGAACTTCGTCCATAGTGGCTTCTCCAGGGACTGGAGTATTACCAAATTGTGAAGGAAGTTGAGAGC
CATGCTCTCGTTCGTATTCATCAGCCTGTTGACTAGCTTGTTCGAAGTGTCTGCGAATTTCTTCGCAACGTGC
TTCATCAGCTTGATCATCGGTTGGTATATGAGCATTCTGTCTATCAGATCTCGGAGGCACCCTTTGCTGGATT
CCTTCTTCGAAGTCCGGCAAGAAAGGTTGTCTCTCGTAATCTCTCAGAACTTTTGAGGAGTGTTTATCCCCAT
AGACGTTATCTGGTCAGTTCGGTACTTTAGTTTCCCGTTTAGACTGTCTAACATTATGTTCTGATTCATCAGG
CGGAGTTACATCACGCCGTTTTCTCCACGTTGGCCGTTCCTGCCGTGGAGGCTGGTTGGACGGGCCGGGAGTT
GCATTACAAGGAGGTGGTGATGGAGCTCTTTGTTGCCGTGGTGGCACAATCGGCTGCGGCGGCGTTGGAGGAC
GCGGCGGTGCTGCCGGTGGAGCTCCATTAATAGGTGGTTGATCCGTAGAGGGACCATTAAGAGAATTAGGGTC
GAGAGTAAAATCGACTTCTCCTCCATTTCTGGAGTTCTCTGATTCCGAGGAATTACTTCGACTCGGGGTATTA
CTCAATCTTCTTTCGAGGTTACCTGGTTTCGATTTATCGCATTTCGGAAGAAAAGTTTCATCGAAAAGGGCTA
TGGGAGAAACGAAAATTACATTTCCGTTTTGGTGTCTCATAAATTGATAATTTGGGCCTTGGTAACCCAAAAA
TATCATAAGCTCGGATCGTGAAGAGAGCTTGTTTTTACGTATTTCTTTTGGAAGATATACATAAGCACCACAG
CCAAACACTCGGATTTTGAACAAGTCCGGTTTGGTTCCATAGAATAGTTCATATGGAGTTTTCCACTTTACTC
GTTGCATAGGCGTGCGGTTGTAAATATAGACCGCGTTATCACGGCCTAAACATATTTAGCCTAGTAATCCCTC
TAGCGAAGGTAGCTTTGACATATTATAAGCCTAGCTACAGCCTAGAAAACCCTTCGCTACATAGAATGACTGA
GAAGTGTAACCACAGGATATCTAAAGGAAGGACACTCGATCTACGCGAGCTCGCATGACAGCCATAGCTGGGG
TCCCCATAGGGAAAACTGTACCGATAGGAGAAATATGACCTTACCAATTGGAACATCAACCATTGGTCTATAC
GCGAAGCGAATACAACGTGTAGCTACGACGCATAAGTACTATTAATTATATTCGCAGACACGGCAAGGTGAAG
GGACTTAGTCGCTACGACGGTCTGCTTACGTACCCTATGTCGCGAATAGGAAGTCCCATCATTTAACAAATTC
GGACGAAGTACGCGAAATGCCATCAAATACGAGTTCCCTTTTTGGTTATACCCAAAGTGTAGCTACGATCGTA
ACTAAAAATATGAAAGAGAGCTAGGAGCTATTAGGAGGAACAGAGATAGGTCAGCAGTCAGTGAACGATTGCC
CAGAAAGAGTAACCGTTAATCAGCGGATTACATGATCTCACGAAAGCTACTCCTAAAACGGGTGGTATGACTC
GAAGGCTGAGTAGCTACAATAGAAAAGATGAGCAAGCTAAGATATAAATACCAGAGTCTTAGTAAACGTAGCG
TACCTGAGCCAAAATAGCTTAGAGAACCATTAGTATAAAAAGCCTGTAGTTAGTGAGCGAGAGGCAAGGAAAT
TGACCTGCCGAAGCTCTGCAATTGTATTTCGCTACTCAGTGAATCCTATTTGTGGTGTTATCTGTGAACTTTT
CCTTCGGACGCTAAGTCCAAACTGAGAATCATACGGACTTGAGAGAATTGTAATCGAAAACCAGAGAGGATTA
TTATCGAGACCTTGAGCTACAACTCGTTGACAGTGTGTGATAGCTTCGTGTTAGCTACTTGGATATCGCAGCT
TGCATCACTTTGGTAGTGGTGAAAGTCGGTGGCCTTGTAGTTTGTTGGTATAAGTCGGTAGCTTCTCGCTAAG

TCGATATCGCAGCTTATATCTACCCACATTGTTTGGTACGCGTCGCCTGCCTTAGCACAGCAGCGTAGTTGAA
CTTCGTCCTGAACAGTCCTTCTCGAATCCCTAGATTACTTACTCTTCGAGTAGCTACAATTGAAAAAGACTTT
AAAACCCGAAAAACAATCTAAAACTTTCAAATCTTCACAAAACATATCTGTCTCCTTCTTGACTTCTGATTTA
ATTCTCCCACGAACTTATAAACTGAGAACAGTTTGTGCGTTAGTGAAATCCTTTTACATTTTTACTTCCGTAA
CTACTGCCACCTCTACAGCGTACGTCATAGAGAAGCCCCACCATGAGTCGGGAAGGCATGCATGCTTGCGCAT
GGTTTGTGATTTCTCGTGCAACGTTCTATTGAGTCTTTCAGCGCGCCCATTTTGTTGGTGAACATTAGGGGCA
GAAAGCTCATGTTTAATGCCGTTGGTGGACAAGTACTCTTGTAGAGCACTATTGGTGTATTCTCCTCCTCGAT
CAGTGCGCAGTCTCTTGATCTTTCGATCAGTGGAGTTAGCGAGCAACGTCATGACTTTCGTCATTTCTCGAGG
TGCATCACTTTTGTGGGCAAGCATTACGGTGAATGCTGTACTGGAATAGTCATCCAATATGGTCAATACCCAT
TTATGGCGGTGATAGGAGATTACCGGAAGTTCGAATAGATCCGAATGGATTAACTCAAGAACTTGTGTTCCTC
GGCTTTCTGAAGTGGGAAAGGGTTTCTGCGCGAATTTTCCTTCAACACACCCTTTACAAACTGGTTTATCTCG
TGGAATACTAATAGGTGGGACACCTAAAGATTCTTTTGAGAATTTACTGAACACGAGATCGGACGGGTGTCCT
AATCGACGATGCCAGGTAGAATAATCAGTGTTTGTGGTGGTATAAGCACCAGCTTCGGGGATTTCCTTAACTA
TCCTGGAAAGGACAGCGGAAATACTGGGAAAGTGTCCCGAGATACCCGAAAGTACAGCATGATTATTTTCATC
TCGAAAGGTGAGATGCTTCGCATTGGCTTCTAATCTTAGTCCGGACTGTAAAAGTTGTCTGACAGAGATGAGC
CGCATGTGCATACCGTTTATGTAGAAGACAGGCCAAAGTTTAGTCCTTTGAACTTCTTTGGTTCCTGTGTTTG
TATCAACAACTAAGTGTTGGATGTGTACTGTTCCTCGGCCTTGGATTTTTAATTGGCCAGAGGCGGTTTGTAC
CATCCCAAAGAAGCCTCGATCGACTGTAGAAAAGTCCGAGAGACTGCCAGTAAAGTGCGCAGAAGCGCCACTA
TCTAGCATCCACTTAATCGGATAAGAATCAGAGTTTGATCTTATGCTCATGATGAATGACTTAAATTCAGGCG
TGATGTTATCGAGAAGAGCGTTCCTGACGCTCAATTCCATCGCATAATTTATATCTGAACCGTTACATGAGGA
ACCAAGACTACTTGTCTGTTCTACTCTTGATCCGGAGCGATTTACATCATCGCACTGAAAAGAAGAATTAGTA
ATTGACTGCGAGTTAAGGGGTAATACTTGCGAGTATGAATCCCCTTCGTTCTCAGCATCATAGTCGATTTCTT
CTTCGTTATCCATCAGGTCTTGCCTGGAATGGATCGAGATCTCACCGTCTTCCTTCTCTTCTATCACAGATAT
AGTGGCCGCGGATCCAAGTGTACGTTCGTCCGTGAGGAAGTTCATTTTCCGTGATCACATCGTCCGAAGAGAA
CAGTAGATCCCAGTGGTGACGGAGGTCGAACGACCAGGTGTACGAGTAGTGCGAGGAAGGGGAATGTGAAAGT
TCATCATTATCCGAAAACATGTCACAATATCCATTGTTAATGTCGGTTAGCCAATGATCGATGGCAGACCCGA
GTTTAACAGAGGCATTGGACGATAGTTCACCTGAGAGGATGTGATCGTCCGATGGTGATTCCCATACAGGGAA
TGGTATGACATTTATGTCGTCAATGGAATATCCCCGTTCAATTAATTCACCCCAAGAGAGGTTTGAAGGGATT
TCCAATGACGTATCATCAATGGGAAAGCGGTTAGCGTTATCCCATGCATCACGAAAGGAAGTGGTAGAAGAAC
TGGCAGGTTCAACGTTCCAGTCCATGGGCCGGGAAAATGGTTCCAGCCATTGGTTATTCCAGTTGTTACCCAT
ATTATCAGTTGTCCATCCATATGCAGCAGATTCAGAAGAATTGCGCGTTATGGATTTGATGTCATCAGATTGG
TTCGAGGGAACCGTGCGTTCGATGTTGTCATTGGGCATACCATATAAGGTCCAAAAGGGTCAATCGCAATCAA
CCGAGATTGATTGTTCCCGAGATCCATCATCGAGCCAGGTTCCATCAGCAGATGGATCAACCGATTCGAGATC
ATTCGGGTTAGATTCGATCGAGTTTTGGTAATCGACGTCGTGACGCCAATCATCAATCCATTTGTTTCCATCA
TGAATGTTTAACATGATGATTTCCATGTGAAGGGGAGAGTGCACAGAAATTTCGTTAGGAGATGATTGTGATG
TAGAATGAGACAACTCACCAATCAAGGGAGTCGGCGGCGTGATAGCTGCTTTCTCATTTCGGATACGATGCAC
CAAAGACCGTGTGGTCGGTGCAGAGAGAGAAGGTGAGCGAGGGTCGTAGGGTTGGGTTTCAATACCCGGAGGA
ATCGCGATCGTAGGGAACGCAATCACAGAAGCTGCAGTGTAATCGGGAGGCGAGGCCTCGACCGCTGCGGCTT
TTCCTTTTCCCTTGCCCTTGCCTTTGCCACGAGTCCTCTTACGCTTCTTATCATCATCAGGTTTAGCCCCTTG
CGGAGCTTGCTGCCTCTGCTGCTGGCCTTGCGGCCGTTGCTGCTGCTGTCCATGAGGACGCTGCTGCTGCTGC
TGATTTTGCCCGGATGTTCCAGGCGTGGCATTCTGCCATCGCGGAGCCTGTCCGGACGGCTTAAACCTGGCGG
CGGCGAGATTCTGAGGGTTTCGCCAAGCCGCGGTGATAATACCTCTCGCTTTCTCAATAGTGAGATCAGCTAC
CTTCGTGGCTGGAGCGAGGATGATTCCGATCGTAGATTCCATACGAGCCGGAAGAGCGGAAAGGAGCATCATG
GTCTGGAGCGAGTCATGAAGAGTCGCCCCAGTGGCAGTGATCTGTGAACAGATCGCGACGAACTCAGCGATTT
GTCCAGATGGATCCTGGGTGTCCTGAACCTTAAAATTTATGAGGGCCTGGAGCCTTTCGTAGACGAAAGTCGG
TCCTCGGACTCCGTAATTCAACTTCATTTTGTCCCAGAGATCTCCAGGGCCGTTGAAATCCTCTCGGATCATG
TAATCCGGGAGAGTGGACTCGATCAATGCAGTTGCCTGCAGGCTGAGAGTCATCCTGATAGCATTTTCCTTAG
CTAGCTCAGAAGGAGCTGTAGGCAAGGGGGGGAGCGGCTGAGAGCTACCGCTAACATCCCATGCGGTCAGGGA
GGAGTCCTTATGTTGCATAAGGATCAGTGCGTTTTTAACGCGACAGGCCCAGGTGTCGTAGTCCTTGGCGCCA
GTGAGCTTGGGAAGAGCATTGGAGTTGGTACCAATATTTGCCCTGCAACCCGGATCGGTCAGTAAGTCTCTGA
TCTAGGGTACGACATGCTATCTACAGGCTATTCTAAATGGGCTACACAATGTCCATCCAAAAGCGGTTGGTCT
AGAGATCAAACGATCATCCATAGATGCACCAACCGGGGCCCATAACCTGTTGCGAAAGCAGCGCACAATCTAC
GCTTAGTGGTAAACCAACTTACATTTGGAGATAGCTTGATAGTAGAGCGAAGCGGTGTTTGAGAGTAAATGTA
GCTGGTTAGGCTAAGAGTTGAGAGCGATTGGGAACTTAAAATTATCGGTAGACTACAGTGCTCTTATATACTA
CAACGATATAGGGTTTAGGGTATAGCTAAAAGAACTCATGAAATCATAGTGCCCTGCGGGCGTAGTCTACGAG
AGGATAATGCATGGAGGCACAGTAATAATGTGTATAATGCATGTATTTGAAACAGGAGTCATTCATACGTCAT
ACCGAGTGTAAGACTCAAGGTATTCATTTCAACAC
>Agabi_varbisH97_2_Cluster10#LTR/Copia

AAAGTAAAGTATGACTCAGTGATAACGTCGTGTGTTGATCACTGATATATTTACCTAAAGTAAAGTATGACTA
TACACGAGTAAGTACAAATAGATCAGTAAAGAAAGTAAAGTAGCTCACCTCAGTGATAACGTCGTGTGTTGAT
CACTGAGGAGCGAAATATAAGGTTTTAGCTATAAGTATGTCGGCCACCTGTGCCGAGGTAATACTGTACAGTA
AATGTATAGTTATAAAGAACGTAGAGCGGGAACGGTCGAAAGAGAACGGGAATGTAGAGAGAATGGTAAAAAT
AGAATAAGGGAAAATATAGACAAAAAAAGAAATGGTAAGTTGGTTGAACAACATACATACGTCCGAGTCTCTA
CTGAGTGTTGAAGCACTCACAACACTCCCCCTGGTTCGACACTCCGTAAACCCATGTCACCGCAAAGCCTCAA
CACCGCGTCACGAGCGAGAGGTTTGGTAAAGATGTCGGCTAGATTCTCGGCAGAGGGAATATACTCGAAAACA
ACGTCTCCCATCGCAGTCCTCTCCTTGATAAAATGGTACCTGATATCAATATGCTTCGTCCTCCGGTGGTTCT
TGTAGTTTTGTGTATTCGCAATAGAACCATCGTTATCGGCGTATATCAAAGTGGGCGTATCCTGTGGTAATCC
CACCTCCTTCAGAAAACTTGCAAGCCAGATTGCTTGTTGAACGGCACGGCTGAAAGCCATGTACTCAGCCTCC
ACTGTGGAAAGTGCCACAGTATCCTGCCGCCTACTAGCCCAAGATACAGGTCCGCCTGCTACGAAAAAGACAT
GTCCCTCTGTGGAACGTCGAGAGTCGATATCACCAGCGAAATCTGCGTCCACGTATCCATAAGGTTTTATGCT
TGCATCACGGTAGTATGTAATGCCGTAGTCCATAGTAGCTTTGACGTAGGCCAGTGTGTGTTTCATAGCTTCC
CAGTGGCTACGTCCAGGATTGTTTGCGAAACGAGAAAGAATGTTCACCGCAAAGGATAGATCGGGGCGAGTTG
CAACTTGAAGCCACATGAGAGAACCGAGGACTTCACGATAAGGCACATTCTTCATGTCGGCCTTTTCTTCATC
AGAGACTGGCGAACCTTCGATGGACAAAGTAGTCCCAACAGGTAGCGGAGTGGAGCGAGGTTTCAAGGAATCC
GAAAATTTGAATCGTTCAATAATGCGCTCACAGTAAGTCCGTTGGGATAACCTCACATCTCCATTGATTCCTC
TCTCAATCCTCATCCCAAGAATGAATTTCGCCTCCCCCAGATCCTTGATCTCATAACTCCTTCCCAACTCGTC
CTTAGCCTTCGCTTCACCATCTGCTGTTGATGAGGCGCCGAGGACATCATCCGTCCAAGTTGATGTGAGGGTG
AATTCATTCTCTTCTACTTTCGATCGCACCTGGGGGTCGGCTTTCGATATATAATATCCATGACCCGCGTAAG
TTCGATCGAGAGTCTGTGCCCAGTCGTGGGCGCCCTGCATCGTCCCATACAAAGTTTTGAGGAGTAACCATAC
ATGGTCTCCCCCCTCCTCGAAACCAGGAGGCTGCTCCATAAATACCTCGAAGGAGTTCTCGCTATTAAGAAAA
GCAGAGACGAAGTCGACCTGCCACAGTCGAAGACCACGTGCGGCAGCAATAGCACACACCATCCGTACAGACT
CCAAGCGCGCCACCGAGGCATAGGTCTCGTTGTAGTCTTCGCCGATGACTTGTGTAAATCCCTTTGCGACAAG
GCGTGCTTTGCGGCTAACCACCAAACCGGCTTCATCATATTTGTTTGCGAACACCCATCGTGATTTGACAACG
TTCCTCCCAACCGGGCGCGGAACAAGTCTAAAAACCTGTTTCTCCGTCATGATATTCAACTCCTTCAACATCG
GCTCAAACCACAGGTCAGGCTTCTTCATCGCTTCATGATAAGTCTTTGGCACATGAAGATCCCCCGAAATTGA
TTCCATCGTATGTGCCTCTGGACGACTCAGGTAATCCAGACTCTCTTTCATTCGTTGGGACGGTTTAGGTATC
CTTTGACTTCTCCTCAGTACCACCGGCGGCTCTTCGACAACTCCAGTAACGGCATCCGTAGCTTTACCCTCAA
AAGTAGCGGATGAATCCCCCTGCACTGGCGTCAGCACGGTCGGCGGGGTTGTCGTAACGCCATTGGCTGTATC
ATGAAGTGTATTGATTGGTTTAGGACGTGGCGCAATCTCTTGCCGTATAGTTGAGGTACGAGAGTCGTCTGAA
GGGAATGTCGTGATGGCGTCGGTAGAGAAATGAGGTCGAGCTTCCTCGAAGATCACGTCTCTTGATTTGTAGG
CGACACCGGTGGTACGATCCAGCAGCTTGTAGCCGGTATGACCGTAGTATCCAATCAATGTGAGCTTCGCTGA
GCGAGGAGACAATTTTGAAGGATGTATTTCGACGGGTATGTGTGCATATGCGGTGGACCCAAAAGGGCGTAGA
TGTGATACGTCCTGTCGTTGTCCAGTCCAACGTTGAGCAGGGATATTGGCGGGGTCACCAGTCGTTGGAGTGA
GATTTCGTACATAGGCAGCAGTTTGGGTGGCATCCGCCCAATACTTCTGTGGCAAACCGGAGTCTGCCAGTAG
GGTACGCGCCATATCCAGCAGAGTACGCATAGAGCGTTCTGCCTTGCCATTTTGCTGGTGGGCGTAAGGTGTG
GTAAAGTTCAGAACGATCCCATGCCCTTTTACGTACGTACTCCAAAGCTCGTTGTACCACTCACGTCCCATGT
CTAGCCGGACCTCCTTTAGCGTTTCACCAGTCTGCCTTTCCGCCTCTTTATGAAACTCCTTGAAGGCCATTAA
AGTGGCGTCCGCTGACTTCGAGGTAAGGAAATACAACTTTCTGTAGGATGTGGCGCCGTCAACAATTAACATG
AAATACTTTGCCCCCCCAGCAGATTGCACTGGAGAAGGCCCCCAAATGTCAATGTATACGCGTTCAAGTGGAC
GTTTCTCCAGGAGTGTAGGATTGTTATACGGGTGTGAGGTGTGTTTGCCGAAAATACAGTCTTCACAGAGTCC
TTCCATCTCCATAGATCCCACAATACTCAAACCATCGACAAGATCTTCACGCAACATCCTCTTTATAACTTCC
ACGCTAATGTGTCCCAGGCGGCGGTGCCAGATCTCCAATGGTACAGCTCTCCTCTTCGACTGCACGGCGTTCG
CATGGACGTTCACCGAAGGAACGACGTATAACCCATTCTCCTTCACGGCTACTAGCGCTACAGCACCACTATG
ATTGCGAACAATTGCTGTGTTCCCCTCAAAACTCACAGACGAACCCCGTGATACGAGCTTTGAAACAGAGATT
AAGTTCGACCTCAGTTGTGGGGTGTGAAGTGCGTCATTCAATAAAATCTTTGTCGAGATTCCATTCAGAGGCG
TGAAGAAGCCTACTTCGCCAGCTCCTTCTATGAAGAATGTGGATCCCTTTCCCGCAGAATTTCCTTGTCGAGG
TGGTGTCATCGGGACATACGTAGAGAAGGATAATTTATTGACGAAACAATGATCGCTTGCTCCAGAGTCGAGC
AGGGTAATCACGTCATATGGTAAAGTGGGAATAGTCGTATTCGAGGCGGTACTAGCACTAACTCTCTGATCCT
TCCCCGTTACGCAGATCTCAGTACGCTGTCTCTCAGAGACACTAGGGGTTGAAATCAAAGAGCCATTAGGATT
GTTGGAATGTATTGGGAGGGTGGATGGTACCTTAGGTTCGTTGCTCTCTGCCTTCTCCGTGGTACTGGCGGTG
GTGTTATTTGCAGAAGCTTGATATCCTCCTGTAGTAGTGTTGTTGGCACTACCTCCAGCTCCCCCACGTCGTC
CGAAACCTGGCGGAAATTGTCCGGCTTTGCCACCACCTGGCCAGTAACAAAACTCGATGGTATGTCCCCTGCG
GTTACAGTTGGTATTGGTGCAAACTAATTGGCTATTTTGATTTCGCTGGCGGTTGCGGCTGTTCGATTGTGTA
GTGGTCGTTTGGAGAGCTGAAGTGGTCTGCGACGTAGTGACCCGGTCTCGGCTTATTCTAGCCCAGTGGGTTA
GAAGTTGGCTTATAGCCTCGCGTGACGTTTTGGTAGTGTATAGTGTGGCGACGATGGAGTCCCAAGAGCGGGG
TAGAGAGTTTAAGATAATTGTCCGGAAACCTTTGTCGTCAATGCTGGCTCCAAGAGCGTTGGCGTTCGCCCAT

TTGGTCCTGAGTCGAGACACGAACTCGGTGAAGTCTTGTCCGTCTGCGTATACCATGTTTCTCAAGTCCATTT
CCGCATTGAGAGTAGCAATCTCGGAGGCTACTTCATAGTTGTCGCGGTACGATTTCCAAGCTTCGGCTGCGCT
CCCACTAATGGAGATTCCAAGGCCAATGGGATCGGTAGTGTTATAGATAAGAAGACCCATAGCCCAGGCGTTA
CGGACTTTCCATTCGGCGGTATTTGGGGTAGTGGATTCCCATGGGGTTTCCAGAGGTGTGGAAGTAGTTGTGG
TTGTAACTGTAGTAGGCGACGGAGGCGTCGTTGTGATAATAGGAATGCTTTGGGCAGGGTTGATAGCTGTACC
GTCGAGGTACCCGGATACACCTCGCAATTCCGCAGCGATGCGAATCATTCCACTCCATGACACCCAGTTGGTG
CCATTGAACTTTGCCGTGTCAGCGAAGATAGGAGGGGCGCTACCACCCGTGTTGCTAGACATCGCAGTATGTT
ATTTCAAGATGTGGTTTGTGTATGAGTGGGTTTCAAAAGAGCTCGGGGCCCATAACCTAAAGTAAAGTATGAC
TCAGTGATAACGTCGTGTGTTGATCACTGATATATTTACCTAAAGTAAAGTATGACTATACACGAGTAAGTAC
AAATAGATCAGTAAAGAAAGTAAAGTAGCTCACCTCAGTGATAACGTCGTGTGTTGATCACTGAGGAGCGAAA
TATAAGGTTTTAGCTATAAGTATGTCGGCCACCTGTGCCGAGGTAATACTGTACAGTAAATGTATAGTTATAA
AGAACGTAGAGCGGGAACGGTCGAAAGAGAACGGGAATGTAGAGAGAATGGTAAAAATAGAATAAGGGAAAAT
ATAGACAAAAAAAGAAATGGTAAGTTGGTTGAACAACATACATACGTCCGAGTCTCTACTGAGTGTTGAAGCA
CTCACAACA
>Agabi_varbisH97_2_Cluster15#LTR/Gypsy
TGTTAAGACTATGACCGGAAAGGTAACGCTAAGCCGGAAAGGACTTAGCGAACAAACGGCTTTGGCCTTAGCG
ACACGGGAACGGTGCGCTATGCCTTCCAACTAAGTTGATAGCCGATTTAGAGACAGAGACGAGCGACCCGCGG
AATGCGGAATCGTTTGAGAGCGAGAGAGAGATCAAGCGAGAGATAGTGAGGAACAATCTAAGTTGATTTTCAG
ACTGAAGTACCGTACAGAATATCTACGGCTTAAATACACGAAGAATGAATAGAATACGGATTCGGAAACATGA
AAGATGATAGCGCAAGGATTGCGCAATAGTTCAGATTGAAATAACGGAAAGGAGTCTTTACCTTAAGGGATTC
TGACGGTGCAACGGAAAGGAGGTCTTGAAGCGGGTAAGTCTTGCGATGGAAATAACAACCCATGTTGCGTTGG
TCGTAAAGCGACCATGGGAGCGCAACCACAATACCGAAGGTATTGCGGCATTCCATCGGTCAACGTGACCAGT
AAGTCGGAATCGATATTGCCGGGATTGATGCGAACAGATTGAGTCGGAAAGGGATATCCGGAACGTACTGGGA
TTGATACCGAGAATGGAAGGTGGTGGAGTTAGCGTGTTCCGGATATAACAGTATCCTCCCCTTAAGGCCGAGC
CCACGAGGCCGGATCAAGAAGCTGCCGGAAATGGATATGTGGACCCAATCGAAGGTAGCTGTCAATGAAGCTT
CGGAAGACAATGGTTCGGATCTGTTCAATAGCGGAGATGGCCATGGATAGTGTCTTGAGTGGCACATAAGAAT
CCGAAACGTTGGAAAGTCACCCATTCTTCCCTTACCCCCGAGATGCCACCACGGAAACCGACCCCAAAGCAGG
ATCTGACGGACGAACAGTTGGAGGCACAAATGATGGCTATGAGTAAGATCATGGCGGAGAGGCGGAAAGCGAA
GGAGAGGGCAGAGATGGCTGAGCTGGTCGACGAGACCGATCAAGAGGTGGAGATCATGGAGGAATCAGCGACG
GAAGAGAAGGGGAAGTCAAAGGAAAAGAAGCGGAAACGAGCCGCGGACGAAGAGCCGCTTCCGGTGCGCCTTA
TTATGAGTCATGTCGAGATTCCAACGTTGAAGACTCCACAGGTGACCGCCGTAATGCACAGCGAACGGTGCAA
ACGGTGCTTGACAAAAAATCTGGCATGTATATCGAGGATGCCGGGTCTCGGATGTGAGGCTTGCAAGTCAGCC
AAGGTTGGTTGCAACCATTCGTCGCAGGGTCCCGCGACGTACAAGATCCGGGCCAAGCTTAAAGTCCCCCCTC
CTCCTCCTACTTCGACTCCTGTTCCGGAAAACACTATGATCGCCCCCATCCGCATCCCACCGACCCCTACAGC
CTTTGTTCGGGAGGTACCGACGCCTACCAATCCATCCCCAATCACACCTGCCCAACCGGTCGCTTTTGTTCGG
CCACCGACACATGAGCGTTCGCTCAGTAGTCCGACCGGAAAGAAGGTGGAGGTCGTTCAGAAATCGCTGGACG
ACGTTACCGAGGCGATGCGCGAGTTTACGATGGAGACTAGATCATCAAGGATGGCAACAGAAAGGTTGACGGA
TGTGGCGGTTCAGTTGACGCAGGAGTTGACGACGGCATGCGGTAGGATCGACGCAAACACGAGGGCACATCGG
AGGTCCGAGGATGCGCAGAAGAAGACGGAGGTGGCGGTTGACGGGTTGCGGAAGGAGATCACATCGCTTCGGA
TGATGATGAAGGAGAATCGCCGGGCAGTGGGGAATGAGGACGACGAGATGGATGTCGAGCCGGAATATGAGGT
GCCGGAATTCGAGGAAGGGAGTAGCTTGTCCCGTCAGCATGGGTAGGTGGCCGGCCTCCTCGTCGTATACTAT
GCAGGACCTTCAATGGTCAACTTGAATGCTCAATATATGTTTTCTTGTTTCCGCTTGAGTGCAACCGTTCGGA
ACCCTAAGAACGAAGAATCAAAGATAAAAGGTAGAAGAAATCATATACTGGAAATAGAGAGGATGAACTAGAT
TTGAAGGAATAGAAGATTAGAATATTAATTAAGAACGGAAAGAGGACCGGGCTTAGCGGGATAAGCGGCATGG
AAGTCGCCAACCAATTCCATTGCATGCTCGAGTTCCGTAGCGGGTAGCCAGGAGAATTCTTCATCGGTATCCT
CATACCCTAACCATATCACCTTGTACAACAGTTTGCAAGCGCGTCGACGATCGATCTTGGAGTCTACTACCCG
AGCAATCTCATACTCGGTTTCGCCTTCGATATCAACGGGAGCTGGAGGTGGTTGATTGCGGTTTGGAATAGTA
TTGGTAGTTGCGGGTTCCAGCATGGAAACGTGGAATACCGGATGAATAGCACGCATCGAGGGTGGAAGATGAA
GAGTGAATGAATGCGAACCCACTTGGGCAATAATCTCGTAGGGTCCGAGATACTTCTCCGATAGCTTCTTCGA
AACACGAGTCGTCTTGAAGAACTTCGCTTTGACGAACACCCGATCACCTATCTTGAAATCCGGAGCCGGTAAG
CGATGAGCGTCGGCTGAAACCTGATAGCGTTGTTGAGCGAGAGCGATTTCGGATTTAAGAGTAGAGTGTAGGT
CGTCAAGGTCGATTGCGAATTCTCGAGCTCGTGAAGAAGCGAGATCCCGTTCCGGATAGACAGCTATAGAGGG
ATGATAACCTTTATTCGCAAAGAAGGGAGAGATACCGGTAGTAGCGCTAGGTGCGTTGTTGAATGCGAATTCC
GCCAACGGTAAGAGTTCCGACCAGTTGTCCTGCTGGTAGTTGCAGTAGATGCGAAGGTATTGTTCCAGAGTCT
TATTTGTTCGTTCCGTCTGACCGTCGCCTTCTGGATGGTAACCCGAAGTGAAGTGAAGGGTCATATCAAGTGC
TTGGCCTAGGGAACGGAAGAAGTGTGAGACGAACTCGGAACCACGATCTGAAGTGACATGGGAAGGAACACCG
TGCTTGGAAAAGACATGGATAACGAAGAGTTGCGCTAACTGAGCGGAAGTGATGGTATCGTGTGTCGGAATAA
TGATTGCTTGCTTAGTGAGTCGATCAACAATAACAAGGATAGCGTCGTAGCCGGATGAAGCGGGAAGTTTCTC

GATGAAGTCCATAGATACATGGAATGCCAGGGGCGATCCGGAATGGGAAGCTGTTTGAGGGTGCTGTAGGGTC
GATGTCGTTGAGGTTTGGATCGCATACAGGTGACGCAGCTTTTGCAGAAGTGTTTGACGAAGTCTCGGATAGC
GGGCCAGGTGTAGTTTCTCCAGATAATCTCCAAAGTCTTGTTTTGGCCGAAATGACCAGCGGTAATGTGGTCG
TGATGATATTGCGTGATGCGGAGACGTAGGTCGTTAGCGTCGGGTACGTAGATCCTACCATCGAGGAGTAGTA
AGCCGTCGGAATTGATGGTCCATCTTCCCTGGGGCGACGCGAGGTTCTGAGCAGCAATGGAATCTTCGGAAAG
AGATGAACGGATGTCGGAATGAAGTTGGAAAAGATCCATGATAGTAGCAGCGCGGAACACAGGGGTCGATAGA
GAAGTAGCACGTAAGGAAGAGATGAGTTGTTCTTGAGTGAATACAGGGCGAAAGTTGTGTGGGTTTGCCGAAG
CGTAGACCTTATCCTCCCCTTTTGGGTACACATCCCAACGCCTTGTCAGAGCATCCGGCTTGGTACCCAGCTT
ACCCGGTCGGAAACGAATGACCAAGTTGAACTGTGAAAGGTATTCAGACCAACGAGCTTGCCGACGAGTGAGT
AGTTTAGTGGTAGCAAAATACTCAAGGTTTTTGTGGTCGGTAACAACGTCAACCGGTAATGAGGCACCTTCCA
GATAGTGCCGCCAGATGCAAAAGGCTTCAAAGATAGCGAGAAGCTCCTTATCATGAGTGTCGTAGTTGAGTTC
GGGAGCTGTGAATGTGCGGGAGTGGAATGCGATAGGGTGGATGTCGCCATCAGAGGTAATGGTGGATAGGATA
GCGGCTAAGGCATAATCCGAGGCGTCGGTTTCAACAATTATCTGCGAGTCGGGAATCCAATGGGTCAGGACTG
GAGCCGTAGTGAAAGCCTTCTTCAACGTTTCGAACGCTTCCCGAGCTTCGGAACCAAAGTTCCATGGTGTACC
TTTGCAAGTAAGGCGAGTCAAGGGAACTACGATGTCGGAATAGCCATAGATAAAGTGTCGGTAGAAGTTAGCG
AAACCCAGGAAAGACTGAACGTCTTTGACCTTCCAAGGTTCCGGCCAGTCTTGGATAGTCTTGACCTTGTCGC
TAGACATAGTTAAACCGCCAGGAGAAAGAATGTAACCCAGATACTCCACAGACTCAGAATTGAATTCGCACTT
GTCAGCTCTAGCATAAAGACCGTGTTCACGAAGACGTCGAAGGACTTCCCGTACATGTTTGCGATGTTGCGAA
GGATTATCGGAATAGATGAGAATGTCGTCAAGATAAATGGTAGCGCAAACGTTGAGTAAGTCTTGAAAAATGT
CGTTCATGAAGCGTTGGAAAGCGGCAGGCGCATTGGTGAGTCCGAACGGCATAACCAGCCATTCAAAGGAACC
ATAGTGAGTACGGAAAGCTGTTTTCCATTCGTCACCATCAGCAATGCGGACGAGATGATAGGCATGCCGGAGG
TCAATCTTGGTATAGATGCGAGCTTTGCGGGGAGAGTCGAGGAGATCAGAAATAAGTGGTAGCGGGTAGCGGT
CTTTCCGGGAAATCCTGTTTAGGCCACGGAAATCAACACAAAGTCGGAGTGAGCCGTCCTTTTTCTTCATGAA
GAGAACGGGAGCGCCATGAGGAGAAGTAGCTGGTCGAATGAATCCGATGGAAAGGTTTTCATCAATGAAATCA
CGTAAAGCATCAAGTTCGGTGCGAGACAAGGAATAGATGGGACCTATCGGTGGTAAAGTTCCGTCCTCTAAGT
CAATCTTGAGATCGTAATGCCGATGAGGCGGTAAGGTGTCTGCACGTTTCTTGCTGAAGACGTCGGTGAAATC
ATGGTACTCATCCGGTATAGCGGAAAGGTCGACGGGTGATTCCGAGATAGCGGTTGCCGCAGCAACAACTGAA
TGAAGGTTCAGAGCAAAGCGATGAGAGCCAGGCAGTCGAGATGCGCGAGCAAAAGCCGCGGCATTGATTAGAC
ATATGTCAGGCCTGGAAGGTGAATCCGAGAGTTTGTCTGTGATGGAAGCTGATGCCGACGTTCGTGGAACAGT
GTCAACAGAAGATAGCGCGCGGAGAGAAGGCGGGACTGCAATGGTAGTGGAACGGAAAGTGATGCGGCCAGTA
ACCCAGTCAATCAACGGATTGTAGCGGGACAGCCAGTCGTGCCCAAGAACCACTGAACACTGCGGGTCCAGCG
AAGTGACATAGAAGGAAAGTGTCATAGATTCACCAGTAGGAAAAAGTACAGGAAGCTTTGCGATCTCGGAAAT
AAAAGAATTGGAAGTCCCATCAAACAAATGTAGCTTTACAGGAGGTACCGGAAATGTCGGAATACCATGTTTG
CGTACAAGCGAACCTTGGATAAAGCAATGAGTAGAGCCACTGTCCAATAGCGCAGAGAATTCGGATAAGGAAG
GGAAATCAGGAGAAGAAAGGGGTATGCATAACGAGTTCGGATCAGAAAGAGCGGAAGCGTTGAGACGGATACT
CTCTGGTGCACAGAGGTTGTCAACGCGGTCTCCCACCTGTGCAGAGTCAAGGGGACTGCGTTCTAATTTCCCG
AAGTGCTAGTGGTAGCACCCGAGGAATCAGCGTTTGCCGAACGTCCCTTTGGTTTGTTTGAAGCGGAATCAGA
TTTCCCAGCCGTCTTCTTGGAGCAGTTGTCAGCTGTATGCCCGGGACCGCCGCAGAACATGCAGAGGTTCTTG
TCCTTGCGACGTTGGCGTTCTTCCGGAGTAAGTTTACCGTTTTTACCGAGGACATTAGCGAGGGGATTAGAAG
ACGAAGAGGATGATTGCGCATTCGAGTTCGAATTTGATTTAGAGTTGGGATTGGAATTGGAATTGGAGTTAGG
AGTTTTGCCGGGTTTGGAGTTATTGTTTCCCGAGGACGATTGCTGGTTATTTGAATTGGAGTTAGAACCCTTT
CCTTTCCCCTTATCCTGTGACGATGCCTCCTTCGAACCCGACTGCTTGGTAGCACGATTCCGTTCACGTTGCC
GTTCCCAGTAGCGGTTATCAATACGCATTACCTGTTCCCACATCTCCCGCATGGTAGGGGGTTTGCCGTTGGG
AAGAGCGGAAAGAGTGTCCTGAATGCGGTCGGGAAGGCCATGATAGTAGCGATAGACGAGTACGGAATCCAGC
CAGTGAAGCCGGCCAGCAAGACGGGCAAAAGAGACGTTATACTTGTTGACATGCTCGGTAGTAGCCATGCGAA
GATGCTCAATTTCTTCAGCAGCCTCAGCTAACGGATCCACAGCACCAAAGTTAGAAGTCAGCTCTTCGACAAA
GAGATCCCAGTCGTTGTACCAGGGGATAGCGTTCCAATCCGGAGTATCCTCGAGGAGAGTCTGGAACCAAGTC
TGAGCGTTGCCGGTGAGGTAGGACAAGGCGAAGGAAACTTTAGTATGATTGGTAGGGAATTGATGCGGATTAG
CGTCGAAATAGATACGGCATTGGAAAATGAATTCATTGAGTTTAAGGGGATCCTTGCCGTTGAATGTGCCGGG
TTCCTTGATAGCGACCTTTGGGGTTTTGACAGGGGGTGGCTGAGCGGAATGACGATTATGAGTTGCTAAGCCA
CCAGCGATGCCGGCCAGGAGGTCGTAAAGGACCCGATCCTGAGGGTTGAGACCACGGAGCGCATCGCGAAGAC
GGGTTTGAGTGTTGTCCGTTGCCACGGATTCATTGTCACCTTCCTCACCATCACCTCCGGGATCCCCACCGTC
TTCACCATCATCACCACCATCGCCATCATTTCCACCATCGCCATATTCCAGTTCGGGTTCTTCGGCTATTTCT
TCAAAACGAGATGAGTCGTCACCTGCGAATCCGCCCGGAACGTTATGACCACGGGAACCAGAAGGTTGAGTAG
ACATAGGAGATATGGAGCCGGGATCAGGGGTTGTCGATGTTGCCGATAGTTGGCCTTTTGGGTGTTTCGGTTT
GCAGGAAGGAGGAAGTGGAATGGTTTCAGGGGAAATACCAGTAAAACGAGAAACACTGCGAGTATGGCTCATA
AGAGCAAAAAGACGTAAAACCTAGTTGGAAGAGTAGTCTGAAAATACTGTTAAGACTATGACCGGAAAGGTAA
CGCTAAGCCGGAAAGGACTTAGCGAACAAACGGCTTTGGCCTTAGCGACACGGGAACAGTGCGCTATGCCTTC

CAACTAAGTTGATAGCCGATTTAGAGACAGAGACGAGCGACCCGCGGAATGCAGAATCGTTTGAGAGCGAGAG
AGAGATCAAGCGAGAGATAGCGAGGAACAATCTAAGTTGATTTTCAGACTGAAGTACCGTACAGAATATCTAC
GGCTTAAATACACGAAGAATGAATAGAATACGGATTCGGAAACATGAAAGATGATAGCGCAAGGATTGCGCAA
TAGTTCAGATTGAAATAACGGAAAGGAGTCTTTACCTTAAGGGATTCTGACGGTGCAACGGAAAGGAGGTCTT
GAAGCGGGTAAGTCTTGCGATGGAAATAACAACCCATGTTGCGTTGGTCGTAAAGCGACCATGGGAGCGCAAC
CACAATACCGAAGGTATTGCGGCATTCCATCGGTCAACGTGACCAGTAAGTCGGAATCGATATTGCTGGGATT
GATGCGAACGGATTGAGTCGGAAAGGGATATCCGGAACGTACCGGGATTGATACCGAGAATGGAAGGTGGTGG
AGTTAGCATGTTCCGGATATAACA
>Agabi_varbisH97_2_rnd-1_family-1#LTR/Copia
AGAGTCACATAACCACCCGTAGCTCGACCTGTAGCCTTGCAGGCTCCGTGAGAGGCGTCCGAGTAGGTAGTGA
AGAGTTCCTCCGAGTCACTAGCTCGATAGACTAGCTTGTAGTGCATAGTGCCAATGAGATAGCGGAACAAGTG
CTTGACTGCAAGCCAGTGAGCCGGAGCTGGGTTCGAGTTGAACCTCCCGAGATAGGAGACAGCATAGGCAATG
TCGGGACGAGTCATAGTGGCCAGATACTGAAGGGCACCTACAGCATTGAGATAGGGAACTGACTTCATCTCAG
CCTGATCCTCGAGTGAAGGAACAATGTCAGAGAGCTCAGATCCCGGAGCGAGTGGAGTGGANAGAGGATTAGC
ATCATCCATATTGAAGCGTTCTAGGAGCTCCTTGATGTAGTGCTCCTGCGAGAGGGAGATGGNGCGATGAGAG
CGGTCCTGCTTCACCTGAATACCCAGCAGAAGTGTAGTAGACCCGAGATCACGGAGTTTGAAGTGTTTGGACA
GCTCAGAGACAGCCGAGGCCATGGCAGCATCACTTTTCGAGACAAGAGTAATGTCGTCAATGAANACTGGCAC
AATGACCTTGATGTCACCTTTGGAGTAGATGTAGATGGAGCTGTCGGAGCGAAGCCTAGNGTAGCCAATGCCT
TCAAGGACACCCCGGAGTTTCTTATTCCACTGCCTGGCAGCCTGCTTGAGGCCATAGAGCGATTTACGGAGCC
GAAAGACCATGTTAGGACTACCAATGTGATAGCCCTCGGGTTGGCGCATATAGATCTCCTCTTCGAGTTCCCC
ATAAGTGAAGGCAGAAGAGATGTCGACTGAGCGCATTTTGTAGCCTTCTCTAGCTGCAAGAGCAACAATGAGG
CGTAGTGACGCAGGACGGAATGTAGGGGCAAAGACCTCGGTGAAGTCGAATCCGGGACGTTGGGAATAGCCCT
TAGCAACGAGACGAGCCCGGTAGCGCTCAATTGAGCCGTCGGCAAGTCGTTTGACTTTGAACACCCAGCCAGA
GTTGACTACCTTCTTGCCTGGAGGCAGTTCCATTAGCTCCCAAGTTCCGTTAGCCTCGAGATTGAANATCTCA
GTGTTACAAGCCTCAGTCCAGCAATCCGAGTCAGGAGACTTCATAGCTAGTCTATAACTCCGGGGATCAGCAC
CCGAAACGGCTCCAGCAAACTCGACTTCACAGTACTCAGAGTCAGCTAGCAGAGCAGCTTCGTCGTCAGAGTC
AGAGTCATTACCAGCTAAGTCTGGTTGTTTGAGCTTCCACCATTGACCAGGAGGCTTCCGAACTCGCCCAGTA
CGTCTAGGAACGTGAGGGGGCTCAAGAGGCGCTCCGGCAGTAGGAGGAGTGACTGGGAGTGGTGCATGGATTT
GTGGAGGAGTGACTGGAGGAATCACGGGAGGAATNACTGGAGGAATCANTGGGAGAGGTGGTAGAGGTAGAAC
TGGCTTCTCTTCAGGAGGAACTGAAGCGGACGGAGTGGGAGTCTTAGGACGAGCAGGAGGGAAGTGATTATCA
CCCCCTTCATCCTGTAGAGGTGGCAGTCCCGAGTGTGACGATGTATCATTAGGAATTGGTAGTGACTCTGGTA
GGCAAGCAAATGACGGGACAGCCTCGAGTTGGGCTTTAGAAGTGCCCGGAAAGTATCTCTCGTCGAAATCAGC
TCTCTCGGAGATCACGGTTCGCTTTGTGACTGGGTTGTAGAACTTCCAGCCTTTGTAGCCAGCCGGATAGCCT
ATAAAGACACACTTCTCGTAGTGAGGCTGGAGAGAATTGCGCTTATCTCGTTGTACATGGACATAGGAAGTGC
ACCCCCATACCCGGAGATGTGAGACATCAGGTTTTCGCTTATAGAAAGCTTGGAAGGGAGTAGTGCCCGAAAG
CGAAGCAGTAGGGAGCATATTCCAGACATGGACCATAGACGAGATACATTCACCCCAGAAAGAAGCAGGTAGC
CGAGATTCCGAGAGCATAGCAGTGATCCTCTCTCCCATAGTCCTATTAGCCCGTTCAGCAAGNCCATTCTGCT
GAGGCCGGTTCCGAGTCGAGTGACGTCTCTCGATGCCAGCAAGCTCAGTAAAGCTTNTGAAAGCNTTAGACAT
GTATTCGCCTCCCTTGTCATCCTGTAGNGCTCGCAGTTTGACTCCGAGTTGAGTCTCAGCCCAGGATTTGTAG
ACTTTGAAGGCTTCAAATGCCTCCGACTTAGCTTTGAGCAGATANACTGCCCGGAAACGCGTCGCGTCGTCAA
TGAAGGTGATCCAGTACTTGTAGCCTGAGTGTGAAGCGACTGGGAGCGGTCCGTGAAGATCTGAATGCACCAG
CTCGAGAGGAGAGCGATTGTGATTCAAAGAGGAGGGAAAAGGAAGGGAATGCATCTTGCCAGCCAGACACGGC
TCACAGATAGAGTCAGGGGACGAGTTAGACTCGAGTTTGAGCCCGGAAACNAGGTCCTTACTGATCATAGACT
TCACATCAGCGTAGTTGTGATGAATGAAGCGACGATGCCATAGCGAGAGGTCAACCGGGAGAGTAGAGACAGA
GAAAGCGGCCTCAGAAGTGAGAGTTGAGCCGTCGAGCTCAGCAGAGTTGTTGGAATGGATAGTGGCCGTGAAA
AGCGAAATCCCTTTGCATTGGAAGTCAATAGANTGAGCGGAGATGGTGATATTGAAGCCCTTGTGTCGGGCAA
GGTAGAGACATGATAAGAGATTACTCCTGAGGTCAGGAACATGAAGGACTCTCGTAAACTCGACCGCTCTAGT
CTCTTTTCCATTCACCACCGGTTGAAAGACAACAGACCCTACACCAGCTGAATAGACAGTAGAGTTGTCAGCA
AGGCGGATAGGGATACGATGAGGGGAGTAGGATTTGAACCAATGGCGGTGAGGAGTCATGTGGGATGTAGCAC
CAGTATCAGCCAGCCAGAAAAAGTCAGCATCAAGCTGTAGTGGCGAGGATGGTGAGGAAGATGATGAGGGAGA
GGATGAGGGAGAGGAATAAGCACCAAGAGCGGACGCTCTCAGTGGAATATCAGAGCCAGGAGAGCCAGAACCA
GTAGAAACATCCCCAGGAGGAGTGGGTGGTGTCTTGACAGCTGGAAGAGCTGCACCTATCCCACTCAGATGGC
CAAGTGGCCCTTTGACATTACTTTTAGATGTTCCAGGCAGTCTGGAGCGCTCCTGAGCGCGTTTTTTCGCGTT
CTTTCGCTTCCTGCACCTCCTAGACATGTGCCCTACCCTCCCACAAAAGGAACAAGCGATTGACGCGTTCTTA
GGGCCGGAAATCGGTCCCAGAGAGTGTGGAGAGGGTGTATGGCTGCCAGAGGGAGTTGTGGTCGATGTAGAGG
CTCTCAGACGCGTCGGAGACGCGCCAAAACGCGTCGAAGTGGTGTCCCAGGCGTAGGGAGCGTGTGTGCGCGA
TTGCGAGGCCATAGACGTCGATTTGAGTGCAGGGGAAGGGATAGAAGTTGAGATATAGAGGGGAAAATGGTAG
AAGAGTGGCGAAAAACGCGTTTTGAAGGCTGTTAGAAGCCGGGGCCCATAACCTGTTAAACGTAGAACGATAG

CGGTTGCAGTGGCGATCCTAACGAGCCTAGAAACGAGTCGAACGGATAGGAACTGACTGAGCAATCCCAAGGA
CANCTGTGGACAGACTGTAAGAAGCTAAGGCCTCTGAAGCGTCCACAGGGGAGTTCAACCATACATGACTAAG
CTATCATGTGACTAAACTCGTCCGCATATATCTAAGTCCACCAACNACACTTGTAGACTGCTTGCTCACTCTA
ACACACCCCCTTGATCCAGCCAGTCTACTCAAGTCCTAATAGAGGAACAGCAAACTCAACTACCTTCTTTGCC
ACAGCCTTGGTAAGGATGTCGGCCACTTGCTCGTGAGTCGAAACATACAAAGGATCTATGAGTCCTTCTTCAA
CAGCCTCCCGGAGCCAGTAGAAGCGCAGGTCTAGGTGTTTCATGCGTCCGTGATGCTCTGGATTCTTGGCTAC
ACTGATTCCTGACTTGTTATCAACGAAGAGNGTCGAAGGAAGTGTCGGAGGGAAGCCAAACTCGGTCAGGATA
TTCCTAGTCCACTTGATCTCCTTGCCAGCCTCGACGGCAGCAACAAACTCGGCCTCAGTGCTCGAGAGAGTCA
CAAATGGCTGGCGCTTAGAAGACCAGCCTACAGCAGCNCCCGAGACTAGAGTCACATAACCACCCGTAGCTCG
ACCTGTAGCCTTGCAGGCTCCGTGAGAGGCGTCCGAGTAGGTAGTGAAGAGTTCCTCCGAGTCACTAGCTCGA
TAGACTAGCTTGTAGTGCATAGTGCCAATGAGATAGCGGAACAAGTGCTTGACTGCAAGCCAGTGAGCCGGAG
CTGGGTTCGAGTTGAACCTCCCGAGATAGGAGACAGCATAGGCAATGTCGGGACGAGTCATAGTGGCCAGATA
CTGAAGGGCACCTACAGCATTGAGATAGGGAACTGACTTCATCTCAGCCTGATCCTCGAGTGAAGGAACAATG
TCAGAGAGCTCAGATCCCGGAGCGAGTGGAGTGGATAGAGGATTAGCATCATCCATATTGAAGCGTTCTAGGA
GCTCCTTGATGTAGTGCTCCTGCGAGAGGGAGATAGTGCGATGAGAGCGATCCTGCTTCACCTGAATACCCAG
CAGAAGTGTAGTAGACCCGAGATCACGGAGTTTGAAGTGTTTGGACAGCTCAGNGACNGCCGAGGCCATGGCN
GCATCACTTTTCGAGACAAGAGTAATGTCGTCAATGAANACNGGCACAATGACCTTGATGTCACCTTTGGAGT
AGATGTAGATGGAGCTGTCGGAGCGAAGCCTAGAGTAGCCAATGCCCTCAAGGACACCCCGGAGTTTCTTATT
CCACTGCCTGGCAGCCTGCTTGAGGCCATAGAGCGATTTACGGAGCCGAAAGACCATGTTAGGACTACCAATG
TGATAGCCCTCGGGTTGGCGCATATAGATCTCCTCTTCGAGTTCCCCATAAGTGAAGGCAGAAGAGATGTCGA
CTGAGCGCATTTTGTAGCCTTCTCTAGCTGCAAGAGCAACAATGAGGCGTAGTGACGCAGGACGGAATGTAGG
GGCAAAGACCTCGGTGAAGTCGAATCCGGGACGTTGGGAATA
>Agabi_varbisH97_2_Cluster33#LTR/Copia
AGCCGAAAGCATTAGACATGTATTCACCTCCCTTGTCATCCTGTAGTGCTCGCAGTTTGACTCTGAGTTGAGT
CTCAGCCCAGGATTTGTAGACTTTGAATGCTTCAAATGCCTCTGACTTAGCTTTAAGCAGATAGACTGCCCGG
AAACGCGTTGCGTCATCAATGAAGGTGATCCAGTACTTGTAGCCTGAGTGTGAAGCGACTGGGAGCGGTCCGT
GAAGATCTGAATGCACTAGCTCGAGAGGAGAGCGATTGTGATTCAAGGAGGAGGGAAAAGGGAGGGAATGCAT
TTTGCCAGCCAAGCACGGCTCACAGATAGAGTCAGGACACAAGTTAGACTCGAGTTTGAGCCCAGAAACTAGA
TCCTTACTGATCATAGACTTCACATCAGCGTAGTTGTGATGAATAAAGCGACGATGCCATAGCGAGAGGTCAA
CCGGGAGGGTAGAGACAGAGAAAGCAGTCTCAGAAGTGAGAGTTGAGCCGTCAAGCTCAGCAGAGTTGTTGGA
ATGGATAGTGGCCGTGAAAAGCGAAATCCCTTTGCATTGGAAGTCAATAGATTGAGCGGAGATAGTGATATTG
AAGCCCTTGTGTCGGGCAAGGTAGAGACATGATAAGAGATTACTCCTGAGGTCAGGAACATGAAGGACTCTCG
TGAACTCCACCGCTCTAGTTTCTTTTCCATTCACCACCGGTTGAAAGACAACAGACCCTACACCAGCTGAATA
GACAGTAGAGTTGTCAGCAAGGCGGATAGGGATACGATGAGGGGAGTAGGATTTGAACCAATGGCGGTGAGGA
GTTATGTGGGATGTAGCACCAGTATCAGCCAGCCAGAAAAAGTCAGCATCAAGCTGTAGTGGTGAAGATGGTG
AGGAAGAGGATGAGGGAGAGGATGAGGGAGAGGAATAAGCACCAAGAGCGGATGCTCTCAGTGGAATATCAGA
GCCAGGAGAGCCAGAACCAGTAGAAACATCCCCAGGAGGAGTGGGTGGTGTCTTGACAGCTGGAAGAGCTGCA
CCTATCCCACTCAGATGGCCAAGTGGCCCTTTGACATTACTTTTAGATGTTCCAGGCAGTCTGGAGTGTTAGG
TGATGACAGGACAAGTCACCTGACAGTTCGGAGCTCTGTGCTCTCACATAATCTGTACTGTGCCTCAGCCCCT
GCAGCAGGCCCTCAGTCGTGGGTTCCACCTCTAGGCCTAAGATGTATACATATAACATCTTAGCTACTATATA
CCAAGATCATATAGCTCAGTGGTTAGAGCTTCCTAGTGCAATTGGGATGGTCCCAGGTTCAAACCCTGGTGTG
GTCACTTTTCTATCTTGACATGGAGCGCTCCTGAGCGCGTTTGTTCGCGTTCTTTTGCTTCCTGCACCTCCTA
GGCATGTGCCCTACCCTCCCACAAAAGGAACAAGCGATTGACGCGTTCTTAGGGCCGGAAATCGGTCCCAGAG
AGTGTGGAGACGGTGTATGGCTTCCAGAGGGAGTTTTGGTCGATGTAGAGGCTCTCAGACGCGTCGGAGACGC
GCCAAAACGCGTCGAAATGGTGTCCCATGCGTAGGGAGCGTGTATGCGCGATTGCGAGGCCATAGACGTTGAT
TTGAGTGCAGGGGAAGGGATAGAAGTTGAGAGTTCAGCGTGTCGCAAATCAGGCTCAAGCATTAGTAGTAGTC
AACAATGATCACAATCTAGAAATTGCAAGCCACACCGCCACCGGCACCGGCGGGCAGGAACAGAGTAACAGGC
CCTCGACAAAACGAGCGCATGTCTATGACTTTGCAGTTAGTTTGAGCCACATACTACTATTCAAAAAAGTTGT
ACTCACCAAACACACATTGCAGGATCCTAATACCCCAACTATACGATCACCAATGGCGAAATGTACACGCAGC
AGCCGCTTTGGTAATTCCAAAGCAAGTGCAAGACAGGTTGTTCCGAAAATAAACTGGAAAAGTCAAGTTGAAC
TCAGCACAAGTGCAAATACGATGAATGTTGAAGCCAGAACAGATGACATGTTTGACTCACAACAGGTTGATAC
GCCGGCGGATCTCTTCCAAGCTGAAGGTGAAGATTCCCAGTTCCGGGTAGATGAACGGGATGGTAGACTTGAA
GGAATGGGCGACGCTGCTGATGACAGACACGATATCAGGGCTAAAGAAAGGGTGAAAACTTCTGATCAACTTA
CCCGGATGGGTGGAAGGGATACAGCACCCAATGCCGCCGTTGAGTCCAATAATGACAAGGCTTATTTGAGCTC
AGAGATATTGAGCTCGGAGTATGAACCCAGTGAATTGGGCAAATCCGAGTCTACCTTTGACGAAATGGACTCA
GACACTGGCAGGCGGAAATCACCAGATGTTGAGCCCATCCCCATTCCCCTTGATCGATGCAATATGAGGTCTC
CGTTTGTCAACGAGCTTTATGATGACTACAAGGAGTGCCATCGAAGGTCAGATGACGAAGATGATCAGAGTAC
ACATTCGACGAGGATATTATGGGGACTCACTACTCGATGGGAGTCAACCTCTTGAAGACTTCGGTTCTCCTGA

ACTTGAAGTAGATGAAGCGCAAGAGTTCGACGTGCTGAATCCTGGCGAGAATGTGAGTACTGACCTCCTGCCA
ACCCAGATCAAAAGCTACAACTTACTTGGTGTTGTGGGAATTCATCAATAGAATGACTCTCGCCAACTGGTCG
CTCGACTCGCAGGAGAACCGAGCTATCATGGACGCCGTCCGCAACCGGGGCATTGCCCCCGCGGTCGCCATCG
CCAACGCCAAAGCTAGTGCGCTAGCTAAAGGTAATGTCAAAGGGCCCTCTGGCCAACCTACTGGGATCGGTGC
AGCCCCCCCGGCTGTCAAGACACCGCCCAGAGTCCCTACTCCCCCTCCCCGTGTGCCGACACCTCCACCGCCG
GTGCCCATTCGTCCCCCTTCCCCTCCTCGCGCACCGTCGCCTTCGGCTTCGGTGCTTTCCTCGAAGCGCCCCA
TCTCGCCGGGGTCTTCATCTTCCGGTCCCTCTGTCGCTGCCGTCTCGGTATGCGGCAGCGACGACTCTTCAAT
GGCCCTTGACTACGAGGACGCCACCACCCCCCGTCCCACTTCTCCTGCCGATGAGGAGCATTTGGACAGTAAA
GAATCCAAAGAAGTTATCATTAACGCTATCCGCACCGCTCTCGGTTTTTTCGAGGCGTTTGCATCTAAGGATT
GGAAGACTGTCGTCCTCGACGTCTTCCCCACACTCATGGACGACGATCTCAACGACGTCCACAAATATTTCAT
TAGGAAAGTACATGATTTCCGATCCCTCCTCCCCCCGGCAGTTGAAGCGCCTGCATCCGCAGCTGACGCACCT
GCCACACGTGTAAATCCGCGGGACGCACTGCCACCCGCTGAGTCATCCAAGGCTAAGCCTCCAAAGAAGAAGG
TTCGGGTCGATCCGGCCCCACAGTCTGACGTCACTCCTCCGGCAACGGCCCCACTCATGGAAGTGGATGAGCC
GGCCGCTCCACCTCCAGCGTCTGGGAAGGCACCCACCAAACCCCTTCCCATCCCAAGTTCAAAGCGCGCGCCT
CCTCCCTCTAGCGCCCCTTCAGTTTCGTTCCGCGCTCCTTCGGCGCCTCGCAAGAGACGCCAAGGCAAGCACA
CAGCACACGGCGCTTCGCGCCGTGCTATAATCCTGACACCGCCTGCGGACTCTCCGGTAACAGCGGCTTCATT
CACTCCCGAGATTATTAATAGTCTCAATAAGCTCATCAAGAATGACATCAAGTCTGATGTCATTCTTACGCAT
GCCTCTGTTGAAGGCAACGGCATCTGTATTGCCGCCTCCCGTGTCCCGTCCCCCGCTGAAGTCTCTTTCGTGC
TCAAGCACGTTCGGAGAATCTTCCCGAGCCCGAATGTCCCCATCGGCCATCTCCCCATCACGTCGACATCTTA
TTTGAAGATCGTCGACATCCCTCACGTGCCTGCTTCCTCCAAAGAGTGGGCACTCAAACAACACGAGGCGTTC
ACTAGCGCTCTCAACAAATCTCCAGTCGGCGCTTCGCTGGCCAAATTAATCAAGCACAAGCCGCGCTTCATGC
GCGCCTCCCCCCATTCCGACTCGTGCTGGGCATGGGTCGATATACACGACACAGTGTCGGGCAGCAATGCTCG
CACTTATATTTCCAAGTTTGTGTCTATTGGCGGCACAAACTGTCAGATCAAGGGCGCTCACCCTCATTCCGGC
TCCGTCCACTGTGCTCGCTGTCAGCGCTGGGGCCATCATTCAGATCAGTGCCGCGCCAAGTGTGCCCGCTGTT
CCCTCTGCTCTGGTCCTCACACCGAGGCCAATCATTTCAAGTCCGTCGACGTGCGCCAATGCGCAAACTGTAC
CGCGGCGAAACACCCAGCGGACAAGCGCTCTCATTCCGCTACGGACTCGAAGGTCTGTCCGTTTTGGAAAAAT
AGGTTCGATCGTGCCTGGCTCAAGTGCCAGTTCCCCGCGCGTTCGACTTAGGGTGTACAGTGAACCGTCGCTG
GGTTGGGTCGGGGATTCTGATGGAGAACTGGGAAGGTTGTGTAGCGGTTTAGGAGGGTGAACGGTAGTGGTGA
GGACAATTTCTACTGAGGATCTAAAGTGACTTCGAGTCTGCCTCAACTTTTTACTTTCATATTTGATATGTTT
GATTCCGCAAAAGGGACTGCGAGTGCTCTAGCGTTACGGTATTATGTAGGGTGTATTGGCTGACTGCTTGGTT
TTATCCCGGGTCCCCTTACGTATGTATCAATGTCTCGATTTGTTTTTGTTTTGTTTCTTTTCTCTCTCTTGAT
AGCGCTTGACCTCGTCGCGCTGGATACGGTTTCGGTGTAGTTAAGTACAGGTGATGTAAAGTACGGGGAACGC
GTCATGGCACCCTTTAAGTAGTTTTGAAATAAACTATAGTGATTAAAAAAAAAAAAAAAAAAAAAAAAAAAGAT
GTAGTGTTAAACGTAGAACGATAGCAGTTGCAGTGGCGATCCTAATGAGCCTAGAAGCGAGTGAGCAATCCCA
AGGACAAACTGTGGATACACTGTAAGAAGCTGTATTGTGATATACTAACTTGTACTCACCTGTGGATACACTG
TAAGAAGCTAAGGCCTCTGAAGCGTCCACAGGGAAGTTCAACCATACATGACTAAGCTATCATGTGACTAAAC
TCGTCCGCATATATCTAAGTCCACCAGTAACACTTGTAGACTTTTTTTTTTTTTTTTTTTTTGTAATCAGTG
GCTGTTCGAGCCTTCTATTCCAGCGATATAAGTACATGCGAACACTATGTACAACATGCTGAGAATACGGTTG
CCATGACGCGTGACCATAAGAAAAAGAAAAACGTATCCTGCGCGACAAGGTCAAGCGCTAGCGTAGAGAAAGA
GAAAAATAAGTGTTTGATCGAAAAAGGATGATACATCAATATGCGGCCCCGGAACGAATCACAAACAGACAGG
CACAGACCATAGAACATATCGGAACGCGAGACCGGAACATAATCAAGCGGAATGTAGACATGAAAAAATGAAA
ACGAAAGTTGGGAGGCCTCACCGGCTACTAACCCCGAAACGCGTTTTGACGCGCGCTGATCACTTCTTAGGGA
ACTGGCGCCTCAACCAGTCCCTGTCGAATCGGTTCTGCCAAAACGGACACGCAGTGTCCGTTGCAGAGTGGTC
GCGTTTAGTCCTGCCTGCCGCACTACAGTTGACACAGGACCGGGCGTTGGGATCGCGCTTCGAGTGGGCGCAG
TATTCGTGATGGGCGGCCTCGGAGTGAGGGAGCCCGCATAGAGGACATCGGATACCTTTGGACCGGCAAACCG
AAGAGTGATGGCCCCACCTCATGCACCGAGTGCACAAGGCAGAACCGGGCCGTGGAGCTGTGCCGCGGATCTG
ACAATTCCGACCGCCGATGACGATCGTCTTGCCGATATAGGAGCGGGCCGAACTGCCCGCAACGGAATCGCAT
ATGTCGACCCACGCGACACAAGTGTCGCTGTGAGCCGAAGTCCGCATGAAACGGACGGCGTGCTTGATGGCGA
TGGAGAGTTCCTTGCCAACCGGCGATATCGATAGCGCGTTGGTGAATGCGCTGCGTTGGGCGGATTGCCAAAC
CTTCGGCTCGGCGGGGATCATGGGGACGTCGATGACCTTGAGATAGGAGGTCGATGTAACCGGAGTGGATTTG
ATGGGCACGACGCCCGCGACGGTGACAAGACGCCGTATGTGTTTGAGCACACACGTGGTCTCGGAGGAGGTGG
GGACTCGGCTCGCGGCAATGAAAATGCCGGATCCGGAGTCCTCGGAGTGCTCAAGAATGACGTCGGAACTGAC
GTCATTCTTGAGGTGGGCGTTGATCTCGGCCAGCATGGCCGGAGATACGTGAGCAGCGCGGATGGAAGATCCG
GCCGGGGGAGTGAGGAACACGCCGCGACGCGAAGCGCCGTGGCATGTGTGACGGGCCCTACGTTTTTTGCCCG
AAGGCTTGGACGCAGGGGTGGTGGGGGCGATAGCCGCGTGAGCGGCGGGAGCCTGGGGACGGGGCGGAGGTTG
CTGCTGAGGGGGACGACGCGCAGCAGCCTTGGCATATGTGTTATTTTTGGAAGGGACAGGGTCCTTTCCAAAA
ATGGGAGGCTGGGGTGGAGGAGGGGGGGTCGGCTGTGCCGGCTTCGCCTTCTTCGATTTTCGCGGGGTCATGT
CCATGTCGGCCGACGGAGACCGTGGCCGAGGGGTGGCTGTACCCGAGGGAGTGGGAGGGGGCGGAGTTGGCTC

GAATGCCCGTGGAGGCGGATCGACCATACCGATTATCTTCGCGAGCTGTTCTGAAAAAGAACCGATGCCCGCG
ACGGAGGAGGTTTTGTACCCGTCAAGATCGCGCCGCTTGATGAGTACATGTGATGCCCGCAATATACAATCCG
TAAACGGTGCATACAGCGGGTGTTCGGGCTCGATATCGTCAAGCATGACGATCGAGGTGTTGACTAATTTGAG
CATTCCCTTCTGACCCGAGGTCAGAGTACGGGAACGCCCAGTGGAGGGGGTGGAAGTCGGAGCCGGTGTCGGA
GTCGACGGAAACAGCATTGGAGAGCGGTTGTCCAGCGGAACGGGGACCGAGGTGTAGGACAGGGGGGTGAGAG
AGGACTCGGAACGAGTCCTAGCAGGATTTGGCGTCGTCATGCGAGACGCCGCCGGGAGTTCGAGCTCGTCTTC
CATACGAGCCGCTGCCTGTAGAAGAGCACGTCCAACAGAACTGCTGTACATACCCTCCGGGATATTCTCATCA
TCAGACCAGTCGCTGTCGTCGCCGGCAGCATCCACGTCCATTGCAGAAGGTTTATTGCTACTCATAACTAGTG
ACCACTGAGACTTCCAAAAGAGGAAGGTAGTGGTACAGGTTCAAGAAGCGATGTAACACTTGTAGACTGCTTG
CTCACTCTAACATGTAGATGGAGCTGTCAGAGCGAAGCCTAGTGTAGCCAATGCCTTCAAGGACACCCCGGAG
TTTCTTATTCCACTGCCTGGCAGCCTGCTTGAGGCCATAGAGCGATTTACGGAGCCGAAAGACCATGTTAGGA
CTACCAATGTGATAGCCCTCGGGTTGGCGCATATAAATCTCCTCTTCGAGTTCCCCATAAGTAAAGGCAGAGG
AGATGTCGACTGAACGCATTTTGTAGCCTTCTCTAGCTACAAGAGCAATAATGAGGCGTAGTGATGCAGGACG
GAATGTAGGGGCAAAGACCTCAGTGAAGTCAAATCCAGGACGTTGGGAATAGCCCTTAGCAACGAGACGAGCC
CGGTAGCGCTCAATTGAGCCGTTGGCAAGTCGTTTGACTTTAAAGACCCAGCCAGAGTTGACTACCTTCTTGC
CTGGAGGCAGTTCCATGAGTTCCCAAGTCCCGTTAGCCTCGAGATTGAATATCTCAGTGTTACAAGCCTCAGT
CCAGCAATCCGAGTCAGGAGACTTCATTGCTAGTCTATAACTCCGGGGATCAGCACCCGAGGCGGCTCCAGCA
AACTCGACTTCACAGTACTCCGAGTCAGCTAGCAGAGCAGCTTCGTCGTCAGAGTCAGAGTCATTACCAGCTA
AGTCAGGTTGTTTGAGCTTCCACCATTGACCAGGAGGCTTCCGAACTCGCCCAGTACGTCTAGGAATGTGAGG
GGGCTCAAGAGGCGCTCCGGCAGTAGGAGGAGTCACTGGGAGTGGTGCATGGATTTGTGGAGGAGTGACTGGA
GGAATCAAGGGAGGAATTACTGGAGGAATCAATGGGAGAGGTGGTAGAGGTAGAACTGGCTTCTCTTCAGGAG
GAACTGAAGCGGACGGAGTGGGAGTCTTAGGACGAGCAGGAGGGAAGTGATTATCACCCCCTTCATCCTGTAG
AGGTGGCAGTCCCGAGTATGAAGATGTATCATTAGGAAGTGGTAGTGACTCTGGTGTGCAGGCAAATGACGGG
ACAGCCTCGAGTTGGGCTTTAGAAGTGCCCGGAAAGTATCTCTCGTCGAAATCAGCTCTCTCAGAGATCACGG
TTCGCTTTGTGACTGGGTTGTAGAACTTCCAGCCTTTGTAGCCAGCCGGATAGCCTATAAAGACACACTTCTC
GTAGTGAGGTTGGAGGGAATTGCGCTTATCTCGTTGTACATGGACATAGGAAGTACACCCCCAGACCCGGAGA
TGTGAGACATCAGGTTTTCGCTTATAGAAAGCTTCGAAGGGAGTGGTGCCCGAAAGCGAAGCAGTAGGGAGCA
TATTCCATACATGGACCATAGACGAGATACATTCACCCCAGAAAGAACCAGGTAGCCGAGATTCCGAGAGCAT
AGCAGTGATCCTCTCTCCCATAGTCCTATTAGCCCGTTCAGCAAGACCATTCTGCTGAGGCCAGTTCCGAGTC
GAGTGACGTCTCTCGATGCCAGCAAGCTCAGTAAAGCTTTTGAAAGCATTAGACATGTATTCACCTCCCTTGT
CATCCTGTAGTGCTCGCAGTTTGACTCCGAGTTGAGTCTCAGCCCAGGATTTGTAGACTTTGAATGCTTCAAA
TGCCTCCGACTTAGCTTTAAGCAGATAAACTGCCCGGAAACGCGTCGCGTCGTCAATGAAGGTGGTCCAGTAC
TTGTAGCCTGAGTGTGAAGCGACTGGGAGCGGTCTGTGAAGATCTGAATGCACTAGCTCGAGAGGAGAGTGAT
AGTGATTCAAGGAGGAGGGAAAAGGGAGGGAATGCATTTTGCCAGCCAAGCACGGCTCACAGATAGAGTCAGG
CCACAAGTTAGACTCGAGTTTGAGCCCAGAAACTAGATCCTTACTGATCATAGACTTCACATCAGCGTAGTTG
TGATGAATGAAGCGACGATGCCATAGCGAGAGGTCAACCGG
>Agabi_varbisH97_2_rnd-1_family-117#TIR/MITE
TAACACGGCGGACAAAACAACTGTCAATTGATTGGCCGGGGTTTGCTGTCAATTGATTTGTCAGTTGATCAGT
TGTCACTCGAGTGTCACGCTAAATTGCTTGCGTGTCACGCTCTGGTACGAGATGTACTGCGCATCATGATGCA
TATGTGAACATGACTACATTACTATGTAGACGAAACTTAGGGGGTAACACCGTAACAACCTCTGTCACATGTG
ACAATAGCTGAATTTGCAATTGACAGATCAGTTGACAGTTAACCCTAACCTATCAATTGACAGTTGTTTTGTC
CGCCGTGT
>Agabi_varbisH97_2_rnd-1_family-107#PLE/Penelope
ACCACCCGTTCTTCATCTGTTCTTCTAGTTTTCGAACGTTAAAAAAGTCCCAGGATATGTTATATTCGCTTTT
TAGCACAAGCACGTCAGCCCGTGGCGGCGAGTTCTCTCTTAATCTCGTATCCCACCGTGTTTGGTAATTTTGT
TTTAACCAAGAGGCGATTAACATAGGAGGATAGCCACGTGCGATGTAAATGTCCGCAACTTCTTTACACGCAT
CCGCAAAATCGTCAAATTGCGAGCTAAGCGTAGCCATCCGGCTCAGCTCACCGATGAATGTGCCTTTTTTGAC
ATACAAAGGGTGAGAACTGATCCAGGGTATCCGTTCAAAGTGGTTCAGCGGTTTCCGGTACGGCTTCCATTGA
AGTCGCTTACTTTTATCGAAGTAAAGTGTCATATCCAGAAACGGCATAAATTGGTTAGACGCCGACCACGTTA
ATGTACAGTCTGTGAACGTAACGCCCTCTCTAAGTAGATTAATAGCGTTGGCTTCGCTTGTAGCGTAGACCAA
CGCAAGGACGTCATCGATGTAGCGTCCATAGAACGCCACATCGGGAGATTGTAATACTTCAGATCTTAATTCG
TGCCAAACCCCATAAAGGTTTGCCAAATCGGGGCTACTAGCTACCCCCATGGCGAGGCCACGTTTCTGTTTGA
AATATTTCCCGTCAAACTGACATAATAGATTATCGTCCGCTGCTGATAGACATTCCCTAAACAAGTGTAAATA
TTCACCCGCTTTGTCTACGCTAATGTTTTCCTGTAAACTAAACCAGTGAGCGAGAGCGGCTAAGGCACAATCA
TGTGCGAGCGGTACATTCACATTGGGATAGAAGGCGACGACGTCACCGGAAACAATATATAAGCGTTTCAGCA
CTCCGTTATCTAACTTTACGTACGGAAGCGATAATTTGTTAAGTTTTAGACTAAGGTCCTTCGTTCCGTGNAT
AACGTACGGAGCCGATTCAATCACCGGCCTTAACATTTTTGATACAAACTTCCCTGCAGGTCCTTGAACTACA
GAGTGGCACGGTATAATTGGCCT

>Agabi_varbisH97_2_Cluster27#LTR/Gypsy
GTTATGATCTCATCGATTATGGATATTCTAATTAGGCTAAACTGTTTCTCCTATTCCCATGCGTACTTCTTTT
CAGTTTCTTTGATCTTCCCCTATCTGTATACTTTCATCTCACGCTTACTCCACTCCCATATGCTTTCACTCCA
CTCTTCTATTACCCCGAGTACTATGACTCACTCTTGTCCTATACTTAGTTACCCTGGGTAGTACTCTTTACAT
ATATAAACCCTATAGGTACTGCTGTACTTCTTTACGCTTTACCAATTGTATCTTTCCCTTTCTCTACTCTCAC
CTCGCTCTCAAGTCTTTGTCACTTGTCTGCAACCTGAGTCTCTCAGACTAAGGTCGTAACATCTGGTAAAGCG
TCTACACCTCACCTTGCAGACAGCAGCTATCGAGGGCTTGCTGTAGTGGTAGTGCATACGGCAGAACCTACCA
GTTGAACGCACTTGTCGCTTTATCGCGTCGAATTTTTTTTACGGAGTAATCACTCGGGCACTATTGCATGCCA
AGTCGGAAACAGGATCTACAGAGAACGATCGAAGAAGATTTAGACATCTACCAACAAGAGACGTTACAGGAGC
TTGAAAAGAAAGCAGAGAACGAGCATTTGAGGATATTACAACAACTTCAACCGAGACCGGAAGAAGACGAGCA
GATCCAATCATCAGTAGAAGAGAATCATCACTCAGAGAAGGAAGACGAAGACGAACCAACCAGTCGTATATCA
CCTGAAGAAGAAGAAGAACTTGCCAACAAACTAGCAAACATGTCGACCAACGATTCTTTCAGAGCTTTGATCC
CCGATCCAGGAAAGTTTGAAGGAAGTAGAGAGAAGTTTGAAGATTGGTGGAGGAGTATCCAACTATTTTTGAC
ATTCAACAAAGTCACCACCGACAATGACAAAATCATCGCTTGCATCTCACGAATGCACAACGGAACAGCAGGA
GCATTAGCCGCTTTAATCGCTGACAGGATCAATGGTAACAACAAGATATCATGGAACGAGTTTGAGAAGGAGA
TGAAGGAAGCCTTTATTGATGGAAAAGAAAAGGAGAAGAGCGAAGTCGCTATCGAAACCCTAGTCCAAGGAAG
GAGGCCCATTATCGACTACCTGGTCGAGTTCAACTTGCTGAAGAACAAAGCGGGCACCGACGACCAACACGCT
GTATTTTTGCTCAAGAAGAACATTCGGAAGGATATTATACGAACCATCTTATCATTCCCACCCACCAATCTAC
CCACCAACCTTGCTGAATGGACCAATGTAATCAAGTCGGTTGGAATAGGAGCCGAAACCATGCACATGTTCGA
CAAACACACCTCTTCTGGAGTAACTTATGGAGGAAGCGGTCAACCAATGGAAATTGGTAGTTCCGAAACCGCT
AGAGCTCAATTCTCTAACAACCAGAAGAAATGTTTCAACTGCGGAAAGTTCGGACACTTCGCCAAAGACTGCA
GGCAACCAAGGAAACCAATGACTTGTTACAACTGTAACCGCATAGGACACATCGCCAAATATTGCAAGGCACC
GAAGAAAGCTAGGACTAGAGAATTTGCCGAAGAAAACCCTCAAGAGGAGGATAAGGCAGCCGAAGAACAGGAT
TTTGTCGACGGGTCAACCTGAGCGCAAAGGATCGACCCCTAAAACGTAGAGTCTCTCCCCATGTATCATCTAT
TTCGAAATCTACCTATATTATCCCTTTCAAACGAGACTCGAAGCGTGAAATCTTGCTGGACGCAAACATCAGA
ACGAAGAATGGAACTAAAACGGTCAAGGCACTTATCGATTCGGGAGCACATATGACGCTAATAGCCAAAGGAT
TGGTTAAGAAGGAAAACTTGGAGAAAACCAAACGGCAACAACCAATTAAGATGAGAAACGCAGATGGAACACC
CAATCGGAACGGAAAACTAACTGAAGACGCCAGGATTCAAATGGATTTAGGAGGACACAAAGAGGAAATCAAA
GCATTAGTATCAGACATATCAAGCGCCGACATTTTCTTGGGATACGACTGGCTAATTCAACACAACCCGGAAA
TTGACTGGATAAATCAAGAAGTCAGATTCTCTAGATGCCCAAGCACTTGCACTGTTCAACATAAAACTATCCC
TTTCGCTCGACGGCTGACCACTGATGTGGAAAGCGCTACGGAGGAAAAAGAAGTGGACCCTACCAATCCGAAA
GATTTACCTGACTACATCCGTCCCTACGCCCATCTCTTTAACAAGAAGAACTTCGACAAACTTCCTGCACGAA
CGGAATGGGATCACGAGATTAACTTTACGGAAAACGCACCAACGGAGATATCATCAAAAGTCTATAGCATGAC
GCCATTAGAAAAAGAGGAACTGGACAAATTTTTGGATGAAAATCTGGCTACCAATCGAATCCGACCATCGAAA
TCACCTTACGCAGCACCATGTTTTTTCGTCCCCAAGAAAGACGGTTCACTACGGTTATGTCAGGATTACAGGA
AACTAAACGATATCACGATCAAGGACAAGACGCCACTACCCCTCATCAGTGAAGTATTAGATCAACTCAAAGA
CGCCAAGGTTTTCAACAAATTGGACATCATCTGGGGATACAACAATGTTCGGATACGAGAGGGAGATGAATGG
AAAGCAGCCTTCTTAACAAATCGCGGATTATTTGAACCAACGGTCATGTTCTTTGGAATGAGCAACTCTCCCG
CAACTTTTTCACGCATGATGGCAACAATTTTTCGAGAAATGATACAAGACGGATCTCTGGCCAACTACATGGA
TGACTTCATTATTCCAGCAAAGGACGATAAAGAATTAGAATCACGGACCACACGTTTCCTCAAAATAGCGGAG
AAACACCATTTATCATTCAAACGAACGAAATGTGAATTCAACGTCTCATCAACAACGGTATTAGGAACGGTTA
TTGGGAATGGAAAGGCAACTATGGAAGAAGAAAAGGTCAAGGCAATACGAGATTGGGCAGTCCCTACCACGGT
CAAACAAGTCGAAAGTTTTTTAGGTTTTGCGAATTTCTATCGACGATTTATCAAGAATTTCAGTACCATCGCA
CAACCGTTAAACGAGCTAAAGTCTAAGAAGGGGGAGAAATGGTATTGGAACGATGAACAACAACAAGCTTTCG
AACAGATCAAACAAGCAATCGCCAGTGAACCGGTCCTCGCATTGCCAAAAGACAATGGACAATTCAGGGTCGA
AGTAGACGCATCAAACTATGGAACAGGAGCAGTACTATCACAGGAACAGGAAGACAAATGGCACCCGATCGCC
TTCATGTCAAAAACACTATCTGAAGCCGAACGAAATTACGAAATTTACGACAAGGAATTACTAGCCATTATAA
AAGCCTTGAAACTATGGAGACATTATCTATTGGACGCAAAGGAACAATTTGAAATATGGACAGATCACGAGAA
CCTCAAGTATTTCCGAGAACCTCAAAAGCTCAACGCTCGACAAGCAAGATGGTACCTCATGCTACAGGAGTAC
GACTTCCTTCTACGACACATTCCAGGAAGGACAAACACCAAAGCTGACATCTTATCTCGCATTGTCAAACCGG
ATATCTCAGAAGACAATAAAGACGTCAAAATGTTCAAAGGAAAAGTTTTCACTCGAGAATTCATAGAAGACAT
ATCAGATTATGAAGAAACGATCTTACTCAACCGACGATTCGAAGTATCAGCGGATCAGAGAATATTGGAAGAC
ATCAGGAACTCGAGAAAAAGAGAGACAATGGTATTACAGGAGATGAAGAAACAACCGGAAAAAGTATGGGAAT
CAGAAGGAATTGTTTTCCGACAAGGAAGGATCTATGTACCAGAAGAGAAGAAACTCAGAGATCGGATTCTTTA
CGATCATCACTACCCACCCGACATCGGACATCCAGGACAGTATCGCATGCTAGAGTTACTAAAACGGACTTTT
TGGTGGCCCACGATCAAGACGGACGTCAGAAAATACATCAGGGGATGTGACATGTGCCAGAAGAACAAAACAA
TTCGACGACCCGATCACATTCCACTTAACCCACTCCCTATCCCCGACAAACCTTGGGAAGAGATATCTATAGA
CATGATTGGACCACTACCGAAGTCAAAGGAGAAGGATGCTATTATTGTTATCGTTGACAGATTCTCCAAAATG

ATCCACCTCGTTCCCACCACCACGTCACTCACATCCATGGATCTCGCGGAGATCTACAAAGAAGAAGTTTGGC
GACATCACGGAATTCCAAAGCGTATTATCAGTGACAGAGGACCACAGTTTGCATCGAAATTCATGGAGTCCTT
ATGCAATGCTCTAGGCATCAAACGAAATCTTTCCACTGCCTATCACCCACAAACGGACGGACAAACAGAACGG
ATGAACCAGGAAATTGAAACCTATCTTCGAGCATTCATCAATTACCGACAAGACGATTGGACAAGATGGCTTC
CTATGGCAGAATTCCATTACAACGACAAAACCCACGCTGCTACCGGACAAACCCCATTCTTCTTGAATTACGG
AATTCATCCATGGAAAGGAAACATCACGGTTGAAACGACAAACCCTACCGCCACCTCCTTGATAGAAGAGCTA
GAAAATGTCAGAAAAGAAGCTAAAGCTGCGATGGAAGCAAACAACGAGATGATGAGAGAAAGAGGAAACAACA
AACATCACAAGGAACCATTTGCCGAAGGAGATAAAGTTTGGTTAGAAGCAACGAATATTCATTCCAATCGTCC
GACTCGGAAACTAGATCACAAATGATATGGACCTTTCGAGATCTTGAAACAAATCGGCGATCGATCTTACAAA
CTAAAGTTACCTGACACTTGGGCGATACACGACGTCTTCCATACTTCGCTCCTAACAAAAGTCCGAGACCCCG
AGTTTGATAGTCAAAAGAAACCCACTCCACCTCCACCTGACATCATCAATGAAGAAGAAGAATATGAAGTTGA
AGAAATCCGAGGACACCGACGAAAAGGCCGAGGAGTACAATTTTTAGTTCATTGGAAAGGTTATGGAAACGAA
GATGACACATGGTTACCACGCTCTGCTCTAACGAACTCCACGGACATCCTAAAAGAGTATCATGAAAGAAATC
CCTGCCTATAAAAGGATATCTGAAACTCACAACCGGTATAGCATCGTCTATTGGCATCTGAAGAAGATCATCA
TGAGCAACCAACAAACCACAACCCGTACCGCCGATTTCGGAGATTCTCTCCAACGACTCTTAACCATTCACAA
TGACATCACCGACCTCTTATTATTCCAGTCCAACGTACAACTTCCTCTTTTCATAAACCCGCCTCCACATATT
GAAATATCAGATGAAGAAAAGAGTCAATTAGAGGAGCAACTCGAGAAGGAGAAGAGGTTAATGAAGCTGGTAG
AAAATCTGTTAAGGATGGCGAAAGCGAACAACAAAGAAGCGAAAGAAGCATTGTCACCTGGATCCACAACGTG
GACTACAAACAATGATGGATGGGAGTAACAAGATCATATCTGGAATCTAGATCCCAAGAGAGGGGATAATTTA
TGTATAAAAAACCCAACTCCCGTTCTCATTTTCATCATCATCTTTTGCCTCTTCTTCCTCGAAAACTCAAACA
TTCACCATGTCTAACATCAACACCCCTATCCCTATCTCCACCAACTTCCTCGACACCGACTTCCACACTGGTT
ATTGCGAATCTATATCATCGATCGAAAATAGACGACGAGCCTTGCTCATGTCCATCCAATCCATAGCAAATAT
CCGCCAATTTGCCAAACATATTTCCTCCACCGTCATTAGCGCTACATCTACTGCCATCAAAGTCAACCCATAC
ATCCAAAACCTAATCGGAAATTCTGCTGAAGCTATGGGATATATGACCGTCATCATGGAGACACTCCAAGCTG
GAACTCAATGGGACATTGAGTATTCTTCGACCAAAGTCTGCGTTACTCTAGCCAAAGCACTCAAACAATTGCC
GGACGTAGGAAACCAAGATGAAATCAACGCAGCCCACTCCATCGCTAACGCTTACTTATCCCTTCCATACCCG
GCTCCTACTCGCAACAACACTCCTGAACCTCTACCTATCCGTTCTCAGCCACCGAGTTCCAACAACACAGATA
TCGACAACAATGCTCCGAAGCATCCCAATACAAACACTCCCGTTGACGACATCGACGAAGAAGTCCATCGCCT
CACCAATTTCTTGAAATCTGCTCCACCTTACCTCATTCCTGACGAATTCCTTCCACCAACCAATTATACTACC
TTCGTTGACAAAGGAGAAGAAAATCTATCTTACTGGAACGACATACTCAAAAGATTGTTCAAAAACAATCGAC
AAGATGGAAAATACAGAGGATACTCGGTTGGATACGCTGAACTAGGTGTTCCGAACGCTCCTCACTTCTTGGG
TATTGACTACAACGGTAAATTCATAATCGCCAACACCAAGAAAGCTCTCTTTGGAATTATCGCTTAAAAAGTT
TCATGTGGACACGAAACCTAATCGGAGGGTATTGTTATGATCTCATCGATTATGGATATTCTAATTAGGCTAA
ACTGTTTCTCCTATTCCCATGCGTACTTCTTTTCAGTTTCTTTGATCTTCCCCTATCTGTATACTTTCATCTC
ACGCTTACTCCACTCCCATATGCTTTCACTCCACTCTTCTATTACCCCGAGTACTATGACTCACTCTTGTCCT
ATACTTAGTTACCCTGGGTAGTACTCTTTACATATATAAACCCTATAGGTACTGCTGTACTTCTTTACGCTTT
ACCAATTGTATCTTTCCCTTTCTCTACTCTCACCTCGCTCTCAAGTCTTTGTCACTTGTCTGCAACCTGAGTC
TCTCAGACTAAGGTCGTAACAT
>Agabi_varbisH97_2_rnd-1_family-113#TIR/MITE
CATCAGAAGTTTGGCAATTAGCTTTTTTTCGCTTGTACAAGCTAAAAAAGCGATAAAAGCGATAAAAAGCGGC
GAAAAAGTTGTTGCCACGTTTTGGGCCCGGATTTGAAAAAAGCTGGCCTCCTGCAGATGCCGTGAAAAACTGG
AAAAAGCGAAAACCTGGAAAAAAAAACTGAAGGTTACAGACGTTACTACTAGATGTACCTAATGTTGAAANGAC
CATTTTATTATTTCGACTTTTGCCCGGCTTTTGCGCNTGTATCAAATTCGCAAAAAGGCCGAAAAGCGACAAA
AGGTAAAAAGCAAATTGCCGACTTGCTGATG
>Agabi_varbisH97_2_rnd-1_family-72#TIR/MITE
CTCAGTAAAAACGAGTTTGCATGTGTTTGACATGCAATTGACATGTAATTGACATGCGATCAACAAAAAGCGC
ATGTCCTGCGCAAGATTCTTGCATAGATGTCGGTCCCTCGCATGGTTTGCGCATACTCTTGCAATTCCATCAA
AAAGTAATGAAGGCGTGAGAAAAGTATCCTCAAGCCCTCGAGTTTCCTCGTTCGAAGAAGGCTCAAGGAACCA
CTTTCCCACGGCCTCAAGCAACTTTGTGGCCATCGAACGTGTTGGTGTACGGCGCCAGTGGACTATAATCTCT
AGATAACTTGGGCACAGTCGGTAACTTTGCTTTTCTTTGTTTTTTCTTTTGAATCAGGATTTATTACGCGACG
CTCGAACACAATCCGCGGTACACTATCGCTAGAGTAGACGAAAGAAGAATGATAGGCGGGGTTGGACCCGGGC
TTCTATGGATACCATGAAGAAGGTAGAGCAAAAACAATCCTTTTGGATGCGACATCTCTTCATTTCCAGGCAT
GCATGGCGATCTCGAGGAAGTGAGGGTCGTCATTTGCATGCTAAATTGCTAATCGCATGTAATTCGCACGTAA
ATGACATCCTCTGAGCGAAAATCTTGCCAAAATGCGATTTACATGTCAATTGCATGTCAAACACATGCAAACT
CGTTTTTACTGAGCGT
>Agabi_varbisH97_2_Cluster54#LTR/Copia
GATGAACAAACTCGCGGTGATGCGAGCGTGGCAGTGCGAGAATGACGAATGAAGAAAATTTCTTTTTATATTC
ATTGTCTCATGTATTATACAAGTGTCGTATGCCTTTGTAGCACCTCAGCTCTCATCATCGAGGCTGAGGTGAG

CCGTCTAGTTTTACTCTCTCTTCCTAAGCTAATTGTACTTGTAGCTACGTTGTAAATCTTTCAGGTTATGGGC
CCCGCCCCATTGTGAGACACTAAACTCGAGAGTGTTGCAAGTGAAAGAAATATAACATGTCAAGTACAACAGC
TATAAACGATGTGCTCCCGCCTACAGTGCCTGCATTGGAGGTTTCCGGATCGAACTGGGCGATTTTCGAGTTG
CGATTTCGGATGGTAATCCAAGGGAAGGGCCTGTGGGGTCATTTTGACGGGACAGTTCCTCGTCCCACACCAC
CGCGTGCTACTACTCCAGTGACACGTCCCTCTACTCCAGTTCCACCTACTTCTTCCACTGCGACTGCAGCACA
AGTAGCTTTACCTTCGACACCAGCTCCTACAACGGCCGATCTTGACGCCTGGGATCGAAACAAAAACATATCC
CTCTCGCTCTTGGCCCAGCGTATTCCCGACTCTACACTTGTTGTCGTTTCAACCCAACCTACAGTTAGAGCAA
TGTGGATGAAGATAGTACAAGAATATACGTACAAAAGTGCCTTTTCGCAGGCAAACCTTTGTCAAGACTTCAT
GTCTTCGCGTTGCCCAATCGGTGGAGATGTACGCTTGTTCTTGAATGACTTGCGAGCTAAGAAAGCAGAATTG
TTAGCCATCGGTGTTCATATTACAGATGATGAATACCGAAGTGCGATTATCCAATCATTACCTCGCTGGTTGT
CAACGTACGCTTCGAATCAACTTTCGGCAGCTCGTCTTCACACCTCCCTTCATAACACAATTGATCCCGATCT
CTTGATTGTCATGATCTGTGACGAATGGGACCGTACCCGTCGCTTCGCTAAGAAGAGTCAGAAGACTGAAGGG
AATGATGCTCTGGGTGTAGAAACAGAAGACAAGAAAAAGGGAAAAGGAAAGGATAAAGCTAAAGGAAAGGAGA
AGAAAAAGGGACCATGTTGGATTTGTGAAGGAGATCACTGGAAGAGAGATTGCCCAAAGCGGAAGAAAGAGGA
GGAAAACAAAGAGAAGGAGAAGTCAAAACCCAGTGCTAATGCCGTAGCTGAAGACGATGATGACTCGTTGACG
GTGGATATTGAGGAGGTAGGGAAGGTTTCTGAGATGACGGAACAAGTTGAGGTTTTTGACTCAGGGTCTTCTC
GGCACATCACTCCCTATCGTAATTCATTCACCACTTTCGAGTCTATTACGCCTCGCACTCTTCGAGCAGCCAA
CCAACAACAATTCAGCGCAGTTGGCAAAGGCGAAGTAAACGTTGACGTACCAAATGGTGACTTTGTATCCCAA
ATTCGTCTCAAAGAGGTGTTGTACACACCTAAGGCCAGCTACACTTTGATTTCAATTGGCCGATTGGACGACG
AAGGCTTTACGACTACATTCCGTGATGGGAAATGTATTATCCGAAACTCGGAGGGTGTTCGTATCGCTGAAAT
CCCTAGAAATGATAAGGGATTATACAAGTTAATCAAAGGCAATGGTGACGAAGTCAATATCGTGACGGAATCA
CTTACAAGGGATGCTCTTCACCGTCGTCTGGGTCATATTTCACCTATCGCCGCTGAGAAACTCGTTAATGACG
AACTCATCACTGGTATAAAGCTCATTGAAGGGAAACCAGATGTTTCTTGTGATTCATGCGCATATGCAAAGGC
TACGAGGCAACCAATCGCTAAAGTTCGAGAGGGTGAGAGGGCAACTCGAGTAGGAGAGGAGATCCATACAGAT
GTGTGGGGGCCAGCCAGAGTTGCTACCAAAAAGGGACGACGTTATTATGTCACTTTTACGGACGACTATTCGA
GATGGACTCACATCGAGTTCTTGACGAACAAATCCGATGTCTTCGATGCATATAAATGTTTTGAAGCATGGTG
CGAAACTCAATTTGATGTTCGTATCAAGGTTCTGCACTCGGATCGGGGAGGGGAGTATACGTCTGAAGAATTT
CAGCGTTATCTCAAATCTCGTGGTACGGAAATAAAACTTACCGTTCATGACACCCCTCAACATAATGGAGTCG
CGGAACGTCGAAATCGTACAATCGCTTGAACGTGTTCGAGCATTACTTCATGCTAGTCGTCTACCCAAAACCT
TCTGGGCAGAGGCCGCGCTACATGTCATGTGGCTGATGAACAGATCGGGTACTAAAGCAGTAAAAGGCAAAAC
TCCTTTCGAAGCCCTCTATGGTCGTAAGCCAAATCTACGGAATGTTCGTGAGTGGGGTGACGAAGTGTGGGCT
CATCAAGGAGGTGGAGATAAGCTAGGAGCTCGAGCGAAGAAGGGGAAATGGCTGGGATATGATTGGGAAAGTA
ATGGGTCACGGATCTTGTTTCCAGATACTGGAACGGTAAAGATTGAGCGAAATTTTCGCTTTATCAATGGAGT
TACTGACCTAGACGTTGAGGGGGAGCATACACCTATCCCTCAGACATCCCAAATCACTCCACCATCCAACCCC
AGTCCTCCAAATACTCCCGAAGTTGCTACTACACCGAAATCACCGAGTGTTGGAACTCATACACCCCAAGAAG
TATCACCAAACACCCAGACACCTCCAGAAATGCCAGATATCAAAGAGAAATCGCTTGAGGATCCTCCTATTCG
CCGTTCACAAAGGATTAGGAACCCTAGTCGCAAGGCCCAAGATATTCTAGAGGGAAAAGCTGTAACAGTGGCT
GAGGAGACAGATTGGGAAGATGTACATGCCTTATCAACGGAAATGGATGAGATGGAGTCCCTTGAACCTAAAT
CATGGCAAGAGGCAATGAGAAGGCCTGATTCGACGTTGTGGAAGAAAGCTATGGAGGATGAGCTGGCAATGCT
GAAGGCATCACGCACGTGGGAACTCGTTGATCCCCCATCAAAAGTCAACATTGTAGGCTCGAAATGGGTTTTC
AAGTGCAAAAAGGATGCAACTGGCAATATAGTTCATTATAAGGCTCGTCTAGTTGCTCAAGGATACTCTCAAG
TTCCTGGTGTCGACTATTTTGACACTTTTGCACCAGTAGCCCGGCTTTCTTCTATCCGAACTGTTCTCGCAAT
TGCCGCTGCTCGTGATCTCGAAATCCATCAGATTGATATCAAAGGAGCCTACCTTAACGGGAAGTTGAACTCT
GATGAAGTAATCTACATGCGTCAACCCCCTGGTTTCATGGATATAACTCATCCTAGTTATGTCTGTCGATTGA
ATAAGACTCTCTATGGTCTTAAGCAATCTGGGAGGCGGTGGTATCAACGTCTATGTGAAATTCTCATGGATAA
CTCGGGTTTCTCGCGCTGTGACGTTGATCATGGAGTCTTCTTCAAGGCTGACGAAAAAGGGCTCATCATTATT
CTCGTTCATGTTGATGACTGTACGTTGGTTGCACCGGAATTTAAGCTGATTAGTGTTAGGGCGACTGTGACTG
AGATGTCACAGGTCACACCTGAACTTAAGAACTAAGGAGTAAGTGACAAAAGTTTTATTAGGCGTGAACCTAA
GAGAACTCGACGGAGTCTTCTACAAGTTTCTACCTACTTTATATACCTAGACTATGGCGCACAGACCAACTAC
CCTAGGTAACTATTTATATATTGACACGAGAAAAGAAGCAACGTATGTGAAAATAAACTAATTATACATTTGA
CATGAGAAAAGAAGAGTGGAGTAAGAAGAAAGTAAACAAGAACAACTTTGATCATAAAAACGTCAACAAGTCC
AAACAAACCATAAAAGTGTCCACAAGTACAAACAACTATCTAAGGACGTAACCTTTCGTACCAGTCCCTGCCG
AAGTATGTTGCCATTACTTCTCCACACGGGCACATCGTAAAGAACCGGACCGGTTCCCAACGACGGAAGTCCG
GGTTATTCCATCGCTGCAACATCCAGGGTCTCTCCAATACTTCGTCCCACATTATCAACGTCACTCTTCCACA
CACTACGCACCTACGATCCTCTAACCTCCATCCGTCTGGTCGGGGTTCTCTTCCGATAGCCGCCGCCGCCCTT
CTCCACTGTACCTGCTCCGCATGGTTCGGAGGCAAGAAAGAAAAGATAAGATCAATGAGTTCAATTGGTAAGT
CTGTAGCTTGCGCCATTTAGCAGTATGCCGATAGTTTAGCCTCCGTCGAACTGTAGCAGCAGAGGGAAATTAT
TATGTAATAAAAAGTGTGACAGATGTGACAGCGTGACGCCGTGACATTCTGGCAAAGTTCTAACAATTAGCTG

GGTAAAAGAGAGGATGAATGAGTTTGTTGAAGTTACTGATCTCGGAGAAATTCACTGGTTATTAGGAATTGAG
GTCAAGAGGAACCGAGAAGAGGGGAAACTAATGTTATCTCAACGTTCCTACATCAACTCATCCCTGCGTCATT
TCGGCTTTGAAGACGCTAAGCCCAGCAGCATTCCGATGGACCCTTCTATCCATCTTTCCACCGACCAATCCCC
AAAATCGACTACAGAGGTTGCTCGTATGCAACAAATTCCTTACCGGGAAGCAGTCGGCACTCTAATGTATGCT
GCCATTGGGACCCGACCAGACATAGCGTTCGCAGTTCAAGCTTTGTCGAAATTTTCCAAGGACCCGGGTGAAG
CACATTGGGAAGCAGTTAAACGGGTATTCAGGTATTTGAAGAGTACTCAAAATTTATGGTTGACATTCGGGGA
CCAGGGAGATGCTTTAAAGGGTTATGCCGATGCTGATGGTAATATGTCAGAGGACCGTCGAGCTGTTTCTGGC
TTTGCGTTCATCATTAATGGTGGCGCAGTTTCGTGGAGTGCAAAGCGACAAGAGATCGTCACGTTGTCTACAA
CCGAGTCCGAATACGTAGCTGCAACTCATGCTACGAAATCTCATCTCACAAATATTCGACACAGTTTTAACGA
CTACTCCACTCTTTTCCGACAACAAGTCAGCCATAGCGCTTACCACGGACCACAAATATCATGGTCGTACAAA
GCACATCGATATTCGCTATCATTTTATTCGCTGGGTAGTCGAAGAAGGCAAAATTCAACTAGTTTATTGTCCT
ACCAAAGACATGGTAGCAGATGTTTTCACAAAAGCTTTGCCTTCTCCAAAGGTAAAGCATTTCGCGTGTGAAT
TAGGACTCACCACGGTTTGAGGGGGAGTGTTAGATGAACAAACTCGCGGTGATGCGAGTGTGGCAGTGCGAGA
ATGACGAATGAAGAAAATTTCTTTTTATATTCATTGTCTCATGTATTATACAAGTGTCGTATGCCTTTGTAGC
ACCTCAGCTCTCATCATCGAGGCTGAGGTGAGCCGTCTAGTTTTACTCTCTCTTCCTAAGCTAATTGTACTTG
TAGCTACGTTGTAAATCTTTCAGGTAT
>Agabi_varbisH97_2_Cluster36#LTR/Copia
gttggaagtaacaaacacgtgtcgatgggttagggtttgtatggagctgaagtcgagattttcgtctgtgcgt
tttgtctcatttgttccatggccattttatgtgcgtgattgtatttaaacacctcagtcttcgcgtttcgag
actgaggtacgtagcatacttgtaaccgaaattcttctattaagtctgtacttgtagcgtaaatctttcaggt
attgaaatgtatcagtcttcgcgtttcgagactgagcgtaaatctttcaggttatgggccccgccccattgtc
acactaaagtaaactagtgctgcaaagtgaaagcgagtaacgctatgtcgaattcgatggctgtaaatgatac
tctaccacccaatgtgcctgcattggacatttcgggatcaaattgggctatgttcgagttgagattccggatg
gtcgttcgagggaaaggcctatggggtcatttcgatgggactactcctcgtcctgctcttcctactcctactc
ctgcacctacaccagcaccacctagtactccggcagctcctacggaatctgctgttcaggcagctttaccccc
atctccacctccagttacaattgatgacatcgagttgtgggaccgaaatgaaagtatttcacaagcgctgctc
gctcagcgaatccccgactccactctcgttgttgttgcaagttACCCCACAGTTCAACAGATGTGGAAAGCGG
TTGTCAATGAATATACTTACAAGAGTGATTATTCACAAGCAAATCTCCATCAGGAATTCATGGCCTCGCGCTG
TCCACCTGGAGGTGATGTTCGAGTATTCTTGACTAACTTGAGGGCGAAGAAGTCGGAGCTACTAGCAGTCGGC
GTTCATATTAGCGATAATGAATACCGAAGTGCCATAATTCAATCCTTACCTCGATGGCTAGCAACCTTTGCGT
CCAACCAGTTATCTGCTGCTCGTCTTCACTCCTCCCTTGGCAAAACTATCGATCCGGACCTGCTCATTATCAT
GATATGTGATGAATGGGATCGTACACGCCGTTTTTCAAAAAAGACAGCTAAGCCGGAAGGGGATGATGCTCTT
GGAGTTGAAGCCGAAAATAAAGGGAAGTCTAAAGCCAAAGAGAAGAGTAAAGGAAAGGAGAAGAAGAAGGGAC
CGTGTTGGATTTGTGGAGGGGAGCACTTGAAGAAGGATTGTCCTAAGCGAAAGGATAAAAAGGAGGAAGGATC
TAAGAAAGATCAATCCACCAGCGCCAATGTTACGACCGAAGGAGGTGATGACGATGAGTCGTTTGCTGTAGAT
GTTGAAAAAGTCGGGAAAGCTAGCCAGTCGACGGCAAGAATCAAGATTTTTGATTCAGGATCTTCTCGTCATA
TTTCTCCCTACCGAGATGTGTTCACGTCGTTCACATCGACTTCACCCTGCATGTTGCGAACTGCTAATCGGCA
ACAATTCAGTGCAATCGGTAAAGGAGAAGTGAAGCTTGACTTACCGAATGGAGACTCAAAGTCAGAATTACAT
CTCAAGGATGTTCTCTATTCACCAGAAGCTAGCTATATGCTGATATCGGTTGGCcGTTTGGACACAGAAGGCT
ATACGACGACGTTCAGGGATGGACGTTGCATAATCCGAGATGCGGAAAGTGTTCGTGTAGCGGAGATCCCTCG
AAACGAAAAGGGATTATACAAGTTAGTCAACGATGACTACGACGAGATCAATGTAGGCGAATTACTCACCAAA
GATGCTCTACATCGTCGCCTAGGTCATATTTCACCTATGGCTGCAGAAAAATTGGTCAAGGATGGGCTGGTTA
CAGGGTTGAAGCTAAGCGAAGTTGAGTCCGGGAATGTGTCCTGTGATTCTTGCACATATGCTAAAGCCACTCG
TTTGCCAATTGTGAAAGTCCGGCAAGGTGACAGAGCAACGAAAGTCGGTGAGGAAGTGCACACAGATATCTGG
GGACCAGCTAGAGTGGCGACGCGCAAAGGACGACGCTATTATATTACGTTCACAGATGATTATTCTCGGTGGA
CGTACGTGGAGTTTCTATCCCAGAAATCGGATGCTTTTGAGGCATACAAGCGGTATGAAGCATGGATGGAGAC
TCAATACGATGCACGTATCAAAATACTTCACTCGGATCGTGGAGGAGAATATACATCTGAGGAGTTCCAGTCT
TATCTCAAATCTCGTGGTACGGAAATAAAGCTCACCGTTCACGATACCCCACAGCATAACGGTATTGCCGAAC
GTCGAAATCGTACAATCGTTGAACGTGCTCGAGCTCTGCTTCATTCGAGTGGTCTTCCAAAGTCGTTTTGGGC
GGAAGCCGTAGCGCACGTAGTATGGTTGATGAATCGAACTGGGACTAAGGCAGTTGAAGGAAAGACGCCGTTT
GAAGTTTTATATAACCGAAAGCCGTATATGGGAAACGTTCATGAATGGGGAGATGAGGTATGGGTTCATCAAG
CTGGACAGGATAAGCTAGGCGCACGGGCGAAGAAAGGTCGTTGGTTGGGTTTTGACATGGAGAGTAATGGATC
TAGGATCTTATTCTCTGATACTGGGGCAGTTAAGATCGAGCGTAATTTTCGCTTCATCAAAGGGAATACTAAC
CTGGAGCTTGAGGGGGAGCAAGTTATTACTCCACAGGCACCTCCTGCATCCGTTCTTGCCCCAGCAAAATCGG
CTAAATCCCCTACAACTCCGGAGTCACCAAGCGTTGGTACTCCAACCCGAACCGAATTGCCATCGCTACCAGT
TGAACCGGATATCCATCCAGAGATCTCGGATCCAGTGTCCGAACCGCCTACAACTTCGCGTCCTGTAAGGAAG
AAATTTCCTAGCCAGAAAGCTCGCGACATTCTTGAAGGGAAAGCTGTTACTGTTGCTGAAGAATTGGACTGGG
AAGAAGTTCATGTTTTAGTAAATGAAACAGACGAGATGGAGGCTTTAGAACCTCGTACCTGGAAAGAGGCTAC

CCAGAGGGATGATTGGCCATTGTGGAAGAAGGCGATGGAAGAGGAACTGtgttagggcgactgtgactgagat
gtcacaggtcacacctgaacttaagaactatagaggaaagtgacaaaagttatattaggcgtgaacctaagag
aactcgacggagtcttctacaagtttctacctactttatatacctagACcACGgcGcaaAGACcaaCgaCaCa
aGGgAACTTaTTgATatactgacatgtgaaaagaagtacgtatgagaaagtagactaattatacatttgacat
gagagaagaagagtggagtaagaagtaagtaaacaaaagtaaattgatcataaaaacgtcaacagcaccaaac
aaaccataaaagtgtccacaagcacaaacaactcctaaaccggtaatctactcacacaaaccaatacgtcccg
aagtagttacctaacatcgcatcacatggacatagcacaaagaatccggctattgcccattgccgcggccccc
agtatccccatctctgtatcacccatgccttctgctcctcatagtcccacattgctaagcataccctcccaca
ccgtctgcaccgatgatccgataggttccaacctaccggtcttgcctctcttcctatagccgccgccgccctt
ctccattgtacttgctccgcatggttcggaggcaagaaagataagattaaatcgataagttcaattggtaagt
ctgtagcttgcgccattttagcagtatgccgatagtttagcctccgtcgaactgtagcagcagagggaaatta
ttatgtaataaaatatgtgacagatgtgacagcgtgacgccgtgacattctggcaaggttctaacagtacccc
ctccttaaagtcggctcctccggagacaacccttcaTGTCCTCTTGGCATCAACGTCGTGGGATCAAAATGGG
TTTTCAAAGCGAAAAAGGACGCTACAGGTAACGTTGTTCGTTACAAtgtaaatcgattaggtagttccctttc
ttggaacccggtgtcacgtagaacgtgctgtatcctattgagttccccatagagttggtggaattcagtaatc
aattctttggcgttagccaagttttcctcgggttcccacgagtctgcttctgccgtataccctttccatcgaa
ctaagtaccGCTCGTCTGGTGGCCCAGGGCTTTTCACAAGTACCGGGTGTTGATTATTTCGACACGTTTGCAC
CAGTAGCACGATTGTCGTCAATCCGAACCGCACTCGCTATCGCTACGGCTCGGAACTTAGAAATCCATCAGAT
AGATGTCAAGGGAGCTTACCTAAACGGTAGACTCAACAACGACGAAATCGTTTACATGCGTCAACCTCCTGGT
TTTCCTGATCCTACACACCCCCACTACGTCTGTCATCTTAAGAAGACGCTTTACGGCCTTAAACAGTCCGGAC
GGCGCTGGTATCAACGCCTCTGTGAAATTCTCATTGACAATCTGGGCTATTCACGCTGTGATGTCGATCACGG
CGTCTTCTTTCGTGTTATTCAAGATGATTTAGTCATCATCCTCGTCCATGTCGATGATTGTACTCTGGTGGCA
ACAAAGCTTGAACTTATCAAAGAGTTGAAAGAGCGGATGAATGAGTTTGTAGAGGTAACTGACCTCGGAGAAA
TTCATTGGCTTTTGGGGATAGAAATTCGAAGGGATCGTAATGAAGGCAAGTTATATATGTCTCAAAGGTCGTA
CATTGATTCGTGTCTCCGACGATATGGTTTCGAGGATGCTAAACCAGTTAGTATCCCTATGGATCCATCTATC
CACTATTCTACCAACCAGTCACCGAAATCAACCGCTGAAATCGCTCGCATGGCACGTATACCCTACCAAGAGG
CCGTAGGATCACTTATGTATGCAGCGATTGCTACTCGACCGGACATCGCCTTCGCAATACAAGTTCTTTCGAA
ATTTTCGAAGAATCCCGGAATTGATCATTGGGAAGCCGTTAAGCGAGTTTTCAGGTACTTAAAAGGTACGAAA
GAATTGTGGTTGACCTTTGGAGGTCAAGGAGATACGTTGGAAGGTTTTGCCGATGCGGATGGAAACATGGCAG
AGGATCGACACGCTATTTCAGGTTTCGCATTCATCATCAACGGTGGTGCCGTTTCATGGAGTGCGAAACGTCA
GGAAATTGTTACCCTGTCCACAACGGAATCAGAGTACGTAGCTGTCACTCATGCTACCAAAGAAGCCTTGTGG
CTTCGTTCTCTCATCTCGCAGATTTTCGGAGCAATCCTTCCCACCACACCACTTTTCTCCGACAATCAATCCG
CTATTGCGCTTGCAAAAGACCATAAGTATCATGGTCGCACAAAACATATCGACATACGGTACCACTTTATTCG
ATGGATCGTCGAAGAAGGAAAAGTCCACCTGGTTTATTGTCCCACTGCGGACATGATTGCGGATACATTGACA
AAAGCTTTACCATCTCCAAAGGTAAAGCATTTCGCATCTGCGTTAGGCCTCGCCACGGTTTGAGGGGGAGTGT
TGGAAGTAACAAACACGTGTCGATGGGTTAGGGTTTGTATGGAGCTGAAGTCGAGATTTTCGTCTGTGCGTTT
TGTCTCATTTGTTCCATGGCCATTTTATGTGCGTGATTGTATTTTAAACACCTCAGTCTTCGCGTTTCGAGAC
TGAGGTACGTAGCATACTTGTAACCGAAATTCTTCTATTAAGTCTGTACTTGTAGCGTAAATCTTTCAGGTAT
TGAAATGTATCAGTCTTCGCGTTTCGAGACTGAGCGTAAATCTTTCAGtaccccacagttcaacagatgtgga
aagcggttgtcaatgaatatacttacaagagtgattattcacaagcaaatctccatcaggaattcatggcctc
gcgctgtccacctggaggtgatgttcgagtattcttgactaacttgagggcgaagaagtcggagctactagca
gtcggcgttcatattagcgataatgaataccgaagtgccataattcaatccttacctcgatggctagcaacct
ttgcgtccaaccagttatctgctgctcgtcttcactcctcccttggcaaaactatcgatccggacctgctcat
tatcatgatatgtgatgaatgggatcgtacacgccgtttttcaaaaaagacagctaagccggaaggggatgat
gctcttggagttgaagccgaaaataaagggaagtctaaagccaaagagaagagtaaaggaaaggagaagaaga
agggaccgtgttggatttgtggagggagcacttgaagaaggattgtcctaagcgaaaggataaaaaggagga
aggatctaagaaagatcaatccaccagcgccaatgttacgaccgaaggaggtgatgacgatgagtcgtttgct
gtagatgttgaaaaagtcgggaaagctagccagtcgacggcaagaatcaagatttttgattcaggatcttctc
gtcatatttctccctaccgagatgtgttcacgtcgttcacatcgacttcaccctgcatgttgcgaactgctaa
tcggcaacaattcagtgcaatcggtaaaggagaagtgaagcttgacttaccgaatggagactcaaagtcagaa
ttacatctcaaggatgttctctattcaccagaagctagctatatgctgatatcggttggctgtttggacacag
aaggctatacgacgacgttcagggatggacgttgcataatccgagatgcggaaagtgttcgtgtagcggagat
ccctcgaaacgaaaaaggattatac
>Agabi_varbisH97_2_Cluster1#LTR/Gypsy
gttagggcgactgtgactgNgatgtcacaggtcacacctgaacttaagaactatagagNaaagtgaNaaaagt
ttattaggcgtgaacctaagagaactcgacggagtcttctacaagtttctacctactttatatacctagacta
cggcgcacagaccaactaccctaggtaacttNtttatatactgacatgNgaaaagaagNNcgtatgagaaagN
agactaattatacatttgacatgagaNaagaagagtggagtaagaagtaagtaaacaaaagtaNattgatcat

aaaaaNgtNaacaaNNaaNcaNaNcaNaaNaNtgNNNaNaNNNNNNaNNNacNNcNaNacNgtaaNcNNNNac
acNaaccNaNNcNNNNNNaagtNNttNccNNNNNNNgcaNcNcaNggNNaNaNNNNNNagNatcNggctaNNg
cccaNtgNgNgNNNNccaNtatNNccNtNNNtNNNtNNcccaNcNttNtNNtcNtNNtNgtNNNNNattgNNN
agNNtacNNtNcNaNNNNNctgNacNNNtgatNNNNtaNNtNNcaNNcNNNNNNNctNNctctcNtcNtatNN
NNNNNcNNNccttNtcNaNtNtaNttgctcNNNNNggttNNNaNNNaNNaaagatNNgaNNNNNNNNNNagtt
cNatNNgtaNNtNtgtagNttNNgNNaNtNNNNcagNatgNcNaNaNttNaNNctcNNNNgNNctNNNgcagN
aNNgNNaNattaNtNtNNaaNNNNaNNtNtNacNNNtNtgacNgcgNgNNgccNtNaNaNtctNgNNNNNNtN
NaNNNNNNNNcNctNcttNNagtcNNNtcNNNNNNNNNNNNNNNcNNcNNNNNctNNNNtNNNNNNttNcgtNN
cNNctNNNNNNgatatNNNgcatNaNacNNNttNtcatNNNagtttccgaataaagtNttggctgtNaatcga
ttaggtagttccctttcttggaacccggtgtcacgtagaacgtgNtgtatcctattgagttccccatagagtt
ggtggaattcagtaatcaattctttggcgttagccaagttttcctcgggttcccacgagtctgcttctgccgt
ataccctttccatcgNactaagtaccgcttctttccgcggagccatttggcgttaaggattttctccacttcc
cactcctgttctccttctatttctacNgccggtgccggtNtactcccttgtccatccacttgttctcgNtata
gtactattcggctgacgttaaccaccggatgNatccgaatagatcggggaagatctatttccactgcgttggg
ggaaatgatcttcttgatggtgtatggtccgatgaacttctccgtaagtttNcgggtaggtcgtccctggatt
tgaaggtcccgggtagccaatagaactttatcNccNactttgtaNttcaccgcttctcctctgcttctatccg
catacctccgcatatcgtcttgtgcctttttcagggcNgcttgagtttcctcgtgttgaagtttcatNcggtc
cgcaaaatcNNctgccgcNggNtgNttgccttgtcgtcggagatcgaaaccagttcttgggtgtcttcccacg
ttgaccataaatggagtttcctttgttgacgattgttctttattgttataggaaaactccgcNgttgctaacc
aatcgggccaatcagtttgtctatgatcgacaaatgatcggagatattgttctagttcttgattggttcgctc
ggtttggccatccgtttgtgggtgaaacgccgtagataacctagtatcaattcccaacattttattcaactcc
tttgtaaagtctgcagcaaattgcggtcccctgtctgagatcaccgtttctggtaacccgtgcaacttccata
catgatccctgaacagtttggctagtcctagcgctgatgttgtttccgtagttgggataaagtgagccatttt
actgaatcgatcNcacaccaccaaaattgagtccattccctgtgattctggtaattttactatgaagtcgacg
gatacgtcggcccatggtttggtgggaatagggttcggcattaatttcctgccggcggNgttggtcgattct
tgtttcgttggcattgatcgcatccttctacgtacttctggacttgcttagtgacgcctggccaccagtaatt
gcgcgtNaccatttctactgtcttccattgtcctccgtgtccagcaatgggNgtatcgtgatgtagtcgNatg
attttagtcctcaacgtttcgtctttcggcacatagacttttccttctttcagtaccaatccttcttccagct
tccactcgtctccccttagcgactttacttttgctcgcttcatctcttccaccgctttcactacatcNtcttc
ttttgcttctgattttcNgatttcctctaatatttcctcttcctccgcattgattaacatatgaccttcttcc
attgctcggattgcgaaatactctggtttNaacagggtttgatccttgttatcgtcctttgttcctNgttcaa
agtcaggtcttctgctcaatgcgtctgccttccccattgtctttcctgggcggaattttaattcgaaattgaa
tctagatagatataaggcccatctggcttgtcgtcgatttagttgttgggctttcgcgaagtattctagattg
cgatgatctgtccaaatttcgaacttatgtttcgccccttccaggaagtgtctccattcttctaaacaccgca
taatccccagcatctccctatcgtgaatgtcgtagtttcgttcNgtctcNgtcattgatttggagatNtatgc
taccggcctccactttccgtcttcacatttcattgccaaNtctccacccgtggcgtaagccgatgcatcagat
tccacctttagNtctttatccaattctggggctactaggattggatgtgacgtgaatcttgcttttagcgtac
caaaggcctcctcttcttcatctccccaactccatttcttgtcctttttagttaatgcgtgtaatggttttgc
aatactggcgaatttttcaatgaaccgtcgatagtaatttgccagtcccaagaatttttgtacatctttaacg
cattttggagtcggccaNtctactacgtctttcactttcctttcctccatcttgattccgttNctccctatta
tacatcccaaaaaNcccatNtcNcttcgtttccattcgcatttttctggttttgcNaatagatcattgtccat
caacctctgcaacacttcttcNaccaccttatcNtgctcttcttcatcgtctgttcctatcaagatatcgtca
atgaatacagctacttttccNgtatttactaagtccctaaaaagttcgttcatcatagtctgaaaagtagcag
gcgaatttgtaagtccaaaaaacataacagtgggttcaaaggcacccatgaaagtagtaaaggctgccttcca
ctcgtctccttcttttatcctaatgttattataaccccaacgtaaatctatcttagagaataatcgtttNcta
cccatagtgtctataagttcagaaattaaaggtaaaggataattgttcttaattgtccaatcgttcaaaaatc
gatagtcctgtaccatccgcttctttccatccttNttNggcacaaaaaataccggtgaggtttggggagattt
cgaaggNctaatatatccctttcggagttggtccttgatgaatgcttggacttcttctctttcgtttggNgac
aaggggtatatNtttcctttcttgggtttgaaatcctcctttaattcgatagcatgatcccatggtttcctag
taggcattctctctgattccttttgtttgaatactttaaggaacttgtggaaccgtcttggtacNacttgctc
tactgagtctatgcttcgaatttcttcttcttccggcatctcttctgtcattcctcttacccacNtctctagt
tgtccttcttcttctctactcctatttttcctttgggcgcttattcgtttaggacgtacctgtccagccNccg
cttgtcccgcggtggctgtcttgctgttatcttcttctagacgttctgccttttccccatttaatcggtcagc
cacgtcaccgggtgattgcgcccacccctcagctttctctttggattgttgcgccaaNccctcagctccacta
ttagcacgtctattacgtgccaattccctggcgaatgctgcttcgactcctaatgcgatcccctcgatgatgt
ttttgggagtgNtgaatctccctaNtgctttgtttactcgNtcgtatctttcttcctcatctcggatttcttc
ctgctcttcttcgtatgcttctatcttggccatggttggNccttcctcttcgtcatcacttccttcgtatggc
atNccaaactcgtctaaggcNatttcctctNttgctgggcatttgtgtttgctcagcaaatGttccgaNgttt

gNattccacactcctcgcatctataNtgcaaNactgttccccaaNttgatttcttNgggaaggaaactaattt
ccaacctgcatattgtttgtgNgacatgtcaggNttttcccttttttcttcctctcgtgcttccttcatcaac
tgcctagttgatttccttggttcttctacccttcgtatttcttcctgtttttcttggtttttcctttgtctt
tgtacccacacccgtctgggcacctggtcatctttacctctcctttctcccaatcgatttccggattatgcat
ctgcaaccaaggcattcctaatatgacNttattctttcctaggccacatatttcgaagtgtactctttcttta
tgccctttgtagtacacgtttgcagcaactcggtgtgtcactgctccagatgcgttgattgtcccatcaacat
tcttgaccgggattggtctttctaaaggttccagcttgaacccttcttcctctgcaaacNccttNtccatgaa
catccccgtNgctccactatccaacaacgcctgtactccNactccatcatgtgtgtccaaNttNtctacccccc
acattcagcttaatttcccttaaaggtcgtattgtccttacaaggaaactctcctcttgtacagtgtccaaaa
cattgatttcgttacatagtacatcaaatttgttgtaggaagtcaaacgaggagactttgcttcctttactct
tttaaattcttctgttcctcctcgaacatcctcttccaatagtcccgctgctcgtccacatctaacgttcgca
catctcgagccggagccttgctccagcaattcttggccatatgNcccaactttccacacttgtaacacttgat
aggacctcgactgggtcctgcNgcggtgtcgacatccatcgcgttgggatctcgacgttgaggctgtggcctc
acgaaagtgttgttggNggtgtgggttcgtagttggggcttNgcttgcccccgccgatagtagtcttcNtcNg
ccttNgcNtgcctccactgcctatccaacttcatgctcctctcgtaccaactcctgatagtagttggtggNtt
NtcNgaNtccatcagcgccttcctaatNcttcctgaNaaacttctcttgaattcttcgatgagggctcgtccc
tcgtatccgctNtctcgtgcNatcctcttNaaNtcctgtacatgctcNtccgcgctNttNcttccttgctcNa
tcaacctcaactttcctactgccgtactctcctcatccgcatctccgaaattgttccgcaNactatcaaaaag
ctcctccgtgtgggctggagcttcttNttcNccNNNttcNatcatctccatNatgttNtccttccaNgaNtcN
gcNgctcctccttgtacataNgaNaNtatccaNgtNatctgatcNccttgtagtctNcctaccattttaaac
tcaNataaatNttgcaNgccgttatgaagccactcagctccttcccatctcctgagaatattttggtcttgc
cacatcgatttgtggcggNactacttggacgatgggagtttctccgtcgtcccctggtggacctNgtggNcct
ggtggacccggttgcgggttgttctgcagctgggcgagttgctgctgcagttgggNgatgagggtgtgctgag
cgttcagctgatNgattatNtcttggggNttggccatgtctggctcgtggtattcgggggaggtggttgata
taggggNagggggggatacggacggttgtcgttgttctggttcggattcctcggattgggattccgaggcggat
tcttcttcttcctcagattcagaggaaggtactcggaatgttccgggcggtaNtcggtgagaaattttgaaga
aggtgagggaggtcttttcctgtcacctgNtNcgtatccaccaNttcaatgaagccaatagcctgcttcactt
gNaNgtgtatacaaaaagaaaaaaagtttttattNaagaaatgccgcactggcgaagggaaNgactactgccct
agataaagctatatacgaagacggatcttcacgctaatctagacacgcacttaatgtggcaatgtcactcctc
tgcgtgtaccacttgacaatgcaagcgcagcacaaggtttatacaNgaagttacaaaNgaggtagtacaggga
taNaaaataacaccacacagtatggtaggtctcgtcttatcNatggtctgtgtcaaaccactgaNgtccacgg
cgcacgttgtgtttatgcgcagacaccccttaagcctagggaccggctatgtgtgtggcggccgcgatggcgc
gcagagagtagagggcgaNNagagagtaaaagatggggagggcactgtgctgtgcgatccccggagtagtcga
gttctactgttagggcgactgtgactgNgatgtcacaggtcacacctgaacttaagaactatagaggaaagtg
aNaaaagtttattaggcgtgaacctaagagaactcgacggagtcttctacaagtttctacctactttatatac
ctagactacggcgcacagaccaactaccctaggtaacttatttatatactgacatgtgaaaagaagtacgtat
gagaaagtagactaattatacatttgacatgagaaaagaagagtggagtaagaagtaagtaaacaaaagtaNa
ttgatcataaaaacgtcaacaNNaccaaacaaaccataaaagtgtccacaagcacaaacaactcctaaaccgg
taatctactcacacaaaccaatacgtNccgaagtaNttacctaacatcgcatcacatggacatagNaNaaaga
atccggctattgcccattgNcgcggcccccagtatccccatctctgtatNacccaNgccttctgctcctcNtN
gtcccacattgctaagcatacNctcccacacNgtctgcaccgatgatccgataggttccaacctaccggNctN
Ncctctcttcctatagccgccgccgcccttctccattgtacttgctccgcatggttcggaggcaagaaagata
agattaaatcNatNagttcaattggtaagtctgtagcttgcgccattttagcagtatgccgatagtttagcct
ccgtcgaactgtagcagcagagggaaattattatgtaataaaatatgtgacagatgtgacagcgtgacgccgt
gacattctggcaaggttctaaca
>Agabi_varbisH97_2_Cluster17#LTR/Gypsy
TGTTAtGaatTTAccaacctANAggaaGgacGtcatcAtGcttaNtcattTccANAtccctGgatatactctg
NtccacctANgcttctcccaTcANtCaTtcccatgtCActtNttctTtgaNgTtaccTtgtttatctttccct
TctcatgatcAttcttaTtgttccCttGaTtgaTccttcatgatacctNttATgtcTttTcNtTcccatgatc
aAtcttctaTtcTcatGattGAtCATcttccttcctaGtTtAtaCtTtccttctcANNAttActcATctacct
cggtcatggttActcagCcNGttctatAgttagtTacctNgGgtAgNtaNtctttaNAtAagtaAacgTagat
actNNatgtAttTcNNtANGctTtAccaattgtatcTttccctttctctactctcaCctcGcTcTcaAgtctt
tgNcaCtTGtctgCaaCCtgagtcTcacagacTAAggTCgTAACATctggtaaaGcgtctAcacCtcacCttg
caGAcAgcAGcTAtcgagGgCttGctgtAGtGgtagtgcatAcggCAgaatCtACcAgTtGAacgCAcTtgcg
TtgAaggTcgCtcTattgcgtcgttatccacattactgaaagcactNgctttgaaggtNgctNtatcgcNtcg
NaNtTtttttAcggagtaNataactcGggcactAttgNaTgccAaGTcgGaaAcaggatcTacagagaacgaT
caaAgaAgaTttaNaCAtctaccaacaggAaAcattacaagANCttgaaAagacggctgAgaacgagCAttg
aggcTaTTacAAcAaCtTcAaccgaNACcgGaAgaAgacgaGcAgatcCaatNGtcagtaGaagaGAatcAtc

ActcaGAGaaggaagAcGaagacgaAccaaccagTcgcatatcAcNtgaagaaGAagaAgaActTgccaAcaa
ACtaGcAAacatGTcgaccaacgattCtTtcAgAgctTtNatcCcCgAtccAggAAagttTgaNggaAgtaga
GaGaAgtttgaNgatTggTGGaggagtatccaaNtatttTtaAcattcaAcAaAGTcaccaCcgacaatGaca
agaTcatcgctTGcNtcTcNcGaAtgcacaatggAacNgCaGgagcaNtagCcgCTttAaTcgCTgacagNat
caatGGTAaCaAcaAGatatCaTgGaaNgagTtTgagaaNgAgatgAaggAAgccTtTATTgaTgGaaaagag
aaggaGaagagcgaagTcgCTatagaaaCcctagtccaaggaaggagGcccATtatcGactacctgGtcGaGt
tcaaCTTgctgaaGaacAAagcgGGcaccgacgaTcaacacgcNgtattTtTgctcAAGAaaaacgtTcgNaa
ggAtaTcatAcGaAcCaTCttaTcaTtcccaCccaCcaatcTacccacCAAccTtgCcgAAtGgaCcaAtgta
aTcaagTCNgttggaatAGgaGccgaAaccatgcacATGTTcGaCaaacacAcCtcttCTggAgtaaCTTatG
gAggaaGtNgTcaaccaatggaGaTtggTagTtcNgaaaccgcNAgagCNcaaTTcNCTaaCAaCNcgaagaa
atgTTtcaattGCggaaagttcgGacatTtcgcCAagGANtgcagGcaAccAaggaaaccaatgACttgttac
aactgTaaCCgcatagGAcacatcgccaaatattgcAANGcGccNaagaaaGctaggaCNaGaGaaTTtgCcg
aagAAtacccTcaaGaggaGGaaAaggcagccGaagAAcaGgaTTtTgtcgAcggGtcaAcctgagcgcGAag
GaTCgaccccTaaaacNTagagtctctccccatgtaNcctcTaTtTcGaaatctacCtaTgttAtcccTTtca
aacgaGaCTcGaagcgtGaAatcttgcTagAcGcAaatatcaGaacAaaGaaNggaaCtaaNACgGtcaAggC
NCTTatcgAtTCNGgaGcacatatgAcgcTagTagccaaAGgatTggttaAGAaggaaaacTtgGAGaaaacc
AaaNGNcaacANccgaTcaAgatgagaaaNGcAgatGgaAcaccNAAtaggAacgGaAaNtTaactgaagaNg
ccaGGatNcaGatggATttaGgaggacacaAagagGaNatNAaagcatTgGtatcNgacataTcaAgtgcNga
cAtTtTctTNGGaTacgaNtggttaaTTcaaCacaaccCggAaaTcGaNTggataaATcaAgaagtcagNtTC
tcTagatgcCcaagcaCAtgcActGtTCaacatgAaactaTCcctttcgCtcgACgGctNaccAcggacgtgG
aaaGcgctacgGaGgaaAagGaAgtagAccCtAcCaacccGgaagattTacctgaNTAcATccgTCCcTaCgc
ccatctcttcAAcaagaagaacTTcgacaAacTTcctgcacgaacggaatgggatcacgAAattaAcTtTacg
gaaaacgcaCcAacggagatatcaTcaaAggttTaTagcATNacgccAttNgaNaAAgaggaaNTgGAcaAat
ttTtggaCgaaAatcTggctaccaatcgaatccgaccaTCgAaatcAcctTatgCagcaccAtgTTTTttcGT
TcccaaNaaNgaCNGttcacTaCggtTatgtcAgGattacaggaAacTgAacgagatcacNatcaaggaCaaG
AcgccactaccNctcatcagtGaAgTacTagAtCAactNaNAgacgCcaaNgTtTtcAaCaaattggacaTtA
tcTggggatAcAacaacGttcggaTacgagaagGagatgAatggAAAgcagcNtTtTtaAcgaAtcgaggAcT
aTtNgaAccaacggtcatgTTcTtTggaatgagcaANTccCccGcNactttTTcacgcATgatGgcaAcaaTN
tttCgagaaaTgatACAaGAcGGatcTttggccaacTacaTggacgaNTTCaTtatNccagcaaaNgaCgaTN
aagaAtTggagTcaNgaactacacgtttcctcaaaatagcgGagaaacaccAtTtatcattCaaacggACgaa
atgcgaaTTcaaTGtNtcaTcaAcgAcggTaTTgggaatgNTtaTTggaAatggAaaggcaaCtaTggaagaA
gaaaAGNTCaaNgcAatacgagattggGcagtccctaccacNGtcAAAcaggtcgagagttTTctaggttttg
cgAAtttcTaccgacgNTTTaNCaagaaTttcagtacCatcgcacaaccNctNaacGAgcTgaagTctaagaA
NgGggagaaaTggtaTtggAAcaAtgaacaaCAAcAagcTTtcgaacaaNtCAaNcAAgcNAtNgccaGcgaa
NNNgNNNNNNNaNtNNNNNNNNNcNNNNNNNNNNNNNNaNNNNNNgNNNNNNNatNNNaNtNNNNNaNNgNNN
NNNNNNNgNNNNaNNaaNNNNNNNaNNNaNNNNNNcNNaNNNccNtcNtgtNNNaNNNNNNNNNNNaNNNNNN
NcNNNatNNNNNaaNNNNNNNcNNgNNaNtNNNNNNNNNNaNaNaaNNtNNNNNNNNNtNNNNaNaNtNcNNN
NtNNNtNNaNaNNNNNNNtttNNNNtNNNNNcNNNNNNNNaNNNNNNcNNNNNNNtNcNNNNaNNtNaNNNNc
NNNNtgNtNNaNaNNNNNgNNNNNNNNNNNtgNtNNaNNaNtNNNNNNNNNNNNNNNNNaNNNNNtcNNgNNNN
NNNNNNNNNNNNNNNNNaNNtNNNNNNNNNcNtNNNNNaaNcgNaNNNNNcNgNNNNNNNNNNNaacgNNNaa
aNNtNNNNNNNcNNaaNNtNNNNNcgaNNNNNNNNNNNNNNtgNNNNNNaNNNNNNNgNNNNaNNattNNtcN
aNNNNcNNtttNNNNNNtNNNNNNatNNNaNaNNNNtNNNNNNNNNNNNNNNNNtNgNNNNNNNNNNNNaNaNt
NNNNNTacaGgaNatgaagaaacaacNggaGAAagTatggGAAtctgaaggaaTTgttttccgacaaGgAaGG
AtctActGTaccagaagAaAaggaaCtcagaGaTcGggTcCttTaCgaTcatCactatccacCcgaCatCgga
caTccaggaCagtatcGCatgNtaGAattacTaaaacgGAcCttTtggTGgcctacGAtcaagAcaGAcNtca
gaagaTacATcagNggAtgCgAcaTgtGccagaaGaacAAaacgattagacgAcCCgAtcAcatTccacttaa
TccActtcNtatcCcCgAcaaaccTtgggaagagaTatctatagaCatgATtggaCcactaCCgaagtcaaNN
gagaAggaTgCtaTTattgTTAtcgtTgaCagaTtCtctaaaatgatcCAcctNgTtcccaCcaccacgTcaC
TCacAtccacNgatCTtgcggAgatCTacaaagAagAagtTtggcNNcatcacgGAATcccaAAgcGtaTtat
CaGTgatAGaggaccNCagtttgCttcNaaaTtCaTggAgtctttaTgCaatgcTCtAGgCatcaaNcgAAat
ctTtctacNGcNtatcacCcAcANAcAgacggacaaacagagaggatgAaccaggAaattgagAcCtaTCttc
gagcatTcatCaattatcgAcAAgacgaTTggaCgagatggcTTcCCatggCagaAttccaTTacaacGacAA
aaCcCacGctgccaCcggacaaacCcCaTTcttcTtgaattacggactTCaTccatGGaaAggaaacatcaca
GTTgaaacgacaaaCcctacCgccaccTCcTTGatAgAAgaNctagAaNaTgtcagAgaagAAgcTaaagctg
cGaTGgaAGcaAAcaacGagaTgatgagagaNagAggAaacAaCaaacatcacaaggaaCccttTgCcgAAGg
agaTaaAGtttgGtTagaagcgaCgAACATtcattccaaTcgtcCgaCTcggaaActaGACcacAaacgATat
GgaCcTTtcgagAtcTTgaaAcaaAtcNgCGatcGAtcttAtaAactGAaatTaCcTgatAcNTgggcNatAc
acgaCgTCTTccatAcAtcNctcctaacNaaAgtccgaGacCCCGagTTtgaCagtcANAAGaaaccCaCtcC

acctccacCtgacATtatCaatgaagaagaaGaataNGaaNttgaAGaaAtccgaggacaccNacNNaaaggc
CgAggAATAcAattTTtagTtcaCTggaaNggAtAtgNaaacgAaGAcaaCTctTgGaTACCacgNtcatccc
tAGagAaTgcAGaaGaagcactctCtGaataTagagcaaaNcTTCcNaAtggaCAattataaAaCcaacCtaa
aaNtGctccaACttTtTcatcccTtcANcNaNcacaaaatgcaagCCAttAccgaatcttttaCaCcNgttTc
aaaagAcatcTTtaTccGTggaGccgacAtggTaTtcAtGaccNacgacggaaCcaaaTTcCacGTccattcc
tAcNTttTcacTCgcgaatctaTctattGgcAacaaaaacTcactggcCaTaNcgaacCTCaccaTccTNtaT
CcaaaCNctataCaNcaaacgaTcCctaccTcaTcCaagaNGTagactctcAtGaTtTTcgAAaAtttctccg
tGtNttctaTaAtacgcgaTaTggcGaTtactcttTcTtTtCcaacctcgacTGggtcgAcaTctTttcaatt
gcNcataaAtgggattTTCCacAcgTcaaAaNtctTNccaaaCgttaTcttggaNaaaTgggctATggagttg
ttgAacGaaattgTggAgttcAcTGtacTCGAaTcGTtGAcgatGaaaTgattgaccgctCTtatcCtcgtCa
agtcTAtCTtaTtTCgtgtgGacacgaaATctgaacagggggtaATGTTAtGaatTTAccaacctANAggaaG
aacGtcatcAtGcttaNtcattTccANAtccctGgatatactctgNtccacctANgcttctcccaTcANtCaT
tcccatgtCActtNttctTtgacgTtaccTtgtttatctttccctTctcatgatcAttcttaTtgttccCttG
aTtgaTccttcatgatacctNttATgtcTttTcNtTcccatgatcaAtcttctaTtcTcatGattGAtCATcT
tNcttcctaGtTtAtaCtTtccttctcANNAttActcATctacctcagtcatagttActcagCcNGttctatA
gttagtTacctNgGgtAgataNNctttaNAtAagtaAgNgtagaNNNNNNaNNNaNNtNNNNaNgNNtNaNNN
NNNNNNNNtNNNNNNNNNNNNNNNNNNNNcNNNgNtNtNNaNNNNNNNNNNcNtgNNNNcNNccNNNNNNtNNN
NNNNtaaNNtcNtaaca
>Agabi_varbisH97_2_Cluster53#LTR/Copia
GTTAGAGTGAGCAAGCAGTCTACAAGTGTAGTTGGTGGACTTAGATATATGCGGACGAGTTTAGTCACATGAT
AGCTTAGTCATGTATGGTTGAACTCCCCTGTGGACGCTTCAGAGGCCTTAGCTTCTTACAGTCTGTCCACAGG
TGAGTACAAGTTGTTAGAACTCAATACAGCTTCTTACAGTCTGTCCACAGTTTGTTCTAGGGATTGCTCAGTC
AGTTCCTATCCAATTGACTCGCTTCTAGGCTAGTTAGGATCGCCACTGCAACCGCTATCGTTCTACGCTTAAC
AGGTTATGGGCCCCGGCTTCTAACAGCCTTCAAAACACGTTTTTTGCCACTTTCTACCATTTCCCCCTCTATA
TCTCAACTTGTATCCCTTCCCCTGCACTCAAATCGACGTCTATGGCCTCCCTACGCCTGGGACACCACTTCGA
CGCGTTTTGGCGCGTCTCCGACGCGTCTGAGAGCCTCTACATCGACCACAACTCCCTCTGGCAGCCATACACC
CTCTCCACACTCTATAGGACCGATTTCCGGCCCTAAGAACGCGTCAATCGCTTGTTCCTTTTGTGGGAGGGTA
GGGCATATGCCTAGGAGGTGCAAGAAGCGAAAGAACGCGAAAAAACGCGCTCAGGAGCGCTCCAGACTGCCTG
GAACATCTAAAAGTAATGTCAAAGGGCCACTTGGCCATCTGAGTGGGATAGGTGCAGCTCTTCCAGCTGTCAA
GACACCACCCACTCCTCCTAGGGATGTTTCTGCTGGTTCTGGCTCTCCTGGCTCTGATATTCCTCTGAGAGCA
TCCGCTCTTGGTGCTTATTCCTCTTCCTCATCCTCTCCCTCATCCTCTCCCTCACCATCCTTGCCACTACAGC
TTGATGCTGACTTTTTCTGGCTGGCTGATACTGGTGCTACATCCCACATGACTCCTCACCGCCATTGGTTCAA
ATCCTACTCCCCTCATCGTATCCCTATCCGCCTTGCTGACAACTCTACTGTCTATTCAGCTGGTGTAGGGTCG
GTTGTCTTTCAACCGGTGGTGAATGGAAAAGAGACTAGAGCGGTCGAATTCACAAGAGTCCTTCATGTTCCTG
ACCTCAGGAGTAATCTCTTATCATGTCTCTACCTTGCCCGACACAAGGGCTTCAACATCACTATCTCCGCTCA
GTCTATTGACTTCCAATGCAAAGGGATTTCGCTTTTCACGGCCACCATCCATTCTAACAACTCTGCTGAGCTC
AACGGCTCAACTCTCACTTCGGAGGCCGCTTTTTCTGTCTCCACTCTCCCAGTTGACATCTCTCTATGGCATC
GTCGCTTTATTCATCACAACTATGCTGATGTGAAGTCTATGATCAGTAAGGAACTAGTTACTGGCTTGAACCT
CGAGTCAAATTCGCCTCCTGATGCTATCTGTGAGCCCTGCCTTGCTGGCAAGATGCATTCCCATCCTTTTCCC
TCTTCTTTGAATCACAATCGTTCTCCTCTTGAGCTTGTGCATTCAGACCTTCACGGCCCGCTCCCAGCCTCTA
CTCACTCAGGCTACAAGTACTGGATTACCTTCATCGACGACTCGACACGTTTTCGGGCAGTGTATCTGCTCAA
AGCAAAGTCTGAGGCATTTGAAGCGTTCAAAGTCTACAAATCTTGGGCTGAGACTCAACTCGGAGTCAAACTG
CGAGCTCTACAGGATGACAAGGGTGGCGAGTATATGTCTAAGGCTTTTCTAGCCTTCACACAGGAAGCTGGCA
TCGAGAGGAGGCATTCTACTCGAAATAGACCTCAACAGAATGGACTTGCTGAACGGGCTAATAGGACTATGGG
AGAGAGGATCACTGCTATGCTCTCAGAATCTTGGCTACCTCCTTCTTTCTGGGGTGAATGTATCTCGTCAATG
GTTCATGTCTGGAATATGCTCCCTACTGCTTCGCTTTCGGGCACCACTCCCTTCCAAGCTTTCTATAAGCGAA
AACCTGATGTCTCACATCTACGGGTATGGGGGTGCACTTCCTATGTCCATATACAACGAGACAAGCGAAATTC
TCTCCAGCCTCACTACGAGAAGTGTGTCTTCATTGGCTATCCAGCTGGCTACAAAGGCTGGAAGTTCTACAAC
CCAGTCACAAAGCGAACCGTGATCTCTGAGAGAGCTGATTTCGACGAGAGATACTTTCCGGGCACTTCTAAAG
CCCAACTCGAGGCTGTCCCGTCATTTGCCTGCACACCAGAGCCACTACCACTTCCTAATGATACATCGTCATA
CTCGGGACTGCCACCTCTACAGGATGAAGGGGGTGATAATCACATCCCTCCTGCTCGTCCTAAGACTCCCACT
CCGTCCGCTTCAGTTCCTCCTGAAGAGAAGCCAGTTCTACCTCTACCACCTCTCCCATTGATTCCTCAAGTAA
TTCCTCCCTTGATTCCTCCAGTCACTCCTCCACAAATCCATCCTCCTCTCCCAGTCACTCCTCCTACTGCTGG
AGCGCCCCTTGAGCCCCCTCACGTTCCTAGACGTACTGGGCGAGTTCGGAAGCCTCCTGGCCAATGGTGGAAG
CTCAAACAACCAGACTTAGCTGGTAATGACTCTGACTCGGATGACGAAGCTGCTCTGCTGGCTGACTCTGAGT
TCTGTGAAGTCGAGTTTGCTGGAGCCGTTTCGGGTGCTGATCCCCGGAGTTACAGACTAGCTATGAAGTCTCC
TGACTCGGATTGCTGGACTGAGGCTTGTAACACTGAGATATTCAATCTCGAGGCTAACGGAACTTGGGAACTA
ATGGAACTGCCTCCAGGCAAGAAGGTAGTCAACTCTGGCTGGGTGTTCAAAGTGAAACGACTTGCCGACGGCT

CAATTGAGCGCTACCGGGCTCGTCTCGTTGCTAAGGGCTATTCCCAACGTCCTGGATTCGACTTCACTGAGGT
CTTTGCCCCTACATTCCGTCCTGCATCACTACGCCTCATTATTGCTCTTGCAGCTAGAGAAGGCTACAAAATG
CATCGCTTCTTGAACCTGTACCACTACCTTCCTCTTTTGGAAGTCTCAGTGGCCACTAGTTATGAGTCGCAAT
AAACAATCTGCAATGGACGTTGACGCTGACGGTGACGAAGGCGACTGGTCTGATGACGAAATTCCGGAGGGTA
TGTACAATACTTCTATTGGACGTGCTCTTCTACAGGCGGCGGCTCGTATGGAAGACGAGCTCGAACTCCCGGC
GGCGTCTCGTATGACGACGCCAAATCCTGCTAGGACTACTGGGTTCCCAGAGTCCTCCCTCACTCCCCTGTCC
TACACCTCGGCTCCCCGACCGCTGGACAACCCTTCCCCGACGCCGTTTCCGTCGACCCCGACACCGGCACCGA
CCTCCACCCCTTCCACTGGGCGTTCCCGCGTTCTGACCGCTGGTCAGAAGGGAATGCTCAAGCTAGTCAACAC
CTCGATTGTCATGCTTGACGATATCGAGCCCGAACACCCGCTCTACGCGCCGTTCACGGACTGCATACTGCGG
GCAACATACACTCTCATCAGACGTCGCGATCTCGATGGGTACAACACTTCCTCCGTCGCAGGCATTGGCTCTT
TCTCTGAGCAGCTCGCGAAGATTATCGGAATGGCCGATCCGCCTCCACGGGCATTCGAGCCTACCCCGCCCCC
TCCTACTCCCTCGGGTACAGCCACCCCTCGGCCACGGTCTCCGTCGGCTGACATGGACATGACCCCGCGTAAA
ACGAAGAAGGCGAAGCCAGCGCAGCCGACCCCCCCTCCTCCACCCCAGCCACCTGTTTTGGAAAGGACCCTG
TCCCTTCTAAAAATAATTCTTACGCAAAGGCCGCTGCTCGCCGCCCCCCTCAACAGCAGCCTCCGCCCCGTCC
TCAGGCTCCTGCCGCTCCCGCGGCCGTCGCCATTGCCCCTGCGTCCAAGCCTTTGGGCAGAAAACGTAGGGCT
CGTCACACTTGCCACGGCACTTCGCGTCGCGGCGTGCAACTCACTCCACCGGCCGGATCCTCTGTCTGCGCCG
CCCACGTGTCCCCGGCCATGCTGGCCGAAATCAACACTCACCTTCGGAATGACGTCATGTCTGACGTCATTCT
TGAACACGCTGAAGACTCTGGATCCGGCATTTTCATAGCCGCGAGCCGAGTCCCCACTTCTTCCGAGACCGCG
TGTGTTCTCAAACACATTCGGCGTCTCATCACTGTCGCGGGCGTCGTGCCCATCAAGTCCACTCCCGTCACCT
CGACTTCCTACCTCAAGGTTATCGACGTGCCCATGATTCCTGCCGAACCTAAGGTTTGGCTATCCACTCAGCG
CAGCGCTTTCACCAACGCGCTTTCGATTTCGCCGGTTGGCAAGGAACTTTCCATCGCCATCAAGCACGCTGTT
CGCTTCATGCGGACCTCGGCCCACAGTGACACTTGCGTCGCGTGGGTGGACATTTGTGATTCTGTCGCGGGCA
CTTCGGCCCGCTCCTATATTGGTAAGACGATCGTCATCGGCGGTCGGAATTGTCAAATCCGCGGCGCAGCTCC
ACGGCCCGGCTCCGCCTTGTGCACTCGGTGCATGAGGTGGGGTCATCATTCTTCCGTATGCCGATCAAAAGGT
ATACGCTGTCCACTTTGTGGGCTCCCTCATTCCGAAGCCGCCCACCACGAATACTGCGCTCATTCCAAGCGCG
ATCCAGAAGCTAGGTCTTGTGTCAACTGTAGTGCGGCAGGCAAGACGAAACGCGACCACTCTGCTACGGACAC
TTCGTGTCCGTTTTGGCAGAACCGATTTGATAGGGATTGGCTGAGGCGCCAATTCCCTAAGAAGTGATCAGCG
CACGTCAAACCGCGTTTCGGGGTTAGTAGCCGGGTCGGCCTCCCGCCATTTGTTTTCATTTTTCTATGACTAT
CCTCAGCTTGATTTTATCTTGATCTCGCGTTCCGGTATTAAGTATAGTTCGTGCTTGGCTGTTTGATTCGTTC
CGGGGCCGCATACTGATATACTACTCTTTTTCGATTGGACTTTTACTTTTCTTTCTTTCTACGCTAGCGCTTG
ACCTCGTCGCGCAGGATATGTTTTCTTTTTCTCTTGGTAACGCGTCATGGCAACCTTATTCTCAGCACGTTGT
ACATAGTGTTCGCATGTACTTATATCGCTGGAATAGAAGGCTCGAACAGCCACTTGATTACAAAAAAAAAAAA
AAAAAAAAAAGAAGGCTACAAAATGTGTTCAGTCGACATCTCCTCTGCTTTTACTTATGGGGAACTCGAAGAG
GAGATTTATATGCGCCAACCCGAGGGCTATCACATTGGTAGTCCTAACATGGTCTTTCGGCTCCGTAAATCGC
TTTATGGCCTCAAGCAGGCTGCCAGGCAGTGGAATAAGAAACTCCGGGGTGTCCTTGAAGGCATTGGCTACAC
TCGGCTTCGCTCTGACAGCTCCATCTACACTCCAAAGGTGACATCAAGGTCATTGTGCCAGTATTCATTGACG
ACATTACTCTTGTCTCGAAAAGTGATACTGCCATGGCCTCGGCTGTCTCTGAGCTGTCCAAGCACTTCAAACT
CCGTGATCTCGGGTCTACTACACTACTACTAGGCATTCAGGTGAAGCAGGATTGCTCTCATCACACTATCTCC
CTCTCGCAGGAGCACTACATCAAGGAGCTCCTAGAACGCTTCAATATGGATGATGCTAATCCTCTTTCCACTC
CCCTCGCTCCGGGCTCTGAACTTTCTGACATTATTCCTTCTCTCGAGGATCAGGCTGAGATGAAGTCAGTTCC
CTATCTCAATGCTGTAGGTGCTCTTCAGTATCTTGCCACTATGACTCACCCCGACATTGCCTATGCTGTCTCC
TATCTCGGGAGGTTCAACTCGAACCCAGCTCCAGCTCACTGGCTTGCAGTCAAGCACTTATTCCGGTATCTCA
TTGGCACTATGCACTACAAGCTAGTCTATCGAGCTAGTGACTCGGAGGAACTCTTCACTACCTACTCGGACGC
CTCTCACGGAGCCTGCAAGGCTACAGGTCGAGCTACTGGTGGTTATGTGACTCTAGTCTCGGGTGCTGCTGTA
GGTTGGTCTTCTAAGCGCCAGCCATTTGTGACTCTCTCGAGCACTGAGGCTGAGTTTGTTGCTGCCGTCGAGG
CTGGCAAGGAGATCAAGTGGACTAGGAATATCCTGACTGAGTTTGGCTTTCCTCCGACACTCCCTTCGACTCT
CTTTGTTGATAACAAGTCAGGAATCAGTGTAGCCAAGAATCCAGAGCATCACGGACGCATGAAACACCTGGAC
TTGCGCTTCTACTGGCTCCGGGAGGCTGTTGAAGAAGGACTCATAGATCCTTTGTATGTTTCGACTCACGAGC
AGGTGGCCGACATCTTAACCAAGGCTGTGGCAAAGAAGGTAGTTGAGTTTGCTGTTCCACTACTGGGACTTGA
GTAGACTGGCTGGATCAAGGGGGTGTGTTAGAGTGAGCAAGCAGTCTACAAGTGTAGTTGGTGGACTTAGATA
TATGCGGACGAGTTTAGTCACATGATAGCTTAGTCATGTATGGTTGAACTCCCCTGTGGACGCTTCAGAGGCC
TTAGCTTCTTACAGTCTGTCCACAGGTGAGTACAAGTTGTTAGAACTCAATACAGCTTCTTACAGTCTGTCCA
CAGTTTGTTCTAGGGATTGCTCAGTCAGTTCCTATCCAATTGACTCGCTTCTAGGCTCGTTAGGATCGCCACT
GCAACCGCTATCGTTCTACGCTTAACA
>Agabi_varbisH97_2_rnd-1_family-102#TIR/MITE
CATCAGCAAAAGGGTTTTCAAAATAGAAGGGCTTAGAGACCTTGTTATGGAACTAGTTCCACCACAATCCATC
AGAAAGGTAACTTTTGGAATATAATATAATCTTATGCAAAGTAGAAAGGCGTAAAATCCTATGGTGCCGTTCT
AAGCCCTTCTATTTTGAAAACCCTTTTGCTGATG

>Agabi_varbisH97_2_Cluster49#LTR/Gypsy
TTAGCCGCACTCGCTAAGATTACATGTCATTCCACGACTGGCCAAGGCTTAGCTTGGTCTACATGCAGAGGCT
CATTATAGCTAGACATATGTTTGTCATAGCTAAAGGCTATAGTTTCCCTAATGGTACAGTTTTCCCATATAGG
AACCAATGATCCCTCATGTAGACTCACAATGAACGGTATACTCTGCTGAGTGACAAGTAAGGGTTATAGGATG
ATGTAGCTAAGCCCATAATGAACTATAATTCATAGCTAAGGCATATTCCAAGCGTAAAACGGCTTGGGCTGTA
ACAGCGCAAACGTCGAGTAAGTCTTGGAAAATGTCGTTCATGAAGCTTTGGAAAGCGGCGGGCACATTGGTGA
GTCCAAACGGCATAACCAGCCATTCAAAGGAACCATAGCGAGTACGGAAAGCAGTTTTCCATTCGTCACCATC
AGCAATGCGGACGAGATGATAGGCATGCCAGAGGTCAATTTTGGTATAGATGCGAGCTTTGCGGGGAGAGTCG
AGGAGATCAGAAATAAGTGGTAGCGGGTAGCGGTCTTTCCGGGAAATCCTGTTGAGGCCACAGAAATCAACAC
AAAGTCGGAGAGAGCCGTCCTTTTTCTTCACGAAGAGAACAGGAGCGTCATGAGGAGACAATTACTGGTGGCC
AGGCGTCACTAAGCAAGTCCAGAAATACGTAGAAGGATGCGACCAATGTCAACGAAATAAGAATCGACCAACA
CCGCCGGCAGGAAAATTAATGCCGAACCCTATTCCCACCAAACCATGGGCCGACGTATCCGTCGACTTCATAG
TAAAATTACCAGAATCACAGGGAATGGATTCAATTCTGGTGGTGTGCGATCGATTCAGTAAAATGGCTCATTT
CATTCCAACTACGGAAACAACATCAGCGCTAGGACTAGCTAAGCTGTTCAGGGATCACGTATGGAAGTTGCAT
GGATTACCAGAAACGGTGATCTCAGACAGGGGACCGCAGTTTGCTGCAGATTTTACAAAGGAGTTGAATAAAA
TGTTGGGAATTGACACTAGGTTATCTATGGCGTTCCACCCACAGACTGATGGCCAAACCGAACGAACCAATCA
AGAATTAGAACAATATCTCCGATCATTTGTCGATCATAGACAAACTGATTGGCCTGATTGGTTAGCAACGGCG
GAGTTTTCCTATAACAATAAAGAACAATCATCAACAAAGGAAACTCCATTTATGGTCAACATGGGAAGACACC
CAAGAACTGGTTTTGATCTTCGACGTCAAGGCAACCACCCAGCGGCAGTAGATTTTGCGGACCACATAAAACT
TCAACACGAGGAAACTCAAGTGGCCCTGAAGAAGGCACAAGACGACATGCGGAGGTATGCGGATAGAGGCAGG
GGAGAAGCGGTAAAGTACAAAGTAGGAGATAAAGTTCTATTGGCTACTCGGGACCTTCAAATCCAGGGACGAC
CTACCCGGAAACTTACGGAGAAGTTCATCGGACCTTACACCATCAAGAAGATCATTTTCCCCAACGCAGTGGA
AATAGATCTTCCCCGATCTATTCGGATCCATCCGGTGGTTAATGTCAGCCGAATAGTACTCTACCGAGAACAA
GTGGAAGGACAAGGGAGTAAACCGGCACCGGCCGTAGAAATAGAAGGAGAACAAGAATGGGAAGTAGAGAAAA
TCCTTAACGCCAAATGGTTCCGCGGAAAGAAGCGGTACTTAGTTCGATGGAAAGGATATACGGCAGAAGCAGA
CTCGTGGGAACCTGAGGAGAATTTGACTAACGCCAAAGAGTTGATTACTGAATTCCACCAACTCTATGGGGAA
CTCAATAGGATACATCACGTCCTACGTGACACCGGGTTCCAGGAAAGGGAACTACCTAATCGATTCACAGCCA
AGGCCTTATTCGGATGGGATGACAAACAGTTTGATGCCGAATATCTTAAGAAGTTAGAACGTAATTGGCGAAC
GTGGAGGTCTTGAAGGGTCGTCTCCAGAGGAGCCGACTTTAAGGAGGGGGTACTGTTAGAACCTTGCCAGAAT
GTCACGGCGTCACACTGTCACATCTGTCACATATTATATTACATAATAATTTCCCTCTGCTGCTACAGTTCGA
CGGAGGCTAAACTATCGGCATACTGCTAAAATGGCGCAAGCTACAGATCTACCCATTGAACTCCTTGATCTTA
TCTTTTCCTTTCTACCTCCGAACCATGCGGAGCAAGTGCAATGGAGAAGGGCGGCGGCGGCTATAGGAAGGGA
GAGTAGACTGGTAGGTTGGGACCTATCGGGTCATCAGTGTAGGGAGTGCAGAAGGATGACGTTAGCCATGTGG
GACGCGGAGGAAGATAAAGTGTGGGTAATACAGAGGTGGTCAGCGTGGGGGGCTCGGTATTGGGAACTATCCG
GGTTCTTCATACTATGTCCGTGTGATGCGATGTTGGGTAACTACTTCGGACATCAATGGTTTGTGTGAGTAGA
TTACCTGTTTAGGAGTTGTTTGTACTTGTGGACACTTTTATGGTTTGTTTGGTATTGTTGACGTTTTTATGAT
CAATTTACTTTTGTTTACTTACTTCTTACTCCACTCTTCTTTTCTCATGTCAAATGTATAATTAGTTTACTTT
CTCATACGTACTTCTTTTCCCATGTCAGTATATAAATAGTTACCTAGGGTAGTTGGTCTGTGCGCCGTAGTCT
AGGTATATAAAGTAGGTAGAAACTTGTAGAAGACTCCGTCGAGTTCTCTTAGGTTCACGCCTAATATAACTTT
TGTCACTATACTCCTTAGTTCTTGAGTTTAGGTGTGACCTGTGACATCACAGTCACAGTCGCCCTAACACCAG
AACAACAGTATTCTATCTACGTATCCAGCGCGACTTGGTTGAGCGCTCTAAGAGTCCAAACAAAAAACAAAAA
CACAAACGAAAAAGAGATATCCAGTTAAGCGGCCCCGGGAACAAGCCAAGCAGTCAGGCAATACAACTTATCC
AAACATCGGAACACTCGATCGAGACCAGAGCTGAAATCAAACATAAAACATATGAAAGAAAAAGAAGCGGGCG
CTATCAGCGAACACCGCGGAACGGGGACTAGAGAGAGCCTCGCAGAACGAGGTACGCAAAGCCCATAGAAGAT
CCGGGATAACTCCAACAGTGGCGGGGTCAGAGTATAGACAATGGACTCGAAGGCAGTCCGGATTATATGGATC
CCGATCTAGTTCTTCGGAAACTGGCGCTTGAGCCAGAGTCTGTCGAAACGGTTCCGCCAAAACGGACACGATG
TATCCGTAGCAGAATGAGAGCGCTTGTCCGCTGGGCGCTTGGCCGCGGTACAGTTGATGCAGTGGCGCGCGTC
GACGCGAGAGGCATCGACACACTTGGAATGGTTGGCCTCAGAGTGAGGCCCGCCGCAGAGGGGACAGCGCGCG
CACTTGGCACGACACTGATCAGAATGGTGACCCCACCGCTGGCAACGAGCACAGCGGACAGAGCCGGAATGGG
GTTTGGCGCCTTTAATCTGGCAGTTCACTCCGCGGATGGAGATGAATTTTGAAATGTAGTCCCGGGCATTGGA
ACCCGAGACCGTGTCGTGGATGTCGATCCATGCCCAGCATGAATCAGAATGAGGGGAGGCACGCATGAAGCGT
GGCTTGTGCTTGAATAGTTTGGCCAGCTCAGTGCCAACCAGAGACTTGTTGAGAGCGTATGTGAACGCCTCGT
GTTGTTTAGTCACCCACTCCTTAGAGGAAGTGGGGACGTGAGGGATGTCAACTATCTTCAAGTAAGAGGTTGA
CGTGGCTGGGGAGTGAACAATGGGTGTGGTTTTGTTCGGGAATGTTCGCCGAACGTGCTTGAGCACGAATTGA
ATTTCGGCGGCGGTAGGGACACAGGAGGCGGCTAAGAATATGCCGTTGCCTTCGACCGTTGCATGCGTAAGAG
TGACGTTGGACTTGACATCGCTCCGGAGGACTTGATTGAGGTTATTGAGAACCCCAGGAGAAAATGACTCAGC
CGTGACAGAAGATCCGGCTGGCGGTGTAAGGATTATGCCACGGCGCGAAGCGCCGTGTGCAGTGTGTTTGCCT
TGGCGTCTCTTACGAGGCACCGAGGGATCGCGAAATGAAACAGGGGGAGCGCTAGCAGGGGTAGGCACGCGCT

TGGAACTTGTTAAGGGAAGGGGTTTGGTGGGCGCCTTCCCAGACACTGGTTGTGGAGCGGCCGGCTCATCCAC
TTCCATGAGTGGGGCCGTTGCCGGAGGGGTGACATCAGAAGAAGCCGCCGGCTCGACCCGGACCTTCTTCTTC
GGCGGTTTGGATTTGGCTGAGTCAGCCTGTGGCGGTGTGTCCCGCGGATTTACATGTGTAGCAGGTGCATCAA
CTGCGGATGCAGGCGCTTCAACTGCTGGGAGAGAGGCACGGATACGTGGGGCGAGTACCTTCATATTGTGCAA
GAAGTGTTTGTGGAGGTCGGTAAGATCGTCGTCCATAAGTGTCGGGGTTTCTTTGAGTAAATGCTCTTTCCAG
TTGGGCTTAGCAAAGAGCGCAATAATGTCGAAAGTGTTGTCGATGGTTTTGTGGAGATCCTTGACAATTGGTT
TGTTGTCGACTGGCATAACGGGGGAACCGGGACGTGGAGTAACAGCCTCGTAATCGAGAGCCATGGAGGGAAT
CCTCACTGTCGCATACAGAAGCGACAGCAACGGAGTGACCAGAGGAAGAGGACCCCGGCGAAAGGGGCCGCTT
GGAGGAGAGTACCGACGCCGAAGGCGACGGTGCGCGAGGAGGGGATGGAATGGGCGGGGCCGGCGCAGGCGCA
GGCGACGGCTCGCGGGTGGGAGGAAGACTGGGCGGTGTGTTGACAGCCGGAGGGGCTGCACCGATCCCAGTTG
AGTGGCCAGAGGGCCCTTTGACATTACCTTTGGCTATAGCGCTAGCCTCGGCGTTGGAGATGGCGACCTGGGG
GGCAATGCCCCTGTTGCGGACGGCATCAAGAATAGCCCGATTCTCATGAGAGTCGAGCTGGAAGGCGGCGAGG
GTGACTAGACCTCCGTACTTGGGGTCTGAAAGAGCTACAATGTCGTCTTCAAGAAGACGACGAGCGGTCGGAT
TGGCAGGCGGTGGCGGCACCTGAGACGGTGGACGGACGGGTATGGCGCAGAGGCCAGTGCGGACTGTTGCTT
GCGACGCGGAGCGGGCATTGGAACGATATATTGCTACGACTATACTCTAGCACTAAGACTTCCAAAAGAGGAA
GGTAGTGGTAAGAGGTCAAGGAGCGTGAAGACTAACTCAACAGTTCGTGTTAAGCGATCTTGAGTTCCTAGCT
GTGCTACCTAGGCTGAACGTTATGCTGATCTATTCTTAGCTGCCGCACGGCAACGCTTAGCTACTATTTCCAA
TTGACCTTTTATGGAAGCAGGTCCATGTGCTGAATACCCAGAGTCGGAGCTAGGGTTTATTATCCCCATATAA
TGTCGGATTTACGGAGCTACCTCTGATCTTCATCAGATCGAAGTGCGATCAAGCTGGATTCTCCATTGGGAGT
ATAGGATCCACATGTTAGCTGCACTCAATTTTCTTTTATCTCATCTATGAACGTTTCTGTTTCTTTGTCTATC
TTAGTGTACTTGTATACAGATTTGAACGGTCTTTGGTTTCTTTTTCTTTCTCTGTTCTTATAGCTTCGACCTC
GTCAGCTGGATACGGACTCGTAGAGTAGTTAAGTACAGGCGATGTAAAGTACGGGGAACGGGTCATGGCACCC
TTTAAGTAGTTTTGAAATAAACTATAGTGATTACAAAAAAAAAAAAAAAAAAAAAAAAAAAAAATGTTAGCCGCAC
TCGCTAAGATTACATGTCATTCCACGACTGGCCAAGGCTTAGCTTGGTCTACATGCAGAGGCTCATTATAGCT
AGACATATGTTCATCATAGCTAAAGGCTATAGTTTCCCTAATGGTACAGTTTTCCCATATAGGAGCCAATGAT
CCCTCATGTAGACTCACAATGAACAGTATACTCTGCTGAGTGACAAGTAAGGGTTATAGGATGATGTAGCTAA
GCCCGTAATGAGCTATAATTCATAGCTAAGGCATATTTCAAGCGTAAAACGGCTTGGGCCGTA
>Agabi_varbisH97_2_Cluster20#LTR/Gypsy
tgttaGggcgactgtgactgagatgtcacaggtcacacctgaacttaagaacaatagaggaaAgtgacAaaag
ttataTtaggcgtgaacctaagagaactcgacggagtcttcTAcaagtttctacctaCtttaTatAcctagac
tAcggcgcacagaccAacTaccCtaggtaactatttatatattgacatgNgaaaagaAgNacgtAtgagaaaa
taaactaattatacatttgacatgagaaaagaagagTggaGtaagaAgaaagtaaacaaNaaaaacNttgatc
atAaaaAcgtcaAcaaNaccaaAcaaaccAtaaaagtgtccacaagtacaaacaactatcaaaggacgtaaCc
tttcgtaccaatccctgccgaagtatgTtgccatNaNctctccacacgggcacatcgtaaagaAccggaccgg
ttcccaatgacgaaagtccgggtcataccatcgctgcaatatccagGgtctctccaatacttcGtcccacAtt
atcaAcgtcactcttccacAtattacgcacctacgatcctctaaccTcCatccgtctggtcggggtTctcttc
cgatagccgccgccgccctcctccattgtacTtgctccgcatggttcggaggtANgaaagaNaAgattaaatc
aatgagNtcaattggtaaatctgtAgcttgcgccatttagcagtatgccgatagtttagcctCcgtcgaact
gtagcagcagagggaaaTtattacataataaaatatgtgacAgatgtgacagcgtgacgccgtgacattctgg
caaggttctaAcAgtAcccccctccttaaagtcggctcctCcggagAcgacccttcaagacctccacgtTcgcc
aatttcgttccaactTcTtaagatattcggcatcaaactgtttgTcatCccatccgaataaNgNcttggctgt
gaatcgattaggtAgttccctttcttggaacccgGtgtcacgtaggacgtgctgtAtcctattgagttcccca
taGagttggtggaatTcagtaatcaattctttggcgttagccAagttttcNtcgggttcccacgagtctgctt
ctgccgtAtaccctttCcatcggactaagtaccgcttCtttccgcGgagccatttggcgttaaggAtcttctc
cacttcccActcctgttctccttctatttctacggccggtgccggtttacttcctTgtccatccActtgttct
cgNtatagtactattcggctgacgttaaccaccggatggatccgaatagatcgGggaagaTcTatttccACtg
cgttgggggaaatgatCttcttgatggtgtaNggtccgatgAacttctccgtaagtttccgggtaggtcgtcc
ctggatttgAAGgtcccgggtagccaataGaactttatctCctactttgtacttcaccgcttctcctctgcct
ctatccgCatacctCcgcatatcgtCttgtgccttttttNAgggCcgcttgagtttcctcgtgttgaagtttca
tgcggtccgcaaagtcNgctgctgccgggtgattgCcttgtcgtcgaagAtCgaagCcagttctTggatgtct
tcCCacgttgaccataaatggagtttcctttgttgacgattgttctttattgttataggAaaactCcgccgtt
gccaaccaatcagGccaatcagtttgtctatgatcNacaaatgatcggagatattgttctagttcttgattgG
ttcgNtcggtTtggcCatccgtttgtgggtgaaacgccgtagataacctagtatcNattcccaAcatTttatt
caactcctttgtaaagtctgcAgcaaattgcggtcccctgtctgagatcaccgtttctggtaatcCatgcaac
ttCcatacatgatccctgaacagtttagctagtcctagcgctgatgtNgtttccgtagttgggataaagtgag
ccattttactgaatcgatcNcacaccaccaaaattgagtccattccctgtgattctggtaattttactatgaa
gtcgacggatacgtcggcccatggtttggtgggaatagggttcggcattaatttcctgccggcggtgttggt
cgattcttgtttcgttggcattgNtcNcatccttctacgtacttctggacttgcttagtgacgcctggccacc

agtaattgcgNgtcaccatttctactgtcttccattgtcctccgtgtccNgcaatgggagtatcgtgatgtag
tcgaatgatNttNgtccttaacgtctcatctttNggcacatagacttttccttctttcagtactaatccttcc
tctaacttccactcgtctcccctNaatgacttcacctttgctcgcttcatctcttccaccgctttNacNacgt
catcttcttttgcttcNgatttttctgatttcNtctaatatttcttcttcctcNgcattgattaacatatgacc
ttcttccattgctcgaattgcaaaataNtctggtttNaacaNggtttgatccttgttatcgtcctttgttcct
tgttcaaagtcaggtcttctgctcaacgcgtctgccttccccattgtctttcctgggcggaattttaattcga
aattaaatctagatagatataaggcccatctggcttgtcgtcgatttagttgttgggctttNgcgaagtattc
tagattgcgatgatctgtccaaatttcgaaNttatgtttNgccccttccaggaagtgtctccattcttctaaa
caccgcataatccccaacatctccctatcgtgaatgtcgtagtttcgttccgtctcNgtcattgatttggaNa
tgtatgctaccggcctccactttccgtcttcacatttcattgNcaactctccaccNgtggcNtaagccgatgc
gtcagattccacctttagNtctttatccaattctggtgctactaggattggatgtgatgtgaatNttgctttt
agcgtaccaaaggcNtcttcttcttcatctccccaactccatttcttgtccttttttagttaatgcgtgtaatg
gttttgcaatactggcgaatttttcaatgaaccgtcgatagtaatttgcNagtcccaagaatNtttgtacatc
tttaacgcattttggagtNggccattctactacgtctttcactttcctttcctccatcttgattccgtttctc
cctattatacatcccaaaaaNcccatttcgcttcgtttccattcgcattttttctggttttgcgaatagatcat
tgtccatcaacctctgcaaNacttcttccaccaccttatcNtgctcttcttcatcgtctgttcctatcaagat
atcgtcaataaatacagctacttttcccgtatttactaagtccctaaaaagttcgttcatcatagtNtgaaaa
gtagcaggcgaatttgtaagtccaaaaaacataaccgtNggttcaaaggcacccatgaaagtagtaaaggctg
ccttccactcgtctccttcttttatcctaatgttattNtaaccccaacgtaaatctatcttagaaaataatcg
tttactacccatagtgtctataagttcagaaattaaaggtaaaggataattgttcttaattgtccaatcgttc
aaaaatcgatagtcctgtaccatccgcttctttccatccttctttggcacaaaaaataccggtgaggtttggg
gagatttcgaaggNctaatatatccNtttcggagttggtccttgatgaatgcttggacttcNtctctttcgtt
tggNgacaaNggatatattttccctttcttgggtttgaaatcttcctttaattcgatagcatgatcccatggt
ttcctagtaggcattctctctgattccttttgtttgaatactttaaggaacttgtggaaccgttttggtacta
cttgNtctactgagtctatgcttcgaatttcttcttcttccggcatttcctctgtcatNcctctcacccacgt
ttctagttgttctccttcttctctactcctatttttcctttgggcgcttatccgtttaggacgtacctgtcca
gcctccgcttgtcccgcggtggctgtcttgctgttatcttcttctagacgttctgccttNtccccatttaatc
ggtcagccacgtcaccgggtgattgcgcccacccctcagctttctctttgggttgttgNgccaacccctcagc
tctaccattagcacgcctgttacgtgccaattccctagcNaatgctgcctcaactcctaatgcgatcccttcg
atgatgtttttgggagtggtgaatctccctactgctttgttcacccgttcgtatctNtcttcctcatctctga
tttcttcctgctcccttttcatatgcttctatcttggccatggttggcccttcctcttcgtcatcacttccttc
gtatggcataccaaactcgtccaaggccatttcctccattgctgggcatttgtgtttgttcagcaagtgttcc
gacgtttgggttccacactcctcgcatctatattgcaacacNgttccccaatttgatttctttgggaaggaaa
ctaatttccaNcctgcatattgcttgtgtgacatgtcaggcttttccctcttttcttcctctcgtgcttcctt
catcaactgccgtgttgatttccttggttcctctatccttcgtatttcttctgctttctttgttttccttt
gtctttgNaNNcaNNNNNgtcNNNNcaccNgNNNNtcNNNNcNNNtccNttcNccNNaNcNNtNtcNNNNtNN
NNNNNcNgNaNNNNNgNcNNtNNNNNNNNgacttNNNNcNtNNctNgNNNNNNtNNNNNgaaNtgtaNtNNNN
NNtNNtgNcNttNgtNNtNNNcNttNNcNNNNacNNNNNNNgNNactgNNcNagatNcNNNNatNgNcNcNtN
NaNaNtNNtgNcNNNNatNNgNNNttNNaaaggttNcagNNNNNNcccNNNNtNNNcNNcNNaNacNttNNcN
atNNNNatNNccNtNNNNNNNctaNNcNacNNcgcNtNNactcNNNcNcNaNcNNgNNNNNcNNNNtNttNNN
ccNNcNNNttcaNNNNaatttNcNtNNaaNNtNNNNtNNNccNtNNaaNgaNactNNcNNNNNNNNNagtgtc
caaaacattgatttcgttacatagtacatcaaatttgttgtaggaagtcaaacgaggagactttgcttccttt
actcttttaaattcttctgttcNtcctcgaacatcctcttccaatagtcccgctgctcgtccacatctaaNgt
tcgcacatctcgagccggagccttgctccagcaattcttggcNatatgccccaactttccacacttgtaacac
ttgataggacctcgactgggtcctgcggcggtgtcgacatccatcgcgttgggatctcgacgttgaggctgtg
gcctcacgaaagtgttgttggNggtgtgggttcgtaNttggggcttagcttgcccccgccgatagtagtcttc
ctccgcctttgcttgcctccactgcctatccaacttcatgctcctctcgtaccaactcctgatggtagttggt
gggttNtctgactccatcagcgccttcctaatccttcctgaNaaacttctcttgaattcttcgatgagggctc
gtccctcgtatccNctgtctcgNgctatcctcttaaactcctgtacatgttcttccgcactcttgcttccttg
ctcgatcaacctcaactttcctactgccgtactctcctcatccgcatctccgaaattgtttcgcagactatca
aaaagctcctccgtgtgggctggagcttcttgttcaccNttttcNatcatctccataatgttctccttccaag
actccgcNgctcctccttgtacatacgataatatccacgtcatctgatccccttgtagtctNcctaccatttt
NaaactcagataaattttgcacgccgttatgaaaccactcagctcctttccgtctcctgagaatatNtttggt
cttgccacatcgattggtggcgctactacttggacgatgggagtttctccgtcgtccccctggtggaccttgtg
ggcctggtggacccggttgcgggttgttctgcagctgggcgagttgctgctgcagttgggtgatgagggtgtg
ctgagcgttcagctgattgattatNtNttggggggttggccatgtctggctcgtggtattNNNgNgNNggNggt
tgaNNtNNgNNNNNggggNNtaNNgNNgNtNNtcNNNgtNNtggNtNNNNttcNNcggNttNNNNttcNNNNN
cgNattcNNNNNcNNcctcNNNNNNagNNNaNggNNNtNNNNatNNNNcNNNNNNNagtcNaNNagaNNNttt

NNNNNaNNNNagNgNNgtNNNttccNNNcNNNtNaNNNNNNNcNaNNNNNNNNNtNaaNNNatNNNNNNNNt
NaNNtNNaNNNgtNNaNNNNNagagaaaNgNNNtNNtNNNNaagNaNNNNNNNNNNNccNaagNgaNagNNNN
agaNgagNggNNNNNaNNNNNaNNgNNtNNNNNNNNaNtNNaNagagNcNNNtNaNNNNNNtNtNtNaNNcNt
NtgcgNNtNcNagcNNaNNatgNNgNcNcNNcNNaagNttgaNaNNNgNNNaaNaNaNNaNtataNaaNNNNc
NccNcacNNNgNgaaNNtcNNNNgtNtaNNtaaNgNNcgtNaNacNNcNNNgtNNcaaNgNNgNNNNttNNNN
NNNNNNcNgaNNNcccNtNaNNcNtNgggacNggNtNNNNNNNNgNNggcNgcNNNgNNNcNNaNNNaNtNgN
ggNNNataagNNaNNaaNNgaNgggNNggNNNNtNNNctNNgNNNNccccggNgtagtcgagttctactgtta
gggcgactgtgactgagNtgtcacaggtcacacctNaacttaagaactaNNNNNNaaNNtaaNaaaNNttNtN
tNNggcNNNaNcNNaaNNgaNNNNNaNNNaNNNtNNtacaNNttNNNNcNNNcttNNtaNacNtNNacNaNgN
NNNNNNNNcNaNctNccctNggtaactttatttatatatgacatgNgaaaaagaagcNacgtatgagaaaata
aactaattatacatttgacatgagaaaagaagagtggagtaagaagNaagtaaacaaNaaNNacNttgatcat
aaaaacgtcaacaaNaccaaacaaaccataaaagtgtccacaagtacaaacaactatctaaggacgtaacctt
tcgtaccagtccctgccgaagtatgttgccatNaNttctccacacgggcacatcgtaaagaaccggccggtt
cccaatgacggaagtccgggttattccatcgctgcaatatccagggtctctccaatacttcgtcccacattat
caacgtcactcttccacaNattacgcacctacgatcctctaacctccatccgtctggtcggggttctcttccg
atagccgccgccgccctcctccattgtacttgctccgcatggttcggaggNaagaaagaNaagattagatcaa
tgagttcaattggtaaatctgtagcttgcgccatttagcagtatgccgatagtttagcctccgtcgaactgta
gcagcagagggaaattattatataataaaatatgtgacagatgtgacagcgtgacgccgtgacattctggcaa
ggttctaaca
>Agabi_varbisH97_2_rnd-1_family-132#LTR/Copia
CTCGAACGCGTCTACATCGATATNTGGGGACCGTCNCCGGTCCAGTCNGCCGGGGGAGCNAAATACTTCATGC
TGNTNATNGACGGCGCCACATCNTACCGAAAGGTATATTTCCTNCCCNCGAAGTCGGCGGACGCGACGCTGAN
GGCCTTCAAGGAGTTTCACGNGGAGGCGGAGCGGCAGACTGGNAAGANGCTGAAGCGGGTNCGNCTGGACATG
GGANGNGAGTGGCACAACGAGNTNTGGGACGCGTACGTAAAAGAGCACGGCATCGTCCTCGACTTCACNACGC
CNTACGCCCACCAACAGAACGGCAAGGCNGAGCGCTCNATGCGNACCCTGCTGGANACGGCNCGNACNATGCT
GGCCGACTCCGGTCTGCCNCAGAAGTATTGGGCNGACGCCGTCCANACNGCCGCCTACACTCGNAACCTCATC
CCNACNNCCCGTAACCCCGCCGNTATCCCCGCTGAACGCTGGTCTGGGCAACGACAGGACGTCTCNCACCTCC
GNCCTTTCGGNTCNACTGCNTATGCNCACATNCCCGTCGAAGTCAGCCCCTCGAAACTGTCCCCNCGTTCNGT
CAAGCTCACNCTNATTGGNTACTATGGTCGTACNGGCTACAA
>Agabi_varbisH97_2_Cluster7#LTR/Gypsy
AGTGTTTTGTTAGGGCGACTGTGACTGAGCTGTCACAGGTCACACCTAAACTCAAGAACTAAGGAGTATAGTG
ACAGAAGTTATATTAGGCGTGAACCTAAGAGAACTCGACAGAGTCTTCTACAAGTTTCTACCTACTTTATATA
CCTAGACTACGGCGCACAGACCAACTACCCTAGGTAACTTTATTTATATACTGACATGTGAAAAGAAGTACGT
ATGAGAAAATAAACTAATTATACATTTGACATGAGAAAAGAAGAGTGGAGTAAGAAGTAAGTAAACACAAGTA
CATTGATCATAAAAACGTCAACAACACCAAACAAACCATAAAAGTGTCCACAAGTACAAACAACTATCTAAGG
ACGTAACCTTTCGTACCAATCCCTGCCGAAGTATGTTGCCATTACTTCTCCACACGGGCACATCGTAAAGAAC
CGGACCGGTTCCCAACGACGGAAGTCCGGGTTATTCCATCGCTGCAACATCCAGGGTCTCTCCAATACTTCGT
CCCACATTATCAACGTCACTCTTCCACACACTACGCACCTACGATCCTCTAACCTCCATCCGTCTGGTCGGGG
TTCTCTTCCGATAGCCACCGCCGCCCTCCTCCACTGTACTTGCTCCGCATGGTTCGGAGGTAGGAAAGAAAAG
ATAAGATCAATGAGCTCAATTGGTAAATCTGTAGCTTGCGCCATTTTAGCAGTATGCCGATAGTTTAGCCTCC
GTCGAACTGTAGCAGCAGAGGGAAATTATTATGTAATAAAATATGTGACAGATGTGACAGCGTGACGCCGTGA
CATTCTGGCAAGGTTCTAACATGTTTCAAACCAAACAAGCGAACAAAAAACGTTCAGAAGAACCACCGATCAA
GGAAGGAGATCTTGTATACTTATCCACAAGAAACCTAAACCTACCAAAGAATAGAACACGGAAATTATGTCCA
AAATTCGTAGGACCTTATAGAATTATAGAAGCACGACCTGAGAGTTCCAATTATAAAGTAGAATTACCAGCAG
CATTGACACAACGAGGAATACACCCTACGTTTCACGTGTCATTACTCAAACCATACTACGAATCACAGGACAA
AATGTTTCCCAACCGGAAACAAGCGGAGCCATACGACTTTGGAGCAAAAGATGACCAAGAATGGCAGGTAGAT
GAGATAACCGATCATAGGAAAGACAAAGGAAAAATAGAATTTCAGGTGCGGTGGTCGACAGGCGACACCACAT
GGGAAACAGCCGAAGTATGCAAGGAATTAATAGCGCTAGATCGGTATCTAGAAATTCATGGAGAAAAAGATAT
ACGCAAACTACCAGAAAGGAAATCGATAAATATAAAAGGAAAGGAAACCTAAACCGTCCTTCCATCACTATCA
TCCCCACTACGATGGATCAGTTTTACAACGAGAACAACGCGAACTACAGAGGAAGAGGGTATTCGAACCCCTA
CTATCAATCTTCAAATAGGAGAGGGGGAAGGAGTAGGCCATCACGCGCACCAGGAAGAGCCGTCGGGCAGACT
ATGGGCGCAGGTAAAGAAATTACAACTAAAAAAATACAAAACAAAGATTGAATAGGAAGAAGTAACGACCGAG
AACAGGCAGAGGAGCCATAATGGCCACTATGACACAACTTACGACGGACCGGACACGCCAGAGGCTGACAGAA
GCGGGAGTAGACCAGCAAACGATCAATTTTCTCAATGAAAATGACCCCGCGGACATATTGGTCATAAACGGAC
TATTGGACATCATGGAGAGAAATAGGAGCTCACCCACATTCAAATCACGACCCGAAACGCGCGCTAACAGTTG
GGGAACTACACCATCCACCGACAACCAAGCAACTAGGTTCGCAACACCGATATCTCGGACTACATCTTCACCA
CTTCCCATACGAGGCGCTGCGAATAACGGAACGATGCCATCTCAACCGGCATTTCCCAAACCGCACACGAATC
AATTCCACCCCTATTCACAACAACCCTTGGTAAAGTACGACCGATTAACTATGATCGAACCTATCGGCGAAAA

AACAAGGGAATCACCAATCCGATGTTAGGGCGACTGTGACTGTGATGTCACAGGTCACACCTAAACTCAAGAA
CTAAAGAGTATAGTGACAAAAGTAATATTAGGCGTGAACCTAAGAGAACTCGACGGAGTCTTCTACAAGTTTC
TACCTACTTTATATACCTAGACTACGGCGCACAGACCAACTACCCTAGGTAACTTATTTATATACTGACATGT
GAAAAGAAGTACGTATGAGAAAGTAGACTAATTATACATTTGACATGAGAAAGGAAGAGTGGAGTAAGAAGTA
AGTAAACAAAAGTAAATTGATCATAAAAACGTCAACAGCACCAAACAAACCATAAAAGTGTCCACAAGCACAA
A
>Agabi_varbisH97_2_Cluster5#LTR/Gypsy
CTGACCGCATCCAATAGCATTGGCACGGCCTGCTTCAAACTGGTACCAACTTTATGGACGGTGCCTCTGGTGT
CAACCAATGCCCTATTGCACCGAATGACTCTTTCCTGTACGACTTCCCTGTTGAACAGTCTGGAACATATTGG
TACCATTCTCATTTCGGTATTATTTCCTTTTGAAGATCGTTTCTTTCCATTACTCACTGGTTACCATCTTAGG
CGTTCAATATTGTGATGGTATCCGAGGAGCGCTGATTGTCAAAGACCCTCATGACCCGTTGCGATTTTTTTAT
GATGGTATGTGAATTGCAATTGCTGCATGAACCGTTGACTGATGGTCTTACCGTAGTCGATGATGGTAAGATA
GTACGTTCGGACCGTACAATATATCCCTAAAGATTTCATTTAGAATCAACGGTGATCACCTTGTCTGAATGGT
AATAACCCTCATGTTCGTTATCAGAATGATTCTCATCCTACTGTATACTTAGGTACCACGCGTCGGCGAAGTC
ACTTAGGTCAACCATTGCCGAGGCCGACAGTACTCTCATTAATGGCAAGGGTCGATATGTCGGAGGTCCCAAG
GTTGACCTAGCTGTCGTCAACGTCCAACGTGGAAAACGCTACCGGCTTCGTCTCGTTTCCATGTCATGCGACC
CAAACTTTGTTTTCTCCATTGACAACCATGACCTTACTATCATTGAAGTCGAAGGTGTAGTAGTCCTTCCTCA
CAAGGTCAACACTATTCAAATGTTCACTGGTGAGTTGTTCATCCTTAAAGGAATCCTATAACGCGTTGACAAT
ACTGGTAGGCCAGAGATATTCTGCTGTTTTAGAAGCCAACCAACGTCCTGACAATTATTGGATACGAGCTCGT
CCAAACTCTGGCCATGGAAACCTCACTTCTACTTTTGAGGGTGGTGTAAATTCCGCCATTCTAAGATACCAGG
GAGCGCGCAATGCGGAGCCTAAATCGCGACAACAAAAGGAACAAGATCTGCTGAAAGAAACTAATCTCCACCC
CTTGTTTGATCTACTTGCACCTGGAAAACCAACACCGGATGGCGCCGATGTAACATTCGACTTGGATTTCTCA
TTCGATCCTGCACAATTCAAATTTCTCATCAATAACAAGTCCTTCGTTCCACCCGACGTTCCTGTCTTGCTTC
AAATATTGAGTGGTGCTAGAAATGCTCACGAGTTACTACCCGAGGGCAGTGTCTACACGGTAGAACGAGGAAA
GACTGTGCAAGTCAATATGCCAAGCAACCTCATCGGTGGACCCCATCCTTTCCATCTTCATGGCGTAAGCCAA
CATTCCTTTTATTCTATCTCTGAGGCTGACAACAAGCACAGCACACGTTTAGCGTTGTCCGGAGTGCTGATGC
CGGACACTTCAATTTCTTTAATCCTCCGAAACGAGATGTTGTTAACACCGGAATGGCAGAAGGAGACTTTGTT
TCTATTCGATTCAGAACTGATAATCCAGGACCATGGATCCTCCACTGGTCCGTAAATCTTCTTTCAACCTGTT
TTGTGATTGGCTTACATGTCGCCCTCTAGTCACATTGAGTATCATTTGGATGTGTAAGTCTCGGTCTTCCTCC
AAGAGCCTATTGTGAAGTAAAACTGATACTTTTCAGTGGTCTCGCAATTGTATTTGCCGAAGCCCCCGATCAA
ACGGCCTCTGCTAATAAGAACATACCAAGTTTGTCGTTTTCTTTTTTTGTGGAGGGACGCGCAGTGATCTTGT
GTGCTAGATGATTGGAAAGAAATATGTCCCCAGTGGAATGCGTTCTCTAACGCTACGGGGCCAGATCTTTAGC
ACATGTAATACCTGCTCATTAAAAGACAGACATTACATTAATCGTCCATCTTACCTACGCCCTTTTATAATAC
CCAGTAACTGGCTTTTGCACGTTTCTTCTCTTGTTTCGTTCAAAACTTCAGAGACGATTATATACTCTTCCAT
ATTGTATTGTACAATGATTTGGAACAATGGATTCTTTTGTTGACTGTTCTTTCGGTAGCACTCTTGATAAAAT
TGAAGGCAGGCAGGGAGAGGCAACGCGGATACCACACTCCGTAGTCCTGACACACCAGGAGAAATAACATGAT
TACATCATCCACTACCATTTCCGTCACCCACGGTGGGTGTGCTCGATGATTTCGCCACTCGTTGTTACATCCA
CCATATTCGATCCAGTTTACAGACCACAAGCAATGTATGAGGTTTCGATTACAGATATTTGCTGTCACATCAG
GACATTTGGAGAAACTGACTATGCATCCTACACCTACAAGAAAGTTGCCAACAGAGTGACAGAGTCAAACCAC
TTGATCGAGTCCACGCATTTCTCACTCCGATGAAGCAGAACTTGAAGCTGGTAGTGCACGAGTCAATACGACT
GGCTCATGAAGAAATGGGTCACAAATGGACCTTCTCAGTCCGTATTCGACTTTTGGAACAACTTTGGCGGCCC
CAGCTCGAACAGGATGTCAAGTGGTTCATCAAGACGTGTCATGAATGCTAAACGCGTCTACGCCATAACTTCC
ACATCCCACCAACCTTCTCCGACTCATTTCGCATTGTTTCGCAAGGTGTATGTCGACGCAATATTTGTGCCCA
AGTAAAATGGATATCGCTACATCGTTCACGCGCGCTGCTAGTGTTCTTCCTACCCAGAGTGGCGAATACTCAG
AGTCGAGACAGGACGAACGCTAGGAGCGTTCGTTTCTAAGGACATATTGTGTAGGTGGGGTGCAGTTGAGGAA
TTGATAACAAATAAGGGGCCGGCATTCATTCAAACTAATACCTCTCAGCTCAACGCAAGGTCAATCACGTTTG
AATTTCCCTTAGCAACCCACAGGCCAATGGTCTCGTCGAACGCCGACACTACAGCGTGCGAGAATCACTTACC
AAAGCTGAAGAAAACAAATGGTTGTAAGTCGCGCCTTGGGTCTTCTGAACGAGTTTCCATTCAAAAATCCACA
GTGTACTTGCCCTACTACATTGCCCACGGCCTCGAACCCTTACTTCCCTTCGACCTTGCTGAAGCCACATACT
TATTTCGCACATGTGCTCACTAAACCCCTATCCACACCCGAACTCATCGTAATCCGAGCGATTCAACTTCAGA
AACGACCGCAGGACTTGGAGACGGTGAAATCGAAAGTGATAAAGGCCCATTTCAATTCTATTCACAAATTTAA
CGAGATGTATTCCAATCTGATTCGAGATTCCAACTTCTCACCCGGTACACTGGTCCTATTCAGAAATTTTTGC
TACGACACGGACGTTGGTAGCAAGATCAAGCCTCGTTATTTCGGACCCACGGTTGTATTGAGACGGACGCAGG
GAGGACCTTACACAAAGACCATTAAATGGTTAGCTCCTACCTGACCCTCCATTCGACAACTCGTCGACAATGA
TCCCCTTCGAACTCGGAAGCTACCTTCATAAAACATGGTAGCAGGGATTGTGCAGTCACCGTTGCAAAGCCTT
GGAGTAGGAGGAATTTTCCATCGTGATCCCAACGTGCCTGACTCAAGCGCAAGTCCAGGAGACGAGTCAAGCA
TTGTATTGACAACTGAAGGATTCGTAATCCGCCGAGCACACTACAACACTACAGCCCCAAGTTTTGCATGTTT
TACAATGCGGATAGGTTCGGTGGTGTACCTTAACAGGTAGCGGGAGCATCTGAAAATGGACATTGATAGGGGA

ACAGCAATCAGGGGGCTGTCATCCGAATAAGCCGTGAAATCGACGCCCAGCAACATCCCCTCCAAGACTTCCT
AAACCTGAATTCTCACGAGGAAATTGCAGATTTATATCCAACCCCCTGGCAGTAGTCTTTACATTCCTTTACG
GTTGTCGCCGGTTGAATTCCGAACGAATCCGGCGCGGGTTACTTGGTACCGTCCTCCATTCAATGGGCAAATC
CAGTCATTGCTTCTACCTTGAACTTGAACAGGTCGAATAGTCGGATAGATATGAATGCCAGGTGTGAGGAAAT
GTTGCCGGAACACACGGAAACACAAGTAGAGATGAAACCATCACGGATGGTTTTCCCTGTCCTAAACCATTCC
CCAGCATCAGCCCCTAACACAGCCCAGGCGTCCGCTGGGCGTCCGTGGGATCGACAATGGGCACCCGGTTAAG
GCAATTATCACCGGCCATCATCGTGGATACTATAAAATACCGTGATGCTTGGTAGTTATGCTTCATCAATCGT
CTCTTCTTCTCTCTTCCGGGCGCTAGCCTCATTCAAAATTTTCTGTCTGTAATATCTTTTTGGAGGATGTCCA
CTTTCTTGGTTTTACTCACCTTGGCGGCGGCTGCACTCGCGACCGGGTCAACGATTGGACCTGAAGGAACCCT
CCACCTCACTAACATGGATATCTCCCCGGATGGGTTCAAAAGAGCGTAGGTGTTTTGGACCTCCGACGGTAAC
AGCTCTGGTTGACAGAATCCCTTTAGAGCCTCAGTTATCAACGGTGTAACCCCCGGACCCGTAATCATGATAG
AGAAGGTATGAGATTAATGAATATTTTGTTTGCGTATTCACTCATTACTAGTTATGAAGGGTGATCGCGTCAG
GATGAACGTCGTCAATCAATTATCTGATCCAAACCAGCATCGAGGGGCGAGTATTGTACGTCGTAGAAAATAT
TTCATTCTGCGAGCTGCTCTAAACTTATCCCATCATTAGCACTGGCATGGTATGCGGCAAAAAGGCACCAACT
TTATGGACGGTACCTCTGGTGTCAACCAATGCCCTATCACACCAAATGACTCTTTCCTGTACGACTTCCCGGT
TGAACAGTCGGGGACATATTGGTACCATTCTCATTTCGGTATTATTTCCATCTGAAAGGCGTTTCTTTTCATT
ACTCACCGGTTGCCATCTTAGGCGTTCAATATTGTGATGGTATCCGAGGAGCGCTGATTATCAAAGACCCTCA
AGACCCATTGCGATTTCTCTATGATGGTATGTCGATTC
>Agabi_varbisH97_2_Cluster8#LTR/Gypsy
NgttatNNNccataNNNacNNcggtNagatcNNNNNNtgNagNtacNNNNNNtNNaattNNNaNNgNgttNNN
aaNNNtaaNNaagtatacNtcNtcNNtNaatNgaNaaNagtaNttcatatgcNcNNNNNNNgNtttaataNNN
NgagNaNNtttNataaNtaNNNNNNtttaattaNNNNgNtcNagNNNNgtNtNtatattaaNgtNacgaaNac
NtctNtNNaaNtcNNNaNttNtNcNtgattNNaNtctcgGtaccttaagaagttgatctaagttatatataga
NtacttgtAaaacatatataagacgctcaactttaccatgtAcaaacaacgtttctacttggttaggtggaga
catgaatacaaagtcttaatctaactatacacttgaaccgtcagacTtaattgctaagctctataaaNcgctc
tcaagctcttagtactaaaaaggcgcatttgaattacaattctcgattcatNGagGAggtctcctttgataca
ctgtaaaactcagagtatttcaatcgacgtaaagccacaacttaggaccgggCcggagacactacaacattaa
gtacaAacgttcctcttccgtaaTcagcacaaAgTAattcgctcgccGagagacccatcgagtgcgcaTaaTA
aaaggtgaacTtgggttaccgcNgaTaaaNcactagactgtcttaaaactactagtgggttatcaacggaaaa
ggatcaactaaaaccggtaactggtggactagttacacgattgcttataacaatNgNagNNNgNNNagaNttt
NNaagccgggtNNNNgNNNgtNNNNNNtNNNaaNactcgaccaataGagggtctNtactcgaaatcagtgaag
actttaaacactcgacaaccaagGgtctgtacttgaagagtcttatagagactaggaatagtgagtccaagtg
aaggagcactaaccacggatttacagaattgtacctgtaaGttttgtacacaacaaaatttaccNttcaaagc
aataccagagatgtGggctggtagtgaacggaaactgccaaaacggagtcattctttagtaacaaatgNaagc
aaacNgcttacaaGgaagacaagacaaagaagtggtactataccaagatcacaGaaggaaaaagctactactt
caaNgacgcagaccagcaacgaattgcatctgagtatcttgaacacgaaacccagaaactctttagagcagca
aacNttccaagaatcagaacaagcagtcaagacgaacacactaatagacaaacctcagaagcaatcagaaacc
tccgatctgctacaCaatcagccgcacaaTctgctacacattctgcaaaatcagatcatcaagaaaaaggaaa
aGataAagaaccttcgNagtCagtttatgactggccagaaacAacagttggagaaGgaaatacagattNtgga
aaaggaaatccaggacctcgagatcaggacacagaaagtccacaggaggaaattaaaactaacagaagtccaa
caggaaatacgaacacaagccctccgaaaagccctcagccaacgccaccaagaacaccaacNccaccaacacc
accagcaggaaacccaggaagtccagcCggaaatatggcacaaccaccagcaggacctcacatcaaataccg
aaAccaaataggtattagggagacccaagattatacagaCcatggatggtatccctcNaactctatttcaaca
gctatgggaatacaacgtNcNactccaaaatcaattacacNcttggattacttgaaggagccgcatccaactg
gaaatcagattttatcgccactcacCcaaattatccaaactggNcagacttcaagaagtctcttgaaCaggcc
attgcaccagcccaacaaggatatgaagcagaacaaaagctacgacgctatagacAaaatggagaatacattg
aacactacattagtggattctcagtattatcctcacaagcaggattaccagacagcacNgcactcagagcata
ctttgcAgaaggactcgataaagacgtccgaatTgaagcaaccagatttaccccaacaacactacaaNagtgg
aaagacgccgcaagaaatgcCttcagaattgttatggaacaacgaaaagcatcaggcaatttcggacaaTcgt
caagatcacataaaCcatataagaaaaataaacaccgatctcaaaagagtattccgagatacagtaatatcta
caacNaaccagtccaacataaacccgcaagaagcgagtgggacatggaTgttgaccagatctacttccaactc
aatcaactcggattggacgacgaaTccgaagatTcagaatcagaatcagaNgaatcagaatccgaagacagcg
atgatgaagaagtcaatttcAgcatacaacgaagaacccaaacccaaacgcctaagcaagcGgtacagggatt
tattaatgcaatcctcacagatgaacaacgaccagctctcaaacNaggagaatgtttcttctgtcacaagaaa
ggAcactggtacagagattgccgtgcaaggaagcTatatattaagcaaggaggaagaacaagcatggatcgaa
atacaggaacagatagaaagaagaaacaaggaaacggaaaacctaagaagcaaaaaaaatggaaaaagaagga
acccatagacccgaatgctatggtctacaacattgaaaaagaagaagacaacaTtgaagaatttctaaagac
ggaaatTtttgagtacttctgaagatccaatNgacgtgatagatcttNagtcacctaaaggaacaacactTct

agcaccgataaaaattcacattgaagaNgataatcctattaaactaaccgcactaatagattcaggagcagca
agagaatttaccgatcacaaactcgtagagaagtattacttaccaaaatttccattgaagaaagtcAaaaata
ccatgaatgccgatggatcaagaaatgcatcaggagtatgcacacactatctcctNctcaacattgaaatcaa
caacaagaaaatgttgatacAagcaagcatagtatcgcttggaaacccacaaactatTcgtaggtatatcatgg
cgaagaaaattcaatccagcaatagattggacaaaacaaactctaaaatggaGagatgaagatgacgactcaa
ctcatacactttctagaggaccatcaagcaacttcattaacaatatcttcgttgaaatcaattCaaaaagcaa
tactccgcaacagctatccaatcaagctgaaaagaagaaaacggaatctgatcctNctaaagcagtaccgaaa
agatttcacaaatttctcaaagtNttcaacaaagtagaatcagagaaaNtaccaccaagacgtccttgggatc
ataaaattgaaaTtcaaacCgacttcaaacccaagagaatgccagtcTacaatctaacacctatagaacgaaa
aGaAttagataaNttcatagctgaaaacctacgaaagggatacatccgacaaTctaaatcaccaaTggcatca
tctttctactttgttggaaagaaagGaggagatcTacgcccatgtcaagattatcgctatcttaatcaaaata
cgattaagaatgctcaccctatTccaaacattgctcNaatcatggataaactaaaagattcgaaatggtttAc
aaagNtagaTgtcagactaggctataacaacgtcagaattcaTGaaggAaatgaatggaAgcagcattcaga
acAcctaatggattatacgaacCTacNgtcAtgttctttGgaatgacaaactcgccagccacATttCaaTcaa
TgatggaTcacattttcaaacaattaaTtgaaAatggaGgagtcattatctaTatggacgatatNtTaatcca
taCaaaaacacaaaaacaacttgaTgaacttaccaaNcaagTtcttcaaatActcgaacaaaatcatctctat
cTcaagacagagaaatgcgaaTTtgaaacgcaacgNctagaatatcttggagtcattataactccagattcta
tcgaaatggatccaattaaactaaaaggaAtcgaagattggcctgTacCtaaaacggtcaagaatgttcgaca
attcattggattctgcaacttctacagaaagttcattcacaattacgccAaaatcgcccaacctctcaactcT
ctTatgaggAagacaaagaaattcgaaTggAatccaaatgcNcaatcagcatttgaaacNctNaaacaacaat
tcctaaaacgaccagttcTacaaaTggtagatcaaAcNAaaccATttgaaATagAatgNgatgcctCtgcatt
tgccacaggagcagtcttacTacaANgagaCacgaATggagataagcaTccggttgcTtattactctaAatcc
cttaatccAgccgaaaggAactatcaNgtttcggatcaggaattcttatcaatcattagagcattAaaagaat
ggagacattatgttgaaggatcaccTgaaccattactcattTggtNggatcacgagaaccttActcggtGgaa
ggaaccccaacaattaaaccgaagacaagcgagatggatgttatatatgacaagattcaattaTGtcatcaaa
catCttccaggcaataagaatgtcttagccGaTgccctgagtagacgTccagaTctaactccagaaggcatag
ataacgagaacttaaTtgctctaccttctaCgaagTttatcaatttctTaacagaagAactagaagagaagat
caaNgacaagaaggacactatNacaccatcagagcagtttactcGagaagaagacatctataCTtaTcaagga
agacttgtgataccggatgaCTcggacatcaagaaggaactcttaacccgAGtccatgaTcatGaaTcagctg
gacaccctggAaTagccgaaacgcttcgaaaattaacgaagGAagtctactggccaggaatgaacacttatgt
ccagaactacgtcaaaggatgccccatAtgtcaacaatacaagatcaacagacatCcactaAaaccgccgatg
caaccgatcaaaggaccttcagtaacgaagccattTacacaaatatctatggatcTtatcacagatctacccc
ctAgtgatggctatgatgcaatattgtccaTagTggaccatggacttacgAaagggataattctaacccctac
TaggaaaacagcaacagctgatgaNatcgccgaaatgctaattgataaggtattNtcTaaataTggaacacct
caNaagatcAtaTcagatcgagacccttgatttgcNgccaaatctatgcaaagactatatgaaaagcttaaca
tcaagcccgcattttcaacagcttatcatccacaaactGatggaactacagaacgctacaatcaagaaataga
attctatcttgcagtttatacttcaagaaatccaaatacatggagacgagcattaccTatgaTtgaattcgtt
cacaatactaaaccacatgcaggaagacaacattcgccttcagaactcatattaggatacaatcctaaagcaT
tcattaacaaagaagaNtcaaatgtaccttctatcgaagagaagacattattcttagaacaagctagagaAgc
agcaatcgaagcacatgagcaagcacgtttgaagatggctagccgaagtaaccgaccccggaatgcacttaag
gttggtgaTaaagtttggttagataaccgaaacttgcccgtgccgtacgcatcaaagaagctcagtcaaagaa
gagaaggacccttcagagtaattagaagaacttctcccacaacatacgagttagaacTtccgaagcgatggaa
gatacacaacaaattccatgcatcNcTNcgtacacccgtagtagagaatgatatctatggaaagcatAatcca
cagccacctccgacattaGtgtctggagaagaggagtatgaagtcgaagcgatccttaatcacagagNaaGga
agaNacgcTtggNaaatggaagactcgcaacaagaacagaatatcttgtcagatggcaaggagatggaccgtc
agaggattcatgggaatcggaagaagatcttgcaaattcaagcgagatacttgaagagtataaaaacctgaag
aaactcagataactccccagttNNaTgagcatcaNNcaagaaNtNNNacaNattctctcaactctcNcatctt
cctccactctNaatgccagcaatcagaNNNatgatNataNccggaagtctgactcgtcagtaccNccaaccgc
tcctcgcaatacNccttgccaagNNgaagNatccNttgataagaatgccaNctttcGacgNgcatttctNNNa
gaNNgaacagccNcaNactatctgtccagtcacNgccgcattcctcgaCtcNNttNaattgtgNacgatatcg
gggctcatattgatgaactcacttctgcaagacgtNcataccttcaaNgaatggaagaaatggacgcccgcct
tNcNNNtctggcacgcttttNcccgaggNctccCNgtcgNaatgaNattttcacNNNtatcgaggaNatcaat
tcctcgccattgctcccaNatgcgtacatcagtcaaccaactccataccgacatgaaNcggttNctcgNagca
cctcccaacCtgacNatttaacaacTtccNcacctcctaTacaacttcNccaatttcatgaattggctttacc
atacgcNNgaatttcgcctcctacaaatNNgtcgaacNaNccagtaNcttatcgaacNcNatNaggtactgct
NcNNgNNNtagatataNaggtaNaacctgcNgaCtatgtggctgaatgggtcatctgcagacNNNttgcccgc
tNtaccactgtaNttattgtaataccNaggNNcctggccaatttgccaaattTtgtNcccgaaaccccttatNc
NggagttgctNgNaaggatctccccccagaagccNTggtataggtcNatNNcNtNgNtttNacNNtcNNNcNc

ccccttactNatccaaNctccgNNaatNNNNcttctgNNNccacaactcccaattccNtcgcNNactcctcta
NtgcctctctgNNaNgNaNatctNccacttccNacacaNccNatgactccNaNttactacagcNNtcNggaca
gctactNNccaaacagtcaNNtataccaaNcggcaccatcNattcctccaaCaccacttcNacttccggtcca
tttaccaccacNcatcttggaccaacctatcaacctatctaccgNccNaaNNccaaNcctaggtccacNNaca
acaaNcaCaattacggaaaNcttgcgatatccatccctccccccctatcgtcactctNgaagaacaactNccag
taaccttNCggcttctagNNaNcctNaacgccaacgatccccgtNgNcagaacattatgatgcaggtgatgaa
tacactTAcNacgAtgtcgacgaatacaacatGagcgctgaaggacaccctGacAtctagaTacTtctggtaa
ActatcttaggaaaaggatagTgtTataaaccgTgacaactacggttagaTctctcactgtaattactNcatc
tgtaattacaattatgtttacaaNtNaaagtAagtatactTcgccNttNaaatgataatagtatttcatatgc
tattgatccgttttAataatccgaaaaNagtttataattaNctttctatAattatNgNgttctagttccgttt
ctatattaatgtgacgaaaacttctttataattcccgagtcgtNcNcgattNNagtctcgGtaccttaagaag
ttgatctaagttatatataagaNaacttgtaaaatatataagacNctcaacttNaccatgtagaaacaacgtt
tctacttggttaggtggagacttgaatacaaagtcttagtctaactatacgcttgaaccgtcagacttaattg
ctaagctctataaaNcgNtctcaagctcttagtactataacggcgcatttgagttactcttctcgattcatcg
aggaggtctcctttgttacgctttagaactctgagtatttctatcgacgtaacgccccagcttaggaccgggc
cggggtcactatNNNNct
>Agabi_varbisH97_2_Cluster64#LTR/Copia
TGTTGTGAATGCTTCAACACTCGTATGAGTATTCATCAGTTGAACTTGTTATTTCCTTTTCGATCTATTTCTC
CTATTCTAATTTTATCGCTCTTAACATTCTTTCGACCGTTCCGCTCTACATTGCTTATAACTATACAGTTCTG
CCTATAACTTATACAGTTTTACGTCGGCACAGGTGGCCGGATTACTTATAGCTAGAACTTTATATTTTGTACC
TCAGTGATCAACACACGACGTTGTCACTGAGGTGAGCTGTCTTTACTTTCCTGATTCACTCGTACTTACTAGT
ATATAGTCTACTTCACTTTAGGTAAATATATATAGTGATCAACACACGACGTTGTCACTGAGTCTACTTCACT
TTAGGTTATGGGCCCCGAGCTCTTTTGAATTTATTCACATACAGACCTCTACTTGATTTAACACACTGTGATG
TCTAACAACACCGGTGGTAGCGCTCCTCCTATTTTCGCTGATACGGCGAAATTTAATGGCACCAACTGGGTGT
CATGGAGCGGAATGATTCGTATCGCTGCGGAGCTGCGAGGCGTGTCTGGGTACCTCGATGGTACGGTCATTAA
TCCAGCCACTGTTAACTCTGCACAAACCATCCCTGTTATCACGACAACACCATCCCCTACTACGACTACAGCT
ACCACTACTTCCACACCTTCTGAGACCCCATGGGAATCCACCACCCCACGCACTGCCGAATGGAAAGTCCGCA
ATGCCTGGACTATGGGTCTCCTCATTTACAACACAACCGACCCTATCGGCCTTGGTATCTCTATCGGAGGAAC
AGCAGCCGATGCTTGGAAGTCGTACCTCGACACCTATGAGGTCGCATCCGAGATTGCTACCCTTAACGCCGAA
ATGGAGTTGAGAAATATGGTATACACAGACGGGCAGGATTTCACTGAATTCATATCACGCCTCAGAACTAAGT
GGTCAAACGCAAATGCTCTTGGGGCGAAAATTGACGATAAAGCATTCCGAACAATTATCTTGAACTCCTTGCC
CCGCTCCTGGGATTCCATTGTTGCTACTCTGTACACCACCAAAACGTCGCGGGAGGCTATAAGCCAACTTCTA
ACCCATTGGGCCAGAGTCAGCCGTGATCGAGTCACTACGTCGCAGACTACTTCAGCTCTCCAAACTACTACCA
CACAGTCGAACAATCGCAACCGTCAACGAAGCCAAAACAGTCAATTAACTTGCACCAATCCCAATTGTGGTCG
TAGGGGACATACCATCGAGTTTTGTTACTGGCCAGGTGGTGGAAAGGCCGGGCAGTTTCCGCCTGGTTTTGGA
CGACGTGGGGGAGCAGGAGGTAGTGCCAGCAACACTGCTACCGGAGGATATCAACCTTCTGCAAACAACGCCA
CTACCAACACCACGGAGAAGACGGAAAGCAACGAACCGAAGGTCTTCGCTCTCGCTGCGATCACGGACACAAT
CGACGATACGCCAAAGGTACCATCTACCCTCCCAGTACTTTCCAACAATCCTGACGGCTCTTTGATTTCAACC
CCTAGTGTCTCTGAGAGACCATATGCTGAGATCTGCGTAACGGGGAAGGATCAGAGAGTTAGTGTTAGTACCA
CATTGAATACAACTACTTCCACTCTACCTTACGACGTGATTACTCTGCTCGATTCCGGGGCGAGCGACCATTG
CTTCGTTAATAAATCACTCTTCGCTACATATGTTCCGATGATCCCACCTCGACAAGGAAACTCCGCAGGCAAA
GGTTCTACTTTTCTGATAGAGGGTGCGGGTGAAGTTGGCTTCTTCACTTCTCTTGACGGGGCGCCAACGAAGA
TAATTTTGTTTGATGCGCTCCATACCCCGCAACTGAGGTCAAACCTGATCTCGGTCTCCAAGCTTGTATCACG
GGGATCATCCGTTAGTTTTGAGGGGAATACAGCAGTTGTTCGTACCCGTAGCGGCGCAGTTGCATTGGTAGCC
ATGAAAGAGAATGGTCTATATGTTGTACCGCCGGTTGACGTCCATGCGAATGCCGTGCAATCGAAAAGGAAAG
CTGTATCTTTGGGAACTTGGCACCGTCGTCTCGGCCACGCCAGCGTGGAAGTCATCAAAAGGATGTTACGCGA
AAACCTCGTAGATGGCTTGAGTGTTACAGGACCTATGGAGTTGGAAGGGCTCTGTGAAGACTGTATCTTCGGC
AAACATACCTCACATCCATACAATAATTCCACATCGCAGGAGAAATCCCCGCTCGAACGTGTTTACATCGACA
TATGGGGTCCTTCCCCGGTTCAATCTGCTGGGGGAGCAAAGTACTTCATGTTAATCATTGACGGCGCCACATC
CTATAGAAAATTGCATTTTCTTACTTCTAAGTCAGCGGATTCAACGTTAACGGCCTTCAAGGAGTTTCATAGA
GAAGCGGAGAGACAGACTGGTGAAACATTGAAGGAAGTTCGTTTAGACATGGGACGCGAATGGTACAATGAAC
TTTGGAGCGCATACGTAAAAGAGCACGGCATTGTCTTGAACTTTGCCACGCCTTACGCCCACCAGCAAAATGG
CAAGGCAGAGCGCTCTATGCGTACCCTGCTGGATATGGCGCGTACTCTGCTAGCGGACTCCGGTCTTCCACAG
AAATACTGGGCAGATGCTGTCCAAACCGCCGCCTATGTACGAAACCTCACTCCTACATCTGGTGACCCTACCA
ATATCCCCGCTCAACGCTGGACTAGCCAACGACAAGACATATCACACCTCCGTCCTTTTGGGTCTACTGCCTA
CGCACACATACCTGTCGAAGTACACTCCTCAAAATTGTCTCCTCGCTCAGTGAAGCTCACTCTGCTTGGGTAT
TATGGTCACACCGCCTACAAGCTGATGGATCGTACCACCGGCGCCGTTTATAAATCGAGGGATGTGATTTTCG
AGGAGGCTCGTCCCCATCTTTCCACTGATACCATCACAACCTTCTCTGGGGACGACTCTCGTAGCTCAAATCG

CCAGGAAATCGCACCACGTCCTTTGCCAATTAATACGCTCCATGGTGTGGACAACAGTACTACTACGATAACG
CCATTGGCTGTACCGGCACCGACACAAGGTGGTACAGCCTCCGCTACGGGAGACGAGGCCTTAGATGTTACGG
AGGGTGTTAAAGAGCCGCCTTTGGTATTGAGGAGAAGCCAGAGGGAATCAAAACCGTCACAACGTATGAAAGA
GAGTCTGGAATACCTACGACGTCCAGAGGCGCATGCAACAGAATCTATTACGGAGGATTCTCGTGTGCCGAAG
AACCATCATGAGGCGATGAGGAGACCGGATTTATGGCTTGAACCAATGATGAAGGAGCTGAATGTGATGACAG
AGAAGCAGGTATTCAGGCTTGTTCCTCGCCCAACTGGAAGAAATGTTGTCAAATCGAGATGGGTATTCGCAAA
CAAGTATGATGAGGCGGGTACAGTGGTGAGCCGAAAGGCACGGCTGGTAGCAAAAGGATTCACACAAGTCATC
GGCGAGGACTATGACGAAACTTACGCCTCGGTTGCACGCCTGGAGTCAGTACGGATGGTATGTGCCATCGCTG
CCTCACGTGGTCTTCGACTGTGGCAGGTCGACTTCGTCTCCGCTTTCCTCAACAGCGAGAACTCTTTTGAAGT
GTACATGGAACAACCTCCTGGTTTTGAGGAGGGGGGAGACCATGTATGGTTGCTCCTCAAAACGCTGTACGGG
ACGATGCAAGGCGCCCATGACTGGGCACAGACTCTCGATCGCACCTACGCGGGTCATGGGTACTACATATCGA
AAGCTGACCCCCAAGTGCGATCAAAGGTGGAAGGTGATGAATTTACACTTACGTCAACCTGGACAGACGATGT
CCTCGGCGCCTCGTCGACAACAGATGGTGAAGCAAAGGCTAAGGATGAGTTGGGAAGAAGCTATGAAATCAAG
GATCTGGGGGAGGCGAAGTTTATTCTTGGGATGAGAATCGAGAGGGGAAGGAACGGCAATGTCAGACTATCAC
AACGAGCTTACTGCGAGCGCATCATCGAACGTTTCAAACAATTGGACTTGAAACCACGTTCTACTCCACTTCC
AGTTGGAGTAACTTTATCCATTGAAGGCTCGCCGGACTCCGACGAAGAGAAGGCTGACATGAAGAACGTACCC
TATCGTGAAGTCCTCAGCTCCCTTATGTGGCTTCAGGTCGCAACTCGCCCCGATTTATCCTACGCTGTGAACA
TTCTCTCTCGTTTTGCGAATAACCCAGGACGCAGCCACTGGGACGCTATGAAGCACACTCTGGGATACATTAA
AGGAACTATGGACTATGGAATCACTTACTACCGTGACGCAACTATCAAACCTTTGGGCTACGTGGATTCAGAT
TTTGCTGGTGACATCGACTCTCGACGTTCTACGGAGGGACATGTTTTCTTTGTAGCAGGTGGACCTATATCAT
GGGCCAGCAGACGACAGGATACCGTGGCTCTGTCCACTGTAGAGGCGGAGTATATGGCTTTTAGCCGCGCCGT
TCAGCAGGCAATCTGGCTTGCGAACTTCCTGAAAGAAGTGGGTTTGCCACAGGATGCACCTACGGTGATACAC
GCTGACAATGATGGGTCTATTGCCAATACGCGAACCTACAAGAACCATCGGAGGACGAAACACATTGATATCA
GATACCATTTCATAAAAGAGAGAACTGCAAACGGAGATGTTGTTTTCGAGTATGTTCCTTCCGCAGACAATTT
AGCCGACATCTTCACAAAACCCCTCTCGCGTGATGTGGTCTTGCGACTTTGTGGAGACATGGGTTTACGGAGT
GTCGAACCAGGGGGAGTGTTGTGAATGCTTCAACACTCGTATGAGTATTCATCAGTTGAACTTGTTATTTCCT
TTTCGATCTATTTCTCCTATTCTAATTTTATCGCTCTTAACATTCTTTCGACCGTTCCGCTCTACATTGCTTA
TAACTATACAGTTCTGCCTATAACTTATACAGTTTTACGTCGGCACAGGTGGCCGGATTACTTATAGCTAGAA
CTTTATATTTTGTACCTCAGTGATCAACACACGACGTTGTCACTGAGGTGAGCTGTCTTTACTTTCCTGATTC
ACTCGTACTTACTAGTATATAGTCTACTTCACTTTAGGTAAATATATATAGTGATCAACACACGACGTTGTCA
CTGAGTCTACTTCACTTTA
>Agabi_varbisH97_2_Cluster67#LTR/Copia
GTTAGAGTGAGCAAGCAGTCTACAAGTGTAGTTGGTGGACTTAGATATATGCGGACGAGTTTAGTCACATGAT
AGCTTAGTCATGTATGGTTGAACTTCCCTGTGGACGCTTCAGAGGCCTTAGCTTCTTACAGTCTGTCCACAGG
TGAGTACAAGTTATTAGAACTCAATATAGCTTCTTACAGTCTGTCCACAGTTGACCTTGGGATTGCTCAGTCA
GTTCCTGTCCGTTCGCCTCGTTTCTAGGACCGTTAGACTCGCCACTGCGTCGCGTACATTCTACGTTCAACAG
GCTATGGGCCCCGGCTTCTAACGCTTAGAAAACGACTATTACTTTATCAACCTCAACTCTTCTCACTTATCTT
CCTATCTCTATCAACTTCTATCGCTTCCCCTGCCCTCAAATCGACGTCTATGGCCTTGCAATCGCGCACACAT
GCTCCCTATGCCTGGGACACCATTTCGACGCGCTTTGGCGCGTCTCCGACGCGTCTGAGAGCCTCTACATCGA
CCACAACTCCCTCTGGCAGCCATACACCCTCTCCACACTCTCTGGGACCGATTTCCGGCCCTAAGAACGCGTC
AATCGCTTGTTCCTTTTGTGGGAGGGTAGGGCACATGTCTAGGAGGTGCAGGAAGCGAAAGAACACGAAAGAA
CGCGCTCAGGAGCGCTCCAGACTGCCTGGAACATCTAAAAGTAATGTCAAAGGGCCACTTGGCCATCTGAGTG
GGATAGGTGCAGCTCTTCCAGCTGTCAAGACACCACCCACTCCTCCTGGGGATGTTTCTACTGGTTCTGGCTC
TCCTGGCTCTGATATTCCACTGAGAGCATCCGCTCTTGGTGCTTATTCCTCTCCCTCATCCTCTCCCTCATCC
TCTTCCTCACCATCATCACCACTACAGCTTGATGCTGACTTTTTCTGGCTGGCTGATACTGGTGCTACATCCC
ACATGACTCCTCACCGCCATTGGTTCAAATCCTACTCCCCTCATCGTATCCCTATCCGCCTTGCTGATAACTC
TACTGTCTATTCAGCTGGTGTAGGGTCTGTTGTCTTTCAACCGGTGGTGAATGGAAAAGAGACTAGAGCGGTT
GAGTTTACGAGAGTCCTTCATGTTCCTGACCTCAGGAGTAATCTCTTATCATGTCTCTACCTTGCCCGACACA
AGGGCTTCAATATCACTATCTCTGCTCATTCCATTGACTTCAAATACCAAGGGAGAACACTTTTCACGGCCAC
TATCCATTCCAACAACTCTGCTGAGCTCGACGGCTCAACTGTGACTTCTGAGACTGCTTTCTCTGTCTCTACT
CTTCCAGTTGACCTCTCGCTATGGCACCGTCGCTTCATTCATCACAACTACGCTGATGTGAAGTCTATGATCA
GTAAGAACCTTGTTTCCGGGCTCAAACTCGAGTCCAACTCGTCCCCTGACTCTATCTGTGAGCCGTGTCTGGC
TGGCAAGATGCATTCTCTTCCTTTTCCCTCTTCTTTGAATCACAATCGCTCTCCTCTCGAGCTAGTGCATTCA
GATCTTCACGGACCGCTCCCAGTCGCTTCACACTCAGGCTACAAGTACTGGATCACCTTCATGTCAAGATAGA
AAAGTGACCACACCAGGGTTTGAACCTGGGACCATCCCAATTGCACTAGGAAGCTCTAACCACTGAGCTATAT
GATCTTGGTATATAGTAGCTAAGATGTTATAGATATACATCTTAGGCCTAGAGGTGGAACCCACGACTGAGGG
CCTGCTGCAGGGGCTGAGGCACAGTACAGATTATGTGAGAGCACAGAGCTCCGAACTGTCAGGTGACTTGTCC
TGTCTTTTTTTTTTTTTTTTTTTTTTTTTTTGTAATCAAGTGGCTGTTCGAGCCTTCTATTCCAGCGATATA

AGTACATGCGAACACTATGTACAACGTGCTGAGAATAAGGTTGCCATGACGCGTTACCAAGAGAAAAAGAAAA
ACATATCCTGCGCGACGAGGTCAAGCGCTAGCGTAGAGAAGAAGAAAAAATAAAAGTTCGATCGAAAAAGAGT
GGTACATGCTTATGCGGCCCCGGAAACATGTCGCAGTCGAACACAGACCATAGTACGTACCGGAACGCGAGAC
TGAAATATAATCAGGCGGAAAATAGACATAGAAAATGAAAATGAAAGTTGGGAGGCCGTTAGGCTACTAACCC
CGAAACGCGTTTTGACGCGCGCTGATTACTTCTTAGGGAATTGGGTCCTCGCTTAACGCTGCTCGAATCGGTT
CTGCCAGAACGGACACGAAGTGTCCATTGCAGAGTGGTCGCGCTTCGTCTTGCCTGCCGCACAGCAGTTGACG
CAAGACCGGGCGTCGGGGTCGCGCTTGGAATGGGCGCAGTAAATGGGGTGGGCGTCCTCGGAGTGGGGAAGCC
CGCAGAGGGGGCAGCGGATGCCTTTGGAACGGCATACCGAAGAGTGATGGCCCCACCTCATGCACCGAGTGCA
CAAAGCGGAACCGGGCCGGGGTGCTGCGCCTCGGATTTGACAGTTTCGACCGCCGATGACGATCGTTTTGTTG
ATGAAAGAGCGGGCCGAAGTGCCCGCTACGGAATCGTGAATGTCAACCCACGCGACACAAGTGTCACTGTGGG
CTGATGTCCGCATGAAGCGGACGGCGTGCTTGAGGACGGCGGATAACTCCTTTCCGACTGGCGAGATTGCAAG
CGCTGTGCTGAAGGCGCTGCGTTGGGCGGACTGCCAAGTCTTCGGCTCGGCGGGGATCATGGGTACGTCGATT
ACTTTGAGGAAAGAAGTCGAGGTGACGGGGGATGATTTGATGGGCACGATGCCCGCGACGGTGACGAGACGCC
GTATGTGTTTGAGCACACATGCGGTCTCGGAGGAGTTAAGGACTCGGCTCGCGGCAATGAATATGCCGGATCC
GGAGTCCTCGGAGTGCTCGAGAATGACGTCAGAACTGACGTCATCCTTGAGGTGTTGGTTGATCTCGGCCAGC
ATGGCCGGGGACACGTGGGCAGCACGGATAGAGGATCCGGCCGGGGGAGTGAGGTGCACGCCGCGACGCGAAG
CGCCGTGGCACGTGTGACGGGCCCTACGTTTTTTGCCCGAAGGCTTGGACGCAGGGGTGGTGGGGGCGGGAGC
CGCGAAAGCGGCGGGAGCCTGGGGGCGGGGCGGAGGTCGTTGCGGAGGGGGACGGCGTGCAGCGGCCTTGGCG
TAAGAGTTATTCTTGGGGAGGACAGGGTCCTTCCCAAAAACGGGTGGCTGGGGTGGAGGAGGGGGGGTCGGCT
GTGCCGGCTTCGCCTTCTTAGATTTCCTCGGGGTCATGTCCATGTCGGCCGACGGAGACCGTGGCCGAGGGGT
GGCTGTACCCGAGGGAGTAGGAGGGGGCGGGGTTGACTCGAATGCCCGTGGGGGCGGATCGACCGAGCCGATA
ATCTTCGCGAGCTGCTCAGAAAATGAGCCGATGCCCGCGACGGAGGAGGTTTTGTATCCATCAAGATCGCGCC
GCTTGATGAGTACATGTGCCGCCCGCAGTATACAGTCCGTAAACGGCGCATACAGCGGGTGTTCGGGCTCGAT
GTCGTCAAGCATGACAATCGAGGTGTTGACAAGCTTGAGCATTCCCTTCTGACCAGCGGTCAGAGTACGGGAA
CGCCCAGTGGAAGGGGTGGAGGTAGGAGCCGGTGTCGGGGTCGACGGAAACGGCGTCGGGGAGCGGTTATCCA
ACGGGACGGGGACCGAAGTGTAGGATAGGGGGGTGAGGGAGGACTTCGAAGGAGTCCTAGCAGGATGTGGCGT
CGTCATGCGGGACGCCGCCGGGAGCTCGAGTTCATCTTCCATGTTCCTCGCAGCACGTAGAAGGGCCTGTCCA
ACCGAACTGTTGAACATACCTTCCGGAATCTCTCCTTCGTCAGACCAGTCGCCCGAGTCCTCAAAGTCCTCGC
GCGTATCCATCGTAGAATGTAATGTATTGCTACTCGAGTATAAAACACTGAGACTTCCCAAACAGGAAGGTAG
TGGTTACAGTTCAAGAAGCGATGTGTGACTTGTCCTGTCATCACCTAACACTTCATTGACGACGCGACACGTT
TCCGGGCAGTCTATCTGCTCAAAGCTAAGTCGGAGGCATTTGAAGCCTTCAAAGTCTACAAATCCTGGGCTGA
GACTCAACTCGGAGTCAAACTGCGAGCACTACAGGATGACAAGGGAGGTGAATACATGTCTAATGCTTTCAAA
AGCTTTACTGAGCTTGCCGGCATCGAGAGACGTCACTCGACTCGGAACCGGCCTCAGCAGAATGGACTTGCTG
AACGGGCTAATAGGACTATGGGAGAGAGGATCACTGCTATGCTCTCGGAATCTCGGCTACCTCCTTCATTCTG
GGGTGAATGTATCTCATCTATGGTTCATGTCTGGAATATGCTTCCTACTGCTTCGCTTTCGGGCACTACTCCC
TTCCAAGCTTTCTATAAGCGAAAACCTGATGTCTCACATCTCCGGGTCTGGGGGTGCACTTCCTATGTCCATG
TACAACGAGATAAGCGAAATTCTATTCAGCCTCACTACGAGAAGTGTGTCTTTATAGGCTATCCGGCTGGATA
CAAAGGCTGGAAATTCTACAACCCAGTCACTAAGCGAACCATGATCTCCGAGAGAGCTGATTTTGATGAGAGA
TACTTTCCGGGCACTTCTAAAGCCCAACTCGAGGCTGTCCCATCATTTGCTTGTCTACCAGAGTCACTACCAA
TTCCTGATGATACATCATCACACTCGGGACTGCCACCTCTACAGGATGAAGGGGGTGATAATCACTTCCCTCC
TGCTCGTCCTAAGACTCCCACTCCGTCCGCTTCAGTTCCTCCTGAAGAGAAGCCAGTTCTACCCCTACCACTT
CCAGTAATTCCTCCATTCATTCCTCCTGTGATTCCTCCAGTCACTCCTCCACAAATCCATGCACCTCTCCCAG
TCACTCCTCCTACTGCCGGAGCACCTCTTGAGCCCCCTCACGTTCCTAGACGTACTGGGCGAGTTCGGAAGCC
TCCTGGCCAGTGGTGGAAGCTCAAACAACCAGACTTAGCTGGTAATGACTCTGACTCAGATGACGAAGCTGCT
CTGCTAGCTGACTCTGAGTACTGTGAAGTCGAGTTTGCTGGAGCCGTCTCGGGTGCTGATCCCCGGAGTTATA
GACTAGCAATGAAGTCTCCTGACTCGGATTGCTGGACTGAGGCTTGTAGCACTGAGATATTCAATCTCGAGGC
TAACGGAACTTGGGAGTTAATGGAACTGCCTCCAGGCAAGAAGGTAGTCAACTCTGGCTGGGTGTTCAAAGTG
AAACGACTTGCCGACGGCTCAATTGAGCGCTACCGGGCTCGTCTCGTTGCTAAAGGCTATTCCCAACGTCCGG
GATTCGACTTCACTGAGGTCTTTGCCCCTACATTCCGTCCTGCATCACTACGCCTCATTGTTGCTCTTGCAGC
TAGAGAAGGCTACAAAATGCGCTCAGTCGACATCTCCTCTGCCTTCACTTATGGAGAACTTGAAGAGGAGATC
TATATGCGTCAACCCGAGGGCTATCACATTGGTAGTCCTAACATGGTCTTTCGGCTGCGTAAATCGCTCTATG
GTCTCAAGCAGGCTGCCAGACAGTGGAATAAGAAACTCCGGAGTGTTCTTGAGGGTATAGGCTACTCTAGGCT
TCGCTCCGACAGCTCCATCTACATCTATTCCAAAGGCGACATCAAGGTCATTGTTCCTATCTTCATTGACGAC
ATTACTCTTGTCTCGAAGAGTGATGCAGCTATGACTTCAGCAGTCACTGAGCTCTCGAAACACTTCAAGCTCC
GTGATCTCGGGTCCACTACACTTCTGCTGGGTATTCAGGTGAAGCAGGATCGCTCTCATCACACTATCTCCCT
CTCGCAGGAGCACTACATCAAGGAGCTCCTAGAACGCTTCAATATGGATGATGCTAATCCACTTTCCACTCCA
CTCGCTCCGGGATCTGAACTCTCTGACATTGTTCCTTCACTCGAGGAGCAGGCTGAGATGAAGTCAGTTCCTT
ATCTCAATGCTGTAGGTGCTCTTCAGTATCTGGCCACTATGACTCGTCCCGACATTGCCTATGCTGTCTCTTA

TCTCGGGAGGTTCAACTCGAACCCAGCTCCAGCTCACTGGCTTGCAGTCAAGCACTTGTTCCGCTATCTCATT
GGCACTATGCACTACAAGCTAGTCTATCGAGCTAGTGACTCGGATGAGCTCTTCACTACCTACTCGGACGCCT
CTCACGGAGCCTGCAAGGCTACAGGTCGAGCTACGGGTGGTTATGTGACTCTTGTCTCTGGAGCTGCTGTAGG
TTGGTCTTCTAAGCGTCAGCCATTTGTGACTCTCTCGAGCACCGAGGCCGAGTTTGTTGCTGCCGTCGAGGCT
GGCAAAGAGATCAAGTGGACTAGGAATATCCTGACCGAGTTTGGGTTCCCTCTGACACTTCCTTCAACACTCT
TCGTTGATAACAAGTCAGGAATCAGTGTAGCCAAGAATCCAGAGCATCACGGACGCATGAAACACCTAGACCT
GCGCTTCTACTGGCTCCGGGAGGCTGTTGAAGATGGACTCATAGATCCTTTGTATGTTTCGACTCACGAGCAG
GTGGCCGACATCTTAACCAAGGCTGTGGCAAAGAAGGTAGTTGAGTTTGCTGTTCCTCTATTAGGACTTGAGT
AGACTGGCTGGATCAAGGGGGTGTGTTAGAGTGAGCAAGCAGTCTACAAGTGTAGTTGGTGGACTTAGATATA
TGCGGACGAGTTTAGTCACATGATAGCTTAGTCATGTATGGTTGAACTTCCCTGTGGACGCTTCAGAGGCCTT
AGCTTCTTACAGTCTGTCCACAGGTGAGTACAAGTTATTAGAACTCAATATAGCTTCTTACAGTCTGTCCACA
GTTGACCTTGGGATTGCTCAGTCAGTTCCTGTCCGTTCGCCTCGTTTCTAGGACCGTTAGACTCGCCACTGCG
TCGCGTACATTCTACGTTCAACA
>Agabi_varbisH97_2_Cluster66#LTR/Gypsy
TCAGCAAAAAGTCACACAACTTATGCAAACTTATGAAAAAGTTGTCTTATGCTTGCCTTGTAAAGACCAACTT
AAGGCACATAAGTCTCCAAAATAGCATAAGTTCATAACTTTTCATAAGCCAGACAAGAAGTTTACACATAAGT
CACACATTAGTTCATAAGTCTATCATAAATTCATAAGTTGTATAACAAGTTATACAAAAATAGCATAAGCACA
GGGCATTTCATAAGTCAGACCAAAGGTTAAATCACAAGTCAGACAAAATTTCATAAGTTTATCATGAATTCAT
AAGGCTACTGACAAGTCAAACAGTATCTCATAAGTGAAACATAGGCTCATAAGTCAGATAACAAGGCAAGAAT
CATAGCATTATAAGAACATTATGCTGTATGTCAAGTTCTACTGCTCATAAAATTCCGGAAAAATCACGACAAA
TCTTAGAATATTAAAAGAACACGAGTTTATATGTATGTTTTTATCGGAAAACAGACCCGTTATGGAGATATCA
AGATTCCATCATGGAGTGGGGGGATCATTGTCAACTGCAGATAGACTCGTCGTAGAGCCTGCTCTACTAACCG
AGAAATTCTGGGAAAATCATGACAAATCTTAGAATATTAAAAGAACACAAGTTTATATGTATGCTTTTGTCAG
AAAACATACCGGTTTTGGAGATACAGCACAATTTTGTGTTTATTAAATGTTAAATGTGCCATATCTCAATAGG
CTTTCATCCAACAAAGAATCTAATTAGATATTCGAACTCCTCGACCTCGAAACTATAGGATTACATTTTTAGA
TCATTAGAATCCATGCAGAAATGACCCGAAAATATCTCCTACCTATGTTGAGGAACTTTGACTCGAAAATCCC
AGATGTCATGTGATGTCATGTGACATGTTGAACTTCTGTTTCGAGCACGAACATTGATCCTTGCTCATCACCA
CCATCATTCCTCCCCTCCCCTCCCTCCCCTTCCCTCCTCATTGCTCTCCTAGGCTTATATTCAAGTAAGTAAC
TTCCCTTCCCTTCCCTAGCTCTCTCCCAGCTAATTCTAAGCTGGTTTAGGACGCTTTTTTTGCCTATTTTGGA
TGCTTTTTTGGGGGATTTTTGGAGGGTTATTTGAGGGGATACTTGAGCAGATAATTGGGGGGATAATTGGGGG
AATAATTGGGGAATGTATGGGGGGATTTTGGGGGGATTTTGGAGATTCTCTGGTCCCTGGTGAGTGCTCTCTA
TCTCTGTATGTTTTCATACTCATTTGTTACTTGTAGACTATTCCTTTTCTCATCATCACTCCCTCATCATCAC
TCCCTCATCTTCCCATCTTATCCTTTTGTATAGATTGTATCCAATGTATTATATTTACCATGCTGATATAATC
ACTTTGTTGACTTATTCACTTTTAAGCTTCTTATTATTGTCTTAGTTGACCTTGAGTGTCTTAATTATGTACT
TGAGTGACTTTAATATGACTTTTTACTAATCTATCTTTATGTCTATTTTTATATCTATGAAAAAGACATCAAC
TAAGTACAGTTTAAGTGACTATTAAGTCATTTCCAAGAACCTTAAGATACACAGGCCTAAGTATCATTAAGAA
GATTCAGAAAGGTATCTGCTCTTAATTGTTTCTTAATGACCAATTAAGAACAATTAAGAAAAAATCTTTTTTG
CTGATGTGCCTGCCTTCTCCATGGGATTTGGCAAACCCAGCAAACTGCTTCAGTGTGCAACTCCATTTGGTTC
TTATCATATTTCTGCTGATGCTCGAGATTATCTAATCGCCATCTCTCAAAGTGGACATTTCCACGGCAGTACC
TTTTCTTTTACAAATTCTCTGAGAGACGAAGATCAGCCAGCACTTTTTTTCCCGATGGGCACCTCTCCAGCCA
TGCCACCCCCCAAAGACCAGCTTCAGGCGACTACTCGAATGCAAATGCCTGCACTTCAAGAGTCAAGCACCTC
TGGTAACCTTCCACGTCCTAGTATCAACGGCATGGAGTCTCCTAGCATCAACGGCATGGATGGGACTGCCAAG
AAGGTGAACCAAAAGGTACAAAGGGGAGGAATGGATAAGGAGCCCACTCTAGTAGCTTTGCAGGACGCTGTTT
GCGACGGTGATGAACCCAAAGACTTTTCTCACGAGCCTGCAATTTTTGCTGTCCTTCCGAAGCCCAATGATCC
AATTTTCACTCCACCTGCTTCACGCCATAATATTCTGGGGTTCAATCCTCTTGATGTCCACTCCATTCCCTAT
TGGAATTGCAACCACATTGATCCTGCATATGAGTCTACCACTACAAAAGACTTGACGCATTTCACTTCTCTGC
CAGGCAATGTTAAATCAAAATTAGCACGTGAGTTGCAACCTCCTATAAATAGATCAAATCACATAGCTGATCG
AGATTTTAACCCTTTGAAGGCAAAGCCCGAGGTGATCTAGATCACTTTGTGCGCCCTATCAATGCCTCGCCGA
TATTGCCCAAGTTCCGAGACATGGTTCAGAACGAAGCAGCATTCCTTCCAGCTGCAATGCTCCACAGCTACCG
GGCAGCCCTCCATGTTCACACAATGAATGCCTGCAATATTTTATGTGCCTACCACAAATTCTCGAAGAAAGCC
AATTTGCATGCTTGGGATGGTGCACAACCTAGCGTGCCCAAGGGGTTTGGTGTAACAGGAGTTCCATATCCCC
GGGCTGTTTCAGCCAAGCTTTCGTATAGTGTTACTCCTCGCCAGCCAGGCCGTCTTCATTGTGGCTGCCCTGA
GAATATTGTCCTAATGGAGGCAATTCTCCGTAAGGTAACACAGGCCACATCTGTGCACTCTAGTACCACCAAA
ACTTGGCAGCATACGTACATGGACCCTAGAACCCGCCTGCTCACAATGACAGCATTTATGGATTGGACCGGGC
TAAGTCTCAGCGACTTTTTCAGGGTAGCCCCTGATGGCCATGTTTGGGTCAATCCATATGCTGAGCTTCTTGA
TCGGCAAATTCTAGTGCTTCAGCAGCGTCGCCAGGCCCTCGGTACCAATTCACCCGTGGACCCACATGGCAAC
ACACCTGTAAATAAGTCTGCTCAATGGATTGAGCAGACTTTGGCCCAACTTGACGATCCCTCTCAATTACACA
ACCTTTATATTTGAATAGTACACATGTCTTATGTAAACTTTATGCAAACATTACCGTAAATATTATTTCAATA

ATACATGTGTACTATTTAAAACATATGTAAATTAAAAATGCAATAAAGCTCAATTTAAGACTTTTGTTTGCAT
AAAGTTTGAATATATAAAACATATTACATATGTACTATGTAAATTAAATTTACGGTAACATTTGCATAATGTT
TACATAATACATGTGTATTATATAAATATAGAGATTGTGTTTACTGATAATGATTAATGAGTAGCAGACCTTG
TGAGTTCGCTATTTTTTTAACTCTCTGCCATTTCATTGATTTTATTGAGAAGCAATACTTATAGTCCTCCACC
AGCAGTCCTTGCTACAGTTTTTATAGTTCATTTTTGGAAATGCTATTTACTTTTGCCTTTCCATAAATTTATA
GTACCCCTAAGCTGCGGCCACTAGCATTTCGAATTGATCAAGAGGGGTCTCTAGGTTTTCTGCGAGAATCGTT
GTGCAGTGCTCGAGCACAATCCATGGCCAATCGGCTCAATTTGGCGAGCTTCTACCCAGCCTCTCGTTGCCAA
GGAACCAAACATGCACAAACCTACCGCGACACGAGCTTCACTTGCTGAGTTGTGCTAGGTGGAAACCTGATCT
AGCAGGAAGCCAGGTTTAAGTGTGAGTTGGTTAACACAAAAGGGAATAATTTGCACAAAGAGATTCCAAAGGT
GGGGGAACACTATACAGTACAACGATTACAGTCAAAAGAAGACACTGAAGAAAAAGAACCGGATCCAACGAAC
CCCGAGGACTTACCAGCATACATGAAACTCTTCGCCTATCTTTTCAACAAAAAGAACTTTGACAAGCTTCCTG
AGCGAATGGAATGGGATCACGAAATCAACTTCACCGAAAACACACCAACAGAGATATCATCAAAGGTTTATAG
CATGACACCACTGGAAAGAGAAGAACTGGACAAATTTTTGGATGAAAATCTGGCTACCAATCGAATCCGACCA
TCAAATTACCTTATGCTACACCATGTTTTTTCGTTCCCAAGAAAGATGGTTCATTACAGTTATGTCAAGATTA
CAGGAAACTGAACGAGATCACAATCAAGGACAAGATGCCACTACCCCTCATCAGCGAAGTATTAGATCAACTC
AAGGACACCAAGGTTTTTAACAAATTGGACATTATCTGGGGATACAACAACGTTCAGACCTAGCACAACCTCT
ATATTTACATAATACACATGTACTATGTAAACATTTTGCAAATGTTACCGTAAACAGAATTTACATAATACAT
GTGCATTATATAAAACTTATCTAAACCTTATGCAAACAAAATGTATAAAATTTAACATTATTGAATATATTTT
TTGCATATGTTTTACATAGTACACATATATTATGGAAATCAAATTTACGGTAATGTTTGCATAATGTTTACAT
AAGTACATGTGTCTTATTTAAATATAGAGACTGTGTAGGTTTCATAATAGTGGTTTAAGGTCAAGGTGATGTT
TGATCAACAAAAAATGATGCAAATTAATATAAACAAAGTCATGTGACACTCTCAAATTTTCCCAGGATTTTTG
CGGTTATTTCTGGAGCTTCAGAGCATGATTTCTGAAAGTTGAGAAGAGGGTCAAACAACTGTCCCAATGATTG
ATTATTTTGTTGATTTAATTTCTCAACCTTTTACTGCTCAAATTACTTCATCTTACATGCGTTCTGAAGAACA
CATGTAGGGGTATAAAATGTATTAATCACATAATCATACTCACAAAACTCATTGTGCTATGACACAAATAGTG
CAAAACCTCGAAACATTGGGACAATTTTTTGAGCTACTGTAGCATACTGTTCCCATGTTGCTAAGGCCAAAGC
TGTTTGCTCGCTAAGTCTTTTCTGGCTTAGCAATCTCTTTCTAGTCATAGTCTTAACACTTTTTCAGGTTACA
TTATTTGGATGAAAGTATGATGGACAGACATCATGGACTGACCACAACCCTAAGCTGATATGTTTTTATTATC
TGGATTTTGTTGAAGAAAAGGGTTGTGAGTCAAAAACGCATCCAGGTTAGGTCACTGACAGCCATAAAGATTC
ACTAAGTACTATCAACTACTCATCAACTTTTCTTGGCACTTTCACATCAATTTTGAATAAACTCCTCTATTAG
CCTCTCATTATACGCCACTTGATCTTCTGTCTCTTTCAGATGTCATTTATTGCTATTACTTGACAGATATTCA
TTTAACATGGCTCATTTCTTCTTGGGAGACAGCCATTGATCTTTTGTGGTTTGAATATGATATTTGAGTACCA
GCAGCAATTCCTCAGTGCGTATTGGAGGTGGATAAAACCATCTTGCATAAGCCTTGCCGTGATCCCCACTGGT
CATATATTGTTGTAATTTTTCTTATTGTGTGTATTCAAGTACCATTTGCACTTAAGGCAGCTGTTTTTTCTGA
CTCCAATGGACACAATTTGACGCTTGCTTGGGGATAGTTGATTAGCTGTTTTCTCCAGAGTTGGCCAAATTTT
AGCAAATTCGTAGGACACTGCAAACCACTCTATATAACCCATGATGTGAAGTTCTGCATGAAAGTGCACTGTA
AACTCCTTGAATTTGTTATGATAAGGATATATGACAACATGTTCCCAATTCCTTTGAAGGACCTGGTGAAGCA
ATTCATTTTGTGAAAACAATTCAAAGGTTGTTGTGCTTTTTAGTCCTCTTCCAAAACACTTAATCTTTCTTGT
ATTTCCTGGATCTCAGTTTTGAATGCATGATTGAAAGGGCCATACACAACCTCTATATTTCAGTAATATACAT
ATATTATGTAAACATCATGATAAAAATGTTTTACATAGTACACATCACAACCTCAATATTTCAATACAACAAA
TATTGTAATATTATTGAAACACTACTGTATTTTTTGTTTTCATACTACTTGGAAAGTATTAAAACATTATTTA
AACATTACCATATTTTATTTCCATATATGAAAAAAAAATACAAAGAAAAATAACCTATTGTTTAAATATTATT
TCAATACTTTCCAAGTACTATTAAAATAAAAAATACAGTAGTGTTTTAATATTATGGCAGCATGGATATAGTT
ATTGATGCTGGGATTGATGAAGGGACTGGTTCTCACAATGACTCGGATGATAGCTCTGATGATGACTTAGAGG
GAGAAAGTAGCAGCAATGAAGAAAGTGAAGATGAAGACATTGATGATAATGATGGCAAAGTACGTACTAATTG
TTGCGCTTCAAATAAACACACTAAAGAGGTGAAGGTGAAGTCAAAATCAAATTCAAACCTGAAGCCGAAGTTG
AAGTTGAAGTTGAAGTTGAAGTTGAAGCGAAAGTTAAAGTCGAAGCAAGAGTCAAAGTCAAAGTCAAAGTTGA
AAAAGCGAAGAAGCCCTTCAACTAGTGGTGACAAGAATGATGCTGAGATTAATACTCAAAGGCCCAAAAAGAA
GGGCAAACATGCCTAGGTTTGAAATGGGCAAACATTATGCAAACATCACAGTAAGTATAATCTAAATATATGT
AAACCAAAAATATAATAAAGCTCATTTTAAGGCTTTTGTTTGCATAATGTTTGAATAGTATATGTATATTATG
TAAATACAGAAGTTGTGATAGTATATGGTTACTTTGTAAACTTTATGCAAATACAGAAGTTCATAAAGTAAAC
TATATTGTTTGCATATTTATCATGTCCTACACAACTCTGGAAATAAGATAATATTAAATGCAGATTTCTAGCA
TAACAATAATAAATGACTACAAGTAATTTTTGTTTTAAAATACATAAGGAAAACATACACCAGCTGTGAACTT
CTTCCATTTTCTGTGAACTTCCCAGATCTATTCAAATTTCCAAGAATCTCTTTGAATCTCTCCAGATCCTCTA
GAACTATTGAAATATTATTGAAACACTACTGTATTTTTTACGAGTGGGATGGTTGGCGGATCCCCATATACAT
CCAGACGACAAACTTGTTCGTCGTCCGCAGAATCGAATGAGGACTGATGGTTCAGACTTCCAGTGTAGACCTG
ATCCATGTACCGTAAGCCGAAGTTATAGGCATGGTCAACCCATGATGGCGGTACACCGTTGTGGGTGAGGACT
TCAGCTATGGTGTCCACGTTCAAATTTGCGATTGACGAGTCGTTGATCTCGAGTGGGCGGAAGTGGTAGAATG
GTCCAGTCTGCGGAATGAACAGTTGATCCGGGTGTCTCTCATTCCATTGCGCAATGAGGTCCCGGTATAACTG

GGGCGATGCAGCCAAGATGGTGAAGCATCGTGTGAATACGACATGGCCGTTGCGGGAACGGCCACGTCCGGTT
TCAGGAGGTCCAAAGAACCTCGCTAAAGTGTAGCCGAAGACGGATCGACAATGCACAGCTCGTCGATAATCAA
ATTGAGTCCCATGAATGTAGTTCTGGGACCCGGGGCGATTATGAGTGAGAAGATATCGACCAAGGAGGTCGAG
ATCAAACAATGCGGCCCCAGTGGGCTGGGCCATACCCATCCCGCCAGTATTGTTGGAGGGGTTGGACAACCGA
GCCGCTGGCCAGTTCCTCAGGATCTCCCCCCAGTTGCGGCGGATATTGCGAATTGGTTCTGGAATTTCGTCTG
TTCTAAACTCTGCCAGAATATATTCCATCGCGTTGTCCGTGTGTCGGGGTGCGATCTGTAAGGCTATGGTGTG
CAGCTCCGTCAAGAGGATGTACGCTTCCGTGCGCACTCGACTGTCAGCTAGATGATCGCGAGCCAATCTGAGG
AGGCGTCCAGCCTCCAGTGGACTTTGGGGAAGACCAGATGGTGCGGCATTGCGGATAGTCCTGGAGCCGGTTT
CCGCCTCAAATCTCTTGTGTCTCAGAGCGGCTTCAGCTGCTCTGATAGCGGTTTGACCAGCATACACTACATT
AGTGTACGGATCATGGAAGACATGTTGGGACAGCATGCCCTGCATTCGGTTATCCTGCATGTGGTCAGGACCG
ATAATCTCATCGGTCGCCATTCCCCACAGGTCAGGGATCACGCCACCTTGTCCAGGGAGGTCACTAATTCCGT
CTAGTGGCGATTCAGTCACCACGACCGGTCCTTGGGATCTCTTCTTCTTGCGTTGGGACTTTCCCTCCAGTTC
GCTTTCTTCGAAACAGGCATTGGAAGGTGTTACCCTATAGTCCTCTGGTTTTGAGCGGTCCAAGGTGACTGGG
GGTTTGGGTGGCAGCTGCCCTGGCATTTTCACCCTAACTAAATCGCCGTAGTTACACGACGGCGAGTTCTTTC
CGATGGGTGCATCCCTGACTGCAGACAGATGCCAGATTGACGCTTGCAGAGTGTCAGTCCATTTGGGATCACC
GTAGTCGGGGGCACTTCAGGATCCTGGGCGATGTTTGAGTTAGTGGTCGACCCTTGTCGATGCCTCTTTGTTG
TTGGCTCCGCAGTAGGGGCCAACTTGGATGAGAGAGGTCGAGCGAAGAACGCCTTTTGTTCGGTGGCAGCTGC
TTTACTGCTACCCGCGACTGACGATGGGGGAGACACTGGTTGCGCCTTCCTCTTTCCTTTACCCTTCACGGGT
TGGCTGGCCACTGAAGGGCCTTGTGCTGGCATTGGTCGAGCTCTTGAAGAGTTCGACAGAGAGTATCCCACAG
GAGTTTTACTCCTGCTCGGTGCTGGCTCGCCTGAGTGGCTGGTTGTTGCCGTTTGGGCATCTAAGAGTCGTTC
AACTGCACCTTGCAGTCGATCCAGTTTTGATTCCATGCTCCTCATCGAGGAGCGTTGTCCTTCGTACTGTTGG
ATCAACTCCTCAGCGGCGTTAATCCCAGTGTGCGTGTAAAGATCTAGGGAGTTAATGATGATATCACTAACTC
CAGCATTGCATAGATGTCTAGCGTACGTCTCTACACGTTGCTCGTGGTATTCTTGAGCCTCGTCTTGCTCAGT
TGCGCGTCGTTTTCCTTTACTACGCGTCATAGGATTGTCATCAGCCTCCATAGGTTGCTGATATGGTGACGGC
GGCGGCGGTGGCGGATGCTGGTGATAACCTCCACCCTGGTGCTGTGGGATGTAGGGTACGTGGCCAATATATC
TGTACGGTACAGTTGTGGTGTGCGCTTGAGGTTGTTGGGGATACGTGGCGTACTGGAACGGTGGTGGAGGCAT
CTGCTGTTGCCTCCACATTTCATAATGTGGTTGCGGGGGGTATTGTGCCAGTATTGACGCGAGAAAACTCATG
TGAGAATTGTGAGCATCAGCAAAAAGTCACACAACTTATGCAAACTTATGAAAAAGTTGCCTTATGCTTGCCT
TATAAAGACCAACTTAAGGCACATGAGTCTCCAAAATAGCATAAGTTCATAATTTTTCATAAGCCAGACAAGA
AGTTTACACATAAGTCACACATTAGTTCATAA
>Agabi_varbisH97_2_Cluster68#LTR/Copia
TGTCAACGATAAGTCGGGTTGATCACTGAGGTGAACCGGTGGCTTTTCGTGCCCTAAATACCCGGCAGTCTCG
GCTACAAGTAACTATACAGTTTAAGGACAAATAAGTATGAAAAGAAATATCAAGAATAAACGTGCCTAAAACT
TTAAACGTTTCTTGGGGAAAATAATAACCAACTGATCTAAGATGCCCATAAAAGTAAAGCGAATATAAAAGGT
AAGAAGGGTCATCAATATCAGACAGAAATGGAAAACAATCTACACGAGATATGTCGGAGTACTCACAACACTC
CCCCTACTTCGGCATTCTCGCTCTTGACTCCCCCTCGAATATCAATCCCTATGCAACAGCGAGTGACTGCATC
CCGAGCTAGAGGCTTTGTTAAGATGTCCGCAAGGTTTGTGGCCGACGGAGTATAGATAAACGTGATGTCACCA
TTGTCGACCTTTTCTTTGACAAAATGATATTTGACGTTGATGTGTTTTGTCCTGCGGTGATTTTTGTCGTTCT
TAGTGTTGGCAATCGCTCCGTTATTGTCTGTGTGGATTGTAGTGGGTCCGTCCTGTCGTAAGCCAACCTCGTC
GAGGAACTTCTCGAGCCATATAGCTTGTTGAGCCGCCCTCGTAAAGCCCATATATTCAGCTTCGACCGTTGAG
ACTGCTACAGTCTCTTGCCGTTTACTCACCCAGGATACCGCTCCCCCTGCTACAAAGAAGACATGTCCCTCGG
TGGATCTCATAGTATCTCCATCACCAGCATAATCAGCGTCCACATAACCTACGGGTTTAAGGCTTCCTCCCTT
CCTGTAAGTGATACCATAATCCATCGTCGCCTTAACGTACGCAAGGGTATGCTTCAGCGCTTCCCAATGCGCT
CGACCAGGGTTGTTGGCAAAATGGGAGAGGAGATTGACGGCAAAGGATAATTCAGGTCTGGTTGCGACTTGGA
GCCACATTAAAGAACCAAGCGCTTCACGATACGGAACTTTCTTCATCTCGTTTGACTCGTAGTCGTTTTGTGG
TGCGTCGTTGGAAGATAGTGATATACCTGCTGGAAGGGGGGTAGATCTGGGCTTGGCATCGGTCATATTGAAA
CGCGAAAGAAGTCGTTCACAAAAGGCTTTCTGAGAGAGGCGAATGTCTCCCTTGTTGTTACGATCGATGCGCA
TACCTAGAATAAAGTGGGCTTCACCGAGATCTTTGACCTCGTAGCTACGAGCGAGTTCATCTTTTGCCTTGAG
TTCACCAATATGAGTGGTTGAGGCGCCTAGAACATCATCGGTCCAGGTCGAAGTGATTGTGAATTCACCGTCC
TCGACTTTTGAGCGGACTTGGGAGTCAGCTTTGGACTTGTAGTAGCCGTGGCCTTGGTAAGTTTTGTCGAGGT
TTTTGGCCCAGTCGTGAGCACCTTGCATGGTGCCGTAGAGGGTTTTAAGCAAACGCCATACTTTTCCCTCCCC
CTCTTCAAACCCTTTCGGAGGTTCCATGTAAACATCGTACTGGCTATCACTGTTCAGGAAAGCCGAAACGAAG
TCCACTTGCCATAACCGCATTCCTCTAGAAGCCGCGATAGCACAAACCATCCTAACAGATTCCATCCGAGCCA
CTGACGCGTATGTTTCATCATAATCTTCCCCCAACACCTGTGTAAATCCTTTCGCAACAATTCTAGCCTTCCT
CGCCGTAATATTGCCGTCTTCATCAAATTTCAACGTGAATACCCATCTAGAATCTACCACATTCCTACCAACT
GGTCGATCCACCACCTCAAACACGCCTTTCTCCTTCATCATCCCAAACTCCTTCACCATCGGTGGTAACCACA
ACTCGGAACGCGTCATCGCCTCACGATAGTTCTTTGGTATCCAATTTTCACTAGAGGTGTCTATCATGTTCGC
TTTGGTATGTTGCTGGTATTCCAGTGTTTCTTGCATTCGTTTAGACGGACGAGAGACTCTACTCAATCTCCTG

ACAGGGACGGATGGTACGACCCTTGTGGCGTCGGTGGTATGATCCTCATCAGCTGTCAAAGCTTCCTCGATCA
ATCTTTCCTCGGCATCACTGTCAACCCTGTCAGGGTCTGGTAGACTAGCATGTTCATTGTCATCATCGTGGAG
CGTTGTTATCTGGTGAGGTCGTGGAGCAATTGCGCTAGGGAGTGTGGTAGGAGGTGGTTGGCTGTTTATAGGA
GTGATAAAATCGTCTGCGGTTGGAGTCGGCCGTGAGGTAAAATGGGTTTTGCCTTCATCAAATATTACGTCTC
GGGAGCGGAAGATTCCTCCCGTCGTCCTATCGAGTAATTTGTAGCCAGTGGTACCGAAGTATCCAATGAGTGT
CAGTTTAACTGAACGTGGTGCAAGTTTTGAGGTTCCCAGATCGAGAGGTATGTGAGCATATGCTGTTGATCCA
TAGGCACGGAGATGAGAGATGTCTTGTCGTTTACCAGTCCAGATTGTAGCGGGAATTTTCCCGGGAACGCGTG
ATGTAGGTAATAGGTTCTTAATATAGCCTGCCGTAGTAAAGGCGTCTGCCCAATAACGTTGTGGAAGACCGGA
TTCGGCGAGCATTGATCTCCCCAACTCAACCAATGTTTGCATGGACCGTTCGGCGACCCCATTTTGTTGATGG
GCATAGGGGGTGGTAAACTCCAAGACTATTCCCTTCTCTTTCGCATACGTCTCCCAGACATGGTTATGCCATT
CCCGTCCCATGTCCATCCTTATCCGCTTCAACTTCTTTCCAGTCTGTAGTTCAGATTGCGTTTGGAACTCTTT
GAGGTGAGTAAGCGTTGTCTCAGCCGATTTATCATGGATAGGGATAACGTGTCGGTACGAGGAGGCGCCATCA
ACAAGCACCATGAGATATCTTGAGCCCCCAGCTGATTGGATAGGGGCCGGTCCCCAGATGTCTACATGAACTC
TCTCCAAAACCTCAGCTTCTACGTCTCGTTTATCGGAGTACGGGTGAGTTGTGTGTTTCCCATAGATACAATC
TTCACACATGCCACCTAATTCGTGTCTGCCATCGATGTCCAGCCCATTGACCAAGTTTAAACGCAACATCTCC
TTTATCGCAGTTTCACCAGCGTGTCCGAGGCGGCGGTGCCAAGTATCAAATGGAACTGCCTTCCGTTTGGCTT
GCATCAGGTGAGATGATATTTGTGATCCACGTTGGATGGTGTTGACGACATATAGACCGTTCATTTTAACTGC
TACAAGGACTGTAGTCCCCTTCTGGTCGCAAACCATCGCAGTATCACCGTTGAAAGTGACGTTCATCCCTTTC
GCTGTCAGCTTCGAGATTGAGATGAGGTTATGGCGTAGTTCTGGGGAATAAAGTGCATCCGAAATGAAGATCT
TTGCGACAGCCCCATCCTTCGGTGTGTATATCTCGACTGTACCGTGTCCTGTTATGTCGAAGACAGCTGATTT
ATTGGCTGAGTGACCGTTCCTAGGCGCTGACAATGGTTTAAACAATGCGAATGATTCACAGCTAGCAAAACAA
TGATCGGTGGCACCGGAATCGAGGAGGGTGAGCAAATCTGCCTTCGTATTCATTTCATGGGTAGACGATGATG
ATGACATTGCCGATATAGTATCTACCGTATCCCCAAACTCATCTCTTTTAGCACCTCCTAGATCATCAGCGTT
GACGGATGGCTTAGATGATCTAGGTGAAGAGTGATGGTCAGGGATGACAGCAGCGGTGATAAGATTGGTGCGT
GGTACCTGGTCTATGTTCGACGTGATTGATGATAGGACATAGACTTGTGGGTCGTCTTGGGTATTATGAGGTG
GGTATCGATGCCGTGTTAAAGGACGTGTTGGATGCTCCATTATTTCCTCCTTTTCCCCCTCCTTTCTTTCCAA
ACCAGGCAGGGAACTGTCCCGCCTTTCCTCCTCCTTCCCAATAGCAGTCAGTGATCAAGTGTCCTCTACAACC
ACAGTTCGGGTTCGTACACAGGAGGTGTTGGTTTTTAGGTGGGTAGCGATTACGCCCATTGTTATTGGCTGTT
ACCTGGAGAGCCGTAGTAGCATTTTGAGGGTTCACGACCCGATTTTTGCTGATGCGAGCCCAATGCGTGGCGA
GTATCGCAATGGCTTCCCTCGAAGTTGTCGTACTATAAAGTGTGGATACTATAGGATCCCATGATCGGGGTAG
GGAAGATAAGACGATGGTTCTGAAGGTTTTGTCGTTTATCTGGGCTCCGAGGGCTGTGGCGGTAGCCCATTTT
GTGCGTAGTCTGGATATGTGTTCGAGGAAGTCATGGTTATCCAGGTAGGTGATGTCTCGCAGGTCTCGTTCTG
CATTCAATATGGCAATCTCTGATACTTTCGCATATTGGTCCTTATAGGACTTCCATGCTTCGGCTGCTGTTCC
TGAGAGAGCGATACCGAGTCCTATCGGGTCTTTGGTGTTGTATATGAGCAGTCCTTTAGCCCATGCGTTACGT
ATTTTCCATTCGGCGGGAGTAGGGGTATGTGATTCCCAGGAAGTGTCAGTTGACGGTGCGGGCTCATCCTTGG
CAGCTGTTGTCCTGCTGCTAGAAGGTTGGGGTATAGTTCCTTCAAGGTAATCCGATACGCCTCTGAGGTCAGC
GGCTATATTGATCAAGCCGCTCCAAGCTACCCAGTTCGTGCCATCGAACTTGTCGGTGTCTGGGAAAATAGGA
GGTGTGTTCTGAGACATTTTAATAATAGTGTGGGGAATGATCGTTCTATATCAAGGTTTCGTTGTTCGTTTTA
ATAGGTCAAGCCGGGGCCCATAACCTAATAAGAATAAGAATCTCAGTGTCAACGATAAGTCGGGTTGATCACT
GAGGTGAACCGGTGGCTTTTCATGCCCTAAATACCCGGCAGTCTCGGCTATGAGTAACTATACAGTTTAAGGA
CAAATAGGTATGAAAAGAAATATCAAGAATAAACGTGCGTAAAACTTTAAACGTTTCTTGGGGAAAATAATAA
CCAACTGATCTAAGATGCCCATAAAAGTAAAGCGAATATAAAAGGTAAGAAGGGTCATCAATATCAGACAGAA
ATGGAAAACAATCTACGCGAGATATGTCGGAGTA
>Agabi_varbisH97_2_Cluster58#LTR/Copia
GGATGGGTATTGAGAATGGCTTCGACTACTTCACTTTCGATCTTGAACGACTACTTTCCTTATTCTGATAAGT
TTCTATTTTTATCTTTATTTCTATTTTCGGTGTTCCTCACCAAGTTACATTTCAAGCCGCTTCCGCATATACT
ACACAGTTTTTCTATTATACTGTACAGTTTATCTTAAGCCGAAATGTGGCCGAGTACTTATATCCTCAGCCAC
CACATTTTCACCTCAGTGGTCAACAAGCCTTCGTTGACGCTGAGGTGAGTAGTTTACATTCGTCTATCTGATA
TATACTTACCCATGTCCGTAGTTTATATTTCTTTAGGTAAATATTATAGTGGTCAACAAGCCTTCGTTGACGC
TGAGTTTATATTTCTTTAGGTTATGGGCCCCGGTCTGTAAACTTATATCAACGAAGATACACACAGCATAAAC
CACTAGCTTACTTTCGACATGGCTGGCACTGGCGGTAGTGCACCCCCAATTTTCGCTGACGCAGCGAAATTCA
ACGGAACGAACTGGGTTGCTTGGAATGGGTTGATTCGGATAGCCGCCGATTTACGAGGCGTCTTTGGTTACCT
CGATGGTACAATCCCCAACCCATCCCCACAGAATAACATTACTCCAACCCCGACTCCCACCACAGCAGCGGAC
GCCGCTACAACAGCCCAAACACCGGTTGATACACCCTGGGAGTCGACTGCTCCCTCGGCTACCAAGTGGCGAG
TTAGGAATGCCTGGACAATGGGCTTGCTCATATATAACACGGCCGATCCGGTAGGACTTGGCATAAACATCAA
CGGATCAACTGCAGAAGCTTGGAAATCATACACAGACACCTACCAGATCACCTCCGAGATTGCCCTTCTCAAC
GCCGAGCAAGACCTTCATAACGTAGTTTTCAATGATGGGGAGGACTTCGTCGACTTCATCTCGCGTCTTCGCA
CAAAGTGGTCGAATGCTACAGCACTCGGTGCTCAAATCAGTGACATCTCGTTCAGGACTATCATCTTGAATGC

ATTGCCGTGTTCATGGGATCCCATCGTCGCTACTCTTTATACCACTCAGACTTCTCGTGATGCTATAAATCAA
CTCATGACTCACTGGGCGAGGATCAGTCGCGACCGCACCAATAACACCCAAACTTCAACCTCGGCACTCCAAA
CCATTTCCAATAACCGTGGTCGTGATCGTCAGCGCTCGCAGAACTCCAATCTTTTGTGCACCAACTCCAACTG
TGGTAGGCGTGGCCACACGATCGAAAACTGCTATTGGCCAGGAGGGGGGAAAGCAGGACAGTTCCCTGCAGGT
TTCGGCAAACAGAGAGGAACGACCAACGCTGCGACTACCAATGCCGAGCAGACATCCGCTAACTCGGCAGCGA
ATACTACTGAGCAAGTTTTCGCGCTCGTTGCCCATGATCAGCACGACGCCGTCTCAGAGGTACCACCTTCCCC
AACCCATCCTTCATCATCTCCTTCTGACAAATCCAATGTCAGGCCGTCCTACAATATCATCCCACTTACATTC
GGGACGATTGAAGCAGATGATTGGAGGGGGAACGACAATGAACTTACACAGTCTATACCTGAGTGCCACAATG
TCGATATAAATGTTAACGATGTTGCTACTTTGCTTGATTCCGGAGCGAGCGACCACTGTTTCGTCGGCAGACC
TCTGTTCTCCAAATACCAAACCATCTCACCACCTCGTAAGGGGAATTCTGCGGGGAAAGATTCAACGTTTACG
GTTGACGGTGCAGGGATGGTGGAATTTACCACTGTCATAGACGGTGTATCGTCGAGGATCGTGCTGAGCGACG
TTCTCCACACGCCGCACCTCCGTTCTAACTTGATCTCGGTGTCCAAGTTGGTCTCTAAAGGAATGAGTGTGAG
CTTTGATGGCGACGTAGCGAGAGTGAAGAGGTCGGGAAATGTTATTGTACTTACAGCAATCAAAAGGAATGGC
CTATATGTTGTACCGACCACGAGTTCCTCGCGTCCTGAGGCGAACATCTCTCAGAGCAAGAAACAGGCAGTGG
CTTTCGACATCTGGCACCGACGACTCGGTCATGCTGGTGTCGAAACGATAAAGAGGATGATAAAGGGAAATAC
AGTATGGTCGATGGTGTCGAAGTTGCTGGTCCTACAGAGTTGAAAGCTTTATGTGAAGACTGCATATATGGAA
CACACACAACTCACCCCTTCCCTGACTCGAGCTCTGCTGAAACTGAAGCCCTACAACGTGTCTACATCGATAT
TTGGGGACCGTCGCCTGTCCAGTCGGCCGGTGGGTTGAAATACTTCATGTTGATCATCGACGGCGCCACATCT
TACCGAATGGTGAATTTCCTACCCACGAAGTTGGCTGATGCTACACTGAAGGTCTTCCGGGACTTCCATCGCC
AGGCAGAACAGCAAACGGGCAAAACGCTGAAGCGAGTAAAGGTGGACATGGGGAGGGAATGGTTCAACGCCTC
GTGGGACACGTACGTAAAAGAACACGGTATCACCCTTGACTTTACCACCCCATACGCCCACCAGCAAAATGGC
AAGGCTGAGCGGTCTATGCGTACGCTCCTTGACACGGCCCGTACGATGCTCACCGACTCCGGCTTGCCGCAGA
AATATTGGGCTGATGCTGTCCTCACTGCTGCGTATATTCGTAACTTCATCCCAAATACCCGTACACCTGATGT
CATACCTGCCGAGGCGTGGACAAACCAGAGACAGGATCTTTCACACCTCCGTCCCTTTGGTTCTGTTGCATAT
GCTCACATCCCCGCCGAAGTCAGCCCTTCGAAGTTGTCTGCACGCTCTGTCAAGCTAGTCCTTATTGGGTACT
ATGATAGGACAGGTTATAAGCTGATGGATCGATTGACCGGCGCCGCTTTCAAGTCGCGCGACGTGATTTTCGA
GGAGGCTAAACCCCACTACTCGACGGATCCCATTGTCACCTATCCTGCTGACGACGCCCAGGCCACAACACCC
CGCTCATCAGTCATTGCACCTCGACCCAAACCAATTTCATCCTTACACCCTACTCCTGCTGCTCCTACTCTAT
CACATAACCCGTTACCTATCACACCTCCAAAGCCGCCTGCCGTTCCACAATCGCAGAAGACTCCCGATATTTC
GGATAGCGAGGATGAGGTATCCAAAGCTGTCATGCCGGACGGTGACACCCCCATTGCTATCCGCAAGCCTAAA
CGGGAGGTTAAGCTGAGTGCAAGGATGAAGGACAGTCTGGAATACATGAATAGGCCTACTGCAAGCATGGTTT
CTGCGTTTGTAGTGGACGACTCCGCAGTTCCTAGCACCTTCAAGCAGGCTATGTTACGCCCGGACCTCTGGAT
GGAGCCCATGTTGAAGGAACTCGCTGTCATGGAGGAGAAGAGTGTCTTTCGTCTCGTCCCTCGGCCAGTTGAC
AAGAATGTCGTCAAGTCTAAGTGGGTCTTTGCGAATAAGTATGACGAAGAGGGTAGGATTGTGACTAGGAAAG
CGAGGTTGGTGGCGAAAGGATTCACACAGATCCTGGGGGAAGACTATGATGAGACTTACACTTCGGTTGCTCG
GTTGGAGTCCGTACGGCTAGTTTGTGCGGTCGCCGCTTCTCTTGGCCTACGCCTCTGGCAAGTCGACTTCGTT
TCTGCATTCTTGAACAGTAAAAATTCCTTCGAAGTCTACATGGAACCACCGCCGGGTTTTGACGAGGGGGGAG
ATCGAGTGTGGCTGCTCCTCAAAACCCTCTATGGGACAATGCAGGGCGCCCATGATTGGGCGCAGACTCTCGA
ACATGCCTACCAAGGACACGGATATTATACTTCCAAAGCAGATCCACAAGTCCGCTTCAAGACTGTCAATAAC
GAGCTCACCTTGACATCTACATGGACTGATGACATACTTGGCGCGTCCTCGACGGAAATGGGGGAGATTAAAG
CCAAAGAGGAGCTGAGGAGTAGCTATGAGATCAAGGATTTGGGAACGGCGAGATTCATTCTGGGGATGAAGAT
TGAACGTAATGACGCCGACGGCAGCATCACCTTATCACAACGGGCATACTGTGAACGAATGCTTGAGCGCTTC
AGCATGAACGACGCAAATCCTCGCCGAACTCCTTTACCTGCTAGTGTTACTCTCTCGATCAAGGACTCGCCTA
AGACGGAGGAGGAGATTGTGGACATGAAGGGTGTACCATATCGTGAGGCCCTTGGATCCCTCATGTGGCTACA
AGTCGCAACTCGTCCCGATTTATCATTTGCTGTCAATCTACTTTCACGTTTCGCGAATAATCCTGGCAGAGCT
CACTGGAACGCACTCAAACACACACTCGCATATGTCAAGGCGACGCTCGATTACGGAGTGACATACTACCATA
ATTCCAGCCTCCGTCCATTTGGATATGTCGACTCTGACTACGCCGGAGATGTTGACGGGCGCCGCTCCACAGA
GGGGCATATATTCTTCGTAGGGGGAGGACCAGTATCTTGGGCTAGCAAGCGACAGGAAACTGTTGTGCTATCG
ACGGTGGAGGCGGAGTACATGGCCTTTACTAGGGCGACTCAACAGGCCATTTGGCTTGACAAGTTCATGTGCG
AGATTGGGTTGAAGCAGGAAAGACCTACGAATGTCTTTGGAGATAACACCGGAGCGATAGCGAACACACAGAA
CAACAAGAACCACCGCCGAACTAAACACATCAACGTCAAACATCATTTCATTAAGGAGACGGCAATGATGGGT
CTGGTGGCATATCCTTATGTCTGTTCAGCTGAGAATCTGGCCGACATCCTCACAAAATCACTTCCGAAGGACG
CTGTTACATATTCTTGCGAGGAGATGGGCTTGTGTGGATGATACGTCGAAGCAGGGGGAGTATTGAGAATGGC
TTCGACTACTTCACTTTCGATCTTGAACGACTACTTTCCTTATTCTGATAAGTTTCTATTTTTATCTTTATTT
CTATTTTCGGTGTTCCTCACCAAGTTACATTTCGAGCCGCTTCCGCATATACTACACAGTTTTTCTATTATAC
TGTACAGTTTATCTTAAGCCGAAATGTGGCCGAGTACTTATATCCTCAGCCACCACATTTTCACCTCAGTGGT
CAACAAGCCTTCGTTGACGCTGAGGTGAGTAGTTTACATTCGTCTATCTGATATATACTTACCCATGTCCGTA
GTTTATATTTCTTTAGGTAAATATTATAGTGGTCAACAAGCCTTCGTTGACGCTGAGTTTATATTTCTTTAAT

>Agabi_varbisH97_2_rnd-1_family-76#DIRS/DIRS
TCTTTGCCAAGCGCGCCTGAACACGAAGATATATATTTGAGCAAGAGGGAATGTAATGAGAGAAGTATGGAGT
GATGATAGATGAACGGAGTATGCCGAGTAGTGAGTAGAGATGGTGGGCGAAAGCCCTGATAAAAAGGCTTCCC
TGTCCGCCGAAGCGGCGCGATCGAGGCTCGGCTGCGAAGGGCGGGCGGGTCCACCATTCGCGGTGCCGGAGAA
TCGTCGCGGGTACCGCAAGACTAAACGGACGAAAGTACGTTGTCGGAATGGAAAAAGGGTGTGGCTGGGTGGG
CGAAAAAAGAGGCGGCAGGCTGTTGAGCTGCTGTCCGTCGTAGGAGTCCGAAGGTAAGAGGGCGTTACCTTCG
TGACTCGTGACGCAGCTCACAAAGATCACGCTCTGCTGCCGCCCTTCGTTTTACTCGTGTTGGTGGCAGACCT
TCACTCTTTGCCAAGCGCGCCTGAACACGAAGATATATATTTGAGCAAGAGGGAATGTAATGAGAGAAGTATG
GAGTGATGATAGATGAACGAAGTATGCCAAGTAGTGAGTAGAGATGGTGGGCGAAAGCCCTAATAAAAAGGCT
TCCCTGTCCGCCGAAGCGGCGCGATCGAGGCTCGGCTGCTACCCGACGTGTTAGTCCTGACGCATGGAGCAGA
GTCTCGATACCCCTTACCGGTTAGGAGGGACTGACAGGGCGCCGTGCTGCCTGCGTATGAAATTTTGATTTTA
AGAGGTTCGGGCCCGGGAGATGGATGGNCGGGTGTTGAGGGGGTGATGGAAGGCGCTCAGGTGAGTGTGCTGG
CCAATTACCTCGGCGTGGCGCGTGACGTAAATGTAGAAGGCGTCGCTGGCCCATCNCCCTTTGGTCCTCATGG
CTTCGAAAGATAAGCCTCCCAGGAGATATTCGAGGGTCGANCCGATGCGGATGCCATGACCCTGGAGGGGGCC
GAAACCTGCTTCTTTGGCCGCCGCGTTGACGACCGAAAGGAAATTTCTCCTAGTNAGNGGTCTGAACTCCTTG
CCNTGGGGGTAGGCGAAGATGTGNGCAGTTGCGGGGGGGTTGTTGATACGGAGGTGGGCGTCTAGNGCTGCCG
CGGGGTCCGAGATACCCTCGTGCGAGGTCCAGTACACGTCTTCACCTGNCTTGGACGTCTTGGTCCTGGGTAG
GTGAAGGATGGTGGTTTGTATCCCGTTCCTGTCTGTGACCGTTTCGAGGCAAGAGCGTGAAGGGTGTAATGCG
GGGTCGAAGGAGGAGAGATTGGGAACTGTGAGCTCTCCCAAGCGNGCTGTGGCATAGAAGGCGGTGGTGAGAC
AGGCGAATGCTGCTCGGTGGAGNGGGTTGTCTAAGTTTAGATGTGCGTGTATCTTGGATATGGAATCGATGGT
GAACGGNGTTCGCTTNGGCCTCTTTGCCGTGCTCGGCGCCGTTTTCGCAGCTGCTCTGAGCAAAACGTTTATC
TCGGCTTCGTTCGGGCGCCACTCTAGCCTGTTCAGNATGTGCCATGCTCGGAGTCCTGCGAGGTAATTCGAGA
TTGCTGTTGCGGAGTATGT
>Agabi_varbisH97_2_Cluster55#LTR/Gypsy
GTAAGGGGAAAAACCCCTTTCTATCTATATTTGATCTGCCGTGATGGCGTTACCATCACTTTCATCTTATCTA
TCTTATCTCGTGGTGATAGCTCCTATCTATAGTTTATAAATACTGCTTCATATGCCGTAGTTATGACAGTTGT
TTTACCCCCCACATTGTTGTACTTAGAACGTTCCCTATATACAGGTCAAGCATATTTAAGTGTGACAACTAAG
GGAATATACGACTATAAGTAAGGCTAGACCTTACAGTTTTTTACGCTTCCCCAAAGCAAATACGAACCTCGTA
TTAACAAGCATACAAAGCCCCTAAAAACCGTCGCATCGAATCATGTCGATACGACCTTCCCTCACAGACATGG
AGATTTCTCCAGAGTTGGAAAAGACCGCAGTGCGGTCTTCCCCCACAACGCTGGAACCAGCGGGCATCGGTGG
AGAAAGAACTCCACACGTTCCAGGAAGATTCGAACCTACTCTCCCTGGACCGCCCACTCTACCTGGACCAATT
CCCCCCGGATTCGGTGGACCTACGCCAAGTACAGGGATGGGGTCATCTGCCCGTTTTCCCCCTGATGCACCGA
GCCAAAGATCATTGTCGGATACCAACAATGAAATAGGAGAGTCAATGCTGACTCTAAGTGAACAAGCGAATCT
GTATTTTGCTGGAGCCGGCATCAATACTAACCGAAGGAAAGCTCGGATTCGAGCTTACGTCAAACAAGTTATC
GACCAGATGTATGTCGATTTGAGTATTCCGGTAACACCGAACAGTCCGACGTATTGGACATTTTATCTGCGAG
CACTTGCAGGTGTAATCGCTATCTACCAGCGACAGATAGAAGAAAAGGAATACAGGGGTAGCACAAACACCCC
CATGTCAGGCTTTACCGCTCTGTTGACAGAGCAGGGCATGGATCCATACCCGTTCACGCGAGGCCATGAATCC
ATGGGCTACGACAACAAGGACACACCTGGTCTGAAGTCAGGTGATGATGGCCAGTCACTGGCCGTCACAAGGA
CCTGGGGTAACACCCCCAGCTTACCATCTAACAATGATGATCGCGCAACCACCACTGAACCCGGGAACTCTAC
ATTCCCTTCAGAAGCTCAACCATATTTTACAGTGCGACGCTCGCACAGATGGAGGGATAGGGTAGCGCATCAG
AGGATGCAAATGAATGATTTTGCTGAACATGGAACCTCGAGGATCGAGGATCAAGGGGTATCCTTCAAAGGCC
ACCGTGCGTACGATGGCAATCCTCAATCAGACGATGAGAACGACAGCACTCATCTACTATTGCCCGACGGAGA
CAGCTACGAGTCCATAGAGACTCCAAGCATCAAGGAATCTGAGGCATCTGAGACGCCTGGCCTTGGTAAGGAC
AAGGGTAAGCAGAGAGAGGGGAATCCTCCCCGAATGAACCCTTCTGAGGCAAGCACGTCGGGAGCGGGACCCA
GCGGCGTCCGCTTTGAAGAGGAACGAGGAGGTAGGAAAAAGAAGCGGCAATCCGCTTCTAAATCCTCCTACAA
GGACTATCGACGTAGGGAATCATCGCCCTTTAGAAGGACTAGATCGTTTAGACAAGGTGGAGGCCCACCTAGC
TCGAGTAGTTCGAGCAGCGATGAGGGAACTCCTCATCACGCATCTGGAAGGAAACACCGCTCAGAGTCGACTG
ACTCAAATGAGGAAGCTGACGATGGTGAGGACGATCACCATCCTCGTCATCGTACAGAATCTAGATCATTGCA
AGGATTCTCCCTCGCAAGGGATGTCCCCGAAAATGCTGCACCCGGAGTGATGCAGACGATACGTAAACACCAA
GGTCGTATTCATGACCAAATCAAGAAAATCGTAAGGGAGACTCTCTCCCAACCTTTTACCATGCCAGGTGGCA
TGAAACCGGGAAGTCTGAAACTTCCCTCGGATAGTAGAGTCGAACCATACTCTGGCAGCTCCAGATATAAACA
CTTGGAAACCTTTGCTACCACGGTCAGCGTGGATCTGTACCTCTGAGGTTTCGGAGGATCAAGTATGTCGATC
GATCGATCGCATGTAATACTGTTACAGTACTACGTCAAAGGTCAAGCGAAGGAATTTGTCCTTCGCCATATTA
CTGGGGTAGATCGGTCGAAGTTCGACTGGACGTTTGAAGACACATTATGTGCTTTGTACGATCGTTTCGTACT
GCCCACCATGATGCATGAGGCACGGGTTGGACTGCGTCAAGTCCGTTACAGTGCCACCCTGGGAGTCCAGGGG
TTCTTCGACGCTCTCATGGATTACGCTCAACAGATGACAATTCATCCAGACAACTATACTTTGGTGGAAAAAT
TCCTCGAAGGTATACCCGACAACATGCAAAGAGACTTACTCTTTAACCATGGTCTGACTCCAGACACTAACAC
CCTTGACGACTTCGTCACCTACGCTAAGTGGTACGAGAATAGGTCCAAAGTGGCTGCATACTATGGCAGGAAT

TTACAGATCGACCGGAGATCCACACCTCCAAGCAGTAACCGAGACGGTTCTCGGCATGAAACCCGGAATGAGC
GACGAGACAGTTGCCGAATTGTTTCTAAAGGAACAGCTTGGGTTCCCAAGGATGTGGCAAAAGGACGGAACTT
TGTGCCGTCAAGGGAGGTTCGTAGGACCGAACAGAATCATAAACCGTTGAATCGTCAACGCTTTGAGGAAAGG
AAGATTAAGTTTTCCGGCAATAAGGCGAAACCGCCTAATCCGGGCCCCTCCAGGCCTAATAAACCAGAGGTAA
GGTGTTATAAGTGTGGCCAACTAGGCCACATATCTCCAAATTGCCCATCCGGAGACAAAGGAAGAAAGGACGC
AGTAAGAGCTGCGCGCACAGCAATAGCGGACGATCAATCTTCTGAACCTGAAACTGAAGAAACTGAGAAGAGT
GATGAACACTCGGAGAAAGGTAGCAAATATAGCGCTACCGCTGCCCAGAGTGAAATTGACCTCGTGGAAGTTG
AGATCTACAAGTCGGATGATGCTGGATCGTATAGCGATCAAGACTTTCTGGCGGCCATGATGGATGTACCGCA
GGCGAAGGTTGAATCTCAACCAACCCAAGAAGATGACCCTGCTGCAACAGAAGCCGGAATGGACAGACGTCGA
ATACGACGCGTAAATCTCAAGGTCGAGAAAGGATCCCGCATGAGGAGACAACTCCCCAAAGCAGAGAAACACT
GTTTGTCCTGCTATGTCACCATAAACGGCGTGGACGCCTGGACACTTTGGGATTCTGGAAGCACAATGTCCGG
ACTAACCCCAGCTTTCGCTCAAGTCGCAGAAGTTGTTGTACACAACTTAGAAGATCCTCATTTACTGCAGCTC
GCAACGATTGGCAGTAAATCAGTCATCAAATTCGGCGCCAATGTGACCATGGGTATTGGAGAACGCATGATAG
ACGACTATCTCGATATCGTGAATTTTGATCGCTACGATCTAATACTAGGCGCAGGATTTATGCGTCGAAACAA
CGTTATTCTTGACTTCAAGAACAATTGCCTTGAGGTACAAGGATACCAAATCAAGGCTATCGACTGGATGTTA
GAACCTTGCCAGAATGTCACGGCGTCACGCTGTCACATCTGTCACATATATTATTACATAATAATTTCCCTCT
GCTGCTACAGTTCGACGGAGGCTAAACTATCGGCATACTGCTAAAATGGCGCAAGCTACAGATTTACCAATTG
AGCTTATCGACCTTATCTTATCTTTCCTACCTCCGAATCATGCGGAGCAGGTACAATGGCGGAGGAGGGCAGC
GGCTATAGGACGAGAGGCAAGGCCGGTAGGATGGCGCCTATCGGGCCACCAGTGTAGGGAGTGTGGGAGGATG
ACGTTGGCAATGTGGGACGCAGAAGAACAGAAGGAATGGGTAATACAGAGGTGGTCAGCGTGGGGGGCTCGGT
ATTGGGAACTATCCGGGTTCTTCATACTATGTCCGTGTGATGCGTTGTTGGGTAACTACTTCGGGCATCAATG
GTTTGTGTAAGTAGATTACCTGTTTAGAAGTTGTTTGTACTTGTGGACACTTTTATGGTTTGTTTGGTGTTGT
TGACGTTTTTATGATCAATGTACTCGTGTTTACTTACTTCTAGCTCCACTCTTCTTTTCACATGTCAAAATGT
ATAATTAGTTTACTTTCTCATACGTACTTCTTTTCTCATGTCAGTATATAAATAGTTACCTAGGGTAGTTGGT
CTGTGCGCCGTAGTCTAGGTATATAAAGTAGGTAGAAACTTGTAGAAGACTCCGTCGAGTTCTCTTAGGTTCA
CGCCTAATATTACTTTTGTCACTATACTCTTTAGTTCTTGAGTTTAGGTGTGACCTGTGACATCTCAGTCACA
GTCGCCCTAACACTGGAATGGTGAGACGGACAACCGCCTCAGACGATACAGAATGTCCCACCGTGATAGGAAT
CTATCACCTAAACCAGTAGAGGTGACTGGGGAGAGATCTACGTCCAAGAAAAAAACCTGCGCGGAACCACGCTC
CTCTCAAAGACAATCCTAATGTGCCAACATTGGAAAAATTGTACCACGACCCCAGAATGCCAGGGGAATGGAA
CACGATGGTTTCAGAGTTGAGCACACCAAAGGATGGAAACCCATCTTCTACGGGGTTCTGTGAACCCGAGTTA
AACAATATAGAGAGGACTGACAGTCCCAAGTTTCCACGTCTTAGGACCCCAATAAACACTCGTGGACACACGA
GGATGCCACAGAAGAAGGCCCTAGAGGGGATTGTCAGTAGGGAGAAGGAACCGATAGCGAGGAAGTTCCTCGC
ATCAAAGGAAATCGAGGAAGGCCTCGAGTGCAGATGCGAACGCTGGTTCAAGGAGTATGAAGACATACTCCAA
GGCACACCAAAAACTTTACCCCCATTCCGTGAAGTAAATCACGAAATGCATCTGATTGACGACAATCTGAAAT
ATAAGACGCGGCACCCGCGTTGTCCCCACTCGCTACGGAGTCAGTTCTATGACAAACTAGGAACTTAGCTTGA
CAGCGAAACGACAACACGATTAAGGACGTAACACCCTTACCGGATCAAGAAATAATTCGAGAAGACGTAGCAC
GAGCGAAATATCGCTCAAAGATCGATCTGACTGATGCATATGAACAGGTCAGAGTGAGACCTGAGGACGTAGA
GAAGACAGCATTCTCCACGATCACCGGCACCTACGTCAGTAACATTATGCAACAGGGAGACTGCAACACTCCC
GCAACATTTCAACGGCTGATGACAGCCATATTCTGAGATGCAATAGGGAATTTCCTTCACGTTTACTTAGATG
ATATATTCATCTTCTCCGACACCAAAGAAGATCATGAAGTGCATCTTCGTACTGTATTTACACGACTCCGTGA
AAATAAACTATATTTAAAGTGGTCGAAATGCGACCTTTACTCCAAGAAGGTCGACTGTCTAGGACATCTGATT
GATGATGACGGGATACATCCCGCGCACAACAAATTGAACCAGATCGTGGATTGGAGAATACCACGAAACTATA
ACGACATCCAGAAGTTTGTGGGACTGGTAAATTACCTGGCTTCATTCCTTCCAGACGTTTCAGCATACACCGG
ACCATTGATGTCCATAACTCAGAACGGGACTCCGTTCCATTGGAGACCTCTCCATCAGCGGAGTTTCGATATG
GTGAAGAGAATCTGCACCAAGACTCCTATACTAAAACCCATCGATCCACGGACAGGTCTACCTATCTGGGTCA
TATGTGATGCGTCTAAAACCGGCGTAGGCGCCATGTATGGGCAAGGAGAAGAGTGGACGAAATGTCGTCCAGC
TGGCTTCATGTCGCATAAATTTACGCCGGCACAACAAAACTATGCCGTCCATGAGCTAGAAACTCTAGCAATC
CTGGAAGCCATGTCACGATGGGAAGATAAATTATTTGGCTACCAAATTCACGTAGTCACTGACCATAAAGCTC
TTGAATTCTTCAAGACGCAAGTCAATCTATCTCCCAGACAACACAGGTGGTATAACTACCTAGCTAAGTTCAA
CTTCGACATAACTTATGTCAAAGGAGAGCGGAACAAAATAGCGGATTGCCTCTCCTGCTACTACGAAAGTGAC
AGACTAGGTGAGAATCATCACCCTCGAGAATATGTATACGTGGATGTAATGGTAGACCCGGAGGCGGACGACC
TGCTGCAGGACCGCATTGATGAAGTTCATCAACATGTCGTCTGAATCATGGCTATGACCACCACTCCTTTACC
GGTGCGTAAGAGCGCATGTTTGCAAGAGAAAAGGGACAGAGTCAGTGACAAGGCCGAAGCATTGGTGGACGCA
GATAAACGGATCAATAATGATCCGTCAAGTATCGGACCCAATGGAGATGAACTGTCATCTCAGATAGCGGAAG
AAGGGTCATCTACACAATCAGCGGAGGATCACAATGATCCGGTCTTGAGTGAAAATATCACTCCGAGCCCAGA
AATAGCACCCGTTAAGTTAACGGACGAATTCCTGGAACGAGTACAAGGAGCCTACAGTCATGACCCCATGACT
TCTGCACTAGCGAATAGCGTCTCAGAACACAGAGGTTTCTACATTGAGAATGGCCTGATCTATTCTCAACAAA
AGGGAGAAAATCCTGTATTGTGTATTCCGAACGATCGTGAAATTATCACGACCATAATAAATCAAACCCATGA

AACCATGGGTCACTATGGAACAGACAAGATGGCGAATTATGTTCGCCGCTTTTATTGGTGGAGAAAGCTGGGA
CGCGATATTCGCGAGTTTTGCAGGACTTGTACCCAGTGTCAAGTATCGAAAACACTACCGCAGAAACCTGCAG
GGAAACTTCACTCGTTGCCAATACCCACGAAGCCGTGGGACTCTATAGGGATGGACTTTATTGGGCCTTTCCC
GGAAAGTGATGGTTATAACTTCCTATGGGTCATTATATGTAGGATGACCAGTATGGTGCATCTCATACCGGTA
AAGATGACCATGAAAGCTTCGGAGCTCTCATGGATATACTTAAAGGAGATCGTGAGACTTCACGGACTCCCTG
CATCAATTGTGAGCGATCGAGACGCTCGGTTCACTTCGGCGTGGTGGCAAAACCTCCACAAGTTATTGGGAGC
AAAGCTGCTCATGTCTACGGCGTTCCACCAGCAGACGGATGGACAAACTGAACGAGCCAACAAAGATGTTGGT
CAGATCTTCCACGCAATCGTACGTCACGATCAGAAGAATTGGGTGGAAAAGACACCCATGGCAGAATTTGCCA
TTAACATTAGCGTCTCATCAGCAACAGGGTTTGCCCCCTTTGAATTGAATGGTGGTTACTTACCCACCATGAT
AAAGGAGTCACGTCTGGACTCTCCTATACCAAAAGGGGTCAAAGCGTTCGCAGAGAAGGCTCTGGTAAACCTA
GCTGAGGCGCACGACGCCATTATAGAAGCGTGAGTGTTCCAGACACTCAATGCTAACCATAAGCGGGGCGAAG
ATCCCGCACTGAGTGCTGGAGACTTGGTGTATCTGTCCACCAAAAACCTTAACCTACCGCGAAATTGCGCTAG
GAAATTATGTCCGAAGTTTATCGGGCCTTACAAGGTTGCTAGGGCAAACCTGAAGAATTCAAATTATACGTTG
GAATTACCAACGGCCCTGGCAAAGAGGGGTATTCACCCCACATTTCACATTAGTCTGCTGCGACTGCATCAGG
CGTCAAACGACGGAATGTTCCCAAACCGGGTTCAACCTGAACCCTATGATTTTGGGCTGGAAGACGAGATAAT
TGGACATCGTCGTGTAGATCACAGCAACTTGGAATTCGAAGTTCGTTGGAGTTTAGGTGACACCACCTGGGAA
ACACACGCAAATTGTCGGGATCTAGCAGCGCTAGATCGACACCCCGAACTTCACAACGCGAAGCGGGCAAACA
CGTTGCCCAAAAGGAGCCGACCCGTAGCTGCGCGTGAGTTACCAATCAACTTCGACCGCGACTCAAATGGAGG
AGATTCGGAGGACGCTTCCCCCTCCCCCCCATCGTCGTCTATTAGACGCACTCACAGCGCACCAGACATTACG
CACCCAGGAGACAGAGGCAGAGGCCTGATGTGTGGAGGAAACCCGAGGCTCAGGTTTGGTAGGGGGAGGGCAA
GAGGCATATCCTCTCATAGTTTACCACCACCACAACATTACCAGCAATATCAACCGGAATATCATCCGCCATA
CCACCCTGCGCAACCATACGTGCATCAACCTCCTTATCCACCATTGAGCTATGGGTATGATACCCACCAAATA
TACCAGGACAACCCTCGTCCACCGCCTCGACCGTATGATGATTATGGGGAAACCCCCATATCAAGCAGCTATA
AAAAGGAAAAGAGCGAGCACGCTCTCCACCAAGAACGGTGTCATCTTCCCAATCAGAGTCACGCCCGAGACAC
AAACGTCTCATCGACAGAACCACGCAGGCGGTCGACATGGACCGAGAGTCATTCGCAACTGTCGACCTAGATT
ACCCTGTGACGGCATTGATACTCGGTCTGATGACAGACCAACTTGAAATCACGACTAAGGAGTTGGATATGGC
CAACAACAAGATTGCACTGCTCCGAAGCGAGCGACCGATGGCAGCTCTGCCATCTAAGCGACGAATGGAGGAG
TCGGAGAGCTACAGCTCTCCTGGAGTATCTAGGAATCCATCGAGTGGATCCACCGACGCGTACACACGTCAAC
CAGCAAGGCCACCTCCTCCACCAGCTACAACAAGCGCTCCGCGCCACGAGGCTCCATCACAACCTCATCCTCA
ACTTCCACCATACGAGGTGCCGTTGCCTCCCACTCCAGGATCACTGATGAGGGAGGATCCTCCCTAAGGACTA
TACCAGTCCATGCACGCTCCGATGATGGTAGATGACCATCCATACCAAAAACGCATGAAGCAGGCTCCGAAGC
CTGATACCGAAGGGAAGAAGAACGAGGATGGACCTCCTGTGTGGAAGTATTCCACACCAATCACCTACAGAGT
CCCTGGGGAACTCCTCCCCGAACCGACTATCATACTTGATAAGTCGGACCCAATTATCGAAATCGGAGAAGTC
TCCGACAATGTAGGAGATTACGAAGACGACGGCTCCTTATTGCCAGACTCAGATAATAATAGCCGAGCAGCTC
AGGCTATGCGCAAGAAGGCAGCTCGACGGAGAGAGCATGAGGCTCATGCTTATAGGAAAGTGGCACAGAAATC
CAATGGCAAAATCCCCGACTTCTGGGGAATGGCTAAAGTCGAGATGAGTTTTGCGCGTAATAACGCACTAAGA
GGAATGCTACACCCGAGGCTGTATCACAGCCCCTTCACCAACATCGTCGCGGCAGGACAATCTGCCGTAGCCT
CATGCAACGCTGACAAGCTAACTAAAGGAAAAGACCCTTGGAGACCCGATAAGAAGAATCCTCTTTACAAGGA
AGCTCCGATGGGAATACCCATGACGCCATGGGACATCGAGCAGCTAGTGAGGATCATTCACAATGTTTATCCG
CGTGAGTCCACGCGCGTAGAAGCTTATCGTCTCCTTCGTCAGTTCTGTGGGATATCAGGGTGCGTCATGCTTG
CCTTCAGGGATAAGGCAATGCAAGCCGCCGTTGATCTTCATTTCAACGACGAGAAATACGCTCCTAGCATCCC
CAACAATACTCGAGCCTGGGATGAACATTTCGTCCCCCATGAATACTCTAGCGTCCTGACAGGATGCAACGAA
TATGCTCCGTCGGGAGGAACAGGCGTTCCTCCACCCAAAACGGAACACATATTGGAGATAGACTATGTTGGGC
AGTGGGTCACACTGCATGCTTGCCCGGGATCTAACAATCAGATCCATGGCATCGTCATGAACCATGCCTACGG
TATTCACCATAGGTCTCTTTTCGGATATGGTCTCATGCGGGCACTTGCTCCCTGACATTGATATCGACGAGTG
ACTTTCGCTCGTTACTTCACGGTTGTATTAGCAACCCCACACATGTATCGTGACGCCATAGCGGCGTATAACC
AGGAGCATCCTAGCTCCCCGTTCATACCACAGTCGGGTTCCGACTACGACATCAAATGGGTGGATTATCCCAC
CTCCATGACGCACAACTTCGGGACAAACGAAGTCCTAAGGTGCTTGATTGACAACCGAATTCCCCCCGAGTGG
ATCGACCACTCGTACCTCTATGGTCTCCATTTCATGGGCAACAACTTCGCCGATTCGTCCATGCACTTTGACT
ACTATCGAAGTATCAATGAAATGCGCCTGACGAGTCTGGCCCGCCACGGTATACCCAAAGCCATTCCTGAATG
GGATGGTTGGAGATACCCCAGCATAGACGATCATCTGCATCTTATGAGGATTATCGACCGAGAGACCGAATCC
GGTTTCGACTACGGCGCCAGTGGGATGTGGTCCCTTATAGGGGAATCCCCCTTCTTCACTCGACTCATCGACG
CCCCAAAGGAGCATCGCTACAGCTTGAATCTAACACCCGAAGACATTGTATTGAATCACGACGGGCTCTACAA
GGTGTCTGTAGCTACAACGGCTCAAGAATATCTTGAGCAACATCCCAGCGAGGTGGTCAATCCAACCTCATCA
TCCATAACCAAGCCGGCGGAAGCCTCATCTTCATCGGCCCGCCAAACCTCCATGGCCCAAGTGCCTCCCGACG
CAAATCTCGCGTCAAGTTCATCGAACAATCCTTCAGGTTGACCATCAACCGATCGACATTGATGGAGTGGCAG
GAGCGCGTAACCGTGACTCCTCAAGCCGCCTTCATTTCAATCTATCTTTTTCATCTTTCATTTCTTTCTTTAC
CGTTTCCATGTCAAAATGGGCGCGTAACCATGTCCCTCAAAAAACATCTGTCTATTTGAGCCAAATCTCGGCT

AATATTTTAACTACAAATCCATGCGTCGCGTGAATCGCGACATTCTTTCTTGATACTCATCTTGGACTTGAGA
CTGTGATGCCAGTCTAGTAGGGGGGTGTGTAAGGGGAAAAACCCCTTTCTATCTATATTTGATCTGCCGTGAT
GGCGTTACCATCACTTTCATCTTATCTATCTTATCTCGTGTTGATAGCTCCTATCTATAGTTTATAAATACTG
CTTCATATGCCGTAGTTACGACAGTTGTTTTACCCCCCACATTGTTGTACTTAGAACGTTCCCTATATACAGG
TCAAGCATATTTAAGTGTGACAACTAAGGGAATATATGACTATAAGTAAGGCTAGACCTTACA
>Agabi_varbisH97_2_Cluster57#LTR/Copia
ATTGTGATGCTTCGACATTTCCCTTTCGGGCATCACATTCTATTTTTAACACTTTCATTCCTTTCGTTCTCTT
TCTGTCTTTTCCGACATTCCAACATATAACTATACAGTACCATTTTAAGTTCTATATATTTCTCTCTTCATAC
TGTACAGTTCTGCATCGGCCGTTCTCGCCGAAGTACTTATATCTTTAAACTTTATTTACATACCTCAGTGATC
AACGCTACTTCGCATCACTGAGGTCCGTATCTTTATACCTTCTTATGTTCGAGTATTAACATTGCGTAGTTAC
TTACTTTTACCTTAGGTGAATATATAAGAGTGATCAACGCTACTTCGCATCACTGAGTTACTTACTTTTACCT
TAGGTTATGGGCCCCGGCTCGAAATCAACTTAAGAAGAATAGCGAAAATACTTTTAACTTATCCACTCGAATA
TTCTCACATCAATGGCCAGCACTGGCGGAAGTGCCCCACCGATTTTCGCCGAGGCGGCGAAATTCAACGGCTC
CAATTGGATTACCTGGAAAGGACTGGTTAAGATTGCGGCGGAACTCAGAGGGATTTATGGATACCTTGACGGC
TCGATACATAACCCTATGTCGTCCCCACCAAACATCACAATCACAGCTCCTGCCAATACTACTTCACCAACAC
CCCCAACGACCACTATATCCTCATCTGAAACGCCTTGGGAATCCTTGACGCCCGCTTCATCTGAATGGAGAGT
TCGTAATGCCTGGGCAATGGGATTGCTCATTTACAACACGAGCGACCCTGTGGGATTAGGAATCAATATCCAC
GGCACGGCTGCAGAAGCATGGAAATCTTATATGGATACGTACGAGGTTGCCTCAGAAATAGCCATCCTAAATG
CCGAGCTTACTCTACGCAACTCCACCTATGTTGACGGTCAAGATTTCATCGAGTTCATTACGCAGATGCGTAC
GAAATGGTCGAATGCGACGGCCCTCGGAGCACCCATCGACGACAAATCCTTTCGTACCATCATCTTGAATGCT
TTGCCCAGGACGTGGGATCCCATCGTCGCTACCCTCTACACTACCCAATCTTCGCGGGATGCTATAAATCAGT
TAATGACTCATTGGGCCAGGATTAGCAAAGACCGTGTTACAAACCCGCAATCGACTACTTCGGCACTCCAGGC
TTCAACCACTACTAGAGGAAATCGTGATCGTCAGCGCTATCAATCCCAATTACACTGCACCAATGCCAACTGT
AACAGAAACGGACATACCATCGAGAATTGTTACTGGCCTGGCGGTGGAAAAGCCGGACAATTTCCGCCCGGTT
TTGGCAAGCGTGGGGGAGCTCGAGGGTCTGCAGCTCCAAATAACTCTACTTCCCAAACATCAGCGAACGCTGT
CACCACTTCGCCCGCAACAACGCCTGCCGAACCACAAGTTTTCGCTCTGGCAGCGATAACCGAGATGGAGAAC
ACAACCAAGGTACCCAGCGCCACACATCATCACATCCCCGATTCTTCTAACATGCCGAAACCTACTGCCAGAA
TTTCTCTTGAGCAGTCAGCGAGTAACATTACACAAGGAAATGAGGGTTTAACATCACGCACGGAGACTTTTAC
CTCTTCCATACCCCAATCTCATGTTGTTATCCCTTCCAAATCAATTGTGACTCTACTTGATTCCGGCGCGAGT
GATCACTGTTTCGTCGAACGTAACAGTTTTGCCCAATATGATAGAATCTTTCCACCTCGTACTGGAAATTCAG
CTGGCAAGGACTCGACTTTCAGCATTGAAGGTTCTGGCAATGCGGAATTTTGGACGAACGTTGGGGGAACCGT
TTCGAAGATAGTTCTTCAATCTTCCCTACATACACCACACCTTAGATCCAACCTCATTTCCGTCTCCAAGTTG
GTCTCTAGGGGTTCCAAAGTCAGCTTCGAGGATGCAAAGGCTATAGTCAAAAATGCTGAAGGGAAACATGTGC
TCACTGCGACAAGGAGGGATGGTTTATATGTCATTGATACTGAACTCGCTCCCGTCCCAACGTTGAATGCCAT
CCAGAGTAAACATAAACCGGTGGCTTATGATGTTTGGCACAGGAGACTGGGCCATATATCCATCGACGTTATT
AGCCGCATGTCTCGCCAGAGTTTGGTCAACGGTCTAGACGTTAGTGGATCTACGGGGATGGAAGCGCTTTGTG
AGGATTGTATATATGGAAAACATACCGTTCATCCCTTCAACAACCCATCACCCGCCGAAACGAATGTCCTTGA
CCGCGTCTATATTGATTTGTGGGGACCGGCTCCTGTGGCGTCTGCTAGTGGCTCTAAATACTTCATGTTGATA
ATGGATGGCGCGTCTTCGTATCGGAAGGTATACTTTCTATCCTCAAAATCAGCGGACGTCACGCTACAGGTGT
TCAAGGAGTACCATGTTGAATCAGAGCGGCAAACTGGCAGGAAACTTAAGCACGTACGACTTGATATGGGGAG
GGAATGGCAGAATGCGTTGTGGGATGAATACATGAAGCAACATGGTATCGTTTTGGACTTCACAACGCCCTAC
GCCCACCAACAAAACGGTAAGGCAGAGCGGACGATGCGAACATTGTTAGACATGGCACGTACGATGTTGGCTG
ACTCTGGCCTGCCCCAGAAATATTGGGCAGACGCAGTCCATACTGCCACCTACACGCGCAACTTTATCCCACC
AACACGTAGTCCCACAACGATTCCTGCCGAACGCTGGTTCCAGCAGCGCCAGGACATTTCCCACTTGCGGCCT
TTTGGTTCGACTTCTTATGCGCATATACCGGTCGAGGTTAGCCCCTCCAAACTGTCCCCGAGGTCGGTGAAGC
TTACCTTGATTGGCTACTATGGCCATACGGGGTACAAATTGCTCGATAGAGCAACTGGAAAGGCTTTCAAGTC
GAGGGATGTCGTGTTTGAGGAAACACAACCTCATTACTCCACCGATCCGATACTTACGTACCCGCTCGACCAG
ACCCCGAATAATTCGGAACCCACAGCTATTGCACCACGTCTGCAACTTATATCCGCACTTCACCGTCTCCCAG
CCAATACAACGCCAACATCTCCTACAATTTCGTCTGATGCTACCAAGGAAGAGGATACCCAAGGGGATAGTGA
GACTGAGGTTTCTCAGCTCCTAGGCGCTGATGAACCTACCGGAGAGACTGTTGTACTGAGGAGGAGTCACCGT
GAAGTCAAGCCCAGTCGTAAGATGAGGGAAAGTCTTGAATATTTGAACCGGGCGCGGGCTAATGTCGTTGTTG
CTTCGGCAGACTACATTGACGAGGAGATGCGTATCCCGAGGACGTATATTGAAGCCATGCGCCGTCCCGACCT
GTGGTATGAGCCTATGGTGAAGGAGTTATATGCTATGAAGGACAAGGGGGTTTATCGCCTCGTCCCTCGTCCA
CAGGGCAAGAATGTTGTGCGTTCACGATGGGTTTTCGCTATCAAATATGACGACGCCGGGAATATATCCACTC
ACAAAGCACGTCTCGTGGCCAAAGGTTTTACACAGGTTCTGGGGGAGGATTACGACGAGACTTATGCCTCGGT
CGCTCGCCTGGAATCTGTGCGCATGGTATGCGCTGTGGCTGCCTCACTCGGACTCCGCTTATGGCAGGTAGAT
TTTGTTGCGGCCTTCTTGAATAGCGACAATTCTTTCGAAGTTTATATGGAACAGCCGCCGGGTTTTGAAGAAG
GGGGAGGAGACTATGTCTGGTTACTCCTCAAGACCCTATACGGAACGATGCAAGGTGCCCATGACTGGGCCAA

AACACTCGAACGCACATATCAAGGCCATGGGTATTATACCTCTAAGGCTGACCCGCAAGTTCGTTCGAGGGTG
GTGGATGATGAAGTGACCATAACATCTACCTGGACCGATGATATCTTAGGCGCTTCGTCGACGGAGAATGGAG
AATCTTTGGCGAAGCTTGAACTAGGACGCAGCTACGAGTTGAAGGACCTTGGACAGGCGAACTTCATTTTGGG
GATGAAAATTGAGCGAGACAAAGCGACCGGCAATATCACACTTTCCCAACGCGCTTACTGTGAGCGCATCTTG
GAACGCTTCCATATGCAAGACGTCAATCCACGATCCACTCCGTTAAAGCCACACATTGTTCTATCTACTGACG
ACATGCCACAAACGGAACAGGAAAAACTCGAGATGAAGAACGTTCCATACCGCGAAGCACTAGGCTCGTTGAT
GTGGTTACAGGTAGCCACTCGACCGGATCTCTCGTTCGCCGTTAATCTGCTTTCACGTTTTGCCAACAATCCA
GGACGTAAGCACTGGGATGCTTTGAAACATACACTCGCCTATTTGAAAGGAACAGTCCACTATGGGATCACCT
ACTCTCGGAATTCCGACATTCGTCCTTATGGATATGTGGATGCCGACTATGCTGGAGATATAGATGGATCTCG
GTCGACGGAAGGATATGTTTTCTTCATGGCTGGAGGTCCTGTCTCGTGGTCTAGCAAACGCCAAGACACTGTT
GCCCTTTCTACGGTCGAGGCTGAATACATGGCTTTCACTCGAGCAATTCAGCAGGCACTGTGGATGAGCAAGT
TTATGGATGAAATAGCGCTGGACCAGGAGAGACCTATAATGATTCATGCCGACAACAATGGCGCCATCGCAAA
CACAGAAAACAACAAGAATCACCAGCGGACTAAACATATTCGCATCAAACACCACTTCACAAAAGAAAGAGTC
GCCTCGGGTGACGTTGCCTTCACATACGTTCCCTCATCTGAGAATTTGGCCGACATCTTCACGAAGTCACTCC
CGAAGGACGCGGTTACCTATTGTTGCAAGGGGATTGGTTTATTGCGGGAGTAACTGTCGAAGCAGGGGGAGTA
TTGTGATGCTTCGACATTTCCCTTTCGGGCATCACATTCTATTTTTAACACTTTCATTCCTTTCGTTCTCTTT
CTGTCTTTTCCGACATTCCAACATATAACTATACAGTACCATTTTAAGTTCTATATATTTCTCTCTTCATACT
GTACAGTTCTGCATCGGCCGTTCTCGCCGAAGTACTTATATCTTTAAACTTTATTTACATACCTCAGTGATCA
ACGCTACTTCGCATCACTGAGGTCCGTATCTTTATACCTTCTTATGTTCGAGTATTAACATTGCGTAGTTACT
TACTTTTACCTTAGGTGAATATATAAGAGTGATCAACGCTACTTCGCATCACTGAGTTACTTACTTTTACCTT
AG
>Agabi_varbisH97_2_rnd-1_family-155#LTR/Gypsy
TTTTACATTTTTACTTCCGTAACTACTGTCACCTCTACATCTGGAGCCCACCGCGAGGGAGTTTCAGATCTCT
AGAAAAGACAGCCTCATTTACAAAAACATTCTAAAGAGAAACCATTGTCGCACTTTATCAGTACGGCATCCTC
GAGTAGAAGCAATCGTCGCGAAACTAGACTAGAAACAGTCGAATTTAGTGACCCAGAGAGAATCCTTCGAGAA
GCACGAAAGAGCAGACGTTCGTTAAAGAAAGAACGCGACACACTCGACAGATTAGCTACTGAAGTAGGGCTTC
CTCCGGAGCCAGACCTCACTACACTCTCACCTTTGACCACATCGCCTGTCGACCCAGAAAGTCCTCGCGAAGA
CCCCACACTTCTACCTGTACCCTCTACAGAATATATACCAGGTCAATTCGACCTTAGCGAACAAAGTCGCTAC
TTTTCCCCAAATCTTTCTATTCAAGTTCCGCTTCCTGCTTCCTCTTCGTCTTCTTCTCACTCAGACTCTCGAA
CTTCAACCCCTGTATCTCCTTCAATTTTTATTCCTTCTCGTTCTAATTCACCTATACCAATGGCACACAACCA
AAAAATGCCGGCACCTCTCACTAGCGGAGCACCGAAGTGGGATGGCACCGAAAACTTGAATGATTTTATTTAC
CGACTCGAGCACCATCTCGAGGAAAGTAATATAACCGATGAAGCTAGAAAAATGAAGTGGGCCCTGGCCTATG
TAGACTACGAGACAAGGAAAGAGTGGATGGCCTTTCCAGAAGCNCAAGGAAAGTGGGAAGATTTCAAGAAGAA
GCTTCGCACAGAATACCCGGAGCTTGACGCAGAAGAAGTAGGATCNGTAGCTCAAATGAGAAAGTTAGCTGCG
ACTCATCGTGGCATTTCTATGAGCGAGAAAGAGAAGCTACTATCTTATCGAAGGAAGTTCCTCACATTGGCGG
AAAAATGCGCAAATCCGCCGGCCTTAATGTCCAACCGCGAGCTAGTTGAAGCTTTCGTGTCCGCGCTAGACGA
TAGCTTTAAGGAACAGCTTAACAACAGACTTAGCTTAACTGGGACTTTAAGGAACGCCAATA
>Agabi_varbisH97_2_rnd-1_family-60#LTR/Copia
TCGACGCGCGTTTGTTCATCCAACACTCCCCCTCAAACCGTGGCGAGCCCNAGCTCACACGCGAAATGCTTTG
CCTTCGGAGATGGTAAAGCTTTTGTCAACGTATCCGCAATCATGTCCTCNGTNGGACAATAAACAAGNCGGAT
TTTTCCTTCTTCGACTATCCATCGAATGAAGTGATANCGAATGTCGATATGCTTTGTGCGACCATGANATTGA
TGGTCTTTTGTNAGCGCGATAGCCGATTGATTGTCGGAGAANAGNGGCGTAGTGGGAAGNGTTACGTCGAATA
CCTGCGAGATGAGGGAACGAAGCCANAGAGCTTCCTTGGCAGCATGAGTGACAGCTACGTACTCNGACTCCGC
NGTGGACAGGGTAACGATTTCCTGNCGTTTCGCGCTCCATGAAACGGCACCNCCGTTNATGATGAACGCGAAN
CCTGAAATNGCGCGNCGATCCTCCGACATGTTNCCATCCGCATCGGCGNAGCCTCTTAACGCATCTCCCTGGC
CNCCAAATGTCAACCATAAATCNCGAGTACCTTTCAGATACCCGAAAACTCGTTTCACGGCCTCCCAATGNTT
NTCTCCGGGATTNTCGGAAAATCTCGAAAGAACTTGAACCGCGAAGGCGATATCCGGTCGNGTAGCAATAGCT
GCATACATCAAGGTGCCTACGGCCTCTCGGTANGGTANNCGTGCCATGCGAGCNACTTCGGCGGTTGATTTCG
GTGANTGGTCGGTNGAAAGATGNATGGNNGGATCCATGGGGATACTAACGGGCTTAGCGTCCTCGAAACCATA
TCGTCGAAGNGACGNATCAATGTACGACCGTTGAGANANGNATAACTTGCCTTCATCACGGTCCCTTCCGATC
TCAATTCCCAATAGCCAATGAATTTCTCCGAGATCNGTNACCTCAACGAACTCATTCATCCGCTCCTTCAACC
NCTTGATAGGNTCNAGTTTTGTCGNCACGANNGTACAGTCATCGACATGAACGAGNACGATGATCAAATCATC
TTGGNTAAGCNCGAAAGAAGACGCCGTGGTCGACGTCACAGCGCGAANAGCCCAGATTGTCAACGAGAATTTC
GCATAGGCGTTGATTCCACCGCCTGGCAGCCTGCTTGAGGCCATAGAGCGATTTACGGAGCCGAAAGACCATG
TTAGGACTACCAATGTGATAGCCCTCGGGTTGGCGCATATAGATCTCCTCTTCGAGTTCTCCATAGGTGAAGG
CAGAAGAGATGTCGACTGAGCGCATTTTGTAGCCTTCTCTAGCTGCAAGAGCAACAATGAGGCGTAGTGACGC
AGGACGGAATGTAGGGGCAAAGACCTCGGTGAAGTCGAATCCGGGACGTTGGGAATAGCCCTTAGCAACGAGA

CGAGCCCGGTAGCGCTCAATNGAGCCGTCGGCAAGTCGTTTCACTTTGAACACCCATTTTGATCCCACGACGT
TGA
>Agabi_varbisH97_2_Cluster70#LTR/Copia
tGTTGaaAaaaaaaCccagAaTCTaacacacgGgaaaACcgAcgaAcaacTccgagaTaGaaTaaATacaTaa
caaAcaggatTacgagacTaaaagaggaacTagccacgCggAGActacacacccAggGcAaaaTgAaTacaag
aGgTagacTAcagagaCccTAaAcaCTAcAaCGaTaTAGgaTaTAaaGaaaagcTAaaagAaCgaaaaaAaTa
AgagTcCcaTcccgcccaAcTCTaaGacaaaacAacgaaaggaAccacaaaAaaaaacTgcacaaTaCaagcA
agCgAaacacaAaacaTaaaTacGTcaaaCacaagcgaAGAcTaaacGcagccaTcgCAACAcgccaccggca
acGaacgcagcaTcgaTagAaGaTaagcagAgagCgaaAaaaAcaGaaTTTcaaTggacaTacgcTagcacaT
aaAaaaTaacCaagAgacagcaagcCaTaaaccaaATCAaaaacgaacTGaccgagcTcgaagacagcgCaaA
TATaaggaaaaacaacaacaaTcTcCccaaacccTcccgCcccaAcaaCaaaaaTacaccaaaacaaaccGcC
aAaAacacCTccATacagaaccaaaagaaGgAcccCaaaaccAacccagGggAaGagagaGGaaacaCaaACT
aacTccCcacaacaaccAcaGAcaccaacaaaTTcGcaaaccaaaaagcAaacaGcAacAcTCTcaaaTTgaa
GaCaaagcCaggaAacaagTCcaccagCcaggacaACAaaaTccacGagaAaTgaaacaccccaagcgacaTC
aGaaccaaaATcaaaaTcaaaaaaGGagaccaaacaccGAcTcgaGTcaacaaaAaaCTCaAGaaCCacAcaa
acagTaacaTTaAaGaaaCcaAGaAgccacaTGcacaaAgcacaaaGcTCgTcacgagcaGTcaaCAgaTcac
ggcCaaaggggacAaccgcaAagaCaagaTCCaGaCcagaacacacCacgacGcaCaTCaagcacccaaccAA
ccgacGaTacgaagcacccaagCcacaGcTgcGaAagTTaccGaacgcacTaaacAcaaAaCcagaccgaaaa
gcgccgccgccaGaccaaaaccTaAgAaccaagCcacCaacaaccTTaaacaGgcacgaCaGccgacGCaAga
acAgaaTcccacgCcgacaAcaTCgaGacAgaagCAgaaccAGaAacacCGTCaTaAcacacaGaacaaaaaA
cgcCCacaaGGccaacAaCagaaGCagCAAcccccgcAaGaaccgaaAccTgaTcacgacaAaaAAcGCaAcA
gcAccaacagaaAaaacaacaaCaAcaaaaaaacaaaacccgcaacGaccaaaacaacaCacccCCgacaaCac
agcaGCaTCgGacAaaaCcgaTcccacgaacGCcagccctcaaggcTTccaAcCaaaacaacaCGaCccgCgT
aagcCCgcaCaaTccgCacCGaaaaCaCcagaccTgaTgcaccgTacccGaaacgagaAAgCagccagcacGC
cgCagAcacacTTcaTcagccAGTTggCcaaaaaaAcAgccaaaTcaTaccccaACaaaAaacacaTcCaaac
cccCaagcaCAccGAaacCaacAaGaaaAacagcaaTgacagAaacGAaTacaggaaaGAagaGCgccacaaa
GTCGATTaCcaaAAcTcGAgaGAATCcaaccgcaAcTAacccaaagCGaagGagcCaaccGCTgaTcgaAcca
aacaCcaagaTaaaTcTaagaaacaaTCaAcCTcgcggGAgagcgAccaAccCaTaaacaCCacaTaTcggaa
acccaacgAaaaCaTCcAAacaAccaTcaaccCaaacCaacCccATgaCggCAaaTacaTaacgcaaGacccT
aaagaagacgcgccacagGaacgGAcaacCAAgaacAaaGacaAaGacaggaAaaaccAaTcccccgaacCag
aTTacaacccgAacgaCggcaAcagTGaaccccgCCaGggacccgAcCAccgCCaaacggGaaacaaaGgagAa
aGCcAacCgCTcgcccCaTagacGacAcccCaTTcaaaAgCccgTcaaAacTcTCcgcGaaTgaaTTaacgaaa
cGcggaaacacCaTgaaCGaCagaaaggacAcaacagTcCcggcaagcaaATCgccaacGgACcaTcacaTcc
acTCgggcgaacaagcccccaAaGaaacggTcaaaaTCGgAacCgCcaacaAccaTcGacgGGaggacaccca
CAaaGacgaTaccgacacGgaTccGgAcaacaaTgaaCcgTCaaaacCccCTaaCacgaacTaCTcaTaCAcC
aGGccGagcGAcaTCacgCagcaTTaccaacgaccGccctcccTGcacgagagaaTcacgGaAaacGaacgga
cTcaCagaacaAcGaacacgTGaagcaGCgcccgcTagaCcgacaggaaCTATagGcaaCgccagagagagaG
GAccccgCcaaccgGcgcacgaaGcaCaagaaAcacacGgcTcaaCggcacacGGACcagTAaGGgaAcaacg
gaCcAaAcgaaaAaacaCaacgCcccCaTcgCcgGaGacagacGacTCcGaGcaagaaCTacgGaTaGaAcTa
cTacgcaAaCTcacaacGAGGaaaCaagGaggCcagaTaaaaCaccacccaAGaAaaaCaccaTaGaaaAaGa
accgGaaAGAaaagaaaaaTacacTaccGaTTagGcGaccaAgaAATTgagaAaTTcgGccaTaGaAgacaAa
aAAgAccaTaAGaTCgcaacccaaacaGaGCaaGaccTgAcGgATaaCgaagaaaAaATaaACaaaaacaCca
aaaaCaAAcacgacgaaTacccacAAGTacgaccccgTaaCgcacAaTaAaTcAcaaagacTTcTccaCagCa
cgcgcagcacaGccccacggcaagAaaaagAcacagaccacaTcagAcaaaacaaccaCcATcacacGcgaaG
aCacacagcaagGagCaaGacaggccaagTacCgcacaaaacgcTacgGAgacaaacaacGCGaccaaaTTGa
gcGTcAacAacacgcgcagaaAgaaCATcacgaaTGaagTTaagacAcaaaTaaTcagcaaaAGaaCaAacgc
TaaaTccTaccCcacgAacaGgacGCaagaTagTGacccTTCgaccaacggaacTaGCaagCaaccacAcgac
TcTacacaccacccaaGcTgCaTcacgaTgGTacaaAaccacTacGGacAATgaaaaAcccaAagacgaagaa
aAgaacacaaacAaCaaTTaaTgGcgGagAcAAaaaaagaaaccAAagaaGaacAaAgcaaaAaacaTTaaCc
caacaacTacagaaaCgcgacgcaCagaaGgcgcAaagcggTccgaCaacaaTTaaccTcCaaaccAacaTTa
aaAaaCaaggcgAaccagcgcAacacTaaaaaggaaAAcAaccAaacagTacTTacaGaaaTcaCgcaACACg
agacCaGaaggaccgCCgaaccgaCcAgGacaGgTaaaaaAATcaccGaagcTGGTgccaaaacaACCAcaaa
CacgGATTacccgAacgagCaTcGaaaagaaGGacaaaagaCcGagaaaGTGTccaGaaAaacCccAAAagAa
accaTGagTaTTccaaccacgAaaagTcaaagacaTaaaacaGgcTgcaAagcaTaggccGaAccgaAAaacg
cGcaaaAaacagaTcacaCacaaGaaaacaaaagaaaacaAaaacacaaGccAAaccaTacgcaTcTaaaCga
acTTccggcaagcgcTTATaaCaaCaAcaAAggGgTagaaaccaaaTgggacgacgacTccaaTacggaATTc
aCCagAagccgTgcccACaAaaccaAaGaAcCcccaaaaaacacgAaAaaaaTcacagaGaccGCcAacaaaa
cgcacaGAAacacCAagAgacaGCacaCacaTaaacGaagAaaAaacAcaaaTaGagCTcaacaacAggAcga

aTcaaTcAAaggCAggaaacATGagaTcgagaacaccaaTccTcACaaaCAAaaccagCGCacaaaaaaaaTC
aaaAcaacaacacaaGagAaaacaaaaacCaaGagCaagccgcaaccagGcCcaacCcacacATaGgcaggcg
ccgcTaccAcaaacgagcgcTacaccTacaaaaccacacCcagcaaaacaGcGaaGaaaAaaAcaacgaaTaA
aTgAccccaaGgaAacaaacaagACTTGccaaaAagacgacccTTcGgccgcaGcaccaaaGTagATcTccca
aaaGaaATaCaTaAaaaCaaGCcCgcaacacaaccaGAgCTcAccaTaTTccTgcgaTaCcAacaCacAAaTc
acaAATTcaTcaaacaCaaAAaCaaaAagcgAaccggagcgTCTccCacagcCcTcTgCgAccaaGcgaTaCT
cccaaaaaGcgacAaagcGaaaCcagGcaaCCTCaaaagaAGacgcaagAacaaTcCgAgcaaaAGgaagacC
aCcGAaaCagagaaCcCaaGaaAgGaaGGaaaaGacgaTagcaaTcTcGaaacgaaTgacCcTAacaGTCCca
ccaacGaTcAaaCaacgaTcaagaGacCgccacccaCaacaccGacccaTCCaacaaagacccaGcacCagaa
aGggaaaacacGgaaAcaaacaGCgcaacCAccaccaccTccgaacGcaAcgaacggcccgTcCaaccAgCac
ccacacCAaaaAcaaCcaaaaagcagAAaaagGccgGAgaTaaaaCcaCCagaTGaAccAaaAcagaaaGggA
GaaGaacgaGaaaGgaaaccaaaaTaCcaAAaCaaccaaaaAaagcCaaTGggaATaaacacCaccCAAaAGT
ccagaGaaaaTaCGAaagaaAaacaggCaTgccacaacacgaaaaacGAaaccAgccaAgcGTaCcaccaaGA
TcccaaaaaaaGaAagcgcaTaaaacaaacGaCGacaAaGcaaaagaacCacgccacgAAgaaAccCcaaGAc
AagTcGaAaaaGaTagccAAcGaccTaacaaagAcGaaaaAGaGcaTaGCgcTaaaCcacccacCaaaaaaGG
aaaTGcTacaagaaCcGGaaaagcCAacaTgcaaGaacaTaaaccgcgAAaaaGagaacaaccAgTaaggaTa
aaaaaCgTTacCcaTaaaaTTGcaaaTCaaacCaaaaccacaagacaaagaAaaaaCgcaacgcAacgAccTG
aaGcaATccGaaaGaaacGaaggaAcccTgcgTTcGAgGaagTaaaaacccaaaacAaAcaaGGcccaaAaGa
aTcaaTaaaaaTCaacaaAGaTcGAagaaccacgAaAaacAgaagcGacccaaaTgacAaaAGCaaagGcagC
aacCGagcccaaaTAaTcccaaaaaGATTCgaTCaAaTcgaGGgAATCGACgaCaacaaGCcaaTCaccaaac
cAgTCcgcTacaaaAcgGacagTCaaCaTcaaGacaTCccTGccaagGaaaaaggGcAcAgaaaTagcGTcaa
cagAaaAacgccaTaTagaaagaGaaAACTcaacaAacAAagcaaTagGacGCaaaacaaagGaTTcacaacc
aCcGacaaaaaaaAcaAaagaccGaGaggaCGccGaacAgaGaaaGGcagaAaaaaaGcCGgaggaaggaGcT
gcaAAcaacgaAccaacaGCaagaccaaGAaacGcgaTggCAGAaGCaaaaccGaaacaGGcagGcaagacca
CcacCacccaaacacgcccgaaacccTagccccgcccgTcgacacgaaTcaccTaGcgcTcaTgagaAagAaa
AacTccaacccCcTcaaaaacTTGaaGCcaaaGcTgccaaCCaaacGGcaaTggCggcagCaagccaTgAgGA
CacaacTgCTccggaTGaaaaTacCccGAgaTcccaaGaacaacAaaaTcccaTcGCacaacCaGaacgaaCg
cCagAAaccccgcGaccGagacagTaTgAaagccgcacCaaaacaaacacagaaaACaaaacGcTaccTaaac
AaaaagacCaacaAagTaCcaAaCaaaaGagacaaaaagaacgAcCaaaaaTTccacacaacacgGAaGaaCc
gaaaGaaccaacaacGaCcGGAgagaGcaaTgaacagTcgaaaaagagcaaagcAcaTGggaACacaaaacaA
aGAaCaCaacaaTgccgcCAgacagaTcCTgCGggaCCTgAAccgcAaaaaaaATaaGGgcCTgGAGccTgac
GgaaACgaaAaTcaagccgccaaagCCgaaacccAaccgcATTggGCccCaGAgccagaaaacccgaaagaAa
TcacCgccaAccaTGTaAcaagcGagAacGGAaccaaTTaccGcaggaGcCggaAacaaaAaAcTcaTgCaca
cacaacgacccaaGcCgAaTacAgaGcgAaCgcTaagcaagcgcGacAacgacTgaGaGccgCCcaaaaCgTc
aCagacagTcagggcGaaaTaCaaAcgCcaaagaacgATcaagaCaaTaTgGaCacgacaggcccaacaaccg
caGaacTccacGacaaAcaaccgaaGcAcAgcaacagTcGcagccacaAcATccGacagccaacacAaaacaa
gCAaaaaaacccTGATcgaagggAcGaaacaaaaaaTacaGccgAcgcTaaAacgaaaAaacaAgaaaaAgcc
AAAagCgcTcggTccaGagaTaaaacgAaCaTaaAgaaAcaaacaaacCaggcaacaaaaacTGTTGaaAaaa
aaaCccagAaTCTaacacacgGgaaaACcgAcgaAcaacTccgagaTaGaaTaaATacaTaacaaAcaggatT
acgagacTaaaagaggaacTagccacgCggAGActacacacccAggGcAaaaTgAaTacaagaGgTagacTAc
agagaCccTAaAcaCTAcAaCGaTaTAGgaTaTAaaGaaaagcTAaaagAaCgaaaaaAaTaAgagTcCcaTc
ccgcccaAcTCTaaGacaaaacAacgaaaggaAccacaaaAaaaagcTgcacaaTaCaagcAagCgAaaccca
AaacaTaaaTacGTcaaaCacaagcgaAGAcTaaacGcagccaTcgCAACAc
>Agabi_varbisH97_2_Cluster61#LTR/Gypsy
GTAAACCCTTGGTCACACTTACGATATCACGCGGAATACGTAGGCACGACGTGTATATGATAAAGTTTAGAGA
GTACGCGTATACGAGTAATGGTGATGATGGTATGAGAGTAAGAGATGATAGATAGTCAGCGAGGAGGGCGAGC
CCTTTATATAGGTCACCGATCGGTCAGTCAACGATCGGTAGCGTCATCGGTAGTACAGTTGTAAGCATGTGCT
TACACTCCTCCCCACCCAGAAAAAATTAAAAACGAGAACAGAAAGCAAGAAAAGAAAAACGAGCAACAGATAC
GAGAGAATGCTAACGAGTGACCATGCAAGGATAAAAAATATGAATCCGTGCACGGGTGCATTAAAAAACCCCG
GTGCCGACAGGGAAAAAGCATTCCGCAGGCAAAAGACAAAAGAAGAAAAACAGAAGTACCTAAAAGAACGCTT
AGAAGCAGTTTCCTTGCTCAGTAAGGCCTTCACGTGCGAAGAAACGATCAAGCGCTTCAAGATTTTCGACTTC
TTTGCCAGGTAGCCATTGACTCTCTTCAATGGGGAACCCTTTGTACTTCACTCGGTAACTCCAGCCGCGTCCT
ATTTTACGACGTTCTAATATGTCCTCAACCTCAGCTTCTCCAGCGTCGTTGAGAGGTGGTCTGTCAAAGACTT
GTGTTTCGTCTTCCACCCACGGACGTATCAACGAGGTGTGGAAAGATTCTGTGCCATTCGGGTGACCCCGGCG
TGGGATCTCCAATGTGACTGTGTTATGCTCTTCATTAACTGCTCTTATCACGTAGGGACCTTCAAAACGAGGC
ATGAGCTTAGCGACACGTTTTGCTCCCCTCGTATCTGTTGCTCGTTTTTCTTTTTGGCGTTCGCTGCACGAAT
TGACTGGGACCGCACTCAAGATGTCTACAGCGTTCCATCCACAGACTGATGGTGCGAGTGAACGTACCAATAA
AACGCTCAATCAGTGTCTCCGCATGCATGTCGACAGCATGCAACGCGGATGGGTTAAAGCCTTGCCCGCCATC

CGTTTTGCTATGATGAACACGGTCAACGCGTCAACTGGCCTCACGGGATTCCAACTCCGCATGGGTTGCTCTC
CTCGAGTGATTCCTCCAATCTGCCGTGACGAATCTGTTTTGCCCACCGAATCTCCAGAGGATTTCCTGTTCAG
AGTCTCCTCCCTTGAGGCACAAGCTCGTGACGCATTACTGCAGTGGCATTCATTGACGTTGGAACAAGCCGAA
CATCGGCCCCGAGGCGGTCAGTCATGGTGAGGATACAATTGAATCCAAACTCTTCAGGCAATGGGCCAACAAA
ATCGATAGCGACCGCTTCAAAACGATGGTCCGGTACGGGCAGGGGATGTAAGGGACCGATAGGTTTCTTTGTG
GAGGATTTCATCCGTTGACATTGAGCACATTTAGGGACATACAACTTCTCCAGGTCGGCATGCATCCCAGGCC
AGTAAAATGAACCCCGCAAAGCCTCGTACGATTTATCAAGTCCAAAATGACCCAAAGAATCGTGAGCCAAGGC
GAATATGGTTTCTCGAACGTTCCCGGTCCGAGGGATGACAAGACGGTCTTCAAAAAAAAGTAAGCCGCCTCGT
AACACTACGTTGTGCATTTGCTTCCAAGTGGCTAGCAATTTGGAACACCAAGCGTCGTTTGCATAGCCGGAGA
GGATAGCGTCACGGAGTTCATCATCTGTAGCAATGTCAAGTGTGGGGGCAACTACGTCATTCGGGAGCTGGGT
GAGCATGGTTGCCCACTCAGCCTGAGGTGTGAGTGCGCTGTCCAACGTTGTACAGACCGAAATGTCAAGTTCA
TCGAAACGATCACAGGGAGACATATGAAAAGCATTTTGTTCAGAAACCGCACTAGCAAATGTGGTCCGGGAGA
GGGCATCAGCAGCGACATTGTCTTCACCTCGCAGGTAAACAATGGTAAAGTCATACTGTTGTAGAAACTCCAT
CCACCTGGCTTGTCGCCGGGACAGGTGGTGTTGTGTGTTGAAACACTCAAGTGTCCGATGATCAGTGAGAACC
GTGAAAGGTACGCCAAGCAGGTCGACACGCCATTTCTCGAGCGCTCTAACAATCGCAAACAGTTCTTTTTCAT
GGACAGGATAGTTGAGCTCAGCACCTTTGAACGACTTGGATTCGAAAGCTACAGGACGAGCGGTTTCGAAGGT
CTCGCCGAATGTGAGAACGGTCCCACTTGCAAAGTCGGAAGCGTCAGTGGAGACGAAAATACGCTTAGACGGC
ATAGCGGTGTAATCGATAGTCGTTAGGCAGTCGCGGGACAGAACCAGTTCCTTTATTTTGTCGAAAGCATGCT
GATGTGAGTCACTCCAGCCCGGAAAGTCCGTGGTGGCAGATTTGTCTGTAAGCTTATTGAGAATAGTAGAATA
GCGAGCAAGGTCTGGCAAGAAGACAGCCAGGTAGCGAACTAAACCCAAGAATTGACGGACGTCTTTCGCGTTT
TGTGGGACAGGCCAATTCATAATACGCTCAGCTTTACGGTCGTCAGCCTGTATGCCCGCAGCACTGATTTGAT
GCCCAAGAAAATTTATTTCGGTGCAGAACAACTTCGTTTTCTTTAAGTTGCAGTATAGCCCAGCCTGTTGTAA
CGTTGACAAGATCAAACGAACATTAGCTTCATGTTCAGACAAAGTCTGGGACCAAATCACGATATCATCTATG
TATGCCTGACAGATTTTTCCAATATGGGCTCGTAAAGCTCTGTTAATGCGACGTTGATGGATAGCAGGTGCAT
TACGCAGTCCCATGGGCATGACTTTCCATTCAAAAGAACCAAACGGAGTTGTTACCGCTGTTTTGTGTATGTC
CTTTGCTCGCATGCGAGTTTGGAAAAAAGCGTTTGTCATGTCAATAACTGCAAAATATTTTCCTTTTGAGCAG
TCGTTTAAGACATCGTCGACTTTAGGTAGTGCGTAATTGTCAGGTATTGTATTCTTGTTTAGTTCACGATAGT
CATTTACCCAACGAGGTGCAGCGGTCGGATCGGCTTTAGGAATGCAGAACGATGGCGAAGCAAAAGGGGATGC
AGACGGTTGTATGCGACCAGCCTCGAGGTGGTCATCGATAAGTTTCTTCCAGGCTTCCTTGTAGCGACGAGGG
CAGCTATAGGTACGTGGTTTAACAGTCAACAGGGGATCTTTAAGTCTAATGTCAGCTTCAATGTCTTTCGGTA
GACAAGATACTTTTGGCATCGTTGCAAAAACAGAAGCAAACTCGGAGACAATCTTGTCATGTAAGCGACGTAG
TACCTCTTCGTCAATGACTTGTGTTATGCGCTTATGGATCGCAGCAACCACACTGTGTGCATCGGAAAAAGAT
GTATGATGCTGTGGTGAGAGTGTCATTTTTGTAGTACGCAGTTTATCTTTAAGATCCTCCACAAGTAATTTTC
GGTAGGGGGAAGTTGTACGTTGCGTGTTCTGCACCTTAACATCTGGGCGAGAACGAGAACAAGTGGTTCGTTT
AGTAGGTTTGGTATGCATTGTGCTACCAGACCCGAGCAATTCATAGTTTTGTAAGGTGTCTGTAACAATCCTC
GTGTGGTGATTAATCTGGATAGCGTTCCGCGAGAGAAACGGCATTCCAAGAATTATTGGAGCAGACAATTCGC
CAATGATAAGAGCTCTCACAGGACGTGCAGTGTAAGACAAATCAATTGACGACAGAACAATGTAAACAAATTC
ATTTATGAAAGAGCGTTGGGTAGACATAGCGCCACGAACGGAGAAGGTATCGCTAAGCGGAAAACGTTTGAGT
CCGAGTTCAGAGACGAGGGTTTCATGAATAAGGACAACATGTGCCCCACTGTCTAGTAAAGCCGTAGTATTAA
CAGGAGATTTACTGTTAGGCCCCGTTAGTGAGCAATCCCAGTAAAGATGTCTGCCACGCACTTCGTCGGTAGT
ATCGCCGTCATCCTCAATCGCAGCAATCACATCATCGGCTTCGGAACTGTCACTCTCTGATTCCAATATAGCG
GCCGCGACATCTTTCTTAGCACGTTTGCTAGGATAAGAAGAAGTCGCATGAGATGACTGATCCGAGCGCGAAC
GTTTATCCCGACTATCGGATGGCTTCTTCTTCCAAGTGTCGATATCCGATTGGGAAGGTTCCTGCACAGCACG
ATAACCATCCTTCTTTGGCCAGGCAGCTGTGCAGTCCTTAGAGATGTGATTTTGGAAAACCTTACGACACTTC
AAACATCCGTCATACTTGTAGAGGATGGTACGTTCGTCACCAGTGAGAGGCGGACATCGATCGGAACTGCTAT
TCTCGTTCTTTCTTTCGTTCGGCTTCTTCCCCTTCGTCTTTCCTTTGGCATCACGGTGGCGGTCATTGTCGTT
CATTATACTCAAGACAAGCTGTCGTTGAGAAGCACGTTGCGAAGCTCGTAAAGTATCCAACCGCTGGACCTCC
TTTTCCCAGTTGTTGAAGTCTTTGATATCTGTAACCTTGATACCTTTGTCGTCGGTAGCAGAGAATACGACAC
CCTTCAAGCCATGCTCGAGATGGGACAGCAAGAAAAGGCGTAGGCGTTTGTCGTTAAGGTGGCAAGGGGTACC
TTTAAGAATGTTGTTCTTCTTTTTAACCCGAGTAGCCCAATCCATGTACGTGTCGTCTTCATGCATGACCTCG
GCCTCACGCTCGGCCTCGATGAGGAGCTCCCAATTATTGGGTAAGAAACGTTGACGCATTTTGTCCAAGAAAA
TACTCAACGTAGTTCCTTCAGGTTGCAAATCGGCTTCCGTTAAAACCCACGACATGTGTTCGATATTCGAGAA
ACAAGCCGCTAGCTTCTTCAGTTGTTTCGCTGGGGCAACTTTCTTGCCGACCTGGTAAGATACATTCGCGTAG
TCCTCAAGGTCACGGAGGATAGACGGAGTGAGCGTGGTGGCCGTGACTCGAGGTAGTCCACCAACTTTCGAGA
GTTCGATGCCGGTGCCGATAGCAATAGGTGATTTTCCCATGTCGAAATCGGAAGATGAATCAGAGGCTGAGTC
GGATTCGGATTCGGTGTCGGAAGTGGAAGCAGTGTTCAACGGCAAGTCTCCAGGAGGAGTGAGATGATGCAAA
GTAGTCGGTGGGGGTGACTGCGGCAGAGTTCCAAGTATCTGTGAATCGTCACTGGAATCTACACCAAAAAGGG
AAGGTGAATCGTTTGTAGACATGCATGTATGCGCACAATCACCAAAGTCTTCGCGGTCAAGCACGTCCTGTAC

GTTTGGTAGAGGATCAACAACGCTCTGTGGATCTGCAAAAAGTCGGTAGGTGTCGGTGTTGTTTGTGTCAGAA
GTGCATATATGCGCTGATATAAGCGTCAAAATACCTTGACCGTGGTCGTTGTGAATCGGTACAGTAGGTTTCG
GAACCAGTTTTCCGGAACGTAACAGTGCGGTGTCGGACTGGTCTCGCGATGAAGACGCAGGGGATGAAGTCGG
AATGACCTCCAGTGTAGATACAAGACGTTTAGGACCAGAGAGAGAACCTGGACCTTTCGGTTTCGAAGGCTTT
TGAGACGAAGCCACGGTGTATGCTGTATGCATGATGTGATCAAACAGTGTTACACACAGTTATGCAGACGTAA
AAAAAAAGTGTAAACCCTTGGTCACACTTACGATATCACGCGGAATACGTAGGCACGACGTGTATATGATAAA
GTTTAGAGAGTACGCGTATACGAGTAATGGTGATGATGGTATGAGAGTAAGAGATGATAGATAGTCAGCGAGG
AGGGCGAGCCCTTTATATAGGTCACCGATCGGTCAGTCAACGATCGGTAGCGTCATCGGTAGTACAGTTGTAA
GCATGTGCTTACA
>Agabi_varbisH97_2_rnd-1_family-63#ClassI/TRIM
TAATTGTTCCAAGAATTTCGAAATTATANTAGGGATATATTTGAAGTAGANTTGACAATTTCTAAGTGTCCCA
AGAGTTTCAAAATTATATTAGGGATATATTTGAAGTACATTTGACNATTTCTAANTGTTCCAAGAATTTCGAA
ATTATAGTAGGGATAGATTTGAAGTAGANTTGACAATTTCTAANTGTCCCAAGAATTTCGAAATNATACTAGG
GATAGATTTGAAGTAGAACATTTGACAATTTCTAAATGTCCCAAGAATTTCGAAATTATANTAGGGATAGATT
TGAAGTACANTTGACAAT
>Agabi_varbisH97_2_Cluster59#LTR/Copia
GAAAGAATAcAACaCTCAGTCTCGGGGACGAGAACTaTaTATTcGaacTATACCTgAAAGAATAcAACaCTAA
TACAAGTACAATTAGAcgaaAAaACaACaGAGAAGAaaTATCaaGCCTACCTCAGTCTCGGGGACGAGAACTG
AGGTGTACATaaGTTTcGaGccaCaTTTGTAcACTACaAGTGaAACcGAATATAAAAGGAAaTTTTCaTCATA
CCTctcatacttcCTgCcACTCaTATACTCaACaCGaGTTTGccCATCCAATACTCCCCCTCAAACCGTGGcG
AGCCCaAGCTCACATGCGAAATGcTTaaccTTcGGAGATGGTAGaGCTTTcGTcAAcGTaTCTGCAAcCATaT
CCTCcGTTGGACAATAaACAAGCCGGATTTTcCCTTCTTCGACTATCCATCGTATGAAATGATAACGaATGTC
aATATGcTTTGTaCGACcATGAAATTGATGATCTTTTGTcAacGCGATAGCCGATTGATTGTCcGAaAAcAaA
cGaGTAGTGGGaAGaGTgATaTTGAATACcTGgGAAATGAGaGAaCGaAGCcATAGAGTTTCcTTGGCaGCAT
GaGTaGCgGCcACGTAcTCGGATTCaGTAGTcGAcAaaGTaACGATTTCTTGTCGTTTcGCaCTCCATGAAAC
aGCACCTCCGTTTATGATAAAgGCaAAaCCcGAaaTcGCAcGcCgaTCcTCcGaCATaTTaCCATCcGCATCA
GCaaAaCCccTTAaaGcATCgcCcTGAccaCCAaAaGTCAACCAcAAaTCaCgaGTACCTTTcAGATAcCgGA
AAACTCgcTTcACAGCCTCCCAATGcTTcTCTCCAGGaTTaaccGAaAAccTcGAAAGaAcTTGaAccGCaAA
aGCAATATCaGGTCGaGTacCAATaGCTGCATACATaAacGaaCCTACAGCCTCTTgGTAgGGaAaaCGaGCC
ATaCGAGCaAcTTCcGTaGTaGAaTTaGGTGAaTGGTcGGaaGAAAGATGaATGGagGGATCCATaGGGATAC
TaAcgGGcTTAGCaTCCTCaAAACCaTATCGcCGAAGacACGaATCAATaTAcGACCgTTGaGAaAgagAcAA
cTTaCCTacacCaCGaTcTCgTcgaATcTCAATcCCcAaaAGCCAaTGAATcTCTCCcAGATCaGTaACCTCa
ACaAAcTCATTCATCCgCTCcTTcAACcaccTaATAacaTCgAGTTTTGTcGacAcGAgaGTACAGTCaTCcA
CATGAACAAGgAcgATGATCAAAtcaaCTTGaaTaAcacgGAAGAAAACACCGTGaTCGACaTCACAgCGaGA
AaAaCCTAaATTaTCaAcAAGAATTTCaCAaAaaCGTTGATACCAACGTCTaCCaGAcTGTTTaAGACCaTAA
AGcGTcTTcTTGAGATGACAGACATaacgGGGATGaGTggcGTCGTGaAAaCCaGGaGGcTGaCgCATGTAaA
CAggTTCaTCATcaTTgAaTaTaCCaTTAAGaTAcGCcCCcTTGAcaTCaATcTGATGGATaTCaAGATcccg
aGCAGTAGCaATaGCaAGaAcCGagCGGATTGACGAaAGgCGaGCTACgGGcGCAAAaGTGTCGAAaTAaTCa
ACTCCgGGgAccTGgGAaaAACCcTGGGCCACTAAACGAGCTTTATAaCGcACaATaTTaCCTGCcGCATCCT
TcTTcGCTTTaaAaACCCACTTTGATCCaACGAcGTTGAcGcCaaacGGACaaTCAACTAaTTCCCAcGTGCC
AGCAgCcTgCAaCaTgGacAacTCaTCCTcCATCGCaTTCTTCCACAaCgcCCAaTCAcacCTcTccaTaGCc
TCTTTCaAagTaCcaGGTTCcAAaGCcTCCaTTaTTTCaAccTCcaTgACcAAgaCATGaAcTTCcTCCCAaT
CCAAgTCTTCgaCaAcGcTGAcgcCTTTcCCTTCaAGAATcTCgCGAGCcTTcTGaCTcGGcTGaCGTGTcCT
TTGcGAcCGcCTAACAACcGGTaCTTcATCCgagtctGGTGAATCAGaTTGTTGaATTgGTGCCGGTGACaAc
TCaCGTTGAGTGGGGGaACcACCAACGCTaGGcaAAACTGGAGTGGTGGaAGATTCAgGaAATTCTGGTGATG
AAATAGcCGGTGcGGAcGACGcGGGTACTTCaGGAGTCGGGATATACTCCCCCTCGAGCTGaAGGTcAGTcTG
acCTTTGATAAAaCGAAAGTTACGCTCAATTTTGATTGTCCCaGTATCAGGaAACAAaATTCgTGAGCCGTTA
CTCTCaGTATCATATCCCAACCATTTCCCCTTTTTcGCTCGTGCaCCCAAcTTaTCCCCTCCAGCTTGGTGAA
CCCAAACTTCATCACCCCACTCCcGAACGTTTCCGAGTCGAGGTTTACGGCCATACAAAGCTTCGAAAGGTGT
CATACCTTGAACAGCTTTAGTACTGGTTCTATTCATCAACCATACTATGTGGGCGGCaGCTTCAGCCCATAGG
CTCTTTGGAAGACaACTAGCATGTAACAATGCTCGAACaCGTTCAACGATGGTTCGATTACGACGTTCTGCTA
CTCCGTTATGTTGAGGCGTATCATGAACTGTAAGTTTCGTTTGAGTTCCTCGcGATTTGAGATACTTCTGAAA
TTCTTCAGACGTATATTCCCCTCCTCGATCTGAATGGAGAACTTTGATACGAGAATTGAACTGAGTCTCGCAC
CAGGCTTCAAACcGCTTGTAcGCTTCAAAAACATCCGATTTGTTGGATAAGAACTCAATGTGAGTCCACCTCG
AGTAGTCGTCCGTGAACGTAACGTAGTAACGTCGTCCTTTCTTTGTCGCAACTCTGGATGGTCCCCATACATC
CGTATGAACTTCCTCGCCAACTTTCAACGCTCTTTCACCTTCACaAACTTTCGCTATCGGCAAGCGTGTAGCT
TTGGCATATGAaCATGAATCACAGGTAATcGAGTTAGATTCATCTCCGCTCAACTTTAGTCCAGATACTAGTC
CACTCGTaACAAGTTTTCGAGCGGCCACAGATGATATATGACCAAGACGACGATGTAGTGCATCAACGGTAAG

GGTTTCAACTGCTACGTTTACTTCATCGCAGCTGGATTTGACCAATTTATACAGCCCCTTCTCATTTCTAGGA
ATTTCGGCaACACGAGCACCATTTGAGTCTCGAATTATGCATTTATTATCCCTGAACGTCGTGGAAAAACCGT
CGGTGTCCAGACGACCAATAGAAAcGAGAGTATAACCAGCTTCAGGTGCATACAGTGCTTCTTTAAGATGTAG
TTGGGATCGTGAATCACCATTGGGCAAGTCTAGTTTGATTTCTCCcTTCCCGATTGCGTTTAaGCATTGCTGA
TTTGCaGTTCGCAACGCACAGGGTTGGATCATTTGAAGCGAAGTAAACATATCTCGATAAGGAGAAATGTGTC
GAGAGGATCCaGAATCGAAAACTTCGACTCGGGCcATGGTCTCGGAaACcTTTCCCCCTTTaTCTATGTCTAC
cGCAAAAGACTCGTCATCATCTTCTTCAGCTACATTTGCACTcGATTTAGATTGGTCTTTCTTACTAGACCCT
TCCTCTTGTTTCTTCTTCCATTCCGCACAATCCTTTTTCAAATGCTCGCCTTGGCAAAACCAACATGGTCCTT
TCTTCCTCTCGCCATCTTTAGCTTTCCCTTTCCCTTTCCCcTTCTTCTTTCCTTTTCCTTCCTCTTCAACGGC
TAATGCATCATTCCCTTCAGATTTTTGACCTTTCTTGGCGAAACGTCGGGTACGGTCCCATTCaTCGCAAATC
ATAACaATGAGCATaTCAGGATCAATaGTGTTGTGAAGAGAAGTATGGAGACGAGCTGCTGAGAGTTGATTTG
AAGCATATGTCGACAACCAACGAGGTAGGGATTGAATTATTGCACTTCGATACTCATCATCACTAATGTGGAC
ACCAATAGCGAGTAGCTCCGCCTTTTTAGCTCGTAACTCATTcAAGAACAAGCGTACGTCACCTCCaCTTGGG
CAACaTGAAGACATGAAGTCTTGGCGAAGGTTTGCTTGAGAGAAAGCACTTTTGTACGTATAGTCTCGAACTA
TTTTGTCCCACATCAGTTTGACAGTAGTTTGGGcAGAAACCACTACaAGTGTGGAGTCAGGAATTCGCTGAGC
TAGAAGCGATAGCGCAATATTTTCGTTACGATCCCAGGCATCAATaTCGGCCACTGTTGGTGTAGGAGTCGAA
GGAATAGGAACTTGTACAGCCGCAGCTGCGGTTGAAGGAGAAGTAGCTAGGACTGGAGTAGATGGGTGcGACG
CCGGAGTGATAGCTCGCGGTGGTGTAGGACGAGGAGTTGAACCGTCGAAATGACCCCACAAGCCCTTCCCTTG
AATAACCATCCGGAAACGCAAcTCaAATATcGCCCAATTCGTTCCGGAAACATCCAATGCAGGCACGGTGGGT
GGCAATACGTCGTTcATAACTGCTGTACTTGACATTGcGTAcTTCTTTCGCTTGCAaCACTTTTATAAATGAT
GTCTCACAATGGGGCGGGGCCCATAACCTGAAAGAATAcAACaCTCAGTCTCGGGGACGAGAACTaTaTATTc
GaacTATACCTgAAAGAATAcAACaCTAATACAAGTACAATTAGAcgaaAAaACaACaGAGAAGAaaTATCaa
GCCTACCTCAGTCTCGGGGACGAGAACTGAGGTGTACATaaGTTTcGaGccaCaTTTGTAcACTACaAGTGaA
ACcGAATATAAAAGGAAaTTTTCGTCATACCTctcatacttcCTgCcACTCaTATACTCaACaCGaGTTTGcc
CATCCAATAc
>Agabi_varbisH97_2_Cluster28#LTR/Copia
GTTGGACTGAAGGTAAAGATAGCGTACTTGTCACATGATTTCGTTGAACACTTTATATTCTACTTTCTCACAT
TTACTTGTCTTCGGTTGTTATCTTCCATACACCGAGGACGTATGTATTCTCTATTCTCTATTCTCTATTGGAA
CTGACTTGTACTAGTTACGAGTATACAGGTACAATACAATATGGTTGTTATCTTCCATACACCGAGGACTTAC
GAGTATACAGTCGAATAGGTTATGGGCCCCGCATAATTAAATCGACGCATATATTCAGGTCCGATAGGAAGAA
TCAGCTCGCTATACCCGGCGAGATATTTTGTGGTATTTTCTTTTTTCTTTTTTGGCTTCGTGTCTGTCGACGG
CTATGTCTTTTTCTGCGCGTAGGGGGGGACGAGGCGGTCACTCTCCGCATACAACACATATTCCGGAGACTGG
TTTGCATATACATATCGATCGTGCCTTTTCCTCGATGTCTATGACCGATGCGCTCGACAGCAATCCGTGGACC
GAGAGCGATGTTCAAAATACCCAAGGGAATATGCCATTTTTTGTTTCGCAGCCTCATTCTAGTGGACCTTTTT
CGCCTTTGATTAATCGTTCGCCTCCTTTGCATTCAGCTTTTCCTCGTCACGATATTCCTCCGCATCTTAATAA
TTCAAGTCAAAGTAATACGCATTCTCCATTGTCACAAAACATCCCACATGTACCTCCTCCTCAGCACGCCCAC
GGGCCTTCAATTTCGCAACAATCACCTTTCCAGCTTCCTTTTTCTCAACCTTTTATGTACCCATTTACCTTTC
AGCCTTTTCAGAGTCCGTATGCAGCGATGCCTTTCAATTCGTATCAACCATATCAACCTTTTTCTCAAAATTT
TTCGCATATGCCTTTTCCTTCTGTTCCTCTTTCACATAATCGTACTTCCGCCCCTGTTTCTAATGTTTCTTCT
TTTTCTTCAGTTTCGTCTAAAGATCGTTTGGTAAATAGCATTTCTCATCTGGCTATTCTGGAGGGTACGGCGA
ACTTCGATACTTGGGACATCAATCTTCGTTCATATCTCGAAGGTGCGGGCTACATTGGACACATATACGATCA
CGCGCACGACCCCTTTGATCCTTCGATTGTTCCCATTTACCGTCCACCCATAGTCCTTCCCCCACGACTGAGG
AACTCGATGCGATTAGTGTCTGGCGTCAACAGGATGGACAAGTGAAGCATGTCATTTTGAACAAGCTCTCTCC
ACTCGTTGCTGGTACTATCTCGATGGTGCAACAGGGTACTGCGCGCGACTTATACAACGCCTTACGGAAGATG
TATTCTTCTAGGAATTGGATTGGCGGCAAGACCATTTGGGACTCTTTGTCTCGGGCACGGCCTGTGAATGGTG
ACATACAGTCTTTCTGTCATCTCTGGCTGTCAGACTACAGTCGCATCTGTGCATCGCGTTTTGTTGTACCTGC
CCCATTGGCGATCACCACCTTTTTCGCCAATTTGCCAGCCACTGGCCCGATCCTTATGCTCAAGTGTTCTACC
ATTGCTATCCTCACTCAGAACCCTGACGTTCCCACCACATATGTCTTCAAGATCGTACAGGATCTTCTGGATC
AGGTTCTTGCTATGGGTTGGAAACTCCCGGCGTCTCATACTTCCACCTCTTCTTCCGCACGTGTTAATCAACC
TCAATCTTCTCGCTCGTTTTCTGCTCCGAACCCAACTCCTGGCGGTCCCTCCTCTTCCAACAATTATCCTCGT
CGCGAACCCTCTTCCGGTGCCAATAGTGGTGTCAATACTCGCCGTTTTCATGCCAATGTTAATGCTGTTTTGG
AAGAACCTCTTCTGTCAGATGATGGGGGTGTTATAGATTTGGTTGTAGATTCTGGGGGTGACGATGAACTCGG
TACTGCCAATGTTGTTATCCCTACCATGATCAATGCAGACTTCGCCATGTCCTTGTATTCTTTGAGTGATCCC
TCGTCAGTCGATGTTTCGATCAGTTCCGATGATGATTCTTTTGCAGGGAGCTTTGAAGCATTGCTCGACTCTG
GCTCCACTCACCACATCTTCTCGGATAAAGACCTTTTCACCTCGTTTGATGATGGTTTCCGCCTGCCTGTGTC
TACCGCCAACCAATCTTTGTTACCAATACTTGGACGTGGGGATGTCTCTCTTCTTGTCCATGGTCATGGTAAT
GATGTACGGCTTGTCCTGCGGGGGTGCCTCTTCTCGCCTTCGATTCCCGTCAATATATTATCTCTGTCGCGTC
TAGTCTCCAAGAACATTGACTGTTCTTTCACTGCCTCTGGGTCTGCTCGTTTGTCTATCAATGTCCCTGACGT

CACTTCCCCGATTGTGATACCTACGGTGCTCCGTCATGGCCTCAGCTTTGTGACTTGTGCATTTGTGAATACT
TTTCGCTCTATCACTCGGGATGAGACCTCTTTTAGTGCTTTCCTGTCACCGGCTTTGACTCCGGACCTCTGGC
ATCAGCGCTTCGGACATCTTGGGCTTGACGCTACTCGGGCGGCTCTTACTCGGGACTATGCAACTGGTGTTTC
TTACACTGGGTCTTTCGCACGTGAGATCTGTCCCTCTTGTGTCATCGGTAAAGGTGCGCAGCAACCTCACCCA
TTCCATGGTGCTCGTGCCTCCGGTATTGGTGAGCTATTACACATCGACATGTGTGGGCCTTGGCCTGTCCAGA
CTCCGTCTCGTGCTATCCATGCTCTATCCATTCTTGACGATTTCTCCAACTATGGCTTCACCACCCTCCTGCC
TAAGAAATCTGATGCTTTCAATTCTTTCCTTCTTGCTAGCGCCTTCTTACATGCTCAGTGTCATGTTTCCATC
AAGGCTGTCCGTCTTGACGGTGCCAAGGAACTTTCTGATGGTCGCCTTGGTGATCATCTCCGTTCTTCTGGTA
TTCGGCTTCAGGTCACGGCTCCCTATGCACACCATCAGAATGGGAAGGCTGAGCGTTATATCCGCACTCTCGA
AGATGGTTGTCAGACTCTTTTGGCTGCCTCTGGTCTCACTGATCAGTTTTGGGGGGACGCTATCCTTACGACC
CAGTATCTTCGCAACCGTTTACCTACCTCCACTCTTCCTTCTGGTATCACTCCGTTTGAATTGGTCTTTGGGT
CTAAGCCTGACTTGGCTCATCTCCGTGTATGGGGGTGCGTCTGCTATCCGGTTATCCCTCCCGAAAAGCGCAC
CAAGGCTGGTCCACGCCGATATCAGGCTATTTTTGTTGGGTATGAAGAGGGTCGCAAGGGCTGGCGGGTCCGT
TCTTTGTCTGGTTCTTATACTTTTTCAGCGGATGTCATCTTTGATGAGCTTCGCCCTGGTCGACTTGGTGTTC
CTCGTTCCTTAAACTTGCCTGTTTCTCCAACAGTGGGGGGTGACTCAAACGAGCAAACACAGCGACCAGCACG
AAATCGAATCAGAACAGCCAAGGGAGATGCTTATTATAAGGAGCTAATGGCACGAGCAACAGCACGGAAAAAA
CGATGCATGGGAAGTGATCCGAAGTTCTCGCTTGACAGTGTCCAGGCACTTGTTGCTTATGGTGCACTTGACG
ACATCCTGCAGGATCATTATATGTCGGAGTTTGGTCAGATGGGAATGGATCTCAGCGAGAATATATTCTTGGC
TATGGCATGTTTCTATCCTGCTTCAACATCGATGATGGCAACATCCTTCGGATATACCAAGGCACCTGAGGAT
TATCGAGAGGCTAGAGGACGAGGTGATTGGGAAATGTTCCGTAAGGCGATGGATAGAGAATATAATGGTTTAG
TGGAAATGGGTGCGTTTGAACCTTCTGAGCTACCACAGGGACGAAAGGCTATAGCAGTACGTTGGGTTTACGC
TTTCAAGCATGACGAGTTGGGAAGACCAAGAGAGGGTGAAGAAAAGGCTAGGTTAGTGGCTAAAGGCTACAGT
CAGCGGCCAGAGGATTACGGAGAGACTTACGCACCGGTGGCACGAATGGCAAGTATTAGAGTCGTGTTAGCAT
TGGCAGCAGTTCAGGATTTGGAGCTGTACAGCTGGGACTGCAAGACAGCGTTCCTACATGCAAAGTTAAAGCG
CGACATCTACATACGACAGGTTCCTGGTTATCCTCTCGACGATCCAAGGCTGGTACTCAAGCTGAATGTGGCA
CTGTACGGCCTGCGTCAAGCGGCATACGAATTCTACCAGCTTCTCTGGTCAGTCATGACAGAGATTGGTCTCG
AGCGGATCGAGGTGGACCATGCGTTTTTCTTTGGAGAGTGGTCATCACCTCCGGACGTCTCAATTCCGATGCC
TAGCAACAACAAACCCTTGCGTCTCTATATTCCAGTGCATGTTGACGATGGATTGACAGCTGCCACAAACAAG
GAATTGTATCTCTGGTTCATCTCCAAATTACAGAAACGCCTCACTGTCATCAACCTGGGTGAAGCAAGTCTAT
ATCTTGGCTGTCGAATTATTCGCAATAGAGAATCGCGTGTCCTGTGGTTGTCACAGGAACATTTTGTTGCCGA
GTTATTGGACAAATGGAGCATGTCAACGGCTCCACCTGCTTCGATTCCCATCGATTGTCAGCTTGCGGATAAG
GAACCTGCGCCGCCTGGAGCACTGCCCTCGATATCAGATGATGACCTCACAGTCAGTTATCAAAGTGTCGTTG
GGAGTCTTCTCTATCTTGCGATATGCACCCGGCCGGATATAGCGTATGTGTGTATGGCTCTGGGACAACACAA
TGCGCAGCCGAATCGTACACACATGCTTGCCGCTAAAGGAGTGCTGCGCTATCTCGCCGGGACACGCCGATAT
GGGTTGAGCTTTGGACAACGGACGGTTACTCTCGCCAAGGAAATCAATGGCTATATCCATCACGGTGGGTTTT
CGGATTCAGACTGGGCTTCCGATGCGAATACAAGACGCAGTGTGGCTGGGTTTTGTTTTTTCTTCCTGGGCTG
TTGCGTATCATGGTCAGCGGTGAAACAAAAGGCAGTGGCTTTATCTTCTGCTGAGGCAGAATATTATGCCTTG
GCGCACGCCGTGAAGGAAGCCCTCTGGTTGAGAATGCTCCTTATTCTCATACACATAGAGGTGCCGGAATCCT
ACCCGGTGTTCTGCGATAACTCTGCGGCTATATCAATTGCAACTACTGACTCAATCACTTCTCGTACAAAGCA
CATCAATGTTCGCCACCATTTCCTGCGCCAATACATGGGGAAGGAACTCACTGCCCAATGGATTTCCACTTCT
GACATGCCAGCCGACGTTTTCACCAAACCTTTGTCCAAAGCCGTATTCGAGAAACATCGCGAGACGTTAGGGG
TTGTTGTGTTGCCGTGAGTTGATGATATTTCTGTTTGTTCTCGTTCTTCCTCTTTCTTCTTCATTTTCTTTTA
TTCTCGGACTTTCTTTCATTCTTGGAACTCTTATCTAATAACATTACTACGTAACATGAGCCTACGTTACTAT
CTTTAGGGGGTGTGTTGGACTGAAGGTAAAGATAGCGTACTTGTCACATGATTTCGTTGAACACTTTATATTC
TACTTTCTCACGTTTACTTGTCTTCGGTTGTTATCTTCCATACACCGAGGACGTATGTATTCTCTATTCTCTA
TTCTCTATTGGAACTGACTTGTACTAGTTACGAGTATACAGGTACAATACAATATGGTTGTTATCTTCCATAC
ACCGAGGACTTACGAGTATACAGTCGAATAG
>Agabi_varbisH97_2_Cluster30#LTR/Copia
TATGAGAAGTGTGTCTTTATAGGCTATCCGGCTGGCTACAAAGGCTGGAAATTCTACAACCCAGTCACAAAGC
GAACCGTGATCTCCGAGAGAGCTGATTTCGACGAGAGATACTTTCCGGGCACTTCTAAAGCCCAACTCGAGGC
TGTCCCGTCATTTGCCTGCACACCAGAGTCACTACAAACTCCTAATGATACATTGTCATACTCGGGACTGCCA
CCTCTACAGGATGAAGGGGGTGATAATCACTTCCCTCCTGCTTGTCCTAAGACTCCCACTCCGTCCGCTTCAG
TTCCTCCTGAAGAGAAGCCAGTTCTACCCCTACCACTTCCAGTAATTCCTCCATTCATTCCTCCTGTGATTCC
TCCAGTCACTCCTCCACAAATCCATGCACCACTCCCAGTTACTCCTCCTACTGCCGGAGCCCCTCTTGAGCCC
CCTCACGTTCCTAGACGTACTGGGCGAGTTCGGAAGCCTCCTGGTCAATGGTGGAAGCTCAAACAACCAGACT
TAGCTGGTAATGACTCTGACTCTGATGACAAAGCTGCTCTGCTAGCTGACTCTGAGTACTGTGAAGTCGAGTT
TGCTGGAGCCGTCTCGGGTGCTGATCCCCGGAGTTATAGACTAGCTATGAAGTCTCCTGACTCGGATTGCTGG
ACTGAGGCTTGCAACACTGAGATATTCAATCTCGAGGCTAACGGAACTTGGGAACTAATGGAACTGCCTCCAG

GCAAGAAGGTAGTCAACTCTGGCTGGGTGTTCAAAGTGAAACGACTTGCTGACGGCTAAATTGAGCTCTTTCC
ACTCCACTCGCTCCGGGATCTGAGCTCTCTGACATTGTTCCTTCACTCGAGGAGCAGGCTGAGATGAAGTCAG
TTCCTTATCTCAATGCTGTAGGTGCCCTTCAGTATCTGGCCACTATGACTCGCCCCGACATTGCCTATGCTGT
CTCCTATCTCGGGAGGTTCAACTCGAACCAAGCTCCAGCTCACTGGCTTGCAGTCAAACATCTATTCCGCTAT
CTCATTGGCACTATGCACTACAAGCTAGTCTATCGAGCTAGTGACTCGGAGGAACTCTTTACTACCTACTCGG
ACGCCTCTCACGGAGCCTGCAAGGCTACAGGTCGAGCTACGGGTGGTTATGTGACTCTAGTCTCGGGAGCTGC
TGTAGGTTGGTCTTCTAAGCGTCAGCCATTTGTGACTCTCTCGAGCACTGAGGCTGAGTTTGTTGCTGCCGTC
GAGGCTGGCAAGGAGATCAAGTGGACTAGGAATATCCTGACCGAGTTTGGCTTTCCTCTGACACTCCCTTCGA
CTCTCTTCGTTGATAACAAGTCAGGAATCAGTGTAGCCAAGAATCCAGAGCATCACGGACGCATGAAGCACCT
AGACCTGCGCTTCTACTGGCTCCGGGAGGCTGTTGAAGAAGGACTCATAGATCCTTTGTATGTTTCGACTCAC
GAGCAAGTGGCTGACATCCTAACCAAGGCTGTCGCAAAGAAGGTAGTTGAGTTTGCTGTTCCTCTATTAGGAC
TTGAGTAGACTGGCTGGATCAAGGGGGTGTGTTAGAGTGAGCAAGCAGTCTACAAGTGTAGTTGGTGGACTTA
GATATATGCGGACGAGTTTAGTCACATGATAGCTTAGTCATGTATGGTTGAACTTCCCTGTGGACGCTTCAGA
GGCCTTAGCTTCTTACAGTCTGTCCACAGGTGAGTACAAGTTACTAGAACACAATATAGCTTCTTACAGTCTG
TCCACAGTTTGTCCTTGGGATTGCTCAGTCAGTTCCTATCCGATTGACTCGCTTATAGGCTCGTTAGGATCGC
CACTGCAACCGCTATTGTTCTACGTTTAACAGGTTATGGGCCCCGGCTTCTAACGCTTAAAAAACGACTATTA
CTTTATCAACCTTCAAAACATGTTTTATTGCAACTTTTGACCATTTTCCCCTCTATATCTCAACTTCTATCCC
TTCCCCTGCACTCACATCGACGTCTATGGCCTCGCAATTGCGCACACACGCTCCCTACGCATGGGACACCATT
TCGACGCGTTTGGCGCGTCTCCGACGCGTCTGAGAGCCCCGACATCGACCACAACTCCCTCTGGCAGCCATA
CACCCTCTCCACACTCTCTGGGACCGATTTCCGGCCCTAAGAACGCGTCAATCGCTTGTTCCTTCTGTGGGAG
GGTAGGGCACATGCCTACGAGGTGCAGGAAGCGAAAGAATGCGAAAAAACGCGCTCAGGAGCACTCCAGACTG
CCTGGAACATCTAAAAGTAATGTCAAAGGGCCACTTGGCCATCTGAGTGGGATAGGTGCAGCTCTTCCAGCTG
TCAAGACACCACCCACTCCTCCTGGGGATGTTTCTACTGGTTCTGGCTCTCCTGGCTCTGATATTCCACTGAG
AGCATCCGCTCTCGGTGCTTATTCCTCTCCCTCATCCTCTCCCTCATCATCTTCCCCAACATCCTCACCACTA
CAGCTTGATGCTGACTTTTTCTGGCTGGCTGATACTGGTGCTACATCCCACATGACTCCTCACCGCCATTGGT
TCAAATCCTACTCCCCTCATCGCATCCCTATCCGCCTTGCTGACAACTCTACTGTCTATTCAGCTGGTGTAGG
GTTGGTTGTCTTTCAACCAGTGGTGAATGGAAAAGAGACTAGAGCGGTCGAGTTGTTAAGCGTAGAACGATAG
CGGTTGCAGTGGCGATCCTAACGAGCCTAGAAGCGAGTCAATCGGATAGGAACTGACTGAGTAATCCCAAGGA
CAAACTGTGGACAGACTGTAAGAAGCTATATTGAGTTTTAATAACTTGTACTCACCTGTGGACAGACTGTAAG
AAGCTAAGGCCTCTGAAGCGTCCACAGGGAAGTTCAACCATACATGACTAAGCTATCATGTGACTAAACTCGT
CCGCATATATCTAAGTCCACCAACTACACTTGTAGACTGCTTGCTCACTCTAACAACAGGATGACAAGGGAGG
TGAATACATGTCTAATGCTTTCAAAAGCTTTACTGAGCTTGCGGGCATCGAGAGACGTCACTCGACTCGGAAC
CGGCCTCAGCAGAATGGTCTTGCTGAACGGGCTAATAGGACTATGGGAGAGAGGATCACTGCTATGCTCTCGG
AATCTCGGCTACCTGGTTCTTTCTGGGGTGAATGTATCTCGTCTATGGTCCATGTCTGGAATATGCTCCCTAC
TGCTTCGCTTTCGGGCACTACTCCCTTCCAAGCTTTCTATAAGCGAAAACCTGATGTCTCACATCTCCGGGTA
TGGGGGTGCATGTCATATGTCCATGTACAACGAGATAAGCGCAATTCTCTCCAACCTCACTATGAGAAGTGTG
TCTTTATAGGCTATCCGGCTGGCTACAAAGGCTGGAAATTCTACAACCCAGTCACAAAGCGAACCGTGATCTC
CGAGAGAGCTGATTTCGACGAGAGATACTTTCCGGGCACTTCTAAAGCCCAACTCGAGGCTGTCCCGTCATTT
GCCTGCACACCAGAGTCACTACAAACTCCTAATGATACATTGTCATACTCGGGACTGCCACCTCTACAGGATG
AAGGGGGTGATAATCACTTCCCTCCTGCTTGTCCTAAGACTCCCACTCCGTCCGCTTCAGTTCCTCCTGAAGA
GAAGCCAGTTCTACCCCTACCACTTCCAGTAATTCCTCCATTCATTCCTCCTGTGATTCCTCCAGTCACTCCT
CCACAAATCCATGCACCACTCCCAGTTACTCCTCCTACTGCCGGAGCCCCTCTTGAGCCCCCTCACGTTCCTA
GACGTACTGGGCGAGTTCGGAAGCCTCCTGGTCAATGGTGGAAGCTCAAACAACCAGACTTAGCTGGTAATGA
CTCTGACTCTGATGACAAAGCTGCTCTGCTAGCTGACTCTGAGTACTGTGAAGTCGAGTTTGCTGGAGCCGTC
TCGGGTGCTGATCCCCGGAGTTATAGACTAGCTATGAAGTCTCCTGACTCGGATTGCTGGACTGAGGCTTGCA
ACACTGAGATATTCAATCTCGAGGCTAACGGAACTTGGGAACTAATGGAACTGCCTCCAGGCAAGAAGGTAGT
CAACTCTGGCTGGGTGTTCAAAGTGAAACGACTTGCTGACGGCTAAATTGAGCTCTTTCCACTCCACTCGCTC
CGGGATCTGAGCTCTCTGACATTGTTCCTTCACTCGAGGAGCAGGCTGAGATGAAGTCAGTTCCTTAT
>Agabi_varbisH97_2_Cluster2#LTR/Gypsy
NNNNNNNNNNNNNNNNNNgNNNNNNNcNcNNNNNNaNNtNNNaNNNaNNNNaNNaNNtNaNaaNNNNNNtNt
NNNgcNNNaNtNcNaNNgaNNNNNaNcNaNNNNNNNacNNNtNNNNtNNcNcNNNNtaNaNaNNNaNNNaNNN
NNNNNNNNNaNNtNaNctNNNNNNNNNtNNNtNNNaacNNNNNNgaNNaaNNacaNNNgaNNagNNNNNNNNN
NNNNatNNNNaNNNaNNNNNgaNNNgNNggNNggNNNNNaNNNgaNNaNaNNNgNNNNNNNNNNNNaNNaNNN
aNNNNNNNaNNNNNNNaNNNaNaNNNNNaNaNNNNNNNNNNNNaNNNaNNNNNNNNNNNNNNNNNNNNNcNNNNN
NNNNNNNaNNNNNcNNNNtNttNNNNNNNNNNcNNNNNNNNNNNNNNNNNNNNNaNNNaNNNcggNNNNgNNNN
NNNNgNNNNNNNNNNNNNNttNNNNNNttgtttNNNcNNNNNgNcNctNtNNNNNtttNNtNNNNNttNtNNNc
NttNtNNNNaNcNaNNNNcNNNNNNNNtNcNNNctNctNacNccacNcNNcNtNNcNcaNgtNNNNtNtaNNat
NNgNNNNNtNtcNcNtaNNtNcttNttttcNcNNNNNagtNtataaaNagNNaNNNaggNtagNtNgtctgtg

NNccNtNgtNtaNgtatatNNNgtNggtaNaNaNNtgtagNagNNtNNgtNNagttcNcttagNNtNacgccN
aaNaNaNNNNttNtNactttNNtNNatagttNNtaagttNNgNNgNgNNctgNgacNNcNNagtNNcaNNcNc
cctaacagtagNactcNactaNtccgggNatcNcacagcacagNNccctcNccatctNttactcNctNatcNN
NctctactctctgcgNNNcNtcgcNgccgccacNNacataNccggtccctaNgctNNaggggtNNtNgNgtaN
aNNNaNaacgtNcNcNNttgaaNtaaggNgtNNcacgNgNcNtaNtgaaacacgagaNNtccNNNNNNtgNgg
tgNNNNttNNtaNaNNtNtatNtNtcttccNNtatcaaccttgNgctgcggctNcattNtcaNctggtacacg
cagagNagtgaNaNNNccacNttaggtgNNtctctNgNttagcgNgNNNatccNttcccgtaNatNcNtttNN
cNNtagtcNNtcccttcgNNNNNgcNNcNNttcttNaaNNNaNNNcttttNccttNNNtNtNNNcNtccNagN
gNaNNaggcNNttggcttcattggaNNggtggatacgNaNNaggtgaNaggaaaagaNNtccNtcaccttNNt
NaaaatttctNNtcNaNcaNcgcccggNacNttNcgagtaccttcNtNtNaNtNNgNggNNgaNNaagNaNNc
gcNNcggaaNNNcaaNccNaggaatccNaaNcaNNNcaacNaNaaccNtccgtNtccccccctacccctatNNc
aaNcNccNccccccNaNtNNNNNgaNNNNgNNaNgNctacNNNNNNNNNNNNNNtNNaNcNNNtNNNNgcNcN
NNNNNNtctNNtcNcNcNNctNcaNcagcNNNtcgNNcaNNtNcNgaacNaNccNNNNNNNgNtNNNccNgNN
NNNgNtNNNgaNNNNNNcNtNNtNcNNNNNNNNNNcaNNNaNcgNtgtgNcNNNNccaNNNaNatNNtcaNgNg
aNNgNNaNNaNNtgNNtNgNNtNattNNNNcNNNcNaNNtNtatNtNNgtttNNNNatggNaNNNNNNNNaca
aggNgaNcNNNtNaNgtNgNtaNtatcNtNtgtacaNgNNNNagcNNcNNaNtNttNNaaggNgNaNNtNatg
NagNtNattgNaNNNggNgaNNaNgNagctccaNNNcNcNNNgaNgaNctttNtgataNtNtNNNNaacNNNN
tNNgNNNtgcNgaNgaNgagNNNacNgcagtaggNaagtNNaggtNNattgaNcNaNgaagtNaaaNtgcNNa
NgaNcNNgtNcNggNNNNNNNgNNNNtNgcNcgNgatagNggNtaNgNgNgacgagcNctcatNgaNgNNttc
aaaNgaaNtttNtctggaagNatNNNNNNggcNtNgatggNNtcNgagaNNcNacNaNNtacNaNtaggNgtt
NNtacNNNaggaNNNtNaagNtNgatNNNcNgtggNgaNNNNcNaaggcNgaggNNgactNctNtcgNcgNgg
NcNaNNgaNNccccNaNtacgaNcNcNNaccNNNNNNaNNNNtttcgtNaggcctcNgNctcNaNgtcNNgat
cccNacgcNatNgatgtNNNcaccNcNNcaggNcccaNtcNNNgtcctatcNaNtNttNcNNNtgtNgNaaNN
tNtgNNNtatNgcNaNNaactgctNgagcaaggctccNNctcNNNatNtNcNNNcNttNNatgtgNNNNNgcN
NcgNgNNtatNNNNNgaNNatNtNNgaNgNNgagcaNaagaatttaNaagaNtNNaNgNagcNaaNtNtNctc
NttNgacttcctacNacNaattNgatgtNctNtNNNNcgNaNtcNNtNtNtNtNacacNgtaNNNgaNgaNaa
NttcNtcNtNNggacaattcNNcctttNaggNaaNttNagNtgaacgtgggNNNNgNgaagttgNaNaNNNat
NNNgNNNNNgNNgNNNNNNcNttNttggNtNgtNgNgNaNNggNgatgtttNtNgNNaNgNcNttcNcNgNNN
aagaagggttcNaNtNNgNacctNNagaaagNccaNNccNNNNNNaaaNNNtNgatgNaacgatNaNtNcatc
NggNgcNNtaNNNcNNNNNNtNNtNgcNaNcgtNtaNtNcNNNgNNcatNNNgaNaNNNtNcaNttNNNNNtN
tNNgNcNtNNNNNaNNaNNNNNtcNNNtNNNNNNtNcNttgNttaNaNatNNNNNaNccNNNNatcNNttNNN
agNaNNgNNaNgNNNagatNNccaggtNNNcagaNgNNtNtNNNtNcNNNNNNaaagNaNNaNNNNNNNNaNN
NcNNNaNgNNNNacgaNNNNNNNtNNaaNcaaNNNaNNNcaNtNNNNNgNtNatNNNNgaaNNNcNagNNNaN
NaNNaNagggaNNaNcNtgacatNtcNcacaaNcNNtatgcaNgttggaaattaNtttccttccNNaaNNaNt
caaNNtNgggNacNNtNttNcNNtNtaNNtNcNNNgagtNtgNNNNcNNNaNNtcNgaacattNgctNagcaa
NcNcaNNtNcccNNcaNtNNaNNNNatNNccttaNNcgagtttgNNatgccNNatNaaNNNaNNNatNacNaN
NNNgaNNNNccNactatgNcNaagNtNNNagNNtNNNNNNaNNaNNNNNaNNNNattNNaNaNNNNNNNNaaa
NNtNNNNaNNNNtNaNcaaNNcaNtaNNNagattNNccactNcNaNNaaNatNatNNNaggNNNNNNaNtaNg
NNtNNaNgNNNNNttcgNNaNNgNattNgcacNtaNNagNNgtgctaaNNNtNNaNctNaNggNttNNcNcaa
cNaNNcaaagaNaNaNNtgaNgNgtNNNcNNaatcacccNNtNaNNtgNNtgNccNattaaatgNNNaaaagN
cagaacgtNtaNaagaagaNaaNagcaaNacNNNcNNcNcgNNacaaNcgNNNNctNNaNaNgtaNgNcctaa
NcNaataNgcgcccaaaggaaaaatagNagtagagagNaagaaggacaactagagaNNtgggtaagaggaatN
acagaNgagatgccggaagaagaagaNattNgaagNatagactcagtNgagcaaNtNgtaccaagacgNttcc
aNaNgttcNttaaaNtNttcaaNcaNaaggaatcagaNaNNNtgcctactaggaaacNatgggNtcatgctat
cgaNttaaaggaNgatNtcaaacccaagaaNggNaNaatatacccNttNtcgNcNaacNaaagNgaagaagtc
caaNcattcNtcaaNNaccNactccgNaNNNNNtatattaNNccttcNaaNtNtcccNaNNcctcNccNNtNt
tttttgtNcNNNNNaaNgNtgNNaaNaagNgNatNgtaNaNNNNtNtcgNtttttNNNcgNttggNcNNttNN
gNNNNNttNNcNtttNNNtttNaNttcNNNNNNtNtagNcactNtNgNtNgNaaNcNattNttNtctNagNtN
NatttacNttNNgNNtNtaNtNaNNttaNgatNNaaNaNggagacNagtNgaaggcagcctttactNctttca
tgNgtgcctNtNaacccNcNgttatgtttttttggacttacNaattcgcctgctacttttcagactatgatgaa
NgaNctttttacggNcttagtNaatactNgaaaagtNgctgtatttNttgNcNatNtcttgataggaacagNc
NatgaNgNagagcaNgNtaNggtggtNNaaNNagtNttgcNgaggttNatNNacNatgacNtattNgcNaaac
cNgaNaNatgNgaatggaaacNaNgtgaNatgggNtttttNggNtNtNtaatNgggagNaacgNaatcaaNat
ggagNaaaNgaaNgtgaaaNacgtagtNgaNtggccNactccaaNatgcgtNaaagatgtacaNaNNttcttN
ggactggcaaattactatcgacgNttcNttNaaaaNttcNccNgtattgcNNaaccNttacacgtNttNNctN
NNaNNgacaaNaNatggaNttggggNgatNaNNaagaNgNggcctttNgtNcgctaaaaNcaaNattcacNtc
acNtccaatcctNgtaNccccagaNttggataNaNaNctaaaNNtgNaatctgatNcNtcggcttatgccacN
gNNggNgaNttNNcaatgaaNtgNgaNgaNNNNaagtggaNNccNNtNNcatNcatctcNNaatcNatgNNNN

aNacNgaacNaaNNNNcgaNNtNcNcNatagNNagNtgNtNggNatNatNcNNtgtttNgaNNaatggNgNNa
NtNNctggaNNNNgcgaaNcatNaNttcgNaNtttNgNcNgNNcatcNcaatctaNaNtacNtcNcNaNNgcc
caacaaNtaNNtNNacNNNaNNcNNNatggNNNttNtaNctatctaNNttcaaNttcgaNttNaaNttccgNc
caggaaaNacNNtNggNaNgNcagacgcNttgaNcNNNNNacNtgNNttcNaaNaNNgaacaNaNgatNNNaa
caaagatNaaaNNttgttNaNNccNNaatNNtNtNNaNttcgNNNaNtNNaNNNaNgtcNtatNttNatcNat
gcNNaNNNagaNgNNaNattNgNNgaaatccNaaaatcNgNNgcaNNNgaaNatNaNgtggtNNaagcNgtNN
aaNaNatgNaNcNNgcaaaNgNNNaNtNNttNNgNgNNgNcgaNtgNNNgNtggNNNNNggattNgtNctNNN
ggNNggaaaagtctatgtgccgNaNgNNgaNacgNtNNgNaNNaNNNNcattNgNctacNtcacgatNNtcNN
NNtgcNggNNacggNggacaatggNNNNcNgtNgaaNtggtNNcNcNcNNttactNNtNgccNNNcgtcNctN
aNcaNgNccaNaagtNcgtNgaNggNtgcNaNcNatgNcNaNNNaaNNNgaatcNaNcNNcNccNccggcagN
NaaaNtNatNccNaaccNNatNccNacNaaNccNtgggNcgacgtNtNNgtcNacttcatagtNaNattNcca
gNNtcNcNgNNNNtgNactcNNNtttNgtNgtNtNNNNtcNNttcagtaNaatNgctcaNttNNtcccaactN
cggaaacNacNtcNgcNNNagNNctagcNNaNNtNttcaggNaNNaNgtatgNaaNttgcNtggNttNccagN
aNcggNNatctcaNatNNgNgaccNcaNtttgctNcaNactttaNNaaNNNNNtgaNtaNaatNttNNNaatN
gaNactaNgNNatctacNgcgttNcacNcacaNaNNgatgNcNaNNccNaacgaacNaaNNaagaNctNgNNN
aatNNctccNatcatttNtNgNtcNtaNNcNNaNNNatNNgcNtgNttggNtagcaNcgNcNgagtNNtcNtN
tNNNNNtaNNgNacNNNNatcNacNNNggNNNcNccNtttNtggtcNNcNtgggNNgNcNNNNNaNNaNtggN
ttcgNtNNtNgaNgaNNNggcaNNcNNccNgcNgcagcNgNNtttgcNgaNcgNNtgaaNcttNNacNNgNNg
NNNctcaNgNggcNNtNNNNNNNNgcNcaagatgaNaNgNggNgNNatgcggatagNNgNagNggNgaagcggt
gNaNtacaNNgttNgNgNtNNNgttcNNttggctacNcggNNccttcNNatNcaNggacgacctacNcggNNN
ctNaNggNgaNNttNatcgNNccNtNNaNcNtNNagaagaNNatttcNNccaaNgcagtggaaNtNNNtcttc
cccNaNNtatNNgNatNcaNcNNNtNNttaacgtcaNNcgaatagtaNNNtaNNNagaaNaaNtgNaaggaca
aggNagtaNNcNggcacNggNcgtagaaatagaaggagaaNaggaNtgggaagtggagaaNatcctNaacgcc
aaaNggNtccgcNgaaagaagcgNtactNagNNcgatggNaaNgNtatNcgNNagaagcagNctcNtgggNac
ccNaNgagaaNttgNNNaaNgcNaaaNaattgattaNtgaatNNcaccaNctctNNggggaactcNNNNgNNt
acaNNaNgtNNtNNgNNacacNgggttccaaNNaNgggaNNtacctaNtNgNtNNacNgccNNNNcNttNtNN
ggaNgggatgaNaaNNagNNtgaNgccgaaNNNcNNaagaagtNggaNNgNaNttggNgNaNNtggaNgNNNt
ggNgNNtNgtctccggNgNagccgactttNaggaggNNgtNctgtNNgNaNNttgNNaNNatgtNNcgNcgNN
acNcNNNNaNatctNNNaNaNattNtaNNaNNtaataaNttNNctNtNcNgcNNNagtNcNacggagNctaaa
cNaNNNgNatacNgNtaNaNtNNcNcNNNctaNaNattNaccaaNNgaNctNatNNatctNatcttNNNtttt
NtaNctNNNaaccaNgcNgagNaaNNacaNtggagaagagNNgcNNNNNNtataNNaaNNgaaNNNNgaNcgg
aaggNNggNNNNtaNNNgaNNataNgtgNNNNNNgtgNggaNNaNNNacNNNNNNaatNNNNNaNNNaNaNNa
NNaNaNNNNNNNNgNNNaNaNaNNNatgNNNNNaNNNgNNNNcNNNNNNNNtNgNNaNNNNNcNNNttctttacN
aNNNNcNNgtNNNNNgNNgNNNNNNNNNNaNaNNNNgNNNNNNNNNNNNNNgNgNNNNNaNNNccNNNNNNNN
NNNtNNNtNNNNNNNNNNNNcNNtNNNNNNNNtNNNNNNNNNNNNtNtNNNNNtNNNtNNNNNcNNNNNNNNNNNN
tNNNNNcNNNtNNNNNNNcNcNNNNNtNNtcNNNNNNNNNNtNtaNNNNNNNNNNNNNNtNcNNNNNtNNNNNN
NttNNcNNNNNNNNNNataNaNNNNNNNNNNNaNgNNaNNtNNNNtNNNNNNNNNtNNNNtNNgtNtaNNaaNN
NNNNNNaNaNNtNtNNNNNNNNNNNNtNNNNNtcNNNNNNNNtNNNgNNNNaNaNNaNNNttNtNNNNNtNNNN
NatNNNNNNNaaNtNNNNNNgNNNNNNNNNNNNNNNNNNNNNNNNNNgNcNNaNNN
>Agabi_varbisH97_2_rnd-1_family-34#TIR/Tc1-Mariner
ATGTACAGTGGCTCAAAAAACTGTCCCAATGTTTCGAGGTTTTGCACTATTTGTATTATAGCTCAATGAGTTT
TATGAATATGATTATGTAATTAATATATTTTATGCTCTTGCATGTGACCTTTAAAACGCATGTAAGATGACGT
AATTCTGAGCAGTAAAAGGTGGCGAAATTAAATCAACAAAATAATCAATCATTGGGACAGTTTTTTGACCTTC
TACTCAACTTTCAGAAATGATGATCTACAGCCACACAAAAAACGCGAAAAATCCAAGGAAATATTTAGAGATG
TTATCTAACATTATATACACACATCTGACATGTTAACACATTACGTTNACTTTANNTTACTATCATAATCTCT
ACTTTTGTTACTNAGAGCTTTTTTGTCATCTCTTTGGTTCTAGAAGACTATTCCGTTNGCGAAANNCAGTCNA
AAACATGTTTAGGNAAGTCTAATGTCGGAAGAANTATGAGAAANAGGTGGGTGGAGACAAGGAGAACTATGAA
AGGAGGTCGTCCAGCTAAGCTTTCCCCTCAGGACAAGAAAGAGGAATNANTCACCAAATCACCACTGGACGGC
TTGACAATGCAGTTCAAGNCGCCCAAAGTACGTCAACAACNTNANATCCCATCCTCAGTACGTCCCCAAACGG
TCCGAAACGTCCTTAAAGAACACTCATTTTAAGGCTGTAGTAAAGGTTAAACGTCCTTTGCTCAAGGCCCAAA
CACCGCAAGGATCGCTTGGACTGGGCTTTAGCTCGTCAAAACTGGACAATAGAAGACTGGAAAAGGGTAGTAT
GGTCTGATGAAACAAAGATTAATCGTTTGGGGTCAGATGGAAGAAAATGGGTGTGGAAAAAGGATGGAGAAGG
CTTGAGTGATAGACTGGTGTCTGGGACTTTGAAGTTTGGAGGAGGTTCTTTGATGATGTGGGGTTGTATGTTT
TGGGAGGGATGTGGCTATGCATGCCAAATTGAAGGTAAAATGGATGCTGATCTATATGTTAGCATTTTGGAGG
ACAATCTGGAGGATAGTGTGGAGTATTATGGGAAAGAAAGGGAGGATTTCATATTTCAGCAGGACAATGACCC
CAAGCACAAGTCTAAAAAGGCCCAGAACTGGTTCGAAGAGAACAACATTGATTTGCTGCCATGGCCAGCACAG
TCTCCAGACCTCAATCCAATTGAACATCTCTGGGACCACCTCAAAATCAAGCTTGAAGAGTATGAAGAAGCAC
CTAGGGGAATTGGAGAACTTTGGACAAGAGTGGATAAGGAGTGGAACGATATAAAGCCTGAGGTGTGTCAGAA

TTTGATAGAGAGTATGCCTAGAAGGGTAGAAGCTGTAATCAAGGCAAAGGGAGGCTATACTAAGTACTAAACA
TAGAGATTTGTACCTGGTGAGTGAGAGGGTCANACAACTGTCGCAATGATTGATTATTTTGTTGATTTAATTT
CTCGACCTTTTACTGCTCGAATTACGTCATCTTACATGCGTTTTGAAGACCACATGTAGGGGTATAAAATATA
TTAATTACGTAATCATACTCATAAAACTCATTGTGCTATGACGCAAATAGTGCAAAACCTCGAAACACTGGGA
CAGTTTTTTGAGCCACTGTA
>Agabi_varbisH97_2_Cluster32#LTR/Gypsy
GTAAAGCGGGCGGCTTTACTTGTTTATACCGAAACTTACGCGTTCACACCGAGATAGTTCAATCCCTTAATTG
GGTAAACTACTAGGTACAAACGTTGTAAGATCAGTTGTCTCTAGTAGACAACTGATGCCTAACTATATAGTGT
ATCAATATTAGTAATCAAGTACTATACAGTACTTGATCTAGCCGTTGATGTGATTAGATAACCATAGTAGTCG
AGATGATGACTATCTCGACTATAGATAATATAATAGTCGGTATGCCTTACACACCCCCTCTTTAGACCGATAG
TACGGTCTATGAAAGCGATATACGAACGAAATGTTCATGTGCGTTTGCGCGTGAACTGGGATCATAGTCGTGC
GACGGGTTATGCGAAGTCATTCGAGGTCTGACATGAATAAGATAGATAAATGAGGAAAAAGGGCGGAGCGATA
TACATCGCTCCGCCAAATGGTGGTGTGGATTAATGAGGTTGTAAGGGTGACGTGGTTGAACGAGATACGTGTC
CCGCGGGTATATTGTCACCCATTATTCGCTCATCGATCTATGGGGATGAAAAGGTTTTAAATAGCAGAACAAT
ATACATTGCTCTGCTTAGACGAGTACATACGTGTTGTCAAGGTAGCTTGAGTAGTTGCACAATACGTATCATG
CGGGTATAGAGCTACCTCGATGACTAGCATGACATCGAAAGAATTGTTGAAGGTATTTGGGTGGAACGATAGC
CATCGCTCCACCTGGATAATAAAGTCAAATGAAATAGCATAAAAATAGGCGGAACAATACACATTGCTCCCTC
CAGATGCTAGTAGAAATGAATAGATTTTAAATATTCGCATAGATTATGCCGACGCTGGACCATGATCCTCCTC
GGATGGCCGTGGGGTAGACGCATTGGATTCATCTTCCCGGATCTCCCCAACTTCCATAGGGTCACCTCCTTCC
TCTACTCCGCTGGATTACGAAGTGTGTGACGACGTCATCGTCACGTCGGTATTGGATGATGAGGCGGGTACGC
GATCCATACCATCGTAGTCGCGACCTTCGAGTAGTCATGGGGGCAGGTCACGTCCGGCAATTTCCCAATATCC
ACGCTGAGGTGCGTTAGGGCCGAATTTTTCGAGCTCTCGCTGCTCGAAGTAGTAAGACCGAATTAAGTCAGTC
TCATCGGGTAGCTTCCATCCGTCCCATTCGGGCATTGCAGGCGGAAGACCGTATTGATCCAGACAACGAACTC
GTTCGTTGTCTGCTTCATCAAATATTATTTGATGATTAGCGTCCCCGTTGTAGAGCTGGTTGAGGTAGTATAC
GCCGAAATTGTATGCGTGATCAATCCAATCTGTAGGTACTCCATTATGTATGAGTACCTCGGCGACGGTATCG
ACGTTTATGTCGTTGATGGATTCGTCGTCCAACAACATCGGTCGGAAATAGCAGACTCGACCAGTCTGCGGAG
TATATAGACGATCAGGGTGTCGCTCGTTCCATTGCTGGATGAGGTCCCTGTACATCTGAGGTGAAGCCATTAG
AATGGCAAGGCTTCGAGTGAACAATACGTGGCCGTTTCGTGAACGGCCACGGCCAGTTTCTGGAGGTCCAAAG
AACCTAGCCAGTGCATAACCGAATATCGATCGACGATGAAATGCTCGTCGATAATCAAATTGGGTCCCGTATA
CGAAATTCTGCGACCCGGGTCGCCCATGCGCAAGGAGATATCGGCCAAGGCGGTCGATATCGAATAGGTCGGC
CCCGGTAGGCTGCGCAAATCCCATCCCTTCTGTCCAATTGCTAGGGTTGGATACCCTAGCCGATGCCCAGTTC
CTGGGTATTTCTGGCGTCCTTCGACGAATACTGTCGAATTGGTCCCCGATTTCTTCAGGATCGAAGTTCGCCA
TGATGTACTCCATAGAATCATCACGAACCTCCGGGATCACAGTCTGAGCTATGTTATATAGCTCAGTTAGCAA
TGTGTACGCTTCAAATCGTACATGATGGTGGGCGTATGTATCCTGGGCCAGTCTAATCAGCCGAGCCGTGCTT
CGAGGAGTGGTGGGTAACCCCCAATGGGTAGCCCGACGAATGGACTTTTTCCCTACATCGCCTTCAAAGTCCA
CTCTTTTCAAAGCGGCATTAGCTGCTTTGATGGCGGTTTGCCCAGCGTACACAATGTTGTTACGTGGGTCGTG
GAAGACATATCTTGATAACATGCCTCGCATTCGGTTGTCCCGAATGTGATCGGGACCTACTGGGGCTGGGTTC
ATGCCCCAGAAGTCGTGAATGATTCCTCTTCGACCTTCGAGGTCGCTAAGGTTGTCGATCGTCGACTGTGAAG
TCGACGGCTCGTTTTGGGCCTTCTTCTTGCCCTTAGACTTCTTCTTACGACGAGGTTCATCGTCGTCGTCTTC
GTCGAGCTCCTCATCAGAGCCTAGCGGTCCATATGCTTCAGGATGCGATTGGTCCCGAAGTACTTCAGTATTT
GGGGGCAAGCGGCCTGGCAGCTGTACCCGAATTTGACCACTGTATCTCCAGGTGGTCGATTCGTCTCCTATCA
GGCCGGTTTGGCCAGCTGGTGGATGCCAAATGGCAGCTTGCTGAGCGTCGACCACAGCTGGGTCAGCGTATAT
TGATGATGAAGTACCCGCCGTCGATCCTTGTCGGCGGTGCTTGGAAGCTGGCTCAGTTGATTGAGCCAGCGAT
GATGCCAGGGGTCGAGCGAAGTGTCCTGGGGTCGAGGAATCTCCTTCCCTCGATTCAGTAGCCCTTGGTGGAG
ACGATTCTCTCCTACGGGATCTTCCTGTGGTCTGTGTTTGAGCTCGCAATGAGCTTGAACTAGCGCTTTGTCG
AGTTCTGGATGAACTCGACGCTCTCGATGGGTTGGTGGATGTTGTGTCCCTTGATGGCTGTCGTGCCTCTGGT
TGTTCAGGTGGCGGAATTCCTACCTGAACGAGTCGTTCGACTGTCGATTGCAGTCGATTTAGTCGTTCCTCCA
TTGTTCTCAGGGACGTCTGGTGCCCTGAGTAGTTACGGATCAACTCCTCAGCGGAGTTGATCCCGACGTCGCT
GTACAGATCCAGGGAGTTGATGATTGCATCACCAACCCCAGCGTTCCTGAGACGTCCTACATACGAAGCGATT
CTCGCTTCGTGGTGTTCCTGAGCTTCATCGTGCTCAGTCATGCGACGTTTACCTTTGGTTTGGGGCGTCGAGC
TTTGATCAGTCTCTGAGTGAGGCTGATAATGTATTGGCGGAGGATGCGGGAATCCTCCGTGAAAGGGGTAGGG
TGCGTGCCCCATGTATCCAAATGGTGAGGCAGACGGTGGCGGCACCGTCTGCATATGTTGTTGGTATGCGTAA
AATTGGTGTGGTGGCGGCATTGACTGCCACTGCTCGTACGCATGTGGTTGATTGTATTGCGTCGGTATCGACG
CGAGTAGACTTCTATGAGGGTCAGCGGCCGTTTGCATGGTGACCGGTGTGTTTGTGGACGTTGCGACGTCCAC
TATATCTGCGGAAGTGTCCATTACGGACCCTCCTCCCCCACCAGGAACCGTGGACGGTTCCGATGAGGTGTTT
TGGGGCGCAGAGCTCGACTCAGAGCCCTGCAAAGGAACTACAGGCGCTGGCACCTGCTGTGAGTTGCTGGGTG
GTGGTACGGGTGGTGGTGGTGGTGGTGGTGGTTCAGACATGGGACGTGGTAAGAATGGTTGGGAGAAAAAACA
CTCGGAGGTTAAAAAGTACGACGAATGTAACCCGTCGAATAGTATAATTGATTGTCAGGGACGCGTCGTGATC

CCGATCGCCGATGTCCTTCCTTTCCTGGCAGCGTGTGTACGCTGCCTCAATTCCTCAACCTTTTTGGCTCCTT
GAAGCTCAAGGTACCTGTCTAATGCTACGAGATCCTTACAGATCTCGTATGGCTCCCATGTGGTGTCTCCCAG
ACTCCACCGAACTTCAAATTCGAGGCTACCAGCATCATGCCAGTGGCCGAGTATCTCGTCGACGAACCATTCA
TGTTCGTCGTCTATACCAAAGTCATATGGCTCGGGTGTGCTGCGGTCAGGGAAAGCCTGATCGTCCGAAGCCA
CATGAGGCCGTAGCAAACCAACATGGAAGGTCGGGTGAATCCTCCGTTTGAGCAAGGCTGCCGGCAGCTCCAA
CTTATAGTTGGATGTCTCAGGTCGAGCTTCTAGGACCTTGTAAGGTCCTATGAACTTCGGACAGAGCTTGCGA
GCTCTGTCCTTCGGCAAATTTAAATTTTTCGTTGACAGAAAAACCAGATCGCCAATTTCAAACCGCATGTCGG
TTTGCCGGCGCTTATTAGCTTGCCATGTCTGTGCCACGCGTGCTTCTATGATGGCATCATGCGCTGCAGCTAT
ATTCAGCAGCGCTGCATCAGCAAAAGCCCGGACACCCTTATGGGCGCTATCTCCGATTTTGCTATCGCGTATC
ATTGATGGCATGAAACCATAATTCAGTTCGAAAGGTGCGAACTTAGTGGCGGCAGCTACACTCGAGTTTATTG
CAAACTCGACTAACGGGCACTTATCGACCCAATTTTTCTGGTCGTGACTAATAGAAGATCGGAATATCTGACC
GATATTCCGATTCATACGTTCGACCTGCCCATCGGTTTGTGGGTGGAACGATGTGGACATGAGAAGATCTGTG
CCCAACATGTGGTGTAACTCTCTCCACCACTTGGACGTGAACTTCGAGTCTCTGTCACTAACTATGGACTCGG
GGAGACCATGGAGGCGTACGATATCCCGTACGTAAATCCACGAGAGGTCAGTGGCTGACATTGTCGTGTGCAC
GGGAATTAAGTGCACCATGCTCGTCATTCGGCATAGGACTACCCAAAGGTAGTTATGTCCCTTCGCTTCAGGG
AAAGGTCCGACGAAATCCATACCAATGGATTGCCATGGTCAGACCGGTATGGGTAATGAATGGAGTTCACCAC
TCGGCTTAGTGGTGGACGTCTTCGATATCGCGCATGTCTGACACGATTTGCAAAAGGCTTTACAGTCCTTTAC
CAAGGACGGCCACCAATACCAACGTCAGATATATTCCGATGTTCGTTGTTCGCCGTAGTGCCCCAACATTTCG
TGACACCTACTGATCACTTTAGTGATCAGCTTCCTATCGTTCGGGATACAAGTTGCTTGTTCCCCAAGGTGCG
TTTCCTTGGTTATCATTCCCTTCGAGTCGACTGAGAAAGCTTTGTATTTCTCAGGGTTCTTTTTTATTATAGA
TAATAACGGGTCGTTGGCTATCCCTGCCACTATTCGATCCGTTATGTCGTCAGACTCCATCCTAAATGGAGTC
ACTGGCTTTGGCATAGTCAAAGATTGCGCTAGTGTCAATTGCTGGGGCCGCTCTCGCCTAGCTGAGTGTCGTC
GAAACTCAGCTGGCTGTTTTGGTTGAGGTATATCCTCAACCATTTTGTTGGCTGCCGCGTCTTCCATCTCGGC
AGCCTCGACTTCACGTTGTTCGCGCTTGTCGTGAAGACGAGCGCTACGTCGTGGCTGTATAGTCGACATTGCT
GCTATACGATCCTGTGTGAGGAGTTGTTCTTGTAACTCCTCCAGTCTCTGATTCGGCAAATCGTCCCCTTCTG
GGTCGATCCTCGCATCAGCTTGCACGTATTCACTGGGGTGATGTCTCTCCCCAGGAAGGTCACTCTCGTAGTA
TCTCGAGAGACAGTCCGCTATTTTATTGCATTCACCCTTAATGTAGGTGATGCTATACTTATATCGTGACAGA
TATTCAAGCCAACGTCGTTGTCGGCTTGACAAATTCACCTGCGAGTTGAAAAACTCCAGGGCCTTGTGATCGG
TGATAATGTTTATTTCATAACCGATCAGCTTGTCCTCCCATTTCTGTAGCGCCTCGAGAATCGCTAGAGTCTC
GAGTTCATGCACAGCGTAGTTTTGCTGTGCCGAGGAAAATTTCTTCGACATAAACCCGGCTGGACGACAATCC
ATCCAGCTGGGACCTTGACCATACATTGATCCAACGCCTGTTTTCGAGGCGTCGCAGATGACCCATATAGGTA
CACCGGATCTTGGGTCAATAGGCCTGATCACGGGTGTCTTATGGCAAATTCTCTTCACCATTTCGAAACAACG
ATCGTGAAGCGGTCTCCAATGAAAAGGGGTACCATTCTGGGTAATGGACATCAACGGTCCAGTATATGCCGTG
ACGTCCGGCAGAAAGTTGGACACATAATTCACGAGTCCAACAAATTTCTGGACGTCGTTGTAGTTGCGTGGCA
CTCGCCACGCTCGAATACGTTCCAGTTTATCGTGATCGGGGTGTATTCCCTCATCATCTATCATATGTCCAAG
ACAATCGATTCGCTCGGCATACAGTTCTACCTTAGATGCCTTAACGTAGAGCTTATGCTCTGCGAGGCGTTCA
AATACTCGCGCAAGAGCATCTTCATGCTCTTTGATCGTTTGCGCGAACACGAATATATCATCTAGATAGACAT
GTACGTGTATACCTATGTCGTCTCGGAAAACCGTGGTCATGAGACGTTGGAAGGTTGCAGGGGCATTACAATC
CCCCTGCTGCATGATATTGCTTATGTATGTTCCCAATATTGTCGAGAACGCAGTCTTGTGTACGTCTTCTGTT
CGTACACGCACTTGCTCGTATGCGTCTGCGAGGTCAATCTTCGATCGATATGGCATTCGAGCTACATCTTCTC
GAATGACTTCTTGGTCTGGCAAAGGTGTTACATCCTTTACCGTGTTGTCATTCCGTTGTCGGCAGTCAATTAC
CGTGCGTAGTCGACCATCTTTCTTAGGAATACACATCAGAGGTGCTGCTTGGGATACCGATTGTGGCTCCCAC
CACTTCGCTGCAATGTATCTGTTGAGCTTCAAGTGAAACTCAGATCGCAACGAGTGAGGACATTTAGGTAGAC
GGTACTGGTATCGTTTACTTTCATCGATGAGATTAATCTCATGATTAACCTCTCGAAATGGAGGGAGCACATC
GGGTGCTCCGTTCATCAAGAATTCATATTTCGTGAACCAACTTTCATGAAGACGTGGTAGGTCTTCCGTAGTG
AAGACCTCCCCTTTTTTCCTTTCGATAGGCTTCATTAGTTGAGATTTTGTCTTCTCAACTGCAGTCGACCGAG
GTCGGTCAGCTTTCCACTTCACGACTCCCATTTGATTTATTCTATCCTGACCATTCCCAATGGTCATAGCTTC
GTGGGCAACCTCGTCTGTTAGGTTGCCAGGGTTGAATCCAGTATTCTCGAATGATGGCTCTACGACTGTAGCC
ATCAGTGTTGAATGGGACGTCATCGCGTTCCATTCGTTGGGCATTCTTGGATCATAGAACCAATCGTAGATCG
CCCTAATTGTAGGGCGATCCCTTAGGTGAGGTCAATTGTCCTCCCCTAAGGTTCCGTCGTTTATTGATCCAAC
TTCCACTCGAGTACTGTTCGGGTGGTCCTCCGACACAGTCTCATCTTTATTATCATTACGCAACGATAGCCAT
CGCTGCTTGTCAGTCTCATTATCTTTTGATCGTGGTTCATTCGATCCGTGATCCCCGATTTTCATACGATGTT
GACTGTACTTATCAGTCGTTCGGTGTTGTCGTATGCGTGAGTCTGCATCGCCGACTATATCCTCGGCTGGTAT
CGGGCAGCCGTCGACCATCACGCTGGATTTTTCGAAATCCAGCAGTACTCCATATTTATGCATAAATGGGGTA
CCTAAGATCATATCGTAGCGATCAAAGTTCGCTACGTCTAGGTAGGTGGTATATAATTTCCCACCAATTTCCA
AGTCGGCTTCAGTACCGAAGTTGACAGTAGATCTACTCCCAACCGTTCCTAGTTGGAGTATGTGAGGGTCAAC
GAGCTCGTTGACTCTGATACCGGCCACGTTGGCGAATGATGGGCTCATCCCCGACGTCGTACTGCCAGAGTCC
CATAGAGTCCATGCCTCTATGCCTTTTACATTTATCATTGAGGCCAGACATTGTTTCTGGCCTAAGGTAATT

GTGGGCGTATAATTTTGTCTTTACCCTTCTTCAGGGTGACTTTTCTGAACCGAACGGCTTTCGGTTCCTTTCC
TACTTCGGTCATAGCCATGACCAAGTTGTTTGTATTAACTGAGGAATTTACTTCCTCAGTGTCTTCCTTATCC
GATTCGTAGTCGGACAATACTTCTATATCTTCCCCATCCATTATCATGGCAAGGAAGTCGTCATCATCTGAGT
AGTCGGATTCGACTACTTCTGTTTCTATTTCTTCATCCTCTGAATCTGAGGAATGATATTCGCTTTCCGTTCG
TTCACTTTTAGCAGACGAGTTTTCGATGACGTCATCGTCGTCGCTATTCGCCTCTCTGATTGAGGCCGATTCC
CCAATTACCGTATGAGCGGCACGAACAAATGATCGCTTGCTCTTATTAGGGCAAGCGTTCGCGTAATGGTTAC
CCCCACAGGAGTAACATTTGACCGACGGTGTGGGTCCTGACGGTCCGCTCTTATTCTGAGGCCTAGGGCCCGA
TGATCCCTGATGTCTTGGTCGATCGTTCGACGCATTTGGTGTAGATCGTCGCTCCGGTCGACGATCCTTAGAT
CTACTAAATGTAGGTCCTGTTGGTTTCGATGGGCCCAATGTGTGGTCCTTCGGGGTCGTATTGGCTCGTTCCG
TTGCGAACCTCTGTGAATTGTGAGGTCGAGGAGTGCTATTCCTCGACGACTGCTGGGGATTTCTCCTGTAATT
AGTATCCCCAGAGAACTTAGACGGAAGATTTCTACTATAGTACGAATAGACTTTCGTCCTACTCTCGTATGCC
ATTGCGAACGATAAGAAGTCTTCTAACTTGCTCATTTCAGGCATGAGACCTTGATTGAGGAATAACTCCTTCC
TCATATCCTCAGGGAGACCTTCTAGGAAGGTCTCGATTAGTGAATAGTCGTCCGGGTGCACAGTCATACACTG
CGCTTTATCCATTATGGCGTCATAGAAGCCTTGAATTCCCGTTCGGGAATTATATCTTATGCGTTTCAGTCCT
TCACGCGCGTCGTGCATCGACGTAGACATCACGAATCTATCATAGAAAGCATGTATTACGCTTTCAAACGTCC
GATCATATTTTGTACGATTGACACTAACTACTTGTCGATCATAGTACTTCTTGGCTTCACCAGCCAAGTAATA
TTGAAGGGTGAATATGCGAAGTCGGTCTGTGGCCGCTTCGCTTCCTCCCAATCCTTTGAGTGACATATCGATA
CTCAAGGTGGTGACAAAGTCCTCCAGTTCACCATATCGTTCCGAGCCTTTATAAGGCTCGATCGATCTATCTG
AGGGAAGCTTCTGGTTCGAGGGTTTATATCCCTCGGGGAATCTGAACGGTTGAGAGAGGTACTCCCTCAATAT
AGCGCTCAGCTTATCACGCATGCCATTGTGATAATGGCGCGTTGTTTGGTTGACACCTAGAGGGTCAACCGAG
GGCGGTACGGGATTCACGATAGAGTGACCCCTTCGTTGACGATGCATATTGCCCCATCCCTCGTCTGGGCGCC
GCTCGTCATCGTACTCCATTTGTCGACCATAATTTTCTGGTCGTCTTTGGTGAGGAGTATATTCCTCGTCCTC
ATCAATACTCCCCACTAGGTCCTGCCACGATATTCTATTATCGTTGCCTCTATCAACCTTTTTTCCTTTTTGT
TTGGCCGACAGTCTTCGGCCATTATCATCCTCTGTTTCCTGTGCTTCGTATGGACGTCCGTTATCGAAACGGA
TGCCCTGGTCATCTATACTTGACCATCCTCGTTCTCGCATGTCTTGATTCCGGAGTCTCTGTGCAGAGGCTCT
CTCGCGTATTGCTTTGTTTCGGCGAGCAACGTCGTCGGCGGGGTTGTATATCGATGATGATGGAGGTCTAAGT
AAAGTTTCGACCTCTTCAAACTTCACAGAGCGACGCTCGTCACTCTGCCGAGGCCTCACTGATTTTAATCCAA
ATGGTGACTTATCTTTATTAAGTCGCCTTAATAGTTTTTGTTGCGCGTCGAGGCGTTTATTGAACGCCTCAGC
ATTTTCGTTATTTTCTCGAGGGGCGAATAGCTCCCTCATAGCTATACTTTCGTCAGCTTCGACGACGAATTCA
GACTCGTCGTCGTCCAGGAAGACGGCTGCGTCGTCGTCTTCCCATATTGTCCCCTTCGGCGTATAGTTATGTA
TAGTCTCGCTATTGCGAGTTTTCTTCAGGTAGGATCTGAATCCTTTTGTCGCCAGGGATTGGTACACCTCATT
AAGCGGTGTACTTGATCTAGGATGCTTATCCGCGCGTACCGATAAGTATCTCGCTACTAGCTTCCTCAGGGTA
GCTAGTATGACTGATTCAATAGCGCCGTCACTCATGGAATCGGACGGTCCGATTACTTCGGTTACGCACTCCT
CAGTGTATAGCCATAATTCTGAACGACGAATCTCGTCGTGTACCTCGTGGGCTGTGAAGATCATATCGATCTT
CTCATCGAGTGACATCTCGCCTTTAAGCCTATCAGTCAGGTCCGTCGGTGCTCCAACGGATTCATTTTGATCT
ATTTCTTGATGGACAGCGTGCTGTCCACCGGGCGACGGGCCAACTCCTGACGGTGCTGGCGCGCTTGACGATG
TTTGGGGTGCAACCCCCGGTTTGTGACGTCCTGAGCCCGTCACATGTGAAGAAGACTCCCCAACGGTAGGCGC
CGTTGGCATTCTGCTTCTTTTGATGTTTGATAAACTTTTTCAAGTGTATTTTTGGGTGTACCTTAGTTCACGT
AGGTTGAACTAAAAAAATGTAAAGCGGGCGGCTTTACTTGTTTATACCGAAACTTACGCGTTCACACCGAGAT
AGTTCAATCCCTTAATTGGGTAAACTACTAGGTACAAACGTTGTAAGATCAGTTGTCTCTAGTAGACAACTGA
TGCCTAACTATATAGTGTATCAATATTAGTAATCAAGTACTATACAGTACTTGATCTAGCCGTTGATGTGATT
AGATAACCATAGTAGTCGAGATGATGACTATCTCGACTATAGATAATATAATAGTCGGTATGCCTTACA
>Agabi_varbisH97_2_rnd-1_family-142#TIR/MITE
TCATNCTCAGTAAAAAATATTNTCAGGATCTATCAGGCTAATCCAGGTCAATTCAACGTAATTCNGGACAATG
CAGGCTGATTTAGGGTCGATTTAGGGTCGATTGGCACCCTGAAGGTCGGTGAGAGTTGAGGTTCTTACACCTT
GAACGATGCCTGAACACAGCCTGAGCTTGGCCCGACAAATGTNCCACATTTAAAGCAACTTGCCTGAACTTTG
GCTGATCATGTTCTCGCATTGTTCGAGAAAGAGGACAATTTTCAGCCTATGTTCAGGGCCTGAGATACCCGAA
AATGCCTGAAAATCTCTTTTTACTGAGCATCAT
>Agabi_varbisH97_2_Cluster22#LTR/Copia
GTTAAACCCAGCAGCCACAGACAGTGGTGAGGGAGACCATGCACTTACTTGGTTGGATGCTTGAGAGAGAACT
GAGATGATAGTGGTAAGAGAGTAAGAGACGAATTGCATAATTGTGTAACTACACTGTCCTTATATACTAGCAG
ACGAGGCTGCGCGTACAACAAGTACATACGAAAGATAAAGCATAGTAAATATAGCATAGCCTCAATGTCCAAT
TGTTATAGATATAATTAGAACAAAGGCATGTCATAATTCACACGGAGCTTGCACAGCATCACGCGCTATAATT
ATTCCAACACTCCTCCATGCTGAAAAGCGGATAGAATTTATGCTGCATTACTGCGCAAAGAATTCCATACCTA
GTAGGGATCGAATACGCTCGAATTTAGTCTTGTCGAGATTTTTGGTGAAGATGTCGGCAGGATTCTTGGTGCC
ATCAACCCAAAATAATTCAATCTTTTTGTCCTCGACATATTGACGGACTATGTGATATTTCACGTCAATGTGC
TTTGAGCGTTTTTCTTGGACAGGGTTAGATGCCCAGAAAATGGACCCTTGATTGTCGCCAATCAAATGAGGAG
TTGGACAACTAAGTCCTATTTCCGATAGCAGCTGGTGCATCCAAACAAGCTGTTTAGAGCAATCTGACAGCGC

CATATATTCGGCCTCAGTCGAAGAAGCGGCCACAGTCTTTTGACGTCTGCTCAACCAGGAAACACACCCAGAA
GCAAGAGTTACGTAGTTACCTGTGACAGATTTGCGGTCAGTGGAATCTTGAGCCCAATCAGAGTCGGAGTAAG
CAACAATTGCCTCATTTGACGACCCGTCAAACACCATTCTGTACTTGCGCGTAGCAAGGAGGTATCGAAGAAC
ATAGAGTCCTGCCTCATAGTGTTCAGGTGCAGGGTTTGCACACTGTTGGCTCAGCTTAACAACGGCGTAAGCG
ATGTCTGGTTGGGAACCGATCGTCAGATACATCAAAGATCCCACAAGTTGTTGGTATTTTTGGCGAAACATTG
GATTAGCCGGTTCAGAATTTGGAAGGAAGGAGAACCCGGACGCGAGAGGAGTCTTCGTCGGCTTCGCAGTGAC
GTTGAATTTCTCAAGGACCTTATCAAGATACTCGACCTGGTCAAGGTATATTTTCTGGTTCTTCCTGTCGCGA
GAGATGCTCATTCCTAAGAACTCTTTGCATGCTCCAAGATCGCGACATTCCCACTTCTTCATGAAGGAGTCCT
TCAAACTCTTGAAGAGCGAAGTCCGTTTGCCAAGGATAGCGACATCGTCAACATAGACAAGTGCTATGATAAG
TTGCGCCGTTTTCGGGCAGCGGTAATAATAGACACCGGCATCTGATTCACAGCGCTTAAAGCCTAGTTCCTTC
ATGGACGTGGTCAGTTCACGCCACCAGGCCAAGCCAGCTTGTTTCAGGCCGTAAAGAGCTCGGTGTAATCTCC
ACACTTTATTTTCTTTGCCTTTCAGGCGAAAGCCTTCAGGCTGCTCCATGTAGATTTCTTCATCCAGTTTACC
GTAGAGATATGCTGTTTTTACATCCACGCTCTCAATTTCCCAATCCTCAAGTGCTGCAACACCGAGTAGGAGG
CGTGCCGTTTCATAACAGACTACGGGAGAAAATAACTCATCGAAGTCAACTCCTTTGATCTGGGAGAAACCTT
TAGTCACGAGTCGAGCACGTTTCCGGCCATCAGACTTCGTGTTAAATACCCATCTATTTTTGATCGCTCTACG
ACCTTTTGGGAGATCAATCAATTCATATACTCCACGTTTCTTAAGAGCTTGGAGCTCTTCAAGGCATGTGTCA
AGCCAAGCTTTGCGATCAGATGGAGATAGATCAGCTAAGTCTTTGAATTGGATTGGTGTTTTGGACTTGGCGA
CGGCATTGGCCATTAAGAATTCGATAAATTCCAGTCCCCCCTCATGGACAATACGTTGTAAGTCCTCCATGGG
ATCTCTAGATTGTCCCATATCTGGATTTGGAGAGCGTTCCTGGGAAGGTTCGCTCGGAGGATGTACTTCAGGG
TTGACTGGGGGCTGTGCTCCTCCAGGATTCCATCGTGGACGCCTTTCATATTCTCTGGCACGACGTTCTCTAT
AGGGGAAGGAGCGACGAAAGCGCTCTTCTTGCGGCCCTATTACCACTCCTTCAGGAGGGTATGGACGTCGGAC
ATCACGTTCACGGGGGTGTCGTTCAGCTTCACCCTGTGCTCAGATCTTATCAAGGAGATCATCAAAGTCATTT
TTGAGATCCTCAACTGGATCAGATCGATCACCGTAAACGTTACCAGGACGCAGTGGGGCACGGCGTTGTCTAC
CAGATCTCCGTGGTTCACTCGGACCAGGCTGAGGAACACGAGGCGGAGGTGCTTCTTGCTGCGGTGAAGGTGG
TCTAGATGGGGTTCTCGGCCTTGAAGGGCCAGAACTCGGAATGGAGGGCGATCGAGCCCTAGGTGGAGTAGGT
GGGAGTGGCTTACTAGGGTCGCCACCAAACCCAAATGGAGCTGGAGCGGAGGGTGGATCCACATCTCGTGAGT
TGTCATCGTCATCATTAGGGACTGACGGAGCTCGTCTCGGATATCCTCTAGGATCAGTCTTGGGACATTTCGG
GAAATATGTTTCTTCAAAAATGGCTGTAGGAGAAACAAACTCGGCATTATTTTTAGTGTGCCTGATGAATAGG
TAGTTTGCTCCTCCAGAAGTGTAACCAACAAAGAGCATTAACTCGGAACGAGGCTCAAGTTTGTTGTTACGTT
TCTCCTCTGGGATAAAAACATATGCCCCACAGCCAAAAACACGGAGTCGAGAGACATCCGGTTTTACACCGTA
CCATAACTCAGATGGTGATTTCCATCCAATCCGTCGCATTGGCGTGCGATTATAGACATGCACAGCCGTTTGA
ATCGCAAATTCCCAGTATGACTCAGGAATGCACGCATTTGTCATCATGGCCCTTATCTTATCGAGTAAGGTGC
GATTCAATCTCTCAGCGCGACCGTTCTGTTGGTGCGCGTAAGGTGCGGTTGTTTCATGGATTATCCCCTTTGA
CTTAAAGAAGTCATCCAATTCCTGAGATACGTATTCGCCGCCATTGTCGGTTCTGAAACGCTTCACCTTAGCA
TCATGTTGGGTGCTAATCAAAGCAACAAAGTCCTTCAGAGCTTTTAGTGCCTGCGATTTTGAACGCAATAATG
CGATGGAGACGGCTGATGAATAATCATCAAGGAATGTTATCGCCCATTTGTATTTATGGTACGATTGGATCGG
GAACTCAAGAAGATCAGAATGAATCAATTCTCCAAAATGAGTTGCCCTTTGGGGATTCTCGGAGAAGGTCTTC
TGGTGCTGCTTACCAAGAGCGCACCCAGGACATGTAGATTTCTTCCGAGGGAAATCGATTTTTACAGGTGTTT
TTGAAGATTCAGGTACCCTTTTCAGGGCGTCATCCGATGCATGCCCAAATCTCTGGTGCCAGATCTCATAAGA
TGGATCTTGAACTGATCGGTTCACATGTCCGGAGTTTTTAATAATCCGACAGAACACGAAGTGGAGATTATTT
TTGCAACATCCTATGCCTCGTTTGGCTGAAAGGATGGGAGATCCTGAGGGATCGAGGAGCGTAGTAAAGTTCT
CAGAGGCAGACATTGTCACACCATTTTGCAGAAACGTATTGGTGGACAGAATGCGGCCCCCCATGCCCTTGCA
ATAATAGACAGGAGCAATTGTCGAAGTCAAAACATGGACTTCACCAGTTCGATGGTTCACAACTTCATGCTCA
AACATGACGGTTCCACGAGCGGTGATCGGAAGGACCTTTCCCGCTTCAGCAGTCCCTACATATCCGAAATTTC
CCATTTCGTGCTCGATGAAGTCATCCAACACACTGGTAAAGTGGCAGGATGCTCCAGAATCGAGGAGCCACTC
AGTTGGATATTTAGTGTCATGAGCAGGCTGTTTCCCACATTTTTTGCACTCAGGTTTATCACACTCGTAAGAA
GGAGACGCATAAGCAAATGTATTTAAACAAGAACAAGTCCGATCTACATGTAGAGGGTTGGAGATGCAGGACT
GTATTGCGTCATTAATTTCACTTCTACGAGGTGAAGTCACATACCAATCATACTCAGAAGCGGACGAACCGAG
CAAGATCGCATCGTCGTCATCCTGAGGAGCGCTGAAATGTTCAGTGATCGCCTCGTCGATGGGATCTACTTCC
ATTGCGTTCTTTTCTTCGATCTCCTTCCCTTTTTGTTTCCAGACCCGAGCCTTGGCAGTTTCGAATCGCCCAA
TAGGATGAGGCTTCGGATAGGGGCTCGGTCTCGGACGAGGGTCACAAGTTGGGGAAGAGATACGATGAAGCAA
TGATGCGGGGGAAGCGAACTGTGTCACGGATTGGGCCGTGTACTCTTCGATGCCAGATGTAGTGGCCTCGTTC
GCATTCTGATTATCGTTCTTACGTTTGCCGCGACGAGTTTTCTTTTTGGGCTGCTCACCCTGAGGCTGCTGCT
GCTGCTGACCTTGCGGTCGCTGCTGCTGCTGTCGTTGCTGTTGCTGCTGCTGCTGCTGGTTGTCTTGCGACTT
CCATTGTGGCTTTTGATTGCCACGCCTCATGCCAGAAAACCTATTAGCTTGCGCTTGAGAAGTGCGGCGCTGA
TTAAATTCAGTCAAGATGGCGGCGCGCACATTCTGATAAGTAACGTCGTTAACCGACGACTTGGCGGCAAGAA
AAGAAGATGCGATGTTCCAAGAAGAAGGAACAGAGCCAAGAAGAGTCATGGCTCGAACGAGGGCCGGAATGTC
AACACCGGCAGACTGGAGGCGGTTGTAAAGCTGCTCGAGATTAGCGATCTCGGCTGACGGATCCTTGTTGCCA

GAAATAGCGAAGCCTAGGGTCTGTTGGTACATTGAATAGATTGCGGCAGGGCCAGCAGTACCATACGTATTCT
CCAGGGCAACCCAAAGGTCGCGCACTGAGGTATACGAGATCGAGATCAAGCCCTCAGCGATGCGCGTGTTTAT
CGCAGCAATCGCTATCTTTTGGACTTGAGCCCTTTTGGCTACTTCGGCAGGATCGTTGGTTGTAGTTGCAGTG
TCCTCGCACATGTCAAGTGCAGAGCAACTAGACCCATCAGCTTGGAGCAAGGTGAGGATGTTCCTCATGTTAC
GCTTCCAAGAGTCGTAATTATCGGCGCCAGTGAGGACCGGAACAGAGTTGAGGAGGGGGAGAACAGCAGAGGC
CATTGTTGATCAGATCTGAGAAAAGATTGATCAGTACCGCGCGTTGTAAGAAATGTCGCTGCTGGTACAGGTG
CAGAGCTCCTCCTCGCCTGCTGTGAGGTTGACCATGAACGGGGAGCGACATAGTTGTCAGCTCAGACGATGAT
GACCAAAGGTCAATCAAGGAGGGGGCAAGCCTCACAACCGTTCAGGCATGGTGATCAACCCTCTCAACGAGTG
CAGCGACGAGCGCACATCGGAGCAGGCAATCGGGGCCCATAACCTGTTAAACCCAGCAGCCACAGACAGTGGT
GAGGGAGACCATGCACTTACTTGGTTGGATGCTTGAGAGAGAACTGAGATGATAGTGGTAAGAGAGTAAGAGA
CGAATTGCATAATTGTGTAACTACACTGTCCTTATATACTAGCAGACGAGGCTGCGCGTACAATAAGTACATA
CGAAAGATAAAGCATAGTAAATATAGCATAGCCTCAATGTCCAATTGTTATAGATATAATTAGAACAAAGGCA
TGTCATAATTCACACGGAGCTTGCACAGCATCACGCGCTATAATTATTCCAACAC
>Agabi_varbisH97_2_Cluster14#LTR/Copia
CCTGCTCTACTAACCAGAGAATTCTGGAAAAATCACGACAAATCATAAAATATTAAAAGAACATAAGTTTATA
TGTATGTTTTGATCGAAAAACATACCAGTTTTGGAGATATAGAAAAACATGTCAAGAATGTCATGTTTTATAC
TGGTTCCTGTGCCGATATTGATGGTTTAAGAGTGTAAGTATATGTATTCAAGGTCCAGGAATCCGAATCTGAT
TTTAGATTTATTATCCGATCAATACTTGAGGAGCTACAGCGCAATCTTGTGCTTATCATATGTTAAATGTGCA
ATATCTCAATAAGCTTTCATCCAACGAAATATCTAACGACATATTCGGACTCCCCGACCTCGAAACTATAGGA
ATACACTTTTAGATCATTAAATGCCATGCAGGAATGACCCGAAAATATCTCCTACCATTGTTTTGAGGAACTT
TGACTCGAAAATCCCAAGTCCAGATGTCATGTGATGTCATGTGACATGTTGAACTTCTGTTTCGAGTATGACC
GTTGATCCTTCCTCATCACCACCATCATTCTTCCCCTCCCCATCCTCCCCCTCCCCCTTTCTTGCCCTCCAGG
CCTGTAATATGGTAAGTAACATCCCCTTCACCTTCCTAGCTCTCCCCTAGCTGATTCTAAGCTGGTTTAGGAC
ACTTTTTTGTCTATTTTGGACGCTTTTTTGGGGGGATTTTGGGGGATTTTGGAGGCTTATTTGAGGGGATACT
TGGGGGGATGTTTGAGGGGATACTTGGGGGGATACTTGAGGGGATATTTGGAGGATTTTGGGGATTCTTTGGT
CACTGGTGAGTGCTCTCTATCTCTGTATATTTTCATACTCATTTGTTACTTGTAGGATATTTCTTTTCTCATC
ATCACTCCCTCATCATCCAATCTCATCCTTTTGTGTAGATTATATACAATGTATTGTATTTGTTATGCTAATA
TAATGACTTTCTCTATGACTTATTCACTCTTAAGCCTCTTATAATTTCGTTATCACTGAGTTTACTTTATTTT
AGGTTATGGGCCCTGGCTCTTTCCAAAATTAAACGAAATCTCATCTTGCAAACTTTTATCTTTGCGAAATCAC
TTGAACTACTTCATACATGGCTAGCACTGGCGGAAGTGCACCACCCATCTTTGCCGATGCGGTGAAATTCAAC
GGTACGAATTGGGTTACATGGAAGGGGCTAGTTAAGATTGCAGCAGAGTTGCGGGGCGTCTTTGGTTACCTTG
ATGGTTTGACCGTCAACCCTGCAGCCAATGTTGTGATACCAAGCCTACAGATCATTCCACCCTCACCTACCAC
CAATGACGCTGCAACTGTTGCCCCACCGGTACTTACCTCGACTGAAACTCCATGGGAATCAACCACACCATCT
CCCTCGGAGTGGAAAGTTCGAAATGCATGGGCGATGTTACGGCCCGAAACGTTTTACGCGAGAAATGAGCCTC
GCTATGGTCGAACTTACTCGATTCACGAGTAGTGACACACTTATGACCCCTTCACTACTAGGAAATAACTTCG
AGAATGGTCACTACCTAACTAGAAGCAATGCAGAATAATACTTGACAGTAGCTACTAAGGTCACCTACAGGGA
AAACTGTACTTGTAGGGAATATCACAGCCTTGGTAGTGAGTAGCGAACACACCATTCGCGTCAACCACTGTAG
TGAACAAGTCTGAGCTCTCGGGAATATGGAATGGTCAAGGGACTTAGTCGCTACGACGGTTTTCTTACGCATC
CTATGTCGCGAAAGCTCCTCGATTACATAATATACCCAATCGAGTCTACCGACAAAAGTTCCCACTTCCCCGA
CCATATGTCCCGAATAGTCCTTCGCGAAAGAAGGAAATAGAACAATCTCGAACCTAGCTCAATTGCCAATGAT
CTGTAAGCAGATAGAAGGTAGCTACCGATAAAAAGGGAAATATGAAATACGATGATGGCTAATGACTCGAGAA
TGAGTAAGCCGTAGCGAAAGGATATACATAAAGAGTTCAGGAGACTCGACGACATTACAAAGATAGGAAGAAT
GACTCGAAAGGATTAGTAGCTACTATACAATAGATGAGTAAGATATGTAGCGACTTAGTAACGAGACTCGGTA
TGTTTAAAAATAGTGTAGCGGTAGTATAAGTAGAGGACCCGTAGCGAGCACACAGGCGAGGAATTGTTTGCCC
AAGCTCTACAGCTGTATTCGCTACTCAGTGAATTGTGATTGTGGTGTTATACGCTATCAGTTTCTTTGGACGC
GAAAGTCCAAAAGTGATATAAACTTGAATCTTATCTTGGAAATTGGGTAGTGAACACCTAAAAGGATTATTAG
CGAGATCTCGAACTACAAACCCGTTGGTAGTGTGTGATAACTTCGTGTTAGCTACTTGGACATCGCAGCTTGT
ATCGTTTTGGTAACGTGTGCAAGTCAATAGCCTTGTAGTTTGTCGGTGCAAATCGGTGCCTTCCTGCTAAGTC
GGTATCGCAGTTTGTATCTACCCGCATTACTTGGTACGCGTCGTCTGCTTTAGCACAGCAGCGTAGTTGGACC
TTCATCGCGAAACTTCTCTATGGTCGGAACTCCCTTTTCTCTAAGGAAAATACCTTTCTCGAAAAACACATAA
AACCAGAAAACACTTTATATATTTCTTTGAAAAACCCTTATAAAAATCTCTGTCCTTTTCTCGAACTTCTGAC
TTAATTCTCCCCACGAACACACAACTGAGAACAGTTTGTGCGTAGTGAACATATATATTTTATATTTTCCCTC
ACGTAGATACTGTCGTCTCTACAGCGATGGGTCTGCTAATCTACAACATGGGTGATCTTATCAGCTTAGGAAT
TAACATTCATGGAAGAGAGAGTGGCACAATGAGAGTTGGGATATGTATGCCAAAAACGAGGGGATTATCCTCG
ACTTTACTACCCCGTACGCTCACCAACAGAATGGAAAGGCTGAGCAGAGTATGAGAACCTTGCTTGAGACGGC
CAGAACCATGCTGTCTGATTCCGGCTTGCCACAGAAGTACTGGGCGGACACAATACAGACAGCAGTCTATACT
CGCAATTTTATCCCACCGGCCAATAATCCCACCTCTATCCCTGCTGAACGTTGGTTGGGGAAACGTCAGGATG
TCTCTCATCTTCGTCCTTTCGGCGCCACGTCGTACGCCCACATTCCTGCAGAAGTCAGTCCTTCGAAATTGGC

ACCACGGTCAGTCAAGCTCACCTTGGTTGGGTATTTTGATCAAACGGGCTACAAGTTGTTGGATAGAATGACT
GGGAAAGTCTACAAGTTGAGGGATGTCATTTTTGAGGAGAGACCGCCTCATTACTCAACTGACCCAATCGTTA
CGTACCTGGCGGACCCTGGCCATGTGGCGTCTGACTTGACAGCGATAGCTCCACGCCCCAAGCCCATTTCCAA
ACTACATCCTGACACTCCTATTTCGACGCCCCAGACCAATCCTACTACCAACCCAACTCCTGCTTTCGGACGC
GTAACTGATGTTGATGATGAGGTTACCGACAGCGAGACTGAGGTCGCTCGCATGCTCGAAGTTACTCGCGAGC
CGGCGTCTGGATTGCCAAACCCCAACAGTGGTGCTGGAGAATCGCTTGCGCAGAGGCGGACTCGACGGGAGCC
CAAAATTAGTCGGAAGATGCAGGAGAGTCTTGAGTACCTCAACTGTTCCAGGGCTAATGTGGCGTCGGCAACA
CCTTCTCTATATGTAGACACCGAATCATACGTCCCCAAATCCTACACCGAGGCGATGCGGCATCCAGACTTGT
GGTATGAGCCTATGCTGAAGGAGTTGCGAGTTATGGAGGAGAAGGAGGTATACCGCTTGATCCCTTGTCCTGA
GGGGAAGAACATTGTTCGGTCTCGGTGGGTCTTTGCTACGAAGTTTGATGACTTGGATAATATTTCTGGGTAT
AAAGCACGCCTCGTTGCGAAGGGTTTCATGCAGGTCCTGGGGGAGGACTACAATGAGACATATGCATCTGTGG
CTCGTCTCGAGTCGGTTCGGCTCGTCTGTGCCATTGCAGCTTCTTTCGATCTTCACCTCTGGCAGGCCGATTT
TGTGTCAGCTTTCCTCAACAGCGACAACAGTTTCGAGGCTTACATGGAGCAGCCGCCAGGTTTTGAAGAAGGG
GGGGACAATAAGGTATGGCTCCTCTTGAAAACCTTATACGGCACCATGCAGGGCGCCCATGACTGGGCTCGCA
ACCTCGAACATACATATCAAGGTCACGGATACTACACTTCTCTAGCTGATCCTCAGGTTCAATCGCGAGTTGA
GAACGACAAGTTCACGCTTACTTCTACCTGGACCGATGATGTTTTAGGCGCTTCCTCTACCAAAGCTGGAGAA
GTCAAGGCGAAAGTGGAGTTGGGAAGGAGCTAAGAGCTCAAGGATCTGGGAACGGCTAGATTTATCTTGGGCA
TCATCAGGAAAAGGTAAAGTGACTTAAATTTGACTTATGCAAACTTATAAAAATCATAAGTCAGTATAAGTCA
TTTTTGCATAAGGCATATCACCGTCTGAGGTTCTGACTTATGCAATACTTAAGTAAATCATAAGTACATAAGT
CACAATATGGTCATAAGATTCAAGATTATCATAAGTCATGAGGATTTATCTTAAGTTGCATAAGTAAGTACTT
AAGACAGTAAGAAGATGTAAGAGAGTATAAGCTCAATCATAAGGAACATCATAATGCATAAGGAACAAAGAAT
TATATTCATATAGTAAATACAATACATCATGTATAATACAGACTTCCTAAAGAGGAAACAGACCCTGAAAAAA
GTGGACAAGAGGGATATGACAGCAGCCAGGGGAGTAGAAATGGAAAATGGGGGGAAAATGATGAGAAATGTGA
TTCTAAAAGTAACAAATGAGTATGAGAAGATGAAGAGTCAAAAAGCACTCACCAAAGACTAGAGATCCCCCCA
AGTACCTGGGGTTAGTGAACTTGAGATTCTCATCGGGAGGTTGTGGTTTTCGAAGGGCAGTATTAAATTCTTT
GAGGTGAAGGAGAGAGCGACAGTTCCAACAACTGCGGCTAGCACCTTCAGGGACGGATCTCTCACACGTTGTT
GAGTACACACGGAGTCATTGATGATCATTACACCACCTAAACATGAGATAGTGCTTGTCATCAACCACCTGCC
ATTCTTCCTCTGTCAGCCTCGCTAGCAACTTGCCCTCATACCACTCCTCTGCTATCTTACGTGGATCTTGTCC
CCCTCCTCCTAAAGCCAGCTTATGTTTAAGCATGGCTTCTATACACGGGTCGTCAGCCCCAGAAAGTCCTATG
CAAGGGATGGAAACCGGTGCCGTAGTCTTCTTGGCTCGCTTCGCTGGCACTGGCGTTTTGGTGGTGTTGAAGA
AGCCCATTGATTGTAAAGTCCGTATGCCTGCTGCTTTCTTTTTCCGTTTTTTCTCTGACATGCAGCCTTTGAG
GTGTTCCCTCCACCTTGTGACATCCAAAGGCCCTTTGACACGAAACTATGTTCCACAAGAGCTATGGCGGACA
CGATACAAACTCTTGGAGTCAAATTCTACATATGGGTTGTTGGCACGGAGATGGCTACGCCAATCCTCGAGTG
CTTGATGGGTAATGTTGTGTTTGCCCTCACGGTACATTTGCAACCTCTTCCGTGCCAAGATACTCGATTTACT
CTTCCCTTGTCCTGCAGGGATGTATTTCCACTTGAAACCGTTGTCCGTACTGCTGGTCTCGCTGCTTTCCTCA
ATCAGTAGCTGAATTGAAGAGAACTCTTCATGGCTGCGCTTGTTTGTCGCCTTCCTCCTTTTCCTCTGCTCCC
ACCAAATTCTTCTTGGTTATCTCCCCCTCCTCACCTAGCTTAACGACATCCTCATTCTCATCGACTGCATTCG
CTTGTTTATCCTTGTCATTGTGACCTCTACAGTAACCATCTACTGGGGCTTTGTCCGTGGCTGGTGTGACTGC
AGCTTGTGCTACATCTTCGTTGGTATCTATCCTGTTGTACCCATCTGTGTCTTCTTCACCCGCCGTTCTTGTT
TGGTACATATTTGCTGACATTATGTTAAACCAGAAAATACGATCAAAGTTCACGTCAGACAAGGAAAATGAGG
TTCACAACAATGTTCCAAGGGTATGGTGATGAATGGTGTTGATGGCCAGGATACCACAACTTGATGTATCCTT
TTGTGGAGCACAGGGTAATGTATTTCCAACAGAATCAAACATTTGGCCCGTCAATCACCTCAACCAAACTACT
AAGGCTTGAAGTTCATCTCGAGGAGGGGTCATGTGGAGCGGATTGCCTTAGATGTTTAGGTAAGTTCACACAT
AGTAAGAGCAAGTGTAGTAAACTCACCATAACTGATTTGAGATTTTGTGAAATAAACTGCCCCAACAATCCAA
TGACAACTGTGTACATGCATCGGAAACACAAGTTTACATGTGCGACTGGTTTGGATCGCCTCCCCTACATGTT
TTACGACCATAGAACTGTCAAATGTGCCTTGTTTTGCACTTCTGAGCAAACTTGACATGAACAGGGAGTCAAC
AAGTAGAATGCCTGCTGAAAGATCATTGTTCATCTCATCAACCATAACCAATATAATCTCGTCATTGATCCAA
ACGTCACCAAGAAGTGTTGTGCAGCTGAACGCGTGGATCGAGCCGCCCTGGTATTTGAGGTGTTCATTCCATC
CCAGCTTGGCCAGTTCCTTGAGAGCTGTCAACGCGATGCCAGCAGCCTGTGTTCGCAATCCCTTTCCTTCCAG
AATGGTTTCAAGCCATTTCTGCTGCTATTCTTGTACAATGGCCACTCGTATCCAAAATGTTATGGCCCACAAC
GGAATGTATGTACTTCCATTGTTATAACGTGGATCTGTGACTGATTTGGCACCATCTAACCAGACCTGGGGGA
GTAGAGTGTAGAGTTCTTCAAGGACCTCGTGTTGAGGTATGGCACAAGAATAGAGAACTTTGGGGTCCACTGT
AGGTTCATTTTTGGAGAACCACAACCTTGCTTTGATAGACGTGGCAGCTGTTTTTTCTTTAGGAAACTGTAGT
GCTATGAACTCGAGGATGGGGACACAACCTCAATATTTGAATAATACATATGTATTATTAAAACATTATGCAA
ACATTACCATAAATAGAATTTACATAATACATGTGCATTATGTAAAACCTATCTAAACTTTATGCAAACAAAA
CATATAAAATCAACATTATTGAATATATTTTTTGCATATGTTTTACATAGTACACATGTATTATGGAAATCCA
ATATATGGTAATGTTTGCATAATGTTTACATAAGTACATGTGTCTTATTTAAATATAGAGGTTGTGGGAGGTT
GTCAGGAGGGAAGTAGGATTGCACATATTGTGGGATGGAAATTGCCATATTTTTGGCATGCTGGACGTATGAA

GGTAGTTTGAATGTGTATTTCTTGCCTGTCCGAATCCACTCCTCGGGTATGAAGACTTGTGTGGTGGAGACAT
TGTCATCGAGATCGAGGACTGAAGACATTGCCAGTGGATAATGTGAAATGTGATAATAGTTAAAATTTTGTAA
TAAAAAAAATGTATTTCGACTGGATTTGAACTCTGGCCCCTGTAGAACGATCAAAAAAGGTCTAAATTTTCCC
TACGCCATGAGGCTCGCAAAGATTAGGTAGTCTGTACAGGCTTAGAGAGACTTAGGTGCCTCTACAACTCTCT
AAGTCACCTTCCGAAAATTTGTCCATATTTGTCCACATGGCCATCTGGCCTACCCCCAAATCAGCCTCCCATG
TCACCAATTTGGAGTCCAGCCTCAGGTGTTTTCAAGCAAAAGTTGTCCGCATCCAGTTCTATCCTATATAGGG
ATTATCGGGGGTCCTGGTTCTACTAACTGGAAAATTATGGAAAAATCACAGCAAATCATAAAATATTAAAAGA
ACATAAGTTTATATGTATGCTTTTGTCGGAAAACATACCAGTTATGGTGATACAGAAAAACATAACAAGAATG
CCATGTTTTCTACTGGTTACTGTGCCCATTTTGATGATTAAAGAGTGTAAGTATATGTTTTCAAGGTCCAGGA
ATTCGAATATGGTTTTAGAATTATTATCGAATCAATATCTGAGGAGTTATAGTGCAATTTTGTGTTTATCATA
TGTTAAATTTGCCATATCTCAA
>Agabi_varbisH97_2_Cluster24#LTR/Copia
ATTGGGATTGTGAATGCTTCAACATTCCGACTGTTCGACTTCATACTTCCTATTTTGGGTTGTTTCTTTTTCT
GATCTTTATATTCTATTTTTACCGTTCTACATTTCTTCTTATCCCAAGTCTTTCCGAAATTCGTTTTACAACT
ATATAGTTTTCGCTTCAATACTGTACAGTTTATATCTCGGCCGTTTTGCCGACATACTTATACGATGTAACTT
TAAATTCGTACCTCAGTGATCAACGTTCTTTCAGAATCACTGAGGTAAGTAGTTTACATTCTATACTATACCT
TTTACTTATGTCGCGTAGCTACTTTTATCTTAGGTAAATATAAAAGAGTGATCAACGTTCTTTCAGAATCACT
GAGCTACTTTTATCTTAGGTTATGGGCCCCGAGCTCAACTAAAACCCCACAAACCATACATTACTACAGACAA
CCACCTTATCTTACACGTCGCAATGGCTACAAACACAGGGGGTAGCGCCCCACCCATATTTGCTGATACTGCG
AAATTTGATGGCACAAACTGGGTGTCATGGAGTGGAATGATTCGTATTGCTGCAGAGTTGCGAGGAGTATTTG
GCTACCTCGATGGTTCAATTATCAACCCAGCTATCACCCCTCCTACTATACCAGCTGGACCAGCTGCGACTCC
ATCCCCGCCGACCATTACTATTACTCCACCATCTTCAACCACAGAGACACCATGGGAGTCTACTACTCCACAC
GCCCCCGAATGGAAAGTGCGTAACGCCTGGGCCAAAGGTCTTCTCATCTACAATACCACCGATCCCGTTGGTC
TTGGTATATCCATTGGGGGAACCGCAGCCGATGCCTGGAAGTCATACATCGACAACTACGAGGTGGCTTCCGA
GATCGCTCTCCTTAATGCTGAAATGGAATTGAGGAACATAACATATAGTGATGGACAAAACTTTACCGAGTTC
ATTTCACGTATCAGAACCAAATGGTCCAATGCAAATGCTCTTGGAGCGAGTATAGACGACAAATCCTTCCGTA
CCATCATCTTGAATGCACTTCCTCGTTCTTGGGACTCCATTGTCGCCACTTTATACACCACCAAGACCTCTCG
GGACGCTATAAGTCAACTCCTGACCCATTGGGCTCGTATCAGCAGAGACCGTGTCACTACATCGCAGACAACC
TCAGCACTTCAGGCTACCACCCAATCTGGCAATCGCGACCGTCAGCGCTCCCAAAATAGCCAACTCGTCTGTA
CCAATCAAAATTGCAACCGGCGAGGACATACAATTGAATTTTGTTACTGGCCGGGTGGAGGAAAAGCTGGTCA
GTTCCCACCAGGCTTTGGAAGACGTGGAGGAGCTAGGGGAAATGCCAATAACACTGCGACCGGGGGGTATCAG
ACATCAGCGAACGCAACTACCACAACCGAGAACGGCGAGAAGAAGGAAGAAAAGGTCTTCGCCCTTGCTGCTA
TTACGGACGAACCTGAAGATGTTAAGGTACCACCCGAAACCCCCATACATCCCAGCACTCCTAACACCTCTCC
AATCCCTAGTGTCTCTAATTCCTTACGCGTTGACGCTAGTAACGGACAAGGGGAGCTGGACGTTGGCATTAGG
ATTACGTCGAGTACATCTGAAACTACCTCACCAAATGATATGATTACTTTGCTCGACTCTGGAGCGAGCGATC
ATTGCTTCGTGAATAGAAACTCCTTTTCTACATACAACTCGATAATTCCCCCTCGCCAGGGAAATTCTGCAGG
AAAGGGATCGACGTTTAGGATTAACGGAGCTGGAGATGCCGAGTTACACACATCACTGAATGGCGTACCGACG
AAGATCATGTTCGCGGGCGCATTACACACCCCAGATCTGAGGAGCAACCTCATTTCAGTTTCTAAACTAGTTT
CAAGGGGGGCATCGGTTAATTTCGAGGGGAATAGAGCCGTTGTTCGTAATGGTGCTGGAAATGTGGCGATTGT
GGCAGTCAAGGAGAATGGCCTGTATGTCATACCGTCAAACAATGTATATGCAAACGCAGTCCAATCGAAAAGG
AAGGGCGTACCGTTAGAAATATGGCACCGGCGTTTAGGCCACATTAATGTCGAGGTGGTGAAGAGGATGCTGA
AAGATAATCTCGTGGATGGATTGAACGTCACTGGAGCGATGGAGATGGAGAGCCTTTGCGAGGACTGCATCTT
CGGTAAACATACTGCTCATCCCTACAACAACCCTTCACCACCCGAGAGCGAAGTACTACAACGAGTATATATC
GACATATGGGGACCATCCCCAGTCCAGTCAGCTGGGGGAGCTAAGTATTTTATGCTTGTCATGGACGGCGCCA
CATCCTATAGGAAAATATACTTCCTCACCACTAAGTCTGCTGAGATGACGTTGACGGCGTTCAAGGAATACCA
CAGGGAGGCAGAGAGACAAACGGGCAAGAAGTTGAAGGAGGTGCGAGCGGACATGGGAAGAGAGTGGCACAAC
GAATTGTGGAGTACGTACGTAAAAGAGAACGGCATCGTTCTCAACTTCACCACCCCCTACGCACACCAACAGA
ATGGGAAGGCAGAACGATCCATGCGTACGCTGCTGGACATGGCACGTACTATGCTAGCGGATTCTGGTCTACC
CCAGAAGTACTGGGCTGACGCTGTCCAAACCGCCGCATATGTGAGGAACCTCACTCCTACTGCGAACGACCTC
ACTGTTATCCCCGCTCAACGCTGGTCTGGTCAACGACAGGACGTCTCTCACCTCAGACCTTTCGGTTCAACTG
CTTATGCGCACATACCCACTGAGATTAGTCCTTCGAAATTGTCTCCTCGTTCATCCAAGCTTACACTGGTTGG
ATACTATGGACATACAGGTTATAAGCTACTCGATCGTACCACCGGCGCTGCCTACAAGTCGAGGGATGTCATC
TTTGAAGAGACACGTCCACACTTCTCAACCGACCCTTCCCCATCCTTCCAAGTTGACGAATCTCATGCCTCAA
ACCCTTCGAGCGCATTTGCGCCACGCCCAAAGCCTATTACCACCCTACATGACCATTCCACAATCACGCCACA
ACCCGTCGACGACGAACCTGCTGCTGTGGCGGAAGGTGTTGTCTCGCCGGAGTTACCTCTTGCTTTGAGGAGG
GGACGCCGAGAGATAAAGCCGTCCCAACGCATGAGGGACACTCTGGAGTACTTGGGGCGTCCAGTAGCCCATG
TTGCGGCATTATCAGAGGACCTTCTCGTGCCGAAGACCTACTGTGAAGCTATGAAGAAACCAGAGTTGTGGCT
TGATCCGATGTTGAAGGAGCTGCAGGTCATGAAGGACAAAGAGGTTTTCCGGCTAGTATCTCGTCCTGTCGAC

AAGAATGTAGTCAGGTCGAGGTGGGTTTTTGCCAACAAATACGATGAGGCGGGTGACGTTGTTAGCCAGAAGG
CTCGTTTAGTCGCTAAGGGCTTCACACAGGTCCTGGGGGAGGACTATGACGAGACATATGCATCGGTGGCACG
TTTGGAATCTGTCCGACTCGTGTGCGCTATTGCTGCAGCGCGGGGCCTCCGTCTGTGGCAGGTGGATTTTGTG
TCGGCTTTCCTGAATAGTGAGAACTCGTACGAGGTTTACATGGAGCAGCCGCCTGGTTTTGAGGAGGGGGGAG
ATAACGTCTGGTTACTCCTCAAAACATTGTATGGCACTATGCAAGGCGCACACGACTGGGCGCAAACGCTCGA
CCGAACCTACGCAGGACACGGTTACTATACGTCCTTAGCCGACCCACAGATACGATCAAGGGTTGAAGACGAT
GAACTAACTCTGACTTCAACTTGGACAGACGATGTACTCGGCGCTTCATCTACAATGGAGGGTGAGGTTAAGG
CGAAGGATGAATTGGGAAAGAGCTATGAGATCAAGGACTTGGGGACGGCAAGGTTTATATTGGGGATGAAAAT
TGAGAGAGGGGAAGATGGCAGTGTCAGGATATCACAACGGGCTTATTGCGAACGAACTATTGAACGTTTCAAA
CTTTCCGACTTGAAACCTCGTTCGACTCCCCTACCCACAGGTACAATACTCTCAATTGAAGACTCGCCGACGT
CCGATGAGGAAAAGGCTGACATGGCGAACGTTCCGTACAGGGAAGTACTTGGTTCTCTGATGTGGCTCCAAGT
CGCTACACGCCCAGACCTTTCCTTCGCTGTCAACCTATTATCCCGTTTTGCGAATAATCCCGGCCGCGCTCAT
TGGGATGCGATGAAGCATACTATGGCTTATGTCAAAGCAACGATGGACTATGGCATCACTTACCATCATGGAA
CAAGCATTCAGCCTTTGGGTTTCGTCGATGCAGACTTTGCGGGTGACACGGATGCTCGACGCTCAACTGAAGG
ACATGTTTTCTTTGTGGCGGGGGGACCTGTGTCTTGGGCAAGCAAGCGTCAAGATACTGTCGCCCTATCGACT
GTTGAGGCTGAGTATATGGCTTTTAGCCGTGCTGTCCAACAGGCTGTTTGGCTGACGACTTTTATGGGAGAGG
TGGGATTGCCTCGAGAGGAACCTATCTTGATCTATGCCGACAATAATGGAGCCATTGCCAATACGCTAAACTT
CAAAAACCACCGACGCACGAAACACATTGACATCCGGTATCATTTTGTTAAAGAAAAGACGGCGAATGGAGAA
GTTGTTTTCGAGTATATTCCTTCGTCGGAGAACTTAGCCGATATTTTTACCAAGCCACTTGCTCGCGACGCTG
TCATACGATGCTGTGATGGTATGGGTCTTCGGAGTGTCGAAGCAGGGGGAGTATTGGGATTGTGAATGCTTCA
ACATTCCGACTGTTCGACTTCATACTTCCTATTTTGGGTTGTTTCTTTTTCTGATCTTTATATTCTATTTTTA
CCGTTCTACATTTCTTCTTATCCCAAGTCTTTCCGAAATTCGTTTTACAACTATATAGTTTTCGCTTCAATAC
TGTACAGTTTATATCTCGGCCGTTTTGCCGACATACTTATACGATGTAACTTTAAATTCGTACCTCAGTGATC
AACGTTCTTTCAGAATCACTGAGGTAAGTAGTTTACATTCTATACTATACCTTTTACTTATGTCGCGTAGCTA
CTTTTATCTTAGGTAAATATAAAAGAGTGATCAACGTTCTTTCAGAATCACTGAGCTACTTTTATCTTAG
>Agabi_varbisH97_2_Cluster23#LTR/Gypsy
ATCAGGAAAAGGTAAAGTGACTTAAATTTGACTTATGCAAACTTATAAAAATCATAAGTCAGTATAAGTCATT
TTTGCATAAGGCATGTCACCGTCTGAGGTTCTGACTTATGCAATACTTAAGTAAATCATAAGTGCATAAGTCA
CAATATGGTCATAAGATTCAAGATTATCATAAGTCATGAGGATTTATCTTAAGTTGCATAAGTAAGTACTTAA
GACAGTAAGAGGATGTAAGAGAGTATAAGCTCAATCATAAGGAACATCATAATGCATAAGGAACAAAGAATTA
TATTCATATAGTAAATACAATACATCATGTATAATACAGACTTCCTAAAGAGGAAATAGACCCTGAAAAAAGC
GGACAAGAGGGATATGACAGCAGCCAGGGGGGTAGAAATGGAAGATGGGGGGGGAAATGATGAGAAATGTGAT
TCTAAAAGTAACAAATGAGTATGAGTAGATGAAGAGTCAAAAAGCACTCACCAAAGACTAGAAATCCCCCCAA
AACTCATCCACATATCATCCTCACATCTCCTCTCAAAACCCTCCCAAAATAGCCCTAAAAAAGCGGCCTAAAA
AGGCAAAAAAGCGTCCTAATTAAGCTGAGAATCAGCTGGGGGAGAGCTAGGAAGGTGATAAGGATGTTACTTA
CTAAGTTACAGGCCTGGAGGGCAAGAAAGGGGGAGGGAGAGGATGGGGAGGGGAGGAATAATGGTGGTGATGA
GGAAGGATCAACGTCGTGCTCGAAACAGAAGTTCAACATGTCACATGATGTCACATGACTGCTGGACTAGGGA
TTTTCGAGTCAAAGTTCCTCAAATCATAGGTAGGAGATATTTTCGGGTCATTTCTGCATGGATTTTAATGATC
TAAAAGTGTATTCCTATAGTTTTCAGGTCAGGGAGTCCGAATATGTTGTTAGATCTTTGGTTGGATGAAAGCC
TACTGTGATATGGCACATTTAACATATGATAAACACAAAATTGCGCTATAGCTCCTCTAATATTGATCAGATA
ATAATTGTAATGACAGATTCGGATTCCTGGACCTTGAAAACATATACTTACACTCTTCAATCATCAAAATCGG
CACAGTAACCAGTAGAAAACATGTCATTCTTGTCATGTTTTTCTGTATCTCCATAACTGGTATGTTTTCCGAC
AATGACATACATATAAACTTATGTTCTTTTAATATTCTAAGATTTGTCGTGATTTATCCGGAATTTTATGAAC
AGTAGATCAAGACATACAGTATAATGTTCTTATAATGCTGTGATGCTTGCCTTGTTATCTGACTTATTCACTT
ATGAGAACCTGTTTGACTTGTCAATAAACTTATGAAATTTTGTCTGACTTGTGGTTTAACCTTTGGTCTGACT
TATGAAATGTCCTGTGCTTATGCTTTCTTTGTATCTGTTAAGACTATGACCGGAAAGGTAACGCTAAGCCGGA
AAGGACTTAGCGAACAAACGGCTTTGGCCTTAGCGACACGGGAACGGTGCACTATGCCTTCCAACTAAGTCGA
TAGCCGATTTAGAGACAGAGACAAGCGACCCGCGGAATGCGGAATTGTTTGAGAGCGAGAGAGAGATCAAGCG
AGAGATAGCGAGGAACAATCTAAGTTGATTTTCAGACTGAAGTACCGTACAGAATATCTACGGCTTAAATACA
CGAAGAATGAATAGAATACGGATTCGGAAACATGAAAGATAATAGCGCAAGGATTGCGCAATAGTTCGAGTTG
AAATAACAGAAAGGAGTCTTTACCTTAAGGGATTCCGACGGTGCAACGGAAAGGAGGTCTTGAAGCGGGTAGG
TCTTGCGATGGAAATAACAACCCACGTTGCGTTGGTCGTAAAGCGACCGTGGGAGCGCAACCGCAATACCTTC
GGTATTGCGGCATTCCATCGGTCAACGTGACCGGTAAGTCGGAATCGATATTGCCGGGATTGATGCGAACGGA
TTGAGTCGGAAAGGGATAGCCGGAACGTACTGGGATTGATACCGAGAATGGAAGGTGGTGGAGTTAGCATGTT
CCGGATATAACAGTATCCAACTTATGAATTTATGATAAACTTATGAACTAATGTGTGACTTATGTGTAAACTT
CTTGTCTGGCTTATGAAAAATCATGAACTTATGCTATTTTGGAGACTTATGTGCCTTAAGTTGGTCTTTATAA
GGCAAGCATAAGGCAACTTTTTCATAAGTTTGCATAAGTTGTGTGACTTTTTGCTGATGAAATCGGAGCGGAA
AGTTAACTGAAGACGCCAGGATCCAGATGGATTTAGGAGGACACAAGGAGGATATTAAAGCATTGGTGTCGGA

CATATCAAGTGCCGACATTTTGGGATACGATTGGTTAATTCAACACAACCCGGAAATTGATTGGATAAATCAG
GAAGTCAGGTTCTCTAGATGCCCAAGCACATGCACGGTTCAACACGAAACTATCCCTTTTGCTCGACGGCTGA
CCACTGATGTGGAAAGCGCTACGGAGGAAAAGGAAGTAGACCCTACCAACCCGGAAGACTTACCTGACTATAT
CCGTCCTTACGCCCATCTTTTTAACAAAAAGAACTTCGATAAAGTTCCTGAACGAATGGAATGGGATCACGAG
ATTAACTTCACCGAAAACGCACCAACAGAGATATCATCAAAGGTTTATAGCATGACACCATTGGAAAAGGAGG
AACTGGACAAATTTTTGGATGAAAATCTGGCTACAAATCGAATCCGACCATCGAAATCACCTTACGCAGCACC
ATGTTTTTTCGTCCCTAAGAAAGACGGTTCATTACGGTTATGTCAGGATTACAGGAAATTGAACGAGATCACA
ATCAAAGACAAGAAACCACTACCCCTCATCAGCGAAGTCCTAGATCAACTCAAGGACGCCAAGGTTTTCAACA
AATTGGACATTATCTGGGGATACAACAACGTTCAGATACGAGAAGGAGATGAATGGAAGGCAGCGTTCTTAAC
AAATCGCAGGTTATTTGAACCAACGGTCATGTTCTTTGGAATGAGCAACTCTCCCGCCACTTTTTCACGCATG
ATGGCAACAATTTTTCGAGAAATGATACAAGACGGATCTTTGGCCAACTACATGGATGACTTCATTATTCCAG
CAAAAGACGACAAAGAATTGGAATCACGAACTACACGTTTCCTCAAAATAGCAGAGAAACACCATTTATCATT
CAAACGGACCAAATGTGAATTCAATGTCTCATCAACAACGGTATTAGGAACGGTTATGGGAATGGAAAGGCAA
CTATGGAAGAAGAAAAAGTCAAGGCAATACGAGACTGGGCAGTCTCCACCACGGTCAAACAGGTCAAAAGTTT
TTTAGGTTTTGCGAATTTCTATTGACGGTTTATCAAGAATTTCAGCACCATCGCACAACCACTGAACAAACTG
AAGTCTAAAAAGGGGGAGAAATGGTATTGGAATGATGAACAGCAACAAGCTTTCGAACAGATCAAACAAGCAA
TTGCCAGCGAACCGGTTCTCGCATTACCAAAGGACACAGGACAGTTCAGAGTTGAAGTGGACGCATCAAACTA
TGGAACTGGAGCAGTACTATCACAAGAACAGGAAGACAAATGGCATCCGATTGCCTTTATGTCGAAAACGTTA
TCTGAAGCCGAACGAAACTACGAAATCTACGACAAGGAACTACTGGCCATTATAAAAGCCTTGAAGCTATGGC
GACACTACCTATTGGATGCAAAGGAGCAGTTTGAGATATGGACAGATCACGAGAACCTCAGACATTTCCGAGA
ACCTCAAAAGCTCAACGCTCGACAAGCGAGATGGTACCTCATGCTACAGGAATACAATTTCCTTCTACGACAC
ATTCCAGGAAAGACTAACACCAAGGCCGACATCTTATCTCGCATTGTTAAACCGGACACCTCAGAGGACAACA
AAAATGTCGAAATGTTCAAGGACAAAATATTCACACGAGAATTCAGGGAAGACGTATCAAGTTACGAAGAGAC
AATCTTACTAAACTGACGATTTGAAGTATCAGCGGACCAAAAGTTATTGGAAGATATAAAGAACTCGAAAAGA
AGGGAAACAATGGTACTACAGGAGATGAAGAAACAACCGGAGAAAGTATGGGAATCAGAAGGAATTGTTTTCC
AACAAGGAAGGATTTACGTCCCAGAAGAGAAGGAACTCAGAGATCGGATCCTTTACGATCATCATTATCCACC
CGACATCGGACATCCAGGACAACATCGCATGTTAGAGTTACTAAAACGGACCTTTTGGTGGCCTACAATCAAG
ACGGACGTCAGAAGATACATCAGAGGATGCGACATGTGCCAGAAGAACAAAACAATTCGACGACCCGATCACA
TTCCACTTAATCCACTTCCTATCCCCGACAAACCTTGGGAAGAGATATCTATAGACATGGACCACTACTGAAG
TCAAGAGAGAAGGATGCTATTATTGTTATTGTTGACAAATTCTCCAAAATGATCCACCTTGTTCCCACCACCA
CGTCACTCACATCCACGGATCTCGCGGAGATCTACAAAGAAGAAATTTGGCGACATCACGGAATTCCGAAACA
TATTATCAGTGATAGAGGACCACAGTTTGCATCGAAATTCATGGAATCTTTATGCAATGCTCTAGGCATCAAA
CAAAATCTTTCTACGGCGTATCACCCACAGACAGACGGACAAACAGAGAGGATGAACCAGGAAATTGAAGCCT
ATCTTCAAGCATTCATCAATTATCAACAAGACGATTGGACAAGATGGCTTCCCATGGCAGAATTTCATTATAA
CGACAAAACCCACGCTGCCACCGGACAAACCCCATTCTTCCTGAATTACGGATTCCATCCATGCATCAGGAAA
AGGTAAAGTGACTTAAATTTGACTTATGCAAACTTATAAAAATCATAAGTCAGTGTAAGTCATTTTTGCATAA
GGCATGTCACTGTCTGAGGTTCTGACTTATGCAATACTTAAGTAAATCATAAGTACATAAGTCACAATATGGT
CATAAGATTCAAGATTATCATAAGTCATGAGGATTTATCTTAAGTTGCATAAGTAAGTACTTAAGACAGTAAG
AGGATGTAAGAGAGTATAAGCTCAATCATAAGGAACATCATAATGCATAAGGAACAAAGAATTATATTCATAT
AGTAAATACAATACATCATGTATAATACAGACTTCCTAAAGAGGAAATAGACCCTGAAAAAAGCGGACAAGAG
GGATATGACAGCAGCCAGGGGGGTAGAAATGGAAGATGGGGGGGAAATGATGAGAAATGTGATTCTAAAAGT
AACAAATGAGTATGAGTAGATGAAGAGTCAAAAAGCACTCACCAAAGACTAGAAATCCCCCCAAAACTCATCC
ACATATCATCCTCACATCTCCTCTCAAAACCCTCCCAAAATAGCCCTAAAAAAGCGGCCTAAAAAGGCAAAAA
AGCGTCCTAATTAAGCTGAGAATCAGCTGGGGGAGAGCTAGGAAGGTGATAAGGATGTTACTTACTAAGTTAC
AGGCCTGGAGGGCAAGAAAGGGGGAGGGAGAGGATGGGGAGGGGAGGAATAATGGTGGTGATGAGGAAGGATC
AACGTCGTGCTCGAAACAGAAGTTCAACATGTCACATGATGTCACATGACTGCTGGACTTGGGATTTTCGAGT
CAAAGTTCCTCAAATCATAGGTAGGAGATATTTTCGGGTCATTTCTGCATGTATTTTAATGATCTAAAAGTGT
ATTCCTATCATTTCGAGGTCGGGGAGTCTGAATAT
>Agabi_varbisH97_2_rnd-1_family-41#LTR/Gypsy
AATCGAAGTCAGTGAAGACTTTAAACACTCGACCAATAGAGGGTCTGTACTCGAAGTCAGTGAAGACTTTAAA
CACTCGACCACCAAGGGTCTGTACTTGAAGAGTCTTATAAAGACTAGGAATAGTGAGTCCAATTGAAGGAGCA
CTAACCACGGATTTACAGAATTGTACCTGTAAGTTTTGTACACAACAAAATTTACCNTTCAAAGCAATACCAG
AGATTTGGTCTGGTATTGAACGGAAACTGCCAAAACGGAGTCATTCTTTAGTAACAAATTCAAGCAAACCGCT
TACAAGGAAGACAAGACAAAGAAGTGGTACTATACCAATATCACAGAAGGAAAAAGCTACTACTTCGACGACG
CTGACCAGCAACGAATTGCATCTGAGTATCTTGAATACGAAACCCAGAAACTCTTTAGAGCAGCAAACCTCCC
AAGAATCAGAACAAGTAGTCAAGACGAACACATTAACAGACAAACCTCAGAAGCAATCAGAAACCTCCGATCT
GCTACACAATCAGCCGCACAATCTGCTACACATTCTGCAAAATCAGATCATCAAGAAAAAGGAAAAGATAAAG
AACCTTCGCAGTCAGTTTTTGACTGGCCAGAAACAACAGTTGGAGAAGGAAATACAGATTCTGGAAAAGGAAA

TCCAGGACCTCGAAATCAGGACACAGAAAGTCCACAGGAGGAAGTTAAAACTAACAGAAGTCCAACAGGAAAT
ACGAACACAAGCCCTCCGAAAAGCCCTCAGCCAACGCCACCAAGAACACCAACGCCACCAACACCACCAGCAG
GAAACCCAGGAAGTCCAGCCGGAAATATGGCACAACCACCAGCAGGACCGCACATCAAATACCCGAAACCAAA
TAGGTATTACGGAGACCCAAGATTATACAGACCATGGATGGTATCCCTCGAACTCTATTTCAACAGCTATGGG
ATTACAACGGACAACTCCAAAATCAATTACACCCTTGGATTACTTGAAGGAGCCGCATCCAACTGGAAATCAG
ATTTTATCGCCACTCACCCAAATTATCCAAACTGGGCAGACTTCAAGAATTCTCTTGAACAGGCCTTTGCACC
AGCCCAACAAGGATATGAAGCAGAACAAAAGCTACGACGCTATAGACAAAACGGAGAATACATTGAACACTAC
ATTAGTGGATTCTCAGTATTATCCTCACAAGCAGGATTACCAGACAGCACCGCACTCAGAGCATACTTCGCAG
AAGGACTCGATAAAGACGTCCGAATTGAAGCAATCAGATTCACCCCAACAACACTACAACAGTGGAAAGACGC
CGCAAGAAATGCCTTCAGAATTGTTATGGAACAACGAAAAGCATCAGGCAATTTCGGACAATCGTCAAGATCA
CATAAACCATATAAGAAAAATAAACACCGATCTCAAAAGAGTATTCCGAGATACAGCAATATCTACAACGAAC
CAGTCCAACATAAACCCGCAAGAAGCGAGTGGGACATGGATGTTGACCAGATCTACTTCCAACTCAATCAACT
CGGATTGGACGACGAATCCGAAGATTCAGAATCAGAATCAGAGGAATCAGAATCCGAAGACAGCGATGATGAA
GAAGTCAATTTCATCAGACAACGAAGAACCCAAACCCAAACGCCTAAGCAAGCGGTACAGGGATTTATTAATG
CAATCCTCACAGATGAACAACGAGCAGCTCTCAAACGAGGAGAATGTTTCTTCTGTCACAAGAAAGGACACTG
GTACAGAGATTGCCGTGCAAGGAAGCTATATATTAAGCAAGGAGGAAGAACAAGCATGGATCGAAATCCAGGA
ACAGATCGAAAGAAGAAACAAGGAAACGGAAAACCTAAGAAGCAAAAGACTTGGAAAAAGAAGGAGCCCATAG
ATCCGAACGCTATGGTCTACAACATTGAAGAAGAAGAAGACAACTTTGAAGAATTTTCTAAAGACGGAAATTT
TTGAGTACTTCTGAAGATCCAATCGACGTGATAGATCTTCAGTCACCTAAAGGAACAACACTTCTAGCACCGA
TTAAAATTCACATTGAAGACGATAATCCTATTAAACTAACCGCACTAATAGATTCAGGAGCAGCAGGAGAATT
TATCGATCACAAACTCGTAGAGAAGTATTACTTACCAAAATTTCCATTGAAGAAAGTCAAACATACCATGAAT
GCCGATGGATCAAGAAATGCATCAGGAGTATGCACACATTATGTCCTACTCAACATCGAAATCAACAACAAGA
AAATGNTGATACAAGCAAGCATAGTATCGCTTGGAAACCACAAACTATTCTTAGGTATATCATGGCTAAGAAA
ATTCAATCCAGCAATAGATTGGACAAAACAAACTCTAAAATGGAGAGATGAAGATGACGACTCAACTCTTACA
CTTTCTAGAGGACCATCAAGCAACTTCATTAACAATATCTTCGTTGAAATCAACTCGAAGAGCAATACTTCGC
AACAGCTATTCAATCAAGCTGAGAAGAAGAAATTCGAAGCATAGACTCAGTAGAGCAAGTGGTACCAAGACGG
TTCCACAAGTTCCTTAAAGTATTCAAACAAAAGGAATCAGAGAGANTGCCTACTAGGAAACCATGGGATCACG
CTATCGAATTGAACTTCGCGGATTTCAAACGCACCAAGAGAGGTATCATCAAAGGTNTATANCNTGNCGCCAA
NNGAAAGAGAAGAACTGGACAAGCATTCNTGGANGANNANCTCCGAAAGGGNTACCAATCGAATCCGACCATC
GAAATCNCCCCANGCNTCACCGNTNTTTTTCGTNCCNAAGAAAGACGGATCACTANGCNNATGGTACAGGACT
ATCGGTNTCTGAACGANNNCACGATTAAGAACAATTACCCTCTACCTCTAATCGCTGAACTNATAGACCAACT
ANGGGNNGCCAAACGGTTTTCANCNAAGNTAGATATTACGTGGGGNTATAACAACGTTAGGATACGAGAAGGA
GACGAGTGGAAGGCAGCNTTCNCGACGNNTCGCGGGTGCNTTTGAACCNACNGTCATGTTCTTTGGAATGACN
AATTCGCCCGCCACTTTTCAGNCNCGATGATGGACGANCTTTTCGGGGANTTAGTANANANCGGANNAGTAGC
CGTNTNCATGGACGATATCTTGATTCCAGGAACGGACGATGAAGAGAGCATGATGAAGTCGCGGAANAAGTNT
TNCAGAGGCTGACGGACAAACACGATCTATCGTTCAAAACCGGACGAAATGCGAATTGGAAACGCAACGTCTG
GAATATCTTGGGGTGTATAATAGGGAGAAACGGATTATCGAGATGGAGGAAAGGAAAGTGAAAGGCGTAGNAG
ANTGGCCGGTCCCCAAAACGCGTCAAAGANGTACGAAAGTTCTTTGGGTTCTGCAAATTNCTATCGACGGTTC
ATCGAAAANTTCGCCAGCATCGCGCAACCACTAAACTCTCTTATGAGGAAGACAA
>Agabi_varbisH97_2_Cluster19#LTR/Copia
AATGGAAACGACTCAGTAACAACGAAGATAGTTGTCCACTAAATGATATTTTACCTAATGGAAACGACTACGC
ACAAACATAAGTATAAAGATATCTACGAAGATAATAAGGCTCACCTCAGTAACAACGAAGATAGTTGTCCACT
GAGGTGTAAGGATAAGGTTTACAGTAATAAATACCTCGGCACAAATGCCGGATGTAAACTGTACAGTAAATCT
GTAGAAATATATAGTTGGGATTTTGGGTTTGAATGTAAAAGACGGATAACCAAAAGACGGAAGAGCAGAATGT
AGAAGATGACGAAAATAGAAAGATAAAAATAGCCCGTGTCTGAAAGAAGTAATATAAGTTGAGAAGAAATATA
CTTCGGTGTCGAAGCACCTCAACACTCCCCCTGCTTCGGCGACGTGTCCTTGATAAGCCCAATACCACCACAA
ATGAATAGCACGGCTTCTTTAGCGAGTGGTTTTGTAAAGATGTCGGCTAAGTTGTCTGCTGAAGGAATGTATG
TGAAGCCGACCTCGCCAAGTGCAACGCTCTCTTTGGTAAAGTGATGTTTGACGCGGATATGTTTTGTACGACG
GTGGTTCTTGTTATTGGTAGTGTTAGCCATTGCGCCGTTGTTATCCGCAAAGATCATCACTGGCCGTTCCTGT
CCCATGCCTAGCTCGTCCAAGAATTTGGTCAGCCAGATGGCTTGTTGCGTAACATGAGTGAAGGCAGTATATT
CAGCTTCAACTGTAGAAAGAGACACTCGTTCCTGCCGTTTGCTCGTCCAAGATACCGCGCCGCCAGCGACGAA
GAAAATATGACCTTCAGTTGACCTGGAAGTGTCCTTGTCGCCAGCGTGGTCTGCGTCGACATATCCAAAAGGC
CGGATACTGGCATTTCTGTAATAGGTTATGCCATAGTCGAGTGTTCCCCGAAGATAAGCCAGAGTATGCTTAA
GGGCTTCCCAGTGAGCATGGCCGGGGTTGTTGGCGAATCTAGATAACAAGTTCACGGCGAAAGACAGGTCAGG
TCGGGTAGCCACTTGGAGCCACATGAGAGAGCCCAAGGCCTCACGGTACGGTACGTCCTTCATCTCTAGGGTT
TCTTCAGCAGAATGTGGAGAGTCGTCAACTGAAAGTACGACTCCAGCGGGCAAAGGTGTCGACCGAACATTGG
CATCAGCCATATGAAACCGTTCAAGGACTCTTTCAGCGTAAGCGCGTTGGGATAATCTGATGCTCCCTGTAGT
GTCGTCCTTCTCTATCTTCATCCCAAGAATGTATCTCGCCGCCCCCAGGTCCTTTAACTCGTAGCTGCTCTTC

AACTCCCCCTTGGCTCTGATCTCCCCCATTTCTGTCGAAGATGCCCCGAGAATATCATCAGTCCATGTCGATG
TTAATGACAGTTCACCATCTTGTAGTCGAAATCGTATTTGTGGGTCGGCTCTGGAAGTAGTGTATCCATGGCT
CTGGTAGGTACTCTCGAGTGTGCGTGCCCAGTCGTGCGCTCCTTGCATTGTTCCATACAGCGTTTTGAGAAGT
AACCACACGGTATCTCCCCCCTCCTCAAAACCTGGCGGCTGCTCCATATACACCTCAAACGTGTTCTCGCTGT
TCAAGAAAGCTGACACGAAATCAACCTGCCAGAGACGAAGGCCGAGGGAGGCAGCTAGCGCACAAACCATACG
TACGGACTCCAGGCGTGCCACAGAGGCGTACGTCTCATTATAATCTTCCCCCAGCACCTGCGTGAACCCCTTG
GCAACCAGGCGAGCCTTGCGCGCCGTAATGAGTCCAGACTCATCATATTTGTTCGCATAGACCCAGCGGGACT
TCACAACATTCTTGTCGGGAGGACGTGGTACCAAACGATAAACCTGTTTCTCTCTCATCACTTCCAACTCCTT
CATCATAGGCGGAAACCATAGCTCAGGTTTCTTCATGGCGTCATAGAAGTTCTTCGGCACGTATGTGTCTTCG
TCGACAATGTTGGTGTCGGCAACGTTCGCTCTAGTTCGTCCCAAATACTCCAAGCTCTCCTTCATCTTCGAGC
TCAGCTTCACCTCCCGTCTAGGTCTCCTCGCCGCTATTGAATCATCGTCAGGATCACGGAGAAGGTGTGTAAC
CTCCCCTTCACTGTCCTGCTTGCCATCGGAATCGGAGTCATCCTTCGAAGTTGCCAAAGGCTTTGTTGGTTGA
ACTGTAGAAGGAAGCGGCGGGGCAGGCGGATGGAGCGTCGAGATAGGTTTGGGTCGTGGTGCAATAGCAGCAG
GATCTCGAGTGACCGGCGCCGAGTCGTCAGGCCGGAACGCCACAATCGGGTCGGTTGAGAGGTGGGGACGAGA
CTCTTCAAATATGACGTCTCTGGATTTGTGTATAGCGCCGGTAGAACGGTCAAGTAGCTTATAGCCAGACCGT
TCAAAGTAGCCAATAAGTGTGAGCTTGAGTGAACGTGCGGACAGCTTGGAAGGGCTTATCTCAGAGGGGACGT
GGGCATAAGCTGTAGATCCAAAAGGACGAAGGTGGGACACATCCTGTCTCTTCTGTAGCCACATTTGAGCAGG
AATATGACTCGGGGAGCGGGTGGTGGGGATGAGGTTGCAGGTATAGGCCGCCGTTTGGACAGCATCAGCCCAG
TATTTCTGTGGCAGCCCAGCGTCGGCCAACAGTGTGCGGGCGGTATCGAGCAAGGTGCGCATGGAGCGCTCAG
CTCTACCGTTTTGTTGATGTGCATAGGGTGTGGTAAAGTCGAGAAGAATGCCGCTCGACTTTACGTATTGATC
CCACGTCGAGTTGTGCCACTCTCTCCCCATGTCCAAACGCACTCTCTTCAATTTCTTCCCAGTTTGCCGTTCA
CTCTCCAGATGATATTCCTTGAAGACCTTCAGGGTCTCGTCCGCAGACTTCGAGGGTAGGAAATAGACCTTCC
TGTATGAAGTGGCGCCGTCTACCAAGAGCATGAAGTATTTCGCTCCCCCAGCAGACTCCACCGGTGAGGGTCC
CCAAATGTCCACGTATATGCGTTCTAGAGGTTCTGTCTCATTCGGAGCGGTATCGCTGAATGGGTGAGTCGTA
TGCTTCCCATAGATGCAGTCCTCACATAACCCATCCAGCTTAGGTTCGCCACTCGTATTCAGGCCGTCAACTA
GGTTCTCTCGCACCATCTTCGAGATGACATCCGTAGGAATATGTCCGAGGCGTCTATGCCAGATATCATACGG
TACTGGCTTGCGCTTGGTCTGGGCCGCGTTCGCATGTGCATTGGGTACATCAGCCGTATCAACCACGTATAAA
CCATTCCTTTTGATAGCGGTGAGTACGTTGTTCCCTGCCACGTCGCGCACAACTGCTGAGCTCCCTTCAAAGC
TAACGGTCGATCCCCTCGAAACCAATTTAGAGACAGATATCAAACTCGAGCGTAATTGCGGCGTATGAAGACA
ATCGACCATCACGATCTTTGATGCGACACCGTTCACGTTTGTCAAGAATTCTATTGTGCCAGAACCTTCAATA
TTGAAGGTTGACCCTTTGCCGGCAGTATTTCCCGTTCTCGGTGGAACAAATGACTGATATTGAGAGAAACGGT
GTCGCTGGACGAAACAATGATCACTCGCACCCGAATCCAACAGAGTGGGGATGTTATTGTGAGAGTCAGGTAT
GTTTAACAATTCGGTATGATACCCATCCACAGCTCCCTCAGTCTGATTCGATCCTTCAGATGTGATATTGGAC
GTAATTCTGGTAGCGATAGGATCAGAATCTGGGGATGATGGAGGGTTGGAGGGTACCTCTTGCTCATCAACCA
CTGTGGTCGCGAGGGCGAAGACCTTGGGTTCAGTATCATTGTTAGGGCTAGGTCCACTGGTCGTAGTGACGTT
GGCGGTAGTGTTTTGGTTGGTAGCCTGATCCGTAGTAGGTGTGTTTTTCGCTCCCCCGCGTCTGCCAAACCCA
GGAGGAAACTGACCGGCTTTCCCTCCTCCAGGCCAGTAACAATCTTCAATGGTGTGGCCACGACGGTTGCAAT
TCTGATTGGTGCAGTGAAGATGTTGGTTACGCTGACGACCACGGCTGGGTGTGGATGCTTGGAGGGCAGATGT
CGATGACTGGGGATTGGTAGGTCTATCTCTACTTATTCTAGCCCAATGGGTCATTAACTGATTAATGGCATCT
CGTGAAGATTGGGTAGTGTATAACGTCGCGACAATAGAGTCCCATGATCGTGGAAGAGCGCTGAGGATAATCA
TGCGGAAAGCTTTATCGTCTATGGTCGCTCCTAGAGCTGTTGCATTCGACCATTTCGTACGCATGCGAGAGAT
AAACTCAACAAAGTCTTGTCCATCAACGTAAGACACATTGCGGAGGTCAAGCTCAGCATTTATGATAGCGACC
TCTGACGCAACTTGATAGGTGTCAACGTATGATTTCCAAGCATCAGCGGCCGTTCCGCTTATGTTAATGCCAA
GGCCAACCGGATCGTTTGTGTTGTAAATCAGCAACCCCATAGCCCACGCGTTACGGACTCTCCATTCAGTCGG
GGTGGGTATAGTCGATTCCCAAGGCGAATCAGGAGTGGTGGTAGTCGTCTGCGTGATAATTGGTGGTGTAGGT
GGTGTATCAGCAGCGTCATCGTTAGGTGGTGGAGGTATAGGCGCGTTTAATGTGTTAAGGGATGGCTGAGGAG
GTTGGGTGACAGTACCATCAAGATACCCTCGTACACCTCGTAAGTCAGCCGCAATTTGTATGAGGCCTCTCCA
ATTGACCCAATTAGATCCATTGAACTTCGATCCGTCAGCGAAGATCGGTGGTGCACTGCCGCCAGTGCCAGAC
ATAGTTGTAAGTAAAGTTGTTTAAGTGCGAGTGGATGATGAAGGTTAATCTTCGTTGTTTAGGCGGGGCCCAT
AACCTAATGGAAACGACTCAGTAACAACGAAGATAGTTGTCCACTAAATGATATTTTACCTAATGGAAACGAC
TACGCACAAACATAAGTATAAAGATATCTACGAAGATAATAAGGCTCACCTCAGTAACAACGAAGATAGTTGT
CCACTGAGGTGTAAGGATAAGGTTTACAGTAATAAATACCTCGGCACAAATGCCGGATGTAAACTGTACAGTA
AATCTGTAGAAATATATAGTTGGGATTTTGGGTTTGAATGTAAAAGACGGATAACCAAAAGACGGAAGAGCAG
AATGTAGAAGATGACGAAAATAGAAAGATAAAAATAGCCCGTGTCTGAAAGAAGTAATATAAGTTGAGAAGAA
ATATACTTCGGTGTCGAAGCACCTCAACA
>Agabi_varbisH97_2_Cluster18#LTR/Copia
TTAGTCACATGATAGCTTAGTCATGTATGGTTGAACTCCCCTGTGGACGCTTCAGAGGCCTTAGCTTCTTACA
GTGTATCCACAGGTGAGTACAAGTTATTAGAACTTAATACAGCTTCTTACAGTGTATCCACAGTTTGTCCTTG

GGATTGCTCAGTCAGTTCCTATCCGATTGACTCGCTTCTAGGCTCGTTAGGATCGCCACTGCAACCGCTATCG
TTCTACGTTTAACATTTTCACGAGGCTACCCCATCGACATATAATGTCTTCGAAGATCCAAGATCCAATAGCT
TTAGCGTCTTCCTTTCTAAGTGCTCTAGCTTCCATCCAAGATGACAACGCACACCTTCCATGAACAATGTATT
TACATCCGTTCGAAGGTGGAGTCATATGAACAGTATCCGCATGCAACACTTGGAATATAGAAGGGGTATGGGT
AACTACTGGTGGCAATTCTAATGCCATTTTCTGTCTGACTTGGCAGAGATGACATGACTTGACGTACCAAGCT
ACATCTCCATCTACTTCTGGCCACCAGAAGCGCTGAGCTATTAGTTTACTAGTCGCATAAGCGCCTCGATGTC
CTAGCTTATCATGGCATTCTCGCATTATTCTCATGCGATCTTCTATTTCGACCACTAGCTGAGGAAATCCGGA
TCCTGCAGTCTTTCGAAAGAGTCTCCCTTCCTTATCTAGCCAGAAGTGGGAAGCTGCTCTCACAAAACGAGCT
GTTAGAGTGTGAGCAAGCAGTCTACACTTGGCCATCTGAGTGGGTTAGGTGCAGCTCTTCCAGCTGTCAAGAC
ACCACCCACTCCTCCTAGGGATGTTTCTACTGGTTCTGGCTCTCCTGGCTCTGATATTCCTCTGAGAGCGTCC
GCTCTTGGTGCTTATTCCTCTCCTTCATCCTCTCCCTCATCCTCTCCCTCACCATCCTTGCCACTACAGCTTG
ATGCTGACTTTTTCTGGCTGGCTGATACTGGTGCTACATCCCACATGACTCCTCACCGCCATTGGTTCAAATC
CTACTCTCCTCATCGTATCCCTATCCGCCTTGCTGACAACTCTACTGTCTATTCAGCTGGTGTAGGGTCAGTT
GTCTTTCAACCGGTGGTGAATGGAAAAGAGACTAGAGCGGTCGAGTTCACGAGAGTCCTTCATGTTCCTGACC
TCAGGAGTAATCTCTTATCATGTCTCTACCTTGCCCAACACAAGGGCTTCAATATCACTATCTCCGCTCAATC
TATTGACTTCCAATGCAAAGGGATTTCGCTTTTCACGGCCACTATCCATTCCAACAACTCTGCTGAGCTCGAC
GGCTCAACTCTCACTTCTGAGACTGCTTTCTCTGTCTCTACTCTCCCAGTTGACATCTCGCTATGGCATCGTC
GCTTCATTCATCACAACTATGCTGATGTGAAGTCTATGATCAATAAGGATCTAGTTTCCGGGCTCAAACTCGA
GTCTAATTCATCCCCTGACTCTATCTGTGAGCCGTGCTTGGCTGGCAAAATGCATTCCCTCCCTTTTCTCTCC
TCTTTGAATCACAATCGCTCTCCTCTCGAGCTTGTGCATTCAGATCTTCACGGACCGCTCCCAGTCGCTTCAC
ACTCAGGCTACAAGTATTGGATTACCTTCATTGACGATGCGACGCGTTTCCGGGCAGTCTATCTGCTTAAAGC
TAAGTCGGAGGCATTTGAGGCCTTCAAAGTCTACAAATCCTGGGCTGAGACTCAGATCGGAGTCAAAGTGCGA
GCACTACAGGATGACAAGGGAGGTGAATACATGTCTAATGCTTTCAAAAGCTTTACTGAGCTTGCTGGCATTG
AGAGACATCACTCAACTCGGAACCGGCCTCAGCAAAATGGTCTTGCTGAACGAGCTAATAGGACTATGGGAGA
GAGGATCACTGCGATGCTCTCGGAATCTCGGCTACCTCCTTCTTTCTGGGGTGAATGTATCTCGTCTATGGTC
CATGTCTGGAATATGCTCCCTACTGCTTCGCTTTCGGGCACTACTCCCTTCCAAGCTTTCTATAAGCGAAAAC
CTGATGTCTCACATCTCCGGGTATGGGGGTGCACGTCATATGTCCATGTACAACGAGATAAGCGCAATTCTCT
CCAGCCTCACTGCGAGAAGTGTGTCTTTATAGGCTATCCGGCTGGCTACAAAGGCTGGAAGTTCTACACTCAT
CAAACACAAGCCGCGCTTCATGCGCGCATCGCCTCACTCTGACTCGTGCTGGGCATGGGTCGATATTCACGAC
ACGGTGTCGGGCTCTAATGCTCGACTCTATATTTCGAAGTTTGTGTCCATCGGCGGCACGAATTGTCAAATCA
AGGGCGCTCGCCCTCATTCCGGCTCCGTACACTGCGCTCGTTGTCAGCGCTGGGGCCATCACTCAGATCAGTG
TCACGCCAAGTGCGCTCGCTGCTCTCTCTGCTCTGGTCCTCACTCCGAGGCCAATCACTTCAAGTGCGTCGAC
GCTAAGCGCGTTGACGTGCGCCAATGCGCAAATTGTACCGCGGCTAAACGCCCAGCGGACAAGCGCTCACATT
CCGCTACGGACTCGAAGGTATGTCCGTTTTGGAAGAATAGGTTCGATCGTGCGTGGCTTAAATGCCAGTTCCC
TGCACATTCGACTTGAGGTGTACAGTGAACCGTCGCCGGGTTGGGTCGGGGATTCTGATGGAGAAATGGGAAG
GATGCGTAGCGGTTTAGGAGGGAGAACGGTAGTGGTGAGGACAAGACTTTCTGAGGATCTAAAGTTACTTCGA
GTATGCCTCAATTTTTCACTTTCATATTTGATATGTTTGATTCCGCAAAAGGGACTGCGAGTGCTCTAGTGTT
CCGGTATTATGTAGGATGTATTGCCTGACTGCTTGGTTTTATCCCGGGGCCTCTTACGTATGTATCAATGTCT
CGATTTGTTTTTGTTTTGTTTCTTTTTCTCTTTCTCGATAGCGCTTGACCTCGTCGCGTTGGATACGTTAGGA
TAGAATACTGTAGTTTGGGGCCTCTGTAAACTGGCGAGTGTAGTAAGCCGCAGGAACGTGTCGTGACACCCGA
CGTACGAGTAGTATTGAAATATACTTACGTTGATTACAAAAAAAAAAAAAAAAAAAAAAAAAAAAAAAAAAAGC
TGGAAGTTCTACAACCCAGTCACAAAGCGAACCGTGATCTCTGAGAGAGCTGATTTCGACGAGAGATACTTTC
CGGGCACTTCTAAAGCCCAACTCGAGGCTGTCCCGTCATTTGCCTGCACACCAGAGTCACTACAAACTCCTAA
TGATACATTGTCATACTCGGGACTGCCACCTCTACAGGATGAAGGGGGTGATAATCACATCCCTCCTGCTCGT
CCTAAGACTCCCACTCCGTCCGCTTCAGTTCCTCCTGAAGAGAAGCCAGTTCTACCTTTACCACCTCTCCCGT
TGATTCCTCCAGTAATTCCTCCCTTGATTCCTCCAGTCACTCCTCCACAAATCCATCCTCCTCTCCCAGTGAC
TCCTCCTACTGCCGGAGCGCCTCTTGAGCCCCCTCACGTTCCTAGACGTACTGGGCGAGTTCGGAAGCCTCCT
GGTCAATGGTGGAAGCTCAAACAGCCAGACTTAGCTGGTAATGACTCTGACTCTGATGACGAAGCTGCTCTGC
TAGCTGACTCGGAATACTGTGATGTCGAGTTTGCTGGAGCTGTTTCGGGTGCTGATCCCCGGAGTTATAGACT
AGCTATGAAGTCTCCTGACTTGGATTGCTGGACTGAGGCTTGTAACACTGAGATCTTTAATCTCGAGGCTAAT
GGGACTTGGGAACTCATGGAACTGCCTCCAGGAAAGAAGGTAGTCAACTCTGGCTGGGTGTTCAAAGTCAAAC
GACTTGCCGACGGCTCAATTGAGCGCTACCGGGCTCGTCTCGTTGCTAAGGGCTATTCCCAACGTCCTGGATT
CGACTTCACCGAGGTCTTTGCACCCACATTCCGTCCTGCGTCACTACGCCTCATTGTTGCTCTTGCAGCTAGA
GAAGGCTACAAAATGCGCTCAGTCGACATCTCCTCTGCCTTCACCTACGGAGAACTCGAAGAGGAGATCTATA
TGCGCCAACCCGAGGGCTATCACATTGGTAGTCCTAACATGGTCTTTCGGCTCCGTAAATCGCTCTATGGCCT
CAAGCAGGCTGCCAGACAGTGGAATAAGAAACTCCGGAGTGTTCTCGAGGGTATAGGCTACTCTAGGCTGCGC
TCTGACAGCTCCATCTACATCTACTCTAAAGGTGACATCAAGGTCATTGTGCCAGTATTCATTGACGACATTA
CTCTTGTTTCGAAAAGTGATACTGCCATGGCCTCGGCTGTCTCTGAGCTGTCCAAACACTTCAAACTCCGTGA

TCTCGGGTCTACTACACTTCTGCTGGGTATTCAGGTCACTCAGGACTCTTCTACTCACTCCATCTCTCTCTCG
CAGGAGCACTACATCAAGGAGCTCCTAGAACGCTTCAATATGGATGATGCTAATCCTCTATCCACTCCCCTCG
CTCCGGGATCTGAGCTCTCTGACATTATTCCTTCACTCGAGGATCAGGCTGAGATGAAGTCAGTTCCCTATCT
CAATGCTGTAGGTGCTTTTCAGTATCTTGCCACTATGACTCGTCCCGACATTGCCTATGCTGTCTCCTATCTC
GGGAGGTTCAACTCGAACCCAGCTCCAGCTCACTGGCTTGCAGTCAAGCACTTGTTCCGCTATCTCATTGGGA
CTATGCACTACAAACTGGTCTACCGAGCTAGTGACTCGGAAGAACTCTTCACTACCTACTCTGACGCCTCTCA
CGGAGCCTGCAAGGCTACAGGTCAAGCTACGGGTGGTTATGTGACTCTAGTCTTGGGAGCTGCTGTAGGCTGG
TCTTCTAAGCGCCAGCCATTTGTGACTCTCTCGAGCACTGAGGCCGAGTTTGTTGCTGCCGTCGAGGCTGGCA
AGGAGATCAAGTGAACTAGGAATATCCTGACTGAGTTTGGCTTCCCTCTGACACTTCCTTCGACACTCTTTGT
TGATAACAAGTCAGGAATCAGTGTAGCCAAGAATCCAGAGCATCACGGACGCATGAAACACCTAGACCTGCGC
TTCTACTGGCTCCGGGAGGCTGTTGAAGAAGGACTCATAGATCCTTTGTATGTTTCGACTCACGAGCAAGTGG
CCGACATCTTAACCAAGGCTGTGGCAAAGAAGGTAGTTGAGTTTGCTATTCCTCTATTAGGACTTGTGTAGAC
TGGCTAGATCAAGGGGGTGTGTTAGAGTGTGAGCAAGCAGTCTACACTTGGCTGTTGATGGACTTAGAGTTAT
CCGGGTGTTGTAGTCACATGATAGCTTAGTCATGTATGGTTGAACTCCCCTGTGGACGCTTCAGAGGCCTTAG
CTTCTTACAGTCTGTCCACAGGTGAGTACAAGTTATTAGAACACAATACAGCTTCTTACAGTCTGTCCACAGT
TTGTTCTAGGGATTGCTCAGTCAGTTCCTATCCAATTGACTCGCTTCTAGGCTCGTTAGGATCGCCACTGCAA
CCGCTATCGTCCTACGTTTAACA
>Agabi_varbisH97_2_Cluster29#LTR/Copia
NttaaacgtagaacNatagNggtNgcagtggcgatNctaacgNNcctagaaNcgagNcgaNcggaNaNgaaNt
gactgagcaatcccNagNNNNaactgtggatagactgtaagaagctgtattgagttctaNtaacttgtactca
cctgtggaNaNactgtaagaagctaaggcctctgaagcgtccacagggaagttcaaccatacatgactaagct
atcaTgtgactaaactcgtccgcatatatctAagtccaccaNctacacttgtagacNgcttgctcactctaac
acacccccttgatcNagccagtCtactcaagtcCtaatagtggaaCagCaAactcaactaccttctttgCcac
agccttggtaaggatgtcggccacttgctCgtgagtcgaaacatAcaaaggatctatgagtccttcttcaaca
gcctcccggagccagtagaagcgcaggtctagGtgtttcatgcgTccgtgatgctCtGgattCttggctaCac
tgattcCtgacttgttatcaacgaagagagtcgaagggagtgtcNgaggaaagCcaaactcggtcaggatatt
cctAgtCcatttNatcTcNttgccagcctcgacggcagcaacaaactcNgcctcagtgctcGagagagtcaca
aatggctggcgcttagaagacCagcccacaGcagctcccgagacNagagtcacataaccacccgtagctcgac
ctgtagccttgCaggctccgtgagaggcgtcNgagtaggtagtgaagagttcctcNgagtcactagctcgata
gacNagNttgtagtgcatagTgccaAtGagatAgcggaacaagtgcttgactgcaagcCagtgagccggagCt
gGgTtcgagttgaaccTcccgagataggagacagcataggcaatgtcgggacgagtcaTagtggccagatact
gaagagcacctacagCattgagatagggaactgacttcatctcagcctgatcctcgagTgaAggaaNaatgtc
ggagagctcagatcccggagcgagtggagtggatagaggaTtagcatcatccatattgaagcgttctaggagc
tccTtgaTgtagtgctcctgcgagagagagatagtgtgatgagagcgatcctgcttcacctgaatacccagca
gaagtgtagtagacccgagatcacggagtttgaagtgtttggacagctcAgagacagccNagGccaTagcagt
atcacttttcgagacaagagtaatgtcgtcaatgaaNactggcacaatgaccttGatgtCacctttggagtag
atgtagatggagctgtcNgagcgaagcctagNgtagccaatgccttcaaggacaccccggAgtttcttaTtcc
acTgcctggcagcctgcttgaggccataaagcgatttacggagccgaaagaccatgttaggactaccaatgtg
atagccctcgggtTggcgcatatagatctcctcttcgagttccccaTaNgtgaaggcagaagagatgtcNact
gaNcgcattttgtagccttctctagCtgcaagNgcaaNaatgaggcgTagtgaNgcaggAcggaatgtagggg
caaagacctcNgtgaagtcgaatccNggaCgttgggaAtagCctttagcaacgagacgagcccggtagcGctc
aattgagccgtcggcaagtcGtTtcactttgaacacccagccagagttgactaccTtcttgccaggaggcagt
tccatNagcTcccaagttcCgttagccTCgagattgaAtatcTcAgtgtTacaagccTcagtccagcaatcNg
agtcAGGagacttcatNgctagTCtataactccggggatcagcaCCcgagacggctccagcaaactcgacttc
acagtActcNgagTcagctagcagagcagcttcgtcatcagagtcagagtcaTtaccagctaagtctggttgt
ttgagcttccaccattgaCcaggaggcttccgaactcgcccagtAcgtCtaggaacgtgaggggctcaaggG
gcgctccggcagtagGaggagtgaCTgggAgtggtGNatggatttgtggaggagtgactggaggAatcacNgg
aGGaatgaatggaggaattacTggaagtggtagNggTagAactggcttctcttcaggaggAacTgaagcggac
ggagtgggagtcttaggacgagcagGagggaagtgattatcACcccccttcatcctgtagaggtggcagtcccg
agtgtgataatGtatcattaggaatTggtAGtgactctggtNggcaNgcaaatgacgggacagcctcgagttg
ggctttaGaagtgcccggaaagtatctctcgtcaaaatcaGctctctcggagatcacggTtcgctTtgtgact
gggttgtagaacttCcagcctTtgtagccagccggatagcctataaagaCacacttctcgtagtgaggNtgga
gagaaTtNcgcttatctcgttgtacatggacataggaagtgcacccccatacccGgagaTgtgagacaTcaGg
tTttCgcttatagaaagcttggaagggagtagtgcccgaaagcgAagcagtNggNaGcatattccagacatga
accatagacgagatacattcaccccagAaagaaNcagGtagCcgagattccgagagcatagcagtgatcctct
cTcCcatagtcctattAgcNcgttcagcaagNccattctgctgaggNcNattNcGagtNgagtGacNNctctc
gatgccagcaagctcNgtaaagcttNNNaaagcNttagacatgtactcNcctcccttgtcatccTgtagtgct
cgcagtttgacTccgagttgagTctcagcCcaggaTttgtagactttgaacgctTcaaatgcctCcgacttNg

CTttNAgcagaTaNactgcCcgNaaacgtgtcgcgtcNtCNatgaaggtNatccagtacttgTagcctgagtg
tgaagcgactggGagcggtccgtgaagNtctgaatgCacNagctcNagaggAgagcgattgtgaTtcaaNgag
gagggaaaaggNagggaatgCatcttgccagcNagNCaNGgcTcacagAtagaNTcaggNgNcgaNttagact
cgagtttgAgNccNgaaacNagcTcCttactgatcatagacttCacaTcagcgTagttgtgatgaatgaagcg
acgAtgccatagcgagaNgtcaacNgggagagtagagaCagagaaagcagNctcagaAgTgaGagTtGagccg
tcgAGctcagCagagttgttggaatggatagtggccgtgaaaagcgaaaNcccttNgNattggaagtcaatag
aNtgagcNgagatNgtNatNttgaagcccttgtgtcgggcaaggtagagacatgataagagattactcctgag
gtcaggaacatgaaggactctNgtaaactcgacNgctctagtctcttttccattcaccacNggttgaaagaca
acagaNcctacaccagctgaatagacagtagagttgtcagcaagNcggatagggatNcgatgaggggagtagg
atttgaaccaatggcggtgaggagtcatgtgggatgtagcaccagtatcagccaNccagaaaaagtcagcatc
aagctgtagtggNgaggatggtgAggaagatgatgagggAgaggatgagggagaggaataagcaccaAgagcg
gatgctctcagtggaatatcagagccagGagagccagAaccagtagaaacatccccaggaggagtgggtggtg
TCttgacagctggaagaGctGcacctatcccActcagAtggccaagTggccctgtgacattactTtagatgt
tccaggcagtctggagcgctcctgagcgcgtttttcgcgttctttcgcttcCtgcacCtcctagaCatgtgc
cctaccctcccacaaaaggaacaagcgattgacgcgttcNtagggccNgaaatcggtccNagagagtgtggag
agggtgtaTGgctgccagagggagttgtggtcgatgtagNggcNctcagacgcgtcggagacgcgccaaaacg
cgtcgaNNtggtgtNNcaggcatNgggagNgtgtgtgcgcgattgcgaggccatagacgtcgaTttgagtgca
ggggaAgggatagaagttgagatatagaggNgaNaatgGtagatagTgNcaaaaaacgcgtttTNaaggctGt
tagaagccggggcccataacctNttaaacgtagaacaatagNggttgcagtGgcgatNctaacgNNcctagaa
NcgaGtcNaNcggataNgaactgactgagcaatccctagNacaaactgtggacagactgtaagaagctgtatt
NagttctaNtaacttgtaCtcacctgtgGacaNactgtaagaagctaAggcctctgaagcgtccaCagggNag
ttcaaccatacatgactaagctatcatgtgactaaactcgtccgcatatNtctaagtccaccaacNaCacttg
tagactgcttgctcactctaacaN
>Agabi_varbisH97_2_Cluster25#LTR/Gypsy
TGCTCAGGTTGCAGCGAAGGCAAAGACTTGAGGGCGAGTAAGGGAAAAAGCAAGTTGGTAAAGCGTAGGGAAA
TACAGAGAGTATTTACGCTTACTTATACTAAGACTATCTACCCGAGGTAACTAACTATAGAACGGGCTGAGTA
ACCATGACCAAGGTAGATGAGTAATCAGTGAGAATGAAAGTATAAACTAGGAAGGAGGATGATCAATCATGAG
AATAGAAGATTGATCATGAGAAGGAAAAGATATAACAGGTAACATGAAGGATCAATCAAGGGAACAATAAGAA
TGATCCTGAGAAGGAAAAGATAAACAAGGTAATGTCAAAGAGAAACACAGATAGAAATAGAATGACATATCCT
GATGGTAGAAATAGAACGGATAAGGTCCGCATCAGATAAGGATTAGATAAGATCAGATAAGATTTAGAATACT
CGGATTTGTTCCACACTCATAACACTCCTCCCGGGGAGTCAGCTTAAGGTTGTGGGCTGTCTCATCACCGGGC
GCATAGGTAACACCAGTGCCCACAGTGGCGTAGTTGCCTCTCCTGTGGTGGCGGTTTTTGGCAGGCAGGTGTT
TCATCCCCTTCACATCCTTGAGCATGAGCTGGTAGAGCTCTGTACAACGAGCTTGCCAGCTAGGATCCACAGG
TCTTCCAACCAACGCACCAAACACCCTCTGGCTACCATCAACAAGTAGAGTGGGATTAATACCTGTCAAGGTG
GTGAGCAACAAATAGCGGAGCATCCAGATTGACTGACCATTCCAGGACAGGGTGCGATACCCTCTGCTCCTCA
AATCCTCCATAACCTCCTGGGCTGGGTTGATAGACGAGCCGTTATAAGCGGATTTGGTCCTCCCCATCATCCC
TTCAATCTCCTCCTCCGTATCGTGGGCAAGAGGGCGGTAGCCGCCCAGCTGGGTCTCCTGGACCAAACTGCTC
TGTACATCAAACTCAAGGAGCTCGCTTGACACCGTGATTGATATTTTGTCCTGGGTGTGGGGTAGTGTGAGCC
GGCCTTGCTCTTTTCTATAGAGATCTCGCTTTGCATCCCGACGCCGCCAAGACCCTGCGTTCTTGGATGAGGG
CCCGGGAGAGGCAGCAGCAGATGAGGCGTTTGGAATGGACGTGGTGCTGGCCAAGGAAGCCCTGTTAGGGTGA
CTGTGACTGTGATGTCACAGGTCACACCTAAACTTAAGAACTAAGAGTAAAGTGACAGAAGTTGTATTAGGCG
TGAACCTAAGAGAACTCGACGGAGTCTTCTACAAGTTTCTACCTACTTCATATACCTAGACTACGGCGCACAG
ACCAACTACCCTAGGTAACTATTTATATACTTGACATGAGAAAAGAAGTACGTATGAGAAAGTAAACTAATTA
TACATTTGACATGTGAAAAGAAGAGTGGAGCCTGAAGTAAGTAAACACGCGTACATTGATCATAAAAACGTCA
ACAACACCAAACAAACCATAAAAGTGTCCACAAGTACAAACAACTCCTAAACCGGTAATCCACTCACACAAAC
CAATACCTACCAAAGTAATTCCCCAACATCCGCATCACATGGGCATAGTACGAAGAATCCGGCTATTGCCCAG
TGTCGTGGTCCCCAGTATCCCCATCTCTGTATTACCCACGCCTTCTGTTCCTCATAGTCCCACATCCCTAAGC
ATACTCTCCCACACCGCCTGCACCGATGATCCGATAGGTTCCATCCTACCGGCCTGTCTTCTCTTCCTATACT
CGCCGCTCTCCTTCGCCATTCTACTTGTTCCGCATGGTTCGGAGGTAGAAAAGATAAGATTAAATCAATGAGT
TCAATTGGTAGATCTGTAGCTTGTGCCATTTTAGCAGTATGCCGATAGTTTAGCCTCCGTCGAACTGTAGCAG
CAGAGGGAAATTATTATGTAATAAAATGTGTGACAGATGTGACAGCGTGACGCCGTGACATTCTGGCAAAGTT
CTAACAGTCTTGTCTTTGATCGTGATTTCGTTCAGTTTCCTATAATCCTGACATAACCGTAGTGAACCGTCTT
TCTTGGGAACGAAAAAACATGGTGCTGCATAGGGTGATTTCGATGGTCGGATTCGATTGGTAGCCAGATTTTC
ATCCAAGAATTTGTCCAGTTCTTCTCTCTCCAGTGGTGTCATGCTATAAACCTTTGATGATATCTCCGTTGGT
GCGTTTTCCGTGAAGTTAATTTCGTGATCCCATTCCGTTCGTTCAGGCAGTTTGTCGAAGTTCTTTCTGTTGA
AAAGATGGGCGAAGGGTTTCATGTATGCTGGTAAGTCCTCAGGGTTCGTTGGATCCGGTTCTTTTTCCTCAGT
GTCTTCGTTTGACTGTAATCGTCGTATACGTGGTTCGAAAGAGATGTCGGTATGGGGAAAAGAACATGATGTG
GGGCACCGGTTGAATTTGATCATTCCTTGTTTCCAATTGATTTCGGGGTTGTGGTTCGTTAACCAATCATGAC

CTAGAAAGAGGTCGGAGGATTGTAATGGAGTGACGACCGCGTCCAGTTGCTCGTTATGACCGTTGATGTTTAG
ATCTATCTTCGCGAATTTGGTGATATCCTTCTTTCCTGCTATCGTTCCATCTGAGTTCCTGCATGTGATCAGC
CGTTCCAATTTCTTCGTTGGGATTTTCTTCTTCTTTACTAATCCTTCGTCGATACATGTGTGTGTGCATCCCG
AGTCGACCATGGCTGAGACTTTGAGTTTCTTTCTGTTTGTTGTGGTGAGGAGGGGTTTTACTTCCAGTTCTTG
CACTCCTAGACGTTTTTGAGGGACAACGTATCGATTGTTCATTTGTCTAGGTACATTGGTTCGCGAGGTCGTG
CCTACTCGGAACCATCGGAAAAACCCTGATCTTTCTTGGAGTCGTCTTCATCGTCAGAATCCTTGTTCATCGC
TCTAATCCTTCCACTCCTGTTGCTTCGGCAATTCTTCGCAATGTGTCCCAACTTTCCACAACTGAAACACTTG
AAGGTTTTCTTCTCTCCGAAATTCTTCTTCTTCTCTTCGCAGTCCTTCCCGATGTGTTCAAACTTTCCGCATT
TGTGGCATTTGGGTTTCCCATCCTTGTCCCATTCGAATTTCTTCCATCCTATCTCCATTGGTTGTCCACTTCC
TCCGTAAGTGATCCCAGTTTCGGTCTTCTGATCAATCCTTTTCATCGTCTCTAATCCTGACCCAACTTGTAGA
ATTGCATCATTCCATTTCTCTCTCGTGTCCGGGATGTCTTTCCTTGGATATCCTAAGATCGTTTTGATAATGT
CATGACGGGTGTGGCGTTTAAGGAGGAATATGGCATGTTGGTCGTCGATTTTGGCTTTGTACATGAGAGCTGT
GAATTCGACGAGGAAGTCCATGGTGTTCCTGTTTCCTTGTTTGAGGGATTCAATTCTTTCTTCTGCTATATCC
TTCTCGTTTCCCTTTCCAAATGAAGTCTTGAGTTCTTCTTCAAAAGTATCCCATGTGTATGTGAAGTTGGTGG
CGATTTTCTCCGTCCATATGTCGGCAAAACAGCTGGGGACTGGTCCTTTCAATTGTACGATGGTAGCGATAAT
CTTCTGGTCGGTGTCCGTGACCTTGTTGTATTTGAGGAAAAGCATCATGCTCCTCCACCATTCTGAAAACTGC
TCTCTATTCCCCCCAAAATCCTTCGGGCTCGGGATAAGTGACTTGAGTGCATCTGCTTGTGCACTAACGGTTA
CGGCGGCGTCATTGCTCATGTTGTCGTTGTCTCGGGTGTTCTGATCGTCTGTGTTGATGGTGTTCTCATTCTC
TGTCTCTGTGTCGACGGGGTTCCTAGTGTCTCTGTAACTCCAAAAGTCAACTGGTTGTCGTTTCTTCTGGTTC
TTCCTGTTCTGGTTTCTCCTAGTAGATCTGGCTACTCGTTCGTAGTGTTCTTCGTCTAGACTGTTGTAGTCGT
TTGTCTCTGTGCTCATACACTACTTCTCTGGTGCCCGTGTGGCTAAAATTCGTCTGATGCCTGTATGGCTAAA
AATTCGGTTCTGCAGCAAGCGTAAGCCGCACAGTACCCTTCCAAGGTACTTACTAGTTGCAAGGTGAGCTAGG
GACGCTTTACCAAATGTTACGACCTTAAAGCGAATGCTCAGGTTGCAGCGAAGGCAAAGACTTGAGGGCGAGT
AAGGGAAAAGCAAGTTGGTAAAGCGTAGGGAAATACAGAGAGTATCTACACTTACTTATACTAAGACTATCTA
CCCGAGGTAACTAACTATAGAACGGGCTGAGTAACCATGACCGAGGTAGATGAGTAATACTGAGAAGGAAAGT
ATAAACTAGGAAGGAAGATGATCAATCATGAGAATAGAAGATTGATCATGGGAACTAAAAGATATAACAGGTA
TCATGAAGGATCAATCAAGGGAACAATAAGAATGATCATGAGAAGGGAAAGACAAACAAGGTAACGTCAAAGA
GAAA
>Agabi_varbisH97_2_rnd-1_family-177#LTR/Gypsy
AGCTTCTTAACTATTCTTTTCTAGATGTAGCTTCTTCTCTTTCGCTATCTACATCTTCTAGATAACCTTCTAC
TAGGTCGTCTGTGTTTACTTCTTCTTCGAGTAGCTTCAGCTCTTCTTCGGTATCGNGTCGAGTAGCTNCTGTA
CCTCTTCGCCGAATTTGATCTTTTCTCTCGCGAAATATGGTACTACCCTAAAGGCAGCTACCTTATTCTGCCA
NACTGCTCCATTGTANTCGGCNAAGATATANGCTCCTCCNGCGAGTCTTTTTACCACTATCACGGGCCCTAGA
TACCGGGGCTTCATTTTCCTGTCCGCCGACATTTCTATCGCAGNATTCTTGCACTAGAGCTAGGTCGCCTGAN
TTAGAGTGATAATTCTTTATCTTGTGTTCATTATCTTCTTGTAGCTNNANTGCCCGTCGAAGCTTCTCGTCTG
TTGGCTCTNTGTCTCATAGCGTCNACATGAGNTACGTGCTTAGCTAGGGCTTGCGCTCTTAGTCCGATTAGNT
CTGAATGTGGTAGCTATTGTGTCTGGGTATTTGACTAGCCANGTCGCTTCTACTATATCTAGAGGCAACGTAG
GGTGCGCACCGGTGATCATGAAATAAGGAGAACATCCCGTNCCCTTCCTNACGGTAACTCTGTCTGCCCANGC
TACGTGTGGNAAGTGCAAGTACCATTTCTTTACGTCTCCTGCNGTAGCTTTGTANAGCATCTGCCTNAGGTCC
CAGTGTGGTCGCTCCACCGCTCCGTTAGCTTGCGAATTGTATGGGGAGATAGCTACTCCTTTAATGCCATACT
TCTCCTCTAGCCACTTCACNGCTTTTCTAAATGGCGATCCATTGTCTGTTACTATCTTAACTATTGTACCCCA
AC
>Agabi_varbisH97_2_Cluster43#LTR/Copia
TGTTCGAATATGTAGTCTCCTCGTTCCACTATTAGATAAGATTCATGGTCACGTGTACGGACACGTAGTTTAC
TTGTTTTTCGTCTTCCTCTTCTTTTCCTCAGTTGTTGTCTTCAACTGAGGTGTGCGTATCGTGTTTCTCCCTC
TACGTTCTAGCTTTGTCTAACAGTAACCTATAGTAACGCATCTGCGTCCTCTATAGGTAATATATTCAGTTGT
TGTCTTCAACTGAGGTTAACGCATCTGCGTCCTCTATAGTTCTACAGGTTATGGGCCCCGCCTAAACAAGACT
ATAGGGATTCGTCGACCTCGCACCAACTCGTTGATTCATTTCTTATATTTTTTTTTTTTCTCGAGGTCATACA
GGCACGTTACCTCGCATACATTCACTCTTTACGGTCACGACATGTCGGACAATATTCAAGATCATATTTTTCG
GGTTACTTCATCGCAACAAGGACCAGGGACTTCCGGACAGGACGCATTGCGGCGAATGGCTATATCCCGTTCC
TCCAGCAGTGCAGGTTCGCAGGATTCCCCTCCAATGCGCAATGTTACTTTCAATTTGCCTTATAATTCGCATA
CTTCCACTTTTCAAACTGTTTATCCGCATGATTCTCTTCCTTTTATTCATAATTCTTCGCATACACCTCATAA
TTCTCCTTTTTTCTTTCCTCCTACTTCTGGTCCAATTCCTTCACCTTTTCTTATTGCTCAACCGCATACATCG
GCCCAAAATATTCAATCTTCTTCTGCTCAACCGATTACGGCTTCTGTTTCTGGTTCTGGTTTCACTCGTCAGT
CACCGCCACATCTTCCTCATCGACGACCGTTACCAGCGGCACCACATCACAAACAACCACATTTTCAACCCAC
TTCTTTTCCTTCTGCGGCACCTATCTCTGTTCCTCCAGCATATCCAATTGTTTCTCAATTCCCTTCTTTTGTT
TGTCCTCATCCGGGTTGTCCATCTCGTATTGTTCCTCAGCCTGTTTCTTCACAACCGATTCATTCTCCGTCTT
TGTCTTCTTTACATCTTCCTTCTCCCGTTTTAGCACCTGTTCCTGCCATTTCTGTTTACCTGGTAACACTGT

TTCTTCCGTTTCGGCTTTACCTTCTGTTTCTCCTTCTGTTTCACGTGTTATTCGTTCTCTACCTTCTATTTCG
CACATTGCAGTATTGGAGGGAGCCTCGAATTTTTGATGACTGGGATTCCAACATTCGCTCGCTCTTGGCTTCC
TTTGGACCTGAGTATCTTGCACACATTTTCGATCCTACCTACGATGCACCTCATCCTCGTATCACACCGGCTT
TTGCACCTATCCTCGGTCCTTCTCCCACACCACAGGAGCTTGTGACGTACGACGAGTGGTTTACAATCGATAA
TCAAATTAAGCACGTCATGCTTAACAAGTTATCGACTGCCGTTTCTGGATCAATCTCCGTCATACGTGATACT
GGTGAAGCTCGTGCGATCATGCAAGCTTTACGCAAGGCTTATTCGTCAAGGGATTGGATCAGTGGCCGAAAGA
TTTGGGAGGCTTTGACCCGGGTTCATCATAACGGTGATCTTGATGGTTATTGTCGTCGCTGGCTCACTGATTA
TGCGCGTATCTGCAATTCCCGCTACCCAATATCAGTGACGCAGGCAATTATGACATTCTTTACTGGTCTCCCT
TCTTCCGGACCGTTACTCTTTGCCAAAGATGATGCATTCCGTGATCTCGAGCGCGATCCTTCCCTCACCACCG
ACTATGTTTTTCTTATGATCAATCAGCTTCGTGATCGAGCTACCCTTCATAACTTCCGCTTGAACACCAGGGA
TACTCCCTCCACTGCCACATCATCTACCATGCCTCGTCCGACTTCTGTCGTTGGTCCTCGTCCTACCACCTCT
AACACTACCAAACCCCATCCCCCCCCTTCTTCCACATTACCTCGACGCGACAATTCTTCGAGGGTTACACCTA
GGGCTAATCTTGCATCTATGCCCACCGTGGATGATCAGGATGATTTGGATGTCGGAATACAGGAGTTGGTGAT
TGACGATTCTGGGGGTGATGATGATAGTGTAACTATTGCGAATGTGATTCTTCCTGCCTCAATTAACGGAGAC
TTCTCCACGTTGTTCTATCCTGCCTGCGTGATGTCTGCTCAGGACTCGGCTGACACTACAGTTGCCCATGATG
ACGAAAAAATTTTCGGGTATTTTTGAAGCGTTGTTGGACTCCGGCACCACCCATCACATTTTTTCTTGCCGAGA
CTATTTTTCTTCGCTCGACTCAAACTTCACTCTGTCTGTGGTCACAGCCAACAATAGTCTCCTCCCAGTTCTT
GGGCGTGGTGATGTTACACTTTTGTTGTCGTTGCCATCGGGCCCTGTTCGGCTGGTTCTTCGTGGTTGCCTGT
ATGCTCCTGATGTCCCCATCAATCTTATATCAGTTGGCTGTCTGGCTCGCAAAGGGGTATCCTGCCGTTTTGA
AGCTGATGGGTCTGCTTCGTTAGTCCTTGCAGCTCCGGGTTCCACATTGCCTCTCGTCTGCCCTACTGTCTTG
CGTGGTAATCTGTCTTACTTGATATGCCAATATGTCAACTCTTTTCGCTCTATTGCTTCCGATGACCCTTCGC
TGGCACTGTTTTTATCTCCGCCCTTGACACCGGATCTTTGGCATCAACGATTCGGTCATCTTGGTCATGATGC
GACACGCGCTGCCTTGACACGTAACTACGTTCTTGGTGTCAAATACGATGGTTCTTTTGACCGTTCTATTTGT
CCTTCATGTGTCATTGGAAAAGGTGCCCAGCAACATCATCCTTACAAGGGTAATCGTGCGGACTCAATTGGCG
GTCTCCTCCATGTTGACCTTTGCGGACCGTGGCCTGTTCGTACACCTTCAAAAGCCCTTTATGCATTTTCAAT
CCTCGACGATTTTTCTAACTTTGGCTTCACTACCTTGCTTGCCCGCAAATCTGATGCATTTGATGCCTTTCGT
CTGGCCGAGGCCTTCCTTCTGAAGCAATGTTCTGCTTCTGTTCGTTCTGTGCGTACTGATGGTGCCAAGGAAT
TTGTTGAGGGCTCTTTTGGCAAGTACCTTCGTACCTCAGGTGTCCGGGTTCAGTTAACTGCACCTTATGCGCA
TCATCAGAACGGCAAGGCTGAACGTTTTATTCGCACCTTGGAAGACGGTTGTCAGACTCTGTTGGCGTCTTCA
GGTCTTCCCGACCAGTTTTGGGGAGATGCCATGCTCACTTCACAATACCTTCGTAATCGTCTCCCGACTTCAA
CACTTCCTTCCGATATCACCCCTCATGAACGTATCCATGGTTCAAAACCTGACCTTGGACACCTTCGTGTATG
GGGGTGCCTTTGTTTCCCTATCATTCCTCCCGAGAAACGTGCTAAGGCTGGTCCTCGTCGTTTTGAGGCTATC
TTTGTGGGATATGAGGAGGGTCGTGTTGGGTATCGTGTGCGTTCTACTTCTGGCTCTTATTCTTTTTCTGCCG
ATGTTATCTTCGACGAATTGCGACCTGGCCGCCTGGGGGTGCCCCGCTCTCTTGTACTTCCATCTCCTACACT
TGATGGGGGTACATCTGCTCCTCGACGACGTCTACGGACAGCTAAGGGTCAAGCATTTTCGGATGAAATCACG
CGCAGAGCAGATGCACGAATGAAGCGAGGACTCGGTAGTCCACCAAAATATTCCACAGCTAATATCACTGCTT
TAGCCAACTTTGGTGCATTGGAGGAGATCGTTCATGAACGCTATCTTTCCGAATTCACAGAGGATGCACTGGA
GAAATGTGAGAGTGTATTGGTTGAATTGGCAGCCTATGAGGATAATCCAGGGGTTACCATGATCGCTACTTCG
TTCGGGTACAGTAAAGCGCCGGAGTCATATAGAGAAGCAAAAGAAAGAAGAGATTGGTCATCATTTCAGAAAG
CAATGGATCGAGAGTATGAGAGTTTGATGAAGATGAATGCGTTTGAGGTTAGCAAGTTACCCCCAGGACGGAA
GGCTATTCAGGTACGATGGGTTTACGCATTCAAATGGGATGATATGGGGGTGCCAAAGGAAGGAGAGGAAAAA
GCTAGGCTAGTGGCGAAGGGTTATAGTCAGAGACCTGAGGATTATGGTGAAACATATGCGCCAGTGGCGAGAA
TGGCGAGTATCCGGATTGTTTTGGCATTGGCAGCTTACTACGACCTAGAGCTCTATGGATGGGACTGCAAGAC
TGCATTCTTACATGCTCAACTCAACCGTGACATCTACATCCGACAGATTCCGGGTTACCCACTCGATGATCCA
AAACTGGTACTAAAACTCAAGGTAGCGCTGTATGGGCTTCGCCAGGCAGCTTATGAGTTTTACCAGCTAGTAC
TGTCCGTGATGAGCGAGATTGGACTTCGTAGGAGTGAAGTAGATCATGCATTCTTTTATGGAGAATGGGAACA
TCCACCCGATCCTTCTATTCCCAATCCCAAGAATGGTCAGCCACTACGTCTTTATGTGCCAATCCATGTCGAT
GATGGTCTTACGGGGGCGACAAGTAAGGAGTTATACTCCTGGTTCATTTCCAAGCTCCAACGTCGGTTGACTA
TCATCGATCTTGGTCCTGCTCAATTGTACTTGGGTTGTCGCATCATTCGTGATCGTGTCAAAGGTCTACTCTG
GTTGTCGCAGGAAGCTTTTATATCTGACTTACTCGATGACTGGAAGATGTCAGATGCTGCACCGTTGACTGTT
CCAATCAAAATGCAGATATCGGATATGAAAGAGGCGCCACCTGGAGCATTACCATCGATCAAAGATGACGAAC
TCAAGGTCAGATATCAAAGTCTTGTGGGGAGTCTCCTCTATTTGGCCTTGTGCACTCGACCTGACATTGCCTA
CGTATGTATGGCTCTGGGTCAGCACAATGCCAACCCCACACGCACACATATGCTTGCAGCCAAGAGTGTATTA
CGATATCTGGTTGGGACACGAAGGCTTGGAATGGGATTTGGTAGGAAATCTGATGTTTTTCCGCCCTCTATTC
AAGGATACATTCGACATGGTGGCTTCTCTGATTCTGATTGGGCATCAGACTTGGAGACTCGGCGGAGCGTAGC
TGGTTTCTGCTTCTTTTTCCTCGGGTGTTGTGTTTCATGGTCCGCGGTCAAGCAGAAGTCCGTTGCTCTATCT
TCGGCTGAGGCGGAGTACTATGCTCTTACTCATGCAGTCAAGGAAGCTCTTTGGTTGAGAATGTTTCTTGTTC
TCCTCAACATTCCACTCCCAACTTCTTATCCCATCCTATGCGACAATTCTGCTGCCATATCCATTGCAAGCAA

AGAAGTGATCTCGTCGCGAACCAAGCACATTGATGTTCGCCATCACTTCCTTAGACAATATATGGGAAAGGAA
CTTAATACATCCTGGTTGCCTACTGCCGAAATGCCTGCCGATATATTCACGAAGCCACTGGTCCAAGCCACTT
TTGAAAAACATCGTGATACCCTTGGTGTAACTCTTCTGCCTCCTTAGCTCATCCTATCTTTCTATTAGAACGT
CGTCGATGGGGGTGTGTTCGAATATGTAGTCTCCTCGTTCCACTATTAGATAAGATTCATGGTCACGTGTACG
GACACGTAGTTTACTTGTTTTTCGTCTTCCTCTTCTTTTCCTCAGTTGTTGTCTTCAACTGAGGTGTGCGTAT
CGTGTTTCTCCCTCTACGTTCTAGCTTTGTCTAACAGTAACCTATAGTAACGCATCTGCGTCCTCTATAGGTA
ATATATTCAGTTGTTGTCTTCAACTGAGGTTAACGCATCTGCGTCCTCTATAGTTCTACA
>Agabi_varbisH97_2_Cluster21#LTR/Copia
gtTgAAgaaaaaaacTAacgagcccaacagaaGagcagacagAacaaaacagacAaaggGaaaagAGcTaaca
aacagacTccTgaGcaaagaAccgcacaagaagaGcacaaaTacacTccGaaaTGcTATacaAGTAAgcgaAg
caaTaaaGaaTAaaTaCTaaAcgaaCTaaagggTccAaaCcaaAGacaaTagAGcAaaTAcaCagaAcacaTc
acaTTACTcgaATAaCAacaCaaAcacgAgggcaaCgaggagacacaaaTgagacccCaaAacAagaacgcgaa
aAacgagcaaCaacaTacacagaaaaaTagacaaaagCacaagaaaccacaaaTAccagcaggaTTaAaaacT
caTccacccCcGAacGaccaagcAaTataTgcAcaCaaaGaAcTccaTacaaAGaaggGATCaGaaAcaTgcaa
aCaTaaagcGaaaaAGAcTaTgCccAaATaTcTCaaCcgggTggTcgacaaCaGagacAggcAaaacAacgAT
TTaCaggccgaaaAaAcaTTcTaaaacaaaaTcATacaaaagAaaaAaGTgTcTAcAGcGaacTgacTGAaCa
gcAaTAcAaacCcAaaaaAcaaagcCTcaagTaacgGCAcTTagacaagcgGaTGcAAgaccgAagccgacag
aaaacaaTAaTacccgcAgcaAaaCaAggTccgagaagcaGaCagaaagacCCacAgAagcAgcccCcgTaaa
aGaccagGacacTggTgaaaacaaAcGacTAAgcCAacTaAcaaAcaccccgGccAgaGcAaCAgAgggaaCg
acgACAGacTccCgcaccgagGaacagagaGaccAAaaaGAagccaaagAgaacaCTAacaaggaaagaGAac
cgCcaTccAaaacaacTaTAgacagGagaaAgcCaaGTAcgaacaaaaGaacaaacaacaccaGaTcggAaaa
cgCTcaaGaacgcTggaaaaaccgcaaacacAaTTacaccaTaGCcgaacCAacGgCaagccaagcaacGaTc
aaaaaaaaCaggAAagacCcaAccacggGTgcgaAagaaccccccAaaaGaGagcGaccaagagcaGcaaAaCg
aaAcaaAGcTaAaaaggaATaagagcGaaaTcccaccccgcgTacAaGcAAcaaCaaaccTCacAacAATTcc
cgaaaaaTaaaaaaccgcaTcgacAaacaTTcccccgaagggccaagcaaAagagaTcaccaTTCagacAaaC
cccTccGcTgaacaaAaacagCGaCAgaCccacaagaacacgaaagcGCaccgAagcTcTagaaacAaacaAG
gCaaTcaaaaGacTacggcgagacCATaaAaagaaaagaagcaaaaGaagAagccaTacaacaacGGacCaCa
ccAAaAacAaaCaCaaacaccccaacAacagCaccaAaAaCaaaaacaccgaAgTGacaCacccaaaaaGaac
aAccagccTaaGaaaaAaaaccaCaaGCaagagAaTGaacggTccAATagaaAgACagcTcaaTggaTggacT
aTacgaaaCaggaaGccaccaccaTagacaccgCcacTgccaaccgacaggacacagaTcaacaTgTAacaga
AacAccaccaaTaaCaCaaacTTCgaaTGagcCAacaCCgAaaaccaaacaaccacTCaCccaGcGcacgacc
aaAaagaaaAaaccacgcaacgcAaTaCagTaaaacgaAcacaaaaacaaaccAaaAAaTCgcGcaCgcaTcC
cGCcaaCaGACaaaaaacTaaaaaccaAgCTacTccgcAccGagcTCaccaaggagcgcAaAaaGaaaaagca
AgAgaagaaaCgcaTcacaagagcaagGAGcgcagcaaaaaaTgCTaCTATCCacaaggggaAacaccagaa
GcaaTacTGaccaAGcaagaGgaacaaaaCGaagaaaggcaaaTgaaCaAaAgAagccgaacaaAaTcCgcaa
CaccagaaccgaaaaaGaccTcCaaaagcgaaaccgaCGaTgCaGgagcaaacaccAaagcagaacagGcacC
AacAccgggcaaTcCgGaaaCCggacCccTaggCcaaccaGaacCTcagaagaccgaaaggAaagcgacGgTT
acgcgcggaccTccaTcgAaccTTGaTGaccTaacacaaaaaTTaAcCcagacCcgaaaaaTacCcgacaaaT
caaAaggagaaacagAgATaTgaagaacTCcGacTGgAGaTaaccaaaagacagTagaccTcacTgccagAaa
TcaggaaGaAaGaaaTAgCcaAcaacaaaAaaGcagaagTTcAGaaAgTcagCaTTgcTCgggaaaacccgGt
tgaatgttacgaccttaaagcgaatgctcaggttgcagcgaaggcaaagacttgagggcgagtaaggggaaaa
gcaagttggtaaagcgtagggaaatacagagagtatctacgcttacttatactaagactatctacccgaggta
actaactatagaacgggctgagtaactatgaccgaggtagatgagtaatgctgagaaggaaagtgtaaactag
gaaggaagatgatcaatcatgagaatagaagattgatcatgggaactaaaagatataacaggtatcatgaagg
atcaatcaagggaacaataagatagaaatagaatgacatatcctgatgatagaaatagaacggataaggtccg
catcagataaggattagataagatcagataagatttagaatactcggatttgcttcacaatcataacaTgaAA
CaTcagcaacgaaTacagcAaAaaaaaaaTaCccaagaaGaggGcgcCgaaAaTcaacagCaTcaggTcaccT
CacAaaaaAccccgaagAagaaAgagaaTaagaggcaacaGAagcCgcaTCAaTaGaaacaGaccaAGaacGA
aagagCccgcAaaTaaccGcagaaGGgTcaGTcaggaTCGaacagaaagcAacTgaagaaGAaaCagaTaaaa
cacgGaaaAaAacAAaTaaaagaaCaaAaccTaaaTacGaaGaagcaggAacaggccgcacCgaaTcccacaT
cagaTccTCaGagGacaacaaagGgaCgaTgGgaagcccgacaccAcacAcaaCaacaTacaaACATaAggga
aagaTCCccaCAgCaaAGAggaccccaacacAaaaGaAcaGTgcACAaTaagTgcTAacaCGaagAaaaAaGa
aAcCAcaaaCTacATAcaaAAcaaaaagcaTaaaCaCagAacaaagcggaaaccaaagaTgTcGacaTacTTC
aGaaAcgaaaacgaaaaaaccACcgaaAaaTACCcgAacAaaAgcAaaaaTacccTcaAaAaCggcaacTagc
TcTccaGGaggTgcCCaaggcAAccaGacCaaaaaAcTgcgcTaccaaAagcccAaAacaGcTaagAacccaa
aagaccaAaaacAaTCgGacaaaCaAGaaTggaaaagcAcagCgTaccaCaaaTccAAgaGaGTaagAacaag
cCgagacGaccaTcCaTcgaacTGcTcaacaTaTcgcccaaaacaaaaATcgaaAacTaCTcaaaCCaTaAAa
aAacgaGccAaTgccaCaGAaagaaagaccaacaCAaaaTcagTaCaGaaCCGcAaacAcaaTGgaggacaaa

agAggTCaGaAcaAgTaaCaaagTccTTaAgGGaaaaAGgaacTacacATgaaaccacaacaacAaAaacccA
cCAacaaaAaGaAaaagcagaaaGaaaaaaTagcACCCaaTTaaAaaaGgcacAaGaaaTaagaggaaAcgCT
aGcaaacCgGAcTccgaTaacgaagcgaccaTaagAaCgccTgTacaagTAgacaAcaaaacccccagGcgCc
GgTTaaaaaGGaaAaagCCcacAcAaacAcgccaaGgTaTaaAacccAagacTaaTcaTcgTacGGTAgcTgg
agGTacggcgTaaagcgcaaTTcCcGAAaaAaagCcaaAgaacaaacgcaaaccgccgTaaGaGaacAacaca
gccGTTggcTATaaAGaaacTGcTaaCaTcacTacaCGccaTAaaAagaAacGTaaACgcTcCgcgTcaccca
caacaaTCTggGaTGaaaGGAcaggcaaaTcATGcccaaAcagaagaaTggacacaacacccacaaCaAgaGa
aCaagaaacAcCaaCaGAcaAgagaAcagccaAcaaGaacaacaacacTaagcaaccgaCagAacaAacgTcT
GggacagcAgTTcaccTagaCacaaggccAcaaGaacCaaaagaAccTacCgccccccCGAgaacGACaaAaC
CaaaaccaccgAgaTaaaaGgcaccTCcagCTacagCaccacCcAaTaAacaGaagTCgcagaaaaaggAcca
gaTaaagTaagaaaacTcggaTccGAaacaAaaaaaaGaacccGAaTcgaGcacCaagCcgacgccGaAaacg
cCaTcTaaacgAacaaaacagcAaGTTcaACacccaacgaaaGAaaAAgGaaaAaTgcCTaAAaggagaCagT
TagaacaTgaGacAacaCcTcCggaaaCAgaTacacaAacAAaacaaAaacgaacacgacaAcCTaCaccCcG
AccTcaaaAaATaTaTcgTcaaacaTcaaAacccaaaGagAcagaaacacaaaaGaTaAAagaagcgccaAaa
cCATCAAcagTaaAacaAaGcaaaaaaccAAacagaaaaaTaaccaaacgaggaacAGcacCaaaaccGaacA
aaGaaaaGGacgCaGaAgcaaaacaTccTGcgaCagccaagcaaaTacccaagaagaCgGaaaCGgacaagca
caaaccacAccgCCgcgcaaacgagccgGgaaaGaAgTgcaccaacaaTaTaTcGgcaAaagcaaggaacccCG
gccacgacCaaagaCTgccacAagAaacCccagcaccaTcaaaaagccgGGATAGaAGaAggcacacaagaac
TCCggaacgcaaacAaaaGaggaggaTacgaaaccgcCgaTcTgacagaaggaaTaAaaaCcaTcaaaAagAG
aTgccgacggAacagcaaGTCgGagaGCcGaAaaaGgcCcaGAcTTgcTccgaacggcagcccccaaacggAa
gGgAgTaacgccaagaagcTggcagcTcCcaaacacCcCgacGaGAcgccccggcacagacTaGGgcgTGcag
CAcgaGAAaaccGgGaaAaaaaaaaTcgTgaccccAaAgcagcAgaacgaaaaacaaaaacaaaAcaGGaaaa
cgagAacaaacacccCggaagGcgcaagAaAagaaAaacAcaaAacaGTaTggagAcTacAaacagCAcTaaac
gGccgGaaGcaaTccggCccAccccGaTgccgCgaaggaaccGcacCAaTgaacgaccgagGcTgTaaccGac
ccTccgcacacaccgGcGaaTagTaTTtacaGaGagCCacaaacaaAgcaacTctgcagCagcgcacgaggcc
acgTagATGaccgacaaacAagCgggaaAaaGaCTgcgcTaacgacAaAcaaTaaacaCaagagcaaaaaaaa
agcaGaaTGaCGagaacTggAagattgaagaatAaCaaAacaGacTccagGAAacaacAcacaacggaagacA
aaaaaaccaacaAgcaaTcaaaAacaaaaaaaccAccgcgaaaaAATTcaTcaGAacagcGacaTTaCaTcaA
aaacaacaaaaAcccCaacaaaATcacgagTcAcCTgacTacaGaTcaaCgcaaaagcagCaacCgCagccaa
acagaagTaacaACaagaaaTacGaaaaTTaacGgaccgaagaAaCaggaagaaaacaGaCaaTGcacaaGCa
AcAccaaAAgTaacccaaaacagcgcaaAcacagCgcaAGaacacTaaaAcCaAagaggaaagagaCcaagag
accTACgaGcaTcgagcaacgaTAgAccgaaacTccAaaGacccTCcaacgaagccCTAGcagcaTaaacaaa
cTcggaTTcagCgaaagaccCaacaaacaGaaAaacTGTaacaGgaaaaTaTGcTaCgaTacCaaaaagcTgc
cTaAaccaaaTTaacCaTaaacaaaaaaaAaccgCaacacCaccgAaaGaaaaTcaaTaTacGGcgagaaacG
aCaaaacaaaacAaaTaacgTgcaTcaaccaAgTAcacaaggagacacgcgTaaacaTTcCAaCacaagaaag
AAaTGCcaaaAaggaAGaaacaaTagacTGGaccTcTAaTcaaGaTCAagaAccaCaCTccAaAaACacggaT
acaggaaATcAggcaaagcgAaAAgaTgTcgaacaaaaGaAAATaGgTcaagTcaaTagaaCaGggaaaaAaa
AaaaaGcaGAcAgATccacGaAaAaTCTcgaaCaaacaAaagaccaAgaTaaCaGATCaaccCTcgcgAaccA
aTgcaaaGacTaaAcAaAgTcagcacgCaaTcaGcacccAgaAccggTgAAgaaaaaaacTAacgagcccaac
agaaGagcagacagAacaaaacagacAaaggGaaaagAGcTaacaaacagacTccTgaGcaaagaAccgcaca
agaagaGcacaaaTacacTccGaaaTGcTATacaAGTAAgcgaAgcaaTaaaGaaTAaaTaCTaaAcgaaCTa
aagggTccAaaacaaAGacaaTagAGcAaaTAcaCagaAcacaTcacaTTACTcgaATAaCAacaCaaAcacg
AgggcaaCgaggagacacaaaTgagaccCaaAacAagaacgcgaaaAacgagcaaCaacaTacacagaaaaaT
agacaaaagCacaagaaaacacaaaTAccagcaggaTTaAaaaga
>Agabi_varbisH97_2_Cluster45#LTR/Copia
GCCTTGGACATGTATTCACCTCCCTTGTCATCCTGTAGAGCCCGCAGTTTGACTCCGAGTTGAGTCTCGGCCC
AGGATTTGTAGACTTTGAAGTCTTCAAATGCCTCCGACTTAGCTTTCAGCAGATAAACGGCCCGGAAACGTGT
CGTGTCGTCAATAAAGGTGATCCAGTACTTGTAGCCTGAGTGTGAAGTGACTGGGAGCGGTCTGTGAAGATCT
GAATGCACCAGCTCAAGAGAAGAGTGATTGTGATTCAAAGAGGAGGGAAAAGGAAGGGAATGCATCTTGCCAG
CTAGACACGGCTCACAGATAGAGTCAGGGGACGAGTTAGACTCGAGTTTGAGCCCGGAAACTAGATCCTTACT
GATCATAGACTTCACATCAGCGTAGTTGTGATGAATGAAGCGGCGGTGCCATAGCGAGATGTCAACCGGGAGG
GTAGAGACAGAAAGCAGTCTCAGAAGTCACAGTTGAGCCGTCAAGCTCAGCAGAGTTGTTGGAATGGATAGTG
GCCGTGAAAAGCGAAATCCCTTTGCATTGGAAGTCAATAGATTGAGCAGAGATAGTGATATTGAAGCCCTTGT
GTCGGGCAAGGTAGAGACATGATAAGAGATTACTCCTGAGGTCAGGAACATGAAGGACTCTTGTAAACTCGAC
TGCTCTAGTCTCTTTTCCATTCACCACTGGTTGAGCGCGTTTTTTCGCGTTCTTTCACTTCCTGCACCTCCTA
GACATGTGCCCTAACCTCCCACAAAAGGAACAAGTGATTGACGCGTTCTTAGGGCCAGAAATCGGTCCTAGAG
AGTGTGGAGAAGGTGTATGGCTGCCAGAGGGAGTTGTGGTCGATGTAGGGGTTCTCAGACACGTTGGAGACGC
GCCAAAACGCGTCAAAGTGGTGTCCCCGGCATAGGGAGCGTGTGTGCTTGATTGCAAGGCCATAGACGTCGAT

TCGAGTGCAGGGGAAGGGATAGAAGTTGAGATATAGAGGGGAAAATAGTAGAAAGTGGCAAAAAATGCGTTTT
GAAGGCTGTTAGAAGCCGGGGCCCATAACCTGTTAAATGTAGAACAATAGCGGTTGCAGTGGCGATCCTAACG
AGCCTAGAAACGAGTCAAACAGATAGGAACTGACTGAGCAATCCTAAGGACAAACTGTGGATACACTGTAAGA
AGCTGTATTGTGTTCTGATAACTTGTACTCACCTGTGGATACACTGTAAGAAGCTAAGGCCTCTGAAGCGTCC
ACAGGGGAGTTCAACCATACATGACTAAGCTATCATGTGACTAATTTTTATTTCAAAACTACTTAAAGGGTGC
CATGACGTGTTCCCCGTACTTTACATCACCTGTACTTAACTACTCAACAAGTCCGTATCCAGCCGACAAGGTC
GAAGCCATAAGAGAAAAAGAAAAAGAAAGAAGAGACTGTTCGAATCTGTATACAAGTACACTAAGATAGACAA
AGAAACAGAAACGTTCATAAACGAGATAAAAGAAAATCATTCTTCTCCTACCCCTTTTCAACACCGCCTGCGA
CACAGGCTTAATCTCTCTCATCATCGCACAGTAGATATCTCTCCATCCATCATTTATCTCCCATATGTTCCAA
TGCCGTACGAACTCGATCGACTCGTGCAAGAAGATCGGTCCTTCTAATAGAGGAAGCCTTCGCTTCTTCCTCC
TTTTGTTCTCTTCATCTCTAGCCAACTCCAACTCTTTCCTATAGGCCGGCCAGCTTTCGTCTGCGTGTCGCGG
AAGTTCCGGTGGTTCGAAACTAAACGCTTGGGGGTTGTCTTGAAGAAAGAGGAAATGTCAGAGAGTTCAGATC
CTGGAGCGAGGGGAGTGGAAAGTGGATTAGCATCATCCATATTGAAGCGTTCTAGGAGCTCCTTGATGTAGTG
CTCCTGCGAGAGGGAGATAGTGTGATGAGAGCGATCCTGCTTCACCTGAACATTGGTCGCATAAGCGAGGATT
ATCCCCTGATGGCAACAGTCGTAAATCGCGATTTACGACTGTTAATCGTCACTATGATAATTTTTAACCCCAA
TTAGTATATATATAATCATGATTATATTTATTATCCGGAACCCAGACTGGTGATTTTGACAGGCATAGAAAAG
AATGTTTAATCACGATTATAATCGTGATTTTTAAATCATGATTATAATCTTGTAATCTTATCTTAAACTATAA
TCATGATTATATTTGTAATCTTATCTACTGTGCGATAATCGTAATTGTGATTATAATCTAATAATCATTTGTA
CTACGTATATAATTAACTACCATGGGAAATACACTACAAACTCTCAACTACTTATATTAGATTGAAGATAGAA
TATAGATGTCAGTGCACACTGGAAATTTGGCGGTCGGCACCACCGGGAATTGAACTGAAGCCTTCTGGACTGG
GACTCAGAACAATGCACCAGTTGTGCCGTTACGCCAGGCGTCCAAGCAAAGGTGTAAAACACTTATGCAATAT
AGGGCATCACAAAAGCAGCATTACACCCTTATAGATTGATAACTGTTGAAACAATAAACGTTTCACATGGTGT
AATGGTAAAATTTCGTAGAGCTATGGTTCTGTCGCGAACGCTTTGGTTCGAGCCCCGCTGAAGCCATCTCCGA
TGAAATTGATTAAGTAAGTTGCAATCATTGGTCAATTAAGGCTATGTTTATTAAACGTAAATTATTTGTAGAT
TAGATGAGTACTGGGGAGCAGTACATACAAAGTACATACGTTTCCTGATGAACATGTCGATTATTTCAATTTA
TCAGTCGCAAAAATCGTGGTTCTAACACAATAATAATATATAAGTACAACAGTAATCATGATATTGCGATTAT
CAGTCACAAATCGTGATTAACGGCTGTTGCAATGGACGCTTATCCTGGCTTACGTGGTCCCTGACCTGAATGC
CTAGCAGAAGAGTGGTAGAGCCGAGATAACGTATTTTGAAGTGTTTCGAGAGCTCAGTGACTGCCAAAGTCAT
AGCTGCATCACTCTTCGAGACAAGAGTAATGTCGTCAATGAAGATAGGCACAATGACCTTGATGTCACCTTTC
GAGTAGATGTAGATGGAGCTGTTGGAGCACAGCCTAGAGTAGCCTATACCCTCGAGAACACTCCGGAGTTTCT
TATTCCACTGTCTGGCAGCCTGCTTGAGGCCATAGAGGGATTTACGGAGCCGAAAGACCATGTTAGGACTGCC
AATGTGATAGCCCTCGGGTTGACGCATACAGATCTCTTCTTCGAGTTCTCCATAAGTGAAGGCAGAAGAGATG
TCGACTGAGCGCATTTTGTAGCCTTCTCTAGCAGCTAGAGCAACAATGAGGCGTAGTGACGCAGGACAGAATG
TAGGGGCAAAGACCTCGGTGAAGTCAAATCCGGGACGTTGGGAATAGCCTTTAGCAACGAAACGAGCCCGGTA
GCGCTCAATTGAGCCGTCGGCAAGTCGTTTCACTATGAACACCCAGCCAGAGTTGACTACCTTCTTGCCAGGA
GGCAGTTCCATTAGTTCCCAAGTTCCATTGGCCTCGAGATTGAATATCTCAGTGTTGCAAGCCTCAGTCCAGC
AATCCGAGTCAGGAGACTTCATAGCTAGTCTATAACTCCGGGGATCAGCACCCGAAACGGCTCCAGCAAATTC
GACTTCACAATACTCAGAGTCAGCCAGCAGAGCAGCTTCGTCATCCGAGTCAGAGTCATTACCAGCTAAGTCT
GGTTGTTTGAGCTTCCACCATTGACCAGGAGGCTTCCGAACTCGCCCAGTACGTCTAGGAACATGAGGGGGCT
CAAGAGGCGCTCCGGCAGTAGGAGGAGTGACTGGGAGGGGTGCATGGATTTGTGGAGGAGTGACTGGAGGAAT
CACGGGAGGAATGAATGGTGGAATTACTGGAAGTGGTAGAGGTAGAACTGGCTTCTCTTCAGGAGGAACTGAA
GCGGACGGAGTGGGAGTCTTAGGACGAGCAGTGTTACGACCTTAGTCTGTGAGACTCAGGTTGCAGACAAGTG
ACAAAGACTTGAGAGCGAGGTGAGAGTAGAGAAAGGGAAAGATACAATTGGTGAAGCGTAAGGAAGTACAACA
GTATCTACTGGTCTTATATATGTAAAGGTATCTACCCAAGGTAACTAACTATAGAACGGGCTGAGTAACCATG
ACCGAGGTAGATGAGTAATCAGTGAGAAGGAAAGTATTAACTAGGGAGTAGGATGATCAATCATGAGAATAGA
AGATTGATCATGGGAAGGAAAAGACATAATAGGTATCATGAAGGATCAATCAAGGGAACAATAAGAATGATCA
TAGAAGGGAAAGACAAACAAGGTAACGTCAAAGAGAAAGTGACATGGGAATGACTGATGGGAGAAGCGTAGGT
GGAGAAGAGTATATCCAGGGATATGGAAATGAGTAAGCATGATGACGTCCCTCCTATAGGTTGGTAAATTCAT
AACAAGCAGGAGGGAAGTGATTATCACCCCCTTCATCCTGTAGAGGTGGCAGTCCCGAGTATGACGATGTATC
ATCAGGAATTTGTAGCGACTCTGGTAGGCAAGCAAATGATGGGACAGCCTCGAGTTGGGCTTTAGAAGTGCCC
AGAAAGTATCTCTCGTCGAAATCAGCTCTCTCGGAGATCACAGTTCGCTTTGTGACTGGGATGTAGAATTTCC
AGCCTTTGTATCCAGCCGGATAGCCAATAAAGACACACTTCTCGTAGTGAGGCTGAAGAGAATTGCGCTTATC
TCGTTGTACATGTACATATGACGTGCACCCCCATACCCGGAGATGTGAGACATCAGGTTTTCGCTTATAGAAA
GCTTGGAAGGGTGTGGTGCCCGAAAGCAAAGCAGTAGGAAGCATGTTCCAGACATGGACCATGGACGAGATAC
ATTCACCCCAGAATGAAGCAGGTAGCCAAGATTCTGAGAGCATAGCAGTGATCCTCTCTCCCATAGTCCTATT
AGCACGTTCTGCAAGTCCATTCTGCTGAGGCCGATTTCGAGTCGAGTGACGTCTCTCAATGCCAGCAAGCTCA
GTAAAGCTTATGCAAGCCTTGGACATGTATTCACCTCCCTTGTCATCCTGTAGAGCCCGCAGTTTGACTCCGA
GTTGAGTCTCGGCCCAGGATTTGTAGACTTTGAAGGCTTCAAATGCCTCCGACTTAGCTTTAAGCAGATAGAC

AGCCAGGAAATGTGTCGCGTCGTCAATGAAGGTGATCCAGTACTTGTAGCCTGAGTGTGAAGCGACTGGGAGC
GGTCCGTGAAGGTCTGAATGCACCAGCTCGAGAGGAGAACGATTGTGATTCAAGGAGGAGGGAAAAGGAAGGG
AATGCATCTTGTCAGCAAGGCAGGGCTCACAGATAGAGTCAGGACACAAGTTAGACTCGAGTTTGAGCCCGGA
AACAAGGTCCTTACTGATCATTGACTTCACATCAGCGTAGTTGTGATGAATAAAGCGACGATGCCATAGCAAA
AGGTCAACCGGGAGAGTAGAGACAGAGAATGCAGTCTCAGAAGTCACAGTTGAGCCGTCAAGCTCAGCAGAGT
TGTTGGAATGGATAGTGGCCGTGAAAAGCGAAATCCCTTTGCATTGGAAGTCAATAGATTGAGCAGAGATAGT
GATATTGAAGCCCTTGTGTCGGGCAAGGTAGAGACATGATAAGAGATTACTCCTGAGGTCAGGAACATGAAGG
ACTCTTGTAAACTCGACTGCTCTAGTCTCTTTTCCATTCACCACTGGTT
>Agabi_varbisH97_2_rnd-1_family-14#LTR/Copia
AGTGTGAAGCGACTGGGAGCGGTCCGTGAAGATCTGAATGCACCAGCTCGAGAGGAGAGCGATTGTGATTCAA
AGAGGAGGGAAAAGGAAGGGAATGCATCTTGCCAGCCAGACACGGCTCACAGATAGAGTCAGGGGACGAGTTA
GACTCGAGTTTGAGCCCGGAAACNAGGTCCTTACTGATCATAGACTTCACATCAGCGTAGTTGTGATGAATGA
AGCGACGATGCCATAGCGAGAGGTCAACCGGGAGAGTAGAGACAGAGAAAGCAGTCTCAGAAGTGAGAGTTGA
GCCGTCGAGCTCAGCAGAGTTGTTGGAATGGATAGTGGCCGTGAAAAGCGAAATCCCTTTGCATTGGAAGTCA
ATAGANTGAGCGGAGATGGTGATATTGAAGCCCTTGTGTCGGGCAAGGTAGAGACATGATAAGAGATTACTCC
TGAGGTCAGGAACATGAAGGACTCTCGTAAACTCGACCGCTCTAGTCTCTTTTCCATTCACCACCGGTTGAAA
GACAACAGACCCTACACCAGCTGAATAGACAGTAGAGTTGTCAGCAAGGCGGATAGGGATACGATGAGGGGAG
TAGGATTTGAACCAATGGCGGTGAGGAGTCATGTGGGATGTAGCACCAGTATCAGCCAGCCAGAAAAAGTCAG
CATCAAGCTGTAGTGGCGAGGATGGTGAGGAAGANGATGAGGGAGAGGGGTACTTGCATTGCCAGCAAACTCG
GTGACTCTCTGCGCTTTTTCGGGAGCCTGAGTCGAGCTAGAGCTCCCAGACTCTTCCTGAGCCTTGTTAGCCC
TCTTGTACGGAGTTCTAGGCTTTTGTGCGTCCTCCTGAGCCCGGGACTTCGCTCTCTCGAAGGTTTTGCATTC
AGNGAACTTGTGGCCCTCTCTCCCGCAGAAATCGCAGGTTTTCGTCGCGNNTTGGGAGCCAGAGCGAGCNGCN
AGNGCNTGGGAGGNGGCAGAGGCGTCCTGAGCGCGTTTTCCGCGTTGTAAGTCCTCAGTGACAAAGGCCTGGT
GGACTGTAGTCTTGTCCAGCTTGTCCATAAGCAATAGGGACGANGTGAAGCCGGAAAACTCGTCCGGGAGCGC
TCTGATGAGAGCCATAGAGGCTAGCTCATCATCTAGCTGCTCTAGAGTGAAGGAGGAGGGGCGGAGTTCCTTG
ATTTTTCGTTTAGAGCTCTCTACTCTGTTGATAAGGGATTGGAGGGACTCCTCCTCGAGTTTTCGGATGGAGA
AGAGGTCATCATAGGCATTGAANCGCATTCCAGCCCTTTTCTGNCGATGTACAGCCTCTAGAGCCTCCCACAT
CTTCACNGGACTGTCCTGGATGCCCTGGAAGTGAATCCTCTGCTCCTGCTCCACCATGAGGTACAGCCAGCCT
GCAGCCTTGTCCAAGCGAGCCTCAAACGCGTCCAGAGCGTCCGCTTCGGCCTGGGTAACAGGGCTAGAGGTGG
AGGGAGCCTTCTGCCTGCCNGAGACAAGTCTCCAGAGCCCAGAGGCTCTCAGCCACGCCTCCATCTCTCCAGA
CCGCGTGGAGTAGTTCTCCTCGTTGAGTTTNGCGAAGGTGCGCGATCCGGAGGCCATAGGNGTCGANATGAGT
GCAGGGGAAGNGANAGAAGTTGATAGGACAGGAAGAAGTGATTGGGGAAGAGTTGNGGTTGNTGAAGTAAAAG
TCGTTTTTTAAGCGTTAGAAGCCGGGGCCCATAACCTGTTGAACGTAGAATGTACGCGACGCAGTGGCGAGTC
TAACGGTCCTAGAAACGAGGCGAACGGACAGGAACTGACTGAGCAATCCTAGNGACAACTGTGGACAGACTGT
AAGAAGCTATATTGNGTTCTAATAACTTGTACTCACCTGTGGACAGACTGTAAGAAGCTAAGGCCTCTGAAGC
GTCCACAGGGAAGTTCAACCATACATGACTAAGCTATCATGTGACTAAACTCGTCCGCATATCTCTAAGTCCA
CCAACNACACTTGTAGACTGCTTGCTCACTCTAACA
>Agabi_varbisH97_2_Cluster46#LTR/Copia
AAAGATAAGACAACTCAGTGACAACGAAGGTAGGATTGTCCACTGAGGTGTAAAATACGGTGGCTGGTGTATA
AGTACGTCGGCAAACGATGCCGCAGAAAGAACTGTATAGTAGAGACAGGAATGAATAGCGCAGCTATGTCGGA
ATGTCAAAATATTGGAAGTGTCGAAAAGGAAAGAATGTAGAATGTTATAAATAGTAATATTTACCAAAAGAGG
AAATATACCAGAAGAGGAAACATAGGTTGAAATAAAAGAAACGAGAAGTGATAGATGTCCGTGTGTACGAACC
AACTTCAATACTCCCCCTGGTTCGGCACTTAGGACTGCGCTATCAGACCGAAATGGAAACAAATACGAAGGAC
TGCGTCACGAGGAAGAGCCTTGGTAAGGATGTCGGCAAGGTTCTCGGAAGAGGGGATATAACTGAAAACGACC
GAGTTGTCTGCGACTTTCTCTTTGATAAAGTGATGCTTGATGTCAATGTGTTTGGTGCGACGATGATTGCGGT
CATTGGTGGTGTTAGCGATAGCACCATTGTTGTCAGCGTAGATAGTGAACGGACGTTGCTGGGCCAAATCTAT
TTCATAAAGGAACTTGTCGAGCCAGACTGCTTGCTGAACCGCACACGTGAAAGCCATATACTCAGCCTCCACC
GTCGAAAGGGCAACAGTTTCCTGATGTTTGCTTGCCCAGGAGACTGGTCCTCCTGCCACGAAGAAAATGTGTC
CCTCAGTTGAACGTCGGGAATCACAATCACCCGCAAAATCGGTGTCGACATAACCATATGGTTGTAGAGTAGA
GTCTGGATAGTAAGTGATGCCATAGTCCAAAGTACCTTTGACATAGGCAAGTGCGTGTTTCATGGCGTTCCAT
TGGGTCCTTCCGGGATTGTTCGCAAACCTAGACAGCAGATTCACGGCGAATGACAGGTCCGGTCTTGTAGCTA
CCTGTAGCCACATTAAAGATCCCAACGCCTCTCGATACGGCACCCCTTCCATCTCAGTCATTTCGTCTTCTGA
CCTTGGTACCGAATCAGCAGTTGAGAGATCTATGCCGGGAGGTAGCAGTGTCGATTTAGGTGTAGCGTCCCCC
ATGTTGAAGCGATCTAGGATACGTTCACAGTAAGACCGCTGTGATAAACGTATAGTACCGTCCTTTTCATCAC
GTTCAATCTTCATTCCCAATATAAACTTCGCAGCTCCCAAATCCTTGACCTCATAACTGTTCCCCAACTCCTC
CTTAGCTTTCTTCTCCCCCAATTCCGTAGACGAAGCTCCAAGTACATCATCCGTCCATGTCGACGTAAGGGTA
AATTCTGTATCTTCAACCTTCGAACGAATCTGAGAATCTGCTCGAGATACATAATATCCGTGGCCTTCATATG
TGCGGTTGAGGTTGAAAGCCCAATCGTGGGCGCCCTGCATGGTACCGTATAAAGTTTTGAGGAGTAACCACAC

ATTGTCTCCCCCTTCCTCAAAACCCTGTGGCTGCTCCATGTATATCTCAAAGACGTTATCACTGTTGAGGAAA
GCAGAGATGAAATCCAGTTGCCACAGCCTCATCCGCCGAGCTGCCGCCACAGTGCAGACCAGCCGAACAGACT
CGAGACGAGCCACAGAAGCATATGTCTCGTCGTAATCTTCACCCAGGACCTGGGTAAATCCTTTAGCAACTAG
CCTGGCTTTGCGATCTATCGCCTCCCCCGCCTCATTGTACTTGTTAGCGAAAACCCATCTAGATTTCACCACG
TTCTTGCCGACCGGACGAGGAACGAGCTTGAAGACTCCCTTTTCCTTCATCATGGTCAACTCTTTAAGCATCG
GCGGTACCCACAAATCAGCTCTCTTCATCGCATCACAATATGACTTTGGTACGATGAGATCGTCGTGAATGGT
AGCGACCCCAGCCATGGGCCGCCCAAGGTATTCCATACTGTCCAACATCTTCTGAGAGGGTTTACGGTTGCGT
TGAGACCGTCTAACAGTTGCAGAGGAGTCTACAACCGGTGTCACATCCGAGTCGCCTTCGGCCGTCGAAATAG
GCACCGCAGGTGTAGTCACAGGTGTGGTACTAGGAGTAGATATGGCAGGTGGGTGTAACAAAGTGATATGTTT
CGGTTGGGGCGCAACGAAAGTATCAGAGGGCCGTGTGGCGTTGTCATTGTCGGAGGGATAGGTAATGAGGGGA
TCCGTAGAGTAATGAGGCTTGGACTCCTCGAATATGACGTCACGTGAACGGAAAGCCTTCCCAGTAGAACGGA
CAAGTAGTTTGTACCCAGTATGGTCAAAGAAACCAATGAGTGTAAGCTTGACGGAATGTGGGGAGAGCTTGCT
GGGACTTGCCTCGACAGGAACGTGGGCATAACATGTGGAACCGAACGGCCGCAAGTGGGACACGTCCTGGCGG
TGCCCGAGCCAACGCTCAGCGGGTATGTGGTCAGGTTGGCGAGTGAAAGGTATGAAGTTGCGCAAGTAAACGG
CTGTCTGAACAGAGTCAGCCCAATATTTCTGGGGAAGACCTGAATCAGAGAGGAGAGTTCGGGCCATGTCGAG
CAGCGTGCGCATCGAGCGCTCTGCTTTACCGTTCTGTTGATGGGCATAGGGAGTAGAGAAGTCCAGAACGATG
CCATAACTCTTTACGTACTCGTCCTGCAACCTGTTGTGCCACTCTCTCCCCATATCCAACCTAACCCGCTTGA
GCCGTTTCCCAGTCTGACGCTCTGCCTCTCGGTGGAACTCTTTGAAGACGTCTAACGTCGCCTCAGCCGACTT
GTTGGAGATGAAATACACCTTACGGTACGATGTGGCACCGTCCATAATCAGCATGAAATATTTCGCTCCCCCG
GCCGACTGTACCGGTGACGGCCCCCAAATATCGATATAGACACATTGGAGAGGTTCAGTCTCGGCGATAGCTT
GGTTGTTGAACGGGTGAGTAGCATGTTCGCCATATATACAGTCCTCGCACATTCCCATCATCTCAGTCGTTCC
TTCAATATTCAGCCCATCCACAAGCTTGTCCCGAGCCATACGTTTGAGGACATCAACGCCAACGTGGCCAAAA
CGACGATGCCAAATGTCAAAAGGAACCGCCTTACGTCTCGCTTGTACGACGTTAGCGCTAGGTGTGTTTGTCC
CCACCGGTATCACATAGAGTCCGTTCGCTTTAGCCGCCTCCAAAACCGTCTCTCCACTAGCACTACGCACGAC
AGCAGATCCGCCCTCAAAGCTAACCTTGAAACCTTTGGCAACAAGTTTCGAGACAGAAATGAGATTTGAGCGA
AGTTGGGGAGTATGTAGTGCATCTCCCAGAAGAATCTTCGAAGTGGATTCCTTTCTGTGCACCCAAAGTACAG
CCGTTCCAGATCCATCAATATTGAACGTTGATTCTTCACTAGCTGAGTTACCAAGTCTCGGTGGCGATATAGG
TTTATATTGGACAAAATAATCCCTCTGGACAATACAATGATCGCTGGCACCAGAGTCGAGGAGGGTTATGATT
TCCCTAGTGGATGCGCTATCAACATCCTCTGGTGTTATGGTTGCTAGAGCGAAAACTGTCGGTTTGTTGTTCC
CATTCCCATTATCACTACTAGCTGTGTTGGCTGACGTTTGACCAGTTGATGCCGCATTGTTCGTCAAGCCTCT
GACTCCTCCACGCTTGCCGAAACCCGGTGGAAATTGCCCGGCCTTTCCACCGCCAGGCCAGTAACAATTCTCT
ATAGTATGGCCTCGACGACCACAATTTTGATTGGTGCATTGAAGTTGTTGATTCTGGCCGCGTGGTCGATCTC
GACAAACGTCTGAAGATGTTTGGAGAGCGGTGGTGGTGGATTGGGAATTTTGGGAGCGGTTACGACTTATCCT
AGCCCAGTGCGCCATCAATTGATTTATTGCCTCATGAGAAGACTGAGTAGTGTATAATGTGGCGACAATGGAG
TCCCAAGAACGGGGAAGTGAGTTGAGTATGATGGTGCGGAAAGCTTTATCGTCAATCTTCGCTCCCAAAGCCG
TTGCATTAGACCACTTGGTACGAAGTTTAGAGATATATTGAACAAAGTCCTGATCATCAGCATAGAGCATATT
GTGTAATTCCAACTCGGCGTTTAGTAGCGCGACTTCTGACGCTACTTCGTATGTGTCCACATATGATTTCCAG
GCTTCGGCGGCACTACCATGTATGTTTATGCCGAGGCCAACTGGGTCGTCGGTATTGTAGATGAGTAAGCCCT
TTGCCCATGCGTTACGCACTTTCCATTCAGCAGTGCTTGGAGTTGTAGACTCCCATGAGGTTTCTGTCGGCGT
AGAAGTTGGAGATGTGGTCTGTGAAGTAGTAGTGTCAGGCGGTGAGGAAGGTAAGGGCACATTCAACGGATTG
CTTGTAGGATTAATGATTGAACCGTCCAAGTATCCAGAAACACCACGAAGCTCAGCAGCTATACGGATGAGGT
TGTTCCAAGAGACCCAGTTATTTCCATTGAATTTCGCCGTGTCGGCGAACATGGGAGGTGTGTTTCCACCCAT
ACCGGACATTGTTGTAGTAATGTGTTATGTGCGTAGTTGGAAAATATATCGAGTGGGACGTTTGTGTAGTGCA
CACACAAAAAAAAATCTTCGAGTTGAGTTTTTGGTATCTATCAGAACAGACTGGGGCCCATAACCTAAAGATAA
GACAACTCAGTGACAACGAAGGTAGGATTGTCCACTGAGGTGTAAAATACGGTGGCTGGTGTATAAGTACGTC
GGCAAATGATGCCGCAGAAAGAACTGTATAGTAGAGACAGGAATGAATAGCGCAGCTATGTCGGAATGTCAAA
ATATTGGAAGTGTCGAAAAGGAAAGAATGTAGAATGTTATAAATAGTAATATTTACCAAAAGAGGAAATATAC
CAGAAGAGGAAACATAGGTTGAAATAAAAGAAACGAGAAGTGATAGATGTCCGTGTGTACGAACCAACTTCAA
TA
>Agabi_varbisH97_2_rnd-1_family-109#ClassI/TRIM
GTACTAACACTACTAAACCAGCCGGCCGCTTGGGCTGACTGCCCGTTCGTGTGTTCCCCCCATATAGTAGGCA
ACCTGGTCTGTTTGGTCATGTTGTGTCGCCGCGAGCGATGGGCGTGAAAAAGGTGTCCACAAATACCTTTCCC
TATACAAGTCCGGCATCCGACCTCATATTTTGGAGAAATAATACCGTAGACACCTAGAATACCATTCCCTTAA
TAGCCTGGTGTATGTATCTCGGCATAAAGCCTTCACAAAACAAGCCGAGGAGCCGGTAAACTTCTCTGCCCAG
TCAACAATTGGTTCCACTAGGACAACCCAGTAACAATATGACTACCATGAGTGTACCAACACTACTAAACCAG
CCTAGACCTTAGTCTGACCACCCGTTCATGTCTTCCCCCCTACGTAGTAACTACCACTATGACTGACCGACCA
TCACAAGGTGTAGTACTAACACTACTAAACCAGCCGACCGCTTAGGCTGACTGCCCGTTCGCGCGTTCCCCCC
ATATAATAGGCAACCTGGTCTGTTTGGCCANGTTGTGTCGCCGCGAGCGATGGGCGTGAAAAAGGTGTCCACA

AATACCTTTCCCTATACAAGTCCGGCATCCGACCTCATGTTTTGGAAACATAATACCGTAGACACCTAGAATA
C
>Agabi_varbisH97_2_rnd-1_family-176#LTR/Copia
GAATTTCGCTTTTTCNAGGTTTTTNGTGAAAACGTCAGCAGGATTCTTTGANCCGTCAATCCAATCAAGAACG
ATTCGCTTTTGTTCAAGAGCTCTCGAACANAATGGTAGCGAATGTCGATGTGCTTCGAGCGTTTTTCCTGAAC
TTCAGAAGTTGCCCAGAATAGACTCCCACATTGTCTCCATAGANNAGGAGTTNGNATATCGAAACCGATCTCG
GTTAAGAGTTGGGACATCCAAACTAATTGTCGNGAACAATCGGAGAGAGCCATATATTCGGCTTCCGTCGANG
AGAGNGCGACNGTCTTTTGTTTACGACTCAGCCAGGANACAGNTCCGTTGGCGATCGTAACAAAGTTGCCGGT
GACNGATTTCCGGTCTNCTGGATCTGGAGCCCAATCAGAGTC
>Agabi_varbisH97_2_rnd-1_family-138#LTR/Copia
TCTGGATGGATATCCGGTTCGACTGGTGGCGGTGGCAATTCGCGTTCGGGTNGGGGAACTACCAACGCTTGGC
GANNCCGGAGTNGTGGGGGATTCAGGCGATTCTGGTGGNGNAATAGCGGTGGATGNNGNGGGTGCCTCGGGAG
TCGGGATAACTTGCTCCCCCTCGAGCTCCAGGTCAGTNTNCCCTTTGATGAAGCGAAAGTTACGCTCGATCTT
GACTGCCCCGGTATCAGGGAACAAGATCCTNGANCCGTTACTCTCCGTGTCANANCCCAACCANCGCCCCTTC
TTCGCCCGTGCGCCCAGCTTATCCCCTCCAGCTTGATGAACCCANACCTCATCNCCCCACTCACGAACGTTTC
CNAGNCGNGGCTTNCGGCCATACAAAGCTTCGAANGGCGTCTTNCCTTCAACTGCCTTAGTCCCGGTTCGATT
CATCAACCATACTACGTGCGCNGCGGCTTCCGCCCATAAGCGACTTTGGAAGACNACTNGNATGAAGCAGNGC
TCGAGCACGTTCAACGATTGTACGATTTCGACGTTCNGCNACNCCGTTATGCTGNGGGGTATCGTGAACGGTG
AGCTTTATTTCCGTNCCNCGAGATTTGAGATAAGNCTGGAACTCCTCAGACGTATATTCCCCTCCNCGATCCG
AGTGGAGNACTTTGATACGNGCATCGNATTGAGTCTCGCATCCATGCTTCANACCGCTTGTACGCCTCAAAAG
CATCCGATTTGTNGGATAGGAACTCNACGTGNGTCCACCNNGAATAGTCGTCCGTGAACGTAACGTAATAGCG
TCGTCCTTTGCGCGTCGCNACTCTGGCTGGTCCCCANACATCCGTGTGNACTTCCTCGCCGACTTTCGTCGCT
CTNTCACCTTGCCGGACTTTCGCNATCGGCAAGCGNGTGGCTTTGGCATATGNGCANGAATCACAGGACACAT
TCCCGGACTCANCTTCGCTCAGCTTCAGCCCNGTNACCAGCCCATCCTTGACNAGTTTTTCNGCGGCCATAGG
TGAAATATGACCNAGGCGACGATGTAGAGCATCNNCGGTGAGTGATTCGACTGCTACATTGACCTCGTCGCAG
TCGATCGTTGACTAACTTGTATAATCCCTTCTCGTTTCNAGGGATCTCNGCNACACGAACACCNTCCGCGTCT
CGGATTATGCANCGNCCATCCCTGAACGTCGTCGTANAGCCTTCTGTGTCCAAACGGCCAACCGANATCAGCG
TATAGCTAGCTTCNGGTGNATAGAGNACNTCCTTGAGATGTAATTCTGACCGTGAGTCNCCATTCGGTAAGTC
AAGCTTCACTTCTCCTTTNCCGATTGCGCTGAATCGTTGCCGATTNGCAGTTCGCAACGCGCAGGGTGAGGTC
GATGTGAAGCGACGTGAACACATCTCGGTAGGGAGAAATATGNCGAGAAGATCCTGAATCGAAAACCTCGACT
CGTGCCGTCGNCTCGGCTAGCTTTCCCNACTTTNTCAACGTCTACNGCAAACGACTCATCGTCATCACCTCCT
TCGGCCGTAACATTGGCGCTGGTTTGGATTGGTCTTTCTTAGATCCTTCCTCCTNTTTATCCTTCCGCTTNGG
ACAATCCTTCTTCAAGTGCTCCCCTCCGCAAATCCAACACGGTCCCTTCTTCTTCTCGCCATCTTTCGCTTTA
CCCTTCCCTTTGGCTTTNNNCTTCCCTTTNTCTTCNGCTTCAACNCCNAGAGCATCATCCCCTTCNGGCTTAG
CTGACCTTTCTTGGCGAAACGGCGNGTACGGTCCCATTCGTCGCANATCATGACAATGAGCAGGTCNGGATCG
ATAGTNTTGCGAAGGGAGGNGTGAAGACGAGCNGCNGANAGCTGGTTNGANGCANANGTCGNCAGCCANCGAG
GTAAGGATTGAATTATNGCACTTCGGTATTCATCATCGCTAATATGAACGCCGACNGCTAGTAGCTCCGNCTT
CTTNGCCCNCAAGTCANTCAAGAACANNCGNACGTCACCTCCAGNTGGGCAGCGCGAGGNCATGAANTCCTGA
CGGAGGTTTGCTTGNGAGNAAGCACTCTTGTACGTATATTCATCGACAACCGCTTTGTCCCACATCNGTTTGA
CNGTGGTTTGGGCAACTTGAAACNACNACGAGTGTGGAGTCGGGAATTCGCTGAGCNAGNAGCGATAGCGNAA
TATTTTCGTTNCGATCCCAGGCAACTCGATGTCGGCCACTGTAACTGGNGNNGGAGTCGAAGGNANAGGNACT
TGTACAGCCGCAGCTGCGGTTGAAGGAGCTGCCGAAGTAGCTAGGACTGGAGTAGATGCTGGTGTAGGCGCCG
GAGTGGTAGCTCGCGGTGGTGTAGGACGAGGAGTTGNNCCGTCGAAATGACCCCACAGGCCCTTCCCTCGAAC
GACCATCCGGAANCGCAACTCGAACATCGCCCAATTCGNTCCGGAAACGTCCAATGCAGGCACGGTGGGTGGC
AGNACGTCGTTTATAGCCGCCGTACTCGACATNGCGTTACTCGCTTTCGCTTTGCAGCACTAGTTTACTTNAG
TGATGTCTCACAATGGGGCGGGGCCCATAACCTGAAAGANTACAACGCTCAGTCTCGGGGCACGAGAACTGAT
ATATTTGACGTATACCTGAAAGANTACAACGCTAATACAAGTACAATTAGACGAAAATACAGAGAAGAGTTAT
CAAGCCTACCTCAGTCTCGGGGACGAGAACTGAGGTGTACATAAGTTTCGAGCCACATTTGTACACTACGAGT
GAAACCGAATATAAAAGGAAGTTTTCGTCATACCTCTTCTACT
>Agabi_varbisH97_2_Cluster39#LTR/Copia
GTTGTGAGTACTTCGACATCTCATCTCAAGTTATCTTTACTCCTTTCGGTCATGATGACCCTTCTTACTTTCC
CCTTATGTTTGTGTTACCTTATACGGCCATTATAGATCAGAAAAACATTTTACCGTTTCGAATATCCAATTTG
TTTCCTTTATTTCATTTCCTATTTCCTTTTTCACTCTATAACTGTACAGTCGAGTCAGCCGGGTATTTAAGGC
ACTAAAAGCCACCGGGACACCTCAGTGATCAACACAATTATTCGTTGTCACTGAGGTGAGTAGGTTATTCTAA
TCCTGTCTTCATTATACTTACAGTAGTCTAGACTTATACTTATTATTAGGTACATAGTGATCAACACAATTAT
TCGTTGTCACTGAGACTTATACTTATTATTAGGTTATGGGCCCCGGCTCAATAAACTAAACACAGCGAAAAAC
TCGATTTGGAACAATCACTACTATTCACACAGAAATGTCGAACACTACACCCCCTATATTCCCAGAAACTGAC
AAATTCGACGGTACAAACTGGGTAGCTTGGAATGGCCTGATCGGTATAGCCGCCGATCTCAGAGGCATTTCCG

GATACCTTGAGGGCACGATCCCTAAACCCCCTGACAATCCACCTATCCTTCTCACGTCACCTGCTTCACCTCA
AGGACAGACTACTACAACGTCTCCAACAACACCACCACCTGACACTCCATGGGACTCCATAAACCCTTCCCTT
GCTGAATGGAAAACCCGTAACGCTTGGACCAAAGGATTGCTTGTATACAATACGAAGGACCCCATTGGTTTAG
GTGTCAACTTATCTGGCACTGCCGCCAATGCGTGGAAATCCTACAAGGACCAATATGCGAAAGTATCAGAAAT
GGCGATCTTGAATGCTGATCGAGACCTACGAGAAATCACTTACTCTGATGGTCATGATTTCCTCGAGCATATT
GCTCGACTACGCACAAAATGGGCTACAGCTACGGCTCTCGGAGCACCAATCGATGACAAGACGTTTCGAACTA
TTGTCCTCTCCTCATTACCACGATCTTGGGATCCCATAGTGTCTACCCTCTACAACACGACAACATCCCGTGA
AGCCATCGCTGTACTCGCCACCCACTGGGCTCGTATTAGCAAGGATCGAGTCGTGAATCCACAAAACGCAACT
ATGGCGCTCCAATTCAACACATATCACAACAATCATAACAGAAACACACCAAAGAATCAACATCTTGTCTGTA
CCAATCCTAACTGTGGTCGACGAGGACACTTGATTGCTGATTGTTATTGGCAAGGGGGAGGCAAAGCTGGCCA
ATTCCCAGCGTGGTTCGGAAAGAAGGGAGGCACTAGAGGAAGCACACCACACGCAAATGGAGGAAACACTAAT
ACTTCTGCCAACTCAGCGTCTACTTCGGACACAACAGCCCAAAGTTCCGAACCTCGTGTTTTCGCACTCTCCA
TGGTCACGTCGGAGATGCCTCAGGTACTGAGCATCAACCCTATAACCCCTATCCATATTCCTGACCACGACAA
AACCTCCAGGATGCTGAAAATAGCCACTAGCGGCATAACACCGAGAGGGGAGCTGGAGGGTGGTGCTCAAATG
TCCTACACGTTACATAATGTCTCAACGTTGTCATCTATAACGCAGACGAGCAATACGACAGACTTGCTTACCC
TACTTGATTCCGGTGCTACCGATCACTGCTTTGCGGAGCGTGAAGATTTCATAACATATAAATCCTTCCAAAC
TCCACGAGACGGCCATCCTGCAAATAAATCTGCAGTTTTCAACCTTATTGGTACTGGAACTGTCGAAATTCTT
ACCCATAGAGACAATGCTACAACCAAGATCCAAATCTCTGAGGCTCTACACTCGCCTGATCTCCGCCATAACT
TGATCTCGGTGTCGAAATTGACTTCTAAAGGACTAAACGTCGTCTTCGATGATAATACGGCTACTGTGTATAA
CAGTCAGGGCATAGCTGTGGTTACCGCCCGTAAGATTCAAGGATTATATATCGTCGAAACCATATCACGCACC
TCGTCGATCCATGTTATGCAAACAAAGAGGAAATCTGTCCCATTCAGTACCTGGCATCGACGTTTGGCCCATG
CAGGAGAAGGAACAATTAAAGAGATGTTACGTTTGGATTTAGTTGATGGCCTAAACGTTGAAGGGCAACATGA
GATAGGAGGCACATGCGAGGATTGTATCTTTGGGAAACATACCGCGCATCCACACTCGGATAAAGGGAAAGTC
GAGGAGGAGGTCCTGGAACGCATCCATGTTGACATATGGGGGCCGGCTTCAGTCCAGTCGGTTGGAGGATCCA
AATATCTTATGGTCATCGTAGATGGTGCTTCATCTTTCCGCACAATATTCCCTCTCCCTGAGAAATCGGCCGC
CGTTACTCTCAACTGCCTCAAAACTTTTCAAGTGCAGTCGGAATTGCAAACGGGCAAAAAGCTGAAACGGATC
CGTTTAGACATGGGACGAGAATGGTATAACAATTTATGGGAGACGTATGCGAGGGAAAGAGGAATTATCTTGG
AGTATACAACTCCATACGCACACCAGCAAAACGGCGTTGTTGAACGATCGATGAGAACCTTGGTTGATCTCGG
AAGGTCAATGCTAGCTGAGTCAGGTCTCCCACAGCGCTATTGGGCAGATGCATTCGTAACAGCAGGTTACATT
CGGAACTGTTATGATTGTGGAACAAATCCGAGTATTCTAAATCTTATCTGATCTTATCTAATCCTTATCTGAT
GCGGACCTTATCCGTTCTATTCCTATCATCAGGATACGTCATTCTATTTCTATCTTGCATTCTCTTTGACGTT
ACCTTTTCTCATGATCAATCTTCTATTCTCATGATTGATCATCCTCCTTCCTAGTTTATACTTTCCTTCTCAC
TGATTACTCATCTACCTCGGTCATGGTTACTCAGCCCGTTCTATAAGTTAGTTACCTCGGGTAGATAGTCTTA
GTATAAGTAAGCGTAGATACTCTCTGTATTTCCCTACGCTTTACCAACTTGCTTTTCCCTTACTCGCCCTCAA
GTCTTTGCCTTCACTGCAACCTGAGCATTCGCTTTAAGGTCGTAACAGGAACCTCCTCCCGACCTCAAGATCA
CCAGGGAAAATCCCAGCTGAAGTTTGGACGGGACACCGCCAAGATATATCTCATTTACGAGCTTTTGGCACTA
CTGCCTACGCTCACATTCCCCTCGACCTCGGAATCTCTAAACTCGCACCTCGTTCCGTCAAACTTACTCTCAT
AGGCTATTATGGGACGTCTGGCTATAAGTTGCTCGATCGTTCGACTGGGACGATATCGCGTTCACGAGACGTC
ATCTTCGATGAAGGCGTAACACACCTTACCTCGACACCATCAACCACATCCACTGATGACAACATTCCTCCTA
CTACCACTTGGCCACATACCACCTCAATGTCCAACGACTCTGCGAACGCACCGCGTCCCCGGGACATGTCATC
ATTACACAAAGATCAGGCAGGGTCAGCGGCACAGCGTGCTCAAACTACTAGGGACATCAATGATGTCGAGGAG
ATTTTGATCGAGGAGCAATTGATGGCTGATGACGCTCAGGGTACCGATACCTCCGATGATCCAACGCCGCCAG
TCTTACGACGTTCGAGTAGGATCTCACGACCTTCAAAGCGCATGCAGGAGAGCCTGGATTACCAACACCGTAC
CAAAGCGAACGTCGCAGATACCCGTATGGAGGAATGGGTGCCGAAATCTTACCGTGAAGTGATGGCTCGTCCT
GATTTATGGCTAGAGCCGATGGAGAAAGAGTTTGGTGTGATGAAGGAAAAGGGTGTATTCAAGGTGGTAGATC
GTCCGCTTGGGAAGAACGTTGTACAATCACGATGGGTATACGCGTTGAAATTTGACGCTGATGGTAACATTTC
GGGTAGGAAGGCGAGGATTGTGGCTAAAGGTTTCACGCAGGTGTTGGGTGAGGATTACGATGAGACGTATGCG
TCTGTCGCTAGGATGGAATCGGTGAGGATGGTTTGTGCTATTGCGGCTTCGAGGGGGATGCGACTCTGGCAGG
TCGATTTTGTTTCGGCTTTCCTCAACAGTGATAACCACTACGAGGTTTACATGGAACCTCCGAGAGGTTTTGA
AGAGGGGGAGGGGAAGGTATGGCGTCTCCTCAAAACCCTCTACAGTACTATGCAAGGAGCGCACGATTGGGCG
CAGAATTTAGACAAAACTTATCAAGGCCATGGGTATTACAAGTCGAAAGCCGACTCGCAAGTACGTTCGAGGG
TGGAAGATGACGAATTCACTCTTACCTCAACATGGACGGACGACGTGCTCAGAGCCTCAACGTCCAAGAATGG
GGAATCAAAGGCGAAAGAAGAATTGGGTCGAAGTTATGAGATAAAGGATTTGGGGGAGGCACATCACATTCTG
GGAATGCGAATCGACAGAGCCGCAAATGGTGACATACGGCTGTCCCAGAAAGCCTTCTGTGAACGTTTACTTA
CTCGCTTCAATATGACCGACGCTAAACCACGATCAACACCACTTCCAGTTGGGATCTCATTGTCCATCAACGA
TTCGCCGCAAAACATCTACGAGATCAAGGAAATGGAAAGGATACCATACAGAGAGGCTCTGGGTTCGATTATG
TGGCTCCAAGTCGCTACAAGACCCGATTTATCCTTCACAGTCAACTTGCTTTCCCGTTTCGCGAACAATCCTG
GACGAGCACACTGGGAAGCACTCAAGCACACTTTGGGATATATTAAAGGAACGATTGACTACGGAATAACATA

TCGAAAGGGAGGCGATCTGAAACCCATAGGCTACGTAGATGCGGATCATGCGGGAGATGGGGACACGAGCAGA
TCGACAGAAGGTCACGTCTTTTCGGTAGCTGGGGGAGCAGTATCATGGGCAAGTAAGAGACAGGAGACGGTAG
CGGTGTCAACAGTGGAGGCGGAATATATGGCGTTTACAAGAGCAGCACAACAGGCACTATGGCTGGAAAAATT
TCTCGACGAAGTTGGATTATCTCAACCCGGACCAACGACCATTCACGCAGACAACAGTGGAGCAATAGCAAAT
ACCAAAAACGACAAGAATCACCGAAGGACGAAGCACATTCACGTCAAATATCATTTCGTTAAAGAGAAGGTCG
ATAACGGTGACATCACTTTCGTATATACCCCCTCTGCTCAAAACCTTGCGGATATCTTCACCAAACCACTTCC
CCGTGATGCCGTTATACGATGCTGTGATGGAATGGATATTCGAGGGAAGTTGTGAATGAGAATGCCAAAGTAG
GGGGAGTGTTGTGAGTACTTCGACATCTCATCTCAAGTTATCTTTACTCCTTTCGGTCATGATGACCCTTCTT
ACTTTCCCCTTATGTTTGTGTTACCTTATACGGCCATTATAGATCAGAAAAACATTTTACCGTTTCGAATATC
CAATTTGTTTCCTTTATTTCATTTCCTATTTCCTTTTTCACTCTATAACTGTACAGTCGAGTCAGCCGGGTAT
TTAAGGCACTAAAAGCCACCGGGACACCTCAGTGATCAACACAATTATTCGTTGTCACTGAGGTGAGTAGGTT
ATTCTAATCCTGTCTTCATTATACTTACAGTAGTCTAGACTTATACTTATTATTAGGTACATAGTGATCAACA
CGATTATTCGTTGTCACTGAGACTTATACTTATTATTAG
>Agrped1_rnd-1_family-33#TIR/MITE
TACTGTACCATTTCGNTCAATTCGTCGCGGCCTGTGACCTGTGACCGCCGCGACGAAATTTTNGTTTCGTCAG
ACGNNNNTTTGACCTGTGACCGACGAAAAATTTCGTCTCAGGGCCTTGTTTTCGGCACGAAACCGACCGGCCT
CTGGGAGAGTCTCGCTGTGACCCCAAAATTCGTTTCGTCGAGGTCGTCTGTGACCCGTGACCNCGNCCATATT
TCGTCNCGTCGCGAGGCCCTGTGACCCGTGACCGACGAATTGANCGAAATGGTACAGTAC
>Agrped1_rnd-1_family-19#TIR/MITE
TACTGTACCATTTCGATCAATCCGGCGGGCCATGTTACGCGTTACACGCCTGCCGAANTTTTCAATTCGGCAG
ACCGAAAAATTTNGCGTTACATACCCNCATTTTCGGCAATTTTCGACCAAAAATAGCTCAAATACGACAAATT
TGGGGTGTAACGGCCAAAAAATTCGGCGGCAAGCTTTTGACGCGTTACAAGCGTTACATTTCGGCCAAATTCG
GCGCCGCGTTACGCGTTACCGCCGAATTGATCGAAATGGTACAGTA
>Agrped1_Cluster22#LTR/Copia
TAACATCAGGAAATTCACTTTATGAGCAAGAGCACTCTATGTGACTTTGGCGATTAGGAAAGACCCCAAATGT
CTCTGTGTGGCTCTAAGTGTCTCTGTGATGCTTTCCCTGAGAGATAACTTACCAATACACTCTGTGTGGCTCT
GTATAAGCTGTCTACTTTGGGTGTGCTTTGCATGTCTTTGGGTGTCTTTCCAACTGACAGGTAAGATCATGTA
TTGCATCCCAGTTTATCAGTTACACTACATGGGAAATATATCCTGCAAAGTCTTGTCAAAATTCATGCAGTAT
TGGTGGTACTGCATGTCTTTCCTTCCTGCTGTACTGGCCTTTAAGTAGATCTCGCTCTTTTGAGGTCAAATTT
GGACTGTTTCCTTAGATATTTGGAGGATCAATGTTTGACCCATCTTCAATTTATCATGCAAGTTTACAAAAGG
TAAGGTCTCCGCGTTCTTTCGCGCTCAAAATTCTTCGAAATTCACTCAAAATTCGCAATATTTATTTCCTTGA
TCATTTCCTGACCGATTTACTTCGCGATTTATGTGCGAATCAATAATTTGCAGTTATAAAACACTATTTGGAA
AATTTCAATTGAAACGATCCAAGTCTGACCAAGTTATGCACCCTGCCTACCTTTCCTTCCTCTCCAAACAAAA
ATTTCGTCGCTCTGAGATCATTACTGCACTGTTTCTTCTGATATTTGGAATTTAGTAAACGACTGTATGTCGA
ATTTTAATTCCTCAATATCAAAAGGTTTGATCCATTCATTGCCTCGCATTAGAAATTCGAAGAAAAATCAGAT
CGTTTATCCATATCACAACTTCTGAATGGAATGTCTTCGCGGTTGGTTTATGCGTTCACATTTTAAGGCCAGA
AAACACTATTCCCAAAAGTTTAGAGTCAATACATGAAATTTTGAATGAGTTCTGACACATTCCTCCCTTTCCT
TCCCCTCAATAGGAGGATTTTGTCATTCTGAGAGCAGTTCTGCACTGTTATATCGGATACTTGGAGGTTGTCA
TTTAAATGTACATCAGAGTTGAAATCCTACACAGTAGTTTGGCACCCAGGTTACCATCAGCTGGGAGCCAAAG
TATGGTAATCATGTCTCTCCCATGCATCACCAGCTGAATGATATCCTGGGGGAATGTTGAATTCGCACTAATA
ACATGCTGGCACCAGGTACACTGGCTAGCTGCTGGAAAAATACCTGACCTTCACCATTTGTGCCATCTGGTGC
TGAGGTATTCACCCCAGTTACTAAATATGACACAGCCTTAGGACTGGGCGGCATTTTACTTCTTTGAATCACA
CAGCAGAGTAGGATGATTCAATTTATATATTATTGCATGTATATAAGCTACTATAATTACTACAATTGAAAGT
ACACAATAAGAAAAGGAGCCAAGGCGAGGGAATTGAACAGTAAGTAACTTAAATTACAGCTGATGTAGAACCC
AACAACACCCATCTTTCTTGAACCAACATGTCAGTCCAGTACTGCACATCTTACTCTGATAAGACCACTTTAC
GCAGCCAATGGCCCCAACTCAAGAACAATTCTGAATCTGCAAATCGAGAACCAAATGAGTAGTCGTTTAGCAA
AGAAGTACTCCAGGCAAAACTTACAAGTTACGGTGTAAGGGACCTATCATGCAGAAAGGGAGTGGAATGAGAC
GTGAAAGTGGGAAGAAAGAAAGGTTAGCTATCTGAGTTGGAATTCTGAGTTTGAAATGATTTGACACATTTTT
GGCATTTGACCAGCCTGAGCCGCAAAGCCTCAACGAAGCCTGGAGCAAAACTAGATACTTGTCCCTATAATAT
GCTCTATACTGTAGTCATAATGGAGTTAGAGGCCTGAAAAATCCTTACGATCTCAAATTTTATGACTTCTATG
AGCAAATAATGTGGGGCAGTTACAATGGTACACAGCTGTTTGACCCAGCTGCCGGGCAGGATAGACTGTGATC
TGGAAACAGGCTGGACATCCGAATGCTGGCACTTGGCTGCCGGCCAGCTCATCTACAGTTAATTATATTGATG
TTGTACAGCAAATTTGCCAACATTAACTATTGGTCTAAATCTGACTGTACCAACACCTTCTGAATATATAACT
CTATGGTCCGCTAGTCTAATTGGCACTCTATAAGGGGTATAATTGCGAATCCAATTGCGGTGTGGGGTCATAT
TAGAAGTGGCACCTGTGTCCATATTCCAATGAAAAACATTAGAAGATGTAGCATTGACTTCATGTTCAGCAAC
AGCACTTGCCTTGCCAGCAAATTCTCTTACTGCTGCAGCCTTCTCTTCATTTGCCTGTTTGGCACTCTCTGTA
TTACGCTTCTTCTTTGCAATCTCGGGGCAATTGTGTACCCAGTGACCCTTTTCTTTGCAATAGCGACATGTAT
CTCTATCCTCCTGTGGCCTATTCCCTCCATTGTATGGCTTCTTCCCCTTGTATCCATTCCCTCCTGACTTGGC

CTGGTTCAGCGACTCTCCTCCAGATTGACGTTGTCAATTTAGTTCTTCACTTCGGAATGCCTGGATTATTACT
GACTTGTCCAGCTTATCCAACAATAAGAGAGGCATTGTGAGGTGACGATAATCCTCTGGCAAAGCACGGATAA
GTGCCATGCACTGAAGTTCTTCATCCAATGTCTCAATCTTGAAGTCAGCAGGACACAGGTTTTGAATTGCCTG
CATAGCTTTCTCTATCCGGGTTCCCAAATCCACCAAAGACTCGTCATCTTGTTTCCTGATGGAGAACAAGTCA
TCATACGCATTGAACCTAGCACCAGGTTTCTTGGATAGGTGTGCTGCTTCCAACAATGACCACATTTTGATAG
GGTCTTCTTCATGCCCTCTGAAATGCACCCTCTGATCACTTTCCACCAACAAGTAGATTTCTCCTGCCGCTTT
TTCTGATTTTATCTCCCATTTCTCTGCTTCCTTTGCCTCAGAAGGCTTCAGTTCCTTGCCAGAGACTAGTCTC
CATAGTCCATTTCGCATTAGCCAAGCCTTCATCTCACCACACCATTCAGTGTAGTTTGAATTAGACAACGGCT
TTACTTTGGCAACCGAGTTGTTGTCTGATGTCGACATATTTGTAGTCCCTTTGTATCAAGTTGTAATCCCTTT
GTAATGTTTTGTAGTCCCAAGTTATAATCCCTTTATAACGTGTTGTAACATTTTGTAGTCCCAAGTTTGTAAT
CTCCCAGTTTCAAAATGTAATAAACAGCAATAAGACTATAATCAAATGTATAGCCAAGTTTATAAGCCGGGGC
CCATAACCTGTTAGAAATTGACAATAGAGTCACTAATAGTCAATAAATAGACTTATAGTTAACAATGTAGTCA
AATCTCAAATGCTTAACAATGACTAGTCAAGTAAGAAAATAGTGTATTGCTTCTATGTAATCCAATGCCCAAT
TCTAGTATCTCGGTTTCCCAGACATGGTAGGAAAAGTGGTTTGTTTGGTCATATTGTACCCAAAATGCCTGAG
GAGTGACTTTAGGTGCCCACTATATCGCGTTCGAAATCGGTGGTACGCAAACATAACTTCTACACTACAAAAT
GGATATCACCGGATGCATATCGGCGAGGAGATGATGAATCTAGAAAGATTATGTAAATAGCACTAGTCTAAAA
GGAGTTATAAGCGAAATACTCGCAGAGAGCCGGCAATGAGAATATCAACTATGTACAATATGGGTACCGTTAG
ATGCAGTTTGAAGAGGAGATTAAGAATCTGAAACAATTACCTAATCTACTTTACTCTATAAATAGATATTAAC
AAAATGGTGTCCAAATGAAAAGTAAAAGTCCAAATTCAAAGTGTTTGATCACCAATTTCAACAACTTCAAGGT
ATTGTGGCGCTTTCAACAACTGAAGCTGAGTACATTACTGCTGTTGAGGCTGGTAAGGAGATTTCCTGGATGC
GCAACATTCTCCATGAGTTTGGCCTTACTATTGACCAACCCACTCCACTCCACATTGACAACCAGTCTGCCAT
TAGTGTCTCCAAGAATCCTGAACACCATGGACGCATGAAGCACTTGGATTTGCGCTTTTACTGGCTTAGGGAT
GCTGTCCATGACAAGATGATCCATCCCCTCTTCATTCCAACCAAAAAACAAGTTGCTGATATCTTCACCAAAC
CTCTTTCTGCTTCTCAAGTCGAATTCTGTAGGACCAATATGGGTGTGTCTGTTTAACTTTGTTTCTTTTCTCT
TATTGTCGCTTTTTCTGTCTTCCATTTTGTAGAAACTTGTCAGATCATGGAGGGGTGTTAGATTTTGCCTTCC
AGTCTCCACACATTCCAGACATCTCAACATTCCATCTATCATTACTCATCATTCCACTTGTCATGACTGATCA
TTCCTTCTGTCATTATTCATCATTCCATCTGTCATGACTGATCATTCCTTCTGTCATTATTCATCATTCCACC
TGTCATGACTGATCATTCTATCTGTCATTAATCATCATTCCATTTGTCATTCTATTCTGTATATAAGTCTGTA
CTCCAAGACCTGAAGTCTCCAGTTACTCACTCTGGACTTCAGCTCTTCATAGCTCCTCAGTCCTTCGTCTTGG
GCATACCATATTAGGTTGCCAGATAGCTCGCGACGTCCTGCATTCGCTCCTTTTAACAACATCTGGAAGAGCA
GAGATGTAGGTGTTATAATCACCTGACACGATCGGGTGACCTTGGAAAAGAATGACCAGACCTGGGATTGAAC
CAGGGACCTTGCCACTGCTAGAGGAATGCTCTAAACCACTGAGCTATCCGGTCTTGTGGTTACATAGGATGCT
TAGGGTGGTTAACCCAATGACCGGGTGTGGAGATGCTCCAGGGACTGTCTGCTGTTAGTAAGCGAACAGATCA
ACTTCTAACAGTAGGCATCTTTGTTAGGCACATCAAGAAGTTCTTGGAGAGCCTGTCGGCCTCTCTCCAAGCT
GGATTGGAATGCCGCTATCTTATCAACAAAAATCTCCAAGTTGGATGGCCAGGCGAAAGGTGCCTGCTGATCT
TGCTGATCGCGGGCAGGGGATGACTGGCGGGACCTCTTTGCGGGGGTGGCTGAGCCAGAGCTAATCCGATCGT
CTTCTTCCTCGTTGATCAGCTGGTCGTTGCTTCCCATCGAGATGTCAGACGGTGCGACGGAGACAGAGCGAAG
TCGTTCAGGGTTTCAAGGCCCCGGAGTGGATTGTTGAGACCCTGCTACCAGTGAAGGAGCAGGGGTTGGAGAG
GGTCTGGCGCCTTGTGTTGACGGTGGTGCAGATCAGCGGTCTGCATCACCCGGGGGGGCTGCTTTTGGCGGCA
TTCGAAGGACAGATTGTTAGATCGATCCTTTAGTGTCGTTACCCGAGTATTAGATACCCAGTTCCAACGTGTT
TGCGAGTAGAAGAGTCGAAGACGATGAGTTGCAAAATGTGATGGTGGTGGTAACGAGTCAAGAAGAGATGTTG
TGATTTTAAGTACCGACATGTATGAAATGCCATTTTAAGATATAAAGAATGATATTAGATAATTGCAACTTGA
TGCTGCAGCAGGACTTAGTCAAATTTCCAGAACACGTCGGATTTCAGGTTTCTAAAAACCTGTACAACGCAAA
AAACCATTGAAAACCGGAAACTAAAATGCCTAACTTACTGATGTTGCAAGAAAATCATTTTGTTACAATGATT
TGTAAGGCTGTGCAATAATTACAGGCCTTGGACACCTTCTATTGCAATAGAAGGTCGGTGCCGTTTTGCTGAT
ATGATGCAGAGCAGAGATTCAAAATGCAAATAAATGTGCATGGAGCGTGGGGCCCATAACCTGTTAAGAGGAG
CGAGTGCGGGACTTCGTGAGCTATCTGGCAACCTCACTCTAGTATGGTATGCCCAAGACGAAGGACTGAGGAG
CTATGAAGAGCTGAAGTCTAGAGTGAGTAACTAGTATACTGTACCAATTCACTCAATTCGGTAAGGCATGTTA
CGTGTTACATGCATACCGAAATTTTGAATTCGGTATCTGCCAGAATTGCCCGTTACGCAGGGAAATTTTTGGT
GTAATATAAGATAAAATATTGTAAATACTCGGAAGAACGAGGGCAAATACATGCACTGTTTGACGTTGAAGTC
ATTTCAAACTCTGCTGGACGCGCATTGCACCCTCACGTGCTGCTGCTGATGCATATGTGACAGATCAACAATG
TGATGTTGACTGTCACACGCATCTCGATAACTTGATCCTCGACCACCATCATGCTCCACCCCACCGCACCCTT
CCCATCCTTTCCACTTCGTTACTATTTGAAACGCACAATGTGATTGTGTGCTTGCTCGTTCTCTCTTCTTCTC
CACCATGATAACTGTTGCTCTCCCCCTACCTCCTTTTCAATCATGGATTTCCCTTCCTACTACTGTCCAGCGG
ACACCGTCTTCGAAAGTAGTACTCCGGTGAGTGATGAGTCTCCTGTTGTCTTTCGCTGATTACTAGTATGTTA
CAGTTTTCAATGAGGTGTGCCTGCACTTGTTGTTGAAGGACGATGAGGTAAGTATCTTTGACTAGGGAATATC
AAGGGTCAGACTGACCCGCACGGGTCATGGGTACGGGTAGGTGTGGGTAGGGGTGCGGGTCAGGATTCTCTTA
CCCATGACCTGTACCTGACCCGTGACTTGATAAGATTTAAGATTAGATCACATCATTACATAATTAGCAATGG

CGTGATCAACTACCAAATACACTGCAAAAAAAAACTTGACACAACTTCTACCATCCCATGGCTGACACCGACA
ATCCCACAGAGCCCAGTACTACATGCTCAGGGTGTACGGTACATATTTCATCAAAAGCTGCTGAGGCTTTCAA
GTCTCTTCTGCCTAACAAGCAACCTAAGTCCAAGAAGACGGAGAAAAAGACCAAGAAATGTACTGCCGACGAA
GTCCAATCCTTGGATAAAGATAGTCTCAACGACACATCAGCTAAGAAGGTAATACTTACTCTATATTAATGAA
TGATTGATGCTGACATGTTTCTAAGCCATCAAAGATAAAGAAAGTCAAGGAATTACTCACAAATGTCACATGG
AATAAGAAAAAAAAAAGAATCTAACAAAACGGTATCAGAGGTATGTTCATGTAAAATGATCAAAACTTAAAACT
TACCTGAGGTATCCAAGGTCTCAACACCGGTATCCAGCATGCCAGCGGCCCCATCCACAGTCCACGATGTCAT
CGAAATAGAATCTGATGATGATGTGGAGGGTGAGACCACTAGTAGTAGCAGTGGCGAAGACTCGGGCACCGAC
GAAGGAGTGGAGTCCGCAGATGAGCCTTCAGATGCCGAGCTAGGTCCATCAGGAAATTCACTTTATGAGCAAG
AACACTCTATGTGTCTTTGGTGATTAGGAAAGACCCCAGATGTCTCTGTGTGGCTCTAAGTGTCTCTGTGATG
CTTTCCCTGAGAGATAACTTACCAATACACTCTGTGTGGCTCTGTATGAGCTGTCTACTCTGGGAGTGCTTTG
CATGTCTCTGAGTGTCTTTCCAACTGACAGGTAAGATCATGTATTGCATCCCAGTTTATCAGTTACACTATAT
GGGAAATATATCCTGCAAAGTCTTGTCAAAATTTATGCAGTATTGGTGGTACTGCATGTCTTTCCTTCCTGCT
GTACTGGCCTCTAAG
>Agrped1_Cluster6#LTR/Copia
TAGCCTATTCACAATATCACTTTTTTCTCCAGCGTATGCAGGATATAATCCTCGTCGTTGTCTGCTGGTTGTC
CGAGCAAAAAGGAAACCCTGTAACTTGTAAGTATATTTAATTACTGGCAGTGGTCCCAATTGAAATAGCCATG
TTTCCAGTGTCCTCCGAGTCAATACCAAAGGGAAGAACGAGACTTGTTAGCATTCACTATTACTAGGTCTGCC
AGTCACTAGTATCCACCGGTGTAAGTATCGCAAAACCCAGCTTGAACCAGCACCCAGCTGGTCAAAATCGTTG
ATCGTTGATCGATTTGATCACCGGCACAGTTGGCTGGATTTGGGAAGCCAAAAGTCACGTGGAGTCAGTCGTA
ACCGAGTCGAGTCGAACCGTGGCAACACTACCTCCCCTGGTCGGGTTCTGCGCATATTGGTGGCGTTGTATGG
TCTGCGTCAATCTGCCTACGAGTTTTATACCTTCCTCTCGTCGTTGCTCCTGGATTTCGGCTTGACACACTGT
GATGCTGACCACGGTGTCTTTGTTGGACGTTGGACAACGCCTCCGCACTCTTCCATTTTGATGCCCACGGATG
GTTCACCACTGACTCTCATCGTTCCGCTGCACGTTGACGATGGCCTTGGAGTCACTAACTCGATTCCTTTGTA
CGACTGGTTTATCAAGATTTTGTCCAGTTGTCTTCAAATCGTGGACCTTGGTGATTGTGCTAAATTCCTCAGC
ATGCTGATTATCCGCGATCGTCCTGGCCGTCGTTTGTGGTTGTCGTCCCATGTGTACGTTGCTGAACTCCTCG
ACGAGTGGAATCTTTCTTCCTGTAAAACAGCGAAGACACTGTTGCCCATGCAGCCTCCGTCGGACGCGGATGA
GCCCATTAAGGACTTTCCTGCTGCAGATCTTACTCAACGATATCAGCGCTTGGTTGGCTGCCTGATGTATCTT
GCTGTTGCGACGAGGCCCGACATTGCTTTTGCGTCCATGTGGCTTGGCCAGTACAGCTCGGCACCTCTTCCCT
CTCACTTTCGTCTAGTGAAACATGTCTTGCGCTATTTGGCTGGGACAAGACACCTTGCTTTGACGTTGGGAAC
TGCGTCTAAATCCACACCAGAATCTTTGGGAGGTTTCATGCGTGCTAATGGGTGCACCGATGCAGACTGGGCC
TCTGACGCGAAAGACAGGAAGAGTATTTCGGGATACGCCTTCTTTTATATGGGCTCACTGGTGTCTTGGTCTG
CGGTTAAGCAGAAGTCGATTGCACTATCTTCAACTGAAGCCGAGTATTATGCAATGTCCCATGCGTTCAAGGA
GGCCTTGTGGTTTTGTGTTTTCTTGCGTTCAATGGATTTTCCTCTTCCACGTCCGTTTCCTATACTCTGCGAC
AATCAAGCTACTTGTTCTCTTTCGGAATCCCCTTCAATATCGCGCCGCTCCAAACACATCGACATTCGTCATC
ATTTCCTTCGCGAACACATCAAAGAGGGCACGTTCTCAACTTCTTGGATTCCAACTCATGACATGCCTGCCGA
CATCTTCACAAAATCCCTTTCGCACGTTATTTTCTCTCGCCACCGGGATGTTTTGGGTTTGATGGTGCCTCCT
CAATAAGCTTCTTGATTTCTTCCTTCTGATTTTTGTTTCTTTTGGTGGTTGTTTCAACAGTCTCTCATGGTCA
CTCTAGTTACTTTATTTCTCTTTAATTGCCTTGTTTCTTGCCTGGTTTCTTTCTCCTGATGGGGGTGTGTTGA
ACGGTGTCCCTTGTTGTCAGGATCACTTCACCAGTCACGTGTTATATCGCGTCCCTTATATACCACGCCTCGT
GTTTCGTTTACCTCAGTCGTCTCTTTTCCTTTCCGACGCTGAGGTAGGTTTTCTTGGTTTCCGTGTTCCTACC
ATCCTGTATTCACACATGTCACCTTAGCTCGCTCTTTGGGCGTCTAGGTGTTCTGTTGACAAGAATAACATAT
CTAGCAATATCACTTTTTTTTTCCAGCGTATGCAGGATATAATCCTCGTCGTTGTCTGCTGGTTGTCCGAGCA
AAAAGGAAACCCTGTAACTTGTAAGT
>Agrped1_rnd-1_family-13#TIR/MITE
TACTGTACCATTTCGATCAATTCGGCAGTAACGCGTAACGGCGGCCCAAAATTTGCCGAAATGTAACGNNTGT
AACGCTCAAAATGGCCGGTTGCCGAATTGATTTTAGAAAGAAATTATGTAATAATTCGTTCAAAATATCAAAA
TTTTCTCCTGGACCTCGAATTTGGCTTTTCCTGCGCTTTCTANAGTGAAATAATTACATTTGGNTTACTTTCN
CCTTGTATTTACAGTATTNTATCTGATATTACGCCGAAAATATTCGTATGTAACGGGCAATTCCGGCGGNTGC
CGAANTNAAAATTTCGGCAGGCGTGTAACGCGTAACATGCCCTGCCGAATTGATCGAAATGGTAC
>Agrped1_rnd-1_family-16#TIR/MITE
TAGNGAATGGCAGGGGTTCGGCGACCCGCACGGGTTTGGCCACGGGTAAGCGCGGGTACGGGTAGGGGTCCGG
ATTCCCGGACCCGTGACCTTTCAAAACGAGCCCNAGAACGTCCAAAACGGATGAAAACTGACCGGGTTATGAG
CCAAACAATGTTTGCGCTCTAANTCNGTCAGTTTTCGCCCATTTTGGACGTTTTNGGGCTCGTTTNGTGCGGA
AAACCCGCGGGTTTCGCGGGTAAGGGTCCGTGGGTACGGGTGAGGGTCGGAAAAATTTACCCACGGGTTACCC
GTGCCATTC
>Agrped1_Cluster17#LTR/Gypsy

AGTACTTTACAGTCCCCCACGTTTCCTTGTGGATTCCTTGTTCATTCCCTGATTCCTTGCTCATTCCATGTTG
ATTCCTTATCCATTCCATACTTTTGGGCTGGTTGGATTCCACAAGGAATCCAGGGAATGGAGCAGGAATGGAA
TGGGAATGGAACAGGAATCCACAAGGAATCCACCAGGAATCAAAACTTTGAACTTGTCAAAGATGAGCACAAA
AGAAATGTGCCGAAATGTGCCCTAAGTAATTTTATTTATTGATGCACATAAATTTATTAAATTCATTATTATT
ATATAAGTACATTAATTACTATATACAGGTATACAAGCAATAAAAGTAATAATAAAAAGAAAAACACCTAACA
TGTTGAAGTTCTTTACAGCTTTCCTTAAGCGGACCATGTCTTTGTAAAAGTAATTAAGCACATTAGTAACGTT
TGACTATCTTGTCCGAAGTCAAGCAAGATATTTTTCAATGACTACCGCTCTTTGAGATAGTACTTACTATAAA
AGGCCTCGACATTTAAGAAAGACATCATGACATGGTCGTTATCAGGCTGAATGTGTGCAAAGTTGTATTATGT
AATCTCGCATTGTGCCTACCAGGCCGTAAAACACTCCATAAAGCATTCATAAAGCAAACCAAAAAGACTAGGT
GTGGTGACCAAAGACAAACTTACGTAGAATTAAAGCATACCAAACACATTAAAGACAGTTAGAAAGGCTCATC
TATCGAATGAAAATTACTGCTCCTGGTGATTCTAGTTACCCCCGCTGAGTCCTGACAGTCGACTCTGCCGATG
CATTGTCCAGGCTGTGGTTACTGGAGGCCGCTCCTCCCTGGGTGTTGTCGCTTCCGGTATGGGGCTTGGAACT
TGAGCTCTGAGACGAGCCTGAGGCAGTGGCTGGAACTTAACAGACTTGGAGGAGCTGGGATCATCATCAATTC
ATCGAACATTGGGTCGTGCAAAGCGCTGTTGTACGCATCTTCGTCAAATGCTGATTCTGATTCTTCTTCTTGT
TCTGGATCAGGCTGAGGTGTGCGTTTCTTCGTAGTATAAGGCTTCGCGGCGTTGATGATAGCAGTCCAACATC
AGGAAAAAATCTCATGTGTATGTACTACTGTACTAAATCGCTCAATTCGTCGCAGCCTGTGACCTGTGACTGC
CGCGACGAAATTTTGGTTTCGTCAAAGGCTCATGTGACCTGTGACTGCCGAAAATGTGCACCCAAAATAATAT
TTTTCTGCGTCTATTAGCCAGATATAAGCAAATATTCATGTTGTACTACGCATATGCTTGCCAATTTACACTG
AATAAACAAAAAAAAAGCAGAGAATTGATTGAAAATCCGAACATCCGGATTTTTTTGAGTTGTGACCGTCTG
ATGGTTTCATCAACTGAAGCTGTGACCCTGTGACCACGACCATATTTCGTCTCGTCAGAGGGGCCTGTGACCT
GTGACCGACGAATTGACCGATTTAGTACAGTACTCAGTAGTTCCAATATGTGACAAAATGGGCTCCTCATGAC
TGTGAACAGTGACAACGAGATGGTTTTGGCACAGAGAGAAAGAGCAATCAAAAGACGGGGTAAAATACTCTTA
AGATTGACTGACTTTCTAGCTACACAGAAGGGTCCTTTATATACTGTAGAACTACGTCGTCAGGTGATTAATT
AAGGGGAAAGTGAAACTAAAGGAATGTGGGGATATGTCAAGGTCTATAGTTAGGGCATATCTAGTACGAGATA
AAGAAAGATAAGATGGGGTAGATAAGATAATAGCATGGGTATAGACGAGATAAGTATGGGTTCGTTACACCCG
CCCTCCAGGCTTTCGCCAGAAAGTCTTCTGATTAATTAGTAGATTCGGTGTCGTTCATGGGAAGGTCCCCGAA
GTCAAGGGGTTCTTCTTCTCCATCGGGTTGAGTGGTAGTTTGGGTGGTGGTAGTAAAGCCAAACAATTGATGG
AGGTCGGCATCGGTGGTAGTGACTCCATTAGGACCAACGACGGGATTGTGAGTGTCAGAAGAGTGAGAAGAGC
CGTCGGCGGATGGAGTGAATGCAATACTGATGGCTGCAAGGGACATAGCGTGGTTGGAGGGTCCAGTGAAGGA
AGTGCTCGAAGATCTGGCGTGAGTTTGATTACGATGGTGGTGTGAGTGGGTGTGGTGGTTTTGAGCGAAGGAT
ACAGTTTCCTGGTTGAAAGCCTGACGCATTTTGCGAGCGGCAACCTCCTCCTTGACGTGTTCCTTCTGGCGTT
TGATCTGTTTGATCCGTGCGGCGTTTTCGATGAGAATATCGTTGACGGCAGCCATGCTGTCTGGATCACCGGT
GATACCATGTTGAGGTGGTGGTGCGAGACCAGTCTGTTCAAAGAGTCCAGGAATTGGAATTTCTGGCACGTGT
TTCCGGCGGCATTACGGCCAGAATAACGCGTCACAGGTAGTCACCACATCCATTTGCAGTGGTTGGGCAGCGA
GCCAGAGATTCCTCACAATCTTTGACCTCATGATTGCTGTTGAGCTTCTCGAAGGCCTCAATAGCCATCCCAT
ACTACGTTTTCTTACTCTCCACAGTGCTGTCACTCCTTCCCACATTCTGAGCCACCTCAAACGCAGCATTTGC
GGGGCATACTTCAACCGCAACCTATCCTTGAAAGCAGCCCATCCCACCTACCTTAGTTCTTGCCTGAGCACAT
CCAGAACTTTGTTCGCGAATCTGTAGACCTACCTGTCTTTGCTGAACTTTGAAGCCAGTCTCAGGTCAACTCA
ATGCCAGTGTACTCGAGAAAAGACTGTGAACGACGAGGGCTAACGACTGCAGATGAGCAACGAGCAATGCGAC
TATCTCTCAAACGATCATAGGTTTCTATGGAGTCGGCTAATGCTATAGAGTAGACCACTATGAGCGCAAGGTA
TGTCGCAGGTCTCAGACTCTCAGTATCCCTATTCATGGACTCTTAGCTTGAAGTTGTTATCAGGGTATTTTCC
GGAGCTTTCATCATTGCGTACACAGGAATCCAGCTCACCAATTCGGTGGTACCTATATCAAAGTCAAACGCGG
CACCCTGTACAAGAAAGTCATTACCGAACTCGAAGATGAGGACTGGCGATACATCACCAAGAAAGTCATGCGC
ACCCGTGCCAATAAGATTTCTCTTCCCCAGAGGCCATGGATAAGGAGAGTATTGATGACGGGGCACTTGAGGA
GGGCTGGTTGACGACTGATAGATAACCTTGTCTGCAGTATCTTGGGTGTACAGTACTTGTGTGTAAATGCAGA
ATTTCATTTTTACATCCAAGTTCCTTATTCTGTGTGCAAGTGCGGACACCAAATAGGTCTTCCAGTGACTACT
GACCACTTTCTATCCACTACAGTTGTCTTGAGCTGTTACATTCAGTACAACTTTCTACAACTTATCCTTACTT
GAATGGCTTGGTAAAGCCATCATCAACACTGTGCTTCTTCCCTTTTTAAGGCATGCGGGCGGCGGAATGTGTC
CGTGCAAGTCCTTGTACTAACAGAGTATGTTCCATACTGATTTAGTACATGTAATGGAAGGATATGATACAGA
GGAATAAGTAATATAGAGGTGCATTGCGAGTTGCACTGTGTACTCAATGTGTACTGATTTAAGTGTGGCGGAA
TTTACCGCCGGAATCACGCCGGAATTTGCACCGGAAATTCCAATTCCCAGATGCACTCAGTCTGTTGTGGAGT
GATTTGCCAGAGCTGCTGGGACATCGGTCGGCCTTCAGTAACAAGGTGCTGTCCCTCAGGGTGGATATAGGCG
AATCGAGCACTGTTTCCGGTCACATGGGCATTGTAGGCGCGTGCGATGAAGTTGTACCCGTATGGCCAATAGA
GAGAATTGGGATCGGGAGGCGGATTCGACCGCAGGAGAGCGTCCATCGAGATGCAGAGCGAAATTTCTGCTGC
AGAGAAGGTATAGGCGAGTGGTACTTCAGCTTTGGTGTTCGTTGTGTCGGTAATAAGAAATAGAGTTGGTGAG
GTTCAACGAACAGCGGTGTTATTGACGGTCGTGGGTGCATTAGATGCTGGAGTGTTTGTCGAGTGATTTTGAT
GATTGCGGTTGCGTCCACGAGCCATGGCCCATGGTATAATTGAGAGGGTGATGATTTGACTGAGAGAGGAGCT
GTTGGGGATTTTGGTGATGAGTGCTAGTTGCGAGGTGTTGGGTTTTATAGATTGTGGGTGACGGCAGACTGTT

GAAACACAAAGCCCCAACGAAAATTTGAGGATCGTTAAAGGGTGGCTTACCTGATCCTTTCTTCAACTGCGAG
ATGTTGTCCGCTCCTTGCAGTTCAAGATATGCCCCTAAGGCTTGTAAACCTTGTATTTGGGAGTACGGCAGCC
AGGTGACATCGCTTGACTTCCACTTCACCTCAAAAATTGCATCTTCTGCTTTTCCATAATGCGAAAGAATTGG
CGCGTTTGGTAGATCTGTCCGTCTGAAGGTGATAGGCACTCGACATTTTGAGTTTGGTTCCTATTAATGAGTG
AAGGTGCTGCCAAAAGGTACTGGTGAATAGCACATCCCTATCACTTATAATATTCTTCGGAATACCATGGTGT
TTATACACCTCTTCATACATAAGCTCTGCAAGCTGCTTAGCTTTGTAGTTAATTCTACTGGGTATCAAGTGAA
CCATACCAGTAAGTAAACAAATTACGACGGTAATAGAGTCGAATGTACCATTTCTATTTTTGGATTCCGGAAG
CGGACCGACGAAATCAATCCCGATCGATTCCCAAGGTTCTCTGGGAATAGATAATGGGTTTAGTAACCCATAT
GGCTTCTGATTGCTAGGCTTACTTCGCCTACATGTCATGCACGTTTCGCAGAACACTTTCGTGTCATTCACCA
TGTCTTTCCACCAGACATGGTCCTTGAGGTAGTCAATAGTTTTATTTGCTCCCAAGTGGGCTAAAAGCGAATG
CGCTTCAGAGATGACCACTTCCTGTATGCTTCTCCCATTGATAATACCTTTAGGTATGCATAGCAGTTTCTTA
TTCTTCTCGGTAAGGTAGACTAAACCTTTATCCACCGTGAAGTTACGGAATTCCTGAGGTTTTACCAGGATTG
AGCTAAAAAAATGTATCCTCGCTATATCTAGCTCGGATTTCCTTAACTAAATCAATGCTGTCGGAAGTGCTCGA
CATGAATTGAACTAGGTCGACATCACGAGGTACCTCACTAAAGTCCGACGGAGGTATTTCCGTATTCGGTTCA
GGGGAAACCCGTTTAGGTGATATGCGAATTGTTAGCCACGGCGCTAATCCGGGTTCAGCAACTGATGAGTTTC
CCAATTTAGTATGTTGAGTTGATGCATCTTGTGCACCCTCGCCCTCCGTTCGTTGTCTAGGGCCTTTGAGAAC
GAACTTCCCATGTACGCGGGCAGCAAATTCTTTGGACGTCTCAGGGCGTCCGGTTTCCGCACCCTCTACAGGT
TTCCACCCACGTTGAGGCCGTACAGCAGCTCAAGCTTCCAATCCTGCTAAAATAGGCAAAGGTTCAATGCCTA
CAGCAGCGTCATCATCCACGACATCAAAATAAGTGTACTCGCTGCGAGCGCGGACTGTTTATGGCGCATCGTT
GGAATAAATACGTGACAGGGCATCCGCCACAACGTTTTCACTGCCAGGAACATATACAACTTAATCAAAGCTC
GATATTTTCTCAATCCAGTGCGCTTGACGGCTGGATAAGTTTTTCTGATTTAAAAGGTGGGTTAACCCCTTGT
GATCGGTTACCCAATTGAAATGAACTCCTTGGAGGAGGTCTTTGTGGCACAGCATAGTTTCAATGCCCGCCAG
CATCTCGATCTCGTGAACAGGATAGTTCTGTTGTGCCGAGTTAAGTTTAGCCAAGTAGAAGGCAGCGATCTTA
GCCGTCTTCCAATCAGGACCTTGACTAACCAGTCCTGAGATTCCAGTCGCACACCCATCGGTAACCATCCAAA
TAGGATGCGCGTCATCTTTATAGCTCAATGGAGACCTATGGTGGTCTCGAGCAGATTGCACAAGAGACTTAAC
ATCTTCAAAAGCATGTTGTTCTGTGTAAGTCCATCTAAATGGTACTGTATCGCCGGTTAAAGCGGTCAGAATG
CCCATAGAAATCCGCACGCCAGGCACATCATCTGCCAAATACCCTACGAAGCCGAGAAACCCTCGAAGTAAGT
CACAATTAGTCGGAGTTTTCCAATTAGTTACTGAATCAACTTTCGCAGGATCCATTCTAATTCCGTCGTTATC
AATAATGCGACCGAGCAACTTCATTTCTGGTGCAATAAAATGCAATTTATCCTTGCTCAGGTATAGTTTCTCA
CGCTTTAATATGTCCAAAACGGTTTTAACATGCATTATATGGTCCTCCAAAGTATTTGAGTAGATAATAATGT
CATCTAAGTAGATGTCCATGTAAATTCCAATATATGCTGAAAACAGGTAATTCATTAGAGCCTGATATGTTGC
CGGGGCATTAACATCCCCGATTTGAATAACAAGACTCACCATGTTTCCATCCGGCGTGGTTACAGTGGTGCGT
TCAACGTGTTCTGGGACTATCCTGATTTGTTCATATGCGCTTTTGAGGTCTAGAGAGGTTCGGAATCGATGCT
GACACGTCCTCCACAGCATGCCATCCATATCCGGCAGCGGCGAGGTTAACTTGTGCGTATTCTTGTTGAGTTC
TCGCAGGTCTACAACTGTCCAGAGTTCGGCAGGCCTACCATCCTTCTTTGGCTTCGGGATGAGCAGCATGGGA
ACTGTATTGCCGGCAGAAGTGATTTTCCATCGGCCAGACTTGAGGTATGCGTCTCGCTTCTCGGCCCATTGCT
TTCTGAACGCCTCCGGGCATCTGGAAGGTCTCCACAGATAAGTCTTGTTCACATCAATGAGTGGTATGGTATG
GTTAATAGCACGAAAAGGAGGGAGCTCTGTTTTGCTCACATCCTTGCAGAGAGGTTCGGCATATCGTCTCAAA
GCTTCCCGAGCAGCAACAACCTCAGAAGTATTCCAAGCCTCGAGGAAAGCTTGTGATAGCACCTTAGACTCGG
TGCCCGACTTGATAGCGAGTGCAATATCACTGCCAATAATAACTCGTGCGGGATTTAGCCCGATACACACCTG
ATGTTGGTACATCCAAGGTGTACCTAAAATAACATCATAGTTATTTAGGTTTATCACATCGAAGGTGCGCTCT
TCGTCTATGTTCTGATACTGAAACCTCGACCTGATTTGTGCATTGATTTGTGACCGAGAACCTTGCGCCGCGA
GTTGTAATGAGATTGGCGCAAGCAGTTCTTCTCGCTTGAGCTTCAGCTGATCAACAAGAGTCGAAGAAACAAA
ATCCCCGAGAGATCCTGGATCTATCAGCGCACGAACAGGCTGGCCGTTCATTAGCACCTTTATTACTACGGGC
TTTGGTAAGATTCTCGCTTTATCTTTCACGGATGCGACATTACGCTGAACGGCCGGGTACTTGCGTCTGTCGA
CCTGTACTCCGTTTAATTCAACCGTCCCTTCGTAGGTATCTGGCTGATTAAGTCTCCAGTCCAAAATATATTG
GGTAGGATCGGGTACATCATCCGCAGGCTCTGTAGACACGGTAGACGATGGTGAGAAGCCTCTTATGCTGGTC
AGAGTAGTCATAACTAATCTCCTTTCATACCACTCAGCTAAATCGAATGAGGGATCGTCGAATAGCTCAATAG
GAGCGTAGGATACAAGATCTTTAAATTTATCTTGAATAATCATATGCTTTGGGTCATTGGGTGGGTATGTGAC
CACAAAGCGTTCAGAATCTCGTTCTGGATCGCTATATTCATCCAGGTACGGAGTGCTGAACTCCAGAATGAAT
TCGGCGTGTTCTTCTCGGGCAGGTCCTGCCAATGTATTGAATAATTCTTGTCCCCTTAGGTAGGTGACGAGAT
GGTCCTTATGCCACTCAATTCCGTCTTTAGAGAGACATACTTGCGCATACCAACACGCGATCGAAAACAGCTC
GTGTTTCATGTAGGAGGAATGCAAGGTAAGATCATATCCTTGAAGATAATCAAAGATCTGATAAAATCCTCCA
TCTATTCGTTGCACGGCAAATCTACCCATGTACTGTTGAGTAACATCCCCTGGATAAGGTTGACAGTTGTGCA
GAACGAGCTCAAGGCGGTCTGCAAGCGGATCACCCATCAGTCTCGACGGTGAGCTGATCCAGATATTGGGATG
TGACCCTTGTGCATTATCCTGAGGTGCAGCCTCTTCTTTTTCTGATTCTTCCAGGCTGTGCTCTGATGAACTA
TCAGAGATACTCTGTAGATCTGGTATCTTGTCTTCATTATCGGTTTCCGGAATAGTTTCTAGACCCGGTAGGT
CATCTTCTGAATCACTTGCGATCGTGTTTTCATTCTCAGAATCGTACATGAGATCGAGTTCAGGAGAAGGACT

CTGTTTCTCTTCTTCCTCAGGCACATGGTAAAGCGAGAACGTGCTCTCCCACAACCTCTGATGGATATAATCA
GTACTTTACAGTCCCCCACGTTTCCTTGTGGATTCCTTGTTCATTCCCTGATTCCTTGCTCATTCCATGTTCA
TTCCTTATCCATTCCATACTTTTGGGCTGGTTGGATTCCACAAGGAATCCAGGGAATGGAGCAGCAATGGAAT
GGGAATGGAACAGGAATCCACAAGGAATCCACCAGGAATCAAAACTTTGAACTTGTCAAAGATGAGCACAAAA
GAAATGTGCCGAAATGTGCCCTAAGTAATTTTATTTATTGATGCACATAAATTTATTAAATTCATTATTATTA
TATAAGTACATTAATTACTATATACAGGTATACAAGCAATAAAAGTAATAATAAAAAGAAAAACACTTAGT
>Agrped1_rnd-1_family-23#TIR/MuDR
TCTTGCCTTTTCATGGGATAGCAGGAAGCTTGCGTCGNACTCTGGCTCGACCATCGTAGTCTGGGACTCGTCT
TCCGGCGAAGTGCTTTCTGGTCCATTTGAAACAGAAGAGTCGGGGNNCTNCGGCTCTCTCGCATTTACACCCG
ATGACTCCAANATCCTGTTAGTTTCTGGCATTGGAATGGTGCACGTTTTGGATGCCCACACTGGATCTGATCT
AGCTGGACCGTTCGAAGTAACCAACGAGGAAGTAGGAACATTGGNNTCAACTGCNATTTCNCCCGATACAAGC
ATGGTTGCCGTCGGTTACGNGGAGGGANCGATATATATATGGGACGTTCACGCGGGGACTACAATTCGTGGCC
CATTCGTTGGCCATAGCGACTGGGTATCTGCTCTCGCATTCTCGCANGATGGCAAGAAACTCGTTTCGGGATC
ACGCGACGCAACCATTCGAATTTGGGACGCTGANACTGGCAATGTCGTGGCTGGACCATTTGAGGGGCACACT
NCGACCATCTCTTCACTGGCNCTNTCGCCCGATGGAACCAAAATCGTATCAGGNTCGNATGACCGGAGTGTGC
GCCTCTGGAATGTATTTACCAATGAAAGCGACATTGCTCCGCCATGCCAAAGTGTCACCGGCTNCGTCTATAC
AGTTGCATGGTCTCCGGACGGTACAAGNATCGCCTCAGGGCAANATGATGGTACCGTAGCTGTGTGGAGTGCA
CAGAACGGCGAAGCAGTGATAGAGCCTTTCCGCCACTCGTCGGNCATCTCGTCAATTGCNTTTTCACCCGACG
GCTCCAGAATAAGTGCAGGTACTGGGGATGGGACTGTTGTTTCATGGGATTCGAGGACTGGGGAAATGGTTGG
AGAGCCNTTCCAGGGTCATTCCGACGNTGTCAACTCGGTTGCGTACTCGCCTGATGGATCCACGATAGCGTCC
GGGTCGGGNGACCGCACCATTATTATCTGGGACTGCTCAACAGGCAAGATGATTGGAAAGCCGCTCCGTCACG
ACGATCGGGTCAATTCAGTGGCTTTCNCTCCTGACGGTAACAGACTCGTGTCGGGTGTGCGAGACGGATCGAT
ATATGTATGGGATGTTCATACTGGTCAAATCATTTTAGGCCCCATCAAAGGACACAAGNAGGCAGTTCGCTCG
GTAGCTTGCTCACCGGACGGGACCCAGTTTGTTTCTGGATCNNNNGACGGAACCCTTCGCGTGTGGAGTCTGA
CCACTGGAGAGATGACTGCAGGGCCATTTGATGCCCACTGCGGTGCTGTTNATTCGGTCGCCTACTCACGAGA
TGGTACAAAGATAATTTCTGGATTCCGAAGCGGAACTATCTGTGTTTTGGACGCAGAAACGGGCGACATCCTC
GCTAGTATTGCTGGACACACCNGCNNTGTCGCATCTGTGGCGGTATCACCAGATGGTAGCAAGTTCGTTTCAG
GATCCGGGGACGGATCTGTTCGTGTATGGCAGTTGGATATCTTAACATCGCCGGAATCGCTTCGGAACGCCCC
AACCATCTGGATGCCAGAAGACGTCATTATTCCGACTATTTCAAACATTGCCAAATACCACGACGATGGATGG
GTGGNACTGGACGTAGGACAACACGCATTTGTTCTGGACNCCGCCGGAATTCCGCGAAGACCTCTGTTACCCN
TATAACCCTATCGCCATCGGCCCGCATGGAACGACACGCATCGATTATTCCNCCTCCAAGCTGTTTATTGGGC
GGANGTGGTCCAANTGTTGGAAATATGGCCGTCAAGACTCGG
>Agrped1_rnd-1_family-97#TIR/MITE
CACCGCTGGCGCGGACACCTTTTTACGTCGTAAAAAGTGCTTAATAAATGTTTTCTATTTGGGTTACCTTTTT
ACGACCTCAAAAGGTGCCCAAAAAGATATCGGGCATACCATATATGGGAAGCCCGATATCTTTCTGGGCACCT
TTTAGGTCGTAAAACGCAAATAGAAAACATTTATCAAAGCGCCTTTTTACGACCGAAANAGGNATTCGCGCCA
GCGGTG
>Agrped1_Cluster18#LTR/Gypsy
GTAATCATGTCACATGATTACTTACTATTTATAGGCTATATCAGGGGTTTTCCCCGCATCGCTTCTCTTCCTT
TTCTACCTACCTTCTCTCTGTATTCTCATACCTCGCTCATATTATCATACCCCGTGCGTGTTCTCTCGGTGCT
CCCCTGAGTTCTCTTTCCCTTTTCCTTCATCTCTCGAGTGTTTCCTCTCGTATGACGAGAGGATTCCACTTTT
TTTTGAACACTCAATTGGAATTGATGATTGAGTGCTATACCGAACTGAAGTAACCTTCAGAAAAACACTGAAC
ACTGCTCAATTAATACCGTTTTCAAATCGTCTCAAAAACCGCTCCCAAAATTTCGTCACCGTACGAAATTTCA
CCGCTCTCTGCTCACTGTCATACTGTTCTGAAAAACTCTCCTCCAGAGTACTACCGTCACTCCCATCGTCACC
GCTCACTTCGCTCTCAAGCCTTTCGTCGAAAGGAAATTCTCCACACCGACAACCCGCGAATACTGCAAACGGG
CAAGAAACTCTCAACTTGCCTCCTCTACCTTCGTCTCCGCTCTCTTCCCGCTCAGAAGATACCTTGTCAAGCG
ACGATTCCTTCTACTGTCTACCCAGTCAACGTACCGGACACACTCAGTAAGCGCATATATGCGCCTTATTTGT
GACTTTAGTACGCATATATTCACTGTTTACTATCAGTTTACTGTCCGTCATGTCGTCTTCCATCCCTGTGGTC
CCCAACGCCACCGTCGAACATCACTCTGTCAAGCACTGCCCCTACCTCATGCCGGGGAAACTCACTCCATCGG
TTCTTCACGACCTGAAGGACGCCCACGCGGAATTCTTCTACAACAAGAATATCAAGGACGAGGAGAAAGTCCT
TCGCGTTCTTTGTGGGTTTAAAGATGTACACATCCGAGAGTGGGTGGAGGGTGACCGCGACAGGCTGGTAGCC
TTGAAATACGATGAGTTCATGAAAGAATTTCAAGATACCTTCTTACCTGACGATTGGGAAAAGACCACGAAGA
CAGAACTGCTAAAGTTGAAGATGGGCAAAACCGACAAGTTCGACGACTACACCCAGCGCGCAAGAGCGCTAAA
CGCTGTTCTGAAAGGAACTCCGTCTCATCTTAGTGCCTCTCAGCTACGGACCCAGCTCGAAGTCGGTCTCGAT
GACGAGTTGAACGACTTCTGTTTTGAGAACGACATCCACCTCGAGACGGACATCAAGAAGTGGGAGCTCGCTG
TCAGGAAAGCCGATTCCAAGCTTCAAGGCGAAGGAAGGCGTCACAAGCGAAGGAATGAAGACGGGGCCTCAAA
GAACAACAAGCGCGCTGCCTATGGTTCTTCAAAACCCTCTTCTTCCACCGCCAACGCGTCCTCTACTTCTAAC
GCTTCATCCGACCGCTGCCCCCAGCTCACCCCTGCCGAAAAATCACTCCTACGGGACAACAACGGTTGTTTCA

AGTGCCGTTGGTTCAACCAATCCCACGCTTCCCGCGACTGCACCAATGGCTTCCCTAAAGGCTCTGGCTACCG
TGAAATCACGATTACACGGGACACTTGTGGCAAGCCCACTCGTGAACGCTCCACGACGTCTTCGTCGAATACC
AAGTCGAAGCCCGTCGCTGCTGTTAGCGAATCCAAGAACGAGAGTCCAGACACTACAGGGGACTCGGATTCTT
TTGTTGCTAGTGTGATGCCATCCGCAGTTTGTGGCGATGGTTCCTCTTCAGAGGGTGATGATGTATGTCCCTT
GGTCTCCAAGCATTTAACTTGCGAGTTTTTCGTTGACGGGCCGCTGCATGAATTCCCAGTAAAGACAAAGGCC
TTGATAGACAACGGCGCACACATGGTTCTCATCCGCCCCGATGTCGTCAAGAACCTGGGTCTCCAACGGTCCC
CTTTGCAGACTCCGATGTCCGTCACTGTCGCTATGTCAAAGAAGAAGAAACGTTACACGCTGTCCCATTTCGT
CAAACTCCGCGTTACCTCTACCGACGGCCTTTTTATTTCGCGCTATGTACATGCTATCATCGCCCCCTATCTG
TGCTCCCTCATTGTACTCGGGCTACCCTTTCTAGAACGCAATAAAATTGTTATTGACCACGAATTGCGGACTG
CTGTTGTTAAGACATCTGGTTACGATATCATGAATCCGCCGAAACCTAAGCTGCCAATACCTGAGTTCTGTAC
TCTATCAAGAAAAGATCAGTTCGCTACTGTCAAGAAATCAAAAAAAGCTGTTCTCGAACAGCTTAAGTTGGCT
ATATCGGACAGAGTACAGGCTAACAAGTGCATTTCTGATCCTGTAAAAGATGTGGACGTCGTTGCTGCTATTT
CTGACCACATTGCGCTCTTGTCTTTCAAACAACGATGCGAGTCAGAGGCAGAACAAGTTACGACATCTTACAC
CGATGTTTTTGACAACTTGCCACACACGAGTGAATTGCCGACAGATTATTATGCTAGAATTAGGCTGAAAAAT
GTTGAAAAGAAAATTGCAAATAGATCTTATCCTTGTCCTAGGAAGTACCGGGAAGCTTTCCAGACATTGATCC
AACAACATCTTGACGCCGGTTGCATCCGTCCTTCTTCGTCTGCTTACGCATCTCCGTGTTTCATTATCCCAAA
GGCAGATAAATCTGTGTTACCCAGATGGGTTAACGACTACAGAATGCTAAACACAAACACTGTAACCGATGCT
CATCCATTGCCCCGAATTGAAGAGATACTGAAAGATTGCGCGAAAGGGAAAGTGTGGTCAAAAATGGATATGA
CAAACTCCTTTTTCCTTACCCCTATGCATCCCGATGACATTCATCTGACCGCGGTAAATACACCTTTTGGGCT
TTATGAATGGACTGTCATGCCAATGGGCCTGAAAAATGCACCTGCTATTCATCAACGGAGGGTAACATCCGCC
TTACGATCCCTGATTGGAAAGATTTGTCACATTTACCTTGATGATATTATAATCTGGTCGAACTCCATTGAGG
AACATAGGAGACACCTCCATCTCATCTTCGAGGCTTTGCGCAATGCAAAACTGTTCTTGAATAAGAAAAAATG
CGAATTCTTCAGAACAGAAATCGATTTTTTAGGGCATCACATCTCCGCAAGAGGTCTTGAAGCCGACACAAAG
AAAGTAGACGCTATACTGAACTGGCCCAAACCAAAAACCTCCACGCAAGCGAGATCGTTCTTGGGGCTAACTC
GTTACATCTCTGCCTTCTTGCCGGACCTGGCAAAACACACAGGTGTTTTAAATGAACTTACCCTTAAATCAGC
AGACAAGCAATTTCCGTCTTGGAATGACCGGTTTGATATTGCCTTTGAGGGGGTAAAATCTTTGGTTCTGAGC
AGAGAATGCTTGACAACGATTGACCCTGAAAAGATGCCCGAGAATAAAATCTTTGTCACAACGGATGCGAGCG
ATTTCAGGACAGGCGCTGTTTTGGCTTTTGGCCCAACATGGGAAACTGCACGGCCTGTTGCCTTTGAGTCATG
CACTTTGAAAGGTGCTCAACTGAACTATCCCGTACATGAAAAAGAGTTACTCGCAATTATCCGCGCGCTAACT
AAATGGCGAGCAGACCTCCTCGGGGTGGAATTTGAGGTGTACACAGATCATAAGACCCTCGAAAACTTCAACA
CCCAGAAGGATTTATCACGCCGTCAAGCTCGCTGGATGGAATTCATGGCCCAGTTTGACGCAAAGATCATCTA
TGTAAAGGGCTCTGATAATGATGTCACTGATGCTCTTTCTAGATTACCAATGGTCGCAGGAGAAACTATGGAC
GATGATGAAATGATTGACAGTATTTGCTTCTCCAAGGGACAAGCCGTGTCACCCTCTTGGATGGTTGCGGAAG
GACTGACTACGTGTTTGCAAAAAGATGATTTTTCCTCTGCGCCTGTGTCCGCAGTACTGCAAGTCACAACGGA
TCAAGCCTTCATTGACGACATTAAGGCGGCATATGCTTCTGACAAATGGGTTGTGGGTCTCAAGGATGTTGTT
CCTAGCATGCCGGAAGTTAGGCTGCATGACGGTTTATGGTACATCGGTCAAAGACTTGTCATTCCCCGTGCCA
ACAATATTAGGGAAACCATTTTCCGTCTTGCTCATGACGCTCTCGGGCATTTTGGATTCGACAAGTCATATGC
AGCGCTGAGGGAATCTTTCTACTGGCCCAATATGCGTAGGGATTTAGAATAAGCTTATGTTCCCTCATGCCCT
GACTGTCAACGGAACAAGTCATCTACGGTAAAGCCAATTGGGCCCTTACATCCTCTGCCCGTACCTGATGGTA
GGGGTAAATCTGTGGCTTTAGATTTCATCGGGCCTTTGCCGAAAGATGGGGGTTTTGATCAGATCCTGACAAT
GACATGTCGTTTAGGCGATTCTGACATCCGCATCATTCCTTGCAACAAAAATGATTCGGCCGAGCGTATTGCT
TCCCTCGTCTTCGAAAACTGGTATTGTGAGAACGGGCTCCCGGACGAGTTCATCTCTGACAGGGATAAATTAT
TCACTTCCGCCTTTTGGAAGGCTCTCCACAAGCTAACGGAGGTGAAACTGAAAATGTCCACAGCGTTCCATCC
GGAATCAGATGGTGCGAGCGAAAGAACTAACAAAACGGTTAATCAAATGATTAGGTTTTACGTTGACAGGAAT
CAGAAGGGCTGGGTGAAAGCCTTGCCAATCATTCGCTTCAATATTATGAACTCCGTCAGTAAATCTACCGGTT
TTTCCCCCTTCCAAATTCGTTTGGGCAGATCTCCCCGCATCATTCCACCGCTCGTACAATCTCCTGTCGAAAG
CTCTACCACTGAATCGCCACACGATTTTCTATACCGCATGGAAGAAATCTCGTGCGAGGCGCAGGACAACTGC
TACAAGCAAAGGTTGCTCAATCCTTGCAAGCCAACAAATCATGTTCGGTGGATTTTCCTTTTGTTGTCGGCGA
CCGTGTTCGACTTTCAACGAAGAACAGGCGTCGAGAATATATGGCTACCAAGTCGGGCAGGGTTGCCAAGTTC
ATGCCTCGTTTTGATGGACCTTACCCTATCACTGCGGTGGATCAACAACACTCGACGGTGACCTTGGATATGC
CAAATAATAACAAAAATAAGGTGAACGTATTTCATACATCTCAAGTATTGCCTTTTAAGGAGAACGATGATGA
TTTGTTTCCTTCGAGGAAGCTAGAAAAGCCTCCCCCAATGGTCATCGACGGGGATGACGAGTACTTCATCAAG
GATATTATCGATGAGCGCCGAAGAGGTCGTGGAGTTCAATATCTCGTCCGTTGGGAAGGATACGGCCCTGAAG
AAAATAGCTGGATTGCCGGTTCCGAACTTAACAACGCCAAAGCCTTGGACGACTGGTTGGCGACGAAGGATTC
AAGCTGAGGTCTTTTTTTTAAGTACCTTTCTTCTTTCAATCTCGTTTGTCGCCGGCTGGTAGCTTTTCCCACT
GGGTTTTTGATGCACCCGCAACTGTGCCCTTTGTTTTGTTTTTCTAGTTTCGTTCACCTCCCTCTTTTCTTTT
TATTGCTTCATATTTTTTGGATTATTTCTTTTTCCAGAGTGGGGGAGGGTGTAATCATGTCACATGATTACTT
ACTATTTATAGGCTATATCAGGGGTTTTCCCCGCATCGCTTCTCTTCCTTTTCTACCTACCTTCTCTCTGTAT

TCTCATACCTCGCTCATATTATCATACCCCGTGCATGTTCTCTCGGTGCTCCCCTGAGTTCTCTTTCCCTTTT
CCTTCATCTCTCGAGTGTTTCCTCTCGTACGACGAGAGGATTCCA
>Agrped1_rnd-1_family-441#SINE/Unknown
TTTTTTTTTTTTTTTTTTTTTTTTGTGGAGCTTTACGAANNTGCGACAGTCGACATAGTCGTCCTCGCATTCNA
GCTNTTCGTGCGGGGGTCTCCCCCTGCTTTGTTACGGTAGATCTCCACCCGGGGAAATGCCATTTACTGGCGG
CACACCCTCTCGGGGTAGTGTGGGGCTCGAGGAGAACCGATAGCTATGTCCTCAGCGGTTTCGNCCNGGCGGN
CGCTCCTCCGTACCCACTAAAACCCCGGTTGTTGGATTCGTNGCCCAGTCGTCCGGGAGGTCCCTTTGGTTTC
TCAGNACTNCCCACCATGT
>Agrped1_Cluster16#LTR/Gypsy
AAATGGGCAGTTGCCCAACATACCTGTAGCATGCTGGGGGGCAGTTGCCTACCTTCCTAAACGGGTACCATGG
CCAACAAGGCAGCTGGGTGTGCTCGTGATGGCCGAGAGGCCTTGAAGCGGACTCAAGGAAAGTCGAAACTATT
CTGAATTGGCCACGGCCAAAGAACGCGACTGAGGCTCGCTCTTTTCTCGGCTTGACGCGGTACATCTCCGCTT
TTCTACCAAATCTCGCCGAATTTACGGGTGTTCTTACAGAGCTTACATTGAAGAGCGCGGAAAAAGATTTTCT
GACCTGGAATGACTCCTTCGAGCGCGCCTTTCAGGGCATTAAAGACTTAGTGGTTGGCCGTGACTGTCTGACA
ACAATTGAGTTAGAAAAGATGCCTGCGTATAAAATATTTGTAACAACAGATGCCAGTGATCTAAGAACGGGCG
CGGTACTGTCTTTTGGTGAATCTTGGGATACCGCTAGACCCGTCGCATTCGATTCATGTACACTGAAGAAAGC
TCAGCTCAATTATCCTGTCCACGAGAAAGAATTGCTTGCTGTCATTCGCGCACTTACAAAGTGGAGGTCTGAT
TTATTGGGTATTCCGTTCGAGGTCTACACCGACCACAAAACGTTGGAAAACTTTGATAAGCAAAAGGACCTTT
CAAGGCGTCAAGCCAGGTGGATGGAATTCTTGTCTCAATTCGACGTTAAATTCGTTTATGTTAAAGGAGAGGA
TAATTCTGTTGCTGACGCGCTGTCGCGGATGCCATCTGACTCCAATGTTTCTTCTGCCTACCATGTGTATGCG
CATGATGATGATGACTTGGACACTATTGCTTCGGTCCATTGTTTAAATACTCCCTGGGCGGGCGTCGCAACGG
CTCTGGCTGAGTTGACCAGTGAATATGATGACAACTTGGCGCCTGTGGCCGCTACCCTGACTGTCGAAAGTGA
CCCCACCTTTGTGAAAATGATAACTGAGGCTTATAAGGACGATAAATGGGTCTGCGACCTCAGATCTCTTACA
AAGAGTATGCCGGATATTAAACAAACTGACGGACTATGGTATATCGGTAACAGACTTGTGATCCCTAGGCAAG
GCCATGTGCGAGAGTCCCTGTTCCGTTTGGCCCACGACACTTTGGGGCATTTTGGTTTTGACAAATCATATGG
CGTACTACGAGAGTCCTTTTATTGGCCAAACATGCGCCGCGATTTAGAAAGTGCTTACGTGCCTTCCTGTGTC
GAGTGCCAACGTAACAAGTCCAGTACTACGAAACCGATGGGACCACTGCACCCACCTGTAGCAGCTACCCTCA
TGTATCATACATGGGCAACTGCCCAACATACCTGTAGCATGCTGGGGGGCAGTTGCCTACCTTCCTAAACGGG
TACCATGGCCAACAAGGCAGCTGGGTGTGCTCGTGATGGCC
>Agrped1_Cluster9#LTR/Gypsy
CTGGCCATCCTTTTCAGCTTACCGTATGCTCTACTTATGTGGGGGTAGGCCTTTTCCATCCACCCGCTCCTAT
GGATGATTTGTCTGATGCCGTTAAACATTCAGAATGCTCGCCTTCCTTGCTGCATTTGCGTCACTTTGTTTCA
AGACCTCCGGTCTGTCGACCCGCATTATAGTGGGATCCTCCTTCCTTATGGTCGCTTGCCTGATTGCTTTGTG
CATCCGCACGAACTGGGATAAGCGTGTTCCTCCCCAACCTCATCCGCTACGACCAGAGTATGCATCTGACTCT
CAAAGCACACTACAATCCTTTAAATCGGATGCATTCCCGCTATCAATCTTGCCGTTTCGTCGATCACGATCTT
CGCAGAATACGGCTGTGAACGTGCAATCGAATGCTTAAGGTGGACTTCTGAAAATGTGAGTGCCCTGTAAAAC
ACTTTTCAACTTGAAGATTAGAGCCTAGTTTCTCGGTCCTGTTATATATGCTGGCTCAGAGTTGCGAGCAAAG
TTGTTGGTTTGAAATAAGTTACAACCGAGAGAAATTTGTAAATATACAAGTAACGCAGAAAGAAGTGAAGGTA
TCTTACACCCTCCCCCCCTTTCAAAAAAAGATATGTTCCCAAAAGCAAAAGAGAAGAAAAGGAAAGAAAATGG
GAGAGGGCAGTAAAAATTATTACTTACAAATGCCTTGGCACGGGTGCATCAAAAACCCAGTGGGAAAAGCTAC
CGGCCGGCCACACAAGAAAGAAAAAACTACCTATGAAAAGATTAAGAATTTTGATTTGTGTTAGCCAACCACT
CGTCCAAGGCAACTGTGTCACTTAATAACGATCCGCTAATCCATCTGTCATCTTCCATGCCCGCTCCTTGCCA
TCTAACGAGATATTGCCAACCACGGCCACGCCTTCGGGCGTCCACGATTTTATCGATATAAAACTCTTCGTGG
CCGTTCAAGATTACGGGAGCAGGTGTAGACACACGACGGCTTTCGAACAATTCGTCGTTATTTTCTTTAAAGG
GGGCAAGCAAGGAAGAGTGAAAGACATTGTACTTTCCCGGGGTGTTGGGCATATCCAGTGTGACCGTCGAGTG
ATTTTCGTCAATTGCCAGGATTTTATATGGCCGTCGTACCGAGGCATGAACTTGGCGACACGGCCAGACTTT
GAAGACATGTATTCTCGTCGTCGATTTTTTGTGGAAAGCATTACACGTTCACCCACAACGAAAGGGAAAGTGC
TCAAACGAAATCTATTCGCCTGCAGGCATTGGGCAATCTTTGCCGACAACAAGTTATCTTGAGCTTCTCGCTC
GAAAAGTACCAGTTTCTTTAGCATTGCGTTAGCATTGTCCGTCTCCGAAGCGCTGTTTCCATCCGAAGGAATC
GGCGGATTAGCAAGTGCAGGGATAACCCTCGGTGAACGTCCTAAGCGCAGTTGGAATGGGGAAAAACCCGTCG
ATGCGTTAACCGTATTCATAATGTTGAACCTCACGAATGGCAAGGCGGATTTCCAACCCTTTTGGGCTCTATC
GACGTGGAAACGCAGGAGCTGATTTATAGTTTTATTCGTTCTCTCGCTCGCCCCGTCAGTCTCTGGGTGGTAC
GAAGTCGACATCTTCAACTTCACCCCAGTTAATTCGTGAAGGGCTTTCCAGAAAGTAGACATAAACACTTTAT
CTCTGTCAGATATAATCTCATCTGGTAGACCGTTCTCGCAATACCAATTGGTAAAAAAAGACAAGGCAACATT
CTCAGCGCTGTCCTTAGTTTTTGAAGCGAAAATTCTGATATCAGCACCTCCAAGCCTGTCTGTGATCGTCGTT
ATCTGGTCAAAACCATCTTCTTCAGGTAGTGGGCCAATAAAATCCATAGCCACAGACTTTCCCCTGGCATCCG
GGACAGGCAGCGGGTGTAAAGGACCAATTGGTTTGGTTGTGCTGGATTTATTCCTCTGACATTCGACACATGA
GGGCACGTAAGCCGATTCCAGGTCGCGTCGCATGTTTGGCCAGTAAAACGAATCACGGAGCGAGGCATAAGAT

TTGTCAAACCCAAAATGACCGAGTGTGTCATGGGCCAGTCTGAAAAGCGCCTCGCGAACCTGGCCTTGACGGG
GAATAACGAGTCTGTCCCCAATGTACCAAAGGCCATCAACCTGCCTAATTTCCGGCATGCTCTTCGTCAGTGA
CTTGAGGTCCGTGACCCACTTGTCACCGCTGTATGCGTCTGTTATGAGCTTTATGAAGCTAGGATCACTATCC
ACAGAGAAAGTAGCGGCAATGGCGTTTTTACACTCCTCAGCACGAACATCGCTCAAGGCTGCTGCCACGGAAG
ACCAAGGCGCATTCAGGCAGTGCACCATCGCAATGCAGTCGTCATCGTCATCATGCGTGAAGACATGGTACGC
GGATTTGTCAATCTCTGGTGTAGGCAAACGCGACAGCGCATCAGCGACCGAATTATCTTCGCCCTTAACATAC
ACGAACTTCAAGTCAAATTGCGAGAGAAATTCCATCCACCTAGCCTGTCGTCTGGACAGGTCCTTTTGCTTAT
CAAAATTTTCCAACGTTTTATGATCAGTGTAGACGTCGAACTTCATTCCAAGCAATTCAGATCTCCATTTTGT
GAGAGCGCGAATGACGGCCAGCAGTTCCTTTTCGTGGACAGGATAATTTAATTGGGCCTTCTTTAGAGTACAC
GATTCAAAGGCCACTGGCCTCGCAGTGTCCCAAGTTTTCCCGAAAGACAAAACAGCGCCCGTTCGAAAATCAC
TTGCGTCGGTTGTCACAAAAATCTTATATTCAGGCATTTTTTCGAGATCAATAGTCGTTAAGCAGTCTCGACC
AATTACAAGATCTTTAATCCCCTGAAAAGCTCTTTCAAATTTTTCGCTCCATGAGGGGTAATTCTTTTCTGCG
CTTTTCAGTGTAAGTTCTGTCAAGACACCTGTGAATTCTGCCAAGTTCGGCAAGAACGCGGAGATATAACGAG
TCAAACCAAGGAAGGAACGAGCCTCAGTAGCGCTTTTCGGCCTTGGCCAGTTCACAATTGCATCGACTTTCTT
TGAGTCCGCCTCCAAGCCTCGCGCAGAGATATGGTGTCCTAGAAAATCAATTTCTGTGACGAAAAATTCGCAT
TTCTTTTCGTTGAGAAAAAGTTTGGCCGCTCTCAGTGCTGCTAGGATAGTCCGGACATGCTTAGCGTGCTCCT
CAATTGAGTTCGACCAGATAATGATATCATCAAGATATATGTGACATTTTCCCAATAAAAGGGCGCAATGCGG
CTGTTACTCGACGTTGGTGTATCGCAGGAGCATTTTTAAGACCCATGGGCATGACAACCCATTCATACAAGCC
AAACGGCGTATTTACCGCCGTCAGGTGAATGTCGTCAGGGTGTACCGGAGTATGAAAAAAGGAATTTGTCATA
TCCATCTTCGACCAGATGCGACCTTTCGAACAATCTCGGAGCACATCATCAATTCGAGGTAAAGGATGAGCAT
CTGTGACAGTGTTTGCATTCAACATTCGGTAGTCGTTCACCCATCTTGGCAGCGCAGCTTTATCGGCCTTTGG
GATTATGAAACAAGGAGAGGCGTAATTTGAAGAGGAAGGCCGGATACGACCTGCATCCAAATGCTGATTTATC
AATGTTTGAAAGGCATCCCTGTATTTCCTTGGACAAGGATAGGAGCGATTCGTGACCTTTCTTTCTATGTCTT
TCAACTTAATCCTAGCATAATAATCCGTAGGCAGCTCAGAAGTGTGGGGAATTTGCCCGAAAACGTCTTTAAA
ATCAGACTTTACAATATCAGCCTGTGTTTTGCAAGTCTCTTTACATGCGAGCCATTTCAATTGTTCGGAGATA
GCTGCTACCACGTCGACAGGCTGCACAGGATCAGAAGTACATTTGCCTTCGCTGACACGTTGTTTCAAAACAG
CTGTTAACTCCTTAATCACGAGTTTAAGTTCGTGTTTTGTCCAGGTCTGTTGAGCTTTACGAGATAGCGTTGA
AAATAATGATTTCGGGGGCGGTGGTTTCAAGGGGTGTAGGAGATCACAATCCGAAGACTTAGGAATAGCGGTC
AACAATTCATGATCAATGACAATTTTATTATGCGTAAGAAAGGGTAGCCCGAAGATTATGGAAGAACATAGAT
TAGGTGCGAGAATAGCCTGTACAACTTTTGAAGTCCATCGTCCGTCAGCGGAAGTAGCGCGAAAGGAAACAAA
TTCAGACAAGGCGACGGCTTCTTTCTTTCCATTACTGAGTGCAACACCGACAGACAAAGGTTCTGAAAGGGGG
GATGGAGTCAGACCCAATTTCGTAACGACATCCGGACGAATAAGTACCAAGTGGGAGCCGTTGTCAATCAAGG
TCTTCAACTTCACTGGGAATTCAAGAGCCGGCGAGTCAATCAAAGCATCCCAAGTGATATGTTTAGCTTTCAA
TGGGGAACGTACTTCGGATTCCGAGGCAGAACCTTCACCCAGGACAGCGGTTTGCATCACACTAGCGACAAAA
TCTTCGGCATCGGAACGCTCCTCCTCTTCTTCGGTAACGGCAGCCACAGCTTTCTTGCGCGTCGCAGTAACGT
TTGTGGAAGACACAGTAACCGCGGTTTGCTGGTTATCCCCACGACGAGGAGGGTTACCCTGAGCATCACGAGA
GTCGGTCAGGACTTGATAGTTCTTCCCCGAGGGATAGCCATTCGGACAGTTCGAGCTTCGGTGTTGGACGTTG
TAGCGACGGCATTTGAAGCAGCCGTGGTACTTCACGAGCAATTCGCGTTCCTCGTTGGTCAAGGAAGGGCAGC
GGTCGTTTGACGAAGTAGCAGAGGAAGAGCGCGAAGTGTTTGCATAACGGGAAGAGGAGGCGAGCGCAGGACG
TTTAGCGCGTCCATTGTTGGAGAAATCGTCGAACATCTCTCGGGCACGCTTCTTGTCCTCCTTGAGATGTTCA
TCGGCCAAACGAACGTTTTCGATCCATTCCTTCAACTTCGTTTCGAGGTGGATCTTTTCGCGGTTGCAGTAAC
GAAAAAGATCGCGGTCCATAGACGTTTCCAGGTGGCGCCGAAGCTGGTCATCGGACATGTGGCTGTTAGTGTC
TTTGAGCAAGAAGTTCATGGACTTAATTTCCATAGCCCAATCGTAGAACTTGTCGGCCGTGAGCATCTTCCGA
TTACAAAGCTCGTTTCTAGTAACCACTTCCCAGTCGCTCGGTAAGAAGAGTTTGCGAAGTTCCTTCATGAAAT
CCTCATATGAGAGAGCTTCAATCCTATCGCGATCATTGTTGATCCACTCCTTAATGTGTTCGTCGAGGAAACC
TCCAAGAATCATACGAACACGAGAGGCGGCCTCGATGGCTTTAACAGAAAAGTACTCTCGGTGAGCATTCGAA
AGTTTGTACAATATGCGGGGATTGACGTCGCCCTCGGTCAAAATGGGGGGTTTGTTCAGAGCGAGGTGTTCAA
CAGTTGCCAGAACCCTAGTGTTGGGAGCCATTTTTGCAGGACGACGACTATATGCGCGTTAAAATGCAGTTAT
ATAGAAGAAAGCTGAATGTATGCGTACGAAAACGCGGGGAAAACAATATCACTGTCCGAAGAATCGGAATCAG
AAGAAGGAGCCTTCGGTTTAGACGACTTTGCAGGTTGAGGGGAATCAATTGGAGAAGCAGAAGTAGCCCCACG
TGGCAAAGGGAGTGCAGAAGGAGAAGCGGGCGTAGGTGAGGGAGTCAGAGGAGCCACAGTCGCAGGGGGGACC
GAGAGTTCGGTATCAGACAATTCGGAATCAGAATCAAGCGAAGTAAGCGGGGAAAGCGAGGAGAGTGGGATAA
GCGGAGACGGTGGGTATTCGGAGCTAATCCAGAATATCAGGTTAAGCGAGTCAAAATAAGAGCGGATTCGTAC
AAAGACGCAATGATGTTCAAGTTGACGAGAAGAAAAACAGTGAAGAGCAATTTGTTTGCGAGCGATAAAGTTC
AGAGCTGATTTTGTTGCGAGCAATCCTGATGCGGTTCAAAAAAAAGTGAAAGCCTCGACCAAAGGGCTCGTTT
TCAAGAGTTCCCTGGCTTAGCCTGGCCAAGAAAAGGTCGGAAAATAGCATTTTCACCCCCAGTATCGATCTAT
TAGGATTCTGTCTTTCAGTTGAAAAGTTTTTCATCGAAGAATTGCGTTTGCCATGCCTTCTATTTTAAGCGCA
GGCACTGCCTGCAAGATAGAAGCTCTGTGAGCAGTGCACATATAAGATATGTGTGCGACCACAGTGCCATTTT

TCAGCTTCACCACGTTCTTTTGCCTCTCTCCCTCATATCTGCCCACACAACTCGCTCGAGATGTCCTCAAATG
CTCCAATTCTTCCATTCCTTCGCTGCCTTTTTGGTTTCTTGCTACAAAGGATGAAGAGGCTCTGTCTGCGCAC
AATAGCGCCTTCTTTGAAAGCGATTGCGGTCTTGCTATCAAAGCTAGGTATCCGCTACAAGGCTACAGCCGAT
GAGTACAAAAAACCGGCGCCAAAGGCAGACGATCTCGACATGGCGACGTTGCGTGTTGAATCGGATTCTAACA
AGCCATACGAGAGCGACATTAGCATCGGATTGCAATCACGGTCTACTATCCCATCAGTACCCATGACTGATAT
TGCATGTTCCCTCTACCCTTACTCGGGTGGGCTCCCCAATGCGTCCCGAACCTCCCAGAATTTACAGGCTTCT
CGATCCGCTCATAATAGCCGAACATCGCTCGCGAGTGGAAGTTTATCTAACGCTGTTAATTTGAGTGGCAATT
TTTCAAACAACCCAGTCTCCAGCTCAAACGTCAGAATCAACGTGCGGCGTCCAACAATCCGGGCCACAGGGGA
TTATGTTGGTCACGACAGCGTCCAGACCGACGCCGAACACAATACAATCCAAATCGAAGGTGTGGTTATCGAA
TCGCCCCAGGATCTACCTCCGCCAGAGCTCGGATTTCCGAATCGTCCGTATTCCGTCATGTCGCATTCCTCGC
ACCCGTCCACGTTGCCTTCAATTTCGTCTAGAAGCCCTTCTCCCCAGCCTCTAGGAGGCATACAGGAACAAGT
TGACGATCCGCCTATATTCCTGTCAGCCCGTCTGGCTAGAAGAGACTTGGTACCTGTCATGCCGATGTCTTCA
AGAAGATATACCGAGAAGCCTCGAATGTATGGAACTCATCCTATCCGTGCATGTGTACATGCTAAGTTTTCCC
CCCGTTCTTTGTGTCTAGAAAAGATCAAATAAAGAAAGTAACGCTCCCGGCCTTGGAAAGATCCTACAACAAG
TGGGTTCATTCACATAAATAAATCTACATACTGATTTCTTGCCTTATCAGCCCTGCGCCTCCGGCGAACTGGA
CAAGATGTGTTCATCCAGAAGGCGCTCGATACTTCATGAAGAAAGGGAGAATCATCGTTAGTTGACATCCATC
TCTCTACCTTCACTCTTACTTATTTATCTGTTAATGGATTCTCAGCAGAAAGTTTGGACGGAATCTGACATTT
TTCGACAAAATATACTCACGCAGCTCGAAGCCCACTTGACCATTATCGAAGATTTTATATCTGAAGTCTGTCT
AGTCGTTCGACCGAAGTGGAACCTCGTTATAAACTATTATGATGACGAAAATTCGCCATACACCGGTTATTAC
TTTGTCGATCAAGAAATGCACAAGGTCTTCTTTTTGGATGATTATGAATCTGGTGCCCTCGATGCGTGGGGCC
AAGCTCCGGGTGCTGAATCGGAATGCCACATCGGTATGTGCTTTTGCCTGGGGCGATATTATTTATTCATTTT
TCTCATCGTCTATTCGGTTGTAGGACATGAAATTGAAGCACAATATTGGTACCGTACCCATTCCTCTTTGTGC
CGTTTGATGGCTGATTCGCGTGCAGGTACTTTATTCTTCTGTACCCGCATGCACTCAACGTGGAAAAAAGACA
CGTTCAAAAGCTTAGAGGCATCATCTTGCATTTCATTGGAGGTGCGCAACTCCGATGATATTACGCTCTGATA
ATCGTAGTAAACCCGGATTGTCAGATTCAACGACTAGTTCTACTTCAATTGCGCCATATTCTCTGGATGAGCT
TTATAGGGTTCTGAGCTTGATTGATCACATGAGCGGTAAGGATCTTTGGAGAAACATTCGTTTGAAAGAAGCG
ACTCAATAATTCTGTAGAGAACGTTGACACCAACGACGAAGGGACGGCGAGCATTCTTGGTACATCGAAATTT
CACTTTATTTTCTCTTGAGTGGTGTTATCTGAAGCCGTATTATACACATGGTGATCAGGCCGCCTTATGTTTG
TTATCAGTAAGCTTTTTGTTGATTTGGGATTAGAAAGAAGTGCTAACTTCTTATGTCATCAGACCGTGAACGA
TTTTTGAATTTTCACGGGGAAGCCCACTCGCGCCTCGAGAGGACGTTCTCAGTCTACGGCACCGCCATTTATG
CGCAAAAGTGGCTTGTCAAATGCATTTCGCCCTTACTCTTTTATGCTCCAGACTTCCACTTGAGGACCCTGCA
AAACATGGGGGTCGATGGAGTCACACACAAGTCTGTCTTTCAAGACTTGATGCAAAAGATGAATGATGAATGG
GAACAGTTTATATTATTTGTAAGTCTCTCTGACTGCCTATCGAGTGTGTTGCAGATTACTTGAGTATATTAGG
GGACCGTGATGTTAAATGCCAATGTTGCTTTCTTGGCTATTCAAAGCGTTGATAATAATGATTCGGACGACCC
GCATCGACTCCCAGCTCAGGTTTTAAGCTATCTTTCCGTCGTTGCAAGTGTTGGAAGCATCCTCTTAGGATTG
CTGCTGGTCAGAGAATTCAAGACCAAGGACAGAGAGTCAGTCGACGAGATAGTTCAGTCACTCTTTTTATTAT
TACTATCTTTCTGTCTTTTCCTGACTACTTGGCACTTGTTTGTGTGTAGCAAGCCTATTTCGCGTCGCGTCAT
CGCCACGCATTAGGACTGGGATCGGAGGTTCTGGCAATCCTTTTCAGCTTACCATATGCTCTACTTATGTGGG
GGTGGGCCTTTCCCTCCATCATCTCCTGGGCATGATTTGTCTGATTCCGTTCTACATCCAGTATGCTCGCATT
CCTTGCCGCATTTGCATCCCTTTGTTTCAAAACCTCTGGCCTGTCGACCCGCATTATCGTGGGATCTGCCTTC
CTTATGGTCGCTTGTTTGATGGCTTTGTGCATCCGCACGAACTGGGATAAGCGTGTTCCTCCCCAACCTCATC
CGCTACGACCAGAGTATGCATCTGACTCTCAAAGCACACTACAATCCTTTAAATCGGATGCATTCCCGCTATC
AATCTTGCCGTTTCGTCGATCACGATCTTCGCAGAATACGGCTGTGAACGTGCAATCGAATGCTTAAGGTGGA
CTTCTGAAAATGTGAGTGCCCTGTAAAACACTTTTCAACTTGAAGATTAGAGCCTAGTTTCTCGGTCCTGTTA
TATATGCTGGCTCAGAGTTGCGAGCAAAGTTGTTGGTTTGAAATAAGTTACAACCGAGAGAAATTTGTAAATA
TACAAGTAACGCAGAAAGAAGTGA
>Agrped1_Cluster20#LTR/Gypsy
CGCAAATATTATGTTGGGGAATTTAATCTAATCTAATGGCCCGCTCTGGACCGATGACTGTAAATGGTTCCGG
CCCAAACCCAATTGCCGGTATTTCCGAAGGTTGCCAACGCGAACTAAATATGGTGCGCTACTAAAAATAATTA
GACCTGCGCGCACGCGTCATTCTTGAAACTATAATTAGACGTCATCCAGTTCACCCATTCAGGATCCATCCCT
TCCCGCCCCCCGACGGCGGGGCAAAGGGCGCATATAGGTCACAAAATATTCCCACATTATTTCCCCGCTTTGT
TCCAGCCCGAACGACGCCGCCAACGACAAGCAAAGGCCAAATACAGTACGCGCGCGCTGTTTTCGGCGTTGTT
GACATCTGTTTACCATCGCTGCCCACTGATGGCCATCATGACTACCCCCCGGCTGCCTCGTTGGCTATGATAC
CCGTTTGGGAAGGTAGGCAATTGCCCACCAGGATATATCAGTCATGTTAGGCAGTTGCCTACATTTGATACAT
GAATATAGCTGCAAAGGCTGCGAAGACAAAGGATTGCATAGGCCTGCTCCTAAAGCGGCCTTCTCCGTATGGT
GGTGGGAAGATGCCATCAAAAAGTCGGCTTATCCAATTATGACCAGAAAACAGAGAGCAGAACAGCAAAGAAC
AGATTTGGCTCAAGCTGGAAAGGACGAACATAAAGACGACGCTGAAAACGGCCCTCTGCCTTTCAAGAATGAT
GATCCCTGGACTTACCCCGTTGGCTGTAAAATCCCGCCTGATAACCTCGAAGAAGATTGGGATAATCGTTCGC

ATCTATTGGTATCAATGAATCCGCAAATCGTCAAAGATTTTATCGCGGGATACCAAGAAGACCCCTTTTTTAA
AACGAGATACCTAGATGAACAGCCTAACCCAAATCAAGTGCTGACTGCTTCGCATTTTCGGAAGGCTGAGAAT
GGACTATTGTACTTCTTAGACGCTGATTGGAATGCTAAGCTATGCGTACCCAAGAATCAAGTCAACTTCGTTC
TGAAATGGATTCATGACTCCCCATATGAGAGCGCTCACGCCAGCTACAAGAGATTTTCTCAGCGACTCCAGGA
GGTGTTCTACTGGAAATCACTGTATCAAGATGCCTCTTCTTTCGCCTTAACCTGTGACGTCTGTCAAAAGATA
AAAACAGATCACAGGAAGAAGACCGGAGCTCTGAGGCCTGCACATATTCCTGCTCGACCTTTCGATACTGTAT
CCTTGGATTTAATCACTGGGTTACCGCCGTCGGGAAAAGAGAAATTCATGGCCGTGCTCGTCATAGTGGATAA
ATTAACCAAGTTCGCTATCATTGTGCCGACCCACAACATGCTAGATAGAGAAGGATTTGCCAAGATCTTTGTT
GAGAAAGTAGCAAACGTTTTCGGACTACCTACGAGAATAATCGCAGACCGAGATAAGAGATGGGCTACCGACT
TTTGGAAGTCAGTCGTATCTTGCTACGGCAGTGTTATGGCTCTGTCCTCATCTCATCATCCGCAAACCGATGG
ACAGACTGAAATCCTGAATGCCACTATAGAACAGATGTTGAGAGCTTATGTAGCAGGCAAAAGAAGCGAGTGG
TCATCTTGGTTGAGCGTGTTAACTTTTTCCTACAATTCCGCTGTTCATTCCTCCACCACCTAATCCCCGAATT
TCCTCCTTTTGGGTTATCATCCTAGAATATCTACGAGCGCCCTCCTGAATGAAACAGACCCTTCGTCTAGGCC
ATTCCTGCCTAGCCAAAGGGCTGAAGAATTCATTACTTCCTTAGACGAGGCCAGAACAGCCGCTCGTAACGCC
TTAGTACTTGCGCAAGAAAAGCAGGCTAAAGCCTACAATAAAGGCCGAAGACCCGGAGACAAAATCGAGGTCG
GCGATCTGGTCCTGATAAACCCTCATACCCTGAAATTAGTAGAAGCCGAGGGCACTGGGAAAAAACTAGTCCA
GCGAACGATAGGCCCTTTCGAAGTCTTGGAGAAAGTAAACCCAGTAGTCTACCGCTTGCGTCTACCAGACTCC
TACCCTATGCACCCTGTTTTCAACGTCAGTCACCTCAAGAAATACCACCCTTCCCCTGCCGAATTCGGTGAAC
GAGAAACCCTACCCACTACTCGCGAATTTCTAGCCAGTCCTGAGTACGAAGTAGAAGCTATCCTGGGCCACAA
GCTCGCCAGCAAAAAAACGGGTAATAGGAGAATGTATAGAGTTAGATGGACTGGCTACGGACCCGCTGACGAT
TCGTGGATTTCCGAATATGACTTGCACAACGCCCCCGAACTTAAACGAGAATACTTGGCTTTACCAGCCATGT
AAGCTATATTTATGTATGAAAGTTGGCATTTTTCCTAACCAACGTGATGTATCAAGTTGGCATTTTCCCTACC
TGCCTCTTTGGGAAGGCTCTCCCTAGCATTTTCGCTGCGGCAGGCAATGGCATAGGTGGGGTCCCCGGTAAAC
ATAGGTAAACAAAGCAGAAAATGGTAGTGCAGGCGGTCATATTGTAAAATGGCTCCCTGCGGGCGGTTTTGGG
CGCCAAAAAAAGCGTACCGGTAGGTTCATATAATATTTACCTACCTAATTTTGGTGTGACCCCTGCCTGATATG
GACGGGACGGGCTGGATGGCTCGAAACCACTTTGGATGTAGTAAATATTTAATGAGCACTATTTTTAGTTTTG
TTTCTAAATGCCCGCAAATATTATGTTGGGGAATTTAATCTAATCTAATGGCCCGCTCTGGACCGATGACTGT
AAATAGTTCCGGCCCAAACCCAATTGCCGGTATTTCCGAAGGTTGCCAACGCGAACTAAATATGGTGCGCTAC
TAAAAATAATTAGACCTGCGCGCACGCGTCATTCTTGAAACTATAATTAGACGTCATCCAGTTCACCCATTCA
GGATCCATCCCTTCCCGCCCCCCGACGGCGGGGCAAAGGGCGCATATAGGTCACAAAATATTCCCACATTATT
TCCCCGCTTTGTTCCAGCCCGAACGACACCGCCAACGACGAGCAAAGGCCAAATACAGTGCGCGCGCACTGTT
TTCGACGTTGTTTACAGGTGTTTACCATGGCCGCCCACTGATGGCCATCACGAG
>Agrped1_rnd-1_family-522#SINE/Unknown
TTTTTTTTTTTTTTTTGTAATGTTGTTTATTGTCCGGTAGCGTACGCTATAATAGACATTGGCTGTTCGGTAN
TTTACAATCTATGGGCCCCAATGCCCGTCTACGTTCCTCCAGCGGGTTTCAGCAGCCGANGAGGGCGTGCCCN
GGACACTCGCTCCCCCNCCGGCCCCGGCACGGAAACGCCGTTTTCGGCCCCGCTGNTGTGAGGAGGACTGAGC
AG
>Agrped1_rnd-1_family-409#TIR/MITE
TAAGGGTCAGTTCAGCAACCCAGATTCTCGCGAGAATCTGACTCCGACTCATCTCAAGGGCTGTGACTGATCC
GCGAGACAGTGTGACNGCGTGATGGTTACANTGTGACTCGACGCGGGCGCGAAAACTTAGACGGCTTTTCTCC
AAACTGAACCCTAATAAATGTGAAAAANATATCAGATTTTAGCTTTATAAACCATATATAGAGCATTTAACAT
GTTTTCGTGGCAGCGGAGGTANTATTCGTGGATCGCGCTATCCTTGCGAGCTCACGCCGCGCGCGCGCGCGTC
GAAGTGACGAGGTGGTAGANTAGGGTTAAGGTCCAAACGCTTATGTAATCGTCACTCTGAGATGAGTCGGAGC
CAGATTCTCGCGAGAAACTGGGTTGCTGAACAGACCCTAA
>Agrped1_rnd-1_family-371#SINE/Unknown
TCATAGTACCGAAGTGGGCTCTTTGGATGGAATCCTCTGAGCACTCCTGCTTGTCAGGATAGGCTAAGTCGTG
TGACGGACCCAGACTAGGCGACTGGTTGTCTTGCGTATGAAGGGAGCTTCGGCGTTTGATCCTTCCCTCTGTG
ACCGGACTGGGCGGATGCCCTTCCATCTGTTCTATCGATTGCGGCGAGGCATTCCTCGCTGATCCATCAACAT
TCAGGCTGCGTAGGCGGCTTACTTTCTTGGCGGGTGTTGCACCCGCCCTCTCTAGACGTAGTGTAGTAATAAA
TCCTGCCTTTCATGTAATAGTAGGGACCGGATTGCTAACGGGCGCCGGAATACAAGTTGGGCCTTCGGGCTCG
TGATTTAAAAAAAAAAAAAAAAAAA
>Agrped1_rnd-1_family-56#ClassI/TRIM
ACGTACCNNCTGGAGAGCAGGAAAGCGAAATGATCAGTGGTGGATTCACTTGATTCAAGTCGAGCAGCGACGT
ACCNNCTGGAGAGCAGGAAAGCGAAATGATACAGCGACGTACTCTCTGGAGAGCAGGAAAGCGAAATGATCAG
TGGTGGATTCACTTGATTCAAGTCGAGCAGCGACGTACNCCCTGGAGAGCAGGAACGCGAAATG
>Agrped1_Cluster4#LTR/Gypsy
AATTTGGCGACGTGGTATGAAGCCTGTCTTCGGTGTTACGAGCTTGAGCAGGAGGATTTTCGATTAGCCTCTC
AACCCAAGCCGCCGGACATAGCAAAAGACCCGACTTATTGGGAACGAATTAAAGGTCTTGTAGGTAACCTGCT

GGCTAGTCCGAAAGCACCCCAGCCGAATGTAGACCTCGATTGTGAAGTCATGTCCGATGAGGAATACCTAGAG
GTATTTGGTGTACAAGTAGATCGAAAACTATACCCTGGGTTACAGCGAAATTCAGCCACTGTTAAAGACAAGC
GTAGAATCCTTCCTAAACCCGTCATAATTAAGATGGAAGTAAATGGGCATCCAGTTCGTGCTTTAATAGACAC
TGGATCGCTAGTTCTTCGGACAGTTCTGAATCAATAAAGAAATCTTCCAAGAAGAAAAAGAAGAACCACCGCC
GTAAGAAACCTAAGCGGCATACCAGTGGAGCGAAAGCAATAAAACCCACAATGTATGATGGCCGCTCTGATGC
GCGCGCTTACCATCGCTTTGTCAAAGAAAGTGCCGCCTATCTCGAAGATAGTGGAATTAAACGAAAGAGACGC
GCGTTCTCGTTATCTTACTTCATGGAAGGACGCGCATATGATTTCTATGTGCAGAAGGTATCCAGAACCGAAG
ACGATTGGGACATGCCCACATTTTACGAAGCCCTATTCAATTACTGCTTCCCAATTGATTATAGGCAAAAAAT
GCATCGTAAGTTTGATAACGCAACTCAGGGACATCGCGCAGTAAGCGAATATGCTCACTACCTTGAAGAGCTT
AGCAATCTTATTGGAACTATAACTACGAGAGACTTAGTTAATAGGTTTTGGAACGGAACTAATGAACCTATTC
AAAAAGCGCTATGGAGAGACGGCCTACACCCAGACTCATCCACTTGGGAAGAAGTTCTGGCTCGAGCTGAGAT
CATTGAAATTTCCGAAAATGTATTCAAGACTCGGGACAACCGTAAAGGTATCAATAGTGACCGATCTTCCCGA
CGAGATCAGCCGTCTTCACATAAGAATGCTCGCTTTGATAAAAAGCATCATGATCGATTCAATCGTAACCAAG
GATTCCAGCGAAGGGATAACAATCCCAACGCGTCTACATCAAACTATACTAAGCCCTCTGCCTACAATGTCAA
TAGATTTACGCGTGGATTGCGACATGAATCTAATAAGAAAGATCATCGCCATCACCGAGACAATAAAGGGCTC
AATAAGAATCCACCTCTAAGTGAAAAAGAGCGCGCTGAACGAGCAGCTGATGGACGCTGCTTCAGATGTAATG
AGCATGGTCACTTGTCGCGTAACTGTCCTCGTAATAACTCAGTTAAACATACTGGAAATAAGCCTCCGGGCAT
ACCTGCGTTCGGTATGACGCTTATAGGCGAGGCCGACCAATCCGTCGAAGTCTTGGATAGCATGCCTGTAGGA
GCTATTTCCTTTGAGCCTGAGACTCGAGCAGAACCAATTGGGTTACCACTACCATGGGAAGAGAACCCTAGTG
ATACGATGGAAGATAGCTATCGAATGAAATTAAAATATCTGCTTACATCCAGAAGCTCTTACCCTGGCGACGA
CAGATATACATATGATTCTGATTTCCCGCGACGACCTGAGCATCGGTTCTCTGTTCATCGACTCAGTAGAAGA
CGAGGATATTCTATCTTTGATCACCTCACAAAATTTGATATTAGGATTTCTATGCACCTATTAGAAAACCCAT
ACTTTGACTGTGTGAACTGGTACTCGAAACGGAGGGCGAAAGCTCTTCGTTTACCCCATATTAAACTTCCCTG
GACAACGTTTGGGTGTCCGTTAACTGAAGTTGCCGTTGCGCGCTTACAGTACGCCGCCTATCGGCATTTCCCG
GCTATACAAGCTGATACCGTCCCTGAAGGGCGCTTCAGTATATTTCCTACGGAAAAGGGATCGAGCACATACT
TAGTTTTAGATCACGATCTTAATCTTACGTTTAAGATAAGTAAAACTCGTTTAGAAGATCCAGACTTTGATAT
AGTTACCTGGTACAGGAAACAAGTAGACGAAGGACAACAATATAATTCGCTATACCTCGCGAAACTATTAGAG
GTAGTACCTTCTACGCCAGAGCCCACCGCACCGCCGGCAGAACCTAAGTTTGAGGCGTTCTCCGAAGCTGAGT
TGAAGAAATATCAAGCTGGAATAGTAGGAGATATATTTGCCAATCATATCACCCGTGTACTTAACCAGTGCGC
TCCCTATCCGGGAGACGAACTAAATCCGTGGGTATCACCTTATCAAGCCAATCACTCCCGTTTCGACGTTGAG
CGTGGAAGGTTCGAAGGCATTTATTGTATCTACGATAGACGGAGGCGAATGGAACATTACATTCATGCTGAGC
GCCTACGCTATGATTTGTTCTCCATTGGAAAGTGGTACGCGGAGCAATGTGCTGTAGATCGTGGGGACGCTTT
CCCAGGTGAAACCGCTATTGCTTGGCTACAAACCAGAGCTGTATCCGATACCCTGATGGGTACAGTCTTAGAA
GAACGAGCCGCAGACATTCTACACTTAGGCGCACCTTATCTAGGTGAGGAAACGAAGGAGGATGATCCTAGTC
CTCGTTTCGAAGTAGAATGCTATGCTGGTGATAAAGATATATTCTTAATAAGTGATTATAAGCGACAGAGCGC
TACACAGTTACCTCGAGAAATGTTAGAGAACCCGTCGTTTAATCTAGCGACGTGGTATGAAGCTTGTCTTCGG
TGCTATGAGCTTGAGCAAGAGGATTTTCGATTAGCCTCTCAACCCAAGCCGCCGGACATAGCAAAAGACCCGA
ATTACTGGGAACGGATTAAAAGCCTCGTAGGTAACCTGCTGGCTAGTCCGAAAGCACCCCAGCCAAATGTAGA
CCTCGATTGTGAGGTCATGTCCGATGAAGAATACCTTGAAGTTTTTGGTGTTCAAGTAGATCGAAAACTATAC
CCTGGGTTACAGCGAAATTCAGCCACTGTTAAGGACAAGCGTAGAATACTACCTAAGCCTGTCATAATTAAGA
TGGAGGTAAACGGACATCCCGTCCGTGCTCTCATTGACACCGGTTCGCTTGGTG
>Agrped1_rnd-4_family-1450#TIR/MITE
CACTGCTGGCGCCGACTTCTGTCAGTGGATCAGACAGACGAATATTAACACGTATTCTGTTAATATTCGTCTG
TTAGGGGTCTTAACAGACTCGAACAGAAAATCTGTTCGGTCATATTCGTCTGTCAGGCCCACTAACAGACGAA
TATNAACAGAAAACCTGTTAATATTTATCTGTTAGTAATCTGTTAGGTCCACTAACAGAAGTCGGCGCCAGCA
GT
>Agrped1_rnd-4_family-180#LINE/I
TGGTTCGATCAAAGGGCCACCCATGACGGGCATCGACGCGAGGAGAACCCTTTCNTCNGTCGCGCTGTTGTTG
GAGAGCTATCGGACAAGCGAAGCTGGTTCGATAACGGAATCCGTCAAGGNCACGGTCACGGAGGCGATGGAGG
AGGCGAAGGACGAGNTGAAGAAGACAGTACGAGAGAGCGTGGAGATGATGAAGACGCAGGCGGCGCGCGGAAC
CACCCACCCAATCCAAACCGACATACAGCAGCGTTGTAGCGCGGGGCGGCNACCGCCGATGCCAGACTCGTCG
CNCAAAAGGCCATCCAGGACCGGCAAGTCCTGCTCCTNATCCAGCAAGGANCGANGGCAAGCTCACACNCGAC
AAGGACAACCAGGCGACGAAGAAGATACTGCAGGAAGAGATCGAGACACGAGGCGGCACACACAAAATNCGAC
TGATCCAGAGCGGACGAAAGACCGAGGTCTNCTCGTGAAATGGCATCGGAAGAAGGGGCAGCATGGCTACGAC
GCGCGATNAAGAAAGGACGTCTTCGAAAGATCTAGGNGCGCAGTAAAGGACAGACACCACCACCTTCTCGTCA
AGTTCGTCCCGATCGCCTTCGACGAGGACACGGAGATTCANGAAGTTCTCGACGTCAACAACATCGACCCCCA
AATTCTTCAGGGCGCGNTGGATCAAACCGAAGAACAGACGCCGACCCGATCAAAAAAGCGGCCACATGGTATT
CTACTTTCAACGACAGAGACCAAGCTAACGAGATTCGCATTAGGGGTNTNATCATCCACTCGCAGCGGCGACA

NGTCGAGCGTCTCAAGGACGAACCGACTCGNTGCCTCAAGTGCCACCGNTACGGGCACATCGCAAGGGACTGC
ANCGAGGAGGAGGANATNTGCGGCACCTGCGGGGAACGCGGNCACCGCGCCGACCGATGCACNGCCAACGACA
AAACCTNCTGCGTNTCATGCAAGACGGACGACCACTGCAGCTGGGACAGAAGNTGCCCAGCATTCGCTACGCA
AATGCGAGGAGTACGACAAGCGACACCCNGAGAACNCACTGCCCTACTTCCCCTCGACGGAGCCCTGGACATG
GGCCGCCGGCAAAGCCCCGACATCTTAACTAAGCCCNTCACNCAACGNGCNAAGAAGCAGGAGATAAAGCGAA
GGAAGAACTGCAGGAGCGAGAGACAANCTGCCCAANCGGCGGCGANGCCAGGGAGGGGACCAACAGCGGTGAC
GTTACNTCTTCTCAATGACNGACACNACTAACGACGNCACACAGACGANACAAACCACCTACGAATNCTCCAA
GCGAACCTATGCCACTCGGACCNNGCACACCTCCACCTACTTAACGACGAAGCGGCCAAGGAATGGACATCC
TCGCGATACAAGAACCNCACACCACCTACTTCAANTCCATACGCACNCCNAACGGNTTCCGACAAGTATACCC
NGACAGCGACTTACGACGACGAGAGAAAGCGNGATCAGCNATCTGGGTCAACGCGGCNATCAGCACGAATTCG
TGGAAGGCNCTCGACATACCAGGACCCGTCGGATATCACTGCCATCCAGATNGAAGGCGAATNCGGGATNCTC
ACTATCTTCAACGTTTATAACGACTGCGAGGACGACGAGGCCGAGAAGGCACTNGACAGNTACCTCGTCACAC
GAACAAGAACCATNTTCGGACCAGGGAAGACGCGTNNTGTGGTGCGGCGACTTCAACNGGCACCACCCACTAT
GGGACAGCGACGCGACGACCGNCTGTTCACNCCNNAAGCNATGGNAGCNTCCGAACGACTAATCCAGCTGATG
GCGAAATGGGACATGACGATGCTGCTNCCGAAAGGGATCCCGACNCTCCAACACTGGGTNACGGGNAAATACT
CGAGGCCNGACAACGTATTCGGCACGAAAGAGCTCGCGGACCTCGTCATCACGATGCGACACCCTACCCGAAC
GGCGACCGGTNAAGACCGACCACATCCCAGTNGCNACTATCCTCGACNTNTCGAAAACGCCAGCCCCTAGCCC
CTACAAGAAACTTCCGCATGACNGACTGGGACGANTTCCGNGACGAACTCGACCAAGAATGCAACCGCCCCGA
CGTGGANCGACATAGGACGAAGACGAGTTCAACCGNCAGGTCGAANCACTGACNANTCTATTACAGGANNCNA
TCGANGAAAGGTCCCGGTCAACAAGCCCAGCCCNCTGTCNAAACGGTGGTGGACGAAGGANCTCGACCGGATG
AAGAAGGAGGACGCGNCTNAGTNGAATTCGTACCGTACCGCGCCCTCCCGGACCACCCNTCACACCGCGANTN
CAAGCAACACCGAAANCNNTTCGCNGACGCNATCACGAAGGCCAAGCGNCAACACTGGGAAGACTTCCTGGAA
GAAGCGACNGAGNGGGAAATGTGGATCGCNAACAAGTTCATTAAGGAACCGATCGGCGACGGCGGACGNCCGC
GNATACCCACTCTCAAGATCACCACGCCNACCGGACCCAAGGAGGTCAACTCNAATGAGGAGAAGGCCGAGGC
NATNGCGGCNTCGTTCTTCCCGCCGAAACCCACGGNGTCGAGTGTTCCGNACGACTNTCAGTACCCCGAACCN
CTCAAGCGGCNCTNCCNATTACCGGCGCAGGCGGCTGAGCATGCNAAAAACTTATCACCTTGGAAGGCCCCAG
GCCTTGACGGTATCCCCAACTCGGTACTCAAAGAATGCGCTGACGTACTCTCCCCNCATCTAGCACGAATATA
CACGGCNGCACTACGACTCAACTTCTACCATCAATCNTGGCGNGACTCCATCACATGCGTACTCAAGAAACCA
GGNAAGCCCGACTACCAAGTACCCAAGGCATACCGCCCNATCGCNCTCCTNTCCACNACCGCAAAACTCCTCT
CTGCNATCGTNGCNGACGACATGTCTCGACTNATNGAACAACACGACCTACTNCCGAACAACCCACTTCGGCG
GACGCCCAGGCCGNACAACCACAGACGCGCTNCACTACCTGGTCCACAGGATCAAAACNGCCTGGAAAAAGGG
GAAGGTNGTCTCGATNCTATTCCTNGACGTCGAAGGNGCCTTTCCCAACGCAGTCACGGANCGAGTCATGCAC
AACCTGCGNAAACGACGAATACCAGAACCATACGTCAAGATGATCGACGAANATGCTGGAAGACAGACGNACG
AAACTCCGATTCGACGACTTCCTNTCAGANTTCCGNNCNATCGATAACGGNATTGGGCAAGGATGCCCACTTT
CAATGATCATCTACATCCTATACAATGCTGACCTNTTAACCATNCCAGAGTCNGANGACGAAGACGCNATAGG
CTACGTGGACGACGCCATACTCATCGCNATCGGCGACACNTTCGAAGACACNGTAGCAACNCTCAAAGACATG
ATGGAACGGGACGGCGGCGGCTTCACNTGGTCCAAGGACCACAACTCAAGATTCGAAATCTCGAAAGTGGCNG
TCATGCACCTGTCGCGAAGGAANCTGGNCCAACGGTAGACGGCAAAACGACCAAGCTATACGANCNCGCGCCA
CCACTCGTACTACGAGGCNCAACAATNGNGGCGGTCAAATCCTACAAGTACCTCGGCGTCATGATNGACCCGG
AACTNCGATGGAAAGCCCAAACCGCGCGAGCGGTCGAAAAAGCAACCAAGTGGGTACTACTATTCAAAAGGCT
AACGAAACCGTCCACAGGCGTCCGCNTGAAGCTCATGCGACAACTCTACCTNGCCGTCGGCATCCCAAAANTG
ACCTATGCNCTGGACGTCTGGTACACCCCCCCTCACAAGACCTAGGAAAGAAAAGNAACAGCGGCTCNGTAAC
GGCCCTNCGCGTCATGGCNAAGGTNCAACGNATGGCNACCATCGCGATGACCGGGGCNCTCCGCTCCACAGCT
ACGGACACTCTCGACGCNCATGCAAACATCCCNCCAACAGAGGTGATGTTCAAATACCTCTGCCACAGAGCGC
TGATCCGCCTNTGCACCCTAGACGACACCGCACCCCCTACACGACGTGGTCGCGCACCCCAAGAAGATCNGNC
CNAAGNCNCAGCCNCACGAACAACCNAATAACAAGACTGCGACGGNTATTCAANATCGACCCACGGCGAGGTC
GAAAAAATAGGACGNAACAAACACCCCGCCTGCAACGGACCGAATCTTCGGCATCACATNCGGATCAAGAGAG
GANTCTATCGAAGCGAAGCCGAGACACCGCTCCNATCAAGATTTTCACGGACGGCTCGGGCTATCAAGGAAGA
GCCGGNGCAGCGGCCGTAATNTACGCTGACGGAGCGCAGCAACCAACCGGNACTCTACGTCCACCTCGGACCC
GCGGAAGANCACTCAACNTACGAAGGCGAAGCGGTNGGACTCATCCTGGCAGCNTGGCTACTACGCGCNCAAT
GCGGAGNAAGGGTAGGCNCCGACGCAATAAGCATATACTCGGACTGCCAAAGCGTCATAACAACNGCGTTCGA
CATGAAACCAGGCCCAGGTCAGTACCTCTACTTCAGCGTTTCACAAACTGATAGCNCCCTTCAGGGACAGNGG
CACCAACGCGCNAAATTCACNATCCACTGGATATCGGCCCACAGCGACGTCCCCGGAAACGAGAAAGTGGACG
AGGAGGCGAAAGCNGCNGCGCAAGGCAACTCCACNCCNAACCCCTTCCTCCCCCAATACCTCNAACGCCCNCT
GCCNATCGGNAAATCCTCGCCCTCCTACAGCTAGCAAAGGAACAANGNANCGACGAATGGAAAANNACGTGGT
CGACNTCCCCGCGACGAAAGAAGNTGAAGAAAATCGACAAGGACTTCCCNTTCAAGAAATTCGGCAAACTAGC
AAACGANCTCACNAGACGCCAAACCAGCATACTGATCCAACTTCGCACCGGTCACTTCCCACTGAACGACTAC
CTCCACAAGCGNAAGCTAGCGCCNTCCCCGATCTGCAGCGCCTGCAATGAACAGCGACGNGAAACCATCGACC

ACCTNATCAAAGAATGCCCCGCATACCAGAACACAAAACGTANNCTCAAGNAAGCAATACAGGCAGAGGCCTC
GCCGACCCTCTCTCNCTNCTATCCGACCCGAAACGCGCCAAACCCGTCATTCANTTCGTAGAGGCAACAAGCA
GATGGACNAACGGACGAATGACCGATAGCAAACCCGGAATCACGCCAGACACTACAATCACTACACACACCAC
TCACACCCTTCTTCAATCAACCATCACCCATNTCCGCGACGACACCTC
>Agrped1_Cluster19#LTR/Copia
GTTGAACGACTAATGTCGTGAGGCTGACTAAGCCCATGTGACTCGCACGTGTGGGCGCAAGTCCCGTGCTTGA
TGTCTAAACTTAGTGTAGTTATATATAGAAGACAGCCACCGCATGTCATGAACTATACAGTAGTTACATTAGT
CACGTAGTAAAACATATATAAGGGCTACGCATTTGCCCACTAAATGCAAGCGGTTTACAACCTCTTAGTAGCT
ACAACTAAGTAGAACACCAACACGACCCACAAGGGTAAGTATTAAGTCAAGTCTCTCGCTGCGCATATACATA
GTCTAGTGTACAAGATGCTAGGGCTTGAGCTCGTGCTCGCCTGGGCATATTTGCTGCTCAACAGGTTATGGGC
CCCGCGTCCCCAGTTACGCTTGAGACTGCTGCCTCCTATCCGGAGAACGCGCTCGCTAACAAGGGTTGATCGC
GATGAGACTGTACTGCCCTTACGTCGCCGTTCACACTCTGCTCCTGTTTGGATAAGGACATCCGAATACTCCT
ATGTGCCTTCGCATAATCAGAGATTCGTTGAAGTCTATAGGGGGCATGATGTCAGGCGACTTAATCAGCCAAG
GCATCATACGCCGAAGACTGATGAAGAAGTCATGAGTTATTATCTCTGACTTCTAGGCCTGACCAGCCGACGT
GCGATTTGAGTTTCATTTCATTTCTCTTTCATCTTTCTATTTTAAGGGTGTGAATAAGTTCGCTGATTAATCT
TCGGCAGAACTTCTATTCTTTGCGATTTCAACATGTCTTCCACAACTCTTAACAGTATCGAAGTCTTCATCTT
CTCCGGTTCTCGATTCCAAGAATGGTCCGTGAAAATGAACCATATCTTCCTCATGAACAAGGTTCAATCTATC
ATTGATGGTACCTTCGGGATGCCCATCGAACCTCCTGCCGCTCAGAACCCTGAACCAACCATGCCAGCGGATG
CTGATACCCGCAGCTGGAAGGTTTATGATGTTCAGTTGAAAAAGTGGCAGAAGAAATATGAGGATTACCATCG
TGATGCGAAGGAATGGTCCGATCGCAATAGCATGGCAAAAGGCATTATTATGTCCGCTGTCATTCCCTCGATT
TGGGAATCTATTGCTGAGAAAATGGCTAGAGCCGGTTGGGCAGATCTGAAGGCCAGATTTGATAAGCCACAAT
TCAATGAGCTGCTTGATTTATATCGCAGACTCCATGGCTTCAAATTTGATCTCTCTGACCCGACTCCTCAGCT
CGCCAATTTCATGGCCATCTTCGGCAAGATTGAGAAAATAAAGGCAGGCCTCCTTCATAAGTCAATGGTCGGC
CTTTTGCTTCTCTCTCGCCTACCGCTTAATACTGCGGTAAATCAACCAGAATCTATCTATCAGCCTGTCGTCA
ACTATTTCAATGACGATAGCAAGTTTAGTGCTTCGGCACTTCAAGATCTGGATGACATTCAGAATCGTGTTCG
CACGGCCTGGATGAACCGGTTTGGAACGTTGAACAAACAGCAGCGCCCCAAAAAGGGCACGACTATTAGAAAT
AGTGCGGTAGCTTCTTCTTCGAAGCAACCCGCTGCTGGTCCTTCCGTTCAATCGGCCGAACACAATTCTGCAA
TTAAGCCAAAAACAAACCATCTGCAGTATATCCCTTTGCAGGCACCTTTCAAAGGAACGCCTCAGGGATCTTC
CGCTCCCCAGCAGAATGAGAAGCCTAAGCGCAAGCACACCCGAAAGGGTAAAGGCAAGGCACATGCCAATGAG
ATTGAAACGGGTATCCCCAATGAGGTCTTCATGGCCTCAACAGCTCAGCCGTCACTTCCTGCTCCCACCAGGT
CCACTGTGATGGAGATTAACGGTCGAGGTTCTATTTCCTCCCGTCAATTTTCTCACACCGGAGCATGGAAACA
CCTGGATAATCGCATGGCTCCCTACCTGGCTCCACAAGCCGCTAGGACACTCGCCAACCGTCTTGGAGCCAGA
CCTACGGGTCCGCTCCTGGGAAGGCTTGAGCAAGTTGCCCACCTGTAGCAGCTACCCTCATGTATCAAAAGTA
GGCAACTGCCTAACATCACTGATTTATCCTGGAGGGCAGTTCCCTACCTTCCCAAACAGGTACTATGGCCAAC
GAGGCAGCTGGGGGTGGTCGTGATGGCCATCAGTGGGTGGCGATGGTAAACAGATGTCAACAACGTCAGAAAC
AGCGTGCGCGCACTGTATTTGGGCTTTGCTCGTCGCTGGCGGCGTCGTTCGGGGTGGGACAAAGCGGGGAAAT
AATCTATGATTATTTCTACACCTATATCCACCCTTTGCCCTGCCGTCGGGGGCGGGAAGGGGTGGATTTCAAA
AAGGTTCACTGGATGACATTTAAAAATAACTACAGACGCGGGCGCAACTGGTGCGTCAGAACGAATTATTTTT
AGTAGCGTACTAATTTTAGTTAAAATCAATAGAACATTCAGTTTTTCGGGTTACCAGTAACAACAAATTCCGA
CCTTTTATTATTTTTGGGAAATGGTCTAGAACGGCCTATTCAATTAGATTAGATTGATTTTGCCGCACCTTGG
TACCACAGACAGGGTTCTAAATCTAATCACATCATCAGTCCCGCCCTCCCAACATGGCATGCCTTCCAAAATG
GGAAGTTATTGCCATTTTGGGCTGGGTCCCTCTTTCCTCTGATACATCTGGGTGTGAGATTTTTTTTGTTCTA
ATTACATACATGAATTATACATGATTAAAAAATAGACATGTAAGGGGAAGGCCTCCCCTCCGATTTGCAGTAA
TAGAAAAGAAAAAAAAAAGAAGAAAAGGAGGAGGATGATGAGAACCGAGGTATTGCTGTCCTCGTCCTCGTCG
CCTGGCCTCACCCTCCTCGTCTCCTACTCTCCCTACCCATATGCTTGCACCCTCGGCATCCTGCCCTCCCCCT
TGTCCCACGAACTGCCGGGCCCTGCCCCCCTCCTCCGCGCCAACCATGCCCTCCCTCTCCTGCTCCTCTCTGT
CGTCGTTGCCATCCTCGCCATGTTACGCCTCCTCCGTTGTGGTGGTGGCGATTGACGGTGTTGCGTGGAATCC
ATCTGTGTCGTTGTTAAGGCAGCCGGCGGCTCCCGGTGGAGAGGGCGGCGATGGCGGCGAAGGTCGTCGTCGT
CGCAAAGGCGGTGGTGGTCGCGGTGACGGCGTTCCTTCCCTTGTCCACTCCTCTGCCTTCGACTCCCCAGCGT
CGTGGGTGCTGATGTGCTGGCTTATGGTGGACTTATGGCACGGGCGGCCGCACTTGTCGTTCGTCGTTGTCGA
CAGGCGGTGATCTCTCGTGGTCTGGTGGCATTGGTAGCGACAACCAAAGGGTGGAGGATGGCGGGGAGCCATG
AGGGGTGTTGGATGGTAGCGGTGGAGGCGCAGGTGGATGGCACAGGTGGCCAGCGAGGGGTGGAGGTGGCAAC
GACGGTGACGGCAGTGCCGGTGGAGAGGGTGGTGGTGACAGATATAGTGGTGGTGGTGGTGGTTACGGTGGTG
GTGATGGTGAACGAGGGTGGTGGTGGTGAAAGAAGGTGGTGGAGGAAGGAGGTGGTGGTGGTAGAAGGAAAGG
GCGACATCGACCCGACGCTTGGTTGATGTCGCCGGATATGGTAATTTCGTGCCGGGCAGTTTTTGCCGGGCCT
CGAGCTCATCGTCGAGGGCGTCGAGCATGAGCCTATGAGGCCATGATCGAGAATAAATAATGTGGAATAAGTT
ATCTACAGTTGAATGAAACATAAATGTCAAAGAAATTTGTGAATACCTTGAGACAGTGCTGAGATATTCCAAA
ACCTTCAAATGAACATGGTTCAGATACATTTTTCAAATCCAATTAGTGAACGTCTTGCCTTTAATTTAAGATA

TTATGAGCACACAAGCTTACTGGCCTAAGAAGATCTCAGAAATGATAACTAATATACTCTTACAGTACTGCAG
AACCCTGTTCTTGCTTGATATACTGGGTTACCGCTCACAACAAAAAGATCTTTTTTGCGTATATGTCCGAAAT
CACCACGTCGAGAACTCAGTCATCTCGGAGATAGTCGACTTTCTTAATCACGCTATATTATGGAACTTATTTT
CATTGTTGCAATTTTTACTTGGTTCAACTGCTACGTGGGTTCTTGAAGTACACTACATATTTTAGCCTAAGGT
CCCGTACTCGTCGTTCTTGACGACGAGGTGCAATTCGGTTAGAGCGATTACCAGAGCCGGAGTTGCCTTATCG
GGCAATATCAACCAACCAACTGGTTGGTTCCAGTCTCTCTCTTCTGTGCCGCCATCCTCTCTGCATACCGCCA
CCCTCCCTACTCACCTCCAGCACCATCCACATGTACCTTCGACACCCCCGTCTCCGTTATCGCCCCATCCACC
CCATTCCACCATCCACCACCCTTCCCCATCAACACCACCACCATGCACAACATTACGCCGCCGTCTCCCACTG
ACCATCCCTTACTCGCCGCTACCGTCACCGCCATTTGCCTGATTGCCACAGTCGCTTACCACCTTCGGTCGGG
CAGGGAGAACGAGGAGGCCAAGGTGAGAAGAGGGCAAGAAGTCGAACAGGAAAGGGGAAGGGGTGAGCGCAGG
GTGGCAGAGGAGAGGGACCTCAGCGTGAGGAGAGGCGGCTCAACTCGACACAAGAGAGCGAGGCAATGGAGGA
GGAGGAGAGGTGGCGAGAGCCTCATCGTGCGAAGCAGGAATGCGAGCACTGGGCGAAGAGGGTGAGGCGAGGG
ACAAGGAGACCAGGGGAGAGAGCCGGGATTGAGGGACGCAGGAAGTGTGCACGAGATGGGTGTGGCAGGACCA
GGGGATAAGAAAGAGAGAGGAGGGGAGAGTCAAACCGAGGATACCGAACCTTGGTCCTCCTCCTTCTTCCTTT
TCTTTTTACTTTTTCTTATCACTAGCCATGGAAGGGGAGGAGTTCTTCCCCTTCGCTATTTTAAGTTTAAATT
AAGTATAATACTCTGTATGTACATAGTTAATAAAGCATGTTTTCAAAACAGTTACACAACAGTGTCATTCACT
TATGAAAAGATACTGAGTATTCCGAAAATGGCAATTAGTGCCGATTTGGGAAGCCATTTATTCTCGTGATCTA
AATCATCTAATCTAGTCAGCCATCAGACATCCGGAATAGTTTCGGAAATCACATTACCGGTATCCGACTCTGA
ACAACCGAATGTTCTATTGATTTTAACTAAAAATAATATGCTACTAAAAATAATTCGTTCTGACGCACCAGTC
GTGCCCGCATCTGTAGTTATTTTTAAACGTCATCCAGTGAACCTTTTTGAAATCCACCCCTTCCCGCCCCCCG
ACGGCGAGGCAAAGGGCAGATATAGGTGTAGAAATAATGTCCCACCCCGAACGACACCGCCAGCGACGAGCAA
AGCCTAAATACAGTGCGCGTGCGCTGTTTCTGACGTTGTTAACATCTGTTTACCATCACCGCCCACTGATGGC
CATCACGACCACCCCCAGCTGCCTTGTTGGCCATAGTACCTGTTTGGGAAGGTAGGGAACTGCCCCCCAGGAT
ATATGAGGCATGTTAGGCAGTTGCCTACATTTCATACATGAATATAGCTGCAAAGGCTGAGTTGCCGAGTCCT
CTGGTTTCTCACAGGCAGTCTCTCTTGGTCTTCACAGATCGAGATCGACGACACCTGTCCCCCCTTCTTCCTC
CACAACATCATTTGATGACCAAATGGTTGTCGATGAGACTGCTCCTCCTCTCACCACTGATGAGGAAGTTGAG
CTATTTGGAGAAGAAACTGCTGGAGCTGAAGAATTCACGGTATTGAGCACAGTGTATGATAAAGTAGAATATT
GGAATGTCTGTTGTACTCGTAGACTACAGGCTTTTATACTACGAAATAAGAATGTAAAGTATTACTAAGTTCT
AATGGAGATGAGTAACAAAAATTCTACGGTAAAACTAAAGGCTCCATGTAATTACTAAGTACATTACTGTGCC
GGAATAAGGATGGCCCGTGCACTGCTAGGCCACTAATCATCCAACACACGGGAAGCGCGGAATATTACGAGAA
TCGAAGACAGTTGGAGGCCATGTCTCCCGAGGAACTTGTCGCTGAGCTACGGCGAAATGCCAAAGAATCTCGC
GAGCCGACACCGGCACTCCGATGGGAAGACGAGGAATCCGACTACGGAGTTGATGACCCCGCTGCTTATGCGG
ACTATTACGTCGATAGGCAAGTAAGTTTTCCCACCATCAGACAAACTATATTTGAAACTGAACCCATATCAGG
GACTACCCTTAACTCCGAATCAACATCCGTCGAAGAAAAGGAGGAATTCCGTGGGGTAAATGTTAATATCGAG
CCATTTGTATTATGTGCCTACGATAAATATTTACATCATTTTCATACTTGTCCTGATTGTAAGGGAAAATTGG
CTGTTAACAATAAGTCAGCCGCTGCCTGGTTGTTAGATAGTGGAGCTTCAATACACTTCACATCCAACCTAAG
TGACTTCGCGACATATCGTGAGTTCACTGACGAAAATAGACCTTATTCGCAAACGGCAACAAAGGTTGAGCCG
ATCCAAGGCTGGGGAACGGTGTTTATTAACATCTCCGCGCCTCGTGATGCTCCCAATGTTGTCCGACTCCACC
CTGTGTTCTATATGGAGAACCTTAATATTAGGCTTCTTTCATTAGGACAAATATTAAGTGAAGGACTTATGGT
TCAAGGCTTGAGTAAAGGCTTAGAGTTCTATGAACAATTTGCTACCCATAGTGAATCAGCCTTTTTCCCTGAG
ACCATGGATGAGATTCACACACGGTTTCCAATGTTTTGGGTCAAGTCCCGAGTAGTCGCTGAGGAGCGACTCT
CGACGTTGATTCCTATTCTTAAGTCTGAAGCACATGATTTGTGGCATCGCCGACTGGTGCATCTGTTCGATGA
CGTGCTAAGACAATTTGGTAAATCCACTAAGAATTTTCCTAAGTTCTCAGTACCTAAGGATCATAAGACCGTT
TGTGAAGGCTGTGCGAAAGGGAAAATATTGGATGTTTCGCACTCCACAAATGACCGACGCGCAAAGAAGCTAT
TTGAACGTGTCCATGTGGACCTTAAAGAATTCTCATGCCTGTCATATTCAAAGTTTAAGTATTTTATTTGCTT
CCTGGACGACTACACGTCCCATGGCTGGCTCTTTTTCCTTAAAAAGAAAAATGATGCCGCAGGAGCTTTTAAT
GACTTTGAGTCCATGGCGTTGACGCAATACAATGCGACCGTCATGTCATACTTCTTTGATAATGGTGGAGAAT
TCGTAAGTGAGGAGTTTCTAAAACATCTTCGCTCAAACAGTACTAAAGCGTGTTTCACCGCTCCCTACTCACA
CCAGAGTAACGGTAGAGCAGAACTGGTCCCTAGATGTCAAGTCCGCATTCCTTTACGGGAAACTTGACGAGGA
GATCTATATGGAACAGCCTGCAGGCTTTACCATAAAGGGACAGGAGAAGAAAGTACTCCGCCTCCGCCGCGCT
ATCTATGGGCTGAAACAAGCCGCCCGAGCCTGGTGGTCCGAGCTAAACGAAAGTTTGAAATCGCTCGGATTCA
CTAAGTTGTAATCGGATGCTGGAATATTCGTCTCACGTCACTCCGACGGCACCATGGTCATCATGCTTGCTTA
CGTCGATGACATACAAGTGACAGGTCCAAACTCTAAATTGGTCCAAGAAAAACAAAAGCTTTTTATGGATAAA
TGGGAGTGCCGAGACCTTGGCGAATCCAGGGAGTTTCTTCGCATGAACATTCGGTATTCTGATGGAAAGATCT
TTTTAGATCAGACCTCATACCTTGAGAAAGTTCTTACGCGATTTGGCATGAGTGATGAGAAAGCAGCCAGAAC
TCCGCTTCCCACGGGCTATAAGCCTAAACCGTTTGAGGGCACATCAACGGTGGAACTTCGCTCAAAATACCAA
TCCGTGATAGGGTCGTTGCTATATTTAATGCTTGACCATTTGAATAAGGCTAAATATATTATGCGGTACTTAG
TCGGTACTCGCAAATATGCCTTGGTCTATGATGGCAAGTCCCGCTCCAGCTTGCACGCTTACTGCAACGCTTC

TTATGGAGATGATCAATCTGATCTTGACCTAAAATGTCGATCGACTCAGGGCTTCTTCTTCTCGCTGGCTAAT
GCCGCGGTCTTGTGGAAATCACAAACACAGAAGTTGGTTGCCACGTCCTCCACTGTAAGCGAATACATGGCCC
TCTCTGACTGCGCCAAAGAATGTGCGTGGTACAAAAATCTATTTGCAGAGCTTGGTAAGCCTCTCAAATACGT
CACCTTGTACGGTGATTCCCATGGTGCGATATTCAATTTGCAGAATCCTGTGACCCAAAAGGGTATAAAACAC
ATCGAGATTTGTTACCATTACATAAGGGAACAAGTTGAGAAAGGCGTGTTGAAGTTGTTCTCGGTACCAACTG
CCGAGAACATTGCCAACATGTTTACCAAGAATCTCGGCCCGCAGGACTTCCTTAAATTTAGGAGTGGCCTAGG
CCTCGAGTTCTATGCATTAGATAATGATGCTTAGTCGTCATTTGACAAGTCGTCAATATCGAGAATGGTTGAG
TAAGCGGCATGATCCGCGACTCCCAGAACAAGATCAAAAACCGCTCTTTTGATTTGAGCTTGGTCTTATCTTA
TCTCGACACCACACAACTTGAGGGGGAGTGTTGAACGACTAATGTCATGAGGCTGACTAAGCCCATGTGACTC
GCACATGTGGGCGCAAGTCCCGTGCTTGATGTCTAAACTTAGTGTAGTTATATATAGAAGACAGCCACCGCAT
GTCACGAACTATACAGTAGTTACATTATTCACGTAGTAAAATGTATATAAGGGCTACGCATTTGCCCACTAAA
TGCAAGCGGTTTACAACCTCTTAGTAGCTACAACTAAGTAGAACACCAACACAACCCACAAGGGTAAGTATTA
AGTCAAGTCTCTCACTGCGCATATACATAGTCTAGTGTACAAGATGCTAGGGCTTGAACTCATGCTCGCCTGG
GCATATTTGCTGCTCAACAG
>Agrped1_rnd-1_family-448#LTR/Gypsy
ATACCTCGTAAGATCGCCCGNCAACTGAAACGNTCTAAAGGACCATCGNTAAAGGACCGGAAGNTTCGAGGGC
TACGCCGGATCTGGAAGCCGAGGGCTGCGACGAAGNTCGCGCCGAAGGATTCAAAGATCCGGNTGGCCTCTCG
NCGCCGNCGGTNGTNATNGTNCCCAAAAGCAGAGCTCTNCCGGAAGGAATGGGATCNCTAGGNTCTCGAGCTC
TNCACATNAAAGCTCGAGTCGCTCGNATCGAACGAGGANCCCATAGTAGCTCGTCTAGATTCCGGNGCNGACA
TCACCCTAATGTCCGAAGAGTACTACCGNTCGCTGAAAGATATGCCNAACATTAAGGAAGGNCTNAGGATGAA
GCTATACCACCTCACNGGCAACGCCAAGGTNCTAGGATACATNCGAACTCNGCTGTTCGCNGAANCTGACGAC
GGTACTTGGCTACAATTCGAACTGGAAGCCTACATNGTCAGAGACATGAAGGTTCCGNTACTGCTGGGAGAAG
ATTTCCAAACCACCTACGAACTNGGNCTNCGACGNTACGCTTCCGGACATAGCGAAGTAGTAGTAGGAAGACG
NGAACTACACGTAATNCCNGCCTCNTCNGCNCAAAGCGTTAATCTCGGNTTCGAAATCCGGCAAGCTTTCACN
TCGCAGTCCTTCGTNCGATCNAGAGCNGTNCGACGAGCTCGAGCTAAGAACCGCGAACAGAGCAAGGACGCTC
CCGTAGTNGCTGCAGCGGACACCCGNATTGAAGCCGGNTCCGTCCGCAACGTCAAAGTTCAAGCNCCNTTCGG
NTCGCAAAAAGAATGGATNGTNGAGAAGGTCATCATTGGNACNGAAACNACNGATATCCTAGCCGCCCCTACT
ACNTGGATNAACTCCTCCCATCCGTATCTNCCCATCGCNAATCCTAGTNCTCGNCCTATGTACATACGAGCNG
GGGACGTCGTNGGNCATCTNCTCGACCCCCACGCCCTAGATAACCCNGAAGGNGANTCCTTAGATCGCTGCGC
NGCTGCNGCNGAAGCCTTGCGTACNGTNATNACCGGATCCTTACGAGATCAAGATCTNGCGCAAGCTNCNGAG
NCNAAGGAACCTAGTTCNGATCGACTNGACGACGACGAAAACTGGGGNCCTAAGACCACCGCTATAGCGGAGG
ANCGAAGTTGGCGAGATATCCGAGCTCGTCAACCTAGGACCAGACATCCCTGANGAATACAAGGGTCCTCTNG
CGGAGGTNCTGAAACGNAACGCGAAAGCTTTCGGAGTNGACGGTCGCCTNGGGCAAGTNCAAGCTCGAGTNGA
NATNCCNCTGAAACCCGGNACTCAACCCATNTCCGTNCCNATGTACGCCGCCTCNCCNGCNAAACGAGAAGTC
ATNGACAAGCAAGTNAAAGCCTGGTTCGAGGCGGGNGTTATNGAGCCNTCGGTNAGCCCNTGGGGCTTTCCNG
TNGTAGTNGTNTACCGCAACGGCAAACCNCGNCTNGTNGTNGACTACNGGAAGCTNAACGCNTCNACNGTCCC
NGACGAATTCCCCATCCCTCGACAATCNGAAATCATTCAAGCTCTATCGGGCTCCAGCGTTTTATCCTCCTTC
GACGCCCTNGCCGGTTTCACNCAACTNGAAATGGCTGANTCNGCGAAGGAAAGAACGGCTTTTCGTTGCCACC
TAGGNCTNTGGCAATTCAAAAGGATGCCCTTCGGNCTNCGCAACGGCCCTTCTATCTTCCAANGAATCATGCA
AGGCGTNCTNGCCCCNTTCCTNTGGCTNTTCGCTCTCGTCTACATCGACGACATCGTNATNTTCTCNCGNAAC
TGGGACGATCATCTNGTNCACCTNGACCGAGTGCTAGGNGCCATTTCNAAAGCCGGCATCACCTTATCGCCNT
CNAAATGCTTCGTNGGNTTTTCNTCTATTCTACTTCTNGGACAGAAAGTNTCNCGCCTGGGNCTNTCNACNCA
CGCGGAAAAGGTCGCCGCTATCATGGACCTAGCCCGNCCNCGGAATACCGCCGACTTGCAAAAATTCCTGGGC
ATGGTNGTCTATTTCTCGCAATACATNCCNTTCTACTCGTTCATTGCCAGTCCNCTNTTCGCTCTNCTNCGNA
AAGGCGTCCGNTGGCACTGGCACGCGGAGCACGAAACNGCCTGGCTNCAAGCCAAGNAAGCCTTGGCGGCNGC
TCCNGTNCTNGGNCATCCGATNGCNGGCGTCCCNTACCGCCTGTACACNGACGCTTCNGACTACGCTCTGGGN
GCTAGCCTNCAACAAGTNCAACCNATNCTAGTNCGCGACCTNAAAGGCACGCCNGCTTACGACCGACTGCGCA
AAGCTTGGGAAGCGGGCGAACCCGTCCCTTCNCTNTTTCCTACCCTNGTCAAGGAAATCGTNGAACGNGCTTC
ACCCGACGAATGGGCCGCCGAATTCGANGACACTCGCGTACACGTCGAACGNGTCATNGCNTACTGGAGCCGN
TCTCTCAAACCAGCGGAACGCAACTACAGCGCTACCGAANGGGAAGCCCTGGGCGCTAAGGAAGCNCTAGTCA
AATTTCAACCNTTCATTGAAGGCGAAATCATCACGCTNATCACCGACCACGCCGCNCTACAATGGGCTNGAGT
NTACGAGAACGCGAACAGGCGNCTAGCGGCGTGGGGGGCNGTATTCGCCGCCTACCCNGGCCTGAAAATCGTN
CATCGACCGGGNAGAGTNCACTCGAACGTNGACCCTCTATCNCGAATGCCCAGAATTCCGCCGCACAATTCGC
CTGCTCGCGACGACNGCGTAACGATAGTNCAAGACGANGCNAAACGAGAGAAAGCNCAANNAGCNGAAGACAA
AGGAACNAATAAACCTGCTNGCAAAGCCGCNTTCTCCGTNATGTGGTGGGAAGACGTCGTNGAAAAATCGGCG
TATCCNATNATGACTCGCAGACAGAAAGCNGAACTGGA
>Agrped1_rnd-1_family-5#TIR/MuDR

GTCAGTCGCTTTCTCTGCAGACGGGAAGTGGCTTGCGTCCGGGCACGNCGATGGCACAGTCCGGATTTGGGAT
CTCAAGAACGGTAGCCAGACTGGGAAAGCTCTCGGTCGACATTCCGACNCTGTTGCGTCCGTCGCATTCTCGC
CAGACAGCAAGCACCTTGTCTCCGGATCNCGGGACNACACCATACGNTTGTGGGATATTGAAAAAATGGAACA
AGTTGGGGATCCATGGAAGGGGCACACCGATNATGTCNACTGTGTGGCGTTCTCGCCTGATGGAACAAAGGTC
GTGTCTGCGTCGGATGACAAGACACTACGNGTCTGGGATCCAGAGACCGGAAAGGAGATAGCTGTTCTGAAGG
GACATGAAGATTGNGTTCGATCAGTGGCATTCTCGCCGGACGGCCTACACATAGCGTCAGGATCTNNTGATGA
NACTGTGCGCATCTGGGACGCTCGCACATATCAACAGGNCGGTGAGCCGCTCAAGGGACACACCGGCTGGGTT
CGGTCCGTCGCATACTCTCCCGACGGCAAGCGCATTGCATCCGGCTCCNGGGACGACACGATACGCATCTGGG
ACGCTGAGACGAGGGTGCAAGTGGGGGAGCCCCTCCAAGGACATTCCGATTGTGTCAACTCTGTCTGCTTCTC
TCCAGATGGGAAGCTGCTAGCCTCCGGATCGGATGATCGCACCATCCGGATATGGAATGCCGATACAGGAGCG
CAGATCGGAGAAGGCCTTCGCGGGCATGGCGATTGGGTGCGGAGTGTGGCGTTCTCGCCTGATGGAAAGCAGC
TGGCTTCGGCATCANATGATGGNACAATCAGGATATGGGACGTAGAAAGCTATGTAGAATTGTNTTTACTTTC
GATTGGGTA
>Agrped1_rnd-1_family-171#TIR/MITE
ATCAGGACTTTGTCNATTTACTTTTTCGCTTATTTCGCTTGTGGAGCTTGCCGCAAGTTACGGCAAGTTCCCA
TACTAAAATCCATAATAGTACTAGCGCAATTACATAACAAAGTCAAGTCGAACCGAAGAGCGAAGCTATGACG
CACGGCATTAGCATAGCAGCTTTTTGAGAAAATGGACCTGGCTTTTTACTTTTCCCTTTTCGAGCTTTTTNAC
GCTTTTTTCGCTTGGCCAAGCGGAAAAAAGTAAATTGNTAAACTTCTGATG
>Agrped1_rnd-1_family-556#LTR/Gypsy
AACACAGGATGTAGGCGCTTCATTGAGAGAGGCAGTTTGAGCCGGTAGGCACTACTGCTGACTTTCTTCTCAA
TCTTGAAGGGTCCCAAGCGCTTGTGAGCCAACTTCCTTGACGGACGAGTTGTCTGGATGTCGTCTGCCTCAAG
ATACACCATGTCGCCTGGCTTGTACTCTGGAGCAGGAGTCCGCCGTTGGTTGTAGTACCTGGCCATGTCATCC
TTTGCCTTCTTCAGCGCTGACCGTGCCTCTTCAAGAGTGTCCTTCATCCGTTGAGTGAACTCGTTGACTGTCT
CCAGTCTCGAAGGAGGCTGGTTTGGCTCAAAGCCCATTCTTGGATGACGGCCAGTGTCGAGGAGGAAGGGAGT
CTGCTGCGTAGAAGAGTGGACGCGGTTGTTGTACGCGAACTCNGCCAGAGGAAGAAGGTCGNCCCAGTCGTCT
TGCCTCTCGCTGACGAAGATGCGGANGTACTGCTCCAGCTCTTGGTTGACGCGCTCGGTCTGACCATCNGTCT
GCGGGTGATAGGCNGTGGATGACGCGACCTGGATACCCAAGAGACGGCAGAGCTCTGTCGTGAACTCTGCAAC
AAACTGAGGGCCGCGGTCACAGATGATCTTCCCGGGCAGACCATGNAGCTTCCAGACGTTGTGGAGGTAGAGA
CGGGCAGCGCCGGCAGCGGTTACCGTTGTGGTCGTTTCGATGAAATGACCTCGTTTCCCAACGGTGTCTACCA
CGACCATGATAGCATCGTGTCCAGCGGACTCAGGCAACTCAACAATGAAGTCAACCGAGATGCACTTCCAGCG
CTCCTCCGCAACCTCTGTCGGAACGAGCTCTCCATATGGTCGACGATGCTGAACCTTGGTCCGCAGACACAAG
TCGCAGGTGGACACATACCGACCAACGTACCCGGACATGTTTGGCCACCAGTAGTTTCTAGACACCAGCTCCA
GTGTCTTGAAACGTCCGGCATGTCCAGCTATCTTGGTGTCGTGGCAGAGAGAGGTGATGCGACGTCGGAGATC
AGAGGTAGGGGGCACGTAGACACGACCACGGAAGTAGAGAAGACCATCTTGCTGAGACCATTCAGCGGAGCGG
ACAGAGCGGTTAGAGGACTTCTTCAACTCCTTCACAGCCTTGGCAACAGGTTCCTCGGTTGGCTCCTTTGATC
CCTTGCGTATATCACGCAGGATGTCAACCTCAGATCCAGAGAACTCCAATCCTTCCATGGCACGTATGGCAAA
GAGCTTGCGGTCCAGAAGAACAATGTTCTTGTTGTCTTCTGAACCATCACCATGGTCTGCACGTCTGGACAGG
GCATCTGGCTTTCCCATGCTCTTGCCTGGGCGGTGATGNAGGATGAAGTCAAAGCGCGCCAGGTAGAGAGACC
ATCGAGCTTGNCGGCGGTTCAGCTGCTTAGCGGTCATGAAGTACTCCAGGTTCTTGTGGTCTGTCCAGATCTC
GAACTGGTGCTC
>Agrped1_rnd-3_family-45#LINE/L1
TTTCGACTAANNTTACTCGTAGNGCTTTCCTTCCGTACCTTNNCGTGTTCGTNTGTAGGCAGTCNATTCGTAG
CCTGCGGTTTATCTGCCTCAACCATGTGTTCTGGTTTCCTGTGCCGTNGGTATTCTTTCCGGTCGCCATTCAG
CGATATTTTCCATCTGCATCGTTGCTTCCANATTAGGTGGGCGGATTCNGANATNAGNATCGCGTANAGCCTG
TTCGTTCCNGGGGACGCCCTTTCCCCGGTTCCGTTCGAAAGCCGCCAGCCCGCATCCGAGNATNANCCCTATC
GTTGGCTCANGCCACGGTAGNCGCTTCTTTTGCCAGAGTTCTTTCGCTAGCTTCCAGATCGTCTCCTGNCCGC
TGGCCTGACACTCNGTNAGGATGTGTTCCATNGTTTCGGTCGCGTCGCACACNTGGCATCGACCTCTGTTCGN
AGTTCGGNATCCTCTCCCACCAGCGCCCGATCTTGTANGCGTTGTGCANTGCCTTCCACAGGAAGCTCNCGGC
CGTAGGCCNAGGTCTTTGCTTCGCAGNGATAGCCAGATTTGTTCGTCGCTCGGTCTTTTNCCAAAGGCGTCNC
CGACCGCATGCCTTATCATGTCTAGGTGAGCNACGGTGTCCCTCTTTACGTGGGCGTCGACAGTGTTNANTAT
NCCNCGGTACAGCAGGCTCTGGGTTGCTACGGGGAGTCTNACGCCTTCCACCCGGNATCGGGCTGGCCGTTTG
GCGGATTGTGGTTTGCTATTCTCGGGGGCGTCGGGNAGGACCATTCGTCGTAGGCGTTCGTAGGTCAAGGTGN
CTGCTGGCGTCGATGTCTCTTGGTGAGTATCGTTGTCTGCTCGCTGTTTCGNAGCTTTGCAACAATCTTCTTA
TACAGGTCNCCTTGGGGTACGCCGATCCACCCTTTATCCTCGAACCTCGTCAGATGTTTGGTNAGCGCTNTCA
NTACTGGTTCGTTCGTNATCCGATTCGTAGCGGGNTTGCGGTATCGGTCGCGTCTAGGACTAGCAGGAGCGCT
TCGCATGTACTTCGNTCGGCTGTNGCGTCCCCCCCGTTGGACATCATCACCGCGTCTCTTTCNCGGTCGTCCG
CGTATCTAATNCCGGCGGCAGTTTTCCCGGAATCGTAGGACACGTTCGTGCTTTGGGTTACGATGGTNACTTC
CACTCTTGTTCTCGGTGGGGTCGGCCTCATATCGCTGGGTCTTGNTCNTCTTCCCCTTCATCCTGATCCCAGG

CCCGGTATAGGCGTTGAAGACTCGTACTTCNTCCAGGGGTGTGGTATNGCGCTTGCGAGGGGGTCAAAGGTAC
GCTGGTCTAGTCCCTCTCCCNTTTCTTCTTCTATGCCCTTTCCGCGCGNTCGTGGCGTCTCGTTATTCTGACT
GTCGTATTNGAGCGCGGATCCCATTCGGGNCGNAGTCCGTCCAGGATCTTTCTNGCTTCTTCCCGGCATATNG
CGGGGTTCTCACAGCCCCGGTGTCTTAGGTTCCGGCATATTGCGCAGGCGCAGTTTCTTCGTCGACGGTGTCG
GGGCCCTAGCNGTGTATTGGCATAGTCTACCATCTGTCCCACNGTNNNTATATCGTGANTNGTCTCTGGCAT
TCCGCCCATTGTTTATTCTCNAGTGCGGCCGNNTCTTCTCTNCGTCCACGGTGGTCCCANATTANCATTTCTC
TCTGTATGTNTGGTCGTATTACAGGTGAGTGTATTGATACTTTATGTTTTTTTGCCGCTGTTAACATNTCTCN
TATGCTNTTNGGNAGNGCGGGGNTNCCTCGCTCGCGCGGNGNCCAGTTTTGTAGGAAGATGTTNNTCTTNACA
TGGGGTCTACTTGATCCCGGCTTTTTGGCACATTCGGCGCTATTAGCCCATCCGCNACTTTCGCCCACTTGGG
TCTATCNTGTCGTGGTGAGGTACCTCTGCAGTTTCATAAGGTCTATNGCCTCGTTCCTNGCTTTTATGTCTAG
CANTCNCTTNCCTCCTTCTNCGTGGTCTGGGACATTAGTGTCGGCGTTNACCATCGGNGNTNCGCCGTCCCAC
ATGAANTTGTTNATCTTTCTNCGTANGACTTCCTCNATCTCGGCGCTCATTCCTTGCACCCTCGTCAGGTACT
GTGTTANGCCTCCTACGACCATGCCNACTACCAGNCGTCGTCCNTCCGGCGTCGGGTGGCCCTTTGACCANCT
GGCGAGCCTGTGAGTTATTTTNTCNANTGTGGGTGTCCANACCGCCGCTTGGTCCACNTTGTTTCCTACATAG
GCTCCNAGNACTCGNACNGGCGTTCCGTCNNCGGCTATNTNNATNTCGGGTGGTANTTGCGCCCCTTGGTCAT
TCAGCTTCCGGCTGCGGANTACATCTCGGCGATATNGTTCGGTTCCGACAGGTANTATNACTGTTTTCGCNAC
GTTGAATTTNGCNCCNGACGCCCGGCACCATGTNTTCAGGATNTCTTGTAGGTCTTCGAAGTCGTCTTCTTCN
GCGAGCCATACGGTTGTGTCGTCCGCGAATAGNGTTGTTATCAGCCGCTCGGCTTCACCTTCGATNNCGAAAC
CTCGTAGGCCGGAGTCCCGTAGCATGGACGCTAGCGATTCTATCGCTAGGTTGAAGATTAGGCANGANAGTGG
GTCNCCCTGCCGTACNCCCCGTGTGACCTTGTAGCCGGTGCTNATAACNCCGTTTAGNATNATCACTGTTTCG
CCGTTTTCGTANAGCGTTTGGATNGTCCTAATGAAGTGTTCCGGGAAGTCGAACTTCCNTAGNGTTNNCCANA
TGAAGTCGTGCCNNATCTTGTCGTANGCTTTCTCTTGGTCCAGGCAGACNATCGCCCCATTTTCTTCGTCAGC
TTCACANTTGTCTATCATCAGCTTGACCAGTTCNGTCTGGTCTTCNATTCGNCGTCCTTTCATAAACCCGGCT
TGGTCCGTGTGNATNAGCGGTNCGGCGACCTTCGANAGNCGNGTGGTNAGCGCGCGTCATTATCTTATAGT
CAGTGTTTAGNACNGTNATNGGNCNGTAATTGCTTATTTCCGTNCNNTCNCCCTTTTTGTATAGGGGGCACAT
CCACCCTTTCGGGAAGTCGTCGGGGTATNAGCCCATATCGCTCGATGTCATTGTANAGAGCTGTGAGGCATTT
TACAACGTCAAAGCGCTCCTCCTGGGGTNTTTGGCTCGTCTCGTATCGCGACACAAGCATTTTCCACAGTTCG
TGCGGTATNCCGTCGATNCCTGCCGCTTTTCCGTTCGGTAAGTCNATTATCGCCTTTTTAACTTCGGCCTCAG
TCAGGTANACTGCTAGAGATTGCTTATCTCTNTGCGTCATTCGGGGTTCNAGGCGGTCCAAGACTTCGTCGAC
TGCTTTCCGGNACGTTTTCTAGNCCCGTCTNCTGCAGTTTGTCGTGGTAGTTTCGTGCCANTTCCGCCATTTT
NTCGGATCGTTTCTCGTAGCCGTCTGGCCTTCTCGTTNTGTTCGTTGTCTGGTTTGCGCAGCATCGTTATTGT
GTCNCGCGCCTTCCGCTCCTTGTTCGCTCTGATCCANCCTTTGCAGAGAGTCTCGCTNTCNAGCCTGCACTTC
GCTTCNAGGTTGTCNCGGAGTTTCTTGTANCGCGTCGCTTCTAATTGCTTTATTCTTTCTTCAATTGCCGCTA
CGTTACACCGGATCTACCTCTCGGGNAGGTCGTTCGTTNAGGGCTTCCTTTCGTCGTTCTTTCAGTGCNTCCG
ATCTTNTTATCAAGCTTNGGGGTTTCAACTTTTGCGAGGTCTCGCGCGAATCGTGTNATNTCTTTCTTGAANT
CGGAGAANTGNNGTTGNATATTATGTGTTCTTCCGCCTTTTCTCGGGTTCGTTAGGTCNCCTAGTTCGTCTTG
NAGGTTTCCAGACTTCTCNTCTATCGCTTGTTTAGCTTTCCGGTTTTGNAGCAGGAACAGCGGNATCACCCAT
CGTCCTTTCCCTATGTAGGGTGTNCCGGGGTTTGTGAATTCCATTGACACTAGTTTATGGTCGGTTGCCACTA
CGGTGTCCTCTATCGACCANTTCCTGCTGTGCCGAAAGACTTCCGTGTTGCGTATATTCGGTCTATNCGNGAC
ATTGAGTNCGTACCGTTAACGTTTGANCTTTGTACGTAAGTGAAGTCTANTTGGTGGGGTTTGTTGTCCTCCA
ACCATCGCTTAANCCGTGANTTGCTTTAAAATNCAGTAAGCTCTNTACCGTTCCGGCGTCGGGGTGCGCGGGC
ATTCGATCAATCATNTCTTCGACCATATTGAAATCCCCNAGGAGTATATCCGGTGGTGGGTGGTTNTCGTTTC
CGTAAAGTNTTCGTTAAGTTTGTCCAAAAGGCGGCATTNTCANCGGNCGCATTCGGTGCGTANACAGCNAGGA
TGTTNACTCTGTGAGTTNTCTGCTTTCCACGGNACCGTTACATGGATNGCNCGTCCCGGGAATGAGTGTTATT
TGCGTNGCCTCATTCCATCGAGTTAGCTGTTTGTTNAGAATTANTGCNACTCCTTTCGCGTTCGGTTGGGTCG
GGTCTTGGGATTTNAGTATACGCATCGCTTGAATCGGTCATGTAGGTTTTCGACGTCGGCGTCTGTTAGGTGN
CCTTCCTGTACGGCGAGTACGGCGATTCTTTTCTCCTTAATNATNCGATTTACGTGTGACCATTTCTCCTGGT
TTGCNTCAGCGAGTGNCCTCCTCTTATGTTTAGAGTTGCTACTTTCAGGGCGGCTCTCGTAGTTTTTA
>Agrped1_rnd-1_family-163#TIR/MITE
ACACGGCGGGCATNAATATGTCAAATGCGGTTACGCGGTACGATGTCAAATGTTTGTCAAATGCAGTGATCGC
AATTAACCTTGCCGAGACCTGCCGTGGACGACTTCCNAGGAGCCATGGTCGGTTTCTACCCCCAGGCGATGTG
CCGACTCACGAAAAAGGTACATATAGTGCTGGTAGCATCTCATGGAAGTTACACCACCGGNACAAAATTGCCA
CTTGCATGTGTAGGGCGGACCACTTGCAACTGACGGACCNGTTAAAGCTCAACCCAGANTTTGGACCGGCAGA
CGNTAGAAAACCGTCGGAGCGNCCTGGNTGGAAAGTANCNATGGAAGNTTTCTTATATATAGGATGTATTTNG
TGCTAATAGACACGTTAAATACCATCTAGAGGTGATACTAAGTGGACGAAATTGAAAAACAGTGGAAAGAGCC
GCAATGCCGGTGCNAATGTTTGCTTTTGATTGCGATCACTGCATTTGACAAACATTTGACATCGTACCGCGTA
ACCGCATTTGACATATTGATGCCCGCCGTG
>Agrped1_Cluster5#LTR/Gypsy

TGTAAAGGGTACGGCTTCTATAACCGTACCCTTGTTTAATCTTATCTTATCTTGATATGCTCTTGTTTGGCAT
CGCCCGAAGGCGATGCCACTATAAAAATTGCTCTATATGTATTCGTGCCTAGTAGTTCTTTATCTTGTACTAT
CTAGGCTACCAATATACCTTGGTCTGGCTTATTTCGTGTGAGGTAATAAGAACCCGTAAAGCCCAGCTTTACA
TTTTTTTAAGCCATCCAAGTATTTGGAAACGCGAGTACCAAAAATACCACGGCAGAAGCTATGCCTTACGGCG
ACACACCACACGACGGCGATAGGGAGGGACAGTTGAACCCTCCCACGGTGCCAGGTCATTCTGCCACCACACC
ACAGTATATCCCCTTCCCGGACGTTGGAACTGGTACCAACGATGGTAATACTGGGAACACCTCCCAGACGACT
ACCACGGAAACCGACGGGGATACAGTTCACCAACACGACCGTAATGATCGTGATAGAACGAGATCCCCGCGGG
CATCGTCGACACCAAGTCGTCGAGACGACGAGGACCATACGGAGGACCGATTCATGTCCATGCTGGAGGAACG
ATCCGACCTCCAGGACGAATCAGATTTCCTCGACAATGACATCGATTTACGACTGGAGCCGATATGGAGGATG
CATGGGGTCAACGATATGGATCGCAGGGACATGATAAAGGAAAAAGCCAGGAAGTTCGTCATTGACGCAATGG
CTGAAGCAGGAATGGACTTACCTGCGTACACCGAACGATACACCGCGTTGATCGTCCATACCGTCCAAAAAAT
CGCGCTGCGATACCACGAGTACCAGGCGAGGAAAAGCACTAGTCGACCAAACACCCCGATGTCACACGTACTA
GCAACGACTGTGTCCGTTGTACCGACCATTAGAGGTGGCGAACCTGTCGTACCAACCGATGCACACCAAGGAC
CAACGAACCGAAGGGTAACAATCGGATCACCTCACATTGACGGTGCTAGAGCCCTGTTAGAGGCACCCCGCGC
CATCGAAGAGAACGACGATCAATATAATCGGCGCCGCGCGGCGGCACTGAGATTGTCCTCGATGGAGGGTCAG
CATGCCCCTAAGGTGGAACGATCCGATCCACCTATACCCCCACCATCATCGTCTGACTCGATCTCAACGAGAT
TGGGCAATCACCCAAATGTCACCGTTCGCGACAAGCGTGAATGGACAGACCGGATTGCGCGGCAAAAAATGCG
AGACGAGGACTTAAGGACCTCGGGCACGTCAATGATAGAAGACCAAGGGGTCAGGTTTGAGGGACGTCGTGCC
TACGAAGTACCCATACCAGAGACCACGATGGCACAAGGCGCACAAGCCCAGCGACCTAAGGTCGAAGTGGGAG
CGACGCGCATCACTAACCCACGCCAACCACAAATTGGAGTAGGTGGTGTCGCACCCGTTGCACGAGAACGCGG
GACTACACCCATGTACGATAAACACCCACGAGGATTTTCAATGCCTCGTGTACAACCGCACTCCTCAGATTCC
CCACCTGGTGTGATAGAGGCATCTAAGAAACACAAGGCGGAGATGGAGGAGAGGCTCAGTAGATTAATACGCG
AGACCTTAGATACTCGCATTGTATATCCTGACGGATTTAAATACAGCCAAAAGATGGACTCTGGGCCATCCGA
TAAATATAACGGTTCGTCCAAGTACTCCGTTTTAGAAGACTGGTTGTCGGCATTATGTTATAGATTCGCAGTG
CGACAGCTAGGAGGACCTGAACTTGACCGCGCCCGAACCATGATAGTCATGGAATATCTCGGAGGCGAAGCGC
TCAAGTGGTACCTCCGACATGTCACCAGCGTCCGTCGAGCCAAGTGGGACTGGACGTTCGAGTCAGTTGTCCT
GGGACTCTATACCCGTTTCGTTCACCCGTCCACGATGCAGGACGCAAGAGAAGGGTTCAAGAATGCCAAATAC
GACGCCGCGACCGGCGTTCAAGGATTCTACGATGTGCTGTTGGATTATGCACAAAGCATGGCGGTATATCCTG
ACGAGTATTCTATCCTCGAACAATTCATAGAGGGTATACCCGACGCCATGCGCGATCATCTCATTCGCGAAAA
TGGCTTATCGCCCGAAGTGAATGACATAGATGATTTTGTTTCCTGGGCAATTCAATATGAAGAGCGCGAAAAA
ACTGGAGACTATTACAAACGACGGAAAGCTGCACGCACCGGCGGTACCAGCAGCACCTCCAACTCACGCCCCG
GCGATAACGCGAGGCGCGTGAAACCTGGTACAAAATTACTGGTACTTCGGTCTAGTATCCAGCATGGAAACGC
TACGCCATCGAAGGTGGTTCCTGAACCCACCCAGAAACCAGCCTTGCCTCGACGAGATACTTATAAGTCTCAA
CCAGGTCCACCGCGAGGATCACCAGGCACCTGCTTTAATTGTGGGAAAAGTGGACACTTCGCTCGCGAATGCC
CGTCACCGGCACGCGACAGAAAGGTTCACGTCAGGGCGGCCCACACGGCTGTACCCGACGAGGGATCTGGGGA
TGAACCCGATGAGGACGCGAACGGCGCTGCCTCCGAAGGTGATGAATCCGCCAACGAGTCAGATGGCGAGGCC
TATACGGAAATCGAAGTGGGCGAGGATGACTGCTATGTATATGATTCGGACTCTCCATCCGACTTCATGCGAG
CAATGACGGAGTTACCTATGTCTGAAAAAGACGATGCAACACCTGCACCGCAAATGCGTAAGGTCAAACTACG
CACCACCAAGCAGAGCCGCACGCGCCCTCAGGTTCCACCTGAGGAAAAAGAGTGCTTGGCGTCTTATGTCGAG
ATTGGCGGTGTAAAGGCGTGGACGCTGTGGGATTCGGGCAGCACAACTTCTGGAATCACACCCACGTTCGCCC
AGATCGCGGACCTACCCGTTGACACCCTACTGGACCCTCATATCCTCCAGTTGGGCACTGTCGGCTCACGATC
CATCATTAAATATGGGCTCAATGCAGCAATAAAGCTTTCAGAACAGAAGACACTGCCTGATGTCTACCTTGAC
GTCGCGAACTTTGATCGCTACGACATGATCATCGGTACGCCATTCATGCACCGCTTCAAAGCCGTTCTCGATT
TCGAGCGCGGAGAAGTACGTGTTGCTGGTGTCCGTATACCTGCCATACGTGTACCTGCAGACCTTGATTCCAG
AATCCATCGCCATAGGCTGACGGACAAGGGTAACAAGGAACAAACGCCCAAGTGACTATCGACGCCCCGGGTC
TCCCGAAGAACAACGATGGAAAACGTTGACGAGCAGGAGGCCCAGCATCGGGGCGAGACGTTGCCGTCGGATG
GTCGATATTCACTCGAGCAGATAGATGGCCTGATAGAGGACCCTACTGTTCCTCTGAAAGGGGGGGAAACGAA
GAGAAACAATCGGAAAAGTACAAAAACACCAAACGCGGATGGACCTAAAGATCCATCTGACCAAAATCCCAGT
AAGAAAAAAGTGACTCCGACATCAAATAGAGCAACCGTTGGAGACAACAGCTCATACTGCCAACCTACCTCAT
CGAAATTCACGATGGAGGACCTAATTAGGGACGATGAATCATCACGCAAACATGATGATCCTCCCTTGAAGAA
GAAACCGAGATTCTGGGACACTCCCATTCCTCCAACAAACATAAGTTGGACCTATGACCCACGAATGCCACGC
GAATGGAACGAAGTAGTGGCCAGCATGCAGGCACAACGGTGGACACAGACACGAGATACCACCGAAAGATACA
GGCAGCCCGACGACGACGAAATATCCGAGGCCGTAGCATTCGAACCTCGACCAACACAACCTCGCCCGTCGTC
TAAACTACCGCCTGTAAGACTCCAACCCAAAGACAGCTCCGAGGTGGCCATAGAATCACTGAAACAAAGATTC
AAGCCTAAGGCAAGACCGCCTGGAGACACGATTCACATCCATAAACCCGACAAGGGAAAGAGAAAGGCCACCG
GGCTCGATTGGGACGACGTACCCAGGCTGAGACAACAATGGCATGACGAATTCATTGACATGGTACAAGGCAC
ACGGAACGAACTTCCCCCGTTCCGAGAGGTCAACCATGAGATTCATCTCATTGACGACAATAAACGGTACACG
TACCACCTACCGCGATGCCCGCTATCCTTACGAGAGGAATTTTATGCGAAACTCAATCGATACTTGGATGCTG

GTTGGTGGAAGGCACAAGCTGCGTCACAAGCAGCACCGCTAATGTGCATTCCCAAGAAAGACGGTCACCTTCG
AACTGTGGTCGACGCCCGCCAACGAAACGATAACACTGTCAAGGACGTTACACCCTTGCCAGACCAAGAAGTT
ATTCGTGAGGATGTTGCACGGGCCAAGATCAGGTCCAAGATCGATTTGGCAGACGCCTACGAGCAGGTTAGGG
TACGCCCCGAGGATGTACATAAAACCGCATTTGCTACGATTTCGGGCACATATGTCAGCAATGTCATCCAACA
AGGGGACTGCAACGCACCCGCTACGTTCCAGAGGCTGATGACCAGCATCTTCCGCAATCACATCGGCCGATTC
ATGCACGTATATCTGGACGACATCTTCATATTCTCGAACTCTATCGAGGAACACGAAGAGCATCTCAGAATTG
TGTTCGAAAAATTGCGGGAAAGTCAACTGTACCTCAAATGGTCCAAATGCGAACTATATGCCAAAAGTGTGGA
CTGCTTGGGCCACATAATAGACGACAGGGGAATACACCCTGACGCGGATAAAATGGACCGTATTCGTGAATGG
CGAGTACCACGCAACTACAACGATATTCAGAAGTTCGTTGGCTTGGTCAACTACGTAGGAAACTTCCTACCGA
ATATCACGGCATACACCGGACCACTACTAGCGATGACTCAAAACGGAGCACCGTTTTTTTGGAGGCCACTGCA
TCAGCAATGTTTTGACATGATCAAACGCATTTGCTGCAAAACGCCAGTCATTCGACCAATCGACCCTGCAAAA
TCCGAGCCCATCTGGCTCATTTGTGACGCGTCTAAGACTGGTGTTGGAGCAATGTATGGGCAAGGGCCCGCCT
GGAATAACTGCCAGCCCGCAGGTTTCATGTCAAAGAAGTTCACCAGTGCACAGCAAAATTACGCTGTGCATGA
ACTTGACTCAGACACTGGCAATTCTCGAAGCAATGCTCAAGTGGGAAGATAAGCTAGTGGGACAAAAGATCCA
CGTAATCACAGACCATAAAGCTCTCGAATTCTTTAACACGCAGTCGAACCTATCCCACCGTCAACGACGATGG
ACCGACTACATGGCGCGATTTGACTTCGATATCACATATGTGAAAGGCGAGTTCAACAAAATCGCAGACTGCC
TATCTCGATACTACGAGAGCGACACTTCGGCAGACGTTCATGAACCACACGACTACGTCCAGGCTGACGCACG
GATAGACCCGGACGGAGAAGATTTGCCTGGCGAACGTTATCGAGAGGTCAAAGAACACGTCGTCGAGATCCGC
GCGATGCAGGCCACTGAGTTGCGACGGAGCCGCAGACTACAGGAGGCACAAGAAGAACGGGAAAAGGAGGCTG
CACTATTGCAATCTCCAGACCACACAGCGCCATCTAAGGACAGTGACCTTCCACCCCATACCGCTGAGGATCA
CGACATTACGCTAGCAGAAGCCCTCGAGGATGGGAAGTTACCGACACATCCTGCACCAGTGCAGGATGATGAT
TTTCTCCAATCAATCCGACAGGCATATGGGGACGACAATACCTTCGCGACCATACTCGCGCAACCATTGCAGC
ACCGGTCCTTCGAAGTGAAAGAGGGTATCATTTACTTTACAAACTCGAAGGGTGTAACCGTTACTTGTGTACC
GCGGGAACGAGCACTCATTACTACCATAATCACCAGCGCCCATGATATTGTGGGACACTTTGGTGATCAACGT
ACGACAGAATACGTACGCAGATGGTTTTGGTGGCCTGGTATGGTCAAAGACATAACAGTGTTCTGCCAAACAT
GTGAATTATGCCAACGATCAAAACCTTCCAACAAGAAGCCTAGCGGGTACCTCCACTCTCTGCCGGTCCCAAC
AAAACCCTGGGACTCTATTGGAATGGACTTTGTAGGCCCATTTCCGGAGTCGCGTGGGTATAACTACCTATGG
GTAATTATCTGCCGCATGACCAGTATGGTTCACCTTATCCCAGTCCACACATCTTGGACGGCGTCAAAACTCT
CATGGATTTACATGCGCGAGATAGTCCGGCTACATGGATTACCTTCATCCATTGTAAGTGACCGAGACTCTAA
GTTTACCTCACGCTGGTGGAAGGAGCTCCACGCTATCCTTGGTTCCAAATTGCTCATGTCGACCTCGTTCCAT
CCACAAACGGACGGACAGACGGAACGGGCCAACCGTAATGTTGGACAGATATTCCGTACGGTGGTACGTCATG
ACCAGAAGGACTGGGTCGACCGTGTCGACATGACCGAGTTTGCAATCAATTCGAGCATCTCCGAAACTACTCG
ATATGCACCTTTCGAGCTAAATGGGGGTTATATGCCATCCATGATAAAGGAAATCCGATCAGACACCGTCGTG
TCCCAGGGCATAAAGTCCTTTGCGAACAAAGCCCTGCTTCATCTGGCTGATGCTCACGACGCAATCATTGAGG
CTCGCGTACGGCAGACAACCGCGGCTAACAAGCACCGCGGTGCTGAGATCCTGTTCACAGAGGGTACACTGGT
ATACTTATCCACAAAGAACCTCAATTTACCCAAAGGGCGCGCTCGAAAACTATGCCCTAAATTCATTGGGCCT
TACAAGGTCCTACGGGCACACCCTGAAACATCTACGTACACGCTGGAACTACCTCCAGCTCTGGTAAAACGTA
GGATCCACCCTACATTCCACGCATCTCTGTTACGCGAATACCACGCCTCCAGCGATGCACTGTTCCCTAACCG
AGCCCAACCAGAACCCTACGACTTCGGCATATCTGACGACCATGAATGGTTCGTGGATGATATTATTGGCCAC
CGTTGGACCGGTAACACAGTGCACACGCTGGAATTCGAGGTCCGATGGAGCCTTGGAGACACCACCTGGGAAC
CCTATGCCACCTGCAAGGATTTAGCAGCCCTTGACAGGTACTTACAAATCCATGGGGCCAAACGACCCTCGTC
TCTGCCGCGACGCGACGGTAACGTGACCACACAGTGATGGGTCGGAGCCTATCGCTTGTTTCAAACTCCGACC
CAACACCCAACATTTTTTCTTATCGCCTCTCGTACCACGTTCCTTCTTCTACATGTCTCGCTCACAACGCAAC
ACCCGTGAATGGAGCCCTCCGCATCGACGACGCGGCCCTTCACAGCCGCGGTGGTCGTCTGGAGGGGGGGGGA
ATCGATCTCACCCACCTATGCAACAGGACAGCAGACATCGCACGAGCCACCACACACACGCCAACGCAAATGT
AGGATCACGGAGCTGGGCCGATGACGACCCAGCCACCTACACCGCAGAAGGCTACCCGTCCACGATGGACGAA
GACGAGTACATGGCCCCTCCCCCCCACGAGGAGCCGAGATACCCAAAACCCACCCGAAGAGACGACGATTCGG
CGAGAGGATCGACGGACCAGAAGCCGTCGCACCCAAAGGATAAGGGAAAAGGAAAGGAGAAAGAGAGGAAATG
GGAATCGGCGGGACAGAGTTCCCTCCAACCCAGCATCAAAGACCGCCTCCTGACGGTCGGAATAGAGGAGGTG
CTCCTCAACGTACACCTGGACGAGGACGCGAGCATGATCGTCGAAGATCTGCTCCTACGTCTCCAGGCATCCC
AGGCTGAGGCCGAAAATTTACGAGCTCGAAACGAGCTTCTCACCAACCAGCTCAACCGCACCAGGAAGAGGCC
GGCCTTGTCAGACAGCGACGAGGACAGAAGGCCACCTCCACCCAAAAAGGCGGCTCCAGTAACAACCATGAGC
AGGACAACGGTACCTGCTCGCAACATCCCCACCAACCACGACGAACCTGCTCGCCGTTGGCCTGAACCGGCCC
AGGGCAGCTCGTCGACGAGAACCATCGCACCTACACCGACATCGTCGGGATATCGTGGTGCAGATCGTGGACC
AGAGCACACACCCGCCAAAACCACGACTACCGCACCACCGATTACCCAAACTGGTCGGCGCATGGCTGAACCG
ACTGTCTTTCGTCAGAGAAAGACCATCACCAAGGTCGCTGAACAGCCAGCACCAGCACCGGCCACGGTAACCG
TGACGGTCCCAGGACCACTCCGCCCATCGCCCCGCCGACAACTCGACCCTTCTCCGGATGACCCGAACCCGCC
TGGCTACAGGGGTGAGGAATTGGGGGAAGGGTTCACCGAGGACGAGGGAGATTCGGATAGCGAGTGGTCCCTA

GGACCCGTCGAAGAGGACGGTAGCAGACGAAGTGCACAGACGTTGGGGGAACGACGAGCCCACAACGCAAAAA
TCGCGAAAGCTAAGGCAGCCAGGATAAGAGCCACAGAGGATGGACGCCGACAGGCCTACCGCGAGTACAACGG
TCGGATCCCTGATATTTGGGGCGTCGTCGATACCCCTCGGTTCGGGACGCAGCGCAACAACTCGTTTAACGGG
ATGTTGACTCGACGCGCGTACTACTCCCAACGGTCGAATGTGGTGTACGTCCGAGAGACCGCCCTGGCTGCCA
GGGAACACGAGCAGAACCACAAATCGGCCTACACCCCCCCAGACTCGCACCCTATCTACCAACGCTGCCCCTT
TGGCGTCCCGCGACACCCAGCAGACGTGGATCGACTTTTATCGATCCTCCACGACAAGAAGGTACCTACGTGG
GCTAGAGGGGAAGCCCACGAGCTCCTATTCGAGTTCCGCTACATCGCGCAGCGAACAGAAACTACATCCATGG
ACCGCGCCATGGAATACGCATTCACCGTCTCCCGCGATGAGCTAGAGGCAGACCTGCCAGAGCGGGATCTGAC
CGTCATAAACATTCCGCGAAATACCTCTAGCATCATAACGCGACAGGCTGAATCGTCGTCGAGAGGCGAGGGC
ATAGGCATACCCATGCCCTCTCGCGAACACTACCTCCACGTCGACGAGGTGGCGCGATATGCGCTCATGTACG
GCAGGCCGGGAGCCAAAAACCGCATCCTAGGCATCGCCATCGACTTCACTTATCGCGTATATCGTCCCACCGC
CTTCGGCTTTGGCCTGTCTCGCCTACTATCACCAGCGGATGCGAACGCACGCGTCGCGTTCGTACGATTGTTT
GCACAGCTATGTGCATCCATCAATCGGTATCGCGAAGCAGTCGAAGACTATGACCGCACACATCCGTCATCAC
CGTTCGTGCCACAAACTGGCCCGACCGTGACCATCACCCGTAACTGCATGGACCCTAACCATGTAAAAAATAT
GTCGATACAAGACGTCATCGACACGCTAATCCATAACTGTATACCCGTCGAGTGGATTGATCATTGCTATACC
TACGGGTACCAGTATTTGAACCAACGATTCAACGGTTCGAAACTGGATATTGACGTCCTGGCACCTGTCGATG
ATGATCGACTCGCCCGGCTCGCCAAGTTTGGCGTACCACCCACGATTCCAGCATGGGATGGATGGTGGACACC
GTCTAGCGACGACGTGCTCCGGGTGACCACAATCATGGCCCAGGAAGAGGATAGGATTCCGCCCATTCTCTCT
CTCGAGGCTCGGGGCTGGTTGCTCGTGGGTGAAAACCCCGTGCAGCGATACCTTGCCATGCGCACCCCGGAGG
ACGACGACACGACTGATAGGGAAATGATTGATGCTGTGGACCCAGACACCACAGACCTTCCCTCTGCTAACGC
GGAGGATGTATCCATGATCATTGACAATGCGTCAGGCGACACTGCTAACGCGGCGGGCGCCTCGATCCCATCT
ACACCAGTCGCGGCTCGTACGGACAATCCAACCACTCCTCCTAGTGCTTGATGAGACCAGCATGGACGCTGGT
CTGAAAGGGGGGTATGTAAAGGGTACGGCTTCTATAACCGTACCCTTGTTTAATCTTATCTTATCTTGATATG
CTCTTGTTTGGCATCGCCCTCAGGCGATGCCACTATAAAAATTGCTCTATATGTATTCGTGCCTAGTAGTTCT
TTATCTTGTACTATCTAGGCTACCAATATACCTTGGTCTGGCTTATTTCGTGTGAGGTAATAAGAACCCGTAA
AGCCCAGCTTTACA
>Agrped1_rnd-4_family-260#LINE/I
AGCAGAAGTATTCAGACTTGTTGTGTTCGAGAACAAGCCCTATGGCAGTAAAGAGGCGGAGGAGAACTGCATA
GGCCGCTATAAGCGTCTCTGCGTTCTCCCCAAGGGTCTTCGATTGGGCAATACAAGTGCCATCGTCGACGTAC
GATAGAAGAGACGTCTTGCGAGATATCTCTTCCATGTGGTAGAGTTTCATCACCGGAGCGATGTATAGGGCTG
ACAGGACAGGGGANAGGGCAGAGCCCTGCCCTACTCCNACGTCTGCCTGACGTGGGTCCGATGTGAACGAGCC
CCACTTATAAGAAGTGTGCCTGTCCACNAGGTATGAGGAGAAGAAGTTCGCGACCATAGGCGAGAACCCCTGT
TTCCGAAGAATGGCCAAGAGCATGTCGTGGTTAAGAGANGGGAAGAATTGTGCGATGTCGAATGCCACCACAC
TTGTCTTAAGTCCCTTGGCCCACCCAGCGCGAACTAGGTGAGTTAGGTACANCCCTGCGTCCTCGGTGGAGCG
CTGGCGCACTCCGCCTACTTGGTTTGGGTGAACAAGATCGTATTTGATCATGTCGAATTGGAGACGATTTGAA
ATCATCTTCTCAATAAGCTTCCCGGTAGTGTTCAGCAGCACAATGGGCCGGAACGCTTTCGGGTCNGAGTAGG
TAGGTTTGCCGGGTTTCGGTATGATGACCGAGACTGACTCCTTGAAATGTTTAGGCCAATGCCCCACNCGGAT
GCAGGCGTTGGCCAAGTCGATCGGAGGTTTTGCTCAGCGNCGGGCAGCCTCACAACTTCCTTCAATAGCGACC
ATGTAATGTGGTCTGGGCCAGGCGATGAACGGCTGGAACAAGACTCGAGNGCCTCAGNGAGCTCGAGTGAAGA
GAACGGAGGCCACTCCCTCTCTATTAGGTTTGGGAGTGGGTCCAGAACTGACATATCTACCACTCGGCCGGAG
GCCGAGTTATATGTGTTGTGGAGTGCATTCCAAAGGGAATCCATTCCATGGCAGGGCTGTCCTTGATATCGGA
TAGCCTCGCATGCTGGTAACTTGCGCTGTTTGACCCACTCCATTAGGTCCCATGGGCGCCTGTTAGCGTATGC
GATTTCTCCGATTCTAGTTTCGAAGAATTCGCGTTTGACACGTTTGACNGTGTCTCGAAAGAGATGCCAATTC
ACATCAGATTTATCGTTCCGATATGTGGCAAGNTTNGCGGAGCACTCNTCATTCCACCACGGTTGAGAGTGCT
TAGTGATTCGGGATACCTTGGAGTGGGCTATCCACGCCTCGGCATAGGCATCGGCGAGGGCTTGCGCCACCCG
CTCAACGTCTTCCCTGGTTTCAAGGTCGCCGACTTGAATGTCAGCGACCTTCTGNGGAAGTTCGGTCCTCATG
GCCTGTTCGGCTTCTGAGTCCCGAGGGATAGTGCGCCTTGTGACGGGTTCAATCTCGTCATCCAGCTTGACAA
CGGTGAAGATCGGAATATGATCCGATGGTCCCTGTCGGACCGGGTCGCGGAAGGTGCAGTCACTTGCTGACTC
AGCGATCCCGTGGAAGACCAGATCTATGACCGAGGGCCGAAGGTCTGGATTGTGAGATATAAATGTAGGGCCT
GGATTGGACGGTTGGGTCCGTTCTAGGTCCAGGTCTGCTGCTAATTCCAGCAGGTTTATGGCATCCCCTCTGT
GGTGCGTGACCTCTGGGTCCCACTCCCGAGAGTGAATGTTAAAATCGCCACCCATGTAATGAAGTTGGGGTAG
CTCGTGAACGTGGTTCGATAGGTATCGAACCGCAGTACCATCAGCGTCATTGTAGACGTTCATGAGGTGATAG
GTCTCGTTGCCCTGAAACAGCGAGAGAATAAATATGTCTCGGTGATCTGCGAGATCCCGGCGCATCGAGGGAC
GAAGCCGTTCGAGT
>Agrped1_rnd-1_family-604#TIR/MITE
CACGGCGGGCATATAGCTGACCACTCGCGTCTTACGTGAGCCTGACCAATCATATGGCCCTTGATATGACGCG
TAACTGACCAGTCGGTAATGCGTGACAGTTAAGTCACGCCTCCTTTTCGACTTGCGTCGTTAATCGCCCTTTT
GCGCATTCCCTTTTTGTTAATACCGACTATCTCGTCGCTCTGGGTAGTTATATGCCTCNTGAGAGGTTTTAGT

GGTGTCTACAAGTGATGAAGTCGCTGAGACGAAGCCAGACATAAAGACTTGTCAAGGAAGAGGGTAATAAACA
TGGAGCGCGCCCTCGGCATCAGTTCCGATGCCGGGCACAAAATCAAATTGGTGCCGGAAGAGTGACCACGCAG
CCAACACGCAGTCGCACGCCTACGGGTTGGATAAATTATACGTTCTACGTTATTACATGGCGGGTAGTGTCTA
CAAGTCGTCTGGCTGTTTTACGAAGACTTTTAAAAAGAATATCGCCATCCATTCGTGTTAAACAAATGCATCT
CGGCACAGTNTTTGACTTGGGACCGAACGCGCGGCTCGTCACGCTGTCACGAATCAGTAGTTGACACATGACT
GGTCACTTACCGGCCAAACCACCGGTCAGGCCCATCCGGAGGCCGAGTGGTCAGGTAAATGCCCGCCGTGT
>Agrped1_rnd-1_family-67#TIR/MITE
TAGTGATGTGCCGGCTCGGCCTGAAAAGCCCAGGCCGAGCCGAGCTGACCCTTCCAGGCCGAGGCCGAGCCGA
GCCGAGCGGTGGGCTTTAGTCGGCCCAGGGCTCGGGCCTCAATTTTGCGAGGCCCGAAGCCGNAGCCCAGGCT
ACGGCCTGCGCCTACCCGATTTGGAAGGTGGCTGTACGCNAAATTCGGCACTCGGCGGGTGAAATATAGGTAG
ATAAATGTTTATATCGTTATTCTATACTACCTAGTTAGTTAAAATATCAATTATTTCGCGTTTACCAAGCGTC
ATCCGAATGTCCGCTGAGCCATCCATCTAATCACGTGGCAAAAATGGCTCGCTACCACCTTCTATGTNGAGGA
AATGGCTGTAAATAGCGAAAGGTGTTTTTATTTCATTGAAAATGGTATTCTACGTGACGTGGTAGTCAATATA
CAGTGCCGCCGACCNTTTCTGGCGATGTTTGCACATGCTTGGCTGNACCATCTGCCAGGCCGCGGCCTGAGCC
GAAGCCGAGCCGAGCCGGGCCGACTTTTGGGCCTGCAGCTCGGGCCTGTGATTTGGTAGGCCCGAGCTGCCGA
AAGCCGGGCTNAAGCCGTAGCTTTCAGGCCGAGCCGAGCCGGCACATCACTA
>Agrped1_rnd-1_family-208#TIR/MITE
AATAGGGGGGGTCAAAGTTGCGGGTTCGAGTCCCGGTTCGAGTTTCGGTTCTCAGAACCGAATCGCAGTTTCG
AACGGTTTCGACTCCGGAGTCGNAACTGCACGAAACCCGGTTCTGTAGCTTTCCCACGGTTCTCTCAGAAACC
GGTTCTGACCATTTTTCGTCGAGAACCGTTGCACGAACCGGGACTCGAACCGACAACTTTGACCCCCCCTAAT
>Agrped1_rnd-4_family-503#LTR/Copia
CCCCCATCAGGAGAAAGAAACNAGGCAAGAAACAGNAATTAAAGAAAAGAAAAAAAGTAACTAGAGTGNCCAT
GAGAGCCGTTNAAAAAACAAAAAAACAAAAACGGAAAAAAGAAATCAAGAAGAGCATACTGAGGCGGNACCGT
NAAACCCAAAACATCNCGGTGGCGAGAGAAAGTAACGTGNGAAAGCGACTTTGTGAAAATGTCGGCAGGCATG
TCATGAGTCGGAATCCAAGTAGTCGAAAAAGANCCCTCCTTGATATGTTCGCGAANGAAATGATGNCGGATGT
CGATGTGTTTNGAGCGTCGCGATATNGAAGGAGANTCCGAAAGAGCACAAGCGGCTTGATTATCGCAGAGAAT
NGGGAAAGGGCGNGGGACAGGGAAGTCCATTGAACGAAGGAANACGCAGAACCANAGNGCCTCCTTGAACGCG
TGNGACATNGCGTAATATTCAGCTTCCGTCGATGATAGTGCAATNGACTTCTGCTTGACCGCCGACCANGANA
CCAGNGAGCCCATATAAAAGAANGCGTACCCNGAAATACTCTTCCTATCTNTCGCATCNGAAGCCCAGTCGGC
ATCGGTGCANCCATTAGCACGCATGAANCCNGCCANCGATTCGGGCGTGGAACGAGAGCAGCTCCCAAGGTCA
ANGCGAGATGTCTNGTNCCAGCCAAGTAGCGCAGGACATGCTTCGCCAGACGAAAATGNGNGGGAAGGGGTGC
NGAGCTGNACTGNCCNAGCCACATGGACGCGAAAGCAATGTCGGGCCTAGTNGCAACAGCGAGATACATCAGG
CAACCGACNAGGCGCTGATATCGTTGAGTNAGATCNGCGGCGGGGAAGTCCTTGATGGGCTCGTCAGCGTCAN
GTGGNGGCTGNATGGGCAAAGGNGTCTTNGCNGTTTTGCATCGACGANAGATTCCACTCGTCGAGCAGTTCAG
CGACGTAGACATGGGAAGACAGCCACAGNCGACGNCCGGGNCGATCGCGAATAATCAGCATGCTNAGGAACTT
GGCGCANTCACCGAGGTCGACGATNTGNAGACGNGNNGACAGGGACTTGATGAACCAGTCGTACAGAGGGACC
GAGTTNGTNACNCCNAGGCCGTCGTCGACGTGNAGCGGGACAATNAGAGNAGNGGTGANCCGTCAGCGGGCAT
NGGAATGGNAGGGTCCGGNGGAGAAGTCCAACGTCCAANGAAGACGCCGTGGTCAGCATCGCAGCGNGTNAGN
CCGAANTCGAGGAANAACGACGAGAGGAANGTGTAGAACTCGTANGCNGATTGACGAAGGCCATANAGCGCCA
CNCGNATNCGNAGGACNTTACCAGGNGTGCGNACNTTCCAGCCAGGAAAAGGNCGAGCGTANACNGAGTGACG
NAGCTTCGCGTGCAAAAANGCNGTCTTGCAGTCGAATTGACGAATTTCCAAGTCGTCGGCNGCAGCANAGGCC
AGNATGGTNCGAACACTGGCAATTTCGAGCCACAGGNGCGTAGGTTTCGTCGAACTGGGANGGNCGCTGACTG
AATCCTTGNGCNACNACACGCGCTTTCTCCTTCCCNGCAATNCGAACNCCCTCGGCATCCGTCTTCANCTGGA
AAACCCACTTCAANCCNACNGTCGGCTCNCCAGGNGGNAGATCGGCTTCCTCGAAAGCGTNCATCCCATCAGG
NCCAAGACTATCCATCTCNCGCCGCATGGCNGCNANCCAAGAAGCAGCGTCAGGCCTGGCGACAGCCTCNGCG
TANGANGANGGCGCCTTCGANAGATCCCAAGATTTGGAGCTNGACTTNGCATNGGCGACGAAGGCAATGGAAG
ACGGGCGTGTCGCGGAAGAGCCGGCGGGAGGTGGANAGTCCCAAAGCAAAGAGGGCTCATGTCNNGCAANGTN
TCGNANTCGACATGAAANNCGTCCGTCGAGAGAGGNAAGTCNGCNAACGCAGCGCAAGACGCCAGTAAGAACT
CAGGGNCAGGCCCGCACATCAACATCCTCCGCCCGCACAACAGCNCCAGAAGAACATCCGCCCGCGCACACCG
CCATCCACGCACTCATCCCCGNCCGCCAGAACATCCGCCCGCGCACGCAATGAACCATCCGCCCGCACACACA
TATCCTGCCACACCCAACATCCACTCCCCAAATCACCAATCACCCCCATCCGCAGCACNGTCAAACTATCCNG
CAGCAGTGAGGCGTTCGTACGAGTAGACGCTNTCGTCGCAAGGCGAGAAGTTCGTCGNANGCACGACCAGCCT
CTGTNCGATTNCGAGCACGCTGTGGAGGNACGACAGCCACGTTTTCGGGCGAANCCGGAGCAGNAGGCGGAGC
AGGATCGCGGTGAACAGNACGAGGAATAGCGAANCGNCCGGACATATCCTCGTTGAAAATGACATCGCGAGAG
AAAGCATACGTGCCGTTGGGCCTCCGAACACGCCAGCCNACACGATGTTCATCGTAACCNACAAAGATGGCCT
CGTACCNNGCCAAGTCACCCTTCTTTCGCAACTCCTTCGGCACGNAGACAAAGCATTGACACCCCCANACACG
CAGATGGGANAGATCAGGCTTCTTGCCNTCGTTCATGACCTCGAAAGGAGTCGTGTCGGCAGGAAGAGTAGAA
GTNGGTTCNCGGTTNGCCAGGTANACGCTCGTGTCGAAACCGTCCGCGTAGAAAGCCATGGACAGACCAGACG

CNGCCAGCAGCGACTGAGTGCGCTCCTCNACGGTGCGAATANAACGCTCAGCTTTNCCGTTCTGAGAGTGNGC
GTANGGAGCNGTTTTCTGNACGGCAATGCCCTGATCGTCCAACCACGTACCNAGNGCACCCTTTATGAACTCC
AGGGCATTGTCGCAGCGNAAGGNGAGGACCGGAAGACCAGTNACGCGAACGAGANAGGCGACGATGCGCTTGA
CGAACTCAGCGACCTCCGNCTTCTTCGNCATAGCCNNCTTGAAGCCCCAGTTNGAGCAATCATCGAGGAGATG
ACAATGAACATNTCGCCGGTGAAGGACATCAGGCAAANGGACCGCANCCGTCCATGTGNAGNAGTTCNCCNAC
GCGNGAAGCGCGATTACCCGGGGTCGCATANGGNCGCTGAGGAGCNTTNCCAATGATGCAAGCGACACANTTA
CCGTAGGAAAGCGTACCGGAGTGCTCAATNCCAGTAGCANAGTCCTTNGTAAGCAAAGCACGAGTAGCATCCT
GCCCAAGATGCCCAAAGCGNCGGTGCCANAGCTCCGGNGTCAAGNTAAGACGNGGAAACGNNGTCTGACCGAC
AGGNACAGCAGGCAAGGCAGACGCAGAAGTCGGAGAAGCAGGGCGAACGAAATCGAGCTTCATGAANGACAGG
CGATGATGCTGATCAGCAGTCATGGCATANCCCGGCAGGGAACGATGAAAGCGATCGAANGTCAATGNCGTGA
CAGGNTTNNCCGAGTCGAAGAAATGACATCAATNCCCTTCTCAAGAAACGCACCAACGGAGAGGAGACCAATA
GGAACATCTGGGACCCAGAAGCATTCCGTCAACTNCATNGTGATAGTACACCCCTTGTACGTGTAGCGGAAGG
ANACGGCGCCNGAACCCCTTGAGCGCATAGTACCACAGTTCGCAGTACCAATNTCGACGTTGTCCCCCTCGTT
CAGGTCGTCGAATAGCGANCGATCCGAGAAAANATGGTGGGTGCAACCGGAATCCAGNAGNGCGTTGTACTGT
GCAGTCAAGGAGACATACGCAGCGTAAGGAATATCACGCAAGGTAGGAGCCGAAACGAAGGCAACAACTTAAG
ACCCGAAGAGTAAAGGGAGAATTCGGNGATNGGATTAGGTGGCACGTAGGTAGAGGTAGGGATACAGAAGGCA
GCTACAGGAGGTACGGANGTATCGGACGTATCAAAAGTAGNAGGGCTATCNGGNCCGTAAGGNGAAGAGGGCG
CAGAGGCAGGAGGATANTGAGAGCGATCACCATCCTCGTCGAATGAGGCAATTGTCTGCAACAATCCTTCCNT
AAACTCCGCNANAAAAGCACGCGCGGTCGACTTCTTCTTCTCCTGACCACCCCCAGGCTTGNAGCATGTNGCG
ATGGTGTGCCCGACAATGTTGCAGTTCGAGCAAGTAGGACGAGCCGTCGCGATCGGAGGAGGCGGTCGCAGCC
GTGGACGTAGGAGCAGCAGCGGCAGAGGAACGCTGAGCAGCATGCACGGAATAGAGGTGACGGGCGCGAGTAG
CGGCGAGGGNACGTTGGAATGCGGAAGGTTTGAAAGCATTGACAGCCTTGAGNATGGCGCGGCACACGGCGTA
GAANNCAGCGATATCGTCCATGCGCATGNACTTGAGNCGGTCGTTCAAAGAGTTACGAAGGCGGCTAAAGGTG
GGACCNGAAGGNAGACCGCGAACCATGCGGTTGACATGNGTGCGGACGTCNCCAGCCTTGCCGTTCGAATACG
CCGNCGTAACGGCNGAGTTCCANCGNGCGACGTATTCGTGGACCTTGTTAGGNTCGCAAGGGGACAACGACGC
GGCGTCNAGCGNCTCAGCGCATTCCTCGAAATCGCGAGCNCCATAGAACTGAGCNAGCTTGTCGTAGAGCANG
AANGCGGTGCGNAGATGCCNGGTGCGTTGGCAGGAGGNAGGAGAGAACGTGCTTCATCACCCAGACGNCCACG
TAGGACATAACAGACNGTCGCGTCATCGNAAGACCAACGGCGNGCAGCATANACGTCGGCGAGCGTAGGGTTN
GCTGGCCANGGAGGAGGCGGCTGAGGACGATCCTCATAGCAGTGNGACGGCGGNGACGCGGGATCAAGAAGGT
GNCCGTANAGATCNAGAAGGCGAATCGTGCTNATGATTCCGTTCTCCCAGGCGAGGAAATCATCCCTCGACTT
CAGGATGGGNATATGCGTCAACGTCGGCGGNGGCAGAGGCTTCGGTTCCGCAGGCGNAGGTGGTGGAGCAGGC
GCGGGGGCAGGCGGAGGAGGCGCAGGCGGTGNACGAGGTGGAAGCGGAGGAGGAGCATGANGACCTAAGTTAC
CACCNAGAGCACCCCCATGAGGAGGACCGACCACGGGAGGAATAGGGGGCGCGCCGCAAGCAGGAGCTTGTTG
ACGAGACGGACGACCTTCGGACAGAGGCACAGGATGACGTAGAGGCGAGGGAACATAAACATAAGAAGATTCA
GAAGTAAAGGAAGAAATAACAGGACTAACGGAACCCAAAGAACTAGAAGAGCGAGAACGNGAAGAGGCTATAT
GCGCGGGAGACAATAAAGGTTGCGAGAGGCGGAACAGAAGGAGCGCGAGNAGACTGGAAGAAGAAGCCCAAGG
ATTNGAAGGNACAGAAGGCGCATAATAGGAAAAAGGAAGAACATTATTCGAAGCGACTGGAGAGGAAACGGCA
GGGGGAAGGTGCATAGGAGTCGGNGTACGAGGCTCATCGAGGTGTGGGGGACGCTGATGATGAAAGGAAGAAG
AATAATCCGAAGAAGTATCGTGTAGGACGATATGAAGTAGGTAAAGGGGGGTTCAGGCAAAGTATAGGAAGAAG
TATATTGGTAAGGGGAAACAGGATGAAAGAGAGGATCCGAATACGAAGCAATAAAAGGAAAAGACGAACGANG
TGGTTGTGGAGTGTGCATGCCCTGAGTCAGAGTCTCTTGATCCCATCCCTCGTCCATCGGAGAGAGAGTTGTG
GAAGAAAACATCGTAGGCGGTCCAACTAGAGTGGGATATATGCGCCAAGGATGATGAAATGAGGAAGATCCGC
AGGATCCTACCGCATATATGCGGNAAACGAGAGAAAGAATAAAGGCGAGACCGTCAGGATGAGCCAAAAATTG
AGTCGCCAACGAGATTAAGCCGCAGTAGTAGTCTACTAGACGGGGCCCATAACCTGTCAACAGAGCACCTAGA
CGCCCAAAGAGCGAG
>Agrped1_Cluster2#LTR/Gypsy
GTAACGAACCCATACTTATCTCGTCTATACCCACGTTCTTATCTTATCTTTAGTAATCTTATCGTCTCTTATC
TCGTATGTCCTAACTATAGTCACTCTTTCATCTAGACATATCCCCACTCTCCTTAGTTCGCTTTTCTTTACTT
ATCACCTGACTAGCTAGTTTACAGTATATAAGGACCCTTCTGTTTAGTTAGAACCTCAGTCAATCTAAGAGTA
TTTTACCCCGTCTCTGGATTGCTCTCTCTGTGCCAAAACAATCTCCTCGTCGCTGTTCGCAGTCACGAGGAGC
CCCTTTTGTCACATCTTTTTGACGCTACACAACTACTCTCTACTCTCTATCTCTTTACTTTCTTAAAACCCCG
TTGCTCGTCGCTTTGGAACCTCCGGCTTGATCACCCGCGAACACCAGCACCAGCAACGACCAAAACTCTCAAA
AATATTTCAGTTAAGTTTCCTATTAAATGAAAATGTCTACTAACGTGCCCAGTACCTATACCCTGCGCCCCCG
CACCACGACTACCGCCGTCGTTGCCCGAAAGAAGACCACCGTCGCTTCCGTTACCGAGGACGACGTGAAGACC
GAACCCGGCACCCCAAGGTCCAGTAGCCCAGACCTTGGCTTTTCGCTCCGTCGCTCGTACAGCGACGTCGCAG
CTAGTAGACCCCCGTCGCCGTTGTTCAGTGAACGCGGCGGGGAGACCACCCCGAATGCCACCGTTGCGCGCGG
TTTGGCTGAGAATACGGGAGAACCTATTACCCCTGTAAGGGATGTTGTTAGTATGCCCGTGGTTAACGGCAAT
AACATACACGGTTCGGAGTCTGAGGACTCACCTGATGAATGGACCACGGTAATGCGCAGACGTGCGCGCAGTC

CTAGAACCCCCCGTAAGTCGGTTAGAAAGGCTGAGACTGGTGTAGTCCACCATGTCGAGTTAGCCACAGTAAT
AAAAGAGGCGGAGAAATCGCTTACCAAGCAGCAGAAAGAACAACTTAAACGACGAAATGATAAGTTGATGAGC
GCAGACCGAGAGGAAGGTCCCTCTCGAGGCAAAGGAAAGGGTGTTGACCCTCGTGAATGGGGCAATCTGCCAT
CCGATGAAGAGCCCGTGAATATCGTGGCTCAGCAAGCTGCATTAGACACTTTCCGTGCACAGCGAGTAGCAGC
AAAGGCAACAACTCACCCTGCTGACATCGACCCAATTCGCGCGTCGGCGCAATTGACAAATCGGGCGCTTGAT
GCGAAAGCAAGACATGCCAAAAGTCTCGCTAACGCAACCCTGCCGGCTGCCGTACAACCTGCGGCACAAATCG
ACGCGAGATCTTATCTCGGTGTCGCATTGGGAAAGCTCGACAAGCCTGCTAGGGCTACGTCTCGTAAATCAAA
AGGCAAAGCTAGGAGAAGTAGCACATCACCTCCATCTGGATCTTCGTCATCCAGCTCGTCTTCTAGCTCAGAT
ACTGAGAGCACGAGTTCGTCATCTTCGGATGATACGGAATCAGACTCCTCGGACTCGTCATCCAGCTCTGGAA
GGAGCAAATCGCACAGGCATCGATCCAAAGGTCGAGGGAAACGGTCTAGGCGTAGACATCACCATAGATCACG
CTCGAGAAACACGTACAAAGTTTTTAGACCGCGTGAGTACGAAGGAAACGCAGATTCACGCGATTACATGAGG
TTCATGAAGGAATCTATCGCATACATCAGAGACAGTCATATTCCTGAAAAACGAGCCTGTGCTCACCTGTCCC
ACTTCATGTCCGGGCCTGCATATGACTTTTATGTCACTAAAGTATCATTGAATGAAGAAAAGTGGACGCTTAA
GCAATTCTTCACTGAGCTATTCAATTACTGTTTTCCACTGAACTACCGGATTAAACTACGCGAGAAACTACGC
ACCTGTGTTCAAGGAAATCGCAATGTTAGCACGTTCTCATACGAGCTTGAAGAACTTCTCAATATGATAGGAA
CTATAGACGAACGTACCAAAGTGCATAGTCTATGGAATGGATTCCGCCAGTCGATACAGCGGTCACTATGGAG
AAATAATCTCAATCCAGAAATCTCATCTTGGGAAGAGGTACTCACCAAAGCCCAAATCATCGAAATATCTGAA
TCAGTCGTGCCTCGACGAGACCGGAATAATGACAGGTCCAACAGACCTTCGATCCATCCAGGAGGATCGCGTC
AGAACAATCACAGTAGTCGAGGATTCAATCACGATGGAAATAATGATGACCATTCGTCGAGGGAGGATTTGAG
ATTCAAGCGTCGACGTTCCAGTGATCGAAAACGTCGCGATAGGCATGATAACGACTCCAAGTCGGGCTACGAT
GGTAACCGTCGTTCGAGATTCTCGTCCAACCGAGGAGATCGCCCTAGCGTAAAGCGTGAATCGATGACGCCAG
TGTTATCCGAGAGAGAATGGGCTGAGCGGTTAGCGTCTGGGAAGTGCTTCAGGTGTGGAGAAGCCGGGCACTT
AGCTAGAAATTGCCCACAAGGCAATACCGTGAAATCATCAGGGAATCGACCCCCTGGTGTACCTAATTTCAAC
GTTGAATTAACCGGCGATGAAGTACCAGATGAAGAAGTCGAGGTTCTCGACAGTCTACCGCTCGGTGCAATCG
AGATTCAACTGCAGGGATGTAAGGATCGTTCCTACCTTCAGCTATGGAATGTTAGTGAGCCAAAATGGCGTAG
ATGGAATCCAGATCACCCAGAGCGAATACTGGGACCCGCTCCTATGCTTATGGCGGAGCTGATTCTTCATCAG
ATGCAACCGTATCCTGGTGACGAATTGTACCTACCTCGTGTATTGAATGGATTTGACTTGAGCCGTCGATTCC
GATTGGTAGAACATGGTAATCAACCTGTGGGACAACCTGAGTACAGTGTTGAAGACAACCTGGCAAGATGTTC
TATCAAAGTTCCTAGTGGAAAGTTATCCAACCCACTGTTTGATGTCGCCGAATGGTATGCTCGCTCGCACCCA
AGACACCGGCGAAATCTTCGAGAAGAAAACTATCTCATTCCCTCGCACTCTATCAGTTGGCCGTGGGCCGACA
TTGCTCGACATATGCTTGCGGATGGCATTACCTCGTCCTACCCGACAGTGAACCCAGATCTACCTCCAGAAGA
CCAATTCAGGGTAGAATATCATGTCGATCCTATATCTAAGCAGGAGTGTTATTTGATCACGGATCACGATCTG
GAGCTGCTCACCAAGATCTCTGTGAGGCAGTTGGAAAATTCTAGGTTGCAATTGATTGCTTGGTACCGTGATA
GACTGCTTCACGACGATTATATCCATCAGAGGTTATGGGAGAGCACGTTCTCGCTTTACCATGTGCCTGAGGA
AGAGGAACAACAGAGTCCTTCTCCCGAACTCGATCTCATGTACGATTCTGAGAATGAAAACGCGATCGCGAGT
GATTCAGAAAATGACCTACCGGGTCTGGAAACTATTCCGGAAACCGATAATGAAGACGAGATACCAGATCTAC
AGAGTATCTCTGATAGTTCATCAGAGCACAGCCTGGAAGAATCAGAAAAAGAAGAGGCTGCACCTCAGGATAA
TGCACAAGGGTCACATCCCAATATCTGGATCAGCTCACCGTCGAGACTGATGGGTGATCCGCTCGCAGACCGC
CTTGAGCTCGTTCTGCACAACTGTCAATCTTATCCAGGGGATGTTACTCAACAGTACACGGGTAGATTCGCCG
TGCAACGAATAGATGGAGGATTTTATCAGATCTTCGATTATCTTCGAGGATATGACCTTACCTTGCATTCCTC
CTACATGAAACACGAGCTGTTTTCGATCGCGCGTTGGTATGCGCAAGTGTGTCTCTCTAAAGACGGAATTGAG
TGGCATAAGGACCATCTCGTCACTTACCTAAGGGGACAAGAATTATTCAATACATTGGCAGGACCTGCCCGAG
AAGAACACGCCGAATTCATTCTGGAGTTCAGCGCTCCGTACCTGGATGAAGATAGCGATCCAGAACGAGATTC
TGAACGCTTTGTGGTCACATACCCACCCAATGACCCAAAGCATATGATTATTCAAGATAAATTTAAAGATCTT
GTATCCTACGCTCCTATTGAGCTATTCGACGATCCCTCATTCGATTTAGCTGAGTGGTATGAAAGGAGATTAG
TTATGACTACTCTGACCAGCATAAGAGGCTTCTCACCATCGTCTACCGTGTCTACAGAGCCTGCGGATGATGT
ACCCGATCCTACCCAATATATTTTGGACTGGAGACTTAATCAGCCAGATACCTACGAAGGGACGGTTGAATTA
AACGGAGTACAGGTCGACAGACGCAAGTACCCGGCCGTTCAGCGTAATGTCGCATCCGTGAAAGATAAAGCGA
GAATCTTACCAAAGCCCGTAGTAATAAAGGTGCTAATGAACGGCCAGCCTGTTCGTGCGCTGATAGATCCAGG
ATCTCTCGGGGATTTTGTTTCTTCGACTCTTGTTGATCAGCTGAAGCTCAAGCGAGAAGAACTGCTTGCGCCA
ATCTCATTACAACTCGCGGCGCAAGGTTCTCGGTCACAAATCAATGCACAAATCAGGTCGAGGTTTCAATATC
AAAATATAGACGAAGAGCGCACCTTCGATGTGATAAACCTAAATAACTATGATGTTATTTTAGGTACACCTTG
GATGTACCAACATCAGGTGTGTATCGGGCTAAATCCCGCACGAGTTATTATCGGCAGTGATATTGCCTTAGCC
ATCAAATCGGGCACCGAGTCTAAGGTGCTATCACAAGCATTTCTTCAAGCATGGAATAATTCTGAAGTTGTGT
ATATTCCCGGTAGTGAAAACGTAGTGGCAGATGCCCTTTCACGTATATATTCCAGCGACGCGCACGGAACTGT
CCATGCTCGCAGCAGGTACACTTATTTCGATGTCGTCGACTACGACGCTGCTGTGGGCATAGAGCTATTGCCT
ATTTTGGCAGGATTAGAGGCTCGCGCGGCTGAGAGGCCCAAGCGTGTACGCAAACCTGTGGAGGGCGCGGAAA
CTGGACGGCCTGAAACTTCAAAAGAATTTGCTGCCCGTGTACGTGATAAGTTCGTTCTCAAAGACCCTAGACA

ACGAACGGAGGGCGAGGGTGCACACGATGCATCAACTCAACATACTGTTTTGGGAAACTTAGCGGCTCGTGTT
AAGCAAACCCGCGACGCGACTCGAAAACGGCAGAACGCGCCTTTCGCTGAAGGCGATCTGGTGTATCTTTCAA
CAAAAAATATATCTTTTCCTAAAGGATTAGCGCGTAAGCTGATTCCCAAATACATCAGACCTTACCGCATACT
GAAAGACTTCAAAAACTTCTCGTTCAAACTTGACTTGCCTACCCAAATGAAGCAGCGTGGTGTCCACGATGTT
TTTCACTCGTCACTACTTCGAATTCACCATCCGAATGATGATCGACTCTTTCCAGGAAGGCTAGACTGGCAAC
TTAACCCAGGACTCGACCCTGAAGGTGAATGGGCCGTCGACTCAATTATTTCGCATTACGGAAAAGGTGAAGA
TGGGATTTTTAAGGTAAAGTGGAAGCCAAGCAATATCACTTGGCAGCCGTACTTTCAAGTGCAAGGCCTACAA
GCCTTAGGAGCCTATCTCGCGCTACAAGGAGCGGATGACATTCTGCAGCTAGCGAAAGGATCAGGTAAGCCAC
CATTCAACGATCCTCAAATTGTCGTTGGTGCTTTGTGCTTCAGCAGCCTGCCGTCACCCAGAATCTATAAAAT
CCAGCCCCTCAATATCAGTATCCGTCACCAAAATCCCCAACCAATCTTCTCTCACTCGAATCATCATCCTCTC
GACTCAAATATGGCTCGTGGACGCAACCGCAATCATCAAAACCATTCGACACCTGCTCCAGCTTCTACTGCAC
CCGCTACCATTACTAATGCAGCTGTCCGTCGCACTTCACCCACTCTATTTCTTATTACCGACTCTACAAACAC
CCACGCGGAGGTTCCCCTCGCATATACCTTCTCTGCAGCAGAGATCTCGCTTTGCATCTCAATGGATGCTCTT
CTGCGATCGAATCCCTCTTCCGATCCCAACTCATTGTATTGGCCCTACGGTTACAACTTCCTCGCACGTGCCT
ACAACGCCCATGCAGCCGCCGGCGGTGCTCAATTCGCTTATATTCACCCCGAGGGACAGCACCTTGTTTCCGA
AGGCCGCCCAATGACCCGGCAGCTCTGGCAGATCACTCCACAACAGACTGGTCTCGTACCACCACCTCAACAT
GGTATCACCGGTGATCCAGACAGCATGGCCGCCGTCAACGATATTCTCATCGAAAACGCCGCACGGATCAAAC
GCCAGAAGGAACGCGTCAAGGAGGAGGTTGCCGCTCGCAAAATGCGCCAGGCGTTCAACCAGGAAACCGTATC
CTTCGCTCGAAACCACCACACCCACTCACACCACCGCCGTAATCAAACTCACGCCGGATCTTCGAGCACTTCC
TTCGCTGGACCTTCCAACCATGCTATGTCCCTTGCAGCCATCAGTATTGCATCCACTCCATCCGCCGACGGCT
CTTCCCACTCCTCTGACACTCACAATCCCGTCGTCGGTCCTAATGGAGTCACTACCACCGATGCCGACCTTCA
TCAATTGTTCGGCTTCACTACCACCACCCAAACTACCACTCAACCCGATGGAGAAGAAGAACCCCTTGATTTC
GGAGACCTTCCCATGAACGAAACCGAACCCACTAATTAATCAGAAGACTTTCTGGCGAAAGCCTGGAGGGCGG
GTGTAACGAACCCATACTTATCTCGTCTATACCCACGTTCTTATCTTATCTTTAGTAATCTTATCGTCTCTTA
TCTCGTATGTCCTAACTATAGTCACTCTTTCATCTAGACATATCCCCACTCTCCTTAGTTCGCTTTTCTTTAC
TTATCACCTGACTAGCTAGTTTACAGTATATAAGGACCCTTCTGTTTAGTTAGAACCTCAGTCAATCTAAGAG
TATTTTACCCCGTCTCTGGATTGCTCTCTCTGTGCCAAAACAATCTCCTCGTCGCTGTTCGCAGTCACGAGGA
GCCCCTTTTGTCACAG
>Agrped1_Cluster0#LTR/Gypsy
TGTAAAGCTGGGCTTTACAGTAGATCTTAAGTCGCACGATATAAACCTGACCAAGTCTATATGGTAGTTATAT
GCTAGTACAAACGAAAGATCTATCTATCTATTTGGTTCAAGCCGGTTAAGTATTGGTATGGGCGAGGCCCATG
CCAAACAACGTGAGGCTTGTAACAAACACGGTCAAACGCCGTAACGGGATGCGGCCCGTTACACATCCCCCCT
TTAAGATCATTGAATGATCTTGACACGACAGCAGCTTTTTGTCGTAGGTAGTGAATAGATTCTTATGCGTCGG
CGGGTGTAGTGTCTTCGTTGGTGGGTGGTGGTGCCTCTGGCTGGTCGTCGACCTGCATGTTCGTTGGCTCGGT
AGCCCCATCGGTGTGATCCTCCTGTGCATCTTCCATTGCGGCGTCCATATCGCGTACCGGTGGCACGTTAGCG
TCACCGAGTGTCGCGTGGGGCTCGTTCTGGGGTGCCACGTTAGCGGCAGTGATTGACGATGTCACGAGGGAGG
TCGATTGCCGGTGGGCGATGCGCGCGTCAATAACTTCGCGAGGTCGATGGTCGAGATAGGTATCGATGGCGTC
TCTCCCAATGGGGCACCATCCCTGAGCTTCGAGGCTGAATATGGGGTTTTTCTGACGCTCTTCGAAGTCGATT
AACAACTTGAGCTTGGTTCGTTCCATGGTCGAAGGCTCCCACCATCCATCCCATCCCGATATGGCAGGAGGTT
CACCGAATGTGGCGAGTCGGGATAAACGCTCGTCATCGACGTCGGCCAGCAGGTCCTCATCAAGGTAGGATCC
TGAAAAGCGCTGGTTCAATGATTGGTATCCGAATGGATAGGCGTGATCGATCCATTCAACAGGTATCCCGTGC
TGATCAACACGTTGGCGATATCGTCCTCGCCGATGTTGTTGCTTTGTACAGCCGTGATGGTACACCTATTGAA
GGTGTACATCGGGCCCGTTTGTGGTTTGAACTCACAATCGGGGTGGCTATCGTTATATTTGTCGATGATCTCG
CGGTACCTGTTAGGCTTGGCTATGAGGTATGCTTGGCTATGAGGTATGCCAGGTTGCGCGAGAACGATGGTCG
TGCGGCCTTGTCCAATGGCGACAGAACGCGACTGAGAGCTACTCCGAATATTGATCTTCGGTCCACCATTCCC
AATCGGTTCATGATAGCGCCGCATGTTGGGCTCAAAGTGCCCGGCCGGGAATGCATGAGGGTGTAGGCCGCGA
GCATGTCGATGTTCATGATGCTGTCGGTAGGGATCATGGCCATGCCCGGCTTCCGTAGTGGGTTGCTGGGCCG
CCCGCCCACATGGATCACGATGCGCTTCCACAGGTGGTCTGGTGCATCGATGTCGGCTTTCTCACATGGTAAG
CTGACGATGTGGCTCATCGCACGATCACGATGGCCTTCGTGTGTACCCTCAGCTATAACACGGAATTCCGTGA
TAAGCGTGTGTGCCTCGATTCTTGCCCAGTTAGGAATCTTCGCGTCCCGGACAATTTCCTGGAGATAGTCCAC
TTCCATAGGCGACCTAGGCATTCCGCGGGGTACTCGTCGATATATAGGGTGGCTATGATGGAGGAAGGGGGTA
CCCCTTACTCTTTCGTGATATTCCGCATCGAGTCCAGTACGTCTCGTGAACACAGTGTTCCGTGCTTTTGAGT
AATACATGGTGATGGGTAGCATGCCTCTGAACGAGTTATCCCGTTCAAAGGAGCCGTCCTCGGCCTTCACCAC
CCCCCATACATCTGGAACCCTCCCAGAGTAGGAGTGTATGGCGAGATTGCGCTCCATTTCGAGCCTGTGCCTC
CGATCTTGCCTTTTCTGATTCCTGTGACGTATGGCGGCTACCTCGGTCATGGATCTCTTCCTTCCTAGCCCAT
CTTCTTCGTCTGTCGTCTCATCGTCTGACTCCTCTGGCTCGTCGTCTTCGTTGTATCGAAGTCTCATTGGTAG
AGGGTGGTCCTCTGCTGGATCCATAGTCCTCGATTCAGGCGTCCGAATCGGGCCAGGTACGGGTATGTTTTTG
ATGCAGGTTTTGTCCTCCGGGGCAGCGGGCTTCATTACAACGTCCGCGGGTGGTGCCTTGGTTGTAGAGAACA

TAGGTTTAGTCACCGGCATTGGGGAGTCCCCCCATGCGGTAGTGCCCTCAATCCTTCTCTCGGGCTCGATAGC
GGCTTCGTGTCGCTTTGTTGGTCGGTCATCATCGGAGTTGGGTGGTGCCGTCCGTTTTCCAGATACGGCATTC
AACTTCTTTCTCAATATGAGGTTTTCGGCGCGGTAGTGGTCTGCCTGGGCTTGTACGTTCACAAAGTTGAATA
GTAAATCGTTGACAATGAGCACAGCGTCGTCGTTCAGATAACCGCTGGCGTGAGGTTCCGTGAGTCCAGCGTT
GAGTAGGCGGATGCGAATAGCCGCTTCCACCGTGTCTTCCTCATCGTCTCTCGGTGCGTTCGACGTTGTCTCA
GGTGTCGAGGGCGGTGGGGATGCCCTTGATCCTTGTGGTGTCCTTGGCGCGTTATCTTCGCGTGCGTGTCGTT
TATTGTATCGGTCTTCGTCGTAACGATGACCTCTTGACGAGCTGTTGTGCTGGTAACGGTCTCGTTCTCGTTG
TTCCCTTTCCCCCCCCCAGCGAGACCGTGGCCGGTCTCGTGAGGAATCGCGGTGACGCGACGTTCGATCTCGA
TCTCGATGTGCCATTCTTATTACAGAGATATCGGTCAAGAACGTTCCTGACCGATGTGGCGTTACTGATTAAT
GAGTGAATGTCCACGACAGATTGTCGAGAAAACCAAAAATTTGACGCGTGGCGGAGTTACAAATTTGGAAGTC
CATCGATCACCTGACCGCGGCAGGTGTGGCGGTCATCCAGATCAATACCGTTTAGGCAATTGGGTCGGTCTCT
CTACCTGATGAAGCAGGAGGTAACGATCCAGCGCGGCGAGGTTCTTGCAGTACGATAGTGGTTCCCAGGTTGT
GTCACCCATGCTCCATCGCACTTCGAGTTCCAAGTTCTTATCTCCGCGCCACCTGTGTCCGATTATGTCGTCC
ACGAACCACTCATTGCTTCCACCAACGCCAAAGTCGTAAGGGCCAGGCTGCGAGCGGTCAGGGAACTGACTAT
CATCGGACTCTCGGTATCTGCGGAGCAGTGATACGTGAAAAGTGGGGTGCACACGTCGCTTCGCTAGGTCCGT
AGGCAATTCTAATTTATATGTCGAGGTTTCAGGGTAGGCGTCCAATATCTTGTATGGCCCAACAAATTTAGGA
CATAACTTCCTCGTACGATTCTTCGGTATAGACAGGTTCTTGGTGGATAAATATACCAAGTCGCCTTTGTGCA
GTAGGGGCTCAGACCCACGGCGTTTATTAGCGTTCCGTGTCTGAAATACTCTTGCCGCGATGATCGCGTCGTG
AGCATCAGCTAAGTTGATCAGTGCCTGTTCAGCAAAGGATTTGATACCCTTGGACAGTGGTGTGTCGCCGCGT
ATTTCCCTAATCATCGATGGCATATATCCGCCATTCAGTTCAAACGGCGCGTATTTGGTTGTTTCCGATACGC
TGGCGTTAATAGCGAACTCGGTCATGTCCACCTTGTTGATCCAGTCCTTTTGGTCCGGTCGTACAAGTGTGCG
AAAAATCTGGCCAACATTGCGGTTGGCGCGTTCCGTTTGACCGTCGGTCTGCGGGTGGAATGACGTCGACATT
AGGAGGCGAGTTCCTAAGATCTTATGTAGTTCACGCCACCATTTAGACGTGAATTTTGAATCGCGATCGCTTA
CTATGCTGGAAGGGAGACCATGCAACCTGACTATTTCCCGCATATATATCCATGATAGGTCGGCTGCTGTCAT
GGTGGTGTGGACGGGTATCAAGTGTACCATGCTCGTCATCCTACATATGATGACCCATAGGTAGTTGAACCCT
TTGACCTCCGGAAACGGGCCTATGAAGTCCATGCCGATCGAATCCCAGGGTTTGGTAGGTACCGGCAGTGGGT
GTAGCTTGCCAGGCGGTTTCTCGTTAGAAGCTTTCGCTCTCTGACAATCTCCGCATGTGCGACAGAACTCCCG
TACGTCTTTAATCATCTGTGGCCACCAATAAACACGGCGTATATAGTCGGCCGTCTTCAGTTCACTGAAGTGT
CCGACCTGCGCATGTGCGCTGGCTAATATGTCAGTTATCAATTGCCTATCCCGAGGAATACACAAGATGTGAT
GTTTTGCTTCTGAATTAAACCAAATGATACCGTCTTTTATCTCAAATGCGGGGTATCGATTTGGGTCCGCCAA
TATCTCGGTGAACAATTTGTCATTCCTGTAGCCACTTGACACCTTCGCGCAAGGGGGTGTCGGGATTAAAATC
CCGGACCAGACGTAGGTAGGGAGGGTCTGGCGGTCATGTACAAAAAATAAATTTATATATAGGTAAATATGTT
CATTTTCGGCGTTTTTTCAACACATTTTTGGCTTTAACACATTCTAACGAGTGCCTGAGGCAAGTAAATGGGG
TATATTACGAGAAAATACTACAATAAAGGCACATAAGGGCAAGTTCTGGAATTGACACAAAGATAAGATCATG
ATTAGATTAGATTAACGCACCACAATTCAAAGTCTGTGATCTTCTGGTAAAACGATTCACACCTAAAAGCTAC
AGGTATCTATGTACACAATACTTATATAGTATCTTCACTTGCGAGCACCGGTGCCAGAGCCTCTATGTTATCG
GCTCCTCTCAACAGAGATCATCGGCAGCACATATAAGGAAATTTGATGGATGACTAAGCGGCACGAGGTAGGT
AGGGTCAGCAAGCAAGCCTTGAATAAAAATCTATACGTCTGGTATTTTTTATTATTCTTCACATCCCCTTGCC
TTCGCGCGGAATGTATCCTCTCTGGATCGGTCAATGTGGTCGGTGTGCGGTTCGGAAGCTTCCAAAGCCTCCC
CCAGGGTCATGCCCTGGTTGCCACCGTTAGATACGGACTGTCCATCACGTTGCGTGCTTGCAACTTCAGCAAT
ATCTGCGTCGGCTTCTTTTAGCATCTGCGCTTCGATGTCTCGCTCCTCAGCTTGCTCTTGTAGCCGCTTGCTT
CTGCGGAGTTCCATTGCTCGCATGGCCCTGAGCTCTATGGTGTGCTCTGTAACCTCTCTGTATCTTTCCATGG
GCAGGTCCTCTCCGGTAGGGTCAAGTCTCACATCAGCGCGTACATATTCATGTGGTTCATATACGTCCGCGTG
TGTGTCGCTCTCGTAGTATCGTGACAAGCAATCGGCTATTTTATTGTGTTCGCCTTTCACGTAGGTTATATTG
AAGTTGAACCTGGACATGTAATCCGTCCATCTATGTTGTCTGTGCGATAAGTTACTTTGCGTCTTAAAAAACT
CAAGCGCCTTATGGTCCGTTATGACATGGATGCGATTCCCTACGAGTTTATCTTCCCACTTTATGAGCGCTTC
CAGAATCGCCAAGGTCTCTAGTTCGTGAACCGCGTAATTTTGTTGAGCGCTGCTGAACTTTTTCGACATAAAA
CCCGCAGGGCGACACTCCGTCCACGATGGACCTTGCCCGTACATGGCACCCACGCCGGTCTTCGAAGCGTCGC
ATATGAGCCATATAGGTTCATCAGTACGAGGGTCTATTGGTTTTATTATAGGTGTCTTGCAGCATATCTGTTT
GATCATATCGAAGCATCTTTGATGTAACGGGCGCCAATGGAACGGTGCGCCGTTCTGGGTCATAGCTAAGAGA
GGACCCGTGTATGCAGATATGTTAGGAAGAAAATTCCCAACATAATTGACGAGCCCAACAAAGCGTTGTATAT
CGTTATAATTTCTCGGTGTACGCCATTCGCGGATTCGTGACATCTTGTCCGCGTCTGGGTGAATCCCCTGAGT
GTCTATTATGTGGCCCAGGCAGTCTACTTTGTCAGCGTACAGCTCACACTTCGACCATTTGAGGTATAATTGA
CTTTCACGAAGCTTTTCGAAGACGAATCGTAAGTGTTCTTCGTGTTCTTCGATGCTATCAGAGTAAATGAATA
TATCATCCAAATACACGTGCATGAACCGCCCAATAGCATCTCGGAAAATACTTGTCATGAGCCTTTGGAACGT
CGCAGGTGCATTACAATCACCCTGCTGAATCACATTACTAACGTAGGTGCCTGATATTGTGGCGAAAGCCGTT
TTATGTACGTCCTCCGATCTGACTCGTACTTGCTCGTACGCGTCGGCTAGATCAATCTTAGATCGATATCTAG
CGCGTGCGACGTCTTCTCGTATGACGTCTTGGTCCGGTAGAGGCGTTACATCCTTTACAGTATTGTCATTTCG

TTGTCGTGCATCGACTACGGTGCGTAAATGTCCGTCTTTCTTGGGTATGCACATCAGAGGAGCAGCTTGAGAA
GCCGCGGCCGCTTTCCACCAACCCGCGTCAATGTATCGGTTTAGTTTGGCATAGAATTCCTCTCGCAATGATA
AAGGACATCGTGGCATGTGATATGTGTATCGTTTATTTGGGTCAATTAAATGGATTTCGTGGTTGACCTCCCT
GAACGGAGGTAATACATTCCTTGTTCCTTGTACCATGTCAATAAATTCATCATGCCATTGTTGGCGTAATCGA
GGGAAATCATCGGCGCATAGCCTATCGGCCTTGCGCTTGCCCTTGTCTGGTTTAGGTGACTCCGAGACTGTTG
GTTTACGGTCTTCGTATTTCTTTGTTAGTCGTTTAATTGCAATGTCCACTTCGTCTCTAACCGGCAGTCGTTG
CTTGACCGTATTATTGCCGGTAGGTTTAGTTCTATTTGCGCCAAGGAACTCGTGGGCGACGGATATTTCTTCG
TCTCGAGGTTGGCGATATTGTTCGCCTTGTAGTCGTACCCGGTCCCATTGTAGCGCTTGCATGCTGGCTATGG
TCTCATTCCATTCCCTGGGCATTCGAGGATCAAATGCCCAATCGTATTTGGGTGGTGGTACTGGTCTATCCCA
GAATGCTCTTCGTGCTTTGTGTATTTGCTCGTCGTATTCCTTAGTCTCTGATTCGTCAGTTACGAGGTCTTCT
GTTTTGATCCGTGACGATGTTGGTTGACAATAGGATGACTCCCCTGCTGTGGCCATCGTCGGTGGATTCTTAG
GCACTTTATTGCTTTGATTTGAGTCCTTCTTATCATTTTGTCGTTTAGTACGATGATTTTTTGGTAATTTCTC
TTCTTTTTTCACGTTATTCTCGTTTACTACTTCCCCCTTGGAGGCTGAGGTATACCCCAGCCTTCCACATCG
TCTTCCGCCACCAATACGTGGCGATGGTCCCGCGGCAGCGTTGGTAGGGACTCAGAATCCGAGTCCTCTACTT
CCTCGACGCTAGCTCGCTGCCGTCGGCGTTGTAGTCACTCAGCTGATTGTGGCTCGACGGGTACGGCGGTGAT
CTTATCAGTCAGGCGCTGCCGGCGAATACGTCCATCAGCGTCTGGACTAACTCGTTCCGCGTCAAACGCAACG
CCGTTGACTATGACACGATTGGTGTCGAAGTCCAAGAGGACCTTGTGCGCCCGCATGAAGGGGGTGCCAATGA
TCATATCGTATCGATCAAAGTTCGCCACATCAACGTACGCTGTCATGGTCTTACCCCCAATGGAGATGTCGAC
GTCGGCGCCGTAGTTAATCTTCGATCGGCTACCGATGGTGCCAAGTTGTAATATATGCGGGTCCAGAAGGGTA
TCGACTTCTATATCCGCGATCTGAGCGAATGTAGGGGTAATGCCCGTGGTTGTACTGCCAGAGTCCCACAGTG
TCCACGCATCCATGCCTGCCACGTTGACATACGTAGCTAAGCACTCTTTATCCTCATGACGCGTTACAGGACG
CATTCTCGATTTTCTGGACGTGCGAAGGCGTACCTTGCGCATCTTTGCCATGGGTGGCTTGTCAGGTATCTCA
GTCATGGCTCTACTTTCCACGGTACAGAAATATCCGTCCCCGTGTTCTTGGGCCATCGAGTCGCCGGCGTCCG
CTGGCTGTACCCTCATTGCTCGCATGTGTACGGATGGTGAATCAGACTCGTACCATTCGGAGTCTAGTGCTTC
CACTTCTGTATACGCGTCGTCCGCTGACTCTCTGTCCAATGTACCTTCTTCGTGTTGGGGCAAGTTCTCGTCA
TCCTCTTCATTAGAGGATATATCGGCGTCGCTAGCGTCAGGCACGGCCGTGTGAGCAGCTCTCACATGTACGC
GTTTGTCCCTGGGCGGCGCCGGACAGTCGCGAGAGAAGTGACCTTCTTTTCCGCAATTAAAGCAAGCGTTAGT
TTGTCGTGGTCTGGGTATCGGCCTTCGTGCTCCGCCGCGGGGATCCTGCGGTGGTCGTTTTGAGCCCAGTGGC
TTGTCTAAGGGACGGTCGTCCCGCTTCGTCGCGTCCTCTTTGGATTTATGCGGTGCGAGGCTGGACCTAAGCA
CCATGATCTTATCTCCAGCCCGCACTCTACGTCCTGTGTCCGTAGTTGTAGGTCTATACTTGGCTGATGTGCG
CCGTTTATAGTATTCATCGGTCTTGCATCGTTCCTCGTACTGAATCGCGTACGCCACGAAGTCCTCTATAGTG
TTAACTTCAGGTGATAGACCATTTTCCTTAATGAGGTACGATCGCATGGCATCGGGTATGCCGTCGTAGAACG
TTTCCAGCATGGAGTAATCGTCAGGATATACGGCCATATTCTGAGCATAATCCAATAACGTATCATAGAATCC
TTGGACTCCAGTGGACGGTGAGTAACGAGCGCTTTTGAAGCCCTCGCGTGCGTCTTGCATCGTCGATGGATGT
ACAAACCGCTCGTACAACCCTATGATAATGCTTTCGAATGTCCAGTCAAGCTTCGCACGGCGCGGGCTGGTGA
CATGCCTTATGTACCATCGCTGTGCTTCCCCATCCAGGTGCTCCATAAGCAGCATGCTCCTCATGCGATCCAG
CTCTTGTCCTCCCAGTTGTCTGACCGCGAATTTATAACATATCGCTGCCAGCCATTCCTCTAAGTCTGAGAAT
TTAGCTGAGCCCGCATACTTGTCAGATGTGTGGGTCTCAATCTTTTGTGTAAATTTTACTCCGTCCGGGTATA
CGATCCGTACGGACAAATTTTCGACTATAAGCCTGGTGAGGCGTTCATGCATTTCGGCTTTATGGCGTAGTTT
CGCTTCAGGTACTCCTGGGTTATGGTCTATGCTTCTCGGCATCGAGAATCCGCGACTCTGACCCCGCATGAGC
GTTTCCTTAATAGGGTCGTGTTCTTGATCATCGTGCATGTAAGTCATAGGTACGACGGTTCGTTCCCCATACG
TCTTACTTCGCCCCTTAGGCTGTGGCCTGGTCATCGGTTCCCGATGTGCCTGTTCTATCGCCTTGGCGATTTC
GTCTGGTCTGGCTTCGCGTGGGATATGGGCGTCGAAACGCACACCTTGATCTTCGATGTGTGAGCTCCCTTCC
CTCTTTATCGCGCTCTCTCTCATGCGTTGGTAGGCCACTCTATCATTCCAGTTCTCTGCCCTTGGCACACCTC
GGCGTTGCGTCACATTTGGGTGGTCAATCATTTCAGCGAACGTGCCTCCGGACTGTCGTCGGTCAGTCGCCAC
GCGATCATGTTGATTAAGGCGCGCCAATGCAGATCTGCGATTGTTGAATTGTTCGTCCGTTTCGCCCGGTTGT
CGAGGAAGCGCCAACGCTTCCCTAGCCTTGTCGATGTGAGGTGATTGTGTCGTCTGGGCACGAGCGATTGGTG
GCTCTCCCACCGATGTGGATCCGGTTGATGATTCCACCTCGTTGTTGTTCGTGGTGATACGTTTTCCCTTGTT
AACTGGTGTCTCAAGAACCTTTGACATAGGGGTGGGGGCCCTAGATCCCCTCATCGCCTTTTGTGACTTATAC
GTATCCACTATGTTTTGTACGGACGACAACAACATTTCAATATATTTCTCGGAGGCAGGATCTATCTCCTGCC
TCGCTATCACCAGTGCATTTTCTACGACCTTAGTAGCTTTCGCGAGAATCGCGTCTCGCTGATCCGGGCTACG
TATATCCCCTAGGGCCCATATTGGCTCTAGGTGCACGTCTATATCCATCTCCAGATTGGTGAGGATATCCTGC
CAATCATCGTCGTCATCCTCCGTTGGTGCGTGTGGCCGTTCGGTGGCGTCGTCAGGGTGGTCCGCATCTCCAT
CCGTCGTCTCGCCACTTTCCTCCGTTAGTTGGATGAGGGGTCCCTCTTCCCGTTCTCCCCGAGGTGGCGTCGT
CAGCGGGTCAAGGTCGTCCGAATCGAATGAGCTGTTGGCTCTCGGGTTGGGAGTGGGGGCCTTGTACGTGCCG
GGTAGCGTGTTCTCGCGGTCCATTGTGGTCGTGAAAAATTTCCTTTTGCGGTGATCTCGCCTGATCCTCGGA
TATTCCGATGACAGGGTATTCTTCCAATATATCTGGTAGGTTTAAAAAAATGTAAAGCTGGGCTTTACAGTAG
ATCTTAAGTCGCACGATATAAACCTGACCAAGTCTATATGGTAGTTATATGCTAGTACAAACGAAAGATCTAT

CTATCTATTTGGTTCAAGCCGGTTAAGTATTGGTATGGGCGAGGCCCATGCCAAACAACGTGAGGCTTGTAAC
AAACACGGTCAAACGCCGTAACGGGATGCGGCCCGTTACA
>Agrped1_Cluster1#LTR/Gypsy
TGGGAGGACCAGTGTCTATAAAAAATTTTCGGCGGGAGTAACGGTGTATCGGCGGACAGAGACGGTGTTGTCG
ATGTCAAAAAAAAAGTGAAAGGCTGCCCGTTTGTGGCAAGCCTCACTGATGTTCGCGCAGACAGACAGGACAA
CGGTATCTTCGTTGCGTATGGTAAGAGATGATGTACAAGTGTAAAAGTGGCTAAGAAAACATACCTTCGTTGC
GTATGGTAAGAGATGAAGAGCAACGAAGGTGAAACTGGAAAAAAGGGCATGCGCGGTCATGTGACGAAAAAGA
GAGAAAAACGCGATGGGCTCCATCGCGATAAGGAATCAAAGAAAACGTGAGATAAAAGAAACAGAAACGTGAT
GCAAAGACGCGTCTCGGGCGCGTCGCGTGGTGAGCTCAGGGGCGTCGGGCTTACACTCCTCCCCCCCCTGAAA
AATTCTCCGTGTGTGACATTGTTAAGGCGGCGGCTCGATGCCGTTGAGACGCTGCCAAACATCCAGCGCCTCG
TTGTTCTCCAGCTCCTTGCCGGACATCCACTGGTCGTGTTCCTTCCCATAACCAACCCACCGGACTAGGTATT
GCATACCCCGTCCTCGACGCCGTTCATCAACAATCTTGTCCACCAAAACTTCCTCCACTCCGTCTTCGGTTAC
CACAATCCCCGGATTAGGCAGCGCGCGGTCGGGGAAGTAATCTGGGTCGTTGGGGTGGAATGGTTTCAGAAGG
CTCGCGTGGAATCCAGGGAACGTCTTGGGGTTGTTCGGCAGACGGAGCGTGTACTCAGACTTCTCTGGGTGTG
CGCGGACGACTGTGTATGGCCCGTCCCAACGGGGCATGAACTTCGCAGCTCTTTTCTTCCCTTTTCGCTTGTA
GTTGCGGCGGCGGTTCGTGGTAGAGAGCATCACTAGGTCTCCTTCTTTGTAAACAATCTCGTTGCCGCGGTGT
TGGTTCGCGTGATACGCCTGTCGAATCTTGGCGGCGGTGAGAGAGTCTTGAGCCTGTTGAACATCGGTGTTGA
TCTGTTCGATAATTTCTCGTGCCGTGATTTGTGCTGGTGAAGCGTCATCAGGCGGTGGAGTGAGTGGCGGGAT
GATTCTCGGTGAACGACCCGTCTTGAGCTGGAAAGGCGAGAAACCGGTGGAGGCGTTTACGGTGTTCATGATG
GCAAAGCGAACACGGGGGAGCGTCTTCTTCCAGCCCTTCTGGTTGATGTCGATATGGTAGCGGATAGCTTGTG
TAACTGTTTTGTTCGTCCGCTCACTGCTTCCGTCCGTTTCGGGGTGATATGAGGTCGACATCTTGAGCCGAAC
CCCAGTGAGCTTGTGCAGCTCCGTCCAGACCTCGGCCGTGAATAAAGGGTCCCTATCTGAGATGAGCTGCATC
ATCAGGCCATTTTCGCAGTACCACTCGTTGAACAATACCTCTGCCACTTGCGCGGCGGTGAAGCTGGAGTGGA
TTGCCGTGATTTGGACGTCCGCACCGAGCATCTCGGTCATGGTAAGGATGGTATCACATCCTTCCTCCTCGGG
CAGTGGTCCAATGAAATCCAGCGCGACACAGCTGAAACGTTCATCTGGAACCGGCAGGGGATGCAGAGGTCCA
GTCGGCTTTGTTGTGCGGTCCTTATTGCGTTGGCACTCGACGCAGGATGGGATGTAGGCAGTTTCGAGGTCCT
TTCGCATGTTCGGCCAGTAGTAAGACTCTCGCAGGGAGGCGTATGTCTTGTCAAACCCAAAATGGCCCAAAAC
ATCGTGTGCGAGGTGATAAAGTGTCTCTCGTAGTCGTGTGTCTTTCGGTATGACGAGACGCCGGCCCACGTAC
AGCAGGCCATTCTCCTCGCGTGCCCCGGCGATGCTGTTTGTGGATATGCCCGCACGCAGTTGTTTTGCAAAGT
CGCAGTGCTCGTAACCCGCACGTATGCTCTCCAAAAACTCCTTATCGGCTTCGATGTTCAGCACGGCGGCCAC
CAGTGCCTGCGTGTCCGTCTCAGGGATCATAGGCGAGGGGTGAGAAGAGGAAGCTGTGCGTGTGTATGCAAGT
GCGAGGGCCGCCATGGCCGGAGTCGGTTCATCGTCTGGCATGCGCGAGAGTGCATCGGCTGCAGAGTTGTCTT
CGCCCCGCACATAGACGATGTCGAAGTCAAAATCGGCCAGGAACATGGCCCAGCGTTTCTGGCGATGTGAGGT
GTCCTTCTGCGTCTGAAAGAACTCTAGCGTCCGGTGGTCGGTGTGTACCTCGAATTTGGCACCAAGGAGGTAT
GTCCTCCACTTCTCGAGAGCCTTTACCACCGCTAGGAGTTCCTTCTCATGCGTGGCATAGTTTCGTTGTGCAG
CGTTGAGTTGATACGAGTCGTAAGCGACCGGCCTCGAGGTTTCCCATGTCTCGCCAAAACTGAGAACTGCTCC
TGTGCGGCGGTTACTCGCGTCCGTGGTGACAAATATCTTCTTCGATGTGTCGTCATAGTCAATGACGGTAAGG
CACTCGGTGCCCGTCACCAGTGTCTTGATGGCATCGAAGGCGGCCTGGTGTTCAGCAGTCCAGGGTGGGAAAG
CCTTCTCTGCCTCGCTTGTTGTGAGGGGTGTGAGGACACTTGTGTGTTCTGCTAGGGCGGGGAGGAAGGCTGA
GAGGTATCGAGTCAGACCAAGGAAACCGCGGACATTTGTTGCTGTGGTGGGGATTGGCCAGTTTGCGACACGT
TCGACCTTTCTTGGGTCAGCCTGTATGCCATTTCCGGAAATGACATGTCCTAAAAAGGAAAGCTCTCTTGCGA
AAAGCTGTGTCTTCGACTGGTTGCAGTATAGCTTCGCAGCACGGAGTGCTTCCAAGACAGTGGCGACATTATG
TTTGTGTTCCTCGATTGTCTGAGACCAAATAATGATGTCGTCGAGGTACACATGGCAGATCTTGCCTAGAAGG
GGGCGCAAGGCATCGGTCATACGGCGTTGATGTGTTGACGGCGCATTCGAACCGCCCATTGGCATGACGGTCC
ATTCGTACAGACCCCAAGGGGTGCGTACTGCGGTCAGGTGGATATCGTCAGGGTGCATGCGTGTCTGGAAGAA
CGAGTTTGTCATATCCAGCTTGCCAAAAATTCTGCCTTTCCCGCAGTCTGCAAGAATCTCGTCGACACGTGGT
AAAGGGAAATTATCCCTGACAGTGTTGGCATTCAGTTCTCGGTAATCATTGACCCAACGTGGTGGAACAGTCA
TGTCTGGTACGCCATTACGGTATTTTGGAATGATGAACGCAGGTGATGTGAATTCAGAGGATGACGGGCGCAG
CCGTCCAGCAGCCAGATGTTCGTCGAGGAGGCTCTTCCACGCGTCGTGATACTTCTTTGGAGCAACATAACCT
CGTCCTCGGACGACCTTGTTTGTGTCCTTGAGCCGGATACGGTGATAAATGTGGTTTGGAAGGTGGTTCAGGT
ATGCGGTGTCCGGGAGACGCGTGGGGAAACAGTCGGCAAACTTCTCCTTCATCTCTGCGTCGAGTTTGGCAAA
TTGTTCATCTTCCGATAACCTCTCGATGCGGTCTCTGACAGCTGCCATCAACTCTCGCGTGAGGTGTGTGCCG
TCTAAAACTGCCGCTGGCACCTCCTCAAAGGGTATGGCCGGTGACTTCTTCGGTTTGGGCGGCGTTGGTGGAG
GCACCACTTTCTCGGGTGCCCATTGTCGGGGAGGTACTTTGGGATTGATGATGTCGAGGCCACTATCCCTCAC
GACGGCTGTCCTCCTCTCTGGGTCTATCACAATGTGTTCGGTGGTAAAGAATGGCATTCCCAGTATTAGCGGT
ACAGGTAACAATTCCACTACTTTTGCATAGTTTGTCCGTGAAACCCAAGCTCCATTTGCGACCGACATCTCCA
ACTTCACATACTCCTTGCTCGACAATGGCGTGGCTGACAGCGATGACAAATTGTCTTCCTTGGCTGGAAGCGG
ATAACGCCTAAGGCCTAGTTGCGAGGCGAGCTGGCCGCTAATCACTGTGCAGGGGCAACCAATGTCAAGCAAT

GCTTTAACTGAAAGAGGGAATTCGGAGATGGAAGGGCCCGATATGCTGAGTTGGGCATGGAGATGGGGCATGG
TGAAAAGAGGGTTGGGCGAGGGGACGTACGTGTCATCGTCAAAATAGTCGTCCTCGGCGGCCGTGTTCACAAA
GGCCGCCGGAAGGCGAGCTTTGGCGTCGTGTGCAGCCTTGGCTGCGAGAGCAGCCTCCTTGGTCAGTGGTTTA
ACGGTGGTAGGGTCTGGGAAAGTGCCCGCTGCTGCCATCGGACATCCTGCAGAGCGGTGTCCTGCGTAGAACA
CCCTGCAGCGGAAACACCCATCATGTTCCGAGAGCAGTTCGCGTTCGCGATCTGTGAGTTTGGCGAGTCGCTG
GCGGGGTGTGTTGTCTGTAGCGGAGCTATTTGCCTGGGCGTTCGTCGAGTTGTTGGATTTCGCATCCGACATG
CGTGAGAGGAGAGACTTCCTCTCGGCACGGTGTTGGTTCCTGTTCTCGGTGATGGTGGCTGCAACGCGCTTCT
CCTCGGCCCTCAGCTCCTCATCCATCTCCTGCACCTTATCTCCCCATTCCTTGTACTTGACAGTTGCATCGAT
AGGCTTGTTGTAGAGGGCGAGTGCGAGAGGCGCGCGGGCGTTGGCTTCCAGCTGGAAGCGGAGGGCTTCCTCG
GTGACGGCATATGATTTGGCGGCGCTGGTGAGGAGAGCATTGAGATTCTCGAGCTCGATCCTCCAGTCCACGA
AGCGCGTGTTCTCCGGCTGACGTGCCGAGATGATCTTCTGACGGATCTGGTGCGCCCAGTTCTTGTCGAGAAC
CAGCGCCGAGAGCTCGGCAATGTATTCGTCCAGAGTCAGGGCGTCGATGCGATCTTGCGAGCCCTGATACCAC
GCCTGCAGTCTCGGTTCCAGCATGGCATCGGCCACAAAAGACACTATCTCCTCCGCCTTCTTCCCCGAGTGCT
TCTTGAAGCGGCGGCAGGCGTGTGTCCATTCCTGGAGAATGAGTGGGGTGATTGTCCCGGCCGTGAGCATGGG
GCAATCTTTTAATTTGGTGACCACGATTGTGGCGTGTCCGTTCTGGCTGGGCATCTTTGATGTGCGAAGTCGA
GTGACGGTGGCTGTGTCTGAGTCGCTAGACGACGATGATGTCGACGAAGGTGTTGATAGGTATGCGTCGACCT
CGTTGTCACTTCCAAATTCGTCTGATGTCGGTGGGCTCGGTGTTTCCTCTTCAAGCAGTGTGGACTGTGGCGT
TGGAGATGGAGGTAACGGTGTCTGGAACGGTGTCGGCGAGGATGGCAGTGGGGGCAGCACAGGCGGCGGCTGA
GACAAACCTGTCGTAGGCGGCGTGGGGAGCTGGATGTGCAGGTCCGGGTCCAGATTCTGGGAGCGAGTGACAG
GCGGTGTATCTCTCGGGGGCGGCGCGGTCTTCCGAGGCAGCGTGTGAGATAGTTGTTGACAGCTGGTGGGAGG
ACCAGTGTCTATAAAAAATTTTCGGCGGGAGTAACGGTGTATCGGCGGACAGAGACGGTGTTTTCGATGTCAA
AAAAAAAGTGAAAGGCTGCCATTTCTGGCAAGCCTCACTGGATCAC
>Agrped1_rnd-4_family-972#LTR/Gypsy
CTGGAGCCCACCGCGAGGGGTTCTAAACTTTGTTTACAACATTATTCCCTATCCTCACCTTCGGTCTTTTAGA
CCATTTTTCATTCCACCATTCTTTAGGTTCGGTTCTGGCAAGCCGAACTCGCTCGTTCTGCTACCATCCACAA
CCTTCCTACGTCGCCATCGTCGTCGCCGTCCGCTCGCCGTCATTATCCGTCGNATTCTAGCCGCCTTACCTCG
CCNTTGTCCCCGGAACCGTATACCATCGCCTCAGCACTTTATTCTGCACCTGTGCATTGCTGCGGTCCGTTGT
GGACGTCATGCAGTTCATGCCAGGTCGCTTTGACCCCGCCTCGCCTCGATTTGACGGANAAGCATCGCACCTC
GCTATATTCCTCGACGAAGTCGAAGCCCTAGCCGCCTCACGTTCCCAGACACCGCAGCAAACCATCCGCGCAG
CTATTCGATACTCGCCAGACGACGCCTACGAGTTATGGTCAGGCATTCCNGAAGCCCGAGGAGAAGACTGGCA
AGCGTACAAAGCCGCACTCTATAGGCTTTACCCAGGATCGGATGGAGCTCGTCGNTACANCATACGTCAATTA
GAGCAGCTCGTCGAACGGCAAGAATTCGCTCCCATAGAAAATCAGGAACAATTCGGCACTTATTACCGGTCAT
TCAGCGTCGTTNCAGANTACCTCCTCGAGAAGGGCAGACTCTCGCGCCGGGAAGCCAGCTCATATTTCCTATC
CGGATTCCACATCGAATTTCGCAACCAGATTCGCCGACAGCTTCGAGCCGAGAACCCGCGACGATATCACGAC
GATTTCTTCACGGTCGAAGAGATTAGAGACGTCGCAGTCTTTCTCCTTGGAGGCGAACGTCATACTAACGCCC
TCACCGTCGATAAGCACCGTCCACAGTCAACTTGCTTCCGTCGCAANCATCGTCCCCGATACGCCTCACACNC
TGCCAGCCTCCGTTCCTTCGCACCCATCTAATTTGTTGCATGAGCTACAAGCCACCGCCATTTCTACATCGCC
ATCGTCTTTCCCTGTCNCAGAAGCAACATCGACGCATAGCGCAACGAGTACAGCAGATCCCTTCGCTGCAACG
ACGCCCGTAGACTCNCACATGCAGAACTTACACCCAGACGACGTCACCATCAATCAAGCGCAATACGAGTCCT
CGCTGTTCGGCAACTATCATCTCAGCTACGAGGAACGGATCTCAGTTGTCAAAGAGATACAGAGAATCCTTCA
GATGTCTACCACCGCTCTAGCCACGAATTTCGAGCAAGAAACCGTTCCTTTCGCCCCAGCCGTCGTACCAGAA
GCCACAAGTACACCTGNTATCGCCTCCTTCGATTTTGTTGAGCCACAAAGCAGCAGCCCATCCGAAGAGCACC
AGCACAGCATCCCTGTCGAAGAAGNCACCTCGCAGGCCATCTCCTTCGTTAAATCAGACATAGACGACGCCAN
TGTTAATTCTTCTNCGAACCCGCTGACAGTCTCTTNCCNANTAGNGGCGCCGACAGCTCACTCAGNAAACGAC
ATCTGCGCCTTCATANCCCCAGGAGTCCAAGAANCGAAGCCCATCTCGTCAACCGTCGNCAACTCGCCAGAAT
TCAATGAAGAGTCGCTTCTAGTCAACGTCACGCAGTCAGANCAGCCNCAGCAGCTCCCCAGGATNGNCTCAAG
CTCGCAGAAANGCTCTGATTCGACCGCNCCGCCCGAGAAGACCACGATAGACGCGAAGAGTGCTCGTATGGAC
GCAAACCAAGAGCCTAGCAACAAGGACGAGCNGGACAGAGTTAGTCGACACCCAGCGCATAGAACCGCGGTTC
CATTCGCCAAAACGCCGCCCGCTGCAGCTANTACAGGCCTAGCCGTCGTTATCAACGCCCTACTCGCTTTTGG
CTGGNTATTGGTCGCATCCTTGATTAGCCTTACTTCCTGCGCATCGTCTCTATCACGNCCTCTCGTCCCTATA
CGTCTATTAAGTCCCTTGCAACCTCTGCACTCGACACCGTCGCTTCTTCGAGCTTATTTGCACTTTACAAGTT
AATATTCAATGGAAATNCNTCGTCNCGGCGCCTGCCTTCGAAGTCAAGCAGGTCACATCCNTCGATACACAGC
CAATTTCGACGAATCACCCCACCAGCACGCTTTCCTCTTTACAGACATGTACGAGCATCGTTCCCNGCCTTCT
CCGCCGTTATCACTCCCTTCTAAAGCACCCCGTCGACNTCAGCAAGAAGCCAGNCACGGATCGGCTCAATTCT
CGACTAGGAAGTGCTCNGGAGCGCCAGNAGCCGCGGAANGAAGCTCAATCGACAGCNCGTACCGAAGAGCGTC
CCCTCACAGCGCCTGCCTCCCAAAACACGCCGGATCTCGAATATAAATCCATATTCACTCGTACCATCACGCG
TCGCCCTTCATACTCGACGAAAACGTACCTCAACCTCGCTGGTGAGGTCGCACCCGGCACTAAAAGACGTCAT
CAAGACCCGTCCTTCGATCCAGCTCGNCCTCAGAAAGTAGCCACCTCGATCACAAGCTCAATCAGCGAACAGC

NTCCTCAGACACACGTCNAGCGCTTACCGGAGACCAAAGCCGTCGAAATCAACACCGCGAATTCCACCGGACG
ACCTCCCAGTCAAGTAATCGCGAACCTACGCCGTACCCGCTCGCATCTCGACGCTTCTTCAAGTCTGCTTCGC
CGNCCANTATTTTTCCGTCCTCTGCNAATCCCCAGCCCCACTCGATCGCCACAGCNATAATTTGACACATAC
CTCGCCTGCGACGCCGTCGATTTCCGCAGCAATTTTGCGCTTCGCCCTTTCGATCTTATCCGCCCTTCCGATC
CTTTCACCGCTCCGTCATCGCCCTCCTCGATCCTCCCNCGCTTTCGCCGTCGTCCTCGTCGTNCCGCTGACCA
CTCGCTATCGCGTATCCATCA
>Agrped1_rnd-1_family-529#LTR/Gypsy
GACGAAGAATGGGATAATCGTTCGCATCTGCTNGTATCCATGAGTCCGGANATNGTTCGAAANTTCGCCGAAG
GATACCNGGAAGACGCCTTCTTCAAATCTCGATACGTGGACGAGCAGCCTAACCCGAACGCNGTGCTNACCGA
NTCNCATTTCCGAAAGGACGAGAACGGNCTNCTGTACTTCCTGGACGCNGACTGGAACGCTAAGCTATGCGTT
CCCAAGAGTCAAGTCAATTTCGTGCTGAAATGGATCCACGATTCCCCNTACGAAAGCGCNCACGCCGGCTACA
AAAGATTCGTCTTAGCGGCTNCAGGAAGTGTTCTATTGGAAAACTCTGTATCAAGACGCCTCCACTTTCGCTN
CNACCTGCGACGTCTGCCAGAAAATCAAGACCGACCACCGNAAGAAGACCGGAGCCCTGAGGCCCGCNCATAT
TCCCGCGAGACCTTTCGAAACCGTGTCGTTGGATTTGATCACCGGGTTACCNAAGTCNGGNAAGGAGAAGTAC
ACCGCCGTCCTCGTCATAGTGGACAAGCTNACCAAATTCGCTATNATNATNCCNACCCACGACACCTTGGACC
GAGAAGGNTTCGCTAAANTGTTCGTNGAACGAGTAGCCAACGTTTTCGGACTACCNACGCGNATNATCGCCGA
TCGAGACAAGAGATGGGCTACCGACTTCTGGAAATCGGTGGTGTCNAACTACGGCAGCGTNATGGCTCTNTCC
TCGTCTCATCACCCGCAAACNGACGGACAGACCGAAATCCTCAACGCCACNATCGAACAAATGTTGAGAGCCT
ACGTNGCNGGNAAAAGAGANGANTGGTCNTCCTGGCTCAGCGTTCTGGCCTTCTCCTACAACTCCGCCGTGCA
TTCCTCNACCACTTACTCTCCNAACTTCCTGCTNCTGGGATNCCACCCTNGAATTTCGACCAGCGCTCTGCTN
AAGGAAATCGACCCCGCTTCTCGNCCNTTCCTNCCCAGTCAAAGAGCCGAAGACTTCATTAATTCCATAGAAG
AAGCTCGNTCGGCCGCTCGAAACGCTCTCGTNCTGGCCCAAGAAAAACAAGCCAAAGCCTACAACAAAGGNAG
ANGACCGGGAGACGACATCCGAGTNGGAGACCTGGTCCTAGTNAACCCTCATACCTTGAAACTAGTNGACGCC
GAAGGAACTGGNAAGAAACTAGTCCAGAGAACNATNGGCCCNTTCGAAGTCATGGAGAAGGTNAACCCNGTCG
TNTACCGNCTTCGNCTGCCNGACAACTACCCNATGCACCCGGTCTTCAACGTCAACCATCTCAAGAAATACCA
TCCTTCCCCGGCTGAATTCGGCGAACGNGAAACCCTGCCNTCNACTAGAGAATTCCTNGCCTCNCCGGAATAC
GAAGTNGAAGCNATNCTNGGCCACAAACTAGCCAAGCGAAAAAACGGTAACCGNCGAATGTATCGAGTNAGAT
GGGCCGGNTACGGNCCCGCCGACGACTCCTGGATNTCCGAATACGACTTGCGAAACGCCCCCGAACTCAAACG
AGAATATCACGCCCTTCACTCCCT
>Agrped1_Cluster14#LTR/Copia
GATGAGGCTATATGCACGGAGCGAGCTCAGAAGTCGGAAAGGGAAATGACAACTAATATATTTACCTATACGC
ACGGAGCGAGCTACATCGATGTGAATAAGTATAACGAATGAACCACGACAAAGGGAAACACAATGCATACCTC
AGAAGTCGGAAAGGGAAATGACAACTGAGGTGAAAGAAAGAGGAAACGTGCTATATAAAGGAACGAGGAGAGC
ACGTGACAATCTCGTGAATGAACCTGACCAATGACTCCGTCCAACACACCCCCATCAGGAGAAAGAACGAGAG
AAAAATAATAATAGAAATAGAAAAAGAAGTAACTAGAGTGTCCATCAGAGTCCATTCAAAAAACGAGAAAACA
AGAAGGAAAACAAGAAAGAAATCAATCCTCGGAAGCATACTGGGGTGGAACACACAATCCTAACACATCACGG
TGATGGGAGAAACAAGGGAATGGTAGCGACTTTGTGAAAATATCGGCAGGCATGTCTGCGGTAGGAATCCATG
TAGTAGAAAAAGATCCTTCGCGTATATGTTCCCGAATGAAATAATGCCTGATGTCAATATGTTTGGAGTGTCG
GGATATTGCAGGAGAGTCGGATAAAGCACACGTCGCCTGGTTATCGCATAAAATGGGGAATGGCTTGGGGACA
GGAAGACCAATGGTCTGAAGAAACACACAAAACCATAGTGCATCCTTGAATGCATGAGACATAGCATAATATT
CAGCTTCCGTTGACGAGAGAGCAATGGATTTCTGCTTGACTGCCGACCAGGAGACAAAAGAGCCCATAAAATA
GAATGTGTACCCGGATACTGTACCAATCCACTCAATTCGGTAGTAACGCGTAACGGCGGCCCAAAATCTACCG
AAATGTAACGCGTGTAACGCTCAAAATGGGCGGTTACCGAATTGATTTTAGAAAGAAATTATGTAATAATTCG
TTCAAAATATCAATTTTTTCTCCTGGACCTCGAATTTGGCTGTTTCTGCACTTTCTAAACTGAAATAATTACA
TTTGCTTTACTTTCACCTTGTATTTACAGTATTGTATCTGATATTATGCCGAAAATAATCATATGTAACGGGC
AATTCTGGGGGTTACCGAATTCAAAATTTAGGTACGCGTGTAATGCGTAACATGCCTTCCCGAATTGAGTGGA
TTGGTATAGTATGCTCTTTCTATCCTTAGCATCGGATGCCCAATCAGCATCTGTGAAGCCACTAGCACGCATG
AATCCAGAAATTGATTCAGGTGCGTCGCTACTAACCACTCCCAAGGACAACGCGAGATGCCTAGTGCCAGCCA
AGTATTGCAGAACGTGCTTCGCAAGCTTGAAGTGTGTGGTCGAAGGTGCAGAGCTGAACTGCCCCAACCACAT
TGCCGCGAAAGCGATGTCCGGGCGTGTAGCAATGGCGAGATACATCAAGCAACCGGCAAGATGATGATACTTC
ACAGTTAGGTCAGGCGGAGCAACAAGTTTATGGTCAGGAGGATCATCGGTACTTGGTTGCTGAGTGGGTAAAG
GAGTTTTGGCCGTCTTGCACTTCGACAAATTCCATTCGTCCAACATGTCTGTGACATAGACATGTGAGGAAAG
CCACATCTGGCGCTTGAGACGGTCGCGGATTATAAGCATGCTCAGGAATTTGGCGCAGTCAGTCACCGAGGTC
CACAATGTGCAATCACGATGATAGAGAATGGATAAACCAGTTGTACAGAGGCACAGAGTTTGTAACTCCCAAG
CCGTCGTCAACATGCAGCAGGACAATCAGAAGAAGCGGTGAACCATCACCAGGCATGGGAATAGTCGGGTCCG
GTGAAGAAGTCCAACGACCAAAGAAGACGCTGTGATCAGCATCGCAGCGGGTGAGCCCGAGTTCAAGGAATAA
ACCCATTAAATAAGTGTAGAACTCGAAGGCTGACTGCCAAAGACCATAGAGCGCCACGCGGATACGAAGGACT
TTACCAGGAGAGGTCATACGTCAGCCAGGAAAAGGTCGAGCAAACACGGAGTGACACAATTTGGCATGGAGAA

AAGCCGTCTTGCAGTCGAATTGACAAATTTCCAGATCCTCTGCTGCAGCATAGGCCAGTATAGTGCAAACACT
GGCCATCTTCGCCACAGGCGCATAAGTTTCGTCGAACTGGGAGGGACGCTGACTGAATCCTTGAGCTACCACA
CGCACCTTCTCCTTCCCTGCAATTCTAACTCCCTCGCTATCAGTCTTCATCTGAAAAACCCATTTCAATCCCA
CTGTGTGTTCTCCAGGGGGCAAATCGGCTTCTTCGAAAGCATGCATACCACCAGGGCCAAGACTCTCCATCTC
ACGACCCATGGCAGCAATCCAAGAAGCAGAATCAGGATGTGCAACAGCCTCCGCATAAGATTTTGGAGCCTTC
GTGAGATCAAAGACCCGAGTTGACTTAACCCCAGCTAGCATCGCTGTTGCAAAGTGACAGTGACTCGAAAAAG
TGTCCGCAGAAGAGCTCTCGACTGAGAGAACATCATCCCAAGACAGACAATGTTCCATGTCCTGCAGCGTCTC
AAAATCGACATAAAAAGAATTCGTCGAAAGAGGCAAGTCAGCCAAGGCAGCATAAGAGATGTAAGAACTCATC
AAGTCGGTGTTGAAAACATTGTATGCACGTAGTGCACCCCCATTGTAAACATCCGCTTGTTCACACATCCACT
CGTTCACACACATCCACTCGTTCACATGCATCAGCCACCCGCTCACACATAACCGCCGCCCGTTCAACATCCC
CAATCTCAGGAAAAACACCAACAGACAGCTGCTCAGCACCCCCATTCGCAGAAGACACATCCACCCGCAACGC
CATTGTAGCAACCTCCGCCCGCACACTATCAACAACCCCTTCTTCACTATCAGCACCCATATCACTGTGAGCA
CTCTCCTTCAAGTGAGTTTGAAGACGGGTACGCTGCGCAGCAATAAGCTCGTCAAAGGCACGACCAGCTTCAG
TCCGAGTACGAGTCTGTAAAGGTCGAGGGGACGGAACAGGGACAACGGGTGGAGAAGCCGCAGGAATGGGACG
TGGGATAGAGAACCGACCGGACATATCCTCATTGAAGATGACATCGCGGGAGAAACCTTCCGTACCATTGGAC
TTTCGAACACGCCAGCCCACACGATGCTCATCGTAACCGACGAATATGGCTTCGTACCTAGCCAAATCACCCT
TCTTTCGCAGCTCATGAGGCACCCAGACAAAGCATTGGCATCCCCACACACGCAGATGAGACAAATCAGGCTT
CCGGCCATCATTCATCAGCTCAAAAGGTGTTGTATCTGAAGGAAGAGTAGACATAGGTTCCCTGTTCTTCAAA
TAGACAATGGTACGTGCCGCATCCGCATAGAAAGCCATCGAGGGACCAGAAGAAGCCAATAAGACCTGAGCGC
CCTCTTCAACCGTCCGAATAAAACGCTCAGCCTTCCCATTCTGAGAATGAGCATAAGGAGCCGTATTCTGAAC
AGTAATGCCCTGTGAAGCGAACCAAGCACCCAAAGCACCCTTGATGAACTCCAGAGCATTGTCGACACGAAAG
GCCAAGACCGGATTACCAGTTTTCTGTGCAACAAATCAGGCTATATTGTTATATTCGTGCATGAGAATATGGT
AACTACCGTTTTTTAAAGTTGCACCTCAACGGTAGTTTACCATTACCGTTTACAGGTGCACCTGTTCGCGAGC
CTGGTGGTATGGGTGCATTTTCGACGGTGATTACCATCTACCGTTAGCTGTGCTCTCCCTTCGGTCCCTAATT
TGGTTTGCTCTTGGGCTACGCCACCCGCCTCTTCCTGAATAGCACTTAGCAACGGTCAACCGCATGGAACACA
TAGTGTCTGATATTAAGGCCGAAGATAAGGGGTGTTAGCGGATGGTGGAGCTCTTGTTAAGTCCAGGTTTAGT
ACCTTATCTTGGTATGTCCCGTTGAATTAATGGTTTCGTAGTTGGTAGTACCTCCTGAGGTGATACTTCTCTC
GTTCAGGTATATAGTATAGTAGAATAAGCCTAGTCTGGTATCAAAAAAAATCTTATAATCATACATTACAGTC
GTATTCTATACATAGTAGAAGCGGCTTTTAGGGGTGCACGTAGGACGGTAGTTACCGTCAACCATTGACGCTC
ACAATTAAAAGTCAGTGCACGTAGGACGCTAAGTACCGTCTACCGTTGAGCCTGTAAGGTCACCCTAAGGTCA
CTCGTATTTTCAGTCATCCTTAGGGTCATCAGTGGGTCATCACAGTGCAAGTCCTGTAAAGGTCGTCCCTTAA
GGTCACTTTCAATGTCAGCACTGGGCATGTGATGCAATATGTATCTTATTTCATGAGAACTATTTGTACCTTA
AACTTTGTATTATCTAGGCTCTATACTTACGTCAACATCTTTTCAGCTGCAAAGCATGAGCAGCCTTTTGGTT
CCCTAGATTTGTATGAAGGTCAACATCCGACGAAGCCATGAAGGAACCGGCAGGGTGCACCCAAATGACGGTG
TTCTATAATTACCATCAAAGGTGCACCCCACCCCGTGCAACGTCAATGGTGGACGTGGATTAGCGTTAAAAGT
GCACTAATCATCAAGGCAACCACGTGAACATATGGGTGTGCACATTTGACGGTAGACGCTACCCAGCATCAAG
GGTGCACCCTGTTGCCAGCGCAACCGTTCCCTTTTGACCCACACAGTCGCTAATAGCTCCCAAAAGCGCCACA
TAGGTGGTAGGCTATGGTAAAGCAACAGGTATCGTGCGTCCTACTGTATACACCCTTGGCCTCAGTGACTCTC
TCAAAATCTTCAGACTTCAACTCAGCTTTCACTGCTACAGCTCACATTTGGGTCTCTCAGACGTGTGGGGAAA
TGAAGAACATGTGCATCAGTGTGCGGATATTTGCTGTGGTCGTGGTATATTTCAAATAATTACCGGCTTTTAT
TCTATTTGTTTTGTCCTAATAGTTTTTTTCCGATTAAATAAACTTGTTTCTCATTAATTACTGATTTTGAGCA
CCGGCTGGGAGGTCGCGGGCAAGGAGGAGTCATGCTCTGGGCCACCAGAGGAAGTAGGCCAGAACCCTGCTTC
GATAATTACCGTTGTGCGTGCACCAAATGGACGGTAAACTACCATCCAGGTGCAACTTTCAAAAACGGTAGTT
ACCGTTATTAGGTGCACGGAACCAGTCTTAGCTGGTGAACAAATGCAACGATATTTTTGACAAACTCAAAAAT
GGCTGTCTTCTTTGACATGGACAAAAAGAACACCCAGTTTGAGGAATCATCGAGGATAATAAGAAACATATTA
TCGGTGTTGAAAGAGAACACCACAGAAGGCCCACACATGTCCATATGAAGGAGTTCTCCGATGCGTGAAGTGC
GATTCCCGGGGTGAGTATAAGGACGTTGTGGAGCATGCCCAATGATACAAGACACACAGTTTCCATAAGAGAA
TGACCCGACAAAGTCAAGACCAGTCGCAAAGTTCTTCGTCAACAAGGCACGAGTAGCATCCTGTCCAAGGTGC
CCAAAACGTCGATGCCAGAGCTCAGGAGTAAGACACAGACGGGGAAATGTTGTTTGTCCGACAGGAACAGCAG
GAAGGGCAATAGGAGCGGAGACAGACAACGGCAACAACGCGGAAACTGCATCGGATGGTCGAATAAAGTCGAG
ACGCATAAAAGAAAGGCGGTCATGCTGGTGAGCAGTCATAACATATCCCGGCAACAGTTGATGGTCATCCAGG
AATGTCAACGTCGTGATAGGCTTCTTCGTGAAGAAATGAACGTCAATGTTCTTCTCCAAAAGGGCACCAACGG
AGACGAGACCAATAGGCACAGATGGCGCATATAAACAATTACGGAGGGTAAAGACCACCGTATAATTGTTATA
TGTGTAGCGGAAAGAGACGTCACCAGAGCCTAGAGCCTGCATAGTGCCACAATTAGCAGTCCCAATATCCACA
GTGTTGGTGCTGTTAAAGTTGGAGAAAAGCGAGCGATCCGAGAAGAGATGATGAGTACATCCTGAGTCCAAGA
GGGCATTGTATTTTGTACTCAAGACTGCAAAAGCCAACGGATGCAGAGACTTGATGGGCAAAGTAAAAGCAAA
ATGCCCAAGAGGCAGGAGCTCGGAGAACTTATCACTCAAAGGGTAAAGGGAAACACTTGCGATGGGATTGGGT
GGCACATAGGTAGAAGGCATACAAAGAGCAGCTACAGGAGGTACGGACATCGTCGTCGTAGTATCAAAAGAGG

TAGGGCAGTTTGGACCATAAGGAGAAGGTGGGCAAGAAGGAGGTGGATCATCATCCTCCATGGACTTCAAACT
CTCCATAACCTCTGCAATAAACGCCTTCACAGCGGTATTCTTCCGTTCCATTCCACCCCCAGGCTTGAAGCAA
GTTGCAATGCTGTGACCTGTATAATTGCAATTCGTACATGTGGCGTCGCGATCTCGAGGAGGTGGCTTTGAGG
CTGCAGTAGGAGCTGATTCCAGAGGCTGGGTCGAAGAGACAGGACGCCCAGTCAGAGGACGAGGGCGGGTGAC
AGAGGGAACTGTAGTAGGAGTAGGACGGAAAGCCTTTTTGAGTGCCAGTACGTCTTCGCATACAGTGAAGAAG
GCATCCGTATCATCCCTGTGCGTGCTCCGAACCCGAGTGGGCAACATTGAGACAAGACTGCTAAATGTGGGGC
CACCAGGAAGGTTCCTAATCATCCGACTTATCAGCTTACGGACGTCACCGACACTTTCCTGCGAGACAGCCAA
GGTAACAGCAGCAGTCCACTTGGAGATGTAGTCGTTCACTTCAGCCTGTCCATGCTTGCAGCGAACAAGACTC
ATGGCGTCCAAGATGTCAGAGCATTCCTGATAATCCCATACTCCAAAGAAGCGAACCAGCTTCGTATAGATAA
TGAAAGTGGTCCGAAGATGTGCTGGAGAAGTAGGTGGAGGAATCAATAAACAAGCCTGATCTCCCAGACAACC
GCATAAGACAAAAGTGACCGTAGCATCATCCATCGACCAACGCCTGGCTGCATAGACGTCCTCCAATGTAGGG
TTGGCAGGCCAGGGAGGAGGAGGCTGAGGACGATCTTCCCAAGTGGTGAACGGTGTATGGGGATCAAGAAGAT
GCCCAAATAGATCCATGTGTCGTACAGTCGCTATGACTCCCGATTCCCAAGCATCGAAGTCCTGCTTAGTCTT
CAAGATAGGTATATGCGTGATCGCAGGTTGAGTAAGGGGTTTTGGTGGCTTCGGCGTATGAGAGCAGGCATGC
GCGGCAGGCTGAGGAATACCAGGTGGCGCATTATAGTGGAATGGAGTCTGCAGTGGAAGAGCTGGATGCTGGG
ACCGACGTCGCAGAGGTGTATGCAGTGACGGCATAGGCGGCGGATACCTAGAGTTCTTCAGGTAAACAGACGG
CGTCTCGGCCAATGAAGCAAGTGTATACACATATTCAGATGCAGTGGAACGAGGCAGAGGCACATTGTGGGGA
GAAATATAAGGATGGGAAGAAGAAGAATTAACAGGAGGAATTGAAACAGATGTAACAGCGGGAGGAAGAAAAG
TAGGGTTGGTTTGATAGTTAGGTGGCGCAGTATGTTGGATAGTAGCAGAAAAACGCGAATGATGAGGAACAGG
AGTAGAAACACCAGGAGGCAAATGGAAGTCAACAGGTACAGGCAAAGGAACCGAAGGCAAATGAGGGGGAACA
TCACGACGAAAGTTATAACTAGTACCTACAGGCGTGGATGAACGACGAGAGTAAGAGGAAGTAAACATGGAGA
CAGGTTGGAGTGTGCATGCCTGTATCCGAGCGAGCCGAGCCTGTATGCGAAGAACCGAGTGAACGGTGAGAAA
AGTGAGATGAGCGAGATGAGCTAGGGGTTTCCCGCCCTTTGCTGATAGGGTCCAGAGCCATTGTATGCGTTGC
GTGTAACCCAAAAGACACCAAGGAATGCAATTCGTGGTCACAAGAAGGACCCTACTGCCGCCGACAGGATATA
GATATATGCGTGAAGAAATGCGCCAGAAAGAAGCCAAAGTAGAACAATGGTGAAAGAAAACAAGGCGAGATTG
TGAAAATGAGCCAAAAAGAAACTGGTGAGTTGCCGTTGTTATCGCAGTAGTAGATTGACTACACGGGGCCCAT
AACCTGTCAGACGATGAGGCTATACGCATGGAGCGAGCTCAGAAGTCGGAAAGGGAAATGACAACTAATATAT
TTACCTATACGCACGGAGCGAGCTACATCGATGTGAATAAGTATAACGAATGAACCACGACAAAGGGAAACAC
AATGCATACCT
>Agrped1_rnd-4_family-103#LTR/Copia
AGCGAGAGCGCGNAGAGAAAAAGGAAAAAGAACAAACANGACCAAACTTGATGATCGGATAAGATAAGGTGCT
AGGGTTCTGTCTTAACACTCCCCCTGAACCCGTAGCTTGTCGCTAGTAAATAGAATGAAGAGTATGNGGTCAA
TGAGAAAGGAANGAAGAGNAAGNTGAAANATAAGAATAGAGAAGGNAAAGTGAAGAGTTTTCGGTTTAGATNT
CGAGGGTTTCCATTCCGAGCATCTTGACGAATTTTTCGTGCTTNGTTCTTCCGAGCGGTTTCGTGAANATGTC
GGCNGGGTTGTCGTCNCCGGCGATGGGAAGCACTTTNATGTCTCCCATTTCGACGGCTTCCCNNATCCAGTGN
GTNTTGATGTCGACGTGCTTGATCCTNGAGTGTCCGCTGGCGTTTTTCGTGAGTGACACNGCGACGTCATTGT
CGCAGTAAATTGTGACAGGTCCGTCGTAGTGCTTGTCGAGTTCCTCGTACCACGACGTTAGCCACTTTGCTTG
CTTCCCGGCCTCGAATACCTCCACATATTCCGCTTCTCCAGTCGATGTGCTGATCCTCCTCTGCGTCTTCGCC
CTCCACCCTATGGCTCCCCCTCCCGAAATGAATACGTAGCCCGCCGAGGATTTTCGAGTATCNACGTCGTCTG
CAAAGCTCGAGTCGGCGTATCCNATGGGTTTTATATCTGCGTCCTTCTTATAGCGGATACCTAGGTTCTGTGT
CGCCTTCAGGTATGCNAGGAGGTGTAATGCCAGTCGCCAATGGACGGGGCGAGGGTCAGCCGTGTAGCGCATG
AGNAGCATTACCGCGAACGCGATGTCAGGTCTGGTGCCGTTTGCGAGGTGGTTGAAAGTNCCGCAGGCCTTTC
GGTAGTCTTTGTCGCGCATGAAGTAGGCGTCGTCNCCTGGAATTGGTTTGGGTTGCGAGTCGCTGAGGTTTAG
NGACGATGGCAGAGGTGTGTACTTTGGGTTGCANTCCNGCATCCCAAAGTCCTCCAGNGCCTTTAATGTGAGT
GATTTCTGGTGAATNGATATATCCCCGTTTTCGTGGTAGACAGTCCCCATTCCGAGTACGACTTTTGGNCGCC
CATGGTCNGTCACCTCGTANGCCTNGCCNATTTCGCTNGTGGTCTGNTTTTCGGCCTCGNCTGTGCTGGACGC
TGTCATAATGTCGTCCGTGTATGTNGCGCTCANGGTGTATCCTCCGTCCTTCGTGTATTGNATGCGCATGCAT
GGGTCAGCCTTGGACTGGCGGTGTCCTAGTTCTCGGAANGTGGTGTTTAGNAGTTTNGCCCAGTTGTTTGCGC
CGTCCATGGTGCCGTATAGGTTCTGGTTTAGTCGAAGGACNGTTCTTGTTGCACCTGGGANTGNGTAGTGGCT
TTGAAAACCCGGTGGGATGGCAACATAGATTAGNGCCTGTGGTTTTGCGTTCAGGAAAGCTGTGACGACGTCG
ACAAGATGAACCTTCAGGCCTAGNGCGGCTGCCAGTCCGAATAGCATTCTGACTGACTCGTACCGAACCACTG
CTGCAAACGTCTCCCAGTAATGCTCCCCCAGCACCATCTTAAAACCCTTCGCNACACATCTCGCTTTTCGCTT
CACCAACTCCCCCACTGCATCCGTCTTCTCCTGGNACCTCCACANGAGATCTATCACCTTCGGCATNTCTTTA
CTCCATTCCACCGGGGTGACTACNCCCTTCTCATCCCATTTCTCTATCTCTGCTTGCATAGCCGGCAGCCATA
GGTGGCGACGCGACAAGTCCAGTGCCTCATTATGGTTTTGAGGGATGTAGCGATTGAGAGGCTCCGGTACAGG
TGCGTTCTGGGCGGCGAGGGCAGTNGCTTCGCTCTCTTCGTCAGGNTCTCGNCGTCTTCTGCNCGNTCGTGAG
ATCGCGTTCGNCTGGGTTATCGATGTCGTCGGTCTGATTAGGCGATGTCGCGTCGGCTGTTGACGGTCTGCCA
AGCGGGCAGAGTGTCGNGATGCCGGTGGGTACACCTTTCTTGGTCTTGGCCTTGANGCTCGCGCTGGTTTGTT

TGGCGACTCGTTGGTGGGTTCTCTGTCTGGTCCGATACTCAGCTNGGCTTCGTCGTCCAGGAAATCGTAGTCC
GCGTCGCCATCGTCTCCCGCGAAGAAATCGGCGGGGTATTGGCCGTCCGCCGTTAGTGTCCGGTGGCCGTGGC
CTTCCTCGAACCGGACATTTCTNGATCTCACGATCTGTCCGGTTTCCGGGATCAGAACCCGGTATGTGCCACG
TCCTTCGATTCCCACCAGCCTTCCTTTCTGACCTCTGTCCCCCAACTTCCCATTCCCCTTCTCCCTAGGAATG
TGCACGTATGCGACACATCCCCAGACCCTGATATGACCCCACGTCCTGCTTCTCCCCAGTCCACCTCTCCTCC
GGTATCGTCTTGGGGTGTCTCGATGATGGTAGCAGGTTCTCCACCATGTCNGAGAACGCCCAAGCCTCCGCCC
ACCACTTCGCGTTCAGACCNGAGTCGAGGAGCATCGCCCTCACCGATCCTGTCGTGATTCCGATCGACCNTTC
TGCCGTGCCGTTCGCGCTCGAGGAGTAGGGTGCTGTCGGTATCAGTTTGATACCGTTTTCGCGCATGNANTCG
GTCCAGAGGCTGCTTAGGAATTCGGGTGCGTTGTCAGTNCGGACGNACTTCATCGNTTTNCCNGTCAGGTTTT
CTGACAGCGNTTTGTAGGTCTTGAGCGCGTGGAGTACGGTTTCGGCNAGTCGGTTTTCGAGGAAGAATGATTT
CCTACGGGANGATCCGTTGTCGTGGAATTTCATAGCGTATAGCGCTCCCCCTGCCGACTTNACCCGTGCCGGT
CCCCATAGATCGATGTTGGTGATTTTTAGGACTTCGTCGGTGGGTGTTATTTCTTCGTCGTAAGGNCGTCGTT
TTTGATTNGCTATTATGCAGTCCTCGCAGTCTCCNTGGCTCTTCCCGTCCACTATCTCCAGCCCGCTCACGAG
TTTTGCAGCCTCNCCTATCCGTCTGACTCCGAAGTGNCCCATCCTNCTATGCCAGGTGTCCATGTCTACAGGT
TCTCTCAACGATCTCGCTGTGAGTGCTGTTGGTAGCTTNGAGACGATCGACCCGCTGATCATGTATAGGCCAT
TCTTCAGTTCTCCGCCGAAGATTTCGTCTTTTCCGTGGAAGAACCNGACCTTTTTGTCACCAAACAGGGCGTG
CCATCCTAGCGTGTCCAGTTTTCCGATNGAAATGAGGTTGGCGGTGACGTCTGGNGCGTGTAGTGCGTCGTTG
AAGGTGAGNGTGTGTTGTTTNCCGTCGTGTACAACGTTTATGACCACCTTCCCGATNCCTAGGACCGCGAAGC
GCGCGTCNGCCTTGGATGATTGACCTGTNAGCTTTATTTCNCGGTACTCNGTGAANGCGCTCTTATCCTTGAA
GAAGTAGGTCGTGCATCCGGAGTCCGCAACACAGGCNGATGTCATGTTTACGCTGGGATTTGCGGCAGCGGCG
AGNGCTACCTTTCGCTCGATATGTGCGTTTTCGGCTATGATCTCATTCAGTTTGNTCATGTCGACTGACGCTG
TTAGNGCATAGCTCAGCCCNGTCACGACCGGGCCGCTGGTGCTCGTNGAGGCGTCCGCTGTTGCCATGTTNGC
TGTTGGCGTCGCCGCCGGTGCTGTGCGCTTCCCCTTCCACCAGTGAGGGTATTGGCCNGCCTTTCCGCCGCCC
TGTTGGAAGCAGTCCGCAATNAGGTGCCCGGTCTTCCCGCATGCTGCGTTGAGCATTTCAGGTTCGAGTTTCC
CCCGCCGCGACGGTTTTCAGTGCGTAGTGATTTTACCTCNGCTTGCAGNGCGGTGATGGTTGCTTCTAGTGCC
TTNACNGCGTCGGATGGTGGNGTCGGCTTCTTCGATTTTGCNTCGCGGATNGATAGGCGCTCGGCGTGGGTGG
TTAGGTTCATGATGATTCTGTTNAGGTCCGATTCTCCGTACATCGGCGTGATGACGGGGTCCCAGGATTCGGG
GAAGGAGTCCAGCAGGCGCGTGATGAACCTTTGGTCACTTATCTTTGCGCCAGCCTCGTTTGCGCGTTGGCGC
AGCTTCCTCATCTTTTCGATGTGTCCGTCCTCGCCAGCCACTTTACCCCCTTCGGCCAGTTTGCAATTCGCTA
GCGCTTCCTCGCGCATGTTCCTTGCGCGGTCGCTAGCCCTGCCGTACTGNGCTTCGAGGGCTTTCCAAACTTG
GTGGGATCGTTTATTTGGGTCGATCCCGGAGCCGTATATGTCGACGACGTTGATCAGTATGGACGATAGNGCC
ACACTCTCGCGCAGCTCGTATTCGAGTAGACTAGGCGTNGTNGAGTGGAGTGGAGTGGTTTTTTCTTTGTCGT
CGGACGGTTTCGGTGCTGGGTTTCGGGCGTTTGCTTGTCGGACTGGTTCTGTGGTTTGTTGCGCAGCNTTTTC
TTCGCTGGCTTCGACGNAGTCGAGTTCGACTTCTTCGGGTTCCTCGTAGTCGGCTGGCACGATGATCTTGTTC
TGCCAGTATTTGTGNAGGCCCTTCGGCTTTCCATGTGCCTCCATGAGGACTTTCCAATGGGCATAGTTCTCGT
ACCCTTTTAGTTTATCCTCTGCTGCGAGGGTGATCGGGTTNGTCGAAGACGAAGATGACATCGTTACGATTTC
GGTTGGCTGTATTTATTAGGTTCAATATACAAACCCTTTTTTGAGTTGTTTCGAGCGGCTGTCTACTTTCTTC
TGTAGCAGTGTGGGAACACGGGGCCCATAACCTATCGGGATAGCGAACGGAGTGAGCTCAGAGTTCACGGTAA
GAACGACTTGATATTAGNGAANCTACCTATCGGGAGCGAACGAAGTGAGCTACGTAGGTAGGGTGAGTGAAGN
AATANAATATAAGNATAANATAAGAAACATACCTCAGAGTTCACGGTAAGAACGACTGAGGTGTAAGGGTGCG
AGGGCGCGGAGAGAAAAAAGAAAAGAACAAACACGACTACAAACTTGATGATCGGATAAGATAAGGTGNTAGG
GTTCATGTCTTAACACT
>Agrped1_rnd-1_family-333#TIR/MITE
TTACAGCTACTTTCATATCGATAGACCAACTTTCTAAAATTATACACACAGGCGGGGAATTGTGGTGATTGAA
TTGCGGTTATGGCATACCGGCACGCCCCCTTTCGTCCTCAGACCAGGGAAGGTGGTGCCATTTTGCGTCTCTA
TAAATGGGTATTTTCTTGTTCGGAACACCCCTCGGATCCCAAATTCCAAGGGGCGCCTGTATATGTACCTTGG
TGCTAAACGGTCTATCGATATGAAAGTAGCTGTAA
>Agrped1_Cluster15#LTR/Copia
GTCAACAGAGCACCTAGACGCCCAAAGAGCGAGCTCAGCGTCGGAAAGGAAAAGAGACGACTGAGGTGAACGA
AACACGGGGCGTGGTATATAAGGGACGCGATATGCCACGTGACTGGTGAAGTGATCCTGACAACTTGGGACAC
CGTCCAACACACCCCCATCAGGAGAAAGAAACCAGGCAAGAAACCAGGAATTAAAGACACAATAAAATAAAAG
TAACTAGAGTGACCGTGAATGACCGTTGAGAAAACAAAGAAACAAAAAACGGAAACAAAGAAATCAGGAAGAA
GCATATTGAGGAGGCACCATTAAACCCAAAACATCCCGGTGGCGAGAGAAGATGACGTGTGAAAGCGATTTCG
TGAAGATGTCGGCTGGCATGTCATGAGTTGGAATCCAAGAGGTTGAAAAAGTTCCTTCTTTGATGTGTTCACG
AAGGAAATGATGTCGAATGTCAATGTGTTTTGAGCGTCGCGAGATAGAAGGTGATTCTGAAAGAGAACAAGTG
GCTTGATTGTCGCAGAGGATCGGAAAAGGACGAGGGACTGGGAAATCCATTGAACGCAGGAAAACGCAGAACC
ACAAAGCCTCCTTGAACGCGTGTGACATAGCGTAATATTCAGCCTCCGTCGATGATAGTGCAATTGACTTTTG
CTTGACCGCCGACCAAGACACCAGTGAGCCCATATAAAAGAAAGCGTACCCAGAATACTCTTCCTATCTTTC

GCATCAGAAGCCCAGTCGGCGTCAGTGCACCCATTAGCACGCATAAACCCAGCCAACGATTCGGGCGTGGAAT
GAGATGCTGTTCCCAAGGTCAAAGCAAGATGTTTTGTGCCGGCCAAGTAGCGCAAGACATGCTTTGCCAGACG
AAAATGAGAGGGAAGAGGTGCAGAGCTGTACTGACCAAGCCACATGGAAGCGAACGCAATGTCGGGCCTCGTA
GCGACAGCGAGATACATTAGACAGCCCACCAAGCGTTGATACCGTTGAAGGAGATCGGCCGCAGGGAAATCCT
TGATGGGCTCATCAGCGTCAGATGGAGGCTGCATGGGTAAAGGTGTCTTCGCCGTTTTACACGAAGAAAGGTT
CCACTCATCGAGTAGTTCGGCGACATAAACATGGGAAGACAACCACAAGCGACGACCAGGGCGATCTCGAATA
ATCAGCATACTGAGGAATTTAGCGCATTCACCAAGGTCAACAATTTGAAGACGTGAGGACAACGTCGTGATAA
ACCAATCATACAAGGGGATCGAATTAGTCACCCCCAGGCCGTCGTCAACGTGGAGCGGAACTATGAGAGTCAG
CGGTGAGCCGTCAGTGGGCATTGGAATGGAAGAGTGCGGAGGAACAGTCCACCGTCCAACGAAGACCCCGTGA
TCAGCGTCACACCTTGTCAAACCGAAATCCAGGAGCAACGACGAGAGGAAGGTGTAAAACTCGTACGCAGATT
GACGAAGGCCGTATAGAGCCACTAAAATGCGCAAGACGCGACCGGGGGAGGAGACTTTCCAGCCAGGAAAAGC
TCGAGCGTAAACAGAATGACGTAGCTTTGCGTGCAAAAAGGCGGTCTTGCAATCGAATTGACGAATTTCCCAG
TCGTTGGCCGCAGCAAAGGCTAAAATGGTACGCACACTGCAAGTACGAGCCACAGGTGCATAGGTTTCGTCGA
ACTGGGATGGCCGTTGACTAAACCCTTGCGCTACAACCCTGGCCTTCTCTTTACCAGCAATACGTACCCCCTC
GGCATCGGTCTTCAATTGGAATACCCACTTCAAGCCAACAGTCGGTTCGCCCGGAGGTAAATCCGCTTCCTCA
AACGCGTTCATCCCTTCAGGACCCAGACTATCCATCTCTCGCCGCATGGCTGCCAGCCAAGAAGCAGCGTCGG
GCCTGGCAACAGCCTCAGCATAGGAAGACGGCGCTTTTGACAAATCCCAAGATTTCGGAGTAGATTTGGCATG
GGAAACGAAAGCGAAGACGGACGACGTCGTTGGCAAAGAGCCAGGGAAGTCGGAAGGTGGATAGTCCCAAAGC
AAAGAGGACTCGTAATCGAGCAAGGATTCGTACTCCACAAGATAATCCGCCGTCGAGAGGGGTAAGTCCGAGA
ACGCAGCACAAGATGCTAGAAAAAGGGATAGACCTGCAACATCAACATCCTCCGCCCGCACAACAGCTCCAGA
AGATACATCCGCCCGTCGCACACTGTCATCCACCACACTCATCTCCGAGTCCGCCAGAACCTCCGCCCGCGCA
CACAATGAACCATCCGCCCATACACACATATCCTTGTTACACCTAACATCCACTCCCCAAATCACGCAATCAC
CCCCATCCACAGCACGGTCAAACCATCCAGCAGCAAATAAGGCATTTGCACGAGTAGAACGCTTTTGTCGCAA
GGCGAGAAGTTCATCATACGCACGGCCAGCTTCTGTCCTATTCCGAACACGCTGTGGACGCACAACTGTCACA
TGTTCAGGTAAATCCGGAGCAGAAGGAGGAGCGGGATCTCGTGGTCGGATAGTACGAGCTATGGCAAATCGCC
CAGACATATCCTCATTGAAAATGACATCCCGAGAGAAAGCATAAGTGCCGTTGGGCCTCCTGACACGCCAACC
AACACGATGTTCCTCGTAACCCACAAAGATGGCCTCGTACCTGGCCAAGTCACCCTTCTTTCGTAACTCCTTC
GGCACAAAAACAAAGCATTGACATCCCCACACACGCAGATGGCCGAGATCAGGCTTCTTGCCCTTGTTCATGA
CCTCGAAAGGAGTTGTGTTGACAGGAAGGGTAGAAGTCGGTTCGCGATTAGCCAAGTACACGCTCGTGTCGAA
ACCGTCCCCGTAGAAAGCCATAGACAGACCAGACGCAGCCAGTAGCGACTGTGTGCGCTCTTCCAAGGTGCGA
ATATAACGTTCAGCTTTACCGTTCTGGGAGTGCGCATACGGAGCAGTTTTCTGAACGACAATGCCTTGATCGT
CCAACCACGTACCCAGCGTGCCTTTTATGAACTCCAGAGCATTATCGCAGCGCAGAGAGAGGATTGGCAGACC
AGTCACGCGAACAAGGTAGGCGAAAATGCGCTTAACAAATTCAGCAACGTCTGATTTCTTCGCCATAGCCTTC
TTGAAGCCCCAGTTCGAACAATCATCGAGAAGGATGACAATGAACATTTCGCCAGTGAAAGACATTAGGGCAA
AGGGACCACAACCGTCCATGTGAAGAAGCTCGCCCACTCGAGAAGCACGATTACCCGGGTTTGCGTATGGCTG
CTGAGGAGCCTTACCAATGATGCAAGCAATACATTTACCGAAGGAAAGCGTACCAGAGTGCTCAATTCCAGTA
GCATAGTCCTTAGTAAGTAAAGCACGAGTAGCATCATGGCCTAAGTGACCAAAGCGACGGTGCCACAGTTCCG
GTGTCAAGTTAAGACGAGGAAATGAGGTCTGACCGACAGGCACAGCGGGTAAGGCAGACGCAGGAGTCGAAGA
AGCAGGGCGAACGAAATCCAGCTTCATGAAGGACAAACGATGATGTTGATCAGCTGTCATGGTATAGCCCGGA
AGGGAACGATGAAAGCGATTGAAGATCAATGAGGTGACAGGATTGACAGAGTCAAAGAAAACATCGATTCCCT
TCTCAAGAAATGCACCCACAGAGAGGAGACCAATAGGAACATCTGGGACCCAGAAACATTCCGTCAACTTCAT
AGTGATAGTACACCCCTTGTACGTATAGCGGAAGGACACCGAGCCCGAACCCCTTGAGCGCATAGTACCACAG
TTCGCAGTACCAATCTCAACGTTATCCCCCTCATTCAGATCATCGAATAGCGAACGATCCGAGAAAATATGGT
GTGTGCAACCGGAATCCAGCAGAGCGTTATACTGTGCAGTCAAAGAGACATATGCAGCATAAGGAATATCACG
CAAGGTAGGAGCTGACACAAAGGCAACCTTAAGACCCGCAGAGTAAAGGGAGAATGCGGAGATTGGATTAGGT
GGCACGTAGGTAGAGGAAGGGATACAAAAGGCAGCTACAGGAGGTACGGAGGTACTGGACGTATCAAAAGTAG
AAGGGCTATCAGGTCCGTAAGGGGAAGAGGGTGTCGAGGCAGGAGGATATTGAGAATGATCCCTATCTTCATC
GAACGAGGCAATTTTCTGCAACAGTCCTTCCTGCAACTCCGCCAAGAAAGCACGCGCGGTCGATTTCTTCTTC
TCCTGACCACCCCCAGGCTTATAGCATGTCGCGATTGTGTGCCCAACAATATTGCAGTTTGAGCAAGTAGGAC
GAGCTGTCGCGTCGGACGAGGTGGTCGCAGCCGTGGACGTAGGAGCCGCTGTGGAAGAGGAGCGATGAGGGGC
ATGCGTGGAAGTAGCAGTTTGACGGGATCGAGCAGTAGCGAGGGTGCGTTGGAATGCGGAAGGCTTGAAAGCA
TTGACGGCCTTGAGAATGGCACGGCAGACAGAGTAGAATTCCGCTATGTCGTTGATGCACATGGACTTCAAAC
GTTCATTCAAGGAGTTACGAAGGTGGCTAAAGGTGGGACCTGAAGGCAAACCAAGCACCATGTGGTTGACATG
TGTGCGAATATCTCCAGCCTTGTCGTTCGAATATGCTGATGTAACAGCCGAGTTCCACCGCGCCACGTATTCA
TGGACCTTGTTAGGCTCACAAGGGGTCAATAACGCTGCGTCGAGAGCCTCAGCACACTCCTCGAAATCGCGAG
CTCCATAAAACTGTGCAAGCTTGTCATAGAGCAAGAAAGCGGTGCGAAGATAACCAGGTGCATTGACAGGAGG
GAGGAGAGAACGTGCTTCGTCACCCAAGCGCCCACGTAGGACGTAACATACTGTCATGTCATCGTATGACCAA
CGGCGAGCAGCATACATGTCGGCAAGCGTAGGATTCACTGGCCATGGGGGAGGCGGTTGAGGACGATCCTCAT

AGCAATGCGATGGCGGAGATGCGGGATCAAGAAGGTGCCCGTAGAGATCAAGAAGGCGAATTGTACTCAGAAT
TCCATTCTCCCAGGCAAGGAAATCATCTCTCGACTTCAGAATGGGGATATGCGTCAACGTCGGTGGGGGCAAA
GGTTTAGGCTCCACAAGCGAAGGTGATGGAACAACTGGCGCTGAGGGGACAGGCAAAGCAGGTGCAGGCGGTC
TGCGAGGTGGAAGCGGAGGAGGAGCATGATGACCTAAGTTCATACCACCAAGAGCACCAGGAGGACCGACTAC
GGGGGGAATAGGGGGCACGCTGCAACCAGGAGCTTGTTGACGAGATGGACGACCTTCGGGCAGAGGCACAGGA
TGACGTAGAGGCGAGGGAACATAAACGTAAGAAGATTCAGAAGTAAAGGAAGAAATAACAGGACTAATGGAAC
CCAAAGAACTAGAAGAGCGAGAACGGGAAGGAGCTATATGCGCGGGAGATAATAAAGGTTGCGAAACAGAAGG
AGCGCGAGAGGACTGAAAAGAGGAAGCCCAAGGATTCGAAGGTACAGAAGGTGCATAATAGGAAAAAGGAAGA
ATGTTATTCGAAGCGACAGGAGACGAAACGGCAGGGGGAAGGTGCATAGAAGTTGAAGTACGAGGCTCGTCGA
GGTGTGGGGGACGCTGATGATGAAAAGTAGGAGAATAATCTGAAGAAAAATATCGTGTAGGGTGATATGAAGT
AGGTAAAGGGGGTTCAGGCAAAGTATAGGAAGAAGTATATTGATAAGGGGAAACGGGATGAAAGAGAGGATCC
GAATACGAAGCAATAAAGGGAAATGACGAACGATGTGGTTGTGGAGTGTGCATGCCCTGAGTTAGAGTCTCTT
GATCCCAACCCTCGTCCATCGGAGAGAGAGTTGTGGAAGAAAACATCGTAGGCGGTCCAACTAGAGTGGGATA
TATGCGCCAAGGATGATAGAATGAGGAAAATCCGCTGGATTCAACCGCATATACGAAGCAAATCTGAAAAAGA
ATAACGGCGAGACCGCCAGGAAGAGCCAAAAATTGAGTCGCCAACGAGAGAAAGCCACAGTAGTAGTCTGCTA
GACGGGGCCCATAACCTGTCAACAGAGCACCTAGACGCCCAAAGAGCGAGCTCAGCGTCGGAAAGGAAAAGAG
ACGACTGAGGTGAACGAAACACGGGGCGTGGTATATAAGGGACGCGATATGCCACGTGACTGGTGAAGTGATC
CTGACAACTTGGGACACCGTCCAACA
>Agrped1_rnd-1_family-388#LINE/I
TTGATAGGTCGAGGACATTCGATCCTCCACCCCTAGATCTCGGTTATGCCCCGTATACCGGGGACACACCGCT
TGCTCCAAAGCGCATTTGGCGCTATCTGGGATTCTACTTCGATCGTCACCTTACTTTCAAGGAACACGTTCGA
TACTATACTACCAAGTCTCTCTCATCGGTGAAAGCCATGAAGATGCTTGGCAGCTCTACCCGTGGCTTGTCCC
CAAAGGAGAAGCGACTCCTATACCGTTCGTGCGTCCTCCCCATCGCAACGTACGGCTATAGACTGTGGTTCTA
CGAGGGTGCCCGCATTAAGGGCGTCCTTGCGGATTTCCGCAAAATGCAGCGTAAGGCTGCANTCTGGATTACG
GGCGCGTTCCGTACCTCTCCCACCGGTGGGGTTGAGTCCCTTGCAGGACTNATCCCCATCCATCTACATCTGA
AAAAGCTTGCCGGGCGCTCTAACTTCCGTGCAGCGACTCTTTCAGACACACATCCGCTTCGGTCNATCCTCTC
CGAGGATCACCGCAAGCAAGCCAGTGCTCACCCATTGGCAATATCTAGGCTGACCCCTAACCAACGCTTGAAG
GTTAAGGGCTCCCTCATGGAGGTGGACCGCCATCTCCCTGAACTCACCGAGTCTTTTGCGCCCGTGGCACCAG
AGGCTCGGCCTGGTGTACGCCTACTCGACCGNTTCCCGGATCGAGTGGAATTTCACGGCACGGAAGGCCTTCT
AGACGAACGTAGTCTGGAGGCATACCGTACCGAACTTAATACAGTTCTTACTGAGGCTCGGGAGCAGCCTGGC
ACTGTGATTTGTGCGGTGGACGCAAGTCTGCCAAACGAAGAGCATCGCCATGCTGTCTCTGCAGCCCTTCTAT
TCCGGGGGGTGGAACGGGTTCATACCGCAATCCACCCAGCCGGGCGAGTTACTGCACCAGACGCAGAACTCTT
CGCGATACGTTCAGCAGTCTGTCTTGCCGTACAGCAGGACAATTGCGAACGGATTGTCGTCTTCACCGACTCG
CTTACATCNGCTAAGCGAGCGGTAGATCCTACACTACATAGCGGCCAGGGTCACTCCCTGGCCGTGTGCAGGG
CTCTCGAGCCATGGTTAGCAGAGGATGCTGACTGCTCAATAACGTTCATTCAGGTTCCTTCGAAGCTGAAATG
GAAAATTCATCAGGAGGCACACGACGAA
>Agrped1_Cluster10#LTR/Gypsy
GAAATGTTAGGGTCTTGCCTGTGACTTGCAACGCAACACGGAAAGAGCCTAGAAACGAAGTTACATTGCAGAA
CCAGGGATGTACCTAGAGTACATACCTAGAAACGAAGTGGACAAAGATAAGGAGTACACTACGAGTAGGCTAC
GAAGTATGTGTACGGTGTGGTCATGTGACCGTATCATGACACCATACCCCCTCCCAAAACGAGGCGAGGATTA
AAAATAGAATGATGCAAAAATAGAAAAGAAAGGAATAAAAGAAACAAAGACGTAAAAAGAAATCGACTGTCCG
CCCAAAAGCCCTGCTGACACAGCAGCCCGCGGGCTATTCCATCTCCTCAACTCCTTCCCTTAGCAGCTCAGCT
TGGTTGTTTGCGAGGTCGACCGCGTTTCCTTCGCGGCGTCTGCAGGGGCACTGACTGTGACCGCCGCTCATCC
GCCTCCTTCCTCTCAACCTCCACCACGCTCTTCTCCCTTGCCACATTCGCCAGTTGCTGTTTCCCTCTCCCAC
GACGTGTCCGACTCTTACCACGCGTGGCTTCGTCCCTGAAGAAATCGGCTATGGCATCCTTCGCATTAGTGAG
TTCGTACTCAGCCCGCCACTCATTCTCGCTCTCATCAAAGCCTAACCACTTCACCAGGAAATGTCTCACACCC
CCTCTCGTCTTCTTCATCTTCAACAGCCTCTCGATCTCGCGTTCCTCCCCTGTTTCCATCATCACAGGCGGCG
GCGGCTCTTGCTTTCGACCCAGAATGGTGTCCTCGCGAGCCGGGATCAAACAGGAGACGTGAAACACGTCGTG
AATTGTCCAGCCATCTGGTAAGTCCAACCTGTATGCCGTATCCGAAATTTTCTGAGAGATCTTGAAAGGACCA
AAGCGGCGGAAGTCCAGCTTGTGGCTAGGGCGAGTTGACCGCAGGTCGGCGCGCTCCAGGTATACACGGTCGC
CTGGGATGTAGTCGGGCGACTCGTCCACCCACACATCATGGAACTTCTTCATTCGCTCCGCCGAAAGTTGAAG
TAGGGACTGGAGCTCCGTCTGCACCTCGCGGAGTCGGTCGAGGCGCTCGTCTGCGGCCGGCACTGACTGCGAG
CTGACGGGATCGATGGAAAAAGTGGGGTGGTAGCCATACTCGGCAAAGAAAGGCGACATACCAATTGCCGTGT
GGAAGCGGTTGTTATACGCAAATTCCGCAAAGGGCAGCCACGAGACCCAGTCGTCTTGATAGTAGTCAACATA
TGCCCGCAGGTAAATCTCCAGCTCCTGATTAACACGCTCCGTCTGACCATCAGTCTGTGGGTGATGTGCAGTT
GACAATGCCCTCTTAATCCCCAACCGCTCCCACATGCGTTTCGTGAACTTCGCAACAAACTGAGGACCCATGT
CCGACACCAACTTCTTGGGAGTGCCATGCAAAGGCCAAACCCGCGACATGAAACTATCACACATATCGGGAGC
GTCGTCAGTGGTATGTGCTGGAAGGAAATGTGCTCTTTTTGTTGACTTATCGCACACCACCTGAATAGCATCG

TACCCCTCAGAAAGAGGCAGCGGCCCAACGTAGTCGGTTGAAACATGTTGCCAAGGGCCCAAGGGAACCTCCA
GAGGTTGCAGCTTCCCATGAGGACGTTTTTGACGTGGTTTGGTCTTCTGGCACATCTCGCAACCCTCCACATA
GCGCGCCACATACTCCGACATCCGAGGCCAATAGTAGTTCAGAGAAACCAACTCCAGAGTATTCGAAATCCCA
GGATGACCAGCCGGAATGGCATCGTGGTAGAGCTGCAAGATCCAGCGCTTCACCTCCTCATCATTTGGCACAT
ACACCAAACCCCGAAAGCGGACTAGCCCATCATCAACCTTGAAGTCCTTGAGGTGTGTTTGTACTAGGGCAGG
TGCAGTCTCGAGGGTCCACATGAGCTCGAGCTTTGGATCCAAACGTGCCACCTTCCTAATCCTGTCCAGAATC
TCCCCATCCGCCCGAATGACCTGCCCAACCTTCGCGACCACATTCGATTCACCACACCACGCCACATCAACAA
CCACCCCTCGCCGACCACCAGCCACCAAAACCTCCTCATCCACCTTCTGTCTCCCAGCCTCTAGAGTCCCATC
ACCCTGCCCAAGTCTGTCTGCTGACAACACACGCTGTTGGAGGTTGTCCCGCGCAGTGCGCAAAACCTCATGG
TCAGCACGACGCGACAACTCATCGGGGCCTGCACTAAGACGCCCTGGCCGGTGGTGTATCTTGAAGACAAAGC
GATTGAGGAACAGACTCCACCGTGCCTGACGACGATTAAGGTCTCGCGAAGTCATGAAGAATTCAAGATTACG
GTGGTCGGAGAAAATGTCCACCAGGTGTGGATTCCCCTCTAGGTAAGGCCGCCAAACCTCGAGAGCCCGAACC
ACAGCTAACAACTCCTTGTCGTGAACATCGTAGTTGCGCTCAGCTGGTTGCATGGCTTTCGAAAAGTACGCCA
CTGGGCGCCAATCACCTGCACCATCCTTCTGCGAGAGGATAGCACCCACTGCAAAATCAGATGCATCCGTCTC
AACACGGAATGGCGCCTCCATCTCTGGCATCATCAACACAGGAGCCTTGGTGAACGCACGCTTGAGAGAGTCA
AATGCGGCCTGCTCAACTTCACCCCACCGCCATGCTACATCCTTCCGCTTCAGGCGTTCCAGAGGTTGGGTAA
TCTGAGCATAGTCCTGCACAAACCGCCTGTAGAAGTTGCAGAACCCTAGGAACTGATTGACATGCCTGACATT
CCGAGGAACAGGCCAGGAGGTCACACCGCTGACCTTGACTGGGTCCATGCCAACATGGCCAGGCGAGATTTTC
AGGCCCAAATAATCCACCTCTGTAGTCCGGAACTGACACTTCTCCGGCTTCAAGAACAAGTCTGCCTCCCGTA
ACCGCCGTAGCACCTCCTTCACCTGAGCATCATGCTGGGATGGGTCCTCCGTGAATATGATGATATCATCCAG
GTATAGCACCACGTTCACATCCACCAGATCATGAAATAATTCATTGACCATCCTCTGAAAGGCAGCTGGTGCA
TTACGCAAGCCAAACTGCATAACCAGAAACTCGAACTGGCCATAGCGAGTGCGAAAGGAAGTCTTCCACTCGT
CGCCTTCGCGAATGCGCACATTATAGTAGCCCCAGCGCATATCCAGTGTGGTGAACACCTTGGCGGCCTTGAG
GCGGTCGAGAAGCTCAGTAGTCAAAGGAATCGGGTACGAGTCTGGCTTTGTGATGGCGTTTAGGGCACGAAAG
TCAACAACCAGGCGTAAGGACCCATCCTTCTTCTTAACAAAGAAGACTGGGGCACCGCATGGAGCCTTCGACG
GTCGGATGTAACCTTTTGCCAGGTTCTCGTCAATGAATTCCTTCAGCGCCCGGAGCTCGCCAGCAGCCATCTG
GTACAACTTTCCCACCGGTGGGGGTGCGTCGCCTTCAAGGTCGATGGCAAGATCATGAGGCTTGTGTTCTGGT
AGGCGCTCCGACTTCCCTTTATCAAAGACATCAAGAAATTCATGAAATTCCGCAGGAACCATGTCCTCCACCT
GCCGCACACTGCTGTCTGCATCAGAGGTGCTCAAAGCCTGTTCCGCCAGAGACTGCGAAAATGAGCGTGCTGC
TGCTGCGCGCACTGAGCGGAGGTTTGAACCACCCACACCTCTGCATGACTCCTCATCCCAGCGCTGCTCCAAA
AACACCCCAGCCCTATCTTCCATCCCAGCAGTGGCACCTGCCAAATCCACCCGTCGCTCATGTGCCTCAGCAC
GGAGCAAGGCCTCCTCAATAACAGCTCGCGCCACACGTACCGCCTCACCCTTCCGAGAGCGGAGTAATGCAGT
TCGTGACGTCCGACTGCGCAGCCATCCCGGAACAGACGTGGGCAGACGAGCTGCCCTGCCAACAGTAGTGTTT
GTTCTCCGCGAGCACCTTGCCCAACCTTTCCCCTGACCCACTGGACCCTTCACCTTCTCCAGCAGCTGCAGTC
GTGCAGCCTCAGTCTCCTCCCTTCCTATCCCACTAAGGCACCTTGCTTTCCTTCTCTCCAACCTCTGTGTCGT
CCGCGCTGCCCGTCGAGCCTTCTGTAAGGCACGCTTACGCAGGCGACGTGGCGACGCGCAAGCAGCTGCAACC
ATTGCCTGCACAGGTGCTGACTCTGCTCTGGGCAGACAATGCTGCTTGCAATGCAACGAGACAAAGTCAACAG
TAGAACGACTCCAACAGATAGCAGGGTCATGTAGACGTAGCCAGTTGATACCCAATATGAGGTTGTCGTCACC
AATATCAGCTACATCACAGACCAGCCTTTCAGAGTGGTCACCTATGGTGAGGCGCAGATGGACCTCATGGGTG
ATTAAACCAGATTTGATGTCGGAGTTATCAATGGTCCGAAGGCGCAGTGGACGTTTCTTCGGAACTGCAAGGA
GGGTATGCAGAGCAACAAAGCGTGGGTTGATAAAACAGCAGCTAGCACCACTGTCCAACATGGCGCGGGTGTC
AATGACAGATGGTGAGGAAGGGGTCGCAGAACTGTGAACTTGCAATGTGATTGGTACTTCGAAAGAAGAAAAT
GAAGAGTGTGGTGATCTCGCATGAATCACTGCAGCGCGACGATGGCGGCGATGCTGGCGAACAGGCACAACTT
CATTTGGACCTAACAAAACACTTTTATTGGCAGGAGCAAGTACACAGAACTTGTTATAGGTTGCAGCCGATGC
GACTACTCGAGGCAACCCTAGACAAAACCCGGCTCCGGAGGAGCAGGTGAAGCAGCACCAGACGCAACAGCAA
CTGATGGCGATACCTCAGCTGCACTCCGACGTAACTCCTCCAAAATGGCCGCCTTCAAACGACGTTCCAGCGC
ACCAGTGCATTGCGAGCGGATGTGCCCAGCAGCACCACAGCGATAACACGTGCGAGATGAACGTGCCCCATCG
ACATCCATGGCATTAGGATCGCGAGGTGGAGCAGGCAAAGAGACCTGCGCTGGACGAGGAGGTGCTGCTACCA
CTGTACTGGACACAGGCGGAGTGTAACGAGCGACATTGGCAGGCACTGCAGGGGACGGAGCAGGGCGGAAAGG
GTTGTTGCTACCACGTTGCCTCTCCTCGGCGGAAATGTCAGCCAAACGAGACTCCCTGCGCAAAAGGATCTTC
TGTGCCTCAGCTGCAGTGGTGAAATCGGAACCGACAAGGTTTTTCTTGGAGACAGTAGAGAGAGACCCTGCCAAT
AACGCTGATCAATGAAGTGACCAGTCTGCTCCGCAGCCGGCAGCTTCGCGACGAGCTCGCGGAACTCGGTGGT
GTAAGCCGTGATTGTCTTTGACCCCTGCGTAAGCTTCATGAGGCGCTGTAGCGCATCGCCCGCCGCATCGCGC
GAGCAGAAGGTTTCCTTGAACGCCTTCAACCAAGCAGCCCAGTTCGTGCGCTCCGCGAGATTCGAATCGCCTT
CGCTCATGGTGATAACGGCAGACCAGGCCTGAGCTTGGCCGGAGAGATAAGTTTGAGCCCAGAGAATCTTCTG
TGTGTCGGATGCGAACACCTCGAACACGGAGGCCACCGTAATCTGCTGCACAAACTGCTCGGCCTTGGCCACG
TCCGCGCCATCGTAAGTCGTCGGTGCGTGCACCTTGGGAAGCCGGACAGAACCACCGCGAGACGCAACCACCG
CGGCCAGATCCTTCCGAAGTTCCTGGTTCTCCTCCTCCAAACGGTTGCAGAACGCCGCTGCATCCACCAAACC

CTGCTCCAGCGCCGCAAAACGCGGCTCCAACGACCGCTCAATCGCGGCAAACAGGCCGTCCACCAAACGCGAA
TCCGGAGTAGACGCCAAGGAGTCAGAAGTAGCGGCGTCGTGAGACGAAATGGGGGCAGGCTCGGACATGGTGA
GGGGTGCGAAAATTTTCAATGTTTTCGCGGACCTGATAAGATCTTTGTCCAAGTGTTAGGGTCTTGCCTGTGA
CTTGCAACGCAACACGGAAAGAGCCTAGAAACGAAGTTACATTGCAGAACCAGGGATGTACCTAGAGTACATA
CCTAGAAACGAAGTGGACAAAGATAAGGAGTACACTACGAGTAGGCTACGAAGTATGTGTACGGTGTGGTCAT
GTGACCGTATCATGACATGAAA
>Agrped1_rnd-4_family-772#LTR/Copia
TCACGACCNCGAAATNCGCCAANTCGACGTCAAGGGCGCTTACCTGAATGGCACCCTGACGGAGGANATTTNC
ATGAGACAACCGCCAGGNTATGCAGCCGCCGGATTCGCGGGCAGAGTNTGCGAGCTATTCGAAGATCCCTGTA
TGGTCTGAAGCAGTCGGGACGACGTTTGGANCGGAGAACTGAACCGAGTACTCAAAGAACTCGGGTTCTTTCA
GCTCAAATCCGATTACTGCTGCTATATTCGACGAAGCGGCGAAGACTTCACTATTTTACTCGTNCGGGTCGAT
GATTTTATTGCCGTATCAACCTTAGACGCCGAAAACGACCGCANAGAAGAAGATCTCAAGCAGCATTTCGAAA
TTAAATCGCTTGGACAACCGTCTATTTTATTGGGCATTAAAGTGCGCCAATCGAATCACCTCATATCCCTCTC
GCAAACNCATTTTATCGAAACTCTTCTGACAAAATTTGGACTAGAAGATGCGAACGCAGTCACTACACCTCTC
GATCCCCATGTAAAGCTTGACGATGAAGACGACGGAGAATTTAATGCATGAAGAAAATATTATTTCNCATGGA
TATGCGGCGCTTATCGGTTCCCTCATGTATCTGGCACACGGAACACGTCCAGACATNNCCTATGCAGTTCACA
AACTCGCGCAATTCACCTCCAACCCGAAACCGAAGCACTGGAANGCCGTCAAGCGAATATTTCGTTACCTAAA
AGGNACTCGGCATCATGCTCTCACCTACGGAGGANAGGATCATTTATTGAAGGACGACTTATATTTGTATTGC
GATGCGGATTGGGCATCCGACGCGGATAGAAAATCCATCAGCGGNTNCGTTATCACGATGGCTGGCGGCGCCG
TCGCCTGGAGCTCGAAGAAACAGGCCACGGTTGCATTATCNACTGCCGAAGCCGAATATATTGCAACTACCCA
CGTTGCGAAGCAGGTNCTCTGGCACCGANCATTATTCACGGAGCTCGGACTACCCCTTTCGAAGACCTCTACC
ATTTTCTCTGATAATCAGGCNGCAATCTCGATNNCTCATCACCCCGAATTCCATGCGCGAACGAAGCATATCG
ACATCGCNTATCACTTTCTACGCGATCTCGTCGACTCAAATACTACTCGACGTTGTCTATATTAAAACNGACG
AGAACCTCGCGGATATNTTTACAAAAGGTCTACCAAAGATNCTACACGAGAATCTCAGCTACGAGATTGGAGT
ATTGCCTGACCAAGGGGGAGTGTTGGAATGATTGGTCAGCCAACTCGGAGAACTGGGTTCGGCGCTTACTCGT
ACGCCCCACTTCGTTATGTAGAATAGTAATCTTCATTTTTCTATTATAAATACTGNACAGTAANATCTACTTA
GTAGTATACATGTCTGTAGTATAAAAGCTCGTCGAAAGCCTTCGACGAGCACTAGCTAATTACATTTACTTTC
TTCTTTATTTAATACACTGCGCTCAATAA
>Agrped1_rnd-4_family-1113#LTR/Gypsy
GTAAGCCCGACGCCCCTGAGCTCACCACGCGACGCGCCCGAGACGCGCCTCGCATCACGTTTCTTTTCTTTCA
CTCACGTTTTCTTTNACTCTCTCATCGCGACGCGNCTCGTCGCGTTTTCTCATCTTTCATCACATGACCGCGC
ATCTAGTTTCTCTTTTCGCCTTCGTTGCTCTTCATCTCTTACCACACGCAACGAAGGTANGTTTTCATAGCTA
CTTTACATTTGTACATGACCTCTTACCACACGCAACGAAGACACCGTTGTCCTGTCTTTCCGCGCGAACNTCA
GTGAGGCTTGCCACAAACGGGCAGCCTTTCACTTTTTTTTTGACATCGACAACACCGTCTCTGTCCGCCGANA
CACCGTTACTCCCGCCGAAAATTTTTTATAGACACTGGTCCTCCCACCAGCTGTCAACAACTACCTCACACGC
TGCCTCGGAANACCGCGCCGCCCCCGAGANCTGCACCGCCTGTCACCCGCTCCCAGAACCTGGACCCGGACCT
TCACATCCAGCTCCCCACGCCGCCTACGACAGGTCTGACTCAGCCGCCGCCTGCCCTGCCCGCTCCGCCGTCC
TCGCCGACCCGGATACCGCTACCACNCTCTCCAACGCCACGGTCCCCGCTGANCGAAGAGGAAACACCGAGCT
CTCCGACATCAGACGATTTCGGCAGTGACGACGAGGTCGACGCATACCTATCCACACACTCGTCGACATCATC
GTCGTCTAGCGACTCAGACACAGCCACCGTCCTTCGACATCGCACATCAAAGATGCCCAGCCAGAACGGGCAC
GCCACAATCGTGGTTACCAAATTAAAAGATTGCCCCATGCTCACGGCCGGGACAATCACCCCACTCACCCTGC
AGGAATGGACACACGCCTGCCGCCGCTTCAAGAAGCACTCGGGGAAGAAGGCGGAGGAGATAGTGTCTTTCGT
GGCCGACGCGATGCTGGAACCGAGACTACAGGCGTGGTATCAAGGCTCGCAAGATCGCATCGACGCCCTGACT
CTCGACGAGTACATTGCAGAGCTCTCGGCGCTGGTTCTCGACAAAAACTGGGCGCACCAGATCCGTCAGAAGA
TCATCTCGGCACGTCAGCCGGAGAACACGCGCTTCGTGGACTGGAGGATCGAGCTGGAGAACCTCAATGCCCT
GCTCACCAGCGCCGCCAAGTCATATGCAGTCACCGAGGAAGCCCTCCGCTTCCAGCTGGAAGCCAACGCCCGN
CCTNCTCTCGCTCTCGCCCTCTACAACAAGCCCATCGACGCNACCGTGAAGTACAAGGANTGGGGAGACAAGG
TGCAAGAGATGGACGAGGAGCTGAGGGCCGAGGAGAAGCGCGTNGCAGCCACCATCACCGAGAACAGGAACCA
ACACCGTGCCGAGAGGAAGTCTCTCCTCGCGCGCATGTCGGACAGCGAAATCCAACAACTCGACGAACGCCCA
GGCGAACAGCTCCGCCACCGACAACGCACCTCGCCCGCGCCTCCCCAAGCTCACGGATCGCGAACGCGAGCTN
CTCAACGAGCACGACGGNTGTTTCCGCTGCAGGGTGTTCTACGCAGGACATCGCTCCGCGGGNTGCCCNATGG
CGGCCGCAGGCACNTTCCCGGACCCAGCCACCTTCAAACCGCTCACCAAGGAGGCCGCTCTCGCAGCCAAGGC
CGCNCACGACGCNAAAGCTCGCCTCCCGGCGGGATTTGTGGNCACGAGCGGTNTCGACGACGACGAAGCCTAC
TTCGACGATGACACGTACGTCCCTCCTTCCGCACCCATCTTCACCGTNCCCCATCTCTATGCCCACCTCGACA
TTTCGGGCCCTTCCATTTCCGAGTTTCCTCTTTCAGTTAAAGCCCTGCTTGACATCGGTTGCCCCTGCACAGT
GATTAGCGGCCAGCTCGCCTCGCAACTTGGCCTNCGACGTTTTCCNCTTCCGGCGACGGAGGACAACCTGTCA
TCGCTGTCATCGACGCCACTGTCGAGTAGGGAATATGTCAAGTTGGAGATGTCGGTGGCGAATGGGGCTTGGA
TNTCACGGACGAACTATGCAAAAGTAGTGGAGTCGTTACCTGTACCNTTGATACTGGGAATGCCATTCTTTNC

TACNGAACACATCGTGATAGACCCGGGAAGGAGGACTGCCGTTGCGAGGGATAGCGGNNTCGACATCGTCAAC
CCAAAAATACCCGCCCGACAGTGGGCACCCGAGAAGGTGGTTCCACCACCAACGCCNCCCAAACCNAAGAAGC
CNCCGCCCGTACCGTTTGAGGAGGTGCCGGCCGCCAGNCTGGACGGCACACACCTGACGCGAGAGCTGATGGC
AGCTGTCAGAGANCGNATCGAGAAGCTGTCGGAGGAGGAGCNGTTCGNCAAACTCGACGCTGAGATGAAGGAG
AAGTTTGCNGACTGTTTCCCCACGCGCCTNCCGGACATCGCATCNCTGAACGACCTNCCAGACCACATCTACC
ACCGCATCCGNCTCAAAGACACCAACAAGGTCGTCCGNGGACGTGGTTACGTCGCNCCAAAGAAGTACCANGA
CGCGTGGAAGAGCCTNCTGGACGAACACCTNGCTGCTGGACGACTNCGGCCATCATCCTCTGAATTCACATCA
CCTGCCTTCATCATCCCAAAATACCGTAACGGCCTGCCAGACCTGACNGTTCCGCCGCGCTGGGTCAACGATT
ACCGAGAACTGAATGCCAACACCGTCAGAGACAATTTCCCNCTACCGCGCGTCGACGAGATTCTCGCTGACTG
CGGNAAAGGAAGGATTTTCGGAAAGCTCGGACATGACAAACTCCTTCTTCCAGACGCGCATGCACCCGGACGA
CATCCACCTCACGGCNGTTCGNACCCCTTGGGGCCTGTACGAGTGGACGGTGATGCCGATGGGNGGNTCTAAT
GCACCGTCNACGCACCAACGCCGTATGACGGATGCTCTNCGCCCCCTTTTGGGNAAGATCTGCCACGTCTACC
TCGACGACATCATCATCTGGTCNCAGGACATCGAGGAGCACGAACGNAACGTCGCNGCCGTGCTGGANGCACT
NCGCGCTGCCAAACTATACTGCAACCGGTCCAAGACACAGCTNTTCGCGAGNGAGCTTTCCTTTTTAGGGCAT
GTCATTTCCGGAAATGGCATACAGGCCGACCCGAGGAAGGTGGAACGCGTCGCGAACTGGCCGATNCCCACCA
CAGCCACCAACGTCCGAGGATTCCTTGGNCTGACCCGNTACCTCTCAGCCTTCCTCCCNGCCCTCGCAGAACA
CACCAGCGTGCTNACACCCCTCACAACNACCGAAGCCGAGAAGGATTTCCCGGNNTGGACCGCCGAACACCAG
NCCGCCTTCGACGCNATCAAGGCCCTGGTGACGGGCACNGAGTGTTTNACCGTCATTGACTACGANGACCCGA
CGAAGAAGATCTTTGTCACGACGGACGCGAGCAACCGACGCACAGGCGCAGTNCTCAGTTTTGGCGAGACATG
GGAAACATCTAGNCCTGTCGCTTACGATTCNTACCAGCTNAACGCCGCCCAGAGAAACTACCCNACGCACGAG
AAGGAGCTCCTCGCCGTCGTNAAGGCTCTCGAGAAATGGAGGACGTACCTNCTTGGCGCGAAATTCGAAGTNC
ACACCGACCACCGNACNCTGGAGTTCTTTCANACGCAGAAGGACACGTCNCATCGCCAGAAACGCTGGGCCAT
GTTCCTAGCGGATTTCGACTTCGACATCGTCTACGTCCGGGGCGAAGACAACTCCGCAGCNGATGCGCTCTCG
CGCATGCCAGACGACGAACCGACGCCGGAGNTGGCGGCCCTAGCGCTNGCGTACACGCGCACGGCGTCNTCCT
NTCACCCNTCGCCAGCCCGGAGCCGCCGACCCTGCGCGGGAGCTGGTAGCCGCCGTNCTGACCATCGAGGCCG
ACAAGGAGTTCCTGGANNGCATTAAGTCGGGNTACGGCACTTGCGAGTTTGCNAAACAGCTNCGNGCGAGCAT
NGCCGCGAAGAGCATCGCGGGTGCACGGGAGGAGAATGGCCTNCTCTACGTGGGCAGGCGTCTGGTCATNCCG
AAAGACGTCAAGGTGCGCGAGACACTNTACCATCTCGCNCACGACGTCCTGGGACATTTCGGCTTTGACAAGA
CCTACGCCGCCCTACGNGACTCTTACTACTGGCCGAACATGCGNCGGGACCTCGAGAACGCGTACATCCCCTC
CTGTGTCGAGTGCCAACGCAACAAAGACCGCACGACGAAGCCNACNGGCCCGCTGCATCCNCTACCCGTCCCA
GACGACCGTTTCAGCTGTGTNGCGCTGGATTTCATCGGACCGCTGCCCGAGGAGGACGGNTGCGACACCATTC
TGACCATGACCGAGATGCTGGGCGCGGACGTCCAGATCGCGGCCGTGCACTCCACATTCACCGCCGCGCAGAT
CGCCGAGGTNCTNTTCAACGAATGGTATTGCGAGAACGGCCTGATGCTGCACCTNATCTCGGATAGNGACCCC
TTGTTCACGGCCGACGTCTGGACGGAACTGCACAAGCTNACAGGTGTTCGACTCAAGATGTCGACATCGTATC
ACCCAGAAACGGACGGAAGCAGCGAGCGGACNAACAAGACAGTTACACAAGCNATCCGNTACCATATCGACAT
CAACCAGAAGGGCTGGAAGAAGACNCTNCCCCGCGTTCGCTTCGCCATCATGAACACGGTCAACGCGTCNACC
GGTTTCTCGCCTTTCCAGCTCAAGACGGGTCGGTCGCCNCGGATCATCCCTCCNCTGNCNCCACCGCCCGGCG
ACGCGTCGCCNGCGCANATCACNGCNCGCGAGGTCATCGAAAAAGTCAACATGGACGTCCAACAGGCNCAGGA
CTCTCTCACCGCCGCCAAGGTCCGACAGGCGTACCACGCGAACCAACATCGNGGCGACGAGATCGTNTACAAA
GAAGGAGATCTCGTCATGCTNTCNACCGCANACCGCCGCCGNAATTACAAGCGNAAGGGGAAGAAACGNGCCG
CCAAGTTCATGCCCCGCTGGGATGGNCCTTACACAGTCGTGCGCGCACACCCCGAGAAGTCNGAGTACACGCT
CCGTCTGCCGAACAACCCCAAGACNTTCCCCGGGTTCCACGCCAGCCTNCTNAAACCNTTCCACCCCAACGAC
CCCGACTACTTCCCCGACCGCGCGCTGCCCAACCCGGGCATTGTCGTNACCGAAGACGGNGTGGAGGAAGTTC
TGGTNGACAAGATTGTCGACGAGCGGCGCCGAGGNCGAGGTATGCAGTACCTNGTCCGNTGGGTCGGNTATGG
NAAGGAACACGACCAGTGGATGTCCGGCAAGGAGCTGGAGAACAACGAGGCGCTGGACGTCTGGCAGCGGCTG
AACGGGATCGAGCCGCCGCCNTAAAACACGGGGAATTTTTCAGGGGGGGGAGGAGTGTAAGCCCGGCGCCCCT
GAGCTCACCACGCGACGCGCTCCAGACGCGCGCACGCATCACGTTTCTTTTCTTTCAACTCACGTTTTCTTTG
ATTC
>Agrped1_rnd-1_family-179#TIR/MITE
TAAGGCTGACCACGAGTGCCGCCAACCCACAGGTACTCGCAAGTAGACCCGTGGGAATACCCGTGAGTATTGC
TACTCGCGGGTCCGGGTCACCCGTGGCCACGGGTCCTTGCGAGTCCGAGTTTGGAAATTGGGACGGCGAGTAC
CCGCGGCAAGTACTTGTGCGTGACTCGCACAGCCGCNTAGAGCGCCNAGGTCACATGTTTCGACCTCGCGCAG
CTNTATTTACCAAGCTATTATGGCTCAGGTGTCTTGAGCGCGAGTTTTCGGGCCTCGNAGTCCGGGGTTCGAG
TCCCGGACGTGCCATTTTGCCACTTGGCAGNGCCCATTGCTCGAAAATTCGGCGCTTATGTCAGCTGATCAGC
CATTCACGTGCCCTCACGGGTTGACCGCGAGTACTCGCCGTACTTGCGAGTACTTGACTCGCGGCTCCGGGTG
ATCCGTGGGAACAAACTCGCACGGATCCGAGTNTGGGTTTGGAACACCCAANTACTCGCGGGTACGGATCCGG
GTCACCCGCGAGTGCACTCGTGGTCAGCCTTA
>Agrped1_Cluster12#LTR/Copia

AACCGCCAGCGGACACTCCAGGATCAAGCACGTCGATATCAAAACCCATTGGATTCGCGAAGCTGTTGAAATG
GGAGACCTACAAGTGCTTCCCATAGCCGGAGAAGACAACCCTGCCGACATTTTCACAAAACCGCTCGGAAGAA
CCAAGCACGAAAAATTCGTCAAGATGCTAGGATTGGAAAACCTCAACATCTAAACCGAAAACTCTTCCTTTTA
TTTCTTCTCTTTCTTTCTTTCCTCCTTTTCCCTCATCTTACTCTTCTTTCCTTTCTCATTGACCGCAGTAGTA
CCCTACATCCTCATTTACTGACAAGCTACGGGTTCAGGGGGAGTGTTAAGACATGAACCCACTATCTTATCTT
ATCTTATCTACAAGTTTATAGTTATTTGGTTATTTTCTTCCTTTTTCTCTATGCACCGTCGCACCCTTACACC
TCAGTCGTTCTTATCTTGAACTTCTGAGGTATGTTTATTCTACACCTATATTTTATTCCTTTACTCACTCTA
CCTACGTAGCTCACATATCTGTTCGCTCCAATAGGTAGATTACTAATTCAGTCGTTCTTATCTTGAACTTCTG
AGCTCACATATCTGTTCGCTCCAATAGGTTATGGGCCCCGTGTTCTCACACTGTCAATAAAACCGCAGAAGCA
AAACAGACACGCGCTCTTAGAAGTACGAGACTTTCCTTACCGACTCAAAGACAAAATAAAATTTTATTTGAAC
CACAAAACACACTATCGAAATGTCATCGACCTCTTCGACCAACCCAATCACCCTCGCAGCAGAGGACAAGCTA
AAAGGCTATGAAAACTACGCCCATTGGAAAGTCCTCATGGAGGCACATGGAAAGCCGAAAGGCCTCCACAAAT
ACTGGCAGAATAAGATCACCGTACCAGCCGGCTACGAGGATGACGAAGAAGAAGTTGAGGAACCGTACCTCGA
GGCAGAGGAAGAACCTGAGCCACAACCACAACCAAAACCCAAAAGCAGTCAAGCTCGAAAGGCATCGACGCAG
TCCGACAAAGCAACTCCACTCCACTCCACAACGCCTAACCTCCTCGAATATGAGCTGCGTGAGAGTGTGGCTT
TGTTGTCCATTTTAATCAACGTCGTGGACATATACGGTTCGGGCATAGATCCCAACAAACGATCCCATCAAGT
GTGGAAGGCGCTCGAGGCCCAATATGGCAGGGCAAGTGACCGCGCCAGGAACATGCGCGAGGAAGCATTGGCG
AGCTGTAAACTAGCCGAAGGTGGAAAGGTGGCAGGAGAGGACGGTCACATTGAGAGGATGAGGAAGCTGCACC
AGCGCGCAAACGAGGCTGGCGCCCAGATAGGAGACGGAAGGTTCATAACCCGCCTGCTGGACTCTTTCCCCGA
ATCCTGGGACCCCGTCATAACGCCTATGTATGGGGAATCAGACCTCAACAAAATCATCATGAATCTGACCACT
CACGCGGAGCGCCTATCGATCCATGAAGCGAAATCCAAGAAGCCGACACCACCCTCAGACGCTGTAAAGGCAC
TCGAGGCCACCATCACTGCTCTACAAGCCGAAGTGAGATCCCTACGCACTGACAGCCGTCGAAGCGGGGGAAA
CCCCAACCTAAAATGCAGCAACAGTGCCTGCGGGAAAACCGGACATGTCATCGCAGATTGCTTTCAGCAGGGC
GGCGGAAAAGCTGGACAATACCCACACTGGTGGAAGGGAAAGCGTACAGCACCAGCTGTCACACCAACAGCCA
ATTTGGCGACGGCAGACACCTCAACGACAGCCGCTGGATCTGTAACTCCCGGATTGCAATTTGCCCTAACAGC
TTCGGTCGACGTCAGCAAACTGGAGGAGATAATTGCCGAAAACGCACATATTGAGCGGAAGGTAGCCCTTGCC
GCTGCAGCAAACCCAGGGGCACACATATCATCAGCTTGTGTTGCTGACTCGGGGTGCACGACATACTTCTTCA
AGGACAAAAGCGCCTTCACGGAATACCGCGAAATCAAGCTGAATGGCCAGTCGTCCAAAGCTGGAGCGTGTTT
TGCTGTCATAGGCATTGGGAAAGTCAGCATAAAGGTTACACACAACGGGAAACAACACACCCTCACCTTTAAC
GACGCCCTACATGCACCAGACGTCACCGCGAACCTCATATCCATCTCTAAGTTGGACACCCTAGGCTGGCATA
CTCTGTTCGGTGACAAGAAGGCCAGATTTTTCCATGGGAAAGACGAAATCTTCGGTGGCGAACTGAAGAATGG
CCTATACATGATCAGCGGCTCTATCGTGTCGAAGTTACCAACCGCACTCACAGCAAGATCGCTCCGAGAGCCT
GTTGACATGGCTACCTGGCATCGCAGGATGGGACACTTTGGGGTGAGGAGGATAGGCGAAGCGGCCAAATTGG
TGAACGGCCTGGAAGTGACGACGGGGGAAAGCAAGGGTCACTGCGAGGACTGCATATTAGCAAATCAAAAGCG
ACGGCCGTACGACGAGGAAGTAACACCTACCGATGAAGTCCTAAAAATCACGAACATCGACTTGTGGGGACCA
GCAAGGGTCAAGTCGGCAGGCGGAGCGCTATACGCTATGAAGTTCCACGACAATGGGTCGTCACGCAGGAAAT
CCTTCTTCCTCGAAAACCGACTTGCAGATACAGTCCTCCACGCACTGAAGACCTACAAAACCTTGTCAGAGAA
CCTAACAGGCAAAACCATGAAGTACATACGCACTGACAACGCCCCCGAATTCCTAAGTGCCACCTGGACCGAC
TATATGCGCGAAGCCGGAATCAAATTGATTCCAACGGCACCGTACTCGTCAAGCGCCAACGGAACGGCTGAAC
GCTCTATCGGTGTTACAACAGGGTCAGTCAGAGCGATGCTCTTAGACTCTGGGCTGAACGCTAAGTGGTGGGC
TGAGGCGTGGGCATTCTCCGACATGGTGGAGAACCTGCTACCTTCATCGAGACACCCCAAATCCATACCGGAG
GAGCGTTGGACGGGAGAGAAACAGGATGTGGGGCATATAAGGGTTTGGGGATGTGTCGCGTATGTGCATGTTC
CAAAGGAGAAGGGGGGAGGAAAGCTAGGTGACAGGGGACAGAAAGGAAGGCTGCTGGGAATTGAGGGACGTGG
CACTTACCGGATCCTCATCCCGGAAACCGGACAGGTCGTGAGATCCAGGAATGTCCGTTTCGAAGAAGGCGAC
GGTCACAGGACGCTGACAGCTGATGGACAATACCCTGCCGATTTCTTCGCGGGAGACGATGGCGACGTGGACT
ACGATTTCCTAGATGAAAGTCCGCCAGCGGACACTCCAGGATCAAGCATGTCGATATCAAAACCCATTGGATT
CGCGAAGCTGTTGAAATGGGAGACCTACAAGTGCTTCCCATAGCCGGAGAAGACAACCCTGCCGACATTTTCA
CAAAACCGCTCGGAAGAACCAAGCACGAAAAATTCGTCAAGATGCTAGGATTGGAAAACCTCAACATCTGAAC
CGAAAACTCTTCCTTTTCTTTCTTCTCTTTCTTTCTTTCCTCCTTTTCCCTCATCTCGCTCTTCTTTCCTTTC
TCATTGACCACAGTAGTACCCTACATCCTCATTTACCGACAAGCTACGGGTTCAGGGGGAGTGTTAAGACATG
AACCCACTATCTTATCTTATCT
>Armme1_1_rnd-1_family-141#LTR/Gypsy
GAGATCCATTGGCCGAAATACCAACGTTATCTCCTACCCCACCACCATTCATTCCTACCGAACGTTATACCCA
AGAACGCAAGGAGTACATCGATTCCATCCATAATGACGGATTTCTTTGGCCAGAAGAAATGAAGCTTATGCAT
CATTTCATGATGGTCCAGGAGAAGGCGTTCGCATGGGANGACAACGAACGAGGCAGTTTTCGTCGAGATTTCT
TTCCTCCAGTCGATATCCCAGTCGTCGAACATACNCCTTGGGTACTACGAAATATNCCCATTCCCCCTGGTAT
CTATGCAGAAGTTTGCCGTATGATCAAGACGAAAATTGACGCGGGCGTATACGAGCCTTCCAATTCTTCGTAC
CGATCGAGATGGTTCACGGTTTTAAAGAAAGACGGCAAGAGTCTTCGAATAGTCCACGGCCTAGAACCGTTGA

ACGAAGTCACTATCGCACATTCCGGATTACCACCAGCCACCGACCCGTTAGCAGAACATTTTGCAGGCCGTGC
TTGCGGCGCTACCTTAGACTTATTCGTTGGATACGACGAACGTTTATTGGCAGAGTCCTCGCGNGACCTCACC
ACCTTCCAGACCCCTTATGGAGCGTTACGCCTAGTCACTTTACCCATGGGTTGGACCAATTCCGTCCCAATAT
TCCATGACGACGTNACNTTCATTCTACAAGACGAAATTCCAGANTTCACCATTCCTTACATCGATGACGTCCC
AGTTCGAGGACCAGAGACTCGCTACGAAATTGAAGGAGGCTACGAAACCATTCCTCTCAATCANGGCATTNGA
AGNTTCGTNTGGGAGCACTTCCAGAACCTNAATCGGATCGTNCAACGCATGAAGTATNGCGGAGGCACNTTTT
CNGGCAAGAAGGCCTACCTNTGTTGCGCAGAGATCACCGTGCTAGGNCACAGGTGCACGTATTCGGGCAGGNT
ACCATTGGAGAGCATAGTCGCATCAATCATGCATTGGGGACCTTGTATTGACGTCTCTGACGCACGCGCATTC
ATTGGTACTGTCGGAGTCCTACGGATATTCAT
>Armme1_1_rnd-1_family-46#LTR/Gypsy
TCCCAGTTCAACCTNGTAATCCGGTTCCGACCGGGTAAACTTGGTGGGAAACCGGACGCACTCACTAGACGAT
GGGACGTCTACCCTAAAGAGGGGGATAGTCAATACGCTCAAGTCAACCCTCACAATTTCCGTCCTGTATTTAC
TCAAGAACAACTCTCCGCTTCTCTCCGCGCTACTTTTCTTGAAGGCCCCGTACTCCGCGCTAGTGCTATCATG
GACGTTGACCAGCTCCACTCCGATATCAAACAAGCACAGCCAACGGATTCCGCGGCGACCGAAGGTTTTCGTC
TTGCTTCTTCCCCTTCCAATTCCTCCCGGTGGTCCGTGGATGAGTCAGGCATNCTCCGTCTTGACAATCGCAT
CTACGTCCCGGACTCCGCGGATCTCCGTCTTCGTGTCCTACAAAACAATCACGACCATATCTTGGCAGGTCAT
TTCGGTCAAAATCGGACACTCGAACTAGTCCGACGGAATTATACCTGGCCACAGATGCGTGAATTTGTTCGCC
ACTACGTCAAGTCCTGCACTACTTGTGGTCGTAACAAAACACCTAGACACCGTCCTTTCGGCTTACTCAAACC
CTTGCCTGTACCGGAACGTCCGTGGGATTCCATCTCCATGGATTTCATCGAACAACTCCCGGAATCCGACGGA
TTCACCGCCATTCTGGTNGTCATTGACCGTGCAAGCAAACAAGCCATTTTCATTCCCACTCACGATACCATTA
ATTCCGCGGAACTTGCCCAGCTATTCGTTACCCATGTATTTTCCAAGCACGGCGTTCCTAATCACATCACTTC
CGACCGCGGCTCCGAATTTGTCTCCCAATTCACTCGCGCCCTTGCTAAGGCTCTCGATATGGAACTTCACTTC
ACTTCTGGTTATCACCCGGAAGCCGACGGACAGACTGAACGCGCTAACCAAACCCTTGAACAATACATCCGTA
TCTATTGTTCTTACCAACAAGACAATTGGGATACCT
>Armme1_1_Cluster58#LTR/Gypsy
ACAATCACTCACCTAACACTCAGCATCCACTTCTTTTACACATGAACTACACAGACAACGTTCTATTCTTAGT
CACTCAGTTTTCATATATAAGTAGTACTGCTCTTGCTATAGTATCCTCAGCTTCGAGCTAAAGTTTATTCATA
CCACACTTTACTCTTGCTGAGAACTCAACTTAACAGCCTAGTCCTTCTTTGCCGAGTCTTAGACCCTTCAGTC
ATTCCCCAGTGATTGTCCAAACATGAGACAGCCAAGGTTTAGACTAAAGTCTTTGATCACGTAAAGTCCAGGT
CTAACAGTTAGCTCGAAGCTTCCCTTCCTGTCTCTTCACCGCCAGTTGTGAAGAAACATGACCTATGAACTAC
TATGTACTTGCCGCTGCCATTGCAATCTCCTTCACTGCTGGGGCTCTAGGAGTCCTCGCCCTCATCGCCATCC
TTGTCTATTATTCAGAAGAAATCAGACTCTGGCTACGCAGTGCTATCACTGTCCATCACAAAACCCACCACAA
TACCACCCACTATGTTCCACCATTTCAAAATGAACAACCCGTTTCGACCCAAGGAAACCTCACCCACAGATCC
CCCTTCACCACCTCCTCTAGTGACAAGTTTCCAGCCCAGGAAATCCCTCCCAGAAATACAACCCCTGGGCCTA
GCAGACCAAGGGGGATACCCAGAACTCCAACACCCCCTTCCCCAGTACTTCCCCAACACAGCAATCAAACCCG
AAGCCCCTGCCTCAGTGCAGAAGAAATTGGGAGGTACATTCTCTGCTTGCTTCAGTTTGACCAGCCCCCTGCC
TACTTTGATGAGGTTGCCAAACCAGATACCAGTCACTGGCTTGACCAATACAAAATCAAAGTGGAGGGGCTGA
ACTGGACGTTACAGGGAGAAACCCCCTACCCCGAGGAGAGCGATGACTCCTCCACTGAAGTTGACTCTGACAA
ACAGTGAGGGGAGGAGCCCGAGCCAGTCAAGCCTGAGCATGTCAGACTTTCTGACCCCCCCCGAGCCACCCAG
GGTCCCCTCTCCCTGGACCGCTGATGACCCCCTACCCTTCACCTTCACAGACCCCCAAGACTTCCACGATGTC
CAAAGGGGAGTCCTGTTGGACCCAGATAACATCATGGCCAGGATTCTTGACCATGAGGTTCCCAATGACCCCT
TCAACCCCAGGGGTCTTGACTACGAGTCGCTCAGCCTTTCCCACCAGGACTGAGAGATATTCAGCCCCACCAG
GGCCCACCGCCTGGATGGTATGATGAACAGATGTCGAGACCAGAACCACTGACCCCCTTCCCCTCAGGTCGAC
CATGGACAACTGGTTGGCCATTGCAAGCGGAGACCCTATCGAGTTAGGAGGCATGGTGGCCAACATCAAGGCC
AGACTTGAGTCCAATTGTGACCGACACCTATACCCTGCAAACACAGGCCCCAGGGTGCACGGACTACGAGCCA
GGATGGTTGAAGAACAAAGCACCTTGGAGCTCATCCTTCTCGACCTCGCCGCTGACCATGAGGACTTCAGGGC
TCAGTGACAGGGTGAGGAATTTAATTAATTTTGATAGCTTTACATACAATATAGACACTTTTGGCAGGGGCAC
AGCATATAATGCCCCCAATATATCCTCTTCCATCTATGACCTGACTGAATACCCAAAACCACTCCCCGACAAA
CCCCCTTTCCCCCCACCATGCGCTGACATGCCAATAAGCTGATATTGGCTATACAGCATGCCACCGACTGGCA
CAAGCCGACTATTTGCTGGAGGAGACCATGACTAAGGAGGAGGAGGAGCTGGACCATCAAATCCAGGAATGCA
GCTCACCCCAGAAGAACAACAGCAGATAGTGATCAAAGAAAACAACACCTGAACAGCAAAGGTCGCCAAACTC
CAAGCCCAGCAAACTGAGCTGGCTTGAAAACTTACTGAAGCAGAAGCAAACCACTCCTGGTTCACCCAATTCC
ATGGGTTCAAAAAAGGAAAAGAACCTGAATGAGGCAGGCTTCCTGTCCCAAACCATCTCCGACGGCTAAATGA
CAGAGACGACAGACACTCAATCCTGTGCCCCCCAGATTTCCCCTATCCCACCATGCCTTACCCGCACCCGATC
AGCAAAGCACCAGCTGATGCCCCCTACCTAAGCCTCAAGCCTCATTGGCAAACCCCCAACCCCTTCCAGGGAG
AACACGACAACATCAAATGATTCCTTGGGGACTGCATCATCTACTTTGAGCAGTTTCAAAGCTTTTTCCCCTT
TCCTTCCCAGAAAGTCACCTGGGCTGCTGCCCTGTTCTCCGGACCAGCCAAGGACTGGTGGGTCTATCAATGA
CAGGAATTCTGGCAGCCCTCTGACTACAATGGCCAACCACCATGATTCCGCTACCCAAGTTGGTAGGAATTCA

CAGATATGGTCACCAGCCAGTTCCGGAGCCCAGCCATAAAGGAAACACATGAACAAGAAATGAACGACCTATG
AATGGAAAACAGGATGGCCAATAAGTATTTCCAGGAACTGGAAAGACTAGCCAAGCTCACCAGGCGAAGAGGG
GAAGAGGATGAGCACGGGACCATGGTCCGAGCATTGAGGAGAGGAGTCCCCTCAAACTACATCAATATGATTG
CCAATATCAGAACAGATGTCCCCACCACATACCCAGCTGGAAGGCCCACATCAGCAAGATGTATGAGGAGCGA
CAAAAACAATACATCATCCAATGAATGATCGCTCCACCCGATGCGTGTGACAACTGGTAGAACACAAAACCTG
GGGGGCATGGCCCTAACCCTGCCAAACCCACAGAGACAGTTCGACACCAGCCAAACCGATGGGCACTGGGCCG
ACTCCAAAATGAGATGCAACCACCGGAAAATGGGTTCCAGTCAAAACGACTATGTACTCAGGACAAGGACAAC
CAATGGAGATCAATTGACTCAGGAAAGAGGGGAGGTGCTACTGATGCAAGGAGAAAGGCCACATGTCAAAGGA
CTGCCCAAAGAGGAAGGAATACAAGGACATTCGCTCAGTAGTAGTCAAACAAGAGGAGAAAATGGAGTTGAAA
GTCGAGGAGGTAAAAGAATAGGTCGCCAAGGCTTGATTCCCCTTTGGTGACCCAGGACTTCACTGGTACGCTC
TCGTCTTCTGCTGGACAGTCTTATGCAGTCTCTTTAGTTACCCCACCTGTTTTATCTACCGCTGCTTTTAATG
TCCACCACATAACCAGCAAACCAGTACCATCAGAATCGAACCGCTACACTGCTCTAGTGAAATTGAGCGACTC
GGACTCAAGCACTGACAAGGCCAAGACCTTTAATGCCGAACAGGGAGCAGGACTTACAGTCCCAGCAGCAAGC
CCCCATAATGACTTGCCAGAACCCCTAAAACAGGAACTGCCTACACATAAACCCCTCAGGGGGAGTGCCTCTG
TAGGACTGGCTAATCAGAGTGCAAAAACCCAAAAGGGAAAAGGCTCTGGAAAGTGACCCCATGAAGTCACAGA
ACCCTGAACCCTTGAACTTCCTTGACCATCACGCCTGTCCCCCAGGGAGGACAACAGCAGGGCCAATTAGAGA
AACAAATGAATCCCCGAGTCGGAAGCACCAAGGGGAGCAGACACAACCTCACCGGCAGCCAGGCACCAGAGTG
CAGGGACAGTTACAGTTCATGTGACAAGAACACGATGTGCACGAATGCATCCCCTGCTGAGAAGGGTGGCCAT
GGGATACCAGACCGATGCGAGCATCCTATCATCACTCCCCTGGCCAACCCCAATGAAGATGCATGACCAGCAC
AGGACAACAAGAAAGCTGCAGGATCGGAAGTTGTTGGCACCCAGACGATAAAAAGGGGGCTTGCTGTTACTAT
AGAAGAAGTGCCCGATGAAGAAGACGAGACCATGTACCAACAGTGGCTGGCAGTGCAAAAGGCAGATCAGAGG
GAATGAGACATTCAGAACGACGATGACTGGGTTCCCACCAAACCTGACACCAGCACACCACTGCACTCACAGG
CCTGGTATAAACCTTTTGAAGTCCACTGGATGCTCCAGGCCATTTGTGAAGCCCAAACCCCCAAGGAAGCCAG
ACCCACCCTGAGCAGCTGGATCCACGAAGATCGACACCATGAAATCATCATGGGCCTAGTCAACAGGCTGAGA
CAAGGGGGAGAGGAGGCAAGGGAGGTTATGCTTGAGCTATGAGAACCCATCTGATACAACATCCGAAGGACCA
AGAGCAGTGGGAGGGAACTCACCATCGACATCAGGAGCTCCACCTTTCCATCGGTGGACACCATGAACGAATC
GACCTAGCCATCACTGACCTCGGCTCAAAGGATCTTTACCTCGGCCATGACTGGCTCAAGCGCCACAACCCAG
TGATCAACTAGAAAACCAGCTCCATCATCTTTGGGTGCTGCCAAGAACCCTTTCCCACTCCCCGACGCAGACC
TAGAGGATCGGTGGGATGAGGAGCTCGAAGAAGGAGAAACTATCCTTGCTGTAAATATGCAGGAAGAATTAGT
CATCCAGGCAGTACATCATGCAAACAACTTAGCGGCTCAGGCCAATGTGGACAAGCCGAAGAAAACCTTTGAA
GAAACGGTACCCAAGCATTATCACTCCTACTGTGACCTGTTCTCGAAAGACAACTTTGATGAACTTCCCGACC
GAAAACCCTGAGACCACACCATAGAACTCATTCCCAACACTAAATCAATGCTAGACTGCAAGGTCTACCCTCT
AAACCGAAGTGAACAGGAGCAACTGGACAAGTTCTTTGATGAAAACCTGGAGACAGGCCGTATTCATCCCTCT
AAATCCCTGTTTGCATCCCCCTGATTTCTGAACTCATTGACAAACTCAGGGGCACCAGGTACTTCACCAAGCT
TGACTTCTGTTGGGGATACAACAATGTCTGGATAAGGGAAGGAGATGAAGAGAAGCAGCATTCAGGACAAATC
GGGGGCTCTTCAAACCCACCATCATGTTCTTCAGGCTCACCAATTCGCCCGCCACATTCCAGTGGATGATGAA
CAATATCTTTAAAGACCTGATCGCTGAGGAAAAGGTCACTATCTACCTCGACGACATCCTTATCTTCACGAAA
GACCTAGATGAGCATTGGTGGGTCGTGCGATGAGTGCTTCAATGCCTTAGGGAAAACAAGTTATTCCTCAAAG
CCGAGAAGTGCAAATTTGAAGTCCTGGAAACCAAATACCTAGGCATCATCATCAGCCAAGATGCTGTCTGGAT
GGATCCTATCAAGGTCGCCAGAATAACAGAGTGGCCCACACTGACAAAAAAGAAGGAACTTCAGTCGTTCCTC
GGCTTCACGAACTTTTATCGCAAGTTCATCCACAACTACTCCAAATAGTCCACCCTCTCATCCAACTCACCAG
GAACACGGAATGGACATGGGGAACGGCCCAGAACCAGGCCTTCCAGCAGCTCAAGAAGCAGATGGCAAGGGAC
GTAGTTCTCACTATCTCCACTGAGACAGGGCAGTTTAGAGTGGAAGCTGACTCAAGTGATGGTGCCATCGGTG
TGGTCCTGAGTCAGAAGCATGATGGGAAATGGCAACTGGTGGCATTCATGTCCAAATCCCTAACCGTCACCGA
GAGAAATTACAAGATCTATGACAAGGAGTTACTTGCCATCATGCTTGCCCTGTCAGAATGGAGACATTATCTG
ATGGGAGCGACCAAAGAGGTAGAAATCTGGATGGATCACCAAAACCTCAGAAGCTCAATCAACGCCAGGCAAG
ATGGGTCACCGAACTGGTGGAATATCATTTCACCCTTCACCACCAACCCAGGACTGCCAACAAGAAGGCTGAC
CTCCTTTCTCGACAAGGTGACCACGACCAAGGGAAAGGGGATAATTGTGATATCCTCGTCCTCCAGCCACACC
ACTTCTGTGCTCTGGGATCAGGAGATAGCCAACTCCCTTGCCCATGAACGAGGAATCACGAGGGAGCATGAAC
TCCTTATCTACAACGGACAGATCTACATTCCAAAAGATCATGCCCTATGAGGAGAAATCATCACCCAGAGCCA
CAACCACATTACTGCTGGACACCTAGGGGATAGAAAAAACCAAGGGGCTCGTCCAGTGAGAATACTGGTGGCC
AAAACTGAAAAAGGATGTAGAAGTTTATGTGAAGGGATGCAAAGCCTGTCCACATGCGAAATCCAGCAATCAG
GTGAAGGCCACTCCCCTCCACCCGAATGTGATTGCCATTGGACCATGGACTCACATCTCCATCGACATGGTTA
CTGGTCTCCCAGAGTCAAATGGCTATGACACCCTCCTCATTGTCATAGACCAATTTTCCAAAGCAATCGTCCC
AATAGCCTGCAACGTCAAACTATCAGCTGAAGGTTGGGCCAAGGCCCTCAGGGACCACGTCTATGCCAAGCAT
GGAATGCCTCAGGTTGTAATATCCGACCAAGGACCCCAGTTCGTCTCCCAGTTTATGAAAGACCTGTACCAAT
TACTCGATATCACCCCGAACGCATCCACAGCGTTCCACCCTCAGACCAATGGGCAAACCGAGCGAGTCAATCA
GGAGCTCAAGAATACCTCTGCCTCTTTGTGGAGTACCGACAAACTGACTGGAGCGACTGGCTGCCCATGGCCG

AATTCGCCCACAACAACCGAGTACACTCCGCCACTGGAAAGAGCCCCTTCATGGTCCTCTACGGCCAAAACCC
CAGACTAGTCCCAGACTCAACCCATCCTTCCCCATTCCTCAATCCCACTGCTGAGACATTCACTGAGACCATG
GCCGAGGTCCACAAGCAGACCCAAAAGGCTCTCGAAAGGGCAGTGTGTGCAATGAAAACACAATACGACAAAC
AGAGGTGAACCACATTTGACTACAAGGTCAGGGACAAAGTATGGCTCGATGCCAGGAACCTACACCTCCCTCG
ATGACAAACGAATAGGCCCCTTTGTAATACTAGAAAAGGCTGGAGCATCAGCCTATAAACTGAAGCTCTCCCC
TCACTGGAAGATCCATCCACACTTCAATGAAAGGCTACTCACCCCATTCATACCACCTGCCTTCCCAAACCAG
CAAGAACTGCCACCGCCACCACCGGCCCTCATTGACGATGAAGAACAATGTGAGATAGAGGAGGTGCTAGACT
CACAGCCATGAAAGGTCAGGGGAAAACAGGGACGAAAGTCAAAAGAAGTCATTGACTACTTCATCAAATGGAA
AGGATGGACTCGAGAACACAATTCATGGGTATGAGACTCCGAGATGGGTAATGCCCAAGAAGCGATACAGGAA
TATGAAGACTGGACATGAGTCAATGCAATCCAAATCGCAACACCCAGACGAACCTTAACAGCAGTCGGGATGC
CTGCCTTAATGAGGGGGTAATGTTACAATTTTCCCACCTATCTTATCTTATCCAACATCTTCCGCTTATCTCA
TCTTATCTCAACACCCCTATCCCACAACTTTAACAGACGGTCTCAAGCCTTGTGCTACGGCTTGCTACAATCA
ATCGCTCACCTAACACTCAGCATCCACTTCTTTTACACATGAACTACACAGACAATGTTCTATTCTTAGTCAC
TCAGTTTTCATATATAAGTAGTACTGCTCTTGCTGTAGTATCCTCAGCTTCGAGCTAAAGTTTATTCATACCA
CACCTTACTC
>Armme1_1_Cluster40#LTR/Gypsy
CATCCAAAGAAAGGTCTGCCTATGATATGGTATATCATGTGATTCTAGCAAATGAAAACAACCTATATGACAC
ATGTAGTCTGTCCAAATAAGTAGATTAAAATATGATTGCAAGGAGAACATTATACTGCCAAAAAGGAAAACCC
CCAAAAAAGAATTTTAGAAACAAGGCAATCAAGATCCAGCATTAGATGAAAGGGTCATACAACAATACAGGAT
CCCATGGAGAAAAAAAGAATGCATGATGCGACTCCCAGAGCTGGTTTAAGAAAACAGCAGCCATCAAATGCAC
CACAACGTAACACAAAACAATCATGGACTGTTTCTACTGCAAAACAGACACCAGTAAGCACAGCTCCACACAT
GCTTTGACAAAACAGCAGCTGGTAACTTACAGATTGCACAGTGAACACCATAGAGGCATTGACCAAAGAAAGG
GCCAGTAAGGATTCACTACACCTAATCAGACTATTTGAGGTAAGTCAATACAGTCAAATGGAGCAAACAGAGA
CTGACAATGGAGGCAGGATCAACATGCCCTTGATGACATATGTGTCATGCATGATAAAGATGACCAAGTGTGG
AGACTGTCAAAAGGTACCAACAAGGGAGGAATAGATGAAATAGTATTCACATTGACTGGTACTCTTATGAGGT
TAGATCTGCCACCAGTACACAGAGAGACATCATGAGTGATCAGACTAATACACAAAATAAACAGCAAGGCTAA
TGAAGGTGCCACAGGGGCCTGAGAGAGAAAATTCACTTCCTCAATCAAGCAGTTACAATAACCAGACTCAGAA
GTGTCATGTTCAGCAAGGCCATAGAGAGTGTGAAAGACATGAGATGTAAGGTGGAGCAAGACTTTCATCAAGG
GCAGTTGGAGGATTAGTTATTGAACCAATTCCAAGGTGAAGACATGCTCGAACTGACTAACAGATACTTCATA
CCACATGGGAAGCTGAAGCAAGATGATGGCATACCTTTAACTGAAGATATAGACCCAAACAGAGTTCTTGCAT
GACTTGGTGGTGCAAACATGATCCACACCAAAGATAATGTCATAAAATATTACAGGGGTATGCTGGACAGAAA
AAAGAAAAGGTGAGCATCAATCTACATTGGTGAACATGCTAACATATATGATGATAATAGGTATATCCCTGTG
AAGCTGCAATCATTCAGACTAGGTGATATAGCGGAAGCGCAATGCTTAGTGGTGTTCGTGAAGAGCAAAGGAG
GCAGCATCAAAACAATGCTGATCCTCAGGGCACTGGCAATGGTGAACTGCAAGCATTCAATGGTAAGCCTGTT
CATATACATGATGTGATGGAGAACATACTAAACCAAGTAACAGAATGTAGACAGAGAACATAAGAGAGATGTG
GAGCAAGAAGGAAATGGGCATGGTGCACAGAAGGTAAAGTGAAAGGTTGGATTCAAGTACAGTGACAAAGACA
AAGAGGAGCAGCCAACATGGAAATGCCAAAAGGCCAGTGAGAAAGCCACAGAAGAAAATGGAGGAGTACAGAT
GGAGGACTGAGGGACATTTGGTGAATGAGTCTGTGAGGATAGTACTAATAGCACCTGTGATGTCCTCCCTCAA
GAGTATGCTATTATGGGGATAGACAAACACAGAATATGTTGACCAGAAACTTTGACTGTTGTCAATGAAAGTA
GAGATCATATAAGACCAATTGAAACCGAGGCTGATGGTGGTGGCAAAAAACAGTATGAAAACAGAAAGAGACA
CAACAGAAAAGCAACAGATTGAAGGAAATCAAACAATAGTTCATACAGAAAGGAAAAAGTCGGACCAAGCAAA
TGGAAGAAAGTGGGCCTGAATAGAAAAGTTCCCATGGCAAGCTCAGGTGAATAGGTCCAAGGGGGAGGCATGG
CTAAAATCCGGTAATAAGCCTCTAGGCTCTGTGTTATGATTTTCTTGCTTATCGCCATCTTATCTTATCCCAA
ATTGTAACCTCGGACATATTCAGCTTGCTTCAACAATGCTACTGCATGTTTCACCCTTCTTTCTCTCACGAAT
GGATGTTCTATTATTAGCTCCTACTCTCCATATATAAGTAGCACAATTCTCGCTGTAGTATCTCCAGCTTCGA
ACTAAAGTTTATCAATACCAATCTTCACCTCTCATGAGAACTCAACTAGCCAGTCCTAGTCCCTCTTCGCAGA
GCCATTAGACACCCCAGCTATTCCCCAGTGATTGTCCAAACATGAGACAGCCAAGGTTTCAGCTTAGTCTTTG
GTCACATGGAGTCCGGGTCTAACACTCTGTATGCTCAAAATTTCGTGGATTTACACTTAATTCCTACACCTAG
TCCCCCTAGCAGACTTTCGGCAAACCAATAATTGCCTACATGGTGATACCACTTTTGTATCACAGCAATAACG
AGAACTCAACTAGCCAGTCCTAGTCCCTCTTCACAGAGCCATTAGACACCCCAGCTATTCCCCGGTGATTGTC
CAAACATGAGACAGCCGAGGTTTCAGCTTAGTCTTTGGTCACATGGAGTCTGGGTCTAACAGTTAGTTCGAAG
CTTCCCTTGCTTCCCTTCACCGCCAGCCGTGAAGAAAGCACCCATATGAACTTCAACGACACCGCATTCGCCA
TCGCCTTTTCTATTGTAGAAGGAGGGCTCCTAGTCGGCCTCACTGCCCTGACAGTATTTACCTACCATTACGA
AATCCAACGCTTTCTGGTGAGACACCGACTCCTGATCCTCCCCTGACAAAGACTACCCCTCCACTATGTCCGA
CCATTCACAACCGCCCCCCCTGCCCCCGAACAAACCCTTACCCACCACACCAGCCAGTACTACACCCCCTGAT
CCAGCGAGGAGTTCCCAGCAAATCAGCAGGAACCCCCTAGGCAGCCCACCCCAGGCCCGTCCCAGGTGGGGCA
ACAACAATTCATTCACAACTCTGTGGAGGAGGAACCGAGACCAAGCCTTGCAACGCAGGAGCTCGGGAGGGAC
CTACGGACTCTTTTCAGATCCCCCCAGCCTGACCAGGACCCCACCCCTCCAGTTATTCCAGAACGAAGATCCA

TCTTCGCCCCCCCTGTTGGGCCGTATGGGACAGCCTTCCTACAATCCGGAAGCTTTCCATCCGATAGCGAGAG
CACTAACTCCACCGACTCCCTCCCCCCCGAGCAGATACGGCTCCGGGGATGATGATCCTTTCAACCCCAGGGG
AGGGGACTATCAGTGGCCAGAGCTCGCCCCAATCGACAGACAGATCATGGGTGAGCCCAGAGCCTGAGCCTGG
GACCGATGCTGGCAGGACTTGGCACACCAACTTGGGAATTATTACACCTATCGAATTCCCATGGACAACCACC
TCGCTATCACCAGTGGAGATCCACTCCCCCCAACCTACCAGCGATGGGCTATCGAGGCCAGAATTCATGCTAA
CCGAGACAGAAGATTATACCGCCCAAATGCAGGGCAAAGGCTCTATGGGGTCAGAAATAGGACTCCCGAGGAG
CAGGCCGAGCTGGAGTGTGACCTCTGCGCCCTCGACCACCAGGCATTCAGGGACTCGGTTGAGAGGAAGGATT
AGTGGGCCGATCCTGACCGGCCAAGGATTTTTTAATGATTTTGATACATTTGTATACGATGCAGAAACCTTTG
GGGGAGCAACTTTGCTCTCCCCCAATATACCTCCTACCGCCTATCCCAGAACCATCGAAAGCCCCCAAAGACT
ACCCGACAAACCCCCGTTCCTCAGCCAAACCCAATACCAGCAACATGGATATCCATACCCGGGAAATAGCTGA
CTCTTGGCAGGGGCAGGAGAAGACCCTCCCCCAGAACCTATAGTGACCGATCCCCCGGTCCTGGGAGAACCGC
CTTCTCCCCGAACCGCCTGAGAACAAACTATCCTACGAAACCAACTTGCACTCGACTGACAGCAACATAAACT
AAGAGAGGCGCTGACTCAACAAATTAACATGCTAAAAGACCAGCTCCGGGACCTTCAAGAGGAGGGCCCTGTG
AATAGGCCTTCCCCTGGAGGCCCACCTGACCCAGGCAGACCGCCCCGGGAGCATGGCTCCTTCTATTTGCTGC
CACGGCCACCTGAAGGCCCAAACTTTCCACAAGACCCTCCCCCCGCACCCACGGGAAGAGCCCCCAAAGATGC
CCCCTTTTGGGGAGTAAAACCTGTCCTCATGCCTCCGCCCAAACCCTTTCAAGGGGACCATGCGGACATCAGT
CGCTTCCTGGGAGACTGCGAAGCCTATTTCGCAATGTTCTCCGTTTATTTCCCCTACCCCTCCCAGAAGACCC
TCTTTGCCACCCATCACCTCAAAGGGCCTGCCAAGGACTGGTGGGTCTATCAATGACCAGAGTTCTACAATGA
AGCCTGGCAAAGGAGCCGATACCCCGATTGGGCTGGATTCATTCGAATCTTCAAAGAACAATTCTCAGATCCG
GCGGTTGAAGAACAACATGAAAAGGCCATGTATAACCTTCGAATGGGTAATGGACCAGCCACCACCTTCTTCG
CCAAACTAGAACAGGAAGCAAAGCTCGCAGGGCTCCACGATAAAGAGGAAGAGAAGGACCACCTCACCCGAGC
AGCGAGGCTAGGGATCCCCGCTTCCATCCAACACGCCATCACAGGAGGAGGATTGAACGTTCCCCGGACCTAT
CTGGAATGGAAGGAACGAGTCATAAAAGCCTGGGAAGAGTGTCAAAGGGATCAAGCCTTCCAGCAAACATTCG
CCCACGCTGGCGAACAACCGAAGGCTGGACCCTCTCCCAAAACAACCAACACCTGGACAAAACCAGCCAGCAA
CGACAAGCGAGGAAGAGATGCGGGCACAGGAAGATGGACAACCTACACAGGACAGGGGCAACCCATGGAGATC
GACCGACTCCGAAAAGAAGGGAGGTGCTACCATTGCAAGGAGAAGGGACATATGTCAAAGGACTGCCCCAAGA
GAAAAGAGATCAAGGACATTCGCTCAGTAATAGTAACTGAGCAGGAGGAGAAAATCGACTCAAAAGTAGAGGA
GGTAAAAGATTAGGTCGTCGAGGGCTCAATTCCCCTTTGACGACCAAGGACTTCACTGGTGCGCTCTCATCTT
CCACTGGCCGGACTTATGCAAACTCTTTAGTTACATTGAATGTCGTATCTACCGCTATATATACCCCTGCTCT
TAACGTCCCCTGCATGACTAGCAAACCAGTAAAAACAGAATCGAACCGCTACGCCGTTCTAGCAAAATTGAAC
GACTCGGACTCAAGCGCTGATGAGACCAAAATCTCTAATGCTGAACAGGGAAAAGGACCAACGGTCCTGGCAG
AGAGCCCCCACAACAACGCAAAAACGTCCAGAAGACCCACACACGAGCCTGTTAAGGGGAGCACTGCTGCCGA
ACCCCGCGAGGGGGCAGGCACTGAACAACAGTCACAGAGAGTCAAAGAGAAGGACTCTATGTTGCAACAAATG
GACACCCGAACCGGGAGTCACGAAGGAGATGAGCAAAGCCTCACTGACTGCCAGGCCTCAGAGCGCAGGGACC
ACCACGGTTTATTCGACAAGAACGAGACGGACATGGGTGCATTCCCCGTCATGCCTGGAGAAGATCATCCAAA
GAAAGGCCTGCCTATGACATAATATATCATGTGATTCCAGCAAATGAAAACAACCTATATGACACATGTAGTC
TGTCCAAATAAGTAGATTAAAATATGATTGCAAGAAGAACATTATACTGCAAAAAAGGAAAACCCCCAAAAAA
GAATTTTAGAAACAAGACAATCAAGATCCAGCATTAGATGAAAGGGTCATGCAGTAATACAGGACCCCATGGA
GAAAAAAAGAATGCATGATGTGACTCTCAGAGCCAGTTTAAGAAAACAGCAGCCATCAAATGCACCACAACAT
AACACAAAACAACCATGGACTGTTTCTACTGCAAAACAGACACCAGTAAGCACAGCTCCACACACACCTTGAC
AAAACAGCAGCCAGTAACTTATAGACTGCACAGTGAACACTATAGAGGCATTGACCAAAGAAAGGGCTAGTAA
GGATCCACTATACTTGACCAGACTATTTGAGGTAAGTCAATACAGTCAAATGGAGCAAACA
>Armme1_1_rnd-1_family-309#TIR/Tc1-Mariner
TCCTTCGCCCATTGTTTTCGCACCTTCCTTTGTTTTGCAGAGAGAAAGGGACGTTTCCGTGCAACACGTCGAT
GCAATCCGTGGTCATCCAAGGCCTTGGCAATAACCTTCTCGCTCGCATCAATACCGACCTCATTCCTGAGGTC
TCTGAGTGGCAGTCGGCGGCTTTTTACAGCTGCACGAGCAAGACGACGTGAGGTACGCTCATCAATCTTCTTT
GGACGTCCACTACGGGGTAGATTTTCGACAGAACCACGCTCCCTGTACTTCCGCCAGATTTTTCGTGCCGTCT
CATA
>Armme1_1_rnd-1_family-15#LTR/Gypsy
TGCAATCCTTGCATGCGCGTATGGAGAAGATGGAGGGCGAGTTGGNGGCCGTCGGAGGCCGCGTGGACGATTT
GGTGGACGATTTCCACCCGGGGATGCNNTTGAATACCCNNAAGATTTCGTGCGCGCGGCATCCGCGGANGAAT
GGGCCTNTCCANGCATGAGTTGGNGGACTTGCAGGGTGCGAATCCCGAGGCCCTCCGNCGGGCGATGTCNATG
CGACTGGATCAAGATGTCGCNCAAGTGTGGCATTCAAGGATCGCGTCCGCGGAGACATTCAACGGTCGCGATC
CNTTCGAGCTGGCNAACATGGAGTTGTGGCGCGGAAGCCGCGGAGAAGGGGCNTTGGAGAAGGCNATNGCNGC
NAGCAAGAAGTTCCGGGAGGCTCGNGAGGAGTTTTATAGNCTCGGAGGCCGNCGGTCGGAGTGGGCGTTGTGG
AAGAGGTTCTTGAAGGGAAGTGAGGAGTTTTTGGTGGAGGATTCCGACGGGGAGTTTGGAGGAGGAGGAGGTT
AGGGAGGAGATGAAGCCGGGCGCGGGATCCGTCGGGATCCCGGAGTTGGATGAGTTGGAGTATGGTGATGGGG
AGCCCGAGGGATCCGCGGCCGCCTGAGNCTCCTTGCCTTGTATAATTTTATCTTGATGTGTATGGACATAGAT

AGTACCGCGCCCGGAGGGGCAAAAATGTATATGATTTTCTTGATGCTTGAAATGGAGTGACGAAAATGAGAGT
GAAGTCAAGAAANAGGCCGACGGAGTCCGTTCCCGATTAGGAAGTGAATTTGCAATAGTTTTCCAGAATCGAA
TTTGAAATTGGGGAATCGGAATCCGCAGGAGGCCGATGGAGATGGGAATCAAGAAGGGATAGGCGTAGGCTTG
GATGTAGCCTTGCGAATGGTGACCTTGGATAGTGGTCCTGGTTTGTCTGGGTATCGACGGTGGAAGTCTTCAA
TGTAATCTTCCGCGGAAAATTCGGTAGCGGGAATCCAAGCAAATTCTTCATCAGTACCTTCGTAACCCAACCA
TTGAACGCGATAGAGGAGAGGGCATCGGAACCGACGGTCGAGTTTAGAGTCNAGGATCTGTGAAATCTCGTAC
TGTTCTTCGCCGTCGACTTCGACCGGAGGTGGAGGTGGTTGGGTACGATTAGGAATGGTATTGGCTTCCACGG
GTTCCAATTGAGACATGGAATACGGGATGAATGGAACGGAGGTCCGCGGGAAGCTTGAGAGTGAAGGAATGGG
ATCCGGGCTGAGCAATGATATCGAACGGTCCGAGGAATTTCTCGGAGAATTTCGCGGTAGGGCGAGTAGTCCT
GATGTGTTTAGCACTGAGGTAGACTTTATCACCTACTTTGAAATCCGGAGCCGGAGACCGTCGCTTGTCCGCT
TGGAATTTGTAGCGAGACTGAGCAAGAGTGATTTCATCGCGTAGGAATTGGTGAAGATCGTCGAGGTTGACTA
CGAAATCGCGGGCCCGAGCGGAGGCGATATCGCGTTCAGGGTATACCGTGATGTTAGGGTGGTATCCCTTATT
AGCGAAGAATGGAGAGATTCCGGTGGAAGCGTTNGGAGCATTGTTGTACGCGAATTCGGCAATGGGTAGTAAG
GTATCCCAATTGTCTTGTTGGTAAGAACAATAGATACGGATGTATTGTTCAAGGGTTTGGTTAGCGCGTTCAG
TCTGTCCGTCGGCTTCCGGGTGATAACCAGAAGTGAAGTGAAGTTCCATATCGAGAGCCTTAGCAAGGGCGCG
AGTGAATTGGGAGACAAATTCGGAGCCGCGGTCGGAAGTGATGTGATTAGGAACGCCGTGCTTGGAAAATACA
TGGGTAACGAATAGCTGGGCAAGTTCCGCGGAATTAATGGTATCGTGAGTGGGAATGAAAATGGCTTGTTTGC
TTGCACGGTCAATGACGACNAGAATGGCGGTGAATCCGTCGGATTCCGGGAGTTGTTCGATGAAGTCCATGGA
GATGGAATCCCACGGACGTTCNGGTACAGGCAAAGGTTTGAGTAAGCCGAAAGGACGGTGTCTAGGTGTTTTG
TTACGACCACAAGTAGT
>Armme1_1_rnd-1_family-0#LTR/Gypsy
TCTTCGTAGTCCCGTATNGCCTCCTGTGCGTTTCCCATCTCCGAGTCTCGTACCCACGAGTTGTGTTCCCGGG
TCCATCCTTTCCATTTGACGAAGTAGTCGGTGACGTTTGTTGGCTTTTGTCCCCGCTTTCCCCTGACCTTTCG
NGGNCGNGAGTCNAGNACTTCTTCGATTTCGTATTGTTCTTCGTCGTCGACGAGGTCNGGCGGTGGTGGTGGT
GGTTGTTGTTGGTTNGGGAACGANGGTGGTACGAACGGGGTGAGTAGCTTTTCGTTGAAGCGCGGGTGGATCT
TCCAGTGCGGNGGNAGCTTCAGTTTGTANGCCGAGGCTCCCGTTTTTTCGAGNATNNCGAANGGNCCNACNCG
TTTGTCGTCGAGCTTTTTCTTGGGNCGGGGGAGGTGTAGGTTTCCGGCGTCGAGCCAGACCTTGTCTCCGACC
TTGTAGTCGACCGCGGNCCGTTTTCGTTTGTCGTACTGGGTTTTCATCGCNTCCGCCGCCTTTTCGAGNGCCT
TTTGGGTCTGTTTGTGGACTTCNGCCATGGTTTCGGTGAAGGTTTCGGCNGCNGGGTTGAGGAANGGGGANGG
NCGGGTNGAGTCNGGGACNAGTCGGGGGTTTCNTCCGTATAGGACCATGAANGGGCTCTTNCCGGTGGCGGAG
TGTACTCGGTTGTTGTGGGCGAACTCCGCCATNGGNAGCCAGTCGCTCCAGTCGGTTTGTCGGTATTCNACGA
AGAGNCGGAGGTATCCTTCGAGTTCCTGGTTGACTCGTTCGGTCTGNCCGTCGGTCTGTGGGTGGAAGGCNGT
GGATGCGTTCGGGGTGATGTCGAGTAGTCGGTATAGGTCCTTCATGAATTGGGAGACGAACTGGGGTCCTCGG
TCGGATATTACGACCTGGGGCATCCCGTGTTTGGCGTAGACGTGGTCCCGGAGGGCCTTGGCCCATCCTTCGG
CCGATAGTTCNACGTTGCAGGCNATCGGGATGATNGCTTTCGAGAATCGGTCTACGACGACNAGNAGGGCGTC
GTGNCCGTTCGACTCNGGNAGNCCGGTGACCATGTCGACCGAGATGTGGGTCCATGGTCCGACGGGGATNGCG
TTCGGGTGNAGGGGGGCNGCCTTNGCCTGGTTGCTGGACTTNGCCCGTTGGCANGCTTCGCATCCTTTTACGT
ANGCTTCTACGTCCTTCTTCAGCTTCGGCCACCAGTATTCTCGTTGGACGAGTTCCTTGGTCTTTTCTATCCC
CGGGTGTCCGGCCGTGATGTGGTCGTGGCTTTGGGCGATTATCTCCCCTCGTAGAGAGGAGTCCGTGNTCCCT
GGTGATNCCTCGTTCGTGGGCGAGGGAGTTCGCGATCCCTTGGTCCCAGAGTTCTTCCCGTCNGAGGGCGTCC
TTGACCTTGTCGTGGGNTTCGGTGGTCGTNGGCATGATNAGGGCGCGGAAGTGNTGGGGTTGGAGGACGANNA
CGTCGTCGTTGTCCTCCTTTCCTTGGTCGTGGTCGTCCCGTCGGGAGAGGAGGTCNGCCTTCTTGTTGGCGGT
NCCGGGNTGGTGATGNAGGGNGAAGTGGTATTCGGCCAGTTCGGTGACCCATCGNGCCTGTCGTCGGTTGAGC
TTCTGGGGTTTTCGGAAGTACTGGAGGTTCTGGTGGTCCGTCCAGATTTCTACGTCCTCGGTNGCNCCCATCA
GGTAGTGCCGCCATTCGGACAGGGCGAGCATGATNGCGAGCAGCTCCTTGTCGTAGATCTCGTAGTTCCNTTC
GGTGACGGTNAGGGATTTGGACATGAACGCTACNGGTCGCCACTTCCCGTCCCGCTNCTGGCTNAGGACCGCG
CCGATGGCNCCGTCGCTGGAGTCGGCTTCTACCTTGAACCGCCCTNCCTCGGTGGGGATGGCGAGGACTACGT
CCTCCGCCATCTGCTTCTTTAGCTGTTGGAAGGCCTGGTTCTGGGCCGTTCCCCANGTCCATTCCGCGTTCCC
GGTGAGCTGGGTGAGNGGGCGGACGATCTTGGAGTAGTCGCGGATGAACTTTCGGTAGAAGTTNGTGAANCCG
AGGAACGACTGNAGCTCCTTCTTCTTNGTCGGCGTGGGCCATTCNGTTATCCCGGCGACCTTGACGGGGTCCA
TTCGTACGGCGTCNTGGCTGATGATGACGCCTAGGTACTCGGTCTCNAGGACCTCGAACTCGCACTTCTCGGC
CTTNAGGAATAGTTTGTTCTCCCGNAGGCGTTGNAGTACGCGTCGTACGATTCGTCGGTGTTCGTCNAGGTCC
TTGGTGAAGATGAGGATGTCGTCGAGGTAGATGGTGACCTTTCCTTCGGCGATCAGGTCCTTGAAGATGTCGT
TCATCATCCACTGGAAGGTGGCGGGCGAGTTNGTGAGNCCGAAGAACATNACNGTGGGTTCGAAGAGCCCTCG
GTTCGTNCGGAACGCCGCCTTCTCCTCGTCCCCTTCCCTGATCCTGACGTTGTTGTATCCCCANCGTACGTCT
AGCTTGGTGAAGTANCGGGCCCCCCGGAGTTTGTCGATGAGCTCNGAGATGAGCGGTAGCGGGTAGCGGTTTT
TGATCGTCATCTCGTTGAGCTTTCGGTAGTCCTGGACCGGTCGNAGCGAGCCGTCCTTCTTTTTGACGAAGAA
GAANGGNGAGGCGAAGGGNGATTTGGAGGGTCGGATTCGTCCCGACTCCAGGTTTTCGTCGAGGAACTGGTCG

AGCTGTTCCTGTTCGTTTCGGTTCAAGGGGTAGACCTTGCAGTCGAGGGTGGACTTGGCGTTGGGGACGAGCT
CNATGGCGTGGTCCCAGGGCTTTCGGTCGGGGAGTTCGTCGAAGTTGTCTTTCGAGAACAGGTCGCGGTAGGA
GCGGTAGTGCTCGGGTACCATTTCTTCGAAGGTCTTCTTCGGCTTGTCCGCGTTGGCCTGGGCCGCCAGGTCG
TTGGCGTGATGTACCGCCCGGATGACTANTTCCTCCTGCATGTTTACGGCGAGGATGGTCTCTCCTTCTTCGA
GTTCTTCGTCCCANCGGTCCTCCGGGTCNGCGTCGGGGAGNGGGAAGGGGTTTTTGGTGCATTGGCAGCGNCC
GAAGATGATCGANCCGGTCTTCCAGTTGATTACCGGGTTGTGGCGTTTGAGCCAGTCGTGGCCGAGGTANAGG
TCCTTNGAGCCNAGGTCGGTGACCGCNAGGTCGATTCGCTCGCGGTGCCCGCCGATNGAGAGGCGGAGTTCCG
CGAATTCGGTGATGTCCCCCGCCTGGTTGCGGGTTCCGTCGGCGTTGTANACNGGGATCGGGGCGACGGTCTT
TCTGGTGTCCAGGCCGTGTTTCTGGACGTAGGANCGGTTNATGGTGCTCCCGGTGCATCCGCTGTCGATGAGN
CCGGNGGCGGATAGGGTNCGTCGGTCGGTGCAGGTGGTTAGGANGATGTCGACGGTGAGTTCCCTTCCGCTGC
TCTTCGTCCTTCGGATGTANCGGATGGGTTCTCGGAGTTCGAACATGATTTCCCTNGCCTCCTCCCCNCCTCG
TCTGAGTCCGTTGACTAGGCTCGCGACGATTTCGCGGTGTCGGTCTTTGTGGATCCAGCTGCTNAGCGCGGCC
CTGGCTTCCTCCGGCGTGCTTCGCAGATGGCCCGGAGGGTCCAGTCGACTTCGAAGGGTTTGTACCAGGCCCG
AGAGTGCGGNGGGGCGCTCGTATCGGGCCTGGTGGGTACCCAGTCGTCGTCGCTCTGGATGNCCCGCTCCCTC
TGNTCNGCCTTCTGCTCCGCCAACCATTGCTGGTAGGCGGTTTCGTCTTCTTCGTCNGGTACCTCCTCCATGG
TNACGGCAGGCCCCCTTTTTATCGTCTGGGCGCTAACGACTTCCGCCCCTGCGGCTTTCTTGTCGTCCGGGGT
CGGTCGCGCGTCTTCGTCGGGGCCGGCNGGAGGAGTGATGGGGGGGAAGTCCCATCGAGGGTACTCACATCGA
TCCGGTATTTCGTGGCCGTCCCGTCGAGGGACGGGGGACGCGCCCGTGCCCGTCTCGTTCTTGTCGAATAAAC
CGTGGCNGTCCCTGCGCTCCGANGCCTGGCCGTCGGTGAGGCTGTGCTCGTCTCCTCCGCGCTCCCCGACTCG
TGGGNCCATTTGTTGCGGCGTGCCTGCTGAGGCCTCGTCCTTCTGCGACTTCCCGGGGTCGCTTTCCGGAGCC
TTTCCCCTTGCGGGGCTTAGCGCTCCGNTTAGCCAGTCCTGCAGAGGCGTTCCCCCCGAGGGGTTCGCGTGCG
GGCAGTTCCCGTATTAGGGGTTCTGGCGAGTCGTCGTGGGGGCTCGCTGCCGGGACTGTCGTAAGTCCTGCTC
CCTGTTCGGCGTCGGAGATCTCGGTCTCGTCAGCGCTTGAGTCCGAGTCGCTCAATTTCGCTAGAGCGGCGTA
GCGGTTCGATTCTGTTGGTACTGGTTTGCTGGTCGTGCTGGGGACGTTAAAAGCAGGGGTATATAAAGCGGTA
GATAAAACACGNTGTAACTAAAGGGGTTGCATAAGACCGTCCAGCGGAAGACGAGAGCGCACCAGTGAAGTCC
TTGGTCGCCGAAGGGGAATCGAGCCCTTGGCGACCTACTCTTTTACCTCCTCNACCTTCGACTCCGTTTCCTC
CTCCTGCTCGACTACTACACCGAGCGAATGTCCTTGNANTCCTTCCTCTTCGGGCAGTCCTTCGACATGTGTC
CCTTCTCCTTGCAGCGGTAGCACCGGCCCTCCTTTCTGAGCCNGTCGATCTCCATCGGCTGTCCCTGTCCGGC
GTACGTCGTGGNTTTGACTGGGACCCACTTCCCGGTGGACGCGTCTCTGCTCGGCCCTCCCCCNCCTCCCGTC
GTCTTTGCCGGCGTCGAACTTGTGGCCCCAGCGGGTTTGCTGGGGCTNTGGTTGGGTGGTCCGGGTTTGGCGT
TTGGTCGGTCGTCGGGGCGGGCGTCGTGGGTGAGCATCTTCTGGATGACGTACTGCTTCTGTCGTTCTTCGTA
CATTTTGCAGATGCGGGCCTTCCATTCCGGGTANGTGTCGGGGACGTTNTCCCCGATGTCGCCGATCATNTTG
ATGTAGGNGTAGGGGACCCCCCGTCTGACTGCTCGGACCATGATTCCGCGGTCTTCTTCCTCCGTCCTTCGCC
CGGCGAGCTTGGCCAATCGTTCCAGTTCCTGGAAGTAGTCGTTGGCGGGNCCGTTTCCCATGCGGAGGTCGTT
CATCTCCTGCTCGTGCTGTTCTTCTACNGCCGGGCTTCGGAACTGGGCGGCGACCATGTCCGCGAACTCCTCC
CAGCTTGGGTATCGGAATCGCGGGGGTTGNCCGTCGTAGTCNGAGGGTTGCCAGAACTCCTGGCGTCGGTAGA
CCCACCAGTCCTTGGCCTTGTCTTGGAACAGCGCGGCGGCCCAGGCGACCTTCTGGGANGGGAAGGGGAAGAA
GCTTCGGAACTGTTCGAAGTAGATGACGCAGTCCCCGAGGAATCGTTCGATGTCGTCATGGTGGCCTTTGAAC
GGCTCGGGNGTTTTCCAGTGCGGTTTGAGGCTGAGGTAGGGGGCGTCNTCGGGTGCTTCCCCGATCGGGTGCG
GGTATGGTGCGGCTGGGTAGGGGAAGTTCGGGGGTCGTGGGATGGAGTGNCGGTCGTCGCGGTCGTTNAGTCG
TCGCATGTGGTTCGGGGGAAGGGGTCTTCCTCGTTCGGGTTCTTTTCCTTTTTCGAACCCGTGGTACTTGGCG
AACCAGGCGCGGTCGGCTTCGGCTTCCGCGAACTNTTGNGCTAGCTCGGCTTGTTGGGCCTGGAGCTCGGCGA
CTTTCTGGTTCGGGCCTCGTTTTCTTCGGCCGCCTGTTGTTTTCGTTGTTCCGGGGTNAGGTGCGTTCCTGGG
TTGGATGGTCCAGACCCTCCTCCTCCGGTGTCGTGGTCTCCTCCGGCGAATAGTCGGCTCGTGCCGGTGGGCG
GCATGCCGTGNAGCCGGTATCGGCTTGTCGGCATGGTGGCGCGTGGTGGGGAAAGGGGGGTTCGTCTGGGTA
CGGTTTCGGGTATTCGATCGGGTCATAGGCGGAAAAGGATATATTGGGGGCGCTATATGCTGCGCCCCCGCCG
AANGTTTCTGTATCGTATGTAAAACTATCAAAATTAATTAAATTCTTCGCCCCGCCCCTGAGCCCTGTAGTCC
TCGTGGTCGGCGGCGAGGTCGAGGAGGGTGAGCTCCAGGTCGCTCTGCTCTTCGGCGGTCCTGNCGCGTAACC
CGTAAACTCGGGGTCCNGCGTTGGCGGGGNANAGGTGTCGGTCCCNGTTGGACTCNAGNCNGGCCTCGATGTT
CGCCACCATNCCTCCTGACTCGATGGGGTCTCCGCTNGCGATGGCGAGCCAGTTGTCCATGGTGGACCGAGGG
GGNAGGGGNCCCATGGCGCGGGCTTCGACGTCTGCCCNGCGGACCATCCAGGCGGCGGCCCTGGCGGGGCTGA
ATATTTCNCTGTCNCGGGCAGAGAGGNCGGCCACTCGTAGTCGAGGCCCCTTGGGTTGAAGGGGTCGTCGAG
GGCGTCGTGGTCGAAGA
>Armme1_1_rnd-1_family-542#LTR/Gypsy
TGCCTACGATATTTTGCTTGGCAGGCCCTTCGACACGTTAACCCAGAGCATCGTACGGAATTACTCTGACGAG
AATCAGACCATCACCATCCATGATCCGAATACCGGAAAAACAGCCACCATCCCGACGATCCCGCGTGGTCGTC
CCCGCATCCTTAATCATGTTCCATTTTCGTGGGACAATTGACTAGAAGAACCCAT
>Armme1_1_rnd-1_family-25#LTR/Gypsy

GACCTGGTGGTAATCCCGCTAACCCTGGCAATGATGACGCCCGCCTCATGATCGAATTCGTAAACCTCCTCCG
CGGCCTGAGCAATCAGGTCAACAACCCTCCTCCGCGGCCAGTCCGTACCAAAGTCAAAGAACCTGATGTGTTC
GATGGCTCGGACCCGCGGAAACTGAAGGCGTTCATTGTTTCCCTCCAGTTGAACTTTAATGACAGGCCCGACG
CATTCCGCGCGGACACCGCCAAAGTCAACTACGCTATCTCTTTCCTCTCCGGTGTGGCCCTCGACTGGTTTGA
ACCAGATATCCTCCATCCTAACCACACCAACCCTCCTGCTTGGGCATATTCCTATGCCGCCTTCCTTTCGGAA
CTCCGGACTAACTTTGGTCCATTCGACGCCGTCGGAGATGCTGAAGATGAACTCGAAAATCTTCGGATGCGCG
ACGGAGACCGGATTATGAAGTATATAGTCCGTTTCAACCAACACGCTGCTGTCATAGGCTATGGTGACAATTC
CCTCCGCCATGCTTTTTACCGCGGACTTTGTACCCGGATTAAAGATGATATGGCTCACCATGGTAAGCCTAAT
AACCTCCATGACATGCGGAATCTAGCACAGGAACTCGACGCGCGGTATTGGACTCGGAAGACCGAGATTGCAC
GCGAAAACCGTGATAAACCTTCCTCCTCTTCCTCTTCTTCCTCCAAACCTAAGGGTTCGGGTTCTAACTCGAG
CTCTTCCTACAATTCCGCGGCGCCAAAGTCTTCTAACTCAGGCTCCGCGGGATCCGCTTCATCCACTTCCTCT
TCCTCGGGCGCCGCCAAGAAGCCCTATGCTGACAAGCTAGGCAAGGATGGCAAGCTTACGCCCGAAGAGAAGG
AACGCCGCCGCACGAACAACCTCTGCATGTTCTGTGGTAGCAAGCACAAGACCGAGGACTGCAACAAGCGCAA
GGCCGCGGCTTCCGCGCGTGGTCGTGCTGCTGAGGTTTCTGAACCTCCCGCCTCTACTGATTCGGCTCCGTCG
GAGTCGGAAAAATAGAGAGCAGCCCTGCTTCCTCCGTGCAAGGACAGGGTTGCGTTGACTGTCACTGCACCAC
GGAGATCATNACCCTCAACGCTGCCGCTCTTTCCAATCATTCACTCACTGTATCCTTATCCTCTGAAGTCGGC
TCCGGATCGGAGTCCGACGGCAGTCCTATATCCATCATCCTTCGTTTTCTCGCACTCATTGATTCTGGTTCTA
GTCATTGTTTCGTTGACTCGAAATTCGTTGAGGACCATGATCTCCCTACCAAATCCATTCCTCCTGTCCAACT
TCGGCTTCTTGACGGCTCCGCCGGATCTCATATTTCCCAATCCATTACTCTCTCCGTACGGTTTCCGTCGGGA
GATGTTATTCCTATTGACTTCTACGTCACACGTCTTGATTCGTCTTGTAATATTGTCCTGGGATACAACTGGC
TCTCCCGCTGCAATCCGTTGATTGACTGGGTAAAGCACAGTATCACTTTCCGTTCAAAGTTATCCGATGTCTC
TTCTTCCGCGGCTTCCGGCGCTTCAGCCCAAGCTCGCATAGACCCTTCCGGTCTTGTCTCAGACGTTTCCGCG
GCTTCCGACCCTCCGGTCTCAGCTCGCACGGACCCTTCAGGTCCTGTCCCATCCTCCAAACCCCCTTCGAGAT
CCCGACGGACTCCGCCGCCTTACGAGCCAATCTACTCCTACCCTTCCATTCCTTCATCTGCTGCATCGACGGC
ACCCAAGATATCTATTATTTCCGCGGCTGCTTTCGTACGAGCTTGCGCAGAGGAGGGTGCACAACAGTTCACA
CTCCAACCTTCTTTGCCAAGTGTTAGTGGCCAGGCTGCCTCCGCGGCTCCGCCGGATTTATCGGATGTGCCTG
AAGAATATCATGACTTCGCCGATGTATTCAGTGATTCCCTCT
>Armme1_1_rnd-1_family-156#TIR/MITE
TAGTGTTGCCGCGACTCGGCTTATCTCGGCTTAACGCCGAAGTCGGTTTTTAGCCGTTTGCGCTTGGTGTGCT
CTGACTGAACAATTGTTCAAAATGGCAGATAATCTGAAGTACGTATTCACTCACAAGAATTGTTGGTAATGAC
AGCGACAGTGTAGCTGTTCCCTCTGNCCTTCTATCCTCGGCGGCTCAAAAGTCGACTTCGGCGTTAAGCCGAG
ATAAGCCGAGTCGCGGCAACACTA
>Armme1_1_rnd-4_family-996#TIR/MITE
AGACTCGAGCCAGTCCATCCGCCACTTGATGACAGTCGATCAAGTCCGAAAAGCGGATTGATCGACTGTCATC
AANCGACGCCGGGGACTGGCTCGAGTCTA
>Armme1_1_Cluster46#LTR/Gypsy
GCTGAAGGACGAGGACATTGTGGTTGTCCTCCTTTCCTTGGTTGTGGTCATCTCATTGGAAGAGGAGGTTGGC
TTTCTTGTTGGCGGTTCCGGGATGGTGGTGAAGGGTGAAGTGGTATTCTGCCAGCTCAGTGATCCATCTCGCT
TGTCATCAATTAAGCTTCTGGGGTTTTCAGAAGTACTGGAGGTTCTGGTGGTCTGTCCAGATCTCCACCTCCT
CCATTGCCCCTATTAGATAATGTCTCCATTCCGACAGGGTGAGCATGATAGCAAGCAATTCTTTGTCATAGAT
CTCGTAGTTCCTTTCAGTGACGGTTAATAACTTGGACATGAACGCTACTAGTCACCACTTCCCATCCTGCTTT
TGGCTCAGGACAGTGCCGATGGCGCTGTCACTTGAGTCTACTTCTACTTTGAACTGTTCTGATTTGGTGGGAA
TGGCGAGGATTATGTCCTCTGCCATTTGTTTCTTCAGCTGTTGGAAGGCCTGGTTCTGGGCTATTCCCCAGGT
CCACTCTGCATTTCTGGTGAGTTGGGTGAGAGGATGGACTATTTTAGAGTAGTCATGGATGAACTTCTGATAG
AAGTTTGTAAAGCTGAGGAAGGACTGGAGCTCCTTCCAGGTCCACTCTGCATTTCTGGTGAGTTGGGTGAGAG
GACGGACTATTTTAGAGTAGTCGTGGATGAACTTCTGATAGAAGTTTGTAAAGCTGAGGAAGGACTGGAGCTC
CTTCTTTTTTGTCAGTGTGGGCCACTCCATTATTCCAGTGACCTTGACAGGGTCCATTCAGACAGCATCCTGA
CTGATGATGACACCTAGGTACTCAGTTTCTAGAACTTCAAACTCGCACTTCTCAGCTTTAAGGAATAATTTGT
TCTCCTTAAGGCATTGGAGTACACATCGTACGATTCAGTGGTGTTCGTCTAGGTCCCATGTGAAGATGAGGAT
GTCATCAAGGTAGATGGTGACCTTCTCTTCGGCAATTAGGTCTTTGAAAATGTCGTTCATCATCCATTGAAAC
ATGGTGGGCAAGTTGGTGAGGCTGAAAAACATGATGGTGAGTTCAAAGAGTCCTTGGTTTGTCCAGAATGCGG
CCTTTTCCTTGTCCCCCTCCCTGATTTGAACATTGTTATATCCTCAGCAAACATCAAGTTTGGTGAAATACCT
CGCGCCCTGAAGCTTGTCGATGAGTTCGGAAATTAACGGGAGTGGGTAGTGGTTCTTTATCATCATCTCATTG
AGCTTTTGATAGTCTTGTATGGGTTGGAGGGAGTTGTCCTTTTTCTTGACAAAGAAGAAGGGTGATGTGAAGG
GGGATTTAGAGGGACGGATTCGTCCTGACTCTAGGTTTTTGTCGAGGAACTTGTCTAGCTGTTCCTGTTCGTT
TCTGTTTAAGGGATAGACCTTACAGTCAAGGGTGGATTTGGCATTTGGGATGAGTTCTATGGCGTGATCCCAT
GGTTTTTGCTCGGGGAGTTCATTGAAGTTTTCTTTGGAAAACAGGTCGTGGTATGAGCAGTAATGCTCAGGTA
CCATTTCTTCAAAGGTCCTTTTTGGCTTTTCCGCATTGGCTTGGGCCACAAGGTCATTGGTGTGGTGTATAGC

CTGGATGACCAGTTCCTCCTGCATGTTTACCATAAGGATGGTCTCTCCCTCTTCAAGTTCCTCATCCCACTGG
TCTTCAGGGTCAGCATTAGGAAGTGGGAAGGGATTTCGGGTGCATTAGTAGTGGCTGAAGATGATGGAGACGG
TCTTCCAGTTGATGACCAGGTTGTGGTGTTTGAGCCAGTCATGACCAAGGTACAAGTCCTTTGAGCCCAAGTT
GGTGACTGCAAGGTCAATTTGCTTGTGGTGTCCACCAATCGAGAGGCAGAGTTCCATGAATTTGGTGATGTCT
CCTGCTTGGTTATGGGTTCCATCAGCATTATATACTGGATTGGGGTGACAGTTTTTTTGGTGTCAAGGTTAT
GCCTTTGGACATAAGAGTGGTTTATGGTGCTTCCAGTGCATCCACTATCAAGGAGCCCAGTCACAGATAGGGT
GCACTGATTGGTGCAAGAGGTGAGGATGATGTCAACAGTGAGTTCCCTCTCACTGCTCCTTATCCTTCGGATG
TACCAGATGGGTTCACGTAATTCAAACATAATCTCCCTGGCTTCCTCTCCCCCTTGTCTGAGCTTATTGACTA
GGCGCTATCGAGGAGCCCAGTCACAGATAGGGTGCACTGATTGGTGCAAGGGGTGAGGATGATGTCAACAGTG
AGTTCCCTCTCGCTGCTCCTTGTCCTTCGGATGTACCAGATGGGTTCACGTAATTCGAACATAATCTCCCTGG
CTTCCTCTCCCCCTTGTCTGAGCTTATTGACTAGGCTCATGATGATCTCATGGTGCCAGTCTTTGTATATCCA
GCTGCTTAGTGTGGCCCTGGCCTCCTTGGGAGTTCATGCTTCGCAGATAGCCCAGAGGGTCCAGTCAACTTCA
AAGGGTTTATACCAGGCCTTCAAATGAGGTGGGGCACTAGTGTCAGGTTTGGTGGAGACCCAATCGTCATCGT
TCTGGATGTCTTGTTCTCTGCAGATAGCCCAGAGGGTCCAGTCGACTTCAAAGAGTTTATACCAGGCCTTCAA
ATGAGGTGGGGCACTGGTGTCAGGTTTGGTGGAGACCCAATCGTCATCGTTCTGGATGTCTTGTTCTCTCTGG
TCTGCCTTCTGTGCCACCAGCCACTGCTGGTAAGCGGTCTCATCTTCTTCATCGGGTACCTCCTCTATGGTGA
CGGAAAGCCCCCTTTTCATCGTCTGGGTGCCAACGACTTCCTTTCCTACAGCTTGTTGTCCAGTGCTAGTCAT
GCGTCTTCATCAGGATTGGCCGGGGGAGTGATGATAGGATGCTCGCATCAATCTTGGTCTCATCTTCTTCGTC
GGGTACCTCCTCTATGGTGATGGAAAGCCCCCTTTTCATCGTCTGGGTGCCAATGACTTCCTTTCCTACAGCT
TGTCGTCCAGTGCTAGTCATGTGTCTTCATCAGGATTGGCCGGGGGAGTGATGATAGGATGCTCGCATCAATC
TGGTATCCCATGGTCACTGCTTTTGGCAGGGGACATGCTTATACATGTCACGTTCTTGTTGCATGAACCATAG
CTGTCCCTGCGCTCTGGTGCCTGGCTGTTGGCAAGGTTGTGCCTGCTTCCTTTGGCGCTTCTGACTCAGGGGT
TTATTTGTTTCTCTAACTGGCCCTGCTGTTGTCTTGTGCTCATACATGTCAAGTTCTTGTTGCATGAACCATA
GCTGTCCCTGCACTCTGGTGCCTGGCTGTCAGCAAGGTTGTGCCTGCTTCCTTCGGTGCTTCTGACTCAGGGG
TTTATTTGTTTCTCTAACTGGCCCTGCTGTTGTCTTCCCCGGGAGACAGGTGTGGTGGTCGAGGGAGATCCAG
AGGGTTGGGGTTCTGCGACTTCATGGGGTCACTTTCCAGAGCCTTTTCCCTTTCGGGTTCTCATGCTCTGTTT
AGCCAGCCCTAAAGAGATGTTCCCCCTGAGGGGTTCACATGTAGGTAGTCCCTGTATTAGGGGTTTTGGCAAG
TCATTGTGGGGGCTCTCTGCCAGGACTGTAGATCCCTTTCCCTGTTCGGCATCAGAGGGTTCGACCTTGTCGG
TGCTTGAGTCCATGTTGCACAATTTTGCTAGAGGAATGTAGCAGTTCGATTCTGGTGGTACTGGTTTGCTGGT
CATGCCGGGGATGTTTAAAGCAGTAGTATATATATCGGTATGTAAAACAGGCAGGTCCCTTTCTCTGTTTGGC
ATCAGAGGGTTCGACCTTGTCGGTACTTGAGTCCATATTGCACAATTTTGCTAGAGCGATGTAGCAGTTCAAT
TCTGGTGGTACTGGTTTGCTGGTCGTGCCGGGGATGTTTAAAGCAGTAGTATATATATCGGTATGTAAAACAG
GCAGTGTAACTAAAGAAACTGCATAAGACCATCCAGCGGAAGACAAGAGTGCACCAGTGAAGTCCTTGGTCGC
CAAAGGGGAATCAAGCCCTTGGCAACCTACTCTTTTACCTCCTTGACCTTCGACTCTGTTTTCTCCTCCTGCT
CAACTACTACTGAGCAAATGTCCTTATATTCCTTCCTTTTCAGGCAATCCTTTGACATGTGGCCTTTCTCCTT
GCACCGGTAGCACTGTCCTTCCTTCCTGAGTCAGTCAATTTTCATTGGCTGCCCTTGGCCCCTGTATATGGTG
GTTTTGACTGGGAGCCATTTTCTGGTGGCGGCATCTCGGTCTGGTCCTCCCCCTGCCTCAGTGTGTTTAGTTG
GTGTTGAGCTCGTGGTTCCGGCGGGCTTGCCAGAGTTAGTGCTCTGCCCCCTGGGTTTAGTACCTAGTCGTTG
GTCAGGGTGCACATCTGGTGAGGCAACTAGTCATTGGAGGATGTATTGTCTCTGTCTTTGCTCATACATTTCG
CATATGTGGGCCTTCCACTCAGGGTACATGGTGGAGACATTCGTTCTGATGTTGGCAATCATGTTGATGTAGC
TGGAGGGGATCCCTCTTTGGAGGGCTCTCACCATTGCCCCTCGCTCCTCTTCTTCACTTCTCTGTCCAGTGAG
TTTGGTCTTAGTCACCAAAGGGGAATCAAGCCCTTGGCAACCTACTCTTTTACCTCCTCAACCTTTGACTCTG
TTTTCTCCTCCTGCTCAACTACTACTGAGCAAATGTCCTTATATTCCTTCCTTTTCAGGCAATCCTTTGACAT
GTGGCCTTTCTCCTTGCACTGGTAGCACCGTCCTTCCTTCCTGAGTCAGTCAATTTCCATCGGCTGCCCTTGG
CCTCTGTATATGGTGGTTTTGACTGGAAGCCATTTTCTGGTGGTGGCATCTCAGCCTGGTCCCCCCTCTGCCC
CAGTGTGTTTAGTTGGTGTCGAGCTCGTGGTTCCGGCGGGCTTGCCAGGGTTAGTGCTCTGCCCCCTGGGTTT
AGTACCTAGTCGTTGGTTAGGGTGCGCATCTGGTGAGGCAACTAGTCATTGGAGGATGTATTGTCTCTGTCTT
TGCTCGTACATTTCACATATGTGGGCCTTCCACTCGGGGTATGTGGTGGAGACATTCGTTCTGATGTTGGCAA
TCATGTTGATGTAGCTGGAGGGCCCTCACCATTGCCCCTCGCTCCTCTTCTTCACTTCTCTGTCCAGTGAGTT
TGGCCAGCCTTTCCAGTTTCTGAAAGTATTTGTTGGCTGTCCCATTGCCCATATGTAGGTCATTCATCTCCTG
CTCATGGGCTTCTTTGATGGCTGGGCTGCGGAATTGGCTAGTGACCATGTCGGCAAACTCTTCCCAGCTTGGA
TAGTGGAATCGAGGCAGTTGGCCATCATAGTCTGAGGGCTGCCAAAACTCTTGGTGTCAGTATACCCACCAGT
CCTTGGCTGGTCTCTGGAATAGGGCGGCAGCCCAGGTGACTTTTTGTGATGGAAAGGGGAAATACCCTTGGAA
CTGCTTGAAGTAGATGACACAGTCTCCAAGGAATCATTTAATATTGTTGTGCTCGCCTTTGAATGGATTGGGT
GTCTGCCAATGGGGTTTGAGGTTCAGTACCCTTGGAACTGCTTGAAGTAGATGACACAGTCCCCAAGGAATCA
TTTAATATCATTGTGCTCGCCTTTGAATGGATTGGGTGTCTGCCAATGGGGTTTGAGGTTCAGGTAGAGGGCA
TTGTCTGGTGCCTCGCTGATGGGATGTGGGTATGGCGCCGTGGAGTAGAGGAAGTCTGGGGGTCATGGGATCG
AGTATTGGTCATCACAATTGTTTAGTTGTCAGAGAGGATTGGGGACAGGGGGTCTCCCATGGTTGAGCTCTTT

TCCTTTTTCGAACCTGTGGTACTCGGCCCACCACTTCTAGGTGGCTTCTGCTTCTACAAGGTCCCTAGCCAAC
TTCTCATGTTGTTAGGTGAGCTCAGCAACCTTCCGACTTCAGAGGTTGTTTTCTTTCTCTGCTTGTTGCTTGT
GTTCCTCAGATGTGGGCTATGCATCTGAGTTCTGGGTACCAGATCCTCCTTCTCCTTGATCATGGTCTCCTCC
GGCAAATAGTCAGCTTATGCCTGTTGGAGGTCGGCCGTGTTGCCAAATTCAGCTTATCGGCATGGTGGCGCAT
GGAGGGGGGTAGGGTGGTTCATTGTTTAAGGGTCTTGGGTTCTGGAGCAGGGAATAGATGGAAAGAGATATAT
TTAGGGCGCCATATGCTGCACCCCTGCTGAAGGTTTTTGTATCAGCTTGGCAGACCACCCAGGCGGCCCTGGC
AAGGCTGAATATCTCTCGGTCCCTGAATGAGAGGCTGGGCCACTCGTAGTCAAGACCTCTTGGGTTGAAGGGG
TTCATGGTTGGGCTCTTTTCCTTTTTTGAACCTGTGATACTCGGCCCACCACTTCCAGGTGGCTTCTGCTTCT
ACAAGGTCCCTAGCCAACTTCTCATGTTGTTAGGCGAGCTCAGCAACCTTCTGACTTCAGAGGTTGTTTTCTT
TCTCTGCTTGTTGCTTGTGTTCCTCAGGTGTAGGCTGTGCATCTGAGTTCTGGGTACCAGATCCTCCTTCTCT
TTGATCATGGTCTCCTCCAGCAAATAGTCAGCTCATGCCTGTTGGAGGTCGGCTGTGTTGCCAAATTCAGCTT
GTCGGCATGGTGGCGCATGGAGGGGGGTAGGGTGGTTCATTGTTTAAGGGTCTTGGGTTCTGGAGCAGGGAAT
AGATGGAAAGAGATATATTTAGGGCACCATATGCTGCACCCCTGCTGAAGGTTTTTGTATCGGCTTGGCAGAC
CACTCAGGCAGCCCTGGCAAGGCTGAATATCTCTCGGTCTCTGAATGAGAGGCTGGGCCACTCGTAGTCAAGA
CCTCTTGGGTTGAAGGGGTCATTGAGGGCATCGTGGTCGAAGACACAGGCGGCAAAGAGATTGTCATTATCGT
AATTGGGGACGGACTCTGTGAAGGCAAAGGGGAGGGGGTCCTCTTCAGTTCAGGGAGAGGCGACCCGTATCTG
CTCGGGGGAGTCAGGAAGCCTGACATTCCCAGGCTCGGGCTGTCCAGGCTCTTGGGGGACATCAGGCTCAACA
TTGGTGTCGGAGTCTGTTTTGTTAGAATAGGGGTTTCCTCCCTGTAGTGTCCAGTTTAGCCCCTCAACTCGAA
TTTTGTATCACTCGCACCAATGGCTGGTGTCTGGTTGAGCAGCCTGGTCAAAGTAGAAGGTAGGCTGGTCAAA
TCGGAGCATGCAGAGAAGGTACCGGCTGATCTCTTCAGCACTTGGATTGGGACTGGGGGTTCAGTTGAGAAGA
TGAACAGTTTCGGGGGCCATTGGGAACGGGGGTGTTGGGGTTCTGGGTACTCCCTTTGGTCTGCTGGGTCCAG
GGGTTGCATTGCGAGGGGGAATTTCCTGATCTGGGAACTCGTTACTAGAGGGGGCGGTGTAGACGGATCAGGG
ACGGAGGGAGTTTTGGTTTGGAATGGGTTGTTCGACCTAAACATGGCAGGCAGCAGCAGGGTTCTGTTCTTTG
ATGTGTTTGATGACACGTAGGAGGGTCTGGCCCAGCCAGTCCCTTATTTCTTCTGCGTAATACACAGAGACAG
AGCTGGTGTCTGGTCGAGCAGCCTGGTCAAAGTAGAAGGGGGGCTGGTTGAATCAGAGCATGCAGAGAAGGTA
CCAGCTGATCTCTTCAGCACTTGGATTGGGACTGGGGGTTCAGTTGGGAAGATGAACAGTTTCAGGGGCCATT
GGGAATGGGGGTGTTGGGGTTCTGGGTACTTCCTTTGGTCTGCTGGGTCCAGATGTTGCATTGCGAGGGGGAA
TTTCCCGGTCTGGGAACTCGTTACTAGAGGGGGTGGTGTAGACGGATCGGGGATGGAGGGAGTTTTGGTTTGG
AATGGGTTGTTCGACCTAAACATGGCAGGCAGCCAATGAGTGTGAGGATCCCTACAAATCCAAAGAAAAGGGT
GATAGTGATGATGGTAGCAAGTATGAAGGGGTTCATGGATGTGTGTTTTCTCATGGCTAGCAGTGGGAAAACA
AGAAGGGAAGCTTCAAGCTAACTGTTAGACCCGGACTTCACATGATTAAAGACTTTAGCCTAAACCTCAGCTG
TCTCATGTTCGGACAATCACTGGAGGAATAGCTGAGGCATCTGAGACTTGGCAAAGAAGGACTAATGGTGCTA
GTCGAGTTCTTTTAAGCAAGAGTAAAGTGTGGTATGAATAAACTTTAGCTCGAAGCTGAGGATACTACAGTGA
GAGCTATGCTACTTATATAAGAAAACTGGTTGGCCAATAATAGAAGAGCATCCGTGTGTGTCACATGTAAAGA
AGAGAAGTTGTGCAGTAGGCAAGTGATAGTAACGAGCCGTAGTACAAGGGCTGAGAACGTCCGTTTAGGGCAT
GGTAAAGGTGATTAGGGGTTTTGAGATAGGGTGAGATTAGCAGGATCATTGACGAGAGATAGTAACGAGCCGT
AGTACAAGGGCTGAGAACATCCGTTTAGGGCGTAGTAAAGGTGATTAGGGGTTTTGAGATAGGGTGAGATTAG
CAGGATCATTGGATAAGATAAGATAGGCAGTAAAATCATAACATTACCCCCTTGTTAAGGCAGGCATCCTGAC
TGCTGTTAGAGCTCCCCCAAGCATTGCGATTTTGACTATATCGACGTATCTGTTCTGTTGGACTTGATTTTTG
TAGTCTTGTATTGCTTTTTGGGTGTGTTCCATTTCTGTGTCTCGAATCCAGGAGTTGTGTTTGTGGGTCAATT
TTGACTATATCGATGTATCTGTTCTGTTGGACTTGATTTTCGTAGTCTTGTATTGCTTCTTGGGTGTGTTCCA
TTTCTGTGTCTCGAATCCAGGAGTTGTGTTTGTGGGTCATTGTCCAGCCTTTCCTCGTACCTTTCGTGTTCTG
GAGTCTAATACCTCCTCTACCTCATATTCTTCTTCATCATCGATAAGGTCGGGTGGTGGAGGCGGCAGTTGCT
GCTGGTTTGGGAAGGTAGGTGGTAGTTGTCCAGCCTTTCCTCATACCTTTCACATTCTGGAGTCTAATACCTC
CTCTACCTCATATTCTTCTTCATCATCGATGAGGTCGGGTGGTGGAGGCGGCAGTTGCTGCTGGTTTGGGAAG
GTAGGTGGTACGAACAGGGTGAGTAGTTTTTCATTGAAGCATGGGTGGATTTTCCAATGAGGAGGGAGCTTTA
GCTTGTATGCTGATGCTCTGGTCTTTTCAAGGATCAGGAATGGTCCCACCCATTTGTCGTCAAGCTTTTTCTT
TGGATGAGGGAGATGTAGGTTTCCTGCATCAAGCCAGACATGATCTCCAATCTTGTAGTCTACAGCTGCATGC
TTCCTTTTGTCATATTGGGTTTTCATGGCATCAGCTGTCTTTTGGAAAGCCTTTTGGGTCTGTTTGTGGACTT
TGGCCATGGTCTCGGTGAAGGTTTCTGCGGCAGGGTTAAGGAAGGGAGAAGGACACAATGAGTCTGGGACAAG
CTGGGGGTTGCAGCTGTACAGAACCATAAAGGGGCTCTTTCTGGTGGCTGAGTGGATTCAGTTATTGTGGGTG
AATTCAGCCATGGGTAACCAGTCACTCCAGTCAGTCTGTTGATACTCAACAAACAGGTGGAGATATCCTTTGA
GTTCCTGATTTACTCACTCAGTTTGTCTGTCGGTCTGAGGGTGAAAGGCTGTGAATGCATTCGGGGTGATGTT
GAGCAGTCTGTACAGGTCTTTCATGAATTGGGAAAGGAATTGGGGTCCTCGGTTGGAGATTACCACCTGGGGC
ATGCCATGCTTTGTATAGATGTGATCCTGGAGAATCTTTGCCCATCCCTCAGCAGATAGTTCAATGTTGCAAG
CGACTGATATGATAGCCTTGGAGAACTGGTCTACAATCACTAGGAGGGCATCATAGCCATTTGATTCTGGGAG
GCCAGTGACCATGTTGACAGAGATGTGAGTCCATGGGCCAATGGGGATAGTGTTTGGGTGAAGGGGTGTGGCT
TTGGCCTGGTTACTGGCCTTGGCCCGTTGGCATGCTTCACACCCTTTGACATAGGTTTCAACGTCCCTTTTCA

ATTTTGATCACTAGGAGGGCATCATGGCCATTTGATTCTGGGAGGCCGGTGACCATGTTGATGGAGATGTGAG
TCCATGGGCCAATGGGGATGGTGTTTGGGTGAAGGGGTGTGGCTTTGGCCTGGTTACTGGCCTTGGCCCATTG
GCATGCTTCACACCCTTTGACATAGGTTTCAACGTCCCTTTTCAATTTTGGCCACCAGTGTTCCCTTTGGATG
AGTTCCTTAGTCTTTTCTATTCCTGGGTGTCTGGCCATGATGTGGTCATGGCTCTGTGCAATTATCTCTCTCC
AGAGGGCATGGTCCTTAAGGACATAGACCCATCTGTCATAGAGAAGGAGTCCGTTTTCCTTTGTGATTCCTCA
CTCATGTGCAAGGGAATTAGCAATCCCCTGGTCCCAGAGTTCCTCCCATCTGAGGGCGTCCTTGACCTTGTTA
TAAGTCTTAGTTGTTGTGGGCATGATCAGTGTATGGAAATGGTGTGGCTGAAGGACGAGGACATTGTGGTTGT
CCTCCTTTCCTTGGTTGTAGTCATCTCATCGGAAGAGGAGGTCAGCTTTCTTGTTGGCGGTTCCAGGATGGTG
GTGAAGGGTGAAGTGGTATTCTGCCAGCTCAGTGATCCATCTTGCTTGTCATCGATTAAGCTTCTGGGGTTTT
CGGAAGTACTGGAGGTTCTGGTGGTCTGTCCAGATCTCCACCTCCTCCATTGCCCCCATTAGATAATGTCTCC
ATTCTGACAGGGTGAGCATGATAGCAAGCAATTCTTTGTCGTAGATCTCGTAGTTCCTTTCAGTGACAGTTAA
TAACTTGGACATGAACGCTACCAGTCACCACTTCCCATCCTGCTTTTGGCTCAGGACAGTGCCAATGGCACTG
TCACTTGAGTCCACTTCTACTTTGAACTGTTCTGATTTGGTGGGAATGGCGAGGATTATGTCCTTGATGA
>Armme1_1_rnd-1_family-179#TIR/MITE
GACTATCAGATTCGGATTCCTACACCCAAAAAACAGTAGAGTCATCTATCATCCATGGGGNTCATNTTTACTA
GTGCGTCATCCCTGTACACCACTATATCTTGTGCACAAGTGAAAGCACNTNCATTACCTCGGAGTTTNGACAC
ATGAACCCCATGGATGATGGGTGACTCTACTGTTTTTTGGGTCCAGGAATCCGAATCTGATA
>Armme1_1_Cluster67#LTR/Copia
TACCTCGAATACTCTGGATACTACAGCTCCCACTATGACGACCCCAGCTCCTGAACTGACTTTGCACCGATCA
GCATGTGTAACATGACCTAGCCCTGAAGCTGCCGAATCTCTATTGTCTGAAGAACATGAAGCCGAGACTTTGC
GCTGATCAGCACATGTAACACAACCTAGCCCTGAAGCTGCCGAATCTCTATTGTCTGAAGAACATGAAGCCGA
GGTGCATTATTGAGTGATTCTGGCTTGGCAGATCGCTACTGGGCAGAGGCTGCATCGATGTCCATATTCACGC
GCAACCTGGCACCTACGAGACAACACCCTGGCGTGGTACCAGCAGAGTCGTTCTCTGCACAACCTGGCACCTA
CGAGACGATACCCTGGTGTGGTACCAGCAGAGTCGTTCTCGGGGCGTCGCCAGGACGTGTCATTCCTTTGAGT
GTTTGGGGCGACGTGCTGGGCCAAGAAACCAGTGTCGAACGGACTTCTGGTGGATGGTGGAAGCAAATTGGCT
GATCGATCGAAGAAGTGTGTCTTTCTCGGTTATGCAGGGTCAAACTACCACGTGCTCAATCAATCGGGCCACA
TATTCGTCTCTCGTGATGTCATTTTTGACGAGGGACCTCCACATCGCACTGTGAGTGTGGGGGAGACTGAGAC
TGATACTCCACTCACTGCTGCTTCGTCTACTGCTTCGTCGGCCCCTACTTCGAATACTCCGGATACTACAGCT
CCCACTACGACGACCCCAGCTCCTGAACCCGACTTTGCACCAATCAGCGCGTGTAACACGACCTAGCCCTGAA
GCTGCCGAATCTCTATTGTCTGAAGAACATGAAGCCGAGGCCTGTGCGAGTGGAAACGAGTGGGCAACCAATA
GCAAGCACCCACGAGCCTTGATGGCAGCATCTGGGAATAGTCCGGATCTATATATCCCACGGAGTTACAACGA
TGCGCTACGCGTTGACGAAGAGCGCTGGAAGGCTGCGATGGATGTCGAGTATGGCATGCACTGAATACTCCAG
ATACTACAGCTCCCACTATGACGACCCCAGCTCCTGAACCGACTTTGTGCCGATCAGCACGTGTAACACGACC
TAGCCCTGAAGCTGCTGAATCTCTATTGTCTGAAGAACATGAAGCCGA
>Armme1_1_rnd-1_family-268#TIR/MITE
TGTTGTAATGTCAAGTGTCGCGCATCACGGTGCAGTCGACGTCGGGATCTTCGGTAGGAGGCACGTGTCCAGT
TGTTCTCCTCCTCGGCTTTCCTTATTACATTGCATTGCTTCTATTAGTGTACAGGCATGTAGATAAATAGGTA
GACGAACGACGATGACCCTCGCCTGTTTGCGAGGGTTCCCTGCTAAATCCGAGTGAGCGGCTGGAGAATTACC
GCTCATTTCAACA
>Armme1_1_Cluster36#LTR/Gypsy
ACCAAGATCTGTTCTCAAAAGAGAACTTTGACAAACTCCCCAAACGAAAACCCTGGGACCATGCTATCAAGCT
CATCCCAAACGCGAAGGCAATGCTGGACTGCAAAGTCTATAAATTCAATACATTGGTAATTAGTGTAGAGTTA
GTGATAGGTTTAGTGTTGTTGGTGTTCATTGCCAGCCACTAGTCCGTTTGCCACTCGCCGTTACCGAGCACAT
CACTGGTTAGAGAAACAAATGAACCCCCGAGTTGGGAGTATGGGAGGAGACAAGCACAGCCTCACTGACTGCC
AGGCCTCAGAGTGTAGGGACAACCATGGTTTATTCAACAAGAACGAGACAGGCATGGATGCATCCCCTGCCCC
TCTACGGGATGGCCACAAGATACTGGATCAATGTGAGTATCCTCCCATCACTCCTCCCACCAACCCTGATGAA
GATGCATGAATGACCTTGGACACCAAGAAAGCTGCAGGAGAGGAAGTCGTTGGCACCCAGACGATAAAAAGGG
GGCTTTCCATCACCATAGAGGAGGTACCTGATGAAAAAGATGAAACCGCCTACCAACAATGGTTGGCAGCACA
GAAGGCTGACTAGAGGGAAAGAGACATCCAGAACAATGATGATTGGGTACCCACCAAACCTGACACCAGTGCC
CCACCATACTCCAGGGCCTGGTACAAACCCTTCAAAGTCAACTGGACTCTCCAAGCAATCTGTGAAGCATATA
CTCCAGAGGAGGCCAGGGTGACGCTGAGTAGCTGGATACATGAGGACCGACACCATGAAATTGTCATGAGCCT
AGTCAACAAGCACAGATGAGGGGGAGAAGAGGCTAGGGAAATCATGTTTGAACTACGTGAACCCATCTGGTAC
ATCCAAAGGATGAAGAGCAGTGAAAAGGAACTCACCATTGACATCATCCTCACCCCCTGCACAAACCAGTGCA
CCCTCTCCACCACCAGACTACTCGACAGTGGGTGCACTGGGAGCACCATAAACCACTCCTATGTCCAGAAGCA
CAATCTTGACACCAGGAAAACCATTGCCCTGATCCCCATATATAATGCCAATGGAACCTGCAACCAAGCAGGG
GACATTACCGAATTTGTGGAACTCTGCCTCTCCATTGGCGGACACCACAAGTGAATTCACTTGGCAGTCACCA
ATCTCAGCTCAAAGGACCTCTACCTCAGCCACGACTGGCTTAGATGCCACAACCCAGTCATAAATTGGAAGAC
GGGCTCAATCATTTTTGGTCGTTGCCAATGCACCAAAAATGCCTTCCCACTCCTGGATGCTGACCCTGAAGAC

CAGTGCGATGAAGAACTCAAAGAAGGAGAGACCATCCTCGCTGTAAACATGCAGGAGGAGATTGTCATCTGGG
CGGTACACCATGCCAACAACTTAGCGGCTCAGGCCAACATGGACAAGCCGAAGAAAACCTTTGAAGAAATGGT
CCCCGAGCACTATTGCTTGTACCAAGATCTGTTCTCAAAAGAGAACTTTGACAAACTCCCCAAATGAAAACCC
TGGGACTATGCTATCAAGCTCATCCCAAACGCGAAGGCAATGCTGGACTGCAAAGTCTACCC
>Armme1_1_Cluster35#LTR/Gypsy
CTGCGACAACCATGACCAAGGAAAGGGAGACAATGATAACATCATCGTACTCCAGCCCCATCATTTCTGAGCC
CTCATTGTGCCAACCACCGCCAAAGTCCATTCCAAAATCAAGAACTCTCTCCACCAAAAGGAACTTTGGGACC
AAGGGATTGCCAACTCCCTTGCCCACAAACGTGGAATTACCAAACAAAGCAGGCTCCTATTCTATGACAGACA
AGTCTATATCTCAAGGGACCATATGCTCTGAGGAGAAATCATCTCTCAGAGCCATGATCATATCACCACCAGA
TACCCCAGAATAGAGAAAACCAAGGAGCTCATTCAATGAGAATACTGATGGCTGAAGCTCAAGAAGGATGTAG
AAACCTACATTAAAGGATGTGAAACATGTCAATGAGCCAAATCCAGTAACTAAGCAAAGGCCACTCCTCTCTA
TTCAAACACCATTCTATCCAAACCATGGACTCACATCTCAGTAGACATGGTCACTGGACTACCTGAATCCGTT
AAAGGATGTGAAACATGTCAATGAGCCAAATCCAGTAATTAAGCAAAGGCCACTCCCCTCCATTCAAACACCA
TTCTATTCAAACCATGGACTCACATCTCAGTAGACATGGTCACTGGACTACCTGAATCCAATGGCTATAATGC
CCTCCTCATAATTGTCGACCACTTCTCAAAAGCCGTCATCCCCATCACCTGTAACATCGAACTCTTTGCTGAA
GGATGGGCCAAGATACTATGGAATCATGTCTATACAAAACATGGCATGCCCAAGGTGGTCATCTCCAACCGAG
GACCTCAATTCATCTCTCAATTCATGAATGACTTGTATTGACTCCTAGACATCACACCAAACACCTCCACAGC
ATTCCACCTGCAAACTGATGGACAAACTGAAAGGGTCAACCAAGAACTCAAAGAATATCTTTGGATATTCATC
GATTACTGACAAACCGACTGGAGCAACTGGCTTACCATGGCTGAATTCACTCATAACAATCACATCCACTCAG
TCACTGGCAAAAGCCCTTTCATGACCCTTTATGGCCAAAACCCTTGACTCATCCCTGACTCTGTCAGACCCTT
CCTGTTCACAAATCCCATGGCAAAAACCTTCACCGAAACCATGACAGAAGTCCATAAACAGATGCAGAAAGCC
TTACAGCGATCTGCCAACACCATGAAGATGCAGTACAACAAAAGAAAATGCCTGGCTGTTGACTACAAGGTTG
GTGACTGAGTCTGGCTCAATGCAGGAAACCTACATCTTCCCTGCCCAAAGAAGAAGCCTGACAACAAGCATGT
CAGACCCTTCATGATCCTCGAAAAGGCTGGAGCATCAGCATACAAGCTAAAGCTTCTACCCCATTGGAAGGTA
CACCCATGCTTCAACAAGAAGTTACTCACCCTGTTCGTACCATCCTCATTCCCAAACCAACAACAACTGCCAC
CACCTCCACCTGATCTCATTGATGATGAGGAACAGTACAAAATTGAGGCAGTGCTTGACTCATGAATGTGCAA
GATCAGGGGAAAGAAGGGGCAGAAGTCCAAAGAAGCCATGGACTACTTCATCAAATGGAAGGGTTGGACCTGA
GAGCATAACTCATGGGTACAAGATTCAGAAATGGAAAATGCCCAGGAAGTGATCCAGGAATATGAAGACAAGG
TCCAGCAAGCCGGATGCATTGACATTGCCAAAATTGTAACCAATGACTCTGACAAAACCATCACCATGATTCT
TGACCATGAATATCGTGACAGCATAGTCTATTATAAAGCCCAACGACAAGACGGATTCCAGAAATGGGTCAGA
AACCCTGACAAACACCGATGGAAAAGCCTCATACAGGAGTATTGGGAGAATGATGTACTAAAACAACTTGACC
ACCCCAAAGAGGGGCCTTGACAGCCAGGATGACTGTCTTAACGAGAGGGTAATGTTATGATTTTTCTGCTTAC
CTTATCTTAACAAGGCGCTTCTTGCTATTCCATCTTATCTCAAGTGCCTATCCACAGTTACCATGACCTAAAT
GGATGACCTCCATGGTTGTGCTATGGCTTGCTACTATTGCTCACTGACAACCATGACCAAGGAAAGGGAGACA
ATGATGACATCATCATACTCCAGCCCCATCATTTCTGAGCCCTCATCATGCCAACCACCGCCAAAGTCCATTC
CAAAATCAAGGACTCTCTCCACCAAAAGGAACTTTGGGACCAAGGGATTGCCAAC
>Armme1_1_Cluster49#LTR/Gypsy
CGAAGGGGGTAAGTAGCTTCTCGTTGAAACGGGGGTGAATTTTCCAGTGGGGTGGTAATTTTAGTCTGTAGGC
GGCGGCACCTGTCTTCTCGATTATGGTGAATGGACCGACTCGTTTATCGTCAAGTTTTTTCTTTGGTTGGGGG
AGATGTAGGTTCCCGGCGTCGAGCCAGACCTTATCCCCTTTCTGATAGTCAACTGCCGGTCGCTTCCGCTTGT
CGTACTGTGCTTTCATCGTTTGCGCCGCTTTTTCGAGTGCCTTCTGGGTCTGCTTGTGGACGGGGGAGATGTA
GGTTCCCGGTGTCGAGCCAGACCTTATCCCCTATCTGATAGTCAACTGCCGGTCGCTTCCGCTTGTCGTACTG
CGCTTTCATCGTTTGCGCCGCTTTTTCGAGTGCCTTCTGGGTCTGCTTGTGGACTTCTGCCATTACTTCGGTG
AAGGTTTCGGCCGCAGGGTTAAGGAAGGGAGATGGCCTGTTGGAGTCTGGTACGAGTCGAGGGTTTCTTCCAT
ATAGGACCATGAATGGGCTTTTCCCGGTGGCGGAGTGTACTCTGTTATTGTGAGCGAACTCTGCCATTGGGAG
CCAGTTACTCCAGTCGGTCTGTCGGTATTCGATGAAGAGTCGAAGGTATCCTTCGAGCTCTTGATTTACTCGC
TCTGTCTGTCCGTCTGTCTGCGGGTGGAAGGCGGTTGAGGCATTGGGAGCTCTGCCATTACTTCGGTGAAGGT
TTTGGCCGCGGGGTTAAGGAAGGGAGATGGTCTGTTGGAGTCTGGTACGAGTCGAGGGTTTCTTCCATATAGG
ACCATGAATGGGCTTTTCCCGGTGGCGGAGTGTACTCTGTTATTGTGAGCGAACTCTGCCATTGGGAGCCAGC
TGCTCCAGTTGGTCTGTCGGTATTCGACGAAGAGTCGAAGGTATCCTTCGAGCTCTTGATTTACTCGTTCTGT
CTGTCCGTCTGTCTGCGGGTGGAAGGCGGTTGAGGCATTGGGAGATAGTACAAAGGGAGTAAGTAGCTTCTCG
TTAAAACGGGGGTGAATTCTCCAGTGGGGTGGTAATTTTAGTCTGTAGGCGGCGGCACCTGTTTTCTCGACTA
TGGTGAACGGACCGACTCGTTTATCGTCGAGTTTTTTCTTTGGTCGGGGAAGATGTAAGTTCCCGGAGTCGAG
CCAAACCTTGTCCCCTATCTGATAGTCAACTGCCGGTCGTTTTCGCTTATCGTACTGCGCTTTCATCATTTGC
GCCGCTTTTTCGAGTGCCTTTTGTGTCTGTTTGTGGACTTCTGCCAT
>Armme1_1_Cluster33#LTR/Gypsy
AGGCTCTGAGGAAGGCTGTTTCCGCATAGTGGATTATTATGACACGCCGGAAGGATGGCCTTACCCCAGGAAA
GAGCCACTGACGTATGCTGATGAATATGTCATCTTGGAAGATCGACACCGAGAACGTTTCGACATCATCTCTC

GATCGTTACTTCGAAAATTCCACTTTGATCTGGACAGATGGTATCGTATTCGGTTGGCCCATGAATTTCGTGA
TGTGAATCGTTGGGATATGTGGACTGATGACGATGACCCAGATGACTCTGATACCGTATGGGGATATCTGTTG
GGAGTTATGGACAAGATTGATACGGATTTGGTTTACTCGTGCTTACGCGCTGAGAGAGCTAGGATTCGTTTGA
ATACTCAACAACAGTTTTTTGACTGCCTCGAGCTCAATGCCACTCAGGCTCAACGAGGCGACTTTTCAGCAGT
GCAGCGTAACTCGGCTAATATCCGTGATGCAAGGAGAAAGATTCCTCGTACTCTTGTAATCGTCGTGAAGATC
AATGGGCACCTGGTTTTTTGACTGCCTCGAGCTCAACGCCACTCAGGCTCGACGAGGCGACTTTTCAGCAGTG
CAGCGTAACTCGGCTAATATCCGTGATGCGAGGAGAAAGATTCCTCGTACTCTTGTAATCGTCGTGAAGATCA
ATGGGCACCCTTGCCATGCCCTGATTGATACTGGATCGATGGGTGATTTCATTTCTACAACGATAGTTGATCA
GTTGGGATTAAAGAGGATCGTTTTGGCCGTACCCTTGCCCTTGCAAATGGCAGTTCAGGGCTCTAGGTCCAAG
ATTAATTGTGGAGTTCGTTGCAATTTTGAATACCAGACGTTGAAAGGTGAACGGTACTTTGACGTGGCAAATT
TATCAAGTTACGATGTAATCCTGGGTATGCCTTGGCTGTATCAACACGAGGTCACTGTTGGATTCAACAGTTC
CAAGGTGTGTTGCAATTTTGAATACCAGACGTTGAAAGGTGAACGGTACTTTGACGTGGCGAATTTATCAAGT
TACGATGTAATCCTGGGTACGCCTTGGCTGTATCAACACGAGGTCACTGTTGGATTCAACAGTTCCAAGGTGA
TCATTGGTTCCATAGTGCCCCGTCTGATGCAGGGTCCTGCAGTTGGAATACTGGAATCCCAGGCTACAGTGAT
ATACGAGGAGTCGTTAGCGAGGTTCCGTGAAGAATTGATTGCATATGCACGGCCTTTGTTCAAGAAGGCCGCC
GAAACTCCATTACCGCCTCTGCGAGCGATAAACCATCAGATCCCTCTCATAGAACCTGAGAAGATTGGGCTAC
AGTGATATACGAGGAGTTGTTAGCGAGGTTCCATGAAGAATTGATTGCATACGCGCGGCCTTTGTTCAAGAAG
GCCGCCAAAACTCCATTACTGCCTCTGCGAGCGATAAACCATCAGATCCCTCTCATAGAACCTGAGAAGATTT
ACCCTTGGCGACCCTCTCGTTGCCCGGAAATGTTTCGCGAGCAATGGGACGCTAAGCGCAAGGCTTATATCGA
AATGGGTCATTGGAAGGTCACCACCGTGACCAATACCATCCCAATGTTATTGATTAAGAAACCTGCCTCGGTG
CTGTTGAGAACTGTCGTCGATTACGTTGCTTGCGCGAAGTATTTCTCATTGATGGACTCAAGCGATGCTTACG
AGCAGGTTAGAGTCGAGCCCGCGCACGTGGAGAGAACTGCTGTCTCGACTCCACGAGCAATGGGACGCTAAGC
GCAAGGCTTATATCGAAACGGGTCATTGGAAGGTCACCACCGCGACCAATACTGTCCCGATGTTATTGATTAA
GAAACCTGCCTCGGTGCTGTTGAGAACTGTCGTCGATTTGCGTGCACGAAATGCAAACACAAAGAAGCTGGCG
TCGCCTCTGCCCGACATTGACGGTATCCTGAGGCGCGTTGCTTGCGCGAAGTATTTCTCATTGATGGACTCAA
GCGATGCTTACAAGCAGGTTAGAGTCGAGCCCGCGCACGTGGAGAGAACTGCTGTCTCGACTCCAGATGGAAA
TATGCTGAGCTTGGTGATGCAGCAGGGGGATTGTAACGCCCCTGCTACCTTTCAGGCCATAATGAACCATATT
TTCTTGTCATATATTGGGAGATTTATGGACGTTTACTTGGATGATATTATAGTGTATTCCGATTCCCTGGAAG
AGCACATGAAGCACGTCAAATTAATTATTGATATCTTGACGAAGGAAAAGTTTTACCTTGGCGAGAACAAGGT
CCACTTTCTTGCTAAGGAGCTGAAGGTGCTCGGTCGCATCGTCGACCACCATGGTATTCGCATGGATCCGGAC
AAGGTTGACGCGATCCTAAAGTGGCCCACCCCTACAAACAAGGACCTACTGTTAAGCTTCCTTGGTTCGTTGG
GTTGGTTGGCAGATAATATAGCGAAGATTCGTATCCCTATGGGCCAGCTGTCAACATTGACTGGTTCAACGAT
GCCGTTTCGCTGGACGTACACGGAACAGCGAGTGTTTGAAGAAACAAAGACCTTGGCGAGCGCGTGTCGTTCA
AATCATAGAGTCCCCATTGATTATTCGAAGAATGCGAAGCCAGTATGGCTGGGGATGATATAGCGAAGATTCA
TATCCCTATGGGCCAGCTGTCAACATTGACTGGTTCAACGACGCCGTTTCGCTGGACGTACATGGAACAGCAA
GCGTTTGAAGAAACGAAGACCTTGGCGAGCGCGTGTCGTTTGAATCATAGAGTCCCCATTGATTATTCGAAGA
ATGCGAAGCCAGTATGGCTGGTATCCGATGGATGTGGCGCTGGGATTGCCGCGTACATTATCCAAGGTGAAAC
CTGGGGAAAAGGCGTCGTTTGTGCATTTTATTCGGCGAAGTTAAACCCCGCGCAGCAGAACTACCCTGTACAT
GAGATAGAGATGCTAGCGGGGGTGGAGTGTATGTTGAGACACAGAGATATTTTGCAAGGTCTGCACTTCACCT
GGATAACAGACCACAAAGGTCTCATACATCTTTACAACCAAAAATCCCTGTCCGGGCGCCAGGCTTGTTGGAT
GGAGAAGCTTGGGGAGTTTGACTTCTCGGTTGAGTATGTCCCTGGGGCACAGAATGTTCTCGCAGATGCGTTG
TCTCGACTCTGGTCAAACGAGTCTCCTGGCACCATTCGTGCTCGTGGTGTGTATACATATCACGACGTTATCG
ACAATGATTCTATAGAGACGCATGGACGCGTTGTCTCGACTCTGGTCAAACGAGTCTCCTGGCACCGTTCATG
CTCATGGTGTGTATACATATCACGACGTTATCGACAATGATTCTATAGAGACGCATGGCGTGTCCATGCCTGT
ATTAGTGGGAATAGAGGCTGCTTGCCTCGTGCCAGAGGGCGTCCGATTGAATGCAATGTCACTTCGTACTAGT
CGTCGGCCGTCTGCGTGAGCGAGGGGTCTTGATAATGAGATCGTTCCTGGAAAACTGCAAAGTCGTGGTGATT
CCGCGTCTAGTCATAGATGACGGAAGGAGGGCGATGGTGCAAATGAAAATGTACCTCAGCCCAAAAGTCCCAA
ATCCAAATCCAAATCCTATCCCAAGTCCAAGCTCAAACAAATGGCAGTACCAGAGTCCGAGATCTTTGAGCCT
GTAAATGAGCAAGGCGACGAGAACGTGCAAGAAGAGACGAAGCTAGCAGAAGCTGTTTCGTCCACGTCGTCGA
TCGAGCATCAGGAACCCATGGCACAGCCACCTGAAGAACATGAATATTCACCGAGTAATACTTCGTTGAACAC
TGGCAATGTGGTATGCGCAGGAGAGAAGCATGATAGCTGGGATTCCATCGTCGGACTGGCCCGCGCAGAGTCA
CTGGATTTCCAGTGGGAAGATGAGAGCACCGTTCCAGTTTGAGTTGGAGAGGCTCTGAGGAAGGCTGTTTCCG
CATAGTGGATTATTATGACATGCCGGAAGGATGGCCTTACCCCAGGAAAGAGCCACCGACATATGCTGATGAA
TATGTCATCTTGGAAGATCGACACCAAGAATGTTTCGACATCATCTCTCGATCGTTACTTCGAAAATTCCACT
TTGATC
>Armme1_1_Cluster63#LTR/Gypsy
TAAGTGTTAGACCTGAACTCTGAGTAAACCAAACAAGAGTAAACCAAGAGCCATCTCGCGTTTGGACGATCAC
TCAGGGAGTCTCTGACTGAATAGTTGACTAGAGCATAGGACTAATGTCTTAGTCGTAGCAAGAGTCAACTAGC

TAAGTTCGGTATGATTAAACTTTAGCTCAAAGCTGGGGAATATACAGTAAGAGTGGTCCTACTTATATATGAA
AGCAAGGGTCTAATAATAGGTATGGGTAGCAGTGTCTGTGGGTGAAACAATGAATACAACAAGGCAGTGGTGC
TCAGATAGTGAGCCATAGTACAAAGGTGAGAGTTGTCCAGGTAACGGTTGGTAACCATGTGCTGATGAGATAG
CAATAGGCATGCTTGATAAGATAAGATTGCCACTGATAGATTAGATATGTGAGAAAATCATAACATTACCCCC
TCATTAAGGCAGTTGTCCTGACTGCCCTAATATTTGTCTCCATTTGGTGGCTCTTCCAGTTCTAGGTTGTGTT
CGTTTGCCCACCAATAATACTCGATGAGGTCTGTCTCATTTGTCAGACTGTCCTCTGCTTTCCATTCTCCTTC
CAACCATTGAAGTTCCATGCCCCCTTTTTCTGCCTGGGCACTAGCAACCTTGATAAGGTAGTACCAAATGTTG
TCCCTGGGTCTGACCGGTTTGTCTTCTTCCCTTTGGTAGTAGGCATCAGTGATTGCCATGGTGTTTTCGTTGT
TTACGACCATGGCGTCGATCGACATTCCTCCTTTCTTGGTCCAGTAATTGGCAATCGCTTCTTTTGTGTTGCC
TAAGTACTTTTCAGCCGTCCACTTGTTGTCTTCTGGTCTGTAACCCTTCCACTTGACAAGGTAGTTGGTGATG
GTTTGCGACTTTTGTCCATTTCGGCTGCGCACTTTGTGAGGTTTGTTGTTGAGGATTTCTTCGACTTCGTACT
CTTCTTTGCCCATGATGATGTTGGGTGAAGGTGGTGGCAGTTGTTGTTGGTTGGGAAAGGCCAGTGGTGTGTA
TGGTGTCAGGAGGGCCTTGTTGAAGACAGGGTAGATTCTCCAGGCAGGGGGGAGCTTCAGCTTATAGGCTGAG
GCTTCATGCTTTTCTTGAATCTGGAAGGGGCCAACGCGTTTGTCGTCGAGTTTCTTCTTTGGATGGGGAAGGT
GAAGGTTGGTCGTGTCGAGCCAGACTTTGTCTCCATTCTGGTACTTGACTGCTTCTTTCTTCTTTTTGTCGTA
TTGGGTTTTCATTTGTTCAGCGGTTTTGGCAAGAGCGGCTTTGGTTTGGGCATGGATAGCCTTCATGTTGTCA
AAGAAGGCTTCCAACATGGGGGTGCAGAGAGGAGCATGGGTATGGGAATCAGGGGCGACTCAAGGGTGTTGTC
CATATAGGACCTGGAAGGGGCTTTTGCCAGTGGCAGAGTGGATGCGGTTGTTGTGTTGGAAGGTGCAGATCGG
TCGCCAATCTGCCCAGTCATTCTGGTGGTGATTAAGGACTATTTGAAGGTATTTCTCAATTTCTTGGTTAACT
CATTCGATTTGTCCATCAGTTTGTGAATGAAAGGCGGTGGAGGCATTGGCCTTGATGTTGAGCATTTTATAGA
GGTCATCCAGGAATTTTGAGATGAATTGAGGTCCCTGGTCTGAGATGATGGTGACGGGCATTCTGTGTTTGGC
ATATACTTTGTCCCAGAGGATTTTGGCCCAGCCTTCTGATGAGAGTTCAACATTGCAGGCAACAGGAATGATA
TTTTTTGAGAATCTGTTGACGATTACCAGGATGGCGTTGTACCCATTTGATTCCGGTAGTCCAGTGATCATGT
CTACTGAGATGTGTGTTCAGGGTTCGGAGGGGATTTGGTTGGGATGAAGGGGGGTGTGTTTGGCCTGGGTGCT
TGACTTGGTTCAGGCACAGGTTTCACATGCTTGGATGTAGGTTTGGATGTCCTTGTGGATCTTGGGCCACCAG
TAGTCTTGGAGGACTAGTTCCTTGGTCTTTTCTACTCCAGGGTGTCCCACAGTGATGTGATTGTGGCAACAGG
AGATGATTTCTCCTCGTATCATGTGATTGCAGGGTACATAGACCCTCTGGTTGTAATAAAGGATCCCATTGGT
TTGCTTAATACCATGTTCATGGTTAAAGGAGTTTGCAATTCCTTGGTCCCACTGGGCTGGGTTGCGGGTGGCA
CTCTTGATGTGCTCTAGGGATTCATTGATGGTGGGCATGATGAGTGCCCAGAAATGTTTGGCTTTAAGGATGA
CGACTTCTTAGTTGTCGTCTTGTCCTTGGTCATGATCTGCACAGTGGGAAAGTAGGTCTGCCTTTTTGTTCAA
GGCACCAGGTTTGTGGTGAAGCATGAAGTGGTATTCTGCAAGCTCAGTGACCCACCATGCTTGTCGATGGTTG
AGTTTTTGAGGTTTGCAGAAGTACTGGATGTTCTGGTGGTCCGTCCAGATTTCAACATCTAGGGTGGCGCCCA
TGAGGTAATGTCTCCATTCCTCAAGGGCAAGCATGATGGCGAGGAGTTCCTTGTCATAGATCTCATAGTTGCA
TTCTGTGGCCATTAAGGCTTTGGACAAGAATGCTATTGGTCTCCATTTTCCTTCCTGCTCTTGAGATAGTATG
GCACTGATCGTGCCTTCACTAGCATCTGCTTCTACTCTGAATCTTCCTGTTTCGGTGGGGATGGCCAGTATGA
CATCCTTGGCGAGGCGGCTCTTGAGTTCGTCAAAGGCCTCCTGCTGAGTGGTCCCCCAGATCCAGGATTTGTT
ACTGGTGAGGCGAGTCATCAGGCGTACCACTGCACTGTAGCCCTTGATGAATCGCCAATAGAAGTTGGTAAAC
CCGAGGAAGGATTGAAGTTCCTTTTTTTTGGTGGGGATGGGCCAGTCGATGATGCCAGCGATTTTGACCTGGT
CCATGCGGACAGATCCTTCGCTTATTATTACCCCGAGGTACTCTGTTTCCAGTACCTCGAACTCACATTTCTC
AGCTTTCAGGAACAGTTTGTGTTTCCTGAGGAGTGTGAGAACTTGGCGAGTGATCTGTCAATGCTCTTCTAGA
GTCTTCGAAAAGATCAGGATGTCATCAAGGTATATGGTGACCTTGCCAGAGGCGATCAAGTCTTTGAAGATGT
CGTTCATCATCTACTGGAACGTGGCAGGAGAGTTAGTGAGACCAAAGAACATCACTGTTGGTTTGAACAGTCC
ATGATTGATTCTGAACATGGCCTTCCACTCGTCATCCTCTTTAATGCGTACATTGTTGTATCCCCATCGGATG
TCTAGTTTGGTGAAGTACTTGGCACTTCCTAGTTTGTCCATGAGCTCGGCAATGAGGGGTAGAGGATATCGAT
TCTTGATGGTTATCTCATTGAGCTTTCGATAATCTTGTACAGGGTGTAGTTTTCTGTCCTTCTTTTTGACAAA
GAAGAAGGGGGAAGCCATAGGGGATTTGGAGGGTCAGATGCGGCCAGTAGTGAGGTTCTCATCAAGGAATTTG
TCAAGTTCCTCTTGCTTGGTGTGGTTCAGGGGATAGACTTTACAGTCAAGGGTATTTTTGGCGTTAGGGACGA
GTTTGATGGTGTGGTCCCAGGGTTTACATGTGGGGAGCTCATCGAAGTTTTCCTTTTCAAAGAGGTCGGCGAA
GTCTCAGTATGCTTCAGGGACCATCTCTTCAAAGGTTTTCTTCTCTCTGCCCTCGTTTGCTTTCATGGCTAGC
TCGTTGGCTTTGTGAACAGTGTGGATTTCAATTGTGTCTTCAAAATTAATGGCTAGAATACAGTCTCCCTCCT
CGAGCTCTTCGTCCCATTTGTCATTGGGGTCAGAGTCGGGAAGTGGGAAGGGATTTTTTATGCAGTGGCAGTG
GGCAAAGGTGATCTTGCCAGTGCTCCAGTCTATGAGCAGGTTGTGGCAAGCTAACCAGTCATGGCTGAGAAAG
ATTTCTTTAGAGCCGAGGTTGACTACAGCAAGGTCGATCTTCTCGCTGTGGTTGCCTATGGTGAGTCTGACCT
CTACATACTTGGTGATTTCCCCGGCTTTATTACACGAGCCATCTGCATTGTAGATAGGGATGGGGCTGGCAGT
TTTGTGAACAGGGAGTTGATGTTCTTGAATGAAGGTGCTGTGGATGGAGCTACCAGTGCATCTGCTGTTGAGC
AAAGCGGTAGCACTGAGGTGGAGTCGGGAATCAAGGGTCTCAAGTTGGAGTGGGATTTTAAGGCCCTGCTTGG
AGGTAGTTACGGATCATACGAGGTACCATTTGGGCTTTCGCAATTCATCTAGCCATTCCAGCACATTGGTCCT
GTTTGCCTGGCGGAGCCCTTCCAAGAGATTGTCGACTACTTCTTTGGCCCTGGTCTTGTGTATCCAATTCAGG

AGCACAGACCTGGTAGCAGATTCATCTTTGGCCTCTTGGATGGCTCTCCAGGTCCACTCAGTCTCAAACAGTT
TTAACCATTCAGTGTGAGGGGCCTTGGGGGTTGTTAAAGGCTGTTGGGCTGTTGCTTGCTTTGCCTGATGGAG
TCGGAAGGTGGTGTCGTCCTCTTCATCTGGTACTTCAACCATGGTGACTTGATGGGGGCTCTCTGCCCTGGGT
TTTGGCCCTAGCAGGCGCTTGAGTTCCTCTGCACTGATCCTTGCGGGGGGCGTCTTGGCCTTCATAGAAGGTG
CTGGATCTTTTGGCTTCAGAGGCCCAAGGGCATCCTGCTTACTTGATCCTGACCCAAGTCCTATTACTCGTTT
TGAGAGCTCCAGTGCTACCCAGCAGGTCCCCTCCCTCACAGCAGGCTTGGATGTCATTTTTTCTTGTGGTTCT
TGGTTATTGGTCTTTTGAACAGGTAAATCAGGTGTATTTTTTTGTATTTTATTCATAACTACAGAAATAGTAT
GAGTCTGACCAGCGATATTGTTCTTCGTACTGGATAAGTCCTTCACCGAGCAAGTGGAACCACTTACTTCTTA
GTGCCATCTTTTACCTCCTTGACCTTTGATTCTGTGGCAGGCTCAGTCATTGTGGCCCTGACTTCCTCCTTCT
TCTTGTTCCAACTTTGTAGCAGGCAATTCTTGCTGATGTGTCCCTTCTGCTTGCACTGGAAACACTTCCCTTC
TGACCTGAGCATGTCGATGTCCATTGGCTGCCCTCGACCTCCGTATGTGGTACCAATGCCCGTTTTTTTGTCT
GTGGCGCCGGCAGTAGTCTTGCTGCTCCCGGCGCTTTGATTGGTGCTTCTGGAGTTCAGAGTCTGGTAGTGGG
GGTGGGCAAGTCCATCTTTGATGGCCTTGCACTGGCGCTCATTGTTCATGACCGTGATGCGGGCTTTCCAGTT
GTCATAGGTGATAGGCATGTTGATTCCAATATTGGAGATCATGTTGGTGTAGGAGTTGGGGACGCCTTTCTTG
ACGGCGGCCACCATGTATCCATGGGGCCCAGTATTGGTGCAGAGGTTGGCTTTTTTGGCAAACCATTCTAGGG
CTCGGAAGTAGTCTTCCGTGTTGCCTTTTCTGAGTCGGAGGTTGTACATCTTCTTTTGTTGTACTTCTTTGAC
AGTGTGGTCTCGGAACTCATCATGGATCATCTGAGAGAAGGCATCCCAGCTGGGGAGGTGGAACTTTCATTAG
GTGTTACTCTGGCATTCCATTAACTCCATGGTCCACCACTCTTTGGCTTTGCCAGTGAAGTGGGAGGTAGCGA
AGGCAACTTGATAGGGGTCAGTGTACATGTAGGGGTCATGTACTTGGAAGTACATTTCACATTGGGTGAGGAA
TCGATCGATGTCCTCAGGGTCGCCTTTAAAGGTGGCGGGCTTTTGCATTAGGTCGGGTTTGATGCTGAGCCAG
GGTGCATCTTCAGCCATCTGACCAAAGGGATTGGGTGGAGGGTAGTCAGGGTCATGAGATTTTTCTGGAGCCC
GAGGCAGCGACCAGCTGTCTGGGTCATGGGCGAGGGTACTGCTATATTGGTCCAGAGGTGGTGAGCCCTTGAT
GGCACCGTCATGTTGGGTCCAACTTGGCATGATGGGGGGTTGGTTGTCAGGGTTAGAAGGCCTGGCCCAGCTT
TGTCTGGGCCGGTGCCGGCAAACATTCGGCTTTTGCCTTTCCTGTGGCTGCTCAGCATGTAGACTTGTGGGGG
AGGGGGGGTTCTAGGCAGAGGCATTTGGGGGAATAGGGGTTTGTAGTCGGGTGGGTCGTCATATGGTCAATGT
GGGTATTTAGCATTAAAATCATATATGGCCTGTTCTCCTGCACAGTACTCAATAATATTCTCCCGCTCTGGAT
CCTCCTCCTCTAGGCTCTTCCAAGATCTCCCCATCTTAGAGGAGAGGAAGGTCGGGTCCAATGTAAGGACCGA
TAAGTGACCTATGTAAATTTTCGTGAATTCTTGTGCCCACAGATCAGGGATCCCCTGGACCAGCATTGACAGT
TCACTGATACTTGGTGCAATGCTGTTTGAGGGTGATGTCGCAGGGCCCCTTCGGATGTGTCGCTGGCAGTTTG
CTTCAAACTGCTCAGCAAAGTTTTGTCCATAGATAAGATGCTCATTGGTGGTGAGTGTGAAGCGTATCCGAAT
ACTGAATGCTTCAAGTTCAATTGATGACCTAGCGATTACGGTGTACTGGTAATTGATAATGGTTGTGGTGTAA
AGGTCTATGAGCAATGATACTGAGAACCTTTAGCAAGGTAAGATAGCAGAGGTAGCGAGTAGTACTAGATTCT
AACTCCTCAGTTCTAGGTCGTGTTTTCTCTGGCCTGACACAGGTCCAATATGAGAATTCACATTTAAGAGTAA
GAGGGTAAGAAAGAAGTATATTTCTTTACTATCTACCTGCTTATATACTACTTAGGTAGGAGTCATAGAAGGT
CATTACGAGTATTTACCAGTCAGTACTGAGTCAATCCTTAATAATATCTCCTAACTCATAGCAACTCACTCTA
AGTTGCCCGGTTAAAATTAGATGGGTAGTGAGGGGCAGCGGCGCTTAGTTGGCATTGAGCTGGTTATTGATCT
GCTGGGGTCTTTGCCAACTCTCATTGTACTGGCAGAACTTGTCTTCCTTGTACCAGTGCTCCTCATCTGCGCT
GATATTATGGAACTGGTTCCAGTGTTGCTGGTGGGCATAGTCCTCATCGTCCTCTCCCAGTAGGTGTGAGCTA
GTTGCTCGGTGGGCATCTCGGGCTGCATGCTGCTGGAACTGCCAGTCATTGTACTACTGCTGATTGTGGATTC
CGAGGTGATGGAGGCGCTGCTTGAGGTAGGTTTGTTCCTTGGAAGTAACAGGGCAGAGGTGAGCTGGGCGATT
GACGGGGTGGGGTTCAATGTTGATGGTGATGCCTTGGAGGTGAAGGAGTTCGAGTTCAGCGGCTCTCTCTCTT
TGCCTCCTGGCCTCTTCGAGATGTCCAAGGGTCGGGGACTGGGGGCGAGTGATAGGAGTGAGCTGGTAGGCCT
CGAACTCACTCTGGGTGGGAGAGTGGGGCTGGTAGGGGGTGTTGTCCTCATTGTCAGTGAGCCAAATGGTGGG
GAGGTCCACGCTGACTGGGTTTGGTACCTCATAGAGCTCAAGGGAGTCTCGGGCTCAAGGAACAGCTCAGGGA
GCGGATAGTTCTATAGTTGAGGAGTCAGCGGTAGTGGTGGCAGCGAGTACGGCAGGGACGGGGGTTCCGAAGG
TGGAGTCGTGAATGAGGATGCCATGTCCTTGCTCATCAATGTCACTTTCAACTTCCTTGACCTCAGTGAGCCT
AGGAGGTTCTGGAGTTTGGGGATGGGGTACGGGTTCAGGCGATGAGGGGAGGGGTGAGTCCTGTCTGTTGGGC
TGGGTGGTGCCATAGCGATGGAGGCTGGAGGGCGAGGGATGGAATGGGGTGTTTGGGATACGAGGGGTGTGGA
AGGTATGGTTAATCCAGTGCTGAAGCTGGTCCTGCTGGTGGTGGTACCAGAAGGTAATCGGCCACCTAGACTG
ATACAAGAGGTAGGAAAGGGTGGGAATCCTGATGAAGAGTATCAGGAAGAAGGCGACAATGGTGCTGGTTTGG
TGGAATGGGTTGGGCATTCGGTTTCGTCAAGCATTTTGGTGGTCGCTTGGAGAAAGGGATGCGAAGCTTCGAG
CTAATTGTAGACCTGAACTCTGAATAAACCAAACAAGAGTAAACCAAGAGCCGTCTCGCGTTCAGACAATCAC
TCAGGGAGTCTCTGGCTGAATGGTTGACTAGAGCGTAGGACTAATGTCTTAGTCATAGCAAGAGTCAACTAGC
TAAGTTCAGTATGATTAAACTTTAGCTCAAAGTTGGGGAATATACAGCAAGAGTGGTTCTACTTATATACGAA
AGCAAGGGTCTAATAATAGGTATGGGTAGCGGTGTCTGTGGGTGAAACAATGAATACAACAAGGCAGCGGTGC
TCAGGTAGTGAGCCATAGTACAAAGGTGAGAGTCGTCCAGGTAACGGTTGGGAACCACATGCCAATGAGATAG
CAATAGGTG
>Armme1_1_rnd-4_family-2537#LTR/Gypsy

TTATCTTATCTTATCTTTTACTACTTAGTTAGAGACTCTTGGACTTTATATGNAATTTGTCTTTTATTCTTTT
ACCGTCTTGTATACGCAAGTAGTCTACCATATACATTGGTCTACACTACCTCGCGTGACGCAAGATNAAGGAA
TCTTTTACACTTTTTTAGTGTACTCAGCTATATAAGCGACTACTGCAATACCAACGAAGTACACCGAACAGAC
AACGATGTCTGGCACAATGACCAGATCGAAGAAGCGGGCGACGGAATTGGCTCGATACCAACCGCGGTTGACC
CCCAAGAACCGCAACAAGGTTCANCCGGAGCAAATGTCGCCCCTGACGACGCCCGGGNTGAGGCGGAACNCAN
TGCGGAGCANCCGCACACTAGCNCANACCCCGATCTGGCTAATNCATCAGGCCTCGACCANGTCGAGGCCGCG
CATATGCCACGATGGTGTGACATGCAAAATGAGTTCNTTAACACCGGCCGAGTCNCCTNCTGCTACGCNTGCA
GGGGATGATGTAATGAACAACCCAAATGTAGAACTGCCTTCCGCNTTCAGCTCAAACAATGACCGTCGACGAA
GGGTTCAAGGTGACAAGCCCAGCGAGGGAGAGTGAGGATGGCGTGAACCGCCACCTACTGTGCCCAACTCNGG
GAGCGACACCATCCGCGACACTGAGGACGANCCNCCGAGCGATCGCCCCCTCGAGTTAGAGGCCGTCCCGGAG
GCGGACATCCGCTGACAGCGAACTCGNNATAGCCGAATTCGAATCGAATCGACCATCATCTCCAGAAGACGAC
ACAATCGACCAAGATCGAAGTCGATCGANCAGACACGTCACCTGATTACTCACAACACCCCGGAAACGATCTT
TACGAACCTAGCNTCGCTAGCGATGACGATCAGAGCGAGAATCGCATGACACGTGAATTNGATANCGACATCC
TGAACATGATCAGGTGGCCTACGACCTAGCCCGCGATAATAACGGNCCATGGAGACCACCGAGCGAAGACGAA
CGATCGGCGCAANTATTTCTTGATGGCCTNACTGACCATCAAAGGGCGGTAACACGCGTATTGGCCCACCGAC
AGGTGGGACAGNTATTCAAATGNTTNGGGTTCGANGACGTCGACCCAGAATCGGATATATACATGGAACAATA
TAACNTNACCATCACCACGATGGTCGGTGGAGGTATGATCATGGGGCACACCCCCAGAACCAGANAACGATAC
TGTGACGAAGTGACTCCAGAACCAACCGCCCNTAACCCGCTACACCGCGTAGCAAGNCGAATTTCGGCTAACG
CCAGAACCGGCAACGTGAGGCCCTTCACGTTTAACCCAAAGCGGGCTCGATCGCGCAGCCCNAACGCACAAGT
NAAGCAAGAGGAGGTAACNCCGGNGCTGCCCGGGCCGATACCGNCNCCTACGAATAATAANCGTAACCTAACA
NTGGTGACAAGCCGAGCGAAACCAAACATGNCAGAGCNGGCACCCTGAACCCCAAGGCCCCAACGTTNCGATA
CGGGAAGACGNTNAAGGACAGGGTNATCCTCCAANAATGGAGGATNCGAGACCTGGCTGACACTGGCCGAACG
GANATAGCCGACCAGGGCATNCNCATCGACGNGAACGGTGACGCGTNCATCATNAAGGGAGGAGTGGCGGACN
CAAAAAGCAAACAGTCCTCAAAGAATCGAAACACCGTACCTAAACACAAAATAGTCCCACCNAAGGNGGCGCC
CCGGGTGGGANCAGCAATGGCCCACCCGGAGGCCACCATGGACGGCGGAGGNCCACCCGGNCACCGGTGGAGG
ACGNCCGCCAACGGGGACCACCAAATGGAGGACGGTCCCCCAAATGGNGGATCCCTCGAGTGGTCCCAGGAAG
TCGAGTACCNCGCNCATCTCGAGGGATCGGNACGCCGGGACGGGGTGGCGCTCCCATCCCACCTAACAGGCCG
CCGCCTCCCCCATCGGGAGGAGGCCCATCCGATGGTGGATCAGNCGATGAGGACGATCCGATGANTCGAAAGA
TGAACACGATGTACNCCGCCTAGGGTATCACGATCCCGTCGTGTCACGGAGATACCCAGCTTNCCGACCGATC
TCATAACCGNGCGGAANGNAATGCNCGAGCACTAGGTGACCATCGCAAGCANATGCATAATCGCATTGAGCAA
TATGTNGACGAGCACCTNCGNGTNCGNGTACACCTACCTGAAGGAGTGAAAGCNCCGNGACTGGACTCAAAAA
ATGTCAGTCCATACGCGGGGAGCCCCTCGGTGGCCGAATTTTGGACTTGGNTCAAGAGTCTGGTGGTATACCT
TGAGGCGTCNCAACTCGGNGGGNTTGATAGAGAGAGAGAACAGAAGCTGCTNATTGAACCAGTACTGNCTGGA
GCTGCGAAGAAATGGTATCACGACCACGTNATCGAAGTTAATGAGTATTCGAACTGGACNTTCGTATCGGTAC
TAATNGGACTGTACGACCGNTTCATACACGACTCGGCNATGCAAGAGGCNCGGTCTAAGTTCGACCGCGCNAC
ATTCGCCGANGGCGGAGGCACCGCNGAAGGNTACCGTGACCTNCTNCAGACACTGATCCGNGATATGACGAGA
AAACCNGACGANTATACNGTGANTCGNCGGTTTGTNACNGGACTNCCGCACGACATGAGGGANGCCGTGTTCG
ANGACCGACTGAATGTNGAGGTCAATNCNCTGGATGAATTCGTAGAATCNGCCAAAGCCTACGAGGTNACCGA
ACGTTCAAAGAAGGAGTATAGCCGGACAAACAACCCTCAGTCATCTTCGAAGGAAAAAACCCGTCCNGTAGAT
AATAAAGAGCGGGCGGGCCCGACGACAAGACCACTCCGNGGCCGTATGTTTATNCGCCAACTGGCGGGNGATT
CTTCAGACCTAACGAGGGCGACCCATCGAGCCGAANCCCTCCACAAANACCGGCTGCACCTCGACCTANTAGC
CNTGCCCGAGTGGTTCCCAACAGCCAGAATGAACCCTCACGACCGTGAAATGGCCANCCGACGGACTTAAGTA
ACATTAAGTGCTACCGNTGTGGTGAGANGGGNCACTACGCATCCCATCATGACAAGGACGCCAACCCGCGGAT
TCGCGCGGCNCACACNACCATAGGCGACGATATTCGTGATGATGGAGAAGATGACGATTCTATCGTCGAAACT
GGAGATATCCGTTNCGGGGATGACTACGAAATTATGACCAGCCAGNGTGGCAGGAGGTGGAATTTGAGGAGTA
TCCTAATGATTACGAGTCAAAACCGACGAGGTCGAATTTATGGGNATGACGAGGATGGGGTGGAGCGGACTGA
GGACCAGGATTCTCAGAGCACCGAATCCGGCCCTCCTGACGACGGTAATGTGGNCNNCGCNACGCGGTATTTG
GCGAGTCGTGACTTTGGANNGGACCACGNCGGTGGTCCCAACTTCCAACATAGGATACATCTTCTGAAGGATA
AATCACCACGTACGCGGATANCCTGACAGCGATGACGGAGACGCCAGAAGAATGGCCATCTACTCGATATAAG
TTGAAAATCGCGACCCAACCGAGGGACAGACCGATGTACTCGGCGGGATAAGATGTGCCTAGCGACATATCTC
GACGTNAACGGGATAAGCGCCCTAACTCTATGGGATAGCGGNAGTACGTCAACCGCTATGTCCCCGCATTTCG
CCGATGTNTCAAAGACNCTGGTCTTTAACCTGANNGAGCCAGTGACCCTGCAATTGGGTACGGTAGGAAGTCG
CTCGAAGATCAACTTCGGGACGATGGCCGATATAANCGTAGCAGGGTTGANTCCCATGGAATATATCGATGTC
GTAAACATCGACCGATACGACGTNCTNATTGGNACCCCGTTTATGCATCGGCACCATAGTCGTNCTCGACTTC
GANAAGAAATGTATACGCGTAAATGGNGCAGGATATTCCGGCCGAGGTTGTACCCACCCGGGGTGCAAACGCC
ATGACGCGCGAAGACACCGNATACGTCGACCGATACCCCCTGNCGGGAGGTCGATGACTCCATGGCAGGCCGA
CGGTGGAGATCATGGGGCGNGAAGTTATGGGGAGGACCNTGAATCCGCTCCCCGACCCGAATCTCCGGCGATN
ATGATGACGGAGAATGAGTATCTGAGCGAAACCGAGTGATTCGAGCGGCCAAGCTGCGAATGAAANCTGGCAA

GCCNCGCANNNGGCGAGACGAGACCGACCANAATAACGANCNACTTCTTGAGGACCGGATGCCTAATACAGCC
ACTAAACCCACNGCTCCACTAANACACCGGTCGANNNCAGCTCGACTGCCGCNGAACCTCCAGGGTCATCANG
GGTGCANGTCAGTGAATGCGAACCTACATCATCACGCGTACTGGTTGAGGACCTNGTCACCGATGAGGACGAC
GATCGACACGACCATGAGTTCCATGANCCCGTAGGTCCAGCCATAGCGCACCCCACGCCGACAAGCCGGCCNG
AACACGACGTGTTTTTGGGACAGACCGGCCCCNTCCNCNGGGGTCAATTGGCCGGGGACCACCGCGCGCCGAN
NGAATGGAATATGGTCATGGGCGAAATGTGGGAAATCGATCCCAGCACAAGGGATCATTGTTCAACTACCCCG
AACTACCGGCGACCGATGATCCGACTGGGATATCAACGATTGATTATCGTTCGTGGTACCTCGCCACGCATTG
AGNCTGAAGGACCGTACGAATTCACCGCTACGATGTATCCCGACGCGCGTCGNCGTGAATTCCTCCGCTGAAA
NACGCCGNTNGTGATACCAGATTACTCTGGAGAACCAGATGCTCGCATCGCGAGNATGGCGGCTCGTAACGAA
TCGAGGAATAACCGTGAGTTTGATCCACATGCCTCGGCGCTCCCCNACAGTAAGAACGAGANAAGGGNAAGAC
AACGGGCAGCCGATTCGCCTNCAGACGATGATATCGAATGTCTACGACAGCACTGGTACGCTGATTACGAGGA
GTTACTACAGGGAGTACCGGANGGNATGCCACCNTGGAGGGTGGTCAATCATGAGATCCCAATTATTGACGCG
GATAAGAAGTACCACTATCACCTGCCACGNTGCCCGAACGCATTNCGAGGCGAATTCGATGAAAAAGTAGCTC
GNTATACGCGAGCTGGATGGTGGGAANTGACGTCTGCNAGCCAAGCGGCNCCNATGATGTGTATNTTTAAAAA
AGATATNCACCTACGAACCGTTGTAGACTGTAGGCAGAGGAACGAAAACACCGTGAAGGACGTNACTCCCATG
CCGGACCAGGACAACATCTGCGAAGACGTGGCCCGAGCAAAATACCGATCAAAGATCGACTTGTCGGACACGT
ATGAGCAGGTGTGCATCGTAGCTACGGATCAGACGTNTGGAAGACCGCGTTCTNCACCGTGCCGTGGTACCTT
CGTTAGCATGTAATGCAGCANGGGGACTGTACAATGCNCCCAGCTACTTCCAACGCCTTCAATGACATCCATT
TCCAGGATGTCATTGGACGTGCCCATGCACGTCTATCTCGACGATATCTTTATNTATTCAGACATACCGAGGA
CCATGAGAAACACGTTGCGCATAATGTTCGATCGACTGAGGGAACAGGCTATACCTCAAATGNTCGAAATGCG
AATTATACGCCGATCGCGTAGACTGCCTAGGNCATATCATCGATGACGAAGGCTCCATGCCGATGGAGACAAG
CTCACCTGTATTTGTGATGGCGCATCCCGAGAANCTACAATGATATCCAACGATTCGTTGGNCTNGTACAGTA
CCTCGCNGCNTTCCTGCCAGACATATCAGCNTACACGGGNCCNCTGNTGAGTATGACACANAATGGNAGTGCA
TTCNACTGGNGNCCGNTACACCAGCGGTGCTTCGATATGATCAAGGCNATATGTTGCAAGACACCNATNCTTC
GGCCCATCGATCCNAGGACGAACCGATATGGGTGATCTGTGACGCNTCGAAGTCTGGTGTNGGTGCGATGTAT
GGCCAAGGGCCAACNTGGCAAACNTGTCGNCCGGCCGGATTCATGTCGAAAAAGTTCACAACGGCTCAACACA
ATTATCGGGTACACGAGCTNGAGACGCTNGGCATNCTGGAAGCCCTGCTCAAGTGGGAGGACAAACTGATAGG
ATACAGGATCCATGTTATCATGGACCACAAAGCCCTAGAATTCTTTAAGACCCAGTCNNACCTNACGGGACGN
CAAATGAGATGGACGGATTACNTATCCACGTTTCGACTTTGACATCACGTACATTAAAGGAGAAAACAATAAA
GCGGCNGACTGCCTCNGCCGATACTATGAGAACGACACTCGGGATGAGGACGTCCATGTCGANGAACATGTAT
GCGCAGATGCACGGATTGACCCGAATGGGGANGACCTCCCNCCGGACNGACTNCNGGAAGTTCATGACAGAGT
CATAGAGATACGAGCNATGCAGAACACCCACGCTGACGTAGNCGTACGCCTCCAGGAGAAAANGGAGCTACGC
GATATTGAAGCATCGGAACTGGCCGAGAGCCATACGGGCGATGCCACAATGGTACACAATGTGCCGGTTANCA
AAGGCATGGACCAGCACCACNCGAAATAACCTTAGATGACGCNCTACTATCTGGCCCNGATTTGACCCCCATA
CACCTGTCGGACGAACGACTTATCGGGGTCGATAAGCGAATCGATACCAGGNTGACTCGTTCTTCAAGATGAT
CGTCGAAGACCCGNCGGCTAACCGTACNTTCGTGGTAC
>Armme1_1_Cluster38#LTR/Gypsy
CCTTGATGACATCCTGATCTTTTCAAAGACCCTAAAAGAGCATTGACAGATCACTCGCAAGGTTCTCATGCTC
CTCAGAAAGCACAAACTGTTTCTGAAAGCCGAGAAATGCGAGTTTGAGGTACTAGAAACCAAATACCTTGGGG
TAATAATAAGCAAAGGATCCATTCATACACACCACCTGCCTTCCCTAACCAACAACAACCACCCCCACCTCCA
CCCGATATCATCAAGAGTGAAGAAGAGTATGAAGTCAAGGAAATCCTCGATGACAAACCATGCAAAATCCATG
GCCAGAAAGGAAAAAAGTCAAAGATCATATGGAGCCTGCCATGGAATCAAAGACCGAAGAGGTAAAAGATAGC
ACCAAGAAGTAGGCAGTTCTACTTGCTTGGTAGAGGACTTATCTGGTATGAAGAACAATATTGCTGGTCGAAC
TCATAGTATTTCTGTAGCTATGTTTAATATCTCGCATTCAAATACACCTGCCTTAAAAATTTCGAAACCAGAT
AAACAAGAACCGCAAGAAAACATGACATCCAAGCCTGCCATGGGAGAGGAGACATGCCAGATAGCACTGGAGC
CCTCAAAATGAGTATTAGGACTTGGGTCAGGATTGAGTAAGTGGGATGCCCCCAGGCCTCTAAAGCCGGAAGG
TCCAGTGTCTTTCACAAAGGCCAAGACACCCCCTATGAGAAACAATGCAGAGGAACTCAAGCACCTGCCTAGG
GCCAACACTGGGGCAGAGAGCCCCCATAAAGTCACCATGGTCAAAGTCCTGGATGAAGAAGATGACACCACCT
TTCGACTCCATCAAGCGAAGCAAGCCAAGGACCAAGAAACTCTGCCACCAGCCCTCAAAGAAACACAGTCAGA
ATGGTTAAAGCCGTTTGAGACCAAGTGGACTTGGAGAGCCATCTGAGAAAAAGCAAGCCAAGGACCAAGAAAC
TCTGCCACCAGCCCTCAAAGAAACACAGTTAGAATGGTTAAAGCCGTTTGAGACCAAGTGGACTTGGAGAGCC
ATCTGAGAAGCGAAAGATGAATCTGCTGGCAGGTCAGTATTTCTTAATTGGATATACAGGACCAGGGCTGAAG
AGATAGTCAACAATCTCTTGGAAGGGCTCTATCAAGCAACTCAGACAAATGTGTTGGAGTGATTAGACAAACT
ATGCAGACCCAAACACTATCTCATCCATTCCCAGACCACCTGTTAGACAAACTATGCAGACCCAAACACTATC
TCATCCATTCCCAGACCACCTCCAAATGGGACCTCAAGATCCCACTCCAGCTTGAGACCCTCGACTCCCAACA
CACCCTCAGCACAACTGCCCTACTTGACAGTGGATGCACTGGTAGTTCCATCCATAGCAAATTCGTCAAAACC
CATCAGATCCCCATCCACAAAACTGCCAGTCCCATTCCTATCTACAATGTGGATGGCTCATGCAACAAGGCTG
GCGAGATCACTGAGTTCACTGAAGTAAGGCTCAAAATTGGTAACCACAGTGAGAAGATCGACCTCACCATAGT

CAACCTCAGCTCAAAGGAGATCTTCCTTAGTCATGACTGGTTTGTCTGACACAACCCACTCATAGATTGGAGC
ATGAGCAAGATCACATTCACCCAATGCCAATGCGTAAAGAACCCCTTCACCCTCCCTGACTCTGACCCTGACA
ACAAATAGGATGAAGAACTCAAAGAGGGAGACCAGATTCTAGCAGTCAACTTTGAAGAAGCCATTGAAATCCA
TGCTGTTCATAAAGCCAACGAGTTAGTCACCAGAGCAAATGAGGGCAAGGAAAAGAAAAATTTTGAAGAGATG
ATCCCTGCCATGTACCATGACTTTGTGGACCTCTTCAAGAAGGAAAACTTTGATGAACTCCCCACCTGGAAAC
CCTGGGACCATGCCATTGCTTTTGAAGAGATGATCCCTGCCATGTACCATGACTTTGTGAACCTCTTCAAAAA
GGAAAACTTTGATGAACTCCCCACCTGGAAACCCTGGGACCATGCCATTGAGCTCATCCCCAACACCAAAAAC
ACCCTCAACTGTAAAGTCTATCCCTTGAATCACACCAAACAAGAAGAACTTGACAAGTTTCTTGACAAGAACC
TTGCCACAGACCACATCCACCTCTCCAAATCCCCCATGGCCTCCCCCTTTGTCTTTGTCAAAAAGAAAGACAG
GCAGCTATGACCTGTACAGGACTATTGAAAACTCAATGAAATGACCATAAAAAATTGATATCCTCTACCCTTC
ATCGCTGAACTCATGGACAAATTAAGGAGCACAAAATACTTCACCAAACTAGATGTCTGATAGAGGTATAACA
ATGTACACATCAAGGAGAACAACAAATGGAAGGCTGCCTTCCACACTAACCACAGCCTATTCAAGCCTACAGT
AATGTTCTTTGGACTCACTAACTCCCCTGCCACATTCCAATGGATGATGAACAACATCTTCAAAGACCTCATT
GCATCCAGCAAGGTTACCATATACCTTGATGACATCCTGATCTTTTCAAAGACCCTAGAAGAGCATTGACAGA
TTACTCGCAAGGTTCTCATGCTTCTCAGAAAGCACAAACTGTTTCTGAAAGCCGAGAAATGTGAGTTCGAGGT
ACTAGAAACCAAATACCTTGGGGTAATAATAAGCAAAGGATCCATTC
>Armme1_1_Cluster37#LTR/Copia
ATTGACTTCCAATCATCAGGATTTGATGCAAAACTTGCATCCGAGTATCCGGTGCTAATTATTTCTCCTTCTT
CATTGGCACAAAATACTAGCTCCAGGTTTCTTGTTCCAGCAAGATATCTGCATACATGCTTCAATGCTGTCCA
ATGTTCTGGTCCAGGGTTTGATGCAAATTGTCCAAGCTCTTGGACTGCATGTGAAATATCAGGCCATGTTCCT
CTTGCTGCATATAGCAAGGAACCAATTACAGATTGATATCCTCCCACCATTGCAGGGTCAGCGACCAAGTTAT
CGGTTCATTTCTTGAGTATCACATTGGGATCAAGTGGGGTTGATACTGGCCATGCATTTTGCATGCTGAATCG
AGTTAAAATGCCTTCGATATACTGCTTTTGAGATAATGAGATGGTACCATTTTCCCTATCTCTCTTGCAGGGT
ATATCTGGTATGCAGTGGTAGACTACTGAGACTTGTAAGTAGGTAGTTGCTTCCCAGATGATGGGAGTTGAGT
AAGAAGAATGAATTATTACTACTGTAGAAACTGTACAGGTATATACACTACTGAGAAATTATCTAGAAAGTTC
TATCTTACCAAGAAACAATCCTTCAAGTCCTTTCTCCTACTCTCTTCAAAGTATGAGCATTGATGCTCAAATA
TTCCAGATGTTACTGGATTGCTGATATGCCCGTTACCCAATATGGTACTTGCAGCATTGTTGAGTCCAATACT
CCCCCTCAAGACAGTAACAGACTAAGTTCTCATACAAACTTCTCATGCTGTGGTCTTGGTAATGTCTTTGTAA
ATATGTCAGCCACCATTTCTTCTGTGGGACAATAGTATAGCTTGATTGTTTCATTCTCCACCTTTTCTCTAAT
GAAATGATTTTGAATGTCAAAATGTTTTGATTGAGCATGGTACTGTGGATCCCTCACAAGCACAATGGTTGAT
TGATTATCTTCAAAGATTAAAGTAGAATCTTCTTGTGGACAGCCCAATTCAGATAGTAAGTTCCTATTCCAAA
TTGCTTGTCTTGTTGCATGGGCTAAAGCTGTATATTCAGCTTCTGTAGATGAGAGCATGACTGTGCTCTGTTT
CTTGGAACTCCATGCAAATGATGCACCACCAATAAGGTACATGTATCCTGAGATTGACTTCCAATCATCAGGA
TTTGATGCAAAACTGGCATCCGAGTATCTGATGCTAATTATTTCTCCTTCATCATTAGCATGAAATACTAACT
CCAAGTTTCTTGTTCCAGCAAGGTATCTGCACACATGCTTCAATTGCTGTCCAGTGTTTTGGTCCAGGGTTCA
ATGCAAATTGTCCAAGCTCTTGGACCACATGTGAAATATCAGGCTGTGTTCCTCTTGCTGCATATAGCAAGGA
ACCAATTATGGATTGATATCCACCCACCATTGTAGGGTCAGCAACCAAATTATTGGTTCGCTTCTTGAGTATC
ACATTGGGATCAAGTGGGGTTGATACTGGCCATGTGTTTTGCATGCTGAATTGAGTTAAAATGCCTTTGATAT
ACTGCTTTTGAGATAATGAGATGGTACCATTTTCCCTATCTCTC
>Armme1_1_rnd-1_family-366#LTR/Gypsy
GAGAGACAAGGAAAGCTTTGGCGTAAAAACGTGGATGGAAGACATCAGCTCGTATTATTTCCAGACCGTCGCA
TTCAAGTGATGACGTCTTGTCACGATGATGTCGCTCACAAGGGTTTCTATGCGACGCACGCGCTGATAAAAGA
ACGTTATTGGTGGCCTGACATGAAGTACGATATTGCCTGGTTCGTCAGGTCGTGTCATCTTTGCCAAATCCGT
CAGACACGTCAAATATACATTCCGCCCACCGTCGCACTCCCTGCTCCTTTGTTTTCAAAAATGTACATGGACA
CGATGATCATGCCAGTCTCAGCACAATACCGNTACATCGTNCAAGGCAGATGTTCGCTCAGCCATTATCCAGA
ATTTCGTTGCTTAAGAAAAGAAACGGCACGAACTCTAGGAGAATGGATTTTCGAGGATATCTTATGCCGTTGG
GGATCATTAGTCGAGATCGTNACCGACAATGGGTCCGCTTTCGTTGCAGCAATAGGNTATTTGAGCAAGAAGT
ACCACATCAACCATATCCGTATCTCNGGCTATAATTCGAGAGCCAATGGGATAGCAGAAAGGCCGCACTTCGA
CGTCCGTCAGGGTCTNTTCAAAGCNTCTGATGGAGACCAAAAGAAGTGGAGTCAGAACGCTTATTCCGTGTTT
TGGGCCGATCGAGTAAC
>Armme1_1_rnd-1_family-271#LTR/Gypsy
TCCCAGTTCGAGGACCAGAGACTCGCTACGAAATTGAAGGAGGCTACGAAACCATTCCTCTCAATCANGGCAT
TAGAAGATTCGTTTGGGAGCACTTCCAGAACCTNAATCGGATCGTNCAACGCATGAAGTATTGCGGAGGCGCA
TTTTCAGGCAAGAAGGCCTACCTNTGTTGCGCAGAGATCACCGTGCTAGGNCACAGGTGCACGTATNCGGGCA
GANTACCATTGGAGAGCATAGTCGCATCAATCATGCATTGGGGACCTTGTATTGACGTCTCTGACGCACGCGC
ATTCATTGGTACTGTCGGAGTCCTACGGATATTCATCGACGATTTTGCAACTCGAGCTCTACCCATCACCAAG
CTATTCAGAAAAGACGTTCCTTTCAAGTTCGAAGCGGTTGAGATTGCCGCACAAGAAGATCTCAAAGAAGCAG

TTAGACACAGTCCAGCATTACGACCTCTCGGTTACACGTCAACTGGAGCTGTCATACTTGCCGTGGATACAGG
GCCTGCCGCAGTTGGTTATTATTTAGCGCAGTGCGACCCTGTCGATCCCAAAATCCGATAT
>Armme1_1_rnd-1_family-206#LTR/Gypsy
CAGTTGGTTATTATTTAGCGCAGTGCGACCCTGTCGATCCCAAAATCCGATATTTCGCCAGATTCGGATCAAT
CACCCTNAACGAGCGAGAGTGCGGATATTCTCAAGCCAAGAAGGAGATATNTGGACTTTATCGAGCGCTAGGA
GATTGCAAGATTTATCTCATTGGCCTTCCAACGTTTATTGTGGAAGTCGATGCGAGTGCTATCAAGGGAATGC
TCAAGAACCCGGATATAGCACCGTCAGCAGCAGTTAACCGNTGGATTATNGGAATCCTNTCGTTTCATTTCGC
GTTAGTCCACGTACCAGGCTCCAAGCATGGTCCAGATGGTCTATCACGACGCCCACCCCAACTAGGAGACCCT
CCACCGCCTGCAGATGGCATGGAAGACTTCTTACACACTCATTACGGGCTTATGCATTTTATTAATTCGACTT
CACCGACAATTCATTCGCCGATCACGTTAATTCAGACTCTACAGCAAGAAGTCACCGTTGATGACACGGAAAT
AGATTCCTACGAAATTGTTCCTCGTACAGCCAAGCAGCAGCGTCTAGACGAACGATTGACTCATCTATTGCCG
TGGCATTTGTACCTCAAACGGCCACCGCAGATGGATGACAACGCGTACCGCAAGTTCATGAAATATGCAGTTC
ATTTCTTCGAGAGACAAGGAAAGCTTTGGCGTAAAAACGTGGATGGAAGACATCAGCTCGTATTATTTCCAGA
CCGTCGCATTCAAGTGATGACGTCTTGTCACGATGATGTCGCNCACAAGGGTTTCTATGCGACGCACGCGCTG
ATAAAAGAACGTTATTGGTGGCCTGACATGAAGTACGATATTGCCTGGTTCGTCAGGTCGTGTCATCTTTGCC
AAATCCGTCAGACACGTCAAATATACATTCCGCCCACCGTCGCACTCCCTGCTCCTTTGTTTTCAAAAATGTA
CATGGACACGATGATCATGCC
>Armme1_1_Cluster61#LTR/Gypsy
AGGGGAGAGCGCCAGAGAGATAATGTTTGAACTCCGAGAGCCCATCCGATACATCCGAAGAACCCAAAGCAGC
AACCGAGATCTCACCATCGACATCACACTCGAGAACTGCACCGACCAACGCATCCTATCCGCCAAGGGACTCA
TCGACAGTGGGTGCACCGGAAGTTCCATAAACCAATCCTATGTCCAAAAACATGACCTAAATACCAGGAAAAC
CATCACCCCCATTCCAGTGTACAATGCTGATGGAACCTGCAATCAAGGCGGGGATATAACCGAATTCACGGAA
CTTATCGTCTTGATCGGCGGACATCGTGAACGAATTGACCTCGTGGTCACCGACCTAGGAACCAAAGATTTAT
ACCTTGGACATGACTGGCTCAAACACCACAACCCGGCGATCAACTGGAAGATGGGATCAATCATCTTTGGTCG
CTGTCAATGCACCAAAAATCCTTTTCCCTTACCAGATGCCGATCCAGAAGATCGCTGGGACGAAGAACTAGAG
GAAGGAGAAACGATCCTGGCCGTAAACATGCAAGAAGAAATAATCATCCGAGTGGTCCACCACGCCAATGACT
TAGCAGCCCAGGCCCACGCAGATAAACCAAGGAAAACCTTTGAAGAGATGGTCGCCGAGCATTATTGCTCTTA
CCGGGATCTGTTTTCCAAGGACAATTTCGACAAATTACCCGATCGCAAGCTCTGGGACCATGCCATAGAACTC
ATTCCCAATGCCAGGTCCACCCTCAATTGCACTCCTCCGGCCAACCCCGAAGACTCCCCCCACTCGGACAACA
AGAAAGCCGTTGAACCGGAAATCGTTAGCACCCAGACGATAAAAAGGGGGCCAGCCGTCACCATGGAGGAAGT
ACCTGACGAAGAAGACGAAACCGCCTACCAGGGCTGGTTAGCGGAGCAGAGGAGTGAACAGAAGGAACGGGCC
ATCCAGAACGACGATGATTGGGTACCCACCAGGCCAGATACGAGTGCCTCCCTGCATTCTCGAACCTGGTACA
GACCCTTCGCTGTCGACTGGACCCTATGAGCCATCTGCGAAGCCCATACTATGGAAGAAGCAAGGGCAGCGCT
CAGCAGTTGGATCCACAAAGACAGGCATCGAGAAATCATCGCCAGGTTAATCAATCAACTCCAGAAAGGAGGG
GAGAGCGCCAGAGAGATAATGTTTGAACTCCGAGAACCCATCCGATACATCCGAAGAACCCAAAGCAGCGACC
GAGATCTCACCGTTGACATCACCCTCAAGAACTGCACCGACCGGCGCATCCTATCTGCCAAAGGACTCATTGA
CAGTGGGTGCACCGGAAGTTCCATAAACCAATCCTATGTCCAAAAACACAACCTAAATACCAGGAAAACCATC
ACCCCCATTCCAGTGTACAATGCTGATGGAACCTGCAATCAAGGCGGGGATATAACCGAATTCGCGGAACTTA
TTGTCTCGATCGGCGGACATCGCGAACGAATTGACCTCGTGGTCACTGACCTAGGAGCCAAAGATTTATACCT
TGGACATGACTGGCTCAAACGCCATAACCCGGTAATCAACTGGAAGACGGGATCAATTATCTTTGGTCGCTGT
CAATGCACCAAAAATCCCTTTCCCCTACCAGACACCGACCCAGAAGATCGCTGGGACGAAGAACTAGAGGAAG
GAGAGACAATTCTAGCCATAAACATGCAAGAAGAAATAATCATCCGAGCGGTCCACCACGCCAATGACTTAGC
AGCCCAGGCCCACATGGATAAACCAAGGAAAACCTTTGAAGAGATGGTCCCCGAGCACTACCGCTCTTACCGA
GACCTGTTTTCCAAGGACAATTTCGACGAACTACCCGATCGCAAGCCCTGGGACCATGCCATAGAACTCATTC
CCAATGCCAGGTCCACCCTCAATTGC
>Armme1_1_rnd-1_family-19#LTR/Gypsy
AGGAATTGGAAGGGGAAGAAGCAAGNCGGAAACCTTCGGTCGCCGCGGAATCCGTTGGCTGTGCTTGTTTGAT
ATCGGAGTGGAGCTGGTCAACATCCATGATAGCACTAGCGCGGAGTACGGGGCCTTCAAGAAAAGTAGCGCGG
AGAGAAGCGGAGAGTTGTTCTTGAGTAAATACAGGACGGAAATTGTGAGGGTTGACTTGAGCGTATTGACTAT
CCCCCTCTTTAGGGTAGACGTCCCATCGTCTAGTGAGTGCGTCCGGTTTCCCACCAAGTTTACCCGGTCGGAA
CCGGATTACNAGGTTGAACTGGGAAAGGAATTCGGACCAACGGACTTGACGTCGAGTAAGGACCTTGGTAGTA
GAGAAATACTCAAGGTTCTTGTGGTCTGTAACGACGTCGATGGATTCCGCGGAGCCTTCAAGGTAATGACGCC
AGTATTTAAAGGCTTCAAAGATAGCTAGAAGCTCTTTATCATGGGTATCGTAGTTGAGCTCGGCTCCGTGGAG
ACTGCGGGACAAGAAGGAAATAGGATGAATCTCACCATCCTCAAGGTAAATAGAAAGGATGGCGGCAATAGCG
TAGTCGGAAGCGTCGGTTTCCACGACAAGTTGACGGTCGGGAATCCAGTGGGTAAGGATAGGAGCGTGAGTGA
AAGCCTGTTTCAACTCGTTGAATGCAGCCAAGCATTTATCGTCGAAATTCCACGGAATGCCCTTGCGAGTGAG
GCGTGTTAAGGGCAGGACAATTTCGCTGTATTCGTGGATAAAACGACGGTAGAAGTTGGCGAAACCAAGAAAT
GATTGGATATCTTTGACCTTCCGCGGGACCGGCCAGTCAACAATGGCTTTAACTTTGTCTTCAGCCATGCGTA

AGCCTTCAGGAGAGAGGATGTAGCCTAGATATTCCACGGAATCCCGATGAAATTCGCATTTGGAACCTTTGCA
GTAGAGCCCGTTGGCTCGGAGTCTGCGTAGGACCTCACGAACGTGTTTTCGGTGTTCCGCGGGATCGTCGGAG
TAGATGAGGATATCGTCGAGGTAGACGACAACGGAGACGTCAAGCATATCGGAAAAGATGTCGTTGACAAAGC
GTTGGAAAGCCGCGGGNGCATTGGTAAGTCCAAATGGCATGACGTGCCATTCAAAGGAACCATAGCGGGTGCG
GAATGCGGTCTTCCATTCGTCTCCTTCGGCAATACGGACTAGATGGTAGGCATGGCGGAGGTCGAGTTTAGTA
TACAAGCGCGCGGAACGCGCGGAGTCAAGAAGATCGGAAATGAGAGGAAGTGGGTATCTGTCTTTTTTCGTGA
TCTTATTCAGACCGCGGAAATCGACGCAGAGTCGGAGTTCACCAGTCTTCTTCTTGACGAAGAGTACCGGAGC
TCCGTGGGGAGAGCGTGTAGGAGAAATGAAACCGTTGCGAACATTATCGTCAATAAATTCGCGNAGAGCTTTG
AGTTCGGATTCCGAGAGGGAGTAGATAGGACCAAGTGGAGGTGAAGTACCTTCCTCAAGGTTGATTTTGAGAT
CGTAAGGGCGATGCTCGGGGAGTTTCTCGGAGAGGGAATCACTGAATACATCGGCGAAGTCGTGATATTCTTC
AGGCACATCNGACAAATCCGGCGGAGCCGTGGAGGCAGCCTGGCCACTAACGCTTGGCANAGAAGGTTGGAGT
GTGAACTGTTGTGCACCCTCCTCTGCGCAAGCTCGNACGAAAGCGGCCGCGGAAATAATAGATATCTTGGGTG
CCGTCGATGCAGCAGATGAAGGAATGGAAGGGTAGGAATAGAT
>Armme1_1_rnd-5_family-4525#LTR/Gypsy
AAGAGGTGGCGCGTCGACCTACTGGGNTCGCATATACACATATTTATACGACCACAAGACCCTGCAAAACTTC
GATACCCAGAACGACTTGTCTAGNAGNCAAGCNCGCTGGATGGAGTTCCTCCAACAGTATGAATATACTCTGC
ACTACGTCAAGGGAGAGGACAATACCGTNGCCGATGCCCTATCACGCTTACCGGTAGAGGACGAGCCATCGGA
TTTCCCCCCTGACTTCGCCGCCATCTTTNCCGTTCAAACTGACCCTGGCATNCTAAAAAGGATCAANCTCGGT
TACTTGGTCGACCCGTTCTGCAAGAGACTNCTGTCGGATTGCGAAGCGGGCTCCCTACACAACGCTNCCNTGT
AATAGNCTCCTCTACCTCGGNGATCGTTTGGTNGTNCCCAGAACNGATGACATCCGGGAGNAACTCTTCGCCC
TGGCGCACGACTCTCTCGGCCACTTTGGTGGCGACAAGTCNTATGGAGCGCTCAGAGACTCGTACTATTGGCC
GAACATGCGGAAGGACCTGCAGGAATCTTACGTGCCTTCTTGCTCATCATGCCAACGTAATAAATCCCGTACA
TCCAAAGTCCCAGGCCCNCTACACTCCCTNCCAGTGCCAGACTCCCGTTTCTCGTCAGTGGCGATTGACTTCG
TGGGCCCACTACCGGAAGAGAATGGGTTCAACGAGATTATTACCATGACAGACAGATTAGGAGCGGACATCCA
GATTATTCCATGGCGCACCAACGGNACTGCAGAAGAACTCGCNCAGATATTCTTCGACAGGTGGTACTGCGAA
AACGGCCTNCCTCTCGAAATCATCAGCGACAGGGATAAACTATTCGTTTCTCGTTTCTGGAAGGCGTTCACAA
GACTTGCCGGCGTTAAACACAAANTGTCCACTGCATGGCACCCTGAGACCGACGGCNCATCNGAGCGCTCGAA
CAAGACGGTCGTTCAGGCCCTCCGTTTCTACGTGGACCGNAATCAGAAAGGTTGGTCGACGAAACTGCCGCTC
GTGCGCTTCACGATCATGAACACCATTAACGCNTCCACCGGTTTCTCGCCGTTCCAACTCAAAATGGGNCACT
CTCCCCGNTTGATTCCTCCTCTCNCTGGAAGGGCTACACGGACGCTCCNTTCGATACTAAAGGCATTCATGCA
AAACCTCGAACTCGACATTCGGGAGGCGTCTGACGCNCTCGCCACGGCCAAAATTAACNAGTCGCATTTTGCG
AATCCGACGTCGACCA
>Armme1_1_Cluster30#LTR/Gypsy
TCGCCGTTTGTACCTGCATATGAATTGCTCCTGAGATTGAATTCTAGATTGCTTCAATATCTCCGCTGCTCGG
TTCAGGTCAGCTGGATGGTGGGAAAGTTGTCGGATGCGCAATGCTAACAAGTCTTCTGTTGACATTCCACTCC
GGAATCCTTCTACCATCAGCTATTTCATATCAACACTAATTCACCAGGTTTGTATTCTGATTTCTGAAGTCGC
CGTTTGTACCTGCATATGAATTGCTCCCAAGATTGAATTCTAAATTGCTTCAATATCTCTGCTGCTCGGTTCA
GGTCAGCTGGATGGCAGGAAAGTTGTCGGATGCGCAATGCTAACAAGTCTTCTGTTGACATTCCACTCTGGAA
TCCTTCTACCATGAATGTGGCTTCTAGCAAGTCTAGAGGAAGTAATGGATCAACCCCATGTAGCAGATAATAG
GGGGTATATCCTGTGACATTGCTTGTGGTTACCCAATCGGCAAATACTGCGAGATCAATGTATTTATGCCAAT
TCTTGATTTTCCCATTTCGTAATCTTAATTCTGAAGGGTTAAGCCATCGCTGTGAGTTGTCCATATATCATCC
ATTTAAAGTAGGGGATGTACCGATTTAATTCCTGCTGCACCACATCCTCCACATCCATACGGACTAGTTCCAG
TGTGGGCTTCTGTGTAAGCATCAGCTTATGCCTAAGGCAGTCAAGGAGTCGTTGGCTGCGGTGAAAGAACGGT
AGGTGGAAGTGCTTGGCTCGTGTGATGAAGGTAATAAGAAGAGGTTGGTGCGGATATCCGACATTCCCAAAAA
TAGCGTTGAACACTACATACTCCCGGGTGGCAAGGAGAATTCGGACAATCACACCATTGCCAAGCCACTCCTT
GTAGTATATGTTCCCAAAGAGGCGCAGCGGTATTGGGTTCTTTATGCCCAGGACAAGCGGCAGGTCGACCCGC
CCAAGTCTCTCCCTGGCAGGTACCACCACAATCCGACTAGGACGAATGGCAGGTCGACCCGCCCAAGTCTCTC
CCTGGCAGGTACCACCACAATCCGACTGGCTGTCCCTGGGTTCTCGCAGGAAGGGAGGGGTACACCTGGACCA
CGCCCATCCAGTACTGGAGATGTAACATGCTGGTCCCATTTCATCTTCTCTAGGTGAATCTCACCACAGAGGC
GAGAGATGGAAAGAGATGGATGTGGGTGCAGCTATCGTTGTAAAGAAGCACTGAAATTGATTACGTAACATGC
CGGTCCCATTTCATCTTCTCTAGGTGAATCTCACCACAGAGGCCATCATAAAGAAGCACTGAAATTGATTATG
GTTAGGCTCCTTTCTCAGTAATTGGGTATTCTGTTGCGCACTTCAACATTCTTTAATGTGATATCTGCCAATC
TGCTCCCTCCCTCTTTTAACTGTGTATAGTCAGGTCTTTCTATATTCCAGTTCCCCGTATCTCCTGCTGCGCT
GGCCACAGTGGGGATGAGTGAGATTTCGGTGAAGTTAGGATGTGTAGCTATTTGTGCCGGGGGTGGAAGAGTA
TTAATGAGCATGTTAGTGGTCATGGTGGGAGATTAGCTGCAGAGCAGGACACTTTCCCAGCAGTGCACTTCAA
CATTCTTTAACGTGATATCCGCCAATCTGCTCCCTCCCCCTTTTAACTGTGTATAGTCAGGTCTTTCTACATT
CCAGTTCCCCGTGTCTCCTGCTGCGCTGGCCACAGTGGGGATGAGCAAGATTTCAGTGAAGTTGGGATACGTG
GCTATCTGTGCTGGGGGTGGAAGAGTATTAATGAGTGCGTTAGTGGTCATGGTGGGAGATTAGCTGCAGAGCA

GGACACTTTCCCAGCAGTGCAACCCTGTTTTAAGCTTGTTTTGCATAAGGCGAGTCGGTTGCCTCCGATTCGT
AGATGGGGTCCATTGATTAGCATGAAGGGACAATTGAATGGCTTACGTTTGTCTTGGAGTGGGAAATCCTTTT
ATGCCATGAATTTCTGTGAATCCTCAACCATGAATTTCTGTGAATCCTCATGATTGATTCCTTTTTGCTACAA
GTCTGGATCGTCATCATCTTCATCCAATAATTCCATCATACTGTCTTCTGATTCAGAATCGTCTAATTCCTCC
TTGTCTCCAACTAGGTCATACTGTCTTCTGATTCAGAATCGTCTAATTCCTCCTTGTCTCCAACTAGTTTATG
CATATCAGAAGATTCTCGGGTTATGTATGGCAATAGGCGAAAGGCAGCGACACTTCTGGAAAGCTCAGTCCCA
TCCAATTCCCGCAATTTGTAAGCTCCTCCATATGTTTTCTGCACTACTTCATAAGGTCCAAGGTATCGAGGAT
CAGTCTTGAAGCGATTCACTGTCATTTCTAGCTGGCTATTTCGTATCAACACTAATTCACCGGGTTTGTATTC
TGATTTCTGAAGTCACCGTTTGTACCTGCATATGAATTGCTCCCGAGATTGAATTCTAGATTGCTTCAATATC
TCCGCTGCTCAGTTCAGGTCAGCTGGATGGTGGGAAAGTTGTCGGATGTGCAATGCTAACAAGTCTTCTGTTG
ACATTCCACTCCGGAATCCTTCTACCATGCCATTTGTACCTGCGTATGAATTGCTCCTGAGATTGAATTCTAG
ATTGCTTCAATATCTCCGCTGCTTGGTTCAGGTCAGCTGGATGGCGGGAAAGTTGTCAGATGCACAATGCTAA
CAAGTCTTCTGTTGACATTCCACTCCGGAATCCTTCTACCA
>Armme1_1_Cluster0#LTR/Gypsy
GTTAATCTATACCACACTTTACTCTCGCAACAGAACTTGACTAGCCCAGTCTAGTCCCTCTTTGCTGAGTCTT
AGACCCCACGACTACTCTCTGGTGACTGTCTGAACATGAGGCAGCTGAGGTTTAGCCTAAGTCTTTGATCACA
TGAAGTCTGGGTCTAACAGTTAGCTCGAAGCTTCCCTTCCTGTTTCCTCACCACCAGCTGCGAAGAAACAGAC
ATCCATGAACCCATTCATACTCGTCGCCATTATTACCCTTGCCCTCTTTTTCGGATTCATAGGAATCCTAGTC
CTCATAGCCATCTCCATGTACTATGCGGAAGAAATAAGGGATTGGCTAGGCTGAACCCTCATACGTGTCCTCA
AACGCATCAAGGAAACGAACCCCAAAAGCACTTGCCATGTTCAGATTGAACAACCCATTCAGACCCCCATCCC
CCTCCAACACCGATCCACTCACACCGCCCATTCCAGCAACGAGCCTATGAGCAGGGAAAATCCCCCTCGCAAT
GCTTCCCCCAGACTGAGCAGGCTTAGGGGAGTACCCAGAACCCCCACTCCTGAGCTGGCCCCTGAATCCATTC
AACCTCGGACCCCCAGCCCCCATCCAAGTGCCAAGGAGATTGGCAGGTACATTCTCCGCATGCTCAGATTTGA
TGAGCCCCCCGCCTACTTCGACCAAGCCGCTCGACCAGACACCGGAAACTGGTGTGGACAACACAAAATCCAT
GTGGAGGGGCCGAATTGGATGCTACGAAGGGGAGACCCCTACCCTGACGAGTCCGACGACTCAGACACTGCAG
TCACCCTCCACACAGAGCCAGTCTTTGACGACAACTACATTCCATACGCCCCCGAGCCAATTCGGGTCACCTC
TCCCTGGACAGAGGAGGACCCTCTCCTGTTCGCCTTCACCAGGCTCATTCCCCCTCTGGTCCATAACACAGGA
CCAGCTCCCCCTCCAGTCTTTGACAATGAAATCCTTTTTCATGCCCGCATCTTCAACCACGATGCCCTTGACG
ACCCCTTCAACCTAAGGAGCCTTGACTATGAGTGGCCTGTTCTCTCTGCCCATGACAGAGAGATATTCAGCCC
TGCCAGGACTGCCGCCTGGATGGTCTGCAGGGCAGACGTTGAGGCCTGTGCCATGGGGCCCCTATCCCCTTGC
TCCTTCATGGACAACTGGCTGGCCATCTCCAGCAGAGATCCCATTGAGTCAGGAGGCATGGTGGCAAACATTG
AAGCGAGACTCGAGTCCAACAGGGACTGATCTCTCTTCCCCACCAACGCCGGACCCCGCCTTCCTGGACTATG
GGAAAGAACCCCAGAGGAGCAGAGCAATCTGGAGATCGTCTTACTCGACCTCGCTGCCAATCATGAAGACCAC
AGGGCTCAGGGGCAGGGTGAAGAATTTAATTAATTTTGATACATTTCCATATGATACAGAAGCATTCGGCCGG
GGCATAGCATACAACACCCCCAATATATCCTTTTCCACTTATGACCTGATTGAATACCCAAAACTATATCTGG
ACGAACCCCCTTTCCCCCCACCACGTGCAACCATGCCGAAAAGCTGATACTGGCTACACGGCATGCCGCCCAC
CGGCACAAGCCGACTATTTGCCAGGGGAGACCACAACACGGGAGGAGGTGAGGCTGGATCATCTAACCCAGAA
GCGCACCTCACCCCAGAAGAGCGCAAACAACGAGCGATCGAAGAGAACGCAGCCTGAACCAAAGCTGCTGCTG
ACCTCGTCACCTAGCACGCTGAACTAACCCAGAAACTCGCCGAGGCCAAAGCCGCATGCTCCTGGTTCGCTCA
ATATCATGGCTTCAAAAAAGGAAAGGAACCTGAAAGGGGAAGGCCCCTTCCTCCCAACCACATGCGATGACTC
AATGACCGCAATGACTGGCACTCCATTCCATGACCCCCGAACTTCCCTTACCCAGCCATGCCATACCCACACC
CGATCGGAGAAGTGCCTGACAATGCCCCCTACCTCAACCTTAAATCCCATTGGAAAACCTCTAAACTGTTTAA
AGGCCACCATGATGACATCGAGCGATTCCTCGGGGATTGCATCATCTACTTCGAACAGTTCAGAAGCTTCTTC
CCCTTCCCATCCCAAAAGGTTGCCTGGGCCACCACCCTGTTCTGAGAAAAAGCCAAGGACTGGTGGGTCTACT
GACGCCAGGAGTTCTGGCAGCCCTCGGACTACGATGGGCAACCTCTGCAATTCTGGTACCCGAGTTGGGAAGA
ATTCGTGGACATGGTCGCCACCCAGTTCCAAAGCCCTGCAATAGAAGAGAGGCATGAGCAGGAAATGAACGAC
CTCTGCATGGGCAATGGGTCAGCCAATGACTACTTCCAAGAACTGGAAAGGCTGGCCAAACTCGCTGGAAGGA
GGACAGAAGAAGAGGAATGCGGGATCCTGGTTTGGGCAGTCTGATGAGGGGTCCTCTACGGCTATATCAAGAT
GATTGGTGACATCGGGGAGAACGTCCCCACCATGTACCCTGGCTGAAAGGCCCACATATGCAAAATGTACGAG
GAACGACAGAAGCAGTACGTCATCCAAAAAAATGCTCACCTCCTCCGACATGCACCCCGATCACCGACAAAATG
TCAAGACTGGACCACCCAACCATAGCCCCAGCAAGAATGCTGGGGCCACAAGCTCGACACCGGCAAAAATGAC
GGGGAGCGGGGGAGGACCAGGCAGAGACACATCCACTGGGAAATGAGTCCCAGTCAAAAGCATCACATACGCT
GGACAGGGACAGCTGATGGAAATCGATAGGCTCAGAAAGAAGGGTCAATGCTACCGCTGCAGAGAGAAGGGAC
ACATGTCGAAGGATTGCCCGAAGAAGAAGGACCATAAGGACATTCACTCAATTGTAGTAGTCGAGCAGGAGAA
GGAGACCAAATCAAAGGTTGAGGAGATAAAAGAGTAGGTCGCCAAGGGCTCGATTCCCCTTCGGCGACCAAGG
ACTTCACTGGTGCACTCTCGTCTTCCGCTGGACGGTCTTATAAAAATCCTTTAGCTACAAAAAAACATGTTTT
ACCTATCACTATACATTCTCCTGCTTTTAACAACCCCAGCACGACCAATACCACTGCTCTTAACGTCCCCAGC
ACGATCAGCAAACCAGTATCAAAAGAATCGAACCGCTATGCCGCTCTAGCGAAAATGAGTGACTCAGACTCAA

GCGCTGATGAGACCAAGATCTCTAACACTGAACAGAGAGCAGGACTTACGACAGTCCCGGCAGCGAGCCCCCA
CAAAGACTTGCCAGAACCCCTAACACAGGAACTGCCTGCATGTGAATCCCTTGGGGGAAACACCTCTGCAGGA
CTGGCTAAACAGAGCGCTAAAACCCACAAGGGGAAAGGCTCTGGAAAGCAACCCTGAGAAGTTGCATCCAAAG
ACGGGGCCTCAGTAGGCACACCGCAACAAATGGTCCCACGAGTCGGGGAGCACGAAGGAGAAGAGCACAGCCT
CACTGACGGCCAGGCATCAGAGTGCAGGGACAGCCATGGCTTATTCAATAAGAACGAGATGGGCACGGGCATG
TCCCCCATCCCTCGATGGGATGGCCACGAAATACCAGATCAATGTGAGTACCCTCGATGGGACTTTCCCCCCA
TCACTCCTCCTGCCAGCCCCGACGAAGACGCAAGACCAATTCTGGATGCCAAGAAAGCCATAGGAAAGGAAGT
TGTCGGCGCCCAGATAATAAAAAGGGGGCATGCCATTACATTGGAGGAAGTGCCTGATGAGGAAGACGAAACC
GCTTACCAACAATGGTGGGCAGCACAGAAGGCGGACCATAGGGAAAAGGCGATCCAGAGTGATGATGACTGGG
TATCCACCAAGCCTGATATGAGTGCCCCGTCGCACTCTCGAGCTTGGTATAAACCTTTCAAAGTTGACTGGAC
CCTTCGGGCTATTTGTGAAGCGCACATGCCAGAGGAGGCCAGAGCTGCGCTTAACAGTTGGATACACAAGGAC
CGACACCACGAAGTCATCATGAGCCTAGTCAACAGACTCAGGCGAGGCAGGGAGGAAGCGAGGGAAATCATGC
TGGAACTCTGAGAACCTATCCGTTACATTAGAAGGGTGAAGAACGATGGAAGGGAACTCACCGTCAACACCCT
CCTAACCACCTGCACAAACCAACAGACCCTGTCAGCCTCTGGCCTCATCGATAGCGGGTGTACGGGGAGCACC
ATCAATTGCTCCTATGTCCAGAAGCATAACCTAGACACAAGAAAGACGGCCGCCCCAATACCTGTCTACAACG
CAGATGGAACTCGCAACCAGGCAGGGGATATCACCAAATTCGTAGAGCTCCGCCTCTCGATTGGCAAACACCA
CAAAAGAATCGACTTGGCAGTCACCGACCTTGGCTCGAAGGACCTCTATCTCGGCCATGACTGGCTTAAACGC
CATAATCCAGTGATCAACTGGAAAACCGGCTCGATCATTTTTGGACGCTGCCAGTGCACCAAAAATCCCTTCC
CACTCCCTGATGCCGATCCAGAAGACAGGTGGGATGAAGAACTGGAAGAAGGAGAAACTATCCTCACTGTAAA
CATGCAGGAGGAGTTAGTCATCCAGGCGGTACATCGCGCCAACGATCTGGCGGCTCAAGCCAACACAGACAAG
CCGAAGAAAACCTTCGAAGAAATGATACCCGAGCATTACCGCTCCTACCGAGACCTGTTCTCGAAAGACAACT
TCGACGAACTTCCTAACCGAAAACCATGGGACCACACCATTGAACTCGTCCCAAATGCCAAATCCACTCTCGA
CTGCAAGGTCTACCCTCTGAACTGAAATAAGCAAGAACAGCTGGACCAGTTCCTTGACGAAAACCTGGAGTCA
GGGCGAATCCACCTCTCCAAGTCCCCTTTCGCCTCGCCCTTCTTCTTTGTCAAGAAAAAGGACGGCTCCCTAT
GACCAGTCCAGGACTACTGAAAGCTCAACGAAATGACAATAAAAAACCGTTACCCGCTGCCACTCATTTCTGA
ACTCATCGACAAGCTCCGAGGAGCCAAATACTTTACCAAGCTGGATGTACGATGGGGATACAACAACGTCAGG
ATCAGGGAGGGAGACAAGGAGAAGGCAGCATTCCGCACCAACCGAGGGCTCTTTGAACCCACCGTAATGTTCT
TCAGCCTCACAAACTCGCCTGCTACATTCCAGTGGATGATGAATGACATTTTCAAGGAAAGGTTACCATATAC
CTCGACGACATCCTCATCTTCACCTGAGACCGAGACGAGCATCGACAAATTGTATGACGCATACTGCAACGCC
TTAGAGAGAACAAGTTGTTCCTCAAAGCCAAGAAGTGCGAATTTGAAGTACTCGAGACAGAGTATTTGGGAGT
TATCATTAGCCAAGATGCCATCCGAATGGACCCCATAAAGGTCGCTGGAATAACGGAATGGCCCACACCGATG
AAGAAAAAGGAACTCCAGTCATTCCTTGGGTTCACCAACTTCTATCGAAAATTCATCCACAACTATTCCAAGA
TAGTTCATCCCCTGATTCAGCTCACCGGAAATGTGGAGTGGACCTGGGGAACAGCCCAGAACCAGGCCCTCCA
ACAACTAAAGTGGCAGATGGCAGAAGACGTGGTTCTCGCCATCCCCAGTGAGACAGGACAGTTCAAGGTGGAA
GCCGACTCCAGCGATGGTGCCATCGGCGCAGTCCTTAGTCAGAAACGTGACAGGAAATGGCGACCAGTAGCAT
TCATGTCCAAATCTCTAACAGTCACTGAAAGGAACTACGAGATCTACGACAAGGAACTGCTTGCCATCATGCT
CGCCTTGTCTGAATGGCGGCATTACCTGATGGGGGCAACCGAGGAAATAGAAATCTGGACGGATCACCAGAAC
CTCCAATACTTCCAGAAACCCCAGAAACTCAATCGACGACAGGCGAGGTGGGTTACCGAACTAGCCGAATACC
ACTTCACCCTTCATCACCAACCCAGTACCACCAACAAGAAGGCCGACCTCCTTTCCCGACGAGACGATCATAA
CCAAGGAAAGGAGGACAACCGCGACATCCTCATGCTACAACCACACCACTGCTGTGCCCTAATCATGCCGACG
ACCACCGAGACTCATAACAAAATCAAGGATGCCACCAGACGAGAGGAGTTATGGGATCAAGGGATCACTATTT
CCCTTGCTCACGAATGAGGCATCACAAAGGAGAACAGAATCCTCCTCTATGATGGACGAGTCTACATCCCAAA
AGACCACGCCTTACAAGGGGAAATAATCGCCCAGAGTCACGACCACATCACAGCCGGACACCCTGGGATAGAA
AAGACCAAGGAGCTGATTCAGCGAGAATACTGGTGGCCGAAGTTGAAGAAAGACGTTGAAGCATATGTGAAGG
GATGCGAAGCCTGTCAATGGGCGAAATCCAGTAACCAAGCCAAAGCAGCTCCTCTTCATCCGAACGAAGTTCC
CATCGGGCCATGGACCCACATCTCTGTCGATATGGTCACTGGGCTTCCAGAATCAAATGGCCATGATGCTCTC
CTGGTCATCATAGACCGATTCTCAAAAGCAATTATTCCAATAGCCTGCAATATTGAACTATCAGCCGAGGGAT
GGGCAAAGGCCCTTCGGGACCATGTCTATACCAAACATGGAATGCCCCAAGTCGTAATATCCGGCAGGGGCCC
TCAATTCGTCTCTCAATTCATGAAGGACCTATACCGACTACTCAATATCACCCCAAACGCATCAACAGCATTC
CACCCCCAGACCAAATGAGTCAATCAGGAACTTGAAGGTTATCTCTGACTCTTCATTGAGTATCAACAAACCG
ACTGGAGCGACTGGCTACCCATGGCCAAGTTTGCTCATAACAACTGAGTACACTCTGCCACCGGCAAGAGCCC
ATTCATGGTTCTATATGGATGAAACCCCCGATTAGTCCCAGACTCAACCCGTCCTTCTCCCTTCACCAACCCT
GCTGCTGAAACATTCACCGAAACCATGGCTGAAGTCCACAGACAGACCCAAAAGGCTCTTGAAAAGGCAGTAA
GCACAATGAAGACCCAATACAACAAGTGAAAAAGGCCCACCATCGACTACAAGGTTGGAGATAGGGTCTGGCT
CAATGCCGGAAACCTGCACCTCCCTTGTCCCAAGAAAAAGCTCAACGACAAATGTGTGGGACCATTTGAAATC
CTCAAAAAGGCTGGAGCTTCGGCATACAAACTAAAGCTTCCCCCACACTGGAAAATACATCCACGCTTCAATG
AGAAGCTACTTACCCCGTTCACACCACCATCCTTCCCTAATCAACAACAACCACCACCACCGCCACCAGACCT
CATTGATGACAAAGAACAATACGAAATTGAAGAAGTTTTGGACTCATGACCACGAAAGATCAGGGGAAAGAAA

GGACAAAAAAATGTGAGAAGTCATTGACTACTTCGTCAAATGGAAAGGATGGACGCAGGAGCACAACTCATGGG
TCCGGGACTCAGAAATGGGCAACGCACAAGAGGTCATAAAAGAATATGAAGACCAGACACGAATCAACTCAGT
CAAAATCGCAGCCTCGGGAGCAGCCTTAACAGCAGTCAGGACACCTGCCTTAACAAGGGGGTAATGTTACGAT
TTTCCCACTTATCTTATCTTATCCAACATTTCTCGCTAATCTCATCTTATCTCGAAACCCCTATCCGTGTTTA
TCACGACCTAGTCAGACGTTCTCAGCCCTGGTGCTATGGCTCGCTACCATCACTCGCCTAACACACAGACTCT
GCTTCTTTAACACACGATCCACATGGACAATGATCTATTCTTAGTTACTCAGTTTTCATATATAAGTAGTGTA
GCTCTCGCTGTAGTATCCTCAGCTTCAAGCTAAAATTAATCTATACCACACTTTACTCTCGCAACAGAACTCG
ACTAGCCCAGTCTAGTCCCTCTTTGCTGAGTCTTAGACCCCATGACTACTCCCCAGTGACTGTCCGAACACGA
GGCAGCCAAGGTTTAGCCTAAGTCTTTGATCACGTGAAGTCTGGGT
>Armme1_1_Cluster6#LTR/Gypsy
TTCTCAGCCCTTGTACTACGGCTTGCTACCATCGCTCACCTAATGTATGGACTTTGCTTCTTTAACGCACGAT
CCACACAGACAACGATCTATTCTTAGTTACTCAGTTTTCGTATATAAGTAGTTTAGCTCTCGCTGTAGCATCC
TCAGCTTCGAGCTAAAGTTAATCTATACCACACTTTACTCTCGCCACAGAACTCAACTAGCCAGCCTAGTCCC
TCTTCGCCGAGTCTTAGACCCCATGACTATTCCCCAGTGATTGTCCAAACACGAGACAGCCGAGGTTTAGCTT
AAAGTCTCTGATCACATGAAGTCCGGGTCTAACAGTTAGCTCAAAGCTTTCCTTCCTGTTTCCTCACCGCCAG
CCACGAAGAAACAAAACCCATGAACTACGCCGCCCTCTTCCTTGCAGTATCCATCGCCTTCCTCGCAGGGGTC
ATAGGAACCCTTGCCCTCGTATTTGCCCTCATCCGCCACTCCGAAGAAGCCAGACTCTGGCTACACAGCACCT
TCACTGTCTCCTACAACACCTACCACAGGATCCCATACACTGATGGCCCACCTGTGGAAGAAGAACAACCCGT
TCAGACCCCAGTCGCCCTCCGTCGAAGACCCTCCTTCATCATTGAAGACGAGAGGACTCCTAGAGAACCCTCT
CCCCCCCACAATGCAACCCCAGGGCCAAGCAGGCCAAGAGGAATCCCCAGGACTCCGACCCCTCCTTCCCTCG
AACATGCTCTCAGCTTCCAACCCGATGCAGAGGAAATCGGGAGGGACATTCTCCACATGATACGACTCGACCA
GCCCCCCACCTACCTCGACTCGATCCGGCTCACCAGACCCCCTACCCCCAACCGTCACTGGGAAATCCACGTC
GAGGGACCCAACTGGACCCTCCAGGGGGGAATTCCATATCCCGATGAGTCCAGCGACTCAGACACTGCGGTCT
CCGATGAGTCCAAGCGAAACGAGTCAGAGGACGTCAGGATTCCTAACCCCCCCGAGCCAATTCGGGTTGCCTC
TCCATAGACAGAGGAGGACCCCCTCCTGTTTGCCTTCGCTGAACCACAGGACTTCTCCATCTCCCAAATTGGC
GATCTGACCCCTGACCCAACATGGGCCAGGAGGCAGGGACCACCTGCTGACCCCAACAATCTCATGGCCAGAA
TCCTCAACCACGAGGCCCTCAATGACCCCTTCAACCCAAGGGGCCTCGACTATGAGTGGCCCAGCCTCTCTCA
CAGGGATAGAGAGATATTCAGCCCCACCAGGGCCGCCGCCTGGATGGTCTGATGGGCAGACGTTGAGGCCTGA
GCAATGGGCTCTGTCCCCCTTCGGTCTACCATAGACAACTGGCTCGCCATCTCCAGCAGAGACCCTATCGAGT
CAGGAGGGATGGTGGCCAACATCGAAGCCTATCTGGAATCCAACCATGACCGACACCTCTATCCCGCCAACGC
CGGTCCCCAAGTTTACGGGTTATGCTCCAGGACCACCAAAGAACAGAGCAACCTGGAGCTCACCCTACTCAAC
CTCGCCGCCGACCATGAGGACTTCAGGGCTCAGGGGCAGGGTGAAGAATTTAATTAATTTTGATACACACGAT
ACTGAAGCATTCAGCCGGGGCGCAGCATATAGTGCCCCCAATATATCCTTTTCCACTTATGACCCGATCAAAT
ACCCAAAACCATATCCAGACAAACCCCCCTTTCCCCCACTACACGCCGACATGTTGAAAAGCCAATACCAGCA
TCACAGCGGACCACCCCTAGGAATGAGCCGACTACTCGCCGGCGGGGATCATGATACAGGAGGAGGTGAGGCC
GGACCATCTAACCCCGAAACGCACCTAACCCCAGAAGAACGCAAACAGAGAGCAATTGAGGAAAATGCGACTT
GCACCAAAGCAGCCCTCGACCTCGCTGCCCAGCATGCCGAGTTAACCATAAAACTCACAGAGGCCAAAGCCGC
TCGCTCCTGGTTCACCCAGTACCATGGCTTCAAAAAGGGAAAGGAACCTGAACAAGGAAGGCCCCTACCTCCC
AATCATATGCGACGACTCAATGATCGCAATGACAGACATTCCATCCCATGACTCCTGGGATTTCCTTATCCAG
CCACACTATATCCACATCTGATTGGGGAAGCACCCAATGATGCCCCTTACCTTAGCCTCAAACCGCACTGGAA
AACACCCAAGCCGTTCAAGGGCCACCACGATGACATCGAGTGATTCCTCGGGGACTGTGTCATCTACTTCGAA
CAGTTCTGCAGCTTCTTCCCCTTCCCATCCCAGAAGGTTGCCTGGGCCGCTGCCCTTTTCCAGGACAAAGCCA
AAGACTGGTGGGTATATCAATGGCAGGAGTTCTGGCAACCCTCTAGCTATGACAAGCAACCCCCGTGATTCCG
ATACCCAAGCTGGGAAGAGTTTGTGGACATGGTCGCCGCCCAATTTAGAAGTCCGGCAGTGGAAGAACAGCAT
GAGCAGGAGATGAACGATCTCCACATGGGAAACGGCCCTGCCAATGACTACTTCCAAGAACTAGAGCGATTGG
CCAAACTTGCGGAATGCAGAACGGAAGAAGAAGACCATGGAATCCTCGTCCAAGCAGTCAGACACAGGGTACC
CTACTCCTACATCAAGATGATTGGAGATATCAGGGACAACATTCCCGATACCTATCCCCGATGGAAGGTTCAC
ATCTGCAAGATGTACGAAGAAAGACAGAAGCAATACATCATCCAAAAAATGCTTACTCATGATAGTCGCCCCA
ACAACCGCCAAAGCTCTAAACCCAGACCACCCAACCACAACCCCAACAAACCTGCTGGGGCCACAGGCTCGAT
GCCAGCAAAGACAATGGGAGGAGGGGGAGGCCTGAACCGAGACACATCCACCGGGAAATGGGTCCCCGTCAAA
ACCACGACATATGCGGGGCAGGGACAGCCAATGGAAATCGATAGGCTCAGAAGGGAAGGCCAGTGCTATCGCT
GCAAGGAGAAGGGACATATGTCGAAGGACTACCCAAAGAGAAAAGATTACAAGGACATTTGCTCGATCGTAGT
AGTTGAGCAGGAGAAGGAAGCAGAGTCAAAGGTTGAGGAGGTAAAAGAGTAGGTCATCAAGGGCTCGATTCCC
CTTCGACGACCAAGGACTTTACTGTTATAAAAAAATGTGCTTTACCTATCGCTATAAATTCCCCCGCTTTTAA
CGTCCCCAGCATGACCAGCAAACCAGTACCATCAGAATCGAACCGCTACGCTGCTCTAGTGAAATTGAGCGAC
TCGGACTCAAGCGCTGATGAGATCAAGATCTCCAAAGCCAAACAGGGAGCAGGGCTTACAACAGCCCTGGCAG
CGAGCCCCCACGACAACTTGCCAGAACCCCTAATACAGGAACTGCCTGCATGTGAACCCCTCAGGGGGAACAC
CTCTACAGGACTGGCTAATCAGAGCACTAAAACCCATAAGGGAAAAGGCTCTGGAAAGCGACCTCAAGAAGTC

ACAACAAATGAAGCCTCAGCAGGCATGCCGCAACAAATGCACCTGTGAGTCGAGGAGCACGGAGGAGATGAGC
ACAGCCTCACCAACGGCCAGGCATCGGAGCACAGGGATGGCCACGGCTTATTCGACAAGAATGAGACAGGCAC
GGGTACATCCCCCATCCCTCGACGGGATGGCCACGAAATACCGGATCGATGTGAGTACTCTTGATGGGACTTT
CCCCCCATCACTCCTCCTGCCAGCCCTGATGAAGACACATGAAGGGCCCTGGACACCAAGAAAGCCACAAGGG
AGGAAGTTGTTAGCGCCCAGACAATAAAAAGGGGGCCAGCCATTACCATGGTGGAAGTACCAGACAAAGAAGA
TGAAACCGCTTACCAGATGTGGTTGGCAGGACAGAAAGCTGACCAGAAGGAGCAGAGTATCCAGAGCGATGAT
GACTGGGTGCCCACCAGACCCAATACGAGCACTGAACCATGCTCTCGGGCCTGGTATAAACCCTTCGAAGTCG
ACTGGACTCTCAGGGCCATATGCGAAGCGCAAACCCCGGAGGAAGCCCGAGCCACGCTAAACAGCTGGATCCA
CAAAGGCAGACACCATGAAATTGTCACAGGCCTAATCAACAAGCTTAGATGGGGAGGAGAGGAAGCAAGGGAA
ATCATGCTGGAACTCAGAGGACCTATTCGCTACATTCGAAGAATGAAGAGCAGCGAAAGGGAACTCACCGTCG
ACATCCTCCTGACCACCTGCACCGATCAACAAACCCTATCGGCCTCTGGCCTCATCGACAGCGGATGTACAGG
AAGTACCATAAACTGCTCCTACGTCCAGAAGCACGACCTGGCCACCAGAAAAACCATCGCCCCAATCCCTGTC
TACAACACAGACGGAACTCGCAACCAGGCAGGAGACATCACTGAATTCGTAGAGCTCTGCCTTTCTATCGGAG
GACACCACAAATGAATTGACCTAGCAGTCACCGACCTCGGCTCGAAGGATCTCTATCTCAGCCATGACTGGCT
CAAACGCCATAACCCGGTAATTAATTGGAAGACCGGCTCGATCATTTTTGGACACTGTCAATGCACCAAAAAT
CCGTTCCCACTCCCCAACACAGACCCAGAGGATCAGTGGGATGAAGAACTTGAAGAGGGGGAGACTATACTCA
CTGTAAACATGCAGGAGGAATTAGTCATCTGGGCAGTACATCATGCCAACGATCTAGCGGCCCAAGCCAATGC
GGACAAGCCAAAGAAAACCTTTGAAGAAATGGTAGAGCATTACTGCTCGTACCACGACTTGTTCTCGAAAGAC
AACTTTGATGAACTTCCCGACCAAAAACCCTGGGACCATGCTATTGAACTTACCCCAAACGCCAAGTCCACCC
TCGATTGCAAGGTCTACCCCTTGAACCGAAACAAACAGGAACAGCTTGACCAGTTCCTCGATGAAAACCTAGA
AACCAGTCGGATTCGACCCTCCAAGTCCCCCTTCACCTCACTGTTCTTCTTTGTCAAGAAGAAGGATGGTTCG
TTGTGGCCAGTCCAGGACTACCGAAAGCTTAATGAGATGACAATTAAAAATTGATACCCGCTACCACTCATTT
CTGAACTCATCAACAAACTCAGAGGGGCCAAGTATTTCACCAAACTGGACGTACGATGGGGGTATAACAACGT
CAGAATCAGGGAAGGAGATGAGGAGAAGGCAGCGTTCCGCATGAACTGAGGACTTTTCGAACCCACAGTAATG
TTCTTCGGACTCACCAACTCACCCGCCACATTCCAGTGGATGATGAATGACATTTTCAAGGACTTGATTGCTG
AAGGGAAGGTCACCATATACCTTGACGACATCCTTATCTTCACCCGAGACCTAGATGAGCACTGACGAATCGT
ACGACACGTACTGCAGTGCTTCAGGGAAAACAAACTATTCCTTAAAGCCGAGAAGTGCAAGTTTGAAGTACTC
AAAACGGAGTACTTGGACGTCATCATCAGCCAAGACTCTGTCAGGATGGATCCCATCAAAGTCACCGGAATAA
CCGAATGGCCCACACCAACCAAGAAAAAGGAACTCCAGTCCTTCCTCGGGTTCACCAACTTTTACCGAAAGTT
CATCCACGACTACTCTAAAATTGTTCAACCCCTCACCCAGCTCACTGGAAATGCAGAATGGACCTGGGGAACG
GCCCAGAACCAGGCCTTCCAACAGCTAAAAAGGCAAATGGCAGAGGATGTAGTCCTTACCATCCCCAGCGAAA
CAGGACAGTTCAGGGTAGAAGCCGACTCCAGCGATGGCACCATCGGCGCAGTCCTAAGTCAGAAGCGCGATGG
GAAATGGCGACCAGTAGCGTTCATGTCCAAATCTCTGACAGTTACCGAAAGGAACTATGAGATCTACGACAAA
GAACTGCTTGCCATCATGCTTGCCTTATCAGAATGGCGACACTACCTGATGGGAGCCACCGAAGAAGTGGAGA
TCTGGACAGATCACCAAAACCTACAATACTTCCAAAAACCCCAGAAGCTTAACCGACGACAGGCGAGATGGGT
CACCGAGCTAGCCGAATACCATTTCACCCTCCATCACCAACCCGGTACTGCCAACAAGAAGGCTGATCTCCTA
TCACGATGGGATGACCACGACCAAGGAAAGGGGGACAATAGTGACATCCTCATCCTCCAACCTCATCACTTCC
ATGCCCTAATAATGCCCACCACCACGGACATCCATAACAAGGTCAAGGACACCCTCAGATGAGAAGAACTCTG
GGATCAAGGAATTGCGAACTCCCTGGCCCATGAACGAGGGATTACAAGGGACCACGGACTTCTTGTCTATGAC
GGATGGGTCTATGTCCCCAGAGACCAAGCGCTCAGGGGGGAGATCATTGCTCAGAGTCACGACCACATCATGG
CTGGCCACCTGGGGATAGAAAAGACTAAAGAACTTGTTCAGAGGGAGTACTGGTGGCCGAAGCTGAAGAAGGA
TGTAGAAGTGTATGTAAAGGGCTGCGAGGCATGCCAACGAGCCAAGACCAGCAATCAAGTGAAAGCTGCCCCC
CTTCATCCAAACGAGATCCCTGTCGGACCATGGACACACATCTCAGTCGATATGGTCACCGGCCTCCTGGAGT
CGAAGGGCCACGATGCCTTGTTGGTCATTGTGGACCGATTTTCAAAAGCAATCATCCCGATAGCCTGTAATGT
TGAGCTGTCAGCCGAAGGATGGGCCAAGGCCCTCAGAGACCATGTCTATACCAAACACGGGATGCCCCAGGTC
GTAATCTCTGACCAAGGACCTCAGTTTGTTTCCCAGTTCATGAAAGACCTGTACCGACTACTTGATATCACCC
TGAATGCATCCACAGCCTTCCACCCACAGACTGATGGACAAACCGAATGAGTCAATCAGGAACTTGAAGGATA
TCTTCGTCTCTTCGTAGAATATCGACAAACTGACTGGAGCGACTGGTTGCCGATGGCCGAGTTTGCCCATAAC
AATCGGGTACACTTCGCAACTGGCAAGAGCCCCTTCATGGTCCTGTATGGGCGAAATCCTAGACTGGTTCCAG
ACTCAACCCATCCTTCCCCGTTCCTCAACCCCACAGCCAAAACCTTCACCGAAACCATGGCCGAAGTCCACCA
ACAGACCCAGAAGGCTCTCAAGAAAGTGGCAGGGGTGATGAAGATTCAGTACGACAAACGGAAGTGACCTATG
GTCAACCACAAGATCAGGGATAGAGTTTGGCTAGACACTGGAAACCTACACCTTCCGCAGCCCAAGAAGAATC
TTGACAACAAACATGTAGGACCGTTCGAAATCCTCGAGAAAACCAGACCCTCGGCATACAAACTAAAGCTTCC
CCCGCACTGGAAAATCCACCCATGTTTTAATGAAAAACTACTCACCCCGTTCATACTACCATCATTCCCTAAT
CAGCAACAACCACCCCCGCCACCACCAGACCTCATTGACGATGAAGAACAATATGAAATTGAAGAGGTACTAG
ATTCATGGCCATGAAGGGTCAGGGGAAAAAGGGGTCAGAAACCGCAAACGGTTATCAACTACTTCGTCAAATG
GAAAGGATGGACCTGGGAACACAATTCTTGGGTATGAGACTCAGAAATGGGAAATGCACAAGAAGCAATACGA
GACTATGAAGAGCGTGTCAATAGTGTCAAAATCGCAACTCCTGGATGGACCTTAACAGCAGTCGGGACGCCTG

CCTTAACGAGGGGGTAATGTTACGATTTTCCCCTTATCTTATCTTATCTTATCCAACATTTCTCGCTAATCTC
ATCTTATCTTGAAACCCCTATCCACTGTTACCATGACCTAACTGGATGTTCTCAGCTCTTGTACTACAGCTCG
CTACCACCGCTCGCTTAACGTACGGACTTCGCTTCTCTAACACACAATCCACATGGACAAAGGATCTATTCTT
AGTTACCCAGTTTTCATATATAAGTAGTGTAGCTCTCGCTGTAGTATCCTCAGCTTTGAGCTAAAGTTAATCA
ATACCACACTTTACTCTTGCAACAGAACTCAACTAGCCAGCCTAGTCCCTCTTTGCCGAGTCCT
>Armme1_1_rnd-1_family-64#TIR/MITE
CACAGCGCGGTGGCGACTATTTTCGGATGGTCAAAATAGAATCAAAATCCGAGATTCCGATTTTGGACGAGGC
AAACCTTCGATATTTCCTTATTAGGGAATTCCCCGGATTTTGGACGGGGCAAAATCCTTCGATATTTTCCTTA
TTAGGGAATTCCCCGGATTTTGACGGGGCAAAATCCAACATTTTGGACTTTGATTCTATTTTGACTGCCCCAA
AATAGTCACCACCCCGCGGTG
>Armme1_1_Cluster66#LTR/Gypsy
CGGTGTTCATCTAGGTCTCTGGTGAAGATGAGAATGTCATCGAGGTAGATGGTGACCTTCCCTTCAGCGATAA
GGTCCTTGAAGATGTCATTCATCATCCACTGGAATGTGGCAGGAGAGTTTGTGAGGCCAAAAAACATGACCAT
GGGCTCGAACAGCCCTCAATTCATGCAGAATGCTGCCTTTTCTTCATCCCCTTCTCTGATTTGGACGTCATTG
TACCCCCAACAAACATCGAGCTTTGTGAAATATTTAGCTCCCCACAACTTGTCGATGAGCTCCAAGATGAGAG
GTAGTGGGTATCAGTTCTTGATCATCATCTCGTTGAGTTTTCGGTAGTCCTGTACAGGTCGGAGGGAACTGTC
CTTCTTTTTGACGAAGAAGAATGGGGAGGCAAAGGGCGACTTGGAGGGGTGGATCTGTCCTGACTCCAGATTT
TTGTCAAGGAACCGGTCTAGTTATTCCTGCTCACTTCAGTTCAAAGGGTAAACCTTGCAATCAAGGGTGGACT
TGGAATTTGGGACGAGCTCAATGACGTGGTCCCAGGGTTTTCAGTCAGGGAGTTTGTCGAAGTTGTCTTTTGA
GAACAAGTCGTGGTAGGAGCGATAATGCTTGGGTACCATTTCTTCAAAGGTTTTCTTCGGCTTGTCCATGTTG
GCCTGGGCTGCTAAGTTGTTGGCATGATGTACTGCCTGGATGGCTAATTCCTCCTGCATGTTTACAGCAAGGA
TAGATGGTCCCAGGGTTTTCAGTCGGGGAGTTTGTCGAAGTTGTCTTTTGAGAACAAGTCGTGGTAGGAGCAA
TAATGCTTGGGTACCATTTCTTCAAAGGTTTTCTTTGGCTTGTCCATGTTGGCCTGGGCTGCTAAGTTGTTGG
CGTGATGTACTGCCTGGATGGCTAATTCCTCCTGCATGTTTACAGCAAGGATAGTCTCCCCTTCTTCGAGTTC
CTCATCCCACTGGTCTTCTGGATCTGCATCGAGGAGTGGGAAAGGGTTCTTGGTGCACTGGCAGCGACCGAAG
ATGATTGAGCTGGTCTTCTAGTTAATAACTGGATTGTGGCACTTGAGCCAGTCGTGGCCCAGGTACAGGTCCT
TTGAGCCTAAGTTGATGACTGCAAGGTCAATTCGTTCATGATGTCCACCAGCTGAGAGCTGGAGTTCTATGAA
TTCAGTGATGTCTCTCGCTTGGTTGCGAGTTCCATCAGCATGGCCCAGGTACAGGTCCTTTGAGCTTAAGTTG
GTGACTGCAAGGTCAATTCGTTCATGATGTCCACCAGCTGAGAGCCGGAGTTCTATGAATTCAGTGATGTCTC
TCGCTTGGTTGCGAGTTCCATCAGCATTGTAGACAGGGATTGGGGCAACTGTCTTCTTTGTGTCCAGGTTATG
TTTCTGGATGTAAGAGCGATTGATGGTACTCTCAGTGCACCTGCTGTTGATGAGGCCAGTAGACGATAGGGTT
CATCAGTTGGTGCAAGGGGTTAGAATGATGTCGACGGTGAGTTCCCTCTTGCTACTCCTCATTCTTCGGATAT
ATCGGATGGGTTTGCATAGTTCGAACATCATCTCTCTCGCGTTCTTCGGATATATCGGATGGGTTTGCATAGT
TCGAACATCATCTCTCTTGCTTTGTCTCCTCCTTATCTGAGTTTATTGACTAGACTCATGATGATCTCATGAT
GCCGGTCTTTGTGGATCCAAGTGCTCAGTGCGGCTCTGGCTTCTTCCAGAGTTCATGCTTCACAGATAGCCCA
GAGGGTCCAGTCGACTTCGAAGGGTTTGTACCAGGCCCATGAAAGGGGTGGGGCACTAGTGTCGGGTTTGGTG
GGAACCCAGTCGTCATTGTTCTGAATGTCTCGTTCCCTCTGGTCCACCTTTTGTGTTGCTAGCCATTGCTGGT
AATGTTACGATTTTCTTCACATATCTAATCTCCAGCGGCTATCTTATCTCTGCTCAGTGCAGCTCTGGCTTCT
TCCAGAGTTCACGCTTCACAGATAGCCCAGAGGGTCCAGTCGACTTCAAAGGGTTTGTACCAGGCCCATGAAA
GGGGTGGGGCACTAGTGTCGGGTTTGGTGGGAACCCAGTCGTCATTGTTCTGAATGTCTTGTTCCCTCTGGTC
CACCTTTTGTGTTGCTAGCCATTGCTGGTAAGCAGTCTCATCTTCTTCATTGGGTACCTCCTCTACAGTGATG
GAAAGCCTCCTTTTTATCATCTGGGTGCCAACGACTTCCTTCCTCGTGGCTTTCTTGTTGTCCAGTGCCGGTT
GTGCATCTTCATCAGGGTTGGCTGGGGGAGTGATGATAGGATGCTCACATCAGTCTGGTATCCCATGGCCGCC
TTCCTTGGTGGGGGATGCATTTGTGCATGTCGCATTCTTGTTGCATGAACCATAACTGTCCCTGCACTCTGGT
GCCTGGCTGCCAGGATATAGGTCCTTCATGAACTGGGACACGAACTGGGGTCCCCGGTCGGATATTGGCGGTG
TTCGTCTAGGTCTCTGGTGAAGATGAGAATGTCGTCGAGGTAGATGGTGACCTTCCCTTCAGCGATAAGGTCC
TTGAAGATGTTGTTCATCATCCACTGGAATGTGGCAGGAGAGTTTGTGGGGCCAAAAAACATGACCATGGGCT
CGAACAGCC
>Armme1_1_Cluster20#LTR/Gypsy
TTATCTTATCTTATCTAAATCATAACCTTGGTCATCATTGGATCGCTTCAACGATGCTACCACATGTTTCTCG
CTTCCTTTACGCACGGATGGCACATTCTATTCTTAGTCACTCGATTTTCGTATATAAGTAGCTTAGCCCTCAC
TGTAGTATCCCCAGCTTCGAGCTAAAGTTTATCTATACCAACCTTTACTCTCGCTCCAGAACTCAACTAGCCA
GTCTAGTCCCTCTTTGCCAAGCCTTAGACCCTTCATCCAGACCCCAGTGATTGTCCGAACGCAAGACAGCCAA
GGTTTAGGCTAAAAGTCTTTGGTCACGTGAAGTCTGGGTCTAACAGTTAGCTCGAAGCTTCTCTTGTTTCCCT
CACCACCAGCTGCGAAGGAAACACCCATATGAACTTTAACGAAACAGCCTTCGCCATCGCCTTCTCCATTGCA
GGAGGAGGACTCCTAATCGGCCTCATGGCCCTTACTGTATTTACATACCACTACGAAATTTGATGCTTTCTGG
TAAGGCGTGGACTCCTAATTCTTCCCCGACAAAGACTCCCCTTCCACTATGTCCAACCATTCACAACTGCTGC
CCCTACCACCAAACAAACCCTTACCCGCTGAACCAGCCAGTACCACACCCTGCGATCCAGTGAGGAGTTCCCA

GCGAATCAGCAGACTCCCCTTAGGCAGCCCACCCCAGGTCCGTCCCAGATGGGGCACCAACAACCTGTTCACA
ACTCTATGGAGGAGGAACCGAGACCAAGTCTTGCAATGCAGGATCTCGGGAGGGACCTACGGACTATTTTCAG
ATCCCTCCAGCCCGAACGAGACCTCCAGCCCCCTGCCGCCCCTGAACGAAGGTCCATCTTCACCACACCCCCT
GGGCCCTATGGGGCAGCCACCCTCCACCTCAGAAAGTTCCCGTCTGATGACGAGGGAACAGATTCCTCATCCT
CACTCCCCCTCCCCCCCTCGCTCCCCCAGCACATACGAGTCGTCACCCCAGAACCCCAGCCCGATCCAGAGGT
ATGGGCTGATGACGACCCTTTTAACCCAAGGGGAGGAGACTATCAGTGGTCAGAACTCACCCCGATCGACAGG
CAGCTTATGGGAGAGCCCAGAGCGCGAGCCTGGGATCGACGCTGGCAGGATCTGGCACACCAACTCGGAAATT
ATTACACCCACCGGATTCCCATGGATAACCACCTCGCCATCACCAGCAGAGACCCACTCTCCCTGACCTATCA
ATGCCAGGCCATTGAGGCCAGAATTCATGCTAACCGAAACTGAGAACTACACCGCCCAAACGCGGGACGAAGA
CTCTATGGGGTCAGAGAGAGGACTCTCCAGGAACAGGCTGAGCTGGAGCGCAACCTCTGGGCCCTTGAGCACC
AGGCGTTCAGAGACACAATCGAAAGGGAGGGCTAGCGGCCCAATTTCAACCGGGGGAGGATTTTTTAATGATT
TCGATAATTTTGTATATGACCCAGAAACCTTCAGGGAAGCGACCTTGCTTGCCCCCAATACATTTCCCCTTGC
CACGTACCCCAGGACCATCACGAGCCTCAACAGGTTACCCAATGAACCTCCATTCCTCGGGGAACCCCGATTC
CGACAACATGGCTACCCATACCCAGGAAGCAGCCGACTCTTGGCAGGGGCAGAAGAAGACCCTCCCCTGGAGC
CTATAGTAACTGACCCACCAATCCCAGAAACCCACCCCTCCCCCAGGACCCTTCGAGAACAGGCTATTCTATG
GAACCAACTCGAACTCGACTGACAGCAGAACGAGCTAGGGCACGAACTGACCTGACAAATTAACGAGCTCAAA
GATCAGCTCCAGGATCTCAGAGAAGACGGCCCAGCAAACAGACCCTCCCCTGGTGGACCGCCTGATCTGGGAA
GACCACCCCGAGAGCACAGCTCTCTTTATTCGCTCCCACGACCCCCGGCAGAACCAAACTTCCCACAGGAACC
TCCTCCAGCACCCACGGGGGTCGCCCCTGAAGACGCCCCCTTCTGGGAGGTAAAACCCATTCTCATGCCCCCG
CCAAAGCCCTTCCATGGGGACCATGCCAACATCGGCCATTTCCTCGGGGACTGTGAAGCCTACTTCGTGATGT
TCTCCATCTATTTCCCCTACCCATCCCAAAAAACCCTCTTTGCCGCCCATCACCTTGAAGGACCTGCCAAGGA
CTGGTGGGTTTACCGATGACCAGATTTCTACGATGAAGTCTGGCAAAGGAGCCGATATCCCGATTAGCCCAGG
TTCATCCGAATCTTCAAAGAACAGTTCTCGGACCCTGCAGTCAAAGAACAACATGAAAAGGCTATGTATAGCC
TCCGCATGGGGAATGGACCAGCCACCACCTTTTTTACAAAACTAGAACAGGAAGCGAAGCTCGTGGGACTCCG
CGATAAGGAGGAGAAGAAAGACCACCTCACCCGGGCAGCGAGATTGGGGATCCCTGTCTCCATCCATCACACC
ATCATGGGAGGAGGATTAAACGTCCCACAGACCTATCCGGAATGGAAGGCGCGAGTCATAAAAGCCTGGGAAG
AATGCCAGAGAGACTTGGCCTTCCAGCAGACATTCACCCACTCCAAAAACTGACCCAATGCCAACCCGTCCAG
CAAGACAACCAATGCACCGCCCCCGGTCAAACCAGCAGGCGACGACAAGTGAGGAAGGGATGCCAGCACCGGA
AAATGGACAACCTATGGTGGTCAGGGACAGCCCATGGAGATCGACCGTCTCTGGAAGGAGGGGAGATGCTACC
GTTGCAAGGAGAAAGGACATATGTCAAAGGACTGTCCAAAAAGAAAGGAATTCAAGGACATTCACTCAGTAAT
CGTAACTGAGCAGGAGGAGAAAACCGACTCAAAGGTGGAGGAGGTAAAAGATTAGGTCGTCAAGGGCTCGATT
CCCCTTTGACGACCAAGGACTTCACTGGTATGCCCTCATCTTCCGCTGGACGGTCTTATGCAGCCCCTTTAGT
TACAAATGATGTTTTATCTACCAAATTGTATACCCCTGCTTTTAACATCCCCTGCATGACTAGCAAACCAGTA
CCAACAGAATCAAACTGCTATGCCGCTCTAGCAAAATTGAGTGACTCGGACTCAAGCACCGACAAGACCGAGA
TCTCCAAGGCTGAACAGGGAGCAGGACCTACAGTCCCAGCAGCGAGCCCCCACGAAGAACCAAAAACCAAAAC
ATCGAGGCCACAGAAGCACGAGCCTGTCAAGGGGACCGCTTCAGTGGGCCAAGTTGACCAAGGCGCCAAGCCA
AGTAGAGGGGGAGACGCCGAAGGACAACTCCAGGGAGTCAAAGAGAAGGACTCCACGTTGCAACAAATGAACA
ACCGAACCGGGAGAAATGGAGGAGACAAGCCCAGCCTCACCGACTGTCAGGCCTCAGTGCGCAGGGACCGCCA
CGGCTTATTCGACAAGAACGAGACAGACATGGGCACGTCCCCCATCCCTCAACAGGATGGCCATGAGATACCG
GATCAATGTGAGTCCCCTCCCACCACTCCTCCAGCCAACCCCGATGATTCCACCCATCTGGATGACAAGAAAG
CTGCAGGAATGGGAGTCGTTAGCACCCAGACAATGAAAAGGGGGCCTGCTGTTTTGCTGGAGGAGATACCCGA
TGAAGAAGACGAGACTGCCTACCAACAGTGGTTGACGGCACAGAAGGCCAAACAGAAGGAATGGGCTATCCAG
AGTGATGACGACTGGGTACCCACCAAACCCGATACGAGTGCCCCCTGACACTCTTAGGCCTGGTACAAGCCCT
TTGATGTTGACTGGACGCTCCAGGCCATCTGCGAAGCCTGCACACCAGAAGAAGCCAGAGCAGCGCTCAACAG
CTGGATCCACAAGGACAGACACCATGAAGTCATCATGAAACTAATCAATCAGCTCAGACATGGTGGGGAAGAG
GCCAGGGAAGTCATGTACAAGTTACGTGAACCTATTTGATACATTCGGGGGACAAAAAGTAGTGGACGAGATC
TCACCATCGCCATTACCCTCGAGACCTGCACCAATTGACGCACTCTATCCACCAGTGGACTTATCGACAGCGG
ATGCACCGGGAGTTCCATAAACCGATCCTATGTCCAGAAGCATGGCTTGGACACTAGGAAGACCATTGCGCCG
ATCCCAGTCTACAACACCGACGGAACCCGCAACCAAGGTGGGGACATAACCGAGTTCACAGAACTCCTTCTCT
CGATCGGAGGGCATCATGAGCAAATTGACCTTGCCGTTACTGACCTAGGATCTAAAGATTTATACCTTGGTCA
TGACTGGCTTAAACACCATAATCCAGTAATTAACTGGAGAACTGGGTTGATCATTTTTGGCCGCTGCCAATGC
GCTAAAAACCCTTTCCCATTACCTGATGCAGACCCAGAAGACCGGTGGGATGAAGAGCTCGAAGAAGGAGAAA
TTATCCTGGCCGTGAACATGCAGGAAGAGATAATTATATGAGCAGTACACCACGCTAATGACTTGGTGGCCCA
GGCCAATGTGGGAAAGCCAAAGAAGACCTTTGAGGAAATGGTCCCTGAGCACTATCATTCCTCTCATGACCTT
TTCTCCAAGGATAACTTTGACAAATTACCCAAACGAAAACCTTGGGACCACGCTATAGAACTCACTCCCAATG
CAAAGTCCACCCTCGACTGCAAGGTCTATCCCCTGAACTGAAACGAACAAGAGCAACTCAACCAGTTCCTCGA
CGAAAACCTGGAATTGGGATGGATCCGCCCTTCCAAATCCCCCTTCACCTCACCCTTTTTCTTCATCAAAAAG
AAAGATGGCTCGCTATGGCCAGTCCAGGACTACCGGAAGCTCAACGAGATGACGATCAAAAACCGGTATCCGT

TACCCCTCATCTTGGAACTCATCAACAAGCTCCGCGGAGCAAAATACTTCACTAAGTTGGATGTGTGGTGGGG
GTATAACAATGTCCGGATTAAGGAGGGAGACGAGGAGAAGGCAGCATTCCGAACAAATCGTGGACTCTTTGAG
CCCACCGTCATGTTCTTTGGGCTCACGAACTAGCCAGCCACATTTCAATGGATGATGAATGATATCTTCAAGG
ACCTAATTGCCGAAGGAAAGGTTACCATCTATCTTGACGACATCCTTATCTTCACTAAGGAGCTTGACAAACA
CCGCCGAGTCGTACGACGTGTACTCCAACGCTTACAGGAAAACAAACTATTCCTAAAAGCCGAAAAATGCGAG
TTCGAGGTCCTACAGACCGAGTATTTAGGGGTCATCATCAGCCACAATGCTGTATGAATGGACCTGATCAAGG
TCGCCGGAATAACTGAATGGCCCACCCCCACGAAAAAGAAGGAATTACAGTCATTCCTTGGATTCATGAACTT
CTATCAGAAATTCATCTGTGATTACTCCAAAATCATCTGCCCATTGACCCAACTGACTGGCAACACAGAATGG
GTATGGGGGGCAGCCCAGAACCAGGCATTCCAACAACTAAAGAAGCAGATGGCTGAAGACGTAGTACTCGCCA
TCCCAATGGAGGAAGGAAAATTCAAAGTGGAAGCCAACTCCAGCAAAGGAGCCATAGGTGCGGTACTCAGCCA
GTGACAGGACGGGAAGTGGCAACCCGTGGCATTCATGTCCAAATCACTCACCGTCACCAAAAGGAACTACGAA
ATCTATGATAAAGAATTGCTCGCAATTATGCTAGCATTATCCGAGTGGAGACATTACCTGATGGGTGCTGCCG
AGGACATAGAGATCTGGACGGATCACCAAAACCTCCAGTATTTCAGGAAACCCCAGAAGCTCAATCGCCGGCA
AGCCTGTTGGGTCACCGAGCTGGCGGAGTACCACTTCTCCTTGCATCATCAGCCTGGGACCGCAAACAAGAAG
GCAGACCTCCTCTCCTGAAGGGATGACCACGACCAAGGAAAGCAGGACAACGAGAACTTAATCATCCTGCAAC
CCCATCACTTCCATGCCCTGATCATGCCCACGACCACGGAGCTCCATGACAAAGTAAGGGATGCCCTTTGACA
AGAGGACCGGTGGGACCAAGGCATTGCAAATTTCCTCGCCCACGAAAGGGGCATTACCAAAGACCAAGGACTG
CTCTTCTACGATGGACAAGTATATGTACCCAGGGATCATGCCCTAAGGGGGGAGATAATTGGACAAAGCCACG
ATCATATCACCGCTGGTCACCCAGGAATAGAAAAGACCAAAGAGCTGGTCCAACGGGAATACTGGTGGCCAAA
GTTGAAAAAAGATGTGGGAGCATATGTGAAAGGGTGCGAGGCCTGCCAAAGGACAAAATCAAGCAACCAAGCC
AAGGCGACCCCCCTCCATCCAAATGAAATTCCTGCTGGGCCCTGGACCCACATCTCCGTAGACATGGTCACCA
GACTCCCCGAATCAAATGGACACGATGCGCTCCTCGTCATCATGGACCGATTTTCAAAAGCAATTATCCCGAT
TCCCTGCAACATAGAACTATCAGCCGAAGGATGGGCCAAGGCCCTCCGAGACCATGTCTATGCCAAACATGGA
ATGCCCCGAGTAGTAATATCCGACCGGGGATCCCAATTTGTCTCCCAGTTCATGAAAGACCTGTATAAGTTAC
TGAACATCACTCCCAATGCATCCACCGCCTTTCATCCTCAAACCAATGGGCAAACGGAGCAAGTCAACCAAGA
GATCGAGGGATACCTCCGACTCTTCGTGGAACACCAACAAACCGATTGGAGTACCTGGTTACCAATGGCAGAA
TTCGCCCATAACAACCGAGTGCATTTGGCCACAGGGAAGAGTCCATTCATGGTCCTATATGGAAGGAATCCCC
GGCTTGTCCCAGACTCGAATCGACCCTCCCCTTTCCTTAACCCAGCAGTCAGGAACTTCACAGAGACAATGGC
TGAAGTCCACAAGCAAACCCAAAAGGCTCTCGAGAAAGCTGCAGAAGCGATGAAAACCCAGTACGACAAACGG
AAACGAGCCACAGTCAATTATAAAGTCAGGGACAAAGTCTGGCTCGATGCCAGAAATCTACACCTTCCCCGAC
CGAAAAAGAAACTTGACAACAAATGAGTCGGCCCCTTCACTATAATTGAAAAAACAGGCATAGCAGCTTATAA
ACTGAAACTTCCCCCTCATTGGAAAATCCACCTGTGCTTCAATGAAAAACTACTCACACCGTTTGTACCACCC
TCCTTCCCTAATCAACTCGAACCACCACCACTGCCGCCTGATCTTGTCAATGACAAAGAATAATATGAAATAG
AAGAAGTGCTTGACTCACGCCCCTAAAAGGTCCGGGGGAAGCAGGGACAAAAGCCACAGACAGTCATCAATTA
CTTTGTCAAATGGAAAGGATGGACACATGAGCACAACTCCTGGGTATGCAACTCGGAAATGGGTAATGCGCAG
TAGGCCATCCAGGACTATGAAGACAAGATCCAACAAACCAGGCATGTAAACTTTATCGCAACCATGCTCACTG
ATCCTTCAAAGGCGGTCATGATGATCCTCGACCACAAATACAATGAACAAGGGAACATCTTCTACAAGGCCCA
GCGAGAGGACAGACACCAAAAATGGGTCAAAAACCCCGACACAAACACTTGGCACGACCTCATCGAAGACTAT
TGGAACAACACCCCTTAATGGCAGTCGGGACGACTTTTTAATCCTTATCTTATCTTATCCGAATCATAACCTT
GGTCATCATTGGATCGCTTCAACGATGCTACCACATGTTTCTTGCTTCCTTTACGCATGGATGGCATGTTCTA
TTCTTAGTCACTCAATTTTCATATATAAGTAGCTTAGCCCTCGCTGTAGTATCCCCAGCTTCAAGCTAAAGTT
TATCTATACCAACCTTTACTCTCGCTCCAGAACTTGACTAGCCAGTCTAGTCCCTCTTTGCCAAGCCTTAGAC
CCTTCATCCAGACCCCAGTGATTGTCCGAATGCGAGACAGCCGAGGTTTAGGCTAAAAGTCTTTGGTCACGTG
AAGTCCGGGTCTAAC
>Armme1_1_rnd-1_family-193#LTR/Gypsy
TTTCACATTCCCAGCCAGTCGTTCGCGTCAAAGAGGAACCAGTCGAGCCACAAGTTAAGATGGAGCATATCAA
TCAGCTCATCAAACTTGGCGAGACGTTATTGCAATTGAACTCGAACGATCGCTTCCAGAAGGCCCAGCAGACA
CGTTTTTCGTCTGGGCCGCCAGCTCAAAGTAATTTCGCGAACGCCGCTACAGGTCAAAGTTGCAGCTTTTGTG
GAGAAATAGGGCATTTTATTCGAGAATGTCCTAAAGTCGAGGAAATGATTCGCGATGGAAAGTGCAGGCGCAA
TATAGATGGGAAGATCGTCCTGTCTACCGGCGCCTGGGTTCCACGGAATATCCAAGGCACTTGGCTTAAAGAT
CGCATCGAAGAATGGCATCGTCAGAACCCTGGNCAGACGGGTATCACTCATATGATGAACGCCATTGTCGAGC
CCAGTTCCAGTCAACCACGTCTTTCTTCGACGCCAGCTCACGTCAATTCACAAATGCTCAATACGCCTGCGCC
CGGCATTGATGCGCGCCAGCGTCTCGAAGCGCTTATTGCCAAATCAGAAGCTATCGGCAAAGAGATTCAGGGC
ATCAAACAGTTTATGAATGCGGCCGAAGAAGAAACCGTTGATCCGAAGAGCGCTGATAGCATGGATACCATGC
TACGTCAGGTCGTCGTCGAGGTCCCGAAGTTAAAACGTGGACCACCCAAGAAGGCTAGCCCCGCAGTAGCTGT
GGAGCCTGCTGTTACTCAGATTCCAGCTAAAGTTGGTCGCCAGACGCGTACCAGTCGTAAAGCTGCAGAGGAA
GCCGAGAATGATGACGTGACCGGAGTTGACGTATCCAAGAGTGTTGATCCGTTCGATCAAGATCTTACCCCGG
ATTATGACGGCATGCCCGCACTCGTCGAGGACTCGGATGATGAAGATATTTCCGTTCCCCAGCAGACCCAGCA

CGACGACGAACTTGCAGCAGCCATCATCGAGTCTTATTCTCCACGTCAGTTCGAAAAATCCAAGACTGTCGTT
CACGACAATAGCAAGAATTCNGTTTTGAAGTTTCCGTCTTATGCGCCGCCAGACCAGTCGAATATAGTCACGG
ATATTCCCGAGCACGCACCAGTCAAGGAGCGTGTAGCTCAGCTTATCCCCGAGGATATTCATCCTTTTGCCGC
AGCAAAGCCAGCCACTTATGCACCGCCAGCAGTTCGTAACGCGGGTGCTAAGCCAGATATCCGCAAGCGTCCC
AAGGACAATGAACCCGCGTATAGAACTAGAGCACCGATAGAAGGATCGCATGTTGCGCGAGAAGTCTATGAGC
GCGCGCTTGACTCCACCATCACGTTATCGCATCAAGAGCTATATTCTTTATCACCAG
>Armme1_1_rnd-5_family-112#LTR/Gypsy
TAAGGAAACGCATGGGTTGTTCACCATACTTCGCCGCAACAGGAGCAGAACCTCTTCTTCCTATGGATATCAC
GGAATCGACATGGTTATTGCCACCTCCAGACAATATTCTCAGTACTACGTCCTTAATTGCACGTCGAGCCATC
AGTCTTCAGAAGCGAGCTTCAGATCTTGAACGTTTAAGATCGAAAGTCTACGAAGCACGGCGTAATGCCGCGG
CAAAGTTCGAGCGAGACCACGAATTCTCGATCAGAGACTTTGATTTCCAGCAAGGCGATCTAGTTTTGATGCG
TAACACGCAAATAGAGAAAGCTCTAAACCGTAAAATGCGCGCCAGATATTTAGGCCCCTTAGTGATCTTGGGG
AGGAATAAAGGCGGAGCATATCTCCTTTGCGAGTTAGATGGATCAACGTTTNCTCGACCGGTTGCAGCGTTCA
GGCTAGTGCCGTACTTCGCGCGAAAGAAGATTGACCTACCGGATATCAATGCGATCATTGATGTACCGCCAGA
GCGGATCAATGACCTCTTCGCTTCGACAGATGATGGAACGCTTGAAGGACTTACGGATACAGAGGAAGGTCAT
GAAGAGGCGGAACTTGTTTGAGTTTTCAGGCGCATCAAGTCACGAAAACACAAAATTTATTTATTTGGGTCCC
ATTTTGGGTACCCCTACACATTCGTTGCAAAAATTTATCTATGGCCGGTATATCCATACCGGCCCACCGACAC
TCCTTTCAAACATATACCCCACGCAAATTATCGTACAACTGCCAGCTATCTACTAAGGAACACGGCATCTAGA
AGCGCTTGCCTTTCTTGGAGCCAGAAGCCTTGGATCCGGACGCCTTAGAGCCCGACGCCTTGGCACGAATAAA
ACCGTTGCCGATGCGGACTCCATCCTGGTAGCGAACGTTATCAGTATGAGCGTCCTCTTCCCAATTACCGTAG
TTCTTGTTGGGACAACCCTCGAATTCGCAACCCCAGCCCCAAGTGTTGCACCCCTCGACACACTTAGTCAAGA
CAGGCTTCGGCACCTCCTTCGCCTTACGGTTATGCTTCTTCCCCTTGCCCTTCTTCTGAGCACGGTCCCGCTT
GTTNGAACGGCGGCCACGCTTATTGTTGNAGAGCCAGTAGGGGAATTGCTTCGGAGCCGAGCTAGGATGAGAG
TTGCGAGCGGACTGAGCCGCAGCATCGGCGCGAGTTT
>Armme1_1_rnd-5_family-1123#ClassII/Helitron
GCTCCAATAGACTGTCTCCTGGCCTGGAAATTTGTCCAAAACCTGCTGATTGACTTCATCAACATCATCGTTG
TTACGTCCAGAGAGAATTGTTCTCTCTAAAAAGAAACGATCTTGAAAATTGCCAGCTTCGATATTTGGATAAA
TGTATCTGATAAGTCCAGAAAGTGTAGAGTCAGTAAGGCGCATGGAAGNAGGAAGGGAGATAGTTCGCTCTTC
TGTAAGGCCTTCGCCTGCACCAACTGTCAGTAGCCAACGTGCAAATCCGACGTTTTCTGGGGTCTGGTCCAGG
CGCATGTTTGTTTTCAAATGGATGACTTGAATCTGAGCCCACAAAGGTGACTGACGAAATGAAGCTGCAACAA
TCTGTTCTCGGGATCCTCGTGGAACAACAGGCAATGTCTGACGAAAGTCNCCTAAGAAAAATGATCAGATGAA
ATGATCTTAGAGACTTGGAATAGAAACACACATACCACCAAATAAGACAGTGATTCCACCAAAAGGAGCATCA
TTATTGCGGATATCATGGAGAGTGCGATCAACTGTCT
>Armme1_1_Cluster47#LTR/Gypsy
TTCAGAATACAAAACCACTGGCTGCAGTAGGAAGATTTACTGGCTTCTTACACTCCAGCTTTGATTTTGCTTT
TCAGAAAATGAATGACCTTTTCGTGTATTTCCGGTGGTATAGGTATGTTCCTGTATTCCCATGGCGTGTGCGA
TACAACAGGCATGATGTAGTCTGAAAAATATTCCTGGCTAAAAGTTCTGCGATCCTTTTCTTCATATGGCAAG
GTTTGCTCATTTAATTTAAGCACATGCTTGAATAGCTTCTCTTCTTCTGGCCACAGGAATTCATCCTGATTCA
CATTCAATTCATGCATCCTTTCTTCAGTTAATCACATGGTAGGCACGAACTCGGGTGGTAGTATCGGCAAGGG
GGCCAATGACAAAAGAGGGTCTCTCAGTATGGTTCTCCTTACCTGAGCTTCCTCTGGAAAGGTGGTGGATACC
GGCTTTACTTTCTGGTCCACCCTAGATACTGGCTTTACTTTCTGGTCCACCCTTTTGTACATGGTGAATGCAT
GCACCCGAGCTTGGTGGATATTGGATTGGATCTCCTCCATTTCTTCACCAGGGACAATCATAATGTCACCGTC
CTCAAAGGGGTAATCGGCTAAGGTATAGACCCTGTACTTGTCATCGTCTGGCAGTTTCTGATCTGTCCATTGC
CTAGGGGGATCCTCTGCTTCCCCTTGTATCTCCATTGAGGTCTGCTTTATTCTCATCCGACATCTTGAAGTCA
TGGTTGGAGGCTTCATTCGGTTGGATTGAGCAGACTTAGACTCATCTTTCAAATGGAGATGGCCAGAGAAAAA
CGACTTCATAGCAGAGTAAGTAGGTAAGGGTGGGTCCAAGGTTGGTACATAAAAGGGGAAGAAGGTATGTGTC
AATTTGGGTTCTTGAAATAAAAGGGGAATGGAGTGGAGTTTGTATGTATCAAAAAGTTTATCACAGATTCTGT
TATATAGGTATTGAGTGGGGGGTTATACTGAGTCAGCACACCCAAACCGTCTCACAGCCTCTCTCTGTGCATT
TCACAGCCATCTCTTGTTCCTCGACCTCGACTCAACAGCTGCAAATACAGTCAGAGAGGATCAGAGTTCTTGG
CCACTTTTCCCGCATGGCGTGGTGCTGATACCATTGGTAGGATGCTTCTAGACGTCTTGAACCACGCATCCAT
TGTGCTTTGTCTCCCATTCTTATTGGTGCAGTCTCGCTTCTTCTGAGCATCAATACATACCCCTGAACCTACA
TGCTGCTGAACAAGGTTCTGTACTCCCCCAGGGCCTAGCCTGATTGACTCCCCGCACTTTGGGCAGGTTGCAT
AGGTGCCATTTACAATAATCTACAGGGTCAAAGGTAAATTATCCATCCTATCACCATTTCATCTATAATTGGC
TTATTGTGTTAAGATCAATATCATCATTCAGTGCTGCCATTGCAATGACTTGATGGTTTGCACAGATCTGATG
GCTGGTGGTGTCAGGACGCGGGTTGTGTCGGGCGGCATTGGATATATTGCCCCTATGCATCCATCTCATTTGG
AATACACCAGGAATAGGTGAATTGGGACCAATTTCATTATATCTTGTCCGATAAATAACTAATTAATGACAAA
AATTTATGCGTGATAATTTTTTTAAGACTACCATTGAGGGAACGGCATATGGGCCGCAATTTCCGGGCCCCC
TTGAATACAGTCATTAAAAAAACAGTAAATATTGGTACCATGTGATCTCCACAATCCTGAAAGCGCTTACTTA

TATGTTATGTTGCAGTCCCAGCTGCGTTGCTCTGCTACTTGGACCAAGTTCATGAGACATTTGCCAGAAATAT
GGATGAAAGTCAGCAGGTACATATCCTATTCTGTCACTAAGTATATCTTCTCACTGAACCCTCCCTTAGTCCA
CTGAAATGGTTCCACCTATTCCATGGTCTCCTCTTAGCTTTGGACTAGTGGTACCCTTCAAATGTTGTCATCA
GAACAATACCACGCCATCTATCCATAATCCTCCTTCCGAGATAATTTTTACCATCTTCGAACTTCTCTGTCAT
GCATTGTCCCTGTTTAGGTTTACGAAAGGCCCCATTCCTCCTCTCTTTTGTCCGTGCCTGCTGGCAATCCATT
GCACTTGGCCGTCCCAGCTCGTGGACGTAAATATCCATTGAAAACTTGTGCGACAAGGACAGGGTAGATGCGT
GTCTCCAACAATCGGGGCACAGTCTTCTGACTATCGATATCTATGTCCCAGATGATGAACTGTTTGAGGATGA
GCGCCTCCAGGCGCCCATCTGGCTCTGTGTTCAGCATCCATATTGTTGGTGCCATTCAAATGTTCATATTTCC
TATGCTTCATGCTTAAAGCTCTCCGATGCAGGAAGACTAGAGCTCCCCGAACTCATCTGGCTTACCATATGAC
ATGGCCTACTCGATACTACCTTGCTAATCGACGAAGCATCAAGGTTTATCAGTGCACTAAAAGCTCCCAAACT
GTCAGTTGTTGAATACAGGGGAATGCCACATAATAACCACTACTCGATTTCGTCGTTGCCATGGAGAGGAATC
AAAGACGCAACTTTCTTTGTGTGTTTTGGTCATTTGATCTCGACCGCTCTTCAACACTTGACGTCTCTTGATA
AGTGCACCATTAAATATCCCACTTCCCGTTCACCGCTGGGAGAGTCGGTGATGTCTCCCCCCTTCACGTCTTA
GAACTAGGATTCATGTGCAAAGGGAGCGATTTCTCACTGATACTTGAAGACATTTCACTCCCAAATATCGAAA
TTCTTTGTGTTGGACAATCCTGCCGCAATCTTACAAAAGTCCTTGTCCCAATCTCACACCCCTCACACTCATA
GACATCTGGAGCTTTTTGGATGTCAACATCCTGAAAATGTTGGAGAACGCACCAGGCCTACAATCATTCGTTC
TACATGAGGTGCACAGAGAGGAGCGACCATCCCGAGTTACAGAAGCACTGTTGCATTGACTGGCCCATGAGAC
GTTTATGACCAAACTTCAGACCATCCCATTTGTTGTCATAGCTGCGATCAGTGGAGAAAGCCTGGTACGCATG
ATTTCGGCGAGGGCTGCTGCATTCAAGGAGATAGAAGTGTATTGGAATTCTAGGTATTGCACAGGGTAGACTC
CAGGCTGGTAAAGCCAGATGTAAATGAGCTTGTATTCTACAGCTCCTACAGAGCTCATATTTATACTACTTCT
GTATTATCACATTAGTCCTAAGGGTGTGTAGAAGGCTGTCAGGTCTCTGGATTTCAGGTCTACTTTATGGGCA
TCAAATCCCCAGAACATGTCAAACATTGTGTAACATTGACATCCTCCTGGAAGGAGAATTTGTATATCCCATG
GGTAGTACAGTGATCCGCATTAGTCCTAAGGGTGTGTAGAAGGCTGTCAGGTCTCTGGATTTCGGGTCCACTT
TACGGGCATCAAATCCCCAGAACATGTCAAACATTGTGTAACATTGACATCCTCCATATGGTTCGATGAATTC
ATCTGGTTCTGGTATTAACCCTGCATCCCGAATAGAGATCTTGTTCAGGGGTTGCAGATCATGAATCAAACGT
AAGGCTCCACTCTTTTTCAGAATACAAAACCACTGGCTGCGGTAGGAAGATTGACTGGCTTCATACACTCCAG
CTTCGATTTTGCTTTTCAGAAAATGAATGACCTTTTCTGTAAGGCTCCACTCTTTTTCAGAATACAAAACCAC
TGGCTGCAGTAGGAAGATTGACTGGCTTCGTACACTCCAGCTTCGATTTTGCTTTTCAGAAAATGAATGACCT
TTTCGCATATCCCCAGTGGTATAGGTATGTTCCTGTATTCCCATGGCATGTGTGATACAATGGGCATGATGTA
GTTTGAAAAATATTCCTGGCTAAAAGTTCCGCGATCCTTT
>Armme1_1_Cluster4#LTR/Copia
AGCAAAGATCTCCTTGCCAGACTGGTCACAAACAGCAGCGCATCCCTTTGAGAAAACAGTGGATAGGCCAGCC
CTGTCTAGGGAGCTAACGGACATGAGGTTTGCTGCGAGGGAAGGGGAATGCAGGGCGTTCTTGAACAACAGTT
TAACGTCCCTCCCATCCAGGTGAAATGTCTTGGAGACCGTTCCCTTCCCAAGGATCTCGAATGTCCCCTTTCC
TTCAATGGCAGAGGAGCCTGTATGGAACGCGACAGGTGTGTAATTTGTGAAGTCCTCACGATTCTTGAAGAAG
TAATCGCTGGCGCCAGAGTCCATAAACATCAGTGTCACGGTGGGGGTGCGGCCTTCGAATGTGGTGGTCACGA
AGGCTCTGTCCTCTGTTTCAGCCTCACAGATCACTTTGACCTCATCATCATCGTCATCGACACTTGTCAAGGC
TGGCATATTGTCAGCCTCATCATCAGAGTCATCCTCCTCATCATCCTCTTCCTCATCCTCATCGTCAACGTTT
GTCAATGCAGGCATATCGTCATCGGTATCCGCCTCAGAGTACTCGGTGGCAGAGCTCACACTCTCAGTGTCAA
AGGTACAGTCAGACACATTGGAAGATAGGGTGGAGTCTGACACATCTCCCTCAGTTTCACCCCATCTCCAGAA
GGAACGACTTCCATCCCATTGGTAAATTCTCTCTCCAGTGTCTCCATCCTCCATGGTGAAGAGACTCTGTGCA
CGCAGGCTGGAAATGCAACAGTGTCGAAGACAAAGGCTCATCAGCAGATTGCCCACTCTGCAAGCCCGTTTTG
CGCAGATGAATGAGAGGCTGTCAACTCGAGTATGATGCTGTGTTCTGCACAGTATTTGGCCCATTTAGTCCCT
GCAAAAGCCCTATCAGCTCGCATGCGCTTGACTTTGCAGCCTGTCTGAGTCTCCGCCCTCTTCCTGAAACGGT
CAAACACTGCCACGGTCATCTCATCTGACTTGTCAGCTAGGAACTCCACCTCCTTGAAGGATGTCCCACTGTC
CACCATCACCATCATGTACGTCTTCCTGCTCACAGATGCAGTCCTCGAAGGACCCCACAAGTCTGTAGTGACT
AGCTCCAGTGCATCTACCTCGGGATCTGTTGGTTCATCGAAAGGACGGCAAGCCTGGCGTGCTAGAATACAGT
CCTCGCATCAACCATGCAAGGAATCAGAGGTAATCTCAAGCCTGTTGATGAGATTCTTCGATGCCATCTCTTC
AATTGTGGTAGGGCTCATGTGTACAAATCAACGATGCCATTCTGACAGTGAGGTAGGGGATGAACGGGATGAC
ATTGCCTGTGGTGAAGGCACCGGATCGAGAACGTACATTCCATCTGAACTGGCCAGCAAAGATCTCCTTGCCA
GACTGGTCATGAACAGCAGTGCGTCCCTTTGAGAAAACGGTGGATAGGCCAGCCCTGTCTAGGGAGCTAACGG
ACATGAGGTTTGCTGCGAGGGAAGGGGAATGCAGGGCGTTCTTGAATGACAGTTTAATGTCCCTCCCATCCAG
GCGAAATGTCTTGGAGACCGTTCCCTTCCCAAGGATCTCGAATGTCCCCTTCCCTTCAACAGCAGAGGAGCCT
GTATGGAATGCAACAGGTGTGTAATCTGTGAAGTCCTCACGATTCTTGAAGAAGTAATCGCTGGTGCCAGAGT
CCATAAATGTCAGTGTCATGGCAGGGGTGCGGTCTTTGAATGTGGTGGTCACAAAGGCTCTGTCCTCTGTTTC
AGCCTCACAGATCACTTCAACCTCATCATTGTTGTCATCAACACTTGTCAAGGCTGGCATATTGTCAGCCTCA
TCATTGGAGTCATCCTCCTCATTGTCCTCTTCCTCATCCTCATCGTCAACATTTGTCAATGCAAGCATATCGT
CATCGGTATCTGCCTCAGAGTACTTGGTGGCAGAGCTCACACTCTCAGTGTCAAAGGTACAGTCAGACACATT

GGAAGATACGGTGGAGTCTGACACATCTCCCTCAGTTTCACCCCATCTCCAGAAGGAATGACTTCCATCCCAT
TGGTAAATTCTCTCTCCAGTGTCTCCATCCTCCA
>Armme1_1_rnd-1_family-6#LTR/Gypsy
CCGTCCTTCTTTTTGACGAAGAAGAANGGGGAGGCCATGGGGGATTTGGAGGGNCGGATGCGGCCCGAGGCGA
GGTTCTCGTCAAGGAATTTGTCGAGTTCCTCTTGCTCGGCGCGATTCAGGGGGTAGACCTTGCAGTCGAGGGT
GTTTTTGGCGTTGGGGACGAGTTCGATGGCATGGTCCCAGGGTTTGCGGGCGGGGAGTTCGTCGAAGTTTTCC
TTTTCGAAGAGGTCGGCGAAGTCTCGGTACGCTTCGGGGACCATCTCTTCAAAGGTTTTCTCCTTCTTCCCGG
CGTTGGCTTTCGCGGCTAAGTCGTTGGCGACGTGGACGGCGCGGATTTCGATGGCGTCTTCGAAGTTGACGGC
TAGGATGCGGTCTCCCTCCTCGAGCTCTTCGTCCCACTTGTCGTTGGGGTCNGAGTCGGGGAGGGGGAACGGG
TTCTTGACGCAGTGGCAGCGGGCAAAGGTGATCTTGCCGGTGGTCCAGTCGATGAGCGGGTTGTGACGGACGA
GCCAGTCGTGACCGAGGAAGATCTCCTTCGAGCCGAGGTTGACTACGGCGAGGTCGATCTTCTCGCTGTGGTC
ACCGATCTTGAGTCGGACTTCGACGAATTCGGTGATTTCGCCGGCTTTGTTGCGNGAGCCGTCCGCATTGTAG
ATAGGAATGGGACTGGCGGCTTTGTGGACGGGGATTTGGTGGGTTTTGACGTATTCGCTATGGATNGAGCTGC
CGGTGCACCCGCTGTCNAGCAGGGCGGTGGCGCTCAGGGTGCGTTGGGAGTCGAGGAGTTCGAGTTGGAGCGG
GATTTTTAGGTCNCGCTTGGAGGTGGTCTGGGAGCGGATGAGGTAGCGCTTGGGTTTGCGCAACTCGTCNAGC
CATTCTAGCGCGTTGGTCCGGTTTGCCTGGCGGAGCCCATCCAAGAGATTGTCGACTACTTCTTCGGCCCTGG
TCTTGTGTATCCAATTCAGGAGCACAGACCTGGCGGCAGATTCGTCTTTGGCGTCTCGGATGGCTCTCCAAGT
CCATTCAGTCTCGAACGGTTTTAACCATTCGGGTCCGCGTGGCTTGGGTGNCGTCGGGGCTTCGGGGGCTTTG
GCGGGTTTTGCTTGCTTGGCTAGGTGGAGTCGGAAGGCGGTGTCGTCCTCTTCGTCCGGNACTTCGACCATGG
TGACTTGATGGGGGCTCTCTGCCCCGGTCTTCGGCCCTAGCGGGCGNTTGAGATTCTCTGCATTGGTCCTTGC
GGGGAGTGTCTTGGCCTTCGTGGAAGGCGCTGAGTCTTTCGGCTTCAGAGGCCCGAGGGCGTCCCGCTTACTC
GACTCTGACCCGAGTCCTAATACTCGTGTTGAGGGCGCCGGCGCTACCCGGCAGGTCCCCTCCCTCGCAGCAG
GCTCGGATGTCGTTTTTTCTTGCGGTTCTTGGTTATCGGTCTTTGGAACGGNTAAAGCAGGTGTATTTTTTTG
CGATATTTTACGGTAGCTACAGAAATACTATGAGTCCGACCAGCGATATTGTTCTTCGTACCAGATAAGTCCT
CCGCCGAGCAAGTAGAACCGCCTACTTCTCGGCGCCATCTTTTACCTCCTCGACCTTCGATTCCGTGGCCGGC
TCCGTCGTCGCGGCTCTGACCTCCTCCTTCTTCTTGTTCCAACTCTGTAGCGGGCAATTCTTGCTGATGTGTC
CCTTCTGCTTGCACCTGAAACACTTCCCCTCCGACCTGAGCGCGTCGATGTCCATNGGCTGTCCTCGNCCGCC
GTAGGTGGTGCCCGTACCCGTCTTCTTGTCTCCGGAGGAACTTGTGGCGCCGGGAGTAGGCTTGCTGCTTCCG
GCGCTTTGGTTGGTGTTCCCGGAGTGCGGGGCCTGGTAGTGGGGGCGGTCGAGGCTGTCTTCGACGGCCTTGC
GGGCGCGCTCGTTGCTCATCTCCGTGATCCGCTCCTTCCAACTGTTATAGTCGGTCGGGAGGTTGGTGCCGAT
GTTGGAGATCATGGTGCGGTAGGAGTCGGGGACTCCCTTCTTGACGGCGGCGACCATGTGNCCNCGGGGNCCG
GTGTCGTGGCGGAGGTTGGCTTTCTTGGCGAAGCGCTCCAGGGCCCGGAAGTAGTCCTCGGCGTTGCCTTTGC
CGAGGCGGAGGTCGTACATCTTCTTCTGTTGTACTTCCTCGACGGCGTGGTCGCGGAACTCGTCGTGGATCAT
NTNGCAGAAGTGGTCCCAGGNGGGGAGGCGGAACTTGCGGCGGGTGCGGCTGCGTCGTTCCGTGAGTTCCATG
GTCCACCATTCTTCTGCTTTGCCGGAGAAGNNGGAGGCGGCGAAGGCGACGCGGTAGGGGTCGGCGAACATGT
AGGCGGCGTGTACTTGGAAGTACATTTCGCACTGGGCGAGGAATCGGTCGATGTCCTCGGGGTCGCCTTTGAA
GACCGCGGGTTTGCGGAATTCGGGCTTGAGTCCGAGCCAGGGCGCGTCTTCTGCCATCTGGCCGGAGGGATTG
GGCGGGGGGTAGTCGGGGTCGTGGCGTTTTTCGGGGGCACGTGGNAGCGACCAGCTGTCTGGGTCGCGGGCTA
GGGGACTGCTGTAGCGGTCCGGGGGTGGCGANCCTTTGATGGGGCCGTCGGCTTGGGTCCAGATGTTGGCGAT
AGGGGGGGGTTCGCCGGGGTTCGAGGGTCCGGCTTCGTCCGGGCCGGCGCCGGCAAACATTCGGCTTTTGCCT
TTGCCGCGGCTGCTCGGCATGTGGACGCGTGGGGGCGGGGTTCTGGGCATGGGCATTTGGGGGAATAGGGGTT
TGTAGTCGGGCGGGTCGTCGTANGGGCGAAGGGGGTATTTTTGATTAAAATCGTANACGGCNCGTTCNACNAT
GCAATACTTAGTAATATTCTCCCGCTCCGGATCCTCCTGCGNCAGGTTCTTCCACGATCTCCCCATCTTCGAG
GACAGGAAGGTCGGGTCCGAGGTAAGGCCCNTCGAGTGACCTATGCAGATTCTCGTGAATTCTTGTTCCCACG
GATCGGGGATCCGNTGGACCAGCAGCGATAACTCGCTGACGCTCGGCGCGATGCTCCTCGAGGGAGATATCGC
GGGCTCCTCGTCGGATATGACGCTGGCGGTTCGCCTCGAACTGCGAAGCGAACCGNTGTCCGTAGANAAGACG
CTCTGCGGGATCGAGGGGCGGGGGCGCGTAGTTNGCGCCGAGTCGGTTGCTGATCTGCTGAGGTCTTCCCCAG
GCCTCGTTGTACCGGCGGAACTCGTCTTCCTCGTACCANCGCTCCGCCTCNGGGCTGATGTTACGGAGGCGGT
TCCAATGCTGNCGCTTCCTGTAGTCGTCGTCGTCTTCTCCCTCCAGCCGAGCGCTACCCGCGCGGCGGGTGTC
GTGAGCGACTCGTTGCTCGTACTGCCACTGCTGGTACTGCTCTCGATTGTGGACTCCGAGGTNATGGAGGCGC
TCCTCGAGATAGGTCTGCTCCTCGGGAGNGACATGGCGGAGGTGAGCTGGGCGATTGACGGGGCGGGGTTCAA
TGTCGACTGTGACGCCGCGGAGACGAAGGAGCTCGAGCTCTGCGGCTCGCTCTCTGTTTCTGCGGGCCTCTTC
GAGGTGACCGAGAGTCGGAGACTCTGGTCGCGAGGGATGCTGGGTGGGCCGGAAGGCCTCGAACTCGCTCCGT
GGTGGGCGAGTGGGGGTGGTAGGGCTCGTCGTCCTCGCTGTCGCTGAGGGTGATGACCGGGAGCTCCTCCTCG
TAGAGCTCGAGGGAGTCTCGGGCTCGAGGAACTCCTCGGGGAGCTGNGCGTTCCGNAGNCGAGGAGTCGGCGG
TAGTGGTTGCGGGGACGACGGCGGGGATGGGGGTTGGGTCGCGGACAAGGACGCCGTGTCCTTGCTCGCCAAT
GTCGCTTTCGACTTCCTGAACTTCAGNGATCCCAGNAGGCTCTGGGCGAGCTTCGGGATCGGGTACGGGGTCG
AGCGATGANGGGAAGGATGGTGTCGGTCGGTTGGGCTGGGTGGCGCCATAGCGACGGAGGTGCGGGAGCTGG

GGCGGAAGGGGATGGCGGGCTGGCGACGTTGGTCTAGCCAATACTGAAGCTGGAGGCGCCGGTGGTGGTACCA
ATCGTGGATCGGCCACCTGTTTCGGTACAGGAGGTAGGTGAGGGCGGGTATCCCGACAAAGACTGTCAGGANG
ANGGCGATGGCGAAGTTTACGGCGGNACTGGGCATTCGGTTTCGACAAGCAATGGTGGTTGCTT
>Armme1_1_Cluster8#LTR/Gypsy
ACAGTGTTACAATTTTTCCCTTATCTTATCTTATCCAAATGTTTCTCGCTAATCTCATCTTATCTCAAAACCT
CTATCCATATTACCATGACCTAACCGGACATTCTCAGCCCTTATACTACAGCTTGCTACCATTGCTCGCTTAA
CATATGGACTTCACTTCTTTAATGCATGATCCACATGGACAGGATCTATTCTTAGTCACTCAGTTTCCATATA
TAAGTAGTGTAGCTCTCGCTGTAGTATCCTCAGCTTCGAGCTAAAGTTTATTCATACCATACTTTACTCTCGC
AACAGAACTCAACTAGCCAACCTAGTCCCTCTTTGCTGAGTCTTAGATGCCTCAGCTATTCCCCGGTGATTGT
CTGAACATGAGACAGCCGAGGTTTAGGCTAAAGTCTTAGATCATGTGAAGTCTGGGTCTAACAGTTAGCTCGA
AGCTTCCCTTCCTGTCTTGCCAGCTGCGAAGAAACAAACCATCCATGAACCTATTCATACTTGCCGCCATTAT
CACCCTCACCCTTTTCTTTGGATTCGTAGGAATCCTCATTCTCATTGCCGTCTCCATATACTACGTGGAAGAA
ATAAGGGATTGGCTGGGCCAAACCCTCATCCGTGTCCTCGAACACATCACAGAACAAAACCCCGACGCCCACC
ATGTTCAGATTGAACAACCCATTCTGACCCCACTCCCCCTCCGATGCCAATCCACCCACACCATGGACAGCAT
GAGATCCATGTGGAGGGGCTGAATTGGATGCTACGAGGAGGGACCCCCTATCCTGGCGAAGATGACTCTGACA
CTGACATCGAGCCCAACATCCCCCAGGAGCTGGAGCGGCTCGAGTCTAGGGATGTCAGGATTCCTTACACCCC
TGAGCAGATTCAGATCACCTCCCCCTGGACTGAAGAGGACCCCCTCCCATTCGCCTTTGCCGAGCGAACTCCT
CCCCTGTCTTCGACGACAAAATCCTCTTCCATGCCCGTGTCTTTAGCCACGATGCCCTCGATGACCCCTTTAA
CCCAAGGGGCCTCAACTACGAGTGGCCTGCCCTCTCTGCCCGTGACAGAGAAATATTCAGCCCTACCAGGGCC
GCCGCCTGGATGGTCCGCTGAGCAGATGTCGAGGCCCATGCCACAGGACCTCTACCCCCTCAGTCCTTCATGG
ACAACTGGCTGGCCATATCAAGTGGAGACCCCATCGAGTCAGGAGGCATGGTGGCCAACATTGAGGTGAGGCT
CGAGTCCAACCGAGACCGATCCCTATTCCCTGCAAATGCCGGCCCCTGCCTTCTCAGGCTACGGGAGAGAACC
CCAGAGGAACAGAGCAACTTGGAGCTCACCCTCCTCGATCTTGCCACTGACCACGAGGACTACAGGGCTCAGG
GGCGAGGTGAAGAATTTGATTAATTTTAATACGTACGATACAGAAGCCTTTGGCCAGGGCGCAGCATATAGTG
CCCCCAATATATCCTTTTCAGCCTATGACCTGATCAAATACCCAAGACCACTTCCCGACAAACCCCTGTTTCC
CCCTCCCCACGCCAACATGCTGACAAGCTGATACCGGCTACACGGCATGCCACCAAGCGGCACAAGCCAACTA
TTCGCCAGAGGAGATCATGACCAAGGAGAAGGAGGGTCGGGAGCAGGAACACAGCCAACCCCAGAGCAACGAA
AGCAACAGGCAATTGCGGAAAACGCAGCCCGAGCCCAAAAGGTCGCAGAGCTTCAGGCCCAGCAAGCGAAACT
AGCCGAAAAATTCACACAAGCAGAATCCGACTGCGCTTGGTTCGCCAAGTTCTATGGGTTTGAAAAAGGAAAA
GAACCCAAATGAGGAAGACCCCTTCCCCCAAACCACATGCAACGACTCAATGACCGCGATGACTGACACTCCA
TTATGCAGCCCTCAAACTTTCCATATCCCAACATGCCATATCCACATCCCATAGGGGAAGCTCCAGAGGACAC
CCCCTACCTTCACCTGAAGCCACACTGGAAGACACCGGAACTGTTCAAAGGCCATCATGACGACATTGAATGA
TTCCTCGGGGACTGTGTCATTTACTTCGAACAGTTCTGGGGTTTTTTCCCCTTCCCTTCTCAAAAAGTCGCCT
GGGCCGCTGCACTGTTCCAAGAAAAAGCTAAGGACTGGTGGGTCTACCGACACCAGGACTTCTGGCAGCCCTC
AGACTACGATAGACAACCCACACGATTCCGATATCCGAGTTGGGAGGAGTTCACGGATATGGTCGCCAGCCAG
TTCCGAAGCCCGGCAATAGAAGAAAAGCATGAGCAGGAGATGAATGACCTACACATGGGCAATGGTCCAGCCA
ACAATTATTTCCAGGAACTGGAATGATTGGCCAAGCTCGCTGGATGACGAATGGAGGAAGAAGACTGCGGCAT
CATGGTCCGAGCACTTTGAAGGGGAGTCCCTTCCAGCTACATTCACATGATCACCAATATTGGAGACAACGTT
CCCACCATATACTCCGGATGGAAGATCCGCATCTGTAAAATGTATGAGGAACGACAAAAGCAGCACATCATCC
AGAAGATGCTCACCTCATCCGACCCACACCTTGACCACCGACAAAACGCCAAGTCTGCACCACCCAACCCTAG
CCCCAGCATTTTTGCTGGGGCCACAAGTCCAACACTGGCAAAAACAACAGGAGGGGGAGGGCCAAACAGAGAT
GCATCCATCGGGAAGTGGGTCCCAGTCAAAACCACTACGTACACAGGACAGGGACAGCCAATGGAGATCGACA
GGCTCAGAAGGGAGGGCCGCTGCTACCATTGCAAGGAGAAAGGACATTTATTGAAGGATTGCCCAAAAAGAAA
GAAGTACAAGGACATTCGCTCAGTAGTTACCACCAAGCAGGAGGAGAAATCAGAGTTGAAGGTTGAGGAGGTA
AAAGAGTAGGTCGCCAGGGGCTCAATTCCCCCTCGGCGACCAAGAACTTCACTGGTGTGCTTTCGTCTTCCCC
TGGACGGTCTTATGCAGTTCCTCTAGTTACAACAAATGTTTTATCTACCGCTATAAAAAACACTGCTTTTAAC
GTCCCCAGCACAACTAGCGAACCAGTACCAACAGAATTGAACCGCTACACCGCTCTAGTGAAATTGAGCGACT
CGGACTCAAGCGCTGATGAGGCCGAGACCTTTGATGCTGAGCAGAGAGCAGGGCTTACACCAGCCCTGGCAGT
GAGCCCCCACGACCTGCCAGAACCCCTAGCACGGGAACTGTCTACATGTGAACCCCTCGGGGGGAACACCTCT
GTAGAACTGGCTAAACAGAGCGCCAAAACCCGCAAGGGAAAGGGCTCTGGAAAGCGACCTCAGGAAGTCGCAT
TGAAGGACAAAGCCTCAGCAGGCATGCTGCAACAAATGGTCCCATGAGTCGAGGAGCGTGGAGGAGAAGAGCA
CAGCCTCACCAATGGCCAGGCATCGGAGCGCAGGGACAGCCACGGCTTATTTGATGAGAACAAGATGGGCATG
GGCACGTCCCCTGTCCCTCAATGGGATGGCCACGAAATACCAGATCGATGTGAGTACCCTCGATGGGCCTTCC
CCCCCATCACTCCTCCTGCCGGCCCCGATGAAGATGCACGAGTGGTACTGGATGCCAAGAAAGCTGTAGGGAA
GGAAGTCGTTGGCGCCCAGACGATAAAAAGGGGGTGTGCTGTTACCATAGAGGAGATCCCCGATGAAGAAGAT
GAAACCACTTACCAACGGTGGTGGACAGAGCAGAACGCTGATCAGAGGGAGCGAGACATCCAGAGCGACGAAG
ACTGGATCCCCACCAAACCTGATACGAGCGCTGCACCGCACTCTCGGGTTTGGTACAAACCCTTCAAAGTCAA
CTGGACTCTCTGCACCATTTGCGAAGCTCACACCTCTGAAGAAGCAAGGGCCGCTTTGAGTAGTTGGATACAC

AAGGACCGGCACCACGAAATTGTCATGAGCCTAGTCAATAGACTCCGACAAGGTGGGGAGGAGGCGAGGGAAG
TGATGATGGAACTCCGAGGACCTATCTGTTACATTCGAAGGACGAAGAGTAACAGAAGGGAACTCACTGTCGA
CACCCTCCTAACCACCTGCACGAACCGATGTACCCTTTCCGCCTCCAGACTAGTTGACAGCGGATGCACCGGG
AGCACTATCAACTGCTCCTACGTCCAGAAACACGGTCTTGACACCAGGAAGACTGCTGCCCCTATCCCTGTAT
ACAATGCCGATGGAACCCGCAATCAGGCAGGAGACATCACTGAATTTGTAGAACTTCGCCTTTCCATAGGGGG
GCACCACGAACAAATCAACCTTGCAGTCACTGACCTCAGCTCAAAGGACTTTTACCTCGGCCATGACTGGCTT
AAACGCCACAATCCAGTAATCAATTGGAAGACTGGATCCATCATTTTTGGGTGCTGCCAGTGCACCAAAAACC
CTTTCCCTCTCCCCGACGCAGACCCAGAGGATCGATGGGATGAAGAACTCAAAGAAGGGGAAACTATCCTCAC
TGTAAATATGCAGGAGGAGCTAGTTGTGCATGTGGTACACCATGCCAATGACCTAGCAGCCCAGGCCAATGCG
GATAAGCCAAAGAAAACCTTCCAAGAAATGGTCCCCAAACATTATTGCTCCTATTGTGACTTGTTCTTGAAAG
ACAACTTCGATGAACTCCCTGACTGAAAGCCCTGGGACCATGCCAACAAGCTCATCCCTAATGCCAAGTCCAC
CCTCGATTGCAAAGTCTATCCCTTGAACTGACACGAACAGGAACAGCTCAACCAATTCCTTGACAAAAACTTG
GAATCGGGACGAATCTGCCCCTCTAAATCTCCCTTCGCCCCACCCTTCTTTTTTGTCAAAAAGAAGGATGGCT
CATTACAACCAGTACAGGACTACCGAAAACTCAATGAAATAACAATCAAAAATCGCTATCCGCTACCACTCAT
TTCCGAGCTCATTGACAAACTCCAGGGGGCCAAATATTTCACCAAGCTAGACGTACGTTGGGGATATAACAAC
ATCAGAATCAGGGAAGGAGATGAGGAGAAGGCAGCCTTCTGTATGAACCGAGGGCTCTTTGAGCCCACTGTGA
TGTTCTTTGGTCTCACGAACTTGCCCACTACATTCCAGTGGATGATGAATGACATCTTTAAGGACTTAATCGC
TGAGGGAAAGGTTACCATATACCTCGATGACATCCTCATCTTCACCAAAGACCTAGACGAGCACCGGCAAATC
ATACAACACGTACTACAACGCCTTAGAGAGAACAAACTGTTCCTCAAAGCTGAGAAGTGCGAATTTGAGGTCC
TTGAAACCGAATACCTAGGCGTCATCATCAGCCAAGACTCTGTCTGAATGGACCCTGTCAGGGTTGCTGGGAT
AACAGAATGGTCCACACTGATGAAGAAAAAGGAGCTCCAGTCATTCCTTGGGTTCACGAACTTCTACCAAAAG
TTCATTCGCGATTATTCAAAAATCATCCAACCACTTACCCAACTCACCGGAAATGCAGAGTGGACCTGGGGAG
CGGCCCAGAACCAAGCCTTCCAACAACTGAAGAAACAGATGGCAGAAGATGTGGTTCTTACTATCCCCACAGA
AGCAGGACAATTCAAAGTAGAAGCCGACTCGAGCAATGGTGCTATCAGTGCAGTCCTGAGCCAGAGACGTGAT
GAGAGATGGTGACCGGTAGCATTCATGTCCAAGTCCTTAACCATCACCGAAAGGAACTACGAAATCTATGACA
AGGAATTGCTCGCCATCATGCTTGCCTTGTCAGAATGGAGACACTACCTGATGGGAGCGATGGAAGAGATAGA
GATCTGGACAGATCACCAAAACCTCTAATATTTCCGGAAGCTGCAGAAGCTCAACTGACAACAGGCACGATGG
GTCACCGAACTAGCTGAATATCACTTTACCCTTCATCACCAACCTGGAACCGCCAACAAAAAGGCTGACCTCC
TTTCTCGTCGAGACGACCATGACCAAGGAAAGGGGGACAATCACAATATTCTTGTCCTCCAACCCTATCATTT
CCATGCCCTCATCATGCCAACAACCACTGAGACTCATAACAAGGTCAAGGATGCCCTCAGACGGGAAGAACTC
TGGAACCAGGGAATCGCCAATTCTCTCGCACATGAGAGAGGAATCATGAAAGAGCACGGACTCCTCATGTACG
ATGGACAAATCTATGTCCCCAAGGACCATGCTCTGCGAGGAGAGATAATCACCCAGAGTCACAACCACATCAT
GGCTGGACACCCAGGGATAGAAAAAACCAAGGAACTTGTCCAACGAGAATATTGGTGGCCAAAGCTAAAAAGG
GATGTAGAGGCCTATGTAAAGGGATGTGAGGCTTGCCAATGGGCAAAGTCTAGTAACCAAGCAAAGGCTGCTC
CCCTTCATCCAAACACTATTCCTGAAGGACCATGGACCCACATCTTCGTCGACATGGTCACTGGCCTCCCAGA
GTCAAATGGCCACGATGCCCTTCTCATCATTGTAGACTGCTTCTCAAAGGCAATCATCCTGATAGCCTGCAAC
ATCGAACTGTCAGCCAAAGGATGGGCCAAGGCCCTTAGGGACCATGTCTACACCAAACACGGTATGCCTCAAG
TCGTAATATCTGATCGAGGACCCCAATTCGTTTCCCGATTCATGAAAGACCTATACCGACTACTTGATATCAC
CCCAAATGCATCCACGGCCTTCCATCCACAGACCGATGGACAGACTGAACGAGTCAACCAGGAACTTGAAGGG
TACCTCCGTCTCTTTGTGGAATATCAACAAGCAGACTGGAGCGACTGGCTACCCATGGCTGAATTCGCACACA
ACAATAAAGTGCATTCCGCCACAGGCAAAAGCCCCTTCATGGTTCTATATGGACAAAATCCCAGACTGGTTCC
AGACTCGACCCATCCTTCCCTGTTCCTTAATCCCACGGCCGAAACATTCACCGAGACCATGGCCGAAGTCCAC
CAACAGACCCAAAAGGCTCTCAAAAAAGCAGCAAGCACAATGAAAACCCAGTATGACAAATGAAGGAGGACCA
CTGTCAACTACAAGGTCGGAGATAAAGTATGGCTTGATGCTGGAAACTTACATCTCCCTCGGCCCAAGAAAAG
CTAGACGACAAACATGTAGGACCGTTCGAAATCCTCGAAAAAACCGGAGCCTCAGCATACAAGTTAAAGCTTC
CCCCACACTGGAAAATCCACCCGTGCTTTAATGAGAAATTACTTACCCTGTTCTTACCACCATCATTCCCCAA
CCAGCAACAACCACCACCACCGCCGCCACCAGACCTCATCAACGACGAAGAACAATACAAAATTGAGGAGGTA
CTAGATTCATGACCGCAAAAGATCAGGGGAAAAAGGGGACAAAAGTCCAAAGAAGTCATCGATTACTTCATCA
AGTGGAAAGGATGGACTCGAGAGCACAACTCTTGGGTACGGGACTCGGAGATGGGCAATGCGCAAGAAGCAAT
ACAGGACTATGAGAAATGTACTCACATCAACAGTGTCAAAATCGCATCTCCACATGAGGCCTTAACAGCAGTC
GGGACGCCTGCCTTAACGAGGGGGTAATGTTATGATTTTTCCCTTATCTTATCTTATCCAAATGTTTCTCGCT
AATCTCATCTTATCTTGAAACCTCTATCCATATTACCACAACCTAACTGGACGTTCTCAGCCCTTATACTATG
GCTTGCTACCATCGCTCGCTTAACGTACAGACTTCGCTTCTTTAACGCACGATCCACATGGACAGGATCTATT
CTTAGTCACTCAGTTTCCATATATAAGTAGTGTAGCTCTCGCTGTAGTATCCTCAG
>Armme1_1_Cluster50#LTR/Copia
AGACCTATGACAAACTTGGATACCGGACGTCTCGTGCAGACCCTTGTGTTCGAACAATGGGCTCACCAGGTGA
GGATTATACTATCATGGACACGTACACAGACAATATATGGGGAGCGTCGTCGAGTGCGACTGAGGCGAGTCAT
CGAAAGAAAGAGTTGGAGGAGAAATGGGAGCTTAATGACGTAGGGGAGAACCACTATTTTCTGGGGATGAAAA

TTGATCAAGACCTCGAGGCTGGCACAATATCGTTCTCGCAGTGCCCATACTGGGAAAATGTCGTACTGGATTT
CGATCTCGGATACCTCACCCCGTGTTCCACACCCTTACCGGTCGGCATCATCCTGGATAACTCGCAGTCACTG
AATGACCCCAGCTCCTGAACCGACTTTGCGCCGATCAGCGCGTGTAACACGACCTAGCCCTGAAGCTGCCGAA
TCTCTATTGTCTGAAGAACATGAAGCCGAGGCCCGTGCGAGCGGAAACGAGTGGGCAACCAATAGCAAGCACC
CACGAGCCTCGATGGCAGCATCCGGGAATAGTCTGGATCCATATATCCCACGGAGTTACAACGATGCGCTACG
CATTGACGAAGAGTGCTGGAAGGCTGCGATGGATGTCGAGTATGGCATGCACATGCTGAAGCATACCGATGCG
CTACGTGTTGACGAAGAGCGCTGGAAGGCTGCGATGGATGTCGAGTATGGCATGCACATGATGCGCTACGCAT
TGATGAAGAGCACTGGAAGGCTGCGATGGATGTCGAGTATGGCATACACATGCTGAAGCATATGTGGGACCTG
GTCGATGCTCCGCAAGGTGCAAACATCATGGACTGCAAGTGGGTTTATGGGATGAAATGGGATGGAGATGGTA
ATTGGCTCAAGGATAAGGCGAGGCTCGTCGGAAAGGGATACACCCAACAACTTGGTCTCGACTATAACGATAC
TTGGGCAGGCATAACTCGCCTTGAGTCTGTCCAGATGTCAGCTGCCATTGCAGCAAAGCTTAATCTTCATCTC
TGGCAAGTAGATTTCGTCTCTGCATATCTAAACAGTGAGACGAAGGAGGACATCTACATGAACCAACCCCCAG
GCTACGTCATCGAAGGCCAGGAAGATAAGGTATGCAAACTCATTCATACAATCTACAGGACCATGCAAGGCGG
CCACGATTGGTTTGAGACACTTGGGAAGACCTATGACGAACCACCCTTAGATGCAGTTGTAGGCCTAAGTCTA
TGGATATATCCCATGGGTCCCTAGGCTCAATTGTGGATATTATCCATAGCATATGAGAGGTCATTCCACTGCC
AATTTGGCCTCTTTATATGGATGCATTGAGCAAATATCCACAGAGCCATGGATATTAGCCACATCGCTGTCCT
CGGTCTGACCCAAAACAGTTCCAGTATTAAAAAACACACAGGACATCCTTATTTGGCAACTATGCAGGTACAT
CATTATCTATGGCCACCTCGGCATCCTTCTCAGTCGTGGTATCTTCCATCGCCTCCATTGCCATTGCCTCCTC
CGCAGAGCAAGGGGGTCATATCACCGTAACACCAGTCTTGGATGCCCACAACGACTTGAACGAGGCCATAAGG
TTCCTCCACATCGCAGCCTGCCTGGATGCATAAGCTTGCCTACCTTCAAGCAGTGCCGCCGATCCCTCCAAGG
CCTCACTGGCCTTTCCCAGCCTTTCCCAATCTTGAGCACGTAACTCCAACGAGATAAGCGTTCGTTCCATTTC
CACCTTGAGGAGCACATATTCTTCATGCCAACGATGCACCCGTGCCCGACTCTTGAGCCATTCCACCCTAACC
ACTGCGTGCAAGCCAACTAACTATGTCGCCGCATCAAATCAACCTGAAACAGACTTACCGCCATTCATTTCGC
TGTTATCAGCATCATCAAATGCGCCCTCCATGGTCCAAATCCAAGACACCGCATGAAATCCCTCAGCCTCACC
AACCACCTGTTGCGGCGCTGTCTTCTTCTTTTTGCGATAGTGTGCCGCATCCTCCATTCCATCCCCCAAATCA
ATCGTAAACATGGCTCCTTCCATGTTTGCCACATCAGCCTTGGTTAGCACACGCAATCTGCTCTCCCAAACTC
CGCTTCCCCGCAACGCAAGCAAAGCGGACCTTGCAGCATTGTACTTGGCCACAGCTGATTCCATCCATCGGGT
CAAGCGATCCATGAATGCTGCAGCCTGAGTCATATGGGCCTGTCCTCTCATATTTGCATCTCGATAAGCGTAA
GAATCTCGCTGGGCCCGCATGATCCCACAGATGGTGTCTAATGCATCCAGGCACTGAGCCTCGCACAACTGCT
CCTCCATATCTGCGACTCCAGCTTTACAGCCATCGATTCTCTGCAAAAGTGTCAATGCAGATGGTAGTAGCAG
CCGTTCAGCCTCTACATCCAACCCATCGATCCTCACTGGTAGTGACTGTAAAACCGACTCCACGCATGGCATA
TATGCTTTTTGCATGTCTCGAAATTGGGTGACCTGTCGTCGTAATGTGACCTGTTTACTGTGTTGTTCCTTGC
ACTCCGTTAGTGATAACGGATTCTCTGTTGTCTCCCGGAATTCCTGTATGAGCTGTCGCCTGCACAACACCCC
CATTAACCCACGTAACTCTGGAATAAATCGAATACGTACTGAGCATCCTCAATCAATAGTCCCATTCCCATCG
TCGCCACAGGACCACACTTATGCAGTTTAACCACACCCTTTGTCGCTGCCAGCCTATCCTGCTCCTTCAAACA
ATGCTTCACTGCTGCCTCTGAAACATCTACACCAAGTCCAGTTGAGCTAAGGCCCCAATACAACAATAGCACA
TACGTTTTGCCTCACGGAAATATGGATTGGGAGCTTCGTTGTTCTTCTCCCATGCTTCGACCTGCACCGTCCA
TTCTTGTGCCCACTTGCAATCGGTTGGTAACGAATCAGTGAAGACGGCGTGGATCGCTGCATGCTGAGCACAC
TGTGCTGCTGCCAGCACCATATGGGTGTGGAGCAAGTCTCCTATGCACAGTCAGTCAAAGATGGCCAACCAAT
AAAGGCCCACTCACCGGTCCTCGTCGTCTTATGCCAATTATGACCTCCAAACTGGTCGTCGATGATGTCGTGG
CGCGCTCCCGGGCCCATCTCCTTTGTAGATGATGCACACGGGTTCATATCATCCCAGGTATGTTCTATCCCCT
CTCCATCTGTCCGCCCTACACCTTTCTTCAAGTTCATCGAAAATTGGCACTGGCATGCATATTTGTGAGCCTT
GCAGTGGAGTTTCGGCACTCCGAAATCGAGTGCTAAGTCGGGCCGGATATGCACCTGGTCGGGGAGCTCCTTC
ATGCAATCACGAAGCTTCGCTCTCCACTGACAAGCAATGTCATATGAGAAAAAGATCTCATTGCATTTGTCGA
ACGTTTGGGCTGCCGAACCCATAATATAGTCCATGTTACAGTATCTAGCCAATGTAAGTCCTTGCACCTCAGA
TAACATACCACTACTCGTACCTTTCCCCGATCTGAAGGTCTCCGGCGGCTAATGGAACGACCATCTCGTGACA
GGCGCATATGCACAGACCGACACCGGTGGCTCACAGACCCTTGTTATTTTTGCTCTCCGCATGTGCCAGGGTC
CTAAAACCGCTGCAACTGCTAATCTGCATTCTGTTAGTTACATCTCACAGAACCTCCAACAAAACTCACATCT
TTCTGCATCGCATACTTGTGAACATGTGCCAAATAGTTCTCATGATCCACATAGTATGCCAGGCCAGTATGTA
AGCCGGGATTGACCTCTTCATCCGATTTCCGTAGATTGCTAAGCCGGAAGTTTGCATCGAGGCCCAGAACAGG
ACGATAAATATACCTGGTGATATGGGTTGGATCATGAGTCTACCATACAGCAGAATCAAATTTTCAACCTACC
ATTCCTCTGGAGGTGCCTTCTCCCACCCATCTGGTAGATTCACACCTGGGAATGGACAAGCAAGACAGCACAG
TGCAAGCTCACCTGGCGCTGTTGTCTTGATACCATTCTCCACATGTCCTCGTCCGAAACGCTTCATCATGCGT
AGATGATGGAATTGCTGAACCATTCGAAAAAAAGAACGATAATGCATCTGCAGAATAATTAGCCCATCTCCAT
GAACAACCTGAAAAGAAGCCTGACCTTGGGCACGTCCACATCGGTGTTGTCAGTCATATGGTTTAATCCACGA
TAGAATTCAAAAACCAATATCTTGCTGGCCAGTGTCTGGGTGTGGAACAGCTGCAAAAGTGCTATTGTCGCGC
AGCTTCGTGGACGCCGTATGGTCGCAGGATACCAAAGGTAGTGTAGACATTGGACGCGAACCAGCATACGCAT
GATACATGCACAGAAGTCGACAGCGACCTTATGGATGCCATTGATGTGGAGCACCGTGAAATCGGCAGGTGCT

CACTCAGGATTGGGACAAAGCCCGGAAACATGACCTAGCTGCACCCTCAGACCACAATCCCTCAGACAATCCT
GAGCAAAGTAGCTTCCGTTCCAGTGCTAGAACTCACTGTCAGCGTAAACAAAGGCTGTGGGAACAAGGCGGTT
TACCTGGATTCGGTGTAATGGCGTACGCTGGTGGCGCAAGACACAACACCGGATACACTCTAGCAGACCCGGA
CCGCATTCATCGCATCTAATGATTGCCGCTGTATCCCAGTCGTGTTCGTCCTCGGCCAGGCTTGCACAACTTT
GGCAGGTGTCGCCCTCAAAATCCCCGTGGCCCTCCGCTCTCAGCAATTCGGCCAGATATTCCTCTTGATACCC
CGGCTCTCCTTCCTTCCCCATCCACTCTATGTGAGGTTTATCCTGCTCCCGCATTAGATAAACCCAGCTACAC
ACTTCACCAATACAACGTACCGAATTGTCATATCTCTTCCATCCCTCAGCCTGTGGTTCCTCTAGATTGACGA
AGGGTTCCCACATGTCGTGATCAGCATACAGATCTTCGGCTGAGAGCGTCTGCCAGTTAGACGACGATTCGTC
GAGGTACTGGACCAAAGGGGGACCCTGAATCTCCCGCTGCCATCGTTGTGCACTGGGGCGTCGATCTGTGGTG
ACTTGAATGGTTCAAGTGGTAGGCAAAGAGAAGGTTGTTTCGGCGAGATGCTGGTTGTATCGGAAGACTTTGG
GGCCACGCCCAGGTTTGGATCGGTGCGACATTTTTGCCTACCGGTTTAGTAGACACATTTGGATTTGGCACTG
TTTGCACCACCCCAGAGCTTATCCGTGGTGCTGATGTACAGCTCTTCATTGACCTTGCAGTCATGTCTAGTGA
GCAATCTTCGAGTATCGAGAGCAATGAACCCAAGTGAGTGAGTGAGTGTATGAGGCTGGGTCAGCCACACTGA
CCATTTGCAGTGCATGGAACAACTCTGAACCCATGGCATCGTAAGTGCCGTGATTGTTCTCGGAAAACTCGGA
ACTGACCTGTCACAGGCCACATGTCACTTTCTACCTCTTGCAGAGATATCAAGTCTTCCTCCCTTTCTCTGAG
ATCCAGCTAGCCTGGTTGGACCTCATACTCCCAGACTTCGAAGAAGCCGCCATCACAGCGAAGGCCAACCGCT
GGCTGGACGGTATCTTGTTTCCGTGTTGGTTTGCACGATGGCCGGAGAACCCCTTCAACATGTTTTGCCAGGA
GGAGGAGAAAGACGACATCGTCACCCGCAAGCGTGTATGTTCTGCTTATTTCGTTTTTGGTGACTCGATCCAC
TAACATTTTAATCCTGCAGGCTTTGCTTCAGATACTTACCATGAGGCTGGAAACACAATGGAGCATGCCTGGT
GGAATGCCGGAGGTCTTTGGCGGGGATCCAGAGTGGGTGATGGAGATTGTGGATAGCCTGCATGATAGGATTC
AGCGTGACTTCGAGTAGGTCTCTACCTCAGCGGCGTCCATGTCTTAGTTTTATTTCAAATGGTACCATTGTAA
TGCAGCATCAAAAATTGTAGAGTACACTACGGGGGAGCCAAAAAATCGATCACGGTATGAGAACCCTCGCCTC
CATCGCCGCGCGTCTCAGCGTCGGTCTGTTCTTCTTCGCTAGGGCATGCGGCATCCAAAGCTGCATACAGCGC
CTTGGACAACTCCGCGCCTCCCAAAGATCTTGGCGGTGCTTTGCCTTGGCGAACACAGGTCCTTGCACTACTA
GCTCAGCTAGCCTCCTGTTCCTCTGCAGCTATGATTGACTCTGCTAGGACCCTCGCCTCCTCCACCTCATGCA
TGTGATTGGCATCCAGGCGGCAGAGACGTGCTTCGTCATTTTCAGGCCCCTCACAGACATCCGCGCATAAAGC
GGTGCCGACGTCAGGCCACATCCAATGGGACCAGTTCAACATTTTGATCAGATCCTGAGCACCGTCAGTCTTG
TCCTTTGTACAAATAAGAGAACTGACCTTTCGACGAGCGGCCTCAGCGGCCTCCAAGTCCAAGCCAGTCAAAG
TCGACTTCTCTGGGTACTCCACAAACCACTCGGCAAAAAATCTCTCCAAAAACCATTGCAGGTGAGGAGCAGA
GATGCGCTTGGCCTTGAGGTAAAATACCACCTTCTTGCGGAGACGCATGTGCTGCTCGGGAGTATACTCTGGT
TGCTCTTCTGCCCATATCTGATGGTAGGGATCGACATAAGTTATGATCAAGGAGTAAATCAAATACTCACTTT
CGGCATCGGTGACAATCAGAAAATAGTTTTCACGGATAAAAAAAGCACCCGATGTGGTTTTCAATCCGGAGAT
TGCCAGGTGGCTGAGTGTGGCTGAATCAACTGTACAGTTGCACCCCGGGGATCAGACTATATTTTTAGCACAA
TTCTAAAATATTTGTGGAGGTATCAAGATGATAAGATTGAAACATGATGCGACAGTTATATTTCAGGGAGAGA
ATTCTGCAAGTCGATCGTAAGAGTGCTGAAGGGGTCGCTGTGACCAGGGTCACTATCCATCTCCCAGTTGGTC
CGACCCCACCAACCCTCAGTCGCCTCGAACGTGTTCGACGGATCACCCCACTTCTCAACAGGCTCCCGATCCC
CCCATGTCATCCCAACTGGCTCCCCCCAGGTTGTATCATCCTCCCAAGACTGAATTTCTCCCCAGGTTGACGT
CCCTGTTTGCATATCAATGCTCTCCTCCTGCTCCTGTACTCGCCACTCCTGCTCCTGCTGTTCCTGCTCTCGG
TATTCCTGCTGAATTTTAGTGGCCTTCAACTCGTGCGCTTCACAGTGGTGGCAACAGTCACGTGCTTCAGTAT
CCAGCTGGGCAAAAGCTGATTGCACGAACTATTACATTACTTTGTTAGCACTCGTCCCCGGCAATCAAGAGTG
GACTTAAACATACCGAGCGACATGTCTCTTCCTTGTGATCGGCAGGGTTGAAGTCAGGGTACTCCCACATGAA
TTCGTCGGTGTGTTCACGGATCCAACGCTTGCGCTCGTCGAGAGGGGTCATGTCATAGGCATTATATCGTGCC
TGAAACCACAGACGTTCACGACATTCCAGGAACTAGCCGAGAACAGGGTCAGAGATCAGAGGGGGGATAAACG
GCATTGCTCACCTCGAGATGACGCATGATGGCTGCAGTACAATAGCCCAGGAGTCTGGGATTGGCCGGCACTC
TCAAAGTCCGATTACGTATTTGACAGACGAAAATGTTTTCATTGCAGTTGGAGATAAGCGAGGGTTCCCTTCT
TCCACGCGTCTCGCAGCTCATCGGCCCCAAACATGGCTGAGCACAATAAGTCTTGAAGTAGTGATACATATTG
TTTAACTTTGTTAACCGCATCATCAATAATTTTCCATCGTGCTGTGCATCCCTCTCTTTGATAGCACCGCTGA
AGCCGACTGCCGCCGTCAAAGTCGAGTTGAGTGAACGGCGCGAGCACCTCACCAACATATATTGCTGGCTCCA
TACAGCTGGCGTCGATGGCATGAGCATAGACACCTGCAAGGAAGAGGGACGGCTCTTGTCCGGAGACGATTCT
GTCGAGCCTCGCTAGGATGGTGTTCGCCGACGAAGTCTTGTCCACTTGAACAGAATCCTTCGGTTTCTCGTTC
GTATGCCTGTAAGTGTTTGGCTTGAACAAGATATTGGCATGAGTGCACATCTGCATACCTGGCTGCTGGTTCG
TCCCCTTGAGTTCGCGTCTTTTTGGGCTTGGTACGCATTTTCTTGCGAGTGTACGTTTTGGCGTTGTCCTCTC
GCTGGTGGTACTGTTGATGTAGTTTACGACAAACTTGGTCTCGATTGCTGAAGTCTGTGAGTGACACACTCGT
CGATGCTCTGGGTCTGCAATAGGACATACCAATCGTAATATGCCTGCTGATTAACAGCATGGGCAGCAGCGCG
TTCCTCCGACGTATTATACTTTTGTGGCCGACCTCTCGAGCATTGTGGGGGTGGGGGAGCAGGATCAGGATCA
GAGGAAGTAGTGGCGGCGGTGTTGGTCATGATTGCTGGATGAGAAGAGAGCAGGAGATGGGTCATGGCTGGCT
GGAAAAGGAAGGAAGGTGCTCGTGCGAGTTCTCCTGATCAATATTGCAATCGGACGTGCAGCCCGTGTCGCGA
GGGATGTAATTAAAATTATAGACGGAATGTTGACCGATGGACATATCCTGCATAGCTGGCAAGCAACAGCTAC

AGTGAGTGCAAGGGAAAGGAGGAAAAGAGCTGGTCATCAGAGGTGGCTGCAGTTGAACTCTTCTTAGGTCCAT
TTCATTAAGAATTGATGAAAGGGTCGACTACTGACTTTTAAATGACTTTAATATGATTTTAATCCCTGGAGTA
TTTGATGGGCCCCCACGTTGCCACAGACCTTCAAGTGTTGAACTACGAGTAGAATAGATCGTATTCTGAACTA
TCCGGAAGGTCCGTGAAGGATCCATATCCTGTAGTGCTGGCTATTGACTGTTGAGATGCTCAAGTCAGGGCTG
GTCCGGAGTTCGGATCGGGCTCCTTTTATGCTCATATGCACCCGAGTTGTTCTTTTTGTTGGACTGTGTGATG
GACATGACCAGCGGATGCAATAGGGTACCAAAAAATTCAATTCTAATAAGACGCTTGGTACTATAACTGGTGT
CTGTTTAAAGTACTACACATGCTCTACAGCCAGGACCTAAAATCGCCTGCTTCTTCTCCATCGTTCCAGCCCA
CACCTCGCACTGGGGCATATACACGATGAGCGTAGAGATGAAGTTTGGGAATGGAGCGAAGGTAGAAGTTTGG
ATCGCAGTGTCTGGCCAATGAGTGAGCTTACTGAACACTAACCAGAGAATGAAGGACTGCTTACAATACGAAT
GGTTTGAAGGTCACCCTGACACAAAGCAAGGAGACGACGTCAGTATAACACAATGACAAACGATTGATGGATG
CTTACCTGGGAGGAACAGGATCCTTGCGGGGTAACCAACGCAAATGGACGAGAGCCAGGGCCGCAGCTAGAAT
GCTGTTCATTGGGATTAGTGGAGGGGGACTTTGCTTGGAAAGAAGAGACTTACTTCTCATCTTCAGTCACTTG
TCAGGGGGCTTTTTCTTGGGACATGGCATGACGTGAAGAGCTTCGTTGAGCGATGGCTGGTGTGGCAAACTTT
CGGAATTGCGCGGCAACTGCATAGTAGCTCGTTTAAGGTTTGATATCCGGATTGAGAATGCCAAAGGCTTCAA
AAAGCTATGCCGCGGAGTTGCGTCCAAGCTTCACAAAATACGTCACATCCCGTTAGCCAAGTTGACAAAAAAC
GCGTCGAGGGAATATGCCTGGCCCTGATTCAGTACGATGGCTGAAGTGAGTGATCCTTTTGGCAATGTCGCAT
GTGTCTGACACGCGGAACATCACATATGATATTGATGCTGACTTCCTTCCCTGCAGGGTCTTCGAGGTCCATC
ATCATTGGGCTAGAGGGTCGCATGAGGTTTGGATGGCAGCATGGATCGTGGGATTCTGTGCGGCAAAGCGGAA
GGGTGCAAGTGGTGGCATGGATTTTGTCGACAAGGAGATGAAGGAATATTACAAGATATTCCATTGGTCGCTG
AATCTGAAGCAGGAGCCACAATTGCCGGCTCGAAACCTGGACAAAGTATTGATGCAGGAGGAGTTGGATTATA
AGGCGGCGCATGAGCATCAGTTGCGTGGGGTATGTGATCACTTATATTTCCAAACTAGTTCGATGCTAAATTG
ATTACAGGTACTGTACCGCTGGTGGGATCGACGCGCCACGTGCATCGACGGATTGCAGACGTTCAAGCCCGAC
TTGCCTTTGGCTGGCTTGTTGGCCGAGGTGTCCGGCACTGGAACCGAGGAGACACGAGGTACACGTCCACACG
CGCACGGCGGCGCAACTGTGGTCGAAAGAACATTTCCAGGGTGCAGTCCAGGAGGAGTTTAAAGCTTGGTTTG
TGGCGAGCGGGCTGCCCGACAAGAAGATGCTGGCTGAGCAAGGGAGATGGACTTTGGAGTTTTTCAAGAACCT
GCCAGAAGATGTCTGCGAGCCATTTGAGCGACAGTTGGAAGTAGAGAGGTTGCCTCCGCCCACGGTTCGCAAG
CGGAATAAGAGGAAGAGGGAGAACGATGACAAGGGGACACAATTGGAGGATGAGCAAGGCGCTCAATGCAGCA
GTAGTTCCGTGGTGCCTGCGGTGATTCCGAGTACGACTCTCTCATCTGAGGAGACTGTCAGGTATGCTTAAAT
TTACATCTGTCATGGTGGGATGGTACTGATTGTTTGGTATAGAGTTCTGGACAACATTGCATCTGTCACGTAT
CCCTGGATAGAGACACTTGGGAAATCGCTGATGGCGAATGTGACGCTGCTGATAGCAGGGCCCCATCCAGGCC
AGGGGGGGCAAATTGATGGTGTATCGTAAGTTTTGACACTGTTGACAACCAAAATCAACGACTTATGTGTTGG
AATTAGATTAAACTACGGCTGGAATAGAGATCCAAGCCCAAAAGACTGGTCAGAAGCAGGGGGGGGGAAAGCA
CATCAGACGGCAGTGGATATGTTTGTTGGGTTTGCAAAGACATGCTATAGTGAGTAAATGGATCATGTGCTGA
AGGGATACTGGACTTACCGATGTCATAGCTCCGGAGGAAATGCAGGCACGGGTGTTACCACAGGATAACGCTG
ATGAAGACGCTCCGGTGGAAACAAGATCCGAGGATGCCGCGGGTCGAGAGGAATTCAATGGCGGGGAGCAAGA
GGAATGCCCGCCTGACACGGGAAGTAAAAAGAAAGGAAAGAGAGGAGTGAAAAAGGGGAAGGAGGGAGCGAAA
GGAGATGGTCCCCACCCAGCCAAGTGACCAAAGACGAAGGCCTTGGCGGATGTCACAAATGTAGACCCGAAAG
AACCGACGGCATCTCCCAAACCCGCACCAAGTAGCGCGCGATCCAGACTGCCTCTGGCGCCTACATCAACCGC
TGGTGGCTTGCCCGTTGCTGTGGGGTTGGTACCAGAGATCGATCCAGCTCTTCAACAACAAACGGCCCACTTC
ACCTTGCCGCCTGGAGCTTCTGGGATGCTGCCGCGTCATTTATATGATATGCTATCCATTGGGGGCTCTAATG
ATCATTCAGCTGAGTGTATTGAAGACGGAGGAAGGCCAGAATCCATGGAGGATGGTGCAGACAAGGAGAGCGA
GGAGACAAGCGCCGACAAGCCCCCCGATATGAATCTTTCACGTGCACCTCTAATGGGTGCACCGTCGTCTCCT
CCATCATCTCCTGAGCCATCATCCCCTCCCTTCCCGTTGACTGAGGCTGATATCCAAATGCTTGAAAGGAATT
TGTGGCCACCTTGGTTCAAGGATTTGATGGAGTACCTGGAGCGCTACGATCTCGGACTTCGTTGGAAGGATGT
GTTAGGGTGTTGGACTGTTTGGGAGGGGCGAGGAGGTTTCCTGGATCACAAGGGAGCAAAGGGAGCGAAGCAC
ACGTTGCCGCCAGCAGGGCATCCAGCGGAGGTAGCCGTGTGGATCAAGAACTACCGAAGACTGAAGACTGAGA
TTTCCGCGCACAATTTGGGAAGGTTCTCGAGCGATTGGTGGCTATGGTGGAAAGGTGTCCAGCCAACATGGCG
TAATGTCGGAGCATTAGATGGACCTCTGGGAATCGAGCACAGATCGCCCATCGTCGGGGGTGATTGGGAGGTG
TTGAGAAAGCGAGGACAGAACGGTTTTGTCTCTGTGCAGGCCTGTTTGGCGTGGTGGGGGGAAGCTGTACAGT
CTCACAGTGATGATGCGGAACTGAGACGGGAATGGGAGTATGTGGTGGAAGATTGCCATCATGTATTATTTAA
TTTGTTGGCAGAAGCTTCGACCTCGGGCTCTGAAGGGTAACACTCTTGTCTATTATCGTAGTCTGTTTACAGG
ATGTATGTAACGATACAGGAGTGTAATGATACGAGAATGTTCCGAGAGAATGACTACATGGCTTAACAGTAAC
TGATTTCTTCAGTTCGAGAAAACAAAGTGCCCTGGAGAAAAAAATAGTACAAGATAACCAAGTGTCCCGGACA
AAAAAAACAAACGGAAAACATATTAGTGGGTTGGAGGTAGTGCTGGCACTTGACGTGCGCTGGCAAGAAGGCA
CGTGTATGGTTGGTTGACGGAATCAGACGGATAAACATCGGTTACGCATGCAAGAAGATTTGGGGCCCAAGCC
GTGCAGCCGTGGTTGCCCTGAGGTATGGCCATCAGAATCATGGATAAATAGCAGCATCCTGTAAAAAATGAGT
GCAGTGGCCCTAAAATCCATGATTTGGGCAAAATTGGGTGCTGAATAATCAAAATTTGAGCTGCGCATGCTTC
TAAACTCGGCCTGTGGATATTATCCATGAGCTAGGCTAACCACTTCATGGATATATCCCTGTACTGTAGGGCT

GTTTCTACATCTATGCCTGGACGAACTTGGATACCGGATGTCTCGTGCAGACCCTTGTGTTCGAACGATGGGC
TCACCAGGTGAGAATTATACTATCATGGACACGTACACAGACGATATATGGGGAGCGTCGTCGAGTGCGACTG
AGGCGAGTCGTCGAAAGAAAGAGTTGGAGGAGAAATGGGAGCTTAATGACG
>Armme1_1_rnd-4_family-70#TIR/MITE
ACTGTTCGATTTCGACCATAGCTTCATGGGGTGTTACGCGTTACGCTTTATGGCAAAATTGTCATAGCTTCAT
TCACCAAAATATGATGAAGAATTTACCATTATATAGTGTCTCCGGCGNCAGCCGGCCGCCAAAANTTACCAAA
TTGGGCCGATACTACCTCTCAGAAGGGCTTGTTACGCGTTACGAATTACTGGGAGCTTCACTTCACAGGGTCC
CGTTACGCGTTACGTGAATTAACAGGTCGAAATCGAACA
>Armme1_1_Cluster54#LTR/Gypsy
GAAAGGTCGGTCTACAGACTGAACACTCAGAACGAAATGAACAATCCAGATATAGGACCAAAGACACATAGAT
TGTGAACAGAACTATGGAAAGCAATAGTATACAGAAAAATAAAGTGGGGGAATGGAAGCAAAAGGTAAAGGTC
TCAAAATAAGTACCGCAGGGTTATATCAGTAGGAACTAGCATGTGATAAAGTATGTGGTAAGCGGGCTTACAC
TCACTCAACCTCTGAAAGAAATGGTAAGGAATAATCAACAAGAAGTAAGATAAAGGAGGCAAGAGGAAAGGAA
GGTAGGTGAACAATATCAACAGGGAGGTGTTAAAAAACCCAGTGGAAAAAAGCTGTTCCCTACCCACTACCCT
AAGTAGACTCCTGAATTGAGTCTTGCAATTTCTCCCAGTCATTGAGTGTGACATTGTCAGCCATTTCGGTCCC
AGAAATCCAATGATCATGTCCAGAGTCATAACCCTTGAAACATACAAGGTATTGTTTGCCCTGACCACATTTC
CACTCATCCACAATGCAATCCACAAAATGTTCTTCAAGGCCATTAGCAGTGAGAATAGGACCGGGTTCCAAAA
TTTCATGAGAAGGGAATAGCAAAGGGTCATTCAGCACAAACTTGTGCAGGAGAGAGGAGTGGAATGTAGGGAA
GATGTCCGAGGAAGGAATATCTAGAGTAAAAGTAGAAAAGTCAGGGTGTGAATCCACAATAGTATAAGGACCA
TCGTAGCGAGGCATGAATTTCACGACACGATTCTTCTGGCCTGCTTGATAATCTCTTCAGCAATGTATGGTTG
ATAACATGACCTGGTCACCTATGGTCAAGGATGGACTAGGTTTTTGGTGTTTATTTGTATGATGTGCTTGCAA
CACCTTTGCAGCAAGAATGTTATCTGTGGCTTTAGCAGAGTTGAGTTCAATCTTTTTGATGGCTGCAGCAGCT
GCATCAACATCCATATCAGTATTTGGCAGGTGGTCAGGGACAAGAGGCTGAATTATTCAAGGAGAACGATCCA
TGCATAGCTGGAAACCAGAGAAACCAGTGGAAGCATTAACAGTATTTATCATGTTGAATCAGATGATGGGTAA
GGCATGAACCCAACCCTTTTGATTATGGTCGACATGGAAGCAAATAGCCTGGTTAACAGATTTGTTGGTGCAT
TCAGATGCACCATCAGTTTGAGGGTGAAATGCAGTAGACATCTTCAATTTAACACCCATCAGTTTATGAAGTG
CTTTCCAGAATTTTGAGATGAAGAGTTTATCACGATCAGAAACAATATCCAACGGCAGGCCATTTTCACAATA
CCATTCATTAAAAAATATAGTCACAAAGTCAGAGGCAGAAATGTCAGTTCTAGTGGGCACAATTCAAATATCA
GAGTTCAAACAATCAGTAAACATGACAATACAATTGAAACCATTATCCTCAGGCAAAGGATCAATAAAATCAA
TGGCTACAGAGTCGCCACAATTCTTGGGGATTGGCAAAGGGTGTAATGGTCCAGATGGTTTTGTTGTGGCAGA
CTTATTTCACATGCATTCTTGGCATGCCAGAATATAAGAGCTTTCTAAGTCATGTTGCATATTTGGCCAGTAA
TAAGTGTCTCAAAGGGTAGCATAAGCTTTATCAGCCCCACTATGACCAAGAGAGTCATGTGCAAGACGAAACA
ATTGTTCATGTAGATCCCCAGTGTGAGGAATCAGCAAGCAGTCCCCAATATACCAGAGACCATTCACTTCACA
GACATTAGGTAAAATTGTTTGACTGCTGACAAACTTTTTACAGAATTCATCAGCTGCATATCCTTTCTTCAGT
TCAGACAGAAAGACAGTATCTGCAGCGATGTGCAGTACAGAATTGACAGAGGATGGATCCATCCAAGAGGACC
AAGGAGGTGATTGTACTTCCTCAGGGTCAACATCAGATGCAGAATCAGGCACTTCAACAGGCATTCAAGATAA
GGCATCAGCAATGGTATTATCTTCCCCTCGAATGTAATTGATAGTGAAGTCATATTGGGACATCAATTCCATC
CATCTTACTTGTCATCTTGAAAGGTCTCACTGACAGTCAAAATTTTCCAGTGTCCTATGATCAGTATAAATAG
TAAATTCAGAACCAAGCAGATCAGAACACCATTTTGTGATGGCACAAATAACAGCTAACAATTCTTTTTCATG
TACCAGATAATTTAGTTGAGCACCTGTCAACAGCACAGAATTGAATGCTATGGGGCATGCAGTTTCCCAAGTT
TCTCCCCAGGAAAGGACAGCACCAGTATGGAAATCACTAGCATCAGTGGTGATGAAAATCTTGTTGTTGCCAG
GTGTTATGTGGTCAATTACAGTTAGACAGTCACGAGAGGCTACCAGATTTTTGATGCCATCGAATGCAAACTG
ATAATTGGGGGTCCAAGGGGGCCAATCAATCTATACAGTGTCTGAAGTTAATGGAGACAATATCATCGTGTAC
TCCACCAAATGTGGGAGGAAAGCAGCCAGATACCAGACCAAACCCAAAAAAGATTTCATTTCACTCACTGATT
TGGGGATCAGCCAGTTCAAGATCTTTTCAGCCTTTTTCTTGCAGGCAGCAATGCTAGACCTGGAGATTCAATG
CCCTAGGAAATCAACTTCCAGCAGGAAAAACTTTGATTTCTTAGGATTGCAAGACAGATGATGTTTCTGCAAG
GTATTCATAATTAAACAAACATGTTTCAAATGATTGTCAAGCAAATCAGACCAAATGATAATGTCATTGAGAT
AAATGTGACAGAAGTCACCCAAGAATTCATGAAGAGCAGCATTGACACACCATTGATGTACAGGGGGGTGAGTT
CTTTAAACCCTGAGGCATGACTGTCCATTCATACAGACCAAAGGGGGTATTAACTGCAGTTAAAGGAATAGAA
GCTTCATCAAGACGAGTGTGGAAAAAGGCATTGGTCATATCAAGTTTGGACCAATACTTTCCATGAGCAGCAT
CAGTCAAAATATCATCCACTCTTGGAAGGGGATGTTGGTCAGAGACAGTATTCACATTTAATTCCCAAAATTT
GTTAACCCAACAGAGTAAAACAGCCTTGTCCGGTTTAGGGATTAAGAAGATTGGCGAAGTATACAAAGAAGAA
GAAGGATGAATGCATCTCACTTCCAAGTGTTGATCAATTAAATCTTTCCATGCTTGTCAGTATTTGCAGGGGC
AAGAATAAGAACGAGAGGCAATTTGCTTATCAGCATTCTACAGCTTAATGGAACACATGATGTCTGTAGGCAA
TTCATCAACATGTTTTGTAGGTTCAAAGACATCCTTATATTCATCCTTAATGTCTTCTCCCATGGCAGTTAGT
CTTGCTTGAAATTCAAGAACCTCAATGCGAGAACAGACAGTGGATACGACATCAGCATCATAGATTGTATTAT
ACATGGAATCATCCTGTACAGTGAATGCAGTATCTATGGGTGTTAAGAAATGGAATGGTAAAACTTTCGAAGC
AGCAAGATTATCTGAAGAAGTCCTTTTACGTGCAAGTACAGGTTGTGGTACAGAGGGAATTGTGGGCAGAGGG

ATATTGGGATGTAAGAGGTCAAAACCGGTCACTTTATCAGTTACAGTACAAAGCACAGGATCAATGATAAGAT
TGTTTGCTGTGAGAAATGGAAGTCCAAGGATGATATTGGAACAGAGATGAGGTGCAATAATGGTGCGAATAGT
GTGGAAGTCCAATATTTGTTATGGTTATAAAGCTTTAATTTAACCCAAGTAGTTAGGGTAGTTGGTGCAGCAT
TTTCAAGGGTGATGATGATTTCTATCAGCTGCAGTAGCTGGCATATTCATAAATTTAGCTTGTTAACAAGTTG
CAGTCATATTAAGACTAAATGAGAATCATCATCAATAAGGGCACAGACGTTTACAAAAACAGAAGGTGAAATA
GTGGAACGATTTTCAATAGAGCAATGCCATATTAAATGCTCCATTGAAAAAGGTACATTTAAATTTTTGACAT
TACTCACCTTGAAAGAAGGTGAAAGTGACATCAAGCAGTTCAATGAGGTGTCCACTTTATCAATGTCTTCATC
ACTATCATTGTCAGACAAGATGTTGATGATGCAAGTGTCATTGGATGGAGGGAAGGTGTTTGTAGAATTAGAT
GAGGTGATAATAGCAACAGATCCAGAAGAAATTAATGTGGAGGTGGTAATAGCATTGACAGAAGTAGCAGCAG
TGTTGGAAGAGGAAGTATTGGGGGCTGGGATAGCCACAGTTGCAGTTGCAGTGAAGGCAGTAGATGGAGAAGG
AGCACCAGCAGTAAGTGTGATGCCATCAGCCAAAGTAATCCCTCGATATATATCAGGGTTCAGAAAACTGAAA
GGACAATCATCAGACATGTGGTCAATGTTGCATCTGCAACATTTGTGGCAACCTCAATGTTTCTGGAGGTATG
CCCTCATGTCATCTGTCAAACCACCAATTCCTTTCCTTTGCTGGGGTGCAGATGAGGATGAAGAGGTAGAGAT
GGGCAGAGGAGTAGGAGTTACAGAACCAGTACAGGAGGGAGGAGCAGATGAAGATCTGGAACATTTGTTTGAT
GTGTTAGGTTGGAAAGGGCCAGGATGTTTCAAAGAAGTTCAGGTGAAGTCGGTGAATTCCTGATATCGATTAT
TGATGTTGGATTCAAGAGTGACCATGGCAGCTGTCCATTTCTTGATCTCCCTAATCTTCCTAATTTCCTTATT
ATTCTTATATGTGTTGAGCAGATGAGGATTCAAGTGGGATTTGAAGTGGTCAAGGAGATGTTCATTGGAGATG
GTGGAAGTGGATTTCAAGAGGTATAAGAATTCTTTATGAAGACAATTTGTCCAAATGGATAGGGATTCCATGG
CAGGTTGGACATCAGCCAAAATCTTTGTACATAGGTCTGTCACCCAATGTTCATCCAAAAATTCAGTGTGCAT
AGTGAAAGCAATTTCAGCAATGGACATCTGTGCAAAATCCTTGTGGTTGGTGTTGTACCAATTCCTCACCAAC
TTGTCCTGGAAGCTAGACACAAGAGTGGGAATTAAGGCCACAGCAGAGATCTTGTCCTTGTTAACTTCGGTGC
AGTTGGTGGCTGCATATTCCCACAAGGCGATGTTTGCCATGGTAAGAGGTTTGGAGGAATCATAAATAATTGG
GATATTTTTTTGGGAAGTATGGACTATGTGTTTCTGTATGTCAGAGAACATGTCAATGTCGAAATTGGAAGGA
GAAGAAGACAAGAGTATGTGGGAAGTGATGACAGGAGTGAGTGGGCTAAAAGAAGGGGATATATGCAGTGGGG
GTGAGGGAGTATGATGGAGCAGTAAGAGTGGTGCATGACATCTCGTTGGCAAGGTCAAAGGAGCTGCAGTGCA
ACCTGGTAATGCAGTAACATTATACAGTTTGAAATTTTTTTAAGTAACAGCAGGCAGTACAGTTGATGGTGGA
TGGTATGGTTAGACTGTGCAGTAAGGTGTCAGTGAAACTGCTGTAAATAAATTTAAAAAAAAGTGAAAGGTTG
GTCTACAGACTGAACACTCAGAATGAAACGGACAATCCAGATACAGGACCAAAGACACATAGATTGTGAATGG
AACTACAGAAAGCAATAGTGTACAGAAAAATGAAGTGGGGGAATGGAAGCAAAAGGTAAAGGTCCTAAAATAA
GTACCACAGGGTTATATCAGTAGGAACTAGCATGTGATAAAGTATGTGGTAAGCAGGCTTACA
>Armme1_1_rnd-5_family-199#LTR/Copia
AAGTCNTCCACTGGCAAATGGTGCTCGAACCCGAATTGCAAGGCCCGCAAACGTTCGACNCACAATATCACAG
ACTGTTACTGGCCTGGAGGNGGGNAGGAGGGTCAGTTCCCTGAGAATTTCGGGAGGAATAGANGGAATAACCA
GGCGAACAACGCAACGTCCGAAACGTACCAGCCACGACNGCGAATCCCANCGCACCCCAGGCAAACNTGACGA
TAGCGCATGNCCCCCATATCGCNTTCATGGCGCTGGAGACGGCCTGGTGAGGAGACTAGCCTGAGGGTATCAT
AGCGAGGAGATACTNCTCGAAGGAGACGTTACCGTGTTTACTGGGGAGAACGATATCGCGTACCCGTTGCAAG
CCGGAGACGAGCTCACCGATGAGGAGATGCTACCCCTTGAAGACGTCGATACCGAGGACGAATTCGAGTTGAC
CCGTACGCCCACCGAGTCGGATTTTGAGGAGATCACAAACTCGTCCACCTCGGATTTCGACATACTCGATCTC
GAGGAAATTGACGACGATGAAGCTCTGAAATTGGATTTTAGTCTCTTCGAGGTCGAAGAATTTATATGTGACA
CACCCGTTGCCGACAGAGCNTACGTTTCNACAACGTTCGAGGGGCGGAAACCNACCAAGTCACTNCACTTCCT
TGACTCGGGCGCTAGCGATCACTTCTTGAAGGATCGTAAGGGATTCACAGAGTACACGCCCGACCATTCCGTA
CNGGNTCCTCCGCGAAGCAGTCAGAAGGTGGATTCGCGATCCTNGGCGTGGGAACGGCCAGCAAGCTCTTTCG
TCGCAGGACTGGAGGGAATAAGTTAGTGAGGGTGACTTTGAAGAACGCGTTATATGCACCCTCACTGGCAGCA
AACCTNATNTCCGTCAGCGCTCTCGATGCAGGGCCGGCTTCTTCACGACGTTTTCTCGGAGGAGGACTGGCTG
TCGTTAAGGATGCGACCGGCGCCGAGGTCTTCGCTGGNCACGGTGAGAGCGGAATGTACGTCTTGGATCCGGT
GCGTTGACGACGAGGGGGATATACCCGCTGGACCCCTACCTCGCGATGTCTTCAAGGAGCGATCCGGCACCGT
CGTTTTGTGCACACTGCAGCCCTAGCACAATCGAAGANATGGCGTCGAAGAACCTCGTCGACGGGCTCGCTGG
GGGAATTACCTGATACGAAGGTCGAGGGGAAGTGCGAGGACTGCCTGATGGCAAAACAGACACGTCGACCTTT
CGATGAGCCAACCGAGCCGAATGTGGACCCCTTGGATCTCGTGGCCACGGACTTGTGGGGACCCAGCCGCGTG
GTATCCAATGGAGGGAAGTCTTACTTCATGCCGATCGTGGATAGCGGGACATCATTCAAACACGGAGTATTCC
TNGCTGATAAATCAGATGAGACGACGATCCAGGCNTTTGACGANTTCCGCGTGATGGCCGAGAATCAGACGGG
CCGAAGAATCAAACGGCTGCGTGCTGATAACGCGTTTGCGGGGTCGAAGTGGGTCGACTACTGCGAGACGCAC
GGCATCATCCTAGAGTTGACCGCNCCNTATTCGTCTGCGGAGAACGGGCTGGCGGAACGNGCTATCCGTACNA
CNATTCAGGACACGCGTGCGCTACTGCGAGACTCGGGCTTACCTCATAAGTACTGGGCAGAGGCAGCGGCTGT
ATCGGTTTACACGAGAAATCTGATACCATCACGTCGCCACCCTGACTGCGTGCCNGCGGAGTTGTGGACGGGN
AAACGGCAGGACGTNTCGCACCTGCGTGCATTCGGTAGTACGTGCTGGGCGAAGATCCCNACGGTGAACGGNC
AGCAGGTTACTGGCGGAAGCAAACTGGACGATCGGTCGATCAGGGCACGNTTACTNGGGTATGCCTCCGGGGG
ACATTACAAATTACTCGATGAGTCCGGGAAGATCTTCGTTTCCCGTGACGTCATNTTCGAGGAAGGNCTGCCA

CGCCGCACATGTCAGCCGTGGGGGAGACTGAAGCGAACGGCGACCTTTTCCCTGATTCCCCTTCGGAAAACCT
GCAACCGACACCAGTGGCACATCGACCGTCAATGATACGCCAGTGAAGTCCAACCCGAGCCAGANGTACCCGA
AGTCCAGGAGCCAGCCGCCAGTCCGGGAACCNGCCACAACCACGTCGTTCAACGAGGATNTCGAATTCGACGC
GAGGAGCTGCCGAATCTCGCGAATATCAACAGTCTGAGAGGCANGCNAGGGATNTGGGGGAGGACTGGGCAAC
CAACTCCGTACACCCATGAGCAGCNGTCGCGCAGATGAGGAAGCTCGGAATGGACCTCGACGAATACGTGGCC
TTCCTTACCGATACCAAGGCATCCCACCACATCCCGAAGACGTACAAGGAGGCTATNAAACTCGATCCAGACC
GNTGGGGAGCAGCGATGGATGACGAGTACAATATGCACATCCGGAAGCATACNTGGGATTTGGTACCGGTAAC
CCCAGAGATGAACGTGATGGATTCTATGTGGGTATACGACATCAAGTGGGATGGCGATGGAAATCGGGTTCGG
GACAAGGCGAGGTTGGTCGGAAAAGGNTACACCCAGCAACTCGGCGTCGACTATAACGAAACCTGGGCAGCCG
TNACGCGCCTCGAGTCNGTTCGNATGACAGCAGCCATCGCAGCGAGCATGGACCTCCATCTCTGGCAAGTCGA
CTTCGTCTCNGCATACCTGAACAGCGAGACNAAGGAGGACATNTACATGCGCCAGCCAGAGGGCTACGTCGTA
CCTGGNGACGAGGACAAAGTCTGCAAGCTAGTCCACACGATTTACGGCACGATGCAGGGAGCACACGACTGGT
TCGAAACACTAGCCAAGACGTACGACGAGCTCGGATACTATACCTCTCGAGCTGACCCNTGTGTNCGAACGAA
GTGGGATACAGACGGATCATTCACTATCACCGGATACATATACGGACGACGTNTGGGGGGCATCAACGAGTAC
TGAGGAGGCGAGNAGGAGGAAAGNNGAACTCGCCGAACGCTGGGAGATAAAAGATGTGGGGGAGAAGAGCTAC
TTCCTGGGNATGCGAGTGGACCAGGACCTGAAGGCCGGCACGGTGCGACTGTCGCAACGGCCATACTGGGAAC
ACGTNATCGAANGGTTTGGACTTCAAAGTATAGCACCNCGNTCGACGCCCATGCCTGTGGGGACCGTACTGGA
CTCAACNATGGCACCNTCNTCAAANGAAGAGGAGGAGGAGATGGCAGGGAAGCCCTACCGCCAGGTNCTTGGA
TCNGTGATGTGGGGTCAGCTTGCNACGCGGCCAGACCTNGCGTACGTCGTCAGTGTGCTATCCCGATTCCAGA
ATAACCCCGGGCTGGCGCACTGGAACTCNCTCATGCACGTCATCGGNTATGTCAAGTCNACTATCGACTACGG
GATCACGTACTCNCGGGACGGACCCNTGAAACCGATGGGNTTCGTCGACGCCGACTACGGAGGNTGCAAAGAC
ACCCTGAGGTCCACCTCNGGGTACGTCTTCATGATGTCTGGCGGTCCCGTCGCGTGGAGTTCGAAACGTCAAG
CCACCATTGCCTTATCTACCGTCGAGGCCGAGTTCGTCTCGCTCACTCGGGCCGCCCAACAACTGAGGTGGAT
GTTCTCTTGGTTGCAGGAGGTCGGGTTGGAACAGGATANGCCGGGGGTGCTCAAGTGCGACAATCGCGGNGCG
GTCTCGCTGACGAATACGACGTGTAATCACCACAAGGTCAAGCATATCGACATCCGCGAGCATTATATCCGCG
AGCTGGTACGCGNTGGCGAGATCAAGGTCGACTTTATCCGTGGGAAGGTCAATGCTGCAGACCTCTTTACGAA
GCCCCTCCACCGCGACCACCATTATCGCTACCTCGGCAGACCTGAACCTTACTGACTGATCTCCCGTGTCTGG
GGGAG
>Armme1_1_Cluster55#LTR/Gypsy
TGGACACTCTATTTTTAGCCATTTAGTTTTGTATATAAGTAGTTCAGCTCTCGCTGTAGTAACCCCAGCTTCG
AGCTAAAGTTTATCTATACCAATCTTTACTCTCGCAAACCAGAACTCGACTAGCCAAGTCTAGTCCCTCTTTG
CCGAGCCTTAGACATCTCAGCCATTCCCTGGTGATTGTCTGAACATGAGACAGCCGAGGTTTAGGCTAAAGTC
TACAGTTAGCTCGAAGCTTCTCTTGTTTCCTTCACTGCCAGCTGTGAAGAAAACACCCACATGAACTTTAATG
AAACAGCCTTTGCCATCGCCTTCTCCATTGCAGGAGGAGGACTCCTAGTCAGCCTCACTGCCCTCATAGTATT
CACATACCACTACGAAATCCAACGCTTCCTGGTGAGGCGCAGACTCCTGATCCTCCCCTGACAAAGACTCCCC
TTCCACTATGTCCGACCATTCACAACCGCCGCCCCTCCCCCCGAACAAACCCTCACCCAACGAAGCAGCCAGT
ACCTCACCCCACGATCCAGCGAGGAGTTCCCAACAAATCAGCCGGAACCCCCTAGGCAGCCTACCCCAGGCCT
GTCCCAGGTGGGGCAACAACAACCCATTCGCCACTCTATGGAGAAGAAACCAAGACCAAGTCTTGTGACTCAG
GACCTCGGCAGGGACCTACAGACTCTTTTCAGATCCCCCCAGCCCGAACGAGACCCCAGCCCCCCTTCCATCC
TAGAACAAAGGTCCATCTTCATCCCACCTCATGGCCCATACGGGGCTGCCATCCTCCACTTTGGAAAATTCCC
ATCAGACAATGAAAGCACCAACTCCTCCTCCTTGCTCCCCCTCGCCCCCAAGCTCCCACAGCACATATGGGTC
GGCACTCCAGAGCCCCGTCCTGAGCGAGAGGGGGTATGGGCCGATGACGATCCTCTCAACCCCAGAGGAGGAG
ACTATCAGTGGCCTGAGCTTGCCCCCATCGATAGACAGCTTATGGGCAAACCCAGGGCACAGGTCTGGGATTG
GTGCTGGCGGGACCTGGCAAACCGACTTGGGAATTATTACACCCACCGAATTCCCATGGACAACCACCTCACG
ATCACCAACGGGGATCCACTCCCCCCAACCTACCAGCATCGGGCCATCGAGACCAGAATTCATGCTAACTGAA
ACCGAGACCTATGGTGCCCAAATGTGGGGCGAAGACTTTACGGGGTCAGAGAGAGAACCCCACAGGAGCAGGC
CAAGTTGGAGTGCGACCTCTATGCCTTTGAGGAACAGGCCTTCAGAGACATGATCGAAAGGGAGGACTAGCGG
CCCAATCTCCATCGGGAGAGGATTTTTTAATGATTTTGATAAATTTATATATGACCCAGAAACTTTTGGGGAA
GCAACCCTATTTGCCTCCAATATACCTACCCCTTCCTCGTACCCACCCTGAGATATTCCAAGCCCTGAAAGAC
TACCCAACAAACCCCTGTTCCTCAGAGAAAACCAATATTGACAACACGGCTACCTATACCCGGGAACTAGCCA
ACTCTTGGCAGGGGCAGGAGAAGACCCACTGCTGGAACCTATAGCGACAGACCCCCCGGCCCCCAGGACCATT
CAGAAACAAACCATCCTGCGAAACCAACTCGAACTCGACCGACAGCAAAATGAACTGGGCACTGAGCTGACTC
GACAAATCAACGAGCTCAAGGACCAGCTCAGGGACCTCAGAGATGAAGGACCCGCCAATCGAGCCTCTCCCAG
CAGACCACCCAACCCAGGAAGACCACCCTGAGAGCATGGCTCCCTTTATTCGCTCCCATGACCACCTGAAGGA
CCAAACTTCCCACAAGACCCACCCCCCACTCCCACAGGAACCACTCCCAAAGATGCCCTCTTTTGGGGGGTAA
AGCCCATCCTAATGCCCCCTCCAAAGCCCTTCAGAGGGGACCACTCCGACATCAGTCGATTCCTGGGGGACTG
TGAAGCCTACTTCACCATGTTCTCCATGTACTTTCCCTACCCATCCCAGAAAACCCTCTTCGCTGCTCATCAT
CTTGAAGGACCTGCCAAGGACTGGTGGGTCTATCGACGACTGGACTTCTATGATGAAGTCTTACAGAGGAGTC

GATATCCCGATTGGTCCGGGTTCATCCAGATATTCAAGGAACAGTTCTCGGACCCGGCGGTCGAAGAACAGCA
TAAGAAAGCGATGTATAGCCTCTGAATGGGGAACGGACCGGCAACCACCTTCACAAAGCTAGAGCAAGAAGCG
AAACTTGCGGGACTCCACGATAAGGAGGAAGAGAAGGATCACCTCACCCGGGCTGCAAGACTAGGGATCCCTG
TCTCCATTCACCATGCCATCATGGGGGGAGGACTGAACGTTCCACGAACGTACCCAGAATGGAAGGTGTGAGT
CATAAAAGCCCATGAAGAATGTCAAAGGGACCTAGCCTTCCAGCAGACATTTGCCCACCCAGACAGCTGACCC
AATGCAAACCCTAGCTCGCCCAATAAACCGACCAATGCAACAACCCCAGCCAAGTCAGCAGGGGGCGACACAT
GTGAAAGGGACATGGGTACTGGGAAATGGATGACCCATGGAGGACAGAGGCAGCCTATGGAGATTGACCGTCT
CTGAAAGGAAGGGAGATGTTACCGATGCCAAGAGAAGGGGCACATGTCGAAGGACTGCCCCAAGAGGAAAGAA
TTCAAGGACATTCGTTCAGTAATAGTAACTGAACAGGAGAAGAAGACCGACTTGAAAGTAGAGGAGGTAAAAG
AGTAGGTCATCAAGGGCTCGATTCCCCTTTGACAACCAAGGACTTCACTGGTATGGGTTCGTCTTCTGCTGGA
TGGTCTTATGCAGTTTCTTTAGTTACACCGCCTGTTTTATCTACTGCTTTATATACCACCGCTTTTAATGTCC
CCAGCATGACTAGCAAACCAGTACCAACAGAATTGAACTGCTATACCACTCTAGCAAAATTGAACGACTTGGA
CTCAAGCGCTGACGAGACCGAGATCTCTAACGCCGAACAGGGAGCAGGGCTTACAACAGCCCTGGCAGCAAGC
CCCCACAATAACTTGCCAGAACCCCTAATACGGGAACTGCCTGCATGCAAATCCCTTGGGGGAAACACCTCTG
CAGGACTGGCTAAATGGAGCGCTATACCCCACAAGGGGAAAGACTCTGGGAAGTGACCCCGAGAAGTCGCAGC
GAAGGATGAGGCCTTAGCAGGCACGCCGCAACAAATGGTCCCACGAGTCAGGGAGCGTGAAGGAGAAGAGCAC
AGCCTCACCGACAGCCAGGCATCAGAGCACAGGGACAGCCATGGCTTATTCGACAAGAACAAGATGGGCATGG
GTGCGTCCCCTGTCCCTCAACAGGATAGCCACAAAATACTGGATCGATGTGAGTACCCTTGATGGGATTCCCC
CCCCCCCATCACTCCTCCCGCTGGCCCCGACAAAGACATGAGACTGACCCAGGATGCCAAGAAAGCCATAAAA
GAGAAAGTTGTTGGTGCCCAGATGATAAAAAGGGGGCACACCGTAACCATGGAAGAAATATCTGATGAAGAAG
ACGAAATCGCTTACCAACAGTGGTGGGCAGCGCAGAAGGTGGATCAACAGGAGAAGGCTATCCAGAGTGATGA
TGATTGGGTACCTACCAAGCCTGATATGAGTGCCTCACCACACTCTTGAGCTTGGTATAAACCCTTCGAAGTC
AACTGGACCCTTCAGGCCATTTGCGAAGCACACTCACCAGAAGAAGCCAGAGCAGTGCTTAACAGTTGGATTC
ACAAAGACCGACATCATGAAATTGTCATGAGCCTAGTCAACAGACTCCGATGAGGCAGAGAGGAAGCAAGGGA
GATGATGTTGGAACTCTGAGAGCCTATCCGCTACATATGACGGGCTAGGAGCAACGGAAGGGAACTCACCATC
GACAACCTCCTCACCACCTGCACAAACCAGCAAACCCTCTCTGCGTCTGGACTAGTTGATAGCAGATGCACCG
GAAGCACTATTAACCACTCCTATGTTCGGAAGCATGGCCTTGATACCAGAAAGACTATCGCCCTGATCCCCAT
CTACAACACCAATGGAACCCGCAATCAAGCAGGAGACATCACTGAATTCATGGAGCTCTGCCTCTCTATCAGT
GGGCACCATGAGCGAATCGACCTAGCGGTCACCGATTTAGGCTCAAAAGACTTGTACCTTGGCCACGACTGGC
TTAACCACCACAATCCTGTCATCAATTGGAAGACCGGCTCAATTATCTTCGGTTGCTGCCAATGCACCAGAAA
CCCCTTCCCGCTCCCTGATGCAGACCCGGAGGATCGGTGAGATGAGGAACTCGAAGGAGGAGAGACTATCCTT
GCTGTAAACATACAGGAGGAGTTGGTCATTTGGGCAATACACCATCCCAACGACCTAGCGGCCCAAGCCAATG
CGGACAAGCCGAAGAAGACCTTTGAAGAAATGGTACCCGAGCACTATCGCTCCTACCGCGACCTGTTCTCGAA
AGACAACTTCGATGAACTCCCCAACTGAAAACCCTGGGACCATGCTATCAAGCTCATTCCCAATGCCAAATCC
ACTCTCGACAGCAAGGTTTACCCCTTGAACTGAAACGAACAGGAACAGCTTGACAAATTCCTTGATGAAAACC
TAGAAACCGGGTGAATTCAACCCTCCAAATCCCCCTTCGCTTCACCATTCTTCTTCGTCAAAAAGAAAGACAG
TTCACTACGACCAGTTCAGGACTACTGAAAGTTAAATGAAATGACAATCAAGAACCGCTATCCATTACCGCTC
ATTTCCAAACTCATCGACAAGCTCCGGGGAGCCAAATACTTCACCAAGCTAGACGTACGTTAGGGATACAACA
ACGTCAGGATCAGGGAGGGAGACGAGGAAAAGGCAGCATTCCGCATGAACCTGTTACGATTTTCCACTAATCT
TCATCTTATCTTATCCCAAATCGTAACCTCGGCTATAAATGGATCGCCTCAATGATGCTACCACATGTTTCTT
GCTTCCTTTACACATGGACAAGACGTTCTATTCTTAGCCATTCAATTTTCATATATAAGTAGTTCAGCTCTCA
CTGTAGTATCCCCAGCTTCGAGCTAAAGTTTATCTATACCAATCTTTACTC
>Armme1_1_Cluster32#LTR/Copia
CTTGACTATCAAAGAAACTTCGGACCCATCAGAGTTGAATACGACATATTTCGAGAGTACGCCATCGCTTCCA
TTCTTAAGTATTAATCTATACATATTGTTCGACAAATACATTGCTGTTGGCCCTTCAAGGTTTTGCCAGAGTG
TGCCATGCTTCAAATCTCTCCTCAGTCGAGCAGTACGCGAGTTTGTGAAGACATAAACTCGGTTCCAAATTAA
GAAGTGTTCTCTGTTCGCAGTACATTTTACCCACAAGAGCGACTCAATACCCACAACTATCTACACGAAAAAC
TGTGACCCCTAACAATGTCGATGTCGAGCCCTTCCATTACCCAACTCCCCGAAGCCTCCCAATTCAACGGGAA
AAACCTTGCCTCCTGGCAGGTTAAGATCACTGAAATTATCAGTGGCAAAGGCCTATGGGGATACATGGATGGG
ACAATACCATGCCCACCTACTGCCCAGGCGACTATGGGCACAGCACCGACCACTACGCTGCTCCCACTGGACC
CCACACCACTCTACTCATCCACACCGTCATATGATGAATGGAAATTCCGCGACTCCCATGTACGCTCGCATAT
CATCCTCAATGTCTCCGACCCCATAGGATTAGGAGTCAAGACAATGGGAACAGCAAAGGAGGCATGGGACTCA
ATCATAGACACTTACGCTGTTCAAAACGATATGGCCCTATCTGAAGCTCAGCGTATTCTCCAGAACATGAAGT
ATACGGAAGGTGATGACATGGACGCCCACATCCAGGACCTCCGCACAAAGCGTACTGCGGTAGATAACCTGTC
CAAAACTGGATGGGAATTAACCAATAAAGACTTTCGAGGAATCATTATACGCTCCCTCCCTCACAGCGCAAAC
TGGCTCTCTATTGTACCTTCCTTATATCAATGCAAGACATCCATCGAGATTATATCCCTCCTACAGACCCACC
ACATTACCCTGTATCCCACCAAAGAAGATGCTTCCCTTGCCCTTAACACGAAGTCATCTACTGGCAAATGGTG
CTCCAATCCAAATTGCAAGGCCCATAAATGTTCGACCCACAATATTACGGATTGTTACTGGCCTGGAGGAGGA

AAGGAGGGTCAGTTCCCTGAAAATTTCGGAAGGAATAGACGAAATAACCAGGTGAATAATGCGACGTCCAACG
TACCAGCCGTGACTGCGCCCAATGCACCCCAGGCAAACATGACGATAGCCCACGTCCCCCATATTGCTTTCAT
GGCGCGCATGGAGATGGCTGGTGAGGGGACTAGCCGAGGTGTTATAGTAGAGGAGATACTGCTCAAAGGAGAC
ATTACTGTGTTTACTGGGGAGAACGATATCACGTACCCATTGCAGGCTGGAGATGAGCTCACTGATGAGGAGA
TGCTACCTCTTGAAGACATCGATACCGAGAAGGAGACGTTACTGTGTTTACTGGGGAGAACGATATCACGTAC
CCGTTGCAGGCCAGAGATGAGCTCACTGATGAGGAGATGCTACCTCTTGAAGACATCGATACCGAGGATGAAT
TCGAGTTGACCCATATGCCCACCGAGTTGGATTTTGAGGAGATCACAAACTTGTCCACCTCGGATTTCGACAT
ACTCAATCTTGAGGAAATTGACGACGATGAAGCTCTGAAATTGGATTTTAGTCTCTTTGAGGTCAAAGAATTT
ATATGTGACACACCTGTTGCTGACAAAGCATACGTTTCGACAACGTTCGAGGGGCAGAAACCAACCAAGTCAC
TTCACTTCCTTGACTCAGGTGCTAGCGATCACTTCATGAAAGATCGTAAGGGATTCACAGAGTATACGGCGCA
ACTATTTCGTACTGGGTTGTCCGTGAAGCAGTCAGAGGGTGGATTTGCAATCCTAGGTGTGGGAACAGCTAGC
AAGCTCTTCCGTCGCAGGACTGGAGAGAATAAGTTAGTGAGGGTGACTTTGAAGAATGCGTTATATGCACCCT
CACTGGCTGCAAACCTGATTTCCGTCAGTGCTCTCGATGCGGCCGGTTTCTTCACGACATTTGGAGGAGGACT
GGCAGTTGTTAAGGATGCAAATGGTGCCGAAGTCTTCACTGGGCATGGTGAGAGTGGAATGTACGTCTTGGAG
TGCATTGATGACGAGGGGGATATACCCGCTGGACACTTCGCGATGTCTTCAAGGAGCGATCTGGCACCCCTGT
GCCAATGGCACCGTCATTTTGTGCACACTGCAGTTCAGACAATCGAATCCATGGCGTCAAAGAACCTAGTTGA
CGGGCTCGCTGGGGGAATTACTGATACGAAGGTTGAGGGGAAGTGTGAAGATTGCCTGATGGCAAAACAGACA
CGTCAACCTTTCGATGAGCCAACTGAGCTGAATGTGGACCCCTTGGATCTCGTGGCCACGGACTTGTGGGGAC
CCAGCTGCATGGTATCCAATGGAGGGAAGTCTTACTTCATGCCGATCATGGATAGCAGGACCTCATTCAAACA
CGGAATATTTCTCGCTGATAAATCAGATGAGACAATGATCCAGGCTTTTGACGAGTTCCGCGTGATGGCCGAA
AACCAAACGGGTTGAAGAATCAAACAACTACATGCTGATAACGTGTTTGCGGGGTCGAAGTGGGTCGACTACT
GCAAAATGCATGGCATTATCCTAGAATTAACTGCTCCCTACTCGTCTGCTGAGAACGGACTGGCAGAACATGC
TATCCGTACCACAATTCAGGACATGTGTGCGCTACTGCGAGACTCGGGCTGCGATGTCTTCAAGGAGCGATCC
GGCACCCCTGTGCCAATGGCACCGTCGTTTTGTGCACACTGCAGTTCAGACAATCGAATCCATGGCGTCAAAG
AACCTAGTTGACGGGCTCACTGGGGGAATTACTGATACGAAGGTCGAGGGGAAGTGTGAAGATTGCCTGATGG
CAAAACAGACACGTCGACCTTTTGATGAGCCGACTGAGCCGAATGTGGACCCCTTGGATCTCGTGGCCATGGA
CTTGTGGGGACCCAGCTGCGTGGTATCCAATGGAGGGAAGTCTTACTTCATGCCGATCGTGGATAGCGGGACC
TCATTCAAACACAGAATATTTCTCGCCGATAAATCAGATGAGACAATGATCCAGGCTTTTGACGAGTTCTGCG
TGATGGCCGAAAACCAAACGGGTTGAAGAATCAAACAACTACGTGCTGATAACGTGTTTGCGGGGTCGAAGTG
GGTCGACTACTGCAAAACGCACAGCATTATCCTAGAATTAACCGCTCCCTACTCGTCTGCTGAGAATGGACTG
GCAGAATGTGCTATCCGTACCACAATTCAGGACATGTGTGCGCTACTGCGAGACTCGGGCTTACCTCACAAGT
ATTGGGCAGAGGCAGCTGCTGTATCGGTTTACACCAGGAACCTGATACCATCACATCGCCACCCTGACTGCGT
GCCAGCGGAGTTGTGGACGGGAAAACAACAGGATGTCTCGCACTTGCGTGCATTCGGTAGTACATGTTGGGCG
AAAATCCCAATGGTGAATGGACAGCAGGTTACTGGTGGAAGCAAATTGGATGATCGGTCGATCAGGGCACGTT
TATTGGGGTATGCTTCTGGGGGACATTACAAATTACTTGATGAATCCGGAAAGATCTTCATTTCCCATGATGT
CATATTCGAGGAAGGTTTGCCACATCGCACAATGTCAGCCGTGGGGGAGACTGAAGTGAATGGCGACCTTTTC
CCTGATTCCCCTTCGGAAAACCACGCAACTGACACCGGTGGCACATTGACCGTCAATGATATCAACGAAGTCC
AACCTGAACCAGAAGTACCCGAAGTCCAGGAGCCAACCGCCAGTGGAAACCTGCCACAACCACGTCGTTCAAT
GAGGATATCGAATTCGACACGAGGAGCTACTGAATCTCGCGAATATCAACAGTCTGAGAGGCATGCCAGGGAT
ATGGGGGAGGATTGGGCAACCAACTCTGTACACCCATGAGCAGCTGTCGCACAGATGAGGAAGCTAGGAATGG
ACATCGACAAATACATGGCTTTCCTTACCAATACCAAGGCATCCCACCACATCCCAAAGACGTACAAAGAGGC
TATGAAACTGGATCCAGACCGATGGGGAGCAGCGATAGATGACGAGTACAATATGCATATCTGGAAACATACA
TGGGACTTGGTACCGGTAACCCCAGAGATGAACGTGATGAATTCTATGTGGGTATACGACATCAAGTGGGATG
GTGATGGAAATCGGGTTCGGGATAAGGCGAGGTTAGTTGGAAAAGGTTACACCCAGCAACTCAGTGTCGACTA
TAACGAAACCTGGGCAGCCGTGACGTGCCTCGAATCCGTTCACATGACAGCAGCTATTGCAGCAAGCATGGAC
CTTCATCTCTGGCAAGTTGATTTTGTCTCAGCATACCTGAACAGCAAAACCAAGGAGGACATCTATATGCACC
AACCAGAGGGCTATGTCATACCTGGTGACGAAGACAAAGTCTGCAAGCTAGTCCACACAATTTACAGAACGAT
GCAAGGAGCGCACGACTGGTTTGAAACACTGGCCAAAACATACAACGACCTTGGATATTATACCTCTCGAGCT
GACCCATGTGTGCGAACGAAGTGGGATACAGATGGATCATTCACTATCACGGACACATACACAGATGATGTCT
GGGGGGCATCAACAAGTACTGAGGAAGCTAGCAGGAGGAAAGCCGAACTCGCTGAACGCTGGGAGATAAAAGA
TGTGGGGGAGAAGAGCTACTTCTTGGGCATGCGAGTAGATCAGGACCTCAAGGCCGGCATGGTGCGACTGTCC
CAATGGCCATACTGGAAACACGTAATTGAAAGGTTTGGACTTCAAAGTATACCACCTCATTTGATGCCCATGC
CTGTGGGAACCGTACTAGATGCAACGATGGCACCCTCATTAAAAGAAGAGGAGGAAGAGATGGCAGGGAACCC
CTACCGCCAGGTACTTGGATCTGTGATGTGGGGTCAGCTTGCTACGCGGCCAGACCTCGCGTATGTCGTCAAT
GTACTATCCCAATTCCAGAATAACCCCGGCCTGGCACACTGGAACTCCCTCATGCACGTCATCGGATATGTCA
AGTCAACTATCGACTATGGGATCACATATTCACGGGATGGACCCCTCAAACCGATGGGATTCGTTGACGCCGA
CTACAGAGGCTGCAAAGACACCCTGAGGTCTACCTCGGGGTATGTCTTCATGATGTCTGGTGGTCCGGTTGCC
TGGAGTTCGAAACGTCAAGCTACCATTGCCTTATCTACCGTCGAGGCCGAGTTTGTCTCGCTCACTCGAGCCG

CCCAACAACTGAGGTGGATGTTCTCTTGGTTGCAGGAGGTCGGGTTGGAACAGGATATGCTGGGGATACTCAA
GTGCGACAATTGTGGTGTGGTTTCGTTGATGAATACGACGTGTAATCACCACAAGATCAAGCATATTGACATC
CACGAACATTATATCCGCCGGTTTCATTGATGAATACGATGTGTAATCACCACAAGATCAAGCATATTGACAT
CCGCGAACATTATATCCGCGAGCTTGTGCGTGCTGGCGAAATCAAGGTCGATTTTATCTGGGGAAAGGTCAAT
GCTGCAGATCTTTTTATGAAGCCCCTCCACCATGACCACCACTATCGCTACCTGGCAGACCTGAACCTCACTG
AGTGATCTCCTGTGTCTGGGGGAGTGTTGAACAAGACACGTGATACCCGACACAGTCCCAACTCTTACCTATA
TTGTATGCCGTTCCTTACCCGTTCTTCCTTACTCCTAAACGAAGCCCCTCCACCGTGACCACCACTATCGCTA
CCTGGCAGACCTGAACCTCACCGAGTGATCTCCCGTGTCTGGGGGAGTGTTGAACAAGACACGTGATACCCGA
CGCAGTCCCAACTCTTACCTATATTGTATGCCGTTCCTTACCCATTCTTCCTTACTCCTAAACTTCATCCCAA
CAGTATATATACCATGTAGTTACTTCCGCACCCTCAGTCATTTCACCTGTACTGTGCTGACTGAGTTCTAATC
ACTTTGTTCTTATTACATTAGTTCTGCCTATAGTTGCCACCTATGGAATGCAGTATAAACATCCGCTTCATCA
TGGCTGGTCTGCATCTCCCCTCCTTAGGCCTCCTTCCTTCGCTGAACGTGTACCTGTCAAAAACCATTTGTGC
CTCATCGCTAGTATTATTTCGCCATTTCCATCAACATTGCAGCAGCTCTGGAGTTGTGCTGTAATGGCACGAA
CTGCGAAGTGATGAAGAGACTCTATGACTTGCAGGACAGGATGTATGCTGGTTACTGTTTTTGTGAAGTAAGT
TGTACTTCTAAGTCCTACAGTGTTGCCAACTGAAAGGTCACGCTCTCTTACATTGACGATGAAGTCTATTTGA
ATTACAGATTTCACTTTCTCAACGTGCAGCCAATCCAACGTGGACTGATTACACCTTATCGCAAGGCTCTCTG
TTATGCCTTACCTAGCATGTACCCAGTAACTTCGCACCCACGAGCTAGGAATGCCTCTGCAGCTTTTCACACC
ATTGCCGCAGAGTAAACACTACCATCCAACCCATTACGATGTGTACGCATGTGTACCGACAGAGCTACACGAC
TACCTTGGTACCCATCGTCTGCAAGGCTCTCTGTTATGCCTTACCTAGCATGTATCCAGTAACTTCACACCCG
CGAGCTAGGAATGCCTCTGCAGCTTTTCATGCCATTGCCACGGAGTAAATGCTACCATCCAACCCATTACGAC
GTGTACGCATGTGTACCGACAGAGCTACACGACTACCTTGGTACCCATCGTCAAGCCTACTGTCCCATGCTCT
TCAAGTGTGTTCAGGAGAATTTTGACCATTCCGAGCTCATTAGGGCTGATATAGAAAACAATTGGGTTGACGG
TATTCTTGGCGGCGAAGAGCAGGAAGAAGGTAAGGATGAAGAAGCCGACGGCCTGGATCCACTTCACAGGATC
TTCGCCGCAAATGCTCCCAGTTCTGACGAACGTGAGGAAGACGACAACTTCCACCCTGATTCTACATACTGAT
CCCGATACCTTGACTATCAAAGAAACTTCAGACCCATCAGAGTTGAATACGACATATTTCGAGAGTACACCAT
CGCTTCCATTCTTAAGTATTAATCTATACGTATCGTTCGATGAATACATTGCTGTTGGCCCTTCAAGGTTTTG
CCAGAGTGCGCCATGCTTCAAATCTCTCCTCAGTCGAGCAGTATGCGAGTTTGTGAAGACATAAACTCGGTTC
CAAATTAAGAAGTGTTCTCTGTTG
>Armme1_1_Cluster48#LTR/Gypsy
CCTGCGGTGATATGGTCGTGACAGCGGGATATGATTTCTCCTTGCATGGCATGGTCACGTGGGATGTAAATTC
TTTGGTTGTAGTATAGGATCCCCTTGATTTCCTTGATACCGCGTTCATGGTTCAAGGAACTTGCGATTCCCTG
ATCCCACTTGGTTGGATTGCGGGTAGCACTCTTGATGTGTTGGAGGGGTTCGTCTACGGTGGGCATGATGAGT
GCCTGGAAGTGTTCGGCCTTGAGGATGATGACTTTGTTGTTGTTGTCTTGTCCTTGATCATGGTTGGCACATC
GTGAGAGGAGGTCGGCCTTTTTGTTCGAGGCACCTGGTTTATGATGGAGGGTGAAGTGATACTCCGCGAGCTC
AGTAACCCATCGTGCTTGTTGACGATTAAGTTTCTGGGGTTTGCGGAAGTATTGGAGATTTTGGTGGTCGGTC
CAGATTTCGATGTCTAGGGTGGCGCCCATGAGATAGTGCCTCCACTCCTTGAGGGCAAGCATGATGGCAAGAA
GTTCCTTGTCGTAAATCTCATAGTTGCGTTCTGTGGCCATTAGGGCTTTAGACAGGAACGTGACTGGTCTCCA
TTTTCCCTCTTGTTCTTGGGACAGTATGGTGCCGATCGTGCCTTCACTGGCATCTGCTTCTACTCTGAACCTT
CTGGTTTCTGAGGGGAGGGTGAGCATGACATCCTGGGTGAGGTGGGATTTGAGTTCATCGAAGGCTTCTTGCT
GTGCGGTTCCCCAGGCCCAGGACTTGTTACCGGTGAGATGGGTCATCAGGCGCACTACTGCACTGTAGCCTTT
GATGAAATGTCGGTAAAAGTTGGTGAACCCTAGGAAGGACTGGAGCTCCTTTTTCTTGGTGGGAGCAGGCCAG
TCAATGATACCAGCGATCTTGACTTGATCCATGTGGACGGATCCTTTGCTTATTATTACCCCAAGGTATTCCA
TTTCTAATACCTCGAACTCACACTTCTCTGCTTTCAGGAACAGTTTGTGCTTCCTGAGGAGCATGAGAACTTT
GTGAGTGATGCCAGGGTGTCCTGCGGTGATATGGTCGTGACAGCAGGATATGATTTCTCCTCGCATGGCATGG
TTGCATGGGACGTAAATTTTTTGGTCGTAGTATAGGATCCCCTCGATTTCCTTGATACCACATTCATGGTTCA
AGGAACTTGCGATTCCCTGATCCCACTTGGTTGGATCGCGGGTAGCACTCTTGATGTGTTGGAGGGATTCATC
GACAGTGGGCATGATGAGCACCCAGAAATGTTCGGCCTTAAGGACGATGATTTCGTTGTTGTCGTCTTGTCTC
TGATCGTGGTCTGCAC
>Armme1_1_Cluster31#LTR/Copia
TCACCAAAGAAGAAAACATCTTTATGAAGCAACCTCCTGGCTTCCAAGCACCAGGCTCGAAATCCCTCATCTG
TCATCTCTTGAAAACATTGTATGGCTTGAAGCAGTCAGGTCAGTGTTGGTACCAAAAACTTGTTGAGATCCTT
GTCGGTAGGATGGGATTGACTCACTGCGATGTTGACCAAGCTGTGTTCTTCAGATGTGATGGAAAGAAGTTCA
TAGTGGTATTGGTCCATGTAGACGACTGCACAATCACGGCCTCGTCACTCATCCTTGTCCACCAACTCAAGCG
ACAAATGGCCTCCTATGTCAAGATCACTGATCTTGGTGAGCTGCACTCCTGAAAGAGTCAGGTACAGACCAGA
AGCTCACCGTGCATGATACTCCCCAACAAAATGGTGTTGTGGAGCAGCGAAATTGTACCATTGTGGAACAAAT
TTGTGCCTTGTTGCATGCGAGTGGCCTCCCAAAATATCTTTGGGGAGAAGCGGCATGTCATGTTATATGGTTA
CTCAACCATACGGCAACAAAAGCTCTTGATGGAAAGACGCCTTTTGAAGCAGCCTATAGTCGCAAGCCATGCC
TTAAGGGGTTACGAGAGTGGGGTGAAAAAGTCTGGGTCCGCACCAAAGGTGGGGACAAGCTTGGTGGTCACGT

GAAAGAAGGCTGTTGGATGGGTTTTGACGAACAGAGCAAGGGATTCCACATCTACTGGCCTGACAAGAAACGT
ATCAGTGTCGAACAAAATGTGTACCATGACAATACTGCCTCACCAACTTGTCATCTCGAGGGAGAGAGTTTAG
ATGACACTGAGACAAAAGCCGATGCTTTCCTGCCTCCCACTGTTCCCCTCGAAGCAGCACCTGCACCTGCAGC
ACACTTGTCAGTTGATCCCGAGCCCATTCATGAGCCAGAACTACCCACCAAACGTGTCCGAAAGCCCAGTCAG
CGCATCCAGGACATCCTTGATAGCAAAGACACAAATTCAGCAAAGCCTGCTGACCCAGTCATAGCCTCAGGTA
TCCAGCTCCCCACCATGCTTGAAGCGGAAGGTACAGCCGAGTGGATGATGGCTGCCAACTTCATCGATGAATA
TGCGATGGCGGCTGAGATGTTGGAAGCTGAAGCTCTTGAACTGAAGTCACTCACCGAGGCCAAGAATTGTCCT
GATTGGCCCCTATGGGAAAGAGCAATCCAGGAAGAGTTGCAGATGTTGAAGGATGCAGGAACATGGGAGCTTG
TCGAGCCTCCCAAGGATGCCAATATTGTTGGATCCAAGTGGGTTTTCTGTGCAAAGAAAGATGCTGCAGGAAG
TGTTGTACACTACAAGGCTTGGTTAGTTGCTCAGGGATTCTCCCAAGTACCTGACTTCGATTACTTCAACACC
TTTGCTCCTGTAGCCAAGCTTGCATTGATCCACGCTGTCCTTGCAATGTCGGCCATCCATGACCTGGAGCTTT
ATCAGATTGATGTCAAGGGTGCATACCTCAACAGTGAACTCACCGAAGAAGAAAACATCTTTATGAAGCAACC
TCCTGGCTTCCAAGCACCAGGCTTGAAATCCCTCATCTGTCATCTCTTGAAAATGTTGTATGGCTTGAAGCAG
TCAGGTCGGCATTGGTACCAAAAACTCATTGAGATCCTTGTCGATAGGATGGGATTGACTCGCTGCGATGTTG
ACCAAGCTGTGTTCTTCAGACGTGATGGAAAGAAGTTCATAGTGGTATTGGTCCATGAAGATGACTGCACAAT
CGTGGCCTCATCACTCATCCTGGTCTGCCAATTCAAGCAACAAATGGCCTCCTATGTTGAGATC
>Armme1_1_Cluster12#ClassI/LARD
CTGACAACTGATCCAACACAAACCCCAACCCCCCAAGCAAACCCGCCAACAGCACGACCCCACACAAATCGGC
AGGGGGAGATAGCCATGGAAGGGACCCAAATACGGGAAAATGGATGACCTATGCTGGGCAAGGACAGCTGATG
GAGATTGACCACCTATCATACCCAGACATCCCGAGCTCCAAAAGATTACCCGACGAACCCCCATTCCTCGGAA
AACCCCAACACCGCCAATATGGTTACCTGTACCGAGGAACTAGCCAACTCTTGGCAGGGGCAGGAGAAGACCT
GCCACTGGAACCCATAGTAACAGATCCTCTGATCCCTGCAAACCAACCCTCTCCTGGTGGGCCACCTGATCTG
GGAAGACTGCTCTGAGAACACAGCTCCTACTACTCACTCCCACGACCACCCGAGGGGCCAAACTTCCCATGGG
ATCCACCTCCCGCACCCATGGGAATTGCTCCTGAAGACTCCCCCTTCTGGAGGGTAAAACCCATCCTAATGCC
CCCGCCGAAGGCCTTCCATGGAGAACACACCGACATTAGCCACTTCCTGGGGGACTGCGAAGCCTATTTCACC
ATGTTCTCCATTTACTTCGCCTACCCATCCCAGAAAACACCCTCTCCTGGTGGGCCACCTGATCTGGGAAGAC
TGCCCCGAGAACACAGCTCCTACTACTCACTCCCACGACCACCCGAGAGGCCAAACTTCCCATGGGATCCACC
TTCTGCACCCATGGGAATTGCTCCTGAAGACTCCCTCTTCTGGAGGGTAAAACCCATTCTAATGCCCCTGCCG
AAGGCCTTCCATGGAGAACACACTGACATTAGCCACTTCCTGGGGGACTGCAAAGCCTATTTCACCATGTTCT
CCATTTACTTCGCCTACCCATCCCAGAAAACCCTCTTCGCCACCCACCACCTCGAAAGACCCACAAAGGATTG
GTGGGTCTATTGACGACCAGACTTCTACAACGAAGCCTGGCAAAGAAGCCAATATCCTGATTGGCCCAGGTTT
GTCCGAATATTCAAAGAACAGTTCTCGGACCCGGCGGTCAAGGAACAACACAAGAAGGCCATGTATACCCTCC
GAATGGGAAACGGACCAGCTACCACCTTCTTTGCAAAATTAGAACAGGAAGCAAAGCTCGTGGGGCTCCGTGA
CAAAGAAGAGGAGAAGGACCATCTCACCTGAGTGGCGAGACTGGGAATTCCCGTCTCCATCCATCACGCCATC
ACTGGAGGGGGACTAAACGTCCCACAGACTTACCCAGAATGGAAGGCACAAGTCATAAAAGCCCATGAGGAAT
GCCAGAGGGACCTAGCCTTCCAACAAACATTTGCCCACTCTGACAACCGATCCAACACAAACCCCAACCCCCC
AAGCAAACCCGCCAACAGCATGACCCCGCACAAATCGGCAGGGGGAGATAGCCATGGAAGGGATCCAAATACG
GGGAAATGGATGACCTATGCTGGGCAAGGACAGCTGATGGAGATTGA
>Armme1_1_Cluster18#ClassI/LARD
GCCAGCAAAAACAACAGGAGGCGGGGGAGGACCGAGCAGAGACGCATCCACCGGAAAGTGGGTCCCAGTCAAA
TCAACAACATATGCTGGACAGGGACAACCCATGGAGATCGACAGGCTCAGGAAGGAAGGCCGATGCTACTGCT
GCAAGGAGAAGGGACATATGTTGAAGGATTGCCCAAAGAGGAAGGACTATAAGGACATTCGCTCAATCGTAGT
AACTCAGGAAGGAAGGCCGATGTTACTGCTGCAAGGAGAAGGGACATATGTCGAAGGATTGCCCAAAGAGGAA
GGACTATAAGGACATTCGCTCAATCGTAGTAGCCGAGCAGGAGAAGGAAATGGAGTCAAAGGTCGAGGAGGTA
AAAGATTAGGTCATCAAGGGCTCGATTCCCCTTCAACGACCAAGGACTTCACTGGTGTGCTCTTGTCTTCCGT
GGGACGGTCTCATAAAAATTCTTTAGTTACAAAAAAATGTGCTTCATCTACTGCTTTATATATCCCTGCCCTT
AACGTTCCCCGCATGACCAGCAAACCAGTACCATCAGAATTGAACCACTACGCCGCTCTAGCAGAATTGAGCG
ACTCAGACTCAAGCGCTGACGAGACTGAGATCTCTAATGCTGAACAGGGAGCAGGACTAACAACAGTCCCGGC
AGTGAGCCCCCACGACCTGCCAGAACCCCTAGCACGGGAACTGCCTACACGTGAACCCCTCAGGGGGAACGCC
TCTGTAGGACTGGCTAAGTGGAGCATCATGACCCGAAAGGGGAAGAACTCCAGAAAGCGACCTCAGGAAGTCG
CATTGAAGGATGAGGCCTCAGTAGGCATGCCACAACAAATGGACCCACAAGTCGGGGAGTGTGAAGGAGAAGA
GCACAACCTCACCGATGGCCAGGCATCAGAGCGCAGGGACAGCCACGGCTTATTTGACAAGAACGAGACAGGC
ATGGGCACGTCCCCCATCCCTTGACGGGACGGCCACAAAATACTGGATCAATGTGAGTACCCTCGATGGGACT
TCCCCCCCATCACTCCTCCTGCCGGCCCTGACAAAGATGCATGACGGACCCTGGATGCCAAGAAAGCCACAAG
GGAGGAAGTTGTTAGTGCCCAGACAATAAAAAAGGGGCCTACTGTTACCATGGAGGAAGTACCAGACGAAGAA
GATGAAACCACTTACCAAAAGTGGTGGGCAGGACAGAAAGCTGACCAAAGGGAGCAGGATATCCAGAGCGACG
ACGACTGGGTACCTACCAAACCCGATACAAGCGCCACACCACACTCTTGGGCTTGGTATAAACCCCTTTGAAGT
CAACTGGACCCTCCGGGCCATCTGCGAAGCGTGCACGCCAGAAGAGGCTAGAGCCGCTCTCAGCAGCTGGATT

CACGAAGACCGACACCATGAAATTGTCATGAGCCTAGTCAACTGACTCAGATGAGGCAGGGAGGAGGCAAAAT
GCCGGCAAAAACAACAGGAGGCGGGGGAGGACCGAGCAGAGATGCATCCACCAGAAAGTGGGTCCCAGTCAAA
TCAACAACATATGCTGGACAGGGACAACCCATGGAGA
>Armme1_1_rnd-4_family-459#TIR/CACTA
ATAACTTACCACCGACATGGTCATTAGTATTGATTTTCTGCAGCGACCCGATGAGACGCTCAAACGGAAAGCA
CCACCAGCCAATGACGGGCCCAAAGGAGATAACAAAGTCGTAAAGATGGAAGGCTGCGTGAACATTTGGTCTT
TTCTTGAGCGTCTGCGTGTGGGGATGCACTGAGTACAGCCTATCAACCCACTTCTTTAGCAGGTTCTTGTACG
TAGTGGCCCGATCAATGTTCATGGTATATCGGCAGACGATAGTGACGGCCTGAAATAGTGCCATCGAGTGGTC
TAATATTGGGAGATGCCAAGAATTATCTGGCGGGGGTTGGCCATTATTATCGCCCCATAAGGTTACCAGTGCG
ATGGGAAGGTAGATGGTGCTAAGAACGTGCCATTCATCAGCTTTGATAGTACCTGCTGAGGCTTCTCCGTAAT
TAGAGGGTACAGAGTTAATCCATGATGGTGTTGAGGTATTCTTGATGACTGATTGTATATAGGCGATGTCATT
CGGGTCGATACACCGCTTGGCTTGTATCTCATCAGACTTATGCCGTTGAATAAGACGCTGCAACCAGAAAGAT
ATGGATCAGACGCAGAGAGCAGCATATAGCAGACGTTTACATACCCAATTAATCAACAAATTCGAGAGGTCGC
CTCTAGTCTTCACTTTGGTCTCAGTCCACATTCCTCCGATTTTAAATCGTTGCATCCACGGNAAATTGAGCGT
GCGACAAACAAACTGTAGGGGCGCAACATGCTTGTTCGCTAACCTCTTTCTCAGTGTCTCAACCGAAACGTCG
ACTATCGTNCCATCCTCTAGCGGCACCCCATCTCCTATCGGCTGTACCAACAATTTGTGAATCGCGGCTATGT
CCTTAGAATGGCTTTTGTCTGGCATCGGACAGCCCGGTTCGGCCGCCTTCTTTACAGAATAAGTTATCCAAGG
GTAGGCGAAAGCTGGAAAGAACCGTCGAGCCTTGGCAGCCTTGGTGAGATCGATTTCCAGGACATTCAGACAG
TGATAATGGACGAGTCCTTCTAATATGCAGTGCATGCTGTTNATNACAAGCATCTGNGNAGGATCCCAATATG
GCAGTCCCCAGAGCTCAGACCAGCGCACGCCAAAATCCTTTAGAATTTTTTCTCTTTCANTGAATGGTTGTAG
CGTCTCGCGAAGCTTCCGTGTATCTCCGCAATTCTCCGATATCTCTGTGACCCCAGTTCTCAGAACAGGTGTC
ATACATGGTATCCGTGTGATATGCATCACATACAGTGCAATACCAGTGCGANGAACGAGCGGCCGCGCCAGAC
AACTTCCGAGCAGCGGGCAGATCATTAACAGAGAGGACAACGGCAANTTCGGCATCATGACCNTCNGGNGCGG
AAGCTGTCCGAGAGACACGAATNCCACGTTCCCANGCNATNNGACAGNTGTCAACGAGTGGCCANATGAAGTC
GGGGATNNCATCNACATCAGGCTCNTCGGGACCAGGNATNATGCCGGCGAGGAAGGTAT
>Armme1_1_rnd-1_family-436#LTR/Copia
ATCTACACACCCATGAATCAAAATCCGAATACCTATCAAAGAAATCGGGTACCTTTGGGTCGTACAAGCGCTA
CGAAACCTGGATCAAGGTCCAGCGTGGTGTTGAGGTCATAAAGATCCTGGGTACGGATCGAGGGGGAGAATTC
CTAAGCGACGAGTTCACTGAGCACCTCAAGGCGAAGGGGACAGAGCGGCACCTTACAGTACATGATTCCCCGC
AGTCAAATGGAATTGCCGAACGTTCCAATGGGTATCTGATGGATACGACGAGAGCATTCCTAATCAGTTCAGG
ATTACCAAAATACCTATGGGCCGAAGCTCACCGGCATGCCGAATGGGTCTACAACCGCACCCCAACGAAAGCA
ATACCATCAGGGAAAACCCCGTTTGAGATGGCAACTGGCAGGAAGCCCAATATCTCCGGACTGCGTCCATGGG
GATGCCGTTGCTGGGTCCGGGTCAAGGCACCGGAAAAGCTTGGGGAACATGCTGTTGAGGCT
>Armme1_1_rnd-5_family-169#LTR/Gypsy
GGAACCCGAGCGAGGTCGGGAACACTGAGGAGGCACGCAACAGAATAGGAACCGACTCTGTTGCGTGGTGGGA
GTCAAAGTACAAGGTAAGGCTACCGATCTGGTTCCGCGGAAGTATCTAGNGNTGCGGGCAAACAGGCGTACAT
AACGCGTCGTAATCCGNNGGGGTTACACCCNCCCCCACTTTAAAAAATTANTGTGGATTCTCCTTTAGCCACT
CGTCGAGNGCCGCATTGTCTTCGAGCTCNCTGCCAGGCAACCAACGATCGCCTTCGGGGCCCTCGTTCACATA
TCTNACGAGGTAACGCCAACCTTTACCCCACCTGCGAGCGTCGACAATCCGATCGACAAAAAATTCTTCTCCG
CCATCGGCGGTTACTACAGGNGCNGGACGTGGAGGCGGCCGCGTGGGAAAAAGNGCGGGGTCATTGGGTACGA
ACGGTTTGAGCAAAGAGGAGTGGAAGGTCGGGTGTATTGAGGATGAAGGCATGTCCAATGTGTACGAGGACAT
GGCCGGGTTTGCGTCGAGNACCGTAAAGGGGCCGTCGAACCTGGGCATGAACTTTCCACATCGCTTTGCTCCC
TTATGGCAATATTCTTTACGGCGGTTCGCCGTCGACAGCATGACCTTGTCNCCAATCTTGTACT
>Armme1_1_Cluster65#LTR/Gypsy
TGGTGTACTAAGAATAGAACGTCCATTCGTGCGATAAAGAAGTGCGAAACATGCGGTAGCATCGTTCAGGTGA
TCCAATTGTGTCTGAGGTTACAATTTGGGATAAGATAAGAAGAGATAAGTGAGAAAATTGTAACATTACCCCC
TCATTAAGGCAGTCATCCTGACTGCCATTAAGGGTTGTTGTTCCAATAGTCATCAATTAATTCGTTCCATGTT
TCAGCGTCCGGATTTTTGACCCATTTTTGGTAACCATCTTCTCGTTGGGCTTTATAGAAGACATTGCCTTGCT
TGTCATATTCATGGGTGAGGATCATGGTGACGGCTTTGGCAGGATCATGGATTACTGCTTTGGTGATGTTGAC
ACGACCGTTTCGCAGGATTTTCTTTTCGTAATCTTTTATCACCTCCTGTGTGTTGCCCATCTCTGAATCTCGT
ACCCAGGAATTGTATTCTCGGGTCCACCCTTTCCATTTGACGAAGTAGTCGGTAACTGTTTGTGCTTTCTGGC
CACGTTTCCCTCTGATCTTTCGGGGGCGTGAGTCCAGCACTTCCTTGATCTCGTATTGTTCTTTGTCGTTGAC
AAGGTCAGGAGGTGGAGGTGGTGGATTTAGTTGGTTGGGGAAGGCGGGTGGCATGAAAGGTGTAAGCAGTTTT
TTGTTGAAGTGTGGGTGGATCTTCCAATGCGGTGGTAGTTTCAGCTTGTAAGCAGCGGCGCCTGTTTTGTTGA
GTATGGTAAATGGGCCGACCTGTTTGTTGTCGAGCTTTTTCTTGGGGCGGGGGAGATGGAGATTTCCTGCGTC
AAGCCAGACCTTGTCTCCGACCTTGTAGTTGATCGCCGTTCGTTTCCGTTTATCATATTGGGTTTTCATTGCT
TCGGCCGCCTTTTCGAGCGCCTTCTGGGTTTGTCTGTGGACTTCTGCCATCGCTTCTGTGAAGGTTTCGGCTG
CCGGGTTGAGGAAGGGGGATGGGCGAATGGAATCGGGTACGAGTTGGGGATTCCTTCCATAAAGGACCATAAA

TGGACTTTTTCCGGTGGCCGAGTGTATTTTGTTGTTATGGGCGAATTCCGCCATTGGGAGCCAAGTGCTCCAG
TTGGTTTGTCAGTATTCAACGAAGAGTCGGAGGTATCCTTCGAGTTCTTGGTTGACTCGTTCCGTCTGACCGT
CGGTTTGTGGGTGAAAGGCAGTGGATGCGTTGGGGGTGATGTCAAGTAGTCGGTATAGGTCCTTCATGAAATG
GAAAACGAATTGGGGTCCTCGGTCGGATATCACGACCTGTGGCATCCCATGTTTGACGTAGACGTGGTCTTGG
AGGGCTTTGGCCCATCCCTCGGCCAACAATTCCATGTTACAAGGGATCGGGATGATAGCTTTGGAAAATCAGT
CCATGATGACAAGCAGGGCGTCGTATCCCTGTGACTCGGGTAGTCCGGTGACCATGTCGACTGAAATATGGCT
CCAGGGCCCGACGGGGATCTCGTTTGGATTTAGAGGGGCAGCTTTGGGCTGGTTACTTGATTTTGCCTGCTGG
CAGGTCTCGCATCCCTTTACATATGCCTCTACATCCCCTTTTAACTTTGGCCACCAATATTCTCGTTGGACTA
GCTCCCTGGTCTTCTCTATTCCTGGGTGGCTGGCGGTAATGTGGTCGTGACTCTGCACGATGATCTCTCCCTG
CAGTTCACGATCTCTCGGGACATATACTCGTCCGTCGTAGAGGAGGAGTCCGTGGTCTCTCGTGATGCCCCTT
TCATGAGCGAGGGAGTTTGCGATTCCCTGGTCCCAGTCGTCTTCTCGTCGGAGAGCATCTCTGACCTTGTCAT
GGAGTTCGGTGGTGGTTGGCATGATGAGGGCATGGAAGTGTTGGGGTTGTAGGATGATCAGGTCCTCGTTGTC
TTGTTTTCCTTGGTCATGATCGTCCCTCCGAGAGAGGAGGTCTGCCTTCTTGTTTGCTGTCCCGGGGAGATGA
TGCAGGGAGAAATGATATTCGGCCAGTTCCGTGACCCAGCGAGCTTGTCGATGATTAAGCTTTTGGGGCTTTC
TGAAGTATTGGAGATTTTGATGGTCGGTCCAGATTTCTATGTCTTCGGCCACACCCATCAGATAGTGTCGCCA
TTCGGATAGGGCGAGCATGATTGCTAGCAGCTCCTTGTCATAGATTTCATAGTTTCGTTCGGTGACTGTGAGG
GACTTGGACATGAAGGCGATGGGGTGCCATTTCCCGCTCTGCTGCTGGCTGAGTACCATGCCTATGGCGCCTT
CACTGGAGTCGGCTTCTACCTTGAAGTGCCCTTCCTCTGTGGGGATCGCAAGTATCACATCCTCAGCCATCTG
CCTTTTTAATTGCTGGAATGCCTGGTTCTGGGCCGTCCCCCATGTCCATTCTGTGTTCCTGGTTAACTGGGTG
AGCGGGCGGACGATCTTGGAGTAATCCTGGATGAACTTCCGATAGAAGTTGGTAAACCCGAGGAACGATTGTA
GTTCCTTTTTCTTGGTAGGGGTGGGCCATTCAGTTATTCCTGCGATCTTGACTGGGTCCATTCGCACGGCGTT
GTGGCTGATGATGACCCCTAGGTATTTGGTCTCCAGGACCCTGAACTCGCACTTCTCAGCCTTTAGGAACAAC
TTGTTTTCTCGCAGGCGCTGGAGTACGCGCCGTACAATTTGTCGGTGTTCGTCAAGGTCCTTGGTGAAGATAA
GGATGTCGTTGAGGTAAATGGTGACCTTTCCTTCAGCGATTAGGTCCTTGAAGATGTCATTCATCATCCATTG
GAAGGTGGCTGATGAGTTGGTCAGTCCAAAGAACATTACAGTGGGTTCGAAGAGTCCACAATTCGTTCGGAAT
GCTGCCTTCTCCTCGTCCCCTTTCCTAATCCGGACGTTGTTATACCCCCAACGTACATCTAACTTGGTGAAAT
ACCGGGCCCCCCAGAGTTTATCGATAAGTTCAGGAATGAGCGGTAGCAGGTAGTGGTTTTTAATCATCATTTC
ATTGAGCTTTCGGTAGTCTTGGACGGGTTGTAGGGAACCATCCTTTTTCTTGACGAAGAAGAATGGCGAGGCG
AAGGGCGACTTGGAGGGTCGGATCCATCCCAATTCTAGGTTCTCGTCGAGGAAGTGGTCGAGCTGTTCCTGCT
CATTCCGGTTCAAGGGGTAAACCTTGCAGTCGAGTGTGGATTTGGCGTTGGGGATGAGCTCGATGGCATGGTC
CCAGGGTTTTTGATCTGGTAGCTCGTCAAAGTTGTTTTTGGAGAATAGGTCCTGGTAAGAGCGATAGTGTTTA
GGGACCATCTCCTCGAAAGTCTTTTTGGGTTTATCTGCATGGGCCTGGGCCGCCAGATCATTGGCGTGATGCA
CTGCCCTTATGGTTATCTCTTCCTGCATGTTTACGGCCAGGATCATCTCTCCTTCTTCAAGTTCTTTGTCCCA
GCAGTCATCAGGGTCAGCATCCGGCAAGGGGAAGGGGTTTTGGCGCATTGGCAGCGACCAAATATGATCGAT
CCAGTTTTCCAGTTGATTACTGGGTTGTGGCGTTTGAGCCAGTCATGACCGAGGTACAAGTCCTTAGATCTGA
GGTCAGTGATGGCTAGATCAATTTGTTCGCGATGCCCCCCAATCGAAACGAGGAGTTCCGTGAATTCGGTTAT
GTCTCCGCCCTGATTTCTGGTTCCGTCGGCATTATATACCAGGATTGGGGCAACGGTCTTCCGGGTGTCTAGG
CCGTGGTTTTGGACGTAGGATCGGTTTATCGAGCTTCTGGTGCATCCGCTGTCGATAAGCCCCTTGGTGACTA
GGGTGCGCTGATTGGTGCAGGTTTCAAGGATGATGTCGACAGTAAGGTCCCGGTCAGTGCTTTTGGTCCTTCG
GATGTATCGGATGGGCTCCCGGAGTTCGTACAGGGTTTCTCTAGCCTCTTCCCCCCCTTTTTGGAGTCGATTG
ATCAGTCTGGCAACAATCTCTCGGTGTCGGTCTTTATGGACCCAGCTGCTAAGTGCCACTCATGCTTCCTCCA
TGGTATGGGCCTCGCAGATGGCTCGGAGGGTCCAGTCGATGGTGAAGGGCTTATACCAGGCTCGAGAGTGTGG
GGGAGCACTCGTATCGGGTCTGGTGGGTACCCAATCATCGTCGCTCTGGATGGCTCGTTCCTTCTGCTCGGCT
TTTTGCGCTGCCAACCACTGTTGGTAGGCAGCCTCGTCTTCCTCGTCAGGTACCTCCTCCATGGTAATGGCAG
GCCCCCTTTTCATCGTCTGGGCGCTAACGACTTCCGATCCAGCGGCTTTCTTGTCGTCCGGGTGGGAGGAGTC
ATCGGGGTTGGCCAGAGGAGTGGTGGGAGTGGACTCACATCGACCCAGCATTTCATGGCCGTCCTGTGCCCAT
GTCCGTCTTGTTCTTGTCGAATAAACCGTGGCGGCCCCTGCGCTCTGAGGCCTGGTGGTCGGTGAGGCTTTGC
TCGTCTCCTCCGTTTCTCCTGGCTCGGGTGTCCATTTGTTGCAACGTAGAGTCCTTCCCTCTGACTCTCTGTA
ACCACTCTTCGGCGCCTTCCCCCTTGCGGGGCTCGGCTGTGGCGCTCCCCTTGACAGGTTCGCGTGCGGATAC
TCTGGACGTTCTTGATTTGTCATGGGGGCTCGCTGCCAAGACTGTAGGTCCTGCTCCCTGTTCAGCATTAGAG
ATTTCGGTCTCATCAGCGCTTGAGTCTGAGTTGCTCAATTTCGCTAGAGCGGCATAGCGGTTCGATTCTGTTG
TTACTGGTTTGCTAGTTGTGCAGGGGACGTTAAAAGCAGTGGTATATAAAATGGTAGGTACAACAGTTGATGT
AACTAAAGGGGGTGCATAAGATCGGCCAGCGGGGGGCGAGAGCGTACCAGTGAAGTCCTTGGTCGTCGAAGGG
GAATCGAGCCCTTGACGATCTAATCTTTTACCTCCTCTACTTTTGAGTCAGTTTTCTCCTCCTGCTTGGTTAC
GATTACTGAGCGAATGTCCTTAAACTCCTTCCTTTTCAGGCAATCCTTCAACATATGACCCTTCTCCTTGCAA
CGATAGCACCTTCCCTCCCTTCGCAGACGGTCGACCTCCATGGGCTGTCCTTGCCCTCCGTAGGTTGTCCACC
TCCCGGTTCCTGCATCCCTTGTTGGTTTGTTGTCAGCTGTCAGTTTGGCCTGGGACAGAGTGTTGGCGGCCTT
CGAGGGTGGATTGGCCTTGGGTGGGTCGCCAGCATGGGCGAACATTTGTTGAAAGGCCTGATCCCTCTGGCGT

TCTTCCCAGGCTTTTATGACTCGCTCCTTCCATTCTGGGTAGGTTCGTGGGACATTTAATCCCCCCCCTGTGA
TGGCGTGTCGGATGGAGGTGGGGATTCCTAGTCTCGCAGCTCAGGTAAGGTGATCTTTTTCTTCTTCCTTGTC
GCGGAGGCCTGCCAACTTGGCTTCTTGTTCTAGTTTAGTGAAGAAGATGGTGGCAGGCCCATTTCCCATGCGG
AGGTTGTACATGGCCTTCTCATGCTGTTCTTCGACGGCTGGGTCCATGAATTGTTCTTTAAAGATTTGAACGA
ACCCGGGCCAATCAGGATATCGGCTTCTTTGCCAGACCTTGTCATAGAACTCTGGTCGGCGATAGACCCACCA
GTCCTTGGCAGGGCCCTCAAGATGGTGGGCGGCGAAAAGGGTCTTCTGGGATGGGGCAGGGAAGTAGATTGAG
AACATTGTGAAGTAGGCCTCGCAGTCTCCCAGGAAATGGTTGATATCTGCATGCTCCCCAGTGAAGGGTTTTG
GCAGGGGCATGAGGACAGGCTTTACTCCCCAAAAGGGGGCGTCTTCTGGTGCAACTCCCATGGGTGCCGGTGG
TGGATCCCGTGGGAAGTTCAGTCCTGGTGGGGGTCACGGGAGCGAGTAGAAGGAACCGTGTTCCCTTGGTGGT
CTTCCCAGGTCAGCAGGTCCACCAGGGGAGGCTCGGTTTGGTGGTCCTTCCTCTCGGAGATCCCGGAGCTCAT
CCTTGAGCTCATTGATTTGCCGGGTGAGCTTGGTCCCTAGTTCATTTTGCTGTCGGTCGAGTTCAAGTTGTTG
GCACATAATGGTTTGGGCTTGAAGGGTCCTAGGGGAGGGTTGGGGCCCTGGAGCTGGGGGATCGGTTACTATA
GGTTCCGGGGGAGGGTCTTCTCCTGCCCCTGCAAGAAGTCAGCTTCTTCTGGGATAGGGGTAGCCGTGTTGTC
GGTAACGGGGTTCCCTGAGGAACGGAGGTTCGTCAGGTAACCAAACTGGGTTTTCAACGGCCCTGGAGTACGT
GGCAAAGGAAGATGTATTAGGGGTGAGCAAACTGGCTCCCCCGATTGTTTCTGGGTCGTATGTAAAATTGTCA
AAATCATTAAAAAACCCCTGTCCCATCAGGACAGGGCCACTAGTCCTTCCTCTGAATCGAGTCTGCGAGGGCC
TGGTGCTCGAGGGCACGGAGGTCGTGCTCCAACTCAGCCTGCTCCTCGGGAGTCCTCTGTCTAACCCCGAAGA
GTCTTTGCCCCGCATTTGGGTGGTATAGCATTCTGTCTCGGTTAGCGCGAATTCTGGCCTCAATGGCTCGGCG
CTGGAAGGTTGGGGAGAGTGGATCTCCACTGGTGATAGCAAGGTGGTTGTCCATGGGAATTCGATGGGTGTAG
TAATTCCCGAGTCGGTGCGCCAGGTCCTGCCAGCGCCGGTCCCAGGCTCGGGCTCTGGGTTCGCCCATGATCT
CCCTGTCGATTGGGGCAAGCTCTGGCCACTGATAGTCCCCCCCTCTGGGGTTAAGAGGATCGTCGTCGGCCCA
GGCCCCATCGCTATCGGGGACAGGTGCTCGCTCACCGACCCGTATCTGCTCGGGGAGGTCGGGGGCGAGGGGG
AGGGAAGAGGAGGACTCAGTGCTCTCGCTGTCAGATGGAAAGCTTCCGGATTGTAAGAAGGCTGCCCCATACG
GCCCGACGGGGGGAACGAAGATGGACCTTCACTTGGGAATGACGGGTGGGGTGGAGTCTGGGTCGGGCTGGGG
GGATCTGAAAAGAGTCCATAGGTCCCTTCTGAGCTCCTGTGTTGCAAGGCTCAGTCTCGGTTCCTCCTCCACA
GGGTGGTGAACGGGTTGTTGTTGCCTCACCTGGGATGGACCTGGGGTGGGCTGCCTAGAGGGTTCCTGCCAAT
TCATTGAGAACTCCTCGCTGGATCGAGGGGTGTGGTACTGGCTGCTTCGGCGGGTGAGGGTTTGTTCGGGGGG
AGGGGTGGCGGTTGTGAATGGTCAGACATAGTGAAGTGGGAGTCTTTGTCTGGGGAGGATCAAGAGCCTTCGC
CTCACCAGGAAGCGTTGGATTTCGTAGTGGTAGGTAAATACCGTTAAGGCGGTGAGGGCGACTAGGAGTCCTC
CTCCTGCGATGGAGAAGGCGATGGTGAATGCGGTGTCGTTAAAGTTCATATGGGTGTTTTCTTCACGGCTGGC
GGTGAAGGAAAGCAAGGGAAGCTTTGAACTAACTGTTAGACCCGGACTTCACGTGACGAAAGACTTAGCCTAA
ACCTCAGCTGTCTCGTGTTCGGACAGTCACTGGGGAATGGCTGAAGTGTCTAGGGCTCAGTAAAGAGGGACTT
AGTTGCAGCTAGTCGAGTTCTTTATGAGAGTGAAGAGTCAGTGTACTAAGAATAGAACGTCCATTCGTGCGAT
AAAGAAGTGCAAAACATGCAGTAGCATCGTTCAGGCGATCCGATTGTGTCCGAGGTTATGATTTGGGATAAGA
TAAGAAGAGATAAGCGAGAAAATCATAAC
>Armme1_1_Cluster21#LTR/Gypsy
gtgagACaaggaccacCacaTcacCAaagaagcgaacCcaAAaccggaaccTCacacaTacgcAcAaTcacca
aagGATGccacaacaggcTaagccccgccaaAcAcAcactagacaaaacaaaTccaaTTCcaaGaCAagaaga
aagAgTagaaaaGaaaaacagcacccccaagaagagcacaagacaccAacaaaaGaacaaCaaaaacAaTaaa
aaCaacacTCaaAGAAcacaACaaccaaaaCaTacTcCaTcTaaccaaaaacacaagacccCaaaaaCAaacc
CcaaTGagTgcCacaATGgcaGAaagcaaAagTTgaGGcaAaaagaagggaGacaCaaGaaaaaagcGTaaaA
aaaccacCTCAaaaaggCacTTGcccTcacTcaCaacaAGccaTGaaagaaaaAcaaaCATgAATcaaaacGa
aaCAGcccTcaacaTCagcaTcTcaaaTccaggaggaagAacCaTCcTcccccTcacCcccgTAaaAaacaTT
aCagaccaCaaCGaaaTCcaaggCgaccagcagaGgcAcccacTcTgaAgccTccccAaacAaaGcCcccaaa
aTcaCgAcaccTcaccaTTCacaaccccTGccCccacaccccaacAaAcCCTcacccaCagaAcCaacCaaca
aaacaCacacCcAgcaagcaaGgacccaccacCaaaccggCaagaacaCgaaagacacaccAaCccaaccccG
ccccaGaccgcacaacaaCaacaCcTTcaacacTCgggaGacgAGgAacccagaaaaagCaTacccacacacG
accTCAacaaGGaCaggcaGacgcggcacaGaccCcaccaGcccGaGgaAaaaccGaGacaCaaaGgcagcca
caAGCgaagcgccaaaacaagccTGaacCacacAacaaATaGGGaGgcTaagcTccaaagCaGaaaggcCccg
TccaacGAgaAgaGcaaaGacgCCaaaaCcccacTCacCcacgccCccccgcgccCcaaGcAgaTacacaTca
CCacCccaGAaCCacaCCcgacCccGAcggaGTAgggaacaacGaTgaaccTaTcaaccccagaggGaGaca
ccATcagcgcCaacaaCTcgcCcagggcgccagacaaCTgaTgGgaGAcccCaGccaacGagcCcggGAacGa
cgCgGccacgacaTgaaGcAacGGagaGGaAaaaacgaaacCaacTaaaagcCaagGaacaacCaCaTagaaa
aCaaTaaacaGaaTCcacTcCccccaacagagcAcTccagTaccATcGagGaaagaagcCAcacaaAaacAAa
TccacaagTacacCgccCaaaagccGaaaaAaaGcTccAcagaccCagaGAGagaacgccTGAcgaGcacccc
aaacccaaacGacaccgaggcaaacgcaaacaacacacgggcAGgGAcaccaacaAGacGagagAcgaccggC
acaAaTTcGaacacGAaagGaggTccTaaggacgaTaacAaTacTggagacaaCCCAgaaacagcaaagaaag
caaCTCgGaggGCcCccaacaaacCccCagCaGacAcgTacaCaagaaaaagcGcaaGcaTGAaaacccTaca

aAAcaAacCTacaaTcaTcAGgaAAgCaccaTACCcAcaacACggcgacacccaaccAacaAgcaccacacac
aTagcacgccaaGGacaaaacccgacccCagacccaagAagaaccGaCaCaccGGcCccgggGcaccaCcccg
CCccccaGaaaaccTcaAcaaCaggcTAcacaccacaacaaaccCgagcTcaaCcgacaaCAaaAagAaccTcG
aAaaaacaagaCccgacaaaTcaacaacCcgaaAgacCaGccccaaaaacgcacAgaacaagGCcCAacaAaC
accCCcTcaaccccagGAcCaCcTGaaaCAgaaagaccaCccccAgAaaAcAGcaccccccTaaTCaCTcccaa
gACCcCcggaagGacCAAaagaccTgcaAaacCCcTcgcCacaacacaccgcccgCcagaccaaAgcccccac
ccTcTcaaGGagaaaaccgaTCcTTagcaccacaagaaaacccaacgccTGGGaccacaccAaaATcaGgccc
TcccTGggcgaCTgccacgccTaaTgggCAacccTccccaaaaacagaccCgacccaTcccacaaaacCCccg
gaaccacacaCaagcaaaAagGacaTgccaaaaACTcgggGacccAcCGacaaccAcAacgaTaaaaTaaagg
cgGccacagGAaacaaTacacggaTTGGccaacggTCcacAaaaccaTcaacgaacaagcacCaaacacGgaA
ggccAacAacaacAcaaaaaGaccATGaaCAcaagcaGcacgggaaaagaaacagCaaacacCcacTTgacGA
aacTaaaacAggaaGacAaGaacacGacacggcaCcATaagcaAgaagagaaggacCacagcACCcccacCaac
GacaCcggcaaTccaagccgCTacaaaTcacAaCagcaCaccggcaGgacgaAagGcaacGagcACcTaTcCA
GaaagcaacgcaTgaaacagaaaaaacgccgaagaccGccacagacacCAaGcCcaCcagcaaaccccccGcCa
ACccaaacAGCgGgCcaAaaacCaagcaacaaaccaaaacGaaaaaTccacaaaacccaacgAAacCcAcaaa
cGATgGcAGCTcaggaagggagGcaaGcACaGGcaAgTcaaCaAcacaTGggacCcaGaggcaacCCATgcAg
AcaGaacgacgcCgaAaGaaaaccacaaGgTACccaggCaacaaGAacgcGaATacGcaaaaggacgccccaA
aGAaaAaggaaTacaaccACAgTcaTTcAAgaacaggacccAaAaaccAacaGaagaCggacacGaacgTaGa
GaAGaTaAaaaaaTaagccAgcaAGagcgcGAccccCCcTccagcaacaaaaaCaaaaaaGaTaccaTTcagc
accCagcggGaaCGggccTagcaAaccacTcaagccaaAcaACaTGacTgagagacCaAAgaacacACccccTGc
TaTgaAaggcCcCcgcaTcaccAgCaaaccacTagacaCaGAagcgaaccgacacaCaacgcaaGcacAaTTg
aGcGAagcAgaagcaaccGcgGAggagacCaAgaaccccaaaaacGAaccaaaaGcaGgAcCgAgggTcaagc
cacagagCccccAcaaggaAgcAagaccagaaaaGgCacaaacagcCAacCcTgTcaagGacAaaaaacaaGc
ccaAccCaaaaaGcGaagaaGaagGgcAAagCaacCacaAggacacacagacaacGAcgaaacagTccAacaa
aTcaacccCaaaaCgGggaaAaagagAaaacaaGaacacaacCgcacaaacgGCcagaccgagGAGcgCaagG
acaaccaCccaggaTTccaaaagaacgAGacaagCAaAGgcGTccccacaaaaCacaaaAaGAaaGGacagaa
aATaCcaaagcaacaTcaccccaaacccgccacTccgCcgGcCaACacgGacgagacCacCCaccaAaacaac
aacaaaaccaaacGaTCGgAaGacgaTagCgcCCaGaaaaTgaAaaGccaGcaTGcTgTcTCccTccaaaAGa
TacccaaCcaAaaaGaCgaaaccacCTaccaaaaGTaaagaaaaacGcAacaaGcTGaAacGacaGaGAccaa
aAaccAcaGagacaacgacccaGTacacaaCaGaccTaagacGagTGagCCaccaaacaCTgaaGaaTaagaC
aagacaccCgacaTCaAcgacAcccagggcaacaTcCacaaaacacaCaCgcaacaAGAaGacaaagcccacc
aaaacaGTTagaTCCacaacgacgggcaacaTcaaATcagCaTgaaAaacagcAATcGacgCAGccGcaggcc
gaaaaacgaaaccaaagTcaTGgacGaAagAcccGaacacAgaccgcaCAgccaAaccaCgcacaacAacgcc
aggaaccgTaagGgcaaaaccATccgcaaAaccTgcaacaaccAacgaaaCcgagacaccaaagcccccAccc
acaGcacaTccaacGgaaccgacagaaaCagaTaaTacaTaCAgaAacacccccgccaCaccaccaaaaCaac
cGacccGATccaacaacacaATGacGATggaAcccggaaacaaggcagaGAcacAACTaaggaccacgaaTcc
cgCcTcccaagcGGgacAcacTgCaaAaAaagCaaccacGccccaacCaAcccaaaAgccaagGaaccagaCC
TcaGacacaacgGGaaCaaacGcaacAagcCaGgaaggaACccaAacAccGgaacaaTcaaTggccccgCTg
TCaaTgcacCaagaacCaccgCCaaCcAccccacAcaGaCacaggaaaCTgCcggGacaaaGAaCTCaaccAa
GaAgAaacCAgcCcccCcaTaaAcaggCaGGacGagaaacaCaTaCaagCaagacaccaccccaaCGaCcaGG
cgGcaaacacCcagGcgGacaaGaaGAagaacAccagcaaacacAcagccacccaGcAccacagcgCCaacCa
ggAgcgGgacgccAaAcACaaCcaTGaCcaaCTcaccaacggaaAacaaaccaaccaaGcCacaGaGCTCgga
ccCaagGccaaaaccaaGccTgagcgcaacgcCTacaccTcaaacgGAaaaGaacaacAgcaacTccAaCAcg
gacTCcaTcacaacgacgacccaggaccaacccaacccgCcaaacCcCccgTgGccccaccaaaCagCgcTcc
CaaaAagAaGcaCaccTaccgaaaACcaGgaCaagacTacCgaaaccTcaagGaaagcaccaggaAaaaccGa
TaCcccccccCTGcTCAgcacaaAacacaTCaacaaacTCaacGcagcacaacaagTCaCTaAacTgcacgaaa
GcgGcagagacaacaaaaTCaGaaTTaAcgaagggGacaagcAaaaGGgaGaagTcaaAAgcAaccGcAgacg
aagcGaaacaaaCAacATgggaagcaaacaaAcaaAcagcacaacaaCaTcCaAggGaagaagaaAcgaTAcC
agTaAaGacCTgagcaaTcaAaGaaaaaTTaacacCTacacaAacgACacccTcAcCTcCaCcAacgaCcacg
acGaacaCCcCcaaagCgcaccgaccacaaccaAGcaaaTcTccgaGaAcaagcTacTCcTaaaacaggacaa
agacaaATgcaaagTCccggaGaaccaaTAaCaacgacccaccaTcaaaaaaaaggCaacaccaATGGaCcca
accaaGccgccggaAaTaAcCcAgcGgCCcacaacTaCcaacaagaagcAgcTGcaGTCGccaacTGGCTTga
cCAagggCTacccAaaagTTacccggcaCTacgcaaaaaTgcTgcGcCcacTaaCcaaGTgaaCcaaaAaTgc
agaaaaaaccTGGgccccagccCacaaTcaccaggcccaaaaGacaaAaaaccacaaggccaaagAgGTccga
cgaACcaTaaccaCacaacaaagGagGggcaaAggaaaaGacGaggccAgCcagaaacccacaaccaaaGTaa
gcaGccagcagcaggccacgaaaTcGTGGaccacCaCGagcaTcgCcaaaTccccCAccaTcaccaAGaAaaa
cgagGaaagCcaagacaaaaaGcaaaggccAaaCATccccgcCccATcCgaaTcgccAaacgaccgGaccaag
aaCaccgacgacAaaaaaaacacaAaccaacacCAAaagcgacAgcaacTCaggaacCacaaaaaGcTgaAga

AcccacaaccTCGaTaGaccaaacAaTcggcgGaAgacCacTcaacacgacagcaacTgaaTaGaaTggcaaa
caAaaaagaaGacaaccacacgaaGAaaaacGagaAcgAccaagcaaAGcaccacAagaAacAcCTGATagcc
ccAcaAcCCCaAcacaaccGcgcccgcagcagcacaAaaAccaccgaaacCCaccAaAAGGgcacagaTgCac
gccaaaaacacaaccacgcgGaccaaGGGacTgcgGccaaccTTGcTCaaGacaGGGaCagcacaAGaGAacA
gaGacacCcaTgcTaccaTGGacGacTacaTGgacCaaGaGaTCaacgacgGcaaGGacaaaTaaccGaaCaA
acCcaCaacaagacCaacaccgGccaTcTGaGcacagaGAaaaccaAGGaaagcgcacaaCcaaaaTagTgcT
GgccgaaaTcaAaaaaGaaTggcgaccaaaacATAaaagGGggCcaaacgggcCaacaGGaaaagTcGaccaa
ccaGaCCaaGGcagCccccacaaaacgcaaTGaGagaccagccgcaCCcgccacccacAacacTcggcagaTg
cccaccaaaagacagcaGTcaaaTcGaTAagagGcTcccCTcaTaaacGgAGacTcaTcggcaAAaacTgcaa
gaCTGagcacaggcaagGgAgAcaTagcaaccaaaaacggagccaGaGCaagCaaaGaCcaTgcacacaagaa
acaTGgaacacacaaaaaaagTaacAaaagaacgAaaAaaCCaaccaccagcCaAAaTcagaaaGcaacTacac
ccaCTcaaTgaTaTcaCgcaaaaCccagccacTGacggccaacaGcagacgcacgaAcacagccaacaccTca
aacagcaaCTcgaaGaagaCcTacaaCccTcaacCgaaaaccaacaGaccGAcggcaccacTTcGcTaagaAT
GaaacaaagCccCcATaacaacacacTGaaagccgccagGaAGaaGAgccCaaccaggGagagagAcGgaaga
aacCacgGacacGcacccaAcgcaGTaaaccCcgagcCggTcCgcaAcccaccacCaGaAaCagTcacaGaaa
CcagcgcgGaGgTCcaCaAaaAgaccCagaaaGaaCgcGaaaAGcTcgcGcaAcTGaTaaacaCCCaaTacaa
caagaAaAGGagaacCGCcAgcaaaTacaaccccgcGaaCaaGGacgGGcgcgaTGcCaccaAcCTGcATCTc
CcTcGacaaaaaaaGaaacacaagGAcaaAgGaagaGGaccCggaaCccTacccGacaAAacaGaacCagCaa
caTacAaacaAaagcgcCCaccaCacTcgaaGaaCcacCcaaAagacaaagaaaaGcTaaggaagcaaagcGg
accaaccgccTccCcCaacCaGcgGGaAccgcaAaaaacaCCacagGacCccccCGAccaccaacAacaaaAg
GcaagcaagcaagTaCTccaCcaaaGacccaaaAAgagcagGgGaaaaccccaaCaaaGaCgGaaaacGGgTa
cccagcacagaaggaaacgGaaaagccGcacgcAaaaacaCaacgcaTgGGgagGcaaggcAgaaacgGccaa
aGCacagaAGGggaTccgaGacgacAaaGacAaaGgccaacaAgccacAgaCaTaGacGaaaccaaGgCcaTa
cacaagaAccaTcagaAagCcacaaCcaacaagcaaGaacagGAaTaCGaaGaGcaAgGcAAacaCTgcTAca
aaGcccAGcaaGAcaAgGagcaCCaaaaagacAacaaAaAaccaaAcaccaAaaccgGgcgCGacaaaaTccA
TaacgaTgggaaCaaCacccCTgaagGgcAagcAaaacaAcgGaagcaacGAGcgaacaaTgagACcaggacc
acCagaTcacCAaagaagcgaacCcaAAacCggaaccTCgcacaTacgcAcAaccacTcaacGATGacgcaac
aggcTaagccccgccaaAcAgggaccAggcaaaacaaaTTcaaTTCcaaGaCAagaagaaagAgTagaaaaGa
aaaacagcacccccaagaagagcacaagacagcAacaaaaGaacaaCaaaaacAacaaaaacgacgcTCaaAG
AAcagaACgaccaaaaCacAcccCaTcTaaccaaaaaaacaagacCccgaaaaCAaacccCcGaTGagTgccac
aATGgcaGAaagcagaagTTaaGGcaAaaagaaggaaGacaCAaGaaaaaaaacaTaaaAaa
>Armme1_1_rnd-1_family-197#LTR/Gypsy
ATACCAGGGGGAATGGGTATATTTCGTAGTACCCAAGGNGTATGTTCGACGACTGGGATATCGACTGGAGGAA
AGAAATCTCGACGAAAACTGCCTCGTTCGTTGTCNTCCCATGCGAACGCCTTCTCCTGGACCATCATGAAATG
ATGCATAAGCTTCATTTCTTCTGGCCANAGAAANCCGTCATTATGGATGGAATCGATGTACTCCTTGCGTTCT
TGGGTATAACGTTCGGTAGGAATGAATGGTGGTGGGGTAGGAGATAACGTTGGTATTTCGGCCAGTGGATCTC
CANNGATATCGCGGATGATNCGAAATTTATCAGGCAAAGTTGCGAGTACCGGCTTTGTTTTCAACGCGACCGG
CTTGTACTTCTTGCCTGCAAATATCGAGACTTTCTTATCGAACCATGGCTCGTAGGATTGATCTCCAATAATA
TTAGATCCGTAAAGACGGTACATATAATCTCGGGGCTTGGTAACGGAAATGGAATCGGAANTATCTGAAGTAT
ACTGGGGTGGAATGGAATATAAAGGTAGCTCTCGCTCGCGATCGCTGGGTAGAGAAAATTCGGCATAAGGACT
GGAACTGTACACGGTTTCTGTCGACCATAGTGATTGAGTAAACGTAGTGGGCTGGATAGGGAATTTATTAGGG
TCGCAGGCTGATAAATATTGAGTCGAAGCTTTGTCTGAGTCAAATTCTGATTTCGAGGCGGCATAGGCAATAA
TTTTAGGTTCTTGATTCACGTCTAATTCGATGACGAGGGCGATCTCCTCGACGTCATCTTCGATGGGTTCTTC
TAGTCAATTGTCCCACGAAAATGGAACATGATTAAGGAT
>Armme1_1_Cluster29#LTR/Gypsy
TACGAATGGACCCCATTAAAGTCGCTGGAATAACCGAATGGCCCACCCCCTCCAAGAAAAAGGAACTGCAGTT
GTTCCTCAGATTCACCAATTTCTACTGAAAGTTCATCTGAGACTACTCCAAAATCATCTGACCCCTCACCCAG
TTGACCGGAAACGCCGAATGGACGTGGGGAACAGCCCAAAACCAAGCGTTTCAACAATTGAAAAGGCAGATGG
CTGAAGATGTAGTACTCGCGATCCCCACCGAAGAAGGACAGTTCAGGGTGGAGGCCGATTCCAGTGAAGGAGC
CATAGGTGCAGTACTCAGTCAGCGGCAGAACGGAAAATGGCAGCCTGTGGCGTTCATGTCCAAATCCCTCACC
GTCACCGAACGGAATTACGAGATCTACGACAAGGAATTACTAGCGATTATGCTCGCCCTATCCGAGTGGCGGC
ATTACCTGATGGGCGCCGCCGAAGATATAGAGATTTGGACAGATCACCAAAATCTCCAATATTTTAGAAAGCC
CCAAAAACTTAACCACCGACAGGCGCGCTGGGTCACGGAGTTAGCCGAGTACCACTTCTCCTTGCATCATCTC
CCTGGGACAGCAAACAAGAAGGCGGACCTCCTCTCTCAAAGAGATGACCACGACCAAGGAAGGCGGACAACG
AGGGCCTAACGGTTCTTCAGCCCCAGCATTTCCATGCCCTCATCATGCCAACGACAACGGAACTTCATGACAA
GGTCAGAGACGCCCTCCGACAGGAAGACTCCTGGGACCAAGGAATCGCAAATTCTCTTGCTCACGAGAGGGGA
ATCACAAGGGACAACGGACTCCTTTTCTATGACGGACGTATATACGTTCCCCGAGACCACAAGCTGCGGGGAG
AGATAATCGCTCAAAGCCACGATCATATAACCGCTGGCCACCCGGGGATAGAAAAAACCAGGGAACTGATCCA

ACGGGAGTACTGGTGGCCAAGACTAAAAAGAGAAGTCGAGGCATACGTCAAGGGGTGTGAGGCTTGCCAAAGG
GCAAAATCGAGTAACCAGGCTAAGGCGGCCCCCCTTCACCCAAACGAGATCCCCCTTGGACCCTGGAGCCATA
TCTCCGTCGACATGGTCACAGGACTACCCGAGTCCAATGGGTACGACGCCCTACTCGTAATCATGGACTGATT
CTCGAAAGCCATTATTCCTATTCCCTGCAACATGGAACTATTGGCTGAAGGATGGGCAAGAGCCCTCTGCGAC
CACGTCTATGTCAAGCATGGGATGCCCCAAGTCATGATATCAGATAGGGGACCTCAGTTCATCTCCCGTTTCA
TGAAAGATCTATACCGACTGCTCGACATAACCCCTAATGCATCCACCGCCTTCCACCCACAAACAGACGGCCA
GACAGAGCGAGTTAACCAAGAACTCGAAGGATATCTTCGACTCTTCATCGATTACCGACAAACCGACTGGAGC
ACCTGGCTCCCGATGGCAGAATTCGCTCATAACAATAGAATACATTCCGCCACTGGGAAGAGTCCCTTCATGG
TCCTATATGGAAGGAACCCACGACTTGTACCAGACTCCATTCGACCATCCCCCTTCCTCAACCCCGCAGCCGA
GACTTTCACAGAAACCATGGCAGAAGTTCATAAACAAACCCAGAAAGCACTCGAAAAGGCGGCTGAGGCAATG
AAAACGCAATACGACAAACGGAAGCGAACAGCGATTGACTACAAGGTAGGAGACAAAGTCTGGCTCGATGCCG
GAAACCTCCATCTTCCTCGACCCAAAAAGAAGCTTGACGATAAACGTGTCAGCCCTTTTATCATACTCGAAAA
GACAGGCGCCGCCACTTATAAACTAAAGCTACCACCGCACTGGAAGATCCACCCGCGATTCAACGAAAAACTA
CTCACCCCTTTCGTACCACCCACCTTCCCTAACCAAGTACCACCACCACCTCCACCTCCTGACCTCGTCAACG
ACGATGAACAATACGAAATCGAGGAAGTCCTTGACTCCCACCCTCGAAAAGTCAGGGGGAAACGTGGACAAAA
ACCACAGATGGTCACCGACTACTTCGTCAAATGGAAAGGATGGACCCGAGAACATAACTCCTGGGTACGAGAT
TCCAAGATGGGTAATGCACAGGACGCGATAAAAGATTATGAAAAGAAGATCCAACAGAATGGGTGCATCGACA
TCGCGGAAGCGGTAATCCATGACCCCACCAAGGTTGTCACCATGATCCTTGACCACGAATATGATGAGCAGGG
TAACGTCTTCTACAAAGCTCAACGAGAAGACGGACATCAGAAATGGGTCAAAAACCCGGACATTGTCACCTGG
AATGAGCTAATCGACGACTATTGGAACAATGCCCCTTAATGGCAGTCGGGACGACTGCCTTAATGAGGGGGTA
ATGTTACGATTTCTCCGTTCCTCCTCATCTCATCTTATCCTAAATCGTAACCTCGGACACAATCGGATCGCTT
CAACAATGCTACCGCATGTCTTGCCCTTCTTTTCGCATACACAAATGTTCTATTCTTAGTACCTCACTTTTCA
TATATAAGTAGCACAGTTCTCGCTGTATTATCCCCAGCTTCAAACTAAAGTTTATTAATACCGACTTTACTCT
CTACTCAGAGCTCGACTACACTAGATCAGTCCCTCTTTTCGAGCCTCAGACGCCTCAGCCATCCCTCGGTGAT
TGTCCGAACACGAGACAGCCGAGGTTTAGGCTAAAGTCTTTGGTCACGTGAAGTCCGGGTCTAACACTTATTG
GGCCTTACATCGGACCCGACCTTCCTCTCCTCGAAGATGGGGAGATCCTGGAAGAGCCTGAGGCAGGGGGATC
CGGAGCAGGAGAATGTTATTAAATACTGTGCAGGAGAACAGGCCGTATACGACTTCAATGAAAAGTACCCAGA
TCAACCGTACGACCCGCCTGACTACAAGCCTCTATTTCCACAAATGCCCCTTCCTCGAACCCCGCCCCCATGC
GTCCGCATGCCAAGCAGCCGAGGCAAAGGCAAAAGCCGAATGTTCACTGGCACCAGCCCAGAAGATGCCGGAC
CCTCGAACCCCAGTGACCCCCCAAATGGACCCCATTAAAGTCGCTGGAATAACTGAATGGCCCACCCCCTCCA
AGAAAAAGGAACTGCAGTCGTTCCTCAGATTCACCAATTTCTACTGAAAGTTCATCTGAGACTA
>Armme1_1_rnd-4_family-1142#LTR/Copia
GGGTCTTGGCCGACACCTAAGAGGAACTGCTCGCATACCCCCTCACCCTGTCGAGCACAAAGGAAATTTTTAC
CGGTCTACCGACACTGGATTTTCGCACCCACTCTCAGATGATGACCTGGAGAAGCTAGAGGATAAGCTAGAGG
AGTACGAGCAGAAAGAGGCAAAGATGCGCTGCATCATTTACGAATCCATCTCCGACTCTACCTTCAACGAGAT
CAAGGGCGAAGCAACAGCTGCTCTCGTCTGGAAAAAGCTCACTGCTGCCATGACTGGCAAGGGCGATCTTGTA
CGTGAACACCTCCTCACCCAGCTCAGCAACATGTCCTGCCCTGATGAAGGCGACATGCGAAAACACCTTGGCG
AAATGAAAATCCTCTGTGAACGTATCGAGGGCATGGGCCTGAAAATCGAGGACAACCAGTACACGTCAATGAT
CCAAAAATCGCTCCCACCTTCCTATGACCATATCCGCCACACCCTCTCCGCTGCCAGCAAATTCACTGGGAAA
TCCCTCACATCAGACATCCTCGTTGCTGCACTCCATGAAGAATGGGACGAAGAAGTGGCATCGAAGGCCACAG
CTGAGAACGTAGCAATGTCTGTTCAGCGGCAGAACAAAGGAAAAGGTCAGGGCTCAAAGGGGACGTCAAAGAA
AAAGCCAGACAGATCCCACCTTAAATGCACGAACCCCGTGTGCAGAAAGACCGGCCATCTCATTGCAGACTGC
TGGTGCGAAGGTGGTGGAAAGGCGGGACAAGGCCCAGCTTCGAAAAAGAACAAGGCTAAAGAAAAAGGGAAGG
CGAAGGAATCGGCATCAGCAGCCATTGATGAAAGCGATGATGAGCACGTCATTGCGGTTGTAGCTGATGACAA
CGATGACTCAAATGTGGCCTTAATGTGCACGTCGGACTACAAGGACGAGGGCGATGCTCACGTAGCAAGGGGA
GAAGAAGTTACAGCAATCGCTGATTGCGGGGCTTCGAAAACGTACTCACCCGAACGTTGGAGATTCAAAAACT
ACACCAACATCGAGCCGAAGCCCATCAAAGCCGCAAATGGTGAAGTCTTCTATGCGGTAGGCAAAGGGGACCT
CCAATTACCATTACCTATGGGTAAGGATCAAAAACCCACCATTGCGAGATTCCGAAATGCATACCATGCACCG
AAAATGGCATACACCCTCTTATCCCTATCACACGTCAGCCGCGCTGGATTTCCCATCCATATCGAACCCACCG
ATCGCACCATGTATATCCGCACACCACGACCCGAGGCTAAAGTCATAGCCAAGATTCCCGAAGCCGGAGGCTT
ATATATCATCTCAACCCCAGGTACACGCCAGACCACGGGAGCAAAACCAGATGTCGCTATGGCGGCAGGTAAG
CTCAAGGTGAACATCACTGAGCTACACAATATCTGTGCTCACCGAAGCCATGCCACTCTCCGTCGCATGTACG
TTGATGGCCATATTGAGGGAATCGAGCTTGACCTGAATTCGAAACCGGAATTTTGCGAGACCTGTGTAAAGGC
CAAAGCGAAGCGCAAACCATTCCCCAAACAAGGCCGATACGAGTACGTTGAGAATACCGGCGACAAGGTTGTT
GGAGACTTAATGGGACCGATGTCTGTCACATCACTAG
>Armme1_1_Cluster5#LTR/Gypsy
GGCAGTGCTTGCTGGTTTGGGAAGGATGGCAGAGTGAATGGAGCGAGCAACTTCTCATTAAAGTATGGGTGGA
CCTTCCAGTGGGATGGGAGTTTCAGTTTATATGCTGATGCTCCAGCCTTTTTGAGGATTAAGAAGAGGCCAAC

TCATTTGTCATCGAGTTTTTTCTTGGGATGGGGGAGATGTAAATTAGCTGCATTGAGCCAAACATGGTCGCTG
ATTTTATAGTCAATGGCCATGTGCTTCTTCTTGTTGTATTGGGCCTTCATGGCATTTGTGGCTTCCTTGAGTG
CTGCCTCAGTCTGTTTGTGGACTTCTGCCATAGTTTCTGTGAAAATTCTGGCTGCAGGATTAGTGAACAGGGA
GAGTCTAACAGAGTTGGATACTAGTTGAGGGTTTTAGCCATAGAGCACCATGAACAGACTTTTGCCAGTGGCA
GAGTGGATGTGATTGTTATGAGCGAACTTGGCCATGGAGAGCCAATCACTCCAGTCAGTTTGTCAGTAATTGA
TGAAGAGGCAGAGATATCCCTCAAGCTCTTGATTGACTCATTTGGTTTGTCCATTGGTCTGAGGGTGAAAGGT
GGTGGATGCATTCAGGTGATGTCGAGTAAGCAATACAAATCCTTCATGAATTAGAAAATAAACTGGGGACCTC
AGTCAGAGATGACTATGGTGGGCATATCATGTTTGATGTAGACATGGTCCTAGAGTATTTTGGCCCACCCTTT
AGCAGATAATTCAATATTGCAGGCAACAGGGATGATAGCTTTGGAGAAACAGTCGACGATCACTAGGAGGGTG
TCATAGCTGTTGGACTCTGGTAGTCCTATAACCATGTTAACCAAAATGTGGGTCCATGGTTCAGATAAGATGG
CATTTGGATAAAGTGGAGTGGCTTTGGCCTGCATGCTTGCCTTGGCATGCTGACATGCTTCACATCCCTTCAT
GTAGGTTTCGAGTAACTAAGAATAGAACTATTGTCTGTGGTTGTCATGTATGAAACAATGAAAACAATGTGGA
AGATGAGTGATGGTCATGAGCCATAGTATAATCATTGAAAGTGTCCAATATAGTTGTGGTGACAGTGAGTAGG
TGGCTAGATAAGCAATAAGATGAAGATAGGTGCCTGATAAGATAAGATAAGCAGAAAAATCATAACATTACCC
TCTTATTAAGACAGTCATCCTGACTGTCAAGGTTCATCCTCAGGGGTGTTGAGCTGATGAGTGAGGTCATTAT
TCCAATATTCTTCTATAAGCTCCCTTCCTGGGTGTGCTCCATCTTAGTGTCTCGTACCCATGAGTTGTGCTCT
TGGGTCCATCCCTTCCATTTGACAAAGTAGTCGGTGATGACTTTGGACTTCTGGCCCCTCTTGGCTTTGACTC
AGCAGATTCTGGAGTCTTCTTCCTGGGTGTGCTCCATCTTAGTGTCTCATACCCATGAGTTGTGCTCTTAGGT
CCATCCCTTCCATTTGACAAAGTAGTTGGTGACGACTTTGGACTTCTGGCCCCTCTTGGCTTTGACTCAGCAG
ATTCTGGAGTCTAGGACCTTCTCCACTTTGTACTCTTCTTCATCTTTGATGAGATCTGGGGGAGGTGGTGGCA
GTGCTTGCTGGTTTGGGAAGGATGGCAGAGTGAATGGAGCAAGCAACTTCTCATTGAAGTGTGGGTGGATCTT
CCAGTAGGGTGGGAGTTTCAGTTTGTATGCTGATGCTCCAGCCTTTTCAAGGATTAAGAGGCCAACTCATTTG
TTATTGAGTTTTTTCTTGGGATGGGGGAGATGTAAGTTAGCTGTATTGAGCCAAACATGGTCGCTGATTTTGT
AGTCAATGGCCATGTGCTTCCTTTTGTTGTATTGGGCCTTCATGGCATTTGTGGCTTCCTTGAGTGCTGCCTC
AGTCTGTTTGTGGACTTCCACCGTAGTTTCTGTGAAAATTCTGGCTGCAGGATTAGTGAACAGGGACCGT
>Armme1_1_Cluster1#LTR/Copia
GGAAGATGTTGATTATAACAATGTGTTACATCACATGCATGTGACTACCATACATAGGTGCCATTTCCTGTTC
TTCTCACCTCAGTCATTCCAACATCCTGGCTGAGATAGTGATTCCCTCTATGTGTACAGATTCTAACAGGTTA
TGGGCATTTGAAGAACTCTTCACCCTGTACACTTCATTTCTTCTGATTACTATGTGTGGCTAGCCTCCTGTTT
CTTCTTTTTTTTGGCACATATACCCAGCTTTTCTGAATGATGTGATGTCCTGCCAGCACCTCTTGTTAAAACC
CTAGCTCCCATTGTCCTTTAAATCATGCCACTGATGACCCTGACATCAACAGTGAGCATACCAATGGCAACAT
TGATCCCAATGATGATGTCGACAACGAGGATAACGACCCCATCAACAGGTTCCCTGACCCCATTCCTCACCCT
CATTGAATTAATACCTTCATGCCTGACCCCATCCCTGGACATCAGGCTGTTGTCCCAGATCCAAACACTGTGA
CCCTTCAACTCTCCGTGGCAGAGATTTTCTGTTTTGACCGGACTCATCCACACATCCATGTCTGCCAAATTCA
TAACACATCATGGCCTTTTGTCCTTGCTCTGGATCTGACCTCCCAGAACTAGAAGGAGTGGTCCTGTATGGTC
AACAACATTTTGAATCTCATAGGCCTTTGCCCACATTGTCAGTGATCCCAAGTTTGTCATGCCTGACAGACAG
CTGGAATCTACCACATATGACAATTTTTTACACAACAACCGTGTTGTTATGGCTTTCTTGGATCTATATCTCA
CCAAGACAGAACAAATGTATACTGTTGGCTCTGCTGATGCTCATTCACTCTGGTCCAGACTTCATTCTTGCCA
TGAGACTCAAGGCATCACTTCACAAGCCTTGCTCATTGAGGAGCTTCTGCAAGTTGTTTACTCTAAGGACACA
ACTTTCGCTGCTACTACTGAGCATTTGCATGAAATTAATAATTGCATTTGGGACATGGGTGCTCCTACATGCA
ACAATTTTCTCTGTATTGCTATTGTCAGAGCATTAACTCCTAACTTCCCCAACCACCATCTCGCTGTCGTACA
TGAGCTTGCTGCTGGCACAACCCTCTGACCTTGTTCATCTACAAATCTGATCAAGATTCTTGAGCTTGACACC
AAGTTTACCTCGAGTGGTCCTGCCCCTTTTGCACTTGCTATCCAACACTTATGCATACCCATAAAGGAAGTCT
GCACCATATGCCATAGATCAACTCATTCTGCTGCATACTGTGTTCATCCAGGAGGTGGCATGGCTGGACAGAC
TGCCCAAGCTGCCCACACAAAGTGTCTCACTGATAAGGGTGTTTTCAATATGTCCTCACCCTCTCCTGCAAAT
ACCATGGCCAATACCAATACAGCAAATACTGCCACATCTTCATCTCGCATCCACTATAATAATCATCTCCTTG
CTATGGGTTCCTGCCTTCCAAACTGAAATCTCTACAAGCTCAACTGCACAAATGTCCATACTGAACATGCCAA
TTTTGCTGCTCAGGCTGTCCCTACTATTGACATCTGGCATCATCTCAGCCATCCAAACAACCATGCTATCCAC
CTCATGGCTACCAAACACCTTGCTGAAGGTATGCCTATCAACCTCTCTTCCCTTCCACCCTCATGTGAATCCT
GTATCCTTGGAAAACAGACTAGGTCATCTGTCCTGAAAGTTCACCAAGGTTTGAGAGCATTGAAGAAGTTGGA
GGTGGTTTCAGTCAATTTGACTGGACCAGAGAATGTGGTGTCTGCATCTGATAACAGATATATGATGTCCATT
GTTGACAAATTCATGTGCATGCATTTTGTCAAGCCTCTCAAATTGAAAGATTCTGCATTTGCACATGTCATGG
CTCGGGAACTGCAAGTTGAGTGTGAGACAGGACAATGTCCTGTGAAGTACAGGATTGACAATGGTGAACTGAA
GTCAGTGCAGTTCGAGAATTGGTGCTCATCCTGGGGTATCACCCTTGAGTACACTGCCCCTTATATGAGTGCT
CACAATGGACAGGTTGAGAGGACACACTGTACAATTATGAGCAAGGCTCATACTATGCGCATTGATTGTGGTA
TGCCCCCTAATAGGTGAGACAGATTTTGTCTTACAGCTGCCTACTTATCTGCTCAGACTCCTGCTGCTGGTGC
TAAGTTCGAGACTCCATTCCAGTGTTGGCATAATCGCTTGCCTAATCTCTCCCACTTATGTGAAATTGGTTGC
AAAGCTTTCGTTTTTATTCAGCCTACTGATAAGAACCTGAAAATTTATCTTAGGTCTGTCAAGTGCATTTTAA

TTGGTTACTCCCACTCTTCAAAAGCCTACCATTGTTTCCACAGGCCCACCAACAAAGTTGTTGAGTCTTTTCA
TGTCTGTTTCATTGAACACAAGGACTCTGTCGAGCAGCCTCTCCAACCTGGTGTTATCAGTTATCATTAATCC
TTTGGACTCTCCTTCCCTGGCTCAGTCTTCACCCCTTCCTCCTGCTACAATTGATCCTGGTCCTGATCCTCTG
CCTTCTCTCAGATGTTCATCTTGCTCTATTATCCCTACTGAACATGCTGCTGAAGCTCAAGGTATTCATCATA
TCCTTGCTGTGCAGGCTGCTACCCTGGCCTCTGCCAAAACTGGAGCATGACTCAAGGCTGAGGCACTGCTGCC
AAAGACCTTCATCACCAAGCTTGACTTGAGGTCTGCACTGCTCTCCTTGATAACTCCAGCCAGACAGGGCCCC
TCCCTTGCTTCCATTGCCAATGACAAAGACATTCTCCTTGATGAAATTCTTGCTGCTGCTGAACTATATGCTG
CTGATGACATTGCTGATGATAATCCCACCACCTTACATGAAGTCCTGGAGTCCCTAGAATGTGCATCTTGGAT
CACTGGCATTCAGGAGAAGCTTTCCAGCTTGAAGAACATGGGTGTCTACAAGCTTGTTCCACACTCCTCCATC
CCTTCTGGTCAAAAAATCCTCTGAGTAAAGTGGGTCTTCAGGCTTAAGTGTGATAGCTTTAATATCCCCATCC
ATCACAAGGCTCACTTGGTTGTCTGTGGATTTGAGCAGATTTATGGAAGGGATTATGACTGTACTACTTCCCC
TACTGCTTGTATGGAATCTTTCCAATTGCTCCTCCATATAGCAGTGATGTGCAACTGGGATATACAACAATTC
AATATTCATATGGCTTTCTTATATGGTCTGCTTGGAGATGATGAAACCCAGTGGATGGAACAACCCAAAGGCT
TTGCTGAAATTGGCAAGGAGGACTGGGTGTGGCAGCTGCAACATGGATTATATGGTATGAAGCAGAGTGGGCA
TATCTAGAACCGAACTCTTAATGAGGCTATGCTATCTTGGGGATTCAAACATCTGCAATGTGAATGGTGCATC
TATTATCGGAAGATGTCCACTGGCATCATTATTTGTGCTGTCCATGTCAACAACTTCCTCTCAGTCAGCAGCT
CCCTAGAAGAGAACAATCTCTTTTTAGCCCAGCTATGGTCCAAGTGGAAAATCTCTGTAGAAGATGTGAGGTT
CATTGTTGGCATAGCCATTGAGTGTGACCATATGTTGTGCTGGATCTACCTCTCTTAGACTGCACTCATCGAC
AAGGTGCTTACCCAGTTTGGCTAGGTGGATGCTGATCCCAAACTGATCCCCATGGACCCCAACATACACCTCA
CATGCCCTAACCCTACTCTAGTCCTACCTCAGGAAGAGTCTGCCTCTCTAGCTCACTTGCCCTACTGCTCACT
TGTCAGCTCCCTCATGTACATTGCCATTGGTACTCATCCTGACATTGCTTTTGCTGTTTCCAAACTCACTCAA
TTCCTTGACTGTTATCACACCTCCCATTGGAATGCTGCAGTCAATGTGCTTTGATACCTCAAGGGCACTTGTA
CCCTTTGTCTGACCTTAGGTGGTGACAGATGCTCCCTTGTTGGTTTCTCGGACTCATCCTATGTGGATTGCCT
TGATGATCGTTGCTTAACCATGGGATACTGTTTCTCGCTAGGCCTTGGGATGGTCACCTGGAGCTCATACAAA
CAGAAGACTATTGTGACTTCAACTACTGAAGCTGAATACATTGCTGCCTCAGAGTCTGTCAAAGAAGCAATGT
GGATATGTCAAGTATTTTGGGAGATTCAACTTGCAGATGCTCAGCCTACCCCCTTGCTCTGCGATAACAATGG
TGCTATGTGCCTCGCTTTTGACCCTGCTTTCCATGCACATGTCAAACACCTCAATGTTCATTACCACTATATC
CATAAGCGCATCACTGAGGGATCCATCAGTATCCATCACATTGCTTCCAAAGAGAATGTGGCTGATGTCTTCA
CCAAACCTCTACCACAAGTTGACTTCCTGCATTGCCGTACAGACCTTGGTTTGATGTGATGTCCATGCAAGGA
GGAGATTTGGCGTTGTTCTCTTTTCTTTGAATGTTTCTTTTTTTTGTGATATTCTTCTTCCTTTTTTTTTTTG
TGTCTGCATTGAGGAGGAGTGTTGATTATAACAATGTGTTACATCACATACATGTGACCACCATACATAGGTG
CCATTTCCTATTCTTCTCACCTCAGTCATTCCAACATCCTGGCTGAGATAGTGATTCCCTCTGTGTGTACAGA
TTCTAAC
>Armme1_1_Cluster64#LTR/Gypsy
GAACTCGACTAGCCAGCCTAGTCCCTCTTCGCCGAGTCTTAGACCCTGTAACTATTCCCCGGTGACTGTCCGA
ACATGAGGCAGCCGAGGTTTAGCCTAGCGTCTTTGATCATGTGAAGTTCGGGTCTAACAGTTAGCTCGAAGCT
TCCCTTCCTGTTTTCTCACCGCCAGCCACGAAGAAACAGACCACCCATGAACCCCTTCATACTCATCGCAATC
ATTACGCTCGCCCTCTTCTTTGGATTCATAGGAATCCTAATCATCATCGCCATCTCCGTATACTACGCGGAAG
AAATAAGGAACTGGCTGGGCCAGACCCTCATACGAATCCTCGAACACGTAGAGGAACAGAACCCCGACGCCGC
CCGCCATGTTCAAGTCCAACAACCTGTTCAGAACCCCATCCCCCTCTGACGCCGATCCATCCACACTGCCCAC
TCCAGCAACGAACCAACGAGTCTGGAAAATCCCCCTCGCAACACTTCCCCTGGACCGAGCAGGCCTAGGGGAG
TACCCAGAACCCCCACTCCCCCGATGGCCACTGAACCCATTCAACCTATTCCCTGAACCCCCAGTCCATGCCC
CAGCACCAAGGAGATCGGCAGGTACATTCTCCACATGCTCAGATTCGACGAGCCCCCCGCCTACTTCGACCAA
ATCACCTGTCCTGACACCACCCATTGGCATGGACAGCACGAAATTCATGTTGAGGGGCCAAACTGGACGCTAT
GAGGAAGAAACCCCTACCCAAAAGAGCTCAGCGAGTCAGACGACTCGGAAACCACAGTCGCCCTCAACACAGT
CCCAGTCTTCGATGACAACTACATTCCATATGCCCCCGAGCCAATTCAAGTCGCCTCTCCCTGGACAGAGGAA
GACCCCCTCCCGTTCACCTTCACCAGACCCACCTCTGTCCCCCCTGATGCTCCACCAGCCCCTCCACCCATCT
TTGACGACGAAATCCTCTTCCACGCCTGCGTATTCAACCACGACGCACTCAACGACCCCTTCAACCCAAGGGG
TCTCGACTACGAGTGGCCCGTCCTCTCTGCCCGTGACAGAGAAATATTCAGCCCCGCCACCTGGATGGTTCGA
CGAGCAGACGTCGAAGCCCACGCCGTGGGGCCCCTACCTCCCCGCTCCTTCATGGACAACTGGCTGGCCATCG
CCAGCGGAGACCCCATCGAATCAGGAGGCATGGTGGCAAACCTCGAGGCGAGACTCAAGTCCAACAGGGACCG
ATCTCTTTTCCCCGCCAATGCCAGACCCCGTCTTCCCGGGCTACAAGAAAGGACCCCCGCAGAGCAGAGTCGG
CTGGAGATTACCCTCCTCGACCTTGCTGCCAACCATGAAGACGATCGAGCAGAGGGGCGGGGCGAGGAATTTA
ATTAATTTTGACACATACGATACAGAAGCATTCGGCCGGGGCGCAGCATATAATGCCCCCAATATACCTTTCT
TCCCTTATGACATGACCGAATACCCAAAACCACTCCCAGACGAACCCCCCTTCCCACCACCACATGCCGACAT
GCCGAGAAGCCGATACCGACATCATGGCGGACCACCCCTAGGAACAAGCCGACTATTTGCTGCAGGGGACCAT
CAACCAGAACCCGGAGGCTCGGGACCACCCAACCTGGAGACACAGTTAACCCCCGAGCAACGGAAGCAACAGG
CGATGGCGGAAAATGAGGCTCGGACACAAAAAGTGGCCGAGCTCCAAGCCCAACAAGCCGAGCTGGCGCGACA

GTTTGCAGAGGCCGAGGCCAACCGAGCCTGGTTCGCCAAGTATCACGGGTTCAAAAAAGGAAAAGAACCCGAA
TGGGGAAGACCTCTTCCCCTGAACCACATGCGGAAACTCAATGACCGCGACGATCGCCACTCCATTCCATGAC
CCCCGAACTTCCCCTACCCAGCTGCACCATACCTGCATCCAATCGGAGAAGCCAATGAAGGCACCCCCTATCT
CAGTCTAAAGCCACACTGGAAAACCCCTGAGCCGTTCAAAGGCCACCATGACGACATCAAAAGGTTCCTCAGG
GACTGCGTCATCTACTTCGAACAGTTCAGAAGTTTCTTTCCCTTTCCCTCCCAGAAGGTTGCCTGGGCAACCG
CGTTGTTCCAAGACAAGGCCAAGGACTGGTGGGTTTACCGCTGCCAAGAATTCTGGCAACCCTCCGACTACGA
CGGACAACCCCCATGATTCCGATACCCAAGTTGGGAGGAGTTTGCTGATATGATCGCCGCTCAGTTCAGAAGC
CCCGTGGTAGAAGAAAGGCACGAACAGGAAATGAACAACCTCCGGATGGGTAATGGACCCACCAACGACTACT
TCCAGGAACTGGAAAGATTGGCCAAGCTTGCCGGAAGAAGAACAGAGGAAGAAGAACGTGGAATGCTAGTCTG
AGCCATCTGACGTGGGGTCCCATACGGCTACATAAAGATGATCGGTGATATTGGGGAGAACGTCCCCACCACG
TACCCCGACTGGAAAAGGCGCATCTGCAGAATGTACGAGGAACGACAAAAGCAGTACATCATCCAAAAAATGC
TCACCTCGTCCGACTCGCATCCTGAACACCACCAGGCCAACAAGACTGGACCACCCAACTCCAACCCCAGCAA
GTCTGCTGGGGCCACAAGTTCGACGCCAGCAAAAACGACCGGAGGAGGGGGAGGCCTGAGCAGGGACACGTCC
ACCGGAAAATGGGTCCCAGTCAAATCCACCATGTACGCCGGACAGGGACAGCCAATGGAAATAGACAGGCTCA
GAAAGGAGGGCAGATGTTACCGCTGCAAGGAGAAAGGACACCTATCCCGGGACTGCCCGAAGAGGAAAGAATT
CAAGGACATTCGCTCGATTGTAGTAGCAGAGCAGGAGAAAGAAACGGAGTCGAAGGTTGAGGAGGTAAAAGAG
TAGGTCACCAAGGGCTCGATTCCCCTTCGGCCGGTCTTATAAAGCTTCTTTAGTTACAAAAAAATGTGCTTTG
TCTACCGCCTTATATATCCATGCTTTTAACGTCCCCAGCATGACCAGCAAACCAGTACCATCAGAATCGAACC
GCTATGCCGCTCTAGCCAACTTGAGCGGCTCGGACTCAAGCGCTGACGAGGCCGAGATCTCTAACACCGAACA
GGGAGCAGGACTTACAACAGTCCTGGCAGCGAGCCCCCACCAACTGCCAGAACCCCTAACACAGGAACTGCCT
GCACGTGAACCCCTCGGGGGGAACACCTCTGCAGGACTGGCTAATCAGGGCGCTAAACCCCAAAGGGGGAAAG
GCCCTGGAAAGCAACCTCAGGAAGTCGCAAGAAATGAGGCCTCAGTAGGCGTGCCGCAACAAATGGTCCCACG
AGTCGTGGAGCACAGGGGAGAAGAGCACAGCCTCACCGACGGCCAGGCATTGGAGCGCAGGGACAGCCACAGT
TTATTCGACAAGAATGAGACGGACACGGTGCGTCCCCCGTCCCTCGACGGGATGGCCACGAAATACCAGATCG
ATGTGAGTACCCTCAATGGGACTTCCCCCCATCACTCCTCCTGCCGTTGGATGACCAGAAAGCCACCAGGGT
GGAAGTTGTTAGTGCCCAGACAATAAAAAGGGGGCCTGCTGTAACCATGGAGGAAGTACCAGACAAGGAAGAT
GAGACCGCTTACCAAAAGTGGTGGGCAGAACAGAAGGCAGACCAGAGGGAGCAAGATATCCAGAGCGACGACG
ACTGGATACCCACCAAGCCTGATGCGAGTGCCACCCCGCACTCTTGGGCTTGGTATAAGCCTTTTGAAGTCGA
CTGGACTCTCCGAGCCATCTGTGAAGCATGCACACCACAGGAGGCCAGGGCCACCCTAAGCAGCTGGATCCAC
CAGGACCGACACCACAAGATCATCATGAGTCTAGTCAACAGACTCCGATGAGGTGGGGAGGAGGCGAGGGAAC
TCATGCTGGAACTCCAAGAACCCATCCGCTACATCCAGAGGACGAGGAGCAGCGACAGGGAACTCGCCGTCGA
CATCCTCCTAAACACCTGCATGAACCAGCAAACCCTATCAGCTGCCGGCCTCATCGATAGCGGGTGTACTGGA
AGCACCATCAATCGTTCCTACATACAAAAACACAGCCTAGACACCAGAAAGACCGTTGTCCCGATCCCCGTCT
ATAACGTGGACGGAACCCATAACCAAGCAGGAGACATCACCGAATTCGTGGAACTATGCCTCTCTATCGGAGG
ATACCACGAGCGAATCGACCTGGCGGTCACCGACCTCGGCTCGAAGGACCTCTGCCTCGGCCACGACTGGCTT
AAACGCCACAACCCAGTGATCAACTGGAAAACCAGCTCAATCATTTTCGGATGCTGCCAATGCACCAAAAATC
CCTTCCCACTCCCCGATGCAGACCCGGAAGATCGGTGGGATGAAGAACTCAAGGAAGGGGAGACTATACTCAC
CGTAAACATGCATGAAGAATTAGTCATCCTGGCGGTACATCACGCCAACGACCTAGCAGCCCAGGCAAACACA
GAAAAGCCAAAGAAAACCTTCGAAGAAATGGTCCCCGAGCACTACCGCTCCTACCGCGACCTGTTCTCGAAGG
ACAATTTTGACGAACTCCCAGACCGAAAGCCTTGGGACCATGCCATCGAGCTCATCCCAAACGCCAAGTCCAC
CCTCGACTGCAAGGTGTACCCCTTGAACCAAAGTGAACAGGAGCAACTAGACAAGTTCCTCAACGAAAACCTG
GAGACAGGACGAATCCGACCCTCTAAATCCCCCTTCATCTCACCATTCTTCTTTGTCAAGAAGAAGGACGGCT
CGCTACGACCGGTCCAAGACTACCAAAAGCTCAACGAAATGACAATCAAAAACCGATATCCGTTACCACTCAT
TTCTGAACTTATCGACAAACTCCGAGGGGCCAAATATTTCACCAAGCTAGATGTCCAGTGGGGATATAACAAC
GTCAGAATTAGGGAAGGAGATGAGGAGAAGGCGGCATTCCGCATGAACCGAGGACTGTTCGAACCAATGGTGA
TGTTCTTCGGACTCACCAACTCACCCGCCACTTTCCAATGGATGATGAACGATATTTTCAAGGACCTAATCGC
CGAAGGAAAAGTCACCATATACCTCGACGACATCCTCATCTTCACCAAGAATCTCGACGAACATCGACGAATC
ACCTGACGCATCTTACAACGCCTCAGAGAGAACAAATTATTCCTCAAAGCCGAAAAGTGTGAGTTTGAGGTCC
TCGAGACTGAGTACCTAGGAGTCATCATCAGCGAGGACTCCGTCCGAATGGACCCCGTCAAGGTTGCCGGAAT
AGCAGAATGGCCCACACCGACAAAGAAGAAGGAGCTACAGTCTTTCCTTGGATTCACAAACTTCTACCGGAAG
TTTATCCGAGACTATTCAAAAATCGTCCGACCACTCACTCAACTCACCGGAAATGCGGAGTGGACCTGGGGAA
TGGCTCAGAATCAGGCCTTCCAGCAGTTAAAGAAGCAGATGGCAGAGGATGTAGTCCTTACCATCCCCAGTGA
AACGGGCCAGTTCAAAGTAGAAGCCAACTCCAGCAATGGCGCCATTGGTGCAGTACTGAGTCAGAGCCGCGAC
AGGAAGTGGCGACCAGTAGGGTTCATGTCCAAATCTCTGACCGTCACCGAAAGGAATTATGAGATCTACGACA
AGGAACTACTTGCGATCATGCTCGCCCTAGCCGAGTGGCGACATTATCTGATGGGAGCCACCGAAGAAGTAGA
AATCTGGACGGACCACCAAAACCTCCAGTACTTCTGAAAGCCCCAGAAACTCAATCGCCGGCAAGCAAGATGG
GTCACCGAGTTAGCCGAATACCACTTTGCCCTTCATCACCAACCCGGCACCGCCAACAAGAAGGCTGACCTCC
TTTCCCGACGAGATGACCACGATCAAGGGAAAGGGGACAACCAAGATGTTCTGGTCCTCCAACCGCATCACTT

CCATGCCCTAATAATGCCGACAACTACCGAGACTCATAACAAGGTCAAGGATGCCCTCAGAAGGGAGGAACTC
TGGGACTAAGGGATTGCGAACTCCCTTGCCCATGAGCAAGGAGTCACCAAGGAACACGGACTCCTGCTCTATG
ATGGATGGATCTACGTTCCCAAAGACCACACTCTCTGAGGAGAGATCATCGCCCAAAGCCACAACCACATCAT
GGCGGGACACCCTGGGATAGAGAAAATGAAGGAGCTCGTCCAACGAGAATACTGGTGGCCGAAATTGAAAAAG
GATGTAGAAGCCTACATGAAAGGATGCGAGGCGTGTCAATGCTCGAAGTCTAGCAACCAAGCAAAGGCCGCTC
CTCTACATCCAAACGCCGTTCCAGCCGGACCATGGACCCACATCTCGGTCGACATGGTCACCGGTTTACCAGA
GTCGAAGGGCCATGACGCCCTCCTGGTCATAGTAGACCGATTCTCCAAAGCAATTATCCCAATAGCCTGTAAC
ATTGAATTATCGGCGGAAGGATGGGCCAAGGCCCTCAGGGACCATGTCTATGCAAAGCATGGGATGCCCCAAG
TAGTAATCTCTGACCAAGGACCCCAGTTCGTCTCCCAGTTCATGAAAGACCTATACCAACTACTCGACATCAC
CCCCAATGCATCCACGGCCTTTCACCCACAGACTGACGGGCAAACTGAACAAGTCAACCAAGAACTCGAAGGC
TACCTCCGCCTCCTTGTAGAATATCGACAAACCGACTGGAGCGACTGGCTACCGATGGCAGAATTCGCCCACA
ATAACCGAATACACTCTGCAACCGGCAAGAGTCCATTCATGGTCCTCTACGGATGAAATCCCAGACTGGTCCC
AGACTCGACACGTCCTTCCCCCTTCATAAACCCCGCTGCTGAAACATTCACTGAAACCATGGCAGAAGTCCAC
CAACAGACCCAGAAGGCTCTGGAAAAAGCGGCAAACACAATGAAAACTCAATACGACAAGTGAAAGCGGCCCA
CCATTGACTACCACGTTGGAGAAAAAGTCTGGCTCGATGCCAGAAACCTACACCTCCCTCGACCAAAGAAAAA
GCTTGATGACAAACATGTGGGACCATTCAAGATCCTCGAAAAGACAGGAGCGTCGGCATATAAACTGAAGCTT
CCCCCGCACTGGAAAATCCACCCATGCTTCAACGAGAAGTTACTGACCCCATTTGTACCACCATCGTTTCCTA
ACCAACAACAACCACCACCGCCACCACCAGACCTCGTCGATGACGAAGAGGAAAGAGGGGACAGAGATCACAA
ACCATCATCGACTATTTCGTCAAATGGAAAGGATGGACCAGGGAACATAATTCGTGGGTACGCGACTCGGAAA
TGGGAAACACACAGGAACCCATACGGGATTATGAGGAACGCATCCACAGGGTCAAAATCGCAACCTCGGGACG
AGTCTTAACAGCAGTCGGGACACCTGCCTATCTCGGAGAATAGCATTTTTGTTTTCAGGCGAGGGACAGCCAC
AGGAGTGACATCGAGCAGGATTTGCATCCATGGCCAACAAAGCCAGGCGCCATGGCTCAATAATTGGAGGCAA
AAGCTGATGTTGAGGATCTTGCAGGCAATTACTGGCTCCACTTTGAACATGTTGCTTCTTTTGATCTGCATGA
GTTATCAGAACCGGCACGTACATGCACTAGCACCATGAATAGATACAATGTTACAATTTTTCCGCTTATCTTA
TCTTATCCAACGTTTCTCACTAATCTCATCTTATGATGAGGGGGTAATGTTACGATTTTTCCGCTTATCTTAT
CTTATCCAATGTTTCTCACTAATCTCATCTTATCTCAAAACCCCTATCCGTATTACCACAACCTAGTCGAACA
TTCTCAGCCCTTGTACTACGGCTCGCTACTATCGCTCACTTAACATACGGACTTCGCTTCTTTACCACATGGA
CAACATTCTATTCTTAGCCACTCAGTTTTCGTATATAAGTAGTGTAGCTCTCACTGTATATCCTCAGCTTCGA
GCTAAAGTTAATCTATACCATACTTTGCTCTCGCAACAGAACTTGACTAGCCAGCCTAGTCCCTCTTCGCCGA
GTCTTAGACCCTGTAACTATTCCCCGGTGACTGTCCGAACACGAGGCAGCCGAGGTTTAGCCTAGCATCTTTG
ATCACGTGAAGTTTGGGTCTAACATA
>Armme1_1_Cluster11#LTR/Gypsy
CGCGATGTTACCTGCTATTGAATTCGCGATCAACTCGGCTCGCTCGGAAATTACTGGTTACGCACCCTTCTTT
TTGAATAACGGACGGATGCCGCGATCGATGATATGGGATAACGCCGCTAAGACTGAATATCCTGGCGTGCGTG
TCTTCGCAATGCGGATGAAGCAAGCAATTATTTCTGCCCACGATTCTGGAAATTACTGGTTACACACCCTTCT
TTTTGAATAACAGATGGATGCCACGATCGATGATATGGGATAATGCCACTAAGACTGAATATCCTGGCATGCG
TGTCTTCGCAATGCGGATGAAGCAAGCAATTATTTCTGCCCACGATTCAATTCTAGAATCACAGGTCAAGCAA
ACACGGGATGCTAATAGGAAACGGCGCACCATTCCGTTCACGGAAAAGGACTTGGTATACATTTCAACCAAGA
ATATATCCTTTCCGAGAGGTCTAGCCCGCAAGCTGAACCCCAAATACATTGGTCCCTATCCTATCTTGAAGGA
CTTTGGGAACGGATCATTTAGAATTGAGCTCCCGTCGAACTTGAAGGCGAGAGGCATCCATGATGTATTCCAT
GCATCGTTGTTGAGGGTCCACATCCCAAACAATGACCGATTGTTCCCAGGCAGAGCAGAAAACCAAATGGGAT
TGACTGACGAAATGTCAGGGGAATGGGCTGTAGAACAAATCATAGGTCATTCTGGTTCGAAGACGGACTCCCT
ATTCAAAGTAAAATGGAAGTCAGGAGATGTGACATGGTTGCCATACGAAAAGGCTGACCATTTAGATGCGCTA
GGAGAATATTTCGACGCGCTAGGGATTTCGGATGTCTCACAACTTAGAGACAGCCGAGCACCAGCTCCTGGGG
ACGATGAATTAGACGTTCTGGGGCTGTCGGAGGTTCGGGTGTCCGCCATGGAAGTCTATCCTGGTTGGGAAAA
TCCCTCCGTTGGTCAGCTCAATAAATTCGAGGAGAAAATGACCACAACGCACCCTAGCATTGGAGGTTTGGGT
GTCCGCCATGGAAGTCTATCCTGGTTGGGAAAATCCCTCCATTGGTCAGCTCAATAAATTCGAGGAGAAAATG
ACCACAACGCACCCTAGCATTTTGCTATTCCCCTCTGCTTCATCTCCATACCAGTCTTACCATTTGTCCCTTT
TCCCATCCTCTGCTACCATGTCTCCCAACCCTCCTGCCGATATGCGCCTCAATATCCTAGGTCTTGATTCCTA
TGGCACTGGACTTTGGACAGTCTGATACGCGCCTCAATATCCTAGGTCTTGATTCCTATGGCACTGGACTTTG
GACAGTCAATACGTGCCTCAATATCCTAGGTCTTGATTCCTACGGCACTGGACTTTGGACGGTCAACGACCGC
GAGGAGAACCAAAAATTCCTCACCAACTCCGAGTCCATCCATATGTGCCTCCTCCTTAGCGAACGTGTTCGCA
AGGGCACCTTCGATATCTGCCATGACCCCATCTCTGCCATTTACTTCCCTCTCACCCGTATCTTCAACAACTC
CTGCGTCTCCTTTGACGTCCCTTATCGCATGGTCTGGCTCCTGACCGACCGCGTCACCAGTAAAGTTGTCATT
GAGGGGGAGCCGGCCAAGTTCAAGCACCTCTTCAGTGATGACGACGAATATGCGAAGAAGATCAGAGGCGGCA
TTGCCATTTACTTCCCTCTCGCCCGTATCTTCAACAACTCCTGCGTCTCCTTCGACGTCCCTTATCGCATGGT
CTGGCTCCCGACCGACCGCGTCGCCAGTAAAGTTGTCATTGAGGGGGAGCCGGCCAAGTTCGAGCACCTCTTC
AGTGATGACGACGAATATGCGAAGAAGATCAGAGGCGGCATCGAAGATGTCGACGACACGAAGTTCCAAAGCA

TCACCACGTACCTTTTCAAGGAACTCGGATTGGGGACTATGTCCCTCCCCGACGCAAAGATCTATATCCATCA
GCTTCGCCAGGCTGGTAAACGTCAGGCGAAATTGACGCGAAGTTCCAAAGCATCACCATGTACCTTTTCAAGG
AACTCAGATTGGGGACTATGTCCCTCCCCGACATGAAGATCTATATCCATCAGCTTCGCCAGGCTGGTAAACG
TCAGGCGAAATTCGAGGAGAGTTGGCAGGCCAGGCAGGCCCACAAGCATTTCAGGGAGGCGACGGGGAGCGAC
GACAAGGCCAGCGGTTCCAAGCCAAAGAAGCAGAAGACCTGAGCTGAGCCCACCATCAAGGGCTTCGACGTGG
CCTTTGTTCGTCCTGTCAAGCCCAAACCGAAGGGCTGGAAGAAGATGTCGATGGTGACGCGGCCTTCATTCGT
CCTGTCAAGCCCAAACCGAAGGGCTGGAAGAAGATGTCGATGGTCGAGAAAGCCGCTTACGTCGACAAGGAGC
AAGCCAGCGAATTGGGAGACGAAGATATGGCTGAGGGAGATAGTGCTTCCGTGGAGGCTGATCGGTTCTGATC
GGAGAGAACTCGGTGATTTCTCTGGAGGGCGAATGTAACGATACCCAGCACTGCCACTCCTTCCGTTCTCGTA
ATCTTTATTCTGAGAAAGCTGCTTACGTTGACAAGGAGCAAGCCAGCGAATTGGGAGATGAAGATATGGCTGA
GGGAGATAGTGCTTCTGCGGAGGCTGATCGGTTCTGATCAGAGAGAACTCGGTGATTTCTCTGGAGGGCGAAT
GTAATGATACCCAGCACTGCCACTCCTTCCGTTCTCATAATCTTTATTCCGCTCACTAACAAGGAGCAAGCCA
GCGAATTGGGAGACAAAGATATGGCTGAGGGAGATAGTGCTTCCGCAGAGGCTGATCGGTTCTGATTGGAGAG
AACTCGGTGATTTCTCTGGAGGGCGAATGTAACAATACCCAGCACTGCCACTCCTTCCGTTCTCGTAATCTTT
ATTCTGCTCACTTTAGTCATTCATTTACTACTCCTTTCTTATTTATCCACGGCTCTCCGCGTCCAACTCGTAC
GACATTCTTTCTATTGATTTATTCCTTATTTTACTCCGTCTCTCTTACATGTGATAACTTACTATAGTATATA
TGTGAGATTATTGGAGTGGTAGAGTATAAGAGTAAAGTGAATTAGCTACGCTTGATGTTACCTGCTATTGAAT
TCGCGATCAACTCGGCTCACTCGGAAATTACTGGTTATGCACCCTTCTTTTTGAATAACGGACGGATGCCGCG
ATCGATGATA
>Armme1_1_rnd-4_family-138#LINE/I
AACCTAAGGCCCTATAGGGCCTATGCGTACATCTTGCGCTTTGTAACACTACATTGTATCTAACTGTATGTAA
GGGTATCTGCGTGACGCGTNGATCTATCTGAGTGGGTGTATCTAACCGTGCTGAGCTGTGGTTGTTGCCTGGT
CGTGCGTGGTTGTGTGCGTCTTGGAGCAGGGAGGAAGCTGCGAGTGGAAGACANGACCGTGTCGTGGACGGTC
GGTGAGAGGGGTGTGTTGGGTGGGGTTCGCGGCGGAGTTTTGGTGGTGGTTGTGTCGCTGTCGCCTGTGGCGT
CGAANGTAGCTGCTTAATTGGGTTGAAACANGGAGTTAGCGAGTGCGGGAGTAAAGCACGAGTTGGGTACTCT
CCTCCATAGCGGAGGTGGGAGGAAAGTCTGCGAACTGTTCCTCCCCCTCGCATGCCCAAGTGAGGCACAGATA
TTTCGGTGACCGTTCATCTTGAGCTACTTTACTATGGGGGAGGTCCGCCTTTGTTGTTGGAGAAGTCGGGGAA
TCGCTTGGAACGTTGCACGTAAGCGAGAGTGGCAAAGATATGTCTTGATGTGGCGGATAGTAGATGTCGGAGT
TGTAGGTTGCCAAGCCTCGTGCGGCGCCGAAGGGCTTGTCTCTCCTCGGAGTAGCGCCTGCAAACTAGTAGGT
AGTGTGTCACTGATTCTGGGACGTTGCAGTGCGAGCAGTTGGGGGACGGTGTTGCCCGTGAGCGGAAGAGGTA
TGCATTGAGCGGNATGTGGCCGGTACGGAGCTGCGTGAACATGCTAGCTTCTGCTCGCGACATGTTCTTGAAG
ATCCGTTGGATTTTGGGCGAGGGCGGTGAGCGGTCGAAACGTTTCGTGAAGCTTCCCTTGGGTGACGTAAGCC
ACAAGTGTGTCCACTGCGCTCTGACGCGCTTCGAGAAGTCTGCTNCTGCNGCNGCCTTGCTGCGTGGGAGTGG
NCNGGCGAGCATCGGAACGCGGTTGTCGNGGAGNGGGGTGGNNGANCCCTCGGCCGCCAGTTTNGCTTCCGCG
TCCACCATTTCGTTNCCGATAACGCCAACGTGCCCCGGCACCCACTGNATCCNGATGCGGAGGGATCTTCTNC
TNTGCCTGCAGNCGGCGCGCTTGGCGGTGGAACTCTTCNACTAGGTGCCNCCCGGACTTNGTCTGGCCCGACT
GGAGGGCGAGNATGGCNGCTTGGGAGTCNATNAGGATCGTGGCTTTGGCGAGTCTGGGNGTGCTTTTGATNAT
GTCCAGGGCCAAGATTGCGCCTACCAGTTCTGCTTCAAAGACCGTGTGGTGAGTTTCTGGGCCNAGGTAGAGG
TGACGGTGGACTTCCCCATGCGGGGCGTTTTCGTAAGTCTGCGACCAAGCAGCCGCCCTGATTCCNGTTTTGA
ANCCTGATCCATCGGAGAAGATGGTGCGNTCTCCTCTCGGGGTCGACGCCGCTCTTCGTTCTGCTTGATCTTT
GTTCGGAGCGATGTCGAATTCGATACAGTCGGCTCTTTGNATCGGGGAAGNGTCGATCCGTTCCCAGTCTTCG
TGGAAGTCTGNNAGGGCATTGAGNAGGTNGTGGATNGGTGAGCGGTGGAANCNCGGCTGGCGTTTGCATCTCC
GAACCGTCTTGAAAAGCGGGTGGGTTGGNGGGAGTGTGCATAGGCAGGCTGCGAACTTGTAAGCCGCTAGGTT
CAGGCGNAGGTGAGTGGGGAGCACGTTGGCATGGTAGTCTAGGGCATCCGTNGATGTTGACCTTAGGCCACCA
GCCATCACCTTGCATGCCAGCCNCTGCGGCTTGCTTAGCTTNCGCGCTACGCCCACCGCNCCGCTTCTCCTTC
CTTCACCCTCCTTCACTGGGTTGTACCACACCGGGAGAGCATATTCCATCCGNGGAGCGACCAGGCCTATGAA
TAGGCGACGGACGAATTTGTGGGGCATGCCGATCTTGGTGTTNGCAAGCCGGGTCAGTGCTACTGCNGCCTTG
ACGCCTTTCCCTACCGCATACTCGACGTGCTCCTTGAAGTTCAGGCGCCGGTCGATGTGCATTCCAAGGTATT
TGGCACTCTCCTTGGGTTTGATCGTGTGTCCCGCTACCGTCATCGTTCTTTTCTGATCATCAACCGAGTCAGA
GCGCCGCGTGTGGTGAACTAGTTGGTACTTAGCGACGTCGAAGGACGACGCGGAGTCCTTTGCCCAAGCGAGN
CCTTCCTCCGCAAGCTTTTGAAGCTTGTCGACGTTCTCTCCCACACTGTTCGAAACCGCCACTAGTGCCGTGT
CGTCGATGAAGCCGTATATTTTGTCGTTCTTCCCTTTGATTTCTGAGCCGATTTCCAGTAGACTTGAGCTGTA
TATGAGGTACAGGATGACGCTGAGTGGCGAGCCCTGTGGGACACCTGTAGGACCGAACAGGGTCAGACGCGTA
GTCGTCGAAGGCTAACGAGGTGGTTCTGCCTGACAGAAATGACTCTGTCCAGTTCACCACCACGTCGCTAAGN
CCCATCNCCTCCATTCTCTCTAGNAGGCGTTCATGTGCGACATTCAGAAAGGCTTGCGAGACGTCCAAGTACA
GGACACTCACTACCTGTTTTCTTCTCCAGGCATCCTTAATCGTCTGGGTTAGCTGCAATAACGGGTCTACCGT
GCATCTCCCCGCCCTCCCACCGTAGTGGTGTTCAGGGAGGCGTCCGTTGGCCTCTAGTATGTCCGTCATCCAG
CCCGCCATTGCCGATTCCANCATCTTTCCTAACATTTCTTCCAGNGCGATCGGTCTGTACGCTTTGGGGATCG

TATAGTCGTCTTTACCTGGCTTGCGGAGCACGATCGTTTTGAAGATCTTGAATCGCGTGGGAAGTGACCTAGG
CNGNGCGAAGCTTTCGGGATCGTATGAAGTATTGGCGCGTAGAAGTCGATTGCTGCTTGGATCGCGATGTTTG
GNATCCCCGTGGGCCCGGTGCCGAGTACGACTTCGCTCGCTTGATCGCGTTGACTATTCGNTCCAGGTCGATG
TCTGGGTCGGGGAGAGGGTTTAGGGGTGCTTGACGTCGCCTTACGAGGTTAGGACGCTCTGGGAAGAATGACG
ACGCGAGGAGCTTAGCTTTTGCTCTAGGGTTTACTTGTACCTGCCCCCCGGAGTCTTTCAACGCAGGCGTTTT
GGTGGGACCGGCGTTTTCGCCCGGTTCCGTGGCGTACTTCGCTGCAGACCAGATGTCCTTATCTAGGATCTCC
GTTAGCCAGCCTTGCCAATGCTCCTTCTTCTGCTTCTTGATCTCGCGGCGGTACCGTTTCTTTAGTTCTATCA
TTTCAGCCCACCATTCCGCCGTCTCTCCACTTAATCCATTGNCGTTGCGCCTTGTGGTAGTGTTTCCATAGNG
TCGTGAGGTCCCTCGTCCACCATCGCTTCGAGTATTTCGAGGGTTTGGACTGCGGGACGACCTGTCGAAGGAT
TTCCTCTGAGCCTGCTATCAGTTTGTCTACTACCTCTTCCAGTCCTGCTACTGTTCGNAGTGCATCCTCGGAG
GTGTCGATCGGGTTCTCCTCGAAGTAGTCGGCCAGTCGTTCCCGGAANTCGGTCCAGTCCGTGTCTCTGTATA
GCGGTCTCGAGGGTGGGAGGTTTCGCGCGAGCTCCAGGTCGAGGTCGACTCTCAGCATTCTGTGCCAGGCTGA
TTCCCCTAGAACGGCGTCACATTTCACCACGCAGGGCGCCAGAACGTCCCGTGTGGCGAAGACGAGGTCAATA
GTTGATGTCGTCTTGTGAGCTGCTGATACGTACGTGGGTGTCCCAGCCGGGAGGCAAAGGTCCAGCCCCAGCT
CTGCTAAGAGTTGTAGGAGGTCTTCCGTGTCGCTTGTCGCACAT
>Armme1_1_rnd-1_family-338#TIR/MITE
GAGGTAGAGGAAGAGGATCAAGCCCCCGCCGCTGCCCCGTCCACTTCTGCTGGCGCGAAGAAGATTTTCGGGA
CGATGAGGAGACCGAATCCTACAGCTAAGAAAGGTGTGAAAAACGCTTCTTANTCTACCTCGTATCTT
>Armme1_1_rnd-1_family-182#TIR/MITE
TGTTGGGAATAGACGTCGGCAGGCACAAAGGACGTGACGGGGATGGCTCGAACGAACGTATATTCGTGGACAG
ATACGTAGTGGTGGTGGTACGAAGCCAAGAAACGGTAATGATCGGTACGAAAATCAAAATAAGGTAAAACAAA
AAACGGCATAGCCGTCGGCCCNGTGGCTGTGAAACCATATCCTCAACA
>Armme1_1_rnd-4_family-460#TIR/CACTA
AAAAAAGTGAGATCGATNCCCAGGACATNGNGGCAGTGGCAGATAGACAACGTCCTTCAAATATGCAATGTGC
ATNCTNCTGATCAAAGCACCTGTGAAGGATCCCAATATGGCAGTCTCCACAGCTCAGACCANCGCACGCCAAA
ATCCTTTAGAATTTTTTCTCTTTCATTGATGGTTGTAGCGTCTCGCGAAGCTTCCGCGTATCTCCGCAATTCT
CCGATATCTCTGCGACCCCAGTTCTCAAAACAGGTGTCATACATGGTATCCGTGCGATACGCATCACANACAG
TGCAATACCAGTGCGACGAATGAGCGGCCGTACCNGACANCTTGCGAGCAGCGGGCAGATCATTAACAGAGAG
GACAACGGCAATTTCGGCATCATGACCTTCAGGNGCGGAAGCTGTCCGAGAGATACGAATTCCACGTTCCCAG
CCTATAAGACAGATGTCGACGAGTGGTCGTATGAAGTCGGGGATTTCATCNACATTAGGCTCCTTGGGACCAG
GNATTACGCCGGCGAGGAAGATATATTCAGGCTGGTAACGAAGGTGTTCCGGCAGATTGAGATTCGCCATCGA
GATCATGCCGATTGAGTCGTGGTTGCCTCGTTTTCGTACACCATTCGGGTTTAAAAAGTCCATTTGCAGAGCG
AGAGCTATGCGAACTTTNCCTTTTCGATCAATGAAAAGGGTTTGGCCGTCAGGNCCTTTAAATTGACGCATGA
AATCGGCCTCGAATACATTTTTCGTATCCCTTCGAGGGTCCCCTGGGTTCTTTAATGATTCCATGGCATCATC
ACAAGCTTTATCGCACAGTATTTCAATATTTGGATCCGCAAGCGTCCGTGCTATGAAGTCCTCCAACGACGCC
ATGAGAAACGGCTTTCGGGGCTTGGGAACGCCGTCCTTCCCGCATTTCAGCAAACTAGCACCGCAAACAACAA
GGCCTGATGGCCCTGGATTTTTTTTCTCGCAATGGCTAGGGTATGAGGGGCTCGAGGCAAGAGGTGTGATGTA
TGTAGGCTCATAGCAGGCGTTACAATCGGGGCATACAGCATACATTGTGGTATGGCCATCTAGCTTCATTGCC
TNCNGGGCGGTATGCAGACTGGATGGTANTTGATCCANAATGATCTCTTGGTTGTGTTAGGGTCCAGGNCGNA
NTCGCCATCAGGATCTTTCTCCGTCAAGTTCGCTGCCATCACTCCTGACATGAGCAANAGCACAATCTAAGAT
TAATGAGATTGCGATAATCATNTTGCACATGACGCGACTCAGTCCCACGATACAATTGCAGGTGACACCNAGC
AACGTGACGAGCTGCGCCACCGCATTGAAAGTATCCAAACGTTGGCTAAAGAGATGATCTAATATAATGATGA
GAACAT
>Armme1_1_Cluster57#LTR/Copia
GATGACCCCAGCGACAGTGACAGTTCTAGCGAAGAAGGGAGTGATGAGGATGGACCCAGACCGCCCAAACGTC
CAAGAATCGATGAATCACAATTCCCATGGCAACCTGTCAAAGAGATCAGTAGAGCACTCCTCTCTCCTGAATT
GCAACTCACCTTGGAACTCATCGAGAACTATATGCTCAACCTCAAGGGAGCATGTTGGAGTCTCATCAACTCA
CCAGAATGTCCTGAGTTCCCTGATGAACAATGGCTTGAGAACTATACGCTCGACCTCAAGGGAGCATGTCGGA
GTCTCATCAACTCACCAGAATGTCCTGAGTTCCCTGATGAACAATGGCTCTACCTCCTCGCCAGTAAAGCCAT
CAACCTCGATGCTGTGTTCGCCTCTGAGCACTCCACCTCAATCAATGAAGTCCACACTCATGAAATATCTGAG
GGGATTTCTATCCGGCTCTCTGAAAACTTGTCTGACCCTGTTGAGCAAAAGAAAACCATCAGCAATGCAAACA
AGTGGACCAGCACCTGGTACACTTACAGCCAGGCTGTACTCATTGCTTTCCCGCATTGTGAGTGAGAGCTTGC
TCTCTACCAAAGGTACATGCAGTCACTCTTCCAGACTACAGCCATCCCACTCCACGGATGCATCTTCATCCTC
GACCGAGTGATTCATAACCGAGCAGGGAGGTGCCAGAATATCCTCTTGTCCGACTCAGAGAAATTTCATAACC
TTGAGCGATCACATATCTCTCCACTCGGCATGGCCTACCAAGAGTTCGAAGATACATGAGCTGAGCAGAGATC
CAAGCATAAGAGCAGCGATCGAGCCCACAGCAGTGATGCCTGCCACCGGTGGAATGCAGGCATTTGTCCGTCC
TCTGATGGAAAGTGTCGTTACAAGCATGTCTGCTCGGGATGCAACAAGGGAGGACACACAGTCTCCACTGACC
TTGAGCAATCACATATCTCTCCACTTGGCATGGCCTACCAAGAGTTTGAAGATACATGAGCTGAGCAGAGATC

CAAGCATAAGAGCAGCAATCGAGCCCGCAGCAGTGATGCCTGCCACCAGTGGAATGCAGGCATTTGTCTGTCC
TCTGATGGAAAGTGTCATTACAAGCATGTCTGCTCGGGATGCAACAAGGGAGGACACACAGTCTCCACTTGAC
CCCAGCAATGGTGACAGTTCCGGCGAAGAAGGGAGCGACGAGGATGGACCCAGACCACCCAAACATCCAAGAA
TCGACGAATCACAATTCCCATGGCAACCTGTCAAAGAGATCAGTAGAGCACTCCTCTCTCCTGAATTGCAACT
CACCTTGGAACTCGTCGAGAACTATATGCTCGACCTCAAGAGAGCACATCGGAGTCTCGTCAACTCACCAGAA
TGTCCTGAGTTCCCTGATGAACA
>Armme1_1_rnd-4_family-446#TIR/MITE
ACAGCTGTTTGCATACAGCTTGACAGTNGCATGCGTTTTGCGCGAGAGAAGAGAGCGATTCGGACTTCGTCAT
AAACCGTGGTCTANGGGCTTGANGACGCGTTTGCGGCTGGTTTATGGCTATTTCGGCGATCTCGGAGGCCCTT
TCGTGCGCTCGAAGTCCCCGAAACGCGGTTTATGACGAAGTCCGAGCCTTCTTTCATCATCCGCGAAAACGCC
CCTGACTGTCAAGCTGTATGCAAACGG
>Armme1_1_Cluster60#LTR/Gypsy
AGGATCTTCTTGGAAAATGGCGTATCCAACTGCTTGTGAGCTTGTATCCACTGCTAGGATAACGGGTGCATCC
GATTTATAGTCTATCGGACGTAGTGCGGACAAATTGATGATTTCTTGCTTCAGCACTTCTTGAGTGGCCTCTT
GATCTGGTCCCCAGAAGAACTCAACTCCTTTGCGGGTCAGGTTTACTAGGGGCCTTGCCAGCAGGCTGTAGTT
GTGGACATATATCTGTAGGATCCCAGCAGTTCCAAGGAACGAGCAGACGTCAGTCAAGTCTAGGCATGGACCC
CAGTTTTTGATTTTATCGACTTTGCCATCTTCAGGTATCCTGCCCTCATATGTGCACTTGTGTCCAACGATGA
TGGCTTCTGGTACACATAGGTACGCCTTCTTACCAGACCAAGTGCACCCAGCATACTTCATCTGCTGCACCAC
CCGATTGAGGTTTTGGAAGTGTTCCCATACAAAACGGTGGATCCCTGAGTTTTCGGGTATTGTTTCGTATCCA
CCGTCCGCTGTTTCATAGCGGGATACTGGTCCTTTCACACCTGCATCATCAACGAAGGGAATTGTTACATCCG
GTATTTCATCTTGTAGCGTGTATGTGATATCTCCCTGTAGGACTTGCATGGAATTTGTCCAGCCCATTGGTAG
CATGCTGAGGCAGAAGGTGTCTAGAGGTGTCTGGAAGGTGGTGAGGTCTCTGCTCTTTACTGACAGCAGCCTT
TGGTCGAATGCCATGAAGAGGTTGAATACCCCATAACAAGCATGGGCGCCGAACATTTCCGCCAGCTGTTCTA
TTAGTGGAGGTACGCCCGAGTTGTGGATGGTGACAGCATTGAGCGGCTGTAAGTCGTGTATGATTCGAAGTGA
GCGTCCATCTTTCTTGAAGACTGTGAACCATTTGGAATGGTAGGAGGAACTAGAAGGCTAAAGCCGAAGTGTG
AGCCACGTATGCCGGCTTAACTCGGCTTTACGCTCTTTTCAGCCACGAGCCAAATTTTCGGCTCGGCTAGGCT
TATAGTGGCTCGGCTTGTCCCGTGTGCCATATTTAGCCGCGCCACGGCAACACTAGGTATGATCTTCTTGGAA
AATGGCGTATCCAACTGCTTGTGAGCTTGTATCCACCGCTAGGATAACAGGCACATCTGATTTATAGTCTATT
GGACATAGTGCAGGTGAATTGATGATTTCCTGCTTTAGCGCTTCTTGAGCGGCTTCTTGATCCGGTCCCCAGA
AGAACTCAACACCTTTGCAGGTCAGGTTTACTAGGGGCCTCGCAAGTAGGCTGTAGTTGCGGACATATATCTG
TAGGATCCCAGCAGTCCTGAGGAACGAGTGGACGTCAGTCAAGTCTAGGCATGGACTCCAGTTCTTGATTTTG
TCGACTTTGCCATCTTCAGGTATCCTGCCCTCATATGTGCACTTGTGTCCAATGATGATGGCTTCTGGCATGC
ATAGGTATGCCTTCTTACCAGACCAAGTGCACCCAGCATACTTCATCTGCTGCACTACCCGATTGAGGTTCTG
GAAGTGTTCCCATACAAAACGGCCGATCCCTGAGTTTTCGGGTATCGTTTCATATCTGCCGTCTGCCATCTCA
TAGTGGGATATGGGTCCTTTCATGCCTGCATCATCAACAAAGGGAATTGTTACATCTGGTATTTCGTCTTGTA
GGGTGTAAGTGATATCTCCTTGTAGGACTTGCATGGAATTCGTCCAGCCCATTGGTAGCATGCTGAGGTGGAA
GGTGCCCAGAGGTGTTTGGAATGTAGTGAGGTCTCTGCTCTTTACTGACAGCAGCCTTTGGTCGAAGGCCACA
AAGAGGTTGAATACCCCATAACAAGCGTGGGTGCCAAACGTTTCCACCAACTGTTCTATTAGCAGAGGTATGC
CCAAGTTGCGGATGGTGACAGTGTTGAGCAGCTGTAAGTCATGTACAATTCAAAGTGAGCATCTGTCTTTCTT
GAAGACTGAACCAT
>Armme1_1_Cluster24#LTR/Gypsy
GAGTGCATCATGGCCATTCAACTCCGGGAGGCCGGTGACCATGTCTACGGAGATGTGAGTCCATGGTCCGGTG
GGGATAGCATTGGGGTGGAGCAGTGCAGCTCTTGCCTGGTTACTGGACTTCGCTCGTTGGCATGCCTCACATC
CCTTGACATAGGCCTCAATGTCTCTTTTCAACTTTGGCCACCAGTATTTGCTACTGGCCGCCACCTTCCATTT
TGTTGTTAGCTTAAGACAGCCCCTATTGCTCCATTACTTGAGTCGGCTTCCACTTTGAACGGAGTTTGTCAAT
AAGCTCGGAGATTAGGGGAAGGGGATAGCGATTCTTGATGGTCATCTTGTTGAGCTTTTGATAGTTCTGTACA
GGTCACAGGGAGCCATTCTTCTTTTTGATGAAGAAGAAGGGGGAGGCAAAGGGTGATTTGGAGGGAAGGATCC
GTCCTGACCCCAGATTTTCATTGGGAAACTGATCTAGTTGTTCCTGTTCATTTTGGTTTAAAGGGTAGACCTT
GAAAACTGATTAACTAAGAATAGAACATTGTCCCTGTCATTCGTGTGTAAAAGAAGCAGAAGCTGCATGGTAG
GCAAGTGGTGGAAGCGAGCCGTAGCATAAGGCTTGAAACCATCCGTTTGAGTCATGGAATAGGGGTTTTAAGA
TAAGATGAGATTAGCGAGAGGTGTTGGATAAGATAAGATAAATGGAAAAATCGTAACAGAGAGCATCATAGCC
ATTGGATTCTGGTAATCCTGTGACCATGTTGATGGTGTTATGATTTTCTTCACATATCTAATCTTCAGCAGCT
ATCTTATCTTATCAGCACACCTATCGTTATCGCTTCAGCATGCGGTTACCAACCACTACCCAAACAACTCTCG
CATTTGTACTCCGGCTCGCTACGAGGGCACCACTGCCTTGTTGTATTCATTGTTTCACGCATGGACACCGCTA
CCCCCCGTTAACCGAATGACTCTCATCTTTATACTCCAGCTTGCTACAAGGGCACCACTCCCTTGTTGTATTC
ATTGTTTCATGCATGGACACCGTTACCCATACCTATGAAAATCATAACATTACCCTCTTGTTAAGGCAGGCAT
CCTGACTGCTGTTAAGGTCCATTCCAGCATTGCGATTTTGATTGCGTTGATTCATGTTCGGTCCTCATAATCC
TGTATTGCCTCTTGGGTGTTGCCCATCTCTGAATCTTGTACCCATGAGTTGTGTTCTCAAGTCCATCCCTTCC

ATTTGACGAAGTAGTCGATGACTTCTTTGGATTTCTGATTCTTCTTTCCCCTGATTCTTCGCAGGTGCGAGTC
TAGTACTTCCTCTATCTCATACTGTTCCTCATCGTCAACAAGGTCCGGTGGTGGTGGTGTTGGTTGTTGTTGA
TTCGGGAATGAGGGTAGCATGAACGGAGTGAGTAGTTTCTCATTGAAACGTGGGTGGATTTTCCAATGAGGAG
GGAGCTTTAGCTTATAAGCCACCGCTCCCGTCTTTTCCAGTATCACGAAGGGGCCTATGCATTTGTCGTTGAG
CTTCTTTTTTGGCTGGGGAAAATACAGATTTCCGGCATCAAGCCATACTTTATCCCTGATCTTGTAGTCAATC
GCAGCTCACCTTCGCTTGTCATATTGAGTTTTCATTGTACTTGCCAACCTTTCGAGAGCCTTTTGGGTCTGTC
TGTGCACCTCGGCCATGTTCTCAGTGAATGTTTCAGCAGCAGGGTTGAGGAATGGGGAGGGATGGGTCGAGTC
TGGGACTAGTCTGGGGTTTTGCCTGTATAGGACCATGAAGGGGCTCTTTCCCATGGTGGAGTCTATAGCCATG
GGTAGCCAGTCGCTCCAGTCAGTTTGTCGGTATTCGACAAAGAGATGGAGATATCCTTTGATCTCCTGATTGA
CTCATTTGGTTTGTCCGTCAGTCTGAGGGTGGAATGCTGTGGATGCGTTTGGGGTGATGTCGAGTAGTTGGTA
TAGGTCCTTGATGAATTGGGAAATGAACTGGGGTCCTCGGTCAGAGATTACGACCTGGGGCATTCCATACTTG
GTGTAGACGTGGTCCCTGAGGGCCTTGGCCCATCCTTCAGCTGATAGCTCGACATTACAGGCTATTGGGATGA
TTGCCTTTGAAAATCGGTCCACAACGACGAGGAGTGCATCATGGCCATTCAACTCCGGGAGGCCGGTGACCAT
GTCTATGGAGATGTGAGTCCATGGTCTGGTGGGGATAGCATTTGGGTGGAGCAGTGCAGCTCTTGCCTGGTTA
CTGGACTTCGCTTGTTGGCATGCCTCGCATCCCTTGACATAGGCCTCAATGTCTCTTTTCAACTTTGGCCACC
AGTATTCTTGTTGG
>Armme1_1_Cluster17#LTR/Gypsy
GGACCAAATGCGACTATCAACCGATGGATAGAGGACATTTTGATGTTTCATTTCACCCTTAGGCATGTAAAGG
GAAGTACTTTCCCTGCTGATGGACTATCAAGGTGAGACCCACAACCTGGCGATGAAATCCATCCTGATTTGGA
GGATTACGAGCTTGAGGACCATGGACCTTTGCAATTCAAAACCACAGAAGGAACTAGTACAGTGCCGAAGGAA
CTAGAGGAGTTTAAGAGCAAAATCGATACTAGAGGCAGCTACATGCATGTACGAGCCACTAGTGTTGAGGATT
TTTCGAAACCGCAGAAGGAACTAGTATGGTGCCGAAGGAACTAGAAGAGTTTAAGAGCGAGATCGATACTAGA
GGCGGCTATATGCATGTATGAGCCACTAGTGTTGAGGATTTTTCTGCAGAATTAGATGAAGCACGCTCTCGTG
AACTTGAATTAAGAAAACTGTATTTAGCACAAAAGAAAACCGATGAATCTGTATGCCAGTTCTTGAAAGCTGC
CCCACTCATTATGAACTTGAATTAAGAAAACTGTATTTAGCACAAAAGAAAACTGACGAATCTGTATGCCAGT
TCTTGAAAGCTGCCCCACTCATTCCTGATCTCGAGTACAAGTTCGATCCGAATAAGCGAGAGGACTATCCAGA
GAAACAACGGAGTAATAGTGGACTAGTGCAAGATGAATGACTGCCGCATGTCAGAACTTGGTTGAGAAATCCT
AGACACCGCCCAGAAGGACTTAATGACAAACAGTACTTGACATTCAAGCAGTACTGCCGCAACTTCTTCTTGG
ACAAGGAAGGATGTCTATATTGATGGAGTACTGGAGGAGACCATTGACTAGTGGTCGACAAGGAGCATCAAAT
GTATATGATGAGATCGGCTCACAACTCGTTAGGACATAGAGGCTTCTATGCGACTAAGTTGTTGTTGGAACAA
CGGTTCTGGTGGCCAGAGCTGGAACAAGATGTCAGTTGGTACGTTAAGACTTGTCATCTATGCCAAGTCCGAC
AGAAGACTATGCTTCGAATCCCACCAGTGGTCACTGAGACACCGTCACTATTCCAGAAAGTACACTGTGATAC
TATGATGATGATGCCTGCGTCTAATAAGTGCAAGTACATTGTTCACATGTGTGACTCGCTATCTTCTTGGCCA
GAAGCTAGGGCATTACAACTAGAGAATGCGAAAGCTATTGCGGTTTGGTTCTTTGAAGATGTGATATGTCGGT
GGGGCTGTCCTGCAGAAATAGTGACAGATAATGGTGGACCGTGGGTCGCTGTTATTCAATGGCTAGAGGACAA
ATCGACTACCACATGTCAGAACTTGGTTGAGAAATCCTAGACATCGCCCAGAAGGACTTAACGACAAGCAGTA
CTGCCGCAACTTCTTCTTGGACAAGGAAGGACGTCTATATCAATGGAGTACTGGAGGAGACCATCGACTAGTG
GTCGACAAGGAGCATCAAATGTATATGATGAGATCGGCTCATGACTCGTTAGGACATAGAGGCTTCTATGTGA
CTAAGTCGTTGTTGGAACAACGGTTCTGGTGGCCAGAGTTGGAACAGGATGTCAGTTGGTACATCAAGACTTG
TCATCTATGCCAAGTCCAACAAAAGACTATGCTTTGAATCCCACCAGTGGTCACAGAGACACCGTCACTATTC
CAGAAAGTACACTGCAATACCATGATGATGACGCCTGCGTCTAATAAGTGCAAGTACATTGTTCACGTGTGTG
ACTCGCTATCATCTTGGCCAGAAGCCAGGGCATTGCAGCTAGAAAATGCTGCTATTTAAGGCAACTAGTGGGA
ATGTAAAGAAGTGGTTTTACTTCCTTCCGCATGTTCTGTGGGCAGACCATATCACTATTAAACGTGGTACCGG
CTGTTCACCATATTTTATGGCACTAGGTGCGCATCCAATAGTACCCCTTGACATGGTCGAAGCGACTTGGCTA
GTCAAGCCCCCGAGTGGAGTCCTCAGTACTCCAGAGCTCATAGGCTTGCGAGCAACAGCGCTAGCCAAGCACG
CTCAGCATGTAATGGCCATGCGACAGAAAGTAAGTAAGAACAAACTAGATACGGTACTGCGCTATGAGTGTGA
GCACAAAGCAACTATAGTCAACTATGACTTCAAGCAGGGTCGACTAGTACTCATGCGCAACACGCAAGTGGAG
GATAGTCTTGATAGTAAGATGGATCCAAGGTATCTGGGACCACTAAGAGTTCGGGTCTAACAAAATGCGACTA
TCAACCGATGGATAGAGGACATTCTGATGTTTCATTTCACCCTTAGGCATGTAAAGGGAAGTACTTTCCCTGC
TGATGGACTATCGAGGCGAGACCCACAACCTGGCAACGAAATCCATCCTGATTTGGAGGATTACGAGCTTGAG
GAACATGGACCTTTGCAATTCGAAACCACAGAAGGAACTAGTACAATGCCGAAGGAACTAGAGGAGTTTAAGA
GCGAAATCAATACTAGAGGCAGCTACATGCATGTACGAGCCACTAGTGTTGAGGATTTTTCTGCA
>Armme1_1_Cluster7#LTR/Gypsy
CCTAACTTCACCCTCCTCACCAAACTCCCTGTTGGAATCCTCAACCACAAACTCATCTGACCCCTTCAAAAAC
CTCTTCCAAAGTCCCCATTCTGACCGTCACCGTCGACTTCAACTGGAGGTGGAGGTGGTTAGGTATGATTAGG
GATGGTATTGGCTTCCACAGGTTCCAGTTGAGAGACATGGAATATGGGATGAATGGAACGGAGGTCCACAGGA
AGCTTGAGGGTGAGAGAATGGGATTCGGGCTGAGCAATGATATCAAATGGTCCGAGGAATTTCTTGGAGAATT
TCGCAGTAGGGCAAGTAGTCCTAATGTGTTTAGCACTGAGGTAGACTTTATCACCTACTTTGAAATCCAGAGC

CAGAGACCATCACTTGTCCACTTGAAATTTGTAGTGAGACTGAGCAAGCATGATTTCATCACGTAGGAATTGG
TGAAGATCATCGAGGTTGACTATGAAATCATGGGCCTGAGCGGAGGCGATATCACATTCAGGGTATACCATGA
TGTTAGGGTAGTATCCCTTATTAGCAAAGAATGGAGAGATTCCAGTGGAGGCAGGCCTGAGCAGAGGCGATAT
CGTGTTCAGGGTATACTGTGATGTTAGGATGGTATCCAAGGTTGACTATGAAGTCACAGGCCTGAGCAGAGGC
GATATCGCATTCAGGGTATACCATGATGTTAGGATGGTATCCCTTATTAGTGAAGAATGGAGAGATTCCAGTG
GAAGCGTTCAGAGCATTGTTGTACATGAATTCAGCAATGGGTAGTAAGGTATCCCAATTGTCTTGTTGGTAAG
AACATCTCCAGTCGAAGTCGATGGCAAAGAACAGTACGAGATTTCACAGATCCTTGATTCTAAACTCGACCAT
TGGTTAATCCCATTCTTTCACCCAGCTTCCTGTGGACCTCCGTTCCATTCATCCCATATTCCATGTCTCTCAA
CTGGAACCTGTGGAAGCCAATACCATCGCTAATCGTACCTAACCACCTCCACCTCCAGTTGAAGTCGACGGCG
AAGAACAGTACAAGATTTCACAGATCCTTGATTCTAAACTTGACCATTGGTTCCGATGCCCTCTTCTCTATCG
TGTTCAATGGTTAGGTTATGAAGGTACTGATGAAGAATTTGCTTGGATTCCTGCTACCGAATTCTCCGTGGAA
GACTACATTGAAGACTTCCACCATTGATACCCAGACAAACCAGGACCACTATCCAAGGTCACCATTCACAAGG
CTATATCCAAACCTATGCTTACTCCTTGATTCCCATCTCTATCGGCGTCCTGCAGATTCTGATTCTCCAATTT
CAAATTTGATTCTGGAAATCTATTGCAAATTCATTCCCTAATTAGGAATAGACTCCATCAGTCTATTTTCTTG
ACTTTACTCTTATTTCGTCACTCCATTTCTAACATTAAGAAATTTCTATACATTTTGCCCTCCAGGCACGGTA
CACTACCTATTCATGCAACATTAAGAAAATCAAAATTATATAAGGGTCAAGGAGTTAGGCAGCCGCGGATCCC
TTGGGCTCCCCATTAGCATACTCCAACTCATCCAGTTCAGTGACCCACTTACTCCTTGATTCCCATCTCCATT
GGCCTCCTGCAGATTCCGATATCCCAATTTCAAATTTGATTCTGGAAAACTATTGCAAATTCACTTTCTAATC
GAAAACAGACTCTGTCGGCCATTTTCTTAACTTCACTCTCCCTTTCATCACTCTGTTTCGAAACATCAAGAAA
ATCATTTATATACTACTGCCCCTCTGGGTTTGATACTCTATCTACTTTCATACACATCAAGATAAAATTATAT
ATGAGACAAGACTTTCAGATGGCCGTGGAGCCCTCGGGGACTTCCCCATCATACTCCAAATCATCCAACTCGG
GGATCCTGATGGATCCCGCAGCTGGCTTCATCTCTGCCCTAACTTCACCCTCCTCACCAAACTCCCTGTTGGA
ATCCTCAACCAAAAACTCATCTGACCCCTTCAAAAACCTCTTCCAAAGTCCCCATTCTGACCGTCGGCC
>Armme1_1_rnd-5_family-464#LTR/Copia
AGGAGACNGCTGGGGAGGATACGGCATGGGTCGTTAATAATCACGATCCTGGGCTAGACACGTCACGGATGTA
CGGTGATTCAGGAGCGTCGACTCACTTCATGCAAAACAGAGANCGCTTCTTCAACTATATCCCGCTTGAAACG
TCTGGAAGTTCNTCGAAAGTTGGCGCCGCATTGAAAGTATTGGGGACTGGAACGGTCGCTGTGAAAACAACAA
TGGAGGNACNGAGAAAATTATCACCTTCAGCAAGACNCTGCACTGCCCAGACGTATCNGCAAACCTGCTNTCG
ATAAGCCGNCTCGACAAAGAAGGGTGGAAAGTAACCTTCGGCGGGGGGAAAGCAGACTTCGTGGACCCGAGCG
GCAANGCCCAATTCACGGCGTCGATGTTCAACGACTTATACATGATCGATTGCGACCTCATGCGCGGCGANGA
ATACACNGCGCTGGCAGCAAGATCCCTGGAAANTTCAGTCCCNATCGAAACATGGCACATGCGATTCGCTCAC
TTCGGGGTTGACCGAATCAAAGCCCTATCGAAGGGAGACCTCGTCGANGGGCTGCACATNAAGCATGANCAGC
AGGCTCCTGGNAGATGCGAACCNTGCATCCTAGGAAACCAAAAGAGACGTCCGTTCGATGCTGATGTTGAGCC
NGAAACTGAGGTTCTTGGAAGGGTCATGCTGGATATCTGGGGNCCNGCACGNGCGCGATCCATTGGCGGTTCG
AGGTATGCCATGATCTTTGTNGACGATGCGTCATCACGNCGTTCGCCATACTATTTGTCGGATCGGAGAGCAG
AGACGACCGTGGAGACCCTNGAACAATTCGCGAGCATGGCGGAGAGACAGACGGGGAAGAAGTTGAAGAAGAT
CCGNTGCGACAACGAATTCGACTGCTTGTTATGGCAAACGTGGGCAGCAAAACGGGGAGTCGTGATNGAACTA
ACGGCGCCATATTCGTCAGCAGCTAACGGGGTTGCGGAACGGACTTTCGGGGTCATNTTTGGATCAGTCCGGA
TCCTGCTACTNGAGGCGAAAATGGCACATGGNTGGTGGGCCGAGGCGTGCGACTATGCTGTTAAGGCGGCAAA
TTTACTACCAACCTCTCGGCACCCGGGGAAAGTGCCTGAAGAGGTNTGGAGTGGGAAGAANCAAACGGTGGGA
CACTTACGAGTATGGGGATCNACTTGCTATGCGAAAATACCGGCTGCAAAGGGACACAGCAAACTAGGTCCNA
GAGGNCAGAAAGGGAGATTCATCGGACTTGGCGGGNCACGGATCATGTACCGAATTGTACTNGATGACGTACC
CGGNAACAAGATCATCGTNTCCCGTGACGTCATCTTCGAGGAGTTGGCTCCGACGCGTACCGTTTCTTCTCGT
GAGGGGGAGATCGCGTACGGTGANCTAGTTGCTCCAACCGNTCAAAGAAACAGCACCGATCAAAAATCCGACC
AAAAATCCTCAACCACACCCTGATCTGCGAGATCGATCGCGAATCCCAGTTCGAGTTGTCCAAAATCAAATTC
GAATAACAAATCCAGACCCACCTCCACGCCAAATCCCAGTTCATGACCGCCCAGACCGTCCAACACGTATCCC
AAAGCCAACACGAGCCATACAAGAATCTACAGAATATCTGAGACGTGAAGAAAATGCCCGAGCCCACGGAAAG
AACTGGGCACATGATGGAGACGTACCACTAGTCGAAGATGAAGACGATGTAGAGGATATCATTGCGATGAAGG
CGTCAGAAATCATGAGCATCGTGGATAGGAAGGATATGTGGGTACCNGATAACTATGAACAGGCCTTGACCAA
GCCGAAGATCTGGACACCAGCTATGGATGCAGAAATACGCCGTATGGAAGAACGAGACGTCTGGCGAGTCGTA
CCCCGCGAACCATGGATGAAAGTNATCGACAATCGATGGGTATTCGACAACAAAATGGACGGAGACACTGGGG
ACCTACTCAAACGCCGAGCACGCCTGGTCGTAAAAGGATTCACGCAGATCAAAGGACTCCATTACTACGACTC
GTTTGCTGCNGTTGTCCGATACGAATCACTACGAATGTTCTTTGCAATCGTCGCAGCCCACGGACTNGANTTC
TGGCTTATCGATTTCGTTGGCGCGTACCTTAATGCGGAGCCGCAAGGAGAGAACTACGTCGCGCTGCCAAAGG
GCTACGAAGACGTCGTTACTCGTGACTACCCGAAGGTCCAGAATATGTCCTACAGATGTTGAGAGCGATGTAC
GGGACCATGGACGCGGGGAA
>Armme1_1_rnd-5_family-1490#TIR/MITE

AATGGAGAAAAGCAGGTACAAAATATTCATTGTACAGGCTCAGAAATAGGTCGAAAAATTGTGAATCCTACAG
TATGTATGTATGATATATCAGAGAATATTTGTAGGAGCAAATGAGCCCTGAGGAATAGGCGCAAAGACAAAAA
CGAAGGAGTGGCAGGTAAGTCTCGGACCGATCGGTGGCAGGTAAGTTCGCTGACAAAGCATGGATAGCATTAT
TTTCTTCATG
>Armme1_1_rnd-4_family-697#LTR/Copia
TGTGCTCCGTGCAGCTGGGTTGTTAAGGATCCGGTAACTCGGTTGAGTAAGTGATGCGATCCGAGGCCGATTT
GTCACGTGGCTCGATTGTCTTCCCACGCCAAATGGGTATCTTTGATTTAGTTGCCAGAACATCAAGGGGCATT
TCCCCTTTTGATTCGGCACTCTCGGCATCCGTGCAACTGTCCAAATATCATTATTTCCGTCCGGAGNCNGCTC
GACTTCATTCCCTNNCTCCCCCTCGAGCTGCGTAGGGNCAGGGACAAAGTCATCTCTATCAGTAGTACTGTCT
GTAGTATTGTACTGAGGAAATATNACATTTCTTGATGTTTGAATAACTGCCTGGAACTGAGAGTTATAGTATC
TCCAGGCCCTAGATTCATCTGTAAGACCTGTAAAAACGAATTCCCTTGATTTCGGGTCGAGTTTTNGGCTTTT
GCCATCCTGCTGAAGTACCCAGCACTTGGTNCTGAATTCTTGGAGGGTCGATATGTCAGGTTTCNTGCCCCAA
AACGCTTCTTCTGGGGTTAGATCATTCTTGATGGCTCTAGTTGGTGATCAGTTCTTCAGGTACGTGGCATATG
CCACTGCCTCGGGCCATAGAAATTTCGGCAAGTGATGTTCTTCTAGCATGGCACGCGCATGCTCCACGAGAGT
CCGATTTACTCTCTCGGCAACTCCGTTTTGGGATGGAGAGTAAGCTGCAGTCAGTTNCAGCTGNATGCCATTG
TCCTNNAGGTATTCCTTNAGCTCCTCACTGACGTATTCCTTCCCATTGTCAATCCTGANGAACTTCAGATCNT
TCCCGGTCTGATTCTTGATGTATNCCTGGTACTGCTNNACTCTCTTNCGATATNTCCGCATCCTNCTTCNNCT
TTAGAAAATACAGCATTGTCCTTCAGGNTTTTCCATCTGTGAAGCTTATGTAATATCTTTCTCCATGGATNCC
CTGAGTTCGGGCTGGTCCCCACAGATCCGAAAATGTCATTTCACCTATTTGGCATAGCCTCTTTCAGCTTCA
TGTGGGAATGGCT
>Armme1_1_Cluster59#ClassI/TRIM
AAATCTGCCATCAACATCATGATCTCACACGACATCGATCTACATGTACTCCACATTCCGGGCTTGGAGAATG
ATGTAGCGGACGCACTGTTGTGATCTCAGTTCTCCAGAGCTTGTGAGTTGGTTCCCCGACTCCTTATCTTACC
GTTTTGACCCCCTAGCAATGCCCTGAGAGTGTCGAAATGTTGATAGACATTACTCTAGGGTCCTGTCAGAGGC
ATCATGAGCTCTGGACCCTGGAGAAGCTTTATCACGAATGGTGCCTCGCCCTTGGCTCGGCCATTGACACCAC
AACTCTCGGACCCTACAATTCTGGCTTAAACTTGTACATAATGTTCTGCAAACTCCACAGATTCCCTGTCGAA
CCCATGCCTGATACCATGAGCTACTTTGTCATCTTCGCATGCTCCTACTTGAAACCAGACACTGTGTCTAGCT
ACCTGTCCGGCGTCTGCAACAGACTCGAGAACTTCTTCCCTGACGTTCGTACAATTTGCAATTCCCTCCTCAT
CAGCCGGACCTTGAAGGGATGCCAATGCCTGAAAGGGTCAGCCATCAACCACAAGGCTCCACTGTCCATTGTG
AACATATGCCATGCTATCAGCTCTTTAGCCAACTCTTCCAACTTCAACGACTCACTTTTCTTGGCAATGCTCC
TCACTGGATTCAATGGCCTCCTATGCCTCACTGAACTTAGCATGCCTGACTCGAAGAAAGCTTGCAACTGGAG
GAAAATCACCTGACGAACGAGCATCAAATAGATTCCCCAAGGTTATGTGTTCTTCCTACCAGGTCACAAGGCC
AATTTCGCTTTGAAGGCAACCGAATCATCATTCCTTCCAACGACGACTCCTCCATCAATCCACTACCAATTTT
CAAATGTTACGTGGCCAAACACAATGCGAAGTTCCTCGTGCACCCTCCTCTGTGGGTCACCGATACTGGGCTC
GTCCCCACAAGATCTTGGTTCATAAAACGACTGTGCCAACTCTTCCCCAGCAAGTCCATAGCCGGTCAATCCA
TGTGAGCAGGTGGTGCAATGTATCTCGCAGAACAGAATGTCCTGCCCTACCTTGTTCGAGGTCATGGCAGATG
GTCTTCGAAGGCATGGAAAATCTACATTCGGAAGAACCCTGTTCTCCTTCAAGCAATGGTTGGCACATGCCCA
TCGCAGCTTTAATCTTTATCATATCGCCATCACACCTCATGTATCACCATCGACCTTATGATCTATTACCTTA
ATAGCACTCATACATACAGTGCCTGGGTAATCTCAATTGGACTTATGCCCTTAGGGGCGATTTTCCATCTAAA
ATCTGCCATCAACATCATGATCTCACATGACATCGATCTACATGTACTCTACATTCTGGGCTCGGAGAATGAT
GTAGCGGATGCACTGTCATGATCTCAGTTCTCCAGAGCTTGTGAGTTGGTTCCCCGACTCCTTATCTTACCGT
TTCGACCCCCTAGCAATGCCCTGGGGGTGTTGAAATGTTGATAGACATTACTCTAGGGTCCCGTCAGAGGCAT
CATGAGCTCTGGACCCTGGAGAAGCTTCATCACAAATGGTGCCTCGCCCTCAGCTCAGCCATCGACACCATGA
CTCTCGGACCCTACAATTCTGGCTTAAACTTGTATATAATATTCTGCAAACTCCACAGATTCCCTGTTGAACC
CATGCCTGATACCATGAGCTACTTTGTCGTCTTCGCATGCTCCTACTTGAAACCAGACACTGTGTCTAGCTAC
CTGTCTGGCATCTGCAACAGACTCGAGAACTTCTTCCCTGATGTTCGTACAATTTGCAATTCCCTCCTCGTCA
GCCAGACCTTGAAGGGATGCCAATGCCTGAAAGGGTCAGCCATCAACCACAAGGCTCCACTGTCCATTGCAAA
CATATGCCATGCTATCAGCTCTTTAGCTAACTCTTTCAACTTCAATGACTCACTTTTCTTGGCAATGCTCCTC
ACTGGATTCAATGGCCTCCTATGCCTCACTGAACTTAGCATGCCTGACTCGAAGAAAGCTCACAACTGGAGGA
AAATCACCTGACGAACGAGCGTCAAATGGATTCCCCAAGGTTATGCATTCTTCCTACCAGGTCACAAGGCCGA
TTTCACTTTGAAGGCAACCGAATCATTATTCCTTCCAACAACGACTCCTCCATCAATCCACTACCAATTTTCA
AATGTTACATGGCCAAACATGATGCGAAGTTCCTCGTGCACCCTCCTCTGTGGGTCACTGATACTGGGCTCGT
CCCCACAAGATCTTGGTTCATAAAATGACTGTGCCAAC
>Armme1_1_rnd-3_family-41#LTR/Gypsy
AAAAAAAAAAAAAAAAAAAAAAAAAAAAAAGACCCNACCTTCGTACTGGATNTGNAGNCTTCCTACGCGNNGGAT
GCCCTACTTCCGAAGGTCCTTACTGGNCCGANCCTTGGACCACCCCCGATTTNCCGTCGGGGATGGNNTGATN
TGGACGGAAAATTCATATAAGCCTGGCGAAAAATTACTATGCATCCCTAAGTCGCCTAAGGGATTCCGTTCGC
TTCACGGAGATATTATCGAACAAGCTCATGAGGTNGTCGGCCACTTCGGTCCCCAACGAACANCCGAGTACAT

TCGACGTTGGTATTGGTGGCCAAGGCTCTATGGCATGTGCGAACTCTACTGCCGNACCTGCGANACTTGCGAC
GAGTCGAAGGGCAACTATAGAGCTCCCTCCGGGAAGCTTCATTCATTGCCCATACCCACGAGACCATGGCANT
CTATCGGAATGGATTTTATNGGTCCATTTCCNGAAGTCAAAGGATGTCAATTACCTNTGGGTAGTTGTCTGCC
GATTGACATCGCAAGTCCATCTAATTCCGATCCACACATCCAATACAGCCACGGATTTGTCCGCAATATATTT
NCGCGAGATCGTACGGCTNCATGGNCTCCCAGANTCGATTGTNTCAGACCGTGACTCNAAGTTCACTTCAGTA
TGGTGGAAAGAACTCCACCGANTGCTNGGTNCNAAGCTCCTNATGTCCACNTCATTCCATCCGCAGACAGACG
GANCCACAGAATGAGCGAATCGATCTATCGGCCAGATTTTCCATACTGCAATACGCCCTGATCAGCGAGACTG
GTTGATCAAGGTACCTATTGTCAAGTTTGCNCTTAATTCGAGCATGAGTNCGNCCACTGGATTCGCCCCNTTC
GAACTCAATGGAGGATATATGCCTTCAATGATGCAAGAGGTTCGACACGATAGACTCGTCCCACCTGGTATCA
ANGCATTTGCCGCTCAAGCTGATGCAGAACCTTTACGAGGCTCACGATGCAATCATCGCCTCCAGAGTATTCC
AGACTCACNANGCCGACAAGCGTCAANNACCCTGAACTTGATATCAAGGGAGGATCGAAGGTNTATCTATCNA
CGAAAAATCTGAATCTACCAAAAGGCTAGNGCAACGAAACTCTTGCCGAAATTCGTTGGCCCATACACTGTGC
TCATGGCAGATCATGCCTCGTCCACCTACGAGTTGGATCTCCCNGAAGAACTCGTGAAACGTCGTATTCATCC
CNGGTTTCACGTCGGCCTATTACGGCCCTACGTTGAAAGCGACTCAGTCCTATTCCCGGACNGGAAGNAGCCN
GAACCNTATGATTTCGGCGCGCCTGACGATGCCGAAGAATTTGTTGAATGCATCGAAGGACACGAATGGAAGA
ATAACTCACTATTCTTCCATGTATGCTGGGCCCTTGGTGATTTAACCGTTGAGCCATACTCTAAGTGCGAAGC
ACTTAAAGCACTCGACGAATACCTAGCTGTTGCTGGTATTTCAGACCCAAAGAACCTGCCAAAACTGTCGGAC
AAAGGTTCTAGACCACGAAAACGGCGACAAACAAGAAATGATACACGTGTTACTAAAATGGATGAGTAACAAC
GTGGTGATCAGATCACTCTGATCGTCAGGGTACAGTGGCAGTGCCGGTTCTACATTTTATGAACGCCGACTCG
TCGCCGCCACACCGATATCGCGACCAAGTTCTAGCACTTGGCCGCTG
>Armme1_1_Cluster16#LTR/Gypsy
ATGATCAGGTTAGCATAGTTCTTGCACCACCATAGTAATACTCATCAAATTTTAGTCTATCAATGTAATTGAG
AGTCCCCATTTGCATTCAATATTTTTTGACACTGTTTTATTTCCAGCCTGCATGCACAGAGTCACTTCAGCAG
GTAGTTTCATCACCCAATCCTTCTGATTGGAAGATACACACTGCCTTAGCATTGCACCAACAGTTTAGTTTGA
TTGTTCTGTCATTCCATCAGTCTCAGGATGATATGTGCTCAACATTTTAAGCTTTGTTCTGATAAGCTCATGG
AGATAAGACCAGAATAGGCTAGTGAACAGCATGTCGTGATTGCTAACAATAGCTTTTGGCAGCCCATGGTACT
TGTAAACTTCCACAAAGATGAGTTCAGCTACTTCCTTAGCCTTGTAATTCATTTGGCTAGGTACCAGATGTAC
CATTCCTGTTAGATGATCAATGATAACCATTAACATATCAAATGAACTGTCTCTATTTGTAGACTCTGGTAAG
AAATCCATAAAATCAATCCCAATTACTTCCCATGAGTATGTGGGAGATTCCAAGGTGTGTAACAGTCCATATG
GTCTCTGGTTATTTGTCTTCATTCACTTGCAAGTCATGCAGGCACTACAAAAAGATTCAACATCTTTAGCCAT
TGATTTCCACCAGACATGACTTTGAAGATAGGATAGCATTTTACAGCTTCCCAAATGAGCTAGCAGAGAGTGA
GCTTCATCAATAACTATCTCTTTGGCACTTTGTCCTTTGTACTCATAGTCTGGTATGCAGAGAAGCTCTCAGT
CTTTCAGTTTGATGAAGATGAGACCATCTCTTTGCTCAAAATTGCAGAATTCCTTCAGATTGGCCAGTATCTT
ATAAAACAGAGGGTCTCTGGAATAGTTATTGCAGAGACAAACAGATAAGTTTATAGCAGAATGTCCCTCAGAG
ACATGTGTGAGCAAAGGCATGTCATTAATGACTTGAGCATTGATGCTCTTAGCTGAAGTCACATGACTTCACT
CAGATATACCAAAGTTAAGTTTTGCCCATGTAGATCATTGTGGGGAAATTCCAAATTAAGGTTTTTCTCCTTT
GCTTCTGCTATTGATGACTCATCTTCAATTCCCACTGAATTCTCTGGATAACTGGTGTTCAACACTGGTTCAT
CTGCTGTGTTCTTGACTGTGAAGGGTTTTGCTCCACTCCCATCCTCCTTTCATCATCCATGGACAATGAAATG
TTGGACTACAAGCTTACCAGGTACCAGCACATTATTGAGGCCCCAAGATTTTGCAGATGCCAATCAATTTGTC
TGCAACATCATTGCTGATGATTCAAGGTTTCTTTTATCAACAGTGCAAGCAGCTTCAAGGCCTACAAGCAATG
GCATGGCAATGCCATGCATGTTGATGGAATCATTGTCAAGTACATCATGATAGGTGTATGCACCCCGACCTCT
GACAGTGCCTGCAGCTTCATTGGACCAGAGGCAAGACAATGCATCTGCTAAGACATTCTCTGTCTCAGGAACA
TAGTGAACCTCAAAATCGAACTTGCTCAATTTCTCCATCCAGCGAGCCTGCTGACCAGAGAGGTTCTTCTGCC
CATACAGGTGAGTCAGGCCTTTGTGATCAGTGATCCACACAAAGTGAACTCCTTGCAGAATATCCCAGTGACA
TAGCATACTTTCTATGCCTGCTAGCATCTCAATTTCATGCACAGGATAATTCTGTTGGGCTGAGTTCAACTTT
GCAGAGAAAAATGCACACACCACACCCTTATTCCAATGAGGGCCTTGTATCACTACAGCCATGACACCAGTGA
AGCATCCATCTGATATGAGCCAGACCGGTTCTGCATCATGTCCATATTTCATAGGTGCACAGTGGTGATCTCG
ACATTGGCTTGTGAGGACCTTACATTCTTGAAAGGCATGTTGTTCCATGGACCCCCCAACAAAACAGAATGGT
GGCACCTGTAACTGCACTAAGTTAGCCCATTGGGACACATATTCATGCAATATCGTCAGCTAACCAGCCCAAG
GATCCCAGGAATCCCATCAACAGGCCTTTGTTCATTGGTGTAGGCCACTTTAGAATCACATTGACTTTATCTG
GGTCCATTCATATGCCATAGTGATTGACTATGCAGCCAAGAACTTTGAGCTCTTTAGTAAGGAAGTGTACCTT
ATTTTCACCAAGATAAAAATTTTCCCACTGCAGGATGTCAATTATGAGTTTAACATGCTTTATGTGATCTTCC
AGTGTTTCTGAATATACAATGAGATTGTTGAGGTAGGTGTCCAAAAATTGGCCAATGTACAGCAAAAAAATAT
GGTTTATGATAGCCTGGAAGGTTACAGGTGCATTACAATTGCCCTGCTGCATGAATAAACTCAGCATGTTTCT
GTCAGGTGTCGAAACTGCTGTACATTCCACATGCTCAGGCTCTATGCAGACCTGCTCCTATGCATTGCTACTA
TCCATCAACAAGAAGTATTTTGTGTAGGCTACACAATGGAGGATTCCATCAATATCAGAGAGTGGTGAAGCTA
GTTTGCGAGTATTTGAGTTTCAGGCTCACAGGTCCACCACTGTCTGGAGCAGCATTGTCCCTGGCTTGCATAT
CAGCAGCATAGGTACAGTATTGCTCATGGTTGTAACCCTCCAGTATCCAGTCTCAATATAAGCTTTTCGTTTC

ATGTTCCATTGTTCCCTGAAGAGTTCAGGGCACTGAGAGGCCTTCCAGTGGCCAGGGCACTATGCTTTCCACA
ACTAGTGTTGCCATGACTTGGCTTCAAGCTGCTGAGGAGCCGAGTTGAGTCAGAGTCACACTAAGCCGAGTCA
TAGGTGACGACTTGGGCACAAGGTGGCTCAGCTTGACTGCCACTCGACTTTTCCCAGCTTGAGTGGACCCAAG
TCACTGACTTGTCCATTTTAGGAAGTTATTTCTCTTTCAGCTACTTTCATCTCATCCATAGCGACACATCAAT
ACTGGTCTCTTTATATCACTGCAACACTTTCTGGAGTGGAGGATGGTGCTTGTTAGAGTACATTTTGGGTCAC
TTTTATGGACTTTAGTACATCTGCTGCTTTTTTGTGTTGACATGGCACTGATCTGATCTGATCTGATAAGGAG
GAGGCCCACATTCAATGCAATGTAAGGAGACTCAGGTGTCAGACATAAGATGTGTACATTTTATAGTATCTAT
CTCCTTAGCTTTCACTGACCCTAGCTACCTCAACTTCAAAGAATCATGGCCTGTTACGTGTTGATCTCTTGTA
TGTGCCGTTTCTCAGTGGGAGTATAGGTTCTGGATGGCCTATCTGTAAGCATGTACAGTTCACTGATGTTTAT
TACTTCCCTTGACTGGCACATAGGTTCTATATCATCCTTTTCACTCTACACACTAATATATGTTGCTCACTGA
TTTTTAAACATGCTACCACACACCATTATTTACATCGTCGCTTAGTCCTGCAATGAAACTCCATTTATCTTTG
CACAAGATATTGTGTTCTGCCCTTCATACCTTGTTTCAGCTATGAGAACATCTCTTGGTAACGTATTATGCAC
ACAGGCAGAGTTCTTCTGTTCTTTGGTTCATTTTAATCTCTTTCCTGGCAGTGGTAGGGCATGCTATATGTAT
TTGAGAGTACTCAAGACCTCGCTTTTAATTACTTGCGACTCTACTTGGCTTCAATACAGATGGACAACAGTCA
GCACTGGTGAAATGAGGCTGCCCATTGACTCAATTATTAACAAATGTTGACATAGTATGTGATTTATTACTTC
TTTTACTTGTGTCTGATGTTTTGCTGGCTAGTATGTCTCTCAGCAAGGCATCACTGCCTCATGTCTAACAGTT
TATAACCTGTAGAGTTTCATGAATGCTATCAAACAAGTGACAACAGCTCATCACAACAAGAATACTACAACTA
CTATTGCTGCACCACCTGTTCATCATACTCAATGTTCGCCTCCAGATCCCTATGTTGAGCCCACAGCACCATT
GTCCACTCGCTCTCACTCTCATATGCATGCTGAAATGCCCACTATGTCAGCCATACCAACCGGAGAGATGATG
CATGTGTCAGCTCCTTCATATGCCGCTCCAGTTTCAGGTATTTAAGCCACATTCCATGTACAGGGTGAGTACA
AATAAATCTATCCTGTTTTCCAAATTTTGAGGCACATGTCAGATTATAATCATTGTTCAGAATCTCCCTTGTC
CCCTGAGGTGCACACCATTTCATTATCACCACCACCACCACCACCAGCTCATGAAGGACCATGAGCAATGGCT
GCCAACTCATTGTACCATGTTGATCATGCTGACAACATAACACAGGCACCAATGCAGACTACCGCAGGTTCTC
GGAGAGCACACTTTGAAGATGATTTGGATAGCTCAGGTGGAGAATTTTCTGAGCTGGTGCCCTCACCATCTTC
ATCTGTGTCATCACATGCAGGAACATCTCCCTGCCAAGCACCAATGCCAAGAGCAAGAGCTGAGAGTCCACAC
ATACCCCCTCAGACACCATCTCCTCTTCCTGTATCTCGACAACTTCTCAAAACACCAGGGACACCACAACAAC
TTTTTCATGCTGCTTCTGGTCTTGGCAAAAAGGGTCACCACACAGCTCAGGATACAGTGCTCATCAGTGCGCA
TTTTGCATGTACATGTCTTATGCATTATCTCTTTGATTAAACTCATAGTTTCTACTTCATATAAGTGTGGCTC
ATTTCGGATCGTTTAGGTATACAGGTCCACTTGACACAAAAACACTACCAAGATGCCATCAGCAAGTTCTACA
TCAAGCATGGCAAGCAGCCAGCAGCCTCATCTTCGAATCATCCTGAGTGCAGCCCATTTTCGCATGAAGCATT
TATTGATGTGCTGATTGCATGGATTGTCACTGATGATCAGGTTAGCGTAGTTCTTGCACCACCATAGTAATAC
TCATCGAATTTTAGTCTATCAATGTAATTGAGAGTCCCCATTTGCGTTCAATATTTTTGA
>Armme1_1_Cluster13#LTR/Gypsy
GACTTATCTGCCACAGATTCCAGATGTTCAATGAAATCAGCGTATGTACAGCGGCACCGAGGAAGATGATTGG
CATGGTTTTGGTCCTCTGAGGTCATCGTCGCGATATGACATGGAGTGTCCATCACAATGACAAGTCGGATCAC
CTGTAATGACCTGGTGGACACATTGGGACCTACCTTGTACAAGAACCTCCGCATCTCACTATTGAGGACCCAT
TTCAACGATTTCTCTCTCAACTCCTCGCACACAACTCAGCCAGGATGACGGATTCCGCTGCACAAATCCCACT
GCCGGAATCTCGAGATTCCTCTCCTCCGCGAGGACCAGCACCGACTACCACTCCATTCGTCTTCCCGGATCCG
TTAGCTCCATTGGTATGTCCCATCGAACCTCGCGAATTGCTTGAGGAAGACACACATGAAGTAGATCGCCCCT
GGCCAACATGTGCCCCGCTTGTTTCCTTTGAACGCCACTCAAACCTGCGAGAGCGTAGGGACCGCGTCATTCA
GATCCTGAAACTCGGCACAAAGATGGGGTATGGCTTACTTTCTGCACGACCATTAACTCATAATCAGTACACA
AAAACCTTCGGCGCGGAGACTGCAATGCTAGATAACCTGCTTACTCGCGTACTAGCTTGTGGAACTGTCACCA
AGGAGACGGCGTACAAACTACGAGAACAAACTTACCAAGACCTGCGGTCCATCCTCCTCACTAAGCATGAGCT
AGCTTTAGATTCGCTTGTAGAATTTGGTTACGACGACTTGGAAGTACCCATTTGGGGTCTACCCCTAGCGAAA
GGGCTGACGGTGAACGACTTTGAGATTTATGCCTTGCACTTTCGTATTCGTGTCGAGCATTTCCTCTATAGGC
TTGACACTGTGTTTGACTGGGAAACCTTCAAAACTCGTCGGAACCGGCATGAAGGCTCCTTAGCAGCGCAACG
GGATGCCCTGAGTGCACAAGTCAAGGTAGAAGACGGCGTTCAAACCCCAGCACTTTCTCCCTCACCACAAAAA
ACTCACAAATCGAAAAACTCTCGCCGAGATGATTTCGACTGGAGTCCAGGGGGGGTACGCCGTGCAGCTACTG
GAGAAAGTAAACGGCACCCAAGTCACAGGCACGCTTCGGAATATTCAACTAGAGCAGCAGAGATATTAGGTTA
TCCGTCGCAAAGGATAGATACGAAGGCTAATAAATCTCACGGTTTCAGTCCCCGAGGGCATAAAAATCTTCGT
GCCTTTTCCTACCAACCAGGATCCTTGACAGCATCGGAGGAAGGAAACCCATCAGAAGAGGAGGATAAGCACG
GCTATAAGGACGGTTCACCAAGCGACCCAGACTCAGATCCTAGTGATACAGATGGAAATCGGCGCTCACCTCG
TCGTCCCTCGCGTAAACCTCAAGACACTAGTCGATCTACTCGTACTGAAATCTCGAGCATTCATTTTGACACC
AAGCTTAAAATATCAGACGTACCTTCCTGGGACGGAAACCAGGACACGATAATGGCTTGGATATTGAAAGTCA
ATGAGCTTGCAGTCATGAGCGACTCCGTATTCAAGGACTTAGGGCGCATAGTTCCTAAACGTTTGACAGGTGA
GGCGGATAGATGGTATTATTCGTTACCCCTTCCTTACCGTCTCGATTTGGAAAGGAATTGGGCTACTCTTCGA
GAGGGCATCTCGGATTATTACCTGAATAGAAGATGGTGGGAACGACAGAAGGATCGTGCTCGTCAAACTACCT
ACCGGCAACAGGGATTCACACGAGAAACCCCTAGTGAGTATTACATCCGCAAAGCCGATCTTCTAAACACAGC

GTTCGACCTCACGGATTCTGAAATGATTACCCAAGTCATGGATGGGGCACCATTGAATTGGAATACAGTACTC
ACCACTCAGCTATACAAGACAGCAGTGGAATTTCAAGCTAGTATTCGTTTCCATGAAGATCAGCTACTCCGTC
TAGACCCAAGTTATCGTAATCGTTCCTTGAATACGTATGAATTGCGTACTAGCCAGGACAAGCAGAGTTTTCC
TTACCAAAATTCACACGTGAGAATGATAGGCGCCTCGAATCAGCCTCTTAATCCGCCTTTTCCCAGAGACGAC
AGCAATAAGACGCGACGTCGCAAGGCACCCAAGGACGTGGGAGCCAGACCGTGCCGTCTCTGCGGCAGTGGAG
AACACTGGGATTCCGAATGCAAGCATCACGATGAAGGCATGAGGACTGCTAGAACTCGCCTTGCCGAAGCCAA
TATCTCCGAACTCCAAGCTCAAGATGAGTACGACGAGCTGTACTATGGTCTCGACGTGGTAGAGGATTTTGAG
CCAGCCCCTCAGATTACTGAGAGTTCATGCAGACTTGCTATACCATGCAAGATCGACTCTGAAGATCTATCAG
CCCTGGGGGGAGTAAGCTGTGAAAATGAGGAATCAGTGGAACCGATCATGGAATCAACTCAAGCGACTCAAAC
CTTCAAAACGGAGACTTCATGCACCAAGTTCAAGGGATCACCTTCTTTTCCACGAAAAGAAAGGCGCCGTTTG
GAAAAGCTACAGAGGAGACTTAAGCGCGTTTTCCATATCACTCACCCATCGACAGGACTCCAAGCACAGCAAC
CAGTCGTGGAACTCAAGAAGCATATGGAGCGACCGGCGGGCTGTACATTTTTGGGAAGCAAAGCGACTACGGC
TTATGCTGGTATCAATGACGTCGGATTAGATATCTTGAAAATCATCATAGATTCTGGATCTGATATAACCTTA
ATATCTCGAAAGGCTTTACAGCAACTGACATCTAGGCCTAAAGTTCGCGTGGGTCAGAAAATAAATCTCGTAC
AAGTAACTGGAAACTCAACTATTTCAGGGTATACTAACTTGGATTTGTTCTTTGAAACTACGGAAGGACCGGT
CAAGATGAATGTCGACGCATATGTGGTTGACGGAATGACTACTCCGTTCATTCTAGGGAATGATTTCGCTGAT
CAGTACTCCATTTCGATTCTACGAAAGGACGGAGCGAGTTACCTCATTTTCGGCGATACAGGAAGAGAACTCG
AGGTAGAGAACTCAACTGCCCCATCAGCCGTGGATACCGAAGGTAAAACCTTTCGAGTTCGAGTAATTCCAGA
AGCAACCTCTCGTTTGGCTAAGCTCCGCAAGCATCGCTGGTCACAGAAGATTCGTCGAAGACAGAGACTGCTC
CAAACTAGCGGAGAAGTTCGTTCTAGTAAACGAGTAGTAATCCCTCCCGAAACAAGCGTCAAGATACCGATTG
AAGCAACGTTTCCGCAGAGAATTGACGCTCTGTTCATCGAACGCCAGATCAGTCAAGACTCAAATCAACGACA
TTTCTATGGCTCACCCGACACGCTCATCTCCAAGACTCAGTCCTTCTTACACATTTCCAATTTCTCGGACACT
CCAGTCATCATAAGCAAAGGCGAGATCGTAGGACGCGCACACAATCCTCGAAATTGGTTAGATGGGAAAAGAG
ACGCTGACGCCATGGAATCAAGGCATGCCCACGCTAACCTGATTCGGCAATTAGTCAAGGCACAGGTTCCTAG
ATCAGATCTCATGGGATCTATGGGACAGATGGTCCAAAGCGAATCCACCGTCACTTCCAAGGCACAAAGAAAC
GCCTCCGAGCCTGATGACCCATCATCCTCTGACCCCATAGAAGGAGGACCTAAAGTTTCCGAGTCTTGCCCAG
ACCCCGTTCCATCGGCGGATATTTTATCTTCGCTTGACCTATCACCCGGTTTGACGGCCGAGCAGAAACGAAG
CCTCGAGACAGTTATTCTCGACAATCAGCTAGCATTCGGATTGGACGGTCGGCTAGGCGTCAACGATGCCAGG
GTAGAAATTCGCCTCAAACCCGGATCGCAACCCATATCCTTGCCACCGTTTCCCGTTTCACCTGCGAACCGAG
AAGTTATGGATAAGCAGATGGATTCTTGGATACAGCTAGGCGTTATCGAGCCCTCTGAAAGCCCGTGGGCAGC
GCCTGCTTTCATAGTATACCGAAATGGTAAACCCCGCATGGTCCTCGACTACAGAAAATTGAACGCAGTCGTC
ATTCCTGACGAATTCCCATTACCCAAACAGGACGACATCATGCAAGCTCTAAGTGGGTCCCAGTGGCTCACCA
CTCTCGACGCGTTGTCCGGGTTTACTCAACTTATTGTACACGAGGATTCTAGGGAGAAACTGGCTTTCCGTTG
TCACCGGGGTTTATGGCAGTTCACCAGAATGCCGTTTGGGTATAGGAATGGGCCGAGCGTTTTCCAACGAGTA
ATGCAAAATATACTCGCCCCTTTCTTATGGATTTTCGCTCTAGTCTACATCGATGATATCGTCATTTTCTCAC
TGACATTTGACGAGCACCTTCAACACATCGATAAGGTTTTAAAGGCTATCCGAGCTTCAGGCATTACACTATC
ATTACCGAAATGCCATTTCGCTTACCAGTCACTGCTTTTACTAGGACAAAAGGTATCTCGGCTAGGCATGTCA
ACTCACAAGGAGAAGGTTGAAGCGATACTCCAGTTAGAAACCCCAAGTAATGTTCACGAACTTCAAACATTCC
TTGGAATGATGGTATACTTTTCAGCTTACATTCCTTTCTACGCGTGGATGGCTCATCCTTTATTCCAGCTTCT
AAAGAAAGGAACGCCGTGGGAATGGACCGAGCTACACGACGAAGCCTTTGAACTCTGCAAACAGGTGCTGTCA
AACGCTCCAGTACGAGGCTACGCGACTCGAGGATTACCGTATCGAGTGTATACGGATGCTTGCGATTACGGTC
TCGCAGGAATATTACAGCAGGTACAACCTATCCACATCCGAGACTTGAAAGGCACCCGCATATATGATAGATT
GAAAGGAGCTTTCGAAAGGAAGGAGGACGTGCCTCAACTCGTTCCTCGAATATCCAAGGAATGTGATGATGTA
CCCGCAGTAGGCAATTGGGCCAAAGACTTCGAAGAAACTATAGTTCACGTTGAACGTGTAGTATGTTATTGGT
CCCGCGTATTAAAATCTGCCGAACGAAACTATTCCCCCACGGAACGAGAGGCTTTAGCACTCAAAGAAGCTTT
GATTAAGTTCCAGCCATACATTGAAGGGGAACAGATCCTCGCCGTCACCGATCACGCCGCCCTTACTTGGAGT
AAGACGTTTCAAAACGTCAATCGTCGTCTGCTAACCTGGGGAACCGTTTTCTCCGCTTACCCTAACTTACACA
TCATCCATCGAGCTGGTCGAGTCCACTCGAATGTAGACCCGATCTCCCGCTTGCGAAGACGACTTCCTCTCCA
GGATGGACCTCTGTCCGATGCAACAGTACCTGTTGATTTGACCAAGGCCGAGAATGAAGACCCTATGCGCAAC
ATGTACCAGGAATTAGGAGAACACTTTGAGGAGAAATTGCTCACCGTGGGATCTAAGTTTGTGGCTACAGAAA
TCACTCATCCCAAGGAGTTTGCAGTAGACCTCGGGAGGATCCCCATCGAGCTCAACGGTGAAGAATTAGCCTC
AATTCAGATAAGTAGCGCACGGAACTACTCAATCTTAATCGGGTTAGCCTCGGAGGAAGTACAGAACTGGAGA
CAGGCATATTTGGAGGACTCACACTTCTCTGAAGTCCTCGCCTCTTTACAGGATCAAAACTCACACGTTACAC
CACGATTCCCTCAGTATCGTTATTCGGACGAAGGATTATTCTATTTCGAGGACAACAATGGAAACAGCCGTCT
CTGTGTACCACGGTCATTACGCGTTGAGCTAATGAGTCAGATTCACAACTCACTTACTGAATCAGCACACGGG
GGCTACCACAAATGCTACAATCGTTTGGCCGCCACCCATTATTGGCCAGGAATGTCTCGCGACCTAAAGAAGT
ATGTAAATACTTGCGACATTTGTCAGAAGGCGAAGCCTCGACGCCATGGACCAGTTGGACTTTTACAACCTAT
CCCAATTCCGACTCGCCCCTTTGAAGTAGTTTCAATGGACTTCATCCCCGAATTACCAAATGCATCGGGATAC

GATAACATCCTCGTCATAGTGGATAAGCTCACCAAGTACGCCATTTTCATCCCATGCTCTACGCACATCTCAG
AAGACGCCACAGCTCGATTATTCTTCCAGCATGTCATTTCAAAATTTGGTATCCCTGCACAAGTCATAACTGA
CCGTGATTCCAGATGGCGACACACCTTTTGGAAAGAAATCTGTACTCTGATGGGAATGAAACGTGCTCTCACT
ACTGCTCATCATCCTCAAGCAGACGGGCAAACAGAAGTCCTAAATCAAGGATTAGAAATTTCCCTGAGAGCGT
ACATCGGCCCTGAAAGGGATGACTGGGATCGGTACCTGGATGGCCTCGCTTTGTCTTATAATTCCACACCGCA
CACGGCAACCGGTTACGCGCCTGCCTTCTTGCTTTATGGTTTTACGCCGACTACCGGATCTTCCCTATTAACT
ATTCCGGAATTTATCCCTAGAGCTTTGGGGGACACGGTGGGAGAGGGCGGTAAGGCGGATCATATACACCTTA
CTCACGAAAATGATAAGGCTGTAGACATCGTGGAACAGTTTGAAGCCGAACGGACTCGTGCCAAAGAAGCCTT
GAAACTAAGTCAAGCATACCAGCAGAGATACTACGACCGAGGGAGGCTAACTTCAGAATTTTATGAAGGGGAC
CTCGTGGTGTTAAATCCACATTCGTTAGAGCTACTAAGAGATGTGCGAGGCAGAGGAAAGAAGCTGCTGATGA
AATATGATGGACCTTTCGAAATTATCAAGAAGATCAGCCCGGTAACCTACCAACTACGACTTCCAGCGTCTTA
TGGCATTCACCCGATCTTGAATATTGCTCATCTTGAAAAATATGATGCGTCTCCGCTAGAATTCGGTGAAAGA
CCTACTAAGAATTTAAACAGAGCCGATTTCGAACAATTACCGGAGGTAGAAGTGGAAAAGATTGTGGCTGAGA
AAATGTTCAAGGCCAATGGGAAGCACCGCAGAGTTAAAAAATACCGGGTACGGTGGAAAGGTTTGCCGGAGAA
CGAGGACGAATGGAAAACAAAGGAAGAATTGCGGAATGCACCAGAGATTCTGGAAACTTGGATGAAAACCCAG
AACCTCGCGACATGACCATATTAATCGTGAACAATCCAACGACCTACTAAATATGGCAACTCATCGGCAATTA
TTTCGTTTCCGCTACGCGACGCTGTCATCCCTTCCTTTGTCTTAACAAGCTGCAGAACCTTCGAACACTTTCG
AGCAACACCCGAAGAAAATAATAATATAAAAATTCACCTGAACGCCGTATAAAAACCTTCGGATCCATCCAAA
GAATTATTCTAGCTTCGCGTCTTCTCTCATTCCAATATTTCGCCCTCCATTCCAAACTCTGAATCGTTTCACC
TTACTTATCTTGGACAATTATATTCTTACAGGTATGTGTAGACTGGGGGGGCTATTGTTATGGAATACTACCA
TGCGTACGAGTCACGCCTACCATAGTACGAGTCACACTTACCTTTTAGAATATTTCCTTTCAAATAAACAATA
CGAACGTTGTAGAAGTTTCTTTTTCTTTCTTTACTTTTCTTTACCTTTCCTAGCACGAAAACCTTCTCTCATC
CACTAGTAGATTCTAGAAGGACTACCCACTATATAGTCAATGTAATACGGTACAAGAACTATCGAATCTTTAT
CTCTTTTGAACTCCACTTAACTGTGTGAACCTACTAAGTGATCGTGTTCGGTTACGCCTAGCGCTTGCCGATT
CACTCGCTACTGATTACGCCAAGCGCTTGTCAGTCTCAACTACGCCTAGCGCTTGTTGATTTCTCTTGGAAGT
TCTCGTCGGTAACTCGAACTCAATTAGATAATCTCTTCGATACGACTTAGATTTTATTTCTCTTGTTAGTACA
AAGTCACGTACTACCAAATCTACTCGTATTCTCAACAGCCTGTACTACTTCATCCAAGGGCTTGTCAGTTCCA
ATTTCTATCGTCATCACCGATGGAACCCAGGGTTACGAGGGACTCAGCTGAAAATGCAATACCCAATCTAAAG
CCTACTGGGCAATTTTTGTACGACTTGAACCCACAACCTCCATCCTTCTGGATTGTGGAAGCGACTGGGACAT
TTCCAAGCCGGGCATAGTGGCTAATCCACTTGAGCCACCGTGCGCCCTCGCCTGTCGACCTTCACCGAGTCCG
AATCGCGCATTTGTATCGAGCACTGAAGGGAAATTAAAATTTTATCCTGACAGGGTGCACTCTATTCGACAGG
TGTCTCTAATCATCCGTTCTGCCACCGTCCACGGCGGTCAACGAGGTGCAGACGTGGTCGACGTCATATACAC
CACCAGGCAATTTCAAGGGGTAACACCTCTCGATATGTTTCGGTACATGTTTGGCCACATTCCCACAGACTAG
TTACACTTAGAGGCTCCTTTCTAGAGCGTCCGGTGGATAATCTGACATCCCTTCATGGCATCACCTTCTCACT
GTTCGCTTACTATTGCTTACTCCACACCATCGCCTTCGTCGCACCGACGGTTCGGTGCTTGGATCGGCACTGC
GACCCCATGACGTCAGGGATGTGGGAGTACATCACCGCTTTTGATAGGCATGGCTGTGCGTCGGGGTCGGTAT
TCACTGCAAACGCTCCTGGTATTCAAGCAAAGTGACTCAGATCAGAGTCATCCATTCAATCTACAGCTTTCCC
TTGATCAAGTCGATCAGTCCCATGTCTTCTTTTGGCAACAACTCCTCCGACTACAACATGACCAACTCCGTAT
ACTCATATGATCCCATGACTTATCCTGTGGATTATCAGCAGGTTCATCAAATGAACAACAACAATACTGCTGC
TCCTTCCATGCAGGTTAACACTTCCTCATATCCCCCTTCCCCTCCAGCATTGATGAACAATGCATACGGTGCT
GTTCAGAAGTGCCACTCTCCCATTACTTTGAGCTCCCTCGGACTTGACGCGCCTACTCAGCCCTGTCGCTATG
TCACTCCTTCTGTCACCTCCAGGAAAGAGATCCCCGCTTTCTTCATGAGGAGGAGGCCCAATCGATCCCATCC
GATGGGGAGGTTGGGAGGAGCTACCTGTAGTGGTGTAGGTGTAGGTGTGGGTGGAATGTGCGGAGGTAGTAAG
AAGTGTGGTTTCCTAATGGAATACCATCAGGAAAAAAAACAGCAAGTCATCGACCTCGACCGCCAAAAAACGC
ATCTCTGCATGCCGCTCTTCTCTTAAAATTATTCCGAGAGGTTTCTTTGTGGTATTCTCCCCCTGACCATGGT
TCGGGGAGATTTTTTTGGTTTTCAGGCATTCAAAAATGCCATTTTTTGCACTTGTCAGTTCATTTTGTCGAGT
AAACTTTACATGTAAAGTATTGTACTTAAAAACTCAATTTTATACCCAAATTCTTGTTCTCTCGTGTGAGGAA
ATGCCTGGAAACAATGGTCAGAAGTGCTTCAAGAATAGTGTGGAGACATGCTGAGCCGTTTCAAGAACCACGT
CGAGAGGATAAGATATTCCTACAAGTCTAAAATCAATTTTAGGATGAAAACTCGGAGAGATCAGAGGGATGTA
CCATTGCTGGACGTCTCCATCCATCCTCCGCCATTTTTTGATAGTCCTGACCTTGCTTGCCGGACGGCATCTC
CATCCATCCTCCGCCATTTTTTGATAGTCCTGACCTTGCTTGCCGGATGGCAATCCGTTGTGTCAGCGCAAAA
TCACACATTTATTGTGTTTTTGTAGTATTTGCATGTGTGAAAGTGAGTGTGCATGAAGAAGAAGACCAACGGG
ATGGACATACCATCACCCACAGAAATCCCTCCCTTGTCGCTTCCTCTCACCCTCCAACAAGGCTTGGCCTTGC
TCACCAGACGATGATACATCGTGTCAGCCCAAAAACACGAGTTCTTCAAGTGAAATTTGAAAAACAAAAATAC
CAGTCAGTACCTGTGAATATGAACTAACAGGATGCACATACCATTACCTGCAGACCTCCCTCCCTCGTCGCTT
CGCTTCGCGTCCTGTCCTTGCTTGCCAGACAGCCATACATCGTGTCAATGCAAAAATATACCCTATTTGAGGT
CTCGAGGGCTCCATTCGAGAGTGTTGCTTCGCTTCGCGTCCTGTCCTTGCTTGCCAGACAGCCATACATCATG
TCAATGCAAAAATATACCCTATTTGAGGTCTCGAGGGCTCCATTCGAGAGAGTGTATGGCATGAGATCACTCT

GGAAGAAAATGAGTCAGTGCGCATGAGTTTGGATAGAGAAGAAGACGTACCTCCAGTCGTATGCATCAATCCT
CCATATGCCCCTGTTCCATCCACTCCCTCGTCTTTATTCTATCAAAGCTCTGGCTCGCTGGACAACGATATAT
TGTCTCGGTGCAAAGTCCGACCTCACCACACTGTTTGAACTTGAGGGTGAGTCCTGGAAGCTTCAAGCTTCAA
GCCTCTCGCCGGACAACGATATATTGTCTCGGTGCAAAGTCCGACCTCGCCACACTGTTTGAACTTGAGGGTG
AGTCCTGGAAGCTTCAAGCTTCAAGCCTTGAAGTGTGAGGGAGAATGTTTGGGGATTCATTCTTGAGACGTGC
ATGAGCGCGCATGAATGCAGAACTGAATGGAGTTGCTTACCATCCCCAAGACATTCCCTTCCATCCATCCCAT
GCCATCATCTCCTCTACTATCCACTCTTCTTCTTTAGACAGTCCAGACATTGCTCTCCGGACGACAATACATC
GTCTTGGGGCAAAATCTTCCTCGAATTGGTGTGAAAAGTATAATAAGGGTGTGGAAGGAGATCAGGTAACCAT
CAATGGTGGGGGTGCTGAAGTGCGAGATCAAGATCTGTGCGTGGGTGAGCACCCCTGAATGCATTCAAAGTGT
GTGCGGACTTTGCACTCAACTTGGCCCGTGCCAATCCCTCAAACCCTCCCAAACCTCCAATATTTGATGGCCG
TTTGTCAGATGGGGAAAGGTTCTGTTGAAGCAGAATCCAGCGGTGAGCAGTGAGATCGCCAATCCCTCAAACC
CTCCCAAACCTCCAATATTTGATGGCCGTTCATCAGATGGGGAAAGGTTCTGTTGAAGCAGAATCCAGCGGTG
AGCAGTGAGATCGTAGAAAAATCTGCAACGTGAGATAAGTGAAGGGGCCCGTTTATATTGAGGAAGACCTACC
TGCTGTATTATCGTGCTGGAAGAAGATTCATTGGAAAGGGAGACCATCGGAACGTCCGCGCATGCAAGAAAGC
AAAGAGGTAAGTAAAAGCTAGAGTGGTGGTGGTGAGAAGGCAAGAAGCATGCACGCACATGTTCCTGATCATC
AAGTGTCCGCTTATGGACCTTGACGCCCACCTTGATCCCACCTCAATCTCTAACACCCATGCACACTCCTGCA
TGCTGTCCAGATTGATGTCCAGAGTACAAATAAGAAGGAGGGCATGACTGTAGATGAAGGCAAGGGAGAGGAA
GAGGGAGGGGAGATGGAAGGGGAAGAATAGGATGACTCTGAAAGTACGTGAATAAAAGGGATTGTTCACTTGC
TACTGATAGTCAATAAACTCTAGACAGCATCACAGGATGCGAGACACACTCGCCTCCATTGACAGATGGCGAC
CGGGACTGGTCCCACATGTCAAAATATTCCTAGACACCACTGACTTGGCCTTCTACTTTCCTTGTTTCAGCTA
CACGATTCGAAAACTCTTTGACTGGAGACCTGTCAGCAAGTAAGACGTAAAGTACCTCAAGTATACAAACAAC
ACATCTCACGGGGTACAAATATATAACCTTTCCTGTTTGTTTCCTCCTGTCACCACCATTCTCGACCCTTCAA
CGGCTGTAGCTGCTTGAATTTTCATCTGGTAACATCCACTTAGAATGCAATGCCATGCTGACTTCTCTTCAGT
TCTAGAACTATTTCAGACAAATATTGTTATAACCTGCGAGTATCATTTTCTTTACTCTAAGTCTTTTCCCTAG
ATGATCGCCTTGCCAGCTACCTTGATGTATGCACAATTTGCTCAAGCTTGGGGTTTCTCTCTTCCAAGCGACC
TGCGCCTGATGCAATCACTGTGTACGTCTTATTTACTATATGTATTCTGATTTCTCGAGTCCAAAGGTGTCTA
TACGTTGCCAGTGAAAATCCCATTGAATACAGTGGACTTACCCCACTCGTCTAGGATCCGAATGACATTCAGA
ACATCTGCAGCAAGTGGTTGAGTAAGCATTATATCGACAGCTTTGATACGTGGTGCATTGATGCGATTTGATC
TCTATCGGGAGATGTGGGATCGTACGCCTCTGAAAGATCAATCAGGCGACTTATCTGCCACAGATTCCAGATG
TTCAATGAAATCAGCGTATGTACAGTGGCACCCAGGAAGATGATTGGCATGGTTTTGGTCCTCTGAGGTCATC
GTTGTGATATGACATGGAGTGTCCATCACAATGACAAGTCGGATCACCTGTAATGA
>Armme1_1_Cluster22#LTR/Gypsy
GTCACGATCTCACTAATATTTCACCATATCACAACCCATGACCACAACATGTTGCCGTGCCAACCGTCTATGA
CCACAAAGTAGCACGATGACCATGACGAATAGTATATTATTAGAACTCTCCAAACCATATATAAACCATGTAG
TAAAAGGCCTTAAGCCTCAGCTTTGGACTAAGTCTATACTTTCTAACTCTGAACTCCTCACTGAGGCTAAGAA
TCTATGATGTGCTTTGCTCAGGTCACAAGTCCCTCGGATTACCGGTATAGGGCAGACTTATTGAGTGTTTGAG
TTAGTCGCTGACAATTAGTCCGAAGCTTGTCCTTGCGTATCCAGGTTCGCAACTGGTCTCAAGTTTCCAGAAA
CACAGCTATAATGCCACCCCGACCTCAACCCATCCCCGCTGGTATACAGCACAAGAACTATGACTGGTACATC
AGCCATGTCACCCCCCACAAAGACCAAAACTGTACTGACAACCGACAACGAGCCTGGGAGCAGAACGTCCTAA
ACGAAGCCCTCGACACCAAAGAAGACTTCTACGGGGCACCTGCCACGATCAGACCTATCGTTCCGGGTTCCGC
CCAATACAAGGCAGAACATACCATTCCCAGAGCCCCACGGCCTTTACCACAGCCACCAAAACTCCCACAACCC
CCGAAACCCTGAGTACCCACGGTACGACTTCCCACCCCGGCACGACACGTACCACACCTGGTCGCAGCCAGCA
GACCACCAGACCCCACCCCCCAACCACCAACCACTCTATGGCGAGATATACCTACCTCCTTGTCATCCCCGGC
ATCATCGTCATCATCCTCAGCCTCCTCACAACTGGCTACTACAGTCGTCGAGCAAGGCTATCCCGCCTACAAC
AATGGGCCCGACGAACACCACGTGTCAGAGGCCCAGAGATACCCATTCCCCGCCCCAGGTCCCTACTCTCACC
CCCCTCCATCCATTCGATGACAAGCCTCAAAGAGTTCCCAATCCAGGCAAACTTTTCAAAATCCAATAGGGCT
CTTCGGGAACCTGAACCCCTCCACCTCCAAGACCACCACCGCACCGCTGCTCAGTACTACTCTGACAGTGCAT
CCTTACTCCTCCTATTGGGAACCACCCCTGATCATACCGGAGACCCCCTCAGGAGGTTCCACACCCCCGGTTG
GAGTCACGTTCACATCTATAGTCGCCCCAGTCCCTACCCGACCATTGGCACCACTATCGATCCTGCCGAGTCC
AGCATCGGTGACACAGGAGTTTGTAACTCCTGTGACACCCTCCCCGGAATGGGAAGCTTCCACGAGTGGCAAA
GAGAGGCAGAAGGGGAAGCGTCTGGCAGCACCAGTCTGGAGAACAGACATGGTGAGCAAGAAGACATATAAAG
AGCTAGTAGACTGGGCAGAAGAAAGACTGCCGATGGAGATTTCCTGGAGGAATCGACCGGCCAGAGAAGAATA
CACCAAGTACGAGGTAGACTACCCTCACTACACACAGTACATACTAGACCGAGAAGCAGAACATGAAGCAGCT
ACCATCTGGAACAAGTGGCACCCATGCAGGGCCATGAAGGTTCCGGAGTGATGGGAAGACCCAACAACAATCA
AACCCATGTGCACCACTCCCCCACAACAGCGATCCCCACCCTGATTCAGGATGCAAGGAGGCCAACAACCAAC
ACCGAAGGGCCAGCTATTTGCCGGAGCAGACGGATCAGGAGCGCCAGGACCCTTGGGCAGTGGAGCACCTTCG
GGAGCACCCCCGAGCAGTGGACCTGCGCCCCTTGTGCCCCAAATGTGGAATCTACCCCAGTTCCCAACAAGGC
CAGACCCACCTCCACCACCAGGACACTGGGTACTCACCACCGGACAGGAGGCCCTGTATGACGGACCGAAGCC

AAAGGTCGCAAGAGAACCTGCACCTTTTGAAGGCAAGTCCGACGACATCACTCGCTTCTTCAACCAGTGCGAG
ATGTACTTCAAAGTACACGGCGGCTACTTCCAACAACACCCACACCGAGTAGTCTTCTGTGTGACCTGATTCG
ACAAAGAAGCCGAAACCTGGTGGGCACTACAAGCCCGAGAATTGTATGACCACACCAATGAGTGCATGAGGTA
CCCTGCCTACCGAGTGTTCAAAGCGGCTGTACAAAAGAGATTCTGGTGAGACTCAGACGCAAAAATCAAACAA
GCGCAATGGGAGAAACTCTGACAGCAGAACTATGCAGATGGAGAGGTGTTTTTCCAAAAATTCAAAGAACTCA
TGCTAGAGGCAAACGTCCTCGCCAACGAGCAGATGATGTGCTCGCAGATCGAGAAAGCCGTCTGCCAAACGTC
CAAGGACAAGATCCTACTAAACGACGTGGAGGGACCAACCACTTACCAAGAGTGGAAAACCCGACTACTTCGC
ATGGACTACGGGTTCCGGCGAAATAGAGCCATCGGCGGCACCACCAATGACCAACCCTCGGGAAAGAAACCTG
ACAGGGGAAACTAGAAGAACTCCGCTGGCTCGAACCAATCAAGCGGCAAGTCGTACGAGCCCAAGAAGACCGG
GACTGGAACAACCTATGGAGGGCAAGGTCAGCCGATGGATCTCGGCAAGCTACGCGCAGAAGGCAAGTGTTAC
AACTGTGGCAAGAAGGGCCATATCAGCAGAGACTGCAAGAAGGAATGACGATGAAAGACCGTGGCTGAGATCA
GAGCGATTCAAGAATACTGGAAGGGACAGCTGGAAGGCATTGAAAAGGAAGACACCGCAAGCACCAGCAAAGT
GGAGGAGGTAAAAGATGAGGCCAGGCAGTAGATTCATCTGCACCGGCCAAGAACTCGGGTACGCACTCTAACA
CCGCTGCGTGGACTAATACATCTTCTGTAGCCATGACATCAAATAAATATATGCTGCTTAATGAGGAAGAGTG
CAACTCAGAAACTGAATCAACCATTTCACCCGGAAGCGGTCCTACCACGATTCCCCCTGACTCAGGGATGACA
ACCAGGACCCGAACTGAGGATGGGAACCTCCAACAGAACACGGGTCACCGCCTGCCAAGAGAGAGCAATGACG
AGGCACGAAGCACGACGACAACAGCACCCTTGGCAAGTGGGATGAAACGAGAAACACCCACAGGTAAGACTAA
CCTAGGAGCCGACAGGGAGGAAGCCGCTAGCATCCAGGCGGTAGAAAGGGGGCCCAAGGAACGACCACCATCA
CCAACAGAAATGGTAAGTAACCGATCTACCACCTCCAGCTCACGAAGCATTTCCCTAGTTGAGGTCCTGGATG
AGGAAGATGACATCTCATACCACAGATGGATGGCTGCTGGAGCCAGGGCCACTACTAGTGACCAAAAGCCAGA
CGCATGCGGGCCATCCCCGAAGCGAGAAAGTCCTCCTAGAGAGGCAAGACGCCCAACCAAAGGACCCAAAAGC
ATGGGAACCGATGGTGGCAACGTCCTGCAGGAAACTGCCGTGTCTCCAAAAGGAGCTAGATGGACACCGATCG
TACACGTTTAGGCCAAACCCTTTATGGCCGACTGGACCCAACGACGAGTAAGGGAAGCCAGGATGCATGAAGA
GCATATGGCAATCCTGAAAGACTGGGTCCACCCGAAGAGAAGGAATGAAGTAGACGATGCAATGATAGAGGAT
GTAGTAGGACCAGAGACGTTCAAGTCTGAACGATACCAAGCAATAGTAGACTGGCTGCATGAGTTGAAACAAG
CGCCGAGATACATCAGGGGAAGGCCCAGGAAGAGCGTAATGGTAAAACTGCGACTAGAAACTACCGACACCAA
AAGGAGTTTCCAGGTCAATGCCTTACTAGACAGCGGATGCACCCACAGCGTCCTCAGCTCTAGATTTGTTCAG
GACAATGAAATGAACACGACACTGAACGAACATCCCACCCCAGTATACAACGCGGACGGAACCCAAAACAAAG
AAGGAGACATCACTAGTCACATAGACACCCGAATCATCATCGGGGACCATGTAGAATGAATGGATCTAGCTGT
CACCAGCCTAGGCACTGCAGACATGTTCCTCGGATATGATTGGCTACAATGACATAATCCTCTAGTTAACTGG
GAAACAGGCGAGGTATCCCTAGTCCGATGCTATTGCCGAACAGTGCCATTCCACCTACCAGACGAAGACCCAG
AAATACAGTGGAAGGAGATCGAGGAAGGAGATCATATCCTCATGATAGACATGCAGGAAGCCATACACATAAG
AGCAAGCGGCTCTAAGGTGAACGACCTAGCGGCAGAAGAGCACAGGAGAAGGAAGGCAAAGATGTACGAAGAG
ATAGTGCCAGAATGGGCAAGAGACTTTGAAGACATATTCTCGAAAGAAAACTTCGACAAATTACCCCCGAGAA
AACCATGGGACCACGCCATTGAACTAATCCCGAATGCCAACGCCAGCCTCGACTGCAAGGTGTACCCGCTGAA
CCCTCGAGAGCAGGAAGAGTTAGATGCCTTCCTAGACAAAAACCTAAAGACGGGCAGGATCCAACCCTCGAAA
TCACCCATGGCATCACCATTTTTCTTCGTCAAAAAGAAAGATGGAAAGCTATGACCCATACAAGACTATCGAA
AGCTGAACAAGATCACGATCAAGAACCGATACCTGCTACCATTGATCAATGACCTCATGGACAAGCTACAAGG
GGCGAAGTATTTTTCAAAGCTAGACGTCAGATGGGGGTACAACAATGTGTGAATCAAAGAAGGAGACGAGTGG
AAAGCGGTGTTCAGAACAAACCAAGGACTGTACGAACCTCTAGTAATGTTCTTCGGGCTTACCAATTCACCGG
CAACGTTCCAAACCATGATGAACGACTTATTCAAGAACCTCATACATGAAGGGAAGGTCACTATTTACTTAGA
CGACATCCTAATCTTCACAAAGGACAAAGCCGAACACCGAAGAATCACCCGACAAGTACTACAGATACTCCGA
GAAAACCAACTATTCCTAAAACCAGAAAAGTGCAAGTTCGAAGTACTAGAAACCGAGTACTTGGGGGTAATCG
TCAGTGAAGGAAACATTCAAATGGACGCTGTCAAGATAGCGGGCATCATGGAGTGGCTGGTACCAGCTAGGAA
GCAACAGTTACAGAGCTTCCTAGGGTTCACGAACTTCTACCAAAGGTTCATCAAGGACTACTCCAAGATCATA
CGACCAATGACCAAGTTGACAGGTAAAGAAGAGTGGAAGTGGGGGAAGGAGCAACAAGACGCATTTGAGAAAC
TAAAAACACAATTCAAGAACGGCGGAGTAGTACTGATGATGCCGACCGATGAAGACAGATTCTGGGTAGAAGC
TGATGCGAGCAAAGGGGCAATAGGAGCAGTCTTGTCACAAGAACAGAAAGGAAAGTGGAGACCGGTAGCATTC
CTGTCAAAGGCGTTAACAACCACTGAATGTAATTACGAGATTTACGACAAAGAGCTGCTAGCAATAATGATCG
CCCTAGACGAATGGAGACATTACCTGATGGGAGCAAGAGAAGTGTTTGAGGTCTGGATGGATCATCAAAACCT
TCAATACTTCTGAAAGCCACAGAAACTCAACCGCTGACAAGCACAATGGGTAACGGAGCTGGCAGAATACCAA
TTCACGCTGCACTATAAACCAGGAACCGCCAACAAGAAAGCCGACTTATTATCGTGACGAGTGGATCATGACC
AAGGGGGAGAGGACAATGAAGGGATAATGATATTAACACCCGTGCATTTTCGTGCCCTAGAAATCCCTGAAGC
AGAAGAAATATACCATAGCATATGAAGTGCAACCAAGGACAAAAAGTTGTGGGACTCCCACATTGCTTCCGCC
TTGACCCACGACAAAGGACTCAAAATGGACAAAGGAGTAATCATATATGATAATCGCATTTACATACCACGAG
ACAGAGAGATAAGGGGGAACATCATAGCCACCTACCACAACCACATGACATCCGGACACCCAGGAGCAGCAAA
AACCAAAGAATTAGTCTTACGCAATTACTGGTGGCCAAAAATGAACAAAGACGTTCTCGACTACATACAAGGA
TGTGAAGTGTGCCAACGGAGCAAAGCCAAGACACAAAAAAGAAAAGCACCGCTATACCCCCATGCCATCCCGA

CCGCCCCCTGGGAAGTCATCAGCACCAACCTAATCACCGATTTACCTGAGTCCAAAGGGTACGACTCGGTCTG
CGTTATCGTAGACAAGTTCACCAAGGCCGTAATCTACGAGCCATGCCATAAGGCACTCACCGCTGAAGGATGG
GCCAGAATTCTATGAGACCAAGTGTTTTGCAAATACAGAATGCCGACAAAGATCATCTCGGACAGAGGGCAGC
AATTTGCATCAAAATTCATCAAGGAACTATATGCTGGATTGCAGATAAAGGAAAACCCCTCCACAGCCTTCCA
CCCACAAACCGACAGACAGACGGAAAGAGCAAATCAAGAACTCAAAAAGTCCCTATGAATGTTCATCAACTGG
CAACAAGATGACTGGGTAGACTGGTTACCGATCACGGCCTTTCAACACAACAACCGAGTGCACTCCACGACTG
GATATTCCCCGTTCTTCCTGAATTACGGAAGGGAAGTGACTATACGGCCCGCGATAGAATACACGGCCACCAG
ACCCACCACAGAGGAGTTCATAAAGGACATGGAAGAGGCCCGCTCAAAGGCAGCCACGGCAATGGAAAAAGCG
GCAACACAAATGAAGAAACAGTACGACCGAGACAACGAAAAGTCACGACAATATCAGATCGGAGACAAGGTGT
GGATCGAAGCAACCAATATCACCACCCACCAACCAAAAAAGAAGTTAGACGACAAACAAGTTGGACCGTTCGA
AGTACTCGAGAAGGTCGGAAACTCCGCTTACCACCTGAAACTGCCTGCCACCTGGAAAATCCATGACATCATA
AATGAAGCATTACTCACCCCGTATCACCCACCTGCATTCACAAATCAAACCATACCACCACCACCACCACCCG
ACCTCGTAGAAGGACAACCCAAACAAGAAATTGAAGAGATATTGGATGCACAAAGCCGGCACAGCGTGTGGTA
CTATTATATCAAATTCAAAGGGTTCCTGTGAGAAGAGGCAGAGTGGATCCCCTACCATCAATTGGACCATGCA
AAAGAATCCATCCAGGACTATTGGGAAGAGAGAGGTGCGATTTGGATAGATGACGAGGGATTTACTCACCCTG
ACTCTGAAGAAACCTATGTCGCAGAACTGGAGGATCCCGACTTATTCCCCAAAACGATAATCAGACACGTAAT
GGAACGGGGACAGTGCATATTTCTCATAGAAAACGGATATGGAACCAGAAAGTGGACAGAAGATTACCCAAGA
CTGATTGCCGATTATGATGACTGAGAAGATGAACAGGAAGAGCTTCGCGAGCTATTGGGATGATAGCCTTAAA
AAGGGGGTATTTGTCACGATCTCACTAATATTTCACCATATCACAACTCATGACCATGACATGTTGCTGCGCC
AACCGTCTATGACCACAAAGTAGCATGATGACCACAACGAATAGTATATTATTAGAACTCTCCAAACCATATA
TAAACCATGTAGTAAAAGGCCTTAAGCCTCAGCTTTGGACTAAGTCTATACTTCCTAACTCTGAACTCCTCGC
TGAGGCTAAGAATCTACGACATGCTTCGCTCAGGTCACAAGTCCCTCGGGTTACTGGTATAGGGCAGACTTAT
TGAGTGTTTGAGTTAGTCGCTGACA
>Armme1_1_Cluster34#LTR/Gypsy
ACACAGTTTCACTGCAAGGTACATAGATGAAGACAGTAATGTATGGTATAATGACAGAATCATATGTGGAAGA
CAATGCATACTCAAAGGTAAAATCAATGCCATAGATATAAAAAATTACCAAGGACGCCTCTTGATCCAATACA
GTGTAATGGGAGCTGGCCTATTAGGCCACATCTTATCTTATCTTTCATCTTATCTCTTATGACTACAAACAAT
ACAAGCAACGAAACGGAAAGAACAGTGGGATACAAGCAACAAACTATGTTGAGCAACAAACCATGTTGACCAC
TATGACTTTCCCTGAGGACGATGATTCAGACATTGACATTATACCTTCAATGATGAAGGAGTACAGAGCTGCT
GCAACGGCACCTCCAGGCATACAAAAGTTTGCCAACCAGGCTCTCGCTAACTTGGCTGAGGCCCATGACTCGA
TCATCAAACATTGAGTATTTCAAACCTTCCAAGCCAACAAGCACTGATCGTGAAAGGTGACCTGGTATTCCTG
TCTACGAAGAACCTGAATCTCCCAAAGGGTTGTGCTCGAAAACTGTGCCCGAAGTTTATTGGTCCCTACAAAA
TAAATGAGGCGTACCCTGAAACATCAACTTACACATTGGAGCTTCCATTATTGCTACAGAAAAGGAGGATTCA
TCCTACTTTTCATGTCAGCCTGTTATGCCCGTATCACGCGAGCAATGATACCTTATTCCCGAATCACACTCAA
CCTGAACCCTATGATTTTGGCGCAGAGGACGATACCGAATGGTTTGTAGATGAGATTATCGGTCACTGTTGGA
TTGACTGGACGCTGGAATTGCAAATCTGTTGGTCCTTGGGCAATACGACCTGGGAACCGCTATCTTCTTGTAA
AGAATTAGCAGCGCTTGACCGATACTTGGAAATTATGGGTGTACGCTACCCTCGACAGTTGTCTAAATGACTG
AGCGATGATGCGCCACATCATATCTGACTAAGATCCATAGCCAACCCGATTTGGCTTGGGAGATCGTGCTGAA
CCTCTGATCATGTTTCACCAGGGACCAACCACTCGGTCCTACCAACCATTCCGCCTCAAATATTGGGTGTACC
ATGGAGGGGTCCCATCCTCCTTTTATTTTCTCCTATCCTGTGCATAATGTTACTGTTTTCACTCGCCTTCATG
GGTGTACTCTGGACCAGCATGTTGAGTAGCGATCCTGGTGGTAGCGGTCCACCTATACCTCGCCCTGGCTCAC
CTTGCCCCGATTCCCCTCAACATGAGTCGCCTCCTGACTGGCAAAATATGTTGAACCCCGCATGGAGTTCTAC
TTGGGGGGGGAACTGGGGATCAGGGAACTGGGGACCCACAGCTCAACAACAACAAGAACCTCCCCAATCCTTT
CAACCACCAATGTGGCCTGGTCAACTCTACAGACAGCACCCAAACCTGGGACCAAGCATGTTTTATCCTCCAA
ATTGGGGAGGATACCCGTACCCAATTCAACAACCTGCATACGTGCACCCAGCATGCGACGACCAAATGACTGT
CGACGACAATATCACTTGTCAATCTAACCCTACCCAGACCGGTCAGTCGGACTTAGACCCGGATGGACCACCA
TTGACTGACAATCTGACAGTCCATACTAACGCATTGCCACAGTCACCACAAGAGCCCAAAGACGAGAAAGGAA
AGAAGAAGGTGTCGGAGGACGAACTGCAGTGGCTAGCTGAAGAGGATGAGAGAAATTGGCAGGATGCCGTAGG
AGACGAAGCAATCCTGCCAGAGGTACTAGCTATTGCATGAAGGATTCACAAGCATGATCACATCGGTGATTTA
GAGGAACGCTTGAAACAAGCTCACGAATCACTGGCTACGGCCCTTCATCATGCTCACCAACTCGAACAAGACC
TGGAGCAAGCAAATAGAGGTACCAAGTGACCCCTCTCCTGCCCACCGGGTGATCCAGCCCCACAACACTCGAT
CAAACATATGAAACCACCACCTACTGGACCCTCCGTGGATTGACAACCACATCGATCTACAGGCACCTCTCAT
GTCAATGACGAACTAGCCGGATACCTATCCGACTCCTCCAAATGGATTATAGCCGACGCTGAGAACCGAGCCT
CCAATTCAAAGTCTGCAAAACCTCGCAAGAAGGAGAATAGCAGTCCTAAGCATCTAACGATCCTACCGTGTCG
TCGTGATTATCTCGCACCAAGAGATGGTGCAGCGTACCTATGAGATAGTAGGGTACATAACGGTACCATATCC
AAAGGATCATCGGTGGAGGACTGGCCAACGTTCTTGGCCGGCACCATGGGGGCCAGGCCCCTACACCCGGATG
AGCCCATGCCCATTAGCACCAAGTGCTACTGAGTCTGATAGTGACTCCAGTGACAGTGATGACGAGGACGAGG
CTGAACTGTGGTATGAAAAATGGCACAAGGCTAATCCATTACCAAAGAACATGCCTAACTATAAGTGATGGGA

ACGTGAGATCTTCTTGAAGGTCGAGCAGAAGAAGCATGAGCAGTATATGTGAGATCACCATCTCAGTCAATAT
AACACTGCCCTCACTTGATGGGAGGCATGACCAGGGAGGATTCCCCCCTCGTTACCGTCTGTGATGATGGAAG
GACATCCTGAATGGGACAATGGGTTCTGGGGCATGCTAGGCCCCGGATTCCTTTATATCCGCAAGTACAATTT
GGTACTTGCCAATACCAAAGCAGCAAATGCCATACGAGCCCTCAATACTAATTGAGGCTTCTGACTCCCCATG
GGTATCGATCAGTGACCCAATCCGCAAGGATTCCCTATGAACATACAGGAAATGCATGGTATGATTTGGGAGA
TTAGGGGAAGGCATCCTCGATGGAGAAGTGACTTATATTTACTTGGGGAGTTCTATAGAATCTCCTTGAACGT
GCGCCTCGAGTATTGCGACTTGGTCATGCAAATAGCAGTGACCTGACTCACTGAAGAGTGGAACGATGTTAGG
GAACGGTTCGAGTTGCTCAAACCACCTGAATTCATCCCCATGCCCCCTGCATGTCTGACCTCCAATCCCCGCA
ATGTGAACCATGGTGCTGGACTAGTCAGGCCGATTAATGGTACTATCGATGAATGGTGCCGCTATATTGCCCA
TCACATGCACCCTGGTGGTCGCAGCACCCCATCTGGGATTGGTATGGACACATCGTTCCGAGTGATGTTGGGG
AGGTCAGCCCCGCATCAGAATTACTACCTTCACCACATCCGAATGTACTCTCAGAGGAGGATGTGGAGATGGA
CGGTCAAGCAGTGGGTAGTATACCCACTGGGGGATCCGGAATTGCCCCAGCAAGTAACTCTTCTCATCAGGAT
GGCGCCATGGCTATCTCTAGTATGGACGTGGCTGGATCCCTAGGCATGGATATTATACAATTATCCATTATGG
TGGATCCTGTGCAAGTCATCAGTGCTAGTATGTCTAACGTCTCTGATGATGACTGAGGGGGCTCCAACATGAC
CGTCACAGCTGATGAAGATATGCCTGCTTCTACATAGTGGATTCCCTTGAGGCAATCCCAACTGGGGGTGTGT
AACGGGAGCTGGCCTATTAGGCCACATCTTATCTTATCTTTCATCTTATCTCTTACGCAGAACAAACATTTCA
AACACAGGTAGATGGCTCAAATGTATCATACAACATGTCTGGAGCTATATATTATGGCATGCATCATTTCACT
GCAAGGTACATAGATGAAGACAGTAATGTATGGTATAATGACAGAATCATACATGGAAGACAATGCATACTCA
AAGGTAAAATCAATGCCATAGATATAAAAGATTACCAAGGACGCCTCTTGATCCAATACAGTGTAACAGGAGC
TGGCCTATTAGGCCACATCTTATCTTATCT
>Armme1_1_Cluster62#LTR/Gypsy
ACGGATGCGTCGAACCACTGAAGAGAAACATACACGTGGTAGCCATGCCTAATTAGGATAGACTGCCTAAATA
ACCAGATGTTGAGTCCAAAATGGGACATCAGCATTGTGTGGCCACGGCCCCAGACACATGTATCTAGTACTGT
AACAGTAAATAGTTATGAATAAATTCAATGCGAATACCTTCAAGTATACATATTACACATCATTATACATGTT
TCTGACCTACTCTGGGGCCTACTTTACTTGTTCATCATTGTATATATGTGACACGCATTGCGAGTTATGCTGA
ACATTACCCATGTTTGGCATCGGATAGGTAAGGTAGTGGGCTCTGCCGCTTGCTGGTGCTACTCTTTATTAAA
TAGTTATGGAAATGCTACCACGCTGTAGTTTTTTGAACTCCACCAATCCATTTGACCAGACCCTCTTTCGAAG
GGATAGCGAGAGCATATCACACATGCATGTCTGACTGTTTGGCATTTTGTCAGAATACTACTGCAATTGTTGC
TGCATCTCCCTGGTGCCGAAGAATGCCGATATCACTAGATTGATACACATGCATGTCTGACTGTTTGGCATTT
TGTCAGAATACTACTGCGATTGTTGCTGCATCTCCCTGGTGCCGAAGAATGCCGATATCACTAGATTGATGTA
GACCTCTTATCAAGCCAAGAAAGCTCACGATCAGTAAAAATTGATTTGGCTAACATATAAGGTGTGAATGGTG
CGGGACACACGGTGGAAATATATAAAGCGACTGAAGAACAAGAGTGTTCATCAATTCATTGGCTTGATAGCCA
ACTGTGAGAAAGAATTGGGGACCTGGTGCTCGCTGCCGCTACCTGCCATCTTATCTTATCAGGCACCAATCAC
TTATCATATCTAATCTCTGGTGCCTACCCCAGTTACTATGACCTAGATGGATGTTCCTAAAAATTGTACTATG
GCTTGTGACCATCACATGCCTACCTTATTGTTTTCATTGTTTTATGCATGGTTACCAGGACCATCATTCTATT
CTTAGCCATTCTGTTTTCATATATTAGTGTAGCTCTTGCTGTAGTATCCTCAGCTTTGAGCTAAAGTTTATTT
ATACCACACTTTACTCTCATCATCAGAACTCAACTAAGAAATTAGTCCCTCTTTGCCGAGCCTCAGATGCCCT
AGTCATTCCTAGGTGATTGCCCAAACACAAGGCAGCCTTGGTTTAGACTAAGTCTTTGATCATGTGAAGTTCA
GGTCTAACAAAGTCATTCTCAACTGGAGATCTTGCAAAGAATTTGGAGACATATGAGCTTTCCTGGGAATGTG
TGGAACATGTTGCATGTTCATTAAAGATTACTCAAAGCTCATGGAGCCATTGACTAATCTTACCAGGTTGAAG
GTACCCTTCCAATGAGAGATTGAATAAGAAGCATCCATGCAAGCCTTGAAAGACATGTTAGCAAAGAGTCTGG
CATTACAATCAGTAAATTATGATGGGCTGATCATCTCATGTGTTGACACTTCTTGGCAGGCAGTAGGATTCTA
CATATGTCAATCTGATCCTGAGAATCCTAAGATTCGATACTACTGCCGATTCAATTCCATTACCTTGAATGAG
TGAGAAGCGAACTTCTCTCAACCTAAAAGAGAACTGTATGGGCTGCACCTGGCCCTGATAGCTTGCAAAAATT
GGGTATGGGGATCAAGAAACTTGATAGTAGAAACTGATGCCAAGTACATCTTAGGCATGTTGAGGAATCCTGA
CAGTGTATCTAATGCAGCAATAAATAGATGGATAGAAGCCATACTTCTATTTCATTTTAAGTTAGTACATGTA
CCTGGTAAAGTTCATGGACCAGATGGGCTTTCTCAGCAGGACTTTCAACCAGGCAATGACAAGTATGAGTGAC
TTGAGGAAGATCTGGGAATTGAGCATGATCCTTTAGAGTATGACACTAGTCAAGAACCAGTACTATTAGAGTT
CAATGAATTCTCTGACAGGATTGATACCTAGCATGGCTATGTCACCATCACCAGGGGCCTGGTAGACCTTGAA
ACAGATTGAAGTTTCCTAGCTAAGAACAGAGGACAGGTGGTGAGCATTTTATATGGTGCACCTGTACTACCAG
AAACAGAGGAATTCAACAAGGTCGAAGAATACTCAGAGGAACACAGAGGCAAAATTGGTAAAGAAATGGATGA
ACAGTTAGCTTTGGTACCAGCCTGGCTTGACCACCCACTTGAAAAGTCAGCCAGCCTGGCAGATGCACAAGTC
TGGAACTTTGTGAGATTTGCAACACATTTCTTCAAGGAGAAAGGACAGCTATACTACAAGAGCCTGAAAGGGG
AACACAAGTTAGTGATTGAGAAGGCAAGATGAATGCATATCATGAAATCCAGTCACAACTATCTGGGACATCA
AGGGGTCTACATGACCACAGAATTGATTAAACAAAGATTCTGGTGGCCTAAAGTAGAAAGAGATGTAGCCTGG
TATGTGAAAATGTGTGAGATTTGTCAGATGAGGCAGATGCTGAAAGTAAAAATCCCACCCATGGTAACACATA
CTCCATCCATATTCCAGGTAATCCATGTAGATACTATGCATATGCCCATGTCTTCAGGATACTGGTACATTGT
GCATGGAAGGTGTGCATTGACCAGTTGGATGCAAGGCAAAATGTTGAAAACTGAGACAGGGAAAGCTATCAGA

GATTGGCTATTTCATACAGTGTTTTGCTGATGGGGCTGTGTCTCATTGATCATAACCAATGATGGGTCTCCCT
TTCTCAAAACCATAGCCTACCTGGAAGCCACTTATGGTATCCAAGGCATTCAGATCTCTGGATATAATTCCAA
GGCAAATGGAAAGATTGGGAGACCCTATCTAGATGTCTGCTAGGTGCTTTATAAAGCCTGCAATGGTGTTGCA
TCTAAGTGGGCTTTGTATTTTCCACATGTCATGTGGTCTGACTGAGTAACTACTCAACAAAGAATGGGTTGTT
CCCCTTTCTCCACAGTAACAGGAGTGCACCCAGTACTTCCTTTAGACATCGTAGAAGCAACCTGGCTAGTAAA
ATACCCAGGTCACATGCTCACTACAGAAGAAGTCATTGGTTACTGGGCTTGTGCATTAGCCAAACACTTGGCA
GACATTGCACAGTTACAAAACAAATTGCATGAAATAAAAGTATGTGATACTCTTAAGCATGCCTGGATGCACA
CTGCAACCATCAAACAATTGAACTTCAAATCTGGTAACCTAGTACTGGTTCACAACATGGCTATAGAGAAATC
TATTAGAGGAAAAATGCACCCCAGATACTTGGGACTAGTAATAGTTGTAAGGCACACCAAGGGAGGATCATAC
ATTGTGGCAGAAATGAATGGAAGCACCTGGGGATTCAAGGTGGCTGCCTTCCAGGTTATTCCTTACATGGCAA
GGAAAAAGATTGACTTACCAGAGAAATTAGAAGCACTACTAGACATGATGAAAGAAGGCTTAGACCAATTAGA
GGATTCTCCTGAACCAGTCAAGGATGTACCTGATGACTTTGCAGATTCTCATCCAGAATATAAGGACTATGAG
TTATTGGATCCTCTTCTTGAAGTAAAGGAAGCATAAAAGGTTTAAGCATATATTTATAAGTCTATGTATGGGC
TGAGAATGCTACAATGCACTTGATCAAAAGCTATCAATGGCATAGCTAAGTTACCTAGATAATGTCTACCAGG
AGTAAAGAGTAACATATAGTAAGATAAGCATATAGTAAGAAGTGCAAGAAGAAGCTATTTACTAGCTTTGCCC
AGTTTATCAGGATTAAGAAGATAGGCTATCTCTCCCTGGACAAGAATCAGCTGCTTGTTGCAAAAATTGTAGG
CTACAACTGTAGCTTTCAGGCAATGAACAAGCAATTCCAGTTCAGTATCCAGATGGCATTTATGTAGCTGGTA
ACCCACAAGGGTCCCATCCTGAACATGGTTGGTAGAGAGTACTTTGAGGGCATCCTAGACCGACTCATATTTC
AATTGAAGTTGAAATTGATTTGACCAGGCTGTGTAAAGACAGAAGTCAGTATACCAGGCATGGGCTGATGCCT
GGATGACTCACCAGAAATGGGAAACACAAGTTGATTATCACCTAGTTTTCCACCACAAGCCAAAAGAGTGACA
TCAGAATGGATGTGCTTATTACTTCTCTTGGCGAGGATCCTATCTTGTTTCTCTAGAACATCGATGGCCTTAC
AGTACATAGGGTCACCTTTTGTAAGCCCTGGTACATTGCAATAAGGAAGTACGTTTACTATAGTACATAAGGA
AAGACCATGAGACTCCTTCCCACACAAATCACATTGTGCAGGATCTGTGGCTTGCTACCAGGAATTCAGCACA
ACTTCATACAAATAAAATGAAGTATTCTTACTGTGACTGGAGCAGGGGAGTGAATAGCAGCATGTTTAGAGAT
CTAGCCACAATGTTTATGAGTGGCCCAGCCTTGGGTTGTTTCTTTGGCTGCAAGGAGAAATGCACCTTTAGAA
AGTCAGCCAGGCTCTGAAAAGCAGTAAGATATCCTCACCAGGCAAGTCTTTGTTGATGCTCATCTATGAAATA
TTAAAAGCAAGTTGAATAATGTGGTCTGTAGTAAGTTCAGGCTATGCATGCTGTCAGATTCTTCACTATCCAA
GTCAACAACCATACTTTCCTCTGCAGGCTTCATCTTGACATCCTTGTCAATAATGATAGTCTCTGAGTTAGTA
TCAAGGCTAACCGTAGCTTTGGATTTGATGGTCTTCATTGTTTTCTTGATTTTGGGAAGCTACAGAAAGAAAA
TCTGGAAATCAGCCAGGAAAAGATAGTCAAGACATAACATGCACTGGAGTCGATGCAGTGGGTTTGGGAGCAA
GCAATGCAGTGGTGGCTTGAGCACTTGCAGCTGCCATGGCCTGTTTCTCTTTCTGGTCAGAATGAATCCAGCC
AGTAGCACAATCAGGAAAAGATGAATTCTCTGGAGCTTTGGAACCATGTAGGATTTTGAGCAGGAGTTTCCTA
TTTTGACAATCTCACTGGGTACCAAAGGCTCCTTTATGGAAAAGAGACAGGTCTTCCTGGTACACCTGCATAA
AATGCTATTTATTATCAAAGAGAGGCATCTAAGAAAACTATGAGCTTGGCATTAAAAAAGCATTCCTGATCAT
GTACCACTCCTTCCAGCTCAATGACTCTGGATACAGAACCTTCCAGACATATTATTTCTAGCCATGCCTATAA
TCATCCTTTCAGCTTACTCAAACCCTTGCCTGTACCAGAGCAGCTATGGGATTCCATCTTCATGGACTTCATT
GAACAACTCCTGGAGTCCAATGGATTCAGTGCCATTCTGGTCATCATTGACTATGCAAGCAAACAAGCCATTT
TCATTCCTACTTATGATATTATTAATTCTGTGGAACTAGCCCAACTATTTGTTACCCATGTATTTTCCAAGCA
TGGCATTCCCAATCATATCACTTCTGACTGTGGCTCCAAATTTGTCTCTCAATTCACTCACACTCTTGCAAAG
GCCCTCAATATGGAGCTTCATTTCACTTCTGGTTATCATCCAGAAGCCAACAGATAGACTGAATGCACTATAT
CTATTGTTCTTACCAACAAGACAATTAGGATATCTTACTACCCATTGCTGAATTTGCATACAACAATGCTCTG
AACACTTCCACTGGAATCTCTCCATTCTTTACTAACAAGGGATACTACTCTAACATCATGGTATACCCTGAAT
GTGACATTGCCTCTGCCTGGGCCTGCAATTTCATAGTCAACCTTGACAATCTTCACTAATTCCTACAAGATGA
AATCACCTTTGCCCAGTCTTGATATAAAGCCCAAGCAGACAAGGGATGGTCTCCAGCTCCAGATTTCAAAGTA
GATGATAAAGTCTATCTCAGTGCTAAACATATTAGGACCACTCACCCTACTATGAAATTCTCTGAAAAATTCA
TTGGACTGTTCCATATCATTGCTCAGCCAAGATCCCATTCCTTTACTCTCAAGCTTTCCATAGACCTCTGTTC
CATTCATCCCATATTCCATGTCTCTCAATTGGAACCTGTGGAAGCCAATACCATTCCTAATTGTACCCAACCA
CCTCCACCTCCAGTCAAAGTTAATGGTAAAGAACAATACAAAATTGCATAGATTCTCAACCCTAAACTTGACC
ATTGGTTCCAATGCCCCCTGCTCTATTGTGTCCAATGCCTAGGTTATGAAGGTACTGATGAAGAATTTGCTTG
GATCCCTGCTACCAAAATTTCTGTGGATGACTACATTGAAGATTTCCACCATTGATACTTAAGTTATGTGATT
TTCTGGAAAATCTTTTAAAGGTTCTGGGTTCAATTTCCTGGGCTGCTAGATTTTTGGTCATCACATGATTTGG
ACTGGGTGCCAGTGCTGATGAATATACCATGCAGTACTGTGAGAATTTGGCCTATAGGCCTGCCTGTCAACTG
ATGCTCTGGATACATTAATTATGACTGTCAACTGACACATTTCAAGCAGAAATCTAGCAGCTAAGGCTGATTT
TGGACTTATCCACAGAACTGCCTCCAATGCCTTACAACAACGGGTCATATCCACACTGTTCAATTGGTGATGT
GATCAATGCCATTGGCTCCAGCAGTGAAGACTCTGATGATGAGGGCAAGCCACTCACCTCAGTCCAATGGGCA
AGACTATTCATCAAATGTAGGAGCTGACAACTGCACCACCCTGGGCGACTACCATTATGGATGGACACCTTCC
AGGCAAATGGAAGTCAAGAGTGGGATAATGGGTTTCAAGGCATGTATGGACCTGGCTTCACCTACAACCCATG
AATGAACATGATGTACATGGACTGAGAGGCCATTGAAGCTGCAAGGAACCTATCTGAAGGATCAGTATACATG

CCACCGGACAGCGAGCAGTGCCGAAATTGCACTCCAGGAGTTAGCCAATGGACTTGACCGTGAGGAGGTGGAA
ACATGCTTGAGAGAGACCGAGGAAAATGGCTCAGGGGAGAAGGATAATGAGGATGGCCTTATTGATGAGCTAG
CAGCTATGACGGTCTCAGAGAGGGAGGAGTGGGAGGAACAAGTACTTCCAGCAAAGTCTGTGTTAGCGAAGGT
ATAACAAATATTCTATGAAAGGATACACACCACTGAAAAGGTCTATCCAGCTCCGTAAACTCTCTTTCAAGAT
CATCAACTCTTTGACCATCTTGCTCCCTGCCTGGAATGCAATTCTCAATGAATTGAAGTTGAAGCTGCACCTG
TAGCAGCCACTTTCATGTATCAAACACTGTCAATTGACTGGTCACTAACCAAAAGGTGTTGAATACTGTCACT
GCCCATGGGGACCATCATGCATCGTCATGGGCCTCCCTCCCGCTCATTGGAGCACAGCCACCCCTCCTCATCA
TCGGCCATGGCTGTCCGCATCATGTGTGCCCATCTACCCATTATGGAGTGGGATATTGCTATTTTGGGATTCA
AAATAAAATCACATTTTTTTCCCACTGGGAAAATTTGTCATCAGATCAGCAGGACATGATAGCACCATGCCGC
TCTTGCACCCACCCCCCTCTTGCTGTATGGAGCGGGAAAACCCTTTTTGGATTGGTCAGCAAAATTTTTTTT
TTGCCAGCATTTAGCAGCTGCTACTGATTCCCGTCAAAACGGCTGATATTTGTGAATTTCAAATTCAATGCAA
TTCCACCCTCATGCCCTCTATCACTGTACTGGGCAGGAAAATCCTTTTTTGGATTGGTCAGCATTTTTTTTT
TTGGCAGCATTTTGCAGCTGCTACCAATTCCCGCCAAAATGCTTGGTATTTATGAATTTCAAATTCAACACAA
TCCCACCCTCATGCCCTCTATCACTGTATGGGGGTGGAAAAATCCTTTTTAGGTAAGTCAGCGATTTTTTTTT
TTTGCAACATTTGGCAGCTGCTACCAATTCCTGCCGAAATGGATTTTTTTTTTTGGCAGTATTTTGCAGCTGC
TACTAATTCCTGCCAAAATGCTTGGTATTTATGAATTTCAAATTCAACACAATCCCACCCTCACGCCCTCTAT
CACTGTATGGGGGTGGAAAAATCCTTTTTAGATAAGTCAGCAATTTTTTTTTTTTGCAACATTTGGCAGCTGC
TACCAATTCCTGCCGAAATGGCTGGTATTTGCCAATTTCAAAATCCATATACTCCCACCCTCGCACCCACCAC
AGCTGTTGCTGCTGCTTTATTACTCAAAGCACTTGAAAAAATGTCGGACGCGTCGAACCACTGAAGAGAAACA
TACACGTGGTAGCCATGCCTAATTAGGACAGACTGCCTAAATAACCAGATGTTGAGTCCAAAATGGACATCG
GCATTGTGTGGCCATGGCCCCAGACACACGTATCTAGTACTGTAACAGTAAATAGTTATGAATAAATTCAATG
TGAATACCTTCAAGTATACATATTACACATCATTATACATGTTTCTGACCTACTCTGGGGCCTACTTTACTTG
TTCGTCATTGTATATATGTGGCACGCATGGCGAGTTATGCCGAACATTACCCATGTTTGGCATCGGATAGGTA
AGGTAGCGGGCTCTGCCGCTTGCCGGTGCTACTCTTTATTAAATAGTTATGGAATGCTACCATGCTGTAGTTT
TTTGAACTCCACCAATCCATTTGACCAGACCCTCTTTCGAAGGGATAGCGAGAGCATAT
>Armme1_1_Cluster39#LTR/Gypsy
GGACTTTGGATGTACGGGATTTATTACATTGGCATGATGAGCAAGAAGGCATGTAAGATTCCTGCAAATCTTT
CCACATGTTTGGCCAATAGTACGAGTCTCTGAGCGCTCCATATGACTTGTCGCCTATACGCGTCTAGGTAATC
CCACTGGGTAGGGTTACACCCACCCCCACTTTAGAACATTAGTGTGGATTATCCTTTAGCCACTCGGTGAGTG
CGATATTATCTTCAAGGTCTCTGCCCGGTAACCAACAATCGCCTTCGGGGCCCTCGTTCACATATCTCACCAG
GTAATGCCAACCTTTACCCCACCTGCGAGCGTCAACAATCCGATCGACAAAAAATTCCTCTCCACCATCGGCG
GTTATTACAGGCGCTGGATGTGGAGGCAGCCATGTGGGAAAAAGTGTGGGATCATTGGGTACAAACGGTTTAC
CTTCGGGGCCCTCGTTCACATATCTCACCAGGTAACGCCAACCTTTACCCCACCTGCGAGCGTCGACAATCCG
ATTGACGAAAAATTCCTCTCCACCATCGGCGGTTATTACAGGCGCTGGACGTGGAGGCAGCCATGTGGGAAAA
AGTGTGGGATCATTGGGTACAAACGGTTTTGGTTATTACAGGCGCTGGACGTGGAGGCAGCCGTGTGGGAAAA
AGTGTGGGATCATTGGGTACAAATGGTTTGAGCAAAGATGAATGGAAGGTTGGATGTATAGAAGATGAAGGCA
TGTCCAATGTGTATGAGGACATGGCCGGGTTTGCATCGACAACTGTAAAGGGGCCGTCGAACCTGGGCATGAA
CTTTCCACATTGCTTTGCTCCCTTATGGCAATATTCTTTACGGTGGTTTGCTGTCGACAGCATCACCTTATCA
CCAATCTTGTACTCGACCTCAGGTGACAACTGTAAAGGGGCCGTCGAACCTGGGCATGAACTTTCCACATCGC
TTTGCTCCCTTATGGCAATATTCTTTACGGCGGTTTGCTGTCGACAGCATCACCTTATCACCAATCTTGTACT
CGACCTCAGGCTGTCGACGTCTATTCGCAAAATGAGCTTGTTCAATCTTGGCTGTGGCTAGTGCGTCTGAAGC
TTCGCGAATGTCGAGTTCAAGATTCTGCATGAATGCCTTTGTATCAAAGGGAGTATCCACCCTTTCCACAGAA
GACAGAGGAGGAATCAACCGGGGAGAGTGCCCCATTCTGAGTTGGAACTTTTCCACAGATGACAGAGGAGGAA
TCAACCGGGGAGAGTGCCCCATTCTGAGTTGGAATGGCGAGAAACCGGTGGAAGCGTTAATCGTGTTCATGAT
CGTGAAGCGCACAAGAGCGAGAAACTGGTGGAAGCGTTAATCGTGTTCATGATCGTGAAGCGCACAAGGGCAA
GAAACCGGTGGAAGCGTTAATTGTGTTCATGATCGTGAAGCGCACAAGCGGCAACTTTTTCGACCACCCTTTC
TTGTCAGTCTCAGGGTGCCAGGCAGTGGACAATTTGTGTTTAACGCCGGCAAGTTTTGTGAACGGATTACGGT
CCATGTAGAAACGGAGTGCTTGAACTACCGTTTTATTAGAGCGCTCTGATGAGCCATCAGTCTCGGGGTGCCA
GGCAGTGGACAATTTGTGTTTAACGCCGGCAAGTTTTGTGAACGCTTTCCAAAAACGAGAAACAAATAGTTTG
TCCCTGTCGCTGATGATTTCGAGAGGGAGGCTGTTTTCGCAGTACCACCTGTCGAAGAATATCTGCGCGAGTT
CTTCTGCAGTTCCGTTGGTGCATTTTCGCAGTACCACCTGTCGAAGAATATCTGTGCGAGTTCTTCTGCAGTT
CCGTTGGTGCGCCATGGAATAATCTGGATGTCCGCTCCTAATCTGTCTGTCATGGTAATAATCGCCATGGAAT
AATCTGGATGTCTGCTCCTGATCTGTCTGTCATGGTAATAATCTCATTGAACCCATTCTCTTCCGGTAATGGA
CCATGGAATAATCTGGATGTCCGCTCCTAATCTGTCTGTCATGGTAATAATCTCATTGAACCCATTCTCTTCC
GGTAATGGGCCCACAAAGTCAATCGCCACTGATGAGAAATGGGAGTCTGGCACTGGTAGGGAATGTAGCGGGC
CCGGGACTTTGGATGTACGGGATTTATTACGTTGGCATGATGAGCAAGAAGGCACGTAAGATTCCTGCAACGG
GACTTTGGATGTATGGGATTTATTACATTGGCATGATGAGCAAGAAGGCACATAAGATTCCTGCAAATCTTTC
CGCATGTTTGGCCAATAGTACGAGTCTCTGAGCGCTCCATATGACTTGTT

>Armme1_1_Cluster2#LTR/Gypsy
AAGTCGTCGACAATCTCTTGGAAGGGCTCCGCCAGGCAAACCGGACCAACGCACTAGAGTGGTTGGATGAGCT
GCGCAAACCCAAGCACTACCTCGTTCGCTCTGTCACCACCTCCAAACGGGACCTTAAGATCCCGCTTCAAGTC
GAGCTCCTAGATTCCCAATGCACCATGAGCGCCACCGCCCTGCTTGACAGCGGGTGCACTGGTAGCTCCATCC
ACAGCGAATACGTAAAAGCTCACCAAATTCCCGTCCATAAAGCCGCCAGTCCTATTCCCATTTACAACGCAGA
CAGCTCCCGCAACAAAGCCGGAGAAATCACTGAATTTGTCGAGGTCCGACTCAAAATCGGAGACCATAGCGAA
AAGATCAACCTCGCCATAGTCAACCTCGGCTCAAAGGAGATTTTCCTCGGCCATGACTGGTTGGTCCGTCACA
ACCCACTTATCGACTGGACCACCGGCAAGATCACCTTCGCCTGCTGCCACCCAATCCCTCTGGCCAAATGGCA
GAAGATGCGCCCTGGCTCAATGTCAAGGTCAACCTTATGCGCAAGCCCGCTGCCTTCAAGGGAGACCCCGAGG
ACATCGACAGATTCCTCACCCAATGCGAAATGTACTTCCAAGTACATGACCATTATCTATATGCCGACCCATT
CCGTGTCGCTTTTGCCGCCTCACACTTCACCGGCAAGGCAGAAGAATGGTGGACCATAGAACTCTCCGAGCGG
CGCAGTCGTACCCGGAACAAGTTCTGCCTCCCCAGCTGGGACACCTTTGCCGAAATGATCTGCCAAGAATTCT
GGGATCACGCAGTTGAGGAAGTACAACAAAAGAAAATGTACGACCTCTGCCTCGGCAAGGGCAATGCGGAAGA
CTACTTCCATGCCCTGGAGCACTTCGCAAAGAAAGCCAACCTCCGCCACGACACTGGTCCTCGTGGCCACATG
GTCGCCGCCGTCAAAAGAGGGGTCCCCGACTCCTACACCAGTATGATCTCCAACATTGGAGTCGGCATGCCCG
TCACTTATAATGACTGGAAGGAGCGCATCACCATCATGAACAATGAGCGCCAACGCAAGGCTGTCGAAGATGG
CCTCACCCGCCCCCACTATCCCAACTCCAGGAACCAGAATCAAAATGCCGGAAGCAGCAAGCCTGCTCCAGGC
ACCACAAGTTCCTCCGGAGACAAGAAGACAGGTACTGGGACCACCTATGGCAGTCAAGGACAACCAATGGACA
TTGACGCACTCTGGTCAGAGGGGAAGTGCTTCCATTGCAAGCAGAAAGGTCATATTAGCAAGAATTGCCCATT
GCAGAGCTGGAACAAGAAGAAGGAGGAGGTCAGAGCCATTGACACGGAGCCCACCACGGACTCTAAAGTTGAG
GAGGTAAAAGATGGCACTGAGAAGTAGGCAGTTCTACTTGCTCGGCGAAGGACTTATCTGGTATGAGGAACAA
TATCGCTGGTCGGACTTATAATATCTCTGTAGTTACACCTGTCGGTTGACATGGAGCCTGCCATGGACTCTAA
AGTTGAGGAGGTAAAAGATGGCGCCGAGAAGTAGGTGGTTCTACTTGCTCGGCGAAGGACTTATCTGGTACAA
GGAACAATATCGCTGGTCGGACTTATAATATCTCTGTAGTTACACCTGTCGCATCAAAAAATATACCTATTTT
ACCTGTTCCAAAGACTGATAACCAAGAACTGCAAAAACCAACCTCCGAGCCTGCTGCAAGGGAGGGGACTTGC
CAGGTAGCACTGGCGCCCTCAAAACGAGTATTAGGACTCGGATCGGAGTTGAGTAAGCGGGACGTCCTAGGGC
CTCTGAAGCCGAAAGACTCAGCGCCTTCCACGAAGGCCAAGACATGCAAAAACCAACATCCGAGCCTGCTGTG
AGGGAGGGGACATGCCAGGTAGCGCCAGAGCCCTCAACACAAGTAATAGGACTCGGGTCAGAGTCGAGTAAGC
GGGACGCCCTAGAGCCTCTGAAGCCGAAAGACTCAGCGCCTTCCACGAAGGCCAAGACACTCCCCGCAAGGAC
CAATGCAGAGAAATCTCAAATGCCTGCTAAGGCCAAAGCAGGGGCAGAGAGCCCTTGTCAAGTCACCATGGTC
GAAGTCCCAGACAAAGAAGATGATACCACCTTCCAACTCCACCTAGCCAAACAAGCATCCACCGTCAAAGTCC
CAAAAGCCTCGCCAACACCAAAACCCACCGGACTCGAATGGTTAAAACCGTTCGAGACTGAATGGACTTGGAG
AGCCATCCGAGATGTCAAAGTCCCAAAAGCCTCGCCAACACCGAAGACCACCAGACCCAAATGGTTAAAACCG
TTCGAGACTGAATGGACTTGGAGATCGACAATCTCTTGGAAGGGCTCCGCCAGGCAAACCAGACCAATGCACT
AGAGTGGTTGGATGAGCTGCGCAAACCCAAGCGCTACCTCGTTCGCTCTGTCACCACCTCCAAATGGGACCTT
AAGATCCCGCTTCAAGTCAAGCTCCTAGATTCCCAATGCACCATGAGCGCCACCACCCTGCTTGACAGCGGGT
GCACTGGTAGCTCCATCCACAGCGAATACGTAAAAGCTCACCAAATTCCGGTCCATAAAGCTGCCAGTCCTAT
TCCCATTTACAACGCAGACGGCTCCTGCAACAAAGCTGGAGAAATCACCAAATTTGTCAAGGTCCGACTCAAA
ATCGGAGACCATAGCAAAAAGATCGACCTCGCCGTAGTCAACCTCGGCTCAAAGGAGATTTTCCTCAGCCATG
ACTGGTTGGTCTGTCACAACCCACTTATCGACTGGACCACCGGCAAGATCACCTTCGCCTGCTGTCACTGCAT
>Armme1_1_rnd-1_family-312#LTR/Gypsy
ACAATAGCAAGAATTCNGTTTTGAAGTTTCCGTCTTATGCGCCGCCAGACCAGTCGAATATAGTCACGGATAT
TCCCGAGCACGCACCAGTCAAGGAGCGTGTAGCTCAGCTTATCCCCGAGGATATTCATCCTTTTGCCGCAGCA
AAGCCAGCCACTTATGCACCGCCAGCAGTTCGTAACGCGGGTGCTAAGCCAGATATCCGCAAGCGTCCCAAGG
ACAATGAACCCGCGTATAGAACTAGAGCACCGATAGAAGGATCGCATGTTGCGCGAGAAGTCTATGAGCGCGC
GCTTGACTCCACCATCACGTTATCGCATCAAGAGCTATATTCTTTATCACCAGCAATCAGGGAAATGATTAAA
GAAGATAACACCAAGCGCAAAGTTCCTAGCACGGTGGAGCAGTTTCAAAAGGAGTTAGCATTGCCGTTCACCG
GACCCGAAACCGTAGAAATCACGGTTAAGTCGTCAGACAAAGAGGAACACGGCAAAACTTCCACTCTTTTATA
CAGCATGCCGTCCACTTTCGTCAACGCGCTCGAGCACACCAGTGACAGTAATAAACGTGCGGATCCGCCAGAG
GGATCCTCAGTCGTCCCTGATCCATATGAAGCTCGGATCAAACGAGGAGAGAAGATTGGCCCCTTTGAAATGCG
CAGCAGATTCCATTTCNTTGCGATCTATCGTACCCATTGTTGATCACCAGCTCAAAGTCGAGTGTATCATTGA
TCCCGGATCACAAGTTATCTCCATGTCCGAAGCTGTTTGCTTACGGTTGGGGTTAATTTACGATCCGACCGTT
GTTCTCGAGATGCAATCAGCCAATGGGACCACGAATTATTCTTTGGGTCT
>Armme1_1_rnd-1_family-283#LTR/Gypsy
GGACATTCTCGAATAAAATGCCCTATTTCTCCACAAAAGCTGCAACTTTGACCTGTAGCGGCGTTCGCGAAAT
TACTTTGAGCTGGCGGCCCAGACGAAAAACGTGTCTGCTGGGCCTTCTGGAAGCGATCGTTCGAGTTCAATTG
CAATAACGTCTCGCCAAGTTTGATGAGCTGATTGATATGCTCCATCTTAACAAANNNNNNNNNNNNNNNNNNN
NNNNNNNNNNNNNNNNNNNNNNNNNNNNNNNNNNNNNNNNNNNNNNNNNNNNNNNNNAAACGACTGGCTGGGAA

TGTGAAACTGGATACGGCATCTCGGATGGTCTAGCTGGCGCCGCCTCACGGAAACTGTGAGCGGGCATGCTGC
TAATATGACTTTCGCCCTTATCCGTGCCTTGTAAGACGAATTGGGCTGCGGCATAAATATCCTCGAGCTTGTA
TGGGTCTGCGGGATGGTGATCTGGTAGCTTGATCTGGAGTCGGCGCGAAATGGAATCCCAAACGTGACTGGGA
AAGCCTTTGCTGAACATATTGCCGGACTCGTGGTCGGACATACGTTTATTGGCGATCAAATGTTTGACTTTGG
GGTAGAACTCCCGATAAAATGCTCCGAGATCTGCGAGATTATCAACCTCGCGCGCGAATTTTCCAATGACGCG
CTCGAGATCGTGTCTCTCGTATTTCCGTGTGGTGCTAGCATCGGGGTACTGTGCGACAACGGCTGTCTTGAAA
TCGGCATAGGTCTTTCTGGCTTCGCTATATTCGGGCAAGCTCTCCCAAAATTCGGAAACCGTTAAGCCAGGGT
AATAGATGAACCATTTCTTCTTCTCCGCGNCGTCTGTGATAGAATGAAGAGTAAAGAGGTCCTCAATGTCATT
GACGTAACGCGCCAATTCGCGTCCTGGACTAATTTCGTCAATGATATTGGGAGCGCTCTTCTCTTTTCTGAAT
GGCATAGGCATGGTGACTGGTGGTACTGGGGGGATAACTGGTGGTGGAACTGGTGGTGTGTTCTGACGAACTG
GTGGTAAAACGGGAAGAATGGGAAAATAAGAGGTAGAGGCTTCNGTAGGTGGTT
>Bjead1_1_rnd-1_family-23#TIR/MuDR
TCTGGATCAACGGNCTGGCGGGGACNGGCAAGACGACGATCGCGTACTCCGTCGCGGAGTGGTGCGAGGAGAA
GGGCATCCTCGGCGCNAGCTTCTTCTGCTCGCGCTCCGACGCCGAGTGCAGCGACCCNAANCTCATCTTCGTC
ACNATCGCGCGCCAGCTCTGCCACTNCCACGCGCCGTTCAAGGAGAAGGTCGANGCGGTNCTGAAGGAGAACC
CCGACGTCGTCNACGCCGGCCCNNNCCGCCAGTTCGAGGAGCTCATCGTNCGGCCCCTGGAGGCGCTGAAGGG
CGCGTTCCCGCGGGCGGTCATCGTCNTCGACGCGCTNGACGAGTGCCGNGACGAGGGCGCGACGTCGACCATC
CTCGCCGTCATCGCGAAATTCGCCCCCCGCGTCGCGCGGTTCCTNCTCTTCGTCGTCACGAGCCGCCCGGAGC
CNCACATCACCGCNCTGTTCGACGAGNCGCGCGAGGAGTCGCTCAAGGACGCGACGACGCTCCTNCTGCACGA
GGTCCAGCTCGAGTCGGCGCCGGGCGACATCCGGCGCTACCTGGNCCACGGGTTCGACGCAAACGTCGGCGCG
TTCGAGGTCCAGAAGGGCTGGCCGTCCGCCGAGGACGTGGACACGCTGGCGGAGCTGTCGCACGGGCTNTTCA
TCTTCGTCGCCACCGCCATCGCGTTCATCATGGACANGGACCATCGCGACCCCGAGGGGCAGCTCGACTCGCT
CTTCGGCGTCCAGTCGGGNTCGTCGGCCTCGGCGCGNTCCTGTACGANCTGTACNCGCAGATCATCTCGGCGT
CGTACGGCAAGGCGTCTCGCACTCTGGCGGAGAAGCTTCCGAAGGTCCTCGGCGCCATCGCCCTCGCGCAGGA
GCCNCTNTCCGTCGCCGCCCTCGCAAGCCTCCTGGGATGCCGCGAGGACGACGTCCGCAACGCGCTGNCGGGG
CTNCGCTCNGTGCTCCACGTCCCCGACGACCGTCNCGAGCCCATCCGCGTCATCCACCCCACGTTCCCCGAGT
TCCTNCTGCGACGCGAGCCGACGCATCCGCTTCACCCGCNACGCCCGACGCGTTCCGCGTCCANCCCGCGGAG
CANCATTGGCTCCTGTTCTCNCGGTGTCTCGAGGCCATGGCCGCCCTCAAGCGCGACATGGTCGGNATCCGGT
ATCCGGCGANGTTCAAGAGCGAGATCGAGGGCTTCCCCGAGCTGACNCTCGAAGCGATCCCGCCGCACGTNCG
CTACGCGTCCCGGTNCTGGGGCGNCCATCTCCGCGANGGTNCGGGGGATCCGAACGAGACCGACGTNTCGGAC
CCGCTCCGCGGCTTCGTGCACGAGCGGNTGCTGTACTGGCTCGAGGCGTGCCTTCTGCTCGACGTNCTGGACG
GCCACTCGCGCGCTNGACGCGGCGCGGAGCGCCTGCCGGGTAGGTGCGCATTGTCGCCATCCCATTCGCGTGC
TCATTCGTTCGTGACAGACNCTGGGAGGANCGTTCGCCGACCTCGCCCCGCTGCTCGCGGACTGCCGCCGATT
CGTGCGGACGTATTCGTCGGCTATGCACGCCGCGCCGCTGCAGNTNTACCACTCCGCNCTCGGCATCGCNCCG
ACCGACAGCCNCGTCAGGGNNNCGTACGCCCGCGAGCTCGAGCACTCCNTCGTCGTGCGCGACGGAGCGCCCT
CGCGCTGGNCGCCCTGNCTCGACGCGTTCGCGGGACACGGCGGCAAGGCGGTGTACTCGGTCGCGTTTTCGCC
GGACGGGAGCTGCTTCGCCACCGGAGGCGGGGACAGCNAGGTCAGGATCCGGGACAGCGNCGCGCGCACNGAG
CTGCAGTCCTTCGACGGGCACACGGGCGGCGTCAACTGCGTCGCCTTCTCGCCGGACGGGGCGCACGTCGCGT
CGGGATCGGACGACAGGACGGTCTGCGTCTGGCAGGTGTCGTCGGGNGAGCGCGTNCGCACNTTGGAGGGGCA
CACGGGCNGGGTCTNCTCTGTGGCGTACTCGGCGGACGGGCGCCGCATCCTCTCTGCTACTNACGAGGGCGAG
GTGAAGCTGTGGGACACCGCCGACGGGCGCTGCGTCCTCGCCCTCGACAAGACGGGNAACTGGACGCGGCAGG
CCGCGCTCTCGCCCGACGGCGGCGTCATCGCNCGCGGCGACGGCGCTTCCCTCGTCCTGCACGACGTGTCTTC
TGCCACGCACGAGGCNCTCGGCCTTCCCGATGNGGTCGAGGCATGCGCGTTCTCGCCCGACGGCCGCTACGTC
GCCGCCAGGNCGGAGCACGCCATCCGAGTATGGGCGACGCGCGGACAAGNCCCTGGTCCGCTCCATCGACGTG
CCCGACGGCGCCTCTCGAGCCGGCTGGCGTTCTCGCGCGACGGCACGCGCATCGCCTGCGGATCCGAGAGCGG
CTCTGTGTACGTGTGGCAGGTCGCGTCGGAGGACCCGCCGCGCGCTCTGCAGGGTCACACNGACCGGGTTCGN
GACGTCGCGTGGAGCCCCGACGGCTCGCAGGTCGCCTCNGCCGCGGACGACGGCACCATTCGGGTGTGGGACG
>Bjead1_1_rnd-1_family-33#TIR/MuDR
GTCTGGCGAGAAGGCGAGGGAGCGGGGCCAGTCGTGGGTTCTGAACGAGAANCCGTGCGTGCGTGCNCCGGTC
GCGACGTCCCAGACGTAGACCCGGTCGGAGTCATCGAGNGCCACGACGTGNTCGTCGTCCGCTGCGAACATCA
CCTCCATGACCCAGTCGTCGTGCGGGCCAAGCACGCGCACGACGCGCTCCGTCGCGACNTCGAANACGCGCAC
GGTCTCGTCGTCGGAGCCCGAGGCGACGAGGCGGCCGTCGGACGAGAACGCCGCGGCGCGGACCCAGTCTNNA
TGCTCNGCGTGGCCGCGGACGAGTGCTCCGGTGGCGGCGTCCCACACCTTGACGGNGCCGTCCTCCGAGCCGG
TGGCGANGCGNGTGCCGTCGGGCGAGAACGCGATGGAGCGTACGTCCTCGGTGTGCCCNNTCAACGNCGCGAC
CACCGTTCCNGTGCGGAGGTCGCAGACGTCGACNACGTCGTCCGAGGTCGGGGANAGGTACGCCANCAGCGAG
CCGTCNNGGGAGATGGCCACNGGGCGCCGACCGAGCTCGGCGTGCCGCTGCCCGGTCAGGAACGCCCAGTCGC
GAGCGCTGTCGCTCGAGGACGACAAGTCGTCGNCAGNCTGCAGCGCGGCGTCCCAGGCGNCGACNACGTCCGC
GAAGAGCGTCACCGTGGGCGCACCCTCGGGCAGTCGGGGGCCGGCGGGGAAGTCGTCGACGCCGAGGGAGGGG

AGGTGGAGGAGGGTCGCCGNGCCCTCGTANCGGACGAGAGCGAANCGAGGNGGNCCGGGAGGGGCGGGCTGAT
GCGCAGATGCGGCGGAGTCTCGGNGGCGGNGGATCGCGGCGCGNCGTCCCACACCCGAATGGTGCCGTCGTCC
GCCGCNGAGGCGANTTGCGGGCCGTCGGGGCTCCACGCGACGTCNCGAACCCGGTCNGTGTGACCNTGNAGGG
CGCGCGGCGGGTCCTCCGACGCGACCTGCCACACGCACACAGAGCCGTCCTTCGATCCGCAGGCGATGCGCGT
GCCGTCGCGCGAGAACGCCAGCCGGCTCGAGAGGCCGTCGGGCACGTCGATGGAGCGGACCAGGGNCTTGTCC
GCGCGTCGCCCATACTCGGATGGCGTGCTCCGNCCTGGCGGCGACGTAGCGGCCGTCGGGCGAGAACGCGCAT
GCCTCGACCNTATCGGGAAAGCCGAGNGCCTCGTGCGTGGCAGA
>Bjead1_1_rnd-1_family-20#LTR/Copia
CGTCATGTTCCTCGACGGCTCGTCCTTTGCCTTTGCCTGCGCGGGAAATGCTGGAGACAGGAGTGGAAGGTTG
GGATAGGGTGGGAATCTGCGAAGGCTTTCGAGCCTGTGCTGCGTAGGAGGTAGCAGGTGCGGTCTGCTGAGAA
GAGGATGGTGGCTGAGTCGGCTGTGTAATTGGTTGAGCTGCTGGGTGCGTTCGCAGCGGTGCCGTTGAAGGGA
CTTGTAGATCCACAGAGTAATAAGTGGACGGCCAGGGCCCGGGGCCCGGGGCTTCNTTCGAGGGTGGGGGTGT
TGGACGGTTGGGAAACACTGTCTCGTCGAATATCACATCTATACTCGNCGTAAGTGTCCGGGTACGTTTGTCC
CATAGCCGGTAGCCCTTTGTACCAGGCTCGTACCCCACGAAGACGCAAGCGTCAGCTTTCGCATCTAGCTTTC
TGCGCTCCGTGCCGTGGATGTGTCGATAAGCNAGGCAACCGAANACGCGGAAGTATGAGACATCCGGTACTGA
GCCTGTGAGAACCTCAGAGGGTGTCCGCCAACGAAGTGCCTGCTTCGGCGACCTGTTGCGAACATGTACGGCA
GTGCGTACTGCCTCCTGCCAGAAACCGTCGGACAAGCCCGCAGCGAGCATCATGGATCTCGCGGCTTCCATAA
GCGTACGATTCATCCGTTCAGCTTTGCCGTTCTGCTGGGGCGTGTGCGCGTGGTGGTCCGTTCGTGGAGAATC
CCNTTCGCCTTGAGCTTCGCCTGGAACGACTCTTGGACATGTACTCGCCGCCGCGATCCGATCGAATGACCTT
AACCGTTGTTTCGAACTGCGTTCTCGACCGTAGGCGCAGAATTCGTCGAACTTCGACTCCTGACTCGTCCTTG
GTGGCCAAGAAGTAGATGAATACCCAGGCATTGTCCGATTTGTCGTCGATGTAGGTCAGGAATGCCGTGTAGC
GCTTTCCTCCGACGGACTGGTTCTGCATGGACCGGCGAGATCNGTGTGNACGAGTTGGAANGGGGATTCCGC
GCGTTCNGNCTCGGAACNGGGGAAGGAGACACGGCGATGTTTACCGAGGGCGCANCCCTCGCAAGGATGGAGG
TGCGACTCGTCGTGAGAGAATTGCTCGAGACCTTTCACCGGTGGGTTGTCTCCAGTAGCGCGAGCTTTTCGAC
GCCTTTCCAGTTCAGATGCCCGAAGCGTGCGTGAACGATGTCCGAGGTAACCGCGTTGTACGAGGTTGCGGCC
ACGTTCCCTGACTGAGTGGCNAGCTCCAGCCAGTAGTGGTCGTTGACGAGTTTGCCTATCGCTAGCGTGACGC
CATCACGCATAACTTCAACTTGGCCTCCGCCGTATAAGAGCTTAAAGCCCTTCTTGTCTGCCACGACTCCGGA
ATAGAGACGATAGTTCATCTCGGGGACGTAAAGGACGTTGTCGAACTTGAACTTGACCCGTTTAGAACCGGAC
GTTGATACTGCGTGGACTGTTCCGACACCGTGAACTCTCCGACGAGTCTGTTCCTTATCCGCCAGGGTGACGA
AACGGAAACTCTTGTGTTCGTGATACTCGTGGAAGTCGGAGATGTTGTTCGTAATGTGTCGTGTGCAGCCGGA
GTCTGCCATCCAGGACGAGAGTCCTCGGACCGACAGCATACGAGGAGAAAGAAGATTCATTGTCCGAGTCTAC
AGTGTGGGGCTACGGCGTGTGCGACGCCAGCGGGTTTCTTGTCCTTGTCCTTCCAGAACCAGAACCAGATTGT
CCTTATCCTTGCCCTTGTCCTCGGACGACCTGTCCTTGGACGCCCCTTGCCTCCTCCGTCGCTGCTTCTTCTT
GCGGCACTCNGCCTCGTAGTGGCCCGTCTTCTTGCAGTAGAAGCACGTGATGTGCGACTTGTCCTTGCTCTTC
TTCTTCTTGTCCGGCTTGGACGAAGGACCTGCGACGGCCGCGATGGAACCGACTCCGTTCGACTCGGACGCGA
CGCCTCATCGATGATCTTAGCNCGAATGGCATCGTACTTGAGGTCCGCAGTCTTGGTGACGGCGAGGATGGGC
TGGACGACGTGCGTTGTACGNCTCGGGGAGCGCAGCGAGGAGACCGAAGACCTTCAGCAANTCCTCGTTCGCG
AGGATACCACTGTCCTCGACCTCCNGCCAGAGACGACCACGCGTCTCTAGCTGCTCCTCGGCGGAGCCGCGNG
CCGTCGACGGCAGTGCGAGAAGTANTGNCGGAACTTGCCGNAGGNCGTGAGNGTGTCGGAGGCTGCGTAGTTC
GGCTCGAGCNCGNCCCAGCATTCCTTGGCGGCGTTGAACCCCTGGAAGAGATGCCAGAGATTCGTCGTCGACC
AGGNGCTGAGGAGGTAGAACTCGATGGTCTTGAGCAGATTGGCCTTGCAGCCGCGCGGTGCCGTCGGCGTTGG
TGAGGTCGGCGGGGACGGTGATCTTGCCGTCGACGAAGCCCTCGGATTCGTTCAGCTCGAGGAAGGCCGTGAA
CAGTACGGCCCAGCTTGCGTAGTTTTCACTACCGCGCAGCTTCTCGATCTCGGTCGATCTTGACGGTCGGGGC
GGTCGACGTGGTGGACGCGAAGGCGGACTTTTGGACGGCGGAGACGGATGTCATGGTGGCAGATGTGGGGGTG
CGTGCGGGGTGCTGATGGTTGTGGAGCGGAGGGACGCGCGATGATCCTGGTCAGAGCCGCTCGGGGCTCATAA
CCTGATGAAGCGACTGCTTCCCGTGTGGAGCCAGGCGCAAGAACGGCGATATATCGCGGTACTCGCTAGACCT
CTGGCGGAATGGGCGAATGCAAGCTCCTGAGATATATCTCGGGGACGCGAGCGGAATGAACGGGAAGGGCTGG
GGCACAGCGCGCTGGCGAGATGACGCAGGATCTAGTACGAGCGCGCGGACGTGAGTACCGCGCGCGGAGGGAA
ACGAAGCGATGATATACNGTACCTAGTACGAGCGCGCGGACGTGAGTACTAGGGATAACGGTAAGCATACGGC
ATAGGGCATAGGGCAT
>Bjead1_1_Cluster13#LTR/Copia
accggacatgtgattggactcatcggacttgatcggctttcctagtatagtgatccttgtattccattttcac
atgctagttgtattagttctcccgcgctgcccattggctggctattttcactctcgtttgtacttgtttactt
ttctcttggcgctctgtagtatatatacctggacgctttgtagtgcacaccatcgcttcgtttccctccgcgg
tggtgtgagtaccctccatgcctcatgccctatgttgtatgcttacctgttatccctagtactcacgtctgcg
tgttcgtactaggtattattatacatcgcttcgtttccctccgcggtggttactcacgtctgcgtgttcgtac
tagatcctgcgtcacctcactagcgcgctgtgccccagcccttcccgttcattctgctcgctctcgggatata
ttccgagtgcctgcattcgcccattccgccagaggtctagcgagtaccgcgatatatcgccgttcttgcgcct

ggcttcacacgggaagcagtcgcttcatcaggttatgagccccgagcagctctgaccaggatcatcgcacgct
ccacacatcgcaccctgcacgcacccccacatctgccaccatgacatccgtctccgccgtccagaagtccgcc
ttcgcgtccaccacgtcgaccgccccgactgttaagatcgaccaaatcgagaagctgcgtggcagtgaaaact
acgcaagctgggccgtactgttcacggccttccttgagctgaacaaatctgagggcttcatcgacggcaagat
caccgtccccgccgacctcaccaacgccgacggcctcgcatggctgaaggccaacaagaccatcaagttctac
ctcctcagctccgtcgacaagaatctctggcatctcttccaggggttcaacactgccaaggaatgctgggaca
tgctcaagctgaactacgcagcctctgacactctcacgtctttcggcaagttccgtcagtacttctcgcacgc
cgtcgacggcaacagctccatcgaggagcagctagagacacgtggtcgtctctggctggaggtcaaggacagt
ggtatcctcgcgaacgaggaattgttgaaggttttcggtctcctcactgcgctccctgagacgtacaacgacg
tcgtccagcccatcctcgccgtcaccaagactgcggacctcaagtacgataccattcgtgctaagatcatcga
gaagttcaagtccaacggagtcggttccatcgcggccgtcgcaggtccttcgaagaacaagaagaagaagaag
gacaagtcgcacatcacgtgcttctactgcaagaagacaggccactacgaggccgagtgccacaagaagaagc
gcgacgagaaggaaggggtgtccaaagacaagtcgtccgaggacaaggacaaggataaagacaaggggcacta
gtggttaacatcttttgaccaaattgcaccactatagtggtttttgcattgtactagcagtggtcagtgtgt
ttttgaaccaaatagtggttaaatttcagtgtgtttacaattaacagtgggccactatgtttttaacatatta
taatagtggtttctgtctgttttatgaagtccaagctagtggccactaattgaatgcaagtaaaatagtgggt
gctgtgtttgttgaagatttttgaatagtggccactaattgaatgcaaacaagatagtggctggtatgttttt
tgtggtttttggatagtggccactaattgaatgcaaacaagatagtggttggtatgtttttgtggattttaag
atagtgAGCAATGGTGTCAGCGACACACTGCCCTGAGACGACCACGACACACTAGGCTGCAACAACGAGGGAC
TTAAGGATCCGAGGGTTGTGGGAAAACACTTGAATTGCGCCTGAATAGGCCACTATCTTAAATCCACAAAAAC
ATACCAACCACTATCTTGTTTGCATTCAATTAGTGGCCACTATCCAAAAACCACAAAAAACATACCAGCCACT
ATCTTGTTTGCATTCaATTAGTGGCCACTATTCAAAAATCTTCAACAAACACAGCACCCACTATTTTacttgC
ATTCAATTAGTGGCCACTAGCTTGGACTTcataaaacagacagaaaccactattaTAATATGTTAAAAACATA
GTGGCCCACTGTTAATTGTAAACACACTGAAATTTAACCACTATTTGGTTCAAAAACACACTGACCACTGCTA
GTacaatgcaaAAACCACTATAGTGGTGCAATTTGGTCAAAAAGATGTTAACCACTAGTGCAAGGGGAAGGAC
AAGGACAAGAAACCAGCTGGCGTCGCACATGCCGTAGATACCATAGACGAGTCAGACAATGAATCTCTTCTTT
CTCCTCGTATGCTGTTGGTTCAGGACTCTCGTCCTGGATGGTAGATTCCGGCTGCACACGACACATTACGAAC
AACATCTCTGACTTCCACGAGTATCATGAACACAAGAGTTTCTGTTTCGTCACCCTGGCGGATAAGGAACAAA
CTCGTCGGAGAGTTCACGGTGTCGGAACAGTCCATGCAGTATCAACGTCCGGCTCGAAGCGGATCAAGTTCAA
GTTCGACAACATCCTTTACGTCCCCGAGATGAATTATCGTCTCTATTCCGGAGTCGTGGCAGACAAGAAGGGC
TTTAAGCTCTTATACGGCGGAAGCCAAGTTGAAGTTATGCGTGATGGCGTCACGCTAGCGATAGGCAAACTCG
CCAATGACCACTACTGGCTGGAGCTAGCCACTCAGTCAGGGAACGTGGCCGCAACCTCGTACAACACAGTTAC
CTCGGACATCATTCATGCACGCTTCGGGCATCTGAACTGGAAAGGTGTCGAAAAGCTCACACTTGGAGACAAC
CCACCGGTGAAAGGTCTCGAGCAATTCTCTCACGACAAGTCACCCCATCCTTGTGAGGGATGCGTCCTTGGTA
AACATCACTGTGTCTCCTTCCCCAGTTCCAAGACAGAACGCGCGGAATCCCCATTTCAACTCGTACACACAGA
TCTCGCCGGTCCCATGCAGAACCAGTCCATCGAAGGAAAGCGCTACTTCCTGACCTACATCGACGACAAATCG
GACGCCGTATTCATCTACTTCTTGGCCACCAAGGACGAGCAGGAGTCGAAGTTCGACGAATTCTGCGCCATGG
TCGAGAACCAGTTCGAAACAACGGTTAAGGTCATTTGATCGGATCGCGGCGGCGAGTACATGTCCAAGAGTTT
CCAGGCAAAGCTCAAGGCGAAAGGATTCTCCACGAACGGACCAACGCGCATACGCCCCAGCAGAACGGCAAA
GCTGAACGAATGAATTGTACGCTTATGGAAGCTGCGAGATCCATGATGCATGCTGCGGCCTTGTCCAATGGGT
TCTGGCAGGAGGCAGTACGTACTGCTGTACATGTTCGCAACAGGTCGCCGAAACAGGCACTTCGTTGGCGGAC
ATCCTCTGAGGTTCTCACAGGCTCAGTACCGGACGTCTCATACTTCCGCGTATTTGGTTGCCTTGCTTATCGA
CACATCCACGGCACGGAACGCAAAAAGCTAGATGCGAAAGCTGACGCTTGCGTCTTCATGGGGTACGAGCTTG
GTACAAAGGGCTACCGGCTCTGGGACAAACGTACCCGGACACTTACGACGAGTATAGATGTGATATTCGACGA
GACAGTGTTTCCCAACCGTCCAACACCCCTACCCTCGAAGGAAGCCCTGGGCTCCAGGCCCTGGCCAACCACT
TATTACTCTTTGGATCTACAAACCCCTTCATCAGCACCGCTGCTCACGCATACAGCAGTTGGTCTATCTACGC
AGCTGACTCAGCCACCATCCTCTTCTCAGCAAACTGCACCTGCTACCTCCTACGCAGCACAGGCTCGAAAGCC
TTCGCAGATTCCCACCCTATCCCAACCTTCCACTCCTGTCTCCAGCATTTCCCGTGCAGGCAAAGGCAAAGGA
CGAGCCGTCGAGGAACATGACGACTCTCCTGGGGCATACAGACCGTTCACCCAGCCGATCCTCGACGCTGAGG
AACACCGACCACGACGCGATCCTTCCACCACTGGCGCCACACGCCGCTCAGAACGCGTTAGGAAACCGCCGGC
CCCGCCAGGGGTGCCCGACGACCCTAAGGCTACTGATAAGGAACTCGCGCAAGGACGTCCTGGAAAGTGGACT
CCCGGTGTTTGGCAAACGGCCCAGCCCGCAAAGCCACGGAAATCTGGTAAAGCCATGAAATACGCTCACCCCG
ACGCGTTGTTCGACGACACCGGCGAGCGGGTAGACACACTTCCTCGAGGCCCACTTGATGACGAGAACGCTGA
CGCACTAGTCGATGCGGTCATTGCTTCAGTCAGCGCAGGTTTGCGCAAGCCAGCGACATACAAGCAGGCTCTC
AAGTCGGACCAACGGGATGAGTGGTTACAGGCTATGGACGACGAGATTCAAGGTCTCGTCAACCAGGGAACAT
GGGAAATCGTCGATCTACCTGAGGGTCGACGAGCGATTAAGTGCAAGTGGGTCTTCAAGTTGAAGACAGACGA
AACAGGAGAGGTAGAACGGTTTAAGGCTAGGCTCGTAGCCAAGGGATTTTCACAGATCCAGGGTATCGATTAC
GAAGAAACTTTCGCTCCCGTCGCTCGTCTAGAAAGCTGGCGCTATTTGGTGGCGTTGGTGGCGCACCTAGATT

GGGAAATCCACCAGATCGACTTCGACCAGGCGTATCTGAACGGAGAACTCGACGAGGAGATCTACATGGAGCA
ACCCGAAGGTTTCGAAGTGGGCGCATCGGGCAAGGTTTGCCGATTGCGTAAGGCCCTGTACGGTCTAAAGCAG
GTGGGACGTCAATGGTTCCTAACGCTGCAGACTTATCTCGAGGGAATCGGTTTTGTTTGCCATGATACCGGCG
ACATTTCCGTGTTTGTCAATCGTCAGGGGGAGAGCGTGGAGATCCTAGTGGTCTACGTGGACGATCTGACAAT
GATGGGTAGCAGCCTCGAACTCATCAACAGGACGAAAGAGGCACTAAAAGAGCACTTTGCGCTGAAGGATCTC
GGCGAATTGAAGCTATATCTCGGCGTTAGGGTCGCGCGAGATAGGGGGAGTAGACTCATCTACCTCGACCAGG
AAGCGTACATACGCTCAGTTCTCAAACGGTTCAACTACGACGACTGCAATCCGAAGCCGACACCACTTCCCGT
CGGCGCCGTCCTCGAACGGAACGAAGAACCTCCAGAATCCTGTCCTCCCGAACGACTCCACGAGTATCGCAGC
CTTCTTGGCAGCCTCATGTATGCGATGCTCGGAACTCGCCCTGACATTGCGTTCGCGGTTTCGCGTCTTTGTC
AGTTCCAGTCTAATCCGACTGAAGGCCACGTTGGCCTGGCGAAACACATacacccaaaTcTTCAaaaaaaaaA
acaaaaGaggccacaacaagagcACaAcaacAcaaacAAaaaTCAaaTaGaaGgaTTcacggaTgcgcATaaa
gCTacgaaaaccGaaacgacacacaCaaccacgagTagGgaacTaCaaagaGcacAaagaGcgGTcagaTagA
GgaaaaacAaacaAaAacaaGTgaCgACAgaTaaaTgcaaagagGaacaaggagcaccAaaagAacCcTcaCa
aaaaCcTcagTgGcgcaacaTcTgagaTaagcaaATcgaaaaacagcgcGaAAAgcaaaaaacAaaCTaCcaC
aAcaaCcacaaaaaaaaaaggcCCaCcaAaaggcCcAaccaGAAgcAcccaAAaaacaaCACcagcATgAaaa
aacAcGCcaccaacaAaTcTggaaAaAacGaAaaaggaaAAaaccacCcacTaacTaCaaAaaaaAaaacacC
acaaaTTTccaaaAAaacaaaacccaAAaAaaTAcACaAaaAaaagAaaaaaagAacccacCaagaacAgTAT
cGaaaccccaAaagGcaaaaaTGaGCccAcgaaacgacCaaaaaaagaaaaACaaATagacacgaAgccgaaA
aaTccGAaaagATcccCacTaaaagaaaAagaATcaaaaaAaTaaagcacCACaaaaTaggcaaagaaaaTag
ccccaccgcacaacTagcgcaaTAgTgccAaacTcgcgaaaAagTgTcgaaaaacaTCaccaCaaagccaaag
aaaaAgaCaggaAaaaaCaccAcTaaAaAaCaaaGCaacgTagacacCaccGGagGTacgaGcaacccaTATG
ccacagGcCaTaagcgGTAaGaTcaCcgcTcacCaCTAGTacgcaCaTcTccGggagacgacaaaggaTTacg
AcaaaaCcagCTcacagtcctttctcaaactgatctttgagagccttgtcgtcccagccggtatgcatctcat
actcttcaaatgctacaatgtactcgtcggctgtcttggtaccttgttggaggttacgaagcttatccacggc
attcagcgagggatcagctgagccaaaggattccttgagcaatgcttcgaactcctgccaagagacggagtag
tggttgagcatctggttgtgttccatgaagcgtttggcccagggttgcgctcggccagacttcatgtacgaga
gtgcgagagtgatgcgattatcttcttctaggtagagatcaggctctgcccagaacaccaaattgagttggtg
gataaaggcgtccagcttagactcagcgccatcgaaagaatcgggttggcgatgtcgaccttgcgggggatg
gctgatgaaagcccaacggtgcagagtagtgtggttgtagcttcgagcttatgtcagcgacacactgccctga
gacgaccacgacacactaggctgcaacaacgagggacttaagggtccgagggttgtgggaaaacacttgaatt
gcgcctgaat
>Bjead1_1_rnd-1_family-36#TIR/MITE
TGTTGAATTTCGAGCTCNAGCTCACGGACTCTGACTCTACACGGACTATTTCCTCTTATCTTATCAGTAGATT
ACTCGTTATAGTCCTCCTTGTTTTTGTATCAGTGATCTTCCTCATTCGGCACGCTCCTTGTAGTTTCCTCTTT
CGGACGATCCCTTGTACTTTGGGTATATAGTCCTGGACTCTCTGCCCTCGCTCCATCGCGCTCTTTCCCTTCG
CGCGCGGAGTGAGTACTTCGTATGCCCTATGCCCTATGCCNTATGCTTACATCCTGTACTAGCTCACGCGCTC
TCGCGCCCTCGTTAGGTACAGAATATCATCGCGCTCGTTTCCCTTCGCGCGCGGACTCACGCGCTTTCGCGCC
CTCGTTAGATCAGACGTCGCACACCCGCCCTCGGGCGTCGCTCTGTCGCTTTTCCCTGCTCCCATCTCCCTCT
CTGTCGGATACCGTCTCGCCTCNGGCCAGACTGGTATTTGGGTTGTCGCTTGCTATAGCCACACCACCGCTCA
GCCGCTTCTGGCCGCTGTTGCTGTTCAGCCTCTACCCTCCCGATAGGTCGGAGGTCCGCAGGACGTAGCTGAA
CGGTCGACGTTCAACA
>Bjead1_1_rnd-1_family-139#LTR/Copia
CGAGTTTCCCAAGGGTGGNGTGCAGCGTGCGGTTCCAGACTCGCCCGGCCTGCTTCAGCCCGTACAGTGNCTT
CCTGAGACGCAGGACGTCGCCNGGATCGCCAAAGTCGAAGCCCTCNGGCTGGTCCATGTAGATCTCCTCNTCG
AGCTCACCGTTNAGGTACGCNTGGNNGATGTCNACAGAACGCNGGTCGAGGTCCTCCAGGGCGGAGAGCGCAA
GCACAACGCGAATGGACGCCATGCGCACGGCGGGAGCGAAGGTCTCNAGGTAATCGTAGCCGGGGCGCTGGGA
GNACCCCTTTGCCACCAGACGAGCCTTGTACCGCTCCAGAGCTCCGTCAGAGAGGAACTTCTGCGCAAAGACC
CAGCGCGAGCCGATGGTCTTCTTGCCAGGAGGCAAGGGACAGGGGGACCAGGTCCCGTTCGTGCTGTGTGCGT
CGATCTCCTCCTGGGCAGCTTTCCTCCACTCGTCTGCGTCTGAACGCTTCATGGCCGCAGTCCAGGAACTGGG
CCAGGCGTCATGTGCAGAGAGAACGCGCATAGCAAGCTCATCCTCGGACTCCTCGTCCAGTTCTGCAGCCACC
TCGTCCTCAGAACGAGGATCTGGGGGCGCAGCGTCTGGCAGAGGCACATCCTCGTCGTCACCATCCTCGTCCT
CGTCCTCTGGAAGAGGCTGGGGAGCGGCGGCAGGCTTGACCTTCCACCACTCGCCCGGAGGCTTTACATTGCG
CGCAGAGCGACGCGTTGCAAGAGGAGGTTCCTGTGAGGGCGCGCGAGAGCGAGAGGGACCAGGGCGTGGCTCA
GAACGAGCAGAGGCCCTGGAATGCGAGCGAGCTGGNGCAGGAGGGTCCTGGACCGGCTCAGAACGAGCAGGAG
GCAGGACACGCGCACCAGGGGCAGGCGCAGCAGGTCTCACAGCGGCCGGGGCTGCTGGAGGCGCTGCCGCAGG
CACAGGAGCTGGAGGGACAGCAGGAGGCGCAGCAGGCGCAACAGGAGGAGCCAGCGCAGGAGCGCCAGCAGGC
TCAGGTGCACCCACAGGCTGATTCTCCCCCCCAACTCGAGGCTGCTGGGCTTCTACTGGGTCCTCGGGCGGCG
CAGGCTCGTCACCAAGGTCAGCGGGCGCACCAGCAGGTGCCTGGGGAGGGAGGGAGCGGACAGAAGGCACAGA

GGACCAGTTCTTCAACCCGGGCAGGACGCGTTCGTCAAAGACAGCACGCTCCGAGATGACCACCTTCTTGGTC
GATGGGTTGTAGAACTTCCAGCCCTTGTAGCCAACCGGGTACCCAATGAAGATGCACTTCTCCATGTGGGACC
CGAGCGCACCACGCTTGTCCTTTTGCACGTGCACGTAGGCCAGACAGCCCCAGACGCG
>Bjead1_1_Cluster30#LTR/Gypsy
GAAGGGCAGGTCGATATGGGCCTTCAGAGACGAGGCGGGTGGAGGCGACGTGAGGGGACGAGCGAGGAGGTCG
ACGAAGGCGTGCGAGGACGTCCAAGTAACGATGCCTAAACTGGTGTTGGTTTCGCAAGAGGTACAAACAGGGC
GGTCGCAGTGATAAGGGAAAGCGAGTCAGGTACAAACGGAAAGCGTGCCGAAGGCGCAAGGCGGGTAAACGAA
CGAGAAGAATGAAAGTAACCGGATCGGGAAGGTAGCTACCCTAAACAGGTAAATGAAAGAACCAGGTTAATGG
AACGTGCTACGATTGGCCGTGGAGAGTCCATGCGAGGCGTACAGAAGTCCGAGAAGTCCGAGAGCGTATCTAG
AGTGTCCGTAAGTGCTAGGAGTGGCCGTGATGTGCTAGATAGTCCATGTAACGGTCGAGCCGTTACATCATCC
CCCTTTCAAGCGCGAGCAGAGCGACGCTTTCGAGTGGGAATGATAGGGTCGAGCTTTTCAGCAGGACGAGAGA
CATGCCCAGAGCGGATCGTGGTCTGTGTGGGTGTGACGTAGTTCGAACGTAGGTTTTCGCAGACGGGCGACGG
CGAGCGAGGACGGGCAGCAGCGACGGGTATGGCGGAGGCAGCGGGCAGCGGAGCGGACGGCGGGACGGGTGAG
GCAGCGACGGGCAACGGACCTGGGGAGCAGAAGGGGAAGAAGGCAAGTCAGTATTGTCTGGAACGGGTGCAGG
GAATGTCTGGTCGAGGACGAGACGATGAGGACGGGGCGCGCGCGGGTGACGGCGGTGAAAGCACTCGATGAGC
TCGTTGGCGGTAGTAGGGATGTCAGAGAGGGGAACCCATGAGTCCTCGTCGTCCGGGAGAGACTTCCAATGGA
CGAAGTACTCGTAGCGATGACCGATCTTGCGGGAATCAAGAATAGAGTCGATAGATTGAATGGATGTGGGAGA
ATGGGAGTCAGGGTGAAGGGCAAAGGGTTCGGGTGGAGCGTGCATGTGAAACTCTGAAGGATCGTGGTAAGGT
TCAAGAAGAGAGACGTGGAAGACGGGATGAAGGCGAGAAAGGTAAGAAGGGAGACGAAGTTGATAGACAAGGG
GGGAGATTTTTCATGAGATGGGAAAGGGGCCGAGACGGCGGTGGTCGAGCTTGTCGGACGGACGTGTAGTCTT
GATGTTGCGACGAAGAAGCCAAACGAGCTGGTCGGGCTTGTACTCGGGTGACGCCTGGACGTGCTTGTTGTAA
TAACGCGACTGGACTTCTTGAGCATGTTTCAGCTCGGCGTGAAGTTCCACTAGTGGTTGCAGTCGCAAGCCCA
AATACCCACCACAAACCCCCAAATCGATATTGATCCAGTCAGCGCTGTTTTAATCAAGGATATCCTAGCATAG
TTTTTGTTCAAATACTGGTCTGGGTTTGCTTTGAGCATGCATATGCTAGTAAGTGTTTTTGTGCTTTTTGTGA
GGTTTTGATGGGTCTTTTCAAAGACACTCTGTATTTTGTATAGTGTATTTACTGAATCTAGGCAGTATTTTCA
TGATGTTTTGATGAATTTTCTTTGATAATTGAAAACACTGGCTGATGCTTTTGACATTAAATTAGGTGATTGT
AAACAGCATTCAAGTAATGTATAGATTATGCACTGATACAATTCTATTATTGGTTTTAAATCATGAAAAATGC
TGTTTGAATTTTGTTCAAATACCAGCACAATTTTCAATCAGTGCAAAAACCTGCCTCGTTTTGAACCAAATTT
TAGCACGATTTCAATTTTTGCACTGATTGAAAATTGTGCTGGTATTTGAACAAAATTCAAACAGCATTTTTCA
TGATTTAAAACCAATAATAGAATTGTATCAGTGCATAATCTATACATTACTTGAATGCTGTTTACAATCACCT
AATTTAATGTCAAAAGCATCAGCCAGTGTTTTCAATTATCAAAGAAAATTCATCAAAACATCATGAAAATACT
GCCTAGATTCAGTAAATACACTATACAAAATACAGAGTGTCTTTGAAAAGACCCATCAAAACCTCACAAAAAG
CACAAAAACACTTACTAGCATATGCATGCTCAAAGCAAACCCAGACCAGTATTTGAACAAAAACTATGCTAGG
ATATCCTTGATTAAAACAGCGCTGACTGGATCAATATCGATTTGGGGGTTTGTGGTGGGTATTTGGGCTTGCG
ACTGCAACCACTAGTGCGGACCCCACTGCAGAGGCCATGGGAGGCCTCTGCACATCCATCTCATCGTCTGCAC
GCCCGTCAAGCGCACACCCGACCCCCTGCCGTCCGCCCGTATGCGCAACAACGCGCGCGACCCGGCCACCCCC
TCGAACGTCCCTAGCAAGCAGGGATCGCGGCACGCCCTCCTGAGGCGACTCACGCAGGCTACCTAGGACTCCA
TACATCCCCTCCGCCATCTCTCCCTGCTCACGCGCGCTCGCGTGCAATCCCTGCAACACCCGGCCTCAAACTG
GTCAATGGGGCACTTCCACGCCTCCCCAAGCCGGGACTACAGTGCGGGGACCCGGGAGACGACAAACACGCAA
GAAAGGATGAATGAGGACACGTCAGGAGGACTGGAGAGGCAGGGGTAATAGGTACGCCCTCATTTTCTTTCGC
TTTCCCCTCCCCCCATCCATTATATTCCTCCGCCGCGGTGCGTCGTACGTGCTGCGGCGGAGCGACCTTTTTT
ACATTCTCGAATGTTCTATCAGCACTAATATATCGTACAGTTTATTTGCGTGCGCACTGGGCCTCGCGCAGCG
TTCGCGCGAACCGGCAGGGTCGCGTATGGCGGAATCGCGCCTCCCGCGACTCTAACTGCAATTGCCGGTAAGT
CCTATGGTACGATTCAGGTGCTGATGTACATCTCTGGATTCCAGGTCCCTCGAGTGGTCCCAGGCGAGTGAGC
GAGGTACCAAGCCTTACTCTCGCCCGGGGTCACCCTCTCGCGCCCGTGCCGCCTCCCACCTCTCCCCCGTCCT
CAAGACTCTTTCACAACACATTCCCTATCATACCCCGCATCTTCCGCCGACCCACCACACTCTCGCACCCCTC
ACCGGCCCCGACGAGCGCGTGCAGCCAGAAAAGCGCACGCGTCGTCGCACTTGCTTGCGCGTCGCGACACGCA
CAACCCAGGCACATCATACGACTCGCGGAACCCCCACTGGCAGTGCTCGTACTGTCTGGTGTATCAACAATGG
CCTCCAGCCCGGTACAACTTTTTTCGATTTTTATATGATTTGTTTTCTGTTTGTTCTCGCTTTTCTTCTGTTT
TAGTCCCCCGCTTCTCGTCCCAGAATGTGTGTACTATATTAATACAAATATATAATTTTTATGAGAGCCCTGG
AAGGTCCTGAGGCCGGGAAAATGTGGATGCGCTGCGCTGTGCTGGACGGCAGGGCAGGCGCCTGACATTTCCC
GATGCGGAAGGTCATCGCCCGTGTGGGTTCTTGATGCACAACTGCACACTGTCCGAAAGGGCGCAGCCAAGAG
AAATCAAATAAATATTGTTACAATTTGATTGGCAACATCCCTATTAGGATGAGTCGCCGGTGGAGTATGGTGC
ACCATACTCAAGAGCTCGGGGCCCTGAGGCGCAAGGCAAGGCTTACCACAAAGTGTGCACAATACTCGGTGAG
AGTTATCATGAGTTTGGTACACCAAACTTGGGCCAGGGGATGTTAATCTCGGCTGGCGTGAGCTTGCGCAAGA
AATAACCGACAGGGTGAAGGAGTCCAGCAGCATCTGGCTGTTGGAGTACACCTGAAATTGCGAAGTCTGAAGC
ATCAGTAGCAAGAGTGCACGGCAACTTTGGATTGGTGTGCCGCAGAACAGGGTAGGATAGGAGCGCTGCCTTC
AGCTCGTTAAAAGCTGAGCGACATGCGTCCGAGAAGACAAAAGGAGTGTCTTTACGTGTCAACGCGTTCAAGG

GCAACGCGATGCGCGCGTAGTGGTCAACGAAGCGACGGTAGAAATTATTGAAGCCAAGGAAAGCCTGAATATC
CTTGACAGATTTTGGCTCTGGCCAATCGGAAACAGTCTCTCGCTTCTTCGGGTCCATTTGAATGCCGTCAGCA
CCGAGCAGGTACCCGAGGTAGCTGACTTGCGAGCGGTCAAACTCGCACTTTTCCGCGTTAGCGTAGAGGTGAG
CTGACTGAAGGCGCTCGAGCACCTTCATGACATGATCGTCATGCTCAGACTGTGAGCGTGAGAATATGAGAAT
GTCATCCAGGTAGACGACACAGAACACGTCGAGGAAGTCTCGGAGTGTGTCCTGGATGAAGGCTTGAAACGTA
GCAGGGGCATTAGTGAGTCCGAAGGGCATGACAAGATACTCGTAGAGGCCGAGAGGCGTACGAAACGCGGTTT
TCCACTCGTCTCCCTCCTTGACACGTATGAGGTTGAAGGCGTTCTTCAGGTCAAGGACCGTGAAGAGAGAACA
GCCTTGAACACGGTCAAGAAGATCGTCGATAAGCGGGAGGGGATACCGGTTTTTCTTGGTCATAGAGTTCAGT
CCGCGATAGTCGACGCAGAGACGTAAGTCTCCGGACTTCTTACAAACGAACAGAATGGGAGAAGCCGCAGGCG
AGGTTGAACGTCGGATGAACCCCTTCTTGAGGTTCGTCTCGATGTACTCCGTCAGAGCACTACGTTCCTCGGG
CGACAGACGATACATGGGACCAAAAGGAGGGAACTTACCTTCATCGAGGTCGATGCCGATGTCGTACGGGCGA
TGCGGCGGAAGGTGGTCGACCTCGATAGGCGAGAATACAAAGGCCCAGGGCATGTACTTCGAAGGGATATGGG
ACGTAAAGTTGCGGGGAATATCCACGGGTGGTGGATCATCGTCAAGCCCCAGATCAGATGCCTTGGTAGACAT
TGACGCAAGCTTATATGACGGCGAGTCTACGGGGTGGAAACAAATCAGCGCAATAGGTGAAGACTTGGAATAC
TTCAGAAAGCGACGAGGGTTTACAACCGCAACGTTGGGGCGCTTGGGCGGAGGAGCTTCTGCAGAGGAGCAAC
CGAGGCCAGTCCATGCGCGAAGTGTCGAAGTGAACGAGAACGGAGAGGGCATCGTAGCAGTCGTCGCAGACTG
CGGGAGTGAGGCGTTGGCCAAACACTCACGAGGGACAGGATCCGGAGGCGGGGTCGGAGTCGAAGTTGAGACG
CGAGTTGCGAACAACGAGCCGTTAAGACCGAAGCCTAGGCCGACTGTCGTCGCGACGGAGTTGAACGAGGTAG
TGAGTGAGGGGCGAGCAAGACCGAAGCCTTTTGCCGAGACGGTGGAGCTGGTGGGGCGAGTTTAATGCACCTT
TGCCTGGTTAGGCTAGGCAAGTTTGTGCATTAAACCCTGATGGCCATTTTCGGGCTTCCCTTTTGGTCAGGCT
TGTCTGGAGGGGGCATGGCCCATCAAACACATCATTCCTACAATAATATATACTCATTTATTTGTTTTTGCGT
GAAGTCGAGCCAGCTCGCGGTGTTTGTTTGGTCCCTGGGGACCAAACAAAGCCCGATTAGGTGCTAAGCATGC
CCGGGCAGCAGCACTGCTCGCACTGGGCGCAAATGACGTCGTGCGACAGCAGGTACGTGTATGTGAGCCCTAT
TACCCATACGGGCCACTTCCCAAGCTGCCGCGCCCTCCCCGCTCGCTGCGCGCCGCCGCGCGCCTTCCATTTC
CCGATTAGGAACGATCCCGCCCTCAGCTCGCGAATCGCTCCAGCGCGGCCCCAGCACGCCCAATAAATCCCAA
AAAAAACGTACAGGACCATGGAAGCCGCTCTATTCCCCTCTAACAAACTCAGCCGGCGATACGTCACCTTTAA
TATCTGACACGACGCAATATTTGTTAATAATAGCCCTGCTGGATAGCTGGTTCTGGGACCCCTACAGCACTCG
TTCCAGCTCCAACAAGCTATAGTACACCTCAATGGTTGCTTCAATGATCCCCCAGGCCTTGAATAGGCCGCTC
CTACGTGTGCGCCATCCGTCCAAATCGGGACGAATCCCGCCCAATGCCGCCCCTCGCTCCAGCGCCGTCAAAT
GGCCACCAACAAATCTGGTATATTGGATATTGCCTCTGCGTTCATACGCGAGCTGGAATATATAAGCCCGTCC
CACAAGTAACGCACCATACGCCCAATAGAAAACACTTATATATAATGGGAGCTACCTGCTCCTAGCCTCTGCA
CGGCCGCTACCGCGTACTATAACGTGTATCATTGACTGCAGTGTATACTAATACCCTATTGAATCCCCCAAAC
TACCTTGATACCTCTAGAAGGGCCTAGGTGTGATTGCGTAAAAACGCGAAATATATTTTCTGTTAATCGTATA
AATATTCCGAGTGGTCTGTGCATTATTCGTACGAATTTAGGGGTTACTGAGAGGTGTCAAGGAGCGCAGGGAG
TCCAATACGGGACGGGGGAAGTGAGCGGGGATCTCGGGAAGCTTCCTTATCGGGTAATTGATGGGAGCGACCG
TCCGCGACATTGCGCGTCGTATGGCTTTCCCCCTCGATAGAATGGCTGATGGCGCGGTATAATATATTCTATA
TAGTATTGGTAGTGCACGGACAGCAAGAAAGCCGGAATATTTGGGCGTGTATTTGAGTTACAATGGTCAGAAG
GCTGAAACATACCAAAGAAGCCTCGCAATGAATTTCCGAGGAGCTAGGAACACCGCCAACGTGCTGATTTCGT
GGCCCATTCCATGCATAACAACTATCCGTATGTATCAAAAATTTACAGACCCGTTTGGGCCGTTACAAACGTG
TGTAGAGCTCGAGTTTGTCAGATATTGAAAGACAGGAACTTGACATAACATATATGTTGTTATTCCTTCTCAC
ATAGACCTCGCAATTTTATGAAAGCGGTACCATTGCATTCCTGGAATTAATCCGGTCCTGTACATATAAGATA
TACCCTAAATAATTCTATTTATGAGGAGATGGCCGTCCGTGCAGGACGTTCGCACTCGACATTCAGCGTAGAA
TCTCGAGCTGAACGCGCTTTTCGCGTTCGGCTGCCCCGGAATGTTTCAGACTCAAAAAGCTCTGGGTTTTAGG
CCTCAAATGTATGCCCCTTTACTAATAAACAATGTCCCGCGAAGTCCGAACATTCCGCGACCAGAACTGAGCC
CGCTATCGTGCACGCGAGCTCGTCGCCGAGGTTGCCGAGAGCGGGCGCCTTGTGCGCGGACGGTCTGCGGCGA
CCTTGATCGCGTTCTCTGTCGTCGCCCCTTCTCCATCCCGTCCCCCAATTCCACTGCAGAGGCCTTCCATGGC
CTCTGCACATCTATCTTGCTGTCTACACCCCCTTCAAGCACAGCCCGACGCCCTGCCGTCCGCCCGTACGCGC
AACAAGGCACGCGACCCGGCTACCCATTCCCCCACCCCTAGGCAGCAGGGATCACAGCACGCCGGCCTGGTGC
TGCTCGCGTAGGCTACCGGGGACTCTACGCACCCCCTCCGCCATCTCTCCTCTGCTCATGCGTGCTCGCGTGC
TCTCCCTCCAAACTCCGGCCTCAAACGCTTGAATGAGGCACTTCGATGCCTCCCCAAGCCGGGAATACGATGC
GGGGACGTGGGGGACGAGGTATACGCGTGAGATGAAGGGTTATCACGTGTTGGGAGGACTGGAGAGGGCAGGG
AAATAGGTACGTTTCCAGCTTTTCGATTTTCACCCTCCCCCTACCCATTCTACCTCTCCGCCGCGCCGCGTCG
TTCGCGCAGCGGCGGAGCCATATTTTTCTAGTCTAGAGATATCCTTCATTCACTAATTTATTGCGTAGTCTCT
CTAGATACTTGCTGGTCCTCGCGCAGCGGTCGCGCGGACCGGCAGGGTCGCGTACGGAGGATCTCCGCCTCCC
GCGACTCTGAGTGCGATTCCCGGTAAGTCCTATGGTACAAACCGGCTACTGATGTATATCTCTGGATTCTAGG
TCTCCCGAGTGGTCCCAGGCGAGGAGGCGAGGTACCCGACCTTACTCTCGCCTGGGGTCACCCTCTCGCGCCC
GTGCCGCCTCCTACCTCTCCCTACAGTCGCAAGTCTCGTTCACAACCTATTCCCCACCATTCCCCGCATCCTA
CCTCAATCCACCGCACTCCCGCACCCCGCACCGACCCTAGACGAGCGCGTGCAGCCCAAAAAGCGCACGTTTC

GTCGTGCTCGCTCGCGCATCGCCACATACCCAGCCCGGATGCGCTACGCGACTCGCCAAACCACCACTCGCTG
TGCCTGTATTTCCTGGTGCGCCAATAAGGGCCCCCAGCCCGGTACAATTTTGTTTTTGATTTTTGTATTACTT
GATTTTCATTTTATTCGGGTTCCTTCCGTTTTCGCCCCCTCTTTCTTGTCTTAGTATGTATGTACCGTATTAA
TACGAATATCCGAATTTTTTCGAGCACCCTGGAAGGCCCGGAGGCCCAGAATATGCTGTCGCGCCGTGCTGGA
CTGGACGGCAGAGCGGGCGCTTGACATTTCCCGATGCGGAAGGTGATCATCCATGTGGGTTTGAACACACAAT
TGCGCACTGCCCGAAAGGATGAGGCCACAATATATAATGTAACTAATTGATTAGCACCCACTCTATTAGGAGG
GTACTTAAAGGGCCTCCCAAGAGTGGTAATGAGTATGAGGCATTAAACAGGCCCAAGTTGAGCCAGGCCACAG
TACCAGATAAACAATGCATCGTATGACCTACAGTAACCAGATCTATGGTGTATTAAACACCTCCCATGGAGTG
GACGGTGACGGGATGCGAGCGCGTGAGGTTGCAGCACGAAAGGGAGAGACTTGCGTCTTCCCAGTCGACATCG
GGGTTGTGGCGTCGAAGCCAATCAAGTCCTAGAATGATGGGATATGCGACAGAAACCACACCGAGAGTTATAA
TCTCAGCGTGTTTGTCTACGGTCAGCTGAGCGACGACGTTGTGAGTAACCAACCCCAAAGCAATGGGGCGGTT
GTCGACGGCAGTAACAGGCGTGGGAACGTCCTTGAGACGTCGGGGTATAGAGTGACGTCGGGCAAATATGTCA
GAGATGCAAGATCCTGACGCACCCGAGTCGACAAGGGCAAAAGTGCGAATAGGCTTCCGGTTGGGAAAGCAAA
GTGAAACATTGATAAAGAAATGGTCAGGAGAATTTAGAAAAGAGTTCGAAGCGAAGACGTAGAGCGGAGGAGG
AATAGATGAGTGTACGGGTATATATCGTCTGTCGGTAGAACGGACGTGGGTGGGCTTCGCGGCACGATGCGAT
AGACGGGTCCCTTCGTCAACCGGAAGCCCAGGGACCAAGGAAGGGCTACTGGTTACGCTTTTCCCGAAGGAGC
AGCGTTGCGAGTGGCGGCGTGTGCCTTGTAGGCAGCCTTCGCCTTCGCAGACATATTGGGACAGTCGTCGATG
AGGTGCTTGCCTTGTCCGCAGTAATAGCAAAGGCCCTCGTGACGGCGGTGAGCCTTTTCGTCAGGTGTCACAG
GGCCTCGACGTACGGCGTCGATTTCCATAGGAACAACCTGCGAGGACGAAGCAACGGAAGGCTGAGGAGCGGC
CGGAGGTGCAGGCGTTGCGCGAGGACGGTTCTTGCGTTCAGAGACGGGGCGATGCGAAGAGTTCTCGTAACGC
TTCTCAACTTCGCGATCGTGGACGCGGTTGTCGATTTCGATGCACTGGTCGACAAACTCGTCAAAGACGGTCG
AGCGGTTGATGGTGATGAGGAGGTCCTTGACGGTGCCCTTGAGACCGGCGTAGAAGTAGTCAATCTTGGAGAA
GTCGTCGAGCGAGAGGTGGTCGTGAAGCTCGCGCGATCTCGAGGCGTAGCCCGCGCATGAGCCCGTTTGTTTG
AGCTCTTTGAGCTTGCGCAGGGCATTGCTGGAGAGGTTGGAATCGCCGAAATGCGAGCGGAAGTCAGAGATGA
CCGCCTCAAAGTCGTAGGTGAGGTGTTGCTTGCTGGCGAGGATGCCGTAGTACCACGACTTGGGCGAACCGTG
CTTGAGGTAGGACGAGAGGTAGATGCATTTGTCGTGGTCGGTGGGAAGGGCGTTGCGCTGGAGGTAGACGGCA
TTCTTGATCTCGATGATAAAGCCTTCTACCTCGCTCGCAGAGCCGCCAAATACGGTCGGCTCTTTGAAGCGCG
GAGTGCCGCGGGGATCCGAAGAAGACGGCGTGGGAGCGGGAGTGTGAACGGGTGGTGGTGCAACAGGAGCCGG
AGCCGCGAGAATGCGGTTGGTGAGAGCCTCGAAGGCCGCGCCGAACTGGCGTTGGGTCTCCCCGAGGGCTTGA
ACCACGGCCCAAAGGGCTGAGCCTGCGTCCAGCGCGGAAGGCGCAGTGTTATCTTGAAAAGCACCCGGGTCTT
CGGGCATGGGAGGTAACGGCGGAGCCTCGCCGGCCCACTCGTTACCTGGGTCCTGTCCAGAGAACATGTTGAT
GAGTAGAAGGGGTAGAAACCCACGAATGCGAAGGAAGAAAGGAATGTACGAAATCCGCGCGAATGCGGAAATA
CAGCGTGAGAGCTGTCCAAACGAGGGATTGTTGGTCGGGTCCGAGTTGGTGGCGAAACCAAACTGTGAAGGGC
AGGTCGATATGGGCCTTCAGAGACGAGGCGGGCGGAGGCGACGCGAGGGAACGAGCGAGGAGGTCGACGAAGG
CGTGCGAGGAAGTCCAAGTAACGATGCCTAAACTGGTGTTGGTTTTGCAAGAGGTACAAACGGGGCGGTCGCA
GTGATAAGGGAAAGCGAGTCAGGTACAAACGGAAAGCGTGCCGAAGGCGCAAGGCGGGTAAACGAACGAGAAG
AATGAAAGTAACCGGATCAGGAAGGTAGCTACCCTAAACAGGTAAATGAAAGAACAAGGTTAATGGAACATGC
TACGATTGGCCGTGGAGAGTCCGTGCGAGGCGTACAGAAGTCCGAGAAGTCCGAGAGCGTATCTAGAGTGTCC
GTA
>Bjead1_1_rnd-1_family-67#ClassI/TRIM
GCCTCCCGCGCCCGCAGCTCGCGCCCNGCCGCCCCGNCGACGACGCGCCGCACGNACGACGCCCAACGAACGT
CCCCGAGGCGTCCGGGACGCGATCCGACGAGGTCGACNTTTCGACGTTTTCATTGGCGTTTTCGACCGACTCG
CGATTTCGGCCCCCCGCGCGCCCGGATCGCGCCCGCGATCGCCGCGACGACGACGCGCCGCACGNACGACGCC
CGAAGAACGTCTCCGNGGCNCCCGGGACGCGATCCGACGAGGTCGACNTTTCGNCGNTTTCATCGCGTTTTCG
ACCGATTTTCGATTTCGGCCTCCCGCGCCCCGAAGCTCGCGCCCGGCCGCCGCGACGACGACGCGCCGNATGC
ACGACGTCCGNAGGACGCCTCCGCGGCGCCCGGGACGCGATCCGACGAGGTCGACNTTTCGACGATTTCGTCG
CGTTTTCGACCGATTCTCGATTTCGGCCTCCCGCGCGC
>Bjead1_1_Cluster12#LTR/Gypsy
TATAGTGTATATCACTAATGTGATACAATGATATGATGATATGGCATCATATAATAATTCTATGACTATAACA
AAAATAAGTACAGTCAATGCAAACATGTCACCATGTGTACACTAAGCAGAATGCACAGTTTGGGACATGCATA
GTATGAACATCAAACACTAGTTGCAGAGTGATTTTTCAGGCATTATTCCATCTCAATACCTATTTTCTATAAG
AAGTTATGGGCAATTCCAAAAAACTGCAGATTTGCAGTGATTTATTTTTCATGCAGCCAGAATTTCTATTTAT
GAGAACTCGTACCACCTAAAAGGAAGTCCTTTCAGCCCTCTACTACACCATATTTATGCTTAGGCTGTTTGTT
GTCCCATGGCCAAGTTATAACCAAAACAAAATCTAGGTTAACACTCATGAGCCAGACTTTTGACTGCCACTGT
ATATTGGGGGTATATTGGAGATATGTTGGAGGCATATTTGAGGTGTTCTGAGAGTGTTCTGAGAGTGTTCTGA
GAGTGTTTTGAGGGTATAGGGGGTGAGCTGGGAATGGTTTCAGAGTGGTTTGGGTGTGTGTTGAGGACAGTCT
GGTGTATGTCAAAAATGTGTTCAAAGTGTGTTTAGGATGTATCTAGGGTGTATTGAGAGTGCGTTCTAGGAGT
GTGTTGGGGTGTTCTAGGACTGTATTGAAGGTATTCTTAGGAGCATTCCTGAGGGTGTACTGGAGTATATTGG

GAGTATATTGGGAATGTATTGAGGGTATACTGGGGTGTTCTGGAAGTAGATTGAGGGTAAATTAAAACAGTCT
GGGGGTAGTTTGAGAGTAATTTGAAAGTTGTTTGGAGCATGTTGAAAGTGTGTTGAGAGTGTGTCCTGGGTGT
TCTGGGGGTGTGTTGAGAATATATTAGGAGTGCGTTGGTAGTGTATTGCAAATATATTGAGAGTGTGCTAGAA
GTATGTTGAGGGCTCATTAGAAATGTATTGAGAAGTGTATTGGGACTTTATCAGGAGTTTCATGAGGGGTTTC
TTGGGGATACTGGTGGTGTATTGAGTGTGTTCAGGGTATGTTGAGAGTGTATTGCAAGTGATTTTGAAAGTAT
ATTGAGGATGTCATAGAGACCTTGTAATTTTGGTGAAACTGTATTGGAAGTGCATTGAAAGTGTATTGGAAGT
GTTGAGAAAGTGTATTCAGTGTTTTTCAGGGGTTTCTTGAGAGTGTCCTGGAAGTGTCTTGTGGATAAATATT
GATAGCCTTTCAGTAAGCATGCAGTAAAACTCTAGAAAAAAATCAGAATAAAAACCAAAACATAAGAAACTCA
GGTGTAAACTCAGAAAACGCCCCTGCCGCTTGACGGCCTGCACTCTCAGGTAAAAAAGAAAGACGGGAAGCTA
CGTTTTGTACAAGACTATCGCAAGCTCAATGAGTTCACGGTGAAGAACCGGTACCCTCTACCCGTCGCCACTG
ACATCGTCAATCAGCTCAAAGGAGCCCGAATCTTCACCAAGTTTGACGTCCGATGGGGTTATAACAATGTCCG
GATTAAACCTGAGGACCAGTGGAAGGCAGCCTTTGTTAACAATCGAGGGCTCTTTGAACCACGAGTCATGTAC
TTCAGTCTTACCAATTCCCCGGCAACCTTCCAGGCCCTCATGAACGTCATCTTTGCAGACTTGATTGCGGCCA
ACAAGGTTGCAGTTTACTTGGACGACATCCTGATCTGGAGCAATGACCTGCAGGAGCATCGAAAGATTGTCCA
TGAGGTGCTAGAGCGACTCCAGAAGCACGACCTCTACCTAAGGCCTGAGAAGTGCGAGTTTGAGAAATCTGAA
GTGGAGTACCTAGGGATGATCATCAGCGAGGGACAGGTTCGAATGAACCCAGCGAAAGTCGAAGCGGTCTTGA
AATGGCCCGCCCCACGAAACCTTAAAGACGTACAGGGATTCGTAGGGTTTGCCAATTTCTACAGACGATTCAT
CAAGGACTTCTCCAAGATTGCTCGACCCTTACACGACCTAACCAAGAAGGACACACCGTTTACATGGGGCCAG
CCTCAACAAGCCGCCTTTGATGCTCTCAAAAAGGCATTTACTGCGCCACCCATACTTGCTATCTGGGCAGCGG
ACAGGCCTACACGGATTGAAGTAGACGCATCCGGGTTTGCAACAGGAGGAGTTATTCTGCAACAATGCGAGGA
TGGACTCTGGCACCTACTGGCCTTCCGCTCCCACATGATGAATGAAGCAGAACGTAACTACCAGATCTACGAC
AGGGAGATGCTAGCCATCATCGAAGCCCTCCGAGACTGAAGACACCTCCTCGAAGGACTCCCTGACAAGTTTG
AAATCTGGACTGATCACCAGAACCTTCAGTACTGGTGAGAAGCACAGAATCTTACCAGGCGACAGGCCCGCTG
GTCGCTAGAGCTTGCAGACTACCACTTCGTGTTGATACACAAACCAGGCAAGACTCACACCCAAGCAGACCCT
CTGTCCAGGTCGGCATTACCAGAAGCACCGTCAGATGCAGACGACAACCAGCAAGTGACCGCCCTATCCCCAG
AGCACTTCCGCATTGCAGCAGCGACAGCCATAGTGGAACCCTCGCTGTTTGACGACCGCATCCGCAACTGCAC
TCAGCGAGAGGTAGAGGTAGCGAAAGCCCTAGATACATTAAAGAAACGAGGGCCCCGACGCCTCACCTCAGGT
CTACTAGAATGGGAAGAGCACGAAGGGTTACTCTACTACCGTGGAAAGTTGTACATCCCGAATGACAAACAAC
TCCGAGCAGACATCCTGCAATCCTGCCATGATGCCCCTACAGCAGGCCACCCAGGAAAGCATGGCACAGTGGA
GCTCCTCTCACGGTACTACTGGTGGCCGCGAATGACTGCAGACGCAGAAAAGTATGCCCTAGGCTGTGACACC
TGTCAACGCTTCAAAGCCGCCGCACACCCAAGATCCACCCTGGAACCGCAAGAGACCCCATCGAGACCCTGGG
AGAACATAGGAGTAGACCTGATTGGACCGCTCACTAAGGACAAACGACACAAGAAAGATACCATTATCACCTA
TGTCGACCATCACTCGGGCCAAGTTCACCTAGTCCCCTGCAACTCGACTATCACTGCAGAAGGAACGGGTGAT
GCACATTACAAGAATATATTCCGTCTTCATGGTATTCCAAAGAAGGTCTTCTCCGATCGAGGACTACAGTTTG
CAGGTTTATGCGAGCCCTATACAAGCGTCTTGGCATTGAAGCCAGTTTCACTACCGCCTATCACCCCCAGGGC
AATGGAAAGACAGAACAGGCCAACCAGGAGGTTGAAAAGTTTCTACGGATGTTTGTTAACCAACGACAAGACG
ACTGGGTAGACTGGCTCCTCATGGCCGAGTTTGTACTAAACTCCCGTGTACACGATGGCAACGGACACTCCCC
GTTTGAAATTGTCTACGGCTACAAACCTGACTTCACTATTCCCGCGGGCAAGCAGTCAACAGTACCCGCCTTG
GACGAGCGACTAGACAACATGGCCAAAGTACGCAAAGAAGCCGAAGCCGCCCTACGATTGACTAAGCAACGGA
TGAAGGCAGACTATGAGAAGGATAAAAGACAGGCACACCAATTTGCACCAGGGAATCTAGTGGGACTATCAGC
TAAAGACATCAAAATCCACCAACCTTCGCCCAAGCTAGGACCCAGGCAACTCGGACCCTTTAAAGTCCTCGAA
CGCGTTGGTGACCTCGATTATCGTCTCGAACTCCCAGTATGGCTCAAGATACACGATATCATTTATGTCAACC
GTCTCAGTCCATGGCGGGACAATGGCATTGATAAACCTCCCCCACCAAAGCCAGTCGAAATCGACGGAGAAGA
AGAATACGAAGTCGACGAAATCCTTGACAGCCGACCGACGACAGCTACAGTACCTGGTCCGGTGGAAAGGTTA
CGACCCAGGAGCAAACAGCTGGGAACCGGCAGCCAATCTAACCAATGCCCAAACTGCAGTCCAAACCTTCCAC
AAACGTCACCCAGAGGCACCACGTGCTCTCAATGCCAAACTCGCAGCCAGCCTCCAAGCCTACCTGCGACCCC
TCTACACTCACACCGAACCCGACAACTTGGCAGAACGCTACCCACTTACGTATGACCTCGAGTGGGAAAACGG
CCGACGCCTCTAGAAACTTATACTCGCGAGGACGCTCGAGTCTAGAGGGGGGGTATTGTCACGATTGTGACCT
GTAGCGCCCAAAGTTTTTGCACGAACGGTCACCCGTGACTAGAGAGAACGCCCACATTGCCCATCAGCTTACC
TAACACTGCGGCGCAGCATGGTCTGTGCACGTTCCCGTGATGCCCATCAACTTACAGTGGCAGTCAAAAGTCC
AGCTCATGGGTGTTAACCTAGATTTTGTTTGGGCTATAACTTGGCTGTGGGACAACATACAGCCTAAGCACAG
ATATGGTGTGGTAGAGGGCTAAAAGAACTTCCTTTTAGGTGGTACAAATTTCCATAAATGGAAATTCTGGTTG
CATGAAAAATGAACCACTGCAAATCTGCAGTTTTTTTGAAATTGCCCATAACTTCTTATAGAAAATAGGTGTT
GAGATGAAATGATGCCTGGAAAATCGCTTTGCAACTAGTGTTTGATGTCCATACCATGTATGTCCCAAACTGT
GCATTCTGCTTAGTGTACACATGGTGACATGTTTGCATTGACTATGCTCATTTTTGTTATATTTGTAGAATTG
TTATAATGAAATGATATAGTGTATATCACTAATGTGATACAATGATACAATGACACGGCATCATATAATAATT
CTACAAATATAACAAAAATGAGCATAGTCAATGCAAACATGTTACCATGTGTACACTAAGCAGAATGCACAGT
TTGGGACATACATGGTATGGACATCAAACACTAGTTGCAAAGCGATTTTCCAGGCATCATTTCATCTCAACAC

CTATTTTCTATAAGAAGTTATGGGCAATTTCAAAAAAACTGCAGATTTGCAGTGGTTCATTTTTCATGCAACC
AGAATTTCCATTTATGGAAATTTGTACCACCTAAAAGGAAGTTCTTTTAGCCCTCTACCACACCATATCTGTG
CTTAGGCTGTATGTTGTCCCACAGCCAAGTTATAGCCCAAACAAAATCTAGGTTAACACCCATGAGCCGGACT
TTTGACTGCCACTGTATGT
>Bjead1_1_rnd-1_family-145#TIR/MITE
CACTGGGTGGAAACCCGGATATGTTNGGCCGNAATATTCAGAATGGTTTGGCGNNAAATTGCATAATGTGTTT
ACCATTTATCGGCCTATAAATGGACATAAATATCGTAAACAATTTCATACCATTTTCGCCCGACAAATCGTNA
TTCACTTTGGTAGTTTGTTCACCCTNTTTCGGCGTAAATCATCGTAGANACACGGAAACATTGTGAACAAAGT
CTGAAATGAGGATNATATNCGTCNCAACAGGGTGAACAAATTACCAAAGTGAATNATGATTTGTCGGGCAAAA
ATGGTATGAAATTGTTTACGATATTTATGTCCACTTATAGGCCGATAAATGGTAAACACATTATGCAACTTGG
CGCCNAACCATTCTGAATATTACGNCCGAACANATCGGGATTTCCACCCAGTG
>Bjead1_1_rnd-1_family-104#TIR/MITE
TAGTGTTGTGCGGGCCCGGCTCGGCTCAGAAGCCCGGGCTTGGGCCCGGCTCGGAGGGCTCAGGGCCTCGCAA
ACGTCTGAGCCGGGCCCTGAGCCCGCAAGCAGGCCCGGCTCAGGCGGGCTTGGGCTCAGGCCCGGGCTTTGTA
GGTGCAGCGGAGGATAGGAATTGGTACTTCGAAAATAATATTAGTGAGTTACATTGAGACGGAGACGTCCATA
TACGCGGCGACGACTTACTTGAAGTGATGCGTTCGTGTTCAAGACGCGTATAGGCGTGTCGCGAAGCCGTCGC
GAAACATTCCCACGGGTGGTCAGAACGGCCGACCAAGTATAACAGTAAGTACTACGTCGCGTAAAGCCAGCAG
AAAAGGGCGGAGAACTGAANTGGGACGTACACAGTGAATATTCTGCCGAGGAGCCGCGAGAAANGGCCTCCGG
AGTGACCGCNCTCTGCGGCGTCCTCAGGCCCGGGCTTGAAGGCCTGCAGGGCCGGGCTCTCAGCTCAAGCCGG
GCCCTGAGCCCTCTCAAGCCGCCACCAAGGGCCCGGCTCGGCCGGGCTGCCCGCGGTCGGGCTTTGCAGGCTT
TTGGGCTTCAGGCCCGGCCCGCACAACACTA
>Bjead1_1_rnd-1_family-234#TIR/Tc1-Mariner
TACACTGGGCGCCAAAAGTAAATGAACACCGCCCGATTTGGAAAGATCTGCTCACCGCGCTGTGAATATATTT
GTTTTCTTCGGATTTCTTGCTGCTATAGGATCACTAGGGTCCCTGTAATCCTGTCTGCATGAGACCGTCCAGA
AAAAAAATTGATGCCCAAAAGCAGTGCCACATTTAAGGGCCAATACTACGCCCACCTCTCGACTTCGGCGATA
CGACGTGTACTCCACCGCCTGGGCTATCAAGCTTATCGACGCAGGAAAGTATCCTTCCTGACCACGCTGCATA
GACGGATCCGGAGGGTGTGGGCGAGGATGTTGCGGGACTGGGATTTTTGGAAGTGGAGGCATTGCTTTTTTTC
CGACGAGTCAAAGTACAACCTCCTACGCTCGGATGGTCGCGACTTTGCTTGGAGGAAGAAAGGAAGGGCCTAT
GACCCCAGATACACCATTAAGACGAAGAAATTCGGTGGAGGCAAGGTTATGGTGTGGGGGTGCATTACGCCGT
GGGGGGTCGGAACACTTCATCGCATCACCGGCACGATGGATCGCAAGATGTACCAGGAGATCCTTTCTGAATC
CCTTCTAGGGACCCTCAGGAACTACGGTACCAATCCTCGTGGCATCATCTTCCAGCAGGACAACGATCCGAAG
CACACGGCAAAGTCGACGCGCGAGTGGTTTGACGAACATGGCTTCATTCTCCTACCGTGGCCGGCCCAGAGCC
CCGATCTCAACCCCATTGAGAACGTCTGGGACCATTTGGCCCGTCGACTTGCCAAGCGAAGCGTTCAGCCTGC
AACGGAGGACNAATTGTGGGAGGCTTTGCAGGAGGAGTGGTACCGGATTGACGTGGCGTATATTCAAAAGCTC
TATCAGAGCATGCCAAGGCGGGTGGAGCAGGTGATTCAGAGGAAGGGGTGGAACATTCCGTATTAGTCTCGGA
AATATACACACTGGTATTGTCAATAAACGGAGACAAATCTTCATGGAGAGTGCTTGATTGAGCGAAGGTTTGG
CGTAAAATTGAGCGTAGCACCCTAAGATTCCTGCAGAATCTTGCAATTTTTGTACCAATAATCTGTGTATATC
GTAGCTAGTATACATAAATATTTTCGCGATTTTATGTGGCCGTTAAGGTAGTTAAACGGGACGCGAGGGCGGT
GTTCATTTACTTTTGGCGCCCAGTGTAT
>Bjead1_1_rnd-1_family-336#LTR/Copia
AGCCAGAGTGGGTGCGCACAGGGAGAGGACCGTGGACGTCCGAGTGGACGAGCTTCAGGGGCCTGTCAGCGAC
AGTGGACGACGAGGGGAAGGGCGCAGCGTTGAGCTTGCCAGAGAGGCAGGGCTCGCAGATGGGATCGGCCTGC
TGAGACGAGGTGAGCCTCAGGCCAGAGACCAGGTCGCTCTGCAGCAGTGCGCGCACTCCCTGGAGGTGGAAGT
GGGCGAGGCGACGATGCCAGAGGGTCTCATCGAGAGGGAGAGTGGAGGAGGCGGACACCTGCGCAGACTCGAA
GGCAGGGACGACCTGGCCAGCGAGGAACGCGGTGTTGGAGCTGTCTATGGGCGCGCAGAACAGAGTCTGGCCA
TCTCGAACGAAATCCATGGAGGAGGAAGAGATGTGGACATTGAAGGCGCATTTTCGCGCGAGATACAGCACAG
AGAGGAGATTGGAGCCAAGCTCCGGCACATGAAGGACTCTAGTGAACTCCACAGACCGCACGACCTCCCCCCT
GACCTCTGGAGCGAACACCACAGACCCTACGCCCGCGCTGAAGATGACCGTGTGGTTGGCTAGACGCACAGGG
ATCTTGCAGGGGGTGTAGTTGCGCAGCCAGCCCCTGTGGGGCGTCATATGAGCAGTGGCGCCTGAGTCTGCGG
TCCAGCGATCAGAGACAGGCTCAGGGGAGGGAGACGAGGTAGGGGGACGAACTGAGGCCTTGCCAGCGGACTC
TGTCGCACCAGAGGAGGCGAAAGCGGCCGCCTCCTGGGCCACAGCAGCAGCAGCGGACGGAGGAGTCGAAGCA
GCAGCAGCAGAGCCGCCAGAGCGTCCTTTGGCTCGCTGTTTGCGCTTCTCCTGGGCCTCCTTCGACGCTGTGT
CCTTCGCGCGTGCGTAGCGCGTGCAGGTCGCCTCAGCGTGGCCAGGGAGCGAGCAGAACGCGCAGGTGAGAGA
GGGATTGGACTGGGGGGCGAAAGCGGAGAGGACTGAGGTGGAACCAGGGGTGCTGGAGCGAGTGCGCAGGGCC
TCCTCGTTGATGAAGGCGGCCTGGAGCGTGTCCTTGTCCAGCTTGTCGAGCAGGGTGAGGCTGGAGGCGAAGC
CGGAGTAGTGCGCAGGCAGGGAGCGAATGAGGGTCATGGAGACGAGCTCCTTGTCCAGATCGTCGATGGTGAA
CGAGTCTGGGCGCAGATTGCGCACCCTGACCATGGCAGCGTCAACCCTGGTCATCAGAGACGACAGGGACTCG
TTCTCCTCCATGTGGATGGCGAAGAACTCCTCCCAGGCGTTGAAACGCATGCCAGGCTTCTTCTGCAGGTGCA

CTGTCTCGAGCTTCGTCCAGATCTTGCAGGGATCGTCCGAGATGTTCTCCAGGTGCGTCTTCTGGTCGTCTGA
GATGTGC
>Bjead1_1_rnd-1_family-137#LTR/Copia
AGATCCTNGTGGTCTACGTCGACGACCTCACCATGATGGGTAACAGCCTCGAGCTCATCAACCGGACCAAAGA
GGCACTNAAAGGGTCTTTCGAGCTGAAGGATCTNGGCGAGTTGAAGCTCTATCTCGGCGTTAGGGTCACTCGG
GATAGGGGGAGCAGACTGATCTACCTNGACCAGGAAGCGTACATCGAGTCCGTCCTCGAACGCTTCGGNTTCC
AAGACTGCAATCCGGAACCCACCCCACTCCCCGCCGGCGCCGTACTCGAACGGAACGAAGAACCNCCAGAATC
CCGCCCCCCCACGCTCCTCGTACCGCAGCGTTCTNGGCTCCCTCATGTACGCCATGCTCGGNACTCGCCCGGA
CNTNGCGTTCGCGGTCGCGCGNCTCGGNCGGTACCAGTCCAACCCGACTGAAGGNCACCTGGCCTGGCGAANC
ACATCCTCCGGTACCTTCGGGGTACCGCAACATCTNCGCCTCTGTCTAGGTCACNACGACACGCGCAACGATC
AGCTCGTCGGCTTCACTGACGCCGATCGCGCCGNNGACACCGATACGTCTCGCTCTACCTCGGGTCGGGTATT
CCNNCTNGGTCGCGGCGCNGTTTGCTGGGCNTCGCGCAAACAACGGTCTGTGGCGANATCTACCTTCGACGCG
GAGTACTTCGCGGCACACGAGGCCTGTCAGCAANTCCGNTGGCTCGACGNCTTTTCTGAGCAAATCGANCACC
CGCTCGAAAAGCCNGTCACNCTCTNCTGCGACAACNNGTCGGCNGTCGGCGCCTCCGAACGGCCTCAACGTGA
AGCACCGCNCGAAGCACACCCGCGTTATGGCGCACGCCGTGCACGAANCTTTCGAGAACGGNCTGGTNAAACT
CGAGCGCGTTGCTACCGAGGAAAATNCCGCCGATATNTTCACCAAACCNCTTCCCAAAGATCTACACGAAAAG
CATACGTTGGGACTCGGTCTAGTCCAGTATCGCTACGCCAAGGGGGAGTGTTGAGCTGATGGCTCGCTCATGG
ACA
>Bjead1_1_rnd-1_family-325#LTR/Gypsy
CTACGTCGGGAATCANGCNCCNTTTGATCTGCTNCTAGGACGGCCGTGGCAGCGNGGNAACTTCGTTTCCATT
GACGAGCGNAAAGACGGAACNTATCTNCNGTTCAAGGATCCGGANTTCGAAGTNCGTTTTGAGATGCTNGCNA
CACCCGAGCACGAANTGGAGGCCAGCATCACGGAGTACCTTGCGAAATCACAGAGCGTGCTTAGCATGTTCTG
CGGAGCATCATCAGGACCGACGACCAAAGAGCTAGACCCAGCTACTATCAATCAGCAAGCCGTTATCAGACGC
CAGCATTCACGCGACCATCTCAGAAATGCAGAACCAGAACAATGGACTCCCCAGATAGGCAGGAACAGTGCCC
AGGAGTCTGGCGTTTTAGGCATTTTAAGCATTTTAGACGTGAATGCACTAACGGCCGAGATGGTGCCTGAGAT
CATGTCCGACGGCAGCGGAATCCAAGAAGTCGACATAGAGGCAGATGAGAACTCAGAATCACGCTCAGACGAC
TCACTGGGCCCAGCGCAAGACAGGTACAGGACCAGCAGGTCAGCTGGTTTGGAAGACAGCGTCCACGACAGCA
GCGAACACATGCCCAGAGCGAACCAGCAAGCGTGGGATCTGCAGAAGATTGACCCTCCTATGCAGAGGCCCAG
GCGACGCAGACTGCTTTCACGCGTGTGGCGCACCCTCAAGAACTCAGTTACACGGCACCATCGACCCTCAGAG
TCAATCCCAGACAGCCAGAGCCAAAGCGAGCTTCAGGAAAGCAATGATATTTATTTCTGTGCTACCAATAGTA
CAGAAGTAGATAGCCTGGAGGACACATTGGCAGCAGTGAGCATAGCAGACAATGATGAGAGGATGGACGACGT
ACCTGAGAACCCGGAACCCAGGACCAATGACGATGAAAGCGAGGACGACGACGACGATCCAGGCGACGGCGAG
CTCAGTGAGCTCAAAATCAGCATTGACACACCAGACAGGCTAGAGACAGACCTTATTGAGCTGGTGTCATGGC
GGAATACCACCCGACACCAGATGGTAAAGGCCCGAGACGACGAACTAAGCAAAGTTGATGAGCATGAGGCACA
AGGACGTGAAGACACGCTTACTAGTCAGCACCGCCGCTGGCTGGCTTGGCGTGAATTCAACTTGAACATGTGG
CAGCTTGGCGAGGAAGTCGAGGGAGCCCTACGTCATATGTTAGATGTCAACCAAGCTATACGAGGAATCGTGC
ACGTACCTGATCAATCGCACACCAATGTGCGAAGTCAAAATTCCCCGCATTCACCCCCACCCGACACACTATT
CATCGAGGTTATAGATGAGCCCTGCGAGCACCTGCAAAGAACACATCAGCGTAAGAAGGATTGGACGCAGATA
CTCACCCGCATTCTTGAGTCCGTACCGGTAGCCTTGGGCGTCGTGATCGTAGTCGCCCGAGTAGCCCAGCACG
CGAGCTTGAGGTGGGGAGCGAGAATCTTCCCAGGCCTCTTGTGTATATTGCAGCAGCCCCGCACTGCCGACCG
TGGCGGTGCGCGAGCTTTGAGAGCTCCAGATCCAGCCGATTTGGGTGACGACGAGACTGTTCAGCCAGCCCGG
AGGCCCGACGGAACAACGGCGANCCGGAGGAAAGTACCAGAAAGCGGCGAAATCTACGGACACGATCTGAGCA
CTCGACGACGCGACAGCGGCGATNTTCACTCACCGATGGAGGGGCAGACCTCGCCCGCGGACGCGCTCAAGTA
CCCAGGAAGTAGCCTGCGACGGGCCAAGTCAGTAAGATTCAGCACGGAGATTCGGACGACCAACGAGAGCAAG
CACGTACCTCAGATCGACGCGCAAGATATGCGCGCAGCAGCAGGATCGAGAGCGTCGACACAGCCCCTCCGTC
CCGAGGAGCTGGTCGATTGGCCGGTTATTCTCTCAGCCGCCGAGCTTTCACGAGATCGCGCGACGACGACCCG
ACGGCGGCGGAAGTCAGCGAACGTTGAGGCTGCACACAGCAAGTCTGGACTCACCTGCGCAGATCCCGAGGTG
CACGGACGTCGAGTGACCCCCGGATCTGGGGGGGATCTCGAGTTCGAGCCCCGCAAATGCACGAG
>Bjead1_1_Cluster31#LTR/Copia
TCTTTTGTTCAACGAGGCGCTCGGGCGGAAGGCTGCGTCGAGGAAGACAATGCCGATGAATTATTATGTGTAC
AGTAGGATATACAGAGGATGTGCGTACCTGCGTCGAGGAAGACAATGCCGATGAAGAGCAAGTCTCCTCGACG
CGCGGTTAATGGACAGACTGATTACATAAACTGTACAACTGTCCGAAATAGGAAACTGAAGTCGAACGGACGT
GTTCAACTTCCAACACTCCCCCCGAACTCGGCACGTTTGAGTTCTTTCAGTCCAAGAAGGGCCTGCATGGACT
CTACTTTCTGTCGCGCCAGTGCTTTCGTAAGAATGTCCGATGGCATATCATTGGTGCTGATGTGTTCGACTCG
AATGGCTCCGCTCGCTACGGTGTCTCGCAACCAGTAGTCGCGCAAATCCATGTGCTTGACACGGCCATGGTGT
TCGGGGTTCTTCGCAACGTCGACGGTGGAGCGATTGTCGATGTAGAGGGTGGAAGGTCCAGGGAGATCATACC
CAAACTCGGTGAAAAGGTTTCGGAGCCATATGATCTCCTGTCCGGCTGATACGGCGGAGATGTACTCGGCCTC
GGTAGTTGATAGCGCCACCACAGACTGAAGGCGGCTCGACCAGCTGATGGCACCAGTGCCTACTTTTACGACG

TAGCTGCTGGTAGAACGGCCGTTGTCTGGGTTTCCACCGTGGTCCGCATCGGAAAACGTCGCGAACGGTTCCA
GCGGGGATATGCGAGAGGATGGGTCTGGCGCGTACACCAGTGCCATGTCTTTTGTGCCCTGGAGGTAGCGAAA
CAAGTGTTTGACCGCCTTCCAGTGTAGGAGACCTGGGTTTTGACTGAAGCGGGCAAGGACGCTCACAACAAAT
GCGATGTCTGGACGCGTCGCAACGGCGAGGTAGGCCAGGGCGCCAACTGCGTTGATGTACGGGATCCTGCTCA
TCTCCTCTTTTTCGGAAGGAGTTGTCGGGGACATGTCCGCGCTGAGTTTGGCCCCCGGGTCGATGGGGGTAGT
GACCGTATCGCAGTCGGCAAAGCCGAAACGCGCAAGGATCTGGATGATGTATTGGCGTTGAGATAGGGAGATG
GATCGTAGGTTGCGGTCGCGTTTGATTTCAACACCGAGTAACCAGGACGTGGGACCAAGATCGCGCAGTTTGA
ATGATCCACGGAGGTCTTCTTTGACTTTCGCCACGTCATCCTTGGACTTTCCAGCAATGATCATGTCGTCTAT
GAACACGGGGACAATGATGCGTGAATCTCCACGGCCGAAAACCCAGACGCTGCTGTCACTGCGGATTCGCTGG
AAGCCTAGCGACTGGAAAGACTCATTGAGTTTCTCATGCCAGCAACGGCCGGCTTGTTTCAGCCCGTAGAGGG
ACTTGATCAGACGCCAGACGTGGCTGCGATCACCTTCTTCGAAGCCCTCGGGTTGCCTCATGTAGACTTCCTC
TTTCAGTTCGCCATTGAGGTACGCTGAGGAGATGTCCACGCTTTCCAATTCGAGATCCTCCAATGCACCGAGC
GCAAGAATGGCACGAAGTGACGCCCACTTCGGTGTCGGCGCGAATGTCTCGGTGAAGTCAAAACCAGGTCGTT
GCGAGAAGCCTTGGGCGACGAGGCGGCCTTTGTACCGTTCGATGGAGCCATCGGCGTTTCGCTTGACTTTGAA
GACCCAGCGACTCCCGATCGCTTTTCGGCCCGGTGGTAGCTGCACAAGCTCAAACGTACCACTCTCGATCAAA
GCCCGTAGCTCGTCGGCCGCAGCGTTGTACCAGTGGGAGCGCTCAGGCTCTGGACGCTGCATGGCGTCACTGA
AACTCCGTGGTTCGCCAGTGTGTGTTGCTGCTTTGTACGCGAGCTCAAAGCCCTGATCGATGGTCATGTAGGC
GCCTGTGTGTGCTCCAAAGACGAACTCGAGCTCATTCGCGCTTTGGGCTCCGTCGGTGTTGACTAGGTCCTCA
ACCTCGTCTTCGCTCTCAAGGGGCGCATTGGGGACTTGTAGTGGCTGCCGTGCACGTGGCATGATTGGCTTCA
GGCCTGACACACTGCGAGTAATGTGGTTGATGTTCATCGTGCCAGGCGGCGGGCGGTTCGTCTGGCGAGAGCT
TCGGCGGAGAGGTGGATCAGTCTCAAGTGGGATAGCTTGTGTGGCAGGCGGGTCAATGGGAACGTCGGAAGCA
ACACCAAGTTCTGGTTCAGGCGGTTCCTCAAGGTCAGAGTTACTCCGGCTAGTGGATGGCGCAGTGGCGTCAC
TTGATGAAGTGTCGGCCTCTGTCAGAGGTGAAAGGCTTTGTGTTGGAGCAAGGTCATCGTCGTCGTCCCCCCA
ATGTGACAGCACGGAGGCTGAATCATCACTAGGCTGGTCCGCAGTTGTCTCTGATGGTACGCTCAGCCCTGGG
AATGAGCGCTCGTCGAAAACTGCGTGGCTGCTGGTGACGACACGGCGGCGAGAAGGATCCCAGAACAGCCACG
CCTTGGTTCCTGGAGCATATCCAATGAAGATGCAACGGCGGCTTCGGGGAGCGAGCGCGCGACGGTCTGACTT
TCGTTCGTGGACATACGCGAGGCAGCCCCAGACCCGGAAATATGAGACGTCGGGTACATGCCCAAACCAGGCC
ATGTAAGGGGTGCCAGAGGGTATGGCCGAGGTAGGGGCGCGGTTTCGAACATGGACATAGGCCGTCACAGCGC
GGGCCCAGAAGCTGTAGGGCAATTTTGCCTCTTGCAGCATGGCCGTAATCGCCTCGTGGATAGAGCGATTCGC
GCGTTCAGCCACGCCATTTTGATATGGTTCATCAGGCTCTGTGTGTCGCCGTAAGATCCCTTCGGCGGCGCAG
AAATCATCGAATTCCTTGCTGATGTATTCCCCTCCCTTATCGTCCTGGAACTCGCCGATACGGACACCAAGTT
CGTTTTCCGCGTAAGCTTTGAAGGCCTTGAATGCAGCGAACGTGTCACTTTTCTGCCGGAGATAAGCGACTGC
CCAGTAATGGGTACCTCCGCAGATGAAGGTGACCCAATACTTGTATCCTTCGGGCGTTGCTACGGGCAACGGT
CCTTTCAGGTCGCTATAGACGCGGGCAAGTGGTCGAGATTTGCGGGGGCGAGCACGGTGAGGGATGTTGTGAC
GATGTTGCTTGCCAAAAATGCACGGTTCGCAAATTGGATCCGGAGCATCAGACGATGTGATGTCCATACCGAC
GACAAGCTTGTCATTTTTCATCTGACGGATAGCCGCATGGTTGATGTGCGCGCAGCGTCGGTGCCATAGACCG
AGCGTAAGGGGTAGTACTGCACTGGCGGAGTGTGCCTGGGTAGTCAGACTGACGATGGTGTGACCGTTGAGAT
AGCCTGAGTTGCCGTCGTTCACTGTGGCTGTGAGGCACGCACGCCCAGCTCGTTCAAAGAGCACAGAGGAACC
GTTGAGGGTGATGGTGAATCCTTCCACACGAGAGAGATGGAACATGGAGAGTAAATTGTTTGCGAGGGCGGGG
ACGTGCAGTACATCACGGAAGACTATATGCGGCCATTTCTCCCCGCTACCACTCTCCGGCTGAAACCAGACCT
CTCCGACTCCCGCAGAGTATATGATGGTGCCATTTGCCAGGCAGACTGGGGTTGTCTGAGGTCGGTAGGTGTG
GAACCAGTGGCGATGTGGTGTCATGTGTTTGGTTGCACCTGTGTCGGTGTTCCAGTTAGCCGCCATAGACGTC
ATGAGATCATGGGGGACCGAGCCGGCGAGGGCGCTAGCGTGCCCGGCATACTCGCAACGTACATTCGCATCGC
TCGCAGCGGGAGGAGCTTGGCTCGGTGTGACGGAGGGTTGGTACAAGGGAAAGGTGACCTGGTTGGCAGCGAC
GGGTATCTGGTTCTGGTCCCGATACTGGCGATGTTGGCGAGAGCGCTCGCGGACATCGGCAAGGGCACGCGCG
TACTTGCGACACTTGTCTTGGCCATGTCCTGGGATGTCGCAGAACGAGCATCGGCCGGTGGATGCGGCACGGG
CGAGTTGTATGGTGGGCGCTTCGGAGACTGCGCGTGACTCGCGCTGGGATTCCTCATTGAGGAATGCCGACTG
AATGGCGGAGAGCGTCAGGGTGCCGGACGCTTGTAGCAAAAGTGAGGATACAAAAGCATTGTAGTCGGGACCG
AGGGAGCGGATGAGTGCCATGCAGACGAGTTCTTCGTCGAGCTTTTCGAGGGTGAAGGCAGCGGGCCGGAGCG
CGCGGATGTCCTGTTGACATTTGTCGGCTCGGGCGAGCAAAGATGACAGTGTCTCCTCCCCCTCCTTGCGAAG
GTTGAGCAGAATGTCGTACGCACTGAAGCGTGCCGCAGGCTTCTGCTGCAGATGGACGCTACGGAGTTTGGCC
CACATGGCTATGGGGTCTCCATCGATGTCCGCGACGTGGACCTTCTGCGAGTCCTCGAGGGCAAGAAAAATCT
CGCCGGATGCTTGGTCCCGTGCAACAAGGTACTTGTCCCACGCAGTGCGTTCTTCGGCTGTGGAGGATGCATC
CACAGGAAGAGGCGGTGTGCTGTTGCCGTCGGGCTCAACATGGCGCCAAAGCCCAGCTCGTCGGAGGTGGGCA
CGGAAGTCGGCGTAGAAGGCGTTGAAGTTGCGTGGGCCGAGCTTGGGGCCTGGGTAGGCCGGTGCGGAGGGCA
TATGGCGGAACAGCACAGGAGTGGGAAGAAGTGTCTGATGGAAATTTTCGCCGCGAGGTCGTCGAGCTTAAAG
GCTCATAACCTGTTCAACGAGGCGCTCGGGCGGAAGGCTGCGTCGAGGAAGACAATGCCGATGAATTATTATG
CGTACAGTAGGATATACAGAGGATGTGCGTACCTGCGTCGAGGAAGACAATGCCGATGAAGAGCAAGTCTCCT

CGACGCGCGGTTAATGGACAGACTGATTACATAAACTGTACAACTGTCCGAAATAGGAAACTGAAGTCGAACG
GACGTGTTCAACTTCCAACACACCAA
>Bjead1_1_Cluster9#LTR/Copia
ATAAGGAAATGGATAATCACAAGGAAAGGGAACGAGTCGGTACGTCCGTATATGGAAGTCCCGCCTCCGGCGC
ATCAGCTCGCCGGGTGGTGCCTAGGCGCGGCTCCACGACACTCCCCCTCAACGAGCACGCAGGTTGACGAGTT
CGACCTGGTGGAAGTGCGCTGGCTTGGGAAGCGGCTTCGTCAAGATGTCGGCGCAGTTGTCTTGAGACGCGCA
ATGGGAAATGGTAATGTCGCCGGCTTCGATGCGTTCGCGCGTGAAGTGATGACGTATGTCGATATGTTTGGAG
GCGCGCGACCGGAGGAACCTGCGACGGGCGAGGATTTTGGCGAGTGCGCCTTGCGCGTTGAAGACACCCGTGA
GCGTGTCCCATGCGTCCTTCGACGTCGGGGAGGACATGATGTAAGTTATGATCGACGAAGTAACGCGGAGACG
GATGATCGTGAGTGCCTTACGATCGGCTTTTACCCAATCGGGCAACTGTGCCTTGTCGCTGGGTTGCGGTTTG
CTTCCGTCAACGTAATCGATGTACCCCATGTCGGTGAGGACGTCTTGGATTTGGACTTGCCAGGAGAGCCAAT
TCTCCGGCCCTTTGAGAGGTTCGATGCGGTACGCGCCGCCGGTACTGGTGTCGTTACTCGACATGTCGATGGG
CGCTAGTCGTCCGATCTAAGATCAAAGTTTAAGTCCGCAATGGACGAGCAGTCAAGCCGGGCGATCTGCAATA
GATCAAGCAGTGGGTAGAGTTCGGGGGCGATTCTAGAGCGCGGGGCTCATAACCTATTGAACTCATAAAACTG
GCTAGACTGGGGATTGAACCCAGGACCTCCCCGTTTGCACTTGGGTGCTCTACCACTGAGCTACCTGGCCGAT
AAGAGTGTGACTTCTTACTTCAACATATTGAACTCATAAAACTGGCTAGACTGGGGATTGAACCCAGGACCTC
CCCGTTTGCACTTGGGTGCTCTACCACTGAGCTACCTGGCCGATAAGAGTGTGACTTCTTACTTCAACACTCC
CCCTAAGTCACACGATGTGCTTAGGTAGCCAGCTTTACAGTTCACCTCACACAGGAGGACCTAGAACCACGTT
CCAGGCTCGCGCTATGGCCATCTTACTACTCAGTGCGTCGAGTAGCGCAGACGCCATGTGGCGGGGCTCATAA
CCTGTTGTTGCGCAGGCTTGGATCACTCGGGGTAAGACTGCGAGAGCGACTGATGATTGAACTACGCTATTAC
AGGTTCTATTTATATTAGTTTAGGATAGGAGGCCGGTAACTCTATATGGGGACTTAGTGGATCAGATGTGAAA
TATAAGCGGGATAAGGAAATGGATAATCACAAGGAAAGGGAACGAGTCGGTACGTCCGTATATGGAAGTCCCG
CCTCCGGCGCATCAGCTCGCCGGGTGGTGCCTAGGCGCGGCTCCACGACATCGGTTTT
>Bjead1_1_Cluster25#LTR/Gypsy
GTAAGGGACGTCAGTACTGCTTACTAACACCCTATGTTTCTTTTCGCAGGTCGTCCTCGTGCGCCCTTCGACA
CTCTCGTTCGCCCTACTGGCGCCTTATCAGTTTCCTTTTTAGTACGATTCCACGTGGCCTAAAAGTAGATTAG
CTCGTGTATAAACTCGCTACTCTACACCCTGTACCTCAGTGATTGCTCTTTCCCCCGAGAATCGAGTCCTTTT
CCCGGTTCTCTCCTCGTTCGAGCTTTCCTCTCTCGTGTGTGGGAACTCCTCTCCGTCCTCGGAAGGCTTTCCT
CACATATTTTGAACCCCTCTCGACTCTTTTCTCTCGACAACCCCGACAACCGTCGACACCGACTTTTCCCCTT
CCACTTTTTCGTTTTTTTTTCTTTTCCCTCTACTCCCCTCTGGCAACCACCTCCGGCTTGATCACCCGCGAGA
AGACCGCCAACCTCTGACCGTATGCGTACGCGACGAGGCACCGAATACCAAAACGACGCCGGCCGGCCTCAAC
CTCCCGCGATGGCTACTCAGTCCAAGGACAGTGCCTCCGCCAACGGGCATGAAGACTCAGGTGAGCGAGTCGC
CTCTTGGTTAACAGAATTGACGAACACGGAGACTGTCGAGTCATCCGCACCTCCCACAGATTCACACGCGGGT
GCGGCTGCTCCCACCGCAGACGACGACCAACAGCGCTCCCGCACCGACGCGGATGTCGCTTTTTCTCGGGCAG
GCCCTCATACAAGCGAGGAGGCCCCCGAATGCTCGCCTTCCCACCATGAAGAGGTGCCGGAAGCCGCGAATGA
GGCTCTGTCTATTGCAAGAGACGTTGAGCATGATGCGCTGCCCTCTGAGGCCAACGAGCCCCGCCCACGCTCG
CGCACAAGCCTGTCATATATTGATGAAGACGGCTTCGTACACCCTGCACGCACGGCTTCGCCTGGCGGTGGCT
CCGCGACTTCGCGCGTGAAAACCGATTGAAAATTTTTCGACGAAAAATGGTTTGGACCACGCGTCCCTCCCGT
GGACCCCATCGATGACACGACGAGCACCACGACGGCCTCCTCGCCCCGTCGTAGCTGGTCGGATATGGTGGAA
GACGATGACGGCCACATAGGAGAGGTGCCCGAGGAGTGGTACACCGCGCCCCCCGTCCGTGGCAACTCGCCCA
CCATAGACGTACCTATCATGTATGACGGCTTTTATGATGATGTTATGCAGCTCCTCAGCCCGTCTGCGCATGA
GTTAATTCACTGCCGCGCCGAGAACCTTCAACACATTCGGGTCACCACCCTGCGGACTTTCTCGGACAGCTCG
CAGGCCCGTGGACATTTTACTGCTAACGTTCGTGCTAGCTCCACGCCTTGGCCGCCCGCACGTATCCCGAGCT
CGCTGAAGGGCAAGGGCCGCGCGACTAGTATTGCGCCGAAGCTGGAGCCCGTCGATGAGCAACTGCCAGCTTC
CAACAATGACGAACATCGGAGGGCGCAACTTGAAGCGGACACTCTGCTTGCCCAACAACTGCAACAACAGCTT
ACTAACGAAGGTGACACTGACTCGTATTTCGAGCCAGCCCCTCGCTACACGCCGTACGAACAGCCAGGCAGCT
CGCGCGCTTGCCCACCTTCCGTTACGCGTGAGCGAGTGGCCGACGATGCCCGCTATGACCGTGATATTGCCCT
GGCCTTACAGCTTTCGTATACAGCCAAGGATAAGGGTGCGAAGGCAGTCCGCATGACGCATGACGACGTGCAA
CGCCCGCGTGAAGATATACCACGGCACACCAGACCGCCGGTCAGCCACGACAAGCCGGTTCCTCTGCGCCGTG
CCACCTCAGAAATACCACGAGCCTCGTTCTTATACAACGCCATGCACTCGCACGACAATGGCGACCCCATGGA
GCCTTCGGACTCCAGCTCGTCAGACTCGTCGAGTGACACCGACAGCTTGACTTCTCCTCGTGCCCGCCGTACA
AGATCCCCGTCGCCGTCTGCCAGCGAGTCTGACTGGAGTTCTCTTAAGTCGGAACCTCCTGAGCCCGATAGCG
ATGACTCCCAACGGACGCGCAAGAAGAAGAAGGCTGCTCGACAGCGTTGGAAGAATAGGGTTATGAAATTCAG
GATGGAACAGTCCAGCGCGAAGGCGGACCCGCCGTTCATTTACAAAGGCGAACCTAAGTACGAACTGTACGAG
AAATGGGTGATGGAGGCACGGGACTGGTGCAAGCTCAGCTTCATCCGCCGCAAGCACCGCGTCGCGCGACTGA
AGAAGTACCTCAGTGGCGAGGCCTTTGCCTTCTACATGCGCGAGGTCTCAAAAGACCCATACAAATGGTCCCT
CAGCCACTTCCTACGGGAACTGTTTGACAACTGTTTCCCCTCGGATTTCCGTTCGACGCAGCGCGCTCGATTC
AACGAGTTCACGCAGCGTGGTCGACCCATTCGCAACTACTGCCGGGACCTTCAGCGCATTGCAGATAGCATCA

GCGATATCTCTCGCCGGTGCGTTTGTGATACGCTTCTGGCAGGGTGCTGACGCCGAGGTCCAGAAACAGTGGA
TCCTGGACGGCTTCTCTGAGGAGGACGCGCACCTGAACGACCTCCTCGCGTATGCAGAACGCTATGAGCGAGC
GGCGAAGCGCGTACAACAGCACCAGCGACTCGATAGGAACAGGGCCAATAGCCCTGTTAACCGGCATCTTCAT
ACGTACCGCCATGATGTCCCGCGCGACAACGGCTCCCGCCATGGACAGCGCTCTCATGGCCATAGGGACCACA
GCCAGACTTCAGGCAGCCGACCACATAGTGGTCAGCGGGGACAGCGCCGTGATGACCGCCGCAACAACGATGA
CCGCCGCCCCCGTAGGGATGACCGCTCTCGCAAGGACAATGCTGGCAAGCCCCGCTCGAAGAACCAGCTCACT
GATGCTCAGAAGGATGAGTATCGTGCACAGGGCAAGTGCTTTGAGTGTGGCGAAAAAGGCCATCTTAGCAAAG
ACTGCCCGAAAAAGCAGACATTACCCCGAAACCGCGGCAACGCGTTGGGCGCAGTCACTCTCGACTTCGACAA
GATCGACAAGTTACAGGCACTGGATACTGCCGCGACACTCGGGCTGTTGGCCATATCCCTTCAGGACGACACT
GATGATACACTCTCGGTGCCCGACTCACATGCCTCAAGCTCCCTAGACTCGGCGGCATGCGAGGCGGAGATCG
TTGGCGTCATCAGGGACCAACTTCTACTTGGTGTCCCGTACAGCTTCGACGACGTAAACGACGCCAACGTGCA
ACATATGTACAGCCCGGATAGGTTCTTTGTTGCACGTTCAGAACGCGCCGCATTCATGGTCTTCGACTCCACA
GCCGGGACCTACTACCGTGTCGACATGGCGGACATCCTTGACGAGGATTTCAACCTCCATGATTTTATTGAAC
GAGGCTACAGCAATGCACCACGGTTCGACTACACACCCGCTGCGGACGACGGGCCTGTCTATGACGCGCCGTC
AGACTGGGACTCTGACAAGTCTCTTGATGACGACGGCGGGTCCTCTGGTGGCTCGCGGTCAGACTGGGATTAT
GTGTACGACGGTAGACTCGACTCACCACCAGCACCGGCCTCCATGCCGCCTCTTGAGGCCTGCGACGACGCTG
ACTCGATATCGAGTTGGTCGTCGATTTCTTCACGCAACTCTGACCCTATTGCCTCTGACGATGACAGTTCTCT
GCCCACGCTTCGCACACCGTCGGACTCGTCGCCTTCTGATGGTGGCTACTCTGAGGTGTCTGGTGATGATGCT
TGGGTTACTGTGAGTGAGGACAACATTGAGCTCGGCTCCATAGCAACAAAGAAGCCGAAAGCTTCACCTACCG
ACTTGCGTGCCCTCGAGCGCAACGCGTCACAGGTGCGCGATTTCCAACGCCGTCTCCCGAAGCCTATCGTGGT
TGCAGTGTTCTTGAGCGGCAAACCGGCTCGCGCCTTGCTCGACTCTGGCTCACTGTTGGACTTCGTGTCAACG
ACTGTAGTTGACCAGCTCGGCCTGACAACGGATGTGCTGGCTAAGCCGATAGCGGTTAAGCTTGCTGTCACAG
GCTCGCGCTCTCGGGTGAATCATTCTGTGATGGTGCCATTTCAGTACCAGACTATCGACGAGCGAAGGCGATT
TGACGTACTCAATCTCGACAGCTACGACGTGATCCTGGGCACACCATTCCTTTTTCAGCACAAGGTATTCATT
GGCATGAACCCGACTCGTGTGGCCATAGGGAGCGACAAGTCGCTGCCGATTGATGGTGACCAGGCTACGACGC
TATCATCGTTGGCTGTTGACCTACTTGAAGAAGGTCTCGAGCACCTCCGCGTACAGCTCCGAGAGTATGCGAA
GCCGATTTGCAAGGGTGCTGTGGATACACCCCTCCCCCCGCTGCGGGCAATCAATCACACAATACCACTGAAG
GATGAGAAGAAAACGTACCCTTGGCGACCTGCTCGCTGCCCAGAGGCATTGAAGCCTATCTGGCGTGCGAAAC
GCGAAGCGTACAGCCGTACAGGTCGTTGGGAGTTTCAGAGCGGCTCAAATTCCGTGTCGCTGTTGATCTTGCC
TAAGCCTGACAAGGGTGACGGCGTCCTCCGTGCCCGCACGACGTTTGACTGCCGCGCGCGGAACGATAACACC
GTTAAACTGGCGTCACCCCTACCCAATATCGAGGACATCTTGCGTAATGTTGCTGGGAAGAAGTTCCGCTCCT
TGATTGACGGTAACAAGGCGTTCGAACAAATACGTGTGACGCCTGACCACGTTCCGCGCACGCTTTTCAACAC
ACCTGAGGGTACTATGGTCAGTCACGTTCTGCAACAGGGTGATTGTAACGGCCCTGCGACCTACCAGGCAGTG
ATGAATCATATATTTGCGCCGTACATCGGAGTCTTCATGGACGTATATCTCGACGACATTGTGATCTACTCTG
ACTCATCTGAGGAGCACATGAAGCACGTCAGGACCGTGCTCGATGTGTTGAAGAAGGAACAGTTTTACCTGAG
CGAGCATAAGCTCAATTTCTTCGCCCGCGAGCTTCGCATCCTGGGTCATGTCATTGACGACAAGGGCATTAAG
ATGGATCCGGAGAAGGTTGACCGTGTGGCGAACTGGAAGACGCCTACGAACCGTGAGCTGCTAAGCGGGTTTC
TTGGTGCTGTGGGATTCCTCGCACCTGATTGCGAGGGTATACGGATCCCCATGGGCGACCTCACCCCGCTGAC
TGGGACGACAACTCCCTGGCGCTGGGGCCCAACTGAGCAGCGCGCATTCGAATCTGTCAAAGCGTCGGTTCAG
CGCTGGCGTGATAATCACCGTGTAAAGTTGGACTATTCACCTGGCGCGCCTCCTATCCTATTGATCACGGACG
CGAGCTTGACTGGTGCTAGCGGAGTCCTGGTGCAAGGAGACGACATGAAGTCTGCGTCAATTGCTGCCTTTTG
GAGTGGGAAGTTCAACTCGGCGCAGCAGAATTATCCTGTGCACGAGCAGGAGCTTTTTGCAATTGTTGAATCC
CTGAAGCGCTTTTCAGGTTACCTATACGGCGCACACTTTCGTATCTTGACGGACCACAAGGGCCTCGAGTTCA
TCCTAACGCAGAAGTCATTATCCCCTAGACAGACGCGTTGGTTAGAAACACTTACAACCTTCGATTTCGAGGT
CCAGTACATACCTGGTGAGACAAATCTCCTGGCAGACGCTCTGTCGCGCATCTACAGCGCTGATCCTGCAGGT
GTTGTGCGTTCACAGGGCGAGTACGCGTCGCTCGAGGATGACGAGCGACATGCCTCGTTGGGCGAGGTATTCG
CGTCGTTCTCGCGCCCTGTGTATACGGGCCCCTTGGTGGACATGCCTGCTGGCCCAGCGGAGGATATCACGTT
GAGCGCAATTCGAACTCGCGCTTCGGGTGTGGCAGGCACTGCTGGTGATGGTGATCTTCCTCTGTATGCCCGC
GCTCGCACTGTGCCCTCTGGGACGGCTGGAGGACGTACACCTGCGCCTGTGACCAGTGAAGTGCGTCAGAAAC
GCGCCCGGGGCAATAAGAAGGCCTCTGGGAGTATTGAGAAACTCACTATCAGGATCCCTGGGGGCCAGATGCG
CCAAGGCAATGGTCCTACGACGTCCCTAGAGCCTGTAGTGGCCCCTGTGTCAACTCCAGCCACTGCGGAGGTG
GCAGAGGCCGTTGGAGGCTTACCCGGTGTTGCTCTACCCATTGGTGTGACTCCACCTCCTTCTGCTGTCTCTG
CTGAGCCATTGGGGGCCCCTGTGCCTGTTGTGAGAGCCGGGGAGCCCGACACGCGTACAGTGGACAAGTTGGT
AGTGGTAGGAGATGTCGGGTTGGGCGAGAGCGCGACCCGAGACGTGGCAGCGCCCGGCGCTTTCATAAGCGCC
GGTGAAACGCCTGAACAGGCCCTCGAGGCTGTCGTCTCTCCTCCTCTCACCACCATTGTCGCGATGGGCAACC
CAGATGTCCAGCTACCGGACTGCCTGCGCGGCCGCTATGACGACGATGCTTTCTTTGCTCTTGTGCTCGCCAA
TCCGGACCACTATGCAAATTTTGAGGTGCACGACGGCCTCATCTACCTACGCGACTCGTTCCGCCGGATTCTA
TGCATACCGGATGTGTCTGTTGGCTCATGAAGGATCCATGAAATCATAATATCCCATGCACACTCCATCCTGG

CCCATCTCGGCTCTCGAAAGACGCTGTATTACCTCAAGGAAAATGTGTGGTGGAAATCGATGGTCGACGATGT
CCGCGCGTTCTGCGACTCCTGCGGCCTATGTGCGACCTCCAAGCCCGCCAACCAGAGGCCCTATGGGCTTCTC
CACCCGTTGCCTGTGCCCTCCTACCCTTGGGAGGTCATTGGCGTCGACTTCGTGGGACCGCTACCAGAGTCGA
AGAACCGCCACGGTGGTTTTGACATGATCACGGTCATCATCGACCATTTTTCCGGTATGGTCCACCTGGTGCC
GTCTAGGCAGACATACCGCGCCAAGGATGTGGCAGAACTAGCTTTCGACCACATCTACAAGCTGCACGGGTTA
CCGCGAGCGATTGTGAGTGACCGGGACTCGCTCTTCACGAGCGTGTTTTGGGACAGGCTACACGCTCTGATTG
GTACGGAGCTACGGATGTCATCGTCGTTTCACCCCCAGTCTGACGGTATGACTGAGCGGGCGAATCGCACGAT
AGAGCAGATGCTCCAGCAATGCATTGCTCCGAACCAGAAGGACTGGGTGACTCGGCTCCCAGGTATTGAATTT
GCAATGAACGCTGCCCGCTCCGAAACTACGGGGTTCAGTCCGTTTTTCCTAAATTCTGGGCGGACCCCACGTC
CTATGATTTGGGACGCGAAATCGGAGTATCCTGGCGTACAGGTTTTTGCGCAGCGGATGAAGGACGCCGTCCT
GGCGGCTCACGACGCCATTATCCACGCTCGTGTGAAACAAACGGTCCAGGCGAACAAACGGCGTCGAGCGGCG
CCCTTTGCCCTTGGAGACTTGGTATACCTATCGACGCAGAACCTCCGACTGCCGAAGAACCGTGCGCGCAAAC
TGATGCCTAAGTATATCGGTCCATACAAGATTCTACACGACTTCGAAAACTTGACATACAAGCTCGACCTGCC
GTCTGATCTGCGTGCGCGTGGCATCCATTCAAATTTCCACGCCTCTTTACTTCGCATACACCGCCCAAACGAT
GACCGCCGATTTCCTAGACGACTGTTGTCGCAGGTAACAGAGTTAGGCGGGACCGAGGATGAATGGGCCGTAG
AAAAGATTATTGCCCATTCTGGTAAAGGCACGACAGCACAGTTCGAAATCGTATGGTGCGGCGGAGACAAGAC
CTGGGCTCTGTATGCGGATGTATCGCACCTTGACGCCCTACGTTCTTACTTTGAAGCGTTAGGAATTTCGAAA
TTGGCCGAATTACCTGCCGGTAAAGCGCGCCCAAGCACGGATTCAGAAGTTTTCGTTGGTGCAATTTTGCTGG
GCAAGCAGGTATATAATGACGGTCCGACAAAGCTTCGGGGCCCACATTCCACCCTACGGCAACTCTGTTGCTA
CGCTGCGATGCCAGTCAACTCTCCGTTCACGCCAGCGCAAGTTGCGCTTTGTGAACAACACGCTGCTGCTATC
AATGATGGCTCCGACGCCCAGGCTCCTCCATGCTACCGCGCGTGGTTTCTCTGGACCCACGAGGAAGCTCGTC
GTGACCCTGCTAGCTTCCCCCGCCTCCCTCCGCCTCCTCGCGACTTCTACGGTCGCCTGCCGTCCGCTGCGGA
CGCGATTACAGAGCTGACAATGCGCCAGGCCGAGCTCCAATCGCAGATGCTGGCGCGCGTCATCGACAGCCAC
CCTGGCTTGCGTACCACGTCGTTACAAGGCTCCACACGGCCCCCTCGTACTCAACGTGGCGGTCCACACCACC
GTGCTCGCGGCTGGAACCGTTCCTGGCACCGCCCGGCCCCCACTCGTGACTACCGCCCTCACCGACGCCCACC
CCCGCGGCGCCCCCGTTACGACGACCGCCGCGTCCACCAGCCTTATGACGACCGCCGTATTCACCGCGACCGC
TCTCCCTCCGTCCCATGCGATGGCCGCCGGAACGCGTCAGCCGGCCCTTCCACTCGTGTCTATGTACATGTCG
CAGACACACTGGAACAGGTGGCAGCGACAATTGCTTCGTCAGACTCTGATGTTGACATGGACGACGCCGTCCA
GCGCATGCCGATGCCCGTTGCAGGTTTCGTGACACCTGTCAGCGTGGTACCCGAGACTGTGGCGGCACACACG
CCAGACGCGGCCGCCGTCCCCGCACCAGTGGTACACGACGCGCCGGCCGCTGTGCCCGCAGTGCAGACCGAAG
GGGCTGACGCCACCACCAGCGGGTCAACAACTGCCGACATGCCAAGCACGCCAGTTGCTGGGCCGTCCAGTGC
GCAACACAACGACTCCCCGCTCGACGCAATGATGGCGAGCTCACTCCGCCTGACGACCGAGGAGCACGGCGGC
GCGATGGAGACTGTCCACGACTTCTTTGTCGCGGTGGCTACTCCAGATGACGCGTTGCCCTTCGCCGCAGCGG
ACTTCTCTGCCCTGTTCGCCAGTACGGGCAATTGTACGGGCAATTGACCCTCGCTTCAACTTCTAAAAGTCTT
CGGTACGTCCTTTCTAGCTAAAGGACGGGAGGGCGTATGTAAGGGACGTCAGTACTGCTTACTAACACCCTAT
GTTTCTTTTCGCAGGTCGTCCTCGTGCGCCCTTCGACACTCTCGTTCGCCCTACTGGCGCCTTATCAGTTTCC
TTTTTAGTACGATTCCACGTGGCCTAAAAGTAGATTAGCTCGTGTATAAACTCGCTACTCTACACCCTGTACC
TCAGTGATTGCTCTTTCCCCCGAGAATCGAGTCCTTTTCCCGGTTCTCTCCTCGTTCGAGCTTTCCTCTCTCG
TGTGTGGGAACTCCTCTCCGTCCTCGGAAGGCTTTCCTCACAT
>Bjead1_1_Cluster24#LTR/Gypsy
TGAAAGGGCGCGAACCCTTGAAGTCCAACGAAGTCACAAACTTCCTAATCAGGACGAAGGAAGCGCTCGGCAC
GCGAGCGCTCGGGGAGTGCCGTAGACACGTGGTGAAAGGCGGGACGTCCAGATTGGGACGAACTGCATAGTTC
TAGCGAGTGGCTGACTATATAGGACTTTTAGAATAGATAGTTAGACACCTTTTATCACTCGCACATACGCTGT
ACACTTTCCGGACGAATATACGTCGTACGGTTCTCACAGCGTGACGCAACGTGAATATCTGGTCTTACACTTT
TTTGAACCCTCGTACTATTTCACTCCGGACTCTGAACAGTCCAAACGACCCCTACCCATCGCTCCGAACACAT
CCCGTTCAACTCCGCCACCCGCCTTGTGACGATGAGCGCAACGGCAACGGGGAGAGTTACAACTCGGAGCGCC
AGCGCCGCATCACGGGCAGCCTCTGATGCGCCTCCTGGCGGCGCGCCGTCGGCCACGAGGCCGGTCCCATCAA
CATCTTCGGGCGCTGCGTCAAACTCAACCAGACCCGCGTCCTCTCTACCCTCTTCCATCCCTGTCCTATCCCT
GAAGACAAAGGCTGCCAAGGCCGAATCTGTCACCAAAACCCGAGCCAACGAGACTACCGTCAGCAATGACGCT
ACTCCTAGTTCGCAAGGGATCCCGGCGGCGACGCCACCAATCACTCAGACGACCAACGACGACCGTGGAAGTA
ACCGAGAGGACCCCACACCCACCAGTGCCGAAGTCGACCCCACTCCACTGTCCGACGATTTGACAAACGCCGC
CACCCTCAGCAATGAGGCTGGCCCGCTTGCCGAAGACCCAACCTCGGAGGATTCCCACAGCCATCGCACTCAG
CTCCGTGAGGCCTACGCACTACTCAACGACCGTGCCGACCTCGCAATAGGCCAATTACAATCTATAGTCGACC
GTTTCGGCGAAATGCGATCCAGTATGCGCGCCGAGATGGACGAACAGGCCGCCGAAATGCACCTGCTAGTAGA
AGAAACAAGCAGATCATTGCGAGAACAAACCGACACGGCCTCCATGCTAGTGCTGGATGCCCTCGAGACCTGG
GACTCGCCGGGGGAATGGGAGAAACGACGAGAGGCGCAGAAGCAGGAGCTAGAACGACGACGTACAGCGCAGG
CTCAGGCCCCGTCCGTGTCCTTCCAGCTCCCGCCCTCGTCAGCCAAAGGAAAGGAGCCGGCACACCGTCCCAT
CACGCCGGTCCACGGGAAGTCTTCGTCTGAACGACTGAAACGGCACGTCTAGGCAACTCACTCAAGAGCGCG

CCACCGATGCCAATAGACACTAGAACGCCACCGACATTTCGCGAAGCGCCGCCACACCTACACACTGAGGGGC
GTCCCAGTCCTTACGAAGGGACAGGAAGGGAGAACAACGAGCCCACAGGGGGAGGCGGAGCGAGCGGAGGAGG
TGGTGGTAGCGGCGGTGGAGGTGGTGGTAGCGGCGGTGGAGGCCCTCCGGGAGGTGGCCCCCCTGGATGGCCG
CTCGGGGATTCGAGCGACGAGGACGAAAGCCATTCAGGCACCGGTTCAGGGCGTGGAAACAACGACGGCGCAG
AAGACCAGCCTGCACCTCGTGGACACCCTGCCAGGCACCCCGCGAGCCTATTTGCATCCTCGATATCCGGAGT
CGTGCAACAACGACGAGATATACAGTCTCGCGGCCCATTCGCCACCGCATCTACATTTCGCCAGTCCCCTTCA
GGGCTCGCACAGCTGGAGACCGAATTCACCAAATGGATCCGACAGCACCTTATGGACACCGTCGGGGTCCCAC
TCTCGAAAGACATACCCTCAAACAAGCAAAGCCTACCCTGCCCTCGCCGTACTCCGGCAGTGACAATATCGAC
ACGTTTACATCTTGGTTCCTGGATCACACCCGTTGGACCCGACTGTCGCACCTATGCGGGCCCAACATGGACA
ACGAGCGTCTCATGTACCTAGGTACAAATGTCAGTGGACGCGCGGCTGATTGGTACCGCGACACAATCGAGAT
TGAGATATCCCGCTGGACGCACGCCGGGGCTATTTGCGCGATGTACAAGCGGTTTATACGCCCAAGCACCAAA
CGCGACGCCGCAGCTCGATTCGACGCAGTACGGTACACCGTTAGTGGCGGCATCCACGGGCTATACCACGACA
TGATCCGATGGGCGAAAAGGATGCCCGTCCACCCAGACGAGTATACAATCGCCCGCAACTTCTACAACAAGCT
ACCGGACGCAGTACGCGAAGTGCTCGCATCCTACCATCGCATCACCCCAGAGGACTACCCCGACATCGACTTT
ATACGCGACATCGCCGTCGATACCGAGGACGGCAAGGCCTATGCTACCAAGAGTCATGCCGGCGAGACACACG
GTTCTCCCAGTACGCGCAACCAGTATACGCAAGGTACCACGCCTAGCAGGTCCCGCGAGGAGTCTAAGTTCTC
CAGGAATAGGGCCTCGAACACTCCACACGGCCAGGATCGACCGGCGCGCGCCAGCGACAGGCCAGCGAGGACA
GTCGCCCCGGGGCTACTCCTGCGGCGGCTGCAAGTCCTAACACAAAGAGGCGAGCTGAGAGGCCCACCGTTAC
CTGCTGGAACTGCAAACAGGTGGGACACTACGCCACGGACCCAGCATGCCCTGCGAATAAGAAACCGGCCCTT
CGACGCATGGAGGCGATGGACGACGGGGACGACGACACCGCCACTGGACAGGACGTCGATCAGCCACCTCAGC
AACAGGAGGAGGACCTCGGGGAAGACGTTGTAGGGCACCAGTACGAGTCATATAGCGATGTTGGCTCAGAATA
TATGGATTTCGATGACACCCATGACGACGCCGGAGAACGCGTGGAACGTATGTATGGCATGTCCATTGCAAGG
TACTACCACGGCGAGGACGAGCCTGAACTCAACGCGGAGCCAATCGCGAGCGATTCCGCCTCGCGCGTAGAGC
GCTATGAGGGGGAGAGCGACAGCGACTACAATGACCTACCTGCTCTAGTGGATGAGGAAGGCCGCCTAGACTA
TGGAATCGAAGGACAGTATTCGGGGCGCACACTCGTGGATGACGGATTCATCGAACCCGGTGTCCCCTTCATC
ACCTCTGACAACTTTGACCGTCTTTCGGGCAGCATATCCCACGCGTCGTTTACTGCAGGCACACGTCATCGTA
TACAGACGCTAGAAAATAGGACTCGCCTTCTACTGGCTATATCCACCGTCGCTGACTCGCCCTCCCCCAACCG
CATCGTCGACGACTTGTCGAGGCTAGGCGCTTCCCACGCCACAACGGCGCTGCCGCTCCAAGGAGCCGCCAAC
GCTTCCGCCATGTTTCAAGCACTATCGACGCAGGTTACAGAGCGCGACCCAGCCACCCAGAATCTCCACGACA
GTCTGATGTTCCACACGGAGACTATTAGCTTGCTGCATCGAGAACTCGGGCTTCTCGGCCGACTATTGAGGCT
CGATGTTGACTTAGACACGGTACGCGAGTACGCCATTCGTAGCCACCGTCGTGCGGCGTACCGAAGGCAAGAA
GAGGAGGAGCACTACGCCTTACAGAGGGGCCTCCTTGACCCCAGCAGGAGCAGCCAGCGTCAACGAAACCAAG
AGCGAATCAACTTTCGCCGACGCGTCCCATACATCGCGGATGAGGAAGAGGCCATCGCGCTATTGGAGGACGC
GCAACACAGACGGGACGAGCAGGATCCCGCCAACGAGCGGCTGTTTTCGCTCATCATGAAGCACGACCGCGGG
TATCGCACCGCACTCACGCCCGCGACCAACTCTCGAGAGCGCCCAGCGGGGCGCGGCCGCTGTATGACCTTCT
ACATTTCCTTTGGAGAATACGATGCACTGGTCCTCGTTGATACGGGGAGCACAGTGGACGCCGTTTCCCACGA
CTTCGCGAAGGTTGCCCGCTTGAAGACGTTCCCTCTGAGCAACCCTATCACACTGCAACTCGGATGTTCCGGA
TCGCGCTCCAAGGTGAATTACGGCATTCAAGGCTCGTTCACCTTGGGCGGGGAACACCACGAATTGTACTTCG
ACGTGGCCAACATCGATCACTACGACGTGATCCTTGGTGTCCCATTCATGGACAAGGTCGGTATGGTCATCGA
CTTCCGCAACTACACCATGCGCATTGGTTCGAGGACGTTTGACGCGTTGAAAAGGATTGACGGTGGCGTAAGC
AAGGTCCGAGCCAGAGCAGGGAAACCTACCGTGGCACAGACGGCGAAGGCGATCCAGCCGGCCAATTGACGGC
CAACAGTGGGTTCAACACCCACCAGGACACAAGACAGATCTCGTGTCCCCGATACCCGCAGCAGTCAATTTAT
TCACTCTCTCGCCGTATCTCTGAGTTCTAACTTGTTTGGTGCAGGCCGATACTGGCAATTTCCGAAACGGAAC
GTGGGGAATGATCCCAGTAATAAAATGAACCTGAAAATGATAAACAAGTGCAACTACAGGAAGTTTCAAGCAT
GATAAAACAGTTGAAATAGGGGCAGTATGACAGCAAAGTGAAAATGAAGGCCTGCTTTCCAAATGCGACATTT
CAAAACCAGTGTCCGCGAATAAAAGTTGACTTAGCGCATTTTCCAGCTCTGGGAGCAACTGAGGGGAGATGGT
GTTTGCAGTGTGGTGCTGCTAGACTGAGGCTGGTGTAGAGTGTTGGCCCTTGGTGTCTGGGGCCCTTGGTGCT
GGGCAGGCACCTTCCAGAAGTGGAAGGCCATTCAGGGTGCACCTGTTTAGCGCCTGTGCGACTGTTTAGCGCC
TGTGCGAAGAACACTGAATTGCCAGCCCTGTCCTCAGTTCCCATTTGTTCTCCATGAGACTTGGCAGGTGCCA
TGGCTGCAGGCACTAGGTTGTACGGTTCAATTCCCCCAGAGAACGGAGCAGTATAGCAGGCCCTGCAAGGGCC
CCTGTATGGGATCAAAACGCAGTAACAATCAGATAAGCGGCAATTGCCGCGATCACAGCAGATGTAATGCCTT
TGTATTCCCTGGCCTGATTCCCACGCTTTGGGCCGTTTTCGTGTCGTTACAAGCGCCCTAAATGCGGCGATCC
CAATTGTACCGCTCTTCACTCACTTCGTTCGTTCCGTTTCGGAATATATCTTCTATATCACACCGCGCCGCGG
CATCGCCATCTATCCAAAATGGACCCGCGACAAAACTATCAGTAAATCTTCTCAATCTTCTCTGCCTTCAGC
TTCCCTCGATATAAACCAGCCATATAAACCAAAAAATATACCTAATTCACTTACCAAAAAGCCTCCCGACCGC
GAACGTGTTATGCGAGAAGTACACGGCCTCGATAAGACCGCGGTACGCGCGAGGTGGTTTGACAAATTCAAGG
ACATCACAAGTGGCGTCCCTGAGGTCCTTCCCCCTTTGCGCGAGATAAACCATCAGATTTCCCTCATAGACGA
GAATGCGAAGTATTCCTACCACATGCCACGATGCGCTGAGTCCCTACGCCCACTTCTCATGGAGAAAATTCAA

CGGTACGGCCGCGCGAAGTGGTGGATTCACGCCACTACGCCACAAGCCGCACCTATGCTCTGCCTACCTAAGA
AGGATGGAGGCCTCCGTACAGTGGTCGATTGCCGGAAACGGAACGAGAACACCGTAAAGGACGTGACCCCGTT
CCCAGACCAGGAACAGATTCGAATGGACGTCGCGCGCGCACCAGTGCGTTCCAAGATAGATCTATTGGATGCC
TACGAACAGATTCGCGTAGAGCCGTCTGACGTTCGCAAGACTGCGTTCGCCACCATTTACGGCACTATGCTAA
GCAACGTCCTACAGCAGGGTGACTGCAACGGTCCCGCGACGTTCCAACGGCTAATGACTCACATATTCCGCTC
ACACATCGGCCTTTTTGTGCACGTATACCTCGACGACATATTCGTATTCTCGGATTCCATCGACGACCATGAG
ATACACCTTGAGATAATATTCAGCACACTGCGTGCCAATCACCTCTTCCTAAAGGCAGCCAAATGTGATCTTT
ATTCTGAACGGATGGACTGCCTTGGCCACCTAATCGACAACCGCGGGCTCCACGCGGATTCGGACAAGATGGC
CCGTGTACGGGAATGGAGGACTCCTCGCGACTACCTCGATATACAACGATTCCTCGGGCTGATCCAGTATCTC
GCGCAATTCCTCCCCGACATCTCTGCCTACATCAGCCCACTCACCTCAATCGAACGAAACGGCCACTTGTTCG
AGTGGCGGCCAATCCATGAGAAATGCTTCGAGGCCATTAAAGCGCTCGCATGTAAAGCCCCAATATTGCGACC
CATAGACCCTCGTAGAACCGAACCAATCTGGCTGGTTTGCGACGCGTCTATATCCGGCATTGGCGGATTCTAT
GGTCAAGGCGAAACTTGGGCGTCCTGCAGACCAGCGGGATTCATGTCGAAGAAGCTTACCGATGCTCAACGTT
CATATACGACACTCGAAGCTGAACTCCTGGCCATAATGGAATCGTTATTGAAGTGGGAAGACCGTTTGCTGGG
GCGACGAATTAGGATAGTCACGGACCATGAAGCCCTGAAATGGTTGAAGACACAATCCAAACTGTCGCGCAGG
CTTACGCGCTGGGTGCAGTACCTATCCCGGTTTGACTATGACATTGAGTACCAACCAGGCTACAAGAACATTG
TCGCGGACAGCCTGTCCCGCTACTTTGAGCAAGACAACGCGGGTGAGACTCACCACTTCGACGAGTACTCTGG
CGCGGATGTACGGCTCGATCCCGAGGGTGAGGATCTTCCCATGGACCGACTCACCGAGTTCCACAAGATACGG
GTCGGCCCAGGGCCCTCAGATACTCCGAAGCCCGCCAGGGGTTTGATAGCCAGCGAGAGCCTCACGGCTATAC
GATCCCTTGCACTACACTCGGAAAATCGCACTGACGAGGACCCTATGGCCATGGTACCGGACGAGGCCCTTGC
GCCGCTAGACGAACGATTAGAAACGGTGCCTACATTCCTCAGCGATGTCAAAGCAGGCTACGAAAGCGACTCA
ACGTTCCGCAAAGTCGCCGCGAATGTAGACCATTACAAGCAATTTTCCAGCGACGACGGCCTTCTGTATACGA
CCAATAGGCTCGGGTCGAAGGTCCTCTGCATACCTAGGGCGTGCCCAGACCGCAAACCGTTGATTGAGACTGT
CATTGAACAAGCCCATGTGCTTCTCGGCCATCTCGGGCATCGTAAGACAAGTGACTACGTACGGCGATGGTAC
TGGTGGCCTTCACTCGGAAAGGACATCGAGCGATATTGCGCAAGCTGCGGGATCTGCCAGACGTCCAAGACGA
TAAATCAATCTCCGGCGGGATTACTACACAGTCTGAACATACCAACGCGACCGTGGGGTTCAATTGCCATGGA
TTTCGTCGGGCCTCTACCGGAATCAGCTGGATTCGACTACCTGTGGGTGGTCATTTGCCGGCTTACGTCCATG
GTGCACCTCATCCCCACCACAACTAAGGTGCGGGCGTCAGAATTGGCCTGGATGTTCGTCCGCGACATTGTGC
GACTCCACGGTGTTCCCGAAGATATTGTGTCAGATAGGGACGCCAAATTCACGTCAAAATTCTGGCGCGAGAC
GCACCGTATCCTGGGAGTAAGGCTGCTAATGTCAACTTCGTTTCATCCGCAAACAGACGGTGCCACTGAACGT
GCGAATCGCTCAGTCACCCAAATCCTGAGAGCGCTAGTGCAACCCGACCAACACGACTGGGTCGCGCAACTCC
CTTTAGTCGAATTTGCGCTGAACTCTAGCACAAGCAAGTCCACGGGCTACGCGCCTTTTGAACTGAATGGTGG
ATTTATGCCGAAAATGACCACTGATTTCGGCAAGCAAGTGGCGGAGATGCCGGGGATACAGGAGTTCGCCGAG
CGCGCCAGGGATAATTTACTGCGAGCACATGATGCCATCCTGGACAGCCGGGTGGATCAGACTTTTCATGCAA
ACAGGAAGCGGCGCGACGAGAACAGTCACCAGGGTAATACGAAGCCAATAATGCCGGGTGACTTGGTATACCT
GTCCACGGCGAACCTGTCACTACCCGGCCAAAGGGCGAGGAAGTTAATGCCGAAGTATATTGGCCCGTACCCT
GTGACCGCCAGCCACCCGGAGACGTCGAACTACAAGCTGCAATTGCCTGAGTCCCTGGCGAAGCGAGGAATTC
ACCCAAAATTCCACATTTCCGTTCTGCGACGCCACGAACCTAACGACTCGTCAGTCTTCCCGCATCGGGATAC
CCAGGTATACTACGACTTCGGAGAAGATGAGAAGATGGAATGGTTAGTGGACGAAATCACAGCTCACCGCTGG
GACGGACAGGAAGTCGAGTTCAAAGTTCGATGGAATGTGGGTGACCAGTCATGGGAACCACTGACGCACGTAC
AAGAATTAGAAGCATTGGACAACTACCTTGACCTGTATGGCGTGTCAAGCTGGAGCAACTTGCCTCGAACAGT
CACAGAGAAGCCACTGCCAGCGACCAGGCGTCGTCGAGCCCCTAAGCGGGCGGACGCCTGAAATAGGGAACGC
ACATCTCGATTCCGGCGGTACTGGCGCTGGACGCCGGCCACTTCGAAATCCGAACCGCCAGCCGCTCGTTTAC
CATTCCTCTCTTTACCACCAACCACACCATCCCTTCCCCTCTTTCGCACACAACACAAGACAACTACATCGAC
ATTGAATCATATACAACATGAGCCGCGACAGATATCGCTATACGCCATTCGACCGATCCAACCGCGGACAGCG
CTACCAGGACGCGGGACAGGGGCCAAGCCAGTACATCACACTCGACGACAACGACCCAATGCTCAAGTGGGGG
TGGGACTCAATTCACAAGATTATGCGCCACATCCGCGGATGCCGTACATGTGACGAGTACCTGCGACATGTCG
AACGGGCGGCGGGCGAACGTACCTTCGAAGACGCTTCGCTCAAGCGCGAACTCAACATTCAGGAAGTTCACCA
GTTCGCATTGGAACGCGGGCAGCGGGAGGCGACGGAACACAAGGTTACCATCCGTCAGCTGCAGGAGGAACTG
GAGCGAGAGCGCAAGGACCTCGCCGACGCTAGGGCCGACCTCGCCACTGTCCGCGAACAGAGGGACAGGCTCG
AAGACGACCTCGAGGACAGCCGCAGAGGGCGCAGCCGGGCCAGAAGCACCTTTTGGACTAGGTATAAAGCAAT
ACTATGTAGGATATTTTACCGTAGGTCATGCATGGTCTAGTAGGAAATCCTACGCACCTCTCTGTAGGGTGTT
TTGGCCTGCGCCCGGCATGCCAGGCCATCAGCTGAGCTCGAAAATATGATGCGAGCCGTGCAGGCCATTCTTA
GAATGTAGAAAACATACCCAAAAGCGGTGAGAAGCCTTAAATCAGCTCGAAAAACCGTGGAAAAAGTTCAATT
ATATTCTGACACGTTGACAGTTTGATCAGGAGCACTTCCCCGCCGTCATAGATGCATCAATGGGGCTTCCGCA
CCCGGTTCCGCATCCCAGAATCCCGCGCAGCAGTAACCGCGCGCTCGCACTTGCCTTTTTTGGGCGGGATATG
TTGCGGCCGCCGCACCATACCGTTACGCGAATGTCGCGGCTGGACACCTTGGGGGGATAGAATAAAACATAT
ATATGTATATAGTACTACATACACACCCCTACGAACGTGCGTAGGGGCGGGGCGAGCCGAAATACAAAACACG

AAATTAAAATTCAAATAAAATGGAATAAAATGGAACCGCGTTGCACGGAGCCCAGGGCGGCGGGAGCTTGGCT
TTTCGGCATCCGTGAAGCGTCCCAATAATAAGTAATACGAGAGGAGGGCCGGGAGGGGCGCGGGGATGGACGG
AAAGCGACGGGCAGGCGACCGCGCTCGGGCGTGCCGTCCTGCGCTCGTTCTGCGGATTCCACGGCCTGGAACA
TGCTACTAGGGCTCTTATGCGCTGGGAAGACGTCTTCTGCGGGGTTCGAGAGGTTCCTAGGCGGTGCGGGGGG
CGTACCATGCGGCACGGCGTGTTCGCGTTGGTAGGGGCACGGCGCGCGTCGCGCGCGAGCATAGGGGCCTTGG
GGGGTATGATATTGGCCTCTGCAGTGGGTGGGCGGTGTGTAGGGCCGGGACTCGCACTGGTTAGCTGCCAGAG
GTGGAAGGAAAGGCGGGAGGAGGTCAAAACATAGAGAATAACGACTTACCCGTTTCGCGTCATCCTACACCCG
TCCCGCGTGCCCATAACCACCCAGCCATCGTGAGAGAAGTGTCCCCCAGTGCGTCCAGCTTGAATGGACGTGC
TGGGGGCCTCCTGGGTCGCGTCGTCGCCGTCTTGCTGTACGTTCTTCGCGCGGGTGATGGTGATCGTGCGGGG
TGTCGTGGACGGCCGTGGAAGAGTGAAAAAGCCGGTGACGGCGCGGCGCAAGGATCCCCCACTCGTCGCAGAC
TTGCGCGACGGCGAGGGGGAGAGGGGGAGACAAGGAACGAGGTCGTCGCGCAGTGAGAACAACCAAACGCGCT
CGGCCGAAGCGCAGGCGCCCGCGTCTCGGCGACCTCGGCGAAGATGAAAACGCAACATACGGGCTCTCCTGTT
GCGCGGAGAGCCCCGGAGACGTTCGGGATGAACTCAGCGTACCTGAAAACAGGTACTCCAGGTCTAAAATCCG
GGGTCCCTGTCTTCTGGCATGCTGGGCACGCTCCGAGGCCATTTCGCGCGGGATTTGCGCGAGAAACTGCGAA
TCCGTACGTCCTCTCTGCGCGTACTTTTCGTTTCCCCTGTTTCCACATCGTACTAATCCCTACATATTATATA
TGTACGGGTAGGTACTGATGATTAGAGTAGTACTACAGCCACTAGAATAAATTACAACCATTTATAAGTGCGT
CAAACCGAACATTCTGTATTGTGATCCCCTCTTGGGTCACCCTGTAAAGGAATCGCACAATCCCCGGCCCCCG
GCTGGTTGGAATCGCGTCAAATTATTCAGAGAGGTTTGAACGTATTATTACACCATAATGGACACAAACATCA
CAGGATTGGACGGGAGGCAGAGGCCGAAATAAATGCGTATGTTTGAATTTGTATGTTTGAAACATAACTTCGT
TATTTCTCTATGGATATTGTATGAAACAGGTTCTAGATAGCCGGAATCAATAGAGGTACATGCCAAAGTATTT
TACAAACAGAAACGACAGGGATACAGGGAGGGGGCGAGGTGTAGGAGCTCCCCGCCACCCGGTCAAATTAAGG
TTTGCTCGGGGTCTCACGCGTTTCCGCGCGCAATTGCGCGGCCGAAAATGCTCCCCCGGGACCAGGAACTGTT
CCATAAATGATATAAATACTCTGGGGACTCCTTGGAATATTTACGCGAATTTAGGAAAATGTTTTATCATCTC
GCATCGCACCTGGAGAATGTAATTATATTTTTTCTAAACCCCATAGGGAGGGTCTCTATACTAATACATATAC
ACTACAGCTCACGCGGTTTCTCCATTTTCGCTCAGTATGTATGGAATTGGGTATTGATCAGCCCATTGAATAT
ATTCTGGCGGTTCGCGCGCGTGCAAAACGGGCAAACTAGCTATTTGTTTTTGTCGCTATGCAATGTCAATCAT
AATGAGACCTACGCACAATTTATCCAGTATTTTTTGGAGGCCGGGGAGGCCCCCTGGAGCGAGCGCAAAGTTG
GAGGGCGGGACGTTTCCCGATTAGGAAATGAAGGGCGGGATCGCGCGCGCTGCGGCGCACAGCGAGCGGGTGA
GGCGCGCTGACCGGAAATGCGATCAGTGACATCAGGTACTGATTTCAGATCCCCTAGATGATGGCCAGGCACC
CACAGAAGCGGCCTGCCAGAGAAGATCTGGATCTGTCAGATCCGGCCCGATCCAGCTTTGTTTGGCCACCAGT
GGCCAAACAAAGACCGTGGGGGATATGTTTTCTGGGTTCGTCAGGGGGGCGGGACGGCTCAGGCTAGATTTCA
AACCCAGCTGATATCTCATCATATTGATACACCCCCAAAACAGCCCAAAGTACAGCCAGTAGGAAATCCTACC
AGACCATGCATGACCTACGGTAAGATATCCTACTAGAAATTACTTCAACCCTGGTCCCACAGCTCCCAGAAGC
CACACTCGTAGCAGGGGATCCAGCTATCGCAGCACCAGCCGGGCGCGTGAACGCCACCGATCGTATAGTGCGC
GCTCCCGCAGCCCAAGCCCTGGCCCCCGGCGCAAGACGAGATCTCGGTCACCCTCGATAGCGCGATCGGCGCA
CAATGACGTCGGAATGCCCGACGCGCCGCAGGACGCTACGCAGGACACGCCGGTGCCTACAACCCCCAGTCTG
TTCGAACGGATGAACATTGAAAGGCCGAACGCGTCACCGAGTCTCCTCAGCAGAATCGCACCCGCAGCTAGCA
GCCCTGAGTGTGCCGCCCCGGCGTTACTTATGAGAATGAGTGACGCGCCGCCGGCGCCGCCAGAGGGGGGGGG
CCCAACGGATAATGGCAACCAGACTCACAACACATCCTCGCTCCCGAGCCCCGACGATTACGAGCCAGGACTC
GGATTCTTACGCATCAACCAGGCGGAATCGACGCCAATCGTAAACGAGTCGCAACTAGGATGGCCAGGAACAC
TAGAGGAGACGCGCAGATTAGCGGCGGAGGCACAGCGGGATGGACACAGGCTAGCCCTCAAGTGGTGGGGCTC
ATTTCTACGGGCCGCCAACCTTAAAGAAGTCCATGGCGAGGCAGCAGACACCGCGCGCGCCATAAGTTGGAAG
AGACCTGCATGGGTGGCGGCTCAGCATGCCCTCCGAGCACAGAAAGCGAAGACCACAGTCACAACTGACTCGG
CGACGCGCGAGGCCGCCAATCAACCACCTCCATTCACGACGACCTACGAGCCGCGATGGAAGGAGTTCCTCAT
CCAGCACCCCAATTTCCGTCTTCCAGGCGTTCCGCGAGACCAAGACGGTGCGCTGGCTACTGGGCAGTCCAGC
TGTTTAACGGGATTTCTCCGCATCATGGAACTAGGACCGGCGCCCTACTATGGTCCGCGCATGCGCGAATGGC
TGCGTATTATTCTGGAAGTAGACTACGAAGACACCTCTCTACTTGAACGCATTCGCGACCTCCGCAACGCACG
ATCCTTCCCTGAAGATCGCCTATTATCCCACGCAAGTGTCCGCGAGTTCCTGATGTCGTTGGCTCCACCCGAG
GACGAGAAGAACAATGTCCCACGCAATGACAAAACTGTCTCCGCTATCGCCGACTGGGCTTACTCGCTATGCG
TTCTTCTCCCCGAACGCTTCGGGCTATCCGACTCGTCCATGAGTTCACTCTTTGGATTCGCACCCAAGTCCTG
ACGCCTACCGTGTACTCGCCGTTGTACCTTATAGCGAACCCCATTTGCAATCTACGAGACCCGCTCAATCACT
GTATATTGTGTGCCTTCACCCATGGTTCCGTTCTACCGGGGAGGGTGAAAGGGCGCGAACCCTTGAAGTCCAA
CGAAGTCACAAACTTCCTAATCAGGACGAAGGAAGCGCTCGGCACGCGAGCGCTCGGGGAGTGCCGTAGACAC
GTGGTGAAAGGCGGGACGTCCAGATTGGGACGAACTGCATAGTTCTAGCGAGTGGCTGACTATATAGGACTTT
TAGAATAGATAGTTAGACACCTTTTATCACTCGCACATACGCTGTACACTTTCCGGACGAATATACGTCGTAC
GGTTCTCACAGCGTGACGCAACGTGAATATCTGGTCTTACA
>Bjead1_1_Cluster2#LTR/Copia

GTTCAACGAGGCGCTCGGGCGGAAGGCTGCGTCGAGGAAGACAATGCCGATGAATTATTATGCGTACAGTAGG
ATATACAGAGGATGTGCGTACCTGCGTCGAGGAAGACAATGCCGATGAAGAGCAAGTCTCCTCGACGCGCGGT
TAATGGACAGACTGATTACATAAACTGTACAACTGTCCGAAATAGGAAACTGAAGTCGAACGGACGTGTTCAA
CTTCCAACACTCCCCCCGAACTCGGCACGTTTGAGTTCTTTCAGTCCAAGAAGGGCCTGCATGGACTCTACTT
TCTGTCGCGCCAGTGCTTTCGTAAGAATGTCCGATGGCATATCATTGGTGCTGATGTGTTCGACTCGAATGGC
TCCGCTCGCTACGGTGTCTCGCAACCAGTAGTCGCGCAAATCCATGTGCTTGACACGGCCATGGTGTTCGGGG
TTCTTCGCAACGTCGACGGTGGAGCGATTGTCGATGTAGAGGGTGGAAGGTCCAGGGAGATCATACCCAAACT
CGGTGAAAAGGTTTCGGAGCCATATGATCTCCTGTCCGGCTGATACGGCGGAGATGTACTCGGCCTCGGTAGT
TGATAGCGCCACCACAGACTGAAGGCGGCTCGACCAGCTGATGGCACCAGTGCCTACTTTTACGACGTAGCTG
CTGGTAGAACGGGCCGTTGTCTGGGTTTCCACCGTGGTCCGCATCGGAAAACGTCGTGCAACAAGGTACTTGT
CCCACGCAGTGCGTTCTTCGGCTGTGGAGGATGCATCCACAGGAAGAGGCGGTGTGCTGTTGCCGTCGGGCTC
AACATGGCGCCAAAGCCCAGCTCGTCGGAGGTGGGCACGGAAGTCGGCGTAGAAGGCGTTGAAGTTGCGTGGG
CCGAGCTTGGGGCCTGGGTAGGCCGGTGCGGAGGGCATATGGCGGAACAGCACAGGAGTGGGAAGAAGTGTCT
GATGGAAATTTTCGCCGCGAGGTCGTCGAGCTTAAAGGCTCATAACCTGTTCAACGAGGCGCTCGGGCGGAAG
GCTGCGTCGAGGAAGACAATGCCGATGAATTATTATGCGTACAGTAGGATATACAGAGGATGTGCGTACCTGC
GTCGAGGAAGACAATGCCGATGAAGAGCAAGTCTCCTCGACGCGCGGTTAATGGACAGACTGATTACATAAAC
TGTACAACTGTCCGAAATAGGAAACTGAAGTCGAACGGACGTGTTCAACTTCCAACAT
>Bjead1_1_rnd-1_family-175#TIR/MITE
ATACTGTATCCGCGCGACCAAACCGGCCGGTCAGGTGTCAGGTCGATCCCAGCCGGCCGGTTTCCGCCCCCAT
GTCAGGTGTCAGGCCGCCGCCAGCCGGTCCTGACATACACCATTGCAGCAAGGTGTACCCTATAATTTTGCGG
CCTGCGGCGCAAACGATGTGTGAGCATNATAAGTCAGTCGATAAACTATAANTGTGATGCTCGCAGTTGGTTA
TTCACTAGTAGATGGTTTGATATCCATCGTACCTGAGCTCTTAGAGAAGGATTGAGGTGGGTACCTCACTACT
TGTGGGCCGGGTGTCAGGTGTCAGGCCCTCGTCGGCCGGCCGGCCGGTTTGTACGAAACGGTCAGGTGTCAGG
TCCCCGGCCGGTTTGGTCGCGCGGATACAGTA
>Bjead1_1_rnd-1_family-318#LTR/Gypsy
GTGCGGTTCGCGCGTTCNGTGGCGCCGTCCGTCTGNGGGTGGAACGCNGTNGACATGAGNAGTCGGGCGCCNA
GNACNCGGTGCGCCTCCCTCCAGAACTTNGANGTGAATCGCGGGTCNCGGTCCGACACNATANCGTCNGGGAG
CCCGTGCAGTCGNACGATNTCCTCGACGAACANCCAGGCGAGCTCCGACGCCCGTACGGTAGTNTTGGTAGGN
ACGAGGTGCACCATGGACGTGAGTCGGCAGATGACGACCCACAGGNAGTCGAATCCNTCGGATTCCGGNAGCG
GNCCGACGAAGTCCATNGCNATCGANCCCCACGGTCGCGNCGGTATAGGNAGACTGTGCAGCAGTCCNGCCGG
NGCCGGGTTCGNCGTCTTNGTGGTCTGGCANACNCCGCACGTCGCGCAGNACTTCTCGACGTCCCGCGCCAGC
GTNGGCCACCAGTACCATCGNCGNACGTANTCGCCCGTCTTCCGNGTGCCCAGGTGNCCGATGAGCGCGTGNG
CNTGCTCGATGAC
>Bjead1_1_Cluster29#LTR/Copia
GTTTAAGTCTATTACACTGATCTAATTGATACGCACCCTCGTGGAATGCGTTCAACTACATAGGCAGCGCCTA
TATTTCCTCGCAGCCGCGAGATGAGCCCAGCGGGCGACGTTCCCTCGCAGCCGCGAGATGAGTCCAGCGGACG
TCCCGGGACTGTTGTGAAGGTTTCGATCGGGTCTGCCGTGGTCGCGGCGGATGGGCTCGATCGAATGGGCTCC
CTTCACAAAGGGCGCAGGAGCAGTTAGGAGGTGCAGCGCACCTTGCCTCTCGACTGCTCACTGCTGCCCAGGT
CCGTATCGTATAGCTGCTGCACCATGCCTCGTATGCTCACTACTGCCATAGCCACGTTATTTCGATCTCCCTG
TCGTACTACTCCCATCTTTCGAATCAACTTCATGACCGGAAGCCCCTTACCCGATGCATCCCAATCATCTTCT
GCCGCGGGCGCGGGATCCGGTACCTCTGGAGGACCGCCTACAGGACCCACCAACGACCTTGACACCGAGTTTG
TTGGGTCGGTTGTCCAGCGATGCGACATCATTGTCGAGGATTTCCGAAAGGGGAAGATCAACAAAGCGAAGGC
CATTATCGACCTCCTCGAAGCATTTGCTTTCGAACGTTCGCGCTCCGCCGAAGAGAAAGAAATCAAGCGAAGC
GCTTTTCAGGCCTACACCGTCCAACTTGACGAAGCGGAGACGGACGAAAGGCGTGCAGCTGAACGAGGGCGAC
AACCTGAATCAACAGGGGCGGGGGTCCGGCCTTCTGGAGAAGCTGCCGAAGCTGGACCTCCTGAGCCGGATTC
AGACGAAGTGGGGCCGAGGGCAATCACCAGACGAACCCGAGGCACAGATGAGTCAGAGGACGATGGAGATGAG
TCCGACGAACGTCCGAGGAGGAAGAAGCAAGTCGACGAGTCGCTCTTCCCCTTCGGGCACGACGGCGCTATCG
GAAACCTATCTCCAGTGCTCCGGAAGACGCTCACGCTCAAGGCCAACTATATGCGAGACCTCCCCCTCTCCAA
GCAGACAGTGCTCATCCAGCCCAATGTCCCGTCACTCCCAGACTCACTCTGGAATGACGTCCTCAAGAATGAC
TACGTCGATCTTGACAAGATCTTCGCGTCAGTCTTCTCCATCAGCTCGGACCCCAAGCAGGTCCTCAAATTCG
GCGATCTTGAACTCGAGGCCGTCGTCCCACGCGTCGAGCGCAAGATTGTTCGTCACGGAGATTGGACAGTCGC
ATGGGCAAAGTACGAGAAAGCGGTCTACTTCACCTACCCCCACCGACTCGATGAGCTGCGCGAGTACTACGAG
CATGTCACCGGACTCTTCACCAGTGTTGACGTCAGCCAAGAGTGGCGTGTCATCAATTACGACAAGGCTATCC
GACAAGAAGCCGGAAAGTCAAACGCCTACTTGCTCACCGATTTCGGACGGTTCAACAAGTATTTCAGCCGCTT
CCTTGTCGGCTCGGGAGGCAGAACTCAAAACACTGCTTCTCAAGCGGAGAGTTCAGGCCGAACTCAGAAGTCA
ACGTCGACCGAGATCTGTCGCAAGTACAACGACGGACGTTGCTCATCTAAGCAATGTCGATATCGGCACGTTT
GTTCCAAATGCGGAGGAAAGGATCACGCCGTCACCGCCTGTTCCACTGCCCGATCTTCTGAAGGGAAGAAGTG
AGCGGCCTCGTTTCCTACGGGGCTTCATCTGGGAAGCAGCAGAGACTTCCGTAACTCCACTTGCCACATTGTC

TGAAACGCTGCGCCCTCTTCCTTCACCTCCCGCAAATTTCTTGAACGATCCGATCACCAACGCTACTCTCGAA
CAAAATCCAAACCTCTTCCGAGTTGTCACTCCCATCAATGTCGACCGATTCGAGGCACTACTCACCGATCACC
CAAACCGTCCCCTGGTCGACTCGGTCATCCGCGGACTTCGCTATGGATTTTGGCCATTTGCTGACACAGAGGG
AAAAGATTTCCCAGAGACTTGGGATGCGGGGAGTCCCGAGTTAGACGACAACGCACAAACATTTGTGGAGCGC
TACGTGCGCGAGGAAGAGATAGCGGGTCGATACTCTGAGCCCTTTGGACCGGACCTCTTACCTGGGATGTATT
CCATGCCAATCCACGCCGTTCCGAAGCCCAACTCCGACAAGTTGCGATTGATCAACAATCATTCGGCCGGAGT
ATGGTCGCTGAACGCCATGATTGACAAGGGCAAAGTGGGAATGCGACCTGATAACGTTCAAGACCTCGGTAGA
AACCTGGTCCGGTTCCGCAACGTACACGGTGACGCTTCGGTCTGGCTTTTCAAGTCAGATGTGAAGAATGCGT
ACCGTTTGCTCCCGATGCACCCGCTGTGGCAGTTGAAACAAATCGTCACGGTCGACGGACAGCGCCGAGTCGA
CCGATGTCTTTGTTTCGGCAGCCGAGGATCTCCCGATATCTGGTGCTCGTTCCAGGCACTCGTGGTCTGGATC
GCGATCCACGTCAAAGGCATCGACGACCTCCTTGCCTACATGGACGATACCTTCGCTCATGACGCATCCCGCC
ACCTATGTCTCTACGAACCGTACCAAAAGCTCATGCCCCCGAAGCAAAGGTCGCTTCTGCAGCTCTGGGACGA
ACTCGGCATTCCTCACACACCGGAGAAACAGCTTCACGGTCGCTCTCTCACCATCATCGGATTCCATGTGGAC
ACCACGACGATGAAGATCACGCTAGCAGACGGGAAGAGAGAGGAATTAGTAGAGGGGATTAGGTCTTTCATCC
ACGATGCTCAGAAGCGTCGCCGCACTCTGCGCGAGTGGCAGCATCTCGTTGGTTGGATTAACTGGGGTCTCAA
TGTCCAACCACTGCTGCGCCCGGCTTTACAGTCAACTTACCAGAAGATCGGAACGTTGAAACGCCCCCATGCC
GCTGTCTGGCTCAACGCAAGAACAACCCGCGATCTACTGTGGATTGCCGACCAGTTTGCGCGTTCAGACGGCA
TCTTTATCTTACGATCTACGGTCTGGCGACAGAACGACGCTGATCTCATTCTCTACTGCGACGCATCTCTGCA
TGGACTCGGTTTCTGGTGCCCCGCCTCCAATCAAGGCTTCATATCCAACCTCCCCGCTGCTCCAAAAGACGTC
GAGGACAACATATTCTGGTTTGAGGCACTCACCGTTCTATCAGCTCTCGAATGGGCAGCGGCTCGCAAGGATC
GCCCCCGTCGACTCATCATATACACTGACAACTTGAACACGGTGCAAATGTTTGACCGCCTACGTGGTGCGCC
AGTCTACGACGAACTTCTACTACTGGCGTGCGACATTCTCATCACGCACAACATTGACCTTCGAGTATGGCAC
ATACCTGGCGTTCTCAACGTCATCGCGGATGCTCTCTCTCGGCAACTCTTCCAGGTGCTCGACAACTACGTCC
CTTTCCTCGATATCAAACCTTTCATACCCCCTCAATTCTCGCTGGGGGCACGCCGCCAATGATTTCTCTCTCG
TCCTCTTCCAGGCAGCCCGTTCGCCCTGCCTGGTCCAAAGAGAAGCTCGTCCGAGAGCGAGCAATCGCACTTG
GTCATGCGATCGACACGTCCACTTTGGCTACGTACACATCTCACCTGCAGTCTTATCTCGCATTCTGCAAGGC
ACATGGATTCCCGATTGAACCCACGCCTGACACTCTATCATTCTACACCGTCTTCATGTGCCATCAAATCAAA
CCCGACTCGGTTGCCTCATACCTCTCCGGCATCTGCAACCAACTCGAACCTTTCTTTCCCAATGTCCGCATCG
CTCGTCTCAATCCTCTCGTTGCTCGCACCCTCGCCGGGTGTAGACGCATGTTCAACTCGCCGACGAAACGCAA
ACGCGCACTCACGGTAGACGATATCGAGCGTCTGCTTAGCACCTACACACCCACCTCCCACGACAACCTCCTC
TTCCGCGCGATGACACTCGCTGCCTTCTTTGCTCTCCACCGACTCGGAGAAGTTGCCTGCCCTGACAACGCAG
CTCTTCGCTCTTCGAGGAAGATCATCCGCCGAACGACACTGTCTGCAGACGGAAACTTCATCCGATACGTGTT
GCCAGCGCACAAGGCTGACCGGTTCTTTGAGGGAAGTACAATCGTGCTTGCTCGACGTCTGGACGCACTTGAT
CCCTACAGTGCTCTCGTCAGTTATGCTCGCTCTCGCGACCACCTCTACCCCTTCCGCTCCGAGCTCTTCCTGA
CAGCCTCCGGCACCGTACCCACGAAATCCTGGTATCTGGCGAAGCTTCATTCGGTCTGTGACGACGATGTCTC
CGGACACTCGCTACGGGCCGGTGGTGCAACTTTCCTTGCATCCTTGGGATGGCCAGACGACCGAATTCAAGCT
CTAGGACGGTGGTCGTCGAGCGCGTTCAAAGTCTATATCCGGAAAAACCCCGTCGTCCTTCAGGCGCTTCTGC
ATTCTCATGCTCACACGCCAGCGGCGTAGTATATGTACTCACTTCCTCTCACCACCAACCCACCCCTCCTTTC
CTACATACCATACACCAATACGGGAGCCCATACACGCCAGCCCCGCGACTCGCGCTAGTCTACATCAATTATG
ATTGACCGACTCTTCGGAGCGGTCGTTTCCGGCTACACGGCAGTCGCGACCTCTCAGCGAGAGCGTCAAAGCC
AGTCTCCTGCGGTGAGGAGTCAATTAGTTCGGGTTTAAGTCTATTACACTGATCTAATTGATACGCACCCTCG
TGGAATGCGTTCAACTACATAGGCAGCGCCTATATTTCCTCGCAGCCGCGAGATGAGCCCAGCGGGCGACGTT
CCCTCGCAGCCGCGAGATGAGTCCAGCGGACGTCCCGGGACTGTTGTGAAGGTTTCGATCGGGTCTGCCGTGG
TCGCGGCGGATGGGCTCGATCGAATGGGCTCCCTTCACAAAGGGCGCAGGAGCAGTTAGGAGGTGCAGCGCAC
CTTGCCTCTCGACTGCTCACTGCTGCCCAGGTCCGTATCGTATAGCTGCTGCACCATGCCTCGTATGCTCACT
ACTGCCATAGCCACGTTAT
>Bjead1_1_Cluster35#LTR/Gypsy
AGTAGGGGCGTCGGAGCGGGAAGGGTCAGGTCGGAGAGAGCGCGGATCGACGGGCCGAGGCAGCCGAGCGTAG
CGTAGAGTGACCGTATTTGGAAGCATATTATCATCACCTCTTCTGCCATCTCACCGTCGGACTGAGAGGCTAC
GGACGCCCAGGCCTCTCCATCTGGTGACGAGAGTCGATTCGACACCACGGCTAAGACTGAGCCAAACAGCTCC
GAGTCCGAGAGGGACGCACCGCGGCCGTATCCGAAAAAGAAACCGACGCTGCGACGACACTCCACATCGACAG
TGGCGACAAGCCGATCGACTCAACCTCGCAACGCTCGCGGACAGTGGGTCCGGAAGTTGACCGCATCTACATC
ATCAGACGAGGTCGAGGACACCCTCACATCGTCAGAACCGTCGAGCGAGAGCAGCATCGAATTATCCGGCAGC
GAATTCGTACCAGAGTTGCTACCGCAGCGCAAAGAACCCACGACACCACCTAAGACCTCAAGGCCAGACACAG
CAGACCAGAAGACCCCGAAAGCTGTACGTATACACAGGAGAAGCACGGTCACCCAACGATCAGCCACGGCGAA
CACCAATCAACCGAGCCCCGCGACGACTACAACGACTCGACGGCGCAGGATGGGAGAAATACAGGACTTTGGA
GGGGGCATCAACGGAGTCGATCCAGAGGCATGGGTTCGGAAGCTCATTGCCTGGTTCGAGCCGGGGTCCAAGG
ACGAGGACAAACTGCTACAGTTTGAGTGCCACCTCATCGTGAACAGCGTGGCTGACGAATGGTACTTGCAGTG

GAAGACCACGAATGCGTCGAACGCGACGTGGAGCGCACTCCTTGGGGACTTCCGAGCGCGGTTTCCCAGCCCA
GGAACTCAACTCTCTCCCAGGGAGGCGCTCGAACGCATCCCCACTCTATTTCCGCTACAGGACGACGACCTAG
GACAGTACATCCAGCTCGCCAACGGGCGGGCAATGAGCAGTCACGAAAAATGGGTTCGAGAAGTTACCGACTA
CGCCACGCAGCAGAAGATACCCGAAGACCGTGCGGAAGACATCAGGGCGTCGGTCATGCCAGACCTTCTGCAA
GACATGGTGAAGGCGTCGTCGGTCACGGGATGGAGCACGCTGAAGACGGCAGTGGAGGCGGTGGATGCACGCG
AGCTGAGAGATAAGGTGCGCAGGAAGCGGGAGGAGGCGGATCGAGTAGCAGAGAAGGACAGGGCAATGGCCAA
TGAGCTCGCCAAGTTGAGGGCGGCAATGAATGCGCTCAGCGTCGCCACGCAGAAGACTCCAGCACCATCGACA
AACCCGGCAAGCCGACAAACTGCGTACACCGCCGCACCAGCAATGGCACGACCCGTGCGTGGCTCGCGCCCGG
ATGCGCAAGCACGATTGGCAGCGATGAACGCGAACGCACCACAGCATCACCCCGACACCGACGCAGGCAGAGC
GGCGTATGCAGAGCAAGTCCGAGCATGGAGGGCGCGCAACCCAGGCGGGAGGCCCTCAGAACATGCTCCGTTC
CCACTCTCACCGGGTACCGCAGGGGCAGGCGAGAATGAGTGCTACAAGTGCGGCGCCGTAGGCCACATCCGTG
GCAGTGGGTGTCAGCGAGCGCACGTACCAGCGGAGGAACAAGAGTGGCGGGCGATTGCAGGGAGTATCTCAAG
GGAGATACGGCAGCGGGAGGCCTCGGGCGTTAGGGCAGTGTGGGAGGACGGAGCGCAGTACACGCAGTACGCG
CAGTACGACGACGTTCAGGGGGACCAGGGAAAAGAGTAAGGGCTGTCTGCCGCGCGAAGCAGACAGCCCAAAC
AACGCCTACGCATACAGCAGAGGTCCCCGGGCGAGGCGCGCCATGGTCTGGGGACAGCGACTGGAAGGCGGCG
AGTATAGACTACAGCGATCAGGTGTATATTGCATCTCTCATGTACGCCGATGATGATTCAATTTCAGACATCC
ATTCCACCTATACCGCCGCGACCAGCCCGTCGGGCCAGCCCGACGACGCCACAAGGCCGGCACGGCCGTTCAT
ACACCATATTGCGCTCCAGGGCCCCAATGGAGAAGCCGTAAGAGTCAAGGTTCTCATCGACGACGGGGCTATG
GTCAGCGTCATGTGCGCCAGCCTATGGGAACGCATCAAGCACCGACTCGGGCGGCTGGATCCTTCGCAGCGAC
GACTACGAATGGCAAATGGGCAAGTCGTCAGATCACAAGGCCGGTGGGTCGGTAGAGTGGAATGGGGAGGCAT
ATATCAGCGGTGCGCTTTCGAAATATTCCAGAGCGGAGGAGCATGGTCATTCCTTTTAGGGAAACCCATGCTC
GCACGGTTCAACGCGTTGCACGACTACACACCTGGCTGCGACCACATCTACGTGCGCAGCGGCGCCAAATGCC
AACTCCTCACGAACCAGCTTGGACGCACACCGGATACTAGCGATGGGGTCAACGTAACACTGGATATCAAGGA
CATACGACGGGCGACGGGCAACACAGAAGAGCCGGTATGGTACATAGGGTCGAACGCGCCACCCAACGTGGAC
ACAGACGACCCCGGCGAAGAGGTACCTGACATGTTTCCAGAAGGGGATAGGACACTGTACACAAGGCGCACGG
ACCCATTCAAGCCACAACGAGTCGAGGCAGTGCTCGACGCGATACGGATTGGAGATGACCTTACCCCAGAGCA
GCGGCAACAGGTACGTAACCTCGTTACCGAATACGCGGACTGTTTCGCGCTCTCAGTCAGCGAGGTTATACCA
GTTCCGGGAGCAATACACCGCCTCAACGTGCCGGAGAACGCCACCTTCTCGAGGAAGGTGCGCCAGAAGACGC
TCAGCCCGCCGCAGAAAGCATACCTACACAAGAAGATCGACGAAATGCTCGATGCGGGTGTTATAGCGCCATG
TCATCCGAGCGAGGTCAAATGCGTTTCGCCGATAACGCTGGCCAAAAAAGCGCACGACAACGAAGGGCTCACA
CTACAAGAACTGCAGTATCGCGTCGAGGAGCAATGCCGGGCAGCAGGGCTACCACCGTCCCCAGAACCAATTC
CGATCCCGCCGCAACGGAGCGAGGCAGCGCAAAAGACACCAAGCGAGCCGAAGTGGCGGATATGCCAAAATTT
CGGCCAAGTCAACAAGGTCACGGAGATTGCGCCGATGCCGCAAGGCAATATTCGAGCAAAGCAGCTCAATCTG
AGCGGACGACGGTGGGTAAGCGTGGTTGACTTCGCATCCGGATTCTACGCGGTAACCGTTGCAGAAGAATCTC
GACCCTATACGGCATTCTATGTAGAAGGGCGGGGTTACTTCTGGTGCGTGCGCATGCCGTTCGGCCTAACGGG
GGCTCCGTCCACGTTTGCACAACTAACAGCGGAACGGCTACACGACCTACTGAGCGACGGCACAATCGAGCTA
TTCGTAGACGATGCTGGAGCTGCGGACGACGATTTCAACAGCCTGCTAGAGAAGATGCGACGCATATTCCACC
GGGTGAGAGAGCGACACCTCTCACTGTCAGCAGCTAAAGCACAGTTGTTTATGACAGATACAGTGTTTGCCGG
ATCGACCGTAGGCACAGACGGAGTGCGGCCGGACCTTACGAAACTCACGGCCATTGTCGACTGGCGACAACCC
CCACACGCACTCAATCTGGCTTCGTTCCTCGGAGTCACAAGCTACTTCAGAGACCTGATACGCGACTATGCGA
AGCACGAGGGCCCATTACGCGACTTGCTCAAGCAAGCTGAGCCGCCGAAAGGTGCTTCCAAGGCGGTGTACAG
ACGAGCACTACAGCAGTTCGCGTTGGACGAAACACGATGGAAGATGGCGCACACGGCCGCATTCCTACTTTTG
AAGAAGAAGTTGACGTCAGAACCGGTGCTACGACGACCACTCTGGGACGGAACACCGTTCATTGTCACATCCG
ACGGATGCAAGGACGGCTTCGGCGCAGTCCTAGCACAGAGGTCGAACACAGTGCTGACAAATGGCGAAATGGT
CAAGCGGGTTCACCCGATCGCGTACGCGTCGAAGAGGACATCTCGATCAGAGGAGCGTTATAAGCCATTTCTT
CTCGAGTTTGCTGCGCTAAAATATGCATTAGACAAGTTTGGCGACACAATATGGGGATACCCAGTCGAAGTGG
AGACAGATTGCTCCGCGCTACGATACGCACTCACAAACGATAAACTCAACGACACACACGCACGTTGGCGCGA
CGGTGTACTAGCACACCAGATCGTCGACGTGCGGCACATACCAGGCAAAACAAATCTTGTCGGCGACGGCCTC
AGCCGGCAGGGGGAGGGACTATCGAAGGAAAGAGGTGACGGTAGCGAGTGGACGGTCGGCGAGGATTGGGAGG
AAAGCCGCGGCCTGGTGAACGACATGTTCGCCATCGAGATTGACACCACGCCGCAGCAGCGATTCGCTAAGTC
ACCGCTTTTTCTGCAAGTAGTGAATGCCCTGGAGGATATTGATGACCCTGGCATACCAGAACGAGCAAAACAG
CGAGCAAAGCACCGTGCAGCGCAGTACATGCTCGTGGATGGCCGACTTTGGCGGCTTCGGGGAGGCGCCGCGG
GACGACCAGCAAGCCGGGTCGAGTGTGTCAACAGCGACGAAGCGAAAGCCCTGGCGGCAGAACAGCATACCAC
CGGTGGACACTACGGACGCGACGCAGTCAAGATCGCACTCCTCGACAAGATCACATGCCCGGGGCTCGACACC
ATCATTCTCGACACGATAGCGGAGTGCGGGCACTGCAAACACTTCGGAGCCCCACATCTTCACGCGCTGATGG
ATCCAATTACTAGACAGCAACCCTTCCAACTCCTCGTCGGGGACTACCTGTCGCTACCCAATGGCAAAGGGGG
ATTTCACACGGTCGGGGTCTACGCTGACGTCTTTGCACAGCGCTGCTGGGCATTCAAATTCAAGACGGCCGGC
AGTGCGAAGACCACGATACAAAGCCTCCGCACCATATTCACGCTATTTCGAGAGTTCAACACATTCATGTCCG

ACGGCGGCTCGCACTTCAACAACCAGGAAGTGCGGGAATTCTGTAGCCAAAACGGTACCAAGACACACGTAGT
GGCGGCATACTCGCCCTGGATCAACGGGCTTGTCGAGAACTTGAACAAGCAGCTACAGCATGTGCTCAAGCGG
CTTTGTGCTCCCGACTTGGATGCCGATCCGCACGCGCAGGCAAAGCCAGGCGCATGGCCAGACCACCTGGACG
AGGCGGTACGCATTCTCAACAACCGGACGCTACCGGGATTGCGACATTCACCAAATGAGCTTCTGTTTGGACT
ACCTCGGTACGCAGCGCCGAATGGGCCGTTGATGCTGACAGCGCCGCCTACAGAGACCGACGCACACATCCAC
ATGATATTTGCAGATCAAGCACGCGTGGATGGCGGTGCTGCAGCAGCGGCGCATGGGAACACCCGCAAAGCGG
CGTTCGACAAGCGTGTGAGAAAGAGTCGAGCCGGGCAAGTAATCTTTGCGGTCGGAGATTTAGTGCAGATTCT
GCGGAGCGACCTGGACTACACGTTCAAAACGGAACGCAAGCTGCTGTCCAAGTGGTCCTCGCCAATCCGCATC
ACGGGCAGTACCGGGAACTCTTATACACTAGCTGATCTCGCTGGCACTAGCATCGCTGGACGGTTCAGCGCAA
GACGACTGCGCCACTTCCATCCCAAGCCAGGATCAGGGCTGGCAGCAATGCAAGGCGAGTTCCTTGCCAACCT
CCGCCAAGCAACTGACTCGGTGGAGCAAAGCAGGGCCGCTCCCGAACAGAGCTCGCGTAACTGACGGCTAGCA
ACGCTTACGACACCGTCGACACGGTGCAGGCCCTGAAGGCACATTACGACACCACGGCATTAGTTGCAATCAC
GGCCACGTCACTGGCATAGCAGCAACCGTACACACTATTTAACGACACTCGTTTAGCTTAGACACCCAAACAC
GCAATGCCCCGCCGCCATATCCGTACCCTCATCCCAGCCAGACCAACTCCCGTCCTAGTTGCACGCACTGCAA
CAGGCAATCCAGTACTCCACAGTCCGTCCTCCTACATCGTTGTCGCCCAGCCGCCTGCCCAAATCGCAACCGG
TGCCATCCTGGCTATCCAACGCGTGGGTAAAGGCAACAGCGCCAAGTACGCGAAGGCACACGGAGCTGTTGCA
TTCAGTGCCAATACGGCGACTTTCAAATGTGCGCGCACGGACGACGGCGAAACACTTTACCTCGCGGTCCCAT
ACGAGTTCGCAACGCTCGACCTGGGAGAGACGCTCCTGCGTTGGGTCACAGCATTTCTGAGGCGCCAGAGCAT
CGAGCTCCCGCCGACACTTCCGGTATGGACGAAGAAGATCGAGACGACGTGGGAGGAGTACGGGAAGGAGAGA
GTTGAGGACGAGAAGAAGCCAAAGGAGTGCGAAGGAAGGAGGTCAGAGGCAACGTTGGTAGGCGACGAGGACG
CCGCCGTTTTTTTAGGGGGGCACATGGAGTAGGGGCGTCGGAGCGGGAAGGGTCAGGTCGGAGAGAGCGCGGA
TCGACGGGCCGAGGCAGCCGAGCGTAGCGTAGAGTGACCGTATTTGGAAGCATATTATCATCACCTCTTCTGC
CATCTCACCGTCGGACTGAGAGGCTACGGACGCCCAGGCCTCTCCACC
>Bjead1_1_Cluster18#LTR/Copia
GTTGAGCTGATGGCTCGCTCATGGACACCGGACATGTGATTGGACTCATCGGACTTGATCGGCTTTCCTAGTA
CAGTGATCCTTGTATTCCATTTTCACATGCTAGTTGTATTAGTTTTCCCGCGACGCCCATTGGCTGGCTATTT
CCACTCTCGTTTGTACTTGTTTACTTTTCTCTTGGCGCTCTGTAGTATATATACCTGGACGCTTTGTAGTGCA
CACCATCGCTTCGTTTCCCTCCGCGGTGGTGTGAGTACCCTTATGCCCTATGTTGTATGCCGTATGCTTACCC
GTTACCCCTAGTACTTACGCCCATGTGCTTGTACTAGGTACCGTTATATTATCGCTTCGTTTCCCTCCGCGGT
GGTTACTTACGCCCGTGTGCTTGTACTAGATCCTGCGTTATCTCGCCAGCGTGCTGTACCCCAGCGCCTCCCA
CTTTTCCCTGCCTCATATTCCTCTTCCCTGTCGGATACCGTCTCGCTCAACGCCAGGCGGTATTCGGGTGTCG
CTTGTGTTAGCCACGCCATCGCTCAGTCGCTCAGGCCACTGTTGCTATTCAGGCTCTACCCTCTCGATAGGTC
AGAGGTCAGCAGGACGTAGTAGAACGGTCGACGTTCTATACGGGAATCACACGCTTCATCAGGTTATGAGCCC
CGAGCAGCTCTGACCAGGATCATCGCACGCTCCACACATCGCACCCCGCACGCACCCCCACATCTGCCACCAT
GACATCCGTCTCCGCTGTCCAAAAATCCGCCTTCGCGTCCACCACGTCGACCGCCCCGACCGTCAAGATCGAC
CAGATCGAGAAGCTGCGCGGTAGTGAAAACTACGCAAGCTGGGCCGTACTGTTCACGGCCTTCCTCGAGCTGA
ACGAATCCGAGGGCTTCATCGACGGCAAGATCACCGTCCCCGCCGACCTCACCAACGCCGACGGCACCGCATG
GCTGAAGGGCCAACAAGACCATCAGTTCTACCTCCTCAGCTCCGTCGACAGAATCTCTGGCATCTCCGACATC
TACCTTCGACGCGGAATACTTCGCGGCACACGAGGCCTGTCAGCAACTTCGTTGGCTCGACGTCTTTTCTGAG
CAAATCGAACACCCGCTCGAAAAGCCCGTCACACTCTACTGCGACAATTCGTCGACAGTCAGTGCCTCCGAAC
GGCCCAAAGTGAAGCACCGCAAGAAGCACACCCGCATTATGGCGCACGCTGTGCACGAATCTTTCGAGAACGG
ATTGGTAAAACTCGAGCGCGTCGCTACCGAGGAAAATATCGCCGATATTTTCACCAAACCTCTTCCCAAAGAT
CTACACGAAAAGCATACGTTGGGACTCGGTCTAGTCCAGTATCGCTACGCCAAGGGGGAGTGTTGAGCTGATG
GCTCGCTCATGGACACCGGACATGTGATTGGACTCATCGGACTTGATCGGCTTTCCTAGTACAGTGATCCTTG
TATTCCATTTTCACATGCTAGTTGTATTAGTTTTCCCGCGACGCCCATTGGCTGGCTATTTCCACTCTCGTTT
GTACTTGTTTACTTTTCTCTTGGCGCTCTGTAGTATATATACCTGGACGCTTTGTAGTGCACACCATCGCTTC
GTTTCCCTCCGCGGTGGTGTGAGTACCCTTATGCCCTATGTTGTATGCCGTATGCTTACCCGTTACCCCTAGT
ACTTACGCCCGTGTGCTTGTACTAGGTACCGTTATATTATCGCTTCGTTTCCCTCCGCGGTGGTTACTTACGC
CCGTGTGCTTGTACTAGATCCTGCGTTATCTCGCCAGCGTGCTGTACCCCAGCGCCTCCCACTTTTCCCTGCC
TCATATTCCTCTTCCCTGTCGGATACCGTCTCGCTCAACGCCAGGCGGTATTCGGGTGTCGCTTGTGTTAGCC
ACGCCATCGCTCAGTCGCTCAGGCCACTGTTGCTATTCAGGCTCTACCCTCTCGATAGGTCAGAGGTCAGCAG
GACGTAGTAGAACGGTCGACGTTCTATACGGGAATCACACGCTTCATCAG
>Bjead1_1_Cluster34#LTR/Gypsy
GAAAGGACGACGGAAACAGCTACATACGCGTGTACCTGTGTTCTACGGACTTATAGGCGCCTGAGCGAACGGG
CGCCCGGCTCAAGCCGAACGCACGTGGCCAGCGCCTGGCGGGATTCCTAATCGGGAAGAACTGCACAGTTCCG
CCCGATTCTGCGGTGGCTGAGTATATAATCCTAGGCAGAGTAGATAGTTAGACACCTTTTACTACTCACGATA
ACGCTGTACTCTTTCCGGACGAATAAACGTCGTACGGTTCTAGCAGCGTGAAGTGAGTTGATATTTGGTCTTA
CACTTTTTTGAACCCTCGTACTTTTTCACTCCGGACTTTGCACAGTCCACACCCTCACCACCGTCCGAACACA

TCCCGTCGACCTCCGAGCCTCACTGTTTCGCAATGAGTGCAACCGCGTCGACGAGAGTTACTCGGAGCGCCGC
ATCACGAATGGCATCTGATGCGCCCCAGGGCGCACCACCGCCGACCGCCACGAGGCCGCTCCCATCAACTTCG
TCGGGCGCTGGTTCTGGTTCGAACAAACCAACCTCTGCTCTACCATCCTCGATCCCCGTCCTATCCCAAAAGG
CGAAGAATGCTAAGACATCCGACAACGCCGCCAAAACTCGCGACCCCGAGAACACCGCCGCTAATGGCGCCGC
TCCCGGGGCACTCGGGAACCAAGGCAATACGCCACCTTCCAACAATTCGACGGACGACTCGAGGAGCACCTCA
ATTCCGGTAGAGACAGCCTCGGCCATTCCCTGCCCCCCCTCACCTCCCGTCGGCGAGCCAACGAACGCATTCA
CTCCCCCTACAGGGACTGACCCGCTCGCTGGTGACCCCGCGCTGGAGGACGCCAACCGAGCCCAGCTACGAGA
CGCATACGCTCTACTCAACGACCGAGCCACCATTGCTATAGAACAGCTTCAGGGCATCGTGTCTCGTTTCGGG
GACATGCGAGCAAGCATGAGGCAGGAGATGGACGCACAGGCAGCCCAGATGGACAGCCTGGTGGAACAAACGC
GGCGATCGCTCCTTGACCAAACCAATGCGGCCTCTAGTCTCGTCATCGAGGCCATCGAGTCATGGGATAGCCC
GGAGGAGTGGGACGTTTACCGCGCCCGGCAACAACAAGAGCTCGAGCGTCGCCGCTCCGCGAACGCCACCAAC
CCCTCCGTAACATTCCAAACGCCGCCAACGACCGCCCGAGAGAAAGGGAAACAGGCCGCGCACCGCCCGGTCA
TCCCCATTCACGGGAAGCCATCAGCCGTTCGAATTCGCGAAGCCCTGGCGAGACACAACGACAATAGAGGACC
GCAGAACGAGTCTACGCCACCAAATCGCGACCCACCACCACATTTGCCCACCACAAGATACGGACACCGAGAG
ACGCCTGAAGGCGGCCTGGGAGAGATGTCTGGCAGCGGAGAAGCCGCTGGAGGATCTGGGGGAGCAGGTGGAG
GAGGCGGAGGAGGTGGAGGTGGCGGCGGAGGACCCCCAGGGTGGCCGCTATGTGACCCCAGCGAGGACGAAAG
CAGCGACGAAGACGAGACGGGAGACGAAGAGGATGAAGGGGAGCCTCAGCAGCCCCAGCAGCAGCCAACACCT
CAAGTTACGAGCGTCCCGTATCGACACCCCGCTAGCATCTTCGCCTCGTCTATCTCAGGCGTTGCGCACCAGC
GATCATTGGCAACCAGGGGTGTCGGGGAACCCAACCCACTGTCAGCGCGTACGTCCGTGAGGTCTGCAAGTGC
CCCCCGCGAACAGAAATTCGAGGATTGGATTAGACGACACCTTCAGGTCACCGTCGGTCGTACGCTTCCAAAG
GACACACCCCCCAACAAGCAGAAACCCGCATTACCCACAGCTTACGCGGGAAGCGACAACATCGACACGTTTA
CGGCGTGGTTCCTGGACCACACCCGGTGGAACCGACTTTCACACTTATGCGGGCCCGCCATGGACAACGAGCG
ACTGATGTACCTGGGAACCAACGTGTCTGGACGCGCGGCGGAATGGTACCGCGACACAATTGAACTCGACATC
GTCAATTGGACACACGCAGAGGCCATATGTGCGTTGTATCAGAGGTTTATTCGCCCCAGCACGAAACGAGAGG
CCGCCCGCAAGTTCGACAGCGTGCATTACACCACGGCCGGAGGAGCCTTCGGATTGTACCATGACATGGTCAA
GTGGGCGAAGAGGATGCCCGTACAGCCCGACGACTACACCATGGCAAGGACGTTCTGCAACAAACTGCCGGAC
GCGACTCGCGAGACCATCACGACGGTGTATCGCATTACCCCGGAAGACAACCCGGATATCAAGCTCATTCGTG
ATCTTGCGGCCGACGTCGAAGATGGCCGGGCTTACATCGCGCGCAGTCAATCCAGCAGTACTGCCGTTACAGC
TGCCCGCCCTACCCAACAGGCAACAACCGGCACTACTGGCCGGACACGACAGTACCCAGACGCTAGTCGACGA
ACGCAGGACCGCCCCGATCGCCCCTCTATGTCGTCGAAGAATCCCAGCCGTAGCGACCGGGCTCAGCAGGACG
CCAAGGCGATGGCCACGCCTCAACTGTCCGGCAACTCGCGGAGGGCTGAAAAGCCTCCCGTCACCTGCTGGAA
CTGCAAGCAAGTGGGCCATTACGCCACGGACGCGATCTGCCCAGCCAACAAGAAGCCCGCGCTTAGGCGCATC
GACACTACGCAGGATGAGTCGGCAGCCATGGAGGCAAACGGCGATGATGGCGAGTACAACGAAGAAGCACACC
AGGTGAACGCGATCGCTCACGAAGAGCCCGAGGACGAGGACGTCGTGGGCCCTCAGTACGAAACCCGTAGTGA
CATAGGGTCAGAATATAATGATTTCGACGATGCACAGGACGAGTTTCAGGAAACCGAGCGTGAGGAACGTATG
TATGGGATGTCAATCGCTGGGTTTTACCATCGCGAGCCGGAGGGACAAGAACCCGATGATGCAGTCAGTTCAC
ATAGCGACGACTCTGATGAGCCGCCAGAGCTCGTGGACGCTGAAGAGCAGTCCGACGGAGAGCCCCAACCTGA
CCACTCCAGCGAGCACAGAGGGGGACGACTCGACGACGGATTTGTACGACCTGGGGTACCATTTGTCTCTGGC
GACAACTTCACCCGACTCGCAGACGAACTGCCCCGAGAAGTGTTCAACACCGGGTCTCACCACCGCATTCGTT
CCTTAGAAAATCGTGTGGACCACCTCCTGGTACGAGTCGCAGCACTCCAGCGACTCTACGGACGCACCAGTAC
CGACAGGCTGCCCAACGACACCGCAGACGAGCTCGGGCGCTCAGCGTTGAACTTGGATTCCCACGCCACAACG
ACTCGCACATCCCATCAATACATAGCGCCGCCCTTATTGCCCAACGACCCGGGATCGGTTATGCTCCGGGAAC
TGGGGCAGGGGATTGACAACCTCAACCAGTCTGTAGCATTCCAAGTCGCCTCGGTGGTACTTCTACAGCGCGA
ACTAGGCCTGTTGGACAGACTAGCTCGCCTCCCTGTACCAAGCTCGACGATTCGCCAATTCACGGAGCGCGGA
CTCCTCCGTGTAGTCCAGAGGATGAAGGAAGAAGAAGAACACTACGCCGCGCAACGGGGATTACTCGACCCAG
ATCCGGCCGACGAACGGCGACGGATCCGCGAGCGCAACGCGTTCTATCGTCAGGTTGGCCACATCGCTGATGA
CGAGGAAACCGTGGCACGATTGACCGACGAACACCGGGAACGTGAAGAGAATGCAGGCCGGGGAGAACTTATG
TTCTCAATGATCGCGAAACATGATCGAGGATACCGTACCTCGCTGAAGGCATCGACAAACTCGCGAGAACGCC
CCGCTGGGCGAGGACGTTGTATGACATTCTATATTTCGTTTGGCGAATACGACGCTCTGGTACTCGTGGACAC
TGGAAGCACGGTCGACGCCGTCTCTCATGACTTCGCAAAGGTCGCACAACTAAAGACTTACCCGCTCAGCAAT
CCAATCACGCTCCAGCTTGGGTGCTCGGGATCACGTTCAAAGGTCAACTACGGACTAACCAGCACATTCTCCC
TCGGTGACGAAAAGCACGACCTTTACTTCGACGTCGCCAACATAGATCACTACGACGTGATCCTAGGCATTCC
TTTCCTGGATCGCGTCGGCATGGTACTCGATTTTAGAAATTATACCATGCAGATTGGGTCGAAAACGTTCAAC
GCCCTACAACGTAACCAGAAGAGCATTACTCAGTCAAGGGCCAAGCCAGTTCGCCCCGCAACGGCGCTCGCGT
CGGGCGCCGCGGTCAACGTACCAACCCCAGGTCCGTCTAATTGACGGCCACGCGTGGGTTCAACACCCACGAC
GCCACAAAGGGACGACAGTGGCGACGATACCCAATCTATCAATTCCTTATCTGTTTTCCCTCCGAGCTCGAAG
AATCATATCAAAATTGATAAAACCCAAGACAATGACCCGCAATTTCGCTCTATATCTACTCATCTTGTGTCAA
AAAATAAAATACCAATACATATACCAAACCCATTGTCAGCTAAGCCGCCCGACCGAGAGTACGTGATGAGGGC

AGTGAAGCAACTTGACAAACCAAAAACTCGCGAGCGATGGTTCGAGCGCTTCAAGGACATCACCAGTGGTGTT
CCTGAGGTACTCCCACCATTGCGAGAAATTAACCACCAGATTACCCTGATCGACGACGACGTGAAATATTCCT
ACCACATGCCGCGGTGTGCTGAAGCGCTACGACCACAACTCATGGAGAAGATCCAACGGTACAGCCGCGCAGG
TTGGTGGGTTCACGCCACGACTCCCCAGGCCGCTCCTATGCTGTGCTTGCCCAAGAAAGACGGCGGATTGCGT
ACAGTCGTTGACTGTCGAAAACGGAACGAGAACACCGTCAAAGACGTGACACCGTTCCCAGACCAGGAGCAAA
TCCGCATGGACGTTGCGCGGGCGCCGGTTCGATCGAAGATTGACCTGTCAGACGCGTACGAACAAATCCGCGT
CGAGCCAAACGACGTCCGGAAGACGGCTTTTGCAAGCATCTACGGCACAATGTTCAGTAACGTACTACAGCAG
GGTGACTGTAACGGTCCTGCGACCTTTCAACGGCTGATGACACATATTTTCCGACCTTACATTGGACTGTTCG
TACACGTCTACCTGGACGATATATTCATCTTCTCAGACTCGATAGCCGACCATGAGATTCACCTCGAGATCGT
GTTCCAGACACTCCGCGAGAACCATCTATTCCTGAAAGCGAAGAAGTGCGACCTCTATTCAGAGCGCATGGAC
TGCCTCGGGCATATAGTCGACAATCAGGGCCTTCACGCGGACTCTGACAAGATGGCTCGCGTACGCGAGTGGC
GCACGCCTCGCGACTACCTCGACATCCAACGATTTTTGGGACTTATACAGTACCTCGCACAATTCCTGCCGGA
CATCTCGGCCTACATCGCACCCCTCATATCCATCGAGCGCAATGGACATACGTTCGAGTGGCGGCCAATTCAC
GCGAAGTGCTTCGAGACCATCAAAGCACTAACTTGCCGCGCGCCGATACTCAGACCAATCGACCCCAGACGTC
CTGAGCCTATATGGCTGGTGTGCGATGCATCAGTATCAGGAGTGGGCGGGTTTTACGGCCAAGGCAAGGACTG
GGCTTCATGTAGGCCGGCGGGGTTCATGTCACGGAAATTGACGGACGCACAACGGTCCTACACGACCCTGGAA
GCAGAGCTGCTAGCAATTATAGAAGCGCTACTGAAGTGGGAAGACAGGCTGCTGGGCAGACGCATCCGGATCG
TGACCGACCACGAGGCCCTGAAATGGTTAAAGACTCAGTCGAAGCTGTCCCGTCGACTTACCCGATGGATGCA
GTATCTCTCGCGGTTCGATTACGACATCGAGTATCAACCTGGATACAAGAACATCGTTGCAGATAGCCTTTCA
AGGTACTTCGAGCAAGACAATGCCGGAGAAGTCCATCGTATCGACGAATATTCAGGCGCGGATGTGCGGCTCG
ACCCAGACGGCGAAGACTTGCCCATGGAACGACTCGCGGAGTACCATAACTTGAGAGTAGGCCCGGGACGACT
TGACAAGCCAATGCCCGCAAGGTCCCTGATCACGAGGGAGGCGCTGCGCGCGTTGAACGCCACACCGCCACCC
GAAGTGACCGCAGAAGACGAGGATCCACTGGCAATGGTACCTGACGAAGCCCTCAACCCACTTAACGAGAGTG
TGGAAAGCAACTCACAATTCATGGAAGCCGTGCGAGTAGGCTATGAGGGCGATTCCACGTTCAAGAAGGTCAT
AGCCAACGTCGATCAATTTAAGCAATTCACGCTCGACGGAGGACTCCTGGACAACGTCCGCGATCTGCGAGGG
ACCGAGTCTTTTCCTAACGACCGTACCATCTCAATCGAAACAGTGCGCGACTTCTTCCTCGCACTGACGCCAC
CGATCGCCGAACGCCTCAACCAGCCGCACCCGGATCGAGCAATGGCCTCGATACGCTCCTGGACGTTCTCCCT
CTGTCTTATGGTGCCTGAGCGCTTCGAAATCAAGGACGAAGTGCTCGACGCAATATTTGGAGACTCGCGCCTT
ACCGTGTAAACGCTGTATCCCTCGCGCATTTCTCGAGATTACCCGTCTACCGTAGAAGTTCCGTGATTAAAGG
ATATCATTACGTTCTTCCTATTTCTCGTGTGCCTCCACCTACGGTTTCGTTCTACCGGGGAGGGTGAAAGGAC
GACGGAAACAGCTACATACGCGTGTACCTGTGTTCTACGGACTTATAGGCGCCTGAGCGAACGGGCGCCCGGC
TCAAGCCGAACGCACGTGGCCAGCGCCTGGCGGGATTCCTAATCGGGAAGAACTGCACAGTTCCGCCCGATTC
TGCGGTGGCTGAGTATATAATCCTAGGCAGAGTAGATAGTTAGACACCTTTTACTACTCACGATAACGCTGTA
CTCTTTCCGGACGAATAAACGTCGTACGGTTCTAGCAGCGTGAAGTGAGTTGATATTTGGTCTTACAC
>Bjead1_1_rnd-1_family-166#TIR/MITE
TACAGTGCCTTGCATATTTCGCTGCGTGCACCGTNCTCGCTCGCGTTGTTTGTTCCGTACAAATATTNCCTAA
CAAGGCAATATCTGATGGGCAGCCTGTCGTCACGCAATCTGCGGCCTCCTCTCGTCGCTTGCTCCTCCCCCTC
GCGCAGCACAACCGACTCCGCTGTCAGTCCGCTTACGTCGATCCAAAGGCCCCCGAGGTGGAAAACACAGGC
CTCAACCGAGGCGGAATCGCGTCGTAAACAAGCCGTTTTGGGGGAGCTGATGGATNATTCCCACGGCTCNCNG
CGGCTTGTNTCCANGCGATTCCACGNACGGCGACCACGTAAAATCGCGATTATATTCCCCGAGGTCATTTCAA
TGGCAGTAGTCGAATATAATTGAATTATNAAGCCGAGATGAGAGCGNANTGGATGCAGGAGTCGTCGCTGGAC
TCGACCGCGACGCTCTGCGACTTTGATGACGCGTCCCACCATATCGGGCAATATTTGTCCAGAACAAACAAGG
CGAGCGAGAACGGTGCACGCAACGAAACATGCAAGGCACTGTA
>Bjead1_1_Cluster32#LTR/Copia
cctTGTTGtTaCGCAGGcTcgGagCACTCGGGGaAaGACTGcaaaAGCGACTGATGATTctaAACTACGCTAa
TACAGGTTCTATTTATATTAGTTTAGgATAGGAGGcCGGTAAcTCcATATGGaGgACTTAGTGGATcAgAaGT
gaaatataagcgggatacgagtgggtgaaacgcgaGGATACAaACGGATACAAACGGaAtaacgcgtggaggt
aaacggGAaAacGAaaTGGaTgtagcgcatgggAAgaAcAAgaaAAagaAacaAaTacGgACaTaCggAaATG
GaAaTCCCGCCTCCGGCGCATCAGCcCGCCGGGTcgTgGCacAGGCGCGGaTCCACGACACTCCCCCTCAACG
AGCACGCAgGTTGaCGAGcTCGACcTGGTGcAAGTGaGCgGGcTTaGGaAGaGcCTTCGTCAAcATGTCGGCG
CAGTTGTCTTGAGACGCGCAATGcgaaATaGTaATGTCGCCgGCcTCGATGcGTTCGCGCGTGAAGTGATGaC
GgATGTCGATaTGcTTGGAGCGCGCaTGGAATTGaGTGTTGTACGAAAAATCGATGGCGGATTGGTTGTCGGT
GATAAGGGGAGTAGGATCGTCGGAGCCGTATCCGAGTTCGGCGAGGAGTGCACGCAGCCAGATTGCTTCCTTG
GCGGCGTGGGCGAGGGCGATGTATTCTGCCTCCATGGTGGAGAGCGCGACCGTGGGTTGTTTCTTTGATGACC
AGCTGATCGTCCCTTCGGCGAGTTTGAAGGTGTAGCCGGAGACGGAGCGTCGGTCCGCAAGGTTTGCCGCCCA
GTCGGCGTCGCTATACCCGATAAGGGAAACGTCGGTGCCGCCGAGAGTAATGCCGATACGGGGTGTGCCTTTG
AGATAGCGCATGACGTGTTTGACGGCGGTCCAGTGCTCCTTTGATGGGCGCGCCGTGAATTGGGATAGGTGTT
GAACGGCAAAGGCGATGTCGAGGCGGGTGGTGACGGCGGCGTACATGAGGGAGCCGATGGCGACGGCGAAGGG

AACGGTGACGATCTCGTCATTCTCGTCAGGGGGGAGCGGAGCGAGCTTGACCTTCGGATCGAGCGGGGTAGAG
GCCGAGTTGCATCGGGACATGTTGAAACGCTCGAGGACGGTTTGGATGTAGCGTTCCTGATGGATAGTGATAA
TGTGTGCCTGACGGTCGCGTTCGATCTGGAGGCCGAGGAAAGATTTGAGTTCGCCGAGATCTGTGATCTCAAA
ATGGGTGCGAAGCGCCGTCTTGAATTCGTCGAGGGATTCGCGAGTGCCGAACATGGCCGTATCGTCTACGTGG
ATAAGGAGGATAAGAATCTTGGAAAAATGTTTATAGTAGAGGCAGGGATCAGAAATAGAGCGAGTGAAACCGA
GAATTGATGTGAGAGTGTAATTGAACTTGTCGTTCCAGGCCTTGCCGGCTTGCTTGAGACCGTAGAGCGCCTT
GATGAGATGCCAAACGCGTCCTGATCCGTCATCGTATCCGGGTGGCTGACGCATGTATATGTCCTCTTTGAGT
TCGGAGTTGAGGTAGGCGCCCTTGATGTCGCAGGAGTCGGCTTCGAGATCTTCGACGGCGGCGATAGTGCAAG
CGACCTTGAATGATTCTGTGCGCATGACCGGCGCGTAGGTTTCGAAGTAGTCTTCACCCGGCTTCTGGGAGAA
TCCTTGAGCGACGAGTCGGGCCTTGAACTTGAGTACGGCGCCTGTGTGGGCGTCTTTCTTCTTGTCGTGGACC
CAGCGGCAACCGATCGCAGAACGTCCCTCGGGGAGGTCGACGAGCTCGTAGCAGTTCTGGGCGCGAATCTGCT
CGATCTCGGCGTCTTGGGCAACCTTCCAATGAGGCCAGTCGACGCTGTTGCGAGCTTGTTCGAGTGTCGGGCG
GTCGATCGAGTCGGGATTTCCTAATTGGGCAGCGAAGCACATGAGCGCATCAGTGTCTTGTGCCTGTTCGATG
ATTGGTGCGCGTTGGCCACTATCGCCTAATTTCTTGTAGTCGATTCGGGAGGAAGTGGTGCGAGGGGGACGCC
GGGCGGCAATAGAAGTGGTAGAGTCGTCCGATGCAGAAGGAGAAGTGGGCGGTTCGAGCTTGGATGGCTGAGG
CGCGGCGTTGTTAATTACGGAAGTAGGAGCGGGAGGAATGATTTTAGTGGGAGCGGACTTAGCGGGAGCGGAC
GCCGCGGCGGGCGCTGGTTCGTTGACTTGCTGCTCCCCCTCAAGCGAGGGTCCGTCCTCTACGAGTGGTGCCG
CTTCTTCTTTCGGCGGGAAAATGACGTTTCGGGAGTGTAGAATGTGGCGCATGCCGGCATCGTAGTAGCGCCA
GCCTGCAGAGTGGTCTGAGATGCCGACGAACGTGTGTTTTTCGGACTTGGGGGTGAGTTTCGTCTGGCGTGCC
TCGGGGACTAGCACCCAGCAGGGCGAGCCGAATTCCTGGGCGGCCTTGATGTCAGGGCGCTTACCCCAGAATG
CTTCGTATGGAGTCTTTCCGTTGAGGGCACGAGTAGGAGCGCGATTCTTGAGATAGCACGCGTAGGCGATGGC
TTCTTGCCAGAGTGCAGTGGGGGAATTGTGTTGGAATAGCATGGCCCGGGCGCGTTCGACGAGAGTGCGGTTG
AGGCGCTCGGCAACGCCGTTCTGGGCCGGTGAATAGGGCGCGGTGGTACGGCAGATGATGCCTCGCGAATCGA
GATATTTCTTGAAATCGCCTTGCGTGTATTCCTTCGCGTTGTCGACGTGGAGGGTCTTGAGGCGAATGTTGAG
CTGTGTTTCGACTAGACGTTCATATTGCTTGAATCTGTCGAGTGCGGCAGCGCGCGATTTCATGAAGTACACC
GCGGAAAAGCGGGCCGCGGCGTCTGTGAAGGTAATCATGTAAACGTTGTTGTTCTGAACAGATGGAGTGCGCG
CCGGTCCCCAGATGTCGGAGAATGTTAGATCGCCGGGTTCAAGATCGTGGTCGGCGGCGCTATCGGGAAAGGA
ATCCCGCGGGTGCTTGGCCTTGATACAGGCTTCGCAGGTAAAATCCTTGGGAGATGATTGGTCGACGTCCATC
CCCTCGACCATCTTCTTGGCGTGAAGCTCTTGAAGCTGCTTCACGTTCAGATGTCCGAGTGCGCGGTGCCACT
CGAGCCATGTGCGCGAGGTCTTGAGTGTGGCGGGTAGTGCGCGAGTTGCGGGCCTCTTGACAGCCATGTGGTA
GAGGTCTTCGACTTTGCGTCCTTTGCCGATAGTGCGTTTGTCGTCCTTGATGTAGATCTTGTTGCCGATGCCG
TGGTAGGTAAGTCCGGCGGAGGTGAGGCGTCCCAGGGAGATGAGATTGTATTCTAGATCGGGCGCGTGAAGCG
CGTCCTTCAGCGTGATGGGGATAGTGCCGGTGTCTGTGGCGAACAGGACGCGGACGTCACCGCGTCCGAGGGC
GAGGCACGATCCGACGCCTTTGATTTCGGCGCTGATGGGTGTGTAAGTGGTGAAAAGAGAGCGATCGCCCACA
ATGTGTTTCTGGCAGGCGGAATCGGCGAGCCATGTCTCCCCCTTAGCGATTAGAGCCTGAATTTCGGGGTTTC
CCTCGATGGTCAGAAAGGCGTAGTCGTCATCCGAATTGTCTGTAGTCGCGAGGTTGACACGGTGCTCGCGCTG
GTGAGGCGCTTGCGTTGCGCCGTTGTTCTGGTTCGGCTTTGATCGGCACTCGGGCTTGATGTGCCCTTCTCTG
CCGCAGTTGTGGCAGAAAACACCCTTGCGGAACTTCGACTTCTTGAAGTTGGCGACGAGGGCGGTTTCTGGGG
AGCGGTCCTTGCGGCGGGCGTCTTCTTGGAGGATGCGTCCGACGAGTTCGGACGACGCGGGCACGACTGTCCC
TATAGAGGATATAAACGAATCCCATGTGCTTGGCAGTGCAGTAAGGATGGTAAGACTGAATTCTTCGTCTTCG
ACGATGGTGCCGAGGAGGGCGAGTTCCTCCTTGAGTCCGCGAATGTTGCGGATTTCTTCTTCCATGTTCGAGC
CCTCTTCGATCTCGTAGCGCAGGAACTTGCGCCGAGCGAGAATCTTCGCGAGGGCACCCTGCGAGCTATACAC
GCCCTTGAGCGTATCCCACGCCTCCTTCGAGGTTTTGGCGGACATGATGTAAGTGATGATCGACGGAGATACG
CGGAGACGGATGATCGTGAGtgccttacgatcggccttgtcccaatcggcttgagcgctcttgtcattgggtc
tcttgttcgacccgtcgatatagtcaatgtagccagtgtccgtgagaacatcttggatttgaacttggaagga
tagccagttttcggcgcccctgAGaGGcTCGATGCGGTAcGaaCCGCcGGTaCTGgTGTCGgTAcTCGACATG
TcGaTcGGCGCTAGTCGTCCGATCtAAGATCAAAGgTTAAGTCCGCaATGGACGAGCAGTCaAGCCaGGCGAT
CTGCaATAGAcCAAGCAGTagacaGaGTgCGgGagaGATTCTAaAGCGCGGGGCTCATAACCTGTTGTtaCGC
AGGcTcgGagCACTCGGGGaAaGACTGcaaaAGCGACTGATGATTctaAACTACGCTAaTACAGGTTCTATTT
ATATTAGTTTAGgATAGGAGGcCGGTAAcTCcATATGGaGgACTTAGTGGATcAgAaGTgaaatataagcggg
atacgagtgggtgaaacgcgaGGATACAaACGGATACAAACGGaAtaacgcgtggaggtaaacggGAaAacGA
aaTGGaTgtagcgcatgggAAgaAcAAgaaAAagaAacaAaTacGgACaTaCggAaATGGaAaTCCCGCCTCC
GGCGCATCAGCcCGCcGGGTcgTgGCacAGGCGCGGaTCCACGACAccc
>Bjead1_1_rnd-1_family-210#TIR/MITE
TACAGTCATTACGCGTTCGATGAGACACCGCACTCAGTGAGACACTACCATATACGGCAGTCGCCCCAACGCT
GTAGCGCCGACTGTCCCGCAGGGAGCAACGACGATTCTGCGATACATAGACTACGTACAGCTAGATTAGCTAC
TGCGCTGAGGATATCTAACATGCTACCGTGTATCCGCAAACAAACGTTCAACGAAAACGTCCTCGAACGTCTA
AACTTTCAAAAGGCCGAATTTCTNGACAATGTTTCCCCCATTCAAGATGTTCAATGTTCTCAGCTATATTCA

TGTTGATTTGACCATAAAAAACCCCTCCATAGAGCTCCGTCGAGCTTTCCGCTGCAAATGGCCTTATTCTAAG
TTTATGTCGCCTTGAGGTGTTCAACAGGCTTCATGCGACACAAATATTACATATCTACTAATATTATGGTGGT
TGGCACTGGTATAGATAGTGCATTTGGTTTGTTAGGATACAATGCGACCAACGACGAAGGAGTCGTGCTCGCT
GGACAGTCGGCGCTACAGCGTTGGAGCGACTGCCGTATATGGTAGTGTCTCACTGAGTGCGGTGTCTCATCGA
ACGCGTAATGACTGTA
>Bjead1_1_rnd-1_family-127#TIR/MITE
TAAGGTAGGCCCGATCCATTCTCGACTACATCACATGACCTTAAACGGCACGTGCAGCCTAGTTTGGCGGAGG
AAGAGTCAAGAAATTCTTTCTCCGCACTCGGTGAGCNTTTTGTCGAAAAACTAAAACTAGCGAATCGTAGGGC
ACACGGATTGTAGAATATCGCTAGTGCCCGAATTCGCCATTATGGCCCGTGGATAAAGTTTAGAGTCGATGAA
GGTGCGCGAAAGAATTAGAATTACCGCTCGCGAAGGGATAACGGTGGTTACCGCTGTTGATGAGGAGGCGATA
TGCCTATTTTTGGGGCTTAGGATACCTTTTCTCACGGATCACGTGCATTATATTAGACGCCGATAGTAGTCGA
GAATGGATCGGGCCTACCTTAC
>Bjead1_1_Cluster33#LTR/Gypsy
TAAGGACCCCGTAGATAGTCACGGTTGAGTATATACGGGCTACTTCTGTACGTTAAGTCCACGAGCGCCCACG
GACAAGGGCGCTCGGGGAATTCCGAAGCCGACACGTGCGTAGCACATGGTGGACTTCCTAGTTGGGAAGAACT
GCATAGTTCGGCTCGAAATGGACGGTGGCTTAGTATAAATACCTAGAAAAGTAGTTAGTTAGCCACCTTTTAC
CACTCGCAAGCACGCTGTACAGTTTTCCGGACGAATATACGTCGTACGGTTCTCTCAGCGTGACGCAAGTTCT
ATCTGGTCTTACATTTTTTTGAACCCTCGTACTTTATTCCTCTGGGCTACCTACAGCCCCTACAAAAAACTTC
CAAGCGACCCCTGACGCCCCTCCGACCTCATCCCGCCGAAGTCCGAAGCTTCCACGCCGACGATGAGTACTAC
GGCGACGACAAGGGTCACCCGGAGCGCATCAAGGGCGGCATCTGATGCGCCAACAGGCGCAACAGCCGCCACG
AGGACGGTCCCATCAGCTTCGTCGGGCGCCACGACCAACTCGAACCGCCCCGTTTCCTCCACTCCCTCTTCCA
TCCCCATTCCCTCTCAGAAGGCTAAGGGTGCGAAACCCGCCGACCCTGCCACCAAAACCCTCGAATCCGCGAA
CACCGTCAGCAATGGCGCTGCTCCTGGACCCTCAGGGACCCACAACTCAACGCCACTAAAGCGAACAAACGAC
GATGTGGCCGTAAACCCCGTGGAGAAGACCCCTAGCCCTAGCGAGACCGCCCCCAACCCCTCCCATCCGGATG
ATTCCACGAGCGGACCCACTCCCCCAGTTGAGACTGGCTCCCTCAACGAATCGTCCTCCCCCACCGACCCCAA
CCGACTCCAATTGCGAGAAGCGTTCTCGTTGCTCAACGAACGCTCGTCTATTGCCATCGAACGGCTACAGTCG
TTGGTGGAAAGATTCAGCGACATGCAGTTGCAGGTCCGTGAAGAAATGGACGAGCAGGCAGAGCAGATGAGGG
AGTTGGTCGACGAAACTCGGAGGTCGTTCAACGAGCAAACGACGAACGCCTCCTCCATCGTCCTCACCGCCCT
AGAGAATCTGGACCAGCCCGATGCCTGGGAACGTCATCACGAAGCCCAACGGCGAGAACTCGAAGCTCGGCGT
GTGGCGTCGTTCATCACACCCTCAGTATCCTTTCAACTCCCGCTCGAAAATACCAAAGGCCCTCGAGACAAAG
GAAAACAGTCCACTCACCAACAGGCCTCCGCGGCACATTCGAGCGCACTTGCCACACGCACTCGGCAGGGTCC
CGCCCCCAACAGCACCTCATTTCCACAACGCGAGCTGCCCCCGCACATGACGAGGATGTCTATGGCCGAGGAT
CGAGGCTATAGAGAGGTGGCTGGGTTCGGTGGGGCCGGTGGAGGTTCGGGAGGAGGGGGCTCTGGAGGAGGCG
GAGGAGGGCCACCCGGGGGACCACCCGGCGGACCACCAGGATGGCCACTCGTTGGAGACGACGAGTCCAGTAG
CGACTCAGACGAAGATACCGACCAGGGAGAGGCGAACGACGACGCTGGCCGACAGACTGCCTCCCCCCGTCAA
GGCGCCCCTCAGCACCCATCGAGCATGTTCGCGTCCTCAATATCTGGGGTCGCTCAACCACGACAGTCGCAAC
GTCAGGCCGGACCGTTCTCGGCCGCCTCGACGCTGCCTGGCATGGGCCCGGGACGCAACCTCCCAGAGTCGAA
ATTCTCTCTGTGGATACGCCAGCACCTCCAAAGCCTCGTGGGATGTGCGCTTCCTAAGGATACGCCACCAAAC
AAGCAGAAACCGACAATGCCGGCGGCGTACACGGGAAGCGACAACGTAGACAACTTTACGGCATGGTTCCTCG
ACCACGCGCGCTGGAACCGCCTCTCTCATCTTTGCGGTCCCCACCTGGACAACGAAAGGCTTATGTACCTGGG
CACCAACGTCTCGGGCCGCGCCGCCGAGTGGTACAGGGACACCGTCGAACTCAACATCTCCAATTGGACGCAT
ACGGACGCCATCTGCGCAATGTACAAGCGGTTTATACGGCCGAGTACGAAACGAGAGGCGGCCCGCCGGTTCG
ACGCAGTGAAGTACTCAACCACTAGCGGCTCCCACGGCCTGTACCACGACCTGCACAAGTGGGGCAAGAGAAT
GCCAGTACACCCTGACTCGTACACCATGGCTCGCGCATTCTACGACCGACTCCCCGACTCAATCCGCGAAGTG
ATCGCCACGGTATATCGGGTCACGCCCGAGGAGTTCCCCGACATCGACCTTATCCGGGACTACGCCGTTGACG
CCGAGGACGGGAAGTCGTATGTCACGCGCACCAACGCAGCCATAGCCCCAGCCACGACCAGCCCTGCTCAGTC
CTCAGGAGATCGGGCCCGCGCGTCTGCATCTCAGGCCCGCGCGAACGTAGCCAATAGGCCGCGCGAGGATCGA
CGCGAGCGCAAGTCATACGAGCCTAGAACTACCGCACCACCAGCCTCGGCACGCACACCGACCTCTGCCCCAG
CGAGCGGGCCGACCAACACTACGCCGAAGCGAGACAAGAGCTCGGTGACGTGCTGGAACTGTAAGCAGACCGG
CCACTACGCCACGGACCCCAAATGTCCTGCGTTTCAGAAGCAGGCATTGCGCAACATCGAGACAGTTGACGAG
GCGATGGGCGAGGACGTGGAGCAGGAAGGGATTGACTACAACGACCAGACGGCCTCGGACCAAGAGCTCCATG
CAATTCGCGTCGAGGACGAGTACACATCAGAGCTAGTAGGAGCACAATTCGAGTCGAGTAGCGAAGTAGATGG
CGAATACACCGAATTTGAGGATGCACAAGACCGCGGACCCGGGTCTGACTACGAAGAGGTCGAATTTATGTTC
GGCATGTCTGTAGCTGAGTACTACCACCGCGGCGAGAACTCAACGGGTCGCGACGAGGATGACATTCCCTCGG
ATACCGACAGTATACCCGACTTGGTAGACGAATACGGACGTATCGTTGAAGACGACGAGCCTAGTCTGCCAAC
TGCACGACTCACAGGGCTCCCCGAAGGGTATGCGGAGCCGGACGCGCTCTACATAACTTCGTCCAACTTCGAT
CGACTCTATCGCGAAGTGTCCCACGCCGCGTTCACCATAGCCACCCGTCATCGTATTCGCACACTAGAGGACC
GGGTCAACGAACTCGTTGGCTTGATCACAGTCAGAGAAATTGCGAGTGCTGTCGAAGAGAGAAACGGGTCCTC

CAACGCGTTCGCGGACGAGCTCGGGCGCTTGGTCAACCTGAGCGACTTTCCAACCCACTATGGCACCCAGTCG
TCCGCGGGTACCCAATCGAACAGGCCAGATGCCCAGTCAACCCTTCAAGCCGTCCACGACACCCTCGTGTTCC
ACGTCGAATCCCTAGGCCTGCTTCAACGAGAGCTGGGGATGCTCGATCGGCTCGCTCGCCTTGATGTAACTAG
CCAGGAGATCCAGCGATTCACAGAACGAGGCCTATTGCGCTCCATACAGCGCATGCGGGACCTAGAGCACCAC
TACGCGACCCAGCGAGGACTCGTCGACCCGGATCCAACTGAGATTAGGCGCGACATACGAGAACGAAACGCGT
TCTTCCGACGAGTTACCCACATCGCGGACGACGAGGAGACCGTGGCACGCATGACGGATGAGTACAATGAACG
TACAGAAGCTCAGGAAGAACGTCTATTCTCCCTCATTGCCAAGCACGACCGAGGCTACCGTACTGCCATGAAA
GCTACTTCGAGCTCACGGGAACGGCCAATCGGGCGAGGACGCTGCATGACGTTTTACATATCATTCGGCGAAT
ACGACGCCCTGGTACTGGTCGACACGGGGAGCACTGTCGACGCAATTTCACACGACTTCGCGAAGGTCGCGCG
GCTGAAGACGTACCCGCTGAGTAACCCTATCGTTTTACAACTAGGATGCGCCGGCTCCCGGTCCAAGGTCAAC
TACGGATTGCAAGGGGCATTCTCCCTTGGCGAAGACACGCATAACTTGTACTTCGACGTCGCCAACATCGACC
ATTACGACGTGATCCTCGGCATCCCATTCATGGACAAAGTCGGGATGGTACTGGATTTCCGCAACTACACGAT
GAGGATCGGCTCGCGCACATTCCCAGCACTTCAGAAGACCGAAGTCAGCGCATCGAAAGCGCGTATGCGGGCG
GCCCGCAACAATTCTACGCCAGCCACGCCCGCTACCCCCAGTCCAGGGGGCGTCGCACCGTCTAATTGACGGG
AAGCCGTGGGTTCAACACCCACAGCGCCACAGGACGTGTCCCGCGGCATAGATACCCCACGAACTATTTCTAT
CCCTTCTCTGTCTATCACTGCGACTTCTCACATCTCTACTCAATCGGCCATGGCCACCGTGGGTGGTGATACC
ATCACGGATCGGCCGAGGCTCTCAAAAATATCGTCCACTACTCCAATTAATGTTCAGAAAATACCAAAAATTA
TACATAATTCACTTTCTGCCAGACCCCCTGATCGCGAACGTGTTATGCGGGCTGTGAGCGAGCTCGACAAGGA
ACAAACACGAGCGCGGTGGTTTGAACGCTTCAAGGACATCACAAGTGGGGTACCCGAGGTACTCCCACCGTTG
CGTGAAATCAACCACCAAATCACTCTGATTGACGATCAGGCGAAGTATGCTTACCATATGCCTCGATGTGCAG
AGGCACTCCGACCCCTTCTCATGCAAAAGATACAGCGGTATGAGCGTGCTCAGTGGTGGTTTCACGGTACTAC
ACCACAAGCGGCTCCGATGCTGTGTCTCCTGAAGAAGGATGGCGGCCTACGAACCGTGGTAGACTGTAGGAAA
CGTAACGAGAACACGGTGAAGGATGTCACCCCTTTCCCTGACCAGGACCAGATCCGCATGGATGTGGCCCGCG
CGCCCGTGCGCTCCAAGATCGACTTGTCAGATGCGTACGAACAGATTCGCGTAGAACCGCGAGACGTGCGGAA
GACGGGATTCGCGACCATCTATGGTACCATGTTCAGCAATGTGTTGCAGCAAGGGGATTGTAACGGACCCGCC
ACATTCCAGCGGCTAATGACACACATATTCCGATCCCATATTGGACTATTTGTCCATGTCTACCTCGATGATA
TATTCACATTCTCGGACTCGATTGAAGACCACGAAAGGCACCTCGAAGCAGTATTCACGACGCTACGCGACAA
TCATTTGTTCTTGAAGGCCGCGAAGTGCGAACTATACGCCGAGCGGATGGACTGCCTCGGTCACCTCATCGAC
AATGACGGACTGCACGCGGACTCGGACAAGCTCGCGCGGATCCGCGATTGGAGGACACCGCGGGATTACCACG
ACATCCAGCGGTTCCTGGGGCTCTTACAATATCTTGCGCAATTCATGCCCGACCTGTCTGCCTACACCACCCC
ACTCACCGCTATTGATCGCAATGGCCACTCATTCGAATGGCGCCCGATACACTCGAAGTGCTTTGAGTCAATC
AAAGCGCTTGTGTGTCGCGCGCCGATCCTACGTCCTATTGACCCCAGAAGACCGGAGCCAATCTGGCTCGTAT
GTGACGCATCAATATCTGGGATCGGGGGTTTGTATGGCCAAGGACCAACCTGGACTAGTTGCCGACCGGCCGG
ATTCATGTCAAAGAAATTGTCAGACGCTCAACGGTCGTATACCACCCTGGAAGCCGAACTACTGGCGATTATC
GAGGCGTTACTGAAGTGGGAGGATCGGCTTCTCGGCCGGCGAATCCGCATTGTCACCGATCACGAGGCACTGA
AGTGGTTGAAAACGCAATCGAAATTGTCAAGACGGCTCACCCGTTGGATGCAATATCTGAGCAGATTTGACTA
CGACATCGAATACCAACCTGGGGACAAGAACGTCGTGGCCGACAGTTTGTCTCGGTACTTTGAGCACGACAGA
GTCGGCGAGGTACACCGCATGGACGAGTACTCCGGCGCAGACGTCAGACTTGACCCCGAAGGCGAAGATCTCC
CCACCGAACGACTCACGGAGTTCAGGAGAATTCGTGTTGGACCGTCGGAGGCACATACCCCGAAGCCGGCGCG
AGGGATCATCTCGCGCCCCGAACTCATGCGGATGAAGGCCCTTACAACCGAGGATGTGCCAGATGATGACGAC
CCTCATGCCATGGTGCCGGACGAGGCGATTGCGCCACTGGACACGACACTTGACAGCAACCCGGAATTTTTCA
CGGCGGTACGATCAGGATACAGCAACGACGCCACATTCAGAAAGGTCATCGACCACGTCGATCAGTTCAAACA
ATTTACGGAGAACAACGGACTGGTTTATACAACCAATAGATTCGGGAACACAGTACTCTGTGTGCCTCGAGTA
AAGTTGGGGCGGAAACCATTGTTCGAGATCGTGATTGAACAGGCCCACACGCTACTCGGCCACCTCGGCCATC
GGAAGACAAGCGAGTATATACGCCGATGGTATTGGTGGCCGTCAATGGGAAAAGATGTGGAGATGTACTGTGG
GAGCTGCGGGGTGTGTCAGACGTCGAAATCATCCAACCAAGCACCGGCAGGACTGTTACATAATCTGGACATA
CCGCGGCGGCCATGGGGATCTATTGCAATGGATTTTGTCGGACCACTTCCCGAGTCAGAAGGGTTTGACTATC
TTTGGGTAGTCATCTGTCGGCTGACATCGATGGTACACTTGGTGCCGGTGAAGACCACGATCCGCGCGTCAGA
ATTGGCCACGCTATTCATCAAAGAAATTGTACGATTACATGGACTCCCAGACGATATCGTGTCGGATCGAGAC
GCGAAATTCACATCGAAGTTCTGGAAGGAAACTCACCGCATCCTTGGTGTGCGACTACTGATGTCCACGTCAT
TTCACCCGCAAACGGACGGCGCCACTGAACGAGCAAACCGTTCGGTCACCCAGATACTCAGGGCACTCGTGCA
ACCAGACCAGCGCGACTGGGTATCACAACTCCCCCTTGTTGAATTTGCACTCAATTCGAGCACGAGCAAGACA
ACAGGGTATGCGCCCTTTGAGCTCGTCTCCGGCAGCCTCCCGCGAATGACCACGGAGTTCAACCGTGAGTTAA
CGGCACTGCCAGGAATTCAACAATTCGCAGAACAAGCCCGAGACAATCTTATACGGGCGCATGACGCTATCCT
CGATAGCCGGGTGGACCAGACGTTCCACGCAAACAAGCGGCGTCGCAACGAGAGCGAGCATCGCGGAGACGTA
AAGCCCATTGAGCCCGGCGATCTGGTCTACTTATCCACTGCAAACTTGTCATTACCTGCCAACCGGGCGCGAA
AGCTGATGCCGAAATACATCGGCCCTTACCCCGTGCTGAATAGCTTTCCAGCGACGTCCACTTACGACATTCA
GTTGCCCGATTCACTAGCCAAGCGCGGGATTCATTCCCGATTCCACGCATCGTTATTGCGACGTCACGAAGCC

AATGACAACGCCGTGTTCCCTCATCGAGACACCAAAGTATTTTATGACTTCGGTAATGACGAGAAGGAAGAAT
GGCTCGTGGATGAAATTGTGGCGCACCGGTGGGACCAGCAGGACATAGAGTTCAAAGTCCGCTGGAACGTCGG
CGACCACACCTGGGAACCGCTCGCACACGTCGAAGAAATCAAGGCACTGGACGATTACCTCGACTTATGCGGC
GTTCGGGACTGGAGAAACCTGCCCCGAACGGACACCGTGGCTCCTCTGCGAGCAAAGCGCACGCACCGGGCGC
CCAAGAGGGCTGACGAAGGGTGGCGCCAATAGCCTCAAACCGAACCATGGGCTGACGCCGGACGTCACCTCTC
TCTGACTTGAACCACATTCTTTACCACACACCGAGACACGCCTCACCACGCTCACTACCGCTGTACGTCTCGG
CCGCGCCGGACTGATCACTTACATAACCTATCGTTACATAAAACACAGAACCGAACGCTCGCCATGCACCGCG
ACGGATATCGATACCACCCGTACGACCGCCGCGACAATCAACCCAGCCGAAGCTCGCGTGGAGATGAACGCGA
CCGCCACCCAGCGACTCTGGTACTCGACAGAGACGACCCGATACTCAACTGGAGTTGGGACTCGCTCCAGAAG
ATACTGCGCCATCTGCGAGGATGCCCCACCTGCGACGACTACGTGAGGCACCTTCAACTGGCGGCAGGGGGCG
CTGCATTTGAACGTGCCGCCCTCCAACGCGAAGTAAACCTTCAAGACATTCATCAACTCGCTGTGGAACGATG
CCGAAAGGAAGCCGACGAGACGATTCGCAAACTCACCAGCGAGCTCGAAGAAGTCCGCAAGGATCTTAAAGAG
ACGCGAGCAGACCTCAATGACACGCAGGACGACCTCGACGACGCTAACGACGAGCTGAAGGAGGTGCGGGCAC
AGCTGGCGCAGGTTAGGGCGGACGCGGCGGAGCGCGATCGGAGCCATACCCCGGACGAGCGAATGAGCGGACA
GGCACGCAGCCAATCCCCCGGACCCCGGCGCAGCGAACGATCTGCCTCGGAATCAATGGAGGATGGGCCTAAC
GGTCGCACGGGATCAACGGGGCCGACGAATGGCACGAACAGCTTATTGAAGCGCATGGGCATCGTTGGGTCCA
TGTCCGCGCCGGCCAGTCTACTCAGTCGATTGTCAGACGCCCAGGAAGCCGCTCCCCGCCCCGCGTTACTCAT
GCGCATCAGTGACGCCCCTTTGGCGACGTCAGAGGGGGAGGGCCCAGCTGGCAAAAACAAGAAGTTGCCGGCT
GAAGACGACCACGAAAGGGACTTACCACGACCCGAGGATTACGAACCGGGACTGGGATTTCTGCGCCTCAACG
CTATGGAATCAAAGCCGATTAAGACCGTGGTACAACTCAGGCGCCCCGACTCCAAGGAGGAAGCGAAGAGACT
TGCCGCCGAAGCTATGCGACCGGGCAACAAACTCGCGCTGATTCACTGGGGAAACTTTCTGCGCGCGTCGAAC
ACTGGAGAAGCCAAAGGACCTGCTGCAGATACCGCTCGCCTCATGAAGTGGAAGAAGCCTGACTGGGTTACCC
AACGCGCCCAAGCTATCGCCTCAACCAGTCAGGAGCCCTCCGCCAGCCAACCGCCCACGGACGCCTCCGACAA
CGCGATGGATATCGTCACCGACGAAGTAATCACAGAGGTACCTCCGTTCACTTCGGGCTACGAGACCAAGTGG
CGCAAGTTCCTCGCGCAACACCCTGACTATCGACTACCAGGCGTTCCTCGTACCACAGACGGCAAGGTGCAGG
ACGGCAAGGAAAGCACTCTTGCTGGGTTTCTTCGCGTCATGGAACTTGGCCCGGCCCCATTCTCTGGACCTCG
AATGCGCGAATGGCTTCGCGTCATCCTGGAGATCGACTACCACGACAGCTCCACCCTAGTCAACATTCGCAAC
CTCCGTGACGCACGATCCTTCCCTGCCGATCGAATTATCTCGCACGCCAGCGCGCGAGAGTTCCTCACATCTC
TGGCGGACCCCGACCAACTCAGCGTACCTCAAGTCGATCGCGCCGCGTCTGACATATACGAGTGGACCTACTC
GCTGTGTTTGATGGTGCCGGAGCGGTTCGAGGTAACCGAGGAGACTATGAAGTCACTATTTGGTTACGCTACT
TAGTGCCACACCCGGATGATCGCGAGGCGCGGATATGTACGCTCGGGACTCGCAATTTTAGTTCATTTTACTG
GCCGAGAATTGGAGTGTCTCCACCGTTGGTTTCGTTCTAACCGGGGAGGGTGTAAGGACCCCGTAGATAGTCA
CGGTTGAGTATATACGGGCTACTTCTGTACGTTAAGTCCACGAGCGCCCACGGACAAGGGCGCTCGGGGAATT
CCGAAGCCGACACGTGCGTAGCACATGGTGGACTTCCTAGTTGGGAAGAACTGCATAGTTCGGCTCGAAATGG
ACGGTGGCTTAGTATAAATACCTAGAAAAGTAGTTAGTTAGCCACCTTTTACCACTCGCAAGCACGCTGTACA
GTTTTCCGGACGAATATACGTCGTACGGTTCTCTCAGCGTGACGCAAGTTCTATCTGGTCTTACAGT
>Bjead1_1_rnd-1_family-96#TIR/MITE
TGGGTGACTTTCCGTAAGCNNGCTGGCGAGTTGCTAGTTGCGCGTTGCCTGNAAGNTGGGGCCGGCACGCTCG
GCANACCAGATATGGTCATGGGCGGGACGCTAGTCTCCCCGCGTGGCAGAACTCGTCGCCGCGTCTGATCTCT
CTGGAGTGCAGCATTTGCAGGTCCCCTTCACCTATCATTTATTCGAGAACAGGTACTATGGATCACTGTAATT
TACCTACAACATATTCTTGAATTTAGGGCGGTGGAACTGACCATTGTGACGCCGGGTCGTGGCATGAGTACAG
CAGTACAGCCGTCTGGATGTGGTAGGGTGTGGTGCACGGTGTGTAGCTCGATTTGACCACAAACCTCACCAAT
AAAGTAGAAGTAAATGTAGCAGTTATAATTGAGACCATTGAAATCGACACACGTCGAAGGACGCGAGATTTTG
CGTGCAGAGGTCGCGCCACCACGGAACCCAGACCACCCCAGACCGTCTCCCAGTCTGTCGACCAGCCTAGCGC
CTGCCAANCAGCCTTGCGCGTCGCAGCTTCAAGNCCGCCGGCGATCACCCCAGCTAATTATTAACTNTCNACT
CGCCACAGGCTTACGGAAAGTCACCCA
>Bjead1_1_rnd-1_family-109#TIR/MITE
TATGGCGGTCCGCTTGTAGATCAATTAGCGCCGCGAGATCATTTCTTGATGCGAGAAANAAATTCGATCTGTG
CGTCGAAAACTCAGGTTTTCGACTGTCCGGCGGTGTCCCTGGTACCACACACCCTCAGATAATATTATCGAGG
TGTTTTGAGTAGTTTACATGTGTATAATGACAGGTAATGGCTCAATCACATCATAAGGGGCTGGTTACAGCCA
TTTTTGGACCGATAAGATATATTCAAAAATGTGTGCTAATTCCCAAATTTTCGTCATTTCCAGTCATTATACT
GCATCATGGGACCCATCCTCTCGTTCACAAACGCAATTGCTCAGCCAGAGCGTGCGTGTTCGCCGCAGAGAGC
TCTCGGCGCCTGCAAGGCGCTCCATCAGTCCCGATAAGGAAGTTCCCGCCTTCAAGCGCTCGGCGGATCATAC
GGAGCGCCCGGCGGCCCGCGCGGGTGCTCGACTTTCACCTTCGTCCGGTGTATGGAGTCAAATCTTTGGCTCT
ACAGACTTGANAATGGTTGTTTGAGGACTTTNGAAGCTACTATCGCTCAGATGAGACTTCGAGGAACACAATA
AGCGGAAAGTGATTTATTGGCCCCTTCGGCACATTTACACACATTTGGCATCACATAGTTAGTGGAACATGAG
GATGGTGGTCCATGATATTTATTTGGGATGGCTAGAGACCTGTAGAGTGCGCCGAACGTCGACGGAGGCCGTG

GAACGAGCAATTCGGCGCGAAAATCAAAATATTTTGTCGCATCAAAAAATGATCTCGCGGCGCTAATTGATCT
ACAAGCGGACCGCCATA
>Bjead1_1_rnd-1_family-120#TIR/MITE
TACAGCAAATCTCAGAACGATAGACACCCCCCGCGTATTTTAGGCGTAGCGAGTTCGGACTCCGAGCCCAGAT
CTACCCGATCGGGAGCAAGTGAGGCCGACAACTTCTCGCAGGCGAGTGGGGCTGGGTCGGTCCGCGGTCCACC
GACACTTGGATGGGAGGCTTGCCCATGTACGAGACTTGGTTGATAACAGTGAGGGAGCTTCACGACATGCCCA
ATAATGATCTCAGAAGGTTTTCGCGCATATAAAACGGTATATTTTCGATGCTCGACGACCTTTCCAGGCCGAC
GGCGGCGGGGCGTTGAGCCGCGGGCGCCCCGCCGGTGGTGCATCGGTCCTTCTATCGTGTTTGGCGACAACAA
TCTCCACTCCCGCACCACCTCATCTGGTAAGATCTGGGTTCGGAGTCCGAACTCGCCACGCCTAAAAAACGCG
GGGGGTGTCTATCGTTCTGAGATTTGCTGTA
>Bjead1_1_Cluster11#LTR/Copia
GTCAGGAATGGACGACTATGAGGTCTATATGAACTTAGTTACTATATACGGACATTCTGATAAGCGCTACGCC
ACAAGGACAGTAGTAGCTAAATATGGAAGTTCTCCTTTCTATGATGTTCCGTTTCTTTCTCTTGGCTTCATAA
TATGCCTCTAATATGCCCTAGTATTAGTAATATCTCTTCCTATGTCTTACAAAGACATTCTAGGACTTTCTAT
CTGACATAGTACGTCGTAGTCATCGTATTACTCAGCGTACTCTCTAGCATGTATATAAACCTGCTCTCTGAGC
CCTCGAATACTTAGAACAGTTTCCTCCATCCTCTCCTTTGTCAAGGGTCGTAACCTGACAGGTTATGAGCCCC
GAGCTCTAACTCATCCTCATCGTCTTGCTCGTCGTCCAGTGTTGATACTTTGTCGACTGCGCCTTTGTACACA
CTAACCTCTACAGCGTCGCACTCCCTTCACCCCTTTCTATTGCTCACTTGAGCGCACATTGACCCCTCTGGTC
GCCGTAGTGGTATACGCGCACAGTCACGCGGTCCAAGTGGTACGGATTCAACCCGTGCTTGCTGTGTATCTTC
GGATAAACAGTGCTCGTCGTTGATTTTGGGGCACCTTACCCTCGAATCTTCGGTGTAGACTTGGAAAGCTGCC
CCTCACCCGGGTAGGAGGCTGTGAAAAGCGGTTAAGCGAAAGCGGGTAGAGCGTCCCTTGGGCTCGTGTCCCT
TGGGATCAAACGGCAGCAACAGGGCGAGGAGCCTGAGCAGACAGCCGTGGGCGTGCATTTTTAGCTGTAGCAG
CGTAGCAGTAGTACTTAGTCGTCCTATCCCAACTCTACCCCAACCGCTGGTCATTTCGGCTGACTAGTGGAAG
GGTTGCACTCAACAGTGCTCCATTAGCAAGACCACTTCATTCGTCGCACCCTGTGCAGCTCCACTCTGGAGTC
TAGCTCGACCCCTGACGATAAACCGAGTTCCTCGACCTATCCTCCCAAACCTCCTCTTCCAAGTGACGAAGAA
GCCAGAGAAATTCTCAAACAATACCACCCTCGATATCCCTCTCCTGCCAACTTCTCTCACCCATACATCGTTG
ACTGCTACCCTAACGGCAAAGTCAACTGGTTCACTATCACCAGCGCGTACGAAATCGGGGATCATCGAGGAAA
ACCATACTGGTATAATTATCAGGAAGATTGGGGACAAACTCTACGTGATTTCTTCGCATTACCCCAGCCTGAA
AAACCAGTCGAGGATTATTCTCTGGAATTACATTCGCTCTTCGCACTTCCGGAACCACCAAAACCGTCACCGG
TAAATTTCGAGGAAATTTATCGTCGAACTCGTCTCCTATATACACATACACCCAACCTACTCCTAAACCACCA
TGTCCTCTGACGCCTCTGGCCCAAAGATCAACCTCGTCACCCTCGATGGCACGAACTGGAATAGGTTCGAAAC
CCCCATGAAGTCGTACCTTCGCTATCATGGTTGCTGGTTCCTCATTGAGGAATACGGGTACACTCCTAATTCT
ACCACGCCACCGACGGGAACTTTGCGACCTACTCCGTCCACCAACAATGCGGAGGAAGTTGCGAAGTGGGACG
AGAAAAACGATCGCGCCATCGGCCTCATTCTCCTCCATCTCGCCGAACACATCATGCACACCGTCGCCCACCT
GAGCACTGCGAAAGCGATCTGGGACGAACTCAAGAAAGTCTATGCCAAACCCGGCTCAGTGGGAGCATTTGTC
CATTTCCAACAACTTTTTAACTCTACACTTTCGGACAAATCCCCGCTTCGTCCCCAGCTCGATGCTATCCAGG
AGTTCGCGAACAGAGTGAAGAATGATGGCATCGACGTCAAGGACAACCTTCTCGCTTTACTCATGGTCAACGC
CCTGCCCGAATCCTACCAGTCCATCGGTGGAACCATACTTGCTACCTTCACCGACATCTCTACGCTCAAGCCT
TCCGACATCATCCAACGCATTCACGAGGAAGAGCAGCGCCGTATCTCGAACAAGATCCAGATTTCTAAGGTTG
CGCTCCAGAAACACTGCGACAAATGCGGTAAAAACAATCACACTACGGAGCAGCACTGGCCGGACGGTAAACG
TCTGACCAAGAAATCTTCGAGCGACGGCAAAGCGCAGCAGACAAATGGTCAGGGCAATCAGGGTGGCCAGGCA
GACCAGGATGGCGGGAAGAAGAAGAAAAAGAAGGGGAAGGGCAAAGGACAAGCGAATGTGAACACCATCCAAG
CGAATGAATTAACTATTGTCGATTTACCCTCTGTACAACAAACTAAGCCCTCCGAGGCTGCGAATACAATTTC
TATTTCTCTTTATTCGCTGGGTGAATCTACGCTATGGATGATGGACTCAGGATGTACAGAACATGTTACGCCG
TTTCAGTCTGATTTCTTGTGGTACCATGACTTTCCGCGACCTGGCAAAGCGCATCTAGCCGGTCAAGGAAAAA
CCATCGACATTCTTGGTCATGGTGAAGTGCGTCTTAAGCATACCACTAGTCCCGGACGAACGGTCGAGCTTAA
GCTTCGTAACGTACTGTATATTCCTCAAGCCGCACATCGCTACTTTGCACCGGGTGTTCCTCACAAAATGGGG
AAACGATCTGACATCGACTCTGCTCGGTGGAAGATCATGGACAAAGACTCGACGTCACAGGCTAACCGAACAC
TAGTTTGCGCGGAGTACGATCCGACAAACAGGCTGTATTGGCTGAAGGCTGATATCTATCACCCAAAGCCCGG
TAAACCTGCTGGCAACGGTCAACACATCCTTTCTGCTACTGTGGATGATTACGAACTTTGGCACCAACGATTC
GGACATGCCGGCAAGAAGATCATCGAAAACTTGCCCGGTCACGTCAAGGGAGTTCCTAAGAAAATTCCTGCTC
CTTCGACATCGTCACCTTGCGAAGGTTGCGAACTTGGGAAATCGAAACGTCTACCCTTTCCTCCCTCAGAATC
GCGTGCCTCAGCTCCTCTCGAACTCGTTCACATGGATCTCGTCGATAAGCCGTCACTGTCAATTGATGGTTTC
CGTTACGAACACACTCTCCTAGACGATTATTCCTCCTTTGGCATTATGTTCTATCTGAAGCACAAGTCTGATG
TGTTTCGCTGCTTCAAGGCATACAAAGCTTGGGCAGAACGTCAGACTGGACACCAACTTAAATGTGTGCGTTC
AGATAGAGGGGGGGAATTTTTAGGCAAGGAGTTCGACGAGTTTCTCGAGGAGAATGGCATCGAACGTCAACTA
TCGATTGCCAGGACACCACAGCAGAATGGCCGAGCGGAACGATGGCAGCAAACCATTGCGAACAAGGCAGAAG
CGATGCGACACTACGCAGGACTCTCATCTGGGTTCTGGAAACTCGCTTCTGAGACTGCCGTTCATGTTTACAA

TCGTCAACCGCTTCGTCGACTCAAGTGGAAAACACCCATTGAACTATGGGATGGTACCACTCCGGATGTATCG
TACTTCCGAGTCTTCGGCTGCTTAGCCTATGTCCACGTTCTCAAGGAACAACGCCAGAATAAGCTGCAGCCTA
AGGCTAAGGCTATGGTGTTTGTTGGCTACGAATCTGGATCAAAGGGGTACAGATTCTGGGATAAAAGTAGCCG
CTCTGTTGTAGCGTCGCGCGACGTGACGTTCGACGAGAGAACCTTTCCCAACCGCGAATCCAAGGCCAGTCCT
TCGGCTGAGCCTCCATCTGCTCCTCCTGTTCTACCCCCTATTGCCGAAGAACCATCTTCTAATCTCCCTCACA
ATCCTATCCCTGATCCACCTCAGCAAAATCCTCCTCAACAACCGGCACTACCACCAGAGGCACCACAGGATCA
ACCCGATGATATCGAGGATATGTACCAAAACCCGCCTGAAAATCATCCACCTCCACCTGACAACGACATCCGT
CCCCGTTCTCCTTCACCTCCGCCGCCGCCGCCACAACCAGTGCCTAATCCCCCACTAGTCCAACGCCCTAGAC
GCGAAAATGCAGGACGTAGACGAATCCCTGACAACACTTATGGGGATGTTCATCCTACACAGGCATGGCGACA
GCAGAATCGCGACGTTCAACGAGACGAACGACAGGAAGCTCGACAGCGACGAAATATGGAGGAATGGCGCCAG
GCCCAACAGCCAGGCCAGGATCCTAGCGAAAGCGAAGGTCCTGTTGCACATGATGACGCTGACAATGAGGAAT
TCTCCCTCGACGCCATAGTCAGTCTTCTGATGCAAGCCATTCACCATGAGCCTTCAACGTACAAGGAGGCAAT
GTTGCGCCCTGATGCAAATCAGTGGGAGCTAGCCATGATCGACGAACTCAAGTCGCATGAAGAGAATGGAACT
TGGATTCTGGTTGATCGGCCTCACGGTCGAAAAATAGTCAAATCCAAGTGGGTGTACGCTATTAAGGCAGATG
GCAGGTACAAGGCACGGCTTGTCGCCAAGGGTTTTACTCAGGTACAAGGGATAGATTATGAGGAAACCTTCTC
TCCTGTAGCTCGTTTTGAGGCTATTCGTTTCTTGCTCGCACATGCTGCACTCGAAAATTGGGAAATCGAGGCA
ATGGATATAAAAACGGCCTTCCTGTATGGCGAGTTAAACGAGGAAATCTACATGGAGCAACCCGAAGGTTTCG
TGGAGAAAGGGCAGGAGAACAAGGTCTGCAAGCTGCGTAAGGCTATCTACGGCCTGAAACAAGCTTCGCGTAC
GTGGAACGAAAAACTTCACAAGACCTTGCTGCGGATTGGATTCACTCGCACGCACGGCGACGCCGGCGTATAC
GTCTACCACCACCAAGAGGGGGATCTGATTCTCATCGTTTATGTTGATGATCTCCTTCCACTTGGCTCGAGCA
AATCGCAGATCCAACGCATCAAGGATACGCTCGCTAAAGACTTCCAAATGCGAGATCTCGGTCCAGTCAAGAA
CTTCCTTGGCATGTGTATTACTCGTGATCGCGAAAAGCGGACACTCGAGATCGACCAACAGTCGTACATCGAG
CAAATGCTAGCACGCTTTGGCATGTCTGACGCTCACGCCAAGATGACCCCATTGCCCGAAGGGATTCATCTCA
CCAGTGGTCCTGAAGGCTACACTGCACCTCCGGCTCTTCTGAAGTTGTACCAGCAGATGATCGGGTCACTCCT
TTACGCCATGCTGGGCACGAGGCCAGACATCGGTTATGCCGTCACTCGTCTATCGCAGTTCATGTCCAATCCA
ACAAACGAACATCTCGCCGCGGCCAAACACCTTTTGCGCTATTTGCGAGGCACAGCTCATCTTCGCATTCGCT
ACAACGGCGCAGGCCACTCTGGCTTGATTGGTTACTCGGACGCAGACTGGGCTGAAGACAAGGACAATAGGCG
GTCAACCACCGGCTTCGTCTTCTTATTCGCCGACGCAGCAGTCACCTGGGTGTCGCACAAACAAAAAACGGTG
GCACATTCTTCCACGGAAGCAGAGTACATGGCTCTGTCCGACGCCTGTCGTGAACTGGCTTGGCTCCGGTCAC
TTCAGAGAGAGATTGGGTTCGACCAGTCGTCGCCTACTCCTGTCGTCGCAGACAACCACGGTGCTATCTTCCT
AGCGGTTAACCCTGCTCATGATCGTCGGATCAAACATGTTGACATCCGCTACCATTTCATCCGCGAGTTCGTG
GAAAGAAAGGAATGCAACATTGTTTACGTTTCCACAAACGACATGATCGCAGACGTTTTGACAAAACCGCTTG
GTCCAAAGAAGTTCTTGAACTTCCGCCCTCAGCTTGGAATGGTTCAACCGACCATCTAGTCGAGTGGGAGTGT
CAGGAATGGACGACTATGAGGTCTATATGAACTTAGTTACTATATACGGACATTCTGATAAGCGCTACGCCAC
AAGGACAGTAGTAGCTAAATATGGAAGTTCTCCTTTCTATGATGTTCCGTTTCTTTCTCTTGGCTTCATAATA
TGCCTCTAATATGCCCTAGTATTAGTAATATCTCTTCCTATGTCTTACAAAGACATTCTAGGACTTTCTATCT
GACATAGTACGTCGTAGTCATCGTATTACTCAGCGTACTCTCTAGCATGTATATAAACCTGCTCTCTGAGCCC
TCGAATACTTAGAACAGTTTCCTCCATCCTCTCCTTTGTCAAGGGTCGTAACCTGACAA
>Bjead1_1_Cluster1#LTR/Gypsy
ccgaggacggcaaagctagcgatggcggcggcggcggcggcgatgtgggcggacgacgagaagttttcgt
cgcccactctgccgctcaccaggacgctgggatctggcctcgagatctcggcaagtcccggcgctcatcctgg
ttcgtctgcgcgtgtgcgtacacgcatacctgccgtgaggttgggtggagtgccagcctaagggctgggcacg
ggacgcgacgtggagcgccgccctcccaaccaccgctgtccctccgggtcagcggaagactgcggcgacaata
aagtagcggctcacccaaccaaccctcagagatcgtagcgctgtgagagattcgccatgtgctgcgacgagat
ttgctcgaacgcccgcacatacgcctcatacgccagtgaacgccaacggcccgccaggcgcacaacctctgga
tctaccttctggcccaaaaagaacgatgctcctccaatgcggaaggagtggccgaatgaggtgccaaacccgt
tccgcacgaagataccgttgatcatcttcatgaccgtccctgtcactgcagggcggatctcgccgttgttgtc
tcgccaggagaataatggatcagaggcccgtgctgggacgaccacagcgtaaagcctggcatgagtcgaaccg
tggttcaacccagaaccaaacccatgggtcggtcgcggttaattattctgacccagagccaccagaggaaccg
tacacggatcacccgcacccatgagtcatgaacccatgagtcatggttcaggtggggtcacggttcaactcat
gggtattgggctggcaatgtgtgcaccaatgtagacatcattcatgtatatataaccgattcattacccagta
catagacaactacacaagtacattatgttcgttctcatcctcatcagtgtcatacaaagtctctagggctggc
atgtcttcatcatctggaagggactcagggccagcgctggatgtgctagacccacgcgtggccgtagccctgc
tgagaggctcttgaggctgtcctccaagtccctatacaaaaatacattagaaaacacgcaaggtatctatgca
gtggtgggtggtgaggacataccatcggagtattaccaagatgtttccccgcacctagatggtcttgtgccac
agtacgaagggtgagcggcgggtcttgttgtgcggcaaggacaggcccatcttcgccttccgaaacggtcgat
gcgacaacgcgcaggtgctgttgaaacacctccagcaagtctcgctgctcaggcgtgattgccccgcagtgct
cgagttcctggagatagccacgaggaagaacccatcgagtctcggagactcgcgggaggaatctgcggcgcag

ctgttgaatttagttcagcgatggtgttcgagttgagatgatgcgtacctgttgcgccgcgtctcgtgtttcg
agaacgttgccactgaacgaacggccgagggtgtccgtatagtcgtcacgaatgagatggccggacacaggga
cggtagaggggagtgtcgtggtattgccgtgtgatagagccattggtgcacgggaagagcgtgggggaaagct
ggaacgagctgggcgagagtaaggtcagcgatcgcacgtttaagctgccggctggacgtaccttaatcgcgtg
catccggccgtacttgccaacggtccgaatgccctgtgcgcaccatcgcgtcaagagtcgcgttgctgtgcct
attgcacctcgtgggacggtgcaatgaacgtccggtcggcggcgcacgtcattagccatggtagagactcacc
taaggggccagcagcgcctttgcagttcgcactgcacggcatgggcttgcgcacggtgcccacgttgctgtgt
ttgcttggaggcctgtaacgagccgttagacggtatccgttgcggggcgggcactgtcgcgatttgcaaatgg
tgggcggcgtgctggcttttccgcgcgggtttgttctgtcttgctggatgtcgggcacggcggtgaacgcgac
gtaagcctaggccctaaagtgagtcataaaactcgaattagttgtaagagagggagggaaaacaacgtacctg
cgctatctgtgcaatctggttccgctcacctcgaggatcgaaccgtgagccgtgtgggcgcctgacgcagagg
acagatcggagtttgaatacaggctggataagtccgcgtctacactgctcgactcactgacaccctccatccc
tcactccttgcggctttgaaacaccccccttcgcacaaagcacgcttgccatgcacctgctccacggaaaaca
agctataccacacctacgggctcctccgacggacgcatatttcaaaccccgccgctttgcaacaccgccgtgg
ttcgccgccctctcgcaatccgttcacgcgatgtgcctggcccactggatgcgttatctcggacgcggcaagc
cttttcacccctcctgagacacataccactttgaggccgcgtccagaagacctatcccaccatctccgtgttc
aaggccatctgcccgatgccagccaaagcgaagcgcaagcaccacgagcgagtcgattcgagcgactcaggct
cgggctcggatagtccggaacattcagcggatgagaagagtgtggcgggcgactccgacgatgccaccacgtt
gaagaagaaggtgttatttgagaagcagtacaaggtggcggaacggacgaatgaggaggttcttggtgcgtag
ctatttctcatactgtccgtcttgtctgatcgtctcttcggccccagaggaacagcgcaagaaatggaagtcg
gcggtgtacagccacttccatgcgccgaagatccaggtcatcaaaggagatgtcaaatacgagttccaatgta
tcaagcagtgcgtcgcactgcactactgtattattctaacctaatggtctacacagctcccaccagaagctcg
tgcgtgcgcgctgggaaggctctaccagcaacctccgccggcatgtcaagaagtgcgataaccagactccttc
cgccggacaaggtcaaatcaccgacttcgccgcgggctcaacatatactcgtgagaagcttcgactgcttctc
gtgcgctggattacacgtcgtttccggccgtacgctatcattgccgacccagagcttattgagatcttcgaaa
tgctctacggacgcgtcgagatcccttcgccgtcgacgctgtcccgcgacgtgcgagaggtatatgacgtaac
gagcgtaaaggttggggaatttctcaaggtgcgtttaccctgactgcggctgattgtccctaacgttgcgtca
cattctaggcctatcccggacgcttcaacctatgcttggacggctggacatccccgaacgtcatctccttcct
cggggtgactgtgcacctagtgcgcgacggcaagatggactccttcatattggattttatcaagtgcgttgcc
atcactcgtgtttagccccacgctaacgtctttcaggcttacgaaggcccatgagggatcgtatttagcgagt
gagctcgcaacatgcttggagaagtatggcattgcacacaaggtacgccactatccaagtcccgaagtacgcc
gctacaactgatacctagatatccgccatcaccgcggataacgcgtcaaacaatgcaacaatggtcagcgaac
tcgagtcgtctcttggagcgcttggtggtgccaacagcgcgaagactcgtgtacgctgtttcgcgcatatact
gaatctcgttgtcaaggtgtgtagggaacaccattactgtgacgtcggagatcatactgacaaccctctcacc
taggccatcatctcacctttcaagcaacgggccacagcgtcagacgtagatgaagagatgactgagctcacgg
tcgagggcttggactttgaagacgacgacgacacaaccggtgacctcgccgcccaacatgagaccatcttcgg
tggtgctgaggacgactatgagctggatgaagatgcggcggccgatgatgataggctcattgagcacattgtt
gcccaggttgagcggacaatgagtctgtctcccaccgagcagaagctgggccgacacgcggtcaccaaggtca
gtcctatcgcttgtccattctaacgtgtcactaacagcagttgcagatcacgcttcttgcgaagcggatcttc
cattcacccaccatccgagttcacctcgaaggctgctgtgcaaaggccaagattgaacccaagggcatggtcc
gtgctgtcgagacacgttggaattcgctcgctgaggcaatccaacgggctgtctacctccgccctgcgctcaa
gctccttttgagctcgtcaaagtattcgaagaaaggccgcaatgggttggagcggttcaagcactccgacgac
gaatggacattgctcgaagaactcggaaatctcttggaagtgcgtgattgataactacagtcataggctagcg
ctgacagcatctacaggcctttctcatggctacgaagcgcatctcggagtccaacactccccttctgcatgag
gttatacctctgatcgatgtcctcactgtcgagttggagaacactgtgtccaactacggcttgatgcttccgg
ttcgtgttgctgccgcacgcggactcaaggtcctcaaccggtattactcacgtactgatgagtctatcatgta
tcgctgtgcgatgtgtgagtcctttgtctgtgctctagactatcttctgactccccctcccccagtgctaca
cccgcggtacaagtccgagtattttcgttttcaaaaattgaCaGaaacaGcGaGTGaagCcacgacgcaGAcc
cGatcctccaCGaGCAagaGcagaaacacacggtCaagcccGTCgcccgcgCTgccgCgcaaaCcgCgACaCC
accaacCaAaaaaAcccaactctctTaacCaacaTacTctcggaaTcaaccaagTccTCcccCAcGaCcCaag
cgacCgCcgcTCacacagccacaatcaCagacaaaaaaGacGTcAAaGCaaAaaccCcgaCCAaGgaCaagcc
CAagcCcaCcaAcTCaaacGaacacCCGCcccaaGcggccGaCTccGgcccgccgcaaaacggccACacccccg
acTCccacGcCaggaaacacgagacctgcacCTGgcaacccccaAAaaaaCTgCAcTCacAcaaacaacaCcA
aagagcccaTGgcacctgcatcaacagagGTCcaaCcAGCacccccAaGcGccGgccgCaCaaTcacgaaGCG
TCagCaTcacacagCacaTcCTcCtcaGacccccCTgCaacagttcgTccATcaccgcGaGaACGccaacaca
gCagTCagaacaGggacaTCCaaaCactCcggcCcaaTaaaaagaaaCcCccCAaaaaggaaAaaaaaacaGCa
aGaAcaccgGaTcTaaccaagaaaGaaGacacggacaacacgcaaGCaCatactcacgcacaTGgaacAAcga
cTGaGtaacattatgtactctgtcctaatacaTGaAgaggtgacttgctttcgaattaaCCaaccgcgCgaCC

cAcacgatcgttactgccgATcCaGaccCCcaaccaaccgtgggaaacggGgcacccacaaccaaGcacCgta
tgacCaAgccaTTgccacccaaagCcAccCAaaGccaccCacgGcgggaccccaCgcatgccagGcgagacaa
cagcGacacTaCGcaATgcTgCCaGcGGcCAcaacGgccacTgccccggcaccAacaccacCcgacGgTcccC
aacaaccGCTaTcTTgcccgaaGcCAccGacacCcTcgcgTaGGccaccaCGgCcAagTccTcgccaaaaaac
tgGCgTccgcTcggGccAcgacGccCgTggcaGaGaaacgacGgCccgacttgacggacacggCcaCaAaGcc
cAccaTcccccaaAagaCGcacgcccCcaTgcCaCaGacaCaTgccccGaaccGcTcgGcaaGgTTCaaGcgc
CaCTacaGccaCgccagcagaGcTAcacTcaccggcgggcgggcaGccacCacccccTgacCaTcccaTggcT
cCgcgaccccacGGaccgaaaagccaaagcccGCgcggcgGaacTCccTCagTgacAggttccgGcaaacccg
gAGCaaAggGCcaacagcgGaTcCcggaTaGATAggTGaacacgcTGGacTGCGGCGGCCGCTCCATGAGGTC
GTACGGAACGCCAAAGGAATGGCGAACCCCGCGTATGTTGCATACCCTCCAGCTGTCGTCGCTGTCATGAAGG
ATGGCTGGTCGGAGCACATTCCGCTCTCCTGCCTCACCGACGCCGCATGCCAGAGCCCGGATAAGGTTGCTCA
GTCCGTTCGAGGGAAGAATTCCGTGCACACCATGCTAGTCAGCGCGCAGGAGCTGTGGCTTTCGACACTTGAT
TTCATCCAGGCATCGGCCCGCCTCATCGCACTTGTCCGAGCGCACTACCCCGATCGTGCGGAGGCAGAAGCCT
TGGCGGATACCTACGCATGTCATTTCAACCTCATTGTCAAGCATCCGAATTTCTCATCCCTCCCCGGCGTCTT
GGTCGAGTACGACGTCAAGATCCGCCGGCAGATGCACGATGACATGGACGTGGATCTTACCGTCTTTCAGGAG
CCGACGTACTCTGCGTTGTACCAGGCCTTCCTCCTCGGGTACACAACAGGGAAGCGCACCGCAGATGGCTACC
CCTTGGTGCCAGGACCGCCCAAAGCAGTACAGCAGCCATTTCGGAACAATGGTGGAGCAGCTCCAGCCGCGCC
AACGGCAGTTGCGGCTCCAGCAGCTGCGGTCGCCCCTCCCTCATCTCTCAAGTGTCTCGGGTGTGGAAATCTC
GGACACAACGCTCAGGCCTGCGCAGCGCCACTCTACCTCGTTCCCGATGCAGCCGGCCGCCGAGTTCTCCCCA
ATGGCGGCGGCCTCGCCTGTTACGGTTTCAACCTACGCGGCTGCTCCCGAGCTCAGTGCACTTGGGTACACGT
CTGCTCCCTCTGTGGGGATCCAGGGCACAATGCCCAGCGCTGCGCCGCCGGGGGCAGGGCATGAGTTATTTCC
TATCGTGACCCCGCTACGCTGGCAGGCGTGGCGGGAGGAGCTCGAGGCAGCAGGCTGCCTCTCAGAGTATGCA
CATGTTCCCGCTGGCATCCGGGACGGTTTCCGCGTTGGTGTTTCGTCACCCATCACTACGACCTTCATTCCTC
CGAACCATCGTTCCTCTCTCTCTGCCCCACATATTATACAGGACCACATAAACACCGAGCTCCGGGCTGGTCG
TTACTCTGGGCCGTTCCATCCGGAGCGTCTGGAGGCGATCATTGGTCCTTTTCGTACATCTCCCATGGGCGTA
GTTCCCAAAGCGACACCCGGCGCATGGCGCATTATTCAAGATTTCTCCTTCCCACGCGACGACCCCGACCATC
TTTCTGTGAACTCCGAGATTGACGCGGACGCCTTTCAATGTGACTGGGGGACCTTCGCAGACTGCGTTCTCCT
AGTTGTCAATGCCCCAGCTGGCACTCAGGTGTCTGTCAACGACGTAGAGAAGGCTTACCGGAACGTGCCGATA
CATCCTCTCGACCAACGGCATCTTGTGATTGTCTGGCTATGTCTTGTCTACATCGATCATAACGCCGCGTTCG
GCACGTCTAGTGCCCCGGGCATCTGGGGATGGATCGCCGACAGCCTACGCCGCATCTTCCTTGCACATGGTGT
CAACGACCTCATCAAGTGGGTGGACGACTTTGTTTTCTTTCGCTATCCCTCGGCGACAAACCCGGACGGCTCT
TTTGCTTATTCTTACTCGGAGGACTTGATTTTCTCCGTGGCAGCGAAGCTTGGGCTGCAATGGTCTATGTCAA
AGCATACCCCGTTTTCAACAACGTTCACCTACATTGGCTTCGCCTGGGACATTCCAGCCAAGACGGTGCAGAT
CCCGGACAACAAGAAAGTCAAGTATCTCGCCCGCCTAGAAGGCTGGGTCAAGGGCGCGCGTTTCACGCTCAAG
GACTGCGAGGTCGTGGTCGGCTCCCTGAACCATTGTTGCCTGGCGATTCCAGATGGTCGCTCGCGTCTCCCCG
CTCTCTACCGATTCTCGGCCTCCTTCTCGTCGTCCAAGCAGAGGTCAACATTTGCGCGACGCACGATTCCTGC
GGCAGTGGTCGACGACGTCGCCTGGTGGCGGGAGCAGCTCTCCTTGCCCTTCTGCGGCTCGTTGCTGGCAGTG
CCGACGGCAGAATACCAAGGCGTGATCTCGGTGGACGCCTCGACGTCGTGGGGCATAGGTCTGGTCATTGACG
ACCGATGGTTCGCCTGGCGCCTTTCTGATTCATGGAAATCGGACGGGCGAGACATCGGATGGGCGGAGATAGT
CGCCATCGAGCTGGCCTTACGCTTCCTCATTGCATCTGGAGTTCACAACATGCGAGTCACCGTTCACGGCGAC
AACCAAGGTGTTCTAGGCGCGTTACGGTCAGGTTGCTCGCGCAGTCGCCAGCAGAACCGCATCCTCCAGCGTA
TCGTCGCGCTATGTGGCCAGCACAAGATTTGGCCGACTGCTGTGTATGTTCGGTCAAAAGACAATCCGGCGGA
CGGTCCCTCACGTGGAGAGCCTTCCCCTGACATGAGTCGCCTGCCATCCGTAGTAGAAGTCCCGACCCACCTA
TCTCCGTTCCTCTCTCCGTACCTGTAGAAGTCTACCCTCCTGCGCTACCCCATTGTGTCCTCGTCTTTATGTT
CAGTCTCTCTCTCTCTCGTCTGTCTTTGTGTACCTGGTCGATAGTTTAGCTCGCCGCGCACAGTGCGTAATTT
TATAATTGTTGTCTGTTGGCATATTGTCGGACACCGCCCGTTTTGGGCGCCCGTGCTCCTTCGACCGCGACCA
CCATGCCTCCCGCCCGCCATGGACGATCTCGCAGCTCGCACCATCTCTCTCACCCGTCCGGAACGACTCAGCC
TGCTCTGGCAGCACGGCATTCCATCATTTTCCATCCGTACCTCGCCCGCCCGCAAGTCAAGCTGTCGGCTACA
AGACAAGACCAGCTGCGCCGGGTCATGCAGAAGTCTCTCGCGGATTCAACACTGTCAACGTATGCCTCTGGCC
TTCGATCGTTCTTTCGGTTCTGTGCGGAGGAAGGACTGCAGTTCATCGACTGTCTCCCTGCCAGTGAGCACCT
ACTATGCGCCTTCGCCGCCTCTGAAGCAGGCCGCTATCAACATCAGACGGTTAGGAACCACATCAACGGTGTC
CGGGCATGGCACATTATTCAAGGTGTCCCCTGGAACGGTGGGAACCGCCTTCAGTACGCCTTGCACGGCGTCC
AAGCTCTCGCGCCGCCAGCCAAGCCGCCGCGACCTCCTGTCTCCCTCGCGATGCTGGAAGTGCTGCATCTCTC
GCTGGACGTATCACAGCCCTTGTACGCCTGCTCCCTCGCGCTGGCTTCGGTCGCCTTTTGGTCTCCATCCCGC
CTTGGCGAGTTCTGCACTACTTCTTCGCTCTCTTACGATCCGAGACACACACCTGCACGCCAGAACCTTGGGC
CCATGTCATCCATGACCGGCTCCTGCAAGATACACCTACCATACACCAAGACCAAGCGCTTCGCTGGCGACGA
CGTCCTAGTAATGCGACACGATGGTCCGTCGGATCCAATGGCGGCGCTGCGTCAGCACCTCCTGGTCAACAAG
ATGCCAGCACACATTCCGCTTTTTTCATACTGGCATGAAGGCAGATGGCGCTGCagGAacaacagGaCCgTCa

cgGacGgtcCgGCAacgcgGtctggacaGcaccgGGacaTcCccGcctcacaggtcattccggcccgaaagcc
GcGGaacaacGgacTcccTccGcCGacgaggggacCcgcccGgccTGCacgTcacgcGgacacTgGgCcTcgG
aTgcagccgcaccGgacggcagccgggcGgacGAGaccccCacGcaGCagGcGcgcGacGgaccCcaGgccgC
gGAccccGtaGaaGggcacaCcgTgcaGaaaccGcaacgcagcaTTCcgcagCGccgcgcgCccggcatgcaa
aaaCacaacTTGaacaaaatcaTacCTccCccGgaaccAaggcgcaGTccGacGTGcGccTggcccaTcTcca
GCgcgtcAgccaacgCggCGTaTcaGggcactcctcaaaAGacgcCcaGGaacaCAaaacgacCcagacCacC
ccccaccGccGcgcggcaacagGcGcgGaCGAcgaGaaggaagcacTCGCCcaCgcgcCccCTCaCaaacaac
aaGccaccggcCagccAcAacaccGcaaaTcgcccaCgagCaacccaacgccaacaagcccagaggcacgagt
tgacagaacccgggcgtgacgcgagttctgggactcgtgacgggcaagagcgtcctgtcacggtcacgtccgt
cgtccgctctttctctcatacctcgacgcctctacgcgacaaaatcctcctctcttgaccagcatttttcgg
>Bjead1_1_Cluster10#LTR/Gypsy
GTAAGACCTAGACTCTTAGTTCACGCCGTATAATCCAGACAGCGTTTATTCGCGTGCTAGGTATACAGCGTGT
ATGTTAAGAGTAGGTAAAAGTCCGTTGTTGATTCGCGTCCGCTGGTGGCCAGCCACAGCGGAAGCAAATATGA
TACTATACAGTTAGCCCAAAGCGGAAGGTGTCGCGTGTCCAACACGTGGGCTCGGGCGCATCAGTGGAACGCG
CGCCCGACGCCTTCCTTTACAGGAAGGCTCGCTGTACGCCACCCTTACACCCTCCCCCTCTAGACGGAACCGT
AGGTGAAGGCGAACAAAATGAAAGAGAAAAGATTGTACAGGGTATAGTCCGTGCTCAGCACTAAGCATCAGCC
ATTTCAACGTCCTCCGAATTGAGCGGGAATCCGAGTAATCCCCGTAGCTCGTCGACGCTCAATGGCGAGAGTA
CCACAGTCTTCCGGAGCGCTCGCGTCCATTCGCGGATAGGCCCGACCACAACGTGTTGGTTGAGGGTACTTGA
GTCGGCCGCCGACTCTTGCGCGAGGCGGGACAAGTGTGCGTGCACATTGTCGCGTGACAATTCCTGGGCTGCC
GGGAAGCACGTTGACGACGCCAGCGAATGGATGTGGTCGAGTACCGATTCCCCGAGTAGTTCGATTTCGAGGA
CGCGAAGCAGCCATTCTTGGGACAGCGGCCCGGGTACCAGTGACGGTCCGAGTTCCATTACTCGAAGGAAGCC
GCGGAGGCACGCAGCTTGACCATCGTCGAGAGGGCGGTCGCGCTGGGGCGCTATTCCAGGGACGTTGTAGTCC
TTGTTTTGATCGAGGAACAGCTTCCACTGCGAGAATGATGCGCTCGTCAGCGGCGGAGGGTCTGTGATCCACT
GTTCCTCATCGGAGTCATGTCCCAGCGATACCTCGTCCTCGTCGTGGACCTTTGGCGACTGTCGCCGAGTGGG
TGTGGTCGGACGCTCAGATGCCCACGCGGGTCGTCTCCAATCCAGGCGCTGCATGACTGTCGCTCCGTCTGAT
ACGATCTGCTTGGAGTTGGCGGCGTGGATAAACTCGGTGACCTTCTCCAGCGCGACCCATTGACCGGGGCGTA
GGGCTTGTGCTACCAATCGGCGAGTTTCCAGCTCGTTTACTGGCCAGCGCAAATCGTCCGTCGACGCGATGAC
CGCCTTGGATTCATCTTTCTTCAGCCAGTCGAGGCCTGGGGGAAAGTCCGAGACGCTGGGCAGTTCGCGATTG
GTCAGCCTTTTCTGCCGTCGTGTAGTTATTTGCGCCTCTTTCTCCCCCTCCCCCGCCAAAGCCGGTGTAGTCC
CTACGGACGCGATGGCTTTATGGGCCTGTTCGACTAGCGTGCGCAGGCGTGCGGGATCGTGTGCACCCTTGCG
TATGCGCGTCATGTGTGAGTGCTCAGAAGTGGCGGCCCACGCGCTGACGGAGTTGGACTTGGGGCGTAGCACC
GGGTAGCGGCCCCATAGCGCCTCGTCGGGCTCGACGTGGGTGGGACGGGGGGGCAGTACTGCGTCCGTGACAG
TGGGTGGCGGAGCGATCCACGGGCCAGCGGTGGGCTGAGGTCCCGCGTTTTCAGGGTCGTCGATGGCAGCGCG
GCACCAGCTTTCAAGCATGTTGCTGCACTGGATCTGGAACGCAATACGTTCTTCAATCCACTTGCGCGACTTG
TGGTGCCAGGTGATGAACTGCTCCGCTGTGCACCCCTGCAGTGGCGGCTCCGGCCGTCCCTCGCAGTCCTCGA
CGCACAACGCGCGCAGCCGCCGGTAGTCCCTCATCGCCGCCTCGAGCCGCGCATGCCATTCGCTGCGTTCCGA
CCATCGTATGTTGGCAGCATGCTTAGCCGGGGCCTTGCCTGTCGCTGATGTCGAGGGCCCGCCGTCGTCGGCG
TCGCTGTTGTCGTCGTGATGTCCCGAGGGCCAGGTAATGCCCATATCCTCGTCGGGGCTCTCGTAGTGGAATT
CCTCGACGTGCTTCTTCCGCCTGGACCGTCCGGTGGGTTCGTTGGAAATGCGTGCTGCCAGCGGGCGAGATGG
GCCAGCCGTAGGTGTCGGCGCACGCGTGGGGGGATTGGACATACGCGCCAGTAGGTCAGTCGGCGGGGCGGCG
CTCTTCACGTCACTCGCCGTGGAAATCCTGGACGACAATGTGGGGGGCCGCGCGTCGTGGCGGGGCCGACGTG
ATGGCGATGCCCTCCGCCGCGGTGGTGACCGAGCTCGTGGCAGCGGTGCCATATGTCCGCGGCGGGGCGACGC
TGATCTGCGTCCCGCTGGAGGCGGAGTGCGACTCCGGGGCCTGTATGAATCACCCCGTGGCGGCAGCCTGGTG
TCCCGGGAAGGTGGTCGTCCCGCATCCCGTGATGATCGCTGCGACTCGCCAGTCCTCCCCATGCCCACGTACG
AGCCCGCGCTGCGATTGACGTTGTGTCTATCCAGCTCCTCGGCGAGGCGGTCCCGCTCTCGACGGAGGCTGCT
GGCTTCTGCCCGTGCATCGTCTCTCTCCCGCCGTGCCGTGGTCAGGTCTTCCCTGACACGCTCGAGCTCGCGA
CGCACCCGCATGAGATCCTCGTTCGATTTGGCAAGTTCGGCCCGGAGCGTCGTGCACTCCAGACTGGACTGGT
CCGAAGTTGTCCCCGAAGGTCGTTGTTGCGCCTGATACCGGGCACGGGTCTCCTCCGCTGCAAGGTATGATGG
CGTCATCGAGCCCGCGGCGATGTGGTCCAGCCAGTCGTGGCAGAGCTGGCACCCCGCGAAATGCCGGATGAGT
CCCGACATGCGGTCGAAATTCCAGTTGTCGGCCATTGTTGGCGATGTCGTGTCGATGCGGCTAGAACGGAGTC
CTTCGCGCGCACGGTGCTTGAGGGTAGTGACAGCACGAGCTAGAACGGGTGTCCTTTGCGTCGTGCGGTCGCG
GAGTCGTGGTGGTGGAAGGAGTAAAGAGAAAATGCGAGATCATGATATGGCTAAATTTTGAGCGCCCGCGCTC
GGGATTGATGGCTGATCTGACGTGAATAAGGAAATGGTCGGCCAGCGCATCAATTGTTGGGATCACTTGCGCG
CCTTCCTGCGCCTCGTCGGGGGTTTCCTGTTCTGGGTTGCCCGCCTGGGTCCCTGAGGCGGCCGGCTCAGGTC
CTGCCAGTCCGCGGCCCCTTGAGTATCCAAGTACTGATCCAGTGCCTCAAGCTCGTTGACGTGGTGGAGTGGT
TCCCACGTCTCGTCTCCGAGGTTCCATTTCACCTTGAACCAGATTCCCGGGCCTTCCCACGCGTGGCTAACGA
TCTCGTCGACGAGATATTCCCTATCGTCAGGTTCGCCGAGGTCGTAGTATACGTGAGCTTCGCGGTGAGGGAA
TATAGTAGAGTCGTTAGGTTCGTGTCGGCGGAGTAGCGAGACGTGAAAGGTAGGGTGAATTCCCCTCGACGCC

AGCTGTGTAGGCAAGTCCAGAGTGTATACGGAGTGTTCGACGTCAGCGCGCGTGATCTTGTAGGGTCCAATGA
ACTTCGGAACTAGTTTCCTGGCTCGTCTTGCGGGTAATGATAGGTTCTCGGTTGACAGGTACACTAATTCCCC
AACCTGCAGCGGCTGTACTTGACTGCGATGCGCTTTCTCGTCACGCCGATGCTTGTTCGCGTGGTAAGTCTGA
TCAATTCGACTGTCGATAATTGAGTCGTGCGCTTGCAGTAGATTGGCCACGGCTCTATCGGCGAATTGCGTGA
CACCAGGGAGTGAACTGGACACTGGGAGGTTGAGCCCCATCCTCGGCATCCACCCATAGTTTAATTCGAATGG
TGCAAAACCCGACGAACTACTGATGCTTGAGTTCAAGGCGAATTCGACCATCGGAATCTTCGTCGCCCAGTCG
GCCTGGTCGGGTTGTACCATACCGCGGAGTACTTGGGTGATTGTACGATTGGCTCGTTCAGTAACTCCGTCAG
TTTGAGGATGGAATGCCGTCGACATGAGGATTCTTGCACCCAGGAGTCGGTGAGTCTCTCGCCAGAACTTCGA
GTTAAATCGTGGGTCTCGGTCCGAGATAATATCGTCGGGGAGTCCGTGTAGTCGAACGATATGTTGTACGAAT
AGCCACGCTAACTCTGAAGCCCGCACCGTAGTCTTGGTAGGAATGAGGTGTATCATAGACGTGAGCCGGCAAA
TAATAACCCAGAGGAAATCAAATCCTTCGGAGTTCGGCAAGGGTCCGACGAAATCCATGGCTATCGATCCCCA
CGGCCTGGATGGGATTGGTAAGCTATGAAGCAGTCCAGCCGGCGGGGCATTGACAGTCTTGGTGGTCTGACAG
ATACCGCACGAATTGCAGAATTTCTCGACATCGCGAGCCAGGGTTGGCCACCAATACCATCGGCGCACGTAGG
CATCCGTCTTCCGGGTACCCAGGTGTCCAATGAGAGTGTGTGCGTGGTCTATGACCCAACCCACGAGCGATCG
TTTCCCAATGTTCACTCGAGGGATGCAGATCACATCCGAGCCTGCGCGGGACTTCATGAACAGTAGTCCATCG
CCGAGTCTGAATTGGGAGTAATGGTCGGGCTTGTCGATAACTTTCTTGAACGTCGTATCCTTGGCGTACCCGT
CACGCACCGTCCTCAAGAATTGATCGTCTCCCTCGGTGACGGTACTGAGTTTGGCAACCCCGACGGGTGTTTC
GAGAGCGAGCGGGTCTTCTGCCGTGGCCTTGTCTCCTCTGGTACCTCCGATGGCGAATAATTGCTCACGATGG
ATGACTTGTCGGGCAGGGAGTGGTACATCCTGGCTGGTGGGGCCTACCCGACGAAGTTCGTGCTCTCGTAGGC
GGTCCAACGGCAAGTACTCCCCTTCGATGTCAATCCTCACGTCCGCGGAGACGTAATCATCGTACGGGTGATG
TTCTCCTTCTCTGTCATTTTCGAAGTATCGGGAGCAGCAGTCGGAGACGACGTTCTCGACGCCCTTCTCGTAC
TGAATGTCGTAGGAGAACCGGGACAAATAATCCATCCAGCGCATCTGACGGTACGAAAGTCGGGACTGCGTCT
TGAAGAACTTCAACGACTCGTGATCGGTGACGATGTGGAAATAGCGACCGAGTAACTTGTCCTCCCACTTAAG
TAAAGCTTCGAGGATAGCCAGCATTTCCAGTTCGAACACGCGGTATGATTTCTGTGCGTCTGTGAACTTTTTA
GACATGAATCCAGCGGGACGGCACGTTTTCCAATCTTCGCCTTGTCCGTAATACGCCCCTGTTCCTGACCGGG
AGGCATCGCAAACCACCCAGATCGGTACTCCCGTACGGGGATCTATGGGTTGTAGTATGGGAGCCTTGCAGGC
AAGTACCTTGATTGATTCGAAGCACTTCTGGTGTATAGGACGCCACTCAAAGGCATGGCCATTGCGAACTATC
GAAGACAGTGGGCTCGTGAACGAGGAGACGTCCGGCATGAATGGCGCGAGGTATTGTACTAGCCCTAGGAACC
GTTGTATCTCTTCGTAGTCCCTGGGTGTACGCCACTCTCGTATGCGGGCCATCTTATCCGCGTCCGCGTGTAG
ACCCTTATCGTCGATAATGTGGCCTAGGCAATCCATTCTCTTCGAATAGAGATCGCACTTGCTTGCCTTGAGA
AACAGGTGGTTGTCCCTGAGTGTCTCGAACACTACGCGAAGATGCCGTTCATGTTCCTCGATGGAGTCCGAAT
ATATGAATATATCGTCGAGGTATACGTGTACAAAGATGCCTATGTACGCGCGGAATATGTGGGTCATGAGGCG
TTGAAACGTTGCGGGTCCATTACAGTCCCCTTGCTGCAGTACTTGACTTAGCATGGTGCCATAAACGGTGGCG
AAGGAGGTCTTGTGAACGTCTTCAGGTACTATTCGAATTTGCTCGTATGCGTCGGATAAGTCGATCTTCGAGC
GTATTGGTGCCCTCGCGACGTCCATTCGAATTTGTTCCTGGTCGGGGAACGGAGTAACATCCTTGACTGTATT
CTCGTTTCGTTTGCGACAGTCCACCACCGTGCGCAGTGAATTATCCTTCTTCGGGATACACAGCATAGGCGCG
GCTTGTGATACGGTGGCCGGAATCCACCATCCCGCCTTCTCATAACGAATAATCTTTTCCATAAGGAGTGGTT
TGAGTGCCTCTGCGCACTTCGGAAGGTGGTATGAGTAGCGAATAGTCGGGTCAATGAGTGGTATTTGATGATT
AATTTCTCGGAGGGGTGGTAGTACTTCGGGGACTCCGCTAGTGATGTCACGGAATTCTTTGAACCAGCGGTCG
CTGCGTCGTACCCGCTCTTCGCTGGTAATGGACCGCAATATGGTGTCTCGGGAGACGGGTCGATTATTGATGG
TATATGGTGATATCTTCTGTTTTTGTATTTCTTGGGAGTCTATTGCAGCGATTCGGGGTATCTTGGAAGACGT
GGTTGTCACCGTCGACCATTTTTGTGAGGGATTTTCCTGGAAGGGGTGGTATACGAGTCCCTGGCTCGTCCCC
TTGGTTGCGGGAGCCTCTTCAGTTCCCGCACGGTCGAGTCACTTGACGTTGGGAGGTTTGGGTCCCTTGGGCG
TGTGCGCTCGATCATTAAGACCTTTGCGTCCACGTCGGTGGTCCCCTCCCCCCTTGAGCGCACTCAGCACGAG
GTCGCCGATCTGTACCGTATAGCTCTGGAAGTTGATGATCATGCCGAATTCGTCCATGAAAGGAATGCCGAGT
ATAACGTCGTAGTGGTCGATGTTTGCAACGTCGAAGTAGATGTCGCGGCGTTCGCCCTGGATTGTGATCGTCG
TTCTGGCGCCGAAATTGACCTTCGAGCGGGAGCCCGAGCATCCGAGTTGCAATACGATGGGGTCGGACAGCGC
AGTGGTCTCGAGCCGTGATACCGTAGCGAACTCGTGTGATATCGCATTAACGGTGCTACCGGTATCCACCAAA
ACCAATGCATCGACCCCGCCTACGGAGCCGAAGAATGTCATGCATCGCCCACGCGCTTTGGGACGTTCCATCG
TGGTTGCTGTAGTACGCATGGCAGTGCGGTACCCTCGATCGTGAGTTGCGACCATGGAATAGAACTCATAGCG
ATTCTCTCGCGCGTATCGCTCCTCGCTCCGGTCGTGCTCGACGGACAGCTCGAACGCGTCGAATGCGTCATTG
GAGTACTCGACGTAGCCTTCTGGGCGGTCAGGCACGTCTTCGGCTGGCGTGGTTCCTTCGGCAGAGGCAACCG
CATACTGCGGGTCAGTGATTTGTCGTTCCACAGAGCGGATTCTTGCTCTAGTAATATCACGCTCGTAGTCCGC
GCGAGTCGTTGCGTTCCGCGAGACTTCATTCTGCCTTAGAATATCCGCGTTCGCCTCGGACAATGTCAGCCAC
AGTTGGAGTTCACCACGCAGGAGGTTGGCTTCTTCATGGTACCTTGTCGCCGTGACGTTGTGGAGGTCACGTT
CCCGTCGATAGCTGTCCAGCATGCGCGCTGTTTGTAGGTCCAGACGGGCACGGTCATTGACTATGAGGAGGCG
TTCGTAGACGGTCATGCCAGTCAGGGTGTCCGTGGTGAATCGGAATTCTTCGAGTTGCCTGACTTGTTCGACT
TGGTTGGGTTGTTCTGCTCTGGTATCGTTGATGAGCTGAATGGCCTCTCCAAGTCGATCGAACTCGCTCTCGA

TCCTATGAAGACGCATGCGCAGCTCGGCATTGTCGGAGTCTTGTTCACGTAGTCGCTGCCTGACTGACGTTAG
GAACGCGTCACGATCCATTGTCTCGATCAGCGCATCCCACGTATGCTGTGTGACGTAAGGGGATGTTGACGCA
GGCTGCGGTGCAGGTGGGGAGGAGTCAACGTAGGGGTCGGTGTGCAGGGAAGGCATGGAGTCGTTGCTGGTAG
TGGACTCAGCATCGCCTTGAACCTCAGCTGCGACATGCGGGCTTTCCACGGGGTCATCGTCACCCTCTTCGGA
TTCCGAGTCGTACCCGTAGAAGAAGCGTCTTATGGACATGCCGTAGAGTCGTTCGTCAGCGGCGTCGGATTCT
GCATCGGTGTACATAGATTCTAAGTCGCTACCCGTGTCGTACTGGGACCCAATAACGCTGCCTCCGTCACCTG
ACACCGAACCGCCGTCCTCGGTCGATCCCTCGCGGCGATACGCCGTCATGACTCGCATTTCCTCTGGCCGTTC
CTCTGCACCCAGTTCGTCGGTGGTGTCCTGAATTGTCTCTTCCGTAGTTCCGTCGAATTCAACCTCATCAGCC
GCAATCTCTATATTGCGCAATGACTGTCTTCCAGCCATCGGGCACTTAGGATCGGAGCTGATGTGGCCGAACT
GTTTACAGGCGTAGCACTGGGGTCCTTGTCCTGAGCGACCATTGTTGATGACCGGTCGGGCAGACGCTGAGGA
GCCTACGTCTCGCGGGTAGGGTGTGTGCGATACTGGGGCCCTAGACTCCGCATTTTGGGAAGAACTACGGTCC
CTGAATCGAACGTGGCGTGAAGACGAGTTCCTGTTCTCCGTCCTCGGCTTCCGCTCATCCGTGCGCGTCGGGG
ATTTCGCAGTGGACGACGTCTTCTTGGATTCCGCAGCCTTTCTCTTCGACTTCTTGTTGACGATGTCCTCGAC
GGTGAGTGCCGCCGACAATAAGTCGCCAATGGATGCGAGTGCCGGGGTGATGCCGTACTTGAACTCGATAGCG
TCGGTGATTTCTTCGGGCAGTTTCTCCCAGAAATGCCGACTGAGTGTGTAGTCATCGGGGGCAATGGGCATCT
GGCGCGCATGACGTGTGAGTCGGTTATACAGTGCATTGGCACCTCCGGAGTACGAGTACGTGACGGCCTTGAA
GGATTCAGCCGCCTCTTCGGCGTTACTGACCCGTACGAATCGAGTGTACAGGGCGCAAATGGCCTCGGTATAT
GTCCACTGCTCGATGTGTAGTTCGACCTCGTCACGGTACCATCGTTCGGCTTCGCCTGTGAGGTTCTGGCCAA
GCCATAGAAGGCGATCTTCGTCGGCGTCGATGCCACATAGCCGGGCGAGCTTCAGCCACCGGATATGTGCCCT
CAGCCATGTATCGAATACGTCATTACTGTTCTTGCCGTCGTAGTCCTTCGGTGGGACGGTTTGATCCGTGAG
TTGCCAGTGTCTTTTCGAGGGGGTCGCCCGAGCATCGCGTGGATGAATTCGCGTATCCAGTCGCAATTGGCTT
GAGTGAACGCCGCGTTGGCTGGGTAGGACGGTAAGCCACCCACGAACGCGCTGGGCGTCGCTCCGAACCCGTT
GTTCTGGTACGAGAGTGCGGCTGTGCTGGGCTGGCGTGCTGGTGGTAGTGTCCTCGGGCTCGCTCGCGCTTGG
TACGCAGAGAACCGCGGTCCCATCCCACGACGTCCTCGGTTGGAGATAGATCCCCCACGACTGAGGTGCGGAG
GAGTCTGTCGCGCATCTCGACGGAGATAGTCGTCGACTTCGTCCTCCTCGTCGGAGTCCTGGTCGTCCGATGA
ATGGGGTTCACTGGGAGGTCCTCCTCCAGGCGGTCCTCGACCGGGAGGACGTCCGCCACCTCCTCCAGCACCA
TGACCGGCACGTTCCGAGCGATGAGACCCTCGAGAACTCGAGTCGTCTCCGGGCTCGCTCGGGGGGTTCGCGA
CCATGGAGTAGTCTGGCCTTGGCGGGGGATTGTTGGGCGTGGGGACTAGACCACTGTTTCGAAGTCGCTCGTG
TGGTTCCTTCCCGTGTACATACTGAATATTAGGATATTCAGCAATGTAGCGGGGTCCCTTGCCCTTGCCGGGT
ACAGGCTCGTCTTCGGAGTCCTGCACCTCGCGCAACCTGGCCCTGAAGTACTCTTCGCGTTGGTTGTAACGCT
TCTCGGACGCTTCTTGACTTTGCGCGATGAGTTTATCCCAAGAGTCCGGTTGTTCCGTCGCGTCGAGGGCACG
ACTGACTACCTCGGTGAGGAATTCGAGTTTAACGTCGATTTCGGCCTGGGATTGTCGAAGCGTGTCGTCGAAT
TCAGTCGCTACTTCCTCCATTTCGTCACGGAGTTTTCGTCGCAGCGACTGGAATCGATTAGCGATCCTGGTGA
GTCCGTCGTTGGCGGTTTCGGCCTGTTGAAAGAATGCTTGATACGCGCTTTGAATCGTCCCGGGGGATATGGC
TCGGGCAGCGGTCCCAGCCCTCTTCGAACTGGACCCGCCTTCGCCTTTGTCGTCTTGGGAGAATGGTCTTGCG
TTGTCTTCTTGCGCGAAAGTCCCATAGTGCCCCACATCCTCTTCGGATGATTGAGGTACTACGGTCGCGTTCG
TCTCGAGTCGGACCGTCGTCTCCGTGCCCGCGTCGCTTCGCCGAGTTTCTGGTCTCGTCCACCTGGAATGCCG
TGTCTCGTGGGTCTGGTGGTGCATCATTGCCTTGCGGGGTCTTGAGTTTTGATGCGGTATTGTTGCGGGTGTT
GGTGGAAGTGGTTGAAGTCGCGCGAGCTGGCTTCGGCGCTGGCGCCTTTCTCGGTGCGCTCGGTTTCTTCGAG
CCGGTCGTTGGCGCCGTCGGGGGCGCGCCATCAGCGCGCGACTCGGCGCGAGACGTGCTTCGAGTAACGACGC
CGGTTTGGGTCGCACTCATGGGCGAGTCGCGTGGGGGAGGGAGATTTTCGGTGTTTGTTGTTTTCTATGTGCG
GTGTATACCGAGTGGAATAAAGCACGAAGGATTAAAAAAGATGTAAGACCTAGACTCTTAGTTCACGCCGTAT
AATCCAGACAGCGTTTATTCGCGTGCTAGGTATACAGCGTGTATGTTAAGAGTAGGTAAAGGTCCGTTGTTGA
TTCGCGTCCGCTGGTGGCCAGCCACAGCGGAAGCAAATATGATACTATACAGTTAGCCCAAAGCGGAAGGTGT
CGCGTGTCTAACACGTGGGCTCGGGCGCATCAGTGGAACGCGCGCCCGACGCCTTCCTTTACAGGAAGGCTCG
C
>Bjead1_1_rnd-1_family-263#TIR/MITE
CACTGGGTGGACGATCCAGTTTACCGTCGGCTTAATCCGTAATAAATCCGGTAATTATTCCGAATAGATTTAT
CGCGGTTTTCAGGTCAACAAAATCTGATGAAAACGGTAATGTTGTTGTAACAAAACCGCCAAATATGACTAAA
CTGTGACCAATCTGGATGTTATATTACGGATTAATCCTATTTTGGGAATGATAAAAACGTGTTAAATCTTTGG
GAATAATTACCGGATTTATTACGGATTAAGCCGACGGTAAACTGGGTTATCCACCCAGTG
>Bjead1_1_Cluster23#LTR/Copia
TATAGTCCTCCTTGTTTTTGTATCAGTGATCTTCCTCATTCGGCACGCTCCTTGTAGTTTCCTCTTTCGGACG
ATCCCTTGTACTTTGGGTATATAGTCCTGGCTCTCTGCCCTCGCTCCATCGTGCTCTTTCCCTTCGCACACGG
AGTGAGTATTTCGTATGCCCTATGCTCTATGCCCTATGCTTACGCCCTGTACTAGCTCACGCGCTTTCGCGCC
CTCGTTAGGTACAGAATATTATCGTGCTCTTTCCCTTCGCACACGGACTCACGCGCTTTCGCGCCCTCGTTAG
ATCAGACATCGCACACCCGCCCTCGGGCGTCGCTCTGTCGCTTTTCCCTGCCTCCCATCCTCCCTCTCTGTCG
GATACCGTCTCGCCTCTTACTTACTAGTATATGCATGCTCAAAGCAAACCTGGGCCAGTATTTGAACAAAAAC

TATACCAGGATATCCTTCATCAAAATGCTCATGGCTGGATCAACATCGATTTGGGGGTTTGTGGGAGGAATTT
GGGCTTGCGACTGCAACCACTAGTGAAACTCAAGGGCTCCGTCAACTACAAGCCCTGGTCGCTCCTGATCCGT
CAATACCTTCGGGTCACTGACACCTGGACCATCGTCGAAGGCAAGCAAGCCAAACCCGCCACGCCCGCCGACG
ACGTCGCCAAGTGGATCCAAGCAGATGCGGCTGCAAAGCTCATCCTGCTTCAGACCATCGACGTCTCCCTCCT
GCACCTGATCGAAGACGACTCGCTCACCTCCGCCGCAAGCTGGACTAAGCTCGAGGCGAACTACGCCGCCGCC
TCCACGCTCACCACGTACTACAAGTTTCGAGACTACTTCTCGACTGCGATCGACGGCTCCGCGCCTCTCGCCG
AGGAACTCGAAAAGCGTGGTCGTCTCTGGCAAGAGGTTGAGAACAGTGGCATCCTCGACAAAAAGGATCTTCT
CAAAATCTTCGGTCTTCTCTCGTCCATCCCCGACTCGTACTCGCACATCGTCCAACCCATCCTCGCCGTCACG
AAGCCTGCGGATCTGAAGTACGATGACATTCGAGCCAAGCTGCTCGATGAGGCGTCGCGTTCCGCGTCGTCGA
ACGTCTCCGCGCTTTCAGTCCCTGGCCGGTCTTCTTCGTCCAAGCCGGACAAGAAGAACAAGAGCAAGAAGAA
GTGTACGTTTTGCAACTACACTGGACACGTCGAGTCGGACTGCAGGAAGAAGAAGGCAGCGTCGGAGGAGGCA
AAGGCTAAGGCTGCTCAGCCGAAGCCAAAGGCAAATGGACAATCTGGTTCTGGTTCTGGTAGTACAACAACAC
CGACTGGCAATGCTGGCGCCATTACGGCCCAGCCCCCACCTGTAGAGTCTGAGAAACCGAATGGATTCGTTAT
GTCCTCGTATGTTCAGTCTGATAACTACTGGATGGCAGACTCCGGGTGCACCCTCCATATCTCTCCTTACAAA
ACCGACTTCGCGTCGTACTCTGAACATCGCACGCCCAGCTTCGGCACTCTCGCCGACAAGAACTCGAGAATCA
ACTTCTTGGGTACTGGCAGCGTTCAGGGGGAGATGAGCGTAGGGACTGCTCAACAGTCAGTGAAGTTGGAACA
CGTCACGCATTCACCAGAGTGCGCACATCGTATGTTCTCTATCAGCGAGGCCGACCGCATGGGCAACACCGTC
ATCTTCGGTAACGGACAAGTGCGAATCCTGAACCGTGCTAGCGACATGGTTCTAGGCATTGGCCACCTCAAAG
GAGGACAGTATTGGGTTAAGTTCGCGCCTTCACCCTCATCCTCCTCATCTTCCAACAATCACGTCGCCGCCGT
CAAGGCCGTCTCTCCTGAGATTGCGCACCACCGTTTTGGACACCTGAACTGGACGGCTATCAGTAAGCTTCGC
GCTACTGGTGACCAGATAGTGAAAGGGCTGGTCTTTAATGACGCGCCGCAGCCTAAGCATATCTGCGAAGGTT
GTGCCCTTGGGAAAGCCGCTCGGCGATCCTTCCCTTCTTCCGATTCTCCACGAGCTGCCGCTCCTTTCGACCT
CATCCACACCGACCTGGCTGGTCCAATGCAGACGAAGTCAGTCTGCGGCGGAAACCTCTACACGGCATCCCTC
CTCGACGACCACTCGGACAATGCTTGGGTGTTCTACATCAGGCAGAAGTCAGACTTTGAAGCGGTCTTCGACC
AATTCATCGCCTACGTCCGCACGCAGTTCGGCGTCACGCCAAAGGGAATCCAGTCAGACAACGGTGGCGAGTA
CATGTCACGCTCGTTACAGGATAAGTTGAAGGCACTCGGAATCGTCCACCGGACCACCACACCGTACACGCCA
CAACAGAACGGAAAAGCGGAACGCTACAATCGGACTCTGTTCGAGGCAACCCGCGCACTCCTACTCGGTGCTG
GTCTTTCGGAGGGTTTTTGGGAAGAAGCTGCTCGCACAGTCGTGCACGTCCGAAACCGGTCCCCTAAACAAGG
ACTTGGCTGGAAGACACCGTTGGAAGCACTCACTGGCAAAGTTCCGGATGTATCCTACTTCCGCGCTTTCGGT
TGCCAGGCCTATGCCCACATCCCGGATAACAAGCGCACAAAACTCGAGCCAAAAGCCGAGGAGTGTGTGTTTG
TTGGCTACGAGACGAATACTAAGGGTTATCGTCTATGGTCCAAACGTAGCCGGACCGTCATCGTCAGAACTGA
TGTAATCTTCTCGTTATAGTCCTCCTTGTTTTTGTATCAGTGATCTTCCTCATTCGGCACGCTCCTTGTAGTT
TCCTCTTTCGGACGATCCCTTGTACTTTGGGTATATAGTCCTGGCTCTCTGCCCTCGCTCCATCGTGCTCTTT
CCCTTCGCACACGGAGTGAGTATTTCGTATGCCCTATGCTCTATGCCCTATGCTTACGCCCTGTACTAGCTCA
CGCGCTTTCGCGCCCTCGTTAGGTACAGAATATTATCGTGCTCTTTCCCTTCGCACACGGACTCACGCGCTTT
CGCGCCCTCGTTAGATCAGACATCGCACACCCGCCCTCGGGCGTCGCTCTGTCGCTTTTCCCTGCTCCCATCT
CCCTCTCTGTCGGATACCGT
>Bjead1_1_Cluster14#LTR/Gypsy
GCTGTGAAGGGCAGGTCGATACGGGCCTTCAGAGACGAGGCGGGCGGAGGCGACGCGAGGGGACGAGCGAGGA
GGTCGACGAAGGCGTGCGAGGAAGTCCGAGTAACGATGCCTAAACTGGCGTTGGTTTCGCAAGAGGTACAAAC
GGGGCGGTCGCAGTGATAAGGGAAAGCGAGTCAGGTACAAACGGAAAGCGTGCCGAAGGCGCAATGCGGGTAA
ACGAACGAGAAGAATGAAAGTAACCGGATCGGGAAGGTAGCTACCCTAAACAGGTAAATGAAAGAACCAGGTT
AATGGAACGTGCTACGATTGGCCGTGGAGAGTCCGTGCGAGGCGTACAGAAATCCGAGAAGTCCGAGAGCGTA
TCTAGAGTGTCTGTAAGTGCTAGGAGTGGCCGTGATGTGCTAGATAGTCCGTGTAACGGTCGAGCCGTTACAT
CATCCCCCTTTCAAGCGCGAGCGGAGCGACGCTTTCGAGTGGGAATGATAGGGTCGAGCTTTTCAGCAGGACG
AGAGACACGCCCAGAGCGGGTCGTGGTCTGTGTGGGTGTGACGTAGTTCGAACGTAGGTTTTCGCGGACGGGC
GACGGCGAGCGAGGACGGGCGGCAGCGACGGGTACGGCGGAGGCAGCGGGCAGCGGAGCGGACGGCGGGACGG
GCGAGGCAGCGACGGGCAACGGGACCTGGGGAGCAGAAGGGGAAGAAGGCAAGTCAGTATTATCTGGGACGGG
TGCAGGGAATGTCTGGTCGAGGACGAGACGATGAGGACGAGGCGCGCGCGGGTGACGGCGGTGAAAGCGCTCA
ATGAGCTCGTTGGCGGTAGTAGGGATGTCAGAGAGGGGAACCCACGAGTCCTCGTCGTCCGGGAGAGACTTCC
AATGGACGAAGTACTCGTAGCGATGACCGATCTTGCGGGAATCAAGAATAGAGTCGATAGATTGAATGGATGT
GGGAGAATGGGAGTCAGGGTGAAGGGCGAAGGGTTCGGGTGGAGCGTGCATGTGAAACTCTGAAGGATCGTGG
TAAGGTTCAAGAAGAGAGACGTGGAAGACGGGATGAAGGCGAGAAAGGTAAGAAGGGAGACGAAGTTGATAGA
GAAGGGGGGAGATTTTTCGTGAGATGGGAAAGGGGCCGAGACGGCGGTGATCGAGCTTGTCGGACGGACGTGT
AGTCTTGATGTTGCGACGGAGAAGCCAAACGAGCTGGTCGGGCTTGTACTCGGGTGACGCCTGGACGTGCTCG
TTGTAATAACGCGACTGGACTTCTTGAGCATGTTTCAGCTCGGCGCGAAGTTCCGCGTGGATGTGGTCGAGGC
GTTGGGCGAGATCGTGAGCAGCGGGGACGGTGGAGCGCTCGGTAACCTGAGGCTCGAAGGTAGGGTGGTAGGC
GTAGTTCGCGTAGAAGGGGGTCTGCTTGGTCGAGGCGTTCTCTGCGTTGTTGAACGCAAACTCGGCGAGGGGA

AGGTAATCGACCCAATCGTCCTGCTGGTACGAGCAGTACGCGCGAAGGTACGTCTCGAGCGTTTGGTTGGTAC
GCTCTGTGAGGCCGTCGGTCTGCGGGTGATACGCTGTCGAAGCGCGGTGATCTACTTGGAGCAAGGCGGTGAG
TTCTTTCCAAAACTGAGAGACGAAAAGCGAGCCACGGTCCGAGATAACAGACTCGGGGAGGCCGTGGTAACGA
AAGATGCGGTCGAGGAAGAGCCAGGCGAGCTCGGAGGCGGTCATGGACTCTTTGCATGGCACGAAATGCGCAG
CGCGAGTCATGCGGTCGCAGACGACCCAGATCGAATCGTACGCGTGCGAGAGTGGAAGCTTCACGATAAAGTC
CATCGTTATCGCTTTCCACGGTCGCGTCGGGAGCTCGAGGGGCTGGAGAAGGCCGTAGGGCTTGTGGCGAGGG
GTCTTGATGCGAGCACAAACGTCGCACGCTTGAACGTAGCGGCGAACGTAAGTCTGCATGCCGGGCCAGGAAT
AGTCACGCTGAACGAACGCGAGAGTGCGATTACGTCCAGGATGGCCGGCAACGATCGCGTCATGGCGGGAGTG
GAGGATATCGGCTCGCAGCGGCGCGGGGACGAAGAGGCGGCCCTTGTGGAAGACCATGCCATCTTGCGCTGCG
AAATCCGAGTTGCCGTGGGCCAGAGCTTCGCGCCACTCGACGTCGTCCCTGAAGGCATTCTGGAAACGCTCCA
GAAGGACGGAGTTGTCGATAGCGAGCGTGGTTAGCGCGGAGATGTGCGTGGGGGCAGAGACTGTTGGCGGAGA
GGATTCTGAATGGGGAAAAAGAAGTTCAGTATGCGTGGCGGTCAAAAGTGTCTGGCGCTGCGTTTCTAGGACG
TCATCCCCCCTTTTAGGCGCGAAGTCTGGCCGTCGGGAAGGAGAGTCTGCAACATTTTTAAGGCCGGGGCCCC
AGACGAGACGAAAATTAAATTCAGAAAGAAACATCGACCAGCGGGCCTGGCGACGATTAAGAACGCGAGAAGA
CATGAAATACTCAAGGTTTTTATGGTCTGAAATGATGGAGACGGGTGGTGTCGATCCGTGCAACCAGGCGCGC
ATGTCGCGGAAAGATTCAACAACGGCTAGGAGCTCCTTGTCGTAAACCTCATAGTTAATCTCGGCTGGCGTGA
GCTTGCGCGAGAAATAACCGACGGGGTGAAGGAGTCCAGCAGCGTCTGGCTGTTGGAGTACACCTGAAATTGC
GAAGTCCGAAGCGTCAGTAGCAAGGGTGCACGGCAACTTTGGATTGAAGTGCCGCAGAACAGGGTAGGATAGG
AGCGCTGCCTTCAGCTCGTTAAAAGCTGAGCGACATGCGTCCGAGAAGACAAAAGGAGTGTCTTTACGTGTCA
ACGCGTTCAAGGGCAACGCGATGCGCGCGTAGTGGTCAACGAAGCGACGGTAGAAATTAGTGAAGCCAAGGAA
AGCCTGAATATCCTTGACGGATTTTGGCTCTGGCCAATCAGAAACAGTCTCTAGCTTCTTTGGGTCCATTCGA
ATGCCGTCAGCACCGAGCAGGTACCCGAGGTAGCTGACTTGCGAGCGGTCAAACTCGCACTTTTCCGCGTTAG
CGTAGAGGTGAGCTGACTGAAGGCGATCGAGCACCTTCATGACATGATCGTCATGCTCAGACTGTGAGCGTGA
GAATATGAGAATGTCATCCAGGTAGACGACACAGAACACGTCGAGGAAGTCTCGGAGCGTGTCCTGGATGAAG
GCTTGAAACGTAGCGGGGGCATTAGTGAGTCCGAAGGGCATGACAAGATACTCGTAGAGGCCGAGAGGCGTAC
GAAACGCGGTTTTCCACTCGTCTCCCTCCTTGACGCGTATGAGGTTGAAGGCGTTCTTCAGGTCGAGGACCGT
GAAGAGAGAACAGCCTTGGACGCGGTCAAGAAGATCGTCGATAAGCGGAAGGGGATACCGGTTCTTCTTGGTC
ATAGAGTTCAGCCCGCGATAGTCGACGCAGAGACGTAAGTCTCCGGACTTCTTGCGAACGAACAGAATGGGAG
AAGCCGCAGGCGAGGTTGAACGTCGGATGAACCCCTTCTTGAGGTTCGTCTCGATGTACTCCGTCAGAGCACT
ACGTTCCTCGGGCGACAGACGATACATGGGACCAAAAGGAGGGGACTTACCTTCATCGAGGTCGATGCCGATG
TCGTACGGGCGATGCGGCGGAAGGTGGTCGACCTCGATAGGCGAGAATACAGAGGCCCAAGGCATGTACTTCG
AAGGGATACGGGACGTAAAGTCGCGGGGAATATCCACGGGTGGTGGATCATCGTCAAGCCCCAGATCAGATGC
CTCGGTAGACATTGACGCGAGCTTATATGACGGCGAGTCTACGGGGTGGAAACGAATCAGCGCAATAGGTGAA
GACTTGGAATACTTCAGAAAGCGACGAGGGTTTACAACCGCAACGTCGGGGCGCTTGGGCGAAGGGGCTTCTG
CAGAGGAGCGACCGAGGCCAGTCCATGCGCGAAGTGTCGAAGTGAACGAGAACGGAGAGGGCGTCGTAGCAGT
CGTCGCAGACTGCGGGAGTGAGGCGTTGGCCAAACGCTCACGAGGGACAGGATCCGGAGGCGGGGTCGGAGTC
GAAGTCGAGACGCGAGTTGCAAACAACGAGCCGTTAAGACCGAAGCCTAGGCCGACTGTCGTCGCGACGGAGT
TGAACGAGGTAGTGAGCGAGGGGCGAGCAAGACCGAAGCCTTTTGCCGAGACGGTGACGGGATGCGAGCGCGT
GAGGTTGCAGCACGAAAGGGAGAGACTTGCATCTTCCCAGTCGACGTCGGGGTTGTGGCGTCGAAGCCAATCG
AGTCCTAGAATGACGGGGTATGCGACAGAAACCACACCGAGAGTTATAATCTCAGCATGTTTGTCTACGGTCA
GCTGAGCGACGACGTCGTGAGTAACCAACCCCGAAGCAATGGGGCGGTTGTCGACGGCAGTGACAGGCGTGGG
AACGTCCTTGAGACGTCGGGGTATAGAGTGACGTCGGGCAAATACGTCAGAGATGCAAGATCCTGACGCACCC
GAGTCAACGAGGGCAAAAGTGCGAATAGGCTTCCGGTTGGGAAAGCAAAGTGAAACATTGATAAAGAAATGGT
CAGGAGAATTTAGAAAAGAGTTCGAAGCGAAGACGTAGAGCGGAGGAGGAATAGATGAGCGTACGGGTATATA
TCGTCCGTCGGTAGAACGGACGTGGGTGGGCTTCACGGCACGATGCGATAGACGGGTCCCTTCGTCAACCGGA
AGCCCAGGGACCAAGGAAGGGCTACCGGTTACGCTTTTCCCGAAGGAGCAGCGTTGCGAGTGGCGGCGCGTGC
CTTGTAGGCAGCCTTCGCTTTCGCAGACATATTGGGACAGTCGTCGATGAGGTGCTTGCCTTGTCCGCAGTAA
TAGCAAAGGCCCTCGCGACGGCGGCGAGCCTTTTCGTCAGGTGTCACAGGGCCTCGACGTACGGCGTCGATTT
CCATAGGAACAACCTGCGAGGACGAAGCAGCGGGAGGCTGAGGAGCGGCGGGAGGTGCAGGCGTCGCGCGAGG
ACGGTCCTTGCGTTCAGAGGCGGGGCGATGCGAAGCGTTCTCGTAACGCTTCTCGACTTCGCGATCGTGGACG
CGGTTGTCGATTTCGATGCACTGGTCGACAAATTCGTCGAAGACGGTCGAGCGGTTGATGGTGATGAGGAGGT
CCTTGACGGTGCCCTTGAGACCGGCGTAGAAGTAGTCGATCTTGGAGAAGTCGTCGAGCGAGAGGTGGTCGTG
AAGCTCGCGCGATCTCGAGGCGTAGCCCGCGCATGAGCCCGTTTGTTTGAGCTCTTTGAGCTTGCGCAGGGCA
TTGCCGGAGAGGTTGGAGTCGCCGAAATGCGAGCGGAAGTCAGAGATGAATGCCTCGAAGTCGTAGGTGAGGT
GTTGCTTGCTGGCGAGGATGCCGTAGTACCACGACTTGGGCGAACCATGCTTGAGGTAGGACGAGAGGTAGAT
GCATTTGTCGTGGTCGGTGGGAAGGGCGTTGCGCTGGAGGTAGACGGCATTCTTGATCTCGATGATAAAGCCT
TCTACCTCGCTCGCAGAGCCGCCAAATACGGTCGGCTCTTTGAAGCGCGGAGTGCCGCGGGGATCCGAAGAAG
ACGGCGTGGGAGCGGGAGTGTGAACGGGTGGTGGTGCAACAGGAGCCGGAGCCGCGAGAATGCGGTTGGTGAG

AGCCTCGAAGGCCGCGCCGAACTGGCGTTGGGTCTCCCCGAGGGCTTGAACCGCGGCCCAAAGGGCTGAGCCT
GCGTCCGGCGCGGGAGGCGGGGTGTTATCTTGAAAAGCGCCCGGGTCTTCGGGCATGGGAGGTAACGGCGGAG
CCTCGCCGGCCCACTCGTTACCTGGGTCCTGTCCAGAGAACATGTTGATGAGTAGAAGGGGTAGAAACCCACG
AATGCGAAGGAAGAAAGGAATGTACGAAATCCGCGCGAATGCGGAAATACAGCGTGAGAGCTGTCCAAACGAG
GGGTTGTTGGTCGGGTCCGAGTTGGTGGCGAAACCAAACTGTGAAGGGCAGGTCGATACGGGCCTTCAGAGAC
GAGGCGGGCGGAGGCAACGCGAGGGGACGAGCGAGGAGGTCGACGAAGGCGTGCGAGGAAGTCCGAGTAACGA
TGCCTAAACTGGCGTTGGTTTCGCAAGAGGTACAAACGGGGCGGTCGCAGTGATAAGGGAAAGCGAGTCAGGT
ACAAACGGAAAGCGTGCCGAAGGCGCAATGCGGGTAAACGAACGAGAAGAATGAAAGTAACCGGATCGGGAAG
GTAGCTACCCTAAACAGGTAAATGAAAGAACCAGGTTAATGGAACGTGCTACGATTGGCCGTGGAGAGTCCGT
GCGAGGCGTACAGAAATCCGAGAAGTCCGAGAGCGTATCTAGAGTGTCCGTAAGTGCTAGGAGTGGCCGTGAT
GTGCTAGATAGTCCGTGTAACGGTCGAGCCGTTACATCCGCGCTC
>Bjead1_1_rnd-1_family-194#TIR/MITE
TAGTGAATAGCAGGGGTATACCCTCCCCTCCGAGGAGTCTACCCCTACCCCTACCCCCCAATACCCCTACCCT
TGGCAAGGGTAGAGGGTNGAGCAAGGGTATGGGGGGTGGAAGGGTAAACCCCTACCCTGGGGTAANTTTCAGC
CAAACCCGGCCTATATTGAAGATATCACTTAATCAATGATAAGTACACCATAATCCACTACTGGCCCCCCTGT
ACTTTACACCATTCATGTTGAGAGCTGTTGGTAATTATTATTACTGTGATATACAAATCTGCATGCTTGAACT
CCTCGGGTCATGCTTGCAGTAGGTTTAGACCGCTTAAACATATGCAAATCGATTTGTCTGAAGANTAAAAAGA
TTCACTACAACACATCTGACATTTGTGCAGGTTNNTTTTTGGATTTGGATTTTTTTTTCGCTTTGAAATTCGA
ATTTAAATGAATTTCTGACATATTTACTATGGAGGGTATGAAGGGTAGAAGGGTACGATTTTGCCTACCCCTA
CCCCTACCCTCTCTACCCCTACCCCCCTACCCTGAGGGTATGCCATACCCCTGCTATTCACTA
>Bjead1_1_rnd-1_family-119#LTR/Copia
GGAAGAGGCTTGGTGAANATATCGGCGGNATTTTCCTCGGTAGCAACGCGCTCGAGTTTGACCAGNCCGTTCT
CGAAAGNTTCGTGCACAGCGTGCGCCATAACGCGGATGTGCTTCGNGCGTCCAAAGTGCTTCACTAGGCGTTC
GGAGGCACGGCGACGGCGACGAGTTGTCGTCCATGAAGAGCGTGACGGGCGAAGAGGCGGGTGGTCAATTTGC
TCAGAGAAGACGTTCCGCAGCCAGGCGAAGTTGCCGGCAGGCNTCGTGTGCNGCCAGGTACTCCGCNTCGGNG
GTNGACTGTCGCCACAGTCTGCNGCTTGCGCGACTCCAGCCGACCGCGCCGCGACCCAGTCTTGACGATGTAG
GCACCCGAGTGGAGCGCGGCGTGTCCGTGTCCCCAGCGTAGTCNGCGTCCGAGTACGTCAGGAACAGCTCCTT
CGNTGCGCGTGTCGTGGTGACCATAGACCAGGCGCAGTGTCCGGAGTGCCCCGCAGATAGCGGAANAGGTGCT
TGACCGCGAGCCAGTGCGGCTCAGCCAGGGTTGGAGCTGGAGCCGGCGAGGACGCGAGACGGCGTAGGCGATG
TCCGGGCGAGTGCCGGTGCATAGCGTACATGAGGGAGCCAGAAGGCTGCGGTACTGAGGTAGCGGGGAGGGCG
GGTTCATGGTGGCCTTGTCGTTCCGTTCGAGTACGGCGCCGGCGGGGAGTGGGGTGGGTTCCGGATTGCAGTC
TTGGAANCCGAAGCGTTCGAGGACGGACTCGATGTACGCTTCCTGGTCGAGGTAGATCAGTCTGCTCCCCCTA
TCCCGAGTGACCCTAACGCCGAGATAGAGCTTCAACTCGCCNAGATCCTTCAGCTCGAAAGACCCTTTNAGTG
CCTCTTTGGTCCGGTTGATGAGCTCGAGGCTGTTACCCATCATGGTGAGGTCGTCGACGTAGACGCACGAGGA
TCCGCACCGGCCCCCCTGCGATNGACGNAGAGCGCAAGCGTCGCCGGNGTCGCGGCAAACGAANCCGATCTTC
CCGAGGCAAGCGTGCAGCGTTNGGNNCCATTGACGCCCGGCCTGCTTCAGCCCGTACAGTGCCTTACGCAGCG
GCAGACNTCGCCGGGATCGCCCACNTCGAAGCCCTCNGGCTGCTCCATGTAGATCTCCTCNTCGAGCTCACCG
TTNAGGTACGCNTGGNCGATGTCNATCNGGTCGATTTCCCAGTCTAGGTGCGCCGCNAGCGCAACGCGAATGG
ACGCCATTTCAGGCGNGCGGCGGGAGCGAAGGTCTCNNCGTAATCGATACCCGGGGCGCTGGGAGNACCCCTT
TGCCACCAGNCGAGCCTTGTACCGCTCGACCTCTCCTGGCTTNGTCGCGTCTTCAACTTGAANACCCACTTGC
ACTTAATCGCTCGTCGACCCTCAGGCGGATCGACGATTTCCCATGTTCCCTGTTCGACGAGACCTTGAATCTC
GTCGTCCATGGCCTTGTAACCACTCGTCCCGTTGGTCTCGACTTGAGAGCCTGCTTGTANGTCGCTGG
>Bjead1_1_rnd-1_family-91#LTR/Copia
TCTCCGCCCTGGAGGACCTCGACCTGCGTTCTGTAGACATCTCCCATGCGTACATTAACGGTGAGCTCGAGGA
GGAGATCTACATGGACCAGCCAGAGGGCTTCCACTTTGGCGATCCTGGCGACGTCCTGCGTCTCAGGAAGTCA
CTGTACGGGCTGAAGCAGGCCGGGCGAGTCTGGAACCGCACGCTGCACACTACCCTCGGGAAACTCGGTTTCT
CGAGGCTCCAGTCGGACGCTTCGCTCTACCTCTATCGCAGAGGCCCGGTGCGCATCATCATGCCCATCTTCAT
CGACGACATCACCATCGCCGCTACTGATGCTGCTGAGTCGGACCGCGTAGTGCAGGAGCTGGGCAACCACTTC
AAGCTGCGCGATCTGGGCCCCACCTCGTTCCTTCTGGGGATTGAGATCACCAGGGACCGCTCCAACCGCCGCA
TCATGCTCTCCCAGCGCCAGTACGTCCTCGACATGCTGGAGCGCTTTGGCATGGCGTCCTGCGCTCCTGTGGC
CACTCCCATGGTTCCCAAGGTCCGCCTCACCACCAGCGACGCGCCCTCGACCCCAGACGACAAGGCCACCATG
TCCGCCATCCCCTACCTCAGCGCAGTCGGCTCCCTCATGTACCTCTGCACCTGCACTCGCCCAGACATCGCCT
ACGCNGTCTGTCTCCTCGCTCGCTTCAGCTCCAACCCTGGCTGGTCGCACTGGCTCGCGGTCAAGCACCTGTT
CCGCTATCTGAAGGGCACTCCGGACATGCGCCTGGTCTATGGGCCCAGCGCATCGAAGGAGCTGTTCCTGACG
TACTCGGACTCAGACTACGCTGGGGACACGGACACGCTGCGCTCCACCGGTGCCTACATCGTCAAGATTGGGT
CTGGTGCGGTCGACTGGAGCAGCAAGCTGCAGACTGTGGTCGCGCAGTCGACCACCGAGGCAGAGTACCTGGC
CGCTGTCAACGCCGGCCGGAACATCGCCTGGATGCGGAACCTGCTCTCTGAGCTCGGTTACGACCTGTCCTCT
TCGCCCTCCACGCTGTTCATGGACAACAACTCGGCCATCGCTGTGGCCAAGAACCCTGAGCACTTTGGACGGC

TGAAGCACATCCAGCTGCGCCTCTACTGGCTGCGCGATGTGATTCAGGACGGGCTCATCGCTCCGGTGTACGT
GCCCACTGCGCACATGCTGGCTGACCTCCTCACCAAGCCTCTGGCTCCGGTTCGAGTCGAGCAGCTGCGACGC
CTGTTGGGCCTGGAGTAGATGCGCTCCATTCGGGGGGAGTTTGGAATGCAGAGACGTGCTGCATCAGGGGGGA
GTGTTGGTTTCGGTTTTTCCTCTCTATTGGATGATGCTATGACCTCTCTTCTTTGTAGACACCCT
>Bjead1_1_rnd-1_family-144#LTR/Copia
CGCCTATACCACCTCCGGCTATACCCACTCCTGCTCCTGCACCGGCACCGGCAACGCCAGCTCCCAAGCCAGC
TCCGCCACCGCCGCCGTCGCAGACTCCCGACTCCATTTCCCAACACGTCGCCCAACAGCGCAGGCCGTCATGG
TTCTTCCCAGAAAGCCAACCTGCGTTGTCGACCGACACATCTGCCTCCGCCTCTACTCCTCCAACTCCCGTCC
CTCCTACGTCCCCTCCGTCTAGACGTCAACGCCGCCGAGCTAGTAAAAGCAAAGCCGTTGCGTTTGATCCTTC
GACACTTCCTCCCTTGGTACCCAATCCTAACCGGGGTCCTCTCGACGATTTGACTGGCGAGTACGCCGCAAAT
CCTGTTCCGCCCACGCCGCCGATCGCGCAACGCAAAGCTAAACGCGATGCGAAGCCTGCTGGTCTACGCGCGG
CTGGGTACGAGGAAGCTACCGAGAAGGAAGTGAAAGCCTACATGAATCGAAGGGTTTCAGCTCCGCTGAAGTA
CGGACATCCGGATAACCAAGGAGTACCAGAGGACGACGACACCGACGAACAAAGGGACGCCAAGGCCGCCGCA
ATNCTCGCCGCGGTGACCATTACTGGTGTTCCTAAGACCTTCAAGCAGGCCATCTCAAGTCGAGATCAGCGGG
ACGAGTGGTTACAGGCCATGGACGACGAGATTCAAGGTCTCGTCGNACAGGGAACATGGGAGATCGTCGATCT
GCCTGAGGGTCGACGAGCGATTAAGTGCAAGTGGGTCTTCGCGNNGAAGNTCCTCTCTGACGGAGANGTGGAG
CGGTACAAGGCTCGGCTGGTGGCAAAGGGGTTTCAACCGGCCCANGGCNNCGATTACGNNGAGACCTTCGCTC
CCGCCGCGCGTCTGAAACATGGGGTATCCGNTCGCGTTGGCTGCGCACTTAGACTGGGAAATCGACCNGCGTT
CTGTNGACATCGNCCANGCGTACCTNAACGGTGAGCTCGACGAGGAGATCTACATGGAGCAGCCNGAGGGCTT
CGACTTTGGCGATCCCGGCGANGTCCTGCCGTCTGCGGAAGGCACTGTACGGGCTGAAGCAGGCCGGGCGTNN
NTGGAACCGCACGCTGCAGCTTGCCTCGGGAAGATCGGTTCGTCTGCCGCGACNCCGGCGACGCTTGCGTCTC
TACCGTCNATCGCAAGAGGNGATGCGCAGATCCTCGTGGTCTACGTCGACGACCTCACCATGATGGGCAGCAG
CCTCGAGCTCATCAACCGGACCAAAGAGGCACTCAAAGGGCCTTTCGAGCTGAAGGATCTCGGCGAGCTGAAG
CTCTACCTCGGCGTTAGGGTCGCTCGGGATAGGGGGAGCAAACTGATCTACCTGACCAGGAAGCGTACATCGA
GTCCGTCCTCGAACGCTTCGGNTTCCAAGACTGCAATCCGGAACCCACCCCACTCCCCGCCGGCGCCGTACTC
GAACGGAACGACGCGGACCCCAGACTGGCCAACCCNTCCACCGTCCCTCTCTACCGCAGCGTTCTCGGCTCCC
TCATGTACGCCATGCTCGGCACTCGCCCGGACATCGCCTGGGTCGTCGCCGTCTTCGTCGGTACCAGTCCAAC
CCATC
>Bjead1_1_Cluster19#LTR/Copia
GTTGGAAGGTGAACGATGCTCGACGTACTCAGTCGGGCGTCCTACTCGACCGGATATGGAATATGGACCCCTG
GTACCTCAGGGACTCCGTGTACCATCGCCCGCACGGTCGCCCGCCTCCCGCCGTGCTATACACATCTACCGGG
CCACTGACGTAAGCCAAGTATGTACGAAGGTTCCAGCACCTATGACATATTATTCTTTGATTTTCGTAGGTCT
TCTTCTATGATGTAACAAGCTGTAGGACTAGGAATATAAATACCGGGAGACTTGGGCTTTGCCTCGTCGTCTC
GCTCTTTCTCACTCATATCTCGCGTTTTAAAGCTCGTCTATACTCGCACTATAGACTACTGTATTCGCTTCTA
CTACAGTACGTCTATTTCGCTTTTCTACAGGTTATGAGCCCCGAAAGGGTGCTGCCTTGATTCTGCACTCACC
TCCTTGTTACCCCATAGGCTCCCATAATGTCCACTGCTGCCAATTCGTCCGAAACCACCGGTAGCGTGTCCGC
GTCGTTCCGAATCGATCCCCTTCAAGGTCCTGCCAACTGGGCGCCCTGGAAGATCAAGGTCAAGGACATCCTC
GCGCAGGCCGGGTGTCTCGAGCACATCAGTGGTACGGCGGGTACTCCGGCTGCACCCGACGAGAAAGCCGACG
AGAACGCCAAGGCCACTTATCAGACCAAGCTAGCGGAGTGGAAGCGCAAAGACTTGCTCGCGCTCACCCACAT
CCGACTTCGCGTGGCTGATTCCCAGCTTGTCTACGTTGGTGGTGCAACCACAGCGAAGCAAGCTTGGGATGCG
CTCGAGGGGGCCTTCGAGTCGCGTGGTATCCAGGCCGAGGTGTTGGCGCGTCGCGAGTTCCTTCGTGCGCAGT
GTCCTCCTGGCGGCGATATCCCCGACCACATCCGAAAGATGCGCGAGAACATGGATCGTGTCCACACGGCCGC
CGGAAAAGAGGTTATCAGTGATGATGAATTTGCTATCACACTGTTGACCTCCCTCCCGGACGAGTGGAACACG
TTTATTCAGACGTTCGATGGGTCGGACCAGAAGCAGCTGACCAGTGCTGGCGTCATCGCGCGGATTTTGCAAG
AGGACACCCGGCGAAAGGCTCGCGAGAACGCGCAAGGGGACTCGGCCATGGTCGCGAAGAACTTCAAGCACCG
CGGAAAGTGCCACAACTGCGGGAAGCGCGGACACTTCAAGGCGCAGTGCAAGGAGAGGGGGGGAGGTGCAGAT
GCCAAGGGTCCGGACGGTGGACGGTCGTCGTTCGGAGGCAAGGCAAACGAGGTTGTCGAGTCCTCTGATGACT
TCCTGCTCGTTGCGCAGGAGCGGTTCGAGGATGAGACCGATCGCCCGGCAGACCAAGTCTTGGTCGTCGCTGT
GGACGAGTCGCGACTCTGGTACGCTGACAACGGCGCGACGTGCCATGTGGCTACTGCCCGAGATCTGTTCTCA
GAGTACGAGCCAGTGAAAGGGCGCGCGTCGATCAGCGGAGTCGGAGGAGACCAGCGAATTGTCGGGCGAGGTT
CTATCAAGTTGCGATGCAAGGTTGGCGATAAGGTACACACGTTGATGATGTCGGATGTTGCTCATGTCCCAGA
TGCTCCGCACAACTTGCTGTCCCTGATTCGCGCAGACGATGCGGGCTGCAAGTACGTCGGCCAGAGCGGTTCT
CTCACGGTGTATGGTCCGAAGGGCAATGTGCTCATGGTAGGAACGAAGCCACCAGGTACGAGGAGGCTTTACC
GGATGCATGTGGCAGCGGTTGGCGCCGATCAGGCGCAGGTTGCTATACTCGGAAAGCGTACCTGGGAGGAGTT
CCACCGGGCAATGGGCCACGTCGGCATCGCCGCGCTGAAGAAGATGAAGCTCGAGGGGCTGGCGGAAGGTATG
TCTATCGACCTCAGTTCAAGTGACGCATTCCAATGTGAACCCTGTATCAAAGCCAAGCAGCACGTCCAGCCGT
TCCCTGAAAAAGCGACGCGCGTCCCGGAGAAACTCGAGATCGGACAGATCGTGGCTAGCGACGTCTGGGGTCC
CGCCCAGGTGAGATCGCTCCACGGCTTCTATTATTACATGTCATTCACCGATCTTTCGACGCGTTTCTCCGCG

GTCTATTTCAGTGCGACGAAGGCGGACTGGGCACTTGGGTCGATACAGGAGTTCGAGTCCTTACTCCAGTCGA
TGAGCAGGACTGGGAATCGAACTACGTGCTTGACCCTGAGGATTGATAACGGAGGGGAGTACATTAGCAAAGT
GTTTCTAGATGCCTGTAAAGCGAAGGGTATCCGAATAGAAACTACGGCTCCCCATTCACCTTCCCAAAATGGT
GTTGCAGAGCGCCTCAACCGCACCCTCCTTGAGCTCGCGCGTGCGATGATGATCGCGCGCGACCTTCCACGCA
ACTGGTGGCCAGAGGCCATAGCGTACGCGAACTTTATCAAGAACCGCTGCGCGACCCGAGCGCTACGCGGGAA
GACGCCGTACGAGGCCCTCACTGGGAGAAAGCCGGATTTGTCTGAGATCCATGAGTTCGGCTCGAAATGCTGG
GTCTTGGATGAATCCGGAACGCGCGGCAAGCTTGACGTGAAATCGAGAGAATGCGCATTTGCTGGATTCGCGG
ATGCATCGAAAGCATTCAAATGCTGGGACCCAGCCAAGCGCACGTTCGTCAAATCACGAAACGTGGTATTTGA
AGCGCCTCACCAACCAATAGAAGAGTTCTCACTTCTCCCCGGGAGCGCGCCTAAGGGGGAGGAGGAGAATGGC
GGTAGTCGCGCTCCAGAGGGCAGTGGAGCGGCGGCAAGAGACCGCAGTGAGCCAAATATGGAAGGCTCAAAGA
CGTCCAGTTCAGGGGGAGTGCCTGAGAACAAGCCGGCCGAAAAGGAGGCACCCGCCATCAACAAGCCCGAGGG
TCAAGGCGCATCAGCTCCCAAACGGGAAAGAAGCGCGCGAATTGCCCGACAGGACCCTCCCGAGTATCTTCAC
ATGAACAATCCTCAGGCCAGAGGGCCGAGAAAGCCCAAGGAGCAGTCAGGTGACACTGAGTCTGTCTCAGTAG
TTGCGGAGCTCGCATATCTGGGCGAAGAACTCTCCGACGAGCCAACTTACCAACAGGCGCTGAAGCGTCCGGA
TTCCGCGCAATGGCGCAGAGCGATGGAAGTCGAGCTCGGCCAGTTGCGACGGATGGGGACCTGGGAACTTGTC
CCGGTCCCTGATGGTAAGAATATCGTTGGGAACAAATGGGTACTGCGCATCAAGCGCGACTCCCAGGGTGACC
TTGTCAAGTACAAGGCTCGTTTGGTAGCCCAAGGATTCACCCAGGAGCCAGGCACAGACTTCACGGAGACATT
CGCTCCCGTGATGCGTCTCGAGTCGCTCCGTCTGATCCTCGCTATAGGCAACGCGCTTGATCTCGAAATCGAC
CAAATGGATGTCGTCGGCGCCTACCTCCATGGTGAACTCGACGAAGAAATCTATATGCGCCAGCCTGCGGGAT
TTGACGATGGATCGGGACGAGTGTGCAAGCTCAAACGTGCCCTGTACGGTCTCCGGCAAGCTGGACGCGAGTG
GAACAAAGTTCTCGACTCTTTCCTCTTGCATTTAAGGTTTCGACGCTGCGCGACGGACATGTGCGTCTACATT
CGCACAGACGACGGATATCCGACGTTCCTCGCTGTGCACGTCGATGACCTGACCATGCTTGCCAAGACTCGAC
AGCTGATGGACAGACTCAAGGACGAGCTCAAGTCCCGCTTTGAGATGACTGACTTGGGGTCTGTCCGGCAGAT
CGTTGGCATTGAAGTCATTCGAGACCGCAGTCAAAGGAAGATGATGCTGCGACAGAGCGCGTACATCAAGAAA
GTCCTTGACCGCTTTCGAATGATGGAATGCAACCCGGTATCCACCCCGCTCGATCTCCACACGCAGTTAGTTG
CAGCGTCCAAGGACGTCGAAGCTGCCAGGGTTCCTTATCGCGAAGCCGTTGGGTCTCTCATGTACGCCGCGGT
CGGAACCAGACCAGATCTCGCGTTCCCAGTACAAGCATTAAGCCAGTACTGCGAGTGTCCCACTGACGAGCAC
TGGGGCGCCATTAAGCGCACCATGCGGTATCTCCGAGGAACGCTAGACTGGGGGATCGTATTCGAGCCGTCCA
ATGACTCCGTTATCCTATCGGGGTACACGGATGCCGATTGGGGTGCTAACCGGGATGACAGGAAGTCCATCTC
GGGGCAGGTCTTCTACCTGTGTGGAGGGCCTATAGCGTGGGCGTCGCGAAAGCAGAAGTCCGTCGCCATTTCG
ACGATGGAGGCCGAGTATATGGCCGCTGCTGACGCAGTCTCCCAGGCCCTGTGGTGCCGCACGCTCTTGGCTG
AGCTGGGGTTCTCACAGAATGCGCCTACTACGCTCCATATGGACAACCAGTCGGCGATCGCGTTCAGCAAGAA
CACGGGCTCGCACGGACGAGCGAAGCACGTCGATATTCGATACCACTTCGTGCGCGACCGGATTTCTTCGAAC
GAGATTTCGGTCGTCCACTGCGCCGGTACCGAAAATCCTGCTGACGTTTTCACCAAGGTGCTTTCGCATTTTA
AATTCGAGGACGCTCGCGCCCTGCTGAACATGTCGGCATCGAGGGGGAGTGTTGGAAGGTGAACGATGCTCGA
CGTACTCAGTCGGGCGTCCTACTCGACCGGATATGGAATATGGACCCCTGGTACCTCAGGGACTCCGTGTACC
ATCGCCCGCATGGTCGCCCGCCTCCCGCCGTGCTATACACATCTACCGGGCCACTGACGCAAGCCAAGTATGT
ACGAAGGTTCCAGCACCTATGACATATTATTCTTTGATTTTCGTAGGTCTTCTTCTATGATGTAACAAGCTGT
AGGACTAGGAATATAAATACCGGGAGACTTGGGCTTTGCCTCGTCGTCTCGCTCTTTCTCACTCATATCTCGC
GTTTTAAAGCTCGTCTATACTCGCACTATAGACTACTGTATTCGCTTCTACTACAGTACGTCTATTTCGCTTT
TCTACAG
>Bjead1_1_rnd-1_family-82#TIR/MITE
TACTGTACAATTTCGACCTATCCGGCGGGGGGTCTGACTGCTGACATTTANCTTCGAAGCGGCAGCGCGGCGG
CGGGCGGGTTGACTGCTGACAAACCGGCGGGTTAGGCAAGCGGCAGCCCAACGGTGCGCCGCAGGCGCGAAAA
TTCGGCGACACCAGTACCTACCATGGGTCTGATCTGGCGGCCGGCGTCGGTCTGACTGCTGACCGCCCGACCC
AAATCCGGCGGCGGCGGCAGTGCTGACTGCTGACCCGCCGGATAGGTCGGAATTGTACAGTA
>Bjead1_1_rnd-1_family-237#TIR/MITE
CACAGGGTGGAAATCCCAGTTTTTGGCGCGGTATTCCCGTTATATTNCCGGTAACATTCCGCATAGAGTTATA
GCGATTTCTTNACCATCAAAACGTGATACAAATACGGAAATATTTTAAGTTAACCGCTATATATGGCATAAAA
CGTGATATTATTTTGGTTGACTTCCTGATTTTGGGCTTATAAAAACGTTANGTTTCTATGCGGAATATTACCG
GTAATNTCACGGGAATAGCGCTCGATAAACTGGGTTTTCCACCCTGTG
>Bjead1_1_rnd-1_family-241#TIR/MITE
ATTACAGCAAGTTGCATATAGCTTGACAGTCAGCGGAGCGAGACGTTCTTTGGAAAAAAGAAATGAGGTCACC
GGAGGCGCGCATCAGACGCCTAATCGGACCGATTAATGCCGCGCGAGAGTTCGAGTCCCGGTCGCGCAGTCAA
CTNAAACAATTTTATTTTCGGTGATATACCACTCGGACCTCTGCAATACCACTACAACTGCTTGGCAAACACT
TGTTCTTGGGGCTTCAGNCATTTCTAGAGCACACTGCATAATTTACACGACGAGCATCATATGAGCTGGGAAA
AACGAGGCCGAAAACGGCAGGTGCCAACAGAGAGCGCCCGGTGACCTCATTTATTTTTCTAAAGAACGTCTCG
CTCCGCCGACTGTCAAGCTATATGCAACTTGCTGTAA

>Bjead1_1_rnd-1_family-90#TIR/MITE
TAAGGTTGACCTCGCGCACTTTCGACCACTCCACGTGGCGCAAATCAGTCACGTGCCATCTCCACTGCCCTTC
GCAAGGCGGGTGAATTGCGGGTTGCGAGCCCCCAAAAAATGCCCATGACGGTCGGATAAGCAATGTTAGCTGA
CAAGCACCTCAATACTCTAGATTCGCACTGTGGTTTGAGACCTGACGAAATTGTGCGACCTNTGAGANTGCCA
TCATATTACATAAGCTGGCCAAAAAACCACCTTCCGCCGTTAATAACTCTCCAACGAGACCAGCAATGGAAGA
TCGGCCACAGTCAATGTCTTTTGTTTGGACTTCCGGAACTTTTGACTGTGGCCGATCTTTTCTACGCCGTTCT
CTGCTTTCGCTATTAGGCGTTTTGGGTGTCTCTCCGCCAAAANGGTCACGTGATGTGGTCGAAAGTGCGCGAG
GTCAACCTTA
>Bjead1_1_rnd-1_family-171#LINE/I
TTTTTTTTTTTTTTTTTAAATTCCGCGTGTTTATATCCCGCCACCCGTAAGGGGTATGCAGCGGCTGGGCTAT
AGCTATCTAGACCGAGAAAAGTACTCGAGCGTCCGTAGTGGTCAGGCCTGCAGGCGCCGTCATTCCAGTACCG
CCATCGCTATTGTCCGAGAGTCGGGCCCTCTCACCGAGACCGAGAGAACGGAGAAAAAAAAAAAGAGGAATAG
TAAACTACTCTGCACCCCCCTGGGCCATCTCCGGCCCGTCCGCTGTCTCCTGGGTGCCGTTCGGGGGTTCTCC
GTCCTCATTTTGTGAAGCCTCCCATGGTTGCGAGTTTTCGGCCGACATAACTCCTTTCAGCCGCTCAATCTCG
CTCTCTAAGCTGACCTTGTCCTCGAACAAGCGCTTGATTATTTCGTTTGGTTCCCACTTGTGCCCTACCTGGC
GTCTGACGGGTAGATGAATGCCCTTCTTAATGTCGTCATCATCACGTGCGCCTAGCTCGCGATGACGTTTGAC
CCACAGCTTCCGGCCCGTGGTCCAGACCCAAATCTTGACATGCGCCTCGCTNTCGTTGTTCGCAGCGTTCGGA
TGTCGTCTTTCGAATTCAAGGGCTTGCTCAACGAGCTCACCCCATTCGAANGTGGCAACCATTGGATTATCTT
CTAGGAAGGTTGCGACTTCGTCTGGGTGGAAGCCGACCGTGCCGGCTTGTTCCCTGATGTCCTGGGAGTCATA
CATCCCTTGCAGCGCGTTGTTCGGGTCGGCGTTTATTTGTGCCGCCCGAGTGTTCGCTTCTCTTCTTTGCCTT
TCGGCGCTCGTCATGTGCCATGTCGAGTTCTCCGGTATGATCCACAGTCTGCACTTTGTCATAATGTGCTCTC
TGGTCTCTAAGCTGGTGCGGTCGCACCAGCATTTTTTGGGCCCATCTTTGTTCGCCCAGTCTCTGTACTCNCC
GCACGGGAAGTGATTTGACCAGAATCGGCACAGTCGTGCCGTTTCTTTGCACGCTTTGATCGTGCCTGCNAGG
AGTCTGGCCCGCCTTTGTTTGCGGTACTCCTTGGCTTCGTCTGAGTCNGAATACTCTAGATCCTCGCCTCCGC
TGCCAATGTGGATGCCTTTGCCCCTGGTCCAGTCGCAGCCTTCGGCCGCCCACTGGCTCAGGAAGAAGTTTCG
TTTGGTGACCTTTCCAAATTTGACGGCCACGTCCGTTTTCAGGAATTCATGTCCCATGTATCCGGCTCCTGAC
TTGATCAGCGTGTTCCAATCGCGATTGGCTTCTTTGCTGATGTCTGATCGTGCTCGTGCGAACGATACAAATT
CCGGGTGGTCGGCTCGTCTTGCCGCCTCCCCGGCTAGTTCATCAACTTCTTCATTGCATTCGATTCCGATGTG
ACTCGGGCACCATTGCAGAATGACCTTCCTTTCCGCGTCTTTGGAAAGGAACTTCCTTATGTGCTCGCACGCT
TCCACAGCAAGTTGCATGGATGGGCCGAGTTTTGGTTTGACGATGGCGTTGGCCGCCGCCACGTTGTCCACGA
ATATTCTAATCGTTTTGGTGCTTTTCTTGGCCCTTCTAACGGCGGTTTCTATGCCGCCCTTCAGGGCGGCCAT
TTCTCCGTCATATGCTGTGGCTCGGCCCAGCTTNATGCTGTATAAGGCAACCTGTTTGCCCTTCTTTGTGACC
ATCCATGCCCCCGCCACCTTACTTGGGTCAGGAGCGAACTCGTCGTGAGTCGGATTGTCGTCTGGGGATACGC
ATGANCCGTCCGTCCACACCACNAGTGTGCTGGGGTCTTTGTCGGTTTGTTCGACTGTCTTTAATATCTTGTC
CACATATCGGACAAACTCGTTGTCGGGGGTGTCCTTCCTCAGCTTCTTCAGCTGTTCTTCCGTTTTTACAACC
TTGGTCGGAACTGTCTTGTTGCGAACTTTCGCATTTTGTTCGCATCCGATCAATTGGACGCGTCCTTCGAACT
CGTCAATGATCCGATAGCCTGGTCTGGCCTCGTCATGAGTGGAGTCAAATACTTCGTTAGACGCGCTAATCGC
AATCTCGTGGATGTATTTTATGGGTGACTTGGCGTCGTTCTGTATTTCCTTTAGAGGGAATGGTGCTTTGATG
TTCGTGTGGTCTCTATTCCACCTCCACAACGGTAGCACTGCTCTTGCAGGGTGTCCGAGGTGCGTCGTGCGNA
TCCTAAGCGCCGCGCGTTTCATGTAAGCGTTGATCTGTTGCCTAATCGGCATTAGNCCCGCCATCGCGTTGAG
GCCTCCCACCGGAGTGGTGCGGAAGCAACCCGTGATCCATCTAGCCGCCCTGTTCTGGACGACCTGGAACTTT
TTATGGAATTCCGTGATGCCCTTCCACTGTGGGTTCCACCACAGCTGGGCGCCGTACGTCATTACCGGCAGCA
CATTCGAGACGTATAGTCGTCGTTTGTTTACTGGGTTGAACCCTCGAACGGAGTTGCCCAGCATGCTTAGCGC
ACCTATGGTGCTCATCGCTTTCGTTTTGTAAAACTTAACGTGCTCCTTCCACGATAGTCTGTGGTTCAAAAAG
AACCCCAGGTATCGCATATCCTTTTTGGGCTGGATGACGACATCCTTGCCCTCCAGCTTGATGGTGAGGTTCG
GTCCGATGTAGTCGGAGTCCTGCGGATTCTTCCGTCTCAGGAAGTGCATGAGCTCGAGCTTATCAGCTTCGAT
GCCTAGGCCCATGGCGTTGAGATCGCTTGCCAGTCCATCGAACACTCTGGCTACCATNCGTATGTTTTGCCCC
AGTTGCACGTCGCGCGATGCACCTTTCGGAGCCGGGGTGGCGCAGTATATTAGNCCGTCGTCCACGAAGAATT
GGACGCCGAGATTGCCCTCGAGTACCTTATCCACCGGNGCCCACTTGTGTAGCAGCGGGGCGATGTACAGCCC
CGACAGGATCGGGGATAGCGCGGANCCTTGNCCNACNCCNGCGTTGAAGTCGGTAGGGTCCATCANTCTCCCG
TTGAACACGAACCGGGTTTTTCTNCCGACGAGGTAGTTGCCGAAGAANCCGCACAGNTTCCTGTCGAACCCTT
GCTTCTCGAGNACTTCCGCCATCGCCTCGTGGTTGATGGACGGGTAGAATTGNGACACGTCCAGCAGCAGCGC
CGTCGACGTCGAGCCCGTTCTTCCATGCCTGTTGGATGTTGTGGACAAGTTGAATGCCCGCATCCGTCGTGCT
GTGTTGGATGGCGCCTCCGAACTGGCNCGGATGCATGATGCCGAACTTCTGGCCGTCAAATTGCATTCTAGCA
GCGACGACTTTCTCCATCAGTTTGCCCATGCAGTTGAGCAGNACTATCGGTCTGTANGCTTTCGCTTTGGAGT
AATCCGGCTTATTTGGTTTCGGAATGACCACCGTNCNTGACCTCTTGAAAATCGAGGGGTAATATCCGAATTC
AACGCANGCGTTGAATANTTTCTGGAAGAATCGCATAGTGGGCGGCACTGCCAGCCCTTGCGCGTCCTTGTTG
AGCTTAATTCGCTCTTCCGCAACCGCGCGTTTTAGCCAGAACCAGCTAAGNCGGTCGGGGCCTGGGACGGACG

TGTTGCTCGTTCTGAGCAGCTGCTCCCCGATTTCGGTGACGCTGATTGGCTCAAACTCGCGTTCGGGCGCCTG
CTTGATCTCGTCGACGACTCTTTCGTCCACTTNNCGCGGGTTGGCGCTGGTGAATTGTTCCTGNAACGCCTCG
GCGACGTCTTCCAGCGTGTCGGCTGCCGTGCCGTCGCTTTTGGTAAGTGCCGTGGTGGCGTCTAGTCTNCGCG
GCTTCGTCCATTGGACNANGTCCCAGATCCGTTTCGGATGNATGTCGGCCATGAACGTGTCGAAGTACTGGCG
CTTNGAGCNTCGTACTGTTCTCTTCATGACGCGGAACGCTTCCTTCTCANTCAGTCTGGCTGCGTCTAGCTCC
GCGCCTGCCTTCAGCATGGCGCTCGGGTTCCTGGTTTGTTGCGCTCGTGGAGCGCTAGCAGTTTTCCACGTGC
GTTCGCGCGTGCTTCTTTGACCGTCGCGTTNGCCGCTGAGCAGTCGGGGTTCCACCACGATTTGGAGTACTTT
GTCTTCCGCGGCTTCTTGGCATGCGTGCGGAACGCTTCGTCCATGATGTCCTGGAGCAGTTCTCCTACTCGCT
CCAAATCATCCTTGTTCTCGATGGGGACGTCTGCAATGCGCGCGATACCCTCCGATATCGCTTCGCTGTATTT
CTTAGCTTCTGGACCCATGGTGGGTATGATGGGGTCCGGCTCGACTTCAGGACATATCGGCATGCACCATCGT
AGGACTGCGTGATCGCTTTTGTGACGGCCATTCATGTCGATTTGCAGTTCGTCGTATTGTCCGAGTTGGTTAT
CAACCCAGACCAGGTCTAGAACCCCTTCCCGTACCTGGGCGTTGTTGGAGCGCCACGTTGGCTTTCCGTATTC
GTCGTTTAGCATTCCCAGGTTAAGCTGAGTCATTGCCAATTCAATGATGTCATCGCCAGCGTTCAAATGCTCG
CTGCGCGTCTTGTTGTTGTTGGCTCTTTCGG
>Bjead1_1_rnd-1_family-250#LTR/Gypsy
CGATGGTGTCGGCGATGTCGGTGAGGGGCGCGCGGACGCGTTCTTCGCTGCAGCAGCACGGTGGATTTCCTTG
ATTTCGCGTTCTAGGGCCAGGATCCGCTCGTTCGCTAGTGAGACATAGTTCTGTGAAGTGTTCCAGCCGAGAG
GGACACTGCGCGACGCAGGGGGCGTGTACGCATTTGCTAGACCAGGCGTGGTGTGGGGAACGCTCGCGACGCG
CTGAAGTGAAGGACCAAGAGTACGCGATCGCGGTAGATGCGGCTCGGAGCGAGCTGGATGCGAGTAGANCTTG
TTCGCTGCAATGCTATGGGTGCCGATGGTTGCTGCGTGATGCTGGAGTGCTGGACGATGTCGTAGCGCGGTAC
GCGAGGGCGTTGGTGAACCGTCGGTGACCGGGGACGCGACGCTGGCTGGACGCGGTCGCGACTCGCGCGATCT
AGCAGCGATAGGGCCAGTGATCTTCTCCAATGACTCGAGGGCTCTGTATAGTGTCGCAACAATTCTGGGGGAC
TCGCCTGGGGCTGTAGAACTTGTCTGATCAAGCTTCGAAATCTCCGGGCTACCACGAAGGATGAACAGCGCAG
CTTGGTACACTGTCTCGAGGTCGAAGACATGGTCTGGGGCGCGATCAGGCTGCAGGATCTCGAGTCGGAAGCG
AATACGAGTGGCGAGGTCTTCGCGAAGGGCCTTGACGAACAAAGGGGTCCGTTCGAATGTCGCGAGTCGGCCG
TTCTTGCAAAGCGCACTTGAGCATGCTAGGAACTCGCGGTAGAAGGCGGCTAGCTCCTCGCAACTCTGGATAG
GTGCGGCGCGACGCGATTCGATGAGCTCATGGAGTGTCGAAAGAGTCATGTCGGAACGCGTGATGCCTGGGTA
GAGCGCAATGATCGCCGCGACGAAGTCGGAGTATGGCGTAGGCGCGTCGAACTCGTCCAATGTCTCGCACATG
TCGCTTTCGCGCAGGGGGAAGTAGCGTAGGACGTATTGCTTCTTATCTTCGTCGTCGGTGATGCTGTGTCGCG
CGAAAAGGTCGTCGAGGTTGTCGAAAAACGAAACGAGTGTGCGGTGATCGCGGTCGTCGAAGATGGGGGCAGC
GCGGGTCCCTGGAAGGGGCATGTCGCGATGGAATCGGAAGGGCATGGTGGTGGTCGAAGTCGAAGCTCGAGCG
CGGGTCGAGGTGCTATCGGGCGACTGGTGGTACTTGCTCGCGATACGGGAGGTTCAACGAGTCGGCGGAATGA
ACTTAGCCACCCCGGCGGTGGGCTCCAAAATGATGCGCTCGCGCGAGCCTGGGCTACAAACCACAACAAGAGG
GAGAGGGTAAGGAAGTAGGCACGCGTACGCTCTGATAGGGTGCCGGGGCGCTAAGGTGCGACGAGCGAACCTG
TTCGCGTCGCGATCGTAGATCGGATCGTCGCGGCCGCAGGCCGCGTCGCGGTGTGGAATGCAAACGATGGGGT
AGAGCCGTCTATCAAGAACGGGCAAGCTGGGGATGAGAAG
>Bjead1_1_rnd-1_family-85#ClassI/TRIM
TGTTGAATGAATCAAGTCACACTCCTAACGGCTAGATAGCTCAGTGGTAGAGCATCCAAGCGTATATTGGTCA
GGTCCTGGGTTCGACTCCCAGTCTAGCCAGTTTTATCGTTCAATAGGTTATGAGCCCCGCCAACGTACATCTG
CAGCGCCTCGACGCACTGAGGTCCAGATCCGTACAGCGCGAGCCTGGAGTGTTCCAGGTTCCTCTGCGATGAG
GAGAACATGAAACTGGTGCCGCCAAGTCCGACGTGTGACTTAGGGGGAGTGTTGAATGAATCAAGTCACACTC
CTAACGGCTAGATAGCTCAGTGGTAGAGCATCCAAGCGTATATTGGTCAGGTCCTGGGTTCGACTCCCAGTCT
AGCCAGTTTTATCGTTCAATA
>Bjead1_1_rnd-1_family-87#TIR/MITE
ACTGGGTGACTTTCCGTAAGCCTGCGGCGAGTAGCGAGTAGGCGATATTGCTGTAAGATGGGGCCGGCATCTT
CGGCAGACCAGATATGGGAATTTGTGCTTGAACCTCCCCCCGCCTGAAGCGACGGTAGGATATGGCATTCTGT
GAGCAGAAAGATGCGAGGAAATTTATATGGCCTGCGTAAGACTGGTCGAAACCTTCAGTGCGCCGATTNACAT
ATTTCTAGATGCAAATGAGGCGTCGGGAGGTCTGCGAGGCCGTTTCCCAGTCTCCCAGCGCGTTTGGCGCCAA
TCAGCTTTGCGGGTCGCAGCTTCAAAATCGACGCATATCACCCCACTAATTATTACTCTCCACTCTCCACTGG
CTTACGGAAAGTCACCCACT
>Bjead1_1_Cluster15#LTR/Gypsy
gtaagaccaaatagaacttgcgtcacgctgagcgaaccgtacgacgtatattcgtccggaaaactgtacagcg
tagttgcgagtggtaaaaggtggctaatctaactacttgaactagtatttatattcagccaccgtccgattcg
agccgaactatgcagttcatcccgtttaggaagtccaccatgtggcacgcacgtggtggcctcggaacctccc
gagcgccctcgtccatgggcgctcgcggacttagaatacagtaagaataggtactcggcctctagctacatac
ctatggggtccttacaccctcccctgttagaacggaaccaacggtgtagacacatatggattgagacaatgat
cgcgtccgggtcttgggatgtacacatcaagagtactcgcgcgccactgcgtactaagtaggaaccccgaaca
actggttcatgtccgcgtcagttacttcgaggcaatccgggaccagtaggcaaagcgagtacgtccattggta

tatgtcggatgcggcgcggtcgggctggggaacgctgagctcgtccttggtagccaatgatgcgaggaagtct
cgagcggtggagtgggatatgacgccgtccgcggggaaggaccgggcttcgcgaaggtttcggatgcctacta
ggatggagctatcgtggtgatcaagttccagtacggcgcgaagccactcgcgcattcgcggtcccgagaacgg
agggggccctagttccatgacacgtagaaacccggcgaatgttgagtgaggaatcgtcgccacctcttctcat
aacccgaggtaaacggcggcgtggcgttgatgacttccccctcatcggaccctttcgtcggtgtactggcgtc
catgggcgttgcgtcgttggtgctctctttctcgttagccgcgtcgccgtccgcctttgccttgtgatcgcgt
gcgcgctgctcttggagggcgactatccacgccggccttctccactttatgcattgtgccgtccttgccgcgg
gtcccgtaatgtctcgttgattggcggcgcgtaagaacagtccccagtgttctagtgcgagatgatttcctgg
cctgttggcgtccgcggcgagtctcctagcctctgccggcgtttgtggccatctgagttggtccctcgatttg
atcggcgttacttcttgcggtagtcgtcgaaggaatcctaggccaggctcatagtcctcgggtcggggcatct
ctcgtgactctgtttcgtcctccgtatggccctccccttctgatctcgccaaaggggagtcactcagacgcat
gaggagctctggccgcttctgtacgtccgagattcggctaagtaggctattcgtccgctgcgtcggaggggga
atgcccattcgctggaggaggctcgtcgtgggcgcggcgcgatcttgagattcgtccgtcgtgtccatgtcgt
cactgggtgccttgcagattggttccgcggatgatcgatcctcgcgccgaggccctggagatagcgtgcgggg
gggcgcgctgcttgatcgagcccgcccgacgcctcggtctcctggggtggcgctcctggtcctctcgtcggcc
ctttccctggcctcctcgaggctcgcgcgtgcgtgcttgaggtccgcgcgtgcatcgtcgaggtcgtcctcgg
tgaggcgtaggtcggcgcgcgtctccttgaggtccttgcgaacgtcctcgagttcctgggtgagcttgcggat
cgtggctaggtgttcgtcggcctccttgcgaccttgctcgagcgcgtactgatgaatctgctgcatattgagt
tcgcgctgcatggaggccctttcgaatccaggttctccggcggcgagttcaatgtggcggaggtagtttgcgc
agatggggcacccccctcaagtgtcgtagaatccgttgtaaggagtcccaggcccagtcgaggatggggtcgtc
gctgtccagcacgaccattcccgcggcgcggtctagtccgctgtgatgcatgctacggttcgagtcgcggtcc
cggcggtcgtagggatgatggcggtagttgtcgcggctcatcgtaggttcgttcttttatgcgaaggataaac
tgttgttgtgagtatcaccgtatgtgcggcgctgccgtcacgtacgcgggtaaagagcgtgctgtgttgtagt
ggtgtggaaagagtgaagttgagtttgcctctggcgcccagcgtcagcttttggtcgcaattcgaaccggtca
agcgtccgccctttgggcgctcgccttaaccgtttctctcgcagcggagcgttcgaacccgtccgcgggagg
ttcttccagtcccgagtgccgcacaggtcaaggtagtcgtccaacgccttcacatcctcgacgtgtgccaacg
gctcccatgaatgatcgccgacgttccatcgcactttaaattcgatttcctgctggtcccacctgtgcgcaac
gatctcatccaccaaccattctccattttcgtcgtcaccgaagtcgtagtatactttcgtgtccctgtgggga
aacacggaattgtcattcggttcgtggcgccgtagtagagaggcgtgaaagcgcgggtggattcttcgtttcg
caagcgattccggaagatggatttcgtatgtggatgtcttggggaagctttccttgacggcgtatggtcctat
gtatttcggcatcaacttccttgctcgattggccggcagggatagattcgcggttgacaggtacaccaagtcc
cccggcaatatagctttaacgtcgccgtgcgagctcttctcgtctctccgtcgcttgttcgcatggtacgtct
ggtcgactcgactgtcaagtatagcgtcatgcgccctgatgagattgtccctggcttgttcagcgaactgttg
aattccgtccatctcggagacttgtcggccaaactccgtggtcatcctgggaagactcccgtttaccagttcg
aatggggcatagcccgtcgtcttactggtgctcgagtttagtgcaaactcgaccaagggtagttgcgccaccc
agtctttctggtccggctgtactagcgctctcaggatctgggtgaccgagcgattggcgcgctctgtcgcgcc
gtccgtctgtgggtgaaatgccgttgacatgaggagtcggacgcccagcacgcggtgaacttcgcgccagaat
ttcgatgtgaatttcgcatctcggtccgatacgatgtcgtcggggaggccgtgtaatcgtaccacctccttta
tgaacaatgtggcgagttccgacgcccgtatggtggtgcgcactggtacgaggtggaccatggaagtgagtcg
gcatatgatcacccacaggtagtcatatccatccgactctgggagcggtcccacgaagtccattgcgatcgag
ccccaaggtcgccgcggaatatcgagattatgcagtagtcccgctgggggctggttggacgtcttcgacgtct
ggcataccccacagctgctgcaatatttctcgatgtccttgcccattgacggccaccagtaccagcgccgaac
atattcgctggttttcctatggccgagatgaccgaggagcagatgggcttgctcgattacaatttccacgagc
ggcttccgcccgaccttcgcccttggtacgcacaggacaagattggcgaacttgttcttggtgtacagtagcc
catcttccatttcgaactgtttgaattgatccacactaccgacgactttcttgaaagtgacgttgtgtatgta
ccccttctgaatggtcgatttgaagtccgggttactgtcgagggccgtgttgagtggcgtcactgtttcccca
gatgccatggcatacgggtcttcagtatcgttcctctccccgtctgccttactctcgcgaatctggttcaact
cgagtcgagtgatgacaccacgcgctggatgtggtaccgcggagggagacgggcctacgcaaattttaagaaa
ttcgttcagcctatccgttgggaggtcctcgccgtctgggtcaagtcgtacgtccgcgcccgaatactcgtct
attcggtgcacttctccttgcgtatcgtgctcgaagtatcgcgacaagcaatcagccgcgatgttcttctccc
cgggttggtactcgatgtcatagtcaaatcggctgaggtattgcatccatctcgtcagtcgtcgcgaaagctt
agattgcgtcttgagccatttaagcgcttcgtggtcggtaacgatccgtatgcgccgtcccaacaaccggtct
tcccacttgagtaacgcctccatgatggcgagtagctcggcttccagggtagtatatgatcgttgagcgtccg
tcagtttcttcgacatgaacccggctgggcggcacgtcgaccaggactctccttggccgtacaatcctccgat
ccctgatatggaggcgtcgcacaccagccatattggttccggccgtcgcggatcgattggctgcaggatgggg
gcacggcatacgagggttttgattgcttcgaagcactttgcgtggagaggtcgccattcgaacgaatggccgt
ttcgatcaatcgcgttgagcggcgaggtgtatgcagatacatcaggcagaaattgcgccagatactgtagtag
ccccaggaaacgttgaatatcatggtaatccctgggggtacgccactcgcgaatccttgcgagtttatctgag

tcagcgtggagcccttcgttgtcaatcaagtgccctagacagtccatgctttcggaatataaatcgcatttgg
ctgctttcaggaacagctgagcttcccttaaggtactgaatacgatctcaagatgtctttcgtggtcgtccac
tgagtcggagaagatgaatatatcatcaaggtagacgtgaacgaagaggccgatgtatgcgcggaacaggtgc
gtcatcaggcgttggaacgttgccggaccgttacagtccccttgctgtaggacgttactgaacatcgtcccgt
agatgctggcaaatccagtcttcctaacgtcgcctggttctacgcggatctgctcgtacgcatctgagaggtc
gatcttcgaacggacaggcgcgcgtgcgacgtccatccgtatttgctcttggtcggggaacggagtaacgtcc
ttgactgtattctcgttcctcttgcggcaatcgacaactgttctcagaccgccatccttctttagcaggcaaa
gcataggcgctgcctgtggtgttgtggcgtgcacccaccaccgcgctctgcagtatcgctggatcttctgcat
aagtagtggccgaagagactccgcgcagcgcggcatatggtaggagtacttcgcactctcgtctatcagggtt
atttgatgattaatctcgcggagtggcggcagtacttcgggcacgccgctagtgatgtctttgaagcgctcga
accaccgtcgtcgggtagcgtctttgtccagtgtgtgaaccgcacgcatgacgcgttccctgtccggaggttt
cgaggatagagatgtagggatatttttatggattttcatgttttatcaatcaaggaatcggtggtagttata
gtattaaggcgcccgcctatggatggtttcgcatccaaagtcggcgcaaccgtggctttattggaatcaatag
agaaaacgatcgtaggtgaaatgaatgaagttgaatgggcaggctggtatgattgtccgtggtggacaaaaat
cgaggttagtgccgtgtacttacatcttcaccctcccgggaaagctctggagctcccgggagctcctggtgtc
tcccgagagctcctgcgacgtttcccgggagctcttgggaactcccgggagctcctgctgtctcccgggagtt
cttgggagctctcgggaggccaacttgttacctttcccgacgcctcccgggacctccagagatctcccggga
gctcttgggagctcccgggaggctgctggaggtcttcattgccttattaggacgatagaaggcgcagggaaca
caacaagatatatctcccgagagctctcaggagctctcgggagccctctggagagcttcccgggaagccattg
gagctcccgggaagccgttggagacggctggagctcccgggaaggctcaggagctcccgggagctacagtcgt
ctcccgggagttccatagacctcccgtcacaaccagcaaaaccatgggacgtcgattttgtccacggtgaac
aatcataggcccatgaacgagccggtggggcgagtttcatgcattatagcctggataggctagggagtatgat
gcattagttgctggtgcccaatctcgggcctccctttcggtcaggctcatctggaatgggtacggcccatcag
aggcatcatatctattataatatatactgttttatttgttttcgcgcgaaaacgagccagctcgcggtctttg
tttggtctccagggaccaaacaaagcccgattaggtgctgagcatgcccgggcagcagtactgctcgcgccgg
gctcaaatgacgtcgtgcgaccgtgggtgcgtgtatgtgagccctattacctctcctggacgcttcccaagct
gccgcgccctccctgctcgccgcgcgccgccgtgcgccttccatttcccgattaggaacgatcccgccctcag
ctcgcgaatcgctccagcgcggccccagcacgcccaataaatccccaaaaaaaacatacagaaccgcgacaca
cacagcattaccatataacgaaatcaggcggcgatacggcacctctaatatccgacgcgacgcgatatttgtt
aataataggcctagtggatggccggttttgggacccctacagccctcgttccagctctaaccagctgaaatac
acctcaaccgttgctgaaacgatcccccaggcctcaaataggcgattcctacatgcgcgccatctgtccaaat
cgggatgaatcccgccagaatcccgcccctcgctccagcgccgccaaatgcccgcgaaaaactccaaataaat
tggggattacttatacgtctatacgcgagctgtgatatacagaaccgccctataattggcgcaccatacaccc
actaggaaatacatttgtataatgatagccaccttgccccagcctctgcacagctgatacagcatattaaatc
atgtaacggtcgtgatatcatatactagtacaatataccagccttcaagacgctccaattccttcataatggc
ccagataagattgcgcgaaaacgcataatatgtttttttttaattgtgaggatattccagaaggtagaggcat
tatttctatgaattacgacgttatttagaagtggctaggtgtacctcgagctgcataaaccgcgaaggaacag
tgcgggaacctcgggaacttaccatattaggctattgatgggaacgaccgtccgcaacatagcgcgtcgtggg
gcttccccccctcaatgaaattgctgatctttctgtcgtatccaatgctacgagtccatagattacataggtca
cgcgaaacccgcgttttcaggacgtccgcgcgaggtatgggagtcataagttcaaaacatacccaggttgcac
cctaatgaatttcccaaagacaacgtgtgatagagattaactgatacaggtaagcattcaatatttcaatatg
tacactacctccatgtaaaatatgaaccagttttgacggctccgaaagctgatatagcccgagaaacgcgaac
agtgtcactctgaaactaaacaaaacatatcggctgctatacgttgtcacagaggccgcgtaattttttggtaa
atagtaccaatgtattcctggaggtcgtccgcacccgtacatatataatatattgaggtttcctccattattc
aagtgcgtgaacgggcgagcagcccgttcgtactcgagactcacagtgaaatctcaagctgaacgcgaaaatc
gcgtccgggtggtttcggatgtttcggacgtcgaaaattcaggtttttgggcctcagaaagttgcccctttgc
tacagcaggtctcagaacgatagtcatcccccgcgatttttaggcgtagcgggtttggactccgaacccagat
cttaccagatgaggtggagcgagagtggagaatattgtcgccaaacagaatagagggcgtgaggcaccaccgg
tgaggcgccgtggttcaacacctcgccgccgacggccttgaaaggtcctcgagcatcgaccagacatgattta
aaatacgcgccaacgatctgaggctattctggggtatcccataaattttccttagcgttttcaatgaagtttc
gtacatgagagagctcctgcataagtggcggcggtctgcgggctgatccggccccactcgcctgcgagaagtt
ggaggcctcacttcccccgatcgggtggatctgcgttcggagctAGTTGTGATCTCCTCGTAACGATCTTGT
AACGAGTGTTTCAAGACGCTTCAAGGGGCGTCAATCACTCCTGCCTTGTAAGGCAGGAGATAATTTTCCGACA
TGACCCGCAAAGCTGATTGGCGCCAAACGCACTGGGAGACTGGGAAACGACCTCGCAGACCTCCCGACGCCTC
ATTTGCATCTAGAAATATGTAAATCGGCGCACTGAAGGCTTCAACCAGTCTTACACAGGCCATATAAATTTCC
TCACATCTTTCTGCTCACAGAATACCATATCCTACCGTCGCTTCAGCCGGGGGGAGGTTCAAGCACAAATTCC
CATATCTGGTCTGCCGAAGATGCCGGCCCCATCTTATAGCAATATCGCCTACTCGCTACTCGCCGCAGGCTTA
CGGAAAGTCACCCAGTGTGTGGCTCTAGAGTGAGCCATGGCAGGGTGAGccagtataattatgttttatatgc

actaatccatcatccagcccctctagatctgtgctggtcctcgcgcagcggtcgcgcggaccggcagggtcgc
gtAgaGAGGagCCacaCCcccagccaCgCTGaGcacgacTCCAcggaaGacccagaggaCGaaaaaAaTacTg
aCGgaTagcaacGCaTTacAaGgccCcaaAggGcgCCaAgcaGacCaGGgGacgcagAacGcCTTaCaagcaa
acacaaaCaCcCgcaaGCcCaccggaacCagcccaaTTaccacCCcgaccacaaaacgaGacaaaaTTggATg
cacCacCAaacccTaCacacTaCcTcaacaCaccccgccaaCagaCccccaAcCGgcccaaaACgaGCGCcaa
caaCcccaaaagcGcAaGccacGaacaccTcccccGacaaaggaCgaaCacGCAacacccaCaaGgaAgaCGA
cTaGaAaacCccaacCcaCaGgaCCCcgaacGcccgcTGggacaacGaacgcccCcaacaaGacacaaaaTaa
caaTacacccgaGcaaTAggTcTccTcagTgcacTcgCccaTgagCaagccagaaTccaCTCgaTTcGagCgc
aGCATGTACATAGTATATTAATACGAATATAAGATTTTTTACAAGTGTTTTGCGAGCTTCCAGGGCCAGGAAT
TCGCCGTCGCGCTGCGCCGTACAGGATGGCAGGGCGGGCGCCTGACATTTCCCAATGCGGAAGGTCATCGCCC
ATGTCGGTTCTTGATGCACAACTGCACACCGTCCCAAAAGGTGTGGCCACAATAAATCAAACAAATATTGTTG
CTATTTGATTGGCAATATCCCTATTAGGAGGGTCTCCAAAGGGTCCCCCCAGAGTGGAAGTGAGTATAATGCA
TCAACCATGCCCAAGTTGAGCCAGGCCACAGTACCAGGTAAATAATGCAACTTAGGGCCTAGCCTAACCAGTT
CTATGGTGCATTAAACACCTCCCATGGGGTGCATGAACGGGATCCCTAGTATGACGTCGTAATGGTTGATATT
CGCCACGTCAAAATAGAGTTCGTAATTCTCGTCGCCGAGGGCAAAAGTGCTGTGCATGCCGTAGTTCACTTTG
GAGCGAGAACCGGCACAGCCTAACTGCAGGGTGATGGGGTTACTGAGCGGGTATGTTTTCAGGCGCGCCACCT
TTGCAAAGTCGTGCAAGATCGCGTCCACAGTGCTTCCGGTATCGACCAAAACGAGTGCGTCGTATTCCCCGAA
GGAGATGTAAAACGTCATGCAGCGTCCTCGGCCCTGGGGTCGTTCTTTCGAGCTCGAGGTTGCCTTCATTGCG
GTTCGGTACCCGCGGTCTTGCTTGGCAATCAAGGAGAACACCTGTTCCCCTTGCCCGGGCTGTCCGCGCTGCT
GGTGCTGCTCACGCTGCTCATCACCTCGTCGCGCAACTTCCTCCTCGTCGTCCGCAATATGACGAACTCTGCG
ATAGAAATTCGCGCGCTCACGAGTCCTGCGGTGCTCGTCTTCCGGGTCATGGTCCAACAGTCCACGCTGTCCC
GCGTGGTGTTCCTCGAACTCGCGAATCCGTTGTATGGAGCGAAGTACGCTACGTTCCGTGAATTGGCGGAACT
CGCGACTTTCGACGTTCAGCCGGGCTAGTCGATCGAGCATGCCGACTTCGCGTTGAAGTAATCCCATAGAGCG
CACATGGAACTGAATAGTGTCATGTGCTTCCTGGAGCGCATTGGCCAGATATCCCGCCTCTGTGGTGTCTGTG
CCCGTGCCCGTCGTCCTTGACGAGGTACGTCCGAGCTGGGCCGTTGTGGCGTGGGACCTTCCTAAAGTGGTAA
TCCTCCCTAGCGCGTCTGCGAACTCGTTGGAGGACGGGCCCGTCCTTCTCTCGAGGCGTTCCAGATACTGTAC
TGTTATCTGGGTGGACAGTTGTTCCACTTCTGCTTCCAGCACTCTGATACGGTGGCGAACGCCAGCGTTGAAA
TGCTCAGTGGGTAGTTCCATGGCCATGCGATCAAAGTTAGACGCCGTGATCTGTGGGCGGCCGAACGCGATGT
AAGAGCTGTCGACGTCGTCCTCGACGATCTGGCCGTTCTCGTCCATGAGTGGCGGCACGTCCGTCCAATCGTC
TTCGACTATGTCGTCGATATCGGTGTCTTCCTCGCTTTCGGTTGCCGGCACAGCGTCTTGCGCGGTGTCTCTG
TAGTAGTAATCCGCAATGGACATCCCGAACATGCGTTCGGCACATTCGTTTGTAGAGTCATACTGGTACTCGT
TCGCGTCGTCGAAATCGGTGTATTCCTGCTCCTCCTCACTATGCGACTCGAACAGTGCGCCATCTACATTCTC
GGGATACTCGTCCTCGACCCGCAGAGCGTTGAGGTCGTGGTCTGACTCGTCCTCGTTCTGCGCGTCCTCTTCG
TTCTCCGCCGGCTCTCTGTCCTCTCCGTCCAGCGCCTCCATCCGTCGAATCGTCTCCTTGCGACTAGCGGGGC
ACTTCGGGTCGGTGGCGTAGTGACCGGTCTGGTTGCAATTCCAGCAGGTAACGGAGCTCTTGTCCCTTTTGGT
GTTCGTCGAGGGTGTGCCATTGGCGGGGTTCGCCGCCTGTGCTCGTGATCCTGCGACGGGGCTTGCTTTCGTC
TCCTGAGTCTTACGGTCGCGTCGATCACCGAGGTCCCTGGGGCGCGGGCTTGTAGTCGGCCGGGTGGAGCTCT
GGTTTGGCCTTGGGCGGTCCCGAGACGCGTAGCTGGGGCTCGATTGGGGTTGAGTGACTGGGGCACCAGGGCG
GGACATATAAGCTTTCCCGTCCTCAGCGTCCGCAGCGAAATCGCGGATGACGTCGATGTCGGGGAACTCTTCC
GGGGTGACTCGGTAGACCGTAGCCACGACTTCCCGGATGTCGTCAGGGAGCCTGCTATAGAACGCGCGCGCCA
TAGTGTATCCATCGGGCTGGACAGGCATGCGCTTGCCCCAACGATGGAGGTCGTGGTACAGTCCGTGAGCTCC
GCCAGTCGTCGAGTACTTGACCGAGTCGAATCTTCGAGCGGCGTCCTTCTTTGTGCTTGCACGAATAAACCGG
TTGTGCATCGCGCAGATGGCGTCTGCGTGAGTCCAGTTCGATATGTTGAGCTCAATGGTGTCCCGGTACCAGT
CGGCTGCCCGTCCAGATACGTTGGTGCCCAAGTACATGAGCCGCTCGTTGTCGAGGTGGGGCCCACAGAGATG
CGATAGTCGGTTCCACCTAGCATGGTCCAAGAACCACGCTGTGAACGTGTCGATGTTGTCGCTGCCTGTGTAG
GCCGTGGGCATGGTAGGCTTCTGTTTGTTGGGCGGCGTGTCTTTGGGTAGTGACTGACCCACCAGCGCGTGAA
GGTGCTGGCGTATCCATTCAACGAATTTCGAATCGGGCAACTTGCGCCCCGATACGGTGAATGGCTGGGGAGG
CGAGCTGGGGGTTGGACCGAGCTGCCTCTGGGTGTGGCGGTGCTGAGCTACACCCGATATCGATGAGGAGAAT
AAACTAGCCGGGTGTGGCGTCGCTGTATGCTGAAGCGGGGGCGACTGAGCGTCCGGCTCGGTGCTGTCCTCGT
CCTCGTCATCGTCGGTGTCGGAGGAATCGTCATTGTCGTCGACCAACGGCCATCCGGGAGGTCCTCCTGGTGG
TCCTCCTGGGGGTCCTCCTCCTCCTGGTCCTCCTCCAGGCCCTCCCTGAGCTGGGTGCGGCACGCCTCCACCA
GAGTCTCTTCCCTCTATGCTACGCCCCTCGCCTCCTAGCACGTCTCGCGGTAGGTGTGGCGGAGCGTCGCGGG
GTCCCGATGAATAGCCAGAGCTGCTAGGCTGCGGCTGTCGATACTGCATGGCTTCTCGAAGGCGGGTTTCTGA
TGACTTCCCATGGATAGGCAGAATAGGGCGATGTTCGGGCCCCTTTCCCTTGTTGCGTGCGGGCAGCGAGTCT
ACGGGTGGGAGTTGGAAGGATACGGAGGGAGCGGGGAGTGGCGTATTGCGGCGAACTTCCAGTTCCTGCTTTT
GAATCTCGGAGTAGCGTTCCCACTCGTCCGGCTTGTCGAACGTCTCGAGTGCGTTgagtacgagtgttgaggc
gttcgtcgtctgctccctgaacgacctgctcgtctcctccaCTAGCTGGCGCATctggctcgcctgttcgtcg
agctcctggcgcacctgcgaatgcatatcgccgaatcgttccacgagcgactggagttggtctatggcgatgg

tcgagcgttggtttaggagggagaaagcttcgcggagttggagacgattggggtcatcatcgagcgggcccgt
cccattgctgggagtgacggtgttcgttgagtcttcgaccgcggGGGAAGGTGGCAGTGGGATAACGATGGgt
tcgagggcctcctctggttcagcacgggccacgtcgtccgtggttcggtttggtggcgtagaactgtgggtcc
ctgagggtccatgagcggggtcgatgacggggccgttcgcggtCTCGGGGTTTTTGTTggtgttgccagtagg
cttcagAGCCTTGGACTTCTGAGTAGGGATGGGAATAgatgatgggatagaggaggtagggcgggttgagctt
aacgcagcgcccggtgatgttgatgggaccgccctcgtggcggcaggcgcatcagatgtcgctcgtgatgcgc
cgctggcgctccgagttatcctcgtcgtcgttgtcGTAGAGCTCATGCGGGGTAGTATGGGCTCAGACTTCGG
CGGGATGAGGTCGGAGTGCGATAGGGGTTTTTTCTTGGGGGCTGTAGGTAGCCCAGAGGGATATAGTACGGGG
GTTCAAAAAATGTAAGACCAAATAGAACTTGCGTCACGCTGAGCGAACCGTACGACGTATATTCGTCCGGaaa
acTGTACAGCGTAGTTGCGAGTGGTaaaaggTGGCTAATCTAACTACTTGAACTAGTATTTATATTCAGCCAC
CGTCCGATTCGAGCCGAACTATGCAGTTCATCCCGTTTAGGAAGTCCACCATGTGGCACGCACGTGGTGGCCT
CGGAACCTCCCGaacccCCTCGTaaagagcgcGcaaccaGacTTacAAgACacTaaaAagaGcgacaatCaCg
cCcgCcagcTAaagaacgAgagaaTccTcacAcatgacccgcaaagctgattggcgccaaacgcactgggaga
ctgggaaacgacctcgcagacctcccgacgcctcatttgcatctagaaatatgtaaatcggcgcactgaaggc
ttcaaccagtcttacacaggccatataaatttcctcacatctttctgctcacagaataccatatcctaccgtc
gcttcagccgggggggaggttcaagcacaaattcccatatctggtctgccgaagatgccggccccatcttatag
caatatcgcctactcgctactcgccgcaggcttacggaaagtcacccagtgtgtggctctagagtgagccatg
gcagggtgagcgagagag
>Bjead1_1_Cluster0#LTR/Gypsy
NNNaNggNgNcNNNNNNNacNNNNNNNNNNNNNNNaNgNNcNNNNgNaNgNNNNcNNNNNNNgNNNNgNNNNNa
NccNNNNNgNgcNNNNgNgNNNacNtNNNNNNcgNNNNNNNtNtNNNgNNNNNNNNNtNNNNNNNNNNNtNgc
tNNNtNNNNNgNaNcNNNNcNNNNNNaNNttNNNNtNNcNacNNNNNNNaNNNNNNNNNNNNNNNNNNNgNNN
NtaNaNNNtNtNtNNNNcNcNcNNNNNNNNNNNaNNNNNNaNNNNNNNNNNNaNNNNNNNNNNNNNNNNNNN
NctNNaNNcNNNNNNNNNNNNNNNNNNaNNNaNcacNcNNNNNNNgNNNNNNNNNcNNNNNcNNaNNNNcNtN
NNNNcNcNtNgNNcaNNtNNNNNNcNNaacNccNNgNcNggNNNNggNNNNtNNcNgNNgaNNNNgNNNNcNN
aNNNNNgNNNaNNNNNNNatNNNNNNgNNNNtNNNNcNgNNNNagNNNNcNNNNNaNNNNgaNNNNNggNNNN
NNaNccNNNNggNNNNNNNNNNNNNNNNNcNNgNNNaNNcNcNNNNNNNaNNNcNNcNNNNccNNNNNNNNNN
gNgNNNNNNNNatNNgNNNNNNNtNtcNNNNNNNNNNNgNNcNNNNNNgNNgacgNggaNgNcNNNgcNNNgg
NNNNNNgNNNNNNNNNNNNNaNtNNNNNNNaNNtNtNNNNtNNtNNNNNNgNNatNcNNNcNacNNaNNNNNN
cNNaNNNNaatNccNNNNagNNNtNNNNgNNgNNNNNgNggaNNNNNggNNNNNtNNNNNgNgggNNNNgNNg
gNNgNtNNgNNgNNNNNccNaNNgNNNatNNNNNNtgNNNNNgNNNNNNNtcNNgNaNNNNNNNNNNNNtNcg
NNNNtaNNNaNgaNaNNNNNNNNNNNNNNgNaNgNgNNNNtccNgNNNNNNcNgNNNNtNNNgNNNNNNNNNaN
NgNNNNNNNNcaNNNNaNNNNNNaNNNcNNNNNNNNNNcNaNNaNNNNcNgNgNNgNNNcNNgNagNNNNNNNc
NNaNNgNNNcNcgcNcNNNtNgNgcNgNNNcgNNNNNNNNNNNNNNtNNNcNNNNNNNaNNNNNNNaNNNgNNN
NgNNNcNacNNNNNNNNNaNNNNNNNNaNgNaNNNNNaNNNNNcgNNNNaNNNNNNNgNNaNNNNgNNNNNgN
NaaNNcNNNaNNNNgaNNaNNNaagNNNaNNNNNNNNNNNNcNgNNgNNNcNNNNNNNtNgcNNgNNNNNgNNN
NNNNcgaaNgaNaNNNcNNNcNNNNNNggNNNaNNNNNcNNNNNNNNNNNNcNNNNtcNNNaNNcNNNNNNNNN
NNNNNNNNNcNNgNNaNNNNNtNNNNNNaNNNNcNNNNcccgNNcNNNNcNNgagNNNaNNccNNNNNNNtNc
gNNNaNgNNNNNcNggNNgcgNcNNNNgNggNcNNNNNgaNNNcNNNNNcagNNNNcNNNNNtNNNgccNatg
NNNNNgNNaNgNNNNNNacNcNcaNNNcNNNNcNNNNcNNNaccaNNcaNNccNaNNNcNNNNaNNaNNNNNc
NNNNNNNgNNNNNNgNNNNNNNgNNNtcgNcgNNNcNNNNNNcaNNNaNatNNaNNNNNNtcNNNNacNNgNc
NNcNNcNtNgNNgNtNNcNNNcNaNgNNcNNNNgNNNaNNcNNNcNNcNNNNNNNNNNNNNNNaNNcNNtNgNN
NNNNNNNgNcNNNNNNNNaNNaNNNaNNNaNNNNNNNNNNgNNNNNcgNNaNNcNgtNNNNNNNNNNNgNNNNNN
NNccNaNNaNNgNNNcNNNNNcNNcNNNtccNNNgcNNNNNNgNgNaNaNNNNNNNNNNNNNNNNNNNNNNgac
NtNNgNcaNNNNNNNNNNNNNNNNNNctcNNNtNNNcNcNNNNNNgcNNNgNNNNNNNNNNNNNNgNNNNNNNtg
NNNNNNNNNgNNNNNNNNNctNgNcgNNcNNNNNNNNgacNNNNNNNNcNNgNNgNtNgNNcNNgNNNNcNtNN
tNNaNggNaNtNcgNNgacNNNNNcNNNNNNNatNNNNgNNNNNNNNgNaNgNNNNNNNNNNgNNgNNNNNNN
NcNgNgNaNggNNNNNcNNNNNgNNNgcNtNcaNNaNNNNgcNNNcNNagNNNgNNNcNNNNcNcNNNNcaaN
NNNNNNNNNcNNNNNNNNNaaNNNNaNgNcNNNgNNNNcNNNcNNNNaNNNNNNNgNNNNgNgNNNcNtNcNNN
NaNNNcNcNNNNNNNNNNNNcNNNNtNNNcgNNNNNNNaNNcNtgNNNaNNNcNNNNNgNNNNNNNNNNNNNNN
NNNgcNNNNNNcNNcNNNNcNNgNcNNaNNNNNtNNNNNcNNNNagNNNNNNNcgcNtNgNNNNgNcNNNNNt
NNNcNaNcNcNNcNNcNcaNtNNaNNNNNNNNgccgNNgNggNNNNNNNNNcNgNNNcgNNcNNNNNNNNNgc
NtNaNNNNNctNcNNcgNNANNcgNCaaTaACgNCgGccgagNacaCcNNcgccggNcANaaNagtcNNcagc
tgNNtNCNNccagaNNNNgGtagagNNNataaNNNNNNNtNNaNNNcaccNNtNaNcagtgNNcgctACNNcN
ccNcNGgtaNtagagacNNcNaatcatccaaGNNtggtggCgCccNNNaNNaNacgacNNNaaggNgNtGNcN
gtNNtgNagggcgNNNNNNNNNaNNNNggNcaNagNaNagNCcaNccaANNNagNtgagaNtNctagNNcNac
NNNNNcNNcNNNNcNGNNNNggNccNccNNtcGcttgagacNNNcNagNNgagacNgtCcaccgNtaagatcN
tNgNgNgNNNGtNNgacGgNgaNgNNNNAGgtNNgtggtNNgaNcctgNcgNNNNNcgctNtNNcNAggNcgN

NgNCgNcNgNNNNgCcCNcgNccNNNNaCgNccccNANgaNggNNNNNgcNNNgNNtNcNaccgNNNCGNNNN
NNNgagaNatNNgNNNNgacNcatNNTNNNNNNNNNNNNNtNNNNNNNNNgNNNaNNgcNNNNNNNgNNNNNN
NaggNNatNNNNNNNgNNNNNNNgNNNcNNNNNNNcNcNgNNNNgacgNNNNgNNcgNNgNNNNNNNcNccNN
NNNaagaagacacacGaCGACCgcccagaAagAaacGaCgaaacaaacacaaaCcaccgacacaaccccaaAG
acAccacaCaTgcaAaacgACGaAaaATAcacgaccacCaTaCcaagGacagATgaCgaGGaaTcaacGaACg
acgacTcaGaagacGacaacGagcgCgGcgCGaaacaCaagTccaaaccacACaTcgAaaaaacGaAcaaGaa
cGcggacgccGaaaaGagcgaaaCGaacaGagcaaggcgagAGcaCaTGacaacaacGccaaaccaaaAaTgc
aaTgaCGcaGaagGcCccaacaaGaaCcagcaGcaaaAcGcaaacaAacGacccccGcCaaaGcCaGaAccGc
aTcaCcAccaaacTaaCgacAagaaccccaaCcggAcacGaaTcaCcAcccCcagccAagCcacaacgcACC
agcaaCaccaacaccaacgaaccgcagacccCacacgaCcaaccAcAaaacaaacaacaTcaacgcgcacagG
caCacgCaaccagTcaacAAgcccAaGAgaCaaagacgcAcacacagcaAaGaccAcccaacCaacCTCAaaa
aCaaacTcCaccgaccccAaCaaGaAcAAcaaaTaccacCaaGagcaCgcGccccacGacGaacagCccaacA
CcgaaaccCaCaaaCGcaccaaaaaCacagccCaaaaccccgGCgAaCaTaCccTcaacacaccgCGacaCaa
caaaaTccaaaacGaTacaacccaTCacTcaAAcTcaCCaTGcaaaCCcacCcaCacTgaacCacacaaaccc
CaaagcgaaACgaCCTcgcAccaaccaACacgacaggagaaaCcAacCccacaaTcacacAgaAaccaCaCGa
GaaaTccccccaaaGaaGCaGaaacccaaaacgacTcacccaCgaagccCccCAGcCacaacgCaaccagacGT
gcaaacgcgcaTccccaaccACaaaacaacgaGcgcaaGagccaacTaccTCCaaaacAaaaggaGaccgcaG
CAacAAgaTgcaAagaaagcCGcGacaccacGaaagaTaccggcaaGgcacaAacaaagaacaacaacAcATa
aTCcacGacggacaaTcaagaCaggacacaccggaCaTCaagaacaaccgccaaTAcccccgaAcaCcagcCa
aaaccaccAgcaCcGcgcTgaacGggcaacaccaATcccagaGcCaTaaaacccaccGcAaTcacaGacACga
caacaaaccgcaACGcaAcgccccaaAaccccgaCgagaaAccaaAaaaCaAcaCgcaTCccCgaaccgaaCC
aagaaaaacAcaAacaGcacaCccGacaacaacAgCaAaaacaAaCgaagaacTaCaaaaAcaacTcCcAcaa
CaacgCaaTaacaaaGacaaacAcggcaacaTcgCCaaccTCgaCcAgcaaaacagcacaaacaaaacaacaa
cacaacaaGaGGgcgaCcCcagacTcaACcccCcCgaCgagAcaacaaCCATaaaagacCacgaaccgaacaC
aaccaaaaggaccaagaaCAccacCaacgcTGCaaaaaaaaaAcCaaGacTaCccTcaggaAaaAaagacaaC
GagAgacGaccGccaaaaccgccAcacaccaaaccagAccACAAgccaCTCaacgacgagGcAaCaCCAacCG
CgaCccaGaaTcAagCaaaGgAagcgaacaCCgaaacaGcAacGaCAaaacaagcaAcaAaagCacCaccTac
aCCcAcaAaGAgcAaacaacaAaaGAaagacaaAaaaaCaccGacaaaaccAagAaTaaaCaaTaccaCaCcg
acGaaCacaacagcagCGAacCGAaCGaacgaccgaaaAcAccGTcgGaaaCGaTggaTgaaacaaTgCgCaG
acaacaacaAcaacaggcccaaaGaAaTaccCCCgCTaCaagacAaCaACcaacGaagcaCGcaTacCGacaa
acaaaaaccaTAcgccTAcaagcaaacCaaGagcacagAcgCAcTgaagCcGacaCgaacGaaGAaaAccaAc
AAcaacgACaAagCggGaTgGgGacgacaCGaaacaacCaAacAagCCgcaaaGagGCTAaGTcTgAcCaagA
aacaaaACGaacgcagcaCCgcaacCaaaTaaccgAaaaccaAcGATaACACccgcAaacAcacaAaaccacc
ccaGgAccaGaaacaaagaCGcaTcGAaacagCaaccaCgcCaaaCaGgTcaaAGaacgagccCaagaacgaG
caCcagcaAagCCGaaTagaaCcacacgacccacacaaaaagcagccagcAacaAgaaacGcAaacaagcccA
aTcGAGgcCcacaaaaaaGcaacgccaacaaaCgagCAAacacccaaCATccAagGaaCCaaccaTcaaGaCc
AcagATccGaccAcgcGcgaaccaATaccgggaagAcaccaccagacTacaccacgaagTGcAGcaacacaaa
GacaaccacCGgAaTGgaagaaaaaCcccaCcaaagaaTcaaTgacgacgaAaggcAccGAaGcaCAaCacgc
acaCcaaacaTacGaAcaccagagGAcagaTaaaagagaaccacgGAcgAaAcGcGaAcaCAaATaAcagGGa
CaaaagacgAcaaTcGccacaaaCcacAaaccacgaaaAaacTcaaaGacgcagGccccaCagcCaaaaccTC
acACcagacagaccgaATTcaTCGcacgacaCaACgcCaCcATCCAacAccgcaaGgaaagcacaaaCAagcg
aGcGGacccCCaaaCaccaaaccagTcgaGaGaCaccCaaGAacaaagccaacCGcaCGaCgAgTacGaGaCa
caggaacCaCccccaGaaCcacacaacaaGGaccgTccGcgaccccgaagacgaaaacggcGacaggcaCgAg
gGGcacccTaCaAccagGaaGaaccaaaaaCaaGCcGcaaccacGTcaaacaaaagCacggaCaaaCaacGcg
CacacaaccgcagcgaaCaaaaaaCcccacccagaCccaaacaacaacacaaaagacaaGaaAaaCgGagGac
cAcGacCacccagaaaAcgACCcaacacgaacaGaTGaaccgcaTcaAaTagcaaccCaaaCTaacGTaaaca
CaaAcGacaaaGAaggaacagCgacagcGaaggccgggTaaaagaaaGaaccaaaaggcGagAaaGaAagcGc
aaaCccAacGCaccaCCcacTAcTcacaGaagcacaaGcCccAagAgacaaaaCgaaCCGaCgaGgACGgcAG
ggCagaTGCacgaacCGacTCaaAAgcaGAacagCgaCcCTgGcaaaGAcgcaaAgaGcgcgcGcaaagcAaA
caAggacccACCgaagaacaCGaCccACTaacaacaacaccaggaGacaacagcGaaGacCgcaaagcaagca
ccAccaCaaCccGcaagGaCGAgcaaGaGaccgATcCCAaAacgCGaaacaCGACaACaccaagaccacaacc
caaCggaagaaaggAcgaaaacaCggCcacCCTccaGaacGacaGcaAcGGaTaccaTaaGaaCGcgcCaaga
ccCaaacacGTcaAaaaTcCTacGcaacgTcCcacGcccaaacacaTCcAacacccaCgcaAcACCAagaAaC
aaGacgacaagcGaGTccgaagTagcccccacgTTaaaacccaaaaaCGagCacccaCaCaacccccCagaga
ACacgacCaCacACccagaccacAcaTgcacacCaaaaaaacGGAgGGaaacacaCcgAaagcgcacgacccG
aCTaCgcaaGcCaAcGaCaTTgaaaaagcagaCAgaacGcGccaccgAgcgaaaaaaaCGaAcTagaGcaaag
accTaaCaacccGgCgcagaaaCaGaCcacaAagACcaaccaGccaagagccCgCTagaccaATgcaCcacAa
aggCCaacgcCaacaaccCcAagGGaTccaCgcCcTgTGaaTgAgTaTaTGgCGAcacaCCcCGaagGaTcaa

agaagCcCcacaGgTaacacgagaCccgcGccggacacaacgcGcagaacaagCaAgaaAcaaccTCcaCgaa
acGcagcaccCcAcaaTcgacaaTCGaaTacagaaaacacacTaacccaaCcGgccgcGaaacaacgaCacca
aAgacccGGCgaGaGAccagccaTggCaAccgcGTgacacCcCaaaaCggaCaaCgaaaaAcaacaCaCgaac
caaacGaccaaCcaaAaaccgCGCGcaCaGGTTaAgacaaaccaaaAaGAcccgcgaaGaaaacacccccTcc
TCgAaaTagccacgaaaaCTacCacCaGaacagaaaccacGTggacgcCaccCcAaaTgccaaaCGgcaCaaa
aCCaaaaacacgccCccAaCgaCcacacaagacaaccgCaCCacGAcaccAAcggcTTccacaaCaaacGaCA
AcCAaaCgaCgaCaAGcacaTcaTccaacAaagcaTaGgacAcaGcaccaaaccaacCacaccaaaaggcGaa
GcaaGGacGAaaacGaacaaCAccacagGGaaaaaccaagAcaacTGcacGaaccTcgCagccacCaACcagg
acccgcaCcaaGccccagaGaAAcaGccccaaccccCgGcCaacaaaacCcagaCcGagCaaacagccacGacC
caaccaCcgacgaaAGcCaCAcGcaCacacacaaggccCAGCCgCAaCaagaccaccgaCcaCcgccgcccCac
cgCccaaaacccCCcacGcCTcaaaGacCcccGcTacAacgaGaTaGcAacccgaAacCCcaTgTccaaccga
aacTcaagccaCgaaaaaGaaAcacGccaCGgacTcagaaagaaAaAacACaaCcaacgCccgAGaccaacca
gcAcTaccaaTcgacaCaTaccagaCaCcCaTgacGaCgcaacacccCccCCaCaccAccaCgCgacCgaaTc
cCccAacTCGagCaaacTCCcgagcacccaCGCAacCacaaTaGcacaaaccCaacCcaGgcCGaccgCGcaa
caccTcaccacaaaccccccaccaccccccccgaGgccaAgGcAaggCacACGaCaCcgaaaCacaCAaCCCcca
aCaCTCccCacaacaagCcGccagCacagCaGaTCccccaccAGaTcCcAcaCCacaCccgcGcaagaccccc
cGaaagaccagaAaCgaaAaaGacacGGcacaagTacCcAcccaaaacaacgcgccGaCcacaaaccGccCaa
cTaaaTgcacCaCcaaaaacaaTGccccATaccgccgGacgaaagCaaacaCagCaaccacAacccaacAaAc
acacCTcaCCcCCGacCaGgTaAaaCaCAaaATcaacgccaGaagcaaCacggcCcaaCaAcGcaGGCagaAa
CcaaaaGagaccAcgATAgcaccacAcCacGacacAcAaGgaaagTACGagaacgaacaccCcaAcGcgAaAG
aaaaCccaagGcaacCaacaaccgacaACGcGaGcgaAGaTaAgcaaaTcaagccCCGccTcaaaagaaaaCa
GacGaacgacAcATGagaaaCgaaaccACaagcccaccAagaccacaaacacgagAAacACCTacACgaGCaa
cgaGgagcTCgAGccccaGaGCTcgacCaaCacCaaTaCAaGaccacccccCGcagCCGaCaagacCacaaaa
aagcacccCCacgcacaccaacGacacaCcagCCgacacccaacagcccaaACgCCcCacaaCaaccAaTccc
aAaaCacGccaaCaGccTcaggacgaacaCAcccacgCgagCGaCcccaggaTcaCaCgcggCAacCaaCCaC
aAccccggCCGaccaccaCcAagAcCacacaTcaCcaaccaccaaaacTccaacacccccGccaACaCcccGC
aCAAaaCACCAaggcCaaTaATcAaaacaAccaTGgccgaCCgCTgcGcacgCaacGccTCccgCacccAcac
ccaaaaCCCcggggaCTaCaagGaTaccaGcACcaacgcccaccaagcagCgCaacGaaCaTGAacgccaaCG
aacagcGCaacaaTGCaCGaaCGacAcCagccacgCcaaGaGaaaCTcgccacaaCcaCaCacaacGCcacca
CcacccaCCaggaGTcCaaaaaTCgAaaTaCaaaCCcgagagTcagccgcGACCggCaAgacCcGacCcacCg
cACgcaaaacgaaccccaggagcaaAGaaagcGgcccaccccaCgccaAacacaaCAacaccaacAacGacaA
caaCgaaTgCGaccAaaCCaccAccaCaAacAaCgacAgcgcaaaAGaGaaaccCcagAGacccaacAcCcCc
aaaggaCaaaaCccaCaaCcccAaaaacTgAaGcccGCgacCGcaAcaCACcaaccaaaaaacaAcaATcGaG
caAacgcGaaagaCagccaAcacaaacgaaagaacgcTcGccgcCgCaaaCcCacaaacaccagGCaacaacc
TCGCGcTaaaaATcgccGccaacccaaTccacagcGCaaaccAccaaagcgaccccaCccgaTaCacGgAaca
gCaCGaagcCCcacccGacaaCcgcccaaacacaccgcaacGcacCaAacaCaccaacTcTaaccaaaccaac
caaCGCACcaagGaaAaaaAaacAcgCccacaccgCccccCaaaacaGcgcacaaGaaCGaaccgCacaaCaa
cccGcccCCCCaGcaCaaTgGacacaTCGTCaaCaGCaGgAcCaacaaaccaCcAGCcGacgCTacaacccaa
ccaGagTGcagaCgGaGaaacaaGaCgaaggcaTaAaGaccAcGcgcCaggcCaAgccAcaaaaacaaacaAG
cACGgacGAGgaccaAaaacgcaaGCcagcccAGaaagccaAacccTaGCcGaCCGaGGAcaaaGacAaaaaA
ccgGacGaaacccAagCaaaacAaTcgcccaaacaaggaaaCgGgcaaacgacgacagagCccacGTcCaacg
CACgaacaCaACcGcaaGaacgGcccCcccaacGCcccacaacaaggaaACccccaaacccaaaaGGccGcac
cTCGcaAGaaCcCcTGcGaacGggcCACaTaaACgacCcgaCCAgaaccGaaTgccaaacgcGagacagccaa
TcGaCaaccaccacccaccaacagacgaaaccaGcCaaccaCaacacCgaCaGcaacAaaccGCaaccgcAaC
acaCTcacTgaacaaccaccgcaacaacaaCgacaCccTGgaCacCcaacCggcaGaacaAGcCaacGcaaGC
agcAGaggaCacGcGaaGagacCCcagcaTacccCcGccCacccgTgaGagcacacagaaCgaagagAaagcc
AcCacgaCCagcCcaaagaaGaagAaCAAaGagcacCaggcgaacgcacaTgTGCacacaCTgaaaaatcgcg
cgcgagaactggttgggctgccgctgcggcatcctgtttcaacgggggaggggacggcgagtactaccttcat
cccactacgaaggaaggttaacgtcaagctgccctcggtcgacgacttcgcatctggcataacctggctacgc
aaggacaggaccatgcgcaaggtcacaaacatccaccaactccgctggccgcgaactgagctggaagtcagac
agctcatcgccgaggcacttcgccctgatcactggatcgcgtacgagaaggtgaaagcgttcatacgtgccac
caatgcgggcgaaatggacaacgacgccacattcgcgatgaaacgcatcgactggaaacgaccggaatggacg
caagaccgggtagcaccccaagaggtgttcgacgaggacaccccaatcaccgaggacgacgtccgccgggcca
taggcgacgagcagctcagcatggaggagggcgaccccgacaccatcgtcaccctacccgctctcacgtccat
ggcctatcggtggtggatcttgtttcttaatcaaaacaagaactacgttatccccggggtagtgggagagcgt
tcccgtgacctcacgcatgccgagatgctggccctcatcggattccttcgagtcatggactttgggccgtcga
ccatccacggacccagttcgtacagttggctgtatcacgtccttctcatcgagctcctcgacggagaacacta
ccttctcgacttccagtccatcgcagccacgcgagtcatgccggctaccactcctgtgaacgctgacactgtc

cgtagccaccttcgatccatggtcgaggagtcgaaagcccacgcgactcagctcaaccagaagttcctcgtcg
ggccaatccacatctgggcggagaacctccgtgacgccagcttcctcctccccagtgagttgaaggacctcgg
tcagtgctttgggccccaggccactcccatggacatcgacggttaatcattcaattgcacactctttcttgtt
taacctcatctcgagactatgattccttaatattcttttgtattccgcctccgctaccgcttcgtctaaaggg
ggagggtgtaagggaggcgtgagcgaccagggcgcggtcacacgcgcctatggaagcacgcgccgactgatgc
gccgcgatccacgtggcgcgcacgcgatgacttcctaatcgggttaactgtatagtttgacatttgcttccgt
ttgtggctggatccacggaaacggagcagaagtagcttaccctttcgacactcacaaaacactgtacttaccc
gtagcgaatatacgctgtctggttcactcggtgtgccgtgagagtcgaatctaggccttacac
>Bjead1_1_Cluster5#LTR/Copia
CGCCTAGACCCCGGCGACTGATGCGCCGGAGGCGGGATTCCCTCCCAGGTTTCTCTTCCTCTTTCCTTATTTA
GTATCCCGCTCTCGTTCCGCCTTCCGCTCCCGCCTGCTTCCGTTTGTATCCTTATCGCCCCACTTATTTCCCC
TTATAGAGTTACCGACCCCCTATCATAAATAATATAAATAGAACCTGTATTAGTGTACAAATCATCAGCTCTT
CTACAGTCCCTCCCGTGTGATCCGAGCCTGCGCGAACAACAGGTTATGAGCCCCGCGCCATAACGGATCCACA
CTCGACGAATTTCCACTGCTCGATCTATTGCAGATCAACTGGCGTTACTGCTCGTCCATTGCGGACTTAACCT
TTGATCTTGATCGGACGACTAGCGCCCATCCAAGATGTCGACCTCAGAGACAAGCGGCGGTTTCTACCGCATC
GACCCCCTCAAAGGGCCGGAGAACTGGCTATCATTCCAGGTTCAGATACAAGATATCCTCACAGATATGGGAT
ACATGGAGTACGTTGACGGGACGAACCCACGACCTTCCGACAAGTCCCTCCTTCCCGATTGGGAAAAGAAGGA
TCGGAAGGCGCTCGTCATCATTCGTCTTCGTGTATCCCCGTCGATAATCACTTACATTATGTCGGCAACATCC
TCCAAGGAAGCATGGGATACTCTCAAGGCAGTTTTTAGCACCCAAGGCGCGTTGGCCAAGATCACGGCCCGAC
GCAAGTTTTTGCGCTACAGCATCGAAGAAGGCGCTAACATGGAAATCGAGATCCGGAATATCCGGATGATCAA
GGAGGAACTCGCACTCCTCGGGACCATCGTGGATGACGAAGAATTCGCACTCACCATCCTCACCGCACTCCCC
ATCTCGTGGGACTCATTCATCTCATCCATAGGCACCACCGAACTCAAATCTAGCGACTTGATTGGACGCATTC
TCCAAGAAGACGCGCGCCGCCAAGAGCGATCATCCGAAGTTGCGCTCGTGGCCAGGCCCGCACAGACCAGATC
CAAATTCCGCAAAGGCGTAATCTGTCACTACTGCGGCAAAGAGGGGCACATCAAGCCCGAGTGCCGCTCTAGA
CAGAATCAGCAGCCCGGTGGGAACCAACAGTTCGGCGCCGCGCCAACGTCCTATCAGCGCGACAACCAGACGC
ATCTCGCGACCTCTCAGGCACCCGAGGACGACTACGCGTTTCTCAGCATCGAGAACATTCCTGAGGTCGCGGC
CCTAATAGCAAAGGGGGAGCCTTGGCTTGCGGATACCGCGTGCCAAAGGCATATAGTCGGCGATCGCACGCTT
TTCACGTCTTACACGCTCACCGATCAGGAAATCAAAGGCGTCGGCGCGTGCCGCGCTTTAGGACAAGGGGACG
TACGAGTCCTATTCAGAACCAAGACGGGCGACGTCCCCATTACTTTGAAGAACGCGCTGCACGCGCCCGATCT
CGAATACAATCTAATCTCCCTCGGACGACTCACTTCCAGCGGACTCACCTTCAGCGGCGCCGGGGAGCATATG
ACGATCAACGATCGAGGACGCATCATTGGTCAAGGCCGCAAAGTCGCGAACCTTTATCACATGGACGTCAAGG
AACCGATCACGCGCGCTCACGCCGCCACCTTTGCAACACCGCGATCGTGGTACGAGTGGCATTGTGCCCTCGG
ACATATTAACCAGAAACAGCTCATCGAAATGTATAACAAGAAGCTTGTCGACGGTATGGAAGTTGACGAAAAT
TCCTCGAAGGACTTCTCCTGCGAATCCTGCATCAAAGCCAAACACGCCAGGAGTCCCATCCCCGATAGCGCGC
TACAGCGCGATCTCAAACCCGGCGATTTAACCGTCTCCGATATCTGGGGGCCCGCCCGCACGGAATCATTCCA
GCGCAACACGTACGTCATCACATTCACGGATATCGGTTCCCGACACTCCGCCGTATACTTCATGAAATCGCGC
GCCGCCGCACTCGATCGCTTCAAACAGTACGAACAGATGTTCGAAACCCAGTTTGGCCGGCGCCTCAAGGTCC
TTCACGTCGATAACGCGAAGGAATACACCCAGGGCGATTTCAAGAAACACCTCGACGACCGTGGCATCGTTCT
CCGTACCACGGCACCTTACTCCCCAGCTCAGAACGGCATCGCGGAGCGCCTTAACCGCACTCTTGTCGAACGC
GCTCGCGCTATGCTTTTCCAGCACAACGCGCCCGCCCATCTGTGGCAGGAGGCGATGGCATACGCATGTTATC
TCAGGAACAGGACCCCCACCCGCTCCATCGACGGAACGACCCCATACGAGGCTTTCTGGGGAAGGCGCCCCGA
CATCAAGGCGGCCCATGAGTTTGGCTCGCCCTGCTGGGTACTCGTCCCGGATAGCCGCCAGACTAAACTAACG
CCCAAATCCGAGAAACACACCTTCGTGGGCATCGCCGACAACTCCGCTGGCTGGCGCTACTATGACGCAGGAA
TGCGCCACATTCTCACTTCTCGCAACATCATTTTCCCTTCCAAAGCTTTAGAAATTACCGACGACGGTCCCTC
GCTTGAGGGGGAGCAACTGGAATCAGCGCCCGCGGCGGACGCAGCGGCTGCTAAACCCGCACCCGCTAACCCT
TCCCCGTCCAAGACCACACCTGTCAATTATCCGCCCGTTGCGCCAGCTTCACCAGGCAAGCTCGAACCGTCCC
CCCCTACGACACCGGTCACAGCACCGTCTACAGATGCTCCTACAACACTCGCCCGACGCTCCCAACGTTCTAC
CACAACTCGCGTCGACTACAAAGCGTACGGCGATACCGGCGCCAAGACGCCGATCGCCCAGGCACCACGCGCT
GATGCGCTCATCTGCTTTGCGGCCCTCACTTCCTCCGATCAACCGACTCTCGCCCAAGCTCGCGATAGCCCCG
ATTGGCCTCACTGGAAGGCCGCCCAGGACGTCGAGATCGAGCAAATCCGCGCCCAGAATTGTTACGAACTCGT
CGACCTACCGGAAGGCCGTACGGCCATTGGCTGCCGCTGGGTGCACAATAAGAAGAAGCACCCAGACACTGGC
GCCGTCCTCAAATTCAAGGCACGCCTCGTCGCGCAGGGTTTTTCCCAGAAGCCCGGCGAGGATTACTTCGAAA
CATACTCACCCGTCATGCGCACGGAATCCTTCAAGGTCGCGTGCACGATTGCCGCGGTCCAGAACCTCGAGGC
CGACTCGTGCGACATCAAGGGCGCGTACCTAAACTCGGAATTACAAGAGGACATCTACATGCGGCAACCCCCC
GGGTACGACGACCAATCAGGACGCGTCTGGCATCTCATCAAGGCACTCTACGGCCTCAAGCAAGCCGGCAAAG
CGTGGAACGACAAATTCAATCTTATCCTCACCTCGACCCTCGGCTTTACTCGATCGACATCCGACCCGTGTCT
CTACTACAAGCACGGCACGCAAATTCTCATGCTGCTCATCCATGTGGACGACACTGCGATCTTCGGCACGCGC
GCCGCCATCGATACTTTCAAAGCCGACCTCCGGAAACACTTTGAGATCACGGATCTCGGCGTTTTGAAGTCCT

TTCTAGGCCTGCAGATCGAACGCGAACGCGACTCACGCAAAATCGTCATTCACCAAGAACATTACCTCCGCAC
CGTCCTCGCCCGTTTCCAGATGGAAACGTGCAACTCCGCCTCTACCCCACTTGACCCAAAGGACAAGCTAACA
CCACTCTCCACCGAGGACGAAGACGAAATTGCCAACGTCCCTTATCCCGTCGCGATCGGATCTCTGATGTACG
CCGCCGTCGCCACACGTCCCGACATCGCCTTCGCCGTCCAACACCTGGCGCAGTTCACATCACGCCCTTCTGA
GGCACACTGGCGCGCCGTTAAACACGTCCTCCGCTACATCAAAGGGCACTTGGGCGTTGGTATCATACTCGGA
GGGCCCGACGTTTCCCTTATCGGGTATAGCGACGCCGATTGGGCGGCAAACCTCGCGGACCGACGATCCGTTT
CCGGATACCTCTTCAAACTCGGCGAAGGATCAATCAGTTGGTCCTCGAAGAAGCAGGCGACCGTCGCGCTCTC
CACGATGGAGGCCGAATACATCGCGCTCGCCCACGCCGCCAAAGAAGCCCTGTGGCTCCGCTCCCTCCTGACC
GAACTCGGGTATAGCCCGGCGTCCTCGACTCCCCTCATCACCGATAACCTCTCCGCCATCGAATTCTCTTACA
ATACGCAGTTCCACTCCCGCTCCAAGCACATCGACATTCGACACCACTTCACTCGCGAACGCATCGACACCGG
CGAAATCGCGATCACGCATTGCGCGTCCCAAGACAATTGCGCCGACATGTTGACCAAGGCCCTGTTCAAGCCC
ACGCACATGCGCCAACTTGAACTGGCCAACATGCTCGCCCATTGAGGGGGAGTGTTGTGGGAACGCGCCTAGA
CCCCGGCGACTGATGCGCCGGAGGCGGGATTCCCTCCCAGGTTTCTCTTCCTCTTTCCTTATTTAGTATCCCG
CTCTCGTTCCGCCTTCCGCTCCCGCCTGCTTCCGTTTGTATCCTTATCGCCCCACTTATTTCCCCTTATAGAG
TTACCGACCCCCTATCATAAATAATATAAATAGAACCTGTATTAGTGTACAAATCATCAGCTCTTCTACAGTC
CCTCCCGTGTGATCCGAGCCTGCGC
>Bjead1_1_rnd-1_family-161#TIR/MITE
TAAGGTAGAGTCGGTCTGATTCCTGTAACACCACGTGGAATGTGACGCGCCGGTGACTANACGTGACNTTNTG
TGACATTGGCGGTGTCCTGGGCGGTGGTGACGNGTGNGTGACATGCCGTGGAGCCCTTAGCGCACGACGTTTC
GGCAACGTNAANCTCTGTCTAGTCGCTCGATTTCGTCCGCAATTCAAATCCGACATTGGGACCCTGTGGTTAT
GGTGCAGACGAAAGGTTTGCCGATTTAAAATTATTTTTACCTACAAGACTCGTCGACTAGATAGACCTACAGT
GTGTTCATGTCACGCACTCCCCGCTAAACGAGTCGAAGCGTGACATTATTTTGCCACGTGGTGTTACAGGAAT
CAGACCGACTCTACCTTA
>Bjead1_1_rnd-1_family-266#TIR/MITE
TGTCGGAAAAAAAGTTTCGCCCTTGAAGCGCCTTGCGATGGCCTCAAGGGCGTCAAGCGGACCGGGGTCTTGC
GAGGCCTTACAAGGAAAGACGGTTCATTACAAGAGGGGGGTCTTGAGACCCTTGTAAGCCCTTGTAACCATCT
TTGATGGCGGTTCAATGGTTGTGACCTCCTCGTAACGATCTTGTAACGAGTGTTTCAAGACGCTTCAAGGGGC
GTCAATCACTCCTGCCTTGTAAGGCAGGAGATAATTTTCCGACA
>Bjead1_1_rnd-1_family-174#TIR/MITE
AGTCCGTGCGACGCTTTATGCCCAATGTTCTCATCAGCTTACTATCGAGTCTCTTCATGTAGACCATTCGGCG
CACGGGTCTCAGGGCTAGGGGCAGGACCCCTCGCCAAATATTGTATCANTTTCGTAGATGCACGCCGCTGCCG
TCAAGATCGCCCCCCGTGCGTAGCACGGT
>Bjead1_1_rnd-1_family-198#TIR/MITE
TACAGTGCGTTGCATAACAAACGCAAATCCGCGCGAGGGAAATTTTGGGCGTCTGTCCCTATTTGGTAGCCAG
ACGCTAATGCACGTGCCACGCAGCTCAGACGACACGCTCTCAGCCTGCTCGCGTTTCTAATGTCAGGAGGTTC
AAAATTCAAAAAACGGGCTTACACAAGTATTGAATACACAGAAAGCCTAAATCTCGATTTTTTAAAGGCGGCA
CTACCTCACCAACCAGTGCGCGAGCCGAAATGCGTCAGAGCCGCCCGCTGACGGCACCTCGGCAGACCTCGGC
CTACTTGCCTGTTATGGGTAGACGCCCAAAATTTCTGTGGCGACGTTTTGCGTTTGATATGCAACGCACTGTA
>Cersu1_rnd-1_family-88#LTR/Gypsy
TCTAGCTCGGCCTCTTCTAGCAAGAATACCGATACCCCGGGTGCTAGCAGCTCTAAGGCATCGTCGTCTAGCA
GTAAGGGAAAGGAAAAGGACACTGGAAAGTCGAAGTACACGAAGAAGAGCGTACGGGAAATGCACGGCAGCGA
CTCCGATAGATTTGAGGAGTTAGATTCGGAGGATGGGTCTGGGTCGAGTTCGGAAGAAGAAACCCCCAAGCCA
AGGAAGAAGAAGTCGTCTTCAGACAAGAAGGCGCAGAGGGTTAACCGCGTGGTTTTCTCGGATGACGAGGATT
TTTTGAAGGGGCCTATGTAAAGTCTGCTACATACGGTCCGTTTTTAGATTTCCTATATCCCCCGGACTCAGTA
GAGTTTGTACAAACAGTATTGGAATCCAGAAAAGTGTTTAGTCTACCGTCTAAATCGAGTTTTTCGCTTCCAG
TTACTGTGAGTCAGGGAAAGGAAACAGAAGTGCAGACAACCGCGTTGGTCGACTCAGGAGCTTATGCTGTATT
TGTCAATCACCGCTTCGTAAGAAGGTGTAAGCTGAAGACAAATGAGCTAGCTCAGGAGATTCATGTCTATAAT
GCAGACGGATCTCGGAACCGTAGCCGTAGTATCAGGCAGTACGTGTGGCTCGACGTAACGGTTGGTGAACATT
GTTCCCGCCAGGCCTGCCTTGTTACAGACTTAGGAGGAAAGGACATGTTCCTCGGTTTTAGCTATTTGAAAAA
GCACAATCCTGAGATAGATTGGCAAAGGGGTGAGCTGACGTATTCACGCTGCCCGGATACTTGCCGTTTTACG
GGACGACGCGCTCAGATAGCAGAAGCAGAACTTGAAGAACCCATAGATGAGGACTTCGAGTTCAGTGCTGAAG
AACCATGGCTGGAGTGGATTGATGCGACCACACCAGAGGGGCGAGTCTTGGCAGAGACTTGCATGAACATTGC
ATCAGTATCGGCGACTCGCGCGCTGGAAGGAATCGAGGGCTGGGAGCAGCTTGTCCCTAAACAGTATCATGAG
TTCGGAGACGTCTTCTCCAAGAAGGCGAGCGAGCGCATGCCTACTCGCAAGCCATACGACCATGCGGTCGAGT
TTACGGAAGGCGCTACCCTACCCCGACCTGCCAGGAATTATTCCTTGTCGCCAGTCGAGAGAAACTCTCTAGA
TGAATGGATACAGGAGAATTTGGAAAAGGGCTATATTNGACCTAGTAAATCTCCGTTGGCAGCGCCAGTTTTC
TTTGTGAAAAAGAAGGATGGAGGACTACGCTTGGTGCAAGACTATCGTAAGCTGAACGAAGCAATCAGGAAAG
ACCGCTTCCCGATACCTAAGATTTCGGATCTTATCGACCGACTGTCGAAGGCTAGTATCTATACCAAGATTGA

CCTTCGATGGGGGTACAATAATGTTAGGATACGGGAAGGAGACGAGTGGAAGTTAGCCTTTACTACGCCATTC
GGTCTTTACGAACCTACGGTCATGTTTTTCGGTCTCAGCAACGCACCAGCTACGTTCCAGAGAATGATGAACC
ATATTCTCGACGACTTGATACGAGATGGTCATGTAATGGTTTATCTGGACGATATCCTTATCTTCACGAACGA
TTTGGACCACCATCGACGCTTAGTCCGAGAGGTACTGCGGCGGCTACGCGATAATGACTTATTCGCTAAGCCT
GAGAAATGCTTTTTCGAGACGAATAGCATTGAGTACCTTGGAATGATCATCAGTAAAGGATCAGTTCAGATGG
ACAAGAGCAAGGTGGACGGTGTTTTGTCTTGGCCCGTACCCCAGAAGGTGAAAGATGTTCAAGCCTTCTTGGG
ATTTGCCAACTTCTATCGAAGATTTATTCGGAATTTTGGGTCAATTGCCCGCCCGCTCTCAGAGCTCACCAGG
AAGGATAAGGAATGGAACTGGGGACCGGAACAGCAGGACGCGTTTGACGCGCTCAAGAAAGCTTTCACGTCCG
CCCCAATTTTGAAGATTCCGGATGATGAGAACCCATTCAGAATGGAGTGTGACTCATCGGACTTCGCGACTGG
TGCAGTTTTAGAGCAGTTAGGCTCTGACGGCCTATGGCACCCTGTAGCATATTACTCAAAGGCGCTTGGTGTG
CATGAGCGGAATTATGAAATCTACGATAAGGAGATGCTCGCCATCATTCGCGGCCTTAAGGAGTGGCGCCATC
ATCTAGAAGGACACCCGTTGCCATTTGAAATATGGTCAGACCATCAGAACTTGACGTATTTCCGAAAGGCACA
AGATTTAACTCGACGACAAGCGCGTTGGAGTTTGTTCCTGACCAGATTCAACTTCCATTTGAAGCACAAACCC
GGAAAGACGATGTTACGGGCTGATCCTATGTCCAGGAGGCCAGATCATGAATTGGGGGTGGATTGCGACAATC
GTGAAAAAATCCTTTTGAAGCCAGAATACTTCGCGATCAGAGCCATGCAACCAACCCATTCTTCGATTGTGGA
CGATAGCAAATTGCTAGAGAGGATCAGGAAAGCACTGTTGGATGACGATGTCACCAAGTCGTATAAAGACCTG
TTGTTGAAGGGACCAAGAGAGTTTGGAAAGGATTTACAAGAATGGCACCTCGAAGACGGTCTTCTGTTACACC
GAGGCAAGGTGTATGTACCTAAAGACGATAAGATTAGGGCAGACCTGTTGAAACTTCACCACGACTTCCCTGG
AGCCGGACACCCTGGACGATGGAAGACGTTGGAGTTGTTGTCTCGCAACTATTGGTGGCCAGGAATGTCAGTT
TACGTCAAGAAGTACGTTGCAGGATGCGACGTATGCCAGTCACACAAGAACGTCAATCAGAAACCCGGAGGGT
TACTACAACCTAACGAGGTTCCGGACCAACCATGGGAGGTTATGTCTTGTGATCTGATCACCGACCTACCCGA
CTCGGACGGATACAATGCCATTTTCGTGATAGTGTGTCGACTACTCAAGCGAGCCCACTTCTTCCCTGTTACC
AAGGAGTTCTCCGCCAAGGATCTCGCCAATTTGCTCTATGAGCGTGTTTGGCCCTTACACGGCCTACCACGGC
AGATCATCTCGGACAGAGGCACGCAGTTCGCAGCGAAACTCTTCCAAGAATGGTGTAAACTCCTGGGCATTAA
GTCGGCAATGACTACGGCATTCCATCCGCAGGCTGACGGTCAGACAGAGAGGGCGAATCAGTCACTAGAACAG
TACTTGCGCTGCTACGCAAGCTTCAGGCAGGACGACTGGGTTAGTCTACTACCCGCAGCCGAGTTCGCGTACA
ACAACCAAGCGCATGAAAGTACGAAGAATTCCCCGTTTTACGCCGAGTATGGCAGACATCCTCGGATGTTCCC
GGAACAGGACCGTGTTACCCGAATGGTAGACCTTGACGACTGGACTAAGACGAGACAGGAGGCGCAAGAGCAG
GCAAAAGCTGCACTTGTTCTAGCATCAGAGCGTATGAAGTGGTACTATGACCAGGGTCATTCTGATCCAGAGT
TCAAGGTCGGAGACCGAGTTTGGCTCGATGCGAAGGACTTGAACCTGCGCGTACCATCAAAGAAATTGGCACC
AAAGCGAGTAGGCCCGTATGAAATCTTGGAGCAGCTTGGACCGGTAACCTTCAAGTTGAAGTTGCCGAAACAT
GTGAAGATTCACCCGGTCTTCCATGCAAGCAAGCTCATTAGGTACACTGAAGACGAGGTTGCTGGACGCACTA
GAAAGCCCCCGCCACCTATTAGGGTTGGCGAAGAAGAAGAATTTGTTGTCGACAAGATCGTGGATTCGGCACC
AGACGAACGCGGACGAGTCATGTACCGTGTACGGTGGAAAGGCTACGATGAACACAGTGACACTTGGGAGTAT
CTTGGGAATCTGAAGCACGCAATGCAGAAGG
>Cersu1_rnd-1_family-162#LTR/Copia
CAACACGCCCACTCAACGGNAGTGGACGCACCCTCAACTAAACCCATCCCCTTCGCGAACCTCTCGTGCTTCT
CNCGCGCGAGCGCCTTCGTCAGCGCGTCCGCGACCTGGTCGTCCGTNGGGACGTACTCNACNGCGACCTTCCC
TTCCTCGACCNTCTCNCGNATGAAGTGGTANCGCACGNCGATATGCTTCGTGCGCGCGTGGAACATCGGGTTC
TGCGCNAGNGCGATNGCCGATTGATTGTCGGCGAGGAGCGGNGTGCCGCTCTTTCGGTTGCGCCCGATCTCTT
CGAGGAGNGCGCGNAGCCAGATGGCTTCTTTCGCGCCGTCGAGCCCGCCACGTACTCGGCCTCGGTNGTNGAC
AGNGCNACGATCTCCTGCTTCTTCGAGCTCCAGGAGACGGCGCCNCCGNCGATCAGGAAGACGTANCCCGACA
GAACAGCGTGGGTTTACGTCGGAAGCC
>Cersu1_rnd-1_family-94#LTR/Copia
CAGAACCCCGCCCGACCGAGCGCGNAAGCCGAGCGCGTACGTNCAGCGCATNCTCGAAGGCGAAAACGCCGAG
AAGATACCGACCGGCCTACGAGTAGCCGCCGAGACNGCAGCGCCAGCCGAAGAGCCGCAAGATTCTGGGGGAG
CGGGNGACGAGTGGGCGATGGCCGCCGCGACATCAGACGCGGAAGGGCTNGAGCCNCGCAGCTTGGCNGAAGC
GAAGAGACGNCCAGACTGGCCGAGGTGGGAAGAAGCCGTCGCCGAAGAGCTAGCGCGNCTCGAGAAGGCGGGA
ACCTGGGAGGTGGTCGANAGGCCGGAGANGCGAACGTCGTNGGNAGCAAGTGGGTNTTCAAGGTCAAGAAGAA
CGCGGCCGGNGAGATCGAGAAGTACAAGGCGAGGCTGGTCGCGCAGGGNTTCACGCAAGTNCCGGGNGTCGAC
TACNNCGACACGTTCGCNCCGGTAGCNAAGTTGACGTCGATNCGCGCNATNCTCGCNCTCGCGGCCCGGAACG
GCTGGCCGGTNCACCAGATGGACGTCAAGAGCGCGTTCCTNNACGGCGANCTNGACGAGAGCGAACGAATCTN
CATGNCGCAGCCGCCCGGNTACCACGAGCCGGACGGCGCCGGGAAGGTCTNCGCTACTCAAGGCNATCTACGG
NCTGAAGCAGAGCGGNCGNCGNTGGTACCAGACGCTNCGGGGNATACTCATCGAGNTCGGNTTCANGCGCTGC
GAGGTCGACCACGCCGTNTTCTACGTCCACCAGCNAAACGGCGACNTANTCGTTNTCGCCGTGCACGTCGACG
ACNTGACNCTCGTTGCCAACGCCCTCGANCTCTTGCTGCGCNTCAAGGGNGANCTCAAGGCNCGCTTNGAGAT
GACNGACCTNGGCGACGTNCGCTGGNTNCTCGGNATNGAGGTCANACGCGACCGNGACGCNCGCACCATCTCG
CTNTCNCAGCGNTCGTACGTCGACNCNATCCTGAGTCGTTTCGGCTACCGACTCGAAGCCCGCCTCGTCGCCC

ATGGACGCCCGCGNACACTCACCAAGTCNCAAGGNCCNNCGACGCCNCGCGAGTTCGGAGAGATGCGNGACGT
NCCGTATCGCGAGGCCATCGGNTCGCTNATGTACGCCGCGCTCGGNACGCGNCCCGACATCGCGTACGCNGTG
GGCGCNCTAGCCCGNTTCTCNGAGAACCCGGGAAGGNCGCACTGGGANGCCGTNAAGCGCGTCTTCCGNTACC
TCAAGGGGACNCGCGACTGGCGGCTCGTGCTCGGCGGGCGAGACCGAGGACTCGCCGGNTACNCCGACGCNGA
CTGGGCATGTCGCACGANGACCGACGCGCCGTCTCGGGNTACGTCTTCCTGATCGNCGGNGGCGCCGTCTCCT
GGAGCTCGAAGAAGCAGGCGACCGTNGCGCTGTCNACTACCGAGGCCGAGTACGTGGCGGCCGCTCACGCCGC
GAAAGAAGCCATCTGGCTNCGCGCNTTCNTTCCGAAGTNTTCAGGTCGCCNGCAGAGGCGCTCACCTTGAAGT
CCGACAACCAATCGGCNATCGCNCTNGCGCAGAACGATCAGTTCCACGCGCGCACGAAGCACATCGACGTGCG
NCACCACTTCATNCGNCGGGNCGTCGAAGANGGGAAGGTGTCGCTCGAATACTGCCCCACCGACGACATGGTC
GCNGACGCCCTNACGAAGCCGCTNCCCAGCCCGAAGGCGAAGCATTTCGCCCGCGCTCTGGGACTGCGCCCGN
CTTGAGGGGGAGTGTTGGAATTCAAG
>Cersu1_rnd-1_family-3#TIR/MuDR
ACCCGCATCGCGTCNGGCTCGGACGACGGGACCATCCGCGTNTGGGACGCGAGNACGGGCGAGGAGGTNNTCG
AGCCGCTCGAGGGGCACACAGACGNGGTCANCTCCGTCGCGTTCTCNCCGGATGGGACCCGCATCGCGTCNGG
CTCGGACGACGGGACCATCCGCGTNTGGGACGCGAGNACGGGCGAGGAGGTCGTCGAGCCGCTCGAGGGGCAC
ACAGGNNGGGTCANCTCCGTCGCGTTCTCNCCGGATGGGACCCGCATCGCGTCNGGCTCGGACGACGGGACCG
TCCGCGTNTGGGACGCGAGNACGGGCGCAGGAGGTCGTCGAGCCGCTCGCGGGGCACACATACGGG
>Cersu1_Cluster33#LTR/Copia
TGTTGTTTTTGTCAGCACGGTGATGAGGATTCACTGGGATTGGACGACGAGCCTCCCGCGACGCTGCTGCACA
GCTGGGGAAGCCACCCGCCGCTTATATAATTTCGTAGAACAGTTTGTCGCGTCTCGTGTATCGCACACCTCAG
TCTTTGACCTTACGGAGACTGAGGTGAGCATCTTATAGTTATCTACCTACTGTACATTGCTGACTGTAGTAGC
CTGGAGAATTCTCTCCGTCAATAGGTTAGCAGAATACATAGTCTTTGACCTTACGGAGACTGAGCCCGGAGAA
TTCTCTCCATCAATAGGTTATGAGCCCCGAGTAAAAAATTGCTACGGGCACCCCTGAACTCTCGCGCACATCC
CTCGCTCGTTAAAAAATGACTCAGCCGACGGCTACAGCACCCGCAGCCACAGGAGATGCTATCCCCTCCTCGA
CAAGCGGAAACGGCGCCCCGCACACAGTTTCAAACAAACTGTACGACGTCACTCCGCTGCAGGACAACGGCTC
GAACTACCAGACGTGGAAGTATCGAGTCCTGAAGGTCCTCACGCTGCGGAAGCTCAACGGTATTGTCAATGGC
AACGACGTGGACCCTGGTGAGTCAGACCTCACACATCACGCAGCCTGGATACAACCCTGAAATTGAGCCAACA
ATCGACTTCGAACACGTCTTCATTGCCGAGGCCCTCGCGAGCCGAAAAGCGCTCTACACCCGATGGATTGTCG
ACTCGGGAGCGTCCGCACACATGTGTGCACACCGTGAGTGGTTTGCAAACTATCGCAAGCTTGAGACCCCACG
TCGTGTATGGTTGGGAGACAATCGATTCATCCTAGCCATCGGGATTGGTCAGTGCATCATTGACTTCGATGTC
GGCGCTGGACACACCCGCCGCTTGGTCGTACAAGGTGTCTATCACATCCCTGAACTTAGTGGCAACCTCCTCT
CCGTGTCGCATATGGTTGCCGCCGGCTACCGTATGGCATTTGACAAGAATGGGTGTGCAATCGTGGACACCGC
AGGAGATGTTCTCGCGACCGCTGATCTCGAGGACAAGCCCTACATCCTCCGAGGTTCGGTTTGCCCACGCGAG
CAAGCCTACATCGCCAGTCTTGCCTCACAATCCCCCGATGATGACACCGACTCTATTCGTCTCCTGGTAGCGA
GAGAGTCCAAGGCAACATTGAATACCTGGCATTGTCGGCTCGGGCACATCAATGTTGACTCTGTCCTCAAGCT
TGTAAGGAAGGGCATGGTTAAGGGTATGAGTATTGTCAGCTCTAAGAGCCCTGACAAAGCTCCCTGTAAGCCC
TGCCTCGAGGGCAAGCAGTCACGAAACATCATCCCCAAGGTGTCTAACCGCGACGACCCGAAAAACGGCATAC
GAGTCTACTCGGATGTCTGCGGCCCTATCAACATCGCCACACGAGCCGGTCACCGATACTTCGTTACCTTTAT
AGTCGGAAAGTCCCCCCATAAGGTCTTCACGAAGTTGATGAAGACCAGGGATGAAGTTTTCGAGCACAGCAAG
ACTGTCGTTACATGGATCGAGGCCGAGACCGGAAAGCGCGTTGTCGAGCTGCATACCGATGGTGGAGGTGAAT
ACGGATCGGACGAGTTTGCTGAGTGGATGAAGTCAAAGGGGATACACCACGAGAAGACGAACGCATATACTCC
ACAAGAGAACGGAGTCGCCGAGCGAATGAACCGAACGCTCGTTGAGACAGCGCGCGCAATGTTGAAGGACGCT
CGCATGCCCGAAAATGTACTGGGGAGACGCCGTACTTCACGCCACGCACGTCGCCAACCTCACTCCAACTCGC
GCACTGGATGGCGACATCACTCCTGAAGAGGCATACTCGGGCAACAAGCCGTCGGTTGCACACCTTCGCACCT
TCGGTTGCAAGGCCTTCATGCACGTCCCGAAGGAAAAGAGGCACAAGTTTGACTCGAAGTCCATCGAGTGCAC
CTTCATAGGCTACGCAGAAAACCGTCAAGCCTTCCGTCTTGTCCAACTCTCCACCGGCAAGCTCTGGGAGTCA
CGCGACGTTGAGTTCTTTGAGGACGAGGAGCCTGGGTCAGACCGAATCCAGATCCAGCTCGAGGTTCCCCAAC
CAGAGTCCTCAACAACGGATACACCACCTTCCTCGCCATGCAAGGATCCACATCCCGTCACAGAGAACCAGGT
CGATGAGGGTGACGTTCCCACAATACACGGCATGTCAGATTCCTCAGACGATGAGCTCGAGGTTGAGGATGTC
CTAAAACCTGACTCAGAGTCCGAAGCATCCCACCTGCGTTCATCGACACCGACACCCTCGCTACGACGTTCCA
CTTGTGAGCGACATGCCCCTGTCCGGGATGACGATCCCAGGTACAGTGTGTCGTCGTACAACAGCCGCAAGCG
AAGATCCGAAGAGGACGCACCTTCAGTGGGCGACGAACATGCTCGTCAGGCAACGCTCTCAGACGAACCACGG
ACCTACAAAGAGGCCATGTCACGCCCCGACGCGGACCAATGGAAGCAAGCTTGTGCTGAGGAGTTGGCTTCGT
TCCTGAAGCTCAATGTCTACGACGAGGTGGAGCGCCCAAGCGGTCGTCGAGTTGTCGACTGCAAGTGGGTTTT
TAAGATCAAGCGAGGCCCAGATGGGGAAGTCGAGAAGTACAAGGCACGACTAGTTGCTCGCGGCTTCACACAG
GTCGAAGGTGTTGACTACACGGAGACATTTGCCCCTGTCTCGAAGTTCACCTCGATACGAGCCTTACTCGCCC
TTGCCGCCAAGCTTGATCTCGATGTCCACCAGATGGATGTCAAGACCGCATTCCTCAACGGCGCCCTCGACGA
GGAAATCTTCATGGAGGCCGCGCCAGGTTTCCAGAAGAGTCGCGATGTCGTATGGAAGCTCAACAAGTCTATT

TACGGACTCAAACAAGCCGGACGTGAATGGTACAAGACGATTAAAGCCAAATTCGAAGCTCTCGGGTTCAAGC
GTAGTGAAGCCAATCACAGCGTCTTCATCAGGCGCCTCGAGGACGGTGTGTTTGTTATCGTTGTGCTCTACGT
TGACGACATGTTGCTCATTGCACCCAAGACAGTCTCATCCGAGCTTGAGGGCGTAAAACGGACCTCAAAGCC
CGTTTCGACATCAAGGACCTCGGAGAAGTTCACTGGATCCTCGGAATGGAGGTTATCCGTGACCGCGACGCCC
GCACACTTGAACTCTCCCAGCGCCGCTACATCCACGACATCCTCGAACGTCACGGAATGGCCGACGCGCGCCC
GGTCAGCACCCCCATGGCCGCAAATCAAAAGCTCATAAAAATCGAGCACCCCGAAGTCGACCCGAAGGCGTAC
CAGTCTGCGCTTGGCTCACTAATGTACGCCATGCTGGGAACACGGCCGGACATTGCGTACGCCGTTGGCGCCC
TCAGCCAGCATGCCGCAACGCCCAGCAAGGAGCACTGGAGCGCGCTTATGCGCGTCTTTCAGTATCTGCGCAA
AACAACCGACCTACGACTTGTCTACAACGGCCGCACTGCCGGCGCTACTGGGCGCCCTCGACGAGGAAATCTT
CATGGAGGCCGCGCCAGGTTTCCAGAAGAGTCGCGATGTCGTATGGAAGCTCAACAAGTCTATTTACGGACTC
AAACAAGCCGGACGTGAATGGTACAAGACGATTAAAGCCAAATTCGAAGCTCTCGGGTTCAAGCGTAGTGAAG
CCAATCACAGTGTCTTCATCAGGCGCCTCGAGGACGGTGTGTTTGTTATCGTTGTGCTCTACGTTGACGACAT
GTTGCTCATTGCACCCAAGACAGTCTCATCCGAGCTTGAGGGCGTAAAACGGACCTCAAAGCCCGTTTCGAC
ATCAAGGACCTCGGAGAAGTTCACTGGATCCTCGGAATGGAGGTTATCCGTGACCGCGACGCCCGCACACTTG
AACTCTCCCAGCGCCGCTACATCCACGACATCCTCGAACATCACGGAATGGCCGACGCGCGCCCGGTCAGCAC
CCCCATGGCCGCAAATCAAAAGCTCATAAAGATCGAGCACCCCGAAGTCGACCCAAAGGCGTACCAGTCTGCG
CTCGGCTCACTAATGTACGCTATGCTGGGAACAAGGCCGGACATTGCGTATACCGTTGGCGCCCTCAGCCAGC
ATGCCGCAACGCCCAGCAAGGAGCACTGGAGCGCACTTATGCACGTCTTTCAGTATCTGCGCAAAACAACCGA
CCTACGACTTGTCTACAATGGCCGCACTGCCAGCGCTACTGACGACATCACTGGTTACATCGATGCCGACTGG
GCTGCAGACGTGACGACTCGATGCTCTGTCACTGGCTATATCTTCCTGGTCTCGGGCGGCGCCATCTCCTGGT
CCTCGAAGAAGCAGCAGACGGTCGCACTTTCCAGCACGGAGGCTGAATACATGGCGGGCACGAACGGCTCTAA
AGAGGCCATCTGGCTCCGCTCACTCTTTCACGAGATCGGTCGCACGCAGGCTCACGGCACCCCACTCCTCAAT
GACAACCAATCAGCGCTCTCGCTGGCGAAGAACCCGATGTTCCATGAACGCACCAAGCATATCGCGGTGCGAC
ACCACTTCATCCGTGAGAAAGTCGAGAGCGGGAAAGTTGCGGTCGAATATGTCCCCACAGGCGACCAAGTCGC
GGACGTCCTGACGAAGGTGCTCCTGCGTGAAAAGCACGAGAAGTTCACGGAGGAGATGGGTTTGGTTTTGGGT
ACGTCCAACACCCATTGAGTGGGCGTGTTGTTTTTGTCAGCATGGTGACGAGGATTCACTAGGATTGGACGAC
GAGCCTCCCACGACGCTGCTGCACAGCTGGGGAAGCCACCTGCCGCTTACATAATTTCGTAGAACAGTTTGTC
GCGTCTCGTGTATCGCACACCTCAGTCTTTGACCTTACAGAGACTGAGGTGAGCATCTTATAGTTATCTACCT
ACTGTACATTGCTGAATGTAGTAGCCCAGAGAATTCTCTCCGTCAATAGGTTAGCAGAATACATAGTCTTTGA
CCTTACGGAGACTGAGCCCGGAGAATTCTCTCCGTCAATA
>Cersu1_rnd-1_family-257#LTR/Copia
GGGCGTCAAACGGGACCTCGGAGCCCGNTTCGANATGACNGACCTNGGCGAGGCNCGCTGGATCCTCGGAATG
GAGGTCATNCGCGACCGCGGCGANCGCACNATCGAGCTCTCNCAGCGNCGNTACGTCGACGNNATCCTCGAAC
GTCGCGGAATGGCCGACGCGCGCCCGGTGAGCACCCCCATGGCCGCGAACCAAAAGCTCATAAAGTTGTCGAG
CACCCCGAGGTCGATCCGAAGGCGTACCAGNCCGCGCTCGGCTCGCTNATGTACGCCATGCTCGGNACGCGNC
CCGACATTGCNTNCGCAGTGGGCGCNCTCAGCCGNCNCGCNGCGACGCCCGGCGAGGAGCACTGGAGCGCNCT
TATGCGCGTCTTCCGGTACCTACGCGAGACNACCGACCTGCGACTCGTCTACAACGGCCGCACTGCCGGCGCT
ACTG
>Cersu1_rnd-1_family-134#TIR/Tc1-Mariner
TACAGTGCCGAGCAAAAGTATTGCAACATCACCTTTTTGGGCATTGTTTGACTTCTAGCTTGCTTGTTTTCCA
ATCAATTTGGCTCAAATAAATTACAGTAGATACCTACACTACTCTAGTTTATAGCTACCGTATACTATGAGGC
AGAAATTAAATTTGCACCTTCAGGAGTGCATCCGCGTCGACGCGCGCTTAGGCGCCCGTTAAGCGCTCGCCAC
GTCACAAAGTTTCGGAATGATCTTCCATAACTCAAACCACTACTATCATCCTCCAGAAAATTATCTGACACTG
CTCATGGGGCGGGCACCCCAATGCACCCCAACCAAAAAGGCTCGGATCCTTACTTTGTACAAGGAAAATCATC
CTATACAAGACATCGCCAATACCTTACAAATCCATCGCTCTACCGTCTGGCGTCAGCTTCGCAACCTCCGTCA
CCATCCCAGCTTCTACGCTGTGAAGCCCCGTCCTGGACGCCCCCGCATCCTTAGCCCACGCTCCCTCCGTCAT
GCTGCAATTGCCATCGAAAGTGGTGTGGCTAACACTGCAGCCGATGTTCAACGCCAGCTCTTTCCTCATGTGA
GCGAAGCAACCGTCCGGCGAGCCCTCCATAGGGTTGGGCTTAGTGTCTACATTAAACGCAAGAAACCGTTTCT
CAAGCCTAGGCACAAGCTGCAGCGTAGACGGTGGGCGGAGTGTCACAGCAAATGGTCACAGGAGGACTGGCAC
AAAGTCATCTTCTCAGATGAGTCAAAGTTCAATCTTTTCGGGCCGGATGGTCGTCAGATTTGTTGGAGGAGGC
GTGGGGAGGAATTCATAGACCGGGCAGTCCAATCGACTATAAAGCATGGTGGAGGGAGCATAATGGTTTGGGG
ATGCATTACTGCAAAGGGCGTGGGTCGCCTTTATCGAGTTGACAGCAACATGGATGCCCGTCAATACTGCAAG
ATCCTTTCTGAAGCACTTCTAGGGACCCTGGCAGACAAGTTAATCAGGGTAGAAGATATGATCTTCCAGCAGG
ATAATGATTCAAAGCACACTGCACGTCTAACTCGGAACTGGCTCAAAGACCATTTCATCCAAGTCTTGCCTTG
GCCACCACAATCACCAGATATGAACATCATAGAACATTGCTGGGAGTTCCTCGAACGCCGTGTACGGGCCAGG
GTGCCTCCTCCTCAAAGCTTGGAGGAGCTATGGAAGGTGATGCAGGAGGAGTGGGGGCAGATCAATGTTGGAC
TCATACAATCTCTGTATGAGTCTATGCCACGACGGGTTGAAGCACTACAGAAGGCCAAGGGAAAGGGCACTAA
ATACTGATTGTCACTCTTTTGTCAAACCTATGGTCTGTGCCAGAAATCGCAACATAGTCTGTTCCGTGCGACG

GGCGCCTCTCGGGCGCTTAAACGCCCGTCGGCGCGGATTTGTTCCTGAGAATGCAATTCTGATTTTTCTCTCA
AGGGACTTCGGTGGTAGACTTGAGAGACATAGACAATCAGACCAAAAAANTTTCGAAGCAGATTGATTGAGAG
ATGAAGTAACTACAACGAAAATAAGGCCGAACCACGTGATGTTGCAATACTTTTGCTCGGCACTGTA
>Cersu1_rnd-1_family-0#TIR/MuDR
CCAGATCGTGCTGCCNTGTCCGCCTTGCGATCCCAACCACGAGTCTCCCGGCGTCACATCCCATACGCGAATT
GTGTTGTCGNATGATCCAGAGACAAGGCGAGTCCCATCGGGCGAGAACGCGACTGAGCGGACCGCATCGCTGT
GTCCCTCGAGNGGCTTCATCACTGGGACCCCGGTCGTCGCATTCCAGAGCCGAACAGTGGCGTCNNNCGAGCC
AGAGGCAATTACTTCCCCATCCGGTGAGAAGCTGACAGACANGACCGNATCGGTGTGTCCCCTGAGAGGCTCC
ATCACCGCGTCACCTGTCCGGGCATCCCAGAGCCGAATTGTGNCGTCCGCCGAGCCAGAGACGATCCGGGCTC
CGTCCGGGGAGAATGCGACGGAGNNGACCTNATCGCTGTGTCCCTTGAGAGGCTCCATCAGCCGATCNCCCGT
CGTCGCATTCCAGAGCCGGAGTGTGGCGTCCGCGGATCCAGAGACGATTTGTGTCCCATCTGGTGAGATCGCG
ACTGACCAGACCGTGTCGCTGTGTCCCTCGAGAGGTTCCATCACCGGCCTCCCTGTCCTGGCATCCCAAATCC
GGATTGTNCCGTCAGACGAGCCCGAAACGACTCGNGTCCCATCCGGTGAGAACGCAAGAGANTGGACCCAGTC
GCCGTGTCCACTGAGAGGGTCCGCCACTTGTCGGCCGGTCCTTGCATCCCAAAGGCGGATCGTCTCGTCAGCT
GAGCCGGAAGCGATGCAGCTCCCATCTGGCGATACNGCAAGGCATGTGACGAGNTCACCGTGACCCTGGAGAG
GATCGAGGACTGGGGCGCCTGTCTGCGCATTCCATAGCGAGACCGTCTTGTCCTCCGATCCCGACACAATCTG
GGTTCCGTCCGGTGTGAATGCGACACACCGGACTCTGCCGCCGTGACCCTGAGGGCTCTCCGAAGGTGCGTTC
CTCGGCTTCACGTTGGTACCAGGAGCCGAATCTTCGTCGTCGACCAAGACTCGAACCTGGGATCCCTGTGGCG
AGGTCCCATCAGGGAGAGCAGTGTCGCTCGGTGCGACGTGAGGGGATCGGTTCGCGTCCATGACATCTGCACT
CCAAAGTCGGATNGTCTTGTCTCCTGAGCCGGAGACAACCGTGCTCCCATCNGGTGAGAACCCGACAGACCAG
ACGTGATCACCGTGGCCCTCGAACGGCTGCATCACTGGGCGCCCTGTCGCTGCATCCCAAAGTCGAACCGTCT
TGTCAGCCGAACCAGAGACGATTCGGGTCCCATCAGGTGAGAATGCCACCGAAAGGACCGAGTCGGTGTGTCC
CACCAGGGGGTCAATGATTGGGGCNCCCGTTCGTGCATCCCAGAGCCTGATNGTGTCGTCGNNTGACCCCGAG
ACGATTCGGGTCCCGTCGGGGGAGAACGCGACAGACCGGACCCGGTCGGTGTGTCCCGNGAGAGGCTCCATCA
CTTCCTCTCCCGTCGTCGCATCCCAGAGCCGGATCGTNTTGTCGTCCGAACCGGAGACCACTCGCNTTCCGTC
CGGCGAGAACGCGACAGTGTTGACGTCACCGGTGTGCCCCTCGAAGGCGTGCAGCAATGGATTCCCGGTCTTT
GCATCCCAGAGCCGCANTGTATGATCCNTTGACCCGGAGACGATCCGCGCTCCATCTGGTGAGAAAGCGACGC
AACGGACTCCGTCGCTGTGGCCCTCGAGAGGATCCATCATCAGCTCTCCCGTCCTCGCATTCCAGAGTCGAAT
CGTCCCGTCCAGGGACCCCGAGACTACCACCGCTCCGTCTGGTGAGAAGGCCACAGAGACGACCGTGTCACGG
TGTCCCTCAAGAGGGTCCATAAGCAGATCGCCTGTCCGCGCATCCCAAATCCGAACTGCCCCATCCCGTGATC
CCGATACCACGCGGGTTCCGTCAGGTGAAAATGCGACGGAGNAAACTNCCCCAGCGTGTCCTGACATCTGCAG
AAGCGGCCCCCGTGACCTATGTATGCCGGTCACATCGTATGTGGGGACATTGCGAAACTTCGGCCAGAANGTT
TTCGCGATCGTGGAAGATGGGCTTAGGGATGGAAGAACCGAGAGGTAGATGTGCGGTGCGCTGAGCTCGATCG
CGTCGTGACCCAGCATGACGAAGTCTTTTGCATCTCGCAGAAATCCGGCCATCTCGGGAGAGAGCCGATCACG
CTGAGGAGNCCGGGTACGTCAATTCGNAGATCGTCGCAGAAATACGGTTTTGCGTACCATGTTCTCTGCGGTC
AACACTCGGTGCAAAAGGCCCANCGCCAGCCTCGCATGTCCGCTCGCGCTGAGCACCTCGAGCCAGAATAGGA
TCTTCTTTCGCAATACCGAATAGACCGATGCGAGCAGTGGTGACGGATCGGGAACNCGGATGAGATGGTGGAC
CCAGTGAAGGCACGAGTATGCGAGCGAAGTCGGGATCGGTGCGAAGNCCTGCTGCGAATTCGGCAGGTAGGAT
GTGCGACANCCCGAGACGTTGAAGTGCAGCGCGTCCGAGGCCATGATCCGGATGCANGCGTCTGCGAGAGCCT
CGTGNCCGTCGGANNGGTCGATCCTGAAGGCCTCCGGCGCGCGNTCAGGGGNCGTGAGGTAGTCTTCGAAGGA
GGCGTGGAATGTNGTNACNGCGCCGCTGTCGTCCTGCGNCGGCGTATATACGACNGCGTGCAGCCGTTCGAGC
ATGCTTCTGACGTCCTGNGCGGGCATGCCGATCAGCTCGCCGAGCGCCGNCACGGACAGGGATTCTCGCAGGA
CGAGAATGATGGCCAGNANCCGCTTCGTCGTCTGGATCTCGTCCTGCGTGCGCGAGTCCGGATCCAGCGCGTT
GNCGAGGACCAACGAGTACATGTCGTCCAACGGCTCCGTCAGNGGCCGCTCGGGGGCGANATCGAGCTCCGTC
ANCCCNNGAAGGCGTTCCACGGGTCGTTTCTCGATGTATTTCATCGCGGTGAACGCGTAGATGAACAGTTTNC
CCGCNCGNTTCGTGACCGTCTCGACGTCTCGNTCCGACGGCCACTCCGGCGGGAATNCGTACGGAGGCTCCCG
NTCTTCGTGAGAAGCACGTATCCGTTGAAGACGTCTGGTGCAATAGAGNCGGATGTCNGCCTCGACGATGCTG
TGCTCGATGTCGTGCAGCCGCAATGCGTTGTGNAGATCGGGCTGCGTCGACTCGAACTGAGACCGGATGTGTC
GCTCNGGNCGGGAGGTGAGGAAGAATTTGATNGGNATGCGGCGCGAGCGGGGGACGAGCGCTGAGAGCATGGA
TTTCGTGGCTTGTCCGTCGAGCATTCGTCGAGCGCGTCNACCACNAACACCAACGTGGGCGGCCCGTCGCCGA
ACGCATCGCGAAGAGGCTTCTCGAGCAGNCGCTCGACCTGNATCCGGACCGTGTNGTGNCCGACGTCCGGNTC
CTCCCGCAGNACGTCCAGTAGAGCCAGCCTGTANGCCGCGTTCCGGCGAGCAAGCGATNCCGCGAGAGTCGGG
ATGATGCGCGTCACGTCGTCNCGGGTTCCTCNGGAACAGAAGAAGCTTNCGCCGAGNANNCCGGCNTCGCGCA
GNTTGTGACANACNGAGCGNGCGATGGCAGATTTGCCNGTGCCNGCCATGCCGTCGAGCCAGAANATNCGAGC
TACACCGGGATCGCGACTCCAGGCGCCGACGTCGTCGAGGAGNTCNACGCGNGTGCCCTCCATGCATGCGTCG
CCGGTCTGNGCGTCGATNCCGGCGCCCGGGACATGNCCGAGCTTCGCGAGGAGAACT
>Cersu1_Cluster8#LTR/Gypsy

TGACGTTAGCATTCTCCCTTATGCGGACATTACCACGGTTCGACTTGTGTTCTCGATTCCTCCCGCTAGTACA
GTTCTCGCTGCCTCCCCCCCTTTGGTTCCCTCCGATTTAAGCCCCGCTCATCTCGTCGTATATAAAGCCCCTC
GTTTGTACGCGTACGCCATCGAGTCTCAGAATACCAGGACCATAACCTGTCTTTCTTGTGTGCTCGTTCCCTC
GTGTGCCGCCACCTGCTGACTAATCAGCAGCGAGCGTCACATATTTTTGACAGTTCGTCCTAGTCTACGAACG
ACCTCTGCTTTTTATTCTCTACTCTCTGGCGACCGCTGTCTCTGGATTTCGTCGTGTCTGATCAACTCGACAT
TCCGAACGCGTCTCGCCATCCACTTCGACAATCTCACCTTCGCAAGAAAAAACCACCCAAGTTATCCAAGAAA
TGCCTGCTGGCTACAACCTTCGTCCCCGACTCGGCCGCGCTACTAAACCGGCTGCGTCGGTCAAAGTCCCCGG
TGCGTACTACGCCTCTCCTAACGTGAACGCTGACGCTCCACCCGATAGTGTCGAGTCCGACTCGGACACCGCG
AGCGTACGCTCTACACGTTCCGCGGTGAGGCCGGGGCTGTCCTACAGCACGGTAGTAACGGCGCGAGTTCCCC
CGCTCGAGCATGCTCGCGAGGCGTCGGGCACTGGGGAAACCCACCCGAAAGGCGATGAGAATTCTAGCCTGAC
CGATTTGTCTAGTTTACTGGATGAATTAAAATTGAACGATTCCTCTGCATTTTCTGTCATGCCTGAGGATTCG
TCCCCCGTTCCCAATGAGAATGACAGCCCGTGGCCTGAGGTGCCGCGTGCTCGTCGCAGCCAGAGTCTCGACG
TCTCGAGCTCTAGGCGACCCCGCGGAAAGGCCATCCGCTGGGAAAGTGATAATCTCACTCCAGAACAACAGCA
TGCTGTGAGAGCTGCTGAGTCTAGACTGACTGCCGACGACCGCGCTCGTATCCAGAAGCGTAGGCAAGTCACC
AATTCCCAGAGCCACGACAGCTCTGACACTGAGTCTCGTGGTGAAGGCCCCTCCACCCTGGAGAAGGGCAAAT
CCGTTGACGGTCGCAATTGGGGCGCCGTAGGAATTCCTGCCGAGGAACTCGACCCCGAATTTCAGCGCCGTGC
GCTTGAAGCTTGTGCCATCGGACCTGCTACATCGGCCGATGGAGGTTCAGATCCGGAGGAGCTTGATCCGGAT
CAGTGGCGCGAAGTTAATCGCGGCCGCAAAACTACTCACGAGAGGAGTAAACGTCGCCCCGACAGGGAGCCCG
TTATCGCCAGCACACCATTGGCTGCTCCTCGAGACTCTAGGTCCTCTCGACGTGAGGCACCTGGAACTCGCCC
CAGTCCTAGGGACAGGTCCCCAGCAGCACCTACGCGCTCCCGCGAGGATGCGCGATCGTCCCGACGAAGTCGC
CCTGACAAGGTTCGCGACCTGCCTCCTCACCGAGCCCGCCGTCGTGACACCTCTCACTCGCCGCCGAGGCATA
GGGGACATGAACGCGAGAGCTCTCGGGCCCCGTCCGCTGCCCTCGTCGACGATGTGTTGGGACACCTTTCCTC
TCGCAAAGGGACCAAGCGAAAGCGCCAGGAACGAAAGAAGGATAAGATCTCTCTACAACCCGTGACCCAGATC
GACCCGCGCAGTCATCTAGGTCAGGCATTCGAAGGTCTGCGAGACCGCGAAAGTGACTCGCCCGATGGCTCGT
CTAGCTCCTCAGGTTCCTCGGAGTCCTCAAGCCCTTCAGTCTCGGATGACGATAGATCTTCGATGTCAGGAAG
CGACTCGGGTAATTCCTCGAGTGATGAGTCGAGCACAGGATCCACGGGCTCTTCTAGTTCGGGAAGGTCCCGC
CGACGACGACGACGACGTCGGTCACGCTCGCGCTCGAAACGTCACTCGAGCCGTTCCCGCCGCCGTAGACGTT
CGCGTCATAGGTATCGAAGTCAGACCCCACCTAAGCTCAGGCCGCGGGACCCCGACACCTACAATGGTAACAC
CGATGTGCAGGCCTTCCAGCGGTTCATGCAACAATCGTCGGAGTACGTGGAAGGTTACGCTCTTCCTGAACGG
CGACAGGTATCGATGTTGTCATATTTCCTGTCAGACAAAGCCTACGAGTTCTATGTTAATACCGTATCTCGTA
ACCCGAAGTCCTGGACGCTTCGGAGTTTCTTCGTTGGTCTATTCAATTACTGTTTCCCTGTTAATTTCCGCGC
GCGGATGAGGGATAAGTTGGATCGATTCAAACAACACGATAAGGCAGTACTCGACTATATCCATAAGATCGAG
AACCTTTTCCTGTCGTCCGGAGTACTCTCCAAGCAGGACAAGGTCGACAAGCTGTGGAAGGGTTTCCGTCCTT
CTATCCAGCAGGGCCTGTATTTGCAGCACCTTACCCCTACCACTGCTAGCTGGAAACGCATCACCCGGGCAGC
ACAGTTCATCGAGCTCGCCGAGGGAGCAGTGGCAGACGTGCCGAAACATGGGGGACAACCTAGTGGTGACCCA
GTTCCCCGGAAGTCCAAATTCCAGCGTTCTGGAGGATCTAACTCCCGGAGTAATGGCGGTACCTCCTCTTCAG
GACAGATTGATAATCGAGGAAAAACGCCCCTGAGGAGCGACAGCTCCAAAGGGGACAAGAAGCGCGGTGACGC
TCCGCGTCCGGTCGCAACTGGATCAGGTTCACGTCAGTTTCCATCGCAATCTGGAAAAGATAGCAACCGGCCT
AGCTCTGGGCTCACTAAGCTCACTGAGCAGGAACGAGCTAGACTGCTCGCGGAAAACCGGTGCTTCCGTTGCC
GCGAAAAGGACCATGTTAGTCGAAATTGCCCTAAAGGCAGCACGGTACAGTCCGATCGTCGCGGGCAGGCTCC
TGGCCTCTCGACTTTCAACATCGAGATTACAGGGCCTGACTCGGAGTCTCTTCGTTCGCTGGCTGAGTCTACC
CAACGGGGAGACTCTCTCGAGCTTCACTCCCTCGAATTGATGAGCGTGAACCTCGGATCCCCTGATTTGATAG
ATGCCGACGTGGTGCAACAATGCACTGATATTCGTCGGGACCTTGGCTTCCCTGTCTACGTGTGGGACAGGGC
CTACCCAGAGTTTCCAAATACCTCCCGTAGAGGGCGGGATTATAGGCCGCTGGCCGACCTTTTCAGCGCTCAA
GCTACTGTGGTACTCGAGAGGCACGCCCCATACGTACCTGAGGAGCTGGTGATCTCGCTTGGGCTTGATGAAG
ATAACGCCGCCTACAGCTTCGCCGTATATCCTGTTGAAGGAGGATATGCAGTTCTTCAGCATTACCTCCCTGA
GGACCCTGTGATCGCAGCTCACCTGATTCAGGCCCCTGACTTCAATGTTGCTCGGTGGTACGCCTCCCGCGTC
ACCAATCATTTCGAGCTCAACCATGAGCACCTTTACTCTGTCGGAGAACCAATGGGAGACGCCATTGCACGGG
CAGTCGAAGCCCTGCTGAATCATGAGGTCCCGTGGCCCTCCCACGTGCTTCGACACGGCGAGGTGCCTCCTCG
TTTCGAGTGTCGATCCAATGAACTCGGTGTTCGCGTGACTGATCACTACATCGCATGGACGGTCCAGATCTGG
GAGCCACTTCTCTCCAATTTCCGGCTCGATCTGTCCAACTGGTACGCCCGTTGGGTGGAGCATCACCATACTG
CCAACACCTGGCTTGATTGGGATGAAGACGAAAGCATTGTCAGCTCGTTGTTCCTTTCCTCAGCGCCCGAGGA
CGAATCCACTACATACCTTCAACTGAACGCGGTGGACTCCAGCTCGCGATCGAGTGACCCGCCTTTACGGGCC
GTGCAGCGTAACGCCGCCACGCCCCGCGACTTCAAGCGTCTTATTCCCGAGCCTATTATCGTGGTCGTAAATA
TCGCGGGACACCCCGCGCGGGCCCTACTCGATTCGGGATCACTGTCTGACTTCATGTCTTCTAAGCTAGCGCA
TCAGCTTCGCATCCAAACTTTCGAATTGGAGAAGCCATTACCTGTACACCTCGCTGTGCAAGGTTCACGTGCT
AAGATTAATCTTGGGTGCACGACTCTGATCGAGTATCAGACTGTGAGGGAAAAGCGCTATTTCGACATCATCA
ATCTGTCGAATTATGACCTAATCTTGGGCACGCCATTTTGGTTTCAGCATCAGCTGACTGTCTCCATGAATCC

CGTGGCTGTGGCTATAGGTAGCGTGGTTGCCTTGCCCATCGAAGGGAAGCGAGTTCGCGCTCTGGAATCCCGA
GCTGCGGATTTGTTCGAGGACCGCCTTGAACAGGCCAGGACCCATCTTCGGGGCTACGCAGCCCCCATCTGTG
TATCCGCGAGCGATACACCACTGCCCCCACTCCGGGTCATCAACCACACGATTCCTTTGAAAGACGAATCGAA
GGTCTACCACTGGCGCCCCTCGCGATGCCCTGACGCGTTGAGGGACGCCTGGAACGAGAAGCGAGACGCCTAC
CTAAGATCGGGCCGGTGGAAAATGTCCAGCGCCCGGAATACTAGTCCTATGTTGCTCTTGACGAAACCGGGTA
CAGGTGTGAAGGGAATACCTGCTAAACTCCGTGTCGTATGCGATCTCCGTGAGCGTAATTCGAATACTCACAA
GGTGACTTCTCCGCTCCCGGATATGGAAGGGATCCTGCGCCGCGTAGCGAGAAAGCCCTACCGAACGCTCATC
GATGGCAAGGATGCCTATGAGCAGATTCGTGTCGAGCCGGACCATGTCCCGCGCACGGCTATGACCACTCCTG
ATGGTAACATGGTCAGTCTCGTGCTTCAACAGGGAGACTGTAACGCCGTCGCGACGTACCAATCACTAATGAA
TCATATCTTTGGTCCTCATATTACCGTATGGATGGACGTATATCTCGATGATATTATTATCTATTCCGATACG
TTGGAGGAACATCTCACTCACGTTAAAGTGGTAGTCGACATCCTCCGAAGGGAGAAATTCTTCCTTGGTGCCG
ACAAACTGAAACTGCTCTGCTCCGAGATGAAGATCCTCGGGCGTGTCGTGGATGACCATGGAATCCGTATGGA
TCCTGACAAAGTCGACTCAGTCAGTAACTGGAAGGTCCCCACAACCAAGGAGTTGCTGCGTGGATTCCTAGGA
TCAGTCGGCTACTTGGCGGATGATGTAGCGACCGTACGCATTCCAATGGGGATCTTGACGGCTCTTACTGGCT
CTGAGTCGAGCTTCAAGTGGGAGCCGTTGCATCAGCGTGCATTCGACGAGATCAAGCGTCTCGTCAGTGCTCA
CCGGGATCACCGTCGGGTGCCCTTGAATTATTCCAAGGAGGCCCCTCGAATATGGCTTGTGACCGACGGTTCC
CATGGTGGGGTGGCCGGCGTGGTGTCTCAGGGTGACGATTTTCGCTCGGCGCGGGTCGCGGCTTTCTTCTCTG
CAAAGCTCTCGTCTGCGCAGTCGAATTATCCTGTGCACGAAATAGAAATGTTAGCCGGCGTCGAGTCTATGCT
GAGACATCGTGATATCTTACTCGGGTGTCATTTCACTTGGCTGACCGACCATAAAGGTCTGGTACACTTAATG
AATCAGAAAGACCTGTCCGGGCGACAGGCCCGCTGGATCGAGAAGATTTCTGAGTACGACTTCACGGTCGAGT
ATGTCCCAGGCGTCGAGAACGTCCTCGCCGACGCCTTGTCCCGACTTTACTGCGCCGACAGTCCAGGTACGGT
CCGCGCGCCGTCGGAATACGTCGAACATGATGATGGTGACGCGTTCGCGCCTCGGCTGGCGGCGCACGCGATT
TCGATGCCAGTATACACTGGGCTGGAGGCCCTCGCAGTGACGACTCGTCGGTCATCGCGTGCAGCTCGACAGC
CGTCAGCGTCGCCTGCATCGATGGCTACTCACTCACCCATGGGTGACTCCGGTTCAGCGGCAAGGCTGGGACA
TCGCGCTGCGATTACCATCGCCGCGGGCCCGCCTCAAGCACCGCTCACTACTGCCACGGACGCTCCAGTCCTC
CGGCGAGCTCGGCCTCCTGTACCTCCAGCAGAGTCGGGTCGGCCTGAGACATCGCGCGAGTTTGCGAAGCGTA
TTAGACGTGTCGTTCTACGCACACCGCGCGCAGAACGCCAGGAGGGCGTGAGCCCTACGGGCCTAGAAGTCAG
CCCCACCCCTGACAACCAACCTTCTACTCAGGGAGGCAATACAGACTCACCTGGGGGAACGTCCGACTCGAGA
CCCTCGCATAGGCTGGAGGGCGCGAGCATGCCAGCGAACAATGGTTCGAATTTGGACGCTCATGCGGGAACGC
CCGGGCGTGGTTCCGGCCAACCCCCCCTAGACGCACAAGGCGCTACACAGCGAAACACCACCGTACCTATCGC
GCAGAGCGATATTCTCTCTATCATCTCGGAGAGTCACGAAGGAATTGACCTACGCTCTGCCCTCCGTGACCGG
TACGCGGAAGACAAGTTCTTCAAGAACATCCTAGACAACCCACGATACTACAAGAACTTCAGTGTCCGCGACG
GCCTCATATACGTGACAGAACGGCAGCACCTGCTATTGTGTATCCCGGACATCCGAGTTGAAGCTCGCTCGGC
ACGCGAACTCGTTATACGACACGCTCACTCTCTGCTCGCTCACTTAGGACCCCATAAGACAACGGGGCTACTA
CGCGATCACGTCTGGTGGAAGACCATGGTCGCGGACGTCCAGAAGTACTGCGATTCGTGCATGACTTGCAAGC
GAAGCAAGCCGTCTAACCAAAAGCCCTATGGGCTACTGAATCCTTTGTCTGTACCCACATACCCCTGGGAGTG
CATCGGGATCGACTTTGTCGGCCCTTTACCCGAGTCGAAGAACCGAGACGGAGTCTTTGATGCTATCACCGTG
GTCATAGACCTACTCACTGGGATGGTTCACCTTATCCCGAGCCGCACTAATTACAACGCCAAACAGGTTGCAG
AACTGGTGTACTCCGAGATATACAAACATCATGGACTCCCGCGTGCTATAGTCAGCGACCGGGACGTCATATT
CACGAGCCTCTTCTGGTCTCATCTTCATCGCCTTCTCGGCGTTGAACTTCGGATGTCTAGCGCTTATCATCCC
GAATCCGACGGTGCTACTGAACGAGCTAACCGTACGATCACCCAGATGCTACGACAATGTGTGTCTCCGGGCC
AGAAGGATTGGGTCACGCGGCTGCCCGCTATTGAGTTCGCGCTCAACCTCGCCCGATCGGACTCTACGGGATT
CGCGCCTTTCTTTTTGAATACAGGCCGAATGCCCCGAGCCATGATTTGGGATAATCCCGCGCCGGACGAATAT
CCCGGCGTAAGGACATACGCTCTTCGGATGAAGGCTGCTATCATCGCGGCTCACGATAGTTTAATTGCTGCCC
GCGTAAAACAGACCCGAGACTCTAATCGCAAGCGCCGCCCGGCTCCCTTCACCGAGGGAGACCTTGTGTATGT
ATCCACGAAGAACATCTCCCTCCCCAAAGGGCTTGCTCGCAAGCTCGCCCCTAAATATATTGGGCCCTACCGA
ATACTTCGCGACTACGGTAATAACTCGTACAAGTTAGACTTGTCGTCAAACCTCCGACGTCGCGGTATTCATG
ACGTATTCCACTCATCGCTGCTGCGAATCCATGAGCCCAACGACGACCGTCTCTTCCCCGGGCGCCTGGATAG
TCAAATCGCCGAATTAGAAGATCGCGATAATGAGTGGGCTATCGACCGCATCTTGTCCCACAAGGGCGTCGGC
GTCGACGCAATTTTCGAGGTAATTTGGAAGTCGGGAGATCGCACTTGGCTCCCGTATGCTCCGCTAGCCGAGC
TGGAGGCCCTGAGGTCCTACTTGGTCCTACAAGGAGTCGATCGTGTTTCGGAATTACGCGACGGTAATGGGGA
TCCTCCGGATGATCCACAGATCTTCCTCGGATCTCTGCTGATCATGTCTCCGCCCGACTCTCACTATAACAAG
GGCGGGGGTTTTTCTCTTCCGCAGGTTTCGACTTCCTCCTCCTCGACGCCTCTCTCCCCATTCACCATGCTCC
ACAATAACAACCTCTTCCGCCGCGGCGCTGACCAAGTCATCCTCCGCGACACCGCCGGTCGCCTCGACTACTC
ATACGTCTACGCCCAACTACGCGATTTCCACGCGTTCGATCGACGCCTCCGCGACGGTCGCTTCAACCCTGAT
CATCACCCGTACCCCGCCGGTTACGAACGCTTCGTTCAACTCTGGAATCAAGACGAGGACTGCCCCTTCGGCT
TCATGGGCTTCGAGGAAGCCTCCGTCAGTCTCATCGCTACCCCGCGCCCCGCTCCCAGCTTGAACAACCTCGC
TCCGACTCCTGCTGCGCCCGCCGCCAATGCCGCGGCTCCTCAGTTCGCTCCCGAGGATCAGGAGGTCTATCAA

GGCGCTCTTATTGTCACCGCTGAGCAGGCCATCCGCAAGCGCCGCTTCGAGAAGAAGTGCAAAGCCGCTCGCG
TCGACCGCCGTGCCCAACGGAAACAAGAAAAGCGCGAGCTCGTAGCTCCGCGCCGCGACTACCCGCGCTCCCA
CTCGTCTTACTCATCCCGTTCGAATGGTCCGGCCAACTCACCTTCCGGTTCTCGCTCGACCTCTCCTCCCGGA
CGACGAGGGCGCTCGCCTTCTTCCTCTCGCAAGGCCACTCGGCCGTCTACTCCTGAACTCAGCGACAAGGATG
CTGAGGGCGAGCCCGACAACACGGAAGACACCCCGATGCTCGAGGCCGGCGTCGCCGCCTCCGAAGGTACGGG
CTCCGCTGGTGGGGGAGACAACACTTCCGGTAACGTGTCGGCTTAGAGGGCGAGGGATGACCATTGCTATGCA
ATGGCCAGAGGGCGTATGTAGCGCTGACGTTAGCATTCTCCCTTATGCGGACATTACCACGGTTCGACTTGTG
TTCTCGATTCCTCCCGCTAGTACAGTTCTCGCTGCCTCCCCCCTTTGGTTCCCTCCGATTTAAGCCCCGCTCA
TCTCGTCGTATATAAAGCCCCTCGTTTGTACGCGTACGCCATCGAGTCTCAGAATACCAGGACCATAACCTGT
CTTTCTTGTGTGCTCGTTCCCTCGTGTGCCGCCACCTGCTGACTAATCAGCAGCG
>Cersu1_rnd-1_family-58#TIR/MITE
TACAGTTTTTTACCGGCCCGTGGGACGCCGAAATCGTGTTCAGCTTTGGAGTAACGCATATCTACATGATCGG
AAAAAAATAGTACAGCGATGGTTAGGAACACGGTTAAACTGTATTTCTGATGTATCAAAAAGACGTATGTATG
ACTGTGCTGTGAGGAGCAAGACCATCTGTCGAAAAGTTCGTTATCAGTCTTATTGCAGTCAGTGGGACGCCTT
CCCGATTTGGCCAATCTGCCGCTCCGGCCCCGCCAAACCACCTTCCAATTTGGGACGTTCTCCGTACCTCTAT
GCTGTGTTTTGACAATTTGTCGCTCGTTCTACTGCAGAACAAAATGGATTTGGCTCAGAAAAATCGGGTGTGT
ACAGGACATACGGTTTAACAAAAAAAGCGTCCTTGCTGCTGAAAATGAAGGGAGAGCATACTTAAAATGCAAG
TTTTCAGATTCAATTCTCAGCCACATCCGTATGTTTTTGGCCGTAGCAAGCTAAGTCCTCGAGGTAGCGCCTC
ATACACATGGGTGGATATCCGTACGAGGAGTAAAAGTGTTCACAAAATTTCTCCGAGCACGTTTTCCTACCGA
TGGTGGTAAAAAATGGCCATTGTCCCACTGGCCGGTGGTGTCCCACGGGCCGGTAAAAAACTGTA
>Cersu1_rnd-1_family-125#LTR/Copia
CGAGGCTGGTCGCGCAGGGNTTCACGCAAGTNCCGGGTGTCGACTACANCGANACGTTCGCNCCGGTAGCCAA
GTTNACGTCGATNCGCGCNNTNCTCGCNCTCGCNGCCAAGCACGATCTGGAGATCCACCAGATGGACGTCAAG
NGCGCNTTCCTNNACGGCGANCTNGACGAGGAGATCTNCATGNGACGCCCGCCNGGACACGACGCCGGGACGG
CAAGGTCTGGAANCTACGCAAGNCNATCTACGGNCTNAAGCAGGCNGGTCGCGAATGGTACAAGAAACTGCGC
AAGGAGTTCGAGNCCCTCGGNTTCANGCGCTGCGANGCCGACCACGGCGTNTTCACCAAGATCGAAGACGGCC
ATCTANTCGTCGTCGCCGTNCACGTCGACGACATGCTAACCGTCTCNGACGATCTCGGAACCGTCGAGCGCGT
CAAGGCCGANCTCGAAGCNCGNTTCGAGATGACNGACCTNGGCGANGTNCGCTGGNTNCTCGGNATGGAGGTC
ANACGCGACCGCGGCGCGCGCACGATCGAACTNTCGCAGCGNCGATACGTCGANGGNATCCTAACTC
>Cersu1_Cluster28#LTR/Copia
TGTTGTAGAAATCACTATATATGGTAATCTTTCGCGTACCGTAGCTTAGATAATCGGGGCGCCTAGCGCACGC
GTCCATACGAGTCGCCGTATGTTCAACTTCCCTCAGTCTCGTATCCCGAGCTGAGGGTAAGTTCATAGTCTAT
CCAGTGTATCTACATACTTACAGTATACCTAGGCAGTTCCCTAGGTATAGTATTCTCAATACAACCAGTCTCG
TATCCCGAGCTGAGGGCAGTTCCCTAGCCGGTTTCTACAGGTTATGAGCCCCGAGTGCCGCTACCAATAGGAC
ACTCGGAACCGTTATCACACGATCGACAGGCCATGTCAAAGCCCGAGTCAGCATCGACCTCGACACGGGAACG
ACGCCACACGATGCACGGACAGTCGGATATGACGCCGCGAGACACGTCTGGTACAGACAGCCGCCAACACCAC
GCGGGCGCTGACGCAAACACCGCGCACGACTCACTGTTTATCGGGAGACTGAATCCGGCAAAATTCCCCGAGA
TCAACATCCCCGACGTCACATTGACACATCCGGGAGCAACCCCGCATTACGACTACCTCCACGGGCAGAACCC
GTACTCGAGCCGACATCCCTCTATGACTCCGGGAGTCGAGCCACACGAATCGCGGAGCGCGCGCGCAGACCCG
CGCCCGAGCGTTGCCTCCCGGAACCGCAAGTACGCTTCGGATTCTGACGATGAGGACGACAGCTCTGTCCACT
GGACTCTCCGCAGGTCTATACGACCAACTAGCTTCACTAACCTGATCGGACGACTGCCGAATCATCTCACGGC
AGAGAACTACGTCTCGTGAGCGACAATGATCCGATCGAACCTCCAGACTGTCGAACTGTTCGAATACTGCTCT
GGCGACTGCCCTGAACCGCTAACCGCGATGGACGGCATATCGCTATCTCAGTGTCTCTGACGTTGGCAGAAAG
CGGACGCACTCGTCCGAACCATCCTGACTACCAGCATGACCGACACAGCCGCCAAGCAACTCGGACATCTCCC
GAGCGCCTCCAAGATGTGGCGAGAAGCCAAGAAGCTCTTTGCTGGATCGACGCTTACGGATTTCACCTTGCTG
ATCACCCAGCTCATCTCGACAAAATACGTCGTGGGCGAAGACATATCCGAACACCTTTTCAAGATGAAAGGAT
ACCGACGCGACCTCATCATGATGGACCGCGACATTCCTGACAACCTCTTCGCGGCATTCCTCTGCATATCGAT
GCCACCAGAATGGAACTACATCTTCTGTGGGCTACCCTCCCGCTACTCTACGACCGATGTCGAACACCGCATC
CTGGACGAATATGGTATCCAGCAGAACCAGAAGGCAACAGCAACAGCATACGCCGCCTCCCAGAACTCGTCAG
GCAAGAAACCTCGATCGAGACGCGAGCCGAAACCAGGCGAGCCAGTCTGCAGCAACTGCAAGGCGATAGGTCA
CTTTATCAAGGACTGCTGGAGCCGAGGGGGTGGAGCCGAAGGCAAGAAGCCTCGCAAGAAGGACAAGCCGAAG
AAGAAAGACAATTCAGATTTGGACGACTCGAGAAGCACAAAGAGCAAGCGGAAGCAGAAGGCGAACATTGCGA
AATCATGCAGTGATTCAAGTTCGTTATACTCATTCCTTCTGACGACACGTACATCATCCCGATCCAAATGGAT
TCTCGACGGCGGATCCACGACGCACATCTGTCGGGACCGTACCGCATTCACCACATTCAAGCCTCTCCGCGAC
ACAATCAGCAGCATCATCGAGGATGCGCGACCGCTGCAAGTGCACGGACGAGGCGACATCCGGATCTCGTGCC
CCATTGACGGCCACGAAGACCGCATCTTCACACTCCGGGACGTATCGTACTGCCCGAATGCCCGCGACAACCT
TATATCCGAGTCTCGCATGGATCTCCGCGGACTCGTCATCACGAAGCACAACGGCACCGTGACAATCGTGAGC
ACAACCAAAGAGGTACTGATGCGTGCGCGACTCGTCGACGGGCTGTATGAGCTTGACGGCAGCACCTTGCGTA

AGCCGCTGGCAGAACCGGCGCCGCACACAGCCCATCTCGGGCAGATCCCCGAGAATCCTGCTTCGGACACTGA
GTCGCACCAGACCGCTCCGAGCGAGGGGGTGAGAAAAACCTCGATTTCCACCACTCGGAGCTACGCTGATAGC
GTACACCTCAACACTGCGCGCACAAGAACGACACCGGCGACCTCAACCACCTCATCCGCCTCCGAGGCAACAA
CCGCCGCGAGCGAGCGCCAAACGCGCCAACGCACAGCTCCCCCTCCTATCCCTCACGCCTCACGGCCGAGCAC
GCCTGACTATGGGTCTCACGAGTCGATCGCTAACGCGTCGATTCACAGCAAGGCACCATCGTCGAGCGGACGC
GCAGCCACTCCACCTCAACCGCACACCCGCATCCACGTTGACGCCCACACTCCTTCCATATCCGGCCACTACT
CCTCCTCGCGCGCTTCGCATGGCCGCCACTCATTTCCCGATTTCTCGTCTCATCTCGAACCGAGCGCAATTAG
CACTATGATTTTATCGGTTTCACTCTCTTTAATTCTCACTCATTACATACTAACTACGAGTTATCGGCACGCT
ACAGCTTAAGGGGGTGTGTTGTAGAAATCACCATATACGGTAATCTTTCGCGTACCGTAGCTTAGATAATCAG
GGCGCCTAGCGCACGCGTCCATACGAGTTGTCGTATGTTCAACTTCCCTCAGTCTCGTATCCCGAGCTGAGGG
TAAGTCCATAGTCTATCCAGTGTATCTACATACTTACAGTATACCTAGGCAGTTCCCTAGGTATAGTATTCTC
AATACAACCAGTCTCGTATCCCGAGCTGAGGGCAGTTCCCTAGCCGGTTTCTACA
>Cersu1_Cluster25#LTR/Gypsy
TGACGCTCGCGATCCCAAAGGGGAAAGTCGGGTCGCCACACGAGAGAGAGAAGAAAGAGCACACGAGATAGAC
TGGGGTAAGTCTTGTATTTCGGAGCTCGATGGCGTACGCCTACACACGGAGCAGTATATATACGAGAGGGGGA
AGCGGACATTGAAAAGACCAGGAACCGAGGGGGTAGACTATTGAGACGACGAGGGGGAGGATAGGATATTCGA
ACCGTGGGAACGCCCGGAATGGAAGGTCTGCTCGCACCCGCTACATACGCCCTCCGGCCATCGTCCTACGATG
GTCCAACGCCTAAGCATCTCCTTCCACCGCACCTGTCTTCTTCTTCTTCTTCTTCGATCCGCCCTCAGTCGGT
CCAGCGCTCGAAACGACGTTACGGGGCTCCTCGGCACCCTCCTTCTCGTCGGGGTCAGGCTCGCCCTCCGCAT
CGGCACTGAGGTACGCATCCATTTCCTCGATCCGCGATGAGGGTCCAGCGCGCGGCGCGGTTATCGCGACCTT
CGGGACAGGGCGCTCCTTGCGAGCACGCTTCTTGGGGCGTCGTCCAGATGCATCCTCGCCCTCGGAAGAAGAG
TCATCCTCCGGGGCAGGAGCACCGAAAGCTCGCTTGATAGCCTTCTTGGCCTTGCGACGCTCGGTGCGGGCGG
CCATGCTACGCGAAGCCATCTGGAGATGACGGACGTGACGGCCGGCAGTGAAGATAAGAGACCCCTGAATGGC
CTGCACCTCCTCCGGGGTGTGCTGTGGCGGCGCCTGAGGAGGGGGAGCGGCAGGAGGGGGAGGAAGAGGCGCG
AGCTCATCGATAGGCAGGGGAGGGAGCTCGGGGATAGAGGTCTCGCCGGTGTCCGGATCGTAAGTGACGAATC
GGTAGCCGCTGTCCTCGTCCTGGTGCCAGAGGACCACAAAGCTCTCGTAGCCGGCGGGATAGGCGTGTTTGTG
AGGCTTGAACCGGCCCGCACGGAGGCGTCGGTCGAACTTCCAGTACGCACGAAGCTGCGTGACCGTAAAAAGG
AACGGGATGCCCTCGTCTCCATTGCGGATTACGATGTCGTTGTTAGCGCGGCGCGAGAGGAGAATATGACGAG
GCATCGAGGGCGACCGAGTGGACTGCGAGGTGAATGGCGGGCGGCGGAGTTGAGCGGTTGACGGGGGGATGGA
GTCTTGACTGACAGTACTTATAGCGGGCGCAGCGGGATAGCTAAGCAAGTCAAGACCTCCGAGGAAGATCTGA
GGGTCGTCGGGTGGTTCTCCAGATCCGTCGGGTAGGCTCGAAACACGATCGACACCTTGTAGGTCCAGGTACG
CGCGAAGAGCATCCAACTCCGCAACCGACGCGTAAGGGACCCACGTCCGATCCCCCGACTTCCAGAGGGCTTC
GAAGACGGTGTCGACCCCCGAGCCCTTGTGCGACAACAGCTTGTCAATGGCCCACTCATTGTCGCGGTCCTCA
AGCTCAGTCAGTTGGCTGTCCAGCCTCCCAGGGAACAGCCGATCATCATTCGGCTCGTGAATACGAAGGAGGG
AAGCATGGAAGACATCGTGGATACCTCGCCGTTTAAGGTCCCGGGAGAGCTCCAACTGATAGGAGTTGTTTCC
GAAGTCCCGGGAGATTCGGTAAGGGCCAATATACTTGGGGGCCAATTTACGGGCCATACCTTTAGGCAGGGAT
ATGTTCTTAGTCGAAACATATACGAGATCACCCTTCACAAATGGAGCGGGTCGCCGTTTCCGGTTCGCGTCAC
GAGTCTGCTTCACGCGGGCCGCGATGAGGCTATCGTGAGCTGCGAGGATGGCGGATTTTACCCGCAGTGCATA
CGCTCGCACACTGGGGTACTCGTCTGAAGGGGGATTATCCCAGATCATCGGACGAGGCATTCTGCCAGTATTG
AGGAAGAATGGTGCATAGCCTGTAGAATCTGAGCGAGCCGAATTAATGGCAAACTCGATAGCAGGTAGCCGCA
TCACCCAGTCCTTTTGATCAGGCGACACGCACTGACGGAGCATTTGTGTCACGGTCCTATTAGCGCGTTCGGT
GGCTCCGTCGGACTCGGGGTGATACGCGCTCGACATACGCAGTTCCACGCCGAGGAGCTTGTGCAAATGAGTC
CAGAATAGACTCGTGAAGATGACATCACGGTCGCTCACGATTGCGCGAGGCATCCCGTGATGCTTGTACACTT
CTGAGTACACGAGCTCAGCCACTTGCTTGGCATTGTAGTTCGTTCGACTAGGAAGGAGATGGACCATACCAGT
AAGTAGGTCAATGACAACTGTGATAGCGTCATACGAGCCATCACGGTTCTTGGACTCGGGGAGAGGTCCCACA
AAGTCTATCCCGATACACTCCCAAGGATATGTAGGGACCGAGAGAGGATTCAGCAGCCCGTAGGGCTTCTGAT
TAGACGGCTTACTGCGCCTGCAAGTCATGCACGACTCGCAGTATTTGTGCACATCATGAACTATTGTCTTCCA
CCACACATGTTCTCGTAGCAGCCCTAGGGTTTTGCTAGCTCCGAGGTGAGCAAGAAGAGAATGAGCATGTCGG
ATGACAAGCTCCCGTGCATTCCGAGCCTCTACAATGATGTTCGGGATGCACAGGAGATAAGACTGTCGTTCTC
GGACAAAGATCAGTCCGTCTCGCACCCGGAAGTTCTTGTAGAACTTTGGGTTGTCGAGGATGTTCTTAAAGAA
CGGGTCCTCGTGGTACCTGTCCCGTAGAGACACGCGAAGGTCAATGCCTTCGACGCTTTCCGAGACATGTTGC
AGTAGGCTTCCGGTAGGAGGTATCGGAGGCGACTGTCCGCTGGTCTGAGCAGCTGAGGGCTCGTAGTGAATTT
GTGCATCCGTACGAGGGTCGTGGTCAGTAAACACGCCCTCCAGGGAGGAGCTATTTGCTGAAGGGGGCTCTGT
ATGGTGGCCAGGTAGGACGGAGTGCGCCTGGCTCGCGCCCTCCTGTCGTTTAGCACGAGGACCGTGCAGAACG
ACACGACGGATTCGTTTGGAAAATTCCCGCGCTGTCTCAGGGCGCCCGGACTCTGGCGGTGCAGGCGCCGTCC
GAGTTCGAGTCGTACTGCCCTGTACTGCTTCGCTGGCAGTCGAGTCCTGCGCAGCCTGGTCGACGACAATAGT
GCGAGGCTGGCGTTGCTGTCGCTTTTTCGAACGTGTCTTCAGTCGGCTGGGTCCTGGCAATGCCGCAACATTC
GGTACCATGGTAGAAGGGTCCCTCTGTGGTCCTGCTTCCAGCGGGACCGACGGTGCAGGAGGCAGAACCTCCT

GGACAAGCTGCTGCCCAGAAGCAGGTGTTCCAGGCTGGGTATCAGGTATGTCATGCAATTGAGTACGTGTCCG
ACCACGGGTAGTGACAGCCAACGCCTCCAAACCCGTGTACACAGGCATGGAGATTGCGTGCACCTGAAGACGC
GACGCAAACGCGTCGTCGTCATCATGAACAACATATTCCGATGGTGCTCGGACAGTACCTGGGCTATCAGCAG
AGTAAATCCGCGAGAGGGCATCCGCAAGGACATTATCAACTCCAGGTACGTACTCGACCTGAAAGTCGTATTC
AGATACCTTCTCTAACCACCTAGCCTGGCGGCCAGACAAGTTCTTCTGATTCATGAGATGAATCAGACCCTTG
TGGTCCGTTAACCAGGTAAAGTGACTCCCGAGCAGAATGTCTCGGTGTCGCAGCATAGCCTCGACTCCCGCTA
ACATTTCAATCTCGTGCACAGGATAATTGGACTGAGCAGACGAGAGCTTCGCCGAGAAGAACGCGGCAACACG
GGCAGTGCGAAAGTCATTCCCTTGGGACACCACTCCCGCAACTCCACCGTGCGAGCCGTCCGTGACGAGCCAA
ATGTGCGGGGCATCCTTGGAGTAGTCCAAAGGCACACGGCGATGTTCACGGTGAGCATGGACTAATCGTTTGA
TCTCATCGAAAGCCCGCTGATGAGTGTACTCCCAACGGAAAGCGACCTCAGTTCCTGTCAGCGAGGTCAGGAT
CCCCATGGGAATTCGCACGGTCGCGACATCATCGGCAAGATATCCGACGGAGCCAAGAAATCCACGCAGAAGT
TCCTTAGTCGTCGGGACCTTCCAGTTAACCACTGAGTCAACCTTGTCAGGGTCCATACGAATGCCCTGGTCAT
CAACAACACGACCGAGGATCCGCATTTCATTGCACAGGAGCTTCAGCTTGTCAGCTCCCAGAAAGAACTTTTC
ACGCTGAAGTATGTCAATAACTGTTTTCACATGCGAAACATGCTCGAGCAGTGTGTCCGAATAGATGATAATG
TCATCCAAATACACATCAAGCCAGACCGTAATATACGGTCCGAAGATATGATTCATGACCGACTGGTAAGTCG
CGACAGCATTGCAATCTCCCTGTTGGAGAACCAAGCTGACCATATTCCCGTCAGGGGTTGTCATAGCCGTGCG
GGGCACATGAGCAGGTTCGATGCGAATCTGCTCGTATGCATCCTTCCCGTCAATTAAAGTACGGTATGGCTTC
CGCGCAACCCGCCGAAGGATTCCTTCCATATCAGGTAACGGGGACGTCACCTTATGGGTATTGGCATTTCGCT
CACGAAGATCACACACCACGCGCAGCTTCGGAGGGATTCCCTTCACCCCAGTGCCAGGTTTCGTCAACAAGAG
CATAGGACTAGTGTTACGGGCACTGGTCATTTTCCACCGCCCAGAGCGAAGGTACGCATCGCGTTTCTCGTTC
CATGCGTCGCGCAAAGCATCTGGACACTTTGACGGACGCCAATGGTGGATTTTCGATTCATCCTTAAGAGGGA
TCGAGTGATTTATCGACCGCAATGGGGGCAGAGGTGTATCACTAGCTGAGACACAGATCGGGGCTGCATATTC
ACGCAAGTGAGTGCGTGCACGCTCAACGTGATCTTCGAAAACATCCGCGGCTCGAGACTCGAGCACGCGGACC
CGTCGTCCTTCTATAGGCAGCGCGGTGGCACTTCCAACAATGACAGATGCTGGATTCAGCCCAACCGTGACCT
GGTGCTGAAACCAAAACGGGGTACCGAGGATCAAGTCATAGTTCAGTATGTTGATAATATCGAAGTATCGATC
CTCGTTAACGGACTGATATCGAAGATTCGCTGTACAACCTAGGTTGATCTTCGCACGGGACCCCTGCACCGCG
AGGTGAACCGGTAGGGGCTTCTCTAGCTCAAAAGACTTCACTCCCAGCTGATGCGAGAGCTTAGAGGACATAA
AGTCTGAGAGTGATCCAGAATCAAGCAAAGCCCGAGCGGGGTGCCCATCAATCTGAACGACAATTACAATAGG
TTCGGGAATCAGACGTTTAGATCCGTGATATGTAGCCCGTTCGCGTCCATATGGCACTCGTAACGAGGCTCAT
TGATATTGTGCCGTGCGGTGTGCGCAGGCCACGGGATCTCTTGCTCCAGCACGTGAGCTGTACCGGAGGCGAG
TAAATTACGCATAGGCTCCCCGTGAACACGACGATGCCAGTGCCCTAGACCAAGCCCCGCAGAGACCTGAGAT
GCATACCACTGTGGCAAGTTGAAGCCAGCTTGGGTAAGCATCTGTCTTGGGATGACGGGATCCTCCGCAAGAT
AATGCTGGAGGATCGCGTAGCCACCAATAGTGGGGTACACGACAAAGCTGAACCCGGCACTGTCCTCGTCAAG
TCCAAGATGGAGAAACACGTCTGGAGGGACATAGGGAGCGTGCTTCACAAGTACCTCTCCTGCGACCTTGCAG
AACAAGTCAACCATAGGCAGGTACTGCCGTTGGGACCGTCCGGCTGCGGGGAACTGAGGGTATTCATAATCCC
ACACATATGCGGGAAGCTGAAGAATAGCCTGCATGTGTTCGGGAGACTCGACAAGTCCCTCGAGGGGAATGTA
CGTCCCTTCACTAGGAGACTGCTCACCCAGTTCAATAGAGTGAAGTCCCAGAGATTCATACTGATTGGTAGAC
TCTGCTCGGTCACGGAGATGCTCAGCATCCGCGAGCACCTCAATATTGAAGTTCGCGAGACCAGGAGCTTGAC
CTGGCCTGTCCGAAGACACGGTATTGTTCTGGGGACAGTTCCGACTGAAGTGATCCTTCCCCTTACACTTATA
GCAGCGTCCCTCGGCCTGAAGCCGAGCACGCTCTTGCTCACTCAGTTTGGGAGGTCCGGAGTTAGGCCGGGGG
GAATCCCGAGCCCTCTGACGATTCCCTGTTCCTGAGAAATTATTATTGGGTGTTCCCTTGCGCTTTTTATCGT
GCTTGCGCGACTGATTATACGAAGACCCTCATGACTGCTGATGGTCAGTACGAGGGGTCGTCGAGGAGCTAGG
CCCGGAGGAGCCAGCTGCAGGGGTCCCACCTCGGTGGTTGGACTTCTGCTTTGGACGCGAGTGATGCTCACTC
TGTCCCGAAGGGGCTCGAGAATAGTCGGGCATTGTTTTCTCGGCCAACTCAATAAATTGAGCTGCTCGAGTAA
CCTGCTTCCAAGTGGAGGTAGTGGAAGTCAGGTGCTGAAGGTACAGGGCTCGCTGAATAGATGAACGAAGCCC
CTTCCAAAGTCTATCCACCTTGTCCTGCTTGGACAGCACTCCAGCTGTGAGGAACATGGTCTCAAGCTGGTGC
ACATATTCACGGACGGTGCGATCACTCTGCTTAAAGCGGTCGAGCTTCTCCCGGGTACGGGTGCGGAAATCAA
TGGGGAAACAGTAGTTGAAGAGGCCCACAAAGAATTTCCGTAATGTCCAGGAACGAGAGTTCCGAGACACCGT
GTTCACATAGAACTCATAGGCTTTGTCCGTTAAGAAATAGGACAAAGAGGCAATTTGACGGCGCTCTTCCAGG
CCATAGCCGTCAACATAATCTGTGCACTGCTGCATAAAACGCTGGAAGACAGTCACATCAGTCCGTCCATCGT
ATGCATCTGGATCCCGCGGCTTGAGGCGCGGGCGAGAACGGCTTCGAGAGCGGGACCGCCTATAGCGTCGCCG
GCCTCGGCCTCGGCGTCGAGAACGAGAAGAGTGGCGGCGCAAACGCCTAGAGCGTCGAGAACGACGGGCCCGT
GACGAACGCGATGAACCCTCGCTACTGTTAGACTCCGAAGAAGAGCTCTCAGAGTCCGAATCATCCGCAGACG
ACTCGGAGTCGGATGGCTCAGAGCTTGACTCGGGACTCTCACTCGGGGAGTTGGAGGGATTAGTGTCCCGCCG
ATTGAGACCCCTGAATGCATGTCCAAGGGTGCTCCTGGGCTCAATCTGATTCGCAGGAGCAAGCCGGTCAGGT
CCTCCCTCATGCCGACGTCGAGAAGACTTGGCTTTCGACCTCTTCTGAGCCAGGAGGTCATCCACCAGGGCCT
CTGAATGCCGGGTAGACTCTACTGGAGCAGAGCCCTCATGCCGAGAGGGTTTGCTCTTTCGGCCATGAGTAGC
ATCACGTCTGCCACGTCGGGCACGACGACGAGCACGATCCGAACGCTCAGACTCGGATGATGATACATCCTCG

GCCGAAGCTTCATTAGTTGCAGGGTCCTGGCGTTCCGTATGCCCAGGCCCCCTGATATGGCTCGATCGAGGTC
TACGCGACCGATCGTGTGGCTGCTCGGCAGGCCCCTGGTGTCTCTGACGACGCTCCTGCTTTCTTCCAGATGA
AGCGCGTTCTGCCCGGGGGGCTGGCGTGCTGGTGACCACGTCTGATGAGTCACCTTCCTCATGATGCATGCGG
GTCTTGGCCTTTCGACGACGCGTGGCCTCACGCACATCATGTCCGTTAGAGCCGGCGGAGTCATCCTCCGCGG
GCTGAATAGAGTAAGCTTCCAGGGCTCGTCGTTGAGCCTCCGGATCAAGCTCCTCAGCGGGAATCCCGACGGC
GCCCCAATTGCGACCGTCAACCGATTTCCCCTTCTCCATCGTGGAGGGTCCCTCACCGCGGGACTCTGTCTCA
GAGCTCTCGGGGCTCTGGACATTCACAACACGCATCCGTTCTTGCACACGGGCATGTTCAGCAGGTGCCAAGC
CTGCTTCAGCTGCTCTGACAACTTGCCTCTGAGTATCAGTCAAGTGGGCAGTAGGCCGCGACGGGACCCCGCG
CTGAGCAGGCCGAGAAGAAGTGATGTCGAGGCTGCGGCTGCGACGGCCCCTACGAACCTCGCGCCAAGGGCCA
GCACTCTCATCACGCGTATCATCCAATGAATCAGAACGCTCCGTCAAACGATCGGAAGTGTCCTCTATTTTCA
TTTCGTCCAGTAAACTAGACAGGTTGGTGAGACTAGAGGTATCTAGCTCAGTCGGGCGGGTTTTCCCAGTCCC
CGACGTCTCGACGACGCCTTGGCGTTCTCGCGAGGTATAACGCGCCGTTACTACCTGGCTGTAAGACAGTCCA
GGCCTCACAGCGGAGCTGGCAGAGCGAATGCTCGCCGTGTCCGAATCCGACTCCTCGGGCTTATCAAGCGAAG
CATCAGCGTTCTGGCCAGGAGGGGTTTGATACTCACCCGGAACGCGGCCAGAGGACGTTGTAGTCTGGGTCTG
GGCGCGACGAGCAGTGGACCGGAGGTTATATCCAGCGGGCATAGCGAGAAGCAAGAAGGTCGAGAATATCTTG
AGGTTGAGTGGTTGGCGAATCGCGTACGGAACTTCGAGTTGATCAGACTCGAGGGAATCCAGAGACAGCGGTC
GCCGAGAGAGTAGTAAGTTTGGTTAAGCTGAGTAAGAGTTAAACACGAAGACTAAGAGTCAAAAATATGTGAC
GCTCGCGATCCCAAAGGGGAAAGTCGGGTCGCCACACGAGAGAGAGAAGAAAGAGCACACGAGATAGACTGGG
GTAAGTCTTGTATTTCGGAGCTCGATGGCGTACGCCTACACACGGAGCAGTATATATACGAGAGGGGGAAGCG
GACATTGAAAAGACCAGGAACCGAGGGGGTAGACTATTGAGACGACGAGGGGGAGGATAGGATATTCGAACCG
TGGGAACGCCCGGAATGGAAGGTCTGCTCGCACCCGCTACAAGGTCCCA
>Cersu1_Cluster29#LTR/Copia
TGTTGGAAGTCAACTAGGCAACGAGAGGTCACCAGGCGTGGACGCGCCACAAGGACATGAGGCTACGGTACAT
GTCTATATGTCGCGAGTCTGCTTACCTAATTCAAAGTTTAGTTTGTCGTGACTCGCGTCTGAGCACCTCAGTC
TTTACCTACACGGAGACTGAGGTGAGTAGATCTATTACACATACCTGTAGAGCCCTGAGCTGATTTACTGTAG
AGAAGTTATTACTCTGTGCAATAGGTTAGCTGCAATTTAGAGTCTTTACCTACATGGAGACTGAGAGGAGTTA
TTACTCCGTGCAATAGGTTATCAACCCTGGTCTGTAGACTGCTCTGAAATGTTTTCTGTCGCTCCCCTTGCTC
GCTACATCACTCAGTCATCATGACCAAAGCCAATACGACGTCTCCAGACCCGAATACCATTTTTTCGGCCACA
ACGAGTGCGCCAATGGTCATAACAGCGTCCTCGATTCCCCCTACCGCCTCGACCAAACTATACGACATCCAGC
CTCTCCAGGATAACGGAGCAAACTACCAGACGTGGAAGTATCATATCCTCAAGATCCTGACGCTGCAAAAGCT
CGACGGTATTGTCAAGGGCACCGACAAGGACCCTGGTGAGGGACATGCCGGCTGAATGGCTTGGCAATCGAGG
AACAAAGAGGCATTAGCACAGATAACACTTCTGCTCAAGGACGAGCCCCTGAACGGAGTCCTACATGACACTG
ACGTGGCCGCTGTCTGGAGCAAATTAAAGGCTCGATACAAGAGTACTGGGCAACAGTTTATCGCTCAGCTCAT
CGCTGAGCTCTTCCGGAGCACGCTCTCCGATGAGTCGCAGCTGGAGCCACAGCTTAATATAATGCTCCAGAAA
AGCTACATTCTGCAGTCGCTTGGTCTTACCCTTGACGACTCGCTGGTAGCAGTTGCGATGATCGTCTTGCTTC
CCCCATCCTACAACACCCTTCGGACCAGTTTAATGCAAAATTTGGGTTCCGGCACGCTCTCCATCGAAACAGT
TATCATGCAAGGTCTCATGGAAGAGAAGCTTCACCGTGGACGTGACACTTCGATCATGGCCATGTATGTGAAG
CATGGCTCATTGGCTCCACCCTCTCTGAAGAACAAGCAGAAACCGAAGCACGACTCTGGTAATGCAAAGAAGC
CTGACAAGCCTGACAAGAAGTGTGACTTCTGTCAAAGGAAGGGACATCTTAAGTTGGTTAACACTCGCCCCTG
CGGTGGTCACCACTTGTGGAGGGGGAGTGCGTTGTGAAAGGCGGAGATTGAAGTTTGCTTCGGGAACCAACCG
TGACAAGCGCGCACTCGCAGTCAGGTGGTCTATAGAAGCCGAGTTAGTTACAATATAATAGCCAATTTTGCAC
CTCAACGTGCTCGCGTCTGTTGGTAATGAAGTGCTTGAGGCCGCTGCAAAACCCGATGGACCAAGCTCTCAAA
CTGCCCGAAGGCGCGGGCTCAGAGTGAAAATGCGAGGGAAAGGGGAATGCGTCCATTTGAAGGCGGGATGGCA
CCTTAAATACACGAGGATCTCAGCTCTCGTGTCTGACTCAGGCCGAGGCTCCCAACCAATGAGAAGACTGAAT
GAGAGACAATGACGTATGTAGTGAAATGAGAGTACAGAAGAAGTGTGGTGTAACGACATAGCGCGTCAGAAGC
TTGCGGATAGTTGCCTAAATAGGCATGCCTCCTTCCGGATTGGGATGCCGGTGTCGGGCTTCTTGCTTGCGCG
TCTTCACCTGTCCGCGATGCCGGGCTTCTGCGTTCACCGGCGTTCGCTGCACTCCCTATTTACGATGCGCAGC
CTCAGTGGCCCAAGCTGAGACACAGGGTGTACTGGTGACAGCGCGCGATGAACTGATCCCTCAGCGGTCTTCA
TGAGGTCTTGATCATGCGCTCGCTGCCAGCCATGCCGCGATGCCCTGTGTACATAGCACAACAGATGATTCAG
TCATAATGTATGATCACAGATAGAATGAAAGAGGGAAAGAAGAGAACGAGCCTGCCCAATGTTCCATCGGCAG
TCTGCGAGTGTTGAACTTGACGAGGTATAATCACGTAATTGTACAATCTTGCTTACGAGCGTGTTGAACGCGT
GGAGATGGTCGGAGGATACTAGGAGGAAAAACTCCCAAAAAAAAGTGTGTGTCAGTCGTCGTTCTGGCGTATT
TCTTACCCTTCCACAAGAGGGGTAGGCTTTTAATCTCGTCCCCGAGATCGGCTACCACCTAGGTCTCCCGGCA
CTGGCCTGCCATTCCAGCGCGCCTTTGATATTGGTGGAGAGAAGTACCGCCGGACCGTCAAATTGCAGCGGCT
GTCCCGCGTTCACATATGCTGCGTCTGGCGCCTCGATGATTGATCCTTGCATATAGTCGATGTACCTGTGGGG
TAACTCAGCGTTAGCCATCTGACCGAGAGTAGAGTACTGCGGGACTATCGGCAACGCGAAGACTGGTTCCAGT
GGGCACCCACTGCTGCAAACTATCTGCATATTTGCATCGGCAACCAGACCCAGCATCTTCATCTCCTGATGGT
CTTCGCGAAAGTAGAATAACTTGAGGCGGCTCGCAGCCACGTGATTGCAAATGTGCATCCTGTCAATCTCTGC

AATTTTGTAAGAGCCCGATGGTAGCTGCCAAACGACCACGTACGGACCCACCTGCCGGAGCGTGCCCTTGTTG
CCCTGCTGCGCGTTGAGCCAAGTCTCGTGCATCAAGACCCAGGTGCCCACTTCAAAGCCGCGATCCCGGATCT
GACGTGCATGGCGCTTCATGTAGTCATCGATGGCCCGCTGACACGTCTTCATTACTGTTGCCTTGGCTACGCC
GATGTCCTCTTCACGCTGTGCGATCTGCTTGATCCGAAGAGCGATGAGCTCCTCCATAGAAGTTACCTTATCC
CGTTTGAGGCATTCCCATGTCCTATCGGCGAGGTTGTACAAGTGAAGACAATGTCGCCCGTAGAGTATGAAGA
ACGGTGTGTACCCCGTCGAGCGCGTTGTTGTGGTACGCATCACGTAAACCCCAGCTGGAAGGTGCAGCGGCCA
GTTGGCAGGCTTGTCACCGCACACCCAGAACAATGAGTTGACTAGCGTCCGACCATCACGTTCTGCGATACCA
TTCCCCTCAGGGTGGTATGGCGTGCTAAGCACCAGATGCACTCCGTGCCTCAGTGAGATTTCCTTGACGGCGC
CCTTGACTTGGGCCCACCGCCACAAACAAAAACCAGCACACATCCATACCTGCATAGGATCTCCTCCCAAATG
AAACGAGCCCAGGCTGTTGACATATTTCGTCGCGAGCCACGAGCTTCGATGGATTTGATGAGGCCCTCCAATG
CGTGCATGAGGTACTCTAAACCCCTGATCCCCCGCGGCATCTTGATGGTATCGAGTACAAAAAGCTGCAGGAT
GCACGGCGCGATGGTAATGTTCAGTTGTTCAATGGACCTCGCTCTTGCCCTGTACTGACACTTGTTGCATGAG
CGAACAAAATACGCGATCTGATCGTACATATTGGGCCAATAATACCTGTCCAAGACGTGCTTGTATATAGCGT
CTCGGCCTCGATGACCTGCTTCACGATGGGCCTCGGCAACGATCTCTCGGCGCCTGTCCAGGTCCGACACTAC
GAGCTGAGGTATGCCATGTTTGCGCCGCCGCCAGAGCCTGCCATCCCGCAGGAAGAATCCCGACACGACATTC
ATGAACGCTCGTATTGACTTGCAGCTTCAGGTGCCAAAGTCGTCCACAATGCCCATCTGATGATACACCCATA
CCGCCAGCCACCACACCCCACCGTTGTGGTCCTTGATCTTAGTAGTGTGTGTTGAGCAGCTCGGAGTGTCCAG
CCAGTGTCCATTGTCGAACTTGTTATAGCGTAAATGATGGTCCTCATGTGCCTGCGCCGAAGTGTCGCCTGGG
AACGAGAATTGATCGGCTCCCAGACCAAAATCCCTGTACGGAACCCCAGTCCCCACGGACGAAGAGCCATCGA
CGGCGAACGCGGAACGGTACTCAACAATTTTGAGCTCCACTCTCTCATCACCGAGGTCAATCTCCTCAACGCG
ACTCCCGGAGACTCGCCGCCGCATGAACTCTGAGCCGATATAATTGACGTCGTTCTCGTTCCTAAGAAGTCCT
GGGTGGAATGGAAAGTCCTCCGTGTCGATCTGATGCCAAAGGAATTCGCGTCGCCCATTCGTGAGCACCCATT
GTGACTTCAAATACCACTGCGGCAAGCCGAACACAATGTTTGCTGGCACACCCTCGGTTCCGCCGCCCTCACT
AATGGCGGTCGCCGGCCGCATATCAAAATCAGCGAGCGTCGATACATGAAAAGAGAAAGGCTCAGCTGGATCA
GCTGGTGCGTCGATCATAGTGCCCCAGCCACCACTCTGCGGTGTCGCGTCACGCACAATTGCCGCCCGCGCTG
TGTGTCGGTCATACTTGTTGTGTAGGTGCTCGAAGGTGATTGCTGTTGTGCCAACACGTCGTTCGAGCTCGTT
CTCACCGTCAGATTCACTGCTGTTCTCTGGTGATGGCCTGCGCCTTGATAGCCCGTCCTCGGCCGCGTGATTT
GCTGCTGGTACATGGTTTAAGTAAAAATTGAACAGCTGTATATAAGCCACCCATCTGGTCATCGGCGCATTTG
GCAAGTCGGGTTCTCAAATCATTTGCTTCAGATACTTAGTGTCCACGTCCAGCCGAAAGCGCACGCCCCATAC
GCGGTGGCGTAGTTCCTTCAGGGCTCGGAACACGCCATATAATTCCACTTTCGGTTGCGAATAGCGTGACTCG
ACCTTGTTGAAGGTACACGATCCAAACAACACTGGGTGCTTGTCTGTCTTGAGGAACTGGTATAACACCCAGC
CGGAGCCGTACATCGAAGAATCAACCGCGACAACGATAAGTCCGTCGTCATCATCTCGAGGCAGTTGCGTGAT
GCGTCTGGCGCGTCCATAGTCGACTTTCTTCAGGATCAGCGCTGTCGACACGAGGGCTTTGAGCGTGTCCATC
GCGCATTGCTGCTCCTGCCCGAATTCGAACACGTAATCAGACTTCTTCAATAAGGTCATGAGCGGCTTAGCTA
TCAGGGCGAAGTTCTTGATCCAACGACGTCTAACGCCTGCTGTTCCGAGAAAGCCTCGCACTTCGGATACTGA
TTCGCAGTACTGCCAGTTCTCGACCTTGGCAATAAGGCGGTGTTCAAGCTGTAGTCCTTCCTTTGATACAATG
ACGCCGACAATTGTGACACGCAGCGTTGCTAGTATCAGCTTCTTTCCCGATGTGGTTATTCCTGCCACTGTGA
ATCTGTGAAAGATGCGATCAACTCTGGTCGCGAATTCGTACACAAATTTGCGGATCCTCGGATTTGCCGATAT
AGTCTCATTGTCATAGTCATCTTTGGGCCCTTGGATGCCTGCATTGTCAACGAAGGCTTTGGCGATCTCCGGC
GCATTCTCTCCCAGCGTATGGTCTACGCAATGCTGGAACTCCGGCATCGCATTACAAGATCCCTGGGGTAGTG
ACATGTTGCGATTGGGCCCACCAACAGGCGCCTGAAACGTCATTAGATCATGGGAGGACTCGTGCAGGACCCT
TGCATCATATCCCGAGAATAGATCCACCACCATATAGATGACACGACCAACGAAGCTTTCCGCAAAGTTGTCC
ATACGCGGTGATAGCGCCGAGTTGCGGATTGTGATTGAATTGAGCTCCTGCAGGTCGTGCACAAGGCGTGGCT
GCCCATTCTTCTTGAGCACCGGGAACACCGCTGATCGATATGAGGCAGATGAGTACTCAAATTTGCCCGCATC
CTGCTGCTCGGTGATTAGCCTGCGGACATCGTCTTCAAGGGCTTTGGGGATTTTTATGATTGGTCGTTGCCAA
GGTGTATGCTCGATATGCGGTATCTGGTAGTCCGGAAAGTACTCGCGTGAAAACGTTCCGCGTTGCGCGTCAG
TAAAGGCTATCGCCTTCTCATTCTTGATGACCACATGTGCCATCAAGTCTATCTCTGCCTCTGATAGGAAGCC
CTCGGGCACGGTCGCAATGATCTCCTCTAATTGCGCATAGGATATCCTCCCGCTCCCCTCAAACTCTCGCCAA
GACGGCGGATTTGTGATGAGCGGAGGTAACTCCCCTACTACTATTGCCTTAAACTTGTGTGCCGATGGGTTCG
GCATGGATGTAGGCACTGGGCGCACCTTTTTCGATACTGGTTTGTAACGTGCGCCGCTCGCGCGACCTTTGTA
GACCGCAAACACTCTTGACACCCACACGGACGTCGCTGGCCTAGACGGCAGAAGCGCCGCTCGAACTGTCTGT
AATAAATGATCATATGACAGAGGAGGAAACAATTACGAGACTAATAATAACTCATCTATGTCTTGACACCTGG
ATGGGTCCACGACTTTTTCTAAGGTCTTGCCGAGCCTAGCTTCCATAACGGACACGCACTCTGCGAGAGAGGT
AGGCTTTGAATGTGTGCCCGGCTTCAGAAACCCTGCTGCATCCCCTCCTTGCCTTCCGATGGCCGCTCGCTTG
CTGCTGCTAGGACCAGCCGATCCGTGTTCCTTGCCAGATCTATACCCGCCGCGCGCAGCTTGATCAATTTGTC
GGTCTTCACCCCAGTGGGCCACGCTTCGACCTCCATAGTGCCGAACCTGTCGTAATCAACACGGCACGAGAAC
CAATGGAACCATGGTTGGCCCAGGATACCGTCATGCGTGCCGGTCTCTTGCGTGCTGATGAAGAAATGATGAT
CAAAGCGGGACCCATCAATCTTGAGTGGCGCCTCTCGACAGCACCCAAGTAGTTTCACGGGCTCACCGGATAT

TCCACGTAGCGACCACTGGGCTCCGTCCTCATCAATGGGTACGTCGGTCTGCTCCCATACTCGACGACTTATT
AGGTTAAGCTCTGAGCCCGTGTCAATTAGGAAGATGATGTGCTCACCACCAAATCTAGCTTTTACGAAACCCG
TAGCCATCGCAAAGAAGCGTTTTGACCGAAGACTCACTGCTGACGTGTAGCGCTGTAGGATCGCTCCAAGATC
CTCCGTTGTACCACACGTTAGCAGCGCTATGCCAGGCTGGCCGCCGCTCGCCTTGCATTGTGGGCCGCCCAAT
TCCTTCTCATCCTCTGTCATTGGGCTCGCCTCCACTTCTCCCGCAATCTCCGTTATAATCGCTCTCGCGCCGA
CCTCACGTCACGTCTTCGTGAGTGATGCGAAGCGCTTCTGCAACTCGGGAGACAATCTGATGATATCGCGCAG
CGGTAGAGTCACCTTCGTTGCTAGTAGCTGTGCCTGTACTTCTTCCACCTGCACGTCGTCCTGGAGAGTCGAA
GTGAATCGATATAGATTGCCTTTGGACTCCAATGGCAGGGACTCAGCTATATGATCTGTATCCCGTTTCTCGC
TCCTCGCCTCTTGCCTTTTCCGTTCCCACTCCCTCCAGCCTTCCTTCATGTTCACTGCAGGCGGTCGCACCCT
TGCCGGTTCGTCCTCCTGCTGTACCTTCCTGTTGCTCAATTTGGCGACCTCCATCAGCAGTTCCTCGAAGCGG
ACCTCCTTGACCACTTCGTTATCTTTGTCAACTTCCTCGCATGTTGTCGTCCACTTCTGCTTTGTCCCTCTCG
TCTGAACCGGGAATGAGTGAGCCGTCTCTGATACAATAGCAAAAACACCTCCCCTTAATACGTGCTCGCCATC
TCTGCATAAACCCATCGCCAACACGCTCATATGCGGTGGTACATCCTTCTTCGCCGGATCGGACCTAGCCCGC
AGCTTGGTGTCGTTCCTTAGCACCCTTGCAATACCTTCTTGACCAGGTAGTGCCCTAGGTAGCTCGGATCCGT
CCGGTCTAACCAGACGACCTGTGGACCCTAACTTGATGAGCCCTTCTTCGACCAGCTTGATGGTCTCGGGGCA
GCTCTTGGTGCCAATCACATGGTCGAGGTCGATACCTTCAGTCTTGTCGCACATCCAACACTTATGAGCCGTC
CTCTGCCCTAGCATGTGGGAACAGCTACCTGTCTGCGTGCACTGCGTGCTCACCGACGCCTGCACAGGTACCT
GTTGGTTTATCGTGAATGCTATCAATTTCTCGAATCGATCAGCGAGGGACTTAGCATGTTCCAACACCTGTTT
GCTCTCGCTATCGCTTAGTGCCTCATGCTTAACCTTCAGTTCCACTCGCGACTTTGCTGGCTCTTTTGACTTC
CTATGCTTCTTCCTCTTTGAACGTGGCTCACTGTCTTCCGAGCTGCTCAAACCATCGGATCCGGATGAGTCAT
CCTTCGATGTCTTGTCGCTACTGCCTCTGGAATCGTCAGAGTCCTCGTCAACGTCCTCCTCACCAAAAAGAGA
CTTAGCTTGCTCGATTGTCTTCTTTATCGGTGGTGGCGAGTGCATCTCAACTGCCACCCCTGCCTTCTCCAGC
TTGGCATGTATCTTCCGCCTAGCACGCTGTGGAATGCCCTTATAAAAGTAGAGGTCACACTCCTTCTCCGTTA
CCATCTTTGTTTCCGTCACATCACCCAGCATCTCAAGGAACTCCCAAAAGTACTTGTCGAAATGCTGTACTGT
AGTCATCCGGCGACCACGCGCCGCTCGATAGAACTTCTGTAGTTTACTGGGCCTAAACTTGGACTCCTCATCA
GTAGACCCGTACAATTGTAAAAGGTTAGCCTTTGCGGCGTCCCAATCCGACGCCGTCGGTTTAAACTCCTGGA
AATGTTTGATGACGCGTCGTACGTTGTTCGCGCAGTATCGTAAGCATTGATTGGGCCACTCGGCGTCCGGCGC
CCCAGCTGCCTTGGCGCAAGTTGGAAAGTCGTCCACAAAGTCCGTGACGTGAGCGCCCTTGAACTTTGGAGCA
CCTTTCGTACCTGGGAGGGGCATGGCAACTGTAGTACCCGTCGGCTGCATCGTCGATACCGTTCGCCGCTGTT
TGTCCATTGTAGAGGCCGACTGCATCGAGCTCGATTATGGTGTCAAGCTTGACGTCATCGAGATACTGACTGT
GTTCTGCACCATCACTCTAGCCATGGACCTTGTGCTCACTCCTGACGTCGAACAGGTATGAATAGAGTACTCA
GCTCTTGTCAGCGGTGCCGTTCAATGCTCGCGCCCTTGCGTGTCGCTTGTTGATATGTCCTGTCCACTATAGA
TCCGCGAGGTAGACTTGCGCTTGCTGCCTACGGTACCGGTACCGGCCTTCCTCTTCTGTGGCGACGGAATCTT
GACTGCTCCCGGCCTTGTTCGTTGCTATCTGACCAGCTGGGATCCGACCAGCCGCTTCGATACTCACCCCCTG
CGGTGTTGAAAGCTCAAAGCAGCAAAGCAAGAACTCGCTAGGGATGGAAAGGACTCATCCAAATCAGGGGACC
TCACTGCCAAACTTGCCACCACGCAGGGAAGCAATTAAGAGGAATTCCTCCAGGTGTTTGTCGCAGAAGCCCT
GGCTGGTTGCCGGGCAATGCTCACCCGCTAGATAGTTGACTCAGGAGCATCAGCTCCCATGTGCTCGCAGTGT
CAATGGTTCACTAATTATGTTCACCTTGACTCTCCACATCGTGTATGGGTCAGTGCGAGATCAACTTCGACAT
TGGCCAAGGACAAACTCGGCACGCCACCATCCAAGGGATTTACCACATGCCTGAGCTCAACAGCAACCTCCTG
TCAGTTTCGCACATGGTCGCACAAGGTCACCGAGTTACCTTTGACGACAATGGCTGCTCTGTTCGCAATGCAT
CAGGGGACATCCTCGCAAATGCCTATCTCGAGGATAAGCTATACATCCTCTGTGGAACCGTTACCCCACGCAA
ACATGCCTACGTGACAACCATTGGACACGACATGCCTGATGACAGCAACATTCAGGCATTTGTCGCACATGAA
CAGGAGTCCAAGGTAACACTTGAAACATGGCACCGTCGACTCAGCCACATTAGCGTTGACTCAGTTCTCAAAC
TTGTCCAGAAGGGCATGGTGAAAGGGATGAACATTGTTGGGTCACAGAAGCCAAAGAGCGGGCTGTGCAAGCC
ATGCCTTGAAGGAAAACAAACACGCAAGCCTATTCCCAAGATATCAGATTGCAAGGATCCTAGCGAGGGAATC
CGTCTCTATACAGATGTCTGCGGGAAGATGCCTGTATCTACCCGTCCTGGCGAGTTGTACTTTGCGACATTCA
TCGTCAACAAGAAGCCACACAAGGTTTTCACGCGACTCCTCATGACCAAGGATGAGGTCTTTGGATGCTGCCA
AGAGGTTGTTGCACAAGTTGAGACACAGACGGGAAAGCGGGTATTCGCAATATACTCTGACAATGGTGGCGAG
TACATTTCCAAGGAGTTCAAGTCATGGATGAAGTTGCGAGGCATTCATCACAAGATGACAAGTGCCTACACAC
CGCAGGAGAATAGAGTTGCTGAGCGCATGAATCGAACATTGGTTGAGATGGCGCGCGTGATGCTCCAGGAAGC
GGAGTTACCCAAATTGTTCTGGGGAGATGCCGTTCTCCACGCAACCAACATCGTCAATCTCACCCCAACACGC
GCATTTGATGGCAATATCACTCCAGGGGAAGCCTACTCCAGTAACAAGCTATCCGTCGCTCATCTCCGGACCT
TTGGGTGCAAGGCATTCATGCATGTACCCAAGGAGAAGAGGCACAAGTTCGACACAAAGTCAATCGAGTGCAC
CTACCTCGGCTATGCGGAGAACCGCAATGCCTTTTGCCTTGTCCAACACTCCACAGGCCGCTTATTTGAGTCC
CATGATGTGGAGTTCTACGAGGAGAACAAGCCCGAGCCTACTTGAGTCCAAATTGAACTCGAGCCACCCGCAC
TAGTCACCCATGCAGAACCCGAGACACAGCCCATGTTGCCCAGCACCACAACTCAAGATTCCAAGGATACAAC
CAAGGATGTAAACAAGAAAGAGGAGGCAATTGACGAACCTCCCCCGCTGGCAACAATAGGTGGTGATGAATCT
GAGTGGTCGGACAATGAGGATGATAAACACCAGGTAGCTGAAGCTCTGGCCACAGACCCCGAACTCAAACCGA

GTCGTACCCCATCACCCCGACCTCGCCGCTCCACACGTGAATGTCATGCACCTGTCTGGGATGACGATCCCAG
GTACAGTGTGTCCTCATATGGGAGCCGCAGCCGCAAGTCGAGGGACAGCACAGCTTCTGTGGGCGATGAGCAC
GCTCACAAAGCATCCCTCACAGATCAACCACGCACCTACGAGGAGGCCATGTCCTGACTGGATGCAGATCGAT
GGAAGGAGGCCTGTGCCGAGAAACTAGCTTCATTCCTGAAACTCGGTGTGTATGAGGAAGTCGACCGGCCGCA
CGATCATCGAGTTGTTAACTGCAAATGGGTCTTTAAGATCAAACACGGCCCTAATTGCAAAATCTAGAAGTAC
AAGGCCTGTCTGGTCGCATGCGGCTCCACACAAGTTAAAGGCGTAGACTATACTGAGATGTTCGCACCAGTAT
TGAAGTTCACATCGATTCGTGTGCTGCTTGCCCTCGTGGCCAAACTCAATCTTGAAGTACATCAGATGGATGT
CAAGATGGCCTTCCTCAACGGGAACCTAGATGAAGAAATCTTTATGAAAGGAGCTCCGGGATTCTAGTCAGGT
CGTGACAAAGTATGGAAGCTTTTCAAGTCCATCTACGGGTTGAAGCAGGCGAGCCGGGAGTGGTACGAGAAGA
TACGTGCAGAGTTCGAGGCACTCGGATGCACGCATTGCGAAGCTGACCACAGCGTGTTCATCAAGCACCTAGA
CAATGGCACACTCCTTATCATTGCCCTGTATGTTGACGACATGCTCCTCCTTTCTGACAAGACCTTCACCATC
ACAAACATCAAGCCCGAGCTCATGCAACACTTCGACATGAAGGACCTCGGCAAAGCTTGGTGGATTCTCGGCA
TGGAGATTATACGCAACTGTGCACGACACACGCTCAAGCTCTCGCAGCATTGCTATGTCAACGACATTCTTGA
GCGCCATGATATGGCCAACACGTGGCCTGTCAGTGCCCCAATGGCTGCCAACCAGAAGCTCATCAAGGTAGAA
CAGCCTGAAGTTGATCCCAAGGCATATCAGTCCATGCTTGGCTCGTTGATGTACGCCATGCTCGGTACCCGAC
CCAACCTTGCTTTCACAGTGGGCGCGCTCAGTCAATTCTCGGCGACACCTGGCAAGGAACACTGGAGTGCGCT
AATGTGCATATTCCAGTACCTACGCAAGACAACCGACCTACGCATCATTTACAACGGCCACACAACTAATGAC
ACTGGCAACATACTCGGGTACATTGATGCCGACTGGGCTTCCGACGTAAACCCACGCTGTTCTGTTACGGGCT
ACGTCTTCATGATCTCGGGTGGCGCAATCTCCTGGAGCTCGAAGAAACAGTCATTTATTGCTTTGTCTAGTAC
CGAAGCTGAGTACATGGCGGGCTCAAATGGCGCCAAAGAAGCCGTCTGGCTTTGTGATCTCCTCAAAGAGATC
GGGTGCAACCAAAAGAGCAGCACTCCGCTGCTCATTGACAATCAATGAGGTATCTTATTGGCGAAAAATCTGA
TGTTTCATGAGCGCACAAAACATATCACCACGCACCATCACTTCCTTCAGGAGAAGGTCGAGGAAAGGGAGGT
CGAAGGGCTTCCGACGTAAACCCACGCTGATCTGTACGGGCTACGTCTTCATGATCTCGGGTGGCGCAATCTC
TGGAGCTCGAAGAAACAGTCATTTATTGCTTTGTCTAGTACCGGAAGCTGAGTACATGGCGGGCTCAAATGGC
GCCAAAGAAGCGTCTGGCTTTGTGATCTCCTCAAAAGAGATCGGGTGCAACCAAAAGAGCAGCACTCCGCTGC
TCATTGACAATCAATGAGGTATCTTATTGGCGAAAAATCTGATGTTTCATGAGCGCACAAAACATATCACCAC
GCACCATCACTTCCTTCAGGAGAAGGTCGAGGAAAGGGAGGTCGAAGTGGACTATGTTCCACCGGAAAACCAG
GTCGCAGATGTTCTGACGAAGGTGCTCTTGCGTGAGAAACATGAGAAGTTTGCAAGGGGTATGGGTTTACTTG
AGGGTACGTCCACATCCCGTTGAGTGGGTGTGTTGGAAGCCGACTAGGCGACGAGAGGTCACCGGGCGTGGAT
GCGCCACAAGGACACGAGGCTACGGTACATGTCTATATGTCGCGATTCTGCTTACCTAATTCATAGTTTAGTT
TGTCGCGACTCGCGTCTGAGCACCTCAGTCTTTACCTACACGGAGACTGAGGTGAGTAGATCTATTAACACAT
ACCTGTAGAGCCCTGAGCTGATTTACTGTAGAGAAGTTATTACTCCGTGCAATAGGTTAGCTGCAATTTAGAG
TCTTTACCTACATGGAGACTGAGAGAAGTTATTACTCCGTGCAATA
>Cersu1_rnd-1_family-42#TIR/MITE
TACTGTAGTCGAGGTAATTATAACAAACGGGTGTGACNGTGTGACCTAACAANTGGGTCAAATCCATAACAAA
CGGCTTCCGACGTGACGCGACCTTTACAAACTTTGGCTCTGACATAGCTAACAAATGGCCAATAACACAGCAC
GAAACCGACAAAATCACTCCAGAATGAGCGGAAACGAGGAGCTCTTGCAATTTCTGGGTAGCTTGTAGCGAAA
ATTTTGGTCAGAAGAGTAAATTTGTCTGTTCCGGGAGCGTTTGAACAGATTTTAGCCCTGGTTTGAGACATGG
TCAGACCCTTAACAANCGGTCGGTCAATTGTGACGTGACATCTCACCGAATTAACAANACAAACCCTGGCCGT
GACGTGACCGTTTGTTATAATTACCTCGACTACAGTA
>Cersu1_rnd-1_family-9#TIR/MITE
TAGTGCTTTGCACGGGTCTACCCCCGGGTGCCGGCGCTATGACCCGTACCCGTACCCGCGGAGGGTACGGACC
CGTGAGCACGGGTACGGGTACGCGAAGCGCTGGTCCGGGGGTGGCCAAGCGCCCTCTAGGACACTGCTGCGGT
GTCAAAAATGGTACGGAGAGTATGCGGGAGCCCTGGTATGGCTCCCGCATGCTCGAATTCGTTCCCAAAGCGC
TTTTTTGGGCGCTTTGGCCGTACCCGCAGGGTTCACGGGTACTAGCACAACGACCCGTACCCGTACCCTCACG
GGTAAGGGTACCCAACCCGCGCGGGTTACCCTTACCCGTGCAAAGCACTA
>Cersu1_Cluster42#LTR/Gypsy
GTGACGATCACGCGTTCTGTCCTGGAGACTGACCGCGCGCTTACACAGATTTGCCGTGACGAGACCTACAATG
AGGCCTCGTCAGCAACCGTTCCTGGATTCTGACACAGCACATCGATGGTGCTGATTTCGTAGATAGCGACTCA
CCGTAGTATTTAGGTAGCTAGAGCTCCCCATGTACAGCAGAGTCGCTTTTCCCCCGTTCGAATCGAGTGTTTA
CGCAGGTTCTGAGCTGGCGTGACGAGTGACTTTCTTGTGACGCGACTTCTCTGACCATTCAGAGCCCGACGTT
ACATATTTTGAACCTTACTCTCGCTTGATTTTCTCATTCTCTCTCTCTGGGCGACGTCTCGCGGCTTGATCAC
CCGTTCGACATTCGCCAACACGACCTTGAATTCGGCATGCCTACAAGACGAGGTACCGAATTCCGGATGGACT
CCAAGTCCAACCGTGACGAGATGGATGCCACTCTCGGCACCTCGACCGGTAATGCCGTAGCTTTGGAACAGAC
GACAGGTGAGTCCTCTGTCTCTGACCTATCGGCACTGACCTCCGTGGGGACGGGTGAGTCTCCCGCGGATTTG
GTCATAGAACGGCCGGCTACGCCGGTGTCCCAAATGGACAAGATACCACGCTCCCGCTCATTTGCGGATGTCG
TGCGAAGCCCCCGCGACCTGCTCCGTGCCGTCGCCAAGGCAGACTCGCGCTCCCGTCTCGCAAATACGGATGT
CGTTGAAAGCTCCCGCTCCCGTCTAAACACGGATGTCGGTGATGACCGCTCCCTCTCTCAGGAGGATGTCGGT

GAAGGCCGCTCCCTCGTAAATGAGGATGTCGGTGAAGCCCGCTCCCTCGCTAATGAGGTTGTCGGAGAAGCCC
GCTCCCTCATAAATGAGGATGTCGGTGATGACGGGTCCCTTTTCCCGAAGGACGTCAATACGGAAGAGCGCTC
CCTTTCGCATAAGGATGCCGCGGTAGTATCCCCGAAAGATACGATGGGTAGTCCCCCGGCAGGGAACTCGGTT
GATGGCCAGGACGAGATAACTAGTAATCAGCTGCCAGGGCCGGAAGGACCTGGGACCACTGAGTCTGGGCCGG
CGGAAGGATCGGTAACATTTGCATCAAGGGACAGCCTCTCTCTCGAGGGAGCACCTAACGAGTTATCTGACGG
CTTTCAGCCGGCTCGGAAGACTGTCCCCGTTGTCAGTAGTATCTCTAATTACCAGAATAAAAATACGGATCGG
AAGTACTTTAGTGATTGGTTTGATCGGTCGGATGATAACGACGAAGAATTCATCTCAGCACAGCGAGGCCTCT
CGCAAAGATGGGCCGACATGGTTGATGATGACGATCAGAATGACATCGGGGAGATACCAGCTGGCTGGCAAAA
GCCAGCGACTCCTCGCTCAGGCTCCCCGGTCATTTCAACTGGAGTACCGAAATTGGCTGATCTATGGGACGAA
GTAATGACCCTGCTCAGTCCCGAGGCTCGGGAGGTTCTCAAGAAGCGAGCTCAGCAAATGGCTAAGATTATCA
TCAAGACTCCTGATACATCTGAGGCCTCGCCAGAACCTGGAGGATCTCCTATACAAGGATCTATGGAAACTGT
CCTCCCAAGTATCCCGGAGCTCACAGAACATCCCGCTGAGAACAATTCCGACTCGGATGGACTAGCAGGGCGC
ATCCCGGCTGCACTGAAGGGTAAAGGCCGGATGGGGTCAGTCATGAAGGCAAAGCAGCCGGAAAAGCCTATCT
CCTCGCTCGAAGAGAGTCAGGAGACACTCTCCCGGCCAATCCAAGTTAAGCTCGACGAGGAGATAGCTCAGCA
TTTGCACGAGCAGTTGAATGGTCCAATGAACGACCTGTATGTTGGTCAGTTTACACCAGAACAGCCGGAGACA
AGGGGGTTAAAGCAGGATGTTAAACCGGCTTACCTCGATTTGTCTGGCAACTGGAAAGCCTATCAACCTAAGG
AGTCTCGGGTGCCTCAATCAATTGCGTCCATAGTACATGCTATCCCTTGTGCAGCATCAACAACAGGGCTTCC
TGCAAGGAAGAAGAACCCTACTAAGCTGAAAGTACATACCTCTCGGGCTACCAAGGACAGAGTTCCTCGGGCA
TCCTCTCAACTTCCCCGAGACAGTGTCTTGAGAGACTTCTTGGACCCTTCGGAGTCGGACCCATCCTCAAATA
GCTCAGATAGTAACTCAGAGCAGTCGGGATCTGCAGGTTCTGGGCCCGGCAGCGAGTCTGAATCAACGGTGTC
AGACTGGAGTTCCATCGGGTCAGAGCCATCAGAGGTGGAGTCAGATGACTCACGAGGTACTAGGCAGCGTAAA
AAGACCGCGCGAAAACAATGGAAGAAGAAATTGTTAGCGCTACGTCTTGAGCAATCCACAGCGAAGCCAGACC
CACCATTCGTGTATACAGGAGAGCCCAACTATGAAACCTTCCAGAAATGGGTTCTGGAGGCTAAGGATTGGCT
CCGCTTGAGTTATATCCGCCGGCAGAATCGAGTCGCTCGGTTGAAGAAATACCTGAGTGGGCGAGCATTTACA
TTCTACATGAGAGACGTGGCTCGCAAACCCCATGAGTGGACACTCACCAAGTTTCTGGACTCCCTATTCAACC
ATTGCTTCCCGACCAATTTCCGTGAAATACAGCGTACTAGGTATAGGGCCTATAAGCAGAATGGTCATCCAAT
TCGGGACTATCAGCGTGGATTGGAGCGACTTGCACAGAGCGTCGGCCGGATTCGTCACCGAGACTTTATCTTG
CAGTTTTGGGAAGGGGCCGACCGTCGAATGCGGGCGCATTGGGCACGCGAGGGTCTCAGTGGTGAACGTAGCA
GCTTGACGAAGTTGGTGTCATATGCGGAACGATACGAGAACGGCCTGGCTATGGAAAAAATTGAGCTGGGTCG
TTCAGAAAGGACACCCGAGAAGCCGTACCTGAAGTCTTCTCAACTGGAGGGCGTAAAACGTGAGAATTATTCA
CAGAGTGGAAGACCTCGGCCAGATGGGCCTCCAGGTGACAGTCATACCAAGAGCCGCAGCAGTGGTACTGAGT
TTCGGTCGAAGAGTACGAGACCAAAGGAACAAACAAGTCAGACACACACGGCATCACATGGTCATAAAACCAA
GTTGACCCCGGCACAGAAACAGGAGTACCGAGCACAAGGGAAATGTTTTGACTGTGCTGAGGTTGGACACCTG
AGTAAGGACTGTCCTCGGAGGAATTCTGCTCGGCCAGGTGCCAGTTTACGCGCAGTGACGGTCGATTTCGAGG
AAGTCGAGCGCCTGCGGGCACTTCGAGACGCTGACACGTTGGGTCTTATGGCGGTTACCTTTGGTTCGTTTTC
CAATACTACCACCTGGGGCGATTCCTCTGGCAATCCCGACTCGCGCTCTGTAACAATCACACACATTCTGGCT
CAACTACACTCTCTGGTGCCTTTCGTGTTCGATGACCACAACGATCCAGACAATTCTCCCTTCGCGACAGACC
GGTTCTCGGTTCTGCCTCACGGGGATACTGACTTCTTGATTGTTGATTCTCATCTTGACGACATGGTGGTTCT
GAGACAATCTGACCTGGCTGATGCAGGATTTGATTTTGCTGGTTGGCTCTTCACGGAGAAGTGTAATTATACG
GGCAGTGGCGATATTGAACACCCATCACGAGTGCCTCCTCCGGGCTACTTTCATCTGATAGAGCCTGACGCAG
GCAACCCCGAGGAGGAGGAGGCATTTAATGAGATGTTCCAACATATTCGGGACATTCTCCTGATGGGCATCCC
CTACTCCTTTGACTCTAACGAGGAGGATGACTTGTCAGATGTTTATTGCCCGGATCGTTTCGACATTGAGCGA
AATGCCCGACGCAATATCCTAATTACAGATCATCTGGTTCGTCAGCAGTATTCATTGCCGTTGCACATGGCGA
TGGATCCCGAGCTTGATTTACAGGATTGGTTAGAGCGTTCTTACCTCCGGGTGTCGCGTGCTCGAGCTGCACT
CGACTCGTCTGAAGGAGAGAATTTCGACACAGATGATGACTCGGACTCATCGTGTTCCTCCTCATCCTCATCC
GAGGGTGACGAGGATCACGGGGAAACTGTACTGGCGGCTGTATCCATGGTGCAAAACAAGGAGCTGTCAACGG
AGTCTATGGTTCTGTCTGTAGCAGGAGTATCGAACCGAAAGGCTTCAGATACTGCACCGGCTCGAGCAATTGA
TCGTACAGCGGCTAAGGTGAAGGATTTCCCTCGCCTGCTGCCGAAGCCAATTGTGGTACGGGTGCATATTGAT
GGTGCACCCGTGCGAGCACTAATCGACTCAGGGTCGTTGGCGGATTTTATGTCCACCTCCCTGGTCGATCAGC
TAAAGTTAACTACGGATGTGTTGGCAAAGCCGTTACCTGTGCAGCTCGCAGTAGCAGGTTCTCGATCTAAAGT
CAACCATTCAGTATCAGTGAAATTTGAGTATCAAGAAATTACAGAATCTCGTCGGTTCGATGTCATCAATCTT
GACAACTACGATCTTATTCTTGGCACGCCATTTATCTTTCAGCACAAGGTGCTTCTCGGGCTTAACCCGTCTC
GAGTTATTGTAGGAAGCAATGTTTCGCTTCCGATTCAAGGTGATCAGGCAACCACACTGTCCTCTCTGACAGT
CGACATTTATGATGATCGTATTGCGACACTGCGAGCTCAGCTAGTGGACTATGCAGCGGATATCTGCAAGAAT
GCTTTGGAAACTCCACTGCCTCCATTGCGTGATATTAATCACACTATTCCGCTAATTGACGAAAACAAGATAT
ATTCCTGGCGACCATCTCGTTGCCCAGAGGCATTAAAGCCGCTGTGGCGCGAAAAACGAGAGGCATATTGCCG
TACCGGACGATGGGAATTCCGGAGTGGTGTGAATGCCATGCCCATGTTAATGCTGAAGAAGCCCGACAAGGGC
GATGGTATTCTTCGCATGCGTTCGGCTGTGGATTGCAGAGAAAGAAATGCGAACACAAGGAAGCTTGCCTCTC

CTCTGCCAGATATCGAGGGCATTCTTCGGAATGCGGCCGCGAAGAGGTATAGGAGCTTGATGGACAGCAAGGA
AGCTTTCGAGCAAATTCGGGTGGAACCTGAGCACGTCTCGCGGACACTGTTCAATACTCCCGACGGTACCATG
GTCAGTCATGTCATGCAAATGGGTGACTGTAATGGGCCGGCTACCTATCAAGCCCTAATGAACCATATATTTG
CACAGCACCTTGGGGTATTCATGGACGTGTACTTAGATGATGTTATTATCTATTCCGACACAGCGGAGGACCA
CGTGCGACACGTTAAGCTTGTCATTAACATTCTGCGACGTGAGCATTTCTTTCTCAGTGCACACAAGCTTCAT
TTCTTTGCGAAGGAATTGAAGATTCTTGGTCATATCATCGATGATAATGGCATTGCAATGGATCCTGAGAAGG
TTGATCGCGTGGCACACTGGAAGCCTCCGACGACTCGGGAACTTCTAATGGGATTTCTTGGAGCGGTTGGGTT
TCTGGCTCCGGATTGTGAAGGCATTCGGATCCCTATGGGTCACCTTGCTCGTCTCACGGGAACGAAGATCCCG
TGGAGATGGGGACCAAGTGAGCAGCGTGCATTTGACGAAGTGAAATCTATCGTTCAGCGACACCGCGAGCATC
ACAGAGTGACTATTGACTACTCTCCGGAGTCCCCACCTATCAATCTGGTTACCGACGCGTCGCTGACAGGGGC
CAGTGGTGTCCTCAGCCAGGGCGTGGACGTTTCCTCGGCACGGATAGTTGCATTCTGGAGTGGCAAATTTAAC
TCTGCACAACAGAATTATCCGGTGCACGAGCAGGAACTGCTCGCAATTGTTGAATCCATGAAGCGCTTCCGGG
ATCAGCTTCACGGTGTTCGTTTTCGTGTTTTGACGGACCACAAGGGCCTGGAATACATCTTGACTCAACGGAA
ATTGTCACCACGACAGGTTCGATGGCTGGAGGTGGTTTCTGAATTTGACTTCGAGATCGTACATATCCCGGGA
AAGGAGAATGTGGTAGCAGACGCGCTTTCCCGTATGTATAGTAATGACGCTCAGGGTACAGTTCGAGCTCTCA
GTGAGTTCGTGCAGACAGACCGTGATCAACCAGCACGGTTGATTCACGCAGTTGCACAGTCTATTTCACGGCC
ACTATTTGTAGGTGCATTGGCCACTGCAAGTAGCCCACCTGGATTGGGTAAGGTCACTTCACGGAAGGCTCCG
CGGGTTCACTTGGTTGTACGTGATCCGAGCTCTGGGGCTATTGTGTCGTCTGGAGGGCGTAGGGTAAATGAAG
AGGTCAGAGTGCCAGCACAGACTACATCATCTGACAATGTGCCTTTACTCGATGCCGACACTGCTTTCTCTGT
GAGCCATGAGGTTCCTCAAGATACGGATGAGGAGTGGCCAGCAGTACAAACTCCTGAGGAGGAACTGGAGGCT
GTTGCGACCCCGCAACTCGTTCCGTTAATTGCGCTGGGTAACCCAGGTTTGGATCTGCCGTCTGGGATACAGG
GCAAATACGGAATGGACCCTTTCTTCAAATTGATTCAGGACAATCCTTCTCACTACAAGAATTTTGAGTGCCA
CGAGGGGCTGGTCTACTTGAAGGACCTGGAACGTCGCATCCTATGTATCCCGGATGTCCTTATTGGGTCGCGT
CGTGCTCGTGAGATTGTTATTTCACATGCGCATTCCATCTTGGCACATCTCGGAGCCAAAAAGACATTGTATT
ACTTAAAGGATAATGTCTGGTGGAAAGACATGGTCGCGGACGTACGAGCTTATTGTGAGTCATGTGCAGTGTG
TGCGACAACTAAGTCCTCAAATCAGAAGCCGTATGGACTCCTCCGACCTCTTCCTGTACCAGGATCTCCCTGG
GAAGTTGTCGGTATTGATTTCGTGGGACCTCTGCCAGAATCAAAGACAAGGCAAGGTACATTTGACATGGTCA
CCGTGGTGATTGACCATTTTAGTGGTATGGTCCATCTAGTGCCCTCAAGACAGACATATCGAGCGAAAGACGT
AGCGGAGCTGGTGTTTGAGCATGTCTACAAGTTGCATGGTCTACCGCTTGCTATTGTCAGTGACCGGGACTCG
CTGTTCACTAGTATTTTCTGGGACCGGTTACATAAGCTCATTGGGGTGCAGCTGCGAATGTCCTCCGCATATC
ATCCTCAGTCGGATGGCTTGACGGAACGGTCTCACCGTACATCTGAACAGATGCTTCGTCAGTGTATCGCGCC
CAATCAACGTGACTGGGCGACTAAATTACCCGCGATTGAGTTCGCAATGAACTCTGCCCGCTCGGAGACAACA
GGTTTCAGCCCGTTCTTTCTGAACTCGGGACGAATGCCACGACCCATGGTTTGGGATGCTCGTTCAGAGTACC
CTGGGGTACGTGTATTTGCGCAGCGAATGAAAGACGCTATTCTCGCTGCGCATGACTCCATTATCAACGCCCG
TGTTAAGCAGGTTAAGCACGCAAATAAACGGCGTCGTGCTGTGCCATTCTCGGAGGGAGACTTGGTTTATCTG
TCTACACAGAATCTCCGCCTTCCAAAGAACCGGGCTCGGAAGCTTGCACCAAAGTTCCTTGGGCCATTTCGGA
TTCTCAAAGACTATGGTAACGAGACATTCAAATTGGATCTCCCCACGGAGCTCCGGCAGAAGGGGATACACCC
CAATTTTCACGCCTCGCTCCTCCGAATCCATCACCCAAATGATGACCGTCGTTTTCCCGGTCGACAATTCACG
CAGCTAACTGGATTTGGTGATAGCTCAGAAGAGTGGGCTGTGGATAAGATCGTATCGCACGCAGGATCAGGCA
CAAATAGTCTGTTTGAGGTACGATGGAAGGCGGGTGACACCGCATGGAGGGCGTATCAGGAAATCGCGCACTT
GGAGTGCTTGCGATCGTATCTGGAGGCGTTTGGCATTAATTCGATCACACAGTTACCGGTCGGAACTGGCACG
CCCCCGGAGGACCCACAGATACACGTTGGTGCTATGACATTGGTCCCGAGCGACATATATAAAGGGCGTGGGG
AGCAAGGACACCGCTGGAGTTCTGCGCGCAGCCTTCGCTTCACTATGCCTACCACCGCTTGGACACCCGCTCA
TCTTCGTAGTTTTGAAGCATATGCTACTGGCATTCGACGTGGCGACCGTGACTTGGTTGGCGATCAGCCTGTT
CGCGCACCATCAGGCTACGTCGATTGGGTCAGAGAACAGTACCTTGGTTCCGCGCGAGGACAAGAGCTCCCTC
CGTTCCCTCCCTACTATACTGGCGACATTCCGCACCGCGGCATCCCGGTTCCCGAAGGACGCCGCCTGGAGGA
CAAGGTTGGAGATGCCTATTTGGGTCTCACGGAACGCCTGTTTGGGGCCTTCATGAATTCGCCATTCTTGGCT
GGCCGGTCGTCCGCTCGGGGTAGGGGCTTCACGCGCTCTCGAGGACGCGGACGGTCTAGTTTCCCATACCGTG
GGCCACCCCACCGTACGGAGGACGACATATACATCCCCTCCTACAGTAGGCGCAGTGAGCGTCATCGCGCTAG
GGACGACGCTCCCCCTCGCTACCGCGAGTCCCACAATCACCGCTCACATCCCCACCGTTCATGCGACGGACCT
GTGCCACGCGAGGCTCGTCAGGAGGATTCCGAGGATGTTGATATGGCGGACGCAGTGGAGGACGAACCTCATC
CTGTCCATAACCCGGAAGTCCGGCCGGAGGAATTGCTTCAGGTGATGAGGGAGCAGTTTGGCAACGACCTGGT
TCTTCGTGTCGCGGCGCGGGCCCTTGAAGAGGAACCGCAGAGCGCCACCAGGGAGACCGAACTTCAGGCTGAG
GTGACTTCGCGAGAGTCACCTGCGGAGTCCTGTGCATCTGGCTCTCGCACCTCAACTCCATCCTCAGTTCACG
AACTCGCCTCGGCCCTGGAGACTCAGTTGCGTACGACGGTGGAGGAGGCCGAGGATAGTGACGCAGCCGGTGA
ACCGGACCCGGATGTGGCAGTGCCTGGGGGCGCTGCTTCCAACGACGCGTTGAACGCGTCTGCTTGACTTTCG
ACTTGGTACGTGCGCAGTTTCTCGCAGCCTCGTAACTGCGGGAGGGCGTATGTGACGATCACGCGTTCTGTCC
TGGAGACTGACCGCGCGCTTACACAGATTTGCCGTGACGAGACCTACAATGAGGCCTCGTCAGCAACCGTTCC

TGGATTCTGACACAGCACATCGATGGTGCTGATTTCGTAGATAGCGACTCACCGTAGTATTTAGGTAGCTAGA
GCTCCCCATGTACAGCAGAGTCGCTTTTCCCCCGTTCGAATCGAGTGTTTACGCAGGTTCTGAGCTGGCGTGA
CGAGTGACTTTCTTGTGACGCGACTTCTCTGACCATTCAGAGCCCGACGTTACA
>Cersu1_rnd-1_family-86#TIR/MITE
AATAGAGCTTATTTGCTGGCACCTAGGTGAGCGATTTTCTGAACCCCAAGCGCGCCACCTCCCGCACTTTTGC
CGAGAGACAAGTGGAGCTCAATTGGTAATTCAGAACTGGCACCTAGGTGCCAGCAAATAACCTCTAAT
>Cersu1_rnd-1_family-12#TIR/MITE
CATCAGGAAAATNTCCATTTGACTTATAGCGTCTTATTTGCCTTTCGAGGTACTTGTCGGGCGCTAACGAGGC
AGAAAGACGCCGCCAAGCCCCGAAAAGGCGAATAAGACGCGTTAAGNCAACNCGAGATTTTCCTGATG
>Cersu1_Cluster39#LTR/Gypsy
GTAGCGCTCGCGACCAGATAAGGCAAGTCGGTGGCACCACACGAGTGATTTGGGTAAGAGCGTCGAACGTTGA
GTAACAGGAGTGAAGACTGATTCTCGTGTACTAGCTAAGGTACAGGGAATACGGAACTCGGTCTTAGTACTCG
ATTCAGCCACTCGGAGCTAACCGACAGACAACTGTATAGTAGCAGACGAGGAGGGAAAAAGGGATAAGAGTAT
ACGACAGAGTGTAGTAAGAAAACAATTGAACGGAATAACTAAGAGCAGGAAGATAAACGATGACACAGGGAGA
GTTCCTATAAAAGGAAGGAGTACGCAACCATACGTAGCGGCCCGTTACATACGCCCTCCGAGTATCGCAAAGC
GGTACTCAATCTACGCGCATAGTAGAATCGCCGGTCCCAGTGTTGTCAACCCCATCTTCTTGAGGGGGGGGGG
CGGCGGCCAATGGGGATGCCTTCCGGGACTTCTTCTTCTTCTCCCGCGGAGGTTGCTCGCGCTTTTTGCCCTT
GCCGGATGACTGGGTTTCACCAGTCTCGGAAGGGGAAGTGTAATCGATGATACCGTCGTCGCGTGAGTTAGGA
TCCTCAGCGCCGGGAGGAGGAGGAGTGGGAGAGAAGTGATGAGTCGCTCGCGCCGAAGGAACTGGAGCCCTTC
GAGGTTGTGACGGGCCCGCAATGGGGACAGAGTTAGAACGGCGATCCCTGACCTGGGCTTGGATTCCAGCGAA
GAGGGAGCTGTAGACGTTGGTCTCGTCAGAATGATCGACCTCCGTGCGGCGCCGCTTGCGATCTTTGAAGCCC
TTGGCTGAGGATTCAACCCGGCGCGCCTCTTTCATAACCGTCGACATCATAAGAGCTTTGAACGCGCCTTCCT
CGAACTCGGAGTAGGGTGATACCTCGACTACTTGTCGACGAGGACGAGGGGCGAGCTCGTCGACGGGGATGGG
CTGACCGGCGAAGGTGATAAGGCCGAGCCGGTCGAACGAACAAAACTGATAGGGGCAAGTGGGGTCCTGAGCC
CAAAGACGCTGAAACTGCTCGTAACCAGCGGGGATAGGGCAACTAGCGAGGTTCGCTCCGCCAGAGCGTAAGG
ACCGGTCGAATTCGGCGTAAGCGCGCAGCTGGGCCGTGGTGTACAAATAGCGACGGCCGGAGCGAGAGCTGCG
GTCGGTTAGAAGCATATCCTCGTTAGGGAGACGCTTCACGCAGGAGTGCTTGACGACACCCCGAGCCATATCG
ACGATGGTGGGGATAAGATCCAGGAGGGGGGCGAAAGGCCAGGTGCTAGGGTGAGAAGCGCGTCGAATGAGAC
AGATAGGACAGCGCTTCAAGGCTGCATCAGGAAAACATCGCGTTGACTTTGACGCCTTCTACCTGCCTTTCGA
GGCTTGCTGGAGTCTTCGCTGCCCTGTTGCGCGCCAAAAAGGCGTCAAAGTCGCGTCCAAGAACCAAGAAGGC
AGGCAAGTAAAATGGACATTTTCCTGATGTGGGAAGCGAGGATTGGGGATGCGACGAGGTGACGACAAAGACG
AGGAGATAGGCCGTCGGCAGAAGGTGCAGACAGGGATTGGGGGACCAGGGGGGAGTTTTGCGAATGAGGAGGA
CTTGAGATACTTGGGCGGCTGGGGCGGTAGTTGACGCTGTTGCCTAGCCGGACGCTTCCCCTTATATGAGAAG
TCGTCGGCCTCCAAATGAGATATGCATCCGAGGAAGATCTGAGGGTCGTCTTCTGGCGGCATACCCGATCCGT
CGCGGAGTTGCTCGATGCGGTCTATCCCCAGAGCTTCTAAATAGGCTCTAAGTACGTCCAGATGGGCGATCGT
GGAGTAGGGTACCCAAGTATGGTCACCCGACTTCCACACAGCCTCAAATATCGCCCCTCCGCGTTCCCCTTTG
TGGCTGATCAATTTGTCGATGGCCCACTCATTGTCCCGATCTTCAAGGTCGAAGACCTGCGTTTCCATGCGTC
CCGGAAACAGCCTGTCATCATTCGGCTCATGTACCCTCAGAAGGGAAGCATGAAAGACGTCGTGGATGCCACG
TTTGCGCAGCCCCTTAGGTAGATCTAATCTGTAAGAGTAGTTACCGAAGTCGTCTAAGACTTTGTAAGGGCCA
ATAAATTTTGGTGCCAGCTTCCGGGCGTAACCCTTAGGCAGCGATAAATTCTTCGTTGACAAGTAGACAAGGT
CACCCTTTTCGAAGGGAGCAGGGCGCCGGCGTTTGTTAGCATCCCTTGACTGTTTCACCCTGGCGGCCAAGAG
GCTATCATGTGCCGCCATGATAGAATTTTTCATTCTCTGGGCGAAAGCCCTCACTCCGGGGTATTCATCCTGT
CTACCGTCCTCCCATACCATAACACGAGGCATACGCCCTGTGTTTAGGAAGAAGGGTGCGAAGCCTGTGCTTT
CCGACCTGGCCAGATTAATGGCGAACTCTATCGCTGGTAGTTTTGAAACCCAATCCTTTTGTTCCGGCGATAC
ACATTGCCGGAGCATCTGAGTCACGGTCCGATTGGCTCGCTCAGTAGAGCCGTCAGATTCCGGGTGGTAGGCA
CTGGACATGCGCAATTCCACGCCGATGAGTTTATGCAGGTGAGACCAGAATAGACTCGAAAAGAGAGTATCCC
TATCGCTCACGATGTATTTCGGCAGTCCGTGGTGTTTGTACACCTCTGCGAATATCAGCTCGGCCACTTGGCG
TGCCGAGTAGTTAGTTCGGCTTGGTACCAAATGTACCATTCCGCTGAGGAGATCGATAATTACTGTGATGGCA
TCGAATGAACCATCCCGGTTCTTAGATTCGGGTAGGGGCCCGACAAAGTCAACCCCTATGGCTTCCCACGGAG
TACCGGGCACCGAAAGAGGATTCAGCAGCCCATACGGCTTTTGGTTATTCGGTTTGCTCCGCTTGCAGGTCCT
GCAAGTGTCGCAGTACTTCTGCACATCCGATGACATGGTCTTCCACCATACATGATCCCTCAGGAGGTGTAGG
GTTTTGTAAGCACCTAGGTGGGCTAACAGCGAATGAGCGTGCGTGATGAGGACCTCACGGATGCTCCTGTCTT
CCCATTTTGCATCGGGAATACATAGTAGTTCCCGTTCTTTGTCACGGATGTACACTAATCCATCCGCCACGCG
GAAATTCTTGTATTGTTTCGGTTTTACGATAATATCCGCGAAAAACTTGTCTAGTTTGAATCTATCGCGGATA
AGGGAGATCAGATCTTGTCCTCCCGACACGTCGGTTAGGATATTGACGAATTGCCCGGCGAGCGCCTCCGGCG
CCTGCTGGTTTGTCTCCGTATCCAGATTAGGACCACCTTCCCCTTTGGGAAGGTCTTCTACCACTTTCCCAGG
CACCGCCCTCGTGTCTGGTTCGAGTTTGCTGGCGGCGTGTTGTTCTTGTCGCGAACTCTCGCCGCTCACGCCC
TCCTGACGTTGTGCACCCGGAGCGTGCAGAACGACACGACGAATCCGTTTAGAGAACTCCCGCGCCGTCTCCG

CGCGCCCAGACTCGGCTGATGCGCCCGCGTTCCTCGAACCGCGCGCACGACTCCCGCGCGACGACGCCGCAGG
CTCAGCGGAAGGGCGCCGCACGCCCTCCGAGCCGGTGTCACCATCGCCTCGCGAGCGGGTAGCACCCCTCAAG
GGTCCCACCTCACCCCGCTGGCCCAATGACTCCGCCGTCCTGGCCGAGCGGCGCAAAGCCCCGGGCTGAGCCG
CCATCGCCTCCAGCCCAACCAGCACCGGCAGAGAGACCGCTGCGGCCTCTAGGTAAGTACGCAAACCCTCATC
GTCGTCGAACACGGTGTATTCACTCGGCGCACGCACCGTCCCAGGTCCGTCGTTCGAGTAGATCCGGGACAGG
GCATCCGCGAGCACGTTCTCAACCCCCGGAACGTGCTCGACGCCAAAGTCGAATTCAGATATCTTCTCCAGCC
ATCTCGCCTGGCGGCCTGACAAGTTCCGCTGGTTGAGGAGGTGGACGAGACCTTTATGATCCGTCACCCATGT
AAACCGACACCCCAGCAGCACGTCCCGATGCCGCTGCATGGCCTCGACCCCTGCCAGCATCTCGATCTCGTGA
ACGGGATAGTTCATTTGCGCTGACGACAGTTTAGCGGAGAAGAAAGCCGCCACCCGGGCGTTGCGAAAATCGG
GACCCTGAGCCACGACCCCCGCAATACCCCCGTGCGATCCGTCCGTCACCAGCCAGATCGGAGGCGCACCCGA
GCCATAGTCCAAGGGTACCCGCCGGTGCTCTCGGTGAGCGTGTACCAACCGCTTGATCTCATCAAAAGCCCGC
TGATGGATGAAATCCCATTTGAAGCTTGCGTCAGCCCCAGTCAGACCCGTGAGAATGCCCATAGGGATGCGGA
CGGTGGCAATGTCGTCAGCCAGATACCCGACAGAGCCCAAGAATCCTCGCAAGAGCTCTTTATTAGTCGGCAC
CTTCCAGGTGAGCACAGAATCCACCTTATTTGGGTCCATTCTGATGCCCTCGTTGTCCACAACCCGCCCAAGA
ACCTTCATCTCGGAGCACAAAAAGTGGAGTTTACTCGTGCTGAGGTAAAGTTCTTGCTCACGGAGGATGTCGA
TCACCTGCTTAACATGCTGTATGTGTTCCTCGCAGGTGTCGGAGTAGATAACGATATCGTCGAGGTAAACATC
CATGAAAACCCCAATAAATGGCCCGAAGACATGATTCATCAGCGATTGGTAGGTCGCCACGGCGTTGCAATCC
CCTTGCTGCAAAACCAAGCTAACCATGTTACCGTCAGGCGTCGTCATAGCCGTGCGCTCAACGTGATCGGGCT
CTATCCGGATCTGCTCGTACGCGTCTTTACCATCCACTAGGGACCGGTATGGCTTCCGAGCCACGCGCCGAAG
AATAGCCTCCATATCCGGCAGGGGAGAAGTCACTTTATGCGTATTCGCGTTGCGCTCCCGCAAGTCACACACC
ACCCGCAACTTGGGCGGCACGCCCTCCGTGCCCGTTCCTGGTTTCTTAAGCAGCAACATAGGAGCGGTGTTCT
TCGCTGCGGACATAAGCCACCTGCCAGACTTCAAATAGGCATCACGCTTCTCGGCCCACGCTGATCGCAATGC
ATCGGGGCACTTCGACGGTCGCCATGAATATACCTTCCCCTCGTCCTTCAGGGGTATGGTGTGGTTTATAGCC
CGGAGTGGAGGCAGAGGCGAATCACTGGCATCCTTGCAGATGGGTGCCGCGTACCGCCTCAATTCCTCCCTGA
CCTCGTCGAGCCGCTCCCCGAACAAATCTGCCGCCCGCGACTCCAAGACGCGAACGTTTTTGCCACTGATGGC
GAGAGACTCCTGTGAACCAACGATCACTTTCGTAGGGTTCAGACCGAGCAACACCTGGTGCTGGAACAGGAAC
GGCGTCCCCAAGATCAGGTCATAGTTCAACAGATTTACGATGTCGAAATACCGGGACTCGCGGACCCCCTGAT
AGTCCACCTCAGCGGTGCATCCCAGATTGATCTTCGCTCTCGAGCCCTGCACGGCCAGGTGCACCGGGAGCGG
TTTAGCGAGCTCAAAAGTCCGTATGCCCAATTGGTGAGCCAATTTGGCCGACATAAAATCCGCCAGAGAGCCG
GAGTCTAACAACGCGCGAGCAGGCTCCCCATTGACACGGACGACCACGACCACGGGTTCAGGCACCAGTCGCT
TGAAATCCCTAGGCGTGGCCGCATTTCTTTGTAGTGCGGGGTAAGCCTCCGGTGCTGCGATTCGGATCGCAGA
CAACTCGATGCCACTAGCCAGCTGTCCCGGACTCCCCAATGCGCCCGAAAACAAGAGCCCAAGCTCCCCGTCT
AATTCGTCCACCGTCCACGGTGCACGATCCAGCCGCATGCGAGTCATTCTAGCATAGAAATTCATAGCGTCGA
ACCTTGGTGTGAGGACTAGGCCGAGGGGTAACTCCGAGTCCAGACAAAGATAGGCGTCCCAGACCCGGACTGC
TTCTCCGTCACGTCGACACACGAAACGCTGGGAGCTGCCACCCGCCGTCACCCATCCACTCGGGGCCGGCTCC
TCCTCGTTCATAACGGCTTCTACGACCCTCGATGCGGCGTCACCCATCCGTCCAACGGAGTCCCCTAGATATT
GATACTGCATCCGGATCTCGGGTTCCACGAGAGACCGGTACCAGGCCAATATATCAAAATCCGGATCACGGAG
CCAGTCACCGTCCAGAAGGACATCGTCCTGCAGGTCACCTCCGACCAGGACATAACGCCGTCCCAGGTCGTCC
ACAGAATACACCGAGCATGACCAATTCGGGTCCCCCTCAGGCCCCTCGCACAGGTGTTTCATCAGTACGGACC
TAGCTTGGGAGGCGAAAACGTCACCCAGCATACAGGCCGGACGCCCCTCCCGTGAACTGGCCGGCAACTCCAG
CGGGCAAGCCGAAGCGGGCGATCGGTGCTCCGAGGCGAGCAGCGTGTCCGTGCCCCAGTCGGTCATCTCGATA
AAGTCGTCCTCGTCGCCCGAGTCAGACACCGACGCCAGCGTTGGCATGCCCTCCGAGTCTGCATCTTTTTCAT
CGTCCGAAGAGCTCCACCCTGAGTCAGAATCGTCGGAGACGGCTACCAGGGCAGGCAGCGGCATCCCAGACTT
CCCCTGACTCTCGCTAGGCGAAGTGCCTCTAGCATATGACAGGGCGCGCGAGTCCGGGGGACCTGCCTCATCG
TCTGTCACGTCCTGCACACTGACGCGGCAATCCCGGGCGCCCCCCAGCTCTATCGAATTCAACTGCAACTCCG
AGCTGGAGACTGTCCGCGATGCGAGCTCCCGTAGCTCCTCGGTATGATCCCGGGCAAACTCGATATTCGAGGA
GTACAACCCCGGGGCTCGGTCCCGTCGTTGGGCTTTCACTTTAGCCCCGTGAAGGCAATTACGCGCAAAGTGA
CCAACCTCCTTGCATAGATAACATTTTCCGTCAGCCGCAAGTTGCGCTTTCTCCTTAGCCGAGAGCTGAGGGA
CATCGGGCCTACGTGCCTCGCTTCTCTCCCCATCCTTGGGGGAGCGCTGCGGCTTGACGCCGCCCCGAGACTC
TCGGCCCGCCGAGGTGCCCTCGCGCTTGTCCTGACCAGATCTCTGGTCTCCCCGGGGCTTGGATTTAGCGTCC
CGGAACTCAGACGGCTTGGCGCCATTGGAGCTAGCGTGCCCCTTCTCAGCCCTCTTATTTGCCCGTTCTTTCG
CCCGGGCCGCCTCTTCCAACACTTCCGCCGCATGCAGCACCGCCTTCCAGGAATCGGTAGCGGGGGACATCTC
CTTGGAAATCAGACCTTCGATAATATAGCCCTTAAATCCTCGCCACAGCTTACGCACTTTCTGCGTCTTCGTG
AAATCATCGACGACGCGGAACATCCGCTGCAGCTCATGCGAGTATTCTCTCACGGACATATTCCCCTGCACGA
AATTGTCCAGCTTATCCCTCATCCGGTCACGGAAATCCGCGGGGAAACAGTAATTGAATAACCCCACAAACAG
CTCATGGAGGGTCCACTCCGAGGGGTTGCCAGACACCACAAGGGTGTAGAAGCTGTAGGCTTTCCCGGCCAGG
AAATGAGACAGAGACGAGGCGTAACGCTTCTTAGATAGACGGTATCCTGCCAAGTATTCCTTAGATTGCTCCA
TGAATTTATAGAACTTTTCGATGTCCGCCTGCCCATTGTAGGTCTCGGGCTCCCGAGGCTTCAGGCGAGGACG

TTTATCCCCGCCCTTCACCTTCGATTTGCGAGAGGAGCCGTCTGCGTCCTCGTCCGAGTTGGTCGAGTAGCCC
TGCGAAGACTCCGAGCTATTGCCATCCGGAGAACCCAAGTCTCCAGTCTCCCTACTGGCTGAACGACGGGGTG
GTCGATGTTTATCAGACTTCAGGTCCCTGAAAGCCCTACCCAAAAACCCGGCAGGCTCCACCTGAGCTATGGG
TTGCAGTGACGAGAAGCGAGGCCGGTTGTCGCGCGCTGAGGGCGGCTTCATCACCGCTTCTACGACGTTATCA
GCCGCACTCCGTCCCGAAACACTGCCGCTGTCTCGGGAGTGCTTCCGCACCCCTTCGGCGCGGGCACCGTCCT
GTTCGTCCGTAATGGCGGAGCGTTCGACCCCGCCGTCTACGTTATGCGGTGGGGGGTTCGTTTTTAGAGCGAC
CACCTTCTCGAACATCAGCCTCAGAGACCTGAGTTCGTCTCGGAGAGCCTCTATCTCGGGCTTATCTCCCGTC
CCGAGGGATTCAACTAAATTTTCATTTTCTGCCATAACCACCGGAGGAGGATACAGTTTATCCTGGGTGTAAT
TATTGAACTCCCTCTGCTGAGCCAGAGGATCCAACTCGGCTGGATCGATATCCAGGGATCCCCAGTTTCTCGC
GTCCACCGCCTTCCCCTTCTCAAAGGAAGACGGGCCTACTACCTGATCCCCCAGCACGGCCTGTGCTATGGTC
TGGGATCTGGCTTTTGAGACGGTCACACTTTCCCCTTCCTCTCGAGAGAGGTGAGGTGGTATGTCTTTGGGTT
CTCTCCAACCATCTCCAGCGCAGCCCGTCGGCTCGCGAAGGTTGGATTTTTCGGCAGCGATATGGGAGAGCCA
ATTCCTTCCGGCCTCTATCACCGCTGACGTGCCCGTATCCGATCGTTCAGAGTGGGCCTCCGAGATGAGGACA
GAGCGGATTATCTCCTCCTCTGCTTCGTCATCTCGGGAGTTCTCCTCAGCCTCAAGGGCCTGAGGGGGTCTCG
GACCAGCGGCGGGGCCGCCGAGCGGCGCCGTTCGCTGTCCGGTGGTGAAGATGTTGGGACGCGCCTCGCCGGG
CGGGTACTCCGACACGCTGTCAAGGGTCTCATCAGATCCTCTGTGCGGAGTAGCGCGGTACTCACCTGGTACC
GACGGCGTCCTCGGTTCCTCGTGTTGGTTAGCTATGTTCCTGGCCATCGAACGAGTGAACATCTTCTTTTTAG
GGTTCTTAGCAGTAGTGCTATCGAGCTCGGTAGAGTTCGTGAGGTTTTTAGGGGGTTTTGTGTGTCGCTTCTT
CACGGATACAACAGGAGTTGATCAGACTCCGTATCTCCAGGAAAAGCGAGCTGAGATTCTTTTTGTAAAGCGA
GAAGTCTTTCGACTGTTAAAAATATGTAGCGCTCGCGACCAGATAAGGCAAGTCGGTGGCACCACACGAGTGA
TTTGGGTAAGAGCGTCGAACGTTGAGTAACAGGAGTGAAGACTGATTCTCGTGTACTAGCTAAGGTACAGGGA
ATACGGAACTCGGTCTTAGTACTCGATTCAGCCACTCGGAGCTAACCGACAGACAACTGTATAGTAGCAGACG
AGGAGGGAAAAAGGGATAAGAGTATACGACAGAGTGTAGTAAGAAAACAATTGAACGGAATAACTAAGAGCAG
GAAGATAAACGATGACACAGGGAGAGTTCCTATAAAAGGAAGGAGTACGCAACCATACGTAGCGGCCCGTTAC
A
>Cersu1_Cluster41#LTR/Copia
TGTCGGAATTCAAGAGCGGCGGCGCCGATCGCGCCGCGCGCACGCTCGCGCCACAGGCGGTGGTGCGCATCCA
AAACGGGTAATCCGCGCCGTCCTAGTTAGTACGTCCGATCCTGACATACGATACTTCCGTGTCCTGTATAGTT
CTTATGTAATTCATTGTCCCTTTTTGCTCGTACTTTTGTAGTAGTCCCTCAGACTTGTTACCGCGTCTGAGGG
TGAGTAGTATACTCTGCGCTACCTAGATACATCCCGCTGACTGCTAGTAGTCTAACGACGTCTCTGTCGATTG
TTCTAGGTACATAAATGATATTAGACTTGTTACCGTGTCTGAGGTCTAATGATGTCTCCGTCGATTGTTCTAG
ACTTGCGCCGCTGTTAGGTTATGAGCCCCACTGCAGCAGAACACGACAACTCCCGTGATGGGATCTCGGGAGT
ACCCCAGTTGAAGGCCAATGGCTCGAATTGGCCTGTCTTCAAGATGCGTATGACGTGGGCTCTGGATGCCAGA
GATTTGCTGGACCACCTCATGGGCAGAGACACCGAACCGACCGATCCCCTTGCCAAGTGGGCACAGGGGAAAG
GACCGCTCCCTTTTGCAATTGAGGAAGTCAAAGAAGGAGAAGAAGTCGCCGTGCCAGCCCACCCCCCGCAGCT
GACACAGAAAGACAAGAAAATCCATGAACAGTATCTCCTGAGACTGAAGGAGTGGCGGAAGTGAGAAGCCAAG
GTGCGCGACCTCTTTGCACGTCACGTGCCCAACACAGTCCTCAGGCGCATGAAGTGCAATGGAGATACCGTAG
CCAAAATGTGGGACTGGATCGTTGGCGAATTCAAAGACAAGACCCCCCTAGTCCAGTCTAATGCCCTCAAAAG
ATTCCAGACCCTTTGCTGCCCCGACAATGGTAACATTGCGAAACACCTTGACCTTCTTGGCAAAATGATAGAA
GAATTGTCGGCAATTGGCGTCAATGTCTCCAACAAGGATTACTCAGCTCAGATTCTGAAATCAGTCCCCTCCC
AGTTTGCCGACTACCTGTCTGGTATGATGAATGCTGGATAGCTCCTTGGCCATGAGATCCCTCCAGATCAAGC
TATCCAGTTCCTCAAGCAGGAATACAAGAGAAAATGTCTCTCAGAAAATACGTCCAGCCATTCAAAGTCCTCC
AAAGACGACAAAGAAGTTGCGTATTCAGCAGACATCGGTAAAACTCGACATGACAACAGGTGAGGGTAAGGAG
CCGGCAATAAGGACGCCGGGAAGAAGGGGCCAATATGTTGGAACTGCAATGGCACGGACCATCGCAAAGACCA
ATGCCTTAGCCCAAAGGACAATGATGAAAAACCGTCCAAGGGGGCACCGCAGAAGAGCTCGGAGACTGCAAAT
ACAGCTGTAGTAGACAATAGTGACCTCGAGTACAGCTGGATGGCCTCTGTAAGTGAGTCAGAGTGCAAATCCA
ACAATGATATGACCTTCTCAACGTATTTCAATGGACCTGAGCCACCCGAGGATAATGATATCCCAAACCTTCA
ACCTGTATCCGACTCCGATAGTGATGAAGACGACTTCCTTGCAGAATTGGATAGTGATGACAATGAACCATTT
GAATCCCTCTATTTCACCACGCCCGCTGAATGGTCAAGCGATGAAGATGACGAGGATGACACCGATGACAAAA
TTGACATTGTCAAAGCCACACTTGTCCATGACACCACATGCCAAGCCCGGGTCAATCTCTATGACTCTGGGGC
AACACGCCACCTGTCCCCCTTCTGGGATAGATTTGTGTCACTCACTAAAATCCCCCCCAAACCCATCAACGCC
GCCAACCAGCAACACTTCCATGCCATCGCCATCGGAGATATGGTAGTTGATATACCGAATGGCTGGAAGACGT
CGTGGCTGCACTTGCGGGACGTTCTATATGCCTCCCAGATAGGGGTGACGCTCGTCTCGGTCAGGTATATTGA
TGCTGCAGGATACTTGGCGCTGTTCGCTAGCAGACACTGCCAGATTCGGAGTAGCTCTGGTGAGCTTGTGAGG
GAGATAGTGTGTGCTGGTGGTCTGTATCGAGTCAAAAATGATGCAAAAGAGATAAACCACGCAGCCCAAGTCA
AAGAAGTGACGCTCAAGGAGCTTCACAGGCGTCTTGGTCACACTGTGCTCGAAGCCTGTCAACAGTCGGTCAG
CCGAGGATTAGTGACAGCGTCAAGCTGGTTGGGAAACAGGACATACTGTTCTGTAAGGCCTGCATTTTCGCAA
AGATCCGCCGCAAGCCCATACCCAGGGAACGCTCAAGCCCTCTTGCAGCCGAAGTCGGGAACAAGGTCCACAC

CGACGTTTAAGGCCCTGCTGACGTTGCCACGTTGGGGGGCAAGAAGTACTTTGTCGGCTTCACCGACGACAAG
AGCCGACTGAGCACCACATACCTTATGCGACACAAATCGGAGGTCAAGCGAATGTACAAGAAGTTTTGCGCCT
GGTTGAAGGCTCGCTTCAAAAAGGCGATACGAACCCTCCAAAGTGATTGTGGAGGGGAGTACCTGGCGGACAA
GCTGCAAGAGATGCTGGAGGAGGACGGCACTCATCACCAGCTGACCATATGCGACACGCCAGAGCAGAACGGT
GTGGCAGAGCGCTTGAACGGGGTGCTCCTTGCACGCGTATGTACGCGCGATGCTACACGATAGCAGGTTACCT
AAATCGCTGTGGGGAGAGGCCCTCATGCATGTGATTTGGCTCAAGAACCGTATGACTACCAAGCCACTAGGCC
CCGAGACCACCCCCTATGAAGCGGTGTTCGGATCGAAGCCAGATATGTTGTTCCTCTGCACTTTTGATTGTGC
GGTGTGGGTGCACGACCCCGAGAGATCCAAGCTTGATGCTAGGGCACGTGAGTGGCAATGGGTGAGTGTCAAT
TTGCAGAGCCAAGGGCATCAACCCTACTGGGTGAAAACGCGAACCGTGACAGTTGAGAGAAGCGTAGTCTTCG
AGCAAAACTCCTCAGGTGTGCTACTCGAGGGGGAGCAGCATACAGAATTGGTTGATCCCATAGAACGCTTCAT
CAAAATAGAGTCAGTCAATGACGCAAACATTGAGCAAGCCACCCCAGACGAGCCCATGAGCCAAGAGCCAGCA
GCATCAGCCAAGTGAGAATCTCGAGTACGGAAACCGAGCGTGTATATTCAATGCATTCTCGACGGCGATAACA
CATCACCCATTCCTGTTGGCATATGAGTCACAGACAAGACAGCAGCACCAGCCACGGATGAATCACAAGCTTC
AGGGGGAGCCTGCAACAAATAGGCCATGGCCGCCGCAGATGCTGAAGGACTCGAGCCCCGCTCACTTGCTAAA
GCGAAGTACTGCCCCGATTGGCTGTGCTGGGAGGAAGCGATTGCCAATGAACTTGGAAGGCTGGAGAAGGCGG
GAACATGGGAGTTGGTCGACAAGCTGATGAACACGAACATAGTAGGGAGCAAATGGGTCTTTGAGATCAAGAA
GAACGCCGCAGGTGAAATCAAGAAGTACAAAGCAAGACTAGTCACCCAAGGATATACACAAGTCCCCGGCATT
AACTACTTCGACTCGGACATGTTTGCGCCGGTAGCAAAGCTGTTGTCGATCTGAACCATTCTCATGCTCGCTG
CTCAAAATGGCTGACAAGAACAAACAAATTCACATGTCACAGCCGCCCGGATACCACGCTCCAAACAGCCAAG
GGAAGGTACTTCATCTCCACAAAGCACTTTACGGCCTGAAGCAAAGCGGGCGCTGCTGGTATCAGACCTTCTG
GAGCATACTCGTTGAAATCGGTCTCGTTCGCTGCGAGGTGGACCATGCTGTATTCTACACCCACTACCCAAAC
AGAGACATTGTAGTTCTTGCAGTGCACGGCAATGACTTGACCCTCGTAGCCAACTTGCTTGCCCTCTTGCTTC
GTATCAAAGAACAGCTGAAGTCTCACCTCCAAATGACTGATCTTGGCGACATCCACTGGCATCTGGGAATTGA
AGTCAAACGGGATACTGACACTTGTATGATATCACTGTCACAGAAGTCCTACATTGACTTGATGCTCGCTCGT
TACGGTCTAGCCGATGCCAAGACAATTTTGACGCCCATGGATACCCACTCACAGCTTAACAAAACCTAATTAC
CTACCACTCCGTGCGAGTTTGCGAAGATGTGCAACGTCCTATACCGGGAGGCTGTGGGATTGTTGATGTATGC
GGCGCTAGGAATGCGCCCAGACATAGTGTATGCAGTTGGCATACTCGCCCGATTCTCTGAGAACCCGGGAAGA
GCCTATTGGGAGGCCGTCAAAAGAGTCTTTCGTTACCTCATGGGAACCAGAGATTGGTAACTCGTTCTCAACA
GAAATGACAATGGCATGGTGGGTTATACCGATGCTGACGGGATGTTGCACAAGGATCGCCACACTATCTCGGG
TTACGCGTTTATGATCGATGAAGGTGCTGTCTCCTGGAGCTCGAAGCACCAAGAGATCATTGCCCTCTCCACT
ACTGAAGCTGAATATGTCACGGCAACCCACACCGCAAAGAAAGCCATATGGTTGCGCTCATTTGTATCTGAGG
TCTTCAAGCCCATGCAAGAGCCGATGCCGATGAAGTCCGACAATCAATCTGCGATAGCTATCACCCAGAACAA
CCAGTTCCACGCTCAGACAAAACATATTCGTGCAATGGGCTATCAAAGACGGCAAGATTTCCCTTGAATATTG
CCCCACTGATGACATGGTGGCAGATACCCTTATCAAGGCCCTGCTTAGTCCAAAAGCAAAACATTTCCCACAT
GTGCTTGGCCTCTGCCCATCTTGAGGGGGAGTGTTGGAATTCAAGAGCGGCGGCGCTGATTGCGCTGCGCACA
CGCTCGTGCCACAACGGTGGTGCACATCCAAAACGGGTAATCCACACCGTCCTAGTTAGTACGTCCGATCCTG
ACATACGATACTTCCGTGTCCTGTATAGGTTCTTATATAATTCATTGTCCCTTTTTGCTCGTACTTTCGTAGT
AGTCCCTCAGACTTGTTACCGCGTCTGAGGGTGAGTAGTATACTCTGCGCTACCTAGATACATCCTGCTGACT
GCTAGTAGTCTAACAACGTCTCCCTCAATTGTTCTAGGTACATAAATGATATTAGACTTGTTACCGCGTCTGA
GGTCTAACGACGTCTCCATCAATTGTTCTAGACTTGCCCCGTTGTTA
>Cersu1_Cluster44#LTR/Gypsy
TGTAATTGCGCTCGGTTACACATCTTGTGCATCCTCCCAGACTCGAACTCTGTCTCTCTGGATTCGGACGTTT
TTTTTCTACGTACATCACATGACTGCTTTCACTTTCCGCTATAACATAACCATGTTCGAAACCTCGTTGCGGT
TCTTTACTTCTTACTATCACCGCAACGAAGGTACGAGACTACGCATATGTGTTCCTCCCCACGTATTGACTTC
TTACTATCACCACAACAAAGATGTCTTGTGCTAGTTCACGTATCAGTTCGATTATGAGTGTTTCCGGCCTTAC
AAGATGGCCAGTTTCCACTTTTTTTGAACTTTGCGCAAGTTCAACTTTGTACACCACTGTAGCTGTTTCCCCG
CCTTTTGCTCTTCACGTCTCGTGAGGAGAACACCAAATTGAAGGCAAACTGTCCCCTTCGCTCTCAATTGAGC
AGGGAAATATCACCGTTGACGCCAGCTGAATCAGAATCCAACCCCAGTGACAACATGAGTGCTTGGAGCAGTC
ATTTCACCGCATATAGTCACCCTACACCATATATATCCACTCCCTGGTTCCAGCTCAGATCCCCTGAGTCTGA
AAATTCCTCCATTGAAGGCTCGAATACAGGTTCAGGGCCAGAAACAACTCACAACTTCCTGGATCCAGTGCCT
GTGCAAGTCTCATCTTTGTCTGTCTATTACTCAGTCCCTCCGTTGATCTCTCCGTCTTCACCCCGAGTATCTC
TGTCACGATCTGTTTCGTCAAACGGCAGCAATCCTCCTCCCAGTAACTCGAGCTCCGCATCTGATTCGTCTGC
CTCTTTGTCCTCCTCTGAATTCCATTTTCATGCTGAGTCTCAAGAAATGTCCGACAAGAATCCTCGCACACTT
TCTATTATCTCTCATCCGAATCCTAAGCTGCCACCAGTGATCTTCAGTGGCAAATTGACCCTTGAGCTAGTAC
TGGACTACGAGAATGCCTGTCGTGTGTTCTTTGCGACCAAGCAAATTCACGACAATGAGCGAGTCAAGTGAAC
CGCTTGTGGCCTCCAAGACACGCTTATGCGTGATTGGTATTTCACCAACCAAGATGCCTTAGACGGGTTGTCT
TGGGACGAATTCACTCGCAAGTTATGTCAGCGCTTCTTGCCAAAAAACTGGGAGGACAAGCTCGTGACCAAGT
TGCTGGGTCATCAACAACGAGAGGACGAAAGCTTTGAAGACTGGGTAGTGAGTATGGAAAAGCTCAACTCCTG

TCTCCGCGGAACCACTTTGCACAAGAATGAAGGCGCGATGCGTGAAGTGTTGACCGCAAACGTGGTGGAAGAA
ATCCGAATGGCGCTCAACGATGATGACTACCAGGGTATAGATGACTACTTGGACTGGAAGAACTCTGTTGTCA
GCTTGGGTAACAAACGGGTTCGTGAACGGCAACAGAGGCTAAATGAAGTCGAGCGGTGTATCAAGGACCTCGA
ACTGATGTTGGCAGCCTCTCGTGTCAAGTCGAATGGACAACCTTCAGGCTTCAAGACCAATACTGGTACCTCC
TCGTCAACAGTGGCTAGTGCTATGAACAAGAAGACCTTCACAAAACTGCCCAAGCTCACGGACAACGAACATC
AGCCCCTGTACGACAACCACGGATGTCTCAAATGTCGCAAGCCCTTTGTGTATCACACCACCCATGAATGCCC
CAACAGGTTCCCGGATGCGACAGGATATAAGGTGTTGACTCAAGTGGACGTTGACAGGATGTGTTCTCAGCAG
AGCAAGTCCCGTACGGTAGCTGCCGTGTCTACCGACGTGGAGAAGGTCGAAAGCATGGCGGTCATAGCAATCA
CGTCTGTGTCGCCTCTCGTGGCAACTACCAGTATCATGGGAAATGGGACGGACTCTGGAGAGGACTCTGACAG
TGATTGTTCTTCTGGAAGGTGCGTGTGTCATATTTGTGCAACTACATCCCCTCCGGCTTCCCGTGTGCCATGT
ACTGATGACGCTGTCGCTGCCCCCCATGTCACAACCTGCCTTCAGATTCTTGGTAGTTCAGTTAGCTGTGGTG
GTGATATGCTAATGATGATCGATACGGGTGCTCCCTCAGTTTTGATTAGGGATGATGTTGTCTCCAAGATGCG
CCTGAAGCGCCTGAAACTTGCTGAGCCAGTCCCATTGGGGAATGCATTGAACTCTAAAGGAGTGATGGCGACA
GAATTTGTCAGATTACGTGTGCGAACCTGTAATTCAGCATGGACTTTGGATACCGTTCCCGCAGTTGTTGTAC
CCTCGCTTTGTGTGCCGGTTATTTTGGGCAACATGTTTTTGTTGCACAACTGGCTCGTGATTGATGTGCGGGG
AGAAATCGTTCTTGACAAGAGAAACAGTTTTGAGCTACTGACCACGTCTTACCTTTCAAATGAGAAAATAATA
CAGAACCTATTACCCCCCAAGGCGCGCCGTCAATTGGCTCGCGAAGAAGCACAACAGGAATCACATTTCCGAT
TGCAGCAACACCGCGCCCTGATCTGGGAGCTGAAGATGGATATCCCTCAAATGCTGGCCTGTAATACAATGAC
CGAGGGTAACACGGTGGCAGCGATTTGCTCGAGCATTGAGCTGCTCGAGTGACTGGCGTTTCTTCAGTCGTTG
AAGGATGAGGACACGACGATGCGTGCCAAATTCACAGACTGACTTCCTGCTGATATTCCTCATGTAGACACGC
TGCCATCGAACGTCTATTACCGCTTCAAATTGCGGGACGCAAACATGTCAATTGCACGCCGTATATATGAGTG
TCCAAAGAAATACCGCGAGGTTTGCAAAGAGATGCTCCAAGAACATCTAGATGCAGGGCACCTGCGTCCATCA
TCAAGCCCTTATGCGTTCCTGGCGTTTTTAATTCCGAAGTCGGATCTGTCAGCGAAGCCTCATTGGGTGAACG
ACTATCGCCAATTGAATGCAAATACCATGCCAGAAGTGCATCTGCTGCCCTCCATTGCGGAGATCCTTAGCAA
CTGCGGAAAAGGGACTATCTGGGCAAAAATAGACATGACTAACACCTTCTTTCAGACATGTGTTCATCCAGAC
AACATTCCCTTGACGGCAGTGACTACTCTGTTCGGGCTCTACGAATGGACAGTAACGCCTCAGGGCTGCCAAA
ATGCTCCCGCGACACATCAACGTCAAATGTTCCAGGCTTTGCGCCCCTACATCGGCTCTATTTGCCATGTGTA
TATGGACGACATCGTTATCTGGTCATAATCGCTTGAGAAACACCGGAAGAATGTGGAGACGGTATTGTCTGCG
CTACATGACCATCAGTTGTTCTGTTTCCTGAAGAAGACTGATCTGTTTTGCGTTGAAATGAGATTTCTAGGTC
ACAAGATCTCTCAGGCCAGAATCGAGGCCGACAACGACAAGGTGGCAAAGATATTGGCGTGGCCTCATCCGTG
TTCGGCAACGGACATACACATATTCCTGGGCCTTGTACGATATATCTCCTCCTTCCTGCCTGCGCTGGTGCAA
CATATCTTGGTATTGAACGACCTCACAACGAAGGATGCGGAACACCATTTCAAATGGACAGACCAACACGATG
CTGTGTTCCAGGCAGTAAAGATGCTGGTGACGTCTCACGAATGTCTGACGGTTATTGATCACCAGAACATGGG
CAATAACCAGATCTTTGTGTCGTGCGATGCCAGCGACTGGTGTACTGGTGCGGTATTGTCTTATGGGAAGACT
CTCGAGAGCGCTCGCCCGGTCGCATTTGACTTGCAACAGTTGAATTCAGCGCAAAAGAACTACCCAGCCCATG
AGAAGGAGCTGCTCGCTATTGTGCGGGCGCTACAAAAGTGGTGAGTAGACTTGCTTGGCGTTCAGTTCACGGT
ATTCACTGACCACCGAACGCTCGAGAATTTCAACACGCAAAAGTTGCTATCGTGAAGGCAGGTGCGCTGGCAG
GAATTGCTCGTGCAGTACGATTTTTTAACATCAAATATATCAAGGGTGAAGACAACTGCGTCGCCGACACCCT
GTCCCGCTTGCCTGCAGAAGATGTGCTGGATATCTCGGTAACGGCGGCACTAGTGCCCCTGTGGTCTGCGACA
CGCGCCCTGCGTGACGTGCACTGGCCCCCCGCCGCCGCAACATGCTTGTGGTCAATGCAGTTACGCGTCTAC
GGGTATCCACGGACCCGCAATGGCTGGAATTGATCCGTAAAGGATATGCCAAGGACGGATGGTGCCGGAAGCT
GCTCGTGTCACTGTCCAATCTTCAAGGTGAAGACGAGGTGGTGGAATCCTTGCTGCATGGTGACCTCAACGGG
AAGCTTCATGCAGGGATTTTGGCACGGGATGGGCTGCTCTACATGGGTAAGCATCTTGTAATTCTGAGAGTAA
CAGATCTGCGGGAGGGTATCTTCAGGTTGTCGCACGACGCCCTGGGCCACTTCATGTTCGACAAGTCGTACGA
ACATATTCGCCACGCGTATTACTGGCCGAAAATGCGCCAGGAGCTCGAGGAGTTATATATTCTATCCTGCAAG
TTGTGTCAATGTAACAAGAGCTCCAACTGCAAACCAATGGGTCCTTTGCATCCGCTACCTGTACCCAATGGAC
GTTGTCACAGTGTGGCCATCAACTTCATCGGCCCTCTACCGCCTGACGGACCGCATAACTGCATCGTGACGAT
GACCGACCATCTCGGGTCAGAGTTTTGTGCCGTTCTAACTCGCACGGACATCACCGCCGAACAATTTGCGGTG
TTGTTCTTTGAGCACTGGTACTGCGAAAATGGAATGCCGGCCGACATTGTTTCCAACCGTGACAAGCTATTTG
TTAGCCAGTTCTGGCGAGCCTTGCACCGCCTCACAGGAGTCAAGCTGAAGCTGTTGTCCGTGTTTCATCCAGA
AACAGATGGTTCGTCTGAACGTTCGAACAGAACACTGATTCAAATGCTGTGGTTTCACATGGAGCGTAATCAA
TGAGGATGGGCGGCGAAACTGCCTTTTGTGAGATTCCAATTTATGAATTCCGTGAATTTGTCTACTGGTTTCT
CCCCTTTCCAGTTGCGTTTCGGCCAATTGCCGCAATTGACACCACCGCTGTTCAGTCAAGACATCGCCTCCGT
GGTGGAAGAGCTCGGGCTCGATGCCAACAAGGCGGCAGATCTTCTGTAACGTATCAAGATGGACGTTATGGAA
GTGCAGGACAACTTGCTGCTTGCGAAGACAGAACAGGCACACCAGGCCAACCGACACCGTGATCGGGATCCAG
TGTTCAGCGTCGGCGACTCTGTGTTATTGTCGACGGTCCACCATTAGCGCGAATATATGCAGCACGGGGATCA
TTGTGTAGCAAAATTCATGGTATGCTATGACGGTCCATACACGGTCACGTGTGCATTCCCTGGGTCCTCCACA
TACGAGCTCGACATACCCGACCACTCCCACGTGTTCCCCACCTACCATGTGTCGTTGCTGAGATGGCATATTG

CAAATGACGTGTCCCTCTTCCCGGGTCACGAACACACCTGTCCTGGCCCACATGTCACCCTCAACGGTCAAGA
AGAATGGCTCGTGGAGAAGATTGTCGACTGCCGATGAGCAGGACACAGCTGGAGATATCTGATACACTGGCAG
GGTTACGGGCCCGAGACCGACATCTGGCTACCTGGCTCTGAAGTGGCCGACTTGGAGGCCCTTGATGTCTACC
TCAAGGAGAATGGTCTCGCAAGTTAGGAGGTGTTTCGCTCTATTCAAGATGGCAGGGAAGAGTGTAACCGTGC
TTGGTTACATATCTTGCGCATCCTCCCAGACTCAAACTCTGTCTCTCCAGATTCGGACAATTTCCTTTTACGT
ACATCACATGACTTCTTTCACTTTCCGCTGTGATATAACCATGTTTGAAACCTCGTTGCGGTTCTTTACTTCT
GACTATCACTGCAACGAAGGTACGAGACTACGCATATGTGTTCCTCCCCATGTATTGACTTCTTACTATCACC
GCAACGAAGACCTCTTGTGCTAGTTCACGTATCAGTTTGATTCTGAGTGTTTCCGGCCTTACAAGGCGGCCGG
TTTCCA
>Cersu1_rnd-1_family-198#TIR/MITE
ACTGGATGACTTTCCGTAAGGATTTGGAGCTCTCTCAGCGACTCTAAATCCCCAGATTCACCTGATGGCCGAG
GTGCCGTCCGCACTTCGAGCTCCGCTGATGGGGAGAGTGCGTCGTAGATGCAGAATCTAGCAAGGGGGTGGGA
CCCCTCTTCCTAGTTCAAAATCGAGCTGTAAACCTCGTAGCCGATCGGCAATCACTACGCTCCAACCAAATTC
GGAGGTTATTCCACATGGTGACTTTCTTTGCAGCTGCACAAACTGGAATTGCCGCAGCTACTCGAGCGTGAAC
ACCCCTCTGAGGCACTCAATTGCTACCATAATGGCTATCTTCCCGTAGCTCATCGTGTGTGCTATAATTTCGT
GTCGCTGTTTTGTGGCATCCCTTCAAAAGAGGTAATGTCGAAACACGGACAACATATCTCAATCTCATACTGC
TACTATCACTGTTCGTGACTTCCAAATATATCAATATCTTCTCAATTACCCTCGTGCACCAAGTCGAATACAA
TTTGAACGTCTTTCAGCAGAAAAGTCGTCTAAGACTGACCCGGGAGATATCGAGTAGTGTGCATCCGGAGAAG
TATCAGATTTAGGACCAAATGCACTACGGATACGGCGCGCGCGGTTCTAAATGCTTTGAAGGCCCGCTCGGCA
GTTTAGAGCGGGCGCTAGGGCTGTAGATCCTTACGGAAAGTCATCCACT
>Cersu1_Cluster45#LTR/Copia
GTCGGAATTCAAGAGCGGCGGCGCCATCGCGTCACGCGCACGCACGCGCCACGGGCGGCGGTGCGCATCCAAA
ACGGGTAATCCGCGTCGTCCTAGTTAGTACGCCCGATCCCGACATACGATACTTCCGTGTGCTGTATAGTTCT
TATGTAATTCACTGTCTCTTTTTTTGTCCGAACTTTCGTAGTAGTCCCTCAGACTTGTTACCGCGTCTGAGGG
TGAGCAGTATATTCCACGCTACCTAGATACATCCCGCTGACTGCTAGTAGTCTAACGACGTCTCCGTCGATTG
TTCTAGGTACATAAATGATACAGACTTGTTACCGCGTCTGAGGTCTAACGACGTCTCCGTCGATTGTTCTAGA
CTTGCGCCGCTGTTAGGTTATGAGCCCCGCTGCTGCAGAACACGACGACCCTCGAGATGGGATCTCGGGGGTA
CCTCAGTTGAAGGCCGATGGCTCGAATTGGCCTGTCTTCAAGACGCGTATGACGTGGGCTCTGGATGCCAGAG
ATTTGCTGGGCCACCTCACAGGCGAAGATGCCGAACCGGTTGACCCCCTTGCAGACTGGGCACAAGGGACGGG
ACCACTCCCTTCTGCTGTCGAAGACGTCGGAGACGGCGAGGAAGAAGTCGTTGCACCTGCACGCCCTCCGCAG
TTGACACAGAAGGACAAAAAGATCCACGAGCAATACATCTTGCGGCTCAAGGAGTGGCGGAAACGCGAAGCAA
AAGCGCGCGATCTCTTAGCGCGTCGCGTGCCTGACACCGTCCTGAGACGCATGAAGCGCAACGGGGACACCGT
AGCCGAGATGTGGGACTGGATCACTGGCGAATTCGAAGACAAAACCCCCCTGGTTCAGTCTAACGCCTTAGAA
CGATTCCAGACCCTTCGATGCTCCGATGGAGGCAACGTAGCAAAACACCTTGATGTGCTCGGCGAGATGGTGG
AAGAGTTATCCGCTATCGGAGTCGACGTCTCCGATAAAGACTATTCGGCTCAGATCCTCAAGTCCGTCCCGTC
GCAATTCGCCGACTACCTATCCGGCATGATGAACGCTGGACGCCTTCTCGGTCATGAGATTTCCCCCGACCAA
GCAATTCAATTCTTGAAGCAGGATTACGAGAGAAAGCGACTCTCATCGAGCGTGTCAGGCCGTTCGAAGCCCG
CCAAAGAAGAGAAGGAGGCCGCGTACTCAGCCGACGTAGGATGCCCGCGGCGAGACAACAGACGGGGGAGAGG
ATCGGGATCCAACCAGAAGGGAGCCGCCAGGAAGGGACCCATCTGCTGGAACTGCAACGGCACTGGTCATCGA
AAGGACCAATGCCCGAGCCCGAAAGACGACAACAAACAGCCAAAAGCTGACACCCCGAAAACCACGGAGACCG
CGAACACAGCCGTAGTAGAGGACAGCGACGTCAAATATGGGTGGCTAGCATCTGTAAGCGAATCGGAATGTGA
ATCCGACAATGACACTATGTTTTCGACGTATTTCGAGGGTGACGAGCCCGAGAGTGACGACGGGATGCCTGGC
CTTCAAGCTGTCTCGGACTCCGACAGTGAGGATGAAGATTTTCTTGCTGGGCTGGAAAGTGACGACGACGAGC
CATTCCAATCTGTATACTTCACTGCCCCGGCTGAATGGTCCACCGATGGTGAAGATGAGGACGAGTCCGACGA
GGACATAGACGTCGAAGAAGCCGCGCTCGTACACAACACTCCATGTCCCTCCCGAGTCGACCTCTACGACTCA
GGAGCCACCCGTCACCTATCCCCTTTCCGTGACAGATTCGTATCGTTCACGGAAATTGTCCCTAAGCCCATCA
ACGCTGCGAATCAGCAGCGGTTCCACGCGGTTGCTGTCGGAGACATGGTAGTCGACGTACCTAACGGTCGTGA
GACATCCCAACTGCGTCTCCGTGACGTCCTCTATGCCCCCCAAATGGGTGTCACCCTTGTCTCTGTCGGATGC
ATTGATGCCGCAGGATATTCTGCACTGTTCGCCAGTGGCCGATGCCAAATTCGAAATGGTTCCGGCGAGGTTG
TGGGGGAGATAGTGCGCTCGGGAGGCTTGTACCGGGTCGACAACGGTGCGGAGGAAGCGAATCAAGCGTCGCA
CGTTGAAGTGGTGACACTCGAGGAGCTGCATAGGCGTCTAGGTCACGCCGCTCCCGAAGCCTGCCAGCGGTTG
GTCAACCGCGGGCTCGTGACAGGTGTCAAGTTGGTCGGAACCCAAACCGTGCCATTCTGCAAGGCATGCGTAT
TTGCCAAAAGCCGCCGTAGACCTATCCCCAAGGAACGCCTCAGCCCACCAGCTGATGAAGTGGGAGACAAGAT
CCATACCGACGTATGGGGACCAGCCGACGTAGCGACACTCGGGGGCAAGAAATACTTTGTCGGATTCACCGAT
GACAAGAGCCGCCTCAGCGCTACCTACCTTATGCGACACAAATCCGAAGTGAAACGCATGTACAAGAAGTTTC
GGGCCTGGCTCAAGGCGCGCTTCAAGAAGACGATACGAGTCTTGCAAAGTGATCGCGGAGGGGAATATCTAGC
CGATGAGCTTCAAGAGATGCTCGAGGAGGATGGGACGCAACATCAGCTCACCGTCCACGATACACCGGAGCAG
AACGGCATAGCCGAACGGCTGAACGGCATACTCCTCGTGCGCGTGCGCGCACTACTCCACGAGGGCAACCTGC

CCAAGTCGCTATGGGGTGAAGCCTTGATGCATGCGACGTGGCTTAAGAATCGCATGACAACGAAGCCTTTGGG
TCCTGACACCACCCCGTACGAGATCGCCTTCGGGGTGAAGCCCGACTTATCTTCGTTACAGACCTTCGGGTGC
ACTGCCTGGGTGCATGACCCCGATGGATCAAAACTTGGCGCCAGAGCGCGCGAGGGGCGCTGGGTCGGCGTCG
ATCTCCAGAGCAAGGGGCACCGCATTTACTGGCCCAGGACGCGTACCGTCACGGTTGAGAGGAGCGTCGTATT
CAAGAAGACCTCCTCCGGTGCGCAGCTCGAGGGGGAGAGCATGAGGCCTGAAAGCAGCACCAGCATCGAGCAG
GACACACCGAGCGAGCCAGTTGCTCCCGCAAGCTCAGAACCAGAACAACGAACGCCACGAGTACGAAAGCCCA
GCGCATACGTCCAACGCATACTTGAAGGCGTGAACACGTCGTCCATACCAGTCGGTATACGTGTCGCAGCCGA
GACGGCATCGCTAGCCATAGATGAACCGCAGACTTCAGGGGGAGTTTGCGACGAGTGGGCTATGGCGACAGCG
ACAGCGGACGCTGAAGGACTCGAGCCGCGATCACTTGCAGAAGCCAAACGCTGTCCAGACTGGCCGCGATGGG
AAGAGGCCATAGCCGAAGAACTAGGGAGGCTCGTGAAGGCGGGAACTTGGGAGCTGGTAGAGAAGCCGTCAGA
CGTCAACATCGTAGGCAGCAAATGGGTCTTCAAGATAAAGAAGAACGCCGCCGGAGATATCGAAAAATACAAA
GCAAGATTGGTCGCTCAAGGTTACACGCAAGTTCCGGGTGTAGACTATTTCGACACGTTCGCGCCGGTAGCGA
AGCTATCGTCTATTCGCACCATACTCGCGCTTGCAGCCAAAAATGGCTGGCCAGTACACCAGATGGACGTCAA
GAGCGCCTACCTGTATGGTAAGCTTGACGAAAACGAACAGATCTACATGTCGCAACCGCCCGGATATCATGCT
TCCGGCAGCCAAGGCAAGGTACTCCGCCTATGCAAGGCCCTCTACGGCCTGAAGCAAAGTGGCCGCCGCTGGT
ACCAAACCTTCTGGGGTATTCTGATCGAGATCGGGTTTGTACGCTGCGAGGTAGACCACGCAGTATTCTACAT
ACACTATCCGAATGGCGACATCGTCATCCTCGCTGTACACGTTGACGACCTAACTCTGGTGGCGAACGAACTT
GCCCTCCTGCTCCGCGTCAAGGACCAATTGAAGGCTCGACTCGAGATGACAGACCTTGGCGACATACACTGGT
TGCTGGGAATCGAAGTCCGACGCGACACCGACGCTCACACGATATCCCTTTCACAGAAGTCTTACATCGACTC
TATGCTTACCCGCTACGGTCTATCTGATGCTAAGCCGCTCTCGACGCCCATGGATACGCACTCGCAGCTCACC
AAGGCTCAATGTCCCACTACACCACGAGAGTTCGCCGAGATGCGCGACGTCCCGTATCGGGAAGCCGTAGGTT
CGCTCATGTATGCAGCGTTGGGAACGCGACCCGACATTGCCTATGCTGTCGGGATACTTGCCCGCTTCTCCGA
GAACCCGGGAAGAGCGCACTGGGAGGCCACCAAGAGGGTGTTCCGATATCTGAAGGGCACGAAGGACTGGTGG
CTTGTACTAGGCGGTGGAGACAATGGGATGGTAGGTTACACCGACGCCGACGGAATGTCACACGAAGATCGCC
ACGCCATATCGGGATACGCCTTTATGATCGATGGAGGCGCTGTATCGTGGAGTTCAAAACGACAAGAAATCGT
CGCGCTATCGACTACCGAGGCAGAATATGTCGCAGCGACTCACGCCGCTAAAGAGGCGATCTGGTTGCGCTCG
TTTGTCTCCGAAGTTTTCAAGCCCATGCAAGGGCCGATGCCAATGAAGTCGGACAATCAATCCGCTATCGCCA
TCGCTCAAAACGATCAATTCCACGCTCGAACGAAACATATCGATATACGCTTCCATTTCGTGCGATGGGCCAT
CGAAGATGGCAAGATCTCACTTGAATACTGCCCCACCGACGACATGGTGGCGGACACGCTTACCAAGGCCCTC
CCCAGTCCAAAAGCGAAACACTTCGCACATGCGCTCGGCCTCCGCCCGTCTTGAGGGGGAGTGTCGGAATTCA
AGAGCGGCGGCGCCATCGCGTCACGCGCACGCACGCGCCACGGGCGGCGGTGCGCATCCAAAACGGGTAATCC
GTGTCGTCCTAGTTAGTACGCCCGATCCCGACATACGATACTTCCGTGTGCTGTATAGTTCTTATGTAATTCA
CTGTCTCTTTTTTTGTCCGAACTTTCGTAGTAGTCCCTCAGACTTGTTACCGCGTCTGAGGGTGAGCAGTATA
TTCCACGCTACCTAGATACATCCCGCTGACTGCTAGTAGTCTAACGACGTCTCCGTCGATTGTTCTAGGTACA
TAAATGATACAGACTTATTACCGCGTCTGAGGTCTAACGACGTCTCCGTCGATTGTTCTAGACTTGCGCCGCT
GTTA
>Cersu1_rnd-1_family-228#TIR/MITE
CAGNGATGTGCGATGGACTGATGGCACCGGGGGTCGAGGCGTGNGAACTCGTCCTCAACGGAACTTATCACCG
ATCTGTTAGCAGGCATCGTACAGNATGCGAAANCCAGAAATNTGACCGCTTCAGGCTTACGAGACTAGNGGNT
GTATCTCCTTCCAGACGTCTGCCGAGTTTCGGGGTCCGGGGTCAAGCACATCACTG
>Cersu1_rnd-1_family-1#TIR/MuDR
TCCTCAGCGTGATCGGCTCTCTCCCGAGATGGCCGGATTTCTGCGAGATGCAAAAGACTTCGTCATGCTAGGT
CACGACGCGATCGAGCTCAGCGCACCGCACATCTACCTCTCGGTTCTTCCATCCCTAAGCCCATCTTCCACGA
TCGCGAAAACNTTCTGGCCGAAGTTTCGCAATGTCCCCACATACGATGTGACCGGCATACATAGGTCACGGGG
GCCGCTTCTGCAGATGTCAGGACACGCTGGGGNNGTTTNCTCCGTCGCATTTTCACCCGACGGAACCCGCGTG
GTATCGGGATCATGGGACGAGGCAGTCCGGATTTGGGATGCGCGGACAGGCGATCTGCTTATGGACCCTCTTG
AGGGACACCGTGACACGGTCACCTCTGTGGCCTTCTCACCNGACGGAGCGGTGGTNGTCTCGGGGTCCCTGGA
CGAGACGATTCGACTCTGGAATGCGAGGACGGGAGAGCTGGTGATGGATCCTCTCGAGGGCCACAGCGACGGA
GTCCNTTGCGTCGCTTTCTCACCAGATGGAGCGCGGATCGTCTCCGGGTCAANGGATCGTACNNTGCGGCTCT
GGGATGCAAAGACCGGGAATCCATTGCTGCACGCCTTCGAGGGGCACACCGGCGACGTCAACACTGTCGCGTT
CTCGCCGGACGGAACGCGAGTGGTCTCCGGTTCGGACGACAANACAATCCGGCTCTGGGATGCGACGACGGGA
GAGGAAGTGATGGAGCCTCTCNCGGGACACACCGACCGGGTCCGGTCTGTCGCGTTCTCCCCCGACGGGACCC
GAATCGTCTCGGGGTCANNCGACGACACNATCAGGCTCTGGGATGCACGAACGGGNGCCCCAATCATTGACCC
CCTGGTGGGACACACCGACTCGGTCNTTTCGGTGGCATTCTCACCTGATGGGACCCGAATCGTCTCTGGTTCG
GCTGACAAGACGGTTCGACTTTGGGATGCAGCGACAGGGCGCCCAGTGATGCAGCCGTTCGAGGGCCACGGTG
ATCACGTCTGGTCTGTCGGGTTCTCACCNGATGGGAGCACGGTTGTCTCCGGCTCAGGAGACAAGACNATCCG
ACTTTGGAGTGCAGATGTCATGGACGCATGCCGTCCCCTCACGTCGCACCGAGCGACACTGCTCTCCCCGATG
GGACCTCGCCACAGGGATCCCGGCTCGAGTCTTGGTCGACAACGAAGATTCGGCTCCTGGTACCAACGTGAAG

CCGAGGAACGCACCTTCGGAGAGCCCTCAGGGTCACAGCGGCAGAGTCCGGTGTGTCGCATTCACACCGGACG
GAACNCAGATTGTGTCGGGATCGGAGGACAAGACGGTCTCGCTATGGAATGCGCAGACAGGCGCCCCAGTCCT
CGATCCTCTCCAGGGTCACGGTGAGCTCGTCACATGCCTTGCGGTATCGCCAGATGGGAGCTGCATCGCTTCC
GGCTCAGCTGACGAGACGATCCGCCTTTGGGATGCAAGGACCGGCNAACAAGTGGCGGACCCTCTCAGTGGAC
ACGGCGACTGGGTCCANTCTCTTGCGTTCTCACCGGATGGGACNCGAGTCGTTTCGGGCTCGTCTGACGGNAC
AATCCGGATTTGGGATGCCAGGACAGGGAGGCCGGTGATGGAACCTCTCGAGGGACATAGCGACACGGTCTGG
TCAGTCGCGATCTCACCAGATGGGACACAAATCGTCTCTGGATCCGCGGACGCCACACTCCGGCTCTGGAATG
CGACGACGGGNGATCGGCTGATGGAGCCTCTCAAGGGACACAGCGATNAGGTCTTCTCCGTCGCATTCTCCCC
GGACGGAGCCCGGATCGTCTCTGGCTCGGCGGACGNCACAATTCGGCTCTGGGATGCCCGGACGGGTGACGCG
GTGATGGAGCCTCTCAGGGGACACACCGATNCGGTCNTGTCTGTCAGCTTCTCACCGGATGGGGAAGTAATTG
CCTCTGGCTCGNNNGACGCCACTGTTCGGCTCTGGAATGCGACGACCGGGGTCCCAGTGATGAAGCCNCTCGA
GGGACACAGCGATGCGGTCCGCTCAGTCGCGTTCTCGCCCGATGGGACTCGCCTTGTCTCTGGATCATNCGAC
AACACAATTCGCGTATGGGATGTGACGCCGGGAGACTCGTGGTTGGGATCGCAAGGCGGACANGGCAGCACGA
TCTGGAGCGCAATCGCGAGCTCCATGAGATTACCGGCGGCGCTGCGACCGGCGCATTCACTCGAGCCCGACNA
CACTGAGACGTCTCAGTCCTCTCCGACGTCGCAAGCAGGGCCCGAAGAACTGCCCGATCCGTCGTATTTTCGC
CTCGAGTCNGGATGGATCAAGGGCCCGCGAGACGAGCTCATCATGTGGGTCCCGAAGGACTACCGNCGCGGNG
TNNTGATGCCGCGCACAAAGGTGCTCATCGGACGATATCGAGCGACGTTGGACGTAAGCAGGTTCGTNCACGG
CGACAGATGGACGGANTGCTATACTCCGAACATGACTTCGGGCGATCCTTGATCGCACCGTTTCGAGCTGTAC
CTCACTCCTATCGCCTCCGTTCTATTTCACAACCTCAGCACGTTCCGTTACATCCCATCAATGGTACATAAGA
GCGACTGNTCTAAATGTTTCTCTCGGACTCGCATGTCTGTCCCGGTCTGACGAGANCCGAATGCTCAGCGAGA
TAGCCGGACATAGCATGTCGTAGCACCTACAGCGCAGCANTGGACGTAGGTACGGTATGCACAGCACGAGCAA
TAGNAGATTCGCTGNAGGACGACNAGAATCAACCACGATGATGAGAACGACCTCGTATTACTCACTTTCCTAA
TCCGACGTGTATGTCCGTGCGCACCAGCGGAAGCGCGGGATGCGAGGGNAATGAGATCTACGAAATGCTAGCT
TCACTCAAGCGCGAAGNCCCGTNGNAACAACCGAATGCGCATGTCGAATCGACGTCAGATATGCGAAGAAGAA
CAAAAGACGGCGCGTACGGAAAATGATGCAGCGACCCGTCCGTCAGGTCTCGGCGAAGGGTATGGTCCGGCCC
TACGCGCATATACCTGGTCAAATGTTTGTGCGGAATGGCACAGAACCAACAATACGTTGGCCGTATGCACCAA
TATAGGACCCGCCAAGTCCTAGATGCGCTCGTACCGTTAATCCGTATGCATTGATGATTGCGTGATTGCGATT
GCGGCTCTACAACTCCGAAGGTCAGCCCTCTCAAGGTCGCGATGAAATATAGCTCAACGAATTGGCGCACTGT
GTGGGATTGCTGCTCAGCGACGGTGCCAAGAACAGTT
>Cersu1_rnd-1_family-30#TIR/MITE
TTACAGCGAGTTCCATTTCAATAGAGAATTTTGGAAAAAACGGCTTCATGTCACGTGCTGATTCCGCAGCACG
CTCTAACCTACACAGGAAACCATTGGAGGAGATGTGATCGTCACAGCTCCACCGAATATTCAGATTCCCGATT
CCCAGCCGTGTCGCACTGTTGCGACCGTATTTCTACTCAGGGCTCAGTTTCTGAAATGCGGTTTTTGAAAGTA
CTGTTAGAGGTCATGCAGACTACCCGTGGGCCGTATATCGAGAGCATAGACGAGGGACAAGGCCAGTGAGGCG
AGCGGTCGCTCTGGGTGTCTAGGCGTCTCAATGTGGGGTTGCGGAGCACATCCTAGCACATCGAGAGATCCTT
CGCACAATCTCGTCACCTGATGTTTGGTTTGCTCCGAAAGTAAACAGGTGTGCCTGAAGGTAAAATTTCTAAA
AGTCTCTATTGAAATGGAACTCGCTGTA
>Cersu1_rnd-1_family-13#TIR/MITE
TAGTGCTTTGCACGGGTCTACCCCCGGGTGTCCGGCGCTATGACCCGTACCCGTACCCGCCGAGGGTACGGAC
CCGTGAGCACGGGTACGGGTACCAAGGGTGGCGCGGGTACNCGGGGGTGAGGGGTTCATATACGGGTTGTCAC
TCCGAGTAGGCCTCTGCTCTCATTCACTGTACAGTACAAGCTCGTACTAAATGCTCGATCACAACACTAAATT
TGATCCGCGTCCTCATCTGCATCCCGTGTGACCTCAAGGATATGTTTGTCCTTGAGAAGCCCGTGGCGATACC
ACTCACCGAGACACAGAAGTGCACGGACAACGAGCCAGCCACAAGGCTAACAGTGTGCCTGTCAGCTTGTTGT
AAACATTCTAATTCAATTATTTACCGTAAGGGTACCCGCAGGGTTCACGGGTACTAGCACAACGGATACCCNT
ACCCGTACCCTCACGGGTAAGGGTACCCCCAACCCGCGCGGGTTACCCTTACCCGTGCAAAGCACTA
>Cersu1_Cluster36#LTR/Copia
TATTGCGAGTTGCGCTGTACCTAAGTACGTACGGAACGGGCCGTATCTCAGTCTCGAGCACGCGAAGCGATGC
AAATCGAACTAGTGTGCATTACCTAAGTACGTACGGAACGGGCCGTATCTATGGTACATCAGCAATGCGTCAA
TCTGTATGTAAACTAGATCTTACCTCAGTCTCGAGCACGCGAAGCGATGCAAATCGAACTGAGGCGCAGAGTA
TCAAAAGAGAAACTTTTGAATACCATTGCTTACGTAACATTACTGCACGTGCGCCCTGCGCATGTCAGTCTCC
GCGACCACGGCCGGTGTATCCTCCCTCGGATTTCAACACGCCCACTCAACGGTTGTGGCTCGCTCACCATTCT
AGACCCATCCCCTTCGCGAATCTTTCGTGCTTCTCCCGCGCCAGCGCCTTCGTCAACACGTCCGCGACTTGAT
CCGCCGTCGGAATGTATTCCACCTCGATGGTGCCTTCTTCGATCCTCTCCCTCGTGAAATGGTACCTCACCCC
AATGTGTTTCGTCCTCTCGTGGAACGCCGCATTCCGGGCGAGCGCCATCGCAGATTGATTGTCGGCGAGGAGC
GTCGTGGCCTCCTTGAAAACCTGACCTAATTCGGTGAGAAGTCCGCGTAGCCACACGGCTTCCTTTGACGCTG
ACGCCATAGCCATGTATTCGGCCTCGGTACTTGACAGGGCCGACGAATTTTGTTTCTTCGACATCCAGCTCAC
CGCGCCACCCGATATCAAGAACACGTGTCCCGTAACCGAGACGCGATCGATCGGGTCGCTGGCCCAGTCCGCG
TCGGTGTACCCCACGAGCGTGTCGTCCCGCGTAGTCCCTCGATAGACGAGTCGCATGTCGGTGGTGCCTCGTA

GATACTTGTAGACACGGACAATAGCAGTCCAATGTTCTTCGCCCGGATTTGCAGCGTGACGGCTCAGAACGCC
CACTGCGAACGCAATGTCAGGGCGTGAGCCGAGCATCGCATACATGAGGGAACCCAGCGCGCTTTGGTAGCGT
CTCGGCTCGATCTCGGGTGCCAACAGCTTTGTCAGCTTTTGGTTCGCGGCCATCGGAGTGCTCACCGGGCGGG
AGTTCTCCAGTCCGAATCGGGTTAAGATACCCTCGATGTACTGCCGCTGCGACAATTCGATGGTGCGCTTGCC
GCGGTCGCGTATAATTTCCATGCCCAGGATCCATCGGGCCTCGCCTAGATCTGTCATATCGAAGCGCTTCCCG
AGATCTGCCTTGACGCGCTTTACGATCCCGAGATCGTCGCATACGGTTAGCATGTCGTCGACGTAGACAGCTA
CAATAATTAGATGGTCGTCCTCGACTTTGTAGAAAATGCTGTGATCCGAATCGCATCTCCTGAACCCGAGTGA
TTCGAACTCCTTACGAAGTTTCTTGTACCATTCTCGGCTAGCCTGCTTGAGGCCGTACAGGGACTTGCGCAGC
TTCCAGACTTTCTCCTTGTCGTCACTGAACCCGGGCGGCACTGCATGTAGATCTCTTCGTCGAGATCTCCGT
TCAGGAACGCCGATTTGACGTCCATCTGATGGATCTCGAGGTCGTGTTTGGCGGCTAGTGCGAGTAGCGCTCG
AATTGACGTAAATTTCGTCACGGGGGCGAAGGTCTCATTGTAGTCTATACCCTCTACCTGCGTGAATCCCTTC
GCAACCAGGCGTGCCTTGTATTTTTCAACTTCCCCATCGGGACCGCGCTTGATTTTGAAGACCCACTTGCACC
CTACGACCTTGCGGTTGCGCGGGCGCTCGACCTCATCATACAAGTTGGCCTTGGCGAACGCTAGTAATTCCTC
GGCGCACGCCGCCTTCCAGCGATCTGCATCGGGCCGTGACATCGCCTGCTCGTACGTCTTCGGATCATCTGCG
ATCATGGCGCGCTTCGCATGCTCTCCGTCTAACCCCAGCCCTGAGTCTTCAGCCTCCTCATTCTCGGCAACGC
CTTCTGCAATCGATGACTCCTCGACTGTGGGACGCCTCGAGTACGAGGTGACCTTGAAGCTTTCGTCGTCGTC
GGGCTTGGGAGCCTTGCGAGGGCGTGTGGACCGCCGCAGTTCGGATGTCGGCACGTACCAGAATGGTCGGTAC
TGTTTCCGCTCGCTCGGCTGTGTGAGTGCCGATGGCGTCGGCTGATGCGATGTGACAGCCGGCGCTGTGATGC
GTGACTGCGCGTCGCCAGACTCCACCTTTGTGTCCTTCGGGCTCGCCTCAGACTTGGATGTGCCCTCCGTGCT
CTTGTCACCAACTTCTGGGGGGACTTGGTCCCTCTTGGAGGCATCTACTTGCATGGGCTCGAGCTCGACTGTG
ACGCGGCTCGGCTGTGCTTCGGTACCCTCATCAAAAATGACATCGCGTGATTCGAACACGCGTCCGGTGGGTC
GGTGCACTAGCCGATAGGCTTTTCTGTTTTCGGCATAGCCCAGGAAAGTGCATTCCAAAGATTTAGCATCCAG
CTTCTTCCGGCGCTCGTCCGGGACATGCGCGTAGGCCTTGCAGCCGAACACCCGAATATGCGCTACCGTAGGT
TTGGTTCCGAAGTGGGCCTCGTGCGGTGTAATTTCCTCTTCGAGGGCACGCGTGGGCGTCCTGTTGATGATGT
ACACGGCGTAGAGTACGGCGTCGGCCCAATACAAATTAGGTAACCCGGCGTCCTTCATCATCGAGCGAGCGCG
CTCCATGATTGTCCTATTCATGCGCTCCGCAAGGCCGTTTTCCTGCGGAGTGTACGCATTGGACATTTCATGG
TGAATGCCCTTGCTCTTGTTGTATTCTTCCATGGCTTTGGAGGTGTACTCTCCTCCGTTATCGGAGCGGAGGC
GGACGGCGCGCTGTCCGAGTTCGGCTTCGGACCGCTCGATGTAGGCGCGCCAGTGACCCGGGACTTCGTCCTT
GGTTTTCATCAGATCCAGTGCAAGATGGTGCGACTTCCCATCAATCCATGTTGTGAAGTAATAGTAGCCGAGA
CGTGACGGCGTCTCCATTTTCCCACAGACGTCAGAATGTGTTCGGTAGAGCACGCGGGGTGACTTGTTGCTGG
TCTCCTTCGGGATCGGCGCGCGGGCCTGCTTGCCTTGAGCACAGCCTGAGCAGACTCTGCCCTCGGTGGCTTT
GGCATCGCCCACTATGGACATTCCGTTCACCATTCCGCTGCGGACCATCTTCAACACGGCGTCGGTGCTGATG
TGCCCGAGGCGTCTGTGCCACACCTCAGCCGAAGCCTTGGACTCGGTTTCGGCGCTCGTCGCTAGTGCGCGCT
CCGTCGGTTGGGTGTCGACGTCGGAAAGATCCGCGCTTGCTACACGCACGCGCTCGCGTCGAACCCTCTCCGT
GTCTAGGTAATACGTGTTCCCGACTTTGTATGCGGTGGCCGCAACACTTCCGTTTGTGCCAATGATTTTGCAC
CCGGAACCGTGGAAGTGAACTTGGAATTTGTCAGTGGTGAGACGGGCTACGGATAGTAGGCTGCCGTCGAGAC
CCGGGACGTGGAGCGTCTGCTGGACTAGTGTGCGCTTGTTCGAGCCGTCGGGTACCTTGAGGATCACGCGCGC
TGTGCCGATGCCGATGGCAGGCGTGTAGCGTTTGTCACCTAATTTGACCTGAATTGGTGGAACGAGCGGTCGG
TACGACAGGAAGAACTCTCGGCAAGAGCTCATGTGGCTGGTGGCACCAGAATCGACGATCCAAGTGTACGGAT
CTTCGGCTGTGGCCAGCGCATGCTCGCTTGCCATGAACACCTGGTCGGCAAACTCCGGATAGGGCGGCCTGTT
CTCGGCAGAAGCCATCTTCGCAGATTGCTCCTCCCTGGGTGTATTGGCAGCAGCGAGATCTGCCTTTTTTCTC
CGACACTCGTCTTCGGTGTGACCCTCCTTGCTGCATGGTTTGTAGGAGCAGCGCTTCTTCTTCCGGGCATCCT
TTTTCTTTTGCCCCTTGTCCTTACTTCCCTGAGCACGCGTGTAGAGCGCGGCGTGACTCGACTGGTTCCGTCG
GGCCTTTTCCTCCGTGAGGATCTGGGCCACGACAGCCGAGGTCGAGAGCTTGTCCTCGGTAGTCATGAGGATC
GTTCTCAGGGTGTCGTAGGAGGTCGGAAGTGAGATTACGATCGCAACAGCGACTAGATCGTCGCCTAGTGTCG
TACCTAATGAGGTAAGGATCCGGGCCTTCTGCCTCATGGCGTTGAGCTGAGGCTCGAGAGGTGCCTCGTCGGA
TAGTGTCCCGCGGAAGAGCTCGCCGATTAGATAGGCGATTGTCTGCTTTCCCTTGCCCTCGTATCGCTCGCAG
AGCCTGTCCCATGCTCCGTGGGCGCTGAGTTCGTCTAGAACGCCGCTTAGCGGCTCATCCTTGAGCGTGAGTG
TGATCTGTGCAAGAGCTTCTTTATTCCGTGCTTCCCACGTGTCCACACTGGCCTGTGTAGGCTGCACCACTTT
GCCTGCGGCGTCGAGTTTGCCGGTCGGCTTGGGATCTGCGCCGTCGACTACAGTCCAGAGATCCCGGATCTGT
AGGACCGTCTTGATGCGGTATTTCCACGTCTGGAAATTTGCTCCGTCATCTTCAAGCGACGGTACGTCGTACA
GGCGGCTAGATGCGTTCCTCGTGGCTGTGCTTGTGCCTGACATGATGCTGGTGGAGTGGGCGGGTTATCTCGT
AGATTAGAGATAGCCGGGGCTCATAACCTATTGCGAGTTGCGCTGTACCTAAGTACGTACGGAACGGGCCGTA
TCTCAGTCTCGAGCACGCGAAGCGATGCAAATCGAACTAGTGTGCATTACCTAAGTACGTACGGAACGGGCCG
TATCTATGGTACATCAGCAATGCGTCAATCTGTATGTAAACTAGATCTTACCTCAGTCTCGAGCACGCGAAGC
GATGCAAATCGAACTGAGGCGCAGAGTATCAAAAGAGAAACTTTTGAATACCATTGCTTACGTAACATTACTG
CACGTGCGCCCTGCGCATGTCAGTCTCCGCGACCACGGCCGGTGTATCCTCCCTCGGATTTCAACA
>Cersu1_rnd-1_family-174#LTR/Copia

TTTTGGGTCTTATCTGGCAGAAATTGAAGAACTATCGAAGAACCCCTGTTGACGACAACATACCCCACTCTAT
ACTCGTACAATGTCTGATACCACATACGAACGTCAGGCTTCCCGGCGACTTAGCGACGCCAACTACGCGGAGT
GGTCCATGCGGATGGAGGCCGAGCTCGTCCGACGGGAGCTCTGGGACGGGGTCATTGCAGTCCCNGCCGCNAC
GACGGAGACCGAGCCGGAGAAGGTAGACGAGNNGACNGGGAAGATTCCCAGTTGTACGCACGCGATGTGCACG
AAGATGGCGGAGGCGCGCGCGGAGATGATCCTGCGTGCAGAAGGTGCAAGTCTCGCAGCTGGCGCACATGCGG
NCACGCGACCCGCGGGAGATCTGGCNGGCGCTCGCGGAGGTCCACAAGGCTCGCGGGTTTGCGACGCGGCTGG
CGATGAAGCGACACTTTCTTACGTCTGGCCAAGAAGCGTGTCGGATCAGACGATGCAGGCCTGGATAGGCAAC
GTAAGGAGCATGGCCTTCCGGATGGGGGAGGCAGATATCGTGGTGAGCGAGCAGGACCGGATTCTCGCGGTTA
CGAATGGGCTTGCCACCATCATACGACGCGGTCGTCATCGCGCTCGACTCGACTCCGACCGAGCAGCTCACAC
TCGAACACGTCGTCTCACGTCTTCTCAACGAAGAAATCCGACAGTCGTCATCGGGTGCCCCTCAGTCCCACTC
TCGTTCCCGCGACCCCGGCGTTGCTGCAGCTACTACGAAGGCGCTTATAAGGGGGGAGATGCCGAAAGGGCGC
CTTATCGACCGGTCAGAGGTGCAGTGCTATTTCTGCGATGATTTCGGCCACTTCAAGTCCGAGTGCCCGGAGA
AGGCGAAGTGGGAGACAGAGAAGGCGAAGACGAAGGAGGCGGAGGCTGCGGGGGGTCATTCGGCAATACTCGC
AACGACCTTTGGACCCGTAGCCGGAGCTTGGTGACTATGGGGGTGCGGACCTTTTTCTCTCGCTCGAACATGT
GCTCGCGCTGAGCTTGTGGGGGTGTGTTGGAACTTGGAATTGGAGTCGGACGATTCGGGTGGTACGGCGGCGC
GACGGAACTGAGCGCGGCGGGAACGGCGGGTGCAAGCTTACGCGCTCGCGTTCATCCGTGCCTGGTAGCTAGA
GTTACCTTAATTAGTGGCGTTCTGTGTATTTAGATACCTCGTGCTCGTCAACGTACGGCGCGCGTTTGCAACG
CGGGCTCACTACATCACCCCGGAACACCTCGCGCGACGGCTTGTTCCAACA
>Cersu1_Cluster35#LTR/Copia
GTTGTAATTCCTGTTTTGGTACGTCTCGCCACCGAGCTCGTTCGGCGTCCGCCGACACGCCACCTATATAAGT
AAAATTTCATGTACCGTGTTCCGTACCGAACCTATCTCGTTCTCGCTCTCCCCGTCCAATTTCAACAGGTTAT
GAGCCCCGCCCGCTGCTACTGAACTCACACTTAATCGATAACCGACGCTCGTTTACCCCCTCTCGTCGCCCTC
GCCTATCTCGGCTCCCGACATGGTCAACATCAACTCCCTTCTCACGAGTGTCCCAAAGCTCAATGGCGAAAAT
TACCACGATTGGAAATTTTCCATGTCGATGATCCTCCGCTGAGCTGGTGCCTGGAATATCATCAACGGCTCCA
TTACGCGACCCGACAACCCCACTGAGGCCATCGCGTGGGATGCGCTCTCCGAGGAAGGCCTCACAGCCATCGC
GCTCACTGTTGAGCAGAGCCAAGTCGCGTACATCCGGGATTACCAGACTGGTCCCGAAGCCTGGGAAGCCCTT
GCTGACATCTACGAGCGCTCGAGCCGCGCAAACCGCATCATGCTCAAGCGCCAGCTGTTCATGGCAACTCACG
ACACCTCGAAGCCCATCGTCGACTTTATCACGGGCATCACGACAACCGCTGCGCGCCTCAAGGCTATCGGTGT
TCCGCTTGACGAAGAAGAGATCACTGACATGATCATCTATGCGCTCGCACCATCATACGCTGGCGTCGCCACT
GCGCTGATGCAAAGTCCCGAAGACGAGCTCACAATTCGCACAGTCACCGCCATCCTCGTTGAAGCCGAGGAGC
AGCTCAAAAAGGAATCCATCGCATATGATGTCGATGGAAAATACGTTCCCGGTGGGAACCTTAATGCCCTCGT
CGCCCACACAAAGGACCGCTCGCCTGCCGCGTGCTACCGCTGCGGGAAGGTCGGCCATATCGCGCGCTTCTGC
CCCGCGCCTGCGCCGCTCCAGACTGGGTCAACACCCGCGTCGAATCAAGACGACACGTCACTCCGCGCCAACG
TCGCCACATTCAACGACGGATTTATGCAAGTCTTCTAGACGCCGTTGTTTCGTCAATCGTCACGTCAAATTTT
TCATTTGGTTCATTTGCTGGGAGGCCGGGCGTTCGCGACAAGTAGCGGCAGGGGGCCGGGAGGGTCGGAGCGA
TGCCGGAAGGTACCAGAGCCTGGGAGGTCAGGAGAGCGCGGCATAGACGAGGGGAGGCTCGGAGGGCCGGCTC
AGCACTGACGTGTTGCGAGAGTTTTTTCATTTGTGTGTGTTTTTCCGCTGTTTTTTGTTTCATTCTCTCGTCG
CTGCTGTCATCGAGTGGGCACTTCCCGCGCTGAGTGGGCGCTTTCGCTTCCGTGCTACACTACGGTACATATT
TGCACTATCACCTTTTCGCTGTGCTCGCTGCCTCGCCGAGTGGGCGTGTTGTAATTCCTGTTTTGGTACGTCT
CGCCACCGAGCTCGTTCGGCGTCCGCCGACGCGCCACCTATATAAGTAAAATTTCATGTACCGTGTTCCGTAC
CGAACCTATCTCGTTCTCGCTCTCCCCGTCCAATTTCAACA
>Cersu1_Cluster34#LTR/Copia
GACGATCGCGCCCTACCAGTATTGTATCGTATCGTACTTGTATAGTACGTGCATGCTACTGAAGGTGAGCTGT
ACAGAAAACGAAATAGAAACATTACTCATTGACCATCATATTCTTCGTCACTCCCACTCGGCACATCCTTCAG
TGCCACTCTCCCACTCCAAGCGCGGTCCGGAACCGTTCGAAGGCGTCGCGGGGAAGCAGTTTGGTGAAGATGT
CGGCGATCATGTCCTTGGTAGAGCAGTAGCGCAAGTCGACTTCCTTGCTGGCAACGTGCTCGCGAATAAAATG
ATGCTGGATATCGATGTGTTTGGCGCATGAGTGAGTGTTGGGGCCGCGGGTGAGGGTGATGCAACCGAGGTTG
TCGCCGTAGATGACCGTCGCGTTGGCCTGCGTGACACCGAGGTCCTCGAGTAGGACGCGCAACCAGATCACTT
TGCGCGTGGCGTTCGATGCGGCCATGTACTCCGCTTCAACCGTCGAGAGCGCCACCGTGGGCTGCTTCTTCAA
CAACCAGGATACGAAGCCGCCTGCAATTTCGAATGCAAAACCGCCCACCGAGCACTGTGTGTCTGTGTCTTGT
GCCCAGTCAGAGTCCGACCAGCTGACCAAATCTACGCCATTGCCATTGCCGCTGAGCCTGAGTGCGAAGTGCG
CTGTACCCTTGAGGTAGCGCAGCGTGCGCTTTAGGGCTTCGAGATGCGGTTTCCCGGGGTTTGCGCCAAATTG
CACGAGAACGCTGACCGCGTACTGTATGTCGGGTCGCGTCTGAGTTGCGTAGAGCACTGCACCGACAATCTCG
AGATATTTGAGACCGCGTGTGTACTCCTCATAGGCTGTGTTCTCCTCCGGGGTGGATGGGGATTGTGCCGCGG
TCAAGCAGTCCTTTGCGGCAAGCGGGGTCGAGACGGGGGCACAATCCTTCATGCCAAATCGCTCGAGAAAGCT
ATTAATGTAGGCGGTTTGGTTTAGGGTGACGGTCTGTGCTGCACGATCGCGGGTGACCTGTATGCCGAGGATG
TATTTGAGCTCACCGAGGTCTGTGATGTCAAACCACTTCGCAAGGTCGGCCTTAAACTGGGTGATGTACTTGA
GCCGTTTTCCTGCGAGCGTCATGTTGTCAACGTAGACCAGGACCAAGAGGATAATGGTGCCGTTCTGCCATAG

AATATAGAGGCAGTCATCTGCCCGCACCTTCTCGAACCTTAATTCCCACATGACCTCGTCAAGCTTCTCGCGC
CATTGGTGTCCTGCCTGTTTTAGGCCATACAAAGCCTTCGCCAGCTTCTAATACCATCCCTCCTTACCCGGCT
CTCTCACTCCCTCCGGCACCTTCATGTAGATCTCCTCCTTGAGATCTCCCTGCAAGTATGCTCCGATGACATC
GACTTGATGAAGTTCCCAGTCTTCACGTGCTATAAAAGCGAGGAGTTCCCGAAGAGTTGTCGTCCGGAAGACA
GTTGAGGACACAAAGAGAGGGTTGAAGTCGCGTCCGGGCATTTGTGAGAAGCCACGTGCAACAATACGAGCCT
TGTGTTTGGTGACATTTCCATTTCCGTCACGCTTCTCTCTGAAAACGATCCGGCTACCCACTGCCTTGCATCC
TTCAGGGAGCTTTGGGACCCATTTGAAGGTACCTGTGCTCTTCAGCTGCCCGTACTCGGCTGCGATCGCTTTC
TCCCACTGCTTCACAGCAGGTGACCGGGTGGCCTCACGGTATGTTGTCGGTTCTCCCAAGGTTGCGATGTAGA
CTCGGTGGCGCTTGGTGGTATGCTCTGTGAGTCCTGCATATCGCTCTGGGGGTCGTCGTTCACATGTCGGAAG
CTCGCGTTGGGGGAGGTTGGCCCATTTTGATGTTCGTGGCGTGCTCAACTCGGGCTTGTCTTTGGAGGCTGAC
AGCAAATCCTCAACGGCGCCCTCTGAGTTAGTCTCGGAGGTAGGTAAGGGTGCTGGCAAAGCACCTTCCGCTA
TCTTTGGGAGATCTGGTGTCAGTTCCTCAATGGCAGCTGCAGTCGGGCGAGTGTCTCCGCTGTCAATGACTGC
AAGATCACCCGGGCGATCATCCTCGATGAAGCGCACATCACGCGAAGTGATGAGCTTCCGTGTTTGCACGTCT
TCAAGACGGTATCCCTTTGTCTCATCAGCCCACCACCCGAGCAGACAGCATTTGACTGCTGTGACCTCTAGCT
TGCCGCCTCGGAGCTTCTTTGGGATATTTGTATAGGTGGTCGAGCCGAAGATTCGCAGGTGTGAGATATCAGG
CTTTCGACCTGTCCAAGCATGCTGTGAGACCTTGTCGTGTAGTGCAGACGTGTAGGTGAGGTTCCGAATATGG
ACTGCGTACATAAAGGCCTCGCCCCAGTACCGCAGGTCCATTTCCGCGGAGTGTAGCATGCACTGTACCATCT
CAAAGTAGATGGCAATGGCGCGCTCGGCAATCCCATTGTGCTCGGGCGTGTACGGGACCGTCATCTCATGTAC
AATCCCATTTCGGCGACACAGTGACTCCAAGATGTCGTTTGCAAACTCTGTCCCATTATCTGAGCGAATGCGC
TTGACCTTGCAGCCTGCTTGGCGCTCCATCATAGCGAGTGTGTCCTTCAGAGCGTCTGCTGTTTCGTCCTTGG
CTGCAAGGAGACTTCCCACTCCATAGTGTGTTCCCACATCAAGCGTGACCAGCGCGTAATTCTTCCCGGTCCA
TGTCGGGTGTGACATAGGTCCCGACAGGTCGACAACGACAAGTTCAAGCTTCTCGTACTTTGTCTTGCTGGCT
GATGGGAAGGGGAGGCGATGTGACTTGCCAAGAACACATCCCTCACAGACGTGATCAAAATTGCGTGGTTCCG
AGATCTTAAGGCTGGTGACGAGTTGCTTGCGGTCCATCTCACAAATCGCATCAAGATGCATATGGCCAAGGCG
ACGGTGCCATACGTGCATGCTCCCACTGGACTTGACACGCATGGCCGCATGGTGTGGTGATGCGAGCCTCTCG
ATATGGTATAGGGTACCCGAGGCGCTGGCGAAGGAGGTGCGGAAGAGTTCATCCTTCCCGTGCTTAACGACAA
TTCCATCTGCAAAGCTGTTGTATGAACACCCGCCTTCGCGCTGCAGCACTCCGAGGCTGACCAGGTTCGCTTG
AAGCGCAGGGACATGTGATACACCATGCAGAACGACAGTCCGGTAGCCACCGGGCAGTCGGCTTAGCCATTGG
ACAGTGCCACGTCCTGTGACAGGCAACCTGTGTCCATCTCCAACAGAGATCTCGTCTCGAGATGTTGACGCAT
GGTACGATGTAAAGAATGAGCGGTCGCGGAACATGTGGGATGTTGCACCAGTGTCGAGAAGCACGTCTGTGTC
CTCTGTATTGCTCTGTTCTGTCGCCATGTAGACTCGTCCAAGTTCGCGGGTACCTAGATCTCGTAGTTCGCCA
ACGGCATGGTTTGCCGAGCCACCGCTGTGCCTTTTCTTGTCCTCGTCATGCTTGCTCACCCAATGCCCCTTCT
CTCCGCAGACACGGCAGATGTCATCAGGCTGAGGACCACGTTTCTTGCCTCCTCGGCCGCTGCTCTTTGTCCC
ATGTGACTTCTTCTCGGCTAGAAGAGCTGTCTGTGTGCCAGTCTTCTCGCGAAGGTGTCGGTTTGCCTCGCTC
TGGAGTGTCGTGCTGACTGTATCCACGGTGAGCTTTACCGGTTCCAATGAGAAGAGCTGGATCTTGATAAGAT
CCCAACTTTGGGTTCGCGGGAGTGACAGGATGAGTACACGCGCGACATGAAGGTCTTCGAGCGTCTTGCCGCT
TTTGGTGATGCAATGCTTGAGGTTGAGCATTGCTGCGATATGATCCGCCATAGGTGAGCCATCAATGTATTTC
CTCGTGTAAAGCTCCTCAAAGAAGTAGTGCACGTTGATGCGTTGCGAGTTCGTGACATGCACCTTCCTCCAGG
TGTCCCACATGTTGCATGCGAATGTCGTGTCCTTGACGTGTGGCCACTGAGAATTCTCGGCAGCACCCTTCAT
CAGACCCTGCGCGGTGCCATCCCACAATAGCCAGTCCAGGTACTCCTTCTTCTCTGCTTTCCACTCCGTGATG
GCTTGCTTATCCGGCTCCTTTAGATCACCCGATGGCAGCGGTCTCTCCGCAGGCTTCAGTGGGCATGTCTCGT
CACCACTGACTATAAGCCAGAGATACTTCGACTGGAGCGCGGCTTGCATGTGCACAGCCCACTCCGCATAGTT
CTGGCCGTTCAGCTTGGGAAAGCGGATCGCCTCGCTGGTATGTCCCGACATGTCCAAGTTCGTGATTCCGGGG
CTCATAACCTGACAACCGACAGGGTTTGTAGACGGCGCACGAGAGACACGTCGGGTCGAGCGGGACTAGCGAG
CGGGCGCGAGCGCTGGAGTTCACGGGGCACACGATAGTGCGAACGTGTATTAGTGTATCGTAAGTACATCAGA
TGGCGGTTGGCATACCTGGAGTTCACGGGGCACACGATAGTGCAAACGTGAACTCCCAGGCTGCGCAACGCCA
GTGCAAAGTCTGTGGTGGCTGACTAATCGCCGATCGCGCCCTACCAGTATCGTATTGTATCGTACTTGTATAG
TACGTGCGTGCTACTGAAGGTGAGCTGTACAGAAAACGAAATAGAAACATTACTCATTGACCATCATATTCTT
CGTCAG
>Cersu1_rnd-1_family-254#TIR/hAT
GGCTCCAACCATTTACTTTACGTTTACAGATTTCGTGTAAACAGACAGTAAANAGTAAATAGATAGTAANTAG
ACTAGAATAAACNGTTTAAACTCTATGGATCGGCGGGAGTCCTGCGACGACGAGCAGTCCCTCACGGATCCAC
TCGCGAGCGCACATCAACTCNGTAATCGTCGCCGCCTTCATCGAGCACCGAGTATCGGCGCATGCATGGCGCG
ATCCCGAGAAGAGCCGCTCNACNGCCACGCTCGCACCCGGGATAGCGAGAAAGTCCTTCGCCATGAGGGCNAT
GACCTCGAATTCCTGCTCATTCTCCTGCATAAATACAGCGTCAGCTTCGATACAAAACCAAAATCTAACGACA
AACCT
>Cersu1_Cluster4#LTR/Gypsy

GATCGGCCCTTCCCGCATACCCTGAACACCCGCCCACCGGTGGCGGTCGCTCTTCGTCTTCCTTTCCCGATCC
ACGGCTTCCCGCTTCTCTGATCCCCGATCCCCTTCTCGGTTCTCGCCTTCCCGTCGCGCTCCCTCCCGCTCCC
GTAGACGCCGGCGCCTTGTGTTTCGACAACGGCCATTGGTAGACTAGTGGACAGCTGACCGACTACGACATAC
CTCTGACAGATGGTACAATACTGCGCACGGGTGGTGTGACAAGTAGTTATTCGACATCGTACGATACGACTCG
GAAAACGACTCGCGCAAGTAGTATAAAAACACGGTCACTGTAGCTACATTCCTTAGTCTTTCGCTCTTTTGTA
CCTCGCCTCGAGCAATACGAAGAGTTCTTCATTGGCTATTGTTTGTCGTGCATTACAACGCTTGTTTCAAAGC
TTAGAGCAGCCCTCTAAGACCCTTTGACAAGTCTAGTAAAGCGACCGAAAGTTGCTCCAGGCACTTTTGTAAC
ATTAGCGAAAGACTCAAATACCCCAAACCCTTGATCCCACACTACGTGCAGGATCTAGAAACGACATCGAAGC
AAAGTCCTCAGACGTAATTGAGGCAAAGCGACAAGCCCTTGCACGCAACCAAGGCCGAAACAACAGAGGCGAC
GCTCGTCTAGACAAGAGTGTAGCGTCGGGATCAACCGTAACGCGCGTACGAGATCGGGATCCGATTAAACGAA
CAATCACCACCTCACTACGGAAGGCAAGACGTCCTACCGCGAACATACACAGACCCTTGGAGAGGGACTCAGG
GACATACTTAGTCAAGACGACCAAGGACGATTCCTAACTCAGAACCAACGAAACACACTCGAAACCACCCTGC
TATCCGTGCAACAATTACACGACGATCTGCTCAACCTTGAAGGAACGAGATTCACAACTCTCATCGAATCCCA
GAACCCGTGGATCAGAAGGCTCAGGGCTGAACAGAACCGAATTCTGCGCTTGATACGTAACCCCGAAACCCTA
GTGGGGATCTCCGCAGGAACCCTGCAAAGAGCGTCGGAAAACGCTCTCATTCTTGACAGATACACACGAGACT
ACAAGGCGTTACCCCCCATTCCTGAGCCAGGGGCAGAAACAACGTGGGAAACCCCACTCGACCCCCACACCGA
GCTACCCGTTGCCAGAGAAGCGTTAGACTTATCCCCCAGCGAACGAGGGCTACGCTTCTCAGCAGCATACTCA
CTATCCGAAGAAGCAGTAACCCCGGATGAACTGTGAAAAGAGAATTCACTTCCGAAACTACTTCTCGGGAAAG
GACGAACACGTACAACGAAATTTCAGCAAACCTGGAGACCCCGTACGAATCTTCAGGTACTTCTATACCTGCA
ATAAGTAACCGTGCCCGAACCGATATAACACGCCCGTCGAAATCTACCAAGCCCTCACCTACCCAGTCAACTA
CCGTATTCCAATCCGCCAAGACACTCGCAGTGAAACCGAAGGAATGGGCACTCAAACTGTGGAGGAAAGTGTC
TCGGCCAGGACCCGAACCGAGATCTTCGATCTGGGAACAAGGGAATACGACGAGGAATTCGCAATCAGTCGAG
CCGCGACAGGAGATCATGGAAGAGATACGGAGATCCCTCCTCCAACCCCTGGAGGAAACTTGGAGACAGTTCA
TCAGCTTGGAGGAAGTGGCCCAAGCAGCTCCGAACATGAGGGACGAGGAATTAGCGAACGCTCTTCTCACTCT
CCCTCCGACACAGCGCGTCCGCCTGACCCCGGAAGCGATCAGGTGGAGCTATCGACAGGTCGAAGCAATACGA
CAAGCGACTACCCAAGCACAGCCGGAGGAAATCCCTACCAGCCCATGGCCGCCCTTGCCGGAGAATTTCAGAC
TCTTTCAATTAGCAGCTTTAATCAACCAACCTATGCAGGTCTCGGTACGGGAGCAAGCGGAAACTGGCCCAGC
AGCACTAGACCGATCATTCCGCTCCAACCAATCTCTGGAGGAGGTAGCGGAGGAGGGACAGGACCGCCCGGAG
GAGGAGCACCGGGGCCGCCGGGACCTCCGGGACCGTTTGGAAACCCCGCGGGAGGGGCGCCGGGAGCGCCGCC
TCCGCCGCCGCCAGGAGGTCCTCCAGGAGGCCAACCGCCGCCACCACCAGGAGGCCCTCCGGGTCCGGGACAA
CCTGGAGGAGGCCCGCCGAATCCCTTCGGAACAGCGGGGCAACCCCTAGGAGGACAAATCAAAATTGAGAAAC
CCTTCCTCTTCACAGGAGAAAGAGAGAAGGCAGAAGACTGGATCAGAGAATGTGTATTGTGGTTCATACTCAC
AGGATATACCAATGACCAGGGAAAGGTACTTTTAGCCTTAGGACGAATCGACCCGGGGGCCAAGATAGCCTAC
GACTGGAAGCAACTGCTCCTCCGGGACATTAGTTCGGGAACTGTCACCTATACCTGGGCAGGCTTTACCCGAA
TCTTCCAAGAACGCTTTGCTCCATTGGACCAAGAGAACGACGCAGCAGCGAAGCTCGAAGCATTAGTCTTTGA
CAAATCCAAACCCATGGACGACTTCATCGCAACTTTCCAGGACCTCGCGCGGAAAGCAGGACATACCCAGGAA
ACGGCATTGATACACACCTTCATGAGGAAAATCCCTGCGACGAGTCGACAGGACATACGCAGACATTATCCCA
ACGCCACACTGGGGGAATGGTACGACCATGCTCGAAGAATCGATAACATCTTCCGCACCGAGCAAACTATGAG
GAGCACAGCCTCTGCCAGTTCGAACACGAACCCGACCACAAACAGGGGGCGAACCCCGAACCGCGGGCAGTAT
CGCCGAGGACGTGGAACGGGGAGGCCGCGAATTAGAGCAACAGAAACCGACCAGGGAGAGAATGATCAGCCCG
AAAAAGGCAATGATTCACAAGTCAATGCGCTCACTGCCAAGGAGCGACAGAACGCCCGAGAGAAGGGGTTATG
TTACAACTGCGGTAAAGGGGGACATCTCGCTGCCAGTTGCCCAGACCGGAAGCACAAAGCCCCGCAAGGAAAG
GCGCGGAGCGCGACCATAGAAGACTCCCCGGAAAGGGGAAGCGAAGAAAACGACGATGACGATGACGAAGGGA
CCATGGTGGGAGTCCGCCATGTGCAAATCCAAAACGAACTGGATTTTTGACGGGGTTGCCTGAACCGACGCCA
GAGCAACCAACCATCTCCGAAACAAAGGTATTTGGAAGTTCGGTAATACTTACATGTTTCTACAAAGTAGGAG
AAAAAGCCGTCGAAATCAAAGCTCTCATCGACACAGGAGCACAAATTAGCTTGATACACCCCCGACTCATCGA
CAAATACAAGATCCCGACAATCTCACTCCCGAGACCGATACCCGTGCGAAATGTCGACGGAACGATGAACAAA
GGAGGAACCATAGAGAAACGAGTGGAGGGGAACCTACTCATAGGCGGGCTAAGCATGCCAACCAACCTCTATG
TGGCGAACACCGGAAGCAACGACGTCATCCTAGGACTCCCTTTCCTTCGTGAAACCCAACCCACCTTCTACTG
GGAAACGGAGCAGATGAAGTGGAACAATAAGGTCATAGAGGATCTGAGAAAGAAACGAGAGGAACAGGAGAAG
AACCCCCCTGAGGATCGACAACTTCGAAGATTCCCTCTCAAAACCGGAGAGTGGGAAGCACTGGTGCAATATG
TCGCGGGCGAGGAATACCCACGAGACCCAGATACGCTGTGGATCAATAAAACGACGATATCCACACAACTAGC
ACAGAAGAACGCCCAAGAGACCCAGCACTGGAAGAACATCGTTCCAGAAAAATTCCATGAGTTCGCCAAGGTC
TTCGAGAAACCGCAGGCTGGAAAACTGCCCCCTCAGCGAGCAATTGATCATGAAATCCAATTACAACCCGGAT
TTCAGCCCAAGCGACTCAAAGATTACCGGACACCTGAACACCAATTGCCGGCCCTGCGAGAGTTCCTCAGGGA
AAATCTGGAGAAGGGATACATCCGGCCATCGAAATCCCCTATGGCCTCACCCCTCTTCTTCGTGCCGAAGAAG
GACGGGAAGTATCGACCCTGTCAAGACTATCGATATCTCAATGAATGGACCATCAAGAACGCGTATCCCATCC
CCCATATAGACGACATCATTGCCGGGATGAGGGATGCCGACTGGTTCACCAAGTTTGACCTTCGAGAAGGGTA

TAACAACATACGAATCAAGGAAGGGGACGAATGGAAGGCTGCTTTTGTGGCGAACGGGGAGCTCTACGAACCC
ACAGTCATGTTCTACGGGCTGTGCAACTCACCCGCGACCTTCCAAGCCTTCATGAACGAAATATTCAAAGACT
ACATCGAAGAAGGATGGTTCCGCATCTTCATGGACGATGGGAACATCGCGACAAAAGGGAAACTCAAACTCCA
CCATGAACGAACACGCAAGGTTCTGCGGCGACTTATTGACAATGATCTCTATGTAAAGGCGGAAAAATGCGAC
TTCGACAAGAAAGAGATAGGCTTCCTCGGAATGATCTTCGGAAAGAATCAGGTCAAGATGGACCCAGTCAAAG
TCAAGGCGATCCTAGAATGGCCAACCCCCAAGAAGGTCAAGGAAGTACAAAGCTTCCTTGGATTTGCCAACTT
CTACAGACGCTTTATACTCAAGTACTCCCATGTCGTGAAACCCCTTACAGAACTCACTTCGAAAGACAAACAG
TGGGAATGGGGCCCCAGGCAGCAAACGGCCTTCGAAGAGTTAAAGAAACGATTCACCACGGCGCCCATACTCA
GAACTCCAGATCCCACGAAGCCGTTCGTAATCGAAACCGATGCCTCTATGAGAGCGACTGGGGCTATACTCAA
ACAACGGGACGATGAAGGAAGATGGCATCCGTGCGCATTTCTTTCACAAGCAAACCAAGCAGCAGAGCGAAAC
TATCAGGTATACGACCTGGAATTGATGGCGATCGTACGAGCCGTCAAGGCGTGGAGCTACCTCATTCTAGGGA
GTCCGCACGAAACAATAATTCGCTCGGACCACATGAACATCACGCACTACCGATCGAAACAATCGATTTCACG
CAGAGCAGCCCGCTGGGCACAATTCCTGCTACCTTACAATCTGAAGTTCCAACATGTGCCGGGAAAACAGATG
GTACAAGCCGACGCGCTCTCGAGAAGAGCAGATCACGACAACGGCAAGGAGGACAACCAGGAAATCACGCTGA
TCCCCCTTGATGCAATCATCAATGCGGTCCTGGCCAACTTACTTACGGAAATTGCCGAGAATCAGGAGAGAGA
CCCCGAGGTGGTCGAGGCCTTGCAATGGCAGAAAAAGAAGGGGAAGACACCCCCATCTTTTCACCCTGAAGAT
TGGAGAAGTGACGAAGGGCTCATCTATTTCAAGAACAAACTCTATGTTCCCCCCGATCAGGAACTCCGACGGA
AGATCGTGGCAATGCATCATGAACCAGAAGTCATGGGACACCCTGGAGTATTCAAAACCACGGAAGAAGTATC
CCGAGAATACTGGTGGCCGAAGATGAAAACTTTCATCCGACAATACGTCCGAGAGTGTGCCACTTGCCAGAGC
AACAAGGTCAACACTCACCCGACCACCCCGGAATCGATGCCGATCCAATCACAGTCCAGGCGGCCCTTCAGCA
CCATTACAGTCGACCACATCACGGATCTTCCCAGATCGAAGGGGTACGACACTATACAAGTCATGGTCGACCA
TGACGTCACCAAAGGCGCAATCTTCGCGGCATGCTCCAAACAAATAGACGCGCTCGGCACCGCGCAAATCATG
GACAAAGAAGTATATCGAAGATTCGGCCTACCGGAACGAATCATCTCCGATCGAGGACCGCAGTTCGCGGCAA
AGGCCTTCCGGGAGTGGAACAAGATCCTAGGAATAGAAACGCGAATGTCTACTGCATATCACCCGCAAACCGA
CGGCGAAACCGAGCGAGTGAATCAAGAGCTCGAAGTAGCTCTGCGTATCTATTGCAATGCGAACCCCGAAACA
TGGGCCGATAAATTGCATCACTTCGAATTTGCACACAACCACAGGACACACGAGGTCACGAAACAACCCCCGT
TTCAACTAATGATGGGGTACCAGCCAAACGCAGTGGGAACTGTGTTACCGGAAGGAAAAGTCCCGGCTACACA
ACAAAGGCTAATTCAGTTGTCCAAAGACAGGGAGGAAGCACGATCAGCGATCAACCTTGCTTCGGAACAAGTC
AAGGAAAGGATCAAGCGGACATCCGTACCATTCACTCAAGGAATGAAAGTCTGGCTCGACGGCCGGAACTTGA
AAACAGGATACCCCCATCGGAAACTAGCCCCTAAACGCCTCGGACCATTCCGAATCAAAAAGGTCATGGGTCG
GACGACGTACCGCCTTCAGCTGCCGAACAAATGGAGGGTTCACGATGTGTTTCACGGCAGCATGCTCACCCCG
TATCATGAGACCACGGAACACGGGAGAAACTACATAGAGCCGCCTCCGGACATAGTAGAAGGAGAAGAAGAGT
ATGAAGTGGAAAACATACTCTCACATCGGATTCGACGAGGAAAACAGGAATATCTCGTCTCCTGGAAAGGCTA
CAGCGCATCGGAAAATGAATGGCTAACCGAAACAAATCTTCGACATGCAAAGGACACCCTCAGGGCATATAAA
AAGAGGCACAAGGTTCGCTGAACCCTCAGTGCCCTCCCAACACCGGATAACACTACCACACCGACTACTCGCA
ATGGCCTACATCCCCGCTCACCAACGCCCTGTGCTCGAACGCGTCTACGTTCCCCGACGATTCGCTGACATGC
GCATCAACTACCACGACCTCACCATCCTCCAGAAACCCACTATCACCATAAGGGTCGGCGACCGGATGCGCAT
CGCCTGGACCCTAGTCTGGGACGACTTTAAACCACAACTCTCTCTGCTGCTACGCCACACCGACCTGTACGAC
CCGGTCCAACAATTTCAACATCATTACGAGGGGTACTCTTCGAGCAGAGCCGCGCTATGGAGGGGAGCGGGTA
ATACCTCAGCGCAAGATGCCCAGATCAGAAACCTATCCTATCACCTAGTACAGATGAACGACACCCTCGACTA
CGTCATCCGCCACGGTATATATTCCTACATCAACAAGCTACTACCCGGTCCATACCAGCATCTCGACAAGAAG
CTCTGGGCAAAGCTCACCGAACTCGAACAACGACACGGAGTACTGATAACAGGAGAGGTCAACCTTGAAGAAC
TCTCGCGACAAGTCGACAGAGAACGAGAAGAAGCCGAAGCCCGCTACGCTGCGCAGCTACTCGAGGACCAGCA
TGCGCAGCAGGTTCTCAACTGGGTAGCGGAGGATCACGGATGGGGGGAAGCTCCGCCAAACGACGAGAACGTC
CCACCTCCGCCCCAGTACTCCCCTCGACACACGCCGCCGTCCACCCCACCACTCGACGCCATCAACGTCCCTC
TCGGCAATCTGCCGGACGGACACGTGACAATCCGAGAAGCAGTTCGTATCTTGCAAGGAGACCGCCCTGATCT
CGCGCCCCTCGCCGAATACCTCATACTACGGAACCTCGCAGAAGGAGGGCCAGACGTTCACCCTGCCGCCCAA
GCAGCCGCACAGATCGCGCTCGATCTTGGCGCCGACCGTTTTCCAGCGCCGCTTGAAAACGGCGCACTCACGG
AAGGCGAATCAGTGGAAGCGCCGATCACCATTGATTGACCACCATGTCGGCCAACACGTCCATTCCGACCACC
TCCCCTAACTTCGGCATCCGGTTTTTCTCTGGCGACGGTATCACCCTTCTCGTCGTCGCCACTCTCATCATGC
TCGGACTCGGATATCTATTCTCTCGGGACCGCTGCGGCGTCCTCCGCAATTGGGGCAGACCAGGCATCGCACC
GAATGGCGAACTCGTAGCCCTCGACGAAGTCGCCACGCACGACATAGAATTCGACCCCCCACAACAGCCACCA
CCTGCGCATCTACCTTGAACTGATAGTCGGGACGACTATCTTCAAGGAGGGGGTACTGTTACGACTTTTCGTT
TTTCTCTTTCGCTACGCTCACCACGTGATGACTCCAGTCACCTGACCCGGTTCCCGGTCCTGAACAGACCCGG
AAGGCGGCAGATCATTGGATCGCCCTTCCCGCATACCCTGACACCGCCACCGGTGGCGGTCGCTCTTCGTCTT
CCTTTCCCGATCCACGGCTTCCCGCTTCTCTGATCCCCGATCCCCTTCTCGGTTCTCGCCTTCCCGTCGCGCT
CCCTCCCGCTCCCGTAGACGCCGGCGCCTTGTGTTTCGACAACGGCCATTGGTAGACTAGTGGACAGCTGACC
GACTACGACATACCTCTGACAGATGGTACAATACTGCGCACGGGTGGTGTGACAAGTAGTTATTCGACATCGT

ACGATACGACTCGGAAAACGACTCGCGCAAGTAGTATAAAAAACACGGTCACTGTAGCTACATTCCTTAGTCT
TTCGCTCTTTTGTACCTCGCCTCGAGCAATACGAAGAGTTCTTCATTGGCTATTGTTGTCGTG
>Cersu1_rnd-1_family-4#TIR/MITE
CATCAGCAAAATCTCGTTTTTGCTTTTGCCGCTTTTACGTCTTTTTCGGTCGGTAAGCGCCCCGAAAGCGGNA
AAAGTGGGAAAATCCCAGTCCCATGGGACTGGGATTTTCCCGCTTTTCCTGCTTTGGGCTACCAAAGCAGGAA
AAGCGGCGCCAAAGCAAATGCGAGATTTTCCTGATG
>Cersu1_Cluster6#LTR/Copia
TaTTGgaaGgcaaGcGacacAgCacCGGGAaaGGaacccaggGCcGacgcacgtggtcgaggagattaggtaa
tcgaggagagaaaacaaacagcaaatgtttcgagtgcctcagtccgtactgctacgctacgcgagcgcgagac
tgaggtacgcccgttttattacttacacaGTCaCgCgagCgcacAGcagAccAGaaCGGaacgacGcaCccga
aaCacagGcaaTgcgaTagccCgcaccGaTaacCTAagCgaccccaacACggacacCaGCaaccccggCCacT
CCCGcaGaTccagCcCacaaCgaaCAAaAcGccaacacacCcGcacaacaCcaaTCaaaaagagaagaCcaaC
CcaaaaGaaaaCCcCaCaTcaacCcCacGacagaaACcaccaacaaacaaaacccCcaCggcacCaGcCGccg
aaAcGaacTCCacacaCaccaaGacGaccgcgaaaaccTCcaGacggaaaaGgaaCccaaCaacaaaGTgCTg
cagaccCgCGgGcaAcgcaacGTcGccaaCGacAGCcATaccCGaccGGagacCcaccaGaaagaTcGgcacc
TgcGaaATaacGagGcacaacccCaaaagaagCgTaCgcacaaAGATaaaCTccTaaaccGcggACgccAcca
caaaAcacccaaaggacccaGgaaacaaTgaaGcGaaCGCcaccagcgcacagGAaaAcagaaCAgCgCCgac
CgCaaccacGAAcacaTcccCcGcaacCgagagaagaacgcaCaaaTCGagcCaCaAcTCAaCacCaacagac
aCaAacacCgcaTCcTCaCGccaCgcgcCaCaacacaaGAcgaccagcTcagcacgccCGCcaacaTaaTcgc
aaaccCaaagaaTgaagccaCaaTGcgCacaacCcgcacgcccacCcaGGacAagaagacCaCacacTcCaaa
ggcGaTcacagccccAcgGaaGAGaAaccCcgaCacGAGcaagacaagcaaaCcgcGaTaacGcccCGaagca
AacTcGGaacCaaagaacagaAgcAgCagaaaaagaacccccATaGGaaaaagcaCaaGcccTGCacgTaCGa
acCcaaaacGAagaAaGaTcaCgacGAcacagaGTcgCgcaGaaAaaacGagGaTacgcccGCaaaaaacGac
cAgaacccCaAagcCGacgaacCacaGCcCcccGacaAGGaaacgcATCaCaaaTccAcggaCcAGaTagaCa
TgGaCaCcccacaaaccgaaaaGacGAcCcGAcaaCgTgaGcAcGTCGaCgacGgAgaCGGCGATTATGATTA
GATGTCCATCTTCGATCTTGGTGAAAATGCTATGGTCGGAGTTGCAGCGCTTGAAGCCGAGGGCCTCGAACTC
CTTGCGCAATTTCTTGTACCATTCGCGACTTGCCTGCTTGAGGCCGTAGAGCGACTTGCGTAATTTCCAGACC
TTGTTATCCCCGGCGTCATGTCCGGGCGGGCATCCCATGTAGATTTCCTCCTCTAGATCCCCGTTGAGGAACG
CGGACTTGACGTCCATTTGATGGATCTCCAGATCGTGCTTGGCGGCCAGGGCGAGTAGCGTGCGAATTGACGA
GAACTTGGTCACCGGTGCAAACGTCTCGTTGTAGTCTATACCTTCAACTTGCGTGAACCCCTTTGCAACTAGT
CTTGCCTTGTATCGTTCGATTTCGCCGTCTGGACCGCGCTTCACCTTGAATACCCATTTACATCCCACAACCT
TTCGATCTCTAGGGCGCTCGACCTCGTCATACAGGTTCGCCTTGGCGAACGCGAGAAGCTCCTCGGCACAGGC
GGCTTTCCACCGATCTGCATCTGGGCGTGACATCGCTTCCTCGTACGTATTTGGGTCATCTGACATCATGACT
CGCTTCGCGTGTTCTGCGCCCACTGCCGGCGCTCCGCCGTCAGTTCCCTCGTTCTCGGTGGCGTCGTCTGGAG
TCATCTCGTTGGCGACTGTGGGACGCCTCGTGTACGAGGTGACTTTGTAGATGTCGTCGTCATCTGGCTTGGG
GGCCTTGCGCGGGCGCGTGGACCGGCGCACATCGGATGTCGGCACGTACCAGTACGGTCGATACCCTTTGCGG
TCACTCGGCTGTGCGCGCGCTGATGATGTTGATTGATGTGATGTGACAGCCGGCGCTGGCGCTTGCCCGTCGT
CGCGGGGCTCCTCCTCGCCCTTGGTATTGCCCTCGGACTTGGTCGCGTTCTCGCTGCCTTTGTCATTCACTGC
CTCGGAGGGGCGATTCTTCTCTGTAGGCTCTGCGTGCACGGGCTCGAGTTCCACAGTGACGCGGCTCGGTTCC
GATTCGCCCCCTTCATCAAAGATCACGTCGCGTGACTCAATGATGCGCCCACTCGGGCGGTGCACTAGCCGAT
AGGCCTTGCGGTTCTCGGCATAGCCAATGAACGTGCACTCGAGCGATTTCGGGTCTAGCTTCTGGCGCTTCTC
GTCAGGGACGTGCACGTGTGCTTTGCACCCGAAAATCCGGAGATGCGCGACCGATGGTTTGGTCCCGTAGTGA
GCTTCGAGTGGCGTGATGTCTCCTTCGAGCGCGCGCGTAGGCGTCCGATTGATGACGTAGGCGGCGTACAGTA
CGGCGTCTCCCCAATATGAATTTGGGAGTCCCGCGTCCTTGAGCATGGCTCGGGCGCGCTCCATGATGGTTCG
GTTCATGCGCTCCGCGAGTCCATTTTCTTGCGGGGTGTATGCATTGGACATTTCGTGGTGCACGCCCTTGGAC
TTGTTGTACTCCTCCATGGCTTTGGAGGTGTACTCTCCGCCTCCGTCCGTACGGAGCTTGACCGCGCGCTGAC
TAAGCTCGGCCTCTGATCTCTCGACGTAATCGCGCCACCATTTGAAGGTCTCGTCCTTGGTCTTCATCAGATC
CACGGTGAGATGGTGAGATTTCCCGTCGATCCAGGTCGTGAAGTAATAGTGTCCCTGTCGGGTCGGTGTCTGC
ATGCGCCCGCATACGTCAGAGTGCGTGCGATAGAGCACGCGCGGGGTCTCCGTCTCCGTCTCCTTTGGGATTG
GAGCGCGGGCCTGTTTTCCTTCGAGACACGCCTTGCAGGCATTGGTCTTAGCCGCATTTGGGTCGCCAGTAAT
GGCCATCCCGTTTACCATTCCTTTTCGCACCATCTTGACGACAGCATCCACGCTAATGTGTCCGAGTCGTCGG
TGCCAGATCTCGATGCTAGCTTTGGATTGGTTCTCGGCTCTTGCTGCGAGGGCGCGCTCGGCGTCTGCCcaAG
acaacgcGcaaacaagacaaagCaacTcaaacGTaaaGacaACaaccacaaGTaTGcACagGTgaCCAaccTc
gcaccaagTcGCacccaacaCgCCacGcccAccAAccACcagccagacaTccaccaaaaaaccaCaacgaac
cggTCcgcaacGaGgaTCgAcAcGaacAaaacaacaccGgacacacgcccGaacagaaaGccGcGgaTaACaA
GcgcggcaaaTgaCGcaacacccgcTAccgcaacaaccAGcCaaGcCGcGaagagaaCaaGaaaggGcacaaa
GaGTTcGacGCCcacccagacaagcGcaGcaacTaCGAcCacagCaTaTgaaaCaAaaTagaagagcgacaac
caaacaTaATcgcaGgaTaCcgaGaCGACaTaCcaAaGaccTCaGaTCacCgtcggaacggccaaccGcGgCa

AgGaagAcCTcGgaacTcaAcccGaGATaacggaCCTacgCaacGcGCgCgGcaaacgCGaaaTgGacagcaT
acgCcaccgcgGCaacaagAcCagCcagcTacCgcaGacACaagaacTCcaaGgaAcCcTaacTCccacacGa
cgCGaAaacGCAccccCTgGgaagcacaCCcATcacaccgaaccaaaaGaTcaTaaaagcaccgGcacCCGAaa
TcgaccCGaacCaacAcCaaaGTgccagccgaccaCTCacGgacGcaaTgCTCggCacTcaacaccacAgCca
agaCGcAagcgGcGcaaagagTcgCCacacaccaaaccaaGaagaaGaaCAggGgccaacaAgaagccGacac
caaaAgcAccaacGCGacaccaacaAcagacgacTCgacgaccGaGcaaaGacaCGgGAaGAccaGcgccTcG
gccagcacGacGcTGAcTgGgGcagCGAgccGgcacacagcagagaGagcaCcGcacAacaaTTCcccaacGa
GagaGGaGaTGgcagcTggTCagcgaacaaccaaGCGcccCaCggaAccagccaCaaccgaagcgaaccaTaag
cccaTcaaaTaacCcacaaAacaGgcaATCTggcacaGgAaGagaaacacGaacaacaaCaaCccaATcgCaa
AcaccaCaAcccgcCccaccGacaaCCaacCGccaATcGCTccCGcccaCaACccgcaaTacCaCGaGcagac
caAGaACagccgcGagacGgaaCacccaaccCgaGAaaagagaaacagCagCgacaaGcaacgGGAcgaCaTa
caaaCGaCgGaacaaGccGcAcacggcgggacgaagcGTgcagcaCagGaGaagcAcGaGaGGcgaaCgcaga
GGaacGgaaaagaaaggaGAgcacaccaccaCcGcgcacagaaCcTaTTGgaaGgcaaGcGacacAgCacCGG
GAaaGGaacccaggGCcGacccaaGTcgcccacgaGagTAGcgaAgCaagaaGaaaaaacaaacagcaaatgt
ttcgagtgcctcagtccgtactgctacgctacgcgagcgcgagactgaggtacgcccgttttattacttagat
tgtcactctagcttacagtagactagaccggcacgacGcaCccgaaaCacagGcaaTgcgaTagccCgcaccG
aTaacCTAagCgaccccaacACggacacCaGCaaccccggCCacTCCCGcaGaTccagCcCacaaCgaaCAAa
A
>Cersu1_rnd-1_family-8#TIR/MITE
CAGCTGTTTTCATAACGGGGGGCGTACCCCGTCGCGAAAAACACGCACATCGGAGTTGACGGAGTTCGAAGGA
TCTAGGTACGATCTGCTGGCCGGCGGCGCGCCCGCTGAGCGCAGCGGAGGCGACGGGCGGGGCGACGTCTGTA
GAACACGTATCTGAGAACTTGTAGCGCAGTGCTCAGGAGGGAAAGGAGTACATGCTTAGAACATCTCGATGAG
AGCTCAAAGACCACAGGATATCTCACCACACCGACGTTCTTTGAAGGTGCGCTTCGAATTACCTGCACGTCCT
GCAGCACCATCGTGATGCCCGGGCACGTTGTTTGCAAGGCCTAGGACTCGNTTTCGAGCTAGGGGNTTCCACC
TCGAAGCATCGCAGAACACTAATATAGATCGCACNGCCTCGTTCCATAGGTGACTGGCCCTTGCTACCCTCTT
CGCGGACGCCGTCGCGATACTTGAGGCTTGGAGTCCTGAACGGCCACCTCACCGATGAGAATCTCGGGTTCTC
GGCACAGGTGCGGCGCTCGAAATCCGTGATCCGACTATGTGTGNTTTTCGCGACGGGGTACGCCCCCCGTTAT
GAAAACAGCTG
>Cersu1_Cluster1#LTR/Copia
ATTAAATGCGCAGATCTAGAACGGGCGACGAAGTCGCCGTCAACACCTCAGACGGATCGGAAAGTCTGATTGC
ATTATGTACCTAGAACGGGCGACGAAGTCGCCGTCAACACTAAGGTACAATCAGTCTCGGTACATGTACTCGT
AGTGTAACTATACTCACCCTCAGACGGATCGGAAAGTCTGAGGGCCTCTACTGTACGAAAGCGAGAAAACAAG
AAAACGAGAGATTACATAAGAACTATACAGTTTCGACGGAAGTATCGTATGTTGGATCGAACATACTGACATA
ACCAGCAAGTTTCCTAATCGGGAGATGGCACGCCGCCACACTCGCGCCGTTGCGCGCCGTTGCGCGGGGCGGG
GTCCGCTCTTGAAATCCAACACTCCCCCTCAAGACGGACGCAGGCCCAGAGCGCGGGCGAAATGCTTGGCCTT
TGGGCTGGGGAGCGGTTTCGTAAGAGTATCCGCAATCATATCGTCGGTGGGGCAATATTCGAGAGATACCTTC
CCTTCTTCTACGACCCAACGAATGAAGTGGAAGCGTATATCGATATGCTTAGTGCGCGCATGGAATTGATCGT
TCTGGGCGAGGGCGATGGCTGATTGGTTGTTGGACTTGAGAGGCAAGGTTTCGGCCGGCACCATGAAGACTTC
GGAAACAAAAGCGCGCAGCCAGATGGCTTCCTTTGCGGCGTGCGTCGCAGCGACATATTCAGCCTCGGTTGTC
GACAGCGCCACGATTTCTTGCCTTTTAGAGCTCCAAGACACAGCTCCTCCACCGACGAGAAAGACATAGCCCG
AAATAGCGTGTCGATCCTCGTGTGACATTCCATCCGCATCAGAATACCCAACGAGCCCGTCTTTGTGCCCTCC
CAGTACAAGCCACCAGTCGCGGGTCCCTTTCAAGTATCGGAAGACTCTCTTTACGGCGTCCCAGTGCACCTTC
CCGGGGTTCTCTGAAAAACGCGCTAGGACGCTCACAGCATACGTGATATCCGGGCGCGTACCGAGCGCCGCAT
ACATCAGTGACCCGATGGCCTCGCGGTATGGAACATCGCGCATCTCAGCGAATTCACGTGGTGTCGTGGGGCC
TTGGGACTTGTCAAGGTGTGCGTGGGCATTTATCGGCGAGGAAACTGGTTTCGAGTCGGCAAGTCCAAAACGG
CTCAAGATCGAATCTACGTATGAGTGCTGCGACAGGGAGATCGTGCCCGCGTCTTCGTCACGTTTGACTTCGA
TGCCGAGTAACCAGTGGATGGCGCCTAGATCGGTCATTTCTAACCGGGCCTTGAGTCTACCTTTAAAGCGTAC
AAGCAGTTCGACGGAGTTTGCGACAAGAGTGAGGTCATCGACGTGCACAGCGAGAGTAATGAAGTCGCCATTA
GGCTGGTGAATGTAGAACACGGCATGGTCAACCTCGCAGCAAGTGAGCCCGATGTCGATGAGTATTGCCCAGA
GAGTTTGGTACCAGCGACGGCCGCTCTGTTTCAAACCGTAAATAGCTTTAAGCAAACGAAGCACCTTCCCGAC
ACTATCGGGTGCGTGGTAGCCGGGAGGTTGGGACATGAAGATCTGCTCGTTCTCATCCAGCTTGCCATATAGG
AACGCGCTCTTTACGTCCATCTGGTGCGCTGGCCAACCATTTTGAGCTGCAAGCGCTAGGATAGTGCGAATGG
ACGTCAGCTTAGCTACGGGGGCAAAAGTGTCAAAGTAATTGACGCCTGGCACTTGCGTAAACCCCTGGGCGAC
TAGTCTGGCCTTATACTTCTCAATATCTCCTGACATGTTCTTCTTTATTTTGAATACCCATTTGCTGCCGACA
ATGTTCGCGCTCTCCGGTCTGTCGACGAGCTTCCAGGTTTCTGCCTTTTCGAGTCGCACTAATTCCTCTGCAA
CGGCTTCTTCCCATCTGATCCAGTCTGGGCGTCTCTTCGCCTCAGCTAAGTTTCGCGGTTCAAGCCCCTCCGC
GTCGGATATCGCGGTGGACATCGCATATTCATCGACCACTCCCCCAGGGTACTGTAACTCTGCTGTGGTGGCT
ATCTCGGCTGGTAACTGTAGGCCTGGTGGTATGTTCTCGGTATTTTCGCCTTCAAGGATCCTCTGGATGTATG

CACTTGGTTTCCGTGTTCGACTTGGGCGGATTGACGGCTGTTGACTCTGTATGCTGTCGCCGGGAGCGCTCTG
CTCGACTGTTTTGCTAGGCTCGACGTCAGACTCGACATCTTCATCGTCGTTACTCTCCCCCTCGAGCTGCGCA
CACGGCGCTTCTTGAGCCTGCAGCTGGAAGACCACCGTCCTCTCCACGGTTATGGTTCTAGTCGTAGGCCAGT
AAATTCGCTGCCCCTGGCTGTTCAAGTCGATCCCTACCCAACGCCCCTCACGCGTGCGCCCGTCGAGCTTTGA
TCCAGAGGGGTCGTGCACCCAGACTTGGCATCCGAACGCGTGCGCGCCAGAGATATCAGGCTTGACTCCAAAA
GCCACCTCGTACGGCGTGGTGTCTTTGCCGAGGGGCTTGGTCGTCATGCGGTTCTTCAACCAAGTAGCGTGCA
TGACGGCCTCGCCCCAGAGGTTCTTAGGCAGACCGCTGTCGTGGAGCATTGCCCGTACGCGGCTAAGCAACAC
GCCGTTCAGCCGTTCTGCGACGCCGTTTTCCTGTGGGGTGTCATGCACCGTAAGCCGGTGCGTCGTACCATCT
TCGTTGAGCATATCCTGGAGCTCATCGGCAATGTATTCCCCTCCGCGGTCGCTTTGGAGGGTGCCGATGCGAC
GCTTGAACCTCGATCGTATCCAGCCGTGATATTTTCTGTACGCGTGGCGGACGTCGCCTTTTTGACGCAGAAG
GTATGTTGCCGAGAGTCGAGACTTGTCATCGGTGAAACTGACATAGTACTTTCGCCCTCCAATAGAGGCAACG
TCGGCAGGGCCCCAGACGTCCGTGTGTACAAGGTCGCCCACTTCTTCAGCAAGCGGGCTGCTACGCTCCTTCG
GTACCGGCTTTCGCCGTGTCTTCACAAAAACACAGGAACTGCAGAACTCGATTTGGGACGCCCCCACGAGTTT
GACACCTGTCACCAATCCTCGGCTGACAAGCTTCTGCACGGCCTCTGGCGACGCGTGACCCAGGCGACGGTGG
AGCTCCACCAGAGTAACTTCTTCAAGGGCGGAGTGTGCCGACTCCCCTTCTTGTTCGACTTTATACAACCCCC
CACTGCGGAGGATCTCCCCCACAAGGGTACCGTCCTTATCCCGAATCTCACACCTGCCACCTCCAAATAGCGC
CGAGTATCCTGCTGCGTCTATGCAGCCCACAGATATCAACGTGACTCCCATCTGGGGCGCATACAAGACGTCT
CGAAGACGTATCTTGGAGAAGCCCGCGCCATTTGGGACCTCGACAACCATATCTCCAATGCCTATTGCATGGA
AATGCTGCTGGTTCGCGGCGTTGATCGGCCTCGGCGGGATCTCGGTGATTGACACAAAACGGTCTCGGTGAGG
TGATAGATGCTTCGTTGCTCCGGAGTCAAACAGATCGATGCATTTGCATCCTATGTCGCCATGCACTACCGCG
GCGATGTCGATCTCGCTGTCGGATTCGTGCGCGCCGCTCTTGTCCATTGAATCTGTACGTTCATCGTCCCAGG
ATTCACCATCAGAATCTGAATCTGACACACTCTGTAGTCCCGGCATGTCGTCGTCAGAAAAGGAAATGGGTGG
TCCATGTCCCTCGAAGTAAGCCGAAAAGATATCATCATTTTCGCTATCCGACTCGGATTCGCTTACAGCTGCC
AGCCAACTATATTCGACATCGCTGTCCTTCTCCAGCGCGGCATTTGCGGCCGTGGCACCTTTTCCCCCGCCGT
CTCCGCTCGAGTTATCGCTCTTGCTTCTGGGACTCGGGCACTGACTTCTGCGGTGTCCTGTACCGCCGCAGTT
CTAGCAGGTGAGTTCCTTTTTAGGACCTGCAGGTCTATCGCTGGCGCTATCTCTGCTCCTTCCAGGGAATCTC
CTACACCCATCCTCCGCGAAGTAAGCGGCGTCTCGGTCGTCTTTGCTGACTTTGGGCTTGGCTGCGCCGCGGC
TAGGTAAGCGCTTCCGTTCGTATTCTTGCTTGAGATACTGGATAGCCTGATCCGGGGTGATGAACGTCCCGAG
TAATCGGCCAGCGTTCATCATTCCGGACAAGAAGTCGGCGTACTGGCTGGGTACGGATTTGAGTATCTGCGCG
CAGTAGTCCTGGTCGGAGACTTCGACGCCTATCGCCGACAGCTCTTCGACCATTTCTCCTAGCGTGTCAAGGT
GCTTGGATACGTCTCCGTGCTCGGAGCAGCGCGTGGTCTGAAAACGTTCGAGCATGTTCGATTGGACGAGCGG
CGTCTTGTCTTCGAACTCGGAGCAGATCCAGATCCACATGTCTGCGACGCTGTCGCCATTGCGCTTCATCCTA
CGGAGAACGCTGTCGGGGACAAGGCGAGCTAAGAGGTCGCGGGCCTTCGCCTCCTTTTTGCGCCAGGCACGAT
AATCCAGAGCATGTTTCTCGTCAATCTTCTTGTCTTTCGAGGGTAGTGGCAGGACTTCCCCTTGAGCGAGTAG
CAACTGCGCGTCCGCGCTCTCCGCGAGCGACGGCGGTGGCGCAGTCCCGGCACGCCAGTCTGCGAGCGGGTCA
ACAGGCCGGGGATCCTCTCCCGTGAGGTGCCCATGCAAGTCTCTTGCATCCAGGGCCCAGGCCAGGCGGGTCT
TAAAGACCGGCCAATTCGAACCATCTGTCTTCAGGTGGGGAATTCCCGAGAGCCCATTTGCATCTCGGGCATC
GTCGCGTTCTGGCGCTGGTTCGGGGCTCATAACCTATTAAATGCGCAGATCTAGAACGGGCGACGAAGTCGCC
GTCAACACCTCAGACGGATCGGAAAGTCTGATTGTATTATGTACCTAGAACGGGCGACGAAGTCGCCGTCAAC
ACTAAGGTACAATCAGTCTCGGTACATGTACTCGTAGTGTAACTATACTCACCCTCAGACGGATCGGAAAGTC
TGAAGGCCTCTACTGTACGAAAGCGAAAAAACAAGAAAACGAGAGATTACATAAGAACTATACAGTTTCGACG
GAAGTATCGTATGTTGGATCGAACATACTGACATAACCAGCAAGTTTCCTAATCGGGAGATGGCGCGCCGCCA
CACTCGCGCCGTTGCGCGCCGTTGCGCGGGGCGGGGTCCGCTCTTGAAATCCAACA
>Cersu1_Cluster0#LTR/Gypsy
TTGGACTAGGGATGGGAGAGCAGGTTGAGGCGAGAGGCTGAGCAGGGGGTTAGCATACAGGAGTTGCGAGGAG
AGAGCCGAGATTGCTTAGTCAGCAGAGCGCCCGAGGAGGAGTAGCGTACGCATACACGTCCTGTACGACACGA
GCACGACAGCGCACTGCATTCGTCGAGTCCATTCTTCGCCCGACCCACTGCTCCATCGTGGTGACGAGAGCGG
CTCGGACTCATCGTTCATTGTACAGCGGAGATCCCTCGAAGATTCCGCCTCGTCCTGCCTCAGCCCGCCCACG
CGACGCCCTCGGTCGTTGGGTCGCCGCAAACCCATCGTCCGCGTCTGGTCCTTCCGCACCCCAAAGACCGCCC
ACCGCTGATACCGACACTGACGACGCTGACGTCGAAGATCTGCTCTTTCCGCGCCCCGTTCCGCCCAATATCA
GCTCCACTGTCTCATCTGTGTCCAGCCTCCCGCCATCTTCATCCCCGTCCCCACCGAACGAGTTCTCCGACGC
AATTACTTATCAGACACCAATAACCTCCCCCGCACCACCTCAGCACCTTCCTGCCCGACCGATCTCTCCGATC
CATCTATCGATGGCGGCAGGCACCATCCCAAAGTTTTCCGGCGACTCTGACCAAGTCGACCCACACGACTTTC
TGAAAGAACTACAGGCGTGGCTTGGTCAGTCTCGTATCAAGGACGAGAAGGAACGCCTTGAGATGTTTCCCTT
GAGCCTGAAGTCCGGTAGCACCGCGGAGGGCTGGTATGATGATCTCCCCGACCCGACCAAGGCCTCCTGGACT
GCTCTGGTGGCTGCCTTTTCCACACGCTGGCCAAAACGTACTCGTGACCCTCTCTCGATATCAGAAGCCGTCG
AGCTGGTGCGTCAACGTGTTCTCCCACGTGATCGTCTCGGTCTGTACGACACCTCCGAAGACGGGGAAACGCC
CTCGCACAGGGTTTGGGCCCGCAAGGTGGAGGTGCTAGTCTCCAAATACAGGATCCCGGATTCCTACGCAGCA

GAGATTCGCAGAATGCTCCCCCAGCCGCTTCTCGGTCTGATCGATGCTGATGTCGAAACCTGGGCGTCTCTCT
TCAAGCAAGTCGAGAAGGTCACAGCGAAGAAGCTCCAGGAGCTTCTCGCGGCTGAAGAAGACTTGCTCGGCCG
TGCCGCATCTCGAGCAGCCTCCAGTGTGCGCACCGATCCCCCCTCTCGGGCCCCTCCCTCCCCTACCGCCGCC
ATTCGGCAGGGCCTGCACCACCTCCGTCTCCCGACTCCTGCCCCACGTGCCTTCGGTGGTTCGCCGGTCGCCA
GCGGTAATCCCTTCCTGCAACCCGCACGCATGCATCCCTCTAATTTATTCGCGGGCATGCAAACGAATTCTCG
ACCCCCACGGCTAGCGCCTTCTGACCGCCTCACGCTCATGCGTAACAACATGCCGCCTCACCGTCCTGATACT
GAGGCCGGCAAACGCGAATACACTGCCGATGTTGCTCGCTGGCATGCTGCCAACCCCGACGGCCGTCCCGACG
AACGCCGCCCATTCCCTCTGACTCCCGGAACTCTTCCTGTTGGCAGCCGCGAGTGCTACGGCTGCGGCCGTGG
AGGACACGTCGGTGCCATGTGCCCTACCCCACAGGCCCTTCCAGATGAGGAGCGGAAGTGGCGCCAGATTGCG
GGGCGTATTGCTCGCGATGCTGCTTCGGCTGCGGGGGCCGGGACCACGGTGCCGGGTGTCCAGTATGTCGGCG
CGCCTGGCTTCGAGTGGGTTCCTGTGCCAACGGGGTACGTGCCGGCCTGGCCAGCCTACGCAGATGCCTACGG
CTCGGACTGGAACCAGGGAAACGAGTAAGGGCTGGCATTGGCCTTGGGATCCCGCCAGCCCTCCGAACATTCC
CCTCTTCCCCGGTCCCTGTGTGTAACCTGCCGCCACCCGGGGACTGCACTGTAGGCCCGGAAGATGTACATAC
CTCCCCCTGTGCTACGGCCTGCCCAGACGACGTACCTACGGACCCTGTTTGTACTCCCGCGCCTCGCTGAAT
GAAGTTCTTGACTTGTATCACGTGCGTCCGAGCCCCAATTCTCCGCCTGAGCTGCAGCCCGCTAAGCCATTTG
TCCATGCCATTTCCATACACAACCCATCCGGAGAAATTGTGCGGGTCAAGGCTCTTTTTGACGACGGAGCAAT
GGTCAATGTGATGTGCATTGACCTTTGGCAGCGTGTTCAACATCGCGTCGGCAAGCTGCAGCCTTCCGGTCGT
CGCCTGCGGATGGCAAATGGGACAATTGAGCCCTCTGCTGGGTACTGGCTCGGGGACATCGAGCTCGGCGGTG
TCCGCGTACGAAGCGCCTTTGAAGTTCTCCCGAGTGGAGGTGCGTGGTCCTTTTTAGCCGGCAAACCGATCCT
CGAGGCCCTACGGGCTGTGCATGACTATGGCCCGGATACTCTCCGCGTGCCGGCTCCATCTGGTGAAGTCGTG
CTGCGTAACAAATATGGGCTTGGCCACGATGTGGCACTTATTTGTGCCGCCGGCCTCAGTCCCGCCATTGACA
TCAAGGCGCTGCCTGCGACAACCATTCAGTACATTTCGCCGTCTATGTGGGCTGCGTCTGCAGCGCCTGCGGC
TGCTTGGCATGTCGGGGCTGATGTATCTCCTTCTGACGATAACCCTGGTGATGAAATTTTTGCAGGACTCTCT
GATATAAGTACACCCACACCTCCTTATTCCCGGTTGACGGACCCCTTTCATGCCGATCGTGTACGCGCGATTC
TTCAGGCTGTCCGGATTGGGCCTGATCTCACCAGTGATGAGCGCAGGCATGTCATTGATCTTCTTTCTGAGTT
TGCTGATTGTTTTGCCCTTTCTGTCAGTGAAGTTTTTCCCGTACCGGGCGCTGTGCACAAATTGCACATTCCT
CCAGGGACCTCGTTTGGTCATCGTGTTCATCAGCGCCCGCTGACCCCTCCCCAACGGGACTTCCTGCACCGGA
AGATCGATGAACTCCTTGCAGCTGGGGTTATTGAGGCTTGCACACCAGACCAGGTAAAGTGCGTCTCACCTAT
GACATTGGCCCAGAAGGCCCACGAGGGAGGAGGGCTGTCGCTCGAGGAGCTGCAACACCGGGTCAACGACCAG
TGCGTGGCAGCTGGCCTCGAGCCAGCATTTGATCTTCCTCCTCGCCCACCTGGTCGTGACCAGCCTGCTAAGC
CTGCTGGGCAACCAAAGTGGCGCATTTGCCAGAACTTTGGTGAGCTCAACAAGGTGACGGGAATTGCACCGGT
GCCTCAGGGCGATCTTCGTGCCAAGCAGCAACGCCTGAGTGGGCACCGGTGGATTTGTACATTTGACTTTGCG
TCTGGATTCTATGCGGTCACCATGGATGAGGAGTCCCGACCGTACACATGTTTTTTTGTCGAAGGTCGTGGCT
ATTTCCGTTGTGTGCGCATGCCATTTGGACTCACTGGTGCCCCGGCAACATTTGCCCAGATGACCATTGACAA
CCTGCATGACCTGCTCACTGAGGGCACCATGGAATTGTTTGTGGATGATGGCGGTGCAGCAGGAGATGATTTT
GACACCCTTTTGGGGAATATTCGCCGAATTCTGAGCCGTGTTCGGGAGCGGAAGCTCTCTCTGTCTCCATCGA
AGTCTCAATTCTTTATGACCTCCGCCGTTTTTGCGGGCGCGACGGTTGGGCCTGGCGGAGTCTCACCTGATCT
GGCTAAGCTGACGGCTGTTGTTGACTGGGAGCAGCCCCGCCATGCCCTCGCGCTCAGCTCCTTTCTTGGTTTG
ACCAGTCATTTCCGGGATCTAATCAAGGGCTACTCGCGTATTGAGGGCCCATTGCGAGACCTTCTGAAGGCTG
TCAACGTGCCGAAGGCATGCTCAACGCAGACCTACCGGCGCATATTGCAAGGTCATTCCCTGGATCCGGTGTG
GACTGCCACACACACTGCCGCTTTTCTTGCTTTAAAGCAGGTCTTGATATCTGAGCCAGTTCTCCGGCGCCCT
GTTTGGGATGGCACTCCCTTCATTATTGCGACCGATGGTTGCAAAGATGGCTTTGGTGCTGTGCTCATACAGC
GATTTGCGTCAGTCCTGCCTAATGGGCGCACAGTCACCAAGCTGCACCCTATTGCATTTGCATCCAAGCGGAC
ATCTCGCTCCGAGGAAAACTATAAGCCCTTCCTGCTCGAGTTTGCTGCTCTAAAGTTTGGCTTGGACAAGTTC
TCAGATATTGTTTGGGGCTTCCCAATCGAGCTAGAGACTGATTGTCAGGCATTGAGGGATGTGCTGCTTAATG
ATAAATTAAATGCAGCACATGCCCGGTGGCGTGACAGTGTTCTTGCTCATCAGATTATTGATGTCCGCCATGT
CCCAGGGAAACTCAATGTGGTTGCTGATGGTCTTAGCCGTGCCCGCGAGGGCCTGCCTCGCACAGACTCCGAT
GGCAGTGCCTGGACAGTGTGCGAGGATTGGGACAGTCAAACGGGCATTGTGAATGATCTCTTTACTGTTGATG
CCGCCACTGATGCTTCCTATCCTGTGTTGCTTCACCGCTTCCGGGATGAGCCCCTCTTCCTTGAGGTGATCCA
GGCTATTCAGGCTTTGCAGGAGTCTCTGGACACCTCTCCAGAAAACCCTGCGCTACGCCGTGCACGTCATCAT
GCGTCCCAATATATGATCGACTCCGGTAAGCTCTGGAAATTGTTTGGTGGTGCTCGCGCACGGGCCCGGCCCA
GGGTTGAGTGTGTTCCCCGGTCAGAGGCTGTTCTTCTGGCTCGAGAACAACACATGACTGGTGGTCACTGGGG
TCGAGATGCGATCAAGTTAGCCCTTCTTGACCGCATCTGGAGCCCAAACCTCGATGCATCTATTCTGGAAGCT
ATCCGGGAATGTGCGCCATGCAAGAACTTTGGCGGCAGCCATTTTCATGCTTTACTCGACCCTATCACGCGTA
GACATCCGGGTGAATTATTAGTCGGTGACTACCTCTCACTTCCTAAGGGTCATGGTGGTTTCTGCACTGTGGG
TCTGTACATGGACACATGCTCTCAGCGTGTCTGGGCAGACAAGTTCCGGACGGCTGGCAGTGCGAAAACCACC
ATTTCCAGCCTTAGCCGTATATTTCGTGACTATGACGCATCGGAGACTTTTATGTCTGACGGCGGTCGGCACT
TCGATAATGTGGAGGTCCGTACTTTTTGCTCATCATGGAATGTGGCAACCCACGTGGTCTCTGCGTACTCCCC

TTGGATTAACGGCCTCATTGAAGGCACTAACCGGTTGCTTCTTCATGTCCTGCGACGCCTCTGTGCTCCTGCC
CTTGGGAAGGATGGGTATATTGATGGCGACTGGAGCTCTCTTCCGGCGTTGTGGCCCAAGTTCCTTGATGATG
CCATTTATTGTCTTAACAACCGCATTTTACCTGCTCTTCAGTTCTCTCCACGGGAGCTGTACTTTGGGCGTGT
GGTGAATACCCCCCACTCTCCGCTCTCAGCCATCATAGATGCTGTGTCAGCGCCTGAAGCTAATATTCACATG
GCATATGCGGCGCAGCAGGGCCTTGATGGCCATAGTGCTGCCGTGGCCCATGCTAACCGCCGAAAGAGCTCAT
TTGACCGTCGTGTGCAGCGTCACTTCCCAGGGGAGGTGATATTCCAGCCTGGTGACCTTGTGCAGATCTATCG
CAGTGATTTGGACTACACTTTCCGAACAGAGCGCAAGCTCTTGCCCAAGTGGTCACCTCCCCGCCGAGTTGTC
TCACGTATTTCTACTTCTTACCGGCTAGAAACTCTCACCGGTGACCCAGTCCCGGGCACCTTCCATGCTCGGC
GCCTGCGGCCCTTTGTTCCGCGGCTTGGCTCACCCCTCGCCCTTCAAGAGGCTCGACGTCTTGTTAACTATTC
ATCTCCTTCAACATCACCATTGCCTACGTCGCACAACACAGTCATTTTGTCTCCTCCTGCAGATTCTGCGCTG
ATTGGGCTTGTTCACAGTTACCCCACGCCACTGGTGCTTCCACGCCACAGCCCATCTGCTGCGCATTGTCCCA
CCAAATCTCGCTTGCCTTGCACTAGCCCTGGCATGTCAGGCCCATGACCGCCCGATCACGGGCCATGGTTGAC
CTGTTCCCGTCTTTCCGCTGCCCTTTTATTTCCCATCATCCGATCACGTCTGCGGTCGTGCTTGACAAGTCAC
CCACCACTGACAGCTGGCATGTCCGCCTTCACTCGTTCTGCCTCCCCACGCGACTTTAACGTCCGCCTGCCCA
CAAAACTCACTCTCCTTTGGCCTTCCTTCCGTCGCGACCACACACTTTCTGACCCCACTGGCTCGGGTGCCGT
CGCACCCATTCCTGTTCGAGGGATGCTACGTCAGCAGAGTGCTTGGAACCAGGCTAGCACCTACATTATCATG
GACTTCACTTCCAGCCCCCATCTCCTTCCAATCCGTGAAGGACAGTGTCTTTGCATCACCAACGAGTCCAATG
TCATTGTCCACCTCGAGAGCGTGTACAAGATCGAGCGCGACTTCATTGTGTTCTACGCACCCATTCACCCTAT
CGGCGATCGCGCCGCCTACATACAGGTCCCCGCACGCTATGCCGGTGTTGGTCTCTGGGCCTACATCTACTTC
CATCTCTTTTATCATCACCTTCCGCACACAGTCCGCACCCCCGGGCCCTATCTCGGCCAACTTGTTGTGCCTT
CGCAGACCAACTTTAGCGCCCGCTTTCGGACCTGGCTGCCCAAGTTTGGCCGGTTTGGAGATCACATGTGACC
CACCCTGCGCGCACGGGGCCATCTCTCCGGGGAACCCCCGACGTATACCCTTCACTTTCATCACATGCCTTCC
GACTGGCTTACACGGTCCCCTTCTTCGCACATCATAGAGGTTCTGCGCGACGAGGACGTCGCATTTTTTTGGT
GGGGGCACATGGACTAGGGATGGGAGAGCAGGTTGAGGCGAGAGGCTGAGCAGGGGGTTAGCATACAGGAGTT
GCGAGGAGAGAGCCGAGATTGCTTAGTCAGCAGAGCGCCCGAGGAGGAGTAGCGTACGCATACACGTCCTGTA
CGACACGAGCACGACAGCGCACTGCATTCGTCGAGTCCATTCTTCGCCCGACCCACTGCTCCATCG
>Cersu1_rnd-1_family-259#ClassII/Helitron
ACCAGCAACGGCGCCCGTGGACACCACGGGTTCGCAATTACAAATAATCATTGTTATATATTAAGTACAATTC
AATCTACATACAATACCATGTGGTGTTGTCTCATTCGCTACAAATGACCTATTAACACAACGATCAAATAAGG
AACAGAAATACCCACCGTAATAGAATCTCAGAGTATACAATATTTTTGGTAGTTGTAGACTCTGAGTCTGAAG
GAAACAACACCTTCATTCTTGCAGCAGATGTTACGCGGGACAGAGCAACATAAAGTTGACCATGTGTGAATAC
TGGCACGCGCAGGTCTAATCCGACTTGCTTGAGTGATTGTCCTTGTGCCTTGTTGATCGTCATTGCGAATGCC
AGGCGAACTGGGAATTGATGTCGCTTGAGCTCATATGGTATTCCGGTGTCTCCTTCTCCGGCAGTGCAGGTCA
TGCGTGGAATAAAGACCAACTTGCCCGCATGGTTGCCTCCAATGAGCCGCACCTCTAATACTCGGTCGGATGC
TCGGGTAAGAATAGCACGGGTGCCATTGCAAACACCATTAGCAGGATCCAGATTACGCAAAATCATGAGTGGG
CAGCCAATTTTGAGGGTGAGCTTAGACAGTGGTAATGCAGAGGCATTGATTGCGCTAAGTAGCTCAGGTGGGT
AAGCATCTTGTGCATACTCGGCATTGGTGTCCTTGTCCACAACCACAGAGTCTGCACTATGAAAGACCAAGTC
ATTGCCAGGCATGCGTTCCAGAACTTTTGCATTAATGTCATGGACATCAGCATTGCGTGAGCTTAGAATGGTG
CGATTGAGAAAGTAGTCATCGTCAGGGGGAAGTGGGCGGTCTTTATCCAGCTCAGGGTAAATGGTATTGATAA
GGCCTTGAAGTGAATTGTTGGCAAGTCTCATATGAGCAGGAAGAGTAATAGAGCCATCCGCAGCAGTATGCTG
ACCATTGCCCATTTCTT
>Cersu1_Cluster16#LTR/Copia
gttggaActtgGaattGgagtcgGacgattcGgGtgGTAcgGcGgCgcgacgGaActgagcNcggcgggaacG
gCGgGtgCaAGcTtaCgcgcTcgcgttcAtcCgtgcctggtAGcTAgagtTAcCTtaattagtgGcgTtctgt
gtAtttaGatAcCTcGtgCtCGtCaacgtacggcgcgcgtttTgCaacgcgGgCtCacTACatCaCcccggaaC
acctcgCgcgaCggNtTgttccaACACaaaCgCgctgTatagcCctctgttcttgacgcatcGcGANAttccc
NtttttgggtcTtaTCtggcAGaaattGaagaactatCaAAGaaCccctgttGacNacaacAtaccCCaCtCt
ataCtcgtaCaatgTctGatacCaCaTaCgaacgaggCttCccgcgacTtaatGactcCaactAtgcggAGTG
GtCcAtgcggatGgAGgccNAGCtCatCcgacggaAgCtcTgGgacgGGNtCNtTacaGtCtctgttgCtacg
AcggagaCCgagccgGaAaaggGtAGacgaGgaGacgaaGaaGgtttgtAcgcacgCgatGtGcagaagatgG
NgGAggcgCGcgcagagatgaTcctgcGtgcaGAagatggtcagctggcgcacAtgcGgTCacgCGacccacg
GgaGAtCtgGcaGAcGctCGcGgaGgtccAcaagGctcgcgggtTtGcaACgcaGctggcgatGaagcGacac
tTtcttACagccaagaagcggtCggatCaGAcGatgcaGgcCtggATaggcaacATaagGagCatggcCtTCc
GgatNGgggaggcagatatcGTggtgAgcgagCaggaCcagattctCgcgGTtaCgAtgggcttgcCaccaTc
ataCgaCaCGgtCaTCatCgcGcTCgaCtcgactcCGaccGAgCagcTcacacttgaacgcgtcNtcTCacgt
CttctcAatgaagaaatcCGacagTCgtCatcgggtgcCcCtcaGtcccactctcgttcCcgCgaCccCggCg
ttgCTgCagcTaCtaNgaaGGcgcttaTaaggGgggagatGcCgaaAggGcgCCttatCgAccGgTcagaggt
GcaGtGCTatttCTgCgatNattTcGgCCACTtcaagTccgaGtgccCggagAaggcgaaGtgGgagacagaG

aAgGcgaagaCgaAggagGcagagGctgcGgGgGGtcatTcggcaataCtcgcaacGacCTTtGgacccgtag
CcggagCTtggtGAttAtgggggtgcggaccttTttCtCtcNctNgaacatgtgctcGcgctgAgcttGtGgg
gGTGTgttggaActtgGaattGgagtcNGacgattcGgGtgGTAcggcGgCgcgacgGaActgagcNcggcgg
gaacGgCGgGtgCaAGcTtaCgcgcTcgcgttcAtcCgtgcctggtAGcTAgagtTAcCTtaattagtgGcgT
tctgtgtAtttaGatAcCTcGtgCtCGtCaacgtacggcgcgcgttTgCaacgcgGgCtCacTACatCaCccc
ggaaCacctcgCgcgaCggNtTgttccaAcac
>Cersu1_rnd-1_family-138#LTR/Copia
ATCAATCGACATCATCGGCGCGCGCACAGCCGAGCGACCGCAAAGGGTATCGACCGTTCTGGTACGTGCCGAC
ATCCGANGTGCGCCGGTCCACGCGCCCGCGCAAGGCCCCCAAGCCAGACGACGACGACATCTACAAAGTCACC
TCGTACNCGAGGCGTCCCACAGTCGCCAACGAGATGACTCCAGACGACGCCGCCGAGAACGAGGGAACTGACG
GCGGAGCGCCGGCAGTGGGCGCAGAACACGCGAAGCGAGCCGCGATGTCAGATGACCCGAACACGTACGAGGA
GGCGANGNCACGNCCAGATGCGGATCGGTGGGAAGANGCCTGTGCCGAGGAGCTACTCGCGCTCGNGAAGGCG
GAACCTGTAGANGNGGTCGAGNGNCCGGAGACCGAAACGTCGTCGGCAGCAAGTGGGTNTTCAAGGTGAAGCG
CGGTCCGGACGGCGAAATCGAGAAGTACAAGGCNAGACTGGTCGCGCAGGGNTTCACGCAGGTNCCGGGNGTC
GACTACNNCGANACGTTCGCNCCGGTAGCCAAGTTNACGTCGATNCGCGCCATNCTCGCGCTCGCCGCCAAGC
ACGATCTGGAGATCCACCAGATGGACGTCAAGNCCGCGTTCCTNNACGGCGANCTNGACGAGGAGATCTNCAT
GGCACGNCCGCCCGGATACCACGC
>Cersu1_Cluster38#LTR/Gypsy
TGTGGCGGTCGCGGTTGGCTTAGTCAGCCGGTGTCGCCACACGAGAGCTCGCACAAGTCGAAAGACAGGTTAT
GAGACTGGGATTCGAAAGTACGATAGGGTATGTAGTACATGGGCTCGGTCTTATATACGCTAGGGAGAGCGGA
CTTCGAACGAGACATGTACAACTAGATATTATCGTAGGCGAAGAGAGTAGATGGTTTTGGGATCGTGGGAAAA
TGTCCGTCTAAGGGAGGTAGAGTCTGGCGCGTCACATACGCCCTCCGGCCATTGCGAAGTAATGGTCCTACGT
GTAATTGATGATACCGTCCGCGTCAGCTTGAGTGGAGAGGGCGGGGGAGGGTGTGACGTCCACAGCGTCGGCG
CGCTGCTTGCCTTTAGAGGAAACCTCGGTGGAGGTAGCGGGGTCGGCCTCGGGCGGCGTGATAGAGTGGCTAG
GGACCGGAGTGGGCAGTGCCGGGTTGCTGCCAGCGACGAGGTCGGCGGGAAGAGTGGGGTCGACGAGCATGGG
GGACGGGGGCGCCATGAGAGTCTCGACGGAGACAGGGAGGAGGGAATCGAATGCGGCTACATCCCCGAAGGTG
GAGGCCGAGGTGGGGTCTTCGTAGCCGCCAGCCAGCATGGCATAGGCGGAGAGGTCGAGCCCAGTATCCCAGG
GGCTGTTTGTAGTGATGGTGGAGGCGAAGGGACCGGCCACGGATGCGACGGGAGGGGTAGAGGCTGTGGAGCC
AGCGGGAGTATGGCGAGGGCGCTTGCGTGGCTTCTGGGATTCAATGAAGCCGGTCATGAGATTGCGGAGAGCG
TTAATAGCAGCGACATTCTCGGCGTAGAGGTTGTGGCGTCGAGCAGGGTTATTGGGTCGTGGACCGGGTCCGT
GCGAACGGCCGCGAGCGGGCGGGCGCTGGGCGCGAGGGGAAGGAGCCGTGGTGGGCGCGGTAGGCACAACGGG
CGTAGGCGGGGGAGGAGAAACCGGAGGAGGAGGAGGAGGAGGAGGAGGAGGAGGAGCGGGATCAGCCGGGACG
AGCAAGCTAATGCGAGGGGAGGATTTCTTCAGTGAGAATGCCCTCCGTAACAAGGGACTAAATCCCGTGAACG
CGTACGGGCTGGCGCCGTCGGCGTTCCAGATGTGTGCAAAGACATCGTACCCACCCGGCATGACGAGCGCGGA
GGGGGTCGTTCTAGCACGTAAGAGTTCATCGAAGCGGATGAACAAGCAAAGCTGGAAGCGCGAGTAGATGTAC
TGCGTGTTATGGTTGTCGGGATCCCGTAGTAACAGGCATCCATCGGGCCCCGGAGACGCGAAGATGTTGACAA
ATTCGTGTTAGTAAGACATCGGTGTCGGCGAGCAGAGGCGCGGTGCGTGCGACGAGCAGAAGGTACAATGAGT
GATGCGAGAAGAGGACTCGGGATGAGCTGAGGAAGGCCTCTGTCTCGGTCACCTCTTTATACGAGTCCGAGGG
AGAGTCAGGCAGCCGACATATACCTGAGGGTCGTCGGGCAGAGAGCCAGAACCATCGGGCAGCGCAGAGATAT
TCTCGATACCTAGTAGTTCGAGATATGCACGCAGCGCATCAAGACCTGAAATGGTTGAGTACGGGACCCACGT
GATGTCCCCAGACTTCCAACGCGCCTCGAAAGTCGCATTGGTGCCTGATCCGCGATGGTTTATGATCGTGTCG
ATCGCCCATTCACCATCGCGTTCCTCGAGGTCTGCTATCTGGCTATCTAGCCGACCCGGGAAGAGTCGATCAT
CATTCGGCTCGTGAACGCGGAGTAGCGACGCATGGAATACATTGTGGATCCCTCGTTGCTTGAGGTTAGCCGA
GAGATCGAGCTGGAAGAAGTTGTTTCCGTAGTCCCGCAAGATCTTATATGGCCCAATATATTTCGGGGCTAGC
TTGCTGTCACAGTTTTTCGAAGCTAAGCGCTTAACCAGCGCTAGAGCAGTGGCAGGCAGTGTGTGTTCCATTC
TCAGAACAGTGACGAGTTAGCTGGTAGTGGCTGCCTGCGTGGCTCCAGGCTAGTCACATGACATAGCATGCAC
GTGATGCGCACATGTGCAGGTGTCCCAGAGTCACATGTCCCAGTCTAGGTCTGGCTAGGCATGGCTAGGTCTG
GAAGGAGTCTAGAAGCGACAGGAAGGTGCTCCCAGGGGGACTGGGGCCCACATGTATATAAGGAGGGCAGCTG
GGGCAGGTATCCCCCAGCTTCAATCTCATCACTTTCTACCAGACTTGTCTTGGCTATTGCTAGGCCTCTTATA
GTCTCTTTGCATCGCCATTAGTCCCCCTACAAGTCCTCGCTTTATTGACCATTAGTGAGTCTTTATTGCTGGT
TGGTAGATAGGCGCTCTGCCAGGCGCATATTTGTGACACTTGCGTGCATAACCTTTAGGCAACGAGATATTCT
TCGTTGACAGGTACACGAGATCTCCTTCACTGAAGGGGGCCGGGCGACGTTTGCGATTGGCATCGTGAGTCTG
TTTTACACGTGCCGCAATGAGACTGTCGTGCGCCGACATGACAGCGGATTTGACTCGCTGAGCGTACGCGCGC
ACGCCAGGGTATTCGTTGGGCCCTGCGTTGTCCCAGATCATAGGACACAGCATGCGACCAGTATTAAGAAAGA
ACGGGGCATATCCCATGCAATTGGACCGTGCCGAGTTCACAGCAAATTTGATTGCGGGTAGGCGGGCTGCCCA
GTCCTTCTGGTGCGGAGAAATACACTGCCGGAGCATTTGCATGACAGTGTGATTCGCGCGCTCGGTGGACCCA
TCGGATTCTGGGTGATAAGCACTAGACATACGCAATTCCATGCCAATGAGGTGGTGAAGGTGGGTCCAGAAGA
GACTCGTGAAAACGACGTCTCGATCACTGACAATTGCGCGAGGCATGCCGTGATGCTTGTAAATTTCGGAGAA

TACCAATTCTGCTACCTGTTTAGCAGTATGGGATGTCCGACTGGGGATCAAATGGACCATACCAGTGAGAAGG
TCGATAGCGACCGTAATGGAGTCGTAGGACCCGTTGCGGTCTCGCGATTCAGGCAGTGGGCCAACAAAATCGA
TACCCATGCATTCCCATGGGTACGAGGGTATAGCCAACGGGTTCAAGAGCCCGTAGGGCTTCTGATTGGCCGG
TTTGCTCCTCTTGCAGGTCATGCACGAGTCGCAGTAGGACTGAACATCCCGGACCAGGGACTTCCACCAGACA
TGGTCTCGTAGGAGCCCTGACGTCTTGTGCGCGCCGAGATGTGCGAGGAGGGAGTGGGCGTGTCGAATGAGAA
TCTCCCGTGCGCTTCGTCCCTCGATACAAATATCTGGGATGCACAGCAAGTTGGTCTGGCACTCTGTGAGAAA
TACGAGACCTTCTTCGAGTGCGAAGTTCTTGTAGTCCCTAGGTTTCTCCAGTATGGCTCGAAAGGATGGATCT
TCTCCATATCAGCCTCGAAGAGAGCCTGATAAATCGAGCCCTTCAGAGCTTTCTGAGACGAGTTCGAGCAATC
CGATGGAGCGTTGGGGCAGTGAGTCGGGCGCTCGTTCAGTTCGGTCAGAAGCGAAGTTCGACCGAACAGCGCT
GGGAGCCTGAGAGCGTCCTCAGGCGGTGGTTCCCACGCTCTGGCTGACGTGGCCTTGCTCATCGAAGAGCGCG
CTGGGGAGCTCCCCCATGAGCTGCTCGTAGCTCGCAGGTGAGTCCTCTGTTGTCTCGTTATAGCTCGTCAGCG
CAGCTGCCGGCGTGCTCTCGCCCTCCTGTCGTTCACCACGCGGTCGCGGCCCATGGAGAACGACATGGCGGAT
GCGCTTCGCAAACTCGCGCGCTGTCTCAGGCCGCCCGCTTTCCGGAGGTGCGAGGGGCGTTGGGGGAGTGCGA
TGCACGGGACGCGAAAGAGGGGGTGGCGCCACGTTCACGGACGCGGTGCGCGACACTGCTGCGAGCGTACGTT
TGCGGCTAGGCTGTGACGTGGCAGTGGTGGCGCGCGTGCCATGCTGTGGGAGCGACGCCGTGCTTGCCGGGGC
AGCAGGCGGTGTGGTTGTAGGGCGCAATGGTACGCCTGCCGGAGCGGTGCGTGATGCGGCGGTAGGTACGTGC
TTGCGGTGAGACCGTGACGTGTCTGCCGCCGCTCGCGCCGCGCGTGGAGGGAGCGATGTTCGCGGTCGATGCG
CAAGCGGCAGGGCGGGCAACCTTGCGCCCTCCGGAGTGCGTACTTGGTGCGGGAAAGATGTCGCCGCCGCTGG
AGTAGTTCGCGCACGCAGGTTATACACGCTGCCGGCCTGAGCCGCCATAGCCGCCAACCCGGTGACGACGGGC
ATAGACACGTCATGCACCGCAAGACGCGACGCAAAAGCGTCGTGGTCATCGTGCTGCGTATATTCCGAGGCAG
CACAAATGGTTCCCAGTGCGTCGGCGGTATAGATGCACGAGAGTGCATTGGCGAGGACGTTCTCGATGCCTGG
GATGTATTCAATGCGGAAATTAAATTCAGAAAGTTTCTCCAGCCACCTGGCTTGGCGACCGGAGAGATTCCGC
TGGTCCAGGAGGTGGACCAGCCCCTTGTGGTCCGTAACCCAGGTGAATTCGCAACCCTGGAGGATATCACGAT
GGCGCAACATTGATTCTACTTCTGCTAACATTTCGATTTCGTGCACAGGATAATTCGATTGTGCCGAACTGAG
TTTGGCGGAGTAGAAGGCGGCGATACGAGCACGGCGGAAGTCAGGCCCTTGCGCGACCACCCCCGCGATGCCA
CCCTGCGACCCATCGGTAACCAACCAAATAGGTTCGGCATCCTTCGAATAATCGAGCGGTACTCGGTGGTGTT
CGCGATGGGCATGAACCAGACGTTTGATTTCGTCAAACGCACGCTGGTGAGTAAATTCCCATTTGTAACTCGA
CTCGACACCCATTAACGAGGTCAGGATGCCTATAGGGATCCGTACCGTCGCGATATCATCAGCAAGATATCCG
ACAGAGCCAAGGAACCCGCGCAGGAGCTCCTTTGAAGTAGGCGCCTTCCAGTTCAAGACAGAGTCGACTTTAT
CGGGATCCATGCAGATCCCGCGGTCATCAACAATGCGGCCGAGAATTTTCATCTCGGAGCAAAGGAATTTGAG
TTTGTCCTCGCCGAGGTACAGTTTCTCTCGCCGGTGAATATCGATGACGGTTTTAGCATGCTCAACATGCTCC
AGCAGGGTGTCCGAATAGATGATTATATCATCGAGATATACGTCCATGAATACCCCGATATAAGCACCAAATA
TATAGTTCATGAGAGTTTGGTAGGTCGCGACTGCATTGCAGTCCCCCTGCTGCAGTACGAGACTAACCATATT
GCCATCAGGTGTGGTCATGGCGGTGCGTTTGACATGAGCGGGTTCAATTTGAATCTGCTGGTATGCATCTTTC
CCATCAATTAGGGACCGAAATGGCTTGCGCGACACGCGGCGCAGGATACCGTCCATATTGGGCAGGGGCGACG
CCACCTTATGCGTATTCGAGTTGCGTTCGCGCAGGTCGCAGACGACGCGCAAGCGGGGAGGTACCCCCTTGAC
TCCGGTTCCCGGTTTGGTGAGTAAGAGCATCGGGCTCGTATTGCGAGCAGTACTCATCTTCCAATGGCCGGAT
TTTAGATACGCATCGCGTTTCTCGATCCACGCAGGATGCAAAGCATCTGGGCATTTAGAGGGCCGCCAATGGT
ACGTTTTCGACTCGTCCTTAAGCGGAATTGTGTGATTAATGACCCGTAGAGGCGGTAGCGGAGATTCGCTCGC
GTTCGCGCAGATAGGTGTGGCATAGTCCCGTAGCAAGGCACGCGCGCACTCAAAATGATCGTCGAACACCTCA
GTTGCTCGGGACTCGAGAATGCGAACACGCTGTCCGGCGATAGGCCGAGCCGCATCGCTGCCAATGAGAACAC
TAGCGGGATTCAGACCCACTGTCACACAGTGTTGGAACATGAACGGGGTGCCCAATATCAGATCGTAGTTAAG
CAGATTCACGACATCAAAATATCGTAGTTTGCTAACTGATTGGTATGTGATCTGCGCTTTACATCCAGTACTG
ATCTTTGCCCGGGACCCCTGCACTGCCAGATGCACCGGCAACGGCTTAGCGAGTTCAAATGTCTGGGCATTCA
GCTGGTGAGCGAGCTTCGAGGATATGAAGTTGGCTAACGAACCAGTATCGAGCAGAGCACGAGCTGGGTGGCC
ATTTATTCCTACGACCATGACTATCGGCTCCGGAATGAGCCGTTTGAAGTCTTTCGGTGCCGCCGCATTACGT
TGGACCACGAGAATGGGCTCGGGCCTGGATGAGGTAACTGCGCTCAGCTCGATCGCATGCAATGCGAGGGTGG
CAGAGCACTTACGGAACAAAGAGCGCAGCTCGCCATGCAGATGATCAATCGTGAAGCATGGATCGCCCAGTGC
GAACGACTTGAGGACCCGGGTGGCGTACATATCCGTGAAGTCGACCTTGGGGTTGTCAAAGACACTAAGACGA
TACTTGGTCGTTAACCCAAGATAGTCATCGTAGACCCAGACCCAGTTATCGCCTTGCTTGTCGCGCCAGCAAC
AGAAACGAGGAGGCAAAAACGACATAAAATCGACATGCCCCGGGTAAGGGAACACGCTTTCAAGTAGGTGAGG
CACTTCGCTAACGAGGGCGTCTCCTAGTCCTCGCTCGCATGGCTCGGAATGTCCGCGTAAGGGGTTCCGAGCT
GATGACACACAGACTCGGCGACCCACCTAGGGAAGTCAAAACGCTCGTTGCGGAGCCATTCCGAGTTAACAAA
CAGCGAGTCTTTAGCATAGAGGTACAGGACTGCATACCCATCGAGAGTGCGGTAAACCACACCACTCGGTTCA
AGATCATCATCAAACGGGATCCCCTTCAGGCAATGGTAGGGCAAGTGCCGCTCAAAGACTGCGGAGGCGTGCC
GTTCCCAGTAATCGCCTTGCGGCATATACCCTCGGGCACGTTGGCGCTCGGGCAGCACAGGCCAAATGCCGCA
GTACCGGTTTAGCTCGCTGGCGAGTAGCTGGGCCACCCATTTCCAGTCGCGCTGATGAATAGCGCATTGAAGT
TCTTCATCGGGTTGCGTCAACACAAAATCCCGGAAGTCGTGCGAGTCAGGGGAGCACGATCGAGGCTCACTCG

CAAGGGAGATACATTGTAGGTCAATCCCCTCCTCAGTGTCCTGAGACTGCTGATGGAGATCTTCTCGCTCAGT
TAATCCAACCTCAAGACTGAAGCTGGACACTCCCGGCCCGTTCGTGCGCCCAGGAGCTTGTCCCGGGTTGTCC
GAACGGACCCGTCCCGCGCAGGGACAGTTACGTGCAAAATGACCTGTTTCTTTGCACTCGTAGCATTTACCGG
CTGCTTGCAGCTCCGTTTTCTCTCGGTCCGAGAGTTGTCTCTTGGGCGCACTGTCCTTCGGTTTACCGAGACG
AGTACGAGAAGGTGTATTAGAGGAGTCCTTTCGGGTTCCAGGTGGGCGCTGACCATCGGGTCTGCGAGCAGCG
GCAGTACCACGACCTCCCTTCAGCTTGGAGCCTCCGGAAGATCCACTGGTACCTCGCCCTCCTCTAGCCCCGC
CTGGGCCTGGGGCGCCACCTCCGCCTTGTTCTGAGCCGCCCTGGAGACCACGTCGAGAAGTGTCCAAGACACT
TTCGGAGATCTCGATGATCTCAGCGGCCTCTACTACAGCAGCCCAGCTAGACGAGGTCGGGGTCAGGCGTTCA
CGCCAAAGGCCTCGCTGAATATAGGAGTTGAGTCCAGTCCAAAGACGTTCGACACGATCCATCTCGGACTGAA
CTCCGGCGAGCAGGAACAAGTTCTCGAGCTCGTGGACATATTTGCGGACGGACTTATCCTTCTGGTACGCTTT
ACGCAATTTCTCACGCATTCTCATGCGGAAATCCAGGGGGAAGCAGTAGTTAAACAGCTCTACGAAGAGCCGT
TCAAGGTTCCAGGTACGTGGGTTCTGTGATACAGTGACCATGTAGAACTCATATGCTCGACCTGTCATGAATG
CAGACAGCGAGCTGGCGTGACGGGAGGGTTCTAGACCGCACCCGTCCACATAGTCCGTGCATTGTTGCAGGAA
CTTGTGAAAAGACTGGATATCCGGGGTCCCACTGTAGGAATCCGGATCCTTAGGCTTCAGAGCAGGAGTCTTG
CGCTTCTTAGAGCGTGTAGACTTCTTACTGTGCTTGCGCTTAAGACGCTTCCTTGCCTGGCTCGCGGAGTCGG
GGTCCTCGGAGCTCGACTCGGAGCTCGAGCTACTGTCCGGCTCCGAGCTGGGTTCCGAACCATCCCCGTCATT
AGAGGGGCCGGAGCTCCGGCGACGGAGGTTCGCGAACATGTGGCCGATATAGCTATTAGCAGCCACCTGAGTT
GCGGGGCGCATAATGCCAGGGCGATCGTTTCCTCGTGCGGAGGCGCCGGTTTCGGGCAGTTGTCCGTGCTGGT
GGTTCAGCACATGGTTTACAAGGCTGCTCGCTGGCTCAAGATGCTCTGAAAATCGAGCACGGTGCTCCGGCTG
GCTATCAGCGTGCCGCTGTGTGAACTCGCTCAAGCGGGGAGCACCTTGCCCTTCAGACAACTCTGCCACGTGG
CGGCGTTGAGTTCCCAAAGCGGAAGGTGTCTCACTTGGTGAGGGGGCTACGTGTGGCGGCTTATCACGTCGAG
TCGCCCTTCCCATGCCGGTAGACCCATCCTCACGGTCCCGAGCGTCGGGGATCCGTGATTCCGTATGCCGGGC
TCTAGCCTTCTGGTATTTGTACTCATCGAGCACCCGGCGCATTTCCTCGGGAGTCAGCTCGACCTCGCCCTCC
GTCTCTTCGTCAGGAGGATCAGGCGGATAGCCTTGCGAGTACCTCTCGAACTCCCGACGTTGTACGTCCGGGT
CGAGTTCTTCTTGGGGGATGTTAGTCGCACCCCAGTTACGGGGGTCAACAGTCTTGCCCTTCTGCCAAGTGGA
GGTTCCTTCTCCATGGGACTCTGTCTCAGAATCCTCAGTGACCCGGGCGGTAGGAACATTGTCTCCAGGGCGA
ACGGAGTGGACGGGCTCGCGAGCTTCCTCGTCAGCTTGTATAGCGTGCAACTGAATCTGCTCAATCACGTCAC
GCTCATCCTGCCTATCCGAGTCGGGCTCTCGCCGACGGGCAGGGAGGTCAAGGCTGCAAGACCTGCGAGGTCT
GCACCGGACCTCTTTCCAGTCCTGATCGCCCTCGATAGTATCAAGGCGCGAGTCATCAGGTACGGGGGAAGGG
GAATTCTCGTTATTAGAATTACTACTACTCTGAATAGATAGGTCCTCCAGGAGGCTTGACAGGGATGTCAAGT
CCGAGGCATTCAGATTTGTGCTAATATCCCCGTCACAAGCCGTCTTGCGAGCGTGGAGCTCAGCGGATACGAC
GTGCTTGGATATGACCGCGCTATACGTCAGGCCGTGCCTAGTGACTGAGGTTGAGCGGGCACTTGCAGTGTCC
GAGGCCGATTCCTCATGGTCACTCAGCTGAGCATCATCGGCTGCAGCTTTAGGTGTGGAGTGAAAGGCACCGG
GCACCGGAACCGGAGGGGTATCGGGTGCCCTTGCTTGAGACCGCGTGAACGGTCTGCGGGACATATCGTAACC
GGATAAGATCACAATCTAGTACCAGGAAAGATAAATGAACTGGGCGAAATATGTCGGGGTTTTGGCGGCGTTC
GTCGGGTCGGAATCTCGAGTTGATCAGACGCAAGGGATCCAGGAGTCGACGATGTCGAACAGAGAGAGAGGAT
GAGTTAGAGAGAAAGCAGAGAGTTGTCGAACAGTCTGGCTGTCAAAAATATGTGGCGGTCGCGGTTGGCTTAG
TCAGCCGGTGTCGCCACACGAGAGCTCGCACAAGTCGAAAGACAGGTTATGAGACTGGGATTCGAAAGTACGA
TAGGGTATGTAGTACATGGGCTCGGTCTTATATACGCTAGGGAGAGCGGACTTCGAACGAGACATGTACAACT
AGATATTATCGTAGGCGAAGGGAGTAGATGGTTTTGGGATTGTGGGAAAATGTCCATCTAAGGGAGGTAGAGT
CTGGTGCGTCACA
>Cersu1_rnd-4_family-462#LTR/Copia
TCTCTGGCAGGAGCAGAGACGGCCCCAGCGACAGGCCCGGCGGCCCCAGGAAGGAGCTNACNTGCTGGAACTG
CGGCGGCACGGGNCATCGCAAAAGCCAATGCCCGAGCCCCAAGCGAAAGAACGACAACCCGAGCGGAGACGGC
GGAGGAAAGGGGACCACGGCCGCGAACGCGGCGCAGGACGAGGANAGCGACGTCGAGTACAGTTGGATGGCGG
CTGTAANCGAATCCGAGTCNGATAGCGAAAATGACATTANTTTTCGACGTACTTCGAGTGCCCGAGCCACTC
CCCGACGACGACATGCCNGANCTGCGAGCCGTGTCCGACTCCGAGTGATGANGAATTCCTGGAGGGTGACGAC
GACGACGGACGACCGACGCGGACGGCGAGGACGANNCCGACGACGAGATCGACATCGCCGCGGTNGTACACGG
CGACNCTGGGTGCAAGCACGTCGACCTNTACGACTCNGGAGCGACGAAACACCTNTCACCNTTCCGAGANCGN
TTCGCGTCGCTCACGGAGATNCCCCCAAGACCCATCAACGCCGCGAACCAGCAGCGCTTCCACGCGATCGGCG
TCGGAGACATGGTNGTCGAGGTACCGAACGGNTCGGGCTTCTCNAAGATACGCCTCCGCGACGTGCTGTACGC
NCCCCAGATGGGNGTCACGCTGGTATCCGTGGGNTGCATCGATGCNGCGGGATACTCGGCGCTGTTCGGNGGC
GGCCGNTGCCAGATACGGGACGGGGACGGNACCGTTGTGGGGGAGATNCTNCGCAGCGGNGGNCTGTACAAAG
TCGAACATGAAGGGGAGTCGGCCCACTCNGCCCTCGAAGAAGTCACCCTGGAGGAGCTCCATCGTCGCCTNGG
TCACGCGTCGCCAGAAGCCGTNCAGCAGCTNGTCAGCCGCGGGCTGGTGACAGGTGTCAAGCTCGTCGGAGCC
CCANATCGAGTTCTGCGACTCCTGCGTCTTCGCGAAGACNCGGCGNAAGCCGGTGCCGAAGGAGCGCGNCAGC
CCNCTCGCNGANGAAGTCGGCGACGTCGTCCACACCGACGTCTGGGGCCCCGCCGACGTCGCGTCCACCGGGG
GNAGGAAGTACTATGTCAGCTTCACCGACGACAAGTCNCGACTCTCCGCGACGTACCTTATGCGCCANAAGTC

GGANGTCCGNCGNATGTACGNGAAGTACCGCGCCTGGNTGAAGTCGAAGTTCAAGCGNCGGATCGGNACCCTG
CAGAAAAAAAAAAAAAAAAAAAAAAAAAAAAAAAAAAAAAAAAAAAAAAAAAAAAAAAAAAAAAAAAAAAAAA
AAAAAAAAAAAAAAAAAAAAAAAAAAAAAAAAAAAAAAAAAAAAAAAAAAAAAAAAAAAAAAAAAAAAAAAAA
AAAAAAAAAAAAAAAAAAAAAAAAAAAAAAAAAAAAAAAAAAAAAAAAAAATGTNATGCATGCGACCTGGCTG
AAGAACCGNATGACNACGAAGCCNCTCGGGAGNGACACCACGCCGTACGAGGCGGCGTTCGGAGCGAAGCCGG
ACCTATCGCGGCGCGCACGCCTTCGGNTGCGANGTNTGGGTGCACGACCCCTCCGGNTCGAAGCTCGACGGGC
GCGCGCGCGAGGGGCGNTGGGTNGGCGTCGACCTGNACAGCCAGGGGCANCGCATCTACTGGCCNAAGACGCG
NACCGTGACNGTNGAGAGGACCGTNGTNTTCAAGCCAAGACGCCGCGTGCGCAGCTCGAGGGGGAGAGAAGCG
ANGATGAAGNCGGCGAGCCGGAGGACGAGCCGAGCGAGACAATCGAGCATAGAGCGCTCCCGGCGACAGCGTA
CAG
>Cersu1_rnd-1_family-213#LTR/Copia
TGCTTTCGGTCGTCGAAGAGCTCGAAGCCCATAATCCAGTTTGGGTCTTCTTCCTTCATCTCGAAGAGGCTGT
GCAGATCGTGACGGGCAGCAGCGAGTTCCTTGCGCGAGCTCGAGAACGTGAGAGCGTTGTCGACATGGATGCC
CGTGATGGTAGTACCAAGCTCGGTGTCACGGATATACACCGCGTTGTCCGTGAATAGCTGTCGGAAGCCGACC
GATTCGAGCTCCTTGCGCAGTTTCNTGTGACGATTCCAGAGTCGTGCCGCTTGCTTGAGNCCGTAGAGGGCTG
CGTAGTAGTCGTACAACNCGATCGGTGCCGTCCGTGAAGCCGGCGGGCTGTCGCATGTAGATCTCCTCNTCCA
GNTCGCCGTNGAGGAANGCGCNNTTGACGTCCATCTGGTGCGCGGTNCCAGCCGCNTGCGGCNGCGAGGGCGA
GGATTGCGCGTAGCGCATCGAGTCGAACTGCGGGTGCGAATGTNTCGNTGTAGTCGACACCNGGAACTTGCGT
GAACCCCTGCGCAACCAGTCTTGCCTTGTATCGCAGATCTCGCCGTCCGGGCCNCGCTTGATTGCGTAGGCCC
ATCTATTACC
>Cersu1_rnd-1_family-23#LTR/ERV
TCTTGGGAATCTGAAGCACGCAATGCAGAAGGTCAATCAGTACCACAGACGGTATCCCGATGCACCTAGAGCT
ATTGCGGCAATCCACAGACATGAGTCTGAGGAGGATGCGTTTTTTGCACGGGAACTTGTGCTGTTACAGTCAT
GGACTCCAGACGACGCATCTGAGACAGGAAACTTAGTCAAGGATACGGAGACCAGTGACAGCGCAGGAATGGA
CCTTTGCGTTCCCGAAGATATTACTCTTGAACCAGGTGCTCACAGTCTTGTCCCTACGGGACTGAAAATGTGC
CTACCACCTAGAACGTGTGCCCGAATCGCACCAAGGAGCGGCCTTGGCCTCAAAGGCATTGTTGTTGGCGCTG
AACGGTTAGACCGTGATTTCCGAGACGAGCTCAAACTCTTACTTATCAACAACTCGCCCAACGCCTTTACGTT
CTACAAAGGTGATTGTGTTGCTCAACTCGTGATCGAGAAAGCCCAACAAGCTCCGCACTCGACAAGTTTTTGA
CCTTGCTGGCTATCGGCTTCGCCAGGAGGCTCAGTTTAAATTGGGGGTATTGTCACAGTTTTTCGAAGCTAAG
CGCTTAACCAGCGCTAGAGCAGTGGCAGGCAGTGTGTGTTCCATTCTCAGAACAGTGACGAGTTAGCTGGTAG
TGGCTGCCTGCGTGGCTCCAGGCTAGTCACGTGACATAGCAGGCACGTGATGCGCACATGTGCAGGTGTCCCA
GAGTCACATGTCCCAGTCTAGGTCTGGCTAGGCATGGCTAGGTCTAGAAGGAGTCTAGAAGCGACAGGAAG
>Cersu1_Cluster23#LTR/Gypsy
GGAACGCGATATGTTGCGAACACTCGGAAAGTCGATTCGACGAAAGAACAGGCAAAGAACTTCATTACTCGTA
AGTTAGTTTATATACATGGTTCGAACCGACTAGACATACCTTCGTTACTCGTAAGTTAGTTGAAGAGTAACAA
AGGCGTTCCAAGCTCTAAGTACCGAGATGTCGTCGGAGCTGTAACTCTAATTGCTCGGGGGTGTCCAATGAGA
AGTGATTAAGTAACCAAAGCAAAAGACCATCGCGGAAGCAAATGTCCGTCACGTGCATATCGTGCGTGCATAA
GGACATCGGTGTCCTTACACCCTCTCTCACCATAAACTCAAGACATCAGGGATGTTCTCGAATCCAGGTGTCA
AGCGCCTCGCAGTCGTCCAGCTCGCGGCCAGGCAACCAGATATCCGCCTCGGGTGGCCAGCCCGTCCAGCGCA
CCAGGTATTGCCATCCGCAGCCCCGTCGCCGTGCGTCCACGATGGAATGTACCATCCACTCCTCCTCGCCATC
CTCGGTGACCACCGGACCGGGTCGAGGACTCTCCCGCAAAGGGTATTTTGTTGCGTCGTTTGGGATCCACTTC
CTCAAGTTACTGGAGTGGAATCGAGGGAAGACGTTCATTGTTGGCGGCAGATCGAGCAAATATATGGAGTGAT
CCAGATCAGCTTCTAGCACCGTGTACGGCCCATCGAACCGTGGCATGAACTTTGCGACGCGCCCGTCGCCACG
CTGCATGTAGTCCCTGCGACGGTGCTTTGTCGCTAGTAAGACACGGTCCCCGACTTTAAAATTTGGCTCATCT
CCCCGGTGTCGATTCGCAGAAGCTGCTTGAGCGACCTTTGCCGCGAGCAAATTGTCCTGAGCGTCGTGCACAT
ACGATTCAAGACGTTGTATTAGATGTGCTGCGTCGTATTCTGCAGACGCCGGTTGCGCGGAAGTCACCAAGGG
CGGAATAACGCGCGGGCTCTGCCCAAATCTGAGTTGGAACGGCGAGTATCCCGTTGACGCGTTGACTGTGTTC
ATGACGCAGAACCTCACGAAGGGCAATCTCTCCAACCATCCCTTTTGGTTCCGGTCAACGTGGTACCGCAAAC
ATTGCTCCACAGTCTTGTTTGTACGCTCAGAGAGTCCGTCCGTCTGGGGGTAGTACGCGGTGCTGAGTTTGTG
TTTGATACCAGTCAGCTTGCAGAGCACCCTCCAGAAATCTGAGATGAACTTCGGATCACGGTCAGAGATGATT
TCGTTGGGCAGCCCGTTCTCGCAGAACCAGTGCTCGAAGAACTCGGCAGCAAACTGTGGTGCTGGCATGTCCG
TTTTCGTTCTCACAAAGCGGTAGTCGGCGCCCATGTGGTCCGACATAGTGACTATACAGTTCTTCCCCCCATC
TGACGGCAAGGGCCCGATCCAGTCTATCGTGACCGATGAGAAACGGCGATCAGGAATTGCCAGAGGGTGCAGG
GGCCCGGTTGGCTTTGTTGTGCTGCTTTTGTTTCGCTGGCACTCCGAGCAGGATGGAACGTATCCCTCTTCGA
GATCTTTACGCATACCAGGCCAGTAGTATGAGTCTCTGAGGGATGCGTAAGACTTGGCAGCGCCGAAGTGGCC
CAAGCTATCGTGAGCGAGTGCGAAGAGACGTTCCCGGAGCTCCAGCACGTTGGGGATGACCAGACGCGATCCA
ACATATAGCAAACCATCCTCAAGATGAGCACCAGGGGTAGATTCCAGCGTTGCCAGCAGCTTTTGCGCAAAGC
GGTCCTTCGCATACCCAGCCTTGATTTATCGACCAGGTCGTTGTCTGAGGAGACCGAGAAGACCGCTGCCAA

GACTCCAAATTCGTCGCGCTCGATTTGTTTGTCAGACTCAGGCGGCAAACGGGACAAGGCGTCGGCCACTGTG
TTGTCCTCGCCCGGTATGTAGCAGACGTCATAATCAAAGTCCGCGAGAGTCTCCTGCCACCGCATTTGACGTT
TGCTCATGATTGGCTGGGTCTGGAAATTCTTGAGGGTCTCGTGATCAGTATAAATTGTGAACCGAATACCCAG
CAAGTCACTCCGCCATTTCTTCAACGCGCGTATGATCGCGAGGAGCTCCTTCTCGTGCATGGGGTATCTGGAT
GGCGTACCAGTGATTTGTTGCGACTCAAAAGCCACCGGCCGTGAGAGCGCCCACGTCTTGCCTACCAACAATA
CCGCGCCAGTCCTCCATTCGCTCGAGTCGCATGTGACGAAAACGCGGTCTTCGCCCAGGTTATTGTGGTCGAT
TGTCGTCAAACATTCCCTTCCTACCACGAGTTGCTTAATCGACTCGAATGCCTTCTGGTGTTGCACCGTCCAG
TCGGGGAACAGCTTATCCGCGTGACGCGTCGTGAGGGGGTTCAGCACCGAGGTGTGATCCGCGAGCTGTGGGA
GAAACACTGCAATTGTTACGAAAGTGCCTGGAGCAGCTTTCGGTCGCTTTACTAGACTTGTCAAAGGGTCTTA
GAGGGCTGCTCTAAGCTTTGAAACAAGCGTTGTAATGCGCGACAAATAATAGCCAATGAAGAACTCTTCGTGT
TGCTCGAGGCGAGGTACAAAAGAGCGAAAGACTAAGGAATGTAGCTACAATGACCGTGTTTTTATACTACTTG
CGCGAGTCGTTTTCCGAGTCGTATCGTACGATGTCGAATAACTATTTGTCACGCTACCTGTGCGCAGTATTGT
ACCATCTGTCAGAGGTATGTCGCAACCGGTCAGCAGTCCACTAGTCTACCAATGGCCGTTGTCGAAACATAAG
GCGTCGGCGCCTACGGGAGAGGGAGGGAGCGCGACGGGAAGGCGAGAACCGAGAAGGGGATCGGGGATCAGAG
AAGCGGGAAGCCGTGGATTGGGAAAGGAAGACGAAGAGCGACCGCCACCGGTGGCGGTGTCAGGGTATGCGGG
AAGGGCGATCCGATGATCTGCCGCCTTCCGGGTCTGTTCAGGACCGGGAATCGGGTCAGGTGACTGGGGTCGT
CACGTGGTGAGCGTAGAGAAAGGGAAAAACAAAAAGTCGTAACAGCAATATACCTGACCAAACCCAAGAATGA
TCGCACCGCCTTTGCGCTCTTAGGCCTTGGCCAATTCAAGATGTGATCCACCTTCCCGGGGTCCGCCTCGATG
CCGCGTTGGGAAATGCGATGCCCCAGGAAGTCGATCTCATCACAAAACAGTAATGTCTTCTTCATTGAGCAAT
ATAGATTCGCTTCCCGTAGCGCCGCCATTACTTTATCGATATTCTCCACATGTTCCTCCACCGATTGCGACCA
TATGATAATGTCATCCAGGTAGACGTGACATATCTTGCCGATGAGATGCTTTAACGCCGCCGTCATGCGCCGC
TGGTGTGTTGCCGGCGCGTTCTTGCACCCCATCGGCATTACCAGCCACTCGTACAGCCCTATCGGCGTGTGCA
CCGCCATATACATCACGTCGTCAGGATGCACGAGCGTTTGGAAGAATGCGTTGGTCATATCAATTTTACCCCA
TACCTTCCCTTTCGCACAGTCCACCAGGATATTGTCGATGCGAGGAAGAGGGAATTTATCGGGCACGGTGTTA
TCGTTCAGTTTGCGATAGTCGTTTACCCATCGGGGCATCGCTGAAGGGTCTGCTTTTGGGATGAGAAAACCCG
GCAAAGCATAAGGGCTCGATGAAGGGCGGATCCTCCCCGCGTCAAGGTGCTGTTGTATGAGAGTCTGCCACGC
CTCTCGATACCTACACGCGCAGGGGTAGGACCGTGTCGCAATAATCTTATTCGGGTCCTTCAGGACAAAGCGA
TGGTATACGTCTTGTGGTAATGCATTGACATGAGGGATGTCGATAGGGAATTGCCCTGCATGCTTCTGCTTTG
CTTCCGTGTCTTTCTCCTTGAGCATTTGCAAATATGCCAAATGTTCTACCCGACGACGAATCGCCGGCGCTGG
ATCATCGAGTTTCGTGCGTTGACAAAGACGATCTCTCGCTGTCGAACCAGTCGACTACGGAGTTTCTCCACCA
TAGTCATTGTGGCTGCCATCACATTGACACGCTTCTGCTTAGGTGAGAGGCCGTATGACGCCGACGTTGCGCT
GGGAGGCGCGGGCACACACGGAGTGAGTAGATCATAGCCGGTCGGTTTCACTATGGCGGTACGTTCGTCGGAG
TCCACAACCACTTTATTGATCTTTAAGAACGGTAGCCCTAGTATTACATCGGTGGAACATCCAGAATCGATGA
AGGCGTTGACAGTCTGGGAGAAGAAGCGAGAATCTAAGCTCTTGAGACGAAGGCGGACATATTCCTGGTGATA
AGTAACCTCCCCAGACCCTCCTGCTTCCATCGCGCGACGAGAGGGTAAGGGTGTTGGCCGGGTACGGACACGT
AGACCGAGTTGTTTAACGAGCTCTTGACTAATCATCACTATATGCGAACCGTCGTCAACAAGCGCTCGGATGC
GGAGGGGGGTGTTGACTAGAGGGCCGTCGACGAGACAATGCCAGAACATGTTCGGAATCGTAAAAACGTACTT
GTCGGTGTCCGGTCCTTCCAAAACCGAGGATGGAAGAACAGCCGCAGTAATGTGATTCTCTTTGTCATCCTCC
TCGGTGTCTTCCACTGAGGTGACGGCAGCCACTTTGCCAGCACGTTCTGCACGGAGCTTCTCCGCGATGTTAG
CCTCCGTAACCGGCGTGAAGGTTTTCGGGTCGGGGAACCCGTTGGGGCAGTTTGGTAAGAGGTGATTCTGTTC
CACTTGTCGGCACTTATAACAACCGTTATGCTTGGATAGCAAACGGCGTTCCTCGTCGGACATCTTCGGAACT
TTATTGCCGACAAAGGTGGTGGATAGCACGTGCTTCTGGGTGTTTGGCTTGGCGGCATTGCTAGTCGTCGTCG
GGGCGGCGGCGCCTGAGGTACGGCTCTGACTAGCGCGCACCTTAGCAATCGCCTGCTTGATACTTTCCTTGAC
GAGTGCCTCATTCTCGCAGCGCTCTTTATCAAGGTGTTTCAAGGCCTGAAGCCACTGCTCGAAGATACCGAGT
CCGTGAAGATGTGGGGAGTTGTGCTGTGAGATCATAAGACGCTTGAGCTCCGGGTGTACCGAGGCAGAGACAT
GTTCCCGGAGGCGACGCTCGTCTAAGGCCTGTGGGGTGTCGGTGAGGTACACAGCCTTGTTGCGGATGTCGGT
CACCCAGTCCCAGAACGGGCGTTGACCTTGTTGACTGCCCAGTATTTCATCGCATAAGAAGATGTCCCAGTCC
TTCTCGAGGTATGTATCGCGGACAAGTTGCATGAAGACGCTGAACAGCAACGCGTCGTATGTCAACTGGTTCG
AGTTGTACCAGTTTTCCATGCGTGGATCTTCCATGCGGGATGTGATCGTAGCGGTTTGTATTTCTTCGGCTAG
TGCAACTCGGCGGAAGTGCCGCTTCGCAGCGTGCTCCCATCGGTACAGGATCTCCGGGGAGACATCTCCCGCA
GTCAATACGGGTGGTTGCGTATCCTTGGGGAATTCGACCTTAGCCAAAGGCACGTCGGACTTTGGGGGCGGCA
TTACTAGGCTAGAGTCGACTATATGCGGTGACTTTGACTCCAACACGGTCGGCGATGGCTCAGTAGGCTGTCA
CGGTAGATCCTCAGTCGGCAGCGGCGACGTGATGGTTGAGGACAGCGGCGAGATTGCTGACCACTCTGAGGTT
GTCGGTGACGGAGGGAACTCCGAGTACGTGGCAGTCGGTTAGGAGGTGGCAGCCTCCTCGAGATGTGCCCGAG
CTCGGGTCCTGAGAGGTGTCAGTGTGGAAGGCTTGCGTCGGGTAATGACGGGCAGGCCCTCGGCGGTTATCCT
CCGCCTCTGAACGTCGGCGCTCAGGCTCAGCGTCAGGTACGCGAGGACGAAGTGTCTGTGCGATGTTCAAAAA
AAGTGGAACGCGATATGTCGCAAACACTCGGAAAGTCGATTCGACGAAAGAACAGGCAAAGAACTTCATTATT
CGTAAGTTAGTTTATATACATGGTTCGAACCGACTAGACATACCTTCGTTACTCGTAAGTTAGTTGAAGAGTA

ACAAAGGTGTTCCAAGCTCTAAGTACCGAGATGTCGTCGGAGCTGTAACTCTAATTGCTCGGGGGTGTCCAAT
GAGAAGTGATTAAGTAACCAAAGCAAAAGACCATCGCGGAAGCAAATGTCCGTCACGTGCATATCGTGCGTGC
GTAAGGACGTTGGCGTCCTTACA
>Cersu1_rnd-1_family-35#TIR/MITE
TAGTGTTGCGCAGGGCCGGGCTTTCGAGCCCGAGCCGAGCCGGGCGGGCCCTCGGAAGCCGGGCCGAGCCGGG
CCCGAGCCGCCTGCAGGCCCTCCNCGAGGGCCCGGCTCGANNTTCCGAAAGCCCGAGCCGAGCCCGCGAGCCC
GGGGCTTCTGGTACTTGTATAATTATTGTANAAGGCAGTAATAGATCATTTGAGACATACCTGNATGTAANTT
TCGCCATCCTAAGGTGGTAAAAAACCNTTCAGAATCAACTCCGGGGTCTCGCTGAAGTACAGATGTGATNTCG
GACTCGTGCAGAAGTCACGTGACACGTTGTACTTGCGTCCCCGCGTACCTTTCGAGGCATTCTCTTGCGACTC
CTCGCGATATTTCGATACCTACAACGTTCGTTCTTATTCGGTAAGTGTTAGTTCAACGCTTTTGCATAGCAAA
AATGATATATTACTTACCTACCACTACATGCCAAAAGCCCGGGCTAAAGCCCGGCCGAGCCCGGCCGGGCCCG
AGCCCTCCCCAGCGGGCCCGGCTCGCGATTTCGAAAGCCCTGGGCCGCCCTAAGCCGAGCCCAAGCCCGGGCT
TTCGAGCCGAGCCGGGCCCTGCACAACACTA
>Cersu1_rnd-1_family-149#TIR/MITE
TACAGTTTTATACCGGCCGGTGAGACGGCCAAATCTTGGGACGCTCCCTGAATTGTTGTGCATCCCGAGCGAT
TCAATCGGGCATGAGCGATGTATGGGGGCATACTCACTNATGTGAAGAAAAATTGAACTGGTTTCGGCCTACG
CAGAAGAACATGAGATCGAGCTCAATCAGTTGAAAAAGCTCTTATCGGCGAGAGAGTACGCCAGTGAGACACC
CCAGATGACGCCGAAAGTTGCTTATTCAAGTAGCTTAGCGCCCCGAAGAGGNGTACGATTGGTTGACGCCACG
TTGACGTGGGGCGCCGACGGCTGATAACGGAGTCTCCTCAGCGGCTTTGGGCTCGANTCGAAGAAAGTACGTT
TTCATGCTTTCACTGCGCGACGAAGCCGTACCACACATGCCGTACATTATAGCGACGCACTTTGCGTACACAT
AGAGGGTAACAATGTGCGGGAACACTTTGAGAGACTTGGCTAGATCTGCCGCCANAAATTCACTCTCGTGGTA
CTCTCCATATAAATCACGAACTTCAGGTCGACGCAACATCTCGTCCGCGTATTCCTTGCAATGCCAGAGTGTT
TTAGCATAGTTATGAATTATCAGATTTTTTGCTGAGGTGAGCTTTGCAGCGTGACGGAGAAAATCCAGTCTGT
ATCACTGGGCCGTAGTGTATCACCGGCCGGTATAAAACTGTA
>Cersu1_rnd-1_family-119#TIR/MITE
TACTGAAGGGTGCTTAATTATAACAATCGAGTGTGACAGTGTGACCTAACAATTAGGTGTAATTGATAACAAT
TGGACTTCACTGTGACGCGACAGGACCTGTGTCGAGTAACAATTGGTCTTTCAGGTGCAGCAGCATAGAATAT
AGGGCGGCGACAGGGACTACAATGAGCTGCAGCATCAAGTTTTTCCTTTAATGTTGTTGTCCAATTGCTTGGG
AAAACCAGTTCATCGAGATTTCTTGAGGTCGATCAAGCAACAGAACAGGCGCTTTCTACCGGCTCTGACTGGT
TTGGAGTGTCGGAATTGGGTGTTTGCACTCCGTGAGGTTTAACAGCTTCTGAAGCCTTTGTTTCACATGCGGT
ATGTTACAGCAAGATAATAGAGCGGTTTTAGAGGGTTTCTGAACAGGTTTGAGCCTGTTTTAAGGTGGTCAGA
CACTTAACAATCGGTCGGACAGCGTGACGTGAAAACCGCAAAAATTAACAATACAATTGAAGGACGTGACGTG
ACGAATTGTTATAATTAAGCACCCTTCAGTA
>Cersu1_rnd-1_family-99#TIR/MITE
CATTCCGCTCGTTCAAGAACAGAACCAGAACCTGTGAAACGTCCCAGAAAGTTCGCGTACCTATCGGCTATGA
GTAAGTCTAACCTAATTGTAGTTTCCCGCGCACTTTTTCGAGACGTTTCACAGATTCTACTTTTTTCTTGAAC
GAGCGGATTG
>Cersu1_Cluster11#LTR/Copia
TATTAGAAGCACAGGTCTAGAACGGGCGACACGGCGCCGTTAACACCTCAGACGATATAATAAGTCTGTGTAT
ATTATGTACCTAGAACGGGCGACACGGCGCCGTTAACACTAGGTAGCTTCAGCATGCTGTACATACTCGGAAC
ATAACATTGTACTCACCCTCAGACGATATAATAAGTCTGAGGGCTACTACCGTAACGGGAGAGAGAAAAACAT
AGCGTCTTACATAATAACTGTATAGTAGACGGAGGCATCGTATGTTGCGATCGCACTCGTAGACATATCGGGA
GACTTTCCTTATTCGGACGTGGCGCGCCCCCACTGACGCCCGCCGACGTGCGCACCCGCACACGCGTCTCAAC
GCGCCCCTTCCTTGATTTTCCAACACTCCCCCTCAAGGGGGGCGCAGTCCCAGAGCGTGTGCGAAATGTTTGG
CTTTAGGACTGGGTAAGGGCTTGGTGAGCGTATCAGCGACCATGTCGTCCGTAGGGCAGTATTCGAGCGAAAC
CTTCCCATCTTCTACGACCCAACGTATGAAGTGGAAGCGGATATCGATGTGTTTCGTACGAGCGTGGAATTGG
TCGTTCTGCGCGAGGGCGATTGCTGATTGATTGTCCGATCGAAGTGTGAGCCTTTGGGCGGGCGACGTGAATA
CTTCGGACACGAAAGCTCTAAGCCAAATAGCTTCCTTCACGGCGTGTGTGGCCGCCACATACTCGGCTTCGGT
GGTAGACAGCGATACGATCTCTTGCCTTTTTGAGCTCCAGGAGACGGCCCCTCCGTCAATTAGGAAAGCATAG
CCTGACACTGCGTGCCTGTCCTCGTGTGACATACCGTCCGCATCGGTGTATCCCACCAATCCTTCGCTTCGCC
CTCCTAGCACGAGCCACCAGTCCCTTGTCGCCTTGAGATAGCGGAAAACTCGCTTCACAGCCTCCCAGTGCGG
GCGCCCCGGGTTCTCTGAAAATCGAGCCAGTACTCCTACAGCGTACGCGATGTCAGGACGAGTTCCTAGGGTG
GCATACATGAGGGATCCGATAGCTTCGCGGTACGGCACGTCGCGCATTTCTCCGATCTCGCGTGCCGTAGTGG
GGCCTTGCGACTTGGATAGGTGGGCATGGGCATCCATGGGTGAGGAGGTTGGCTTTGCGTCTGTCAGACCGAA
ACGATTCAAGATGGAGTCTATGTACGAGCGTTGACAAAGTGAGATGGTCTTCATGTCTTCGTCTCGTTTGACC
TCGATCCCTAGCAGCCAGTGTATATCTCCCAAGTCGGACATTTCCAAGCGGCCCTTAAGTTTCCCCTTGAAGC
GCAACATGAGTTCAAGAGTATTCGCAGCCAGCGTAAGATCGTCGACGTGGACAGCCAAGACAAGAATGTCGCC
GTTTTGCCGGTGGATATAGAACACCGCGTGGTCGACTTCGCATCGTGTCATTCCTAGCTCGATGAGTATACTC

TAAAGCGTCTGATACCATCGTCGTCCACTTTGCTTCAGGCCGTAGATAGCCTTGAGCAAATGGAGTACCTTTC
CCGTGCTATTCGGTTCGTGGTACCCTGGCGGCTGCGCCATGAATATACGTTCACCTTCATCCAGCTTTCCGTA
CAGATACGCGCTCTTCACGTCCATCTGATGAACAGGCCAGCTGTTGCGTGCCGCGAGTGCCAGGATGGTGCGT
ATAGAGGTCAGTTTGGCTACTGGTGCGAATGTGTCGAAGTAATCGACTCCTGGTACCTGTGAAAATCCCTGCG
CCACTAGCCTGGCCTTGTATTTTTCGACGTCTCCTGCTGCGTTCTTCTTCGCTTTGAAAACCCACTTGCTGCC
GACCACGTTTCCTCTCTCTGGTCTCTCTACTACCTTCCATGTCCCTGCCTTCTCGAGGCGCGCCAACTCTTCT
GCGATTGCTTCTTCCCATCTTGGCCAATCCGGTCGTCTCTTCGCTTCAGCTAAGCTGCGAGGTTCTAGCCCCT
CTGCATCCGACATTGCTGTGGCCATCGCCCATTCGTCTCTTTCTCCCCCAGAATCCTGCTGTACTTCGACTGA
CGCCGCAGTCTCGTTGGCGACTCTTAAACCAGTCGGTATTGTCTCGATGTTCTCTCCTTCTAGGATGCGCTGC
ACATACGCGCTCGGTTTACGCTCTCTGGTCGGGCGGCGTTCTTGTGGTTCGAGAACTTTGGTATCTTTCTGAT
GTGCGTTGTCGCTCGTGTTCGATTCCAACGTGCTCGGTTTGACGTTCGGCTCGTTTCTGACTTCATCTGTGCC
TCTCTCCCCCACGAGCTGAGCGTCTGGCTTGAATACCACTGTTCGTTCCACAGTTACTGTGCGCGTCTTCGGC
CAATAGATACGGTGGCCCTGGCTGTTTAAGTCAACGCCGACCCACTTCCCCTCGCGAGCACGCCCGTCGAGCT
TCGAGCCTGAGGGGTGATGAACCCATATGTCGCAGCCGAAAGTGTGCGCACTCGACAGGTCCGGCTTGGCTCC
GAAAGCGGCCTCATACAGCGTAGTGTCCTTGCTGAGGGGTTTTGTGGTCATGCGGTTTTTCAACCAGGTGGCG
TGCATGACTGCTTCGCCCCATAGGCTCTTCGGCAGCCTGCTGTCATGCAGCATCGCTCGCACGCGATTGAGCA
ACACGCCATTCAATCTCTCTGCCACGCCATTCTCCTCGGGGGTGTCGTGCACAGTCAACCGATGCTGGGTGCC
GTCCTCATCTAGAACGTCCTGTAGCTCTGTCGCAAGGTACTCGCCGCCTCGGTCGCTCTGTAAAGTACCGATT
GTTCTGTTGAACTTTGACTTCAACCAGGCGCGGTACTTCAGGTACATTCGCCTAACATCGGACTTCTGGCGCA
TAAGGTACGTCGCGGAGAGTCTAGACTTGTCGTCGGTAAAACTGATATAGTACTTCCTCCCACCGGTGGACGC
AACATCAGCGGGGCCCCAGACGTCGGTGTGGACAACATCGCCGACTTGTTCTGCGAGGGGACTGTCACGCTCC
TTCGGGACCGGCTTCCTCCGAGTCTTCGCGAAGGCGCACGAGTCGCAGAACTTGATATATCGGGCTCCAGTGA
GTTTGACACCTGTCACAAGTCTGCGGCTGACCAACTGTTGTATTGCTTCTGGTGACGCATGACCCAGGCGACG
ATGGAGTTCTTCCAGGGTTACTTCTTCGTGGACCGAGTAGGCCGACTCGCTGTCATGTTTGACTTTGTAAAGG
CCTCCGCTGCGCAGTATTTCTCCCACAACTGTCCCACCCCCGTCTCGTATCTGGCATCTGCCGCCGCTGAACA
GCGCGCTGTATCCTGCCGCATCGATGCACCCCACTGAGACCAGAGTAACGCCCATCTGTGGTGCGTACAGCAC
GTCACGGAGGCGTATCTTTGAGAAACCAGATCCATTCGGTACCTCAACCACCATGTCTCCGACCCCAATCGCG
TGGAAGCGCTGCTGATTTGCGGCATTAATGGGCTTGGGTGGTATCTCCTTGAGCGAAGAGAAGCGCTCTCGAG
CAGGTGAGAGGTGTTTTGTAGCTCCCGAGTCGTATAGGTCAACGTGTTTGCATCCCGGGTCGTCATGCACTAC
AGCAGCGATGTCAATCTGGATTTCGTCGCCTGTATCATTGCTATCCCAGCCAATATCTGAACCATAGTCGGGT
TCGTCGCTGGTGTCGTCGTCCAGCCCCTCCAGGAAATCTTCATCCTCGGAGTCAGACACGGCCGCCAAGTCTG
GCATACTGTCGTCTAGGTCCGCCAGAAGGTACGTCGCAAAATCAATGTCATTTTCGGTATCGGACTCAGATTC
GACTACAGCCGCCATCCAACTGTATTCAACGTCGCTCTCCTCGTCCTGCGCTGCATTTGCTGCGACCTTGTCC
TTCGAACTCCCCTCCGTGACTAGGCTGTCGTTCTTTCGTTTGGGGCTGGGGCATTGGCTTTTGCGGTGGCCCA
CTCCCCCGCAATTCCAGCAGGTCAGCTCTCGCCGCGGGCTACCGACCCTGTCTCCCTGGGCATCTCGGCTCTT
GCTAGCGGTTCGCTTGTATCCGTCATCTACAGCGTATGCAGCGTCACGTTCGTCTTTGGCGTTTCTGCTCTTT
GTCGACCCTCGACCAGGTACGCGCTTACGCTCGAATTCCTGCTTGAGGTACTGAATGGCCTGATCAGGCGAGA
TGAGTGACCCGAGGAGGCGACCGGCGTTCATCATGCCTGACAGGAAATCGGCATACTGGGCCGGGACCGACTT
GAGAATTTGTGCGCAATATTCTTGATCAGATACTTCGACGCCGATTGCTGACAGCTCCTCGACCATTTCACCT
AGGGTATCGAGGTGTTTCGACACATCCCCATTCTCGGCACATCGCATTGTCTGGAATCGCTCCAGCGTGTTGG
ACTGTACGAGCGGTGTCTTGTCCTCGAATTCCGAACAAATCCAGGCCCACATGTCGGCTACAGACTCGCCATT
TCGCTTCATTCTCTGAAGAACAGTGTCTGGGACGAGCCGAGCGAGCAGGTCCCGCGCCTTCGCTTCTTTCTTG
CGCCAGGCTCGGTAGTCGAGAGCATACTTCTTGTCGAGTTTCTTGTCTTTCGATGGCAAGGGCAGGACTTCCC
CTTGAGCGAGCAACAACTGTGCGTCTGCGCTTTCCGCAAGCGACAGCGGTGGTGCGGTTCCGGCGCGCCAGTC
TGCGAGCGGGTCTTGTGGCCGGGGATCCTCTCCCGTGAGGTGCCCATGCAGATCTCGTGCATCCAGGGCCCAG
GCCAGGCGGGTTTTGAAAACGGGCCAATTCGATCCGTCTGTTTTCAGGTGAGGAATCCCCGAGAGCCCGTTGG
CATCTCGGGTGTCGTCTCGTTCTAGCGTTGGCGCTTCGGGGCTCATAACCTATTAGAAGCACAGGTCTAGAAC
GGGCGACACGGCGCCGTTAACACCTCAGACGATATAATAAGTCTGTGTATATTATGTACCTAGAACGGGCGAC
ACGGCGCCGTTAACACTAGGTAGCTTCAGCATGCTGTACATACTCGGAACATAACATTGTACTCACCCTCAGA
CGATATAATAAGTCTGAGGGCTACTACCGTAACGGGAGAGAGAAAAACATAGCGTCTTACATAATAACTGTAT
AGTAGACGGAGGCATCGTATGTTGCGATCGCACTCGTAGACATATCGGGAGACTTTCCTTATTCGGACGTGGC
GCGCCCCCACTGACGCCCGCCGACGTGCGCACCCGCACACGCGTCTCGACGCGCCCCTTCCTTGATTTTCCAA
CA
>Cersu1_Cluster10#LTR/Gypsy
TGTAACGGGCGCGTCTTGTTCGGGCGCGGAGACGGTCGAGAGAGAGCGAATCGGGCGGGCGCGGTGTGAACGA
AGCAGCGACAAGTACTGGCGTTGGTAGTACGAGGGACGACGACGACCCGCGCGCGCGAGGAGGCCGCGCGCGC
GCGTCTACATGTACAAATCGGGAAGTCTACATATCTAAACTATGGCCACGGGTGCGTCAAAAACCCCAGTGGG
GAAAAGCTACCGGGCAGCTACTCTAGTCCTCGCGCGGTGTAACGGCCTGAGCCGTTACATCATCCCCCTTCTA

GCGCCGGAGGTACGACGTGCGCGAGTCGGAACGGGAGCGTGGAATCGTGTCGGCTTGCGGGCTTGAGGGAGCG
CGAGCGCGCGCGGAGCTTTGTGGCGCGGGAAAGCGTCGGGGGGACTACGTCGGGTCATAGCGAACGGGGGGC
GCGCGACGCGGCCGGAGCGCGTAGTCGTCTGTTGAGGCGGCACGTAGTGTGTGCGCAAATTCTCACGCTCGGG
CGGCGGAGACGGCGTGCGGCGTGTCGCCGACGGGATCGTGGGAGCGGGAGCGAGCGCGGGGGCGCGGCGCGTT
GAGCGCGAAGGCGCGGACGGCTCGGGGAGCTCGGGGGCAGCTGTGGGAACGGGCGGTGCGTCATTCGGGGACA
CGGGAATAGACGCGGAGGGGACAGTCACCGTGTATTCGCGCTGGAGCGCATCGGGCGGCGGGCGAGGCGCGCG
GGGGTGGCGCCGATGGAATCGCTCGATGAGCTCGTCGCAAGTGTGAGGGACATCGGACAGCGGGATCCAGGCG
TTCTCGTCCTCGGAGCGGTCTTGATAGCGCACGAGGTACTCGAAGCGGCGCCCGGTCTTGCGCGCATCAAGGA
TCGATGTGATCTGGGTTGCCGGGTCGTCGGTGGGTTCGAGCTCGAACGGTTGCGGCTCGGCATGCGCGTGGAA
AGCAGACGGGTCCTTGTAGGGCTCGAGGAGTGAGACGTGGAAAACGGGGTGAAGTCGGGAGAGATAGGCGGGG
AGAGTGAGCTTGTAGGCGCGAGGCCCGCAAGCGTGAGCGATGGGGTATGGGCCGAGACGGCGGTGGTCGAGCT
TGTCGGAGGGGCGCGTAGTCTTGACATGGCGGCGCAGGAGCCAGACGAGCTGACCGGGCTTGAGGTCTGGCAT
GGGATCATGCCTGCGATCATATGCGCGGGCTTGAATCTCTTGCGCGTGCGCGAGCTCGGCACGGAGCTCCGCG
TGAATGCGCTCGAGGCGCGCAGACAGGTCGGCGGCGGGGGGAACAGGGGACGTGACGGGCTGAGTGAGCTGAG
GGGCTGAGATGGGATGGAAGCCCGTGAGCGCGAAGAACGGGGTCTGCTTGATTGAGGAATTCTCCGAGTCATT
GAACGCGAACTCGGCGACTGGGAGATAGTCGACCCAGTCATCCTGCTGATAGGAGACATAGGCACGGAGGTAG
GTCTCGAGGGTCTGATTGGTACGCTCTGTGAGGCCGTCTGTCTGCGGGTGATATGCCGTAGAGGTGCGCGCAT
CAATCTGCAGTAGACGAGTGAGCTCGGACCAAAATTGAGAGATAAATACCGATCCGCGGTCGGAAATGATGTT
CTCCGGGAGACCGTGATAGCGAAAGATTCTGTCGAGAAAGAGCCAGGCGAGCTCCGGGGCTGTGATAGTCTCG
TTGCAAGGAATAAAGTGAGCAGCACGAGTGAAGCGATCACAGACAACCCAAATAGAATCATACCCGTGGGACT
TCGGGAGCTTGACGATGAAGTCCATGGTGATGGAGATCCAAGGGCGAGACGGAATCTCAAGCGGTTGGAGTGG
ACCGTAGGGCCGATGACGAACAGCCTTGATCCGGGGACAGACATCACATGCCTCAACATACCGACGGACATGA
GTGGCAATGCCAGGCCATGAGTAGCTGCGCTGGATGCTTTGCAGAGTGCGAGTGCGTCCGGGGTGACCTGCAG
TAGGAGCATCATGACGTGAGTGCAAGATATCTGTTCGGAGAGTGGGGGGAACATAGAGTCTTCCTTTATGAAA
GACCATACCGCCTTCAGCAATGAAGTCGGAGTCACCATGAGCGAGTGCGTTGCGCCATGTCTTGTCGGACTCC
CACTCACGCTTGAAGCGGGCGAGTAATTCTGCAGAGTCGAGGGTCAGCGTTGTAAGAGCGGATATGGACATGC
CTGAAGAGCTGGAGGAATGAAGAACACGCTCGCTTGAATAGAGCAGTTCTGTGTGATACGGCTTGAGAAGCGT
CTTGAACTGCGCTTCGAGAGCCTCATCCCCCTTCTGAGGAACGAAGTCGGGACGACGAGAGGGCGCATCAGCA
ACGTTCTTGGTGCCAGGGCCCCAGACGAGCTTGAAGTCGAACTCGGAAAGGAACATCGACCAGCGAGCCTGTC
GACGATTCAGTACACGAGTAGACATGAAATACTCAAGGTTTTTGTGGTCAGAAATGACCGAGATTGGCTGTGC
AGTGCCGAGAAGCCAGGCGCGCATGTCCCGGAAGGACTCGATGACAGCAAGGAGTTCCTTGTCATATACCTCA
TAATTAATCTCTGCGGGGGTAAATTTCCGTGAGTAATAGGCGACAGGGTGAAGAATACCGTCATCGTCGGGCT
GTTGAAGCACACCGGCAAGCGCAAAGTCCGACGCATCAGTAGCAAGAGTGCATGGACGGGAGGGATCGTAGTG
GCGGAGGACAGGATACGATGCGAGGGCGGTCTTGAGGCTGGTGAAGGCGTCACGCGCAGCCTGCCCAAAGTTG
AACGGTGTATCCTTCCTGCAAAGGGAGGTCAGCGGTAAAGCAAGAGAAGAGTAATGATCCACAAAGCGTCGAT
AGAAGTTGGTGAAACCAAGGAATGCCTGCACGTCGCAGACATTGCGAGGCTCCGGCCAGGTGCGGATAGTGTC
CAGCTTGGTTGGACTCATCTTGATGCCGTCAGTACCGATAACGTAGCCGAGGTAATCAACCTCGGAGCGGTCA
AACGAGCACTTCTCTGGATTGGCAAAGAGACGAGCTTCCTTCAGACGGTTGAGGACCTGCATGACATGGCAGT
CATGGTCCGCCTGGGAACGAGAGAAAATCAGAATATCATCGAGATAAACAACACAGAAAACATCAAGGAAATC
ACGCAGCGTATCCTGGATAAACGCCTGGAAGGTTGACGGGGCGTTAGTGAGACCGAACGGCATGACAAGAGTC
TCAAAGAGACCGAGTGGGGTACGGAAGGCCGTTTTCCACTCGTCACCCTCTCTGACTCGCACAAGGTTAAATG
CATTCTTCAGGTCAATGACCGTGAAGCGTGTGCATCCCTGCGTCCTATCAAGGAGGTCATCGATGAGCGGGAG
TGGGTAGCGGTTCTTCTTGGCGATGGAGTTCAGCCCACGGTAGTCGACGCAGAGCCGGAGCTCGCCAGACTTC
TTGCGTACGAACAGGATGGGTGATGCTGCGGAGGAGGTCGAGCGACGAATAAAGCCTTTAGGGAGATTTTCGT
CAAGGTACTTAGCTAGAGCCTCACGCTCGTCCTGCGAGAGACGATACATCGGACCAAACGGCGGGGACTTGCC
CTCTTCGAGGTCGATGGCGATATCGTATGGGCGATGAGGAGGAAGCAAGTCGACCTCAGAGTCTGAGAACACC
GAATCCGCCCACGGCATGTACTTGGCAGGAAGAGAGCGAAGCACGTCTTGACCGTGAGGTTCATCGGGCCCGA
GTTCCGAGGTGAGAGAGGAAGGCGTGGTGCAGAGCGAGGCGACATGGAGTGGAGAACCTAAAGGGTGGAATCG
GATCAGTGAAATATCAAGGTTCTTGGAGCATTTCGCAAAGCGACGAGAATTGAGGACACGAATATCCATACGC
GGAGGCGGAAGCGCACGGGAGCAGTCTAGCATTGAGCGACCGAAGCCAGTCAACGCAGCCTGACGAGAAAGAA
AGGAGACAGAGGGACGCGTCAGAGAGGAAGACTCCGAGTCCGCAGACAAGGGAGCAAGAGATCCGCGGGCCAA
GCGGTCATCGTGTGAAGGAATAAATTCGTTTGAAGAATGGAAGGGACAACGGGTAGATACACGAGAGTGGAAG
CGAGAAGGAAAAGGAGAACGAGAAGGAAGACACTTGCCGAGTAGACCGGAGCCTAAGCCGACAGTGGCGACCG
AGACAGGATGGGCCGCCGCAGACCGAAGACCGGAGCCTTGGATCACGGAGGTGAGAGGAGGAGACTTGGAGAG
ACCACAGCACGACAGCGAGAGGTGCATGGCGGTCCAGTCAATCTGTGGGTTGTGGTTACGGAGCCAGTCCAGA
CCGAGAATTACCGGATAAGCAACAGACACAACTCCGAGGCGGATAATCTCGGAGTGGTCACCAACCACGAGCT
GGGAGACAACGTCGTGTGTCACCAGACCTCCCGCAATTGGGCGGTCATCAACAGCGGTAACGGGGACAGGAAT
GTCCTTGGGTGAGCGTGGAAGGGAGTGGCGCTGTGCGAAACGGTCAGAGATACAAGAGGAGCTCGCACCGCTA

TCGACCAGCGCAAACGTGTGGACCGACTTGCGATTTGGAAATGAAAGAGTGATGTTGATGAAGAAATGATCTG
GATGATTAGTGTCAGTATTCGTAGCGAAGATATATGAAGGAGCGACGTAGTTTCGGATTTTGTATTGTCCGTC
CAATTGGCAGACGAACGGCGGAGGCTCCGGAGCCCTAGCAGCGCCCGGGATCTCCTGGGCTGGAGAGGATGTC
CCGGTTAGGCTTTTCCCGAAGGAGCAGCCTTGCGTGAGGCGTCGCGAGCCTTAGCCTTGGCGGACTTGTTCGG
ACAAGTGTCCGCGCCATGGCCAGACCCTCCGCAGTACAGGCAGAGGTTCTCCCGCTTACGGCGATCCTTCTCG
GCTTGCGTCAAGGGACCCCGGAAGCGGACGGCATCGACCTCCATCGGCACGACGTCAGAGGAGGAGGAAGCAG
GGGCGAGAGGTGCGGAAGGCTGCGAGCGGGGCTGCGGGAGCGAGCGGGGAGCGGGGCGAGACTGCGAGTGATC
GTGACGAGAGCGGTCCTTGCGGGCCAGCTCATCCTCGTGCAGCTCGTTGTCGAACTCGATGGCGAGCTCGACA
TACTGGTCGAAGGTGGTGGGTTTGGGACGGACCCCGCGGACCTTGTCGAGGACGGTCTCCTTGAGTCCCTCCT
TGAACTGGTCGAGCTTGGTGGTCTCGGACCAGTCCAGGTGCGGGAGGAGCTCTCGGAAGCGCGCCGCGTAGGC
AGCGCACGAGCCCGTCTGACGTAGTGCTCGGATCTTGCGGAGCGCCGTGCGCGAGACGTTCGGGTCGGCGAAA
GCACGGCGGAGTTCCAAGACGAAGGCGGGGTAGTCGGAGAGGAGATGGGAGTAGTGGGTCTTGACCGCGTAGT
ACCACGTCTTCGGGTTGCCGTCCTTCAGGTACGAGGCAAGATAGCTCGTCTTGTCAAGATCGGCAGAGATCTG
CTGCATGCGGATCCCGTTGTCGACCTCATCCAAGAACGGCTCGAGCTCCGCGGTCGCGCCGGAGAACAAGCGC
GGCTCGCGGAACTTCGTGATTGGGCGGTCGAAACTGGACGGAGCAGGAGCAGCAGCTGGTGCCTGGGAAGACA
GTGGAGGCGCGGGCGCAGAGCCGAGAGGAGGCGCCGTGGAAGCAGGGGCAGCAGGAGGCGTCGCAGACAGGGT
TGCCAAGAGGTTCTGGAAACCCGTGAGCGCGGCGGTCATCGCGTTGAGCGCGGTGCTCGTGGCATGGGACTGA
GCCTCCGTGCGGGCACGAGAGTCGGCGCTTTCGCGGCGCAGGTCGTCGAGCATGCGGATGATGACCGCCTCGG
GGGGCTGAGACGGCGCGGGTGTTGACGTGGCATAAGCGCCCGCATCCTGCGAGGCATAACCCTCGGGCGTGTA
TTGCGTGCCCCAGGCGGCCTGCTCAGAGGCGTCGTACGGAGCATCGTACGGATGACCCTGGAATGACATTGTG
AGGTACCGAAAGGTAAATCTCTCGGAAATAACGGTGGGAGGCGGGGAAATGAACGCGGGAAATTTTCGAGGAC
TCAGCTGATGGGACTAAAACGTCCGAGATTTTTGGTACTACCAAACTGTAACGGGCGCGTCTTGTTCGGGCGC
GGAGACGGTCGAGAGAGAGCGAATCGGGCGGGCGCGGTGTGAACGAAGCAGCGACAAGTACTGGCGTTGGTAG
TACGAGGGACGACGACGACCCGCGCGCGCGAGGAGGCCGCGCGCGGGCGTCTACATGTACAAATCGGGAAGTC
TACATATCTAAACTATGGCCACGGGTGCGTCAAAAACCCCAGTGGGGAAAAGCTACCGGGCAGCTACTCTAGT
CCTCGCGCGGTGTAACGGCCTGAGCCGTTACA
>Cersu1_Cluster9#LTR/Gypsy
AGGTAGCTAGGAAGACCGGAACAATGTACCGTAGGAATTCCGTGCAGTATCCAAGTCCGGTGCTATAGTATAG
TCCGAGGGTGAAGAGTAACTAGTGGGATCGAGCTAAGGTACAGTAGGAATATACGATCGAGGGTAGCTAGAGA
TCTGATCTTCGTAACTAAAGTGACCATGAGGCGCGTGACAGCGTGGCCATCACACTACCTCCTCCTCAAGGGA
GAGCGCGATCCACAGCGGTCTGGGATCACGGGTCGTAACGCGTGGCCGGTCTAGACACACGACCAGAGCGCGT
CGTAGTCTGTGTAGGCGGCGTATAGAGAACGCGGGGGTTCTCACGGATGGGCACGGGCGACGCAGGACGCGGG
GCAGCAGGCGGGACAGCGGGCGCGGAGGCAACAGGCACGGAGGACGCGGAGGGCACGGACGAGGCAGCGGGAA
CCTGTGGAACAGAAGAAGAAACTGTGTCAGTAGATAACGAATGGAAGTCCTTGTCAAGGACGAGTCAATGAGG
GCGAGGAGCGCGGGTATAACGATGGTGGAAGCGCTCGAGGAGCTCGTTAGCGGGTGTAGGGATGTCGGAGAGG
GGAATCCAGGAGTCTTCATCGGGTGACAGGTTCTTCCAGCGCACAAGATACTCGTAGCGCTGGCCGAGCTTGC
GGCAGTCAAGAATTGCTTGGATAGAGTCGGGAGGGGAGTCGGCGAGGATGTCAAAGGGTTTGGGAGTGGCATG
GGAATGGAATTCGGAGGGGTCGTGATAAGGTTCAAGGAGGGAAACATGGAAGACGGGAGTCGGGAGAGGTACG
AGGGAAGTCGGAGGTGATAAGCGGAGTCGGAAATAGCATGATCTATGGAGAAGGGGCCGAGACGACAATGATC
GAGTTTGTTGGATGGACGGGTAGTCTTGATATGGCGACGGAGAAGCCATACGAGCTGGTCGTGCTTGTACTGT
GGGCTTGCTTGAACATGTCGATCATGGAAGTGCGCCTGACGAGTTTGCACTTCCTTGAGCTCGGCACGCAATT
CATTGTGCAGTCGTGAGAGGCGGGTGGCGAGATTGGCAGCAGCAGGAACAGAAGAGCGCTTGGAGAGATGAGG
CTCGAAGGTAGGATGGTAGGCGTAGTTGGCAAAGAAGGGGGTTTGCTGAGTCGATGCATTCACGTGATTGTTG
AACGCGAACTCCGCGAGAGGTAGGTAGTCAACCCAGTCGTCCTGCTGGTATGAGACATACGCGCGCAGATATG
CTTCGAGTGTCTGGTTGGTGCGCTCGGTGAGACCGTCAGTCTAGGGGTGATAGGCTGATGAGGCGTGGAAATT
CATCTGAAGGAGAGCGGCGAGCTCGCGCCAGAAACGCGAGACAAATGTGGAGCCGTGGTCGGAGATGATCGAG
TCGGGAAGACCGTGATAGCCGAAGATGTGGTCAAGGAAGAGCCATGCAAGTCCGGGAGTATCGAGGGACTCCT
TGCACGGGATGAAATGGGCGGTGCGGGTCATGCGGTCGCAAACAACCCAAATGGAGTCATAACCGTGTGACGC
CGGAAGCTTCACTATGAAATTCATCGAGATGGACTTCCAGGGACGCTCTGGGATGTCGAGAGGCTGGAGTAGG
CCATAGGGCTTGTGACAGGGTGCCTTGATGCGACTGCAGGTATCACACGCAGTTACATAGCGGCGAACATATG
TCAACATGCCTGGCCACGAGAAGTCATGTGAGACCAGTGCAAGCGTGCGAGCACATCCGGGGTGACCTGCAAT
AATGGAATCGTGACGGGAATGCAAGATATCAGCGCGAAGGGACGGGGGAACATATAGAAGACCTCGGTGGAAC
ACGAGGTTCCCTTGAAGCTCAAAGTCCCTGTCTCCGCGGGCGAGAGCTTCGCGCCACTCGGAGTCGGCTTGTA
GAGCTGTCTTGAAATGCTGTAGCTGCTCGGAGTTGTCCAGCACGAGCGTTGTGAGTGCCGAGATGTGCTGAGG
ACGTGGGGAGTTGGACAATGAGGGGAAAAGTCGCTCTGTATGATGTGAGGTCAGTAGGACTTTTTCCTGAAGC
CGGAGGATGGTATCCCCCTCTCGAGGTGCGAAGTCTGCAGGTCTCGAGGGCGCATCCGCGGGGTCTTAAGCCC
GGGAGCATAATCAAGATGGAAATTAAATTCTGAAAGGAACATGGACCAACGCGCTTGGCATCGGTTGAGGACA
CGAGAGGACATAAAATACTCAAGGTTCTTATGATCGGACACGACAGCGACAGGTGTGTCAGTTCCAATCAGCC

ATGCACGCATGTTGCGGAAGGACTCCACTATTGCAAGGAGCTCCTTGTCGTAAACTTCATAGTTGATCTCGGC
TGGCGAGAGCTTGCGGGAAAAGAATGCGATGGGATGAAGGAACCCCTGGCTGTCGGGTTGATGGAAAATACCT
GCAATGGCAAAATCGGAAGCATCAGTAGACAGGGTGCATGGGAGCTGCGGGTCGAAATGGTGGAGCACGGGAG
AAGAGGTAAATGCACGCTTCAGCTGCTCGAATGCATCAGCGGCTTCCCGTGGGAATGTGAACTCATTGCCGCG
TTTGTCCTTGCGCGTAAGTGCATTGAGCGGGAGGACAATGCGGGCATAATCCGAAATAAAACGGCGGTAGAAA
TTAGTGAATCCGAGAAATGACATCACATCCTTGACGGAACGTGGCGTCAGCCAGTTGGCAGTGGTGTCGAGCT
TGCGGGGATGCATCTTGATACCGTCAGCACCAATGAGGTAACCGAGGTACTCGACTTCAGAGCTGTCGAAGAT
ACACTTCTTCGCGTTGGCAAAGAGCCCTGCTTGCTATAGCCGTTCGAGGACCATGCTGACGTGCCTGTCGTTT
TCTTCCTGGGACCGAGAGAAGATCAAGATGTCATCAATATACACGACACACACGACATCGAGAAGGTCGTGTA
GCGTGTCCTGAATGAATCCTTGAAAAATACCAGGGGCATTAGTCAGCCCAAAGGGCATTACTGTATATTCGAA
GAGTCCGAGATGGGTGCGAAAAGCCATCTTCCATTTGTCGCCTTCCCGAATCTGAATCAGGTTGAAGGCATTC
TTTAGGTCAATAACCGAGAACACCTTGCAGCCCTGAACTCGGTCGAGAAGATCATCAACGAGCGGGAGAGGAT
ACCTATTTTTCTTCATCATGGCATTTAAGCCTTGATAGTCGACACAGAGACGAAGCTCGCCGGTCTTCTTCCG
TACGAAAAGAACGGGCGATGCAGCGGGTGAGGTTGATCTGCGGATAAAGCCTTTACGGAGGTTAGTCTCAACA
TAGTCAAAAAGGACTGCTCGCTCCTCGCGCGAGAGGTGGTACATCGGACCAAATGGAGAAGTCTTCCCCTCCT
CGAGCTCGATGGCAGCGTCGAATTGAGGACGATGTGGAGGCAGATTCTCGACTTCCGTAGGTGAGAAGACTGT
AGCAAAGTCGTGATACTTCACGGGAACATGCTGCAGGTGGTCTTCTTTTACTAGAGGTTCCGGGGGAGAGGAC
GAGGACGGAGGAGACAAAGATGGAGGTTCAGGAATGATAGAGGCATCCGCATACATCATGGAAGCGAGAACGT
AGCCTGCGGAGCCGCGTGGGTGATACCAGAGTAGTGCTGGAGAGTTATTCCGGGCATACTTGCGGAATCGCAT
GGAGTTGATGAATGCAATGTTGAGGGCTGGGCGAGGGGAGTCGGAGGGAACTGGGGGTACAGGGGGCGAGGGA
CAAGATGTCGCGGTTTGACCAAGGCCAATGCCGGACGCAAGGCGAGGTAGGGTCGAGGCCGAGAGATATGAGA
TACCGCGGGAAGCGGGGTTGCGTCTCGCTTGATCAGAGCGAGTTACGCTATCAGAGTGTGCGGGGACAAGAGA
TCTGCCGTGTAGACCGAGGCCTAGACCGATAGAAGTTGCAGGGCAGGACTGGGGCGAGGTGACAAGAGCGGAA
ACTCGGGCAAGACCGAAGCCTTTCCCGAGCGCAGACACAGGATACGAGGGATTGGCACCACAGCATGACAGCA
CGAGCCCTTTGCGGGCCCAATCGATAGCGGGATTGTGGTGTTCAAGCCAATCGAGACCGAGAATTAGCGGATA
CGGGACGGAAACAACTCCAAGTGCGATAACCTCGGAGTGGGAACGGAGAGACAAAGAGGTAATAATGTCGTGC
GTGACTAGTCCCGAAGCTATCGGGCGTTCATCTACCGCAGTAATAGGCACCGGCACATCCTTGAGACGGCGCG
GGAGCCCATGTCGGTTAGTGAAGGTTAGGGATATGCATGATGCAGTAGCACCAGAATCAACAAGGGCGTATGT
ATGTATGGGCTTTTTATCGGGGAAGTGAAGAGAGACCGAAACGAAGAAGTGATCAGAGGCGCGAGGGGAATTA
TGAACATGGGTAGAAAGAACGAATTTGGGGGCACCAGCAGGCGAGGGTATCCACGTGCCGTCATTAAGACGGG
CATGTGAGGCAACTCCGGACGCAGGTGCCTGAGTCCCAGAAGAGGTGTCCAGAGGCCTTGGGACGAGGGGACC
TCCGGTTAGGCTTTTCCCTTAGTGGGAACAGCCTTTCGGTGCTTCTTCGCGTGCTCCGACATGTTCGGGCAGT
CAGACGTGAGATGCTTGCCCTGTCCGCAGTAGAAGCAAAGGCCCTCCTTACGGCGGCGCTCCTTCTCTGCGGC
ACTGACTGGGCCTCGGCACATAGCGTCGATTTCCATGGGGACAACGTCGGCCGAGCCGGAGGGTGTGAGTGTA
GACGAGGACGTGGCAGGAGTTGCAGGGGAGGCTTGAGGACGCGGAGTGCGGTCGGACCGCACCGCTGGTGGCT
GCGTCTTGTGCGGATGCTTCTGCTTCTGCTCGAGCTCGAGGTTGTGAAGGCGGTTGTCGGCCTCGACAACAGT
CGGGATCCACTCGTCTAGGGTGTCGGGGCGATCCGTGTTGATGAGGACCCTCTTGAGCTTGGCTTTGAGGCCG
CGGTCGAACTGGGTGATCTGCATGAAGTCTGTCCAATCAAGATAGTTCAGTAGTTCCTGGAATTGGTTAGCAT
AGGCAGCAGCTGAGCCCGTCTGCTCGAGCTTTTCAATCTTATGAAGGTTCTTGAGGACTAGATCCTGGTCCTC
AAAGCGTTTCCGAAAAGCGTCCTGAAGCTTCTCGAAGTCATGCAGGAGCATCTCCTGCTTGAGCTCGAGAGGA
TTGTACCACGACACGGGACTTCCATCCTTGAGATACATAGAGAGGTATAGGGACTTATCGTAATCGGTCGGAA
GCTCGCGCGTTGTAGGTGAGTTGCGACGCGGATCTCCCAAAGGCACGGCACGACTTCTGCGGCCTTGCCGCTG
AACATGCGCGGCTCGCGGACTTTGGGGGCGCTGGGGCGCGGGGCATGTACAACGGGCGCGGACGCGGGTGGTG
CTGCGGACGCAGGAGTCAGGGCGGGTGGCGCCATGTGTGACTGCGCCTCGAGTCGTCGAGTGAGAGCTTCGAG
CATCTTGCTTTGCTGCTCTTGGATCGTCTGAAACGCGTTGGCGTTCTGGCCGAGAGCGTAGACGGCTTGGTAG
AGCGCTTGGACGGCCACATCTGCACTCGCGAACGCGGGGTAGGGCTGCTCTGGAGAGCCCACGTTGGCGGGGC
TAGAGTACGAGGGCTGCGACTGCGGCTGGGATGCCACGTCGTCCCACGTCTCCATGCCCTGGTCGAGGTTACC
TGGAAGAGACATAACTAAAATTTATAAGCAGTAGACGCGCTTTCGCACGTAATACAAGAGACAAAGGGGAAAA
ATACCGTGAGAGCGGAAACGTCAAAACGCTTCCGTTCGAATCGAGGACAAGGTCGCGACCAAGAGAGGGTGGT
CGAGTCAAACTGTGATGAGCACGTCGGATGAGAGCGCGGGCGAGAGCGAGAGCGAGAGAACGCGCGCGAGAGT
GTTCCGAATGGGCAAGTACTGGCATTGACTCGACAGGGATACAGGACGCGCGGCTCGAGTACTAAGGCTCCAA
CCATTTTCTTTAGTTTATGTTTATAAACTGTTTAAACCCTCTAAAATTACTTTGTATTTAATTCTCAGCTGTA
CAAAGTAATAACAAAGTTAAAAAATGTTTTACATTGAAACTTCTTATACTGTATGCTGAGCTACTACAGGACA
ATTGTGTGATATTCAATACAGCACAAGACTACAAGACACCTCACATAAACACCAGCACAGGTTGTGGTCCTCA
GTTAAAGCAAACATGAGGTCATCATATGCCTCACGTGCATCATAATACCTGTCACATGACCATATATCTTTGC
AGCACGCACACTCAAAGTCAGCTGCATGGTCTGTATCTGTTGTTTAGGCTCAATATGGCTCTCAATTCTGCAA
AACACAGTACATATAACATGGTGGGCTTGCTAGAAATGCTTCTCACTATGAAGAAGAAATTCACATTGTAAAC
CATTTATTATAGTCTAATTACTTTGTATTTACTCTTTACTGTTTGTTTACAGAAAATTTGTAAACGTAAAGTA

AATGGTTGGAGCCTTATCGAGTACATAAGGTAGCTAGGAGGACCGGAACAATGTACCGTAGGAATTCCGTGCA
GTATCCAAGTCCGATGCTATAGTATAGTCCGAGGGTGAAGAGTAACTAGTGGGATCGAGCTAAGGTACAGTAG
GAATATACGATCGAGGGTTGCTAGAGATCTGATCTTCGTAACTAAAGTGACCATGAGGCGCGTGACGGCGTGG
CCGTC
>Cersu1_Cluster13#ClassI/TRIM
CCGAGGTGGAGAGTGCTCTCCTTCTGGATGTTGTAGTCCGAGAGCGTGCGACCATCCTCAAGCTGCTTACCAG
CAAAAATGAGACGCTGCTGGTCGGGCGGGATGCCCTCCTTGTCCTGGATCTTCGCCTTCACGTTATCAATCGT
GTCGGAGGACTCGACCTCAAGGGTAATGGTCTTCCCCGTGAGAGTCTTGACGAAGATCTGCATACCACCGCGG
AGACGCAACACTGGACTTACATGTTAGCAGGAAATGAGTGGCTGTATGCGTGATACACATACCCAAGTGAAGG
GTGCTCTCCTTCTGAATGTTGTAGTCCGAAAGGGTACGGCCATCCTCGAGCTGCTTGCCTGCAAAGATGAGGC
GCTGCTGATCCGGAGGAATGCCTTCCTTGTCCTGGATCTTCGCCTTGACGTTGTCGATAGTATCAGACGACTC
GACCTCGAGAGTGATAGTCTTGCCGGTCAGGGTCTTGACGAAGATTTGCATACCACCACGCAGACGAAGAACT
GGATAGCATTTAGGATTGCGTCGCATTGATTATATGCGCGAGGGAAACGTACCCAAGTGAAGGGTACTCTCCT
TCTGGATATTGTAGTCAGAAAGAGTGCGGCCATCCTCCAGCTGCTTGCCGGCAAAGATCAAACGCTGCTGGTC
GGGCGGGATGCCTTCCTTGTCCTGGATCTTCGCCTTGACGTTGTCGATCGTGTCAGAGGACTCGACCTCCAGC
GTAATGGTCTTCCCCGTCAGGGTCTTAACGAAGATCTGCATACCACCGCGCAGACGTAGAACTATGATTGCGG
TATTAGCCACAGTACCACCAACAAGGACAATGTAAGCTTACCCAAGTGAAGGGTGCTCTCCTTCTGGATGTTA
TAGTCGGAGAGGGTGCGACCATCCTCAAGTTGCTTGCCCGCGAAGATGAGGCGCTGCTGGTCTGGCGGAATGC
CCTCCTTATCCTGGATCTTGGCCTTGACGTTGTCAATGGTGTCCGAAGACTCCACCTCGAGGGTGATCGTCTT
GCCCGTCAGGGTCTTGACAAAGATCTGCATACCGCCACGGAGACGGAGGACTGAACATCATGCTCAGAGACCG
GTAACTCAGTAGGGATAGTAAGAACGCTTACCAAGGTGGAGAGTGCTCTCCTTCTGGATGTTGTAGTCGGAGA
GGGTGCGACCATCCTCAAGCTGCTTGCCAGCGAAGATAAGGCGCTGCTGGTCCGGCGGGATGC
>Cersu1_Cluster14#LTR/Gypsy
TGTTATGAACGCCCACGCGAGCGCCTGATCAGGTGACCTGGCCGGATGAGGAAGGCACCAGCCACAGAGGCGG
ATCCACAGCCACCCAGAGCTCGACGCCTGCAAGAGGCAAGGAGGAGGACGGCAGATATGAATACCAGGACAAG
TAGGCATGGGAACGGCGAGGGAAAGGACAAGAGGCAGTGAGTGACAGGCGCAGGTTCGATAGGGGCACCATAG
GTAGTGGACCGGACAATTGTTTTCTTTTCTTTCTCGCATAGCTACGCGCTGCCGCACGCAGCCTCTGTACATT
AGTATATAAGGAGGACCTCTGAGAGCTCAGAGTCCTCAGAACAATTCTAGTTTGTACTTGGTCTAAGCCACTA
CAAGCCCTAGCACGAGTGCAATCCGCATAGCGCGTCCATTGCTCATGATAAGCTCTCACGTGACATTCTGAGA
TTGTAACAGAAGAATTGTTCTCTACCCCGACCACCTACAATCCCATCCATATCCATCCATTCTCCACACCATC
CCACACTCCATCATTCCAACCCCGTGAGTCGTGCTCCAACTTAAACTGAGCGTATAGCCGGACCGTAGGCTTA
AGCGGCAAACACGAACCCTGAAAGCCGGTGCTACCGGTGGCATACAGACAAGTCCTTGACAGGCAAGGCGTAG
ATACACAGAGGTCTACCATGAAACGAACTCTTGATAGGCGCACCTTCAGTGGTGGTCTATTGGTGTTGTGATA
CACCCTTGTGCGCAGATTCCTGACGGATAATAACCAAAACCGTTATTGGGTCCACGAGCAACTGCGAATTGTG
GTATTCTGCTCTCCGTAGTTAGATTAGTACAATTAGCTACGACGACGAACGGGCACGAGCACTAAGAAGGGTC
AGTGTACTGCCGACGAGAGAAGACCTCGTAGCTCCGCAGAGTACGAGAAAACGTGATCACACCCCCTCCGAGT
AGTAGGGAATCACGGATTTCGGACCTTACCGAGAACGCCCGAATAGCGAATCATCATCCGAGGCTACAACCCC
TGTCAGCGGACTGCCCATCATTACCATTACCCCTGCCCACCCCGAACCAATCACACTCCAACCCCCGGTCCTC
ATCTGCCAACCGCCTCAGGAAGTGCACAGACGGCAACCCCGGCTCCGGCCCCCGCACCACGCAGCAACGGAAG
AGTGACCTTAGCCGGGTCGCCAAGCGTCTTTGACGGTAACAAGGAAAGCTTCCAGAAGTGGTGGAAGCAAGTT
AACCTGTACATTAGCGCGTACGAGGATGACTTCGATGTTGAGCCAAGCAAGGCTGACAGACAAAAAGTAATCT
TTGCCCTCTCGTTCACGACAGAGGGGCAGGCCAAGCTCTGGGCTGATGAATACTGAGAGCAAGCAATTGCGCA
GTTCCCCCCAAACTGGGGCATGTGGGACGTCTTTGTCACAGCGCTCAAAGGGTGTTTCCAGGACACGCAGCTC
AAGGAGCGTGCACAGTGGAAGCTTAACACCTTCTGCCAGAACAAGATGACGGCAGAGGAGTGGTTCCAGGGGA
TGGACCTTCTCCAGCGACAAGCCGGATACACGACGGCTCATGATTCAATCATGATTGCCAACATCCGCATCAA
CGGCAACGCCACGCTCGTTGATAAAGTAATCGGGCTCGATGTGATGCCCACAACGTACATCGACTGGAAGAAG
AAGATGATCCAGTTCGACAATCAGTGGCGGACACGCGAGGAGAAAAAGAAGCTCTTCACCCAAGGACACGGCA
TAGCAATCCCACGGTCGTACACGCCACAACAGAACAGCGCTCCCCACCAAGAGAAGCAGGCAGAGCGCCCAGC
AGCAGTCCCGGCATCCCAAGGAGGAGGGAGCCGCATGGACATTGACAGGCAGAGGCAGCCACGAGGAAGAAAC
TGCCACCGATGCGGCAAAGAAGGGCACTTCGTGCGAGACTGCACAGAACTGAAGCCCAACAAGTTCGCCAAGG
TACGAGCCCTTGGCATGACAAAGGAAGACCTCGAGAACTTCCTCAAGGAGGGTTTTCAGAAGGATCAGTGAAG
GAGCGGCGCTCCGCTGATCAGGACTCGGACAATGTTGTTACACCAACTGGAAAATATGTAGTACCCACGAAAC
AATCTAAGGTATCTATTTTCCGCGTAACACATCGACCAAGTCAAAGCTGCATACACCCTCAGAACGGAGGGCA
AGGAAGAAAGTGTCTAATGAAACGATAGCACTTCTTCGGAGCATGCTCAATGACGAGCCAGAGGAGCAGGGCA
AGATCGAGGAGGTGAAGGAGGACGGCACCTCTCTCACGGATCTAGAATCAGTGATTGAGCTGTCTGAGGAACC
GCAGACAGGGGCTGCAGAAGAATCAGATGAAGCTCCACTGACGGATCAGGAAGTCGACCACGAGGAGTGGTTT
GACGCTGAAGAAGACCAGGAGGAGAAAGAACCCGAGGAGTTCTTCGATGCACCGGAGATACCCGAGAAGGAAG
GACCTCGGCCAGAGCAACAACGGTATGCCGCTTGGACATGCGGCTGGATGGCAAAGCAGATCGACTCGGACAA

GGACTACGTACTCGCACAGTTCAAAACCTGGTGCCACGAGGACCGACGGCCCGAAGCAATGATTGAGACGATT
GCCCAAATTCGACGACTTAGCTGGGACCACAGGATCGCGGCACTATCAGAACTTCGAGAACAGCCAAAATACG
CAATGGGACGAAAGAACCAGTGCGAACTCGAAGTAAACTGCACGCTCCAAACAGTCAGCGACGGACGGACCCT
AGACACCAAGGCGCTCATTGACTCCAGATGCACCGGCACAATGATAGATAGCAAATACGTTGAGAAGCACAGA
TTAGAAAAGGATCCAGTGCTCCGACCAATTGCAGTCTATAATGGCGATGGCATGGTGTCCAAAACAGGAGTCA
TCAGGTACACGGTCACATTGTGTCTACGCGTCGGCGAACACAAGTCATGGGAGATCTTCGGAGTCACCGACCT
GGGAAAGTCAGACGTCTTCATCGGACATGATTGGTTATCGTTCCATAACCCAGAGATCGATTGGGCCACGCAA
GAGATTAAGTTCTCCAAATGCCCCGAACTCTGTGGTTTCAAACCAGAATGGAACCGCACCATCGACGAAGACA
ATGACGGGGATCGACTATTCCTGTTCAATGTACCCGGATACTGTCAGCGTGAGGAGATCATCCGAAGAGTCAC
CACGTCTTCAGAATTGGCACAAGCGGTGAATGCGACCAAACCGAAGCGGCACTGGAAGGACATCGTACCCAGT
CGTTATCATGAGTTTGCAGATGTCTTCACCAAGGAAGGCTTTGACAAACTACCACCACATTGGCCATGGGACC
ACGCTATCGAGCTTAAACCGGGAAGTGAACCACATGACTGCAAAGTCTACCCAATCTCCCCGAGCGAGGACCA
GGCGCTGAGAGATTTCATTGACAAGAACCTGAGAACGGGACGCATTCGACCGAGTCAATCACCAATGGCCTTT
CCTTTCTTCTTCATCAAAGAAGGATGGATCACTGCAACCAATTCAGGATTATTGGAAGCTCGATGAGATGACA
ATTAAAAACCGTTATCCTCTTCCGCTGATCTCAGAGATCATGGATAAGCTCAGCGGCGCTAAGTACTTCACCA
AGTTTGATATCCGGTGGGGGTACAACAACGTGCGGATCAAAGACGACGTTGTCTGGAAAGCGGCCTTCAAAAC
AAAGTATAGATTGTTTGAACCGCTGGTCATGTTCTTCGGCCTATGCAACGCCCCGGCCACGTTTCAGTCCATG
ATGAATGGCTATTTCCATGATATGATCGCTAGCGGTGAGGTCCTTGTGTACATTGATGACATCATTGTCCTTG
GCAAGACGCTGGAAGACAACCGCTGGACCACACGCAAAGTCCTAGAACAGCTCCGAGAACACAATCTCTTTGT
CAAACCAGAGAAGTGTGAATTTGAGATGCAAAAGACGGAATACCTCGGCCTAATCATCTCAGAAGGACGACTG
GAGATGGACCCGGTTAAGATTTCTGGTGTCCGCGACTGGCCCATGCCCACGTGCAAGAAGGAAGTACAGGCCT
TCCTAGGCTTCTGCAACTTCTACCGACGGTTCGTCAAAGGGTATTCGAAGGTCACTCGCCCGCTCAATGACCT
CACTGGCAACGTCCCCTGGGAGTGGACTCCTAAGGCGGAGATGAGCTGAAAAAATGGCTTACGGAAGCACCTG
CGTTGGTCATCCTGAACGAGAGTGACCCCTATTGAGTCGAAGTTGACGCATCAGATTATGCAATTGGGGGAGT
TCTGAGCCAAAAACAGGATGGCGTCTGGCATCCAGTAGCATACATCTCACACTCAATGACACCAGCAGAGCGG
AACTACGAGATCTATGACAAGGAACTGCTCGCAATCATGATCGCGATAGAACAGTGGCGACACTATCTATTGG
GTGCGAAGCACACGTTCGAAGTCTACACGGATCACAAGAACCTCGAGTTCTTCTGGCAACCGCAGAAGCTGAA
CCGACGACAAGCGCGATGGCTTACGGAGCTGGCAGACTACGATTTCGCGTTAGTCCACAGGCCTGGGTCAACC
AATACCAAGGCCGATCTCTTGTCCCGTTGAGCAGATCATGCAGACGGAAAGGAAGACAACCGAGAGGTGGCAC
TACTCCGCGAAGAATTCTTCAGAGTCCGACACGCGATTGACGATGTCAACTGGGTAGCTTCGATCACAAGGAA
GCGGCTGGATGAGTGCTTGGTGCAAATCCGCAATCAGGAAAAATGGATACTGGAGAAAGCCGATCAGAATCAG
GACAGATTCTCATGGGAAAACAGATTTGTAATGCTCAATGAGCGCTACGTCATCCCGAAGGACTGGAAATTGC
GACTTCAGATCATCTAGGATCATCACAACACCCCTGAGGCGGGACACCCGGGACAAGCACGCACATGGGAACA
GGTCTCACGATCGTATTGGTGGCCAGGAATGCGGGCACAAATCAAGCAATATGTCACCACTTGCGTCACGTGC
CAGCAGAACAACACCGTGAGGAAAGATTGAAGCACGCTCCGCCCAAATGAGGTGCCCAGATTCCCATTTGAAG
TGATCACGCTCGACCAGGTCACAGGCATACCGGTATCACAAGGATACGATGCGCTTCTAAACTTCGTGTGTCG
GCTGACAAAGAACCTCATCTCCGTACCATGCAACAAGGAGCTCTCGGCGGAAAACACAGCTAGGCTCTACATG
AAGCACATGTACTGGCACTACGGCCTATCCCGCAAGGTGATCTCAGATCACGGACCGCAATTCATTGCCGGCT
TCATGAAGGCGCTATTCAAATCCATCGGCATCACGGGAAATCCCTCCACGGCGCACCACCCTCAGACCGACGG
TCAGATGGAGCGAGCTCATCAGGACATTGAGAAGTACCTTCGCACATTCTGCAATTACCAACAGAACGACTGG
GCGGAATGGCTCCCGATCATGGAGTTTGTGTACAATGACTCAGTCCACACGAGCACAGGGTATACACCACATT
TTCTGCTGCAAGGGTTCCATCCGTGCAAAGGAAACGCACCGGTACAAACGGGACTTGAGTGGACTGATTCGTT
CTTGAAAAAAGTCAAACGGATCAGGGAAGATGCCATCGCCACGCTGGAACACGTGAATGAAAAGATGCGAAAG
CCAAGCACCGGCCCGAAGTACAAGCCGGGTGACAAGGTCTGGCTCAACACAAAGCACGTGAACCTTCACCAGC
CATCCAAGAAGCTCAGTCCGCAGCGAATCGGACCCTTCGAGATCGTGAAAGCCGAGGGGAGCGAGGAGGTCGC
GTGGAGATTGAAACTCCCCGCGAAATATCAGATACACCCGGTGGTCCACGAATCCCGGATCCTCCCATACATT
GCGGATCAAAGCGAAATCCCACGCCCAGGGCCACGCAACATGGACAATCTCATGGATGAACCATCAGACGCCG
AATCCATTGCCGAGATCGTGGGACACCGTGTGCACGAGACCCCAGATGGACGAAAATTGGTCCAGTACCTTTG
CCAATGGGCCGGAAAGTCGGAAAGCGAAGCAACGTGGGAGGTTTGGGATACCCTACGGGCGCACAGCGAGCTG
GTTCAGGAATATCACCGGCAACATCCTGATGCGCCCAAATTCCGCATATGGATCCCCGACCGCGCTATAAGAC
CCAATGCGCGCCCGAATCATCTCTATGACCCGCACACCGGCGATCTACACCCCGCATAATGACCACTGCTGCC
CACCGCTTTTTTGGCCCCAAGGGCATCATTTTCGTTATTCTGGCCGGCATAATTATTATCCTTGTTGTCACCT
TCCTAATCTATTTCTGCGACTAACTCCCCTGGCACGCTCCCCCCGGCAATATCCACATCATCGCGACCCGCCC
AGCGACCCCGCTCGCCTTCGCCCCTCCACACACGTACCCACTGCCCGCACACACCGTGTCGCCCCGCGCCCGT
ACCGCCCCTCTCCGGGATGCCCACCTCTACGCCGCTCGCCCCTTGCCCCCGCTCCCTGATCTCATGCATCCGG
AGGCTGCATCTAAGGGGGGGAGTGATGTTATGAACGCCCACGCGAGCACCCGATCAGGTAACCTGGCCGGATG
AGGAAGGCACCAGCCATGGAGGCGGATCCATAGCCACCCGGAGCTCGACGCCTGCAAGAGGCAAGAAGGAGGA
CGGCAGATATGAATACCAGGACAAATAGGCATGGGAATGGCGAGGGAAAGGACAAGAGGCAGTGAGTGACAGG

CACAGGTTCGATAGGGGCACCGTAGGTAGTAGGCCGGACAATTGTTTTCTTTTCTTTCTCGCATAGCTACGCG
CTGCCGCACGCAGCCTCTGTACATTAGTATATAAGGAGGACCTCTGAGAGCTCAGAGTCCTCAGAACAATTCT
AGTTTGTACTTGGTCTAAGCCACTACGAGCCCTAGCACGAGTGCAATCCGCATAGCGTGTCCATTGCTTGTGA
TAAGCTCTCACGTGACATTCTGAGATTGTAACA
>Cersu1_Cluster3#LTR/Copia
NNNNNaNNNNNNaNNNNNaNNNNNNNNNNNNNNNNNtNNNgNcNNtNNgaNNgNaNtNcNNNNNNNNNNtaN
NNNNtNcNNNaNgNNNNNNNNNNNaNNNNcNNNNNNNNNNNaaNNNNNcaNaNNNNNNNNtNNNNNNaNgCt
TaccCGtcAgtCcgagcGtagtgCGtcgAgGacTgaggggaaAgCgtagcTACgTggcGtTgAaTatgGAcgc
gcgtGagattaTgtaagCNtggtagaAgGTAttgtAcgaAtatagtaAtGgCTatgctacgagTgaaCGgCtt
actgNAaattcttcaAcacacccccttaaGccgGaacNAgtcCNaAAgagtCgCggaGgCgctcgaaaTNCTC
gCGTTcgagcgGTttcgtCaaGatatCggcaaCCaaTtggcgCgagggNtgGtgTtCgAcaaCgaaggtGCCg
tCcGagacacgttcNCgcacgaaGtggtgatgCAcgtcgAtgtgctttgtgcgtgcgtgatgtcgNgcGtCCT
tcgttaggtCgatCGaacCttgGttgtCgccgcggattGtgGTtGgcGcccTctcgtGCggaCaGcCcatttC
cgtaagTaggcGgCGcatccacaNAcCttgtttcgCaGCgAacgagagggCcAtgtattcgccctCggtagac
GaGgcagcgACaGtgGtCTgcttCTtgCtgctccacgcgaCgGtcgcCttgCcGatCttgaAgacatatccgG
ataCggAgcGccggTcNtcaAtattGcgtgcCcAgtCcgaGtcggAgtAgcCagcGagTgtGtcgaacgtgtg
agTgtCcgcgtCgtcaccNccGagtNcGAgtcgatAGttGcgcgttcCcgcgaggtattgCaggaTgcgtttG
gctgcgcgccagtgctTCGggCctgggtcCGCGtTgaATtgNgCgaggttGttgactgcgaatGaTAtgtCgg
gtcgcgagatGAccgaGAtCcacgtgAgGgCtccgaggagTtcacgNtAaGggcGcCgttTcatgtCGtCtGc
CtcctgagcattCttcGggCcatcNTccTttgtgaGGacatagctcggCcgCatcggGaTtACctcgggagtg
cagtccTCcAttttGaATcTCcgTaGaataGAaaTtATGtaCttctcaTgtgAtATagAtattgTaCcctGCt
tTCggttGtcgAaGagctcNaAgcccataatCcAGtTtgggtCtTctTcCTTcatctcgaagAgGCtgtgcag
aTcGtgacggGcagcagCGaGttcCttgcgcgagctCgagaacGtGagaGcgTtGtcgacatggaTgccCGtg
atNGtaGTaCcaagCtcNgtGtcacgGatAtacAccGCgttgTcCgtgaatAgctgtcggAAgccGacCgAtT
tgagTTgcgcagtcatgtGacgattCcAGagtcgtgctgCttgCTtgaGtcNGtAGaGgCtgcGtagtagtNg
tacaACgCGAtcgGtGcNgtccgtaaAgccaacGGGCTgtCGcatgtagatctcgtgatcNAgttcGctGtga
aggAAGGcgccagtgacNtcgtcttgtgcgcggtaccagccgcatgcagccgcNaggtggaggattgcgcgca
gcgcatcaagtcgaactgtgggtgagaatgtgtcgttgtaatcaacaccgggaatttgcgagaatccctgagc
aacgagccgtgccttgaatcgcaggatctcgccgtccgggcctcgcttgattgcgtaggtccatctattacaG
GACAAGAAGGACGAGAAGAGCAAGTTCTCGAAGCCGAAGAATCGCGCGAACGAGGCCGTTGAGTCGAAGTCTA
CCAAAGCCGACACGACGGACTCGGAGCACTCATCTTACATGGCCGCTCCGCGCTCGGCATCCCGCTATGCCTG
GATCCTCGACAGTGGCGCTACGACACATATCTGTAACGACCGCACGGCCTTCACGACATTCACTCCATCTCGC
AGCTCGATCGGTGGCATTCTTGACGGCGGACCTCAACTCGAGGTTCTCGGACGTGGCGACATCAACCTCGTCT
GCTACGTCACAAGCCGTCAAGACCGAGTCATTACTCTACGCGATGTCTCATACTGCCCGAACGCGTGCGACAA
TCTCATCTCGGAGAGCCGTATGGACAAGCGAGGTCTTACAATCACCAAGTCCAAGGGCCGCGTGAAGGTCATC
AACTCATCTGGAGAAGTTGCCATCGAAGGCAGGCTTCGTAATAGCCTTTACGAGCTTGACTGCGCGACGGCTC
CGCGCTCACTGTCCTGTCCACCCCACCTTGCTTTCACTGCCCGTTCTTCCGACCTCGATCGTGTCGATCTATG
GCACCGACGTCTCGGTCACATTAGTGAGCGAACCCTCCGCCACATGGCGAAGAACCATATAGTTACCGGACTC
AGTTCTCACCTTGTAGGTGACTCCGGCCCGTGCGACGGCTGTGCTAAGGGAAAGCATCACAAGGCTCCATTCC
CCGCACAAGcCAcacaacgCcacaAcaaacCTGcagAaCGacaTCagATcGacccacaaaacCccgcaGaagc
cTaTGgaaCcgcTccaccCaaaaaCcccaccacgGccaaTgaaCacTCccgCaaGGgcggGAaaGcaTaCaac
aaGaacaagaaCgagGCcaccgaCGaaaccaTcacacgcGcTaaccccagcgaGTCTCgcaCcagCAaaAaag
ccaaagaTagccgaagCcAcGgcGGacGcGAGaTgagcgcaaAGcAaCgcaaaaaaggaggaGacGaacacGG
CAcCTcacaaGaaacGgcgccaNaTaNNagNaNtNgatggaatNGgggNNcGgAtggtttcNcaggtNNatc
NgNtaNcaTcNCNggtNcATcaNgNgTcaTgNcgGgcTcNNNNNNNNcNNNNaNcNggggtNatgtgaNaCN
CNcNNgNttNgcgCaAcNtcgaatNaaNtgaCcccctNgNgtagNgcNNcNtNgaNNANaNCgTCtNcNGagN
NNaNNNNNNtNNNNcNaNNNNNNNNNNataNctTatatgActtggaAcCgGgcTTaaaGccCAtaaagaTccC
gtcaagGgagtgaggatCaagcttGccAcggcctTGatCgggAAcaCgGaTatggcaTttagaAccaaaatc
cgtagggTgGagacAtcgggcTtagagCcgtAGAaGgccTcgTttggagtGCATTTCGACAGCGCGTGAGTAG
AACAGCGGTTGCGaATAAATGATGCGTACTCGTGTGCCTCaGGCCAGAAGCAGCTGCGaAGTCCaGCATCaTG
TAGCATCGCAAGaGCATGcTCGTGcGTcGTTTGGTTGAAACGTTCGGCAACACCATTTTGCTGAGGTGTATGA
GGTGCCGACATTTCTTGTGAGATGCCGCGCTCGTcGAAGAACTTCTTGAGaTCCTTCCCGATaAAcTCGCCTC
CACCGTCGCTTCGAATATACTTCACCTTGTTGCTGGTGAGAGACTCGATGCGGTTAACGAGTGTGATAATCTC
GTCCGTGGCGTCGCTCTTGTTCTTGAGGTATGCCTTCCAGCCCTTGCGCGAGTGATCATCGACCATGGcaAGG
GTATAGCGTAGACCGGTGACaGAGGAGTCAAACGGGCCTTGCAaATCGATaTGaAGTCGCTCGCCAGGTTTCT
TAGCaCGTTGCGTGGCgTGTGcGGGGAAcGGAGCCTTGTGATGCTTgCCCTTaGCACAGCCGTcGCACGGGCC
GGAGTCACCTACAAGGTGaGAACTGAgTCCGGTAACTATaTGGTTCTTcGCCATGTGGCGGAGaGTgCGCTCG
CTAAcGTGACCaAGACGTCGGTGCCATAGATCGACACGATcGAGGTCaGAAGAAcGGGCAGTGAAAGCAAGGT

GGGGTGGACAGGACAGTGaGCGCGGAGCCGTCGCGCAGTCaAGCTCGTAAAGGCTATTACGAAGCCTGCCTTc
GATcaCAACTTCTCCAGACGaGTTGATGAcCTTCACGCGGCCCTTGGACTTGGTGATcGTAAGACCTCGCTTG
TCCATACGaCTCTCCGAGAcGAGATTGTCGCGCGCaTTCGGGCAGTACGAGACATCaCGTAaaGTAATGACTC
GGTCTTGACGGCTTGTGACGTAGCAaACGAGGTTaATGTCaCCACGTCCGAGAACCTCaAGTTGAGGTCCGCC
GTCAAGAATGCCaCCaATCGAaCTGCGAGATGGAGTGAATGTcNtGaAgGCNGtgcGgtcgttAcAGAtaTgt
GtcgTagcGCcActgtCGaggatCcAGgcataGcGggatgcNGagcGcggaGcgGCcatgtaggatgaGtgNt
ccgagtccGTcGtgTcggctttggtAgaCttcgaCtCAacNgcctcgttcGCGCgattCttcagCtTCNAgaa
cttGctcttcTcgtcCTtcTtNtcCtggtccttgttgtctttactgtCCTTatcgccCttCttctTcttcgta
cgacgtccNcgtCGggAGCCCtgctcNTgggaaccaccCcCttttCGgGTgcagttctgtgccCaNtGaCccT
tCtcgCcgcAgttcGagcagaatgacTcGctcgtgCgNggcgtgctcgAaaatgAcTtGccCttgTTNaNgtg
ctTggcttgGtAagctGCcaTagcTgcCgactgCtggttGgcccGaACacCgtgctCGtcaAggatacggcgg
gAaaCctcCtCagaggtGtagcgatcgggGagtCcCGagaacaCNtagttCcAggacggggGCaTtgagatcN
gcAAGaAgcANGcgaacAgCTcgtcgggtataTcgcgctgCaTaagCacgaGAtcGCggcGgAatcccTtcAT
cTtcgAgagatgGgtCATgAcgTCatcgCcgtcGAcATaCttcgTcGAGacgatgttggtgatcGTgAgCgtc
caGtcGgtcgctgtctgCCcgcagtAtaAgcgctGtgCctcGcgccagatctcGaCgGcCGtatccAggtGGC
NgaCcTggttgatAacctcttcgGtCaTgttggcgGTcaaGaTCgcgcgtacgagtGcgtTAgCgcggcgcca
tccCgggtatGtctcATcaAgCGtGtggggccGgtCGAgttcgccagtgcagtaCtcgaaaAgatcGacggtT
tcgAgcGtcgatttCatCTggatNaTCcagggCacgtaattCGtgctaGNgAgcttgtgagggagCcgAtaCa
acGaaTggTaTttCgtcGcaTcGattgaCtggcTgttgtAtgtcCaaTgaGctgCgtcggNgtagTcgtcGta
CtGgccGtaatCaGcgcgGcctgcgtGtgAGccaaAGcCtcCcGTgcggtaCgCtgtattaGcatagcGcgtt
cTcGgtgagaacgTtGCcGgtcggagAGaTggtcccgcaCGgCctggAggcggaGgcggaGcCgGttgGCtat
caAcgcCtgCgACgcCttcggcgttcGggttCtgcGatcCGtagGcatctggcgagGgctggtcgacatcata
gtcgggctgctgagTgacatTGgNatcgtgcgggagcgGgttgttgccgNCaGgTGGcatgcgaGcgaTgGgA
gcCatcgtgagGtcgttAagGGcgtgaGcgagCgaGNgttgACgAtcgtAAcggGAGcaagcgacagGtCcga
GaGcgcgTTaNGcgtTgcgcCtctCggggctcataaccTgttgaacGcTAaNGtNcggaCGcgaAgcgtcccg
TcAGtCCGagcGtAgtGCgtcgaggacTgaggggaaAgCgtagcTACgTggcGtTgAaTatgGAcgcgcgtGa
gattaTgtaagCNtggtagaAgGTAttgtAcgaAtatagtaAtGgCTatgctacgagTgaaCGgCttactgca
AatgcTtcAaNN
>Cersu1_Cluster18#LTR/Copia
GTTGGAAACGTAGGCAAAACGAGACGATTTTTATTCGTGCGCGAGCGCGAGACATGTCTCTTCAGTTACATAA
TCAGGTACTATATAACTCTTCGAACCTTAAGTCGGGCGCGCGGAGACTTGCCATATACGGCAGGTCTCATATT
CTGACACATCCCCTCAAGTCTAGCGCGCCGAACATGCAGCGGCACATCCCCTCAAACCAAGCCAACCTTAGAA
GAAAACACATAGCCATGTCACCTAGACCCTTCGCGAGAAACCTAGCATCACTGCCCGACCACCTAGACCCCAT
GCCAATTCCGGGACACCTAGATTCCCATGCCAAGCGTTCAAAACGCGTAATCGAGATCGTCGTTGTCGGTTGT
CGCCATCCCTGCGTTCACCGGCGTCTTCGTCTTGTCCCGCGCCACAAGTTCAGGGCAGTCCCGCACGATATGG
CCCTCCTTCTTGCAGTAGTAGCATGTCCGCTTCCAGCTCTCCGACCGTGTTGGTCCAGATGCTCTTGCTGCGA
AGGCAGAGTCCTTGTGACTGCTGTCCGTATGTGATCCACGTCGCGCCTCCTCGTCCACGAGAGTGCTCTTGAT
ATCCGAGATCGAGAGTTCCGATTGCGAGGCTGTAAGCGAGCCGGCTATGTTTGACCACGAGTCGCCGAGGTTG
AATATGAGCACATCCGTGAGATCGTCATCATCAATGGTGATACCGATAGCCTGTAGCCGAGCGGCCAGGTCTG
TAATGCCGGAGATGTAATGGTGTATCGGGGCCGACTCATCGTGCTTGAAACCGTAGAATTTCCTCTTCAAGGC
AATTCTGTTTGCACGGCTGTTCTTTTCGTAGACATCCGCAAGAGCTTGCCAGGCTTCCGCACCATCCGCGCAG
TTCCAAATGTACTGGTACTGCGTAGGTTCGACCGTGAGCGCTATCGTCGTCAGCGCTGATTGGAGCTTCCTCG
CATCCTCAGCCGCCGTAGCCGAGGCCTTCCGAGTTTCCGTGCCCTCGTGTCCCTTTCCTTTTGTGCCGAGCGC
GGCCTGCACGGCGTCCCACATGTCTTCGCGCCGGAGCGCCATCGATACCGCGAACTTCCAATCATGGTAGTTC
GAGCCATTCAGTTTCGGAACCAAAGACAGGACTGTCGAAGGTGACATGTTGACCGATACGAGAGTGAGCAACA
AATGAAGCTGTCAGAGGTCGGGGCTCATAACCTGTTGGAAACGTAGGCAAAACGAGACGATTTTTATTCGCGC
GCAAGCGCGAGACATGTCTCTTCAGTTACATAATCAGATACTATATAACTCTTCGAACCTTAAGTCGGGCGCG
CGGAGACTTGCCATATACGGCAGGTCTCATATTCTGACA
>Cersu1_rnd-1_family-161#LTR/Gypsy
GGGACAAGTGTCTTTGTAACACCGCTGTCCTTAGTTTGCCGATCCACAGGCGTAAGTGGGCGCGATACCTTTC
TAGGTAACGTGGGGATTACGTGGCGTAAAGTCACCCCAAGAGGGCCTAGGCCTTGTGACGGGTAAACCCCTGC
GGTCGCGTTTTTAGGGCAAGAGTGTAATACTTGAGCTCGACAAGTTTTAGCTGTGTGGCGTGGAAACCCACAG
TAATACCCCTATTCCCCCCCTGACCTACTGAGAGAGAGTAGGCAGACGCCGGCACTACCCCCCCTTCTTTTGG
AGTTTTAGCTTTGCGCAAGCATTGCTGATTAGTTTTTGCCAGTGCCCAAAGCCTCCCGTGTCGTCTTCGCCAA
GAACGAGAGTACTGGAAAGTCTCTCTGGGAGCGTATTGACATCTCCGACCCGCACCGCTTCGATCACGTCGGC
AAGCCTCGTCGCGTGATCAGACCCCGCACTACTCCGTACACCCCTGTCCCTGGACGTCGTGAAGAACTAGCCC
GTCAGGCTGCTCTGAACGCGTTCAAGACCCAGCTCGAGCGAGAGGACTACACGAACGAACAGCGCAAGCGCAA
ACTCGCTGATCGTCTCACTACCCCTCCCCCGCCGCTCACCGATCGCATCAGCTCTCCGGAACCCCCCGTGCTT

GACATCAAGCCCTTCCCCTCCGACCTCCATTTCCACAAGAACGTTCAGGTCAACCAGCTGCGTGATTACAAGC
GCAAGTTTGGAGCGACTGCTACGCGTCTTACCCCGTTCTTCGAGTTATTAGACAAGGCGCGTGCAAGCATCGA
TCCGCAGGTTGCCACACAGTTGTGGGTACTTGGACAGAGGTTCAATCGCCTTTACGACAACCTCACTACGCTT
GTCGAGAAGAAGGCCATTTCGACCCGCGAGTGGAAGGACATTTCGCGTGACTTGACCACGGTTGGCAAGCACA
GTTTTAAGGCGCTCAAGCCTCGTTTTGTCGAGCATTGCCAGGAGTTGGTGACTGAATTGGGTCCAGACTGGTT
GAAGATTGCGGAGGAAATAGAAGTAGTTCTTGTGTAACCGCGTTTTGAATCATAATGAGCTCTACTCCCTTTG
CGAGCGTACAACCTTCCCCCTTCCATCAGAGTGATATGTTGGGACCCTTGAGCGACATCCCCTTGGGATCTCT
GGCTGAGGATTCCCCTACGCCGCCCGCTGTGCCTGTTCCCTTGCCGCATTCTTACCCGGAATACAGTGAACAT
TGGGTTTCCGCTGCGCAACAGAGCATGACGCCTGTTGACGTCAAGAACCTGGTGGACTCTCTGTATAACCAGT
TTTGGCATGGAATGGAGCGTATGCGGAACGAGATGAAGGAAGGAATGCGTAAGCGCAAGAACGAGGCGCGTCA
GCTGCAGACGCAGATTGACAACCTCGAAGCAATTAGACGGGAGCAGGCTGCACAGATTGCGGATGCACAAGCA
ATGAACAGACTTCTCCGTGAGAAGTTAGAACAAGCGGAAAAGGCGTATCAGGAGACTCAGACTGCGCTATTGA
AGGCAGAGAAGGCGTACACAGAATTGAGACTTAAGGTCGACACCATGGGCACAGGTGGCAGTGTTGGACTATC
GCAGTCCCTTTTCCCAACCATGAAGGAGCCCGACACCTTTAACGGTGATAAGGCGAATAAGTTGGATGACTGG
CTGGAATCTATGGCCTTGTGGTTGCGTCACCGCGGCGTAACCCAGGACGAGAAGAAGATCGAGACAGCGATGA
CTTATCTCCGAGGGACGGCGAAGAAGGTCATGCAGGGCTACTTTGACAAGGTCAATGACCAGGAATCGCTTGG
CGCGTATGCTACCTTCATCGAGGAATTGAAGAAGGGTTTCCAGCAGACGGACAAGAAGGATCGTGCAATTGCC
GAGTTTAACCAACTCGTCGCCAAGAAGGGTACTAACGCCCAGAACTTTGGCGAGTTTTCTGCGCGATTCCGGA
CTCTGGCCAAGCAGACAGGCTTCTCCAATGCGGAGTTGTTGTCAAAGCTCTATGCGCATGTTCCTAAGCCTGC
GGCCGACGTTTTGATCACTCGTGGTCGTGAGAAGTGGGCCAAGACCTGGGATGAGTTCATCAACGAGGTCCAG
GAAATTCTCCAGGACTCGCGTCTCTTGAATGGAACCGTGCTTACGAGCCAGATTGCCAAGGATCCGAACGCTA
TGGACGTTGATGCTGTGAAAGGCAAGAAGTCCTCGGATAAGGCAGACGGCAAGCAGGCGAAGGTTCGGAAAAT
GGCCATGTCATTCGAAGAGGCTAGACGCAAAGGATGTTGCGCTAAGTGTGGTGGTAAAGGTCATATGCAGAAA
GAGTGCCCGAACGGAAAAGAGAGTAACTCTAGCTCGGCCTCTTCTAGCAAGAATACCGATACCCCGGGTGCTA
GCAGCTCTAAGGCATCGTCGTCTAGCAGTAAGGGAAAGGAAAAGGACACTGGAAAGTCGAAGTACACGAAGAA
GAGCGTACGGGAAATGCACGGCAGCGACTCCGATAGATTTGAGGAGTTAGATTCGGAGGATGGGTCTGGGTCG
AGTTCGGAAGAAGAAACCCCCAAGCCAAGGAAGAAGAAGTCGTCTTCAGACAAGAAGGCGCAGAGGGTTAACC
GCGTGGTTTTCTCGGATGACGAGGATTTTTTGAAGGGGCCTATGTAAAGTCTGCTACATACGGTCCGTTTTTA
GATTTCCTATATCCCCCGGACTCAGTAGAGTTTGTACAAACAGTATTGGAATCCAGAAAAGTGTTTAGTCTAC
CGTCTAAATCGAGTTTTTCGCTTCCAGTTACTGTGAGTCAGGGAAAGGAAACAGAAGTGCAGACAACCGCGTT
GGTCGACTCAGGAGCTTATGCTGTATTTGTCAATCACCGCTTCGTAAGAAGGTGTAAGCTGAAGACAAATGAG
CTAGCTCAGGAGATTCATGTCTATAATGCAGACGGATCTCGGAACCGTAGCCGTAGTATCAGGCAGTACGTGT
GGCTCGACGTAACGGTTGGTGAACATTGTTCCCGCCAGGCCTGCCTTGTTACAGACTTAGGAGGAAAGGACAT
GTTCCTCGGTTTTAGCTATTTGAAAAAGCACAATCCTGAGATAGATTGGCAAAGGGGTGAGCTGACGTATTCA
CGCTGCCCGGATACTTGCCGTTTTACGGGACGACGCGCTCAGATAGCAGAAGCAGAACTTGAAGAAC
>Cersu1_Cluster15#LTR/Copia
GTTAGATATTAAAAGCCTAGAGCACGCGACGATGTCGCTGTCAACACCTCAGACGGATCAGGAAGTCTGTATT
GCTATGTACCTAGAGCACGCGACGATGTCGCTGTCAACACTAAGGTACAATCAGTCTCGACGCATGTAGCTGT
AGCGTAATGTCGTACTCACCCTCAGACGGATCAGGAAGTCTGAGGGCTTCTACTCTACGAATGCGAGAAAACA
AAGAAACAGAGGATTACATAAGAACTATACAGTTTCGACGGAAGCATCGTATGTTAGATCGAACATACTGATA
TAACGGGCGGGTTTCCTCATTAGGCAATGGCGCGCCGCCACAGGTGCGCCGTTGTGCGCCACCGCGCGAGCGC
GGGCGAGCCCGCTCTTGAAATCCAACACTCCCCCTCAAGACGGGCGCAGGCCCAGGGCGCGGGCGAAGTGTTT
AGCCTTCGGGCTGGGCAGCGGCTTCGTGAGAGTGTCGGCGATCATGTCGTCGGTGGGACAGTATTCCAGAGAC
ACCTTCCCTTCTTCTACGACCCAGCGGATAAAATGGAAGCGAATGTCGATATGCTTGGTACGGGCGTGGAACT
GATCATTTTGTGCGAGGGCAATGGCCGACTGGTTGTCTGACTTGAGCGGTAACGTCTCGGCAGCCACCATGAA
GATCTCCGAAACGAACACGCGCAGCCAAATAGCTTCCTTCGCAGCATGCGTCGCGGCCACATACTCGGCTTCG
GTCGTCGACAACGCCACAATTTCTTGTCTCTTGGAGCTCCACGACACAGCGCCTCCACCGACGAGAAAGACGT
AGCCTGATATGGCGTGCCGATCTTCATGCGACATTCCGTCCGCGTCGGAGTACCCTACCAGACCGTCGTCTCG
TCCACCCAACACAAGCCACCACTCGCGAGTCCCCTTCAGGTAACGGAAGACCCTCTTTACGGCATCCCAGTGC
GCCCTTCCAGGATTTTCTGAGAACCTGGCCAGAACACTCACAGCATATGCAATGTCCGGGCGCGTGCCGAGTG
CAGCGTACATCAAAGAGCCAATGGCCTCGCGATAAGGGATGTCACGCATCTTAGCAAACTCGCGTGGCGTCGT
GGGACCCTGAGACTTGCTAAGATGGACATGAGCATCCATTGGCGACGAGACGGGCTTCGAGTCCACAAGTCCG
AAACAACTTAGGATAGAGTCCACGTATGAGCGTTGTGAGAGAGATATGGTTCCCGCGTCTTCATCGCGCTTGA
CCTCGATACCGAGCAACCAGTGGATAGGACCCAGATCTGACATCTCTAGCCGGGCCTTCAATTTCCCTTTAAA
GCGCGCGAGTAGATCGCCAGAGTTAGCAACAAGAGTCAGGTCATCGACATGGACAGCAAGAGTGATGAAATCG
CCGTTCGGCTGGTGAACATAGAATACGGCGTGGTCGACCTCGCAGCGCGTGAGACCGATGTCGATGAGTATAG
TCCAGAGGGTTTGGTACCAGCGGCGACCACTTTGCTTCAGACCGTAGATGGCTTTAAGCAGCCGGAGGACTTT
TCCGATACTATCAGGCGCGTGATAACCAGGAGGCTGCGACATGAAAATTTGCTCATTTCGTCCAGCTTGCCG

TATAAGAAGGCACTCTTCACGTCTATCTGGTGTGCGGCCCATCCATTTTGAGCTGCCAGCGCTTGGGCAACCA
ATCTCGCTTTGTACTTCTCGATATCTCCGGACGCGTTCTTCTTAATCTTGAACACCCATTTGCTGCCTACAAC
GTTCGCGTTCTTCGGCTTGTCGACCAGCTTCCAGGTTTCCGCTTTCTCGAGTCTTGCTAGCTCTTCTGTGACG
GCCTCCTCCCATCTTGTCCAATCTGGGTGCCTCTTTGCTTCTGCTAAGCTTCGTGGTTCAAGTCCCTCCGCAT
CGGATATCGCGGCAGACATTGCGTATTCATCGGCTACTCCCCCAGAATGTTGTGACTCTTCTGCAGTGGCGGC
TACCTCGGTTGGTATCTGTAGGCCTGGCGGCATATTCTCGGCGTTCTCACCCTCAAGGATTCTTTGGATATAC
GTGCTGGGCTTTCGTGCTCGACTAGGGCGGATATAGGGCGGTTTGTCTGGTACGCTGTCGCCGGGGGCGCTAT
GCTCGATAAGACTTGTATCTGGCTCGGCAACTTCGTTCTCTGGCTCGACGTTTTCGTCATCGCTTCCCTCCTC
CACGAGCTGCGCACGCGGCGTCTCCTGCGGCTGGAAGATGACGGTCCTTTCCACAGTCACAGTCCCCGTCTTG
GGCCAATAGATTCGCTGCCCTTGGCTGTTCAGGTCGACGCCAACCCAACGCCCCTCACGTGCGCGTCCGTCGA
GCTTTGAACCTGAGGGGTCGTGCACCCAGGCTCAGCATCCAAAAGCGTGTGCGCCGGAAATGTCAGGCTTGAC
GCCAAAAGCCACTTCGTACGGCGTGGTCTCCTTTCCGAGAGGCTTTGTCGTCATGCGGTTTTTCAACCAAGTG
GCGTGCATGATCGCCTCGCCCCAGAGAGTCTTTGGTAGGCCGCTGTCATGGAGCATTGCTCGCACACGACTGA
GCAGGACGCCGTTCAACCGTTCTGCGACGCCGTTTTCTTCTGGGGTGTCGTGGACTGTGAGGCGGTGTGTCGT
ACCATCCTCGTCGAGCATGTCCTGGAGCTCTTTTGCGATGTATTCTCCTCCGCGGTCGCTCTGCAAGGTCCCT
ACCCGACGCTTGAACCGGGACTTCATCCAACCACGATATTTCCCATACGCACGGCGGACGTCCCCTTTTTGGC
GCAACAGGTATGTTGCTGAGAGTCGAGACTTGTCGTCGGTGAAGCTAACATAATACTTTCGCCCCCCGATGGA
TGCGACGTCAGCAGGGCCCCAGACATCCGTATGGACGACCTCACCCACTTCCTCGGCAAGTGGGCTGCTGCGC
TCCTTCGGCACCGGCTTTCGCCGTGTCTTTGCGAAAATGCAGGAATCGCAGAACTCGATTTGGGAGGCCCCCA
CGAGTTTGACACCTGTCACCAGTCCTCGGCTGACAAGTTTCTGCACGGCTTCTGGCGACGCGTGGCCCAGGCG
ACGATGAAGTTCTTCCAGGGTGACTTCTTCGAGGGCGACGTGTGCTGACTCCCCTTCCTGTTCAACCTTATAC
AGACCACCGCTGCGGAGAATCTCCCCCACCACGGTACCATCCTTGTCCCGTATCTGGCATCTACCGCCCCCGA
ACAGCGCTGAGTATCCGGCAGCATCTATGCAGCCAACAGATATCAGGGTGACTCCCATCTGGGGAGCGTATAG
CACGTCGCGGAGGCGAATTTTGGAGAAGCCCGCTCCATTAGGGACTTCGACAACCATGTCCCCAACGCCTATA
GCGTGGAAGCGTTGCTGGTTCGCTGCGTTGATAGGTCTTGGCGGTATCTCGGTGAGTGACAGAAAACGATCTC
GGAAGGGGGATAAATGTTTCGTCGCTCCAGAGTCGAATAGGTCGACATGCTTGCACCCTATGTCGCCATGTAC
GACCGCGGCAATGTCGATCTCGCTTTCTGAGTCGCACACGTCACTTTCTGCCTCTGCGTCTGTGCGTTCGTCA
TCCCAAACTTCATCATCAGAGTCTGAGTCTGATACGCATTGCAGTCCAGGCATGTCGTCGTTGGGGAAAAGAA
GTGGTTCAGCGGCCTCGAAGTAGGTCGACAAAGTAATCTCATTTTCGCTAACTGACTCGGATTCGTCTACAGC
TGCCAACCAACTGTACTCGACATCACTGTCCTCCTCTAGCACGGCGTTTGCGGCTGTAGTTCCTCTTCCTCCG
CTATCCCCGCTCGAGTTTTCGCTCTTGACTCTAGGACTCGGACATTGGCTTTTGCGGTGTCCTGTACCCCCAC
AGTTCCAGCAGGTGAGTTCCTTTCTGGGTCCCGCCGGTCTGTCACTAGCGCCGTCTCTGCCCTTTCCAGAGAG
CCTTCTACGCACATCCTCCGCAGAGTATGCTGCATCTCGCTCGTCCCTGCTGACCTTAGGCTTGGCCGCGGCC
CGACTAGGTAGGCGTTTTCGTTCGTACTCCTGCTTGAGATATTGGATGGCTTGATCCGGGGTGATGAGCGTGC
CGAGCAATCTGCCAGCGTTCATCATTCCGGATAGGAAGTCAGCGTACTGGGCAGGGACGGATTTGAGGATCTG
TGCGCACATCAGGAAAGCGTCCAGTTGGTCTGACACGTCTGAAACGTCTGGATCAGACATTTTCAGACGAAAA
GTCGATCAGACACTGTTGTCTGAATCCATAAGGTTCATTTCTGAACCAGATGGTCGGTATCTTATTCAGACTA
CCTCAGACACTATCCAGACGCTTCTCAGGATTAAATCAGACATCTGTAAGACACAAGATATATTACAACTATC
GTGATGGTATATACATTTGCGCCCAATACGTTTGCGAACAGGATAGTGTGGTGCCCAACTTAGCTGCTACCGG
GATACGATTGGTCTTACCGACATCGCAGATATTTACTGTGAGGAGCGAGAACACAGCGAAGCCCGGCACTGTG
AGTTCGAAATGTACTGGGGACCAAGAAGATGTATATCTCGTGAGTGCAGATTCTATTGAGGGAGGTGGTAGCG
ATCGGGTGACATGTTACGAGGTGCGCTGGGGGAGAAGATTCATGTGCAAGGTGGCAGGAGAGCCAAAGGAGGG
TGAGCGGAGAACGAAGGCACAGACAAGGGAAGGTAAACGTACAGAGTGCACACTGAGCTGAATGTACTTGATG
CAGACAGACGGCGTCTCTGGACCAGGTGTCAAGGGCTTTTAATAATTGTCCGGAAGAGCCAGCGGGAGAGGAA
TGTTAGCGTGCTCGTTCAACAACATTGGGAAGTTCGCAAAGATGAAGAAGTAAATATCGGAAGCATACTCTGA
GGGCCTTCGCATGCGCCAGCTACATACCATATACGTGGCGAGCACAGTGTACCACAAATAAACAATGTATCCG
CAATGATACATTTGTAGATCGAGTTACCACACTCTGAGAACCGCAATGATACTTGCGACCAATTTCACGGGGC
ACAGTGTCTTCCGACTCACCTAACACGATCCAAACGCGGCGTCCCCGTGCAGTCTGCTGTGAACGATCGAAAG
GTCCCGCACAGCATCCACCGGCACACCCGGACTGCGAGCTGTCGACACACTAGAGATCACGACTATTTGCTGA
GAACATCAGACTCACCATAGTTCCAGTACTTGAAGCCGTTGCCCTTTCCGACCCGTTGCTGTTTGCCCAGTCA
CGCAAGGGTACACACGAATCACCCATGGCGCCATAGTAGAACGCAATGCACATACCAGACAACCTGTGACAGC
CTGTATGAGCTAGATAAGGGAGATGTATGACCAGGCGGTGGGGCAGTAGTTTCGTGTTTGAGGACTTCAAGGA
CTGAAGATGAAAGAGAACAGAGTGGTCAGCGAGCAAAGAACTCCGGCTAAGAGACCGTGGGAACGTCTTGCTA
TCGTTCAACATAATCACGTGATATGAAGACTTGAAAAATTAAACGTCTTACCACAATGCCGCCTCCCCATTGC
ATGAGGCAAAATTTATCAAGCACCCGTAACAACTGTTGTCTGCAGCGCAGCCTCATAAGATCCATTCGTGCAT
CTTCATCACTATACAGAGCGGAACTGATGGAAATCCGCATAATTCTACGAGATTGGTGCGGATAGAAATTTAA
GGTGTGGGCGCGGATCGAACGACGGTGCTCCGCAATGGAGAAGCGCTCAAGACCCCTCAGAGCGAGGTCAGAC
CCTCAGAAGGATCCAGGACGGTTTCAGACGCGTCAGACGCGTCCAGAGAGTGGACACTTTCCTGATGCAGTAG

TCCTGGTCGGATACTTCGACGCCTATCGCCGACAGTTCTTCGACCATCTCTCCGAGCGTATCAAGGTGCCTTG
AGATGTCACCGTGTTCGGTGCAGCGCATGGTTTGAAAGCGCTCGAGTGTGTTGGACTGGACGAGCGGCGTCTT
GTCCTTGAATTCAGAGCAAATCCAAGTCCACATGTCCGCGACGCTGTCACCGTTGCGCTTCATCCTGCGAAGG
ACGGTGTCGGGGACAAGGCGAGCAAGCAGGTCTCAAGCCTTGGCTTCTTTCTTGCGCCAAGCGCGATAGTCAA
GGGCGTATTTTTCGTCTAGTTTCTTGTCCTTCGAGGGGAGAGGCAAAATTTCTCCTTGCTCCAGCAGCAGCTG
AGCATTAGCGCTCTCCGCGAGCGACGGCGGAGGTGCAGTCCCGGCGCGCCAGTTTGCGAGCGGGTCAACCGGC
CGGGGATCCTCTCCCGTGAGGTGCCCATGCAAGTCTCTTGCATCCAGGGCCCAGGCCAGGCGGGTCTTGAAGA
CCGGCCAATTTGATCCGTCTGTTTTCAGGTGGGGAATTCCCGAGAGCCCATTGGCATCTCGGGCATCGTCGCG
TTCTAACGCTGGCGCCTCGGGGCTCATAACCTGTTAGATATTAAAAGCCTAGAGCACGCGACGATGTCGCTGT
CAACACCTCAGACGGATCAGGAAGTCTGTATTGCTATGTACCTAGAGCACGCGACGATGTCGCTGTCAACACT
AAGGTACAATCAGTCTCGACGCATGTAGCTGTAGCGTAATGTCGTACTCACCCTCAGACGGATCAGGAAGTCT
GAGGGCTTCTACTCTACGAATGCGAGAAAACAAAGAAACAGAGGATTACATAAGAACTATACAGTTTCGACGG
AAGCATCGTATGTTAGATCGAACATACTGATATAACGGGCGGGTTTCCTCATTAGGCAATGGCGCGCCGCCAC
AGGTGCGCCGTTGTGCGCCACCGCGCGAGCGCGGGCGAGCCCGCTCTTGAAATCCAACAC
>Cligib1_rnd-1_family-144#TIR/MITE
TTACAGCGCTTTCAGAAAACGNTGAGACAAGCAAAATTTTAAAAATCGGACGCGCGCCCTCGCACCCCGCGCG
AAATCGGCGGTGACGTCATCGCCCCAACTGCGTTCTGCTACATTTTGCTCGTCCGGTTCCACCCGTGCAACTA
TCAACGTGTAATTACCAGATGTCCAACCAAAGGTGTGACGTCATGTGGACGTCGCGCGGTTTTGCGCGGTGCG
CGCACACTTTTTCAAAAACTTGTTTGTCTCAAGGTTTTCGGAAAGCGCTGTAA
>Cligib1_rnd-1_family-171#LTR/Copia
TTCTTAGAGGACGACGTCTGGAGNGCCGTGACGGAACTCCCGGCCGTGTGGACCGTCTTNCCGNGNGTCATCC
TNTCCCAGTGCGTGCCGANCCTGTCGATAACGGCGTCCGACGNCTTGGTATCGTATAGNGTCGCGACGATCGN
GTCCCAGGACGGGGGTAGGGATGCGAGCACGATGGAGCGGAAGTCGGGATCGGTGACTTTCCCTCCCGTCGCG
TTGGCTTCGAGCCACTTNGCCCGGAGCTTCTCGATGTGNGTGGGCANGTCCTCGCCGTCGCGGTATAGGCAGG
TGCGCAGGGCCTGTTGGGCGCGGACCATNGCNAGGTCGTTGTCCACGCGGTTGTACCGNGCGGTGAGCGTCGT
CCAGACTTCGGCCGCCGTTCCATCTAAGTTAACCCCTAAGCCGACGGGGTTGATCANGTTGTCNATAAGTAGC
GTCATNGCCCACGCGTTNCGCATNTTCCACTCGTTGATGGAGGGGGTAAGTGACCACCAGNGGGTGGCCGCGG
CGGTGTCTGGGTCAGGTGGGGGAAGTGGGGAAGTCTCTGCGTCCTCAAGCGTAGGTCTCTCCGCGGTTCTTTA
ATGGTGCCGTCTAGGTAGCCGNCGACGCCGCGAAGTTGGGCGGCTCGGAGCACGGCGCTNTTCCAGACGATCC
AGTTGGNGCCGTCGAATTTGTCCTTCTCGGCGAAGAGGGGCGGGTGGGAAGACGATGTAGTGGAAGTCATTTT
GGGTAAGTATGAACGTATTGAGTTTGCGCGTGTAACGGTATCCGTATTGTTCAGGCTGCAGGGCCCATAACCT
GTTGACAACGGAAGCTCAGACGCCGTCACACGAGTGAGAGCAAACTAATATATTAGCTACCTGTTGACAACGG
AAGCTANATAGGATANATAGTCAGTCGAGTGTCCGTGTAGATATGTATAGAATGTACTAACCTCAGACGCCGT
CACACGAGTGAGAGCAAACTGAGGTGCGTAGGTACGAGCGNGAGATATATAAGGGAAAGAAAATAGTGGGGCG
CGGCGTGTTGAACCCGCGCCNGTGATGTGTGAAACGAGATGTAAGTGAAACGGTAGAGACGATATGAAATAGA
GTAAAAATGAAATAGAGTCGCGCTGGCGCGCGCGCCGAGCGGGATGCCATATTTGGTATCCGGCGGCGTCACG
GGCGTTACCTTCAACA
>Cligib1_rnd-4_family-6014#LTR/Gypsy
AGAAGAAGTCATGATGTTCAAGAATCATGGGTTTGGTATTCCTTCAGAAATGGAGGAAGACGATGAAATTTCG
GACTTTGGCATACGGTTCTTCGGAGAAGAACTACCTGAAGAGGAGGTGATTCTGGACACATCGGAAATTTCCA
TCTCAGCTCTCACTGAATTCGGCAATGAAGATCTGGAACAGCGCATCAGAAACCACCCACGCGTAGAATCAGA
AGTCCGATTCGGCATGAAGGAAAGTCCACACTTCTTCAAAGAAGCTGATGGCATCATCACAGTCGGAGACAAG
GTCTATATACCCCCAGACAAACAACTACGTGAAGAATTAATCAACGAACATCATGATTCACCACTAGCAGGTC
ACCCAGGGATCCACAAAACGAATGAACTCATTGGACGCACACTATGGTGGCCAGGGATGAGATCAGACATCAG
AGATTATGTCATGGGATGCGAAACATGTCAAAAGAACAAGATCAATCGAGAAGCACGCCATGCGCCACTCTAC
CCCAACCCGATACCCACGCACCCCTGGGAAGAGATCTCAATCGACATGATCGGCCCCTTACCACCGTCTAAAG
GTTACGATGCTATTTTGGTCATTGTGGACAGGCAATCGAAGATGATCAGGGTCGAACCGACACACACCACCCT
GACGTCCAAGGGACTTGCAGAAATCTATCGAGACACCATCTGGAGACAACATGGAATACCCAAGAAGATCATT
AGTGATCGTGGACCACAGTTCGCATCGAACTTCATGAAGGACCTATGCACCCTCTTAGGGATCAAACGGAATT
TATCAACGGCTTATCACCCGCAGACAGATGGACAAACAGAACGCATCAACCAAGACGTCGAACAGTTCCTTCG
GATCTTTTGCAACTACGATCAGGACGATTGGGCCTCATGGCTATCAATTCTAGAATTCCAGTACAATGATAAG
ATCCACTCTAGTACCGGACAAACTCCGTTCTTCCTGAACTATGGCCGACATCCATGGAAAGGCGACATCTCCA
TGGAACCGGTAAAGAACGAAGCCGCTCAAGAATTCGCATCACGCATTGAAGAAGTCCGGAAGAAAGCTATCAC
CGCCTTAGAAGCATCAGCCGAACGAATGAAGAAACAATATGACCGNCACAAGAAGGACTCGCTTAATCTCAAA
CCCGGTAACAAGGTGTTTTTAGAAGCCAAGAATATNACAACCACCAGACCATCCAAGAAGTTATCAGAACGAC
GATTAGGACCATTCGAAGTCATTGAAAAGGTAGGCCACAGTGCCTACAAACTCAAGCTCCCCGCCAACTGGAC
GCTCATCCACCCGGTATTCAATGAGAACCTACTAACACCTCTCAAGACAGCACGCTTCGAATCACAACAGAAG
CCATCACCTCCGCCACCCGATGTCATCGAAGGTTCAGAAGAATACGAAGTAGAAAGCATCCGAAAGGTCAAAA

AGAAGAATGGAAGACTTCTATTCTTCGTCCATTGGAAAGGGTATCCGGAAGAAGAAAACACCTGGGAAGTCCG
CGCAAACCTCAAACACTCCGCGGACCTGGTTAAAGACTTCTACCGCCGACATCCCAACGCACCACGATAAATA
CNACACTCGTTTCGACCACTCTACTTTACCCCCACATCTTCTAANTCGCATTCCACTCACAAACCATACTCTC
CACCATGTCCATCCCCGCCACTCAACTCGCCTTGGCCATCGCCGCCCTCTTCCCCACCCTCGAACTCCTCCAC
TCCACCCCCATCCATATTCGAACAATCAATGACTGGATCAAAGCGCTGGACAACATTGCGCGCACCCTATCNG
AAGACGACGTTACCATGCTGCAACCCCTCCGCGACCAACGGGCTCAACTCCACAATCTGCGACAAAGTCTACT
GGACTACGAGAAGGACCTACATGCTCATGTCCAACGGGTGCTAACCCANCACGCNCCTGGTCTCACCGACGGA
GACATGACTACGCTTCGTCAACAAATGACGGTCAACGAATGGGGTGTTCCCATCGGCCCCACGGAGGATCCAC
CCGGAGTACCGNCTCCCTGTCATTCCCCCTCCCCCATCTTTAGGCACTCCCACTCACTCCGCCTCGTCTAGCC
CCCAACCTCTCCAACACAACTCTCCCCCACAGGAGAAGACGCCATCGCAGGCCCCGGCCACCTNCCACCAACA
CCACCCACGTCCACCCCCATCGGGGTCCTCCCACTACACCGGTTGTGCACCGTCAANATCCCCNNAACGTTCG
NNCCCACNGANCCCTACAACGGNACNCACNCGTACAGCGGNNAGNCGTTCCGGTACTGGGTGNGCGGACCGGA
CGACNCGGCCGCCTTCTTTACGCGCANNGGCGAGGAGTACGTGACNGCNANAACGAAGNAGGANCTCGGNAAG
GTGCTGGTGGACGNTGATTCTGGTTTAGAGTTTCGGGACGAAACCCCTGGAGGGAAGGTCATGTTACGATCCA
TTGCTTCTCTGNTCATCATCATCGGNCACCTGACTGATCCCATCTTTCATAGACGCTTCTATTACTTCCACCA
TCATCCATCATCATCTGACATGCTCCACATGCTCCATCATCAGATGATACATCATGCGATCCCTTATCTACTT
CTCTACCGCATGGTCGCCGTTCTATCTTTGTTATCTTCGTTGTTCTTACTCAGCTGCTCAGTCAGCTCGCTTC
TATNTCAGTCATCATNCTCCATATGTCCTACTATACTCACGGACNTAGTCATTATTGCGTAGTCATAGAACTA
CATACACGTCTATATAAACGTAGCTACATACTCCTGTATTCGCTACGTTTAATCGTTTATCANTTTCCCCCTC
TACTGCCTCCTGACCCAGGAGACTTCAGACCTTGCTAAGTGTCCTTGAGTCTTTACTGTCGACTCATTGACTT
GCGTTAGCAGGACGTAACA
>Cligib1_Cluster23#LTR/Gypsy
TGCTGTTATGTCCTGCTAACGCAAGCCAATGAGTCGACAGTGAAGACTCAAGGCACTTAGCAAGGTCTGAGTT
GCCTTCTGGGTCAGGAGGCTTGTGAAGGGGGAAACTGATAAACGATTAAACGTAGCGAATACAAGGGTACATC
AGCAAAACATGGATTTTACTTAAAATGACTTTGGAGCACTTTGAGGCACTTTCAAAGTGCGAAAGTGCAATTA
GTATTTCTGACCATTCTTACTTTGGAAAGTGACCATTCTTACTTTGGAAAGTGAATGTACTTTGAGAGAAGTA
CCGTGAAAGTGCTCCAAAGTACATTAGCAGCCATGGCAGGGTAGTTATTAAGCTCAGAAGTGAGGGAAAGTAA
TAAGCAATGAAGGCTGCAAGAAAAACTTGTATTCAATAAGTATTCAAGTACATGGGTATGTCGAAGTGAGGAA
TAAACATTTAATGATGTCAAATCTACTCTTCTATCAACACCTATATTCCACACCTTTTCAAGCGCTCAGCATC
TCGCAGCTCACCCCGACTTCAAACTGTCACCGATTGCGCATGATGCAGCGCAGCAGGAGCGGAATGATGGACT
GGACAAGTGGTATAGAGGAGGAAGCACGGACTGAGAGGGCGAAGGCCATGATGGCTGCTTCCAAAGTGTTGTG
TGGGGTGCCGGGTGCCAAAGGCGCAAGACAAGAATGAGCAGTGTCCAACATGAATGCTATGCACTATAATCAA
ATTAGGGCATACTGAGAGCTGAAGACTGAAGTGCTTACAATGGTGATAAACAGGGTATACTCTCGCCTGTGTC
GTGCACCGTCAATGGCTCAGTATCCTCCCCCTCGCGTGTTCCATGTCCAGTAGGAGCGCAAAGGCATTTGCGG
GAGGTTGGAAATCAAAGAGGGGATTGAGGGCGCCTAAGCACTACCTATTCTCGATGATCGTATAGGTCAGCAT
GATACATGGATGGGAGATAAGCATTACTCACCCCGAGCTGGACTCCCATTGCCTGCGCACATGGGGGTTGAAG
ATTGCCGCAAGCGAGAGGAGAGAGAACATGGGGGAGCGAAGCAAGGTGGTGGAGCAAAGGCGAACATGACGTG
GCCAATGATGATGTGGCTAGTTTTTCAAGCGGAGTTTAAAACAGACTTGGGGAGAATTTCCATCCATCGACCG
CCAACACATCTGTTAGGGCGTGAGCCTTTACAACTGATACAAGTCTGTCGTTCGTTCTGGTTCTATCTTGAGA
GTAGTTTCAAGATGTCTCCTGTCCAATGACAAAGGAGTTCAAGTCACTCTGAGCTGGTCTAAGCTCAGGTTCT
GAGTAAGAGAGTAAAGGCTGTAGATTGCCTAGGTCAAGATAAGGCTCAGCACGAGCACGGGTTACCTGTGGGT
ACCTGTGGGTAGACCCGTGGGAATACTTGCAAGTATTGGCCTAAACAGGTACTTGTACTCATGGATCTATTTA
CTTGCGGATCCGAGTCAAGCATTTGGACATTGCAGGTACCTGTGACAGGTACTTGTGACTCATACCGGACCCT
CAAGGACCCTTAAAGTATAGTTCCCACAGTACATACAGGCTCAAATTCCATTCTAAACCAACAAAATCCTTGT
AAATACCCATAAGTACTTGTGAAATACTGTCCCTGATGACTAATTGCTGACAAATGACCTGTGACCTGTGAGT
AATCATGAGTAATCCATGAGTAATCAGGATCTGTGACAAGTACTTACTGAGTTGTAAGTACTTGCCAAAAAAT
ACCCTGAGTACAAGTACTTGCTGACTAAGCTACCCGCAGATCTGGGTAGTGACCTTGTGTGACCAAATACCCA
CGAGTACGGATCCGGATCACCCACGGATCTACCCATGCTGAGCCTTAGGTCAAGATGCTAATACAGGATGTAC
AAGAGAGGTATATATACTAGAGGGGAGTGACCATGAGGGGGATGCATGTTGAGGGACTCTACAGCAATATATA
CTAAAGGGGAAGAAATGCTGTAGACCATGAGGAAACCGAGAGGCGGCAGTAAGTCAGAGGATGCCCATGAGGA
GTCCATGAGGCAAACACATGTACAATATGTGTTGAAGAAAGAAGCGTCTGTGTATGATGTCAGAAAAAGAGTA
AGCCCCCTAGGAGCGCTGTAACATTACCCCCTGTTTATCGTTGAAGTCCCTGACTTCAATCTTGGTGGTACGG
TTCGTCGCACATGTTAGACATGGCGGTATTGTCAAAATCGAGATCCCCATCAGTATAGGGATGGTAGCTGGCA
TGGGAAGTGGAGGATCGGTAGCGACATCTATCTTGGAGGTGTCCTGGTGCGTGGTTTCCACAGTGTCGACAGA
AGTAATGCTCGCAGTTCTTTATGATATGCCCAGGTAACTTGCATTCCCAGCATACTTTGGGGTTGGGTGGGA
GGATGTTACCTAGGTCCAGTGGGGGTACGAGTGCGAAGTGGTGTGGGAACGGGTTCAGGGGATGAGATTCTTT
CTACGAGGGGTCTTCTTCCAGCATGACTGAGGGGGTTGATGGTAATGGTGTGTCCACGCGATCGGCGGTTTCG
GTGATTAGCGATGGGTGCAGAGTTGTGTCTGGGAGGGAATTCTACGATCTCTTGAGGGACAGGGACTCTTCTG

ATGATTCTTTGTGGGCGGATTAGGGGAGTGTCAGGGGCGGGTGGGGTGGAGGGAGAGGTGGTTTCAGAGGAGG
GTCTAGAGGAGGGTAATCTGATAGTAGGTTGGGGAAAGGGGGTTCTTCGATATGCGGTCTGAGATGGGGGTGT
CTGTGTCATGGCTTCGGCAGTCGTTCTGGTGGTTTGTGTAACCAGGATGGCAATGTCTGGGGTAGGAGAGGAG
AGTTGGATGAGGTTTTGTCGGGATATAGGAAGGGGAGAGATGTTGGGAGAGCAGGGATTCAGGATTTGTTCAA
TTCCATGTTTTAGGAGTAAGTAGCTGAGGCTACCAGCCAAGGCTTCTTGTTGTTCAGTGAGGCGGTTGACTGA
AGAGGTTATCTCCAGTAATTCGTTATGGATGTTTTGTATTGGGACAGTGGTTTGGTGATTCTCATAGAATGTG
AGTAGGAGGTCTTGGAGGATGGGATAATCGTGGCAGCTATGATACATAGTGGCGAGATCGGTAGTCTGGGTGA
GATGTTGGATGAGGTCCTCTTGGGAGAGTAATCCATCAGGAGAGTAGGGTCGTTGGTATTGAGGAGGCGTGCC
CAGATGGGCATTGATAAATTGGATAAGGTCTTCTTCAGATCCAGAGTGAGGAGTAGGAGACTGGGGAACGGAG
GATGGTTCAGACGGAGGATAGAGAAGGGAAGGGGTGGTGACTGGGGATGGTGCAGGGGAAGAGAAGGCAGAGG
TATACTGGGTGGAGGCAGGGGAAGACATGTTGATTCTGTTTCTGAGGCTGTGGTAGTAATCAGTTGAGTACTG
AGGATTTTAGTCTAGAGAGTGTCGCTTTTTATAAGCTTTGAGGACGTCGGATGCATTACCAAAGTCGCGGGTT
GGCAGCCACATGTTGTCTGCGGAAGGGTAACCCTTCCATTTGACAAGGTACTTGGCGCCTCGACCTTTTCCTT
TGTGTTTCAGGATGGCTTCTATTTCGTATTCTTCTTCTCCATTGATTAAATCAGGGGGGTCGCGGAGGTAGTT
GGGGCCATGAGCTTCGGTTTCTTTGTAGGGAGTTAGGAGGCATGCATGGAACACAGGGTGTATCTTCCATTGT
TCTGGGAGTTCTAGACGGTAGGTGACAGGTCCCAAGACTTTGGTAATGGGGAAGGGCATCAGGAAAAAGGCAA
AACACTCATAGATCTCCGGTTAATTATGTAGTCATACAAAGTCTATATTAAGGCTCAGCATGGGTAGATCCGT
GGGTGATCCGGATCCGTACTCGTGGGTATTTGGTCACACAAGGTCACTACCCAGATCTGCGGGTAGCTTAGTC
AGCAAGTACTTGTACTCACGGGTATTTTTTGGCAAGTACTTACAACTCAGTAAGTACTTGTCACAGATCCTGA
TTACTTATGGATTACTCATGATTACTCACAGGTCACAGGTCATTTGTCAGCAATTAGTCATCAGGGACAGATA
TTTCACAAGTACTTATGGGTATTTACAAGGATTTTGTTGGTTTAGAATGGAATTTGAGCCTGTATGTACTGTG
GGAACTATACTTTAAGGGTCCTTGAGGGTCCGGTATGAGTCACAAGTACCTGTCACAGGTACCTGCAATGTCC
AAATGCTTGACTCGGATCCGCAAGTAAATAGATCCATGAGTACAAGTACCTGTTTAGGCCAATACTTGCAAGT
ATTCCCACGGGTCTACCCACAGGTACCCACAGGTAACCCGTGCTCGTGCTGAGCCTTAGTCTATATAGTTGTA
TGTAGACATGTGACATGCACACATATACCCCATAGACCTCCATAGACCTCATAATGTCTATATAGGTCTATAT
ATACACCCTGTATGTCTACATACGTCTATATATACACCTGTAGACACTCTGTATGTCTACATACATCTATATA
TACACCTGTAGACACCCTGTATGTCTACATATGTCTATATGTACACCTGTATACACCCTGTATGTCTACATAC
ATCTATATGTACACCCATATACACCCTGTATGTCCACAGAAGTCTGTATATACACCACATAGATCTACATAGA
TCTATGTAGTCCATGCCCAGGTCTATCAGACAACCATAGACGTGCAATAATATCACACGGGTTCACCGCAATC
TTCGTGAATTCCCATGCTGGCTGCGTAAGCTGTAGTTTACTCCCCTCATTCCCATCACGACACACGGCCGATG
TGGCGAGTGCAATGCAGGGATGAGAATCCAGTGGTGGCTGCATAAGCTACCGCTCACACCCCCCATTTAGCCT
CGCGATACCACTGCACACGTCAAATTGGCACGGCGAGTGCAATCGCGAGGGTGGAGGAGAGTATGAGCCGGGA
GCGAGCCATCCCACCCTGTAAACATAGAGGGTGATAATAGGGTGCTTACTCAATGGGGTACTTGAGTATTGTA
CCTCCCTGGCACTATGCCATCCCGTTTTCAGATGGCTATCACCGGCACGGTGGTGTCATGGTCTTCCCACAGG
TGGTGCGCCGACGGCGCCATGTCACTTTCATATCGTTCGCCTTCCCTGGTCTTGCCCTGGTGGTGACACACGC
GATGATCATGCAGCTCCACAGCTGAAGCAGCAGGGTATTCTCGGACGCACTGGAGTGAGGGAAGTGGGCGCGG
GTCGAGCTGTGAGTGGACAGGGAAAGTGGATGGAGACGCAGTAGTGGGTGATGAATCGTCAACACATCCGCGT
TAATGTTGATCGTGGCTGGTGGAGCTGTGGATGCGCCTCGACATGAGTGACAACAGCACACTATAGACAGGTG
CACCCGTTATCCCATTCATTCCGTCTGTCCTGCTTATCCGCTATCCTGCTTGTCTGTTATCCTGTTGTCTGCC
ATCGCATTCATTTGTTGTCTTGTTAGTTTGGTTTTCATTCATCATGGGCGGGATATGCGTGTATGTGAGCGCG
TGAATATGTAGGTTCATTCGTTCGTTTGTGAATGAATGGGTTGAAGGAGGCATAGTGCTACCAGGTGCGAAGG
TGCGCATGTGGACGGTATTAGTGAACTTGGTTTGAGAGGTTCGCAAAGCCGATGTAGCTCACCGATTATTCGC
AAATAACAGGGGATGGCAGAGTTGTGGCCGGTTCGGGAGTGCCAGGCTGGGCTATGAAACCGCTTATATTAGT
GAACTTAGTTTGAGAGGTTCACAAAGCCGATGTAGCTCACTGATTCTTCGCAAAAACAGGGGATGGCAGAGTT
GTGGTCGGTTCGGGAGTGCCAGGCTAGGCTATAAAACCGTTTACTGAGGCACACCGTCGAAATCCAACATCTA
ACAAGGCGTGAAATATGAGGGGCAACAAGAGAGAGAAGCAGAGCACCTTCCGCACGGGTATCCCTTACCCTGC
TTGCTCATCCTTAGTACGTTTCCTGCACGATTTTGTTGTGGAGTGTCGGCGGCTATCGTCAAGAGAGAGTACG
AGTCCAACATCTAACAAGGCGTGAAATATGAGGGGCAACAAGAGAGAGAAGCAGAGCACCTTCCGCACGGGTA
TCCCTTACCCTGCTTGCTCATCCTTAGTATGTTTCCTGCACGATTTTGTTGTGGAGTGTCGGCGGCTACCGTT
GAGAGAGAATACAAATCCAACATCTAACAAGGCGTGAAATCGAGGGGCGACAAGAGAGAGGAGAATAGCACCT
TCCGCACGGGTATCCCCTACTCTGCTTGCTCATTTTTAGTACGTTTCCCACACAATTTTGTTGTGGAGTGTCA
GTGGCTGCTTGTGGCGCGTTCGTGGCTGGGAGAGTGGCTGGGGCGGGTGGAGCGGGGGTTGTAGCAGCTGTGG
CGGTTGTGGCAGTGGTTGTCGGTTGTGGGAAGAGGGAACGTTTGGGCGCCCGCCGAAAGTCGTCGAGTGAGCA
GTGATGTGGTTAGGGCGGCTGGTGGTGTCTTGGTAGGCTAGGGGGTGGGTATTGCTACAGGGATAGTGGCGTC
TTCGGGGTGTTGTATTTGATGGACAAACATGGAGAATTCAAGCTACGGAAGGTCATTAGTCCGAAAATCAACT
GTGAGATGGACTAAATCATACCGTTTCGTAAACAAAGAGGATGCATGCCAAGGATGAGGGAGCGATGAAGATC
TCCCCTCGACGAGGATAGCAAACGTACGTGGATAATGATGGTCTTGATATGACCCACAAGTGAGACAGGATGA
TGATCAGTGGACCAAACGCTGATGTGACACTGCGGCCAGGTGGCAAAACCAAACCTAACCTGTGTGTTTCGCA

GTTTGCGAGTGGGTGTCTGTTTAGCAAGAGGTGCCTAGCCCAAGAGTCATGCTTTCAATGCGAGGGCACAGCC
CTATAGAAGAGAGTGGACGCGCCAATGCGCGACTGAGATGCGAGCCAAAAGCTTCTTATTCTAGGTGTCTACT
GAGGTCTCTATATATCGGAGCTAAACGGCCATGAGGTATAGTAGAATGTAGGTAATACTATGTAATGAGGCTG
CGGACCGATGAGGAACTATGCGAGAGACATGCGGAAGATACGGCCAGAACCGGAATCGAAGCGCGATCTCGAC
GCGTTCGGAGCGGAATGGGTGCGATCCATACTGTAAGAGTGCGATGAATGCGCCTAATGCGCCCAATATCAGT
AGGAGATGTGAGATGCGAACCCCTAAACGATAAGCTTGCTTATCAACGCTGATGTGTCACAAGTGTGACGTAG
GAAAAGAATCTTTGAGTGATCAATTAATGTTTACAGTCGTAAACATGTCTACGTGTATGTATGGATTTAAGTA
AAAATTCTTTGCCCTCTCGAGAACATTGTCAGTAGTCCATCCCTTAGCGATTGCATTACCAAACTGCCCCATC
AAATGGCAACTGCGAACGATACGGGCGGTTGAAATAACGGCGCCGTTTGGTTGATGCTGGCGCGTGAAACGGT
TAATGACATACATACCTGTTTGAGGATCCGGAGCGCCTAATGAGGTAAACCATTCCACATATGCAAGTGGATG
CGGATATGCGCCGAAGTATGGTGGCAGTAGGAAGATCACGCGCACTTGCGCTACACGCAGACCTATAATCGAT
ATCAGGCAACTAGGATTGAGAAAAGGGTAGGAAGAATATTACCTGCAAGCCCACTGCGCGATCTATATTCCAT
TGGATCGTCGATGATGAGAGCAGTGTCAAAGACACCTGGCAATCCTTGTTTGTGGCCACGAGGCGGGACTGCT
GGTGATGTATGGATGTGATTGAAAATTTTCTGACTGCTAAGAAATGTGTTGGGTGGGAGAGTAATGATGATTT
GTTTGTACAGGTCAAAACGGTCGGACGTATTTGGGAGGAGTGGGTAGGATTGTTGTTGCTGGAGGAAGGATGT
TAACGCTGGGATAAATTTTGTGGCGCCGAAATGTTCGATTAAGCGTTGGGCGGTCACCCCCCGGAGTGGTGGT
GCTTTAGCTACGCGGTATGATACAAGAGCCGTACCGGAAGTGTTGTAGGCATTGGCTCGGTCATTGGTAGGGT
GTGGCACGGGTCACACCGCGGGTCACATTTATGACACCACACCCGCACCCGCTGAAACCGCACCCGCAGACAC
ACCGTGCCCGCGGGTACGGGTTTCAACCACGGTGTAAGGCACAGCACCGCGGCTGTGCCACACCGCGGGGTAC
CGCGCTACGCTGCAGGGCTCAGCGTCGTGCCGTGGGGTGCTGCCGTGACCGCCGGCCCTTCTACAATGCCCTC
GCACAGTGTCGCGCGCATCAATTGATGCGGCTGGCACCGTACGAGGGGTGCGTAGCGATGGGGGGTCAACGGA
TCCCAGTGTGGCACTGCAGCCCCCGCCGACAATGCCCTCGCATGATGTCGCGCGCATCGATTGATGCGGCTGG
CGTCATGTGAGGGGTGCATAGGGCGGGGGTTAGCTGCCAGCACTCCTGCGTGAGGGAGAACGGATGGGGGCTC
ACCATTTGAGCTCCTGGCCCTCGTATGTGAGGGGTGGCAAGGTGGGCCGATGTATTGCGTGGCATGGGCGATG
GTGGTGCAGGCGAGAGTGGTGCAGGTGAGGGACGCAGGTGAGGGATGACATGTGGAGGTGGGGCGCAGCAGGG
AGTGACAGGGTAAGAGTGTGGGGGAGGGTGTGGGAGGGCGGGGGAGCTGGAGGTGCGGGTGCAGTGGTATGGG
CGAGGATGGCGTGGTGCGGGTGCAGTGGTATGGGCGAGGATGGCGTATGTGAGGGGCGCGGGCGAGGGATGGC
GTATGGAGGTGGGCGATGGGGTACGAGTGTGGGGGTGTGCAAGACGCGAGAGGGCGAGGCGGCTGGGGGCGCA
GATGCAGGCAAATGAGGGGGTGGGTGAGCGAGCAGGCAGGTGTGGGATGTGAGCTAAAACTCACCAGTTTAAG
CCCCCGAGCTCTCTCAAGCTGGGGGCGCAGGCCCAAGGGCTAAAACGATGCCCAGCTCACATTTGCAAGTGAG
CTGGCAGTGCAAAGAAAAGCTCGTTTGCGAGCGGTCCCCGGCCTGCTTGTTTGTGAGTGGTGGGGCTAGGATG
AACGGCACTCTCGTTTGCGAGAGTGGGGAGTGACGGTTGCGTCAAACGATTGGAGGGCGGCAGGTGGGTTGAA
CAACGATGAGAACATTGAAAACAACGTTCCGATCGTTGGCCGATCGATGTGTCAGGGAGCCGAAAACGGCCCG
CAGCCACCCGAGGAAACCCGCCACACCGCGGGAACGGGTGCGGTTTTGGCGCGGTGTCCATTTTTGGACACCG
CGCAAACAACCGTAAACACCGTAGCCCGAATACCGCGGGTTTACCCGTACCCATGCTATTCCCTAGTCATTGG
CGTCTAGGTCATCAGAGTCATCGGAGTCGTTGGGGTTGTTGGGGTTGTTGGGGTCGCTTGTGTTACCGAGTTG
TTCAGCTGCAGCCTGTTGTTGAGCATTGAGCCATGTGATGTACGCTTCACGTTGCCACATTGCCTCGCGGCGC
TGAAGCCACAAGGTCATCTGCTCATGGTAGTCCTTCTTGTTGCTGGCACGGAAGGCGTCCTTGGCGAAGTCAA
TATGGAGCCGTTCGGGTGATTCGGAGTTGTACCCGTCGTTGCTCCCAAGTGCCTGGATGGACGCGAAGTAATG
AAGGAGAGCGTGAATTTTTGGAATATTGAAGTGTTTACGAATCTCGTATTTGATAAATATATCTTTGAAGCGG
TGAAACGTCGCAAGGCAGGCCTTGAGAGCTGCGAGTGTTGTTGAGGTTTGCATCTGGAGCTGTGTGTAATAAA
TAAAATCGAGCGCCGCCCGCACGACGGTCAGGACATCCTGGTCCACGGATCCGGCTATGACCCCAAGGAAGAC
GCGTTGCATCTCTCAATGCTCCATGCCCGTCCATTGAGAGATGGACGAGATACCTTTTGAGAAATGTCGCAGG
CCTGGGTACCCCGACATTGCCTTAAAACGAGCGTCGAGCTCGTCCTCGCCAATGAGGGAAGTGCACCACTTGA
CAATATGATCCTTGAAAAATCCCTTGTGGAGCTGATGAAGGAGGTCGGGGGTGAAACAACCGAAAATGTTACA
GTGCGGGAGATTGCACCAGAATGGTTCATAAATAGCGCGAATGCCATCGTTGGTGAATTTTTCGGGCTCGCAA
CCGTGCTCGTGGGCGTAGAGGGTTTCAAGCGTTGACGGCACATTGCGATCGGGAGATGGAGTAGGATCACCAC
AATCGTTGGGATCCACAGTACAGCGCGGGCAGCGGTTCTCCATGCAACACGATACCAGACACTGTTCAGGGTA
GTCTGCCACGTATGCTGCGAGGATTGGGTAGATGAGCCGGACAAAACCGTCCGCGCAAACCATCTCGACACCG
GTTTTGCCAGCCTCGACGAGTGATGCGAGGATCTTACCCATACAGTAGTGGAAGAGGTGGTACCCAGCAAGTG
AACGTGTTGCATCGGAGTAGCAGCTGAGTTTGGGCACAGGAAGGTAGCCGAGGAGGACAGTGGCATGCGCGGA
GGGACAGCGCCGAATCTCTTTCGAGATGTTGCCGAGGGTGAGGTACACAGGCCACGCCTTCTTGTCGCCTTGG
AATTGCGATAGTTGCGTCTTGTCCGATGCGAGGATAAGCGGCGCGATGGCTGCACCATCTGGTAGCTTAGTCT
ATTAGAATCATGAATTAGTGAGATTGTTCGTTAAGCCCAGAATCACATACCTGCGTATCCCACCACCAGTCGC
CTGTCCACATCTCGTCGTAAATCCGATCATCACCGTCACTTGAGGTATAAACACGTTCGGGTACATATGAAAT
TGAATCCTTGAAGGCGGGGTTGCTGAGAAGCTCCTTGATGCATTCGACCGGATCGCGATACCATAACTCCATG
TCTTCCCCGACGATTTCCCCATCCTCCCCACACTGGTCCCCCGGCGCCGTCACGATCTCGCACTCCCAATTAG
GCCCAGTCGGAAGGGTGTCGACTCGCTTGAGGAAGGAACGATTATTGTGAAATGATAAGTTCATTCGATCACG

CATCTGAGACCAAAGGTACGTCAACAACAACTTCCGCGTGCCGTTGAATGCTCACTCACTGCTGGGAGCTTGA
GAAATTCATCCGTCGAGGTCTGGCCTACGTTCTTCATCAGCCATACCGCAAGTCCCCATTCCTCCTGGCTGGC
AAAGGGAGCCCACTGGTTCATCCCATCGTGTATCTGATTTTCAAGCAGTACCTCAAACTTTGTTTTTTCTTTT
GAGGTATGCACTGGAGTTGCAACTGGCCGGGGAAACGGCTCAATATGCCGCTGAAGTGTTGGCGGTTCGTCAA
TATCAATCTCTTCAAGTGTCAGCATAGGCTTTGTTCTTGGGCTGTGGTGGATAGGCTCGTGATCGACGTATGT
CCCCCCCCAGTCTCCGCGTCATGATTCTCACCATCTGAGATATCGGATCCATCGCTCTTGGATGCGACCTGCT
GCCTGCCTGCCCTCCCTTGGCGCAAGATCTCTTCGAATCGGTTGCGGCAGCTTCCAGCATTTCCGATGTGTCG
TCAGACTGCTGATTCAGTAGAAAAGCGGCGACGGCAATATAAACAGGTGAGGTGTCGTTCTGGGGGCATTTTC
TGTTGCTGATAAGTAGCACCATTTATCTATTACATTTTATGGATAACTATTGTATAGAGTCAGGCCCCGACGG
AAGATACGTGGACTCACTCACGACGACACCCATTGGTGGCCGGACCAAGTGGATACGTCAAACTAACACCCCT
CAACCACGCTGCTAAAAAAAAAATACGGGTGCTCAAGCCATATGTCGACTAAGCGGGTGCGTGTAACCAACACC
TGCCTGACCGCATTGTGCTAAAACATGCTAACAGCAGGCGATTCCCGGGTGTCTAAGCCGCATGCCTCATCCG
TTGGCCTACAAAATACCTTCTTCGATGGTTTTTATCCCATCAACTCAACGACACATACCTGCAGTGATGGCCG
AAGTTCGACATTCTGGGAGGAACATAATCCCAACGGAAAAGATTGCTGCACGCCATAAGTTCTTATTCACTAG
AGAGTGTACGATGTACTAACTTTACTCATAGAACGGATGAAGCCGATGCAGTTGATGCTTTGGAGAAAGTGGT
GAAGAAAAAGGCTGAGAAGGGGAAACGCAACCAACGCAAGATGGATTATGCCGCTGGGATCATCCCTGAGGGT
AATGGCGAGCGCAGAGATCGCGCTTCAGAGGTGTGTTAAGCTACTACTATTCTCTATTAGATCTAATCCATTA
CCTAGTTTACCTCTCGATCTGTCATGTCCAAGCCAAAGGCAGTAGCCAACCGCGCCGTGCGTGCAGGCAAAGT
CCCAGTGCCGCCTCAACCCGAACGCCCGCTCCGCATTACCCCGCCAGCCGGCCACAGAACACCCGCACCGACG
GTGGCTTCGGGTTCACATGCAAGTCAACAACGCCACTCTTCCACACAGGGATCTCGTATTCCTGATGGCTCTG
CATCACAGCGCCCCTCCAGGCCGCAGTCTCATACACCTGAGAACCCACCGGTAACAGTCCCGCAGCCTCGTTC
CTCCAGGACCCCACTGCCGCAATCCCTGGTACCTGCTGTACCCAAGCCACAGCCGGCTTTACGGTCCCCCTCG
CAGCCTTCGCGAGCTTCACGAACTCCATGGTCTCCTTCACGAACTCCACGGTCTCCTTCACGATCCTCGGGGT
TCTCACAATCTCCGCAGCCTGATGACAACCTCCCCCCGCATCCGTGGCAGTCGCCTCGGCTTGAACACTCGCC
AACCCCAGATATTGGAGACAATCATAACCTACCCCCAATCCCCTGAGCGGACCAGGACCAAGCGGAAATATGT
ATCGGACCAAGTGGTAGCTTCGGGCCATGGCAAAAAAAAACGAAAAGGGCGCCCGAAAGTCTCGGATTACGCG
GAGACGGAGCAAGTTGTTCTCACATTGGCTAACACAATTTATAGGTGCCTTCTTTCCATGGAGAATGTGTTTC
CTCTCCGGGAGGACGAGCTTGAGCTTGCGCAACGGGCATGGGAGCATGCATTCAATTCCAAGGACCTCGATTT
CACAGTGATGGACCACGACATGTTTCGAGTGGTAGGTCCACCTTTATCATACCATCTATATATCCAACTCTCA
CGGCTTAAGGTCCGAGGACGTGGTCCCCAAATCCGAAGTGAAGCAAAAGCAAAAATTATACCACTAGTCGCAT
CAACCTATGGTTTCGACGACAGGCGCAAGCATCGAATCATCAAGGCCAATCGCGAACTCGCTGCGACACTGAA
GCACGAGAAATGCTTCTTATACGAAGTAAATAAGGATCCCTCGTGAGCATGTGTAACTACTAATATTTCCTAG
TCACTCGGCGTCGGAAACCAAGGCCTATATCATAACCCCATCATCCAGAAGAGCATCAATGCCATGTGGTTTA
CGGATACCCAGGATGAAGGTGCCGAATATCCTCTCGCCTTTGGACCTGATGGCATGATCCCATTACCTGCCAT
TGCTTTTGTCCTTACCGTGGTGAGTATTACTGTTATTATTCAATATACTCCTTAATTTATGTTTTTTGGCCAG
GTTGAGTGCGGGATTGACATGTGACTCACTGGGATCAAAAAGGACATCACGTTTTGAAGTGGTGAATATCGCA
CAGTGTACCAGACCCATATCAACTTACTCCACAAGTATGAAGAAGAAACCAAGGAGCTCAATTTATTTAATAA
GCTTCATCGTAAGTTATATCGGAGAGCTGCGTGGGTCATTATTTTATTCTTGTTGTCCCTTATTAACCCTGAA
TATGTTAGGATTTCAGCTGGTGTGAACCTACTGTCCAACAATCCTCACCCCATTATCCCAGCTTCAGCATATA
CCAATGCAATGAAGCAACACAGAGATAATCCACAGGAGTCAGAGTCAGATGACTTATATGATGGTTAATTACA
TGGCTCTTGATGTTGTGGGCTCCGAAAAAATTCATTGATGCATGTTTTGGAATACTATTACTGTTTGATTATA
AGTCTATGAGTTGCTATGGTGTTCATATATGACCATGGAAGTTATGGTACTATACAATAAATCATATACCATC
AATGTCTATGTGGCCTTTATAGCCATGTCTGAGGTCTATAGTGTCTATGGATGTCTGTGGGAGCTATGGTACT
ATACAATAAAGCATATACTTCCAATGTCTATGTAGTTCTTATAGTCATAACTGTTACGTCCTGCTAACGCAAG
CCGATGAGTCGACAGTAAAGACTCAAGGCACTTAGCAAGGTCTGAGTTGCCTTCTGGGTCAGGAGGCTAGTAG
AGGGGGGAACTGATAAATGATTAAACGTAGCGAATACAAGAGTATGT
>Cligib1_rnd-1_family-24#TIR/MITE
TAAGGCTCAGCATGGGTAGATCCGTGGGTGATCCGGATCCGTACTCGTGGGTATTTGGTCACACAAGGTCACT
ACCCGGATCTGCGGGTAGCTTAGTCAGCAAGTACTTGTACTCACGGGTATTTTTTGGCAAGTACTTACGACTC
AGTAAGTACTTGTCACGGATCCCGATTACTCGTGGATTACTCACGATTACTCACAGGTCACGGGTCATTTGTC
AGCAATTAGTCATCAGGGACAGGTATTTCACAAGTACTTACGGGTATTTGCAAGGATTTTGTTGGTTTAAAAT
GGAATTTGAGCCTGTATGTACTGTGGGAACTATACTTTAAGGGTCCTTGAGGGTCCGGTGCGAGTCGCAAGTA
CCTGTCACAGGTACCTGCAATGTCCGAATGCTTGACTCGGATCCGCGAGTAAATAGATCCGCGAGTACAAGTA
CCTGTTTAGGCCAATACTCGCAAGTATTCCCACGGGTCTACCCGCAGGTACCCGCGGGTAACCCGTGCTCGTG
CTGAGCCTTA
>Cligib1_Cluster51#LTR/Gypsy
GTAACGGACACGTTTGTACTTATTTACCTACGCCTTCACCGGAGTGACTTCATAAGGAGTCACCCGTTACATG
TAACAATAAGCTCAATTTACACCCGTAGTACCACGTTACCAATAGACCTGGTCAAACGTAGTACTGCACCATA

AGACCGAGTCTTTGTAACAGCACGATCTGTTACATTTTTTATGTTCTCAAGCTATTGAGAACTTTGCGACTA
CGGAACCCCTCAGCACCAACACCCTCGCAAAGCACCAACCCACGCACAGCCACCCATCAACCATCAAACCTTA
CACACTGCAAATCACCCCAAAAACGGCACTGGTACCCTAAATGGATCAAGACTTCACACCAGATAGGGTATCT
GGTCGGCAACTGGACGAGCCGACTGTCAGTCGGCATCAGACACCCCAACGTCGCCCTGGACATGCCACGTCAC
CAGCCAGGACAAGCGAAGGGAGGACCCCTGGTCCTTCCACGGTCAGATGACGACCCATTAAGACCGAACTGCT
ACAAGAAGAGCTCCTAAGAGCTCAAAAAACGAGTCATGCGAGATCAATATCTCGCGAAGGATACAACGGACTG
GAGACATCGGAGGAGGAAATGGAAGACAGGGAACTGGAGATGTCGATGTGGGAGAAGGAGATGCTCGACAAAT
ATAGAATACGGAACGATAATAGCCGTGAGGATAGCACATAACCTAGCGCGGAAAGCGCTAGGAATAATATACG
AAAGAGATGATTTCGTATGGACTGAGGAATACGTCAAGAAATATCTCGACCAACTCAAGAGGGCATGCGAAAC
TCTGGCGGAAAACACAGAAGAACACCGCCAGTCCCGCCAATCATCGAGAGTTATCTCTCGAACACCAACGCTG
GAACCCGAGACGAGAGGCATAGAAGTGCCTTGACCAAGACCAATCGAAGAAGAACCACGGGAAAATATCCCCG
TGCAAGAACAAAGGGGTATACAAACCCCCGACTACGCCATCGAGAAGGCAAGGGAGCAACTACGAGCTCCCAA
ACGAAGAGACAAATCTGAGGTAGAGTATATTCAGAGACAATGGGCGATGAAAAGAAACAATGAGCCCCCCCAC
CAACCACACTCGATGGACCCAGCTGGATGAATACCAATGGCAGAAAAATTCTCTGACAGAAAAGTATACCAGC
AATGGCGGAACAGGGAAATCGCCGAATATGGGCACTCAAGATACGATGATCAAGGGATCATCTTCATCGCGAC
ACAACCAGGCGACCCAGCGAAAGCGGTTGATAAAACAGAGTACATGACCTCTGTCGCAGGGGAGGAAGGAAGG
CGAATAACAACTCGCCTGCAAGGAGAGGCGAACAAGTTCGCCATCCACAAGCCAACACCACGATGTCCGAAGG
ACATCAAAACAGAGAAGAAACCAGGAGCATCAGAGGATACTCCATGCCGAGAGCAACGACACTGGCGAGACAT
AATACGCCAGGAATAATTGAGGCGGCGGAGAACCACTGCTCGACGATGCACGAGAGACTTCTAACTCTCATAC
ATGAGCGATTGAATGTGAAGCTCACAATACCGGAGGGATGCAAGTTGAGGAAGGCTGACGGACAAACGTCGGC
GAATATAAAGGAGGACCAAGTTCAGCGACTTGGAGGAATGGCTAACGAGCCTAGTAATATACCTCGAAGCTAG
TATGCTTGGAGGACGCGATAGGGATCGCGAGCGAATGCTCGCAATATCAGAATTTCTTGGAGGCGAAGCCAAG
AAATGGTTCAACCGGCACGTAATACATACGAACCGGACTCAAAAGCACTGGACGTTCGAGAACGTCGTAATAG
GCTTGTACGATAGATTTGTACACGCGTCCACGATGCAGGACGCGAGAGAAGAATTCATGTCTTCAAATATAAG
CCAGAAACAGGAATTCAAGGGTACTATGATACCCTACTGGACCACGCGCAGAACATGGCAGTGTATCCAGACC
AGTATGCAATACTGGAGAGATTCCTAAACAGAATAACACCGCAAATGAGAAATGAGTTATTAACTCAGAGCAG
ACTATCACCTGAAATCAACACAATAGACGATTTTGTGGCGGAAGCGAAGTCTATAGAGACTTCACAGAAAACA
GCGGAGCATTACGCCCGCAAAGTAAGAACAAGCACGGCCGATAGACCGTACAAAAACAACGAACCGAGACAAC
CAGCTCACAGAGCTGATAAAGTATATAAGAATGAACGGTATAACCGTCCAAAGGAACCCGAGAGGTACCGGGA
CAACAGGGTACAACAGGACAAGACATACCAGAAGGATCGTAGACCCTCAAAACCGCGCGAGATAACTCGCGCC
CCATACAAGGATACGCGGAACGACGACAAACCGTCGAGGAAACCAGTGATCCAACAATCAACAGCAACGAACA
ATTGCTTCAATTGCGGACAACCAGGCCACTTCTCGAAGGACTGCCCACGTCCACGAAGGGCGCGAGACCAGCT
GAAAGCAGCGCGCACGGAGGCTCAGGACTCCGAAGAAGATGTCGACGATAACCGACCCCAGACCAGCGAGTCT
GAAGGAGAAAACGAACGAGACCGAGTATCAGACAACTCGGTCAAAGGAAACAAATCAGACAACGATCATGAGA
ACATCGAAGTCGAAACCGGTACCAATAACTGGTACGAAGAAGACTCAAACTCGGACTACATGCATGCATCGAG
AGTAGTCCAAGCAGAGGATACGAACAAAGTCCGTATGAGAAAGGTCACCATACGTGCTGCGAAAGAAGCGCGG
GACAGACCAATATACAACCAGGCAATCAAGAACTGCCTGGTAACATATACCAAGGTCAACGGAATGGACGCTT
GGACGTTGTGGGACTCGGGGAGTACCACAACGGGAATAACACCAGCGTTCGCTAACATAGCGAACATCCCGGT
ATTTCCACTAAAGGAGAAACACATTCTCCAATTAGGAACAACTGGAAGTCGCTCCATGATAAATTTTGGAGCA
AACATAGAACTGGAAGTAGCAGGTACGACATTTAATCGTACGTAGATGTAGCGAACCTGGATCGCTACGATAT
GGTAATGGGAACCCCATTCATGAGAACGAACAAAGTGTCGCTCGACTTCGAAAATAATGAAGTCATGATAAAC
GGAACAAGAGTAATAGCTGAACCAGCTATAACAGCGGATACCGACGCGCGAGCGCGTCGTCACAGATCCGTAG
ATAAAAAGGGAGAATGACTACATGACAAGCGAAAACCATGATCGAAACCACGAGTGATGACGAGGACAATAGA
CCTCACAAAGTACTCCCGGAGGGATCAAAATCCCTATTAATGACTGAAGAAGAGTATATAAATACTCTGGGAA
CGGGGGAAAAGCTGCCCGCACCCGAACAACACCAGAAGAAAAAGAAAAAGGTGGCATACGTCGAACCAGACGT
ACTGGAATGCCAACCAACATCGTCAAAAATCAAGGTCGAAGACCTTGTGACCAATGAACAAGAAAAACGACTG
GAAGAATCCAGCCACCTAGCGGAGCGCGGAACAACCACGAACAAGAAGAAACGGACATTTTGGGATAGACCCA
TCCCAAGGCAACGATTCACATGGGACCATGACCCGCGAATGCCCAAAGAATGGAATGAAATGGTGAGCGCCAT
GCGCTCATACGAGGAAGACAAGACAAGAGTCATAGAGACTCTATACCGGAACCCACTACCGTCTGAAATATCG
GACAAATACGAGTTCACGAACTCGTATAAACCCAGCATAAGAAACAAGGATTGGATGAGGAAACAATCCTCAG
TAAAGCCGGCGTATGACTACCCGCCGAATCCCAAGCCCAAGAAACCTGACAAAGGAAAAAAGCCAATAAAAAG
CTTAACCAAGGATGATTATCCTTGGCTTAGGCAACACTGGTATAACGAATACCAGGATATCATGGGAGGAGTG
AAGAATCAACTCCCCCCTTGGCGCGAAGTGAATCATGAGATTCACTTAATCGATGAAAACAAGCGGTACCACT
ACCACCTGCCGAGATGTCCGAACTCGTTACGAGACGAGTTCCACGAAAAAAATCAACAGATACGTTGATGCGGG
GTGGTGGAGACCCAAATCGGTGAGCCAAGCTGCACCATTGCTATGCATTCCCAAGCGGGACGGAAGACTACGC
ACTGTCGTGGATGCACGACAGCGGAACGAGAACACGGTAAAGGACGTAACGCCTTTACCAGACCAAGAAGTGA
TACGTGAAGACGTATCAAAAGGAAAAATCAGGTCGAAAATAGACCTGACAGATGCGTATGAACAAGTACGCAT
TATCCTAAAAGACATAGATAAAACCGCGTTCGCAACGATCGCGGGACATACATAAGCCTAATCATGCAACAA

GGGGACTGCAACGCCCCCGCAACATTTCAGAGACTAATGACGTCCATATTCAGAGACGTCATTGGGAAATTTA
TGCATGTATATCTAGACGATATATTCGTCTACTCAGATACAATCGAAGAACACGAAGAACATCTTCGTGTAAT
ATTCGAACGACTAAAGAAAAATGTTCTTTGCCTCAAATGGGCGAAATGCGAATTATACGCAGACAAAGTAGAT
TGTCTGGGTCATATAATCGACGACCAGGGTATACACCCTGATTCAGATAAACTAGGAAAGATCCGGGAGTGGA
GAATTCCCAGAAATTATAACGACGTCCAACGGTTTGTAGGACTCGTGAACTACTTGGGGAATTTCCTCCCCGA
CATCACGACATATACTGGACCGTTGATGTCCATGATGCAGAACGGCACCGCGTTCCTATGGCGCCCAATACAC
CAACGATGCTTCGATATGATAAAGCGCATCTGTGAAAAGACGCCCATCATACGACCCATCCGACCAGACAAAG
AGGAGCCAATATGGCTCATTTGCGACGCCTCTAAAACGGGCGTCGGAGCAATGTACGGACAAGGACCTGCGTG
GCAACAATGTCGCCCCGCGGGATTCATGTCCAAGAAATTTACCACAGCCCAGCATAACTATGCTGTGCACGAG
TTAGAGACACTCGCAATCCTGGAAGCACTCCAGAAATGGGAAGACAAGTTAATAGGATATGATATCCATATCA
TAACCGACCACAAGGCGCTTGAGTTTTTCAAAACTCAAGTAAAATTGAGCCCGCGTCAAAGACGCTGGATGGA
CTATATGTCGAGATTCAATCTCGACATCACGTATATCAAAGGCGAACTCAATAAAGTAGCGGATTGCCTATCA
CGCTACTACGAAAATGACAACATTAGGGACATATATATGCCCTACGAATACGTGAGCGCTGACAAGCGCATGG
ACCCGACGGGTGAAGACCTACCCGGAGAACGATACAAGGAAATCACAGAGAATGTGATTGAGATACGCGCAAT
GCGCGAAACCGAACGAAGACGTAGCAAACGTCTTCAAGAAAGAAAAGAGGACAGAGAGATTGAAGCTCTCATA
ATGGAAGAGGCCAACGAACGGCATAACAAGGATAATATGAGCATGAACAATGGTTCATCCTCAGAAAGGGGAA
GAGACAACATCCCCGCGATCGCGGACGAGGACATAACCATTGGTCAAGCGACTATAGGGATAGTGGACGTGGA
AAAAACCACCGATCAGAGCGATGAAGAGTTCAAAAGAACTCTAAAACAAGGATACCTGAAGGACGAATTCTTC
GAGAAAATACAGAAGAATCCACAAGGAGACCCTAGCTTCTCCATACACGAAGGAATCGTGTATACACGCAACG
CGAATGATAAACGAGTGGCATGCATACCACGCGACAGAGAAATCATAACGCGCATATTGGAACAAGCGCACGA
AGTGATAGGACACTTCGGATTTCAACGAACGTCAGAATACGTTCGACGATACTACTGGTGGCCAAAAATCACC
AGCGACGTTAAAGAATTCTGCAAGACCTGCGAGCCTTGCCAAGTAGCCAAAGGAGCCAATAAGGCTCCACCAG
GGAAATTACATCCCCTACCGATACCTACAAAACCATGGAACTCAATCGGAATGGACTTCATTGGTCCATTCCC
GGAATCCAAAGGATTCAACTACCTATGGGTAATCATATGCTGGATGACCAGCATGGTGCATCTAGGGCATGTC
ACGAGTTAACCCGCAAGTAACCTGGATCTGCAGTCACAAGTACTTGCATGTGAAAAACTGATACCTGGATCTG
CACAAGTTTTATACCTGTGAGATCTGGATCCACAGGTCATTTACTTGTGAGTATCCAAGTACTTATGAGTTCT
AAATACTTGCAGGTACTCGCAAGTTACTCACAGGTTACTTATTGGCTTTTGGAAACTGGCATACCCTTGAAAA
ACCCGCCCCTAACAAGCTATCACATAAATAACCATCAGTTCCGCACCAAATCGGGACTTTGAGGGGTCTATCG
GACACTCCTAAAGGCCCTTATAAATGTCCAAAAGACCCGACAGGGCTACCTATCCCCATTCTCAAGTTATAGG
GTGCGCGCTCCAGGGGGAGGAGGAGAAGGTCATGTCATGGAGTCAATTCTGCATCGGAATGTATAATTGTGGG
ACGCATTAAACACTAATTCATAAGCTAAATCCGATGCGCATAGCGCGAGAGACATCGGTATCTCCGATCCTAA
GATACAGAATGATAGTCTGCGAGATTGCGCGTACCGTGGGATCGAGGATATCGATGCCTCTCGCGCTATGCGC
ATCGGGTTCTGCTCAGAAAATAGTGTTTAATGCGTTCCACAATCATACATTCCGGTGCAGAATTGACTCCATG
ACATGACCTTCTCCTCCTCCCCCCTGGAGCGCACACCCTATAACTTAAGAATGGGGATAGGTAGCCCTGTCGG
GTCTTTTGGACATTTATAAGGGCCTTTAGGAGTGTCCGATCGGCCCCTCAAAGTCCCGATTTGGTGCGGAATA
AGGTCACCCACAAGGGTATAACCAAAAACCTGCACCAACCCACTGATTTTAAACCTGTGGGTGGACAGGTTAC
AACCCATATGGGTGCAGGTATGGGACACCTTCTGGATACCCACAGATGTACCCATGCCATTGCCTATTACAGG
GTGGCCCAGGTAGCTACATAGTCCACATGGAAGATGACAGCATCTGAGCTGTCCAAATGGATATACCAAGACT
GCCAGAAATCGTGCGCCTTACCACGGATTACCAAGCCTCAATCGTGAGCAGACAGCCGGACTCCCAAGTTCAC
GTCCAGCCAAATGGTGGCGCGAACTCCATAAAATATTAGGAGCAAAGCTCCTAATGTCAACATCATTTCACCC
ACAGACGGATGGACAAACCGAGCGCGCCAACCGTAGCGTTGGGCAGATATTCAGAACGGTCGTGAGCCACGAC
CAGAAGAATTGGATCGACAAAGTCGATCTCACGGAATTTGCAATAAATGCAAGCGTATCTGAAACCACCAAAT
ACGCACCATTCGAACTGAACGGAGGGCATATGCCTCTATGATCAAAGAAATCCGGAACAGCGAAGCAATCCAC
AAGGGAATTAGGAGCTTCGCCGAGCAAGCACTGGAAAATCTAGCCAGTGCACACGACACGATCATCGAAGCGC
GAGCTATGCAAGCTCGCAAGGCAGATGGCAAACACGGACCGGAATTGGATACCAAAATAGGCGACTTGGTATA
TGTATCCACCAAGAACTTTAATTGCCAAAAGGGAGAGCGCGAAAACTCTGCCCAAAATATGTAGGACCATATA
AGGTCCTACAAGCATCACCTGAAACGTCTACCTACAAGCTGGAACTACCTACGACGTTGAAACAACGCAGAAT
CCACCCAACGTTTCACGCGTCACTATTGAGGCCATACCACGCGTCAAACGACGCGTTGTTCCCGAATAGGGTT
CACCCTGAACCCTATGACTTTGGAACACCAGACGAACACAAATGGTTTGTCGAAGAAATAAGAGGGCACCATG
GAAGAACTCGAGTCTAGAACTCGAGATTAGGTGGAGCCTAGGAGATACGACATGGGAGCCGTATGAGAACTGC
AAGCGCCTTGAGGCGCTTGACCAGTATCTTGAATTACAGGGCGTAAAACGCCCTGCATCGTTACCAAAGAAAT
CCTGAGTGACGCATCACTCAGGTATGAAGATGGGTTAGGGCTGCTTACGTCAACCTGAACTCAGGTAGATCAC
GCGTGATGGGATCAAAATATTTGGAGTCATGTGATCTCCAAAACTTTGAGTGGAATGACTGTCAGACGCGACC
CTTAAACGCACCTTAAACATGTCTTACAACAACCGCAACACCAACACCAGCCAGCAACCAGAGGACAAGGCGG
GAACAATCGTTCCCGCCAAACCGGACATGCGGTGATAAGCATGGTAATGGTTTAGCATCAGTTCGCATCTATT
TCTAGTCATTGTAGCTAATTTCCGTACTTACATTCGCATCGCGCGACTAATGCGCGATCACTGATCGCTTCGT
ACGCGTGGAGATCGCGCTTTCGATGCGATTCAGATCGCATCTTTACCATAGTGATCGCATAGTTTCTCAGTAT
CTTACAGATTATCTTACATTACATACATACTGCCTCATGCCCGTGTAGCTCCGATTTATATAGAGTCGCATGT

AGCTCGCATAATACAACTCCCCCTTCGCATTAGAGCTCTCGAGTCTCGCGTTCATCCCTTAGCTTGCGAAGGT
CTCCACAGTTCTTGGAGATTCAGGCCTTCACTGCACTCAAGACACCCACTCGCAAACTGCGAAGCACTTGGTT
TGTTGGTTTGCCCCACAGGCCGCAGTGTCCTATCAGCGGAGGACAGGCAGCCAAGAGCCGCCTCCGAAGGAAG
GAGGGATGGAGGACAAGCGCCCCCTCCATATATACCACAGGGACCGTACCCTCAATACGGACAAAACCCGGCC
AGGGGTCCGCAACCCCAATACCAACAAAACCCGACCTGGGGACCAGAGCCCCAATATGAACAAGAACCGATAT
GGGGACCAGAACCCCAATACGAACGACCGATGGCGCAACCAACGATGCCACCACCCATGAGGACTCGTCAGGA
GAACTTCTACCAACGACAATGGGGCAGGCATCCGGGAGCAGCACCCATCTACACCCAAGGATGGGCAGGGGCG
ATGACCCCTGACGAACTCACATACGAAGAGAATAGGATGGAGATCATGATCTCCCCCAACCACCACCTCACGC
TGAAGCGGAAAGGCGACACGACACGCGCGCCCCTCAACAAAGACCGCAATACGCGGCACCACCGGGACTCCAA
AGGGGTCCACACATGCAACCACGTGGCCGGCACGAGAGAGAACTCGCGCCGAAACCCAACAAGGCCCGCACTG
AGTCGCGCAGCTCGTGCGAAGAACAATATGAGGCTCGACCTCAAATGAAGAATAAGCAAAAGGCTCAGGACAA
AGAACCTGAGAATGAGGACGAGGGAATCAATTTCATCCAAGCACAACTGGAGTCGCTTGGAATCGACGCAAGC
TTCACGGGGTTCCTGAAGGACCCCACCCCCAATACGTGGTATCAAAACTCGTCGACATCGCGACGAGTGAGAA
GCAAGAAAGAGAGAGATTGGAGTCCAGAAAGGGAACATTGGACTCGCTCCAAGAAAAACCTTGAGGGCCAGCT
GACAACGGCCCTCGCAAAAAGACAGATCTCCCCACCAATGGGCGAAATCGACGCATGCCGCGCAAAGAAGAGC
CGCGAGGCGTCACCAAGGAGGGAAATCATTCCCCTACCACAAATGAGACCGAGGTCTCAACCACCACAACATA
CCAGCAGCCAATTGCTGGACCATCAATGACGAGGATCACCTCGTCACAATCAGACAACATCCCGAGGAGAGAA
CCACCTCTCCCATATAACGAAGTAGCAGTCCCACGAGGACTGGGACCTAAGGTCCCAAACAATAGGGTCAGCC
GGAAACCATCCGCCCCTACCCCGAGGATCGAAGTATACTGCAAGATCCCCAAACATTACCCCCGGGAGCAGAA
AATAATCCCACCGGAGATCCAAACACGGAGGAAAACGCTCCGAAAACAAAACAATCGGACGAATCTACGTCCG
ATGAAGAAGGAGAAATCTGGAGCGCATAAGCACTCCAGAACACGAAACCCAAATGAAAAGCGTTTGAGAAATC
AGAAGAATGTGGATCAAGAAGCCAAAAAGGCAAGGCTCGTAAGAGAAGGAAGCAAAGACGCGAGAAATACGAA
AAATCGCAACCAGGACGAATCCCTGACGCCATAGGCGTCATCAAAAACAACTCCAACGGACAGTTCGAACGAG
ACAACTGGCTGCGGGGATCATCAGATCCCGAATTCTATTATTCGAGAATATCCAACACAGTGTACGTTGGACC
ACAAGCCCAGGTAGCAGCCACAAGCGAGGGACTCAAAGTACCTCCAACATATACCAACAACGGCGGTGTACTA
TAAAGCACCCAGAGGGTTCCCAATGAACCCTCAACAAGTAATAGACGTACTCAAATTGATACGGGAGACCAAA
GTGTCTCCAAGAGAAAGAGTAGAGGTGTTTCGACTGATACAGGAATTTAGGCGAATTGCATACGCCTTCCTAC
CAAGTCAGCGGGATCTAGCAATGCACAAAGTGCTTGAAGATTACCCGTACCAACAAGTAAAAGAAATGATCAC
GAACCAAAGGTTCCGTGATTACTACGCGGAAAAGTTTGAGCCATTCGGCTCAGAAGACGCGTATAAAAAAGAC
AACAACGGCCGAACTAAGGCCGGGGTGAGCCAACTCCATGAGGATCAAACCCTCAACATAGACGCGTGGGCGC
AATATATCGTACACCACTGGCGCCCAGGAGGCATAACCCGCTCAACGGAGCAGCGATAAACCGCGCATATCAC
GCCAACCGCCGAAGCATATTCGGCTACCTCATAGCGAAGGCCTTAGGCCCTCGAACACAAGAAAAGGCAGTAG
CACGATACCGAAGTCGCACTTTACGCGACAATATGCGATACTCGTGGCAAAACCCACAGGCCTATCACGAGGCC
ATCACCCAATGGGAACAAGAGACGGGGAACAAGTTTTCCCCAATAAACACAATACCGAGCAGCGTAACGCTCA
AACGATTCCAGCGGAGCAATGCTCCGCAAACATAACACAAGACGACATAACTCGTCTACTCATAGAAAACCGG
ATACCACCAGCGTGGATAGACCACGCCTATACATTCGGCATATACTATCTAGATCATCAGATCTGGAACGCGC
TGAATAAAGAAGCGTATATTGAAGCAGACGACGAACGGCTTCGTCGTCTGGCAGAACATGGTCTGCCGCCACC
TATACGGGCCTGGGATGGATGGTATATCCCAACCGAGGAAGACAAAATACGAATTCGTTACCTGATAAGGGAC
GAAGTAAAAAACAAAAACATAAATGCCTGGAGAGCAATGAATGGCTATTCATTGGGGAGGAGGTACACCCCAT
CTACCTCCGAAGCCGCAGCGCCGAGAGCGCTGAATACCTATATGACACGGAGCGGCAACGAATCGACGCGTTA
AGACACGCCGAACAAGCTGTCGGCGAGAACCCACACGATCCAGAAGGAGTACTAGACGCGACTAACGTCGCGC
CAGGGACGACTAACGTCGTGCCAGGTACAACTGAGGACGAAAAAATGATAGAGGATACAGACGAGAATCCTCC
TAGCGCCGAACCATCGGAATTAAACGCTCCGAAGCAATGTCCTCAAGCGCTGGGGACGAACCAGCCGCGACAT
CCAGCAGTGAAGGGTCATGAACCCTACATAAAAAACAAAAATAAAAAACGGAGTACTAAGTACTCCACGACAA
CCAATACGCATCCACGTACGCAACATGGAATACCAGGTACTCCAACACGAAGCACAGGAATCACGCGCAATAA
ATACTACGGAGTACAAGGTACTCCACATAACATAGCGGGGCGGATCTTCAGATCCCGCCCTTATTCTTCTTC
AATATTCCTTTACATATATACGTATCAACTTTTGAAAGACCGCCATTAGCGATCTGAAAGGGGGATATGTAAT
GGACACGTTTGTACTTATTTACCTACGCCTTCACCGGAGTGACTTCATAAGGAGTCACCTGTTACATGTAACA
ATAAGCTCAATTTACACCCGTAGTACCACGTTACCAATAGACCTGGTCAAACGTAGTACTGCACCGTAAGACC
GAGTCTTCGTAACAGCACGATCTGTTACAG
>Cligib1_Cluster83#LTR/Gypsy
GTAAGGACACGCTAAACACCAATGAAGTGTGCGGTCTCTCAAGATGTCTCAGAGGAAGTTGGATGTGTGAAAG
CCGACTGACGATGGAGAATCCCACTGTGAATAGCGGAAAACCAAAGAGAATCAGATTGAGAAACGAATCATAC
AAACGACAAGACTATATACATAGGTAAGAGTCCGTGAATATAATAAGTACAAACAACATGTGTTTACAATGAG
GTGAAACCACGGTTCGCTTACATAATCAGTCACGTGATTAGTTATGTGATACTACAAACGAAAAGAGAAAAGA
AAAGAAATTACAAGTCCTGACACTACTCCCTCTTTAGGGATTTTCTCTCCGGGAAAATCTTGAGCGGTTCTTC
ATCATCCCTGATAGTGCGTCTCGCATTCAAAAGAATGCGCACATATCAATTCGAAGCACTTGAAGCGTTCAAT
TTTGAAAATAATGCCGAATTTGATGGTGGATCGGTGAAAGTTTCATATGGAGCGAACAAAGAAAGGAATTCGT

CCTGAGTTACATTGAGGCGAACCTTTCGTGGAGCTTGTGGATTTGTTCGATGAAATTTGTTGACGAGATCCAT
GGCATTTTTCAAATTTGATAATGGTTCCCAAGTATTATGTTGCGGCGTGTATCCTTCCCATTTAACCCAATAT
TCAAGACCTTTACCATTTTTGTGAGGCGTGAATTTTGAATTTCTTCGACGGTATACTCGAATTCGCCATCAAT
TTCTACTGGTGGTGCAGGAGGGATTTGTTGTTGGGGAATTTTGGGTGATTGGTATGGTCGTACTCGTGAAATA
TTGATGACGGGGTGAATTTTGATCGTAGATGGAAGTTTGAGTTTCACCGCGTTAGTTCCAATGAGTTCGAGGA
TTTTGAACGGACCAACTCGTCTTTCGGTGAGTTTCTTTGATGGTCGTTTGAGATCAAGATCTTTGGTTTCTAA
CCATACCATTTGTCCAACTTGAAACGATGGGGTTTCACCGTGATTTTGATCAGCGTATCGTTTCATTTCGTCG
CGTGCTTTGACAAGGGCTGATTGTGCTTCTTCTTGAATTTTCTTCATCCGTGTAGCGAATTCATTAGCCGACT
CATTTGTTGGTTGTCGTATTGGTTCAGCGCCCATTCGTGGATGACGACCGTAGACGTTAAAGAAGGGGGATAT
TTTCGTGGAGGAATGAGTGGCGTTATTGTATGAGAATTCAGCGAGAGGGATCCATTCAACCCAGTCGGATTGA
AGGTGCGATACGAAAATTCTAAGATAGGCTTCGAGATCTTGATTAACCCGTTCTGTTTGACCATCGGTTTGAG
GATGATAAGCGGTGGAAAGTTTAGTTTGGATATTCAATAATGAATTTAATTCTTTCATCATTGAACTTGCGAA
TTGTGGTCCTCGATCGGAGATAATAGAATCAGGGAGACCGTGGAGCTTCCAGACGTTTTGCAGGTATAATTTT
GCAAGACCCGTCGTAGATGTTTGAGAAGTAGTAGGAATGAAGTGCGCCATTTTTGATGCGCGATCGACAACAA
CAACTATTGCGTTGTATCCATTACTCTCAGGAAGTCCGGTGATGAAATCGCAGGATATCGCTTTCCATGGTTC
GGAAGGGGTAATCATTGGAACAAGTTTTCCAATAGGAGGAGCAGGGAAGTTTTTGAAACGTTGACAAGAGTCA
CATCCGTCGATGTACGAATGAACGAATTTTGTCATGCCAGGCCACCAGTAGTTTCGTGCGATTAATTCCAGAG
TTTTGTAGTGTCCTGGATGACCGGCAATACTGGAATCATGGTGGAGTTTAACGATTTCTCGTCGAAGTTGGAT
GTCGTTGGGTACGTAGACTTTTCCTCGGAACAATATAAGATCTTGTTCCTGAGACCATTCTTCAGATCGTAAA
CGTTTCGTGGGTGATCGTTTAAGTTCTTCGATCGCTTTGACAACGGATTCATCATAGTCTTTCGATTTGCGGA
TCTTCGAAAGTACTCCCTCTTCTTCGCTGTTAACAAGAACATGTCCTTGTTGTAATGCTTTAATGGCGAAAAA
TTCCGGTTTAAGGAGCGTGATGTCGTCGTTATCAGTTTCCACTCCCTCTTTTAGATCTGTCCTCCTGGACAGT
GCATCCGCTTTTCCCATTGAGGATCCTGCCTTGTGAATTAGTGTAAATCAAATCGTGATAAGTAAAGAGCCCA
TCGTGCTTGTCTACGATTTAACTTTTTAGAAGACATAAAGTATTGTAAATTACGATGATCAGACCATATTTCA
ATCGTATGTGAACTACCTTCTAAATAATGGCGCCAGGCCTCCAACACGTATAATACTAAGCATTTCCTTGTCA
TGTACATCGTAGTTACGTTCAACGTCAGATAAAGATTTAGAATAATAGAACATGGCCTCCATTTATCGTCGGC
ACATTTCATAGATAAATACCTCCCGTAGCAAAGTCCGAAGCGTCCGATTCTACTCGAAGGGGTTTTGTTGGAT
CTGCTGTAACCAATATTGGATCGGATGTAAATCGGTTTTTAAGCATATCAAATGCCGCTGTTGTTTCTCTGTC
CAATCGAATTTAGAATCCTTTCGTAAGAGATTATTGAGGGAGCGGCAAGTTTGAAAATCCTTCGATGAATCGT
CGATAGAAGTTAGCGAAACCTAAGAATTTGCGAACGTCGCGGACTGTTTTAGGAACCGGCCATTGTTTTACTG
CGTCGGTTTTGTCGTCCATCATGCGTACTTCTCCATTTCCAAGTATGAGTCCAGGAATTCGACTTGTTGAACC
GAGAAGGAACATTTCTCTGGTTTAAGGAATAAATCGTTATCGAGGAGGATTTGGAGAACGCGTTCGGCAATCG
TTCGTTTTCTTCTTGAGTATCGGTAAATATTAAAATATCGTCTATATATACTACCACATGTCCTTCGTCTATA
AGTTCTCGTAGAAGGCATTCATCATGGTTTGGAAAGTGCCGGTGAATTGCATAAGCCAAAATACATAACGAGT
GGTTCGAAAGACCTCTATGGTTGTGAATGCTGCTTCGGTTCATCTCCTTCACGCATGCGAACATTGTTGTATC
CCCATCGTAAGTCTAGTTTTGTAAACCACTTGATTGCCGCTTTTCATAAGATCGGAGATCATGGGAGGGATAA
TTGTTTTGATTGTCCAAGCATTGAGTTGTCGATAGTCTTGGACCATGCGTTTCTTTCCGTCTTTCTTGGGTAC
AAGAATACGGCGGATGTTTGAGGAGATTTGCTTTCCCGAATATAACCCTTAGCTAGTTGATCCGCTAGAAAGT
CGTCGACTTCTTTCTGTTCGGTAGGGAAAGATTGATGACTTTGGCTTTCTTAGGTTCGTAGCCATCTTTCAAT
TCTATAGCTGATCCCAGGGTTTTCGTTCAGGCATGCGTTCGATGCTTTCTTTTCAAAGACTTTGAGCCAACGT
CTAAATCGTCGAGGAATTTTATTGACGAGTTCGGGTTCCTCTGTTTCGATATGTATGTTGTCATGGTCTTCAG
CTCGTTCTATGGATCGGACAAAGTGTTCATCTACTATTTCATGAACCACTTCATCATAATTAATTTCTTCAAG
TAATGCGCCTTCTTGTTCAGTAATAAGGCCACCAATCACTCGATCTGCATCTTCGTCATCGATCTCTTCAATC
GTGGGTTGGTATTTCCATCGATCGGCTAGAGCTTTACGCTTCTTTTGCTTTTTAATAGCGCGTACTCTAACAT
TACACTCTGGGGGCATCGAGTAAATGTAATTTCACCGGTTGACCAATCGATAGATGGATTGTGTTTCTTTAAC
CACGTGTGACCGATTATGATCGGAACTTTTCCTAAGTCCGTGACTTCGAAGAGCATCTTTCTCTTATGTCCGT
TATGAACCATCATTAAATAGACTTCTTCCGTGATCGATCCTCCTTGATTTAAGGTACCGTCCACGTTAAATAC
TTTAACCGGATATTTTAAAGTTTGGGTATTCCATCCCATACGTCGTACATATGCTCGATCAATAAAGGAACCG
GTTGCACCAGAATCTAGAAGCGCTTGACATGATGCAGTTTCATGAGTATCAACTGTTGTTACTGAGATTGATA
ATTTTGTCTCACGTTCCAAATTAAGCGTGCGAATAAGAAGTCTTTCTCTATAAATATGACCAGGACATTCCGA
AGATACGTTACCGCCGGGACCCACAGAGTTACCGACTCTGGGACGGACGGATGTTCCATTTAAGGAATCCTGG
TGGGTTGACATGACCATAACGCCGGGACCCTGATTAACAGGACGGACGTCTATCTGATCACCAAAACCGCTTA
TTTTTGTCTCCACTGATTTTTCTGTCGTTTTAGATTTTTGAAAATGATAAATTGGGGTGGGAATTAAGCACGC
TTCCTGAGCGCCAAGCTCCTTATGGGAGACCCCCTGAGATACGATTACATTGGAAGGCGTATCTATGTTTTTG
ACCGTGTCGACAGATAAAAGATGAAATTTATTTTTAGGCAACGCTAGTGGTGGAGATTTCACTGCGCGCCTCC
TTGAAAAGACGATTGGGCGTTAGATTGCCCCTGAACTTGTGAATCCGGATTGACTTCCGTAATTCCTGATGTT
GACGGCGTATCCTGCCAAATCAACCCTTCTTCGATTGCGACCTGTAAATAATGGTCGCGTGCTTCATCATGTG
TGGCCGAGTTTAAATTTATATTTGCCCTGCAATCTCGCATCATGTGGCCGGGTTTACCGCATCGATAACAAAC

TCCGGGTGGGCGACGATTTCCTCGATCAACATCCATTGCATCTGGAGCACGTGTTGGAGCCGGTGGATTAGGA
CTAAGGCATGTCACGAGTGCGAGTTACTTGTGAGTCACTGCGGGTTGACCCGTGGGAAAACCCGCGAGTATGG
TGACCCGCGGATCCGGATCACTCGTGGCCGCGGGTTGCCGCGGGTCCGAGTTTCAATTTTTCAACCACAGGTA
CTCACGGCAAGTACCTGCCGTGACTCGTGGCCTGTACTCATCGCAATCTCCGCCACATCTACATACAATCCAT
CCCACTGCCTCGCATCTACGCGTATACACCAAACGGACGGCCGCCTTTGTGCTTCGGATTTTCAGTCAATTCT
AGGGATGAGTTGGGAACTTAGGTAAGTCAAAGTTTTTTATATCGATAACGCAGCATAGACGACATCCGGGCG
AGCTGGTGATGGGCAACGGCTCACGGCCCCACATCAATGGCACCACCATTGTCGCAGCACACCGCTGCCAGCC
ATAGCATGGCGCTTGCACTCACGCCGCTACCTCTCACCTCAATTGCCGACACCCCACTGTCTCGCACCGCCGC
TCTACATCGTTCCGCCACGATGACCCGTTCTCATTCCGCTGCACCATCGCTACCACGACGCATTCACAGCGTC
TCACACAGCCTCACCGCTGCCACATCATCATCATCGCCATGCCATCATCAACCGCAGCATGGCAGCAAAACGT
CACACAGACATCGCACCCACCTTTGACGGCTCTCCAACATCATCATCAATCGTGACTGGGCTTGCCAGCACCC
CATCCCCCTTGCTCCCGCATGAATGGAATCCATCAGCGCCACCGCTACCCCGCTCTCTTGCAAACAGAGAGCT
GGAGATGGCCCGTCGGTTTTACTCCGCCGCATCCCACCAGTACGTCAGCATCGCTGCCACTCCGTTCTTTGTC
AGTCCCCCCACAGGATCCAGAGCACTACCCACTCGTTCATTGTCGACCCCTTACACTTGTGTCCGACGCGTCA
CCATCAATCATCGTGCATCCCCACCACCACCACATGTCTTCACCACCTCGCGCTGTCAGAAGATCGCTTGGGT
GAACCGTCACTTACGGGAATGAACCCGACACCGCCGTCGTCACTACATTCCGCCCCTGCCACCACCCATCCTG
CCACCCCACCCGCTCGTGCATTGGTAACACCGCCGCTTGCCATCCCGCCGCTTGCCATCCTGCTAGCACACCC
CCATCCTTCGTCCCCACCCCCAACTGGTCCACACCACTACCGCCGCCGTATCCTCTTGCAAATGAGAGCAAAT
GGGCGGGGATTCGATGAGGTATGTACCATCTACCCTCTCGTTGGCTGGAGCGGCTTGGGCCCAGCTCGCGTGC
CCCCTCCACCCTCGCGACTGCGTTCGCCGTGTCAATTGACGCGCGCAACGATGTCGCGAGGCTAAACGGGCGG
ACGTGGGCTGTGGTTTCCGCACGGGTAGTACATGCGAACGGATGCGCCGAGGGACTGGCGCGTGCATGCCCTC
CAAGCCGGTGAGTAACCTGCAAGTAACTTGTAAGTACCCACAGGTATTTAACACTCACGAGTACTTGGATACT
CGTGAGTAAAATACCTGCGGATCCGGATCTCACGAGTACAAAACTCGTGCAGATCCAGGTATCATTTTTTCGT
ATGCAAGTACTTGTGACCACGAGTCCAGGTTACTCACGGGTGCACTCGTGACATGCCTTAATTAGGACGAGCT
TGTTGGGCAGGTCGAGTCGGTTGTGCTGATTGACCGAAAGCCGGGCGATTGAAACCAAATGTAGGCCGAGCGG
GTTGTGCCTGTTGATTAGATCCCATATTTCGGTGGGACCTTGTGTCTGTGTGGTTCCTGTTCCAGTTTGCCGT
GGGTTAGACGATGAAAATGCCGGGCGAGAGAATGTCGGTTGATATAATTTTTTCTCTTCCTGAGATTGTCGGT
ATTGCCTGTCCAGATGTGATGCTCGTGTGTACCAGTCCTCATAGTATTCGGTTGATACTCGGATTCCATTAGC
CGTTTCCGAAGACCTTTGTTTAGAGACCGTTTGAATTCCTTAATTAAAGGATATCCACTATAGCCAGAACCGT
CGGCCGTTTTCTTAAACTCTTGGATGTGTTCCTCTGCCGGTAAAGTTCCCTGTTTCATGGTGCATAATTTAAA
CGCTTTAGTTCCCTGTGGGTCGTGGTCGGAAAACGCCAGTTTGAATTCTTTCCAGAAATCGTTAATAGTATAA
TCCATTGTTATTGGATTCCGGTTAAATTCCTTGATCTTTAGATCACGCATGTCGCTGCTGAACCTTGAGAACA
GAGATGTAAAACCCAATATACCGCGCTAGTGACCGATGAAAATCTGCGTGTCGAACTTGTAGATAAATCCGGC
ATGCATTTTCGAAGGTATCCGCATTAGTCATTGTACCATCGAACGGTGTAGGATTTTGGAGTTTAGGCGCACG
AGATCCTTTAGCCGCGTTAGAGTATGAACCGGGGAGGGGATCAAATCCGTTGAGAGGAGAAGGTTTAGCGTCG
CGTTCCCGTTTGTCCTTGACCAAATCTGTGAGGAGTGTTTCGAATCGATTAAACCGAGAATGGAGTTCGTGAT
TTTGTTCCCATAGTCTCGAGTTTTTGATCCTGGAGAACCTTGACGTGATTTTCTAGAGTTTGAATCCGTTGGA
TAAATTCCGTGCTCAATTCCGTGACGTGTTTCATTTGACCATAAAGTTCCGTAAATTTCTCAGCGTCCGTTTG
ATAAGTGACCGTGTCGGGGTTGACCTGAGGGATGGCGGGTGTGTGGAAATAGTTTCCATCGAACGGATTGATT
AAACTGCCGCGAGGTGTCGTCTGTTGCGATCCTCCCCAGTTTGCTGAATCTTCAGACATGACGGGTGTTTTGG
AGAAAATAGGAGAGGGGTGGGCGGAGGAGTATGGTCGGAAATGTGACGGAATTTTGAAAATGAAGGGGGCCGC
GGGTATGGACTTAATCCAGTAGACCGGCGGTGATCACCTGCTTCAAGAGTGGGGACGGGACAAAGGTGAGATT
CGAAATTCTCGCCTGCTCAAACCGTGTAACGGTAGCAAATAGGATGAGAGGTTGCAAGCCTACTCAAACAGGA
ATGGGAATGGTGATGCCGCGAGAGCCTGTCCAGGCGTCTGACTGATACTAGTGGCTAACCCTGAAACGCTTAT
CCTGCGCCAGGTGTGGGCTGAACTAGCAGTGAGAGGATGTCGGGGGAATTCTGTGTTCTGGGGACTTGCAGAC
CGGAAAGTCAAGCAGCCGAGTTAACTCAGATGAAGACCGGTTGTTGCCAGAAAGAGAAGGGAAAGCAGCCGAG
TTAAACTCAGATGAATCCCTTCTTCTGAGTGGGTAGAGGTGGTGTTAATGACTGAGGTGGTGGGTGGCTTACT
CCGATAGACTAGACTCCTTTGAGCGGCGATGTTGTTTCGCGCGCTTCGATGTCTCTTTGGAGAAGCGGTGTTT
GAGTGTTGGTGGAGGAAGAGAGAGGGCACGATAAGAGAATCTGATTCTTCTGTAAGGACACGCTAAACACCAA
TGAAGTGTGCGGTCTCTCAAGATGTCTCAGAGGAAGTTGGATGTGTGAAAGCCGACTGACGATGGAGAATCCC
ACTGTGAATAGCGGAAAACCAAAGAGAATCAGATTGAGAAACGAATCATACAAACGACAAGACTATATACATA
GGTAAGAGTCCGTGAATATAATAAGTACAAACAACATGTGTTTACAATGAGGTGAAACCGCGGTTCGCTTACA
TAATCAGTCACGTGATTAGTTATGTGATACTACAAACGAAAAGAGAAAAGAAAAGAAATTACAAGTCCTGACA
G
>Cligib1_rnd-1_family-9#LTR/Gypsy
GGGAGTAATGGATTTTATTTTATATTTCTCATAACATTGTGTAGCGAGCAGACGATTCGAGTTTGACGAACCA
TGGATACTCTCCAAAGCGTTCTCCTCGTCGTACTGTGTACGCCCCTTTGTTTGCCGCACTGGAGTCAATCTTC
GCTTGAGGCGATGCGGAGTTTTTCTTTTCTCTTTTTCTTCTTCTATCATTTTCTTTATTTTTCCGGAGGAAAA

ATTCTAGAAGGGGAGTTATGTCACGACTCGCTCTTTTCTTTTCTTTCCTCCAAGTCTTTCTCATCTTATCTTA
TCTTATCCCCTTATCTTATCAGAGCTTATCATATCTTATCAGGGGTTTATCTTATCTTTCTCAAATCCTTATC
GCAGATCATCACGTGATCCATTATGTAAGCCAAACCGCGGTTTTGCATCATGGAGTACGCACGCTTTAGGATA
TTACTTTACTTTGTTTTCTTTCTCTTTTATTGCCAAACACTCTAGAAGTACTTAAGGCTTTTCACTTGTATTA
TGTACGCTAATTCGATTCTCTTTGGTTTCCGCCTTAACAAGTGGTTNCTTAAGTCAGTCGTATTCATCTTTCT
TTCTTTNTATTCATTCTACTTGAGAGACTGCCGCACCCTTTTGGGTGTTTTGGCGTGTCCTTACGATCTACGA
TGGGGATACAATAACGTTCGNACGAAGGAAGGCGACGAATGGAAAGCGGCGTTCACNACNCATCGCGGTTCTT
TCGAACCACTCGTCATGTATTTCGGGCTCGCGAATTCACCGGCTACGTTCCAAACCATGATGAACGCCTTACT
ACGAGAACTTATTGACGAAGGACACGTNGTAGTATATATCGACGATATTCTNATCTTCACCGACACNCAAGAA
GAAAACGAACGGATCGCCGAACGNGTTCTCCAGATCCTACTNGACAACGACTTATTCCTGAAACCGGAGAAGT
GTCCTTTCAGTACCCAGAACAGGTNGAGTTCCTGGGGTTAATCTTGGATAATGGAGNNGTACGGATGGCGGAG
GATAAAACCGACGCGGTTAANAAATGGCCGGTCCCGAAGACGGTCCGNGANATNCGAAAATTCCTGGGATTCG
CAAATTTCTATCGACGGTTTATCGAAGGGTTCTCNAAGCTNGCCGCGCCTCTNAACAATTTATTACGAAAGGA
CTCGAAGTTCGAATGGACNGANAAGCAACAGGCCGCNTTCGATACGCTNAAAAANCGTTTCACGTCGAACCCG
ATCTTGGTTACGGCAGATCCTACGAAACCTCTTCGCGTAGAGTCGGACGCTTCGGACTTTGCTACGGGNGGTA
TTTTATCTATGTTATGCGACGACGGTAAATGGAGGCCGTGCGCTTATTATTCCAAATCTTTATCTGATGTTGA
ACGTAATTACGATGTACACGACAAGGAAATGCTNAGTATTATACGATGTCTGGAAGCNTGGCGNCATTATTTA
GAAGGCGCCGAACATACTGTAGAAATATGGTCGGACCATAGAAACTTACAATACTTTATGACTAGTAAAAAAC
TNAATCGTAGACAGGCACGATGGGCTCTTTATTTATCACGNTTCGATTTACTTTAATTCACAAAGCNGGATC
NNCNATGGGAAAGGCGGACGCNCTGTCCAGGAGGACAGATCTAAAGAGGGAGTGGANTCCGATAACGACGACG
TCACGCTCCTNAAACCGGAATTCTTTTCNATTAAAGCNCTNCAGCAGGGTCATGTNCTCGTTGGACGGCCATG
AAGAAGAGTTNCTNNCGAAGATTAGGAAGTCGAAGGANTACGATGAATCGGTGGTCAAGGCGATCGAGGAACT
NAAGCGNTCGCCNACNAAGCGTTTGCGTTCNGANGAGTGGTCCGAAGAGCAGGATCTNATACTGTTCCGAGGA
AAAGTCTACGTNCCGAAGNCGCCGATCTCCGNCGAGAGATCGTTAAACTCCATCACGANTCCGGTCGTTGCCG
GACACCCAGGACGNNACAAAACNCTNGAATTAATTACGCGTAATTATTGGTGGCCCGGNATGGCTAAATTTGT
NNNTTCNTACGTCGACGGNTGCGACAAATGCCAANGGCACAAAAACTTCCCCGCCCCCCCTGTCGGAAAGCTC
GTACCGATGANTACCCCGGTCGAACCNTGGAAGGCTGTNTCTTGCGACTTCATTACCGGACTNCCNGAAAGTA
ACGGNTATGACGCTATCGTTGTNGTCGTTGATCGCGCTTCGAAAATGGCNCATTTTATTCCTACNACGTCTCA
GACGTCTNCGACCGGTCTCGCTAAACTATATCGCGAATNTGTCTGGAAGCNTCACGGTCTCCCGGATTCGATT
ATCTCGGATCGAGGNCCTCAATTCGCGTCATCCGTGATGATGAAAGAGTTGAACTCNCTACTGGGCATTCAAA
CTAAATTATCGACGGCTTATCACCCGCAGACGGACGGNCAAACGGAACGNGTCAATCAGGATATTGAAGCNTA
TCTNCGACTTTTTATTGATCACATCTTCAAACCGACTGGNCCGAATGGATCCCTTCAGCGGAGTTCGCACACA
ACAACGCNACCCACTCNNCCACGAAGATATCCCCTTTTATTATGTCAACGGCCGNCACCCGCGCATGGGCGTC
GAACCGATNCGTCAACCCACAAGAATGAGTCGGCNANCGAATTCGCCGNGCGAATGAAGAANATTCAAGAAGA
AGCGCAGGCNGCCCTTATTAAATCGCGCGACGAAATGAAACGTTNCGCCGATCAACATCGTGGAGAAAGCCGA
TCAGTTTAAAGTCGGACGATAAGGTATGGCTAGAAACCAAGGATCTNGACCTCAAACGACCGTCGAAGAAACT
CGCGGAACGNCGAATTGGTCCGTTCGAAATTCTCGAACTCGTNGGAACCAATGCGGTNAAACTNAAATTCCCC
GCCTCGATTAAAATTCACCCCGTCGTNAACGTCTCNCGNATCCGNCCNTACACCCCTCACTATTCCTGATCAA
CAACCCGCACCACCCGCTCCAGTAGANATCGACGGCGAATTTGAATACACGGTAGAAGAAATTCTCGATTCGC
GTCTGAACGGNAAACGACTTGAATACTGGGTTAAGTGGGAAGGATATACCGCCGAACATAATACTTGGGAACC
GATTTCGAATTTGAAGAACGCCATGGATCTNGTCAACGATTTCCATCGNAGAAATCCATCGGCGCCGCGAAAG
ATACAGCTCGCCGTTACTCAAGACGAATTCCTTTCTNTGTTCCAACCATACGANAATTTCACCGTTCCAGTCG
AAAAGAGCTTATTGTTTTCATAGATAGTGATGCGCGCCCTTAGGGGAGTGTTAGCGCCTATCTGGCCGATGAA
GAAACAGTGCATCTCGTTATTTTCCTCGGAGAGAAAAATCCTAAAGAGGGAGTTATGTCAGGACTTGTTCTTT
TCTTTTCTTTTTCCAACTCTTTTTCATCTTATCTAATCTCTTATCTATAGGTTATCTCATCTTATCTCTGTAG
TTATCTTATCAACATCACGTGACTGCTTATGTAAGCCGAACCGCGGTTTCGCATCATGTAGTACACATGTCGT
TTGTACATATTATATTCACGGGCTCTTAGCATTAGTATATAGTCTCGCTTGTTTGTATGTTCGTTTCTTAACT
TGATTCTCTTCGGTTTCCGCTATACACAGTGGGACTTAAGTCGTTCGTCTTCATTCATTCTTTCCNCTTGAGA
TCTTCTTGAGAGACCGCACGCCATTTTGGTGTTTTAGCGTGTCCTTACAACCTCCCCCTACCTCTGATTAAGC
CCGACATNAGGATACCGTGCAAGGAGGAGGANANCACACTGGGAAAACCCGCGCTGAACCAGAGGGTGGCCAC
GAGGAAGATCTCGAGCACCAAAGGCAGCTACTCGAAATACGCAAGGAGGAACTGCGGTGCGCAAAAAGGAGCT
GCAACTACGTAAGGAAGAGTTGGAGCTTCACGTACGCATTCTAAGTCCTGGGCATTTTTCATTCATTTAACAG
CAAACAGTCCAGCTGGATCTTATCAAGACCAAACGCCAGAAGACAGGGGTCAAACCAGAACCTCGCCCTCCTG
TGAACCTGCAACTGGATACAGATATCATTGACCTTACTTAAATGAATCTTTCCTTGCTGATTATTTTGTTCCG
CTACTCCCCAACACCGATAATTTGATTCTTTGGCACACCGAACCAGCCAAGCTATGGCAACCTGAGTGCGGCC
GCCTCCACTGGTCTATGGTCGGGATCGCTTGATTGTATCCCCAAGAGTTCAGTGATGCTAGCAAGATGTGACA
TGATCACACCCAGTGATAAGATGGCGCTGTTCATTACCAACCTCGACGGTGTTGAACGATCTCTTTCGTCTTC
CAGTTGTGGTCGAGACACCACAAACTTGACGGTCAGACGGGTGCAGTGGCGGCGCTGTCGGCAAGCGGGCTTC

CATTACATAATGATTTACACACGATCAATTAATTAGGTTCTCATCAACTTGAAAATGTATATCTTTAGCTCGG
GCCCAACACCACCAGTGCTGGTGATGGCCGGATGCTGACCCCCTTGACGGATTAGCGGGTCTGACGGACCGAC
GGGTACCGCTCTTGGAGGTCGATGGATCTGACCCTGTCATAGCCATCACTGAACACCGCACCCCGCAGGCTGC
AGCATGGAACCCGAATTCATAGATGGCTATTCCGCTTGGATTTCTATTAACAATGCTACTCTCCCTCAATATA
AGAGGACAGTTACGGTCGCAGCTTCGGGGAAGAAAGAGGTCACTTGCTGGATCGTGTCGGAGGTTGGTAAAGT
GGGTTGACTTGAGTTCTATTTTCGTGTATTCGTTGATTGATTAACATGGCTCGAATACGAAAGAAATTCTCAG
TACACTGGACTCTTCCCCCATCCCGGGAACGATTGGCGGGGCAGCTATGGATTGATGGGAATGCTATGACGAA
AGCATCAAAACCCGGTCGACTCGAGCCCATTACCATAACCAAATCGGATGAATATA
>Cligib1_rnd-1_family-394#TIR/MITE
TAGACTCGTCCCCAGTCGGCTCACAAGCTCTGGCGTGACCGTCACCCGTGAGCGTTACTAACCACATGACAAG
ACAAAAAAGACGGGCGAGCTTGCGAGCCGACTGGGCGGNCTCGGGCCTGTTTTGGCGGGAAGTGGATTTCCCC
GCCATTCTTGATGGGCTGACTTCGTCGAAAATAGTGGCCACATCTCGAAAAAAAGTGCTCAAACTTGGTATAT
ATAAAATCGTTTTCGAGAGAAACGGGATCGAATGGTATAGGCAAGTTTTCTACTTTTTTTTCGAGAATANTCC
CCTCAAACCGACACCACACTCGCACTCAAGTTCGTCAAAATTTTTGTAAAAAANTTGTATACTCCATTGTATT
CTAATGGCTTAAATTTTGACTTTTCGTATACCCATCTTGGGGACTTTTTTTCGAGGTTGAGGCCGCTTTTTCG
ACGAAGTCGGCCCATCAAGACTGGCGGGGAAATCCACTTCCCGCCAAAACAGGCCCGAGACAGCCCAGCCGGC
TTCGCAAGTGTGCTCGCCAACCGTGTTTATCACGGGAGCACATTATGTAATTGCGAAGCCGGATGGGGACGAG
CCTA
>Cligib1_Cluster43#LTR/Gypsy
TGTTACAACGGTCCCCACCCCTATCTTTTTCCTTTGTTCACCGATTCCCTTTGTTTAACTATGCTTACATTGT
TCTCTGATTCTACATTGTTCTATCGCATGTCCAACACATGCCTCTATTCTTAGATACTGACTCATCCATGATG
TCATAGCTGACTGAGCTTCAACAGCTCTTTTACCTTACATTGTCTTCTGTTCCCATTGTACTATATTCTCGCG
CCTCATAGTCTCAGTATATAAACCCTAGGTACATTGTCTGTATTCCCTATCTTGACCTAGGCAATACTACACC
CTTTACCTTTGAACCTTGATCACTGAACCATCGATACCCAAGTCAAAGCCTGACTTGGAGTGACTATAGTCTT
GTTGGTATTGACCCAAGACTCTGACTTGAACCTCTGGACGATATTCGACGCTTGAACCTTGGTAAAGGCACTC
CGCCTTAACAAAAGGTCAAGATCTACCGATTGCCAACCTCTTTAGGTTCTGGCACCCATTGATTTAAACCGAC
ACCCCTCCGATCACCGTGGCTCCCCACTCCTTACAGGTTCTTCCTAGAGGAGCTGTCCAGAACGACCAAGGAA
CTTGGCTAGAAGGTACACCACTCTTCTTTGACAACACCGCCTTCCGCTGGCACTACAACATTGATCCCTATCG
AGCGGTATCAGACTCATATTTTACCGAACCATACTCCGCACCCCCACGACAACCTCGAAACACCTTCTCTGCC
AACACTTCTGATAGTTCTTCCTCCGAAGCTTCCACTCCCACACAATCACATCAATTCCAAACCCCTGAACCAG
AACCAACCCCCGCACCAGCACCCAAGATGACTACTGTCGCACCCATCACTACTGCCACCAAGACCCCCAAACT
CAGAACTCCCGCCATATTCAAAGGAGAAAGGGACCGATGCTCACCCTTCATCAAAGCGGTCAACCTCTACCTC
AACCTGAATACCTCCGTTTATGACTCCGATATAGCAAAGATCAGTTTTACCCTTTCATATATGGATGAAGGAA
CGGCAGGATGGAAGGAAGCGGAAATGGAGAGACTCGCAAACTTAGAAGGAGGATGGGGAACATATCAGGATTT
CATCACACGTGTAGAAGAAACATTTTCTCCGGCAGACGCCACGGGAGATGCCACCCACCAACTAACCGTTCTT
CGACAAACGGGAACAGCAGAAGATTACGTAGGCCTCTTTAGACAAGCGGCCCTTCGAAGCAAGATCACAGAGG
ACGGACCCCTCATCGTGTTCTTCCAAATGGGACTCACTAAAGCTCTCACTGATAAGATTCACAATCTTATTCC
GTTACCCTCCACCATTGATCTCTGGTATGAGATGTCCATCCGATTCGATAGCCAATATCGAAGAAGCAAACAA
ATCCACCAAAGAAACAACCCGAACCAATACACCAAACCTAAGGGTCCCAATACCCCACGATATATCCCACCAA
GAGACCCATTCGCCATGGACGTAGACCATCTCACGGTTGAAGAACAGAAGGAACACATGCAGAAAGGACAATG
CTTCGTTTGCCACAAGATTGGCCACCGCGCCAGAGATCACAAGAACGGAAGAACTCCACCAGAAACCTCACAA
AATAATTACCGCATCCAGAAGATTGAAACTCCGTCCACTTCCGGAATTGTTGAAGTACCACCTGATGGAACCA
TTGCATTTACACGCATCCAGGCCCTCATGGCTAGCCTATCCCAAGAAGAAAGAACGGTAGCTATAAAGAAGAT
GGAAGAAGAGGGTTTTTAGATCGGCGAACTGAATCGACGTCAGTATCGCCAGAATTGTTCATTTCCCCTGTAC
TAGTCACGGTTACAGATAAACAATCTATGCATTTGCCTATTTGCATCGAAGGAAATGGAAACATCGAAACTCA
AGCCCTCATCGACTGCGGTGCAGGAGGGAAATTCATCAACTCCACATTCGCCGAAAAACACCACCTACCACTG
GAAGAATTACTACAACCGATCTCCGTATTCAATGTTGACGGAACCCGAAATAGCAAAGGACAGATCACCCTCA
GTAAAAAAGGAAAGTCACATGTTCGTCATCGTGGTCACAAAAATGTTAAAATTTTGTCCTAGGTAGTCAAGTT
CAGAATTTATGGGGATTTACATGACTATTGCCTGTCATTTTTTTCATCCCAAACTCATATTTACACCTAAAAT
ATGGGTGAATTTTTCATTCGCTTATTAACTATAGCTACTATCACTGATAAGACGGTCCAAATCATCAATTGCA
CTAACTAAATGAAACTTTGAGATGAATAAAACAACAGGCAATAGTCATGTAAACCCTGAACTTGACTACCTAG
GACAAAATTTTAACATTTTTGTGACTGCATGACAAACATGTGACTTTTCGTTTTTACTGAGGATCACCCATTA
CACCTGGGTAAACCTTCGACTCCAAGGACAAACTCTCAAGACACGCCTACTGGTTACCGGATTAGGAAAGGAA
ACAGTTATTTTAGGACTCCCTTGGTTAAGGGAAATCAACCCGATCATTGACTGGAAACATGGGACTATGGAGA
TAGATCCCACCAAATTCTCCCTCAACGCCACCAACATCATTCGCACTATTCGGGAAAAAGACATGAAACCTAA
AATCAAACCCCGAAAAGTCACCATCGAAGATGTCACTGAGGAAGAAGACGAGATCGAACCCCACCATCTAAAC
CAAATCGACCCTGATGAACCGGATACCATATACTGTCTCCCAGGGTATTCCCCTATCCATGACCCTACACCTC
CTATCTCCGACCCTACTTCCCCAGAAGGAGATCTCATCATGGCATACCTACAAGGAGAACCAGTAATAGCGAT

CTTTGAACCCACCATTGCCAAAATCACACCGGAATATACTGGACCACGCTACTTTTACAAACCCACCCAACGA
ATCGGAATCACCACTCAAGACCACCGGTCCAAACTGATGTCCACAGGACAAGGAGTCCGCATTCGTGTAAAAA
TAACTAGTTCCACTGCCATGGCCCAGGATTCCCTCTCTGACAAACCGAAAACGATTGAAGAACTCCTTCCTGA
ACCTTACTTGAAGTACCGACAGGTCTTCGAAAAGAAAGCCGCCGAACGCTTCCCCGAATCCCGAATCTACGAT
CATGCCATAGACTTGAAACCAGATTTTGTACCCAAAGACTGCAAAATTACCCACTCACACCAGGAGAACAAGT
CAAGTTGGACGAATTCCTCAAAGAAAACTTGGACAAGGGATACATTACTCCATCCAAATCTCCGATGGCTTCT
CCATTTTTCTTTGTAGGAAAGAAGGACAGTTCATTACGACCGTGTCAGGACTATCGTTACCTAAACGAAGGAA
CCATCAAGAACTCCTATCCCCTTCCCCTTATCCAAGAACTCGTCGATAAACTCCGAGGATCCACAATCTTCAC
CAAATTGGACCTCCGCCAGGGATACAACAACGTCCGACTCAAAGAAGGTCATCAATGGAAGGCTGCATTCGAA
ACCAACCGCGGACTATATGAACCACAGGTCATGTTCTTTGGAATGTGCAATTCTCCTGCCACCTTCCAAATGA
TGATGAATGAACTCTTCAATGACATGATTAACGAAGGATGGATTGTCATCTACATGGACGATATCTTAATCCA
CTCCTCAGACTTATCTGAACACCGACAAAGAACCCTACGAGTGCTCCAACGACTTCAGGAGAACGACCTCTTC
CTGAAACTGGAGAAATGCGTCTTTGAAACTACAGAAGTAGAATTTCTTGGAATGATTATCCGACCAAATCAAA
TCGCCATGGACCCCACCAAACTCAAAGGAATCAAGGAATGGCCAACCCCCTCTACCGTAAAAGGTGTACGCTC
CTCATTCTTAGGATTTGGTAATTTCTACCGGCGATTCATTGGAAAATACTCGGATATTGCAAAGCCACTCAAC
AACCTCACCAAGAAAGATCTCAAATGGGAATGGACCCCCGAATGTCAAGAAGCATTCGACACGTTGAAACGAA
AATTTGCCGAAGCACCAGTCCTTCTCATGCCAGATCCCACGAAACCTTTCACTATTGAGTCCGATGCATCAAA
GTTTGCCACAGGAGCAGTACTCCGCCAACGTGACGTGAATGGAGACTGGCATCCGTGCGCCTACCTTTCACAA
TCCTTTGACGCCACTCAACGAAATTACGAAATCTATGATCGAGAACTCCTAGGAATTGTACGAGCTCTGGAAG
CCTGGAGACACTACATCCAAGGCTCCCCACATCCAATTACCATATTCTCAGATCACAAGAATCTCACCTACTT
CCGCACCGCCCAAAAACTCAATCGACGCCAAGCACGATGGAGTCTCTTTCTCTCCGAATTCGACATCAAGTTG
GTCCATGTCCCTGGAACACAAATGGTCCAATCAGACGCTTTATCAGAAGAGAAGACCTAAACCTGGAAGAAGA
TACCGATAACGAAGACATCACATTACTCCCAGAGGAGCTATTTGTGAAGACTATTGACACCGACCTCCGTGAT
CAAATTCTCGCCTCCTCCTCCAAAGAGGATGTGATTACCCAGGCCATTGAAGCACTCAAGAAAGGGGGTACCC
CCCAATGAAATCCGCACTTTCCGATTGGAAAATCGAAGAAGAACTGGTCTTCTACAAAGACCGATGCTATATT
CCCCCAGACGAAACCCTGAGAAGGAACATCACATCAAGATACCACGACTTGCCAATGATGGGACACCCGGTCA
CATGAAAACCCTAGAACTCATCCGTCGCATTACTGGTGGCCTGGAATGTATACTTTGTAAAAACTATGTTGAA
GGATGCGCTACCTGTCAACAAACCAAAGTAAACACTCACCCCACCGCACCACCTTAATGCCGATAAAACCCCG
ACGGACCCAGACCTTCTCACAGATCTCCATGGACTTTGTCACGATCTACCGAAATCCCATGGATATGATTCAC
TCTTGGTCGTGGTAGACCAAGGGCTTACAAAGGGGGTAATTCTAATTCCTTGCAACAAAACGATCGATGCCTT
AGGAACTTCAAATCTTTTATTCCGAGAAGTCTACAAACGATTTGGTCTCCCAGATAAAATTATCTCCGATCGT
GGTCCCCAGTTTGCCTCAGCAGTACAAAGGGAATTAGGACGACTCCTTGGCATCAAACTTGCATTCAGTACGG
CGTACCACCCTCAAACTGATGGAGAAACGGAACGTGTCAATCAAGAAGTAGAAGCTTACCTTCGGATCTTTTG
CGTTAATCACCCCGAAACATGGGTGGAATTCCTCCCTACCGCCGAATTCACCCACAACCAACGCCTACACTCC
GCACGAAATGCTTCCCCATTCTTCCTCATGATGGGCTATAACCCTATTGCCGTTCCCCTTCCTTTCCTCAAAT
CTGACACACCCGCTGTACAAGAACGCCTCCAATCCCTTCAGAAGACACGAGATGAAGCCCTCGCTGCACATGA
ACTGGCTAGCCAACGTGTAGCGGAAAGAACGACACGAAACTTCACCCCTTTCAAGAAGAACATTAGTATGGTT
AGAATCTACTAACCTCAAACTGAAATATGAAACCAGAAAGATGGCACCAGACGAGAAGGCCCCTTCCCCATCA
CCGAGGTGTTAGGACCTCTCACTTACCGGCTACAATTACCCGATCAATGGAAAATACATCCGGTGTTTCACGC
ATGCCTTCTTACCCCATACAAGGAAAACGAAGTCCACGGACCTAATTATACCAAACCACCCCCGGAACTAATA
GAAGGAGAAGAAGAATATGAAGTCGAAGCCATCCTCAAACACCGCAAACAAGGAAAAGGCATGATGTACTATG
TGAAATGGAAGGGTTATCCTTCTACCGACAACACATGGCTTCCCACACGCAGCTTTGGGAATGCCTCCGACAT
TCTCAAAGCTTATAAGAAACGACACTCCCTTTGATTCCCCCATCTTTTGAATCCCCACATTCGAAAATCTAAG
TTCACTACCATGTCTTCTTCTAACGCCTCCACCACTTCCACTGACTACGCTGTCTCCTCTGACTCCCCCTCCA
AACCGCTTCCTCCTCATCATCCCCGACTCATGCGACCGCTTCCACCTCCTCTTCCTCATCATCACTTGTGGGT
GAGGGCCACCGCTTACGCGTGGCATGACCGAGTCCAATACTTATTGATAGAAATACTTATCATCTTTAGCATT
ACTTCAACCAACTTTGAACTTGTTTGTGTAAAATGTTCAAGAATTGTCTACAATCGTACGGGTGTACTATCGA
TATTAATACTAAACAGGTGTGGCAGACGTAAATGTGATGGGCCAGGGAGTCAACAAGCATAGGATGTCAATAG
GTAGAGGCGGGAGGGTGGCTGAGGTCAGACAGAAGCATGACAGCTCTACGCAACTGGCAGTGCCCAAGGGTGT
TGTGCTGGGGCTGGGGATGGGGATGCGAGGGGCATTAGAGCTAATACACGTAGGAATAGGGGTAGCCTGTATG
AACCAGAGGGTGGATGCGGCGGGGCCAGGCACACGGCGGCGTTGATCGTAGCGTAGTAGCATGGTGGTGGTGG
GCAATGGAATGGTAGGCGATGTCGCGAGAGAGTGTGACAGTTGATGGATGCGCGGGTGACGAGGCCGCGGGGT
TGTAACAATGTTCGTGCTGCCTTCGTAGTGTGCAATGCGAGTGCGACAGTGGCTGAGAGTGATGAGCTAGATG
AGGCGCAACAGCGGATGGATATGGGTGGGGCCGGATGAACGCATGACAGGGCTCGTGGTGCTCGAAGGTGATA
GGGACGCAATAAGAGAGGGAGGATGCGACATATGATGAAGCTGTGAAACGGATCAGTACTGATGAATAAGGAA
GATGGGGAACTCACTCGATAGGAACGCAAAAGGGCATAGAACGAGTCACACTATTGGAGGTGGGAGGCGACAT
AGAGACAACATTGGCGGAGGCTATGGCGACGTGGATAAGGATGACATGAAGTGAATAGAGGGGAGTGCACTCG
CCCGCCCAACGTAGGCATGCGTGTGTCGTCCGTTCCCCTGCCCATTTGTCTGGTACAGACATGCATCCATGCG

CTCGTGCACCTGCGTCGCTCTTTCGCCCACCTGTTCATCCAATGCGGGTACACATCCGTGTGCCTATGCATCC
ACGTTTTCCATTCACCCATTCATCTGACACGGATAACATTTGTGCCTCAGAAAATATGGGAGGCGGGGCTTGG
GATTTAGAGACAAGATTGTTTCATCTGTATATCATGTGTTAGAGAGTATATTGGGGGAGACGCAGGAATACAT
ACTGAAAATTGAACTAGCATAGAACAAGCAGTAGCGAACTGATAGATGTGTTCATATGGAATGTCGAGAGTGA
ACAATCCCCAACTTTGTCTGCGAGAGTTAGATTGGGGGGCGGGTAGCAATCTTTAACCCTCTCGCTTGTGAGA
AGGACGTCAGGCGGCGGTGGTGGCGAGATCGAAGTGGCAGAATGCGAGGGAGGAGTCTGGCAGCGCGAGCCAT
CCGGCCTTTCTCGTTTGCGGTGAAGGAGCTTTGCTTGTGCCATTTTCAATCTCAGCTCACGGCCATCATTCCG
ATGCGTATCAAATTTCGAGCTCAGACCGCGGCTATCACCGCAACGACAGAGCTTATTTTCGTGGCGGACTCGA
CAGGTAAAATAACCGAGTCCTAAAAATATACATTTTTGGACTCGGAATCTTCTGCCCCCACACGACGCGAACC
CCTTCAACCGATCCCCGAAAACGAAACTGAAAACATCATCAACCCGAATGAAATCCCGAAGGACTTGGAAGAT
TTCTACAATCTCCTCATCACCATCAAGGACCCTGAGTCCTTCGTCACTTCCATGAGAAAGTATCCTATCCTTC
ATGACTTCATGACGGCGTTCTACGACATCAACGACCTAGATGCGCCCATTCGAAGCCTCTTTGGAACCATGGG
CCAGGTTCAAGCCCAGCTCACTCGACTCAAAGAAGCCCATGAATGCGCCCAGAAGACCCTGTTCCACGCTGGT
AGGAAATGGGGTCTAGACGCCCTTATCGGATTCGATGGCCCACACGGATACCCTCACCCCTTAAGAACCGAAC
ATATTCAGCAACTGCTGAACGACCAACAGATGAACCGAATCCCTGGAGTAGTAACTCCACGACTCACAAACGA
CCCATGGCCTCTCACCAATCGAACCATGGACGAACTCACCTCCACACCCTCCATCGTCCTTACCGACCTGGAA
GCCGAAAGACCCCCAAACCCAACCACTCTCATGGAATCTACGTCCTCCAGAACAGATTCCCGAGGAGCAACAC
CATTTCCTCGATCACCCATTAACGATACATCCTCCGCCCCTTCTCCTGAACCACTCTCTACCACCCCTCTTCG
CAGACCGTCCTCTCTACCCGTCGCCCACTTACCTACCCCTACACATAGTACTACTCCTCCACCGAATCCTGAA
CGACTAGCACCCCCTTCTTATGACCAAGCCCATGATAGAACCCCTGTTATCCGAACCCCACGACGACAACGAC
AACGCCCACAAACCATCTTTGTCACCCCTCTTTCCCATGCCGGTCGACCACGACACATCCAATCCCCCGACGC
CGAAAACGTTCCCCCTCCTCGGACTGTCTTCTCTCGACCGGAACGACCTCGCGTCCGATACTGTTCCTTTTGC
CATGGCACTGGACATGCATTCTCCACCTGCCCAGATTTTTATTGTAATTTCTGTAATTGGCAAACCCCTCACG
TTACTTGCGCCCACAGTCATTACGGACCCCGACAACCCGATTTCGATGAAGAATTTTACTTTGATGACTCTGC
CATCTCCAATATGATCGACGAACCCTACGGCAGTTTTGCAGGGTAACTTGAAGTCAGGGGACTTCAACAATAA
ACAGGGGGTAATGTTACAACGGTCCCACCCCTATCTTTTTCCTTTGTTCACCGATTCCCTTTGTTTAACTATG
CTTACATTGTTCTCTGATTCTACATTGTTCTATCGCATGTCCAACACATGCCTCTATTCTTAGATACTGACTC
ATCCATGATGTCATAGCTGACTGAGCTTCAACAGCTCTTTTACCTTACATTGTCTTCTGTTCCCATTGTACTA
TATTCTCGCGCCTCATAGTCTCAGTATATAAACCCTAGGTACATTGTCTGTATTCCCTATCTTGACCTAGGCA
ATACTACACCCTTTACCTTTGAACCTTGATCACTGAACCATCGATACCCAAGTCAAAGCCTGACTTGGAGTGA
CTATAGTCTTGTTGGTATTGACCCAAGACTCTGACTTGAACCTCTGGACGATATTCGACGCTTGAACCTTGGT
AAAGGCACTCCGCCTTAACA
>Cligib1_rnd-1_family-26#LTR/Gypsy
TTGTTAAGGCGGANNGCCTTTACCAAGGTTCAAGCGTCGAGTATCGTCGAAGACGTCAAGTCAGAGTCTTGGG
TCAATACCAACAAGACTATAGTCACTCCAAGTCAGGCTTTGACTTGGGTATCGATGGTTCAGTGATCAAGGTT
CAGAGGTAAAGGGTGTAGTATTGCCTAGGTCAAGATGGAGAACAATGACAATGTATCTAGGGTTTATATACTA
GGGACCTTGAGGCGCGAGAACATAGTACAATGGGAAGNAAAGACAATGTAAGGTAAAAGAGCTGTTGAAGCTC
AGTCAGCTATGACGTCATAGATGAGTCAGGTATCTAAGAATAGAGGCATGTGTTGGACATGCGATAGAACAAT
GAAGAATCAGAGAACAATGTAAGTATNGTCAAACAAAGGGAGTCGGTGAACAAAGAGAAAAAGATAGGGGTGG
GAACCGTTGTAACATTACCCCCTGTTTATCGTTGAAGTCCCCTGACTTCAAGTTACCCTGCAAAGCTGCCGTA
GGGTTCATCAATCATATTAGAGATGGCGGAGTCGTCAAAGTAAAACTCCTCATCGAAATCGGGTTGTCGTGGT
CCGTANTGACTGTGAGCGCAAGTGACGTGAGGTGTTCGCCAATTGCAGAAATTGCAATAAAAATCAGGGCAGG
TGGAGAANGCGTGTCCAGTACCATGGCAAAAGGAACAATATCGNACGCGAGGTCGTTCGGGTCGAGAGAAGAC
GGTCCGGGGAGGCGGGACATTTTCGGCGTCGGGGGATTGGACGTGTCGTGGTCGACCAGCATGGGAAAGAGGG
GTGACAAAGATGGTTTGTGGGCGTTGTCGTTGTCGTCGTGGGGTTCGGATGACGGGGGNTCTTTCGTGTGCTT
GGTCGTAGGATGGGGGTGCTAGTCGTTCGGGGTTCGGTGGAGGAGTGGTACTGCGAGTAGGGGTAGGTAAATG
GGCGATGGGGAGAGAGGATGGTCTGCGGAGAGGAGTGGTAGAAGGTGGNTCAGGAGAAGGGGCGGCAGANGTG
TCGTTAATGGGTGAGCGAGGAAANGGTGTCGCTCCTCGGGAGTCTGTTCTGGAGGAAGTAGACTCCATGAGAG
TGGTCGGGTTCGGNGGTCNTTCGGTTTCCAGGTCAGTGAGGACGATGGAGGGNGTGGAAGTGAGTTCGTCGAC
GGTTCGATTGGTGAGGGGCCAGGGGTCGTTCGTGAGTCGTGGAGTTACTACTCCAGGGATTCGGTTCATCTGT
TGATCGTTCAGCAGTTGCTGAACGTGTTCTATTCTTAATGGGTGAGGGTATCCCTGTGGACAGTCGAATCCAA
TAAGAGCGTCTAGACCCCATTTCCTACCTGCGTGGAATAGGGTCTTCTGGGCGGATTCGTGGGCGTCTTTAAG
GCGAGTGAGTTGGGCTTGAACCTGGCCCATGGCTCCAAAGAGATTTCGGATGGGAGCGTCTATTTCGTTGACG
TCGTAGAACGCCGTCATGAAGTCGCGGAGGATAGGGTATTTCTTCATGGAGGTGACGAAGGACTCGGGGTCCT
TGATGGTGATGAGGAGATTGTAGAAGTCTTCTAGATCCTTCGGGACTTCGTTGAGGTTCGGGATATCTTCGGT
TTCGTTTTCGGGGATTGGTTGGAGGGGTTCGCGTCGTGTGGGGGCAGAAGATGATGATGAGGAAGAGGAGGTG
GAAGCGGTCGCATGAGTCGGGGAGGATGAGGAGGAAGTGGATTGGAGGGGGGAGTCAGAGGAGACAGCGTAGT
CGGTGGAAGTGGTGGAGGCGGTAGANGAAGACATGGTAGCGAGTTAGATTTTCGAATGTGGGGATTCAAAAGA

TGGGGAAATCAAAGGGAGTGTCGTTTCTTATAAGCTTTGAGGATGTCGGAGGCATTTCCAAAGCTGCGTGTGG
GAAGCCACGTGTTGTCGGTAGANGGATAACCCTTCCATTTCACATAGTACATCATGCCGTTTCCTTGTTTGCG
GTGTTTGAGGATGGCTTCGATTTCGTATTCTTCTTCTCCTTCTATTAGTTCCGGGGGTGGTTTGGTATAATTA
GGTCCGTGGACTTCGTTTTCCTTGTATGGGGTAAGAAGGCATGCGTGAAACACCGGATGTATTTTCCATTGAT
CGGGTAATTGTAGCCGGTAAGTGAGAGGTCCTAACACCTCGGTGATGGGGAAGGGGCCTTCTCGTCGTGGTGC
CATCTTTCTGGTTTCGTATTTCAGTTTGAGGTTAGTAGATTCTAACCATACTAAGTCGTTCTTCTTGAAAGGG
GTGAAGTTTCGTGTCGTTCTTTCCGCTACACGTTGGCTAGCCAGTTCATGTGCAGCGAGGGCTTCATCTCGTG
TCTTCTGGAGGGATTGGAGACGTTCTTGTACAGCGGGTGTGTCGGATTTGAGGAAAGGAAGGGGAACGGCAAT
AGGGTTATAGCCCATCATGAGGAAGAATGGGGAAGCATTTCGTGCGGAGTGTAGGCGTTGGTTGTGGGTGAAT
TCGGCGGTAGGGAGGAATTCCACCCATGTTTCGGGGTGATTAACGCAAAAGATCCGAAGGTAGGCTTCTACTT
CTTGATTGACACGTTCCGTTTCTCCATCAGTTTGAGGGTGGTACGCCGTACTGAATGCGAGTTTGATGCCAAG
GAGTCGTCCTAATTCCCTTTGTACTGCGGAGGCAAACTGGGGGCCACGATCGGAGATGATTTTATCCGGGAGA
CCGAATCGTTTGTAGACTTCCCGGAATAAAAGATTTGAAGTTCCTAGGGCATCGATCGTTTTGTTGCAGGGAA
TTAGAATTACCCCCTTTGTAAGCCCTTGGTCTACCACGACCAAGAGTGAATCATATCCATGGGATTTCGGTAG
ATCCGTGACGAAGTCCATGGAGATCTGTGAGAAAGGTCTTGGGTCCGTCGGGGCTTTTATCGGCATTAGAGGT
GGTGCGGTGGGGTGAGTGTTTACTTTGGTTTGTTGACAGGTAGCGCATCCTTCAACATAATTTTTGACAAANG
TATACATTCCAGGCCACCAGTAATCGCGNCGGATGAGTTCCAGGGTTTTCATGTGNCCAGGGTGTCCCATCAT
TGGCAAGTCGTGGTATCTTGATGCGACGTTCCTTCTCAGGGTTTCGTCTGGGGGAATATAGCATCGGTCTTTG
TAGAAGACCAGTTCTTCTTCGATTTTCCAATCGGAAAGCGCGGATTTCATCGGAGGGGTACCCCCTTTCTTGA
GTGCTTCGATGGCCTGGGTAATCACATCCTCTTTGGAGGAGGAGGCGAGAATCTGATCACGGAGGTCGGTGTC
AATAGTTTTCACAAAAA
>Cligib1_rnd-1_family-145#LTR/Copia
AAGGAAGACGGACCAGAGTAAGCTGGACCGGAGTAAGAGAAGCTGATCCATTTATGATGGGATCAGCTGTGAA
CTGCTTTCCTTAATTGGAAGCCGGTTTAGGGGTAATTATGTAAGCAGGGCACTTACAGTTAGAGCGTTGAAGT
TTAACACTCCCCCTCGCTTAGNACAGCGGATGCAAATAATCAACCTTTGCAGGAGCTATAAGCGATACTACGA
CTTCAAGACGAGTAAAACTCAAGTCCCAGTTGAGGTACGAATTCTTTGAATTTGATATGCCCCAGACTTTTCG
TAAGAACGTCTGCGGGGTTGTTCTCTCCTTCAGTGAAAAGTATGGNGACTTTTCCTTCGTCANNACATTCGCG
AATGTAGTGATGACGGATATCAATATGTTTGGTTCGTCTTTCTTGGACCGGATTGGAACCAATGAAGATTGAT
CCTTCATTATCGCCGACAATTGGTATTTTGTCGAGTTTGATTCCGAGTTCNCCAAAGAGNGACCGTAACCAAA
CNACTTGTCGACANGTGTCAGACATGGCNATATATTCNGCCTCGGTNGANGATNGNGCGATTGTTTTCTGTNG
TCTGGACGTCCANGTCACAGCACCGGAAGCAAGNNTTACGAAGAAACCGGAGGTAGATTTCCGAGTATTAGGG
TCAGAGGCCCAGTCGGAATCTGTGTGNGCAATTAAGCCTTCGCCCGAATCNCCATTGTANACNAGGGCATAAT
CTTGAGTACCAGCTAAATAGCGGCATATGTTGAGGGCCTTTTTCAGGTGCTCTTCAGTGGGATTTGCGCTCAT
TTGCGACATTTTAATCACAGCGAAGGCAATGTCGGGNCGAGTTCCTAACATTAGGTAAAGCAGGGATCCTATG
ACGGATTGATATTTGCTTCGTAGGGTGTGATCTATTGGACCCCATTTTTCATGGCTGGTAGATATCCNTGAGG
TAGGGGGGTTTTGGCGGCGGATGCATTAAGCATTCCAAAGCGCTCTAATACTTTGACCAGATAAGGTTTCTGA
TCAAGTTTGATCTGGTTTTTCTTTCGCCAGATGCGCATTCCGAGGAATTCTTTAGCATCACCAAGATCCCTGC
ATTCCCATTGTTGCATGAATTCTGCTTTTTTCTTGATTACGTACGACCGATTAGGCCCCAAGAATAAGGCATC
GTCAACGTAGACTATCGCAATGACAATCTGATTCGAGGATGATCGGTGNATGAANATGCCTGCATCTGAAACA
GTGCGTTGAAAGCCCATTTCTTTCATNGACGCAACGAGAGCTCTCCACCATTCAAGAGCTGCTTGTTTCAGAC
CATATAAGGCACGTTTGAGNCGCAGGACTTTGCGCTCATGGCCTTTTCGGACGAAGCCTTCTGGTTGGTNCAT
GTATATCTCGACGTCCAGTTTGCCATANAGGAAGGCAGTTCGAACGTCAAGTGATAGAATNACCCAATTTTTC
AGGGCTGCTGTTGCAATCATGAGACGCACAGATTCATATCGGACNACNGGTGAGAATANTTCGTCGAAGTCNA
CACCTTCGACTTGTGAGTAACCTTTGGCTACGAGGCGGGCTTTCTTTCGNCCATCAGATTTGACGTCGAAAAC
CCAGCGATTGCGAATCGCCTTTCGTCCTTTAGGTAGATCGCANATTTCATANACGTCGCGATTNTTGAGGGAG
TCTAATTCCTCTTCGCAGGCGTTCATCCACTCTTTTTGAAGCTTTTTATCGAGCTTGAATATGTCTCGGAAGT
GAGTAGGAACTTCGTCTTTGACNGGAACGGCTTTGGANAGTAAGAAATCCATTATTTCTCCCCCTTCCTCTCT
ACATATNCGNATGAGNTGTAATTCTTCATCGTCGGTAGAGGGGTTCGGAAGATCCCTTTCTCCAACATTTTTA
CGCCAATNTCGNTCACTAGCAATGTCNCGTTCTATTTCAGANGGTAGTTTCTTTTCTCCATAAATATTTCCAG
GACGGTTNGGNACTTTACGTGCTCGGCCAGATCTTCGCGGGACAATGATTTCTGGCTCGGGTTCATCCTCATC
ATCAACTGGTTCAGGTGTTGATTCACGAGGAGCTATTGATCCCGTGCCATCGGATTCCGATCCTACTTCCTTC
GGTACCTTATTTCTGATAACATCGTCATCATCAATTTCCTCCCTGACATTCAGATCGTCGTTGTCTGAATCTG
AGTCAGGGGTAGAAGGAGGTGGAGANGGAGGTAGATAATCNTCGGTTGGAGGAAGNAGATCTCTAGGGGTNGT
ATTGTTTGGACATTTCGGGAGTGTTTCTTCATCGAAGATAGCNGTNACTCCGATGAACAATGCGGTGTTTTTA
ATTCGCATAAAGCGATAACCCTTTAATTCCNTCAGCGTGACCGAGGTAAATCATTAGCTCGGATTTGGGCGCA
AGTTTATTGGCACGTTTTTCNGNTGGNAGATANACGTATGCAGCGCAGCCAAANACACGAAGATGTGTTAAGG
AGGGTCTTTCACCCTTTAAGACTTCATATGGAGTTCGCCATTNCAGGCGCACCATAGGAGTACGGTTATANAT
GTGAACCGCTTGTTCCACAGAGAATTCCCACCATGAACTAGGAAGGCATGCGTGATGNCGCATAGACTCTGCC

TTATCCATAACGGTTCTATTGATACGTTCAGCNCGCCCATTTTGTTGTGGTGTGTGGGGNGCCGATTGTTCGA
TTACAATGCCCGATGTTGTCAATTCATCAATAAATTCCTGGGATTTAAATTCTCCCCCTGCATCAGAACGCCA
NCGTTTGATTTTNGCGTTATATTGGTTTTTAACAGCTGCATTGAAGTTTCGCGGATGGCGCCATTGCGTCTGA
TTTATGTTTTAGGTGTGAGACCCAAACATATGACGTGTAGTCGTCAAGGAATGTNATGATGTAATGAATACCC
GTGATAAGATTTCACGGGGAAGCTTTTTAAATCCGAATGAATCAATTCGAATGGCTTCTTGGCGCGTGAGGCG
GANGGTTTAAAAGATGAAGANGGCATCTTTCCGTATGCGCAGCCAGGGCANATAGGTGGGTTCTTCGGGATGA
TTAGTCCCTGTGGGAAGTCTTTAATGTGTTTNCGAGATTTGGAGACGACCTCTTTCGAGGGATGACCNAGGCG
TCTATGCCANATGTCATAGCGATCGATNGTATTGACAAATAGGGATGTTTTAATCCGNTCGGTTTGAACACGA
TCTGCGTCGACCCAGAACGTNGTGTCCCACGCAAAACGCTTTTCAGCAGAGGTTAATCGTCGTTTGTCCCTTT
GTTGAATATNAACGCNTTTGATGANGCATTTAACGACANTCCAGAATTNAGTAATTCTCCCATGGATATAATT
CGGGAGTTCAACTTTGGATGGTGATAGACAGGATGTAAGCGCGTGTTGCGTTTTTATANGATCCATCTGTTTG
TAGGACATCGTTTTCAAGGATAACGGTTCCTTGTGTNTCAATTTCGATNATCTCNGTCGCGGTAGNAACAGGA
ATNGGGTGTTCGATTNTTGAACGTTCCACGTAATCATCCAANATGCCAGTGAAGTGCATGGATGCNCCTGAGT
CNATTAACCATTGGTGTTCGACGTGTTTCGCTTTTCCCTTACAAGATGCACAGNNAGCANAATGATTATTTAT
TTCGGCCATTTGGCCGCGAGACAAAGACACATGATCTGCATATTTANATACAGGGGGCAATCCGATGATTTAA
TCGAAAAGCGCCCTGCAGAATTGATGCGGTTAGTCTCATATATNGGAAGTGAAGGAGGCGGTGACTGACGATT
GACCGTCAAACCGGGTNCCCCAACTCTCAGGATCGAATTCGGGTTCTTCACCCATCGANACTGGATCCTCGCG
ATCGTCCTCTTCAGGAGACCGGAGAGACTTTGCGCCGTTTCTTAGGCGGAGGGNNCCAAGCCTCNNCGTCGTA
ATCGACGTATGCGGCGCCTTCATCNTCACTGCCAC
>Cligib1_Cluster50#LTR/Gypsy
CAGTACTTCCTAACAAGAGACCCTAACGCGCCTCGACCCACACCGACTGCGACACGTACCAACATCACCGCGA
GAGCCTCCATGAGGCCCGGAGACCTCGACAATGTGCCCATTAGGTGACCCGAGCAAGACGCGGACGCGCGATT
GCGCCGGGTTTGTTGCATATTCTCTTATTTCACTTTAGATTACCGGACTCAGGACATGATCGGCGGTCCATCG
CATCGTACATAGACCCTTACATACGCCGCGACTCGCCCACAGTATATAGGCCATGTACATACTTCGCTTTCGA
CAGAGAGAATCTAGCAGAGTTTCCCCCTCAGAGTTCATAGCTCAGTTTCGCAGTCTCAGAGTAGACTATCCAC
GTTCCCTACAGGAGTCGCACGTCGCACTCCTCCACGTTCATTCGCATTCACGTAGTCGGTCTGTGACCAAGTC
GCCCGATCGCAGTCAAAGAAGTCTTGCGTATTGGTGTACGCCGTCTCGCGTCGCACCGTGCCCGAAGATTTGA
GAGCGCTACTCGCGTCGCACTCATTCTACGCTAAGTTCCCAGTTCCCGGACGCCCTCGCGTCCATTCCATTCG
TTCATCTCCCCTCTCGCGCGTCGTACGCGTCGAGGTCGCATCCAAAGCGGGTCCCCTCGCAGTCTTTGAGAAC
TCTTCTAGAGTCCTTAGGAGGCTTTGTTTGTTGTTAATCTAAAACACTCGCTCACTCAACATCTTATCTAGGT
GTTGTTTACTTAGTTGGTCTTTGTGGTTTGTTTTGGCCACAAGCCTCTTTGCATATCACCGTACGCGTTGTCA
ACGTGTCATTGATCAAAAAAATTTGGCCAACTTACCTAGGCAGCCCATTGATTGATACCTGCCGACATCCTCTG
CATTTTGATGTAGCGAGCAGGTTGAGTAGCGTTGACGGGGCGCGTGGGGAGAGACGGTGGCCCAAAGGAGAGT
GTCGGTTCGAGGTGGTGGCTGGTGATGGCAATGATCTCACCAGATCGAGCCACGAGGGGGAATGGCAGTGACG
TTGAGGTTGAGCCGTTCGCCCTTGTTCTGTCACCTGAGATGGTGTGGCTCGTTGTTGGCTGTAGTATGTCATT
GTCAGGCGGAGCGGGTTTTGCTGGAAGACGTTCTGGCTCACCTAGCCCATCCCTAAAGGGCTGAAACTCCGAC
TTTTGTCGCGTAAATCCATGCGCACGATACAGCCGTTCGTTTGGTGTGTGCGGATGCAAGAGAATGGGGTGGG
TCTTGGTGTGTGAACGTGAGGAAGAGCGGGATCCCAATATGGAGTGTGCCTGCGGGTTGCGGCAAGTACTTGC
CGCGGGTACCTGCTGTTCAAAATTTGAAACTCAGACTCGCAATGACCCGTGGCTGCGAGTGATCCGGATCCGC
AAGTCACCAAACTCGCGGGTTTTCCCACGGATCAACCTGTTGCGACTCACGAGTTACTCGCACTCGTGACATG
CCTTAATACGGTCTATACTTCCTAGACTATCACATCAAACGCGTCTCACTAGACGTACAAATGTTCATTGAAG
CAGACGACGAACGACTTCGTCGACGAGCTGAACATGGGGAACCCCCCGCAATACAAGCATGGGACGGATGGTT
ATATACCATCCGAACACGACAAACTCCGTATTCGTCATCTATTGGATGACGAAAGAGAAAAGAAGAAAATCGA
CAGCCTAATAGACGATGATTGGCTGTTCATCGGGGAGGAGGTATTACCCATATACCTCCGTCACCGCGACGCA
AGACAAGCCGCACATGCGTATGAGGACGAGCTGAATCGGCTAGCCGCCCTACGGCACGCCGAGGCAGTCGTCC
GCGAAGGACCACCTACTCCACAAAAAGAAACTGGGACAAGTAACATCGCAACAGGATCCAACGTGGAGGACAC
AACTATGGGCGACGCGGAGGATAACTCCTCCATCGTCGAATCCTCGGGGCGTAACCTCCCAGAGGAGATACCA
TCAGACATTGGGGGGGAACTAAACGACGCGGAAATATAAATGCGCCCGACCATACGAAGAATCAAAGCCGCGG
GAACACAAGGACCCCGCGCATACACAATATGGAGTATAAAGATACTCCACAGAACAACATATCAGATATGGGG
TGGAAAGCATTAGTCCACCCCCGCATAATACCACGCACGGACACAATCTACCTACATACATAAATAGATCCCA
GAATGCGATCTAAGGAGGGGGTAATGTAACAGGCGTATTTGTAACTATGTACCAACACCTTTACCAAGGGTGA
CTTCATAAGGAGTCACCCGTGACTAGTAACACTTAAGCTTATTTTCACTACCGTAGTACAACGTTCACCAATA
GACTGGTCTAACGTAGTGCTGCATTGTGAGATAAGTCTTCGTAACAATAATAATTGTTACACCAACACAACGG
CGATACGAGATCTATGACAAGGAACTCCTATCCATCATGGAAGCACTGCGCATTTGGCGACAATACTTGGTGG
ACGCACAGGAACCGTTCGAGCTATTGACAGATCACAAAAACCTGACATATTTTAGGAAAGCTCAGCGGTTAAA
CAGTCGACAAATGCGGTGGTATTTGAAACTACAAGATTACGATTCGAAATGCGACACATACCGGGCAAAACAA
ACACGAAGGCAGACATCCTCTCAAGACTGGAATGGTACGAAGAAGACACTGAAGAAGAAGAAGTCATGATGTT
CAAAAATCATGGGTTTGGTATTCCTTCGGAAATGGAGGAAGACGATGAAATTTCGGACTTTGGCATACGGTTC

TTCGGAGAAGAACTACCTGAAGAGGAGGTGATTCGGGACACACCGGAAATTTCCATCTCAGCTCTCACCGAAT
TCGGCAATGAAGAACTGGAACAACGCATCAGAAACCACCCACGCGTAGAATCAGAAGTCCGATTCGGCATGAA
GGAAAGTCCACACTACTTCAAAGAAGCTGATGGCATCATCACAGTTGGAAACAAGATCTATATTCCCCCAGAC
AAACAACTACGTGAAGAACTGATCACAGAACACCATGACTCGCCACTAGCAGGTCACCCAGGAATCCACAAGA
CGAACGAACTCATTGGACGCACCCTATGGTGGCCAGGAATGCGATCGGACATCAGGGACTATGTCATGGGATG
CGAAACATGTCAAAAGAACAAGATCAATCGAGAAGCACGTCATGCGCCACTCTACCCCAACCCGATACCCACG
CACCCCTGGGAAGAGATCTCCATTGACATGATCGGCCCCTTACCACCGTCTAAAGGTTACGATGCTATTTTAG
TCATTGTTGACAGACAATCAAAGATGATCAGGGTCGAACCGACACACACCACCCTGACATCCAAGGGACTCGC
TGAAATCTATCGAGATACCATCTGGAGACAACACGGAATACCCAAGAAGATCATCAGTGACAGAGGACCGCAA
TTTGCATCGAACTTCATGAAGGACCTATGTACCCTCTTAGGCATCAAGCGGAATTTATCAACGGCTTATCACC
CGAGGTCTTTACACTGCCCCACGGATTCCTAGTCAGTTTTCCAGAATCCGAGTCGGATTACTAGGATTTTTTC
GGACAAAACTCGGAAAATTTTGGCTGAGAGCCCAGCCAAAAGTCAAATTTTATCGGAAAATTTTCCTAGTAAA
TTCCTAGTAATCCGACTCAGAAACTCGGAATCCTAATGAGTCCGAATTCCTAGTAGCCAAACTGGCTAGGAAT
TTACTAGGATTCCTAGAAAATTTTAGATTCATAGAGAATTTACTCAGATTCTTTAGGAATTCACTCAGATTCT
TAGGAATTTACTCAGATCCTTAGAAATTTACTCAGATTCTCACTCAGATTCTTAGGAATTTACTTGGATTTTT
AGGAATTTACTCAGATTCTTAGAGAATCCACTCAAACTTTCTGTAGGCTTTAAGGGTTCAAATGAGTATTCAT
ATTTGTAGATTCACACAAATAGTAGTTAGCAGTTAGGGTGAGATTATTAGGGTAAGGCATAGCATAGATTAAT
CCATGGGTAGATCTGGATCTGCATTCATGAGTAGTTGTTTAGTCTAGGGCCCAACTTGGATCTATGAGTAATT
TGGTCTGCAAGTACTTGAACTTTTCAGAACCAGTTGCAACTAGTTGAGACTGCACAAAAAGACTGGTTACACC
AGTCACAACCAGTCTCAGTCACAGCCTTGTTGCAGTTTTTTACAGTAGACTCTCTGGTCTTAAGGCCAGTTTG
GACTGGTTTGAACTGCAGTTCAGGTATTTCCAAATATGGAAAGACCAAGACTGCAGTCCAGTTTTGAGTTTCC
GCCCTGAACTTTAAATCAAATTTGGAAGGGATCTTATTTAGCCGTTGATAAGCATGCCGCGCACTTATGGGTT
CATAGGCCACATCTCGCACCAAGATAAGATCAATGCGACTCGCCAGGCTCGACATGTGCACCCACAAGCCCAC
ACAACGGCCTCGAACCGCATCAACACCCATGATGATGGCCAGGATCAGCTCGCCACATCTCATATATACAATG
CGAGGCCGTCACACTCGTGTAGGACCCTCTCGCACATCAGCAGATCGCCCTCCCAACTCAGCACATACGGGAT
TGCACCTCAACACAGCTCCGCATGCACTCAGTACTTCCCAACAAGAGACCCTAACGCGCCTCGACCCACACCG
ACTGCGACATGTATCAACATCACTGCAAGAGCCTCCATGAGGCCCAGACACCTCGACAATGTGCCCATTAGGT
GACCTGAGCAAGATGCAGACGCGCGATCGGCGCTGGGTTTGTTGCATATTTTCTTATTTTACCTTAGATTACC
AGACTCAGGACATGATCAGTGGCCCATCACATCATACATAGACCCTTACCTACACCACAACTCAT
>Cligib1_rnd-1_family-79#TIR/MITE
TAGGGCATGCCACGAGTGCGGGTCACTCATAGGTACCCACGGGTTGATCCGTGGGAAACCTGCGGGTATTCAC
GACCCATGAGTTCCCAACTTATGAGTTAACACACTCACAAGTCATTTTACTTGTGGATCCAAGTCCCCACTGC
GGGTACCCACGAGTCTTGTTTGACTCGTCTCACTGTGTTTTCTGACCCACCCCCTTGTCACATACTCATGAGT
ATGTGACTCACGGATCCGAGTCTTTCTGTGAGTTTTGAACTTGCAGTGATCTGTGGTGGTGAGTACACAAGTC
GCGGGGATCCGGACCCGCGGGTTTTTGTTCCCACGGATCCGGGTCGATCCGCGAGTGCACTCGTGGCATGCCC
TA
>Cligib1_Cluster31#LTR/Gypsy
GTTACGATTCCTTCCTTCTCTGATCATCACCACTTGGACATCTGACTGATAGCATCATTCATAGACACTTCTA
TTACTTCCACCATCATCCCATCATCATCTGATATGCGCCATATGCTACATCATCAGATGATACATCATGCGCA
CCTTATCTACTTCTATACCGCATGGTCTCCGTTCTATCTTTGCTATCTTCGTTGTTTCTTACTCAGCTGCTCA
GTCAGCTCGCTTCTCGTATCAGTCATCATCCCCCATATGTCTTACTATACTCACGAACTTAGTCACTAGTGCG
TAGTCATAGAACTACCTCACTTGTATATAAACGTAGCTACATACTCCTGTATTCGCTACGTTTAATCGTTTAT
CTCTTTCCCCCTCTACTGCCTCCTGACCCAGGAGACTTCAGACCTTGCTAAGTGTCCTTGAGTCTTTACTGTC
GACTCATCGACTTACGTTAGCAGGACGTTAATAAGCATGGTAATGGTTTAGCATCAGTTCGCATCTATTTCTA
GTCATTGTAGCTAATTTCCGTACTTACATTCGCATCGCGCGACTAATGCGCGACCACTGATCGCTTTGTACGC
GTGGAGATCGCGCTTTCGATGCGATTCAGATCGCATCTTTACCATAGTGATCGCATAGTTTCTCCTTATCTTA
CAGATTATCGTACATTACATACATACTACCTCATGCCTGCGTAGCTCCGATTTATATAGAGTCGCATGTAGCT
CGCATAATACAACTTCCCCTTCGCATTAGAGCTCTCGAGTCTCGCGTTCATTCCCTTAGCTTGTGAATATCTC
CACATTCTTTGGAGATTCTGGCCTTCACTGCACTAAAGACACCCACTCGCAAACTGCGAAGCACTTGGTTTGT
TGGTTTGCCCCACAGGCCGCAGTGTCCTATCAACGTAACATTCGATTAAACGTCTAGGGTCACCAGGCAACCC
TGCAACGAAACAGGACAACCCCTAGGGATAGGGACGACATCGATATCAATCACAGTTGGGAGTATATCACTCC
ATATAGGGGCTCATTGCCAGTAACAGCATCGGAACTCGAACTACAGAGATTAGGCGACGAAGCGACCGTCACA
CGCAATAGGTATCACAGGACGTCGAAAGGATCGACAAAAAAGGAGTGGATTAACCTCCACAGACAGCATCAAA
CATTTGAGAAGCTCCACAACGAAAGATTAGCCGACATCAGGAGAAACATTCGTCGGGCGAAGGAACAAGAAGA
AGTCACACGCATCATTCAGGACAGTCGTCGTCCTCGAGAAGAGAAGGGAGTAGCCACAGATCGCATCACACTT
CCCACCCCACAACCCACAGTTCACGGGTCGCGCCCCCCGTCAGAGAAGAGTCAATCACGGCAGAACCAGAAGA
CGAATACGCCGCATATCACCAATCCCACCCCACACCAACAACTAGCCAATACACCTCCGTCCCCCGTACCCCA
GTCACTACCCCCTCACACTCCCACAGCCTCGCATCAGGGTTCCAACCGTCTCAGGAAGCGCCGTCCTCACCAC

CATACACCTTTGGTTCAGAGTGAACCAGTACCGTTCAGCCAGTCGGACCCATCCAACCGGAGCGTACAGCTTC
CTTCACTGAATATCAAGACCCCACTCCCCCTATCCCCTCAGACGACGAGGACGAACCGTACGGAACAATGGCT
ACAGAGACAACGACCCCTAAGCGCAACGACAATGGAATCGCAGACCCCGGCATGTTCGACAGGGATTGCAACC
TTTTTGACAGTTGGTGGCGTCGCATGCAACTTTACGTCAGAGGGAACGCTGTCCTGGACACGGATGAAAAGAA
GATTTCGGCCGCGCTCTCTAGATGCTACAAGAGGGCCGAGAACTGGGCGGCAATGAAGACAGATGCTGCAATG
AAAGCAAACAATTTCGGCACATGGAAGGAGTTTGTACCCGAAATCACTAAACAATTCTTCCACGGTCAACGAC
AAATACTGGCAGAAAATCGGATCAGGGTGATGACACAAGGATCTCAATCAGTTGAAGAATGGATTGCAGACTT
CGACGCACAAAAACACATTGGTCAAGTTTCAGACGAACAGGCCATCTTCCTAATTCAGCGAAACACCAAGAAC
TCAGTCATCGATAAATGCCGTATCCAGAATGGAGGCACAGCCCCATCATCTTATGACGACTGGAAGAAAGCGA
TCCTCTTATACGGCAACATTGAGGAAGAACTCAACCTCATTCGGAAAGGAGTACAACGCGCATCCACATCAGG
AGACCGATACGATGGTACAGGTGTCACCTATGGTGGCAAAGGACGCCCCATGGACATTGGACAGTTAGAAGAT
ACCAACATTAATGCGGCCAACTGGAACGCAGCAGGAAGACCCAAATGTTTCAATTGCAACAACTACGGTCATT
TTGCGCGCGAATGCCGAAAGAAAAAGACAAACATCAAAATCAAGATCAGGACAGGAATGAAATGCTGGAAATG
CAACAAGGAGGGACACATGGCAAAAGAATGCCGGAGCTCAGGGATGGTAATCAAGGGCAAATTCAGGGTCCTT
GATGATGATGAAAGCGACGATGAAGGTAAAACGGTTAAGGGAGATGACGATGAAAAGAAGGGTTTTCAGGAAG
GGTCAGACTGAGCACGAAGTCTCGACCCAAGGGTTCGTCGAATCATTCTACATCTGTACCACCACTGAAGAAT
AACAAATACATGATTCCCCAACGACGAACTAGTGCACCTGAGCTTGCAACTCTGCATCTCGCCACTCTCAAAA
CTAGAAAGGACAAAACAATGGTCACGGTCAAGGTTATAACCACGGACAAACGAAAAGCTAAACTCGACGCACT
CTTGGACAGCGGATGCACCCACCTGGCAGTCAATCGACGGCTGGTGGAAGCAGAAAAACTCCCCACGGTTGAT
TTACCTACTCCTATTCGGGCATTTAATGCGGATGGTTCTCTCAATGCGGATAAACTAATCACCAAAAGCGTCA
GACTCCAGATGCAAATTGGCGATCACAAAGAAGAAGCAACAGCACTGGTAGCGGACTTAGGCAAGAAGGACCT
CTTCATCGGACATGAATGGCTGCACAAGCACAACCCCTTCATCAACTGGAAGACTGGCAAGATCAAATTCCCA
GACTGCCCCCCAGAATGCGCCAAAGTAAGCGTCAGTACGCTACCCAAGGCTAAAGTTCGGTCAACAGACATGG
TCATGGATCTAAAACTCCCGGAGTACCTCATGACTTGGAAGGATCGATTCGAGAAGAAAACCTTTGACACACT
TCCACCGTACAGGAAACATGACCACGAAATCAACCTGAAACCTGATGCACCAGACGAATTACCAGCCAAACTA
TACCCGATGACGGCAGCAGAAATGGAAGAAATGGACAAATTCCTAGAGGAGAACTTGGCTTCGGAACGAATAG
TACCGTCCAAAAGCCCATACGCTGATATGCAAAGAGGCTTGTGGCCAAAACAAACCACAAAAACCAACTAAGT
AAACAACACCTAGATAAGATGTTGAGTGAGCGAGTGTTTTAGATTAACAACAAACAAAGCCTCCTAAGGACTC
TAGAAGAGTTCTCAAAGACTGCGAGGGGACCCGCTTTGGATGCGACCTCGACGTGTACGACGCGCGAGAGGGG
AGATGAACGAATGGAATGGACGCGAGGGCGTCCGGGAATTGGGAACTTAGTGTAGAATGAGTGCGACGCGAGT
CGCGCTCTCAAATCTTCGGGCCCGGTGCGACGCGAGACGGCGTACACCAATACGCAAGACTTCTTTGACTGCG
ATCGGGCGACTTGGTCACAGACCGACTACGTGAATGCGAATGAACGTGGAGGAGTGCGACGTGCGACTCCTGT
AGGGAACGTGGATAGTCTACTCTGAGACTGCGAAACTGAGCTATGAACTCTGAGGGGGAAACTCTGCTAGATT
CTCTCTGTGGAAAGCGAAGTATGTACATGGCCTATATACTGTGGGCGAGTCGCGGCGTATGTAAGGGTCTATG
TACGATGCGATGGACCGCCGATCATGTCCTGAGTCCGGTAATCTAAAGTGAAATAAGAGAATATGCAACAAAC
CCGGCGCAATCGCGCGTCCGCGTCTTGCTCGGGTCACCTAATGGGCACGTTGTCGAGGTGTCCGGGCCTCATG
GAGGCTCTCACGGTGATGTTGGTACGTGTCGCAGTCGGTGTGGGTCGAGGCGCATTAGGGTCTCTTGTTAGGA
AGTACTGAGTGCGCGCGGAGCCGTGTTGAGGCGCGATCCCGTATGTGCTGAGCTGTGAGAGGGTCCTATACGA
GTGCGACGGCCTCGCATCGTATATATATGGGGTGCGGCGAGCTGATCCTGTCCGTTATCATGGTGTTGATGCG
GTCCGAGGCCGTTGTGTGGGCTTGTGGGTGCGCGCGTCGAGCCTGGCGAGTCGTGCTGATCTTATCTTGGTGC
GAGATGTGGCCTACGAACCCATAAGCGCGCGGCGCGCTTATCAACGCGTCACCTTGCTTCTTCATTAACAAGA
AAGATGGATCTAAACGGTTGGTACAAGACTATCGAGAGCTGAACAAATGGACAATCAAGGATAAATTCCCCTT
ACCTCTAATCAACGACATCATCGATAAACTGAAAGATGCAAATTTTTTCACCAAGTTGGATCTCATATGGGGC
TACAACAACCTGCGCATTGCACTCTACCACGAATGGAAGGCAGCTTTCCTAACACCACGCGGATTATTCGAAC
CACGGGTCATGTTCTTCGGACTCTGTAATTCCCCAAGCTCATTCTGCCGATGGATGGCCGAGATATTTAAAGA
TCTCATATGACAAAACAAGATGATCATCTATATCGATGACATTATCATCTATGCCAAAGACAAAGATCAGCTT
AAGAACGTAGCCTCGAGGTCATGGAAATCGCACGCGCCAATGGGATCTCGTTCAAGGCCAGTAAATGCTTATT
TGAAGTCCCTGAGGTACCATTTCTAGGTGTTATTGTTGGCAATGGGAAGGTCCGCATGGAGCAGGAAAAGATC
GAAGTCATCCAGACTTGGAAACGCCCAGAAAACAAGAAAGGTGTTCAGAAATTCCTAGGCTTCGCAAATTTTA
CCGCCGCTTCATCAAGGATTATGGAAAATGGCCGAACCCCTCACACGCCTCACAGGGTCTAGCGAATGGTCTT
GGACACCAGAACAAGAAGTCGCTTTTGAAAGTCTGAAATCGGCGATATGCTCAGAACCAATACTGGCACTCCC
CAGATCCAAGGGACGTTTCAGGGTCGAAACAGATGCGTCAGGATATGCTATAGGAGGTGTCCTCTCTCAAGAA
CAGGATGGTAAATGGCACCCTATTGCCTTCATGTCCAAAGTAATGAACCCAACACAACGGCGATACGAGATCT
ATGATAAGGAACTCCTGTCCATCATGGAAGCACTGCGCATTTGGCGACAATACTGGTGGACGCACAGGAACCG
TTCGAGCTATTGACAGATCACAAAACCTGACATATTTAGGAAAGCTCAGCGGTTAAACAGTCGACAAATGCGG
TGGTATTTGAAACTACAGGACTACGATTTCGAAATGCGACACATACCGGGCAAAACAAACACAAAGGCGGACA
TCCTCTCACGACTGGAGTGGTACAAAGAAGACACTGAAGAAGAAGAGGTCATGATGTTCAAGAATCATGGGTT
TGGTATTCCTTCAGAAATGGAGGAAGACGATGAAATTTCGGACTTTGGCATACGGTTCTTCGGAGAAGAACTA

CCTGAAGAGGAGGTGATTCGGGACACATCGGAAATTTCCATCTCAGCTCTCACCGAATTCGGCAATGAAGATC
TGGAACAACGCATCAGAAACCACCCACGCGTAGAATCAGAAGTCCGATTCGGCATGAAGGAAAGTCCACACTA
CTTCAAAGAAGCTGATGGCATCATCACAGTTGGAAACAAGATCTATATTCCCCCAGACAAACAACTACGCGAA
GAACTGATCACAGAACACCATGACTCGCCACTAGCAGGCCACCCAGGAATCCACAAGACGAATGAACTCATTG
GACGCACCCTATGGTGGCCAGGAATGCGATCAGACATCAGGGACTATGTCATGGGATGCGAAACATGTCAAAA
GAACAAGATCAATCGAGAAGCACGTCATGCGCCACTCTACCCCAACCCAATACCCACGCACCCCTGGGAAGAG
ATCTCAATTGACATGATCGGCCCCTTACCACCGTCTAAAGGCTACGATGCTATTTTGGTCATTGTTGACAGGC
AATCGAAGATGATCAGGGTCGAACCGACACACACCACCCTGACGTCAAGGGACTTGCTGAAATCTATCGAGAC
ACCATCTGGAGACAACATGGAATACCCAAGAAGATCATTAGTGATCGTGGACCACAGTTCGCATCAAACTTCA
TGAAGGATCTATGCACCCTTTTAGGATCAAACGGAACTTATCAACGGCTTATCACCCGCAGACAGATGGACAA
ACAGAACGTATCAACCAAGATGTCGAACAATTCCTTCGGATCTTTTGCAATTACGATCAGGACGATTGGGCCT
CATGGCTATCAATTCTAGAATTCCAGTACAATGATAAGATCCACTCTAGTACCGGACAAACTCCGTTCTTCCT
GAACTATGGCCGACACCCATGGAAAGGTGACATCTCCACGGAACCGGTAAAGAATGAAGCCGCTCAAGAATTT
GCATCATGCATTGAAGAAGTCCGGAAGAAAGCTATCACCGCCTTAGAAGCATCAGCAGAACGAATGAAGAAAC
AATATGACCGCCACAAGAAGGACTCGCTTAAACTCAAACCCGGTAACAAGGTGTTTTTAGAAGCTAAAAATAT
CACAACCACCAGACCATCCAAAAAGTTATCAGAACGACGACTAGGCCCTTTGAAGTCATTGAAAAAGTAGGCC
ATAGCGCCTATAAACTTAAACTCCCCACCAACTGGACGCTCATTCATCCGGTATTCAACGAGAATCTATTGAC
ACCACTCAAGACGGCACGCTTCGAATCCCAACAGAAGCCATCACCACCGCCACCCGATGTCATCGAAGGTTCA
GAAGAATACGAAGTAGAAAGCATCCGAAAGGCCAAGAAGAAGAATGGAAGACTTCTATTCTTTGTCCATTGGA
AAGGATACCCGGAAGAAGAAAACACCTGGGAAACCCGCGCAAACCTCAAACACTCCGCGGACCTGATTAAAGA
CTTCTACCACCATCACCCAATGCACCACGATAAATACAACACTCATTTCAATCATCCTACATTACCCCACATC
TGTCTAATCCCCTCCCCACTCAACACCAAGCACCATGCCCGCAGTCATCACCGACATCATCAACCTCATCCTC
CTCTCCCTCTCCCCAGTCTTCACCATGCTACGCCTTATACCCAATTACTTGTCCAACATTCGACGACACATCC
GCGATCTCGACCAACTCACACAGCTCTTCCCTACCGGCGCCCCCACCCTGATCGTCCGACTAAGCGAAGCCCG
GGAACACCTCATTGCCGCCCGTCAATCATTCCTCATTTTAGAAACCCAAATGCATGACCACATCACCACTACG
GTGCAAGGGCTTCTTCCAGATCTGACAGATGACGACCGACGGGCTCTGGAACGAGCAGTACATGTCAACGAAT
GGAACGTGCCCATCGCAGAAGATGCCACCTATGAGGAGTACAGAATCCCAGTTACTACGTCGTCCTCCTCAAG
TGATTCCGAATCTCATCCCCCCCTCCTAGCATTCCCCAGCCGACGAACAACGAGCCCATCGGCTCCTGGCCCC
CTTCCTCACTACCCACGGGATCCATACCCCTTGGAGTACTCCCTCTCCATCGAGTGTCCCACATCCGCATCCC
CCTCAACTTCGAGCCCACGGAACCCTACAACGGGACATACTCATACAGCGGCAAGACGTTCCGGTATTGGGTG
AAAGGACCGCACGACTCGCCCACCTTCTTTACATGCATTGGCGAGGAGTACGTGACAGCGAGGACGAAGAAGG
AACTCGGGAAGATGTTGGTGAACAATGATCTGATCTAAAAGAAGTTTCGGGACGAAACTCCTAGAGGGAAGGT
CATGTTACGATTCCTTCCTTCTCTGATCATCACCACTCGGACATCTGACTGATAGCATCATTCATAGACACTT
CTATTACTTCCACCATCATCCCATCATCATCTGATATGCGCCATATGCTACATCATCAGATGATACATCATGC
GCACCTTATCTACTTCTATACCGCATGGTCTCCGTTCTATCTTTGCTATCTTCGTTGTTTCTTACTCAGCTGC
TCAGTCAGCTCGCTTCTCGTATCAGTCATCATCCCCCATATGTCTTACTATACTCACGAACTTAGTCACTAGT
GCGTAGTCATAGAACTACCTCACTTGTATATAAACGTAGCTACATACTCCTGTATTCGCTACGTTTAATCGTT
TATCTCTTTCCCCCTCTACTGCCTCCTGACCCAGGAGACTTCAGACCTTGCTAAGTGTCCTTGAGTCTTTACT
GTCGACTCATCGACTTACGTTAGCAGGACGTAACA
>Cligib1_Cluster29#LTR/Gypsy
GTTACAGCGCTCCTAGGGGGCTTACTCTTTTTCTTACATCATACACGGATGCTTCTCTCTTCGACACATGTCA
AACATGTGTTTGCCTCATGGACTCCTCATGGGCATCTTCTGACCTACTGCGCCTCATGGTTTGCTCATTGTTT
ACAGCATATCTTCCCCTTTAGTATATATTGCTGTAGAATCCCTTAACATGCATTCTCCTCATGGTCACTGTCT
CTTAGTATATATACTCCTTCTGTACATCCTGTATAGCATCTTGACCTAGGCAATCTACCGCCTTTACACTCTT
ACTCAGAACCTGAGCCTAGACCAGCTCAGAGTGACTTGAACTCCTTTGTCATTGGACAGGAGACATCTTGAAA
CTACTCTCAAGATAGAACCAGAACGAATGATCGACTTGTATCAGTTGTAAAGGCTCACGCCCTAACAAAAGGT
CAAGATCTCAACCCCCCCCAACCTCCTTAGGTTCAGGCGCACCACCACCCCACCAGACTCCCCAACAGAAACA
CCACAGTGGCTTCCCAAACCTTCCTGATCAAACCCTTTGGCACCACCGTCGAACAGAAAAGAGGCGAAAACCA
ATTTTGGCTCCTTGGAAAACAGATCGCCTTCCACCCAGACGAAAGACAGTGGTACTTTATTGAAGCCAACGAA
CAAGTACCACACACCACAGACTATTTCCATATTGAAGACGTTGCACCAGAAACTTTCACCAATTCCACTGAAC
AAATCCCTGTTCCCCCAGGCACAGAAACCACTTACATACCAGTAGAAGAAGAACTCGAATATACCGACACCCC
TACATGACTACAGAAACCAACACCCGAAGCTTCCTCTGTGCACCCACCAAGTATGATGGAGAACGCACCAAGA
CTAGCACCTTCATCAATGAAATCCTGCTACACATAGCAGTCAATGACGGACTACTCAACACCGACAAGAAGAA
GGTAGCATTCATGCTATCATATATGGTTGGAGGAACCGCTACTTCCTGGAAGGAAGCATCTATCTCCTCCTTT
ACCAACTCAACCACTGGCTGGCCCACCTTTGACAACTTCCTAGCTGCTTTCAAAATCGCTTTCGCCCAGACAG
ATGTAGCAGGATCCGCCCGAACCACCCTACGCACACTCCGACAGACCGGAACAGTCGACGAGTATATCGCCAA
ATTCCGAGTCCCAGTATCCCTTTGCGAAATCACTGAAGATGCCGCACTTATTGAATATTTTGTGGAAGGATTA
CATCCCGAAGTAATGAAGAAAGTCTACACCATGGAAAACGTACCATCCACCTTGGAGAAGTGGTACTCAGCAG

CCTCCAGAATGGACAACCAATACCGAAGAGCCAGAGCCATTGCCACTCGCTTTTCCAGCAGACCTTCCTCCTC
CCCCCAGAAGAAATTCCAAACCCCCAGATACGTGGCACCCAGAGACCCTGACGCCATGGATGTGGATCGACTC
TCCACCGAACAACATGCTGAACACATGAGAAAGGGAATGTGCTTTGTATGCCACAAGACTGGCCACCGTGCCA
TAGACCACAAGAATGGGAAACTCCCAGAAGAAAAGAAACCCTTTAGGGTTAGAAAGATTGAACCCACCCCACC
AGAATCCTCCAAGGACGGACCTACCACCTTTGCCAAAATCCGCGCCTTGATGAAAGATCTAGCAGAAGAGGAG
AAAGAAAAAGTGTTAGAAAAGATGTATGAAGAGGGTTTTTAAAGAGGCGAACTGCGTCAACGTCAGTCTCGCC
TCCCATCTCAATTTCTCCTGTATTAGTGACCGAAAATGATCGCCAATCTATGCATTTACCTATTTGCATATCG
GGAGAAACAACCGTTGAAACCAAAGCCCTCATCGACTGCGGAGCAGGAGGCCAATTCATTGACTCCTCCTTTG
CCAAAGAAAAACAACTCCCACTTGAAAGACTCTCCTCCCCCATCGCTGTCTATAATGTTGATGGCACACGAAA
CAAAACCGGACAAATTACCCACTACACCTGGATTGACATCCAGCTAGGTGAACAGAAACTCCACACCCGCCTA
TTGGTCACTGGCTTAGGAAAAGAAACCATCATCCTTGGACTCCCCTGGCTCAGGGAAATCAACCCCATCATCG
ACTGGGTGAAAGGAACTCTTGAAATCAATTCAGCAAACCTTGCTATCAAGAGTACTCAGATCTTCAAGAAAAT
CCGAAACAAACTCCGAACCAGGAAACACACATCTCCAAACCTATCTCTACACCTTCTTTTCCCAAACCCACCC
TCGAAGACATCACCGATGAAGAAGAAGAAACCCCTACCAACGAGATAAAGACTGAGACTCCGGACACCATCTA
CTGCTTACCTGGCTACACCCCATCGGACACCCTCGAACACCCGCCCATCCTCGAACCAGATGAACTAATCTTG
GCATACATCCAAGGCGAACCTGTCATTGGTATCTTTGAACCTATCCCTGAAGCTCCCCTCACCGTAGAACATG
ACGCACCCTCCTACTTCTACAAAACCATAAATCGAATAGGCATAGTCAACAAGAGTCTACATTCTAAACGATA
CTCTTACGGACAAGACATTCGAATTGGAGCAAAGATCACTTCATCACAGACTTTTGCCCAGAACCATGAACCC
CCTGACAAACTGAAAACCACTGAAGAACTCCTCCCCGAACCCTATAGGAAATACCAACACGTCTTTGAAAAGA
AAGCTGCAGAACGGTTCCCAGAATCCAGAATTTACGATCATGCAATAGATCTAAAACCTGACTTTATCCCAAA
AGACTGCAAAGTATACCCTCTCACACCTGGAGAACAGATCAAACTGGACGAATTCCTCAAAGAAAACCTGGAT
AAAGGATACATCACTCCTTCAAAATCACCTATGGCATCTCCATTCTTCTTTGTTGGCAAAAAGGATGGTTCTC
TAAGACCTTGCCAAGACTACTGGTATCTCAATGAAGGAACAATCAAGAACGCCTACCCCCTTCCTCTCATCCA
AGAACTTGTGGATAAACTCAAAGGGGCTACACTGTTTACCAAACTGGATCTCCGACAGGGATACAATAACATA
CGGATGAAGAATGGACACCAGTGGAAGGCAGCTTTCAAAACCAACCGCGGACTCTTCGAACCACAAGTAATGT
TCTTCGGAATGTGCAATTCACCAGCTACATTCCAGATGATGATGAACGAACTCTTCCGAGACATGCTTGACAA
AGGATGGATTGTCATATATATGGATGACATACTAATCTATTCCGATAATTTAGAAGAACATAGAAAACGAACC
CTCCGAGTCCTACAAAGACTCCAAGAAAACGACCTATTCCTGAAACTAGAAAAAATGCGCATTCGAACAAGAAG
AGATCGAATTCCTGGGCATGATCATCCGACCCAACCATATTGCCATGGATCCTGCAAAATTGAAAGGAATCAA
AGATTGGCCCATCCCAACCAATGTTAAAGGTGTCCGATCCTTCCTAGGATTTGGAAACTTCTATCGAAGATTC
ATTGGCAAATATTCCGACATCGCCAAACCACTCAATAACCTGACCAAGAAGGATCTAAAATGGGACTGGACAG
AAGAATGCCAAACAGCATTCGACACCTTAAAAGCCAAATTCGCAGAAGCACCCGTACTCCTCATGCCCGACGC
TACAAAACCATTTGTAATAGAATCTGATGCCTCCAAATTCGCCACAGGAGCAGTCCTCAGGCAAAGGGATGTT
AATGGAGACTGGCATCCCTGTGCCTATCTCTCCCAATCCTTCGACGCAACCCAACGCAACTACGAAATCTATG
ATCGAGAACTACTAGGGATTGTACGAGCCCTGGAAACCTGGAGACATTACATCCAAGGCTCCCCTCACCCCAT
TGTCATATTCTCTGACCACAAAAATCTCACATACTTCAGGACAGCTCAGAAACTAAATCGCCGACAAGCCAGC
ATCAGGAAAAAAAAATCTCTCTACATAAAGCTACATAGCCGGGATAGCTGTCATAGCTCCATTGCGACCCCCC
CCTTTATATAGCATTACATAGCGGCCCCACCTTATTATGTAGTGTTATATAGCAGTATATAACTCTCATAGCT
CCCTGCCAGACCTATATATTATTAGGGGGCTGCTATATGGCCCACATAGCTCACATAGCTCACATAGCCCACA
TGGCCCATATAGCACTATATATCCAGTAACGAAACTACACTTATGGTTGAGCATAAAGCTTGCTGCTCAACGT
CGTACAGTTGTTATTTGGTGATAAATCTGATGATCATCGCAGTAAGTGGTTCTGGTACGGATGATTATAAGGT
AGAGGAAATATACCTTGGCACGGCAACTGGGGTGCATTAGGCTAAGAGAGGTGGATGCATCACACATCAAATC
ACTGCGGAACATATCATATAAAATTGCAGGTTGGCGCAACTGAGGGAAGCCTGCAAGCGATCAGGCGAACTCA
GATGGACCACATGTAAGTTACTCACATATGCAGGCACCGAGTTTGAATGCGGGGCTCCAGCTCAGTAATGGAG
GCGCTACGTGGGTGAGGCGTCGAGTGCGGGCCTGCTTCAAGTGCCCTAGGGGCTGGGCTCAGAGTTAATTAAT
CCATAATGATTTGCGTGTATAGCGACGTATATGAAGGATATTATGGCGTGGCATTGGCACGGGTGAAGGACGA
GGTGGTGGGGCAGAGTGAAGACACCAGTTGGGGATGCGCAAAGATTTTCACAAGTCAATTCTAACTCCGACTA
AAAATCGGACCCACGCACGGACCCAGCGCTCCAGATTCTCCACCGATGATATCACAACGTCGTCACCCACCCG
CCGTGAATGGTCTGCCCATCTGAGTCCTCACCCACCCGAGGTAAGCAGCTGAGCCACCCTGTTTTGTGCGACA
CTCACTCATTTTGTATTTCCTCCGCTTTGAATGCTCTAAACATCGAATGTCCCGCTCAAGCCATCGCTCCAGA
TTCGTTGAGATCAACGCTCGATCCTGCTCAGAACGGTGAGCGGTGAACGACAAACAGCCCCAGTGCGTCCGTG
GGTTCTCGTGCTTTCGAGCGCGATTGATAGGCCTCTCAAGGTGCGTATTCCTCTTTTTCGTCATGCAAACCTG
TTCTAATTTGGTTGTTTAGCATAGTACGAACGATAATTGCCGGGTCAAAGCCGACGCCACCGCTGCACGACCG
CAACCACAACCATCTCTCCCGTAACCCCAGGCATGCGCCGTTAGCGACGATCACGAATGATGCACAAGCGAGC
CAACAATGGATACACGCTATTTGTGCTGGCCGGTGGGTGCTCGCCATCGACGACGGCTGCGAATGACGCGCAG
GCGAGCCAACGAAGGGCGTGCAATATTCGTGCCAGCCACCCCTCCCTTGCAAACGAGGGAGTGTATCATAGAC
CTATTAAGGTAAGTCCGTACTCGTACGTGTATTAACTAGGTTACCTCGTCCACCCCCTGCACCCTCGTGGCTT
CGCTCGCCGCATCACTCGACGCACGTGCAGCATGGTCGCGAGGCAAATTGGGGTGTATGGCGAGGGACCCGCG

GTTAATTCACGGTCGTCGTTGAGCGAGGCCATGCAGATCTCCAATCTGACAGTGAGCTGCAGCCCAAGCTGAG
CCCCGGCCAGCCTGAAGCCAGCTGTGTGCCATTGCCTACATGTGCGAGCCAATACCGATATATGCAGCATTGA
AATTGAACAGTATGGCACGGGCGCGGTGGAGGCAAATGGGGAGATGGTTGAACGTGGGTGCGGCCCATCTATA
ATTTGCTACGCTCATTTAAATATATTACTATTCTACACTCCTAAGGCATCTTTTAAAACAAGTCCGACAAAAT
TTATATAATTTTACCGAGGCTAGTAGGATGATATCAATGTTGTAGCTCCATAGCCTCCATAGCCCTACATAGA
CACTGGTGGAGGCTATATAGCTATATATGGCAGACGGCTATTGCTATATAGCTATCAATGCTATATATAGCAG
ATGGCTATTGCTACATTACCCTCTATAGCATTAGCATGAGGCGATAGAGTACTATAAAAGGTATTATAGATCA
AGTATCTAGTTATATCACGTAATTTGGGCTATGTATCGCTCCATACTCAACCGGCCTGCTATGGCCGAATTTG
GTTTTTCCTGATGAGATGGAGTCTCTTCCTATCTGAATTTGACATGCAACTGATCCATGTCCCTGGAACCCAG
ATGATACAATCTGATGCCCTCTCTCGACGAGAAGACCTAAACCCCACCGAAGATAACGACAACGAAGACATCA
CCCTTCTTCCTAATTCCCTTTTTGTGAAAACCATCGACACGGATCTTAGAGATCAGATACTCTCCTCCACTTT
CAAGGATGAAATCATCACACAAGCCCTTGAAGCGATCCAAAAAGGAGGTACTCCCCCCATGAAATCCGCTCTT
TCCGACTGGAGAACCGACGAAGGACTTATATTTTATAAAGACAGATGCTACGTACCTCCAGACGAAGAACTCC
GCAGAAATGTGGTAAAGAAATATCATGACCTCCCCTCCATGGGACACCCAGGACGGTTCAAAACACTTGAACT
AATCAAACGAGACTACTGGTGGCCCAGACTATACACCTTTGTGAAGAAATTTGTAGAAGGATGTGCTGTCTGT
CAACAAACCAAGGTTAACACTCATCCAACCACCCCACCCATAATGCCTATCAAAGGACCCTCCAACAACCGAC
CCTTCTCCCAAATCTCTATGGATTTCGTTACCGACCTACCCAAATCTGACGGATTTGATTCTTTATTGGTCGT
GGTAGACCAAGGGCTTACAAAGGGGGTAATTCTCATCCCTTGTAACAAAAACATCGACTCTTTAGGAACTTCC
AACCTCATCCTCCAAAATGTATACAAGCGATTCGGACTCCCAGACAAGATCATCTCTGACAGGGGTCCCCAAT
TTGCATCTGCAGTCACCAGAGAACTTGGAAGACTCCTTGGCATCAAACTCGCCTTCAGTACTGCCTATCACCC
TCAAACCGATGGAGAAACAGAACGAGTTAACCAAGAAGTCGAAGCATACCTTCGGATCTTCTGTGCAAACCAC
CCTGAAACATGGGTATCCATGCTCCCAACCGCAGAATTCACACACAACCAACACATTCACTCCGCATGAAATG
CATCCCCATTTTACCTAATGATGGGATATGACCCTAAAGCCATCCCTCTCCCCTTCCAGAAATCTGATATACC
CGCAGTAGAAGAACGTTTCAAGAACCTCCAGAAGACTAGGGATGAAGCCTCCGCAGCACATGAACTGGCACGC
CAACACATGGCCGAAAGAACAACATGAGGCTTTACCCCTTTCAAGAAAGGAGACCAAGTATGGCTTGAATCCA
CAAACCTGAAACTCGGATATGAAACCCGGAAGTTTGCACCACGCCGAGAAGGCCCCTTCCCCATTACCGAAGT
CTTGGGACCTGTCACCTACCGTCTAGAACTCCCAGAACGATGGAAGATACACCCTGTGTTCCATGCATGCCTC
CTAACTCCCTACAAAGAAACCGAAGCTCATGGCCCCAACTACCTCCGCGACCCCCCTGACTTGATCAATGGAG
AAGAAGAATACGAAATAGAAGCCATCCTGAAACACAAAGGAAAAGACCGTGGCACCAAGTACCTTGTCAAATG
GAAGGGTTACCCTTCCGCAGACAACATGTGGCTGCCAACCCGCGACTTTGGTAATGCATCCGACATCCTCAAA
GCTTATAAAAAGCGACACTCTTTAGACTAAAATCCTCAGTACTCAACCGATTACTACCCCAGCCTCAGAAACA
GAATCAACATGTCCTCCCCTGCCTCCACCCAGTATACCTCCGCTTCCTCTTCCCCTGCACCATCCCCAGTCAC
TGCCCCTTCCCTCCTTTACCCTCCTTCTGAACCATCCTCCGTTCCCCAGTCTCCTACCCCCCATTCCGGATCT
GAGGAAGACCTCATCCAGTTCATCAATGCCCATCTGGGCACACCTCCTCAATATGAACGACCTCGTTCGGCCG
ACGAACTACTCTCCCAAGAGGAACTTATCCAAATTCTCACTCAAACCACCGACCTCGCCACCATGTATCACAG
TTGCCACGACTACCCAATTCTCCAAGATCTTTTACTCACGTTCTATGAAAATCACAACACAGCAGTCCCCATT
CGAAACATCCATAATGAACTGCATGAAATAGCCTCCTCGGTCAACCGCCTCACCGAACAACAAGAAGCCCTGG
CTGGTAGCCTAAGCTACCTACTCCTGAAACATGGAATTGAACAAGTCCTGAACCCCCGCTCCCCCAACATCTC
CCCTACTCCCATCTCCAGACAAAACCTCACCCAACTCTCCTCCCCCACCCCAAACATTGCCATCCATGTCACC
CAGACCACCAGAACGACCGCAGAAGCCATGACACAGACACCTCCATCTCAGACCGCATATCGAAGAACCCCCT
TTCCCCAACCCACTATCAGACTACCCTCCTCCAGACCCCCCTCCGAAACCACTTCTCCCTCCACTCCACCTGC
CCCGGATACCCCCCTAATCCGCCCACAAAGAATCATCAGAAGAGTCCCCGTCCCTCAAGAAGTTGTAGAATTC
CCTCCCAGACGCAACTCTGCACCCACCGCTAACCGCCGAAACCGATGATCGCGTGGACATACCATCACTCTCA
ACCCCCTTAGTCATGCTGGAAGAAGACCCCTAGAAGAAAGAATCTCCTCCCCTGAACCCACTCCCATACCGCT
TCGAACTCGTACCCCCACCGGACCCAAGGTAGCCTCCTCCCCTCCCAACCCCAAACTATACTGGGAATGTAAA
TTACCTGGGCATATCATAAAGAACTGCGAGCATTACTTCTGTCACACTGTGGAAATCACGCACCAGGACACCT
CCAAGATAGATGTCGCTACAGATCCTCCACTTCCCATGCTAGCTACCACCCTATACCGATGGGGATCTCGATT
TTGACAATACCGCCATGTCTAACATGTGCGACGAACCATACCACCAAGATTGAAGTCAGGGACTTCAAACGAT
AAACAGGGGGTAATGTTACAGCGCTCCTAGGGGGCTTACTCTTTTTCTTACATCATACACGGATGCTTCTCTC
TTCGACACATGTCAACATGTGTTTGCCTCATGGACTCCTCATGGGCATCTTCTGACCTACTGCGCCTCATGGT
TTGCTCATTGTTTACAGCATATCTTCCCCTTTAGTATATATTGCTGTAGAATCCCTTAACATGCATTCTCCTC
ATGGTCACTGTCTCTTAGTATATATACTCCTTCTGTACATCCTGTAATAGCATCTTGACCTAGGCAATCTACC
GCCTTTACACTCTTACTCAGAACCTGAGCCTAGACCAGCTCAGAGTGACTTGAACTCCTTTGTCATTGGACAG
GAGACATCTTGAAACTACTCTCAAGATAGAACCAGAACGAACGATCGACTTGTATCAGTTGTAAAGGCTCACG
CCCTAACAC
>Cligib1_rnd-1_family-314#LTR/Copia
CCANATTTGGTATCCGGCGGCGTCACGGGCGTTACCTTCAACACTCCCCTAACCCCGTGGCGTCGCCGCCTAC
GATGTCCAGGGTGCGGCAGATGTGTTGGTGNGCAACGCGTGGNAGNGGCTTNGTTAGGACGTCGGCGAGGTTG

TCCGCCGATGGGACGTATCGACGGAGATGTCGCCNTCCTCGACGCGTTCGCGAATGTAGTGATAGCGGACGTC
GATGTGCTTCGCGCGCGCGTGNCCTTTNGTGTTGAGNGTGAGNGCGATGGACGCCGCGTTGTCGGCGTTGAGC
CTNGCCGGCGACTCCTGCGGCATGCCGATCTCGCTCATGAANGAGTACATCCANAGCGCTTGTTGGGATGCCC
GCGTCAGACCCATGTATTCNGCCTCNGTCGTGGATAACGCGACTGTTGGCTGGCGCTTCGANGCCCANGAAAC
CGGNCCNCCGCACATNGTGAAGACGTANCCCGAAGTCGAGCGCCTGGTGTCCGGATCCCCGGCGTAGTCNGCG
TCGACGTACCCGACGGGTCGCATTCCGGTAGCGGTGCCCGGCGTGTAGGTGATTTTGTAGTCCAAGGTNCCCT
TGATATACGCGAGGACGTGGAGGGCCGCCTTCCAGTGNGCTGGGCCAGGGTTAGTCTGGTGACGCGAGAGCAG
GTTGACCGCGTAGGACAAGTCCGGCCGGGTCGCCACCTGNGCCCACATNATGCANCCCAGCGCCTCGCGGTAG
GGCTTGTCCGCCATGAANCGNGCGTCTTCNTCGGACGTCGCGCGGTCNGCTTCTGTGAGCGTCAAGCCNACTG
GGAGCGGTGTGGACTTTGGGTTGCACGTCGACATCTTGAAGCGCTCCAGGACGCGCTCCAGGTACGCGCGCTG
TGANAGGGAGATGGCGCCGGTGGNCTCGTCGCGGTCAATGCGAATACCNAGGATGAANTTGAGCTCCCCCAGG
TCCTTGACCTCGTAGCACTTGGAGAGCTCTTGCTTGGCGAGTGCAGCGCCGGCGTCGGTGGANGAGGCTCCGA
AGACGTCGTCGGTGTACGTGCTGGTGATGGTGAGCTCGTCGCCGANCTTCCGNGAGCGGACGCACGAATCCGC
GCGCGACTGGTAGTANCCCAGNCCGTCGTAGCTGCGGCTCAGCTCCTCGGCCCANGCGTTCGCGCCGTTCATC
ATNCCGTAGACCGTTTTATCAGCAACGTANACCTTGTCCTCCTCCCCTTGCTCGATAAAGCCGGGCGCCTGCT
CCGTGTAGACGGTGTACTTGTTCTCGCTGTTGAGGTANGCGGCGATGAAGTCCACCTGCCAGATCTTNAGCCC
GAGCTCGGCNGCGACNGCCGCGACCATGCGCATNGACTCCAAGCGGACNACGGANGCGTATGTCTCGTCGAAG
TCCACGCCGGGGATCTGCGTGNANCCCTTTGCNACCAGGCGGGCCTTCCGCTTNACGATGTTCCCGTCGGCGT
CGTATTTGTNNTCGTANACCCAGCGNAGNCCGACGACGTTGGCNCCCTCGGGGCGCGGTACGAGCCTGAAGAC
CTCCTTCTCCTTCATCACGCGCATCTCNTCCTCCATGGGTCCCCTCCAAAGGTCAGGGCGCCTCATGGCCTCC
TTGAAATTCCGGGGCGCCACGGCATCAAAATCACCAGCGTCGCTGCCAGCGACGAGCGCGTGCGGGGCGTCGG
TAGCCCAGTCCTCAGAATTCCCGGAGGCCGCNGCCACCCGGTCCTCGGACTCCTGNGATTCGCGCAANGCGCG
CGATGGGACGTGGGCGCGNTCGCTCCTGCGCGGNGGAGGCGCTGGTGGAGGTGGCGTNGTTCTGCGCCCGGTA
GGCCTCAACCGGGCGNGCAGGGTGCAGNGGAGGGTCGGTAGGGCGCGGGCGGGCNGCCACCGGTCGTGCATCC
TTGACGACAGCGGTCGGTTCAG
>Cligib1_Cluster55#LTR/Copia
AAAATGTTGATATCTTGCCTCCCAGTGCAGCTAGAGGCTTAATTGTTGAATATTCTAAGTCCAGTATTTCAGT
CGCGATATAGTGGACACCTGAGTTCACGCCTCGGGTGAAACAGGCCAAACATGTACAAAGGAAGTGTAATTCC
TACTATGTCCCGGAGTAGTGTTTGCTACTCCCTTGACAACAGTATTAGACAATCATATACTTGCATTCTATTA
TCAATATCAAGTGATTAGAGCGTCTATCAGAGGGTTATAAACTGTGTCTGGCTATACTCTATAGCAGATCAGT
CAGTGAACACAACTTGTCAGTTTATTATCTTCTTGGTATGCAACTCCCCTCTATCGGACCGGATCCACCCAAC
ACGTTCACCAGACTCATTCTCCGCACGGCGTGCCCAGGAAATGAATGCATCAGCAGCATCAGATTTCAATTTC
AAGGGGATAGACCAAGGATAACTAGAGAAGTCGTCGAGAATATTCATAATGTACCGATTGCCTGACGCAGAAA
CCACCGCCTCGGGTCCCGTGAGATCCACCCACACTATACCCAATCGCCGATCACTCCACGGGCCCGACCGCAC
CTTAGGGACAGGGGACTTAGACTGTTTCCCAAGGATGCAATGGTCACAAACGGGCGGAGCGACGGAGAGGTCG
ATGGGCATACCTGTGGCAAGGTTCTTTTTGGATAAATCCACGACAGATTGGTTGTTGGCGTGACCCAAACGGA
AATGCCAGGTGTCGATTGAGGGCTTGGGGGAGGCAAGGAAGGCGTGGTCGATGGAGAGGCTACCAGAGAGGCG
GTAGAGGTTGCGAGAGGGCATACGGGTACCAGAGGCGATGACAAGGCCAGACGATTTTGCAATGGAGACGGAA
TCAGAAGTGAAGGTAACGGAACAATCAAACGCGTCGGTAAGGCAAGCAATGGAGATAAGGCGCACAGTTGAGG
TGGGGATGTAAAGAACATTATCGAGGGTGAGAGAACCACCTCCGGAGACCCGAACACGAACAGAACCAATACC
GACTGTGGAGATGGAAGATCCGCCAACACCCTTGACATCACGACCCGAGATGGGGGAAAGATGGAAGAAGTCA
CTGCGATCAGGGGATATATGCGTTGAACAGGCGCTATCAAGAAGGAAAGGGGCGGAGTCCATCGGGACAGCCA
GTGCCGGACCAGTGAGAGAGTGGAGGGCGACGGGGCACGAGTAAGAATCCCAGTCAACAGAGGCGACGACATC
ATCAACGGCGCAGGCATACTCGGCGAGGTCATCGGCGGACATGGAAGAGGCAACGAGTGGGGAAATGAGCAAA
GAGATATCATCCATCGCTAAGGCTGAAAAATGGGACGTTGTGGGCTCAGTGGAAGCTGGGGGTACTGTGGGCG
CATGGCCTCGACCCCGTGGAGATAGTAGGCCTGACCGGAGGCCGCATCCACCGCAAAGGCACGACCACTCTGG
TCCCAAAGGACGGAGGCCGGCGTAGCAGCACTAGTCGTCAAGGGCTTCTTCCCTTTCTTTTCTCGGAAGGCAG
CCTGGGCCTCGGTTACCGACTTACCCGCCATGCCACCACCGGGCTTTATACAGAATTCGGCTTCGTGGCGTGG
ACGATTGCAATTGGTACAAGCCAACTTGCGAGCTGAAGGGCCTGATTGGGCGACATGGGCCTCGCCAGTAGGG
GATTCCGAGGCAAGAATCTGCTGCTCAGTGTCAAGGCGAGCAATGATGTGTGCAGATGTGTAAGGGTTTTCTT
TTGTGCTAGAAGTAAGACCATTTGCCACGGCGTCGTGAACCCCTCGCAGTTTGGTGTTACGGAGGCCAAAAAG
CATCATCATAGAGAGGTAGTTGTTGATGGAAGGAGGGCCCATATCCCATATGCGTTGGTTTAGCCTGCGATAT
TTCTCTGTAGTCTCGGAAAACGGCACGGACGGGGAATACTCCAAAGTGAATGCCTCCTTCATGAGGAGCACCT
GCGAGATCGGGCCCTGGTGCTCATGGCGCTGTCGCAGGATAGACCACATCTGGACGGCCGTAGTACAGTCCTT
GATGAATGGGAGTTCCGCAGGCGCGCATTTAGTCCCAATAAATGCACGGATGACGCGATCATTTATGCGGTAG
TTGTTCCAGGCGCGAGGTTCAGTCGTCTTGTTGGGCACAGTAAGCCCCGGGACAAGGTACTCGTCCATGGCGG
AGGACATCGCGAGGATGCGGCATACATGGTCGCTCCAGGTGTGCCAATTGTTTGCGGCACGGTCGAGCAGAGG
AAGGGTACCCCAGGCAGTGTCGCGAACATGTAGTAGAGGGACCGACGGGTGTGCTGCATCAGAAGGAATGGGA

ACCAAAGGAGATGGGGCCGCCGGGGCAGGCGTAGGCGTTGGGGGCCTGGGCGTCGCAGTGCTGGATGCCATAG
ACATATCGGAGTTGGTGGAGGGACTACGTGCAACAGGAGCAGGAGCGGTGCGAGTGGGAGGGCGAGTGGGAGG
GCGAGTCTTATCGCCAGGGGCCATGAAACAACAGGGAGTAAAGGTCGGAAAGACACAGTGAGCCGAAAAACCT
AAGCAAGGAAGCACAGATAGTATACGAGATCTAGGGTCGAGAAAGGCCCATAACCTGTTAGAATCTAGATATG
ATACCTCAGTCGCGTGTAGGTCAACGACTTTATTATTCACTATACCTAGATATGATACCTATATGTATAGTGA
GTATATTGTAATGTATGTAGCGGAATGTTCTTACCTCAGTCGCGTGTAGGTCAACGACTGAGGGAAAGATTGG
GGGGATGGGAAGAATGGTTTATATATGTTTACGTAAAGTTTAGACGGAGCGCGAATCCAACAGATAGGCAACC
AAGAGCCGCCTCTGAGGGAAGACGGATGGGGGACAAGCACCCCCTCCATATATACCACAGGGACCGTACCCTC
AATACGGAATAAACCCAGCAAGGGGCCAACAACCCCAATATGAACAAAACCCGGTATGGGGACCAGAGCCCCA
ATACAAACAACCAACGGCACAACCAACAATGCCACCCCCAAGGACCCGCCCGGAGTCCTTCTACCAAAGACAG
TGGGGTACGCACCGGGTGCGGCACCGATCTACAACTCGGGATGGGCGGGGGCGGTAACTCCAAACGAGTTAAT
GCACACATCGGACAGGATGGAGATCGACAATCTCCCACAACCACCACCCCACACGGAAGCAGAACGAAGGCGA
GACATGCGCACTGTCCAACCAATGTCGCAATACGCAGCACCACCAGGATTACAGCGTGGGCCACAACTAACTC
ACTTAGTTCACAGGAAGGGGAAGCACAAATGGGCTTCCAACACAAACCATGCGCTGAATCACGCAGCTCGCGA
GAGGGAGAGTATGAGGCGAGACCTCAAAGCAGTGGCAAACAGAGAGCCCAGGACAAAAAACCTGGAAACGATG
GAGAACTTAAGTTCCTCCAAGCACAACTCGAATCCCTTGGGATTGATTCAAGCTTTACGGGGTTCCTAGAAGA
CCCCATAGCTCATTACGTAGTATCGAAACTAGTAGACATTGCAACGAGCGAAAGGAGAGAAAAAGAACAATTA
ATAGAGGAGAGAGAGCACCTGGCTCACTCCAAACAAGAAATGGAGAGTCAATTAACGACGGCTCTCACGAAAA
GACAAATCTCACCGCCAATGGGTGAGATCAACGAGCGTCGTGCAAAGAAAAGCCGCGAGGCAACACCCTGAAG
GGAAATACTTCCCTTACCACAATTGAGACCTAGGTCTCAGCCACCACGGCAACACCAGCAACCTACGGCAGGC
CCATCAATGACGAGAGTCACCTCGTCACATTCAGACACCACACCAAGGAGAGAACTACCTCTCCCATATAACG
AGGTGTCAGCACCGCGAGGAATGGGACCCAAGGTCCCAAACACAAGGAGGAATACAGCCACAAACCCTCCAAG
AATTGAAGTCCATTGCACGGTCCCCCACAGCTACCCACGGGGAGAACAAAAATTGATCCAAGATCCAACACCG
GAAGAGGATCAAACAACAGAAGAGAATCCCCTGGAAGAATCACAGCCAGGGGAATCAACGGATGAAGAAACAT
CCGAAGAAGAAGAACTGGAGCGCCTAAGCACTCCAGAAAAGGAGATGCCGAACCAAAAGCGTCTAAGAGATCA
GAAAAACACTATGCGCGAGGCAAAGCGAGCAAGGCTCGCTGAAGAAACAGCTCAGAAACGGGCGAGGCACAGA
AACTCGCGACCAGGAAGAATCCCTGACACCATAGGCGTCGTAAAAAACAATACCAACAGCCAGTATGAACGAA
ACAACTGGCTACGGGGATCATTAGATCCCGAATTCTATTACTCAACGGTAACCAACACCATATATGTCGGGGA
ACAGGCCCAGGTAGCGGCCATCAGCAGGGACTAAAGGTACCCTCACAACATATACCGACAACAGCGGTATACT
ACAAAGCACCCCGGGGGTTCCCAATGAACCCCCAACAAGTATTAGACGTATACAAACTGATACGGGAGAGCCG
AGCATCTCCAAAAGAAAAAGTAGAAGCATTCAGACTTCTACAAGAATTGAGGCGTATCGCATACGCCTTTCTG
CCCAACCAACGGGACCAAGCAATGAAACTAGTGCTTGAAGAACAACCGTTCGAAGAAGTGCGGCACATGATAA
TGCACCGGAAATACCGAGACTGGTACGCAGTATTGTTTATGCCGTTTGGCTCAGACGATGCGTACAAGAAGGA
CAACAATGGCCAGACTAAGGCCGGACTATCTCAACCAATAGATGAACAAGCCTTGAACGTGGATGTATGGGCA
CAATTTATTCTACACCATTGGCGACCAGGAGGCGATAACCCGATCAGCGGAACCGCAATGAACCGCGCCTACC
ATGTCAACCGCCGTAGTATCTTTGGGTACCTACTAGCGAGAGCTTTAGCTCCTCGAGTACCCGGAGGGAGCGG
AACTGTCCCGAAAACACATTTTGTGAGGCAATACACATTACTTGTAGCAAGGCCACAAGCATACCGTGAATTT
ATCATGCAATGGGAACATGAAACCGGGGAGGAATTCATCCCCGTAAAAACCAAACCGAGCAACATAGTGCTCA
AACGCATCCCCGTGGAACAATGTTCCGCGAATACAACACAAGACGACGTGGCGCATCTATTAATTGAAAACAA
AATACCACCAACATGGATAGACCACGCCTACACATTTGGTCTATACTACATAGACCATCATCTACGGATTGAC
TCAATGATCAACAACATATACATCGAAGCAGATGACGAACGGCTTCGTCGACTGGCAGAACATGGTCTGCCAC
CACCCATACGCGCTTGGGACAGATGGTACATCCCGAGCAAAGAAGATAAAATCCGTATCTGTTACATCATCAT
GGACGAAACGGACCACAAACACATATATAGTCTGGAGAGCGATGAATGGCTATTCGTCAGGGAGGAGGTACAC
CCTACATATCTTCGAATCCACAGCGCAGAACGCGCTGCACACTTATATGACACAGAGCGGACCAGGATTGACG
CTCTGAGGCACGCCGAGCAAACTGTTGGAGACAATTCACACAACTCACAAGTGGTACCAGAGGCGACTAACAT
CGTGCAAGGGACGACTAACATCGCACCGGGTACCACGGAAGGAGACGTGAGGATGTCCGAAACAATGGAGGAT
GACCCCTCCAACGCCGGCCCATCAGGAAGCGCCCAACCAGAGGAAGCACCCTCTGGCACTGAAGGGGCACCTA
TCAGAACACTCAGTGACGAAGAAATGTAGAACCCGCACAGAACAATAATTACGGAGTACAGGGAACTCCAACT
CGACGCGTGCAATGCCACGTGAATATACCACGGAGTACATAGTACTCCACACAACATCACGGGACAATAAAAA
ATGCCCCACAAAATCATTACCACAATTGGGCGAGATCAGAAGATCTCGCCCCTTTTAACTACTTCACCTACCT
TGCTATTCTCGGGGAAAAGATAGCTGTTAGCAATCTAAAGGGGGGATATGTAACAGACGTGCTTGTACTTATT
TACCTACAACCTTAGTCAGGTGACTTCATAAGAAGTCACCCGTGACCCTCAGTAAAAAAGGAAAGTCAGACAA
AAGTCATGATTGACTTTTACACCTCATCCTGGGTCAAAATTCTGACTTGAGAAGTCAAATAAAGTCATAGGGG
TCAAGAATTTGACATATTCAGGTTGAATGGCATTGACTCTGGGGTTGTTTATAGATCAATAAAATAATTATTG
TTTGATAGTGTGCTAGCTAATTAATGTGCTAAAATTTTGACTAACTGTGACTTTCATCTGACTGCTAGAGTCA
GGATTCTGACTTAGGATGAGGTGTAAAAGTCAATGTGACCGTTTCTTGACTTTCCTTTTTTACTGAGCGTGAC
CTGTAACACTCAGTAAAAAAGGAAGTCGAGTTGGGGTCAGGTGATGTCAGGTGATGTCACATATGAGTCACCT
TGGGTCAGGCTCAGTCGTGTTGTAGTCTCAAATTGACCAGTGGAAATCTTTCCAAGTCCATTCAAACTCCGCT

TGAAAAAAATGACCATGTCGTCACTTGGTTGTTGCACCTGTTCGTCCAACTCCTCCTACACCTCTGTTTGAAA
GCCCCTCCAAAATTCTGCCCGCACCAAGCTTGCCACTTGCATTACCTTCTAGCACCCACATCAGAACATGAGG
GATGGACTGCTGTAACAATTATTATTGTTACGAAGACTTATCTCACAATGCAGCACTACGTTAGACCAGTCTA
TTGGTGAACGTTGTACTACGGTAGTGAAAATAAGCTTAAGTGTTACTAGTCACGGGTGACTCCTTATGAAGTC
ACCCTTGGTAAAGGTGTTGGTACATAGTTACAAATACGCCCGTTACAACTGCTGCCAGTTGGAGGATGATGAG
ATGGGTGCCAGACCTGGTCATCTGCCCAGAGGAGAAAGGTAGGATGGTTTTATGTACTATTGGTCCCCAGACT
GACACCCTCCATATCAACCCTCTCTTGTAGCTTGGTAGGACCTTGCATGTAGTATGTTTCATGCCCCCCTCCC
CCCCCCCCACCAACCTCATGCCCTGTAGTATCCAGCATATTTTACAGTAGATATATCAGTAGTACTATTACTA
CTACTTGATACTAGTATTTCATGCTATCTGAATACAATTGGCTTTAACATGACTTGTGGTTGACCTGATTCAA
ATGACTGTTGCCTGACATTAGAATGACCCCCAATTGAACAATTGCTTGACGTCAGGATGACCCATACATGACT
GTGACAAGACATTCCCCTGACTATTGTAGTTGGCTGTAGTTGGCAAAATGTAAAATAAGCCGCAAAAAATGAG
CGGCAATGGTCATGTTGAGTCTTATTATACATTTGCATGACATAAGCCTGACTTCCTTTTTTACTGAGTGTAA
CACCTAAGCCTTATTTTCACACCCGTAGCACGACGTTCCAATATACTTGGTCAAACGTAGTGCTCACCGTGAG
ATAAAGGTCTTCGTAACAATATAATTGTTACATAGTAGGAGTCCCCACAAGTCCTGATAGGAGTTCTTGTGAG
TCCTAGTAGAAGTTCCCACAAGTCCTGGTAGGAGTTCTTGTGAGTCCTGGTGGGAGTTCCTAGGATTCCCAGG
AGGAGTCCCTGTGAGTCCTAGTTAAGGTTTAACACAGTGATAAGTGGTACCCAGGATAGACCCCCAAAGCAGG
TTGACATCCTGGGATCCGCAGCAAACACCACACCTGCCCCTGATCATTGTGGGTTTAAGGGTCCTCAATTTAC
TGCTTGTGCACTGACCTTGGGGTCAAGTTCTGCACCAAATCGGGTCAATGAGGGGCCGATCTAACATTAATTT
AGTCCCTGAACATGATGCACATGATGTGGGAGGGATCGGGATCTCTGATCCTTCGATATATATGGAAAACTCG
CAAAACCCTCGATTACTTGCAACCTGTGGCCTATCTGGGCAAGAACCCGCATGCTATCAGACCTGTGGGTGCA
GGTCCCGTGGGAAAACCTGTGATGTACCTGTGCCATTCCCTAATCATGCCTGAGTAAATGTAAAATATAAAAT
TCAACTTGTGGACTGGAGGTGTGATAGCACCAACAGGCAGGGGTTTGGGGGCACAGCCCCCAACAAGCTGTTG
ACCCAGGTGTGCAAGGGTAGCAAGTGTGTGGCACAGCCACACTTCCAGCCCATAGCAAAGTCACAGGTCACAA
AGCTTGTGATTTCATTACAGAATATAGAATTAATGATGATGTAATAGGAAATATAGTTGAATTCTTAGAGGGA
CTCCTGGTAGGGTTCCTAGGGATTCCTGGGGGAACTCTTGGTGGGCTCCTAATATAGCTGAATGGGGACTCTT
AGAGGGATTACAGGGGACTCCTGGTAGGGTTTCTAGGGATTCCTGGTGGGATTCCTGGGAGAACTCCTGGTGG
GCTCCTACATGGATTCCTGGGAACTCCTTGTGAAATTCCTGGGGGGACTCTAAGGGACTCCTGGTGGACCCCT
GATGGACTCCCAGCAGGACTCTTGGGGACAAGTCCCAGGGAATCCTTAGGATTCCTAAGGATTCCTTAAGGAT
TCCCTGGGATTCCCTCTGTTCCTGATGGAAAATACACAGAGGCTCCTGCCAAAAGTGGTCCTAGGATTCCCCA
GAAATCCCTGGGAATCCCTGGGAATCCTGGTGGGAATCCTAGGGAATCCACAAACTTAGGTGGGGGAGTGTAA
AGACCTCAGTTTTTTCCTTCCACACAATACCTTCCGAAGGTCGCTACAGTGAGGATCTGTGATACTGCAAACC
TTGAGTGCAATGCGGGGCCCTACCAAGGACCCCAGCGGCATCTCCCAACATCTCCCCTTGCATTCCTGTTGTT
GGGGTGTGGATGATAGGCGAAGGCCTAGCATTTGTATTTGGAGCTTATTTTGAACTTGAATTGGAATTATTTC
ACCCGATTGTGGGCTCAAATACTATAAAGCAAAGGCCCATTAATCCGCCATTGAATTGAAAGTAGGTGAGGCT
CACGATGCGTGCACAATCCATGATGAATTTTTCGAAGTGTGTTGGAGAGTCAGGACATATTGATATCAAAAGT
ACTACATGTTCCGGAACTACAGAATAATTTGTTAGCTGTACTATACCTCACTAGACACAAGGGATTCACTATA
CAGATAGATAGAGACTGCATGGAGTTCAAGCTTAATGATAGAATTGTCTTCACTGCTTCTATTGGTGTGAACA
ATGTTGGCTATCTGGATGGCATCACTGTAGACAACTCTGAGAGAGCACATCTATTGTCAACTCTGCCTCTTGA
TCTTGCTTTGTGGCATAGGCGGTTGTGTCATCACAATCATGATGGAGTAGCTAAGCTACTGAAGGACGGGTTG
GTGACTGGAATGGTCATCAACTCGAAGGGCAAGCCTGATCCTGTGTGTGAGCCATGTCTGGGAGGCAAGATGC
ATGCCAATCCATTCCCACCAAGTGAGCACAGAGCATCACAGCTGTTGGAGCTGATCCACACGGATGTGCACCA
GGTTGGAGTGCCATCCATGTCCGGATTCAAGTATTGGGTCACATTCATTGATGACTACTCAAGGTTCAGAGTG
GTTGTGCCAATGAAGGCAAAGTCAGACACATTTGGGGCATTCAAGCGGTTCAAGGCATTTGCTGAGAACCAGC
TGGGACACAAGATCAAGGCCATCCGAGATGACAAGGGAGGGGAGTACATGTCCAAGGAGTTCCAGGCATTCAC
TGATGAGCATGGGATCCTGAGACAACACACTGTGCAGAACCGACCCCAACAGAACGGTGTGGCAGAGCGGTTG
AATCGTGTCCTGGCAGAGCGGATCACTACAATGCTGATTGAGAGTGGGCTACCAAAGTCATTCTGGGGTGAAT
GTCTGGCATCATTGATCCATGTGTTGAACATGTGTCCAACATCAGCTGTGAAGGAGGGGACACCCTATGAGCT
GTGGCATGGGCGGAAGCCTGACATCTCGCACCTGCGGGTGTGGGGATGCAAGGCCTATGTGCATGTGCAGAAG
GATAAGCGGTCACATCTGGGATCACACATGGAGCTGGCAACCTTCATTGGATATCCTGAGGGATACAAGGGAT
GGAAGTTCTACAACACCAAGACCAAGAAGGTAGTGATCAGTGAGAGGGCAGACTTTGATGAGAGATATTCATT
GGGCACCATCAGTCATGCACCTATACCAACTCATGCTGTACCAGAGGCCACTCATCCGGGCAAGCGCACTGTA
CCAGAGAGCACAGTGTTGGAGAAACACACTGATGATGGATCAGATGATGAATCAGATGATGGATACACACCCA
ATGTGCCAGATGATGATGATGGAGCTGATGCTCCGGGTGCACCGCCTGCAGGGGAAGCAGATGCGCATGATGG
CAATGAGGGGAATGTGCAACCAAAGGTTGACAAAGGGAAACAACCAGCACGGGGGCAGAGCAACGCACCACAA
TCACAGGCAAAGAAGGTCAAGCTGAAGGTGCCAAAGGCGAAACCACAGACACAGGTGCAAGTGGAAGTGGATG
AACCACCAGTTGACCCACCAGTTGAAGTACCAGTTGAACCACATGTTGAACCACCAGTCCAACCACCGCAGGT
ACAACCATCATCAGAGGATGAGGATGATGAGGATGATGATGTCCCAGTGGCACCAAGGAGATCACACCGTGAG
CGGAAACCACCAGGGGAATAGTGGAAAGTGCACAGAGAGCCAACACCAGTCATTCAGTCTGATTCTAAGGATG

ACAAGCCAGTAGCTACACCTGCAACTGGTGATGATGACTCTGATGATGATGATGAAGAGGCACTAGCCACAGG
CCACAATGCAGAACCTGTGACCTACAGACAGGCAATCAACAGACAAGATGGAGAGCAATGGCGTCAGGCAGCA
CAGGAGGAGATCAATGCACATCTGGAGAATGGCACATGGACAATTGTGGATCTCCCTCCTGATGCAAAGGTCA
TTGGATCAAAGTGGGTGTTCAAGATCAAGTACAATGCAGATGGGAGCATTGAGCGGTACAAAGCGAGATTGGT
GGCAAAGGGATACAGTCAGAGACCTGGATTCGACTTTGCAGAGACATTTGCACCAACTACTCGATCAGCAACA
CTCAGACTAGTCCTGGCCATTGCAGCACTTGAGGATCTGGAATTGCGGTCTGTAGACATCTCTCATGCCTTCA
TCAACAGTAATGTGGACACTGTGATCTATGTACATCTCCCAGAGGGTGCACATCCTGAGAATGAGGTGAAGAA
GGTGGTGGTCACACCTGGATCCAAGCCTCAGAGGAGGTGGGTGGCTAGGCTGAACAAGGGATTGTATGGGCTG
AAGCAATCACCTAGACTGTGGCATGAGCGCTTGTGGGAAGTACTCAAATCAATGGGATTCAGGAAGATCCGAT
CTGACCCATCCCGTTCATGTATGAGAATGATGGTATCAAGCTGATTGTGCCAGTGTTTGTAGATGATATCACA
ATTGCATCCAAGTCCAAGGTGGCCATTGACAAGTTTGTCAGGGATCTGGCCAAGCACTTCAAGTTGAGGGATC
TGGGAGACACCTCATACTTGCTGGGCATGGAGATCACTCGGGACAGGCCCAATCGGAAGCTCAGCTTGTCACA
GAAGCAATATGTGATCAACATGCTGGATGGATTTGGAATGTCAGACTGCAAGCCTGTGGATACACCCATGAAT
CCAGGAATCACTCTGACTCGAGGACAAAGCCCTCAGACAGGTGAAGAGAAGGATGAGATGGCAGAGATCCCCT
ACATGAATGGAGTGGGGACAGTGATGTATCTGGCAGGAATGACCAGACCTGATATTGCATTTGCTGCAGGGGT
TCTTGCCCGATTCAATTCCAATCCAGGCATGATGCATTGGAAGGCTTTGAAGCATTTGATGCGATATCTCAAG
GCACAATGGACATGAAGCTTGTGTATGGTCCGGAGGATGGGGCAACAGAGCCATTCACCACATTCTGTGATGC
AGATCATGGGGGCAACAAGGACAATGGGAAGTCAACCACTGGATATCTGGTGCGGATGGGGACTGGAGCTGCA
ACCAGTGGTAGCACTGTCAACCACAGAAGTGGAGTATGTGGCAGCTGTGGCAACAGGAAAGGAGATCCTATGG
ATGCGGAAGATGTTGACAGAGATGGGATATGCAATGGGATGTGCATCCACATGTCACATTGACAATCAGTCAG
CAATCACAGTGGCAAAGAATCCGGAGCATCATGGATGGATGAAGCACCTTGACCTCTGCTTCTATTGGCTTCG
AGATCAGGTTGGGGCCAATGCTATTAGGCCAATATATTTGGAGACTGCTAAGATGCCAGCAGACCTCCTAACA
AAAGCACTACCTCGAACCAAAGTGCATGAGTTCCGAAAGCTTATGGGACTTGTGCAGGATTGAGGGGAAGCAC
TATGATGTGTTGATGATCTATTTTCATCAATGCACTATCAAGGGGAAGTGTTAGAAGTGGGGATAACATGTTA
GAAGTGGGATGACAGTACATTGGTACTGACAGATAATCACTTCATCTGCGCTTCCCAGCACTCCCTAGTCCAT
CTATGATACACAGGTTACATAGATGGACACCCAATAGACCGCATTAGACCATTAGTATGGTGGTTCATAATGC
TGTATATTCATTTTGTTGATATCTTGCCTCCCAGTGCAGCTAGAGGCCTAATTGTTGAATATTCTAAGTCCAG
TATTTCAGTCGCGATATAGTGGACACCTGTTTTCACGCCTCGGGTGAAACAGGCCAAACATGTACAAAGGAAC
TGTAATTCCTACTATGTCCCGGAGTAGTGTTTGCTACTCCCTTGACAACAGTATTAGACAATCATATACTTGC
ATTCTATTATCAATATCAAGTGATTAGAGCGTCTATAAGCGGGCTATAAACTGTGTCTGGCTATACTCTATAG
CAGATCAGTCAGTGAACACAACTTGTCAGTT
>Cligib1_rnd-1_family-195#LTR/Gypsy
GTTGNTGTTGCGGTTGTTGTTGCAAGACATGTTTAAGGTGTGTTTAAGGGTCGCGTTTGACAGTCATTNCGCT
CGAAGTTTCGGAGNTCACGTGACTCCAAATATTTTGATGCCATCACGCGTGATCTACCTGAGTTCAGGTTGAC
ATAAGCNACCCTAACCCGTCTTCATACCTGAGTGATGCGTCACTCAGGATTTCTTTGGCAACGGCGTAGGGCG
TTTGACGCCCTGCAATTCGAGATACCGGTCAAGCGCTTCTAGGCGCTTGCAGTTCTCGTATGGTTCCCAGGTC
GTGTCTCCTAGGCTCCACCTAATCTCGAATTCCAAGTTCGAGTTCCTCCAGCGGTGGCCTCTTATTTCTTCGA
CGAACCATTCGTGTTCGTCTGGTGTTCCAAAGTCATAGGGTTCGGGATGAACCCTATTCGGGAATAACGCGTC
ATTCGACGCGTGGTATGGTCTNATTAGCGATGCGTGAAACGTCGGGTGGATCCTNCGTTGTTTCAGCGCCGCG
GGTAGTTCCAGCTTGTAGGTAGACGTTTCAGGTGATGCTTGTAGGACCTTGTATGGTCCTACGTACTTTGGGC
AGAGCTTCCGCGCTCTCCCTTTCGGNAAGTTGAAGTTCTTGGTGGATACGTACACCAAGTCGCCTGTTTTGGT
NTCCAGTTCCGGTCCGCGTTTGCCGTTNGCCTTGCGAGCTTGCGTAGCTCGCGCTTCNATGATTGCGTCGTGC
GCGCTGGCTAAGTTTTCCAGTGCTTGTTCGGCGAAGCTTCTGATTCCCTTGTGGATTGCTTCGCTGTTCCGNA
TTTCCTTGATCATGGAGGGCATATGCCCTCCGTTNAGTTCGAACGGTGCGTACTCGGTGGTTTCGGATACGCT
CGCGTTGATCGCGAATTCCGTTAGATCGACTTTGTCGATCCAATCGTCCTGGTCGTGATTGCAAAAGATCCGA
AGGAACTGTTCGACGTCTCGGTTGACGCGTTCNGTTTGTCCGTCCGTCTGCGGGTGATAAGCCGTCGATATTA
GGAGCTTCGCTCCTAATANTTCGTGGAGTTCGCGCCANCATTTGGACGCGAACTTGGAGTCCCTGTCGCTCAC
GATTGAGCTCGGTANTCCGTGTAAGCGCACGATTTCGCGCTTGTATATCCATGACAGCTCNGACGCTGTCATT
TTCGTGTGGACGGGTACGAGGTGGACCATACCGGTCATCCTGCATATGACTACCCATAGGTAGTTGTATCCTT
TGGACTCCGGGAATGGGCCNACGAAGTCCATTCCGATTGAGTCCCAGGGTTTCGTNGGTACCGGTAGGGGATG
TAATTTCCCTGGTGGTGCCTTGTTGGCTCCTTTGGCTACTTGGCAAGACTCGCAGGTCTTGCAGAATTCTTTA
AC
>Cligib1_Cluster118#LTR/Gypsy
TCAGCTAGCGTTAGCAGGACTGTTACCACAGGTATGAGGTAAATCCAAAACCCGCAGTGATTGCGGGTTTTTG
GCAAAACCCGCCATAAAATCCGGATTAATCCAACCCGTGGGGCGGGTACAGGTTTCCCACGGGTATGGGTACG
GGTCATGGGAAATTACCTGCGGTAAACCCGTGCGGATCACTAGCAGGACGTAACAGTCATCCTCAGAATAAAG
TGAGACTGTGACTGGCAGTCGCAGTCTCATTCAGTCCGGTCCGGTGACAGTCACGGTCTTTTTTCGGTCTTGT
GACCGGACTTTCGAACACTGGTAGTCAGCCACTAGGAAAAAGGTATTACTAATCTTGTGTTCCTGGCTATGAC

AATTGTAAAAACATGTGAAACATAAGACATGATCCTGGCTATGGCAATTGTGAAACATAAGGATGCAAATGAG
GAGGAATGGGTGGGCACAAATGAATCTTCTGACTATGTGGCAGAAAGTGCCCCCTGGAAGGTCCATCAGCCAG
ATTCTCCTCAGAAGGAATCCACGTTGTCGACAGCCATAAACTGTACTCATGAAAAATGTCGACCACTTTCCTC
AAGATCGCATTCTGCTGAACACTCCTAGAGAGGCCTGCTTTGGTAGCCCCAACAACCCCACTGTTATCTGAGA
AAATGTGGATGTGGGCATCGTGGATGCCCTGGTGGACCAGGGTGTGAAGAGCAAGTTCCATGGCCACCATCTC
AGCCCAACCAATATCATGGCCTTCACTCTTCCAGCTGGGATGTAGAGGCCAGGCGAGCCAATGGTCTCCCACC
ATGAGGCCAATGCCCCAAGAAGTTGAGGCATCCATGTACACTGCCAGATCCAGGATTGGTGGGGGGACCTGTA
TGAAAATGCCACACCAGGGAGAGTGGAAACGCTCTCTCCACCAATCGAGATCTTCTGCCAAGGCACTGCTGAT
GGAGTGCTGAGAAAAGAAAGACGGATTCAAACCAAAGGAGGAGCATAGGTGATATAAATTTGGCAGATGCAAG
CAGCCATCTGTAGTGTTTAAAAGTCCGGTCAGGTCCGGTTATTTGCCTAATTTGGAAAAGACCGTGACCGTGA
CCGGTTACATCATCACCCAAAAAGGAAAAAGACCGGACCATGACCGGTTACCGGTCACAATTATTTCAAGCTT
GTTTGAAGACCATCGGTCACGCAAAGACCGGTGTGACCGGTCATAACCAGTCCTAACCGGTCGTGACCGATAA
TTTTACCATATATGGATGTCACACAGGGTCCGCGACAGAATATTTCATGTGTAAAAGTGAAATATCCATGTAA
AAAGCAGCTCTCAGAGCCATTTTTGATGGTTTTTGATGGTATTTTGGGGTGTTTGGTGGGTGCGCATGTGGCT
GTGCAATAACCGGTCTATAACCGGTTATGACCGGTCACACAGGCTGTCACAATCCAGGAAGACCGGACCTTGC
GCACCCCAAAATCAAAAAGACCGGACCTGCGGTCACTGTGTGACCGGTCATTTTTCGGTCACGGTCCGGTTCG
GTCACGGTCACATGGACCGGACCTTTAAACACTAGCCATCTGGAATTACCAAGGTGCAGTGATTGAGAGTACC
TATCAAGGATTCAACCGCTTTGTGAGCAATGCGCGAGCCCTTCACCCAGCCATCCAACTTCCGAAGGTATTTG
GCTTGTTTGGCAGGTGGGAGCAAGGCAGTTTTGGCCATGAGGTCCCAATGGAAATGATGTAGGTGAAAGAAGT
TGCAAAAGGGAATGTCTTCTCCGGGGCCCAAGGCCAGCCCAAGCCCTCAGCCACCGCCATAATGAGAGATTCA
TCGTAGGAGAAGGAAACGGCATCCGCCACGGATAGTGTTTCAAGGTCCGGTCTATGCAACCGCAAAAAGACCG
CAACCGGACCGGACCACCCGGTCCGGTTGCCCTGCCCTGGGGCAACTGCGAAAAAACCGGTTTAAACCGGTTG
CAACCGGTTCTCGGCCGAACCGTGGGAAGGGACCCTTCCGCCCTGTGCGCGTGGAGATCGCGTCAAAAAATAG
CACCTGATCGCCCCCAAAACGACCATATTGTGTGAAAAACTCACTCTAGAACACCATGTTCTCCTCCTCCCCC
CAAGTGCGCACCCTATAACTCTAGATCAGGGACAGGTAACCCTGTCGTCTCTTTTGGATCGTATTTGGCACCT
TCAGGGGTGTCCGATCGACCCCTCAATCATCCAATTTGGTGCAAAACTGCGGTCCGGACCGGTTTTTTTCAGG
TTGCAACCGGTCCGGTTGCCTTCCTAAAAAGGCAAAAACCGGACCGGACCGGACTTTAAAACACTAGCCATGG
AAGATTTGGGATACTAGAAAAATACAAAATCGTCCACCCACTTGATAACATCGTCTACACCTTTAGCATGATA
GATGTCCGATATTGCATCACCCAGCCTACCAAAGATGCCGGGCATGCTGGCGCTGCCAAATGAAAAACAGGCG
TCAATGTGAACGCTGCCGTGCCAGAGAATGCAGGTCAGTGGGTGATCCACTGGAGCCATAGGAAGACGACGGA
AAGTGCTTTTGACATTGAACACTGCGGCTTCAGATCCAAGGGGGGCCTGACAAACGAGAAGGTAACACTGGGC
AAACGTGCCCCAGCCACATTGGTACTTAGAAGAGTCTATTTGTGAATTTATCGATTGGACATTCAGATCATTG
TATGGGTAGGAAAAGTTTTGAGTGAGATGGAATTTACTGGAACCGGGAACTTTCTCGACGAGACCCAAAGGAC
TCGTCTGGAAAGGGCCTATTAAATTGTACAAGTCCGCAATCTTGTAATGTGTGGACAGCCGTCCTAGAGAAAT
TTCATTTTGAAGGATAGCATTGACAGCTGCCATGTTGGCAAGAGCCGAGTGGGCATTGGGGGGAGTGAAGAGA
ATGTTGCTAGGGGATTCAACCCCACACCAAAAACCATCCCTGATACCAGCAGGGATATCAGAGTAAACTGAAT
GGAGGCCCCCCTCCTGTAATGCCGACACTAAGGCATAGCATGGATCGATCCATGGGTAGATCCGGATCCGCAC
TCACAAGTTAGTGTTTCAAGGTCCGGTCAGGTCCGGTCTTTTGCCCTAATTATGAAAAGACCGTGACCGTGAC
CGGTTACATCATCACCCAAAAGGGAAAAGACCGGACCGTGACCGGTTACGGTCTTGTTACGATTCATTGCTTC
TTATTCATCACCCATCAGTCACTTGACTGATACCATCACTCATAGACGCTTCCACTTCCTCCATCATCATCTG
ACACTTAGCCATATGCTCCATCATCAGATGATACATCATGCGCACCTTATCTACTTCTCAACCGCATGGTCTC
CGTTCTATCTTTGTTATCTTCCATTGTTCTTACTCAGCTGCTCGGTCAGCTTGCTTCTTGTATCAGTCATCAT
CCCCCATATGTCTTACCATACTCACGGACTTAGTCACTAGTGCGTAGTCATTGAACTACTTCCATACTCCTGT
ATTCGCTACGTTTAATCGTTTATCAGATTCCCCCTCTACTGCCTCCTGACCCAGGAGACTTCAGACCTTGCTA
AGTGTCCTTGAGTCTTTACTGTCAACTCTTTGACTTACGTTAGCAGGACGTAACAGGTCTCAATTTTGTTCAA
GCCTGTTTGCAAGACTGCAGTCACCAAAAGACCGGTGTAACCGGTCCTAACCGGTCCTAACCGGTCGTGACCG
ATAATTTTACCATATATGGATGTCACTCAGGGTCCACGACATAATATTTTAGTGAAACATGCATCAAATACCA
CTAAAAACCAGCTCTCAGAGCCATTTTTGATGGTATTTTGGGGTGTTTGATGGGTGCGCATGTGGCTGTGCAA
TAACCGGTCACACTCGGTCTGGTCACGGTCCAAAAAGACCGGTCACGCACACCCAAAAATCCAAAAGACCGGA
CTGCAGTCACTGTGTGACCGGTCTTTTTTCGGTCACAGTCCGGTCTGTGACCGGTCACAGAGACCGGACCTTC
AAACTCTAAGCAGGGAAACTACATCCCCTACCGGTACCAACAAAACCCTGGGACTCAATCGGAATGGACTTTG
TAGGCCCATTCCCGGAGTCCAAAGGATATAACTACCTATGGGTAGTCATATGCAGGATGACCAGCATGGTCCA
CCTTATACCTGTACATACAAAAATGACAGCTTCAGAACTGTCATGGATATACAAGCGCGAAATTGTACGCTTG
CACGGATTACCCAGCTCAATCGTGAGCGACAGAGACTCCAAATTCATGTCCAAATGGTGGTGTGAACTCCACA
AAATACTAGGAGCAAAGCTCCTAATGTCAACATCATTCCATCCCCAAACGGACGGACAAATGGAACACACGAA
TTGTAGCATAGGACAGATATTCAGAACAGTCGTGAACCACGACCAGAAGAATTGGATCGACAATGTCGATCTA
ACGGAATTCGCGATTAACACGAGCATATCCGAAACTACCAAGTACGCGCCGTTTGAACTCAATGGAGGACATA
TGCCCTCCATGATCAAGGAAATACGAGCGAACGACCAATGCACACGACACAATTATAGAAGCATGAGCTACCC

AAGCTCGCAAAGCCAACGGGAAACGAGGACCGGAACTGGAGACCAAAGCCGGCGACCTGGTGTATGTAGGGAA
TAGCATGGGTACGGGTAAACCCGCGGTATTCGGGCTACGGTGTTTACGGTTGTTTGCGCGGTGTCCGAAAATG
GACACCGCGCCAAAACCGCACCCGTTCCCGCGGTGTGGTGGGTTTCCTCGGGTGGCTGCGGGCCGTTTTCGGC
TCCCTGACACACCGATCGGCCAATGATCAGAACATTGTTTTCAATGTTCTTGTCATTGTTCAGCCCACTCCCT
GTCGCCCCCCGACCGTTTGACGCAACCGTCACTCTCCACTCTCGCAAACAAGAGTGCGGTTCGTCTCTACCCC
GTATCCGCTAGCCCCCTCACTCGCAAACGAGTGAGCTGGATGGTGAGTCCTAGCTCACACCGCATGCCTGCCC
GCTTGCTCACCCCCCCTCCCCATTTGCCTGTATCTGTGCTCCCACCCCCACCCTCCCACCGTCTTGCTCACCC
CCTGAACCCACACCCTGTCGCCCCCCGCCGCGCCCTACCTGCCATCCCCATCTCCTTCACCCACACCATCTTA
CTCACCCCCGAACTCGAACCCCGTCGCTACCTGCTGTGCCCCATCCGCTGTGCCCCACCTCCACATACCATCC
CTCACCCATGCTATCCCTCGCCCATGCTATCCCTCGCCCATGCTATCCCTCGCCCATGCCATCCCTCACCCAC
ATCCTCCCTTGCCCGTGCCACGTGATATGCCACCCCACCTTGCTGCCCCTCACGAATGAGCACCAGGAGCTCA
AACAGTAAGCCCCCATCCCTCAACGGTGAGCCCCCATCCCTCCCATCTCTCTCACGCAGGGGTGCCGCCAGCT
AACCCCCACCCTGCGCACCCCTCGCACGACGCTAGCCGCATCAATTGATGCATGCGGCATCATGCGAGGGCAT
TGTAGGTGGGGGCTGCAGTGCCCCACCGGGATCCGTCGACCCCCCATTGTTGCGCACCCCTCGTATGATGCCA
GCCGCATCAGTCGATGTGCGCAGCACTGTGCGAGGGCATGTAGAAGGGCCGATGGTCACGGCGGCACCCCGTG
GCACAACGCTGAGCCCTGCGGTGTAGCGCGGTGATAAGCGCACCGCGCGCTTATGGGTTCGTAGGCCACATCT
CGCACCAAGATAAGATCAGCGCAACTCGCCAGGCTCGACGCGCGCACCCACAAGCCCACACAACGGCCTCGGA
CCGCATCAACACCCATGATAACGGCCAGGATCAGCTCGCCGCACCTCATATATACGATGCGAGGCCGTCGCAC
TCGTATAGGACCCTCTCGCAGCTCAGCACATACGGGATCGCGCCTCAACACGGCTCCGTGCGCACTCAGTACT
TCCTAACAAGAGACCCTAACGCGCCTCGACCCACACCAACTGCGACATGTACCAACATCACCGCGAGAGCCTC
CATGAGGCCCGGACACCTCGACAACGTGCCCATTAGGTGACCCGAGCAAGACGCGGACGCGCGATCGCGCCGG
GTTTGTTGCATATTCTCTTATTTCACTTTAGATTACCGGACTCAGGACATGATCGGCGGTCCATCACATCGTA
CATAGACCCTTACATACGCCGCGACTCGCCCACAGTATATAGGCCATGTACATACTTCGCCTTCCACAGAGAG
AATCCAGCAGAGTTTCCCCCTCAGAGTTTATAGCTCAGTTTCGCAGTCTCAGAGTAGACTATCCACGTTCCCT
ACAGGAGTCGCACGTCGCACTCCTCCACGTTCACTCATATTCACGTAGTCGGTCTGTGACCAAGTCGCCCAAT
CGCAGTCAAAGACCCACGAGTACTGGTGTACGCCGTCTCGCATCGCACCGCGCCCGAAGACTAGAGAGCGCGA
CTCGTGTCGCACTCTTTCTACGCTAAGTTCTCAGTCCCCGGACGGCCTCGCGTCCATTACATTCCTTTGTTAT
CACTCTCGCGCGTCGTACACGTCGAGATCGCATTCAAAGCGGGTCCTCTCGCAGTCTTTGAGAACTCTTCTAG
AGTCCTTAGGAGACTTTGTTTGTTGTTAATCTGAAATGCTCGCTCACTCAACATCTTATTTAGGTGTTGTTTA
CTTAGTTGGTTTTTGTGGTTTGTTTTGGCCACAAGCCTCTTTGCATATCAGCGGTACCCCGCGGTGTGGCACA
GTACCACGGGTGTGCCTTGCACCGTGGTTGAAACCCGTACCCGCGGGCATGGTGTGTCTGCGGGTGCAGGTAT
GGTGTCACAAATGTGACCCGCGGTGTGGCCTGTGCTACACCCTAGGTGTATGTATCCACCAAGAATTTCAATC
TACCGAAGGGAAGAGCAAGGAAGCTCTGCCCCAAGTATGTAGGACCATACAAGGTCCTACAAGCATCACCGGA
AACATCGACATATAAGCTGGAACTACCCACTGTACTGAAACAGCAGAGGATTCATCCAACGTTTCATGCATTG
CTAATACGACTGTACCATGCATCGAATGTCGCGTTATTTCCAAACAGGGTCCATCCCGAACCCTATGATTTTG
GAACCCCAGACGACCATGAATGGTTTGTGGAGGAAATACGTGGACACAGATGGACGAAATCCTCGAAACTAGA
ATTCGAGGTTAGATGGAGCCTGGGGGACACGACCTGGGAACCATACAAGAATTGCAAGCACCTAGAAGCGCTT
GACAGATATCTCGAATTGCAGGGTGTTAAACGCCCTACGCTGTTATCAAAACAAGCCTGAGTGACAATGCACT
CAGACATAAAGACCGGGTCTGATTACGTCAGCCTGAACTCAGGTGAAATCACGCACAAAAACATACATGATGG
CATCAAAATATTTGGGAATCACGTGAGTGCGAGACCTCAGCCAAAAAAACACCCCACCGCAAACCCTTTACCA
TACCACAACCACGATGTCTCGCAACAACAACAGCAACAGCAACAACTTCAACAGAGCAGCCGGGAGTAGTGGC
GGTAGTGTTTAAAGGTCCGGTCCATGTGACCGTGACCGAACCGGACCGTGACCGAAAAATGACCGGTCACACA
GTGACCGCAGGTCCGGTCTTTTTGATTTTGGGGTGCGCAAGGTCCGGTCTTCCTGGATTGTGACAGCCTGTGT
GACCGGTCATAACCGGTTATAGACCGGTTATTGCACAGCCACATGCGCACCCACCAAACACCCCAAAATACCA
TCAAAAACCATCAAAAATGGCTCTGAGAGCTGCTTTTTACATGGATATTTCACTTTTACACATGAAATATTCT
GTCGCGGACCCTGTGTGACATCCATATATGGTAAAATTATCGGTCACGACCGGTTAGGACTGGTTATGACCGG
TCACACCGGTCTTTGCGTGACCGATGGTCTTCAAACAAGCTTGAAATAATTGTGACCGGTAACCGGTCATGGT
CCGGTCTTTTTCCTTTTTGGGTGATGATGTAACCGGTCACGGTCACGGTCTTTTCCAAATTAGGCAAATAACC
GGACCTGACCGGACTTTTAAACACTAAGTGGCGGGAACAGTCATTCCCGCCAACAAGGATACCGAGGAGACGG
ACAATACAGGGCTGTCTCAGGAGGAAGGGGTGAGGGGGACCAAGCACCCCCTCCATATCCAATGCCAATCTGG
AACCCACCCCAGTACGGCCCACCAATAGTCGCGCAGCCGACAAACCCACAACCACCACGGACTTTCCCAGAGT
CCCGCTACCAGTGGGCATATCAAAATGCCCCAATGCCATTCTTTACCGGGGGATGGGGAGACCCCATGAACAA
CAACCAACTAATCCGTGAAGCGGATGGAATGGAGATTGATGATCTCCCACCACCACCATCATCAGCAGAACTG
GAGAGATGGCGCGACAATGGCGCCAATCAACAACCACAGCAGCGATATGAACGCGCTGCACCCACACGAACCC
AGCGTTACGAACACGCTGCACCACCACCGAGACCACAACGCGAGAATCATCATAACTCACGTGAAGATAGTGA
GGAAGAAAGAAACTCGAGGCGTCAGGGGAAACAAAGGGCACGAGACCCAGAACCCGCGCCCAACACCAACCAA
ACATTGAATCGGAAAATACGAGCCCCAGAACCCGAGCCCAGCAACAAGGCCGTGGAACGTATCTGGACAAGAC
TGGAGTCGATACAGATTGACTCAACACTGACCGACTTCCTGGATGACCCCCAGGTGAGATTCATAGTCTCAAA

GCTTCTCAATATGAGAGAAAGCAAGAGACAAGAAAAAGAGAGATTACAAGAATCTCTGGAGGAAACCAAGAGC
CAGCTGATGACGGCTCTCAAAAAGAGGCAAGCATCCCCCCCTACAAGGGAAAATGAGGATCGCAGCACCAAGA
AAAGTCGTGAAGCAACACCAAGGGATATAATGCCCTTGCCAGAACAACAACAAAGGCTGAAATTCCGACCGGC
ACAAAACCAGCAACCAATTGCTGGACCATCGATGATAAGAACGAGATCATCGACATCGAGTGAATTCGTAGCA
AGGGAGGCACCCCTTCCCTATGATGAAGAAGGAGCCAATGGGAGAGGAAGACCAGGCTCCAACATGTGAGGAC
CATCCCCTCACAAGGACAGAAACGACATGTCTGATTCCACAGAGCTACCCCACAGCCCCGTGGGTAATAGTGG
ACCCAACCCTGGAGGAACAACCTCTGAGAAAAATAGATCCGGCGGACAAATCGGACGAAGACTACGCGTCCGA
TGAGAAACTGGAGTGTGAAGACACTCCAGAAGAGGAAGAAACGACGACAGAGAAACGCGCCAGACAAAGGCGC
AACAAGAAAAAAGAGAAAAAGCGAGCAAACCTCGCTAAAGAAAACAAAAGGAAACAGGACATGTATATAAAGA
CACAACCTGGAAGAGTACCCAACGCTATAGGCGTTGTAAAAATCATGGGGAAATACGAATGAGATAACCGAAT
GAGGGGTACTCTGGACCCCGAATTCTATTACTCAAGAATCACCAACATAGTATATGTTGGGAAACAGGCTAGG
GTGGCCGCAACGAGCGAGAATTTAAATCCTCCCTCAGTACACATGCCAACAACAATGGCGTATTACAAGGCAA
ACCGAGGGTTCCCCATGAACCCTATCGAATTATCAGAAATGATAAGAGCCATCCTAGGGGACCAAGGAACCCT
AGAAGAAAGAGTCGAAATATTTCAACTCGTGCGAGAGTTTAGGCGTATCTCATACGCCTTCCTACCAGACCAA
CAAGACATAACAATGAAGAAAGCGCTCGAAAACGAACCATATGAGACGGTTCAAAACAGAATATCGAATCAAA
CGCTGCCCGAAGTGTTTGCAGACAAATACGCGCCATTCGGTGCGCGATCTGCATTTAAAATAGAGGCAGGGAC
TGGTTGGACCAAAGCGGGTCTGACACAACTGGACATGGAGTATACACTCCACTAGTGATCCGCACGGGTAATG
CAACCCGTATGGGTTTTCCCATGGTGATATGCTGCGAGGCTTGTGGCCAAAACCAACCACAAAACCAACACAT
AACACAAGTACCTAGATAAGGTCTTGTGGATGCGAGTGTTGTCCGAAATCAGTGAGACTTCCTAGCCCAACTG
TGGCGAGATCAACGCAAGGGCTCTGCCCTAAAGGAGTGTTGCATGCGAATCATGCGAAGAGCGAACTGAGAGG
TGAAGACTTTTCTTGTTGATCATGGTAGCTACCGAGCTCTCTAAATATCGGAGCTAAACGGCCATGAGGTACA
GTAAATAGGTAAGTAAACTATGTAATAGAATGCGATCCGCGTGAGGAGTGATGCGATAAGGATGCGAAGACTA
CCACTAGAATCGGAATTGAGACGCGATCTCGACGCGTTCGGAGCGGAATGGGTGCAATCAGTACTGTACGAAT
GCGATATGTGCGCCTAAAGCGCCCAATATCAGTAAGAGATGTGAGCTGCGAACCCATAAGCGCGCGGCGCGCT
TATCAACGGGTGTGGGTAGGGGTTACCCGCGGGTACACTTTTCGCAAACCCGCAGAAAACCCGCACCCGCACT
GCGGGTAGGGGTTTTCCCACGGGTTTTTCACGGGTACGGTTGGCACTATTTGGCCCTATTTTATGCTAAATCT
GCAAAAAAAATCATACATTTTGACTAAATATTCGCCAAAACAAGCCAAAAACCGTAACTCGCACCACTTGCGG
ACATATTACTCAGCTGATATTGATCGTAGAGCTTAGGTTGTTATCCCTGATTGTTGCCCTGACTATGGGGCAC
GTTGGACACTAGTTTCTGAGGCACACGGGTACCACGGGTATGAGGTAAATCCGAAACCCGCAGTGATTGCGGG
TTTTTGGCAAAACCTGCCATAAAATCCGGATTAATCCAACCCGTGGGGCGGGTACGGGTTTCCCACGGGTACG
GGTACGGGTCGTGGGAAATTACCCGCGGTAAACCCGTGAGGATCACTAC
>Cligib1_Cluster71#ClassI/LARD
GTTGGAGTGCAAGCTCACTCAGTATTTAGACTTAGAATAATTTGCGCTTTACTGATTATGTAAGCGCGTCCAA
TTGTTACATTCGAGGATTGTTCCTCATACAACACAATAATACCTATACCATTCTAATAATATCTAATACCCCC
CATCTGCCTCATCCACTGCCAACAGGTTATGGGCCCTGGGCAACCTACGATGTCATCTCTCGAAGTCCCGAAT
CTCGAAGATACTGGTTCAAACTGGGTCATTTACAAGAAAAGGGTAATAAACCTTTTGACTCACGAAGGACTCG
AACAGCACCTGTACGGAACCACGCTGAAGCCACAGGAGCTTCGCGAGGTGGATGGAAAGTGTGTCCTTGAAGG
AAACCCCACGAAACCGCTCAACAACGACGAAATCGAAGAGCAGAATACAAAAATTGACATTTTCGAGCAAAAT
GATGCCATAGTCCGAGGAGTTATCTATGCAACAATCAGCAGATATACTCACCTTCGAATATACAAGGAGAAGA
CCTCCGCGAAGCTCTGGAACAAACTTGTGAGCATTTTCGAGAATGATGAAAACAAGTCCTTTATGGCCGCCTT
GCGATCCGTTCGAATGAATGCCGAAAAACGCGTCCAAAACGCGATGGTAATAGTGGAGAAGCGAGGGGAAGAG
CCTGAGGAGAAATTGAAGGAAAAGAATAGGGAAGCGCACACAAGCGCAGGGCATGCTCGCGACGAAGGATGCT
ATAGGAGAGGAGGAGGAAGAGAAGGTCAGGAACCACAGGAAGAGAAAACGACGGACACCGACGAAGAGTCGGA
TATACCCTGCGCGAAGACCTCATACCCCCGTGAAGACACCCCTGCCCTATCCACTCCACGACCCCCGCCATGC
ACTGTCGATCTTGCAACTAAACCGCCCGAAATATACCTTGAAACCCCAATTGCGAGCGAGATGTCGACGAAAA
ACGCGAATCCACCCGAAATCACAACAGAAAATGAAGACCAACCAAATTATATTGAAGAAAAAGCATATACAAT
ACAAGACGAACACCCTATACCCCGAAAAACCGAACCCGAAGTGGTTAAAGGCCATAAAACCGATATTGACAAA
GATTCCGCAAGCTATATGATCTGTGGGACAGAAAAGCGATCAGGAGCGGTCGAAGGGAACGCTTACCATTGTA
GGAATAGCCTTCCAATAGCCCGAACCGTCCAGATCGAGGGGAAGAACACGCCCCACATCTCCAGCCCCAGCCG
AAGCACCGGAAATAAAGTTGTCGAATCACCGCGCGCTAATAGGCAAGTTTTGAAGGCCTTGGGAACCGCTCCC
GAACGGTCCAACAATCAGCCAAAACCGCCGGAAAATGATCGCGCAACATACCTATCTTTGTCCAACCCACTTA
GGCCAGCCACCGAAAAAGACCCGACCGAATCGCCGCGCACGAATAGACGCGAATTGAAGCCCTTGGAAACCGC
TATCGAACGGTCCAGGAATCAGCCGAAACCGTCGAGGAACGACCGCGCAACGGACGCACCTTCGTCCAAGCAC
AAATCCATCACATCCAAAGTTCCACCAATATCCCCCAACCCAAAAGGCCCTCGAGGCGACATCCCAAGCGACC
TAGAATCACATATCCGTCGCATCCCTCATGCCAGGAGGCCCCCAGGAACCTGGAGTCAGTCCATCAGGCAAGA
CCCACCTATCTTGGAGCCTGGACCAGCAGAAGGAATCGACTATTGGGATTCCAGAGACAAGCCTAGGGAATAT
AAAGGGTTAGCAAGCCATCTAGGAGGACCAGGTTACCTATTTGCGATGGCCATAGATAGGGCATGCCACGAGT
GCACTCGCAGATCGACCCGGATCCGTGGGAACAAAAACCCGCAGGTCCGGATCCCCACGACTTGTGTACTCAC

CACCACAGATCACTGCAAGTTCAAAACTCACAGAAAGACTCGGATCCGTGAGTCACATACTCATGAGTATGTG
ACAAGGGGTGGGTCAGAAAACACAGTGAGACGAGTCAAACAAGACCAAACAAGACTCATGGGTACCCGCAGCG
GGGACTCGGATCCACAAGTAAAATGACTTGTGAGTGTGTTAACTCATAAGTTGGGAACTCATGGGTCATGAAT
ACCCGCAGGTTTCCCACGGATCAACCCGTGGGTACCTATGAGTGACCCGCACTCGTGGCATGCCCTAGCCATA
GAAGTTCAACCTCGAGGAAGCGCCCAAGAAAGATCAACAATCGCTCGGTCAGCATACCAACGTCCAAATCGGG
ACATTTCGACATCAAAAATCATCAACACTCGTTTTGACCACTTCAAGACAATAATTCAATATATTCTTCTATA
CTATGAGGTATATCTACTTACTTACAAGCTCAAGACCCTTCGTTTCGCTTGAGGGGAAGTGTTGGAGTGCAAG
CTCACTCAGTATTTAGACTTAGAATAATTTGCGCTTTACTGATTATGTAAGCGCGTCCAATTGTTACATTCGA
GGATTGTTCCTCATACAACACAATAATACCTATACCATTCTAATAATATCTAATACCCCCATCTGCCTCCTCC
ACTGCCAACAG
>Cligib1_Cluster109#DIRS/DIRS
CCATTACCATGCTTATCACCGCTTGCAAGCGATGTGGGAACCCCCCGCCAGGCCCGCCACCCGCCACGACAGC
CCAAATGTCCCGGGTGGGGCGAGGCAACTTGCTCCTTAACCCAGTGATGGGCATGGTGGATCCTACTTTGTAA
GACGGGGCGCCGCCCATTCGGCGCTTACGCTGAGGGGGACCACCGTGTAATGTCCATCAGACATCTCCCCGGG
TGTGGTCGAGCAAGGTCTCCCGCAGTCGTGGCAGCCACCTTGGGGGGCCCGCAGCGGACCCATGTTCTTTCAT
CTGTGAGCCGCCTTTCGCATCGTTGCTTGACTTTGGCTGACTTATCGCCCCGCCTAGTTTCTTTTGATTTGTG
GGCCGCCTTTCACATTCTTGGGCGACTTCAGCTGACTCATCACCCCGCCTAGTTTCTTTCTTTTGCTTCCTTC
CCCGCTTTGCTCCTTGCTGTTGTTGGTGTTGTTTTCGGGTGTCGTTGCAAATGCTTCAGGTTCCAATCCTATC
TCTAGCCCGGGAGGGGTTCATAGGTAAGCAATATGTTATTCCTGGTTGTAATCACAGTTGCTCACAGCCTCAA
TGCTCTCCTTTCTGGATCGTGGTCGTCCCTAGTGGGGCGGCCCCCCGGGGCCCGTGAGTTTCTCTCTTGTTCT
CGCTGTTGCCACTGTTAATGGATTCTACATGAGGTTGCCCTGGCGGGATTGGCGCTGGATGTTGGGACGGAGC
ATTGAGGGGCCTGTGTCGGTGAATTGATTACTTGGGGACATTCATCACCATCATGTAACTCCATAGTTTTAAT
GATCATTCATTGCACAATTCAACAATCGGTCCGTGGCGTGCTCTTAACCATGTGTGGATCGAGAATACCCTAA
GAATTCTCGTCACTGCAGTGAACAAATCTTGAATGAGCGGAGGTGTGAATTGGGGCTGCCAGGTGCTGGGAAT
GTACAAGGCGGTCCGTGCAGCCGTGTGGAGGAGACCTTCAGAAAGATAATGGACCATCTATTCGATTTCCCAT
CACCGCAAACGAAACGGGTGCTCATCCTCCGAGATTAGGGATTGCGGCAGGGTTAACTCTTGCGAGGGACGCG
ATTGGTTGCCATCGCGCCCAGATTCGCCTTTCCACCCTGCAGCTCGTGCACATCGCAATCCTGGAGAAAGAAC
TTATCTAAATTAAGTACATTCAGTAGCCGCGAATGTTACAAATTTCGCACGAGGGCGGTAACGACATGCGATC
CCCACTTCTCCAGTTGAAGCATGCCGTGGCTGATCAGTGCAATCGATAACTCAAACCTCAACGTACAACATGG
CTCCAACAAGCCATGGCGGCCATTTTCCCGCTGTGTGGCAGATAATGAATGCGCGTGGACTCTTGACTCTTGA
GCGTTTTCAACACTCCCTATATCTCCCTCTTCGAGAGTTATAATTCATGTATACCTTTTGCTAATGGTGCTAT
ATGCAAAATAATAAGACTTGGATACTTTGAGTGCACAGCTGCAGAACTGCAACTGGCGGGGATTGAGATTATG
TAAAGGGGGACTCCATGACGAGCAAAAATCCTGTGACGGCCAACTTGAACACTGCAACCCCACTTCAATTGAT
CAATCATGTCCGAACTCCAGAGGGTTAAACATAAGCTGGACAAAACGCTTGAATCGCTTCCGGGTATCTCTGC
GCCCTTTAGCTCCCTTGAAGCCTGGAGCGAGACTTGCTAGTACTTTTTCGTATGTGGCTTCATGTTTGTTCGT
GCCTAACCTGAGCTTAGTAGCACAGATACACGACACTCCTAAATGGCAAGGTGCCTGATCCAACGGCCACCTC
TTGAATGATGGTTGTTAAGTGCGTGTGGAAAAGAAAACTGAGACGCCCTCTCATACGCCTCTGAGACGTTGGG
TTCCATAAGCGCTGCTTGTGCCGACCCAAGGAAGCTGAAGGGAAAGGGGAAGGGTCTCAGTGCCGGAGACAAG
GATGATGCCGTCAAGAGTGAGGGTGGTGATGAGGAGAGCAAGGATGATGATGAGGAGAATGAGGAGAACGAGG
ATGACGAGGTCGAGGACGAAAAAGTTACAAAGACGCACCAGCCCTCACACTCCATGGGACACCCGCAGCGGGG
TGAAGAGACAAAGAGCTGGAAGTTTAGCGTCAGTGAGGATGAACCCGCGAAGAGTCACGCAGAGATAAACGCA
TGGCTTTGTAGGCGTGGGAATCTCTCACTCCTGTTCCTCAACCACGTCCTACAAGCCATTCCTCAGCGTCATT
TTCTTTATTTTAGCTTCGCGTTCTTGATGAAATACATCTCATGGCTACCGCGGAATCTGGATCTGAGGGACAC
CTCCAGTCTGAAGAAATGAGAGATCGATTAGATCAGTACAATATCTCGAACCTGGATCCGAAGAAACCACTAT
CACAAGGACAAAGAACTTATTTCCTGACATCTGCTCTTCCACGCGAGACGCTTCTCTCACTCAACAATGCGAT
TCTGAGCCGGAATGCCGATAGGATCTTGTTCGCATCCAAACCAGAATGTAGGACAGAACTCTTAGCTTTGGGC
CCGGAGGAAGCAACGTTCTTCGTGCCTCCGCGCTTACCTGTCACTCCTCAACAACCCCCTAGGCCCCACCTCG
TCCGCCAGCGACTCTACAAGGTTCTGGTCAGGTGTAGTCTGGACATTGAGGCTGCCTATCGCACTATACCCGC
CTTCCCGGGACACAAGCGTGTCATGGCAGTCCAACACCGTGGTTGCTTCTATGTGGAGCATTGTATCACCTTT
GGCATCGCGTCGGCCCATGGTCTTCAAGGTGAGGTCGCGGATTGCACGGTTGCCATCTGGAAGGCCAAAGGTT
ATGGTCCCTTTGCCAAATGGGTGGACGACATTGTTCTCTTTCGTTCTCCGGTTGATGGAGGGCATTTTTTCTC
TGATCATTCAGGTCACACCTTCTCCTATGATCGCACCTCGGCTTTGGCGGCCATTGAACACTTGCGTGTTCCC
TGGCACTCGTCCAAAGGTCACGACTTCAGTCCTGAGTTCACCTTTGTCGGCTTTGTATTTTCCCTCTCTGGGC
GCTATGTCACCATTGCTGAGTCCAAACGTCTCAAGAATAAAGGGAAGGTTGACTCAGCCCTGGCTGTTTTCTC
CTCTGGACGGGTTTCCCTGAACGAGGTACAGAAAATCTTCGGTTCTCTTTCACACCTAGCCTTTGTCTACCTC
GAGCTTCGCACCTTTCTTCCTCCTCTTTCCGCCTTCGCATCCTCCTTTGCAGGTAACAAATTTGCTCGTCGCT
TTGCTCCCCCTCGGTTCTCTCGTCCCTGAAGGCGTGGTCAGCAGCACTCTCACTGCCTCCTACATGCCGTTCC
CTGATTCCTAAAGAGCCTGCTATCGACATCAACCTCTGGGTGGACGCCTCCACCTCATGGGGCATAGGCATTG

TCCTCGGTCCATCCTGGATCGCCTGGAAACTTCGTGATGGTTGGAAGGGGCCAGCACAAGATATCGGATGGCT
GGAGACTGTGGCGGTTGAGTTAGCAGCTGAACTTCTCATTTCTCTGGGCCATCAGAATGCACATATCCTCTTA
CGATCGGACAACCAAGGTGTTATCGGAGCTCATGCGAAGGGACACAGTCGCAACTTCGAGAGTAATCTCTCAA
TCAGGCGTACAGGCATCAGTTGCGCTGTGGCTAATATTTCTTTCTTCTTCCTTTATGTTGAGTCTTCCTCCAA
CCTAGCGGACCCTATTTCACGAGGTATTCTGGGTCCCCCACATCTGAAACTTCCTTACCCCTCTTCCCTAGCA
GACGCCCTCATACCGTTTTTCACATGTGTTTAATATTGATTTATATACTCCTTTTTCATTGATCAGTGTCTCG
CAAGTTCATTGATCATCCTCACGCGCTCCTTACTGTCCCATGCCAAACTCCAAATTCATCACGCCTTCCTTAT
GGCGCCCTCAGGGTAAGGTGGAGGATTGTCTCGCCACATGGACAACCCCCTTTGCAACCCAGCACCAACGAGC
AAGTTCCTCACTGTTCAACGAGGCGGCCTATCACACCATGCTGGATGTTTCTCTTGCGAGTCTGGCTCCTCAA
ACTCGAAAGACTTATGCTGCTGGTCTCCTTCGATTCAACCAATTTTGCGATGCTCTCAACATCGCCGAGGACC
TTCGCATGCCGGCTTCGTCCAAATTGATTGCCATGTTCCTCGCCAACACTGCAGCAGGTCAGGTTTCTCATGA
CTGTGCTAAAAATTGGCTCTCTGGCTTGCATTTTTGGCATAATCTCCACGGTGCAGAATGGCACGGCGACACT
GATCAAATCGATGGCATTAAGAAAGGCATCAAGAAGATGGTTCCTGCCTCCTCAAAAAGATCACCACGCTCTC
CCGTCTCTATGAAACACATGTATGCTCTTTGTGAGCGTCTCTCTACATCAAACTCAAGAGACGTCGCTCTTTT
GGCATTGGCCAAGGTGACTTTCTGGGGGACTTGTCGTCTTGGAGAAACAGTTCCCCCTGCAATTGACAGATTT
GATCCTGCCTATCATGTCTCTCGAAGTTCGCCAATCACACATGGCCGAGCACGGAATGGCACCGCATTCATCT
CCATACACATTCCGTGGTCCAAGACTACAGGCTTTCAAGGAGCCGACATCACTCTCATTGATCGAGACGACTT
CAGCAACCCAGTTCAATCATTTCAGTGGCACATGACAGTTAACTCTTCAGTACCTGGTCACGCACCACTCTTC
GCATACGAGACAGCCTCAGGAAGTTTCGACTTCTGGACGCGGGAGTCATTTATGGAAGCATGCAATCAAATAT
GGCTTTCAGCTGGGCTAGAAACGCTCCATGGTCACTCCTTTCGGATTGGAAACGCTACAGAACTTTTATCTCA
AGGAGTTCCCCCAGACGTCGTTGCGGCCCAAGGCCGTTGGAAGTCACACGACAGTTTTCTACAATATTGGAGG
AGGATTGAGGTCATTCTCCCCATGTTTTTGGGTTCGAATAAGCCCGCAACTCTTGCTAACATTCGCCAGTCAA
TGGATTCCTTCTTCCGGAATAGTCATTAATTGTATTCATATCTCTCTTGCATATACGCCAGTATCATTATGGT
CTTGGGATTTCTAATCTACTTTTGCTTGCTTGATTTGGATGAGTCGTTCTCTCACACACGACCGTTGTAAGCT
GCGCTCCGTATGGTTGGTCTGTGTGTTCTACCTCCAGACTTTAGTCTTCTCACGGCCCCTATTTCGGTGCCTG
GACACATCAAGACCTTCCTACACTCAAGAGGCTGATTCTTCAGTCATAATCAGCCCTTTCCCCAAACCACAAA
ATAATGATATTTTGCTAGATCTAAACTACATCCAGTATAGCTCTCCCTGTATGTACTTTGTCTAGAAAGCCGA
TTCTCAGCTGGATGCTGAAGCCAAAGGGACACCAACACTATCCGCCATCGATTCGTCCGCAAAGATCAAGAGG
CCCAAGACACTGGGTGGCTCAAGGCTGGAGCCCGGGGTAAGCTTTGCATGTTCTTGGAGGCAGCTTTCTGACG
CGTTGGTCCAGGTATGGCCCGCCGAGACTGCAAACATTGACGAACCTGCATGCGAGCTCCTGGGCGCTTCCAA
CCCCTCCTATATCTCGCTCTTCAAGAGCTAAAATTCACTATGTTTTCTATTAAAGGTGCCATGGATACGTAAA
AATTTGAACAGTTACTCGGGAAAAAGGAGTTGGCAGACCTGTTCGACGGCATGGTAAGTACTGCCCTTGGAGA
CCACGCACCTGCGGTGTTCTCACCACGCCACAGCCAGGTATTTGACCCTGAAAACTTAAACCCTGCACTACCT
CGCTCCTTCAAATTGATCAACCATGTCCAACGTGGCACCCGAAACCCCCTCATCCACCCTTGCGACGCTGCGT
TCTGTGCGCTCCACTCGTGCCCAACCGGGGGAGCAGAAATGGAAGGGACGCCCGAGTGAGGGGGCCTTCGGTG
CTAGAAACAATGATGATGGTGACGACAACTACGACATCGCAAAGATGCACCAGTGCCCACAGGGCGTGAAGAG
ATCAAGAGCTGATTCAGGATTGGAGGCCAAAACTGGAGAGGTGCCCGTACCACGCACCATTAGCTTGGTCTCG
AAGATGAAGAGACGCAAGACACTGAGTGATTTAGAGCAGGAGCTCAGGGTGAGCTTCGCGTATCCCTGGAGGA
AGCTAGAAAGTTCTGATGTGTTTGTCCAGCGTAACCCGGACACTGAGGACAATGAAGTGCTGCGCCGATGCAA
AATACTGATGGACTTGAAGCCGGACAGGGCGAGCTTCATGTGCTCCCGGAGGCAGTTTCCTGGCGTGTTGACT
CAGCGCGACCCGCGCACTTGGGCGACAAGCACCAGGAAATACACTCAGTTCCTTAGGAAGGGGGGGCACTCCA
CTGACTCAAAGGATGCCAGTATCTGGAAGGTATGCATGCTGGTGACATGATGCTGCACCAGGAATCTGATTCC
TCGGCTGGACAGAAGGGACGTGGTGGTGCGGGAACACTGGGAAAACCCGTTGAGGGAAGCACGCGCGGACTGG
GGGAGACTTGGGGCGGAGGTGGGCAGGCGTCGGGTGGAGACAGCGGTGGTAGAAGGTCTGAACACACTCAACT
CTCCGGGAAGGGAAAACACGCCGAGGATATTGGCAGTGGGAAGACGCACGCAACGCTGGGCGACATGGTACCG
TACCAGGGGTCTGGTGCCTCGGCTGGGCGGTGGAATGCGGCAGCATGGTGATGCTGGGGAAATCTGTCGAGG
AAAGCACACGTGAGAAGAGGGCATTAGATGGCAAGGCGACACTGTCCCAGCGCACGAGTCCAATCCCAGGATC
ATCACATCGATCAGAAGATGATCGGGTGATTATTGCAGCCGAGCTGGCTCTTACGAGGGATGCGCTCTCTCGC
CATCGCGCCCAGATTCGCTTTTCCTCGAGCGAAATCTCGCACCTCGAAGCTATGGAGAAGGAACTATGCGCGA
AGATGGATACGCCTCGAGGAGGGTACTAGGGAGGAGTGAACTGATACTAAATGTCAGAGCGCGCGGAGGGGCG
TTCGGTTAGGGGCTCGTTCTGCATTGGGTTTATACTAGAGCACTGTCATTCAACGAGCATGTGGAGACTTCGG
ATATTTCGACACAGATTATGTAAAGGGTTCTTGACGGGGTCTTGTCACGGAGTTCACACCTCTTCAATTGATC
AACTATGTCTGAACTCCAGTGGGCTAGCTCCATCCGACTCCCCGGGTGACTTTCAAACCCTCGTGGATCTACA
CAAAAGGCCTGAATTCTATAAGACGGATCCGTCGTGGACCGCGATTGACCCTGTCCCTATAATCACTACACCG
ACTTACGACGATCTCACCGCTCCGGCTGTCGTGACTCATGAAGGAGCTGGAGATCCAGCCCGAGTAAGATGTA
AGATAACTCCCACCACCCAGCATACCTCAGGCTCCCCCTCCATTGTGCAAGATATTACAAAAATTATTCGGCT
ACATGCCAATCCTGTTAGCTTCAATCATCGAGTGGTGCTGGAAAAGCCGCTATTGGATGAGCAGGCTGTTCAG
AGCCCTGGACATCCATGAAATAAATTCCCTATGTAGCTGGAGTGGGACGAATACCTCGGGAGTTGTGGTAGGC

GTCTCAATCGATCTATGCCTTCGACTGCTTCAATGTTAATACTGGGTAGTGGGGAATAGGGCACAGTGCCCCG
CGTGAGTGTGTCGATTTTTAATTATATGCACTAGCCTGGCGGAACATGGTGTGAAAACGCAAAAATGACTCGC
GGTAAATTGTGGTCCAACGTTGGGTAACTATTCATTCATGTGATTCGCCGTCGCTGAATATCAAACGATGTTT
TCCAGCCGGGGCGCCGTGTAGACCGGTGATAGCTGATTGTGGGGATTCCTCAATATTTTGCCGTGCCACCTAG
CAAGCAGTGGGATCGAGTGTCTACTTATGGTGACGTCACGTTAAAGGGAGGGCATTCACCTCGAGTCCAAATA
TGGTACCGGAATAACCAAACCCGGTGATCGATCAACTCAAACCCATTTATGGAAACTCAACATCACATATAAA
CCACCCCACCACCTCAAATCCCCCCATTCTTCCTTTCCCACCTCCCACTTCCTCCTGGCTTGCCTCCGCTTGC
AAGCGATGCGGGAACCCTCCCCCAGGCCACCACCCGCCACGACAGCCCAAATGTCCTGGGCGGGGCGAGGCAA
CTCGCCTTCGCCCAGTGATGGCATGGTGGATCTAACCTTCATAAGACGGGGCGCTGGACATTCAGCGCTTACA
CTGAGGGGGACCGCCGTTTAATGCCATCAGACATCTCCCCGGGTGCGGTCGAGCAAGGTCTCCCGCGGTCGTG
GCAGCCACCTTGGGGGGCCCGCAGCGGACCCATGTTCTTTCATCTGTGAGCCGCCTTTCGCATTCTTGGTTGA
CTTTGGCTGACTTATCGCCCCGCCTAGTTTCTTTTCATTTGTGAGCCGCCTTTCACATTCTTGGGCGACTTCA
GCCGACTCATCGCCCCGCCTAGTTCCTTTCTTTTGCTTCCTTCCCCGCTCTGTTCCTTGCTGTCGTTGATGTT
GTTTTCGGGTGTCGTGGTCAACGCTTCAGGTTCCAATCCTGTTTCTCGCTCGGGAGGGGTGCATAGGTAAGCA
ATATCTTATTCCTGGTTGTAATCGCAGTTGCTCACAGCCTCAATGCTCTCCTTCCTGGACCGTGGTCATCCCT
AGTGGGGCGGCCCCCTGGGGCCCGTGAGTTGCTTTCTTGTTCTCGCCGTTGCCACTGTTAATGGGTTCTACAC
GAGGTCGTC
>Cligib1_Cluster110#LTR/Gypsy
GTAAGGACACGCCAAACAACACCCTAAAGTGTGCGGCAGTCTCTCAAGACGAATGAATACAGAGAAAGAGAAA
TGAGAACGAATGACTTAAGTAACCACTTGTTAGGCGGAAACCAAAGAGAATCGAATTAGCGTACATAATACAA
ATGAAAAGCCTTAAGTACTTCTAGAGTATTTGGCAATAAAAGAGAAAGAAAACAAAGTAAAGTAATATCCTAA
AGCATGCGTACTCCATGATGCAAAACCGCGGTTTGGCTTACATAATGGATCACGTGATGATCTGCGATAAGGA
TTTGAGAAAGATAAGATAAACCCCTGATAAGATATGATAAGCTCGATAAGATAAGGGATAAGATAAGATAAGA
TGGAAAAAATTTGCCTCCGGAAAAATAAGAAAAGAGAAAAAGAGAAAAAGATAAAAAGATAAAAGAAAAACTC
CGCATCGCCTCAAGCGAAGATTGACTCCAGTGCGGCAAACAAAGGGGCGTACACAGTACGATGAGGAGAACGC
TTTGGAGAATATCCATGGTTCATCAAACTCAAATCGTCTGCTCGCTACACAATGTTATGAGAAATGTAAAATA
AATCCATTACTCCCCTTCTAGGGTGCAAGTAAGAGGGATGCGTCCGGCACAGTGAAATTGTTGTAAGGTTGAA
ATAAATTATCAAATTGTGTTTGAGAGATACCCTGTATCTTTCGTGGTGCCGATGGATTCCTGTGATGAAATTC
GTTAACCAGATCCATGGAGTTTTTGAGATTCGCAATCGGCTCCCAAGTAGAATGTTCTGGAGAATAACCTTCC
CACTGAACATAGTACTGAAGTCGACCTCGTTCCAGACGCGAATCAAGAATCTTCTCAACATGGTATTCAAACT
CGCCGTCGATATCGATTGAAGGGGGTGGTGCGGATGTTTGACCAGCAATAGGAGGGGTGAAACTTGTAAGGCA
TGTCACGAGTGCACTCGTGGGTGATCCGGATCCGCAGCTATGGGTACCCACACTCATGAGTCAGTTACTCATG
AGGATCTGGGTACTCACAAGTTACTTGTGGATTTATGCTCTGCACCAAATTTCAACATTGAGGGGTCGATTTG
ACACTCCTAAGGGTGCTTATTAATATCCAAAAGAGACGACAGGGATACCTGTCCCTGATCTTGAGTTATAGGC
TGCGCATTCTAAGGGGGGGAGGAGGAGAAGGTCATGCTCCGGGGTCAATTCCCCACGGAATTACATGATTATGA
GACGCATTTAACACTATTTTACGAGCCGAACACGATGCGAATGACGCGAAAGGGATCAGTGTCCCCGATCTCA
CAATATGTTCAATTTGGCAGATTTTTACTCTGTAGCTCAAGATCAAGGTCACCGATCCCTCTCGCTGTATTCG
CATCGGATTAAGCTCATAAAATAGTATTTAATGCGTCCCACAATCATGTAATTCCGTGGGGAATTGACTCCAG
AGCATAACCTTCTCTCCCTACCCCCCTGAAATGTGGACCCTATAACTCCTAATTGGGGGCAGGTAACCCTGTT
TTCTCTCCCAAACAAGGGTCCACAAGTACCCATGAGTAGATCCGCCAAAGACTCGGATCCATGAGTGTAAATA
CCCACAAGTATCCCACGGACCTACTCATGAGTACTCATGAGTTACCTGCACTCGCGACATGCCCTAGGTGAAA
GGGCAGATACAGGAAACGTTCACAACCGGGTGAATGCGAATCGAGCTGGGGAATTTTAATTTAACCGCATGTG
AACCAACGAGTTCTAAAACTTCGAAGGGACCAATACGATGTTCCGCGAGTTTCTTCGACGGACGTTTGATGTC
TAGATCTTTAGTCTCTAGCCATACTTTATCGCCTACTTTATACTTCGGCATATCTCCATGATGTTGATCAGCA
TAGCGCTTCATTTCGTCTCGGGATTTAATAAGGGCGGCCTGAAGGGGTGGTTCCCCCTTCACGCGAGGAATGC
TCTTGATGCCGGCAACCCATCGTCGGCCGATGACATCACAACGTGCCGCACCAGCCCGGCCAAGCACGCTCGT
CGATCTACTCCTTCGACTTAAGCATGGCGACCTCATCATCACTCGTCTTGGACTCAATATCTACAATATCTTG
GACCCCCTTTCCTTCTCATTCTAGTCACCGTTGGCCGTCACCGTTTGTATAAATATCGTACGAATTCCACTCT
TTTAGGACAGTTTTCACTCCAGTTAATCTACAGTACTACTCTCGTTTGTAATCTCGTCGAACATCTCGAATCT
ATCGCGCTCCCTCGTTTTCTTGCCTTCTTCCTTTGTCCCTCCTCGAACTTGCCCGGTCTGAGCTTGCAAGAGC
TCGTAGCCATTCAGGCCTGCGCTTCTTCTTGAATATGTTTCATTCGCTCAGTGAACTCGATTGCTGTCTCATT
CTTCTGGGAACGGATCGGTTCGAATCCCATGCGCGGGTGCCTGCCGTTGGTGAGATAAAAAGGGGAGACCTTC
GTGCTTGAGTGGATTGCGTTGTTGTGTGCGAACTCCGCTGAGGCTAACCATTCTGACCAGTCGGTTTGACGAT
GATCAATAAAAAGTCATAAATAAGCTTCAATATCCTGATTGATGCGTTTGGTTTGCCCGTCCGTCTGCGGGTG
ATAAGCTGTCGATAATTTAGTTTGAATGCCCAGCAGAGAGTTCAACTCTTTCATCATCATGGATGCAAATTGA
GGACCTCGATCTGAAATAATTGAATCCGGGAGACCGTGATGCTTCCAGATAAAGTCGCGATATAATTTAGCGA
GTCCAAGTGAAGACATCTCAGACATAGTAGGAATAAAATGAGCCATCTTCGAAGTACAATCAACAACAACAAC
GATAGCGTTATATCCGGACGATAATGGTAGTCCAGTAATAAAGTCGCAGGACACAGCCTTCCAAGGTTCGACT

GGAGTAACCATCGGTACAAGCTTTCCAACAGGAGGGGCGGGGAAATTCTTGTGCCTTTGACATTTGTCGCACA
CGTCGACGTAAGAAAATATAAATTTAGCCATTCCGGGCCACCAATAATTACGTGTGATTAACTCTAATGTTTT
ATTTCGTCCTGGGTGTCCTGTAACGACCGACTCGTGATGTAGTTTGACTATCTCTCGACGGACATCCGGCAAC
TTCGGAACGTAAACTTTTCCTCAGAAAAGTATTAGATCCCGCTCTTCGGACCACTCTTCTGAACACAAACGCT
TAGTCGCCGAATGCTTCAACTCCTCAATCGCTTTAACTACCGCTTCATCATAATCCTTAGACTTCCTAATCTT
CTTCAACAACTCTTCTTCATGGCCGTCCAAGAGCACATGACCCTGCTGAAGGGCTTTAATAGAAAAGAATTCG
GGTTTAAGTAGGGTGACATCATCGTTATCGGATTCTACCCCCTTCTTTAGGTCTGTTCTCCTGGACAGAGCGT
CGGCCTTTCCCATAGCCACTCCGGGTTTGTTAATAAGTAAAAAGTCAAATCGTGATAAATAAAGAGCCCATCG
TGCCTGTCTACGATTAAGTTTCTTAGACGTCATGAAGTATTGTAAGTTTTTATGGTCCAACCATATTTCTACA
GTTAAGTCCGCTCCTTCTAAATAATGGCACCATGCCTCGAGACATCGTATAATACTTAACATTTCTTTGTCGT
GTACATCATAATTACGTTCAACATCAGATAAAGATTTGGAATAATAAGCGCACGGTCTCCATTTACCGTCGTC
GCATAACATACTTAAAATACCACCTGTAGCAAAGTCCGAAGCGTCCGACTCTACACGTAATTTTGTTTAGGAT
CTGCCATAACCAGGATCAGGTTCGACGTAAAACGTTTCTTTAGCGTATCGAAGGCAACCTGTTGCTTATCAGT
CCATTCAAACTTCGAGTCCTTTCGTAATAAATTGTTTAGAGGCCCTGCTAACTTGGAGAACCCCTCGATAAAA
CGTCTATAAAAATTTGCGAATCCCAGGAATTTTCGTATTTCCCGGACCGTCTTCGGGACCAGCCATTTACTAA
CCGCGTCCATCTTATCCTCTGCCATCTGTACAACTCCCTTATCCAATATTAACCCTAGGAATTCCACCTTCTG
GGTACTAAAAGAACACTTCTCCGTTTCAAGAATAAGTCGTTGTCTAATAGGATCTGGAGAACCCGTTCGACAA
TCTTTTCGTTTTGTTCTTGCATGTCGGTGAAGATAAGAATATCATCAATATATACGACCACATTGTCAGGACT
TGTCTTTTTCTTTTCTTTTTCCAACTCTTTTTCATCTTATCTAATCTCTTATCTATAGGTTATCTCATCTTAT
CTCTGTACTTATCTTATCAACATCACGTGACTGCTTATGTAAGCCAAACCGCGGTTTTGCATCATGTAGTACA
CATGTCGTTTGTACATATTATATTCACGGGCTCTTAGCATTAGTATATAGTTTCATCGTTTGTATGTTCGTCT
CTTAACTTGATTCTCTTTGGTTTCCGCTCATACAAGTGGGACTTAAGTCAAACATCTTCATTCTTTCCTTTTC
CCTTGACTATTCTTGAGAGACCGCACATCCTTTGTGGTGTATTAGCGTGTCCTTACACACATGTCCTTTGTCG
ATAAGCTCTCGTAGTAATGCATTCATCATCGTTTGGAAGGTAGCCGGTGAATTACATAAACCAAAATACATAA
CTAATGGCTCGAAGGAACCGCGATGCGTGGTGAACACTGCTTTCCATTCGTCACCCTCTTTTGTCCGTACGTT
ATTGTAGCCCCAGCGTAGGTCGAGCTTTGTAAACCATTTAGACTTTCCTGCTTTATCGATCAAGTCGGCGATC
ATCGGTAATGGATAATTATTTTTAATAGTCCAGGCATTGAGGAACCTATAATCCTGAACCATACGCTTCTTGC
CATCCTTCTTTGGTACGAAGAAGACAGCTGAAGTTTGCGGAGACTTGCTTTTGCGAATATAACCTTTAGATAA
CTGGTCCTTTAAAAAGTCGTCCACCTCCTTCTGTTCGGTAGGGGATAAATTAATAACTTTGGCCTTTTTGGGC
ACAAATCCTTCCTTCAACTCGATCCCATGATCCCATGTCTTTCGCTCGGGCATGTGCTCCGACGCTTTCTTTT
CAAACACTTTTAACCACCGCCTAAACCTGTGGGGAATCTTTTCTTCTAGACTGGGTTCGGTAGGCTCCGGTAA
TCCACTTATCAACGGGGGGGCGTCAAAGCTCTCCTCGATCTCCACTGTGCGCATGAAGTGTTCCGCAACTACT
TCGTAGACCATTTCGTCAAAACGAATCTCCTCTAAGAGGGCTCCTTCTTGTTCCACTATGATCCCGTCTATAA
CACGGTCGGAATCCTCATCATCGACCTCCTCAAGCATCGTTTTATACTGCCAGCACGAAGCCATGGCCTTACG
TTTCTTTTGGGTTTTAGCTGCACGGATAGCCACGTTGCATTCTGCCGGGCATCTATTCATAGAGACTTCCCCA
GTCGTCCAATCAATCGTGGGGTTATGCTTCTTAAGCCATGTGTGTCCCAAAATAACTTTAGTTTTGCCAAGTG
TCGTCACTTCAAACAGCATGCGTTCCTTATGGCCCTTGTGGGAAACCATCAAAACAACTTCTTCCGAAATAGA
ACTGGCTGCGTTTAAAGTACCGTCGACATTATATACTTGTACAGGGTGACGTAGCGGTCGCGTAGTCCAACCC
ATCTCTTTTACATATTCTTTGTCAATGAATGAACCGGTAGCTCCGCTATCCAATAGGGCCTGTGTGTTGGCCG
TTGCGTGTGTATCTAATGTCGTCATCACGATCGACATTTTAAGCTCTCTAGCCAAATTAAGTGTGCAAATGAA
ATAAGTCCCTGACAAAGGGAAACCAGGACGGGCCGAAGCACCGATACTGCCGGGACCCACGAGTTCACCAGAC
TTGGGACAGACAGATGTACCATTTAAGGCATCCTGATCGGTCAACAGGTTCGTAACACCGGGACCCAGCTTCA
CTGGACGGACGTCAGATGGAACCCCAAGACCCTTACTATGCTCCACCTTCCTTGCTTTGAGCGACTGCATAAA
CGTGGACAATCACTTCTCCAGAGCACGTCCCTGAGCATTATCCGCAATCTTAAAAATAGGGATTGTCTTGTTG
GGACCGACCCGTGCCTTTTCCGGTAGCATAACCTTTGTAATATTAGGTAAATTGTCTAATTTGGAAATGTCCA
TGGGAGAGTGAGAAGGGGAAAAGAACCGTCCGCCGACTTTGACCTTGCCGTCATTGTCGACTTCCATACTATC
TACTGGAAGAGAAGAAAAAGAATTATTAGAAGCAAAATTGTTCGTAATTGGAACTACGGGTACAACAGGTATT
GGAGAAACTAATTTTACAGTAGAAGAAACGGGCGTAATTATTTTTTTGAATTTTTCTGTGGCTTTGTAATTTT
TATTTTGTTTTAGGAATTCTTGTACGAAATATTTTTTTAATTGTTCATGAGTAGCACTACGAAAGTCTACACT
ACTAGTACAGTTTTTCATCAAATGACCGGGCAAGCGGCACTTGTGGCATGTTTTTGGTTTTTTGGTTTTCTTT
TGTTTTTGTTGATTTGCTAAAGCCCGACGAGCAGTCGTGACTTCTAATTTTCTGATGTCGAGGCGTGGAGAAA
TATTTACTGGGGTTGACATCCCCGATGAGCTTTCGCTCAGTGCTGTATACAAATAAGGACGCGCTGGTGTAAT
AGGCGCAGCGGCCATGGATTGTGGGAGTGGCGATAATCGTGGAATGATAACTCAGCCGACCGAAATGCATGGC
CAGACGCTGGAGGAGTCCATCCCCATTCTCGATATTTGGATGCCGGGGAACATTCCGGACTTTTCTTAACAAT
GTTTAGCGGTTTGGTATTCCGCTCTAAAGGAGTAGGTGGCGGGTTGAATCCCGCAATATCGCGTCCAATTCAG
ACCGATACTGAGACATGCGTTTTTCAAGATATGCTATCTTATCATCGGATACCCACGTGTAGGGCACCGACAT
ATTGCCTACTTAGGACATGGGGCGGCGATACCCACGAACAGAATCCAAGAAGAGGGACGGCCGGACTCAGGCC
AGAGTGTTCCTTATGTAATAGGAGCCTGTCCAGGCGTCTGACTGATACTAAGCGCTAACCCTTAAACACTGAT

CCTGCGCAAGGTGTGGGCGGACTTAGCAGTGAGAGGATGTCGTAGGAAGTTTCTGTGTTCTGGGGATTTGAAG
ACCCGCCTCCTGTGATCGCCTAAGCAGCCGTGTTAAACACAGATGAAAGGGACCCAAGGGATTGGGTTGTTGC
CAAGGAGAGACGACCAAGCAGCCGCGTTAAACGCAGATGAAGGTGTCTTCTGTTTGGGAAAAAGGTAGTGGGT
TATTTTAGAAACTGACTTACTTGTTCCAACTACAACTTTTGAGCGGTGATGTTGTTTCACGCGCTAGCGATGT
CGTTGGACAAATGAGAGAGGGCCGCGTTGAGAATTCGATTCTTCTGTAAGGACATGCCAAACAACACCCTAAA
GTGTGCGGCAGTCTCTCAAGACGAATGAATACAGAGAAAGAGAAATGAGAACGAATGACTTAAGTAACCACTT
GTTAGGCGGAAACCAAAGAGAATCGAATTAGCGTACATAATACAAATGAAAAGCCTTAAGTACTTCTAGAGTA
TTTGGCAATAAAAGAGAAAGAAAACAAAGTAAAGTAATATCCTAAAGCATGCGTACTCCATGATGCAAAACCG
CGGTTTGGCTTACATAATGGATCACGTGATGATCTGCGATAAGGATTTGAGAAAGATAAGATAAACCCCTGAT
AAGATATGATAAGCTCTGATAAGATAAGGGGATAAGATAAGATAAGATGGAAAAGACTT
>Cligib1_Cluster68#LTR/Copia
GTCGAATGCACCGCGATACATACATAAACATTACGTCATCATATATATATCTACCCGACCCTAGGTTCAAACT
TACCTCAGTCCTTATCCCATATAGGCGACTGAGGTAAGTATAATCACCATCGCGATACATTACCTCACACTCA
TCTTGTATCTAGATACACATTCTAGGTACAGATAATGATATAGTCCTTATCCTATATAGGCGACTGAGATACA
CATTCTAGGTTTTTAACAGGTTATGGGCCTTCTCGCCTAAGACTGTATCAGAACCGCCAGGATCCGACTCATC
CACTCTCTTCTTTCCACCCTACTTCTTATTATTATTTCTTCCATGGCCGGCGACGATCCTTCCGGTAGCGTGA
TTGGCGTGAATGGCTCGGGCGGCGATGATGTGCCAATCAGACCGCGTACCCCCGTTCCAGTTCAGCCTGCAGT
TCTACAGACGGACGCAACCCACCCTTCCATCCCTTCTCTCTACGTCAGCGAAGGTCCTTGGGATGGACAGGCT
GTCTTGAAGAAAAGTTCGAATAACTGGCCCGCGTGGAAAGAACGCATGGTGGACGTCCTTACGCTATCAAAAG
GGATGAACTGCTATCTTACCGGTACCGTCCCTCGCCCAGATCCCGTTCGAGAACCGCGAGCCACCTATAATTA
CGAACTCAATGATGAATCGGTACGTGCATATATTCGCACGAAACTCTCCCCAGAGGAACTCGCACACGTAAAA
CAATGCCAGTCGTCCAAAGAAGTGTGGTCAATCTTAAAGGATCGCCACGAAAGACAAGGTCCCATCGCCCAAA
TTCTTCTTATGCAAGAGGCGTTCAATATTCGCTATTCCCACTCGGTTCCTTTCGCGGAGACAATTGCATGTAT
TGCCGACATCAATGATCGTATATGGAACATGGGTGCCCCCATGCGCGAACGGTTTCACGTCATTCTTCATCTC
TTAGCCCTGGCCCCCCCGCAAATGCACAACGTCCGCGACGCCATCACCAATGGCCTTTCTACCGCAACACATG
AACATCCATATGCCGTCCAACAGATCGTCGCACGCATCGAAATGGAGCAGAAAGTCCTAGCATCCGATGGTAA
TGCCCCAGGCGACGCACTCATGGTCGATGGCACGTCTAAAACCCCTCGGAAACCCATGACTTGCTCCAACTGC
CATCGCTCACACCATGAGGCGAGGTTCTGCATCAAGCCTAACGGCGGAATGGCTGGGAAGACGGTTGCTGAGG
CTCAAGCTGCCGCGAGAACCGAACGCGGATTACCACCGAAGAAAGCCCCCTCGTCCGCTTCCTCTTCTACCAC
TGCCCCTGCTACCCCCGCCGCTTCGACGTTCGACATCATGCGGGATGCTAAGGGACGTGCCTATGCTGTCGAC
ACCTCTTCTGGACAGGCATACTTCCTCCAGCCCGATCGGCCTGCCCTGCCTCCTGCGCCGGCTGCTGAAGCCC
ATTTCATGGCGATGGAAACCGACGATATTGAGACGCTGGTGTCGCCCTTAGTTGCCAACTCCATGACCAATGC
CGATTACGCCGAGGTGGCTTGGCTTGCCACCCAGGACGACGTCTCTACCTCAGTAGACTGGGAGGCCTATTCT
TCTGGGCCTGCCGATTTCAGCGTGTGGCTTACGTCTCCCATTGCCAACGGTGCGAGGAAGGTGCCCATATCTC
CGGACTCAGCCCCCTTTCTCATTGATAGCGCTTGCACTACACACATCTCACCCGAACGCGCGGACCTCTTCTC
CTTGAGTCCCGTCAACAATCGCTCCGTTTTAGGGCATGTCACGAGTTAACCCGCAAGTAACCTGGATCTGCAG
TCACAAGTACTTGCATGTGAAAAACTGATACCTGGATCTGCACAAGTTTTATACCTGTGAGATCTGGATCCAC
AGGTCATTTACTTGTGAGTATCCAAGTACTTATGAGTTCTAAATACTTGCAGGTACTCGCAAGTTACTCACAG
GTTACTTATTGGCTTTTGGAAACTGGCATACCCTTCAAAAACCCGCCCCTAACAAGCTATCACATAAATAACC
ATCAGTTCCGCACCAAATCGGGACTTTGAGGGGTCTATCGGACACTCCTAAAGGCCCTTATAAATGTCCAAAA
GACCCGACAGGGCTACCTATCCCCATTCTTAAGTTATAGGGTGCGCGCTCCAGGGGGGAGGAGGAGAAGGTCA
TGTCATGGAGTCAATTCTGCACCGGAATGTATGATTGTGGGACGCATTAAACACTAATTCATGAGCTGAATCC
GATGCGCATAGCGCGGGAGACATCGGTATCTCCGATCCTGAGATACAGAATGATAGTCTGCGAGATTGCGCGT
ACCGTGGGATCGAGGATATCGATGCCTCTCGCGCTATGCGCATCGGGTTCTGCTCAGAAAATAGTGTTTAATG
CGTTCCACAATCATACATTCCGGTGCAGAATTGACTCCATGACATGACCTTCTCCCCTCCCCCCTGGAGCGCG
CACCCTATAACTTAAGAATGGGGATAGGTAGCCCTGTCGGGTCTTTTGGACATTCATAAGGGCCTTTAGGAGT
GTCCGATCGGCCCCTCAAAGTCCCGATTTGGTGCGGAATAAGGTCACCCACAAGGGTATAACCAAAAACCTGC
ACCAACCCACTGATTTTAAACCTGTGGGTGGACAGGTTACAACCCATATGGGTGTAGGTATGGGACACCTTCT
GGATACCCACAGATGTACCCATGCCATTGCCTATTACAGGGTGGCCCAGGTAGCTACAGATATATGTACATGT
GAGATTAGGTGGAAATTTTTCACATGGAAGTGAGTGGGAAGCAAGGAATTGCACCCAACATATGGCTACAGGT
CATGGCAAGTACTTGCCACAAGTACCTGTTGTTCAAAATTTCAAACCTGGACCTGCAAGGACTTGTAGCCACA
GGTGATCTGGATCTGTGAGTCGCCATACTCACAAGTATTCCCACGGATCAACTTGTACTGACTCACAAGTTAC
TCACACTCGCGACATGCCTTACTCCGTTTGGGGTGTCGGTGGATCCTCCGTCCATGCCGTTGGCATCGGATCA
ATTCGCCTATCAATTGGGAAAGGAAATGCCATCACGCTACACAATGTTCTTTATATTCCGTCTTCCACTGTCC
GGCTCATCAGTGTCGCCTGCCTTACCGATTCCCTTCAGTGTTGTGTGGTTTTTGATTCCAGCTCAGTCTCCAT
CCTGCATCGCTCTGGCTCTGCTTTCGTTTCTGGTACTCGTCTCCCTGCCCGTAATCTCTATCGTCTCACCACA
TCTGGCGCCTCCGTCGACCATGCCATGCTGTCCACGCCCGTCCCCACCCTAGCCACCTGGCACCTCCGATTGG
GCATGTGAATCATCAATCTATATACAACATGGCCACAAAGAAATTAGCATCAGGTATGCCGATTGATCTTTCG

ACGTCTCCACCCAAATGTGAACATTGCATCCTTGGTAAACAGGTCTCATCGCCTGTCCCAAAGGTCCGTTCAG
GGGAGCGTTCGAAGGAGAAATTAGGCATAGTTTTGTTGATTTGACGGCCCTCAGGATGTTGTTGCTGCTTC
GGGCGCTCGGTACATTATGCATATTCTGGATGACTTCTCTAGTTTCTCTTGGTCTATACTTCTGAAATCGAAA
GAGGACGCTGTCCCAAAATTTCAAGCATGGGCTAGGACTGCTGAGACGGCCTTGGGAGCGAAACTGAAGATCA
TCCGGTCCGACAGGGGTGAGCTCAAAACGAATGAGATGGACGCATGGTGTGCCTCTCGCAGTACTGTTCACCA
GTTTACCGCTCCCTACACTTCGGCCCACATCGGGCGAGGCAAGCGCCTGCATCTCACAATCATGAACAAGGGA
CGCGCAATCCGTCTTCAAACGGGCCTACCCCCGAATTGATGGGATGAATGCGTCAACTCCGCAGGTTACCTCC
ACGTTCGCACCCCATCAGTTTCAGCGGGCGGTCGCACCCCGTACGAACTGTGGCATGGTAAGCTCCCCGACTT
GTCTCACCTAAGGGAACTAGGTTCGCGGGTGTTTGTCTTGACTCAGAATCGCCACAACCCTAAGATATACGCA
CGCTCTTTTGAATGCATCCTCATAGGATATTCACCTTCGTCCAAGGCTTACCAGTTGTATCACCCCCCTTCCC
ATAAAGTTGTTGAATCATTCCACGTTTCTTTTATTGAATCTCTAGATAGTACCCCTTCTACCCTACACCCTGG
TCGCGTGCTCGACAACCCTACAATTCCCGACGCGGCCTCCCCCTCCTTACCTACTTCCACCCACCAAGCGGTG
CCACCTCCTGGCGTCACCCCAGCCCCTTCCTCACTTTCTGGGGTGCCGCCTACCCCTACCTACTTACCCCCTT
CCTCTTCCCCCATAGTTAATGACCTCCCTCGCTCCCGTCGGGGAGTCACCGTTGAAGAAATACCCGACGAGGA
TGCACCTCCCCCTATCACCCCTTCACGTAGTGCTTCCCCCTCTCCCTTACCTACTGCCCCCAATTCCCCCTTA
CCTACACATCCCCCTCCGGTGGAGCCTCTCCGCCGTAGTTCTCGTACTCACACCTTGTCTGAAAAGAAGGCTG
CCGTCGACGGTCTTCTATTCTTACCGGCTGTCTCACGGGCGCGTGAGGCATCCAAGGCCAGCGCCGCGCGGCT
CAAGGAGGAGCGCCGTTTGGAGAAAGACCTGCGCCGGAAGTCAAGGCTCGAGATGCGTGCAGCGATGGCGGAC
AACTCTCGTATTTTCACGCATACCTCTGCTGTGGCTATTCTTGCTGACTCCTTGCCTTTCATGTCCCCCGAGG
AATTCAACCAGTTTGAGGATGCTTTCGCTGCCGTGATAGACCCTTCCCTCATGACTGGAGGAGTGGACGATCC
CACATCCCTTTGCAAAGCTCTAGACTCCCCTGACGGTGATGCTTGGCGCTCTAGCATTCATGAAGAACTCACT
AGCCTATGGGAGATGGACGTCTACAAGCTTGTCCTGCGGGCTTCCGTTCCCAGTAACCGGAAAATCCTGAAAG
GCAAGTGGGTATTCAAACTCAAACGCGACGAGAATGGGCTGCCGTCTCGCTTCAAGTCACGGCTGGTAGTCAA
AGGGTACGAGCAGGTCTTCGGCCAGGATTATGTTCACACGACGTCTCCGACCGCTCGCATGGAGTCCCTTCGC
CTGCTCTTGCATATCGCCGCCACAAATGACTGGGAGGTAACCCAAGTAGACGTCAAGACAGCCTTCCTCTATG
GTTGCTTGTTGGCTGACGAGGTACAATTCATGGAACAGCCCGAGGGTTTCGCCGAGCCCGGGAAAGAAGATTG
GGTCTGGCAATTACAGCGTGGCCTATACGGGATGAAACAGAGCGGACGCATCTGGAACAAAACTATGACCTCG
GCGATGATATCCTGGGGTTTCAAGCGCCTAGACAGTGACCCGTGCGTATTCTACAGGACGACCTCATCTGGTA
CTATACTAGCGGCTGTTCACGTCGACGATTTCCTCATCGCCTCCTCGTCCCCCTCTGAAGCTGATTCTTTCAA
GGACCAGTTGCGTGCGCTCTGGTCGATCACGGACCTGGGTGAGGCGAAATTCTGCGTTGGCATCGGCATCACA
CGGGATCGAGCAAAGCGCACCATCTCTCTATCTCAAACCGCTTTGATCGACCGAATTGTTACCCAGTTTGGCC
AGGCTGACGCCGACCCTATCTCAACTCCTATGGATGATGGACTCAAACTTTTTCGCCCCGACGCCAGCGTTCC
CCTATCCCGAGAGGTGACTGAAGACCTAACAAAGTTGCCCTATCGCTCGCTTGTGGGGTGCCTTATGTATGTC
GCTGTCGGTACACGCCCCGACATCGCCTTTTCAGTTTCCAAGCTCGCCCAGTTCCTGGACTGCTACCGTCGCG
AACACTGGAACGCTGCCATACGAGTCGTACGCTACTTAAAGGGATCACGCTTACTCGGTCTTACCCTGGGTGG
ACTAGACAACCCATCGTTATTCGGATTCTCTGACTCATCATTTGCCGACTGTCTTGATTCCTGCAGATCTAGC
ATGGGTTATTGCTTCTCACTCGGAAAAGGCTTATTTTCATGGTCATCGCGTCGCCAACAGACAGTAGCGACTT
CATCTACGAACGCAGAATACGTTGCAGTAAGTGAATCCTGCAGAGAAGCGCTATGGCTCCGCCAATTCTTGCG
GGAAATCCGAGTGCTTGATAACAGCCCCACACCAGTCCTCATAGACAACGACGGCGCGCGCGCACTAGCTAAC
GACCCATCACATCACTCCCGTAGCAAACATATAGACATTCGCCATCACTTCATTCGCGAACGCATCAACGATG
GAAACATATCATTATACCGCGTCCCATCCTCAGACAACATAGCCGACTGTCTAACCAAACCACTACCTCGTCC
CGCGTTCCTACGGATTCGTGCTCTTATGGGCATCGAATAATTCCCTCAAATCAGGAGGAGCTCACATACCCAT
ATTTACATTGTTTGTCCTTTTTATTTTCTCATTCTCTATTCAAGAAACTTCTCCTCGTGGTGAGGAGGAGTGT
CGAATGCACCGCGATACATACGTAAACATTACGTCATCATATATATATCTACCCGACCCTAGGTTCAAACTTA
CCTCAGTCCTTATCCCATATAGGCGACTGAGGTAAGTATAATCACCATCGCGATACGTTACCTCACACTCATC
TTGTATCTAGATACACATTCTAGGTACAGATAATGATATAGTCCTTATCCCATATAGGCGACTGAGATACACA
TTCTAGGTTTTTAACAT
>Cligib1_Cluster16#LTR/Gypsy
gTGTCACaACTCGCTCTTTTCTTgTcTTTCcTCcAAGTCTTTCTCATCTTATCTTATCTTATCCCCTTATCTT
ATCAGAGCTTATCATATCTTATCAGGGGTTTATCTTATCTTTCTCAAATCCTTATCGCAGAcCATCACGTGAT
CCATTATGTAAGCCAAACCGCGGTTTTGCATCATGGAGTACaCAcGCTgTAGGATATTACATTACTTTGTTTT
CTTTacCTTTTATTGCCAAAaACTCTAGAAGTACTTAAGGCaTTTCAcTTGTATTATGTACGCTAATTCGATT
CTCTTTGGTTTCCGCCTTAACAAGTGGGATctgaggttcatgtcacgtcctgctaacgcaagtcaatgagtcg
atagtaaagactcaaggacacttagcaaggtctgaagtctcctgggtcaggaggcagtagaggggggaatctga
taaacgattaaacgtagcgaatacaggagtatgtagctacgtttatatacaagtgaagtagttcgatgactac
gcactagtgactaagtccgtgagtatagtaagacatatgggggatgatgactgatacgagaagcgagctgact
gagcagctgagtaagaacaatgaagatagcaaagatagaacggagaccatgcggtatagaagtagataaggtg
cgcatgatgtatcatctgatgatgtagcatatggcgcatatcagatgatgatgggatgatggtggaagtaata

gaagcgtctatgaatgatgctatcagtcagatgtccgagtggtgatgatcagagaaggaaggaatcgTaAcaG
cTCAGTCGcaATTCATtacTCTTTCTTTcaTATTCATTCTACTTGAGAGACTGCCGCAcaTTTatagGTGTTT
TGGCGTGTCCTTACAGAAGAATCGAATTCTaAaCGCGGCCCTCTCTCGcTTggcCaacgacacatcgCAAGCg
CGcGaaaCaACaTCAaCacTCAAcAaccgTaGaTgcAaCAGgcaAGcaacgaTcaAAaTaAcCcaCaacCTTg
ggcCcaAAcagaAgacgcttACAaCccaGTTTaAaACaGaaaCttcgctgtctctccttgGCaAcaAcCcAac
gaaTgGcaCccaTgaCcacTGTGcgaAACACagaGgacgTTagTgcccaCAgTacCgGgaCTcCAAgTCCccA
gaacacagAaAgcCaAaCGACACcCTCcCACTGCaaAgTCccCCCACACCTTGCGCAGGATCAGCGTTTAAGG
GTTAGccccgaGTAaCaGaCagaccCcccgACAGGCaCccaTTacataaGGAACACaCTGGCCTGAGcCCGGC
CGTCCCTacTcCCgGgaTTCTGTTCGTGGGTATCGCCGCCCCACGTCCTAAGTAGGCAATATGTCGGTGCCCT
CCATGTGGGTATCCGATGATAAGTTAGCGCATCTTGAAAAGCGCATGTCaCAGTATCGGTCCCAATTGGAcGC
GATATTGCGGGATTCAACCCCGCcCCCTACTCCTcTAaAGCGGAAcACCAAACCGCTAAACATcGTTAAaAAA
aGTCCGGAAaagTcaCCGGCATCCAAATATCgAGAATGGGGATGGaCTCCTCCAGCGTCTGGCCATGCATTTC
GGTCGGCTGAGgagTCATTCCACGATTTtcCacCcaTaCCACAaTCCCTGGCCGCTGCGCCTATTACACCAGc
GCGaCCTTATTTGTATaCAGCACCGAGcGAAAGCTCATCGGGGATGTCAACCCCTGTAAATATTTCCCCACGT
CTCaGAcATaAGAAAAcTaGAAGTCGCGACCGCTCGTCGGGCTTTAGaaAAcAAAcAAAAACAAAAGaAAAaC
AGAAAACCAAAAACATGCCACAAaTGCCGCTTGCCCGGTCATTTGATGAAGAACTGTACCAGTAaTaTAGATT
TTCGTAGTGCcACaCATGAaCAATTAAAaAAATATTTCGTACAAGAATTCCTaAAACAAAATAAAAATTACAA
AGCCACAGAAAAATTCAAAAAAATAATTACGCCCGTTTCATCTCCTGTAAAAATAGTTTCTCCTATCCcTGTT
GTACCCGTAGTTCCAATTACGAACAATTTCGcTTCTAAaAATTCaTTTTCaTCTTTACCaGTAGATaATATGG
AAGTCGACGATGACGGCAAGGTCAAAGTCGGTGGACGGTTCTTTTCcCCttCTCAcTCTCCgATGGAcATTTC
TAAATTAGAcAaTTTACCTAATATGaCGAAGGTTATGCTACCGGAAAAGGCACgGGTCGGTCCTAAcAAGACG
ATcCCTATTTTTAAAATTGCAGATAATGCTCAGGGACGCGCTCTGGAGAAGCGGTTGTCCAGTTTTATGCAGT
CGcTcGAAGCTAGGAAaGTGGAGCGcAGTAAGGGTCTTGGGGTTCCGTCTGACGTCCGTCCaGTGAAGCaGGG
TCCcGGCGTTAaGAACCTGTCGACCAATCAGGAGCCCTTAAATGGGTCATCTGTCCGTCCCGAGTCTGGTGAA
CTCGTGGGTCCCGGCAGTAtCGGTGCTTCGGCCCGTCCTGGTTTcCCTTcGTCAGGaAaTTAcTTcATTCGCA
CACTTAATTTAGCcAGAGAGCTTaAAATGTCGATCGTGATGACGACATTAGATACACACGCGACGGCCAACAC
ACAGGCCcTATTGGATAGCGGAGCTACCGGTTCaTTCATTGATAAAGAGTTTATTAAAGAGATGGGTTGGACC
ACaCGACCGCTACaTCACCCTGTACAAGTATATAATGTCGACGGTACTTTAAAcGCAGCCGGTTCCATTTCGG
AAGAAGTaGTTTTaATGGTTTCCCACAAGGGCCATAAGGAACGCATGcTGTTTGAAGTGACGACGCTCGGCAA
GACTAAAGTTATTTTGGGACACACATGGCTTAAGAAGCATAACCCCACGATCGACTGGACGACTGGGGAaGTC
TCTATGAATAGATGTCCgGCGGAATGCAACGTGGCTATCCGTGCGGCTAAAACcCAAAAGAAACGTAAGGCCA
TGGCTTCGCGCTGGCAGTATAAAACGACGCTCGAGGAGGTCGATGATGAGGATTCCGACCGTGTTATAGGCGG
GATCATAGTGGAACAAGAAGGAGCCCTTTTAGAGGAGATTCGTTTTGACGAAACGGTCTACGAaGTaGTTGCG
GAACACTTCGTGCGCACAGTGGAGATCGAGGAGAGCTTTGACGCCCCTCCGTTGATAAGTGGATTACCGGAGC
CTACCgAACCCGGcTTAGAAGAAAAGATaCCCCGcAGGTTTAGaCGGTGGTTAAAAGtTTTTGAAAAGAAAGC
GTCGGAGCGAATGCCCGAGCGcAAGACATGGGATCATGGGATaGAGTTGAAaGAAGGATTTGTGcCCAAGAAG
GCCAAAGTcATTAATcTATCaCCTACCGAACAGAAGGAGGTcGAcGAcTTcTTAAAgGATCAgTTATCTAAaG
GTaaaaTCGCGAAAGCAAGTCTCCGCAAACTTCaGCTGtTTTCTTcGTACCaAAGAAGGATGGCAAGAAGCGc
ATGGTTCAGGATTATAGGTTCCTcAATGCCTGGACTatgAAAAATAATTATCCATTACCGATGATCGCCGACT
TaATCGATAAAGCGGGAAAGTCgAAATGGTTcACAAAGCTcGACCTAcGCTGGGGCTACAATAACGTACGGAC
AAAAGAGGGCgACGAATGGAAAGCGGCGTTCACCACGCATCGCGGTTCCTTCGAGCCATTaGTTATGTATTTc
GGACTTTGCAATTCaCCGGCGACGTTcCAGACCATGATGAACGCGcTaTTACGAGAGcTgATTGAcGAAGGcC
AcGTcGTcGTATATATTGATGATATccTaATCTTCACCGACACGCAAGAAGAAAACGAaAAGATcGTCGAACG
GGTTCTCCAGATCCTAcTAGACAACGATTtGTTCTTGAAGCCaGAGAAGTGcTCGTTCAGTACCCAGAAGGTG
GAGTTCCTAGGGTTAATATTaGATAAaGGAGTTGTACGaATGGCTGAaGATAAGACGGACGCGGTTacTAAAT
GGCCGaTCCCGAAGACGGTCCaGGAAATACGGAAaTTCCTgGGATTCGcCAATTTTTATAGACGaTTTATCGA
aGGGTTCTCCAAGtTAGCGGGACCcTTAAACAATTTATTACGGAAGGACTCGAGGTTCGAATGGACTGATAAG
CAACAGGTTGCCTTCGATACGCTgAAGAAACGTTTTACGTcGAACCCGATCCTGGTTACGGCAGATCCTAAAC
GaAAaTTACGTGTAGAGTCGGACGCTTCaGACTTcGCTACaGGTGGTATaTTAAGTATGTTATGcGACGACGG
TAAATGGAGaCCGTGCGCaTATTATTCcAAATCTTTATCTGATGTcGAACGTAATTATGATGTACACGACAAa
gaGAagcTGAGTATCATaCGATGCTtcGAAGCaTGGCGTCATTATTTAGAAGGaGCGGACgaAACaGTAGAAA
TATGGTCGGACCATAAAAAcTTACAATAcTTTATGAcGTCtaAaAAACTTAATCGTAGACAaGCtCGATGGGc
tCTTTATTTATCAcGaTTTGACTTTTTACTTATTAACAAACCCaGAGCGGCTATGGGAAAGGCCGACgCTCTG
TCCAGGAGAACAGACCTAAAGAGGGGTGTAGAATCCGATAACGATGAcGTCACaCTacTaAAACCcGAATTCT
TTTCTATTAAAGCCCTTCAGCAGGGTCATGTGCTCTTGGACGGCCATGAaGAAGAgTTaTTGAAGAAGATTAG
GAAGTCGAAGGATTagGATGAAGCaGTGGTTAAGGCGATCGAGGAaTTGAAGCATTCGGCGACTAAGCGTTTG
CGTTCAGAAGAGTGGTCCGAAGAGCAGGATcTAATACTGTTCaGAGGAAAGGTTTACGTTCCGAAGTTGcCGG
AcGTCCGTCGAGAGATcGTcAAGTTACATCACGAGTCGGTcGTcACTGGACACCCAGGACGAAACAAAACaTT

AGAGTTAATTACGCGTAATTATTGGTGGCCTGGTATGGCTAAATTTATATTTCTTACGTCGACGGGTGCGAC
AAATGTCAAAGaCACAAGAACTTCCCCGCcCCTCCTGTCGGAAAGCTCGTACCGATGAGTACCCCGGTCGAAC
CCTGGAAGGCTGTaTCCTGCGACTTTATTACTGGACTACCaTTATCGTCCGGATATAACGCTATCGTCaTCGT
CGTTGATCGTACTTCGAAGATGGCTCATTTTATTCCTACAACGTCTGAGACGTCaTCaCTTGGCcTcGCTAAA
TTATATCGCGACTTTATcTGGAAGCATCACGGTCTcCCaGATTCAATTATTTCAGATCGAGGaCCTCAATTCG
CATCcGTGATGATGAAAGAGTTGAACTCTCTGCTGGGCATTCAAACTAAATTATCGACAGCTTATCACCCGCA
GACGGACGGGCAAACCGAACGCGTCAATCAGGATATTGAAGCTTATTTACGACTcTTTATTGATCATCGACAA
ACCGACTGGTCGGAATGGCTCGCTTCAGCGGAGTTCGCACACAACAAcaCAATCCACTCgAGCACGAAGGTCT
CaCCTTTTtATCTCACCAACGGCAGGCACCCGCGCATGGGATTCGaAACCGATCCGTTcCCAGAAGAATGAGA
cGGCAATCGAGTTCGCTGAGCGAATGAAACATATTCAAGAAGAAGCGCAGGCCGCCCTTATTAAATCCCGAGA
CGAAATGAAGCGCTTTtGCTGATCAACATCGTGGAGATATGCCGAAGTATAAAGTAGGCGATAAAGTaATGGC
TAGAGACTAAAGATCTAGACATCAAACGTCCGTCGAAGAAACTCGCGGAACGTCGCATTGGTCCCTTCGAAGT
TTTAGAACTCGTCGGTTCACATGCGGTTAAACTAAAATTCCCTAGCTCGATTCGTATTCACCCAGTTGTGAAC
GTTTCCCGTATCCGCCCTTTCACCCCTCCTATTGCTGGTCAAACATCCGCACCACCCCCTCCAATCGATATCG
ACGGCGAGTTTGAATACCACGTTGAGAAGATTCTCGATTCGCGTCTGGAACGAGGTCGACTTCAGTACTTTGT
TCAGCATCAGCAAGATCACATCCCCcTGGGAGTGGTAGGGCCAGGACACCCAGCTCACCCAGTCCTCCAGGAC
ATGGAATGGAGCCAGCTGGACTTATGCAACTCCAGGACCATCTCTCTAGCAcCAGCAGCCCCAGTCCTACACC
AGCAACATCCTACCAATCTACCACCCCATTCCCAGCATGATGCACACCTAATATAGCCTTCACATGGACAGTC
ACAATGATGGATTGAAATTCATTCATTCAAATTTTACAGGCTAGTAGTAATAATAAAATTCGAGAACCCTCAC
TTGAAACGCGGCTCTCAGCATTCCACCTACTATACAGTCGACATACCTCGCCACGGCAGGGgTGCGCACCAGT
CAcCCCCAAGCGCCATTGTTCGTGGCGCATCCATTCCGTCGCATTCCTACTGTCCGGGTCGCGTTGTTGTTGC
TTTCCGATGCAGACATCTATCATGTTAGTGCTAGCGGAATTCAGCTTACCCGTCGTCGCCCTCGACATCGCGG
CGATCTCATCGCACCATCGTTTGTCAGCACAGCGGCCCCTGGCGCTGCCGAACTTGGCCTGGCATGGCATGGC
ATGTCCTTACCCAGCAGAATCCCGCTCCTTGAATGTGCGGTGATGCGTATGTGCGCGTGGACGGATGAGGTGC
GAGTGGTAGGCCGACATGAGCCACGTATGTGACAGAGCATGATTGTACGTGCATCAGATGGATACATGAGTGT
GTGTATTAAGATCGGGGCAAGCGAGCACGTACTAGGGTAGGTTCACGCAAGAAGGCGCCGTTTGGTAGTGTGG
GCATGCATTAGGGAAATCCGCTAGTCGGAGATGCTCACTTGTTAGTATGTATATCATTTTTGGGCTGCATACT
CACTGGGgCGGTTTTTCACAGCATTGGatGTCGAGCGAAGACTGCTAAAATATTGAGTGAGTTTCAATGTTGG
AGTTGTGGGAAACGGGATACGCAGCGTTGTTATCAAATGTTGAGCACACAAATGGTGGAATACCCGTAATCGA
GAGGGCCAAGCAGTCTGGACGCGGAGCACATTGAAGTTTGCGGTGGGGACTCGGCAAGTGGGTGAAAATTGTG
CTGGGAGAACACGGAGCCGCCCGGAGAGGTCACGTGAGCTAGAACGAAAATTTTCGTCAAGACATCCAGTATC
TGATATCCAATCTGCCACAACCCCTGGTGAGTACTCCACTAGCTCCCTCCGTTGTATGcACTAATGAGATGAT
CTAGAAAAACAATGAATGAGATGAGGATGGGGCAAGGTgGGGGACAAAAATGGACGATGACGGCCCCACGGC
TAGGTAAGCTCTACTGTCCACCCGCCCAAGCCCCcTCCTCATAGCCTGTACACCCAAGCCGCCCTCAACCACA
CTcCCCCCCTCGCATGCGGCCTCACACCGATCCATGCCCCCACCCACTTATGTGGTGCTTATGCGCTCCTCTT
GTATGGTGCCTGCCCTATATCTTTCCAGGTGTCGTACTCATGCACACACCCTCCTCCCACCCTCTCGCACAAA
ACCCGCCAAATTGGATTCCCCGGCTCCTTCGTGCCCATGTCCTCCCCTGGAGTCCCTCGCCATCCATTGCCCA
CGCGCCACGTATGTACCTGTGCTCTCCACCTGTATTTTTAGGCCCGCGCTTGGTCGCAAACATATTCAACATG
CCGCAAGCGATGAGGGGTCTTAGTCGGGATATAGATCATGTACATAgaaATATTATCAATTTAAATGTAATTT
TCGACGTTTTCGGCCCCATTCTCAGCATGTATTCAGGATATTATAAGACAACAATAAaTATGTAGTTTTTCTG
ACGTGGTGCACTGGTAATGGTAGGTACCTCCTGGGTGtTTATTACAGGCCCTGGAGAGTTGGGAGTGCTGGGG
GTTCACCCAGTGCTGGAGATGGCTGGGACACACACTGGCCCAGGCTAAGCCAGCACCCCCAGTGCCTCCTACC
ACTCCCAGACTGTCCAGCACTGTGCAATCTTGCTGATGAGTGGGAAGGTTATTCTCCGGAACACTCTACTTGG
GAaGCCGATAGCGAATCTCAAAAACTCCATGGATCTGGTTAACGATTTTCATCGCAGGAATCCATCGGCGCCA
CGAAAGATACAGGGTATCTCTCAAATTCAATTTGATAATTTATTTCAACCGTATAACAATTTCACCGTGCCGG
ACGCATCCCTCTTACTTGCGCCCTAGAAgGGGAGTAATGGATTTATTTTATATTTCTCATAACATTGTGTAGC
GAGCAGACGATTCGAGTTTGACGAACCATGGATACTCTCCAAAGCGTTCTCCTCGTCGTACTGTGTACGCCCC
TTTGTTTGCCGCACTGGAGTCAATCTTCGTTGAGGCGATGCGGAGTTTTTCTTTTCTCTTTTTTTTCTTTTAT
CATTTTCTTTATTTTTCCGGAGGAAAAATTCTAGAAGGGGAGTTATGTCACGACTCGCTCTTTTCTTTTCTTT
CTTCCAAGTCTTTCTCATCTTATCTTATCTTATCCCCTTATCTTATCAGAGCTTATCATATCTTATCAGGGTT
TTATCTTATCTTTCTCAAATCCTTTATCGCAGATCATCACGTGATCCATTATGTAAGCCAAACCGCGGTTTTG
CATCATGGAGTACGCATGCTTTAGGATATTACATTACTTTGTTTTCTTTCTCTTTTATTGCCAAACACTCTAG
AAGTACTTAAGGCTTTTCATTTGTATTATGTACGCTAATTCGATTCTCTTTGGTTTCCGCCTAAcaagt
>Cligib1_rnd-1_family-684#ClassI/TRIM
CCCCGGAGACAACAGTCATGAGCGCGGGAATGAATCGACCTCGCCCGGCGGAATAGAGGGCATACGGCCATGC
TCCCGCCTCGACGTGGTGTGCGGTGCAGCGCTACAAGAATGAAGGTGTCGTGCACACCAGAGGTCTCTTCGGG
GGTCGGGACCAGCGCCTGGGTGGCCCCTTGCCCTTCTGGGTTGCTCATGATTGTCCCTTGGTGGGGAATATCG
GCGTGTTGGCGTGGCCGTGGGCGGGATCGATTGGCGGGGTGCTACCCGGGAGCATGTCGGGTGGCATAATGTG

ACGGCGACTGACCCGGATCGAATCTGAACAACGGCGGTCAGCCACATCGACGAGTAAACCGANTCTACTCACC
TTCTACCTCGAGATGGCGAACGGCGGGCACCCCCCGAGACAACGAGCACGGACACGGGAATAAATCAACCTCG
CCCGGCGGAATAGAGGGCATACGGCCATGCTCCCGCCTCGACATGATGTGCGATGCAACGCTACAAGAATGAA
GGTGTCGTGCACATCAGCGGTCTCTTCGGGGGTCGGACCAGCGCCTGGGTGGCCCCTCGCCCTTCTGGGTTG
TCCGCGATTGTCTC
>Cligib1_rnd-1_family-426#TIR/MITE
TACTGTNCCATTTCATTCAATTCGACGGTCACGGGTCACAGGGCATCTGTCGGCGACGAAAATCGGCNCGNTG
TCACGGGTCACGGCGGGTTCGCCGAATAGAAACGAGGTCACACAAAATAAATTTTTTGAAAGGTGCCCNAAAA
CGCCTTTTTTGGTGCCAAATATCAAAAAAATTTCGCGGATCCGTCGAAGTTTTTCCCGCTGTCACGGTCACAT
CGGGTGGTGTCGAATAGCTAAAAAAAGTCGAATAGGTTTTCGAAGGTCACGGGTCACACCGGTGTCGAATTGA
ATGAAATGGGACAGTA
>Cligib1_rnd-1_family-91#LTR/Gypsy
TGTCAGGACTTGTATTTTCTTTTCTTTTCTCTTTTCGTTTGTAGTATCACATGACTGATCACGTGACTGCTTA
TGTAAGCCGAACCGCGGTTTCGCATCATTGTAGTACACATGTCGTTTGTACNTATTATATTCACGGGCTCTTA
GCATTAGTATATAGTCTCGTTGTTTGTATGTTTCGTCTCTCAACTTGATTCTCTTTGGTTTTCCGCTATAACA
CAGTGGGACTTAAGTCGTCAGTCATCTTCATTCATTCCATTCCTCTTGAGTATTCTTGAGAGACCGCACACCA
TTTTGGTGTTTAGCGTGTCCTTACAGAAGAATCAGATTCCCTTTCGTGCCCTCTCTCATCGCTCAACACTTTC
GCTTGTCCAACGACACCTCGAAGCGCGTGAAACAACATCACCGCTCAAAGAGTTTTAGTCGTTTGGTGTAAGC
ACATCNCCGCCCGTTCATCATTAATCCCCNCGACTGTNNCAGANATCAATCGGGCGGTCGCGATCTAATCGCT
CCGCCTGNGCGATNTTTCTTTGGCAACAACTCTTGCGACGCGGTTGAGATCTAATTTCTCCGTTAGCATTATA
AGAGTCAGCAAGTCCCATCCCCCCGTTGCGCTTGGATTGGCTGTTGGCCAAGGCAACCTCGACACTCTCTCAC
TGCTAGTTCGGCCCACACCTNGCGCAGGATAAGCGTTTNAGGGTTAGCCGCTAGTAACAGTCCGCCCGGTCAG
GCTCCCGCGGACCACTCTCGCATCACCATTCCCATTCCTGTTTGAGTAGGCTTGCAACCTCTCATCCTATTTG
CTACCGTTACACGGTTTGAGCAGGCGAGAATTTCGGGTCTCACCTTTGTCCCGTCCCCACCCTTGAAGCNGGT
GATCACCGCCGGTCTACTGGATTAAGTCCGTACCCGCGGCCCCCTTCATTTTCAAAATTCCGTCACATTTCCG
NCCATACTCNTCCGCACACCCCTCTCCTACCTTCTCCAAAACACCACCCATGTCTGAAGACTCAGCAAATTGG
GGAGGATCGCAANCGGCGACACCTCGTAACAATTTTGCGGGATATTATAATCCGGAATACGAACCCCCCAACA
CTTCTCAGGTCAACCCCGACACGGTCACTTATAAAACGGACGCTGAAAAATTCACGGAGATCTACGATCAAAT
GAGACACGCCACGGAATTAAACACGGAATTCATCCAACGGATCCAAACCCTCGAAAATCACGTTAAGGTTCTC
CAGGATCAGAATTCGAGACTATGGGAACAGAACCACGAACTCCACTCACGGTTTAATCGGTTCGAGGCACTCC
TCACGGATTTGGTCAAGGACAAACGGGAACGNGACTCTAAACCATCTCCCCTCGACGGATTTAACCCCCTCCC
CGGTTCATACTCTAACGCGGCTAAAGGATCTCGCGCGCCTAAACTCCAAAATCCGACACCGTTCGATGGTACA
ATGGCTAATGCGGACACCTTCGAAAATGCATGCCGCATCTACCTACAAGTTCGACACGCGGATTTCTCATCGG
TCACTAGCGCGGTATACTGGGTCCTACATCTCTGTTCTCAAGGNTCAGCAGCGACATGGCGCGATCTTAAGAT
CAAGGAATTCAACCGGAATCCAATACAAATGGATTATACTATTAACGATTTCTGGAAAGAATTCAAACTGGCG
TTTTCCGATCACGATCCACAGGGAACCAAAGCGTTCAAACTTTGCACCATGAAACAGGGAACTTTACCGGCAG
AGGAACACATCCAGGAATTTAAGAAAACGGCCGACGGNTCAGGATATAGCGGATATCCTTTGATCAAGGAATT
CAAACGGTCGTTAAACGAAGGTCTTCGCAAACGGCTAATGGAATCGGAATATCAACCGAATACCATCGAAGAT
TGGTACACACGGGCATCACATTTGGACAGGCAATACCGACAATCTCAGGAAGAGAAGAAGTTGTATCAACCGA
CATTCTCACGCCCGGCATTTTCATCGNCTAACCCACGGCAAACCGGAACAGGAACCACCCAGACACAAGGTCC
CCACCGAAATATGGGATCTAATCAACAGGCACAACCCGCTCGGCCTACNTTTGGTTTCAATCGCCCGGCNTTC
GGTCAATCAGCACAACCGGCCCGACC
>Cligib1_rnd-1_family-103#TIR/MITE
CACCTAAGGTTCCCGCGTGATGTCACTATTTAGTGGGGTAGAACTGATTACGTTAACCCCACTAATTTGTGTG
AATTTTCGTTGTTTCCCATACATGCGAATAATTTTCTCAATAACTATTAACTTAGTACCCGAAACCGAGCGCT
TGGTGACGATAAGAGTAGATTACATCAGCGCCGCCCACCGTCATCAGGGTCCGTCCTCAGGGCACGCCACATG
CCGCCCACCGCCCTTGACCCTGTGCTCGCTGTCTGGCACAGTGAGCTCAACCTCGGCCTCAATTATGTAGCCT
ATCCGCCTATTTCTTTTGCAGATAGAATGTACAGAAGTAGGTATTATTAATAGANACATCCCCCTGTATTTTG
AAGCCTGAATAAGCGTCCAAAACAGGAAATTGGCCTGGGGGNAGGGGAGGATGGCGTCCCGTATGGGAACCGT
GGAGCGGAATGCTATCGCGTTTGCTCTAGCATGTTTNCAATATATATGGGAGTNTGGGATCCAATTTTTAGGG
GTATTGGCCGAGNGGATGTAGTACAGTGGGCGGATCAATGGGGGGGAGGAGGAGGGTGTCCAGGTATTTCCAC
AGCGGTTCCNATACTCATCGGGCGGCCTGGGCATGTTCTAATATATATATATAANTGATTNTATGCCGCGCAT
TCGCCCCGCCGCGTCCCAGGGAGACAGCAGCAAGGTTGGGGAGGGGGGGTGTGCGTAAATCAGAGATTACTGT
ACTGAATTGGCTACAGTGGNCGGAATATCATCGGAGGGCCCGGAAATAAGAGCGGTATATAGTAGTATGAGGT
CTCAGAAGAGACGNAAGAGATCCGCCCGGAGGTGGTGAAGATATGTAAGAAAATGTGTTCATGGTGAGTCCAA
TTTGGATNTGTCACACATGTCGTACTCAGCCCGGCCAAATACCACATCTTANGGTATATCTCCACCACCTCTG
GGCGGATCTCTTNCGTCTCTTCTGAGACCTAATACTAGTATATACCGCTCTTATTTCCGGGCCCTCCGATGNT
ATTCCGNTCACTGTAGCTAATTCNGTACAGTAATCTCTGATTTATAACATCCCCCCCTCCCCAACCTTNCTAC

TGTCTTCCCGNGACGCGGCGGGGCGAACGCGCGGCATGNAATCAATTATATATATATATTAGAACATGCCCAG
GCCGCCCGATGAGTATNGGANCCGCTGTGGAAATACCTGGACACCCTCCTCCTCCCCCCCATTGATCCGCCCA
CTGTACTACATCCCCTCGGCCAATACCCCTANAAATTGGATCCCAAACTCCCATATATATTNTAAACATGCTA
GAGCAAACGCGATAGCATTCCGCTCCACGGTTCTCACNCGGGACGCCATCCTCCCCTNCCCCCAGGCCAATTC
CCTGTTTTGGACGCTTATTTNGGCCTCNAAATACAGGGGGTATGTNTGTAATAGTAATACCTACTTCTGTACA
TTCTATCTGCGGGAGGGACAGGCGGGAANGCTATCATAATTGANGCCGGGATTGAGCCCACCGTGCCAGGCGG
CGGGCACAGGGTGACGGGTCCAGGGGCGGNGNGCGGTGGTATTCCGCATGTGGCGNGCCCTGAGGACGAGGCC
CTGATGGCGGCGGGGGGCGNTGAGGTAATCTACTCTTATCGTCACCAAGCGCTCGGTTTCGGGTACTAAGTTA
ATAGTTATTGAGAAAATTATTCGCATGTATGGGAAACAACGAAAATTCACACAAATTAGTGGGGTTAACGTAA
TCAGTTCTACCCCACTAAATAGTGACATCACGCGGGAACCTTAGGTG
>Cligib1_rnd-1_family-1031#LTR/Copia
CCGCGCGCATGGATTGTCCACCAATATTTGGTGGGAAGAGTTCGCGCAANCGTTTCATGAAAAAAGATCGGGT
TGGGACTTGTCCGGCTGACGTTAGCCAGAGGGGAGAGCTAAAAGGGAACAAGCGGTCGCGGGATTTTAGGTAT
CGGATGAAGTGATGGTGGGGGTCGCATGGGCGGTTATTTTGGCGAATGATAATGCGGTTTCCTTCGAAGAACT
TGTCNGCTTTGTGGGCGGGTAGGTGGAACTCGTACGNGCCGCGGTGGACTTTGACTGNGAGGCGTTTGGTTAC
TTTGCGATCGTCNCGAATTGATGGGTCGTCGGGGTAGGTCATTTCACCCAGCCTCATGAGGGCGAAGAAACCC
GATAAGATCATGGCGACGAAGAGGNGGTCGTCATGTTGCTGCGATCGCTCGTAGTGGCGAGCTACCATATCGA
GGTCNGCTAGTGATAAAGCACTTTTGCGGGAGACCGCCACACCTCGAAGACGGCGGCAACCCTTGAGGGTGCG
CCGAACCAGACTGGAGCGACGGTTAGCGCGGACATCGGGGAATTCGGATTCGAGTTGGTGGCAGATTCCGGAG
AGGTAAGCNTCGACTGATTTGGGATTGATGTGATGGGACATGTAGACGGTGAAAAGGCTGAGGGTGTCGGGGG
TGGGGTCTGGGTTCAAGTTGTGCGAGTAAATGAAAGAGAGATAGGAGTTCAGGGCCGAACCATAATTTTTGGA
TGTTGAAAGGTCAATTGTCAGACCAAGAGCGATTGCCCGTTCACGAATTAGTCGTTCATGGACCAGGCAGCA
CGGGCGGGCTGCCGGGGCCGAACGGTACGTCGAGTCATTTGTTACCNGCCCCCAGCGCATCTCGAGGGGGTGA
AAAATGGGAAATTTCGAGNCCCGGTGACTGTGCGATTGCGTTGTGTAGTAGGCCTCGAGAGAGGGCATCCGCG
ACTGAATTCAGTGGGCCNGGGATGTGTAAGACGCGTAGTTGGTAGTCTCCCTCCATAAGGCGGTCCACCGACG
ACTTGAGGATGTCGTTGTATGCAGGCTTCGCTTTGAGAGAGTTAAAAATGTTCACCGTATTTAGGTTGTCGGT
GAAGATCACGATGCGCGAGTTTGCNGGTGCGGAAGCGTGGATGTGGGCAATGGCGGAAGCGACGCAGAGAGCT
TCAAAGAAAAAAATGCAATCAGAAGGGGGATCTGAGGGAGTGTCTGATACAAAGCCTTCGTTTGTGGTCGGGT
ACCAGAAACCCATACCGTCCAAGCAGGCGTCGCAATAAATGGTGAAATCGGCCAAGTTGGGGTCCCAATGAAG
NGCGCGGATGAGGAATACTCCCGGATCACGTTGGATGTGGTTCGCGGCCCACATGAGGTCGTCGCGTACCGCA
TTGTTCACCCACACTTTTTGATTAACGCCTTCCTTGCCTGAGATCTTGGCGTAAACGTTGTTCAGGCAAGGCC
TCAGCAATGGGAAGACGTTGAAGGACCAGTTCATCCAGCCCGCGAGGCGTTGCCAGGCCTTTAGCGGGAAGCT
AGAACCGCGACGTTTGGCCGCGGGGCGAGCGAAGCGATGGAGTTCTTCGACCAATTCCGCGCGCTTTTCGGTT
GACATGGACAGGGTGAAGGTCGTGGCGTCGACTTCAATTCCGATCACGGTCAGGCGAGACCCAGACACCTGTT
TTTTCTCCTTGTGCGGAATNCCGATCTCGTCCCACAAGGAAAGGAGGGTAACCTGATTCGTGGGCATGTATTT
GTGATATGGGGCGTACCAGGAAACGCGCTTATCGGTGTCAAGGCCGAATGTGTCGTCACAGTAGGTACCGAGA
TGACGCAGGCCTCGGACGTTTTTGGCGGCCCAGGTGACNAGCGAGTGGGTGGAAATAAACACGTCACCGGAGC
GCCGCCCACCAAAAGAAATGTTTCGGTCAACATGATACTCCCCTTCGACTTTCACGACTTGCTTCAATTGCCA
GGCTGGGTGGGCAGGCATCGTCCGATATGCCTCGGACACGTCTGATTTGAAGATAATGATATTGGTTCCGGGG
GGAGTATTTTCCCGGAGGTCCAGGAGCATTTGGCCGAGGTGACGCATGTCGTCCAGAGGGTAAAAGTCATTCG
GTGAGTGGGGAGTTACCATGGAGTTGAGTGAGAAGTTGCCGAAACTTTGATTGTTCACGAGGCGGAGATCGGA
AGTTCCGGGTTTGGGTACCGCGTGAATTGGCATACAATACATACCNGGTAGGAGACATTTGAAGCCGGCTGAG
AAACGGTCTTTCTCGATTTCGTGGTCCCGTTGATCCTGAAGAAATTTTCTTTCCGCAGGGTCGGTGGGGATGG
GGAGCGAAGCGTCGAGTGTGTCCGGGTATGTTCCAACGCGAGTATCGGCCCACGGCCAAAAACCTTCGCGAAG
TCCCACGCAAATAGAGTCGACGAAGGCTTGATTCGGATGTGTTGAGAGGAGGTCATGGAAACGATCAACGAGC
AAGGGAGAAACGATACGGAAGAGATCTGGACGAACTGAGGAAGAATAAACGAAGCTTGGGTTTTGAATTTCCT
GTAACGGGGGGCGTGGAAGTGGGGACGCGATTTCGGTCCAATCAGACGAACACATAAGAGGGAAATCGTTTGN
GCTCCAAAGGTTGAATCGTAAATACTTAGGTCGAATTCCCATTAGCATGAGTTTCCTTGCTTTCGCACCGGGA
TTTCCCATGTCCGTAGCGTTTACATGACGCGCAGGCGTGCCGGTATTTGCATGAGGAATTTGAGTTCGGGCAT
CCGGACAAGGAGTTGAAGCGGCGGCAGATATCCGTGGACGGTTTGGCTGAGGACGAGGTTCCACGGACTTGCG
GGCTTTCGTGTTGGCTTTTTTGGGAATATTCGATCCCGTCNGAGCAGACGATTGCCGCGTAGAGTCGATCGAC
GTCGGACCGACCTGGGAGGAGCTTAGACTTTCCGCTCCCAATGTCCCGACGGACGGCCTCGTCATAACGGATG
AGCCGGTGGTGAGCGGACTCGACTTTGGATGAGAACTCTCCGCAGATGTATTGGCCGTATTCGACGAGCTCCT
GTCTGCGATGNGGGAAGACAAACGCGATCGCTTCGGTGGCAACCGTCCAAGCAGCGAACCAGTCTCCACTGGT
CCGGATCTTTTTCGCGGGTTCAACCTGTCCGAGTGTGAATTCAAGCTGTCCCACGCGTCCAAGGTCC
>Cligib1_rnd-4_family-1794#TIR/MITE
TAGTGTTTAAAAGTCCGGTCAGGTCCGGTTNTTTGCCTNATTTGGAAAAGACCGTGACCGTGACCGGTCACAT
CATCACCCGAAAAGGAAAAAGACCGGACCGNGACCGGTTACCGGTCNCAATTATTTCAAGCTTGTTTGAAGAC

CATCGGTCACGCAAAGACCGGTGTGACCGGTCATAACCGGTCCTAACCGGTCCTGACCGATAATTTTACCATA
TATGGATGTCACACAGGGTCCGCGACAGAATATTTCATGTGCAAAAGTGAAATATCTATGTAAAAAGCAGCTC
TCAGAGCCATTTTTGACGGTTTTTGGTGGTATTTTGGGGTGTTTGGTGGGTGCGCATGTGGCTGTGCAATAAC
CGGTCTATGACCGGTTNTGACCGGTCACACNGGCTGTCACGATCCAGGAAGACCGGACCTTGCGCACCCCAAA
ATCAAGGAGACCGGACCTGCGGTCACTGTGTGACCGGTCATTTTNCGGTCACGGTCCGGTCCGGTCACGGTCA
CATAGACCGGACCTTCAAACACTA
>Cligib1_rnd-1_family-109#TIR/MITE
CACCTGACATGCCCATCGCAAGGGAAGCCTCATATTAGGCTAAGGCGATCACGGGTTGCCTCACTATTATAAG
GCTCGTTTGGACTTCACGTCTACACCGGCGTTAATCCTGTCACTTATCCCTTAATAATTACTTAGCATTTGAG
AGCACTGCCTGGACCCTTGACTATAATTGGGAGATAAGGGTTCGTCATCACGTGTCGGCTGCGGCGGCGTCAC
TGCCCGCCACATCGGCAGGGTCCCCGCCATCAATTACTAATGCTCTGCTCACTGTCCCACACAGTGAGCTCAA
CATTAGGCTCAACATTGGACGCTTTCCTTGAATTTCTTTTGCGTACAGGATTNTATATATTATTTAATGTAGG
TACATATACATTTATATAATTTTAATAGCTCTGGAAGCGCCCGGAAAAGGAAACCCCCCCTGGGGGTGACCAT
CTGTGGACGGAGTATCTGGGTAGAGCGGGGCCTAGCGGGGGGCCCTGTGAGGGACCTATTATGATATTATATG
TGAGTATTAAAGCGCGGAGATCATTGGTGTACGTGGGGGGAAAGGGTGGGGGGATGTGTGCAGGGAGGGCGGG
GAGGATGGTGTCCCGTATGGGAACCGTGGAGCGGAATGCTATCGCGTTTGCTCTAGCATGTTTNCAATATATA
TGGGAGTNTGGGATCCAATTTTTAGGGGTATTGGCCGAGNGGATGTAGTACAGTGGGCGGATCAATGGGGGGG
GAGGAGGAGGGTGTCCAGGTATTTCCACAGCGGTTCCTATACTCATTGGGCGGCCTGGGCATGTTCTAATATG
TATATATAANTGATTNTATGCCGCGCATTCGCCCCGCCGCNTCCCAGGGAGACAGCAGCAAGGTTGGGGAGGG
GGGGTGTGCCGTAAATCAGAGATTACTGTACTGAATTGGCTACAGTGGNCGGAATANCATCGGAGGGCCCGGA
AATAAGAGCGGTATATAGTATTAGGAAGTCTCAGAAGAGACGNAAGAGATCCGCCCGGAGGTGGTGAAGATAT
GTAAGAAAATGTGTTCATGGTGAGTCCGATTTTGGATGTGTCACACATGTCGTACTCGGCCCGGCCAAATACC
ACATCTTANGGTATATCTCCACCACCTCTGGGCGGATCTCTTNCGTCTCTTCTGAGACTTCCTAATACTATAT
ACCGCTCTTATTTCCGGGCCCTCCGATGNTATTCCGNCCACTGTAGCTAATTCAGTACAGTAATCTCTGATTA
CCGCACACCCCCCCTCCCCAACCTTNCTACTGTCCCCCCGGGNTGCGGCGGGGCGAATGCGCGGCATANAATC
ANTTATATACATATATTAGAACATGCCCAGGCCGCCCGATGAGTATAGGAACCGCTGTGGAAATACCTGGACA
CCCTCCTCCTCCCCCCCATTGATCCGCCCACCGTACTACATCCCCTCGGCCAATACCCCTANAAATTGGATCC
CAAACTCCCATATATATTNTAAACATGCTAGAGCAAACGCGATAGCATTCCGCTCCACGGTTCCAATCCGCCA
CGCCCTCCTCCCCGCCCCCCTGCACACATTCCCCCACCATTCCCCCCCACGTGCTCCAATGCTCTCCGCGCTT
TAATCCTCACATATAATATATGATAGGTCCCTTATAGGACCCCCCGCTATGCCCCGCTCTTCCCAGATACTCC
GTTCACAGATGACCACCCCCCGGGGGAGTTTCCTTTTCCGGACGCTTCCAGNGCTATTATAATCATATAAATA
TACATGTATTCCCACTANATAATATATTAAGTACTATNCGCAAAAGGATTGAGGGAAAGCGCCCANNGTTGAG
CCCAATGGTGAGCTCACTGTGTCGGACAGTGAGCAGAGCATCAGCACANTGATGGCGGGGGACCCTGCCGATG
ATGGCGGAGCAAGGGGCGCCGNCGCNGCCGACAGGTGATGACGAACACTTACTCCCAACGATAGTCAAGGGTC
CAGGCAGTTNTCTTAGATGCTAAGTAATTATTTGGTGTGTTTATAAGTGACAGGATTAGCGCCGGTGTAGACG
TGAAGTCCAANCGAGCCTTACAATAGTGAGGGAACCACGTGATCGCCTCAGCCTAATATGAGGCTTACCCTTG
CGATGGGCATGTCAGGTG
>Cligib1_rnd-1_family-142#LTR/Gypsy
TCTAACAATTCCTTTTCTTCTCTNCCTGTAGATAGTATGGAAACCGATGATGACGGCAAGGTCAAAGTCGGTG
GACGGTTCTTTTCTCCTTTAAACATTCTCCATGGACATTTCTAANTTAGATAGTTTACCTAATGTGNCGAAGG
TTATGCTACCGGAAAAGGCACGGGTCGGTGNTANCAAGCCGTTCCCAGTTATTANGATTGCAGACGATGCTCA
GGGGCGCGCTCTGGAGAAGCGATTGNCCAGTTACGCAGCTCGTTTCGAAGGAAGGCGGGTGGAGCGTAGTAAG
TGGTCTTGGNGNTCNGNCNGACGTCCGTCCTGTTAANCAGGGTCCCGGCGTTACGANCNTGTCGACCCACCAG
GATTCCTTAAATGGAACATCCGTCCGTCCCGAGTCTGGTGAACTCCGTGGGTCCCGGCGGTATCGGTGCTTCG
GANCGTCCTGGTNNTACTTATAGGGAAAGACTTATTTATTCGCACGCTCAATTTGGAACGAGAGCTNAAAATG
TCGATCGCGATGACGACANTAGATACACATGAGACGGCCGNCACGTCAGGCCTTATTGGATNCCGGAGCNACC
GGTTCATTCATCGATCGAGCATATGTNNAACANATGGGNTGGAACACNCGANCGCTACGTCACCCTGTACAAG
TATTTAACGTCGACGGTACCTTAAACGCAGGCGGTTCCATTTCGGAAGAAGTCTATCTTGATGGTTTCCCACA
AGGGCCATAAGGAGCGNATGCTNTTCGAAGTGACGACACTCGGNAAAACCGAAGTTATTNTNGGACACACATG
GCTNAAGAAGCACAACCCNNCNATCGACTGGNCGACTGGGGAAGTCTCTATGAATAGATGTCCNCCGGAATGC
AACGTGGCTATCCGCGCGGCTAAAACNCAAAAGAAGCGTAAGGCCATGGCTTCGATCGCTGGNAGTATAAANC
CACGATCGAAGAGGTCGATGATGAGGATNCCGACCGAGTNATNGGCGGNNTCATAACGGAACAAGAAGGCGCN
CTNCTNGAGGAGATTCGTTTTGACGAAACGGTCCACGAAGTNGTTGCGGAACACTTCGTGCGCACAGTGGAGA
TCGAGGAGAGCTTTGACGCCCCTCCGTTGATAAGCGGATTACCGGAGCCTACCGAACCCGGTTTAGAAGANAA
GATTCCCCGNNGGTTTAGACGGTGGTTAAAAGTCTTCGAAAAGAAAGCNTCGGAACGNATGCCCGAACGCAAA
ACCTGGGATCACGCNATNGANCTGAAACCAGGNTTTGTACCCAAGAAGGCCAAAGTATTAATCTATCACCTAC
CGAACAGAAGGAGGTCGACGAATTTCTCAAAGANCANCTGGCCAAAGGNTACATTCGCGAAAGCAAATCTCCG
CAGGCNTCCGCNGTCTTCTTCGTACCAAAGAAGGACGGTCAAGAAACGCATGGTCAGGACTATCGNTACCTNA

ACGNNTGGACNATCAAGAACAATTATCCCCTNCCNNTGATCNNNGACTTGATCGACAAAGCCGGAAAGTCCAA
ATNGTTCACCAAACTNGACCTNCGCCGGGGATACAACAACGTNCGNACGAAAGAAGGCGACGAATGGAAGGCG
GCNTTCACAACCCACCGCGGNTCNTTCGAACCACNGGTCATGTNCTTCGGACTNTGCAATTCNCCGGCTACNT
TCCAAACGATGATGAACGCGCTCTACGAGAGCTGATTGACGAAGGACGCGTTGTNGTATATAT
>Cligib1_rnd-1_family-260#TIR/MITE
TAAGGTAGGCGAAGTTCATACTCATCTACGTCACATGACCAAATGTGACTTTTTCGTGACAAACGCGAGAAAT
CGAAATGACGATCTCCGTGTCCACCTTGCGTAAAATGTGCTCCCACCAAAATTTTTCGAATTTGAAAAGAATT
TTGGTGGGAGCAGATTGTTGTCCCGGATCTCGTGGGGCAGAGTCATCTCATCGAACGAGACAAGTAGCTGGTC
ACGACCCCTCAAACTCGCTCCCGGGACCGCGGGAAGTTTGGCCATTTGTGCCCATTTATGGCAAATCACGGGA
CGCTAACCCCCGCTAACCCCATGTCACAGGGAAAGTAACATCACCTGACGTAGATGAGTATGAACTTCGCCTA
CCTTA
>Cligib1_Cluster81#LTR/Gypsy
ATACCCCACTAATAAACTGACAAGTTGTGTTCACTGACTGATCTGCTATAGAGTATAGCCAGACACAGTTTAT
AGCCCGCTTATAGACGCTCTAATCACTTGATATTGATAATAGAATGCAAGTATATGATTGTCTAATACTGTTG
TCAAGGGAGTAGCAAACACTACTCTGGGACATAGTAGGAATTACACTTCCTTTGTACATGTTTGGCCTGTTTC
ACCCGAGGCGTGAAAACAGGTGTCCACTATATCGCGACTGAAATACTGGACTTAGAATATTCAACAATTAGGC
CTCTAGCTGCACTGGGAGGCAAGATATCAACATTTTGAATATACAGCATTATGAACCACCATACTAATGGTCT
AATGCGGTCTATTGGGTGTCCATCTATGTAACCTGTGTATCATAGATGGACTAGGGAGTGCTGGGAAGCGCAG
ATGAAGTGATTATCTGTCAGTACCAATGTACTGTCATCCCCACTTCTAACACCCACCACGTGCATTGCCCAAA
TCATTTGTGCAATCATCATATGCACAATGCTAATAATACATATATTAGCTAGGAAAAATAAATATAACTATGA
TAATACTCACTGTTGGTCCCATCACAATTCCATCAAGTACTACCATCTTCACAGGTGCATAATCAGCATCTAC
ATCATTTGCATAATTATTGGCAATAGCACTTGGCTCAATATTATTAGAATTATTCTCTGAAGCTGGATCTTGA
ATAGCTAGAGGAGGTACAATACCATTCTCATCTACACCAACAACAGCAGCAGGGTCTTGCTGTGGAATATACT
CAGGAACAGCAGAGAATGGCTGTGTACACTCAGAGCATGCATGTCCATCTGCAGGTCTAATGCGCCCACCCTC
ACCAAGAATTGAAAATGCCTGAGTTGTAACTTCATTGATTGGGATGTTATTATCCAATTCCAAGTGAAGCTTT
AATTCAAGTGCAATTGATCTTGTTGATTCCTGGATAAAAGCTTGCCATATATGTCGACGTGTGATTCTAAGTC
CATTTGTAGAGTCTGCATTGCCATATGCATCATTCCAGAAATCTGTGTATACAGCAGCTGATGCATGGAAGCT
ATACATTCCACCAATGACAGCATTTGAGAATACACGATCAACCCAAATCTCACGCCCAATCTTTAGGTACTGT
GCAGTATTCAGATATACTCTAACTGAATTATCATTAGACTTCATGACACTCTCATGATCTGCATAGTATATGG
ATTTGCATTTGGAGCATTTTCCTGAGAGCACAGCAACATTATAATAGATAGAGGTTCCTTTGATGAGTTTGAC
TGTTGGAATATCTCTGATCTTTGTTGCTTGTAAGAGGCCACGTGGCTCACATTCTGTATCTTCACACTCAAAT
GATGCAGGACAGATTGTCTTTACTGATTGGATAGCATTAGATGAGACAGGATGCAATTTGTTTTGAAATGTAC
CATGAGTATCATAAGGTGTGAGATCTAATACCTCAGCAAGTACATCAAGCTTGGATGCAAGAGATGTGATAAT
ATCATCTTCCTTAGTACATTGAGCAAGCTGGAATTTTGTAAATAATATCTTGGTATTGCCCTGAAGTTGGGCT
TTAATTCTACGTGCAAGAGATTTTATCCATCCAGTAGTATTAGATGCATATTCACCATCACAAGTTGGATGCT
TCTGACGTGCATGCAAATTGAGAAGATCTGTGTTATCTGTCATAGTAAAGAGCACCATTAGAACAGTTGCTAA
TGAGATCTTGTCATCAAAATTTGCATTCACAAATTGATTAAATGCTTTGAAAGTCACATCAGGAAAGCTGTCT
TGCACTGATGCAGCATTGACTCCAGGAATTATAAACATAAGAGCTAAGATCCATAGAATCTGTGGTATTCTCA
TAGTCCAGAGACATATGGAAACCAAGTAAGAACATAAGACTAAACTGAAAATGGAAAAAAAAACCAAGTCAAA
CATTGTATAAAATATATTACAAGTCCTTGGGCCTACCTCCCACCACCACCTTTCTTATGAGACCTAGTCTCAT
CAGCCTCCTTGGCTTTCCCTTTTCCCTTTCTATCAACCTTAAGTGTGCCACCATTGGCCAACCACTTTATCAA
ATCAGTGAAACTATATTCCATCTGAGACACCCCCCAGACCCCAAGATTGACCTCTGTATCCTGCTGCTTCTCC
ACCTCCAACCAAGATACCATATCTGGGTACTGAGAAAGATAAGGAAATGTCTTCTCATAATATTTCTGCCACA
TGGAGTGGGAAATAAAAATCTCAACCAAGACTCTCTTGATTGGACGCCTGCCCACCCAGTCACTAGCAGCAAT
CCTGGCATTCAAACTGCTAATCAGGATCTGATATGCCTTATACTTGGCATAGGCACTGAAAAGTGTGCCAGGA
ATGCGGAGTCTGATGGAATCCTCAATATCAAACATCTCAGACAAGATATCCTGGGGTGATGGAGCAGGTGGCT
GTGAGGGTCCAGGTGCAGGTGGCTGTGAGTCTGGATTACCGAATGAACTCTAAATCAAATATTAGAAAGATTT
AAGAATATAAATGAAATTTACCAAAAGGAGATGCAAGACTTGGTTATTGCCCTCCCTAGACCAAGAAATGCTA
TCATTAAATGAAATAGCATTTTCGAATCGTCCCATAGGACTCCAATATCGCCCATCCAGTGCTTGAAGAACAG
GTGCAGCATTTGAAACAACAGGATGGTTCATAGAGAATGAAGATTTCATCTCTATAAGATCCATCTCTGCAGG
ATCAGCATTGGATGTATAAACCCTAACCTTTCCCTGAGGGTCCATTTCAGGGTAATCACTGAGATTAGGGGTA
TTCCCCAGTGCTTTACCCCTCAAAATCCCACCTACAGTCCTTGATGCTCCAAAAGCCCTAGGAGGACTATCAG
GGGGACTAGTCATATGAACATCTAATTCTGAGTTAGAATTGAGATATATAAATAAAAATAAGTTACCTTGAAT
TTCCTCTGGAATAGACTTAGGAGTTTTGCTTGATTTTATAATTTTCTGGTCTAATAAAACAACTGATATCCCA
AATAATTGATTAGGATTGAAACAATAAAGGTATATTTACAAATACCTGGGACCCTAGGGATGGTATGAGTGGA
CTTGAGTGATTTTCGGGATTTTCGGGGATCCTGAACTGGTTCTAACAGAATCAAGTTAGAATTGGGATATATA
AACAGAAATAAACTACCTTTGACTTCCTCTGGAATGGACTTGGAATTTTTTAAGGCTCAGCACGAGCACGGGT
TACCCGCGGGTACCTGCGGGTAGACCCGTGGGAATACTTGCAAGTATTGGCCTAAACAGGTACTTGTACTCAT

GGATCTATTTACTTGCGGATCCGAGTCAAGCATTTGGACATTGCAGGTACCTGTGACAGGTACTTGTGACTCA
CACCAGACCCTCAAGGACCCTTAAAGTATAGTTCCCACAGTACATACAGGCTCAAATTCCATTCTAAACTAAC
AAAATCCTTGTAAATACCCATAAGTACTTGTAAAATACCTGTCCCTGATGACTAATTGCTGACAAATGACCTG
TGACCTGTGGGTAATCATGAGTAATCCATGAGTAATCAGGATCTGTGACAAGTACTTACTGAGTTGTAAGTAC
TTGCCAAAAAAATACCCGTGAGTACAAGTACTTGCTGACTAAGCTACCCGCAGATCTGAGTAGTGACCTTGTGT
GACCAAATACCCACGAGTACAGATCCGGATCACCCACGGATCTACCCATGCTGAGCCTTAGAATTTTTGCTTG
ATTTTGTACTTTTCTGGTCCAATAAAACAACTGATACCCCAAATAATTGATTAGGATTGAAACAATAAAGGTA
TATTTACAAATACCTGGGACCCTGGGAGTAGCATAAGTGGGCTTAATTGATTTTGAGAATTTTCGGGGATCCT
GGGCTACCCCTAACAGAATTTAGAGTTATTTTATGTTATTTACAGTGATATTTACCTGTTTGGGGGTTTAAAG
TATCTATTAGACCTATGGTTATTAATCAAGGTATGAAACAATGATAGGTACTTACTTGGATTGATTTGAATTT
CTGAATGTTTGAGGGTTTGCGCGCTCGGCCAAGATCCCCGAGATCCCCGAGATCCCCGAGCGTCAGCCTGCTG
GACATCCATGGCAGATGGGGATGGGGATGGGGAGGATAGGGGGCGGGAGGAAATTGATGGGGAAGTAGTGACT
GGGGGGGTGGTGCCAGGTGAAATCCCGTCTGCATCGTTCTCGTCCGCATCGTACGCATTAGCGTCGGCATCGT
TCTCATCCGCGTCGTACGCATTCGCGTTGGCATCGTTCGCGTCCGCGTCGTACGCATTAGCGTCGGCATCGTT
CTTGTCTGCGTCGTATGCATTCGCATTGGCATCGTTTGCGTCCGCGTCCGCAATGTTCCCGTCCGCGTCGTTT
GCGTCTGCGTCATCCTCACCTGTGTTGCCCGCAGCCGCGTTGTTCTCGTCTGCGGCGTTTGCTTCCCCGTCGT
TCTTGCTGGCATCCTCCCCGTCGCTCTTGCTGGCATCCTCCCTGTCGCTCTTGCTGGCATCCTCCCCGTTGCT
CTTGCTGGCATCCTCCCCGTCGCTCTTGCTGGCATCCTCCCTGTCGCTCTTGCTGGCATCCTCCCCGTCGCTC
TTGCTGGCATCCTCCCTGTCGCTCTTGCTGGCATCCTCCCCGTTGCTCTTGCTGGCATCCTCCCCGTCGCTCT
TGCTGGCATCCTCCCTGTCGCTCTTGCTGGCATCCTCCCCGTCGCTCTTGCTGGCATCCTCCCTGTCGCTCTT
GCTGGCATCCTCCCTGTCGCTCTTGCTGGCATCCCCCTCGTTGTTCTCGCTGGAGTCACACGTGGCGGGGGAG
CCACGGCAATCCCGCTCGCTTCAAGACGAGCCTCTGTACTGATAGGGGGGGAGTCAGCGCCAGTTGAAGGGGG
GTGATGGTGACTCACCATGTGAGGTGGCTGGGAGTGCCAGCCACCTCCGCTGCAGCATCCACTGCAAGGCGCT
GGGCGCCAGTCATCCTAATCGTTGTCAGTAAACGTTGGCGCCAAGCCGGCACCACGCACCTTGCTGGGGCCTT
GCCCCTCGAGCCGCGTCCCCTGGCCCGCCCCCTCCCCCCTCTTTTCCCCCTTCCGTTGGTCCCCCTGCCGTT
GGTCCCCCTGCCGTTGGTCCCCCTCCCGTTGGTCCCCCTCCCGTTTGCTTGCCCGAGTCGTCATTGCTGTTGT
CGTCGACACGAGGCCGCTTTTGAGCAGGCACCTCGGCAGTGTTCGATTCAGCGCGTTTGCGGCGGGATGAGGG
CTTGACCGCGGGAGGCTGCCTGATAGCCCCGCTGCGTGTCGTTGAGGGGGCGGTTGTGTTGCTGCGAGTGGTC
GGCATTGTCTGTTGTCGTTGTCGTTGTGCGTGGTGAAAGACAGAGGAACCAGCCGGCCAACGATCGAGAACCG
GTACCGAGTAGCCGGTACCGAATAGCCGTACCAAGTAGCCGGACCCAGGTCCGAGGCAGCTGGAAGCGGGATA
TCTTCCCACGCTGACACAACCGGTGTATTGCCTTAATCTGAAGATAAGATAATGATTATCTTCCCACACTACA
TACGACCGTTGATACCGCCATAATCTACAGATAAGATAACGATAATGGTAAGATAAGATATAGAATGCGGAAA
ACAGGCTAATCAATGCCAAAAAATAGCAGAAAAATCAGGAAAAATCAGGGAAAATCAGGAAAAAATAAGAAAAA
ATTATAGTAAATCAGGGAAATATTTAATCATTCAAATAAATTCAAATAAATGCAAATAAATAATGGTATGAGA
TAGTGATTTGCATGGGTCTACATACCCGTACCTGCAGAAACCTGCACCCATGGTAAACCCACAGTAGGCTGCA
GGTTTGCAATATCCTGTAGGATGTTTGTTTACTATTATTTGTTGTGAAATTCCATTGCCTTCCCATAGTCTGA
TTGCCATAAAAAATAGAGTTTAAGTATGCTAATAGTATGAAATGTTGGCCGAAATGTTGGTTTTAAGGTTTGT
GGAGTCATATATTTTTAGGTGAGCAGGAATCTGTACCCTGGGAAACCTGCTATACCTGCACCAAAGTATGACC
TGTACCCACACCCATGACTACCTATAATGGGAACTTATGGGGCTTCATTACACATGCTTGCAGATTTAATATA
AACATGGCCAAACAATGACAATCAGGCCTGTGCAAATCTATATAAAGTCCATGGACTCCACATAAAGTCCATG
GAGTCTACGTAGAGTCCATGGAGTCTACATAGAGTCCATGGAGTCCACATAAAGTCTATGGAGTCTACGTAGA
GTCCATGGAGTCCACATAAAGTCTATGGAGTCTACGTAGAGTCCATGGAGTCCACATAAAGTCTCAGTCCATG
TGGACTTTATGTGGACTAAAATGCCCAAATGGCAGGGCTCTCACCCAAAAAAAGTGTACATAAAGTCCATGGA
AGTCCATGGAGGTCCATGGAGTCCACGTGGATCCACGTGGATTAGGTGGGGCACTGTAAAGACCTCATACCCT
GGGGTGACTTCTACCCATATTGTTACTCTTTTTGTGGCTAAGTCCACATGGCATGAGAATCATAGGAGGGCTT
TTTCTCAGATTGGACCCCATCCTCTCTTGAGGCAGTGGTTGGAGGGTGATAAGGACCCTGACCTTGCTACTGC
ATTATGGGGTATAAAGAAGAAGATTGGGTTTAAGGCTTTAATGAAATGGTTGGGTATTAAGAAGAGGGATGGA
GATAAGAATAATTTGGATGATAGTGAGGAGGAAGAAAGTGAGGAGGAGGAGAAGAAGAAAAAGAAGGGAAAGG
GAAAGAGCAAGGAAAAATCCCACAAGAGGAGGTAGGTTGAGTTAAAGTTTGAAATTACTTGATCAAATATTTG
TATTAGTATCTGGATGATTGATATTATGTTTGTCTTGGCTCTCATGATGAGAGTATCAATGGCTGCCATTATG
CTTGTCTTTGCTCTTATGATCAGAGCTCCATATCTGATCTGGTTAATTTGGATTATGTGTTTTGGGATTTCCA
GTGTCAATGCTATACCAACTGAGGACCCATTCCCTAATATTTCATCTAAAATCCTAAATGAATTTGTGGTTGC
CAATTTTAGCTCAAAGATTTCCTTGGCAACTACAATAATGGTCCTCTTTACATTGATGGATAATCCAGATCTC
CTCAATTTGCATGCACGTCAGAAACATCCAACACAATATGGAGAAAATAGGGTTCGAGTGTCTGGATGGATCA
AATCTTTGGCACGTTAGCTGCAAAATCAATTGGGAGATAAAACACAAATGTTATTCAAGTCTACAGAGGGATA
CTCTCAAATGAAGGAGGACAATTCAATTACTGCTCTTGCAGTCAAACTTGATAAGTTGGCTAAAGTATTGAAA
CTTATGCCACAGTATAACAAAAGAGGCCAGTTGCAAAAAACTTACAGTCAGTATTCTATAAGTCAATTTAATC
TGTCCGTGCAATTTGTCCATCATCCATTGTTTGTGAAGATTTGAATTGCCAGCCACGTGGCCTCTTGCAAGCC

ACAAAAACACGAGATATACCAATTGTGACACTTATTAAAGGATCAACAATCTATCAGAATGTTATTGTCCTTG
CTGGGAAATGTCAAATATGCAAGAGCTTGTACTATGCATATCATGAACAGTTTGCATCAGGGCAGGATACATA
TAAGGTTTATCTAAACTCTGCCCAATATCTGAAAATTGGCCAGCAAATGTGGGTTGATCGCACATTCTCTACT
TCAGCCTCAGCATACACTGAGTTTTGGAATCACAGCTTTGGAAATTTGCAGTCCAAAGGCAAGCCTATATTAA
CTCGACATCAGATTTGGCAGGCTTTTGTACAAGAATCAGTGAGATCAATTGCCTCTGTTTCTGGTTTTAATTT
GGAGTTAAATAGTAGTCTTTCAATTGATGAGGTGACAGCAGAGGCTTTTACTAGGCTAGGGCAAGGTGGCCAG
ATCAAGGCTGCTGATGGTCATGCATGTTCTGAGTGTACACAAGAGTATAAAGATCAGCCTGAGTTTATTCCAC
AGGGGGATTCTGCTGCTGTTGTTGGAATTGATGAAAATCAGCATACAGCTCCAATAGTAGCTAAGAGCAGTAA
TCAGGATATAGATATGGATCATGCACCAGTAAAAATGGTGGTTATGGATGGAATAGTAATGGGACCAACAGTA
AGTTTGACCCCTTATTTAGTATAACATTTTACTAAGATATGACCTCAGCATTGTGCATATGATGGTTGCACAG
CAGATTTGGATAATTCTGTAAGGGTTCATTCTGTGCATATCACAATAAGCATGTCAAGTCTATATGATGACTC
ATCGCAGTCAAATATCACGATCACAGGCAATATAGATCCACAAGTAACTCATGGTACTCATGATGCAGGTAAT
ATACTCATAATCACAAGTATGTGAGTACTGAATATGAGCATAATGCATATTAAGTCATAAGGTCCAAAATCGC
AAGTATCAGTGATACTGTGATCGTGATATGAGATCAAAGCAATACTCAGACTACATGATATTGAGCGTGAGTA
GATCCACGAGTCGCTTACTTGTGAGTATTCCACGGTCAATATCACAATATTGCATTTGGGGCACAATGTCGTG
TGCGTAATTGTGATGGTCAAGTTGTACTGCCAACCCTGGCATGTGAAGACCATCAGCGTGAATGGAATAAGCA
CACTCAGACTCATAATCGGCAAGCCATGGTAGGAGTTCGGAGAATGTGCAGCGACCTGGTGGTCCAATGCCAT
GGGAACCAGCTTTAAGCAGGAATACCCAGCCCCATGATGAGGATACACAAGAGCTTGAAAGAAAGACATACTT
TAGCCCCTCTCGTTTTTACTGTGTGGAAACTATCTGTGCACCATGTGGTGCAGTGATTGCCTGGACTAAATTT
GATAAGGCTGAATCACCTACCAAGATTCTGCAGTTCCTTGAGGAGGTGTATCCTACAGAGGAATCACGTCCAG
CTTATATCTGCATTGATAAGGCATGTCTTGTATTGAGATCAAGCATTGCAAATAAGAGCTGGGATATGTGGAA
ACAGACCAGCCGATTTGTTGTTGATTCCTATCACTATCTCAACCATCGCACAACAGATCGACTTTGCAGAAAG
TGGTGTAACCCAGCTCCTATGGATGGGTCTGCTCCTAATCTTGTTCTAGTTGCTCAGGACAATCAAGGAAGGT
TGTATTATAAAAGGGCATTCAACACACAAGCCTGTGAGCAATTGAATGCATGGCTAGGTGGATTTGAGTCTAT
ATTAAAACGGATGACACCTAGCAATTTCAACTGGTTTCTGCATGTGATGTTATTTTATCACACAAGATATGTG
ATTGAACAGCAAAAGCAAAAGCAGAAGCAAAAGAAGAGTAGAAATAGTGAATCTGGTGAATCTGGTGATGATG
ATGATAGTGAAGATGATATGTAGTTTGGTTCCTCCTCATACTTTGTAATTGTAAGATGTATATATTTATGTGA
AATGATACAATTTGATAAAATATTCAGATGAGGATAGAGTTGTAGCAGCAGAATATCCAGATAGTAGTAGAAA
AATATATAGATTTTACACAGAGTCTGCCCAGAGTCTGCATGGAGTCTGTCCGGAGTCTGCATGGAGTCTGTCT
GGAGTCTGTACAGAGTCTGTCCGGAGTCTGTACAGAGTCTGTCCAGAGTCTGCATGGAGTCTGTCCGGAGTCT
GTATGGATTCTGTACAGACTCCAGCAGACTCCACGTGAATTCTGCACAGACTCCAGTGGACTCCACACAGATT
CCGCACAGACTCCAGCAGACTCCACACAGATTCCACCGGACTCTGCCAGACTCTGCACGGATTCCACCGGAGT
CCACCAGAGTCTGCCAGAGTCCACCGGAATAGGTGGGGGAGTGTCAAGTACTGAAGAGGGTGCATAGGTCCTT
CATGAAGTTTGATGCAAACTGTGGTCCACGATCACTAATGATCTTCTTGGGTATCCCATGTTGTCTCCAGATG
GTGTCTCAATAGATTTCTGCAAGTCCCTTGGACGTCAGGGTGGTGTGCGTTGGTTTGACCCTGATCATCTTTG
ATTGCCTGTCAACAATGACCAAAATAGCATTGTAACCTTTAGATGGTGGTAAGGGGCCGATCATGTCAATTGA
GATCTCTTCCCATAATAAACTGACAAGTTGTGTTCACTGACTGATCTGCTATAGAGTATAGCCAGACACAGTT
TATAACCCTCTGATAGACGCTCTAATCACTTGATATTGATAATAGAATGCAAGTATGAGATTGTCTAATACTG
TTGTCAAGGGAGTAGCAAACACTACTCCGGGACATAGTAGGAATTACACTTCCTTTGTACATGTTTGGCCTGT
TTCACCCGAGGTGTGAAGTCAGGTGTCCACTATATCGCGACTGAAATACTGGACTTAGAATATTCAACAATTA
GGCCTCTAGCTGCACTGGGAGGCAAGATATCAACAATTTGAATATACAGCATTATGAACCACCATACTAATGG
TCTAATGCGGTCTATTGGGTGTCCATCTATGTAACCTGTGTATCATAGATGGACTAGGGAGTGCTGGGAAGCG
CAGATGAAGTGATTATCTGTCAGTACCAATGTACTGTCATCCCCACTTCTAACATGTTT
>Cligib1_rnd-1_family-685#LTR/Gypsy
AGGGATGCTGGACAGACACGGAATAAGGAACGATAACAATCGTTCCAAAGCAATCAAGATGGCGCACAACCTG
ACTCGACATGACCACGGAATCGCATACGAANGAGACGACTTCGCATGGACGGAGGAATACGTCGAGACATACC
TCGANCANCTGAGGAGGGCATGCGAAACACTGGCGGAAAANACAGAGGAACGCCGCCAGTCCCGNCAANCATC
GAGAATACCTTCTCGAGNANCAACGCCGGAACCCGANNCGGNGGGTATCGAAGTACCNCGACCAAGANCGGTC
GAAAGNAGCGCGCGGGAAAATATNCCCGCGCNAGAACNNAGGGGTAGATACACTCCCGACTACGCCATNGAGA
AGGCNGGGGAGCAACTACGTGCTCCCAGACGGAGGGACGAACCCGAGATAGAATACGTCCAGAGACAANAAGC
GGCGAGAAGGANCGAAGAGACCCCACGCCGGACGACACCGTCCGGACCTGCACGACGAATACCGATGGCAGAG
AAATTCTCTGACAGGAAGGTATACCAACAATGGCGGAACGGGGAACTCGCCGAACACGGGCGCTCGAAATACG
ATGATCAAGGGATCATCTTCATAGCGACCCAACCAGGAGAACCGGCGAGACCGGTCGATAGAACAGAATATAT
GGCATCGACAGCNGGCGATAACGGAGGGGCGAACACTCGTCCAACCATAAGGATAGACGATCAAAATCGTCAA
TCCACAAGCCAACACCACGATAGAACNTCAGAGAGAAANGAAAGAAGGAGCGTCAGGGGATACTCCATGCCTA
GGGCAACGACACTGGCGAGACANAACACGCCAGGAATAGCCGAAGCCGCAGAGAACCACCGCTCGACAATGCA
TGAGAGACTACTCACTCTCATACATGAACGACTNGACGTGAAGCTNACAATACCGGAGGGNTGCAAGCTGAGG
AAAGCCGACGGACGAAACGTCGGCGAATATAAAGGAGGCCCCAAGTTCAGCGACTTGGAGGAATGGCTAACAA

GCCTCGTAATATACCTCGAAGCTAGCATGCTTGGAGGACGCGANAGGGATCGCGAGCGNATGCTCGCGATATC
GGAATTCATCGGAGGAGAAGCNAAGAAATGGTTCANANGGCACGTGATNCACACGAACCGGACCCAGAGGNAT
TGGACGTTCGAAGACGTCGTAATAGGNTTGTACGACCGNTTTGTACACGCGTCCACNATGCAGGACGCGAGAG
AAGAATTCATGTCTGCGCGGTATAGGCCGGAGACAGGAATNCAAGGGTACTATGATACCCTACTAGACCACGC
CCAGAACATGGCGGTGTATCCGGACCAGTNTGCAATACTGGAGAGATTCCTCAACGGAATCACATCGCAAATG
AGATATGAGTTGTTAACTCATAGTAGACTATCGCCCGAAGTCAACACGATAGACGACTTCGTGGCTGAAGCGA
AGTCCATAGAGACTTCACAGAAAACNGCGGAGCATTACGCCCGCAAAGCAAGAACGAACACGGNCGACAGACC
GTATAAAAACAACGAGCCGAGANNACCGGCTCATAACAGAGCTGATAAAGNATACAAGGACGAACGGTACNAC
CGTCCAAAGGANCCCGGGAGGNACCGGGAGAACAAGATACGGCAAGACAAGCCGTA
>Cligib1_rnd-1_family-761#TIR/MITE
CACTGCTGGCGCTGGGTTATTTTTCAGGGAGAAAAATGGGTGGGAAAAAAATGTTTCATATCTTCACCAGGGTT
TTTCTTGTAGANAAATACAACATATTTAATTCAGAAAAATACCTGAAAAATAAAGACTTTTTCCGAAGTATTT
TGGGCCGAAAAATCTGATAAAATACCATATGTTTTTCTTCAAGAGACCTGGAATAGAAATACTCTATTTTGCA
TTTATATTTCTGTGGGAAAAATCATTGATTTATTGATTCATTTTTCTTGATGAATGGTTTTTCTCAAATAAAA
ACCCATGGGAGACAAAGGGAAAAAACCCTTAATGGATTTTTCCCCAAGAAAAACGGACTAGTAAACCAAACAC
ATTTCTTACAGAAAAATAAGGGAAAAATAAGATATTTTTATTCNAAGTCTCTTGAAGAAAAACATATGGTATT
TTATCAAGTTTTTCGGCTCCAAACACTTCGGAAAAAGTCTTTGTTTTTCAAGTATTTTTCTGAATTAAATATA
TTGCATTTGTTTACAAGAAAAACCCTGGTGAAGATATGAAACATTTTTTCCCACCCACTTTTCTCCACAAAAA
ATAACCCAGCGCCAGCAGTG
>Cligib1_Cluster11#LTR/Copia
TTAGGAGGCTTTGTTTGTTGTTAATCTAAAACACTCGCTCACTCAACATCTTATCTAGGTGTTGTTTACTTAG
TTGGTTTTTGTGGTTTGTTTTGGCCACAAGCCTCTTTGCATATCACCGATTAGGCCCCAAGAATAAGGCGTCA
TCAACATAGACTATCGCAATGACAACCTGATTTGAAGATGATTGTTAAGGCGGAGTGCCTTTACCAAGGTTCA
AGCGTCGAATATCGTCCAGAGGTTCAAGTCAGAGTCTTGGGTCAATACCAACAAGACTATAGTCACTCCAAGT
CAGGCTTTGACTTGGGTATCGATGGTTCAGTGATCAAGGTTCAAAGGTAAAGGGTGTAGTATTGCCTAGGTCA
AGATAGGGAATACAGACAATGTACCTAGGGTTTATATACTGAGACTATGAGGTGCGAGAATATAGTACAATGG
GAACAGAAGACAATGTAAGGTAAAAGAGCTGTTGAAGCTCAGTCAGCTATGACATCATGGATGAGTCAGTATC
TAAGAATAGAGGCATGTGTTGGACATGCGATAGAACAATGTAGAATCAGAGAACAATGTAAGCATAGTTAAAC
AAAGGGAATCGGTGAACAAAGGAAAAAGATAGGGGTGGGAACCGTTGTAACAATGATCGGTATCTGAAAGAGT
GCGTTGAAAGCCCATTTCTTTCATTGATGCAACGAGAGCTCTCCACCATTCAAGAGCTGCTTGTTTCAGACCA
TATAAGGCTTGTTTGAGACGCAGGACTTTGCGCTCATGGCCTTTTCGGACGAAGCCTTCTGGTTGATGCATAT
ATATTTCAACGTCTAGTTTGCCATAAAGAAAGGCAGTTCGAATGTCGAGTGATAGAATGACCCAATGTTTCAG
GGCAGCTGTGGCTATCATAAGGCGCACAGATTCATATCGCACAACCGGAGAGAATAATTCATCAAAGTCAACA
CCTTTGACTTGAGAATATCCCTTGGCTACAAGACGAGCTTTCTTTCGACCGTCTGATTTGACGTTGAAAACCC
AGCGATTGCGAATCGCTTTCGTCCTTTAGGTAAATTGCAAAATTCATATACGTCGCAACTTTTGAGGGAGTCT
AATTCCTCTTCGCAGGCGTTCATCCACTCTTTTTGTAGCTTTTTATCAAGCTTAAAAATGTCTCGGAAGTGAG
TAGGAACTTCGTCTTTGACAGGTACTGCTTTGGATAGTAAAAATTCCATTATAGCTCCCCCTTCTTCCTGTAC
GATTTGTAGAAGGTGTAACTCTTCTTCATCGGTGGAAGGATAATCAGGAAGGTCTCTTTTGGGACCTTTTTCT
ACATTTTTACGCCAGTATTGTTCACTAGCAATATCTCGTTCTATTTCAGAAGGAAGTTTCTTTTCTCCATAAA
TATTTCCTGGATGGTTAGGCACTGTCTTTCGACGTGTGCTTCGTCTAGGTAATTCATTCCATTCTTCCTGATC
TTCGCGGTTTGGAGTAAACGCACGAGTGTATGGTTCAGGAGAATGACCTCGCATAGGAGATCCTGGCGGAATG
GGAGAATGTACTTCTTCTTCTGGTTCTTCTTCAACCGTAGGAGCCCATGGATGAGGTTCCTTAGGTGGAGAAG
GAGGAAGAGAGAATCATTCCTCTACCGGATGAATTATTTCTTCCGTCTCCACCGCAATCAGAAATACATCTTC
AATGATATTTTGTTCAGGTACTGTAGTTTTTGGTTTGCATTTCGGCAAAGTGTCTTCATCGAAAATAGCAGTA
ACTCCAGTAAAGATAACAGAGTTTTTGATTCGCATGAAGCAGTAACCTTTAATACCAGCAGTGTGACCGAGGT
AAATCATTAGCTCGGATTTGGGCGCAAGTTTGTTGGCACGTCTTTCCGAAGGAATATAGACGTATGCAGCGCA
GCCAAAGACACGAAGATGTGTTACGGAGGGTCTTTCTGTTACAACGGTTCCCACCCCTATCTTTTTCTTAATT
CACCGATCCCCTTTGTTTGACTATACTTACATTGTTCTCTGATTCTTCATTGTTCTATAGCATGTCCAACACA
TGCCTCTATTCTTAGATACCTGACTCATCTATGATGTCATAGCTGACTGAGCTTCAACAGCTCTTTTACCTTA
CATTGTCTTTACTTCTTATTGTACTATGTTCTCGCGCCTCAAGGTCCCTGGTATATAAACCCTAGATACATTG
TCATTATTCTTCATCTTGACCTAGGCAATACTACACCCTTTACCTCTGAACCTTGATCACTGAACCTTTGATA
CCCAAGTCAAAGCCTGACTTGGAGTGACCATAGTCTTGTTGGTATTGACCCAAGACTCTGACTTGACGTCTTT
GACGATACTCGACGCTTGAACCTTGGTAAAGGCTATCCGCCTTAACACTTTCACCCTTAAGGACTTCATATGG
AGTTCGCCATTGCAGGCACACCATAGGTGTACGATTGTAAATGTGAATCGCTTGTTCCACAGAGAATTCCCAC
CATGAACTAGGAAGGCATGCATCGTGTCGCATAGACTCGCCTTTATCCATAATAGTTTGATTGATATGTTCAG
CGCGCCCATTTTGTTGTGGTGTATGAGGCGCTGATTGTTCAATTAGAATGCCCGATGATTTCAATTCATCAAT
AAATTCCTGGGATTTAAATTCTCCCCCTGCATCAGAACGCCAACGCTTAATTTTAGCGTCATATTGGTTTTTA
ACTTCTGCATAAAATGTACGGAAGGCACCAATTGCGTCTGATTTATGTTTTAGATGTGAAATCCAAACATAAG

ACATGTAATCGTCGAGGAATGTGATGATATATGAATACCCATGATATGATTTCACGGGGAAGCTTTTTAAATC
CGAATGTATCAATTCGAATGGCTTCGTGGCTCGCGAGTTAGAAGGTTTAAAAGATGAAGAGGGCATCTTTCCA
TACGCGCAGCCAGGGCAAATAGGAGAATTCTTCGGGATGATAAGTCCCTGTGGGAAGTCCTTGATATGTTTTC
GAGATTTGGAGATGACCTCTTTCGAGGGATGACCAAGGCGTTTATGCCAAATGTCATATGAATCGACGTTGTT
AAGAAATAGAGATGTTTTTAATTGATCAGGTTTGACCACATCTCCAGTGACCCAGAATGTTGTGTCCCATACG
AAACGCTTTTCAGCGGTAAGTTTTCGTTTGCCATTTGAACTGAATATAAGCGTGTTGGATGACGCACTCATAT
TTAACCCAGAATTGAGTAATTCTCCCATTGAAATAATGCGGGAGTTCAGTTTCGGATGGTGATAGACAGGATG
TAAGCGGAATGATTGCTTTTTATAAGATCCATCTGTCTGTAGGACATCGTTTTCAAGAACAACTGTTCCTTGT
GTTTCGATTTGAATAATCTCGGTTGCGGTAGATACGGTGATGGGATGATCAATAGCTGAATGTTCCGCATAGT
CATCCAGTATTGTGGTAAAGTGCATAGATGCACCTGAGTCTACTAACCATTGGTGTTCGACGTGTTTTCCTTT
TCCTTTACAAGATGTGCAAGAAGCAAAATGCATATTTATTATGGCCATATTGGCCGCAGACAAAGGTTTACAT
GATGTGTAAATATTATACAGGGGGCAACTAGGATTTAATCGAAGAATGCCCTGTAATTCATCATTAGTCTGAT
AGAAAGGAAGAAAGGAAGCGGTGACTGATGATTGTCCATCAAACCGAGCACCCCAACTCTCAGGATCGAATTC
GGGTTCTTCACCCATCGATACTGGATCCTCGTGATCGTCATCTTCTTCGATCGGGGAGAGTCTCTGTTTTTTA
GGAGGCGGGACCCAAGCCTCCATGTCATAATTGATGTAGCGTGTTTCGGCGTCTTCTTCTTCAGAATCGCCAC
AGTATCGGTCAACCCAATTTTCGTATTCCTCTTGTCCCATGAGAATATCATTAAGACCATCGGGTGTGGTCAA
GCGGGGCAGAGATGCCACAGGCTCAGGAGTGGGTGCGCGAAACGACGAAGTCGAGGCGCGAGGAGGAGAAGGG
AGAGGACAGATAGGTCGCTCAGCAGGTGGTGGTAGGACAGTTATGTCATGAGCCACGTGGTCCAAAAGAACTC
GCGACTCCGGGAGCGCCATTTGTTGAGTCTCTAAAATCTTGAGAGTCTCAGTAGAGGGCGCAACACCGATGCG
CAAAGCGAGAGACCAAGCTTTTTCGTAAGTCGGGTACGTGCCAGGTAATTGACCTGGGGTTGCTCCTTGGAAT
GTTTGGGCCGGTTTGTCGTGAAGACGTTGAAGGAGGGAGCACTCCTTTGGCCCTAAAGGTTTCGAACGAGTGA
CCTTGGGTGGTGAGGGTGGAACCTCGACAATGAATGCTGAGGAGACGAGATGTGAATTCCCATATTGGGAAGT
CCCGTCGGCGGATCCAGAGTCGGAAGCGGCTTCTTCAGCCTGCTTGGCTTGGCGCTTTTTACGGCGCTGATAA
GCAAGCTTATCGTCTAGGGGTTCGCAGCTCACATCTCTACTGATATTGGGCGCATTGGGCGCACTCTTACAGT
ACTGATCGCACCCATTCCGCTCCGAACGCGTCGAGATCGCGCTTCAATTCCGATTCCAGCCGAAGTCTCCGCA
TGTTGATCGCAGCACTTCGCACTCGGATCACATTACATTACATAGTTCCCTTACCTATTTACCATACCTCATG
GCCGATAGCTCCGATATATAGAGACAGTAGCCACAACCATTAATAAAAGTTCCCATTAGAGCTCGAAGCTCAT
CTCGCATGCTTCCGCATTCCACTCTCGTTTAGGGCAGTGCCCTCTTATGATCTCGTCGAACTTGGGCTAGGCA
TCTCTTGCTAAACAGACACCCACTCGCAAACTGCGAAACACACAGGTTAGGTTTGGTTTTGCCACCTGGCCGC
AGTGTCACATCAGCGCTCCTCACGGTTTTCTCCCTTAGAGGATCGTGGTTTGGATTTGTCATTATTGGTCTCC
TTGGCCTTACCCTTCAACGAACTTGTCTTTTCAGACTTGTTCTCAGGTGCCAATTCCTTGGCCTGCTCCTTCC
AGTTGGGGTTCTTGCCTTTTCATTTGATCCCAGAGATTTTATTCGCTTTGACGGGACCTGAAATGGCAGTATG
GCGCTGGTATTCCATAACGAGAGTGGTGTGAACAATGTCGAATGTGAGATCCGCTTGTTTGTTACTTTGGAGG
ATTTGAGAAGCGATGTTGTCCCATTTGGAAGGTATGGCGGCTAGGAGGATCATAGTTTTTACGAAAGGAGGGA
GTACGACGGAGTTGCCGTCGAGACGTGAGAAAAATGTGGACATGGCCATTATTTCAGGCTCTGGATTATTGTT
TCCACTAATTTTGACAGCGAGGGCAGATCGGAAGTCTAGGAAGATTTGAGAGGGGTCGGCAACGGAATATTTG
GCAACGAGTGCTGCCCATAGTGATCGTGCAACAGAGTAGCCCGAATAGGCAGCACGAATGTCAGGTGAGAGAC
AAATAAGGATCGAGCTGAGAGCTTGATTGTCTTTTGTGAAATAGTTGTTGCGAACCTTGTTCCAATCTGTGGG
AGAAACTCCGGTGGGAGGAGATTCAGAGGGAATGGTTTCAGTGCCGTGGATATTGGTGATTTGCCAAAGACAG
TTGAACCGGAGGAAATTCTTCATGTCATCGGACCAGTTGGCCCAATCGATGCCGTTGAATTTCGTGATGGAAG
AAATGTCGAGTTTTCCGGAAGACATGGTTATCAGATCAAAAGTTTGATGAGGGTGTCTAATAAAGGGGGCGAC
GTTAGCCTCATGTTATGATGATGAGATATAAACCATAACGCCTTGTGCAGGCACATCAACGGTTTACCCAACA
TCTGCTTTATTCAAGCATATGTGGTAGAAGATGCATAGAGCTAGGACGTAAGACGTCCATAGCATAGCAGAGC
TTCGGGAGGTGCAAGGAGCAAGAGCGTAAGATGTTCATTGCAACGAGCAGAGCTCTCATCCAGACTGTTAGCT
GCAGCCTTGTTCAAGCGTGGCAAGTCGTCTGGGGTAACCCAGTGGAAGTGCATTTCATGCAGCCTTATGCAGG
CACGTGTAATGGCGACCAAGTGACCAGTCTGTCGATTGCAGCCTTATTCAGGCGCGGTTGACAGTGGCACCAG
GGCCCATAACCTGTTGAGTGGCACCCCTAAGGCACAGCGGAACTGGTTAACTTACTTGGTAGACAGTAGAGCT
TACTGAGTGGGTGTTTAGGGATTAGCACGGGTGTGGGAAAACCCGTGGTGTATGGTCATGGGTGTCACAAGTG
CGGGTGCCGGTGTCCCGATTAGGCAATACCGCGAACACCACACCCATACCATGGTTACCATGGGTTGCAATAA
TAGCCCTCTCTCACCTCTATCTCAGTGAGTTCCCATCCGTTTTGCTTCATCCTGGGGTTCACCAGGACCTATT
TTTCTCCTAGATTACGAATCCGATGAGACGACAGGGCCCCGCATGACCATTCCCACATGTGAGGCCAATCTTT
TGATTCTTCACCAGATATGGTATCTCAGTGAGTTCTTGCTTGTTTTCCTTCATCCTAGGGTCTAATAGAATCT
ATTTTACTCATAGATTACGAATCCGACGAGACGATAGGGCGCGGTGTGACCGTTCACAGACGCGAAGCCAAAC
ATATGATTTCAAGCAGAAACACTACAACAACTTGGTCAGTTCTTGTTCGTTTTCCTTCATCCTGGTATTCACC
AGGACCCATTTTACGTCTAGATTATGAATCCAATGAGACGATAGGGCTCCGCATAACCACCCGCACATGCGAG
GCTGATTTTATGATTTTACCCACAGATATTGATGTATCTCAGTCAGTTTTTATCCGTTTTTCTTCATCCTGGT
ATTCACTAGGGCCTATTTTATGTGTAGATTAAGAATCCAATGAGACGACAGGGCTCCGGTCAATCATTCACAT
ACGCGAGGCTGGTTTCATGTATTTCCTATGCAAATATGGCCATATCTCAGTGAGTTTTGGTCCGTTTCTTTTC

ATCCTGGTGTTGAATCTGACCTATTTACTCATAGATCAATAATCCGACGAGACGAGAGGGCTCCATGCGATC
GGCCGCACGGAACCACGGCTTTTCACTGTATCCGCGGATGTGACCCGTGACGGGTACGGTGTGACCGTCTGCG
GGTTCCGGTGTGACGTCTGTCACCTGTGGTGTCACCCGTGCTAAACCCTATGGGTGTTGAGAGCAAAAGAGTT
ATATTACTCTCTTATTCAGAGAGGTCTTATATACTACTAGAGTGTGCACAGACCATGTGGCTGTGCTAAAGTC
CTAACTCCTATCTATAATCTAATCTACTGATAAGATAAGATAAGATGTTATCTAGGGTGCTTATATAGACACC
GGATTATGTAAGCAGGGCACTTACAGTTAGAGCGTTGAAGTTTAACAGTACATACTTCACCTTACACAGAGAG
AATCCAGTAGAGTTTCCCCCTCAGAGCCTGTAGCTCAGTTTCGCAGTCTCAGAGTAGACTACCCACGTTCCCT
ACAGGAGTCGCACGTCGCGCTCCTCCACGTTCCTTCGCATTCAAGTAGTCGGGCTGCGACCAAGTCGCCCGAT
CGCAGTCAGAGTAGCACACATATTTCCGTACGCCGTCTTGCGTCTAGTGATCCGCACGGGTTTACCGCGGGTA
ATTTCCCACGACCCGTACCCGTACCCGTGGGAAACCCGTACCCGCCCCACGGGTTGGATTAATCCGGATTTTA
TGGCGGGTTTTGCTAAAAACCCGCAATGGCTGCGGGTTTCGGATTTACCTCATACCCGTGGTACCCGTGTGCC
TCAGAAACTAGTGTCCAACGTGCCCCATAGTCAGGGCAACAATCAGGGATAACAACCTAAGCTCTACGATCAA
TATCGGCTGAGTAATATGTCCGCGAGTGGTGCGAGTTACGGTTTTTGGCTTGTTTTGGCGAATATTTAGTCAA
AATGTATGATTCTTTTTGCAGATTTAGCATAAAACAGGGCCAAATAGTGCCAACCGTACCCGTGAAAAACCCG
TGGGAAAACCCCTACCCGCAGTGTGGGTGCGGGTTTTCTGCAGGTTTGCGAAAAGTGTACCCGCGGGTAACCC
CTACCCGCACCCGTGGGAAAACCCGTACGGGTTGCATTACCCATGCGAATCACTACTTGCATCGCACCGCCCC
CGAAGATTTGAGAGCGCACTCGTGTCACACTCATCCTACGCTAAGTGCCTAGTTCCCGGACACTCTCGCGTCC
ATTCCATTCATTTCGTTACCACTCTCGCACGTTGTATGCGTCGAGATCGCATCCAAAGCAGGTCATCACGCAT
CCGTTGAGAACCCTTCGCAAGTCCTACAGAGACTTTGCTTGTTGCTAATCTGAAACGCTCGCTCACTCAACAT
CTTATCTAGGTGTTGTTTACTTAGTTGGTTTTTGTGGTTTGTTTTGGCCACAAGCCTCTTTGCATATCAACGG
TCTTGG
>Cligib1_Cluster12#LTR/Gypsy
GTCACGACTCACTCTTTTCCTATTTTCCTCTAAGTCTTTTCATCTTATCTTATCTTATCCCCTTATCTTATCA
GAGCTTATCATATCTTATCAGGGGTTATCTTATCTTTCTCAATCCTTATCGCAGATCATCACGTGATCCATTA
TGTAAGCCAACCGCGGTTTTGCATCATGGAGTACGCACGCTTTAGGATATTACATTACTTTGTTTTCTTTCTC
TTTTATTGCCAACACTCTAGAAGTACTAAGGCATTTCACTTGTATTATGTCGCTATTCGATTTCTTTGGTTTC
CGCCTTAACAAGTGGGATTCATTGCTCAGTCGCAATTCATTCTTCTTTTTTATTCATTCTACTTGAGAGACTG
CCGCATCCTTTTGGGTGTTTTGGCGTGTCCTTACAGAAGAATCGAATTCTCAACGCGGCCCTCTCTCGCTTTC
GGCGTCAAGCTCGCGCCTTACTTCAACATTCAACAACCTTGGATTCACAATTGCAGTTTCGATCTAATTGATC
TGCATCTGAGAGTCTCAAGTTCAACAACTCAGTTTCAAACAGAAATGCCAGTTGCGATCTAATTGCTCTGGTG
CCTTTTGTGCGAACATATAGTACGTAGTTTCCTCCATTACCTGCACTCCAATTCCGTAAGAGCCAATTGACAC
TCTCCCACTGCACGTCTGCCCCACACCTTGCGCAGGATCAGCGTTTAAGGGTTAGGAGCGTGTAACGGTCAGA
CGCCTCCTACAGGCACTCTTTGGAACACCCTGACCTGAGTACGGTCGCCCTTTCCTGGATTCTGTTTGTGGGT
ATCGCCGCCCCACGTCCTAAGTAGGCAATATGTCGGTGCCCTCTATGTGGGTATCGATGACAAGTTAGCACAT
CTTGAAAAGCGCATGTCTCAGTATCGGTCCCAATTGGACGCGATATTGCGGGATTCTACCCCGCCCCCTACTC
CTCTTGAGCGGAATACCAAACCGCTAAACATTGTTAAGAAAAGTCCGGAACGTTTCCCGCATCCAATATCAAG
AATGGGGATGGACTCTCCAGCGTCTGGCCATGCATTTCGGTCGGCTGAGTTGTCATTCCATGATTTTCCGCCC
TTCCACAGTCCCTGGACTGCGCCTATTACACAGCGCGCCCGATTTGTATTCAGCACCGAGCAAAGCTCATCGG
GGATGTCAACCCAGTAAATATTTCTCCACGCTCATATCAGAAAATTGAAGTCGCGACTGCTCGTCGGACTTTA
ATAAATCAACAAAACAAAAGAAAACTAGAAAACCAAAAACATGCCACAAGTGCCGCTTGCCCGGTCATTTGAT
GAAAACTGTACCAGTAGTGTAGATTTCCGTAGTGCTACCCATGAACAATTAAAAAATATTTCGTACAAGAATT
TCTAAAACAAAATAAAAATTACAAAGCCACAGAAAAATTCAAAAAATAATAACACCGTTTCCTCTCCTGTAAA
AATAGTTTCCCCTATCCCTGTTGTACCCGTAGTACCAATTACGAACAATTTCGCTTCTAAAATTCTTTTTCTT
CTTTACCTGTAGGGTGTGGCACGGGTCACACCGCGGGTCACATTTATGACACCACACCCGCACCCACTGAAAC
CGCACCCGCAGACACACCGTGCCCGCGGGTACGGGTTTCAACCACAGTGTAAGGCACAGCACCGCGGCTGTGC
CACACCGCGGGGTACCGCGCTACGCTGCAGGGCTCAGCGTCGTGCCGTGGGGTGCTGCCGTGACCGCCGGCCC
TTCTACAATGCCCTCGACAGTGTCGCGCGCATCAATTGATGCGGCTGGCACCGTACGAGGGGTGCGTAGCGAT
GGGGGGTCAACGGATCCCAGTGTGGCACTGCAGCCCCCGCCGACAATGCCCTCGCATGATGTCGCGCGCATCG
ATTGATGCGGCTGGCATCATGTGAGGGGTGCATAGGGCGGGGGTTAGCTGCCAGCACTCCTGCGTGAGGGAGA
ACGGATGGGGCTCACCATTTGAGCTCCTGGCCCTCGTATGTGGGGGTGGCAAGGTGGGCCGACGTATTGCGTG
GCATGGGCGATGGTGGCGCAGGCGAGAGTGGTGCAGGTGAGGGACACGGGTGAGGGATGACATGTGGAGGTGG
GCGCAGCAGGGAGCGACAGGGTAAGAGTGTGGGGGAGGGTGTGGGAGGGCGGGGGAGCTGGAGGTGCAGGTGC
AGTGGTATGGGCGAGGATGGCGTGGTGCGGGTGCGTGGTATGGGTGAGGATGGCGTATGTGAGGGGCGCGGGC
GAGGATGGCGTATGGAGGTGGGCGATGGGGTACGAGTGTGGGGGTGTGCAAGACGCGAGAGGGCGAGGCGGCT
GGGGGCGCAGATGCAGGCAAATGAGGGGGTGGGTGAGCGAGCAGGCAGGTGTGGGATGTGAGCTAAAACTCAC
CAGTTTAAGCCCCCGAGCTCTCTCAAGCTGGGGGCGCAGGCCCAAGGGCTAAAACGATGCCCAGCTCACATTT
GCAAGTGAGCTGGCAGTGCAAAGAAAAGCTCGTTTGCCGAGCGGTCCCCGGCCCTTGCTCGTTTGTGAGTGTG
GGGCTAGGATGAACGGCACTCTCGTTTGCGAGAGTGGGGAGTGACGGTTGCGTCAAACGATTGGAGGGCGGCA

GGTGGGTTGAACAACGATGAGAACATTGAAAACAATGTTCCGATCGTTGGCCGATCGATGTGTCAGGGAGCCG
AAAACGGCCCGCAGCCACCGAGGAAACCCGCCACACCGCGGGAACGGGTGCGGTTTTTGGCGCGGTGTCCATT
TTTGGACACCGCGCAAACAACCGTAAACACCGTAGCCCGAATACCGCGGGTTTACCCGTACCCATGCTATTCC
CTATTTACCTGTAGATAGTATGGAAGTCGATGATGACGGCAAGGTCAAAGTCGGTGGACGGTTCTTTTCCCCT
TTCCATTCTCCATGGACATTTCTAAATTAGATAGTTTACCTAATATGACGAAGGTTATGCTACCGGAAAAGGC
ACGGGGTCGGTCCTAACAAGACGATCCCTATTTTTAAAATTGCAGATAATGCTCAAGGACGCGCTCTGGAGAA
GCAGTTGTCCAATTTGATGCAGTCGCTTGAAGCTAGGAAGGTGGAGCGTAGTAAGGGTCTTGGGGTTCCGTCT
GACGTCCGTCCAGTGAAGCTGGGTCCGGCGTTACGAACCTGTCGACAATCAGGAGCCCTTAAATGGGTCATCT
GTCCGTCCCGAGTCTGGTGAACTCGTGGGTCCTGGCAGTATCCGGTGCTTCGGCCCGTCCTGGTTTCCCTTCG
TCAGGGACTTATTTCATTCGCATGCTTAATTGGCTAGAGAGCTTAAAATGTCGATCGTAATGACGACATTAGA
TACACACGCAAATGGCCAACACACAGGCCCTATTGGATAGCGGAGCTACCGGTTCGTTCATTGATAAAGAGTT
TATTAAAGAGATGGGTTGGACCACGCGACCGCTACGTCACCCTGTACAAGTATATAACGTCGACGGTACTTTA
AATGAGCCGGTTCCATTTCGGAAGAAGTAGTTTTGATGGTTTCCCACAAGGGCCATAAGGAACGCATGCTATT
TGAGGTGATGACACTCGGCAAGACTAAAGTTATTTTGGGACACACATGGCTTAAGAAGCATAACCCCACAATC
GACTGGACGACTGGGGAGGTCTCTATGAATAGATGTCCGGCGGAATGCAATGTGGCTATCTGTGTGGCTAAAA
CTCAAAAGAAACGTAAGGCCATGGCTTCGCACTGGCAGTATAAAATGATGCTCGAGGAGGTCGATGATGAGGA
TTCTGACTGTGTTATAGGCGGGATCATAGTAGAGCAAGAAGGAGCCCTCTTGGAGGAGATTCGTTTTGACGAA
ACGGTCTACGAGGTTGTTGCGGAACACTTCGTGCGCACAGTGGAAATCGAGGAGAGCTTTGACGCCCCTCCGT
TGATAAGTGGATTACCGGAGCCTACCGAACCCGGTTTAGAAGAAAAGATTCCCCGCAGGTTTAGGCGGTGGTT
AAAAGTGTTTGAAAAGAAAGTGTCGGAGCGCATGCCCGAACGAAAGACATGGGATCATGGGATTGAGTTGAAG
GAAGGATTTGTGCCCAAGAAGGCCAAAGTTATTAATCTATCACCTACCGAACAGAAGGAGGTGATGACTTTTT
AAAGGATCAACTATCTAAGGTTACATTCGCGAAAGCAAGTCTCCGCAAACTTCGGCTGTCTTCTTTGTACCCA
AGAAGGATGGCAAGAAGCGCATGGTTCAGGATTATAGGTTCCTGAATGCCTGGACCGTGAAAAATAATTATCC
ATTACCGATGATCGCCGACTTGATTGATAAAGCGGGAAAATCCAAATGGTTTACAAAGCTCGACCTACGCTGG
GGCTACAATAACGTACGGACAAAAGAGGGCGACGAATGGAAAGCGGCGTTCACCACGCATCGCGGTTCCTTCG
AGCCATTGTTATGTATTTCGGACTTTGCAATTCGCTGGCGACGTTTCAGACCATGATGAACGCGCTATTACGA
GAGCTTATTGATGAAGGTCACGTCGTGGTATATATAGATGATATTTTAATCTTCACCGACACGCAAGAAGAAA
ACGAAAGATCGTCGAACGGGTTCTCCAGATCCTATTAGACAACGATTTATTCTTGAAGCCAGAGAAGTGCTCG
TTCAGTACCCAGAAGGTGGAGTTCCTAGGGTTAATATTGGATAAAGGAGTTGTACGGATGGCAGAGGATAAGA
CAGACGCGGTATCTAAATGGCCGGTCCCGAAGACGGTCCGGGAAATAGAAATTCCTGGATTCGCCAATTTTAT
AGACGTTTATCGAGGGGTTCTCCAAGTTAGCGGGGCTCTAAACAATTTATTACGAAAGACTCAAGTTCGAATG
GACTGATAAGCAACAGGTTGCCTTCGATACGCTGAAGAAACGTTTTACGTCGAACCCGATTTGGTTACGGCAG
ATCCTAAACGAAACTACGTGTAGAGTCGGACGCTTCGGACTTTGCTACGGTGGATTTTAAGTATGTTATGCGA
CGACGGTAAATGGAGGCCGTGCGCTTATTATTCCAATCTTTATCGATGTGAACGAATTATGATGTACACGACA
AGAAATGTAGTATTATACGATGTCTGAGGCTGGCGCATTATTTAGAAGGGCAGATAACAGTAGAAATATGGTC
GGACCATAAAATTACAATACTTTATGACGTAAAAACTTAATCGTAGACAGGCCGATGGGCCTTTATTTATCAG
TTTGACTTTTTACTTATTAACAAACCCGGAGCGGCTATGGGAAAGGCCGACGCTCTGTCCAGGAGAACAGACC
TAAAGAGGGGAGTAGAATCCGATAATGATGACGTCACTCTCTTAAAACCGGAATTCTTTTCTATTAAAACCCT
TCAGCAGGGTCATGTGCTCTTGGACGGCCATGAGGAGGAGTTATTGAAGAAGATTAGAAAGTCGAAGGATTGG
GATGAAGTGGTGGTGAAGGCGATCGAGGAGTTGAAGCATTCGGCGACTAAGCGTTTGCGTTCAGAGGAGTGGT
CCGAAGAGCAGGATCTAATACTGTTCAGAGGAAAAGTTTACGTTCCGAAGTTGCCGGATGTCCGTCGAGAGAT
TGTCAAGTTACATCACGAGTCGGTCATTACTGGACACCCAGGACGAAATAAAACATTAGAGTTAATCACGCGT
AATTATTGGTGGCCTGGTATGGCTAAATTTATATTTTCTTACGTCGACGGGTGCGACAAATGTCAAAGGCACA
AGAACTTCCCCGCCCCTCCTGTCGGAAAGCTCGTACCGATGGTTACTCCGGTCGAACCCTGGAAGGCAGTGTC
CTGCGATTTTATTACTGGATTACCTTTATCATCCGGTTATAACGCTATCGTCGTCGTCGTTGATCGTACTTCA
AAGATGGCTCATTTTATTCCTACAACGTCTGAGACGTCTTCACTTGGACTCGCTAAATTATACCGCGACTTTA
TCTGGAAGCATCACGGTCTCCCGGATTCAATTATTTCAGATCGAGGTCCTCAATTCGCATCTGTGATGATGAA
AGAGTTGAACTCTCTGCTGGGCATTCAAACTAAATTATCGACAGCTTATCACCCGCAGACAGACGGGCAAACC
GAACGCGTCAATCAGGACATTGAAGTTTATTTACGTCTTTTTATTGATCATCGACAAACCGACTGGTCGGAAT
GGCTCGCTTCAGCGGAGTTCGCACACAACAACGCAATCCACTCGAGCACGAAGGTCTCTCCTTTTTATCTCAC
CAACGGCAGGCACCCGCTGATGTGACACTGCGGCCAGGTGGCAAAACCAAACCTAACCTGTGTGTTTCGCAGT
TTGCGAGTGGGTGTCTGTTTAGCAAGAGATGCCTAGCCCAAGTTCGACGTGATCATAAGAGGGCACTGCCCTA
AACGAGAGTGGAATGCCAAAGCATGCGAGATGAGCTTCGAGCTCTGATGGGAACTTGTATTAATGGTTGTGGC
TACTGTCTCTATATATCGGAGCTATCGGCCATGAGGTATGGTAAATAGGTAAGAAACTATGTAATGTAATGCG
ATCCGAGTGCGAAGTGCTGCGATCAATATGCGGAGACTTCGGCTGGAATCGGAATTGAAGCGCGATCTCAACG
TGTTCGGAGCGGAATGGGTGCGATCAGTACTGTAAGAGTGCGCCCAATGCGCCCAATATCAGTAGAGATGTGA
GCTGCGAACCCCTAAACGATAAGCTTGCTTATCACCGCGCATGGGATTCGAACCGATCCGTTCCCAGAAGAAT
GAGACAGCAATCAAGTTCGCTGAGCGAATGAAACATATTCAAGAAGAAGCGCAGGCCGCCCTTATTAAATCCC

GAGACGAAATGAAGCATTATGCTGATCAACATCGTGGAGATATGCCGAAGTATAAAGTAGGCGATAAAGTATG
GCTAGAGACTAAAGATCTAGACATCAAACGTCCATCGAAGAAACTCACGGAACGTCACATTGGTCCCTTCGAA
GTTTTAGAACTTGTTGGTTCACATGCGGTCAAACTAAAATTCCCCAGCTCGATTCGTATTCACCCGGTTGTGA
ACGTTTCCCGTATTCGCCCTTTCACTCCTCCTATTGCTGGCCAAACATCCGCACCACCCCCTCCAATCGATAT
CGACAGCGAGTTTGAATACCACGTTGAGAAGATTCTCGATTCGCATCTGGAACGAGGTCGACTTCAATACTTT
GTTCAGTGGGAAGGCTATTCCCCAGAACATTCTACTTGGGAGCCGATTGCGAATCTCAAAAACTCCATGGATC
TAGTTAACGATTTTCATCGCAGGAATCCATCGGCGCCACGAAAGATACAGGGTATCTCTCAAACTCAATTTGA
TAATTTATTCCAACCTTATAACAATTTCACCGTGCCGGACGCATCCCTCTTACTTGTGCCCTAGAAGGGGAGT
AATGGATTTTATTTTTATATTTCTCATAACATTGTGTAGCGAGCAGACGATTCGAGTTTGACGAACCATGGAT
ACTCTCCAAAGTGTTCTCCTCATCGTACTGTGTACGCCCCTTTGTTTGCCGCACTGGAGTCAATCTTCGCTTG
AGGCGATGTGGAGTTTTTCTTTTCTCTTTTTCTCTCTATCTCTTTTCTTTATTTTTCCGGAGGCAAAATTCTA
GAAGGGGAGTTATGTCACGACTCACTCTTTTCCTATTTTCCTCTAAGTCTTTTCCATCTTATCTTATCTTATC
CCCTTATCTTATCAGAGCTTATCATATCTTATCAGGGGTTTATCTTATCTTTCTCAAATCCTTATCGCAGATC
ATCACGTGATCCATTATGTAAGCCAAACCGCGGTTTTGCATCATGGAGTACGCACGCTTTAGGATATTACATT
ACTTTGTTTTCTTTCTCTTTTATTGCCAAACACTCTAGAAGTACTTAAGGCATTTCACTTGTATTATGTACGC
TAATTCGATTCTCTTTGGTTTCCGCCTTAACAAGTGGGATTCATTGCTCAGTCGCAATTCATTCTTTCTTTTT
TATTCATTCTACTTGAGAGACTGCCGCATCCTTTTGGGTGTTTTGGCGTGTCCTTACACAGGTA
>Cligib1_Cluster14#LTR/Gypsy
TCCGAATCCCGAGCGTCGGGTTTCGTCGGACTTGGCTAGGCGGTCTGGGTCGTGGCTCTGATGTTGATTCATT
AGCGTAGACAATAAGTGCTAGTTCTTGGCGATAGTATAAAATGAACGTACCGTAGGATAGTTTCGGGGACGAA
TTCTATCGTGTTGTGAAACGGTTACTCTCGTTTGAATCGTGTCTTCGTGCTCAAGTCTAACTTGTCACGAGTG
TCACATTTTTTAACCAAAGAAGACAACAAAACTATCTACACACTCTCGAGTGCCGCGTCGACGGATCCTTCT
GGCCTGATCACCCGGAAATTCCTGACAAGGTCACTCACACTTTTCTCTATTTCAACTCACTATTTTTAAGACT
TTCAAAATTGTTAAAACATAAGTACCATCGAAATATATTCGTTGATTACTGAAATGGCTCCTAAGAAATCCGC
AACTACGAAAACAACCAAGCTCAATCTCCGTACGGCTTTAGCGCCTACGTTGAGAACCACCCGCAGTGGCCGT
GTAACCGGCAGTGTGATGGAGCATTCCGATGGCGAGCGACGTCGTTACAGTGATGTCGCCGCGTCGCGTTCCC
CTACTCCAGTAGAGAGGGAGACAGGTAGCCCTCGGGCTGTAATGACTGTCGATTCCAATCCAGTCCCTGTAAT
CAGAAAAGCCCCAGATGACGATTTGAGGGCACGAACCGGCGAAGCGGAAAGTGCTGATGAACAGCATGGTAGT
AAGAACCCCAACGAAGGGTGGACTCTCGTCCGGCCGCGTCGTACCCGTAGCCTGGACTCGATTGGTGGGATGT
CCACTGGTCGAGCGAACCGTAAGAGGTTTGCTACGCCTAAGAATAGTAAGCATGAGAAGCTGCGAGTCATTCA
GGCCGCAGAAAATCAACTTACTCCAGCTGAAAAAGAGCGCATCAACCTGAGGTTCGCGCGCGTGAACACCCAG
CCCTCATCTGAATCGGATTCCAATGGCGAAGGGCCGTCTCAGCCTACTAAAGGAAAGGGTATTGACCCCCGTG
AATGGGGAAATATTGATATGTCAGATACCGAGATGAATCTTGAAAATCAACAACGCGCCTTGGATGAATATAA
CAGGGCAAAGAAGCGCCGTGATGATAAACAGAGTCCCAAAGCGAGGGATGCTAAGAAGAAAGGGAGTAAGCAC
TCCAGCAAGAAAGAGAAGCGAGCTGAGCCAATCTCGATCTCAATGGCGCAACGGGTTGCGGACGTGGCCCACG
GGCGCCCGGCAGGAAGTGTTCATCCTAAACGCAAGATGAAAAAATTAGAATTGCGCCCAGTCGAGCAATTAGG
AGCAAAAAGTTACCTAGCTCAAGCACTTCGCAGCACTAAGAAGTCAAAGAAGGGTCGTAAACATCGAGGACGA
GCTCCATCGACCAGCAGCAGTTCTGATCCGTCTTCGTCTTCATCCTCGTCTGATTCGTCGTCGTCATCTGACT
CATCGTCAGACTCAGAAAGCGAGACTTCGACTTCAGATTCATCGAGCTCATCAGGATCAAGCTCGGACTCATC
GTCATCTGGAAGTTCTACCAGTAATGCACGCCGGAAGAAATCTCACCGAAGCCGCAAACGTGGTCGTAAAGGT
AATAGGAAACCGCATCGGCGATCAACGTTGAAACCAGTACCACCGAAAGATTATAACGGGGAAGCGGATGCCC
GACTGTACTATCGGTTCGTTACGGAAGGGAGTGCCTACATCCGTAATGGCCGGGTGGGGTCGAAACAGCAAGT
CTTCGTGCTATCATACTACCTAAGTGGGAAAGCGTATGATTTCTTCACACAAAAAGTTGCATCAACAGTTGAG
AAATGGAGACTTACTGAGTTCTTTGAAGAGCTCTTCAATTATTGTTTCCCTGTGGATTATAGGTTGAGACAGC
GGGATAAGCTGCAACGTTGTTACCAGAAAGAGAAGACCGTCTCTGAATACGTCTATGAACTTGAGGAACTGTA
CAACATGGTTGGTACAACTGATGACCGGGAGAAGGTCACCAAGTTATGGAAGGGCTTACGCACCAGTATTCAG
CAAGGTCTTTGGCGAGACGGATACCATCCCGAGGTCTCAAAATGGACAGAGGTTGCAGATGCCGCTCAGATAA
TTGAAATATCTGAAAATGTGATGAACAACGTTCACTCGCGGAATAAGAATCCAAAAGAAAACACACACCGAGA
TGACGAGGGACGGCGGGATAAACATCGCTCCCGACGATCTCGTAGACATTCACACCAGCATGGCCGGGACAAT
CAGTCGGCTGGTGCAGAGCGCAGTGAATCAAAAGCGCACTCAGCTGGGTCAACATCCAATGCTAAATACCAAT
CCGGGCAGAACCGCGATCGGGGAGGATCCTACAGAGGGCAAGCAGGATCGAGTGCGCGAGGACGACCTTGGCG
TCCACCTGCCGGGTCCCGACCTACTCCTGCAATGGGAGAAAAAGAACGCGCAGAGTTATTAGCTGCTGGGAAG
TGCTTCCGTTGCAAGGAGACAGGGCATATGTCTCGAAACTGCCCAAAAGCGAATGTAGTTTCGTCCAAGGGTA
ACAAACCACCAGGGATGGCTGCGTTCAGCATGGATATCAACTTTGAATCTATAGAGGAAGCGCAAAATCTTGC
GGATACCACAGCAACTCTCTGTGCTATGAGCCTGTCTGAGATCAGTGAACCCAATGGATCTTGGGAACGGCGT
TACCGTAAAGGTGAGAAGGATAGCGATGGCTACCACGCTGGCCAGATGGGAGATGAGGCCGTGCGAGCCGCAG
AACGCCTGTTAGACATGGTGGACGAGTATCCTGGTGATGATGGGATGCTCACTGTGGTTGGTCCACGTTTCAA
ATTGACGAAGACAGACGCATCACCGGGTACCTATATCGTAGAGGATCGGTTAGTAATGCATGAAGGCCCGGAA

ATACCTGCCGAATTCCTGATGAGCGAGGAGTTTGATTTGATTGGCTGGTATTGGGATTATAAGGCCGACGTGT
TTGCCGAGCAGGATCGGCCGTTCCCCAGGGACATGGAACACATCACAGATCCGTTGCCTAGACGTGTTGAGCA
ATTACTAGCACTGGCTGCTGAGAACATCTTACCGTATACCCCCTCAGGGATTAGACCACACCGCGGTTCAAAT
GTGTACCGAATCTTGATGGTGGCTATCAGTTAATGGATCACAGTGAAAGCATTGTGGAATTACTGACCAGGGA
GCAGTTGTCGAATCCTTACTTCAACGTGGTGAACTGGTATAGGAAGCGGTTAGTACGGATCTTCGGGCCTAAG
CTCGTGGAACGCGACCCCAATTACTTGGACCATCGCTTTGGCAATTACCTAGGTGATGAGATCGCCCAACTCC
TTAATGATATGAATAATGACAAGGAAAAACACGCACCTACCAAAACTGGACCTGCCCGGTTTGAGGTTTATCC
GAACAGCGCGTTGACGCACGAGATTTGGGACAGCGCTACAGGAAGTGGCCTGAGCGTGTCAACTTCGTTCCTA
AGGGATCCTGACTTACGAGTCGGCGAATGGTTTGCGCGAGTGAAGGAATGGGACATCCACCCGAACTCGTCGA
CCAATCAAATCGCCCGGGCTAGGGTTGGGGAAATTCAAGCGGAACGAGTACAACAATTACTCACTACATATTC
GCCTCCACGAGGAGGGCGGAGAGCAGCTTACCGGTTCTCAGTGATCGATGAGAATGATGGGTCCGATATGCTA
ACGATTGCCGATCATCACCTGAAAATTGAGTGTCATCTGTCAAAGAAGGATACCGAGCATGAGAACTTCGACG
TAGTAGCTTGGTACGCCCGCGAAATATGGGATATGGATAATTATTCCAGCGGGGATGGCTTGGGCGATGAGGT
ACTCTTCGAGAATAATTTCCTGAGGCTGTTAAATCCAGATCAGGAGCAATCAGTTGAGAAGGAAATTGGACGG
ATACTCCAAGAGATCATTGATGAATGCACCGAAATGGCGGATGCTGAGGCAGAAGCTCTACAGGAGACGGAGG
CTCCGGAGCAACAGCAGGTCGAGGATGAAATACCCGAAGTGGATGGGGACTCCGTTTCCGATTGTGAGGAGTT
TCGGTTGTGTGGATTAGAACCTGCAATGTATGGAGTTCAGATTCCAAAAGGATCACTCGCAGCAATGGAACGG
AACTCAGCTGTACCAAAGGATTTCAATCGGGTGGTCCCTAAGCCCTTGGTAATCGTCGTACGAATTAACGGAC
ATCCAGCGAGAGCGCTGGTTGATACCGGATCTTTGGGGGACTTCATGTCAACAACGCTCGCAGATCAGTTGAA
AGTAAAAACCTTTGCCTTGGCAAAACCGTTGACGCTACAGTTAGCGGTCCAAGGATCACGCACAAAAGTGAAC
TCGGAGCGACGGTTGGTTTTGAGTACCAAGGGATTTCGGAAGACCGATACTTTGATATCACAAACATTTCGAA
ATATGACTTGATTTTGGGTACACCATGGCTATTCCAGCACAAGGTGTCGCTTGCAGTGAACCCGGCAGCCGTT
GTGGTGGGGAGCGATGAGGCGGTTCCGATTCGGGGCCCCACAGTGGCGAAGATAGCATCACAGGCCGCTGACA
TCGTCTGCGAGAAGGTGGATGAGGTCCGTGAGAAATTGCTCCAGTATGCGGAGCCTATCTGCAAAGAAGTGCA
TGAGACAGAGTTGCCTCCGTTTCGGGCCATTAATCATACAATACCATTGATTGATGAGAATAAACAGTACCCA
CATCGTCAGTCTAGATGTCCGGAAGCTCTTATGCCGCAGTGGGTAGAGAAACGCGACTCATATCTGAAGTCGA
AACGCTGGGAGGTCACTAATGCTGCAAATACGGTGCCCATGTTGTTCATTCCGAAGCCGCGGAAACCTGGGAT
GCCAGTGGAGCTGCGAACGGTGGTAGACCTACGAGAGCGTAATAAAAATACAGTAAAAATGTCCTCACCGCTG
CCAGATATCGAAGGCATCCTCCGGAGAGTGGCAAAGGCCAAGTTTCGGTCCTTGATTGATGGCAAGAATGCCT
ATGAACAGATCCGGATCATCCTGGAGCATGTTTACCGTTCGACGGTTACCACACCTGATGGAAACATGGTGAG
CAACATAATTCAGATTGGCGATTGCAATGCGCCGGCCACGTACCAGGCGTTGATGAATTACTTGTTCTCAGCA
TATATGGGACGTTGGATGGATGTCTACCTGGATGATATAATTATTTACACAGACACTCTTGAGGAACATGTCG
ACGGGGTGCAAACCGTTATTGATATCTTGCATCGCGAGAAACTATACTTGAGCAAGAATAAACTGCAGTTCTT
ACCCAAGGAATTGCGTATCCTGGGGAGGATCGTGGATGATAAGGGCGTGCGCATGGATCCGCCAAAGTGGACT
CCGTGTTAGCATGGAAAACGCCCACGAATCGGGACTTACTACGTGGATTCCTGGGATCCGTGGGGTACCTCGC
TGATGATGTGGCGGGGGTGAGAATTCCTATGGGGATTTTGCACGGATTGACTGGGGACACTGTTCCATTTAGA
TGGGAATACACTCACCAGCGGGCCTTTGAGGATGTCAAAACGGCCGTGCAGAAAGCGCGGGATCACCGTCGAG
TGCCGTTGAAATATGGGGAGAATGAAGACCCCATCTATATGGTAACTGACGGGTGCGCTACCGGGATATCAGG
ACTAGTGAGCCAAGGGGGAGATTGGAAGGAAGCACAGATTGCAGCCTTCTACTCGGCTAAACTCAACTCTGCA
CAGCAAAATTATGCAGTGCACGAAATCGAAATGCTCGCCGGTGTTGAGACTATGCTACGGCACCGCGATATCC
TGCAAGGAGCGAAGTTCAAATGGATTACAGACCATAAAGGGTTGATTCACCTACTGAACCAACGGAATCTGTC
AGGGCGACAAGCTCGATGGATGGAAAAGATTGGGGAATTCGACTTTGAAGTGGTATACGTTCCCGGGGTGGAA
AACGTCGTAGCAGACGCCCTATCGCGGATGTATTCAAATGATGCCCCAGGTACAGTAAGGGCGCGTGGGGAAT
ACACGTACCATGATGTGATTGATAATGATATCCTTGGGGAACATGCAATTACGATGCCGTTGCTTGCTGGATT
GGAGGCTCGTGCGGAAGCGCCAGGTATGATGACAAGGAGTCGGACGGCGCGCGCGAATAATCCTGAGGCTCCG
GCTGGGCGACCCGAGACGAAGTATGAGTTTGCGAAGCGAGTAAAGGATCGTTTCGTTCTGCATGGACCGAGGC
CCGAACGAAAGGAGGGCGGGTCAGCAGAGAAAACACCTAATACGGAAGATGACTTACCAGATGATGTACCACC
TTTAGAAGAAGAGCCGGAGGAGGCGCCTGAGGTAACGCAGGGCGCGTCGGCTCCTCGGCTAGTCCACGAGGAA
GTTCCGTTTGGGCAGACGCTGGCAGAAGGCGTGGACGGCATTGACCTGGAAAATGCGATACGGAATCAGTATC
ACACTGATCCGTTCTTCAAGCCAATTGTGGAGAAACCTACCGAGCATAAGAACTTTGAGGTTAGCGATGGGCT
GGTCTATCTGCGAGAGGCAAACAAGCAACGCACCTTGTGTATTCCACGAGTAATCAGTAATGGCCGGAGCGTT
CGCGAGATTGCTATAGCTGAGGCGCACTCAGTACTGGCTCATCTAGGAGCTAGTAAAACGGTCAACTACCTGA
AGGAACACGTTTGGTGGAAATCGATGGTTAGTGATGTCAAACAATACTGTGAGAGCTGTACGACGTGCATGCG
AAGTAAACCGTCGACTCAGAAGCCGTATGGGTTGTTGAACCCACTGGATCGGCCTAGCCGCCCATGGGCATCG
ATTGGAGTTGACTTCGTCGGCCCACTTCCTGAGTCCAGCAATCGTGATGGAACATTTGATTCTATTACAACGG
TTATCGACCTATTTACAGGAAGGACTCATTTAATACCTAGTAAAACGACCTACAATGCAAAACAGGTCGCGGA
GCTGATGTTCGAGGAGGTCTATAAACTCCATGGACTGCCAGAATCAATTGTTAGTGATCGCGACGTGTTGTTC
ACCAGCGAGTTCTGGAGAAGACTCAACGCATTAGTGGGGACAGAATTGCGCATGTCAAGCGCCTACCACCCCC

AGTCGGACGGAGCAACCGAACGCGCGAATCGAACGATTACCCAAATGCTTCGGCAATGTATCGGTCCTAATCA
GCGCGACTGGGTATCTAAATTGCCATCGATTGAATTTGCGATTAACTCTGCGAGATCCGAGAGTACTGGGTTC
GCACCTTTCTTTTTGGATACAGGGCGCATGCCTAGAACGATGATTTGGAACTCGGCAAGCCAGTCAGAATATC
CGGGAGTCCGAAATTTCGCATTAAATCGGAAACTTACCATTATGGCGGCTCACGATTGCTTGCTGGCAGCGCG
GGTGAAACAGACAAGAGACGCAAACCGACACCGTCGCGCCGCGCCGTTCCAAAATGGAGACCTCGTGTACCTA
AGCTCCAAAAACATTAGCTTCCCGAAAGGCTTAGCGAGGAAATTGGTACCTAAATATATTGGCCCCTATAAAA
TAATTAAGGATTTTGGGAATCAGTCGTTTCGGCTAGAGTTGCCAAGTGATCTGAAGAAACGAGGGATACATGA
TTCGTTCCATTCGTCATTGTTAAGGGTGCACATACCGAACGATGACCGACTCTTCCCAGGAAGAGGTTACGAG
CAGATTATTGGCGAAGATGCCAAGGGCGCGGAGTGGTCAATAGACCGGATCGAAGGCCACGACGGTGTGAGAG
AAGACGCGCGATTCCGCATAAAGTGGAAAAGTGGTGATACCACATGGCTACCGTTCCATCAAATTAAGGGGAC
GGAAGCAATTCGAGCGTACCTTGAATTACTGGGGATTAATCAGATCTCTCAACTCACAGCCGGGAATTTGAAA
CTCACGGAAGAGGTGAACGATCCGCAGATCTTGTTGGGCAGTCTGGACGTGATCCATGAGGAGTCCAGTTCCG
GGTATATAACCCGAGGTGGGTTGCTGTCCACCACCCAGCAACACCACTCCTTCCCTCCAATCCAACCCGACAC
TTACTCCTTCGCATCATCCCCCATCTCTACTTTACCTGTTATGTCAACCGACCAGCCGCCCGATCCTCTCACA
GACGCTCCACCAGACCCCACTTCTGTCGACGCAGTACCTACAGACGACCAAGATGCCGACACCGTCGATAATC
CCGGAACTCCAGCCGATCGTGACGGTCACCAGTTCTTCCGGGTGGAGCATAACCACGCCATCCTCGAGCATCC
GATAGATGGTGGCTACATCGTTGAAGTTACCTTCGATCAGGTAACGAGATATTGTCAACACGATGAGGCGCTC
AGGTCGGGACGACGCCCTACTCGGGTACCAGGGGGGTATGATGAGTTCGCGACGATGTTTAATAACGACGCGA
CTTACCCCGTGAGCTTCGCTGTCCTGAATGACGGGGAATTGGTAGCCGAGGGACCGGTTGCACGCGAAGACTC
CATGCTCGCGGAGCTATGTGACGACCCATCGGATTCAGATATGGAAGAGGTCGCTATTGATCACGCTCACTCA
CCCGAAGCACGGTCTCCATCCCCTGCTGCGCCCCCATCTCACCATGGGTCCATTCCTGACCGTCATGGTTCTC
CAATGCGCGGGACATCGCCTGCTCGCCCTGATGCTACTCGCGCCGACTCACCTTGTCGCAATGTCCACAGCCC
CCGTGAGCCCGCATCCCGTGGGACACATCTTTCTTCCCGATCGCGTTCACGCCATGGCCCGCGAGTCAAGAAG
CACACCGGCCCTTATCCAACCACCAGGCCTGCCCAACAGGAGGGCTCGTCTTCGTTAATGGCCCAGCGTGCTG
CCGAAGAAGATGCAAGACTTAACTTAAGCCTCTACAAAGAAGTCGCTCGTAACTGGGCTCGGATCGAGGACAG
GCGTCAGGTTGCTATCCTCAGACGACGGGTCGAGCAGGAAAGGAGATCCGCTCTGACTCCAGAAGAGCGCGCT
GAAGAAGACCAGGCTCGTGAATTGGCAGCGAGATTGAGTCGCAAAGGCGGGTACGAGTTTGGAGGTCGGGGTC
GAGGCGGTCGGCGTTATTGATCGCGCCCCACTGCGAGCGCGATGGAGGGCGCATGTAACGGTCCGAATCCCGA
GCGTCGGGTTTCGTCGGACTTGGCTAGGCGGTCTGGGTCGTGGCTCTGATGTTGATTCATTAGCGTAGACAAT
AAGTGCTAGTTCTTGGCGATAGTATAAAATGAACGTACCGTAGGATAGTTTCGGGGACGAATTCTATCGTGTT
GTGAAACGGTTACTCTCGTTTGAATCGTGTCTTCGTGCTCAAGTCTAACTTGTCACGAGTGTCAC
>Cligib1_Cluster4#LTR/Gypsy
TCGACCGACATCACCAACCCACTCTCGCGCAAACGACCACCACCCCGTTAGTACCTGGCATCCTGCCCTCCAG
ACCCCACTCGTCTTCCCCCCATCCTCCACCTGTTCATGCCGCTCCGTCACCAATTCTCCCATCACCATGCTCG
TTGTCATGTCCGCTGCCTGCCAGTTTGCCCCGGCAAGCTCTCCTGCATACCTCGAGGAGACTCTGCCTGGCCG
CGCGGCGGCCTCCTGCTCCCCTCGCCCTCGCCTTGTTCCCTCAATTTTATAAAGAACAATGACACAACCTTCC
GTTCTTTCCACATCCCATCACTCTCTGAACGCAAAGCTTCTCAGAACCGCTCCGAAACAACCACCTTCTTTTC
CAACATGCCCCAACACATCGCTATGGACCCTGCAAACGAAGTCTGCCCTGACTTTTCACTTCCCATCTATGAC
CTGGCCAGGGACAGCATGATAGCAAACCCAGGGTGCCCACAAATCCAGACCCACGAAGAAGCAGCCAAGGCAC
TTGCCGGTGCCTGGGCAACCGACAATGCTGAGAGATGCAAGCAATATGCAAGGCAATTCGACGCGCAGGAAGA
CAACAACCCCCCACCGGGAGCAGACCTGGGGCGCCCACCGCAGCAACGCCAAGAAACCCCGCGCCACGAACCC
TCCCCGCGGGCGGCCCAGCGCCGCGAGCCAGAACATGAAGACCAGCCGGCGCAGCCACCCAAGTGAAGACCAG
CCCCACCGATCGTGCGGGACGACCTCATGATCTCAACTTCTCTCCCAGATCGCCCCGCCCAATTCGCCCTCAA
CAAACTCGACACCTACGAATATGTCGACCTGTGGTACTTCACTCCCGAGGGCTGCGCGGAGGCGGTGAGACAG
GTGAGAGCCCTGGACGGCGAAGGTTTTAGCTTCTCAGGGGGACTGAACGGTGAGCGTCTAGCCTTCAAAGCAT
CATCCACCGCCTCCCCCTCCAGGAATGCCATCCTCGACGAAAACCTACCATGGCACGTCTTCACAGAGGCAAG
ATACGGGTTCATCAAGTACATGGGCATCGCAAAATGGCCCGCGAAACTGTACAAGGATCTGTGCGCATTGTTC
GTGCGCATTGAGTCCCACCTGCTACACAGAACGGACGCGGGCAAGAGAGCCCTCCTCGAGTACCAGGCGCGTG
CCCGGCGAGACTGGCACGACACCATGAAAAGGGATGGCGAAACCTTCAACATCGCCGCGATAAACGAGGATCT
AATCACGATCATCGCGCGCGAGCAGCAGGCGAGGGATGTAGAATCCGTAAGTAAAACTCCCATGCACTGTAGT
AAATGTACTGACGCTCCCCATTCCCCCTCTCCCAACCCCCCCCCCCTTCCCCCCTTCCTAACTCAATCCTGAA
CTCCACCCATGAATGCTCACCCACCATGCCGTTGCAACAACGCGTGGTGGTCCGTGTCGATGTCCAGGTCCCT
TGGCGTAAGGCATAGCACGGATCGATCCGTGGGTAGATCCGGATCCGCACTCACGAGTAGTTGTTTGGTCCAG
GGCCCGACTCGGATCTGCGAGTAGTTCAGTCCACAAGTACTTGAACTCGCGAGTAGTTTTTACAAGTACTTGT
GAGTCATATGGGTACTTGTGACGAGTCACAAATACTTGTCACTACTCACAGGTACTCGTGGGTCACGGGTCAT
TTGTCAGCAAGAAACAAGTATTTTATGGGTATTTTACAAGTACTTGTAGGGATTTTGTTGGTTTAAAATGAAA
TTTGAGTGTATATGTGTCATGGGAAAGACCCTGTAAGGGTCTAAACGAGTCAGCAAGTACTTGCCACAAGTAC
TTGCAAGGTCAGGATGCTTGACTCGGATCCACGAGTATTTGGACCTGCGAGCTTTTGGCAAAACAAGTACTTG

AACTCACGAGTATTCCCACGGGTCAACTCGCAAGTACTTGCAGGATACTCATACTCATGCTGAGCCTTACCTT
GGCGACCTGTGGACTTCAACGACACAACCAACTCCCGGCGAACGGTCCCCTCGCGACCCGCCGATGGCCGCTG
GCAATGCTGCCTGCCAGGTCCCAACGCCGTGTGCTCGGCTGATGACCAAATACCCCCCAATAACGGCCACTGC
GTGCTGCATGCCGGGACGGAACTGCCGCGCCGTCTGGGATCCCTCAAAACGCAGCTCCACTCAGACAATGCAC
AGCTACGCGCTGAGATGAGAGCCATGTGCTCGCAGGAGGCGCACTCAAACGCCAGGGGAAGACACCAGAGGGG
GAACCGTTCCAAATCCCCACCACGCCGGAGAGACCACTTGCGGTCCAAATCCCTGGCCTCAAGGTACCAGAGG
GCAAAATCCGGGGCATCGGGTGATGCTGATGGTTCACAACAGGCACAACCCTTCCTGCCTTGCGCAGTCTGCC
TCGGAAGGGGCAAGCACGATGTCAGCAAGTGCAACGCCAAGACGATGTGGGACACCACCACCCCAACATGGTG
CCGACAAGTCGACAAGAAGATCATCAACTCACAGGGGCAGCCCCTCTGCGTCAACTTCCAGCGCCGCGGGGGA
TGCCTCAGCAGATCGCACGAGGACAAGCACGAGTGCTCAGGATGCGGGAAGCGCGACCACGGAGCTTTCAAAT
GCCCTCTTGCGCAGAGCCCATAAAGTAGATACACCACTCAACACGGAAGAATGGCGCAGGCAACTAGTACAGA
CTGGGCTAGTCAGTAAATACCCAGATGTCCCAGAGGCCCTAACACGAGGCTTCGACACCGGTATACCTACAAT
CCTTTCCACCTCCACCCCCCCTAATAAATTAGACACCACCATAATGACAAGTGCGTTCGAGAAAACCGCAGCG
AAAGAATTCGACAGAGGTAGATGGCTAGGCCCATACATGTGCGAGGTAATTGAGCAGGTCATGGGGCCTTTCC
AGACATTGCCCATCTCCATGCGCCCGAAGCCAGGAAAGCAAGGCACATTCCGCCTGATCCAAAACTTCTCATT
CCCATACACCCCATCCCCATCGCACACCTCAATAAATCATGCAATCAGATCAGACGACTTCCCATGCACATGG
GGCACATTCAACACTATCTCGCACACCATATTCAACCTCCCACCGGGTTCACAGGCGGTGGTGAGGGATGTCG
CGGAGGCATATAGAACGGTGCTGATCAGGCCGGCGCAATGGCTGGGGCTGGTGGTCAAGCTGGAGAAACCGAA
CACGTTCGCGGTGGACACGCAAGACGCCTTCAGGCTAGCCTCTGGAGCAGGCGTGTTCGGATGCGTGGGGGAC
GCGGGTGCGGACCTGTTCAGGGCGGCGGGACTGGGACCGGTGTCGAAGTGGGTCGACGACCACATATTCTTCA
GGATCCCGCAGGCGCACATTGAAGAATACAACGCGTGGAGGGCCAAGAATGCCGGCATAATAAGACAGAATGG
GGGACGAATACATCAGGGAGGAAGAATCTGGTACCGCGGCGCTGAGAGGCTGGACGGCACCATCGAGGAATAC
GACAAAGACCTCACCTTCCCGCTGAAGAACATAGCCACGGGGCGCAGCAGCGAAGACGCGCCCTTCATGTATG
GGATGGAAGACATAGACGCCGTCTCCAACGCGCTGGGGTATCCATGGGAAAGGTCCAAGGACGTCCCTTTCAC
AACCAAACCAAAATTCAACGGCTTCGAATGGAACCTCGAGGCCAAAACAGTGCACATACCCAATGAGAAGTGC
CAAAAATACACCGCCGAGATCAAGGCGTGGCAAGCAACACGCCTACACACCCTGGACGAAACACGGAGGCTAT
ACGGGAAGCTCCTGCACGCATCCCGGGTGCTCCCACAGGGCAGGGCCTACTTGACAAACCTGGAAACCATGAT
GGCCACGGGATCAGCCTCGCCTTTCGTGCCACGGATGCTGCCTCGCGGCACCCAGGAGGCTTTAAAATGGTGG
ATGGACAAGCTCACCACGTCTTTCCCCTCACGAGACATACCAGGACCAACCGACATACTCGACTATGGAGCCT
TTTCCGATGCCAGCTCTGAAGTGGGGATCGGTATCACAATCGGAGGACACTGGCGCGCATGGATCTTACTGGA
CGGATGGAAACACGATGACAGGGACATCGGTTGGGCGGAAGCGGCGGGCCTCAAGCTCCTCATTCTTACCCTA
CTTCGATCCCTTCGGCGAGGTGAGCACACCCGGGTTTTCGGAGACAATCAGGGAGTCGTTGAGGGGTGGTGGA
ATGGGCGCAGCCGGAACAGCCAAACAAACGGCATATTCAAGAGAATCCACCAGTACCTATCCGATGCGCAGGT
CACGCTCATCACAAGATACGTACCCAGCAAATCCAACCCGGCAGACGGCCCTTCTAGAGGCATCTATCCCCCA
GCCAGATTACTTCTCCCGGCCTGCCCCATCCCAGATGACCTACAGCCGTTCATCGCAGACTTCGACGACAACA
GAGCCCTACTCCACGCTGCTGGAAGAGGTCGCCATCCAGCACCAAAGCCCCCAAGATCATCAAACCATGAGGC
AGAGGCCCGCAGATGCTGGACCGACACGCAGCGGTCTGACGACGCATTCCTCCAGGAGCGAGACTGAGGGGAA
GCGCAGGAAGGCATCTCGTCTCAGGCCGCTCCCGACGAGGCCCGTGGGCAAGCCGGCGCCGTATAAAGCGGAC
CGGACACCAATCCCACACGAATCCAGACCACACTGCCTGGCGCGTGAGCGACTGAAGAGGTGGATCCCCACCA
CAGCTCAAAAGCCCCCGGAGCAAGAAGGGCACTGGGAGTTATCGGAGGAGGACAACCAGTTGATCACCAAGGT
CATAGGTGAGGCGTGGGCGGAGTCAACAATTCAGAATCCCAGCCACGAAGCTTGACCAAACCAAAGGGGAGGA
CATCTTCTGGGCATGCCAGGAGGGCCCGACAGATCCCGAAGCCACCATGGCGGCGCACATAGCGATAAACAAC
CCCACACCCAGGGAACATCTATTCGCATCACGCACGAGCAGGGAGACCAACGCCCCCTCACAAAATATGCATT
CAACGCGCGGATACATCTAGCGGCCACGGTCGCAGGACAGCCAAAATTGAAGGTGCACGGCCTACACATCGGA
GGCACCCTAGAATACTTGCTAAGGGGGATCGACTTCGAAGTCATGAAGTCCCTCGGAAGGTGGCAAGGCGACT
CATTCCTCAAATACCTGAGACAGCATGCACAGATCACCGCCCAATATATACAAATGGACTCAGTGGTACACAA
TCGCTTCCTACGCAACATCACAATCCCACCTGTACGCTGAGGCAAGGTAGGCAAACCAATTTCAATGCGCACA
CTGTTCCTGACATATGGGTACCGAGCCAGCCCGACGTGTCTCAGCGTGCGCGCCAGAGACTGTAAAGATGGGT
ATCGCGGCTGGCTCGTCCGGGCCCCCAGGGCTGCACCGCCGGAGCCGGGAGACGCTTCGCTCTTCACCCATTA
TCTAGTACAATTAATTATAGATCTACATACACTACATACTCTATGGAATCACCAGTTGTTCACCCCAACCACG
CGCATCGTGGGGCGGGACCGCCCCACGATGCACACGAGCCAGATTGGAGCTCCTTGGCAAAGATCACCAACCC
ACTCTCGTGCAAATGACCACCACCCCGTTAGTACCTGGCATCCTGCCCTCCAGACCCCACTCATCTTCCCCCC
ATCCTCCACCTGTTCATGCTGCTCCGTCACCAATTCTCCCATCACCGTGCTCGTTGTCATGTCCACTGCCTGC
CAGTTTGCCCCCCGAGACTCCCACT
>Cligib1_rnd-1_family-407#LTR/Copia
CCCCCAAAGGCTTAGGTGGTGATATATAACGGGTAAAATGAGCATATGTTCAAACCCTACCATTCCACCTTTC
ATAATTTGGAGATACAATTAGTGATAATTAGTGTCAATTAGCACCTGGGGGTTGGATATTTAGTGATAAAAAC
CCATAATGATAATTTTCGATAATTTTTGATAATTGACAGCAATTATGACTAATTAGGAAATGTAGTTTTTCCT

GATGATGTCTGCGAGGAGCCTCCGGACGGCGCTCCCTNTTGACCCACGGCGTGTTCCNTCCTACCCNCCGGCT
GTCNCACGGGACTCATCCCCCCAGGAGATNGCCGCCTNCGAGCTTTGGTGGCGCCGCGATGGTGCCGCTTTCC
ACATCCTTGCCTCGCGTCTNTCNCCTACGGTNCGCAATCAACTACCGGGTGCTGGTTCNCGNATGGACCCCGA
CTTGNCCGCTCGCGAGGCTCACCGCGTCCTGAGGGCGACTTATGGTGTTGGCGACTACATCACGGCNCTCGAC
GTTGAGACCGGCCTNCGCGCTCTTGCCTGCGGCCCNCGNGTNCGCGAGTATGTCTCNCAGTGGCGNTCGGGCC
TCAATCAGCTGGAGGCTGCCGGTTTCCCCTTGTCNCNTCGCACTGCCTTACAACTNTTTGTCGACCGCCTGCC
CTCTGGGGCGTCCTACACCAACATTCGCGACAAAGTCATGTCGAGTCTCAAGCATAAGCCGCATCACCTCCCG
TCTTTCGAGCAGATTGCTGATAGGGTTCTCGATGCGGAGGCCACTATNCGTCGGACGGCCCCTACCCGTCGAT
GCCCGCGTGATCCTACTGGNTCTACCACTACAGTCGCGANATCCTCCACTCCTTCNGCTGCCCCTACGACTAC
GTCTGTCACNGCCTCGTCGCCCCAGNTACCACGCGCGTCTCTGACTTGTACCCACTGCAAGCAGAATGGCCAC
CTGGTCGACACNTGTTGGCGNGCGGGGAACGGNAGAGGGAAGCCNCAGGCNCACTTGGCTGGTGTGGATGCGG
ANNCNCCGAGTGTCGAGGNGGTTGAGGTNCCGCCGGACGATGCNCCGCCTTTACCCGATGAGGATCTTACNAA
CTTCTCCGCGTGTTCCNTAGCCACGCCGCCAGCCATCAATATCAATGACGATCTCCATTTCATGACCGTTATG
ACNTGCGTGATTTGCACCCGGCCGGGCAATGTTGCGCTCNTCTGACNNGCTTGGNCCNATCGCNTTGGTGGCG
GTCGACAGGAGAAGCTGAACACCATNCTNGACTCCGGNTGCANCAACCATATCATTCGGGATAGGTCGNTGTT
CTGGACGTATCATGAGGATCGNGCCCTNGATGTCACGACNGCGAATTGTGGTACTCTGACNACGTTGGCGCGT
GGTATGGTGAAGCTTCGCGTCGGCTGTGGNAGTCGNTCNGTGGTCGTCGCNTTGCATGATTGCCTTCACTCCC
CGGACGTCCCCGTCAACCTGCTCTCGGTTGGCGCGTTCCAGGAGAAGAAGATGGNNCTGACCTTTGGCCGTGG
CTCCACCAGCGTTCGNTTTCCTGACGGCTCCTCCNTTGCTCGCCTTTCNTTCGACGCGGCCGTNCGNCAACGC
TTATCCTGGCTGGACTGCGAATTCGTCCCTGCCCCTCCGTACTCGGCCATCGCCGCTCCGTNTTCCTCAAGCC
CGACCGGTCGCCNGATCTNTGGCATAGACGCTTTGGNCATCTTGGGATGGACGCTACGCGCGCGGTGCTCACC
AAGGATTACGCCACGGGNGTTGAATGGGAGGGNGCGTTCACGNCTTCGCATTGCATACCATGCCTTATTGGTA
AACGTCCNCAAGCGCCTTNCAATCACCTGGGGCACCGGGCTGCTGAGATTGGTGAACTATTGCACATGGACAC
CTGCGGACCTTTTCCCGTNCTCACGCCGCAGAAGACGGTCGATGTTTTGGACGGTCNTNGACGATGCNTCCAA
CTATGGCCACACTTCCTTGCTCGCATCTGCTAAAGAGCGACGCTTTCCGTGAATACCTCAAGGTNGAGGCGTC
NTGGGAACTGNNGTCCGGCAATCGTATAAATGCCTTCGTTCCGATGGNGCCAAGGAGCTTACNCTNGGNNCTT
TCGGGGANCATCTCNCGAAACGNGGNATCGCGCACCAGGTCACTGCNCCGTACGCGCACCAACAGAATGGCAA
AGCGGAACGGTATCTCTACGTCCTAGAAGACGGNGCNCAGGTCCTNCTTGCNGAATCCGGNCTGCCTCCNTCN
CTNTGGGGCCATGCCGTCCTCGCGACNCAATACCTNCGCAACCGNCTACCNACNNCAACGCTGCCCAAGGACG
CCACCCCGTATGAACTCATGCATGGGANGAAACCNGACTTATCNCACCTTCGCGTATGGGGGTGTCAATGCTT
CGTNTTCATATTCCACCGGAGCTACGCACGAAGGGTGGTCCGAGGCGNTTTGAAGCAACCTTCGTGGGCTATG
AGGAAGACCGTATTGGCTGGNCGGTTCGCGACCTAAGCGGNAAATTCCACTTCTCGAGGGACGTCATNTTCAA
TGAGGACACGCCTGGGCGGCTTTCGGCACCGCGGCCCTCGACAGCTCAAGACGATCCAACAGGAAACTGCCAC
CGCATCCCGGCCTCGTCGNGNACTATCGCTCAGACGCCNGCGGGNCGNGANTNCNCTGAGGGCATNCGCGCCA
GGAATGANCGNCTGGCGCGNCTACGCGCCACTCGTGGGGGTGTACCACCCGCATTACCNGTGGCAGATTTCGT
CGCGTTGGGCGCCGCGGATACGGCGATATCACCCGATGAGTCTACTACGTTCACGTCGGANGAAACTTACTAT
ATCGCAGAACACTATCTCCTTGCAGCTCATGCTGGCCCCACGCGTTTCATACGCGATCGCATCTTCGATCTAT
CGAAGGCNCCAGAAAACTATGCNGAGGCNGTGGCNCGTCCGGACTACCCAGTTTGGCGTGCGGCCATGAAACG
CGAATACGACAGCCTNGAGGAGAAACAAGTGTTCAGACGNACCCATCTACCNAAGGACCGCAAGGCNATTGGA
GTCAGATGGGTGTACGCTTATAAGTACAACGCGGATGGGAGCATCGTTAGAGGNAAAGAGAAAGCGNGNTTGG
TCGCNCAAGGNTTCAGTCAAAGACCGGAAGACTACGGCTCGACGTATGCACCAGTTGCCAAGATGACCAGCAT
TCGGGCAGTCCTGGCCTTCGCGGCGTTTCACGATCATGAAGTCATGAGCTTCGACGTCAAGACGGCTTTCCTA
CACACCCTACTCGCNATGATNATTTACGCCAGGCAAATNCCNGGCTTTCCAGAACCGGACCCGACAGCTGTCC
TCCAACTTATCCTCCGAGCNCTCTATGGCCTCCGACAATCATCATACGAGTTCTACATGCTNCTGTGGAAGAT
TCTAGCGCGNCTGGGTCTGACGCGCTGCGAAGTTGACCACGCCGTTTTCAAGGGGACNTGGACNACTCCCCCG
CATNCNTCGATNNCCATGCCGGTCAACGGCGAAGCNCTTTTCCTCATCGTNCCNGTNCACGTCGATGATGGNC
TTGTNGTCACNAACTCCATTCAACTCTACAAATGGTTCNTGGTNGAAGTGGGCAAAGATCTCGAGGTNGTTGA
TCTTGGCGCCGTNTCGCTGTACCTGGGNATACGCATCACAAGGGATCGGACCCAATCGGAAATTATGGCTGTC
NCAGAAATCGTTCATCACCGATCTCCTTTCGCACCTGGGGTATGNCCGACTGCGCNCCTAATGCGCCTATNCC
ACTACGCANCAAACTACATGCNCTNCCGAANCCACCGCCTAATGCACTACCGGACATACCGGATGACGTACTT
AAAGCGAAATACCAGAGCCTCGTNGGTTCCCTGATCTACCTCTCGGTNTGCGCNCGNCCAGATATTGCCTACA
TCGCGTGCGCNCTTGGCCAGTTCAATGCNAATCCTACACGCGCGCACATGCTNGCGGCAAAAGGNGTACTTCG
CTATCTNTCGGGCACCATAGATT
>Cligib1_Cluster92#LTR/Gypsy
ATCGCGCCGGGTTTGTTGCATATTCTCTTATTTTACCTTAGATTACCAGACTCAGGACATGATCAGCGGCCCA
TCGCATTGTACATAGACCCTTACATATGCCGCGACTGCGCACAGTATATAGGACATGTACATACTTCACCTTA
TGACAGAGAGAATCCAGCAGAGTTTCCCCCTCAGAGCTTGTAGCTCAGTTTCGCAGTCTCAGAGTAGACTACC
CACGTTCCCTACAGGAGTTGCACGTCACACTCCTCCACATTCCTTTGCATCCAAATAAGTCGGGCTGTGACCA

AGTTGCCTGATCGCAGTCAAAGACACACGCGTATCTCTGTACGCCGTCTCGCATCGCACTGGGCCCGAAGATT
TGAGAGTGCGACTCGCGTCGCACTCATTCTACGCTAAGTGCTCAGTTCCCTGATGCCCTTGCGTCCTTTCCAT
TCATTCGTTACCACTCTCACGCGTCGTACGCATCGAGATTGCATCCAAAGCGGGTCCTCTCGCAGTCTTTGAG
AACTCTTCTTGAGTCCTTAGGAGGCTTTGTTTGTTGCTAATCTGAAACACTCGCTCACTCAACATCTTATCTA
GGTGTTGTTTACTTAGTTGGTTTTTGTGGTTTGTTTTGGCCACTAGCCTCTTTGCATATCATCGTTACCACTC
TCTAGGGCATGTCACGAGTTAACCCGCAAGTAACCTGGATCTGTAGTCACAAGTACTTGAATGTGAAAAACTG
ATACCTGGATCTGCACAAGTTTTATACTTGTGAGATCTGGATCCACAGGTCATTTACTTGTGAGTATCCAAGT
ACTTATGAGTTCTAAATACTTGCAGGTACTCCTCACAGGTTACTTATTGGCTTTTGGAAACTGGCATACCCTT
GAAAAACCCGCCCCTAACAAGCTATCACATAAATAACCATCAGTTCCGCACCAAATCGGGACTTTGAGGGGTC
GATCGGACACTCCTAAAGGCCCTTATGAATGTCCAAAAGACCCGACAAGGCTACCTATCCCCATTCTTAAGTT
ATAGGGTGCGCGCTTCAAGGGGGAGGAGGAGAAGGTCATGTCATGGAGTCAATTCTGCATTGGAATGTATGAT
TGTGGGACGCATTTAACACTATTTCATGAGCTGAATTCGATGCGCATAGCGCGAGAGGCATCGGTATCCTCGA
TCCCACGGTACGCGCAATCTGGCAGACTATCATTCTGTATCTCAGGATCGGAGATACCGATGCCTCTCGCGCT
ATGCGCATCGGATTCAGCTCATAAAATAGTGTTAAATGCGTCCCACAATCATACATTCCGATGCAGAATTGAC
TCCATGACATGACCTTCTCCTCCTCCCCCTGGAGCGCGCACCCTATAACTTAAGAATGGGGATAGGTAGCCCT
GTCGGGTCTTTTGGACATTCATAAGGGCCTTTAGGAGTGTCCGATCGACCCCTCAAAGTCCTGATTTGGTGCG
GAATAAGGTCACCCACAAGGGTATAACCAAAAACCTGCACCAACCCACTGATTTTAAACCTGTGGGTGGGCAG
GTTACAACCCATATAGGTGCAGGTATGGGACACCTTCTGGATACCCACAGATGTACCCATGCCATTGCCTATT
ACAGGGTGGCCCAGGCAGCTACAGATATATGTACATGTGAGATTAGGTGGAAATTTTTCACATGGAAGTGAGT
GGGGAGCAAGGAATTGCACCCAACATATGGCTACAGGTTATGGCAAGTACTTGCCACAAGTACCTGTTGTTCA
AAATTTCAAACCTGGACCTGCAAGGACTTGTGGCCACAGGTGATCTGGATCTGTGAGTCACCATACTCACAAG
TATTCCCACGGATCAACTTGTACTGACTCACAAGTTACTCACACTTGCGACATGCCTTACCACTCTCACATGT
TGTACACGTCAAGACCACATTCAAAGCAGGTCATCTCGCATCTGTTGAGAACCCTTCGCAAGTCCTTCGGAGA
CTTTGCTTGTTGACTATCTAAAATACTTGCTCACTCAACATCATATTTAGGTGTTGTTTACTTAGTTGGTTTT
TGTGGTTGGTTTTGGCCACAAGCCTCTTTGCATATCAAATATGATGTTGAGTGAGCGAGTGTTTCAGATTAGC
AACAAACAAAGCCCCCTAAGGACTCAAGAAGAGTTCTCAAAGACTGCGAGAAGACCCGCTTTGGATGCGATCT
TGACGTGTACGACGCGCGAGAGTGGTAACGAGTGAATGGAATGAACACGAGAGCATCCGGGAACTGAGCACTT
AGCGTAGAATGAGTGCGACGCGAGTCGCACTCTCAAATCTTCGGGCCCAGTGCAATGCGAGACGGCGTACAGA
GATACGCATGTGTCTTTGACTGCGATCAGGCAACTTGGTCACAGCCCGACTTATTTGGATGCAAAGGAATGTG
GAGGAGCGCGATGTGCGACTCCTGTAGGGAGCATGGGTAGTCTACTCTGAGACTGTGAAACTGAGCTACGAGC
TCTGAGGGGGAAACTCTACTGGATTCTCTCTGTGTAAGGTGAAGTATGTACATGGCCTATTATACTGTGCGTG
AGTCGCGGCATATGTAAGGGTCTATGTACGATGCGGTGGGCCGCCGATCATGTCCTGAGTCCAGTAATCTAAG
GTAAAATAAGAGAATATGCAACAAACCCGGCGCGATCGCACGTCCGTGTCTTGCTCAGGTCACCTAATGGGCA
CATTGTCAAGGTGTCCAGGCCTCATGGAGGCTCTCGCGGTGATGTTGGTATGTGTCGCAGTCGGTGTGGGTCG
AGGCGCATTAGGGTCTCTTGTTAGGAAGTACTGAGTGCACGCGGAGCTGTGTTGAGGCGTGATCCCGTATGTG
CTGAGCTGCAAGAGGGTCCTATACGAGTGCGACAGCCTCGCATCGTATATATGAGGTGCGGCGAGCTGATCAT
GGCCGTTATCATGGGTGTTGATGCGGTCCGAGGCCGTTGTGTGGGCTTGTGGGTGCGCGCGTCGAGCCTGGCG
AGTCACGTTGATCTTATCTTGGTGCGAGATGTGGCCTACGAACCCATAAGCGCGTGGCGCGCTTATCAACGAT
TTGGTCTCCCAGATAAAATCATCTCCGATCATGATCCCCAGTTTGCCTCAGCAGTACAAAGGGAATTAGGACA
ACTCCTTGGCATCAAACTTGCATTCAGTATGGTGTACCACCCTCAAACTGATGGAGAAACGGAATGTGTCAAT
CAAGAAGTAGAAGCCTACCTTCGGATCTTTTGCGTTAATCACCCCGGAACATGGGTGGAATTCCTCCCTACCG
CCGAATTCACCCACAACCAATGCCTACACTCCGCACGAAATGCTTCCCCATTCTTCCTCATGATGGGCTACAA
CCCTATTGCTGTTCCCCTTCCTTTCCTCAAATCCAACACACCCGCTGTACAGACTTTCTCGCCTCGTGGGGGT
TGTTGTCATCTTCCTCCAAGTCGCTCTCTGACGACAACGATTCAGCCCCCCGTTTCCAACTTTTGCCCTTCAA
TGTGCTAGTGGCTGTCAAGGGCTTTGCCTTTGGTGTGGGTCCCTTCCGCCCTCGACTAGACTTTGGCTTGACT
GACTTGGGCTTCATCAAGGAATCAGGGAGTTTTCGGAGTGGGCCAGTCACAGCAATGACACAGAAAAGTTGGG
GGGGGAATGAGTAAATTTATATCACCCAGCCTCATGACTTGTCGCATGTCGACTTGGACACCTTACTGCACAA
TATATTTTTTTTTCTTCGGTAGAGTATAACTTGAGGTCCGGTTATGACCAGTTTAGGACTGGTTTTGACCAGT
CACCCAAAATTCACCAATGGTAGGTGACCGAGACCGGGGAGCAGCCAATTTTGAAAGACCGAGACCTGAGGTT
TGGTTTTTTGGCTGCGGTCCGGTCCAGTCACGGTCTTTTTGCAGTCAGCGTGACCGGACTTTGAAACACTAGC
TGAACGACCAACAGATGAACCAAATCCCTGGAGTAGTAACTCCACAACTTACGAATGACCCCTGGCCACTCAC
CAATCAAACCATTGATGAACTTACATCCACACCCTCCATTGTTCTTACTGATTTGGAAACCGAAAGACCCCCG
AACCTGACCACTCTAATGGAATCCACTTCCTCTGGAACAGATTCCCGAGGAGCGACACCATTCCCTCGCTCAC
CCATCAATGACATATCTACCACTCCTTCTCCTGAGCCAGCATCCACCACTCCTCTTTGCAGATCATCCTCTCT
CCCTATCACTCACCTACCTACCCCTCAGCACAGTACCACTCCTCCACCAAACCCTGAACAATTAGCACCCCCT
TCCTATGACCAAGCCCATGACAGAAACCCCGTCATCCAAACCCAATGTCGACAACAACAACGCCCCCAAACCA
TCTTTGTCACCCCTCTTTCCCATGCCAGTCGACCACAGTACACTCAATCTCCTGATGCCAAAAACGTCCCACC
TCCCCGGACTGTCTTCTCTTGACCTGAACGACCCCGCGTAGTGTTGCCACGGCTGGCTAGCCACCAGCCACAG

CTGACATAGCCATGACTTGGATTTGTCAGCTCAGACCATGCCACCGGCTAATCATATATCTTCAAACTCAGCT
GATCCCGTGACTTCGAGCCATGTTTGTCATGTGTCGACTCCAGCCGGCTGAGCGCCATTTGCATGGCTGGGGT
TTGGCTCACAGCCGGCTTCAGCCTAATTGTCAACTCCATTTCTTGCTTTTCCCGCTGGCTGGGAGTCCTTAGA
CCCCACTCCCCCTCAACAAAGGTACGCGAGCTCGTTTCCGAGTTGTTTCGACTGACTAACACTCTGACTTGTC
CGTCGCGACTCGCCACCGACTGCCCATCATCATGGAATCGACGATGCGGGCCAATCACCTACCTGATCTAGGT
GGATCACGGTGATCGCCACTGGAATAGAGGTATGGACAGTGGGGGAGACCTGGGAAGCAGGAGGGCCACCAAG
TAAGTAACACCATTCTAAATGTCTGGCAATGCCTGACCTGCATCCCGCCTCACTCATACCCAGGCATTTGACG
CGTGGCCTGAGGGTCTCAAGTATTCTGGACAGCACCACGGCATATTTTCATTATTGAATTATCATGATGCCAT
CCCACCTGGACGCCTATCCTTCCACCTCTTGCTCAGAGTATCCTCGAAGTCAGTTCCCCGAGCTTCATGGGCT
ACAGGGCAGACATGTGAGTGATAGTCGGCTGGAGGGTGGTCCAGATAACTAATTTGTCTGGCTTGGCCTCGCT
ATCCTTAGACGACATCTTGATCATTGAACACCACATGCCCATTGAGAGTCTATAATGTTCGCCCCCAGAACCT
TGTCCGGCATTCAGTAAGCTCCTTATTTGGTCCCATATTTCTCCACCGCTCACTAAGTTTCACGCGGTTTGTC
ACATTTCACCTAGACTTATGACCACTTTCACATTCCATCCATTTTTAGTGGCGCCCATCTGTATCTTCTTGGT
ACCGACTATACTCCAACTCACGAGTATAAGTATGTATCCAAAATGTACTGACCACTAACTTTCAGTTCCAATG
CCTCCTAAGTCTCGAGCAGCATGAGCAACTGCTACCAACCCATTGCCGCTAGCAGCTACATCATCTCGAGCAG
CATGAGCAACTGCTACCAACCCATTGCCGCTAGCGGCTACATCAACCACTCCTGATCCATCACCCACGCCCAC
CCACCCACGCGGACAGCATACTGCGCCCGCAACAGAAACTATGATGTGGACGCCTGGGGTCCCTACCAAGTTG
TCGGTAAGTGTTTTTTGCTTTTATGAGTAAGCCTAAGACTAATTATAATTTATAGCTACTCCAAGTCCAGGTA
AGTCTTGCATATATTATTGTTATATTCAGTTTAAGGAACATCAACTTTAGTCCCAGTGGGTGAGTAGTGCCTC
CAATGTCTACATTTAATTACCGGTGAACAATCTTTATTCAAGCAGCCACCCTACGCAATCTACCATAGCCACA
GCTCGAGCCGGGCAGCGAATGATGTCAATCATTTCTTTCCAAGGCACTCGAAGACCATCAATGTATTTGTATA
GTGTGTGGGTAAGTGAGTACATTTTCGTTTCCAGTTCTACGATTGAATTTGGGTTTCTAGGCCACAATATGAT
GTGTTGATGACAAATCTGAGTGGCCCAAAAATAAGACTTTCTCATTTGGGCCCACAATGGCAACTGGAAATAT
GGGCACCACCTCGAGAAATGGCATTCAAAAGCCATGGAAAAAATATCGCTGGAGCGAGGATGGCCAATCGTCC
TTGCTGGGTACAAGGAAAGGTCCAAGAACACTAACAGCATCACCTTCTCAGCCGATGTTGTCCTTGATCGACT
GATCAAATTCATTTGCATTAATGATCAGGTGAGCATCAAACAAATATCTATTACAGCAAACCCAATCCTTTTA
CAGTCTATATATGTTGTCGAGAATAAGCAATTTTGCGAACTACTCAGTGTATTTCGAACAGACGACACTGAAA
GGAATGTTCCCCACCGCACAAAAGTTCGGTCTGCGATCATTGATGCATGGGCCAACCTCTTTTCGCAATTGAA
GGAGGAATTGAAGGTATGCATTTCTCACACATATATATTGGGCTGAATACCTTGCAGAACGCTGCCAGGAGAC
CTTCTTTCACGGCTGATGTGTGGTCTGCGCAAATGCGACGACCTTATCTCTGCATTACTGCACATTGGCTCGC
CTACCAGAACTCCACTGACAAGGAGGGCCTATACCTCAAATCTGGGATCATCGCTTTCCACCACCTTCCTGGG
CAGCACACAGGTGCACTCCTTGCCAGAGCCATAGACCGCCTACTCGAGAGAGCAGATATAACTCTAAGTAATG
TAAGTCGATTCTTCCATTTGAAAGTATAGGGCTGATGACTATACACAACTCAAGGGCTGTTCTGGACGCTCGA
CAATGCCTCGAACAATACAACCTTTCTTGAATCCCTCGAAAAACTTTACGCGTCACGTGGGATCCAGTACTCT
GCCAAGGAGGCCCGTGTGGGTTGCTTTCCGCACATGATCAACATCATCTGCCAGCATATGGTGATAAGCACGC
CGCGTGCTTATGGGTTTGTAGGCCACATCTCGTGCCAAGATAAGATCAGCATGACTCGCCAGGCTCGACGCGC
GCACCCACAAACCCACACAACGGATTCGGACCGCATTAACAACCATGATAACAGCCAGGATCAGCTAGCCACA
CCTCATATATACGATGCGAGGCCGTCGCACTTGTATAGGACCCTCTCTCACATCAGCAGATCGCCCTTGCAGC
TCAGCACATACGGGATCGTGCCTCAACACGGCTCCGCGTGCACTCAGTACTTCCTAAGAAGAAACCCTAACGC
GCCTCGACCCACACCGACTGCGACATGTATCAACATCACCGCAAGAGCCTCCATGAGGCCCGGACACCTCAAC
AACGTGCCCATTAGGTGACCTGAGCAAGACGCGGATGCGCGATTGTGCCGGGTTTGTTGCATATTCTCTTATT
TTACCTGAGATTACCAGACTCAGGACATGATCAGCGGCCCATCGCATCGTACATAGACCCTTACATATGCCGC
GACTCGCGCACAGTATATAGGACATGTACATACTTCACCTTATGACAGAGAGAATCCAGCAGAGTTTCCCCCT
CAGAGCTTGTAGCTCAGTTTCGCAGTCTCAGAGTAGACTACCCACGTTCCCTACAGGAGTTGCACGTCACACT
CCTCCACATTCCTTTGCATCCAAATAAGTCGGGCTGCGACCAAGTTGCCTGATCGCAGTCAAAGACACACGCG
TATCTCTGTACGCCGTCTCGCATCGCACTGGGCCCGAAGATTTGAGAGTGCAACTCGTGTCGCACTCATTCTA
CGCTAAGTGCTCAGTTCCCGG
>Cligib1_rnd-1_family-382#TIR/MITE
ACTGGGTGACTTCCCGTAAGCTTTTATTCAAGAGAATAGAATAAATTTGGTCTTTAGCGAGATCGCGCCGAGG
TGCCGACGACGATTAGGGACAAAAGATACGATGGATCTCGCTTAGACCCTCGAATAGATGAAAGTCTGTTGAT
AGGGTGTCTACCCCTCCTACAGATTAAAAGTGGTCGAATAAACGCTTTTCAAACTCCGTAAGTCCCCATCCGG
ACACAAATCCGAACGTCGGTGCGGTAAAATTTGCCGTCCAGCCGCCGAAATTACTGTCTGCCATGATTACGAC
CCAGAGCTCATCAATATGTGTGCGCAAATTCCCCGTGAAGTAAATTCGGTTGTACCTACTTGCACCAAGCGAC
TACAGCTGCCACAACCAGCTTTCCAGCCTAGGAATTGGCCTCAGGACGAGGGGAAAGCCCGATTCTATAGGCG
TATTGTCAGTAACTTGATGTAATAGTAAGAACTAACAACTTACAGTTGGATACTGTTCCCTGCTATGCCGCGA
AAGAGCCCGAATGCGATGTTTTCGCCGAGTCCCACCAGCCTTGGGACATATTTAATCTATTATATTCGGATGG
CTTACGGTTTAGTCAGCAACATATCATATCGCATTAAACCCTGCGCTGCCTTGCCGGTTTTGATCGAAATACG
ATATGTTTATTCGAAAACCTTACGGGAAGTCACCCATT

>Cligib1_Cluster103#ClassI/LARD
ggagcaAAGGaggaaaagcaacaaGcaaggcaaaAaAccaGcgTccAGacgacaaaaTaGagacaagcagaag
cgagGccagaccgacGGagaggaaaaaCCcaaccgacacCaaaaaagagaaccacagCcAaccgccacgCTca
AacCcactttTgTcgaaaacccCaaccacacccgaaaCCaaagagaaTTcCAgCcccagcacGaaTcgcGacag
cCaccaacaaaaaagaAaCcaAAAggTcgcaGGgGcAaccCaAGCaTcAcaacaGaCcgcaACgccgacACca
aaaaaaGCCGaacacacacCAacAacaaaacccacCcaaGcaacaGcaacGagcAcccAaCAaaaTccaagag
aaacGccaaATgccacccGgGaaccaTacCCTTcccATGaagcaccaacacagCgaccaaAaggccCgcAaaT
aaacagcaTaaaacagccaCgccaaccccacccCgacGCacaCcAgcaccaGGggaacccCgTgaGCCAGGaA
GaAagaGaaaCaaCcaagcCgcaaGcAGcGacaaAaacgTcTCCCaaaaacaaAccaGCgGacaaagacgTTa
accCGggTcacAaagcaaGcaccCaCcATgggccTGAgcaaaCcTcTacaaTcTcTaCaacaGAacacCGgaT
cggaTgccccaaCaGccaCaaccTagGcCAgacaccggacAaCaTgaacTTcCgCacaaggcccCTCcCcgca
TggcaagcaAaaagcgcagaagTcagCCCacTccccccgTgaTcgCAacgGGaaAcTccAaaccaACagaccc
TcTTacCaccaaagcCgGaacAaAcACAgcATgcTagccgaTaagacTTaTacacgagcacaaTcaggacaca
GAgccaaaaGaaACagcaacgaaAagcaTcaTTccccAaAaaccACAgaaaGCgAaaGacCACAcacaccaaT
agAagcggaaTgATagagaCaaccgAcagagcAGcaCacgcaaaaaGggagTccTcaaaaaggGgagaAggcc
accCgTgCGaaCacTcagaagccagaCaaGaCcacccacaaaaCaGcaaAagcGaaaacagcccgaGgCcaAc
AagagagcagaacaAaCgccggaGagGggCcaacccaaAAcaGCcgAgAaAgacggGcGcAccaaAaCaaCac
AaacggggaCaaaacgGcaGCcgacaggacAagcgTCgAGacCAaGaAGcGCcaccacaccacccCgCCcagaa
CaaGaacgaaaGcaGCAacacaAaaCaagCacaaacacaacaaaaccacaccaaTGaGaaaagGcaacAGAAa
AgGagAaacagcaGcaGGaacgacCcgcacagggcacgcCccagcAcaaaAGCTcccgacaCacgaccacaga
GCaaaTccagTaCTcgagcaccAagaggaGacaaTgGgcaaacgcccAcgCCaaacCaACaGcccagccGcca
cCaacAaaGccaaaaCGGTCcaAaTccAcaaacTagGAGcTGcAaacacaTccaaaacgacgCaaaccaccca
caaagcacgTccGATggcgaaAaaGgccgcacCgaaaAggCAaCcGcaAaccGaacgcGAccaCaaccGgGAc
CccacagcaTcccgaccaAaccAaaacCaaGacTgaaaAagccgggcgaacaccaagcaGagaccacTaTgac
gccaacgGGaaACcacGaacaAaAcccacaaaaccGcccaGagaaAcaaaagcCcAgacATgcaccagCgagc
aaagATacgaCcacggcaCaaaaAccTGaaccaaaGCTAGaAcaTccTaAacAccCTagaGcagcaTacacgc
caCcCagCgccaccAAaagccaggacacaccaacaTcAcgacgCaaaagcgGTaaaagccaACaaGgAAcGaA
cGGcCaaaaacacccTTgaaaagaCcgaaccaGagCccTacCcaaaaAaacagaTcgAcTgCgAaaaccaCaa
acCaaCagagaTgGAaaacccaTTcagacacgacTACaaaaCagcaaaccaAccgcCaGaCcGCgAagcaaaa
acTTAcagTcacaCCgAgCcCaAAaaagcTacTcGcacccccCcaaCaacaccaaTcgacTgGGgTccgaaac
cccAggcagaaaaaccgcGccaaaccCaagAagaaaaaTagcagCcaaaacAcaaaacaaTgcaggCacgaaa
gGcGagcaacagTgcagccgACTgaccgaaAaCaagcgGCaaacgagaagAAacaccaggCaaaagcCcgTaT
CaGaCcaaagacgcaGaAaaaacaAaaaaccacacCacagaaaCTgaacGCaTcacgccCacaaAaCCcaacC
aaaacaaTcacCcacgCaTgcAcgagaaaacAcaggcTTGaaaCgaTCgcGcaggGaaGTgaaaccTcacTgC
cAgccaaagCccagTcGaaGCaaaaacacaTaTGaaacccgaaAaaaacacaaaccccccgcaaTgccccaccg
aacccCaAGagcccCaacgAaTgacagaaAaTCaggcTcccaccaAgccaTgccaccaAaaacGAgTacaaag
aAaTaaccGaccaaCTgGacaacacagccaaaAcgcacaaacgcccacgagaTgcaaacgagCAaAgccacaa
cgGCccCaAagacGacagcaaTcGaAagcAaaccgaACaaCaagcCaaGAaaGaccCAAaaacaTccacaaca
cTaaAgGaagaGcgcAcacagccGacgaGGgTggcaGccaacaAgGCagcaAGccgacacGagaaccaaggTc
acagcTcCgcaacacggcGgCaGAgAaaGcaagagGaacacccaTTaccagaaggcGCacaagGcTgcaaaat
aaggttcgatacgggtctacccgcgggtaaaaaatgatataccatacctgctacctgtaataccgcacccgtt
ttgacaccgcacccgcgggtacgggtaaaaaccgcggttccatacgaacgatcgcacggagccctctcgtctc
gtcgaattcgtgatctgtgggtaaaatgggtcatgttcaacaccaagatgaaagaaaacggaccaaaattgac
caagatatggccatctttgcttgagaaacccaagaaatcggcctcgcgtgtgtgaacggctgtccggagccct
ctcgtctcgtcggattcttaatctatgcattaaatgggtcctggtgagtatcaggatgaaggaaaacggataa
gaaatgactgagattcagccaatctactcataaattcataatattggcctcacatgtgaaaacagtcatgcag
caccctgtcgtctcaccagattcataatctatgagtaaaatggaacctggtgaataccaggatgaagggaaac
ggataaaaactcactgagataccacattctgctgttaaaaagtctaaaatcggcctcacacagtgtgcaggca
gatgcggagccctatcgtttcatcagatctataatatacatataaatgggccctggtggataccaggatgaat
aaaacagacaaggactgaccgagatattgtggtatatctgcttgaaaatcataagatcagcctcgcgtctatg
aacagttatgccgcgccctatcgtctcaccggatttgtgatctatgagtaaaatgggttcttgtgaacccaag
gatgaaggaaaacgggcaagaactcactgagatatgatatgtggtgaaaaaccacaagattggccttgcatgc
gggaacggtcatgcggggccctgtcgtctcgtcggattcgtaatctaggagtaaaatggattctagtcaaccc
caggatgaagcaaaacggatgggaactcactgagataggatggtgggggggcacattggaacctgtggttcat
gcgggtaagggttgcggtatttgcggtgttgcctaattgggacaccgtacccgtacctgtgacacccgtacct
gtgacaccgcgggtttacccgcacccgtgtcgaaccttatttccaataAGTaccagcaAaacaccacccCacc
cacgGaagcaAagcagagcTcgcggcGagcaaAagacaagTacaGaacaaacCcaaacaccgaccaaTaaccc
caaccAaCCaAacacAgaccgaaaGggcGaaaaagcCcAacAccaccTTcGaGaTaGGaagcAagaTAAaaca

ccaagTaGCaGcCaGcaacTgccccccagGcaacGTAagaagccacaAAgaaagcgTCaAaagagGcgGagcc
GaaacagTaGaAaTaaAgaaaggTgagcaGccAaaGagCgaaccacGaagcggaAAccacacggcaGcCCaTc
CcTTaCgcGTgccaaccgACaaaacagGaccaaTcAcaggaagaaacaagcaacgTTcccccaGacGaGaaaa
aaagAaaaccCcaaAGaaTcgTgAccAgcTgCTAGCccaaaagCAaaTcccaGccaaagcGgaagATccccca
caGagccaaaATccaTcGaaggcgcTgcaaCgaccCcaggggaccaaTgTccagCcgcGTgcCCacaaaaaac
aAcAccacgacCcaacccccaccacaaaacagTggcaAcgCaaGaaacTaccTgcaaaAccAgcaacgccGaT
CaCacccGaccTCcaacgCccaTgaCaGcTGaaTgggaGaacaccaCcaTccacaaATCaaAaccaacgacca
caaaccaaGaacacccagccAcaacgcTaCAaaTCagAaccccTccAaTgcGACCGgaggccCcaTcagGaga
aCcaagacaCcGTaGcaccGGacTcaaaacaccaCaAccagCgaaaaaTcaccagacAGgAaaacAacgGCaa
gcccccaagGcccaaaaAGCTgcacTcagacGaaagaaaagcaaacGcgaaggCCccCgcaacgaTaaCaTaT
TCTacgaCaaaagCcCAgcccccgaagcgcaacagcgcccaGacgGccTccccaTGCgcCacgacagCgcGaa
accGGaGccgacgaacccaGCaCTcCaTGcaCTaGAacacTccaacGgacGCacCaccccaGaaaaaaaaTcg
GaaGgTgcacTcCcCaaacaTgTaTaaGCacTTcgcacagaGaaCacCgaacaaGgTgaggaccaaacacTcT
ccGaCgaaaaacacaacCaaaTcaaCcGggcccgacagaGcCgaGcaaacaacaaaAcgGaaCGaAaGcaacc
aaTcagaCaaccacgcAccAacaaCaagagcacaGccCTcagaacTacacgGgacagATaAcgaacaaTagAa
gcaaagaTGTGagCgaTaGCggcaTGTaccaTaTccaaAAacAgaagTggaagcaaaGTggCcgaaccaTCgc
gagaagaAaacgaacaaccaAcAAagaccAcaccagAaaATcaTGTcTcTagcCaGcacaccccGagAAaAaa
caaaGTccccgTacAcTaaCCaacTGaaAaaAaaccccaAccGGGagaAaagaaaaaaggaTgcagaaaaAca
aaGcGAGaaaaagaagGaGcAAacaAaGcTcgaaaaaaaaTGcCaaAacggGaacCcGcgcaaaaAaaacAca
CGcgaaTCacggGgAaAaAcacgAaAaGggaaaTCAccaaaATcGaGaagaCcaaagcTcaaAaaCGcaacAA
aacaaggcaCaGCgccTggcgcgggGGGAcAacacTcgccCaCTcCaacaGaacgcGcgGcgcCcaaTgCTaC
CTGGCcaAaGaacaaaaCCcccaaaTcGgccGCcaaGcaagcaaacgcaaGgccagcgcccAgcaacccgAaa
TaaGccaacTcgaccaGaacgcaacccaacaCATccaAAGGcaAccaagCaCcaagacaATccccCcgccaaa
cgcaAgcgTGgTcaaTaagCaccccCgaacaCgccGcgcacagggcaTccTGaaagaccagCGGCgggagcgcG
ccgaccaGTacaaGcCgcagcTcAcGCTcGaccTcCaCCccaaAaaTTTaaGcTcaaaagccaggccgGaaaa
aCcaaAgcCaccaccaaGaTgaAGgAgcaAAGGaggaaaagcaacaaGcaaggcaaaAaAccaGcgTccAGa
cgacaaaaTaGagacaagcagaagcgagGccagaccgacGGagaggaaaaaCCcaaccgacacCaaaaaagag
aaccacagCcAaccgccacgCTcaAacCcactTgTcgaaaacccCaaccacacccgaaaCCTTagctaa
>Cligib1_Cluster100#LTR/Copia
GTCGAATGCACCGCGATACATACGTAAACATTACGTCATCATATATATATCTACCCGACCCTAGGTTCGAACT
TACCTCAGTCCTTATCCCATATAGGCGACTGAGGTAAGTATAATCACCATCGCGATACGTTACCTCACACTCA
TCTTGTATCTAGATACATATTCTAGATACAGATAATGATATAGTCCTTATCCCATATAGGCGACTGAGATACA
TATTCTAGGTTTTTAACAGGTTATGGGCCTTCTCGCCTAAGACTGTATCAGAACCGCCGGGATCCGACTCATC
CATTCTCTTATTTCCACCCTACTTCTTATTATTATTTCTTCCATGGCCGGCGACGATCCTTCCGGTAGCGTGA
TTGGCGTGAATGGCTCGGGCGGCGATGATGCGCCGATCAGACCGCGTACCCCCATCCCAGTTCAGCCTGCAGT
TCTACAGACGGACGCAACCCACCCTTCCATCCCCTCTCTCTACGTCAGCGACGGTCCTTGGGATGGACAGGCT
GTCTTGAAGAAAAGTTCGAATAACTGGCCCGCGTGGAAAGAACGCATGGTGGACGTCCTTACGCTATCAAAAG
GGATGAATCGCTATCTTACCGGTACCGTCTTCCGCCCAGATCCCGTTCGAGAACCGCGAGCCACCTATAATTA
CGAACTCAATGATGAATCGGTACGCGCATATATTCGCACGAAACTCTCCCCAGAGGAGCTCGCACACGTAAAA
CAATGCCAGTCGTCCAAAGAAGTGTGGTCAATCTTAAAGGATCGCCACGAAAGACAAGGTCCCATCGCCCAAA
TTCTTCTTATGCAAGAGGCGTTCAATATTCGCTATTCCCACTCGGTTCCCTTCGCGGAGACGATTGCACGTAT
TGCCGATATCAACGATCGTATATGGAACATGGGTGCCCCCACGCGCGAACGATTTCACGTCATTCTTCATCTC
TTAGCCCTGGCCCCCCCACAAATGCACAACGTCCGCGACGCCATCACCAATGGCCTTTCTACCGCGACACATG
AACATCCATATGCCGTCCAACAGATCGTCGCACGCATCGAAATGGAGCAGAAAGTCCTAGCATCCGATGGTAA
TGCCCCAGGCGACGCACTTGCGGTCGATGGCACGTCTAAGACCCCTCGGAAACCCGTGACTTGCTCCAACTGC
CATCGCTCACACCATGAGGCGAGGTTCTGCATCAAGCCCAACGGCGGAATGGCTGGAAGACGGTTGCTGAGG
CTCAAGCTGCCGCGAGAACCGAACGCGGATTACCACCAAAGAAAGCCCCCTCGTCCGCTTCCTCTTCTACCAC
CGCCCCTGCTACTCCCGCCGCTTCGACGTTCGACATCATGCGGGATGCTAAGGGACGTGCATATGCTGTCGAC
ACCTCTTCTGGACAGGCATACTTCCTCCAGCCCGATCGGCCTGCCCTGCCTCCTGCGCCGGCTGCTGAAGCCC
ATTTCATGGCGATGGAAACCGACGATATTGAGACGCTGGTGTCGCCCTTAGTTGCCAACTCCATGACCGATGC
AGATTACGCCGAGGTGGCTTGGCTTGCCACCCAGGACGACGTCTCTACATCAGTAGACTGGGAGGCCTATTCT
TCTGGGCCTGCCGATTTCAGCGCGTGGCTTACGTCTCCCATTGCCGATGGTGCGAGGAAGGTGCCCATATCTC
CGGACTCAGCCCCCTTTCTCATTGATAGCGCTTGCACTACACACATCTCACCCGAACGCGCGGACTTCTTCTC
CTTGAGTCCCGTCAACAATCGCTCCGTTCGGGGTGTCGGTGGATCCTCCGTCCATGCCGTTGGCATCGGATCA
ATTCGCCTATCGATTGGAAAAGGAAATGCCATCACGCTACACAATGTTCTTTATATTCCGTCTTCCACCGTCC
GGCTCATCAGTGTCGCTTGCCTTACCGATTCCCTTCAGTGTCGTGTGGTTTTTGATTCCAGCTCAGTCTCCAT
CCTGCATCGCTCTGGCTCCGTTTTCGCTTCTGGTACTCGTCTCCCTGCCCGTAATCTCTATCGTCTCACCACG
TCTGGCGCCTCTGTCGACCATGCCATGCTGTCCACGCCCGTCCCCACCCTAGCCACCTGGCACCTCCGATTGG

GGCACGCGAATCATCAATCTATATACAACATGGCCACAAAGAAATTAGCATCAGGTATGCCGATTGACCTCTC
GACGTCTCCACCCAAATGTGAACATTGCATCCTTGGTAAACAGGTTTCATCGCCTGTCCCAAAGGTCCGTTCA
GGGGAGCGTTCGAAGGAGAAATTAGGCATAGTTTTTGTTGATTTGACGGGCCCTCAGGATGTTGTTGCTGCTT
CGGGCGCTCGGTACATTATGCATATTCTGGATGACTTCTCTAGTTTCTCTTGGTCTATACTTCTGAAATCGAA
AGAGGACGCCGTCCCAAAATTTCAAGCATGGGCTAGGACTGCTGAGGCGGCCTCGGGAGCGAAATTGAAGATC
ATCCGGTCCGACAGGGGTGAGCTCAAAACGAATGAGATGGACGCCTGGTGTGCCTCTCGCGGTACTGTTCACC
AGTTTACCGCTCCCTACACTTCGGCCCATATCGGGCGAGGCGAGCGCCTGCATCTCACAATCATGAACAAGGG
ACGCGCGATCCGTCTTCAAACGGGCCTACCCCCGAATCGATGGGATGAATGCGTCAACTCCGCGGGTTACCTC
CATGTTCGCACCCCATCAGTTTCAGCGGGCGGTCGCACCCCATACAAACTGTGGCATGGTAAGCTCCCCGACT
TGTCTCACCTAAGGGAACTAGGTTCGCGGGCGTTTGTCTTGACTCAGAATCGCCACAACCCTAAGATATACGC
ACGCTCTTTTGAATGCATCCTCATAGGATATTCACCTTCGTCCAAGGCTTACCGGTTGTATCACCCCCCTTCC
CATAAAGTTGTTGAATCATTCCGCGTTTCTTTTATTGAATCTCTAGATAGTACCCCTTCTACCCTACACCCTG
GTCGCGTGCTCGACAACCCTACAATTTCCGATGCGGCCTCCCCCTCCTTACCTACTTCCACCCACCAAGCGGT
GCCACCTCCTGGTGTCACCCCAGCTCCTTCCTCACTTTCTGGGGTGCCGCCTACCCCTACCTACTTACCCCCT
TCCTCTTCCCCCGTAGTTAATGACCTCCCTCGCTCCCGTCGGGGAGTCACCGTTGAAGAAATACCCGACGAGG
ATGCACCTCCCCCTATCACCCCTTCACGTAGTGCTTCCCCTTCTCCCTTACCTACTGCCCCCAACTCCCCCTT
ACCTACACATCCCCCTCCGGTGGAGCCTCTCCGCCGTAGTTCTCGTACTCACACCCCATCTGAGAAGAAGGCT
GCCGTCGACGGTCTTCTATTCTTACCGGCTGTCTCACGGGCGCGTGAGGCATCCAAGGCCAGCGCCGCGCGGC
TCAAGGAGGAGCGCCGTTTGGAGAAAGACCTGCGCCGGAAGTCAAGACTCGAGATGCGCGCAGCGATGGCGGA
CGACTCTCGTATTTTCACGCATACCTCTGCTGTGGCTATTCTTGCTGACTCCTTGCCTTTCATGTCCCCCGAG
GAATTCAACCAGTTTGAGGATGCTTTCGCTGCCGTGATGGACCCTTCCCTCATGACTGGAGGAGTGGACGATC
CCACATCCCTTCGCGAAGCTCTAGACTCCCCTGACGGTGATGCTTGGCGCTCTAGCATTCATGAAGAACTCAC
TAGCCTACGGGAGATGGACGTCTACAAACTTGTCCCGCGGGCTTCCGTTCCCAGTAACCGGAAAATCCTTAAA
GGCAAGTGGGTATTCAAACTCAAACGTGACGAGAATGGGCTGCCGTCTCGCTTCAAGTCACGGCTGGTAGTCA
AAGGGTACGAGCAGGTCTTCGGCCAGGATTACGTTCACACGACGTCTCCGACCGCTCGCATGGAGTCCCTTCG
CCTGCTCTTGCATATCGCCGCCGCGAATGACTGGGAGGTAACCCAAGTAGACGTCAAGACAGCCTTCCTCTAT
GGTTGCTTGTCGGCTGACGAGGTGCAATTCATGGAACAGCCCGAGGGTTTCGCCGAGCCCGGGAAAGAAGATT
GGGTCTGGCAATTACAGCGTGGCCTATACGGGATGAAACAGAGCGGACGCATCTGGAACAAAACTATGACCTC
GGCGATGATATCCTGGGGTTTCAAGCGCCTAGACAGCGACCCGTGCGTGTTCTACAGGACGACCTCATCTGGT
ACTATACTAGCGGCTGTTCACGTCGACGATTTCCTCATCGCCTCCTCGTCCCCCTCTGAAGCTGATTCTTTCA
AGGACCAGTTGCGTGCGCTCTGGTCGATCACAGACCTGGGTGAGGCGAAATTCTGCGTTGGCATCGGTATCAC
ACGGGATCGAGCAAAGCGCACCATCTCTCTATCTCAAACCGCTTTGATCGACCGAATTGTCACCCAGTTTGGC
CAGACTGACGCCGACCCTATCTCAACTCCTATGGATGATGGACTCAAACTTTTTCGCCCCGACGCCAGCGTTC
CCCTATCCCGAGAAGTGAATGAAGACCTAACAAAGTTGCCCTATCGCTCGCTTGTGGGGTGCCTTATGTATGT
CGCTGTCGGTACACGCCCCGACATCGCCTTTTCAGTTTCCAAGCTCGCCCAGTTCCTGGACTGCTACCGTCGC
GAACACTGGAACGCTGCCATACGAGTCGTACGCTACTTAAAGGGATCACGCTTACTCGGTCTTACCCTGGGTG
GACTAGACAACCCATCGTTATTCGGATTCTCTGACTCATCATTTGCCGACTGTCTCGATTCCCGCAGATCTAG
CATGGGTTACTGCTTCTCACTCGGAAAAGGCTTATTTTCATGGTCATCGCGTCGCCAACAGACAGTAGCGACT
TCATCTACGAACGCAGAATACGTTGCAGTAAGTGAATCCTGCAGAGAAGCTCTATGGCTCCGCCAATTCCTGC
GGGAAATCCGAGTGCTTGATAACAGCCCCACACCAGTCCTCATAGACAACGACGGCGCGCGCGCACTAGCTAA
CGATCCATCACACCACTCCCGTAGCAAACATATAGACATTCGCCATCACTTCATTCGCGAACACATCGACGAT
GGAAACATATCATTATACCGCGTCCCATCCTCAGACAACATAGCCGATTGTCTAACCAAACCACTACCTCGTC
CCGCATTCCTACGGATTCGTGCTCTTATGGGCATCGAATAATTCCCTCAAATCAGGAGGAGCTCACATATCCA
TATTTACATTGTTTGTCCTTTTTATTTTCTCGTTCTCTATTCAAGAAACTTCTCCTCGCGGTGAGGAGGAGTG
TCGAATGCACCGCGATACATACGTAAACATTACGTCATCATATATATATCTACCCGACCCTAGGTTCGAACTT
ACCTCAGTCCTTATCCCATATAGGCGACTGAGGTAAGTATAATCACCATCGCGATACGTTACCTCACACTCAT
CTTGTATCTAGATACATATTCTAGGTACAGATAATGATATAGTCCTTATCCCATATAGGCGACTGAGATACAT
ATTCTAGGTTTTTAACAG
>Cligib1_rnd-4_family-368#LTR/Copia
ATTTCTCAGCCAAACGAGGTGACGCGCGGCTTCGGCCCATAGGAATTGGGGCAATCCGGACGAGATGAGCATG
GCCCGAACGCCATCAAGGTGCGTTCGGTTTGCTCTCTCACTTTTGCCATTTGATTGAGGGCTGTCGTGCACAG
TTAGGTGCCGGACGGTCCCTGCCTTCTCCAGGTGCTCCGTAAATTCATCCGACATGAATTCTCCCCCTCGGTC
AGTGCCGAAGATCTTTATTTTCCCATCGCGTTGCATCCTGACCCATGCCTCGTATTTCTTATAGATTTCGAAG
CATTCAGACTTTTTCGTAAGGAAATAAATTCTTTCCTCGTGCGAAAACTGATCCTGGAAGCATAAATAGTATT
TCCGGCCGCCAAGGGACGTCGTTTGAGCAGGACCCCACACGTCGGAGACGATTTTATCACCGTAAGCCTGGGC
ACGTTCGCTTGTGCTGATTTTAGGGAACGGTTTGCGGGTTGCCTTTGCTTGGACGCATGTTGGGCAAAAATCG
GGCTTAGATTCGAGATCGAGCTCTATTCCGGTAACTATTTCATCTTTGACCATACGACGTAAGTCTTCATGAT
TGATATGACCCATTTTTCGATGTAGATCATTAATTGTCATGATTTTAGATGCTGTGGACGCTTCGAGAGGGGT

TTTGCATGCGAGGGATACACGATATAGACCATTGGTCTCGGGGATTCGACCGATAACGCGAGCTGGAGGTCCG
GGCTTGGAGATTGTGCATNCCCCNTTGAGGAACGAGACTGAGAANCCCTTCCGGTCCATCTGGCTGACGGAAA
TGAGGGTGAATGCCATTNTGGGTGAGTAATAGACGCTTCGGAGCGTAACGGTGGTAGNCTTATTGTCCTTNCC
CATTGGAAATAGGACTTTGATATCNCCTCGACCNGTTGCATAGAAGACGCGNCCGTCGGCGGCNCGGATCGGC
TCTGGCTCTATTTCGACGAAATTGGTGAATGCTTCACGACTGGGCGAGAAGTGGCGGCTTGCTCCGCAATCAT
ATGAGCACGCCGTGGGGATCTGAGGAGAAGGATAGGGCAGATGCTTCGTTGTGGAAGTCGGAGGTGACGACGA
GAGCAACATTGTCTTCTTCCTCATCATTATCTTCCTTGGTGGCTGCATTTGCGCTCGCGGTTTTGTCTTCCTT
CTTCTTCTTGTCCCAAGGCGATTGACCTTCCTTTCCTCCCCCTTTCTTATAGCAATCTTCGATGACATGTCCC
GAACGACTGCAATTCGAGCATTTCTTATCGTTTTTCGCCTTCGACTTCCCCTTCTTCTTTCCAGATGCTGACG
ACGCACTGTTGGCACTCTCGACACTTTTCTCGACTCGATTATGGATCATTTGCGAATCGGCTTCCTCGAAGAT
GGTTTGGACGAGCTGTGCTGGTCCCGAGCGGCTTCCCGGNGACGAGCGTCGCGGCGCTTAGTGNTTGNAGGAG
GGGNCGGTAGGATGTAGGGAGGGATCTTCGGATGAGTGCAGCAAACGTCCGGTCGTCGACTGCAAGGCTCCCA
TNCCNGCGAGCNCNTCGCGGGTTTCGAGCATCTCCGTAATGTGTTTCCGAACGTCCGCGGATTCGGCGCACTG
CATGTTGTGCAGTCGCGTGAGTGTGTCNACCTTCAC
>Cligib1_rnd-4_family-1097#LINE/L1
AATTTTTTTTTTTTTTTTTTTAGTGGAGCTGTGTATAATACGACAACCAATAAAATTGGCCTCGNATATAGCT
TGAGTAGTGGGGGNTCGCACCCCTCCGTTAGGAGTCCACNACGGGGAAATGCTTTACGCACGCCCTTCGTTTA
TAGGCAGTGTGGGACTCGGANGGGCCGTATCAGCGGTTGCGCCCCGGNGGGCGCCCCTCCGTACCCACTAAAA
CCCCGTGGTTGTATTTGACCCAGTCGTCAGGTAGCCGNTCTTCGTTCAGCAGCGTTCTCTTCCACGTGTTNCG
CACGATGCGATGCGGGGTCGCTTTNGAGCCGTATCTGCGGGCGTCCGCCGTCAGGCAGTCTAGTTTNAGGCGT
CTGTTTATGGNGTGTTCCCACTTGTTCCCTAGTTCNCTCCGGGTGTGTAGCTTGGTTCATGGTCCTCTTCTCT
ACTGATTCTCCATTCGCATCTGGTTTTCCANATCAGGTGNGTCGTCTCGGACACNAGGATGNTGTATAGGCGG
TTNGCCCCCTGATCCCGCTTATCGTCGTTTGTGAAGGCCGCNAGGCCGCANCCCAGCNGNACTCCGAATTCCG
GTTTNGGCCACTCGTGGCCTTTCTTCTCCCATAGCTTCTTNGTGAGTGCCCATAGCGTTTCTTGGCCCGACGC
TTGACATTCCGTCAGGATATGTTCCATGGATTCTTCNACNNCACATACNGGGCATGTGGCTCTTTGCTCGTAC
CCGGGTATGTTNTCCCAGTAGCTGCCGCATCGGTAGGCGCCGTGTGTCGCCTTCCACAGGTAGGCCCTCACGT
TCTTCGAGATTTCCTTCTTCCTTTGGGANCNCCAGATTCTCCCGTTCGTCGGTAGGGTNCCNCACGCGTCTTT
GATGGCATATCTTATCCTATCCAGGTTTACGGTNGTGCCTCCTCGCTTGGTCTTACTTCCGTTTTGTTGTACT
CGCTNATCCCTCTGTACAGGAGCGNTTGNGTCATGGACGCCAGCTTGGCNCCTGTCAGATTCCACTCGTTGGG
NATNGCGAGGTCNAGTTGGTCTTCNGCATCTTTCGCAGCGCCTTTTGCGGCTTCNGCGTCCGCTCCATCATTT
CCTACGTCNCCACTGTGNCCTTTGACTTTNTGGAACGANGCTTGGCCTTTGCGGCTNCGNAGCGCGCTTACGG
TTGCTTTCATNAGNCCCCGGTTCGCGGTCCCGATCCANCCGCGGTCCTCCCACGNNTGGAGATTCTTTGTCAG
CCCCTCCAGTACTAGTAGTGAGTCCGTCTTTATCAGTANGTCAGCGTCCGCGCGCTCGTTCTGCACGGCTANN
AGGATNGCCGCGGCTTCGCCCGTGTTGTTGGATTGCTCNATCGTTCTGGGGAGCCTGTGNGCCCGGTTNCGGG
CGTCNTCNGGTGCGTACCATATNCCGCTGCCTGCTCGGGCGTCCTCGTCTCCGTTATTTATACATGATCCGTC
CGTGTAGATCGTGTTNGTTTGGGTTCCTNGCCCTCGTGCGTTTGTCCGTGGTGCGTTGCGGGGTGTATACTNC
GNACTGTGGGGTCCGTGAACACCCGGAACCCTTCTTTNAGGCTGCCNCGCGTNGTGACGTCTTTNTCGAAGCG
GACTATTCGTCTNTGGTCGGTTGATTTCCTCGGGTTGCGTTCGTCTCTTCGGCTGGGTCTGNCANGGGGTTCC
ATTTCGGNGGNAGGNCCNTCAGNGTGGNTGCNCCTTTCCGGCGGCACTTCGNCGGGTTCTCGCACCCGCTCCT
CCTCTCGTCCTTGCAGGATTCACATGCGCAGTTCCTCCTCGTTTTGTGGCGTGGCGGCATCGCTTTCTGGGCG
AANTCTACCATGTCTTTCACGGTGCGTATGCGGTGGTTCGTTCTTTGGCATTTGGCCCAGGTATCATTGTTTC
GCGATGTCGCCCGCTNCCCGNTTCGCCGATGTGGTGCCACACCGGCATTTGGCCCTTGAGCTCTCTGCTCAGG
GAGACCGGCGCGAAGGCNACTTTGTATTTTTTCGCNGTTTTTAGCATCCTNACTAGCGANTCCGGGAGTTTCG
TNCGGCCGCGTCGTGTTGTCGCCGACCAGTTTTGTAGGAACATGTTCGTTNTGGATGCCTTGTCGCTCACCCT
GTGTGAGTCNGGTATGCTGTCGCCCATGAGCCCGTCTGCGACTTTTGGCCACCTCCCCCGCTTGCTATCCAGT
TGGAGGTATGTTTTCGCTTTCATTAGTTCTATCGCTTCGTTGCGGCTCTTGATGTTTAGGAGTCTCCTTCCNC
CTTTCCCGGTTTCGTCGTGTAGGATGTCCATGCTCACGGCGGGCGTGCTGTCTGGCCACATGAAGTCCCGTAT
CGTTTTGGTGAGTCTCNNCTCTACTCCGGGTGGCATGCCCTGNACCCTNGTNAGGTACTGCGTNCGNCCTCCN
ACCTCCATNCCCACGATCAGGCGTCGTCCTTCTATTGTGGGGCGGCTTTTACTCCACTGTTTGAGTACTGAGG
GGGTCACCCTGCCTGACTCCTCGGATCACGTTGTANGGTAAGCTTATCTCGCCGTTTATTATTACGACNGTTT
CGGCATNTTGATACAGCGCCTTTACCGTGTCTATGAAGTGNTTCGGGAAGTGGAATTTCTCGAGGGTTTTCCA
NATGAAGTCGTGATTTATTTTATCGTAGGCCTTCTCTTGGTCCAGGCATACGATTGCTCCGTTCTCCTCGTCA
GCCTCGCACTTGCTGATCATTAGGTTGACGAGTTCTGTCTGGTCTTCGATTCTNCGNCCTTTCATGAAGCCNG
CTTGGTCCGTGTGNATGAGGTTTGGTACCACCTTCGTNAGCCTCGTGGTNAGCGCTCTCGTGAGGATTTTGTA
GTCTGTGTTGAGGACCGTTATGGGTCTATAGTTACTGATCTCCGTCCTGTCCTTCTTCTTGTACAGTGGGCAC
ATCCACCCTTTNGCGAAGCCCGGTNCCGGCGCGACCCCGTGTCTTTCTATGTCGTTGTATACNGCGGTGAGTG
CCCCGACGACGTCGAAGCTCTTNTTGGGGTTTGGGTCTTTCTTCGCGTCGTCNTCATATTTATTCATCAGNGT
CCTCCACAGCTCGTGTGGNATTCCGTCTATNCCCGCGGCCTTGCCGTCCGGTAGGTCTTTCATTGCCTTTGTG

ACTTCGCGTTTTCTCATGAGCTCGGCCAGTTCCCTTCGTTCTCGTGGGATAGTGTCGGCGTTAGGTGTGGTAG
GACCGCGTCTATATCTGTTCCTCTCTGTTTGTGGTCCGGCTNACNCCTTCCAGTTGTAGAGCTTCATGGTANT
CTCTGGGCGATCGCGGCCATTCCTTGGGATCGCCTTTCGTANTGAGGCGGGTCTCGGTTCGGTNTCTTCAGGG
CCCTTATCGTGTCTCTGGACTTGGGCTCGCGGTTCGTCTTNATCCAGTATTTCCCTATNGTCTCANTTTCTAT
CCGGCTTCTNACTGCNATTTTGTCTCTGGTTCTCGGGTGTTGTACGTTCGCGAGTTGTCTGATACGTTCCTCG
ATNAGGGACGCGTTNATCTGCTTTGTCTCACGTCCTGCCTCATCATCGTTAANGGTGCTTGCCAGGTCTNGTT
CTAGTTTTTCNATNCTCTTCTTNATCTTGGGGATCGCCGCGCGCGCGTGANCCCTCGCGATTTTAACTATNTC
GTCCTTGAAGGTTTTGAAGAGCGTTTGCGCGTTCCTCTCATCGGTACGGTTATATCTCGTCCTTTCNATGTTN
TCCGTCAGCTCATGCCCAANTCTNCTATGGTTTGTAGGATTTTTTTGTCTTTCAGNACGAAGAGNGGGATCGT
CCATCNTCCTTTGCCTACGTATGGGGCTTCGGGGTCTAGTATTTTCACGGAGACCATNCGGTGGTCNGTGTTT
AGNGGNGCNGACTGTATTTTCCATTCGCAGCTCACGTCTAGTACGGCGTTGGATACGTATATCCGGTCTATCC
TGGATTGGATNCCGGNGTTTGNTTGTCGGTACGTGTATCCCAGTGTGTGNGGGTTTTCTCGCCTCCANCCATC
CATCAGGTCGAGGGATGACTTGAGGTCGTGTAGGGCTTCCGTTTGGTCCGCGTGGTCCGGGTGGGGCGGGAGC
CTGTCGAGGCTTTCTTCGACCATGTTGAAGTCGCCCAGCATCAGGTCTGGTTTCTCGNGTCCTTCGCTCGTCC
ACCTCTCATTNATCTCCTCCCAGAAGTCGGCGTTNTCGCGGGGGTTGTTCGGGGCGTAGATGGCCAGGATNCG
CANNTGGTTGTTCTCTTTCCGTGGGACTGTTACCAGCANGGCGCGGCCTTGGACTATTACGTTTACGGTGGCC
TCTCTCCATCTNGTGGACTGTTTATTTAGTACTATTGCNACACCTTTCGCGTTCACGTTGTNNGGGTCGCTGC
TGTTGATAATGTGGACTCGGCGNTCGAACTGGTCGTGTAGGTCTTCTACTCTTTGGGCCGAGAGGTGGGTCTC
TTGGACCGACAGTATGTCAATCCTCTGATCNCGCAGGACTTGATTTACGTGCTGCCACTTGTCTTGCGTTGCG
TTCGATCCCCCNCCTTTAATGTTGAGGGNNGCGATCTTNAGCGCGCTCGTNGCTGTTTTGGGGCGGTTTCGGT
CTNCCCCCCGGTTCTGGGTTGGGGGGTTGGTTGCGTGNGGACTGGTGGTTGTCCTTGCATNTGCGCCGTGGTG
GGGCGCGCTGCGTTCGCGGTTCGCGGGTACATGGGGCCCGTTGTGGGGGGCTGGGGACGTATCCCTTGGGGCG
GGTCCGGGNTGGTCCCGGGGCTCCATAGTCGTGTNTGTGTGACTACCACTTTCGCTGGTACTGTGCGTTCGCG
TGCGGTGGCGGGGCGGTCGTCATTACGCACGGAAACGGCGTTGCTNGTAGGTGTCGATGCTCCTCGGCGTTCG
CTACTAGTGCCAAATCCCGTTGTGGTTCCGCCCGCCCCGNGCCCATCGGTGGTTGGGGGGGTCCGTCGCCGTT
CTCCCTAGGNTCNTCATCGGAGGGGCTGGCGACGGCTNGTCCAGCCCCTCCTCCGATCCGGGGGAGTCGTGTG
CCGTAGCCTGTTNTCTGTATCCTGGGTTAGTAGTTGGGTGGGTTAGTAGAGTTAGCGGTTGGATATNCGGGCT
TGACCTTCAACCAGNGGTTAAGTCGGACGCCAATTGTGACCGGGCGTCGGCGGGTGGACCCGTCGAGGGATTC
GCCTTTCTTCCCGTGTATGGGCAGTCAGANCGGTTTCACCGNAGACTTCAGCATCTCTGCACGGTCGGTCCAG
GGCTAAATAGCCCTCCGTTTGTGTGTTTTTTCGGGAGTGTTTGTGCTCCCTTCAGGCGNCGGCTTTCCTGTCC
CGCNTGCGGTCAGTATATTTGGGCCNTCGCCTGCCCGCCGCCCTCGACTTAGATTGCNTTCACGGCGGGGGCC
CGTTAAATTTGGNTACCTCTCCATACCGAGGGTGTTGATCTGATGCAGGGGTAACCAGGTCAGTTTATCTTAT
CAGGGATCAAGTGTGTGGCACTGTTCATTACACTCCGTTGGGAGCTGGAGCCTTGTGTTCTTCGACTCCTCCT
CCCTGCGTGTTTCGTTGGTTTTATACCAGGCATCTCACATTCATTTTTATGAGCGCGGGGTCAGTTTGATATA
GGTTCGTTCTATTGGTAGGTACCAGGCGTGTACCTATTGAGTACTTTAGATACCACGGCCCCTTGCCCGACCG
CTTTCGCCGCCACGGTTCGTCCGTCCTCCTCTGCCTCTTCCCCCCCTGCCTCGGCCGCGGGTATTTGAGGTGT
TTGAGGTTTGAGCCGCTTCGACGTCGTGTGTCGCTAGGGCTTGGTTATTGTTCCAGCCCGGGATCTTGAGGTA
TTGGCATAGGCCAGCGGGGTGGTCTCGCGCTTTGCAGCAGGTGCAGTGGAAAGGAGTTCGAGGTTTACCTGTG
CCGAAGATGCAGGTGTAATCGAGGTTACGGAGTATCGAGATCCACGTCTCGAGGGCGTTAGTGTTTAGGGTGG
GGGGATGTATGTAGACGTTGAAGACCGTGGACGTCGTTCTTCTTTGTACGATGGAGAGGTTCGTGACCATTAC
TGAGTCCGCCACGTAGCCTAGGAGGTCGACGAAGTCGTAGTCTTTCAAGATATTGTCCCGATTTACGGCAATG
AAGTTTAGAATTTCGGGGGTTTGCAGGATCTTTTTCCGGACCGTTTGCCTGACGATGTTGTTGTTTTCTTCNG
TTGCGGCAAGCGGGAAGTTCTCGAGTGACATGAAAAGTAGGATTTCTAAATATCTGACTAAAACCCTACCCTN
NNNNNNNNNNNNNNNNNNNNNNNNNNNNNNNNNNNNNNNNNNNNNNNNNNNNNNNNNNNNNNNNNNNNNNNNN
NNNNNNNNNNNNNNNNNNNNNNNNNNNNNNNNNNCCCTACCCTAAGCTGAACCTATTTACAAAAGGCATATAG
ATAATAAATAATAATAATAATAATAGTAATAGTAATTAATAGACAGTGTGGAGTAATCAGACTGGGCGGGTGA
TGCGCCATTTGCTCGGGGTGTCGCGGCGTGATGGTATCTGGGCGAAGTCTAATTGGCGTTTTGATCGTCACTT
GTGATCGGCGGGCCGGACATGGTCTGTAGCTGTCGGGATGCGTCGTACGAAGTGCGGGTGAAATGACGGGGTT
GTGGAGAATAGCGGAGGAATAGCGAAGTGGCGAAAGACGGCCATCGGTCGCTGAAGAGCGGCGGAATAGCTGA
TATAAATGCTCTGGTGGACTATAAGAGAAGCCGTTAATTAAATGAGCTGTGAAGCTAATGACAAACCGTTAAT
ATGTATGCAGCCGCTGGTGCGGCTCATCATCTCCTCTCCAGTTAGATATTGACTGTCCTGGTCAATCCATCCT
GTGGCATTCTCGTGGCGTACTAGTAATAATACGAAGTCCCCGATCAATCGTGTCGTAAGGTCCCGCGCCGATC
TTCTTCTTGAACCACCATGTCCGACGCCGTGTCCCGAGCCAAAAGCGACCTGAAGCGCCTTTTCACGCAATAC
CCCAAACCTGACGCCAATCCGTCCGAGCTTCGCGAGTGGGCCTCCGTTTCTTGACCGAATTGGTAAGTGTCGC
GGCGCTCCTGACTGGGGGACGGCTGACGAGAAAAATTTTCAGGCTGCCTATGACTCGGCGCTAAACGGCGCCC
CGCGCGATGACGAAGTCGTAGGGTGGATGATGGAAATTCGGCCTCGGATGGTCGACGCATTCGGGCCCAATTG
GCAAGCGGCGGTTCAAGCTGCTCCAACTGCGGGCGCCGCCGATACTCCTGCGGTACCGGAGCCAGCAGCTGAG
ACGAGTGGAAGCGCCCGGCGAGTAACGCGGGCGACATCGGCGAAGAGCAAGGGAAAAGAGCGCGCCACTACTG

CGCCAGTACGGTACGGGGGCGCTATCGCCACCTAGGCTGCAGCAACGAAGGAGCCGTCGGCCTCCGCCCGTCC
CAGGGCGAGACCTGCGAACCCAATACGACGCAGCAACCGCGCCCGGGCCATGGTGCCGAACCCAGGTCCCAAG
GTAAGGTGCCCAGACGGCGCGCCAGCCCGTACTAAACAGCGACAGTGTGACACCTGCGCCGAGCCGGAGGACT
GCGAATGGGACCCCCGGCGCGGTCGGTGCGACAGGTGCTTCGACGCCAGGCGAGCGTGCCTCTTCGACGACGT
GAACAGACGCGAGGCGTGGGTAACGGGGCAAGCGGGTACCCAAGCGGACCAGGACACGACGCCGGCCCAACCA
ACCGGGATCGCGACCGCGGCTGAGAGCGTCGCTGCGCCAGTACCGCCAAGCGATGCAGCGCCCGCTGAGAGCG
ATAACGAGGGCTCGGAGGAAGCGGGCGCGAACCAGGACGAGCATAGAGCGGGCGCTGGGAGAGGGAGCCAGAG
GAAGAGCGAGGACGAGGAGACGGAGATGGAGACGGACGACAGTGTGNCGGTCAAGCTNAAGCCGCCAACAGCC
GCGGACCGCGCCAGGCACGCCAAGCGAGCACGCCGGGACCAGGCCCTCGGGGACGAACGGGTAATAAAGACTC
CGACACCTACGCCCAGCCAGAGCGACGACGAGAACAGCGACGGGGGGGAAGGAAAAGGAAAAGGCAGGAAGGG
AGGGGAGAGTGAGCGCGTGGCGAGGCCGGAGGCCTTAGCGGAGGACGCGGGGGGCACCGCAAACGAGACTCGC
GGCCCTCGCATGGAGTCGCTTCCTCGGGTCGGAGCCCGGGGGGACGCAGGGAAACACCGTGTCGTGGAGAGGA
AAATCCCCAGCCGATGCCAGTCGCCGGACCGTCGAGGTTTCCTGGAGCGCTGGGGCACGCGAGGACCGACTCC
GGGGCGGGACGCTCGCGAGACGACATCGACCACGACCGCCGAGTCGTCCAACAGCGAATCGCCGAGTACAGAG
CCTACATCCGGGTGTTAACGACGACGTTGGCAATAACGGAGCAATACGCGCGGGCTCTCGAAGCCGAAGCCGA
CAAGACTAGGGAATCGGAGGACAGCGAGTAGAGCCGGGAGGAACAGGAAAGGAGGAAAGAGGAAAAATGTATC
ATACATCTCTTATGCACGTAGTATCTTGTGCTTGTCTTTTCTTGAAGTCGAAATAAAGTGTCTATGAAGATGA
CGGGACTTTGAGGACTAAGGGAACGGTACGGTAAACGGAGAAACACGTAGGGGAAAAATAAGNAGGAAAAACG
AGGAAAGGCTCAGTANATCATCATAGCCGGTAAAACCCCTGGGGTCTTGCGCGCTCGNTCGTANGGANTTGAC
GCGGCTGTAAACTATAGAAAAAGAAAAGAGATTAAGTGCGGGTATATGTNCGGTAGTTAAGATG
>Cligib1_Cluster46#LTR/Copia
gttaaaattcaacgctctaactgtaagtgccctgcttacataattacccctaaaccggcttccaattaaggaa
agcagttcacagctgatcccatcataaatggatcagcttctctactccggtccagcttTACtctggtccGtctt
cCtttacTctgATccctttActctgggtcagcttccTtgtAtttgtacatacgtagtTtActctaTtctAccc
tgGattaccccgggtatgTAgtaTatAaaccccctctGAAcaaGAggCAaaTAcaAgttGtTtcaCacatctt
acCcagtagcAgTatcgtctCaGTcaActaaccAagtaagtAGCcaGttcCagctgtgcAcActGggtgcagc
TcAacaggttatggGCccTGgtgccaCATtcTgagcGCcTgAAacatgGCTgcatAgcgcctgAaATatggct
gctcActGTctgAaacattgAcagcagAatggcTggcActtggttgccattattatgcgccTgaAATaTggct
gcaaattAtggCTTgacTAtgggtTtccaaGacaaaTTgccgcgcctgcaatAtggctgcagctaactgtCTg
gaaggAaAtAtTgtCaAcctTAccacacatcacacgtAtcATtTTgcaTgattaaaaaTgtgtaAatggtgGt
cCtctTaActccGcActCTTGcagtTaAtgAccAtacatccgTTgacggtGgTatcgttgtAcaatAaatccG
cggacttGgtctCAccaacCaatgtccacTcTtTgAcAtATcagggatggcTtctaTaaccgGttatcaccat
tAtagaaTTtCacaacctGatcacTgtcatTgACAcCcaCcataGactagaTctCAtcaAtctatGTCtTtat
cTgCCtgaaatatGgcactGgtccgCcGattGAtgCgcctgaaaCAtggctGgaTggcatTTcTTtgattaTG
atGAaatgaGgcTAatgTcactcccttaCTAgAcAcCctCAaANcccNcaaatcttcaaatttacCatcctta
TcatcgTcctcatcgttctcccaatcatcacaAtgtCgtcctCcAccaCcGtcgataTcaaatctgTtcctcA
attcAccGgcaCaAaCtggaaTgagTggTcccgcaaaAtGactGccTTccTtcGctaccgtgcActttggcaa
gtaacTCaagGaGctgacattcgccctGTtGctcctagAccNACagTgggAAccgacaaTgctgtcaTcccgc
cNactcaaactgaAatcgatgatcatcactacaaacTcgtcgAatgGctgAagtccAatgAcatggcacatgg
atCcAttgacCTcactctCtctcctgacATtAgtgCtgccaacgcctctcaaAccacTtCcCgtgccttatgg
gAtgcgctctCtgagcgctattccAaAcaaGgAgcttCacagATatttGcgGacttcaaATctgcccttgcaa
tcaggctTtcaggAAcagaaaaccctgagcctgaaaTaATgGcTatggCaAccctgttTcaAtCgccttgatgC
ttCaTcagTtatcCtgccaccatttgtcaaagGcatgAtcctccTtgctgCcatcCCTccTaGatgGgAtaCT
gttGccTcccAagccctccagaaCctaagcActgacaacatcAatcttGattcggtccgatctgcaattATgA
ctgaatAccaacgtcGgatagCtAtTaatAaCcgaccaattgtcaACaaggtttcagggAttaagcgtAAagg
caataaccctaattggaaacaacaggcacAggAaCaggcacCTgagAactcccCcTcaCaagggaattcgtgg
aagGgtAaggccAAagCccgcAactcCcgcaGtggtaaCcgCgAcaAtcgcaaaCaaagAcgaCAggcaaaag
aaGtTgctGaaaattcagatgcTgaatCaaacacctcacatcagcacCATcaTcaccagaacTTcttctCcTc
ggcATataTCacggAGgtAaTcGagcCacCtcCcatcACcatAAacaCcaagccGctcgaacccaAggcgcgc
TCTCttCtgaagcgCaTACAtgatgGtcctgcCcagACcTacatggGtgctacaccTggaaaggTCCctagca
cctaTcCaAcattcacaaaagcgCgcTcaCtgGcTtcAcgccTatcttcgAcacCcactaCcCAgAccctcaa
aagGcTtgagTccatttatatgGccaaaccggAatctgaaGtccgactcccggaaCcGGaagaGcagCAagCT
CcTCcTCAACcTacTaCtccccgatcaTCttctcctAcCccTgGAcCatcTaatcggGctccgacTCCagaac
gtgcacCatcACCTccacgaTcgactACccCtgatggccttAgcactctcttGAtgCaagaAgatgaGTTtga
gaAttggGttgataactactgtGGcaGTGatgAtgaAggcgTcGaatatgtcgctTacgaccatGaGgcttGg
aaccctCcgccTGaGAaacggcgcaaAgtctctccAtCtccTGcaaaggatgAtgGgaatacAgTTTccATgg
GtgaAgAatccgAggaTTgggacaacaggtttggcggtcaaTcgtcagTaAtCcCtccTccacttccaAtaTa
tgaGACtgaccacTCAatTctgcagggcgctTttcgactAaatCatcgTtgcCcccTGTatatAgaatataaa

gaTTgtgtgTcggtGtCTcgtggccaAaTggcCNAaatatatcatcaCtTtGctGgctGtaataAatGcaaAg
gaaaggaaattgagattaATcaCaAatggttgaTtGactcaggaGCaTccctcCattTcactggcatCTTaga
tgattttGTggaaTgGtccaaaatgGaaCgTCCAaTTccTgtctaTactgcGattGagcgcatcgagATTgac
AcaaaagGaaccGTtAtcctAgaAAatgAagtCctacAaacAgATgggaCataTaaAatAcgcaaCacgcgtc
tgtccgtctgtccccTGtcTatTaTcaTccaaagTtgaAcaccCgcattctaTctatgGgTGaatTccTGaaT
CaagggatgacaTtAaatGctAcAtCaAaAgcatTaAtaTtcAacaAggAcaaAcaacgcTGcatctcTgctg
aaaagcgatttgcaggggAcaccAcaTTcTgggtTCaaggTcgtgtttTaCaAAccgaccagattaaaacaTc
cCtctttgtcaatActactgAcagctatgaaaTaTggcAtaGacgccttgGtcacccgtcTaaagaagtgctc
tccaAagcTCgCaaAcacAtTaaagAcTttccacaGgGActaaTcatcccaaataaaccAcctatctgccCtg
gctgcgCacTcggAAagatgccatccTCAtcttTtaaacgatccGccAcacgcgccaagaagCcaTTcGaAct
gattcAttcgGAtttaAagAgcTtcccTgtgaaatcttatcacgggttccAttacatCATgacatTcctTgaT
gacTacAcaTcAcatgTcTgGgTcTctcaccTtAaaaggaaatcaGAcgCAatAcacCAgCtgcgaAacttca
atGcagcTgttAaaaatcAatAcAacAccAcaatcaAacgGtgGcgttcTgaTgcAgggGgagaaTtTaaaTc
ccaggaaTttaTTgaTgaatTAACaacaCtgggcaTtgaAATCgaAcaatctgCtccCcacacAccacaAcaA
AatGGAcGagCagaacgaatcaAcagaACtattatggacAAGgcNgagtctAtgcGtcatcAcgCatgccttc
CtAgttcttggTgggaATtcgcagtggaacaagcggttcacCtaTataacCgcacNcCtAtGgtgcGcctgga
aTgGcgaAcTccatatgaAGtCttaaAgggtgAAaaaCcctcactTgAtcAcAttcGagtgtttgGcTgtgca
gcAtAtGtttatctGccAgcTGAaaaacggGTCaataAacTTGcccccaaAtccGagATgAtgatatAccttg
gtaaccctgaaggaattaAAgggTttcgcTttatgcgaATtaAAAaTaacacactgTtcattggaaCAacAGc
TatctttgatgaagaaacacTCcCAaaatGtcccaataAtatcaCccctagagAtcttcttcctcCaAtcgaT
gattatctACctCcttctccacctccttctaccccctgactCagAtTcagacAAcgacgaTctgaatgtCAggG
AGgaaatTgatgatgacGatGTtatcAgaaataAGGTaccGaaggaagtaGGAtcgGaAtCCgatggcAcggG
aTCaAtaGctcctcgtGAatcaacacctGaAccAGTTGAtGAtGAGgaTGaacCcgagCCaGaaatcattGTC
ccaCgaagatcTggccgagcaCgtaAagttccaAacagAcCtggaaatattTaTGgGgaaaAGaaaTtaccct
cAcaaaTagAaCgtGacAttGCtaaagatcGtgatTgGcAagacattgttGgAgaaccaaAccccccctaacCa
ggatgAagTacagctcatcCggatATGTAgggagggagGggGAGAattaatgTctttcctactCtcaaaaGct
gTTccaGTccatGacGaaGttcCtAcTcaCTtccGAgAcATattcAaGctcccAAaagagcttcAaaATGagt
ggatgaacgccTGccaGgaggAattagactcccTcCgtaAgCgcgacgttttTGaaaTtTgcGaactaccTcc
TgGacGaagggcCattcGcaaTcgatGggTTtttgctgtCaaatCtgatggcCgaaaGaaAGcccGccTtgtA
GccaaagGtTtCTcacaNatNgagggtgtggactttgatgaaataTTctcacctgTtgtccgctaTgagTctg
tgcgtctcatGaTtgcAcTaGcagcCCtgaaaaatTgggagaTcATagcActtgacgtccgaactgCcttccT
ctatggcaaacTggatgtGaCaatctacatgTaccAacctgaaGgcTtCgtcCaAaaAGGccaTGagcgcAaa
gtccTgcgTCtcaaacgtgccctctatgGtctgAaacAaGcaGctcttgaatGgTgGAgagctcTaGttgcGt
CtATgAaagagatgggctttcaacgcaTTgtttcAgatgcaggcatcttcctccATcgctctaCcaCtggaAT
catgGttatTgCCatTaTctatgtagacgaTGccttattcTtggggcctGatcGaGgAtactTaaacaaGaaA
aaGagAGagtTcATGcagcaatgGgaatgcAGggaccTTGgTgatgctaaagaaTtccTtggcaTgcGCatcT
ggcgaAagaaaaaccaGatcaaaatagaTcagCaaccttatctgatcAaagtcttAgaGcgctTTgaCAtGaT
aaaTgcatctaCcACcaagacgcccctacCTCaggGAtaTctaccagccATTaacaatggtCcagtgAATcCc
acCtTacgCagcaAatAtcaATctgtGaTaGgGtCccTactcTatctcAtgtTaggaAcTcgacCtGacaTtG
ccttCGctgTgaTcAaaATgtcGcaAcacAgcgcaAatcccActgatgaGcacctgaaaAAGgccctcaacaT
cTGccGtTaTtTAGctgGtactCaAgAcTAtgCcttagTCtaCAgtggtgAaaAAgaagaAggCctaatTGca
cacacaGatTCcgactGgGcctCtGactCtaccactcGGaAatctAcatCtggttTctTGtaAcacttgCtt
ctGgtgcTgTgAcatggAcatcaCgacaacaGAAaacaatTgcgttatcctcCactgaAgctgAatacatggc
aATgTcTgAcAcAtgtcgacaaGtcattTGgttacggtcaCtCtttggtgaacTtggcattaAaCttgacaAA
atccctatTatTggTgAtaatCAAGgaTcCatctTcAttgGctCCAatgccgtccaAgaaagAcgaAccaAAc
ATatTgatatcagatatcacTAtatTcGNgAATgTgTtgaagaAGggAaaatctccatattcTtcactgaagG
aGagAAcaaccctgctgatgttctaacaaagaacttgggacatattaaatttaaacaatttatcccacaactg
ggtcttgaattttattcacctagtcaaatatcgctttagcgcctgcaatatggctgcataaaaagcaccgctt
tatgcctgtaatatggcagtacaaagcgaggggggagtgttaaaattcaacgctctaactgtaagtgccctgct
tacataattacccctaaaccggcttccaattaaggaaagcagttcacagctgatcccatcataaatggatcag
cttctcttactccggtccagcttacNctggtccgtcttcctttacTctgATccctttActctgggtcagcttc
cTtgtAtttgtacatacgtagtTtActctaTtctAccctgGattaccccgggtatgTAgtaTatAaaccccct
ctGAAcaaGAggCAaaTAcaAgttGtTtcaCacatcttacCcagtagcAgTatcgtctCaGTcaActaaccAa
gtaagtAGCcaGttcCagctgtgcAcActGggtgcagctcaaca
>Cligib1_rnd-1_family-1161#TIR/MITE
AGAGTGTATCAGTTTCAATAAAAGTCGAGAATTTTCAATAAATGCTAGCGACATCTCAATANATTTGAAACTG
TGGAGCGGGTGGGGCAAGTCGCCTGCAGTGACGAAATTGGAAGCCTTCCCAGGCCCTTTGGCGTTCGTCCAAT
GCCCAATCCACTCGAACACCTACCCGGCCACTGGGAACTCAAGCTATGGTTGGTGTGCTCAGACTGCACCTAA

ATTTCTCCCTTAACAGTGGTANTATGAGTAGAACAGCAGTGTCAACAATGAAGGCAAGAGCGACGGCGGAGAG
CGGGGCCGGGGTGGCGCCAGGGCATTGCGTCACATGNCGCAGCCCGTCAACGAGAGAGCGCGCCGCANCTCAA
TCAAAGAATTTCCAATAAAAGTGTTAACTGATACACTCTA
>Cligib1_Cluster98#LTR/Gypsy
GTGTTAGAAGTGGAGATGACAGTACATTGGTACTGACAGATAATCACTTCATCTGCGCTTCCCAGCACTCCCT
AGTCCATCTGTGATACACAGGTTACATAGATGGACACCCAATAGACCGCATTAGACCATTAGTATGGTGGTTC
ATAATGCTGTATATTCAAATTGTTGATATCTTGCCTCCCAGTGCAGCTAGAGGCCTAATTGTTGAATATTCTA
AGTCCATAATTTCAGTCGCGATATAGTGGACACCTGAGTTCACGCCTCGGGTGAAACAGGCCAAACATGTACA
AAGGAAGTGTAATTCCTACTATGTCCCGGAGTAGTGTTTGCTACTCCCTTGACAACAGTATTAGACAATCTCA
TACTTGCATTCTATTATCAATATCAAGTGATTAGAGCGTCTATCAGAGGGTTATCAACTGTGTCTGGCTATAC
TCTATAGCAGATCAGTCAGTGAACACAACTTGTCAGTTTATTAAAATGTTCCCTAATTTTGAGCTTAATGAAG
ATATCTTTATGCGTGTGGAATGTCTCTAGGCAGTTTCGTAGGGCCGAAAGGGTTTTTGAGGTATGTGATTGAA
GCTGTGCATAGTAGATGAAGTCGATGAGGGCCCGGATAACAGTAAGAACGTCATCGCTGACTGCGCCGGCCAT
TACCCCAATGAAAACTTTCTGCATTTCTTTAAACTCCCCCCCTGTCCACTGAGATACTGACGAAATCCCTTTT
TTGAAGTGGCGTAATCCAGGATAACCATTCATTGCCTTGAATCGCGCATCCATCTCATCCACCCCGATTATGC
TTGTGCACCACAAAACGAGATGGTCTTTGAAGACGCCTTTGTGGAGTTGGTGTAGGATGTCAGGTGTGATGCA
TGAAAATATGTCTGTGTGAGGAAGGTCTGCCCAAAATGGATTGTAAACAGCACGCAAGCCTTGTTCTTTGAAC
TCTGCAGGTAAGCGCCTTCCCTCGCGATGTCTCCGTCTGTGTTTTTGCAAAAGATTGATCGTTTCGTCCTCTG
TGCGCCATAGGTTTTCGAGGAGTTCTCCGCTCTTCTTAGGGTCAACTTGACATCGCGGACAACGATTCTCCAT
GCAACAGGTAATCAAACACTGCTCGGGATAATCTCCAAGGTAGGCTGCAAGGATTGGAAAAATGCGACGGATG
TACGAGTCACTGCAAACCATCTTGACTCCATTTTTTCCGGCTTTGATAAGTGGCGCAAGGAGCTTTGACATGC
ATTGGTGGAAAAGACGGTAACCTGCGAGGGACCGAGATGAATGCTCAAAGCATTCGAGTTTTGTAACCGGTAG
GTATCCAACGAGAACTGTGGCATGAGACTTGAGCTCACGTCGTTTTTCTTTAGAGATATTCCCAATGGTCAGG
TATACAGGCCATGCTGATTTATCGCCACGAAATTGCGTAAGTTGTGTCTTGTCAGATGCGAGGATTACTGGCG
CAACAACCGCTCCCTCCGGCAGTTTTTCCTATGGCAAGATTTCTGTAAGCATCCGCAACCCCAGCACTAATTA
ATATTCCATTTACCTGCGTATTCCACCACCAGTCGCCTGTCCACGCTTCATCAAATATTCGAGTTTTCCCAGC
TCGGTCTTTATAAGCACGTTCCGGTTGGTAGGCAATATGTCCATCAAATGCTGGATTTCCCATAAGCTCCTGG
ATACATTCAACAGGATCTCGCCGCCAAAGTTCCACCTCTTCAACCATTAACGACCCATCCTCTCCTTTTCTAT
CGCCTGTGGTCGTAATCATTTCGCAGACCCACTCTGGGCCTGTGGGAAGCTTGTCGACTTTTTTCATGAAACT
GTATTTATTGTGGAATGATGGACTGGTGCGTTTGCGAGTCTGTCAAAGTTTATAGATTTTTATCGTTTAATTG
GATATTTTTCTATGACTCACAATGGGTAATTTCAAGAACTCATCGATCGACGTCTGGCCAACATTGTTCATCA
ACCATTGGGCTAAATCCCATTCCTCCACATTGTCGAAAGGTGCCCATGCCTCCTCATGCTCCCTCTTTTGGTG
ATCATGCCATTCCTGGAATTTAGTTTGACCTCGACCAAATGTTTTAGCGGCTGTTGGGTAATGTTCAGTCCAC
CGGCTGGTATTTCGTGCGTCGTCCTCATTTTCAACATCCTCGATTTCAACTCGGGTACGTTTGGTAGGTGTGG
TTGGGTTGTGGGATGTATTTGGTGGATTGAAATTGAAGTTAGCCTCCAGAATCGTGGCCCCATCGTCGTACAT
CTCATTACTGGGGCATCCATCCTCTCCATCATAGGTAGCATTTGGAGCATTTGGTGGTGCGCTGGCGGGTATT
TCTTCAAGCTTGGCAAGTTCTTTTTGCCATGCGGCATAATATGTTGGTGAGTGGCTGATGTGTCGATTGACTG
CACTGAGTGTCGATTTACTCTGGTAGCAATATTTACACCGCGGTGCATTTGGGGCCCGGGGCATTGTGGTATT
CGTTTGGACAGTTGAAGGTTTGACTCCTATCACTCTGAATGTTTTGTTGAATTTCCAATGATACGGTTATCTT
GTTGCCGTGATTGTACAGATCAATCAATAGGAATAAATTGACATATAAACACAGATGCGCGGTGAAACCATCC
TGCTTCGGACGCCCACATTCCAAGTTTTATTTTTCCTCAGCTGCCCTTAATGTCAAGCACACCAACCTCAGCT
ACTCAACGCAAACATAAATCTAATACAAGCCGAGCCCCAAGCAGCCGAACGTCCCGCGCTACTGAAAAAATCA
AACATCAAAGTATGTTTACACCATTCGTCCAGTCCTTACTAGTTCCCCGCTGACAATTATTTTTTAGTTGCTC
AGGAGGAGGAGGCCGCTTCTCAACGTAAAGCTGCATTGGTCAAGAAGGCTCAGTGAGCCAAACGCCTCCAAAG
CAGTGATATCAATGAGCCAAACTGTTCTGATCAAGACGATAACGAAAATCAGCTCGAGGTACTCCTAAAAAAA
TTTTCATTGATGTTTATACATGTTTCTTCTCCAGATCACCTCATGGATTGTCACCACCAGGACATCCCAGAAT
CAGAATAAGTCTCTGACGAAGTCCAACAGCCGTGTTCCGCTGGCTGATACCCATCGACTTAATCTGGGCAACG
GTTCAGATGACGATGGCCTCTCTGGCTCCGATCACAAGGTGGGTACTCTTGTTTGATTCCATTTCTAAGCCGA
TACACTGACAAAAAACTATTACCAGCGTGGCAGGCCAATGGCTCGCAGCCAGCGTTTTACGCATGAACCTCGG
GCCAACTCTCAGCCTCCAGGTGGCCGCCCTACACAAAGCGTTTCTCCCAACCCATCTCTCGACCGTGACGACG
AATCCGTGGTCATTACTGGCTCCAAGCGTCATGCGACCTCAGCCGACCCCCCTCAAGTAACCAAGACTCCACG
GACTAGTGCCACTGGCAGCCGCCCAAAGGCTGCTGACTATGACAATGTAACACAGGAAGTACTAACCCTTGCC
CAGAAAATTTACCGGTGCATGATCTGTGCCCAAGAGGGGTTTCCCGACCACACCGCCAAGACTACTCTTGCCC
GATCCGCCTGGGCTGAGAGTATCAAGCAGACTGGAATTGACCTACACCTAACACCAGGCCTTGCAAAACTTGT
GCGTGCAGCACACCCAACTTTACCACCACCGCTAATTACAGTTAATTTACCAGATCACCGCTCGTGGCTCACA
AGTCCGCGGTGAGTTGAAAACCAAGACCCGCCCACTTGTTGAAGCATTCTATCGTTTCGAGAGTGGAGAAAAC
AGAAAAATTATCAAGTCGAACCGTGAGATTGCTGAGAGACTCAAAGACGGTCGTGGCTTTGTCTATAAGGTAC
ATCCCCCATCATTCACACAACACTCTTCCTCAACTCCTTTATCAGGTCCTCAGAAACAACGGTAACGAAAAAC

AAGGCCTCTATCAAGCGAAGATTTTCCAGAAGGTCGTCAATACGATGTGGTTCCACAACCGCCAGGATGAAGG
TGTAGCCTTCCCAGAATACTTTAGGCCTTTCCCACTCCACGGTTTAGCTATGGTCATCACCGTGGTATGTGTT
CCAATTCAGGTGGACGTTATTTAACATCAACTAACATTATCCCTACTTCATGAAAGGTTGAATGCTGCATCGA
CGAGTGGATCACCGGCATCCGAACTGATATCAACTTCAGCGGCCTCGAATATGGGCACATCTATGAGGGCCAC
GTCGCCTCACTCCAGGCGTTTCAACGCCGCGCTGGAAATTTGGGTATCATTGATAATATCTGCATTAAATTAC
ACAACCGTGGCTGGTATGTTACATTTTAATATGTAATTCATCCACGTCTGACTATGAAAAGGTTCCATGCCGG
TGCACAACCCATCACCGCAGTCAAGGAGTTGCCTATTTCCGATGCCGCCATGGATGCTGCTATTGCTGAATAC
AAGGATGACCCAAACACAGAAACCGAAGCCGAGAATGGATCCGACAGTGATGAGTAGTTACCATTGAGCTTGA
TAGTATGCTGGCATTTGTTATTATTAAATACTATTTCAATCGAAATCTGAATGTTCTGAATACCGATTGTTGG
ATATCCATGGCCTCGCTCTCAATGAGTATATATTAATACTGCCGATCCCGTTTGAGCATTGTAGCCTATAAAG
ACAATATCATCATTCCAACCTCTGAGAGCACTCATATACATATGGTAGGACCACTCGGATACATATTCTATAT
GTAGATATACAACTATGGAACTTCAGGAGCTCTATAGTGTATTGAAAGACTATAGCATATATTGTAGTCCTAC
AATATTGCTTTATAGCGGGTGGTGAGAGCTACAGTGAACTAAATAACAGGGGGAAGTCTACCATGCAACATTT
ACAACATTGTCTATAAAGTACTACCTCATCATCTCACTTTTTCCTGATGTAGACCAAGGGCTTACAAAGGGGG
TAATTCTAATTCCTTGTAACAAAACGATCGATGCCCTAGGAACCTCAAATCTTTTATTCCGGGAAGTCTACAA
ACGATTTGGTCTCCCAGATAAAATCATCTCTGATTGTGGTCTCCAGTTTGCCTCAGCAGTACAAAGGGAATTA
GGACAACTCCTTGGCATCAAACTCACATTCAGTATGGTGTACCACCCTCAAACTGATGGAGAAACGGAACATG
TCAATCAAGAAGTAGAAGCTTACCTTCGGATATTTTGCATTAATCACCCCGAAACATGGGTGGAATTCCTCCC
TACCACCGAATTCACTCACAACCAACACCTACACTCCGCATGAAATGCTTCCCCATTCTTCCTCATGATGGGC
TACAACTAAGGCATTCCACAAGTGCGGGTTACCTGCAGATCCCTGCGGGTAGACCCGTGGGAATACTCGTGGG
TATGCCTACTCACGGATCTGGGTCATTGGTGGGTCCGGATCTCTGCGAGTCTGAGTATTGATTAAGGCATTCC
ACAAGTGCGGGTTACCCGCAGATCCCTGTGGGTAGACCCGTGGGAATACTCACGGGTATGCCTACTCACTAAT
AAACTGACAAGTTGTTTCACTGACTGATGTGCTATAGAGTATAGCCAGACACAGTTGATAACCCTCTGATAGA
CGCTCTAATCACTTGATATTGATAATAGAATGCAAGTATGAGATTGTCTAATACTGTTGTCAAGGGAGTAGCA
AACACTACTCCGGGACATAGTAGGAATTACAGTTCCTTTACATGTTTGGCCTGTTTCACCCGAGGCGTGAACT
CAGGTGTCCACTATATCGCGACTGAAATGATGGACTTAGAATATTCAACAATTAGGCCTCTAGCTGCACTGGG
AGGCAAGATATCAACAATTTGAATATACAGCATTATGAACCACCATACTAATGGTCTAATGCGGTCTATTGGG
TGTCCATCTATGTAACCTGTGTATCACAGATGGACTAGGGAGTGCTGGGAAGCGCAGATGAAGTGATTATCTG
TCAGTACCAATGTACTGTCATCCCCACTTCTAACACGGGACAGTGGGTATCTCGATGAAATGCGGGGTGTCTG
GCTCCTGGATCGTGATCATCTGCTCCTCATTGCTGAAGTTTTTGATGATGCTACATGTCAAGACGTCGAAGGG
GCGACCTAGGAGGAGGTCATACGTGGGGTTTCTGATGACATGGATCTGCATGTAGAGTTTGATGTCCCCAACT
GATATGGGAACATTCCGCACGAGACCTAGAGACCTGTCGATGATTCCGTTGGCCAATTGCATGTTGAGCTGAA
TATTCTTGTCATAGACAAGACCTAGATTGACCATGACATCCTCGGTAGTGATCCGCACGGGTCTACCGCGGGT
ATTTTCCCACGACCCGCACCCGCACCCGTGGTGCGGGTGCGGGTACGGGTACGGGTTACCCACGGGTTTGCCC
AAAAACCCCAATACCTGCGGGTTTCGGATTTATCCCACGGGTATGGTACTTGCAGACCTCAGGGACTAGTGTC
CAACATGCCCCCTAGTTATGGGAACCTTCAGGGCTATCAACCTAAGCTCTATGACCAATATAAACTTAATAAT
ACCTCTGCTGCAGGCTCTGTGAGTGGTGCGAGTTACTGTTTTTGGCTTATTTTGGTGAATATTTAGTCAAAAT
GTATGATTTTTTTGCAGATTTAGCATAAAACAGGGCCAAATAGTGATAATCGCACCCGCGGAATAATAAACTG
ACAAGTTGTTTCACTGACTGATCTGCTATAGAATATAGCCAGACACAGTTGATAACCCTCTGATAGACGCTCT
AATCACTTGATATTAATAATAGAATGCAAGTATGAGATTGTCTAATACTGTTGTCAAGGGAGTAGCAAACACT
ACTCCGGGACATAGTAGGAATTACACTTCCTTTGTACATGTTTGGCCTGTTTCACCCGAGGCGTGAACTCAGG
TGTCCACTAAATCAGACTGAAATACTGGACTTAGAATATTCAACAATTAGGCCTCTAGCTGCACTGGGAGGCA
AGAGATCAACATTTTGAATATACAGCATTATGAACCACCATACTAATGGTCTAATGCGGTCTATTGGGTGTCC
ATCCATGTAACCTGTGTATCATAGATGGACTAGGGAGTGCTGGGAAGCGCAGATGAAGTGATTATCTGTCAGT
ACCAATGTACTGTCATCCCCACTTCTAACACGGAATTACCCGTGGCATTACCCGTTACCCGCAAAACGGGTAC
AGGTTTTTGTGGGTTTGCAAAAAGTGTACCCGCGGGTAACCCGTACCCGTACCCGTGGGAAAACCCGTACGGG
TTGCATTACCCGTGCTAATCACTAATCCTCGGACATTGCGATGATCTGCAAGCCGGGATCTCCTATGGCTGCG
ATGGTCTCCTTACCATCTACAAGAACTGGAACGGCACGGAGTGTGTGTGATTCCTTCGCCACTGGAGTGTTCT
CAGGCGGACTGTCCCTGATGTGTGCGAGGAATATATCATTGGCTATTAAATTAGCTGGAGGATCCACGGGTTG
GCTTGGCTCTTCTGGGAATTGCTTATCCAGTTCGTCGAGCATGCGAGCCGCCATATTATTATCCGTGGACCCA
CCCCGATGCGGGGTGATGATATCCTTAATGGTGGCATGAAGAAGTGGGGAGGCCGTGAACAGATCTCCAATCG
TTAGCTCAATTTTGGTCGCCAAGATGCTCCACACTACTTTGTCTGCGACATTCGGTTCCTGCTTAGGCGTTCC
GCCTCTCCCCGCTTCCAAAGGCAAGGCACCCGCATCTGAGGCCCCCACATGCAAGGTGGGACCTCCTAAGAAA
GTCGCGACTCCCTCCACCTCTTGCATTGAGGAAGTGCCTGATGTTGATGCGCCTGAGCGTCCCCTCCACCCGT
TTGCCAGTGTACGCGAAGCCACGTACACCCCACCGCATGAGAAAGCCGCTGTCTCAGTCGCAAAGAAAGCGAG
GGATGTGCCCCCCGCATTACTCCATCAAATAATTGTCTTGACCATGTTGCAACAGCCAATGTATCTCGTTGCA
GAATCTGCACATCTCCAGGAACCTTCTCGACAACAGGGGTGGACGGCACTACTACCGACATGAGGACTTGGTG
CGGGGTGTCCGCATTGCTTGACAACTGCGTGGATGCCTGCTGGTTGGCGTTGCTATGGTGCCATGCATCGACA

TGTTCCTTTAGTGAGCGTCCCCTACCATCGGGGATCTCGGCTCCTGTCCCTAAGATCAAGAAATTATCGCCAC
TGCGCCGACGAACTTTGCTGTTGTTAATATATTGGCTTAGGATGTCGCATCCAGCCATCCAGTGTCCGTTTGC
TCCACAGAAATTACAGCCTCGCGCCTTTGGTGGCTGACGCGAGTAGGACATGGACACTGTGGGAGACGCGGTG
GGCATCTGCACGGGTGTCTGCGCATTTGCCTGTACTGACTTCAAGAGGGTCACAAACTGGGAGACCAGTGTGG
TCATATCGTCACTACTGACTGCAATGGATCTGGGCTCAATGGGAGTGATGCTGACAGACTGCGCGCACTGCAA
AATATATTTCGCAGCATTGTTGATGTCCTTGGTGCTGGGGAGGTCATCGACTGCACGGCTCTGATATTTTTCT
GTGGCATCAAGGCGATCTAGTACCTTGTTCCATAATTTCGTTGGGAGGCCACGAGCGAAATTGTGACTCTCTT
CGCGCGTGGATAGCCTGAGTTTCCCCATCAGGAACATGGATGCGAGCTTGAAGTTGCGATAGTATGCAGCTAA
CTCAGTGACGGATGTGATCGGAGACTGTGACTGGTCCATGACCATCTGCTCGAGCTCTTTGACGGAATAATGT
TTGTCTTCACTGGCGCCTGGATACAATGCAATAACAGCCTTAGTGAAGTCCTCATATGGCCCTGTCTTGTAGG
ATGCCAATCCTTCCCATAATTCAGCTACCTCGTGGCCAACGTACTTGGTCGCTTTCTCCTTCATGATCTGCAG
GTCATCGATCTTAGCTTCTTCCAAAAGATTGCACAAATCTTTGAAATAGGCACAAAGCGAGTGGGGCTGGTCA
GGATCGAAACATGGTGTGCGCTTCTCGTACTTTCCGGGCATCGGGATGGCGGAACGCTGATAAGCAAGCTTAT
CGTTTAGGGGTTCGCATCTCACATCTCCTACTGATATTGGGCGCATCAGGCGCATTCATCGCACTCTTACAGT
ATGGATCGCACCCATTCCACTCCAAACACGTCGAGATCGCGCTTCGATTCCGGTTCTGGCCGTATCTTCCGCA
TGTCTCTCACATAGTTCCTCATCGGTCCGCAGCTCCATTACATAGTATTACCTACATTCTACTATACCTCATG
GCCATTTAGCTCCGATATATAGAGACCTCAGTAGACACCTAGAATAACAAGCTTTTGGCTCGCATTTCAGTCG
CGCATTGGCACATCCATTCTCTTCTATAGGGCTGTGCCCTTGCATTGAAAGCATGACTCTTGGGCTAGGCACC
TCTTGCTAAACAGACACCCACTCACAAACTGCGAAACACACAGGTTAGGTTTGGTTTTGCCACCTGGCCGCAG
TGTCACATCAACACGGGCTGGCTGACATTTTTCGAGGAGTGTAGGGGGATAGACGTGGTGGTGGTTTGAAAAT
GGGATAGTCCAGGAGCTTCATGTTCCAAGGATCCGGGCCGTCTGCCTCATGTTCTCACTTGACAATCTCTGGG
AAGGGTTCATCTGACCACGAGGCTTCGGTACTGAGTGGAGTGCCAGCCTCGAGGGCGGCTGCAACTAATTTTG
TCTCCAGCTTAACAGGCACTGAATCGGCTATTGGGCTATACTTAGCCGACAAGGGTCCAGCTATTGGACTCTG
CTTGATTGGGGTCGAAGCGACCATGGGACTGGTCTGCTTGGGGGTCCCTTTTGATACTACCTTCTTTACCTTT
GGCTTGGCCATGCCTGAGATCTAAAACCCTCGCAGTGGGCTCCAAAAATGATATGCAAAGAGGCTTGTGGCCA
AAACAAACCACAAAAACCAACTAAGTAAACAACACCTAGATAAGATGTTGAGTGAGCGAGTGTTTCAGATTAG
CAACAAACAAAGCCTCCTAAGGACTCAAGAAGAGTTCTCAAAGACTGTGAGAAGACCTGCTTTGGATGCATTC
CTGACACGTACAACACACAAGAGTGGTAACGAATGAATGGAATGGATGCAAGGGCGTCTGGGAACTGAGCACT
TAGCATAGAATGAGTGTGACATGGGTCGCGCTCTCAAATCTTTGGGCCCGGTGCAACACAAGACAGCATATAG
GAATGTGCAAGTTTCTTTGACTGCGATTGGGTGGTTAGGTCACAGGCCAACTACTTGAATGTGAAGGAATGTG
GAGGAGTGCAATGTGCGACTCCTGTAGGGAATGTGTGGTAGTCTATACTGAGACTGCGATCTGAGCTACAAGC
TCTGTTAGAAGTGGGGATGACAGTACATTGGCACTGACAGATAATCACTTCATCTGCGCTTCCCAGCACTCCC
TAGTCCATCTGTGATACACAGGTTACATAGATGGACACCCAATAGACCGCATTAGACCATTAGTATGGTGGTT
CATAATGCTGTATATTCATTTTGTTGATATCTTGCCTCCCAGTGCAGCTAGAGGCCTAATTGTTGAATATTCT
AAGTCCAGTATTTCAGTCGCGATATAGTGGACACCTGATTTCACACCTCGAGTGAAACAGGCCAAACATGTAC
AAAGGAAGTGTAATTCCTACTATGTCCCGGAGTAGTGTTTGCTACTCCCTTGACAACAGTATTAGACAATCTC
ATACTTGCATTCTATTATCAATATCAAGTGATTAGAGCGTCTATCAGAGGGTTATCAACTGTGTCTGGCTATA
CTCTATAGCAGATCAGTCAGTGAAACAACTTGTCAGTTTATTAAGCTCTGAGGGG
>Cligib1_Cluster72#LTR/Gypsy
catccattgcttctcattcatcatacatcagtcacttgactgatcccatctttcatacgcttctacttcctcc
atcatcactgacaccatccacatgcccatatcagatgatacatcatgcgaccttatctacttctcacgcatgg
tcccttctatctttgtatcttctgttcttactcagctgctcagtcagccttcttatagtcatcatccccatat
gttatatactcacggaccttgtcattactggtagtcatagaactacatacactctatataaagtagctaatac
tcttgtattcctacgtttaatctttatcttcccccgcctcctgacccagagatcagaccttgctaagtgcctt
gagtctttactgtcgactcatgcttgcgttagcaggagtaacattcgattaaagtctagggtcaccaggcaac
cctgcaacaaacagacaacccctagggatagggacacacgacacatcacagttgggagtaatcactccatata
ggggctcattgccagaacagcatggaactgaatacaagactaggcgacgaagcaccgtgccgcaataggatac
agacgtcgaaaggatcgacaaagaaggagtggattaacctccacagacagcatcaacatttgagaagctccac
gacgaaagattagccgacatcaggagaaacatgtcgggcgaaggaacaagaagaagtcactcgcatcattcag
gacagttgtgtcctgagaagagaagggagtagccacagatcgcatcacacttcccaccccacaacccacagtt
cacgggtcgcgcccccccgtcagagaagaacaacacggcagaaccagaagacgaatagcccatatcgcagtccc
atcccacaccacactagccaatacaccccgcccccgctccccagtcactacccccctcgactcccacagcctcg
catcagggttccaaccgctcaggaaggccgtcccacccaatacaccttcggttcagaggaacataccgttcag
cagtcgcccatccaaccgagcatcagcttccttcactgaatatcgagaccccactcccccctatcccctcagac
gacgaggacgaaccacggaacaatggctacagagacaacgcctaagtgaatgacaatggaatcgcagaccccg
gcatgttcgacggggattgcaacctttttgacggttggtggcgtcgcatgcaactttacgtcagagggaacct
gtcctggacacggatgaaaagaagatttcggcccgctctctagatgtcacaagagccgagaactgggcgcaat
gaaacagatcaactaacaaatgagcactggaaaattgtacgaaatacaaacagttcttccacggcaaccatac

tgcgaaaaaaatcaggatgaccaaggatctcaatcggtcgagaatggatcgcagattcgagccaaaacaatgg
tcaagtcgagattagcatattcctatccaacgaaaaccaaaactctatgacaatgtcgcttccaaaaggaggc
tgtccagcactaagatggaagaagcatcctacctatggcaaatgagaagaactgcctatcgaaaaggagtcaa
cggctccacatcggagatccagatggaacaggtaccactatggtggcagaggacgaccatggatatggacatg
aagaaacaaatgacagcaatggaacgcaaggaaacccaatgtttcaatgaacaataggcacttgccgaatgcc
gaaagaagaagacaacataatcaagatcaggacagggaaatgctggaaatgcaacaaggagggacaatgcaaa
gaatgccggagttcagggatggtaatcaagggcaaattcagggtccttgatgatgatgaaaggacagaagtaa
aagttaaggagatgacgatgaaagaagggttttcaggaagggtcagactgagcacaagtctgacccaagggtt
cgtcaatcattctaaatctgtaccaccaccaagaatgacaaatacatgattcccaacgacgaactagtgcacc
tgagctgcaactctcatctcgccacctcaaaactaaaaggaaaaacaatggtcacggtcaaggttataaccac
ggacaaacgaaagctaaactcgacgcactcctgacagcggatgcacccattggcagtcaatcgcgactggtgg
aagcagaaaaactccccacgttgatttacccactcctattcggcatttaatgcgaggctctctcaatgcggaa
aattatcacaaaagcgtcaggttccagatgcaattgggatcacaaggaagaagcaacagcactggtagggact
taggcaaaaaggatctcttcatcggacatgaatggttacacaaacacaatcccttcatcaactggaagactgg
caagatcaaattcccagactgcccccccagaatgtgccaaagtaaggtcagacactacccaaggctaaagttcg
gtcaacagacaggtcacggatctaaaactcccaagtacctcagattggaaggatcgattcgagaaaaagacct
ttgacacactcccaccgtacaggaacatgaccacaataacctgaaaccagagcaccggacgaattaccagcca
aatttatccaatgacagcgcagaaatggaagaaatggacaaattcctagaagagaatctagcctcggaacgga
tatcccatccaagagtccatagcatcaccatgcttcttcatcaacaagaaagacggatccaaacgattggtac
agactatagagagctaaacaagtggacgattaaggacaaattcccccctacctctgattaacgacatcatgaca
aactgaaagacgcaaatttttttcaccaagttagatctaatctgggggatacaacaacttgcgcatcgcacttta
ccacgaatggaaggcagcttctacaccacgcggattattgaaccacggtcatgttcttggactctgaatcccc
aagctcattctgcgatggatggcgaatattaagatatcgaaaaatgatcattaatcgatgacattatcatcta
gccaaggacaagacagctaggaacgagcatgagtcatggaaatgcacgcgccaatggaatctcttcaaggcca
gtaaatgcttattcgaggtccctgaggtaccatttctaggtgttattgttggcaatgggaaggtccgcatgga
caggaaaagatcgaagtcatccagacttggaaacgccagaaaacaaaaggttcagaaattcctaggcttcgca
aatttttaccgccgcttcatcaaggattatggaaaatggccgaaccctcacacgcctcacagggtctaggaat
ggtcttggacaccagaacaagaagtgctttgaaagtctgaaatcgcgatatgctcagaaccaatactggcact
cccaggtccaagggacgcttcagggtcgaaacagatgcatcaggatatgctataggaggtgtcctctccaaga
acaggatggtaaatggcaccctatcgccttcatgtccaaagtaatgaacccaacacaacgcgatacgaaatct
agacaaggaactttcatcatggaagccctcgcattggcgacaatattggtgacgcacaagaaccatttgaact
gttaacggaccacaagaacctaacatacttcaggaaagcacaacgattgaacagtcgacaaatgcgatggtac
ttaaacttcaagactacgatttgaaatgcggcatatacctggaaaaacaaacacaaaagcggacatcctctca
cgactggagtggtagaagagacaccgaagaagaagaggtcatgatgttcaaaatcaggtttggtattccttca
gaaatggaggaagagatgaaatttcggactttggcatacggttcttcggagaagaactacctgaagaggaggt
gatctggacacatcggaaatttcatctcgctctcactgaattggcaagaagatctggaacacgcatcagaaac
cacccacgcgtagaatcagaagtccgattcggtaaggaaagccacactcttcaaagaagctgatggcatcatc
acagtggagacaagtctatatccccagacaaacaactacgtgaagaattatcagaacacatgattcaccacta
gcaggcacccaggatccacaaacaatgaactcattggacgcaccctatggtggccaggatgcgatcgacatca
gagatatgtcatgggatggaaacatgtcaaaagaacaagatcaatcgagaagcagcacgccctctacccaacc
cgatacccaccacccctgggaagagatctcaatgacatgatcggccccttaccacgtctaaaggttacgatgc
tattttggtcattgtggacaggcaatcaagatgatcagggtcgaaccaccacaccaccctgacatccaaggac
tgctgaaatctatcgagacaccatctggagacaacaggaatacccaaaagatatcagtgacagaggcccaatt
gcatcgaacttcatgaaggacctatgacctcttaggatcaacgaattatcaacggcttatcaccccagacaga
tgacaaacagactatcaacaagatgtcgaacattccttcggatctttgcaatagatcaggatgatgggcctca
tggctatcaattctagaattccatacaagataagatccactctagacggacaaacccgttcttcctgaacatg
gcgacatccatggaaagggaatctccatggaaccgtaaagaagaagccgctcaagaatttgcatcaccattga
agaagtcggaagaaagctatcaccgccttagaagcatcagcgaagaatgaagaaacaatatgatctcacaaga
aggactcgcttaatctcaaacccggtaacaaggtgtttttagaagccaaaatatcacaaccaccaggccatcc
aagaagttatcagaacgacgattaggaccattcgaagtcattgaaaaggtaggccacagtgcctacaaactca
agctccccaccaactggacgctcatccacccggtattcaatgagaacctattaacacctcaagacagcacgct
tcgaatccaacagaagccatcacctccaccacccgatgtcatgaaggttcagaagaatacgaagtagaaagca
tccgaaaggtcaaaaagaagaatggaagacttctattcttgtccattggaaagggtatcggaagaagaaaaca
cctgggaagtcgcgcaaacctcaaacactccgcggatctggttaaagacttctaccgccgtcaccccaatgca
ccacgataaatacgacactcgtttcaaccacttactttaccccacatcttttaaattgcattccactacaaac
catattctccaccatgtcatcccgccactcaacttgccttggccattgcgccctcttccccaccctcgaactt
cttcattctacccccatccatattcgaacaatcaatgactggatcaaagcgttggacaacattgcacgcaccc
tatcagaagacgagttactatgctgcaacccctccgcgaccaacgggttcaactccacaatctgcacaaagtc

tactcgactacgaaaaagactcatgctcatgttcaacgagtataacccaccacgctcctggtctcactgacgg
agacatgactacgcttcgtcaacaaatgacggtcaagaatgggggtccatcaaccccacgaggtccaccggag
taccgcctccctgtcattccccctcccccatctttaggcaccccactcactctgcctcgtctagttctccaac
ctctccaacacaactctcccccacaggagaagacgccatgcaggccccagccacctgccaccaacaccaccca
cgttcaccccccatagggggtcctcccattacaccggttgtgcacctcaagatccccaaaattcacccccaccga
cccctacaatggtacccacatgtacagtggacacccttcagatattgggtagcgaccggacaacgaagggttt
ctttacgcgcaagggcgaggaatatttgacgcttataaaaagcaggatctcggtaaagtgttgctagaagctg
tttgttggtttagagtttcgggacgaaacccctggagggaaggtcatgTtgagaTacaagctNaNctacagta
tttgaaTtaggatTaaTtgatcattaatgatTataTaatcgcaTccagtctTttacattagtagattgtctct
cattcaacaacatcacaatagAtcCtataatcatCaaATaATacctAttcaatctCccatctacttCaTccac
tgccaataggttatgggccctgggcAacctacgatgTcgtatcttaNtctcaaagtNcctgaatctNgaagac
tatggttcNaaNtggctNacctataaggaaaggNtaataaNgaNtNtgactcacgaaggactcgaacgacacc
tgtatggaaccgcgcagaagcNagaggagcttcgcgaggtagatgggaaNtggttcaacaaagaagaacctac
gaaatcgctcaNcagaaaagaaatNaNagagcacaaNacgaaaatNgacattttcttgcaaaaNgaNgcatcN
gNtcgNNNNatNatctatNaaacgatcagccaatacactttccttcgaatatNcaaggaaNagacctNcgtga
agctatggataaagctNNccagcNttttcgaacgaacctttcNaggccaNctNgcgatccgtccNaatgcNtN
ccgaaaaacgcgccgaaaacgcggtggtaatagcNgaaaagggaggggaagaatccaaggggaagccaaagga
aaagaaNaagNaattgtgctcNNgcacggggNatgctcgcgacgccgaatgctNtaggagaggaggagaaNga
aatggtcaggaaccacaggaagagaagacgcgagcacgaacgctgccacctcgagtgataccagcNaagacgt
agcNcgcgcNaaNagctcataccccccgtgaagaaacccctgcccaatccactccaNgaccctcgccgcgcacc
gtcgatcgaatccccgagagtcgtaacNtaNatttcgttgattNtNctaataaaccgtNggaaatccNtcttg
aaNNgtcgaatgcatgcgaagcgttgtcgaaaaacgcgagNccgtcggaaatcacagcaNataaNgaNgaNga
aNtaacttttattgaaNaNcNagaatatacaatacagtatcaatgccccggatctcgNaaaagcgaaccagaa
aNggctgaaggccataatacaggttttgatggagattcNgcggataacaggagctgtaggccagaaaagcgat
cagtaacggtcgaagggaacacctaccttgataggaataacctcccagaagctcgaaccgtccagatcgaggg
gaagagaatgcccaagatctccaataccccccaggccagccaccgaaaatgagaccatcgaatcgccgcgcgct
aataggcacNtcttgaaggcctcgagaaccgctttcgaacggtccaacaatcNgccaaaaccgccggaaaacg
gttgcggaacggacNNNNcttcgtccaagctactcagNcNaaccaccaaatcNaNNcgaccaaaccgccggac
NtgaatagNcgcgatcggNagcccttNggaactgNtatcgaacggtctagcgatcagggaaaacNgccggaaa
acgattgcggaacggacgtaccttcgtccagaccacccaggcaaaagaccgaaatccgttcggacgaatcccg
gacgtgaataggcgcgaattaaagccctataggaccgctcccaacgcgcNacaatcaccaaaacggataaaaa
atggtcgtgcaacggacacactttcgtccaagcacaaNNtctccatatccaaaaNcccaccaatatNccccag
ctcaaaggatcatcgaagcgacatcccaagcgacctagaatcgcatatccgtcgcgatttccgtgccagaaaa
cNcccaggaaNctgNagtcagcctatccagcaagactcaccatcttggagaccaggagcaggaatcgatNatc
gggatcccagaagcatatctggggaagatagagggttagtaaccaatataggaggaccaactcacctatccaa
tatggtcaacgatgttgaacctcgaggaagcgtccaacaggaaccaaccatcgctcNgtcagcagtcaaacgt
ccaaatcgggacatttcgacttcNaNaatNattcacactcgttttgctcatatcaagatgattcagcaataca
ttcttttactatatcaggtatattctacctacttacaatctcaaatacctccatttcgcttgaggggaagtgt
tgagataccagcttatctacagtatttgacttagattaatNtccgctttatgattacataagcgtatccagtc
ttttacattcgtagattgtctctcattcaacaatatcacaatagatcctatactcatctaataatatctattt
aaNctcccatctactNcatccactgccaata
>Cligib1_Cluster99#LTR/Gypsy
GATGATACGCTGACTGGACGTCTGAGTGCCTTGAGGCAAAACCAAACACCAGGCAGAACGGAGTAAGACTACT
CCGGGGACGAGTGAGTGAAGCGGCGGTTAGTTAATCAAAGCCTTAGCTAATTGGCAGTCGGGGAAACGGAATG
GCGGAATGGCAACAATGACGCGGGGCTAGAGGGACGCAAAACCACGCGAGACGCGAAACGCTGGACGGAGACG
GATACGCACGCACGCAATGACGGAGACTTTGGATGTAACGCAGATGGCAACTACGGTTTGAGGCTTAATGACG
CTAAACGCAGATTTTGAACGCAAGACGGAGAGGTTGGCGATAAGCCAAAGAGGTTAGAGCGGACTTAGAGCTG
AGACGAAGAGGGCGCAATTGAGAGGTGAACTAACTTGTTGTTTTTGCTAGACTAAGTACAACGGGTTTTATAC
TAAAGTGGGAGATGGCTAGGATCAATGAATGGCTAAGTGAAACGCGGTAAACGGGTTAGGCGGAGAGAACAGT
AAAAGACTAATATTGTCCATGATGAAGGGTAAACATGCCGTTGGCGCACTGCCGGGCAACTCGGACGATGCCT
AATAAATAGCCGAGAGGTCGGGCGTCGGGATTGACGTCATGGCTCTGATGTGGTGTACTGAGTGATTGAAACA
GTCGGCAATGGCGTGGAGGATCAGGCTGGGCGGATGATGCGCTATTTACTGGGGGTGTCGCGGCGTGATGGAA
ACTGAGCGAAGTCTAATTGGCGTTTTGATTGGTACTGTCGATCGACGGAACCCAGACCTGGCCTGGAGCTGTC
GGGATGCGTCGTTTGGAGTCACGGACCGGAACTAGACACGGGAAGAGTGGCGGAGTTGTGAGGAATAGCGGGG
GAATGGCGGAGTGACGAAGGGCGGCCAGTGATCACTGACGAATGGCGGAATAGCTGATCTACGGGCTCTGGTG
GACTTAGAGAAACCGTTAATCAATTGAGTTGTGAAGCTAAAGGCAAACCGCTAATAGGTATGCAGCTGCTGGT
GCCGCTCATCATCTCCTCTCCACTTAGATATTGACTGTCCCCAGTCAATCCGTCCTGTGGCGTTCTCGTGACA
TAATAACACAAGGTTCCCGATCAAGGTGTCGCAAGGTCCCCGTCCTGATCTTCCTCTCGAACCATCATGTCCG

ACGCCGTATCCCGTGCCAAAATCGTCCTGAGGGGCCTCTACACGCAGTACCCCAAGCCAGACGCCAAACCGTC
CGAGCTTCGTGAGTGGGCCTCCGTGTTCGTGGTCGAGCTGGTGAGTGACCTGGTGACCCTGAGGCCGAAACAG
CTGACAAGGAAAAAAATTTTCAGGCTGCCTATGACTCGGCGCTAAACGGCGCCCCGCGCGATAGCGAAGTCGT
GGGGTGGATGATTGAGATCCGGCCTCGGATGGTCGACGCTTTTGGGCCCAACTGGCAAGCGGCAGTCCAAGTT
GCGCCAACTGCGGCCGCTGCTGACGCACCTATGGAGCCGGGGGCAGTCGTTGAGGAGAGTGCCAGTGCCGGGA
GAACAACGCGGGCGAGAACGGCAAAGGGCAAAGGGAAGGAGCGGGCCACTACAGCGCCAATACGGTACGCGGG
GGCGCTGTCGCCACTTAGGCTGGCCAAACGTCAGCGGACCAGTCCACCAATACCAACGAAGCAGCCGTCGGCC
TCCGCCCGTCCCAAGGCAAGGCCTGTGACTGCAACACGGCGCAGCAACCGCGCCCGGGTCATGGTGCCGGTCC
CAGGTCCCAAGGTACGGTGTCCAAACGGCGCTCTCACCCGCGCTGACAGGCGACAGTGTGATACCTGCGCCGA
GGCGTCGGACTGCGACTGGAGCCCCCGTCGCGGCCGCTGTAGGAGGTGCTGTGACGCCAAGCGAGCCTGTCTC
TTTGGCGAGGTGAACAAGCGCGAGGAGTGGATAGAGGCGCAAAGGGGCGCCCAGCCGGGTCAGGGCATGACAG
CGCCCCCGCCAATGGGGGCCGCGGCGACGGGTGACAGTACCCCTCCGCCACCACCACCAGTCGAGGAAGCGAC
CGCTGAAGGCGAAGGCGAGGGGCCCGAGGAGGCTGACACGGAGGCGAACGAAGACGGAGCGGGCGTGGCAGGG
GTGGGGAACAGGAGCAGCGACGACGAGGAGACGGACTCGGACGACCATGTGGTGGTAAAGATGAAGCCACCAA
CAGCTGCGGACTTTGCCAGGCACGCCAAACGGGCACGCCGGGACCAGGCCCTCGAGGACGAGGCAGTGATAAA
GACACCCCCGCCCGTACCCAGCGACGACGGAAGCACCGACGGGGAGGCGAAGAAAGGGAAGGGCAACGCGGCA
GGGGAGGGTGGGCGCGAAACGAGTGCAGGGGCCCCGCCAGCGGAGCCGGGCAGTACCACAGACGCGGCACGCG
ACACCCGCATGGCGTCGCCTCCACGGTCCGTAGTCCGGGGGTCGCAAGGGAACAGTGCCAGGGCGATCCCCCA
GCCAATGCCCGTCGCCGGGCCGTCAAGGTTTCCTGGGGCGCTGGGCCACGCGAGACCGGGATCTGTGGGGCGG
CGCCCGCGCGCTGACATCGACCAGGACCGCCGAGCCGTTCAACAACGGATAGCCGGGTACCGGGCGAACCTTC
GGGTGCTGGCGGCGACACTTGAAATGACGGAAGAATACGCGCAGAAGCTGGAAGCCGAAGCCAAGAGGGCGGA
GGAGTCGGAGGACAGCGGGTAGAGCTGGACGGATGAAGGAATACAGAGGGCGAGAAAATGGGGAGGGGAAAAG
TAACATAAAAACTAATATCTTGAGTCCGGTTTCTTTTCTTGTCTTTTTGAAATAAAATCAAGTGTCTGTGGGA
TACGACGGAGACTTGAGAATAGAGGGAACGAACGGATAAACAAGGGAACACGTAGGAAAAATAAGCAAGGGAA
ACGAGGAAAGGCTCAGTATATCATCATAGCCTGAAAACCTCTACGGTCTTGCGCGCTCGCTCGTACGGAGGTG
ACGCGGCTGCGAACTATACAGAAAGATAAGAGATTAAATGGTAGTGTTCGGCAGACAGTAGATAGAAGGGAAG
GGGACTCAGGCAGTGACATCCCCGTCGTCGTCGTCGCCACTGTCGTGGGCTTCCTCCATTTCTCGGAGGCGAG
CAGTGGTGATGTCGGCGAAACCGCTGGGGATAGGAATGTGAGCGCGGGCAAAATAAGGGATAACACGAAACGC
AGCAGTTGGTCGATCGACTGTGGCCCCATCGAGGCCAGCGAGGAGGTAGGCGCCACCGCGGTTCCGCGAGATA
ACGATGTAGGGTCCGTCATAACGGCGGCGCATTTTGCGGTTTAGTGACTTCTCGATCTGGGTGTGGCGCACGA
GGACAAGGTCCCCGCGCTGGAAGTTGTAGTCGCGGATCGTGTGAGCGTGGTCTTCCTCAAACTTGATAGCGGC
TCGGACGCGCGCCTCGTAGATCTTGGACGAGAGAGCTTCTAGGGCGGCGCTCCGCTTTTGGAGGGCAATGGCA
CGGCGGGCGATCAGATCCGTGGAGGACATGACGGAGTCGGGTGGAGGCTGGAGGTAGGTTGCCTCCGAAACAT
CGAAAGGGAGCAGCGGGTGAATGCCTGTAACAGCGAAGTAGGGTGAGCAACCCATGCGACGGCGAATGGTAAC
GCGCTCAGCCCAAAATACTGAGTGGGCATGCTGGGCCCACTTCCGCTGGTCTCCATCTGCGGCCTTATACAGC
GACTGGCGGACGTCGAAGTGGGGGCGCTCGACGATACCATTGGCGCGGGAGTTGTAACCGCTAATGCGGATGT
GGGTGACATGATATTGTTTAGCGAGGTAGGCCATAGCTTTAAGGAATGGAGCGCCGTTATCAGTAACAATCTC
CTGAAGCCCGCCCCATCTGCACAGGATGTCTTCAAAGATCCAGTCACCAAGGGATTTGCTGTTCTCGACGCGC
AGCATGCGGAACTCGGGGTAGTGAGACAGGGCACAGCGGCCGTGAACGATGTACTTGAAGCCAGACGACGGAG
GCATGTGCATGGAATCGATGTGAACTTTGGCGAATAGCGGTGCTGGCGTTGCAACGGTGGGCGGTATGAGAAC
TTGGCGGATTTGACGCTCTTGGCAGAGGTGACAGGTTTTCACGAACCAGGCGATGTCAGTGCTAATCTGGGGC
CACCAAAACCGCTGGGTGAGCAACGCCCGGGTGGCATAAAAACCTTTGTGCCCAACGTCGTCGTGGGCGGCCT
GCATAATAGCGAACCGGCGGGGAGGCGGGATGACGAGTTGGTGGGCGCCGTTAATGTCCTTCCGCCAGAGTCG
GCCGGAGTCCTTGAAGAATCCCAAGGCGTAGCGTACGAACGATTGGAGCTCTGGGGTGGACATGTCAGTGGGC
CGCTGAAGGTCGTCCAGGAACTTCTCAACACGGAGTAAGCGGTGGTCGTCTGACTTGGCGGTCTTGCTTCGTG
GCACGTTGTCGTATGGATCCGGATCAGCGGGTGCCTCCTCTCCACTTGAGACATTTGCGCCCATGAACGTGGC
AATCGGTGTTGATGAATCCGTAATGGCGATCGTATTGGCGAGTGTGGCGGGCGGTGTATTGATGATGTGGACG
AACCCGTAGAGCCGGTCGATCCAGTCATCCGTATCGTTGTCTGGGGGTGGCCCGTCGCCTGGTTGCTGAGGCC
TCCTGGAGAGACCGTCGGGGCCGTGGTTAACGCCGGGGACGTGGACGAGTTCAAAATGGAAGGTCAGGATGGC
GATAATCCAGCGATTAATACTGGCGCTGGGCATGATGTCGGGGTTCTGTAGCATACCCTTGATGTAGCGTGCG
TCGACTTCTACCACGAGGTTCCGGACGCCAATGATGTAAACCTTGAAGGCCAATAGCGCACGGAAGAGGCCGT
ATAGCTCTAATTTTGCCTGGGAAAAACGTCGCTCTCGGTCGTTCAGCGTGATGGAAGAGAAACGTGAATAATA
GCGGATGCGTGGATCGTCCTCGTCGCACTGGCAAAGGTAGAACCCGACGGCGATGGGGGCAGTGTCGACAGCG
AGGATAACAGGGGCAGAGGACTTGTAATCAATGGCGCGGAGTGCGGGGGAGTTGAGTAAGGCGTCTTTGAGGT
CTTGCTGGGCCGCTAGCTGTTCTGGGCCGAAAACGAACTGCTCGTCCTTGCGCGTGAGCTTGACCAGCGGGCT
GGCGCGTTTAGAGAAGTTCTTGATAAATATTCTGACGACGCCAATGGTACCTAGGAATGCACGGAGTTCTGAC
AAGTCTTTGCATGGCCCCCAATTGACTATAGCGGCGACGCGGGAGGGGTCTGCTTGCCGTCCGAGTGGGGTGC
AGCGGTGGCCTACGACGGTGAATTCTTCTGCACAGAGAGCGAGCTTGTGACCGGAGAAGGTACCTCCGCAGTA

CTTCATGCGTTGAGCGACACGGTTGACATTTTGGAAGTGCTCCCACACGAATCGCCGGATGCCGGGGTTCTCG
GGGATGGTCTCGGGTGTCCCATCCGGGAGCGGGTAAGTCGTTGTCGGGCCCTTGATAGGGACATCGTCTATAT
ATGGGATTGTAATGTGAGGGATCTCCGGGCGTAGGATATGGGTGACGTCGTCGTGGAAAATAGGCACTGAGTT
AGTCCAGCCCATTGGAATTTTGGTGAGGCGCATCGCACCGAATGGCGTTTGGAAGGTCGTATAATCGCGTGAG
GCAATGGCGGTATCGCGTTCATCGTATCCAACGTAAAGGTCGAGCATCCCTCCACAGACACGTCCGCCAAACT
GTTCGGCGAGGTGTTCAGGCATGGGGGTTACACCGGAATGCCGGATAGTGACGCGGTTTAGCGGCTCAAGACT
ATGCACAATACGGAGTGACTTGCCGTCCTTTTTAACGACTGTGAACCAGCGAGAGCGATAGGAAGAATTGGAG
GGCTCGTAGACGCCGGCGTCGATCTTTCTTTTTATCAGCGCACAGACCTCGTCGAAGAGGCCGGGGGGTATGG
GGATATTCTTCTGTACCCAAGGGGTGTGAGGGATAACGGGGAATGAGACAGGGGGAAAGAAGTCGGGGCGGAA
GCAGCCTCTCTCGGTATCATCCCAAGCGAAGGCTTCATTCTGTTTGCACATAAAGTCATGCATCAGCGCGCGT
TCAGCGGGAAGTAGGAAGTCACCGGGGTGATACTGGTCCGTAAGATCTCGCCGTTCGAGGGTATAGCGGCCGG
TTGGCTGAAACGGTGGAGGATTGGGGTCAAGAGTGGGGATGGAGTCAAGGGGATCGCCGATGATGTTGCGGAT
GATGCGGAAGTGCTCGGGGACTTCTGCCAGGATTGAGCGGACCTTCTGGGCGACTGGCTTGTACTTCTTTTTA
GCGAATATACTGATTGGTACTGCGGAGGATGACTGGGGGCGCGTAGGAGACACCGAGGTACCGGGGAAACTGG
TGAGATTGAGAAATTGGGTGGAATGGTTAATGGCGGGGACTGAATCGCAGGACTGTAAATAGAGATAAGAGAG
AGACTGTGGATCGAGAGACAAATCAGCGGAAATGGGGGATAAAACATTATATTTTACGATGCGCGGCTTAGTA
GGGTTTCCGTCGAGGATCAGCGCGACAGTGCCTTGATCAGGAATTACATCCTCGACTTCTGAAAATCCGGGCT
TTGATGGCTATCTTGATTGGCGTTGCTACGGGGGAGTGTCTGGAGGGTGGAAACTTGTCCTGTGTTCGGGTCT
TTGAGCGTGATAGTTTGGTCTTCATTGGAATAGTTCTTGACGATACTGCCAGTTAAAACATCGAAGGGTCGGC
CAAGGAGGATGTCGTAGGCGGGTGCGCGAATAACGTGGATCTGGACAAATAGCGTGATATCGCCAATACGCAG
GGAGACATTTCTTGCTAATCCCAGTGATAGGTCGACGTCGCCATTGGCTGATTGCATACTGAGTTGGACGGTG
GGGTCGTAAGTGAGGCCGAGACCATGGCAAACCTCCTCTGACATGGCGATTATCTGAGAGCCAGGGTCGATGA
TCGCCTCGATGTATTTTTGGTTGTCGACCAGGACCATTAGCGAGCGAAGCGCGTGGGATTCTTTGGCTACCTT
CAAGTAATCTTCGTGTCCGCGAGACCCATGGGTAGTGATGAAAGTGTCGTACTTGTCGGGTATAACGATACTG
TCGGGGGGCGGGGGCGCTTGGCCTGTTGGTGCGTCAGGGTGATTACTGACGGGGAAAGGGAGGAGTTCGGTTT
CTGCGAGCATCAGCGTGGATTCATCAGGTGTGGCAATGCGGCGGGGGGTTAACATGTCTTTGAGAGAGGAGCG
CGCTTCCGGAGATGCCGCCAGCAACTCTTTTAGCGTGAGCATTACCGAGGAGCCCAACATGCGTTCTAGGACG
CCGTCGTTGGTAGCGGAGTCCTGGACTGGAGCCGAAGTCTTGTAGGCTGGAGCCGCTGGCTTACTGGGTTTTC
CCGCTGGGGCGCCGAGGTTCCGCTGTTGAGGTGGGGCGTAGGCGGCTTCCTTAACGTGGCGAAACGGGTGCAA
GTCAGTGCCGTCTTCCTCCGGAGGTGTGATCGTGACTTCTGGTGTTGGGCCGGGTACTGCCAGCTCTTGAACG
GGGGACTGAGGCGTGTTTTTAGCAGCAGAACTGGTGGCACTGGTAGAACTGGGACGCTGGAGGACCCGGGGGA
CGTCGACTGACTCCATAAATCGTCGTGGCTGTTTGAGCGCGTTGACCTCTCGGCCGTAGTGGTAGGTATTAGC
GGAGCGCGGGGTGGAAACAGTGTTAATAGAGTAGATCATTTGACTGGTCGGGTTGGCGTTAGAGGAGAGTCGT
CCGGCTGCCAACTGACCGGGGTTACGGCGGTGCCACTCGTCGACGCGTTCCTGCATGGTTCGGCCCTGGATAG
GGCCTGGGATATAGGCACCAGTGGACAACACTATGCGGTTTTCGTGGTTGCGCCTGATCTTACCATCCTGAAC
ATATTGTTCCGCGACGGGGCAGCGGGCGACAAAGTGTCCTTCAAGTCCGCAAAAGTTGCAGTTCCTATTGGCA
ATGCTGACGAAGGTAGCGGAGTTCCCAGGTTGCGGCGCAAGGGCTTGGATGAGAGACTGTTGGAGATCTTTAA
AAATGGATGCCATGTCTGTTGCCGTGACTGGGGCCGGAGCTGACGAGACAGCTGGTCGTGACATAGCGCGGCG
GAGTACGATACGGGCGGCTTTCTGGACATCCTCGAAGGTATCGGAGTCTTCGTCTTCGCGCTCTGGAAACATC
ATACGGATTTGTTGGTCAACTTCGCGGGCAAGGGAGGGCGGGAAACCATCCAGGAATCTTCTGCCGAGATCTA
GCTCGTGCATGCGTTTGGCGGAACGGAGGTAGTCGGTGATCATTTGAAATTTGCGTGCGTAGTCAATGAGGTC
GTCCAAAGACTTGATCCCGATACGTGCCGTGCGGCTGACTAGCGCGTCGAGGTCACCACGCGTATAGCGGGGG
GAGTTATCGCCTCCAGGGTAGTGATGGAGTACTTTCTTTTTAAATTCAGTGTAAGTGCCCGTCTCGTATACAT
CATAGCCTTCCCAGGTTTCTCCAGTCTCGAAGTCCACGTAGGTAGTGGCGGCCTTCTTCATTTTTGCGTCATC
TTTGAGGCCAGCTTCGGTGAATAGCTGTTCAAGGTGCTTGAAGTAGCCGTGGATGCTGCGGGCGTATTTGGGG
TCGAAGACTGGCGCGGCGCGGTCGCCTTTACGGGGCATTTCTTTAAAATTCGAGTCTGGGTGCATTGCTGATG
GAGATTCGGTGATGGGGTCTGACATGCTAGGTAGGAGTGGTTGTAGTAACGCTGGGGACACGGGGCGGGAGTT
ACTGTCACTTTTGGCGGGGGAATCGGAGACTAGGGCGGGGATAGGGGCGAGGTCGAAGGTTTCGTAGGAAGGA
GATTTGGGTTGTTTGGTTAGGAAGGGATCGTTGGACGTAGAGGCGACGGGTGTGGACGGCGTGATTCCAGCAG
CGATAGCGGCGGATTCTAGTTTCTGTTGGAGTGGGGTACCGATGGGTGTGTCGGGGAACTGGGAGGGACTTTT
GGCGATACGCTTATTACGGCGGCGTTTGGGCTGCTTTGAGTTAGCAATTGTGCTTCAAACCCTCGCGGTGGGC
TCCAAAATGATACGCTGACTGGACGTCTGAGTGCCTTGAGGCAAAACCAAACACCAGGCAGAATGGAGTAAGA
CTACTCCGGGGACGAGTGAGTGAAGCGGCGGTTAGTTAATCAAAGCCTTAGCTAATTGGCAGTCGGGGAAACG
GAATGGCGGAATGGCAACAATGACGCGGGGCTAGAGGGACGCAAAACCACGCGAGACGCGAAACGCTGGACGG
AGACGGATACGCACGCACGCAATGACGGAGACTTTGGATGTAACGCAGATGGCAACTACGGTTTGAGGCTTAA
TGACGCTAAACGCAGATTTTGAACGCAAGACGGAGAGGTTGGCGATAAGCCAAAGAGGTTAGAGCGGACTTAG
AGCTGAGACGAAGAGGGCGCAATTGAGAGGTGAACTAACTTGTTGTTTTTGCTAGACTAAGTACAACGGGTTT
TATACTAAAGTGGG

>Cligib1_Cluster102#LTR/Copia
GTTGAGCGGCACCCCTAGGGCACAGCGGAACTGGTCTACTTACTTGGTTAGAACGGTGAGCTTACTAGGTGAG
TGTTGAGAGCAAAAGAGTTATATTAGCCTCTTGTTCAGAGGGGTCTTATATACTACAGTAATGTGCACAGACC
ATGTGGCTGTGCTAAAGTCCTAACTCCTATCTATAATCTAATCTACTGATAAGATAAGATAAGATGTTATCTA
TGGTGCTTATAAAGACACCGGATTATGTAAGCAGGGCACTTACAGTTTAGAGCATTGAAGTTTAACACTCCCC
CTCGCTTAGAACAGCGGATGCATATAACAACCTTTGCAGGAGCTATAAGCGATACTTCAAACTTCAAGACGAG
TAAAACTCAAGTCCCAGTTGAGGTACGAATTCTTTGAATTTGATATTACCCAGACTTTTGGTAAAAATATCTG
CAGGGTTTATATCTCCTTCAGTGAAAAGTATAGTGACTTTTCCTTCATCATAACATTCACGTATGTAATGATG
ATGAATATCAATATGTTTGGTTCGTCTTTCTTGAACTGGATTAGAACCAATAAAGATTGATCCAGCATTATCA
CCGACAATTGGTATTTTGTCAAGTTTGATTCCAAGTTCTCCAAAGAGAGATCGTAACCAAACTACCTGTCGAC
AAGTGTCAGACATGGCAATATATTCTGCCTCGGTAGATGATTGAGCGATTGTTTTTTGTAATCTGGACGTCCA
AGTCACAGCACCGGAAGCAAGGCTTACAAAGAAACCGGTGGTTGATTTCCAAGTATTAGGGTCTGAGGCCCAG
TCGGAATCTGTATGAGCAATTAAGCCTTCATTCGAATCTCCATTATAATTCAGGGAGTAATCCTGAGTACCGG
CTAAATAGCGGCATATATAGAGTGCCTTATCCAGGTGCTCTTGTGTGGGATTCGCACTCATTTGTGACATTTT
AATCACAGCGAAGGCAATGTCAGGACGAGTTCCTAACATTAAGTAAAGCAAAGATCCTATGACGGATTGAAAT
TTGCTTCGTAGGGTGTGATCTATTGGTCCCAGATTTCTTTCGGGTAGATATTTGGGTGGTAAAGGGGTTTTAG
CAGCGCGTGCATTAATCATTCCAAAACGCTCCAATACTTTAACCAGATAAGTTTTCTGGTCAAGTTTGATCTG
GTTTTTATTTCGCCAGATGCGCATTCCGAGGAATTCTTTAGCATCGCCTAAATCCCTGCATTCCCATTTTCGC
ATAAATTCTGCTTTTTTCTTGATTACATACGACCGATTAGGCCCCAAGAATAAGGCATCATCAACATAGACTA
TCGCAATGACAATCTGGTTCGAGGATGATCGGTGAATGAAAATGCCTGCATCTGAAAGAGTGCGTTGAAAGCC
CATTTCTTTCATTGACGCAACAAGAGCTCTCCACCATTCAAGAGCTGCTTGTTTCAGACCATATAAGGCTCGT
TTGAGGCGCAGGACTTTGCGCTCATGGCCTTTTCGGACGAAGCCTTCTGGTTGATGCATGTATATTTCGACAT
CTAATTTGCCATAAAGAAAGGCAGTTCGAACGTCGAGTGATAGAATAACCCAATGTTTCAGGGCAGCTGTGGC
TATCATAAGACGCACAGATTCATATCGTACAACCGGAGAGAATAATTCATCGAAGTCAACACCTTCGACTTGT
GAATATCCTTTAGCTACAAGGCGAGCTTTCTTTCGACCGTCAGATTTGACATTGAAAACCCAGCGATTGCGAA
TCGCCTTTCGTCCTTTGGGTAGATCGCATATTTCATATATGTCGCGATTTTTGAGGGAGTCTAATTCCTCTTC
GCAAGCGTTCATCCACTCTTTTTGTAGCTTTTTATCAAGCTTAAAAATGTCTCGGAAGTGAGTAGGAACTTCG
TCTTTGACGGGTACTGCTTTGGAAAGTAAAAATTCCATTATAGCTCCCCCTTCCTCTTGTACGATTTGTAGAA
GATGTAATTCTTCTTCATCGGTAGAGGGATAATCAGGAAGGTCTCTTTCGGGACCTTTCTCCACATTTTTACG
CCAGTATCGATCACTAGCAATATCTCGTTCTATTTCAGAAGGAAGTTTCTTTTCTCCATATATATTTCCTGGA
CGGTTAGGCACTATCTTTCGACGTGTGCTTCGTCTAGGTAGTTCGTTCCATTCTTCCTGATCATCACGGTTTG
GGGTAAACACGCGAGTGTATGGTTCAGGAGAATGACCTCGCATAGGAGATCCTGGTGGAACAGGAGAATGTAT
TTCTTCTTCTGGTTCTTCTTCCATCGTAGGAGCCCATGGATGAGGTTCTTTAGGTGGAGAAAGAGGACGAGGG
GAAGATGTGTTTCTGATTGCGGTGGAGATGGAGGAAATAATTCGTCCGGTGGAGGAATAGTAATTTGTTCGGG
TACCATAGTTTTTGTTTTGCATTTCGGCAGAGTTACTTCATCAAAGATAGCTGTAACTCCTGTAAAGATGACG
GAGTTTCTGATTCGCATGAAGCGGTATCCTTTAATTCCAGCAGTATGACCGAGGTAAATCATTAACTCGGATT
TGGGCGCAAGTTTATTGGCACGTCTTTCTGGTGGAATATAGACGTATGCAGCGCAGCCAAAAACACGAAGATG
TGTTACGGAGGGTCTTTCACCCTTGAGGACTTCATATGGAGTTCGCCATTGTAGGCGCACCATAGGTGTGCGG
TTATAGATGTGAACCGCTTGTTCCACAGAAAACTCCCACCATGAACTAGGAAGGCATGCATCGTGTCGCATAG
ACTCGCCTTTATCCATAATAGTTCGATTGATGCGTTCAGCGCGACCATTTTGTTGTGGTGTATGAGGTGCTGA
TTGTTCAATAAGGATGCCCGAAGATTTCAATTCATCAATAAATTCCTGGGATTTGAATTCTCCCCCTGCATCA
GAACGCCAACGCTTGATTTTAGCGTCATATTGGTTTTTAACTTCTGCATAGAATATACGGAAGGCACCAATTG
CATCTGATTTATGTTTTAGGTGTGATACCCAAACATATGACGTGTAATCGTCAAGGAATGTGATGATATATGA
ATACCCATGATATGATTTCACGGGAAGCTTTTTAAATCCGAATGTATCAATTCGAATGGCTTCGTGGCTCGTG
AGTTGGATGGTTTAAAAGATGAAGAGGGCATCTTTCCATACGCACAACCAGGGCAAATAGGAGAATTCTTTGG
GATGATAAGTCCCTGTGGGAAGTCTTGATATGTTTACGAGATTTAGAAATGACCTCTTTCGAGGGATGACCTA
GGCGTTTATGCCAAATGTCATAGGATTCGATACTGTTAAGGAATAGAGATGTTTTTAATTGATCAGGTTTGAC
CACATCTCCGGTGACCCAGAATGTTGTGTCCCATACGAAACGCTTTTCAGCAGTTAATTTTCGTTTTCCATTT
GAACTGAATACAAGCGTATTGGATGATGCGCTCATATTTAACCCAGAATTGAGTAATTCTCCCATAGAAATGA
TTCGGGAGTTCAGTTTTGGATGGTGATAGACAGGATGTAAGCGAAAGGGTTGCTTTTTATATGATCCATCTGT
CTGTAGGACATCGTTTTCAAGGATAACTGTTCCTTGAGTATCAATTTGAATAATCTCGGTTGCGGTAGCAACT
GTTACGGGGTTTTTTATAATTGAATGTTCCGCGTAATCATCCAATATAGCGGTGAAGTGCATGGAGGCACCAG
AGTTGACTAACCATTGGTTTTCGACATGCTTTCTTTTTCCCTTACATGATGCACAAGAAGCAAAATGTTCTTT
TATATTGGCCAAATCGGCCGGAGACAAAGAAGTACATGTGGCATATTTATCATATAGGGGGCAATCAATGTTT
AATCGAAGAGCGCCGATTGTCCTTCAAACCGAGCACCCCAACTTTCCGGATCGAACTCAGGTTCTTCACCCAT
CGATACCGGTTCTTCTCGATCGTCGTCTTCATCGATCGGAGATAGGCGCTGTTTTTTAGGAGGCGGAACCCAT
GCCTCTACGTCATAATTGACGTAAGCAGTACCTGCGTCTTCTTCTTCAGAATCCCCGCAGTACTGATCGATCC
AACTCTCATATTCCTCTTGTCCCATGAGGATATCATTTAATCCATTGGGTGTGTTTGAGCGGGATGGAGAAAT

TACCGGCACAGGAGTGGGTGCACGAAGCGATGAAGTCGAAGCTCGAGGAGGAGAGGGAAGAGGTCGAATAGGG
CGTTCAGCTGGCGGTAAAACAGTGATGTCATGAGCCGCATAATCCAGAAGAACGCGTGATTCTGGAAGCGCCA
TTTGTTGAGTCTCAAGAATTTTGAGAGTCTCAGTGGAGGGCGCAACTCCAATGCGTGAAGCAAGAGACCGAGC
TTTTTCGTAAGTCGGGTACGTGCCAGGCAATTGACCTGGCGTGGCTCCTTGGAATGTTTGGGCCGGTTTGTCA
TGAAGACGTTGAAGGAGGGAGCGCTCCTTTGGGCCTAATGGTTTCGAACGAGTGACCTTTGGTGGTGAGGGTG
GAACCTCTACAATGAATGCCGAAGAAATAAGGTGTGAATTCCCATATTGGGAAGTTCCATCGGCGGATCCAGA
GTCGGAAGCGGCTTCTTCAGCCTGCTTGGCTTGGCGCTTTTTACGGCGCTCCTCACGGTTTCCTCCCTTAGAG
GAACGTGGTTTGGATTTGTCGTTGTTGGTTTCTTTGGCCTTACCCTTCGACGAACTTGTCTTTTCAGACGTGT
TCTCAGGTGCCGAATCCTTTGTTTGCTCCTTCCAGTTGGGGTTCTTGCCTTTTCGTTTGATTCCAGAGATTTT
GTTAGCTCTGGAAGGACCAGAAATGGCAGTGCGACGCTGGTATTCCATAACAAGAGCAGTGCGAACAATTTCA
AAAGTAAGGTCCGCTTGTTTATTGTTTTGGAGTATTTGGGAAGCAATATTATCCCATTTAGAAGGGATAGCAG
CGAGGAGAATCATGGCTTTGACGAAAGGCGGGAGGACGACGGAGTTGCCATCAAGGCGTGAGAAGAATGTGGA
CATAGCCATAATTTCAGGCTCTGGATTGTTATTCCCACTGATTTTGACGGCGAGAGCAGATCGAAAATCTAGA
AAGATTTGAGAGGGATCGGCAACAGAATACTTGGCAACGAGTGCTGCCCATAACGAACGTGCTACGTTGTAGC
CAGAGTAGGCGGCACGAATGTCAGGAGAAAGACAAATGAGGATTGAGCTGAGGGCTTGATCATCTTTAGTGAA
GTAATCGTTGCGGACTTTGTCCCAGTCTGTAGAAGATACTCCGGTGGGAGGAGTCTGAGAAGGAATTGTTTCA
GTTCCTTGGATGTTGGTGATCTGCCAGAGACGGTTGAAACGGAGGAAGTTTTTCATGTCGTCAGACCAATTGA
CCCAGTCGACGCCATTGAATTTGGTGATGGTAGAAATGTCGAGTTTTCCAGAAGACATGGTGATCAGATCAAA
ATTTGATGAGGTTGTCTAGTAAAGGGGGCGACGTTAGCCTCATGTTATGATGATGAGATATAAACCATAACGC
CTTGTGCAGGCGCATCAACGGTTTACCCAACATCTGCTTTAAGGCATGTCACGAGTGCACCCGTGAGTTACCT
GGATCCGTGACCACAAGTACTCACATGTTTGAAAGGCATACCTGGATCCGCACGAGTTTTATACTCGTGGGAT
CCGGATCCGTGAGTTAATTACTTACGAGTGTGCAAGTACTCACAAGTATCCAGGGCCCAGTGCAGGTCTGAGG
CTGTGTGTGCCGGTCCGTCGATCCGTGCATCCGCCGATCCCAACGCGCGTCCGTTTGCGGGTATCACCCATGC
ATCACCCACGCAGAAACCGCAGTTCACGTCCCCCCATTTGGCCTCGCGATGTCGTTGCGCACGTCAATTGACG
TGGCTCACGCGCAGTCGCGAGGGTAGAGGGGGTAAGTGAGCTGGGCCCAGGGGGCTCCACCTCCGCCAACGAG
AGGGGGTAAGTATAGTATTACGCACCTTATCAAGCCCCTCGGGCTCCCGTTTGCAAGATTCTGTGGCCCCAAA
CTTGTTTGCGAGAAGGGACGGGCAAGGAGTGGGGTTGCCGATGTGTATGCATGCATGCGATCGCAGGGTGATG
TCCAGCCGTGTTTCGAGACGCGAGGTGGTGATGGGGACGTGACGTGGCGATGAGAATGCGTAATGTGACATGC
AAACTAGTGATGGCCGTGTGAGCGAGTCCGTAGACGTGGTAGCAGGTGGCCCGGGTGGTTGAGATGCATATGA
GGGTTGGATGGTGGCCGAGAGGGTGGTGTTGGCTCTCTCAATTGCAATGGGTGGAAAGCGGTTGGCGATGTGA
TGGCGGTGATGATGACGTGGCGTTAACTCTCTCGATTGCGATGGTGGAAGCGGTTGGCGATTGGGACACGGCG
TGGTGGCAATGACATGTGGCAGTGAGGGCATATGGCAGCGATAATGACGTGGCGATGGTGAAGCTGTGTGATG
ACGTGAATGCGATGTGGTAGTGATGGTTGGCATTCTCATTTGCCGCGGGGTGGTGGTGCAGTGGAATGGCGTG
GCAGAACGATGTGGAGCAGCGCTTGGAGAAGTGGTGGTGGCGGGATCGGATGAAAACCAGCAATAGGCTCATT
TGCAAGAGCGGGCGGCGGCCCATCGCAGACATCCCCTCTGTATTTTTGACATGATGAGGGGGCAATATGGCGG
CGTCGACAATTGAGGTATGAAGGGTGGCGGAGGGTGGCGACGTGAATGCAAATGGACGATGGCAATGTTGAGG
CGGAGCCGTGAGTCGTTGTCCATCACCGGTTCGTCTAGGTGTCGTCTGCGCTGTGTTGTGATCGATGAAAAAA
CACTTTGACTTACGTGTCGGCGATTAAGGTGTGATCGATGAAAAAACACTTTGACTTACGTGTCGGCGATTAA
GGTGTGAATGGTGGCGGAATCGAGCGCCACGTTGAGGGGCTATGGCTGGCGGCGGCATGTCACGACGATGGCG
GTATTGAGGAGGGGCCGTGAGTCGTTGCCCATCACCAATTTGCCCAGATGTCGTCTACGCTGCGTTGTCATTG
ATGAAAAAATACTTTGACTTACCTAGAACTCGCTGAAAATCCCAAGCACAAAGGCGTGCATGCGGTGTGGCCG
TCCGTCTGGTGTGCACAGCGGGGTAGTGGGATGGTAGATAGGCGGAGATTATGATGAATACACACGATACAGG
CCACGGGTCACGGCAGGTACTTGCCGTGAGTACCTGCGGTTGAAAATTTGAAACTCGGACCCGCGGCAACCCG
CGGCCACGAGTGATCCGGATCCGCGGGTCACCATACTCGCGGGTTTTCCCACGGATCAACCTGTAGTGACTCA
CAAGTAACTCGCACTCGTGACATGCCTTAATCTGCTTTATTCAAGCATATGTGGTAGAAGATGCATAGAGCTA
GAACGTAAAACGTTCATAGCATGGCAGAGCTTCGGGAGATGCAAGAAGCAAGAGCGTAAAACGTTCATTGCAA
CGAGCAGAGCTCTCATCCAGACTGTTAGCTGCAGCCTTGTTCAGGCGCGGCAAGTCGTCTGGGGTAACCCAGT
GGAAGCGCATTTCATGCAGCCTTATGCAGGCGCGTGTAATGGCGACCAAGTGACCAGTCTGTCGGTTGCAGCC
TTATTCAGGCGCAGTAGACAGTGGCACCAGGGCCCATAACCTGTTGAGCGGCACCCCTAGGGCACAGCGGAAC
TGGTCTACTTACTTGGTTAGAACGGTGAGCTTACTAGGTGAGTGTTGAGAGCAAAAGAGTTATATTAGCCTCT
TGTTCAGAGGGGTCTTATATACTACAGTAATGTGCACAGACCATGTGGCTGTGCTAAAGTCCTAACTCCTATC
TATAATCTAATCTACTGATAAGATAAGATAAGATGTTATCTATGGTGCTTATAAAGACACCGGATTATGTAAG
CAGGGCACTTACAGTTTAGAGCATTGAAGTTTAACA
>Cligib1_rnd-1_family-305#LINE/I
CACCGGGGAGGCCAACCGNGAATACCGGNCGGCCATCCGAGCCGCACGCAAGANTTTCTTCGANGACCGCATA
CAGGAGACCGTCGNNAANCGCCGCGCGTGGGACCTCATGGGATGGACCAGACCCCGNNCCGCCCCNGACTACA
ANGCGGTNAAAGTCGACGGCGNCCCNGTCGCCTCCCTCGCNGAACTATGGCCCGGNCTNCAGCGGGCNTTCGC
CTCCGCCGCNGACCGGCCCATCGACCTNNCCCACCTGGACGCCGCCCCTNCNTGGGAGGAGCGNCCGTTCCCC

CCTTCTCGTCGGTTGAGCTGCGNGANGCCCTCCGGCCGACGTCCAACTCCAGCGCCCCNGGNCCNGACCACGT
CNCNTGGCGCCTCCTCAAACTCGCNCTCGGCGAGCCCGGCTCCCCCGAGGCCATGTCCCTCATNCGCCTNTNC
GACGCCTGCGTTCAGCACGGCGTCTTNCCCGACTACTTCAAGGAGTCGCTCTCCGCNATCATCCCCAAGCCNA
AGAAACCCGACTACTCGCTGNTCAAGGCCTATCGNCCNATNGCNCTCCTCAACACGCTNGGCAAGCTNCTCGA
GAAGNTCATCGCCAACCGNATGCAATTCGACGGCGTCCGCCNTGGCTCTTCCATCCNTGCCAGTTCGGCGGCA
CNCCTCAGAAATCCACCGAGGACGCCGGCGTNCGCCTGGTNCACATGATCCGNCAGGCNTGGGCNCGNGGNGA
GGACACNTCCTGCCTCGCCTTCGACGTCTCGCAGTTCTTCCCGTCGGTCAACCACGGNGCCCTGATCCGNATT
CTNCGCCGGACGGGCTTCNCCCCGNCNCTCGTCCGNTTCTTCGAGTCTTACCTCGTCGGNCGNNCCACCAGGT
TCAAATGGGGNCCGGCTATNTCNGGNCCCCACCCCGTNANCGTCGGCGTCGGNCAGGGCTCCGCCCTGTCCCC
CGTCCTNGCTTCCCTNTACGTCGCTCCGCTGCTGCACATTTTGCTNCCGGCGAATCGCGCCAACCCCGTCGAC
TTCTCGGTNTACGTCGACGACGGNGTCTTCGTCGTCTCCTCGGAGTCCCTCTCGTACAACACCGANNTCCTNT
CCGGATTCTACNGGCATATGAGCGNCGGCTTCGCGGCGCTCGGCCTCGAAATCGANCATGACAAGACCGAGTT
CATCCACTTCACCAAGGANGGCCTCGGCCCTCCATCGACCTGGGCGAGGCCCCGTACACGGCGACAACTCCGC
TCCGCCCTCAAGAGGTNTGGCGGTACCTAGGTTTCTACCTGGACCGGCGGCTCTCGTTCCGAGACCACGTCNA
CTTCTACGCCACNCGNGCTATNTCCACCGTCAGAGCCATGCTTATGCTCGGCAACTCCGTCCGNGGCCTCTCC
CCCGCCAACAAACGCCTNCTNTACCGCTCGTGCGTCGTCCCNATNATGACNTACGGCTTCCGNCTCTGGTTCA
GNCCCAAGGGCTACCGNGTCAAGGGCCTTNTCAAGAGGCTTCAGATCGTNCAGAACGCCGCCGCCNGATGGAT
CGTCGGCGCNTTCCGCACCACTCCGTCCACGGCNGTCGAAGCCCTCGCCGGCCTCGTCCCCGTNCACCTCTCC
CTTCAGCGNCTCTTCGCGCTGTCCGCGTTCCGGGCCCGGGTCTCGCACGGTCNCACCCCACNCNGTCCATGCT
CCCGNCCGCGTGGCGAGCCTCAGGGCTCGCCATCCGATCCACNCACTCCATGCGGCCCGANGACGCGTATCCG
TCCCCCATCCGTTACATCGACGACTCCGCCAAGGCGGTCACCGAGCGGTTCGCCCCGTTCCACCCCCTCTCGA
GNCCNGGCGACCGCGTCCTCGACCTNTTCCCGGACCGCATCCATTTCGACGTCTCNCATCCTCCCAAGGGCTC
GGACCNNTTCCCNCANTGGCTCGACGGATTTCAGACGTCCCTNCACGCTNACAAGATCGACCCGACCGCTCTC
GTCNTCTTCACGGACGGNTCGGTCATCGACAACGACAACC
>Cligib1_rnd-1_family-355#TIR/MITE
CAATAGGGGGTTTCACGATAGAGAATTTTACCCCAACTTTGTCTCCAACTTTGTCCTCAACTTTTTCCCGGAC
TTTTGAGTTATTTATTCTAAAAATCGAGTAGAAATTTAGTTGAGCTGCGAATCGTGGGGGAGGGCACGATTCG
CAGCTCAACTTATTTATTCCTTAATTTTCTGCTCTCAACTAAACTTGGAGTCCGACAAATTTTGACAAAAGTT
GAGCGTNAAGTTGGGNCAAAAGTTGGGGTAAAATTCTCTATCGTGAAACCCCCTAATG
>Cligib1_rnd-1_family-242#TIR/MITE
ATATACAGTNGTGCCCGTAAATATTGCATGTGGAGCACCGGAGATAAGAGCTCCACCAATGCAGCGTCGCCCA
AATTGGAGCGATGCGGGGCCACGCCATAAGAATATAACCGACTAGGTATGCCGGTCGAGATTAATATTTTGTA
GTTCTTCCTTGTGTTTTTTTGCAACGGGAATGATCAGATACCATAGTCAATTGATACTATGGATTTAGAATGG
ATANTTCGGTGTCAAAGCAACTGTGATTCATACTACAGTACACTACNACAGTCTGGCGGCGGAGGGATGACGT
CAATCGCTGTAATTGTATCCAAAATTTTGAGATCTTATCTCCGTGCTCCACATGCAATATTTACGGGCACGGC
TGTA
>Cligib1_rnd-1_family-291#ClassI/TRIM
TTTATCGTCCAGGAGATTGAAAAGTGCGCTGAATTGGTCACCGGTAAAACGATNGCCTAGCTGCCAGTTAGTA
CGCTCGCTAACTAGCTGCAATGTAATTGTACCCCGGCGTGCTAGTGCTGCCTCGCAAGGACGTCGCAGGTAGC
GATTTTCACGCCTGCGTCTTTATCGTCCAGGAGATTGAAAAGCGTGCTGAATTGGTCACCAGTAAAACGATGG
CCTAGCTGCCAGTTAGTACGCTTGCTAACTAGCTGCAATGTAATTGTACCCT
>Cligib1_Cluster104#LTR/Gypsy
ACGAGTCCGGGTAACTCGTGAGTCACTGTGAGTTGACCCGTGGGAATACCCATGAGTATGCCTACTCACGGAT
CCGGATCACTCACCACTGAGGATCCCTGCGAGTCCGAGTCCAGATTATTTTATTGTGGGTACCCATGTAAGTA
CTTGTGAGTAATGATGCAGTACTTTAATTCTTTGAAGTTCACCATGGGTCCATGCATCAGCAAAATGATCGAA
TCTGGCGCTAGAGAAAAATATGGTGAAGTAATGGTCCTGAATGACATTGTGAACTTACGAGGAATGGATCTGT
GGTATCACCGTCATCAAAGACTTGCCACCCTACACCATCCTGAGCACTCTAAGGCGATATCAAATCTGAAACC
GGAGCAAGATATGACGAAGTGAGATTAGAGAGTAGAGTACGCACACTGCCTGGGCAGGGCCTCCAGATTGAGG
GTACTGGTAGCCAAGGGGGGCGGGCTCCAGGGGGACAAGAATGGCGACTGGAAAGTACAAAGAAATGAGCCAA
ACCGGTTAGGAGTTAGGAAAATGTAGGAGAGCGGTTAGGCCATTGAGGTCGTTGAGCAACCGGGTAGTCGGTT
GGAACTTGGAGCGGATGATGGGTCTTGCTGATGACATTGACAATGGCGGGCCGCGGATCCAAATGTTGATCGA
TAAAGATCACATGACTAGGAACATCGGCTCTCCACCAAAAATTCTTGTCAAGTCCCCGTCACCAGTGATTGCT
CTCCATTCGCCCTTATAACTATAACTATGCCTACACCTTCACTCCCTAACTTACGATTTACTCGCCATGAGAC
CCCCCGCCCGCTCCCTCTCTCCGCTCGATGTCGAGAATTCTATTGACGGCAGCCCTGAGCGCCCACGAGCCCG
ACGCCAATCTCGACACCAGATAGATGCGACGAACAATGCGATGGAAGAGCGTGCTGATAGAGATGATAGGCGA
GCTAGAGCCACGGCGCGGGCAAACGCCCGAGCCCAGAACAGGGTGGCCGCCAGAACCGTTGAGCAGGCAGGCG
ATGATAATGAAGTTCAGCAGCTCAGAGGTAAGCTATTCTCATAACTCACTCCTATGCCAACCGCTAATAATAT
CTTCTGAATCTTAGCACTCTTGTATGCTGCTGAAGCAGAGCGCGACGCCGCCGAGCGGGAACTGTTGACGGTA

CAAGCCGAACTGGATCGTGATGCCCCCGATATAGATGATGAACCAATCCCCCGGCCCTCAAGAATTAGCAATT
CCTCTGTTCGTCTCTTGCGCCATCACATGAATCTCGTGGGTTCCGAGCATGACATTGAGTGGATTCGTATGCG
GGTATGTTTTGCTTCCACTTCCGTCCCCCCTGCTAATGCATCATAAAGTCCGTAATTCGTGATTACTTAAGGG
CTGGCCGTATTCAATGGGGCCTCCGCTGGAAAGAACAACCAACACGAAGGCTCTCAAAAATATATGACGCGGT
ATGTTCAGCCGGCATATTTTATCCTAAACAATTGATCTCCATCCTACAGATTGAGGATGATTTGCCAGTATTA
CGACGTTTCCAGAATAGTTGGGCAACGGAATACTTAGCCAAGAACTCCTTCAACGGTCACAAAACGTATGAAA
GATGTAAAGACGATGGATCAAGATATCGCGGAAAAGTTAAACGGATTTATAAGAACGCTGTTGCCTTGCACAA
GCGGCGCGTTAATTTGACCATTGAATCAGATGAACCAGAAGAGGAAGAGGGTACAGACCTAGAGTAGGAGATG
CCGATATTTAATACACCCAATGTGATTGAACCCCTTGGATTATGGGCCGAATTTTGAGTGAGCCACTTCCGTC
GCCCGCGCCTTATATACTTATTGGTGATTATAATTATAGAGTTACCATCTAGGTTGCCAGTGTGTGACCCCTC
AACTGATGAGCTGGACTTAACCCTTCTTCCAGCAGTTGTATTCATTTGACGGAAGAGTGGCCATACCACAGGA
TCGTCCTAAAAATGTGGAGGGGATCTCTACGAACGGCAAAAATGATATATGATTACAAGAAACGTTCGCATTT
CAATTTCACAATGGTACCCCATACTGCTCGACGTCCAATAACCTCCCGTTTTGCTGCCATCTCCCACCGTTCC
TATACAAAACCGATCGCCCCCGCGGGTAGATATGTGCCGTGGCAATGACGGGTCTATCAACGGTGGCCCCGCG
TACCGTCCAATGTTTGCTGCGGTCGGACGGTGAGGCGTGAAGGGGACCCCTTGTAACCAGGAAGATAAGTGAA
TCTACGATAAATAGCACAGAGAGACTTACATCAACATCCACCGCATCTCCCATTGCGGATTTCGCGCTCTTTC
AGCCTCGGCTATCCTACAGATCTTTTGGCGTATCATTTCGCGAGTCCTGCTTTGTGGTTTGTACGAGCGAATG
CAGTAGATAGCGGCATTCATATTGGCGGTGAGGGCGAGTGTGTACTGGGTAGGAGTTGGCGCTGACAAAGTGA
GAAGGTATGCTGAATATGAAGATATGTGCTCTCAACACACAAACACAAACGCGCAAATGTAAGTGCGGTGCTC
TGCTCTGGCCTCATCATAATTTGACCAATATTTTTTCATTCCCGCGAACACTACTCAAGATCGTTGAACGACA
CCATGCAAGAGAACGACATGGCGCTTGAGCGCGCTCAGGAGCCCGATTCCCGTTATTGATATGTAAACCCTTG
GTGCAATAAATTTTTTCTTACCTCGCTTTACAGGTAAAGCGGATCACCACAACTCCAAGTTGCGTAAGTTGGA
TCCCCGCAGTTCCAACTCCATTATCACCACTCGTTCGTCGCCAGCCTCCCACGCACCCACAAGCCCGCGTCCA
CCACCAATCTCCAGCATGTCGCTTAGGCCGCCTACATGCCTACAGGTGCGATCTACCCTTGCCCAGCCTTTCG
GGGAGGACGGTGACACCCTCCAGTATGTGCGCGCTTTACACTTTTGAGTTTTTATTCAACTAGAACCACAAAC
CACCACCCTGCCCCTCACCCGCCCTTTTCCCTATCGCCCCCGCTCTTGCCATGCCCATCCGCTCCCCCTTTCC
CATCCCCAGCACAGGATTGCAATTCCAGAAGGAATCGATGCTCCATCCCACTTCTTAACTCTATGGTGCTATG
CAAACTCTTGTCAAAGCTCTCACCAGCGAGACGATTGCGCCAGAGGTCGGGCTGTCGAACAACGTGAGCGAAT
CAAGGTAAGGAGATTCCACCTGACCAACAACATCTGATCTTTGCCAGAAAACAGCTCGAGGACGGGCGCATCC
TTTCCGATTGCAATACGCGGACGGAATCCACTATCCCGTTCCCCGACCCCGTGGCGGTATGCAAATTTTCGTC
AAAGCGCTCACTGGCAAAACGATCGCGCTAGAGATCCCTCAACGCACAGCCGCCGGTACAGCCCCTTTATCGC
GCTGGCCCACGGGCACTCACCAGGTATATCACCTTCTTTGATGATTATCTGCTAACCCCTTTCGCCTCCCAGC
GCGATCCCGCTGGTGCTTGCCCCTTCCATCCGCACTCGCATGTACCTTCTCTGTTGCCGCCTGCCCCCTCCAT
TAGTATACGTCCCCCCATCAACATTCATCCCATTAGTGCTTGCCCTCTGTTATCTTACACCTTTGCTACCTTT
TGCTGGTCCATTTTCAAAGTAGATACAAATGTTTCAGTACATAAATAATTTATTTCATTTCAAAAGCAATCCA
CCCCAAGTGAAAGTTCCAATGAAAATCACCTCAATAGACATGGATAATGATGGGAGAACATAACAATAATCCA
GGTCTTTTATTGAGAAACTCACTCAGAATACAATGAAAGGGAATGAGTGACCCCTGGACAGATAGTGTTACGT
CCTGCTAACGCAAGCCGATGAGTCGACAGTAAAGACTCAAGGCACTTAGCAAGGTCTGAGTTGCCTTCTGGGT
CAGGAGGCTAGTAGAGGGGGGAACTGATAAACGATTAAACGTAACGAATACAAGGGTATGTAGCTACGTTTAT
ATAGACATGTATGTAGTTCTATGACTATGCAATAATGACTATGTCCGTGAGTATAGTAACACATATGGAGTAT
GATGACTGATACAAGAAGCGAGCTGACTGAGCAGCTGAGTAAGAGCAATAGGTGATAGCAAAAATAGAACGGG
AACCATGCGGTGGAGAAGTAGATAAGGTGCGCATGATGTATCATCTGATGATGTAGCATATGGCGCATGTCAG
ATGATGATGGGGTGATAGTGGAAGTAATAGAAGTGTCTATGAATGATACCATCAGTCAGATGTCCAAGTGATG
ATGATCGAGAAGGAAGGGATCGTAACAGATAGTACAGGAATCAGATTCTCTCTATTTTCATTACAGATGGTGA
TATTCACTCTGAATCAAATGAATATATCTCATAATTAAGCAAATATAACTCATTTCTGAGTGAGAGAATTAAT
GGCTTTCATTATCTATCCCTTGTCCAGTGTGTGTGGTACAGTGGAGAATTCTAGTGTACACTACTGCTGGGCC
CCTGCCTGATGCATTGATACATCGGACCCCACCACAATCATGACACATATCAGCTCTTCGTGAGGCCCGGGGG
TATGAGCTATCGTTTTTTTCGTGTTTGCGTGGATGGTACATTTTATATAAGGGGCTATTCCAGTATGTATATT
GATATGTTTTATGCGAAAAACTAGATTTACCTTCGGTGGGATCTCAGGTATTAACAGGCCTGCTAAAAACGGG
CGTGCTAAAAGCGGAAGCGCGGCGTATACATATGTGTGTATGCGACGATTCAGTTTCCCGATGTCTACCTATA
ACAGGATGAAGGCGGGGTAACGGGGCGGGAGGGGCGCTACGGGGTAGAATCAGCGTGCATGTGCATAGACAGT
GGTGGAGCCTAAAAGAAGGCTGATATTTATAGATGTGATGTATCACTCCTGTCAGCAAACTAGAGTTATTGAT
ACAACAGGGCTGGGCTCTCACTGTAATAGAATGATACATCATACTGACACGATTGCCTGCTCAAGATGCTACC
CAGGTACCCGCGAAGCGGCGCTTGAGACCATTTCTCACTGGACTAGGGGCCTCAGGCCTCACACTGTCTCTGG
TTGCATGGCCCAGCTGTTATGATCCATTGCTTCTCATTCATCATACATCAGTCACTTGACTGATCCCATCTTT
CATAGACGCTTCTACTTCCTCCATCATCATCTGACACCATCCACATGCTCCATCATCAGATGATACATCATGC
GATCCCTTATCTACTTCTCGACCGCATGGTCACCATTCTATCTTTGTTATCTTTGCTGTTCTTACTCAGCTGC
TCAGTCAGCTTGCTTCTATTATCAGTCATCATCCTCCATATGTCTTATTATACTCACGGACCTTGTCATTACT

GCGTAGTCATAGAACTACATACACTCTATATAAATGTAGCTACATACTCTTGTATTCGCTACATTTAATCGTT
TATCTCTTTCCCCCTCTACTGCCTCCTGACCCAGGAGACTTCAGACCTTGCTAAGTGTCCTTGAGTCTTTACT
GTCGACTCATTGACTTGCGTTAGCAGGACGTAACATTCGATTAAATGTCTAGGGTCACCAGGCAACCCTGCAA
CGAAATAGGACAACCCCTAGGGATAGGGACGACACCGACATCGATCACAGTTGGGAGTATATCACTCCATATA
GGGGCTCATTGCCAGTAACAGCATCGGAATTCGAACTACAGAGATTAGGCGACGAAGCGACCGTCGCACGCAA
TAGGTATCACAGGACGTCGAAAGGATCAACAAAGAAGGAGTGGATTAACCTCCACAGACAACATCAGACATTT
GAGAAGCTCCACGACGAAAGATTAGCCGACATCAGGAGAAACATTCGTCGGGCGAAGGAACAAGAAGAAGTCA
CTCGCATCATTCAGGACAGTCGTCGTCCTCGAGAAGAGAAGGGAGTAGCCACAGATCGCATCACACTTCCCAC
CCCACAACCCACAGTTCACGGGTCGCGCCCCCCGTCAGAGAAGAGTCAATCACGGCAGAACCAGAAGACGAAT
ACGCCGCATATCACCAGTCCCACCCCACACCAACAGCTAGCCAATACACCTCCGTCCCCCGTACCCCAGTCAC
TACCCCCTCGCACTCCCACAGCCTCGCATCAGGGTTCCAACCGTCTCAGGAAGCGCCGTCCTCACCACAATAC
ACCTTCGGTTCAGAGCGAACCAGTACCGTTCAGCCAGTCGGACCCATCCAACCGGAGCGTACAGCTTCCTTCA
CTGAATATCGAGACCCCACTCCCCCTATCCCCTCAGACGACGAGGACGAACCGTACGGAACAATGGCTACAGA
GACAACGACCCCTAAGCGGAATGACAATGGAATCGCAGACCCCGGCATGTTCGACGGGGATTGCAACCTTTTT
GACGGTTGGTGGCGTCGCATGCAACTTTACGTCAGAGGGAACGCTGTCCTGGACACGGATGAAAAGAAGATTT
CGGCCGCGCTCTCTAGATGTTACAAGAGGGCCGAGAACTGGGCGGCCATGAAGACAGATGCTGCAATGAAAAC
AAACAATTTCGGCACATGGAAGGAATTTGTACCCGAAATCACTAAACAATTCTTCCACGGTCAACGACAAATA
CTGGCAGAAAACCGAATTAGGGTAATGATGCAAGGATCTCAATCAGTCGAAGAATGGATTGCAGACTTCGACG
CACAAAAACACATTGGTCAAGTTTCAGACGAGCAAGCTATCTTCTTGATTCAACGAAACACCAAGAACTCAGT
CATCGACAAATGCCGCGTCCAGAATGGAGGTGCAGCCCCATCATCTTATGACGATTGGAAGAAAGCGATCCTC
ATATACGGCAACATTGAGGAAGAACTCAACCTCATTCGAAAAGGAGTTCAACGCGCATCCACATCTGGAGATC
GACATGATGGAACAGGCACCACCTATGGTGGCAGAGGACGCCCGATGGATATCGGACAGGTAGAAGAAACAAC
ATCGACGCAGCAAACTGGAATGCACAAGGAAACCCAAGTGTTTCAACTGTAACAAGTATGGACACTTTGCACG
AGAATGCCGAGGCAAGAAGAAGACAGACATTAGGATCAAGATCAGGACAGGGGCGAAATGCTGGAAATGCAAC
AAGGAGGGACACATGGCAAAGGAATGCCGGAGCTCAGGGTGGTAATCAAGGGCAAATTCAGGGTCCTTGATGA
TGATGAAAGCGACGATGAAGCTAAAACGGTTAAGGGAGATGACGATGAGAAGAAGGGTTTTCAGGAAGGGTCA
GACTGAGCACGAAGTCTCGACCCAAGGGTTCGTCGAATCATTCTAAATCTGTACCACCACCGAAGAATGACAA
ATACATGATTCCACAACGACGAACTAGTGCACCTGAGCTCGCAACTCTACATCTCGCCACTCTCAAAACTAGA
AAGGATAAAACAATGGTCACGGTCAAGGTTATAACCACGGACAAACGAAAGGCTAAACTCGACGCACTCCTGG
ACAGCGGATGCACCCACTTGGCAGTCAATCGACGACTGGTGGAAGCAGAAAAACTCCCCACGGTTGATTTACC
CACTCCTATTCGGGCATTTAATGCGGATGGTTCTCTCAATGCGGATAAATTAATCACCAAAAGCGTCAGGTTC
CAGATGCAGATTGGCGATCACAAGGAAGAAGCAACAGCACTGGTAGCGGACTTAGGCAAAAAAGATCTCTTCA
TCGGACATGAATGGTTACACAAACACAATCCCTTCATCAACTGGAAGACTGGCAAGATCAAATTCCCAGACTG
CCCCCCAGAATGTGCCAAAGTAAGTGTCAGCACACTACCCAAGGCTAAAGTTCGGTCAACAGACACGGTCACG
GATCTAAAACTCCCAAAGTACCTCACGACTTGGAAGGATCGATTCGAGAAGAAGACCTTTGACACTCTCCCAC
CGTACAGGAAACATGACCACGAGATTAACCTGAAACCAGATGCACCGGACGAATTACCAGCCAAATTATATCC
AATGACAGCCGCAGAAATGGAAGAAATGGACAAATTCCTAGAAGAGAATCTAGCCTCGGAACGGATAATCCCA
TCCAAGAGTCCATACGCTGATATGCTGCGAGGCTTGTGGCCAAAACCAACCACAAAACCAACACATAACACAA
GTACCTAGATAAGGTCCTGTGGATGCGAGTGTTGTCCGAAATCAGGGAGACTTCCTAGCCCAACTGTGGCGAG
ATCAACGCGAGGGCTCTGCCCAAAAGGAGAGTTGCATGCGAATCATGCGAAGTGCGAACTGAAGGGTGAAGAC
TTTTCTTGTTGATCATGGTAGCTACTGAGCTCTCTAAATATCGGAGCTAAACGGCCATGAGGTACAGTAAATA
GGTAAGTAAACTATGTAATAGAATGCGATCTGCGTGGGGAGTGATGCGATAAGGATGCGAAGACTACCACTAG
AATCGGAATTGAGACGCGATCTCGACGCGTTCGGCGCGGAATGGGTGCGATCAGTACTGTACGAATGCGATAT
GTGCGCCTAAAGCGCCCAATATCAGTATGAGATGTGAGCTGCGAACCCATAAGTGCGCGGCGCGCTTATCATC
TCCTCCTCACTTGAAATAGTGACTGTCCCCAGTCACTGCGGGGCTCCGGGGGGCTGTTGAGGTGCTAAATAAT
GTAAACTAAAGGGAAATTATACTAATATGACTTGGAAATAAACGCGGGCTCATCGGAGGGGTCGGAAATAGGG
TGGGGCCATCCAGGCATATCTGCGGAGTGTGTAGGAATGAGTGCGAAGGGTCCGGAAAAAAATGCGAGGCTGAG
AGGGGTGACTGAGGAGATCATATGCGGGGTTTCTGATGACATGGGTAGGAGTCGCATGAAGTGCGTGCAAATC
CTTCGCAACTGGGGTGTTCTCGGGGCCGTCCCTGATGTGTGTGAGGAAAAAATGGGGTCCACGGGTTGGCTTGG
CTCCTCTGGAAATTGCTTCTCCTGTACTGTTTTCTTTCGTGCCTTCGCGTAAATCCTGTGTACTGCGAAAATG
ATGCGGGGATGTGGGGGAGTGTCGTATGAATGGTGCGAGTCGATGAGTGCGAGCGAGGGGGGCTATAATACTA
TTCTAAAGGATTATGAAAGCTATCCTACTGGGAATTATCCGGAACGCGGTGGCTCGTCGCAGGGGTCGCAAAC
CATGCGGATACGTCGGGGCATGCCTGCGGAGCATGCGAGAATGGGCGCGAAGGGCCTGGGATAGGTGCGAGGT
CGATAATGAGCTGGGGGTAACCTGTACTGCTGAGGACAACTGGTCCTGTCTATTGTTGCTGATCTTCGCGTGT
CTTCTGCGTGCTGCGGGACGGTGCGGGGAGGTGCTAGGGTGTCGAATAATGGTTGCGATGCGGCTGTGACGAG
CGGGTGCGAGCTGGGATACTAAACTAGGGGATTATATGGAATTACTACTCTATGGGGATGGAATGGATGCGGA
TGCTCTTCAGGGTTTCGGGACATGTCCAGTAGGGTCCTATCGTGTCTGCGGAGGGCGCGGGAGTCGTGCGATG
CGATGCTGAGGCGGTCGCGTTTTTACTGGAGGGGGTTGTTGGAGTGCAAGCTCACTCAGTATTACTGACTTAG

AATAATTTTCGCATTACCGATTATGTAAGCGCGTCCAGTCTTACATTCGAGGATTGTTCCTCATACAACAATA
CATACAACATATCTATCTAATAATATCTAATACCCCCCATCTGCCTCCTCCACTGCCAACAGGGGTATGACAT
GGGGTGCTTGCAATGAAAGCGCTGGTACTCTGACCAACTCGTACCTTCAATGCGAACACCATGGCGCAATGGC
CATACTGCTGTAGCTGCTGGGTAACTACCCGAGCACGTACTTCCTATAGTGTTCCCATCAGGGATAGACTGTC
AATGCTACTCCTGTTAAAAATGCGAAATGCTCAACAGTGAGCTTCGCGTCGATTCTGCGGACCTGCGACTGAT
GCGAGAGGGTGCTGGTGCATGGAAAATGAAGTGCGATGGATGCGGGGGAGGCGGGACGAGGCGACTAGGACTG
GGTGATGGTGGACGATGACTGGGCGGTCTGGGCGCGTTTCAGAGCACCTGAAGGGTTCCCGGCGATGCGAGAG
CCTATCGGGTGATGCGAGAGGGCTTCAGAGCACCCCAATGACTCCTGGTAAAACAGGGGCCACCTGGTGATGA
AGTGCGGAGGTGCCAACTGGGCGACCTTTGGTGCTGCTGCATTTCTGCGAGTTGCGACCAACGCGATCCTAGA
GCGCATCAGTCAGGGTCAATACCATGTGTCTAAGTAAGGCATGTCACGAGTCCGGGTAACTCGTGAGTCACTG
TGAGTTGACCCGTGGGAATACCCATGAGTATGCCTACTCACGGATCCGGATCACTCACCACTGAGGATCCCTG
CGAGTCCGAGTCCAGATTATTTTATTGTGGGTACCCATGTAAGTACTTGTGAGTAATGATGCAGTACTTTAAT
TCTTTGAAGTTCACCATGGGTCCATGCATCAGCAAAATGACAG
>Cligib1_Cluster57#LTR/Gypsy
gGaaCAcGgCGcagCagCgCccaGaaCaaaCaAgCacaagaccgacaccaaCaaacgcGaacCaaCcaGaTGc
aaaagAacaGacgaGacTcGTacaagACaAaaCaaaaCcacTacccgaCaacaaGagGccaCaaccccGcgcc
cccaaccaaccTGaTCGccgGagCcccGaGagGaccCGaCcTGccCaaccgGccaTgagaccgcAAcgcacCc
TaacCgcacCaaaccacCcaccaaTcaCccCAaAacccCaaaCcccaaaCCgcaaacCccaaaaccaaaAcCc
caCcccaaaAaGAAcCccaccaaaCaacgGccaaCcacaTCcacccaacaacCacaCGgaagccTcacccaac
cCAgcccaccaaCaaaaacCacccCcaCCaCAccCCaacCcaGTgaaaaGagCcAgaGCaCcgaccgaaCcac
CAgcTCGcTCaCCTGacggGcTaCAAaaaCGcGGAaTagCCcccccacaaaccCcacGTaTacaGaCcCCcaa
TCaAcagcaaaCaaaccacAaGaaccaaCAacGcgcaCcaaaAccccCCTagcaGagcaCaaCcgCagcccaa
cgcTgcgaaccacaCcaaCGcTAaccccaaggcCaaccCGaTacAacaccaCaaaCcCaccccagaCcaacaT
CACgcagaTcacacCaccgcCcagcAcaaaaaaaaCcAaGaacgCcaacacccgcTcCccGTgCaGgGccGaa
acaaaccaTcccgccaaGCcagaCgaCTccccaaAaGaCaTcaaGAacaccaTccacGaAgacaGCAacaTga
acaagaccgaTccaATcgaaagccgCGccaaaaacaTGAaaAGCCcCacaagCaaCgacgacgGcgacAACga
cacaaccGcCaaaAcCaaacacaacaGGagcGAGaTccaGCaggCAaagggcaaacGCcagccaCcgGgAaGa
agaTCgAacaaaaCAaccgcccAGcaacacccGcacagcTCaGcCgcaaAaagaacaaCaacAaaATagaGcA
aaAaggTaacggaTcCTgcaaagaAaCcaTgcaCCCACaggGaGTacGCagACAaGacGcgcGaCcTGGcGca
caacGCcGcgaaccaagaaacgaacgcaaaCGccTgGaaagcgaggcaccaGccccacaacaacaTcaacGaa
GacGcaAAaGAGgaGaAaacGaaacGcAaaGAaTTgagcGccGcaaTcGaGcaGaCaTcgcacTcgaaaATcc
accaccaaacGgcaaaGcaacAaGcGcacaaaaAaaaaCcaaCaGaaggccAaCaaCcacgacacaaccgCAa
ccacagccAagcaacaaccAccAaCaccAaGacggccccaCgcccAacaaGcaaCacGacaaaccagAccacc
aCaaCgaacAaaagaccacaaaaaaagCaagCaGcaacgcCacgcaaaAgacaccCaccaaccgagGaGcaac
GaaaGCgcgcCagcacgcacacggacacacgacCcagGcacCAGgacaCgcccgaagGaaaaaCagcaTGGGa
agcgcaaagGccggCaTcgaaaccgaaGcaCgcccaaaaTGcgaGaGcaacgaCcagaaccccCcaaCCcaTG
cGCCacaGcggccCacCcTcaaTcgCagaacagaGcgacagATGccggaaaacgcgCcagcgcCgcacagaTT
GaacaccaaCacAGccaAccAGCGCAaaaaaaaccgaaggaaacAgaagcgCGgcccaGaacccTGgcCaCaG
TGccagGacgcccGTcaAgaCagcgGGcGaacaaacagcagATaaaTccgccacaaaGaacagacaGGcCacc
CaagaaGaccAgacaaAccaAGaGcgGCCaTCAcccccaccgaTacGcaggccgcCccaaaGcCaGcTgacac
caccagCaAaaaTgagaAccaaaacaAcGaGaCccaCaacGAaaaTaaGccaGgacaacAccCCaacaagaaa
accggcaTgaacTgcgaaTcggTcaCccaTaAgcAagccGaaaCcgAaacccccgcaaaaaacCcaaacaTcc
aacacGAaacAaaAgacaagaTcccaGaacaaacCCaGacaaAaggTGaaacaagcAaaccacccaaagaaac
cacaGTaaTccaacacggcaaaaaacGcgcccggGaagGcaaAagcgacacGaaGTAcccaCGcagggcaaaT
ccaaCcagGcaacGTgaccaaGaCaaacgGaGAGaacaaacagacaaaGcCagcaTTaaaCcaAaagGggaag
aCaAaaaaTggaacacCcaaGaaaaCgGGccCgcacaacaccGgaaaacacaCcacaAagCgTTcaaacccaA
ccAAaaccTcGcaGacgaCaaGcccaCAGgcagCcCaaAaCcTacagaCccgCaTgaaacGcTcacAaacaGC
acgaacgaaacCacaacaaagaTaCAcccaccgcaacaGacaacacAaCacgggcgCaagcTcacccgAcaca
cccAacacgTCaaacaaGaaaccagagAaCacaagcaaccagacaaaAacAgacGCacagAcagcTcaaaGca
aGaaCaacaCcacgggaaGcaacgaaagGccGaaTgTGacGaccGCggggCacaaccacgcacccaaaaAaAC
TcccTaccccagaaAaaacacaAGcTgAacagaccAcAAaaaaaccaaTTcaCaAcGcacgcGggccAaaacc
caaTgaAcCcccagaAacccGcaTaCcaacacgcaaaccgAaagaaaaccaTcGacaACGaaaaacTaaacAA
gacccaTccCaaggAaGTCAaGTacaTaGcaccaAcccacgTaccaAaaaacaaacagcaCgCccGTgaccca
aaacggcATcaGacaaaaaaacaAGGTaaaCaAagacTgCaaCacaaATgGaTaAccaaGggcaccacaCaTcc
caaagcGTaCAaccacaaaAcCaggCaaCaaGGgcGAAGacccGaacAaaTcgAgaacCgGacAaaacgacga
gcagAgCaagaAaaTCaCCaaGAggcaaccaagccaTCagGGcGAcaTaagaGaTaaccaacaacgcaTagCa
GagcaTacAcaTccgCacagCTTCcaCCacaccGaagGaTccgagccgTGgACaagagcacAgGacgcccaaa
CggacTaAgCaATcaaCGagGaAcgagTcGgggACCTgacgcaccagCgcaTccacTgcgaaacaACaGGaGg

caccccccagggTgcccAacacccTgcagaCaAgccAcaTGACgTaacgGcaaggGacaTcccggAaaagacC
GTccaCcAgcagggcaggTacccggaaacacagGaAgaaGcaaTaagcaaGcTcaAggcAccgcTcaaaCaac
caCaTcGcaAAaaacgTgccccTcccaCAgTaAaCTacgccaaTgcaccacagAcccAGTgTacgccaccaa
aagagaagGaaaaGCaagCcgCAaACcCaaCaacgacaacCcaaacccaGgcgcGgaCcgcCcCaaAcagcTa
gacGCcgaccaTgagaaagcacacaaacgGcTaaaCccgcaCcgcagaGAacgaaTcaaaagaTagccaaAaa
acGaAaaaacacTccaCgacCaccacaacgcacTGaAgagcccCaaaGcCgcacggaagGCaacgTaccAaCa
ccaaAgGaaaacacaaAaGgTTgGcGagcaCTGgcacCggGaacaCacCaAaacaTTcaTcaacccaaAAaGa
TTacaaGaaTcggagaCcaaacaAcaaGcaCCaGccgcCgagGcaacaaaAaacaTcGaaacaaCagaTgaGc
ccAAgcacGcCaagccagacgacccCTCcCaacggGgcgcccgaACccacCaccGcgccAagaaaaagaCGca
ccTACacCcaagacaaTgacCagcCaaccaaaCagcaccacaacaagacaAacACaaaccagcacagaccgac
cgTcagcccccCAaagTcgaaCaGGacaacaaCccAgAcagcagaTacTcggTCccagaacAaaacgaGacgg
acgaacaacaccTaaGggaCacacTcaTgAcaaGcaAaacAgcacaAacaaATcaaAaaggcaggcacGGgcT
caacgaCcacCggAgCgTCaacGgccGcCaCcTaccaggaaTgaaaAacgTgGagaacaccagcAaCaGaCag
gacGaccGaacaCccAaacaccacacTGAcGGCacCaCTccaTgcccaTcacCcgaccGccaGcCcacaacag
gcacCcaaCacaAcccATaccagccaaccaCcCCaaacGccTccACGcaaaaCaaacCaCgGaacaCAacagc
CaaccaCGaaaccaTacgcaaggaGcggaccaacGCACTcacTacCTcgGacacccagCAAgaccgaaGaaca
caGacacgcgcaacaCATagagaaCaagcacaTGacAaaaAgGaTGccaaaCcagGGCacAgaGGcgaGccAa
cgaaaagacgcaCcCgacCAcgcccccaGaaCagaaccaaacAgGaaccCagagcacaggCCCAcaagGaaca
cCaggacaaaGgacaCTGccaCagcGacaagaccaaaccccacccaagaacaGCgccCaTacCcCaaaccac
aacGacaacaTCggcacgGaaacaaCaGaTgGagcgaaggccaaaaacgTcggagtgGggGcacaccTagacc
cacccacGaacCcaacTaccaGaccacaaccCcTccaGcTcaTcccgcggaacTacagaccaaTGaccggagc
gaaaaGcgGcTacaacaCcGcgcGcccgTaccccgacaCaTcgacCcagcaCacaccagcccacATgcgaaAA
acacAaGgCacCgCaAaaaCaaCaaaaAAagCTcTcCacgaCaccTgCcaCTCaTTaaCccCagCagcaAcca
ccacggcaaaCgccgcaagacaacgCaAaaCagagaaagcaacgcaggcaagacCcGCccacgcCaCCAaGaC
caaCagcgTGTaaGCaaACgCaacGTGGGacaAgaGcTcagcccaaGcAaaaaacAaacTacTcccaaATccT
ccaGcccgaaagTgcaaacCaGAaagcgcccacgacgaCTcccaAcccGcccgccTaGAaaacCgTaCacGca
aaaaGCcCgacaaaaacGaacGCgcaAcCTCaagCCacaacgcgagGaTaggGcCaacgcacaaGTTcaacCa
caacgaGgcGacgcaGcGGCTggTCAacaagacacCGaacacgaaaATccaAgacacagcGccaAaacTGCcc
caTaCagccAaccacaTCcacAcGgCaTacacaaAGcaGcaacGCcgaaacGaaacaccgcaggCacCaCgGa
ACccGcAaacccgcaaaGCacgCaTcGacaaaCgacTGagcGaaccgGcgGaaAaagacGTGAcaTcgGaccc
aaacGaGgggGgccaagTcgAcCaaagcGaaaggacaacTAcCGcaaGcacaaacacaAaacacacaacGaTG
ggGacacCccaaAGGcaacTcccaGaaacCcgTGacaacgCgTagcacgcGgccaaccTgGAagcGGaGgCcc
aAgACcgcGagggcagcccGaGGagAcTCCaCGggaTcGaAcCaacgcacGgGAcCGAaaTGgcaGagaaGca
aGcGCgaTaGagCacggaACgcGaagGaccTaaGGacGGacaGcaggcgGgaccgacGaTGacggccagcAcc
accaCGgaaGaaccagcccaaGAaacaGagaACacGgGggaaagccGaTTCaTcaaccaGgacGaTgagcagg
aaacaagcgaagaagagcaGcaaGcaagcgTGccGgcAagacgcGcgacgagGcgcaGcggAcGcccccTcca
acgagaAgcaCaaaagGaaCAcGgCGcagCagCgCccaGaaCaaaCaAgCacaagaccgacaccaaCaaacgc
GaacCaaCcaGaTGcaaaagAacaGacgaGacTcGTacaagACaAaaCaaaaCcacTacccgaCaacaaGagG
ccaCaaccccGcgcccccaaccaaccTGaTCGccgGagCcccGaGagGaccCGaCcTGccCaa
>Cligib1_Cluster105#LTR/Gypsy
GTTTAATCGAATGTTACGTCCTGCTAACGCAAGCCGATGAGTCGACAGTAGAGACTCAAGGCACTTAGCAAGG
TCTGAGTTGCCTTCTGGGTCAGGAGGCTAGTAGAGGGGGAAACCGATAAACGATTAAACATAGCGAATACAAG
GGTATGTAGCTACGTTTATATAGACATGTATGTAGTTCTATGACTACGCAATAATGACTATGTCCATGAGTAT
AGTAACACATATGGAGTATGATGACTGATACAAGAAGCGAGCTGACTGAGCAGCTGAGTAAGAACAATAGATG
ATAGCAAAGATAGAACGGAGACCATGCGGTGGAGAAGTAGATAAGGTGCGCATGATGTATCATCTGATGATGT
AGCATATGGCGCATGTCAGATGATGATGGGGTGATGGTGGAAGTAATAGAAGTGTCTATGAATGATACCATCA
GTCAGATGTCCAAGTGATGATGATCGAAGAAGGAAGGGATCGTAACAGAAAGTCCACACTACTTCAAAGAAGC
TGATGGCATCATCACAGTTGGAAACAAGATCTATATTCCCCCAGACAAACAACTACGTGAAGAACTGATCACA
GAACACCATGACTCGCCACTAGCAGGTCACCCAGGAATCCACAAGACGAATGAACTCATTGGACGCACCCTAT
GGTGGCCAGGAATGCGATCGGACATCAGGGACTATGTCATGGGATGCGAAACATGTCAAAAGAACAAGATCAA
TCGAGAAGCACGTCATGCGCCACTCTACCCCAACCCGATACCCACGCACCCCTGGGAAGAGATCTCCATTGAC
ATGATCGGCCCCTTACCACCGTCTAAAGGTTACGATGCTATTTTGGTCATTGTTGACAGACAATCAAAGATGA
TCAGGGTCGAACCGACACACACCACCCTGACATCCAAGGGACTCGCTGAAATCTATCGAGATACCATCTGGAG
ACAACACGGAATACCCAAGAAGATCATCAGTGACAGAGGACCGCAATTTGCATCAAACTTCATGAAGGACTTA
TGTACCCTCTTAGGCATCAAGCAGAATTTATCAACGGCTTATCACCCTCAGACAGATGGACAAACAGAGCGTA
TCAATCAAGATGTCGAACAATTCCTTCGGATCTTCTGCAACTATGATCAGGACGACTGGGCCTCATGGCTATC
AATTCTAGAATTCCAATACAATGACAAAATCCACTCTAGCACCGGACAAACACCATTCTTCTTGAACTATGGC
CGACACCCATGGAAGGGTGACATCTCCACGGAACCAGTAAAGAACGAAGCAGCTCAAGAGTTCGCATCACGCA

TTGAAGAAGTTCAGAAGAAAGCAATCACCGCATTAGAAGCATCAGCAGAACGAATGAAGAAACAATATGACCG
TCACAAGAAGGACTTGCTTAATCTCAAACCCGGTAACAAGGTGTTTTAGAAGCCAAAAATATCACCACCACC
AGACCATCCAAAAAGTTATCAGAACGACGACTAGGACCCTTTGAAGTCATTGAAAAGGTAGGCCATAGCGCCT
ACAAACTTAAACTCCCCACCAACTGGACGCTCATTCATCCGGTTTTAACGAGAACCTATTGACACCACTCAAG
ACGGCACGCTTCGAATCACAACAGAAGCCATCACCTCCACCACCCATGTCATCGAAGGTTCAGAAGAATATGA
AGTAGAAAGCATCCGAAAGGTCAAAAAGAAGAATGGAAGACTTCTATTCTTCGTCCATTGGAAAGGATACCCG
GAAGAAGAAAACACCTGGGAAACCCGCGCAAACCTTAAACATTCCGCGGATCTGGTTAAAGACTTCTACCGCC
GACACCCTAACGCACCATGATAAATACCACGCTCACTTGGATCATTTTACTCTACTCCCACATCTGTCTAATC
CCCTCCTCACCCAACACCAAACACCATGCCTGCAGTCATCACCGACATCATCAACCTCATCCTCCTCTCCCTC
TCCCCTGTCTTCACGATGTTACGCCTTATACCCAATATCTATCCAACATTCGAAGACACATCCGTGACATTGA
TCAAATCACACAACTCTTCCCCACGGTGCCCCCACTCTAATCGTACGACTAACCGAAGCCAAGGAACACCTCA
TTGCCGCCCGCCAATCATTCCTCATCTTGGAAACCCAAATGCATGATCACATCTCCACCACCGTGCAAGGGAT
CTCTCCAGAACTCACGGATGACGACCGACAAGCATTGGAACGAGCAGTACATGTCAACGAATGGAATGTGCCC
ATTTCAGAAGATGCCACCCATGAAGAATACAGAATCCCAGTTACTACATCGTCCTCATCAAGTAATTCCGAAT
CTCATTCTCCCCTTCCTAGCATTCCCCCCCGACGAACAACGAGCCCACCGGCTCCTGGCCCCCTTCCTCACTA
CCCACGGGATCCATACCCCTTGGAGTACTTCCTCTTCATCGAGTATCCCACATCCGCATCCCCCTCAACTTCG
AACCCACAGAACCCTACAACGGGACATACTCGTACAGCGGCAAGACGTTCCGGTACTGGGTGAGAGGACCGCA
CGACTCGCCCACCTTCTTTACATGCATTGGCGTGGAATACGTGACCGCGAGGACGAAGAAGGAACTCGGGAAG
ATGCTGGTGAACAATGATCTGATTTAAGTTTCGGGACGAAACTCCTAGAGGGAAGGTCATGTTACGATCCCTT
CCATCTAATCATCATCACTGGGACATCTGACTGATGGCATCATTCATAGACACTTCTATTACTTCCACCATCA
CTCCATCATCATCTGACATGCGCCATACGCTACATCATCAGATGATACATCATGCGCACCTTATCTACTTCTA
TACCGCATGGTCTCTGTCCTATCTTTGTTATCTCCATTGTTCTTACTCAGCTGCTCAGTCAGCTCGCTTCTTG
TATCAGTCATCATCCCCCATATGTCTTACTATACTCACGGACCTAGTCATTAGTGCGTAGTCATTGAACTACT
TCACTTGTATATAAACATAGCTACATACTCCTGTATTCGCTACGTTTAATCATTTATCTCTTTCCCCCTCTAC
TGCCTCCTGACCCAGGAGACTTCAGACCTTGCTAAGTGTCCTTGAGTCTTTACTGTCGACTCATTGACTTGCG
TTAGCAGGACGTAACACTTTTTCTGAACTTCTTCAATGTGTGATGCAAATTCTTGAGCTGCTTTGTTCTTTAC
TGGCTCCATGGAGATGTCACCTTTCCATGGATGTCTTCCATAGTTCAGGAAGAATGGGGTTTGTCCAGTGCTA
GAGTGGATCTTAACGTTATACTGGAATTCTAGAATTGACAGCCATGAGGCCCAGTTGCCCTGATCATAGTTGC
AAATGATCCAAAGGAACTGTTTGACATCTTGATTGATATGCTCTGTTTGTCCATCTGTCTGAGGGTGATAAGC
CATTGATAAATTCCGCTTGATGCCTAGAAGGGTACATAAGTCCTTTATGAAGTTTGATGCAAACTGTGGCCCT
CTGTCACTGATGATCTTCTTGGGGATTCCATGTTGTCTCCAGATGGTGTCTCGATAGATTTCTGCAAGTCCCT
TGGACGTCAGGGTGGTGTGTGTTGGTTCAACCCTGATCATCTTCGTGTAAGGACAGGCCAAAACACCCAATGG
ATGCGGCAGTCTCTCAAGTAGAATGAATGTAAAAGAATAGTTTTGAAGACATTTGACTTAAGATCCCACTTGT
TAAGGTGGAAACCAAAGAGAATCAAATTAGAGTACATAATACAAATGAAAAGCCTTAAGTACTTCTAGAGTAT
TTGGCAATAAAAGAGAAAGAAAACAATGTAAAGTAATATCCTACAGCGTGCGTACTCCATGATGCAAAACCGT
TGATGAGCACAGTGCTGCTCGAGTGCAAGATGCACAAAAACAACACAAATGCCTTCTCGGTAGTGAGTTGGTA
CCCAAGGGCCAGGTCACAGCTGAAAAGCGCTAGCGCGGAGCAATCAAAAGCTCAGAATGAAACTTATAACTTG
TATTGTGTGGTGTCTACTGGGGGACTATATACTAGTGAGCACGAGGTAGCAACAAGGATATGTATGTAACTGT
ATGTAAAACAAGTCATGTGCGGTGAGTACTGCACCGATACTGACGTGTAAGCTACGGTGATAAGCATGGTAAT
GGTTTAGCATCAGTTCACATCTATTTCTAGTCATTGTAGCTAATTGCTATACTTACCTTCGCATCGCGCGACT
AATGCGCGACCACTGATCGCTTCGTACGCGTGGAGATCGCGCTTTCGATGCGATCCAGATCGCATCTTTACCA
TAGTGATCGCATAGTTTCCCATTATCTTACAGATTATCTTACATTACATACATACTACCTCATGCCCGCGTAG
CTCCGATTTATATAGAGTCACACATAGCATGCATAATACAACTTCCCCTTCGCATTAGAGCTCTCGAGTCTCG
CGTTCATTCCCTTAGCTTGTGAAGATCTCCACATTCTTTGGAGATTCTGGCCTTCACTGCACTAAAGACACCC
ACTCGCAAACTGCGAAGCACTTGGTTTGTTGGTTTGCCCCACAGGCCGCAGTGTCCTATCAACGGATCAACTC
GGAATGTGCGCGCGATCTCCACGCGTACGATGATGCTAGCGCACGGATGCCGCGCAGCGAGTCGAGCGCTGGG
TGAAGCGCGGGAGAGTGCTAATGGTACGCCGTGTGTATCACCGTGGTTTGGCTTACATAATGGATCACGTGAT
GATCTGCGATAAGGATTTGAGAGATTAGATAAACCCTGATAAGATATGATAAGCTCCGATAAGATAAGGGGAT
AAGATAAGATAAGATGAAAAAGACTTAGAGAGAAAAAGAAAAGAAAGAGCAGTCGTGACACTTCGATTGTTTG
TCAACAATGACCAAAATAGCATTATAACCTTTAGATGGTGGTAAGGGACCAATCATGTCAATTGAGATTTCTT
CCCAGGGGTGTGTGGGTATTGGGTTGGGGTAGAGTGGTGCATGACATGCTTCTCAGTTGATCTTGTTCTTTTG
ACATGTTTTGCATCCCATGACATAGTCTCTGATGTCCAATTGCATTCCTGGCCACCATAGGGTGTGTCCAATG
AGTTCATTCGTCTTATGGATTCCTGGGTGGCCTGCTAGTGGTGAATCATGGTGTTCCATGATCAGTTCTTCAC
GTAGTTGCTTGTCTGGGGGAATATAGATCTTGTTTCCAACTGTGATGATGCCATCAGTTTCTTTGAAGTAGTG
TGGACTTTCTTTCATGCCAAATCGGACTTCTGATTCTACGTGTGGGTGGTTTCTGATGTGCTGTTCCAGATCT
TCATTGCCGAATTCAGTGAGAGCTGAGATGGAAATTTCCGGTGTGTCCCGAATCACCTCCTCTTCAGGTAGTT
CTAGGGAAAGTCAGGGGTTATCCATGGGTTTTTTTGGCCTACCCATACCCACACCCGCCTAAACCCCTACCCG
CACCCTCAGATAGGATAGGATAGGGATTTCCCATTGGATAGCAGCGGGTTCTTGTGATTTGGCGGGTTTTGGC

GGTTTTACTATAAATATCTCCGGATTGACAATCCCGATCCCTCTCGCGTCATGCGCATCGTGTTCAGGGCCCA
ATTTAGTGTGGAATTCGTCCCTGGTTGACCCGATTTGGCCCAGAACTCGCCCCACAATGACATGTTCTCCTCC
TCCCCCCTGAAGCGCGCAACTTATAACTCAAGATCGGCGATAGCTAGCCCTGTCGTCTAACTTGGAGCTTATT
AAGGACCTTTAGGAGTGTCCGATCGACCCCTCATCGGTCCGATTTGGTGAAGATTTCGCCCCTGGGGAGCAAC
CATGAAAAACCCACCCAAACCTGCGGTTTACCACCCAGGGATAAGGATAGTGGTCAGATAGCGATTCCCCTAC
CTGTACCCGCATCTGGGCCTACCTGTATCCCCTACCTGCATGGTTTGTACTATCCCAGGACATTCACTAGGTA
GTTCTTCTCCAAAGAACCGTATGCCAAAGTCCAAAATTTCATTGTCTTCCTCTGTTACAACGGTTCCCACCCC
TATCTTTTTCTTCTTTGTTCTCTGACTACCTTTGTTTGATGATACTTACATTGTTCTTTGACTTTTCATTGTT
CTATTGCATGTCCAATGCATGCTGCTATTCTTAGATACCTGACTCATCTATGACATCATAGCTGACTGAGCTT
CAACAGCTTAATTACTTTACATTGTCTATTCTTCCCATTGTACTATGTTCTCGCGCCTCAAGGTCCCTAGTAT
ATAAACCCTAGATATATTGTCATTGTTCTCTATCTTGACTAGGGCATGCCGCGAGTGCACTCGCGGATCGACC
CGGATCCATGGGAACAAGTACCCACAGGTATGGGTACTCACCACCACAGATCAATGTGAGTATGAAAACTCAC
AAAGACTCGGATCTGTGAGTCACATACTGATGAGTATGTGACAAGGGGGGAGGTGGCAAAACACAGTGAGGGG
AGCCAAACAAGTCAAAACAGACCCACGGGTACCCACAGCAAGTACTCAGACCCATGAGTCAAATACTCATGAG
TTTAGATACTCATGAGTCAAATACTTGTGGGTTAGAAAACCCGTGAGTTTTCCCACAGGTCAACCCGTGGGTA
CCTGTGGGTACCCAGACTCGTGGCATGCCCTAATCTTGACCAGGCAATACTACACCCTTTACCTTTGAACCTT
GATCACTAAACTATTGATACCCAAGTCAAAGCCTGACTTGGAGTGACTATAGTCTTGTTGGTATTGACCCAAG
ACTCAGACTTGAACCTCTGGACAATATTCGACACTTGAACCTTGGTAAAGGCACTCTGCCTTAACAAAAGGTC
AAGATCTACCAATTGCCAAACCTCTTTAGGTTCCTGGCACCCACCACCAATAAACAGACACTGATCCAATCAC
TGTGGCACCCCAATCCCTACAAGTTCCTCCTCAAGGAGCTACTCTTGACAATCAGGAATACAGACTTGAAGGA
ACTCCCCTCTTTTTCAATAACACTGCGGCCCGTTGGTATTATGCTGTAGAACCCTATAATGCAGTACCCAACT
CCTACTTCACCGAACCATACTCCCCTCCAGTCCGCCAATCTCGAAACTCCTTCTCTGCTAACACCTCCGACAG
CTCTTCCTCTTCTTCCAAAGTATCCACTCCCACACAGTCACATCAATTCAAAACCCCAGAACCAGAACCAGCA
CCAACAACCAGAATGACTACTGTAGTATAAGGCTCAGCACGAGCACGGGTTACCCGTGGGTACCTGCGGGTAG
ACCCGTGGGAATACTTGCAAGTATTGGCCTAAACAGGTACTTGTACTCATGGATCTATTTACTTGCAGATCCG
AGTCAAGCATTTGGACATTGCAGGTACCTGTGACAGGTACTTATGACTCACACTGGACCCTCAAGGACCCTTA
AAGTATAGTTCCCACAGTACATACAGGCTCAAATTCCATTTTAAACCAACAAAATCCTTGTAAATACCCATAA
GTACTTGTGAAATACCTGTCCCTGATGACTAATTGCTGACAAATGACCTGTGACCTGTGGGTAATCATGAGTA
ATCCATGAGTAATCAGGATCTGTGACAAGTACTTACTGAGTTGTAAGTACTTGCCAAAAAATACCTGTGAGTA
CAAGTACTTGCTGACTAAGCTACCCGCAGATCTGAGTAGTGACCTTGTGTGACCAAATACCCACGAGTACGGA
TCCGGATCACCCACGGATCTACCCATGCTGAGCCTTACTGTAGTACCCACCACCACTGCCACCAAGACCCCCA
AACTCAGGACTCCCGCCATATTCAAAGGAGAAAGAGAACGATGCTCACCCTTCATTAAAGCGGTCAATCTCTA
CCTCAATCTAAACACCGCTGTCTATGATACTGATATAGCAAAAATTGGTTTTACTCTTTCTTATATGGATGAA
GGAACGGCAGGACCCTGGAAGGAAGCAGAGATGGAAAGACTTGCAAACCAAACAGGTGGATGGGGAACCTATC
AGGAGTTCATCACATGAGTAGAAGAAGCATTCTCCCCCACAGACGCCACAGGAGATGCTACCCACCAATTAAC
CGTCCTCCGACAAACAGGATCAGCAGAAGATTATGTGGGCCTCTTTAGACAAGCAGCCCTTCGAAGTAAAATC
ACAGAAGATGGACCCCTCATTGTGTTCTTCCAAATGGGACTCACCAAGGCTCTCACTGATAAAATTCACAACC
TCATACCCCTACCATCCACTATCGACAGCTGGTATGAAATGTCTGTTCGATTCGACAGCCAATATCGGAGAAG
CAAACAGATCCATCAACGAAACAACCCGAACCAATACCCCAAGAATAAAGATCCCAGTACCCCAAGATATATC
CCACCCAGGGACCCATTCGCCATGGACGTTGATTGTCTCACAGTTGAAGAACAGAAGGAACACATGCAGAAAG
GACAATGCTTCGTCTGCCACAAGTAGTGTTGCCGCGGCTCGGCTAACTGGAACCGGTTCCGGTTAGCCGAGGA
AAGCCGCTATTTCGGCTCGGTTTAAGGAGCCAAGCCGCCAGGGTGTCCTCGGTTACGGCTAAGCCACGGCTCG
CAACCAGCTCGAGCTGAGGAATGGTGCAATCCCACCAGAAATGTCCAAAACTGCGGCCATACTCCCCTACTAA
CCTTCACAAAACGTCGCACACGTCAACTGACGCAGCAGATGTATATGTGAAGGTTTTGATGGGGAGTGCCACC
CTCAGCTCCTCCAAAAATGAACGAGGGGGCGGCAACGGCATGCAACTTATCTTTGAGGAGTAGTATAGTATGT
ACCATAAATTGTAACAGTGATGCAATAATCACTGACAAAAGTTTGGGCAGCGAAATTAATCTGACCTTTGAGT
GAAATTCGCTATTCACTCAGTTTCATAGCGAAACCGCAATTCGCTCAGGGTGTGAAACATATCAAATTTTGAG
CAAATTTCACTTTGGCCCACAGCTGCCATAGGTGAATATGATTCACTAGAAACATCATTCATTTTTAATTGAG
AAACCATGTGAAACCAGAAATGCGAATATGGTGTGAATGCCAATCACTGAAATCATCTGAAAAGTCGTGTCTA
TTGTTACGTCCTGCTAACGCAAGCCGATGAGTCGACAGTAAAGACTCAAGGCACTTAGCAAGGTCTGAGTTGC
CTTCTGGGTCAGGAGGCTAGTAGAGGGGGGAACTGATAAACGATTAAACGTAGCGAATACAGGAGTATGTAGC
TACGTTTATATAGACATGGATGTAGTTCTATGACTACGCAATAATGACTGTGTCCGTGAGTATGATAGGACAT
ATGGAGTACGCTGACTGAGATAGAAGAGAAGCTGACTGAGCAGCTGAGTAACAACAATGAAGATAGCAAAGA
>Cligib1_Cluster65#LTR/Copia
gtgagaAAacCaacaacaagAcaaaCAcgcTCaaacaaaacATaAcGaCaTcaTaaaTATAcCaAcaacAcca
GGaaaaggaTcggAcCaaaaaccTaacaaacgaaaggaGaCgGaGacaAacTcacCcCaGcCcaaaaaaagag
aaaAcaacagaGaTaacAaaaccagCCacaTaggcTaGaTATAcaaAgaAaGaCcTaATcacggacagGAcac
TGagccTaggagccgaGaaTTcaaaACaccTcaTcacCccgaaaaGaAccaTaGaAacgaaAaAcaAcgcaac

agaaaacGAcTcaggcaaccacgTcagcCaacgTgTCcagagcaccccggaaTacgCagGaAgaGcGaaccAa
AAgAaAGcaaaaccaaaaaCaAaaaacgaaGaTaaGcccaCgCccaaCaaGaacgccaaacgcaacAcCcaga
agcgCcCggacGacTaggaacaTcccaCgcgGcaacggTTagccaaAaacGaCacCaaCggggccgGccaaac
acaccccAgaaAaaaTgacGacaacggcaaCgaTgcGagCgCgaaTaaacgcaaagcgcaCAcccaTgcGaaT
GcTGcgCgaaagcgaTaccgcaaCAacAccGagagcacgaccaacaccGcaAccaAgcaGaagaccAcgTacA
aagacacGcagaacaagGAaTccGcgcAcGaGTTcaccaAgaCaaAacGCgaCaccaCagaacaCGccgaCag
caAaagaTgcacCAccgcaaaacGaGgcaGcgacagTCTcagaaCGaccaaagaaCaAcaaaaaCaaacCaCT
CaaaagagacaAacaaaagaccaTgccacaAccccgTaagcCcAagCaaTCacaaTagcAGaAacCacaaaaa
ccAaaTaaggaCgcAaTcgcaGCAcccccaacaggaacaCcaaGaacGCccacGGaTaTGAaggaGcccaaaA
ccccaaTGcCagcaGacCAccGaaaAcGacGaaaGcaagCaAcaaagTgacaaAccgTagagacaGagaAacC
aAgGaCagccCGaaTaCCGaCacccAaaTaCAcgagcaaccccAcaaGcaAcaGAaAGgaCacccTccTGAGa
gcgcaaacccaagcGaGgcacAcaccGaCcGagGTaccaagcCgCcgGaGCgcaAcacgGTcAcCcacaGcaa
caGaaAacGccccaaccacGaccccGacaaaTggATcaAcaacccTGgaaaTaaGacccGgaaGacagaacaa
GAcgcTCaGaccaaCaCgagcccGaagaAagcCaagcCCcaagCaaaaaagacCCcCaCcaaGGccccaaaTc
cccCaGAccggccgCaacacGccaagaaaaccccaagaccccgcaaaacagGaAcaGGCcaTGagaAagTCcT
cGAcGcgGAaccccgcgaccacGCccCcGacGgcccTgGTccTGccgaaaaaGaaAccGcaaCgaTcaAGaCc
CTcGaagCcCCaggaaaCcaccacTcgaggaacacgcgTGacCcagaccaagacaCgcgGcTgcaccCCcaag
acaCCcccccacaaccGccaaAcccaaccacccacTCcaGccaCGccaAagcccgcgGcTgGacccAcGaaCc
aaacCTCCcCacaaGacacGCaCCcaTagaagaaCgCcaTgTTcaCaTccacTTaGacaaTgcCccaAgCacg
caaagCgcaaaTaagcaaaaGcAgaccgaGgcaCTcCATcCcagacagcagcGAaGcagTcacaaCaTaGgcg
gaaCcaaaAaCCaagccaTaGccaTccaCGaCaacaaGagaTccaTaGcaAacaGcaGTgcCgTGagcaTcGa
gaaTgaccTgaAaaaCCCacctTgCCacccaccaCcTcaccagcagaccggaCcgaacaGacTccaaCaAGcgT
cgagaCACccaTcacgCggaaaCTGccaagaTcCTCcggaaTgCTGGccccCcaagcGcCaCTagaaAacGac
TCcagGcCCgcaaTaacgaaCaccgcacCaccgcCagcaCTAcaGgCcAAaAcccagTCcagcaCacCccCaa
acccaccCTCaAaaacgacCAcCgcCgcCTgGGaCAaGcaAagaAccaAacCAgaaAgaAcagcGaCGgCAaA
GaTcTgacgaaAaacAcGccaATCcaaCTCgcCAcccaaccCCccaaacaTGaaaAggacAgcCcgacGaaaa
AgaTcccacccCaTaTgaCGaacacACgTcaGacGaacaGcGCgAccaaGAaaggcGgcacaagCaagagacA
ccgaAcGGaCcaaCaaGAgGccaTccaaacCgccggTccTCgCTaaGggAgGcaaGaaAcaaaTGaaTacacg
gGcggcacGagGgCcagaacccaTaaaacaaaaGaCcaagccCaccccGaTacCaacgccgGaaaAaCCaGAG
ccGaGcgcGacaacagcaGcAAcCTcaaccaTaaacacccCcGagCGgGcTaagcggcGcTcGaaCGCcAgGaa
aaaCaGGTaTaccagccacGGgAggaagaaaaaaagaAcGaaaaCcaacaaGacagcaCagaacgagcGgaTa
GaaaGccaAaagcgaacgaaaaagaaTAaaacgaGGgacAgTaGaTaGaAaaagcGaCaaccCacaaacaGcg
GGcacaaagGggggCgaAcaGCaAgCaaaagcAaGGcgaGaAcgacaaaaaGTaccTaAaaaacacGaacCac
CgacaAcCcgcccgagGggaagaaaCcTGaCcaaacCaaccacacggacaaTGaagcgaaaacggaaggaCTc
aaaaAcaaccggcaacacCcGAaGacacAgcCgcgaacCcTTcaaaacGgccgaaTaaccgAcaaccaCacac
ccAAaGcagacaGcaTaaagCaTCcGcccgcaaacaagcTggaccAGaaagaaaacgcgaaagcCAagcaaac
gaaaaagGcaaaaGcCAaGcccaaaaaCacaccaacGcTccaaGagGcccCcgccgccTgAcccACcggcgcc
acTcCgacCCgTcaggcaGaTaGCacTgaGaaaaCacccaccCCacCcccccgGccgaGcCcacgaTgCaAc
CgAaTcgaCCgaccgaTAccataataccgtcccctttacctaccgatccaaaaaaacggaaacgcaacCgatc
tacacgcccgatatgggccgacgtgtagGgagcCcTaaaccgacgagaaaTaCCcacagacaAcACCaGgca
ccCaTcGCGcccCcAcCaaacaccAcCaCGagCcGacccaagAaccagaAGcTTcCaaacagacaCcacCcCca
CTCgGGaTgccCaccacagGaaAaCccaaaGaaagacGcCcccgaaaccAacaagcaaaGaCaaCcacaacCa
aGacgAgtCAgccaTcaCaccCaTaaCgcAGaGAaaAaaacaGccagcaAcaaCaTCcgGagcGcCCcTcaaa
TcaacagaGaccacaaCcaaccTCgcccTaGCcCgcccCccCgaAaGcaccagCGgaAgAgGcaaaaaAcaT
gaggCccaaGaaTacaaTggaCAcacggGcGGccggaccTGaaGAGaccGATgccCaTaccTgcaccaAcAcC
TcTGaCGgCcacaTcaTagaTGacTcaaTcacTTaCgTGaCCaAGaaGaaGcTGccacgagTcGAGcacaccg
agGccGccGgcaacGAacccaTgTTcGaCacTAGcaacGcacagGaacacaaGgcacagAagaaGGcCagcGA
caCagTccacAGaGaCaaaaaGcacaCCAGaAcgaacGAGaAgagcacCAGaggaaGaaacAaaaGTGaagac
AaaCTgGagccAcgCgcggaaGcaaccaacaaccacaAaGcccacaaaGGaAacaGGGacaTaaAacaaAaga
gCcAgcagCAaGaaACgcCcccTccCaAgaaAaacCgccagGgCGccaAgccCaAcaaacaGGaTggaaGaca
aaaCgAcGcccacAacaCgTCaaccaccgaacGcATGaAGccaCaacaaaTcCgggcaaacAgggcaGaaaca
aagGaTgcaGaaAccacCcAAcacaAaGgcAAaAccGaGcgacgaaAccGGaGCcccgCcgccGaGGAGaacC
aGagCaTcaaaCaAaCaaacaaaaTcccCGaccCgaGAaaaaagcaaccaaTcaacCaacgccaacaaGGacc
acacGGcagcaAaCaaaGcagacgcaaacaGaaCAacacgaggaaaaaAcccagcGaccaagGGaGagaCaAG
cGaCTcaAcaGggaCccTggCcagagacaCAacACaAcccaCGcCcGcaGCagacgcaacacagTCaaCaTCa
AcGAagTaagCAcaGCCacTgCacccaccaaccacaaaaccccgaccaaacaCcacgcGaacaaccTCcGaac
cAgcacacaCCgacGaGaGGcgaagggcaGaggcGGcaaaGcaaTcacgCGcaagaGaGgcaccCgGAcaaTC
ccccaagcgCaaaCcacccCaaAccgaCAccaggcTcaATaaAaaacgCcacccCaacacgaacccCcaTcgCaa

cccCcacaGaTaACaacaTcaaGCgCaaGaGcacacacACaaCcGgCccccTagcaCgCcaaaccccaaaca
cCTCAaaAaccaGcaCTaTCgcTccCacaTccaacccagaaTGaAcaTcccaGgCGGaAcaCGccaaGaCaTc
GcgTgaaCcagaaacAaccTcgccaAaggccTcGagaaCaacCccCaTgacCacTGagCcaagGcCAaccaca
TcgaccaCgaacTCAaAaCCacccGCcgcCggcGgcccaaccgcccaaaTGCgaacAaTcaGgaaagAaaTac
gggCgccGacTcCTcaaAagcgGAcgGaaTaGaaacAaaccaTgcaaaaAgccgacagcaaTacgaaaaaggG
AGaGacggGgccccaTgGccagcgccgCGccaacAGAaaagacCaaaCcCcacgaccaaTGccaaAaacgTca
acGaaacCGaaccacGacagGaaGCacTacGgaTgcaacAACgCcccaaCaaAaTCaaccaaaacCcaaaaac
TccAacTcccacccCgcaTcaaTaaCaagagcacgaacaagagCacTaaTGaccaaacAaacaaggaGacCAcC
AaacaaAacaaTGacaaagaccaccacAcaaGaGaaCcaAgaGaacaaagcagCcgaAgccTcgaTcaaaacc
ccaccaCaaaaaaaGgcGccGaCcgcTagacaaAAaaccgaCacaGacccAcaagaagAcgCagaaGcGaaaa
cagcGaTcggaacaaaccacgcCggGcgacGaGgaccCggAcCagaaccacTgGggggacccgccccCTgTaTT
aTcaccCaCcaTaCaaGaaaAccaaacaacaacagcGAaAacagGaaaaAacgcaccaaaccAcTCaacacag
ccacacTaaTgTcgaacTcCgAgacTcCagaaaccGcccAgaAccaGTgagaAAacCaacaacaagAcaaaCA
cgcTCaaacaaaacATaAcGaCaTcaTaaaTATAcCaAcaacAccaGGaaaaggaTcggAcCaaaaaccTaac
aaacgaaaggaGaCgGaGacaAacTcacCcCaGcCcaaaaaacgagaaaAcaacagaGaTaacAaaaccagCC
acaTaggcTaGaTATAcaaAgaAaGaCcTaATcacggacagGAcacTGagccTaggagccgaGaaTTcaaaAC
a
>Cligib1_Cluster10#ClassI/LARD
tgttgaGataccagcttAtctacagtatttgActtagNattaatgtccgctttatgattacataagcgcgtcc
agtcttTtacattcgtagattgtctctcattaNNatcactataNatctatacNcatctaataatatctattta
AtctcccatctacttcatccaCtgccaataggttatgggccctgggcaacctacaatgtcgaatctcgaagtc
ccgaAtcttgaAgActatggttcgaatTgggtcAcctaTaaggaaAgGgtaatAaagaNtNTGactcacNaag
gactcgaacgacacctgtacggaaccgcgccgaagccagaggagcttcgcgaggtagatgggaagtggttcac
tgaagaagaacccacgaaaccgctcaccagaaaggaaatcaaagagcatgaNgcgaaaatcgaCattttcgtg
caacaggAggcaacggttcgagaagttatctatcaaacggtcagccaatatacctttcttcGaataTacaaag
aagaaacctccgcggagctctggataaaactcatcagccttttcgaggatgaagaaacctttgcggccacctt
gcgatccatccgaatgcatgccgaaaaacgcgtcgaaaacgcgatggTaattgctgagaaaggaggGgaagaa
tccgaggagaagctgaaggaaaagagtgaggaatcgcgcTcgagcNcggggcatgctcgcgacgccgaatgct
ataggagaggaggagaaggaaatggtcaggaaccacaggaagagaagaccacgaGcacgaacgctgccacctc
gagtgataccagcaaagacgtagctcgCgcNaagacctcataccccgtgaagaaacccctgcccaatccact
ccaggaccctcgccgcgcaccgtcgatcgaatccccgagagtcgaaactcataTtgcgtcaatcttgctaata
aaccgttggaaatccgtcttgaaaNgtcgaatgcaTgCgaagcgtcgtcgaaaaacgcgaatcCGtcggaaat
cacagcNgataaagaggacgaaccaaattttattgaaNaacaagaatatacaatacagtatcaatgccccgga
tctcNgaaaagcgaaccagaaacggccgaaggccataatNcaggttttgatggagattccgcggataacagga
gctgtaggccagaaaagcgatcagtaacggtcgaagggaacacctaccttggtaggaataacctcccagaagc
tcgAaccgtccagatcgaggggaagagaatgcccaacatctccaataccccaggccagccaccgaaaatgag
accaTcgaatcgccgcgcgctaataggcacgtcttgaaggcctcgagaaccgcttTCgaacggtccaacaatc
NgccNaaaccgccggaaaatggttgcgcaacggacNaaNcTTtgtccaagcNactcagNcaagccAccaaNat
cNNttcggccaaaccaccggacacgaatagacgcgatcggaagcccttNgGaaccGctatcgaacggtctagc
gatcaGGgaaaaccgccggaaaacgattgcggaacggacgcaccttcgtccagaccacccaggcaaaagaccg
aaatccgttcggacgaNtcNccggacgtgaataggcgcgaattNaagccctataggaccgctNtcgaacgcgc
NaacaatcagccaaaaCGgatNaaaaacggccgtgcaacggacataCcttcgtccaagcacaatatctcCata
tccaaaaccccaccaaactctcccagcTcaaaggatcatcNaagcgacatcccaagcGacctagaAtcgcata
tccgtcgcgatttccgtgccagaagactcccagaaatctgtagtcagcctatccagcaagactcaccgAtctt
ggagaccaggccagcaggaatNgAttatcgggatcccagaNgcatNcctNgggaagataaagggttagtaaNc
gatataggNggaccgactcacctatccaagatggtcaacgatgttgaaccTcgaGgaagcgtccaagaggaac
caaccatcgatcagTcagcagtcaaacgtccaaatcaggacatttcgacttcNaaaatcattcacactcgtTt
TgctcataTcaagaTgattcagcaatacattcttttaNTaTatcaggtataNtctacctacttacaatctcaa
atacctccatttcgcttgAggggaagtgttgaGataccagcttAtctacagtatttgActtagNattaatgtc
cgctttatgattacataagcgcgtccagtcttTtacattcgtagattgtctctcattaNNatcactataNatc
tatacNcatctaataatatctatttaAtctcccatctacttcatccaCtgccaata
>Cligib1_rnd-1_family-5#TIR/MITE
TAAGGCATGTCACGAGTGCACTCGTGGGTGATCCGGATCCGCGGCTGTGGGTACCCACACTCATGAGTCAGTT
ACTCATGAGGATCTGGGTACTCACAAGTTACTTGTGGATTTATGCTCTGCACCAAATCGGGACNTTGAGGGGT
CGATCGGACACTCCTAAAGGCGCTTATTAATATCCAAAAGAGACGACAGGGNTACCTGTCCCCGATCTTGAGT
TATAGGGTGCGCGCTCCAGGGGGGGAGGAGGAGAAGGTCATGCCCTGGGGTCAATTCTCCACGGAATTACATG
ATTGTGGGACGCATTCAACACTATTTTACGAGCTGAATACGATGCGCATGACGCGAGAGGGATCGGTGTCCCC
GATCCCACGGTACGCGCGATCTGGCAGACTATCACTCTGTATCTCAGGATCGGGGACACCGATCCCTCTCGCG

TCATGCGCATCGGATTCAGCTCATAAAATAGTGTTNAATGCGTCCCACAATCATGTAATTCCGGTGGAGAATT
GACCCCAGGGCATGACCTTCTCCTCCTCCCCCCTGGAGCGCGCACCCTATAACTCAAGATCGGGGACAGGTAN
CCCTGTCGTCTCTCTCGGACAAGGGTCCACAAGTACCCATGAGTAGACCCGCCAAAGACCCGGATCCATGAGT
GTAAATACCCACAAGTATCCCACGGACCTACTCATGAGTACTCATGAGTTACCCGCACTCGCGACATGCCCTA
>Cligib1_Cluster24#LTR/Gypsy
gttacgtcctgctaacgcaagNcNatgagtcgacagtaaagactcaaggcacttagcaaggtctgaNgNctNc
tgggtcaggaggcagtagaggggNaaNNgataaacgattaaacgtagcgaatacaagNgtatgtagctacgt
ttatatagaNNtgatgtagttctatgactacgcaNtaatgactaNgtccgtgagtatagtaagacatatggag
NatgatgactgataNaagaagcgagctgactgagcagctgagtaagaacaatgNagataNcaaagatagaacN
gagaccatgcggtNgagaagtagataaggNgcgcatgatgtatcatctgatgatgNagcatatggcgcatgtc
agatgatgatggNgNNNNgtNgNgNaNtagaagNgtctatgaatgatNNcatcagtcaNNtgNcNNaNNgatg
atgatNagaagNaaNgNatcgtaacatgaccttccctcNaggNgtttcgtcccgaaactNaaaNcaNaNcaNN
gtNNaccaacaNcttNccgagNtcctNcttcNtNNNNgcNgtcaNNtactccNcgccNNtgcNtgtNaagaaN
NNNggNNagtNgtgcggtcctcNcacccaNtaNcggaacgNctNNccNctgtacNagtNNgtNccgttgtaNg
gNtcNgtgggNNNgaaNNtgNNggggatNNNgaNgNNgNaNacNcgatgNagNggNagNacNccNaNgggNat
ggaNcgtgggtNgtgNNNgNNNNNNNNNNNNNtNgNtgNtNgNNggctggggNNtgcNNNNNNNNNgNNNNtNN
gNgNNNNNNNcNtaNNNggNtggcNtcttcNNaNgNNNNNNaNNNtNNNNNNNtgNNNNNNNNtNNNgNNNNN
NNNNattNNNNNNNNttNNaNNNNNNNNNNagaNNNNNNNNNaNNNNNgNcccNgtNggNaagagNtgNgtga
ttNgNtcNatNNcacggatgtgtcNNcgaatgttggatagatagttgggtataaggcgtaNcNtcgtgaagaN
NggggagNgggagagNagNaNgagNttgaNgatgNcgNNgatNgcNgcaggcatggtgtNtggtNtNNaNNNN
gNaNggaattNgaNagNtgtggggNtaNagtaNNatggttNaaNcgagtgtggtatttatcgtggtgcNttNg
gNtgNcggcggtagaagtctttaaccagatccgcggagtgtttNaggtttgcgcggNNttcccaggtgttttc
ttcttcNggataNcctttccaatggacaaagaatagaagtcttccattcttctttttgacctttcggatgctt
tctacttcgtattcttctgaaccttcNatgacatcgggtggtggaggtgatggcttctgttgtgattcgaagc
gtgctgtcttgagNggtgttaataggttctcattgaataccgggtggatgagcgtccagttggtggggagctt
gagtttgtaggcactgtggcctaccttttcaatgacttcNaatggtcctaatcgtcgttctgataacttcttg
gatggNctggtggttgtgatatttttggcttctaaaaacaccttgttaccgggtttgagattaagcgagtcct
tcttgtgacgNtcatattgtttcttcattcgttcggctgatgcttctaaggcggtgatagctttcttccggac
ttcttcaatgcgtgatgcaaattcttgagcggcttcattctttacNggttccatggagatgtcNcctttccat
ggatgtcgccatagttcaggaagaacggNgtttgtccggtNctagagtggatcttatcNttgtaNtggaattc
tagaattgatagccatgaggcccaNtcgtcctgatcNtagttgcaaaagatccgaaggaaNtgttcgacatct
tgattgatacgNtctgtttgtccatctgtctgNgggtgataagccgttgataaattccgNttgatNcctaNNa
gggtgcataggtccttcatgaagttcgatgcaaactgNggtccNctNtcactNatgatcttcttgggtattcc
atgttgtctccagatggtgtctcgatagatttcNgcaagtcccttggacgtcagggtggtgtgtgtcggttcg
accctgatcatcttcgattgcctgtcaacaatgaccaaaatagcatcgtaacctttagacggtggtaaggggc
cgatcatgtcgattgagatctcttcccaggggtgcgtgggtatcgggttggggtagagtggcgcatgNcgtgc
ttctcgattgatcttgttcttttgacatgtttcgcatcccatgacatagtcNctgatgtcNgatcgcattcct
ggccaccatagtgtgcgtccaatgagttcattcgttttgtggatccctgggtgacctgctagtggtgaatcat
gatgttcgttgattaattcttcacgtagttgtttgtctgggggtatatagaNcttgtctccgactgtgatgat
gccatcagcttctttgaagaagtgtggactttccttNagaccgaatcggacttctgattctacgcgtgggtgg
tttctgatgcgctgttccagatcttcattgccgaattcagtgagagctgagatggaaatttccgatgtgtcca
gaatcacctcctcttcaggtagttcttctccgaagaaccgtatgccaaagtccgaaatttcatcgtcttcctc
catttctgaaggaataccaaacccatgatttttgaacatcatgacttcttcttcttcagtgtcttcttcgtac
cattccagtcNtgagaggatgtccgccttcgtgtttgttttgcccggtatgtgtcgcatttcgaaatcgtagt
cctgtagtttcaaataccaccgcatttgtcgactgtttaaccgctgagctttcctaaaatatgtNaggttttt
gtgatcNgtNaaNagctcNAacggttctgtgcgtccaccaagtattgtcgccaaatgcgcagNgcttccatga
tggaNagNagttNcttNtcatagatctcgtatcgccgttgtgttgggttcattactttggacatgaaggcgat
agggtgccatttaccatcNtgttcttgagacaggacacctcctatggcNtatcctgatgcNtctgtttcgacc
ctgaaNcgtcccttggaNctNGggagNgccaatattggttctgagcatatcgccgatttcagNctttcaaatg
cgacttcttgttctggtgtccaagaccattcgctagaNcctgtgaggcgNgtgagaggttcagccatttttcc
ataatccttgatgaagcgNcggtaaaaatttgcNaagcctaggaatttctgaacNcctttcttgttttctgga
cgtttccaagtctggatgacttcgatcttttcctgttccatgcggaccttcccattgccaacaataacaccta
gaaatggtacNtcagggacctcaaataagcatttactggccttgaacgagatcccattggcgcgtgcgatttc
catgacctcgatgctacgttctctaagctgatctttgtccttggcatagatgataAtgtcatcgatatagatg
atcatcttgttttgtcgtatgagatctttaAatatctcggccatccatcggcagaatgagcttggggaattac
agagtccgaagaacatgacccgtggttcgaataatccgcgtggtgtNaggaaagctgccttccattcgtggta
aagtgcgatgcgNNagttgttgtatccccagattagatctaacttggtgaaaaaatTtgcgtctttcagtttg
tcgatgatgtcgttaatcagaggtaggggaatttgtccttaatcgtccacttgtttagctctctatagtcct

gtaccaatcgtttgGatccgtctttcttgttgatgaagaagcatggtgatgcgtatggactcttggatgggaN
tatccgttccgaggctagattctcttctaggaatttgtccatttcttccatttctgcggctgtcattggataN
aatttggctggtaattcgtccggtgcatctggtttcaggttaatttcgtggtcatgtttcctgtacggtggga
gtgtgtcaaaggtctttttctcgaatcgatccttccaagtcNtgaggtacttcgggagttttagatccgtgac
cgtgtctgttgaccgaactttagccttgggtagtgtgctgacgcttactttggcacattctgggggcagtct
gggaatttgatcttgccagtcttccagttgatgaagggattgtgtttgtgtaaccattcatgtccgatgaaga
gatcctttttgcctaagtccgctaccagtgctgttgcttcttccttgtgatcgccaatctgcatctggaacct
gacgcttttggtgattaatttatccgcattgagagaaccatccgcattaaatgcccgaataggagtgggtaaa
tcaacNgtggggagtttttctgcttccaccagtcgtcgattgactgccaagtgggtgcatccgctgtccagga
gtgcgtcgagtttagcNtttcgtttgtccgtggttataaccttgaccgtgaccattgttttNtcctttctagt
tttgagagtggcgagatgNagagttgcaagctcaggtgcactagttcgtcgttgggaatcatgtatttgtNat
tcttcggtggtggtacagatttagaatgattcgacgaacccttgggtcgagacttcgtgctcagtctgaccct
tcctgaaaacccttcttNtcatcgtcatctcccttaaccgttttagcttcatcgtcgctttcatcNtcatcaa
ggaccctgaatttgcccttgAttaccatccctgagctccggcattcNttTgccatgtgtccctccttgttgca
tttccagcatttcgctcctgtcctgatcttgatcctaAtgtctgtcttcttcttgcctcggcattctcgtgca
aagtgtccatacttgttNcagttgaaacacttgggtcttccttNtgcattccagtttgctgcNtcgatgttgt
ttcttctacctgtccgatatccatNgggcgtcctctgccaccataNgtggtgcctgttccatcNtgtcgatct
ccagatgtggatgcgcgttgaactccttttcgaatgaggNtgagttcttcctcaatNttgccNtaNNtgagga
tNgctttcttccaNtcgtcataagatgNtggNNcNgNcctccattctggaagcgNcatttgtcgatgaNtgag
ttcttggtgtttcgttgNatNaagaaNaNNgcttgttcgtcNgNaacttgaccaatgtgtttNtgNgcNtcga
agtctgcaatccattcttcgactgattgagatccttgNgtcattaccctgatccNNttttctgccagtaNttg
tcgttgaccgtggaagaaNtgtttagtNatttcggNtacaAaNtccttccatgtgccgaaatcNtttgtNttN
aNNaNNgcatctgttttcatNgccgcccagttctcggccctcttgtNacatctagagagcgNggccgaaatct
tcttttcatccgtgtccaggacagcgttccctctgacgtaaagttgcatgcgacgccaccaaccgtcaaaaag
gttgcaatccccgtcgaacatgccggggtctgcgattccattgtcNttgcgcttaggggtcgttgtctctgta
gccattgttccgtNcggttcgtcctcgtcgtctgaggggataggggggagtggggtctcgatattcNgtgaagg
aagctgNacgctctggttggatgggNccgactgNctgaacggtaNtggttcgctctgaaccgaaggtgtattg
Nggtgaggacggcgcttcctgagacggttggaaccctgatgcgaggctgtgggagtgcgagggggtagtgact
ggggagcgggggNcggaggtgtattggctagNtgttggtgtgggatgggactgNcgatatgcggcgtattcgt
cttctggttctgccgtgNttgattcttctctgacgggggcgcgacccgtgaactgtgggttgtggggtgggaa
gtgtgatgcgatctgtggctactcccttctcttctcgaggacgacgactgtcctgaatgatgcgagtgacttc
ttcttgttccttcgcccgacgaatgttTctcctgatgtcggctaatctttcgtcgtggagcttctcaaatgtc
tgatgctgtctgtggaggttaatccactccttctttgtcgatcctttcgacgtctgtgatacctattgcgNgc
gacggtcgcttcgtcgcctaNtctNtgtattcgagttcNgatgctgttactggcaatgagcccctatatggag
tgatatactcccaactgtgattgNtNtcgatgtcgtccctatccctaggggttgtcctgtttcgttgcagggt
tgcCtggtgaccctagacgtttaatcgaatgttacgtcctgctaacgcaagtcaatgagtcgacagtaaagac
tcaaggacacttagcaaggtctgaagtctcctgggtcaggaggcagtagaggggggNaaNagataaacgattaa
acgtagcgaatacaNgagtatgtagctacgtttatatacaNNtgatgtagttctatgactacgcaNtaatgac
taNgtccgtgagtatagtaagacatatggNgNatgatgactgataNaagaagcNagctgactgagcagctgag
taagaacaatgNagataNcaaagatagaacNNNgaccatgcggtNgagaagtagataaggNgcgcatgatgta
tcatctgatgatgNagcatNtggNNNNtgtcagatgatgatgNNggNaNtagaagNgtctatNaaNgatNNNa
tcagtcaNNtgNcNNaNNNatgatgatNagaagNaaNgNatcgtaaca
>Cligib1_rnd-1_family-252#TIR/MITE
CAGGCCCCCGTGAGCTCCCCTATAGGTGCGGCCGAAAATTGGGGTTTTGAGACCCCAAAAATGGCATTTTCCT
TGACCGGAATGGGGTTTTTGACGCCAAATTTTGGCCAACCCTTGGCCCGTATTGGGGTTTCGAGGGCCCAAAT
TGGGACATCAGGCGACGAAACGTCGTCGAGGAGAAGCCTCCTGTACTTGGGGCGGGGGCGGATTTTTTTTCGN
ATTATGTTGCTTGAATCCGGGTCAAACGGCCTCATAAACCTACCTCCTGGAAAATGCTCACTCCCAAGTGTTT
GTCGGCCGGTCCGCGCAATTTTTTTCGCGCAAATATATGTTTTTTTCCGTATGTTTTACGTCACGTGCGGTAC
AACCTTGACCGGTATTGGGGTTTTGGGGCTGAAAACATTGGGTTTTCGAGGCCATTTTCGGAGCTTTTTTCGG
CCGGACCTATAGGGGAGCTCACGGGGGCCT
>Cligib1_rnd-1_family-818#ClassII/Helitron
CTCAAATTCAGTCAGGCGAAGTGAGANTAGTGGACAAGTCNTACCTNGAACGCTCGTATTGCTCCCCTCAATC
GAAGCATACAATGGCCGNGGTTGTCGAGGAATACGGCCTNAACGAAGAGCAGCGAGGGCGTTCCGNATTGTCG
CGAACCACGCNTCGAGCCCNNATNCNGANCAATTGAAGATGTATATTGGAGGGATGGGNGGTACCGGAAAATC
GCAGGTGCTGCGNGCNTTGGTGCAATTTTTCAANNTGAGGAACGAGTCNCATCGNTTCGTTGTNGTCGCGCCA
ACGGGTAGCGCCGCGGCNTTGTTGGGCGGTTCAACGTACCACTACATGTTTGGCATNAATGAAAACGCGGACG
AGAAAGTTTCNGGNACTCGATTGGCGCAGNTGATGTCNCGGCTNGANGGNGTAACCACGTATTCTTCGATGAG
GTTTCGATGCTGTCCTGCCGTGACCTNCATCGGATGAGCGCNAGGCTGTGTCAAGTCTTNAATGAGCCNGANT

TGCCNTTTGGCGGTNTGAACATNATNTTCGCGGGCGATTTCGCTCAGCTNCCNCCNGCGATCGGNGGNGAGAA
TGCNTCCTTGTATAGCCGAACGGTNGGGTCAGGTCCACAAGNCGGTCCGATCAAGAGGCCGCNCTNGGNAAAG
CNNTATGGCATCAAGTCACCACGGTNGTNATCCTGAGGNAAAATATGCGGCAGCGGAGTCAANCNNAGGACGA
CGCAAGCTTCGGNAGGCNCTNAGTAACATGCGNTACAAGGCNTGTACGCCGCAGGACCTTGCNTTCTTGCGNT
CGCGNATNTCTACNAACGTACANGGGCGTCCGTCGGTGAAAGACGANGNGTTCAGGAATGTTTCNATNATNAC
CGCNTTGAACGTGCACAAAGACGAGATAAATCGNCTGGGNAGTCTGCGGTTCGCNGAAGAGTCCGGNCAGNCN
TTGGCCGATTTCTATTCCGAGGACTCACTAGCTGCGCAAGAAAAGACCGGCTGAAGACCCGGAGATGGAGGAA
CCCTGGTAACAANAAATCGTTATTGGTGATATCCGACAACTTGCAGGAATNTTGTGGGANCAGCCTCACNGCG
CTAACGCGAAACACATACCNGGGAAGCTNTCACTATGTATNGGGATGCCNGTAATGATCAGGACTAACAGCGC
AACCGAGTTGTGTATTACCAGAGGNCAGGAGGGNTNNGTATATGGGTGGCAGGCGGANGTCGGNTCCCGCGGT
CAAAATCTTCTTGAGGTGCTGTTCGTGAAACTGGCGAACCCNCCNCAGTCAGTTAAATTTGATGGCCTACCNG
AAAATGTCGTNCCGNTGACGAGAACAAGCGCGAACACNCTATGCCGCCTGCCNGATGATTCAACCGTTAGTAT
CNCACGNTCTCAGGTNGAGGTGTTGCCNAATTTCGCNATGACAGACTACGCATCGCAAGGCAAGACAAGGGCG
TACAATGTCGTNGATCTGAACAATNGCTACCTNCACCAATCNTATTACACTGCNCTNTCCCGNAGCGCTTCCG
CNGCTGGNACNATNATNTTGCAGGGCTTTGACGCAAAGAAGATCACNGGNGGGGCGTCTGGGGCGCTACGTCA
GGAGTTTAGNGAGCTNGAACTNCTGGATGAAATANCGCGGCTGACNTACGAGGGTAANCTACCTCCTCGTGTG
ACTGGNGATCGNAGAAACACGCTAATANCCGCGTATAGANCGTTGAAGGGCGAANNTTACGTNCCTNCGACCG
TCCACAACGCGATNCGNTGGAGTAAGTCCGATCCNTACCT
>Cligib1_rnd-1_family-1163#TIR/MITE
TACTACAGTAGTAAATATTGATTACAATACTTCGGGCATGCCAAAAGATGTTTACTCCAACAATAGTCAATTG
TATATTTTGAATACATCCCTGGTACATATCCGCCCAGGAGCCAATCAGAAGTGTCCATTTCGGGTATTAGTCA
GGGATATATTCAGAATATAAGAAACGGCTATTGTTGGTTTCGACATCTTTTGGCATGCTCGAAGTATTACAAT
CAATATTTACTACTGTAGTANGGATTT
>Cligib1_rnd-1_family-332#TIR/MITE
CAAGGGGGTNCCAAAATTNTAAAAATCCACCGGGGTCGATAAANNTTTTTAAGGCTTGAGAGCCCACCCCCCC
CCCCCCCTCCTTCCTAATNCGGTATGTTTTCGGCCGAATTTGGTATGTCTCCGAAACATAAGGTCTCTTGTTT
TTCCAAAACCTCTCAATCTTGGTGCATGTACACAATTTTGATAATAATATTGTAGTTAAATCAACTAGTCATT
GAATTTTCATTAGTACCTGAGGTCAATACCCATCTGTGAGACTCATAAACACTGTAGGAGTGTTCTAAAACAC
CAAGTTTCGGGCTCTGCACCAAGGAATGAGGTCAAATCATGATTATTTAGAGAAAATATAGTGATAATGTAGC
TACAAATGACAAATTTACCACTGAAAACATTGTACGAACCTCGGAAGCCGCCAACAAAAAGTCGTAACAAAAT
TTTTTTTAGGCCTNGCAGGTGACCCCCCCCCCCCCACCTCGGNTGACGGCTCGCAATCCCGGNACCCCCTTG
>Cligib1_rnd-1_family-930#DIRS/DIRS
TCCTGGGGCCNTTCCAATCGTCCCCGCTCAGNNTCATCCCGAAACCAGGNAAGCCAGGAAAATTCCGNNTCGT
NCAAAACCTCTCGTTCCCCCACTCCCCCCGCGCAGGCATCCCTCAATCAACAGCGCCATCGACTCGGACGTCT
TCCCATGCACCTGGGGCACATTCGACACAATNTGCCTTCTCATCGGNAGGCTNCCACCNGGCTCNCAAGCGGC
NGTNCGNGACGTCGCGGAGGCCTACCGGACNATCCCGATCCACCCGGCCCAATGGCCGGGGATGGTGGTCCGN
CTCGCCGACGGCGAGTTCGCNATCGACACGAGCGCGCGCTTCGGCCTCTCAGCCGCTCGGGCGTGTNCGGCCN
CGTNGGGGACGCGGGCGCCCAGCTCATCCGGGCGCATGGCATNGGCCCGTTGTCCAAATGGGTCGACGACCAC
ATCTTCTTCCGGCTCCCNAGCGAGCACNTCGNAGAATACAACGNGCGCCGCGCCGTCTGGACAGCGGAAATCA
AAGCAGCGGGGGNCGGNTNCACGACGGAGGGNGNATCTGGTTCAAAGGGNAGACGACGCCAGACGACNCCCCC
GAAGAATNCGACGAAGACGCCTCCGTCGCGCTNAGGGACCTATCAGGTGAATCGCCCAGATCGGCTCATGACG
CGCGNTTCACGTACGNNATGGNAGACATAGACGCCGTCTCCGACGAGCTGGG
>Cligib1_rnd-4_family-2607#LTR/Gypsy
TGTAGCGATCACGTTGCACGCGACCACATACCACCCCCTTATTTCCCTTGGCATGACTTATCTATCCCATGAC
CTACCTTAGCCTCTCCTTTCTGTCTACCTCTGACAACTTCCCGCCGTTTGCGATCGTCTCTCACTTAGTTACC
ATCACTCCTTTCACTCTTATCTATATTGACTCCTATGGCCTGACCAGTATAAATACCCGGTCCCCTTGTACTG
TAACGGGGACACTCTCTGCAATCTACCTGGCCTCTAAACCGTTATCTTATCGTCCGAACCGGCTATCTCGTTT
ACCGAACCCGAAACCATCGACCGTGTGTCGTTCTTGCTGACGTAGGTCGGCCGAACGCCACATATTTTTTAAC
GGTTCTAGGAAACCTTTGATTTAACATACCCGACTTGCCGTCTAATCGGATCCTCCTGGTCTGATCAACCTGG
AATTCCGTGCCGAAGGTAAATCATCTCATTCTCAATTTAACTAACTTTATTCTCTAGTAACATGACGGCGAAA
CGCTCCAGTCGCGTAACGCTCAATACCCCCAAGATACCCAAGCCGGCGGGCAAGACCACCAAGCCGCCGGTCC
CTGAAACTCTCAAGCATGTGACGGTCTCCATTTCTGAGACTAGACCCTCCGCTCTCAGTCCAGAGGCGCGCCC
CTTGGGCGCCTTGGACGAGGCGAGACCCTCCGTAACTGGAGAGACCGCCGCTATTAGTAGCATGCTTGAAATA
CCTACACCTTCTGTTAATACATCAGATGAAATGAATAATACTCAGGTACAACCTGAGCTTGAGAACCTGAACA
CTGGAATTAAGAATGGGGTCGCTCCCCAAGACTACCTGAAGATTGAACTTCGAGAGACCGAAAGCGAGACTCT
CACTGAATTTACCGAACAGACCCTTGGCCCGAACGAGGGCACGAGCAAGGGGAAAGCCGTAGATCCCGGAAAC
TGGGGGAACTTAGCATTTGAGAAATCAGAAGTAGACCCGCTCGAGCAATTGCGGATTCTTACAGAATGTAATC
AACGGCTTGAAACTAGAAATCGTCAATTGACTCAGAAGGACAGAAGAAGCGTCGAGCGCGAACTTTCGCACCC

GAATGACCTGCGAATCCAGGAACTGGAGGCTGAGTTGAAGCGCCTTCGTAAGGACCACAATAAGCGGGCGACT
CGGGAAGAGCGTAAAGCGGCGGAAGTTCGTCGTGGAAGGCGTAAAACACCTGTTCGAGAGAAATCAACTCGAC
CTATTAGTCAAATAGCTCCCAATAGTTACCTGGGGATTGCCCTGGCTAGGGGCGGGCGAAAGATCTCTAATAA
GAAGAAAGGGGACTCATCAGATCCCGATGAACCGAGTGACTCTGACCCCAGTTCATCAGATGAGACAGGAAAG
TCTCTGTCGTCCTCTACAGAAAACTCCTCGTCGGAAACGGATAACCCTCGTACGACGAAACCTAAGAAGCGTA
AGATTAAGAAATCAAAGTCCGTTGACAAGGACTTGAGAACAATAATTAAACCCGTGCCCCCTACGAAGTACGG
TGGGGATACGGACCCATACAATTACCACCAGTTCGTGACCGAAGGAATGGACTACCTAACCGAGGGACGAGTC
AGGGAGGATCGACAGGTGTTTACCTTGTCACGCTACCTTACCGGACGCGCGCGGCAGTTCTATGTACAGCAGG
TTGCGAGCAATCCATATGAGTGGAACGTGGACGACTTCTTTGAGGAGTTGTTTGATTATTGTTTCCCCCTGAA
CTTCAAGACGAGTCAGAGGGAGAAACTACGTAAGACCTATCAGAATGAGAAGTCTGTAAAGGAATATGTTCAC
GCACTCAACGAGCTATACAACATGATTGGGCGGGAATCTGAACGCAACAAAGTAATAAAGCTTTGGGACGGGT
TTCGACCCGAGATCCAAGGAGCTTTGTACAAAGAGCGCCTGAATCCAGAGACGTCGTCCTGGAAAGAGGTGAT
TACCACCGCGGAAACGCANGGAGATCTCGCAGGAAGGGTGTCGTCCAGCGGNCCGAAACTCTAGGCGGAATCG
AGACAAAGACCGTGGGTCTGGTACAAGAAATCCTAGCCCGAGTCACACGACTCGTCATCAGAATATCCCGCGT
CATAAGCGTAGGAACTACTCACCTGAGGGGGGCCGGAAGGGAAACCCACCTGGTTCGTCTCAGGCTTCTGGAA
CTCCCGGTGGATTCCACCATAACCGCAGTAGCGCGGCAGGTACTGGAAATAACCAGAAGGACAAAGGGCGCGA
TAGCCAGAGCCTCACGAAGCTTACCGACAAACAGCGCGATGAATATCGGGCTGAGGGAAGGTGCTTCATGTGC
CGGGAAAAAGGCCATATGGCCCGGCAGTGCCCTAAAAGCAACTTCGCGGGCAAACCGCGAGGTCAAACTCAGA
AACCCTCGCCTCAGGCCAATAAGATGGGTCTCTCTGTCGCCGCAGCCGGATTGGATTTGGCTCATACGTCGCA
GTTGGTGGAGTTAGCAAACTCGACTGAGACNACNACCAATCTGATCCTNGGGTCAGTTCGACTAGAGCTGGAT
GCAGGNGACAGCGACGGACCGGTNATAACCNACGGATNCCCCAGCCGATGACGAGGAGGAAAACGACTCTGAT
AAGGAGAACCAGTTCACGCGCAGCTTTGAGCGGGTGGACACCCACTTCGAACCTCCGGACGAGGAACCNGNAT
NNTCCGTCGACCTAGGAAATGATCCNGTCTGGAACATGATGGAGCATGACTTGCAAGATGNNCGACGAGCGAT
GATGCCGGATTACGANCCTGACGGGATTCTAGGGGACCCGTTTGCGCGGATCGCCCAGTTCAGGCTCGAGCTT
GAGGANGACTACCCCGGGGATAAGGACTCGGATGNTGACGGAGTGACCGACCTTCACCCTCGGTTCCAGGTGT
ACNGGATCGAAGGCGGANAGCATGCCATCTTGGACGCGCTTCGNCGGGATCGNGAGGCGGTCCTGGTGGATAG
TGACCTGTTGCGGGATCCGACCTTCTGCATNACCCGTTGGTACGCCGGCATCCTGGGACGCGGNGCCGGCCTG
TCTACCTTTGAAATGGGACGGTGGTTGAATGACCCCGCTCGCTTTGATGGATGTATGGGCGACGCTCGCCAAG
AAGCAGTATCCATGGTCTTACTCGACTTCTTTGAAGACCAGTCACCAAATATGGAATCCGGCATTGAAGAAGC
CGATGAGTTAACCGATGATTTGTACCTAAATTTGGAAACAAATACGGGAGAACCCGACTGGAGACCGTTCGTC
AAAATCGAAGCCGCCACTAGGGAACTAGATGACGGCTATGAAGAGGGATATAATATAACCCACCGTGACTGCG
ACTATAGCGTNTTCTTGCCCAGTNNGCTCCTCCGGGACGACAAGTTTGACCTGGGGGAGTGGTTCGTNAGGGG
CTCNGTTGAATTTGAGGAAGCCACCNANCGCACCATGNTGNGGGACACGCGACGGGATGATGACAAAGGCTCC
CGGGGNCTTAGAACTGGGGGGTACCTACATTGAGGAAGCCGAGCTGTNCGCNCTNCAGCGGAATGCGGCGGCN
ACGCGGGACTTTGGCCGNGAGATCCCGAAGCCCATAGTGGTGGTCGTAATGATAAATGATCACCCTTGTCGTG
CGCTGATCGACTCGGGNTCGCTGGCAGATTTCATCTCCACGTCGATGGCTGATCAATTGAAGCTCAAACGGGT
NGAACTCACTAAGCCGTTACCCGTCCACCTTGCCGTCCAAGGGTCGAGGACGATGGTGACGTGCGGGACCAAA
GCCCACTTAGGCTATCAGCACATCAGCGAAGAGCGCTACTTTGATATAATNAATCTNACCAATTATGACCTCA
TTCTNGGGACGCCGTTCATNTTCCAGCACCAAGTNATGTTGGGNCTAAACGAGTCTCGTCTGATNGTCGGGAG
CGATGAGTCCCTNCCNATNGNCGGGGAGAATGTGAAGTTCCTGTCGTCGCGGGCGATGGACTTAGCNGAGGGC
GGGCTNGAACAAGTNCGAGCCTACATCTTGAAGGCGGCCGGGNCTCTCTTCGCGAACCCNGGAGAGGCGCCGC
TACCCCCGCTAAGAGCTAT
>Cligib1_Cluster7#LTR/Copia
gtttgaAgggaacgCcCgtgacaccgCagAatAccaaatacagtatcccgcttgacgcgtgcgcctatAcgca
cgattTcatttttacgctatTtcatatcggctttacagtatcacttacaacttgtctcacCaacatcaatgaC
AcacgttcaacaggacgtgtctcacTatagtctttaccgtatAtatttcacgcaccNgtAccgacacacctca
gtttgctctcacTcgtgtgacggCatctgaggtgagtaaatgctatacataTcgaaacggaNgcacaActaaa
aacatatactatctagcttccggtctcaacaGgtagCtaatatagcagtttgCtcTcactcGtgtgacggcat
ctgaccttccgTtctcaacacgctatgcgccctgtagcctgaacaatCcggatatcgttacacgcgcaaaaca
caatacattcAcatctactaaaaaatgactTcgactactacatcGtctgcccacccacCcctcgaagccaaga
aggacaaAttcaacgctcCcaactggatcctctgaaaaAgcgccgggcgtCgagctgcccagcttcgcGgcag
catcggctacctggacggcacaaTgaaagaaacggcggagaggccgacgcttgaggacccaGagacatCccca
ctacccatatCtgaCcCagacaccaccacggcCAcccactgggcgtcacttagccccTCcatcaatgagtgga
acatgCggaacgcgtgggccatgacattactcatagacaacttgatcAacccagtNggattaggcactaactt
agatggaaccgcagccgaagtctggacGacgctcaccactcggcacAaccgcatggacaatgaCctagctaac
gtccgcgcccaacaggccctgcgcacctGcctAtaccgcgaTggagacgAcatgcttacccacgtagagaagc
tccgGgccaaatgcctcgaagccaacgcGacgggcggaaGagtcacgaacctaGactaccgctctagcgTAcg
cgctgcCtgacccccatcttagcatacaatagttgaaacaatacacaacactaaGacgtcagacgctggcatt

GAcagggtgaccatgcactgggagaGgatGaccctcggtaaagcgagcCaaacgGctggGaggTcGgtcacag
tgcgcaagaCattgtctaAtaccaagaaacctgaacagctcgcttgcacgaacccaaactgcaagcgaaaggg
acataccatcgagaacggttactgcaAaggcagaggaaaagaaggacagttcCccccccgctgccggcaacgg
ggaggaacgtcgacaacgtccacaacgacc Ccacagac Ggg cacggccaccccgggcatagccgacTcctcaa
cacggccgcgcagccaacagcggataaggtatacgctttagAcacctctgccatcgAcaagactaccacTgtc
ccggcaccgatggtggccccttccacgcaaatcccaagCTccGaagacaccaactcaCcagCccacagccatg
tcctcacgtatcctgactcggggtaaagtgActgctgTctcgtgaaCCgcgaggattttgacaccgatactcc
catGaatacgcccgtactTggacgagcggcgagtaagagGagcagttgcAagataatgggCagggcatacgtc
actaagaaGcTtgcgggcaatggccagaccacgaaGatcacattCaaaaacgagtatcatacccccgacctca
tctcaaacCttctatccattgggaagttcgacccggccGgatagcccgtcgTTttcgagtccagcgtgccgaa
gtgctatgactcTaaaaataacataatactgcttgggaaggacagTaatggaagcgacctggtcgactgcctt
gaaccaccactggcgcttacggcgtgccagcatgagaagcccaccgcCatggataacggccacacgcgtctTg
gacacacaggcgtcaccgccagaCgcaacatggAaaagaagaaCttagtcgatgGtcttagtatcacGgGcga
ctcaacgactcaaggactctgtgaggactgtatccacggcaaacacgccgcccgaccatatgaagagaagctc
actcacgaAaccaccgtggtggaacgCgtccacatagagctctgGggaccagcccgggtGCaatcgatCNgaG
gcccacgttacatgcacctGctGacggacggcGaatcgtcgtaccGgaaGGcctaggtcctcacgaacaaGga
cgcggagactaaccacgaagccctccatgatgttcGcgttgaggcggAgcgacaaaCggagaagaagctgcgc
atagtccgacccgacgggggaaNGgaGTGgtacaacaactggtgggcggaCcacctgaggAagaaagacatCg
tcaCcgaaatcaatGccgcGtacgcacactcacagaatggcgtccctgaacgcggcAtgcgcacagtctacga
aagtgtaaggacCatgttgccagagtatgggctccctCcatctctctGggctgagcccgccgcgaccaccgct
taccctaggaacttcAtcccgtcatcCcgacacCcGgaCcggctaCcggcagaggtgtggacccgccagcgcc
aagacgtaTctaATattaggcctaggggttgcaccgccttcgcgaagatCccggcggaagtggGtgcttcGaa
atgagacccgagGtcgataaagcatgagctcattggagactatcaacctggggcgtacaAaccttatgatcgg
gctacggggaacattatcaagagcCGggacgtcaccttcNacgaggggacgggccaccgaactggTactactc
cgcgCcCacctcatcgaacacttcgacctcggccc Tcacgatcaattgc Ggccc Gaccctggtactatcaacc
ctagaccgcgcgcggtcatcaagcacgcacgaCcgctggcagatcatccacgccccaCggaccctcctttgca
tcctgcacgcctcgatgtgGacCctccaaggaCgaaTacagcgccacCgccCataccagcaccgcgcagaagc
gatcgcccacatgtcccatcacgcgcgctgcacgaaactcaggaatccgaggaacgactggccgcagactCcg
acaattctgaggactgggcgacccaccccccgcacacgcgcgatgCtggcagtaatgctggTcacttagaaGc
cgtGgcgccccggaacttcgacgaggcCatgaagcctcccgacctttggaggaagccgatggaggataagatg
ccGcttaggaaagaaaaggaggtattcaagctagtaccacgccctcAaggcgCtaacgtCctcgcactccgat
aggtctaccagtacaagaatgatgcgcaccgaaacAtcgtaaaacgaaacgctcccctacttacaaaagggtt
cacgcagattccaggcgtGgacTgagaTgAGacatacgcctccgtgggccgtctggAAtcgatgcccatggtt
gcggcagtcaagaccgaacgtagacgcaaaatctccCaggtacacttcattgacgcNtaTctCaatagcgaca
aCaaatacaccgtctacacggagcaggcgccgggctgtatcgagcaaggggaggagaaCaaggtctacgttgc
cgagAagaccagctacgGgatgatgaacgatgcgaacgcatgggcagaagaacacagccacaGctatgatgga
ctGggccaggaccaatcaCGccagcactcatgcggcagttctcggaagcttgggcacgagCTcactatcacca
ccacgtagactcAccacctcttcggagcctCgtccaccgacgcaggggctgcactggccaaacaagaaCTccc
cagacgCtacgacgtcaaccAtttaggggagctcaaatccaTCCTgggcatcccCatTgatagcgaccagact
aaTGgcgctatCtcaTtgtcTcaacgcgcGtactgggaacgcgtcctggagccaTtcaaaAtgtcgaagtGca
acccaaaacccaaaccgctcCcagtaggctTGacgctcacagaagcggaccacgCgAcgtCagatgaagacgt
TcgcttcatggcagacaaaccccttccgcgagacattgggatGtagaatgtagcctaaagcaGccacccGGcca
gaactaNaNtctTatacgGgaaacttactctcGcgtcaTcaaactaacccCtcGTccagcTcactgcaaagCac
tcctccacgtcctcgcNtagatcagggataccttcgacttCaaaaccacctAcacgccgGgcaccaccacgcg
aaggcctcatctggGatatgttgatgcagactacgctggagagcctGacacgaggcGccCgactccggggtaC
ctctccActaaatgcgggggacaAgtctcgtGggcagcgaagcgtcaacccAcGgtagccctatccacgaccG
aggcagagaaTatgggcctgacgcGggctgctaaacacgagttaTGGatGtactcattcaTGagcgaGatcGG
catgccgcaagagtcaccggcgaaactcaacgcgGacaaccaggcggccatagcactcaccctcaacaccAag
gaaCaaccacgcgcAaaacacagcgacGttcgctatcattacatccgacagccaatcaaGgaggatgaaaTca
acgTcgactacgtaccatcagcaGacaacctcgcagacaacctaacgaaaccgctcccgcGcAttgaccaaca
aaacatctgccgcgcactgaatgttgtaggcggcgacgccacGgggttaggggagtgttgaaggtAacgcccg
tgacaccgCcgaatAccAaatacggtAtcccgctagacgcGtgcccctgTacgactcgaagtcAtttttactc
tattgcataTcgtctttacagtttcacatacatcttgtttcaccAacatcaAtgaCacgggttcaacacgacg
tatctcactattgTattTacctgagagAatacAcgctccagtacctacacaccaaagcgtgctcTcaatcgtG
tgacagcaactgaggtgagattagtctaaacagAtagacaagaaagcacAactaAcaaaatatactatctagc
ttcccgcgtcaacaggtacctaAtatagtAgtctgctcTcactcgtgtGacggcatctGagctTccctTgtaa
aca
>Cligib1_Cluster128#LTR/Gypsy

CTGCCACGATGGAGACGGCTGATCTGCCGACGCGCGCTGAACACGGCAGAAGCACGGCGTAATGATAAGGTGA
TCTGTCTACCACAAACGGTCTGTCTCTACTGGCCCAACGGTCATGTGATCTGCCCGTCTCCGCTCTCGTCTGA
TGCCGTGTCGTGGCCGGGTTCCCCGGCTACATGTTCCCCCCCCTCGTGCGGCGGCGTCCTCGCTGCCCCGCTC
TCTAGGTCCACGGTATCTTGTCCTTCCTCCTCGTTGTCTGCCTCGCTGCTGGGTGTGTCTTCTTCCCTGTCCC
AATCTCCTAGTGCTTCCTCCATTGCCTCTTGAGCCTCCTTGAGGTGTGTCCCATCATTCGGTATGAACCTCCT
CAACCTTCGCGAGCTGAACCTTCCTGATAGTGGCAAGCCTCCAGGCTTTCTAGCTTATACGAGTTCCTGTCAC
GGCTGGAGATCCGCCTTGGTGCCGACCATTTAGGAATGAGTTTGCGGATTGCAGTGTGGGTGAAATCGAGGTC
ATTGCGATAGACTTGTACTAGGTTGCCCGTTTTGAATATGATCTCTTTGGCTGTTTTTTGTTGATCTTTTTGT
CGAAGGCGGCCTTCCGTTTCTCTGCATGATCCCAGATTTGGAGTATCCGTCTATTCTCTGTTGATTGACATAG
GCCATTTGGAGTAGGACTTCCTCTTTGCTGGTTCGCCTTCGGCCTCGTTTGATGGTGTTCGTGGGGTGTTGAT
CACAAGTCCGAGTAGCAGTTCTGTTGGGAGAACCCTAGGCTTGGTAGGATCCTGTCGTTTAGGGACTTGACGG
CTTCGTCTAGATAGTCTGGCCAGTTGTTGGGCATGTCTGACTCCGTGTTCCCTTCAACGTCGTCGTCTCCGCC
CAGTCCGGTGCGCATAATCGTTTCAGCCGTCCAAGTAACTTGCTATTCATCCCTTCGACCAGCCCGTTGACCC
ATGGGGAGTATGCTGGGACCACTCGCAGCTCCACCTTTTTTCGCATTGCACAAGTCCTTTAGTTCTTTGTTCT
TGAACTCGGTCCGCCGTCTGCCATAGTGTTTCGGTGCTGTGTATGCGTTGCATATTTCCGTAAACGATTTGCA
AGTTGACTTAGCAGTAGGTGCATTTTTCAGTTTCGTGGCCCACACGTGTTGCGAATACACGTCGACATATAGG
GAGATTTTCGTGAAGCCTCCCTTCCCTTTCGGCATGTGAGGGTGTCTGTTACCATCAACTCGAAGGGGTGGCG
CCGCGTGATAGGCTCGAGTAATGAGTGAAGATGGTAGGTCCGAAGCATTTGCATTTACCGCAGGTCATGATTG
CTTTTACGATTGACCTGTCGAGTTTTGGGCTGGTGATCATGTCCATCATTTTGATCTTTATGTTGTCGCGATG
GAAGTGTCCGTTTTCGCAGTGGATCTTTGATGCCATTGCTTCCGCTTCGCCTTTGTGATGCATTCTACCTTGC
TCTTGCTCTCACTGACTTCGTATCTCCCACCTCCATAGCCTTCCTTCCTCTATCATATATCCTTCCGCCCGGT
GCGCGCCCTCTTTTTCTCCTGAGGCTTTTCCCTTGGTCTATCTCGCCGATTGCCTCGACCACCCCTGAACATT
GGTTCGTCGTGGAACGTGCCTTTATGTCTCGATCTCTCTGTGCTTGTGTGTCCACATGGAAGATGTCATTCTG
TAGGCCTGTTCTGACTTCCCAGTCTTCGCTGACCGTCCAAGTATGCCCATCGTCCTCCTCTTTGGGGTATTGA
TGTATTTTCGGCTCAATCCGTCGCCACGGGTTCAGCCGTCCAGGCCGATGCGCACGTCTGTTATTTGGTAAGC
TAGTACCCCATCCCCCAGCGCGCGTGTGTTGAATTCAATTTGTCGCTCAGGAGGTGATGCGTAGGGCTGGCAG
TCTGTTTCGAGCTCTACTGGGAATCCCCAGATGATGTCTGAAAAGCTGTCAATGTTACTTGACGGCGCCGAAT
CTAGGAGGTATGGTTTGTATTTTTCTTCCGTGACTGATGTTCTTTTCCCTGTGAATGCGACGGGGTGGATTCT
TGTGACTTCCTTCCCATCAGGTAAGACTGTGTATACCGCTGTGATAGCATTCCTGCAAAGCCATACTTACACC
CATCTGTGTCACGATGAAGGGGTTCCGTCGAACTTAGGGCCTTTCATACGGGTTCGTTGTTAGCGCGACTTTT
AGGGCTAGAAAGCTTTGTCGTGCTCATGGTTCCATCGACCGTCCAGTGAGAAGTCTTTCATTGCTTTCCGATA
TGCGTACTTCCCTTTTTTCTTAGGAATTCCAACTGTCGTATAAGGTCCGTCAGGTTGTGCAATGGTGCATAAC
CTTTGACCAGTGACCGGAAGTACCCTGTTAGGCCTGTAAAGGAGGCAAGTTCTGGAGGTTTTTCGGTGTTTTC
CAGTCCACTATAGCGGTAGTTTTGTTAAATCTGGTTTTATTCCTGTGGGCCTATTCTGCTCCTGCGAAGACGG
CTTCTGTGACGAAGAAGCTTGATTTTGCTGCTGATAGTGACAGGCCCGTCTCCCTTACTCTGTTAGGAAGATC
TTCAATTTATTGTATGTTTCAGAGAATGTGTCGCCTGCTAAGCTCCGTCGTCCACGAATAGTTCCATGAGTGT
GCCGACAAGGTCGCCTAATTGTCTGCTGTCATCTGTGCAAATGTCGATGGTGCGCCCGTCAAACCAAATGGCA
TGCGCTTGTAACAGAAATATCCTCGGCCTTCGACGTAGAAGCATACGTATGGTTGGTCCTCTCCGCAAGTTGG
CATGCGTAAAAGCCTGCGCCAAGTCGAACACCATAGATATGGTGTCCACTCAATCGCTGTTGTTTTGCTCTTA
TGTCCCTTGGGCATGGGTGGGACTTTGTGACCTTGTTAAGCGCTGCAAAATCCTGACAGACACGCCATTTGTT
TTCGGTGGTGTTTGCCGTTTCGTCATTCTCGCCTGAGGTCTAGGTGGTAAATTCTCATATCCGATGTCTGGTA
ATCCCGCTGCAATACACTCATCATTGATGCGATGTTGGAGCTCTTCAATTGAGAGCCCGCCCCCTTGATGCGC
TTTGTGGCCAGGGTTGTCGCGCCGCAGCATTTTACGTCTTTGTGGTCTATTGGTCTGACGATTCCTGCCTCTA
GCATTTTGTCCAGGACACCGTTGTAGTATTCTTTCTGTGGTGATGTAAGTCTTCGCTGTCCGATGCGTGTGTT
GAACTTGGTCCCTTCAGGGATATTTAGCTTGTGGACAGCGCCTTTGACCGGAGTGACCTCGCTCAGCGAGAGT
GCAAAGCAATCTGCATGTTCGCGCAACAGTTGGACCACTGCTTCGCGTTCTTCTCCTACAACGTCTGGTCCTA
TGGTTATTTCCTTCATAACTGCATCAACCCTTTTAGTATTGAAGGGGTCAGACGCGCGTGTCAAGACTGATGG
GGATAGTTTTGTTGGCATCACTGGCTGTTCTGTACCAGGGTCATCATTGACCCCCGTTCCGTCTTCGCACACA
ATCCATATGTTATCTGGGATGTCTGTAGTAACCAATGTGTCAACTACATTTGCCAGGTGTACTGGTTGAGTTT
CGTTTAGGACTTCCCTCGAGGGGGATCGCGACTCCCCCACTGAGTTTGTACGCGCTGGCTCTGAGTTTCCCCT
TTGGCCATTGTTTGCACCTTCCTCTACTTCTTGTGTATCTGACGCTACTCTGCTACTGGCTTCCTCACTTGTG
TCTGCTCCTTCGCTTGGATCATCTATTCCACTCTGTTACGATGTGGGACTTGCCTGACGGGGGATAAGCATTG
ATCCCCCTCCAACGATATGGTCTGTTTGATGTCGGTTGTCAAGCCGATCAGGGAGCCTGGGGGGTGGGTTGCT
GGGAGGACACGGTATTGGTTCTTCAGGATTGCCTCCATGCCATCCGTCTGCCTGACTCGAATTGTGTCTTTTT
CGTAGTCATGTTGCGCTTTGAATGCTTGGAGCAGCGGTTTCCCGAACAACAGCCCCCATGCTCCACCGCTATC
AAATATTTCGAATCTCCCTTGATTCTTGACGCCTGCCACGGTGACTCCTCCCTCCCATATTCCGGCTGATGGC
ACTAATCGCCCATCTGCCATGCGCAGCACTTTGGATGATGGTTTGGCCATGGTGAGTCGCTCTTTGATTTTTG
TATGGAGTTTTTTGTCAATCGCATTAACCATTGCTCCATCATCGAATACACTCCTTATTCGCACGATTTCTCC

GTTTGGTCCTTCCAGCTCGACTCCATGCACGAACGGGACACTATTCTTCCTCACTGTTGAATCCACCGTGTAC
AGGTCAAGAGTGAATGTATTTGTGGGTACGGAATACATTTTGTTACTAGCACTGATGCATGAAGTTTTGTTCG
AGTCCACTAGGGTTGAGGATGATTGTCCCCAGGCGCTATCTCCACCTGCCTGGGGACTGGCTGTTCTTGGGTT
GTGGTTATGGTACGGGTCGCCGACTGATTGACTAATCCGATGTCGACGACCCTTGACCTTTTCCCGGTTCCAA
GGCCATTCTCTCGAGGAACCTTGTTATAACCTTTGTTTCCTCTTCGTCGAATTCAGCTATGACGTTGACTGGG
GCTGCGATGGCCTCTGCGCGGCGCCGGATAGACGATGCTATTGAGCGCCACTTTTGCTCTAAGGTTGGGACTT
GTGGTTGGGTGCAATCCATGCCACGTGACCAGGTCTCCCGCACTTCCAGCATTCGCCCGATGCGACAGGGGTT
GTTCCGGGTGTGAGTGGGTATGGTCTTAGCTCGGTTGGCCCTGCCGTGCCGTGTTTGCTTGTCCAATCTGAAA
TTTGTCTCTCGTACAGTTCTCTCCCTGCAGGCGTGTTGGGGTGTTGCTGTAGCGCTTTGGTTGTGACGTCCAC
GTATCGATCTGCCTCGGGCCTGAATGATTGGATCGGTCTGATCTGGTTGGGGATTATGCTAGAATTCGGTGTG
AGGGGTGTCGTTGGGCGGATTGCTGTGGTAGGTTGGTTGAATGTTACTGTGCGCATGATGTCCCGAATGCTCT
TCCCCGACTTGTCCGCTTGCTCCATCCTCTGGATCGTCTCCTTCATTGCCCTGAGCTCCTCCTGATCTTCCTG
CGCGTCCTTTATTCCCGGCACTGATGCATCCCTGACAGCTTCGCAAAACTCCTTCCACGTTTTGAAGTTGGTT
CCGATGACCGTTTTCAGGGCTTTTGGCATTTTTTTGATCGTGGCTGGGAGGAGGAGGTTTGCGCTATCTGGGA
CTGCTTTCGCTAGGCGCTCCAACTTGTCTGCCCATGCGACGTGGGCCATCTCCTCCACGCCATTGATGATTAC
CTTCGTTCCTGGCGCAGTCTCCGAGAGGGTGGCTGCTGTGAGTGCTGCTACCTTTTCCTCGGTTGTTTTTGCT
GCGATGATCTTTTCTGGCCACCTTTCTTCGAATGCAATCAGCAGGTGGTCCCAGGAATCTTTCTGTGTGGACC
CGTCGAGATCGTTCCACCATGCTTTCGCCACACTACCTGCTTTGAGGCAAAGCTGGAAGTGCCTCAGCTTTTG
ATCTTCTGAGAGCGAGGTTCTCCCGAGGAAGCTGCGCTCGATGGAGTTGATGAAGTCCTGTGGGTTATCATCC
TTACTATCATCGTCACCAGCAAACAGTTTTACGGCAGTGTTTGACATGTCGTCGTCAATAATTGGCGCTGGAT
ATTTGAATGTCTCTAATGGATTGGGATCTGGTGAGGTTATTGGGGATTCGATGGTGTTTTCTCTTGGGCTGCC
GAATGGTGAGTTCTGAAGTGTGCTGAGTGCTGAGGCTGATATGGATGATACGGAAGATATGGGGCTTGCTACA
GTTATGGTTGCAGGAATGAATCGTCCCTGTGAGTCTCTCGGTGTAGTTGGCTTCCTTGGTGCCAGTTTGCCCC
AGCCCGCGGTGTCTATGGCTATCTTGTCCAGGCTGTGCAACTCAGAGTTTAGTGGAAGTTGTGCGGTTGGTGT
TTTCCTGTTGCTCCGTTGTTGGTCGTCGTATGTTCTTTTATTGCTTCTATCAGACGTGAGCCGAGTCGCACTG
TTGTCACCAGATGGAGACGGCTGATCTGCCGACGCGCGCTGAACACGGCAGAAGCACGGCGTAATGATAAGGT
GATCTGTCTACCACAAACGGTCTGTCTCTACTGGCCCAACGGTCATGTGATCTGCCCGTCTCCGCTCTCGTCT
GATGCCGTGTCGTGGCCGGGTTCCCCGGCTACAA
>Cligib1_Cluster127#LTR/Copia
ATCGGCCTAGATCTAAGACACGGTCAGGGTAATCCTCAGTGGTGGTAATCAACTATATTCAGTACCTAAGACA
CGGTCAGGGTACTGGATGATATCAGTAATGTAGAATATGAAGTGTAGAACTGTACTCACATCCTCAGTGGTGG
TAATCAACTGAGGATGAGAGACGACGCGTGGACACTTATATAGTCTGGTATCATGTGTCTGCTCTGTATCATA
TAACACACCCCCATAAGGGCAGAGGAGTGTAAGTAAAGAAGGATAAGGAATTAAAAGGAATATCTTAGATTGA
AATATTAACCAGGCCCAAATTTTCACAATGTTTGACGAATAGGATGTCGAGTAAAGGTTTTGTCAAGATATTG
GCGACATTGTCACTTGTGGGGATCCAGATTGTAGAGAAATTCCCGGAAGCTATGTGTTCGCGGATGAAGTGGT
ATCAGATGTCTATATGTTTGGAGCGTGATGAGGAAGATGAAGAGTTAACAATGCAGCGTGCTCCTTGGTTGTC
GCATAGAAGTGGGAAGGGTTTTGGTACAGGGAATTCATGTAAAGTGAGGAAGAGTCTGAGCCAAATCGCCTTC
TTGATAGCATGCGACATCGTGTAGTATTCCGCCTCAGTTGACGAGAGTGCGACTGTCTTCTGTTTGACCGAAG
ACCAAGATATCAGTGAGCCTAGGTAGTAGAAACAATATCCAGTAACACTTTTGCGATCTGTTTCATCAGTTGC
CCAGTCCAAGTCTGTCATCCCATAGGCTGTGCCCTTTGCCACAATTTTAATCGTTTGTTGTTCTTCACCTTGT
CGCGATCCATATTCCAATGCTAGATCTGAGGTGCCCGAGAGATAGCATAAGACACCTTTTGCTGCCAACATGT
GCGCTGATGAGCGAGACCTAGGAAAGATGCCTGTGGCAAACCAACCCAAACCTCTACCTCTCTAGTGACGAGT
GGGCGCTTCAGGTCACTGCAATAGGCCACAAAGTCGAAGTACGTCGAAGTCGAACTCGAAGTCGACGAGGTCG
AAGTGCGAGGCGCAAACTCGAAGTGAAAGGGGAAACTGTTTCGTTTTGATCTAGGTACATGTTCGACAACTAT
ATAGTAGTGCTCAGGGTCGAGTAATGTGTCTACAGTGTGTCTAATTACATCTATGAGCATGAGGTCGACTATG
ATGTCATCAAAGTCGAAGTCGAAGTCGAAGTCGAGGTCAAAGGTCAAAGTCGAAGGTCAAAGTCGAAGGTCAA
AGTCGACAAATTGAGGTCGAAGTCCAGGATTGGAGGTCTATCCTGACCACCGCTCATTAGCGCACATGTGGGA
TTAGCATTGAACTGACCAAGAGCGCATGCAATGTAGGCAATATCTGGACAAGCGCATACCGAGAGGTAGATCA
AGGAGCCAACAAGACTCTGGCATTTCCTTTTCAAGTCATCATCAGATATGTCCGGTAGTGCATTAGGTGGTGG
CTTCGGAAGAGCATGTAACTTGTTGCGTAGTGGGATAGGTGCAGGTGCGCAGTCGGTCATACCCCAATGTGAA
AGAAGATCAGTGATGAACAATTTCTGGGACAACCATAGTTTCCGATTAGGTCTATCTCTTGTGATGTGTATAC
CCAGGTAAAGCGAAATGGCACCAAGGTCTACCAGTTCAAGATCTTTGCCCACTTCAACCAGGAACCATTGGTA
TAAAGGGATGGAGTTGGTGACTACAAGGCCATCATCGATGTGAACAGGTACAATGAGGAAAAGCGGTTTGCCG
CTAGTTGGCATGGTGATTGAAGCATGTGGGGGAGTTGTCCATGTCCCTTTGAAAACAGCATGATCAACTTCAC
AGCGCATCAAACCCAGTCGTGCTAAGATCTTCCACAGTAGCATGTAGAACTCGTATGATGATTGTTGGAGGCT
ATAAAGGGCTCGGAGAAGTTGGAGGACAACAGTTGGATCTGATTCTGGAAAGCCTGGGATCTGCTTGACATAA
AGTATAAGGCTCAGCATGGGTAGATCCGTGGGTGATCCGGATCCGTACTCATGGGTATTTGGTCACACAAGGT
CACTACCCAGATGTGTGGGTAGCTTAGTCAGCAAGTACTTGTACTCACAGGTATTTTTTGGCAAGTACTTACA

ACTCAGTAAGTACTTGTCACAGATCCTGATTACTCATGGATTACTCATGATTACCCACAGGTCACAGGTCATT
GGTCAGCAATTAGTCATCAGGGACAGGTATTTCACAAGTACTTATGGGTATTTACAAGGATTTTGTTGGTTTA
AAATGGAATTTGAGCCTGTATGTACTGTGGGAACTATACTTTAAGGGTCCTTGAGGGTCTGGTGTGAGTCACA
AGTACCTGTCACAGGTACCTGCAATGTCCAAATGCTTGACTCGGATCTGCAAGTAAATAGATCCATGAGTACA
AGTACCTGTTTAGGCCAATACTTGCAAGTATTCCCACAGGTCTACCCGCAGGTACCTGTGGGTAACCCGTGCT
CGTGCTGAGCCTTAATAAAGTATCATCACGAGTAAAGTGTGTAGGAATGCGGTTTTGACATCAAAACTCATGA
TTTCGTGATTGTGATACGTCATGAAGGCCAGTACTGCTCGGATGCTAGTCATCTTGGCGACTGGGGCATATGT
CAAACCATAGTCTTCTGGTCTTTGACTGAAGCCTTGAGTGACTAAGTGTGCTTTCTCTTTTCCTCGAATGATA
GAGCCGTCTGGGTTGTACTTATTAGGGCATGCCACGAGTGCGGGTCACTCATAGGTACCCACAGGTTGATCCG
TGGGAAACCTGCGGGTATTCATGACTTATGAGTTCCCAACTTATGAGTTAACACACTCACAAGTCATTTTACT
TGTGGATCCAAGTCCCCACTGCGGGTACCCACAAGTCTTGTTTGGTCTTGTTTGACTCGTCTCACTCTGTTTT
CTGACCCACCCCCTTGTCACATACTCATGAGTATGTGACTCACGGATCCGAGTCTTTCTGTGAGTTTTGAACT
TGCAGTGATCTGTGGTGGTGAGTACACAAGTCGCGGGGATCCGGACCTGCGGGTTTTTGTTCCCACGGATCCG
GGTCGATCCGCGAGTGCACTCGTGGCATGCCCTAGTACTTATAGGCATATACCCATCTGACCCCAATCGCTTT
GCGGTCTTTGGGTAGATGAGTACGTTTGAAAACTTGTTTTTCCTCTAAGCTGTCGTACTTGTGTTTCATGGCC
GCATACCAAACTGGATAGTCGGGCCATGCCACAGCTTCAGCATAGTTTTCAGGTGCCTTCGATAGATCAAAGA
TGCAATCACGTATAAAACGGGTGGGTCCAACATGAGCTGCAAGAAGATAATGTTCTGCAATATAATAAGTTTC
ATCCGACATGAATGTAGCAAACTCATTGGGTAATATTGCTGTATCTGCAATGCCCAACGCGACGAAATCTGCC
ACTGGTACGGGTGGTACACCCCCACGAGTAGCGCGTAGATGTGCCAACCGTTCATTCCTAATGCATATGCTTT
CAGTGTAATCACGCCCAGCCAGTGTTTGGACAATAGTGCACCGAGATCGGGATGCAATGGCAGTTTCCTGTTG
GATTGGTGTTGGTGTCAATGACCGTGGAGACGAAAGATGCCCAGGTGTGTCCTCATTGAATATGACATCCCTT
GAGAAGTGAAATTTACCATTTAGGTCGCAGACTGTCCAACCTATACGATCTTCCTTATAACCCACGAAGGTTG
CTTCAAAACATCTAGGCCCACCCTTCGTGCATAGCTCCAATGGAATATGTACAAAGCACTGGCACCCCCATAC
TCGAAGGTGTGATAAGTCAGGTTTCTTCCCATGCATGAGTTCATACGGAGTGGTGTCTTTGGGGAGTGTTGTA
GTCGGCAGATGGTTGCGTAGGTATTGAGTCGAGAGAACCACATGACCCAACAGTGAAGGAGGTAGTCCCGACT
CAGCAAGTAGAACCTGCGCGCCATCTTCTAGTACATAGAGATACCGTTCGGCTTTGCCATTCTGTTGATGCGC
ATATGGAGCTGTAACCTGGTGAGTAATCCCCCGTCTTATGAGATGCTCCCCAAAGGGACCAAGAGTAAGCTCC
TTAGCTCCATCGGAACGAAGACACCTGATACGATTGCCAGATAACAATTCCCAGGATGCCTCTACTTTGAGGT
ATTTGTGGAAAGCATCGCTTTTAGCAGATAGCAAGGAAGTGTGGCCATAGTTGGACGCATCGTCGATGATCAT
CCAAAACATCGACGTCTTCTGCGGCGTGAGAACAGGAAAAGGTCCACAGGTGTCCATGTGTAAGAGTTCAAAC
GTTCACCAATCTTAGTAGCCCGCTGTCCCAGGTGATTGAAAGGCGCTTGAGGGCGCTTACCAATAAGACATGG
TATGCAATGAGAAGACGTGAACGCACCTTCCCATTCAACTCCTGTGGCGTAGTTCTTAGTGAGCACTGCACGC
GTAGCATCCATCCCAAGATGTCCAAATCGTCTGTGCCAAAGATCTGGCGATCGGTTTGGTTTGAGAAAAACTG
GAGCAGCAATGGTTGAGTCCATAGGTGGCAAGATGAATTCGCAGTTCAGCCAAGAAAGTTGTTGACGTACAAC
TGCATTGAAGGAAAGGCCGGACAATGGAGAGTCATCAGGAAAACGGATGCTGGTGAAGCCATGGCCAAAGGTC
AATTCCATCTTCTTCTCCTGGAATGTGCCAACAGAGAGCAGGTTGACGGGGACGTCCGGGGAGTGGAGGCAAT
CATGCAATGTGACTACCACTGAACGACTTCCACAGTCGACATGAAGCTTTACCATACCACGTGCCAACGTCGT
CAGAGTACCACAATTGGCCGTCATGACATTAAGGGCTTGGTCCTCATGATATGTCCAAAATAACGATCTATCC
CAAATGATATGGTTGTTGCACCCGGAATCCAGAATGGTATTCAGCCTCTCTTTGATCACTACCAAAGCGATAG
GACCAAGCAAGTCAGATGAAGCCATCCCGGCAAGGTGTAAATCACACATATCATAACAGTCGAAATGAAGATC
GTCATTGGGGTTCGACGTGACTATGGAACATGCAGAGAAGTTCGTAAGATCCTCATCGGGCGGTGGTGCCTCG
TCTGGCGGAACCTCAACTTCCTCGACAGGGGCATCCACGTCCACACCAGCCAAATGCGCTTGTGGTCTTCCTT
TACCATTTCCTGCATGCCAACATGTATCAATCAGGTGGCCATTCTGCTTGCAGTGGGTACAAGTCAAGGTCGA
GCGTGCCTTCTGAGGCGGAATGACAGACGAAGGGGTAGCGCCGGAAGAAGTAGATGTCATAGATGTGGAGGCT
CCAGGAGTAGTAGTAGAGACGGTAGGTGGAAATGTGCATTGATGGGTAGGGGCAGTCCGACGAATGGTAGCTT
CCATATCGAGAACCCTATCAGCAATTTGCTCAAAGGACGGAGTTGATACGGCTTATGTTTGAGGTTCGACAT
CACTTTGTCACAAATGTTGGTATAGGACACCCCAGAAGGCAAGCGGTCAACAAACAACTGTAAGGCAGTGCGA
TGAGATAAGGGAAAACCGGCAGCTTCCAACTGATTAAGGCCTGATCACCATTGTGAGACATACTCGCGCACTT
GTGATCCACAAGTAAGAGTGCATAAACTAGTTTCAATGTCAAGGGCCGTGATATAGTCGCCAATACCATAAGT
CACCTTTAGAACACGGTGGGCCTTGCGAGCAGACAGATCGGAGTCCATGCGTGAACCAGCACCCGGTAGTTGG
TTGCAAACCGTAGGGGAGAGTCACGAGACCAGGATATGGAAAGCAGCACCATCACGGCGCCACCAAAGCTCAA
AAGCAGCAATCTCCTGGGGAGATGAGTCCCGTGAAACAGTTGGAGGATATAATGGAACATGCCATGGGTCATA
GGGAGCGCCATTCGGAGGATCATCGCAGATATGCCTAGTAAGTCCATGGGTCTTGATTGCGGCAGCCACAGCA
TCGGACCATGGTCCCCAGTTGGAACACCCAGTTAAAATAGGTATATATGTAAGGGAGCCAATAGAAGGAACAG
TATCAGAGGAAGAGGGCAAGGGTAATGCATCGTTGAAGGCAAGAGGAGTAAAAGGACGTGGCGATGATGATGG
AATAGGCACCGCTGGTGGCACCAGTATAGTAGGCGAAGGAAGCGGCACTGTAGGTAGAGGAGGCCCCGCAGGA
GGCGGAGGAGGATACACAACGTAATGTGTATCCGGCAGCGGATAGTATTGAAAAGGGTATGAAAAATATGTAT
TATCGGAGTGTGGCATGGAGGGCTGTGGATTATAAACTGGAGGATAAGAGGAAAAGAAATGTGGGGGCAATTG

CCGACCGTGTTGTTTTATCAAAAGAAAAATTACGGTGATTATCAGAAACAGAATGATGATTGCGATGCGGAAA
AGATGCAGATTGTGGTATAAAGGGTGATCTAGGTTCGTTTGGTAAAGTATGATGGGGGTTTCACTGGTTTTCA
CTGATGGTTCGAGTTTGTGGTTCGCCTGTCGTTGGCGCGAATGAAACCCGCACGGGTTTTTGTCACGGGTAGT
CATGGGTATGGGTACGGGTCACACTTTGGTGACCCGCAGCAAACCCGTACCCGTGGTGCGGGTTTGGCGGGTT
TCCCACGGGTACAGATTCCCACTCACTGAAAAATATATGACTCCGTAAACCAACCTTAAAACCAACATTTTGG
CCAACATTTCATACCATTGGCATACTCAAACTCTATTTTTTATGGCAATCGGACCATGGGAAGGCAATGGAAT
TCAACAACAAATAATAGTAAACAAACATTTAGTGTCATTGGCAGTGATTGCATGCAGCAACAGTAGATATGAG
TGATTTAATAACATATATTATCATGTTTATATAGTTTATAGAGCTTAGGTAAACATCCCTCATGGCTATCCTG
AGTGTACATACTAAACCCTGAGGTCTCACAGGTGGAATACCAAATTACCCACAGGATATTGCAAACCCGCAGC
CTACTGCGGGTAATACACAAACCCGTGGGTAAACCCGCACCCGTACCCATACCCGCATCACGGGTGCGGGTTT
CTACGGGTACGGGTACGGGTCGACCAAAAAAACCCGCGGTAGACCCGTGCGGATCACTATTGAGGAGGTATAG
GCGCCGTAAACGACATCAGTACACTTTGACCGGAGGATGAAGGTGTAATGTATGGCCGTTTTCCTGCTGATCT
GGATGATTCAGCCATTGTGGAAGGTAACTATATGCGTTATGTGTGATATTGAAAGAATGAAAGAATAAGGCAA
TGAGTGGGAATGGGAATGGAATGTATCCGTCATGATGTCGTGAGTGAGTTGGACTTCACAAGATGATTCCTGA
GAGCGCCGTCGAAGCTAGGGCCTTCGTTCAGGGCCCATAACCTATTGGCCTAGATCTAAGACATGGTCAGGGT
AATCCTCAGTGGTGGTAATCAACTATATTCAGTACCTAAGGCACAGTCAGGGTACTGGATGATATCAGTAATG
TAGAATATGAAGTGTAGAACCGTACTCACATCCTCAGTGGTGGTAATCAACTGAGGATGAGAGATGACGCGTG
GACACTTATATAGTCTGGTATCACGTGTCTGCTCTGTATCATATAACA
>Cligib1_Cluster62#LTR/Copia
gttgaaggtaacgcccgtgacgccgactgatgccaaataaggaatgccgactctgcacgcattctTcgaacaa
gcaAgcTaAgaaCGTagATAcggcagcacagacacCAcggacacaaacacaaaAaGcacTaaCCccAgaTacc
aTCacaCgggagAaAGaAaaCTcacccGcgTAaaCaccgcAGcgTgCTaTaaaaaGgaaGAaaacAaaTaaGG
gcAcgaCgTgacaaaccCacacaaaTGaacacacaaaaaaacTATaTAcGaaaTgAaaTacagaaaaaaaAaa
aaTacccaaaaAaTcaGTaTgCcaTaacccGcaTcAcgccaccTcAccggCcTTacccaaaAcacTacccgaa
CcccagaacGaacaagaccaagacgacCAaacgcccAaAcacAagacGgTcAcaaagacacaaAAggaccTcg
aTcAcaacGTCGgCcacacaccCacCAcaCagcaCcgAgaaGaaaaaAaTcaagccaTCcaaaacGagCgTcc
aGAaGaacgccgaGCGgacAgCcacacaacTcccccGTGaaagccccTaacTAgacaGcacaAgTaaaaaAca
ccCggaaaaGcCgaCccTgaacgacacacagacatccccacttcctccacctgacccagatactgccgcggcg
accccttggtggtcacttacaccctccatcaacgaatggaacatgcgcaacgcttgggcgatgacgctactta
tagacaacttgatcaaccctgtcggattaggggtaaacttagatggaacggcggctgaagtctggtcgacgct
taccacacgatacaaccgtatggacaatgacctagcaatggtacgcgctcagcaggccctccgcacttgctta
taccgcgAagcaaaaGacaccccaAcAcAcGacaaAAaAcgcaGcGCaaaacgcCaagaGGcaaaTcCaacag
gaGgaaaAagaaccGAcagCaaaTTcacTgCgaaAGacagcgCagcccTaccCcCgaCCTGaGaCaacATaaT
ccagaCCaTaaaTgacaccaAaaccTcagaCgcCaTaaacaagAgAgcagcTacGcAcgggGaaaGGacaaCa
cccGgaAagGccaacCacaCgGccacaGGagcgGaCacagctctccaaacaaacacTTcgAaaaacaaaaGca
aGCacccTAggTacaCGaaCcccaAcaGcaaaaggaaAccGcacaacaTcgaGaaaTcaaacTGaAaaccGaG
aagaAAagaGaGccaaccCacccCcgggacCgcacAaaagggcGaaagcTCgacgacGcCaacgGCAaacCCg
acgaCggcccAcaaaGCaaaaAaaaCaaCcgcaGcgaacGcacacgcaAcgaaaacaaCaaccCaAaaCacca
cgGAgaGaacGcAcGaAccagaaacagaCagaaAggATccaagaaCGcccgAACcaacagccAgggCCaCacC
cCaCaCGcagaCcCaaagCaTcaaacagaagacagcggcGcaagaaAaacAcacCcTGaCaTcCgCccaCTcc
ggaaCAAaacaggGcgcCTgcgccaagcaTaaGGaaTaCgagaccTagacaccgagcgACaCacCAATgAagg
gGAacgcacacAccaagaaaaccAcgTccccaaccAcaGcGacGacCacacacAcCaAaaCCaaCccaacTaa
gGacGcAaaGaccaCGacgAAGTcCACcaccGcgagCcacacacaCcaCcTcaccaCCAacCgAgcaTcaagC
cgAAaGagggacccCGgCGGccTcccGggTagTcgTggAaaCacaGagcacgaaaaTcaaggacGacCacgcca
aCcgcaggacaacacgAAAaacCacCaaCgcgaTcaaCgTacgCaaCTcacacaaCacgcccaCaacaCacAG
cgCacGcccccacaaaaaAcCaacaaaaaTCcacacCgccaacacaccaaaagcaCaaGCaggTaTgcCaGcc
AcaaGCaacagcGaGcGcaaGaaAccccacgaCgcagaAcaccTTaaAGaCgaCGCaACaCcTgacgcAcaaT
cTgaAGAcTccagcaacGcaaaacAcacaAcacGgcCGcaagagcaacTacgcaCTcacGaaaccaccGgccg
gaaaCccGTTcaCcTagaCcgcgGGGgccCggCaaGggcagcgcaaccaacGGaGcaccTgaaagGCgaggG
cgaaCgGacagcGcaTccgcgTaTagaAaGaCggacggccgacCgaaTAaGaaCgCGgacgcaaCGccccCgg
cCcgaCgcgaagacCaaacgGaaaaTgaacaacaaaCggccAaaggCcgGaaaagaGTaccacCgGaacGagG
gccgGaaccgTaaaacAAcaTAacagcccaacaCTTGAaGaaacaccccaTaGcGGcGgaAaacAccacggaa
gaccCaCaaTCaaAGaAgcccaTaacccaacaTggaAcccaCacaGgATcCaAgggcGagacAcCcAggcccG
CaGaaaccggaccaaacccCagcccCaAccccacGagccaacATcCaaAACggccTacCgccGaaacgTCaaCaC
aTCCgCccGcaaaCcGgaCcGaggaCcaGaGGAgGgGagGAaaggTCaaaGccAaaaaGTCgcccATaccaca
cCagcccaTaGcaaaGcaggaacgaaaaaccCgGCcaaaaTaGaTcgaaccaaaTcGaATaCaagGcccaTga
aAcacccacggAcaccaTaCTgcGAgcGcGcagacgaaaaacTcaacgaCaCCaCGAccccaaagaCCaTCAA
GaaaccacaCcaTAgcTTagaagAacaTaCaccaCacCcgaCaGagcgaACcacgcgCacGaagaTacaaGac

gaacgcgaagcccCaacagGaaagaacaGgaAGcaaGcaaacGcccacgaCGaGaacgTAccaccAacaaaag
aaGcCgTcaaagAacaacaCcCaacagCaGccaaCaagaGCacgaaaaaccCcCCAccacaCcagacaaaaCg
gGaaGacccCCaaaaGcagAcGgaAACacGCgcaacaaCaccaccTcCaccAGcaacaAagacagaacaCGca
CGaaTgccaTcgcgacCgcaaagccAaTCTcaAcAgacTcaggaAcGgGTcGCGgaGgCcgcAAccaacaagc
aGgacacaacTacCgacCcCcCcaacgcGCgcaTcaCaGcaAgAcagGCagGccaaaaacaccccggccaGcc
cccGAagaccaaaGaagcTacaaaccAcCccaacCTAaGgacgGaaccCAcGgacgagcAGacGaccAcGaac
AgacaGaaGcaGcccTTTacgcacagaaccaGGccacacgGcgCaaaagacAacggacccagCccgGaaTacA
acAacAaaCacaAccCCaaCGacaaaaaCcTcacaagaaAgacTaGccTaGacacGAaGgcGgagacacaGaa
acCccgaGTGaAcTTaaaCGacacCgaTcCacCcgcAacTccgaaGGagTcGaTGaccaaGcCCcTccCCaTc
accccGaAacTgcgcccaaaacTcgccaaagTCcaaaTaATTGccGcGTacagaaacagcaAaaaaaaGaAaa
CCgcAcaaaccaaaaAGcaaCaagcgcTTGacaaccAGgcaGaGaAaGaCAAGacaTaggTagagGaCaaaac
ggaagaagcgaccAcGaacggcGcaaaCGcGgGcGcgGaGGaaaTaacccgaaGTTacGaCGgaacaccaTaa
TagaaGgagcgggaaaaTgCcTacaccCgaTCacgaaaGagTgGgGagGagccgacaaTcaacacgaCaaaca
CcaacGacGgcgTTGgcGcggccTccacaGaaaccGGcGccGCacagGaCAaacaaGAGacccCaaaacgcTa
acaGaaaaaGGaccgaaaggaaCcCaagTTaaccCccccgaTcCacaagcAccgCgacAaGgccacCccTgca
agccccCacTcacaGcgCacacAaacAaaccGCgccCgggacCgcgTaaAAaaaTccacgTGcAacGCaaaGa
CaaaaCCgccccCaGcagGaaccaCacTcaCaaagGccaaCCgcgCaaaGTcCaaAaagaaTaTaccaacgAc
aaaGgaaaAcccTTaccgcgaagacAgGGgagcaAcaATcggGgCCCAGGaaGcGaccaaAcacGacaggaCT
aaaGaAcacAAgccGagcTCacggaaTcaaaCcaaTcccGcAccgaCCcaaaGcaaagcgGCcCacaaTgTTc
ccaCaTaTaTaaacagaaCaccacaaTagaaGaaCAaGcAaacGCcaaGaaCcggcaGaGcaagaacaaccac
CaGGTaagTagaCacAcagTacgacgGaGacgTGaaTacCaacCaGaCgaCagCcGcagacccaTcTAagaTg
acCccTaacCCGacagcGTgGacaaaGaaaccaaaaacgAccGTcaccgcacaaaCGacggacacGgaccATa
caggaaTcaaGcGgGcaacccAaCAccaacgAgggaccgacgCcgTaacgaGcgaaagcGGcagaacacaGGA
ggcCcCgGcaaacccaAacGcaGaCaacgaaGcAgcTaccgcAaTaacGCccaaTAcgaAaccGaaaaCACcc
ccaAagcacaccGaccgaCGaaaccaccaCaTCCacaaaagCcaaaaaGAgccagacAaTacagccgaaTaaG
ccaaGaccgcaGagaagcacGacGACAacaTaacaaAaccaaTTccGcaccaagcTcAaCAaaaTaacTgTcc
gacgaTGagaaaaacccgagccgaCaggacacaGgaccagAaccTggaaaacAAcGaaccTgacgccgactg
atgccaaataaggaatgccgactctgcacgcattcttctttttctctatttaAgaaCGTagATAcggcagcac
agacacCAcggacacaaacacaaaAaGcacTaaCCccAgaTaccgTCacaCgggagAaAGaAaaCTcacccGc
gTAaaCaccgcAGcgTgCTaTaaaaaGgaaGAaaacAaaTaaGGgcAcgaCgTgacaaacccacacaaaTGaa
cacacaaaaaaaacTATaTAcGaaaTgAaaTacagaaaaaaaAaaaaTacccaaaaAaTcaGcaTgCcaTaacc
cGcaTcAcgccaccTcAccggCcTTgttcaacac
>Cligib1_rnd-1_family-522#ClassII/Helitron
TGGCCGCGGCTACTTACCCGCGTTTTCTNCATGACGACACAAGGGGCGATCAAGTTCTTGGGGATACGTCAAN
ATTTCCNCCANCNCCGCTGACCAAAGTCANCGCGGCCACCATCATANCAGGNTTCTGCAAAGACTCGTCNCCC
GAGCGTTTCGAGGAGGCNGGATGCGCGGTNTGTGGTCAACTGGTCCCATTAAGCCAACTNAGCCCGCTGAAAA
GCGTCAGAGGNATGTTGAANGTCCTTGAAGCGCCTGGNGTGACCAGGGTTGAGCGNAAGACGATGACCGAAAA
GGTNCGAGAATNCAAAGGNCCNGTGCTAGATTACGAGTGCGGCCGAGTNTGCCTATCGTGCCGCGCTTCNNTA
NGAAAGGGCAAGGTCCCTCGCGGNGCNCTGGCCAATGGNCTNTGGCTGGGGAAAGTCCCGATCGAGCTNNCNC
GGCTTCGNTTTGTCGAAAAGNTGCTTGTNGCGCGCGTNCGNCACAACTGCTGCTTCGTNAGAGTCGCCTCNGG
TNTGAGGAAAATGGTNTCNCACGTTGTTGCGTTCGAATCGCCCGTGCCAAAGGTATATAACGTACTGCCNCCA
CCNATCGAGGACNTGGACGATGTNCTCGCNATCCTNTTCACNGGNCCGTGCAAACCNACNGAGAAGGATCTCG
AGCGGACGCCNTTGCTGGTGNGGAGGAANTANGTTGCCGNTGCGCTGGAATGGCTGAAACTNAACCATGCCGA
TTACGNCGACCTAGAAATATCNCATGAGAATCTNGCGNAATACCCNGAGGACGCGCCGCCNGTNTCCGTNGAA
TATCGCGAATCAACNACGAACAAGGTCGCNGAGGGNACNAGCGTGTTTGACAANGANGATGAGGACGGNACGG
ACGCNGGCGATTGTCCNTTCATTGTNCACGGNCTGACNGGCGAGCAGCTNGATACNAAANCCGCGAACGCATT
GAAAGGNATCGCGCTGAGGCACCTNAATAGCGGCGGGAAAGTNCTNGCGATTGGNCACTCNTCCAAANTCGAG
TCNATNTACAANAACCCNGAGCTNTACCCGCAAATGTTCCCGTGGCTTTTCCCGTATGGTTTGGGCGGNATCG
GNGCCACGGGTCTCTCNGACNCNGAGCACAAGCGGCGNCTNCTGATGTACCATGATAAGCGNTTCCAAATTGA
TGTCAATTTCCCCTTCGTCGCGTTCAGCCACGAGCAGGTCAAGGCGAGCNCNACGGGNAGCTTTCTNCTNGCC
GAGACGAAACGATTCCACGANATAACNAATCGGCTGCTCAATGTNGACCAAGANGTGTTGGCCGCTCTAGCGA
CGAGAATGGCGGAGGGCGAGATCGTCAAACCGACGACGCAGGAGGAGAAGGANTGTTTCCAGGTNATTCGCGA
CCTGGACGGCGTCGGCGGCAAGGTNAATGGNTCAACCACAAGTAAGAAGTACATGCGGAACGAGATTTGGTCN
CTGATNTCTCATATCGGTGCGCCGTCGTGGTATATTACGCTNTCGCCTGCCGATGTCAAGCACCCNATATGTT
TGTACTACGCTGATACGGGTGAGAAATTCAGTCCAGACCTGAGACCAAATGATGAGCGATTCGCGCTCATTGC
CAGAAACCCAGTCGCGGGNGCGCGTTTCTTTCATTTNATGGTTAGTTTATTTATTAAGCATGTNCTAGGCGTN
GGCGAATCGCACCTTGGNGCTTATGGTGAGACNTCNGGATATTACGGNACCGTCGAGCAACAGGGCCGNTTGA
CGCTNCATCTNCATCTNCTGCTNTGGATACGNGGNTGCGTATCCCCGCAGGAAATTCGNGANCGTATNCTNGA

CCCCGANTCGGATTTTCGNCAACGNATGGTCGCGTACCTNGAANCCGCTCACAGGGGTGANTTCCTGACGGGA
NCCCANGAGGAGGTNGCGGAGCGGGTAGAAGCTGCANTCGATCAGGAAGGATATNCGGACCCNACNCAAACAC
TCCCCACGGCTCCGCCAGCGCCCTGTCGTCGAAGCTGTGGGGCTTGCGAGGANTGTANTGCTTTGAGCGCGTG
GTGGACNNCATTCGATNCGACCGTCGACGATTTACTGCTGAAATCAAATGTGCACCGNTGCAGCACGAATAGA
AATAAGGATGGNACNACGAATAAANCAAGGCAATATACNGGNTGCCTNGACAACNAGTGGGGGACGTGCAAAG
CGCGTTTTCCCCGNCAGCTACGCGAGGNAACGACAGTCGACGCCGAAACNGGTGCTATTGAAATGAAGAAANG
CGAGGCAATGATNAATACCGTNACNCCNTTGGTGACNTACCTCNTTCGNTGNAATNCNGATGTTACCNGCCTG
NAGTCGGGTACTGCCCTCAAAGCCGTNATNANGTACGTCNCNGACTACATCACNAAAATGTCTTTGAAAACNC
ACGTAATNTTTGATTCGGTTCGNGCNGTGTTCANCCGTAATGCGGAAATGATTGGNGGGAGTACGNCTCGTAA
GGAGAAGGCGCGNCGACTGATGACGAAGATNGTCAATANTTTGAGCGCGAAGATGGAGATGGGNTCACCNATG
GTGTGCATGTACCTGCTGGGNAATCCTGATCACTACAAGAGCCACGAATTCGCGNTATTCTACTGGCAAAGCT
ACGTCCGNGAGGCGAGGAAATCCTGGCATGAGGATGACGCTCACTGTGAGACGACGCCGCCNGANAAAGTTGC
CTTGATNAAGCGNCANGGNAGGATTGTNGGGCTCTCACCTGTGTATGACTATATNTATCGCCCNGTCGAGCTG
TCGGATATGTGCCTNTATGACTGGGTNCGGAGATGCCGGCGAGTNAAGCTCCCAAACAA
>Cligib1_Cluster137#LTR/Gypsy
AGAGGTCTTTACACTGCCCCACCTATTCCGTTGGAATCCGTACGGAATCTGCTGGACTCCGTACGGATTCCGT
GTGGATTCCATGGAGTCTATCTTATTTTTTTGGCAGAGAATCCTGCTAAATCCCTCAGAATTCTGTATGGATT
CCATGCGGATTCCACCGGACTCTGCCAGACTCCGCCAGACTCTGTGCATGGACTCCGTACGGAATGGTCCATG
TGGAATCTGTGTGGAATCCATATGGAGTCCATGTGGAATCCGTGTGGAATCTGTGTGGAATCTGTGTGGAATC
CATGTGGAATCCATATGGAATCCGTGTGGAATCCATATGGAATCCGTGTGGAATCCATGTGGATTCTCTCTAG
ACTCTGTGCAAGTCCATCATTGCATCACTGCCTAAAAATGAGTGGCAATAGCCTGAGTTGAACCCACAACCTC
AGCAATCTAAACCAGCATCACAACCACTTAGGGAGGAGCATGGGTACCCATGTGGGTAATGCTTACAGGTATG
TGCGGGTATGGGTACAGGTACAGATCTCTGTACCCTTGTCAAACCTGTACCCGTTCTTGTGGGTTTGTGGGTA
GTGTGGCCTGTATTACATAAATAAGGATGTTGCTGTCAACTTTGCTTTGTCTTGAATTGCAAGCACTCAATGA
TTGCGGATGACAACTGGCCAACAGAATAATGTATCTATGCCAAAAGCAGTACTTTACAGTCCCCTACCTACCC
TTGGGACTCCCTGGGAATCCCCATGAGTCTTATAAATTTCTGGGAAATCTATGAAAGTCCCTCAGGGAATCTC
TGGGAATTCTGTAGGATCCTTAGTAGAATTCTAAGGAGATTGATTTTCACCTGATTACATAAGATTATATAAT
TATATCTATACTCATCATCATACTTACTTTGGATATATTATCCCTCATATCATAATAAGACATAAATTATAAT
AAACAAGTCAATCTTATCTTTTACTACTCTCCTAATTCATTCTTATTTGCTACTTAACTTGCCTTATTTTATC
TCTACTCAGGTCTTTAAATGACAAATAAGACCCTGGAAATACTAATTTAGTTATTTATAACAAAATCCACAAG
GATTTTCTTATAGATATTATATATATTTTAGAGTTTATAGTGAATTCCCAATAGAATTCACAAGGAATTCACA
GAGAATCCCAGGAAATCCCAAGGAATCTCAGGGATTCCATATAAATTCCCTTGGAATTCCCAAATCCCAGGAA
TTTCCAAGCAATTCTAAGCACTCCCAAGCAATTCCTTATAGAATCCCAGAAGAATCCCTAGGGATTCCCAGAT
TCCCAGGAATTCCCAAGAAATTCCCTGAAAAAAGTTAAAATTAGCAGAGTACATGCTAGAAAATCCCTGGGAT
TCCCTGGGATTCCCTGGGATTCTCTTGAGACTCCAAGGGAGTAGGTGGGGCACTGTAAAGACCTCTATGGTGG
GAGCGTGTCAAAGGTCTTTTTCTTGAATCGATTCTTCCAAGTCATGAGGTACTTCGGGAGTTTTAGATCTGTG
ACTGTATCTGTTGACCGAACTTTAGCCTTGGGTGGTGTACTGACGCTTACTTTGGCACATTCCGGGGGGCAGT
CTGGGAATTTGATCTTGCCAGTCTTCCAGTTGATGAAGGGATTGTGTTTGTGCAACCATTCATGTCCAATGAA
AAGGTCCTTTTTGCCTAAGTCTGCTACCAGTGCCATTGCTTCTTCCTTGTGATCACCAATCTGCATCTGGAAC
CTGACGCTCTTGGTGATTAATTTATCCGCATTGAGAGAACCATCCGCATTAAATGGAGGTCTTGACACTCCCC
CACCTATTCCGGTGGAATCCATAAGGAATCCGCCGGACTCCGTACAGATTCCGTGTGGATTCCGTGGAGTCCG
TGTTATATTTTTGGCAGAGAATCCTGCTAAATCCCTCAGA
>Cligib1_Cluster136#LTR/Copia
GTTAAAACCTGATCTATAGATTAGATAGAGTAATCCTCAGTCACGGTATAATCGACTGTATTTGTTTGTATGT
ACCTATAGATTAGATAGAGTAATCTAATCGGTAATCAGTATGACGTCTAAATATTATAAGTGTAAGTGGCTCA
CCCTCAGTCACGGTATAATCGACTGAGGGTGGGCGTATGTACTTGAGTCGTATATAAACTATTCTGTATCAAA
GGAAATATATGAAAGGAAATATATGAAAGAGAATCGTACGTATTGAGGGAGGACGCAACGTGCATGCTGAGCG
CTTACACGTGATTCCTAACACTCCTCCTCACGAGAAGCGCTCCTCCTGATGGGTGATGGGGGATTGTTATTTT
AGTCCGAGGAACCCCCGTAGACGAGTGAATTTCTTGTTGTCAAGGGCTTTAGTGAATAGATCGGCGATGTTGT
TGTTGGTGGGAATATATGTGAGATCGATTTCTTTTGATTGGACTCGTTCACGTAAGAAGTGGTATTTGATGTC
GATGTGTTTTACTCGGGCGTGGAGAAGAGGGTCTTCGGAGAGATTGATAGCAGAATTGTTGTCGCATAGTATG
TTAGTTGGTCCTTCTTCAAGGTAACCAATAGCATGTAGTAGCAATCGTAGCCAAATGGCTTCTTTTGCAGTTT
CGCACGCTGCGGTGTATTCGGCCTCGCAAGAGGATGTTGCGACGGTTTTTTGTTTGCGAGTGCTCCATGATAG
TATTCCAGACCCGAGTGACCATGCGTATCCCCCCACACTTCGGCGCGTGTCGAGACAATTTGCCCAATCTGAG
TCAGTATATCCGATTAGGCAGACGGGGTTATTACCCCCGAGGTGTAGTTTAAATTCGCGCGTTCCCTTTAGAT
ACCGGACTAATCTGATGGCGGCGTTCCAGTGAATAGTGGAGTAGGAGTCGAGATATTGAGACAGTTGTTGAAC
GGCGTAGGCTATGTCTGGGCGGGTGGATATGGCGAGGTATAGGAGACATCCGACGAGTGAGCGATAAGGGAGT
TTAGAAAGGGAGTACTTTTCCTCAGGTGATAATGAGGTTGGGTCCACGCATCTAAGCTTAAGGCCAGGTTCCA

TTGGTAGGGAAAGGGGGTGAGAGTCGCGTTGTCCGAATTGAGTGATTATCCTGTCGATTAGGGCGGTCTGTGA
TAGCTTAACTGTGTTATTGGGGCGGTCCCATTCGACGGCGATCCCTACGACGAAGCGGACTGTCCCTAGGTCT
GAGATAGCCCATACGGACTTCATTTGTTCCTTGAACCTTTCGTTTTCTTCTTTGGAGCTCGCTATGGTCAGGA
AATCGTCGACATGCACAACAGCAATAGTGATACCTGTCTCGGATTTCCTGAAGTAAATGCAAGACTCACAGGC
AAGACGGGTGAAGCCCCAGGAGATCATATTGACATTCAATGTTTGATTCCATATGCGCCCACTTTGTTTCATC
CCGTATAGCCCCCGCTTCAATCGCCAGACCCACCCTTCTTTTCCTTCTTCCTCGAATCCCTCCGGTTGTTCCA
TATATTGTGTTTCATCAAGTGGGAGTAGTCCGTAGAGGAATGCAGTTTTGACGTCGATTTGTTGTGCGTCCCA
TCCAAGTGATGCGGCCAAATGTAGTAATATTCTCCAGGATTCCATACGAGCAGTAGGTGATGTGGTTTTCGTG
TAATCTTTTCCATATATTTGTTCAAAGCCCTTGAAGACGAGCCGTACCTTATGACGTGTGGGTTTACCGTCCT
GGTCGCGTTTTATTTTGAATATTGGGTGCCCTTTTCTGACCTTGGATCCCTGGGGGACTTCTTCTCTGGGGAC
TAATTCGTATACTCCCATATCTTTGAGTGATTTGAGTTCTTCGCGATAGGCCTGCTCCCAAGCTTTCGAATCC
GCTGAGGCAAGGGCCTCTTTCCAAGTGTGGGGTTCGTCTTCCAATTCAAGCGATGCAGGGTCTATCCCGCCGG
CTTCTGTGATCGCGGCTAGGATCCGATCATGATCGTCAGTGTCATCTTTCCCGTTTAGGTTCAACTTAGCGAA
TGCTTGTCGCAGTTCGTCTACGACTGGTTGGGGGTCGGGGTTTGGCTCCGCTGGTGCTCCAGGTGGGTTTACT
GTCTCCATCGCTAGTCTTGCTTGACGTCTCTCTTGTTTAGCAACATGTATGCGTGTAGCGCTTTCTTTTGACT
CCTGTATGGCATTCTCAAGACGCACGGATTCGGACGTTGGATTGCGAGTCGACTTTCGAATTATTGGTGGTTC
TGGTGGATTTTCGGGTTGTGTTTCTGGAGTTGGTATGATGGGGTCCTCGTCGTCGTCATGTAGGATAATGGGG
TTTTGGGAGTTAATAAGATTGTCGGCTTTGTTCAGTGTTTCCGTACTCATTTTTATCACGTCCTTGGGGAGAG
GATGAGATACGGGATGTCCGTCGTGACTCTCGAGGAAGCGGACATGGTACGATCTGTAGACATTTCCACTCTT
TTTGTCGTAGCACCGATATGCTTTAGAATTGGCGTCATATCCGATTAGGACACATTCAATGGATCGCTCGTAT
ATTTTTGGGTTGTGGCGATTTTGGATTAACACGTAGGCCTTGCAACCGATCTCACGGATGTAGGAATAGTCGG
GGCGTCGGCCATACCATAATTCCCATGATGTAGTTTCACTGTTTAGGGATTTGGTTGTGGTTTTGGCATGAAG
GTGGGTTGCCGTTAGGTAGAATTTGTCCCACATATTTGGGGGTAAGCCTGCGTGTATTCACATGGTCCGTGCT
TTTGCCATGAGGGTGCGATGCATCCGTTCGACCCGTCCCATGTGAGCCGATGTATATGGAGCTCCAAATTGAT
GGTCGGTCCCGCGTGATTTTAGCCAGGCCTCCATTTCATGATTTTTGAGTTCGCCGTCGTGCCCTGTTCTGTA
GGTGCCGACTTTGAGTCCAGTTTCGTTCTCGCGCGTAGTTTGCCATGCTTGTAATTCGCGGAATGCGTCATCC
TTTTTCTTAAGCGGGATAGACCATGGCATGTTGGAAAAATCGTCCACGATGTTCATAATGTATATACTATAGA
TTCCCTTGTCTAGATGGTACAGGGCTAGGTCCAGCAAGATCTACCCATACAATCTCTAATTTCCTAGTAGCCC
GCCTCCCCTCTTTGCGCGTCTTTGGAACTGGTTTCCTGGTCTGTTTTCCAAGGATACATGATTCACATTTGGA
CGGCTTTAAGGGGAAGGATTTGGGCATACCTGGAATGAGACCGTTGCGCGCCATGTCGGTGATCACTTGGTAA
TTGGCGTGGCCGAGGCGACGGTGCCAGGTGTTGAGATCAGGGGTATGTTGGATATGGTTGGCGTGTTCGACTT
GTGGAGAATGTAAAGATAAGGAATATAATTTCCTGGAATCGTTCAGGGTGCCATTCGCGATTAGGGCCCCAGT
GGACTTGTCGGTTAACCAACAGATTTGATCGTCAAAATGGGACGTTATACCAGAGTCGACAGCGAGCGAGCTG
ACAGAAATGAGGCGGACTGTTGCAGTTGGTATGTAGAGCGCGTCGCGGAGTGTAATGTATTTTCCCCTTGCTA
TATGGATTTTGATGTCACCCATTCCGAGAGCGGTAATGGACGATCCCCCAACCCCCCGTACCGTTCGAGGTGG
AATGGGTCTGAGACTAATAAAGTCTGTAATTTCTGGTGATATATGTACGGATGCGCTGGTGTCTGCTAGGAAC
GGGCATTTATCCAGTGAGATTGCCGGAGTACGGCGGGACTGCTGGAGTGGCGCGATCATATGAAAGACATCGT
CGTCGATGGGACGCGTATTGGTGGTCCAGTCGACGTTGGTGTTAGGGTCGTTTATGGCCAGCCATCCTTCGTA
TTCAATGACGTCGGCTGTAGTTTGGAATGCGGAAGGTAAATCGTTTGCAGCGAGCCCGGCAAAAGAGGGAGGT
GTCTCGGGGGGTGGTGTAGATAGATCATTCGAGTTCACGTCGAGGAGATATGCTCGGCCATTGTTATCTTTGA
CGGTGACTTGGACTCGGTTACCCGGCGTGCTATTAGAGCTGCCACCTCCTCTCTTCGAGTCGCGATCCTTCTT
GCGTGCTGCCCGAGATTCGTCAATCGTCTTCCCGGCCATGTTACCACCCGGGCTAATGCAATATTGAACTTGA
TGTCCAGATTTCTTGCAGTTGCTACAGGTGGGGATATCGCCTCTGGGTTTGCCGGTTGCTGCGAAGGCGGTGG
CGGTGTCAGAGGAAGCTTTTCCCTTTCCTTCGATGAGGGTTTGTTTGTTCTCAAGGAAGTGACAGATGTCAAT
TGACGTGTATGGTTTTTCTTTGGTTGAACCAGTGATGTCGCGGGATATTATGGATCGTGCGACGTCGTATGAA
CTGTCGTGATTGAGTGCATTTAGGAGTGCAATACATTTCAGCATATCTGCATTTATTTCCCCGGCCGCGAATG
CTTGGTCGATGGCGGTGCATATGACATTGGCGGTTTTAGGAAGGGGAGTGGATTTCTCGAATTTAGTCGACAA
AGCCGTTTGAAGTAATTGGACCTGTTTAACGGGGCCCTGGTCAATGTGACGCTTCGATAGTGCTATCCAGCAG
GCATAAGCGCCTTTGGATGTGTCGATATGTTTCTGTTCGGTCTTCGCCAATGCAGATTTGATGAATGCTGCAG
CTAGACGGTCATTCGCCTTCCAATTGGTGATTGCTATGGAATCCTTCGAGTCCGAAGGTGGTGTCGGTTTCTC
GAGAACATATTCATAGAGGCTATTAAGGGTAAGGGATACCTCCATCTCCTCGCCCAGTCAGTATAATTCCCGG
CGAGTTTGTCGAGTTCATCGCCGGTGAATTGGCTGGTAATTCCTCGAATCCGTGTGACGGATGGGGTGGGGTG
GTGGTGGCTTCAGCGGTCATAGTTAGATGTTATTTGCGGGGATATGATTCCTAAAGTTCGAAGCAGTTCCTCA
GATGGTGGGGAACAAAGGGAATATGATAGGCGGCGCGTCGTCCCAAAATGGTAGTCGCGGGCGCGGGAATATC
AGATCAGGTGTTTAATTCGTCAATAGTATGATGCAATGGCCCATAACCTGTTAAAACCTGATCTATAGATTAG
ATAGAGTAATCCTCAGTCACGGTATAATCGACTGTATTTGTTTGTATGTACCTATAGATTAGATAGAGTAATC
TAATCGGTAATCAGTATGACGTCTAAATATTATAAGTGTAAGTGGCTCACCCTCAGTCACGGTATAATCGACT

GAGGGTGGGCGTATGTACTTGAGTCGTATATAAACTATTCTGTATCAAAGGAAATATATGAAAGGAAATATAT
GAAAGAGAATCGTATGTATTGAGGGAGGACGCAACGTGCATGCTGAGCGCTTACACGTGATTCCTAACA
>Cligib1_Cluster133#LTR/Gypsy
CAACGGTTCCCACCCCTATCTTTTTCTCTTGATTGACCGATCCCCTTTGTTTGACCGTACTTACATTGTTCTC
CGATTCTTCATTGTTCTATCGCATGTCCAACACATGCCTCTATTCTTAGATACTTGACTCATCCATGATGTCA
TAGCTGACTGAGCTTCAACAGCTCTTTTACCTTATATTGTCTTTACTTCCCATTGAACAATGTTCTCGTGCCC
CAAGGTCCCTAGTATATAAACCCTAGATACATTGTCATTATTCTCTATCTTGACCTAGGCAATACTACACCCT
TTACCTTTGAACCTTGATCACTGAATCCTGATACCCAAGTCAAAGCCTGACTTGGAGTGACTATAGTCTTGTT
GGTGTTGACCCAAGACTCTGACTACACGCTCTCGACGACATTCAACGCTTGAACCTTGGTAAAGGCACTCCGC
CTTAACAAAAGGTCAAGATCTACCGATTGCCAACCTCTTTAGGTTTCAGCACCCATTGATCTAAACCGACACC
CCTCCGATCACCGTGGCTCCCCACTCCTTACAGGTTCTTCCTAGAGGAGCTGTCCAGAACGACCAAGGAACTT
GGCTAGAAAACACCGCACTTTTCTTCGACAACACTTCCTCTCAGTGGCATTACAACGTTGACCCCTATCGAGC
GGTACCAGACTCATACTTCACAGAACCATACTCCTCTCCGCTCCGCCCACCTCCACGAAACAGATTCTCTGCC
AACTCCGCCGATAACTCTTCCTCCGAATCCGAAGCTTCCACTCCCACACAGTCTCATCAATTCCAAACCCTGA
ACCAGAACCAACCCCTGCACTAGCACCCAAGATGACCACCGTCGCACCCATCACTACCGCCACCAAGACCCCC
AAACTCAGAACTCCCGCTATATTCAAAGGAGAAAGAGAGCGATGCTCACCCTTCATCAAAGCGGTTAACCTCT
ACCTTAACTTAAACACCACCATCTATGACTCCAATATAGCAAAAATCGGTTTTACCCTTTCATACATGGATGA
AGGAACAGCAGGACCCTGGAAGGAAGCAGAAATGGAGAGACTCGCAAATTTAGAAGGTGGATGGGGAACATAT
CAAGATTTCATCACACGGGTAGAAGAAGCATTCTCCCCAGCAGATGCCACAGGGGATGCCACTCATCAGTTAA
CGGTCCTCAACAAACGGAACAGCAGAAGATTACGTAGGCCTCTTCAGACAAGCGCCCTTCGAAGCAAGATTAC
CGAAGATGGACCCTCATTGTATTCTTTCAAGGAGGACTAGCCAAAGCTCTCACCAACAAAATTCATAACCTCA
TTCCTTTACCCTCCACCATTGATAGCTGGTACAAATGTCTATCCAATTCGATAGCCAATACGAAAGAAGTAAA
CAAATACACCAACGAAATAATCCAACCCAATACACTAGACCCAAGGGTCCCAGCACCCCACGATATATCCCAC
CAAGGGACCCATTCGCCATGGACGTTGATCGTCTCATGACAGAAGAACAGAGGAACATATGCAGAAAGGACAG
TGCTTTGTCTGCCACAAGATTGGCCACCGTGCCAGAGATCACAAGAATGGAAGAACCCCACCAGAAACCTCAC
GAAACAATTACCGCATCCAGAAGATTGAAACTCCATCCACTTCTGGAATTGTTGAAGTACCCCCGATGGAACT
ATTGCATTCACACGCATCCAGGCCCTCATGGCTAGCCTATCCCAAGAAGAAAGAACGGTAGCTATAAAGAAGA
TGGAAGAAGAGGGTTTTTAGATTGGCGAACTGAATCGACGTCAGTATCGCCAGAATTATTCATTTCCCCTGTA
CTAGTCACGGTTACAGATAAACAATCTATGCATTTACCTATTTGCATTGAAGGAAATGAAAACGTCGAAACCC
AAGCCCTCATTGACTGCGGAGCAGGAGGAAAATTCATCAACTCCACATTGCCGAAAAACACCACCTACCACTA
GAAGAACTACTACAACCGATCTCCGTATTCAATGTCGACGGAACCCGAAATAGCAAAGGACAGATCATCCATT
ACACCTGGGTAAATCTCTGACTCCAAGGACAAACTCTCAAGACACGCCTACTGGTTACTGGATTAGGAAAAGA
AACGGTTATTCTAGGACTCCCTTGGTTAAGGGAAATTAACCCAATCATCGACTGGAAACAGGGACCATGGAGA
TAGATCCCACCAAATTCTCCCTCAACGCCACCAATTCATTCGCACCATTCGGGAAAAAGACATGAAACCTAAA
TTCACCTCCTGAAAAGTCACCATCGAAGATGTCACTGAGGAAGAAGACGAGATCGAACCGCACCACCTAAACC
AAATCGACCCTGATGAACCGGATACATATACTGCCTTCCAGGGTATTCCCCTATCCATGACCCTGCATCCCTA
TCTCCGACCCTACTTCCCCAGAAGGAGATCTCATCATGGCTTACCTCCAAGGAGAACCAGTAATAGCAATCTT
CGAACCCACCGTCGCCAAAATCACAATCACCGGAATATACTGGACCACGCTATTTTTACAAACCCAGCCAACG
AATCGGAATCACCACTCAAGACCACCGTCCAAATTGGTATCCACAGGACAAGGAGTCCGCATTCGTGTAAAAA
TAACAGTTCCACAGCCATGGCCCAGGATTCCCTCTCTGACAAACCGAAAACGATTGAAGAACTTCTTCCCAAA
CCTTATTGGAAATACCACAAGTCTTTGAAAAGAAAGCCACCGAACGCTTCCCCGAATCACGAATCTACGATCA
TGCCATAGACTTGAAACCGGATTTTGTACCCAAAGATTGCAAAATATATCCACTCACACCGGGAGAACAAGTC
AAATTGGACGAATTCCTCAAAGAAAACCTGGATAAGGGATATATTACTCCTCGAAATCCCGATGGCCTCCCCA
TTTTTCTTTGTAGGAAAGAAGGACGGTTCATTACGACCGTGTCAGGACTATCGTTACCTAAACGAAGGAACCA
TCAAGAACTCCTATCCCCTTCCCCTTATCCAAGAACTCGTCAATAAACTCCGAGGCTCCACAATCTTCACCAA
ATTGGACCTCCGCCAAGGATACAACAACGTCCGACTCAAAGAAGGTCATCAATGGAAGGCCGCTTTCAAAACC
AACCGCGGACTATACGAACCACAGGTTATGTTTGTTGGAATGTGCAATTCTCCTGCCACTTTCCAAATGATGA
TGAATGAACTCTTCAATGACATGATTAACGAAGGATGGATTGTCATCTACATGGACGATATCTTAATCCACTC
CTCAGACTTATCTGTACACCGACAAAGAACCCTACGAGTACTCCAACGACTCCAGGAGAACGACCTCTTCCTG
AAACTGGAGAAATGCGTCTTTGAAACCACAGAAGTAGAATTTCTTGGAATGATTATCCGACCAAATCAAATCG
CCATGGACCCCACCAAACTCAAAGGAATCAAAGAATGGCCAACCCCCTCTACCGTAAAAGGTGTACGCTCATT
CTTAGGATTTGGTAATTTCTACCAGTGATTCATTGGAAAATACTCGGATATTGCAAACCATTCAAAACCTCAC
CAAGAAAGATCTCAAATGGGAATGGACCCCGAATGTCAAGAAGCGTTCGACACGTTGAAACGAAAATTTGCCG
AAGCACCAGTCCTTCTCATGCCAGATCCCACAAACCTTTCACTATTGAGTCCGATGCATCAAAGTTCGCCACG
GGAGCAGTACTCCGCCAACGTGACGTGAATGGAGACTGGCATCCGTGCGCCTACCTTTCACAATCCTTTGACC
CACTCAACGAAATTATGAAATCTATGATCGAGAACTCCTAGGGATTGTACGAGCCCTGGAAGCCTGGAGACAC
TACATCCAAGGCTCCCCACATCCAATTACCATATTCTCTGATCATAAGAACCTCACCTACTTCCGCACCGCCC
AAAAACTCAATCGACGTCAGGCACGATGGAGTCTCTTTCTCTCCGAATTCGACATCAAATTGGTCCATGTCCC

TGGAACACAAATGGTCCAATCAGACGCTTTATCAAGAAGAGAAGACCTAAACCTGGAAGAGGATACCAATAAC
GAAGATATCACATTACTCCCGGAAGAGTTATTTGTGAAAACTATTGACACCAACCTCCGTGACCAGATTCTCG
CCTCCTCCTCCAAAGAGGATGTGATTACCCATGCCATCGAAGCACTCAAGAAAGGGGGTACCCCTCCGATGAA
ATCCGCGCTTTCTGACTGGAAAATCGAAGAAGAACTGGTCTTCTACAAAGACCGATGCTACATTCCCCCAGAC
GAAACCCTGAGAAGAAACATTGCATCAAGATACCACGACTTGCCAATGATGGGACACCCTGGTCACATGAAGA
CCCTGGAACTTATCCGTCGCGATTATTGGTGGCCTGGAATGTATACGTTTGTCAAAAATTATGTTGAAGGATG
CGCTACCTGTCAACAAACCAAAGTAAACACTCACCCCACCGCACCACCTCTAATGCCGATAAAACCCCCGACG
GACCCAAGACCTTTCTCACAGATCTCCATGGACTTTGTTACAGATCTACCGAAATCCCATGGATACGATTCAC
TCTTGGTCGTGGTAGACCAAGGGCTTACAAAGGGGGTAATTCTAATTCCCTGCAACAAAACGATTGATGCCCT
AGGAACTTCTAATCTTTTATTCTGGGAAGTCTATAAACGATTCGGTCTCCCGGATAAAATCATCTCCGATCGT
GGCCCCCAGTTTGCCTCCGCAGTACAAAGGGAATTAGGGCGACTCCTTGGCATCAAACTCGCATTCAGTACAG
CATACCACCCTCAAACTGATGGAGAAACGGAATGTGTCAATCAAGAAGTAGAAGCCTACCTTCGGATCTTTTG
CGTTAATCACCCCGAAACATGGGTGGAATTCCTCCCTACTGCCGAATTCACCCACAACCAACGCCTACACTCC
GCACGAAATGCTTCCCCATTCTTCCTCATGATGGGCTATAACCCTATTGCCGTTCCCCTTCCTTTCCTCAAAT
CCGACACACCTGCTGTACAAGAACGTCTCCAATCCCTCCAGAAGACACGAGATGAAGCCCTTGCTGCACATGA
ACTGGCTAGCCAACATGTAGCGGAAAGAACGACACAAAACTTCACCCCCTTCAAGAAGAACGACTTAGTATGG
TTAGAATCTACTAACCTTAAACTGAAATATGAAACCAGAAAGATGGCACCACAACGAGAAGGTCCCTTCCCCA
TCACCGAGGTGTTAGGACCTCTCACTTACCGGCTACAATTACCCGATCAATGGAAAATACATCCGGTGTTTCA
CGCATGCCTACTTACCCCATACAAGGAAAACGAAGTCCACGGACCTAATTATACCAAGCCACCCCCAGAACTA
ATAGAAGGAGAAGAAGAGTATGAAATCGAAGCCATCCTCAAACATCGCAAACAAGGAAACGGCCACTGAGCAG
AATTTTGTCAGTGAAGTTCACTTGATGTCTCATCTCAATGCAAGTGAAAGCTTTTCAATTTTCAATCAATTTT
GCTGCATTTTGAGTTATTTTCATACGTTGGAGTGAACTGGATTCACATAAACGCTGTCATCCATGTCTCACTG
TGTTTTAAATGAAGTTCATGCTGGGGTGCCAAGGTTTTTAGTGATATTAACTCCCATTAAAGAAATGTTCACT
CCATTGAGGTGATTTCACAAATGAATTTCAATATGAAAAGAAATGAATTGTGCTTAAATTACATGGAAGGTAG
GTAAAAGACAACATCAAGAACCAACATTGTATATCTACAATGCAGGAGCTCCTGGTGGCGGATGCGACACGCA
AGGAGGCCACAAGCGCGAGCCCAAACACAGTCTCGCGCTTTGGCTTAACATTGAGGGTGGGCGGGGAGAGGAG
CTTTGACGCACCGAGAGCCAGGCGCAGGTTGTGGGATATCCAAGTTTGTGGATGCCTGCCGGGGAACAGGTGG
CGCACAAGAGACGCGCCCGTCGTCTGTTAGATGTGTCCACCTGTCGTTCGTAAGATGCATTGACTGCCCACGG
GATGCGCCCGAGTTCGGCATTCGGGCCTCGCGGCACGTTGACGGATGAGCAGTTGGAGTACTCCCCTGTTCGT
GGGGAAGGGAGGTGGTGGCGATGGTGAAGTCGAGCCCTGTTTGCAAGGGTGGGGTGGCGATGGCGCCAGGGCA
CTTGTCCGCCGCCCGTGCTATGTCGCCAATGATCATAAACGAACACGGAGGTGACATTGGCGAGGCCCAATCG
CGGCGATGAGGAGGGGCAGTGGCGGGCAGTTGTATGATGTATTTGTCGGTTGCGTCCACTGTCCGCGCCGTTG
GTGACCAGAGAAATTTTTCGGCTTACCCATTTACCATTTGGCACGGTTGTGGGTTTCGTCTACCGCTGGCTGT
GGTTTCGTCGTCTACTGCTGGGTGTGGGTGTGCGAGTGCAGAGCGCTGTGTAGAGATAGAGGCACGAGCGAGA
GGTTGAGGGGGTGGGGGATCAAGAAGTGGATGAGCACTGTTGAAATCCTGGGAAGTCAGTGCGCAGGAGGGCC
TAGGAGAAGAGCGCACCTCATGCCATTGAGAACTCTCCATGAAAAACAAAAACAACAGCACCCTGCCGTTCTT
GATGCGGGCGCCAATAAGGAGGGCGTTGAGCCTGTCCGCTCACTATTCGTGATAGTCCTGGTGCATGTCCCAC
CACTGCGTGTCCTGCAGATATCTGATGGTCATCATGAGTGCAAGGTGCACCTGCCATTTGAGATGCTGCGCTC
TCGGCTCCGGGGCCTGGGGGATACAATGTTGGCTGGGTGGTGGAAAGTCGAAATTGGGTCTGTTAGTGTTAGT
GGTTGTAAGCAGGTCGAGGTGGCAAACATCATGGGGATTCTCGATGTGAAAACGTGCAGGGATATTAGGGTGA
GCAGGTTTGAATTTGCAATTTCGTGATTACTTACAAAGCTTATAGATTGGCATAATTCCTGGAGCCCGGGATG
CAAATGCCGTCGCGTGATGTCGGCTGGATGGTGCTCGAGCCTGTCGATGTCAAGCGTGGTGGGGGTTGTAAAC
AACGTGTGTGATTGCGCCACCGCGGGAAAGAAATCGCCAAAGAAGAGTCATGTGATTGCTCCAGCGTGGTTTT
GACGGTTCTAACTTGTCCTCGTTCTCTGTGCTTGCCCAACTAAGTCCCAAGGTTATGCTACGCTCATTTGACA
ACGAAGACACCTCCCTAGATCCGGATGCGCCACAAAAACGGGTGATGCCCCGCCGTGGGATTGATGAACAACA
ATGGGGAGGAAATCAAATTGGGCTGCCAAGGACCCACTAAGGTCTCGAGCCGCAGTGGAGGGCAGAAGATTTG
ATAAGGTGCGCCAACTGCCCCTAGCCGATCTGAATTGCACCAACACAGCCTACGGCAGCCCCCAGGCCCATCA
AAGATGCGACCGCATGTCCATTTGAACGTGCGCACATGCCTCCTCCCTGACCTCTACACAACCTCTGTGGTAC
CTGGTAGGTGCTATGCCTTGCGAAAAACACGAGTCAGCAATCACGCCGCCCATTTGGAGGTTGCATAACCGCC
CATTCAACTCAACACCCACCTCCTCGCATAACTGCCCGTTCGCATGCTCACCAGCTTGCGCCTCAATCCCACA
GCCGTCGCTGACCCGCGCTGCAACTAATTTGCCCACGTACGCCCGTCAGCCCACTTCCTTTCAAATGAGAGAT
GTTTGAGGTCTACGTACGACATACACACTTGAATCTCCCTCTTGCAAACGAGAGTGTCGGAATGCTAGCCGTC
TGAACCGGAGACGGGGGAGCATGCAAGGTACGTGCCTACTCACATTCATCGCCCCCCTCTCTCGCAAACGAGA
GAGTTGGAGGTGGAACAGTTTTTTTCTGCCTCATGCTCTCCCTCTACCCTCGCGGCGCCGTTCGCCATGTCAA
TTGACGTGTGCAATGGTGTCGCAAGGCTAAACGGGGGACTGTGGACCCAGATCTGCGAGGGTTGCGGGAATCT
CCCGCCTCATGCGCCCCTGCTCCTTCTCCGCCATCACAATCATTGAGGAATGGCGGCGCGAATCCACAATTGA
CACCACCGAAGTGTCGCGTCTTGCGGGCCTTCGTCCTGGGGGTGGGCCTGGTGCATTGAATAGATTTCTTATT
TCAGTTTCACACATTTCTCCATCAAAATAAAGGGTTCCTTGGTGATCAGTATTCACATCATACTCACTTGCTA

GTTTCACACAATTTTTCAATTAAAAATGAATGACTTTTATTAGTGAATCATATTCACCTGTAGCAGCCATGGG
CCAAAGTAAAATTCACTCAAAATTTGATATGTTTCACAGCTTGAGTGATTTCTGGTTTCACTATGAAACCAAG
TGAAAAGCATATTTCACTCAGAGTCAAATGAGTTTTGCTAACAGAATTTTTGTCAGTGGGCATGATGTACTAT
GTGAAATGGAAGGGTTACCCATCTACCGACAACACATGGCTTCCCACACACAGCTTTGGAAATGCCTCCGACA
TCCTCAAAGCTTATAAAAAACGACACTCCCTTTGATTTCCCCATCTTTTGAATCCCCACATTCAAAAATCTAG
TTCTCTACCATGTCTTCATCTACCGCCTCCACCACTTCCACTGACTACGCTGTCTCCTCTGACTCCCCCTCCA
ATCCACTTTCTCCTCATCCTCCCCGACTCATGTGACCACTTCCACCTCCTCTTCCTCATCATCATCTTCTGCC
CCCACGCGACGCGAACCCCTTCAACCGATCCCTGAAAATGAAACCGAAAATATCATCAACCCGAACGAAGTCC
CGAAAGACCTGGAAGACTTCTACAATCTTCTCATCACCATCAAGGACCCTGAGTCCTTCGTCACTTCCATGAG
AAAGTACCCTATCCTTCGCGACTTTATGATGGCGTTCTATGACGTCAATGACCTAGACGCGCCCATCCGAAAT
CTCTTTGGGGCTATGGGCCAGGTCCAAGCCCAACTCACTTGTCTCAAGGAGGCTCACGAATGCGCCCAAAAGA
CCCTGTTCCACGCTGGTAGGAAATGGGGTCTAGACGCCCTTATTGGATTCGACGGTCCACATGGATATCCTTA
CCCACTAAGAACTGAACACATTCAGCAATTATTGGAAGACCAGCAGATGAACCGAATCCCTGGAGTAGTGACT
CCACGACTCACGAATGATCCCTGGCCTCTAACCAATCGAACGGTCGACGAACTCACATCCACCCCTTCTATTG
TTCTCACCGATTTAGAAACCGAACGACCTCCGAACCCAGCCACACCTATGGAGCCCACATCCTCCAGAACAGA
CTCTCGAGAAGCAACACCTTTCCCCCATTCTGCTATAAACGACATTTCTGCCACCCCTTCCCCCGACCCTCCT
GCTACCACTCCACTCCCACGATCCCTAGCATCTCACTTAACCTCTCCCGATCCCGAACCCTTTATTCCACCCC
CGCCGAACCCAGAACGACTAGCACCCCCATCCTACGACCAAGCACATGGGAGAGCCCCCGTCATCCGAACCCC
ACGACGACAACGACAACGCCCACAAACCATCTTTGTTACCCCTCTCTCCCATGCTGGTCGACCACGACACATC
CAATCCCCCGATGCTGAAAATGTCCCGCCTCCCCGGACTGTCTTCTCCCGACCCGAACGACCCCACGTTCGAT
ATTGCTCCTTCTGCCATGGTACTGGCCACGCATTTTCCACCTGCCCTGACTTTTATTGCAATTTCTGCAACTG
GCGAACACCGCATGTCACTTGCGCCCACAGTCATTATGGACCACGACAACCCGATTTTGACAAAGAGTTTTAT
TTTGATGACTCCGCCATTTCTAACATGATTGATGAACCCTACGGCAGCTTTGCAGGGTAACTTGAAGTCAGGG
GACTTCAACGATAAACAGGGGGTAATGTTACAACGGTTCCCACCCCTATCTTTTTCTCTTGATTGACCGATCC
CCTTTGTTTGACCGTACTTACATTGTTCTCCGATTCTTCATTGTTCTATCGCATGTCCAACACATGCCTCTAT
TCTTAGATACTTGACTCATCCATGATGTCATAGCTGACTGAGCTTCAACAGCTCTTTTACCTTATATTGTCTT
TACTTCCCATTGAACAATGTTCTCGTGCCCCAAGGTCCCTAGTATATAAACCCTAGATACATTGTCATTATTC
TCTATCTTGACCTAGGCAATACTACACCCTTTACCTTTGAACCTTGATCACTGAATCCTGATACCCAAGTCAA
AGCCTGACTTGGAGTGACTATAGTCTTGTTGGTGTTGACCCAAGACTCTGACTACACGCTCTCGACGACATTC
AACGCTTGAACCTTGGTAAAGGCACTCCGCCTTAACAGGAGGTC
>Cligib1_Cluster135#LTR/Copia
CTCAGCACGAGCACGGGTTACCCACAGGTACCTGTGGGTAGACCCGTGGGAATACTTGCAAGTATTGGCCTAA
ACAGGTACTTGTACTCATGGATCTATTTACTTGTGGATCCGAGTCAAGCATTTGGACATTGCAGGTACCTGTG
ACAGGTACTTGTGACTCACACTGGACCCTCAAGGACCCTTAAAGTATAGTTCCCACAGTACATATAGGCTCAA
ATTCCATTCTAAACCAACAAAATCCTTGTAAATACCCATAAGTACTTGTGAAATACCTGTCCCTGATGACTAA
TTGCTGACCAATGACCTGTGACCTGTGAGTAATCATGAGTAATCCATGAGTAATCAGGATCTGTGACAAGTAC
TTACTGAGTTGTAAGTACTTGCCAAAAAATACCTGTGAGTACAAGTACTTGCTGACTAAGCTACCCACAGATC
TGGGTAGTGACCTTGTGTGACCAAATACCCACGAGTACGGATCCGGATTACCCACGGATCTACCCATGCTGAG
CCTTAATCTATATGGAGCACAATGTCATGTGCGGGGATGTCGGAAACAGAGTGTAGATAAGACCAAAGCATGT
GATAAACACCAGAAGGAGTGGTTTAAATATACACAGCAACATAGCCGCCAGCACTTAGCAGGTGTACGTCGGA
TGCTACAGAGACCTGGTGTATCTATGCCATGGGAGGAGACTGGTATCAAAGGAAATATCCAGCCACATGATGA
GAACTTAACAGAGCCTGAGTGAAAGAATTACTTTGGCCCTGGACGTTTCTATTGTGTTGAAACAGTTTGTGCA
CCTTGTGGTGTGGTGATTGCCTGGGCCAAGTTTGCTAAGTCAGAATCACCCACCAACATTCTGCAGTTTCTGG
AAACTGTGTATCCTACAGAGGAATCACGGCCTAACTATATTTGTATTGATAAGGCCTGTCTGGTGTTGCAAAC
AAGTGTCGTAAATGGTAGTTGGGATATGTGGAAAAAAACAAGTCGATTTATTGTTGACTCATACCATTATATC
AATCACAGAACTACTGATAGTCTTTGCCGGAAATATTGCAACCCTGCACCACTAGATGGGTCAGCTCCAAATC
TTGTTGTTGTGGAATATGATAAGGATGGACAGCCATACTATAAGAGAGCATATAACACACAAGCCTGTGAGCA
GCTAAATGCATGGTTAGGTGGATTTGAGTCTATTCTCAAACGTTTGACACCTGGGAACTTTAGCTGGTTCATA
CATGTGATGCTATTCTATCATACTAAGTATGTATTACAGAGGCAAAAACAGAAGGGTAAAAGAGTGTCTGATC
CTGAATCTGATAGTGATAATGAAATGTAGTTGTAGGAAATACCAATGTTCATATATAGTGATAATGATAAGAA
TGTAGAAAATGTGAAATTCTGAGCCAGTAAGTGAATTCTAAGAATTTACATGGAATAAACTTGGACTGTCCAT
GGAATAAACATGGATTCCATGGACTGTCCATGGATTCCACACAGATTCCACATAGGTGGAATTCCAAGTTAGT
AAAAATTTACATGGAATAACCTTGGACTGTCTATGGAATAAACACGGATTCCGTGGACTATCCATGGATTCCA
CACGGATTCCACACGGATTCTGTACAGATTTTCGGATTCCACACGGATTTTAGGAATCCATGGGGCAGTGTTA
AGTACTGACACAAGTGTTGAAATAATCGACTCCATACACCTGTGAGTAGCTTTTTGCCACGAGACGAGCCTTG
TACTTAACGATATTCCTGTTTTGGTCGCGTTTATGCTTGAAAACGTAGCGACATCCTATAACATTGGCATCAT
CAGGTTGCTCGACGATCTCCCAGGTCTTGAGTTTTTTGATTTGATCAAGTTCAGCTTTGATAGCCGCTGACCA
CTGGCAAGCATTGGATTCATCACCATTGAGCGCTTCTTCGAGGGTTTGAGGTTCACCACCAGTGGCCATTGCA

AAAAGGTGAGTATTCTCTGCCATATGGGCTTCTAATTCCTCATCTTCAATAGATATATCCCCACAGTGATCCA
GGGAATCAGCTCCTTCAAAGACTGCTGCAGTTCCATGCTCAGATGGGGGAATGGTCCCATCATTGAGATGCTT
GTATTGGCCTGGGGGCTTCCTAGGACGAAAACCGCGACCAGTATTTGGTTCGGGCTCGATTAGGCCATCGCTT
CAAGGGGCTTTCTTGTGGGGGGGAATTGGTGGAGAATTGGTGGAGGAAGATTTTGGGCTCGACAAAGGTTCGT
CCGTATCTTGCTCATTTTCTGGCCGATTTGGCTGATTGTTGGACCGTTCGAGAGATGACTGCGAGGGCTTCGA
AACGTGCCTATTCACGTTCGCTAATTCGTTGGTCGGATTTTCGATGTTTTGGCTGGGGGGCTGGAGGAGCTGG
TCATGTTCTCCCCCTCAATCAGGACGGTTTCTGGGGCTAGGGAGTCATTCTTATTGAAGTATACATCCCTCTC
AATCATTACTGATCGCTTTTTGTCCCAGTAGATCCAATAGCCTTTTAACTCTTCATCAAAACCAACAAAACAG
CCTTCGACAGTTTTCGGCTCGAGTTTTCCAGATTCAGTGTGTTTGACCCATGTCTTACATCCCCATTCCTTAA
CATGGCTTCGGTCAGGCTTTTCCCCAGTTGCGATTTCGAGAGGGGTCGCATTTTTCGCTAGCGCTCGAGTTGG
GGCTTGATTTCTCAGCCAAACGAGGTCCCATAGGAATTGGGGCAATCCGGACGAGATGAGCATGGCCCGAATG
TCATCAAGGTGTGTTCGGTTTGCTCTCTCACTTTTGCCATTTGATTGAGGACTGTCGTGCACAGTTAGGTGCT
GGACGGTCCCTGCCTTCTCCAGGTGCTCCGTAAATTCATCCGACATGAATTCTCCCCCTCGGTCAGTGCCAAA
GATCTTTATTTTCCCATCGCGTTGCATCCTGACCCATGCCTCATATTTCTTATAGATTTCGAAGCATTCTTTT
TTCGTAAGGAAATAAATTCTTTCTTCATGCGAAAACTGATCCTGGAAGCATAAATAGTATTTCCAGCCGCCAA
GGGACGTCGTTTGAGCAGGACCTCACATGTCGGAGATGATTTTATCACCGTAAGCCTGGGCATGTTCACTTGT
GCTGATTTTAGGGAATGGTTTGTGGGTTGCCTTTGCTTGGACGCATGTCGGGCAGAAGTCGGGCTTAGATTCA
AGATCGAGCTCTATTCCAGTAATTCTTTCATCTTTGACCATACGACATAAGTCCTCATGATTAATGTGACCCA
TTTTTCGATGTAGATCATTAATTGTCATGATTTTTGATGCCGTGGATGCTTCGAGAGGGGTTTGCATGCGAG
GGATACACGATATAGACCATTGGTCTCAGGGATTCAACCAATAACGCGAGCTGGAGATCCAGGCTTGGAGATT
GTACATATTCCTTTGAGGAACAAGACTGAAAACCCTTTTCGATCCATCTGGCTAATGGAAATGAGAGTGAATG
CCATTTTGGGTGAGTAATAGATGCTTCGGAGGGTAACAGTGGTAGTCTTATTGTTCTTTCCCATTGGAAATAG
GACTTGGATATCACCCCGGCCAGTTGCATAGAAGATTCGTCCGTCAGCGGCTCGAATTGGCTCTGGCTCTATT
TCGACGAAATTGGTGAATGCTTCACGACTGGGCGAGAAGTGGCAACTTGCTCCACAATCTATGAGCATGCCAT
GGGGATCTGAGGAGAAGGATAGGGCAGATGCTTTGTTGTGGAAGTTGGAGGTGACAATGAGAGCAACATTGTC
TTCTTCCTCATCATTATTCCTTGGTGGCTGCATTTGCGCTCGTGGTTTTGTCTTCCTTCTTCTTCTTGTCCCA
AGGTGATTGACCTTCCTTTCCTCCCCCTTTCTTATAGCAATCTTTGATGACATGTCCCGAATGACTGCAATTC
GAGCATTTCTTATTGTTTTTCGCCTTCAACTTCCCCTTCTTCTTTCCAGATGCTGATGATGCACTGTTGGCAC
TCTCGACACTTTTCTCGACTCGATTATGGATCATTTGCGAATTGGCTTCCTCGAAGATGGCTTGGACAAGTTG
GCTGGTCCCAAGTGGTTTCCTGGAAATGAGTGTCATGGTACTTAATGTTTGTAGGAGTGAACAGTAGGATGGA
GGGAGAGATCTTCGAATGGTAGCATTGAATGAGTTCTCGTCAAGCAGGGTTCCCATCCCTGCGAGCTCATTGT
GGGTTTCGAGCATCTCCATAATGTGTTTCCGAACATCTGCAGATTCGGCACATTGTATATTGTGAAGTCATGT
GAGTATGTCCACCTTCACAAGGTCTCCTTTATCCTCGAATAGGCTAACAAGTTTGCACCAGAGTTCTGTGGCA
GTCTTTTCACCTCTAACCTGAAGAAAAACAGTTTTACTGATTGTTTCATAGATTACTTCGTGGACCAACGCCT
CTTTTTGCATGTAATCGTCAAACTCCTTCCCATATTCTTTGAGAGCCACTTTGTTGAGTGGCTTTCCTGGAGA
ATCGGTGCCTTTGGTTTTGATACTCCATTTTCCGTCCTTTTCAACAAGCTCTTCGGGCTTGTGTGCAATTCCG
TACAAATGTCGATGAAGTCCTTTATGAACAATAGTATTTATTGTTCTGTCCTTGTAGGTAACCCAATTCAACC
CATCGTCTTTGAGTTTTAAGAGAGATTGGACTTTGATTGTGTTTGATGAAGACATTGTAGGTCCAGGGCCCAT
AACCTATTGGCAGTGAAAGAAATGAACGGAACGAGATCTGACAATAAATTAAGTCTCAGTGGATATATTAGAC
ACTGAGAGACATGAGAGAAAATCTACAAAGGTAAACTGGACGTGATTACGTAATGATAAAGCAGAGAATTATA
CTAAGTCCATAATGTAAGTGAGGCTTGTAACTCAACATTTTTAGCACACTATAGACCCACCAAATACACCATC
ATTGTGACAATTATATCATCAACTCATGTGGTTAGTCATCTCAGGTAAAATATTCTTGTGTGCAGAGTCTGTA
ATACAGTGAAATCCTATGAATTCTTACCAAATTGTGAGATTTTTGTACATTTTTACCACATTATATGCTATTT
ATATGTGTTTTGTGGCTGTGCGGTGGCTGTGCGGTCTCAACCGGTTGAGGACTGGTCCAAGACCGATTTTTTT
GGCCTGAAAATAAAGTGTGACTGCGACTGGTCATCCTCAGAATAAAGTGAGACTGTGACTGGTGGTCGCAGTC
TCATTCGGTCTGGTCCGGTCACAGTCACGGTCTTTTTTCGGTCTTGTGACTGGACTTTCGAACACTACTGCAG
TTATGCATTAGAAAAAAAGACCGCAGGATATATCACCATATAAGGGCCAATGTTAAGGCAGATGGTCTTTACC
AAGGTTCAAGTGTTGAGTATTGTCGAAGATGTCAAGTCAGAGTCTTGGATCAATACCAACAAGACTATAGTCA
CTCCAAGTCAGGCTTTGACTTGGGTATTGATAGTTTAGTGATCAAGGTTCAAAGGTAAAGGGTGTAGTATTGC
CTAGGTCAAAATGAACATCAGCAAAACATGGGCTGACTTAATGGAACTCTGAGGACTATTTTCGACTTAAGGG
CTTATTGATACTATTTGTAACTATGTAAAAGTGCGATTACAAGGCAAAAAGTTAGAATTTTAGTTGGAAGTAT
ACAAGTTTGCGAAAAGGCATAAAAAGTCAACACTTAGTATTCAAGGTGAAAAGCAATTCTATCAATTATTAGA
TGGAGTTGAATAGTATATCAAATGCATTGTGATCTGCATGTTTACTAACATAATATGATTTGAACATGTCAAG
AGTCTGGGAAAAATGGAGTCCAGGGGGTACAAAGTGTGAGCCACAGATAGGTAATAAATGGACAAGACGATGA
ATGGTATCAAGATGGATTACCTGTAGTAAGGGCGTTTCATCAACAAACTCAGGCTCAACGATCCACATACCCG
TGTCCAGGTCAGGCGCATCACCAAGGGGGAGGAACCAGTGAACGAGCACGCATGGATATGTGGCATTGCGATC
AGCGTAGGAAAATAAGAGCATTACCTGTGCAATGCTCATCCCCTTTATTGTGTTACCCCCAGTGCTCACGAGA
ATGCAATCATGCTGCGGGCCATCCATCCACCATGAAGGTGTGGATCATATATACTCATGATGCATACCACGAT

AGCCTGAAGGGTCTGATGGGGCATGGAAAACTGCAACAGCAGAGTGGTAGACTTTGGTGCGGCCATAGAGAGG
GGGGAATATGGTTTGGTTGCTTGAGGGAGTGTTTGGGTGGCGATGAGTAAGGCTCAGCATGGGTAGATCCGTG
GGTGATCCGGATCCGTACTCGTGGGTATTTGGTCACACAAGGTCACTACTCAGATCTGCGGGTAGCTTAGTCA
GCAAGTACTTGTACTCACAGGTATTTTTTGGCAAGTACTTACAACTCAGTAAGTACTTGTCACAGATCCTGAT
TACTCATGGATTACTCATGATTACCCACAGGTCACAGGTCATTGGTCAGCAATTAGTCATCAGGGACAGGTAT
TTCACAAGTACTTATGGGTATTTACAAGGATTTTGTTGGTTTAGAATGGAATTTGAGCCTGTATGTACTGTGG
GAACTATACTTTAAGGGTCCTTGAGGGTCCAGTGTGAGTCACAAGTACCTGTCACAGGTACCTGCAATGTCCA
AATGCTTGACTCGGATCCACAAGTAAATAGATCTGTGAGTACAAGTACCTGTTTAGGCCAATACTTGCAAGTA
TTCCCATGGGTCTACCCGCAGGTACCCGCGGGTAACCCATGCTCGTGCTGAGCCTTAGCAATGAGAGTGGATG
AATTCTTGCATGAGACATGGGAGGCCAACTACACCTATTTGATTTCCAAGATCATGGAAAGATCTTGATGGGT
AAGCCATTTCTAATAATAATATATTAGATGTATTGTATTGGTGATTGGACAGTTGAAATAAAGACATACAGTG
CATTTTGGTAAGAAATACTTTGTTGAGTGCCTCCCCTTCAATTGCCTTTCACCATTGTCCACAGAATTTTCAT
CTGAGGTTGGTTGAGTCTTGGTTGGTTGAATCTCGGGAGCACCAGAGCTGGGGAGGTCACCTCCCAGAGTAAG
GCTCAGCACGAGCACGGGTTACCCACAGGTACCTGTGGGTAGACCCGTGGGAATACTTGCAAGTATTGGCCTA
AACAAGTACTTGTACTCACAGATCTATTTACTTGTGGATCCGAGTCAAGCATTTGGACATTGCAGGTACCTGT
GACAGGTACTTGTGACTCACACTGGACCCTCAAGGACCCTTAAAGTATAGTTCCCACAGTACATATAGGCTCA
AATTCCATTCTAAACCAACAAAATCCTTGTAAATACCCATAAGTACTTGTAAAATATCTGTCCCTGATGACTA
ATTGCTGACCAATGACCTGTGACCTGTGGGTAATCATGAGTAATCCATAAGTAATCAGGATCTGTGACAAGTA
CTTACTGAGTTGTAAGTACTTGTGAAAAAATACCCGTGAGTACAAGTACTTGCTGACTAAGCTACCTGCAGAT
CTGAGTAGTGACCTTGTGTGACCAAATACCCATGAGTACAGATCCGGATCACCCACGGATCTACCCATGCTGA
GCCTTAGCTCGA
>Cligib1_rnd-1_family-327#LINE/I
CTTCTTGAACTCGAACCACCACCTGCGGTTTTCCCGAGGTTATCCCGACGAATCAAATTGTTTTACACACCGT
GAAAAGACCCATTTTGAAGTGTTAGCGAGCATCTGAAGCAATATGAATGAACGGAGGGCTGGGCTCGAGCCCG
AGCCTCCACCACAGCAAGCTTCCGGTCTACGATTTTNCTTCCAAAATGTTAATCGGAACAATNTCCTTGTTCA
CGACATACTCGATTCGTGTGTGTCNAATTACGATATCCTTTTCCTNCAGGAGCCGCGATACCCTGATACGAAA
TATCCCNTCNTCTACCCGCGGGGATGGGGACCCCATTCGCGGCTTCCCCATCCATCCNTCNTGGATCCTCGTG
GAGCGTGGCGGCCCGTCCAGGGTCGTCGTCTACGTCTCACGCACCATCGCCTCGTCCAACCCGCGATTGTGCN
CNCACATCGTGGACCACCCCGACATCCTNCTGGTCTCCCTNTACCGTCATGGGCAGACCGACCCNGTCTATTT
CATGAATGTCTACAACGATGACCGAGGATCCGCGGTNACGCACATCCGGGACATCGCCCATCGNCTGCCNCCT
TTGCTGGTGGTCCAGGGNGACTTCAACCTNCACTCGGCCCTGTGGGACCCCACGGTCGAGCCTGGTAGGGAGG
ACGCCGTTGCCGTTCTNGCCGACGAGACGCTCACCGCGAGGGGGCTNTCNCTNGTCAACACGGACCACGGNTT
CACTTTCCACAGCCGNATNGCNGGNTTCCGGTCCTCGGTCATCGACCTCGTGTACATTTCCAGCGACGCTTTC
CAGGCGAACACGTTCGCCTTCGAGAACCGCCCTGACCNCAGGCATCCGTCGGATCACTCCGTTCTCACGTTGA
GTATCGACGTCGACTTCGCCCCCCCCCCGCCCCCCCCTCGNATCCCTCGNGACTCNGAGATGGAGGCCCAGTT
CATCTCCGACATTCGGGCGGCNNTCCTCGACATCCCCGNCGCTATCGATTCCGCCGAGGAGTGCTCGGCCGCC
GCGGAGTCCCTCCGTCGCGGCCTCGAGCTNTCCTTCGAGTGGTGCTCNACCACCCCGAAGCCNGGNGTTCCCC
GCCAGAGTTGGTGGACCCCTCGNTGCTCCGAGGCTCTCCGTGAGTTCCGCGAAAGCGGGTACTCCAGGGAGGC
CAACCGNGAATACCGGGCNGCCATCCGANGCGCCCGCAAGANTTTCTTCGAGGACCGCATACAGGAGACCGTC
GNNAANCGNCGCGCGTGGGACCTCATGGCATGGACCAGACCCCGTGCCCCACCTGATTACAANGCGGTNAAGG
TCGACGGCGTCCCCGTCACCTCCCTCGCNGANCTATGGCCCGGNCTNCAGCGGGCNTTCGCCTCCGCNGCNGA
CCGCCCTATCGACCTGACCATCTGGACGCCGCCCCTNCNTGGGAGGAGCGNTCNTTTCCCCCTTTCTCGTCGT
TGGAGCTGCGNGANGCCCTCAGCGGCCACGTCCAACTCCAGCGCCCCNGGNCCNGACCACGTCNCNTGGCGCC
TCCTCAAACTCGCTCTNGGCGAGCCCGGCTCCCCGGCGGTCATGTCCCTCATNCGCCTGTTCGACGCCTGCGT
TCGGCACGGCGTATTCCCCGACTACTTCAAGGAGTCGCTCTCCGCNATCATCCCCAAGCCNAAGAAACCCGAC
TACTCGCTGNTCAAGGCCTATCGNCCNATNGCNCTCCTCAACACGCTCGGCAAGCTNCTCGAGAAGNTCATCG
CCAACCGNATGCAATTCGACGGCGTCCGCTTCGGNCTCTTCCATCCNTGCCAGTTCGGCGGCACNCCTCAGAA
GTCCACCGAGGACGCCGGCGTCCGCCTGGTNCACATGATCCGNCAGGCCTGGGCNCGGGGNGAGGACACNTCC
TGCCTCGCCTTCGACGTCTCGCAGTTCTTCCCGTCCGTCAACCATCGNGCCCTGATCCGNATTCTNCGCCGGA
CGGGCTTCGCCCGGTCCCTCCTCGNCTTCTTCGAGTCTTACCTCGTCGGNCGNNCCACCAGGTTCAAATGGGG
NCCNGCTATNTCNGGNCCCCAGCCCGTNAGCGTNGGCGTCGGNCAGGGCTCCGCCCTGTCCCCCGTCCTCGCN
TCCCTNTACGTCGCTCCGCTGCTGCACATTTTGNTGCCGGCGAATCGCGCTAATCCGGTCGACTGCTCGGTNT
ACGTCGACGACGGNGTCTTCGTCGTCTCCTCGGAGTCCCTCTCGTACAACACCGANNTCCTCTCCGNATTCTA
CGGGCGCATGAGCGCCGGNTTCCGCGCGCTCGGCCTCGAAATCGANCATGACAAGACCGAGTTCATCCACTTC
ACCAAGGACGGCCTCGGCCCGTCCATCGACCTGGGCGAGGCCCCGTACACCGGCGACTCTCCGCTCGCCCCCA
AGAGGGTNTGGCGGTACCTAGGTTTCTACCTGGACCGGCGGCTCNCNTTCCAAGACCACGTCNACTTCTACGC
CACCCGNNCTATNTCCACCGTCAGAGCCATGCTTATGCTTGGCAACTCCGTCCGCGGTCTCTCCCCGNCCAAC
AAGCGCCTNCTCTAT

>Cligib1_rnd-1_family-159#LTR/Copia
CCTCTTTCGAGGGATGACCNAGGCGTCTATGCCANATGTCATAGCGATCGATNGTATTGACAAATAGGGATTG
TTTTAATCCGNTCGGTTTGTAAACACGATCTGCGTGNACCCAGAACGTNGTGTCCCACGCAAAACGCTTTTCA
GCGGAGATTAATCGTCGTTTGTCCCTTTGTTGAATATNAACGCNTTTGATGANGCATTTAACGACANTCCAGA
ATTNAGTAATTCTCCCATGGATATAATTCGGGAGTTCAACTTTGGATGGTGATAGACAGGATGTAAGCGCGTG
TTGCGTTTTTATANGATCCATCTGTTTGTAGGACATCGTTTTCAAGGATAACGGTTCCTTGTGTNTCAATTTC
GATNATCTCNGTCGCGGTAGNAACAGGAATNGGGTGTTCGATTNTTGAACGTTCCACGTAATCATCCAATATG
CCAGTGAAGTGCATGGATGCNCCTGAGTCNATTAACCATTGGTGTTCGACGTGTTTCGCTTTTCCCTTACATG
CGCCACAGCCGGCANAATGATTATTTATTTCGGCCATTTGGCCGCGAGACAANGACACATGATCTGCATATTT
ANATACAGGGGGCAATCNATGATTTAATCGAAAAGCGCCCTGCAGAATTGATGCGGTTAGTCTCATATATNGG
AAGTGAAGGAGGCGGTGACTGACGATTGACCGTCAAACCGGGTNCCCCAACTCTCAGGATCGAATTCGGGTTC
TTCACCCATCGANACTGGATCCTCGCGATCGTCCTCTTCAGGAGACCGGAGAGACTTTGCGCCGTTTCTTAGG
CGGAGGGNNCCAAGCCTCNNCGTCGTAANCGACGTATGCGGCGCCTTCATCTTCACCGCCGCAGTAGTNATCG
ANCCAATTCTCATACTCGTCTTCTTGCATTAGGATGTCGTTAAGGCCATCGGGGGTGTTNGAGCGGGGNGGNG
ATGNTACAGGCTCNGGAGTGGGNGCNCGANGNGATGGTCCAGGGGTAGGAGAAGATGATCGGGGAGTGGTAGG
TTGAGGAGGAGCTTGCTGNTCCTCCGGTTCCGGGAGTCGCTGANTNCGCGATTCNGGTTGCGCCATTNGNTGG
ACTCAAGNNTTTTGAGGGTCTCGGTGGTGGGCGTCGAAGATAGGCGTGAAGCCAGTGANCGCGCTTTTTCGAA
NGTTGGATAGGTGCCAGGNANCTTNCCNGGTGTGGCNCCCATGNAGGTCTGGGCAGGNCCGTCGTGTAGGCGC
TNAAGGAGGGAGCGCTCCTTGGGGCCGAGNGGTTTGGTGTTTATGGTGATGGGAGGTGGCTCGATTACCTCCG
CGATGNATGCCGAGGAGAAGAAGTTCCGGTGATGATGGTGCCGATGTGGGGTGTTGGATCCAGCGTCTGAATT
TCAGCGGCTTCTTCGGCCTGCTCGGCTTGGCGCTTTGCGATCGTCGCGGTTNCCACCGCGGGAGTTGCCGGCC
TTGGCCTTACCCTTCGACGAACTTGCCTCGTGAGGGGGAGTTCTCAGGTGCCTNNTCCTTCGCCTGTTCCTTC
CAGTTAGGGTTCTTGCCTTTACGTTTGACGCCNGAGACTTTGTTGGCCAATNGGCCGGCTATTGATGGCGGTG
CGACGTTGATATTCCNTNACGANGGCAGATCGNACCGAATCGAGGGTGATGTCGTCGGTGCTGAGGTTCTGGA
GGANTTGNGAAGCGACATTATCCCATTTGGGAGGGATGGCGGCGAGGAGGATCATGCCTTTGACGAAAGGCGG
NAGGACGACTGATGAAGCNTCAAGGCGGGAGAAGAAGGTNGACATAGCCATTATTTCAGGCTCAGGGTTNTCC
GTTCCTGAAATTTTGACTGCAAGGGCAGANCGGAAGTCCGNAAATATTTGNGAGGGGTCGGCGACCCCGTATT
TGGCGTTGAGCGCTGCCCAGAGAGCGCGTCCCATAAGCCACGGGAGTGGTGTGNGTNGCGTGGGCGGCGCCGA
TGTCAGGAGAGAGAGNGTGAGGNGAATGGANCCANGNGCCATGATCATCTTTATTGAAGAGTTTGTAGTGATGAG
CGTCATCTCAGTTGAGTAGGCGGGATGACAGCATTGTCGGTGCCAACCGTGGTGCTGGTGGGTCGACNNCGCG
ACCGTCGGCNGCTTGAGTGATTTGCCAGAGTGCGCNGTAGCGGAGGAAGGCGGTCATTTTGCGGACCACTCAG
TCCAGTTGGTNCCGNCGAGTTGAGGAACAGANTTGATATCGACGGTGGCGGAGGANGACATTGTGATGTTGGA
GACGATGAGGACGATGATAAGGATGGTAAATTTGAAATTTGAGGTTGAGGGTGTCTAGTAAGGGGGCGACGTT
AGCCTCATGTCATNATGATCAGAGAAATGCCATCCAGCCATGTTTCAGGCGCATCAATCGGTNGACCCAACGC
CATATTTCAGGCAGATAAAGACATAGATTGATGAGATCTAGTCCATGGTGGGTGTCAATGACAGTGATCAGGT
TGTGAAATTCTATAATGGTGATAACCGGTTATAGAAGCCATCCCTGATATGTCAAAGAGTGGACATTGGTTGG
TGAGACCAAGTCCGCGGATTTATTGTACAACGATACCACCGTCAACGGATGTATGGTCATTAACTGCAAGAGT
GCGGAGTTAAGAGGACCGCCATTTACGCATTTTTAATCATGCAAAATGATACGTGTGATGTGTGGTAAGGTTG
ACAATATTTCCCTCCAGACAGTTAGCTGCAGCCTTATTGCAGGCGCGGCAAGTCGTCTGGGAAACCCAGTGGT
CAAGCGCATTTCACGCAGCCATATTTCAGGCGCGTGTAATGGCGACCAAGTGCCAGCCATTCTGCTGTCGATG
TTCAGACTGAGCAGCCTTATTTCAGGCGCGTGCAGCCAGTTCAGGCGCCAGAATGTGGCGCCAGGGCCCATAA
CCTGTTGAGCTGCACCCAGNGTGCACAGCTGGAACTGGCTACTTACTTGGTTAGTTGGCTGAGACGATACTGC
TACTGGGTGTAGATGNGCGAAANAACTTGTATTNGCCTCTTGTTCAGAGGGGGCTTATATACTAGATACCCGG
GGTAATCCAGGGTAGAACAGAGTAAACTAAGTATGTACAAATACAAGGAAGCTGACCCAGAGTAGATGGATCA
GAGTAAAGGAAGACGGACCAGAGTAAGCTGGACCGGAGTAAGAGAAGCTGATCCATTTATGATGGGATCAGCT
GTGAACTGCTTTCCTTAATTGGAAGCCGGTTTAGGGGTAATTATGTAAGCAGGGCACTTACAGTTAGAGCGTT
GAANTTTAACA
>Cligib1_rnd-1_family-1086#TIR/MITE
GTACTAGCGGTGCCCGAAAGTATGGAAACACGAACACACAAAAATGACATTTTACCCTTGCAATCTTCAGTAG
CGCGTTTACAATACATTATGAGGGCAACTTATATAGGTATTTTTGAAAGCTATACATGTCTAGTATCTGGTCC
TACAAGGGCTTGATTCTCCCTGATGTGTAGGTGGAGAAAACTGTGATTTCCTGATACACCCTAAATTCACACC
TTTGTGCAATTATCTGCTTGGAGAGCAAATTTCAGAAGAACTTTGAGTGTCCAAATATACTCCTTAGGTCATG
ATAAATTAAATGTAGCCCATGTCAAATTATTTAAATGCCCAGTTTCTAAGATGAGTAATAACAGATTCTCAGC
ATTTCACTCCCTATGTCCACTGCAGGCAGCAGAGGATGAATTCAAATCAATGCTTTTAGGAGTGGATAGAATA
CCCTAAATACTCTGTTTTGTACCAATATACCATATGCCAATTCTTTCTCTACGTTGAGTTACACCATGAAGAA
TGTCGTGACTTGGTGTTTCCATACTTTCGGGCACCACGAGTACA
>Cligib1_Cluster131#LTR/Gypsy

AATGCACGTGCTAAACCACGCACGTGGTCAAGCACACGGATTCGCGACGCGCCCGTTCCACGACCTTTGCGGC
TCTATCTCCTACCACGACCTACTCTAGGTTTCTACCACTCAGCTGGACGGTTCTCTCCTTAGTCTTCGTTCAC
CTCCATTTCTCTTTGTTTGAGTCTAAGTTTTCGGCTTCCTGCCTGGTTCCTTCCCGATTTTGGTTCTCGGCGG
GGTCAACTTAACGCATATAGTCGCCCTCCGCCTTCCTGCTTCATGTCGCGAGCTCGCCGCGGTGGGGCTCGAG
GGGCGGCCCCCGCTGGACCTTCGCGAAGGGACTCTGCTCAACCTGCTGCCGTCGTTCCGCCCGTTCTTCCCGC
GATAGCGGAATCTCTCCTTCCGTCTTCGAGCCCGCCGAACCCTGCGGCCTCTGATGGTCCCGATCTGGCCGGG
GGTGGCACGCTTCAGCCTTCGAACCGGCGTCCTAGGGACTCTTCCCAGTTGAGTTCACCCATTACCCCGATCT
CGAAGCGGGTTCGACTGGAGCTGCCCTCTGGGGCGTCTCTGTCCTCACCCTCCGCTCCGCCGCCTTCGATTGC
CCTACCTTCCCCCTCGCCTTTCTTGGTTCCGCCTTCTCCACTGGCGGCGGTCATTCCAGGGGGGGGATTGGCT
CCTCCCACCCCCCGTTTACGTGGTCCTGCTACTCCTGGTCCGACTTCTTCCCCCCTTCTTCCTGCAGCTTCTA
TTCGCTCCTCCGCGCTTCCCCCTTCTCGACCGCATTCAGCTCAGGCGGCTCCTCGCCAGTGGCCCCACCCCCT
AGGTCCTAGCCCCTTGACGCGGGCGCCGGTCTTCCCCCCTCCGGTTGGGGCGGCTTCCCCGGTTGTCCCGGCG
CCGACCTTCGCCCTGACGCAGGCCGATATACTAGCGCTTCAGCGGGACTTTCTGGATCAATATCTGAGGTCTC
AATCGGCCCCTCCTACCTTGGCCCGTCCCGAGTTCCCGGGTCCTGCTGGTGGGTCTTCTTACTTGACTACGGG
AGGGGAGGGCTGGCTGCCCTGGTACCCTGTTGTTCCCCATTGCCACTATATATATATATTTGCCCTTCGAGCC
CCTCCTCTGAGGCGGCCCGTCGGCCCTTTCCCTTTTGACGGTCGCCTGATGCGGCGCTGTCCCTTATTTGGCG
GCGTGATGCTTATATGCGTTCCTTTCCAGGCTACCTCCCGCATTCTAACGCGGCGGGGGCTTTTATACCGACC
GCGGCTCCTCCCTCGGCTCTTCATCCCCTTTCCGGCGCGGCCGCGGCCACTTCCCGTCCGCACCACGTCCTAG
CCTCCAACCCGGTACAACTCGACCCCCTCTCCGCCGTCCACCCCATCCATGAGGTCGCTATCCTCATGGAGGG
CTGGAAGCGCCACCTTTCCTTTGGTAATCTCACCAACAGGAAGCGTGCGCTCGCGGACAAGTCGGGCAACACC
CTTACCAACACTCTGGTTATGGGCCCCGGGGGTCTGGAGATTCAGCCGGCCACATCCAATCCTGCAGCAGAGG
AGTCACTTTCGATGGAGGAATTCATCCAGGCCTCTAATTGCCTGGTTGATATGATTCCACTCTACCTCAAGGC
CGGAAACCCTCCAGAAATCGGTGGCCGGCAGGCCGCTCTTATCGGCGCGGCATGGAGGACTCATTACGACAAT
CTTCTCCGCCGACCTGATTTCACGGAGATCTTCCATGTCTACCGGCGTTACGATCTCCATCTTCGCTTGAAGT
GGCTCACGCAGTCCCAGACGTTCTCTCCGGCAGATTTCCAGGAGGGTATCTATGCGCTCTACGTCAACCGGGC
TCGGGATGAGTCCATTAACGCCATCCTCGCTCAGCAGACCACCCGTGCGGCTCAGGTTCCCGCCATTGCATCC
TCATCCGCCTCTACCTCATCTCAAGCTGGCAACGCCAAGGGTTCAGATAATCGCGCGGGCTCTTCTAAGTCCT
CCTCGAAGTCGTCCTTTCGTAGGTTTTGCCTCTACTGCGGGGACACCGGTCACTGGGCCAGGGAATGCGACGC
CAAAACGGCTCCCTTCCTCAAGAAATCTCCAGAGGATAACCTCTGGCGAACTCCGGGGGGGCTCCAGATCTGT
TACGCACACAATGGCCGCAATAGCAAATGCTCCAAGTCCGCCACCTGTCCCTTCGAGCACATCTGTTCCCTCT
GTGGCTCTTCAAGCCATTCTGCAGGATCCTGTACGAAGGCATGAGCTCTTCCCCATCGTTACGCCCCTCCGTT
TCCACCGCTGGGCGTTGGTGCTGCAAGAGGCGGGCCTCCTTTCCCTCTATTCAGATATCCCTGCTGGTATCAG
GGATGGTTTTCGTTGTGGCGCCAATTCCCCCATTAGTACTCTTTTCACTCCCCCAAACGCCCACTCTGCTCTT
AATAACATGGCCGCTGTCGATGCCATCCTCCTCAAAGAAATTTCGCTCGGTCGTCTTTCCCCTCGCTATGAAA
TCCCAGATCTCTTTGCCTTGATAGGCCCTTTCCAGACGAGCCCTCTGGGCCTCGTCGAAAAAGTTCCCGGTTC
CGGTAAATTTCCTCTCACACAGAACTTTTCTTACCCATACAACGATCCCGATGTGCAGTCCATTAATTCACAG
ATAGATACTTCTAAATACCAGTGTGGCTGGGGCACGTTTGCCCAGTGTTATCTCCTCGTCTGCCAAGCCCCCT
TGGGCTCTGAGGCCGCGGTTTTTGACGTCGAAAGCGCTTTTCGTCGGCTTCCAATGGCGCCGGTTGACCGTCC
ACTCACCTGCATACTCTGGCGCGGTGGTGTTCACCTCGACGCTTGTTTTTCATTTGGTGGCGCCAGCACTCCT
GGCATTTTCGGCAGGCTAGGGGACGCGATAGTCGATATCTTCCGCTTCAAGGGCGCGGACGACGTCATCAAAT
GGGTCGACGATTTTGTCTTCTTCCGCTACCCTTCCCAGTCGGAGGCACAGCCCTTTCCTTCCTTTTCTTATGA
TGAATCTCTGATCACTTCCGTGGCCGAGGATCTGGGGTGGCCCTGGGCTCCTGAGAAGACCTCTCCTTTTGCG
ACTTCCTTTACATATATCGGTTTCCGGTGGGACCTGGGGTCAAAAACCGTCTCTCTGCCTCAGGCGAAGCAGT
TGAAGTACTTGCTCAAGCTAGAGGGCTGGGTAAAGGGTCACCGCGTCGCTCACAAGGCAGTCGAATCGTTAAT
TGGTACCCTCAACCACTGTACCTTGGTAATTCCGGATGGTCGATCACGTCTCCCAAATCTCTACCGTCTTGCA
GCCTCCTTCGGCCAGAATCCTTCCACATTCACCAAGCACTCCATCAGCGCCCCTTTGGCCGATGATCTTGACT
GGTGGAGGGGTCAATTCCGCTTGCCGTGGTGTGGTATTTCTATCCGTTCCCCCCCGCCGATTCTTGACCTCCC
TGTATTTGTCGACGCCTCTACTTCGTGGGGCATTGGGTTGGTGGTAGGGGACCGTTGGCTCGCATGGCCGCTA
CGCCCCGGATGGAAGAGCGAGGGCCGGGATATCGGCTGGGCCGAAATGGTGGCGGTAGAATTGGCCCTTCATA
CCCTAGTTCACAGCGGCATCCGGGACTCCCACATCCACATTTACTCAGACAATGGCGGGGTGGTGGGAGCGAC
TAAAGCGGGCCGTTCGAGAAGCGTCCAACAGAACGCGATCTTGAGGAAAGTCGTCGACTTGTTCCACGACTAT
GGTCTGTGGTTGTCAACAACTTGGATCCCGTCAGAGGAGAACCCGGCTGACGGTCCCTCCAGGGGTGAATTTC
CACCTCGTAGTCAGAAAATACACTACTGTCCTCCAATTCCGTCCCACCTAAAACCTTATGTCGCCCAGTTACC
CTAATTAGGACGCTTCGATTAGTAATACATGTCTTTGAGGCTGACGCCGGTTCTATCTTGCTGTCCTGAACTA
CTTCAACGTCACCTGTCCACAACCTATCAATCATGTCGTTCCCTCCTTCCCTGCTGCATGCGGCCTCAGCTAA
AATATCGGAAGTCTTGGAGAACGCTTGGGCCCCGTCAACCAGAGCAAAATACGACAATTCCGTAACGCTCTAT
CTACACTGGTGCAACTCAGAAGGCTTGCCGTCGGATAAGAGGCTCCCAGCGTCGGAGATCACTCTTTGCGCCT
TCGTTGCTCATCTGGCTGGAACAATGTCAGGCAGTTCAATCCGCTCCCACCTCTCAGGCCTCAAAAGATGGCA

CATTCTGGCCAACATGCCTTACACGTCCAGCCCGCGCTTGTCTTACCTCCTGAGAGGCGCCGACAACATGACG
CCGGGTTCCTCTAGGCGGGCACCCAGACCCCCCGTAACTCTTGCCATGATTCGGGTTCTACATGATTCTCTCG
ACCTCCAGAACAGTCTCGACGCGTGCGTTCTGGCCACTGCTACTACGGCATTTTGGGCCCAGGCGAGATTAGG
CGAGCTGCTCTCACCATCGGCAAAGTACTTCGATCCGTCTCGTATCCCAAGACTGGCCAACTTGGACAAGGCC
TCAACATCGGCGGGCTCACGCAAGCTGCACCTTCCGGCAACGAAGGTCGCTGGTCCGCGGGGCGAGGATATCT
ACATTTGCAAGCAGAACGGTCGTTCTGACCCCATAACAGCATTAGCTCGCCACATCTCTCGCAACAGCTTGAC
GGCCGACCTCCCTCTGTTCTCGTACGATATAGGAGACGGGAGGCACCTTGCCCTAACGCGGACGAAGTTCTTG
AACCGCTGCAATGAAATCTGGACGCGTCAAGGTTGGCCGAGATTGACTGGCCACTGTTTCCGTATCGGAGGAA
CGACCGAATTACTCGTCGCGGGGGTCCCGCCGGACGTCGTTAAGGCCATGGGAAGGTGGTCTTCGGACTCCTT
CCTCAGATACTGGAGGTCCCTCGAACTCATTGCCCCGAAGTACGTCGAGCGATTGAGCAAGACCCATACACAC
TCGCTTCCCTAGGCAGGACTTGGTCCTGTTGGGCGGTGTCCCGTCTCCGTCTGGTACGCGCCTAGGGCTTCTT
CGGTTGGGTTTCTCCTATCCGTTTCCGGTTCACGCCCCCAGGATAGGAGTCGCAAGACTCCTTCCCCTATTCC
CAATTCCCTTAGACAGTATTCACCTTCCTGTTGGTCTACATAATACATTATCACTATTTCCCCTTCTCTATCA
CACGAGGGGAACCAACACAAGTCTCGAATTGCACGTGCTAAACCACGCACGTGGTCAAGCACACGGATTCGCG
ACGCGCCCGTTCCACGACCTTTGCGGCTCTATCTCCTACCACGACCTACTCTAGGTTTCTACCACTCAGCTGG
ACGGTTCTCTCCTTAGTCTTCGTTCACCTCCATT
>Cligib1_Cluster130#LTR/Copia
GTTGAGCTGCACCTACAGTGCACAGTGGAACTGGCTAACTTACTTGGTAGACTGATGAGCTGAGAACACGGGT
GTAGAAAGCAAAAGAACTTATATTAGTCTCTTGTTCAGAGAGGTCTTATATACTAGTAAATGGTGCACAGACC
ATGTGGCTGTGCTAAAGTCCTAACTCCTATCTATGATCTAATCTACTGATAAGATAAGATAAGGTGTTATCTA
GGGTGCTTATATAGACACCGGATTATGTAAGCAGGGCACTTACAGTTAGAGCATTGAAGTTTAACACTCCCCC
TCGCTTAGAACAGCGGATGCAAATAATCAACCTTTGCAGGAGTTATAAGCGATACTACAACTTCAAGACGAGT
AAAACTCAAGTCCCAGTTGAGGTACGAATTCTTTGAATTTGATGTGTCCCTAGACTTTTGTAAGAATGTCTGC
AGGGTTTACATTTCCTTCAGTGAAAAGTATGTCTACTTTTCCTTCGTCATAGCATTCACGAATGTAGTGATGA
CGGATGTCAATGTGTTTGGTCCATCATTCTTGGACCGGATTAGAACCAATGAATATTGATCCTTCATTATCGC
CAACAATTGGTATTTTGTTAAGTTTGATTCCTAGTTCTCCAAAGAGAGACCGTAACCAAACCACTTGTCGACA
AGTGTCTGACATGGCAATATATTCTGCCTCAGTAGATGACTGGGCGATTGTTTTTTGTAGTCTGGACGTCCAA
GTCACAGCTCCGGAAGCAAGATTTGTGAAAAAAACCGGAGGTAGATTTCCTAGTATTAGGGTCGGAACCCCAAT
CGAATCTGTGTGAGCAATTAAGCCTTCGCCCGAATCCCCTTTATATACAAGGGCATAATCTTGGGTTCCAGCT
AAGTAGCGACATATTTGAAGTGCATGTTTAAGATGTCTTCAGTGGGGTTTGCGCTCATTTGTGACATTTTAAT
CACAGCAAATCCTATGTCGGGCGAGTGCCCAACATTAAGTAAAGCAATGATCCTATGACGGATTGATATTTGC
TTCGAAGGGTATTATCTATTGGGCCCTCATTTTTCAGAGGTAGATATTTGTAAGGCAAGGGGTTTTGGCAGAT
TTAGCATTAAGCATTCCAAAGCGCTCTAATACTTTGACCAGATAGGGTTTCTGATCAAGTTTAATCTGGTTTT
TATTTCGCCAGATGCGCATTCCGAGGAATTCTTTAGCATCACCAAGATCCCTGCATTCCCATCGTTGCATGAA
TTCTGCTTTTTTCTTGATAACATACGACCGATTAGGCCCCAAGAATAAGGCATCATCAACGTAAACTATCGCA
ATGACAATTTGATTTGAGGATGATCGGTGTATGAATATGCCTGCATCTGAAACAGTGCGCTGAAATCCTAATT
CTTTCATAGACGCAACGAGTGCTCTCCACCATTCAAGAGCAGCTTGTTTCAGACCATATAAGGCACGTTTGAG
TCGCAGGACTTTGCGCTCATGACCTTTACGGACGAAGCCTTCTGGTTGACTCATATATATCTCGACGTCTAGT
TTGCCATATAGAAAGGCAGTTCGAACATCCAGTGATAGAATTACCCACTTTTTCAGGGCTGCTGTTGCAATCA
TAAGACGCACAGATTCATATCGAACTACTGGTGAGAATAATTCGTCGAAGTCAACACCTTCGACTTGAGAGTA
ACCTTTGGCTACAAGACGGGCTTTCTTTCGTCCATCAGATTTGATGTTGAAAACCCAACGATTACGAATTGCC
TTTCGTCCTTTAGGCAGATCGCATATATCGTAAACGTCGCGATTCTTGAGGGAGTCTAATTCCTCTTCGCAAG
CATTCATCCACTCTTTTTGAAGCTTTTTGTCAAGCTTAAATATGTCTCGGAAGTGAGTAGGAACTTCGTCTTT
GACAGGAATGGCTTTGGATAGTAAGAAATCCATTATTTGTCCCCCTTCCTCTCTACATATACGAATGAGTTCT
AATTCCTCATCATCGGTAGAGGGATAGTCCGGAAGATCCCTTTCAGGACCCTTTTCAACATTTTTACGCCAAT
GTCGTTCACTAGCGATGTCACGTTCAATTTCAGATGGTAATTTCTTTTCTCCATAAATATTTCCAGGACGGTT
AGGCACAATCTTTCGACGAGTGCTACGTCTTGGTAATTGATTCCATTCTTCTTGATCCTCACGATTGGGCTCA
AAAGCACGCGTGTAAGGACCAGGTTGATTATCACCCTTTCGGGAGATTCACGATTGTCTGCCATTTCTGGTGT
AGATTGTCGTGATGGAATTGGAGATGGAATTTCTTCCGGTTCCTCTTCAACTGTGGGTCCCCAAGGATGTTTC
TTCTCATTTTTCGGAGAAGAGGGAGTTGAGAGTGTACCGGATGAATATCTGGTGAACTATGATGTGATTCCGG
TGGGGCAGATTAAGCCAATCTTCAATTGGAGGAATTATAGGTTGTTCTGCTGTCGTAGTTTTTTGTTTGCATT
TCGGCAAAAGATCTTCATCGAAGATTGCAGTAACACCGATAAAGATAATCGGGTTTTTGATTCACATAAAGCG
ATAACCTTTTATTCCATCTGCATGACCGAGATATATCATTAACTCGGATTTGGGCGCAAGTTTATTGGCACGT
TTTTCTCGAGGGAGATTGTTGAGATAACAAGCTCACTCAGATTACTGACTTAGAATAATCTCCGCTTTACTGA
TTACATAAGCGCGTCCAGTTGTTACATTCGAGGATTGTCTCTCATTTTACACTAATACTCAATATCTATCTAA
TAATATCTAATACCACCCCATCTGCCTCCTCCACTGCCAACAGAGATATACGTATGCAGCGCAGCCAAAGACA
CGTAGATGTGTTAAAGAGGGTCTTTCACCCTTTAAGACTTCATATGGAGTTCGCCAATTAAGGCGTACCATTG
GAGTATGGTTGTAAATATGAACCGCTTGTTCCACAGAAAATTCCCACCACGAACCAGGTAGGCATGCGTGATG

TCGCATAGACTCTGCCTTATCCATAATGGTTCTATTGATACGTTCAGCACGCCCATTTTGTTGTGGTGTGTGA
GGTGCCGATTGTTCAATTATAATGCCATTTGACTTCAATTCATCAATGAATTCCTGGGATTTGTATTCTCCCC
CTGCATCAGAACGCCAGCGTTTAATGTTCGCTTTGTATTGGTTTTTTACCAGCACATAAAATGTGCGGAATGC
CCCAATGGTGTCTGATTTATGTTTTAGATGTGAAATCCAAACATATGACGTGTAATCGTCAAGGAATGTAATG
ATGTATGAATACCCATGATAAGATTTCACGGGAAAGCTTTTTAAATCCGAATGAATCAATTCGAATGGCTTCT
TGGCACGTGAGACGGAAGGTTTAAAAGATGAAGAAGGCATCTTTCCGTATGCACAGCCAGGGCATATAGGTGG
GTTTTTCGGGATGATTAATCCCTGTGGGAAGTCTTTAATATGTTTTCGTGACTTGGACATGACCTCTTTCGAG
GGATGACCTAGGCGGCGATGCCAGATGTCGTATGACTCAATTGAATTGATAAATAAGGATTGTTTTAAACCGT
TTGGTTTAACAACGTCTGCATCTACCCAGAACGTAGTGTCCCACGGAAAACGCTTTTCAGCGGTTAATTGCCA
TGTCCCTTTGTTACTAAATATGAACACATCTGATGATGCATTCAGCGATAATCCAGAATTAAGTAATTCTCCC
ATGGATATGATTCGCGAATTTAATCGGGGATGGTGATAGACAGGATGTAAGCGCATGTGTCGCTTTTTATAAG
ATCCATCTGTCTGTAAGACATTGTTTTCAAGGATAACAGTTCCTTGTATTTCAATTTTGAGCATTTCTGTTGC
GGTAGCAACATTGAGTGGAGTGTTGGATATTGAATGTTCAACGTAATCATCCAATAATGCAGTAAAGTGCATA
GAGGCGCCTGAGTCTATTAACCATTGGTGTTCGACATGTTTCGCTTTTCCCTTACAAGATGCACAGAGAGCAT
AATGATTATTTATTTGGCACTTTGGCCGGCAGACAAGAAGTACATGATGCATATTTATAATACATGGGGCAGT
CCAAATTTAATCGAAAAGCGCCCTGCAAAATGATACTGTTAGTCTCATAATAAGGGAGAAAGGAGGTGGTGAC
TGACGATTGTCCATCAAATCGAGTTCCCCAACTCTCAGGATCGAATTCGGGTTCTTCACCCATCAATACTGGA
TCCTTGCGATCGTCTTCTTCAGAGACCGGAGTGAGTCTTTGCTTCTTAGGCGGAGGTGACCAGGCCTCCACGT
CATAGTTGATGTATGCGGTACCTTCATCTTCAGGTCCGCAGTATGATTCGAGCCAATCATCGTACTCGTCCTC
TTGCATTAGGATGTCTTTGATACCATCCAGAGTGTTAGAGCGGGGCGGAGATGCCACTGGCTCAGGAGTAGGT
GCACGCGGTGTTGGCGCACGACGAGGAGGAGTAGGGAGTGGTCGAGTAGGGCGTGGTTGGAGAATTTGAATAT
CCTCCGGCGGGAATTGCCTGAGTATGCGATGCTCAGGCTGCGCCATTTGTTGGACCTCAAGAGATTTGAGAGT
CTCGGTGGTGACAGGGGCACCAATACGCTGTGATAGTGTTAACGCTTTTTCAAACGTTGGATAAGTACCAGGC
AGGATGCCCGGTGTAGCTCCCATGAAAACCTGGGCAGGTTTGTCGTGTAGCCTCACAAGGAGGGAGCGTTCCT
TGGGGCCGAGAGGTTTGGATTTACGAACAAATCGAGGTTCCTCAATGACCTCTTCGATGAAAGCAGATGAAAC
TAAATGAGAATTCCCATATTGGGAAGTGGAGTCGGGTGCGGATCCATCAGATGAAGCGGCTTCATCAGCCTGC
TTGGCTTGGCATTTCTTGCGACGCTCGGCGCGGTTGCCACCCTTAGAAGAGCGGGCTTTGTCGTCCTTGGGCT
TATCCTTCAACGAGCTTGCCTCAGTAGGCTTGCTCTCGGGTGCCTTATCCTTCGCTTGTTCCTTCCAGTTGGG
GTTCTTGCCCTTCCGTTTGACACCAGAGATTTTGTTGGCCTGAACAGGCCCTTTGATGGCAGTGCAACGCTGA
TACTCTTTTACAAGAGCAATGCGCACAATTTCGAATGTGAGGGCAGATTGGGAGTGAGTCTGTAGGAGTTGCG
AACCCACATTATCCCATTTGGAAGGGATGGCTGAAAGGAGAATCATCGCCTGTACGAACGGCGGAAGGACAAC
TGAGTTTCCCGTAAGATGGGAGAAGTATGTGGACATAGCCATTATTTCAGGCTCCGGATTGGAATCCCCGCTG
ATTTTGACTGCAAGAGCAGAGCGGAAATCTAAGAATACTTGAGAGGGGTCAGCAACTCCGTATTTGGTATTGA
GGGCTGCCCAGAGAGTACGAGCCGTGGTTAGGGCTCTCCACCGTCACCGTATATATACGGTTTACGGCTGCCG
TAATACGGGCACCTGGGGACTTGGTTGACGGGACGGTATACGGGCCACCGGATACCGTCACAAGCTGTATGTG
GACGGCCGTCATACGGCCGCCTTCGTTTACCCTCGCCCCTGATGGGTTTTGTGGCTCAATGTATGGTGATAAT
GTGTCAATCGCATTCCGGTTTGCCCCAGGGGTCGTCCGAATCGTGGAATTAGGTGTATATGATTGAAACTGAT
CCAAAATCAGCAGTCGATGAGTTACATAATAGCCGCGGAATCACGACCGGCTCTACCAAAGGTGAAGTTGCAT
GCTTCTCATACTAAGGCTCAGCATGAGTATGAGTATCCTGCAAGTACTTGCGAGTTGACCCGTGGGAATACTC
GCGAGTTCAAGTACTTGTTTTGCCAAAAACTCGCAGGTCCAAATACTCGCGGATCCGAGTCGAGCATCCTGAC
CTTGCAAGTACTTGTGGCAAGTACTTGCTGACTCGTTTAGACCCTTACAGGGTCTTTCCCACGACACATATAC
ACTCAAATTTCATTTTAAACCAACAAAATCCCTACAAGTACTTGTAAAATACCCATAAAATACTTGTTTCTTG
CTGACAAATGACCCGTGACCCACGAGTACCTGTGAGTAGTGACAAGTATTTGTGACTCGTCACAAGTACCCAT
ATGACTCGCAAGTACTTGTAAAAACTACTTGCGAGTTCAAGTACTTGTGGACTGAACTACTCACGGATCCGAG
TCGGGCCCTGGACCAAACAACTACTCGCGAGTGCGGATCCGGATCTACCCACGGATCGATCCGTGCTATGCCT
TATCTCATACTGCCACAGGCTACAACCATGGCCCAGGGCCCCCCTTTGTCCATGGTTGCTCGCAATTTTGCCT
TTCTGCGCGAAAACGATTGTGCGCCACCAGTTACGGCCGTAAAACCGTTGACGGCCGTAAAACCCACCGTACC
GTACCGTAAACCACCGAAAATTTTACCAGTACGGTACGGGGTACGGATAGTCCGTAGCCGTATCTGAACCGTG
ACTATACGGCCATATACCGTTGACGGTACGGTGCCGAGCCCTAGCCGTGGTGTACATTGTATGGGCGGCGGCA
ATGTCAGGTGAGAGTGTGAGGAGAATGGCTCCAAGAGCCTGATCATCTTTGTTGAAAAATTTGGTGAGGGCAT
CCTCAGAAGGTGGGACGATGTCGGTACCATCCATAGTGCTGGTGGGCTCGATTTCGCGGCCATCAGCAGCGGT
GATCTGCCAGAGGCTACTGAATTGGAGAAAGGTGCGAACTTTCTCTTCCCAGTCACGGTAGTTGGTTCCATCG
AGTTTCTTGATGGAGTTGATGTTGAGTTTACCGCCTGAAGACATTGTGATATCAAGGTAAATTGTTTGAGGTT
GTCTAGTAAAGGGGGCGACATTAGCCTCATGTTACAATGAAGAGAAATGTATGCCTTTGCAGGCACAGTAACG
GTTAACCCAACAACATAACCTTTTCAGGTAGTTGTGGTGAAGTTGCATGACGCAAGCATGTAAAACACGCATT
GTGCTCTGCGAGCTTCTAGGAGATGCGCATGAAGCAAGCATATATAACATGCATTGCATCGAGCAGAGCTCCT
CCAGACAGTGAGCTGCAGCCTTATTCAGGCACGGCAAGTCGTCTGGTACACCCAGTGGAAGCGCATTTCACAC
AGCTTTTTCAAGCGCGTGTAATGGCGACCAAGACAGCCAGCTGGTCGGTTGCAGCCTTGTTCAGGCGCGGTTG

ACCAGGGCGTCAGGGCCCATAACCTGTTGAGCTGCACCTACAGTGCACAGCGGAACTGGCTAACTTACTTGGT
AGACTGATGAGCTGAGAACACGGGTGTAGAAAGCAAAAGAACTTATATTAGTCTCTTGTTCAGAGAGGTCTTA
TATACTAGTAAATGGTGCACAGACCATGTGGCTGTGCTAAAGTCCTAACTCCTATCTATGATCTAATCTACTG
ATAAGATAAGATAAGGTGTTATCTAGGGTGCTTATATAGACACCGGATTATGTAAGCAGGGCACTTACAGTTA
GAGCATTGAAGTTTAACA
>Cligib1_rnd-1_family-124#TIR/MITE
CATCAGCAAAACACAACAACGCTATGGACTACTCTATAGTAGTCCGTAGTATATGTCTATTTGTTTATAGTGN
ACCATAGTGGCCATAGTGGCCGTAGTGATGNTTTGTTTTGCTGATG
>Cligib1_Cluster122#ClassI/LARD
AGATATGTAGGTTGTATTATTGTTGTTGTATGAGGAACAATCCTCAAATGTAAAAGACTGGACATGCTTATGT
AATCAGTAAAGCGGAATTATTTTAAGTTAATATCCTGAGTGAGATTGTTATCTCAACACTTCCCCTCAAGCGA
AACGAGGGATATGAGCTTGTAAATAGGTAGATATACCTCATATAATAAAAGAATATACTGTTTGATTGTCTTG
AAGTGGTCAAAACGAGTGTTGATTGTTTTTGATGTCAAAATGTCCTGATTTGGATGTTGGTATGCTGACTGAT
CGTTGGTTGACTCCTCTTGGGCGCTTCCTCGAGGTTCAACATCTATGGCTGGCACGAGAAGGTGAGTACCCTC
TTCTAAAATGCCTTCTGACTTCCCCTTTCCATCAGGTATTCCTTTGCAGTCTCGGAGATTGATTCCTTGGAGG
GCTGGTGCCACTCCCCCTGGTTCAGGTGAGATATCGCTTGCTGACACTCTGTAGGATCCTGGAGGCCTCTTGC
CACGAAAAGCGCAATGACTGGGCGATTCCGGGTCGCTTCTGGGGTCGCTTTGATAGTCCCTTGAGCTGGGGGA
ATCTGGTTGAGATCTGGCAGGGTTGATGTTTGGGCTGGATGAAGGTGCGTCCGTTGCACAGTCGTTCCTCGGC
GGTTTTGGCTGATTCCTGGACCGTTTGATAGCGGTTTCAATTCGCGTCTATTCGCGCGCGGCGATTCAGTCAG
ATGTTTTTCGGTGGTTGGCCTAAGTGGGTTGGATGAAGATCTATCCGTTGCGCGATCAGTTTCCAGCAGTTTC
GGTCGATTGTTGGACCGTTCGGGAGCGGTTCCCAAGGCCTTCAAGACGTGCCTATTAGCGCGCGGTAATTCGA
CCGGTTTATTTTCGATGCTTTGGCTAGGGCTGGAGATGTGGGGCGTGTTCTTCCCCTTGATCTGGACGGTTCG
TGGTATTGGGAGGCTATTCCTACCATGGTAAGTGTTCCCTTCGACCGCTCCTGATCGCTTTTCTGTCCCACAG
ATCCTGTAGCTCGTGGAATCTTCATCAATATCGATTTTACAGCCTTTATCCACTTCTGGTTCGGTTTTTCGGG
GTATAGGATGTTCATCTTGTATTGTATATGTTCTTTCTTCAATATAATTTGGTTTGTCTTCGTTTTCTGGTGT
GATCTCGGGTGGATTCGCATTTTTCGTCGACATCTCATGCACGATTGGGATTTCAAGATGGTTTTCGGGTGTT
TTATGTTTGGAATTGATTTGGTATGTATTTCGACTCTCGGGGGTTCGATCGACAGTGTGCGGTGGGGGTTCAA
GAGTGGATAGGGCAGGGGTTTCTTCACAGGGGTATGAGATATCATGGGGTATATCCGACTCTTCGTCGGTGCC
CTTCGTTTCCTCTTCCTGTGGTTCCTGACCTTCTTTTTCTCCTCCTCTCCTATAGCATCCTTCGTCGCGAACA
TGCCCGGCGCTCATGCGCGGTTCACTATTCTTTTCTTTCATATTTTCCTCAGGCTCTTCCCCCCGCTTTTCCA
GTATTACCATCGCATTTTCGGCGCGTTTTTCGGCATTTCGAAGGGATCACAATATGGCCATGAAAGATTTGTC
TTCATCATTCTCGAAAAGGCTAATGAGCTTTGTCCAGAGCTCCGCGGAGGTCTTCTCTTTGTATATCCGAAGG
TGGGTGTATTTGCTGATTGTTGCATAGATAACCCCTCGGACTATGGCCTCCCTTTGCTCGAAAAGGTCTATTT
TCGTTTCATGCTCTTTGATTTCGTTTTTGCTGAGCGGTTTCATGAGGTTTTCTTCTAGGAACCTCTTTCCATC
CACCTCGCGAAGCTCCTCTGGCTTTGGCGTGGTTCCGTACAGGTGTCGTTCGAGTCCTTCGTGAGTCAAAAGG
TTGATTACCCTTTTCTTGTAGATGACCCAATTTGAACCAGTATCCCCGAGGTTCGGAATTTCGAGTGACGACA
TTGTAGGTTGCCCAGGGCCCATAACCTGTTGGCAGTGGAGGAAGCAGATGGGGAAGGCTCAGCATGGGTAGAT
CCGTGGGTGATCCGGATCCGTACTCATGGGTATTTGGTCACACAAGGTCACTACCCAGATCTGCAGGTAGCTT
AGTCAGCAAGTACTTGTACTCATGGGTATTTTTTGGCAAGTACTTACTGAGTTGTAAGTACTTGCCACAGATC
CTGGTTACTCATTGATTACTCATGATTACTCATGGGTCACGGGTATTTGTCAGCAATTAGTCATGAGATATAG
GTATTTTACAGGTACTTTACAAGGATTTTGTTGGTTTAGAATGGAATTTGAGCCTGTATGTACTGTGGGAACT
ATACTTTAAGGGTCCTTAAGGGTCCGGTGTGAGTCACAAGTACCTGTCACAGGTACCTGCAATGTCCAAATGC
TTGACTCGGATCCGCAAGTAAATAGATCCATGAGTACAAGTACCTGTTTAGGCCAATACTTGCAAGTATTCCC
ATGGGTCTACCCACAGGTACCTGCGGGTAACCCGTGCTCGTGCTGAGCCTTATTATTAGATATGTAGGTTGTA
TTATTGTTGTTGTATGAGGAACAATCCTCAAATGTAAAAGACTGGACATGCTTATGTAATCAGTAAAGCGGAA
TTATTCTAAGTTAATATCCTGAGTGAGATTG
>Cligib1_Cluster117#LTR/Copia
GTTGAAACCTGATCTATAGATTAGATAGACAATCCTCAGTGACGGTATAATCAACTATATTTGTTTGTATGTA
CCTATAGATTAGATAGACAATCTAATCGGTAATTAGTATCTGATGTAAGTATTTATGAGTAAAGATGCTCACC
CTCAGTGACGGTATAATCAACTGGGGTGAGCGTACGTATGCAGGCTATATATAAGGTATTCTAGATGAAAGGG
AGAAAACATGAAAGAGATAATATGAAGGGGAATTGTACGTATTAAGGGGGGGGGACGCAGAACGCGTGTTGGA
CGCCGAGTGCATGTTGAGCGCTTACACATGATTCCCAACACTCCTCCTCACGAGGAAGTGCTCCTCCTGATAT
GGAGCGAGGATTATGGTTTGAGTCCGAGGAAGCTTCGGAGACGAGTGAATTTTTTGTTATCAAGGGCTTTGGT
AAATAAGTCGGCGATGTTGTCGTTGGTTGGAATATATGTTAGGTGGATTTCTTTAGATTGGACTTGTTCGCGT
AAGAAGTGGAATTTGATGTCGATATGTTTGACTCGGGCATGTAGTAGAGGATCTTCGGATAGGTTGATGGATG
AGTTATTGTCGCATAGTATATTTGTGGGTCCCGTTTCTGAGTGTCCGATTGCATGGAGTAAGGATCGTAGCCA
GATTGCTTCTTTCGCGGCTTCACAAGCTGCAACATATTCGGCTTCGCAAGATGATGTTGCTACTGTTTTTTGT
TTTCGCGTGCTCCATGATAGCATGCCTGAGCCTAGAGACCAAGCGTATCCCCCGACACTTCGACGCGTATCGA

GACAGTTAGCCCAGTCTGAGTCGGTATATCCGATCAGTTGGATAGGGTTGTGTCCACCTAGCGTGAGTTTGAG
TTCACGTGTTCCTTTAAGATAGTGAACAAGACATATTGCGGTGTTCCAATGAGTAGTGGAATAAGAGTCGAGG
AATTGGGATAGTTGTTGAACGGCGTAGGTGATATCAGGGCGTGTTGATATGGCGAGGTAAAGGAGGCATCCTA
CGAGTGACCTGTATGGTAGTTTAGCTAGGGTGTCGCGGTCCTGGGCCGTGAGGGATGCGGGATCGACGCATCG
GAGTTTTAGTCCAGGTTCCATAGGTAGCGAGAGTGGTTGGGCATCCCGTTGTCCAAACTGTTGGATAATTTTG
TCAATGAGAGCTGTTTGTGATAATTTAACCGTTTTGTTAGGGCGGTCCCATTCAACGGCGATCCCCACAACAA
ATCGAACGGTCCCCAGGTCTGAGATGGCCCATAGCGAATGCATTTGGTCTTTGAATCTTTCATTTTCTTCTTT
TGAGCTGGATATGGTGAGGAAATCATCGACGTGTACGGCCGCTATGGTGGTCCCGGTTTCTGTCTTGCGGAAA
TAGACACAGGACTCGCATGCAAGACAGGTGAAGCCCCATGAGATCATGCTATCGTTTAGCGTTTGGTTCCAGA
TTCGGCCGCTTTGTTTCATTCCATATAAGCCCCTTTTAAGACACCATACCCACCCCTCTTTTCCGGCTTCCTC
AAATCCTCGAGGTTGTTCCATGTATTGTGTCTCATCGGCCGGGAGTAAGCCATAGAGGAAAGCGGTTTTGACG
TCAATTTGTTGCGCGTCCCATCCCAACGACGCAGCAAGGTGGAGTAGGATGCGCCATGATTCCATGCGGGCAG
TAGGTGACATTATTTTGGTGTAATCTTTTCCATAGATTTGTTCGAAGCCCTTGAAGACAAGCCGTACCTTGTG
ACGCATGGGTTTCCCGTGCTGATCGCGTTTAATTTTGAATACTGGGCGGCCTTTCCTGACTTTTGATCCTTGG
GGGACATCTTCCCTGGCTACTAGTTCATATACACCCATGTCTTTTAGAGATTTGAGTTCCTCTCGGTAGGCCT
GCTCCCAGGCTTTTGCGTCGGCCGAGGCGAGAGCTTCTTCCCAAGTTCGTGGTTCGTCTTCTAATTCGAGGGA
TGCAGGGTCTATCCCGCCGGCTTCGACGATCGCGGCCAAAATCCGATCATGATCGTTATTGACGTCATTACCG
TTTGAGTTTAGGTCTTCAAAGGCCTGTTGTAATTCTTCTGCGGCTGTTGTGGGCTTGGGTTGTTGTTCTGCCA
TCGGCAGGTCACTTGCCTCAACCTCTGTTTGACGTTTCTCCAGTCGGGCTGTGCGTATTCGTTCTGCGCTCTC
TTTTGACTGTCGTATCGCTTCTTCAAGGCGTGCGTGTTACAACGGTTCCCACCCCTATCTTTTTCCTCTTTGT
TCTCTGACTACCTTTGTTTAACGATACTTACATTGTTCTATGATTCCTCATTGTTCTATCGCATGTCCAACAC
ATGCTACTATTCTTAGATACCTGACTCATCTATGACATCATAGCTGACTGAGCTTCAACAGCTTTATTACTTT
ACATTGTCTTTCTTTCTCATTGTACTATGTTCTCGCGCCTCAAGGTCCCTAGTATATAAACCCTAGATACACT
GTCATTGTTCTCCATCTTGACCTAGGCAATACTACATCCTTTACCTTTGAACCTTGATCACTGAACCATCGAT
ACCCAAGTCAAAGCCTGACTTGGAGTGACTATAGTCTTGTTGGTATTGACCCAAGACTCTGACTTGAACCTCT
GGACGATATTCAACGCTTGAACCTTGGTAAAGGCACTCCGCCTTAACAGTGCGGATTCGGATGTGGGGTTGCG
AGTCGATTTACGGGTTGCTGGTGGTTCTGGTGGATTTACGGGCTGTGGTTCGGGGATTGGTATCATGGGATCC
TCATCGTCATCATGTAAAATAATGGGATTTTGGGGGCTGATGAGGTTGTCGGCTTTGTTCAATGTTTCTGTAC
TGGTCTTTATCACGTCTTTAGGGAGTGGATGAGATACGGCATCAGGAAAACCTGAATCCGCCAAATAGTTCCA
ATTTATTGATATTCCGAGTAATCACATAAAATCCGGTAATTGGCTCACAAATACTGAGAAATATCCACTACTG
ATATTTTATGGCTATTTACAAGTTGAGTACTGCTAAAATATTTATATCACTAATATATTGGTGGGGTGAACTA
TTTACTAATATTCAAAGTCTCAGTATTTATTTCCTTGGAATGTCCAGGGATATTCAACACTATTTAGTGATAA
ATAATTCAACCAGAACTAAGTAATATTGGTGCACTTGGAGTCGAAAATCTTCCAAGTCATTTCAATCTCAGTT
GAGCCCGATGCCAAACAAGCGACATCGTTCCACTTTCTTCTTTTGACAACGCGTCCTCCTCCCTGCTCGTTCC
TCATCGTCGTCAATGCCTGTTTCCCAGCCGCATCACCTCCCGCCTCCCACCTCCCGCCTCCCACCTCCCGCCT
CCTGCAGTGCAATGTACCACATCTTAACGTGAGTCATTTCTACCTCGATGCTCACGCTTCTAGTTGCGCCACG
TCATCACCTCCAACTGTGCCACGACATTCTCCACCGCTTCAGGCTCATCATCGTACCTTCAACCGCGCTCTCC
CCCTTCATCTGCATCATTCGCCATTGTTCTACGCAGGCAAGTTGTTTCCTCGCTCTTCCATATTTGTGGGCTC
TAACACCTCCCTTGTCTTCATTGCCAACATGACATCCTCCATTGTTGTATCCGTCTAGCCCTCACCGCCTCCT
CGCACCCTCTGCCACTGTCATGTCTTCACCACATCATGCCCATGCCCATCGACTCCCTATCCCCGCAACCCCG
TTGTTCCACCTTCTGACATTGTCACCCCTGCCGCATTCTCACACCACACTGTACGTCTAGCTCACCACCCCCG
TCTAATGCTTTCTAACGCCCCCCACACTCTAGCCCCCCGGTCCCCCTGTTCAACTATTGCACACCCACCAACT
CCCCGGCCTTTTCGCGCTGTCCCCAAGCAACCATGACGTTGCACCCTTGTTTGGTCTCGCTCTCGCTACCCCC
TTGCGCACACCGCCGTACCTATGACATGTCACCCACAGGCGCCATTGTACCCCCGCCTAGCCCCATCAATTTG
CACCCTCCGCAACCGCTGGATGCCCGTCGGCTCTCCGGGGCCCACCACGCTCATCAACTGTCACACGCCCATT
ACAGCACTTCACGGCTGTGCTGTTGTGCCATCCCTCAGTCAAGCGCTACCATAGGTTTTTCCACTGTTATTTA
TGCCCTCTGCCACCATCACACCTTTGCAACTTCTATATGTAATAATGTAGCTGTTGAATACATGTATGATTAG
ACAATGAACCATAATATATGATATTTCTTGGATATTTTGACATAAACTGGCCTATAAGATGATATGAGAGCGA
TATTACAATAATATTGGGACACATTGGTGGATATACTAAGGATATAATGGTGCTAAAAACAGGTAAAATATTG
CTTGGGGGATAATAGCCAGATAAATAAATATTGAGACTTTGAATGCTGCTAAATAGCAGGTGTTGATATATAC
TGGTATTTACTGATACATATGGATATATGGCCAGATCGGGTTTTCCTGATGGGGGTGTCCATCGTGGCTTTCG
AGGAACCGAACGTGGTATGACCTGTAGACGTTTCCACTCTTTCTGTCGTAGCATCGATACGCTTTGGAGTTGG
CGTCGTATCGGCCTTTTCGAGCTCTAGGTTCTCGACGCCGACTTCAAATACACCGCTTCTTTTCGCCTGTTTT
CGTCTTCACCTTCCTTTCACCTTTTATCGCTTTTCTACCAATGGCGGGTTCGGATAATCCAGCGCCCGCTTCT
GGTTCTGGCACACGCACGCGAAATCAAACGGCGAATGGTGTCGCCGCTCCCATTATCCTTCCCTCTGATTCCT
CACATCCCTCAGTACCCATTCTAGTCATCAAGGATACCGCTTGGCTCGGCCATGCGCTCTTGAGCAAGAACGC
GGACAACTGGCACTCTTGGAGCACTTGTATGCGCAACCTTTTAACCCTTTCCAATGGCGGCCTTCATCGCTAC
CTGAACAACACAGCCCTCATCCCGGATCCTGTCACCGAACCTCGAGCGTCCTATAATTATGCTCTCAACGACG

AGTCCGTTCGCGCATTTATTCACACCAAATGTGATTTTTCGGAACATTCTTATATCGAGGAGTGTGCCACTGC
CCACGAGATGTGGTCCGTTCTCAAGACGCGCCACGAGCAGCAGGGTCCGATCTCCCAAATTACTTTGCTTCAG
GAAGCTTTTACCATTCGTTATTCTCACGCCGTGCCTTTCGCTGAGACGGCCGCGCGCATTTCGGTTATCAATC
GTCGCATTTGGGCCATGGGCGCTCCGACTGCTGATGGTTATGAATGTGTTCTCAATCTTATTGCCCTCAGCTC
GCCCGAAATGAGGAACGTCCGCGACGCCGTTACCAATGGAATAGCCGCCGCCAGCAAAGATAAACCCTATGCC
GTTTCCCAGATCCACGCTCGCCTTGACATGGAGCAGAAAGTCTTGACTTCGGAGGGGTCTAAGCCCCTAGAGG
CACACGCCGCTCGCAACTCCACGGCTCCTGCGCGGAAGACCGTTACCTGTAGCAACTGCAAACGTCCTCACCA
CGACGCGGAGTTCTGCATCAAGCCCGGTGGTGGGATGGCCGGTAGAACGGTACAGGATGCTCAGGCCGCATCT
CGCGCTAAGAAGGGTTTACCCCCCAAGGCTTCCACCTCCGCCACGCCGGCGACTTCTTCCTCGGATGTTCTGC
GTGACGCTAAGGGCCGTGCTTTTGCTGTTGATGCCAGCACGGGCCGCGCTTATTTCCTCGATGCTGATCCTGC
CGCTCCTGCTGCCCCCGCCCCGGATGTTGCCCATGTCGCTTCTCTAGCAGCTGATCCGGTCGATACCCTTGTG
TCTCCCCTGGTCGCGGATTCTATGACCCCAGCCGACCTCATGGAGTACGCTTGGTTAGCGGCTCATGATTCTC
TTTCGGCGTCGGTTGACTGGGCGGTCAACTCTCGCTCTGCGGACTTTGGAGGTTGGACAGGAGCCAACATTGC
CGCTGCCGCTGGCCGTTCTCCCATTTCCTCGCTTGATGTTCCTTTCTTGCTGGACAGCGCCTGTAGCACGCAT
ATATCGCCCGATCGAGCTGACTTCTTTTCACTGCACCCCGTTTCGGGTCGCTTGGTCCATGGCGTCGGTGGCT
CGTCCATTCGTGCGGTCGGTATTGGTTCCATCAAGCTTTCTCTTGGGAAAGGCCGCTCTCTTAGGCTCGACAA
TGTTTTATATATCCCCGATTCTACCGTGCGCCTGATCAGCGTCGCATGCCTCACCGATTCTCTCCAATGCCGC
GTTGTTTTTGATTCTAGTTCTGCCTCGCTTCTTGCCCGCTCTGGCGCCCTCATTGCCTCCGGTAAACGCCTCC
CTGCTCGCAACCTCTATCACCTCTCCACCTCGGGAGCCTCTGTTGAACATGCTCTCTTCACCGCCCCGCTCCC
GACGCTCGACACCTGGCATCTTCGCCTTGGCCATGCGAACAACCAAGCTATCTACGACATGGCCGTCAAGGAT
CTTGCAACAGGTATGCCCGTTGATCTTTCCACATCCCCTCCCAAATGTGAACATTGTATTCTCGGGAAGCAGG
TTTCATCGCCTGTTCCAAAGATACGTTCGGGGGAGCGGGCTACGGAAAAGCTGGGCATAGTTTATGTTGACTT
GACGGGCCCTCAGGATGTCGTTTCTGCCTCTGGTTTTCGCTACATAATGCATGTCATAGATGATTACTCTGGT
TTCTCATGGTCTCTACTACTCAAATCGAAAGACGAAGCTCTCCCCGTTTTCCAGTCCTGGGCGACCCGTGTGG
AGTTAGAGTCTGGTTGCAAGCTCAAGATCATCCGGTCAGATCGTGGTGAGTTGCGCTCGAACGCAATGGATGC
TTGGTGCTCCCTCCGTGGTGTTACCCATCAGTTTACGGCCCCCTACACGTCGGCCCACATTGGGCGGGTGGAA
CACTTACATTTGACCATAATGAACAAGGCGCGGGCGATTCGACTGCAAACCGGGTTGCCTCCGAACCGGTGGG
ATGAGAGCGTCCTGACTGCGAGTTATCTTACGGCCCGAACCCCCGCAACTGCCTCGGGTGGCCGGACACCTTT
CGAACGCTGGTATGGCTCCCGGCCTGATCTTTCTCACCTTCGTGAGCTCGGGTCTCGAGCTTTCGTTCTGGTG
CAGAACCGCCACGATCCGAAGATCTACGCTCGCTCTTTTGAATGTGTGCTTATCGGGTACTCCCCTAACTCGA
AAGCTTACCGGTTGTGGCATCCGACGTCCCATAAGGTAGTTGAATCTTTTCATGTGAAGTTTATTGAGAGAAT
AGACGCTGCGCCTCGCCCCCTTCACCCAGGAAGGGTTCTCGACTCCCCTTCTCCGTCCACTATACCTTTACCT
ACCCCCCTTTCCACTTCTTCTACCACTCCCTCTTTACCTACTTCCACTCCCCCGTCTGTACCACCCCCTCTTT
CCTTATCTACCCCCTCCTCTGATACCTTCGAGCCGTCAAATATACCGCCAATAATACCGTCACCCTTACTTAC
TTTACCTACCGAACCGTTACCTACTGAAACAACGTCGCAATCGTCTCCATCAATACTGTCAAATTCATCTCCT
TTACCTACTTTACCTATTTCACCTACCTTACCTACTTTATCTTCGACACCCCCTTCACCCTCTCCCCCTCTGC
GGCGTGTTTGGATCGAGGAGGTTCCGGATGTTGATGCTCCCGGTCCTTCAGATGCCCGCCCTGCCCAGGATCC
CCCTCTGCGCCGTTCTGCGCGCGTCTCGCCTCCCTCTGAGAAGAAAGCCGCCCTCGACGGCCTACGGTTTGTT
CCTGCGGTTTCCCGTGCTCGTGAGGCTTCCCGCGCGAGCGCTGCTCGGCTCAAGCGTGAACGCAAGCTGGAAA
AGGACAATCGACGTCGCTCTGTTCTTGAGATGCGTGCCGCCCTGGCCAGCGAGTCGGATTCCCGGGTCTTCAC
TCACACTTCCGCCGTTGCCATCCTTGCCGACTCCATTCCCTATCTGTCTCAGGAGGAGCTTGCCATTTTTGAA
GATGCCTTTGCTGCCGTCATCGATCCTGTCTTGGCCACCAATGACGCCGAGGATCCGAAATCTCTCAAGGAGG
CGCTTGATTCGGCCCACAGCGATGCCTGGCGCGCCAGCATCCAGGAAGAGCTCACCAGTCTTCGCGACATGGA
CGTCTATAAACTGGTCCCGCGCAGCTCCGTCCCGTCTAATCGCAAGGTCCTGAAGGGCAAATGGGTCTTCAAG
CTGAAGCGTGACGAGCACGGCACCCCTGCCCGATACAAGTCCCGCCTTGTCGTCAAAGGATATGAGCAGGTGT
TCGGCCAGGATTATGTACATACGACATCGCCGACGGCACGTATGGAGTCCCTGCGCTTACTGTTGCACATTGC
CGCGTCAAAGGATTGGGAGATTACCCAAGTTGATGTTAAAACTGCATTCCTATACGGGTGTCTATCCGCCGAC
GAGGTACAGTTTATGGAGCAGCCAGAGGGTTTTGCGGAGGAGGGTAAGGAGGACTGGGTCTGGCAGTTGCAGC
GGGGATTGTACGGGATGAAGCAGAGCGGGCGGATATGGAATAAGACGATGACAAAGGCGATGGTCTCCTGGGG
GTTCAAACGACTGGACAGTGACCCGTGCGTTTTCCATAGGACGACCGCCTCTGGGAGTATCCTTGCAGCCGTT
CACGTCGACGATTTCCTCATAGCTTCGTCCTCGACCGCTGAATCGTCTCGCTTCAAGGATCAGCTCCGCACGC
TCTGGGCCATCACAGACTTGGGCGAGGCTAAGTTCTGCGTTGGAATTGGCATTACACGGGATCGCATCGCCCG
GACAGTCTCCCTATCCCAGACCGCCCTGATTGACAGGGTTGTCGATCAGTTTGGCCAGACGAACGCAGATCCA
ATTTCCACCCCAATGGATGACGGTGTCAAGCTTCAGCGTCCAGATCCCAATTCCACCTTGTCTCGTGCTACCG
CTGACGAACTGAAGAAGCTACCCTATCGCTCGCTTGTGGGGTGCTTGATGTACATCGCTGTCGGAACCCGTCC
AGACATCGCGTTTTCTGTGTCCAAGCTCGCCCAGTTCCTCGACTGCTATCGCCGCGAGCACTGGTCGGCCGCT
CTTCGTGTAGTCCGCTACCTCAAGGGCACACGACTCCTCAGCCTCACTCTTGGGGGTGCTGAAAACATGTCGC
TGTTTGGCTTTTCAGACTCATCATATGCTGACTGCCTAGACTCACGTCGCTCAAGCATGGGCTATTGCTTCTC

CATGGGTAGCGGCCTGTTTTCATGGTCGTCCCGCCGTCAGCAAACGGTTGCCACTTCATCCACGAATGCAGAG
TATGTTGCCGTCAGTGAATCATGCCGCGAGGCTCTGTGGCTTCGCCAACTCCTTCGCGAAATATGTCAGCTCA
ACAATGGACCAACTCCAATTCTCATCGACAATGATGGTGCCCGCGCGCTCGCAAACGACCCAACGAGCCACTC
TCGCAGCAAGCATATTGATGTCCGTTACCACTTCATTCGTGAACGCATTGACGACGGCAACGTGGCGCTTTAT
CGAGTCCCTTCCGCCGATAATGTTGCTGACGGTCTTACCAAACCATTACCCCGACCCACATTCCTACGCATCC
GCTCTCTCATGGGCGTGCAATGACCCATCCGTGCGAGGAGGAGTTTGCCTATTCTTTTATTTTCATTTTCCCG
TTTTTGTTACACTCACTTACTTTATCATTCACATATCACGACACCTCTTTTGCGACACCCGTCCCTGCGACAC
CCCAGCTGGCCGTGTTTACACCCTCATCTCCGTTATCCCACTTCCACTTCGATATCCCTCTTCGCGGTGAGGA
GGAGTGTCGAATGCACCGCTTGAAGTAAACACGGCACAGCCAAACATTACGTAACATTGCGTCACACTATATA
TATCAACCTCATCGGGAAGACGTTCTTGCCTCAGTCCTTATCTTGTATATGAGACTGAGGTGAGTTCGTCCCC
GCCCATACGCGTACAGTACATTATTGACCATATACCTAGCATCTTGTCCTAGGTATACTAATATAGTCCTTAT
CTTGTATATGAGACTGAGCATCTTGTCCTAGATTCTTTAACAGTATGACCTGTAGACGTTTCCACTCTTTCTG
TCGTAGCATCGATACGCTTTGGAGTTGGCGTCGTATCCGATTAGGACACATTCAATGGACCGCTCGTATATTT
TTGGGTTGTGACGTTTTTGAATTAATACGTAGGCCTTGCAACCAATTTCACGAATATAAGAATAGTCGGGGCG
TCGGCCATACCATAGTTCCCATGGCGTAACCTCACCATTTAGGGATTTCGTTGTGGTTTTGGCGTGAAGGTGG
GTTGCCATTAGATAAAACTCATCCCACATATTTGGGGGTAAACCCGCGTGTATTCGCATGGTCCGCGCTTTGC
CATGAGGGTACGGTGCATCCGCTCCACTCGTCCCATGTGGGCAGATGTATATGGTGTTGGGGTACCAGCTTAT
CTACAGTATTTGACTTAGAGTAATGTCCGCTTTATGATTACATAAGCGTGTCCAGTCTTTTACATTCGTAGAT
TGTCTCTTGTTTTACCACAGATCATATACTCATCTAATAATATCTATTTAATCCTCCCATCTGCTTCATCCAC
TGCCAATAGGTTATGGGCCCTGGGCAACCTACAATGTCGAATCTCGAAGTCCCGAATCTTGAAGACTCTGGTT
CGAATTGGGTCACCTATAAGGAAAGGGTAATACAGACTCTGACTCACCAAGGACTCGAACAACACCTGTACGG
AACTGCACCGAAGCCAGAGGAGCTTTGCGAGGTAGATGGGAGGATTAAATAGATATTATTAGATGAGTATATG
ATCTGTGGTAAAACAAGAGACAATCTACAAATGTAAAAGACTGGACGCGCTTATGTAATCATAAAGCGGACAT
TACTCTAAGTCAAATACTGTAGATAAGCTGGTATCTCAACATATGGTGCTCCGAATTGATGGTCAGTTCCACG
TGATTTGAGCCAAGCCTCCATTTCGTGACTTTTGAGTTCACCGTCGTGTCCCGTTCTGTAGATGCCCACTTTG
TGTCCGGTTTCGTTCTCGCATGTAGCCTGCCATGCTTGTAATTCACGGAACGCATCATCCTTTTTCTTAAGTG
GAATAGACCACGGCATGTTAGAGAAGTCGTCCACGATATTCATGATGTACAAATTTCCTTGTCTAGATGGTAC
AGGGCTGGGTCCAGCAAGATCTACCCATACGATCTCTAATTTCCTAGTAGCCCGTCTCCCCTCTTTGCGCATC
TTTGGAACCGGTTTCCTGGTCTGTTTTCCAAGGATACATGATTCACATTTGGACGGCTTTAGAGGGAAGGATT
TGGGCATACCTGGAATGAGACCGTTGCGCGCCATGTCGGTGATCGCTTGGTAATTGGCGTGGCCAAGGTGACG
GTGCCAGGTATCGAGATCGGGGGTATGTTGGACGTGATTGGCGTGTTCAACTTGTGGGGAATGTAAGGATAAG
GAGTATAACTTCCTGGAGTCATCTAAGGTGCCGTTCGCGATTAGGGCTCCAGTGGACTTGTCGGTTAGCCAAC
AGGTTTGATCATCGAAATGGGATGTTATGCCGGAGTTGACGGCGAGCGAGCTGACGGAAATGAGGCGGACTGT
TGCAGTTGGTATGTAAAGTGCATCGCGGAGCGTTATATATTTCCCCTTTGCTATATGAATTTTAATATCGCCC
ATTCCGAGAGCGGTAATGGATGATCCCCCAACCCCTCGTACCGTTCGAGGTGGGATGGGCCTGAGACTAATAA
AATCTGCCATTTCAGGTGATATGTGCACTGATGCGCCGGTATCTGCTAAGAATGGACATTTATCCAATGAGAT
TACCGGGGTGCGACAAGACTGTTGTAACGGTGCAATGGTGTGAAAGGCATCCTCATCGATGGAGCGGGTGTTG
ATGGTCCAGTCAACATTCGTGTTGAGGTCATTCATGGCCAACCAGCCCTCGTATTCAATGACGTCCGCAGTTG
TTTGGAGCGCAGATGGTAAGTCGTTGGTGGCTAGTCCGGCGAAGGTGGGTGGTGTGTCTGAGGGTAGCGTGGA
TAAATCATTCGAATTAACGTCCAGGAGATAGGCCCGGCCGTTATTGTCTTTGACGGTGACTTGGACTCGTTTC
ACAGGCGTGGGGTTATTGTAGCTCCCCCCGCCCTTCTTGGTCTCCCGATCCTTTCTTCTGGCCGCCCTTGATT
CATCTATGGTCTTCCCCGCCATTCCTCCTCCTTCACTAATGCAATATTGAGCTTGGTGATGAGGTTTCTTGCA
ATTACTGCAGACAATGGGGTCGCGCGCTTTGTTCGTGGATGCTGTGAAGGCGGTGGTGGTATCGGTAGAGGCT
CTTCCTTTTCCTTCAATAAGGGTTTGTTCATTTTCGAGGAATTTATAAATGTTGGTAGACGTGTATGGTTTGT
CTTTTGTGGAGCCAGAGATGTCGCGGGATACAATAGACCGCACATTGTCGTATAAACTGTCATGATTAAGCAC
ATTCAGGAGAGCGATACATTTCAGTAGATCTGCATTGATTTCCCCGGCGGCGAATGCCCGATCGATGGCGGTA
CAAATGTCGTTGGCGGTTTTAGGAAGAGGGGTAGATCTCTCGAATTTGGTCGACAGTGCGGCCTGTAGTAATT
GGACTTGCTTAACAGGACCTTCGTCTGTGTGGCGTTTTGATAGTGCCGTCCAGCATGCATACGCGCCTTTGGA
AGTATCGATATGTTTTTGTTCACTTTTGGCCAGTGCAGATTTAAGGAATGCCGCGGCCAGTCGGTCGTTCGCC
TTCCAATTGGCAAGCGCGGCAAGTGCCTTTGAGTCCGAAGGTGGTTTTGGCTCCTCAAGGACGTATTCATAAA
GACTGTTAAGGGTGAGGGATACTTCCATCTCCTCGACCCAGTCAGTATAGTTTCCGACGAGTTTGTCGAGTTT
GTTGCCAGTAAATTGGCTCGTAATTCCTCGGATTCGCGTGACGGATGGGATAGTTTCAGTAGTCATGGTTGGG
TGTGGTTTGTGGTAGGGAAGCTTCTAAGATTTCTAGTAGTTCCTCAGATAGTGAGGAACAAAGGGAATATGAT
TGGCATCGCATTGTCCAAAAACGGTAATCGCGGTGCGGAATTTCCAGATCAGGTGTTCAATTCGTCAATAATA
TGACGCAATGGCCCATAACCTGTTGAAACCTGATCTATAGATTAGATAGACAATCCTCAGTGACGGTATAATC
AACTATATTTGTTTGTATGTACCTATAGATTAGATAGACAATCTAATCGGTAATTAGTATCTGATGTAAGTAT
TTATGAGTAAAGATGCTCACCCTCAGTGACGGTATAATCAACTGGGGTGAGCGTACGTATGCAGGCTGTATAT

AAGGTATTCTAGATGAAAGGGAGAAAACATGAAAGAGATAATATGAAGGGGAATTGTACGTATTAAGGGGGGG
GACGCAGAACACGTGTTGGACGCCGAGTGCATGTTGAGCGCTTACACGTGATTCCCAACA
>Cligib1_Cluster119#LTR/Copia
GGAGATGGTGGGTGGGTAAGGCGAAAAAAAGCGAGAGGCCGCTAGGGGGGTGTTGAGTCAAATGAGAACCGCC
CAAAAGTGCGATGTGGGCTTACCTAGGGGGGTGTTGAGTCAAATGAGAACCGCCAAAACGGAAATGGTGGTTT
CATCAAGACTTATCAATGACCCCCGCTAGGAGGATGTCTCAGGGCAGGCTGAGACATCGGTGACCTCATTAGC
TGGCTCGGAACGGGGGCCGAGAGCGATGATGAGGGGCGCCTGTGAGAGGCACGAGATAAAAAATCAACACACC
GCGGGGCGGCATATAGTGTTGACAAGAGTGAGTGGCATTACCTTGGAGGGTCATAACACACTGTCATTGAACT
CGCGTAAACAAAATTTTAAGCCCGACCAATGACCAGTGTTAGACGCAGTTCGAGGAAATGAGGACCCAAGGTT
AGCCGAGGAAAGGGAGCGCGCAGCAGCGGGCGCGTCCCAAAAATCGAATTTTACAAATGAGGTGTTACAGAGG
TGGAGCTGAGACGGAAATGGGTGTTGAGGTACCGCTAAGACCTAGAAGATTTTTTTTTTTTTTTTTTTTTTG
ATGAGATGAGTGTGGCGAAACGATGGGAGGAGCGACAACCATGGACCATTATTGAAGAATAAGCTGCCGTTTT
TTGCGCACGATTTCGTCGATGAGGAGAGGATGCTTTCGGATATAAATTTCAAAAGCTGAGGATGACCAACGCC
CGCTGGCTTTGATCAATTGTTCTGAGACACCTAGCTCGGCGAGGTAGGTAGCGCCTCCGGCGCGCATCGATTG
ACCGCCTATGTTTGTGGAGAATAAGAGGTGTAATCGTTGCATAAAGAAACTCCGGGTAGGTATAAATCCCGAC
GAAGTTATCCATACATCAGGAAAACTACATATTTACTTATGGCCTGAAATGGTCTGAAATGGTCTGAAATGGT
CTGATCACCTGGATTTGAGCTGTGGCACTGTGTAAGGCAGATCTGCTGGATTGCCATAAGTCAAAAAAGCCAG
CAGTTCAGATAAGTGTTTTCAGCTCTCTCATAAGTTCCCATTGCAGATCATAAGAAATTGAAAATGTTGTCAG
AAAAAGGGTATAATATATAAGTTAGGTAGGAATCAGTAAGTTTAGTCAAGGCAACCAGAAAATTTTAAGTTCA
CCAGGAGAATAAGTGATGTAAGTGATCCAGCCCATCCAGAGCAAACCCCCTTGTGAAGTCAGACAGTATCTTA
ATCAGGCATGTACCTCTCTTATACAGAAAGTCATGTTCTGGTGTCAGCCATTAAGTTGACCACCTGGTATTCA
GAATAAATGACTTGTGGAAGTTATATTTCAGAATATTAGTTTATTATTTCAGAACATTTGTTTCTTATTCCAG
ACATTCATTTCTTATATCAGACACTGTTCTCTTATTTCAGTCACATCATCTAATACTGGTAGCCTTAAATGTA
ATTAAGAATCTACTGATAGACGAGAGCAGCTATACCACCTCCTCTGGCCCCCAGCACCAACGCCGCCCAACGT
GTAAGCAAACGCGCCCACTTGTATGAATATGAATGCCCCGCCCTTCCACCCCTCGTGTGCGGGCGCACCGTCC
CCTCACGGTGATGTACAGTATGCACCCGTCTACGCAAATACGATGCGCCACTTCTCCACGTACAGGTGCATCG
TCCCCCCGCGCGGATGTACTTTTCGCGTGCGGGTGCGCCATCGTTCCGTGTGCGGTCCACAGATGTACCAGCT
CGTCGCATACAGGCGAAGCGTGGCACACCGCCGTGGATCTTCGAGGGGTATACTAATAGTGGGGAGGGGCGCA
CCGGCGTTTTCAGGACACAGATGGACAGGGCGAGGATGGAGACAGGTTCAGCAATGCTGGACAGACGTTCGCG
AGTTCTAGTTGGATTGAGAGGTATCATTGGATGTCAATGAAAGTGTTGGGAGACGGACCTTCATAGCCGCTTG
CAATTTGCGGCGTAAGGGCGCGCGATACGTTGCTGAGGCCTTCGGGGTAGCTTCATGCGGGGTGAGTGATGGA
GGTTTGAGGAGCGGTTCCTCAGTTCCACTCGGGGAGAGACAGCCGGAGGTGCGAGCGTGAGTGGGTCCCAAGA
AAGAGCTTTTGCATAGCCGGCCCGACTCAAGACGCATGGTTGTGGATGCGGGGTGTGGCTCGTGGGGGTGAAG
GCGGGCCAGCTTAGAAGGCGCGAGCGGCAAAGAGGGGTGGACGTACCGTGCCCACCCTTACACACACCGAGAG
TCCCACTGCACACGAATTGCCAGCGCGAGCACCAAACGGAACGGGAGCCTGTCGCGCTTGCCTCTGCCTGCCC
CTCTCAGGGACCCATCCGCTGACCCGCATGACCACGCCTATTCCGCCAAACCATCAGCTGGCCATGCACAAAG
GCGGGCGGACAAACATACCAGACGCATCCACGAAGACCGTTGTACCACCACTGCTCATCACCCAGCTAATCTC
CCAACCGAAACTCCTATCGCCCGGGGGCCTGCGTTGGCGGGGCGCTACTCGCCGAGAAGGGGCACCACGGTAA
GCGCGTCGAGAGGCGATAACATTGACGGCCAGCGCCGGTTGGATGGGCAAAAACATTCCAAGACAGTTTGAAC
TCCGCTCTAAATAGTTGCCACGTCGTTGGTTGATTTTACAACTGACAGTCAACACATTGCCGCCGTCGTTGAT
ACCAGCGTCAACGCCTCAAACACTTCTATCCCTCGGGCCCTCACCCGTGCAACTGGTACGTATTTCCCCAATC
TCATTAGCATTCCCTAACTAATAGTAACACGCTGGTAGTCAACTCCCGCTGGATGATATGGCGGCTCACTTTG
CCTCGCCCCTTGCTGTGCCCTTGAATCCGTCACCCCCACTGGTGCTAGGGGCCCCACCTCATTCATGCCATGA
ATAGGGCCTGGTGCAACATCGAGGATATGGTAAGTGTCTTATGGCCTGGTTTCGCACACACAGCTCATTCACC
ACTTCCCGCAACTACCCCAGCATGACCGGCTTAGGTATGTATCCCATCCCATCCGCGCATGGGGAGCCCGCTC
AACCATCACCCCATCGACCTCTTCGGGACAACCGCGGTGGTGGGCGCAGACGCTGGGAGTACGCCAGAACCCT
CGCTGAGTAAATACTGCCCAATTCTTCATGACCGCACCTGATTTTCACTACCAGAACGGGCTTTGCCACCGTG
GATGTGACTGAGCAAGCCCTCCCGAGTCCCCCTGCCCGCTGACGACATTATCCCAGGGGCCACTTCGATGCAG
CCGACGTGGATGGCGGGGGTTAGGTTTCCGTCGACGGAAACAATAGCGCGAACGAGGACGCGCTCGCGGCGCA
CTGGCCAAGTTCAACAACACTAGGGGCCGTGGTGCCAAGGAACAACGGCACGACAGGGTGGTTCCCCAGCGTC
GACGGACGATAACACAATAGACTGCGCGCCGCCGCTGTGGTCGGGGGCGGCGACCACACCATTGGAAGGTGTG
ACCCACGGGCGGCAAAGTACGATGGGGTGGGTGCACTACCGGTGATGGTGTCGGGGGGTGGCGACGTGATGAG
TGCGCACCGCTGCTGCTGCAAAGTACGTTGATCTGGTGACATCTGACTTGAATGGTGGCTGGAACACTGGACG
AGAATCTTAAGTACGACTGGTTACGACTTGAATGTAACTGAGATTGTACTTTGAGCTGCTTAAATATAATTGA
TATAAGACTACTTGAATATGACTGATACAAGACTGAGTGGCTTAAACATAACTGAAATTAAATATGAGAATGG
CTTAAATCTGACTGGTATTGGATGAGCTTTGCTGATGCATGACTGAGATAGGCCTGATAAAAGAAACTACTAA
GGTCTGAAATAAGAAACCAAGGTTCTGACATATAACTTCCACAAGTCATTTTTCTGAATACTGGCTGAATCAG
AACATGGCTCTCTGTATAAGCAAAGTACATGCCTGATTAAGAGATAGTTCTGACTGACTGAATCAGAGGATGA

TGTTCTGTATAAGAAAGGTACATGCCTGATTAAGAGAAAGCTCTGACAGCTTATTTTGTCCTGTTTGTCTTAT
GTAAGTCTATAAGTTCATATAAGCAAATGTGCGTTTTTCCTGATGTAGGCCAGAGGTGAAAGGGAAAATACGA
TCACGTGATTTGATGTATCGAATAAAATGCAGCTGTGGGTCGCATCAGTGAGCATTTTGGCGAATGATGATAT
GGTTTCCCTCGAAGAACTTGTCGGCTTTATGGGCTGGTAGCCTGAACTCGTACGACTCGGGGTTTATTCTGAC
ACTGAGCCGTTTAATGACTTTCCGGGGATCTTGGATTGAGATATCATCGGGATAACAGAGCTCGCCCAATCTC
ATGAGGGCAAAGAACCCGGTGAGTAGCATGGCGATAAATAATAGGTCATCGTGGCTGGACGAACCATTGTAAT
AGTCAACGGCTAGGTTTAGGTCAGGGAGTGACAACGCATCTTTTCTGGAAACTGCAACCCCTCGCATACGTCG
GCATCCTTTCAAGGTGTGATGGACGAGAGTGGATTGTCGGCTACTGCGAACATTGGGAAAGAAGGGTTCGAGC
TTTTGACAAATGCCGGATAGGTATGACTCGACGGATTTGGGGTTGATATGACAAGCCATGTAAGCGATGTAAT
AACTCAAGGTTTCAGGGGAGGGGTTGGTATCGAGATTGTGTGCGTTGACAAAGGTAAGATATGAGTTAAGGGC
CGAACCGTATGATTTGTGAGTGGATAGATCAATGGCCAAACCAAGTGCGAGATCCCGTTGGTAAACTAAACGG
TTATGGGTCCAGACAGCACGGGCGGGTTGCCTGGATCGAACCCCGCTTTGAATCATTTCTTAACGGCCCCCAA
CGTAACTCGAGGGGGTGAAAAGGAGTTGATACGGATTCCAGGGGATAAGGCACTGGCCCTAGCGAAGTCACAT
CGAGAAACATCAGCAAAGCAGGTAATACCTAGGGAGAGCTCAGGAGTGCCCCATAGTAAATTATAGTGCTGTA
GTGCCATAGTATAGGTCTATCTTTTTTTAGCACTAGGTAGTACCAGCAATGATCTATGGAGTTCTATGGAATG
CTATATAGTGCTCATATTTAGTATTCACTGCTATCAGACTATCCCTATACTCTATTCAAATTTCGTTCGAAAA
ATGTGGCAGAAGACAATAGTAATGTATTGCTAGCCACTCTAAATCTACGTGATACAACAGGAGTTGAAAATAG
CGGCGGTCGGAAAACTTCGCCGCTGTTGCGTGGTACAATCAATCACCTCCCATTGCTCACATGCACAATATGC
CAAGCTTACGTGGCATGGCAAGAAGCTTACTGGTGGCCACGCAGTTCCGGGTCGTCGAACCATGCCCTCGTGC
GCCTTCGAAGTGACGAATAGGTTGGCTACACCTCACATCACAGCGCATTACTTACGGATCGTGTGGGGGGTGG
TAGGTATGAGGGCGGAGGTGATACGGCTGCCGGGAGAACGGGGCGATGGGGGTGAGCACCACCAGGGCATGAA
AGAAGGGCCACCGTACTCAGCTTTGCAAAACATTTTAGATGAGCGCAGGAGGGGGTGTTCTGTTGTGAGTTGA
GGGAGCGGCCGCGTTGGTGTAGTTGAGTGGGGTTCGGGAGTGTTGGCAAAAGGGATTCTTCAAGTATCGAGAG
TCAAGAGTGTGGTTAGGGCTGAACGGTGGAAAAACAAATACTCATCGGCATCAGTGGTAGGGTTGGATGAGCT
GGGAGGAACGAGGAGTGACGGAATACATGGGTGTTGAGATGGGAGCGGTGTGGGGCTTGGGGCAGAATTGAGT
GTCGATGATAAATGTGCGCGCCTATCAGTTCCCATAAACGAAACCTTGCGGAGTCCGCACAGACCATTTGTAC
ATATTTGTTCGGAGTTTGAATTGACTTGGAGAAGTCTGCCCCCTCGTTGCCCCCATTTGCGGAACGTATCCCG
GGACCCGCGGACCAGCTGAGCCCCTCACGCTCGGACGTTTACCCTACCACGCACATCCATTTCCACACACATC
CATTTCCCTTTCGGCCACTCTCGCCCTCACGCCTCGCAGTATACCCACCTGCATGCCAACCAGCCCCAGAATG
GCGCGCCCGCTCGCATTTTCACATCACTGGATGGAGGGGTGAGGACGGGAGAGAGTGATGGAAATAACGAAGG
AGATCACACAAGCTTTTCCTCATGATTCATCCCCATCTGTGTTATGTGTATGGTGCGTCGCCTTTCGTTTCGT
TTTCCCTCTCACGATACCTACCTGCCTTGCTACCTGTATGTGGCGGCGCATGGGCCCTGTAGTATTTGTTTCC
ACTGTTCGTATGTGCCACCTCGTTTTATACACCATCACATTGGCATTGATGAACTCTTATAGATTCTTTTGAG
ACCTCCGCACCTTTGGATGTCCCCCGACCCACGGCACACAGCCGGCAACCAAACGCGTGTCCTCCCTCAACAG
ATCACCGACCACAGCAGGGCTTGCGCATGGCAGCCGCACATCTGCCTCCTCCTTCGGCGTGAGCAAACAAGCG
GAACCTCACATGCAAGACCTCGCGAGCGAGACCCCACACGATGATGAACAGGTATATTTTCATTGATATATGC
TTTTACATGCCCGCCACCTCCATCACTCATAAATGAGAGTGTCGGGGGTGGAAAGCTCGCGCCCGCCTCACAT
GCCTCTGCCACCCTCGCGGCGTTGTTCGCCGCATCATTTGATGCGTGCAATAGTGTTGCGAGGCAAAATGAAG
GCTGTGAGCCGCGGTTCTTGTAGCTGCTGCGGGTTTGCGCGAGAACCATGATAAATCCGTGCGGTTTTGTACC
CGCACCCGTTGCGGGTGCAGTACGGGTGTGGTGGTGGCTTCCCACTACCGCGATTTACCCATGCTCAACCCTA
TCCGGGTTTATTGTACTATGACTTACTATACCATCTATAAAATACTATAGTCATCTATAAAAATTGATACTAC
ACCTATAATCCTCTATAATCCTCTATGATCCTCTATGACCTCTATGACAAATATTTGGTTTTGCTGATGAAGT
GCATCCGCGATAGTGTTTTCAATCCCAGGGATGTGAAGAACTCGAAGTTGTAGATCGTTAGCTATGAGAATAT
CAACGGCAAACCTCAGGATCCCATTGTATAGCGGTTTTGCTTTGAGGGAGTTGAAGATGTTGACCGTGTTGAA
ATTGTCGGTGTATATGACAATTTTTGATGACGGTGGCGCAATTTTGCTGATGTAGGATAGCGCCGCTGCGACA
CACAGAGCTTCAAATATAAAGATGTGATCATGTGGCGTGTCGGCTGGAGTATCAGAGTAAAACCCATAATTTG
TATCCGGGTCCCAAAAACCCATGCCCTCCAAGCATGTGTCACAATAGAAATGGAAATCGGCTGAGGGTGAGTG
CCATTCGAGAGAGCGGATGAGGAAAACACCTGGGTTCTTTTTAATGTGGGACACCGCCCACATCAAATCGTCG
CGTACAGCATTATTGACCCAGATTTTTTGATTTGGAGCTAGTTTCCCCGCGATTTTTTCATAGACGTTGTTGA
GACAAGGGCGAAGTAGCGGGAAGACGTTAAATGACCAATTGAGCCAACCTGCGAGACGTTGCCATATTTTAAG
GGGGAATGCAATTCCACGCCGGTTGGCAGTGGGGCGAGCAAATCGCTGGAGCTCGTTGATGAGATCAGATCGC
TTTTCATCAGATAATGAAAGAGTAAGCTTATTGGTGTCCACATCAATGCCGATAATCGTTAACCGCATTCCAA
AGAACTGCTTCTTCTCTTTGTGGGGGATTCTGATGTCATCCCAGAAAGATAGCAATGTTGCTTGATTTGTGGG
TAACATCTTCTTGTATGGTGCATACCAAGTGAGTCGCCCAGCAATGTCCAAACCGAAAGAGTCGTCCGAGTAT
GCTGCGAGGAAAGGGATATGGTAAATGGACTTGGCTATCCACGTAGAGAGGGCCTTGAAGGAAATAAAAATGT
CACCAGATCTTCTGCCACCGAAGGTAATGTTTCGGTCAAGATGGTATTGTCCTTCTATTTTGTTTGCTTGTTT
TAATTGCCATGCAGGATGGGCAGGCATGAGGCGGTAGGCTTCGGCCACGTCAGATTTGAACGCGACGAAGCAT
GTATCTGGGAATTGGTTACGGAGATCGAGAATCATTTGGCCGAGATGACGCATGTCGTCAAGTGGATAGAATT

CAGTAGCGGAGTGACGCGAGACCATAGAGTTTAAAGAAAATTCGCCACAGCTCTGGTCTGTAACAAGTCGGAA
ATCGGACGAACCGGGCTTTGGGACCGGATGGACAGGCATACAATACATTCCTGGGAGTAAGTGAGAACCGAAG
CTTTGAGAAAAATGACCTTTTTTAATTTCAATATCTCGTTGGGCGACCAAAAAATTTTGTTCAGCGAGATCCT
TTGGTGGAGGCCTCGACACGTCAAGTTCACTTGGATATCCATTGCGGCGAGTGTTAGCCCAGGGCCAAAAACC
TTGTCGGAGTCCACGGCACACCGACTTGACGAAGGGTTGATTCGGGTGTTGAGACAGCAAATTTTCAAAACGA
TCCACGTGAATTGGTGTAACAATACGGAAAAGATCGGGATAATGAGACGAAGTGTGTACGAAACTAGGATTGT
TGGTTTCAGCAAGAGGAGGGCATGGTAATGGGTCAGCGACCTCGGTCCAGTCAGCAGAAGTCATGATGGAAAA
TTCGTTTGGGTTCCATAAGTTGTATCGTAGGTACCTAGGATGATCCCCTGTCAGGAGCGTTTCCTTTCTTTTC
ACATTTACCCTTTCCGTGCCCGAATCGTTTACAGGACAAGCAAGCGTGGCGGTATTTACAGGCGGCATTGGAG
TTAGGGCAACCGGTGGTGGAATTGAACCGGCGGCAGATCTCACTTGACGACGTGGATTCTCCACTAGACGATT
TGCGTGCGATGGTACCACTCCTTCCAATCGGGTTTTCAACCCCGTCGTAGTCCATAATTGCTGACCGGAGTTC
TTCAACGTCATCCCTGCCTGGGAGGAGGTGTGTTCGTCCATTCCCAATCCGACGGCGCAATTTGGCATCGTAC
GCGATAATTTGACCGTGGCTGGATGCGTGTTTCGCGGCAAAGGATGCTGAGATACGCTGTCCGTATTCCATGA
GTTCGGACTGTCGGTGGGGGAAGACAAACGACGTCGCTTGAACGACGTCGAACCAGGTCGAAATCCAGTCTCC
ACTGGTCTGCACCTTTTTGAGGATTTCGGGCCTTCCAATAGAGAACTCAAGCTCTCCCACGCGTCCAAGGTTC
TCCTTAGGAGGTCCAATGCTGTATCAGGCGGAAAAGACGGTGTCAAGACAGACTGGATATCCGCGGATGATGT
TGTCCCATTCTGAGATTGGGAAGATGGGGGCCCGAGGATCCGCCAAGATCCATTTGCGTGTAGCAATGGGGTC
GAGGTTGACGAGTTTGTAGATTCGTTCTGTCTCTTGGCAACTAGGGGGTAGACAACACCGTCGATGGGTTCGT
CATCGTCTCTGTAGAACGGCATCGACGATTTGGCAACCACGACTCGCTTGTTGGTTTCGACGAACCGTCCTTT
GGAATCAACAAAGGGTTCTCCCGGAGAGTCGGAATCGATAGATGACTGGCGAGATCTGGCTCTTTTTCGTCCA
ACCCTGATGACATCGCGTCCAGATTGAGCAGGTTGTTGGGTGGAAATGGGGAGTCTGTCTCCTTCCGTCGGCG
ATTGGACGTTGGCATCTTGACGGGAATCGATTCTATCTTGGAGACTGAGGGCTGTGGTCGCGCCATTTCGATC
ATGCTCGACCGCGCGGGATTCGAGGAGGTTGAGGGTGCGTAAGTATCCGAGGAATGCTTGGTATTTGACGGAG
TCATCATCATCTCCGTCAGTGTCGAGGTGGGATGTGATAATTGCAAGTGCTCGGGTTTTGCTGATTTCACCTT
TCCGGTAGTCTTCAACCGCGTCCTCAATCCGCGCGACGGTATAAGCAGAGGGGAGCTCATAACTCTCTGGAGC
GGGTGATTGCGTCGGCCCCGCGTCATGTCGATCACCGGATTCGCGGATTTCACCGACGGCGGAGCCTTGGGTT
GTATCTCGTGCAATGGAGCGAGCGTGAGGTCGAGGTACACCTGCTGAAGTATGCAAAGACTCGAAATTCGCTG
AAGTATCTTGTGTCTCAGATTGGTTCGGCATGAGACGAAGTCGATGAAATGTGTGAACTGATTGGCAGATCCG
AGAATATATTTAGATGTAAGGCGAAAAAAAGCGAGAGGCCGCTAGGGGGGTGTTGAGTCAAATGAGAACCGCC
CAAAAGTGCGATGTGGGCTTACCTAGGGGGGTGTTGAGTCAAATGAGAACCGCCAAAACGGAAATGGTGGTTT
CATCGAGACTTATCAATGACCCCCGCTAGGAGGATGTCTCAGGGCAGGCTGAGACATCGGTGACCTCATTAGC
TGGCTCGGAACGGGGGCCGAGAGCGATGATGAGGGGGCGCCTGTGAGAGGCACGAGATAAAAAATCAACACAC
CGCGGGGCGGCATCATCAGC
>Cligib1_Cluster64#LTR/Gypsy
gtcaggaCTTgTatTTTCTTTTCtTTTCTCTTTTCgTTTGTAGTATCACATGACTgATCACgTGACTGATTAT
gTAAGCGAAcCGCGGTTTCaCCTCAttGTAaACACATGTtGTTTGTACTTATTATATTCACGGGCTcTTACCT
ATGTATATAGTCTtGttGTTTGTATGtTTcgTTTCTCAATCTGATTCTCTTcGGTTTTCCGCTatTaACAcaG
tGNgatcgtccgtgtcagtcataTtCattcactccaacttctCTGAGtatTCTTGAGAGACCGCACActtatt
ggtGTTTAGCGTGTCCTTACAGAAGAATCAGATTcctTcTcGTGCCCTCTCTCATCgcTCAacactttcgCtt
gtccaAcgacatCTcgaagcgcGtgAaAcaacatcaCcgctcaaAgagctttAgTtGttTggtgtaAgcacAt
CccCgcCCgTTCATCATTAAtCCcaCGaCTgtccCAgAtAtcAAacgggcagTtacgtTcTAaacttNcNNac
ttgNaagtttTttCTTggCaAcaAcTCttgCtaaacgGttgagATcTtAtTTCTCcGtTtcgcaTtaTAAgAG
TCagCAAGTCCCCctcctgttgcgcttggattggctgttggcCAaggCaaccTCagcACTCTCtCACTGCTAG
TTCAGCCCACACCTggCGCAGGATAAGCGTTTcAGGGTTAGCCACTAGTAACaGTtcaaccgACcCCgGacCA
ctCTtGaATCAccATTCCCATtCCTGTTTGAGTAGGCtTGCAACCTCTCATCCTAtTtGCTAcCGTTACACGG
TTTGAGCAGGCGAGAATTTCGAATCTCACCTTTGTCCCGTcCCCAccCTTGAAGCAGGTGATcACCGCCGGTC
TACTGGATTAAGTCCATACCCGCGGCCCCCTTCATTTtCAAAATTCCgTCaCAtTTCCGaCCaTACTCaTCCG
CaCACCCCTCTCCTACTTTTCcAAAACACCaccCATGTCTGAAGAcTCAGCAAAtTGGGGAGGATCGCAANCg
gCGACACCTCGtaaCAaTTTtgcgggatatTataATccagAaTACgAacCccCCaacActtCTCAGgttAaCC
CGACACGGTCAcTTAtaAAACggAcGcTGAaAAATTcACgGAgaTcTAtGatCAAATGAgACAcacCACGGAa
TTaAacACGGAATTcATNCAACGgATcCAAACccTcGAaAAcCAcGTtaAagtTCTcCAgGATCAgAAtTCgA
GAcTATGGGAACAGAAcaAtGAActcCacTCaCGGTTTAATCGgTTcGAgGCACTcCTcAcgGATTtGGTCAA
GGAcAAACggGAACGcGActCTAAACCATCcCcCTCaACGGATtTAAccCccCCcGGTTCATAcTCtAAcGCG
GCTAAAGGATCTcGtGcgCCTAAACTCCAAAATCCgACACCgTTCGATGGtACaATGGCtAATGcGGAcACTT
cGAAAATGCATGtCGcaTaTacCTACAAGTTCgaCACGCGGATTTcTCATCGGTCACcAGCGCGGTATAcTGG
gttCTACATCTcTGtTCTCAAGGtTCAGCAGCgACATGGCGtGATCTtAAgATCAAaGAATTtAACCGGAAtC
CAATACAAATGGATTAtACTATTAACgATTTCTGGAAAGAATTCAAAcTGGCgTTtTCCGAtCAcgAtCCACA
gGGAACcAAaGCgTTCAAACTTTGtACcATGAAACAaGGAACcTTAtCGGCaGAgGAACAcATcCAaGAaTTt

AAGAAAACGGCCGACGGtcaGGaTAcAGcGGATATCCttTaATcAAGGAATTCAAACGGTCatTAAAcGAaGG
TCTTCGcAAACGgcTAATGGAATCgGAaTAcCAaCCgaAtACcATcGAggAtTGGTAcACACGaGCATCACAT
TGGACaGaCAATAcCGACAATCtCAGGAAGAaAAgAagTtgtatCAACCaACATTcTCacgCCCGgCATTtTC
ATcGatAACCCACGGCaAACCGGAACAGGAACcaccCAgACACAAGGTcCCCACCGAAATATGGGATCTAATC
aACAGGCACaACCCGCTCGGCCTACgTTTGGTTTCAATCgCCCGGCtTTcgGTCAAtcAgCaCaACCGGCCCG
ACCtgccCAACAagCccGTCCtAAtCCAcCgGCtCCAGCAcGTGCcccaGATGCaATGGAtGTcGAtCGAGGA
AAtCGTcGcCCACCcGGAGTcTGTTATCGATGcGGTAAACCCGGcCAcATgATGcGAGAcTGCAGGGCCAATA
TAAAttTAAAtTCGGCCACaCAtGAtGAAGCACGCGAcCAtTATtTACAGGTtGCAATcGAAGAAGGGTTGAT
TTGGCAAGAcAcgCCgTCAACATCAGGaATCACGGAAgTcAATCCGGATtCACAAgTTCAGGGGCAATCCAAC
gCCCAATCGTCTTTTCAAGGAGGCGCGCAGTGAAATCTCCACCACcagcgTtgCcaaAAATAAATTTCAtCTT
TTATCTGTCGAcACgaTCaaaAAaaTAGATAcGCCTTCCAATGTAATCgTATCTCAgGGGGTCTCCCATAAGG
AGCTTGGcGCTCAGGAAGCgTGCTTAATTCCCgCCCCAATtTATCATtTTCAAAAAcCtAAAACGACAGAAAA
ATTAGTGGAGACAAAAATAAgTGGTTTtGGTGATCAGATAGAcGTcCGTCCTGTTAATCAGGGTCCcGGCGTT
ATGGTCATGtCAACCcACCAGGATTCCTTAAATGGAACATCCGTCcGTCCCAGAGTcGGTAgCTCTGTGGGTC
CCgGCGGTAAcGTATCTTCGGAACGTCCTGGTCATATTTATAGAGAAAgACTTCTTATTCGCAcgCTtAATtT
gGAACgCGAGACAAAAtTATCNATcTCAGTaACaACAGTtGAtACTCATGAAACTGcGTCaTGTCAAGCGCTT
CTAGAtTCTGGTGCAACcGGtTCctTTatcGATCGAGCaTATGTACGACGtATGGGATGGAAtACCCAAACtT
TAAAATATCCgGTTAAAGTATTTAACGTGGACGGTACCTTAAATCAAGGAGGATCGATCACGGAAGAAGTcTA
TTTaATGATGgTtCATAAcGGACATAAgGAgAAgATGCTcTTCGAAGTcACgGAcTTaGGAAAAGTtCCgATc
ATaATcGGTCAcACgTGGTTaAAGAAACAcAAtCCATCtATCGATTGGTCaACcGGtGAAATcACATTcACTC
gATGCCCCCAGAgTGtAATGTTAGaGTACGcGCTATTAAAAAgCAAAAGAAgCGtAAAGCtcTAGCCgATCga
TGGAAATAcCAACCcACgATTGAAGAgATCgATGACGAAGAtgCAGAtCGAGTgATTGGtGGcCTTAttACtG
AACAAGAAGGCGCATTAcTtGAAGAAATtAATTATGAtGAaGTGGTTCATGAAATAGTaGATGAACAcTTTGT
CCGATCCGtAGAACGagcTGAAGACCAtGACAACATACATATCGAAACaGAGGAACCcGAACTcgTtAATAAA
ATtCCTCgaCGATTtAGACgTTGGCTCAAaGTCTTTGAAAAGAAAGCaTCGGAAcGcATGCCTGAACGAAAAC
CcTGGGATCAcGCTATAGAATTGAAAGAcGGCTAcGAACCgAAGAAAGCCAAaGTCATCAATCTTcCCCTACC
GAACAGAAAGAAGTCgAcGAcTTtcTAgCaGAtCAAcTaGCtAAgGGTTATATTCGaAAgtAAATCTCCtCAA
ACATCCGCCGTATTCTTtGTACCCAAGAAaGAcgGAAAGAAACGCATGGTcCAAGAcTAtCGaCAACTCAAcG
CTTGGACaATCAAaAAcAATTATCCcCTCCCaATGATcTCCGAtCTtATcGAcAAAGCtGGcAAgTCcAAgTG
GTTtACaAAAcTaGAcTTACgATGGGGATAtAACAATGTTCGcATGCGcGAaGGAGAtGAACCAAAaGCaGCa
tTtACGACaCATAGAGGaTCTTTcgAACCACTcgTcATGTATTTcGGCtTaTGCAATTCaCcGGCtACTTTtC
AAACtATGATGAAtGCtCTttTACGAGAACTTATAGACGAAGGACAcGTaGTaGTATAtATAGAcGAcATtTT
AATATTTACCGAcACTCAAGAAGAAAAcGAACgaATGCcGAACgcGTTCTCCAAATcCTcCTCGATAACGAcT
TATTCCTtAAACCAGAgAAATGtTCcTTCTCgGTTCAACAAGTcGAATTcCTGGGacTaATACTTGGAAATGG
AGAAGTACGaATGATGGACGAcAAAACCGAcGCAGTAAAaAATGGCCGGTTCCTAAAACAgTcCGCGAcGTTC
GCAAATTcTTgGGTTTcGCtAAtTTcTATCGACGaTTcATcGAAGGaTTTtcaaAAaaTgccgCCCaCTcAAt
AAtCTCTTAcGAAAGGATTCTAAATTCGATTGGACAGAGAAACAACAagcgGCATTTGAttTGCTTaAAAACC
GATTtACATCCGAtCCcATAcTGGttACaGCaGAcCCaACAAAACCCCTTCGAGTaGAATCgGAcGCTTCgGA
CTTtGCcACgGGAGGTATatTATCTATGAAATGTgCcGACGAtAAATGGAGGCCaTGTTcCTatTacTCTAAA
TCtTTATCTGAcGTtGAACGTAAtTAcGATGTACATGACAAGGAAATGCTTAGTATTATACGatgttTGGAGG
CCTGGCGCCATTATTTAGAAGGTAGTtCaCATACgatGAAATATGGTCGAtCATCGTAATTTACAATACTTTA
TGtCTTCTAAAAAgTTAAATCGTAGACAaGCACGATGGGCaCTtTAcCTATCaCGATTTGAcTTTACacTAAT
TCACAAggcaGgaTCcTCAATGGGAAAAGCGGAtgCaCTGTCCAGGAGGACAGAtCTaAAaGAGGGAGTgGAA
ACTgATAAcGAcGACATcACGCTCCTtAAACCgGAATTcTTcGCCATtAAAGCatTACAACAAGGACATGTtC
TTGTTAAcGGCGAAGAAGAGGGAGTACTtTCGAAGATCCGcAAATCGAAAgAcTAtGAtGAATCcGTTGTcAA
AGCgATtGAAGAACTcAAacgatcgCCcACGAAACGNTTAcGATCtGAAGAATGGTCtCAGGAACAAGATCTc
ATAtTGTTCcgaGGAAAAgTCTAcGTACCcAACgACAtCCAACTTCGACGAGAaATcGTTAAaCTCCACcatg
aTtCCAGcATtGCCGGTCAtCCAGGACAcTACAAAaCtCTGGAAtTAATCGCgCGAAAcTAcTGGTGGCCtGG
cATGACAAAATTcgTtcaTtcTAcATcGAcGGATGCGAcTCTTGcCAACgTTTCAAAAAtTTcCCcGCTCCCC
CTATTGGAAAACTTGTTCCAATGATTACcCCTTCCGAACCaTGGAAAGCAATaTCTTGCGAtTtcATcACcGG
ACTTCCtGAGAGTAAcGGATAcGAcGCAATaGTtGTtGTtGTCGATCGCGCATCAAAAATGGCgCAtTtattc
CcactACaTCTCAAACaTCTACGACgGGTCTTGCaAAAcTttaTcTGCAAAACGTCTGGAAgCTCCATGGTCT
CCCCGAtTCAATtATCTCtGATCgAGGACCACAATTCGCAAGtTCAATGATGAAAGAATTAAAcTcATTATTG
AATATtCAAACNAAACTTTCcACcGCcTATCATCCaCAAACtGAcGGtCAAACGGAACGgGTTAAtCAAGAtc
TcGAAGCcTAtCTtaGAATTTTCGTATCaCAtCTTCAATCCGAtTGGGTTGAATGGATTCCtctgccGAATTC
TCATACAAtAAcGCcACTCATTCaTCcACGAAAATATcCCCcTTCTTtAAcGTcTAcGGTCGtCAtCCtcGAA
TGGGCGCtGAACCAATACGaCAACCAACAAATGAGTCaGCTAAtGAATTcGCaACACgGATGAAGAAAATtCA
AGAAGAAGCACAATCtGCCCTtGTtAAAGCgcGcgAcGAAATGAAACGATAcGCCGAtCAAAAtCGtgGcGAA

aCcCCATCaTTTCAAGTTGGACAAATGGTATGGcTNGAAACaAAaGATCTtGATCTcAAACGACCATCAAAGA
AACTCACcGAAaGACGAGTTGGTCCaTTtAAaATtCTcAACTcaTtGGAACtAAcGCGGTGAAACTcAAACTt
CCaTCaACaATcAAaATTCAcCCcGTcATcAAtATtTCACGAGTtCGACCATAcCAATccCCtAAAATtCCcC
AacAacAAatCCctcCcGCtCCtCCAGTAGAAATCGATGGCGAAtTTcAATAtACcGTCgAAGAAATtCAAAA
cTCACGNCTtACcAaaAAtGGcAAaGGttTaGAATAcTGGGTTAAATGGGAAGGATAtACGCCgCAACAcAAc
ACtTGGGAACCatTATCaAAcTTGAAgAATGCCATGGATCTcGTcAAcAAaTTTCATCGAACAAAtCCaCAAG
CtCCAcGAAAaGTTCGTCTCAAcgTaACtCAAGAcGAATTtCTtTCtTTGTTCGccCCaTAtGAAACtTtcaC
cgatCcaCCATCAAATTCgGCAtTATTTTCAAAgtTgaAcGCTTCAAGcGCTTCaAaTTgAtATGTGCGCATT
CTTTtGGATGCGAGACGCACtaTcaGGgATGATGAAGAACCGCtCAAGATTTTCCCGGAGAGAAAATccCTAA
AGaGggagtagTGTCAGGACTTGTAtTTTCTTTTCTTTTCTCTTTTCGTTTGTAgTATCACATGACTgATCAC
gTGACTGaTTATgTAAGCGAACCGCGGTTTCaCCTCAtTGTAaACACATGttGTTTGTACTTATTATATTCAC
GGGCTcTTACCTATGTATATAGTCTtGttGTTTGTATGTTTcGTTTCTCAATCTGATTCTCTTcGGTTTTCcg
CTaTtcACAGTGgGatttcNNtcgtcagtcagNttccatNcNtNcNaNttcccctgagNNNtNNtNNNNNNNc
NNNNNNNNNNNtNgNNNttNNNNNNNNct
>Cligib1_Cluster120#LTR/Gypsy
GTCAGGACTTGTAATTTCTTTTCTTTTCTCTTTTCATTTGTAGTATCACATGACTGATCATGTGACTGATTAT
GTAAGCGATCCGCGGTTTCACCTCATTGTAAACACATGTAGTTTGTACTTATTATATTCACGGGCTCTTACCT
ATGTATATAGTCTTGCTGTTTGTATGTTTCGTTTCTCAATCTGATTCTCTTTGGTTTTCCACTATTCACAGTG
GGATTCTCCATTGTCAGTCGGTTTCCATATATCCAACTTCCTCTGAGACATCTTGAGAGACCACACACTTATT
TGGTGTTTAGCGTGTCCTTACAGAAGAATCAGATTACTCTCTTGTGCCCTCTCTCATCGTCCAACACCTCCGC
TTGTCCAAAGACACTTCGACACGCGTGAAACAACATCACCGCCCAAAGAGTTATTAGTCTTTAGGAGTAAGCC
AACCACCACCTTCAGTCATTAACACCACCTCTACCCACTCAGAAGAAGGGATTCATCTGAGTTTAACTCGGCT
GCTTTCCCTTCTCTTTCTGGCAACAACCCGGTCTTCATCTGTGTTTAACACGGCTGCTTGACTTACGGGTCCT
CAAGTCCCCAGAACACAGAACTTCCCCGACATCCTCTCACTGCTAGTTCAGCCCACACCTGGCGCAGGATAAG
CGTTTCAGGGTTAGCCACTAGTATCAGTCAGACGCCTGGACAGGCTCTCGCAGCATCACCATTCCCATTCCTG
TTTGAGTAGGCTTGCAACCTCTCATCCTATTTGCTACCGTTACACGGTTTGAGCAGGCGAGAAATTCGAATCT
CACCTTTGTCCCGTCCCCACTCTTGAAGCAGGTGATCACCGCCGGACCCCGGTCTACTGGATTAAGTCCATAC
CCGTGGCCCCCTTCATTTCAAAATTCCGTCACATTTCCGACCATACTCTTCCGCCCACCCCTCTCCTACTTTT
TCCAAAACACCCGTCATGTCTGAAGATTCAGCAAACTGGGGAGATTCGCAACAAACAACTCCTCACGGCAGTT
TGATTAATCCATATGATGGAAACTACTTCCATACACCCGCCATCCCCCAGGTCAACCCCAACACAGTCACTTA
TCAAACGGATGCTGAGAAATTTACAGAACTTTATGGTCAAATGAAACACGTCACGGAATTGAGTACGGAATTT
ATCCAATGGATCCAAAATCTCGAAAATCACGTTAAGGTTCTCCAGGATCAGAACTCGAGACTATGGGAACAAA
ATCATGAACTTCACTCACGGTTTAATTGGTTTGAAGCGCTCCTTACGGATTTGGTCAAAGACAAACGCGAACG
AGATGCTAAACCCTCTCCTCTCGATGGATTTAATCCCCTCCCCGGTTCATACTCTAACACGGCTAAAGGATCC
CGTGCGCCTAAACTCCAAAATCCGACACCGTTTGATGGTACAATGGCTAATGCAGACACTTTCGAAAATGCAT
GCCGAATTTATCTACAAGTTCGACACGCGGATTTTTCATCGGTCACTAGTGCGGTATATTAGGTCCTACATCT
TTGTTCCCAAGGCTCAGCAGCAACATGGTGCGATCTTAAGATCAAAGAATTTAACCGGAATCCGATACAAATG
GATTATACTATTAACGATTTCTGGAAAGAATTCAAACTGGCATTCTCCGACCATGACCCACAAGGAACGAAGG
CATTCAAACTGTGTACCATGAAACAGGGAACCTTACCGGCAGAGGAACACATCCAGGAATTCAAGAAAACGGC
CGACGGTTCAGGATACAGCGGATATCCTTTAATCAAGGAATTCAAATGGTCATTAAACGAAGGACTTCACAAA
CGTCTAATGGAATCGGAATACCAACCGAATACCATTGAGGATTGGTACACACGAGCATCACATCTGGATAGGC
AATATCGACAATCTCAGGAAGAAAAGAAGTTGTATCAACCAACATTTCACGCCCGGCATTTCATCGAGTAACC
CACGGCAAACCGGAACAGGAACCACCCAGACGCAAGGTCCCCACCGAAATATGGGATCTAATCAACAGGCACA
ACCTGCCCGACCTACCTTTGGTTTCAATCGCCGGCTTTCAGTCAACAAGCACAACCGGCCTGACCTACCCAAC
AATCCCGTCCTAATCCACCGGCCCCAGCACGTGCCCCAGATGCGATGGACGTCATCGAGGAAATCGCCGCCCA
CCAGGAGTTTGTTATCGATGCGGTAAACCAGGCCATATGATGCGAGATTGCAGGGCCAATATAAATTTGAATT
CGGCCACACACGACAAAGCACGCGACTATTATTTACAGGTCGCAATTGAAGAAGGGTTGATTTGGCAAGATAC
GCCGTCAACATCAGGAATCACGGAAGTCAATCCGGATTCACAGGTTCAGGGGCAATCCAACCCCAATCGTCTT
TTCAAGGAGGCACGCAGTGAAATCTCCACCACCAGCATTGCCTAAAAATAAATTTCATCTTTTATCTGTCGAA
ACAATCAAGAAAGTAGATACGCCTTCCAATGTAATCATATCTCAGGGGGTCTCCCATAAGGAGCTTGGCGCTC
AGGAAGCATGCTTAATTCCCGTCCCAATTTATCATTTTCAAAAACCAAAAAGACAGAAAAATTAGTGGAGACA
AAAATAAGTGATTTTGGTGATCAGATAGACGTCCGTCCTGTTAAACAGGGTCCCGACGTTATGGTCATGTCAA
CCCACCAGGATTCCTTAAATGGAACATCAGTCCGTCCAAGAGTCGGTAGCTCTGTGGGTCCCGGCTGTAACGT
ATCTTCGGAACGTCCTGGTCATATTTATAGAGAAAGACTTCTTATTCGCACGCTCAATTTAGAACACGAGACA
AAACTATCCATATCAGTGACGACAGTAGATACTCATGAAACTGCGTCCTGTCAAGCGCTTCTAGATTCCGGTG
CAACTGGATCTTTTATTGATCGAGCTTATGTACAACGTATGGGATGGAACACCCAAACCTTAAAATATCCTGT
TAAAGTATTTAATGTGGACGGTACCTTAAATCAAGGAGGATCGATCACGGAAGAAGTCTATTTAATGATGGTT
CATAACAGACATAAGGAGAACATGCTCTTCGAAGTCACGGACTTAGGAAAAGTCCCAATTATAATTGGTCACA

CGTGGTTAAAGAAACACAACCCATCTATCGATTGGTCAACAGGCGAAATCACATTCACTCGATGCCCCCCAGA
GTGTAATGTTAGGGTACGCGCTATTAAAAAGCAAAAGAAACGCAAAGCCCTAGCCGATCGATGGAAATATCAA
CCCACGATTGAAGAAATCAATGATGAAGATGCAGAACGAGTTATTGGCGGCCTTATCACAGAACAAGAAGGCG
CATTATTAGAAGAAATTAATTATGATGAAGTGGTTCACAAAATAGTAGATGAACACTTTGTCAGATCCGTAGA
ACGTGAAGAATCGCACGATGATATCTTCCCAGAAACCACAGAGCCAGAATTATTAAACAAAATTCCACGACGA
TTTAGACGATGGCTCAAGGTGTTTGAGAAGAAAGCGTCGGAACGAATGCCCGAACGAAAACCCTGGGATCACA
CTATTGACCTAAAAGATGGCTACGAACCTAAGAAAGCCAAGGTTATCAATCTTTCTCCTACCGAACAGAAAGA
AGTCGATGATTTCTTAGCGGATCAACTGGCCAAGGGTTATATTCGTGAAAGTAAATCTCCACAAACATCCGCT
GTATTCTTTGTACCCAAGAAAGACGGAAAGAAATGCATGGTTCAAGATTATCGTCAACTCAATGCTTGGACGA
TCAAGAACAATTATCCTCTCCCTATGATTTCCGACCTAATTGATAAAGCTGGAAAATCTAAATGGTTCACCAT
TGAAATTGGACCTACGATGGGGATACAACAATGTTCGAATGTGTGAAGGAGATGAACCAAAAGCGGCATTTAC
AACTCATAGAGGTTCTTTCGAACTGCTCGTCATGTATTTCGGCTTGTGCAATTCACCGGCCACTTTCCAAACC
ATGATGAACGCTCTTCTACGAGAACTTATAGATGAAGGACATGTGGTAGTATATATAGACGATATTTTAATAT
TTACCGATACTCAAGAAGAAAACGAACGAATTGCTGAATGAGTTCTTCAAATCCTCCTTGATAACGACTTATT
CCTAAAACCAGAGAAATGTTCCTTCTCAGTTCAACAAGTCGAATTCCTGGGACTCATACTTAAGGTTCGATAC
GGGTCTACCCGCGGGTAAAAAATGATATACCATACCTGCTACCTGTAATACCGCACCCGTTTTGACACCGCAC
CCGCGGGTACGGGTAAAAACCGCGGTTCCATGCGAACGATCGCACGGAGCCCTCTCGTCTCGTCGAATTCGTG
ATCTGTGGGTAAAATGGGTCATGTTCAACACCAAGATGAAAGAAAACGGACCAAAATTGACCAAGATATGGCC
ATCTTTGCTTGAGAAACCCAAGAAATCGGCCTCGCGTGTGTGAACGGCTGTCTGGAGCCCTCTCGTCTCGTCG
GATTCTTAATCTATGCATTAAATGGGTCCTGGTGAGTATCAGGATGAAGGAAAACGGATAAGAAATGACTGAG
ATTCAGCCAATCTACTCATAAATTCATAATATTGGCCTCACATGTGAAAACAGTCATGCAGCACCCTGTCGTC
TCACCAGATTCATAATCTATGAGTAAAATGGAACCTGGTGAATACCAGGATGAAGGGAAACGGATAAAAACTC
ACTGAGATACCACATTCTGCTGGTAAAAAGTCTAAAATCGGCCTCACACAGTGTGCAGGCAGATGCGGAGCCC
TATCGTTTCATCAGATCTATAATATACATATAAATGGGCCCTGGTGGATACCAGGATGAATAAAACAGACAAG
GACTGACCGAGATATTGTGGTATATCTGCTTGAAAATCATAAGATCAGCCTCGCGTCTATGAACAGTTATGCC
GCGCCCTATCGTCTCACCGGATTCGTGATCTATGAGTAAAATGGGTTCTTGTGAACCCAAGGATGAAGGAAAA
CGGGCAAGAACTCACTGAGATATGATATGTGGTGAAAAACCACAAGATTGGCCTTGCATGCGGGAACGGTCAT
GCGGGGCCCTGTCGTCTCGTCGGATTCGTAATCTAGGAGTAAAATGGATTCTAGTCAACCCCAGGATGAAGCA
AAACGGATGGGAACTCACTGAGATAGGATGGTGGGGGGGCACATTGGAACCTGTGGTTCATGCGGGTAAGGGT
TGCGGTATTTGCGGTGTTGCCTAATTGGGACACCGTACCCGTACCTGTGACACCCGTACCTGTGACACCGCGG
GTTTACCCGCACCCGTGTCGAACCTTACTCATACTTGGAAATGGAGAAGTAGGTATGATGGACGATAAAACCG
ATGCAGTAAAGAAATGGCCGGTTCCTAAAACAGTCCGCGACGTTCGCAAATTTTTGGGTTTTGCAAATTTCTA
TCGACAATTCATTGAAGGATTCTCTAAACTCGCTGCTCCCCTTAACAATCTCCTACGAAAGGATTCTAAATTC
GATTGGACAGAGAAACAACAAACGGCGTTTGATTTACTTAAAAACCGATTCACATCCGATCCAATATTGGTTA
CAGCAGATCCAACGAAACCCCTTCGAGTAGAATCGGATGCATCGGACTTTGCTATGGGAGGAATCCTATCTAT
GAAATGTTTGGACGACAAATGGAGGCCTTGTTCATATTATTCTAAATCCTTATCTGACGTTGAACGTAATTAC
GATGTACATGACAAGGAAATGCTTAGTATTATATGTTGTCTGGAGGCCTGGCGCCATTATTTAGAAGGTAGTA
CTCATATGATAGAAATATGGTCAGATCACCGTAATTTACAATACTTTATGACTAGTAAAAAATTAAATCGTAG
ACAAGCACGATGGGCTCTTTACCTATCACGATTTGATTTTACATTAATTCACAAAGCAGGTTCCTCAATGGGA
AAAGCGGATGCACTGTCCAGGAGGACAGATCTAAAAGAGGGAGTGGAAACTGATAACAACGACATCACACTCC
TTAAACCGGAATTTTTCACCATTAAAGCATTCCAACAAGGACATGTTCTTGTTAACGGCGAAGAAGAGGGAGT
ACTTTCGAAGATCCGCAAATCGAAAGACTATGATGAATCTGTTGTTAAAGCGATTGAAGAACTCAAACGATTG
CCCACGAAACGTTTACGATCTGAAGAATGGTCTCAGGAACAAGATCTCATATTGTTCCGGGGAAAAGTCTACG
TACCCAATGACACCCAACTTCAACGAGAAATCATTAAACTTCACCATGATTCCAGCATTGCCGGTCATCCAGG
ACACTACAAAACTCTGGAATTAATCACACGAAATTACTGGTTGCCTGGCATGACAAAATTCGTTCATTCATAC
ATTGACAGATGCGACTCTTGCCAACGTTTCAAAAACTTTCCCGCTCCTCCTATTGGAAAACTTGTTCCAATGA
TTACCCCTTCCGAACCTTGGAAAGCAATATTTGCAACTTCATCACCAGACTTCCTGAGAGTAACGGATATGAC
GCAATAGTTGTTGTTGTCGATCGCGCATCAAAAATGGCGCATTTTATTCCTACTACATCTCAAACATCTACGA
TGGGTCTTGCAAAATTATATCTGCAAAACGTCTGGAAGCTCCATGGTCTCCCCGATTCAATTATCTCTGATCG
AGGACCACAATTCGCAAGCTCAATGATGAAAGAATTAAACTCATTATTGAATATCCAAACTAAAATTTCCACG
GCTTATCATCCACAAACTGACGGCCAAACAGAACGGGTTAATCAAGATCTCGAAGCATATCTTAGAATTTTCG
TATCACATCTTCAATCCGATTGGGTTGAATGGATCCCTCTTGCCGAATTCTCATACAACAACGCCACTCACTC
CTCTACGAAAATATCCCCCTTCTTTAATGTCTATGGTCGTCATCCCCAAATGGGCGCCGAACCAATACGTCAA
TCAACAAATGAGTCGGCTAATGAATTCGCAACACGGATGAAGAAAATTCAAGAAGAAGCACAATCAGCCCTTG
TTAAAGCGCCCGACGAAATGAAACGATACGCCGATCAAAATCGCGGCGAAACTCCATCGTTTCAAGTTGGGCA
AATGGTATGGCTTGAAACAAAGGATCTCGACCTCAAATGACCATCAAAGAAACTCACCGAAAGACGAGTTGGT
CCGTTTAAGATTCTTGAACTTGTCAGAACCAATGCGGTGAAACTTAAACTTCCATCAACGATCAAAATTCACC
CCGTCGTTAACATCTCACGAGTTCGACCATATCAATCCCCTAAAATCCCTCAGCAACAAATTCCACCCGCTCC

TCCAGTGGAAATCGATGGTGAATTCGAATACTATACTGTCGAAGAAATTCAAAATTCTCGCCTTACGAAGAAT
GGTAAAGGATTAGAATATTGGGTTAAATGGGAAGGATATACCCACAACATAATTGGGAACCTCTATCGAATTT
GAAAAATGCCATGGATCTCGTCAATAAATTTCATCGAATACATCCACAAGCCCCACGAAAAGTTCGTCTCAAT
GTAACACAAGATGAATTCCTTTCACTATTTGCTCCATATGAAACTCTAACTGATCCACCATCAAACTCGGCAT
TATTTTCAAAACTCGATGCTTCAAGCGCTTCAAATTGACATGTGCGCATTCTTTTGGATGCGAGACGCACTGT
CAGGGACGATGAAGAACCGCTCAAGATTTTCCCGGAGAGAAAATCCCTAAAGAGGGAGTAGTGTCAGGACTTG
TAATTTCTTTTCTTTTCTCTTTTCATTTGTAGTATCACATGACTGATCATGTGACTGATTATGTAAGCGATCC
GCGGTTTCACCTCATTGTAAACACATGTAGTTTGTACTTATTATATTCACGGGCTCTTACCTATGTATATAGT
CTTGCTGTTTGTATGTTTCGTTTCTCAATCTGATTCTCTTTGGTTTTCCACTATTCACAGTGGGATTCTCCAT
TGTCAGTCGGTTTCCATATATCCAACTTCCTCTGAGACATCTTGAGAGACCACACACTTATTTGGTGTTTAGC
GTGTCCTTACAC
>Cligib1_Cluster52#LTR/Gypsy
gtggcgttcggccgacctacgtcagcaagaacgacacacggtcaatggtttcgaggtcggtaaacgagatagc
cggttcagacgataagataacggtttagaggccaggtagattgcagagagtgtccccgttacagtacaagggg
accgggtatttatactggtcaggccataggagtcaatatagataagagttaaaggagtgatggtaactaagtg
agagacgatcgcaaacggcgGgAacgagcaaGagGcAcaCaaaaAcaaaacaCcaacgTAggTCacggGacAg
aaaagacaTacCaaagGAaaTAacggaGccgcaccTggccGccTaCaaCggaaaaccTaCaaacGcccgCcAg
cGgcccTcaaaaaagTaCcAacaacaagggaaaacTaAacTaTaggGaaTCcgaTGaccgaaccAagaaaAag
AccacgcCccCaTGgaaTgaaAaGccgacaCcCcccgcaaaCTacCgacccgagaaaCcGgTaccgTaTcagc
caAAcacacTaTcgCaggaAcccaaCcccaaaCaaacaaCcgaGgcTccgTCTTcCTcaccTaGGccGcccGa
aCGcCaaacacggcggaacgGgaagaGaaaAcacgcGaccgaaCagacCccaCTTcccgcCgaagcacaaaCc
CcTccTccGAgaaacccggaagTccccGCccaaggTaaaccaTcccagccaaaATggaAagaAcgTTacgccc
cagcaaCacGaCGacGAaAaaCTccaagaaCGTAacGcccaaTacCCCCAaGacacaCAacccacCGGGcAaG
aaCaccaacCCcacGagccagaaaacgcTcAaacacccgaCccccgcaATaacggaGaCcaGacCcTCCcaaC
gcaaaaaacAaacGccCcCcgagaccgccggagacaaagccaaAacaTCcGcaAaTgcaaaGAaCcCCGCgag
cAggaGcATGccggaaATaCcTacaccgTCgGTTaacAcaaaaGacgaaaaGaacAagAcgaacgTaaaaccc
gacCgaGaGAAccccaacacaacaagTaacaaggcGgTcacgaCCaaaaacaaCccGaaGaaagaaCTgcgaa
aGAaCGaaaacgacaccaaaacgaaaaTaacCaaacAgaaaaTaaGcacGaaccaacacaggAGcaaGgGGaA
AcacgTaGaacacGGaAacgGacGGaAaaaaGcaaTggAGAAagcaGaaaaacAcacGaTcgAaaaATTccGc
agaCcgacaGaagggaagcaacgGcgcgaaaCTaGaaagCcccaacTcacTcagaagcaCagaAcaagcGaCg
aGcgCcaaCTTgcGcaCCccaacGacaggcgaAaCcaGgAacTccacccgaAgcgaaAgacCccccaaaAagA
cCacAagaaccgcccGAcgcggcaacagcccaaaccGcaacaaGgacaTcggagaaaccgcaAAacaCccaag
agAcaGaaaaaaacgcaacagATgAgTcaAaTaaccacaaagaagcaaaggacaaTcgCCagacCgacaggCa
accgaAAaaaacccaagaagaacaaAGccGacgcaacAgaTcaccagGaacacaGccaacccGaaCcCaggac
accaaAaGacaCAaaaaaGgcTcTcaaGaCcTCcAcaGAAaAcaCcgcagccaaaAcGcAcAacaaTCcTaaG
aaaAaAccaaaGAAacacAacATgAAgAaaTCaaaGTcccagaacaaGcacgaGagaAaaacaATaaaAcccG
TgCcccaaaccaaccAaaaTacGaaaacGcaCaaagaaaaTgaccACCAGTTcccGacCcAAgcaaTgcacgA
acgAaacaagcgaCGaggaaagGaaaAcCaAcaccccTTgaccgaatcacGaTaCcTgaCcGcgaCGagcacc
GcaccgCgaTggacacaaccgaGCGaccAAgacacaagaGTGaaaacTcaaaaaacTagccgagaagcgcTTg
GagaAcgcccTcaCcCcGaacTaCaaaAaaacccagacaGagaaaccaCagAacAcagaTCagaacaacaacc
CgggAaaaaaaaAgaatcacGaacacaaaGaGcTagAcaAcaggaccGaacgGaaagCaaAacGaAaCaaAGg
aAgaaaGCTgacccAcgcgTTccGaccaGagaccaaacaagcgcggaaCAaagaGcgcaTGaagCcagaGaCG
cccTCaggaaAacaagccaTcaccaccgcagAAacaaTcGAcATCTcGaaggaGGTGcCaacCAaggTcCaAA
aCTcTaccCaGAAgagacacaaAaAcaagacaTCacGgaCaagaAaaacaagCccGaGcaACaCGacgcGcCa
gcAGAacacCcaGaaTaaTAAcccaacGAacTacTcacCcgaGgaGgCcGgaaGacAaaccaaCagGgTgacc
aTcaccc Tcaggaaacccacga TgaaTgCaAcaaaaaCcgaaGaaccacgcCaGgcAcacGAaacaaccAaaa
GaaCaAaaaacgCaATacCcAgaGacTCaCAaGcTTaaggacaaACaGcgaaagGaaTaaagaacccAgacaa
ggTccggcaacTccccggaaaaaGgccacAaGgCacGcaaGTccCccAaaAGCaaaTgaacgcgcaaaaCacg
acgTcaaACcaaaaAaccccCgccacagaccaATAaGacggcTcTccccccaGaTGCaccaacAccGaATgTc
ccTCaTacaTcacaaTTgcccgaaTgagcaaaCccaaaTGAaaCaacaaCcaaacgcATaacaGccgcagacC
gacTagaaCcaGaTAaAGaaGaCaaccAccGACcgcaAAgTacggaaaTcCCagccGacGaccaAaaagAcTc
TgaaAaAaaaaATCagcccaaaaGaAccTgTgacccaagaGAcaCccaacgccaAcccCaccaCgagCCaccA
cAcccaaTaGaccTaaGaaAgGagccaaTcaacaacaaaagaaagCagGAggcGcaacaTaaaccaCGaccga
caAggccagacTAggaCcCgGaccgGaTacgacgGGacCagTacgcccGaacTcCccacgTTaagcTcGagCT
gcAGGAccacTAacaacGaaAcaAcGATTcaacaaTcaCgaaGcAAacgaaCgaCacccTcccggcCaccccg
aCacaaccGaaaccggaCaacaccccAaCTTGGagccgccccgccGccaaacgGAaAaGGgCaTaGTcaagAg
TgaCccaaaccGaaaaCCcaccccCTccAgaaCcccgTccaaagCaaGcAagcgagaaccAagaGccGacCgg
aCaacccccGaaaccgcAcgGgaGTagAaccaccacAacccaTcTGaaaGaacaaTcgcaGaCcCacGCaaag

AAgcaacagacAaagTcaaacccGaaaTCTTTgaaaaCCAaccacCaaagagGcaagacaGcaccgaagaAgc
CaAcaaGTgaAccaAccaTTTGTacccAaaaTgccAaacaAAcaccaaacaacccaacaaGacaccaTaccgc
AaaATcGaagCCaCcaCaaccaAaaTagacaacgcaTacaaAGaagaaTacaacagaaCacaacgTaACTgcG
acgAgaacAacgcagcgaCaaacaCccTccTcCaagacGacaacTgaaacaTgacGcaagccacccCcCagGag
acaaccggaAgTgaacAaagccACccaaccaaCcacccTgacGaaCACcaAgGaTcaccacaAAgacTcacGG
gGaccagaAgTAaaaaccaccaacacTgaGaAaGCccagccaTCTacGcagCacCaGaagaacGccGagaccc
ggcaCgagagccgagagAaaaCcaacaccagTaTccgcacCgTaacaATAaaggaccacaagTagCggaCacT
aacCaAcaacgGAacGCagacagacTTgaTcgcaacagcaaTccaagagaaaaccaaGccCAaacgGGcaGaa
ccCAccaaaaccccaccagcccaaaTgaCccgTCaaaGgacgaggAcGagcGgaaaGagCaaaacaaAagccc
acTcAagCaAgaAaaAcaTCacCcaacacaGaaaaccgaATAcaagaaagCaaacaaagaaagaacccaccaa
gGgaaCacaaTaaATaaccaaacaacaaAggaaaacGgcaaTAAAcgaacCgagcaTgacaccaaacAGggAa
aacaccaaTcCTagaaaAGgaGaGaacGcaaaacacccgcCaacgcGaATaAcagaccTAaCcaaGggaccAc
caGaaCaaGTaaaaGCagacaTCcaGAaGgCCccTaGgTcaaTacggaccaacccCagaaGaagCgccgCcAcC
aaCaagaagAccgAaCaagcaccgaacgaaCcggacaaacaaaaCaaagaggTaTccCaaccGaaacGaaAga
cGcaCaaTcgccgcAagccaccagcGcaaagacAAaAgcaTCcTcaAcacCaaaaCagcccgCgGcgaaCCaA
CCaggGcCaCAaAgAccATcccgATaaTATggGcacCGaaGGTTGGCaaaCagaagcaagcaccGaaATagAa
GacaaggTTggaaaccCaagcccccaaTaaAaaaAcgGgACaaaTaaCacccCcacaGccAaAaacggacGAC
acaccAacaaaagcaacaCaGgcaaaaaTCccCacgagTGgaGaaaGaaaaGaCgCgcaaGaAcaaaaaaaagA
ggGaaCccGccGaCgTCaaGAaaaaaGCccgTACaaGccCAaacccgaacacaGcTAaccacaTcacaCaaaa
cGaGaaaaacaaCaCAccCgCaaCgaaaaaaGcaagaaagaaaTacCTcccgacaaacAaAagaGacaaGTTc
AgGcaCaacaaCacggaggaCagaaacATgaAcgccGacaCCagaaaGaagcaagaagcacacaGaggaaaaa
TaTcaaaaaaagCgcgcaacGcaaAaaTgCTgacaGaccaAGaaGaagTgacaaccGaaagcTaCcagCcTaag
aaaaCcggGaacaGcAAgaacaaaaaaaaaaaagccaaaTaaaccCaaCcaaagTAaaCaacaagggaaaagcc
AacaaaccgaacAacAAagaacccaGgcaaCacgacTcaagaAccGcaaGacAaggGgcAgaagacATcaggA
aGacaacCAcaacaagcggaGgGaTacaaaGcacaaaGgccaacacagCcaCcgcacgacGcgGTaaaccGgA
caAccccGCgTcCcaAaaaaaaaaACaccacAcTgagaagcaCCaGaaacaaTaaccaaaaCcgcgcGTggcc
cGcAaaagGGcacaGccAGTccaTacgacAAaTgaaagagagaccacgacGAaccacgcCccCagaacaaAgg
ggcCcgaCcGGAaaAccgcaCgcGgcaacaacaTccaaTaagCGaAATcAaagccgGcaaaacaGaAccaccC
cgcAaAgcaaacAgagATgTcgccaGgcGcaGaaACaAcgcTaacacacaagcaCcaGccGcAagcAccccca
TccacaTccaAaacaaagcAcAAAgcATcGaTaaACTcAccaaaTcagaaAaacCcagcagcCcgacagGcgc
agTGGacggaAAAGAacgCaaaaaagAacaTcgagacgGaTTaCgcaCcaGcaaCacaaAaacaTCcaccaaA
cccaccagcgagaagaaaCcaCaagcagacgcCaaaaacaGTaaGGcCcgCaacgcaacaCAccaTaCacGag
aAccAacacGaTaTGcAGaacaaCaGaaCcccCaaaagggaaaTaacaACaAgGacaaccaCTcgagccCacc
aaacacccgCCAAaaTaacaccTaggaaGgcacCaacccaGaagacgcGcaaccaAactaGaaGagaAgTcaC
caaaccaacagccgAacCcTcaTccCcggcgTAAaccTcCTcaAGacCcAaacgaAacaaaGcaagcCagaaa
aAagGAgCCacaaGaAcCacaaCaGagcacTAacaaaGaaTcagaagaCGaccaccAccagacaacTCagGac
ccaacaaAaaccacAcccaaaCaTgacccAAcgcgATgaaagaTacaGGttcacaagccgGaaaTacAaagac
aGaaTCcacccacaaAaAacTaCgagaaaaacGaTaagcccTacaAaAaTagttttccaccacAaCaaCacaa
gaaacgcaaaaaaTaacaaGTCcaTcacgGaCTaCTacAcCcaGTgaaAcaaGaaaaaaccCTagaATcaaaa
ccGgaagcGaTTCaggaaaaccGaaaaagaaaacaAAccccaAcaCAgGAaccAaATccaaTccgaccGaagC
gacaaacAaacaaGAccTagGAccaccaacaGaaccaTgccgGGTcgaAagaaaTGgggcCacacagaAggca
CTacTccGacACggcCagGaCaTacaaaCgcaccaaccccAcaaAaaacAaaccccaTcaacTagagAacCaa
cgacCcGTaccgacgcaAcCcTtGgGAaacgaTaGcgATTGaccccgTgGgcCcaCTaaccaagcCcacgGaC
aGcaacGcaacagacGacgcgaTTGcAgcaAcaagcGaccccagaAcgcgaAgaggaaaCagaggaacaTccC
gcaCcGcaCcaCcaaGccaAagAcaTcccccagTTAggAcgCgcGaaGaTCTaaaGaCaaaaCGGaTgaCcCa
aGaaTagagTaagcGaaCcaaATccCCgaTcTaccaccagATTccGacaAcAGcTTcGcAaacTGaaCGaCAC
CaaaccCaAGATcTCaAgaaccTaCcacccccagAccaacgcaTccaaaGAgcaCGcCaaccGaacCAgcacC
cacaTGaaaaaacaacccggaagacCgaagCaaAAacacTcccacAGCcggaTaaCTggGaTaCagTcaGaca
cCaaccCaacaagcTccTacAcCaCaggcaacgCcccaacaTaCcccaacaaaGaccgcacaccaaGAaagaT
caTccGgaaagcacCccgaaaacaCgTccaGaAggcCaGcGgcAaacaaTaagCaAAcaaagcacaaaagAag
acgaccAcggccCACgaaaacagaTTaccCGCaaccggcaaaaaAaaGaacaaCccgaagaGgcCcGcaAaGa
AgCcccacaaaagcAAcaccacTcGaTccaCacagaaaCGgacaacagcTaagTacccaAagcaTTcGccCac
aAACTGGTaccaAagaaaagGGcCcgaacCccAcCcTacaacacagcaaGaACaacTccaATcacagTgagC
aaaccCcgcgccacaaACaacGAgggaTTaaTcaTacacTTCacacaTcTCggCgcAcGAggcaTaTcCcTAa
ccaacaacGacTacTCccTGGAcaaCgacAaAcaCaggccTGacaCaTcgaAgaaaagGaGgcCaaaggcaCc
cgcagCagcagcAacTcacaaaaCccCTcacgcaccagcacagaacTTgaacccGaaccgaGccagGGaaAcc
ccaagggaaccacagagaacaAgTgcaaTaATagcacccaacgaaaaCacTagaacaaGgcTcTgaacccgga
caacAccaaCaaccgcCccaaaacaaccggcaacacTCcccaccacacTcaGaTacTcacaaaaGcgaTgcaa

aTcaaccaccAaaTaccaGaTgaaaagccCaagccGGaaCcaAAGgccGaaCaaGaCaaccccTcagGacacaa
gaAagcAacgcacGaagcCTAGccaaaacCaaggaaggAaaggCgaccaGaaacCacAATagcTaaAgCGgcT
ccaccgCccaaTTCTaaAacaCcaagTTCCccCaacTcCCagcacCgAacgcTTaCCcCcaaCTCccccccc
cccccgcCcaAgggcaccgacaccaAacccaaagagaaacaaacacaCGgTCcccacaAGggaaacCccCaaG
cTcccccgGGgaaccAaCcaaaccaaAaacccacaacgcacaTTCaCaaacgaacagagcAaccgaaTaaTag
cCaacgaagCggaccTacgAACaGAacccccAcGcATcgaaacCATaCcaaTaacaGCGGcGgTCacgcaAgT
caCaGAgcgTcgcaaccccccacaacaacccaaaaGcGcaCcccTccggacccaGagcGGAcGagCaaGgCaaa
caaATgcacaccGcaacaaaccTcAcaaccgcagccacCagcGGcggcacagCCaTcCCCGcccacaTGgaca
aCcTGGGGaaAaccaaCccTACGcgacgccAGcgTaTCccCGaCaaGacaacgcgccgcaacatccttaccat
gttcgccagcaccgccgaaacccaGCacaaAagcaaaaagagaTCacAccAaGaGgaccccaagaGaacccAA
GaccGcgagGagaaagCaaagaTcacCaagaaCaagcaaaaaacacgcGcCGggCcccCcaCCaAcaacAGcA
AGAacaAcaCaacGacaacgGgagcGAGcaccсTaaacGacAaAcccgcTTcaaaCaAgAacacAaCaGccga
gaccccaGccAGaaccaacaGaGccgaccCgacaaAggaCAaGacGaacgaaTagTgacacTacccacacCAc
acGAgaCaacAcAcaTaAaagaTaaaggaggaaTaGgAaccaagccAaaaaCcaTcGaaaaCagcgGgAacga
gcaaGagGcAcaCaaaaAcaaaacaCcaacgTAggTCacggGacAgaaaagacaTacCaaagGAaaTAacgga
GccgcaccTggccGccTaCaaCggaaacGcgACa
>Cligib1_Cluster18#LTR/Copia
TGTTaAaCttCAatNCTctaaCtgtaaGtgcCctGcttACaTAaTccGgTgtCtatataagcACccTAgataa
caccttatcttAtcttaTcagTagaTtagaTtaTagatagGagtTaggActtTAgcacAgccacatggtctgt
gcacatTattgTAgtaTataAgatCCctctgAacAagaggctaAtatAactcttTtNtgctctcaacacccacT
AGtGTaagctaAccGctAtCcaAgTAaGTctaCcaGttcCgctgtGccctAGcGgTGccGcTcAACAggttat
gggcCcTgGtgCcaCtGtctaccgcgcCtgaaTaaGgctGcaaccgAcagacTggTcacttggTCgccAttAc
aNgcgCctgcaTaaggcTgcAtGaAatgcgctTccactgggttacccAgaCAactTgccGcgcctgAataagG
CtGcagcTAacagTCtggatGaGAgcAaTgctcgttgcaaTgaacgtTttacgatcTtgCaTcTtgcAcctct
cttgaagTAatgcTctTgctgTgaacgTTTtacGAtccaGcaTgttgCtAcTtcaccacATatgcCtgaATaA
ggcAgAtgTcGggTTaaccattGaTgcgtgcAtaacgCtNAtgGtttataTctCatcAtTAtaacatGAggct
aacgTcgCccctttacTAgaCacCCTCatcAaATtttgatctGATcacTaTgTccAccggTggaaAacTCgAt
aTCAactCtattcccaaActcgatgggActAActgGcgaGactgggaagaNaaaaTgtctaCcttcctCCgcT
ttagNTCTcTcTGgcaaATtAcNgctgaagaTggcCgaGAAgtcgaAcccTtAgcCActactgTCgGcaatNC
taCTActcttCctgAcccAgAtgcaGtcaCCAaGTtCTacaacagGGatgaccaAgcaAcTgGTtctATTcTt
cTCACtctctctcCcgacatcGccgccGcattcAAttcttAtacgActgcccgTaaacTatgGGccgctctca
aagaaaaatacGgcaccTCTgaTccatCCagaTctTtcTGGactttcggTctGccctcgccGtaaaaaTtag
tGgcaaTtCcaatcctgagCCtgAAataatggcTAtgTcAACCtacTtctcccgtctNNaggccaacgcCgtt
TcTcTcctgcctttcgTtaagcaatGAttctTTtagcaGCcattCCatccaaatgGgATaatgtcgcatctca
AatcctgcaggTgcatgctcagtCcgcCctCaccTTcGaaatagtacgcAtNGCcatcgtcAgggAattccaa
cgAcgcACtgcCatcTctGgccccGtcaaagccAacAAAATatCTGgcatcaAAcGAaaGgGcaagAANCCaA
acTggaaggaacAaGCtAaagaAaCggcaCctgAgaacAcgtCTGaaaagacaAGttCgTcgAAGGtaaggc
cAaagaNAccAacaacgaCaaATccaaaccaCGttCctctaagggaGGaaaccGtgaggagcgccgNaAaAag
cgccatGCCAagcAGGctGaagaagCcgcttcCGAcTctggatccGcCgatggaacttcccaatatgGgaatT
caCAccttAtNtcctcggcattcATtGtAgAggttccaccctcAccaCcaaaGgtNaCtcgttCgaaaccNTT
ggGcccNAaggagcgctccCtccttcAaCgTctTcatGaCaaaCcGgcccaAacaTtccAAggagccaNgcca
GgtcaatTNcctGgCacgTAcccgAcTtaCgAAaaagcTcggtcTCttgcttcatgcAttGgaGTtgcgccCt
cCactGagActcTcaAaattcttGagacTcaAcaaaTggcgCttcCagAAtcAcGcgTtcttctgGAtTAtGc
ggcTCaTgacatcactGtTttacCgccaGctgaacgcccTaTccgaccactcCCctCTcCtcCtcGTgCttcg
acTtcgtcgctTcgtgCacCcaCtcCtGaGccGgtaatTtcTCcaccccactcaaacacacccGaTggactAa
ATgaTatCctCAtgggacaagAggAgtAtgAgagttggaTcgaTCAGtActgcGgggaTTcTgaagAagaAga
cgcaggtactGctTAcGTcaatTaTgaCGtaGaGgcaTggGTtccGcctccTaaaaaacagCgcCtatcTCcg
atCGaTgaaGAtgAcgatCgaGaGgaaccGGTatcGatGggTGAagaacCtgAGttcgatCcGGaaagttGGG
gtgcTcggtTTgaaggaCaatcgtcagTcaccgCtTcctttCttccNttTtatGagaCTaAacatGtcatttt
acAGGgCGctcTtcGAttaaaNaTTgattgccccсTGtatgataAAtAtgccAcatGTaCttctttGtctccg
gctgaTtTggccaatataaAaaacatttTGcttcTTgtGCAtcaTgTaagggaaaAAgAaAGCAcgtcgAaaa
ccaAtggTtAgtNgacTcTggtgcCtCcatgcaCttcAcCgcTatATtggAtgAtTaTGcggaAcATtcAatt
aTAAaaaaccccgtaaCagttGcTaccgcaacCGagaTTattcAaatcgaTActCaaggaacggttAtCCttg
AAaACGAtGtccTacagacagatGgaTcatAtaaaaagcaACccttTcgcttacatcCtgTctatcaCcatCC
AaAacTGaACtcccGaAtcatTtctatGggaGaattACtcAattcTgggttaAatAtgagcGcAtcatccaaT
acgcTtgtATtcAgTtcaaaTggaaaAcgAaAaTTaaccgctgAaaagCgtttCgtatgggacaCaacatTcT
ggGtcaccggagaTGTggtcaaAccNgatCaatTaAaaTCAtcTCtattccttAacaaTatcgaaTcAtaTga
CattTGgcatAaAcgcCtAggtCaTcccTcgaaaGaggTcaTttcTaaatctcgtaaacatatcaaggacttc

ccacagggacttaTCatcccaAaGaacTctcctatttgcCCtggttgcgcgtaTGgAAagaTgccctctTcat
cTttTaaAccAtccaaCtcGcgAgccacgaAgccattcGaaTTgatAcattNggatTtaaAAagCttcccNgT
gaaatcaTatCatGggtatTcaTaTAtcAtcacAttccttgAcgaTTaCAcgtcatatGtttGGGtAtcacac
ctAAaaCaTaaatcaGATgcaattggtgccttccgtaTattctatgcagaagttaaaaaccAatAtgaCgcTa
aAatNAaGcgttgGcgtTcTgatgCAggggGagAatTcAAatcccaggaaTttattgatgAatTGaaaTcttc
gGgcatNcTTattgaACAatcagcacctCatAcAccacaacaaaatggtCGcgctGaacgTatNaatcgAacT
aTtaTggataAaggcgAgTcNATgcgAcaNgaTgcatgccttCctagtTcaTggtgggAGttTTctGTggaaC
aagCggTtcAtatcTataaccGTacAccTatGGtgcGcNtacaatgGNgaactccatatgaagTcctcaaggg
TgAaAgaCcctcNgtaacAcatCtTCGTgTTtttGGctgcgctgcatacGTcTaTattccatcAgAAagaCGt
gccaataaacTtgcGcccaaatccGAgCtAatgaTttAccTcggtCATAcTgcTggAaTtaaagGaTaccGct
tcatGcgaatCaNaAactccgTcAtCtTTacaGgagttacaGcTaTctTTgatgaagtaActtTGCcgAaatG
caAaAcaaaAacTacggTAcccgaAcAaattaCtattcCtccaccggACgaatTattTcctccaTcTcCAccg
caatcagAgacacatcTtccAcctCGtCCTNTttcTCcaCcTaAagagccTcatccatggGctccNAcGattg
aAgaagaaccaGaagaagAAAtacAttctCCtgttcCaCcaGgatctccTatGcgaggTCAttCtcCtgaACc
atAcaCTCGCGtNttTaccCcaaacCGNgaNGAtcagGaagaatggaacgAaNtacCtaGACgaAgNaCacgt
CGaaAgAtagtaCcTAacCgtccagggaaTaTataTggagAaAAgaaNCttccTtCTGAaatAgaacgagaTa
tTgcTagTgaTcgatacTGgcGtaAaaATgTggagaAagGtccNgaTagagaccttcctGAttATccCtctaC
cGatGaaGaaGaattAcatcttctAcaaatcGtaCaAGagGaAGggGGagcTataatGgaAtttttattNtcc
aaaGcagtACccNtcaaagAcGAagttCcTAcTCacttccgAgaCaTctTTaaGcttGataaAaagcTacAaa
aagAgtggaTgaAcgcTtgCgaaGagGaattagACtCccTcaaaaAtcgcGAcgtatAtgaaaTatGcgAtct
acccAaagGaCgaaaggcgattCgcaatcgctgggTTttcAacgTcaaatCtGacgGTCgaaAgaAaGcTcgN
cttGtagcCaaaggaTattCacAAgtcgaagGtgttGacttCgatGaatTattctctccggtTgTACgATatg
aatctGtgcgTcttaTGatAgccaCagctgccctgAaacattggGttattctatcAcTtGacgttcGaactgc
CtttCTttaTGGCaaattagaTgTTgagatataCatgcatcaaCCagaaggcttcGtcCGaaaaggccATgaG
cgcaaagTccTgcgCcTcaaacgagCCTTatAtGgtCTgaaacaagCAGctcTtgaaTggtGgagaGctctCg
TtGcgTcaatgaaNgAaaTGGGcTTtcaaCgcacTCtTTcAgaTgCAGgcATTTTcaTtcaccgatcAtcctC
aaAccaGattGTcattgcgatAgtcTatgTtgatgatGCctTattcttGgggcCtAAtcggtcGTatGtaatc
aaGaaaaaagcagaatTTatGcgAaaAtgggaATgCagggatttAGGcgatGCtaaagaattccTCagaatgc
gcAtctggcgaaatAaaAaccagatcaaaCttgaccaGaaaacttAtctgGTtaAagTAttggagcgTTttGG
aatgcttaatgcacgcgCtgctAaaacccCtttaccAcCcaAgtatctAcCcgAGAgaAaTctgGgaccAata
GatcacaccttacgaagcAaattTcaatCCgtcAtaGgAtcTttgcttTacTtaatgttaggaaCtcgtCctg
acattGcNttcgctgtgATtAaaATgtcaCaaAtgagtgcgaatcCCacAcaagAGcAcctGNaNaaGgCacT
cTacNtaTgccgctAtNtagCcggtactcaggattatTccNtgaattaTaaTggaGaTtCgGNgaAggcttTaA
TcgctCAtAcAgatTccgANtGGgcNtcagatcctAataCtcggaAatcaaccACCggtTtctTtgtaacctT
gcttccggtGctGtaActTgGAcgTccagAttAcAaAaaacaATCgctcaAtcATcTaccGAGGcaGaaTAta
ttgccatGtctgacacttgTCgacaggTagTttgGttAcaatctCtcTtNgGagaacTtggaatcaAaCttga
caaaAtaCcAatTgTcggTgATaatgctggATcaagctttAttgGttctaAtCcAGtNCaaGAAAgacgaAcc
aaAcatAttgatattcgtcatcATtacAtacgtgAatGttatgaNgaNggAaaagtNactatActttTCaCtg
aagGAgataTAaatcCtGCagacaTttttacCaaaAgtcTGggTaatatcaaattcaaAgaattcgTaCcTca
ANtgggActtgaGTttacTcaTcttGAagtttgaAgtAtcgctTatagctCctgCaaAggtNGttatatgca
tCcGctGttCtAaGcgagggggagTGTTaAaCttCAatNCTctaaCtgtaaGtgcCctGcttACaTAaTccGg
TgtCtatataagcACccTAgataacaccttatcttAtcttaTcagTagaTtagaTtaTagatagGagtTaggA
cttTAgcacAgccacatggtctgtgcacatTattgTAgtaTataAgatCCctctgAacAagaggctaAtatAa
ctctTtNtgctctcaacacccacTAGtGTaagctaAccGctAtCcaAgTAaGTctaCcaGttcCgctgtGccc
tAGcGgTGccGcTcAACa
>Cligib1_Cluster115#LTR/Gypsy
ATGGTGGTTCATAATGCTGTATATTCATTTTGTTGATATCTTGCCTCCTAGAGCAGCTAGAGGCCTAATTGTT
GAATATTCTAAGTCCAGTATTTCAGTCACGATATAGTGGACACCTGTTTTCACACCTCAGGTGAAACAGGCCA
AACATGTACAAAGGAACTGTAATTCCTACTATGTCCCGGAGTAGTGTTTGCTACTCCCTTGACAACAGTATTA
GACAATCTCATACTTGCATTCTATTATCAATATCAAGTGATTAGAGCATCTATCAGAGGGTTATCAACTGTGT
CTGGCTATACTCTCTATAGCAGATCAGTCAGTGAACAACACTTGTTAGTTTATTATGTTCTATACTAGCTGAA
TCTTCTTGAGATGACCTTTGACACCATGACCCTTGGGCTGGTACCCTTGGGCTAGTACTATAGGCAGAAACCA
TAGGTGAGAATGGGAATGACACATGAATGGTACAGGAGTCATTTGTGTATTGGCAAGATGCCCCAGAATTTGC
ATCACACATTGAAGAAGTTCGGAAAAAAGCAATCACTGTGTTAGAAGCATCAGTAGACTGCATGAAGAAACAA
TACTACCACAAGAAGGACTCGCTTAATCTTAAACCTAGTAACAAGGTGTTTTTAGAGGCCAAAAATATTACAA
CCACAAGACCATTAAAGAAGTTATCAGAACGATGATTAGGACCCTTTGAAGTCATCAAAAAAATAGGTCATAG
CACCTACAAACTCAAACTCCCTGCCAACTGAATGCTTGTCCACCTGGTATTCAATGAAAACCTCCTCACCCCC
TTTAAAGTAGTACACTTCACCTCACAACAGAAGCCATCACCACCACTACCCAATGTCATCAAAGGTTTGGAAG

AATACAAAGTAGAAAGCATCTGAAAGGTTAAAAAGAAAAATGAAAGACTTTTATTCTTTGTTCATTGGAAAGG
ATACCCAGAAGAAGAAAACACCTGGGAAACCCATGCAAACCTCAAATGTTCCACAGATTTAGTTAAAGATTTC
TACTGCTGACATCCTAACACACCACGATCAATATCACACTCGTTTTGACCATTCTACCTTACCCCTACATTTA
CCCAACTCACTAAAACACATCCATCATGCCTGCAGTCATTACCAATATCATCAATCTCATTCTCCTTTCCCTC
TCCCCAGTCTTTATCATTAAGACTTATACCCAACTATCTGTCCAATATCTGGTGACACATTTGTAACCTCAAT
CAGATCATGCAACTCTTCCCTACCAGCTCCCACCCTGATTGTTCAACTAAGCATGGCCAAAGAACATCTCATC
ACAGCCTGCCAATCATTCCTTATTCTGGAAACACAAATGCATGACCACATCACTACCACAGTGCAAGGTCTCC
TCCCTGAACTCATGGATGAAGACTGACAAACCCTGGAATGAGCAGTACATGTGACTGAATGGAATGTGCCCAT
TTCAGAGGATGCCACCTATGAGGAATACAGAATCCCAGTTACTACATCGCCCTCCTCCAATAATTCTGAGTCT
CATCCCTCCCCTCCTAGCATTCCCCAGCCAACAAACAACAAACCCATTGGCTCCTGGCCCTCTTCCTCACTAC
CCACAGGATTCATACCCTTTGGAGTACTTCCCCTTCATCGAGTGTCCCACATCCGCATCCCCTCAACTTCGAG
CCCATGGAACCTTACAACAGGACATACTTGTACAGTGGCAAGATGTTCCAGAACTGGGTGAGAGGACCGCACG
ACTCACCCACTTTCTTTACATGTATTGGCAAGGAGTATGTAACTGTGAGAATGAAGAAGGAACTTGGGAAGAT
GCTGGTGAACAATGATCTAATTTAAAGTTTCAGGATAAAACTCCTAGAGGGAAGGTCATGTTACGATTCCTTC
CTTCTTATTCAATCATCATCACTTGGGGACATCTGACTAATAGCATCATTCATAAACACTTCTATTACCTCAA
CCATCATCCTATTATCATTGCTTCTTGCATCAGTCATCATCCCCCATATGTCTTACCATACTCACGGACTTAG
TCACTAGTGTGTAGTCATTGAACTACTTCACTTGTATATAAATGTAGCTACATACTCCTGTATTTGCTATGTT
TAATTGTTTATTGGATTCCCCCTCTACAGCCTTCTGACCCAGAAGGCAATTCAGACCTTGCTAAGTGCCTTGA
GTCTTCACTGTTGACTCATCAGCTTGTGTTAGCAGGACATGACAAATCCCAAGGCAGCAAGCAGTTCACGTGG
GATCACAACCCATGAATGACCAAGGAATGGAACGAAATGATGAGTGCTATGTGCTCATATGAAGAAGACAAAC
TCTATATCAAGACCTGTTATCATTGGAAATATCGGACAGACATAAGCTAACAAGCTTATACCAACCAAGCATA
TGAAGCAAGGATTGGATGAGGAAACAATCCTCAGTAAAACAAACAATGAATAAATATTCGTTGAATTCTAAAC
CCAAGAAACCAGATAAAGGAAAGACACCAGCAAGAGGTTTAACCGAGAATGATTATCCTTAGCTTAGACAACA
TTGGTATGACAAATACCAAGAAATAATGGGAGGAATAAAAAATGAACTTCCCCCCTGGTCAAAGTGAATCATG
AAATTCACTTGATTGATGAAAACAAGCAGTATCATTACCACTTGCCCGGGTATCCAAACTCACTATGAGATGA
GTTCCATGAAAAAATCAACAGATATGTTGATACAGGATGGTAGAAAGCCAAATCAGTGAGCCAAGTGGCACTG
CTACTATGCATTCCCAAGCTGGAAGGCTATGCACTGTTGTAGATGCACAACAACAAAACGATAACATGATAAA
GGATGTAACTGTTAGAAGTGGGGATGACAGTACATTGGTACTGACAGATAATCACTTCATCTGCGCTTCCCAG
CACCCCCAAGTACATATATGATACATATATGGACTCCTATAGACGCCCTATGGACCACATTTAGTATCTACAA
TAGCAGTTCATAGCGCTTGTTACTCATTTCGTTGATATCTTGCCTCCTAGAGCAGCTAGAGGCCTAATTGTTG
AATATTCTAAGTCCAGTATTTCAGTCGCGATATAGTGGACATCTGAGTTCATGCCTCGGGTGAAACAGGCCAA
GCATGTACAAAGGAAGTGTAATTCCTACTATGTCCCGGAGTAGTGTTTGCTACTCCCTTGACAACAGTATTAG
ACAATCTCATACTTGCATTCTATTATCAATATCAAGTGATTAGAGCATCTATCAGAGGGTTATCAACTGTGTC
TGGCTATATTCTATAGCAGATCAGTCAGTGAAACAACTTG
>Cligib1_Cluster113#LTR/Copia
GTTGACAGTGGAAGATAGACGACGGGAGATCTAACAGATAAGTAAGATCTCAGTGGATAATAATAGGCACTAT
ATTGTAGTAATACCTAACAGATAAGTAAGCTACGAAATATCAGTAAGGTAGCTACAATGAATATAGGAATGTA
CTCACATCTCAGTGGATAATAATAGGCACTGAGATGCGAGATGAAAAATCGTCGAAGAGATAACAGACGTGAT
TACGTAAGACTAAGCGAAATAATACCAAGTCCGTAGAGACGCTTGTACCTCCAACACTCCCCCTCAAGCGGAA
CGCGGGAATTCAAGTCCAAGCATTAATCGAAAACGTTCGAGCTTGAAGCGACTGAGTGATTTCGTGAAGATGT
CAGCAATCATGTCATCGGTAGGGCAATATGGAATTTTGATGTGATCATGTTCAATTGATTCACGTACGAAATG
GAAGCGGGTATCAATGTGCTTGGTGCGCATGTGGAAAGCGGCTTCCTTCGAGAGAGAAATAGCCCCTTGATTA
TCACAGAATAGAGTGGTGGGAGAGTCGGAAGGTGAGAATAGGAAATGGAATTCAGCGTGGAATTTTCTATGCC
ATAGAGCATCTTTGAGAGCGTGAGTGAGAGCAACATATTCAGCTTCGGTACTCGAAAGCGTTATGATAGGTTG
CTTCTTGGAGCTCCATGAGACGACACCAGCACCTGTAAAGAATGCAAAACCGGAAATGGAGTGGCGATGAAGT
TGGGATGCCCAATCGGCATCAGAGTAGCCAATAAATGAGGTATCTGTACCTCCGAGAACTAAACGAAGATCGG
AAGTGTGAATGAGATACCGAAAAATGCGTTTAACAGCATTCCAGTGAGTGGTATGGGGATCCTCCATGAATCG
CGCAAGGCTCGAGACGGCAAAAGAAATGTCGGGACGAGTTACAGTGGCACAATACAGAAGTGAGCCGATTGCC
TCCCGATATTTTGATTTTTCGGTAGGCGTGAGTTTCGTGGATGAGACAGATGGCAAACCTGGGCTAAAGTCGG
CGCCAGGCTCCATTGGCGTGAATGCAGGGCGAGCATCTGCGAAACCAGTCCGCATGATGATTTCTTGGATGTA
GGCCTGTTGTCCAAGAGAGAGATAATGTTGCGATCGAGATCGCGAGTGATGTGGACACCTAATAGCCAGTTAATC
TCACCAAGATCAACGAGCTCAAAGTGCTCATTGAGTTGCTTCTTAATGAGTTCGACGGATGCGAGGGAATTTC
CAATGATCGTGAAATCGTCCGTGGCTGCAGCAACAATGGTGTAATCTCCATCATCGGAGATCTTGTAGAAAAC
AGCAAGATCAGAAAATGATACCTTGTACCCGAGGGCCTCGAAGGTTTCCTTGAGTTTCAAATACCATCCTCGA
CCAGCTTGTTTGGTGCCGTATAGCCCCTTTTTCAAACGACAAACAGGACGCCTCCATCTTTTGTGTCGGGGAA
TTTTTCGGAAGAGGAGATAACCAGGAGGAAGGTGCGTGAAGATCCTTTCCTTGACGAGAGAATTTAGGTAAGC
GTTCTTGACATCAGCCTGGTGGATAACGGCAGATTTGGCAGCGGCGGCACTCAAGAGGTTGCGGATAGTCTCC
CAACGGACAATCCATACACGAGTGTCGAAATAATCAACCCCATAGACTTGTGAGTAACCTTTTGCGACGAGTC

GAGCTTTGTGTTTGATGATTTTCCCATCTTTGTCACGTTTATATTTGAAGACATAGCGACAACCAATAACGTT
GGCGTCGTCGGGCTGCTCAACGATTTCCCAAGTCTCAAGTTTTTCGATTTGATCAAGTTCAGCCTTGATGGCC
GCTGACCAAGCGTCGGCTTCATCTCCCTCGAGCGCTTCTTCAAGGGTTCGTGGTTCACCACCTGTAGCCATTG
CAAGGAGGTGAGTATCCTCTGCTAGGTTCGCTTCTAACATCTCATCTTCAACAGATATGTCTCCATGGTCTAG
GAAATCCACTCCTTCAAACACTGCTGCAGCATCAGGCTCTGAGGGGGGCATGGTTCCCTCATGTAGGTGCTTG
AAGAACCCTGGGGGCTTCCTAGGACGAAAACCGCGACCTGTATTCGGCTCAGGCTCGATAAGACCATCGCTTC
GAGGGGCCTTTTCGTGGGGAGGAATTGGTGGAGTTTTGGTTGAGATGGGTTTTTGGCTGGGCAAAGATGAGTC
CGTATCTGATTCGTTTTCCGACGGATTTGGCTGATTGCTGGACCGTTCGAGAGCGGTTCCCGAGGGCTTCAAG
ACGCGCCTATCTGCGCTAGGCGATTCGACCGGTTCATTTTCGGTGCTTCGGCTGGGGGGGGTAGAACTGTTGG
GCACATTCTCCCCTTCGATCTGAACGGTTTCTGGGGCTAGAGCGTCACCCTTATGGAAGTACACATCTCTTTC
CACCGTTACTGATCGCTTTTCAGCCCAATAGACCCTACAGCCCTTGGATTCATCATCAAAACCCATAAAACGA
CCCTCAACAGCTTTCGGTTCGAGTTTTCCCGAATCGGTACGTTTGACCCACACAACACATCCCCATTCTCTGA
CATCGCTTAGGTCAGGCTTCTCTCCAGTCGCGATTTCGAAAGGAGTTGCGTTTTTCGCCAGTGCACGGGTTGG
GACTCGATTTCGCAACCATACGACATGTCGTGCAGCTTCAGCCCAGAGGAATTGAGGTAGGCCAGAGTCGATT
AGCATTGCCCGGACGCCATCCATGTGAGTACGGTTTCCTCGCTCACTATTTCCGTTTGATTGAGGGCTATCAT
GGACGGTTAGGTGCCGAACGGTCCCTGCCTTCTCCAGATGGTTCGTAAACTCATCCGAAGTGAATTCTCCCCC
TCGGTCAGTGCCGAAAATCTTTATCTTTCCATCGCGCTGCATATTGACCCATGCCTCGTATTTCTTATAGATT
TCGAAGCATTCCGATTTCTTGGTATGAAAATAGATCTTTTCCTCATGCGAGAACTGATCCTGGAAACAGGTAT
AGTATTTCCGACCACCAAGGGATGTCGTTTGGGCGGGTCCCCACACATCAGCTACGACTTTATCACCATAGGC
CTTGGCTCGCTCGCTCGTACTCGTTTTTGGGAACGGTTTGCGTGTCGCCTTTGCCTTGATGCATGTAGGGCAA
AAATCAGGCTTTGATTCGAGATCGAGGTCAATTCCGGTCACGATTTCGTCCTTAACCATGCGACATAAGTCTT
CGTGGTTTATATGGCCCATTCTCTTGTGTAGATCACTAATTGTGACTGATTTAGATGCAACTGATGCCTCGAG
AGGGGTTTTCGGCTGTTCATCTGCAAGAGAGACTCGATATAGGCCATGGGACTCAGGGATTCGGGCGATAACA
CGAGAAGAGGGTGCGGGCATGGTAATTGTACAGGTCCCATTGAGGAATGAGACGGAATAGCCTTTCCGATCCA
TTTGGCTAACGGAGATGAGGGTAAATGCCATCACGGGTGAGTAATAGACATTCTGCAGCTTAACTGTGGTAGG
TTTTTGATCTTTGCCCATCGGGAATAGGACTCTCATATCCCCTCGGCCTGTTGCATGAAAAATGCGGCCGTCA
GCAGCGCGGATGGGTTCTGGCTCCATTTCGACAAAATTAACAAATTGTTTGTGATCAGGCGAGAAATGGCGGC
TGGCTCCACAATCGATGAGGACGCCATGGACTTTCGAGGAGGATGAGAGGGCGGTTGCTTCATTATGGAAATC
GGAGGTGACGACGAGTGCGACATCATCTTTATTGTCACTCTTGGTGGCATTATGTGCGTTCGACTCTTTATTC
TCCTTTTTCTTCTCCCATGGAGCTTGACCTTCTTTCCCTCCCCCTTTCTTATAGCAATCGTCGCGAGTATGCC
CGAACGGCTGCAATTTGAACAGGTCTTGTCACTTTTCGACTTCAATTTCCCTCCTGACTTGCCCTTTCCCTTT
CCGCTTGCCAACATTGCATTTTCGGCATTCTTCTCAGCCCGAGCATGAACCGATTGTGAGTCAGCTTCCTCCA
AAATAGCTTGAACAAGCTGATTGGTTTCCATAGGCTTTTTCATGATGAGAGTAGCGCGCTGAGTGATTGGAGA
AAGGGCGGTAGGATGTGGGAAGTGATTTACGAATGAGGGCAGTAAAAGACCCTTCGTCGACTGTTGTACCCAT
ACCTGCGAGCTCTTCGCGAAGCTCTAACATATCGGTGACATGTTTTCGAACATCCCCGGATTCTGCGCAATGC
ATATTATGCAGTCGGGTGAGCGTATCGACCTTTACAAGGTCACCTTTATTCTCGAAGATGCTGACGAGTTTGT
CCCAGAGCTTGGAGGATGTTTCCTCGCCCTTAATTTGGAGAAGGGTTGTTTTGCTAACGGTTTCGTAGATTAT
CTCGCGAACCGATGCTTCCTTCTGCTCAAAATCATCGACCTTTTTCTCGTGCTCTTCGAGTTCCATCTCGCTC
AGTGGCTTTGAGTCCGGAGAGGCCCCCTTGATCGTCCACTTGTCGCCTGACAACTTGAGAAGCTCAGATGGCT
TCCGCGCGGTTCCTAGAATGTGCCGTCGAAGTCCTTTGTGGGTTACCATGTTTAGGATGCGCTCCTTGTAGGT
GACCCAATTAGATCCATCATCCTTGAGTTTCGGGAGATTAAGGATCTTGACAGAAGAGGATGATATCGAGGAC
ATTGTAGGTTGAGCAGGGCCCATAACCTGTTGACAGTGGAAGATAGACGACGGGAGATCTAACAGATAAGTAA
GATCTCAGTGGATAATAATAGGCACTATATTGTAGTAATACCTAACAGATAAGTAAGCTACGAAATATCAGTA
AGGTAGCTACAATGAATATAGGAATGTACTCACATCTCAGTGGATAATAATAGGCACTGAGATGCGAGATGAA
AAATCGTCGAAGAGATAACAGACGTGATTACGTAAGACTAAGCGAAATAATACCAAGTCCGTAGAGACGCTTG
TACCTCCAACA
>Cligib1_rnd-1_family-169#TIR/MITE
CAGGCCATGCTGTCATNTTGATATATNAAGTGTCTGTAATTACAGACAAGCCGTGATGTATTAACATCTGTGG
CTCATGGTGCATCACGTGACCAATTACGATGCACATGATTANTTACATGATTACATCACCTGATATGTGCTGA
CATAATCAGAACTTTATGTTTTTTTTGGCTCCAAACACAGGCAAATGTTTCACTCTATGTTTTTTCTGGCCTC
AAATTGCAACATATATATATCTATTGCCATCCCCACCCCAAACCTAACTCTAGCCGAACCCTAACCGAACCCT
AACTCTAACCAAACTCTAACCAAACCCTAACCGAACTCTAACCAAACTCTAACCGAACCCTAACTCTAACCGA
ACCCTAACTCTAACCAAACCCTAACTCTAACCAAACNTAACCAAATAATAGCTGATGTAATTGTATCAATTGG
GGGGGGGCANGTGACGTGTTAGATCACATGACCTGATNACGTATCATATTGTCAAACGATAGTTGATGNATAT
GTANTGTATTGGGGAGATGCATTACATGATGNATCGCGTGACGTATCAGATTATGTAATACATCACAATAGAT
GATGCAATCACGTGGTTGGTCATGTGATTTGATCATGTGCTTTGATCATGTGATCTGGTCATGTGGTCAGGTC
ATGTGATGCATCACGTAATACATTGTGTGTATACTGTAGGNGATGCGTCACATGATGCACCATGAGCCACAGA
CGTTAATGCATCATGGCCTGTCTGTGATAACAGACACTTTATATATCACCCTGCCAGCATCAGGTG

>Cligib1_Cluster125#ClassI/LARD
ATTGGCAGTGGATGAAGTAGATGGGAGATTAAATAGATATTATTAGGAGAGTATATTGAGTAGTGATATATTG
ATGAGTGAGAGACAATCTACGAATGTAAAAGACTGGACACGATTACATAATCAGTAAAGCGCAAATAATCCTA
AGTCAAATACTGTAGGTGAGCTTGTATCTCAACACTTCCCCTCAAGCGAGATGGAGGTATTTGAGCTTGTAAG
TAGGTAAAGTATACCTGATATAATAAAAGAATGTATTGCTGAATCATCTTGATATGAGCAAAACGAGTGTGAA
TAATTGTTGAAGTCGAAATGTCCCGATTTGGACGTTTGACTGCTGACTTAGCGATGGTTGGTTCCTCTTGGAC
GCTTCCTCGAGGTTCAACATCGTTGACCATATTGGATAGGTGAGTTGGTCCTCCTATATTGGTTACTAACCCT
CTATCTTCCCCAGATATGCTTCTGGGATCCCGATGATCGATTCCTGCTGGCCTGGCCTCCAAGATGGGTGAGT
CTTGCTGGATAGGCTGACTGTAGCTTCCTGGGGGTTTTCTGGCACGGAAATCGCGACGGATATGCGATTCTAG
GTCGCTTGGGATGTCGCTTTGATGATCCTTTGAGCTGGGAGAGTTTGGTGGGGTTTTGGATATGGAGATATTG
TGCTTGGACGAAGGTATGTCCATTGCACGGCCGTTTTTCATCCGTTTTGGTTGATTGTTAGCGCGCTGGAGAG
CGGTCCTATAGGGCTTTAATTCGCGCCTATTCATGTCTGGCGATTCGTCCGAACGGATTTCGGTCTTTTGCCT
GGGTGGTCTGGACGAAGGTGCGTCCGTTCCGCAATCGTTTTCCGGCGGTTTTCCCTGATCGCTAGACCGTTCG
ATAGCGGTTCCCAAGGGCTTCCGATCGCGTCTATTCGTGTCCGGCGGTTCGTCCGAACGGATCTTGGTGGCCT
GGCTGAGTGGCTTGGACGAAGATTTGCCCGTTGCGCAATCAATTTCCGGCGGTTTGGGCCGATTGTTGGACCG
TTCGGGAGCGGTTCCCAAGGCCTTCAAGACATGCCTATTAGCGCGCGGTAACTTAACCACTTTATTTTCGATG
CTTCGGCTGGGGCTGGAGATGTGGGGCGCGTTCTTCCCCTCGATCTGGACGGTTCGGGCTATTGGAAGGCTAT
TCCTACAATGGTAAGCGTTCCCTTCGACCGCTCCTGATCGCTTTTCTGTCCCACAGATCATATAGCTTGCGGA
ATCTTCGTCAATATCGGTTTTATGGCCTTTAACCACTTCGCATTCGGTTTTTCGAGGTTTAGGGTATTCTTTT
TGTGTTATGTATTCGCATTTTTCAATATAAGTTGGTTTGTCTTCTTTCTTTGGTGTGATCTCGCGCGGATTCG
TGTTTTTCGTCGACATCTCGCACGCAATTGGGGTTTCAAGGTATATTTCGGGCGGTTTATTGGCAAGATCGAC
GGTGCGCGGGGGTCGTGGAGTGGATAGGGCAGGGGTGTCTTCACGGGGGTATGAGATATCACAGGGTATGTCC
GACTCTTCGTCGGTGCCCTTCGTTTCCTCTTCCTGTGGTTCCTGACCTTCACTTCCTCCTCCTCTCCTATAGC
ATTCTTCGTCGGCAGGCCTCGCGCTCGTGCGCACTTCCCTATTCTTTTCCTTCATCTTTTCTTCAGGCTCTTC
CCCTCGCTTTCCAGCTATTACCATCGCGTTTTCGGCGCGTTTTTCGGCATGCGCGTGGACGGATCGCGAGGCG
GCCTTGAAAAAATTTATTTTGTTCTCGAACATGTCGGCAAGCCTTGTCCAGAGCTGCGCGGAGGTTTTCTCCC
TCCTCGTCTGGAGGAAAGTAACCCTGCTGATTGTTGCATAGATCACTTCTCGAACCATGGCCTCCCTTTGCTC
GAAAATATCGATTTTCGTGTAGTGCTCTTCAATTTCGTCGTCGTCGAGCGGTTTCGTACAGGTGTCGTTCGAG
TCCTTGGTGAGTCAGAGTACTTATTACCCTTTCCTTATAGGTGACCCAATTCGAACCATAGTCTTCAAGATTC
GGGACTTCGAGATTCGACATTGTAGGTTGCCCAGGGCCCATAACCTATTGGCAGTGGATGAAGTAGATGGGAG
ATTAAATAGATATTATTAGGAGAGTATATTGAGTAGTGATATATTGATGAGTGAGAGACAATCTACGAATGTA
AAAGACTGGACGCGATTACATAATCAGTAAAGCGCAAATAATCCTAAGTCAAATACTGTAGGTGAGCTTGTAT
CTCAACA
>Cligib1_rnd-1_family-267#TIR/MITE
TACTATCGTTCCCTGTGAGTTGCAAGATCCCTGGCTAATGCAAGATCCGTTTTTGCAAAACCAAATCCCCCTC
TTTCCTCTCCGGTCTTTTACGGGATTCTATTGTAGACTACTCGTACATACGCTCATTTAATATCTAAGAATTT
GATCCCTNTTCTCGATGTCCGAGACGACGACCGGTGATGGGAATTTGATCAATTTCTAAAGAATGTGCAATTC
CTCAAGTATGGGGCCTTTTCGTGCTCTGAAAAAAATCATGGGCATTACACAGGTACATATATATATCATGGTG
TATTTATTTCAGCTCCCCGGTGTGGTGGTCTCGCAGAGGGAGCTGATGAAGAGGGACGGCGCGGTGGGTCACG
AAATGTCACGAAATGTTTCAAAGCCCGCTACGGCATAATCGGCAGCCTGCTTGAGAACGTGGGAAATCTTTAA
ATGAATGCGCCAGTTTTGGTAATTTACAGTTGTGCCCTGGAACGGAGATTGATTTGACCTGGCCCCCGTCACG
TCTGGACTTCGTTGGCTTCCTCTGTAAGCCCGCCACGCCGGGGAGCTGAAGTAAATACGCCATGATGTNTATA
CGTACCCGTANAATGCCCATGATTTTTTTCGGAGCNCGGGAGGGCCCCGTACTTGAGGAATTGCGCATTCTTC
AGAAATTGNTCAAATTCCCATCACCGGTCGTCGTCTCGGACATCGAGAACAAGNATCAAATTCTTAGATATTA
AATTAGCATATGTACGAGTAGTCTACAATAGAATCCCGNAAAAGACCGGAGGGGAAAGNGGGNGATTTGGTTT
TGCAAAAACGGATCTTGCATTAGCCAGGGATNTTGCAACTCACAGGGAACGATAGTA
>Cligib1_rnd-4_family-7832#TIR/MITE
CACACCGGTTGCCCCAGAGTTTAAATGTGCTTTCTACGATCGTTTAAACAACAAATAACAACTTTAAGTTTAA
ACCCAGGTAGAAACACAACTCTCACTTGAAATAAAATATTTAGACAATGCCTTGGTTTCAACTTCAAGTGTCA
GGAGGGGAGCAATCTGAATTTATTCATAAAGTATAAAATTGATTCTACATTTTGCATCACACATAATGAAACA
TCCAAGGTGTAATATTCAACTTCCACTTCAAGTTTAATANAAGGTTTAAATTTTTTTACACTTTAAGTGGAA
TTCACGTTCAAACNGGCAAAAACAGGCCAAAAGGGGAAATAATACTGTGTTTGAACGCGAATTCCACTTAAAG
TGTAAAAAAAATTTAAACCTTTTATTAAACTTGAAGTGGAAGTTGAATATTACACCTTGGATGTTTCATTATG
TGTGATGCAAAATGTAGAATCAATTTTATACTTTATGAATAAATTCAGATTGCTCCCCTCCTGACACTTGAAG
TTGAAACCAAGGCATTGTCTAAATATTTTATTTCAAGTGAGAGTTGTGTTTCTACCTGGGTTTAAACTTAAAG
TTGTTATTTGTTGTTTAAACGATCGTAGAAAGCACATTTAAACTCTGGGGCAACCGGTGTG
>Cligib1_rnd-4_family-4302#TIR/MITE

GTACGGGGATTTAGTGGGAAGTTAAGAACCCCTGTTAATAACACGTGATTAATTACCGGTTAATTACCAATTT
TTGGACCAACCCAGAATTTTGGTGGGTAATAACACGGCACGTGATATCAGCGCCCAACGGTCTGCCCTTACAT
AGGCTACCATTAAGATGCTCGCAAAAGGCACTGGCTTCAATTTGATCTACAGGAACAGTCTCGGGGCTCTTTC
CCAGTTGTTTTGCGATGCCATTGGTGCAACTGGGGGCCGGAGGAAAAAATCAAAAAAATCGCAGCGGAAATAT
CACGTGATTGGGTCACGTGCGGGTATTAATTACAGTTAATATCCCAAGGGTGTAACCAACCGCTGGGGGGGTG
GGGTGTTTAACCCAGTTACTAACCCCCAAAAGTAGTTAATCCCCGAACCCTA
>Cligib1_rnd-1_family-185#LTR/Copia
TTCGCGTTGGAATACGGGTCGCAAGGCATTGGAGATCAACAGACNATTAAAATCGCGGCAAAAGGCACGGCNT
GCGGGATGACGGACTCGGACTGGGCGACCGACGAAACGGATCGCAAAAGTGTTACGGGATATTGCTTCTACTC
CTCGGCTCACTGATCTCNTGGTCNTCCGTCAAACAGAAGACGGTCGCGTTATCGTCNACTGANGCNGAATATT
ACGCGATGTCGCACGCGATCAAGGAGGCGNTNTGGCTCCGACTCTTCCTCACCCTACACGANTTCCCCGTNCC
CAAACCCTTCCCCNTCCTCTGCGATAACCAAGGCGCNCGCTGCATCGCCGACTCCTCATCCNCCTCTTCACGC
TCCAAACATATCGACATCCGATACCACTTCATCCGCGAACGCGTCGCGNCCGGCAATTTCTCGACAGTATGGA
TTCCCACCAGCGACAACGTCGCCGACGTCTTGACNAAACCTTTACTCGACATTCTCTTCGTCAAACATCGCGA
AGGCCTAGGATTGGTTGACATGTTGACCAACCGATAGAGAACTGCTCTTATGGGGGTGTGTTATACGATACAG
AGCAGACACGTGACACCAGACTATATAAGCGTCCACGCGTCGTCTCNCATCCTCAGTTGNTTACCACCACTGA
GGATGTGAGTACGGTTCTACACTTCTATATTCTACATTACTGATAGTCATCCAGTACCCTGACCGTGTCTTAG
GTACTGAATACAGTTGNTTACCACCACTTGAGGATTACCCTGACCGTGTCTTAGATCTAGGCCGATAACCCCT
GTCCGTAATCGTTACTCACCAGGCGGCGGAAGCTCTACCGCTGCTGGATCATCTGGGATAGGCGATGGGCGGT
TGTTGCCGTCCCAGGATTCCTTGCGCTTGTANCGCTTGGTAAAGGATTCGAGAATTCCATCCAATCACTTACA
ACCCTGCATTTCAGGCGGGGGACTCGCGCTTGCTGGTCTTACTCAACCCCTCGGTCGTACGACCCTAACCACT
GGGCTGGGATCGCGGCATACTCCATTTCGCTCGCCGTCAACGCTCTCACCACGTCGCTAATCGTGACCAAGAT
CCTCTTAACCTCACGGGAAGTGCGCCCCGTCTTGGGTTCCGACTCGCATCGGTCTCTCCGCATCGTCGCCGCT
ATGCTGATCGAATCCGGGCTCCTCATGTTCGCGTTCCAACTCGTTTTCGTCGTCCTATTCTCCNTCCGACNCC
CAGCGTTTGGTATCGTTNCTGGCCCAATCACTCAAATATATGTACGTGGCTTCCCGCTGGAACTCGGCGGTGG
ACGAAATTTTAATTCTGGATGCGCAGGGAATTACTCCAACGCTTCTCAATATCCGGGTCGTCATGGGTTCCGC
GTACGACAAAACCACGGAGAAGACCCGCTCCCTGAGATTTGCCCACTCGGGAGGAGCCGCCACTCAGACCACC
GGCCCAAGCATGAGCGCGGCCGGAGTCCAATCCCGGGGTATTGATACCGAACCCGATGAAAGCACTGTTTCGT
TTAAATAAGGAAATAGTATACATCTCCTGGGCGCCGTTTACCGTCATCGGAATCTNAACAGAATNTTTGCCCG
CGAAACACTACGTACCTATTTTCATGGAAGTTANTGGGTGACTTCCCGTAAGGTTTTCGAATAAACATATCGT
ATTTCGATCAAAACCGGCAAGGCAGCGCAGGGTTTAATGCGATATGATATGTTGCTGACTAAACCGTAAGCCA
TCCGAATATAATAGATTAAATATGTCCCAAGGCTGGTGGGACTCGGCGAAAACATCGCATTCGGGCTCTTTCG
CGGCATAGCAGGGAACAGTATCCAACTGTAAGTTGTTAGTTCTTACTATTACATCAAGTTACTGACAATACGC
CTATAGAATCGGGCTTTCCCCTCGTCCTGAGGCCAATTCCTAGGCTGGAAAGCTGGTTGTGGCAGCTG
>Cligib1_rnd-1_family-12#ClassI/LARD
TGTTGAGATACAAGCTCACTCAGTATTTGACTTAGAATAATTTCCGCTTTACTGATTACGTAAGCGCGTCCAG
TCTTTTACATTCGNGGATTGTCCCTCATACANCACTACTCANTATACACATCTAATAATATCTANTTAACCCC
CCATCTGCCTCNTCCACTGCCAACAGGTTATGGGCCCTGGGCAACCTACGATGTCGAATCTCGAAGTCCCGAA
TCTCGAAGACACTGGTTCGAATTGGGTCACCTACAAGGAAAGGGTAATAAACCTCCTGACTCACGAAGGACTC
GAACGACACCTGTACGGAACCGCGCCGAAGCCAGAGGAGCTTCGCGAGGTGGATGGGAAGTGGNTCACTAGAA
GGAGACCCCACGAAACCGCTCGACGACGACGAAATCGAAGAGCACGANACGAAAATCGACATTTTCGNGCAAA
AGGAGGCNACGGTTCGAGGAGTTATCTATGCAACAATCAGCAGATACACTTTCCTTCGAATATACAAGGAAGA
GACCTCCGCGAAGCTCTGGANAAAGCTCGTCAGCNTTTTCGAGAACGAAGAAACCTTTACGGCCGCCTTGCGA
TCCGTCCGAACGCATGCCGAAAAACGCGCCGAAAACGCGATGGTAATAGCTGAGAAAGGAGGGGAAGAGCCCG
AGGAGAAGCTGAAGGAAAAGAATGGGGAATCGCGCACGAGCGCGGGGCATGCTCGCGACGAAGAATGCTATAG
GAGAGGAGGAGAAAGAGAAGGTCAGGAACCACAGGAAGAGGAAACGACGGGCACCGACGAAGAGTCGGATATA
CCCCGCGCGAATACCTCATACCCCCGTGAAGAAACCCCTGCCCTATCCACTCCAGGACCCTCGCCGCGCACCG
TCGATCGAACCCCCGAGAGTCGAAACNCATATCGCGTCGATCTCGCCAATAAACCGCCCGAAATCCGTCTTGA
AACCCCGATCGCGCGCGAGATGTCGNCGAAAAACGCGAATCCGCCCGAAATCACACCAGATAAAGAAGACGAA
CCAAATTNTATTGAAGAACGAGAATATACAATACAAGACGAATACCCTAAACCCCGAAAAACCGAACCGGAAG
TGGCTGAAGGCCGTAANACCGATTTTGATGAAGATTCCGCGAGCTATAGGATCTGTGGGGCAGAAAAGCGATC
AGGAGCGGTCGAAGGGAACGCTTACCATGGTAGGAATGACCTCCCAGCACCCCGAACCGTCCAGATCGAGGGG
AAGAACGCGCCCCACATCTCCAGCCCCAGCCGAAGCATCGAAAATAAAGTCGTCGAATTACCGCGCGCTAATA
GGCACGTTTTGAAGGCCTCGGGAACCGCTCTCGAACGGTCCAACAATCGGCCGAAACCGCCGGAAAACGATCG
CGCAACGGACANATCTTCGTCCAANCCACTCAGGCCAGCCACCGAAANCCGCCCGGCCGAATCGCCGCGCGCG
AATAGGCGCGANTTGAAGCCCTTGGGAACCGCTNTCGAACGGTCCAGCAATCAGCCGAAACCGCCGGAAAACG
ATCGCGCAACGGACGTACCTTCGTCCAANCCACCCAGGCAAACCACCGAAATCCGTTCGGACGAATCGCCGGA
CGCGAATAGGCGCGAATTGAAGCCCTATAGAACCGCTNTCGAACGCGCCAACAATCAGCCAAAACGGACGAAA

AACGGCCGCGCAACGGACGCACCTTCGTCCAAGCACAAATCCACCATNCCCAAAACCCCACCAATATCCCCCA
GCTCAAAGGACCATCGAAGCGACATCCCAAGCGACCCAGAATCGCATATCCGTCGCGATTTTCGTGCCAGGAG
GCCCCCAGGAACCTGTAGTCAGCCTATCCAGCAAGACTCACCTATCTTGGAGACCGGGCCAGCAGGAATCGAT
TATCGGGATCCCAGAGGCATGCCTGGGGAAGATAGAGGGTTAGTAACCAATATAGGAGGACCGACTCACCTAT
CCAGCATGGCCANAGATGTTGAACCTCGAGGAAGCGCCCAAGAGGAATCAACCATCGCTCGGTCAGCAGTCAA
ACGTCCAAATCGGGACATTTCGACTTCGAAAATCATTCACACTCGTTTTGACCACCTCAAGACGATCNANCAA
TACATTCTTTTACTATATCAGGTATATCTACCTACTTACAAGCTCAAATACCTCCGTTTCGCTTGAGGGGAAG
TGTTGAGATACAAGCTCACTACAGTATTTGACTTAGAATAATTTCCGCTTTACTGATTACGTAAGCGCGTCCA
GTCTTTTACATTCGNGGATTGTCCCTCATT
>Cligib1_Cluster21#LTR/Copia
tGTTGAGAGTAACACCCGTGACAGCGCTCAACACCTTATTTAGGAAACGCGCGCACGCATCACTGGTGCGCGT
ATACTATTTCACTTCTCTATTTCATTCTGTCTCTATCGTTTCATCTACATCACTCACATCACGGGCACGGGTT
CAACACGCCGTGCCCCTATTTATTTTCTCTTCGTATAAATATCTCGACTCAGTAAATACACACCTCAGTTCGC
TTTCACTCGTGACGGCGTCTGAGGTTAGTACACATCTACACTACCCACAGACACTTTACTAATTATCTACCTA
CTATAGCTTCaACGTATTTAACAGGTAGATAATATATcAGTTCGCTTTCACTCGTGACGGCGTCTGAGCTTCA
ACGTATTTAACAGGTTATGGGCCCTGCAGCTTGAATAATACGGACGCTGTTACAATCCCCTATATCGCGAATT
CACCCCTTTGACCAAAATGACGACCGTGCAAACTTCCTCGTCGAACCCGCCCCTATTCGCTGAAAAAGACGTG
TTTGACGGCAACAATTGGACGACTTGGAGTAATAAAATTATCATGGCGGCTGAGCTACGCGGCGCGGTCGACT
ACCTTAACGGAACCGTCAAGGAACCTAAGGAGGAACCTGACGAACCTGACACTACTACTTCCCCACTTCCACT
TCCACCTCCGCCACCTACCACCGACCCAGACACCGCCCCAAGCACACACTGGTTCTCCCGTAAACCCTCCTAC
AACGAGTGGAGGATGCGCAACGCTTGGACGAAGACTTTGATAGTCAATAACATGTCCGATCCTATTGCTCTAG
GGGCTGACATAACGGGTACGGCCGCTGACATCTGGGCGACACTCACCGCACAATATAAACGCAACAACGACCT
CGCGATGATCCTCGCGCAACAGAATTTACGCAGTCTCCGATACCACGATGGTGAAGACTTGCCTACGCATATT
GGGAACCTCCGAAAGAGgTGGCGTGAGGCGAATGCAATAGGCGCGAACGTTACGGATGCGGACTTCCGTCTCA
TAATGCTTGCGTCTCTACCAGCTTCCTGGAACCCTATTATCAGCACACTTTACGACGCCAAATCTTCACTTGA
CATCGTTACGCGACTTGACCTGCATTGGAACAAGGTCGTTCATGATAAGTCGGTCAACACACCCAACTCGTCA
GTCACCGCCCTCCAGACGACCACCAGAAGACGCGAGCAACTCGTCTGCTCAAATCCAAACTGTAAACGTAAAG
GTCATACGGTGGACAAATGCTACTGGCCGGGTGGTGGTAAGGAAGGACAATTCCCTCCTGGTTTCGGCCGAAA
GGGGGGATCCAACAACGCGTCGACTGCCTCGGCGACACTTACCCCAACAGTTGCTGCTGCGGACACCAACAGT
ACCATTGTACAACCCACCGACCGGGTTTATGCCCTCTATACCATCACAGATGACGCGACCCCTATCCCGTCAG
CCATGACTACGCCCGTGCAGCAGACCGCGACTGGAGATGGAGTTATGACAACTCATGTGCCAGTTCGCACCTT
CGCGGATTCTGGCGCGAGCGAATGTTGCTTCGTGAACCGCGGCGAcTTTGAGTCGTATACCCCCCTCGACCCG
CCAATCGTTGGGCGAGCTGCGAGTAAGGGGAGCTCCTTCCGAATTATGGGGAGGGGCACTGTCACCAAGATCT
TCTCAGTCAATGGTAAAACTACGACAATCAAATTTACCAACGCGTGCCATACCCCGGATCTCATATCAAACCT
ACTGTCCATCGGAAAATTCGACGCCGCTGGTTATCGTGTAGTTTTCGCCGCGGGCGCCGTTCACTTCTATGAC
GCGCAGGGTGAATTGATGCTCCTTGGAAATGGAAGTAACGGCATGTACCTTCTTGACGCCATCGACCCCCCGA
CAGCTTTCACCaCGCGCTCCCACGAAAAGCCCGTGAATATAGATACCTGGCACCGTCGTTTCGGCCATGCCGG
GGTCGATGCCATCCGCAACATGGAAAGGAAGGGTCTTGTTGACGGTCTCACTATTAAGGGTGGACCGGCGCCG
GAGGGACTCTGTGAGGATTGCATCTACGGTAAACACACTGCCAGACCGTACGATGAAGTGGTGACTCACGAGT
CCACCGTCCTGGAACGCATTCACATAGACCTATGGGGACCGGCGCGTGTCAAATCAAATGGTGGAGCGGTGTA
TATGTTCTTACTCACGGATGGGGCATCCTCGTATCGCAAAGCGTACTTCTTGGGGAACAAGAACGCTGAAACG
ACGCTCAAAGCTTTTCGCGACTTCCACGCTGAAGCAGAATGCCAGACGGGCAAGAAGCTTAAGAAGGTCCGCT
CCGATGGGGGGGCGAGTGGTACAATGACCTGTGGGCCGAGTACTTGAGCGAGCGCGGGATCaTCTCCGAAATC
ACTACGGCATACGCACATGGCCAAAACGGGGTACCTGAGCGAGGGATGCGCCTCGTCTTTGAGGGAGTTAGAG
CCTTGCTGGCTGATTCAGGTTTGCCTCCCTCACTCTGGGCAGAGGCGGCAGCGACCATCGTTTACGTTCGCAA
CTTCATCCCGTCCTCACGACACCCAGACAAAGTGCCGGCGGAGGTTTGGACCGGCCAACGCCAAGATGTCTCC
CATATTAGGCCGTTCGGCTGCATCGCCTTCGCGAAGATCCCGGCGGAAATCAAGGCCTCGAAGCTGGACCCTC
GATCGATCAAGTATATCCATATTGGTTGTTTCGGGCGTGGTGCTTACAAATTGTATGACCCCGCATCCGGCTC
TATCATTAAGAGTCGTGACGTTATTTTTGAAGAGGGGACAGGCCACAGGACGAGGGGAGCTCGAGAGACTGGT
CACCTAGAGGAGGACTTCGAATCCGGCCCACCGGCACAAGCCAACACAGGCGCGGACACCCCTGGGACGGCTG
TCATAAAGGACACACGACCCGTGGCAGCTCGTCCACGGGCTACCGACCCTCCTTTGCATCCTGCACGCGTCGA
CGATGATACCACCCGTGTCGAAAACACACCGCAAGCCCCGCCCGTACGCCGTACCGAACGCACTCATATCCCT
TCACGCGCGTTACGCGAATCCAAGGAGTCAGAGGACAGGATAGCAACGGCCTCCAAGAATTCTGAGGACTGGG
CTACCAACGcCCCGCACGCGCTCGTCACTGGCAACTATGCTGGTGATTCTGATGCCGTGGCGCCGCGGAATTT
CAAGGAGGCCATGAGGCGTCCTGATCTATGGAGGAAGCCCATGGAGGACGAGATGCGCGTTATGAAAGAAAAG
GAGGTCTTCAGACTGGTCCCTCGACCGGAGGGCGCTAACGTTGTTGGCCTCCGCTGGGTCTACACCAACAAAC
TCGACGTCGACGGAAACCTTTTGAAGCGTAAGGCTCGCCTGGTCGCAAAGGGGTTTACTCAGATCCCCGGCGT
GGACTTTGACGAGACATACGCCTCCGTCGTCCGCTTGGAGTCAATGCGCATGGTCGCGGCGGTCGCCGCCCAG

CTGGGACTTAAGATCTGGCAGGTGGACTTTATCGCGGCTTACCTTAACAGCGAGAACAAATATACGGTGTATA
CGGAGCAGGCGCCCGGTTTCGTCAAGCAAGGGGAGGAGGATAAAGTATATGTTGCTGACAAAACGGTCTACGG
GATGATGAACGGCGCTAATGCTTGGGCTGAGGAACTAAACCGCAGCTATGATGGACTTGGCTATTATCAGTCC
CAGGCTGATTCATGCGTCCGGTCGCGGAAAATCGGTGACGAACTTACAATCACCAGCACGTATACTGACGACG
TCTTTGGAGCCTCATCTACTGATTCCGGTGCCGCACTTGCCAAGGAGGAGCTCTCCAAATGCTACGAGGTTAA
GGACTTGGGGGAGCTCAACTTCATACTCGGTATTCGTATCGATCGCAACCCTACTACTGGCGCCATTTCACTT
TCACAGCGAGCTTACCTGGAACGCGTCCTTGAGCGCTTCAACATGACGACTTGCAACCCCAAGTCAACACCAC
TCCCAGTAGGCCTGACCCTCACCGAAACGGATCGGGCATCATCCGAGGAGGACGCACGTTTTATGGCAGACAA
ACCGTACAGGGAGGTACTAGGTTGCATCATGTGGGCGCAAGTAGCAACGCGCCCGGACTTGTCATACGCAGTT
AATCTCCTCTCGCGCCACCAGACGAACCCCGGTCCCGCGCACTGGAAAGCGGCCCTCCATGTTCTCTCCTACA
TCAAGGGCACTCTGGACTACAAGATCACTTACACGCCAGGCACCAGCAGCGGAATGCGACCCATTGGTTACaT
CGATGCCGATTACGCGGGCGACTTCGACACCAGACGTTCGACTTCCGGGTACGTTTTCACCATGTGTGGAGGT
CCCGTGTCGTGGTCCTCCAAACGCCAGCCGACCGTTGCACTCTCCACGACTGAGGCGGAATATATGGGTCTTA
CGCGCGCATCCCAACAGGCGCTTTGGATGTACTCGTTCATGGACGAAATCGGCATGCCGCAAGAGTTACCAGC
TAGGCTGAACGCTGATAATACAGCTTCCATTGCACTCACACTTAACACAAAAGGCCACGCGCGGGCCAAACAT
ATCGATGTCCGCTATCACTACGTACGTGAACGGGTTAAGGAAGGCGATATCTCGGTCGAGTACATTCCATCCG
CAGACAACCTCGCCGATATCCTCACCAAACCTCTTGCACGCCCGGCGCACCAGTATATCTGCCGCTCTTTAAA
CATTCTAGCGGGCGGCGATGCCACGGGGTTGGGGGAGTGTTGAGAGTAACACCCGTGACAGCGCTCAACACCT
TATTTAGGAAACGCGCGCACGCATCACTGGTGCGCGTATACTATTTCACTTCTCTATTTCATTCTGTCTCTAT
CGTTTCATCTACATCACTCACATCACGGGCACGGGTTCAACACGCCGTGCCCCTATTTATTTTCTCTTCGTAT
AAATATCTCGACTCAGTAAATACACACCTCAGTTCGCTTTCACTCGTGACGGCGTCTGAGGTTAGTACACATC
TACACTACCCACAGACACTTTACTAATTATCTACCTACTATAGCTTCAACGTATTtAACAGGTAGATAATATA
TcAGTTCGCTTTCACTCGTGACGGCGTCTGAGCTTCAACGTATTTAACA
>Cligib1_rnd-1_family-271#TIR/MITE
TAAGGTAGGCGAAGTTCATACTCATCTACGTCAGGTGATGTTACTTTCCCTGTGACATGGGGTTAGCGGATCG
TGATGAACCCACACACACAGATTTGTGTGTTTTGTTGTGAAGGGCTCATCAGGTGACTCACGTCCGATTTCAC
ATGCGAGCGACTTCCCCCCGCGGCNCTGGGGTAAGTTTCAGGGGTCACATGTNTCCAGCTACTTATCTCGTTC
GATGAAATAACTCNACCCCACGAGATCTTACAACAATNGCTCCCACCAAAAAATCTCNTTCCAAATTCAAAA
AATTTTGGTGGGAGCACATCTCACGCAAGGTGGACACGGAGATCGTCATTTCGATTTCTCGCGTTTGTCACGA
AAAAGTCACATTTGGTCATGTGAGCGTAGATGAGTATGAACTTCGCCTACCTTA
>Cligib1_Cluster42#LTR/Gypsy
ACTCCAAGTAAGGCTCAGCATGGGTAGATCCATGGGTGATCCGGATCTGTACTCGTGGGTATTTGGTCACACA
AGGTCACTACCCAGATCTGTGGGTAGCTTAGTCAGCAAGTACTTGTACTCATGGGTATTTTTTGGCAAGTACT
TACAACTCAGTAAGTACTTGTCACAGATCCTGATTACTTATGGATTACTCATGATTACCCACAGGTCACAGGT
CATTTGTCAGCAATTAGTCATCAGGGGCAGGTATTTCACAAGTACTTATGGGTATTTACAAGGATTTTGTTGG
TTTAAAATGGAATTTGAGCCTATATGTACTGTGGGAACTATACTTTAAGGGTCCTTGAGGGTCCAGTATGAGT
CACAAGTACCTGTCACAGGTACCTGCAATGTCCAAATGCTTGACTCGGATCTGCAAGTAAATAGATCCGTGAG
TACAAGTACCTGTTTAGGCCAATACTTGCAAGTATTCCCACGGGTCTACCCGCAGGTACCCGTGGGTAACCCG
TGCTCATGCTGAGCCTTAACTCCAAGTCAGGCTTTGACTTGGGTATCAAAGGTTCAGTGATCAAGGTTCAGAG
GTAAAGGGTGTAGTATTGCCTAGGTCAAGATGGAGAACAATGACAGTGTATCTAGGGTTTATATACTAGGGAC
CTTGAGGCGCGAGAACATAGTACAATGAGAAAGAAAGACAATGTAAAGTAATAAAGCTGTTGAAGCTCAGTCA
GCTATGATGTCATAGATGAGTCAGGTATCTAAGAATAGTAGCATGTGTTGGACATGCGATAGAACAATGAGGA
ATCATAGAACAATGTAAGTATCGTTAAACAAAGGTAGTCAGAGAACAAAGAAGAAAAAGATAGGGGTGGGAAC
CGTTGTAACATTACCCCCTGTTTATCGTTGAAGTCCCCTGACTTCAAGTTACCCTGCGAAGCTGCCGTAGGGT
TCATCAATCATATTAGAGATGGCGGAATCGTCAAAGTAAAATTCCTCATCAAAATCAGGTTGTCGTGGTCCAA
ATTGGCTGTGAGCACAAGTGATATGAGGTGTGCGCCAATTGCAAAAATTGCAATAAAAATCTGGACATGTGGA
AAAAGCGTGTCCAGTACCATGACAAAAGGAACAATATCGTACACGAGGTCGTTCGGGCCGAGAGAAGACAGTC
CGGGGAGGTGGGACATTTTCGGCATCTGGTGATTGAACATATCGTGGTCGACCCACATGGGAAAGTGGCATGG
AAAATATGGTTTGCTGTTGTCGACGTCGAGGGGTGCGGATGACCGGTGTTCTACCATGTACTTGGTCGTAGGA
GGGGGGAGCTAGTCGTTCGGAGTTTGGTGGTGTGGGGTTACTTGGTCCCGGGGTAGGTAGTTGGGCGAGAGGA
AGAGAGGAGGGTCTGCAAGGGGGAATGAATGGTTCCAGATCTGGCGAAATTTGACGGGATGCGGGTCGCGGTA
ATGGAGTAGTAGCAGGAGGATCCGGGGAAGGAGTGGCCGAAATGTCATTGATAGGTGAATGAGGAAAGGGTGT
TGCTCCTCAGGAATCGGTTCTGGAGGAGGTAGAATCCATGAGGGTAGTAGGATTCGGTGGTCGTTCAGTTTCC
AGGTCAGTAAGGACAATGGACGGGGTGGAAGTGAGTTCATCAACGGTTCAGTTGGTTAGGGGCCAAGGGTCGT
TCGTGAGTCGTGGAGTTACTACTCCAGGGATACGATTCATCTGTCGATCGTTCAGCAGTTGCTGAATATGTTC
TATTCTTAATGGGTGAGGGTACCCCTGTGGACAGTCGAATCCAATAAGAGCGTCCAGACCCCACTTCCTACCT
GCATGGAATAGGGTCTTCTGGGCAGATTCATGGGCGTCCTTAAGGCGAGTAAGTTGGGCCTGAACCTGGCCCA
TAGCTCCAAAGAGGTTCCGGATGGGAGCATCTATCTCGTTGACATCATAGAATACTGTCATGAAGTCGCGGAG

GATGGGATATTTCTTCATGGAGGTGACGAAAGATTCGGGATCTTTGATGGTGATAAGGAGTTGTAGAAGTCTT
CTAGATCCTTCGGGACTTCGTTGAGGTTCGGGATATCTTCGGTTTCGTTTTCGGGGATTGGTTGGAGGGGTTC
GCGTCGTGTGGGGGCAGAAGATGATGATGAAGATGAGGAGGTGGAAGTGGTCGTATGGTCGGGGAGGATGAGG
AGGATTGGAGAGGGGGAGTCAGAAGATACGCATAGTCGGTGGAGGTGGTAGAGGCGGCAGAAGAAGACATGGTA
GTGAGTTAGATTTTGAATGTGGGGATTCAAAAGATGGGGAAAATCAAAGGGAGTGTCGTTTCTTATAAGCTTT
GAGAATGTCGGAGGCATTTCCAAAGCTGCGTGTGGGAAGCCATGTGTTGTCGGTAGATGGATAACCCTTCCAT
TTCACATAGTACATCATGCCGTTTCCTTGTTTGCGGTGTTTGAGGATGGCTTCGATTTCGTATTCTTCTTCTC
CTTCTATTAGTTCCGGGGGTGGTTTGGTATAATTAGGTCCGTGGACTTCATTTTCCTTGTATGGGGTGAGAAG
GCAGGCGTGAAACACCGGATGTATTTTCCATTGATCGGGTAATTGTAGCCGATAAGTGAGAGGTCCTAACACC
TCGGTGATGGGGAAGGGGCCTTCTCGTCGTGGTGCCATCTTTCTGGTTTCGTATTTCAGTTTGAGGTTAGTAG
ATTCTAACCATACTAAGTCATTCTTCTTGAAAGGGGTGAAGTTTCGTGTCATTCTTTCCGCTACACGTTGGCT
AGCCAGTTCATGTGCAGCGAGGGCTTCATCTCGTGTCTTTGGAGGGATTGGAGGCGTTCTTGTACAGCGGGTG
TGTCGGATTTGAGGAAAGGAAGGGGAATGGCAATAGGGTTATAGCCCATCATGAGGAAGAATGGGGAGCATTT
CGTGCGGAGTGTAGGCTTGGTTGTGGGTGAATTCGGCGGTAGGGAGGAATTCCACCCATGTTTCGGGGTGATT
AACGCAAAAGATCCGAAGGTAAGCTTCTACTTCTTGATTGACACATTCCGTTTCTCCATCAGTTTGAGGGTGG
TACGCCGTACTGAATGCAAGTTTGATGCCAAGGAGTCGTCCTAATTCCCTTTGTACTGCTGAGGCAAACTGGG
GACCACGATCGGAGATAATTTTATCAGGGAGACCAAATCGTTTGTAGACTTCCCGGAATAAAAGATTTGAAGT
TCCTAAGGCATCGATCGTTTTGTTGCAAGGAATTAGAATTACCCCCTTTGTAAGCCCTTGGTCTACCACGACC
AAGAGTGAATCATATCCATGGGATTTGGTAGATCGTGATGAAGTCCATGGAGATCTGTGAGAAAGGTCTGGGG
TTCGTCGGAGCTTTTATCGGCATTAGAGGTGGTGCGGTGGGGTGAGTGTTTACTTTGGTTTGTTGACAGGTAG
CGCAACCTTCGACATAGTTTTGACAAAAGTATACATTCCAGGCCACCAGTAATCACGTCGGATGAGTTCCAGG
GTTTTGATGTGACCAGGGTGTCCCATCATTGGCAAGTCGTGGTATCTTGATGCGATGTTCCTTCTCAGGGTTT
CGTCTGGGGGAATATAGCATCGGTCTTTGTAGAAGACCAGTTCTTCTTGATTTTCCAATCGGAAAGTGCGGAT
TTCATTGGAGGGGTGCCCCCTTTCTTGAGTGCTTCGATGGCCTGGGTAATCACATCCTCTTTGGAGGAGGAGG
CGAGAATTTGATCACGGAGGTCGGTGTCAATAGTCTTCACAAAAAGCTCCTCTGGGAGTAATGTGATGTCTTC
GTTATCGGTATCTTCTTCCAGGTTTAGATCTTCTCTTCTTGATAAAGCATCTGATTGGACCATTTGTGTTCCA
GGGACATGGACCACTTGATGTCGAATTCGGAGAGAAAGAGACTCCATCGTGCTTGGCGTCGATTGAGTTTTTG
GGCGGTGCGGAAGTAGGTGAGATTCTTGTGATCTGAGAAGATGGTAATTGGATGTGGGGAGCCTTGGATGTAG
TGTCTCCAGGCTTCCAGAGCTCGTACAATTCCTAGGAGTTCTCGATCATAGATTTCGTAATTTCGTTGAGTGG
CGTCAAAGGATTGTGAAAGGTAGGCGCACGGATGCCAGTCTCCATTCATGTCACGTTGGCGGAGTACTGCTCC
CGTGGCGAACTTTGATGCATCGGACTCAATAGTGAAAGGTTTCGTGGGATCTGGCATGAGAAGGACTGGTGCT
TCGGCAAATTTTCGTTTCAACGTGTCAAATGCTTCTTGACATTCGGGGGTCCATTCCCATTTGAGATCTTTCT
TGGTGAGGTTGTTGAGTGGTTTTGCAATATCCGAGTATTTTCCAATGAATCGCCGGTAGAAATTACCAAATCC
TAAGAATGAGCGTACACCTTTTACGGTAGAGGGGGTTGGCCATTCTTTGATTCCTTTGAGTTTGGTGGGTCCA
TGGCGATTTGATTCGGTCGGATAATCATTCCAAGAAATTCCACTTCTGTGGTTTCGAAGACGCATTTCTCCAG
TTTCAGGAAGAGGTCGTTCTCCTGGAGTCGTTGGAGCACTCGTAAGGTTCTTTGTCGGTGTTCAAATAAGTCT
GAGGAGTGGATTAAGATATTGTCCATGTAGATGACAATCCATCCTTCGTTAATCATGTCGTTGAAGAGTTTGT
TCATCATCATTTGGAAGGTAGCGGGAGAATTACACATTCCAAAGAACATAACCTGGGGTTCGTATAGTCCGCG
GTTGGTTTTGAAAGCGGCCTTCCACTGATGTCCTTCCTTGAGTCGGACATTGTTGTACCCTTGGCGGAGGTCC
AATTTAGTGAAGATTGTGGAGCCTCGGAGTTTGTCGACAAGTTCTTGGATAAGAGGAAGAGGGTATGAGTTCT
TGACGGTTCCTTCGTTTAGGTAACGATAATCCTGGCATGGTCGTAATGAACCATCCTTCTTTCCTACAAAGAA
AAATGGGGAGGCCATCGGAGATTTCGATGGAGTAATGTATCCCTTGTCCAGATTTTCTTTGAGGAATTCATCC
AATTTGACTTGTTCTCCCGGTGTGAGTGGGTATATTTTGCAATCTTTGGGTACAAAATCCGGTTTCAAGTCTA
TGGCATGATCGTAGATTCGATCTGCTGAGGCATCCCCCGCGGCGCTGGAAGTTGACGCAGAGGGGCTGCCCCT
GTGGGTTGACGATCTTCTTGTCAACTCGTCGGCACCACGTTGGGGTGGTGGTGTCCCACATCGTCTTGGCATT
GCACTTGCTGATGTCGTGCTTGCCCCTTCCGAGGCAGACCGCGCAAGGCGGGAAGGGTTGTGCCTGTTGTGAA
CCGTCAGCATCACCCGATGCCCCGGATTTTGCCCTCTGGTACCTTGAGGCTGGGGATTTGGACCGCGAGTGGT
CTCTCTGGCGCGGTGGGATTTGGAACGGTTCCCCCTCCGGTGTCTTCCCTGGCGTTCGAGTGCGCCTCCTGCG
AGCACATGGCTCTCATCTCAGCGCGTAGCTGTGCGTTGTCTGCACGGAGCTGCGTTTTGAGGGATCCCAGACG
GCGCGGCAGTTCCATCCCGGCATGCAGCACGCAGTGGCCGTTATTGGGGGGTATTTGGTCATCAGCCGAGCAC
ACGGCATTGGGACCCGGCAGGCAGCATTGCCAGCGGCCATCGGCGGGTCATGAGGGGACCATTCGCTGGGAGT
TGGTTGTGTTGTTGAAGTCCATGGGTCGCCAAGGGACCCAGACATCGACACGGACCACCATGCGTTGTTGCAA
CGGCATGGTGGGTGAGCATTCGTGGGTGGAGTTCGGGATTGAGTTAGGAAGGGGGGAAGGGGGGGAGGGTTGG
GAGAGGGGGAAAAGGGGAGCGTCAGTATGTTTACTACAGTGCATGGGAGTTTTACTTACGGATTCTATGTCCC
TCGCCTGCTGCTCACACGCGATGATCGTGATTAGATCCTCGTTTATCGCGGCGATGTTGAAGGTTTCGCCATC
CCTTTTCCTGGTGTCGTGCCAGTCTTGCTGGGCACGTGCCTGGTACTCGAGGAGGGCTCTCTTGCCCACGTCC
GTTCTGTGTAGCGGGTGGGACTTGATGCGCACGAAGAATGCGCACAGATCCTTGTACAGTTTCACGGGCCATT
TTGCGATGCCCATGTACTTGATGAACCCGTATCTTGCCTCTGTGAAGACATGCCATGGTAGGTTTTCGTCAAG

GATGGCATTCCTGGAGGGGGAGGCGGTGGATGATGCTTTGAGGGCTAGACACTCACCGTTCAGTCCCCCTGAG
AAGCTAAAACCTTCGCCGTCCGGGGCTCTCACCTGTCTCGCCGCCTCCGCGCAGCCCTCGGGAGTGAAGTACC
ACAGGTCGACGTATTCGTAGGCGTCGAGTTTGTTGAGGGCGAATTGGGCAGGGCGATCTGGGAGAGAAGTTGA
GATCATGAGGTCGTCCCGCACGATCGGTGGGGCTGGTCTTCGCTTGGGTGGCTGCGCCGGCTGGTCTTCATGT
TCTGGCTCGCAGCGCTGGGCCGCCCACAGGGAGGGTTCGCGGCGCGGGGTTTCTTGGTGTTGCTGCGGTGGGC
ACCCCAGGTCTGCTCCCGGCGGGGGGTTGTGGTCTTCCTGCGCATCGAATTGCCTCGCGTATTGCTCACATCT
CTCGGCATTGTCGGTTGCCCAGGCACTGGCAAGTGCCTCGGCTGCTTCTTCGTGGGTCTGGATTTGTGGGCAC
CCTGGGTTTGCTATCATGCTGTCCCTGGCCAGGTCATAGATGGGAAGCAAAAAGTCAGGGCAGACTTCGTTTG
CAGGGTCCATAGCAATGTGTTGGGGCATGTTGGAAAAGAAGGTGGTTGTTTCGGAGCGGTTCTGAGAAGCTTT
GCGTTCAGAGAGTGATGGGATGTGGAAAGAACGGAAGGTTGTGTCATTGTTCTTTATAAAATTGAGGGAACGA
GGCGAGGGCGAGGGGAGCAGGAGGCCACTGCGCGGCCAGGCAGAGTCTCCTTGAGGTATGCAGGAGAGCTTGC
CCGGGAGTTTCCGCACTGGCGAAGACCCTGCTCTCGCCCTTCCCTTGTTCCTCACTATGTATGAGACCTGAGA
ACTGTGAGGTTAGCTCCTCCTCCTTCATTTACGGGGATTCAGGTGATTGTTATTTCACCTGCATCAGTTGGTA
TGGGTGTGAATGTTTCACCCGTGTCCTGTTCCCATTCCATGATGTATTCATGGTATAGTTGTGGCTTTGCCAC
CAGTATTGCAAACAAGCGCAAGAAGTTCGTTCTTGTTGATGTTACGCCTTTGTTTAGGGTGGGTCCCCCATTC
TTTACTTTGCAAGGAGCCATTGCTCTCGCTAGTGTGTAACCAAATGTACTTTGGTGGTGTACGTGGAAGGCGC
GGTTTACCGCGATTCCTCGCACTTGGTTGTCCCCTCCCAGGCGTCCGTGGTGTACAATGTACTGTGCCCATAC
GTCCACGTCCGGTTGTGTCAGACCCACTTTGGTCTGACCAGTTCCTGCCTCTATTTTAAATGCAGATCATGCA
CCAAATGGCGCATATTTGTCTGCAAACGCTTTGGGCTGCTGATAAGCAAGCTTATTGTTTAGGGGTTTGCAGC
TCACATCTCTACTGATATTGGGCACATTAGGCGCATTCATCACACTCTTACAGTACGGATCGCACCCATTCCA
CTCCGAACACGTCGAGATCGTGCTTCGATTCCGGTTCTAGCCGTATCCTCCGCATGTCTCTCGCATCGTTCCT
CATCGATCCGCAGCTACATTACATAGTATTACCTACATTCTACTATACCTCATGGCTGATTAGCTCCGATATA
TAGAGACCTCAGTAGACACATAGAATAACAAGCTTTTGGCTCGCATTTCAGTCGCGCATTGGCGTGTCCTCTC
TCTTCTATAGGGCTGTGCCCTTGCATTGTTCTCATGATTCTTGGGCTAGGCACCTCTTGCTAAACAGACACCC
ACTCGCAAACTGCAAAACACACAGGTTAGGTTTGGTTTTGCCACCTGGCCACAGTGTCACATCACCGTGTTTG
ATTCAATATTCTGTTTTGAACTGTCTCATACGGTTTGTTTTTGAGCACTTTCTTCATTGCTATGTCTTGTTGG
TCTGGTAGGAAGGCGTATGAGATATGCCTAAACTCTCGCATGAGTCGGAATATTTCGACTCTTTCTTCTAGGT
GTTACATCCTGCTAACGCAAGTCAATGAGTCGACAGTAAAGACTCAAAGACACTTAGCAAGGTCTGAAGTCTC
CTGGGTCAGGAGGCAGTAGAGGGGAATCTGATAAACGATTAAACATAGTGAATACAGGAGTATGTAGCTACAT
TTATATACAAGTGAAGTAGCTTGATAACTACACACTAGTGACTAAGTCCATGAGTATAGTAAGACATATGGGG
AATGATGACTGATACAAGAAGTGAGCTGACTGAGCAGCTGAGTAAGAACAATGAAGATAACAAAGATAGAACA
GAGACCATGCGGTATAGAAGTAGATAAGGTGCGCATGATGTATCATCTGATGATGTAGCGTATGGCGCATGTC
AGATGATGATGGAGTGATGGTGGAAGTAATAGAAGTGTCTATGAATGATGCCATCAGTCAGATGTCCCAGTGA
TAATGATCAAGGAGGAAGGGATCATAATAACACTAGGGTTCCTTGGTTCCCTAGGATGGCTTTTATCATTTCT
GATAATTTGACAGGGTTCATGGGGAGCCCTCGGTTTGCCTTGTAATACACCATTGTTGTTGGTGTGTGTACTG
AGGGAGGATTTAAATTCTCACTTGTTGTGGCCACCCTCACTGAGCAAAATGGAGTGGGTGGAAGTCATTCATC
CTGAGGTGGAAATCCATTTTCACTACTTTACATAGTGAAAGCAAAATTCACTCATGTTGACAAATACAGTGAA
TTGCAGTGACATTCAACTAGGCTATCTTCAGAACATTAATTCACTCAGAAATGACTTAATTTCAAAGGAAATA
TTATAGTGAAATGGAGAGCCACTGAAGGTGTATCAATTCACCCGAAAACCATTGTAACTTGAAGGAAATTATT
TCGTGATCCAGGAGCACTCGGGGGATCAAATTCAATTCATATAATCACTACACACTACACCAATCCATTCTCA
ATGATGTAACTTGAAGACACAGCATAACTGGGGTTTGACGTGAGTGGGTTCTGGGCAGATGATGACAGCACAC
ATGAAGCGATAGAAAGGTGGCGTGCACCAGAGTGTTGCCATGGATTGAATCATGAGACAGCGAAGGACATGAA
GAGCATCTGGATTTTGATACACCGGGTGTGAGCGCTAGGGGCAGGAATTGCAGGAAAGATGTAAAGTACAGCT
TAAGCTCTGAGCCATCCTGGACCATGAAGACCCTCACTTCAGGCAGTTGCATGCCTCAGTTGCAGTGTGAAGC
TCTGGCGTGGAGCTCGGCAGCGTTTATGTGTAGTATCTTCTGTGAGGGCCACTTGGTGGTGAATGTGAGCACT
GAGCAGTGTGGGGGTGGACTCGGAGGGGCATGAGTGCAAGCATTGTGGGAGGGTTTAAGAGCTCCTGAGGTCT
GGGAGAGACAAGGGGTGGTTGTTGATCCACAAAAAAAGAGTCAGCGCGGGAGAAACAGGCAGGGCAACATACC
ATGCAATATGGACTTGTATCTTGTAATGTAGAACTTGAATCTTAAACTTAGTAAACATTGACGACTTTGAACT
GGGAACACATTGAGGGATGAAATCCTCTGAGGAAAGTGGGTGATTAAAAGATTCAATAATGAGAGAAGATGTG
CAGGCCATCTTTTTATATGATACAAACAGTCCTGTGTTGCGCGCATGCTCATACACTGGCAGGTCTCGAATCG
ACAAGATAAATGAAACAAAGGCATGTGATAATGAAAATGTTGGGGTCCATAGATTGTGAATGTCCAATAATGG
AAAGATTGGGGATGGGGAATTGGTGTTACAATCCCTTCCTTCTTGATCATCACCACTTGGACATCTGACTGAT
GGCATCATTCATAGACACTTCTATTACTTCCACCATCACTCCATCATCATCTGACATGCGCCATACGCTACAT
CATCAGATGATACATCATGCGCACCTTATCTACTTCTATACCGCATGGTCTCTGTTCTATCTTTGTTATCTTC
ACTACTCTTACTCAGCTGCTCAGTCAGCTTGCTTCTTATATCAGTCATTGCCTCCCCATATGTCGTATCATAC
TCATGGACTTAGTCACTAGTGCGTAGTCATAGAACTACCTCACTTGTATATAAACGTAGCTACATACTCCTGT
ATTCGCTATGTTTAATCGTTCATCAATTCCCCCCTCTATTGCCTCCTGACCCAGGAGACTTCAGACCTTGCTA
AGTGTCCTTGAGTCTTTACTGTCGACTCATTGACTTGCGTTAGCAGGACGTAACAATTGGGGATAAACGACCG

ACAATCCTCGAATCGCATGAGTCTGCCATACCAGGAATGACTTGGGCCCTCTATTGACATACAATGATCGCCC
CCTTCAACCACGGAGCGCCCGGATTGATGCCTGTATCACCCTACTGTCCGGTATGTTGAAACCTGTTGACGCA
CAGACCAATGATCCCATGTAAAAGGATATATGAGCACTAAAAACGGAAATGGAATTACAAATGACGAGATCTA
TGATATCATCAAGCATCTGATACCAAAAAGGGTGCCCACTGTGCAGTACTTTACAGTGCCCCACCTAGTCCAT
GAGAGTCCATGGAGTCCACATAAAGTCCATGGAGTCCATATAAAGTCCATGTAAAGTCCACATAAAGTCCATA
AAGTCCATGGAAGTCCATGGAAGTCTATGGACTTCCATGGACTTCCACAATGGACTTTTAAAAAAAGTCTCTA
TAAAGTCCACATAAGAAAAAGTCCACGGAAGTCCATGGACTTTTGTGGACTTAGCACTTGTATTCATGCAGAG
CTGATGACATTACATACAAACAAATACAAACAAACAAAGCAAAGAAATACAGATACATCTACCAAGGAAATTA
TTAGCCCTGGATGATACATATGACCCTGCAGGGTGCAGATGCCCCCTTGATTATTTCCTAGTTGAGCTGAAAT
ATTTCCCTAGATAAATATAAACAAATATAAACAAATAAAGCTAAGCCCCTCATTATCTGTCAGTTTCACATTG
CAATGTTTACTCAGGGTGGAAACCAACCCTGGATGACAAATATAACCCTGAAAGACCCGCTATACACTTGATT
TACTCATGAAGTCTATATGAAGTCCATAGACTATCATGGACTAATCCACATCAAGTCCATGGCCTTTATGGGA
GTCTCATGGAGTTTATGTGGAACTAGTCCACATAAAGTGTCAGTTCAATGTGGACTTTTCTCAAATTTAGGCT
AAAAAGTGACAAAAGTCCACATAAAGTCCACATAAAATCCATGGAGTCCATGTGGATCCACGTGGATTAGGTG
GGGCACTGTAAAGACCTCCCCACCATGATACATCATATAGACAAGAAAAAATGTTGGGCACAATGTAAGGACAT
GCTAAACACCAAAATGATGTGCGGTCTCTCAAGAATACTCAGGAGGAATGGAATATAATGAATATGACTGACA
ACTGAAATCCCACTGTGTTATAGCAGAAAACCGAAGAGAATCAGATTGAGAAACGAAACATACAAACATTGAG
ACTATATACATAAGTAAGAGCCTGTGAATATAATAAGTACAAACAACATGTGTTTACAATGAGGTGAAACTGC
GGTTCGCTTACATAATCAGTCATGTGATACTACAAACGAAAAGAGAAACGAAAAGAAATTACAAGTCCTGACA
CTACTCCCTCTTTAGGGATTTTCTCTCTGGGAAAATCTTGAGCAGTTCTTCATCATCCCTGACAGTGCGTCTC
GCATCCAAAAGAATGCGCACATGTCAAATTGAAGCGCTTGAAGCATCGAGTTTTGAAAATAATGCCGAGTTTC
ACTGCAGGCGGCCAAACATTTCTATGCCGGAAAAATAACACGGGGGAAATGTCTTGTAATTCCCTGGCCATTT
TCCTAGGGCTAAAATGTTAAATTTATGTGCAAGTAAAATATCAGAAATACAATAAAATTTTTCCCCTTTGTCA
AATCAGTATTTAACAAAGGGAGGGAAAATGTGTTATTTTGATAGAATTTTGCCCAGGGGAAAATATAATGTTT
TTCCTCAAGAAGAATGATCAGAGAATTACAAGACATTTTTCCCTCAGCATTTTTTTCGTGTAAAAATACCTGG
CCGCCTGCAGTGTTTGATGGTGGATCGGTTAAAGTTTCATATGGAGCAAACAGTGAAAGGAATTCGTCTTGAG
TAACGTTGAGATGAACTTTTTGTGGCGCTTGTGGATTTGTTCGATGAAATTTGTTGACAAGATCCATTGCATT
CTTCAAATTCGATAGGGGTTCCCAAGTATTGTGTTGCAGCGTATATCCTTCCCATTTGACCCAATACTCTAAA
CCTTTGCCGTTTTTGGTAAGACATGAGTTAAGAATATCTTCGACGGTGTATTCGAATTCACCATCGATTTCTA
CTGGTGGAGTGGGAGGGATTTGTTGTTGGGGGATTTTCGGAGATTGATATGGTCATACTTGTGAAATATTAAT
GACGGGGTGGATTTTAATTGTTGATGGGAGTTTAAGTTTCACCGCGTTAGTTCCGACGAATTCAAGAATCTTA
AACAGACCAACTCGTCTTTCAGTGAGTTTCTTTGATGGACGTTTAAGGTCGAGATCCTTTGTTTCAAGTCATA
CCATTTGTCCAACTTGAAATGATGGAGTTTGACCTAAGGCTCAGCATGGGTAGATCCGTGGGTGATCCGGATC
TGTACTTGTGGGTATTTGGTCACACAAGGTCACTACCCAGATCTGTGGGTAGCTTAGTCAGCAAGTACTTGTA
CTCATGGGTATTTTTTGGCAAGTACTTACAACTCAGTAAGTACTTGTCACAGATCCTGATTACTTATGGATTA
CTCATGATTACTCACAGGTCACAGGTCATTTGTCAGCAATTAGTCATCAGGGACAGGTATTTCACAAGTACTT
ATGGGTATTTACAGGGATTTTGTTGGTTTAAAATGGAATTTGAGCCTGTATGTACTGTGGGAACTATACTTTA
AGGGTCCTTGAGGGTCCGGTGTGAGTCACAAGTACCTGTCACAGGTACCTGCAATGTCCAAATGCTTGACTTG
GATCTGCAAGTAAATAGATCCATGAGTACAAGTACCTGTCTAGGCCAATACTTGCAAGTATTCCCACGGGTCT
ACCCGCAGGTACCCGCGAGTAACCTGTGCTCGTGCTGAGCCT
>Cligib1_Cluster45#LTR/Gypsy
GTAAGGACACGCTAAAACACTCGAAGAAAGTGTGTGGTCTCTCAAGAATAGCCAGAGGAAGTTGGAATGAATG
AATATGAATGACAACGGACGTTCACACTGTGTTAGAGCGGAAAACCGAAGAGAATCAGATTGAGAAACAAAAC
ATACAAACAACGAGACTATATACATAGGTAAGAGCCCGTGAATATAATAAGTACAAACAACATGTGTTTACAA
TGAGGTGAAACCGCGATTCGCTTACATAATCAGTCACGTGATCAGTCATGTGATACTACAAACGAAAAGAGAA
AAGAAAAGAAATTACAAGTCCTGACACTACTCCCTCTTTAGGGATTTTCTCTCCGGGAAAATCTTGAGCGGTT
CTTCATCATCCCTGATAGTGCGTCTCGCATCCAAAAGAATGCGCACATATCAATTCAAAGCACTTGAAGCGTT
CAATTTTGAAAATAATGTCGAATTGGATGGTGGATCGGTGAAAGTTTCATATGGAGCGAACAGAGAAAGGAAT
TCGTCTTGAGTTACGTTGAGACGAACTTTTCGTGGAGCTTGTGGATTTGTTCGATGAAATTTATTGACGAGAT
CCATGGCATTCTTCAAATTTGATAATGGTTCCCAAGTATTGTGTTGCGGCGTGTATCCTTCCCATTTAACCCA
GTATTCTAAACCTTTGCCGTTCTTGGTAAGACGTGAGTTTTGAATTTCTTCGACAGTGGATTCGAATTCGCCA
TCGATTTCTACTGGAGGAGCAGGAGGAATTTGTTGTTGAGGGATTTTAGGAGATTGATATGGTCGAACTCGTG
AGATGTTAACGACAGGGTGAATTTTGATCATTGATGGAAGTTTAAGTTTCACCGCATTGGTTCCAACAAGTTC
AAGAATCTTAAACGGACCAACTCGTCTTTCGGTGAGTTTCTTTGATGGTCGTTTGAGGTCGAGATCCTTTGTT
TCGAGCCATACCATTTGTCCTACTTGAAACGATGGAGTTTCGCCACGATTTTGATCGGCATATCGTTTCATTT
CATCGCGCGCTTTAACAAGGGCTGATTGTGCTTCTTCTTGAATTTTCTTTATTTGTGTTGCGAATTCATTAGC
CGACTCATTTGTTGGTTGTCTTATTGGTTCGGCGCCCATTCGGGGGTGGCGACCATAGACGTTAAAGAAAGGG
GATATTTTCGTGGAAGAGTGAGTGGCGTTGTTGTATGAAAATTCGGCAAGGGGAATCCATTCAACCCAATCGG

ATTGAAGATATGATACGAAAATTCGAAGATAGGCTTCAAGTTCTTGATTTACCCGTTCCGTTTGACCGTCTGT
TTGAGGATGATAAGCGGTAGAAAGTTTGGTTTGAATATTCAATAATGAATTTAATTCTTTCATCATTGAACTT
GCGAATTGTGGTCCTCGATCAGAAATAATTGAGTCGGGGAGACCGTGAAGCTTCCAGACGTTTTGTAGATATA
ATTTTGCAAGACCCGTCGTAGATGTTTGAGATGTAGTAGGAATAAAATGCACCATTTTTGATGTGTGATCGAC
AACAACAACTATTGCGTCGTATCCATTACTCTCAGGAAGTCCGGTGATGAAGTCGCAAGATATTGCTTTCCAA
GGTTCGGAAGGGGTGATCATTGGAACAAGTTTTCCGATAGGGGGAGCGGGGAAATTTTTGAAACGTTGGCAAG
AGTCGCATCCGTCGATGTATGAATGAACGAATTTTGTCATGCCAGGCCACCAGTAGTTTCGCGCAATTAATTC
CAGAGTTTTGTAGTGTCCTGGATGACCGGCAATGTTGGAGTCATGGTGGAGTTTAACGATCTCTCGTCGAAGT
TGGATGTCGTTGGGTACATAGACTTTTCCTCGGAACAATATAAGATCTTGTTCCTGAGACCATTCTTCAGATC
GTAGTCGTTTCGTTGGTGATCGTTTGAGTTCTTCGATCGCTTTGACAACTGATTCGTCATAGTCTTTTGATTT
TCGGATCTTCGAAAGTACTCCCTCTTCTTCGCCATTTACAAGAACATGTCCTTGTTGTAGTGCTTTAATGGTG
AAAAATTCCGGTTTAAGGAGCGTAATGTCATCGTTATCAGTTTCCACTCCCTCTTTTAGATCTGTCCTCCTGG
ACAGTGCATCCGCTTTTCCCATTGAGGAACCTGCTTTGTGAATTAAAGTAAAATCAAATCGAGATAGGTAAAG
AGCCCATCGTGCCTGTCTACGATTTAATTTTTTACTTGTCATAAAGTATTGTAAATTATGGTGATCTGACCAT
ATTTCTATTGTATGAGTACTACCTTCTAAATAATGGCGCCAGGCCTCCAAACATCGTATAATACTAAGCATTT
CCTTGTCGTGTACATCGTAATTACGTTCAACATCAGATAAAGATTTAGAATAATATGAACAAGGCCTCCATTT
GTCATCCGAACATTTCATAGATAGGATACCTCCCGTAGCAAAGTCCGATGCATCCGATTCTACTCGAAGGGGT
TTCGTTGGATCTGCTGTAACCAATATTGGGTCGGATGTGAATCGATTTTTAAGTAAATTGAATGCCGTTTGTT
GTTTCTCTGTCCAATCGAATTTATAATCCTTTCGTAGGAGATTGTTAAGGGGAGCGGCGAGTTTAGAGAATCC
TTCGATGAATCGTCGATAGAAATTTGCGAAGCCCAAGAATTTGCGAACGTCGCAGACTGTTTTAGGAACCGGC
CATTTCTTTACTGCATCGGTTTTATCGTCCATCATTCGTACTTCTCCATTTCCAAGTATAAGTCCAAGAAATT
CGACTTGTTGAACCGAGAATGAACATTTCTCTGGTTTTAGGAATAGGTCGTTGTCGAGGAGGATTTGGAGAAC
ATGTTCAGCGATGAATCGCTGACATGTTCGCTGCGATTCGTTCATTTCTTCTTGAGTATTGGTAAATATTAAG
ATATCATCTATATATACTACTACATGTCCTTCGTCTATTATTCTCATAGTAGTGCATTCATCATAGTTTGGAA
GGTGGCCGGTGAATTGCATAGGCCAAAATACATAACAAGTGGTTCAAACGAGCCTCTATGGGTTGTGAATGCT
GCCTTCGGTTCATCTCCTTCACACATGCGAACATTGTTGTATCCCCATCGTAAGTCTAGTTTTGTAAACCACT
TAGACTTGCCGGCTTTGTCGATAAGATCGGAGATCATTGGGAGAGGATAATTGTTCTTGATTGTCCAAGCGTT
AAGTTGACGATAGTCTTGAACCATGCGTTTCTTTCCGTCTTTCTTGGGTACAAAGAATACAGCGGATGTTTGT
GGGGATTTACTTTCACGAATATAACCTTTGGCCAATTGATCCGCTAAAAAGTCATCGACTTCTTTCTGTTCGG
TAGGAGAAAGATTGATGACTTTGGCTTTCTTAGGTTCATAGCCGTCTTTCAATTCTATAGCGTGATCCCAGGG
TTTTCGTTCAGGCATGCGTTCCGACGCTTTCTTTTCAGACTTTGAGCCAACGTCTAAATCGTCGAGGAATTTT
ATTAACAAGTTCAGGTTCCTCTGTTTCGATATGTATGTTGTCATGGTCTTCAGCACGTTCTACAGATCGGACA
AAGTGTTCATCTACTATTTCATGAACCACTTCGTCATAATTAATTTCTTCAAGTAATGCGCCTTCTTGTTCAG
TAATAAGGCCACCAATCACTCGATCTGCATCTTCGTCATCGATCTCTTCAATTGTGGGTTGATATTTCCATTG
ATTGGCTAGAGCTTTATGCTTCTTTTGCTTTTTAATAGCACGTACTCTAACATTACACTCTGGGGGCATCGAG
TGAATGTGATTTCACCGGTTGACCAATCGATAGATGGATTGTGTTTCTTTAACCAGTGTGACCGATTATGATC
GGAACTTTTCCTAAGTCCGTGACTTCGAAAAGCATCTTCTCTTTATGTCCGTTATGAACCATCATTAAATAAA
CTTCTTCCGTGATCGATCCTCCTTGATTTAAGGTACCGTCCACATTAAATACTTTAACTGGATATTTTAGAGT
TTGGGTATTCCATCCCATACATCATACATATGCTCGATCGATAAAGGAACCAGTTGCACCAGAATCTAGAAGC
GCTTGACATGATGCAGTTTCATGAGTATCTACTGTCGTTACTGAAATCGATAGTTTTGTCTCGCGTTCCAAAT
TAAGCGTGTGAATAAGAAGTCTTTCTCTGTAAATATGACCAGGACGTTCCGAAGATATGTTACTGCCGGGACC
CACAGAGTTACCAACTCTGGGACGGACAGATGTTCCATTTAAGGAATCCTGGTGGGTTGACATGACCATAATG
TCGGGACCCTGTTTAACAGGACGGACGTCTATCTGATCACCAAAACCGCTTATTTTTGTCTCCACTAGTTTTT
CTGTCGTTTTTGGTTTTTGAAAATGATAAATTGGGGCGGGAATTAAGCACGCTTCCTGAGCGCCAAGCTCCTT
ATGGGAGACCCCCTGAGATACGATTACATTGGAAGGCGTATCTATGTTTTTGACCGTGTCGACAGATAAAAGA
TGAAATTTATTTTTAGGCAATGCTAGTGGTGGAGATTTCACTGCGTGCCTCCTTGAAAAGACGATTGGGCGTT
AGATTGCCCCTGAACTTGTGAATCCGGATTGACTTCCGTGATTCCTGATGTTGACGGCGTGTCTTGCCAAATC
AACCCTTCTTCAATTGCAACCTGTAAATAATGGTCGCGTGCTTCATCGTGTGTGGCCGAATTTAAATTTATGT
TGGCCCTGCAATCTCGCATCATGTGGCCAGGTTTACTGCATCGATAACAAACTCCGGGTGGGCAGTGGTTTCC
TCGATCAACATCCATTGCATCTGGAGCACGTGCAGGAGCTGGTGGATTGGGACGAGCTTGTTGAGCAGGTCGG
GCCGGTTGTGTTTGTTGACCGAATGCCGGGCGATTGAAACCAAACGTAGGCCGAGCAGGTTGTGTCTGTTGAT
TGGATCCCGTATTTCGGTGGGGACCTTGCGTCTGAGTGGTTCCTGTTCCGGTTTGTCGTGGGTTACTCGATGA
AAATGCCGGGCGTGAGAATGTTGGTTGATACAACTTCTTCTCTTCCTGAGATTGTCGGTATTGCCTGTCCAAA
TGTGATGCTCGTGTGTACCAGTCCTTGATGGTATTCGGTTGATATTCTGATTCCATTAGCCGTTTTCGAAGAC
CCTCATTTAACGACCTTTTGAATTCTTTGATTAGAGGATAACCGTTATATCCTGAACCGTCGGCCGTTTTCTT
GAATTCCTGAATGTGTTCTTCTGCTGATAGGGTTCCTTGTTTCATGGTACAAAGTTTGAACACTTTGGTTCCC
TGTGGATCATGGTCGGAAAATGCCAGTTTGAATTCTTTCCAGAAGTCGTTAATAGTATAATCCATTTGTATTG
GGTTCCGGTTAAATTCCTTGATCTTAAGATCACGCCATGTCGCTGCTGAACCTTGAGAGCAGAGATGTAGAAC

CCAATATACCGCACTGGTGACCAATGAAAAATCTGCGTGACGAACTTGTAGATAAATACGGCATGCATTTTCG
AAAGTGTCCGCATTAGACATTGTACCATCGAATGGTGTCGGATTTTGGAGTTTAGGAGCTCTAGATCCTTTAG
CCGCGTTGGAGTATGAACTGGGGAGGGGATTAAATCCGTCAAGAGGGGATGGTTTAGCATCTCGTTCACGTTT
AAGGCATGTCACGAGTCCGGGTAACTCGTGAGTCAGTGTGAGTTGACCCGTGGGAATACCCGTGAGTATGCCT
ACTCACGGATCCGGATCACTCACCATCGAGGATCCCTGCGAGTCCGAGTATGGATTATTTTCTTGTGAGTACC
CATGCAAGTACTCATGAGTACTCACCCACTTATTCCCACACCAGAGGCTAACAAAATGTTCACGAAAAACATC
CAATTTTGCAGTCTGTTCAAGCCTCAGGTACATTCAATTCCTCTATCTCAGTGAGTTCTGATCCGTTTTCCTT
CATCCTGGGATTGATCATGATGGATTTTACCTGTAGATCATGAATCTGACGAGACGACAGGGATAGGACATTC
CGATTGCACCTGTGAGGTCGATTTTTCGGTTTTCAGGTGGATATGACCATAGCTATGTGAGTTCTCATTTGTT
TTGCATCATCCTGGGGTTCTCCAGAACGATTTCATGTGTAGATCAAGAATCCGACGAGACGACAGGCATAGGA
TGTACCAATCACAAATGAGGGGCCGATTTTGGGGTCTGGGGTAGATGTATCATAACTCTGTGAGTTCTCATTC
GTTTCTCTTCATCCTTGTATTCACCAGCATCGATTTCACGCGTAGATCACGGATTCAACGAGACGACAGGCAT
AGGGTGTGCCGATTACAAGTATGGGGCCGATTTTTGGGGTTTAGGGTGGATATATCATAACTCTGTGAGTTCT
CATTCGTTCTCCTTCATCCTGGTGTTCACCAGAATCGATTTCACGCGTAGATCATGAATACGACGAGACGACA
GGCATAGGACATGCCGTTTGCACATGTAGAGCTGATTTTCCGGTTTTATTATGGAAATATCACCTAACTCTGT
GAGTTCTTATTCGTTCTGGGTCATCCTGGTGTTCACTAGGATTGATTTTGCATGTAGATTATCATTCCAACGA
AACGACAGGTGTATGACATGTTGACCATACACTCGGGGCCATTTTCCGGTTTTCAGGCTAAAATGCATCATAT
CTGGGTGAGTTCTCATCCGTTTCGCATCATCCTGGTGCTCATAAGGGCCATCTTTATCTCTAGCTCATGAATC
CAACGAGACGACAGGCCTAGGATGTGCCAATCACACACGCGAGGCTGTTTTTCTGGTTTTTATGTAGCTTTCA
TATGTTTTTACCAGGTAAATTGCAGTAAATATGTGTATATAGATAGATACTTGTGAGTACCCGAATACTTGTG
AGTAATATACTCACGGATCCGAGTCTCTGTGAGTACGAAACTCATGCGGATCTGAGTATCAAATTTTGACTTG
TGAGTACTTGTGACCACGGATCCGAGTCACTCACGAGTAGACTCGTGACATGCCTTATTCACGTTTATCCTTG
ACCAAGTCTGTAAGAAGTGCCTCAAACCGATTAAACCGAGAGTGGAGTTCATGATTCTGTTCCCATAGTTTAG
AATTCTGATCTTGGAGAACTTTGACGTGGTTCTCGAGGGTTTGGATCCGTTGTATGAATTCCGTGCTCAATTC
CGTGATGTGTTTCATTTGACCATAAAGTTCCGTGAATTTCTCGGCGTCCGTTTGGTAATTAACCGTGTCGGGA
TTGACCTGAGGGATTGCGGGTGGTTGGAAATAGTTTCCATCGAATGGGTTGATCAAACTGCCGCGAGGTGTCG
TCTGTTGCGATCCTCCCCAGTTTGCTGAATCTTCAGACATAGTTGGTGATTTGGAAAAAGTAGGAGAGGGGTG
GGCGGAGGAATATGGTCGAAATGTGACGGAATTTTGAGATGAAGGGGGCCGCGGGTATGGACTTAATCCAGTA
GACCGGTGGTGATCACCTGCTTCAAAAGTGGGGACGGGACAAAGGTGAGATTCGAAATTCTCGCCTGCTCAAA
CCGTGTAACGGTAGCAATTAGGATGAGAGGTTGCAAGCCTACTCAAACAGGAATGGGAATGATGATTCAAGAA
TGGTCCGGGGTCGATTGAACTGTTACTAGTAGCTAACCCTGAAACACTTATCCTGCGCCAGGTGTGGGCTGAA
CTAGCAGTGAGAGAGTGCTGAGGTTGCCTTGGCCAACAGCCAATCCAAGCGCAACAGGAAGGGGGATTTGCTG
ACTCTTATAATGCAAAACGGAGAAATAAATCTCAACCGCTAAGCAAGAGTTGTTGCCAAGAGAAAAATCACAC
AGTCAGAACGTTTAGAACGTAACTGCCTGTTTGATTTCTGCGACAGTCGTGGGGGATTAATGATGAACGGGCG
GGGATGTGCTTACACCAAACAACTAAAACTCTTTGAGCGGTGATGTTGTTTCACGCGCTTCGAGATGTCGTTG
GACAAGCGAAAGTGTTGAGCGATGAGAGAGGGCACAAGAAGAGAATCTGATTCTTCTGTAAGGACACGCTAAA
ACACTCGAAGAAAGTGTGCGGTCTCTCAAGAATAGCCAGAGGAAGTTGGAATGAATGAATATGAATGACAACG
GACGTTCACACTGTGTTAGAGCGGAAAACCGAAGAGAATCAGATTGAGAAACAAAACATACAAACAACGAGAC
TATATACATAGGTAAGAGCCCGTGAATATAATAAGTACAAACAACATGTGTTTACAATGAGGTGAAACCGCGG
TTCGCTTACATAATCAGTCACGTGATCAGTCATGTGATACTACAAACGAAAAGAGAAAAGAAAAGAAATTACA
AGTCCTGACAG
>Cligib1_Cluster73#LTR/Gypsy
NgttagggcctgagccttcacaactgatacaagtcggtcgttcgttcttgTtctatcttgaaagtagtttcaa
gatgtctcctgtccaatgacaaaggagttcaagtcagtctgagctgGtctaggctcaggtTctgaGtaagagt
Ntgaaggcggtagattgcctaggtcaagatgctattacaggatgtacagaagaagtatatatactaagtcaca
gtgaccatgaggagtatgcatgttaaggaattctacagcaataTatactaaagggaagatatgctgtagaca
atgagtaaaccatgaggcgCagtaNgtcagaggaTgcacatgaggagtccatgaggNaaacacatgTgcaaca
agtgttgaaaagaaaagNgtccgtaNatgatgtcagaaaaagagtAagccccctggGagcgccgtaacattac
ccactgcttatNgttgaagtccctgacttcaatcttggtgNaatggctcatcgcacatgtcagacatggCggt
attGtcgaaNtcNagatccccatcagtatagggNtggtaactggcNtgggaggtggaggatcgaNagcgacat
NtNtcttgaaggggtaaaggggcNtgatttccacagtgtcgaaaaaagtaaTgctcgcagttcNtcatcatat
gtccaggtNatttgcattcccAgcatNacttgggagtgggaggNgaggaagNtaNNctgagtccggtgggggt
acgagttcggagNggtNtagNggtgggttcaggggaggagaagctttcttcaaagggtcttcttccagcatga
ctaaggggggttgatggtgatggtgtgtccgcatgtNcgacgatttcggcggttagNNgtgggtgcagagttgc
gtctgggagggaattctacaacttcttgagggacagggactcttctgataattctttgagagcggattagggg
gGtgtctggggcaggtggggtggagggagaagtggttacNgagggggtctagaggagggtagtctgatagtc
ggttggggaaaggggNttcttcgatatgcggtctgagatgggggtgtctgtGtcatggcttctgNggtcgttc
tggtggtctgggtgacNtggatcgcaatgaNtgggggggagaggagagtggagNgaggTtctgtcNggagat

NggagtNggggaaatgctgggNgagcNagggttNaggacttgttcaattccatgttTcagGagtagatagctt
aggctaccNgcNagggcttctTgctgttcggtgaggcgattaNcgaggaggctaaNtcNtgcagctcgttatg
gatNtttNggaaNgggacagcNgtgtggcggatctcNtacaAtgtgagtaacaggtcttccagaatgggtagt
cgtggcagctNcgatacatNgtggcagttcggtggtctgggtgagaatttgNatgagttcctcNtggcagagt
aNccatcggcgagtgacgttgttNatattgaGggggggtgccgaggtgagcgttgatgaaNtgNatgaggtct
tcNgcagatcaNgagtgggggaggagactggggaaaggaggatggttcNgaaggaggatagaggagagaNggN
gcggtgactggNatggtgtaGgggaAgaggacgcggaggtatactgggtggaggcaggggaggacatgNNaa
ttctgtttctgaggcNNgggtagtaatcggtggagtactgaggatttagtcaaNagagtgTcgctttttata
agcNttgaGgatgtcgGatgcattaccaaagtCacgggttggcagccacatgttgtctgcggaagggtAaccc
ttccatttgacaaggtacttggcgccacggNNtttgccNgtgagttccaggatggCttctattcatattctt
cttctccattgattaaatcaggggggtcgcggaggTaNttggggccatgagcttcggtttctttgaagggNgt
tagNaggcatgcatggaacacagggtgtatcttcaatcgttctgggagttccagNcggtaagtgacaggtccc
aNgacttcggtaatgggaaaggggccacctcggcgtggTgcaaacttccgggtttcatatccgagtttcaggt
tggcggattcaagccatacttgNNNNccNNtNttgaaaggggtaaaacctcgtgttgttctttcagccatgtg
ttggcgtgccagttcgtgtgctgcagaggcttcgtccctagtcctctggaggatcttgaaacgttcttctacc
gcgggtatNtcagatacCtggaagggNagagggatggctttagggtcNtatccaatcattAggtagaatgggg
aTgcatttcatgcggagtgaatgcgttgNctgtgtgtgaattctacgggagggagcatggatacccatgtttc
agggtggtttgcacagaagatNcaaaggtatgcttcgacttctggtNacgcatactgtttctccatcggtttTg
aggNgNNNgcagtactgaaggcgagtttgataccaaggactcTtccaagttctctggtgactgcagatgcaaa
ttgggggccNctatcagagatgatcttNactgggagtccgaatcgctagtaacattttgaaggatgAgtctgg
aagttcctaNagaatcgatgtttttgttacaagggatgagaattacaccctttggaagNccttggtccaccac
gaccaataaagaatcaaatccNtcggatatgagtagatcggtNacNaaatccatggagatttgggagaagggt
aggttgttgaagggtcctttgataggcattatgggtggggtNgttggataNgtgttaaccttggtttgttgac
aNatNccgcatccttctacaaatttctttacaaAggtggatagtccgagccaccagtagtCtcgtttgatNaN
ttctagtgttttgaaccgtcaagagtgtccaatggaggggacgtcatgatatttctttacNacatttctgcgg
agttcttcNtctggagataCatagcatctgtctttataaaatatgagtccttcNtctgttttccaatcNgaaa
gagccgattTcatNgggggggtaccccctttttggattgcttcaaaggcttgtgtGatgatttcatccttgaa
cgtggagcagagtatctgatccctaagatcagtgtcgatggttttaNaaaaaggaaattaggNagNAggGtga
tgtcttcattgtcNttNtcttccgtgggatttaggtcttatcGtcgNgaNagggcaTcagattgtatcatNtg
agttccaggNacatggaccagttgcatgtcaaattcggataggaagaagctccaactggcttgtcggcgattt
agtttctgagctgttctgaagtatgtgagAttNttgtggtcggagaatatgacaatggggtcaggggatcctt
ggatgtaatgtctCcaggcttccagggctcgtacaatgcctaatagttctcgatcataaatttcNtagttgcg
ttgggttgcgtcgaaggattgggagagataggaNcagggatgccagtctccgttaacatccctTtgNctaacg
actgctccNgtggcNaAtatggaggcatcagattctattacgAatggtttagtagagtcgggaatgaggagta
cgggtgcttctgcaaatttggctcttaNggtgtcNaAagctattTggcattcttctgtccactcccatttag
ctccttcttggttaggttgttgagtggtttggcaatgtcNgagtatttgccaatgaatcttcgatagaagctt
ccaaatcctaagaaggaacggacacctttAacattggttggggtgggccaatctttgattcctttcagttttg
cagcatccatagcaatatggctgggacggaagatcatgcccaagaattcaatctcttcttgctcaaatgcNca
tatttctaatttcaggaataggtcatttcttggagtCtttgtaNgactNgaagggttcgtttacNatgttct
tctaNattatcNgaatAgaaTagaatgtcatccaTatatatgacaatacatccttcatcaagcatgtctcgga
agagttcgttcatcatCatctggaatgtagcNggtgaattgcacaatCcaaagaacattactgtggttcaaag
agtccgcggttgNtttagaaAgctgcttccacaggtgNccattcttcatccgtatgttattgtatccctgtcg
gagatcaagtttggtNaaNagtgtagcccCtttgagtttatccacaagttcttggatgaggggaaNgggNtag
gcactcttgAttgttccttcaatgagataccggtaatcttggcaaggtctcagagaaccatccctcttgccaa
caaaaaagaatggagataccataggtgatTtcgagggNgtNatNtatcctttatccagNattcctttaggaat
gcatccaatttaatctgttaaccaggtgtNagaggctatactttgcaNtctctagggatNaaatcaagtttta
gatcaattgcatgatcataNatcctagaTtctgggaatcgttCtgcagctttcttttcNaagacatgttggTa
tttNatatatcgttcNgggaggAgttcttcNNtggtttcggtttatcNgggggttcacggttNcgggcaaaNg
tttgNgatgaNgtgattcatgctcCNatccgaatgtttgcccataNgagtatcgtttagAatgtaNNcgccag
ttgaccatacctattctgttgatggttttgtagAagtaggcgggtaNgtcgtgttcNacggtgagggggcttc
ccgagcNggttcaaagataccaataAcaggttcGccttggatgtaNgccaagattagttcatccggttcNagg
atggaNaggtNttcgagggtgtccaatgaagtgcagccaggtaagcagtagataNtgtccagggtctcagtct
ttatctcattgatNgggNtttcttcttcttcatcggtgatatcttcgagggagggttcgggaaaagNaggtgt
agagaaaggtttNggaataNatgtTtcctgttcggagattgtctcagattctcttNaagatctgagtactNtc
gatagcaaggtttgctgaattgatttaaagagctactttNaacccNtcgataaagaggttgatttctctgagc
caggggagtccNaggatNatggtttcttttcccaagccagtgaccaatagacgggtgtggagtctctgttccc
tNactggatgtcaaNcNNgtatagtgGgtaatttgtccNgttttgattcgNgtgccatCaacattatagacag
cgatggggaggagagtctttcaagtggagttgtttgtctttggcNaaggaagaatcaatgaattggcctccag

cgccgcagtcgataagggctttggttacaacggttgtttctcccgatatgCaaataggtaaatgcatNgattg
gcgatcattttcggtcactaatacaggagaaattgagatgggaggCgagactgacgatgatgcagttNgcctc
attaaaaaccatcttcatacatcttttctaacacatttNNcttctCctcttctgctAgNtctttcatcacgca
cggatTttggcaaagctggcaggtccgtccttggaNgattctcgtggggtgggttcNatatttctaaccctaa
agggtttcctttcttctgggagtttcccattcttgtggtctatgNcaNNNtggccactcttgtggcataaaaa
gcacattcccttattcatgtcttcagcacgttgttcggtggagagtcgatccacatccatggcatcaggNtCt
ctgggtgcacNtatctgggggTttgNaAtttcttctgggggaNNaggaNggtctgccggaaaagcgaatggcN
atggatcaggctattcggtattggttgtccattctggaggctgatgagtaccacttctcNNcNgtggatagNa
cNtttccatggtgtagactttcttcattacttccggatgtaatccttccacgaAtattcaatgagtgcggcat
ctNcNgtNatttcNcagagggatactgggactcggaatTtggcNatatactcctcaactgttccNgcttctcg
gagtgaccgNaggtgcttctggcNcctcctgctacaactgtctgggagaatgcNactttgaaagcggctagga
attgtcaaaNctgggcaagccNtggttgagttggtNaggaagagatagatgcttcttccaggaagtagctgtt
cctccaaccatacatgataccatgaatgctacCttcttCctgtcNgtattNagcagtccatcattcactgcta
tNtgaaaNagaatttcattNataaaggtgctagttttggtgcgttctccAtcctacttggtaggtgcNcatag
gaacattcgagtgttggtttcNgtagtcatNtcgNNggtgtcggtNtattNgagttcttattcNaNtggtatg
tAagcgNctgtaNctgggcgaacagggatttctcccgcggtgtcNgtgaaagtttctggtgcNacgacttcNa
tatggaaatagtNtgtgatgtgtggtactNgttNgttNgcNtaaatgaagtaccactgtctttcgtctgggtg
gaaggcgatctNtNttccNNNgagccaaAattggtNttcNcctcttttctgaacNacggtggtgccgaagggt
tTgatcagGaaggactgggaagccacNgtgatgttNNtgttgagNtgtctggtggggcggtggtgcgactgaa
cataaagaggttggcggggtttaagatctggaccttttgttagggcgtgagccttcacaactgatacaaatct
gtcgtacgttctggttctatcttgagagtagtttcaagatgtctcctgtccaatgacaaaggagttcaagtca
ctctgagCtgggctaggctcaggttctgagtaagagaataaaggcggtagaTtgcctagaTcaagatgctatt
acaggatgtacagaagNNgtatatatactaaNagacAgtgNccatNNNgNNNatgcaNgttNagggatNNcac
NNNNatataNacNaNNNgNgNagNtNNgcttNNNacNNtgaNNNaNaNNNgagaccNNgtaggtNagaNNaNg
acNNNgaNgNNtccatNNggcNaacaNNtgNaNaNcaNNNgNNNaaNagaaNaNcNNcNNtNgatNNtgtNNN
aNNaaNagtNaNNccNcNNgNNNNNNcNNNacN
>Cligib1_Cluster36#LTR/Gypsy
GTCAGGACTTGTATTTTCTTTTCTTTTCTCTTTTCGTTTGTAAGTATCACATGACTGATCATGTGACTGATTA
TGTAAGCGAACCGCGGTTTCACCTCATTGTAATCACATGTAGTTTGTACTTATTATATTCACGGCCCTTACC
TATGTATATAGTCTTGACGTTTGTATGTTTCGTTTCTCAATCTGATTCTCTTCGGTTTTCCGCTATAACACAG
TGGGATTTCATCCATCAGTCATATTCAATATTCCTTCTATCCTCTGAGTATTCTTGAGAGACCGCACATCATT
CTGGTGTTTAGCGTGTCCTTACAGAAGAATCAGATTTGTTTTTCGTGCCCTCTCTCATCCTTCAACACTTTCA
CTTGTCCAACGACATCTCGAAGCGCGTGAAACAACATCACCGCTCAAAGAGCTTTAGTTGTTTGGTGTAAGCA
CATCACCACCCATTCATCATTAACCCCCCACGACTGTTGCAGAAATCAAACGGGCAGTTTCGTTCTAAACGAT
CTGACTGTGCGATTTTTCTATTGGCAACAACTCTTGCTTAGTGGTTGAGATTTATTTCTCCGTTTCACATTAT
AAGAGTCAGCAAGTCCCCCTTCCTGTTGCGCTTGGATTGGCTGTTGGCCAAGGCAATATCGACACCCTCTCAC
TGCTAGTTCAGCCCACACCTGGCGCAGGATAAGTGTTTCAGGGTTAGCCACTAGTAACAGCTCAACCGACCCC
GGACCACTCTTGAATCATCATTCCCATTCCTGTTTGAGTAGGCTTGCAACCTCTCATCCTATTTGCTACCGTT
ACACGGTTTGAGCAGGCGAGAATTTCGAATCTCACCTTTGTCCCGTCCCCACTCTTGAAGCAGGTGATCACCG
CCGGTCTACTGGATTAAGTCCATACCCGTGGCCCCCTTCATTTCAAAATTCCATCACATTTCTGTCCATACTC
TTCTGCCCACCCCTCTCCTCAATTCTCCAAAACACCACCAATGTCTGAAGACTCAGCAAATTGGGGAGGATCG
CAATCGGCGACACCTCGTAACAATTTTGCGGGATATTTTAATCCAGAATATGCACCCCCTGACACTTCACAAG
TTAATCCTGACACGGTCACTTACAAAATGGATGCTGAAAAATTCACGGAGATCTATGATCAAATGAGACACAC
CACAGAATTAAACACGGAATTTATTCAAAGGATCCAAACCCTCGAAAACCACGTCAAAGTTCTCCAAGACCAA
AATTCACGATTATGGGAACAGAACCACGAACTTCATTCTCGGTTTAATCGGTTCGCAGCACTCCTTGAAGATC
TGGTCAAAGATAAACAAGAACGAGATTCCAAACCCTCTCCTCTCGGTGGATTCGACCCACTTCCCGGCTCTTA
CTCTAACACAGCAAAAGGATCTTGTGCTCCCAAACTCCAAACCCCGGCACTGTTTGATGGTTCAATGGCAAAT
GCGGACACATTTGAAAATGCATGCCGTATTTACATGCAGGTTCAACACGCAGATTTCTCATTGGTCACAAATG
CGGTATATTGGATTCTCCATCTCTGTTCTCAAGGATCAGGATCTACATGGCGTGATCTTAAGATCAAAGAATT
TAACCAAAACCCAATTGATTTAGACTATACCATCAATGATTTCTGGAAAGAATTCAAACAGGCATTCTCCGAT
CATGATCCACAATCGACCAAGGCATTTAAACTTTGCACCATGACTCAGGGAACTTTACCGGCAGAGGAACACA
TCCAGGAGTTTAAGAAAATCGCTGATGGATCAGGATATAGCAGTTATCCATTGATCAAGGAATTCAAACGGTC
TCTAAACGAAGGACTTCGCAAACGATTAATGGAATCGGAGCATCAACCAATAACGATTGAAGATTGGTACAAA
CGGGCATCTCACTTGGATAGGCATTACCAACAATCCCAAATCGAGAAGAAATTATACCAACCGACATTCTCTC
GTCCGGCATTTTCATCGAGTAACCCACGGCAAACTGGAAACGGAACCACACAGACACAAGGTCCCCACCGAAA
TATGGGATCTAATCAACAGGCACAACCCGCTCGGCCTACATTTGGTTTCAATCGCCCAGCATTCGGTCAATCA
AATCAACCGGCCCGACCTGCTCAACAGGCTCATCCCAATCCACTGGCTCCAGCACGTGCTCCAGATGCTATGG
ATGTAGATCATGGAAATCGCTGTCCACAAGGAGTTTGTTATCGATGCGGCAAACCCGGCCACATGATGTGAGA

TTGCAATGCCAGGATAAATTTAAATTCGGCCACACATGATGAAGCATGCGACCATTATTTACAGGTTGCAATT
GAAGAAGGGTTGATTTGGCAAGACATGCCGTCAACATCAGGAATCACGGAAGTCCCTCTGGATTCACAGGTTC
AGGGGCAATCCAACGCCCAATCGTCTTTTCAAGGAGGCATGCAGTGAAATCTCCACCACTAGCGTTGCCTAAA
AATAAATTTCAACTTTTATCTGTCGAATCGATCAAGAAAGTAGATATGCCTTCCCATGTAATCATATCTCAGG
GGGTCTCCCATAAGGAGCTTGGCGCTCAGGAAGCGTGCTTAATTCCCGCTCCAATTTATCATTTCAAAAATCA
AAACACGACAGAAAATTTAGTGGAGACAAAAATAAGTGGTTTTGGTGATCAGATAGACGTCCGTCCTGTTAAT
CAGGCTCCCGGCATTATGGTCATGTCAACCCACCAGGATTCCTTAAATGGAACATCTGTCCGTCCCAGAGTTG
GTAGCTCTGTGGGTCCCAGCAGTAACGTATCTTCGGAACGTCCTGGTCATATTTATAGAGAAAGACTTCTTAT
TCGCACGCTCAATTTGGAACGCGAGACAAAATTATCAATCTCAGTGACGACAGTAGATACTCACGAAACTGCG
TCATGTCAAGCGCTTCTAGACTCTGGTGCAACAGGATCTTTTATTGATCAAACTTATGTACGATAAATGGGAT
GGAACACCCAAACTTTAAAATATCCCGTTAAAGTATTTAACGTGGATGGTACCTTAAATCAAGGAGGATCGAT
CACAGAAGAAGTCTATTAATGATGGTTCATAACGGACACAGGGAGAAGATGCTCTTCGAAGTAACGGACTTGG
GAAAAGTTCCGATTATCATTGGTCACACGTGGTTAAAGAAGCATAACCCATCTATCAATTGGTCAACAGGCGA
AATCACATTTACTCGATGTCCACCAGAGTGTAATGTTAGGGTACACACTATTAAAAAGCAAAAGAAACGCAAA
GCCCTTGCCAATTGATGGAAATACCAACCCACAATTGAAGAAATCGATGACGAAGATGCAGATCGAGTGATTG
GCGGCCTTATTACCGAACAAGAAGGCGCATTACTTAAAGAAATTAATTATGACGAAGTGGTTCACGAAATAGT
AGATGAACATTTTGTCCGATCCGTAGAACATGCCGAAAACCATGACAACATACTTATCGAAACAGAGGAACCT
GAACTTATCAATAAAATTCCTCGACGATTTAGACGTTGGCTCAAAGTTTTTGAAAAGAAAGCCTCAGAATGTA
TGCCTGAACAAAAACCTTGGGATCATGCTGATGAGCGGTGGTCAGGACAGACCTCCAATCCTGGACTTCGACC
TTGATTTGTCAACTTCGACTTCAACCTTCGACTTCAACCTTCGACTTTGACTTTGACTTTGACTTCGATGACA
TCATAGTCGACCTCACACTCATAGATGTAATTAGACACACTGTAGACACATTGCTCGACCCTGAGCACTACTA
TATAGTCGTCAAACATGTACCTAGATCAACACGAAACAGTTTCCCATTTCACTTTGTGCCCCTTCCACACCCC
TTCGACTTTGTCGACTTTGAGTTCGACTTTGACGACTTTGAGTTCGACTTTGACGTACTTCGACTTCGTGGCC
TATTGCAGTGACCTGAAGCACCCACTCGTCACTAGAGAGGTAGAGGTTTGGGTTGGTTTGCCACAGGCATCTT
GTTACGTCCTGCTAACGCAAGCCGATGAGTCGACAGTAAAGACTCAAGGCACTTAGCAAGGTCTGAGTTGCCT
TCTGGGTCAGGAGGCTAGTAGAGGGGGGAACTGATAAACGATTAAACGTAGCGAATACAGGAGTATGTAGCTA
CATTTATATAGACATGAATGTAGTTCTATGACTACGCAATAATGACTGTGTCCGTGAGTATGATAGGACATAT
GGAGTATGCTGACTGAGATAGAAGAGAAGCTGACTGAGCAGCTGAGTAACAACAATGAAGATAGCAAAGATAG
AACAACAACCATGCGGTAGGAAGGTGATAAGGAAGTGCATGATGTATCATCTGATGATGGAGCATGTGGAGTA
TGTCAGATGATGATGTAGGAAGTAGAAGCGTCTATAAAAGATGGGATCAGTCAAGTGACTGATGAATGATGAA
ACAGAGAAGCAATGAATCGTAACACATCTTTCCTAGGTCTTGCTCATCAACGCTATAGAATTGAAAGACGGCT
ATGAACCTAAGAAAGCCAAAGTCATCAATCTTTCCCCTACCGAACAGAAAGAAGTTGATGATTTCTTAGCGGA
TCAATTGGCTAAAGGTTATATTCATGAAAGTAAATCTCCACAGACATCCACCGTATTCTTCGTACCCAAGAAA
GACGGAAAGAAACGCATGGTTCAAGATTATCGTCAACTCAATGCTTGGACAATCAAGAACAACTATCCCCTTC
CAATGATCTCCGATCTCATCGATAAAGCTGGCAAGTCTAAGTGGTTTACAAAACTTGACTTACGATGGGGATA
TAACAATGTTCGCATGCGCGAAGGAGACGAACCAAAAGCGGCATTTACGACCCATAGAGGTTCTTTCGAACCG
CTTGTCATGTATTTTGGCTTGTGTAATTCACCGGCCACTTTCCAAACTATGATGAACACACTTCTACGAGAAT
TAATGGAGGAAGGACATGTGGTAGTATATATAGACGATATTTTAATATTTACCGATACTCAAGAAGAAAACGA
ACAAATTGCCGAATGCGTTCTCCAAATCCTCCTTGATAACGACTTATTCCTTAAACCAGAGAAATGTTCTTTC
TCGGTTCAACAAGTCAAATTCCTGGGACTTATACTGGGAAATGGAGAAGTACGCATGATGGACGATAAAACCA
ATGCTGTAAAGAAATGGCCAGTTCCCAAAACAGTCTGCGACATTCGCAAATTCCTAGGTTTCGCTAATTTCTA
TTGACGATTCATCGAAGGATTCTCTAAACACACCGCACCACTCAATAATCTCTTACGAAAGGATTCCAAATTC
GATTGGACAGAGAAGCAACAAACGGCGTTTGATTTGCTTAAAAACCGGTTTACATCCGATCCTATACTGGTAA
CGGCAGACCCTACAAAACCTCTTCGAGTTGAATCAGACACATCGGACTTCGCTATGGGAGGTATCTTATCTAT
GAAATGCGCCAACGATAAATGGAGGCCTTGTTCGTATTATTCTAAATCTTTATCTGACGTTGAACGTAACTAC
GATGTACATGACAAGGAAATGCTTAGTATTATACACTGTTTGGAGGCCTGGCGCCATTATTTAGAAGGTAGTA
CGCATACAATTGAAATATGGTCGGACCATCGTAATTTACAATACTTTATGTCCTCTAAAAAGTTAAATTGTAG
ACAGGCACGATGGGCACTTTACCTATCTCGATTTGACTTTACATTAATTCACAAAGCAGGATCTTCAATGGGA
AAAGCGAACGCACTGTTCAGGAGGACAGACCTAAAGGAGGGAGTAGAAACTGATAACGATGATATCACACTCC
TAAAACCGGAATTCTTTGCCATTAAAGCACTACAACAAGGACATGTTCTTGTTAACAGCGAAGAAGAGGGAGT
ACTTTCAAAGATCCGTAAATCGAAAGACTATGATGAATCGGTTGTCAAAGCGATTGAAGAATTAAAACAATCG
CCCATGAAACATTTACAATCCGAAGAATGGTCTCAGGAACAAGATCTTATATTGTTCCGGGGAAAAGTCTATG
TACCCAACGACATCCAACTTCGACGAGAAATCGTTAAACTTCACCATGATTCCAGTATCGCCAGTCACCCAGG
ACACTACAAAACTCTGGAACTAATCGCGTGAAACTATTGGTGGCCTGGTATGACAAAATTCTCCACTCCTATA
TTGACGGATGTGATTCATGCCAATGTTTCAAAAACTTCCCTGCCCCTCCTATTGGAAAACTTGTTCCAATGAT
TACCCCTTCCGAACCATGAAAAGCAATATCTTGCGACTTCATCACCAGACTTCCTGAGAGTAATGGATACAAC
GCAATTGTGGTTGTTGTCGATCACGCATCAAAAATGGCGCATTTTATCCCTACTACGTCTCAAACATCTACGA
CGGGTCTTGCTAAACTTTATTTGCAAAACGTCTGGAAGCTCCATGGTCTCCCCGATTCAATTATCTCCGATCG

AGGACCACAATTCACAAGTTCAATGATGAAAGAATTAAATTCATTATTGAATATTCAAACAAAACTTTCCACC
ACCTATCATCCCCAAACGGACGGCCAAACAGAACGAGTTAATCAAGATCTCAAAGCCTACCTTAAGATTTTCG
TATCACACCTTCAATCTGATTGGGTTGAATGGATTCCTCTTGCCGAATTCTCATACAATAATGCCACTCATTC
CTCCACGAAAATATCCCCCTTCTTTAACGTCTACAGTTGTCATCCATGAATGGGCGCTGAACCAATAAGACAA
CCAACAAATGAGTCGGCTAATGAATTCGCAACACGGATGAAGAAAATTCAAGAAGAAGCACAATCAGCCCTTG
TTAAAGCGCGCGACGAAATGAAACGATACGCCGATCAAAATCGTGGTGAAACCCCATCGTTTCAAGTTGGACA
AATGGTATGGTTAGAAACCAAAGATCTCGATCTCAAACGACCATCAAAGAAACTCACCGAAAGACGAGTTGGT
CCGTTCAAGATCCTCGAACTCATTGGAACTAACACGGTGAAACTCAAACTTCCATCTACGATCAAAATTCACC
CCGTCATTAATATCTCACGAGTACGACCATACCAATCTCCCAAAATACCCCAACAACAAATCCCTCCTGCACC
TCCAGTGGAAATCAATGGCAAATTCGAATATACCGTCAAAGAAATTCAAAACTCACGTCTTACCAAGAACGGC
AAAGGTTTAGAATACTGGGTTAAATGGGAAGGATATACGCCGCAACATAATACCTGGGAACCCTTATCAAATT
TGAAAAATGCCATGGATCTCGTCAACAAATTTCACCGAACAAATCCACAAGCTCCACGAAAAGTTCATCTTAA
TGTAACTCAAGACAAATTCCTTTCTTTGTTCGCACCCTATGAAACTTTAACCGATCCACCGTCAAACTCGGCA
TTATTTTCAAAACTCAATGCTTTTAGTGCTTCAATTTGATATGTGCACATTCTTTTGAATGCGAGACGCACTA
TCAGGGATGATGAAGAACCGCTCAAGATTTTCCCGGAGAGAAAATCCCTAAAGAGGGAGTAGTGTCAGGACTT
GTATTTTCTTTTCTTTTCTCTTTTCGTTTGTAAGTATCACATGACTGATCATGTGACTGATTATGTAAGCGAA
CCGCGGTTTCACCTCATTGTAATCACATGTAGTTTGTACTTATTATATTCACGGGCCCTTACCTATGTATATA
GTCTTGACGTTTGTATGTTTCGTTTCTCAATCTGATTCTCTTCGGTTTTCCGCTATAACACAGTGGGATTTCA
TCCATCAGTCATATTCAATATTCCTTCTATCCTCTGAGTATTCTTGAGAGACCACACATCATTCTGGTGTTTA
GCATGTCCTTACAG
>Cligib1_Cluster40#LTR/Gypsy
CTTGTTACGTCCTGCTAACGCAAGTCAATGAGTCGACAGTAAAGACTCAAGGACACTTAGCAAGGTCTGAAGT
CTCCTGGGTCAGGAGGCAGTAGAGGGGGGGAATTGATAAACGATTAAACGTAGCGAATACAAGAGTATGTAGC
TACGTTTATATAGACATGAGTGGAGTACGATGACTACGCAATAATGACAATGTTCATGAGTATAGTAGGACAT
ATGGAGGAGGATGACTGATCAAGGAAGCGAGCTGACTGAGCAGCTGAGTAAGAACAATAGAGATAACAAAGAT
AGAACAGAGACCATGTGGCAGAGAAGTAGATAAGGTGCGCATGATGTATCATCTGATGATGTAGCATATGGCG
CATGTTAGATGATGATGGAGTAATGGTGGAAGTAATAGAAGTGTCTATGAATGATGCCATCAGTCAGATGTCC
AAGTGATGATGATCAAGAAGGAAGGAATCGTAACATGACCTTCCCTCTAGGAGTTTCGTCCCGAAACTTAAAT
CAGATCATTGTTCACTAGCATCTTCCCGAGTTCCTTCTTCGTCCTCGCTGTCACGTACTCCTCGCCAACGCAT
GTAAAGAAGGTGGGCGAGTCGTGCCGTCCTTTCACCCAGTACCGGAACGTCTTGCCGCTGTATGAGTATGTCC
TGTTGTAGGGTTCCGTGGGCTCGAAGTTGAGTGGGATGCGGATGTGGGACACTCGATGAAGAGGGAGTACTCC
AAGGGGTATAGATCCCGTGGGTAGTGAGGAAGGGGGCCAGGAGCCGGTGGGCTCGTTGTTCGTCGGCTGGGGA
ATGCTAGGAAGGGGGGGATGAGATTCGGAATCACTTGAGGAGGACGACATAGTAACTGGGATTCTGTATTCCT
CATGGGTGGCATCTTCTGCAATGGGTACATTCCATTCGTTGACATGGACTGCTCGTTCCAGTGCCTGTCGATC
GCTATCTGTGAGTTCTGGAAGAAGTCCCTGCACAGTGGTGGTGATGTGGTCGTGCATTTGTGTTTCCAGGATG
AGAAATGATTGGCGGGCTGCGATGAGGTGTTCCCTGGCTTCGTTTAGGCGGGTGATCAGGGTGGGAGCGCCAG
TAGGGAAGAACTGCGTGAGTCCATCGATATCGCGGATGTGTCGACGAATGTTGGACAGATAATTGGGTATAAG
GCGCAACATGGTGAAGACTGGCGAGAGGGAGAGTAGTACGAGGTTGATAATGTCACTGATGACTGAAGGCATG
ATGGTAGTATTGAGTGGGAGGTGAATTAGGCAGATGTGAGGATAGGATAGAGTAAACGAAATGAGTGTGGTAT
TTATCGCGGTGCGTTAGGATGTCGGTGGTAGAAATCTTTAACCAGGTCCGCGGAGTGTTTGAGGTTTGCGCGG
GTTTCCCAGGTGTTTTCTTCTTCCGGGTATCCTTTCCAATGGACAAAGAATAGAAGTCTTCCATTCTTCTTTT
TGACCTTTCGGATGCTTTCTACTTCATATTCTTCTGATCCTTTGATGACATCGGGTGGCGGCGGTGATGTTTT
CTGTTGTGATGTGAAGCGTGCTACCTTGAGGGGGGTGAGGAGGTTTGTTACGTCCTGCTAACGCAAGTCAATG
AGTCGACAGTAAAGACTCAAGGACACTTAGCAAGGTCTGAAGTCTCCTGGGTCAGGAGGCAGTAGAGGGGGAA
AGAGATAAACGATTAAACGTAGCGAATACAAGGGTATGTAGCTACGTTTATATAGACATGTATGTAGTTCTAT
GACTACGCAATAATGACTATGTCCGTGAGTATAGTAACACATATGGAGTATGATGACTGATACAAGAAGCGAG
CTGACTGAGCAGCTGAGTAAGAACAATAGGTGATAGCAAAGATAGAACGGAAACCATGCGGTGGAGAAGTAGA
TAAGGTGCGCATGATGTATCATCTGATGATGTAGCATATGGCGCATGTCAGATGATGATGGGGTGATGGTAGA
AGTAATAGAAGTGTCTATGAATGATGCCATCAGTCAGATGTCCAAGTGATGATGATCAAAGAAGGAAGGGATC
GTAACATGACCTTCCCTCTAGGAGTTTCGTCCCGAAACTTAAATCAGATCATTGTTCACCAACATCTTCCCGA
GTTCCTTCTTCGTTCTCGCGGTCACGTACTCCACGCCAATGCATGTGAAGAAAGTGGGCGAGTCGTGCGGTCC
TCTCACCCAGTACCGGAATGTCTTGCCGCTGTACGAGTATGTCCCGTTGTAGGGTTCTGTGGGTTCGAAGTTG
AGGGGGATGCGGATGTGGGATACTCGATGAAGAGGAAGTACTCCAAGGGGTATAGATCCCGTGGGTAGTGAGG
AAGGGGGCCAAGAGCCGGTGGGCTCGTTGTTCGTCGGCTGGGGAATGCTAGGAAGGGGAGAATGAGATCGGAA
TCACTTGACGAGGACGACATAGTAACTGGAATTCTGTATTCTTCGTGGGTGGCATCTTCGGCAATGGGCACAT
TCCATTCGTTGACGTGTACTGCTCGTTCCAAGGCTCGTCGAT
>Cligib1_Cluster39#ClassI/LARD

GTTGGCAGTGGAGGAGGCAGATGGGGGGTATTAGATATTATTAGATAGATATGTTGTATGTATTGTAAAATGA
GAGACAATCCTCGAATGTAACAACTGGACACGCTTATGTAATCAGTAAAGCGGAGATTATTCTAAGTCAATAT
CTGAGTGAGCTTGTTATCTCAACACTTCCCCTCAAGCGAAACGAAGGTATGAGCTTGTAAATAGGTAGATATA
CCTATAATATAAGAGAATGTATTGCTTGATTAACTTGAGATGGTCAAAACGAGTATGAATTTTTGTTGAAGTC
GAAATGTCCCGATTTGGACGTTTGGATGCTGACCGATCGTTGGTTGATTCCTCTTGGGTGCTTCCTCAAGGTT
CATCTTCTATGGCCATCACGAATAGGTAAGCCAGTCCTCCTAGATGTCCTACTGACCCTCTATCTTCCCTAGG
CATGTCTCTTGGATCCCGATCATTGACTCCTTCAGATGCTGGCTCCAAGATAGGTGAGTCTTCCTTGATAGGC
TGACTATAGACTTCTGGGGGCCTCCTGGCACGAAAATCGCGACGGATATCCGATTCTGGGTTGGTTGGGGTGT
CGCTTTGAGGGCCTATTGGATTAGAAGATATCGGTGGGGTTTTGGGAATGATGGATTTGTGCTTGGACGAAGG
TATGTCCATTGCATGGCCGTTTTTTATCCATTTTGGCTGATTGTCGGCGCATTGGAAAGCTGTGATATAGGGC
TTTAATTCACGTCTATTGACGTCCGGGGGTTTGTCCGAACGGATCTCGATGGTTTGTCTGGATGGTTTGTGTT
GAAGGTAACGCCCGTGACACCGCCGGATGCCAAATATGGTATCCCGCTTGGCGAGCGCGCCAGCGCGACTCTA
TTTCATTTTTACTCTATTTCATATCGTCTCTACCGTTTCACTTACATCTCGTTTCACCAACATCAATGACGTG
GGTTCAACACGCTGTGTTTCACTATTTTCTCTCCCTTATATACTTCACCCTCGTACCTACACACCTCAGTTTG
CTCTCACTCGTGTGACGGCATCTGAGGTTAGTTTATTCTATACATATCGACACGGACACTCGACTAACTACAT
ATCCTATATAGCTTCCATTGTCAACAGGTAGCTAATATATTAGTTTGCTCTCACTTGTGTGACGGCATCTGAG
CTTCCATTGTCAACAGTTTGGACGAAGATATGTCCGCTGCGCATTCGTTTTCTGGCGGTTTTGGCTGATTTTG
GGACCGTTCAAAAGCGGCTTCCAAGGGCTTCAAGTCGTGCCTATTTGCGCGTGGTAATTCGACCACTTTGTTT
TCGATGCTTCGGCTGGGGCTGGAGATGTGGGGCATATTCTTCCCCTCGATCTGGACGGTTCGGGGTGCCGGAG
GGCGATTCCTACTAAGGTAAACATTCCCTGTAACCGCTCCTGATCATTCTTTTGCCCTACAGGTCCTATCATT
TGCGGAATCTTCATCAAAATCTGTATAACGGCCTTCAACCACTTCCAGTTCGGTTTTTCGAGGTTTAGGGTAT
TCGTCTTGTGTTATATATTCGCATTTTTTAATATAAATTGGTTTGTCTTCTTTCTTGGGTGTGATTTCGCATG
GATTCGTGTTTTTCGACAACATCTCGCGCGTGATTGGGATTTCAATATGGATTTCGGGCGGTTTATGTTCGCA
ATTAGTTTGGTATGTATTTCGACTCTCGGGGGTTCGATCGACGGTGCGCGGCGAGGGTCTTCGAGTGGATAGG
GCAGGGGTTTCTTCATGGGGGTATGAGATATCTTGGGGTGTATCCGACTCTTCGACGGTGCCCATTGTTTCCT
CTTCCTGTGGTTCCTGACCTTCTTTTTCTCCTCCTCTCCTATAGCATTCCTCATCGCGAACATGCCCAGCGCT
CATGCGCGGTTCACTATTCTTTTCTTTCATCTTTTCCTCAGGCTTTTCCTCAGGCTCTTCCCCTCGCTTCTCC
AGTATTACCATCGCGTTTTCGGCGCATTTTTCGGCATGCGTGTGGACGGATCACAAGGCAGCCTTGAAATTTT
TTATTTTGTTCTCGAACATGTCGGCGAGCCTTGTCCAGAGCTGCGCGGAGGTTTTCTCCCTCCTCGTCTGGAG
GAAAGTAACCCTGCTGATTGTTGCATAGATCACTTCTCGAACCATGGCCTCCCTTTGCTCGAAAATATTGATT
TTCGTGTAGTGCTCTTCGATTTCATCGTCGTCGAGCAGTTTCGTGGGGTTTCCTTCTAGGACCCACTTCCCAT
CCACCTCGCGAAGCTCCTCTGGCTTCGGCGCGGTTCCGTACAGATGTCGTTCAAGTCCTTCGTGAGTCAGCAG
GTTTATTACCCTTTTCTTGTAAATGACCCAATTTGAACCAGTGTCTTCGAGGTTTGGAATTTCGAGTGATGAA
ATTGTAGGTTGCCCAGGGCCCATAACCTGTTGGCAGTGGAGGAGGCAGATGGGGGTATTAGATATTATTAGAT
AGATATGTTGTATGTATTGTAAAATGAGAGACAATCCTCGAATGTAACAACTGGACGCGCTTATGTAATCAGT
AAAGCGGAGATTATTCTAAGTCAATATCTGAGTGAGCTTGTTATCTCAACA
>Cligib1_Cluster101#LTR/Gypsy
ttaaggcggagtgcctttaccaaggttcaagcgtcgaatatcatccagaggttcaagttagagtcttgggtca
ataccaacaagactatagtcactccaagtcaggctttgacttgggtatcagggttcagtgatcaaggttcaaa
ggtaaagggtgtagtattgcctaggtcaagatagagaatagtgacaatgtatctagggtttatatactaggga
ccttgaggcgcgagaacatagtacaatgggaagtaaagacaatgtaaggtaaaagagctgttgaagctcagtc
agctatgacatcatagatgagtcaggtatctaagaatagaggcatgtgttggacatgcaatagaacaatgaag
aattggagaacaatgtaagtatggtcaaacaaaggggatcggtcaaccaagagaaaaagataggggtgggaac
cgttgtaacattacccctgtttatcattgaagtcccctgacttcaagttatcctgcaaagctgccatagggt
tcatcaatcatgttagaaatggcagagttgtcaaaataaaactcttcgtcgaaatcgggttgtcgtggtccat
aatgactatgagcgcaagtgacatgcagtgtttgccagttgcagaaattgcaataaaagtcagggcaggtgga
aaatgcgtggcctgtaccatggcaaaaggagcaatatcgaacgcaaggtcgtttgggtcgagagaagacagtc
cggggaggcaggacattttcagcatcgggagattggatatgtcgtggtcggccagcatgggagagaggggtga
caaagatggtttgtgggcgttgtctttgtcgtcgtggggttcggatgacggggtttctcccatgtgcttggtc
gtaggatgggggtgctagtcgttctgggttcggcagtggtggaataaaaggttcaggattgggtgaggttaag
cgagatgccagggattgtgggagtggagtggtagccggaggatcgggggaaggagtagcggagatgtcattta
tgggagagtgtgggaagggtgtcgctcctcaagagtctgttctggaagaagtgggttccattggagtggccgg
gtttggaggtcgttcggtttccaggtcagtgagaacaatggagggggtggatgtgagttcgtcgacggttcga
ttagtgaggggccaggggtcatttgtgagttgtggagttactactccggggattcggttcatctgttggtctt
ctagtagttgttgaatatgttcggttcttaatggatgagggtatccatgtggaccggtaccgtcgaatccaat
gagagcatctaggccccatttccttccagcatggaatagggtcttctgggcgcattcatgggcttctttgagt
agagtgagttgggcttgaacctggcccatggctccaaagaggtttcgaataggtgcatctaggtcgttgacat
catagaacgccgtcatgaagtcacgaaggataggatactttctcatggaagtgacgaaggactcagggtcctt

gatggtgatgaggagattgtagaaatcttctaggtcctttgggatttcgttcgggttgatgatattgtcggtt
tcattttcagggattggttgaaggggttcgcgtcgagtgggggcagaagatgatgatgaggaagaggaggtgg
aagcggtgatagaacactgcggcctgtggggcaaaccaacaaaccaagtgcttcgcagtttgcgagcgggtgt
ctttagtgcagtgaaggcctgatctccaaggaatgtggagatcttcgcaagctaagggaatgaacgcgagact
cgagtgctctaatgcgaaggggggagttgtattatgcgagctacatgcgactctatattaaggcatgtcacgag
tgcgagtaacttgtgagtcagtacaagttgatccgtgggaatacttgtgagtatggcgactcacagatccaga
tcacctgtggccacaagtccttgcaggtccaggtttgaaattttgaacaacaagtacttgtggcaagtacttg
ccataacctgtagccatatgttgggtgcaattccttgctccccactcacttccatgtgaaaaatttccaccta
atctcacatgtacatatatctgtagctgcctgggccaccctgtaatagacaatggcatgggtacatctgtggg
tatccagaaggtgtcccctacccgcacccatatgggttgtaacctgcccacccacaggtttaaaattggtgag
ttggtgcgggtttttggttatacccttgtgggtgaccttattccgcaccaaatcaggactttgaggggtctat
cgaacactcctaaaggcccttatgaatgtccaaaagacctgacagggctacctattcccgttcttaagttata
gggtgtgcgctccagggggggaggaggagaaggtcatgtcatggagtcaattctgcatcggaatatatgattgt
gggacgcatttgatactatttcatgagctgactccgatgcgcatagcgcgagaggcatcagtgtcctcgatcc
ccaggtacgcgcaatctggcagactatcactttgtatctcaggatcggagacaccgatgcctctcgcgctatg
cgcatcggattcagctcataaaatagtgttaaatgcgtcccacaatcatatattccggtgcagaattgactcc
atgacaggaccttctcctcctccccccctggagcgtgcaccctataacttaagaacggggataggtagccctgt
cgggtcttttggacattcataagggcctttaggagtgtccaatagacccctcaaagtcccgatttggtgcgga
actgatggttatttatgtgatagcttgttaggggcgggtttttcaagggtatgtgggtttccaaaagccaata
agtaacctgtgagtaacttgcaagtacctgcaagtatttaggaactcataagtacttggatactcacaagtaa
atgacctgtggatccagatctcacaagtataaaacttgtgcagatccaggtatcagtttttcacatgcaagta
cttgtgactgcggatccaggttacttgcgggttaactcatgacatgccctactctatataaatcggagctacg
cgggcatgaggcagtatgtatgtaatgtaagataatctgtaagatactgaggaactatgcgatcactatggta
aagatgcgatctggatcgcatcgaaagcgcgatctccacgcgtacgaagcgatcagtgatcgcgcattagtcg
cgcgatgcgaatgtaagtacggaaattagctacaatgactagaaatagatgcaaactgatgctaaaccattac
catgcttatcagcggttgcatgagttggggaggatgaggaggaagcagtttggagggggggagtcggaggagac
ggcatagtcagtggaagtggtggaggcagtagaagaagacatggtagtgaactagattttcaaatgtggggat
tcgaaagatgggggaatcaaagggagtgtcgtttcttataagctttgagaatgtcggaggcatttccaaagct
gcgtgtgggaagccatgtgttgtcagtagaaggataacccttccatttcacatagtacatcatgccgtttcct
tgtttgcggtgtttgaggatggcttcaatttcgtattcttcttctccttcaattaattccgggggtggctttg
tataattaggtccgtggacttcattttccttgtatggggtaagaaggcatgcatgaaacaccggatgtatttt
ccattgatcaggtaattgtagccggtaggtgagaggtcctaacaccttggtgatggggaaggggccttctcgt
cgtggtgccatctttctggtttcgtattttagcttgaggttagtagattctaaccatactaagtcgtttttct
tgaagggagtgaagtttcgtgctgttctttcagccatacgttggctggccagttcatgtgcggcgagtgcttc
atctcgtgtcttctgaagggataggaggtgttcttgtacagcgggtgtgtcagatttgaggaagggaaggggа
acggcaatagggttgtagcccatcatgaggaagaatggggaagcattttgtgcggagtgtaggcgttgattgt
gggtgaattcggtggtagggaggaattccacccatgtttcggggtgattaacgcaaaagatccaaaggtaagc
ttctacttcttgattgacacgttctgtttctccatcagtttgggggtggtatgctgtactgaatgcgagtttg
atgccaaggagtcgtcctaattccctctgtactgctgaggcaaattgggggccatgatcagagatgattttat
ctgggagaccaaattgtttgtagacttcccggaataaaagatttgaagttcctagggcatcgatcgttttgtt
gcagggaattagaattaccccccttagtaagcccttggtctaccatgaccaagagtgaatcatatctaggctgt
agcatgggtttcgtcacaccgcggttttccggtcacgggtgtcacagtttcaatggacaccgcggcaagaacc
gcacccgtaccgcggtaacgacgggttccttggtgtggctgcgggccgtttttggctccctgacacaccgatc
gatcaacgatcggaacgttgtttccaacgttctcgtcgttgttcaagccacccccccacccccccaccccccctca
catcgtttgacgcaatcgtcactccccactctcgcaaacgagagtgccagggatgctttcccgcatgcatcca
tcagcccacactcgcaaatgagttgctcttggggaacacaaccgttcccttgactttttggaggaggaaaaaa
gtgagtacccccctcgctcgggacttcagccgctgctcacatatcacccccggggcccctatttgctccccaac
gctctcctgcactcccccgcacccctcgcacccttccacaccccaccctccctatctctcacccacacaacac
gacacgctgccccatcctgtcgcccccgcttgcaaactgagccctacactcccactctcgcgctggggctaaa
acgataggcccgccccatccgtgtgccccggcccttgcaaacgagggctggggtctcaaatggtgagtcccca
cctggatccgccggcgcccatccctgtacaccctcagacgatgccagccgcatcaattgatgcgcgcgacatc
gtgcgagggcactgcaggagggtctgtcatggcggcaccccacagcaccaccgggatccgtcaacacccatcc
ccgcgcactccccataaatgccaggagcccaaacggtgagcccccccttccctcccatcccctcacgcagtggt
gctgcctgctaacccccgccatgcgcacccttcgcatgacgctagccgcatcaatcgatgcgcgcgacatcat
gcgagggcattgtaggtgggggctgcagtgcccaccgggatccgtcaacccccatcgctgcgtacccctcgta
tggtgccagccgcatcaatcgatgcgcgcggcactgtacgagggcattgtagatgggccggcggatgcggcgg
caccccacagcacgatgctgagccccgcagcgtagcgtggtaccccacggtgtggcacagcaccgcgggtgtg
ccttgcaccgtggttaaaccccgtacccgtgggtgcagtttcggcgggtgcgggtccagtgtcacaaatgtga

cccacggtgtgacccgtgccataccctaatcatatccatgggatttcggtagatccgtaacaaagtccatgga
gatctgtgagaaaggatttgggtctgtcgggggttttatcggcattaatggtggtgcggtggggtgagtgttt
actttggtttgttgacagatagcgcatccttcaacataatttttgacaaatgtatacattccaggccaccaat
aatcgcgacggataagttccagggttttcatatgaccagggtgtcccatcattggcaagtcgtggtatcttga
tgcgatatttcttctcagggttttgtctggggaatatagcattggtctttgtagaagaccagttcttcttca
attttccaatcagaaagcgcggatttcatcggagggtgcccccctttcttgagtgcttcgatggcctgggtaa
tcacatcctctttggaggaggaggcgagaatctggtcacggaggtcggtgtcaatagttttcacaaataactc
ttccgggagtaatgtgatatcttcgttatcagtatcttcttccagatttaggtcttctcttcttgataaagca
tctgattggaccatctgtgttccagggacatggaccaatttgatgtcaaattcggaaagaaagagactccatc
gtgcttggcgtcgattaagtttttgagcggtgcggaaataagtgagatttttgtgatcagagaatatggtaat
tggatgtggggagccttggatatagtgtctccaggcttccagagctcgtacaattcctaggagttcttgatca
taaattttgtaatttcgttgagtggcgtcgaaggattgtgaAacaTaGGcgcaggcacaCCaagcTccgccca
TcaCacGTggcacGagTaCgcaaCccccGTccaaaacTggaaacCAccacAagaAacccaaaaagaccgCGgg
aacaacGGcatgagaagcacCGGTGcgccGacaAattttcgtttcaatgtgtcAaaaaaaTccagaaAcTCca
gGgTCcaagaaaAacacaacccaccacTAGaaaaacTAcTaaaaccgaTcgCaaTATcCAAggacgaTccAAc
caaaaacaGaaAaaaacaaacaAacCaTaAgAacgaGcgaAaaCccagaacccaAGaAcaaaccagCaAgaCa
cgaaTaCcggTgAccggagTaGGAaaaaaaaCaaTcaggcTaGGaCgcacgagcaTaacaaaAAaTaaCaCaa
ccaTaGacTcGAAaaagcaaacCaccaagaTaaagaacAccaaATTcTCCcgcAaTcccaccAaCAcaaaTaG
caccaTTcGggaaaaagacaagAaacCTaAAaaaaccaccaGaAaagccaCCATcgAAaaaacaAcccAgcaa
gaAgaaaacATggaaccgaAcaacccaaacaaaATCAacccgaAcGaAcCaGaaaaaaTAcACagcCcaaaaa
aaaaaaCCCcgagccaggAcacTaCAccaccgaTcTccaAacCgaccTcCCaagaAgGacaTccCaTcAgggc
aaaacTacaagaacaaccacgaAgagCgAacTTaGaaaacAccGTcGacaaaagcAcaccGgaaaaaAcTgca
cgaagaaaaggaaacaaacaaaaccaacgaATcGgaaccaCcacTaaagAaCaaTaaTCCaaaCagagaTccA
agGaACaagcagTCcgcacTacaaaaAAAAaaacgaaggCcAccGcaaaggcCaAgGaagcaaTcTaTcacaa
acCaAaAacaacTgaaaaaaaTccgcCcaAaccgaaTTggaagcaCgGacaaagcggcgAaAagaaacaagCc
gaacGcacaaaagaagcccgaAacTacaagcaagcaacagAcaTgaaacaggaggggaaaCcaaaaGacgcca
aaaTTTaaaaaaccacaCcagaagaaaAAgaaaagTcgGacaaAagccccaaAaaaaaacсTcGacaaGgcAgA
cAggaaacCaTcaaaaacgcCaaTGGagccaccaaTTTTcacaggAagaaaGAagcacgGTcCagTacaaacc
aGTcaGGAccAgTGgTaccgAaacGaaagaaCcATcaagaaacccgaTccccaaaaccagagccaacaAcTgg
acgacaaacTcagaggcgCcAcaaTcggcacaAAaaTcGacaTcagCcaagcacacaacAaagacccaaTcAa
AaaAgcTCaTcaagGGaAaacaacgTccaAaAcaaaacGcGgAggaTaggaaacacaGGagaagcccggaaGa
aaGTacAacgcacCCacTaCATcacaaacaATgagGaaCAaacgaTgaaaTgaaATgacaaaTgaagcaTcaa
TgacaaccTaaATGGacaaTaTccgaagaaacaccTcAGaccTaacagagcacccaCaAAgaaccCgAagaaT
GcTccaAgcacTcaaGGAaAAcgaccTcggaccgAaacccaaaaaaccaagcTTccAaaccAcaaaaGgagaa
aTTccccgaAacaagagcaaaCCgAAaaaAAcCGccacggaTCCcAacaaacTcAaaaGaAgcaaaGaaTgGa
caacccccccCTagaagaaaAaGagTaacCcaagTcTaAgGagTgagcaaTcccTacCgacgacgcaTTccAaa
AaaaTcGaATAcgGacAacccacgTAacAAccccaCcAagaaaaaccccaaaTgggaagcGAacgccGAaTgc
aaaaAagcacccccAGcaccaaacacaaggagcGccacaccaccGacgTccgcacacccgTcgaaccaaaTaG
caAaagGcaTacATTggTgaaCacTaacgcaaaatagtACaGcacaaaTaaagaagTaacaacgggttccgCa
aTacggacgtcgattcagttcgccgatctaaaaccctcttcttccatcctctttatagctaccgttctttct
tcttgggataggctggccatgagggcctggatgcgtgtaaacgctatggttccatcagtgggtacttcaacaa
ttccagaagtggacggggtttcgatcttctggatgcggtaattgtttcgtgaggtttctggtgggttcttcca
ttcttgtgatctctggtggggttcttccattcttgtgatctctggcgcggtggccaatcttgtggcagacaaa
gcactgtcctttctgcatgtgttccttctgttcttttactgagagacgatcaacatccatggcaaatgggtcc
cttggtgggatatatcgtggggtgctgggcccttgggtctggtgtattgggttgggttatttcgttggtgaat
ttgtttactccttcgatattggctatcgaatcggatagacatttcgtaccacagatcaatagtggagggtagt
gggatgaggttgtggattttgtcagtaagggctttggctaatccccttggaagaacacaataagaggtccatc
ttcagtaatcttgcttcgaagggccgcttgtctgaagaggcctacataatcctctgctgttcccgtttgtcgg
aggaccgttaactgatgagtggcgtcccctgtggcatctgctggggagaatgcttcttccacccgtgtgatga
aatcttgatatgttccccatccaccttctaaatttgcaagcctctccatttctgcttccttccagggtcctgc
tgttccttcatccatgtatgaaagggtaaaaccgattttcactatatcagagtcatagacggcggtattcaag
ttgaggtagaggttaaccgctttgatgaagggtgagcatcgctcgctttctcctttgaatatagcgggagttc
tgagtttgggggtcttggtggcagtagtgatgggtgcgacggtggtcatcttgggtgctagtgcgggggttgg
ttctggttcggggtttggaattgatgtgactgtgtgggagtggaagcttcggaggaagaactatcagaagtgt
tggcagagaaggtgtttcgaggttgtcgtgggggtgcggagtatggttcagtgaaatatgagtctgataccac
tcgatagggatcaacgttgtagtgccaacgggaggcagtgttgtcgaagaagagtggtgtgttttctagccaa
gttccttggttgttttggacagctcctctaggaagaacctgtaaggagtggggagccacggtgatcggagggg
tgtcggttttaaatcaatgggtgccagaacctaaagaggttggcaatcagtagatcttgacctttttgttaagg

cggatagcctttaccaaggttcaagcgttgaatatcgtccagaggttcaagtcagagtcttgggtcaatacca
acaagactgtagtcactccaagtcaggctttgacttgggtatcaaaggttcagtgatcaaggttcagaggtaa
agggtgtagtattgcctaggtcaagatagagaataatgacaatgtatctagggtttatatactagggaccttg
aggcgcgagaacatagtacaatgggaagtaaagacaatgtaaggtaaaagagctgttgaagctcagtcagcta
tgacatcatagatgagtcaggtatctaagaatagaggcatgtgttggacatgcaatagaacaatgaagaattg
gagaacaatgtaagtatggtcaaacaaaggggatcagtcaatcaagagaaaaagataggggtgggaattgttg
taaca
>Cligib1_Cluster53#LTR/Gypsy
AGTGTAGCCGGCGAGCCGGCCACAGGACAGAGCGGACTCGGGGAAGGCAGTGACAGTGACCACGTGATAGACA
GCGAACCACATGTAGAGTCGGAATCACAGTAGACACGGAATTATCATCACACTGCGCTTTTGCCGTGTTCAGC
GTGTCTCGGCGGATCTGCCGTCTCCATCTGGTGACAACAGAGCGATCCGTATCCAACACGGAAGCGATAGCAA
GGGAACTGGAAAAGAACTGGACGCTACACTAAACCAACCATCAGGCTGGGACAAACTGGCGCCGCGTCGCAAA
TCAACCACCTCAAGGGACTCGCAGGGCAGATTCGTACCAGCGACAGTACCAAGTACACCCACATCAACGACAT
CATCAGCTCCATCACTCAGCATACTCAACACAAGCGTGCTAAACACACCATTTGGCAGCCCAAGGGACACCAT
CAGCCTGAATTCCCCGATAACCTCACCAGATCCGACACACGAAGGCCAAGACTCCATCTTCCCAGATAGTTCG
CAGACATACGGACAGATTGACGACGACATGTCCAGGGCGCCGAAACCATTTTGGGGGGACGATGAACATGACG
AGGGTGAACCACAGGATTTCATAAACGCAATTGAGTACAGTTTCTTTGGGAGAACGCTTGGGGATGGTGAAAA
AACACGACAGTTCCAACTCTGTTTGAAATCAAACGGCACAGCAAAACTATGGTTCGACGCTCTCGAACCAGAG
GAACACGATACATGGAGCCATTTGCAACAAGCCTTTTCGAGACGATGGCCCGTCACTATAGTCGTACCAAAAA
CCAAAGTGGAGAGGATCGAAGACCTCACTAGCACCATAATAGCTGAGAGTGCGCTCGGAACCAAGGTGGTAGT
CAAAGAGGTCGAAGAGCATGCTCATGTAGCCTGGGCAAACAAGGTCGAACGGCTAGCGAAGGCAGCCCAGGAT
AGCGGAGCCTTCCTACTCCCTGAAGTCATGGCGAAAATGCCGAAAGCACTGAAGAAAGCCGCAGGCGTAGACC
ACACCACCTGGGACGGGTTTTGCACAGCAGTACGCAGAGCATCGCTGGTCAGCATCAAGGAGGCCCAATCGGA
GGAAAACGAGAGGAGGGCACTTGAGGCGAAGGTCGCAAGAATGGAGCGGGAACAAACACCTGGCCGCGGGATT
CGTGACGTGTTGAGAGCGGCGAGTCTTGGGCAGTCAGCCGGAATTCCAAAACCATCAGGATCACCACCAATAA
TACCGTTTACCACAGTAGCAAGACCAGCACCCAATCCAACACGACAATTCCGGCCAGATACTGAGCGATTTGC
CGACATTCGCAAACTTGCGCTCCCACACCACCCAGACACACCAGCAGGCCACGAGCTGTATAAGGCGCAACTC
ACGGCATGGGAAGCAAAACACGGGACCGCAGCACCCAACGAATTGAGGCCATACCCACTATCACCGGGAACGG
CTCCAGCGGCCTCAGATGAGTGTTGGACCTGTGGCAGGGAAGGGCACCGTGGCGGTGGGCGATGCGACAACAC
ACCACTACCAAATCCAGAGCAGAGGTGGAGATCCATAGCAACAATAATCCGTCGGAAGGCAGAGACAGTAGCC
CAACCAGTCAGCCTCGTGATGGACCAATTCGAATCACACGCACATCAACTCGAGGACCACGACATCATAGCCC
TTGCAGCATATTTCAACGACTTAGCCATGAACCGGGGAAAAGCGGAAGGGTTGTCAACGAGCTGAGCAAACAG
TTGATGACCCTCAGAACCACCCCAAACATGGACCATGCGGTCCCTAGGCAAAGGGAGATATTACCTAGGGACA
AGAATAACAAACCCCACGCGAATGCCACCCGAGTTCAGACCCAACAACCACACACCATGATCCAAGCCGTCGG
AATTTTAGATATTGATACAGAAATGTACCCAGTACCAGAAAATACATGCATTGTTGACTTGTATGCCGTGGAC
ATCATAGAAAAAAAGAACAGTTTACCATTCGTGCATGGAGTGGAGTTGAAAGGACCCAAAGGCGAAGTGGTCA
GGATACGGAGCGTGTTCGACGATGGGGCAATGGTCAATGCAATTGACCTAAAATTGTACAACACCGTCAAAGA
CCGGCTGGCAACATTGGGACCATCATCCAAGGTGCTCAGGATGGCTGATGGCCGATTGGTCGTATCGGCAGGG
ACATGGGAAGGGGAGATGACAGTGGCTGGGGTGGAAGGTGCCGGGCAGTTCGAAATCTTTGATAGTGGAGGTG
CATGGGGGTTGCTATTTGGGAAACCATTGCTCCGTGCATTCAAGGCCCAACACTACTACGAGAATGACACCAT
CATTATTCCATCCACGACGGCCAGCACAGAGGTCACCCTAACCAACCAATACCACATCCTTCCCGCAACCCAC
CTTGCCGGCTCACTTGTTGGACTAACAACAGATATCAAACAGCGCAAAGAAACATGGGGGGATCAGCAGATAT
CCCCCGTTAGGCGAGTACCCCACAGAATAGAAAATAAACGTGATAACCCAAACACATACACAGCTAAGCTCAA
AGAGAGTGAGAAGGCGGTTGATGAGGGGTGCGAGAAGGAGGACAACATGGAGGGCAGAACAAGTCGGACACAT
TATGGCAAGGGGGAAACCCTGGGGCCAATGCGCACAAACTCTATGGGGGATTCGCACTCCCCCTCGAGGGAAG
TCCAACAACAGGCCTATATCCATGAAATAACAAGCGCAACTGACCAATCAAATCCAGAGGTTACGGAGAATGT
ATGGGTGATTGATCACGACAGCAGAGAGCTAGATAGTACCGACGAGGGTGTAGAACAGCCAGTTATACCATCA
AATCTCCCACCATCAACCTTGACCCGAAAAACCGCCCCCTTCAGCAAGGAGCGTGTGGACGCAGTCATGGCGG
AAATAACAATTGGTCCAGACATCAAAGGAGAGGAGAGAGAAGCAGTCGTGAAACTACTACGTGAACATGCCGA
CTGCTTTGCATTATCACTCAGCGAAGTTATACCTGTCGAAGGAGCAATCCACAAACTCAACATACCAGAGGAT
GCGAAATTCAACACACGGATGGGACAACGACGCTTCACCCCACCACAGAAGGAATACTATAACGGCGTACTGG
ATAAAATGCTGGAAGCAGGCATCGTCAAACCTATAGATCATAAGGACGTCAAATGCTGTGGAGCAACCACCCT
GGCAATGAAAACCCACCAAGGCGAAGGACTCACGATAGAGGAGCTACAGCACCGTGTGAATGACGAATGTATA
GCCGCAGGGATCCCAGATGCATACGAAAACCTCCCTCCTAGGCCTGAGTCCAGAGAACTGTCGAACAAGGTAG
AAGGCCAGACAAAATGGCGTGTATGCCAGGACTTTGCAAACCTCAATAAAGTTACAAAAGTGCCACCAATGAA
CCAGGGCGATATACGCGCAAAGCAGCAGCGCCTCAGTGGGCACCAATACTTGTGCGTCTTCGATTTGGCAGCA
GGATTCTACGCATGCTCGATAGCAGAAGAAGATCAACCATATGTCTGCTTCTATGTGGAAGGGCGAGGCTACT
TCAGATACGTGAGGATGCCATTCGGACTTACTGGGGCACCATCTACCTTTGGACAAATGACGGCAGACAAACT

GGGGATCTGGTGGGAACATTAATCGAGCTCTTCGTTGATGATGGAGGACTCGCAGGGGACTCATTTGCGGAAA
CCTACAGCAAACTAAAAACACTGTTGACGAGAGTCAGAGAAACGGGCCTATCCTTGTCCGCAGCAAAATCAAG
CTTCTTTGTAACAGAAGCAGTATTTGCAGGCGCAAGAGTTGGACCATCAGGCATAAAACCAGACCTAACGAAG
CTCACTGCATTAGCAGACTGGAAAACCCCTACGACGCTACAAAACCTAGCAGCATTCACTGGACTTACAGGCT
ATTTCCGATCCCTTGTGAAGGGCTATGCCACCATCGCCCAACCGCTAACAGACCTGTTGAGAAAGTTGGAAAT
GCCAAAAGGCAAAGGGAAACATGCGTACCGTAAAGCCATGAAGGATTTCAAGCTCGATGGACTATGGGGACCG
ACACAAAATAAATCGTTCCTAGCACTGAAGGTTGCACTTACGAATGAACCAGTCCTAAAAGGCCCCAAGTTCG
ACGGAACCCCATTCATAGTCACAACGGATGGGTGTAAGTATGGATTTGCCGGAATGTTATCCCAGCGACACAC
AACTATCCTACCAAATGGAAAGGAGGTCACCCACGTCCACCCAATAGCCTTCACGGGGAAGCGAACATCACCA
ACAGAGGAAAAATATAAACCATACTTGCTGGAGTTCGGAGCGCTGAAAGATACACTGGACAGACTTTCGGACA
TCATATGGGGCTTCCCAGTTGAGATCGAAACGGATTGCCAAGCGCTGAGGGACCATCTCCTGAGCGACAAACT
AAATTCAACCCATGCTCGCTGGAGAGATGGGGTCCTTGCCCACCAGATCGTGGACGTGCGACACCGGCCCGGG
CGGCTCAACCCAGTAGCGGATGGACTAAGTCGGAAGTACATAAATACCCCAGTAGAGGACAACGATGGACACA
CCTGGACAGTAAGCGAGGACTGGGAGGTACGAACAGGCCTCCAAAACGACATATTCCAAGTAAATGTGGCCCC
GATAAATGACCTAGAAACCCTAAAGGAACGATTTGTCAACGAACCAATCTTTCGGGGAATCATTGAAGCGATT
GGGGAGCTGGACCACGGGAAGAGCTTGAGAGAAAAGAAAAGAGCAAGACATAGGGCAGAAGGGTACATGGTCG
AGGGCGCGAAGTGTGGAAGGTGGGAGGTGCAAAGTCAGTGAGGGCGAGAGCGCGGGTAGAGTGCATTACAAAG
ATGGAAGCCGAGGCAATGGCATCAAAAATTCACGCGGAGAACGGTCATTTCCACAGAGACAACATCAAGATCA
AGATGTTAGACCTAATAGCAAGCCCCAAGTTGGATCAATCAATCGTGAAAGCGATAATGACGTGCGGCAAATG
CAAATGCTTCGGACCAACACACCTACATGCACTCATGGAACCCATCACACGCAGACACCCATTCGAACTGATG
GTAACCGATACCTTAACCATGCCAAAAGGAAAAGGGGGATTCAACAAAATATCCCTCTATGTCGACGTATATT
CGCAGCACGTCTGGGCAACTAAGCTCAAAAACGCGCCAACAGCCAAGTCAACATGCAAGTCATTCGGCAACAT
ATGCAACACATACACACCTCCGGAAACACTGATGGCGGACGGAGGACCCGAGTTCAAAAACAAAGAATTAAAA
GAGCTGTGTAATGCGAGAGGCACGGACTTGAGGGTGGTACCAGCATATTCACCGTGGGTGAATGGCCTGGTGG
AAGGAATGAATAGCAAGCTCCTCGGAAGATTGAAGCGGTTATGCGCACCGGATTTAGGCGGCAACGACGAAGA
CACCGGAACCTCGGAGGACATGCCCAGCGAGTGGCCCGAACATCTGGACGAAGCGATCAATGCACTCAATACC
AGGATATTACCCAACCTAGGATTCTCCCCAGTCGAATTGCTGCTTGGACTAGTTGTGAACACCCTGCGGACAG
CAGCTGATGATGCAACCAAAGAGCCGGACAGGGCAGACGTGCAACTCCAAATGGCCTACGTAGATCAACAACG
CATGGACGGATACTCCCAGATTGTCGACCATGCGGAAAAACGAAAGCTTGCATTCGACAAGAAGGTCAACAGG
AGAGAGCCGAAAGAAGTGATATTCAAGACCGGTAACCTAGTACAGGTCTATCGTAACGATCTCGACTTCACAC
ACACAGCAGCTCGGAAAATGGTCCCTAAATGGTCAGCACCAAGACGAATATCCAGCCGAGACAGGAACTCCTA
CAAAATAGAGACACTGGAGGGATTGCCAATAGCAGGGCGATTTAGCTCCAGACGGCTGAGACGATTCATACCA
TGTAACGGAACTCACTTGAAAGAGGCACAAGAAGCGCTAGAGGAAGCGCTAGGGGAATGGGACGAGGAAGCGG
ACATGGTGGAAGAAGAGGAAAGGGACGGGGGGATACAGGCTGAGGGAGCAACGAGGACGTCGCTGCACGGGGG
GGGGGAACATGTAGCCGGCGAGCCGGCCACAGGACAGAGCGGACTCGGGGAAGGCAGTGACAGTGACCACGTG
ATAGACAGCGAACCACATGTAGAGTCGGAATCACAGTAGACACGGAATTATCATCACACTGCGCTTTTGCCGT
GTTCAGTGTGTCTCGGCGGATCTGCCGTCTCCACCAG
>Cligib1_rnd-1_family-413#TIR/MITE
TGGGTGACTTCCCGTAAGGTTTATAGAAATCTGTAGCATCCCAAATGTTCATAAATTGCCATCAGCCGAGATG
CCGATGCTCCATTTGGGTCATTTTCAATTCGATTGCGAGATTTTAGCGTCGGCAAGGTCAAGCCAACGTCTGA
GGAGGCCTCCCGACCCCTCCTCAGACTCAGAACGCGCCTCAAAACCCTTAAGCGAGATCCCATCGACATTTTA
AGCCCCGCTTTATAGCGCTCCAGATTTCTATAAACTTACGGGAAGTCACCCACT
>Cligib1_rnd-1_family-101#LTR/Gypsy
GAATTCACCCACAACCAACGCCTACACTCCGCACGAAATGCTTCCCCATTCTTCCTCATGATGGGCTATAACC
CTATTGCCGTTCCCCTTCCTTTCCTCAAATCCGACACACCCGCTGTACAAGAACGTCTCCAATCCCTCCAGAA
GACACGAGATGAAGCCCTCGCTGCACATGAACTGGCTAGCCAACGCGTAGCGGAAAGAACGACACGAAACTTC
ACCCCTTTCAAGAAGAACGACTTAGTATGGTTAGAATCTACTAACCTCAAACTGAAATACGAAACCAGAAAGA
TGGCACCACGACGAGAAGGCCCCTTCCCCATCACCGAGGTGTTAGGACCTCTCACTTACCGGCTACAATTACC
CGATCAATGGAAAATACATCCGGTGTTTCACGCATGCCTTCTTACCCCATACAAGGAAAACGAAGTCCACGGA
CCTAATTATACGAAACCACCCCCGGAACTAATAGAAGGAGAAGAAGAATACGAAATCGAAGCCATCCTCAAAC
ACCGCAAACAAGGAAACGGCATGATGTACTATGTGAAATGGAAGGGTTATCCNTCTACCGACAACACGTGGCT
TCCCACACGCGGCTTTGGAAATGCCTCCGACATCCTCAAAGCTTATAAGAAACGACACTCCCTTTGATTTCCC
CATCTTTCGAATACCCCACATTCGAAAATCTAACTCGCTACCATGTCTTCCCCCGCCTCCACCCAGTATACCT
CCGCCTCCTCTTCCCCCGCACCATCCCCANTCACCGACCGCTTCCCTCCTCATCCTCCTCCGAATCATCCTCC
GTTCCCCAGTCTCCTACCCCTCACTCCGGATCTGAGGAAGACCTCATCCAATTCATCAACGCCCANCTGGGCA
CACCTCCTCAATACCAACGACCTCACTCCCCGANGGATTACTCTCCCAAGAGGACCTCATCCAACNTCTCACC
CAGACCACCGATCTCGCCACCATGTATCACAGCTGCCACGACTACCCCATCCTCCGAGACCTGTTACTCACAT
TCTACGAGAATCACCAAACCGCNGTCCCAATCCGAAACATCCATAACGAATTACTGGAGATAACCTCTTCGGT

CAACCGCCTCACCGAACAACAAGAAGCCCTAGCTGGTAGCCTAAGCTACCTACTCCTGAAACATGGAATCGAA
CAAGTCCTGAACCCCCGCTCCCCCAACATCTCCCCCACTCCCATCTCCAGACAAAACCTCGCCCAACTCTCCT
CTCCTACCCCAGACATCGCCATCCACGTCACCCAGACCACCAGAACGACCGCAGAAGCCATGACACAGACACC
CCCATCTCAGACCGCATATCGAAGAACCCCCTTTCCCCAACCCACTATCAGACTACCCTCCTCTAGACCCCCC
TCCGAAACCACTTCTCCCTCCACCCCACCTGCCCCAGACACCCCCCTAATCCGCCCACAAAGAATCATCAGAA
GAGTCCCCGTCCCTCAAGAGGTTGTAGAATTCCCTCCCAGACGCAACTCTGCACCCACCGCTAACCGCCGAAA
CCGTCGATCGCGTGGACACACCATCACCATCAACCCCCTTAGTCATGCTGGAAGAAGACCCCTTGTAGAAAGA
ATCTCCTCCCCTGAACCCACTCCCACACCGCTTCGAACTCGTACCCCCACCGGACCCAGGGTAACATCCTCCC
CTCCCAACCCCAAAGTATGCTGGGAATGCAAATTACCTGGGCATATCATAAAGAACTGCGAGCATTACTTCTG
TCGACACTGTGGAAATCACGCACCAGGACACCTCCAAGATAGATGTCGCTACCGATCCTCCACTTCCCATGCC
AGCTACCATCCCTATACCGATGGGGATCTCGATTTCGACAATACCGCCATGTCTAACATGTGCGACGAACCAT
ACCACCAAGATTGAAGTCAGGGACTTCAACGATAAACAGGGGGTAATGTTACGGCGCTCCTAGGGGGCTTACT
CTTTTTCTGACATCATACACGGACGCTTCTCTCTTCGACACATGTTGCACATGTGTTTGCCTCATGGACTCCT
CATGGGCATCCTCTGACCTACTGCGCCTCNCGGTTTACTCATTGTCTACAGCATATCTTCCCCTTTAGTATAT
ATTGCTGTAGAGTCCCTTAACATGCATNCTCCTCATGGTCACTCTCNCTTAGTATATATACTTCTTCTGTACA
TCCTGTATTAGCATCTTGACCTAGGCAATCTACCGCCTTTACACTCTTACTCAGAACCTGAGCCTAGACCAGC
TCAGAGTGACTTGAACTCCTTTGTCATTGGACAGGAGACATCTTGAAACTACTCTCAAGATAGAACCAGAACG
AACGACCGACTTGTATCAGTTGTGAAGGCTCACGCCCTAACA
>Cligib1_rnd-1_family-104#LTR/Gypsy
CCAGGAATTCGACTTGTTCTGACTGAAAAGACATTTCTCCGGTTTCAGGAANAGGTCGTTNTCCAGTAGGATT
TGGAGNACCGTTCGGCGATTCGTTCGTTTTCTTCTTGGGTATCGGTGAAGATTAGGATGTCGTCNATATATAC
GACAACGCGTCCTTCGTCAATNAGTTCTCGAAGTAAGGCGTTCATCATCGTTTGGAANGTAGCCGGTGAATTG
CACANTCCGAAANACATGACCNGTGGTTCGAANGANCCGCGGTGGGTTGTGAANGCCGCCTTCCATTGGTGCC
TTCCTTCGTNCGNACGTTGTTGTATCCCCGGCGGAGGTCNAGTTTNGTGAACCATTTNGACTCTCCCGCTTTG
TCGACGAGNTCNGNGATCANNGGNAGGGGATAATTGTTCTTGATNGTCCANGCGTTNAGGTANCGATAGTCCT
GACCATGCGTTTCTTNCCATCCTTCTTNGGTACGAAGAAGACNGCGGANGCCTGCGGAGATTTGCTTTCGCGG
ATGTATCCCTTGGCCAGNTGNTCTTTNAGGAANTCGTCCACTTCNTTCTGTTCGGTAGGTGAGAGATTGATAA
CTTTNGCNTTCTTTGGGTACAAANCCTGGTTTCAGNTCNATNGCGTGATCCCAGGTTTTCGTTCGGGCATNCG
TTCCGANGCTTTCTTTTCGAAGACTTTTAACCACCGTCTAAACCGNCGGGGAATCTTATTCNTCTAGACCGGG
TTCGGTAGGCTCCGGTAATNCACGTTATCAACGGAGGGGCGTCAAAGCTCTCCTCGATCTCCGCTGTGCGCAC
GAAGTGTTCCGCAACNACTTCGTGGACCGTTTCGTCAAAACGAATCTCCTCNAGNAGNGCGCCTTCTTGTTCC
GTTATGANNCCGCCNATNACTCGGTCGGNATCCTCATCATCGACCTCTTCGATCGTGGNTTTATACTNCCAGC
GATCGAAGCCATGGCCTTACGCTTCTTTTGNGTTTTAGCCGCGCGGACGGCCACGTTGCATTCCGGGGGACAT
CGGTGAATTCATAGAGACCTCNCCAGTCGNCCAGTCGATNGNNGGGTTGTGCTTCTTNAGCCATGTGTGTCCN
ANAATAACCGGAGTTTTNCCGAGGTCGTCACTTCGAANAGCATNCGCTCCTTATGGCCCTTGTGGGNNACCAT
NAGATAGACTTCTTCCGAAATAACCGGCCGCCTTGATTTAAGGTACCGTCGACGTTAAATACTTTAACGGGGT
GNCGTAGCGNTCGNGTGTTCCANCCCATTCGTTCGACATATNCTCGATCGATGAATGAACCGGTNGCTCCGGN
ATCCAATAAGGCCTGTGNGNTGGCGCGTCTCATGTGTATCTANTGTCGTCATGATGATCGACATTTTNAGCTC
TCGTTCCAAATTNAGCGTGCGAATAAATAATCGTTCTCTATAAATAGGACCAGGACGNTCCGAAGCNCCGATA
CCGCCGGGACCCACGGAGTTCACCGGCTCCGGGACGGACGGATGTTCCATTTAAGGAATCCTGGTGGGTCGAC
ATGATCGTAACGCCGGGACCCTGATTAACAGGACGGACGTCTATCCGANCACCAAAACCACTTAATATTTTTG
TCTCCGCTAATTTTTCTGTTCTTTGGTTNTCGAAANNGATGAATTGGGGCGGGAATTAAGCACGCTTCCTGAG
CCGCCAAGCTCCTTATGGGAGACCCCCTGAGATACGGTTACATTGGAAGGCGTATTCTCTANATTNTCGACCG
ANAGAGCAGAAAAGATGAAATTTATTTTAGGCGACGCTAGNGGTGGAGATTTCACTGCGCGCCTCCTTGAAA
AGACGATTGGGCGTTGGGAACTTCTACAATACCCGACGTTGAATTGGCAACGTGAGTAAGGGTGGAGGGATTA
CCGCCGCGTGATTCGGTAATATTGAACTTGCGTTTGAAATAGTCTTCAAGTTCGGTGTACGTTCCTCTAACGT
TTAGCGATGATGGACAGTCTCNCATCACGTGGCCGGGTTTACCGCATCGATAACAAACGACCGGGCGNCGANT
TCCTCGATCGACATCCATAGCATCCGGGNCGCGTGTAGCCGTTGTAGCGGGTACCGNATTTGCAGGAGCTTGG
GTTTGTTGANNAGGTCGGGCCGGTAGTTTCGTGGTGCAGGGACCGTTGACGATGACGTTGTCGTAGTTCGCCG
TCTAGCCGGTTGTGCCGTGTTGAATCTGCCGGTTGACGTGGTTGCCGTTGAGTANAGCTTTTGTTCNCTCTGA
TGTTGTCGCCATTGTCGATCAACCAGTTTGATGCGCGTTCGTACCAATCNTCGATNGTNTGGGGTCGATATTC
GCCGAGTGATATTTTGTCCCGNAGTGCCTTGTTGAGTGACCGTTTGAATTCCTGAATCAAGGCTACCTCAACA
TAGCCAGATCCGTCGGCGATACGTTCGAATTCNTGGACGTGNTCNTCCGCCGNTNNAGTTCCTTGNTTGATNG
TNCGTAGTTTAAACGCTTTNGTNTCTTGTGNATCGGGATCTCCGAATGTTCTCCTAAAAAGGTCCCAGAAGCC
GTCCATAGTGCTATCTGGGGAGACGCCGCGTTCCGGTTGAGNTGTTTGACCNTTNGGTCACGCCATGNNGCCG
CTGNGCCCGTTTGACATAACGAAAGCACCCAGAAAACCTGATGCGCGTGACCGATGGGAANTCCTGATTGTCG
CACTTGCCATATAAATTCGACATGCGGTTTCGAAAGNTTCCGCGTTTGCCATTNTACCATCGAAAGCCGGGGG

TGTAGCCATCTTTGGCATACGAACGCGAGATCCGAAATTTTTAAGGGCCGGATTACCCAAATNAACCGGGGAA
GGGGGAGGAGG
>Cligib1_Cluster37#LTR/Gypsy
GGAAACCCGTACTCCTATTTGGAGATTGGATAACACTCACGTCaTTACGGGAACAGAAAGTGATCAACGTTTA
TTGATGTAAGATCTTGATATCAAGTAAAGTAATTACGAGAAGATGATGGTAAAAGGTAAAGATCGCCAAGTCG
GCTGGGCGCCTTATAACTGGCGCCCAaATGACAACATGCGCAAACGGTGATGTAAGCGTGATGTAAGCGTCGG
GCTTACACCCTCCCCTGCTCTAGaAAAAAAAATCCCGCCCAAAAATTAGTATCTAAGTATCGTAAAAGCTGCT
cGGACACGGGTGCGTTAAAAACCCCGTAGGGAAAAGCTACCGGTCGGCAGGAACATTaAGAAGATGAACTGAA
CATATCCCCAAACCTTGCTTCTCCTGCCATCGaTCGAGTGCTTCGTTGTCTTCCATTTCCcGGCCCGGTATCC
ATCGGTCCTCTTCCGGACCGCACCCTGTGAAACGCACCAGGTATTGCACACCCCTACCCCTCCTACGCTCGTC
GATGATGCTGTCTACCAGGTATTCCTCCATCCCATCTTCAGTCAACACTGGGCCCGGCCGCTCGAGTGTTCTC
GATGGGAAAAGGCTGTCGTCGTTTTCTCGAAAAGGCAACAGCTCCGAcGCGTGAAACaTGGGGAAAACGTTGG
GTGAGTTTGGCATGTCCAAGGTATAAGTCGAaAATTCTGGGTGGCTGGCCACCACCACGTACGGACCATCGTA
TCGCGGCATAAACTTGGCCACCCGTTTCTCCCCTTTTCTTTTGAAATCTCGCCGCCTGTTAAAAGTcGACAGC
CGCACCCTATCCCCGACCTTGTATTCCCTTTCCGGGCCTCGCGACTTATTCGCTTGTTCCGCCTGTTTTACCT
TCGCCATTAGTAAGTTGTCCTGAGCCTCCATAACGTCcAATTCCAGGTCCCTGATAATCaCCCTCGCGTCaAC
CGCTTCTCCTCCTAGGTCCCGCCaTGCATCTACATCCTCGATTGGTGGTAGGATCCTcGGGCTTCGTCCCATC
CGCAGTTGAAAAGGAGAAAACCCCGTCGACGCGTTGATGGTGTTCATGAGGCTGAAACGCACACGCGGTAACG
CCCTCACCCAACCCTTTTGATTCCGCTGAACATGATACcGCAGTGTCTGGTTCACCGTCTTGTTcGTCCTCTC
GCTcGCCCCGTCCGTCTGCGGGTGATAAGCCGTCGACATCTTCAGGTCGACCCCCGAAAGCTTGTGCAATGCC
CGCCAGAACTTTGAGAGAAACAGTTTGTCGCGGTCTGAGACaATGTCTCGTGGGAGGCCGTTTTcGCAATACC
ACTTGTCGAAAAAGAGGACAGCTAGCTCCTTCGCaGTCATGTCCATGCaAATGGGAACCAGTTGGATATCGGC
GCCCAACCGGTCAGTCATGGTCATGATCCCGTTAAATCCATTGTCCAGTGGCAAAGGGCcGATGAAATCGATT
GCGACGGAGgCTCCCCTCTcGTCCGGGACAGGTAGCGGGTGTAACGGTCCTGCTTTCCGCCTCGTCGTGCCCT
TGTTCCTCTGGCATTCCCGGCAAGCTGGGATGTAAGTTTCTTCTAGATCCCTCCTCATCATCaGCCAATAATA
TTCGTTCCTCAGCGACGCaTATGTCTTGTCaAAGCCGAAATGGCCCAAGCTGTCGTGCGCCGCCCTGAAAAGC
TTTTCGCGGATGTCCCCCACCCTCGGGATCACCAACCTGTGATCcACATACCACAGCCCCTCGACCTCCTCCA
CGTTTTGCATACCAGAGCCCaCCGATTTTAATTTCCGGCACCACTCATCCTCTTGGTATCCGTTCCTGATGTC
GCGCAGCAAATTCTCcTCAGCCCCAATcGTGAAAACCGCCaAAGCTGCTGcCACATCCTCATCCCCTCTGGAG
AGTGCCCGGCAGGGCTCCATCTGCGAGTGCTCCCCTACCACGAGGCAGCAATAACTCGGCCCGCGATCCTTCG
CGTGCTCCTCGTATGCCCCGcGTGCGGCCGCTTCTGCTTCGTGGCTGTCCAACACCCCcTCCGGCAGTCTAGA
CAGcGCATCCGCGACCGTATTATCCTCTCCTTTGACGTATATGATCTTACAGTCGTACTGGGACATGAATTCC
ATCCAcCGCGCTTGCCGTCTAGACAGATCCTTTTGCTTATTGAAATTCTCTAGTGTCTTGTGGTCTGTGTAGA
cGAGAAAAGGAGACCCCAGTAAGTCCACCCGCCACTTCTTCAACGCTCTTATGATCGCCAGCATTTCCTTCTC
GTGTACCGGGTAGTTCAGCTCTGCGCCTTTGAATGTCACCGAGTCGTACGCcACCGGGCGTGCCGTTTCCCAT
GATTTACCAAAGGAGAGTACCGCCCCCGATTTCCTATCGCTCGCATCGGTTGTCACGAAAATCTTGTTTTCAT
CCAGCTTAGAGTGGTCGATCGTGGTGAGGCACTCCCTACTTGTCACAATCTCCTTGATTTTCTCAAACGCcAT
TTGCTCTTCTCTCCCCCATTTTGGGAAACACCTCTCGGCTTCTTTCGTCGTCAGTTTGTTCAGAACGTCCGTG
TGTTCCGCGAGGTTCGGCAGGAAGGCCGCCAGATAACGCACCAGACCCAAGAATTGCCGCACGTGTGAGGcGC
TTCGTGGACGTGGCCATGAcACAATCTTATCCGCCTTCTTGGGGTCCGCTTCCACTCCACGTGCCGAAATTTT
GTGTCCCAAGAAGTCCACTTCGAAACAGAAAAGGTGCGTTTTCTTTGGGTTGACGTACAGCTTTGCCTCCCTC
AACGCCTTCAGGACTTTGCGTGTATTCACCACATGCTCTTCCACTGAATCTGACCAgATGATGATGTCGTCCA
GGTATATATGACAAATCGACCCCAGCAGTGGCCGTAGGGCAGCAGTCACTCTACGCTGGTGAATcGAGGGCGA
GTTCTTGAAGCCCATCGGCATCACCAACCATTCATAGAGGCCAAACGGGGTCGTCACTGCTGTCAAAGGTACG
TGGTCCcTCCGCATTCTTGTCTGAAAAAAACTATTTGTCATATCAATCGTACCCCAGATCCTCCCCTTCGCGC
AaTCGTTCAGGATGTCATCGATGCGAGGGAGGGGGTGACTaTCCACCGTGACGTTTTGGTTCAACTGGCGGTA
GTCTATAACCCATCTGGGTAGAGCCGTCTTGTCCGCCTTCGGGATTATGAAAGAGGGTGATGCCCATGGTGAG
GATGAAGGTTGGATCCTCCCCGCATCTAGGTGCTGTTGTATCAGCGTCTTCCACGCTTCGCGGTACTTCCGAG
GGCATTGGTAAGTTCTCGAAGCAATTGTCTTGTTTGCATCTTTCAGGTTGATTTCGGCCAGGACGTCGTGCGG
CAACTCGTCGATGTGTGGTATGGGTTCGAAAATGTCCCGAAATTCTTCCTTCAGGCCCTCACCCAGCTTGTGA
AGCCTGTCCTCAACAGTCAACTCCTCAATCCGaGCCCTGATTGCTGCGGCCACCaGAGGGACCGCGACCTTCT
CAAAGGCACCTGATATTCGTAGCATCTGTTGCCTGATAGCGCACACCTGTTCTAGTTCCGTCAGCATTATCTT
ACGGTTCGCTTTcGTCTCTGCAATCTTGGCTTTAAGTCCCAACTTCTTGCGCTCCTGcACCAGTGGCTTCGGG
TTCAGTAGGTCATACCCACTCTTTTTATCGATACAAGAACGTTCTGCATGATCAATGACAATATTATTGTGGG
cGAGAAAAGGCATCCCTAGTAATATGGTGGTACACAACCCCGGCGCGAGTACAGCTTTAAACGATTTAGAGGT
CCATTGAGCGTCCGTAGAGGTGACAGACAATTTCACGTATTCCGAAAGTATTCTCTCCTTCTTTGAACCATCC
TGCAGTGCAACATCGATCCTCAACGGTTTTCCTAGCTTTCGACGTCGCAAACCCCATTCATCAACGACTTCCg
gcttgatcAAAACTAGATGAGAGCCGTGATCAAGCATAGTCAGTATAGTGACGGGGAAATCCCCCCTGcCTCT

GACGAGGCAATTCCACATCAGGTGGGGGGCGGTGAGAGGGGAACTGACCGATTCCTCCTCCTCAGAGTCTGAG
CCATCCAGGACACACGACGGGAAGCCGCTGGAATTTGGCATGACGGCAGCGACGGTGTCTTCATCCTcGCTAA
TGTGAGCGGTTACTGCGGACACGGCCTTCTTCTTGcTCGCGTTTGGGGTGCTCGTGGATTGTTGACTCTGTGC
CGCTCTGACGTCTGCAGCCGTAAGCGTCTTGTAATTGAGGCCAGATGGGAATTCTGCCTTGCAATCCCaCGAC
GTGTGGCCGGCCATAAGACGTCTGCACCTGAAACACCCGTCATTATCCCTCAATAATTCCCGTTCACTGTCCA
ATAGCTTTGGGAGTTTcACGAAAGTCCCGCTCGTGTTCGCCGCGGACCCTTTTCCACTCGGGGTATTCGTGCG
CCTGcTCCCATCGCCCAAAGCCGGTCTCTTGCTGTGTCTcGCCAAGCTGTCATCGACAATATCGCGCATGCGT
TTGAAATCGGCCCTCCGAATTTCGTCCACaTCCTTGACCTCCTGAAGCCAGGTCCGGAAATCGGCCGCGTGGT
CAACTTTTTGCCTGTTGCACCTCaTTGCGAGGTCGTCATCCATGTTGGCacGCAATTGGTTTCGCAACTGTTC
CTCCGAGAGGTGCACCGTAGTTCCAATTAACAGGCTGTTCGTATTCTGCAATTTTGTTGCCCAGTCCCAAAAG
GCACCGCGGCTGTCCATCTTcGATCCCAATAGTTTACGACACaCatcgtctttagtgttggcaggtctggtta
atgtaaccggattttagaccggaaccggaccggaccagaccgcataagaccggaccaccggtctcggtctacc
ctggtcttggcagggtccggtccaggtctttccaggatctcaaatccgctttaaccaggcaaaaccagtctag
accggtcctaaaccagacccgcggtccatcactgatgaggggtagtcagggggggtctagggcgcacagggtcc
gcctatggtagccaggaggcgcgggtatgcgcaggtatatgcgcatttctgcgggttttcactccatatctct
agatcggggacgccgatcccccccgcgttgtgcgcatcgtgttcagctcttaaaatagtgttagatccttccc
agatccatgcattccggtcaagtattaagcccaggacaacaccttctcctcctccccccggaagcgcgcaccc
tataagtccggatcggggacagctagccctgtcgtctcttttggacattgataagggactgtaggggcatccg
atcgaccctacaatcatccgatttggtggggaactcaccctggagcaagcccagacttgtttctttggcaaaa
aaagaccctgagatggctcgggaccgatctcggatccgtttttacagggtcccggtcctgaaccggtcaggac
tgctttcaggacccgacgaaaccgcccctgaccggtctaagccagtccggtctaggtctcttccggatttggc
tttcccttaaccggtcagggtcccggtctttccatattcgggtcaaataaccggactggaccggacctcggaa
cactaATCGTCTTCCCAGTTCTTTGGGAGGTAGAGAGAGCGCATCTTTGCCATAAACTGTTTGAACGAGAGcT
TCTCTAGTTCTTCGGATTCCATCATTATCCATCCGCaGATACGCTCATCGTGTAGGCCGCCAAGGATTCTGCG
TACTCGGTCCGCCTCTTTGATATCTTTTTGATTGAAATAGTTCATGCAACCAAATTCGAAATTCCTCATTGAT
TCTGGGCTAATATCGCCCGCAGTCATTATCGGCGGCTTGCCGCTTGAGCTcATCTCCACGGTGGCCTGACCAG
TTGGGTTTGAAGGCATTCTcAGCGCGTGACAGGTACTGTAAGAGTCTGAGTCGGAAGAATCGGTAGATGTATG
CGTGCTTGCAGGCGAATCGGGTACCGGGGAAGGACTGAGAGAAGATAGTGGGGAAGGCGTTCGTTGCTCGGTT
TCCCCAGCACGCAGTTTGTTCCCGaAGTTCGGTACAGAGGTGGAATCCGGCAAAGAGTTGTCAAGAGGTGAGA
CGCCTTTCTTGGCCGGTCCCTTCTTTGTTTGcGCGAGCGGTTTCTTTCTGGTTACAGCTACCGACCTCGGTGC
TGATGTAGGTagcgtttgggcCGCCaCTTTCTGCGTTTGCGTGACTTTCGTTGCTGCGAGcGTTTTGCTCCTG
GTGACCGGCAGCGGAGGCAGTTTGACAATACCGCTTGCACCCTGCATGTTCAGGGACAGATCAGTTACGGCTC
GGCAATGTAGATAAGACTAATATAGTCAAAGGCGGTGACAGTTcGGGATGATCTCTCAATGGAGAGGCaGTGA
TAACGGAAGATTGAAACGTTGGAAACAATTTCAAAAAAAAGTGGAAACCCGTACTCCTATTTGGAGATTGGAT
AACACTCACGTCGTTACGGGAACAGAAAGTGATCAACGTTTATTGATGTAAGATCTTGATATCAAGTAAAGTA
ctaATTACGAGAAGATGATGGTAAAAGGTAAAGATCGCCAAGTCGGCTGGGCGCCTTATAACTGGCGCCCAaA
TGACAACATGCGCAAACGGTGATGTAAGCGTGATGTAAGCGTCGGGCTTACAc
>Cligib1_Cluster60#LTR/Copia
GAtGTgAATACAGAtGCTCAGACGCCGTCACaAGAGTGAAGCAACTGATCATTAGCTAACCTGAtGTgAATAC
AGAtGCTAATGATatAaGTAAGTAggTGTCCgTAAGGTAGaagTAATAgAtGtGCATACCTCAGACGCCGTCA
CaAGAGTGAAGCAACTGAGGtATGCGaatGcGaaCGTAgGaTATTTAtCATACGAGGTAGAATAGGGGTCGGC
GGTTGAACGCCGTgTGATGGATGTGAAACaTAGaTAAtAAaGtGAAAATGAAATAagAGAAAAGATATGCGCG
CGGatGTGATcgCGCGCGCACaAGTGtTTGActGcCaAAtTgaGTAtgcGGCGCgTCACGGGCGTTAcGCTCA
ACACTCCCCTAACCCCGTGtGCTCGCCGACGAGaAGGCCTAGGgTGCGGCAAAGATGCTGgTGaGcAACaCGt
GGtAacGGCTTGGTgAGGATGTCCGCaAGGTTgTCgGCCGAgGGCACgTAGTCCAGGGATATgTtGCCGTTCT
TCACatGGTCTCGAATATAATGATAaCGTATGTCtATgTGtTTcGCGCGTGCATGCCCCTTgGTGTTCAGCGT
AAGgGCGATgGCTGCGGTGTTGTCtCCgCGGAGCCGAGCGGGGgAaGCCTGGGGTAaaCCGATcTCGCCCATG
AAcGAGTGCATCCAGAGtGCTTGcTGGGCtGCCCGCGTCAgACCCATGTACTCaGCTTCgGTtGTGGACAACG
CGACTGTTTTCTGGCGCTTcGAtGCCCATGAgACcGGgCCcCCGCACATCGTGAAGATATAgCCCGAaGTCGA
GCGCCTGGTGTCGcTaTCGCCGGCgTAGTCtGCGTCtACATAcCCaACAGGcTTCAgGCCaTtCCCAGCgCCG
GGGGTGTACGTGATTTTGTAGTCAAGcGTgCCCTTGATGTACCCGAgGACgTGGAGcAtAGCCTTCCAGTGCG
CTGGtCCAGGGTggATcTGGTGACGTGAGAGCAaGTTCACCGCgTAtGACAAGTCCGGCCGTGTCGCacCcTG
CGCCCACATGATgCATCCCAGtGCCTCCCtGTAgGGCTTGTCCGCCATGAAccgtgCGTCCTCTTCtGAAgtC
GCCTGGTCGGCCTCTGTgAGtGTCAAcCCGGTTGGGAGTGGCGTGGAcTTtGGGTTaCAGTTTGACATCCCAA
AGcGcTcAAGaGTGCGCTCaAGgTACGCGCGCTGTGAGAGGGATATGGATCCaGTGGtCTTaTCgtGaTCaAT
ACGAATACCaAGTATGAAtTTgAGTTCGCCcAGATCCTTGACCTCGTAGCACTTGGAcAGCTCTTGTTTTGCc
AGcGTCGCGCCCGCgTCAGTGGAGGAGGCGCCAAAgACaTCATCcGTGTAGGTgCTaGTGATgGTGAGCTCGT
TGTCGAtgAcgCgtGAGCGGACACACGGgTCCGCaCGcGACTGGTAgTATCCAAGGCCGTCgTAGCTCTTgCt

CAaCTCCTCAGCCCAGTCGCTCCCGCCGTTCATCATGCCgTAgACCGTcTTATCAGcgAGGTACACCTTGCCC
TCCTCCCCTGGCTTGATgAACCCGGGCGCcTGtTCAGTGTAGACgGTGAACTTgTTCTTACTGTTCAggTAaG
CaGCaATGAAGTCCACCTGCCAtATCTTCAacTTgAGCTCcGCgGCGACcGCaGCaACCATCCGCATGGACTC
CAgGCGgACCACgGATGCGTAgGTtTCGTCGTAGTCCtCaCCgGGGATCTGCGcGTAtCCtTTtGCgACCAaG
CGCGCCTTaCGCTTgaTGATgTTCCCGTCgGCaTCGTACTTGTtaTCatAaACCCAGCGtAgACCAAtGAcgT
TgGCCCCCTCgGGgCGCGGcACGAGCTTGAAGACCTCCCTCTCCTTCATCATCCGCATtTCCTCCTCCATAGG
gGCCCTCCAAAtGTCCGGGCGCTTCATGGCCTCCTtGAAacTCCGGGGCGCCACGGCATCAaAaTCACCAGTG
TtACCaGTGACGAGCGCGTGCGGGGCaTtGGTAGCCCAGTCCTCAGAgtTCtcCAGAGCCGCCGCCTCcCGGG
CCTCgAAtTCCcTCGccTCcTGtaatGCgCGaGATGGGACATGcGGGCGgGtaGTACGcCGAACcACaGGCac
tGCtGCtgtcGtcGttGtGGGTGaaGCatcGGtCgCAgGgTGTaAAGGGGGaTCAGTaGGgCGcggcCGGGGg
GCgCAgGGCCTAGCATCCCTGATGACAGCaTCggcaGCGTTcaTcTCgGCCgTcGGcNccgCtTCCTGaGCaG
GtGGGTCcAGGTCcAGGTCAAAGTCGTCaCCAGggACtacCGGACGcGTGCGGTGtCCcAcaCCCTCCTCgAA
AATCACGTCCCGGCTCTTcAcaATctTgCCAGaGGCaGaGTCgTAtAacTTGTAGCCACCACGTCCgTAGAAA
CCGATCAGCTTgTACTTGcgAGAtCTAGGGTCCAaTTTCGAGgCCCcGACCTCCGccGGgAtCtTCGCGtAcG
CATCgCTGCCGAAGGGTCGAAtGTGaGAGACGTCtTGaCGgcaCCgataCCAaACtTCtgCGGGcACgGCATC
CGGgTGTCgaGAGGAAGGtATGAAGTTaCGtAaaTATACCGCtgTtGagGCtGCCTCaGCCCAtAGgGACGGG
GGCAAtCCcGAgTCcGCAAGCATGCATCGgACgCCcTCgAAGATCGTGCGCATCCCtCGCTCaGctACcCCAT
TCTGgCCaTGtGCGTAgGCgGTaGTgATcTCTGCCACaATaCCgTaGCtCcTcAaaTATTCtgtCCAGGCATT
GTTGTACCACTCtCtACCACCGTCGGAgCGCACTATGCGaAGCtTtTgGCCaGTgaGgCGtTCGGCCTCgGCG
TGGTAaTCtTTGAAgGCCTGcAGtGTcGaCTCGGCgTTCTTtTTGGCaAgaAAGTAaACCTTtCGGAACGAAG
AtGCCCCgTCcGTGATcGACATCAAGTAGAGaGCGCCGCCGTTgGAcTtCACaCGgGCCGGGCCCCAGAGgTc
tatgtgNacccgNtcNaagacggNaNtNtcgtgagtgacNtcctcatcNtatggcctggNagNNtgtttacCa
TAAATGCAGTCcTCgCATAagCCCTCCGGtgCGGAGTCGTCcGTgATcTGgAGaCCaTCGACtAGGCCCTTcC
GCTTCATGTTGCGAATTGCGCCAATACCtGCaTGaCCaAAgCGtCGGTGCCAGGTTTCCAAcGGggCcGGcTT
tTCGTGAGAtCGaGCaGAGAGaGCCaTcGGtGGaTCaAGGCAgTCcACgcGaTACATGCCgTTACTTCCCCTg
CCgGTgAGtATCAGATTATTaTGcCcGTCATAGAACTTGGCCtctCCcCCGCCGAAAACGACtTGGTAGCCCG
CgGtGTCGAAtTTtGCGATGGAtAgGAGGTTGGAgATGAGgCCgGGGGTgTGGCATGCaTTTGTGAAGGTAAC
tTGgGTtGATTGcgcaCCGactTTgAAtAttTTGGTGACGGAaCCcCTGCCTAggATCTcAAAaGTGCTCCCC
TTgCTCGCCGCTCGgCCAAcGATTGGtGGGTTgAgtGGGGTgTAgGTCTCaAAGTCtGCGCgGTTCACgAAAC
AGTAATCGCTCGCGCCAGAGTCTGCgAAgGTGgGGACAGGgGTgTATTCGGcGAaGTtcTGCtCGGTGGAtGG
TGCAATcAGCGCCTCtGGGACcGTTGGcTCcTCgTCAAGGGAgGTGTATAACGCGTAaACCCGgTCCGGcGtg
GtaCTcGCGGtgTCAGCgGTcGCGgCCGTaGGCaTcGcCaCgGGtgCaGTCgtatTGTTGGTaGgcGCCgaAa
catTCCTTCCtCCTCCCCTcTGNCCGAAACCCGGGGGgAACTGgCCtTCcTTTCCTCCACCgGGCCAaTAgCA
CTTtTCaATGGTaTGcCCTCTCCTcTTGCAGTTtTGATTtGtgCAaACtAaCTGtTCgCGcTTCcTgGGGcCt
GCCGTTTGGAGGGCTGTaACCGACGtTCCcGcGGTGCtcACaGTtTGtCCcCGtTTCACcCGCGTCCAaTGCG
ACATaAGCTGGGCGATGACaTCGTCtGAcGAcTTGAAaATATGCATGGAGCCGACGACtGAgTCCCAGGAAGC
AGGgAGGGAATATAGGAGaGTGGAcCGAAAATCaGCGTCaTCgATGtCcGCTCCGGCCTCGTTGGCgTCAGCC
CACTTGGTTTGtAAGtTCtcGATgTGcGtGGGGAGGTCCTCGCCGTCgTGaAACATCAGCGTGCGGAAGTcGC
GTTGAGCTACGCTtTTGGCAAGaTCGCTGGTAACGAGGtACCTgTCGTTGAGTTTCTTCCAaGCTTCcGCgGC
cGTtCCTTTaATGTTAATCCCCATGCCaATgGGaTTcTTAATGTTGTAGATAAGTAGgGCCATTACCCAcgCa
TTACGcGCCatCCAcTCCTCCAAaGtaGGgGTaGTaGACCACCAGgGcGAAGcGcTgGCtTtGGTagCGGTGT
cGGGgTCtGGCGGtGGAAGTGGGGAAGTaGTtGTGTCGGGtTTCTTTATcGTgCCGTCTAggTAtCCaTGaGC
gcTgCGAAGTTGGGCaGTtAtaAGcACtGTTCCGCTCCAcGaGGCCCAaTTtttGCCGTCGAATTTgTCgtgG
TCGGCGAAgAtGGGgGGatTgGAaTTaGaCgaGTTCGACATTTTcaGaaTgTTAAGTTagCaGTTGaGCGTAT
aaaaTaTTGCaACGGTATCTGATTCCTTTAGGCTACAGGGCCCATAACCTGAtGTgAATACAGAtGCTCAGAC
GCCGTCACaAGAGTGAAGCAACTGATCATTAGCTAACCTGAtGTgAATACAGAtGCTAATGATatAaGTAAGT
AggTGTCCgTAAGGTAGaagTAATAgAtGtGCATACCTCAGACGCCGTCACaAGAGTGAAGCAACTGAGGtAT
GCGaatGcGaaCGTAgGaTATTTAtCATACGAGGTAGAATAGGGGTCGGCGGTTGAACGCCGTgTGATGGATG
TGAAACaTAGaTAAtAAaGtGAAAATGAAATAagAGAAAAGATATGCGCGCGGatGTGATCGCGCGCgcACaA
GTGtTTGActGcCaAAtTgaGTAtgcGGCgCGTCACGGGCGTTAcGCTCAACA
>Cligib1_Cluster48#LTR/Copia
AAGAGTGAAAGCGAACTATATATTATCTACCTGTTGAACAAGCGGAAGCTACAGTAGGTAGATAATCAGTAAA
GTGTCTGTGAGATATATATAAGTATGTACTAACCTCAGACGCCGTCAAGAGTGAAAGCGAACTGAGGTGCGTA
TCTACGAATCAGAGATATATATACAAAGAAAATAAATAGGGGTGCGGTGTGTTGAACCCGCGCCTGTGATGTA
AGTGAAACGATACGGACGAAGTGAAATAGACATAATGAAATAGAAAGTGTAGCAGGCGCGTACTAACCGATAC
CGTTTTAGGCATTAGGCATTGTCACGGGCATTACCCTCAACACTCCCCCAACCCTGTGGCATCGCCACCCGTG
TTGATGTTCAACATGCGACAAATATGTTGATGCGCCGGACGTGGTAGGGGTTTTGTAACAATGTCAGCAAGGT
TGTTGGCTGAGGAGATGTATTCGACCGAAATATCCCCTTCCTTGACACGTTCGCGCACGTAATGGTAGCGGAC

ATTGGTATGTTTTGCATGCACGTGACCCTTGGTGTTGAGTGTGAGTGCAATGGATGCTGTATTGTCGGCATTC
AGCTTGGCTGGGAGTTCCTGTGGCATGCCCATCTCGTTCATGAATGAATACATCCACAGCGCTTGTTGGGACG
CGCGTGTGAGTCCCATGTACTCCGCCTCGGTCGTGGACAAAGCGACAGTTGGCTGGCGTTTCGACGCCCAAGG
AACCGGGCCCCCACACATCATAAAAATATAACCAGAAGTTGAGCGCCTGGTGTCCATATCCCCAGCATAATCT
GCGTTGACGTACCCGATGGGTTGCATCCCATTCGCGGTTCCCGGTGTATAAGTAATTTTGTAGTCCAGAGTCC
CCTTGATATATGATAGGACATGGAGTGCCACCTTCCAGTGCGCTGGGCCAGGGTTCGTCTGGTGGCGCGAGAG
GAGATTGACAGCGTACGATAAATCTGGGCGCGTTGCTACCTGTGCCCACATGATACAGCCTAGCACCTCACAA
TATGGTTTGTCCACCATGAATTGCACATCTTCTTCAGTCGACGCCTGGTCTGCTTCAGTGAGGGTTAGGCCAA
CTGGAAGCGGTGTCGACTTTGGGTTGCACATTGACATATTAAAACGCTCGAGGACGTGCTCCAGGTAGGCTTG
TTGCGACAGAGATATAGCACCAGTTCCTGCATTGCAGTCAACACGTATCCCAAGGATAAAGTTGAGCTCCCCC
AAGTCCTTAACTTCATAGCATTTAGATAGCTCATCCTTGGCAAGTGCAGCGCCAGCATCAGTCAATGATGCCC
CAAAGACGTTGTCGGTGTAGGTGCTGGTAATAGTGAGCTCGTCACCAATCCTCCACGATCGGACGCACGAGTC
CGCCTGAGACTGATAGTAACCGAGGCCTTCAAAGCTCCGGCTCAGCTCCTCGGCCCAGGCGTTTGCACCATTC
ATCATTCCGTAGACCGTTTTGTCAGCAACATATACCTTATCTTCCTCCCCCTTCTTGACAAAACCGGGCGCCT
GCTCCATATACACTGTGTACTTGTTTTCGCTGTTGAGGTAAGCAGCAATAAAGTCCACCTGCCAGACCTTTAG
CCGGAGTTCAGCCGCGACCGCTGCGACCATGTGCATGGAATCCAAACGAACAACAGAGGCATAAGTCTCGTCG
AAATCCACCCCAGGGATTTGAGTGTAACCTTTTGCAACCAGGCGAGCCTTACGCTTCAGAAGTTTCCTGTTGG
CGTCGAGTTTGTTGTCGTACACCCAGCGAAGTCCAATGACATTAGCACCAGCCGGGCGAGGGACCAACCTGAA
GACCTCTTTTTCCTTCATAATGCGCATCTCCTCTTCCATGGGCTTCCTCCATAGGTCCAGGCGCCTCATGGCC
TCTTTGAAATTCTGCGGCGCCACGGCATCAGAATCACCAGCATAACTGCCAGTGACGAGCACGTGTGGGGCAT
CGGTAGCCCAGTCCTCAGAATTCTCCGAGGCCACTGCTACCCGGCCCTCCGAGGCCACTGCTACCCGGCCCTC
CGACTCCTTGGATTCGCGCAACGCGCGTGAAGGGATGTGGGTGCGGCCGGTACGGCGTATGGGTGGGGCTTGT
AGAGTGTCTCCAGTGTGGACTACACCGGGTTCAATGTGAGTAGGGTGTAGGGGAGGGTCAGTAGGCCGCGGAC
GAGCTGCCACAGGTCGTGCATCCTTGATGACAGCTGGGCCAGGGGTGTCCACGTGTGGGTCTGTCTGTGCTGG
CGTACCGGGGTCAAATTCCTCATCCGCCCCAGCGAGCTCCCGTACTCCTCTCGTCCTATGGCCAACGCCCTCC
TCGAAGATAACATCACGGCTCTTAACGATGTTGCCAGATGTGGGGTCATACAATTTGTAACCCCCACAACCAT
AGCACCCGATATGGATGTACTTGATCGATCTAGGGTCCAGCTTCGAGGCCTTGACTTCCGCTGGGATTTTCGC
GAAGGCAATGCAGCCAAACAGCCTAATATGGGAGACGTCCTGACGTTGGCCAGTCCACACTTCCACCAGTACC
TTGTCTGGGTGTCATGAAGATGGGATGAAGTTTCGCACGTAGATTGTCGTCGCCGCCGCCTCTGCCCACAGCG
AAGGGGGCAAACCAGAGTCAGCCAGTAGGGACCTAACTCCCCCAAAGACAAGGTGCATCCCACGCTCAGGAAC
GCCATTTTGACCATGTGCGTACGCCGTAGTGATTTCCGAGACAATCCCCGTTCTTTCAATACTCTGTCCACAG
GTCATTATACCACTCGCCTCCCCGTCAGACCGGACCTTCCTAAGTTTCTTGCCCGTTTGATGCTCGCTTGGCG
TGGAATCACGAAAGGCCTTGAGTGTCGTTTCAGGTCCTTTTCCCCAGAAAGTATGCCTTATGATAGGATGATG
TGCCATCCGTGAGTAGCATCATGTAAATTGCTCCGCCATTTGACTGATGCGCGCTGGTCCCCATAAGTCTATG
TGAACGCGTTCCAGAACTGTGGATTCAGGTGTTACCACCTTGTTGTACGGTCTGGCTGCATGTTCCCGTAGAT
GCAGTCTTCACAAAGCCATCCGGCACTGGCCTTCCTTGATGGTGAGACCGTCCACTAAACCCTTCTTTGCCAT
ATTACGGATGGATCAATTGCAGCATGGCCGAAACGATGGTGCCAGTATCTATTGTGGCTGGTTTCTCGTGGGA
TCATGCGGCAAGAACTGTGGGGAGTTGATGGCATCAAGAGGTACATGCCATTACTCCCATTGCCGACAAGCAT
CAAGTCACCCTTTGCGTCATAGAAGTGAATGGCACCCGCAGCAAAACTACACGGTAACCGGCGGTGTCGAACT
TCCATGGACACAAGGTTCGATATGAGTCTGGGTATGGCACATGTTTTAAAGGTAATCTTGTGGTTTTTCCTTC
AAACATGAAGTTTTTGTGATGTCCCCCTTCCCATAATCCGGAAAGGCTCCCCTTGCTGCCGCTCGTCCAACAA
TTGGTGGATAATAGGGGTGTATGTTTCAAAGTCGTCGCGATTTACGAACAGCACTCGCTCGCCCCGGAATCCG
CAAAGGTAAGTACTGGGGTATGCTTTGCATCAATGTCGGCTTCATGTCCCATCTGCTGCGTGGTTGGTGTGGT
CATGACTGACAGGATAGCACCGCCCGTCCTGAAAGTGGAAAGAGTGTAAACCCGGTCTGTTGGTTGCACGTCA
GCGACCGCAGCCATTGGCATAGATGCCCCGCGCAGTTACGGGTATGTTGACACATTGTTGTCCCGCCCCTACG
ACCGAAACTGGGGGGAACTGCCCCTCCTTACCACCTCCCAGCCAGTAACACTTATCCACCATATGTCCTTTGC
GCTTACAGTTTGGGTTTGAACAGACGAGTTGTTTGCGCCTCTTAGTCGTTGTTTGGAGAGCAGTGACAGATGA
GTTGGCTGTGGTAACTGTCTTGTCTAGAACGACCTTATTCCAGTGTAAATCAAGCCGTGTAACAATGTCAAGT
GAGGACTTGGCGTCATAAAGTGTACTGATGATGGGGTTCCAGGAAGCTGGTAGGATGCGAGCAAGATAAGTCA
AAGCCGCATCTGTAATGTTTGCCCCTGTCGCATTCGCTTCGCGCCATCTCTTGCGGAGATTCCCAATGTGCAT
GGGCAGATCCTCACCGTCGTGGTATTGAGGCGCGCAGATTCTGTTGCGCGAGGATCATTGCGAGGTCGTTGTT
GCGCTTATACTGTGCGGTGAGTGTCGCCCAAATGTCAGCCACTGTGCCCGTTAAGTCAGCTCCTAGAGCAATA
GGGTCGGACATGTTGTTAACAATAATGTCTTCGTCCAAGCATTATGCATCCTCCATTCATTATAGGAGGGCTT
GCGCGAGAACCAGTGCTGCTCGGAGCGGTGTCTGGGTCAGTGGTTGGCGTTGGTGTTGGTGCAGTGGAAGTGG
GGAAGTGTGTTATCAGGTTCGTCATATTCCCCTTTGGCTCCTTGACGGTCCCTTTAAGGTAGTCGACCGCGCC
GCGTAATTCGGCCGCCATGAGGATTTTATTGCTCCAAGCCGTCCAGTTGGTGCCATCGAACACGTCTTTTTCA
GCGAATAGGGGCGGGTTCGACGAGGAAGTTTGAATGGTTGACATTTTGATCAGTGGGTAAGTTTTAGTGGGTT
CGCGATATAGGGATGTTAAAACAACGTCCGTATTATTCAAGCTGCAGGGCCCATAACCTGTTGAACAAGCGGA

AGCTCAGACGCTGTCAAGAGTGAAAGCGAACTATATATTATCTACCTGTTGAACAAGCGGAAGCTACAGTAGG
TAGATAATCAGTAAAGTGTCTGTGAGATATATATAAGTATGTACTAACCTCAGACGCCGTCAAGAGTGAAAGC
GAACTGAGGTGCGTATCTACGAATCAGAGATATATATACAAAGAAAATAAATAGGGGTGCGGTGTGTTGAACC
CGCGCCTGTGATGTAAGTGAAACGATACGGACGAAGTGAAATAGACATAATGAAATAGAAAGTGTAGCGGGCG
CATACTAACCGATACCGTTTTAGGCATTAGGCGTTGTCATGGGCGTTACCCTCAACA
>Cligib1_rnd-1_family-709#SINE/Unknown
TTTTTTTTTTTTTTTTTATAATCCATTTATTTAACGACGGACGCGGGGGTACCCGTCGACCGAGGANCCGAGC
CTCGGTTCCGCTACAAACGNATGTACTACGGTATACTGTTAGGCATATTAGCTATTNTAAGGGAGGTGTCTAG
TGAGACGAGTAATTA
>Cligib1_rnd-1_family-84#TIR/MITE
TAGTGATCNGCACGGGTAATGCAACCCGTACGGGTTTTCCCACGGGTGCGGGTAGGGGTTACCCGCGGGTACA
CTTTTCGCAAACCCGCAGAAAACCCGCACCCGCGCCGCGGGTAGGGGNTTTCCCACGGGTTGTAATTCCTGAA
AATTTGTAAAATTTTGTCTTAGAAATAGCATTTTTACTGCATTTGATGAGGAAAGGGTCCGTGGGAATCACAA
CATAGACATGTTTTCATTCCCACGACCACTCAAAAATGATGTAATTATTGAATTTGAGGTGGTTTGCATGTAG
AATAAATACCCGCGGGTAACCCGTGGGTAAACCCGTACCCGCGGCGCGGGTACGGGTTTTCCGCGGGTACGGG
TACGGGTCGTGGGAAAATACCCGCGGTAGACCCGTGCGGATCACTA
>Cligib1_Cluster17#LTR/Gypsy
GAAGGGGTGGTTCCCCCTTCACGCGAGGAATGCTCTTGATGCCGGCAACCCATCGTCGGCCGATGACATCACA
ACGTGCCGCACCAGCCCGGCCAAGCACGCTTGTCGATCTACTCCTTCGACTTAAGCATGGCGACCTCATCATC
ACTCGTCTTGGACTCAATATCTACAATATCTTGGACCCCCTTTCCTTCTCATTCTAGTCACTGTTGGCCGTCA
CCGTTTGTATAAATATCGTACGAATTCCACTCTTTTAGGACAGTTTTTCACTCCAGTTAATCTACAGTACTAC
TCTCGTTTGTAATCTCGTCGAACGTCTCGAATCTATCGCGCTCCCTCGTTTTCTTGCCTTCTTCCTTTGTCCC
TCCTCGAACTTGCCCGGTCTGAGCTTGCAAGAGCTCGTAGCCATTCATCTGGAGCCCACAGCGAGGGGATAAC
AGGTGTTCTGCCACCTCGTTCCCATTCCCTACGCCCGTTTCACCCTCAACTCGGTAGATTCGTCTACCATTCA
ATCTCATTGTCACCACGCCGTCCGGATCGTCAAAGAAAGCGTGGCATAACCGCGAGAAGTCACCCCCAAAGTC
CAAAGCACCTACGCCGAAACCAGCGCCACCTGTCATAACACGAAGCCGATCGACAGGGACGCTACCAGATATT
CCACTACCAACGACAATGCGAAGAAAGAAAAATCCCCAGACCATTATTCCCCTTTCCATTCCACCAATCGTCG
ACTCTACCCCGAAATCATGTCCACCAAACCTTCCAAAGGTATCATCGCGATGCCAGGCGTGGCTTCAAACAAG
GCGCCTACCTTTTCCGGTGAAACATCTGAATTGCTCGAATTTCTTGACTACTTTGAAGAACTTGCAGATGGAT
ATCAGCTCTCGGAGCCGGAGCGCTGCAAGAGTCTCGTACGCTATGTGGACCAGTCCACCAAACGCTTCTGGAT
CAGCCTCGAGGGATTCGGAACACACAATTATCAGAAATTTAAAGAAGCCATCCTCGACCAATACCCGGTGCTA
GCAAAGGTATCAAATATACAATCAGGGACCTCGAACGGCTCACGAAAACCTCATGCGATGATGAGATCACTTC
GGAAACGGAGCTTAGGCAATACTACAGGAAGTTTCGACCCATGGCGGTTTGGCTCATAGCCAACAACAAGCTC
AGCGAGGGTGAGCGTAATCGCTACTTCTGGTATGGTCTGCACCCGTCCACTCGCCGCCAAATCGATCGTCGCC
TGGAGTTGAAGGAGCCCAATTACACTCGCGCCGAAGCCACTGATATGGAAAAGGTGATGGCGGCAGGCCGTTT
CGTATTTTCGGATGAGGCTTTCGATGCGGTATTTGATGATCCCATTGCGAACCGGTTGAAGAAGGATAAGGAA
GAAGAGAAAGGAAGAGGTCGAGGGAGGAAGAGTCGAAGAGTGGTGAGGGATTGGGATTCGGAGGACTCGGAGG
ATTCAGAGGAGGAGGAAAGGCCAAAGAAAAAGAAGGAAACGGTGGAGAGGGAGGTTAGGACTCGGAAAGTGGA
GTTGGAAGGGGGAAAGGACACCCTGGACGAGGTAGAGGAATTGGCGAAGAAGCTTCACGGTCTCCAAGTGCAC
GATGTGGCCTACTCAGGCTACTATGCTCGTCTTGTCTGCCTTGCACCCGCTGTCGCCCAACATTGGAAGGCAC
CCGGATCACATCAAAGCACTGTGCCTCCAGCGGGGAATCCAAATTCAATCCCCATTCAAAATTCGACGTCGCT
ACCCTATAATGGCTATCAACTGCAACCCTACAAAACTATCAGCGCCCACCTCAGGCTCATGATCCCAATGCAC
CATTCGACCCCTCATGCCATTTCTGCAAAAACCTTGATCATCGCCTACGTTTCTGCCCCACGCTGCGGAGTAT
GTCCGAGCAAATCGTCTTCGCATCGACAATGGCTACCATCTTCATTTTGATGGGTCACGAATCCGCCGCGACG
ATCGTGGAATGAAGGCAGTAATCGACGAACGGTTTGGAGGCCCACTGTTGATGCCACAAGCAGCGGTTGGCCA
ACGTGACATGCCACCCCATCTTGCGACGACCTCAGCCTTCTTCCAGTGTGACTTGGTGAACAGCAATGCGGCA
GTGTGGGAGGTAGGAGACAAGGAGGGAGATGGAGAAGCAGGAGAAGAATTGGAAAGCGGAGGCCTTGGCGCAA
TAACGAGAGCGCAGGCAAAGGAAGGAGAGAAGACGGAGGAAAAGAGAAGGGGAAAGGGTCGGGTCGAGGAGCA
GGAAAAGAAGAGTGAAGAGGTGAAGAAAGCAGTGAATCCAGAGTCTCGAGCCGACCCATCCCCCCTACACGTA
CGATGCAAGATTGCCTCTCCGGAAGCTGCCCGGACCCTCCATGAGCGCATCCTCAGTGCAGTTGTCCCCAATG
TCACTGTCGGGGAGCTCCTTGCGCTCAGTGGAGATCTTCGTAAGGAAACTGTCGAACAGTGCCGCACCACCCG
GATTCCCAACAATATAAGCACCGCAGCAATCACCAACAATGAACCCGGTGGTCAGCCGCCAGTTGACATCCGC
GTCGACCATGCCACAGCGCTTCGGGAACTGGAAGTCATCCTTAGTGGCGGTCATGTGGAGAAAGCCCTCTTGG
ACGAAGGATCAGAGATTGTTGTCATTCGGAAAGATCTACGGGATGAACTCAAGCTAAAAACCAATGGGCAGGT
ATCTATGACGATGGAAACGGCAAATGGAGGCTCAGAACAGATGGGAGGATGTGTTGAATGGATGGAGATCAAG
GTAGATGGCCTGACGACCTGGGCGCATGCCTATGTAGTCCCCAATGCGCCATACCGCCTTCTGCTTGGACGTC
CATGGCAGCGTCATGTTCTCCTCAAAAAGGTTGAGGACTCTAATGGGAATGTATTCATCACTCTCTCCGACCC
AACTAATCGTTCCAACACTCGCACGGTGCCAACCACTGCCCGTCCATGGAGCAAGAAGAAGGGAACATTTGCT

ATGACGGTGGGACAGATCAATGACGCATGGAGTGCGCTTGGAGGAACAGCGGTAGTGACATCAGTATGTAACA
CCATCTCTGTACCCCCGCCAAAACTGCCCCCTCCCGCACTAGCCATATCTAATACCACCCTCGCCGAATATAT
CCTCCGGGAGAACTTTGCTTATGACACGGTGCGACACATTTTTGTTTACAAGAAAGTGGCTAATAAAGTACGG
CCGGTTGCAACCACCATGCCTGAGGATGCACGTATCCACCGCCGCTTTCCTGAAGACCCACTCCTTTCCCTTC
CCGAGCTCTCACCTCACCCCCCCGACTTCACACCAGGCATTCGTTTATCTCAAGAACGCTTGGATGGGCTCGG
CATCTTCAACAACAAATTTCTATTACCCGAAGAACAGAAGCTCACGGCACACGTTTGCAAACTCAATGAAATG
GCTCTTGCCTGGGAGGAATCAGAAAAGGGGCGGTTTCGTGATGACTACTTCACACCCGTAGTCATCCCCACAA
TCGAGCATACCCCCTGGTCTTATCGTAATCGCCCAATTCCACCTGGGATTCGCGATGAAGTCATGAAATTGAT
CAAGGATAAGATTGCCTCAGGAGTCTATGAGCCGTCAAACAGTTCGTACCAGTCACGATGGTTTTGTGTGGCC
AAGAAAGATGGCAAGGTGCGCATTGTCCATGACCTCCAGCCGCTCAATGCTGTCACAATCAAAGATGCAGCCA
TGCCACCCCACGTTGAACTCTTTGCGGAACAATGTGCGGGACGCTCAATCACCACGGTGATGGACTTATTTGT
TGGGTATGACCACCGCAGCCTAGCTGAGGTCTCGCGCGACATAACCACATTCCAAACGCCCTTGGGGACAATG
CGGCTCACTTCCTTACCAATGGGCTGGACAGGATCTGTCGGGATCTTTCAAGGCGATGTAGCTTTTATTCTGC
AACATGAATCTGACATAGCACCAAACTTTGTAGATGATGTGGCTGTTCTGGGCTCGCGAACGCGCTATGAGCT
GGAAGGGGGAGGCTATGAGGTCATTCCAGAAAACCCAGGTATTCGTCGTTTTGTTTGGGAACATTGCATTGAC
GTCAATCGTATCATGCATCGACTCAAGCACGCGGGTGCCACTATCTCAGCCAAGAAGTTCTTCATATCTCAAC
CTGAAGCCATGGTGGTAGGACATCGTTGTACGTATGAGGGCCGAATTCCCAATGAAGACAAGGTCACGAAGAT
CAAATCATGGCCCCCTTGTACCAACACAACACAGGTCAGAGGATTCTTAGGTACAGCGGGAGTCGTTCGGATT
TGGATCAAAAATTTTGCTGCCACCGCCCGCCCGCTTATTGACCTCACGAAGCATGGAGTCCCCTTCGAATGGA
CATCTGTGCACCAGCAGGCTATGGAGGAACTCAAACATGCCATCGCAAACTCGTCAGCAATCCGCCCTATCAA
CTACAAATCTGATCATCCCATCATCCTCTCTGTCGACTCATCAATTATCGCTTGTGGCTACATTCTTTCACAA
AATGATGATCTTGGACGCCGCTGCCCTTCCCGGTTTGGATCGATCTCATGGAATGAACGGGAGGCACGGTACT
CGCAAGCGAAAATCGAACTTTATGGTTTATTCCGCTCCCTCAATGAAGTCAAAATCCATATCATTGGCATCAA
GAACTTCATCATCGAGGTGGACGCTAAGTACATTAAAGGCATGCTGAATAACCCAGATATTCAGCCCAACGCT
GCCATGAATCGATGGATCGCTGCGATCCTGACCTTTGACTTCAAACTCGTGCACGTCCCCGGTGACAAGCACA
AGGGACCTGATGGGCTGTCAAGGCGGGCGAAGAGTGAGGAGGACGACGAATGTGAGGAAGATGATGGGCAGGA
ATGGATAGAGGAAGTGTTGGGATGCGGAGTCTGGGTAGCAGGGGGTTTTGGAAGCGCATTTGACAAAGAAGAT
GTGGCAGGAACGGCTCACTTGGTATTGGCAGCAACAACTTCAGACGACCCCCTACCAACATCGCCGGCAACTA
CCAAGCGCGATGCAGAACTTGGGAACATTCAAGAATACTTATCCACTCTCCAGCGCCCCCCTAACCTCACCGA
AATCGCGCTCCCTCATTTCCTCAAAAGAGCGTCGAAATTCTTCATCAAGGGCACCCGATTATGGCGTCGGGAT
CCAGCTGGTCGTCACCAAATCGTCTTGTTCACCCCAGACCGATTCCGGGCCTTGCGTGATGCTCACGACGCCT
TGGGACACCGAGGATTTTATTCAACACGTCGAACAATCGCCGACAGGTTCTGGTGGCCATCCCTCGATGACGA
TCTCAAATGGTACCTCAGGACATGCCACCAGTGCCAAATCCGCTCAGTTCAGAAGGTAATCCTCCCACCCACC
ATCGCTATCCCCGCACCCCTGTTCCGGAAGGCGTATATTGATACCATGCATCTTCCAAAGGCAGCTGGCTATC
ACTATGTCGTTCAGGCACGTTGTTCTCTATCTGCCTGGCCAGAATGGCGCATGCTTCGTACAGAAACTGGCCG
TACCATTGGATCTTTCATTTTTGAAGAAATACTCTGCCGCTGGGGAGGACTGGAGGAGATTGTCACGGACAAT
GGGACCCCCTTTGTTGCAGCGCTTGACTGGCTGGCGGATAAGTATGGAATTCGCCACATCCGCATTTCAGCAT
ACAATTCCCGCGCTAACGGTCTTGCGGAACGCTCCCACCGCACCATCCGAGACTCGCTCGTCAAAGCATGCAA
TGGCGACATCACGAAGTGGCCATCCATCATTCCCCACATCTTCTGGGCAGATCGGGTTACCACACGTAAGTCA
ACAGGGTTCTCGCCGTATTATACCGTCCACGGTGTGGAACCATTGCTCCCCTTCGACATTTCTGAAGCAACAT
TCATGCTGCCCGACATCACTCGCAAGCTCACAACAGAAGAACTTATCGCTATTCGGGCACAACAGCTGCAGAA
GCGTGACGAGGACCTTTGTCTAGCTCATGACCGTCTCCTGGCCAGCCGGTTCGCTTCGGTCTCAGATTTCCAG
AAACGCCACGGCAATACAATCATTGACTATGATTTTAAACCTGGTACTTTAGTCCTCGTTTTAAATAAGAAAA
TTGATTCGCTCGCAGGTGCCAAATGCAAGCCTCGATATTTTGGACCCATGATGGTGGTATCTCGTGGGCGAAG
CGCTTACCGACTAGCTGAGATTGACGGGACAGTGTCGAAGCTATGTTTTGCTGCCTTTCGACTCGTCCCATAT
TTTCCACGCTCACTTAAAACCTTAGAGATCACCGAATTCCTGGACCCCGCCGAACTTTCGGGCATCCACGACG
ACGCAGAAGACGAGTCCGCTGATCAACCAGGGATGGTTGATATTTAAGTGGGGGGTGGATGAAGGGGTGGTTC
CCCCTTCACGCGAGGAATGCTCTTGATGCCGGCAACCCATCGTCGGCCGATGACATCACAACGTGCCGCACCA
GCCCGGCCAAGCACGCTCGTCGATCTACTCCTTCGACTTAAGCATGGCGACCTCATCATCACTCATCTTGGAC
TCAATATCTACAATATCTTGGACCCCCTTTCCTTCTCATTCTAGTCACCGTTGGCCGTCACCGTTTGTATAAA
TATCGTACGAATTCCACTCTTTTAGGACAGTTTTTCACTCCAGTTAATCTACAGTACTACTCTCGTTTGTAAT
CTCGTCGAACGTCTCGAATCTATCGCGCTCCCTCGTTTTCTTGCCTTCTTCCTTTGTCCCTCCTCGAACTTGC
CCGGTCTGAGCTTGCAAGAGCTCGTAGCCATTCA
>Cligib1_rnd-4_family-4414#LTR/Gypsy
GAAACCCGTACTCCTATTTGGAGATTGGATAACACTCACGTCGTTACGGGAACAGAAAGTGATCAACGTTTAT
TGATGTAAGATCTTGATATCAAGTAAAGTAATTACGAGAAGATGATGGTAAAAGGTAAAGATCGCCGAGTCGG
CTGGGCGCCGTTATAAGGCGCCCAGATGACAACATGCGCAAACGGTGATGTAAGCGTGATGTAAGCGTCGGGC
TTACACCCTCCCCTGCTCTAGAAAAAAAAATCCCGCCCAAAAATTAGTACCTAAGTATCGTAAAAGCTGCTCG

GACACGGGTGCGTTAAAAACCCCGTAGGGAAAAGCTACCGGTCGGCAGGAACATTCAGAAGATGAACTGAACA
TATCCCCAAACCTTGCTTCTCCTGCCATCGGTCGAGTGCTTCGTTGTCTTCCATTTCCCGGCCCGGTATCCAT
CGGTCCTCTTCCGGACCGCACCCTGCGAAACGCACCAGGTATTGCACACCCCTACCCCTCCTACGCTCGTCGA
TGATGCTGTCTACCAGGTATTCTTCCATCCCGTCTTCGGTCAACACCGGGCCCGGCCGCTCGAGTGTTCTCGA
TGGGAAAAGGCTGTCGTCGTTTTCNCGACGGGAAAAGGCTGTCGTCGTTCTCCCGAAAAGGCAACAGCTCCGA
CGCGTGAAACGTGGGGAAAACGTTGGGCGAGTTTGGCATGTCCAAGGTATAAGTCGAGAATTCTGGGTGGCTG
GCCACCACCACGTACGGACCGTCGTATCGCGGCATAAACTTGGCCACCCGTTTCTCCCCTTTTTTTTGAAAT
CTCGCCGCCTGTTAAAAGTCGACAGCCGCACCCTATCCCCGACCTCGTATTCCCTTTCCGGGCCTCGCGACTT
ATTCGCTTGTTCCGCCTGTTTTACCTTCGCCATTAGTAAGTTGTCCTGAGCCTCCATAACGTCTAATTCCAGG
TCCCTGATAATCGCCCTCGCGTCGACCGCTTCTCCTACCTAGGTCCCGCCGTGCATCTACGTCCTCGATTGGC
GGTAGGATCCTCGGGCTTCGTCCCATCCGCAGCTGGAAAGGNGNGAANCCCGTGGACGCGTTGACGGTGTTCA
TGAGGCTGAAAACCNNACNCGCGGCAACGCACGCGCCCATCCTTTCTGGTTTCGCTCTACGTGGTAACGTAGC
GCC
>Cligib1_rnd-1_family-325#LTR/Gypsy
CGCTTGTATATCCATGACAGTTCNGANGCTGTCATTTTCGTGTGGACGGGTATGAGGTGNACCATGCTGGTCA
TCCTGCATATGACTACCCATAGGTAGTTGTATCCTTTGGACTCCGGGAATGGGCCTACGAAGTCCATTCCGAT
TGAGTCCCAGGGTTTCGTNGGTACCGGTAGGGGATGTAATTTCCCTGCTGGAGCTTTGTTGGCTCCTTTGGCC
ACTTGGCAAGACTCGCAGGTCTTGCAGAATTCTTTAACGTCGCTGGTNACTTTTGGCCACCAGTAGTATCGTC
GGACGTATTCNGACGTTCGTTGAAATCCGAAGTGTCCTANCACTTCGTGCGCTTGTTCCAGTATGCGCGTTAT
GANTTCTCTNTCGCGTGGTATGCATGCCACCTGTTTNCCATTCGTGTTGCGTGTATACACGATTCCTTCGCGT
ATGGAGAAATTAGGGTCTCCTCGCGGGTTCTTCTGTATTTTCTCGAAGAATTCGTCCTTCGGGTATCCTTGTT
TTAGAGTTCTTTTGAACTCTTCGTTGCTCTGATCGGTGGTTTTTTCCACGTCCGCTGTCCCTATAGTCGCTTG
TCCGAGGGTTATGTCCTCGTCTGTGCTTGCGGGGGTGTTGTCTCTCCCCCTTTCTGAGGATGAACCGTTGTTC
ATGCTCGTATTATCCTTGTTGTGCCGTTCATTGGCCTCTTCCATTATGAGAGCTTCGATCTCTCTGTCTTCTT
TTCTTTCTTGAAGACGTTTACTACGTCTCCGCTCGGTCTCGCGCATTGCGCGTATCTCAATCACGTTCTCCGT
GATTTCCTTGTATCGTTCTCCGGGTAGGTCTTCACCCGTTGGGTCCATGCGTTTGTCNGCATTCACATATTCG
TAGGGCATGTATATGTCCCTAATATTGTCGCTTTCGTAGTAGCGTGACAAGCAGTCCGCTACTTTGTTGAGTT
CGCCTTTGATGTACGTGATGTCGAGATTGAATCTCGACATGTAGTCCATCCAGCGTCTTTGACGCGGGCTTAA
TTTTACTTGAGTTTTGAAAAACTCAAGTGCCTTGTGATCGGTTATGATGCGAACATCATATCCTATTAGCTTG
TCTTCCCATTTCTGGAGTGCCTCCAGGATTGCGAGCGTCTCCAACTCGTGCACAGCGTAATTATGCTGGGCCG
TCGTGAACTTCTTGGACATGAANCCNGCGGGGCGACATTGTTGCCACGTNGGTCCTTGTCCGTACATTGCTCC
GACGCCCGTTTTGGAGGCGTCGCAGATGAGCCATATTGGCTCCTCCGTGTCCGGTCGGATGGGTCGGATGATG
GGTGTCTTTTCGCAGACGCGTTTNATCATGTCGAAGCATCGTTGGTGTATTGGGCGCCACAGGAACGCGGCGC
CGTTCTGCATCATGGACATCAACGGTCCGGTGTATGNCGTGATGTCGGGGAGAAAGTTCCCCAGATAGTTCAC
GAGTCCTACGAANCGTCGGACGCCGTTAACATTAGTTTCTGGGGGTTGGCCACTCCCGGATCTTTCCTAGTTT
GTCNGGGTCCANGGCGATATGNCCTGGTCGGATGATCATGCCCAGACAATCCACTTCGTCTGCGTATAGTTCG
CATTTCTCCAGTTTCAGGAAGAGGTCGTTTTCCTGGAGTCGTTGGAGCACTCGTAGGGTGTTTTTGTGTTCTT
CAAATGTGTCTGAGGAGTAGATTAATATATCGTCCATGTAGATGACAATCCATCCTTCGTCAATCATGTCNCT
GAAGAGTTCGTTCATCATCATTTGGAANGTNGCGGGNGAATTGCAGTCCCCTTGTTGCATGATCAGGCTGATA
TATGTNCCCGCGATCGTTGCGAACGCGGTTTTGTCTACGTCCTTTAGGATGATGCGTACTTGTTCGTACGCGT
CCGTCAGGTCTATTTTNGACCTGATTCTTCCTTTCGATACGTCTTCGCGTATCACTTCTTGGTCTGGTAAAGG
TGTTACGTCCTTTACCGTGTTCTCGTTCCGCTGTCGTGCGTCCACGACAGTGCGTAGTCTNCCATCCCGCTTG
GGGATGCATAACATCGGTGCAGCTTGGCTCACCGATTTCGGTTTCCACCATCCCGCGTCAACGTATCTGTTGA
CCTTTTCGTGGAACTCGTCTCGTAACGAGTTCGGACATCTNGGCAGGTGGTAGTGATACCGCTTGTTCTCATC
AATCAGGTGAATCTCATGATTCACTTCGCGCCAAGGGGGAAGTTCATTCTTTACTCCTCCCATTATGTCCTGG
TATTCGTCATACCAGTGTTGCCTGAGCCGAGGATAATCATCCTCGGTTAGGCCCCTTGTTGGCGTTTTCCCTT
TGTCNGGTTTCTTGGGTTTGG
>Cligib1_rnd-4_family-1594#LTR/Copia
GTTATGGGCCTTTNAGCCTAAGACTTGGTTTATCNCAACCGTCGGGATCCGACTCACTTTCTTCACATCTCTT
CTTTTCGCGCTACTTCTTATTATTCTTTCTTCAATGGCCGGCCCTGGCGGGAGCTCTCCTTCCGNTACCGTGA
CGGCGCGAGTGGCCCGNGCGGCGATGGCGTCGAAGCAGGGACGCGTCCCCCGNGCNAGNACATGCCGTTNCAG
TTCTAGCGACCGACGCGACCCACCCTTCCGTCCCCGCGCTCTACGTCCTCGATAGTGCNTGGGACGGACGAGC
CGTTNCTNAGCAAAGTCCGAAACAACTGGAACTCNTGGAAGGCACGCATGCTNGACGTCCTTATTCTNTCAAA
NGGGNTGAATCCTATCTCGACGGCACCGCCCGTCGCCCAGATCCCGNTCGAGAACCGCGCGCCACNTACAATT
ACGNCCTCAACGACGAANCGGTACGCGCATATATTCGCACGAAACTCTCCAAAGAGGAGCTCTCGCACGTAAA
GTCGTGCCAGTCGGCCAAAGANATGTGGGATGCGCTCAAAGGATCGCCACGAGAAGCAGGGNCCCATTTCTCA
AATTCTTCTTATGCAAGAGGCCCTCGCTATTCGCTATTCTCACTCCGTTCCCTTCGCNGAGACNATTGCACGT
ATTGACGANATCAACGANCGTATNNGGGACATGGGTGCCCCCACGCGCGAATCCNTTCACTGCATCATTCATC

TNTTCGCCCTTGGCCCNCCCGAAATGCGNAACGTCCGCGACTCCATCACNAATGGCCTTTCTACCGCCACCAC
CGAANACCCGTATTCCGTCGAACAGATCGTCCACCGCCTCGAGACGGAGCAGAAGATCCTCGCNTCCGATGGC
AATGCCTCGGGAGACGCGCTCGCGGTCGACGGCACTCCACAACNCCGAAGAAGNCCGTNACTTGCTCCAACTG
CCACCGCTCNCACCATGAGGCGAAGTTCTGCATCAAGCCNAACGGCGGAATGGCNGGGAAGACCGTTGCCGAG
GCCCAAGCCGCCTCGAGANCCGAACGCGGNCTACCNCCGAAGAAAGCCCTCGTCCGCTCCTCTACGCCGCCCC
TGCTAATCCCGCCGCTTCGACGTTCGACATCATGCGNGACGCCAAGGGNCGCGCCTNTGCTGTCGACACCTCT
TCTGGNCAGGCATACTTNCTNCAACCCGANCGNCCTGCCCTGCCTCCTGCGCCGNCCGCTGAAGCCCATTTCA
TGGCNCTGGAANCCGACGACATTGAGACGCTGGTCTCGCCCTTAGTCGCCGACTCCATGACCGACGGCGGATT
ACGCCGAGGTGGCTTGGCTTGCCACGCAAGACGACGTCTCCGCGTCGGTNGACTGGGCGGATTNCTCCGNGCC
TGTCGACTTCAGCGCGTGGCATTACTGTCTCCCGTCGCCGACGGTGCTCGTCGGGCGCCCATATCTCCGGACT
CGGTCCCNTTCCTCATTGACAGCGCCTGCACNACACACATCTCCCCNGAGCGCGCNGACTTCTTCTCCCTGCG
CCCCGTTAGCAATCGCTCCGTTCGGGGTGTCGGCGGNTCNTCCGTCCATGCCGTTGGCATCGGNTCTATTCGC
CTATCNATTGGNAAGGGTAATGCCATCGCNCTGGACAATGTNCTNTATATTCCGTCTTCCACCGTCCGNCTCA
TCAGCGTCGCCTGCCTTACCGACTCCCTTCAGTGCCGTGTCGTTTTTGATTCCGGCTCCGTCTCCATCNTGNN
TCGCTCCGGCTCTGTTTTCGCTTCCGGTACTCGCCTCCCCGCCCGTAATCTGTACCGNCTCANCACGNCTGGC
GCCTCTGTCGACCACGCCNTNCTGTCCGCCCCCGTCCCCACCCTNGCCACCTGGCACCTCCGNTTGGGNCACG
CNAACAACCAATCCATATACAACATGGCCGTNAAGAAATTAGCATCAGGTATGCCTATCGATCTNTCGACGTC
TCCTCCAAAATGTGAACATTGCATCCTTGGCAAACAGGTCTCGTCGCCTGTCCCAAAGGTNCGTTCGGGGGAG
CGTTCGAAGGAGAAATTAGGCATTGTCTTTGTTGATTTGACGGGNCCNCAAGACGTGGTCGCGGCCTCGGGCG
CTCGNTACGTTATGCACATNCTTGATGATTTTTCTAGCTTCTCTTGGTCTATACTTCTGAAATCGAAAGACGA
NGCNGTCCCAAAATTCCAAGCATGGGCTCGCGTTGCTGAGGCGGAATCGGGAGCGAAACTGAAGATCATTCGN
TCGGACAGGGGNGAGCTCAAAACGAATGANATGGACGCTTGGTGTGCCCCTCGCGGTATTGCTCACCAGTTTA
CGGCGCCNTACACNTCNGCTCACATCGGGCGTGTGGAGCGTCTGCATCTNACNATCGCGGGCAAGGGNCGCGC
GATTCGCCTTCAAACCGGNCTACCCCCGAACCGGTGGGATGAGTGCGTCAGCNCCGCGGGNTACCTCCACGTC
CGCACNCCATCNGTTTCTGCCGGCGGTCGNACCCCGTACGAACTNTGGTATGGCAAGCTCCCCGACTTGTCTC
ACCTNAGGGAACTAGGCTCGCGTGCGTTTGTCCTCACCCAGAATCGCCACGAGCCGAAGATCTACGCACGNTC
NTTTGAGTGCGTCCTCGTGGGCTATTCACCNTCTTCCAAGGCCTATCGTTTGTACCACCCNCCNTCNCATAAA
GTCGTTGAATCNTTCCACGTNCGTTTTATTGAATCTCTAGATAGTACCCCCTCTACCTTACANCCTGGCCGCG
TNCTCGGCGNCCCTACATTCCCGACGCGGCCTCAATCCCCNCCTTACCTACTTCCACCCGCTCCAAGCTGTGC
CATCCTCCGCGTCACCCTAGCGCCTTCCCGACTTTTGGGGGCCGCCTACCCCTACCTNCTTACCCCCTTCCTC
TTCCCCCATAGTTACCTACCTCACCTAGCTCCCGTCGGGGAGTCGCCGTCGAAGAAATACCCGACGAGGACGC
NCCCCCCCCCATCACCCCATTCACGTACGCTTCCCCTTCTACTTTACCTACTGTTACCCNCAANTNCCACCTA
CCCACACATCCCCCTCCGTCCTCNCCGCCGTAGTCCCGTCCTCACACCCCGTCGGAGAAGAAGGCNGCCGTCG
ANGGCCTTCTNTTCNTNCCCGCNGTCTCNCGGGCNCGCGAAGCGTCCAAGGCCAGCGCCGCNCGGCTCAAGGA
GGAGCGCCGTTTGGAGAAGGACCTGCGNCGCGAGTCAAGGCTGGAGATGCGCGCCGCNATGGCNGATGAGTCT
CGCGTTTTCACCCACACCTCTGCCGTTGCCATCCTNGCTGACTCCCTACCTTTCATGTCCCCCGAGGAATTCG
GCCANTTTGAGGACGCTTTCGCCGCCGTNATTGACCCTTCCCTCATGNCTGGAGCGGCGTNGACGACCCCNCN
NCCCTCCGCGAAGCCCTAGACTCTGCCCTGACGGTGACTCNTGGCGCGCCAGCATTCAGGAGGAACTNACTAG
CCTACGGGACATGGGCGTCTACAAACTTGTCCCGCGGTCCTCCGTNCCGAGCAACCGNAAGGTCCTGAAAGGC
AAATGGGTATTCAAGCTCAAGCGTGACCAGCATGGTGCTGCCGCTCGCTTCAAGTCGCGCCTGGTNGTAAAAG
GGTACGAGCAAGTCTTCGGNCAGGACTACGTCCACACAACATCTCCCACCGCCCGCATGGAGTCATTGCGCTT
ACTCTTNCACATCGCCGCCGCNAANGACTGGGAGGTAACTCAAGTCGATGTGAAGACNGCTTTCCTCTATGGT
TGCCTNTCGGCCGACGAGGTNCAATTCATGGAGCAGCCNGAGGGCTTTGCNGAGCCGGGGAAAGAAGACTGGG
TNTGGCAACTACAGCGTGGCCTATATGGGATGAAACAGAGCGGGCGCATTTGGAACAAAACNATGACCTCGGC
GATGATNTCCTGGGGTTTCAAGCGCCTNGACAGCGACCCGTGCGTNTTCTACAGGACGACCTCATCTGGNACT
ATNCTNGCCGCTGTCCACGTCGACGACTTCCTCATCGCCTCNTCNTCCCCCGGCGAGGCCGATNNTTTCAAGG
ACCAGCTNCGTGCNCTCTGGTCGATCACGGACCTGGGCGAGGCGAAGTTCTGCGTNGGCATCGGNATCACNCG
CGATCGCGCNAAGCGGACCGTTTCTCTATCNCAAACCGCNCTNATCGACCGNATCGTCACCCAGTTTGGCCAG
GCNGACGCCGACCCTATCTCCACNCCTATGGATGACGGNCTAAAGCTTCGGCGCCCGGACCCCGGCGTTCCNC
TATCCCGNGAGGCGGCCGACGACCTATCCAANTTACCCTACCGCTCGCTTGTGGGGTGCCTTATGTACGTCGC
TGTCGGNACACGCCCCGACATCGCCTTCTCAGTCTCCAAGCTCGCCCAGTTCCTGGACTGCTATCGTCGCGAA
CACTGGAACGCTGCCATNCGAGTCGTACGCTACCTAAAGGGCTCACGCTTACTCGGTCTCACCCTGGGTGGAC
CAGACAACCCATCGTTATTCGGNTTCTCCGACTCATCATTTGCCGACTGTCTTGATTCCCGC
>Cligib1_Cluster107#LTR/Gypsy
gTtacgTcCtgctaacgcaaGccgatgagTcgAcagtaaAgactcaaGgcaCttagcaaggTctgaGttgcct
tcTgggtCAggAggctagtAgAgggggaactGaTaaAcgATtAaAcGtagCgaatacaggagtaTgtagcTa
cgtttTAtatagacaTgGaTGTagTtctaTgaCTAcgcaataatgacTgtgTCcGtgagTatGatAGgacaTaT
ggagTaTgctgactgAgAtagaAgAgaagCTgaCtgAgcagCtGAgtaAcaacagtgaagaTAGcaAagatAg

aACAaCaAccatgcggTaggaaggtgataAggaagCgcatgatGtatCaTcTgATgaTggAgCAtgTggagta
TgTcagAtgaTgaTGtaggAagtaGaAgCgtctataaaagatgGgatCagtcaagTgactgaTgaAtgatgaa
aCagagaagcaatGaAtcgtAacatgaccTtccCtcCagGGGTttCgtccCgAaaCTctAAaccAACAatcCA
gCttCTAGcaacActttaccgAgatcctgcttttataagccGtcaAatattCctCgcccttGcGCgtAaaGa
agaccgCttCgTtGtccggtccgCgtacccaATatctgaAcGggtgtcCacTgtacatgtgggtaccattgta
gggGTCGgtgGGggtGAacgttttGGgGatctTgacggTgCACAacCgGtgTaatgggaggacccctaTgggg
GtgaAcgtgggtGgtGttggtgGtAGgtggcTggGgCctGCgAtGGcgtcttctcctGTgggggagAgttgTg
ttGgagaggtTgGagaaCtAGAcGAGgcaGagtgAGtaggagtgCctAaagatgggggagggggAatgaCagg
gaggCggtatTCcgGgtggaccTCcGtGggGttgatagggacaCcccAttcGttGACcgtcAtttgttgacga
AgcgtagtCAtgtCtCcgtcagtGagAcCaggagCgtggtgGgttagCactCgttgaaCAtgAgCatgtaGNt
CtttttCgtagTCgagTagactTTgtcgtagatTgtGGAgttGaacccgTtggtcgcggAggggTTgcagcat
agtaacgtCgtcttCtgatAGggtGcgtGcaatgTtgtcCaacgcTtTgatcCagtCaTtgattgtTcgaata
tggatgggggtagaaTgaagaagttCgAgGgtGggGAAgaGggCaGcaatggccaaggcaAGttgAgtggCgg
ggAtaGacAtggtggaGaatatGgtttgtaaGtggaaTgCaAtttaaAagatatgggggTaaAgtaagatgGt
tGaaacgaGtgtcgTatttatcGtGGtgcgttgGggtgatggcgGtAgaAgtctttaAccagatCCgcGgagT
gTttgaGgtttgcGcGgaCttcccAggtgttttCttcttccggAtAccCttTcCaatggACgAAgaATagaaG
tcttccattctTcTTttTgACctttcggAtgctttctactTcgtATtcTtctgaaCctTcgaTgaCAtcgggt
ggtggagGtGatggcttctgttGTgattcgaaGcgtgctgttttgagaggtgTTaatAgGtTCtcAttgAata
ccGggtgGaTgaGcgTccAgTtGgtGgggagcTtgagtttgtAggCACtgtggcCTacctttTCgAtaAcTtc
gaaTggtcctAatcgtcgttctGataActtcttggatggcctggtggttgtNatattttggcttctaaaAAc
AccttGtTaCcgggtTtgaGattaAgCGAGtccttctTgtGaCggtcatattgcttctTCattCGttCggCtg
atgCTtctaAagcGgtgattGctttcttCcggACTTcTtcaatgcGtGatgcaaattcttgagcgGcttcatT
ctttaccggttcCATgGAgatgtCgccTTtccAtGgAtgtcGgccAtagttcaGgaagAAcgGagtttgtccg
GtaCtagagtgGatCttATcattgtActgGAAttCtAgaATtgAtAgCcAtgaggcccaatcgtcctgATcgT
aattgcaaAAgAtccgaaGgaACtgttCgAcgtctTGgttGAtacgttctgTttgtccatctGtCtgcgggtG
ataagccGtTGATaAattccGtttgatccctaagAgggtGcaTAaGtccttcatgaAgttcgAtgcgaacTgT
ggtccAcGaTCActaatgatcttcttgggtattCCAtGttgTCtcCagATggTgtctcgATagaTttCtgCAa
gtcccttgGAcgtcagggTGgTgtgtgtcgGttcgACcctgatcatcttcgattgcctgtccacaaTgaCcAa
aaTagcAtCgtatcctttagAcggtggtaagggGccgAtcaTgtcgAttgaGatCtcttcccAGGggtgcgTg
GgtatcgggtTGGggtagaGtgGCgCatggcgtGCttctcgattgatctTGtTcttttgAcAtgtttcgCatc
CcaTgacaTaAtCtctgatgtcTgatctcaTcccTGgccAccaTagTgTgcgTcCaatgAgtTcAtTCgtttt
gtGgaTCcctgggtgacctgctAgTGGtGAatcatgatgttcgttgAttaattcttcAcgtagtTgtttgtct
ggGGgTaTaTagAcCttgtcTccgactgTgatgAtgCcatcagCtTcttTgAAgaaGtgtggactttcctTCA
tgccgaatcggacttCtgattctacgcgtgggtggtttcTgAtgcgctgttCCAgatCTtcAtTgCcGaattC
AGtgAgaGctgaGatggaAAtttcCgatgtGTcCagaAtcACcTcctcttcagGtagttcttctccGAagaac
cgtAtgCcaaagtCcgAaAtttcatcGtCttCctccaTttCtgaAggaataccaaACCcatgattTTtgaAca
tcAtgacTtcttctTcttcagtGtcttctTcGtAccAtTccAgtctTgagagGAtGtccgcCTtcgtgtttgt
tttgcccggtATgtgtcgcatttcgaaatcgtagtcctgTagTttcaAAtAccAccGcatttgtCgActGtTT
aaccgctGAgctTtcctAaaatAtgtcagGtttttgTgAtctgtcAaTagttCgAaCggTtccTgtgcgtcca
ccaagtAttgtcgccaAatgcgcagTgcTtcCatgaTggacaggAgTtccttatcAtAgatctcgtatCgcCG
ttgTgTtgggttcATtactttggaCaTgaaggCgatAgGgtGccatTTaccAtCttGttcttgagaCaggacA
CctccTAtggCgtatcctgatgcgTctgttTcGAccctgaAGcgtcccttgGATCTggggaGggccaAtAttg
gttctgagcATATCGccGAttTcAggcTtTcaaatgcGacctcttgTtctggcgtccAAgaccactcgctaGa
tccaGtgAggcgcgtgagaggTtcagcCattTTtccgtaatccttgatgaagcgacgGtaaaaatttGcgaag
ccTaggaaTtTctgaacacctttcttGttttctgGacgtttcCAaGtttggATgacttcgAtttttttctTGtt
ccatgCggaccttCccattgccaacaataAcAcctagAaatggtaCctcagggacctcaaataagcatttact
ggcCttgAAcgagatcccAttggcgcgtgcgaTttCcatgaCctcgatGctacgTTctctaagctgAtctttg
tcCttggcatAgAtgaTaAtgTCatCgatATagatgatcatcttgTTttgtCgtatgAGaTCtttaaatatCT
cggccaTCcAtcggcAgaatgagcttgggGaatTAcagaGTccgaaGaacatgaccCgtGgTtcgAAtaatCc
gCgTggtGttaggaaaGctgccttccATtcgtggtAaAgtgCGatGcgcaagttgtTgTatccccagAtTaga
tctaActtgGtgAaaAaatttgcgTctttcAgttTgtcgatgatgtcgttAatcAgaGgTaGgGggAaTttgt
ccTtAatcgtcCacttgtTtagcTctctAtagTCctgtaccAAtCgtTtGgatccgtCttTctTgTTgatgAa
gAagcatggtgatgcgtatggactcttggATgggactatccgttccgaggctAGattctCtTCtaggaAtttg
TccAtTtcTtCcAtttctgCggctgtcAttggatAcaatTtggcTggtAattcgtccGGTgcatctggttTca
gGttAatttcGtgGtCatgcttccTgTacGgtgggaGtgTgtcaaaggTCtttttctcgaatCgatccttccA
aGTcgTgagGtACttNggGagttTTagatcCgtgaCcgTgtCTGttgaCcGaAcTttAGcctTGGgtagtgtg
cTgAcgcttactttggcacattctgggGggcagtctgggaattTGAtcttgccagtcttCcagTTgAtgaAgG
gattgtGttTgtgtAaccattcAtgtccgaTgaAgAgAtCCtttttgcctaAgtccgcTaccagtgctgTtgc

ttcttccttgtgaTCgCCAaTctgcatctggaacctGAcgCttttggtGAtTaattTatccGcAttgAgAgaA
ccaTccgCAttaAatgcccgAataggagtgGgTaAatcaAccGtggGgagttttTcTgcttccAcCAgtcgtc
gattgactGCcaggtggGtGCatCcgctgtCCAggagtgcatCgAgtttagcctttcgtttGtcCgtggTtat
aAcctTgacCgtgaccattgttTtatcctttTctaGtTttgAgagTGGcgAgatgcagagttgcaagctcaggt
gcactaGTtcgtCgttggggaAtCAtgtattTGTcattcttcggTggtGgtAcagATttagaAtgATtcGacG
AacccCtTGGgtcgagacttcgtgctcagtcTGAcccTtcctgAaAacccttcTtttcaTcgttatctccctTA
acCgtttTagcttcatcgtcgCtttcaTcATCAtCaagggccCtgaatttGcCctggattaccAttcctgaac
TCcggCAttcttTtGctatGTgtCcttcctTgTTgcacttccAgcATtcgctccTgtcctgatcTtGaTcct
aatgtctgttTTcttcTtcttgcctcgacAatctcgtgcaaagtGtcCatacttgctgCagTTgaaaCacttg
ggTcttCcctctgCaTtccAatttGctgcgTCgATGtttgtttcctctActtgtcCgatatccatcgggcgtc
CtCtgccaccatAAGTggtacctgttcCgtcgtgccgaTctccAGaggtgGaaGcgCGttgGActccttttcg
aatgaGactgAgttcttccTCaaTattaCcatAggtgaGgAtcgcctTtTTccagtcgtcatatgttgccgga
CcagtNcctccaTtttggaAgcgAcAtTtgTCaAtgAtcgAGttcctgGTGttTcgttggattAagaacaCtg
CtAaTtcatcCgCgaCttgaCcaatgTgttTctGAgCaTcgaAgtctgcgatccAttcttcgaCcgattgagA
cCcttgtgtcatcaCcCtgatcctatttctGctAgtaAtcGtcgttgNccgTggaAgaactgtttagTaatt
tccactAcaAAtactttccatgtgCcagcAtcNtTTGtgttgaGtAttGtgTctgtcttcatcGctgcccagT
tctcgGccCtCttgtgaCatctAgAgagCGcGGcCgaaaTcttctttTcaTcCgtgtccaggaCagcgttcCC
tCtgacgtaAagttgCatgcgaCgcCaCcaaccgTcaAaaaggttgcaaTccccgTcGaAcatgcCggggtct
gCgattcCattgtcaTtgCgcTtagggGgtcgTtgtCTCtgtagccattgttCCGtaCggTTCgtCctCgTcGt
ctgaggggAtaggggagtggggtctcgatAttcagtgAaGgaaGctgtAcgctccgGttggAtgggtCcgaC
tggCTgaaCGgtacTGgTtcgcTCtgaaccgAAggtgTattgtggtgaggacggCgctTCCtgAgacggttgg
AAccctgatgcGAggcTGtGggaGtgcgAgggGgTagTGaCtgggGagcGggggaCggAggTGtAttggcTag
ctgTtggtGtggggTGggactGgcgatatgcggcgTattcGTCttCtggTtctGccgTgattgactCttCtCT
GAcggggggCgcGAcccgTGaactgtgGgttgTggggtgggaagtgtgaTgcGgtctgtggCTacTcCcttCT
CtTCTcGaggaCgAcgactgtCctgAatgaTgcgagtgACttcttcttgttCCtTCGCccGaCgaAtgtttCT
cCtgatgtCggCtaatcttTCgtCgtggAgcttctcaaatgtttgatgCtGtcTGTGgAggttaAtccaCtCC
ttctttgTCgatCCtttCGACgtcctgtgatacctattgcgtgcgAcgGtcgcttCgTcgcctaaTctctgta
gttcgagttccgAtGctGttacTggCaaTgaGCcCctatatGgaGtGataTactCccaactgtgattgatatc
gaTgtcgtcccTatccCTAGggGtTgtccTGTTtcgTTgcAGggTtGcctgGtgACCCtaGacGtttAatcgA
atgTtacgTcCtgctaacgcaaGccgatgagTcgAcagtaaAgactcaaGgcaCttagcaaggTctgaGttgC
cttcTgggtCAggAggcTagtAgAggggggaactGaTaaAcgATtAaAcGtagCgaatacaggagtaTgtagc
TacgttTAtatagacaTgGaTGTagTtctaTgaCTAcgcaataatgacTgtgTCcGtgagTatGatAGgacaT
aTggagTaTgctgactgAgAtagaAgAgaagCTgaCtgAgcagCtGAgtaAcaacaatgaagaTAGcaAagat
AgaACAaCaAccatgcggTAggaaggtgataAggaagCgcatgatgtatCaTcTgATgaTggAgCAtgTggag
taTgTcagAtgaTgaTGtaggAagtaGaAgCgtctataaaagatgGgatCagtcaagTgactgaTgaAtgatg
aaaCagagaagcaatGaAtcgtAacaa
>Cligib1_Cluster1#LTR/Gypsy
gttaaggcggaNtgcctttaccaaggttcaagcgtcgagtatcgtcNaNNacgtcaagtcagagtcttgggtc
aataccaacaagactatagtcactccaagtcaggctttgacttgggtatcaNNgttcagtgatcaaggttcag
aggtaaagggtgtagtattgcctaggtcaagatNaagaataatgacaNtgtatctagggtttatatactaggg
accttgaggcgcgagaacatagtacaatgNgaaNtaaagacaatgtaaggtaaaagagctgttgaagctcagt
cagctatgacatcatagatgagtcaggtatctaagaatagaggcatgtgttggacatgcgatagaacaatgaa
gaatcagagaacaatgtaagtatNgtcaaacaaagggaatcggtgaacaaNNaagaaaaagataggggtggga
accgttgtaacattacccccctgttTatcgttgaagtcccctgacttcaagttaccCtgcaaagctaccgtagg
gttcatcaatcatattagagatggcggagtcgtcaaagtaaaactcctcatcaaaatcgggttgtcgtggtcc
atattgactgtgagcgcaagtgaCNtgaggNgttcNcCaattgcagaagttgcaataaaaatcaggacatgtN
gagaaggcgtgtccagtaccatggcaaaatgaacaatacCgaacgcgNggtcgttcNggccgagagaagacgg
tccggggaggNgggacattttcggcgtcggggggattggatgtgtCgtggtcgaccagcatgggaaagaggggt
gacatagatagtttgtgggcgtagtcgttgtcgtcgtggggttcggatgacaggggNtctttcgtgtgcttgg
tCgtaggatgggggtgctagtcgttcagggtttggtggaggagtggtactgtgNgtaggggNaggNaaNgNgc
gatgggNagagaggatggtctgcgaagaggagtggtagaNggtggttcaggagaaggggcagtagatgtgtca
ttaataggtgagcgaggaaaNggtgtcgctcctcgggagtctgttcttgcagaagtagactccatNagagtgg
tcgggttNggaggtctttcggtttccaagtcagtgagaacgatggagggNgtagaagtgagttcgtcaatggt
tcgattggtgaggggccaggggtcattcgtgagtcgtggagttactactccagggattcgattcatctgttga
tcgttcagcagttgctgaacatgttctattcttaatgggtgggggtatccNtgtgggcagtcgaatccaataa
gagcgtctagaccccatttcctacctgcatggaatagggtcttctgggcagattcgtgggcgtctttaaggcg
agtaagttgggcttgNacctggcccatNgctccaaagAgattNcggatgggagcatctatttcattNacgtcN
tagaacaccgtcatgaagtcgcggaggatagggtatttcttcatggaggtgacgaaggaNtcggggtccttga

tggtgatgaggagNttgtagaagtcttctagatccttcgggacttcattgagattcgggatatcttcagtttc
gttttcaggaattggttggagNggttcgcgtcgtgtgggggcagaagatgatgatgaggaagaggaggtggaN
gcggtcgcatgagtcggggaggatgaggaggaagtggattggaggggggagtcagaggagacagcgtagtcag
tggaagtggtggaggcggtagaNgaagacatggtagtgaactagattttcgaatgtggggattcaaaagatgg
ggaaatcaaagggagtgtcgtttcttataagttttgagNatgtcggaggcatttccaaagctgcgtgtgggaa
gccatgtgttgtcggtagatggataacccttccatttcacatagtacatcatgccgtttccttgtttgcggtg
tttgaggatggcttcgatttcgtattcttcttctccttctattagttccgggggtggtttggtataattaggt
ccgtggacttcgttttccttgtatggggtaagaaggcaggcgtgaaacaccggatgtattttccattgatcgg
gtaattgtagccgataagtgagaggtcctaacacctcggtgatggggaaggggccttctcgtcgtggtgccat
ctttctggtttcgtatttcagtttgaggttagtagattctaaccatactaagtcattcttcttgaaaggggtg
aagtttcgtgtcgttctttccgctacacgttggctagccagttcatgtgcagcaagggcttcatctcgtgtct
tctggagggattggagNCgttcttgtacagcaggtgtgtcggatttgaggaaaggaaggggaacggcaatagg
gttatagcccatcatgaggaagaatggggaagcatttcgtgcggagtgtaggcgttggttgtgggtgaattcg
gcggtagggaggaattccacccatgtttcggggtgattaacgcaaaagatccgaaggtaggcttctActtctt
gattgacacgttccgtttctccatcagtttgagggtggtatgccgtactgaatgcgagtttgatgccaaggag
tcgtcctaattccctttgtactgcgaggcaaactgggggccacgatcggagatgattttatcNgggagaccNa
atcgtttgtagacttcccggaataaaagatttgaagttcctagggcatcaatNgttttgttgcagggaattag
aattacccccttttgtaagcccttggtctaccacgaccaagagtgaatcatatccatgggatttcggtagatcN
gtaacaaagtccatggagatctgtgagaaaggtcttgggtcgtcgggggtttatcggcattagaggtggtgc
ggtggggtgagtgtttactttggtttgttgacaggtagcgcatccttcaacataattttgacaaagtataca
ttccaggccaccagtaatcgcgacggataagttccagggttttcatgtgaccagggtgtcccatcattggcaa
gtcgtggtatcttgatgcNatgttccttctcagggtttcgtctgggggaatatagcatcggtctttgtagaag
accagttcttcttcgatttccaatcgaaagtgcggatttcatcggaggggtaccccctttcttgagtgcttc
gatggcctgggtaatcacatcctctttggaggaggaggcgagaattgatcacggaggtcggtgtcaatagtNt
tcacaaaNaactctctgggagtaatgtgatgtcttcgttatcggtatcttcttccaggtttaggtcttctctt
cttgataaagcatctgattggaccatttgtgttccagggacatggaccaatttgatgtcgaattcggaNagaa
agagactccatcgtgcttggcgtcgattgagtttttgNgcggtgcggaagtaggtgagattcttNtgatcNga
gaatatggtaattggatgtggggagccttggatgtagtgtctccaggcttccagagctcgtacaattcctagg
agttctcgatcatagatttcgtaatttcgttgagtggcNtcaaaggattgtgaaagataggcgcacggatgcc
agtctccNttcacgtcacgttggcggagtactgctcccgtggcaaactttgatgcatcggactcaatagtgaa
aggtttcgtgggNtctggcatgagaaggactggtgcttcggCaaattttcgtttcaacgtgtcgaatgcttct
tgacattcgggggtccattcccatttgagatctttcttggtgaggttgttgagtggttttgcaatatccgagt
attttccaatgaatcgccggtagaaattaccaaatcctaagaatgagcgtacaccttttacggtagagggggt
Nggccattctttgattcctttgagtttggtggggtccatggcgatttgattcggtcggataatcattccaaga
aattcNacttcgtggtttcaaagacgcatttctccagtttcaggaagaggtcgttNtcctggagtcgttggag
cactcgtaggttctttgtcggtgttcaNataagtctgaggagtggattaagatatcgtccatgtagatgacaa
tccatccttcgttaatcatgtcgttgaagagttcgttcAtcatcatttggaaggtagcgggagattNcacatt
ccaaagaacatNacctgNggttcgtatagtccgcggttggttttgaaagcggccttccattgatgtccttcNt
tgagtcggacNttgttgtacccttggcggaggtccaatttagtgaagattgtggagcctcggagtttgtcgac
NagttcttggataagaggaaggggNtatgagttcttgatggttccttcgtttaggtaacgataatcctggcat
ggtcgtaatgaaccatccttctttcctacaaagaaaaatggggaggccatcggagatttcgatggagtaatgt
atcccttgtcNagNttttctttgaggaattcgtccaatttgacttgttctcccggtgtgagtgggtatatttt
gcaatctttgggtacaaaatctggtttcaagtctatggcatgatcgtagattcggNtNcgggaagcgTtcggc
ggctttcttttcaaagacttgtcggtaNttcNaataaggtttNggaagaagttcttcaatcgttttcggtttg
tcagagagggaatcctgggccatggctgtggaactcgttatttttacacgaatgcggactccttgtcctgtgg
ataccaNtttggatcggtggtcttgagtggtgattccgattcgttggctgggtttgtaaaagtagcgtggtcc
agtatattccggtgtgatttggcgacggtgggttcgaagatcgctattactggttctccttggaggtaNgcc
atgatgagatctccttctggggaaatagggtcggagataggaggtgcagggtcNtggataggggaataccctg
gaaggcagtatatggtatccggttcatcagggtcgatttggtttaggtgNtgNggttcgatctcgtcttcttc
ctcagtgaNatcttcgatggtgacttttcgggagNtgNNtttaggtttcatgtctttttcccgaatggtgcga
atgatattggtggcattgagggagaatttggtgggatctatctccatggtccatgtttccagtcNatgatcgg
gttaatttcccttaNccaagggagtcctaNaataaccgtttcNtttcctaatccggtaaccagtagNcgtgtN
ttgagNgtttgtccttgaagtcgaagatttacccaggtgtaatgggtgatctgtcctttgctatttcgggttc
cgtcgacattgaatacggagatcggttgtagtagttcttctagtggtaggtggtgtttttcggcgaatgtgga
gttgatgaattttcctcctgctccgcagtcaatgagggcttgggtttcgacgttttcatttccttcgatgcaa
ataggtaaatgcatagattgtttatctgtaaccgtgactagtacaggggaaatgaataaTtctggcgatacTg
acgtcgattcagttcgccgatctaaaaaccCtcTtcttccatcttCtttatagctaccgttctttcttcttgg
gataggctggccatgagggcctggatgcgTgtgaatgcaacagttcCatcNggtggtacttCaacaattccag

aagtggatggagtttcgatcttctggatgcggtaAttgtttcgtgaagtttctggtggggttcttccattctt
gtgatctctggcacggtggccaatcttgtggcaNatNaagcattgccccttctgcatgtgttccttctgttct
tctgttgtgagacgatcaacgtccatggcgaatgggtcccttggtgggatatatcgtggggtgctgggaccct
tgggtNNggtgtaNtgggtNggattatttcgttggtgNatttgtttactccttcgNtattggctatcgaatcg
gatggacatttcgtaccagNgatcgatggtggaNggtaNNggaatgaggttNtggattttgtcggtNagagct
ttggctagtccNccttgNaagaacacaatgaggggtccgtcttcNgtNatcttgcttcgaagggccgcttgtc
taaagaggcctacataatcttcggctgttcccgtttgtcgaaggacNgttaactggtgagtggcatccccagt
ggcNtctgctggggagaatgcttcttctactcgtgtgatNaaatcttgNtacgttccccatccacctgttaag
ttcgcaagcctttccatctctgcttctttccagggtcctgccgttccttcatccatataNgaaagagtgaaac
cgatctttgctatatcggtatcataNacNgcggtgtttaggttgagNtagaggttgaccgctttaatgaaggg
tgagcatcgttccctttctcctttgaatatNgcNggagtcctgagtttgggggtcttggtggcggtagtgatg
ggtNNNacggtagtcatcttgggtgctggtgcgggggtttcttctggttcNggggttttgaattgatgtgact
gagtgggagtggaagcttcggattcggaggaagagttatcggcggagttggcagagaatctgtttcgtggagg
tgggcggagtggagaggagtatggttctgtgaagtatgagtctggtaccgctcgataggggtcaacgttgtaa
tgccaccgagaggaagtgttgtcgaagaaNagtgcggtgttttctagccaagttccttggtcgttctggacag
ctcctctaggaagaacctgtaaggagtggggagccacggtgatcggaggggtgtcggtttagatcaatgggtg
ccgaaacctaaagaggttggcaatcggtagatcttgaccttttgttaaggcggatagcctttaccaaggttca
agcgtcgagtatcgtcNaNNacgtcaagtcagagtcttgggtcaataccaacaagactatagtcactccaagt
caggctttgacttgggtatcNatggttcagtgatcaaggttcagaggtaaagggtgtagtattgcctaggTca
agataNagaaNaatgacaatgtatctagggtttatatactagggaccttgaggcgcgagaacatagtacaatg
agaagNaNagacaatgtaaggtaaaagagctgttgaagctcagtcagctatgacatcatagatgagtcaggta
tctaagaatagaggcatgtgttggacatgcgatagaacaatgaagaatcagagaacaatgtaagtatNgtcaa
acaaaggNNNNNgNgaacaNNNNagaaaaagataggggtgggaaccgttgtaaN
>Cligib1_Cluster8#LTR/Copia
GTTGACAAAGGAAGCTCAGATGCCGTCACACAAGTGAGAGCAAACTAATATATTAGCTACCTGTTGACAAAGG
AAGCTATGTAAAGTAGATAGTTAGTCGAACGTCCGTGTCGGTATGTATGAATAAACTAACCTCAGATGCCGTC
ACACAAGTGAGAGCAAACTGAGGTGCGTAAGTAAGAGAACGAGATATATAAAGGGAAAAGAATATGAGACACG
GCGTGTTGAAGCCGTGTCCGCGATGGTAGTGAAACGATGGATCAGTATGAAACAGTAGATACGATGTGAAATA
GAGTAGAAAATGAAATAGTATCGCGCTTGCGTGCGCCGAGCGGGATACCGTTTTTGGTAGGCGACGGTGTCAC
GGGCGTTACCTTCAACACTCCCCTAACCCCGTGGCGCCGTTGTTCTCGATACCCAAGGTACGGCAAATGTGTT
GGTGTGCGATACGGGGTAGAGGTTTAGTTAGAATGTCGGCGAGGTTGTCAGCTGATGGGACATATTCGACGGT
GATGTCGCCCTCCTTGACCCGTTCGCGGATGTAATGGTAGCGTACGTCAATGTGTTTCGCGCGTGAGTGACCT
TTAGTATTGAGGGTGAGCGCTATGGATGCCTCGTTGTCGGCATTGAGTTTCGCCGGAGACTCCTGTGGCATGC
CTATCTCACTCATGAATGAGTACATCCATAAAGCTTGCTGTGACGCTCGCGTTAGGCCCATGTATTCGGCTTC
CGTTGTAGACAAGGCGACCGTAGGTTGACGTTTCGATGACCAAGAGACTGGCCCTCCGCACATAGTAAAGATA
TAGCCGGAGGTCGAGCGTCTGGTGTCCAGGTCTCCGGCATAGTCAGCGTCGACGTATCCGACAGGGCGTATTC
CGGTGGTAGTACCCGGAGTATAGGTAATCTTGTAGTTCAAGGTGCTCTTAATATACGCTAGAACATGGAGTGC
CGCCTTCCAGTGAGCCGGGCCGGGGTTGATCTGGTGGCGAGATAGCAGATTGACAGCGTAAGATAAGTCGGGT
CGGGTGGCTGCTTGAGCCCACATGATACACCCCAAAACCTCGCGATAGGGCTTATCGGCCATAAATAGAGTGT
CCTCCTTGGACGTCGCGCGGTCAGCTTCTGTGAGCATCAATCCCACTGGGAGTGGTGTGGACTTTGGACTGCA
CATCGACATTTTGAACCGTTCCAGGATGCGCTCCAGGTACGCGCGCTGTGACAAGGAGATGGCGCCAGTGAGT
TCGTCGCGGTCAATACGAATGCCCAGGATGAAGTTGAGCTCCCCTAAGTCCTTAACCTGATAACAGTCGGAGA
GTTCCTGTTTGGCGAGTGCAGCACCGGCATCGGTGGACGAGGCTCCGAAGACGTCGTCAGTATACGTACTCGT
GATAGTGAGCTCATCCCCGATCCTCCGAGAACGAACGCAGGAGTCAGCATGCGACTGGTAATAACCCAATCCA
TTGTAGCTGCGACTGAGCTCCTCAGCCCACGCGTTTGCACCATTCATCATGCCATAGACGGTCTTATCAGCAA
CGTAGACCTTGTCCTCCTCCCCTTGCTTGATAAAGCCCGGCGCTTGCTCCGTGTAAACGGTGTACTTGTTCTT
GCTGTTGAGGTACGCAGCAATAAAGTCCACCTGCCAGACCTTAAGGCGGAGCTCGGCCGCGACCGCTGCAACC
ATGCGCATCGACTCCAGACGGACGACAGACGCGTACGTTTCATCGAAGTCCACACCCGGAATTTGAGTGAACC
CCTTTGCAACCAATCGGGCCTTTCGTTTAATGATGTTTCCGTCAGCGTCATGTTTGTACTGGTACACCCAGCG
AAGTCCAATGACGTTAGCCCCCTCGGGGCGCGGTACGAGCCTAAAGACCTCCTTCTCTTTCATTACGCGCATC
TCGTCTTCCATCGGTTGTCTCCAGAGATCGGGCCGCTTCATAGCCTCCTTGAGATTCCGGGGCGCCACGGCAT
CAGAATCACCAGCATTGCTGCCAGTGACGAGCGCGTGCGAGGCGTTGGTAGCCCAGTCCTCAGAATTCTTGGA
AGCCGCGGCAACCCGGTCCTCAGACTCTCGGGATTCGCGCAAAGCGCGCGATGGAACGTGTACGCGGTCACTC
CGGCGGGCCGGTGGTGGGTTTGTCGCCGAAGGTGGTGCTAACGCTGGTGGATCTTCATTGAGGTGAGCCGGGT
GCAGGGGGAGGTCGGTGTGCCGCGGCCGAGCTGCTACTGGCCGCACATCCCTAACGACAGCGGGCGGGCCGGA
GGGACCAGCATCGGAAACGGTTTCTTGTTTGTCATCACTGGCCTGGAAGTCAATAATATCCTCGATTGGAGGA
CGCGCGGTGGTTCCAGTCCGGTGGCCGGTGCCCTCATCGAAAATAACGTCGCGACTCTTGACAATTTTCCCGG
ACGCACTGTCGTAGAGTTTGTAACCCCCACGGCCATAGTAACCAATGAGGCGATATTTTCTTGATCTCGGCTC

AAGTTTCGACGCTCCAAGTTCCGCCGGGATCTTCGCAAAGGCTGTGCAACCCCACGGCCTAATGTGGGAGACG
TCTTGGCGCAACTTGGTCCACGCCTCCGCTGGGACCTGGTCCGGGTGTCGAGACGATGGGATAAAATTCCGAA
GATAAACAATCGTGGAGGCGGCCTCAGCCCAGAGGGATGGGGGAAGTCCAGAGTCCGCCAGCAGGGACCGTAC
CCCCTCGAAAACCACACGCATGCCACGTTCAGGGACGCCGTTCTGGGAATGTGCGTATGCCGTGGTGATCTCC
GATACGATTCCATACCCCTTTAGGTAATCGTCCCATAGGTTGTTGCACCACTCGCCCCCACCGTCTGACCGCA
CTATCTTCAGTTTCCTGCCTGTCTGTCGTTCAGCCTCGGTATGGAAATCACGGAATGCCTTGAGCGTCGTTTC
GGCAGCTTTGTCGGCGAGGAAATAGGCTTTTCTGTACGAAGACGCGCCGTCGGTTATCAATAGCATGTAAAGC
GCTCCCCCAATGGATCTGACACGCGCGGGTCCCCAAAGGTCTATGTGCACGCGTTCCAGGATTGTCGTTTCGT
GAGTGACGATTTCGTCGTACGGCCGAGTGGTGTGTTTACCATAGATACAGTCTTCGCATAAACCTTCAGGTTC
TGAGTCGTCTGTGATGTGAAGGCCGTCTACTAAGTCCTTCCTCTCCATGTTGCGGATAGCGCCAATGCCGGCA
TGTCCAAAGCGCCTGTGCCAGGTGTCGATGACGGTAGGTTTCTCATGCGAGCGTGCGGTGAGAGCCGCGGGCA
CGTTCAGGCATTCAACAAGGTACATCCCGTTGCTCCCCTTTCCAAGTAACATCAGGTTATCCTGAGCATCATA
GAACTTGGCCGCGCCGGACTCAAAAACTACGCGGTAGCCGGCCGAATCGAACTTGCCAATTGACAACAGATTC
GAAATGAGATCGGGGGTATGGCAGGCGTTGGTGAACGTGACCGTCGACGTCCCACTATCGGTGGTAAAAACCT
TAGTGACGGAACCCCTCCCCACGATCCTGAAAGTGCTCCCCTTACTAGCCGCTCGCCCGAGAACCGGTGGATC
GAGAGGAGTATAAGTCTCAAAGTCTGCGCGATTCACAAAACAACAGTCGCTGGAGCCCGAGTCCGCAAAGGTG
AGTACTTGATTATAGGGTGGCAAGTTTACGTCTACTGGGATTGACGCGGGTGGTGTGAGCATCACCGCCGGCG
AGGGGGTATCGCTATTAGCTGAAGACGTGTAAAGGGCATACACCCTGTCCGTGGTCGATTGCGCGGTCGCACT
CCCGGAATCGGCCACAGCAGCCGTTGGCGTTGCAGCCCTAGGTATGGCCTGAGTCGTGGGTGTTGTAGCGACT
CCTCCCCTCTGTCCGAAACCAGGTGGGAACTGACCCTCCTTTCCGCCCCCTTTCCAGTAGCATCTCTCGATGG
TGTGACCCTTTCGCCTGCAGTTGGGGTTTGTACAGACAAGTTGTTCGCGCTTCTTTGTGGACGACAAGGTTTG
GAGTGCCGTTACAGATCCTGCGGCCGCGTGGACTGCCTTGCCCTGGGTCATCCTTTCCCATTGTAAGGTAACC
CTATCAATAACGGCATCCGACGTCCTGGTGTCATACAGGGTGCCGACTATCAGATCCCAGGAAGGTGGTAGAG
ATGCGAGAACGATGGAGCGAAAGTCGGCGTCGGTGACCTTTCCCCCCGCTGCATTAGCCTCAAGCCACTTAGC
GCGGAGCTTCTCGATGTGAGTAGGCATATCCTCGCCGTCGCGATATAAGCAAGTGCGAAGAGCCTGTTGGGCG
CGGACCATCACCATGTCGTTGTCCACGCGATTGTATCGATTGGTGAGCGTAGTCCATACCTCGGCTGCGGTTC
CATCTAGATTAACCCCTAAGCCGACAGGATTTACCAGGTTGTCTATAAGTAGCGTCATGGCCCACGCATTACG
CATGTTCCATTCATTTGTGGAGGGGGTGAGCGACCACCAAGGTGTGGCCGCGGCGGTGTCTGGGTCAGGTAGG
GGAAGTGGGGATGTCTCTGTGTCTTCAAGTGTAGGTCTCTCCACGGGTTCTTTGATAGTGCCTTCTAGGTAGC
CAACAACGCCACGAAGTTGGGCGGCCCGGCGCACGGCGCTCTTCCAAACGATCCAATTGGAGCCGTTGAATTT
GTCCTTCTCGGCGAAAAGGGGTGGGTGGGAAGACGATGTAGTGGTAGTCATTTTGTGTAGATAATAATGTAAC
GAGTTTGCGCGTGTAACAGTATCCGTGTCGTTCAGGCTGCAGGGCCCATAACCTGTTGACAAAGGAAGCTCAG
ATGCCGTCACACAAGTGAGAGCAAACTAATATATTAGCTACCTGTTGACAAAGGAAGCTATGTAAAGTAGATA
GTTAGTCGAACGTCCGTGTCGGTATGTATGAATAAACTAACCTCAGATGCCGTCACACAAGTGAGAGCAAACT
GAGGTGCGTAAGTAAGAGAACGAGATATATAAAGGGAAAAGAATATGAGACACGGCGTGTTGAAGCCGTGTCC
GCGATGGTAGTGAAACGATGGATCAGTATGAAACAGTAGATACGATGTGAAATAGAGTAGAAAATGAAATAGT
ATCGCGCTTGCGTGCGCCGAGCGGGATACCGTTTTTGGTAGGCGACGGTGTCACGGGCGTTACCTTCAACAC
>Cligib1_Cluster32#LTR/Gypsy
GTAAGGACACGCTAAACACCAATGAAGTGTGCGGTCTCTCAAGATGTCTCAGAGGAAGTTGGATGAATGGAAG
CCGACTGACGATGGAGAATCCCACTGTGAATAGCGGAAAACCAAAGAGAATCAGATTGAGAAACAAAACATAC
AAACAACAAGACTATATACATAGGTAAGAGCCCGTGAATATAATAAGTACAAACAACATGTGTTTACAATGAG
GTGAAACCGCGGTTCGCTTACATAATCAGTCACGTGATCAGTCATGTGATACTACAAATGAAAAGAGAAAAGA
AAAGAAAATACAAGTCCTGACACTACTCCCTCTTTAGGGATTTTCTCTCCGGGAAAATCTTGAGCGGTTCTTC
ATCATCCCTGATAGTGCGTCTCGCATCCAAAAGAATGCGCACATATCAGTTCGAAGCACTTGAAGCGTTCAAC
TTTGAAAATAATGCCGAATTTGATGGTGGATCGGTGAAAGTTTCATATGGGGCGAACAAAGAAAGAAATTCGT
CTTGAGTTACGTTGAGACGAACTTTTCGTGGAGCTTGTGGATTTGTTCGATGAAATTTGTTAACGAGATCCAT
GGCATTCTTCAAATTTGATAGTGGTTCCCATGTGTTGTGTTGTGGCGTATATCCTTCCCATTTAACCCAATAT
TCTAAACCTTTGCCGTTCTTGGTGAGACGTGAGTTTTGAATTTCTTCGACGGTATATTCAAATTCGCCATCGA
TTTCTACTGGAGGAGCAGGAGGGATTTGTTGTTGAGGGATTTTAGGAGATTGGTATGGTCGAACTCGTGAGAT
GTTAATGACGGGGTGAATTTTGATTGTTGATGGAAGTTTAAGTTTCACCGCATTGGTTCCAACAAGTTCAAGA
ATTTTAAACGGACCAACTCATCTTTCGGTAAGTTTCTTTGATGGTCGTTTGAGATCGAGATCCTTTGTTTCGA
GCCATACCATTTGTCCAACTTGAAATGATGGAGTTTCGCCACGATTTTGATCGGCGTATCACTTCATTTCGTC
GCGCGCTTTAACGAGGGCAGATTGTGCTTCTTCTTGAATTTTCTTCATTCGTGTTGCGAATTCATTAGCTGAC
TCATTTGTTGGTTGTCGTATTGGTTCGGCGCCCATTCGAGGGTGACAGCCATAGACATTGAAGAAGGGGGATA
TTTTCGTAGATGAATGAGTAGCATTATTATATGAGAATTCGGCAAGAGGAATCCATTCAACCCAATCGGATTG
AAGATGTGATACGAAAATTCTAAGATATGCTTCAAGATCTTGGTTAACTCGTTCCGTTTGACCGTCAGTTTGA
GGATGGTAAGCGGTAGAAAGTTTGGTTTGAATATTCAATAATGAGTTTAATTCTTTCATCATTGAACTTGCGA
ATTGTGGTCCTCGATCGGAGATGATTGAATCGGGGAGACCATGGAGCTTCCAAACGTTTTGCAGATATAGTTT

TGCAAGACCCGTCGTAGAGGTTTGAGATGTAGTAGGAATGAAATGCGCCATTTTTGATGCGCGGTGATAAGCA
CGCCGCGCGCTTATGGGTTCGCAGCTCACATCTCATACTGATATTGGGCACTTTAGGTGCACATATCGCATTC
GTACAGCACTGATCGCACCCTTTCCGCTCCGAACGCGTCGAGATCGCGTCTCAATTCCGATTCTAGTGGTAGT
CTTCGCATCCTTATCGCATCACTCCCCACGCGGATCGCATTCCATTACATAGTTTACTTACCTATTTACTGTA
CCTCATGGCCGTTTAGCTCCGATATTTAGAGAGCTCGGTAGCTACCATGATCAATAAGAAAAGTCTTCACCTC
TCAGTTTACATTTCGCATGCTTCGCATGCAACACTCCTTTAGGGCAGAGCCCTCGCGTTGATCTCGCCACAGT
TGGGCTAGGAAGTCTCTCTGATTTCGGACAACACTCGCATCCACAAGACCTTATCTAGGTACTTGTGTTATGT
GTTGGTTTTGTGGTTGGTTTTGGCCACAAGCCTCGCAGCATATCAGCGGTCGACAACAACAACTATTGCGTCG
TATCCGTTACTCTCAGGAAGTCCGGTGATGAAGTTGCAAGATATTGCTTTCCACGGTTCGGAAGGGGTGATCA
TTGGAACAAGTTTTCCAATAGGAGGAGCGGGGAAGTTTTTGAAACATTGGCAAGAGTCGCATCTGTCGATGTA
CGAATGAACGAATTTTGTCATGCCAGGCCACCAATAATTTCGCGCAATTAATTCCAGGGTTTTGTAATGTCCT
GGATGACCGGCAATACTGGAATCATGGTGGAGTTTAACAATCTCTCGTCGAAGTTGAATGTCGTTGGGTACAT
AGACTTTTCCTCGGAACAATATAAGATCTTGTTCCTGAGACCATTCTTCAGATCGTAAACGTTTCGTGGGTGA
TCGTTTAAGTTCTTCGATCGCTTTGACAACGGATTCGTCATAGTCTTTCAATTTGCATATCTTCGAAAGTACT
CCCTCTTCTTCGCCATTAACAAGAACGTGTCCTTGTTGTAATGCTTTAATAGCGAAAAATTCCGGTTTAAGGA
GTGTGATGTCGTCGTTATCAGTTTCCACTCCCTCTTTTAGGTCTGTCCTCCTGGACAGTGCATCCGCTTTTCC
CATTGAGGAGCCCGCCTTGTGGATTAAAGTAAAGTCAAATCGTGATAAGTAAAGTGCCCATCGCGCTTGTCTA
CGATTTAATTTTTTACTAGTCATAAAGTATTGTAGATTACGATGGTCTGACCATATTTCTATCGTATGAGTAC
TACCTTCTAAATAATGGCGCCAGGCCTCCAGACAACGTATAATACTAAGCATTTCCTTGTCATGTACATCATA
ATTACGTTCAACATCAGATAAGGATTTAGAATAATATGAACAAGGCCTCCATTTGTCGTCCGAACATTTCATA
GATAATATACCTCCCGTGGCAAAGTCTGAAGCATCCGATTCTACTCGAAGGGGTTTTGTTGGGTCCGCTGTAA
CCAATATGGGATTGGATGTAAATCGGTTTTTAAGCAAATCAAACGCTGTTTGTTGTTTCTCTGTCCAATCGAA
TTTGGAATCCTTTCGTAAGAGATTATTGAGTGGAGCAGCATGTTTTGAAAAGTCTTCGATAAATCGTCGATAG
AAATTAGCGAAACCTAAGAATTTGCGGACGTCGCAGACTGTTTTAGGAATCGGCCATTTTTTTACTGCATCGG
TTTTGTCGTCCATCATACGTACTTCTCCATTTCCAAGTATGAGTCCTAAAAATTCAACTTGTTGAACCGAAAA
TGAGCATTTTTCTGGTTTCAAGAATAAGTCGTTATCGAGGAGAATTTGGAGAACACGTTCAGCGATCCGTTCG
TTTTCTTCTTGAGTATCGGTAAATATTAAGATATCGTCTATGTATACCACTACATGTCCTTCATCTATAAGTT
CTCGTAAAAGAGCGTTCATCATAGTTTGAAAAGTAGCCGGTGAATTGCACAAGCCAAAATACATAATGAGCGG
TTCGAAAGAGCCTCTATGGGTTGTGAATGCTGCTTTTGGTTCGTCTCCTTCACGCATGCGAACATTGTTGTAT
CCCCATCGTAAGTCTAGTTTTGTGAACCATTTAGATTTGCCAGCTTTGTCAATAAGATCGGAGATCATTGGGA
GGGGATAATTGTTTTTGATTGTCCAAGCATTGAGTTGTCGATAGTCTTGGACCATGCGTTTCTTTCCGTCTTT
CTTGGGTACGAAGAATACAGCGGATGTTTGAGGAGATTTGCTTTCTCGAATATAACCTTTAGCTAGTTGATCT
GCTAGAAAGTCGTCGACTTCTTTCTGTTCGGTAGGAGAAAGATTGATGACTTTGGCTTTCTTAGGTTCGTAGC
CGTCTTTCAATTCTATAGCGTGATCCCAGGGTTTTCGTTCAGGCATGCGTTCCGACGCTTTCTTTTCAAAGAC
TTTGAGCCAACGTCTAAATCGTCGAGGAATTTTATTGACAAGTTCGGGTTCCTCTGTTTCGATATGTATGTTG
TCATGGTCTTCGGCTCTTTCTATGGATCGGACAAAGTGTTCATCTACTATTTCATGAACCACTTCGTCATAAT
TAATTTCTTCAAGTAATGCGCCTTCTTGTTCAGTAATAAGGCCACCAATCACTCGATCTGCATCTTCGTCATC
GATCTCTTCAATCGTGGGTTGGTATTTCCATCGATCGGCTAGAGCTTTACACTTCTTTTGTTTTTTAATAGCG
CGTACTCTAACATTACACTCTGGAGGGCATCGAGTGAATGTAATTTCGCCGGTAGACCAATCGATAGATGGAT
TGTGTTTCTTCAACCACGTGTGACCGATTATGATTGGGACTTTTCCTAAGTCCGTGACTTCAAAGAGCATCTT
CTCCTTATGTCCGTTATGAACCATCATTAAATAGACTTCTTCTGTGATTGATCCTCCTTGATTTAAGGTACCG
TCCACGTTAAATACTTTAACCGGATATTTTAAGGTTTGGGTGTTCCATCCCATACGTCGTACATAAGCTCAAT
CAATAAAGGATCCAGTTGCACCAGAATCTAGGAGCGCTTGACAAGACGCAGTTTCATGAGTGTCTACTGTCGT
CACTGATATAGATAGTTTTGTCTCGCGTTCCAAATTGAGCGTGCGAATAAGAAGTCTTTCTCTATAAATATGA
CCAGGACGTTCCGAAGATACGTTACCGCCGGGACCCACAGAGCTATCGACTCTGGGACGGACGGATGTTCCAT
TTAAGGAATCCTGGTGGGTTGACATGACCATAACGCCGGGACCCTGATTAACAGGACGGACGTCTATCTGATC
ACCAAAACCACTTATTTTTGTCTCCACTAATTTTTCTGTCGTTGTAGGTTTTTGAAAATAATGAATTGGGGCG
GGAATTAAGCACGCTTCTTTAGCGCCAAGCTCCTTATGGGAGACCCCCTGAGATACGATTACATTGGAAGGCG
TATCTATATTTTTGACAGTGTCGACAGATAAAAGATGAAATTTGTTTTTAGGCAACGCTAGTGGTGGAGATTT
CACTGCGCGCCTCCTTGAAAAGACGATTGGGCGTTGGATTGCCCCTGAACTTGTGAATCCGGATTGACTTCCG
TGATTCCTGATGTTGATGGCGTATCTTGCCAAATCAACCCTTCTTCAATTGCAACCTGTAAATAATGGTCGCG
TGCTTCATCGTGTGTGGCTGAATTTAAATTTATATTGGCCCTGCAATCTCGCATTATGTGGCCGGGTTTACCG
CATCGATAACAAACTCCAGGTGGGCGACGGTTTCCTCGGTCAACATCCATTGCATCCGGGGCACGTGGTGGGG
CAGGTGGATTAGGGCGAGCTTGTTGATTAGGTCGGGCCGGTTGAGCTGATTGACTAAAAGCCGGGCGATTGAA
ACCAAATGTAGGGCGAGCGGGTGGAGCCTGTTGATTAGATCCCATATTTCGGTGGGGACCTTGTGTCTGCGTG
GTTCCTGTTCCGGTTTGCCGTGGGTTTGACGATGAAAATGCCGGGCGTGAGAATGTTGGTTGGTACAACTTTT
TCTCTTCCTGGGATTGTCGATATTGCCTGTCTAGATGTGATGCCCGGATATACCAGTCTTCAATGGTATTCGG
TTGATATTCCGATTCCATTAGCCTTTACGAAGACCTTCGTTTAGAGACCGTTTGAATTCCTTAATCAGTGGAT

ATCCACTGTATCCTGATCCGTCGGCTGTTTTCTTGAACTCTTGGATATGTTCTTCTGCCGGTAAAGATCCCTG
TTTCATGGTGCATAATTTAAATGCTTTAGTTCCCTGTGGATCGTGATCGGAAAACGCCAGTTTGAATTCTTTC
CAGAAATCGTTAATAGAATAATCCATTTGTATTGGATTCCGGTTAAATTCTTTAATCTTCAGATCACGCCATG
TTGCTGCTGATCCTTGAGAACAGAGATGTAAGACCCAGTATACCGCGCTAGTGACCGATGAGAAATCCGCGTG
TCGAACTTGTAGGTAGATGCGACATGCATTTTCGAAGGTGTCCGCATTAGCCATTGTACCGTCGAACGGTGCC
GGATTTTGGAGTTTAGGCGCGCGAGATCCTTTAGCCGCATTAGAGTATGAACCAGGGAGGGGATTAAATCTGT
TGAGGGGAGATGGTTTAGAGTCGCGTTCCCGTTTGTCCTTGACCAAATCTGTGAGGAGTGCCTCGAACCGATT
AAACCGTGAGTGGAGTTCATTGTTCTGTTCCCATAGTCTCGAATTCTGATCTTGGAGAACTTTGACGTGGTTC
TCGAGGGTTTGGATCCGTTGTATGAATTCCGTGTTTAATTCCGTGGTGTGTCTCATTTGATCATAGATCTCCG
TGAATTTTTCAGCATCCGTTTTATAAGTGACCGTGTCGGGATTAACCTGAGAAGTGTTGGGGGGTTCATATTC
TGGATTATAATATTCCGCAAAATTGTTACGAGGTGTCGCCGCTTGCGATCCTCCCCAATTTGCTGAGTCTTCA
GACATGGGTGGTGTTTTGGAGAAAGTAGGAGAGGGGTGTGCGGATGAGTATGGGCGGAAATGTGACGGAATTT
TGAAAATGAAGGGGGCCGCGGGTATGGACTTAATCCAGTAGACCGGCGGTGATCACCTGCTTCAAGGATAGGG
ACGGGACAAAGGTGAGATTCGAAATTCTCGCCTGCTCAAACCGTGTAACGATAGCGAATAGGATGAGAGGTTG
CAAGCCTACTCAAACAGTAATGGGAATGATGATGCCGCGAGAGCCTGTCCAGGCGTCTGACTGATACTAGTGG
CTAACCCTGAAACGCTTATCCTGCGCCAGGTGTGGGCTGAACTAGCAGTGAGAGGATGTCGGGGGAATTCTGT
GTTCTGGGGACTTGAGGACCCGTAAGTCAAGCAGCCGTGTTGAACACAGATGAAGACCGGGTTGTTGCCAGAA
AGAGAAGGGAAAGCAGCCGAGTTGAACTCAGATGAATCCCTTCTTCTGAGTGGATAGAGGTGGTGTTAATGAC
TGAAGGTGGTGGATGGCTTACTCCGATAGACTAAACTCTTGAGTGGCGATGTTGTTTCGCGCACTTCGATGTC
TCTTTGGAGAAGCGGTGTTTGAGTGTCGGTGAAAGATGAGAGAGGGCACGATTAAGGCATGTCACGAGTGCAC
TCGTGGGTGATCCGGATCCGCAGCTGTGGGTACCCACACTCATGAGTCAGTTACTCATGAGGATCTGGGTACT
CACAAGTTACTTGTGGATTTATACTCTGCACCAAATTTCAACATTGAGGGGTCGATTTGACACTCCTAAGGGT
GCTTATCAATATCCAAAAGAGGCGATAGGGATACCTGTCCCTGATCTTGAGTTATAGGGTGCGCATTGCAGGG
GGGGAGGAGGAGAAGGTCATGCTCTGGGGTCAATTCCCCACAGAATTACATGATTGTGAGACGCATTTAACAC
TATTTTACGAGCTGAACACGATGCGAATGACGCGAAAGGGATCAGTGTCCCCGATCCCAAATTATGTTCAATT
TGGCAGATTTTTACTCTGTATCTCAAGATCAAGGTCACCGATCTCTCTCGCTGAATTCGCATCGGATTAAGCT
CATAAAATAGTATTTAATGCGTCTCACAATCATGTCATTCCGTGGGGAATTGACTCCAGAGCATAACCTTCTC
TCCCTACCCCCCTGAAATGTGGACCCTATAACTCCTAATTTGGGGCAGGTAACCCTGTTTTCTCTCCCAAACA
AGGGTCCACAAGTACCCATGAGTAGACCTGCAAAAGACTTGGATCCATGAGTGTAAATACCCACAAGTCTCCC
ACGGACCTACTCATGAGTACTCATGAGTTACCCGCACTCGCGACATGCCTTAGGCACGATAAGAGAATCTGAT
TCTTCTGTAAGGACACGCTAAACACCAATGAAGTGTGCGGTCTCTCAAGATGTCTCAGAGGAAGTTGGATGAA
TGGAAGCCGACTGACGATGGAGAATCCCACTGTGAATAGCGGAAAACCAAAGAGAATCAGATTGAGAAACAAA
ACATACAAACAACAAGACTATATACATAGGTAAGAGCCCGTGAATATAATAAGTACAAACAACATGTGTTTAC
AATGAGGTGAAACCGCGGTTCGCTTACATAATCAGTCACGTGATCAGTCATGTGATACTACAAATGAAAAGAG
AAAAGAAAAGAAAATACAAGTCCTGACA
>Cligib1_Cluster30#LTR/Gypsy
GTTACAGCACTCCTAGGGGGCTTACTCTTTTTTCTTACATCATATATGGACGCTTCTCTCTTCGACATATGTT
GTACATGTGTTTGCCTCATGGACTCCTCATGGGCATCTTCTGACCTACTGCGCCTCTCGGTTTCCTCATGGTT
TACAGCATATCTTCCCCTTTAGTATATATTGCTGTAGAGTCCCTCAACATGCTTCCCCCTCATGGTCACTCTC
CTCTAGTATATATACATCTCTTGTACATCCTGTATTAGCATCTTGACCTAGGCAATCTACCGCCTTTACACTC
TTACTCAGAACCTGAGCCTAGACCAGCTCAGAGTGACTTGAACTCTTTTGTCATTGGACAAGAGACATCTTGA
AACTACTCTCAAGATAGAACTAGAACGAACGATAAACTTGTATCAGTTGTGAAGGCTCACGCCCTAACAAAAG
GTCAAGATCTCAATCACCGCCAACCTCTTTAGGTTCAGGCGCACCACCACCCCACCAGAAACCCCAACAGAAA
CACCACCGTGGCTTCCCAGTCCTTCCTGATCAAACCCTTCGGCACCACCGTTGTTCAAAGAAGAGGCGAAGAC
CAATTTTGGCTCCTTGAAAAACAGATCGCCTTCCACCCAGACGAAAGACAGTGGTACTTCATTGAAGCTAACG
AACAAGTACCACACACCACAGATTATTTCCACATTGGAGACGTTGCACCAGAAACTTTCACCAATACCAATCC
CTGCCCCCCCAGGCACAGAAGTCACCTACATACCAGTTGAAGAAAAACTCGAATATACCGACACCCTCGAAAT
GACTACCGAAACCAATACTCGAAGCTTCCTATGCGCACCCACCAAGTATGATGAAGAACGCACCAAGACCAGC
ACCTTTATAAACGAAATCCTATTGCATATAGCAGTCAATGATGGACTACTAAACACAGACAAGAAGAAGGTAG
CATTCATGCTATCATATATGGTTGGAGGAACAGCTACTTCCTGGAAAGAAGCATCTATCACCTCCTACACCAA
CTCTACCATTGGCTGGCCCACATTCGACAACTTCCTAGCCCACTTCAAAACCGCATTCGCCCAGACAGATGTA
GCAGGAGCCGCCAGAACTACCTTGCGCACACTACGACAGACTGGAACAGTCGACGAATATAACGCCAAATTTC
GAGTTCCAGTATCCCTCTGTGAAATCACCGAGGATGCGGCCCTCATTGAATACTACATGGAAGGACTACACCC
CGAAGTAATGAAGAAAGTCTACACTATGGAAAATGTAATAAACTGACAAGTTGTTTCACTGACTGATCTGCTA
TAGAGTATAGCCAGACACAGTTGATAACCCTCTGATAGACACTCTAATCACTTGATATTGATAATAGAATGCA
AGTATAGGATTGTCTAATACTGTTGTCAAGGGAGTAGCAAACACTACTCCAGGACATAGTAGGAATTACACTT
CCTTTGTACATGTTTGGCCTGTTTCACCCAAGGCGTGAAGTCAGGGGTCCACTATATCGCGACTGAAATGATG
GACTTAGAATATTCAACAATTAGGCCTCTAGCTGCTCTAGGAGGCAAGATATCAACAAAATGAGTAACAAGCA

CTACGAACCGCTATTGTAGATACTAAATGCGGTCCATAGGGTGTCTATAGGAGTCCTATGTATCATATATGTA
CTCGGGGGTGCTGGGAAGCGCAGATGAAGTGATTATCTGTCAGTACCAATGTACTGTCATCCCCACTTCTAAC
AAATGTTCCATCCACCCTAGAAAAGTGGTACCAGCAGCCTCTAGGATGGACAACCAATACCGAAGAGCCAGAG
CCAGAGCCACTCGCTTTTCCAGCAGACCCTCCCCCTCCCCCCAGAAGAAATTCCAAACCCCCAGATACGTGAC
ACCCAGAGACCCTGATGCCATGGATGTGGATTGACTCTCCATTGAACAACATGCTGAACACATGAAGAAGGGA
ATGTGCTTTGTATGCCACAAAACTGGCCACTGTGCCATAGACCACAAGAATGGGAAACTCCCAGAAGAAAGAA
AACCCTTTAGGGTTAGAAAGATCGAACCCACCCCACCAGAATCCTCCAAGGACGGACCTACCACTTTTGCCAA
AATCCGTGCCCTGATGAAAGATCTAGCAGAAGAGGAGAAAAAAAAGGTGTTAGAAAAGATGTATGAAGAGGGT
TTTTAAAGAGGCAAACTGCATCAACGTCAGTCTCGCCTCCCATCTCAATTTCTCCTGTATTAGTGACTGAAAA
TGATCACCAATCTATGCATTTACCTATTTGCATATCGGGAGAAACAACTGTTGAAACCAAAGCCCTCATTGAC
TGCGGAGCAGGAGGCCAATTCATTGACTCTTCCTTCGCCAAAGAAAAACAACTCACACTTGAAAAACTCTCCT
CCCCCATCACTGTCTATAACATTGATGGCACCCGAAACAAAACTGGACAAATCACCCACTACACCTGGATCGA
CATCCAATTAGGTGAACAGAAACTCCACACCCGTCTATTGGTCACTGGCTTAGGAAAAGAAACCATTATCCTC
AGACTCCCCTGGCTCAGAGAAATCAACCCCATCATCGATTGGGTAAAGGGAACTCTTGAAATCAATTCAGCAA
ACCTTGCTATCAAGAGTACTCAGATCTTCAAGAAAATCCAAGACAAACTCCGAACCAGGAAACACATATCTCC
AAAACCTATCTCTACACCTTCTTTTCCCAAACCCACCCTCGAAGACATCACCGATGAAGAAGAAGAAACCCCT
ATCAACGAAACAAAGACTGAGACTCCAGACATCATCTACTGCTTACCTGGCTACACTCCATCGGACACCCTCA
AACACCCACCCATCCTTGAACCAGATGAACTAATCTTGGCATACATCCAAGGCGAACCTGTCATCGGTATCTT
TGAACCCACCCCCGAAGCTCCCCTCACCGTAGAACACAACACACCCTCCTACTTCTACAAAACCATAAATCGA
ATAGGTATAGTCAACAAGAGTCCTTATGGACAAGACATTCGAATTGGAGCAAAGATCACTTCATCACAGGCTT
TTGCCCAGAACCACGAACCCCCTGACAAACTGAAAACCACTGAAGAACTCCTCCCCAAACCCTATAGGAAATA
TCAACATGTCTTTGAAAAGAAAGCTGCAGAATGATTCCCAGAATCCGGAATTTATGATCATGCAATAGACCTA
AAACCTGACTTTATCCCAAAAGACTGCAAAGTATACCCTCTTACACCCGGAGAACAGATCAAACTGGACAAAT
TCCTCAAAGAAAACCTGGATAAAGTATATATCACTCCTTCAAAATCACTTATGGCATCTCCATTTTTCTTTGT
TGGCAAAAAGGATGGTTCTCTAAGACCTTGCCAAGACTACCGGTATCTTAATGAAGGAACAATCAAGAACGCC
TATCCCCTCCCCCTCATCCAAGAACTTGTGGATAAACTCAAAGGGGCTACACTGTTCACCAAACTCGATCTCC
GACAAGGATACAATAACATACGAATGAAAAATGGACACCAATGGAAAGCAGCCTTCAAAACCAACCGTGGACT
CTTTGAACCACAGGTAATGTTCTTTGGAATGTGCAACTCACCTGCTACATTCCAGATGATGATGAACGAACTC
TTCCGAGATATGCTTGACGAAGGATGGATTGTCATATACATGGATGACATACTAATCTATTCTGATAACCTAG
AAGTACACCGAAGACGAACCCTCCGAGTCTTACAAAGACTCCAAGAAAATGACCTATTCCTTAAACTGGAAAA
ATGTGCATTTGAACAAAAAGAGATTGAATTCCTGGGTATGATTATCTGTCCTAACCATATTGCTATGGACCCT
GCAAAGCTAAAAGGAATCAAAGACTGGCCTACCCCCACCAATGTCAAAGGTGTCAGATCCTTCCTAGGATTTG
GAAACTTCTACCGAAGATTTATTGGCAAATACTCCGACATTGCCAAACCACTCAATAATTTAACCAAGAAAGA
TCTTAAATGGAACTGGACAGAGGAATGCCAGACAGCTTTCGACACCTTAAAAGCCAAATTTGCAGAAGCACCC
GTGCTCCTCATGCCTGACACTACAAAACCATTTGTAATAGAATCTGACACCTCCGAATTTGCCACTGGAGCAG
TCCTCAGGCAAAGGGATGTTAACGGAGACTGGCATCCCTGTGCCTATCTCTCCCAATCCTTCGACGCAACCCA
ATGCAACTACGAAATTTATGATTGAGAACTCTTAGGAATTGTACGTGCCCTGGAAGCCTGGAGACATTACATC
CAAGGATCCCCCCACCCCATCGTCATATTCTCCGACCACAAGAATCTCACATACTTCAGGATAGCTCAGAAAC
TAAACCGACAACAAGCCAGATGGAGCCTCTTCCTATCCGAATTTGACATGCAACTGGTCCATGTCCCTGGAAC
TCAGATGATACAATCTGATGCCCTATCCTGATGAGAAGACCTAAACCCCACGGAAGACAATGATAACAAAGAT
ATCACCCTTCTCCCTAACTCCCTTTTTATAAAAACCATCGACACTGATCTTAGAGATCAGATACTCTCCTCCG
CCTTCAAGGATGAAATCATCACACAAGCTTTAGAAGCAATCCAAAAAGGGGGTACCCCACCCATGAAATCCGC
CCTTTCAGATTGGAGAACAGACGAAGGACTCATATTCTACTAAGGCATGTCACGAGTCTACTCGTGAGTGACT
CGGATCCGTGGTCACAAGTACTCACAAGTCAAAATTTGATACTCAGATCCGCATGAGTTTCGTACTCACAGAG
ACTCGGATCCGTGAGTATATTACTCACAAGTATTCGGGTACTCACAAGTATCTATCTATATACACATATTTAC
TGCAATTTACCTGGTAAAAACATATGAAAGCTACATAAAAACCAGAAAAACAGCCTCGCGTGTGTGATTGGCA
CATCCTAGGCCTGTCGTCTCGTTGGATTCATGAGCTAGAGATAAAGATGGCCCTTATGAGCACCAGGATGATG
CGAAACGGATGAGAACTCACCCAGATATGATGCATTTTAGCCTGAAAACCGGAAAATGGGCCCCGAGTGTATG
GTCAACATGTCATACACCTGTCGTTTCGTTGGAATGATAATCTACATGCAAAATCAATCCTAGTGAACACCAG
GATGACCCAGAACAAATAAGAGCTCACAGAGTTAGGTGATATTTCCATAATAAAACCGGAAAATCAGCTCTAC
ATGTGCAAATGGCATGTCCTATGCCTGTCGTCTCGTCGTATTCATGATCTACGCGTGAAATCGATTCTGGTGA
ACACCAGGATGAAGGAGAACGAATGAGAACTCACAGAGTTATGATATATCCACCCTAAACCCCAAAAATCGGC
CCCATACTTGTAATCGGCACACCCTATGCCTGTCGTCTCGTTGAATCCGTGATCTACGCGTGAAATCAATGCT
GGTGAATACAAGGATGAAGAGAAACGAATGAGAACTCACAGAGTTATGATACATCTACCCCAGACCCCAAAAT
CGGCCCCTCATTTGTGATTGGTACATCCTATGCCTGTCGTCTCGTCGGATTCTTGATCTACACATGAAATCCG
TTCTGGAGAACCCCAGGATGATGCAAAACAAATGAGAACTCACATAGCTATGGTCATATCCACCTGAAAACCG
AAAAATCGACCTCACAGGTGCAATCGGAATGTCCTATCCCTGTCGTCTCGTCAGATTCATGATCTACAGGTAA
AATCCATCATGATCAATCCCAGGATGAAGGAAAACGGATCAGAACTCACTGAGATAGAGGAATTGAATGTACC

TGAGGCTTGAACAGACTGCAAAATTGGATGTTTTTCATGAACATTTTGTTAGCCTCTGGTGTGGGAATAAGTG
GGTGAGTACTCATGAGTACTTGCATGGGTACTCACAAGAAAATAATCCATACTCGGACTCGCAGGGATCCTCG
ATGGTGAGTGATCCGGATCCGTGAGTAGGCATACTCACGGGTATTCCCACGGGTCAACTCACACTGACTCACG
AGTTACCCAGACTCGTGACATGCCTTATATTCTACAAAGACAGATGCTATGTACCTCCAGACAAAGAACTCCG
CAGAAATGTGGTAAAGAAATACTATGACCTCCCCTCCATGGGGCACCCTGAACAGTTCAAAACACTTGAACTA
ATCAAACGAGACTACTGGTGGCCCGGACTATACACCTTTGTAAAGAAATTTGTAGAAGGATGCACCATTTGTC
AACAAACCAAGGTGAACACCCACCCGACCACCCCACCCATAATACCTAGGACCCTCTGACAACCGACCTTTCT
CCCAAATCTCTATGGATTTTGTTACTGATTTACCCAAATCCGATGGATTTGATTCATTATTGGTCGTGGTAGA
CCAAGGGCTTACAAAGGGGGTAATTCTCATCCCTTGTAACAAAAACATCGACTCTCTAGGAACCTCTAGCCTC
ATCCTTCAGAATGTATACAAGCGATTCAGACTCCCAGACAAGATCAGCTCTGACAGGGGTCCCCAATTCGCAT
CTGCAGTCACTAGAGAACTTGGAAGACTCCTTGGCATCAAACTCGCCTTCAGTACCACTTACCATCCTCAAAC
CGATGGAGAAACAGAACGTGTTAACCAGGAAGTCGAAGCATACCTTCAAATCTTCTGTGCAAACCACCCTGAA
ACATGGGTATCCATGCCCCCTACCGCAGAATTCACACACAACCAACACATCCACTCTGCACAAAACGCATCCC
CATTCTACCTAATCATGGGATATGACCCTAAAGCCATCCCCCTCCCTTTCCAGAAATCTGACATACCCGCAGT
AGAAGAACGTTTCAAGAACCTCCAGAAGACTAGGGACGAAGCCTCTGCAGCACATGAACTGGCACGCCAACAC
ATGGCCGAAAGAACAACATGAGGCTTTACCCCTTTCAAGAAAGGAGACCAAGTATGGCTTGAATCCACCAACC
TGAAACTCAGATATGAAACCCAGAAGTTTGCACCACAACAAGAAGGTCCCTTTCCCATTACCGAAGTCTTGGG
ACCTGTCACCTACCGTCTAGAACTCCCAGAACAATGGAAGATACACCCTGTGTTCCATGCATGCCTCCTAACT
CCCTACAAAGAAACCGAAGCTCATGGCCCCAACTACCTCCGCGACCCCCCTGATTTAATCAATGGAGAAGAAG
AATACAAAATAGAAGCCATCCTGAAACACAAAGGAAAAGGTTGTGGCGCCATCAAATGGAAGGGTTACCCTTC
TGCAGACAACATGTGGCTGCCAACCCGCGACTTTGGTAATGCATCTGACGTCCTCAAAGCTTATAAAAAGCGA
CACTCTCTAGACTAAAATCCTCAGTACTCAACCGATTACTACCCCAGCCTCAGAAACAGAATCAACATGTCCT
CCCCTGCCTCCACCCAGTATACCTCTGCCTCCTCTTCCCCTGCACCATCCCCAGTCACCGCCCCTTCCCTTCT
CTATCCTCCGTCTGAACCATCCTCCGTTCCCCAGTCTCCTACTCCTCACTCTGGATCTGAAGAAGACCTTATC
CAATTTATCAATGCCCATCTGGGCACACCTCCTCAATACCAACAACCCTACTCTCCTGATGGATTACTCTCCC
AAGAGGACCTCATCCAACATCTCACCCAGACTACCGATCTCACCACCATGTATCATAGCTGCCACGATTATCC
CATCCTCCAAGACCTCCTACTCACATTCTATGAGAATCACCAAACCACTGTCCCAATACAAAACATCCATAAC
GAATTACTGGAGATAACCTCTTCAGTCAACCGCCTCCTGAACAACAAGAGGCCTTAGCTGGTAGCCTCAGTTA
CTTACTCCTAAAGCATGGAATTGAACAAGTCCTGAATCCCCGCTCTCCCAACATCTCTCCCACTCCCATCTCC
AGACAAAACCTCGCCCAACTCTCCTCTCCTACCCCAGACATTGCCATCTTGGTTACACAAACCACCAGAACGA
CTGCCGAAGCCATGACACAGACACCTCCATCTCAGACCGCATATCGAAGAACACCCTTTCCCCAACCTACTAT
CAGATTACCCTCCTCTAGACCCCCCTCTGAAACTACTTCTCCCTCCACCCCACCTGCCCCAGACACTCCCCTA
ATCCGCCCACAAAGAATCATCAGAAGAGTCTCTGTTCCCCAAGAGATTGTAGAATTCCCTCCCAGACACAACT
CTGCACCCACCGCTAACCGCCGAAACCGTCGATCGCGTGGACACACCATCACCCTCAACCCCCTCAGTCATGC
TGGAAGAAGACCCCTAGTAGAAAGAATCTCCTCCCCTGAACCCGCTCCCACGCTACTTCGAACTCGTACCCCC
ACTGGGCCCAGGGTAACATCCTCTCCCCCCAACCCCAAAGTATGCTGGGAATGCAAATTACCCGGGCATATCA
TAAAGAACTGCGAGCATTACTTCTGTCGACACTGTGGAAACCATGCACCAGGACACCTCCATGATAGATGTCG
CTACCGATCCTCCACTTCCCATGCCAGCTACCATCCCTATACCGATGGGGATCTCGATTTTGACAATACCACC
ATGTCTAACATGTGCGATGAACCATACCACCAAGATTGAAGTCAGGGACTTCAACGATAAACAGGGGGTAATG
TTATAGCGCTCCTAGGGGGCTTACTCTTTTTTCTTACATCATATATGGATGCTTCTCTCTTCGACATATGTTG
TACATGTGTTTGCCTCATGGACTCCTCATGGGCATCTTCTGACCTACTGCGCCTCTCGGTTTCCTCATGGTTT
ACAGCATATCTTCCCCTTTAGTATATATTGCTGTAGAGTCCCTCAACATGCTTCCCCCTCATGGTCACTCTCC
TCTAGTATATATACATCTCTTGTACATCCTGTATTAGCATCTTGACCTAGGCAATCTACCGCCTTTACACTCT
TACTCAGAACCTGAGCCTAGACCAGCTCAGAGTGACTTGAACTCCTTTGTCATTGGACAAGAGACATCTTGAA
ACTACTCTCAAGATAGAACCAGAATGAACGATAAACTTGTATCAGTTGTGAAGGCTCACGCCCTAACAA
>Cligib1_rnd-1_family-711#LTR/Copia
ATCCCTTCCAAATGGGATAATGTNGCTTCNCAANTCCTCCAGACTCACTCCCAATCCGCCCTCACCTTCGAAA
TCGTNCGCATCGCCNTCGTNACGGAATATCAACGTCGCACCGCCATCAANGGCCCGNCCAGGCCAACAAAATC
TCNGGCGTCAAACGTAAAGGCAAGAACCCTAACTGGAAGGAACAGGCAAAGGANCAGGCACCTGAGAACNCGC
CCGCACGAGGCAAGTTCGTCGAAGGGTAAGGCCAAGGACCGCAAATCCCGCTCCTCTAAGGGTGGNAACCGCG
ACGAGCGTCGCAAAAAACGCCAAGCCAAGCAGGCTGAAGAAGCCGCTTCATCCGATGGATCCGCACCGATTCA
GCNTCACCANCACGGGAACTCCACTCNTCTCCTCGGCATNCATCGCGGAGGTAATCGAGCCACCTCCCATCGC
CATCGACAACCAAACCNCTCGGCCCCAAGGAGCGCTCCCTCCTTNAGCGCCTACACGACGGNCCTGCCCAGAC
CTNCATGGGNGCCACACCNGGNAAGNTNCCTGGCACCTATCCAACNTTCGAAAAAGCGCGNTCACTGGCTTCA
CGCCTATCGGCGACGCCCACCACCGAGACCCTCAAAANNCTTGAGGTCCATACANATGGCGCAACCNGAATCG
CGNANTCCGACTCCCGGAACCGGAGGAGCATCCAGTTCTCCGCCACGCCCCANCCGACCACTCCCCGATCATC
TTCTCCTCCCCGTGCGCCANCTAATCGCGCGNCCCACTCCNGAGCCTGTANCATCNCCNCCCCGCTCNAACA
CCCCCGATGGCCTTAAAGACATCCTAATGCAAGAGGACGAGTATGAGAATTGGNTCGAATCACTACTGCGGNN

CTGAAGATGAAGGCGCCGCATACGTCGATTACGACGTNGAGGCTTGGNNCCCTCCGCCTAAGAAACGGCGCAG
ACTCTCTCCGGTCTCTGAAGAGGACGATCGCGAGGATCCAGTNTCGATGGGTGAAGAACCCGAATTCGATCCT
GAGAGTTGGGGCAACTCGGTTTGACGGTCAATCGTCAGTCACCGCCTCCTTTCTCCACTTCCATATATGAGAC
TAACCGCATCATTCTGCAGGGCGCTTTTCGATTAAATTCGGATTGCCCCCTGTATTATAAATATGCATCATGT
ACTTCNTTGTCTGCCGGCCAAATGGCCGAAATAAATAATCATTNTGCTCTTTGTGCATCTTGTAAGGGAAAAG
CGAAACATGTCGAACACCAATGGTTAATNGACTCAGGNGCATCCATGCACTTCACTGCATATTGGATGATTAC
GTGGAACGTTCAANATCGAACACCCAATCACTGTTNCTACCGCGACNGAGATNATCGAAATTGANACACAAGG
AACCGTTATCCTTGAAAACGATGTCCTACAAACAGATGGATCNTATAAAAAGCGACACACGCGCTTACATCCT
GTCTATCACCATCCAAAGTTGAACTCCCGAATTATATCCATGGGAGAATTACTNAATTCTGGATTATCGCTGA
ATGCATCATCAAANGCGTTNATATTCAGTAACAAAGGGACACGGCAATTAACCGCTGAAAAGCGTTTTGCGTG
GGACACNACGTTCTGGGTCGACGCAGATGTTGTACAAACCGANCGGNTTAAAACAATCCCTATTTGTCAATAC
NATCGANTCATATGACATNTGGCATAGACGCCTNGGTCATCCCTCGAAAGAGGTCGTCTCCAAATCTCGNAAA
CACATTAAAGACTTCCCACAGGGACTAATCATCCCGAAGAACCCACCTATNTGCCCTGGCTGCGCATACGGAA
AGATGCCNTCTTCATCTTTTAAACCNTCCGCCTCACGCGCCAAGAAGCCATTCGAATTGATTCATTCGGATTT
AAAAAGCTTCCCCGTGAAATCTTATCACGGGTATTCATTACATCATNACATTCCTTGACGACTACACGTCATA
TGTTTGGGTCTCACACCTAAAACATAAATCAGACGCAATTGGGCATTCCGCACACTTCAATGCAGCTGTTAAA
AACCAATATAACGCNAAAATCAAACGNTGGCGTTCTGATGCAGGGGGAGAATTTAAATCCCAGGAATTTATTG
ATGAATTGAAATCAACACTGGGCATTGTAATCGAACAATCGGCTNCCTCACACACCACAACAAAATGGGCGNG
CTGAACGTATCAATAGAACCGTTATGGATAAGGCAGAGTCTATGCGNCATCACGCATGCCTTCCTAGTTCATG
GTGGGAATTCTCTGTGGAACAAGCGGTTCACATNTATAACCGTACTCCTATGGTGCGCCTGNAATGGCGAACT
CCATATGAAGTCTTAAAGGGTGAAAGACCCTCTTTAACACATCTCGTGTNTTTGGCTGCGCTGCATACGTNTA
TCTNCCANCNGAAAAACGTGCCAATAAACTTGCGCCCAAATCCGAGCTAATGATTTACCTCGGTCACGCTGAN
GGAATAAAAGGTTATCGCTTTATGCGAATCAAAAACCCATTATCTTTATCGGAGTNACNGCTATCTTCGATGA
AGAAACACTCGCCGAAATGCAAACAAAAACTACGGTACCNGAACAACCTANAATTCCTCCAACCGANGANTNG
CTTCCTCCGTCTCCACCGGAATCA
>Cligib1_rnd-4_family-133#LTR/Gypsy
GAATGGACGCTTGGACGCTGTGGGACTCGGGGAGTACCACAACGGGAATAACACCGGCGTTCGCTAACATAGC
GAACATACCGGTATTCCCACTAAAGGAGAAACACATTCTCCAACTAGGAACAACTGGAAGTCGCTCCACGATA
AACTTCGGAGCAAACGTAGAACTAGAAATAGCGGGTACGACATTTAAATCGTACGTAGACGTAGCGAACCTGG
ATCGCTACGACATGATAATGGGAACCCCATTCATGAGAGCGAACAAAGTGTCGCTCGACTTCGAAAATAACGA
AGTCATGATAAACGGAACAAAAGTAATAGCTGAACCAGCTATTACGGCGGATACCGACGCGCGAGCGCGTCGT
CACAGANCCGTAGANAGAAAGGGAGAATGACTACATGGCAAGCGAAAAGCCATGATCGAAGNCGCGAGCGACG
ACGAGGACAACAAGCCTCACAAAGCACTCCCGGAGGGATCAAAATCCCTACTAATGACGGAAGAAGAGTATAT
AAATACTCTGGGAACGGGGGAAAAGCTACCCGCACCCGAAC
>Cligib1_rnd-1_family-599#TIR/MITE
CAGTGAAATTCATTCGACTCCGAGTGAAATATGCATTTCACTCGGTTTCATAGTGAAACCAGAAATCACTCAG
GCTGTGAAACATATCAANTTTTGAGTGAATTTTACTTTGGC
>Cligib1_rnd-1_family-899#TIR/MITE
AAAAGTATTGCACACCTCTCGTCACAGCGATAACTTTGGAAGGACAAAAGATGCNACCGAGATAAAATTCNAT
AAAATAGAGGACCTTGTTGAGTATCTNTNATGATTCAGCCAATGTTCCAGGGTTCANACACGAGGCAGAAATC
GGATAAGTTCTNAAGGGTGCCAAATTCAACTCATGGAATAANGATCCTATTGCGATGATTAAATTATCANAGA
TAGCCCTGGATGAGTAGATCACGTAGCATTTTGAATGGTTNGAAAAGATGTTCATTGGTTCTGAAGANATGAC
CTTCACGAAGNTGTCAAATTTTGCCANTTTTCGGCATTTTCAAGTGTTTTTACCAAANNTTGCGACTTTCCTT
GGCAAGACCATATCTTCGGAACCANTGAACATCTTTTCNAACCATTCAAAATGCTACGTGATCTACTCATCCA
GGGCTATCATCTGATNNTTTTTCATAGCACTAGGATCTTTATTTCATGACTTGAATTTCGCACATTTGAGAGT
TTNCCCCATTCCTAGCTCGTATATAACCCTGGTATATTGGATCACCCCTCAAAGATACCTAATAATATACTCT
ATTTTATNCAATTTACATCTCGGNGANATGTTGTGTCCTCGCAGAGTTATTGCACGAAAGGTGTGCAATACTT
TT
>Cligib1_Cluster26#LTR/Gypsy
GGAGACGGCCGAGGCTCGGGAGATGCCCAAGACGACTTGGGCGCAGCGTCGAACAAGCAAGCGTTTGTCTACT
AAGAAAGCACAAAAGAACACTACTACAGGTTGGACTTGTGCACTACCGTGGCCTAGGTCACGTGACTCCACGG
AGTCGGGCGCTCCGCGCCCTACTCCATCTGCCCCCCTTCTTGGAGGCGGCGTCCTCGTCGCCTGCCCGCTACT
CGCCTCGCGATACTCTCCTCGCCTCCCTCCTCGTCCTCCTCCTCCTCGTAGAAGAAGCCGTCTTGGTGGGCCA
CCTCATTGTCTTCTTGTAGCTGATTGTCGTTTTCTTCCACAGGCTCCGAGTCAGCGGCTGCACCCGCACAGCA
CGTCGGTGCAGCCTCGTCCCCCTTGGCTGCTTCACTGACTGTTGTGCCTGTGTTGTCCGGTGGTGGGGTTTCT
TCCGCTCCCGCGTGCTTCTTCGACTCCTCTATCCATGCTGCTTGTTCTTCGGCCAGTTCAGTTCCGGCTCGCG
GGGTGAAACGCCGCAGCCGTCTTGCGCTGAACTCTCCTCCGAGTTTCTCGCCTTCAAGGGTGGTTAACCGGTA
TGAATTGACGAGGCGTTCCGCAACTCGCCGCGGAACTGACCACATTGGGGTTAATTTCCTGTTGGTGCTCATG

GTAGTGGTTAGGTCGCTTCGATAAACTTGTACTAGCTCGCCTGGATTGAATGTAACCTCCCCGTCTTTTGATG
CTCGCACTTTCTTGTCGAATAAGGTCTTCCTTCGTTGGGCATGGCGGGCTGCCTCCGATTGGCCGTCGAGGCG
TTGTTGGCTCGCATACGCCATGTGGGTGTCCAATGCTGACGGTGGCAAGGCCGACAAGATGTCATCCATCTGG
CTGCGTGGGGTGTTGATTACAAGCCCCAAGAGTAGTTCCTTGGGGGAGAATTTGAGGGCCGGGAGTATTCTCC
AATTGAGTATGGAGATGGCGCGTTCGAAGTGGTCAGGCCAATTGCGCGGTACATTTTGGGGTCCAGTCATCTC
CCAGCCATTCTCACCCAGTTCGGGTGCGCATAGTCGGGCTAGAACATACATGAGTAGCTTATGTGTCCCTTCG
ACGAGGCCATTCACCCACGGAGAGTACGCCGAGACGATGTGTGTCTTGGTTCCCTGCGCTGCGCACCAAGCAG
TTACTTCTTCTGCGTTGAAGTGTCGACCGCCATCGGACATGAAGGTTTCTGCAGGCGTGTATGCATGGAATAT
GTCATGCAAGGACTTGACGGTTGTCTTCGCACTCCCATGGGTCTTGAACATGTGGCCCCAGACATGCTGGGTA
CATGTGTCTAGGTACAGCCCGATAGTGTGGTATCCGCCTTTTCCCGTCGGCATGGAAAGGTAGTCTCCGACGA
TGAGTTCGAATGGGTGTCGTCTAGTGATGGGGTTTAGGAGTGCGTGGAGATGGGTGCCGCCGAAGTTTTTGCA
TTTTGCGCAATCCTGTATTGCCGTGACAATGGACTCGTCCAGCTTGGGGCTGTGGAAGCGGTCCAGCAGGGAC
ATCTTGATGCTGTCGCGATGCCAGTGACCGTTTTCCTGGTGTTCCTTCCGTGCTTCTTCGATTGCCTCCTTCT
TGCTGATGCACTCGCGCCGGGGCCGCGCCCGCGCTTTTGTTCCTCCGCCAATGAACCACAGCTTCCCTTCATC
TATAGAGTACCCAAGCGCCCGGTGCCTGGCACGGCTTTGGGTTTTGAGGTCCAGGTGTTGATCACACTTGGCG
AGTGCATCAATCACCTCTGTGAAGAGGGGTTCGTCCTTGAATCTGGTTTTTAGTGTTTGCATTTCCGTGGACA
TGTCTGGGGCACTCAGCTGGTATAGGTTGTGCACTAGGCCTGTGCGATCTTCCCAATCGGGCGAAACGGACCA
TTCACTTCCGTCGTTTTGCCCTTTTGGTGTCCCTTCGTACTGTCTGCTAATTCCGTCCGCCACGTTGAGGACT
CCAGGAACGTGGCGCACGTCTACAATCCTGTGCGCCATCACACCGTCGCGCCATCTTGCATGCGTAGCAGACA
GTTTGCTTGTTGTTAATACATCCCTTAATGCTTGGCAATCAGTTTCTATCTCTATAGGAAACGAGTATACGAT
GTCTGAGAACTTATCAAGACTGAATTTGAGGGCTGCAAATTCCGTCAAGAATGGCTTGTAGTTTTCCTCTGCT
GGTGAGGTGCGTTTTGAGGCGAACGCTAGAGGGTGGAGTCTCGTTACTACCTTCCCGCCGGGTAAGGTGGTCT
TGACACGTTGGGATAGGACACCTGCAAAGGCATCCTTGGAGCCGTCTGTTGTTATGATGAATGGGGTTCTGTC
GAACACTGGCGCCCTCAGAACGGGCTCTGACACAAGAATTCTCTTGAGTTCAATGAATGTCCGGTCGTGTTCT
GTTCCCCATGCGTTCCCAAACTTGTGTGCCTTCATCAGGCGCTGGTATGTTGCTTTCCCAGCGCCTTTGGGAA
TTTGCACTGCGCGTAACAGATCCCGGAGTGGTTTTTCTATCTTTGCATACCCTTTAATCAGATCTCGGAAGTA
CCCTGTTAGTCCCAGGAATCCTTCTAGATGTGACGCGTCTTGTGGTTTGGGCCAGTCGACAACCGCTGTCAGC
TTTGAGAGGTCCGGGGTGACCCCTCCTGGTCCAACGGTTGCGCCTGCGAATACCGCTTCTGTCATGAAGAGGC
GTAGCTTGCTGGGTGACAACGACAGTTTTTCATGATGTACCCGTTCTAAGAGAGTCCTCAACTTTGCTATTCC
GTCTTCAAATCCGTCCGCTGCCATTCCACCATCGTCTACAAACATTTCTATGATTCCGCTTGCCATTAAATCA
TGCAGCTTGTCCGCTGTGAGGTGGGCAAACTCGGAGGGTCCGCCTGTGACGCCGAAGGGCATGCGGCAGTAGG
CGAGGTACCCTAGCCCTTCTACATAGATTGACAAGTATGGTTGTGATTCTTCTGCAACGGTGCATGCGTAGAA
TCCAGCTGCAAGATCAAAGGTGTGAACGTATCGATGCCCTGACAATCTGAGTTGCTTAGCTCTTATGTCCCCC
TGCGGCATTGGTGCGATTTTTGTGACCTTGTTGATTTCGTCGAAATCATGGCAGATCCTCCACTTCCGCGGTG
CATCCGCCATTTCGACCGCTCTTGGTTCCGGTCGGGGGGGCATGTCGAATGCACTGGGCAGCCCGTTGGCGAT
ACACTCGTCATTTACCTTATGTTTGAGTTCGTCCAGTGTTAGTCCCCCTGTGTCATGCTCCTTAGTTGCTAAC
ACTGTTGGCGCAATACACTTAACGTCTCGTGGGTGGATCTTCCTCAAGATGCCCTCCTTCAACATGTCGGTGA
TCTTCGTATGCAGGTATGCCCGTTTCTCTGGGTTCATGGAACGTTGGCCTACGCGCATTGCAAATTGTGCATC
CTCCGGGACATTGAGTTTGTGTACCGCTCCGGGTACCGAATTTACTTCACTGAGGGACAGGGCAAAGCAATCT
ACAAATTCGGCAATGAGATCTCTCACCCGTTTTCGTTCCTCTGGGGTGATATCGCTTCCTATGTGAATTTCTT
TTAGGATGCGTTCCACCCGCTCCGGTTTGAAGGGTTGGGTGTGGCGCGTGAAGACTGACTCTTCGGGCGCTAC
CAGCAGGGTTTGCAGCTGCTCCCCCTTATTGGTACCCTTCGCGTCGTGATTCTTGAGCATTACAGGTGGCGTC
GTTGGGCTTGGTTGCACCATGACCGTATTATCTGCGAAGTTCTGGTTAGCTCTGAAGGCTCTGAGGAGTGGTT
TACCAAGTAAGAACGACCAGCCCCCTTGGCTGTCGAAAACTTCGAACCCTCCTTGCACTTTTGTCCCTGCTAG
TTCCACCGTTCCTTCCCATCTGGCCTCGGATCGGACTATCATCCCGTTGGCCATTCGGAGGTACTTGGTTGAC
GCAGTCCAGTTATTCAACTGACTCTTCACGTGTTTGAATACTGTTGAGCACATGGCGGCCACCATAGCTCCGC
CATCAAACAGTGCCTCCACTGACGTGGTGGTGCCGTTTATGCCATGCAATGTGATTGGATGGGTGAAGGGCGT
TTCCGCATCGATTTCCTTATGGGTAACAGAATACAAGTCAATCACACGGTTATATTTGGTATCTGCTACTGGC
TCAGCTTCTTGTGAATTCTCGTGATGATTGGGAACTGGTTCCGGTGGGGCCGCCTGCATGTCCTCTAGGCGGA
CGGCCCTTCCCCGTTTCCCTGGCTCCCTGACGTGCCAACAGTGTTTTCCCATGCAAATTCGTCCACTGCGTCT
CCGCCCACGAAATTGATGCGAGATGCTCCCCGGTGCCCTACCGCTGTTCCCGCTATCCTGCGCCAATCACGTT
CTTTGGCCTTGAGGAGCTCCTCACCCGTTGCTGTGCAATCCTGGCGGCGATGGCCAGTTTTGCCGCATTGGTA
ACACTCGCCTGAGAAGAGTGGTGCTCCGCCGGGGCGTAGGGGGAACCCGGTTCTCGGGCTTACCCTCTCGTTC
TCGCCAAATTCTGCCCTCCAGGCCCGCTTTTGCTCCTCCCATGTCGTTCTCCCTTCCACCGTGTCGGGCTGAA
GGGGGTAGGCTGCGGCCCGCCTGCGTATCATTGCGCGTTCCTCATCCGTCAGCTGTGGCCTGGGTCGTTGCGC
TCCAGCTGGATTCCCCGTCGCTTGAGTTGCTGCGGGAGGGGTTGGGTTCCTGGAATTGCTGTTGCTGATTCGC
ATGCGGCTGATTTGGCTCTCAAGCTGGTCGATCTTGGCTCGTGCTTTTTCTTGCTCCTCATGTTGGGCAATTC
CGTCTCGGATACGGTAGACCGGCACCTTCTTGATTGCCTCGCAGAACGTCGTCCAGTTCTCTTGTTCTTCATC

AACCTCCTTTTTGATGATGTTGGGGAGGCTATCCCGCACGGCCCATACACCTACACCAGTTTTTGCAACGCCA
GCTTTATGTGCGAGGTTGAGTAGTTTGTGGGCGAATGCTACGTGGGCCCAGACTTCTTCGCCTGCATACGTTA
TCCTTTTTCCCAACTCGGCTGTCGTCAACTTCAGTTCCCCTAGTTCACGTTCGTATTCCGCTTTTGTTTTCTT
TGATCTCTCGATGGGCGGAAAGCGCCTGAGGAAGGCACGTTGGACTTCGTTCCAGTTGATCTTGGTATCCGCG
ACTAATTCGCGGTATTCGCAGAGCCAATCTTCTGCCTCGGAGCGACTTTTTAAGGAGTTTTGAAAGAGATCCA
CTCGGCCGGTGTCCGTGGTGATGTTGGACCAGGCGAGTGCATTGTTGAAGTCCTTCAGGAAGTCGTCGGGCTG
GGTGGAGTCGCCTGCCTCACCACTGAAGCGGGCTAGATTCTGGTAGTGCGCCATAGTTCTATTTCCACGCGAT
GTAGCTTCGATCTGCGGAATCGCTGTTTGGGAGTGGAGGGGGGGGTTGGAATGGTTTAGGAGCGGGGTTAGGA
TAGGTGTTTGTCCAAGCGATGTGGCGTTGGCGGTCTCCGACAGAGAGCTCTCGGAGGTTGGAGATGGCGCGTG
GGATGTGCTTGCAGTTGAATCAGGATCTGAGTATCTGGGTGGTGTACGCGGAATGGTCCAGGTCCAGGTTGCT
TCAGAGAGTGAGATGTTGGTTAGATCGGGACTATAGGTGTCGTTGGCTGGGGCAGTGGTTGGGTCGTTGTTTT
CTGGTGGTGGTACGACGTGCATGAGGTTTCCACGAGGCTGTCGATGGGACACGGTTGGGTTCACAGGCGGTTG
CGGTTCACGGTTCACGGTTGCGGTTCACGGTTTGCTTCACGGTTTGCGGTTTACGGGTTGTTCACGGGTTGCG
GTTCACGGGTCGTTTACGGGTTGCAGTTTTTGTTGTTCAGGTTAGTTGTGGCAGTGGGATCAATCGCTTCTCA
TCACCAGATGGAGACGGCCGAGGCTCGGGAGATGCCCAAGACGACTTGGGCGCAGCGTCGAACAAGCAAGCGT
TTGTCTACTAAGAAAGCACAAAAGAACACTACTACAGGTTGGACTTGTGCACTACCGTGGCCTAGGTCACGTG
ACTCCACGGAGTCGGGCGCTCCGCGCCCTACTCCA
>Cligib1_Cluster79#LTR/Gypsy
GGAGACGGCACAACGGCAGGACGCGCGGAATGGCAAGATGATGGAATGAATCTATCTACTACAGTCCGTCTGT
GTCTACTGCGGTCCGATGGTCATGTGATCTGCCTGTGTCCGCTCCGTCCGAGTCTGCGTCGTGGCCGGGTTCC
CCGGCTACATGTTCCCCCCCCTCGTGTGGCGGCGTCCTCGTTGCCCCGCTTTCTAGGTCCTCCCCTGGTTCTT
GCTCCTCCTCCTCATTCCCCTCGTCTTCCACGTTGTTGTCAGGCGCATCTTCCTCCTTGTCCCAATCCCCCAG
TGCCTCCTCTAGTGCCTCCTGGGCCTCCTTGAGGTGAGTCCCATCGTTCGGGATGAATCTCCTCAGCCTTCGT
GAACTAAACCTTCCGGATAGAGGCAAGCCTTCCAGGGTTTCTAGTTTATATGAATTCCTGTCACGGCTGGAGA
TCCGTCTTGGTGCAGACCACTTGGGGATGAGCTTGCGAACTGCAGCATGGGTGAAATCGAGGTCATTACGGTA
GACTTGTACCAGATTGCCTGTTTTGAATATGACCTCTTTAGGCTGTCTTTTGGTAACCTTTTTGTCGAAGGCC
AACTTCCGCTTTTCCGCATGATCGCAAATTTGAGAGTATCCATCTGTCCTCTGTTGATCGACGTAGGCCATTT
GGAGTTGGACGTCCTCCTTTACGAGTTCCCTCTCTGCTTCGTTCGTTGGTGTCCTTGGGGTATTAATTACGAG
CCCAAGCAATAATTCTGTTGGAGAGAACCCGAGGTTTGGTAGGATTCTGTTATTCAGAGATCTGATGGCCTCG
TCTAGATGATCTGGCCAATTACTTGGCATGCCCTCGCTGGTTCCTTCATCATCTGCGTCTCCGCCCAGGTCTG
GTGCGCATAGCCGTTTTAGCCGGCCGAGCAGCTTGCTGTTCATCCCTTCGACCAGCCCGTTGACCCATGGTGA
GTATGCGGGCACCACCCGTAGCTCCACCTCTCTTGTGTTGCATAGGTCTTTTAGTTCTTTGTTCTTGAACTCT
GGTCCGCCGTCTGCCATCAGTGTTTCGGGTGCCGTGTATGTATTGCATATTTCCGTGAATGATTTGCATGTGG
ATTTTGCCGTGGGTGCGTTTTTTAGTTTTGTAGCCCAGATGTGCTGTGAATACACGTCCACGTAGAGCGAGAT
TTTTGTGAAGCCCCCCTTTCCCTTTTGGCATGGTGAGGGTGTCGGTTACCATTAACTCGAATGGGTGACGGCGT
GTGATAGGTTCGAGTAGGGAATGAAGGTGAGTGGGTCCAAAGCATTTGCATTTCCCGCAAGTCATGATCGCTT
TTACGATTGATCTATCAAGTTTTGGGCTGACGACTGTGTCCATCATTTTGATCTTGATGTTGTCCCGATGGAA
ATGCCCGTTTTCACTGTGGATCCTCGATGCCATTACCTCCGCCTCGACCTTGGTGATGCACTCTACTCTTGCC
CTTGCTCTCACTGATTTCCCATCCCCCACCTTCCACAGCTTTCCTTCGTCTATCATATATCCTTCTGCTCGGT
GCCGCGCTCTCTTCTTCTCCCTGAGGCTCTTCCCGTGGTCTATTTCCCCAATAGCCTCAACCACGCCTTTAAA
CACTGGTTCGTCATGGAAGCGCTTTTTCAGTGTGTCGATCTCGTCTAAATTCGGAGCGTCTATGTGAAATATA
TCATTTTGCAGGCCTGTTCTGGCTTCCCAGTCTTCGCTGACTGTCCAGGTATGCCCGTCGCTCTCCTCTTTTG
GGGTATTGATATATTTCCGGCTCAGTCCATCTGCTACTGGGTTTAATCGTCCTGGTCGATGCCGCACATCTGT
AATTTGATATGCGAGGACCCCATCTCGCCAGCGTGCATGTGTGGAATTCAATTTGTCACTCAGGAGATGATCC
CGTAAGGCCTGACAATCTGTTTCGAGTTCGACAGGAAATCCCCATATGATGTCCGAAAAGCTGTCGAATGTGT
ATTTGACGGCACCGAATTCCAAGAAGAATGGTTTATATTTCTCCTCGGTGAGTGATGTTCGTTTCCCTGTGAA
TGCAATGGGATGGATTCTAGTAACTTCCTTCCCATCGGGTAGGACTGTGGTGTGTCGTTGTGATAGCATCCCT
GCAAAGCCATATTTACACCCATCCGTGGTTACGATGAAGGGTGTTCCATCGAACTTAGGTCCTTTTAATACTG
GTTCGCTGGTTAGGGCGACTTTCAAGGCCAGAAATGCCCGGTCATGCTCTTGGTTCCATTTGCCGTTGAGCGA
GAAGTCTCTCATCGCTTTCCGGTATGCATACTTTCCTTTTTTCTTGGGAATCTCTAGCTGACGTATGAGATCT
GTCAACGGTTGCGCTATAGTTGCGTAGCCTTTGACTAAGGACCGAAAGTACCCTGTTAAGCCGGTGAATGCAG
CAAGGTTCTGTAGGTTTTTTGGCACTTTCCAGTCTGCTATTGCGGTTAATTTTGTCAGGTCTGGCTTTATTCC
CGCTGGGCCTATCCTCGCTCCTGCGAATACGGCCTCTGTGACGAAGAAGCTTGACTTGGCTGCCGATAATGAT
AGGCCTGTCTCTCTTACCCTTGTGAGGAGGGTCTTCAGTTTATCATAGGTTTCGGAGAACGTGTCTCCTGCTA
GGCCTCCGTCGTCCACAAATAGCTCTATGAGTGTGCCAACCATGTTACCCAGTTTATCTGCCGTCATCTGTGC
AAATGTTGATGGTGCGCCTGTCAGTCCAAATGGCATGCGCTTGTAGCAGAAATATCCTCGGCCTTCCACGTAG
AAGCATACATATGGCTGGTCTTCTTCTGCTATCTGACATGCATAGAACCCTGCGGCCAAATCGAACACACATA
GATACAAGTGGCCGCTTAGCCGCTGTTGTTTTGCTCTTATGTCCCCCTGTGGCATGGGTGGAACCTTCGTGAC

CTTGTTGAGCGCGGTGAAGTCTTGGCAGACACGCCATTTGGTTTGTTCAGCTGGACTCATCGTCTCGTTCATT
TTCGCCTGAGATGATCTTGGTGGTAGGTTTTCATATCCAATGCTTGGTAATCCTGCCGCAATACATTCATCGT
TGACGCGATGTTGTAATTCCTCGATTGAGAGCCCATCTCCCTGGTGTGTTTTCATGGCCAGAGTTGTGGTGCC
GCAGCATTTTACATCTTTGTGGTCAATTGGTCTAACAATTCCCGCTTCTAGCATTTTGTCGAGGACACTGTTG
TGGTATTGTTTCTGTGGTGGGGTCATTTTCCGCTGGCCAATGCGTGTGCTGAATTTTGCTCCTTCGGGAATAT
TTAGTTTGTGGATGGCACCCTCGACTGGCGTGACTTCGCTCAGTGAAAGTGCGAAGCAGTCTGCATGTTCACG
TAAGAGTTGGACCACTGCCTTTCGCTCCTCCCCTGAAATGTCCGGCCCAATAGTCACTTCTCTCATGACTGCG
TTGACCCTTTTGTCGTTGAAAGGGTCCGATGCTCGTGTTAAGACAGATGAGGGCAGAGTTGTTGGTGTTACGG
GTTGTTCTGCGCCAGGGTCTGCATTGGCCCCCGTCTCCGAGTCATCTTCGTATACAACCCATATGCCATCTTG
GTCATCTGTAATTGGTGTGTTAATTACGTTTGCCACTATTTCCAGTTGGATTTCATTCAAGACTTCCCTCGAG
GGGGAATGCGACTCCCCCGTTGAGTTTGCGCGCGGTGGCTCTGGGTTTCCCCTTTGGCCGATATCTGTTTCCG
GCCCTGGGCCTTGCACCCGTGGTGCTGGCACCCTACTGCTAACCATCTCCTTCCGCCTCCTGCATTGCTCCTT
CTCACTGCTAGCTTCTGTCTGTCTGCTTGGATCATCTGTTTCGTTCTGTTCAAGACATGGGACTTGCCTAACG
GGGGATAAGCTGTGATCCCCCTCCGACAATGCGGTCTGTTTAATGTCTGTTGCCAGTCCGACCAGGGAGCCGG
TTTGGTGGGTTGCAGGTAGGGTGTAGTATTGGTTTTGGAGTGTGACTTTGCCGTCCCCGGTACGTGGTATGAT
GACTATATCGTCCTCGTAATGATGTTGGGCTCGGAATTCTTGTAACAGCGGTTTCCCAAACAAGAGTCCCCAT
GCTCCGCCACTGTCGAATATTTCGAAAACTCCGTGTCCTCCGACCTTTTGGATGGTGACTACCCCTTCCCATG
TTCCCACCGATGATACGAGCCTGCCGTCTGCCATCCGAAGCACTTTGGTTGATGGCCCCAGTGCTGCAAGCCG
GTCTTTCACTGTCTCATACAGTTTTGCGTCAATGGCATTGACCATAGCCCCGTCATCAAACACACTCCGAATT
CTGACCACTTCCCCATTTGGTCCTCCCAATTCTACCCCATGGACAAATGGAATACTTCTTTTCCTTGTCGACG
AACCCACGGTGTACAACTCACAAATCACTGTATTTCCTGGTACAGAATACATGCTAGACTTATTGTATATGGT
GGGCTCGACTGTTGGATGTTCCAGATCATTCGCATGGGGTTCTAGATGGCCGTCCCCGAACAACACCTCCACC
TGTCCGGGGACCTCCTGCGCCTGCATTGCAGCTGTATCTTGGGTCGTCAACTGGATGCTCAGTTTGTTGTTGA
CGACCCTTGGCCTTTTCCCTGTTCGAGAGCGATTCTTTCCATGATCCGAGCGATGAACATGGCTTCCTGGTCC
TCCTGGTATGCCATGTCTTGTTCCGAGACGATGTTGACTGAAGGAGGTTGGCTTGCTTCTGCGCGGCGTCGAA
TGGTTGATGCAATGGATCGCCATTTTTGCTCTGCACTAGGGACGAGTTGTTCGCATTCTAGACCACGGTGCCC
AGGCTTCCCACACTTCCAGCATTCACTTGACGCGACAGCAGTTGTCCCGGGTGTCAATGGATATGGTTTCGTT
TCGTTGGGTCCAGCTGTTCCGTTCTTGTTTTCCCAGTCTGTTATCTGTCGTGCGTACAGCTCCCTTCCCGCTG
GTGTGTCGGGATGATGAGGTAGTGCTTTCGATTTAACGTCTGTATATCGCTCGGCGTCTGGTCTGAACAACTG
GGTGGTCGCTGGTGTTCTTGGCTGTGCTATTGTTGTGCCCGCACTTGCTGTGGTAGGTGCTGCTGCTCTCGTA
ACTGTGGTAGGTCGATTGGTGTTCGCCGCTTGGATGATATCGCGGAAATTTCTTCCTTGTGCGCTGGCTCTTT
CTATCCCTGCCATCCGGTCCTTCAATGCTCTCAACTCCTCCTGATCCTTCTGAGCCTCTCTCACTCCTGCTAC
TGAGGCCTCTCTTACCGCATCACAGAACACCTTCCAACTCTTGTGTTTCGTCCCGACAACGGCTTTCAGTGCC
TTTGGCATTTTCTCAATGGTTGCCGGTAGTAAGAGGTTCGAGTCATCGGGAATTGCCCGCGCCAGGCGCTCCA
GCTTGTCCGCCCAAGCTACGTGCGCTAATTCCTCTGTACCGTCGACTGTGACTCTAGTTCCGAGTATGGCATC
CTCCAGTGTTGCTGCTGCCAGCGCCGCCACCTTTTCTTCCATCGTTTTCACTGTGGCGACCTTGCTCGGCCAC
CTTTCGTCGAAAGCTGCCTGGAGCTGTGTCCAATTTTCCCGTGAGGTTTTTTCGAGTCCGTCCAACCATGCTT
TCGCTAAGCCGGTCGATTTGAGGGAGAGCTCGAATTGCCTGATTTTCTCTGGCTCTGTGAGTACTTTCCCCAG
GAAGCTCCGCTCCACAGCGTTAAAGAAATCCTGTGGGTTGTCGTCATTATGCTCATCGTCGCCCCAAAAGAGC
TTGAAACTTGACTGTGACATGTCGTCGTCGTTGCTCGGTCTTGGTTTGGGGTATTCGTATGTTTTTATGGGAT
GTGGGTCAGGGGATGTTACTGGTGATTCAATGGGGTTTGGTTCTCTCGGGCTGCCAAACGGCGACACGTTCAG
TGAGCTAAGCGCTGATATTGATGATATTGATAAGGACGGGCTGCTAGCTGTGGTGGTGGGTGCAAATCTGCCT
TGGGGGTCTCTATTCACGGCTGATTTCCGTGGTGCCAGCTTGTTCCAGCTCGCAGTTTCAGTTGATGAGGTGT
CCAGGCTGTAATACCTGCGGTGCCGTTTCTCTTAGTTCTCTTGTCCGTTTGTCTCTTTTGTCTCTTTATCAGT
TATCGATTATCGCGTGAGTCGTATGCCACTGTTGTCACCAGATGGAGACGGCACAACAGCAGGACGCGCGGAA
TGGCAAGATGATGGAATGAATCTATCTACTACAGTCCGTCTGTGTCTACTGCGGTCCGATGGTCATGTGATCT
GCCTGTGTCCGCTCCGTCCGAGTCTGCGTCGTGGCCGGGTTCCCCGGCTACA
>Cligib1_Cluster78#LTR/Copia
GTTGAATACAGCAGCTCAGACGCCGTCACGAGTGAGAGCGAACTCATATATTATCTACCTGTTGAATACAGAA
GCTATAGGTAGGTAGATAATCAGTAAGGTGTCTGTAGGTAAACGAAGTATGTACTGACCTCAGACGCCGTCAC
GAGTGAGAGCGAACTGAGGTGCGTATGTATTGAGTCAGAAATATATAAAGGAAAAATAAATAGGGGGCGCGGT
GTGTTGAAGCCGCGCCTGTGATATAGACATTGATGTAGATGAAACGGTAGGAGTAAAGTCAAATAGTTAAAAA
TGAAAGAGATAGCCGCACTAAGCGCGCGCGTAGATTGCAGTGCCACATATGGTATCTGGCGCGGTCACGGGTG
TTACTTCAACACTCCCCCAACCCCGTGGCGTCGCCGCCTGCCTGGATGTCCAGGGCGCGACAGATATTTTGAT
GCGCCGGTCGTGGTAAGGGCTTTGTGAGAATATCGGCAAGGTTATCAGCGGATGGAACATATTCGACCGAGAT
GTCACCTTCCTTGACGCGTTCGCGTACGTAGTGATAGCGGACGTCGATATGCTTGGCATGCGCGTGTCCTTTT
GTGTTGAGAGTGAGGGCGATTGCCGCTGTGTTATCGGCGTTCAACTTGGCTGGCAATTCCTGCGGCATACCGA
TTTCATCCATGAATGAGTACATCCAAAGCGCTTGTTGGGATGCGCGCGTGAGACCCATATATTCGGCCTCGGT

TGTGGATAAGGCGACGGTTGGCTGGCGCTTGGAAGCCCAAGACACTGGGCCCCCACACATTGTGAAAACGTAG
CCAGAAGTCGAACGTCTTGTATCGCTGTCCCCCGCGTAATCGGCGTCAACGTACCCGATTGGTCGCATCCCGT
TACTGGTACCCGGCGTATAGGTAATTTTATAGTCCAAAGTCCCTTTGATGTAAGCGAGGACATGAAGGGCAGC
CCTCCAGTGCGCGGGGCCTGGATTCGACTGGTGACGCGAGAGGAGGTTGAGTGCGTAGGATAAATCCGGACGT
GTTGCCACTTGCGCCCACATTATACACCCTAGCACCTCTCGGTATGGCTTATCTGTCATATACTGTATGTCTT
CCTCGGACGATGCCCGATCCGTTTCTGTGAGAGTGAGCCCTACAGGGAGTGGTGTCGACTTGGGGCTGCATGT
TGACATCTTGAAACGTTCGAGGATGCGTTCCAGATAAGCTCGCTGTGAAAGGGAGATAGCGCCAGTGGCCGCG
TCTCGATCAATGCGAATACCAAGAATAAAGTTGAGCTCCCCCAAGTCTTTAACCTCGTAACATTTAGAGAGTT
CCTCCTTGGCGAGTGCAGCGCCAGCATCAGTTGACGAGGCTCCAAAGACGTCGTCAGTGTACGTGCTCGTGAT
AGTGAGTTCGCCGCCGACTCTCCGTGAGCGGACGCATGAATCTGCTTGGGATTGGTAGTAGCCCAGCCCGTCG
TAGCTACGGTTCAGTTCCTCGGCCCAAGCGTTCGCGCCGTTCATCATCCCATATACCGTTTTGTCAGCAACGT
ACACCTTGTCCTCCTCCCCTTGCTTGACGAAACCGGGCGCTTGTTCCGTATAAACGGTGTATTTATTCTCACT
ATTGAGGTAAGCGGCGATGAAGTCCACTTGCCAGATTTTAAGTCCCAGTTCAGCGGCGACAGCGGCGACCATG
CGCATCGATTCCAACCTAACGACAGAGGCATATGTCTCGTCGAAATCCACGCCGGGGATTTGAGTAAACCCCT
TTGCCACAAGCCTGGCCTTTCGCTTTAAGAGGTTCCCCTCGGCGTCAAGTTTGTTGTCGTAGACCCAGCGGAG
CCCGATGACGTTGGCCCCCTCTGGCCGCGGGACAAGTCTGAAGACTTCCTTCTCCCTCATAACGCGCATCTCC
TCTTCCATGGGTCCTCTCCAGAGGTCAGGTCGCCTCATGGCCTCCTTGAAATTCCGCGGCGCCACGGCATCAA
AATCACCAGCATTGCTGCCAGTGACGAGCGCGTGCGGGGCATCGATAGCCCAGTCCTCAGAACTCTTGGAGGC
CTCTGCTACCCGGTTCTCGGATTCCTGGGATTTGCGTAAGGCCCTCGATGGAACATGCGCGCGGTCACTGCGG
CGTACGGTCGGTGCTTGTGGTAGTTCAGCAATTGGGGCTCCCCTTTTGACATGGGCCGGGTGTAGGGGAGGGT
CAGTAGCGCGCGGGCGAGCTGCCACAGGTCTCGCATCCCTGATGACAGCTACACCAGGGGCGTCCGCGCCCAT
GTTGGTTTGCGCCAGAGCGTCAGGTTCGAAGTGTTCAAGAACATCCTCAGACGCCTCAAGGCTTGAAGGAGCC
CTCGTCCTGTGTCCTGCGCCTTCCTCGAAGATGACGTCGCGACTCTTGACGATGGATCCCGACGCAGCATCGT
ATAACCTGTAAGCACCGGGTCATAATACCCAACAAGACGATACTTAAGTGACCGAGGGTCCAGCTTCGAGGCC
CTAACTTCTGCTGGGACCTTTGCGAACGCGATACAGCCGAACGGCCTGATGTGGGAGACATCCTGGCGTTGAC
CGGTCCAGACCTCCGCTGGCACTTTGTCTGGATGTCGTGCAGAAGGGATGAAATTGCGAAGATAGACCGTCGT
CGATGCGGCCTCAGCCCAGAGATAAGGGGGTAAACCAGATTCCGCCAGCATGGATCTAACGCCATCGAAAACG
GTGCGCATCCCACATTCAGCGACTCCATTTTGGCTATGTGCGTAAGCCGCTGTAATCTCCAGATAATACCATG
CTCCTTCAGATATTCGGCCCATATGTCGTTATACCATTCGCCGCCGCCATCAGAACGGACCCTCTTAATTTTC
TTGCCCGTCTGGCACTCTGCCTCGGCGCGGAAATTGCGGAACGCCTCCAGTGTCGTCTCCGAGCTCTTGTTCC
CAAGGAAGTAAGCCTTGCGATACGACGATGCCCCGTCCGTAATTAGAAGCATATACAGTGCGCCTCCATTTGA
CTTAACGCGAGCCGGGCCCCACAGGTCTATGTGAATGCGCTCCAGAACGGTGGATTCGTGAGTTACCACCTCA
TCATATGGTCTGGTAGTATGTTTACCATATATGCAGTCCTCGCAGAGTCCTTCTGGCACTGGTCCTGTGATGC
TGAGGCCGTCCACTAACCCCTTTCGCTCCATATTGCGGATCATGTCGACTCCCGCATGTCCAAAGCGCCGATG
CCATGTGTCCAAGGTCGTAGGTTTCTCATGTGAACGTGCAGTGAGAGCAGTGGGGGGTGTAGCGGCATCCAGA
AGGTACATGCCGTTGCTCCCCCTTCCAAGAAGCATCAAGGAGCCCTGTGCGTCGTAGAACTGGACAACCCCTG
AGGCGAAGACCACGCGGTGACCAGCCGCATCGAACTTCCCTATGGATACGAGGTTAGAGATGAGATCCGGGGT
ATGGCACGCGTTGGAGAATGAGATCTTCGTAGTTTTGCCATCGATGATGAAGTTTTTGGTGACCGTGCCCCTT
CCTATGATCCGGAAAGTACTCCCCTTGCTCGCCGCCCGCCCGACAATTGGTGGATCGATGGGCGTATACGTTT
CGAAATCCCCGCGGTGGACGAAGCAGCATTCACTTGCCCCGGAATCAGCGAAGGTGGGTACTGGGGTGTCATC
GGACGTATTGCTGGCTTCAGTCTTAGCCTGCTGGATGACTGGCGTGGTCATGACTGACGGAATGGGCGTCGAA
TCATCCGTGGACGTGTAGAGAGCATAGACTCGCTCCGAAGCTCCAGCGTTTGTGGTGCCCGCTACTGCGGCTG
TGGGTGTGGTTATTGCTGGCGCAGATGACGAGTTCGGCGTGGTGTTGGATGCTCCTCCCCTACGGCCAAAGCC
GGGGGGAAATTGACCTTCCTTTCCGCCGCCCGGCCAGTAGCACTTATCCACTGTATGCCCCCTTCGTTTGCAG
TTTGGATTAGAACAAACTAGCTGTTCACGCCTCCTAGTAGTCGTCTGGAGTGCGGTGATGGACGAACCGGCTG
TAGTAATCGTCTTGTCGTGGACGATCTTGTTCCAGTGTAGGTCGAGACGTGTAATAATATCAAGTGAGGACTT
GGCATCATAGAGTGTGCCGATGATGGGGTTCCAGGATGGTGGTAGAGACGCGAGCATGATAAGTCGAAAATCT
GCATCCGTAATGTTCGCGCCCATCGCGTTCGCTTCGCGCCACCTTTTTCTAAGGTTCCCAATGTGAGTGGGCA
GATCCTCACCGTCCTGGTATCGGAGGCTGCGTAAATTTTGTTGTGCGAGGATCATCGCGAGGTCGTTGTTACG
TTTGTATTGGGCATTGAGCGCTGCCCAGATGTCGGCCGCCGTGCCAGTTAAGTCCACACCCAGGGCGATAGGG
TCTGACATATTATTGACAATCAATGTCTTTGCCCATGCGTTCCGCATCCTCCATTCGTTATAGGAGGGTTTCC
GGGAGAACCAGTGTGTGCTGGGGGTGGTATCTGGGTCGATGGGTGATGTGGGTGGAAGTGGGACCATAGCTGG
GTCAGCGGGTGATGGAGATGGAAGTGGGGAAGTAGTATCGTTGTGTTCAGGATCTTCTGCTGGCTCTTTGACG
GTTCCTTTAAGATAATCTACCGCGCCGCGGAGCTCAGCCGCCATGAAGATTTTGTTGCTCCAAGCCGTCCAGT
TGTAAGGCATGTCACGAGTGCACCCGTGAGTTACCTGGATCCGCAATCGCAGGTACTTGCGAGCACCAAAGCT
AAACTTGGATCCGCACGAGTACGGATCCGTGGGAGACCTGGATCCGCGAGTAAAATACTCGCGAGTGCACAGG
TACTCACGAGTTACGATGGCACGCCGGTCCGTCAATCCGCGTGCCTGTTAATCTACCTGCGCATCCGTTTGTG
TCAGGTGGAAACCGCAGCCCACATCTTCCCATTTAACCTCGCGACATCGTTGCGCACGTCGATTGACGTGGCG

AATGCAGCCGCGAGGGTGGAGGGAGAGGTGGGCTGGGCACCGTGACTTCCGTCAACAAAAGAGGGGGTGAGTA
TCGGACATACCTCATCATATCTCCCATTTGCGAGAGAGTGGGGCGGTGGCAACGAGTCGGGCTCCTGGGGGGC
AAAACGAGTGCTTGCAAGGAAGGGAGACACGGAGCGTGAGGGGGCAACACGAGGGAATCATTCACGGCCCTCT
CGTTTGCGAGAGCAAACGAGAGGGAGGGTCGTGGGGTTGGAAAGAAAACAGCGGGGTGGCAGAATGAAGAAAG
GCCGTCCCGCACTCTCATTTGCAAGAGAGGGTGATGTGGAACAAGAACGAGTCCCCGAGTCTCCCGTTTGCGG
GAGAGTGGCGTGTCGGAGTGGTGAAACGACACAGCGGCGGATGGTGATGGCGAGAACGAATGAAAAATGGCGG
TGGTGGGGGTGTGGAGTGGGAATCGTTGGGAGGAGGAGAGATTGGTGGAACGGCACGGCGGTGGTGGTGGTGA
GATTGAATGAAAAACGGGAGAGGAGGACAGCGGTGGTGTTGAGGGTACAGCATGTGTCGTTGTTATCAGAAAA
ATGTTGACTCACCTAGCATGGAAAAAGCCTGAGCCATCATCTCTCTCGTTCGCGATAGCGTTAGTGTCGGTTG
GTGTCGGCGAGCCCAACGTGATGGTGGCATTTGCGCTGGGGGCGGAGGTGGGGGGGTGGGTAGTGGGTAGCAG
CAGATCGGGTGGCGGGTGTGGGGACCGGCCGAGCTGCCGGGTGGGTGGGGTGCAGGCGAGGACGAAGGCGGGT
GAATCCGCAAGGAAAAAATGATGCAGGCTGCGAGGTGATGTTGATCGGCGACGGTAGGGGGACGGATCGCGAA
GTGGGGAGGGATGGCGATGAGTATGAGGCGTTAAAGTATTGTTGATTGATCAAATAACAGCTAACTCACCAAT
CCCACAGACATCCCCTCTGTATTTTTCATACTGAGAGTGAAGTGACGTGGCGGAGCCGGCCGGAGGACTTTCG
GAGACTCGTCTATAAGAGGAGAATGCGGGGTGACCGGTGATGTGGGGCAGAAGACATATGGGGCGGGTGGAGC
AAGAATGAGTGGAACGAACAGATGAGAATTGCGATGTTGGTGCAGCCAACACAAAGTCGGCATGATGCCGCGG
GTCACGGCAAGTACTCGCGGCGAGTACCTGCTGCTCAGAATTTGAAACTCAGACTCGCGGCAACCCGTGGCCA
CAAGTGATCCGGATCCGCGAGTAGCCATACTTGCGGGTTTTCCCACGGATCAACCTGTTGTGACTCACGAGTT
ACTCGCACTCGTGACATGCCTTATCCAGTTGGTGCCGTCGAAAACATCTTTTTCAGCAAATAGGGGCGGGTTT
GATGACGAGGTGTGTATGGTTGTCATTTTGACCAGGAATAAGTTGTGGTGAGTTCGCAATATACGATAGCTAC
GATAGTGTAACGGTGTCTGTATTATTCAGGCTGCAGGGCCCATAACCTGTTGAATACAGAAGCTCAGACGCCG
TCACGAGTGAGAGCGAACTCATATATTATCTACCTGTTGAATACAGAAGCTATAGGTAGGTAGATAATCAGTA
AGGTGTCTGTAGGTAAACGAAGTATGTACTGACCTCAGACGCCGTCACGAGTGAGAGCGAACTGAGGTGCGTA
TGTATTGAGTCAGAAATATATAAAGGAAAAATAAATAGGGGGCGCGGCGTGTTGAAGCCGCGCCTGTGATATA
GACGTTGATGTAGATGAAACGGTAGGAGTAAAGTCAAATAGTTAAAATGAAAGAGATAGCCGCACTAAGCGCG
CGTGTAGATTGCAGTGCCACATATGGTATCTGGCACGGTCACGGGTGTTACTTCAACAC
>Cligib1_rnd-1_family-678#LTR/Copia
TTTCGTCCTTTAGGTAGATCGCANATTTCATANACGTCGCGATTNTTGAGGGAGTCTAATTCCTCTTCGCAGG
CGTTCATCCACTCTTTTTGAAGCTTTTTATCGAGCTTGAATATGTCTCGGAAGTGAGTAGGAACTTCGTCTTT
GACNGGAACGGCTTTGGANAGTAAGAAATCCATTATTGCTCCCCCTTCCTCTCTACATATNCGNATGAGNTTA
GTTCTTCATCGTCGGTAGAGGGATAGTCCGGAAGATCCCTTTCGGGACCTTTTCAACATTTTTACGCCAATNT
CGNTCACTAGCAATGTCNCGTTCTATTTCAGANGGTAGTTTCTTTTCTCCATAAATATTTCCAGGACGGTTAG
GCACNATCTTTCGACGNGTGCTNCGTCTNGGTAATTCGTTCCATTCTTCCTGATCCTCGCGGTTGGGCTCAAA
NGCGCGNGTGTAAGGNCCAGGTTGATTATCATCCCTTTCGGGAGATTCACGGTTGTCTGCCATTTCTGGTGTA
GATTGTCNTGGTGGAACGGGAGATGGAACTTCTTCCGGTTCTTCTTCAACCGTGGGAGCCCANGGATGTTTAT
TCTCATGTTTCGGAGAAGGGGGAGTCGAGAGTGTACGCGGATGAACATCTGGTGAACTATGATGTGATTCCGG
TGGAGACGGAGGAAGCNANTCNTCGGTTGGAGGAATTNTAGGTTGTTCNGGTACCGTAGTTTTTGTTTGCATT
TCGGCAGAGTGTTCTTCATCGAAGATAGCNGTNACTCCGATAAAGATAATGGGTTTTTGATTCGCATAAAGCG
ATAACCTTTTATTCCNTCAGCGTGACCGAGGTAAATCATTAGCTCGGATTTGGGCGCAAGTTTATTGGCACGT
TTTTCNGNTGGNAGATANACGTATGCAGCGCAGCCAAANACACGAGATGTGATTAAGGAGGGTCTTTCACCCT
TTAAGACTTCATATGGAGTTCGCCATTCCAGGCGCACCATAGGAGTACGGTTATANATGTGAACCGCTTGTTC
CACAGAGAATTCCCACCATGAACTAGGAAGGCATGCGTGATGNCGCATAGACTCTGCCTTATCCATAATAGTT
CTATTGATACGTTCAGCNCGCCCATTTTGTTGTGGTGTGTGGGGNGCNGATTGTTCGATTACAATGCCCAGTG
TTGATTTCAATTCATCAATAAATTCCTGGGATTTAAATTCTCCCCCTGCATCAGAACGCCANCGTTTGATTTT
GCTTTATATTGGTTTTTTACCAGCTGCATTGAAATGTGCGGAANGCCCCAAT
>Cligib1_Cluster22#ClassI/LARD
NgttggcagtggaggaggcagatggggtggtaTtagaTAtTaTtagaTatatatNttgtatgtattgttgtat
gaggaacaatcctcgaatgtaacNgactggacgcgcttAcataatcNgtaaagcggAaattATtctaagtcaa
aatactgagtgagcttgcactccaacacTtcccctcaagcgaaacgaaggGtNttgagcttgtaagtaNgtaa
AtatacctcatAatataatagaatgtattgtttaattgacttGagNtggtcaaaacgagtgtNaattNttgtt
gaagtcgaAatgtcccgatttgGacgtttgNNtgctgaccgagcGattgttGattNttcttgGgcgcttcctc
gaggttcaacttctatggccatactaaataggtaagNtggtcctcctaNatgNcctactaaccctctatcttc
cctaggNatgtctctgggatcccgataatcgactcctgctgNccNNNNctccaagataggtgagtcNtccNgg
atagNctgactaNaggctcctgggggcctcctggcacgaaaatcgcgacggataNNcgattctgNgtcggttg
gggtgtcgctttgagggcctattggattgggagatatcggtggggttttgggaatgatggatttgtgcttgga
cgaaggtaNgtccgttgcacggNcgttNNtcgacggtttNggctgattcctNGagCgttcgaNagcggttNNc
NaaggcttcaattcgcgtctattcgcgNgcggNgattcggtcggaNNgNtNtcggtggctNgcctaagtgggt
tggacNaagataggtccgttgcgcgatcattttccggcggtttNggctgattgttggaccgttCgggagcggt

tcCcaaggcCttcaaaacgtgcctattagcgcgcggtaattcgacaactttattttcgatgcttcggctgggg
ctggagatgNtgggcgcattcttcccctcgatctggacggttcggggtgttgggaggtcattcctaccatggt
aAacgttcccNNtaaccgNtcctgatcgcNtttctgccctacagatcccataactcgcggaatcttcatcaaa
atcggttttANggccttcagccacttccagttNNNtttttcggggtttagggtgttcgttttgtattgtatgN
tcttgttcttcaataaaatttggtttgtcttctttNtctggtgtgatttcgcgcggattcgcgtttttcgNcg
acatctcgcgcgcgattggggtttcaaNatggatttcgggcggtttatgNNaatNNNttgNNNNNNNtNNctc
NcgNNggNacgatcgacggtgcNcggtgggggtcctNgagtggatagggcaggggtNtcttcacaggggtatg
agatNtcgcggggtatatCcgactCttcgtcggtgNccNtcgtttcctcttcctgtggttcctgaccttctNt
tcctcctcctctcctatagcatccttcgtcgcgagcatgccCNgcgctcgtgCgcgattcctcaNtcttttcc
ttNNNcttNtcctcaggctcttCccctcgcttctccactattaccatcgCgtttttggcgcgtttttcggcaN
NgttcgaagggatcgcaaNatggccatNaaggaattgtcNtcttcattctcgaaaaggctaacgagctttgtc
cagagctNcgcggaggtcttctctttgtatattcgaaggtgggtgtatNtgctgattgttgcatagattactc
ctcggactatggcctcgttttgctcgaaaatgtcNattttNgtatNctgctcttcgatttCgtcgtcgttgag
cggtttcgtggGgtttccttcNaggaNccacttcccatccacctcgcgaagctcctctggcttcggcgcggtt
cCgtacagatgccgttcgAgtccttcgtgAgtcaggaggtttattaccctNttCttgtaNatgacccAaTttg
aaccagtgTcttNgAggtttggaaNtTcgagtgatgacattgtaggttgcccaggGcccataacctgttggca
gtggaggaggcagatggggtggtaTtagaTAtTaTtagaTatatatNttgtatgtattgttgtatgaggaaca
atcctcgaatgtaacagactggacgcgcttAcataatcNgtaaagcggaaAttATtctaagtcaaaatactga
gtgagcttgcactccAaca
>Cligib1_Cluster77#LTR/Gypsy
GTCACGACTCGCTCTTTTCCTTTCTTTCTTCCAAGTCTTTCTCATCTTATCTTATCTTATCCCCTTATCATAT
CAGAGCTTATCATATCTTATCAGGGGTTTATCTTATCTTTCTCAAATCCTTATCACAGATCATCACATGATCC
ATTATGTAAGCCAAACCGCGGTTTTGCATCATGGAGTACACATGCATTAGGATATTACTTTACATTGTTTTCT
TTACCTGTTATTGCCAAATACTCTAGAAGTACTTAAGGCTTTTCACTTGTATTATGAACGCTAATTCGATTCT
CTTTGGTTTCCGCCATAACAAGTGGGATCTTAAGTCAAACGTCTTCACTTCCATTTCTTTTATATTCATTCTA
CTTGAGAGACTGCCGCATCCTTTTGGGTGTTTTGGCGTGTCCTTACAGAAGAATCGAATTCCTTCTCGCGGCC
CTCTCTCTCTCAAGAACGATCAGCGTGGTCAACACCACCTCTCTAGTCGATTACCTGGGATAGTTAAGCCAAC
CAATACACCTTCTTACTAACATCATTATTTCTCCCTACCATCATCTATCCCAGCCTTCAGTCGTCGTTTCGAT
GCTGACCGCGCTCGTTCAGATCTTGGCAACAACCACTTATTAGTGGGTCGTCGTTTTTGACGTCCATTGGTAT
CTGGTCAGCAAATCCCCAGAACACTGAATTCCTTCAACACTCTCTCACTGCTGAGTTCCGCCCACACCTTGCG
CAGGATAAGCGTTTAAGGGTTAGCGCTCAGTAACAGTACAGACGCCTTGGACAGGCACGTGATAACAAGGAAC
ACGCTGACCTGAGTACGGTCGTCCCTCTTCCTGGACTCTGTTCGTGGGAACTGCCGCCCCACGTCCTATTTAG
GCAATATGTCGCACCCCTACACGTGGGTGTCGATGATAAGATAGCGTATCTTGAAAAACACATGTTAATATGT
CGGTCCCAATTGGATGCGATATTACGGGACACATCCCCGTCTATCTACTCCCGTACCTATCCCTACCCCTATC
CTCCAATTACAACATCAAATCGAGATGTTAACAACGTTAATGGAAGGAATGATGAAAGATATTCAAATCCGAA
ACCAAAGGATTGAAGATTTAAGAAACGATTGGAAGAAGATAAATCACTTCCATCCTCAGGTCTGGCCATGCAT
TTCGGTCGGCTGAGTTATCATTTCATGATTCCCTCCACTTCCACAATCCCTGGCCGCTGCGCCTATTACACCT
AGCGCGCCCATATTTGTATTCAGCACCGAGCGAAAGCTCATCGGGGATGACAACCCCAATTATGAAAACCATC
TCTCCACAACTAGATATCAGAAAATTAGAAGCTGCGACTGCTTGTCGAGTTTCAAAGAAAATACAAAAACAAA
AGAAAAAAATAAAACAAATTATGTTATCATTGTAGATTACCAGGACATTTATGTAAAAATTGTCATACTATAA
AAAACAAAAAAATTTTCACTTCTTCTCCTGTAAAATAGTTTCTCCTTTATCCTCTGTACCAAGGTATCCAAAC
CGAACAATTTCTTTTCTTCTCTTCTGTAGATAGTATGGAAACGATGACGAAGGCAATGTCAAAATCGGTGGAC
GGTTCTTTTCTCCTTTAAATATTCCTTAGACATTTCTAATTTAAATAGTTTACCTAACGTATCGAAGGTTATG
CTACCGGAAAAGGCACGGGTCGGTGACATCAAACCGTTCCCAGTTATTATGATTAACGGTGCTCAAGGGTCTG
CTCTGGAGAAGCAACTTAACGAGTACAAAGCTCATTTCGAAGGAAAACGAGTAGAGCGTAGTAAGGGTCTTGG
GGTTCCGTCTGACGTCCGTCCAGTGAAGCTGGGTCCCGGCGTTACGAACCTGTCGACCAATCAGGAGCCCTTT
AATGGGTCATCTGTCCGTCCCAAGTCTGGTGAACTGTGGGTCCCGGCAGTATCGGTGCTTCGGCCCGTCCTGG
TTTTCCCATAAGTGGAAATTACTTTATTCGCACACTCAATTTGGATCGAGAGCTTAAAATGTCGATCACAATG
ACGACATTAGATACACATGCGACGGCCAACACACAGGCCTTATTAGATAGTGGAGCTACTGGATCATTTATTG
ACAAAGCCTATGTAAAAGAAATGGGTTGGACTACACGATCGTTACGTCACCCTATACGAGTATTTAATGTGGA
CGGTACCGAAAATGCAGCGGTTTCATTTCCGATGAAATTACCTTGATGGTTTCCCACAAGGGCCATAAAGAAC
GCATGTTATTTGAAGTGACGACACTCGGAAAAACTAAAGTTATTTTGGGACACACATGGCTTAAGAAGCATAA
CCCCACAATCGACTGGACGACTGGGGAGGTCTCTATGAATAGGTGTCTGGCAGAATGCAATGTGGCTATCCGC
GCGGCTATTGAGGAGGTCGATGATGAGGATTCCAACCGTGTTATAGGCGGAATCATAACGGAACAAGAAGGAG
CCCTTTTAGAGGAGATTTGTTTTGACGAAACGGTCTACGAGGTTGTTGCGGAACACTTCGTGCGCACAGTGGA
GATCGAGGAGAGCTTTGATGCCCCTCCGTTGATAAGTGGATTACCGGAACCTACCGAACCTGGTCTAGAAGAA
AAGATACCCTGTAGATTCAGACGGTGGTTAAAAGTTTTGAAAAGAAAGCGTCAGAGCGTATGCCCAAGCGAAA
AACATGGGATCATGGGATCGAGTTAAAAGAAGGATTTGTGCCCAAGAAGGCCAAGGTTATTAATTTATCCCCT

ACCGAACAGAAGGAGGTAGACGATTTCTTAAAAGATCAATTATCTAAGGGCTATATTCGCGAAAGCAAGTCTC
CGCAGACGTCCGCTGTCTTTTTCGTACCAAAGAAGGATGGCAAGAAACGCATGGTACAGGACTACCGATTCCT
TAATGCGTGGACGATCAAAAATAATTATCCATTACCGATGATCGCCGACTTGATCGATAAAGCGGGAAAGTCT
AAATGGTTCACAAAGCTCGACCTACGCTGGGGCTACAATAACGTACGGACAAAAGAGGGTGACAAATGGAAAG
CGGCGTTCACCACGCATCGCGGTTCCTTCGAGCCATTAGTTATGTATTTTGGACTTTGCAATTCACCGGTGAC
GTTCCAGACCATGATGAATGCGCTATTACGAGAGTTGATTGACGAAGGCCATGTGGTCGTATATATTGATGAT
ATTTTAATCTTCACCGACACGCAAGAAGAAAACGAAAAGATCGTCGAACGGGTTCTCCAAATCCTATTAGACA
ACGACTTATTCTTGAAGCCGGAGAAATGTTCGTTCAGTACCCAGAAGGTGGAGTTCCTAGGGTTAATATTAGA
TAAAGGAGTTGTACGAATGGCAGAAGATAAGACGGACGCGGTTAGTAAATGGCCGGTCCCAAAGACGGTCCGG
GAAATAAGGAAATTCCTGGGATTCGCAAATTTTTATAGACGATTTATCGAGGGGTTCTCCAAGTTAGCGGGGC
CCTTAAACAATTTATTACGAAAGGACTCGAAGTTCGAATGGACTGATAAGCAACAGGTTGCCTTCGATACACT
AAAGAAATGTTTTACGTCGAACCCGATCCTGGTTACGGCAGATCCTAAACGAAAACTACGTGTGGAGTTGGAC
GCCTCAGACTTTGCTACTGGTGGTATATTAAGTATGTTATGTGACGATGGTAAATGGAGGCCGTGCGCGTATT
ATTCCAAATCTTTATCTGATGTAGAACGTAATTATGATGTACATGACAAAGAGATGCTGAGTATCATTAGATG
CTTGGAAGCTTGGCGTCATTATTTAGAAGGTGCGGACGAAACTGTAGAAATATGGTCGGACCATAAAAACTTA
CAATACTTTATGACGTCTAAGAAACTTAATCGTAGACAGGCTCGATGGGCTCTTTATTTATCACGTTTTGACT
TTTTACTTATTAACAAACCCGGAGCTGCTATGGGAAAGGCCGACGCTCTGTCCAGGAGAACAGACCTAAAGAG
GGGGGTAGAGTCCGATAACGATGATGTCACCCTACTCAAACCGGAATTCTTTTCTATTAAAGCCCTTCAGCAG
GGTCATGTGCTCTTGGACGGCCATGAGGAAGAGTTGTTGAAGAAGATTAGGAAGTCTAAGGATTATGACGAAG
CGGTGGTTAAGGCGATCGAGGAGTTGAAGCATTCGGCGACTAAGTGTTTGCGTTCAGAAGAGTGGTCCGAAGA
GCAGGATTTAATACTGTTCAGAGGAAAAGTTTACGTTCCAAAGTTGCCGGATGTCCGTCGAGAGATAGTCAAG
CTACATCACGAGTCGGTCGTTACTGGACACCCAGGACGAAATAAAACATTAGAATTAATTACGCGTAACTATT
AAGGCATGTCACGAGTGCACTCGTGGGTGATCCGGATCCGCAGCTGTGGGTACCCACACTCATGAGTCAGTTA
CTCATGAGGATCTGGGTACTCACAAGTTACTTGTGGATTTATGCTCTGCACCAAATTTCAACATTGAGGGGTC
AATTTGACACTCCTAAGGGTGCTTATTAATATCCAAAAGAGACGACAGGGATACCTGTCCCTGATCTTGAGTT
ATAGGCTGCGCATTCTAGGGGGGAGGAGGAGAAGGTCATGCTCCGGGGTAAATTCCCCACGGAATTACATGAT
TATGAGACGCATTTAACACTATTTTACGAGCCGAACACGATGCGAATGACGCGAAAGGGATCAGTGTCCCCGA
TCTCAAATTATGTTCAATTTGGCATATTTTTACTCTGTAGCTCAAGATCAAGGTCACCGATCCCTCTCGCTGC
ATTCGCATCGGATTGAGCTCATAAAATAGTATTTAATGCGTCCCACAATCATGTAATTCCGTGGGGAATTGAC
TCCAGAGCATAACCTTCTCTCCCTACCCCCCTGAAATGTGGACCCTATAACTCCTAATTGGGGGCAGGTAACC
CTGTTTTCTCTCCCAAACAAGGGTCCACAAGTACCCATGAGTAGATCTGCCAAAGACTCGGATCCATGAGTGT
AAATACCCACAAGTATCCCACGGACCTACTCATGAGTACTCATGAGTTACCCGCACTCGCGACATGCCCTAGT
AACTATTGGTGGCCTGGTATGGCTAAATTTATATTTTCATACGTCGACGGGTGCGACAAATGTCAAAGGCACA
AGAACTTCCCCGCCCCTCCTGTCGGAAAACTCGTACTGATGAGTACCCCGGTCGAACCCTGGAAGGCTGTATC
CTGCGATTTTATTACTGGATTACCGTTATCATCCGGTTATAACGCTATAGTCGTGGTCGTTGATCGTACCTCG
AAAATGGCACACTTTATTCCCACAACGTCTGAGACGTCTTCTCTTGGACTCGCTAAATTATATCGCGACTTTA
TTTGGAAGCATCACGGTCTCCCGGATTCAATTATTTCGGATCGAGGTCCTCAATTCGCATCTGTGATGATGAA
AGAATTAAATTCTTTACTGGGCATTCAAACTAAATTATCGACAGCCTATCACCCGCAGACGGACGGACAAACT
GAACGCGTCAATCAGGATATTGAAGCTTATTTATGACTTTTTATTGATCATCGACAAACCGACTGGTCGGAAT
GGCTTGCTTCAGCGGAGTTCACACATAACAACGCAATCCACTCTAGCACAAAGATTTCTCCTTTTTATCTCGT
CAACGGCCGGCATCCGCGCATGGGTTTTGAACCGATATGTTCCCAGAAGAATGAGACGGCTATCGAATTCGCC
GAGCGAATGAAACATATTCAGGATGAAGCGCAAGCCGCCCTTATTAAATCCCGAGACGAAATGAAGCGTTACG
CTGATCAACACCGTGGAGATATGCCGAAATATAAAGTAGGCGATAAAGTATGGCTAGAGACTAAAGATCTAGA
CATTAAACGCCCGTCGAAGAAACTCGCGGAACGTCGCATTGGTCCCTTCGAAGTTTTAGAACTCGTCGGTTCA
CATGCGGTCAAATTAAAATTCCCCAGCTCGATTCGTATTCACCCGGTTGTGAACGTTTCCCGTATCCGCCCAT
TTATTCCTCCTATTGCCGGTCAAACATCCGCACCACCCCCTCCAATCGATATCGACGGCGAGTTTGAATACCA
CGTTGAGAAGATTCTCGATTCGCGTCTGGAACGAGGTCGACTTCAGTACTACATTCAGTGGGAAGGATATTCT
CCAGAACATTCTACTTGGGAGCCGATACGAATCTCAAAAACTCCATGGATCTGGTTAACGATTTTCATCGCAG
GAATCCATCGGCGCCACGGAAGATACAGGGTATCTCACAAACTCAATTTGATAATTTATTCCAACCATATACC
AACTTCACCGTCCCTGAGGGATCCCTCTTATTTGCGCCCTAGAAGGGGAGTAATAGATTTTATTTTATATTTC
TCATAACATTGTGTAGCGAGCAGACGATTCGAGTTTGACGAACCATGGATATTCTCCAAAGTGTTCTCCTCAT
TGTACTGTGTACGCCCCTTTGTTTGCCGCACTGGAGTCAATCTTCACTTGAGGTGATGCGGAGTTTTTCTTTT
CTCTTTTTCTCTTTATCATTTTCTTTATTTTTCCGGAGGAAAAATCCTAGAAGGGGAGTTATGTCACGACTCG
CTCTTTTCCTTTCTTTCTTCCAAGTCTTTCTCATCTTATCTTATCTTATCCCCTTATCTATCAGAGCTTATCA
TATCTTATCAGGGGTTTATCTTATCTTTCTCAAATCCTTATCACAGATCATCACATGATCCATTATGTAAGCC
AAACCGCGGTTTTGCATCATGGAGTACACATGCATTAGGATATTACTTTACATTGTTTTCTTTACCTGTTATT
GCCAAATACTCTAGAAGTACTTAAGGCTTTTCACTTGTATTATGAACGCTAATTCGATTCTCTTTGGTTTCCG

CCATAACAAGTGGGATCTTAAGTCAAACGTCTTCACTTCCATTTCTTTTATATTCATTCTACTTGAGAGACTG
CCGCATCCTTTTGGGTGTTTTGGCGTGTCCTTACA
>Cligib1_Cluster76#LTR/Copia
GTTGAGCTGCACCTACAGTGCACAGCGGAACTGGCTAACTTACTTGGTAGACTTAGTGAGCTGAGAACACGGG
TGTAGAGAGCAAAAGAACTTATATTAGTCTCTTGTTCAGAGAGGTCTTATATACTACAATAATGTGCACAGAC
CATGTGGCTGTGCTAAAGTCCTAACTCCTATCTAGGATCTAATCTACTGATAAGATAGATAAGGTGTTATCTA
AGGTGCTTATATAGACACCGGATTATGTAAGCAGGGCACTTACAGTTAGAGCATTGAAGTTTAACACTCCCCC
TCGCTTAGAACAGCGGATGCAAATAATCAACCTTTGCAGGAGTTATAAGCGATACTACAACTTCAAGACGAGT
AAAACTCAAGTCCCAGTTGAGGTACGAATTCTTTGAATTTGATGTGCCCTAGACTTTTCGTAAGAATGTCTGC
AGGGTTTACATTTCCTTCAGTGAAAAGTATGTCTACTTTTCCTTCGTCATAACATTCACGAATGTAATGATGA
CGGATATCAATGTGTTTGGTTCGTCGTTCCTGAACTGGATTAGAACCAATGAATATTGATCCTTCATTATCGC
CAACAATTGGTATTTTGTTGAGTTTGATTCCTAGTTCTCCGAAGAGAGACCATAACCAAACCACTTGTCGACA
AGTGTCTGACATGGCAATATATTCTGCCTCAGTAGATGACTGGGCAATTGTTTTTTGTAGTCTGGACGTCCAA
GTCACAGCTCCGGAAGCAAGATTTGTGAAAAAACCGGAGGTAGATTTCCTAGTATTAGGGTCGGAACCCCAAT
CGGAATCTGTGTGAGCAATTAAGCCTTCACCCGAATCCCCTTTATATACAAGGGCATAATCTTGGGTTCCAGC
TAAGTAGCGACATATTTGAAGTGCATGTTTAAGATGTTCTTCAGTGGGGTTTGCGCTCATTTGTGACATTTTA
ATCACAGCAAATCCTATGTCGGGGCGAGTGCCCAACATTAAGTAAAGCAATGATCCTATGACGGATTGATATT
TGCTTCGAAGGGTATTATCTATTGGGCCCTCATTTTTCAGAGGTAGATATTTGTAAGGCAAGGGGGTTTTGGC
AGATTTAGCATTAAGCATTCCAAAGCGCTCTAATACTTTGACCAGATAGGGTTTCTGATCAAGTTTAATCTGG
TTTTTATTTTGCCAGATGCGCATTCCGAGGAATTCTTTAGCATCACCAAGATCCCTGCATTCCCATTGTTGCA
TGAATTCTGCTTTTTTCTTGATAACATACGACCGATTAGGCCCCAAGAATAAGGCATCATCAACGTAAACTAT
CGCAATGACAATTTGATTCAAGGATGATCGGTGTATGAATATGCCTGCATCTGAAACAGTGCGCTGAAATCCT
AATTCTTTCATAGACGCAACGAGTGCTCTCCACCATTCAAGAGCAGCTTGTTTCAGACCATATAAGGCGCGTT
TGAGTCGCAGGACTTTGCGCTCATGACCTTTACGGACGAAGCCTTCTGGTTGACTCATATATATCTCAATGTC
TAGTTTGCCATATAGAAAGGCAGTTCGAACGTCCAGTGATAGAATTACCCACTTTTTCAGGGCTGCTGTTGCA
ATCATAAGACGCACAGATTCATATCGAACTACTGGTGAGAATAATTCGTCGAAGTCAACACCTTCGACTTGAG
AGTAACCTTTGGCTACGAGACGGGCTTTCTTTCGTCCATCAGATTTGATGTTGAAAACCCAATGATTACGAAT
TGCCTTTCGTCCTTTAGGCAGATCGCATATATCGTAAACGTCGCGATTCTTGAGGGAGTCTAATTCCTCTTCG
CAAGTGTTCATCCACTCTTTTTGAAGCTTTTTGTCAAGCTTAAATATGTCTCGGAAGTGAGTAGGAACTTCGT
CTTTGACAGGAATGGCTTTGGATAATAAGAAATCCATTATTTGTCCCCCTTCCTCTCTACATATACGAATGAG
TTCTAATTCCTCATCCATCAGGAAAACCCGATCCGGCCATATATCCATATATATCAGTAAATACCAGTATATA
CCAACACCTCCTATTTAGCAGCATTCAAGGTCTCAATATTTATTTATCTGGCTATTATCCCCCAAGCAATATT
TTGCCTGTTTTTAGCACCATTATATCCTTGTATATCCACCAATGTGTCCCAATACTATTGTAATATCACTCTC
ATATCATCTTATAGGCCAGTTGATGTTAAAATATCCAAGAAATATCATGTATTATGGTTCATTGTCTAATCAT
ACATGTATTCTACAGCTACATTATTACATATAGAAGTTGCAAAGGTGATGGTGGCAGAGGGTTGGATGGGGGC
ATAAATAGCGGTAGCAAAAACCTACAGTAGCGCTTGACTGGGGGACGGCACAACAGCGCAGCCGTGAAGCGCT
GTAATGGGCGTGTGACAGCTGGCGAGTGTGGTAGGGCCCCGGAGAGCTGATGGGCATTCAGCGGTGGTGGAGG
GTGCGAATCGATGGGCCGGGCGGGGGTGGGACGGCGCCTGTGGGTGACATGTCGGGCCCGCGGCGGGTATGGT
GGTGTGCACAAGGGAGCGGCGGACGGCGCGAAAAGGCCGGGGAGTTGGTGGGCGTGCAATATTTGAACAGGGT
GGCTGGGGGGCTAGAGTGTGAGGGGCGTTAGAAAGCATTAGATGGGGGTGGTGAGCTGGACGTACGGCGTGGT
GCGAGAATGCGGCAGGGGTGACAATGGTGGAACGACGGGGTTGCGGGGATAGGGGGTTGATGGGCATGGGCGT
GACGTGGTGGGGACATGACAGTGGCAGAGGGTGCGAGGAGGCGGCGAGGGCTAGACGAGGATACAACAGCGGC
CCAGCGGGAGGACGTCGCGCTGGCAATGAAGACAAGGGAGGCATTAGAACCCACGAATATGAAAGAGCGGGGG
AAAAACTTGCCCCCATAGAACGATAGCGAATAATGCAGAGGAGAGCGTGGCTGAAGGTACGATGATGAGCCTG
ATGCACTGGAGAATGACGTGGCACAGCTGGAGGTGATGATGTGGCGCAGCAAGAAGCGTGAGCATTGAGGTAG
AAATGACTCACGCTTAGATGAGGTACGTTGTGCCGCAGGAGGCGGGAGGCGGGAGGCGATGCGGCTGGAAAAC
AGGCATTGACGACGACGACGACAACGAGCAGAAAGGAGGATGCATTGTCAAAAGAAAAAAGTGGAACGATGTC
GCTTGTTTGGCATTGAGCTCAACTGAGATTGAAATGACTTGGAAGATTTTCGACTCCAAGTGCATCAATATTA
GTTCTGGTTGAATTATTTATGACTAAATAGTTTTGAATATCCCTGGACATTCCAAGGAAATAAATGCTGAGAC
TTTGAATACTAGTAAATAGTTCACCCCACCAATATATTGATGATATAAATATTCTAGCAGTACTCAACTTGTA
AATAGCCATAAAATATCAGTAGTGGATATTTATCAGTATTTGTGAGCCAATTACTGGATTTTATGTGATTACT
TCAAATATCAATAAATTGGAACTATTTGGCAGATTCGGGTTTTCCTGATGTTGTCGGTAGAGGGATAATCCGG
AAGATCCCTTTCAGGACCCTTTTCAACATTTTTACGCCAATGTCGTTCACTAGCGATATCACGTTCAATTTCA
GATGGTAATTTCTTTTCTCCATAAATGTTTCCAGGACGGTTAGGCACAATCTTTCGACGAGTGCTACGTCTTG
GTAATTGATTCCATTCTTCTTGATCCTCACGATCGGGCTCAAAAGCACGCGTGTAAGGACCAGGTTGATTATC
ATCCCTTTCGGGAGATTCACGATTGTCTGCCATTTCTGGTGTAGATTGTCGTGATGGAATTGGAGATGGAGTT
TCTTCCGGTTCCTCTTCAACTGTGGGACCCCAAGGATGTTTATTCTCGTTTTTTGGAGAAGGGGGAGTTGAGA
GTGTGCGCGGATGAACATCTGGTGAACTATGATGTGATTCCGGTGGAGGCGGATTAAGCCAGTCCTCAGTTGG

AGGAATTTTAGGTTGTTCTGCTACCGTAGTTTTTGTTTTGCATTTCGGCAAGTGATCTTCATCAAAGATTGCG
TAACACCGATAAAGATAATCGGGTTTTTAATTCGCATAAACGATAACCTTTTATTCCATCCGCATGACCGAGA
TAAATCATTAGCTCGGATTTGGGCGCAAGTTTATTGGCACGTTTTTCTCGTGGGAGATATACGTATGCAGCGC
AGCCAAAAACATGTAGATGTGTTAAAGAGGGTCTTTCACCCTTTAAGACTTCATATGGAGTTCGCCAGTTAAG
GCGTACCATTGGAGTACGGTTATAAATATGAACCGCCTGTTCCACAGAAAATTCCCACCATGAACCAGGTAGG
CATGCGTGATGTCGCATAGACTCTGCCTTATCCATAACGGTTCTATTGATACGTTCAGCACGCCCATTTTGTT
GTGGTGTGTGGGGCGCCGATTGTTCAATTATAATGCCATTTGACTTCAATTCATTAATAAATTCCTGGGATTT
ATATTCTCCCCCTGCATCAGAATGCCAGCGTTTAATATTTGCTTTATATTGGTTTTTTACCAGCACATGAAAT
GTGCGGAATGCTCCAATGGCGTCTGATTTATGTTTTAGATGTGAAATCCAAACATATGACGTGTAATCGTCAA
GGAATGTAATGATGTATGAATACCCATGATAAGATTTCACAGGGAAGCTTTTTAAATCCGAATGAATCAATTC
GAATGGCTTCTTGGCACGTGAGACGGAAGGTTTAAAAGATGAAGAAGGCATCTTTCCGTATGCACAGCCAGGG
CATATAGGTGGGTTTTTCGGGATGATTAATCCCTGTGGGAAGTCTTTAATGTGTTTTCGTGACTTAGACACGA
CCTCTTTCAAGGGATGACCTAGGCGGCGATGCCATATGTCATATGACTCAATTGAATTAATAAATAAGGATTG
TTTTAAACCGTTTGGTTTAACAACATCTGCATCTACCCAGAACGTAGTGTCCCACGGAAAACGCTTTTCAGCG
GTTAATTGCCGTGTCCCTTTGTTACTAAATATGAACGCATCTGATGATGCATTCAGCGATAATCCAGAATTAA
GTAATTCTCCCATGGATATAATTCGCGAATTTAATCGGGGATGGTGATAAACAGGATGTAAGCGCGTGTGTCG
CTTTTTATAAGATCCATCTGTTTGTAAGACGTTGTTTTCAAGGATAACGGTCCTTGTATTTTAATTTGAATCA
TTTCTGTTGCAGTAGCAACATTGAGTGGGGTGTTGGTTGTTGAATGTTCAACATAATCATCCAATAATGCAGT
AAAGTGCATGGAGGCGCCTGAGTCTACTAACCATTGGTGTTCGACATGTTTCGCTTTTCCCTTACAAGATGCG
CAAAGGGCATAATGATTATTTATTTCGGCTCTTTGGCCTGCAGACAAAACAGTACATGATGCATATTTAAAGT
ACATAGGGCAGTCCAAATTTAATCGAAAAGCGCCCTGCAAAATGATACTGTTAGTCTCATAATAAGGGAGAAA
GGAGGCGGTGACTGACGATTGACCATCAAACCGGGTTCCCCAACTCTCAGGATCGAATTCGGGTTCTTCACCC
ATCGACACTGGATCCTCGCGATCGTCTTCTTCAGAGACCGGAGTGAGTCTTCGCTTCTTAGGCGGAGGTGACC
AGGCCTCCACGTCATAGTTGACGTATGCGGTACCTTCATCTTCAGGTCCGCAGTATGATTTGAGCCAATCATC
ATACTCGTCCTCTTGCATTAGGATGTCTTTAATACCATCCGGAGTGTTAGAGCGGGGCGGAGATGCCACAGGC
TCAGGAGTGGGTGCACGCGGTGTTGGCGCACGACGAGGAGGAGTGGGAAGTGGTCGAGTGGGGCGTGGTTGGA
GAATCTGGATATCCTCCGGCGGAAATTGCCTGAGTACGCGATGCTCAGGCTGCGCCATTTGTTGGATCTCAAG
AGATTTGAGAGTCTCGGTGGTGACAGGGGCACCAATACGCTGTGATAGTGTTAACGCTTTCTCAAACGTTGGA
TAGGTACCAGGTAGAATGCCCGGTGTGGCTCCCATGAAAACCTGGGCAGGTTTATCGTGTAGCCGCGCAAGGA
GGGAGCGTTCCTTGGGGCCGAGAGGTTTGGCTTGTCGAACGAAGCGAGGTTCCTCAATGACCTCTTCGATGAA
TGCAGACGAGACCAAGTGAGAATTCCCATATTGGGAAGTGGAATCGGGTGCGGATCCATCGGATGAAGCGGCT
TCATCAGCCTGCTTGGCTTGGCGTTTCTTGCGACGCTCGGTGCGATTGCCACCCTTAGAAGAGCGGGCTTTGT
CGTCCTTGGGCTTATCCTTCGACGAACTTGCCTCAGTAGGTTGTTCTCGGTGCCTTGTCCTTCGACTGTTCTT
TCCAGTTAGGGTTCTTGCCTTACGTTTGACGCCAGAGATTTTGTTAGCCTGACAGGCCCTTTGATGGCGGTGC
GACGTTGATACTCTTTTACAAGAGCAATACGCACAATTTCGAATGTGAGGGCGGATTGGGAGTGAGTTTGCAG
GAGTTGCGAACCCACATTATCCCATTTGGAAGGGATGGCAGAGAGGAGATCATGCCTGTACAAAGGCGGAAGG
ACGACTGAGTTTCCCGTGAGCGGGAGAAGTAGTGGACATAGCCATTATTTCAGGCTCCGGATTGGAATCACCG
CTAATTTTAACTGCAAGGGCAGAGCGGAAATCTAAAATACTTGAGAGGGGTCAGCAACCCCTATTTGGTATTG
AGGGCTGCCCAGAGAGTACGAGCCGTGGTGTACGTTGTGTGAGCAGCGGCGATGTCAGGTGAGAGTGTGAGGA
GAATGGCTCCAAGAGCCTGATCATCTTTATTGAAAAATTTAGTGAGAGCGTCCTCAGAAGGCGGGACGATGTC
GGTGCCATCCATAGTGCTGGTGGGCTCGATTTCGCGGCCATCGGCAGCAGTATCTGCCAGAGGCTGCTGAATC
GGAGGAAAGTGCGGACTTTCTCTTCCCAGTCACGGTAGTTGGTTCCATCGAGTTTCTTGATGGAGTTGATGTC
GAGTTTCCGCCTGAAGACATTGTGATATCAAGGTAAATTGTTTGAGGTTGTCTAGTAAAGGGGCGACATTAGC
CTCATGTTACAATGAAGAGAAATGTATGCCTTTGCAGGCACAGTAACGGTTAACCCAACAACATAACCTTTTC
AGGTAGTTGTGGTGAAGTTGCATAACGCAAGCATGTAAAACACGCATTGCGCTCTGCGAGCTTCTAGGAGATG
CGCATGAAGCAAGCATGTATAACACGCATTGCATCGAGCAGAGCTCCTCCAGACAGTGAGCTGCAGCCTTATT
CAGGCGCGGCAAGTCGTCTGGTACACCCAGTGGAAGCGCATTTCACACAGCTTTTTCAAGCGCGTGTAATGGC
GACCAAGACAGCCAGCTGGTCAGTTGCAGCCTTATTCAGGCGCGGTTGACCAGGGCGTCAGGGCCCATAACCT
GTTGAGCTGCACCTACAGTGCACAGCGGAACTGGCTAACTTACTTGGTAGACTTAGTGAGCTGAGAACACGGG
TGTAGAGAGCAAAAGAACTTATATTAGTCTCTTGTTCAGAGAGGTCTTATATACTAAATAATGGTGCACAGAC
CATGTGGCTGTGCTAAAGTCCTAACTCCTATCTAGATCTAATCTACTGATAAGATAGATAAGGTGTTATCTAG
GTGCTTATATAGACACCGGATTATGTAAGCAGGGCACTTACAGTTAGAGCATTGAAGTTTAACAT
>Cligib1_Cluster0#LTR/Gypsy
cccgggAagaaccaaaccccaGaaaaaagCCcaaacaaaACAaCAaacacgcttcgacgacaTCgACcacGA
cagCGaccTCaaaccacGAccaCgaccaaccaagaCGaagaaGaaCTcaaCTCccgactttgacctcaactct
cgaactcaacgaccAacggcaAagacGAcgTCGACTagaacaaaCGAaccgccaaagCGAcggCGAacgTcca
agccaaaacgcGacCgaGaaggacaaagagcaaaacaaccAagaacaacaAcgcGaaaagaacGAGaGaACcA
acAaaaaaACcAacTCcaAGaccaaGaccgaacaTCacCaacAaagcaacgaaAcGcgaaAacaGaGcTaaAa

gAaagcacgaggcgCaaaGacacaGcGaaagGccAacaacAcaacCTaTacACgcgccaCaCAacacaaaaGA
AgGGaaacgaaaaaCgAcaacaaaAcaaaGaagCaaaCgcaaacggcaAcagcGcaaaaaagTaaaCAacaCa
cacacaaaacaaaccgacGaccTGTacAagcagggcCcagcTacaaaagcaaaaagGaaaCacgaTcaccggc
TaGagcagaacgaCCaTTCcgagcaagTGcGcccaaaGTccAgAGccgcTaaaaacaaCccgcCcccGgagCg
gaGaaaacaacaaggTagTccAcCgCacgTgaacCaTcgaagccagaTcgaaaaGAacgaaaacCgacaaCTa
cGAagTcGTcggccTccTcGTcCgCTCaccaaagcCGacTacgccgcacTcgcgaccgccagagaCcaacccC
gaGttctcgtcctcgtcctcgttctcgtcctcgtcctcgttctcgtcctcgtcctcgttctcgtcctcgcgCg
aGgccccaagcacAacgTCGacgTCGagaccaaacctcgagtcacaaAGTCGAaaTCGaagTccaaagTccaa
agcacGAGacaAGggCgaacgCGacgcccagaagaGaaaTCaaacgccGAaagCGagaTCcaaGTaGAacgcg
aaGgTGTgggaccacaGGaaccaacCacaacgccccccaaTaacTccgcacCaCacaaagacgGacgggaagC
AcaaacccaacaGacaAccggaAaaacAAGCaagCcaggaAaacAaaCAaccTCGcgaCgAacgCaaggcCca
ccAaaCTccaagacAaacacGaagAagaaacCacAaaagaagaaACaccaccCTcggTccacgTgccagCAgC
aaCCaaaccCcaaCccAAaccCgaacgagacacTccgccaGaGacacaagccgaCccaacCccaGaaaCGcaa
cgcaccTcGcTCgaGTcTagTccCacgaccTcAccAgcCaaacCagaaCcacccaaaacacCaacaCcaaCAc
CcccCTcaTcAcccgTcaccaaaacaaaacgcaaCagcTcaGaagcaCCTgcGcCcggacggAgcccaccAgc
ggCgaTcCacGCaaaAccaacccccGcggCcaaCcAccccgacgaCaaaaaccCCaGCacccgccAcccaaag
ggcaGGgaccTCcaaGccaTagacCcaaCCacacgacTCgacGGcagcgaCTcaGaaagcaaaaacaagccag
TccaagaccccccaCgccgacAaggcCgaAacgTGcaaacGCagCgaCagCTaCaaaAaCcGCaCCgaCaccGA
CggCaacAgcaaagcTcaacCaTAaaacCcaGgcGaaaagaaaTcACcaCGagTCaaccccggaaAccccaAC
caGccGaccGaaaAgacagcagccccaGgCaccgaCaaccTCaaacACCTCaaccagTaaagTaagaaccaaa
AagCaagcaaaGaccagTaGaGcTgGcaaaaccAaAgGcAaaaacaaGaGcaaaGacgTaaagccccCaacgG
acaTcaAAccaaGccgacTaGacaTcgaaaTcaccaccTacGAacCacGTacccacAccggcaccccaAGaAa
CccTaTccaTgggaGaaaaTcaagcaAaaGgcTaagAaaggaccgCaaagGAacaggccagGcaaTcagaGTc
CcacaaaagcCcccaaacTCacagTcGaCGACacGGagaacaaCaagAaGaACcgaaAgaagAaGcccGcccA
agaGcccGagcacaAggCcCacacaAacCCGAacagcggTACgcAggCaGaaAcaGgcgaTGcCCcgacgCcc
cGcAaccGcaacgTccGTccaaacccccGgcacaACaTcccgcGagGCcccacaGccgaagaGTacAgcCGca
acaacaagaTTcccaGAaacgCAcagaacacgGgcacccTccccACaccaGacgacaTccaGcccGAccgcca
aGcagccaaacTGAaacaaacacgccAccaaaGccaCagCcccaacgCacaGcaagaaccaaaaggacaGaac
AacagcaaCGaaGAcacaaacaaacAaaaGacCgaggccaacaAcCcgTCacagAacacacaaCcaCcaagga
GaaacacacCccTacCacagccGaaaAaCTAcCacaaccccaTcTaTGaaacaaAcgaccACaaagCgcaTcT
cGaaccacaacgccgAgcAAcGaaaGaaAgTcgaaaAcaaAcgGaaaaTaTcTcaaacaTGaGcCGAaccAaa
GcaaccaAgGaAgccCcaaAcaAgaTaagaaAAgacgGaAaagTTaaaacAaTcgaTaCTCaAaagAAagaaA
agaAaaaGccgAaaaaagcaaaacagCaaaaccaacaGaaaAaCaacgaTaaacCgaaCGaggagccaCgcaA
gcagTTCGacaagGCTaaaaaaGcaTccaaAccAgcacTTacggAgaaAgaaaGAcaaaacGaGaAAaAaaaa
cGcaaccAaaacacaaccgcAcCcaacGaagagcCacaacccccccaacaaagccTacaCcCcagggcTagagg
aacCaaccaTTaCGCgcAccacccccGaacTcaTGTCgGTcaaAccagcgagaaGcacaaTAgcccgaaGTGa
aaacaaGccaAcgacaacgaaTcacaaaACgGgcCgggCaaaCaacGaACcgcCcagTcCagCGAcaaaggAC
accaaCCCagaaaTcaGgcAaagTaCcaTgcagccgcccgCGaaCacaagcccAaAcgaccgagaGagagACa
TccaaacccaTGaaaccacgacaaacaagaacgccCcaAcagTgaacaaaaAGTCGaaaacacaacGaGCGcg
cCaagcacaGaacgagAaccaGGcacTaacccaAgCTacaaacaacCcCGaCaCaaagagTagAacaacgTCa
gaaacTaTCTGCaaaaaGaTcGcgCgcaGGacaagaGaaCcaTacAGCTcAGGATccAGTCcGccaagGGag
agagcgAcgTAgCTccaGggaTagCGaagaagagCacaaggcGaGTcccTGgcAccaCaaaaTAcGgccAaca
cCacATaagcCGgaGaacaGcAaaccaTacAccaCacaaTacacaGTacaaCacccgTaacCaaagcgTagcc
agcaacTccGagaaCgaGgAgaaaaacagcaccAcccaagAaccaaaTacagcGagcAggcccacgacaaaAa
aaaTgcaaCccaccAcaccAaaAcGaaaaaaagaaTaTcacagcTCcAGGagcTaGacccCgaccAaGaAcaa
gTcGCcgTgATCcgagAcGaagaCcCacaccagcCcCcaacgacaaAagAgagTCTacaAcacaCaaacTCcc
aTcGTaccgCcTgcgaCcaagcGaaGcagccgaAaCTacaaGcaTcTGaTaacagacCAcCcaCagTagACaa
cacacgcgaAacagaaTcAcccacgcacaTGcATcGAcgcGacccGaacCcaGacgaaaAccccccaCgGagc
aGCGaCcgcgccaaGAAacAcaAaCcaGcgcaGCaGaaGgTcaaccCaAaCaTcGCaCaaaccaaCaaAcaaT
TcaagaTCcAacacaAgaTcacgccAcaacCacccaTCGAccaaCagagAcacgacaccaacaaCaCacccaT
cgaaCaGacCGgacgacacacTgTaGAacaTcgaGAcgaagcaaacCGGcAccATcaacaacaggTcagcacc
CcTGaaGaacgcgCaccaAacCacGaCTTTaTcTgTaAAcAaaaccaaacagaagCagacGAAGacaaCGacc
gCcgaaaaTacaaaaccgaacgcaacCcccaaGgacagaacaaggCggccagcAgACTcacgcGaTacacag
caaCATcccaATaCcacCggcaaggcaAgacaaccTaaGcgcccgcagcaaGaacaagaTCcaCgCcacagga
gAccaaacagcaaaCacagcgaacaaGcaccaaTgGgTcaccGTcaTCaAAaagGAgGGaaAggcaccaccca
ccGgCCacagTcTCcaacaAcTcaacAacGacaaTaTgcGaaAagcaGgaGaaaCaaccAacCCAgaacacaa
agcCGaacaagTcagcgGacGcacaTgCGgCGcaagGCTcgacTgacacgcaaGagAgaacaaacGcaaCacc
caccaagCCTccaacGagcaCaacaccaaacaaaaAccCgacGacccAAgcaaCcaaCcaagcaTcCaagcG

cagGGaaaAAgaccGgAacgaTcgacCATaaccaTGacAcaTacagccTccaCcccaAcaacCaaaacaccaa
cacCcaTcAgaTacAcgaTcaTcccGccaAGaGcaCcGaAaagaCcaaacaccTacaGcaTcGaaccGgaGAa
aCGcacCccGacaaCcccGaGagTcaaCGAagCaaAgcGcaccaagTggCcaaccTTaagagGaTccaAcacc
gaaTGaaAaAgggaGaacccaacTgCgaaagcCaaaaaGTCaaacgaTacGacaAaGaaagaacgGgaCTaag
AaaCcagTGcaCgCagTgagGacGAcaccacGAcAgcacgcgaGaggcTccCaTgccCaaCTgcggccccTGc
aacaaacacaaagaagTCagcgcacTTggacaaaccagaaaGggcGTaCGaAacacgagCaAAaacTTcGaaa
aacaTgCGcACcaaCccaagaagcccaaaAAaaaaaagGAaccacgTaaagccaggcCAcaacaacaAGcgGc
caAaGacAcaaaCaaaaagcaTcTCagccacTCccCagaAagaaGaaCAaccGagTacaAGaaCaacaacccC
aTcagCccAgaaggcGACaccgcGcacagaGaAcgcaccgcCgaacTaTgcCAacaacacTgcaAgCacacaA
AaacccGaaAagaccccCcaTacGaaTCGaccAacCcCaacGaacgCaaGcaaCGacTCgaCcaacCaAacCg
cgAacgcgaacGcagcTgCcGgaCcCTacaCaccAacaaaaTcgAcaTaAgaGaccgTcaaaagggGaaccTc
aaaGcccAcaaacgAcaTaaCAacagcATGcaacAgaagTaCcaaaTTagcCCaacCcacaGcAacaacaGgg
aGaccaaTTcaacCcaaacaTgccacgaaaagcgcGaaaaCacgccAGGaagccgccacGgccCagacagcaT
agCccaacgacaAagacAGCacacCGaCGaAgaccCccCagacaAagacgaCGaagagacagaaTGGaaccag
cGcgaCgaCgcagTggCaaAgAgcaaCaaTacgcAcTgacccGAaAacacGGaCaaaacacAaacaaTcgCCa
TCaaaTTcGAcaACaaaaAaCaAgcaagaCcaagaacagaaGcgaaaaagcacaTagaaaccaGaGacGAgac
ccagCaccaccaaccacCagAccaccaaTccaagccaaGcaaGaATgcccATGcacaccacaaaCcaagcAGT
aTcagccaaGaaggcaaagacaaCggacaGcaacaagcacacacgcaAgccaacGaaccCGagacCTTCccaa
GagaagccaccagacTcTTcAaAcAcAAAGaCcaGcaTcaaaGaaaaaaCgaCAaGacacaacAaaacaccgg
cATacTcCCGGaccaacgaaTCgacAccaTCaAaAacacacacgaCGaaCgGggacAaaaagGgGggaacccg
acaaaaTagagGgaccTCGAacaagGcggcTccacacAcaTgaCcaTcGAcacgaAAggaTacGggacgacGg
GCcAcGaTaGccagcAacgaCaGCgccaCcaGaTccTaaTTCcgccaaaaGaCGCaaacCaGgccccccTcgc
aacCaaGaggCacagCaacTCGATaCAgaaaacaacaTcAggagaaTaaaaaaacaggGTacAaaGacGaTGc
gaacccAcCAcAgaCctgttacgtcctgctaacgcaagccgatgagtcgacagtaaagactcaaggcacttag
caaggtctgagttgccttctgggtcaggaggctagtagagggggaactgataaacgattaaacgtagcgaat
acaagggtatgtagctacgtttatatagagtgtatgtagttctatgactacgcagtaatgacaaggtccgtga
gtatagtaagacatatggagggtgatgactgataatagaagcgagctgactgagcagctgagtaagaacagca
aagataacaaagatagaatgatgaccatgcggtcgagaagtagataagggatcgcatgatgtatcatctgatg
atggagcatgtggatggtgtcagatgatgatggaggaagtagaagcgtctatgaaagatgggatcagtcaagt
gactgatgtatgatgaatgagaagcaatggatcgtaacaataccccgAGgTaaacaccCggCaaggcGaaaaA
cGaacgACaAgcaGAaaaaGgagaTacgAGAacaTacTaaccaaagcGcGcagccgccGcGaaacCaTaaacG
ATaAacGCaAaccaTcCcTcaaaGcacaCgAaaaCCTcGAaaacaaAaAacacaccaaaCaTagTcGcaTcag
ccGCgaaAaggcTcggacaaaaaccaTcagCcagcgaccccagTaggacaTacgaaaagcccgaTaCaaGgca
gCcaAcggcaaaaaaAaaaagcGagcAaaaacGgcaaACgcgcgAcccaGgacTaaAccaaaTaCaagaCaaC
GaaggATagGaaccTcaaCaTAgagaGcaaTcaCAccaACgCaaacacaGaccccacgCGagAgaaCcaaAGa
TAacTacacacagCCaccacCcGACTccATCCTaAGCTcaAcaGagCgaaccgaCCgaaGacCcAgCgcgccG
CAGAaAcgagacGAaaacccTaaAagacgcGaGcCGcGcaccaagaaAGaTccaacgAaacCCgccaTaaaca
cCgaaaaaaGaaaCGCcCaaaccacaggCGACTccaacgcaAaacTgaGcaacccagcccggccaagaaaacC
AaaaaccgaaAaGTaaacCaagaacaaaTcTcacacccgGaaCGaaccaccagaaAccGgAaccTcaCgaAac
gaTGGGaaaccGTaaccacTcGaaGcAaacGaaaGTgCaaCaagcGaaaGcaCaGTggccgcAggacgacgaa
TaccCgacacagCACgcaaaaacAggcacCgaacaaagaaTgacAcaGACAacAcaaCacgacgacTcacCaa
AAcgGaaGccgaaaacAaaaaacGcGacgAcaaagacacaGcaaacgaaGaagaggCaacaGaTcaaaagcac
gaaTcagaaCcccgcTcTTcCgCcgccaaTCgaaacagaTaaagaAccaaTaaTagcaAgaCcaccacAGCag
cagCGAAagcaTgaaGaccacaagCGcaaGacccacaaggagTgaaCccgccaagacGagaggccaagcccTT
accCgccTaaaTccaTTTagcTgGAGcAcacAcTcgagaAaaagTaCacgaTTcaacaAcaTaaTaaaGcaac
gCaTaccaacCcgcacgTCGaCgCAaaaacaAcAcAcggaCaaTgaacgacaaAaaccCacgCaTTaaTaaca
ccaaacacaCaAcAacaGaaagGcacaacTaaaccaCAgAcAaccaacacgGaTAGcTggaCaaaaccGacAc
aGacacaaCgCaaaaccGgcGgccccaccTagacGAacGaTccaccaaaaaGacCaccTcgccagccaacTCa
gCGgccagcgTaacCaaaaaagcgaccaacaaCcccGaaagaccGagGgccCaaccaTcaaccccaccacTCA
acgccaagcCggcaaaaTCaccCgcaAccaacgCgccaGTccacAaaacaaCaccaaaaccTGaaggTCcacc
AAcagcaccagaaCGaaTcgACgaggaaaCaaaaacGCaaCcacaaAcGTacccaCcaaccccgccaCaaAaa
gaaCaagTaCcacGaacacGGcCaaaaaCcaTcgCaGaaTcaGTAaaaaaacTCGGcgTacgcgGcGaaTgag
caaCcaaCgaaTcaaCcacCgTacgaTgaacaTcCcTaacGaaccaagCCacGTCagCGAaaccGAcaggaca
GaaggaccccGaACcaaaAagaCacaaaagccTCagccGaaagaaacTacgAcaCgcTccaccaAaagcCgac
aAccaAcAacGaTcCTccacccaaaacTcacacaACaaGaaaCagAacaccccAacgacAaccCaAccaaaCg
gacTTgcAaacCaacaGaacaacgcAaacCCgacCcCgaccccgTaCaTcTaccccacCcAcCgCaAacaccC
ggcaacgGacTggaacacaccAagacAacccaaGAaGTcgccGAaacgGgaaacGaCgacccaacaacgaTcc
gCaaaCacCgcGTccaccTcgaacaacgcGAaaCGacGTcAgcaagaacCaaCacGacaaTAgGaaacAaacc

ccaTccaGaaGTCaacagaGGcGccGcTggaGcAGaaGaaGagGCagaccCAaacTgaGaaagTccaGaaGac
caaagaaccaAacccaagccagaacaggcCacAGcaaagcCCgcaGAcaaccacGGcgaGaaagAaaaCgccG
AaacaCCgcaaacagccaTcaacaccGCgGGcacaaccGgaagaaaaaTcGggGGaacCacaaacgccTcgaG
CagGcAcaGaaaaTcagccaaTaacacaaGaCcaAGGaaacgCTcAcaaaacaacacccagccCaacaAaacc
TcAgcAGaacGcTGccgGgcgaGcgcagcaccagaGgaacGccgaGaaTcaTcAaacaaaGccAagAaACaaG
AcCaAcaacaaaaaCGaacCaccGccgacGcaaaacccgcaCaCcacaacAaacgagaagcaGcagTcacGaa
aGccccaGGaaGaTGaaacaAaaacaAaacAGcacacaGTccagcagccTcgGccggagTagaCaacgaTcga
caaaAGgaTcaacGaacaCGaccTcGaaaCGAccaTGgaTcccTaaagcGagcGCTTccgcTaaacaTgagCg
acgacaagacTGaggaaaacCacaagcgaaGgGcacaaaAaagAagaaccaaaccccaGaaaaaagCCccaaa
caaaACAaCAaacacgcttcgacaacaTCgACcacGAcagCGaccTCaaaccacGAccaCgaccaaccaagaC
GaagaaGaaCTcaaCTCccgactttgacctcaactctcgaactcaacgaccAacggcaAagacGAcgTCGACT
agaacaaaCGAaccgccaaagCGAcggCGAacgTccaagccaaaacgcGacCgaGaaggagaaagagcaaaac
aaccAagaagaacaAcgCGaaaagaaCGAGaGaACcAacAaaaaaACcAacTCcaAGaccaaGaccgaacaTC
acCaacAaagcaacgaaAcGcgaaAacaGaGcTaaAagAaagcacgaggcgCaaaGacacaGcGaaagGccAa
caacAcaacCTaTacACgcgccaCaCAacacaaaaGAAgGGaaacgaaaaaCgAcaacaaaAcaaaGaagCaa
aCgcaaacggcaAcagcGcaaaaaagTaaaCAacaCacacacaaaacaaaccgacGaccTGTacAagcagggc
CcagcTacaaaagcaaaaagGaaaCacgaTcaccggcTaGagcagaacgaCCaTTCcgagcaagTGcGcccaa
aGTccAgAGccgcTaaaaacaaCccgcCcccGgagCggaGaaaacaacaaggTagTccAcCgCacgTgaacCa
TcgaagccagaTcgaaaaGAacgaaaacCgacaaCTacGAagTcGTcggccTccTcGTcCgCTCaccaaagcC
GacTacgccgcacTcgcgaccgccagagaCcaaccccgaGggCgaGgccccaaccacaagcTCGaccTCGaga
tcgaacttcgagtcTCaaacTCGaacgCaaaTTccCaagcccaacgaccGagtcgaggtcgaagtcgaggtcg
agagtcgaagtcgagggtcgaagtcgaggtcgaagtcgaagtcgaagttgtgggatttggggatctatcctgg
acgccgctcatta
>Cligib1_Cluster75#LTR/Copia
GTTGGATTCGCGCTCCGTCTAAACTTTACGTAAACGTATATAAACCATTCTTCCCATCCCCCTGATCTTTCCC
TCAGTCGTTGACCTACACGCGACTGAGGTAAGAACATTCTGCTACATACATTACAATATACTCACTATACGTA
TAGGTATCATATCTAGGTATAGTGAATAATATAGTCGTTGACCTACACACGACTGAGGTATCATATCTAGATT
CTAACAGGTTATGGGCCTTTCTCGACCCTAGATCTCGTATACTATCTGCGCTTCCTCGCTTAGGTTTTTCAGC
TCACTGTGTCTTTCCAACCTTTACTCCCTGTTGTTTCATGGCCCCTGGTGATAAGACTCGCCCTCCCGCTCGC
CCTCGCACTCACACCGCTCCTGCTCCTGTTGCACGTAGTCCTGTTCGGGTCTCCGGAGGTGGTTTTCTCACCC
TTGATAATGTTCTTTACATCCCCACCTCGACTATGCGCCTTATCTCCATCGCTTGCCTTACCGACATGTTTGA
TTGTTCCATTACCTTCACTTCTGATTCCGTCTCCATTGCAAAATCGTCTGGCCTTGTCATCGCCTCTGGTACC
CATATGCCCTCTCGCAACCTCTACCGCCTCTCTGGTAGCCTCTCCATCGACCACACCTTCCTCGCCTCCCCTA
AGCCCTCAATCGACACCTGGCATTTCCGTTTGGGTCACGCCAACAACCAGTCTGTTGTGGATTTATCCAAAAA
GAACCTTGCCACAGGTATGCCCATCGACCTCTCCATCACTCCGCCCGTTTGTGACCATTGCATCCTTGGGAAA
CAGTCTAAGTCCCCTGTCCCTAAGGTGCGGTCGGGCCCGCGGAGTGATCGGCGATTGGGTATAGTGTGGGTGG
ATCTCACGGGCCCCGAGGTGGTGGTTTCTGTGTCAGGCAATTGGTACATTATGAATATTCTCAACGACTTCTC
TAGTTATCCTTGGTCTATCCCCTTGAAATTGAAATCTGATGCTGCTGATGCATTCATTTCCTGGGCACGCCGT
GCGGAGAATGAGTCTGGTGAATGTGTTGGGCGGATCCAGTCCGATAGAGGGGAGTTGCATACCAAGAAGATGG
ATGATTGGTGCAAACTGCACGGATATATTCATGAGGTCACTGCCCCTGACACATCGGGACATATCGGGCGTGT
GGAATGTTACCATCGGACTAATATGGGCAAGGCTCGGGCTATGCGCCTACATTGCGGTTTGCCTCCAAACAGG
TGGGATGAGTTTTGTCTAACCTCCTCATACCTTTCTGCCCGAACCCCAACACGGTCCCTATCAATTACCCCAT
ATGAGGCTTGGTTTGGAGTGAAGCCTGACTTGTCTCACCTGCGGGAGATCGGCACGCGTGCGTTCGTGCTTGT
TCAAGGGAATGAGCCGAAAATTTATGCCCACTCTTTTGAGTGCGTCCTCATTGGGTATGCGCAGAACTCCAAG
GCCTATCGCCTTTACCATCGCCCCACTCACAAGGTTATCAAGTCTTTCCATGTCCGTTTCATTGAGCGTTGTG
ATAGTGCCCCTACCACTACTACTACTTTACCCCCTTTCCCCCCTTGCGCCACTCCTTGCCCTGCCCCCCCCGT
ACCTATTGCTTCCCCTTCCATCCTCTCCCCTGTATCCCCCGTTGATGCCATTAACTCTCCCCGCCCCCTTCCT
CCCCTGTCCACCCCTTTCCGCTGTCCTCTATTCCCTCTCCTGTTGCTACACCTCCCTCCCCCCTGCGTCACGT
TGTTGTTGAGACTGTTCCCAATGTGGACGCGCCTGGCGTTCCCGATCTTCTCACACCTCTGGGCCCCCTCCTA
GAGGCCGTGGATGTTCCAGTGAGTAGCCCACCTGCCGCGCCCGTCCGGAAGTCCTCTCGCGTCTCCACTCCAT
CAGAGAAGCGTACTGCTGCTGAGGGCATTCTCCACTTACTGTCTGTTGCCAGGGCCACTGCAGAGTCCCGCGA
GAGTGCTGTCCGTCTCAAGGCAGCTCGCTCCGCCGCCAAGGATGAACGTCGTCGTGCCATACTGGAGATGAGG
GATGCCTTGCGGGAGGACGGACCCACCTTCACTACCCCGCCCGATTCTGCCACCCCTGCTTTCTCTGATGAGG
AGATTGCCTTACTCGCCAAGGCCTTTCCTGCGGTCATTGATTCGACCGTTCTGGACGGTGGCCCTGACGACCC
TTGAACGCTTTGCGAGGCACTGAACTCTCCGGACTCGGCTTCGTGGATCGCTGGCATTAAGGAGGAGCTAGCG
AGTCTTCGGGACATGGGGGTCTATAAGTTGGTTCCCCGTTCCTCGGTGCCCGCTAACTGTAAAATCTTGAAAG
GGAAGTGGGTTTTCAAGCTGAAGAGGGATGGGGATGGGAAGTCGGTCCGGTATAAGGCTAGGCTCGTCGTGAA
GGGTTATGAGCAAGTATTTGGCCAGGATTATGTTGACACCACCTCACCTACAGCCCGTATGGAGTCGCTTCGC

CTTCTGCTCAGTATTGCTGCCGCGAATAACTGGGACGTGCACCAGGTCGATGTCAAGACTGCCTTCCTATATG
GCTTACTCTCCCTCGAGGAACCCCAGTGGATGGAGCAGGCGGAGGGCTTTGCTGAGCCTGGTAAGGAGGATTG
AATGTGGGAGTTGCAGTGTGGCCTGTAGGTATGAAGCAGAGCTGAGAGCGGGCAACTGGCCCAGTTCTTGGAC
TGCTTCGGCCTGTGCACTGGGAAGCGGCCCTGCGCGTTGTACGCTACCTCAAGGGTACCCGCTCGCTGTGCCT
CACTCTTGGGGGTAAGGAGGAGCAGAACCTTATCGGCTTTACGGACTCATCTCATGCGGACTGTCTGGATTCA
CAGCGCTCAAGCATGGGCTACTGTTTCTCAATGGGAAAGGGTATATTTTCGTGGTCTTCATGACGTCAACAAA
GCGTAGCCACATTGTCAACCAACGCGGAATATGTCGCAATTAGTGAATCGGCTCGAGAAGCACTATGGCTCAG
GCAATGGCTCATGGAGATCGAGGTTCCCCCTACCAGTGCTACACCCATCCTCGTAGACAACAATGGTGCATGG
ACCCTGGCTAATGACCCTCTTCACCACTCCCGCAGTAAACACATCGACATACGACACCATTTCATCCGTGAAC
ACATCGAGAATAACAACCTACTACTCTACCGTATACCCACCAACGACAACCTTGCCAACACCTTCACTAAACC
CTTACCACGCCCTGCATTCATACGTTTCTGCGACCTCATGGGACTTCACTAGGACACCCGCGTGAGGAGGAGC
TCCTGTTACGCCCATTTCCATCCTTTTTATTTCTATTTCGTTTTTCTTTCTATTTTCACATAAATCCTTCTTT
ATTTCACAACTACATGATACACGCGATACCCACAGTGACACCCACTTCACTTCAAACTGTTCCAAGTTCCTCC
GTGTCGAGGAGGAGTGTTGGATTCGCGCTCCGTCTAAACTTTACGTAAACATATATAAACCATTCTTCCCATC
CCCCCGATCTTTCCCTCAGTCGTTGACCTACACGCGACTGAGGTAAGAACATTCTGCTACATACATTACAATA
TACTCACTATATGTATAGGTATCATATCTAGGTATAGCGAATAATATAGTCGTTGACCTACACGCAACTGAGG
TATCATATCTAGATTCTAACA
>Cligib1_rnd-4_family-356#TIR/CACTA
AGCACAGTGCTATAGTCCGGAGAGTTTGGTTGGCATAAAAGATGATAGTCCTAGAAATATTCTCCTCGGCGAG
TGAGTCTATCCGCCTGATCTCGACACCGTTTACCTTGTCCGCACCAGGGGATTGGACGACAACATGGGTGGTG
CACTTCTGGACAAGATCTCACTGGCCCCCTTAAGGTAAATGCGATAGCACCCATCGGGAAGCTCAACGACGAC
ACCCATCGCCCTGCGCTCGCTAGAGAATGGGATCATCTGATGACGCGGGCGTTGTCCCTGGTCATCTTAAAGC
CCATCCAGCCTACATCCTGGAANNNNNNNNNNNNNNNNNNNNNNNNNNNNNNNNNNNNNNNNNNNNNNNNNNN
NNNNNNNNNNNNNNNNNNNNNNNNNNNNNNNNNNNNNNNNNNNNNNNNNNNNNNNNNNNNNNNNNNNNNNNNN
NNNNNGGCACTTTGGGCATGTTTTCTGATGAGGTTAGGCGAATTGACGGGTTGGGGGTGACGGAGACGCGTCC
AGACAATGCCCTTACGGTGCCGGAACGACTTGGGGCCGAGGCGAAGTTGTAGCATTCCGGATTTCGTGCGGTT
CGACGCGGCGTGAGGGGCTAATCGCTTTGGGCGACTTCTGTACCAGTAGTTGCTGTGGGGGGTAAGGCGATTG
TTGGTACCTGTTCCGCGGTGTCCCAGCTATCTATTCCAAGTCTCCCCTCGTAGGATTCCCAGTAGCCTCCAGT
TCCGAGTCATCGAAGGTCTGGATGATGGAGTGGCGCTCGATGATCGTCCACGTATTGCGATCTTTCACCCTTC
CAACGACACATTCGATTGTAGAAGCATCGACTACTTCGATTGGTCCAAGCGCCGTGTAGGACGGCATTCCAAG
CTCGTTGACTGATGGTCGCTTGATGGGAGCAATCGCAGCCANGATCACGCTTGAGGGTCGTCCCGCAGCGCGA
GTTGGATTGGCCTGGATAAATGGGCAAGTAGAGGGCAGGCTAAGAACCACGAAATGANTGACTTGTCCGTAGT
ATGAACGTTCTTCAAGAGCTACAGGTCGGTTTCTGTAGCGCGCGTTTCGGTCGACCTCCAAGGTATACTATCA
TCTTTGAATGAGATCAAGCGGGTAGTATTAATGTGGGCTGGACGCACCTTGACGAACGATTTGTCACGCCCCC
GGGCTGTATTACGAGAGAAGCCGCAGGAGCCTATGAGGTCTCCACCGTCTAGTANTTTAAGCTTTCCACATTC
GGATACGGCGCTGGGTACGAATTTCAGTGCGATTGCGTCCGTGGTTGAGTATCTCGTTGCGAGATGGGACGCG
ATTTTCTTCCGAAGCGCGAGGCTGATTTGAGTATCGCGAAACGGCTTGACCATCACAATCTGAGGGTCTTAGA
TTCCCGCNCGGAGTCAGCGAGGGGAGATAGTATGCAATCGGGCGACTTACATCCCGCGTAATGAATGCCGCTT
CTCTCGACGTCTCGATTGTCGCTGAGATCGAGTTCATGCGCCAGGCTGTAGATAGTTTTGATCTGGTTGAGGA
AGGCGACGTCGCGGATTCGATGTGCCAGGCTCGTGTACGGCCACTTCCTGCTTTTGACAGATGGTAAGAGCGA
CCCACAGAACCTTTCCATTACAAACGCCCAAAAACACCAGACAGGCCCCATGCGTCGAATATCGTCCGCGATG
TGCAGAAGCGCGTGGATTGGGAGCGTGCAAAACGGGAGGCGGTGGTCGGAATATTGATAATAAATCCTATAAA
TGTAGGCTCAGAATTGATTTAGTCAGGCATGAATATTGTAAAAAACCTACCGCTCGTAATCAAGAACCCATTG
CGCAAACCCCTTTTCCACTTCATCGATGTCGGAATCCGTGAGTTCGAATTGGAGACATTTGTTAATGAGTTTT
ATGAGAGAAACGAAGTGTTGGTAATAGATTTGTTTTGAAAATCGATGCCGGAGGAGTACAGGTCCTAACAGGG
TAGCAAATAAAAGCCACGACTCCGCGATAAAATAGTGTTGCTCCGTAGCGATGTTGGGGATGCGGCATCCGAA
GGCGGACGGGATGGTGTGTCCCGACTGTCCGCAAGCTTCTCCAATCGCTTTCCAGACCGAATTCGGAATGATA
TAATCCTCGCTGCCGCTGTCGAGGTTCTTGTAGTTACCGCACCACGCCTTGATTAATGTTTGAAGGACGTTTT
CTAGGACGGCATGCATAAAATCATGCGGGAACGACGCGGGGAATTGAAGGCTTGAAAGTCGAGTCAGGATGGG
GACGCCATTAATACCGAAACGTTTTGCGCGGCGGTTCTGCTCGGCGTCGCTTGGGGCCGTCATGACTTGAGTT
GCTTGACGAATGAACTGATCGTGGTTCCGGAGTGGAAGTGCCAGCGGGTCGTACGACGGTGTCGAAGAGCGGT
GAAGTGGTGTATAGTGCGCGTGGCCAGGAGATTGTTTGTCCTCGATGCCCCGGATGGTACAAGCGCGACATGG
GGAGAGTCCGTTATGGCCTTTCATCCGCATCAATTTCGCAATTGCGGGCATGTCCCCGAAAACGAGAATGAGA
TACACGTGTAGCACGAACATTCGCATGCGCCGCTTATCAAATGCGGATACCCCCCGCTCCAATTCCAAACACT
CCTCGACGAAGGGGATAAGGAAGGAGTCGAGCTCCTTCGGTTGTTTGGGACCAGGAACCACTCCTAGACATAA
TATATTCTTGAGTTGGAACCGGACCTCGGGGGGAAGGTTGTAATTGAAACATATTAGAGGCCAGCATGTTTCT
TTTCGACGTTTGAAAGGGCAAAATCCATCTGTCGAGAGGCCGATGGCAATGTCGGTGGGCTGGGAAAAGAATT
TATGGCCAATGTTTTCGCCGTCGATGGTAACGTGAGTCGAACGCAAGTTGCGATAATGCTCCCCGTCGAATAC

ATCGGCCGTTCGCCCAGGCTTGTGTTCATAATTATTGCGGTAGTCCATCGCGTTGGCAGTTTCCTCATTGAGA
AAATAACTGACGAGGCGGGGGATGAGCGGGAAGTAGGTAAATGTATTCCGAGGTCCTCCGCTGGTATTGTATC
GCGGTTCGTCGCAGTAGGGACAAGGGTCCATCGACTCATACGCGCCTGTGAAGCAACAACAAGAGTTGATGCA
ACAGTGATAATGTACGGGCTTGACCCCAGCTAAAAAGGCGATGCGGGAGCGAAGCCGTTGAAGAGAGAGCATT
CCTGACAGTTTCGGGAATGCCCTCGGCAGCTTCTCGTAGGACCGGGAGCCCAAGAGGGTGTTGACCTTGTAAT
TATGGGCACGGATGGTGTCCATGTCGTCTTCAGATAAGCGATAACCTGGAGGGATTATTTCACAACCGTCGAA
GGGCCTTACGGACGGGAAGTACGTACCTCGAGCAGCGGCCTCCATTTCGAATTCCCCTTCAAGGATATCCCGG
GCCGACAATCCCTGCAGCCAAGGCGCTACAGGAAGCCCATCTGGGATCAAATACATCCTCCTCATCGGAACTC
TCATCCCCACCCCAGTCGTCGTCATGATAGGTCCCCGCTTCTAAACCGCCTATATCTCGGCCGATACCGTCGT
CACGCGATGAGGTGTTGAGGTGTTGACGGGAGTCCGGGATCGGCCCACTATGCTGGTCCACGGCGGTGCCGCG
CATGTCGACGTCTTCGGGCGTAGAAGGCCGATCTGCAGCGTCGCCCACTGGGGCGGGTGAGGGGGAAACAGAG
TCGCGGATAGACGCTGCACGGGGTGGTGAATGACGACGGTGAGAATGGCGGAGATGATGGACGCGTTTTCGTC
GAAGGGCGCGTTGCTGTGCGATTATGACCGCTGTGGTCCCAGTCCCTGCGAGATGGCGTATGACTGTTCGCCG
TGAGACGTCCTTTTCGCAACCGCAGGTACAANGCATTTTCTGACTCAAAAGACATCAGTATTACCATCCCACG
GACTAAGAATATCACTCACGTCGACGCACAATCTGGATAGTTAGTGGGAGTCTTACAATCATTTGTTTCAAAA
GGGCCGTTACTTATACTTACGAGTTCCACACAAGGCGGGGAATCCCCATACAACTAAGTTGCGGACGCGCGCT
AGGTTGAAACCACATAGAATTCAGAAAATAGGACAGCGGGGATCTGGTATAGTACAAAGTATATCTGTCCGTA
AGGTTCAAAGCTAATATTCGAAATGTAGCAAGATTCATACGTTGAGACCCGAGATTTAAATGGCCGGCACTGG
CTCAGCGTTGTAGGTCCCCCATGTCTTGCCGTTCAGAGCGCGATCCCACCGTGTCAAGATATGATGTAGGAGT
TTAGGAACAAGATCTTCCGGCATCTCAAGGTTCTTTTCCATANACTCAATATGCAAACTAGTAGCAGCTTCCT
CGAGATCGTCAAACCCGCGTTGCAAAAGGGCGTCCTTATACTGCAAAACNTTGAACGGAGGGTCGGTCTGGTC
CCGAGCGTCGAAGTCCTTCAAGACATCCTCGATAGGGATGTGGGGGAGCGGCGTCTCCCCATTTGAGGTGTCG
GCGGAGCGGCGAACCCGCTCCTCCCCAGGTTGGGCGGAGTGCCCGGGCGTGGCGCCGATCGCTCCGCGATGGC
GCTTTTTTGGGGGCGAGGAACTCCTGGGCATCTGACGCGCGTAGCTTTTAACCTGGGATCAGATACGTCAGTA
TAAAGTTCCCGAATGCCAACCACACTGGACATGCCTGCGGCTCGGGGTGGCCCCGCTTGGTCCTAATCCTACC
GCGTTCGACTATCTCTGGTTCCGAATCCGAATCACTACTGATGTCGATTACAGTTGGCTCTCTAGCCTTGGGG
GCGGTGGCTGGACGGGCGGTATTGTCGCTCCGCCGTAGATCCAGGCCGATGGTGGCGGACTTGGAGTTTCCAT
TTCCGGTGGCAGGCGTAGGTTGGACCTAGGAAGATAACGGGTCAATATCGGAANGAAGACCGAATAAATTAAA
TATACTACATGGGCGTTGATGGTGACACCATTGTCGGTGATAGAGATAT
>Cligib1_Cluster59#LTR/Copia
ggTgaaAAcCacaTcTAcacacTaaAcAgaCGaATCcccacccaCcccAaaAcagaCgaTaTTcTcgTacaTA
aCaacacaTTaaaaaaacgaaTcTaAcaTagAATaagaaaaAgAccaaacgAgTAcAaacAagaACCcCacaC
aaTcAgaCacgGaaTaAggGacaGcGGTgaaTacaTaTAaTacagacaTaTaaaaacaATTacAgaTgAaAgg
aaacaaacgAAacgaaaGgTAcaaAaaAcAAaCaGcaaaTaaTaccGgaacacacGATaCGcaTaTcgAacc
caTAcAccgGaTTCcaAaaacTcaTccgCaacgGcaacacaTCagccacaaacgaAAacccgTGATcaTAgca
cTAcgCcaaGcaacccCccaAagacGacacaagccgccgcaaaaaacgccTTgggcaaaaAcaacacaAaccag
aacagcgaTcGaaaCAcAagcgAgaaagaaacaaaTcaagaGcACccacTCcaccAAcaaGTcaaaTcaGagc
gccagcaccTcaaCgaagcccTgcacaaGGGcaaagccgGACAaGcTcAaTGcaaAaTaaacGaaacagaccA
gAgaaaTgaccCaagccaccAcaagaccaaaacccTacaAaaaCGaccgTaaccAgacagCccCaccccgCgGc
aaaacAgGaacCaAaagaaccgGcaaagcagaAaGaGaaCGaaacCgTcgcCgccgcGCgTaTgCaacacGaa
agTAgccaaGaTcaGagcGAcCATaccgAcacCaCcAaAcgcccaCaCGcaTccaGGaAcacTcccaAaaCTa
aGcaaaccaAccaaagcaaccgAAaacaagTacaaCaCCcAaaaacAacTcTcacaaCcaaggTccaaagTAa
caaaCcGaccaaCaccAccGCcGCagccaagTGgAcGGcaaaagAacaggCGagcgacgcaGagaagagcacg
aCaGagcaaccaAaGTccagcccgcTacacAacGCgagAaATaaTaacaaccCaaagaaaGacccccacgcga
ggaTagaaaGaGAcagcacCgcTTCcaaaaaGacgaAacccgacaCaaCaccCcacAgaTgcaccccacgCga
aAacGaaaagaaaaggagcaaaGaaaacaaaTcccCcaaCaccaaaAccacCaacgCaAcgaccgccacagca
aacAaCaaTAcGacgcgcgacacgagaaCCcaTaccAacgccAagaagaCaaaaaAaCgaaCgccAcacaGaa
ccaaaaaaGgacacacaaagcccagcaagaCacTaaAccaaTCGaacgCcgCCaGAaAGaaCGacAaGGaaaG
aAAaaaagcGacagGaAccgCcGccAcTgcaacGCCaGgaaaGcgagTgCaGaacTaAacaaagGACaaaaAc
GccaGGccagagaaaCcccAcGaAAacAcaaaggacTTaacCaTaccaccaaaaaccccacCaCccTccTTCa
caccacaTacCcCgaacgacaaaCaacacaCcCaCCagaCaacgcCaGacgcCaccaAgcaccaGGGgTgGTc
aaccaaaaaaGaTccagacaCcaacagcaagccGaacAacAAgaCccgTcccAcggagaacgacgcagcacaC
aacaccaaccacccacaaaaaTccaaaaaTAccCcCaaAGaCTCCaTacaAcaagggccagaccTaGcagaga
ccgacgCaTccccacAaATcTcccCgaAacccacGaAcacaAgcaGTacCagAccaagCccacccCgaaCGgg
acGAgcgcgcgggagaTcaaaCAcgacaccgCcCggaaTaagcgacaacaaggggcacAccgCaacgcgaaag
aacAgaaCacacacacaCaTaccCTaaAgacaaaccacgaCggccCGccacaccTcCgcCaaagCgcTcGaca
caGaCgacGcaAgcacaTCccaCcacGaacaacaaaCATcCTccagaaCcaaCaAaacaacGgcaacccCcGac
cGCaaacGgTAccccaaacaaaacCaGaaaAcgATacTCcccaTcacTaCagTCCaaAcaaaTagaacacaac

aaTcaccccaaacacaCCacCaaccgcgagacaTgccaccgacGcccgGGTaaaGccaacgacCaGgccagcA
TTgacacgccacccaaacGCCgcacgacaggcacGccAaaaacccgcccgccaaaGcCgTccaAaTGcgaaac
AgcaacTccGaGgaaacaGaacccgaAaacaGggccaaaGacacacaaGcaGacAAcgaGgGcTaCcaGaaaa
TTcGaaaccggaTGggcagagaTgGcTagGcccacaccaGTAccagcgAgAcacgcaaaaTcaTaggcaaggA
agATaaTcGaagaTTgcaacaaaATGCcATgGTCcacTcCacTTAacAaaaacGaccacGcTTTTcgCaaAcc
gcaaGcaaGccaaacaaaaaccGaaaaTcaGaCggGaCTCaaGaggaacacgaaaaGAaacGaaCacgAgaca
gaaCcgaaaagcCaagaAaaGcAaccaTagcccaaagaacgcGcGaCggAcCaaaacaggagAcaacCaaaga
aaccggCGcAcATgggacGAgTcgaaCGcaTccacagcAaaaccaaGGCcaaacaaCcaAcCacaCGaATAcA
aGaGgaaTcacccCccaacaTGTaccAcaaaTcCcAccggacGaccaCcCaccTaaacGaCaaaacTacAacc
aaGgCccgaAacgGcGaaacCacaacaTaGaAaccaTcccACagccgccGccccaAccaCgCgTaTATaCGca
aGaTccGagccaacGCCaacagagTaagacacAacCGccacAaTCcaaaaATaTaCGaacaggcaaacGaagc
TacaaacagggGaTaaGaCcCcaacgacaaaacacAaccgTcCaaTgagaGaaagagaaGcaacccaTacaGg
cCcgaTCaagaacgcccaacCgaGAGcCaaGaCgcAcaccCacgagcacaccccCaccCaaaaaaTgcGacAA
AAaCcaccaCcgagaTccTAAaaGaAaccGACaATcGCATgaacgacaAAaagcCcAcgATacTaaagaaagA
caAggaacacccTaTaaacccTACaccacaaccCacAcCaAgagaaaaaCAaaaaacccCcAAccccCcaGaA
cCaCcacTTccCccCaaacccacCcggaaaaCaagaTcagaagcCacccgaCcCacAaaacaTaaacaaCAaT
caaAaGaCagaacaacaaaaaaaacgTacCacaaaacgaaaaaGGCcgcaaccaacaacagaAATaaacaaCcG
acaagcacCgagACCaaaaCcgcaccccaAgccacaaacagcGcgGGaagagcgaccacaAgcagccgccgag
AcgaaTggGGAagGgAagaAcaacGAcgATaaTgacCgGaaacccgccacaaTAaCcgaaGCcagCaGcacgc
aCccgaacTcGcccGacagccAGcATGagccgccgaCcaaGgaaGAaaccaTgcCcgaGgCcaacgCcAaaGa
gcgGaaGcAaaccaacCGcaaaGaacccaaagccaTgaAGaacAcGgcgcgcaacGaaaTaaaacccacacaa
GacaTcccaaaaggaaccaaaaccAgaaaGaGaccgccaaTGgacaAgacaaaaacacaTCacaaCGgaaGac
caacGaggcaTaaGagACgaaTgaacTcCaaaaccTcaGaccAaATgTacaccaAgGaccacacaaaaaCgAc
aaaaccaaacgcTcccAaGGAGaCTagGaGaaTAcgacgacAccgagcaaccgccgcaagaggGgagaaacaa
cacacagaaaTcaaAacaGcaTTcgTagaCcaaccaaccccccGccaagaaAaCgcaaaacacgaAaCAaCCCa
cagaaTccaaGaaagCaGcaaaaGaagGGaGcGcagaggGcagaaaaagaGcGccAaacccaacGAAacAaaG
cGacacaacggggaaccagAcGcacAAcgaaaacaTaaTTaCcTccaGcaaaaacccCCgcccCaTgggaGTCa
ggcacTgAcacCcGaAaaacCgagaCgGGCacgaaAaTacCaGaacTcCacccCcacgacTTcCcaaCCaTag
CGagCTcTCaGGacGacaaCGAaacaTccAagGaacacaccccaaccccccccgaCcacaccaaagCaagaTcc
aGccaGaTgaggaGgaggaaTccacgTccaAcaGGagagacccaacaaaacCcTAaaGgTagcacaaaccaac
ccaaTaGacagGaacaTaaaccaGgccGaacAacgcgaggCacacacaagggcacgacCaaTggaGacgaacc
aaaAaaTcacGcccGagGaccaaacaTcaccCacagccGAAcaGaaaaacTCcCgcgacAaaaaacaccaccc
cgCaccagagGcacgcccAcgATaTaaaGCcaTaaccAcaccaacacACaTgaccaaaaCcGgacaaaaacca
cCgcaagacaaCGaaccaTagacaaCCaTaCAaagcaagGCcGCcaTccgGggaagCcGagacgTAaaagcaA
aacgcGaacacAaAccTcaccgTaGGgGcaacAacgacaTTaccggcaccgcaTacacggaCccAGacTgaac
AaacTgCCaccAaaCGcGcccaacTgTaGgGagaTacacATGcTCaacCgcAgCagcaaTAaggcCagagaGa
AcAaGCaaacaGaaaAaGcgggCGacCgCaTcacaagagcCaaaagacgTcGcggCaTccgaaaCaGaaaagG
aaGacagaTccccaaaaaCGcaaTcacAcaccaggcacaagcTaaaaaaggGaccaAcaaaTaTaccacacga
CaaaaAaTcccCAaTcAaCcTGTCaaaagaCcCCCcacgcaAcacacgaGccaAacacagaaacagCaaAacc
cACggcTTacgcGAaccaaTcCaaaccAaggaaagagagaTaaaaTaTGagcCcAcaaagaagaacagTgcgc
accTgagaaaaaAAacacacgagaaaaacaaagccaccCcaaaTcaaGacaacCggcGacTAaaaaTAaaATC
AacccaTTcaacaccacaagGAgcgcaccCacgaGaccAgaAccggTgaaAAcCacaTcTAcacacTaaAcAg
aCGaATCcccacccaCcccAaaAcagaCgaTaTTcTcgTacaTAaCaacacaTTaaaaaaacgaaTcTaAcaT
agAATaagaaaaAgAccaaacgAgTAcAaacAagaACCcCacaCaaTcAgaCacgGaaTaAggGacaGcGGTg
aaTacaTaTAaTacagacaTaTaaaaacaATTacAgaTgAaAggaaacaaacgAAacgaaaGgTAcaaAaaAc
AAaACaGcaaaTaaTaccGgaacacacGATaCGcaTaTcgAacccaTAcAccgGaTTCcaAaaa
>Cligib1_rnd-1_family-8#TIR/MITE
TAAGGCATGTCACGAGTCNACCCGCGAGTGACCCGGATCCGCGGTCACAAGTACTCGCGNGTCANAAAGCGAT
ACCCGGATCCGCACGAGTNTTGTACTCGCGGGTACTCGTGGGTACCCGCGGGTGTNATATACTCGCGAGTACT
CACAAGTTACTTGCGAGTTACTTGCAGGTATGAGCCATATATGGCATATTAAGGGTCCATAAGGGTCCAAAAC
TCGCAAGTACTCAGGTGAGTACTTGTGACTCACAAGTATGAGGATCCCAAAACCAATACTCAGACTCACGTGA
GTATTTGAGCTGTGAGTACTTGGATCCACGAGTCGCTTACCTGCGAGTATTCCCACGGGTCTACTTATGGGTA
CCCGCGAGTAACTCGGACTCGCGACATGCCTTA
>Cligib1_rnd-1_family-311#TIR/MITE
TACTATCACTTACCGTTAGTTCGGAGGGCCCTACAAACCGGAGACTTCCCAGAACCAAACCTCCTCCCGCATC
ACTTCCCTCTATACTCAACGGGCAGTCCTATATGCCAATAAAAATGAGTTCTACCAAGTNTTGTGGTGATCTA
TGGATTATTCGTCGAGTCCCGTCTTGAGGCGGTTGTCACGCTCGAAACCCGTTGCCGTAGACTGCCGCATCGG

AATTTGAATTCTAAGTGCCTCCAAGCGCGTCTCACGTGCCTCTCCGGNTCCCACGCGTCCCGCCTTCCCATCC
TCGGTGCGTTACGTTTTTGTGACATCCGAAAAAGAGGGCCCTCCGAACTAAGGGTANATGACAGTA
>Cligib1_rnd-1_family-116#TIR/MITE
CACTGCAGGCGCCCAAAAATTTTCCCCCGGGAAAAATGCAGTATGAAAAATGTCTTGTGTTCTCTGAGTCATT
TTTCCCAAGGGGAAATTCCAAATTTATATACAAGTAAAATAGAATTAATATAAATCATTTCTCCCCCCTTTGT
TCATGGGGAAAATGTGACATTTTGCTGCCCATTTTCCTCAAAGTAGAATAGATGAAAAATGCGAAATTTTCAT
CTCATTTCCTCCAGAGTAATATAATACATTTTCATAAGGAAAATCAAGGAAAATCCAGGGAGACATCGCATTT
TCCTTGTGAAAATGTATTATATTACTCTGGAGGAAATGAGATGAAAATTTCGCATTTTTCATCTATTCTACTT
TGAGGAAAATGGGCGGCAAAATGTCACATTTTCCCCATGAACAAAGGGGGGAGAAATGATTTATATTAATTCT
ATTTTACTTGTATATAAATTTGGAATTTCCCCTTGGGAAAAATGACTCGGAGAACACGAGACATTTTTCATAC
TGTATTTTTCCCGGGGGAAAATTTTTGGGCGCCTGCAGTG
>Cligib1_rnd-1_family-659#TIR/Tc1-Mariner
TACAGTGGCGCTCGAAAGTAATGGAACGCGTGACTTTNAAAATCAACGCTTCGATATCTCAACGATCACACAT
TTCCACGGTTAATTTTGGCTGGTGGGATTCATTTATAGTATCTACAGTTCACAATCATATTTTAGTTGGGGGG
ATTCGGACTGGTTTTCATCGAGCTATGGGTGTAATAATTCTGAGCCCGATATTTTGTGATGTAAATATCTGTA
AATATGTGAACCATCTCCTCGTTATCATACCCTGATCTTAATCACTGTCCATCGAAATCCATACCGCCTGCCC
CCCAGCCTATGCCGTACACTACACCGACTAAAAGGGCACGTATCGTGCACATGAGAAGCAATGGTGCCACGTT
CAGGGAGATCCAGGAGGCGCTTTCAGTCCCAGCAGCCACTGCATGCAGAATCATGCAGAAGTATCGTGAACAT
CCCGATTTTTATTATATAAAACCCAAGCCAGGCCGTCCCCGTGCCATCTCTCCTGCCGCAAAACGATTTATCA
CTCGCAAGCTCCGTGCCCCTGGCTTGGTAAAGGATGGTGCTGACCTGAAGCGTCAGTACTTCCCTGATGTTGG
GGCCAGTACCATCCGTCGAACCCTTTTCCAGATGGGTTTCAAGGGTCGTAGACGTCGAAAGAAATTCTTACTT
ACGCCGCGNCATATCCGGGCACGAAAGAAGTGGGCAGTAGATCACGAAAATTGGGGAAGGGCTGCTTGGAGGA
GGGTAGTATTTTCGGATGAATCAAAATTTAATCTTTTTGGCTCGGATGGGGCCCGGTATTGCCGGAGAGAGGA
CGGGCATGCATTCGACGCCCGATTTGTCAAGAAGNNGGTCAAACATGGGGGTGGGAGTATCATGGTGTGGGGN
TGCATCACACCTGNTGGGTTTGGCAGGCTTCATCGCGTCGTGGGCCGCATGAACGCTACTCAATATACCGAAA
TCCTCTCNAAATCACTCCTTGGTACCCTNAGTGACCGCTCTCTCGCAAGATCCGATATTATTTTCCAGCAAGA
CAACGACCCGAAGCACACTTCGAAGCTTGCAAAGAGGTGGTTTGNCGATCGTCGCGTCAAAACCCTACCATGG
CCTGCCAATAGCCCNGATATGAGCCCCATCGAGAACGCATGGCGTATGTTGGATGTCAGGGTTCACTCTCGTT
CACCATTACCTCGGAATCTCGACGAATTGTGGGCTGCCCTGCAGGAAGAGTGGGCCAAACTGGGCAAGGAGTA
CTTGGAGGCCTTGTACGACAGCATGCCTCGACGAATCCTTGCACTTTCGATGGCAAAGGGGAGCTACACTGAA
TACTAATCACCCCGGTATTCTCATNTTTTTTTCCGTGAGTTATTCCGCCCACCGAGCCCATCTTCCTCCTCCT
CGAAGCGAATGTTTACATATGTTTATAGGCCAACACCAACACTCACATTTCTTTTGCCCGATATCTCAACAAG
GATGGCCACAAACTACTGTAATAAAAATGTATTTTCAAACTGTAGATGCTGTAGATAGATCTCATCAGCCAAA
ATTAGTTACAGAAATGTGTGATCATTGAGATATCGAAGCGTTGATTTTNAAAGTCACGCGTTCCATTACTTTC
GAGCGCCACTGTA
>Cligib1_Cluster89#LTR/Copia
AGTATATTCTATTATACTCTTTCCCTCGTGAACTCCAGTGTGCCCTCGCGAGACCCTCTCGGCTATCACCCGC
CTTCCCTGCGCAACCGGCGCTTCTTTTGGCGCTTCGCTCGTTAGGTTATGGGCCCTGCCAATGAATCTCTCAC
GATGTCAACTTCCACCGAATCGTTTAAGTTCCCGAAGCTAAACGGGGAGAATTACTTCATATGGGCGCAGCAC
ATGGAAGCAGCGCTCCAGGGGCGCTATCTTTGGCTCATCGTCCGAGGAGATGAGACTGATCCTGGTGCCCCAT
CTGGAACCCCCCCAACTGATGCCGAAAAGCTTAAAGATTATAAATCCAGACGGAAGGAACATCTTGATTGGTT
GCGGAGTGATCAAGCTGCTCAGGGTTTTATGAAGAGTGCAGCTGATCCCACTCAGTGGGCACATGTCGCGGAT
TGTGTGTCGTCGAAATCGATGTGGGAAGTGTGGCAGAAACTTTATGTCACCAATCTAAAAGGTCTAAGCGTCC
ATTATTATTTCGAGGAGTTGTACACGAAAAAAATGGGACGAGGCTACCCCAATCACCGATCACATTGCCGCCAT
GCAGGACTTGGGACGAAAAATCACCGCAACAGGTGAAAAACTTGATGATCTTCACATCACCCGTGCCCTCGTC
CTCTCACTTCCACGCAACCCTACATGGGACACTATTAAGGTTCAGCTTTTCAGCAAGGAAAAGCTTACGATCG
AGGATGTCACTTCTCAGCTCCACGTAACTGCAAATCGTCTGTCGACCGAAAAACAGTCGGAGACTGCCCTCCT
CACCACCCAGAAAAAATCCAAGAAGGCTCGCAAGCCTGGAAAGCCCAAATCGGATGACGAATGTCGTTATTGC
CATCAAAAGGGCCATTGGATTAATCGCTGCCGCAAACGGGAGGAGGATGAGAAGAAAAAGAAAGATAATGGTG
GATCTGCGAATCTCACCATTGGGGGCCTCCGCGATCTCGGCGATCGGGAAGTGGGACAAGTTTTTGTAGCTGC
GGGATCGCTTCGAGGGAGTGTTGACCTAATTTTGGATTGTGGTGCAACCTCTCACATGTTTTGCAATGTCGTT
TCTTCACCACATACTCACCCCCAACATCCCCCGAAACCATTTCCGTTGGTGATGCCCGCAACATTCCTGTTGC
GGGGCATGGTTCAGTTCGCTTTCGCACCCTCCTTCCCGATGGTTTCCACACTGTCACTCTCCATGGCGCCCTC
CACGTTCCCAAGCTTGCCACGAACCTTGTCAGTCTCGGGAAGCTTCAACGAGTGGGCGCGCAGTACCAGAGCA
TCAAGGGTGGGCTATCAGTTAGTCATGGTCCTGGGGAATTATTCCGAGCAAATCTTGTTGGAGATTCGGGTAT
GCTGTATCATATTAATTGTGCTGAAATGAACAACTCGGATTCTGCCTATGTTGTTTCGAGTGGGAGTCTTCGC
CTTTGGCATCGACGTCTTGCGCACACCAATCTTGAAGCCATTCGTACCATGCATCGCAAGAACCTTGTTGATG
GTCTCGCAATTTCGCGCCTGCAGGATTACAATCACCTTTGCGAGGGATGCGCTCTTGGAAAGTCGCATCGCCT

CCCATTTCCAAAATCGAGTACCACCACGTACGAAAAATGCGATCTTATTACGGTCGATTTAACGGGGCCTATG
TCGACCCCTACGTGGACTGGTATCTTGTATGCCCTCGTCGTAGTGGAAGTCAGTTGCCGATATCCAATCGGGC
GACTCGTACGGTCCAAAGATGAGGTGCCGAAAGTCCTCAAGGAGGTAGTAGCCATCTTGGAGAGACAATCAGG
GCAAAAAGTCAAGCGGATTAGGAGCGATAATGGGACTGACTTCGTGAATGGTGTGTTGAAGGACTTTTGTAGT
CGCAATGGCATCATCCACGAGACAACAAACCCCTACACCCCTGAACAGAATGGAATCGCGGAGCGCGCGATTG
CTATTTTCTTCGAAATGGTTAGGTGCATGCTCCATACTTCGGGGATGGACCTACGCTATTGGGGTGAAGCATT
TTTATATGCGGTCTACATTCGGAGCTGCGTATATTCATCCGCACTCGATGATATCGTTCTGTTTCACGCTTGG
ACTGGCCGAAAACCAGATATTGCCCATCTTCGCATTTTTGGCTCAGTGGGCTATGCCAATATCCCGAAGAAGA
TTCGGGGTGGTAAATTGAAAGTAACCTCTGTTAAGGTTCGTCTCCTTGGATGGTGGACGGACGAGACGAAGGG
TTATCGATTAGAGGATGTTGAGTCGGGGAAGATGATAACGTCGCAGGATGTACAGTTTGCAGAGGATGATAAC
CCTGGGGACCTTGCAGTCATCGAGACGCGTGGTTCTACACCAGCAAAGGAGGCTGTCGATCGCCTGGTGGATA
GGGCCGTGGGGGCTGCAGGTCCAGTCGCTCAAACCCCCCTCCCCACCTCGGATCAACCCCTCCAGCCGTCTGG
TGCCCCGGAACCTCTGGCAATCACATCGTCGGACGCTATGGAAGCTGAGCAGTCAGTTCCCGCTCCTGTCAAA
ACATCAAAATGGGCAAACTTACCCCCTCGAGATCACCCACAACGGAATCGGGGCGCTCCGCCTCGATTTGAAG
ATCAATTTGCATCCACTGAAGATGAAATTGATAACGCTATCCGTTTGCCTGCAACTGGTCAGAATCGGGCATT
CGTTACCTTTTCGAATGAGCCACGGACATTTGCGCAAGCGATGGCGTCACCTCTCTCGAAGCAGTGGGAGAAG
GCCATAGCGACCGAGTACGAGCAGCTGAAGAAGACCGGGACTTTCGAATGGGTTGCCGATGTTCCTGAAGGCC
GCAAGCCTGTTGGGAGTCGTATTGTCTTCAAAGAAAAACGTGATGGGGAGGGTAACCTTCTCAAGCACAAGGC
TCGTATCGTCGCGAAAGGCTTCTCGCAGGTGCCTGGAGAGGATTTTAATGAAACATTTTCGTCTGTTGCGAAA
TTCACTACCTTGCGCATTTTCCTATCCCTTGTCGCCCATCTTGACCTCGAGTTGCACCAGATCGACGCGATCG
CAGCCTACCTTCAAGGTTTGCTGGACGAAGAGATCTATGCGATCGTTCCAGATGGGGTGAAAGAGCCAGGGAA
GAAGGGCTGGTTTTGGAGATTGAGAAAGCCGTTGTATGGTTTGAAGCAAGCGGGACGGCAGTGGAAGTTGAAG
CTCGACGAGGTGATGAGGAGTCTCGGCTTTGTCAAGAGCGCCCCGACGACTGCCTATACCTCCTCCGTGAGAA
CGATGAGGTCATCCTCATGGTATTGGTCTATGTCGATGATATGGCCGTTGCTGGGAAATCCACCGCTCGTATT
GCAGAATTCAAGCGGGATATCGCCAAAGATGTCGAAATCACTGACCTTGGGGAGCTAAAATATATCTTAGGCA
TCCAAGTCACTCGCAATCGCAAAGCACGTACTATTTCCCTTGACCAAACCGCCTACATCCACCAGGTACTCGC
GCGATTCGGTATGGCCGAATGCTCGCCCGTATCCACTCCCGTCGCTGTCAAACATGACTTATCAGTCTCGCAA
TCACCCAACACAGATAAGGAACGCACCGATTATCTCAAATATGCGAACGGTCTTCGATATTTGGAGCTAGTCG
GCTCGCTCCTCTATGCCACCCAAACGCGACCCGATATTCAATTCGCGGTCGGCCTCATTGCTCAATTTGGTGG
CAATCCCGGAAAGCCCCATCTTGATGCAGCAAAACGCATTCTGCGGTACCTAAAGGGCACCGCGGAATTCTCC
CTCACCCTCGGGCGTCACGGCGAAGGCGCTGTTGAGCTTGTTGGGTGGACTGATTCCAACTGGGCTCAGGACC
CAGATTCGCGCCATTCTGTCGGCGGTTTCGTTTTTGATATAGCTGGTAGCTCAGTTTCGTGGTCGTCGAAGAA
GCAACCGACTGTCGCATTGTCCACCGTCGAAGCGGAATACATGGCGGCCTCGAATGGACCAAGGAGGCCATCT
GGCTCCGAACATTGCTTGAAGATCTAGGGTACTCACAGACATCAGCAACACTTATCCATGCCGATAACCAGGG
CTGCATCGCCCTCTCTCGCAACCCCGTCGCTCACTCTCGTGCGAAGCACATCGATATTGTCATCACTTCATCC
GAGAGCGCATTGAAAATCAAGAGATCAAATTAGAATTTTGTTCTACCAAAGACATGGTTGCAGACGTGTTTAC
CAAAGCCCTCCCTCGTGAGGCCTTCCAACGATTCCGATCTGCCCTAGGTGTCGGTTCTTAATCCAGTGGGAGA
ACTTGCCGAGTGGGAGTGTCGAATGAAATCCCCAAGTTGAGTTGTCGGCTCGTTCTCAACATTCCCGTTTAGG
AAGTCTGGAGGTCGTAGACAAACACGCGCAACTAATAGAGATTATTTTCGTTGATCTGTAGATAGTGGAGTTC
ACTCTTTCCCTCGTGAACTCCAGGTATGTTTAGTATATTCTATGTTACTTTACTTATCTCAATATATACTCTT
TCCCTCGTGAACTCCAGTGTGCCCTCGCGAGACCCTCTCAGCTATCACCCGCCTTCCCTGCGCAACCGGCGCT
TCTTTTGGCGCTTCGCT
>Cligib1_rnd-1_family-630#TIR/MITE
CACCTGAGACAAGGGGTATTTTTTCCCCCGAAAATAGTCATTACAAAACTCGCCGAATATTTAACAAGTTAAT
AATCACTATTGTCGTTGCCCCACATTCNAAGGGAAACTCTAAATATCTTCAAGGCTTTGAACCCTGACAACCG
TCGAACTAACCTATTTAGACAATTATCTAGGTACATACCCGCCATCAGCCAATCAGAGCCGCCGATTTCAGGA
TTTTGACCGGAAATATCCACAAAATAAAAAGCAGATAATTGATAGGGTNAAAATTCTTNGAAGCGCTTAGGGT
TTGGCAATGGATATGTATGGTAATAATTGTGATTGTTAAGTTGTTAAATGTTTGGCGGGTTTTGTAATGACTA
TTTTCGGGGGGAAAAATGCCCCTTGTCTCAGGTG
>Cligib1_rnd-1_family-519#LTR/Gypsy
CACCAAAAGAAAAAGAAAAAGGTGGCATACGTCGAGCCAGACGTATTAAAANGCCAACCAACATCGTCGAAAA
NCAAGGTCGAAGACCTTATCACCGACGAACAGGAAAAACGACTGGAAGAATCCAGTCACCAAGCAGAACGCGG
AACAACCGCGAGCAAGAAGAAACGANCATTCTGGGACAGACCNATCCCAAGGCAACGATTCACGTGGGACCAC
GACCCACGAATGCCCAAAGAATGGAACGAGATGGTGAGCGCCATGCGCTCATATGAAGAAGACAAGACAAGAG
TCATAGAGACTCTATACCGGGACCCGCTACCGTCNGAAATATCAGACAGATACGAGTTCACAAGCTCGTACAA
NCCCAGCANANAGAACAAGGATTGGATGAGGAAACAATCCTCATTAAAGCCGGCGTACGAGCATCCGCCGAAT
CCCAAACCCAAGAAACCNGACAAAGGGAAAACGCCAACAAGGGGCCTAACCGAGGATGATTATCCTCGGCTCA
GGCAACACTGGTATGACGAATACCAGGACATAATGGGAGGAGTAAAGAATGAACTTCCCCCTTGGCGCGAAGT

GAATCATGAGATTCACCTGATTGATGAGAACAAGCGGTATCACTACCACCTGCCNAGATGTCCGAACTCGTTA
CGAGACGAGTTCCACGAAAAGGTCAACAGATACGTTGACGCGGGATGGTGGAAACCGAAATCGGTGAGCCAAG
CTGCACCGATGCTATGCATCCCCAAGCGGGATGGNAGACTACGCACTGTCGTGGACGCACGACAGCGGAACGA
GAACACGGTAAAGGACGTAACACCTTTACCAGATCAAGAAGTGATACGCGAAGACGTATCAAAAGGAAGAATC
AGGTCNAAAATAGACCTGACAGACGCGTACGAACAAGTACGCATCATCCTAAAGGACGTAGACAAAACCGCGT
TCGCAACGATCGCGGGNACATATATCAGCCTGATCATGCAACAAGGGGACTGCAATNCNCCCGCNACATTCCA
AACGACTGATGAACGAACTNTTCAGAGACATGATTGGCGAAGGATGGATTGTCATATACNTGGACGATATATT
NATCTACTCAGACACANTTGAAGAACACGAAGAACACCTTCGGTAGTACTCCAACGACTCCAGGAAAACGACC
TNTTCCTGAAACTGGAGAAATGCGAATTATACGCAGACAAAGTGGATTGTCTGGGTCACATGATCGACGACCA
GGGTATACGCCCTGATTCAGACAAACTAGGAAAGATCCGGGAGTGGAGAACTCCCAGAAACTATAACGACGTC
CGACGNTTCGTAGGACTCGTGAACTATCTGGGGAACTTTCTCCCCGACATCACGNCATACACCGGACCGTTGA
TGTCCATGATGCAGAACGGCGCCGCGTTCC
>Cligib1_Cluster93#LTR/Copia
TAGTGATTAGCACGGGTAATGCAACCCGTACGGGTTTTCCCACGGGTACGGGTACGGGTTACCCGCGGGTACA
CTTTTCACAAACCCGCAAAAACCCGTACCCGTTTTGCGGGTAACGGGTAATGCCACGGGTAATTCCGCGGGTG
CGATTATCACTATTTGGCCCTGTTTTATGCTAAATCTGCAAAAAAAATCATACATTTTGACTAAATATTCACC
AAAATAAGCCAAAAACAGTAACTCGCACCACTCGCGGAGGTATTATTAAGTTTATATTGGTCATAGAGCTTAG
GTTGATAGCCCTGAAGGTTCCCATAACTAGGGGGCATGTTGGACACTAGTCTCTGAGGTCTGCAAGTACCATA
CCCGTGGGATAAATCCGAAACCCGCAGGTATTGCGGGTTTTTGGGCAAACCCGTGGGTAACCCGTACCCGTAC
CCGCACCCGCACCACGGGTGCGGGTTCCCACGGGTACGGGTGCGGGTCGTGGGAAAATACCCGCGGTAGACCC
GTGCGGATCACTACTCATCAGTGAATGCCTGGACCTTCTTGGACATGTACTCCCCTCCCTTGTCATCTCGGAT
GGCCTTGATCTTGTGTCCCAGCTGGTTCTCAGCAAATGCTTTGAACCGCTTGAATGCCCCAAATGTGTCTGAC
TTTGCCTTCATTGGCACAACCACTCTGAACCTTGAGTAGTCATCAATGAATGTGACCCAATACTTGAATCCAG
ACATGGATGGCACTCCAACTTGGTGCACATCCGTGTGGATCAGCTCCAACAGCTGTGATGCTCTGTGTTCACT
TGGTGGGAATGGGTTGGCATGCATCTTGCCTCCCAGACATGGCTCACACACAGGATCAGGCTTGCCCTTCGAG
TTGATGACCATTCCAGTCACCAACCCGTCCTTCAGTAGCTTAGCTACTCCATCATGATTGTGATGACACAACC
GCCTATGCCACAAAGCAAGATCAAGAGGCAGAGTTGACAGCAGATGTGCTCTCTCAGAGTTGTCTACAGTGAT
GCCATCCAGATAGCCAACATTGTTCACACCAATAGAAGCAGTGAAGACAATTCTATCATTAAGCTTGAACTCC
ATGCAGTCTCTATCTATCTGTATAGTGAATCCCTTGTGTCTAGTGAGGTATAGTACAGCTAACAAATTATTCT
GTAAATCCGGAACATGTAGTACTTTTGAGATCAATATGTCCTGACTCTGTCTCCCATTTACATATGGTCTAAA
CAGGACATTCCCAACACCTTCAGAGTAAATAACCCGGTGATCTGCGAGCCTGATAGGCACCCTATAGGCCTTG
TAATCACGGATCCAGTGTCTGTGTGGTGTCATATGAGAAGTGGCCCCTGTGTCTGTATTCCATTCAGAGATGT
GAGTTGACATTGCATGAACACCATCTGAAGCACTTGCATTTCCAGCAGATTCTGTCACTGACTCTGCACTCCC
TCCCTCTGCCTTGTTGGCACTTGCCCCCTTGTTTCCACTGGTCCCCTGACCCTTGCTACCCGCATGCTTCTTT
GGACATGCAAATGCGCGATGTCCTGTCTCCCCACAGACATAGCATCCAGATCCTGATGATCCACCTCCAGTGC
TTGATGATCCAGACTGGTTGCCAGACTTCTTGCCACCCCGTGCGTGATAGGCCTTGGCTTGGTTTGCCACCTC
GCCCTTGCACTTGCGATTGAGCTCCTCAGCACGGAATGCTTGCAGAATCACCTCTGGTGTGAGTTTGTCTGTC
AAGAGCAGTGATGATGACAGGCTGGCATACTCCTCAGGCAATGCCCGGATCATAGCCATGCACTCAAGATCAT
CATACAACTGCTCAAGAGTGAACTCTGCAGGAGTGACATCCTTGATGTTTCCTACATTCTATGCAATCCTTGC
CCCCAACTCTATCAGTGATTCGTCACCGGTTACAATTATTTCAAGCTTGTTTGAAGACCATCGGTCACGCAAA
GACTGGTGTGACCGGTCATAACCAGTCCTAACCGGTCCTGACCGATAATTTTACCATATATGGATGTCACACA
GGGTCCGCGACAGAATATTTCATGTGTAAAAGTGAAATATCTATGTAAAAAGCAGCTCTCAGAGCCATTTTTG
ATGGTTTTTGGTGGTATTTTGGGGTGTTTGGTGGGTACGCATGTGGCTGTGCAATAACCGGTCTATAACCGGT
TATCACCGGTCACACAGGCTGTCACAATCCAGGAAGACCGGACCTTGCGCACCCCAAAATCAAAAGACCGGAC
CTGCGGTCACTGTGTGACCGGTCATTTTTCGGTCACGGTCCGGTTCGGTCACGGTCACATAGACCGGACCTTT
AAACACTAGTGCTAATATACAGTTCAATTAACACTTAGATATTTAATAATTTTGGTGAGGTGGAATGACATAA
ATGTTACCTAGGAGTCGCGGAATAAGTAAAAGTCAGACAATCCAAACCAGTGTTAGCTTTGCACTTGTAAATA
GTACGAATGGGTAACTAGAACTAGCCGGTACATTTAATCGATTGCCAAACAACATATAAGGCATACGAAATTT
ACAATCACAAGCATCCGGTTGCCAGCTAGCATTACGGGAATTCGAGGGCCACAGGCTCGGAGGGGATATTCAA
TGGGCGGCAGTGGCATGTCTGCTGTGATTCACATGTGTCTATGTTTTGTATTACAGACCCAATCGTATTGTAT
GTGTTTTGTCCAACAAGTGAGCATGCACAGCATCTGTACTAAGGGCAGAAGGATATTCAATGGGCAGTGCACT
CAACAATGAGCAACAGTATGTTTTATTTGGAAAACATATGTATGATTCCATAAGACAATATGAGGGGGCCCGA
CTGCCAGGCCCACTCTACCAATACTTAGCCTGAAGTCTGTGGACTTTGTTTATAAAATATCTTGACCATATGA
ACTGCTATGTATATTGTATTTACAATAACTAATCTCACACAGGGAGTTTGTGCATTGAGTGCCCATATATGGA
TAATTCCCACGGTACCCAGCAACCACTTGGGGATCCAAGAGCCCCAAACTGTGTGCATGCCCAGTGGTCAGAT
GATTAGATTCCCACGGTATCCAGCAACCACTCGGGGATCGAGGAGACCCCCAAGGACTTGAAAGGAGGCAAGT
CACAACACTTTGGTGACCACCCGCGTGCCGCAAGCAGGAAGTCACAACTGATTGTGACCTCTGTTGGGGTTCG
CTGTGAGCCGTCTCACTTCACCTCCTCGCGCGGGCTACCTTTCCTCCTCGGTTTCTCCTATCGTATATGTCTC

TCTAATCAACCATCCTGTATCTACCATTCGCGTTTTCTATAATATGCCTTCTACAAATCCAAGTTCTAATAAT
CAAAGCGTTATCTCCATCGACCCGGCCCTCTTAGCGCTGAGCGCCCGCTCCGAGGTCGATATTCAAGTTCCCG
ACTTCGAAACCAATCATTCACCATTGCCCGCGAGAATTCCTGAGCCTACAGCATCTACAAGTTCAAGTCCTGA
TAATCGATGTGCTATCCCTATTGACCCGGCCCTCCTAGCGTTGAGTGTCCGCTCCGAGGTCGATATTCCCCAC
CACAAAGCCACTCATTCACTGTTGCCCGCATTAATTCCTGAGCCTAAGGTGCGGTCTTCAGCGAGTGGATCGT
TTCGCTTAGCAACTCCCACCAAGAATGCGACTGGTGAGATTATCTCAACCCGAGACCAATTTGAGACCTACTT
GTGCGTGTTTTTTATTTCGTATTTCACTATTTTAGTACTCAAGACCGCTTAGGAAGTCATTCAAGAAGAAGGA
CCCCCTCCGCGAGGGTTGTGTATTTCTCGGTCTTAAAGCCCCAAAATCAGCAAACCTCGAAACTCTCAGAAAG
TCCCTCCTCGATTATTGGTATGTTGTATTGGATTACAGTGTATTATTTTATGCCTTTGGATTTAGGTACCCAA
GTGCCCAAGCCCAAGTTGATAAGGTAAGGAGGCCAGCTGGCCGCCCAACCTAGCACTCATTCTTGGCCGGTAG
ATTTCATCCTCGTGCATACACCTCCCGCTTAGCACTGATGAACACGAGGCCACACAAACGGAACTTCACCCAG
TCTACCCATCCACCCCACAGTCCCAGGCTTCTTCACAAGCACACATTTCGCATGTTCAAGCACCGGTTGATGC
AACAAATGGAAGTGACGACGGCGACGATGAAGCAGTGTTGGTTAGAGCTTATAACATCGACGGCGCAAATGGG
GATGAAATCCTTGGCTATGATGGTGAAGGGGATGACGATGACGATGATAATGTCGATAGGGACCCAGTAGAGG
ATGAGGAGGATGAGGGTGCAACCCCCGCATCCAAGGGATTTCGCGATTTCCAAATTCGTGTCCGGATGGATGC
GGCCAAGCGTGCGGAAGAGAACCGACGTGCAGGCGGCTTGAAGACCCAACGAGCCATGGCGAAAGCTTGGAAT
GTAAGGACCTGCATTTAATAATTTACTAGCTAACTTCTGGTTGCAGGAATTCATTGAAGTCTCGCTCGCTAAG
AAAGAAGTCAAAGACAATATTGTTGACGAACATTCACTCCTTTTATTTATCAAATATTTTGCTGAGCGGCCAA
AACGAACCCAGGCGGGATTGGAGATCCCAGGTACATTTGTTGGTGCATCACAACTCAAAAAACTTTTCTTTGG
GGCCCTCCGCATTCGCAAGGAACAAGATGCAGCGGACCCAGGGCTAGCGAAACGGCGTCGCGCTACATCAATC
ATTGCCTGGGAAGCAATTAAGAACCGGATGGATGAGTCTCTGGAAAGGGTGCAGTGGTGTGTTTAATCCTTAC
CTACACTTGGAGATACTGAGTATACGGTATCTAATTTCTCTAGTGGACTTGTACCCAGTGAGGATGCGCCCGA
TATTGTCGCAAACACCTTTCTAGCTGAAATAAGCGAGGAACAGCTACAACATGTTGGTATTGCGTTTCTTGAG
CATCATGAATGTTAGTCCACGCTTGAGGTAGTTTCAACAATATTGTTACGGCGCCCCCAGGGGGCTTACTCTT
TTTCTGACATCATCTACGGACGCTTTTCTCTTCAACACATGTTGCACATGTGTTTGCCTCATGGACTCCTCAT
GGGCATCCTCTGACCTACTGCGCCTCTTGGTTTACTCATTGTCTACAGCATATCTTCCCCTTTAGTATATATT
GCTGTAGAATCCCTTAACATGCATTCTCCTCATGGTCACTGTCTCTTAGTATATATACTCCTTCTGTACATCC
TGTAATAGCATCTTGACCTAGGCAATCTACCGCCTTCATACTCTTACTCAGAACCTGAGCCTAGACCAGCTCG
GAGTGACTTGAACTCCTTTGTCATTGGACAGGAGACATCTTGAAACTACTCTCAAGATAGAACCAGAACGAAC
GACAGACTTGTATCAGTTGTGAAGGCTCACGCCCTAACAAATATCATATTAATTATGTGTCTTCGCAGTACGT
TCGGTTATCAATGGGCACCTATCTTGGACGGCCCAACATGCAACTGGCAATCGCGGTGATGATTTTTGGGCAC
TCAAACTCACAGAGCTGCAGCCATATACTATGCTTCACCCTAACAAGGAGACAGCAATCTACTCTGTTCTCGG
GCTCCAAGGCGAGGAAAAGGCTGGGCGTCGAGGAATGAAGACGGTAACATGAATTGTCAGCCTATCACATTTA
ATCAAACTGATATTAATAATTCTGTAGACGGTAAACCCTGTGTATTCCGTTTTCATTGCACATCTTCGACCTG
AGATGTGCCCGCTGGGTGCATTTGCGTTCTACCACCATTATATGCATGACGTCAAGGATATTACCACATTGAT
GGAAATCGATTGGAGTGTCAATAAAAGCTGGCGGCAGGTGCATGGTACTAATACAGTTGTAGGCTTATTCTCA
ATGGTTTTATATCCTAGATCCGAATATTACATGGTCCGAAGTCACCAAGAACACCTTATAATGAACAAAATCT
CTACAACCTCTACGTGAAAGCATTTGCAAAAGCCGGGTTTCATTCAAAAATCAAAGTACATCTCCCGCGGCAT
ATCCTTGGATATAAGCAGGAAGCTTTGGGGTATGCTACTGTTCACTGGTAACGTGCCCCCTCATTAATTTTAT
CTCAAGCGTTGATCCAAAGGATACCTCCAAATTGGGGTGGGTTCGAGGCGAGACATACTTCGACACATACGCA
CCTGCATTGCCAAAGAATGTAAGATATTATACATTACTTTGGATGCTGAGTCACTGATTACCTCGGTAAGGCA
ATTCTCGGTGCACATGGCTTCAAGAGCCACGAGGTTTATGACCCCATTTGGCGGCATGTGCATGTCCCAGAGA
AATTTCAACTCTTGGTCTGTCCTATGGCTGAGAATATATATGCGGAGATCGTTGGTGCGTGAATTCTGAGGTT
CAAACCAGTGAAATTCTAAACTTTCCAATTTTGGGGCCAGGTCGTGCAAATTTGTCAGGTGTGGCTAGTGATT
AGCACGGGTAATGCAACCCGTACGGGTTTTCCCACGGGTACGGGTACGGGTTACCCGCGGGTACACTTTTTGC
AAACCCGCAAAAACCCGTACCCGTTTTGCGGGTAACAGGTAATGCCACGGGTAATTCCGCGGGTGCGATTATC
ACTATTTGGCCCTGTTTTATGCTAAATCTGCAAAAAAAATCATACATTTTGACTAAATATTCACCAAAATAAG
CCAAAAACAGTAACTCGCACCACTCGCGGAGGTATTATTAAGTTTATATTGGTCATAGAGCTTAGGTTGATAG
CCCTGAAGGTTCCCATAGCTAGGGGGCATGTTGGACACTAGTCCCTGAGGTCTGCAAGTACCATACCCGTGGG
ATAAATCCGAAACCCGCAGGTATTGCGGGTTTTTGGGCAAACCCGTGGGTAACCCGTACCCGTACCCGCACCC
GCACCACGGGTGCGGGTTCCCACGGGTACGGGTGCGGGTCGTGGGAAAATACCCGCGGTAGACCCGTGCGG
>Cligib1_Cluster35#LTR/Gypsy
agagactgaaGccagCacGcgaCTaACacGacTcGaacgCcacgaGAcaagcaCccaagagaTaacagcTccc
aGagaTccaGaaTCaacctagTgaTaTcaaaAcaagccAaacaccaaggcagaTAaaccaGacaagaCaTaGg
acaCaAaTcTaaaaaaacaaaaaaaagCaGcaaccgaaaaggaagcAaAcTaAcagaacaaagcaccgccaccT
gaAacAccaTAcagAaTgcacTGaTaagaaaaCAcacagaaagaTACcaGAccaCAaacaGaTAacacagaca
agcTgAgagTaaCGaccccgAacaGTcagaacAaaCaccTGAacgaaacaTaTGcccgccAcaaaaTcTcgaA
caGggcCaaaaaaAaccaAcgaaacaAaaaAccaGacTaAaccggcGAAcGGagacccaggGgcgaaTcaacc

aaaacgacCaaGAcaaaggaaaacTCaacGccgccagCcTcaccacTaacagcgaCcccaGAAgAAaTacaga
gaccgaacaCagcaTaTaCcagggCcaTGCgcaCTacccccgCaagcGcGacaccGTccaaaaaacCcaaaCc
gGcggcgaggCaTcCccCaCCaaCGGccTacgaaccCagcccgAAaccCgcGGaTggacCcgccacgGacccc
cccgCccgcacCgCcggacGcGGagcGaaacCggCcaCaccGGccacCaggcaaaacacccCCgacacaGccgC
aATcaaTgAccgaTaaaaCaGaGacGaaaaaGAccacgaCcAggAcacTaaaaaaaaccgcgaaaaccACaaC
gGccTcgGaacaaGAaGaaaaCgTcaCTaagaAGcaaacCacTagaaccaaGAaaaacccgcgccacacCAcc
aaaGaaaCaaggaaAGaacacacaCCgGaGATgaGcgccccTaGacaAaCCCaacgacagacacAAAcGcCcG
aacaAacaGacccccaaaccgcccacaTCGaccaaaaagccaaaaaaaCTGaaagCacaaCcccGaccccggG
AacaacaaAgcgCAAaaGagaagaccaaggaAacagcgcaaaaacagaacggcaaccaaAaaaaagacaacag
cccaaAcaGcaaccgAcaagcGccaaagcGaacgaacaaaTcaaaaacAaGaacaaGgccccgaCcaGaaCaa
AgcacgCcaacgCcgagAaGgagAacAaGaacCccAAcaaGcgccGgAcgaaGGaGcgaGAagcGGaccaGAa
acGgcgccacaAgacaCacaAcaAGAaGccGGaccAGaacGcgcacAaaCcCgacGGcaaGaaagcgAcTcGa
aAcacaccccgacaCGaaaTcgaCATccgAacTgTgaacCaaATcacccggaAgAGcTacccccaaaaTacaC
TagcgaAcAaaaaaccCggcaaAcTaAagAaacccTcAGacacccCGgacgaaCcgAagaaaTCgGaaaaaAc
CaacTCcaccaaGTCtagggCcacgtcgtcgtcgtcgtcttcGaaaTCcgccccAcacgcgaCTacGgaCaCC
acgcCCgcaAGcaaGgaaaacacGacgcacAaaGaaaaacagaAaCaaaacCGcaaaaaaGacTCaGGAAaga
aaccaacaAAaaacaTGacgAaAcaagcCCagccCgcgaaagacAagGGcaAgGcagacaccacCacaTAgca
ccaaTgagTaacgGaCggGaaGcaCcagcaCcaaaaacagcgcgcaaacaAacaCcGAaaAgCcgaCaacaTg
GgccgacACCaTACagGGaCcgcaAGacAaTccTAccTacagaagGcccCaaggcagcCcaACAaagaGaaac
cgaaaaacaTCaccacTgcgcTaacgcaccaCccgTTcccccgcaagTccaaaAcGCacaagaaTcaaaaGCc
ccGTaacAccagccAaAaTaaaCaAaCggcaAaggCaaAcgTcCacaaAcccaaTGGgaTcTaCAacATgagc
gggaAcaAcTccaacagCAaaAAcgTcaTCaaaaTaagagacGGcacaccTacacaGaccCaacgggCcacGT
acAacaAccacccaaaCccaaaGacGaacgccTgggcAaaAaacgcaaaaGcCGcagaaaaTagTgacacGaC
caaggaaGacgcaTCcccgacacgcgcagCccgCacggCgaaAcccaaGcaaccCgggaacgCacCaaGGcCa
caAaGCCCTacGaagaacaAaacCcgcTagAacAacagCccGccgaATaaGaGaCcCaaCcacgcACccccaa
gccacaaCaaaGGcAcgccaccCagCCCCcAccccGaaccagCCTCaaCGCcaaccccaaGcGGaATGaacag
aAcCcCcGcAaGgTcacaaaaCAagcCcgccaacaaaccTgaaaaAgcGcGTaacaaccAgaaaCgCcccaaa
CgCACaGagaaacaacccgaggaggAccacacAcagGaaaaagccTccaacaGcaaagaaaaaGaaCaaagca
CacagcaaacccacAAGaacAACTacGCGacgTcacCcagCaAccgCaCCccgccaCCCaACaAagCccccaG
acacaaaagGaccacgacacCCgccgGaTccGagTgccaaaaGaaagcgAcGccgggaaaaagCaCCaacTcc
acgaaaAcCacaaCaAacCcaacCaacaacTCAaccccaTgCaaccgggAcgaagGagaCGacGGcgagaGcc
caGcAcAcaacAaTGaCaCcAccggCGaAGaagacaAGgccTCGccTgcgcggaaCAcaccAaCcaCaCCaGa
acaacAaaCggcagcgaTCAgggGGaaTaGcGaCAcccTagccaccaaaAcaaaCgcagaTcaagGagaaacg
cTgaacagcgaaGaccaggaccTGagCTccgaaaACcCcaGccCCcacAGGcacaCacaccccaacGACgACg
gcgacccgGaaacggacacCaacacGcaaAaCGacacacagaCacTaGCGagGacacaTGacagggAacaaaa
gcgcCccAgGaacgaaaGacAaagagaaccGaagGgcagCgTcggccAaccGaccaCTagAgcaaCgcaaccA
caGcccaaccagacCCccgagTaacCaagTaacccTcaaaGcaaacacaACaCcTgGGcaaccagcTggCAgg
TaTacccgacCaGgaAgaaaTaCcaccGcaTaAgGaaCacaaAcaccaGGcagCaagaAaacacaTgagcCga
aAGgGagcccCcacaaaAagaCcaacTcggacagaaacacaTccaagaaccaccagATagacccccCcaAcAag
gaGaaaacaaacGagTgGgAaGccgcccgaaaaaaacAAccCCCaaacaGaATGacagacccaacCagcgaaa
CagcaaaaaTaGTcaaCccgGaCTTCCacaaagacccccgacCaaagcagaaaaGcgaacacccAcCcgTTaC
aaacaccacGcagCaggacggccCcCgaaCcAcgacTaaCaaGAgacccTaaGgGaagGgCaAAccaGacaaC
agTcaaacaGgGaTCaaacaccacgaacacGaagcaaAcAgcaTCaccaACccaGaTgccgacTaaaGTcTCc
ccaccccagcagCGCagaaaAgGGaaaaTaaaTTcaaagcacgcacaaGcTcCCacCgcaaCgcaTcaaaaTa
gGaGgaaacCgTcaCacTccacgGCccccaGaccgCggAcGacaaacaaaaGccTcagCccgGGgaagTcaca
cTcgcAacaaccaaAacTaagcaAccagagaTGcCacccaaGcaaaGgAaTGccGCCgCAaCacGagacgaGA
acaGGCacgcacCaaacaacaTaaccagcGTCcgCaccaTaaaaaaCcaacCaTcacccaacccaATaGaccc
cGaggaCaaagcggacaccaTaTCCaCgTcaATgGcaaaccAgccaaaacTgaaaacaATcaaaCgcaacaaA
ccaAgcCCcagcaacaTcgcaggccaaaaaacaaaCacgaTgcccAacAcGaGaaCAaAGgcGgacagcaaca
acaaAaaaAagaAacaggaaccaTaaaTggaTggcaaaaacagcaaCaacTaTGacCccaTcgTcacaAcacc
GcgcaacccccaaaaccagAgcaggacaGGAaTCaacgaagccaGaccaATccgcggaAGcaagaaaTccccT
cCaATaaAcGgGGAaAaaaacaaacagagGgaacCAaaGgCaATgaacgcaccacAcGacgaGccagacAaca
aGacGACaaacaccAacggcccgGcgaacgGcCgccTCaaaaacCcaGgTGaGGCacCAcTaCcaccCaaacc
cGccAgaaaccaTacaacAaaTcgcacagaTcacAccaacAcgcaCCaaaGaacAacCgcgacgcgccCcTaa
GaacgCcccaaaCaagccAaaGaaAaGaggaaCaCaaAcccgaAaaaGggcaGcaGGaAcCacaccaccaagg
caAAggcAacgcCCaTgagaaGCGaacaCaaaagcaaGAaACcgaAagaccaAcCacaGTgacgGAccaTaTa
ccacaCcccagaccccacagcGAaCaCcagaccAagGgcagCcccTTcGaCaaaCaCagAGaacaTCcaGgcA
cgacTcGcgaGcGccaacaaccGaacCcgccaagagGgAAacGaTaccgaagaaCaaacaccagagGagccac

aagacgccaGGAAGaCaacGagcACcAggccaaagGgcaacagGcgcaccaaaccCATTCagacaGGGaaTTc
caacgcaacccaaaCcTaCaAcgCcgcgaggaaCaaacTaTccGcccaagaaATagccaaagcaaaccAccgc
cacacAgaaaAcaccGgaaTccacacGaaaaCaCTagaGaacaAgacaacccaccGaccCCgacgaTaccaGa
cCaTgAgcCaagaGaAaccaTcaCcGaggcaaaAgAaaTGaaAcagcaggcCCaAGGaGTcaccgaccCgggc
AcaagcaacaGaggAagaaaGGaaacaaagaaaCccTaccaaaGccaaTaaAGccaaCaagTccaacAaGacc
aCaaaCaGagaaaggTaCCcacaacaCaTacGaacagcgaccTaaaacgCAgagaaCaaCGCCAagGcaccca
gccCGaTcgcCcacccGcacaaaagCcCaaccaacaaGTaGTCacaccaccagGgagaCCTcgaGGacCgcGc
GTaccaaaAGacCAaagaaaacaCcgaaaaaggaAcaaagcacCaaacAaagcaGccgcTggcaagcaagAac
aTGgCcCcAgcCCaaaTGAcaaaccaaGgaTccacaaCaGGcGgcgCTacacTGacaacacaAacCGaacaCT
gaaagaCTgCGaacaTcGcGgCAcTcgaaTcTaCcaaaCcaaacCCcgCGcagCagaacTaaacagcGaAgca
aacTGAcaccagGaccaGagTgGaaacaaTgccccgccAcagGGagaggcccaaaaaCcTacacacaaggccc
aAaaCcaAcCacaaGGcacaaacccaCaggCccaacCaaaaaGacTgGTcGGGaccacaGGcGaacTgGagcG
agaAaagCGCcgAGTTcaaCTTcgcacaggaacagGacacggGgcCggacaaaggcCaagccaAccagcggcC
accaacccagTaaaaagaaccgcccggaaCgGTGaGggcAccGaGggaaTacccaccACaCcaTcgagaAcaa
gacgcccaaGccaTGggggccaAaCcTcCaGaGgTgagcacCATgCCACcgaccccccGCGacGAGGacccag
CaaacTcGGcaagaaaCaaagTcCCcccGgcagacacgccacGccaagaGacGcGcCgCgaaTgacccCaaGag
aaTcgTaaAaaaacgcTgcgcaccCaaAGGGCcTaaaGaCCcaGaaGaacgaaaGcaggcCaaaccTcaaGaa
aaGaacGaacacaagccaccaGAgaacaCcgaacaaGaCaacgaAagAaacgaaaCGcCgccaaacacTgcTa
aaaAcaacgaCacCccgccGgagacccccTcacTagACagaGaaacccGaaaCgccagAcacagaCCcgacaa
gAgcagGAacAacTTaaTGagcgacaAcTcaTgaaaaaaaATccTGgAcAaacCaaaaaaagacaGcAacTag
GaaaTgaaaGATcGcccAcTcTaaaacaTcaaACacaGCaagccacgacgacGCaTccccaAagccaaccacA
AgcaGcaaacaaacAaacaAacaaaGAaccccgaaaaccaCcaagTGTaaGcacaaagcGcaaCaacaaaaAc
aTAcaAcTacCTGcaaGaCCacGTAaGGTagaacaCaAgcggGcaaGacagTCaGcccTaccgaaaAacaccc
accacGacaaaGccCagCaaGcCaagcAagaAcaaaccaTAgcgGcaccgaaaacCaTcaCCagTccagacac
aGccagGGaaggccgTaagCAacGAcTTcGggccaccacTcCCaGAccCgTccgacCGcaaagGcTcacacga
agagaTaccacgCacaacagaCcccCTcaaGGaaaagGgGaaCcaaGaacaAgaccaaaacGAagaCcaaGaa
aaGGACacacccGAccaGcTGgaggcagAgacagTaaGacaccacGaccaccCTaAaaagacaaTgAaTgaca
cGcaagcgcTcTccaagAGaacAcgccccaaacAaCTaTgaaaaccgATcgGaacaAaacgcaaaATGgacAg
cgCGcaccaccccccaaacagacgggtccacggagcgcgcgaaccgtaccataacgcagatgatccgccagtgt
gtaggccccaaacaaaaggattgggtcggccgcttacctgggatccaatttgcaatcaattccgcgcattcat
caaccacgggatactccccttcttcttgaatcacggacggatgcctcgtaccatgatctgggagacccctgg
taaggatttccaatatgcgggagtccgcgcctttgccaagcgtgtccagacgagcatcatgaacgcgcacgac
agcatcttggctgcgcgcgtcaagcaaaccaggacggctaacaggcgccgccaagcctcacccttcaagcaag
gtgacttggtatacatctcaacggagaatatctcctttcctaggggcttagcacggaaacttgtgcccaagtt
catcggtccctacaaaatcttagaggacttcaataataactcctatcgtgtcgagctcacgccgcggctcaag
cagcgtggcattcacgatgtctttcattcctctctcctgcggatccacatcccaaacgatgaccgcctgttcc
cagggcggttagagacccaggtctgggactttgacgaccccgagtccgagtgggccgttgaacgcatctgctc
acacgctgggacaggagaccgagctctctttgaaattaagtggcgctcaggagatctcacttggctgccatat
gataagtgcaaccatttgattcaaatgaaacagtacctagaagctttaggagtggaccacatctccaagcttc
cgagcgggacaggagaaccgcctgacgaccctcagatctttgtcgggggtatgtcaatgggagcggaagtctg
ggaccaacagaggagggatgatcgccccgccaacttaggcgaatccacgacgagaggtgccaggtataaaagc
aaacccTcacTcgCcAcTacaaacCaaaCcacaGaGcaTcacaaCcgTCTcCgCCgcCcTCTacTacaGccca
TcaccaaaaCcccTCaccacCaacTCaaaaTCCccaaTaCacaaCccaTTcaGcagaaTcgTacaCgTacacG
aaaccGaccaaTccGagCcaGcCcacaaCcccgcCaTacAcagcAgcacggaccgCcacaccaCcagcaTaca
cacCacTcCacaagcgccgagccccTcccaCccaaaccagaggcagcacaaaaTaaacgacgaaaacCcaTTG
acaTcgcgccgTCaaaccaaCaaaGcaagGcccgCAGgccgcacaaagagcgcGGCgAgaaccaaccagaccc
ccaCccgTgcggCcGaCcTTTcaccaaccaGggGGGaTgccCaAacccacCcaATCcCaGGgacagcgaCccc
aaacggaaCacaaaTGagcgcaagacaaaccaTCacccggaaaacGaTcgaCTcagcAGacAaGgaccCaTCa
aacgCaaAacCaGgaGTcaaCggaaacaaccgaggcAacaTaCTcGaaagaaTCacagcCcaCgCcGgggcAg
aaaTaAacaATgGaaaCacCacaGGcaacaaacccaaGcgaaCCacaGgcGaaGcggaCcaaCCaCccaacGa
GcaaagcGaacaCacccCacgcaacGagaaAgCCaaGaaccTaacaccGaagaaaaAAcCCGgcGGcGccGcA
gGaaaaaaacCacGcacaGacaacggcaACacgcgCcCcagcGaccaaccAGgaaGCAacGaaagaGcagAgc
aagcaaaaaaccgagccagTTaTTCcaagaTacacgccAacacacAaGaaacaaGCaggGAcgagaaagcacT
CacGcaaagcacaccccAacaaaCaaacccgaaCCaTTCaaaccaTaAcaCgccTacaTgaaagacTcacgTa
aacagGaaaCgcTgaaAaAagaccTaaaaaaCAgAgacagacgTCAcccagcTcacaacACgggcacaccccCG
agAagaTaAcagcacagcacaTAaCccccTGAaaTCccGTAgacgCaacagTacaggacaAaCAggcaAaTac
cTAcgcTaaTaaAcccacacaacacacaggagcaAaTcTaaaaaacaaaaacaacCaGcaaccgaaaaggaag
cAaAcTaaacaaCaaaGgacgcgCgaccgTAaaaaaagaacaaaaTcaacgaTagacAcAgaAcaccgGAaaC

cacaacaCagcaAaagaagAgagacaacGcgaaaaTCacagcGaccgCaAacCagGTgAaaaaCcaGaacaCg
aaAGTcgccgtttgttaca
>Cligib1_Cluster88#LTR/Gypsy
GGACAGGCGGGATCTCCAGAGGCGTCTCGCAAGCACGCCGCGCAGAATGCACAAAGAAGCTGAGTTGTTGTCT
GTCTACGTGTCGCATCCGCTACTCTACACCGGCTAGGTCACGTGACTTGCCGGAGCGGGCGCTGCCGCGCCCG
GCTCCATCTGCCCCCCTTCTTCCAGGCGGCGTCCCCGCCGCCTCGCCGCTACCCTCCCAGCTACGGTCGCTTC
TCCCTCCTCCTCCTCCTCCTCCTCCTCCTGCTCGCTCTCCTCTAGGGGTCCCGTCTCCTCACTCTCAGGCTCC
AGCTCTGCCCCCACCGCTTCCTCCATGCTCCCACTCGGCTCTGCCCTCTCCCTCGCTACCTCCCTCACTCCTC
CCGACAACTCTACCTCCCTTCTGCATCTCGACTCCCACTCCTGCTGCTGTGCCTCCAGCTCTGTCCCCGGCCT
TGGCCGAAATACCCGCAGCCTCCGCGCACTGACTTCCCCCTCAAGGGGCTCCCCCGCCAACGTCTCGAGCCGG
TATGAATTGACCAAGCGCTCTCTCACACGCCTCGGCTCTGACCACCTGGGTGTAAGTTTCCTGTCGTTGCTTA
CAGTAGTTGTTAGGTCACTACGATATGTTTGTACCAACGCGCCTTTTTCAAATGTCACCTCCCCTGCCCTTGA
CTGAAGGACCCTCCGGTCAAATACTCTCTTCCGCTTCGCTGCATGCGCAGCTGCCTCCGCTTGCCCGTCAAGG
CGCTGCTGCATCGCATATGCCACGTGAGTCTCCACTGCCCCTCCGGGGAGATCCTCGACGATTTCGCCCAGGC
TTGGGCGCGGGGTGTTGATAACCAGCCCCAGCAACAACTCCTTTGGCGAAAACTTGAGGGCTGGTAAGATCCG
CCAGTTCAGGATCGCGATCGCGCGCTCGAAATGATCTGGCCAGTTGCGGGGCATATTCTCGAGCTTGGCCGCC
TGCCACCCATCCTCCCCCAATTGCGGTGCACACAGTCGCGCTAGAATGTACAATAGGAGCTTGTTAGTACCCT
CCACTAATCCGTTTATCCAAGGAGAATACGCCGCCACTATATGTGTTCTTGTCCCCTGGGACTTGCACCATTG
TTCTACCTCCTCCGACTTGAAGTGCCTCCCCCCATCCGACATGAACGTCTCGGGTGGGGCGAAGTTGCGCACA
ATGTCTTTGAGCGACTTAATGGTCGTTTTTGCGCTCCCATGGGTCTTGAACATGTACCCCCAAACGTGCTGGG
TACATGTGTCCATGTACAGTCCAACGGTGTGATACCCCCCCTTCCCAGCTGGCATCGAGAGGTAATCCCCTAC
CAGTAGCTCGAATGGGTGCCTTCGCGTGATCGGGTTGAGCAGCGCGTGTAAGTGCGTTCCTCCGAAATTCTTA
CACTGCGGGCAGTCTAGGATTGCCGTGACTATTGACTCGTCCATACCAGGACTGTGGTACTTATCTATCAGCG
CCATCTTGATACTGTCCCGGTGCCAGTGGCCGTTCTCCCGGTGCTCCCGCCTCGCCTCCTCCATGGCCTCACT
CCGAGTGATGCATTCTCGCCTCGGTCTCGCCCTCACTCCCGTGCCCCCCCCGATATACCACAGGCGACCACCC
TCAATGGCATACCCCATGGCCCGGTGCCGTGCCCTACTTCTTGTCTTGATGTCCAGGTCTCCATCTAGGCCCG
TCAACGCCTCCACCACCTCACGGAACAGCGGCTCCTCCTTAAACCTCTCTCTCAGTGCTCGCTCCGACTCCGA
GAGCTCAGGAGTACTCAGCTGATACAGGTCGTGCACTATACCCTCCCGTATCTCCCAGTCAGGTAATACTGAC
CACTCGCTCCCGTCTCCTCCCCCCCTTGGGCGTCTCCTCATACTGGCGGCTGATCCCATCCGCCACGTTCAACA
CCCCAGGCACATGGCGCACATCCACAATCCTATGCGCCATCACACCGTCTCTCCAACGCGCATGGGTCGCATT
CGGCTTCGCAGTTGTCAGCACATCACGCAGTGCCTGACAATCCGTCTCAATTTCTATGGGGAAGCCATAAACG
ATGTCAGCAAACTTGTCTAGAGTGAATTTGAGCGCCGCGAATTCCAACAAGAACGGCTTGTATTTCTCCTCAG
CCTCAGAGGTTCGCTTTGAGGCAAAACCTAACGGGTGAAGCTTCGTGACCTTCTTCCCTCCAGGAAGAATTGT
TGTCACTCTCTGGGATAGTACTCCCGCGAACGCATCCTTCGAGCCATCTGTTGTCACTACGAACGGTGTTCCG
TCAAACACCGGCGCGCGTAGGACTGGCTCCGACACCAGTAGTGCCTTCAACCGCACAAACGCCTGGGTATGCT
CCTCCTTCCATACCGGCGCGAGTTTGTATGCCCTCATGATCCGCCGGTACGTACTCTTGCTCGCTCCCTTGGG
TATGACGACTCCTCGCAACAGGTCTCTAAGCGGTCTCTCCTCCTTCGCGTACCCCTTGATCAGGTCACGGAAG
TATCCCGTGAGTCCCAGGAACCCTTCCAAATGTGACGCATCGCGGGGCTGAGGCCAGCCCACTACTGCCTCCA
GCTTGGCCGTATCCGCCGTCACTCCTCCCGGCCCCACCATACTCCCCGCAAACACCGCCTCTGTCTTAAACAA
CCGTAGCTTACTAGGGGCCAATGATAATTTTTCACGACGCACCCGATCAAACAGCCTCCTCCACTTCACCATT
CCTTCTTCGAATGTGTCCGACGCCATCCCCCCGTCGTCCACGAACATCTCCATCATTCCCTCAGCCACCAAGT
CGTGCAACTTCTCTGCTGTGAGGTTTGCAAACTCGGACGGCCCCCCCGTTACCCCGAACGGCATACGGCAGTA
CGCTAGATAGCCCATTCCTTCGACATATATGCACAGGTATGGCTGTGACTCCTCCGCTACTGTGCACGCGTAG
AAGCCGGCTGCGAAATCGAAAATGTGAACGTATCTGTGCCCTGAGAGTCTGAGTTGCTTAGCTCTAATATCGC
CCTGTGGCATAGGCGCTATCTCCGTCACTTTATTGATCTCATTAAACGCGTGAACAATCCTCCAGCGACGCGG
TACCTCGCTATAGTTGACTTCCCTAGGTGCTGGACGCGGCGGCAAGTCAAACACGGGCTCCATCCCATGCGCT
ACACACTGGTCATTCACCCTGTGCTGTAGCTCCTCGATGGTGAGTCCACCCGCCTCAGGATCCTTCGGCGCCA
GGATAGTTTCTGCTATACACTTCACCTCCCTGGGATGGATCCTTCGCAACACTCCCGCCGCCAACATTTCCTC
CACCTTCTTGTGCAGAAATGCCCGCTTCTCGGGGTTCCACCGCTGTTGCCCTACCCTCATCGAGAACTGGGCG
TCCTCTGGCACTCTCAGCGTGTGGACTGCTCCTTCAACCGGGTTCACTTCGCTAAGGGAAAGAGCACAACAAT
CCGCGTATTCCGCAACTAGCGCTCTCACCTTGCCTCTCTCCTCCTCCGATAAATCTTCACCAATCTGGATCTC
GGCCAGGATACGACGTACCCTCTCCTCGTTGAATGGGTCTGAGAGCCTACAAAACACTGAATCTGCCACCACT
GCTGCGATGGGGACCTCACCTGGATTGCCAATCGGGGCCCGGTTATACAAGGTGCACGTCGACTCCCCCTCCT
CCGAGCCGATCTCGACCACATCCTCCGCAAAATTCTGCTTAGCTCCGAACAACCTGAGCAGAGGCTTGCCGAA
TAAGAACTCCCATCCCCCCGCGCTGTCAAACACCTCAAACGTCGCATCCCGCTCACTCCCCCCCAGCGAGATA
CGCCCCGCCCACGTCGCCTCCGATCTCACCACCACCCCGTTCGCCATTCTTAACAACTTATCTGATGGTCCCC
AACTCCCCAACCTGTCCTTGACCCTGGCGAATACTCTTGAGCACATGGCCGCCACCATCGCGCCACCATCGAA
CAAGGCCTCGATGGGTAGCTCTCCCGTGCTCCCTACGATTCCTATCCGGTGGACAAATGGCCTCTCGGGGCCA

GGAACCTTAGGTGAGACAGAGTACAGGTCAATCAATGTGTTATTGTTATCCACGGACTTCACCCCCTTCATAG
ATTCAGTGCTAATGTTTGATGCGAAAATAGCTGGGGCCGTCCGCGCATCCTCTAAACCGGCGGCCCCTCCCCA
TTTCCCTCCTCCGAATCACCCGGTCCAACATAATCCATCCACGCTGTCTCGTCCTCCTGCTCTAAGCCGACTG
CATTGATGCTTGCGGGTACCTCCACCCCACGCCGGCCCAGGATGCTCCCCGCCACCATCCTCCACACGCGCTC
CTTCCCCGGGATCCGGTCTGGTTGCGCCACCGTACACGCGTCCGCGCGGTGGCCACCCTTCCCGCAGGTGTAA
CACTCCCCCGATCCTACCGGCAGCGTGCCCGGCTTCAGTGGGAATCCCGTGTCCTCGCTGGGCCTCGCCTCCC
CATATTGCCCTCTCCATGTTTGCCACTGGACTCGGTACGCCGCCCTTCCTGCCTCCGTATCCGACTGCAAGGG
CCAGGCCGCAAGCTGCGCTCGAACTGCTGCTCGCTTCTCCTCAGGCGTCCGTGGTGGGGCTGGTGCCAGCGCT
GCTGCGGGGGGCATAGGTACAGGGAGTGTCGCCGCCTGTATTCCCCCGGTGAGCTACCCCATGGTCCCCTGC
TCGGCCCGTTCCACCCTGCTCCACCCCTCGCCCACGCCCCCATAGCTGGGGGGCTCGCTACTGCCGTGGCCTG
GCGCTGTAGGGTCAAGCTGCGCATCGAGCTCTGCATGCGCTGCATCTCATCCGCCAGCCTCGTGACCATCCTC
TCGCGCTCGAGCTCTGCTGTAATGTAGGACGCCGGGATGGCCTTAACTGCCTCGCAGAATTCATCCCAGTTGG
AATACTTGCCATCCAGCCGGGTGAGGATTATATTCGAGATCTTCCCCTCCAGCCTCCACACCGAGACTGGTGC
TTCGCTGATCCCCGCGCGCTCCGCTAGGTCCAATGCTCTCTCGGCGTATTCGGTATGTGGCCATCTCTCTCTA
CCCTCGTACTTCCTCTTCTTCCCCAACTCCTCCGACGGCAGCCTCATCTCCCACAATTCCCGCTCAAGCTCGT
CCCTATCCTCACCCTTCTCCTTACGCGGAAACCGCATCAGGAACGCCTCCTCCACCCGGGTCCAAGCCGTCTT
CGACCTATCCTCTTCCCTAAACCACCTCTCCGCCGGCGAGCGGCTCTTGAGGTTGTCCTCGAACGCCTCCACC
ATCGTCTGGTCAGTCGGGAGGGTTCCACCCGCGCCTTTCCTCATTGCTCTCCTGAACGCCTTCACGAATTCAT
CCGGCTGAACTGGATCGCTCCGATCGCCACTGAACAGTGGGACCTCGTCGGGTGACATTGATCTCCCGTAAGT
TACAGCTGGGGTGGTTGCGGTTGCTACTCTCGGCGATGTGGGTGTCGGTGGTGGTGCAGACGTCCCGATACGT
CCAAACTGGCGGTTGTCTACTGCTGGTGGAACACAGGCTGATGTGCTTGGTCCCGCCTCGGCTGAGCCTTCTG
ACGATGCTCTCGATGGTCTCCGGTTGGTCTGAGTCTGCGGCAAAGCGTCCGCTCTCACTCGCTGCGCTCGCCT
CGTGTCCTCGTTTCCCCACTCAACCTCAGCGCTAAGTGGAAATTGAACTTCTGGCCCCCTAGCTTGGGCCGTG
CTGACTGTGCCGGTGTTGTGGTGTTGGTCGACTCCTAAGGTGCCCTCTGGCACTGAACTCGCGTTTTTATTCA
TATGCATCGTTTTCGAACACGTTGTATCCTGCAGGCAACTGGTCAAACTCTAATCGATTATCCACGAATCCAA
TCCAATAAGCGCTGGTTCGATCGCACGCTCATCACCAAGATGGACAGGCGGGATCTCCAGAGGCGTCTCGCAA
GCACGCCGCGCAGAATGCACAAAGAAGCTGAGTTGTTGTCTGTCTACGTGTCGCATCCGCTACTCTACACCGG
CTAGGTCACGTGACTTGCCGGAGCGGGCGCTGCCGCGCCCGGCTCCA
>Cligib1_rnd-1_family-501#LTR/Copia
CGTCGGTGGANGAGGCTCCGAAGACGTCGTCGGTGTACGTGCTGGTGATGGTGAGCTCGTCGCCGANCTTCCG
NGAGCGGACGCACGAATCCGCGCGCGACTGGTAGTANCCCAGNCCGTCGTAGCTGCGGCTCAGCTCCTCGGCC
CANGCGTTCGCGCCGTTCATCATNCCGTAGACCGTTTTATCAGCAACGTANACCTTGTCCTCCTCCCCTTGCT
CGATAAAGCCGGGCGCCTGCTCCGTGTAGACGGTGTACTTGTTCTCGCTGTTGAGGTANGCGGCGATGAAGTC
CACCTGCCAGATCTTNAGCCCGAGCTCGGCNGCGACNGCCGCGACCATGAGACGCACCGACTCCNAGCGGACN
ACGGGNGCGNATGTTTCGTCGAAGTCCACGCCNNNGACTTGTGNGTANCCCTTNGCTACNAGGCGGGCCTTTC
TTTCGAGAGTTCCCGTCGGCGTCGTATTTGTACTGATAAACCCAGCGAAGNCCGACGACGTTGGCNCCCTCGG
GGCGCGGTACGAGCCTGAAGACCTCCTTCTCCTTCATCACGCGCATCTCNTCCTCCATGGGTCCCCTCCAAAG
GTCAGGGCGCCTCATGGCCTCCTTGAAATTCCGGGGCGCCACGGCATCAAAATCACCAGCGTCGCTGCCAGCG
ACGAGCGCGTGCGGGGCGTCGGTAGCCCAGTCCTCAGAATTCCTGGAGGCCGCNGCCACCCGGTCCTCGGACT
CCTGNGATTCGCGCAANGCGCGCGATGGGACGTGGGCGCGNTCGCTCCTGCGCGGCGGAGGCGCTGGTGGNGG
TGGCGTNGTTCTGCGCCCGGTGCGGCCTCAACCGGGCGNGCAGGGTGCAGNGGAGGGTCGGTAGGGCGCGGGC
GGGCNGCNACCGGTCGTGCATCCTTGACGACAGCGGTCGGTCCAGCGTCAGGGTCGGCCCCTAANTGACCGCC
AGCCTCGGCGTGGAGGTCGAAGTCCTCGTCAATAGGCGGACGCGCGGCGGTCCCCGTCCNGTGGCCCGCNCCC
TCNTCGAAGATNACGTCCCGACTCTTGACGATNCTCCCAGTGGCGCGGTCGTANAGTTTGTAAGCCCCACGNC
CGTAGTANCCGATGAGNTCGTACTTAACCGACCTNGGGTCCAATTTCGAGGCCCCGACTTCCGCCGGGATCTT
CGCGAAGGCNGTGCAGCCCCAGGGCCTAATATGGGAGACGTCTTGGCGCTGGCCGGTCCACACCTCCGCCGGT
ACTCGGTCCGGGTGTCGGGANGANGGGATGAAGTTNCGAAGGTAGACCGTCGTCGANGCNGCCTCNGCCCAGA
GAGACGGGGGNAGTCCGGAGTCCGCCAGCATGGACCTNACGCCCTCGAAGACCGTGCGCATGCCACGCTCAGG
GACGCCATTCTGNGNGTGTGCGTACGCGGTGGTGATTTCCGCCACGATNCCGTGCTCCTTCAGGTATTCGTCC
CACAGGTTGTTGTACCACTCGCCNCCNCCGTCGGACCGGACTATGCGCAGCTTCTTGCCCGTCTGNCGTTCNG
CCTCGGCGCGGAAGTCGCGGAAGGCCTCGAGCGTCGTCTCCGCGTTCTTATTCGCGAGGAAGTAAGCCTTNCG
GTACGANGATGCGCCGTCCGTNAGCAGGAGCATGTANAGNGCGCCNCCGNTCGACTTNACGCGGGCCGGNCCC
CAGAGGTCTATGTGGACGCGTTCCAGGACGGTGGTTTCGTGAGTGACNACCTCATCGTACGGCCGGGTAGTGT
GTTTACCGTAGATGCAGTCCTCGCAGAGTCCCTCCGGCGCCGAGTCGCCCGTGATGCGGAGGCCGTCGACTAA
GCCCTTCCNCTCCATGTTGCGNATNGCGCCGACGCCNGCATGNCCAAAACGCCNGTGCCAGGTNTCCATGGCG
GCGGGTTTCTCGTGCGAGCGCGCGGCGAGNGCCGCCGGCGNGTCCAGGCAATCGACNAGGTACATNCCGTTAC
TCCCCTTTCCGAGNAGCATCAAGTTATTCTGNGCGTCGTAGAACTTCGCCGCGCCCGACTCGAAAACCACGCG
GTAGCCGGCCGNGTCGAACTTTCCGATGGACAGGAGGTTNGAGATGAGGTCGGGGGTGTGGCACGCGTTGGTG

AAGGTAATCTTCGTCGTTTCGCCATCGACNGCGAACGTTTTGGTGACGGAGCCCCTCCCCATGATCCGGAAGG
TGCTCCCCTTACTCGCCGCCCGCCCGANAACCGGCGGGTCGATAGGGGTGTAGGTCTCGAAATCCTCGCGGTT
CACGAAGCAGCAATCGCTCGANCCNGAGTCCGCGAAGGTGAGGACATGGTTATAGGCCGGCGAGTTGTCGTCC
GCGGAGGGNGCGATCATCGCCGCCGGNACGGGGGCGGCATCGTCAACGGTGGANGTGTANAGGGCGTACACCC
TGTCCGCGGCTGGCTGCGCGGTCGAGTCGGCNACAGCNGCGGTNGGCGTCGTCGTCTGGGAGGCGTTCGAGCG
TTTGTGGGCGCTGCCGNCGCTCCTCCCCTCTGNCCGAAACCGGGGGGGAACTGGCCCTCCTTTCCTCCGCCTT
TCCAGTAGCACTTCTCGATGGTATGCCCCTTNCGCTTGCAGTTNGGGTTCGTGCAGACGAGCTGCTCGCGTTT
CTTAGAGGACGNCGTCTGGAGNGCCGTGACGGAACTCCCGGCCGTGTGGACCGTCTTNCCGNGNGTCATCCTN
TCCCAGTGCGTGCCGANCCTGTCGATAACGGCGTCCGACGNCTTGGTATCGTATAGNGTGCGACGATCGNGTC
CCAGGACGGGGGTAGGGATGCGAGCACGATGGAGCGGAAGTCGAGA
>Cligib1_Cluster84#LTR/Copia
GATGGAAATACAGAAGCTCAGACGCCGTCACAAAGTGAGAAGCAACTGATCATTAGCTAACCTGATGGAAATA
CAGAAGCTAATGATACGAGTAAGTAAATGTCTGTAGGGTAGGAATAGACGAGCATACCTCAGACGCCGTCACA
AAGTGAGAAGCAACTGAGGTATGCGAGTGGGAGCGTAAGATATTTATCATACGAGGTAAGATAGGAGTCGGCG
GTTGAACGCCGTGTGATGGATATGAAACGTAGGTAGAAAAATGAAATAGAAAAGAAACATGCATGCGTTTACG
ATCACCGACGCGCAAGTGTTTGACTGCCCGTTTTAGTAATAGGCGCGTCACGGGCGTTATCCTCAACACTCCC
CTAACTCCGTGAGGCGCAATAAGTGGCAAAGATGCTGGTGTGGGACACGCGACAAGGGCTTGGTCAGGATGTC
CGCGAGATTGTCGGCAGAAGGCACGTATTCCAACGAGATATGGCCGTCCTTAACACGCTCCCGGATGTAATGG
AAACGTATATCGATGTGCTTTGCGCGGGCGTGCCCCTTTGTATTGAGTGAGAGGGCGATAGCGGCGGTGTTGT
CTGCACGAAGTCGAGCCGGAGAGACCTGGGGCAACCCAATCTCGCTCATAAACGAGTGCATCCAAAGCGCTTG
CTGTGCCGCTCGTGTAAGTCCCATGTACTCAGCCTCGGTTGTGGACAGTGCAACCGTCTTTTGCCTCTTCGAC
GCCCAAGAGACCGGACCACCGCACATCGTGAAGATGTATCCAGATGTCGACCACCTAGTATCAGTATCGCCTG
CGTAATCCGCGTCCACATAGCCAGTGGGTTCGAGACCGTTCCCAGCACCGGGGGTGTAGGTGATTTTGTAGTC
GAGCGTGCCCTTAATGTAGGCAAGGACATGTAGGATGGCCTTCCAATGAGCAGGTCCTGGGTTCGTCTGGTGG
CGCGAGAGCAAGTTCAATGCATACGACAGGTCTGGGCGCGTCGCTCCCTGAGCCCACATTATACATCCAAGTG
CCTCCCGGTACGGCTTGTCTGCCATGAAGCGCACGTCTTCCTCTGAGCTTGCCCGGTCCGCTTCTGTAAGCGT
CAGGCCGGTTGGGAGTGGTGTGGATCTGGGGTTACAGTTGGACATCCCAAAGCGTTCGAGTGTACGCTCGAGG
TACGCGCGTTGTGAGAGAGATATAGAACTGGTGAGTTCGTCGCGGTCAACCCGAATGCCCAGTATAAAATTCA
GCTCGCCCAGATCCTTGACTTCATAGCAGTTGGATAGCTCCTGCTTTGCTAGTGCCGCGCCCGCGTCAGTGGT
AGATGCGCCGAAGACGTCGTCCGTGTAAGTGCTTGTGATGGTAAGTTCGTCATTGACAACTCGAGAGCGGACA
CACGGATCGGCGTGCGACTGGTAGTACCCAAGGGCATCGTAGCTTTTGCTCAGCTCCTCGGACCAATCGTTCG
CGCCATTCATCATGCCATATACCGTCTTGTCAGCAAGATATACCTTGCCCTCCTCCCCTGGCTTGATGAATCC
GGGCGCTTGCTCCGTATACACGGTGAACTTGTTCTTGCTATTCAAATACGCCGCAATGAAGTCCACTTGCCAT
ATTTTGAGCTTCAGCTCCGCGGCGACAGCAGCAACCATCCGCATGGATTCCAACCGTACCACGGACGCATACG
TCTCGTCATAGTCCACGCCCGGGATCTGCGTAAAGCCCTTCGCGACCAGGCGCGCCTTGCGCCTGATGATATT
CCCATCGGCATCATGCTTAAAGTTGTAGACCCAGCGCAGGCCTACGACGTTCGCCCCCTCAGGACGCGGCACA
AGCTTGAAGACTTCCCTTTCCTTCATCATACGCATCTCCTCTTCCATAGGGGCCCACCAAAGGTCCGGGCGCT
TCATGGCCTCCTTGAAACTCTGGGGTGCCACGGCATCAGAGCCACCAGTATTACCAGTGACGAGCGCGTGCGG
GGCACCGGTAGCCCAGTCCTCAGAGTTCTCCAGAGCAGCAGCCTCCCGGGCCTCAAATTCCCTCGACTCCTGT
AAGGCGCGGGATGGTACGTGAGGGCGGGCAGTGCGACGAACTGTAGGTGGTGTCGCCGCTGGTGCGGGCGCCG
TCGGTGGTGCAGCAGTGCCGGCATCGGGGTGCAGGGGCGGGTCAGTGGGGCGTGGCCGAGGGGCGAAGGGTCT
GGCATCCCTGATGACATTTGGGGCGCCCTGGTCGGCCCCTGAGTCCGCCCCCGGAGCGGTTGTATTCGGCTCG
AAGTCGAGGTCAACGTCCTCGTCAGGTGCCACCATCGGACGAGTGCGATGCCCCATCCCTTCTTCAAAAATCA
CATCCCGGCTCTTGATGATCCTCCCCGAAGCTGGGTCATAGAGCTTGTAGCCGCCGCGCCCATAATGGCCGAT
TAACTTGTACTTGACCGACCTAGGGTCCAATTTCGAGGCCCCGACCTCCGCCGGAATCTTCGCGAAGGCGTCG
ATGCCAAAGGGGCGAATATGGGAGACATCCTGTCGTTTCCTATTCCAGATTTCCGCGGGCACTACGTCTGGGT
GTCGAGAGGAGGGTATGAAGTTGCGAACGTAGACCGCCGTCGCCGCTGCCTCGGCCCATAGAGACGGTGGTAA
GCCAGAATCGGCGAGCATGCACCTAACGCCCTCAAAGATAGTGCGCATCCCGCGCTCGGACACACCGTTCTGG
CTATGTGCATAGGCCGTGGTAATCTCTGCCACGATACCATGGCTCCGCAGGTACTCCGTCCAGGCGTTGTTGT
ACCATTCTCCGCCGCCGTCTGACCTTACTATGCGTAATTTCTTTTCAGTCTGGCGCTCGGCTTCGGCATGATA
GTCTTTGAACGCTTTGAGTGTAGTCTCGGCGCTCTTGTCCGCCAGGAAGTATACCTTACGGAACGAAGACGCC
CCGTCCGTAATTGACATCATGTAGCGAGCGCCGCCGTTGGACTGTACACGAGCCGGACCCCACAGGTCGATGT
GGACCCGTTCCAAGACGGACGTCTCGTGAGTGACCTCCTCGTCGTACGGCCTGGTAGTATGTTTACCATAAAT
GCAGTCCTCGCATAACCCTCAGGAGTAGCATCGTCCGTAATTTGGAGCCCATCGACTAGGCCCTTTTCTTCAT
GTTGCGGATGAGGTGACACCCGCGTGGCCAAAACGTCGGTGCCAGGTCTCCAGTGGGACTGGCTTTTCGTGGG
ATCGGGCAGAGAGGACTGTCGGCGGGTCGACGCAGTCAACGAGGTACATGCCATTGCTCCCTTTCCGGTGAGC
ACCAAGTTGTTGTCCTGGTCGTAAAATTTGGCTAACCCCCCACCGAAGACGACCTGGTAACCGGCAGCGTCGA
ATTTTGCGATAGACAGGAGGTTGGAGATAAGGTCAGGGGTATGGCAAGCGTTGGTGAAGGTAACTTGCGTCGT

CTGCCCGTCCACCTTGAATAACTTGGTGACTGAGCCCCTCCCAATGATTTTGAAGGTGCTCCCCTTACTCGCC
GCTCGGCCAATGACTGGGGGTTCGACTGTGGTGTAAGTCTCGAAGTCCGCACGGTTCACGAAGCAATAATCAC
TCGCGCCAGAGTCTGCGAAGGTGAGGACGGGAGTATACGTAGTGGTGTTCGAGTCCTCAGTGGTCGGCGCGGC
AAGCGCCCCGGGGATAACTGACTCCTCGTCAAGAGAAGTGTACAGAGCGTATACCCGGTCTGCAGCAGGAGAG
TTCGTGGCGTCAGCAGTTGCGGCCATGGGTGTCGTCGTGCCATTGGGAGATACTGGTACGTTCCTCCCGCCTC
CCCGCTGGCCAAAACCAGGGGGAAACTGCCCCTCCTTTCCTCCGCCCGGCCAATAACACTTCTCGATAGTATG
CCCCCTCCGCTTGCAGTTCTGATTCGTGCAAACTAACTGCTCGCGCCTCTTAGGGGCTGCTGTTTGGAGCGCG
GTAACCGACACTCCCGCTGAGCTCACTGTCTGGCCGCGTTTCACTCGAGTCCAGTGCGACATGAGTTGGGCGA
TAACGTCGTCTGATGACTTGAAAACGTGCATGGTGCCAACGATCGAGTCCCAGGAAGCAGGGAGGGAGTATAG
GAGAGTTGAACGGAAATCAGCGTCGTCGATGGCCGCTCCGGCCTCATTTGCCTCAGCCCACTTCGTCTGCAGG
CTCGCGATATGCGTGGGGAGGTCCTCGCCGTCGTGGAACATCAGCGTGCGGAAGTTACACTGAGCTACGCTCT
TGGCAAGGTCGCTGGTGACGAGGAACCTGTCGTTGAGTTTCTTCCAAGCCTCCGCAGCCGTCCCTTTGATGTT
AATCCCCATTCCAATGGGATTTTTTATGTTGTAGATAAGTAGGGCCATCACCCATGCGTTACGCGCCGACCAT
TCCTCCGAGGAGGGAGTGGTGGACCACCAGCGTGAATTGGGGGTGGCATCGGTGTCGGTGTTGGGATCAGGCG
GGGGAAGTGGGGAAGTGGTAGTGTCGGGCTTCTTTATTGTGCCGTCTAAATAACCTTGGGCGTTGCGGAGTTG
GGCGGTGAGTAGTACCGTTCCGCTCCACGCGGCCCAGTTCGAACCGTCGAATTTATCGCCATCGGCGAAGACG
GGTGGGATAGAGTTGGGCTGGTTCGACATTTTAGAGGTGTTAATGTTAGCACTTGAGCGTATGTTATGTTGCA
ACGGTTTCTGATTTCTTTAGGCTACAGGGCCCATAACCTGATGGAAATACAGAAGCTCAGACGCCGTCACAAA
GTGAGAAGCAACTGATCATTAGCTAACCTGATGGAAATACAGAAGCTAATGATACGAGTAAGTAAATGTCTGT
AGGGTAGGAATAGACGAGCATACCTCAGACGCCGTCACAAAGTGAGAAGCAACTGAGGTATGCGAGTGGGAGC
GTAAGATATTTATCATACGAGGTAAGATAGGAGTCGGCGGTTGAACGCCGTGTGATGGATATGAAACGTAGGT
AGAAAAATGAAATAGAAAAGAAACATGCGTGCGTTTACGATCACCGACGCGCAAGTGTTTGACTGCCCGTTTT
AGTAATAGGCGCGTCACGGGCGTTATCCTCAACAC
>Cligib1_rnd-4_family-1068#LINE/I
AAAAAAAAAAAAAAAAAAAAAAAAAAAAAAAAAAAAAAAAAAAAAAAAAAAAAAAAAAAAAAAAAAAAAAAAA
AAAAAAAAAAAAAAAAAAAAAAAAAAAAAAAAAAAAAAAAAAAAAAAAAAAAAAAAAAAAAAAAAAAAAAAAA
AAAAAAAAAAAAAAAAAAAAAAAAAAAAAAAAAAAAAAAAAAAAAAAAAAAAAAAAAAAAAAAAAAAAAAAAA
AAAAAAAAAAAAAAAAAAAAAAAAAAAAAAAAAAAAAAAAAAAAAAAAAAAAAAAAAAAAAAAAAAAAAAAAA
AAAAAAAAAAAAAAAAAAAAAAAAAAAAAAAAAAAAAAAAAAAAAAAAAAAAAAAAAAAAAAAAAAAAAAAAA
AAAAAAAAAAAAAAAAAAAAAAAAAAAAAAAAAAAAAAAAAAAAAAAAAAAAAAAAAAAAAAAAAAAAAAAAA
AAAAAAAAAAAAAAAAAAAAAAAAAAAAAAAAAAAAAAAAAAAAAAAAAAAAAAAAAAAAAAAAAAAACAAAGC
AATCAGGATGGGACTNTTCATATGCGGNAAGAGAACACCGGTACGCAAATCACTCAAGGAACCCCTCCGCTGC
CTGAAGTGCCAGAGGGTGGGCACGGGCCACCTCGCAGCGACNTGCAAGGACACGAAGGACACGTGTCCGNGGT
GCGGGGGGGAACACNGAGCGGTTGGTTGCACCGTCGCGGCGGACAGCCTGAGATGCAGCAATTGCAAGGAGGG
CGGGCACGGGGCAGCTGACAGGAATTGCCCNTACTTCAGGAAACGAGCNACGCAACACCTCGAAAAAGACCCN
CNGAACAAGTTCCTGAGATNCTTCCCNACGTCCAGCGNNTTCACNTGGGAGACGTGACGCGATGNCNANAAGC
ATGCAAGATAAAGGAAGGCGGNCCAGGGAGCGAACAGGCGGGGCCAGAGGTAAGANACCACGCGCCAAAGGTA
AAGGAAGGCTGGCAGGGGGCGATCAGGTGATGGAGGCGGGATGCCCGTACCGAAGGAGCAGAATCCAGAAGAG
AGAGAGCAAGGAACGGTGGATAAGTGGCTGGAGGGGCGGTCCCCCCCAGGGGCAGGCTAGACCAGGAAGAGAG
GCGGCGAACACTAGGANCAACACCAACACAACGGGCNCGCGGGACATCCCCCCGCGCGAACGGANTTAGATGC
CTCCNAACCNNAACCCANCGGCGGTGAGGCAAGGTAACAAGCTACGCATCTGGCAACAAAACATCAACAAGTC
GAACGACGGNACGCAGGACCTGCTNCAAAAGCTGGGGGAAGAGGACGACGCGTTTGACATCGTAGCAATCCAG
GAACCCTACATAGACCACTGGGGCAACACGAGAGCCAATCAGTCATGGCGGACCTTCCGCCCAACGGGATACC
GGGACAAACCCAACGANNCAAGAGCAGTGATACTTAGTAAACTCNAAGATTGACACAAACACTTACGCGACGA
TACCGATTGACTCACAGGACATNGTTGGGATCCAACTCCAAGGGGAGCNCGGGACGATGCGGATCATCAACAT
CTACAACCCATGCGANCNGAACTCAAGCCTGGACGCACTAAGGGAGTACATGCAGAGTCCACAGAACAGGANC
GTCGCCAGGGCCCCACTNCAGTACATATGGCTGGGGGACTTCAACNGGCACCACCCGCTGTGGGATGAGGACC
GGAACGCGCACCTGTTCACGCCAACGGCCCTCAACCTCACACAGCCGCTGCTNGACCTCATAGCCAGATACAA
CATGAAGATGGCGCTCCCCAAGGACCTNCCAACGCTGGCGTCAATGGGAGCGTCGAAAAACTGGACNAGGCCN
GACAACGTGTTCTGCTCAGACACGCTATTGGACGCCTTCATCGCCTGCNCCACNCAACCCGATAAGAGACCTG
TNAAAGCCGATCACCTACCGATCACCTGCCTAATCGACGTATCGACAGATATCAAACAGTTCGAGCCNAGGCT
CAACTACCGGGGAACGAACTGGGCGGAGTTCAAGAAGGCAGCCAAGAACGAGCTGAGTAAAATCCCGCGCCCA
AAAGAGATNGATGACANAGGGGAGTTCGATACAGCCCTCGNCGACCTGGAACGNGCCGTCCAACGGGCGACAG
NNCTGCACGTCCCAATGACNAGACCGTGCCCGTTCTCAAAACGGTGGTGGTCGGCGGAACTATCAGCCAAGAA
GAAAAGCGTCCAGAGAATCACGCACGAATCCTATAATGCAAGAGCCATCAAAACCGACCCAGTTCACGAACGG
CTACGCGTAGCACGGAACGAGTATTCCATGCAAATACGCAAAGCTAAAGCGGATCATTGGATGGACTGGCTCG
AGCACATAGATTCGTCTAGCATTTGGACGGCGAGCAGACTAGTAACCGGATCCGCCTCTGACGGAGGCAGGGC
CAGAGTACCAGACCTCAAACTGAAGGACCCAGTGACGGGCGGGACCACCAAAGAAGCGAGGGACAACGCTGAA

AAAAGCGAAATGTTCTTCAAGGCGTTCTTCCCGCCGAAACCCGCGACTGAAGAGGACTACGGTGACGTCGTAT
ACCCCGAACCCAGATGGAAGTTCCAACCCATCACTGACGAACAAGTAAGCAGGGTTATAAGCAAGCTCAAACC
CTACAAAGCGTCGAGACCGGGCACGATACCGAACTCGGTCTTCGTCAACTGTAGTGACATGATGGTGAAGTAC
CTGGCACCACTGTATAGGGCGACTGACGTACTCGGTTACTACCCAGACAAGTGGAAAATCACGGAACCCGTCC
TAAGAAACCGGGCAAANGCGACTACGCGGCTCCAGGCGCNTGGCGCCTGACAATCGTNTTGTCAGACGGATGC
GCAATCCTAAACGCNGTCAAAACGGAAGACATTAATATTGTCAACAACCTTGAACGGCACGGTATCCTCCCAG
ACAACCATTTCGGNGGNTTACCAGGCCGNGCAACAACNGACTCCATCCGAGTACACCTCCTAGTCAAGACGGT
CAAAGACGCGTGGAGAAGGAAGGAAGTNGCATCGGCACTGTTCCTGGATGTCAAAGGAGCATTCCCTAGCGTC
GCCGTCGATGTCCTCCTNCACGAGATGCGCATGAGAGGGATACCNATAGAATACACAGAATGGCTGAANAGAC
GCCTGGACGGCCGGAAAACNATGCTAAGTTTTGACGANTACAAATCCGCNCAATTCTCTATTGACAATGGACT
AGACCAGGGCGATCCNTTCTCNTTGATATGCTACCTNATCTACAACGCAGGNCTGCTCAGCATACCCCGGAAA
CGCTAAGCGGAGAGATGGGGCTACTATACGTGGATGACGCGGCTCTCGTGGTCGCAGGTCACAGCTTTGCGGA
CACGCGCAGGAAACTCATCGATNTCATGACAAGAGAAGGCGGGGTGTTGGAATGGGCGCGGACGCACAACTGC
GAATTCGGCGTCGACAAATTCCAGCTGCTCGACTTCACCAGAAAGACGGTGCCCAATCCAACAGCGCAGAACA
AGCGCATCCCGCNGCCCACCGGTCATATCCGCGTCAACGGTCACAAAATAGAAGCTAAACCAAGTGTGAAGTT
CCTAGGNGTCCACATCGATCAAGGTTTACGATGGAAAATTCACTGCGCANCGGCNCTGGCCNGGGGNCAAGAT
TGGGTCATCCAATTCGGCCGGTTGGCACGNNCCTCCGGTGGCGTTGCATCAAGGCACCTGCGGAAAGCGTACA
CGGCTATCTGCGTTCCCAGAATGCTATACGCCGCAGACATCTTCCTGACGCCNGCNNGGAGGACAACGCAAGA
CGCCACGACCANAAGGGACAACCGAGCCATNGTACGACAGCTGACGACGGTACAAAGGAAGGTAACANTAGCA
ATAACAGGGGCAATGAGANCAACAGCAGGTGATGTACTGGACGCNCACNCAAACCTCCTACCGATGCCGCTTC
TCATAGACAAACACCGNCAACGAGCCGCGCTGAGGCTGGCGACACTCCCAACCTCCCATCCGCTCAACAAAGC
AGTCGCGAAAGCGGCCAAGAGTTATGTCAAGAGACACGCCACGCCCCTCCACAACCTGATGCACACTTATGAN
CTTAAACCAGACAGAATCGAGAAGATAGAGTCGGTCAGGCAGAGCAACAAGTGGAAGCCTAGGATAAGNATCA
GGATCGCTGACTCAAAAGACGTGGCAATAAGGGAGGACCGGAGAAATCGCCCGCGTATCAGAATTTACTCAGA
CGGCTCAGGTATTGATGGGAAAATCGGCGCCGCGGCAGTTTTATACCGGGATGGAATCCCCAAACGCACTCTC
AGGTACCGACTGGGCTCAGACCACCATCACACCGTGTACGAGGGAGAAGGCGTAGGTATCATCCTCGGNCTGG
AACTGTTAAGAAAGGAAGCAAAGGGNTCAGTCGCGAGAGTTCGGATGGGGGTAGACAACCAAGCGGCAATCAC
GNCCACTGCCAGCATCAAACCTAAGCCCGCTCATTATTTATGGGACATGTTAGACAAACGGCTAAACTTAGTT
ATGAACAGACACCCCAACGCAAAGCTAGCCATCCGCTGGACGCCGGGTCATGAGGGGATCGACGGCAACGAAT
GGGCGGACGAGGTNGCGAAAGAAGCGTCCCAGCTGGGCTCAAGCCCCAGCGAGGACCTNCCAGCGACGCTCCG
AAAGAGGCTACCGTGGAGCAAGTCGGCCATCAAGCAAAGATACCAGCCGAAGCTCAAAGCCGCGGCNAAGCGT
ATGTGGGAGAGGTCGCCACGCCACGGAAGACTGAAACNGTTCGACCCCACNATGCCATCCANGCGATTCGCAG
AACTGGTGGANCAGATGTCAAGAAAGAATGCGAGCATCATGATCCAGCTAAGGACCGGNCATGTCCCGCTGAA
CCAGCACTTACACCGNATCAAAAAAACAGATAGCCCGCTATGCCCGAACTGCGGGGTATACGAAGAAACCGTC
AGACACTTGATGTTGGACTGCTCGGCAGCTACGACGCGGCAAGACGACGACTGAGACAGAAAGGAGGCGCCAA
AANCCGCAACCTGCACGCGCTGTTAACCGACAAAACGCTCATCCCNCTGGCGTACCAGTTCATCGACCAACCA
AACGATTACGCAGNGTCTTCGGAGCAATCACACCACCCGAGTTCGAGGAGGAAGAATAGAAGGACGGACCAGG
ANCCATACAAAAACCCACCTTATAATTATATACCTACGCACNCCGACTCGCCGTCGCAGCACGACGGGTCCAT
AATTCCGGCGGGTTTACCGCTCAGAGGANCACTTAGTGATCCTCCCGGCACCAGAAGAAGAAGAAAAGGGATT
ACGTTTCACNTNTATTTTGACCCAAGGTAGNCACACGACGTT
>Cligib1_Cluster47#LTR/Gypsy
caagtgtcatgaacctccggaagaACacaacaaacaaaaGaaaaaaagaCaAaacaacaaaCGcaTCcacaaa
cccaCaacagTcaaaggcCcaagacATgacccaacacaaaAaTcaccAaGagaacgagATaaAGaacaacaca
agTaAacacAcTgcgTTGaAgaTaCAAaaaAatgcAggTAaacgaCgacgGcccaaacacAGTCaacagAgca
accTgGaacacGagaAacgaaacGccaaCaaaaaagCgaTGaaTCgccGaaacccTcaggaGTcTcgcagaaT
ggccggccGTccacgAggTCagACAaGagggaaacgACaaCaaaaCcTCccaaagAgcgAAaccGcgcagcCc
gacGAacgCagagCaagCcCcaagcccCaaGAcaAcgTcctcgaTgGacaacgCcGcTccgaacCCCgacaaC
aaGGcccagcAGTgcGtcacaAccaaCgcaGcgcacCccaacgacgaCcagagggttcaaaggcacccacAAc
caAcTcacacaaGAccgaGccgggagagacaCgAaGacGaAaCaaaaacaaagaaaacggcAcaaAaaaccac
cgccaAacccagCaAcAacTTCAgaaAaacCaaTacgaaTTcCcgaaaAaggcaaccaacActcaTCcgacga
aGcTcaAacagcaCaacgaacaaAAaaacCaTaaaacccccCaACccTCcTacaaTCAAcGaTAaggaaaacg
aCacGaccccgaTCaggaaCaAaATcaAccgcagcTgCGAcTgaaaTaaGgacgCcgTCGGcAaTgaaccAcG
accaaGTaaacggaTcgaGGaaacaAaCgcaaaaaacacagcccagaCGaGagaaagaagTaagcaagagcAg
aaaAaaccCcCgcgaccCaacgaTagcAAcGaTTTccaaacGGCCgacacgaaGcgaaTCaacgacacccGag
gaTCcaccaccccaGATcTcaaGcccGAcaAaCcaAAccAaAcCgccaaTaccaAAgacgaccgCacaGaaTg
acgCgaaccagaggaccaggcaaaaaaaaCggaGcAaaccaAGAgcaCgcccTTcAgcaaggcacgaAaGacc
gaaAggaaggacGCaagTgccagcGcTcccGaacgccccacaacTcccGaaaGcTcGGAaccCAaaGTCAgca
ccaTagaCaacaaTagccAacAAcGcaaAgTcaaacaacGAaccGaaaccaaaacTaagaAaaaacaaTaCac

cGacccCcacagaAcCgacaCaacCAcaTccacGgcaacaaCAGTaGcCgCgaacgcaGacgTcGaccctTag
agaaGTacaTgCaGTgaGcaCggcagcCcgcgcacGGTAAGCacaccaagGccaaAcccgaacaCCaCaTaAa
gaaaaaaccTgGgcaagaaaggaGcAaAcaAaaaCacAaacaCGaccacaGacaaacgagcaacGcaAAcCAc
GGcGACgcaaGAaaaGacaaacagAAaaacGcccAAgccaAcaagCgTCcacGaaggaAcGAaTaGaaaTcaa
ccggcCcgCccaccgcagcCgccaGaacacGacCcaaAaaGacaCaaaTcccgaagacAacgcgaGTacTacg
AgGaaacCCagggaaAaggaacaccaacaacggccagccaacccCgaaaaacaGaacgaagccaTacagTTcGa
gagcccTaTcaTTgaaccgcaaAaAAcccaccaAaaaCgagaCATaaccccaaagcgaacccacCAaccCcca
CaacgacgAaaCacgaggCaaaaGacaaTaacTcCcGgaccGgccggccaagaCagacaCAacccaaacgTag
acAaaagacaagacTgaccAgcaaaagcacGacgcaaaacacAcGaggcCATTGccaAATaagAaagTacctc
agaATgcaaAacATaCacaaccagggagaacagCcacGCcacATcCaGTaTcacTTcGGaaCGaaggaaTcAA
gcaacaaAacacaGagaaagaaTcGacaccaaagcaGgagacgccagaaagcgacGgaGcaaaccacaaagcc
ccaaacTcaGcgacTcGcaGaaaaaagaggccCaGaaGgcaaTTaGcaacgaaacCCTccacTTcgaGcAaga
ccaCagcagcaccagcccTacGaacaaacgccagGCccccagaTcaGcCgccgcCaGGaTcTcTcTcacagag
caCaaaagaCagccacaaacTgaaccaGGAgAcaaacaTagaaaaGCcagaTccaccgCagcccacagcccgc
cTccaaaTgaacgaggCcagAgTcgcaacGaacgacaTTacagaaaggcaccTccAccacCccagcagCCacc
CGcccAcGgccCgaaacccaaacacAaTcacaaggaATTcaTcaacgcaGaacgCaTaAggAAgcacaccAaa
caaaAGgaaacgacaagaaTagGcaAcacacccAAccagaaacTTgaCGccaGcaTgAAgacgAaaccaAcaa
acGaaaaaccaccaaGaaccgGTGAcAacGgcGgaacGcaaCccGaaacTaaaccaTCgGAgaaCccgccagg
caaaGaagaCAAcaaaGaccaTgcCaaaGgaaacgacacCAcaaaaGaaGagcaCAacaaagTacccccTAag
gaccaTTccaaccaagacaagcaaaacaacagcGAaGTCaaAAaaaTacccaaacAaacGcCggTAcaAgaTc
agcAaccacAggaAagaagGaAaGaCTcccaCggaaggaccgAgggaccaccTggacaTGGaagaaaGaTccg
cccgaTTCaaaTagAcacaAgaccaacaaacaAaAgaacaaaaaTacTCaAccaaagacccagCcaaaTCcCa
gaTcTTagcaaTcaaaAagAAaacaGcccaaCgcaGaCgaTccagaaaTgacaGTacaagcaaagCaagaaCc
aaGaaAGaccgAgAacaaaTgacaaTcaCcTCaaaccaaaccaaaCagGagaaagccaGaaacccccgaAccG
TaaccgaaaTaAAGaacgcgaaCaacccaagccccgTgaagaAgcccaAcGaaagGAaaacGgacTcaaaaAa
aCaCagGgGagTacgaaAagaCaccgTCaagcCcTgcccaTcaacaAaccccacaacaacGaaacaagcCTTg
AggcaaaagACccTgaacaaccggCaccacagaaCacGggccaggaaccccGAcGacAggcgaAgagagTaTa
aacCTggCcaaGagaacGaggccagcccACgcgaAgcaacacAaaGaCgaaGagcccCcaacTTacTcgcaAa
aaacAAaAaagGcGAagcgGacaaagaccataaTcGAcgAacagagcaTaTgacgacggccaaTgcaaTacAg
acGAAcaccaaaaAgCgcgacaCCAgcgaaAaagaacaaaTaccgaagaaaGgcaaAGaCaTgCagcaaTcaa
TcgccaTcaccAaaTccTAaGcCcggTcgggcaccAgcaTTccGACaTCaaccggcccgaaaacaaacaaacg
acAAaaacaaaTAaaacaaAatagccTGgaaaCgaCgacccTGcccgaaacgaCgacGaaAaacgCaagcTTG
caggCaccaaagccaccGgaaTCaGAcacgggAagccCcaGagaCcggaCaaCaaTaTCACaaacgCcaccga
caacgcCcTcgcTacaaaTaaaaTTaAacCgaaCGaaAAccccacccTTcaccccaTacCagAcaaaacaaaa
aaaCTggccccTcccaaaaTTaaTgAaGaaagCcaaaacaAaaAATgaaacaccAaTagcgaaaccgccaGca
aCaTacaCGccaGGagcagaaGcaccTcaAcaccaacagagagAAccgaagaCcacaaaAaTagcGaagacgc
cAaggccAccaccaaccacaaacGacagcaAgaacGCagggacAgccgccTaTCCgaaaaaAacggcaaaagG
gacaacGccacgaccAcAaAaAcCCgGcaaaGcCccAgacaaacGCacccacaccagaCcaAaacaaaagcag
aaaGaaccaaTaCaacAAcaaaAGGcgccacacaCgAAcgaaCacCcGaaacgacagcccCccCcAaaaaCgc
AaaccaaaaaacccgaaacgcCaaCacaaaaggacacacaaaaCacaccgccgccCcAaaaaacaaCgcgccT
ggaagaaTTcAgcaaggCGagCCcAAccaAcACcGaATagcGCaaaGaaacaaaaaaaaaacagGaATcaTca
aaATgaccacagAacTTgaATTccaCAATGaaagCcTccacgaacacgCcgcaggacaaTccaaagccccaga
cTcgacAaaactccacTGgcgaaGgacacccaaCacgCaGcAcagAggacCgcggGaaaaacGagTacgaaag
GcccGacaTGcgcacgcacaaacccacgaATGcaaaGgccTgcaacacTTgTccaacaagaAAgaAcAccaaa
aaCaAAaccaAaaaacgaccaCaaccacgaGacacccacaaaacaccagaaaaaaaaaTaaaaagGGacgcCa
TacgaaaACcacacaTccaccagccAgacGacgCcaTgacGaaCaggagCgacaGcagaacccccGacGccaa
cgccAgacCaAaTCcTgaagcCaccGAaGaccccgaaaTTaccCagacTTagcccgacCgcgTCcccaGTCaC
CacGcTTgcCcacaagaaaccGaCacgcacCGacgcTcaGgccTccTcTcCcaaagcagaaacaAacacagga
agTgAaGCGGaaccaacaTgaaaCacgccacaGCTTACCaccacaGaaccaaGgaCgaACgGaAccaACcaac
aaAcccaCaAaacCgaaagcaGaGcCgACTGgcagaaCaacaAgaTGaggGgcgaGaTaaccgcGcaccCac
cGaAggcgcccTcaacAaagccaccagCccaTCGaccaaccAaTcccCcTTcaTcaacAaccgcGgagAccac
caTGACgaTGaaaTCCcAcCccaCaaaAcggaccaccaacccCcacAaCcaccaaAaggGCagaacgaaTcgac
aaCaTgaagcaagaaaTcAAaccaGaaaTCTcccacTcCaaaGacaagacgaaaaaacaagagAaccaGaaga
AacCccaGacccacaAgTgacCAgaacaGaTcTccTTgaGcTccTCcacCgaaAgATCaacacaacacGAcaa
aCcaagaaacgcGAaaacCgaacgcaaGGCCcCgacGAAAgCcTTcaaAaacgcGacgacaGcGcagaTaaaa
TcaaagaGaccAaacaacaaCcCGaaaccaaccCcaccccaaaGgTgcacaCCcacAaacaagACgCccccCcT
caTcAgTcCCGAacGaagaCaaAcggaagcCGaAcaccaacTcaATaTcGccaaTGAaaaaccgCacGaaaca
cccacTAcCaTTGAaaacAcGAaaGTaGcacaccgggAcGagaaTTccacccaaaGgaaacaTgcAcCcgacg

acGAaacTcgaaaTacaATTccGGAAgcaGgAcaGaacTcgacGAAaTccTcGaccaagTccaaacaaaaaaT
ccTcaaagcccaacccaccccGaTgCaaCgTaGacaacacaaaaaccaaAcCaccgaGAgTccGcTgcccAgc
aaccgcaggaccagcaagccccaaCcgaGgccgcccccCcaACTcagaacCCaaGgcaaaGGGaggaccAaCc
GacgcacCaAaaaccAaacgcTCagGccaaaccGcGagcGaCacGacaTCaaaaGaaaaacCaaacTacaTaa
aaaaGAaGggggGagGTaaagaaaaaCcTGTaaGAaaTaaccaACacaaacaaACaaacacAaACgaacaAcc
cacccCaaGAcaCacaGacagcgaGacgcCgaacagaTgagCcaacaCaAGaaccaTaaagaGAcTcaaaaaa
agCacaAGaagagTAaaTacacTgcgTcGaAgaTaCAAaaaAaTcaagTaAaccaccagcggCTcaATagaag
caaCaTaagcAcTcggaacagcacaaATcaaagcGcaaccaaAaaccccGacacacccgcGAgaCGccgagacg
cTcGgagaaggggCgggccgcgcacaaGTcagaaaaGAGcTgaacgaaacatt
>Cligib1_Cluster82#LTR/Gypsy
GTGGTCGTGTGACTTGGGTCACACATGAACTCAACGCTTTTCATTGTTTTCTCACTGACCGTATGTTTGTCAG
ATCCGCTCGCCTTCACTCGCGCCCAATACCTTTCTCGTCTATATGACGTACCCGACGGATGCCCTTTCTGGCT
TCGGACGCTTAATGATATCATGATCACGTCATTTGTAATTTCATTTCTCCTCTTCAAGTCTCATATATCCTTT
TATGTAGGACCGTCGATCTGCAAACAGAGGGTTCATGATGCGATTGGACGGTAGGGTCGACGCGACGCCGATC
CAGGCTCTCCGTGGTCGTTGGGGGGCGATCATCGTACGCAAATAGAGGGCCTGGATGCGTCTGCATTCCCCGA
GTCATCCCCAACGCGTCGGGTTCCAATGTCCAAGGATCGTCGGTCGCGCTTCTTCCCCATTCCCCACGTCATT
TCACAATATATCCCATTCCGGTCTCTCACTATGACGCAATCCCCAACTTATTCCAGTATCACATGTTTCCGCT
ATATTCATCTGACTGTTTTTGGACCTGTCGGCGCAAGAGTACGCGCGCGACATGGGGCTGTTTGTATTGTATA
AAAGGGCGGCCTTCGCGCTCTTCAATCCATCGAATTCTTGATCACTTTTTCCCCCGAGTGTCTCAGTTTCTCG
ACGCGTCCATTCCCAGTCTCAAGATCTCAAGTCAACCAAGTCTACAATAAGAAGTTCTAAGTTACAAGATTCA
AGCTCTCATCGCGTGGTTCGTTCCCCCGCCCGTTTCTCTCGTGCTGACAATTTTGTTGTGGGTCGTCGACCGC
CCCTTGTCTCCCCCGGACCTTAGGAGCTCATAAACCCCTCCACAACTGGTGACCACCGCAGGGGGGGCGGTAT
CCTGTTCGACTTCTTGCTCGCGCTCCTCTTTGGTGCGTACGCATATAATCACCGAGTTTCTGATTCCTCGGCC
ATAGAATCCTCCGCGCCCTTTTTAAATCCATTATCATCATTCCAGCATCAACCGTCGACCGAAATGGCCACGT
CGACCCCTCTTACCCAAACACCAACCCCAATGCCCACACCGCGCTCTCGCGGCGCGCCCTACTTCAAAGGTAA
AGACGTGAAGACTTTCATCAAGACCCTCGAGACTCTCGCAAACGCGGCACATATAACCCATGATAATCTCCCA
AGTCTGGTTTTGGGATACTGTAGCAGAAAAGTCCGACATATCTTAGAAGAATCGATCGAGTGGAACAAACTCA
AAGATTGGGCTAAGGCCTGTGAACATCTGCTGGACCTCTACGAATCGAACGATCGCGCCCCACGTCTTTCCGT
GGACAGGTTTCGCGTGTGGACGAAGAGACACGCGAAGGAAGGTAGGTTTGATAAATTGCAAGACGTTGATGAA
TATTATAGAGACTTTATCGCAATTTCGAGCCGACTTCTCAAAGTGGCGAACCCTCGTATTAATAAAGCGGAAG
TCGACATCCTATTTTACAGGGGGATCCCCAATCCCATTAGGCAGAAAATCAAGCGGAAGTTGCCTGAGAAGAA
CCTGAAGTCTTCAGCTCCACCCCCACTGAAAACGGTTCTGCGCCTCTTACGCGAACATTTCGACGATTCGGAC
ACAATCGAAGATGATTCATCCGGTATCTCGGATATCGGTAGCGATTCAGATGATCCCCTTAGCGACTCGGACG
AAGATATATCGCCCACACCCCCAAAAGCGAAAAAGAAAACGGTTAGGTTTATCCCACCATCGCAACCAACGCC
TCCATCAGTCGAGCCCCCAACTCCTCAACCGATCGATGAGCTCACTAAGCAAATGCGGGATCTTGTGATCTCT
CAGCTCGCGGACTTCAAGCGTGATCTTCAACGTGACATAGTTGCCCAAGTAGCAGGCCCATCGAACTCGACGA
GCAACATGAATGGTAAATGTTTTATTTGTGGAACTTCTGATCATCGTTTAGGTCTTACGAATTGTCCGGAAAC
GAAGAAGCTCGTGGAAGAGGGCCTTGCCATCTTTAATAGCGAAGGTAGGCTGAAACGTTTCGACGGTAATGAC
CTCCCTAGGGCACCAGCAACCGGAGGTGGTGTCGCCAAGGTCCTGAGAGACGAGATTGCGACTTTAAAAGGCA
AGGCCAGGGAGACACGAGACCGACCTCCCCATATGGCCAACGCAGCGGGTCTTCAAGTAGACGGTGAAGATGT
CCTAACCGACGAGATCCAAGGCATCGAGGATCTACAACCGGCCTCTCAATTCAACGTGGCAACTGGGAGTTCA
TCTTATTCTCAGTCATCATCGTCACGACCTCCTTCGAATTGGATGAGGAGTCCATATGAAACGTTGCCTGTGA
CGCGTTCACAGACTCAAAAAGACGGTAGGGTTAATCCGTATCAGCGACCTGATAAACCTCAGGAAAAGACACC
ACCGGTCCCTCAGTCCTCGCGACAAAAGCCTGACGCTAAGAAGAATGAACCAGGTCCTCAACTGGTACAACCC
CTTCCAATTCAACCATCAACAAAAGTTCCGCCCAAGGTTACCGAGCCGAGAGCTAACCAGAATCCAGGCGTGC
CAGTTGGCTTAGCTCCCCATCCACTTCCACCAGTTCCGCGCCCAGTCGCCCCGGTTCAACGTCCAGTCCCACA
CCCCGCAAACACCGAAGAAGCTTGGCGACAAAAGAAGTCTATGCCAAAAGTACCCGTATATCAAAAAGAAGAC
GCGGATATGCACGACGGGACTTCAAAGACGAAACCATCCTCGGGTTATCATTTCTCTTCCTTGATACAAGATA
TGACCAACGGAGACGAGGTCCAAGAATATATTCTGAACACGTCAATCACGATACCACTGAAGCAGCTCATTGG
ACTCTCGCCCGATATGCAGAAACGCTTCACGAACCTCACGAAGACTAGACGCGAATATGCATCACAGGCCGTC
GTCGCTCAACCTGATCCCGAAGACGAATTCGACGACTGCGATGGGTACATGAATAGTTTCGGCAATCAAGACT
CCGACAACTTCGGAACATCAAGGGTTCAGTTAACTTACGCAGGCGACAGGGAGGATGTTAACGACATCCTCAT
GCGGTATTCGTCAGCCGTCAAAGTGCATACTAACCCGCTGTTCGCGATGACAACAGGAAGGTTCGAAGGGACG
CTAGGCGGAATTACCGTCACCTTTATGGTTGACACAGGTTCCGAGTTAAACCTGATCTCCCAAGAATTCCATA
GCCAAACGTCTCTCCCCCTTGATATGGATGGCGCACGTTGGTCTCTGAAGGGAATTAATGGCGGACCGGTACC
TTTGGTAGGTTGTGTGCGTGACGCTGATATACAGATTGGTGGACACCGGTTCGACCACCATTTCTTTGTGAGT
TCTCAAGGCGCAGGCAAACAAGACGTTATTTGGGTCAACCGTGGCTCCAATGGTATTCGGGCGCCATATTCT
ACTCTCGGACGGGTAGGGTGAGAATGAATGTATGGGCTAACGGAGACGCAGACCCAACGGAAGATGGAAGATG

TCGCCGTCCAACTCTATCGATTCAACTGTGTGCCGTCGATGCTTCAAGGAACGTGGATCGTCTCGTTATGAAG
GGCCAATGTCCAGGTCATCACCAAGCAAGGATCGAAGAAGTCGATTCTGATGTGTCGGAAAACTGGTAGTGGG
TGAACGTCCCGAGGCGTGCACCATGCACGGTCCACCCATACCTACTCTCGGGGGATCCTTAAGATGTCTTGCT
GGCGATGTTTTCTTTGACGATGATTATAATTCCCTATTTTCTGCTTATCCACCTGATACTTTTGATTCCCTTC
TTAGATCAACGGCTCTTGTTCTACCATCAAATATGAAACCGCAATTGTCACCTTGGACCTCACGCGCCCTCTC
ATCAAAACCTATTGTTTCAAGCGCCGTGGGTGTGGCAAAGCGAAAATATAAACCTGTCGACCGCAAAGTGCGT
CCCGTCCCTTCATACATGCCTGACCCGCGAGGACAGGTATTTCTGCCTGTCATCATTCAAGACCCGCTTCCGC
TTCCAACGTCCCCGCGGCCACTTTCACAATTTATTCCCTCAACACGTCTCACTCAAGAACGTTTGGACAAAAT
ACTCGGTTCTATTCCTGATGGTTTCCTTACCCAGATGGAAACCAATCTCCTGGTTCACGTCCTGAGCTCACGC
GAGAAGGCCCTTGCATTTGATGATTCAGAACGAGGTACGTTCTCGCCTAAATATTACCCTGATTATGAAATTC
CAGTCATTGAACACACTCCATGGGTGCAACCACCCATTCGGATCCCAAAATCGATTGAAAGTACTGTCCGACA
AATGCTCGAGGATCAAAAAGCGGCCGGTAAATATGAATACTCAACCGCATCTTATAGGTCCAGAATTTTTGCA
GTTGCAAAGAAAGGTGGGGTAAGAATTGTTGCAGATGTCCAGGAAATGAATAAGGTAACCGTCCGGGATTCCG
CACTCCCCCCACGTTGCGATGATTTCGCCGAGGACTTTGTAGGTTATTCAGTTTATGGTCTCGCTGATTTGTT
TTCAGGTTATGATGGCCGTCGCCTGGCCGTGGGTTCCCGACCCATGACCACATTTAACAGTATCATAGGCCCT
CACCGTTCAACAGTCTTACCTCAAGGCGCAACCAATTCACTTCCGGAATTCCAACGATGTACAACCCACATGC
TGAATGAAGAAATACCGGCTTACGGCGGCGTTTTCGTGGACGATGTTGGTATCAAGGGTCCAAGGTCGACATA
TGACGACGCGGAGATCGCCCCTGGCATCAGAAGATATATTTACGAATACGCAACAACACTTGACCGCTTCTTT
GCTCGGTTTATTGAAGCCGGAATTACAGCTTCAGGAAAGAAGCTCATCCTTGCAACCCCACGCCTCAGAATCG
TTGGAACCATTGTCTCACGCGAAGGGTGGCATCTTGAGCACGGGATCGTCTCAAAGATTATTAAGTGGCCCAT
TCCTGAAAGTATTACTGATGTTCGCGCATTCTTGGGTACAGCGGGTGTAGGGCGAAAATGGATCAAGGGGTTC
TCGCTGATCGTGAAGCCCCTCACGGAACTAACACGCTCTACAGATAGAGACTTCGTCTTCGACGAATTCGCAT
TTGAGGCATTCAATATCCTCAAAGAACGGATAACATCTGCCCCAGTCTTGGTAAGAATTGATTACGAACGCGC
GAAGCTGATTACTCCGAAGCCCAGACCCTCTGATGATGGTCTTCTCGTGGTTGGTATCGATTCATGTTTCAAC
GGTGCTGGATGGATTGTTTCACAAGTCTCGGGCAAGGACACGCGACCAGCAATATTCGGGTCGTGCACTTATA
ATGAAACTGAAGCACGTTATTCACAACCCAAGGCCGAATTGTATGGTGTGTTTCGAGCGTTGAAGGATCTACG
ACACCGCGTTTGGGGAGTTCATTTCCGAATTGATGTGGACGCTAAGTTCCTTCTCGAAATGATCCATTCCCCT
GATCTTCCCAATGCCCCCATGACTCGCTGGATTCTTTATCTTAGCCTTTTCGACTTTGAACTCAACCACGTTC
CCGCTGAAAAACACGCCGGTCCTGATGGCCTATCACGAAGACGGGCCTCACGCGAAGACTCAGACGAAGAAGA
CGCTGAGGAATATCTGGACAATTATATTAACGCAGCTGATCTCTATCCCCATTCCCCGTGGACCTCGCTGAGT
TCACGTCTCTTGCCCAATATGTTGACCACTCTATTAGGCCGTCGCCGTCCAATCCCACTCCGGCCTTATGCGA
CCTATATCTCGAATACTGAAGTGGGCGAGGTTTCATTTTTGAACGTCACACCAGCCAACGACTTCAGAGTTGC
AAGAAGACCTCTCGAGAATGCCAACGCCGCCTCTTTCACAACCTCTTCCATGGACAGACAAGTTCCCGGTTCC
TTGCTGAACCATAGCCTCTTGAAGTTGGCAAACGGGGATCCCTATATTGGCCACGAGTTCTTCCATAGGAAAG
TAACCGAGATGAAGACTTACGTTTTTGATCTAGGCGGCGAGTTTGTTACCCTCGAAACAATTGGAACCAAGCG
AGAATTTATGGCGTCGCCTGGTACCGCTGATGAAGAAGATGAGGACCGCCAAGGCTTCGAGTGGATCCAAACG
GAGGATGGGCCTGAGGTAACGTGTGCCACGCATCAGTTTGGGACTAAGGAGGAGGAACCGGCTGGAATGTGGG
ATGAGATCATGCGCTATTTGGAGGATGGTTCAATCCCGGACTCTTGCGCTGACAACAGGAAAGCATTGAAGAG
GTTCACTAACCGTTGCAGGGTCTACATTCTACACGACGGCCGCCTTTGGCGCATCCACAAGAATCAGTCGCCC
AGACTTGTAATTCAGGACGTTGCTCGCAGGGTGAGTCTTATAGCCGAGGCGCACAACGACGTCGGCCACCGCG
GCCGCGATTCAACTTACAAGACCCTTTCCGATCGTTACTATTGGCCGGGTATGTATGACGAAATTGCTTACTT
TATTAGGTCTTGCGATGCATGCCAGTTGCGTTCGAGGGCGCGGCCGAAGGTTCCGTTTTCAGCAACATGGAAC
ACTACAATTCTACGCCGGTTCGACTTGGACACCGTCCACATGCCAGATGGTTTCGGGGGTAAGAAATACTTAT
TGCAAGCCATGGAGCCCGCGATCGGCTGGCCAGAAGCACGCGCTACGTCCAAGAACGACTCCAAGACTTGGGC
GAAGTTCATTTTTGAAGAAATAATTTGCAGGTTCGGTTGCATCCCTTTTTTTCTTACTGACGGGGGTGCGGAA
TTTAAAGGCGCCGTCGAGATTCTTTTCAAGCAGTATGGCGTGATCGCAATCATGTCATCCCCCTACCACCCGG
AGGGGAACGCCGTCTCTGAGCGTGCTCACGACACATTGGTTCCTTCTATATTCCGCGCCTGTGGGGAGGACAA
GGGTAAATGGCCTTTGTACCTTCACTCATGTTTGTTAGCGATGCGCTCTACGACCAGCCGATCGACAGGCTAC
TCCCCCTACTACTTGCTCTATGGACGACAAGCGCTTCTCTCCTTCGATTTATATGACAAGACTTGGGAAACTC
TAAATTGGGATACCATTCGCTCGACGGCCGACCTTATCGCAATCCGGGCCGAGCAAATTCTTCGCCGCGACAG
GAATGTTGTCATCGCACTTGAAGCTCAGAAGAAAGCGCGCCAACGCGCTGTTGATGATTTCAACAAGAAGCAC
GCTAAGTACCTCTCTTCCGGTGATTTTGAAATTGGAGTATGGGTGTTAGCGCACGAGACATGGTTGGATGGAC
AGAAAGGCAATAAAGGCGCTTTCAGGTGGTCAGGTCCTTTCATTGTGCATGAGAAGCGATTGGGCGGTTACCG
TCTGCGAGAATTGGATGGAACGGTCAAGCAGGAAACGTACTCACCTAACCGTTTGAAGTTATTCTATTACAGG
GATCACCATCAATCTATTTGGTCGGTTTCACGTCAAGAATTCGCCATTCACGCGGCTTCAGCTTCACGTTCGG
GTCCAGCAAGCGATTTTGTCCAACATCTGTTCCCAGCGGTCGGTAACAGGGTTCTTGCGACTCACGCAATCCA
CGGGTCCACGCCATATGTCTCTCCGTCTAACGCTTCGCTTACCCACTACCCGATAACGACGAAGCCCCTTCAA
TATCTCTCTCGACGACATATGCTCATTGAAGAATTGCGCAACGCCGAGGCGAACAATGTCCGGCTGTTTACGG

CTCACCTTACGCCACCACGCCCGACGGTTTACTTGGGAGGATCAAACATCAAAGACTTGGAGAGGTGGACACG
CGAGGCGTCCGCCTAATGGTGTTACGCGCTAATTTCTTATATTTCAGCTCTGTGTCATTGAGGAGGGCCTCAA
TTTAAGCCTCCCCCGATTGTGGTCGTGTGACTTGGGTCACACATGAACTCAACGCTTTTCATTGTTTTCTCAC
TGACCGTATGTTTGTCAGATCCGCTCGCCTTCACTCGCGCCCAATACCTTTCTCGTCTATATGACGTACCCCG
ACGGATGCCCTTTCTGGCTTCGGACGCTTAATGATATCATGATCACGTCATTTGTAATTTCATTTCTCCTCTT
CAAGTCTCATATATCCTTTTATGTAGGACCGTCGATCTGCAAACAGAGGGTTCATGATGCGATTGGACGGTAG
GGTCGACGCGACGCCGATCCAGGCTCTCCGTGGTCATTGGGGGGCGATCATCGTACGCAAATAGAGGGCCTGG
ATGCGTCTGCATTCCCCGAGTCATCCCCAACGCGTCGGGTTCCCAATGTCCAAGGATCGTCGGTCGCGCTTCT
TCCCCATTCCCCACGTCATTTCACAATATATCCCATTCCGGTCTCTCACTATGACGCAATCCCCAACTTATTC
CAGTATCACATGTTTCCGCTATATTCATCTGACTGTTTTTGGACCTGTCGGCGCAAGAGTACGCGCGCGACAT
GGGGCTGTTTGTATTGTATAAAAGGGCGGCCTTCGCGCTCTTCAATCCATCGAATTCTTGATCACTTTTTCCC
CCGAGTGTCTCAGTTTCTCGACGCGTCCATTCCCAGTCTCAAGATCTCAAGTCAACCAAGTCTACAATAAGAA
GTTCTAAGTTACAAGATTCAAGCTCTCATCGCGTGGTTCGTTCCCCCGCCCGTTTCTCTCGTGCTGACAATTT
TGTTGTGGGTCGTCGACCGCCCCTTGTCTCCCCCGGACCTTAGGAGCTCATAAAACCCCTCCACA
>Cligib1_Cluster80#LTR/Gypsy
GTCAGGACTTGTATTTTCTTTTCTTTTCTCTTTTCGTTTGTAGTATCACATGACTGATCACGTGACTGATTAT
GTAAGCGAACCGCGGTTTCACCTCATTGTAAACACATGTAGTTTGTACTTATTATATTCAAGGGCTCTTACCT
ATGTATATAGTCTTGTTGTTTGTATGATTCGTTTCTCAATCTGATTCTCTTCGGTTTTCCGCTTTAACACAGT
GTGAAGTTCCGTTGTCAGTCGTATTCAATCCATACAACTTCCTCTGAGTATTCTTGAGAGACCGCACATCATT
ATTGGTGTTTTAGCGTGTCCTTACAGAAGAATCAGATTCCTCCTCTTGTGCCCTCTCTCATCGTTCCAACACT
CGTATCGGCGACAAGAACGCGCCCTCATCAATAGTTACACAGACGCTCTCAACAGAGCTCTCTAGTCGATTAT
TCATAGGGATGTTAAGTCTCCTCACCAACCGTTCATCATTAATCCCCTCGACTGCATCAGATATCAATCAGAC
AGTCGCGATCTAATTGCCCTGCCTGAGCGATACTTCTTTGGCAACAACTCTTGCGACACGGTTGAGATCTAAT
TTCTCCGTTAGCATATAAGAGTCAGCAAGTCCCCCTCCTGTTGCGCTTGGATTGGCTGTTGGCCAAGGCAACC
TCAGCACCCTCTCACTGCTAGTTCAGCCCACACCTGGCGCAGGATAAGCGTTTCAGGGTTAGCTACTAGTAAC
AGTTCAATCGACCCCGGACCACTCTTGAATCATCATTCCCATCCTGTTTGAGTAGGCTTGCAACCTCTCATCC
TAATTGCTACCGTTACACGGTTTGAGCAGGCGAGAATTTCGAATCTCACCTTTGTCCCGTCCCCACTCTTGAA
GCAGGTGATCACCGCCGGTCTACTGGATTAAGTCCATACCCGCGGCCCCCTTCATTTCAAAATTCCGTCACAT
TTCCGACCATATTCTTCCGCCCACCCCTCTCCTACTTTCTCTAAAACACCCGTCATGTCTGAAGACTCAGCAA
ACTGGGGAGGATCGCAACAAACGACACCTCGCGGCAGTTTGATCAATCTGTTTGATGGAAATTATTTCCACAC
ACCCGCCATCCCTCAGGTCAACCCCGACACGGTCACTTATCAAATGGACGCTGAAAAATTCACAGAACTATAC
GGTCAAATGAAACACGTCACGGAATTGAGCACGGAATTCATCCAACGGATCCAAACACTTGAAAATCACGTTA
AGGTTCTTCAAGATCAGAACTCGAGACTTTGGGAACAGAATCACGAACTCCACTCTCGGTTTAATCGATTTGA
GGCACTTCTTATGGATTTAGTAAAGGACAAACGCGAACGAGATTCTAAACCCTCTCCCTTGACGGATTTAATC
CCCTTCCCGGTTCATACTCTAACATGGCTAAAGGATCTCGTGCACCCAAACTCCAAAATCCTACACCGTTCGA
TGGTACGATGGCCAATGCGGACACATTCGAAAATGCATGCCGTATTTATCTACAAGTTCGTCACGCGGATTTT
TCATCAGTCACCAGCGCGGTATATTGGGTCCTACATCTTTGCTCTCAAGGTTCAGCAGCGACATGGCGTGATC
TTAAGATCAAGGAATTTAACCGGAATCCAATACAAATGGATTATACTATTAACGATTTCTGGAAAGAATTCAA
ACTGGCATTTTCCGATCACGATCCACAGGGAACTAAAGCGTTTAAACTATGTACCATGAAACAGGGAACTTTA
CCGGCTGAAGAACATATTCAGGAATTCAAGAAAACGGCCGACGGCTCAGGATACAGCGGATATCCTTTGATCA
AGGAATTCAAACGGTCATTAAACGAAGGTCTTCAAAAACGATTAATGGAATCCGAGTATCAACCAATACCATT
GAGGACTGGTACACACGAGCATCACATCTGGACAGGCAATACCGACAATCTCAGGAAGAGAAAAAGTTGTACA
ACCAACATTCTCACGCCCGGCATTTTCATCGAATAATCCACGGCAAACCGGAACAGGAACCACTCAGACACAA
GGTCCCCACCGAAATATGGGATCTAATCAACAGGCACAACCCGCTCGGCCTACGTTTGGTTTCAATCGTCCGG
CATTCGGTCAATCAGCACAACCGGCCCGACCTGCCCAACAAGCTCGTCCTAATCCACCGGCTCCAGCACGTGC
CCCAGATGCGATGGATGTTGATCGAGGAAATCGTGCCCACCCGGAGTTTGTTATCATGTGGTAAACCCGGCCA
TATGATGCGAGATTGCAGGGCCAATATAAATTTAAATTCGGCCACACATGATGAAGCACGCGACCACTATTTA
CAGGTCGCAATCGAAGAAGGGTTGATTTGGCAAGATACGCTGTCAACATCAGGAATCACGGAAGTCAATCCGA
ATTCACAATCTCAGGGACAATCCAACGTCCAATCGTCTTTTCAAGGAGGCGCGCAGTGAAATCTCCACCACCA
GCGTTGCCTAAAAATAAATTTCATCTTTTATCTGTCGAACGGTCAAAAACATAGATACACCTTCCAATGTAAT
CGTATCTCAGGGGGCTCCCATAAGGAGCTTGGCGCTCAGGAAGCGTGCTTAATTCCCGCCCCAATTTATCATT
TCCAAAAATCTAAAACGACAGAAAAATTAGTGGAGACAAAAATAAGTGGTTTTGGTGATCAGATAGACGTCCG
TCCTGTTAATCAGGGTCCCGGCATTATGGTCATGTCAACCCACCAGGATTCCTTAAATGGAACATCCGTCCGT
CCCAGAGTCGGTAGCTCTGTGGGTCCCAGCGGTAACGTATCTTTGGAACGTCCTGGTCATATTTATAGAGAAA
GACTTCTTATTCGCACGCTTAATTTGGAACGCGAGACAAAATTATCAATCTCAGTAACAACAGTTGATACTCA
TGAAACTGCGTCATGTCAAGCGCTTCTGGATTCTGGTGCAACCGGTTCCTTTATCGATTGAGCATATGTACGA
CGTATGGGATGGAATACCCAAACTTTAAAATATCCGGTTAAAGTATTTAACGTGGACGGTACCTTAAATCAAG
GAGGATCGATCACGGAAGAAGTTATTTAATGATGGTTCATAACGGACATAAGGAGAAGATGCTCTTCGAAGT

CACAGACTTGGGAAAAGTTCCGATCATAATAGGTCATACGTGGTTAAAGAAACACAACCCATCTATCGATTGG
TCAACCGGTGAAATCACATTCACTCGATGCCCCCCAGAGTGTAATGTTAGAGTACGCGCTATTAAAAAGCAAA
AGAAGCATAAAGCTCTAGCCGATCGATGGAAATACCAACCCACGATTGAAGAGATCGATGACGAAGATGCAGA
TCGAGTGATTGGTGGCCTTATTACTGAACAAGAAGGCGCATTACTTGAAGAAATTAATTATGACGAAGTGGTT
CATGAAATAGTAGATGGACACTTTGTCCGATCCGTAGAACAAGCTGAAGACCATGACAACATACATATCGAAA
CAGACGAACCTGAACTCATCAATAAAATTCCTCGACGATTTAGACGTTGGCTCAAAGTCTTTGAAAAGAAAGC
ATCGGAACGCATGCCTGAACGAAAACCCTGGGATCACGCTATAGAATTGAAAGATGGCTACGAACCTAAGAAA
GCCAAAGTCATCAATCTCTCCCCTACCGAACAGAAAGAAGTTGACGACTTTCTAGCGGATCAACTAGCTAAGG
GTTATATTTGGGAAAGCAAATCTCCTCAAACATCCGCCGTATTCTTCGTACCCAAGAAAGATGGAAAGAAACG
CATGGTCCAAGACTATCGACAACTCAATGCTTGGACAATCAAAAACAATTATCCCCTCCCAATGATCTCCGAT
CTTATCGACAAAGCTGGCAAGTCCAAGTGGTTTACAAAACTAGACTTACGATGGGGATATAACAATGTTCGCA
TGCGTGAAGGAGACGAACCAAAAGCGGCATTCACAACTCATAGAGGTTCTTTCGAACCGCTCGTCATGTATTT
CAGCTTGTGCAATTCACCGGCTACTTTCCAAACCATGATGAACGCTCTTCTACGAGAACTTATAGATGAAGGA
CATGTGGTAGTATATATAGACGATATCTTAATATTTACCAATACTCAAGAAGAAAACGAACGAATTGCCGAAC
GAGTTCTCCAAATCCTCCTCGATAACGACTTATTCCTAAAACCAGAGAAATGTTCCTTCTCAGTTCAACAAGT
CGAATTCCTGGGACTCATACTTGGAAATGGAGAAGTACGTATGATGGACGACAAAACCGACGCAGTAAAGAAA
TGGCCGGTTCCTAAAACAGTCCGCGACGTTTGCAAATTCTTGGGTTTCGCAAATTTTTATCGACGATTCATCG
AAGGGTTTTCAAAACATGCAGCCCCGCTCAATAATCTCTTACGAAAGGATTCTAAATTCGATTGGACAGAGAA
ACAACAAGCAGCATTTGATATGCTTAAAAACCGATTTACATCTGATCCCATATTGGTTACAGCAGATCCAACA
AAACCCCTTTGATTAGAATTAAGGCTCAGCACGAGCACGGGTTACCCATGGGTACCTGCGGGTTGAGCCGTGG
GAATACTTGCAAGTTCAGGTCAAAAATTTCCATCTACTCATATGGATCATACTTGCACGGCTCCGAGTTCAAA
ATTTCCATATTATGAAACCCACTGCAAGTACTTGGGACTTGTAGCCCCCAGAAAAAAAATTTAGAGGTGGGCAG
ACCAAACTATTGGGTGTGGTAAGGGGTGATGCATTGATGTAGCAGTCCAGTTATGTGCACAATTAGATTATCA
TTGTTTCAATCATGCAACCATGTAATCAAAGTGATAGTAGGCTACAAGTATACAAAGGGTCTGGAAACCATAG
CTCGGAGGGTCATCAAACATTGTAATATGAAATGCGGGTGGGTGGAGTTTGAGCAATCAAAGGTGTCACTGTG
GATACAGGTGGACACAGCTTTATTTGACAGATAGCATTACAAGCTGTGGATAATGAAGTTAGGAGATGGACGG
AAAAAATGAGCGTGCGACGCCCTTCAACCTTGTGCATGGCACTCTTGGATTTCCGGGCTCCGTACCCGAGGCC
GAGAGGTTGGTGATACCAGCAATGAGTAGGCGGGGGGCTGGGGGTGGTGTAGATATGACAATCCGGAATGGAG
GACTGGTTTTAGGAGTAGGACCAGGGGTGCGACGTACGTGAGATCGAGTGGCAGGAGGGGGGTGGCATGGACA
GAACAAGGGACATAGGGCTGTCCGTCAATGGTGGGACAACGAGGACGAGCCGATATGGTGATTGCAAAATCGT
CGCGTGAACTGGAGTAAGGGGAAAACAAGTTTCCGTTAGCATTTGTTGAAGAATGGGAAACAGGAGTGGTACA
TACAGTGGGACGACGAGGACGAGCCAAGATGGGGCCGTGATTGCTAAGATCGTCGTGTGAACTGGAGTAGAGA
AAACGAGTTTCCGTTAGTATTTGTTGAAGAATGGGAAAGGTACATACAGTGGGACGACGAGGACGAGCCGAGA
TGGGGCCGTGATTGCTGAAATCGTCGTGTGAACTGGAGTAGGGAGAAAACGAGTTCCGTTAGCATTTGTTGAA
GAATGGGAAACAGGAGTGGTACATACATATGTGAAGCCCGCACTCTGCGACTGCTCTTGGTAGATGGCCATCC
CACTCCGATACAAATACGGCTGGGGGTTTGAACCGCTCATGCTGTGGGACGACATCGGGACCAGACGGTGGTT
GGTGTCCAGCCGCTGGCATCATTGTGTTCGATGAGCAGTTGCCGGGAACGTCGCCTGGGCCAGGGGTGGCAGG
GAATCCGACGGGGTCAAGTTGTGGTTGTAGACGCGGGCAGGCCCCAGCGTAGGGGATGGAGGGACCGAGGCCG
AGTCTGCAGATGAAGAGGCGGTGCTCGCACGCTTGCCAGGGCCAGACAAGTTATTCATGGCCCGTGCGATGGC
AGTGCGGGTGGAACTTCAGTGAGATCAGGCAACAAGCGGGTGCAATAACTGAAAAAAGCGATCAACAGATGAG
TCTTGGACGGGTGATGTGAAACATACTGTTCTTGTGGTTCCTTTCGCTGGTATGGACGCGGGACTGGCGTGCG
AGATGGGTCGCAGGTAGGGCAGACATGTTTTTTCTTTGCAGGAGTGGGTGCGGGGGATGGCTGGGACGATGCA
GGGAGCTGTGGAGAGGCCGAGTCGCCTGGGAGTGGTAGAATTGGGTGGCGCCTGCACGAGTCGCGGTCCGGCT
TCTTTGCATTCTCATTGGAGTTGTAAGCGGCGTGGGGACGACAATGGATGGGCACAGGACGAAGTGAGAGCGG
ATGAGGGGCGGGGAAAGGGTAGGGTGCGAGCGGGTAAGGTGCGAGCGGGTAAGGTCAAGCGGGTAAGATGCAA
GCGGAAGGGTGAAACGTAAGTGAAGAATGGGATGAGAAGGGGCGGAGTGAACTTACCGTTGCAGAGGAATGCC
TCGGGGTTCGAGATGTGGGCAAACCGCGGAGGTTGGTGGCCTATTCGAGACCGGGAGTTAGTGACTGGTGTTG
TTTGCTGGCGGGCAGTTGCATGACGCTAGCCACATGGCGTGAATTGAAGCCGTTGGCGTTGATTCCGAGTCGG
CAAGGTGCTGCCCAGTGCCAGGCTTGATCAGCTGCGCCGCATCATTGAACAATCGGCATTTGCAAGCGGCGCG
GGGGCGGTGGTGGATGAGTGCGGAGCGAAGTGAGAGGGAACAAGGTGCGAGTGAAAAGGTGAGACGGTGTAAG
TGAAAAAGAACAATGCGAAGGGGCGGAATGAACTTACCACTGGAAACCGAAAACTCATCGTGGTGTTTGCTCG
CAGTGACCTAAACTCTGGAGCGGGTGGTTCAGTGGAAGAAAACAGCGGTGGGCATTGATGGTGGCCGTTGTGT
GCGACGTGCGGATAGGATTAGTGACGGTGGCGGATGCGGCGTGAATTGATAGTGGGTTGAATCTTCTGATTCA
TGCATGAGGCTCGGCTAATAGGGCAAATCCGACAAAGAATCAACGGAGTGCATTGTTCAATTGTTTGCGTCTG
TGTGGTTAAGGTTCGAAAATCAACTTCCGTTACCCGCCGACTCGCCTATCCATCAACTCGCTCTCAACTCGTG
CCAAACTCGCGTCCACAGCCCATATTTTCAATATTCTGCTTGCCGATTCCTGCGATCGACGACGGTGACCTCA
GCCATCGAGGGCCCAAGGATCTGGCACTCATATTTTCGGTGAGGTCCCTGTCATGTGGCTTTTTTTATTTAAA
CCACCACTGCCTCACCACAATTCTAACAACTTGCCCCCTCCTCATGGCAACTCGAGCCTCTCGAAAACGACCA

CGACCGTCCTCCCCTCTTCCTCCATCCTCACCCACACCATCTTCACCACCACCCCCCTCCTCAGATGTCCCCC
TCTTCATTAGCTCTGACTCGGAAGACCTCCCGGCCATGACTTTCAAATCTGCAAGGGCTCGGAACACCAAAAA
GGTGAAATTTGAAGGGATGACCGAAGAGGAGATCCTCGGTCTGTCATTTCCTGGTCCTCATTTTAATTCTACT
AACAATTCTTTCAGCGGAGCACCAGAAGACATGGCGATCATCATGCTATGCCCACTTTTTGCCTCCGCTCATC
GTTCGGGTGGGTACAAAAATTAAGTACAAATTTATCTGCAAACGGTCAGTATGGATTCATGCCTTTATCTCTG
ATTTATGTTAACCATTATGCACAGGCATCCAACCCAATCATGTGTGCGCGATCGAGAGGATAATTCGACATCA
AACCTCATCCGCCACGTTGAATCATGTAGTCCCCAGAATGTTGAGGCCACGGAGAAAATAATCTCATTTGGTA
GTGGTGTTACTTATTCTGCACCGCGTTTTCGATACCTCCTGGCACTTTGGTGTGCATGACACCATCGCCCCTT
CCTCATTGTGGAGGATGAGGAGCTCGTCGAAATCTTCAAGATGCTTTATGCCCGTGTCGAGATCCCCCACCCC
ACCACAATTTCCCGGGACATTCAGGACATGTTTTCGTTATGCCAAAACAATCTTATTGGAATTCTAAAGGTAT
GTGTATTATTGCTCTCAATTGATGTGACTAAATGTGATTAGGGATACACAGGTAAGGTACACATTGGTGTTGA
TGGCTGGTCATCACCGAATGTATTCTCATTCTTGGGTATCACGATCCATCAATTTGTTGGTGGGCGAATGTGC
ACGTATATTCTTGATTTCATCAAGTAATTATCATTATTATGAAATCTTGATGGGGCTAATATTGTCCTCTGGG
TTGACGCAAGCCCACACTGGGAAATATATGGCAGAGAAGCTTGCTGGTTGTCTGAAGACATACGGGATTGAGA
ACAAGGTTCATATCCTCCATCTTTTGATTCGCTTAACCATGGGCTCACTCGGTTGGATATAGGTACTGGGTAT
TGTCTGCGATAATGCTGAGAACAATACTACAATGGTTCGTGCCCTTGCCAAGCATATTCCGGGGATTCGGGGA
GAGTCTATTCGCATCCGATGTTTTGCCCACATTATCAATCTTGTTGTCAAGGTTTGTCTGTTTGTTTGTTTTT
GGAGCTACACGACACTCATTGTGCCACGTAGGCAATATTATCTCAGTTTGCTCGCACTCAAGGTTCCGAATCA
AATAACGATGATGATGCAAATGATGAAGCCCTCCTCCGCGACATTGATGAGCCTCTTGATGATGAGGATTTGA
AGGGATTGGAAGATGAGGATGCTGATCGGGAGGTGTTTGATGCAGGACTTATCTCGGAGCTGGACGTGGAAGA
AGATGATGATCTTGTACTCACTTGGGAGGACCACAACCTCGGGAGGTTTGCAATTCATAAGGTAAGACCTTCT
ATTCTTTCCTGCGCCTGCTCACATAAGTAAACAGATCACCTCGCTATCAAAAAAGGTCTTCAATTCACCTACC
ATCCGTTCGGAGCTTGCAAAGCTTTGCATTGCTCATGAGCTCAAGGCCCAAGTGCTTCTCCGCTCAGTTCCAA
CTCGATGGAATTCCGTTGCTGAAATGCTCGAGAGAGCCCTTGACCTCAAACCTGTTCTTACTGAGCTTTGTGA
GCTTACACAGTTCAATAAACGTGATGGAGTTCGATTGCGACGATTCAAATTGGATGGGGAGGAGTGGACTCTA
TTGGAACAACTCTCCCCTTTGCTTGATGTGAGTTCTGGTTTACTTGAACCTAGTTTATAACTGATATTTCTCA
TAGCTCTTCTTGTTTGGCACGAAGGAGGTGTCGAAGACCGAAACACCCCTTGTTCACCAAGTCATCCCATTTA
TTGATCAGATGGATCGTGCTCTTAATGATTTCGCTGGTGATATTAAACTTTTACCAGCTGTTCGGATGGCTGC
AAAACGAGGCAAGGTTATGCTGAACAAATATTACAGCAAGACAGATGAGTCGATAATTTATCGTATTGCTATG
GGTGAGGTTTTTCTCTTCATCATTTGTATTTAATCTCACTGGATAATGCAGTTCTTCATCCAGCCTACAAGAC
AACATATTTTCGTAAGCACAAATGGCCAACTGAATGGATTACAACTGCAGAAGACCTTGTCCGTGAAGAATGG
AAAGCACGATATAAAGAAACAAGCCCCATACTCCCGGAGGCACCCCCAGCTGTAAGTGTTGACTTGTATAGAT
TGTTTTATATACTTATTAATTTATGTTTTAGAGTTCGATCCGTCACAAATACTTTGCTGGAATCATGAATCCC
ACGGCAGGTCACGATCCACTGGACTTATATCTTGCAGCACCTGTGATTGAGTCTGTCACCGATCCACTTGATT
ATTGGAAGGCCATGGATCCCAATAAGAACCCACTTGCGCAAATGGCTATTGACTTTCTATCCGCCCCTGGTGA
AGTTATTTCATTTTTTTGATTCAGAGTTTATGAATATGATGTCCAGCTGCATCTACAGATGCTGAGAGATCAT
TCTCACGAAGCAGGCTATGTGTTTCTCGCCTACGACACTCACTTAATTGTGAGTCCACTCGTTCAGCAACAGT
TCTTGGGTCATGGGCAGGGGTTCCTGGTTTGGTGCCAAGAGATGACTTGATTGAAAATATTAGGAAAAAGCAG
TTCCATTGGGGGAAGGAGGATGTGGTGCAGAGTAATAGCATTCACAACTCAGAGGTACCTACAATTTGAGGTA
CCTACAATTAATAATAAATACTTGTTCTGGTGCATCATAAATCCTGAGTACTTGCTGAGTTGTATGATTAATT
GTTTACAAAGTTACCTATGACCTGTGATTACTTGTGAGTAATCATGGGTAATTGTGATCTGTGGCAAGTACTT
ACTGAGTTGTAAGTACTTGCCAAAAAATACTTGTGAGTCCAAGTACTTGCTGACTAAGCTACCCACAGATCTG
GGTAGTGACTTTGTATGACCAAATACCCATGAGTACAGATCCAGATCACCCACGGATCTACCCATGCTGAGCC
TTAATTAGAATCGGACGCTTCGGACTTCGCTACAGGAGGTATATTATCTATGAAATGTTCGGACGACAAATGG
AGGCCTTGTTCATATTATTCTAAATCTTTATCTGACGTTGAACGTAACTACGATGTACATGACAAGGAAATGC
TTAGTATTATACGCTGTTTGGAGGCCTGGCGCCATTATTTAGAAGGTAGTACACATACGATTGAAATATGGTC
TGATCATCGTAATTTACAATACTTTATGACTTCCAAAAAGTTAAATCGTAGACAGGCACGATGGGCACTTTAC
TTATCACGATTTGACTTTACCTTAATTCACAAGGCGGGTTCCTCAATGGGAAAAGCGGACGCACTGTCCAGGA
GGACAGATCTAAAGGAGGGAGTGGAAACTGATAATGACGACATCATGCTCCTTAAACCGGAATTCTTTGCCAT
AAAATCACTTCAACAGGGACATGTCCTTATCAATGGCGAAGAAGAGGGAGTACTTTCGAAGATCCGCAAATCG
AAAGACTATGATGAATCCGTTGTCAAGGCAATCGAAGAACTTAAACGGTCGCCCACGAAACGCTTACGATCTG
AAGAATGGTCTCAGGAACAAGATCTTATATTGTTCCGAGGAAAAGTCTACGTACCCAATGACATCCAACTTCG
ACGAGAGATCGTCAAACTCCACCATGATTCCAGCATTGCCGGTCATCCAGGACACTACAAAACTCTGGAATTA
ATCGCGCGAAACTACTGGTGGCCTGGCATGACAAAATTCGTTCATTCATACATCGACGGATGCGACTCTTGCC
AACGTTTCAAAAACTTTCCCGCTCCTCCTATTGGAAAACTTGTCCCAATGATTACCCCCTCCGAACCATGGAA
AGCAATATCTTGCGACTTCATCACCGGACTTCCTGAGAGCAATGGATACGACGCAATTGTGGTTGTTGTCGAT
CGCGCATCAAAAATGGCGCATTTTATCCCTACTACCTCTCAAACCTCTACGACGGGTCTTGCTAAACTCTATT
TGCAAAACGTCTGGAAGCTCCATGGTCTCCCCGATTCAATTATCTCTGATCGAGGACCACAATTCGCAAGTTC

AATGATGAAAGAATTAAATTCATTATTGAATATTCAAACAAACTTTCTACCGCCTATCATCCTCAAACAGACG
GTCAAACGGAACGGGTTAATCAAGATCTCAAAGCCTACCTTAGAATTTTCATATCACATCTTCAATCCGATTG
GGTTGAATGGATTCCTCTCGCTGAATTCTCATACAATAACGCCACTCATTCCTCCACGAAAATATCCCCCTTC
TTTAACGTCTACGGTCGTCATCCACGAATGGGCGCTGAACCAATACGACAACCAACAAATGAGTCGGCTAATG
AATTCGCTACACGGATGAAGAAAATTCAAGAAGAAGCACAATCGGCCCTTGTTAAAGCGCGCGACGAAATGAA
ACGATACGCCGATCAAAATCGTGGCGAATCCCCATCATTTCAAGTTGGACAAATGGTATGGTTGGAAACCAAA
GATCTTGATCTTAAACGACCGTCAAAGAAACTCACAGAACGACGAGTTGGTCCATTTAAAATTCTCGAACTTG
TTGGAACCAACGCGGTGAAACTTAAACTCCCATCAACAATCAAAATTCACCCCGTCATTAATATCTCACGAAT
ACGACCATATCAATCCCCCAAAATTCCCCAACAACAAATCCCTCCTGCACCACCAGTAGAAATCGATGGCGAA
TTCGAATATACCGTCGAAGAAATTCAAAATTCACGTCTCACCAAGAACGGCAAAGGATTAGAATACTGGGTTA
AATGGGAAGGATACACGCCGCAACACAACACGTGGGAACCATTATCAAATTTGAAGAATGCCATGGATCTCAT
CAACAAGTTTCATCGAACAAATCCACAGGCTCCACGAAAAGTTCGTCTCAACGTAACTCAAGATGAATTTCTC
TCCTTGTTCGCCCCATATGAAACTTTCACCGATCCACCATCCAATTCGGCATTATTTTCAAAATTGAACGCTT
CAAGTGCTTCGAATTGATATGTGCGCATTCTTTCGGATGCGAGACGCACTATCAGGGATGATGAAGAACCGCT
CAAGATTTTCCCGGAGAGAAAATCCCTAAAGAGGGAGTAGTGTCAGGACTTGTATTTTCTTTTCTTTTCTCTT
TTCGTTTGTAGTATCACATGACTGATCACATGACTGATTATGTAAGCGAACCGCGGTTTCACCTCATTGTAAA
CACATGTAGTTTGTACTTATTATATTCAAGGGCTCTTACCTATGTATATAGTCTTGTTGTTTGTATGATTCGT
TTCTCAATCTGATTCTCTTCGGTTTTCCGCTTTAACACAGTGTGAAGTTCCGTTGTCAGTCGTATTCAATCCA
TACAACTTCCTCTGAGTATTCTTGAGAGACCGCACATCATTATTGGTGTTTTAGCGTGTCCTTACAC
>Cligib1_Cluster87#LTR/Copia
ACGGGACCTGTTGAAGGTAACGCCCGTGACGCCGACTGATGCCAAATAAGGAATGCCGACTCTGCACGCATTC
TTCTTTTTCTCTATTTCATATCGTCTATACTGTTTCACTTACATCACTGGCACGGGCTCAACACGCCGTGCCC
CTATTTATTTTCTTCCTTTATATAGAACGCTCCCCCGTTTACGCACCTCAGTTTGCTTTCACCCGTGTGATGG
CATCTGAGGTTAGTACTTTACATATTCACATACATGGACACTCAACTGACCTATCTACGTACTTAGCTTCCTT
GTTCAACAGGTAGCTAATAAATTAGTTTGCTTTCACCCGTGTGACGGCATCTGAGCTTCCTTGTTCAACAGGT
TATGGGCCCTGTAGCCTGAACGATACGGATTCTATCACACGCGCAAACTCAATACGTTCACATTGATATAAAA
TGACTTCTATTACATCGTCGTCCTCCCACCCACCACTCTTCGCCGAGAAGGATAAATTCAATGGCTCCAATTG
GATCGTCTGGAAGAGCGCCGTGCGTCGAGCCGCCCAACTTCGCGGTGTTATTGGCTACCTAGACGGCACCATT
AAAGAACCCCCGGAGAAGCCTACCCTTGAGGACACACAGACATCCCCACTTCCTCCACCTGACCCAGATACTG
CCGCGGCGACCCCTTGGTGGTCACTTACACCCTCCATCAACGAATGGAACATGCGCAACGCTTGGGCGATGAC
GCTACTTATAGACAACTTGATCAACCCTGTCGGATTAGGGGTAAACTTAGATGGAACGGCGGCTGAAGTCTGG
TCGACGCTTACCACACGATACAACCGTATGGACAATGACCTAGCAATGGTACGCGCTCAGCAGGCCCTCCGCA
CTTGCTTATACCGCGACGGTGAAGATATGCCTACACACGTCGAAAAACTCCGCGCAAAATGGCTTGAGGTCAA
TGCAACAGGAGGAAAAATCACCGACCTCGACTTCCGTTCTATAGTGCTCGCATCCTTACCCCCGTCCTGGGAC
ACGATTGTCGCGACCCTATATGACACCAAAACGTCCGACGCCGTTGTCGATAGAGTCGTTATGCACTGGGAAA
GGATGACACTCGGAAAGGCGGTCCACGCGGCCGCAGGATCTGTCACAGCTCTCCAAACGACTTCTTCTACCAA
GAAACGCGAGCAGCTTATTTGCACGAACCCCAACTGCAGACGTAAAGGGCACACCATCGAGAAATGCTACTGG
AAAGGGGGAGGTAAAGAGGGTCAATTCCCCCCCGGTTTCGGTCAAAGGGGCGGAGTGTCGACGACGCCCTCGG
CAAACCCTATTACTTCCCAGACCGCAACGACGCCAACCGTGGCTATTGCCGACTCAACGAACGCAACCACGCA
ACACACCACTGATAGGGTGTACGCACTTTATACATCCATCAATGATGGTGTTACGCCCGAACCAACAGCTATG
GCCACCCCGCCCACGCAGACGCAAAGCGTGGACGATACTTGCTCGGCGCACTATAACCACGTCCTGACATTCG
CTGACTCGGGATCAAGCGATTGTTGCTTCGTGAATCGTGAGGATTCGATACCTATACACCTCTCGACCCACCA
ATTATTGGGAAGGCAGCCAGTAAGGGAAGCACGTTCCGGATCATGGGGAGGGGCTCGGTTACCAAAACCTTCG
CGGTTGACGGTGGAACGACGACGATTAAGTTCACTAGCGCTTGCCACACCCCCGACCTCATCTCCAACCTATT
ATCTATCGGAAAGTTTGACTCGGCGGGCTTCCGGGTAGTCTTTGAATCAGGGATGGCGAAGTTCTATGACGAC
CAGGGTAACTTGATGCTATCAGGAAAAGGCAGCAACGGGATGTACTTAGTCGACTGCCAGAACGCGCCCACAG
CACTCAGCGCGCGCTCGCATGAGAAACCAACAGTCATCGAGACCTGGCACAGGCGTTTTGGGCATGCCGGTAT
TGCTGCCATACGCAACATGGAGCGCAAGAAACTTGTCGACGGCTTACAGGTTACAGACGACGCGACACCTGAG
GGACTATGTGAAGACTGCATCTACGGTAAACACACCACTCGGCCGTATGATGAAGTAGTCACTCACGAAACCA
CCGTCCTGGAGCGCGTTCACCTAGACCTCTGGGGGCCGGCCCGTGTCAGGTCGACTGGCGGAGCACTTTACAT
GCTCTTGTTGACGGACGGCGCATCCTCGTATAGGAAGGCGTATTTCCTAGCGAATAAGAACGCGGAGTCGACG
CTTGCGGCCTTCTGCGACTTCCATGCTGAAGCTGAACGTCAGACTGGTAAATTCCTGAAAAGGGTTCGCTCTG
ACGGAGGGGGGGAATGGTATAACAACCTATGGGGTGATTACTTGAAGAAACATGGGATAGTGGCGGAAATCAC
CACTGCGTATGCACACTCACAGAATGGCGTCCCTGAGCGTGGCATGCGCACAGTATTCGAGGGCGTTCGATCT
ATGCTGGCAGAATCCGGACTTCCCCATCCCTATGGGCCGAGGCGGCATCCACAACTGTCTACCTTCGAAATTT
CATCCCATCCTCCCGCCATCCGGACCGTGTACCAGCGGAGGTGTGGACGGGTCAGCGCCAAGACGTCTCCCAT
ATTAGACCCTGGGGTTGCACAGCCTTTGCGAAAATCCCGGCGGAACTGGGTGTCTCCAAATTGGATCCAAGGT
CGGTTAAATATCGGCTTATCGGCTACTTCGATCGCGGGGCTTACAAACTCTATGACACCGCAACGGGGAAGAC

CATCAAGAGTCGGGACGTTATCTTCGATGAAGGTACAGGACACCATACTGGTGTCACTACGCGCCCGCCGATG
GACGATGACTTCGAGTTCCCCATTGGATCTAACAGTCAGCAAGGGGAGGCTCCTGACGAGAATGTATCACGAC
CACCTGCAGTCGTTAAGGAACCACGCCCAATAGCAGCTCGCCCTCGCCCTACTGACCCCCCATTGCACCCGAC
ACACCTTGAAGAGGTCCCACAGCAGACGGAAACAGGCGTACCACCACCACCTCCACCAGCACGAAAAAGCGAT
CGCGCACGTATTCCATCGCGTGCTCTACGCGAATCTCAAGAGTCTGAGGAACGGGTTGCGGAGGCTTCAAGGA
ATTCTGAGGACTGGGCTACCGACCCCCCGCACGCGCTCGTCACTGGCAGAGATGCTGGTGATTTTGATGCCGT
GGCGCCGCGGAATTTCAAAGAAGCTATGAAGCACCCTGACCTATGGATGGAACCCATGGAGGATGAGATGCGC
ACGATGAGAGAGAAGGAGGTCTTTAGGCTCGTACCGCGGCCGGAGGGCGCCAACGTCATTGGACTGCGCTGGG
TATACACCAACAAACACGACGCCGACGGCAACCTCCTTAGGCGTAAGGCTCGCCTGGTCGCGAAGGGGTATAC
ACAGATCCCCGGGGTGGATTTTGACGAGACCTATGCGTCCGTAGTTCGTTTGGAGTCGATGCGTATGGCCGTT
GCCGTCGCTGCGGAACTTGGGTTAAAGGTCTGGCAAGTCGACTTCATTGCCGCGTACCTCAACAGCAAAAACA
AGTACACCGTATACACGGAACAGGCGCCCGGTTTTGTCAAGCAGGGGGAGGAAGACAAGGTCTATGTTGCTGA
CAAAACTGTCTACGGTATGATGAACGGGGCAAACGCGTGGGCTGAGGAACTGAGCCGAAGTTACGACGGTCTT
GGATACTATCAGTCGCGTGCTGATTCCTGTGTTCGCTCCCGAAAGATTGGGGATGAGCTTACAATCACCAGTA
CGTACACTGACGACGTTTTTGGGGCTTCGTCCACCGACGCCGGCGCTGCACTTGCCAAACAAGAGCTCTCCAA
ATGCTATGAGGTCAAGGACCTGGGGGAGCTCAAGTTTATCCTCGGGATTCGCATTGACCGCGACAAGTCCACC
GGTGCCATCTCCCTCTCACAGCGCGCATACCTAGAGCGCGTCCTGGAGCGCTTCAAAATGTCTACGTGCAACG
CAAAGTCCACACCGCTCCCAGTAGGATTGACACTCACCGAGGCTGACCGCGCAACGTCCGAAGATGATATCCT
CTTTATGAAGGATAAGCCTTACCGCGAAGTCATGGGATGCATTATGTGGGCCCAGGTAGCGACCCGACCGGAC
CTGTCTTACGCAGTCAATCTGCTCTCACGTCATCAAACCAATCCCGGACCTGCCCATTGGAGAGCGGCCCTCC
ATGTTCTCTCATATATTAAGGGGACACTTGATTATAAGATCACGTATACGCCGGGCACGGTTAGTGGAATACG
ACCCATCGGGTATGTTGACGCAGATTACGCCGGAGACTTGGATACCAGGCGGTCGACTTCTGGATACGTCTTT
ACTATGTGCGGTGGCCCGGTTTCGTGGGCATCGAAACGACAGCCGACGGTTGCGTTATCTACGACTGAGGCGG
AGTATATGGGACTCACGCGGGCGTCCCAACAGGCTCTATGGATGTACTCCTTCATGAGCGAGATCGGCATGCC
ACAGGAGTCCCCGGCAAAGCTCAACGCCGACAATGAAGCATCTATCGCACTTACGCTCAATACTAAAGGGCAT
TCACGCGCGAAGCACATCGACGTCCGCTACCACTACATCCGCGAGCGCGTTAAAGATGGCGACATTACTGTTG
AATACGTCCCGTCCTCAGATAATCTCGCTGACATCATGACCAAGCCACTTCCGCGCGTTGCTCACCAATATAT
CTGTCGTACGATGGGCATCATGCGCGGCGACGTTACGGGGTTAGGCACTGACAAGAATTCTGTCAGTGAAATT
CATTTGACTCTGAGTGAAATATGCTTTTCACTTAGTTTCATAGTGAAACCAGAAATCACTCAGGCTGTGAAAC
ATATCAATTTTTGAGTGAATTTTACTTTAGCCCATGAGTGCTACAGGTGAATATGATTCACCAACAAAAATCA
TTCATTTTTGATTGAAAAATCATGTGAAACCAACAAGTGGTATGATATGAATACTGGTCACTAAGAAAAACCG
AAACAAGAAATCTATTCAAGGCGCCAAGGCACGGGGCACATCCTCAGGATGGAGGCTTGCAAGACGCAACACT
TCGGCGGTGTCAATCGCGGATTCACGCCGCCATCATTCTTCAATGATTGCAAGGGCGGAGAGGGAGCAGGGGC
GCATGATGTAGGATGCTTCTGCAACCCTCGCAGATTGTCGTGGTCCACACTCCCCCGTTTAGCCACGCGACAT
CATTGCACGCGTCTATTGACGTGGCGAATGGCGCCGCGAGGGTATAGGGGGAGCATGAGGCAGAAAAAAACTG
TTCCACCTCCAACTCTCTCGTTTACGAGAGAGAGAGAGGCAGAAAGTGTGAGTAGGCACGTACCTCGCATGTTTC
CCCGTCTCCGGTTGACGCGGCTGGCACTCTGACACTCCCTCGTTTGCGAGAGAGAGAGGCTATGGGTTCGAGC
GTGTATCCTCCAACATCTCTCATTTGCGAGGAGGTGGGCAAATGGGTTGCAGCGTGGATCAGCGACGACTGGA
TTGAGGCGCAAGTGGCGAGCGTGCAAATGGGTAGTTATACAAGTAGGTGGGTGCCGAGTGAATGGGCGGCATG
ATTGCTGACTCGTGTTTTTGCAAGGCATAACACCTGCCAGGCACAGAGGTCGTGTAGTGGTCAGGGAGGGGGC
GTGGCGCGTGTGCACGTTCAAATGGACATGCGGTCGCATCTTTTGGTGGACCTGGGGGCTGTTGCGGACTATG
CGAGTGCGGGTCAGGTCGGCCAAGGGCAGGGGGCGGCGAGGCAGCAGCACGAAAGGTATCTATACCCGCAAGT
GTGATTAGAATGGGTTTGCACAGGAAAAAAAGGGGTACTCACCTTACGGAACCTTTCAATCGCACTCAAAAGT
GCGAGAGCCCATTTCCTGCTGATCATTCATGTCGATATGTTCACTGGGGTCAAGTCCATCATCCGTCGTTTGT
GCTGTTGTGCCAGAGAGGGTGTGATCTTATCACCCGGCGACCTGAGACCACTGTCGTCGTTCGCTAATTCTTG
TGTGAGGGCGTCGTCCGAAGGCTGTGGTTTTTCGTGGCCAAATAGGGAGGCGGTATGAGGAAAGCGTCGCCAT
CGGGGATAAATGAGAACTCAGCAACAGAAACATTGATCAATGTTGGGTACTCACGTGACCCATGACATCAATG
CTTTGTAAGTCCCCTAGTGTCAGTGATTTTCTGCCAAGTCCCACACGTTGCTTACGACCAGCATGCCCCGTGT
CGACTCGTCCGCAGGCTAGTGTTCACTTATCCGTCGCTGAGGCCACCGCGGGATCACAAATAACTCGAATGGA
CCCGAAGACCCCAAAGTCCATCTGGCACAGATCGCGTCAATTTTTCCCTGTTTTCCCCTCCTCTCCTGATCAT
TCTTACAGATGCTCGATGCGCTCTCGCTCACTTCGCAGCTCCATTTACGGCCTAATCCCTTGACAAGATTTCC
CGCACCGTCTCCAAGCCGGCCCTACTCAGGTCGTTTCAGGCCTGCACCCTCACTAGAGACCTTGGTGAGTCAA
GCTGGCTCACATACTACCATTTGCTCACCCATGCTTCACCCTTCATCCTCGCTCGAACACCGTCCCCTTTCTC
GTCCGCTCACGCCCAGCTCTTTGTTTGCACCCACCCGGATTCTGGACGGGGCTATGCTCAGTATGTAACCGCG
CCTTAGCTCATCGTGCGCGCACTCAAGATCATGCGCCGCAGCATCAAGCCCGAGAAAATTCGAAGAACTAGCC
GCCCCGCGCTTGCCGGTACGAGACTCGCTGTGTACTTCAGCACCGGGGCCGGCGCGTGTAGTATTAACACACC
TACCGGGTACTGGGGGCAGGAGGGCGCGGCGATCGTGCGTCAGGAGCATGGTAGGATCTTGAGGGCCCAACAC
GGCAAGTCGCCCTTGCTGAATTTGCCGCACTCACTCAGCTTGTAACCCAGTGCAGAACCGTGGTCTGCTTCCA

TCGAAACCACATCCTCGCCGCGTATGCCCCTGTACTTACCCCACCAGACCCCATGCCCTCGGGCATGCATTCT
CAAAACCGCTCCATTGTGATCAACCTTTGCACGCCCCACAAACGCCACGAGGATCTGCACACGTGTGAGTGAG
CACATACCAGCATGAGCTCTTTTCCCCCGAGCGAGGCGCCCCGACCCGAGTTTTAATTGTGGCGCAAAGGTGC
ATGGCCCTCTCTCTCCCCTCATTCCTCCCTCTCACCAGCCGACTCTAGCTTGTACGCTTGCAGGGCACCGCCA
CCCCAGACGACGTGTGTATATCATTGGATGGCACATGCTCGCATTCCTGCAAGATGCGGCGGGGCTGAGACGA
GTGGGAGGGGGTACAATGATTACGCATCAGAAGATCAAGCCCCAGGGCATAACATGGCAAGTCGCTCTCACCA
AGTTTACCGCACTCACACAGCCCATAATCCAGCACAGGGTTCCCATCAGAACCGCACCCTCGCCGCGTGCACT
CCGCACCCAGTCCCGCCGGACCCCACACCCTCAGTTGTGTGTTCCTGAGACCGCTCCTTCGCCTGAGCCCCCA
TATCTGGTGATGCCTGACCCCCATAGCCGGTTGTGCACGTCGCCTGTGCCCCAGTAGGCCCATGAATCCGAAT
ATCCCGCCACTCATATCCAGCCTTCGATGTACTGACTCCCCTCCCTATCTACCCTCGACATCGGGCCGGGGCG
CGAGAACACATTGGGGCTTGTATCCGTGGCCCGCCCGTGCGCCGTGTCCACCACCAGGAGGAGCATGCTGGGT
AGTCGAGGTGCAGCACTGGTAAGCCTTTCAGCGATTAGCAGGAGAATGTGTCCCGAGGATCAATTGATGTAGG
CTCTATGACCTTGCTCCTATGTTGGTGTAGATATACAACCTTGGTTCTTGATGCTGTCTTTTACCTCCTTTCC
ATGTAATATAAAAACAATTCATTTCCTTTCATATTGAAATTCATCTGTGAAATCACCTCAATGGAGCAAACAT
TTCTTCGATGGGAGTTAATATCACCAAAAACCTTGGTACCCCAGCATGAACTTCATCCAAAACAGAGTGAGAC
ATGGATGACAGCGTTTATGTGAATCCAGTTCACTCCAACATATGAAATTAACTCAAACTGCAGTGAAATTGAT
TGGAAATTGAAAAGCTTTTGCTTGCATTGAGGTGAGACATCAAGTGAACTTCACCAACAAAATTCTGCTCAGT
GAGGGGAGTGTTGAAGGTAACGCCCGTGACGCCGACTGATGCCAAATAAGGAATGCCGACTCTGCACGCATTC
TTCTTTTTCTCTATTTCATATCGTCTATACTGTTTCACTTACATCACTGGCACGGGCTCAACACGCCGTGCCC
CTATTTATTTTCTTCCTTTATATAGAACGCTCCCCCGTTTACGCACCTCAGTTTGCTTTCACCCGTGTGACGG
CATCTGAGGTTAGTACTTTACATATTCACATACATGGACACTCAACTGACCTATCTACGTACTTAGCTTCCTT
GTTCAACAGGTAGCTAATAAATTAGTTTGCTTTCACCCGTGTGACGGCATCTGAGCTTCCTTGTTCAA
>Cligib1_Cluster85#LTR/Copia
TTAGAGTCTTGGGTCAACACCAACAAGACTATAGTCACTCCAAGTCAGGCTTTAACTTGGGTATCAATGGTTC
AGTGATCAAGGTTCAAAGGTAAAGGATGTAGTATTGCCTAGGTCAAGATAAAGAACAATGACAGTATATCTAG
GGTTTATATACTAGGGACCTTGAGGTGCAAGAACATAGTACAATGAGAAAGAAAGACAATGTAAAGTAATAAA
GCTATTGAAGCTCAGTCAGCTATGACATTGTAGATGAGTCAGGTATCTAAGAATAGTGGCATGTGTTGGACAT
GCTATAGAATAATGGTGAATTGGAGAACAATGTAAGTATCATCAAATAAAGATGTAAATGAATAAAGGAAAAG
ATAGGGGTGGGAACCGTTGTAACACTTCACTTGTATATAAACATAGCTACATACTCCTGTATTTTCTATGTTC
AATTGTTTGGTTTCCCCCCTCTACAGCCTCCTGACCCAATCTCAGACCTTGCTAAGTGTCTAGAGTCTTTACC
ATTGACTCTTGTGTTAGCAGGACATAACATTTGATTAAACATCATAGGGTTACCAGGCAACCCTGCAATGAAA
TAGGAAGATCCCTAGGGACAGGGACACTGTCAACACTGATTGCAGTTGGGAACATATTACTCCATATAGGGGC
TCATTGCCAGTAACAGCATCAGAACTCAAACTACAGAGACTAGGTGACAAAGCAACCATCACATGCAACAGAT
ATCACAGGACATTAAGGCATGTCACGAGTGCACCCGTGAGTAACCTGGACTTGTGGTCACAAGTACCTGCATA
CGAAAAACTGATACCTGGATCTGCACGAGTTTTGTACTCGTGAGATCCGGATCCGCAGGTATTTCACTCATGA
GTATCCGAGTACTCGTGAGTGTTAAATACCTGTGGGTACTTACAAGTTACTTGCAGGTTACCCACCGGCTTGG
AGGGCATGCACACACCAGTCCCTCGGCGCATCCGTTCGTGTGTACTACCCATGCGGAAACCACAGCCCGCGTC
CGCCCGTTTAGCCTCGCGATATCGTTGCACACATCAATGACGTGGCGAACACAGTCGCGAGGGTGGAGGGGGC
ATGCGAGCTGGGCCCAAGCCGCTCCAGCAAATGAGAGAGAACAGACATGGTACATACCTCGAGTCCCCATCCA
TTTGCTCTTGTTTGCAAGAGGATACGGTGGCAATGGTTGAAATGTAGTCAAGTGGGCCGGTTGGGGTGGGGAT
GAAGTCGAGAGTAATGACAACTGATGTGCTGTGCAAGCAGGATGGCGAGCAGCGGTGTTACCAATGCGTGAGT
GGGTGGAGTGGCAGGATGGGATGGGATCACCAGCTCATGAGGGTGGCGAGGGGGTGGTGGAAGGAGGCAAATC
GGTTGATGATCGCTTTGCGAGAACACGGCGTGGGCGATGTGGTGATGTGTTCGAGGTGTCTGCCCATTACTTG
TGCTATGTTTTTGATATTGGAGTGGCAGCGACTCACCTAATCCAAGGAATCAAATGTCTCCTACATTCCGAAA
GTGCCAGGTGGGATTCAAAGCAAAATAGTGGCGGGGACGAGGTGGTGGCGGGGACGGAACATAGTTACGGCAA
TGGCGGGGTGATGACGTGGCGGTGGTGTGAGAGTAGGCTGGTGGGGTTGGGTTGAGCCACAATCAGTCTTGTT
TGCGAGCTGATGTCGATGGCTACAGCCAGCAACTGCGAGTCATGACGACGGTCTGTGGGTTGTGGCTCCTGGT
CCCTTGCGCCGCGCTGTTAATGATGGAAAAAATGTTTTGACTTACCTAGCTCCCAATCTACCTGCTAAAGCAT
GAAAGCGTGCATGTGATGTGGCCGTCCGTCTGGTGTGAGTGCGTAGATATGAAGTAGTGGGGGAGATTATAGA
CATGAACGCAGGTGCGGAGATCGGGATATGAACATAGACAATACAACTCAGCAACAGTAACAGCATGAATGTG
AGCGCCATACTATGTCTGGCAGCGGTGTGTTGCGACGATGATGGTGTTGATGTGGGGCCATGAGCCGTTGTCC
ATCACCAGCTTGCCTGGGTGTTGTCTATGCTGCGTTATTATCGATATAAAAAAACTAACTTACCTAAGTTCCC
GGCCCATCCCTAGAGCTAACTGAAAATCCAAAGCAAGCGGCGTGCGTTGGCTGTCTGTTTGGTGTATACGCGT
AGATGCGAGGCAGTGGGATGGATTGTAGATGTGGTGGAGATTGTGATGAGTACAGGCCACGGGTCACGGCAAG
TACTTGCCGTGAGTACCTGCGGTTGAAAAATTGAAACTCGGACCTGCGGCAACCTGCAGCCACAAGTGATCCG
GATCCACGGGTCACCATACTCGCAGGTTTTCCCACGGATCAACCTGTAGTGACTCACAAGTAACTCGCACTCG
TGACATGCCTTAGTACAATTAAAGTGATTTGCCAGAGTGGCAGGAAAGAAGCCTGGTGGGATGAAACAGCTGG
TTTAGCTATTGCCTTCTTTTCTTCATCCTATGATAGTTGGAGTTAGACGAGCAATGAGATAAATCCAAAACAT

TGGGCTACCTGGGTGGGCTTCCAAACTTTGATCTCGAAGTCTTCTGTGTCCGACAATCCACTCACTATGATAC
ACTCGCCACCCCATGAGTATGTTAAACAGATACCTCAACAACTTGGACAAGTGAACCAACTAATTCGCCATCA
GATCCGAAAAGATGGGACAAGAGGGGACAACGCACCTTTACCGTGATGCAAGATGCTGGATAATATACCGAGC
CGCTGGTCATAAAGTCGAACCGAGCCATCATCGCCACCCACAACTGTCAGGGAGCCTTCAAGGGCTGATGAGA
CTAAGAGTGGGACATTGTGGTGGATTGGTTGGCGAAAGGACCAGACCAGTTGGCAAGGTGGAATTGCATATGT
ATCAATGCCATCTACAAGATTTGAAACAATGAGTGAACATTGATCAGCATGCAACACGATGGGAAAATAAAGA
CATTCGGCACTGACTGAGGGGGAGAATTCATGTCAGATGAATTTACAGAGCACCTGGAGAAGGCAGGGACCAT
TCGGCACCTAACAGTGCATGACAGCCCTCAATCGAATGGCAAAAGCGAGAGAGGAAACTGAACGCACCTTGAT
GGCATCCGAGCCATGCTCATCTCGTCCGGATTACCCCAATTCCTATGGGCCGAAGCCGCGCGTCACCTCGTTT
GGCTGAGAAATCGAGCCCCAACTCGAGCACTAGCAAAAAACGCGACCCCTCTCGAAATCGCGACCAGAGAAAA
GCCTGATCTAAGCGATATTAAGGAGTGGGGATGTAAGGCATGGGTCAAACACACCGAATCCGGAAAACTCGAG
CCGAAAACCGTCGAAGGCCGTTTTGTTGGTTTTGATGAAGAATCAAAAGGCTATCAAATCTACTGGGACAAAA
AGCGATCAGTAACGATTGAGAGGGATGTATACTTCAATAAGAATGACTCCCTAGCCCCAGAAACCGTCCTGAT
CGAGGGGGAGAACATGCAACAGTCCCTGTAGCCTCATTATTATTTCATGGGGAAATTCCAGCTGTGCATGTTA
TAATGTACCACCATGTCTGCCATAGCTTGGGGGAATTATAATGTGCACTCAGAATACTGCAAGCAAATGGAAA
TTATTGCTTCTAATAATAATACATACATGGCTGGACACATTATAATGAGGGGGGTCCTAGGGGACCATTATTA
TTTGAGCTGCTTCTTTTTGACATCCCATCAACATCTGGACTTCCTTTGACATCTCATCACTCCCCAGACTTCT
TTTGGGTCCCATCATAATTAAATATCATATCCCAAACCAATATGCTTATTTTATTGGGATAACATATGGATAT
TTTGCCCACAGAACATCATCCTCCCTACTAAATACCATATATAAACACACAAAACCTAGTATATAGCACCCAT
GAAGCCATCTACTGCTTCTGGGAAGTAAGCAGAGTATATATTATAAAAGTCAGATGAAACTGTATTTGGCTTG
AGCTTGGAGTTAGTCTAGCCTATGATTATCTTGAAAGTCTTGGGTACTGGGTTCATCCACTGCCAGAATAATT
TCATGTGGGCCCCATGGGAGATGAATAAGTGATCCATGACCCTCATAGGATATGCTAAACCTTTTATAAGCTT
ATTGGTTTGTGTATTGTCTCAGGAAGGGGGTTATCTGGGGATGGGCGGTTTGTGCAGAAGCCATTTCTCCACA
GAATAAGATGAGACAGGGGCGGATCGAAAGCGGCTTGGAATCGCTTTTAGACGCGTCCCTTACCGCTAGAATT
CACTGGTTCAGTAATTTTATATAACAAAAACGGTCCAAATGCATGTTTATTTTAAAATCACTCGATCCTAGCC
ACTTCTCCACGGAATTGCTCGAAAATTAGCGCGTTTTTGAGCGCATTGTACACGCTTTCTAACGCATCCCTGC
GCGCAAAGATCCGCTGGGAGCGGGAGAGCCCGACCCCTGGAGGGCGGGCTGGGGGCGGGCAATGCTGGGATTT
GATCGGATTACTAATGGGCGGGTCAGGGTCCGCTCCTAATGCGGGCATACTACCAGTGCTGCTGATAATTCAA
TCATTATAATTCATCCCTAGTGTACATTATAATTTGCCCTAGCTATGGTAGAGATGCAGACACATTATAACAG
GCATGGCTGGAATTCCCCTGCAAAATTATAATGGGGCTACATGGATACCTCGAACATGACTAGCTCCTCCAGC
CCCCCCAGCCAAAACATCGAAAATCCAACCGACGAATTAGCGAACGCGAGTAGGCACATTTTGAAGCCCCCGC
AGTCGCCTCTCGAACGGTCCAACAATCAGCCAAATCGGTCGGAAAATGATCAAGATATGGACATACCTTCATC
AAGCCAAAAATCCTCCTCAACCAATCCTCAACCAATTCCCCCCACAAGAAAGCCCCTCGAAGCGACGGCCTAA
TCGAGCCTGAACCGAATACCAGTCGCGGTTTTCGTCCTAGGAAGCCCCCAGGCCATTATAAACATGCCAATGA
TCATCAGGAAAAAAGCACTTGTCTACGTAGCTCTATGTAGACTTACATAGACCGTATAGTGGCCATATACCCA
TATACAAGTCCCTAGTTATGTAGACCCTATACAGTCTACATAACTCATCATTTACCATTATTTACTACATAGA
CACCTGCCATAACTATATATACCCAGGTCATGGCCACATAGACCACCATAGACCACCATAGACCACATAGACA
TTGATGGTATATGGCTTCTTGTATAGTACAATAGCTTCTGTAGACATGTATATACACTGTAGACCTCAGTTAT
GTCTATAAAGACTATATATGTTACGGCGCTCCCAGGGGGCTTACTCTTTTTCTGACATCATCTACGGACACTT
TTCTCTTCAACACATGTTGCACATGTGTTTGCCTCATGGACTCCTCATGGGCATCCTCTGACCTACTGCGCCT
CTTGGTTTACTCATTGTCTACAGCATATCTTCCCCTTTAGTATATATTGCTGTAGAATCCCTTAACATGCATT
CCCCTCATGGTCACTGTCTCTTAGTATATATACTCCTTCTGTACATCCTGTAATAGCATCTTGACCTAGGCAA
TCTACCGCCTTCATACTCTTACTCAGAACCTGAGCCTAGACCAGCTCAGAGTGACTTGAACTCCTTTGTCATT
GGACAGGAGACATCTTGAAACTACTCTCAAGATAGAACTAGAATGAACAACCGACTTGTATCTGTTGTGAAGG
CTCACGCCCTAACAATATAGACATTGCCAGTATATAGTTTATTATATAGTACCATAGACTCACATAGACATGT
GCAGACACTATAGACCTCAGGCATGACTATCAAGGCTACATAGATATTGAGGGTATATAGTTTATTGCATAGT
ACCATAGCAACTCATAGACTTATGACAAACAGTAACATCATTCCAAAACATGCATCAATCAATTTTTTTGAAA
CCCTACAACATCAAAAGCCATGTAATTAATCCTCATATAAATCATCTGACTCTGACTCTGGATTATCTCTGTG
TTGTTTCATTGCATTGATATATGCCGAAGTTGGGATGATGAGGCGAGGATTGTTGGACTGCAGGTTCACACCA
GCCGAAATCCTAATATATTCAGAATTAATAAGGGACAATGAGAATAAAATATAAAAGGACACACGCTGCTCTC
CGATATAGCTTTCGACAAAGCTTGTTGAATAGATCAAGCTCCTCAGTTTCTTTTTCATACTTGCAGAGCAAGT
CAACATGGGTCAGGTACACTGTGTGGTATTCACTGCTTCGAAATGTGATCTCCTTCTTGATTCCGGTGAGCCA
CATATCAATCCCACACTCAATCTGGCAAAAAATGTGAATTAAGGAAAATAATAGTAGTAATACTCACGACTGT
AAGGACAAAAGCAATGGCATGCATCGGGAGCATGCCATTGGGTCTGAATACCAGAAAATATTGAGCACCTTTG
TCCTGTAGATCCGAAAACCACAGAGCATTGATGCTCTTCTGGATGATGGGGTTATGGTATAAGCCTTGGTTTT
CGACGCCAAGCGACTAGGAAATATTAGTAGGTATACAGCTCCGAAGGAATCCTTGCTTACTTCGTATAAGAAA
TTCTTCTCGTACTTCAGTGCTGCGGCGAGTTCACGATTAGCCTTGATGACTCGACACTTGCGCCTGTCATCGA
AACCATAGATTGATGCGACCAGTGGTATAATTTTTGTTTTAGCTTCGCTGCAAATTTGGGGACCACGTCCTCG

GACCTCAAGTAGCAAGAGTTAGTCATGTAGATGGCATGATATGGGTGGACATACCACTCGAAACATGTCGTGG
TCCATTGTCGCGAAGTCAAGGCTCTTGGCCTCATACGCAAACTCCCATGCCCGTTGCACAAGCTCGAGCTCAT
CCTCGCGGAGAGGAAATGCATTCTCTGTGGAAAGAAGGCACCTATAAACCACGTTAGCTAGTGTGAGAAGTAC
TTGATCCGTCTTCTCATAATCTGCGACTTTCGGGCACCCTTTTCGTTTTTTTTTTGCCGCGCCCCGAAGCTAC
TAGTTGATCCGACACGAATTTCTGTTTGGCTGTGGTCCGCTTGGGGGATTGGGAAGGGAGGCCATGGTTGCCT
CCAATGTCCGGGGTTGGTGAGCGTTCAACCCGAGGCGACTGCCACGGATGTGGGGGCTCATTGTCGAATTGCG
CGTAATCCCGTGAGGACCTGGAGGATTGTGAGGAAGACCGTGGAGCCCGTGAAGATTGCAAAGGCTGCGAGGG
GGGCTGTAAAGTCGGCTGTGGCTTGGATACAGCAGGTACCGGAAGTTGTGGTAGTGGGGTCCTGGGGGAACGA
GGCAGCGAGACCGTTGCTGGTGGGTTCTCAGGCATACGAGATCCCCGTGTAGAAGAGTGATGCTGTTGACTTG
AATATGAACCCAAAGCCGCCGTTGGTGTGGGTGTTCTGTGGCCGGCTGGCGGGGTAATGCGGAGTGGGGGTTC
GGGTTGAGGCGGCACTGGGACTTTGCCTGCATGCGGGGCACGGTTGGCTATCGCTTTTGGCTTGGACATGACC
GGTCGAGAGGTGAACTAGGTAAAGGATTAGACCTAATAGAGAACAGTAGTAGCTCAACACACCTCTGGAATGC
GATCTCTGTGCTCGTCATTGTCCTCAGGGATGATCCCAGCAGCGTAATCCAACCTACGTTGGTTGTGCTTCTC
CTTCTCAGCCTTTTTCCTTGCAGCTCTGTCCAGAGCATCAGCTGCGTCAGCTTCATCCTGTTCTATAAGTAAC
ATTAGCGCATCATACACTCCCTAGTAAATAAAAACTTACGACGCGCAATAATCTTTTTTGTTGGGTTTGTGCT
TCGCTTGGAACGTTGAACATCAGCCATCACTGCAAGTTTGCGTCATTGAGTTGATGGGATAAAAGCTATCAAA
GAAGACATTTTATAGGCCACTGGATGGGACACGCGGTTTAGATATCCGGGAATCACCTGATGATAGCTCATTT
TAGCACAATGTGGTTGGGCAGGTGTTGGTTACACGCACTGGGTAAGTCTATATGTGGCTTGAGCATCCGTGAA
TCACCTCCTATTATGGTGTATTTTAGCAGCGTGGTTAAGGGGTGTTGATTTGATGTACCCACTTGGCCCAGCT
GCCAACGAGCAAGTCCACGTATCTTCCGTTGGGGCCTAACTCTGTAGGACGTTTTATATAAAATGTAAGAGAT
AAATGAAGGTATTATAGACGGAAAATGCCCCCAGAACGACACCTCACCTGCTTATATTGCTGTCGCCGCTATC
CCACTGAATCAGCAGTCCGACGACACCTTGGAAACACTGGAAGATGCCGCGACCAATTTGAAGAGATGTTGCG
CCGTGGGAGGGCGAACAGGCGGCCAGCCATGTCCGAGAGCGATGGATCCGATATATCAGACAGTGAGAATCAT
GATATGGAGACTGTGGAGGGGACATACATTGATTGTGAGCCTATCCATCGCAGCACAAGAGCACAACCCATGC
CGACACTTGAAGAGATTGAAGATATTGATGAGCCGCCAAAGCTTCAGCGATATATCGAGCCGTTTCCCCGACC
GGCTGCAACTCTGGTGCGAACCTCAAAAGAAAAGACAATGTTTGAGGTACTGCTTGAAAATCAGATACGCAAT
GGGACAAACCAGTGGGATCCATTTGCCAGCCAGGAGGAGTGGGAACTTGCAGTATGGCTGATGAAGAACGTAG
GCCAGACCTCGACAGACGAGTTTCTCAAGCTTCCAGTGGTGAGTGAGCATTCAATGCCATGTGAAAGTGGTGT
TGACGTACCTTTGGTCTTAGATGTGTGATCAGATGAACTTATCATTTCGCAACAACCGTTCCTTCCTCAAGCA
AGTCGACGCCCTTCCAACTGGGCCTAATTGGGAGTGCGAGATCGTGACAGCGCCGGGGGACCAGTGTGGGGAG
GATGGGGAAATTGTCGGGGAGGACATGGAGCTATGGTATCGCGACCCGGTTGAATGTATTAAGGAGCTTCTCA
GCAATCCTGCTTTCAAGGATTTGATTTCGTATGTGCCCGAGCGTGTTTATACCTCAAGTGATGGCGACGATCG
CATTTATGACGAGATGTGGACAGGCGATTGGTGGTGGGATACGCAGGTACGTAATCCCAGTTTAACAAACAAT
CTCACTAACTCATACTCTAATAGACTAAGCTACCAGATGGCGCAGCCATCGCGCCGCTTATTCTCGCATCGGA
CAAGACGCAATTATCACAATTCCAGGGTGACAAGAAGGCGTGGCCCGTATATCTCACCCTCGGCAACATCTCG
AAAGAGATTCGACGCTGTCCCTCTGCGCATGCCACCGTCCTCCTAGGCTACCTTCCCGTGCCCAAACTTAGCT
GCTACTCTGATGCGACACGCTCGCTCGCCGGATACTGCCTCTTTCACTACTGTATGGGCAAGATTCTCGCATC
ACTTGTCGAGGCCGGCAAGCCGGCGTCGAGATGGTTTGCGTGGATGGTTTTGTCCGGCTCATCTACCCGATTC
TCACAGCATACGTGGCCGACTACCCTGAACAGTGTCTGGTATCATGTTGCATGGAGAACTGCTGCCCGCGCTG
TACAGTGGATCCCAACGATTGCGGGGATCCTACTCTGTTCCCCAATCGCGATATGCTGTCGACACTTGAAACT
CTATATGCCCATGAACGTGGTTACGAGCCCGAGAAGTTCACCAAAGATGGAATTCACGCTATTTATGAGCCAT
TCTGGCGCAATCTCCCGCATTGCAACATTTTTGGGTGTTTCACCCCCGACCTCCTCCATCAGCTCATAAAGGA
TTCTTCAAGGACCATATCGTCAAGTGGTGTACTACTCTCATTGGCGAGGATGAGCTCAACGCTCGCTTCAAGG
CAATGTCGGGGTACCCAGGCCTACGACATTTCTCAAAAGGTATATCATCTATCTCTCAATGGACGGGCACGGA
GCATCGGGAGATGCAGCACGTCTTCCTTGGGATCATGGCTGGATCTGTGGACCAGGATGTCCTGACCGTCGTG
CGGGCAGCGCTCGATTTTATTTATTACGCACAGCTCCAGATGCAAACTTCAACAACGCTCGCAGCTCTCAAGG
CCTGCCTCACGACATTCCACTGCTTCAAAGATATATTTATCAGATACGAGATTCATAAACACTTCAATATTCC
GAAACTTCATGCTCTCCTTCATTACTTCGCATCTATCCAGGCACTTGGGGGCAACGACAGGTACAATTCTGAA
TCGCCTGAACGGCTCCATATCGACTTCGCCAAGGATGCCTTCCGCGCCAGCAACAGGAAGGACTACCACGAGC
AGATGACCTTGTGGCTTCAGCGCCACGAGGCGATGTGGCAACGTGAAGCATACATCACATGGCTCAGCACTCA
ACAACAGGCTGTGGATGAACAACTCAGTAACGCAAATAACCCCAACGACTCCAACGACTCCAACAATTCCGGT
GACCTGGACGCCAACGATCGAGCGAATGCGCATGACACTTCTGGTGCGAATCTCGTATCATACCACATAGCTA
AAACACCACCACTCCAAGGGGTGACCGTCCAACACCTAATCGAAAATTTTGGTGCCACTAATTTTATCCCAGC
ATTAACATCCTTCCTCCAGCAACAACAGCCCCCCCACCCGCTCCTTCCGAATATGTCTGACTGTTTCAACCTG
TATAAGCAAATTATCATCACTCTCACACCCAACGCCTTTCTTAGCAGTCAGAAAATGACTAATCGCATCCGTA
CATCACCAGCGGTCCCACCTCATGGCCACAAAAAAGGGTTGCCAGGTATCTTTGATACTGCGCTCATTATTGA
CGATCCAATGGAATATCGATCGCCTGGTGGACTCACAGGTAAGATTTTCTTCTTGCCTTTTCTCAATCCTAGT
TGCCTGATATCAATTACAGGCCTGCGCGTAGCACAGGTGTGTGCGATATTCTCGCTGCCATCATACTTTGGCG

CGCATCCGCATCCGCTTGCATACATTGAATGGTTTACCCCATTAGGCGTTCCAAATCCTCAAACAGGTATGCA
TGTTGTTAACCGTTCCACGCGCCACCATCAGCCAAACAGCGCCGTTATTTCAACCACCCATATCGTCCGTGGT
TGCCATTTAATGGGGCAGTTTGGAAATACAATCACTAAGGGATGGACTACCGACAATATTCTCAAAAAGGCAA
AGGACTTTTATTTAAATCCATACATACATGTAGACACATTTACGATTGTAAACATCAGTTAACTGATCAAAAG
ATCCCTTTGAATATCAGCGCGTCACACCTGTGACACATCAGTGTTTGCCCCATCAATGGTTGATCTTATCCTC
TGATGCTCTTCCCCTCATTCTTGGCGTGTATCCTCTCACAGTTGATTGTTGAACTAATCGGTAAGCCTTCCAC
AGCAATTCTCCATGCCCGTCCTTTAAATACAGCTCCCCGAAGATGACACTGTCCCTGTAGCAATACCCACCCC
CCAGCCTGTCGAGACCCACTGGCCGTCCTAATCACGTCACCGCTCACTCAATGACTCCCGGCAGGCGCCCAAG
CGTCCCCTCCTACAACCACTACAACCCCCCACTCCACCCGCTGCAACCACCACCACTCCGCCAGCCGCAAACG
CGCCGCAAGCAGCCGCCTCACTATCATTCTTTGTCCTTTTCCTCCATCATGTTTGGTCGGAACAACGGGCGGG
CTAGTCGACCACCCTACTCACACCCCAGCGCTTACGCGACACCCACCCAGTGAACCAAACGAACCCGAACTCA
AGCCTATGTCAACTCCGCAACAAAATCATGTGGGAAACGTACTAAAAATGTGCAAGCAGAGTAGGGGATATCC
ATGTGAAGGCTGCCCCTCTTCTCCCTCTTATTGCCCCTCAATTTTTCTCTCTGTTAGATGTCGGACTCGTACA
CACCTTTCTCAACAGTGATGCCTCAGAAAATGGTTTCGTGGCCCCAGCCCACCGCTCCCTTGAACCGGCCACA
ACTCTGCCATCCCACCCCCGTTATTCGCATGGTTTCGTGGCCCCAGCTCACCGCTTCCTTGAACCGGCCACAA
CTCTGCCATCCCATCCCCCTGTTATTCACAATACATGATTTCATGGCCCCAGCACTCACCGCTCCCTTGAACC
GGCCACAACTCTGCCATCCCACCCCCCTGTTATTCGTATCGTTTCGTGGCCCCAGCGCTCCCTTGAACCGGCC
ACAACTCTGCCATCCCATCCCCCTGTTATTCGCAATACATGGTTTTCATGGCCCCAGCGCCCACCGCTCCCTT
GAACCGGCCACAACTCTGCCATTCCCCTCTTATTTGCAAATGATCGGTGAGTTATCTTGGCTTCGCGAACCTC
TCAAATCAAGTTCACTAATGCTGTTTACACGAGCAACTTCGCATCTAGTAGCACCATGTCCCTTGGGGGATAG
CAGACGAGCGGGACAGATGGACAAATGGGATAATAAGTACACCCACAGCTCCACCACCCGCGACTGACGCTAA
TGCGGACATGTCCGACGATTTGTCATCCGTCACCTCCCCGTCCACCTTCCTCACTCGCAACCCGACCTACACC
CACTTTCCTTACTCCAATGCGTTCGGGAATACGCTGTGCTGCTTCAACTGTGGGACTGCGCGATCATCGCATG
TGCCGCTACCCAGGGCAAGGCCAGGGAAGACGAACGACACGAAAACGACTAGGACGAAAAAAATGGCACGAAA
ATGACATGGCATCGCCATTTGCGCCACCGCTAGGAAGACCAGGACATCACCACGCCGGGGATACTAACATGAA
GACAGGACGGCATCGTCGCACGCGCCACCACCCAAGACAAAACTGGGAAGACGAGGACACCGCCGTGCTGGTG
ATAACTATGTGAATACGAGACAGCACAGTGTCAGGGAGGTGTAACATGTAAGTAAGAACATTCAAGTAAGTGC
CCCCACTGTCATTCTTTGCGTTTATGAGGTGGAGTGGCTCGCTCCCAGCTCATGCCCTCCTCCACCCTCGCGA
TTGCACTTGCCATGTCAATTTGATGTGTGCAGTGATACCGCGAGGCTAAATGGGGGGCATCAACTGTGGCTTA
TGCGTCCACCACCGGGTTTTCATCATCGCACTGTACCTGCCACATCAGCTGTGTGATGCAATGAGGGGAATAA
CTTACGGTTTACATGGCTAGCATGGGAATTTGCGAAGATGTAGTGAACCCGCGTGGTGTTCTTACATGTCTAT
GATTGTCTGGATAGACCCAAGCACGAACTACATAGCCTTATGTAGACATACAGAGTGTATATATAGACATATG
TAGACATATCATATGTATATGTGGACCTATGTAGACATACAGGGTGTATATATAGACTTATGTAGACATGCAG
TGTGGATATGTAGCCTACATAGACGTCTAGCATGTCTATGTAGGTCTATAAGGTCTATGGAGGTATATGGGGT
ATATGTATACATGTGATATGTCTACATATGGCAACATATGGCAACATAACTAACCAGGGGTCTATGTACGTTT
GGCCTGTTTCCTGATGATGGGACCATTCCCCCATCTGAGCATGGAACTGCAGCAGTCTTCGAAGGAGCTGATT
CCCTAGATCACTGTGGGATATATCTATTGAGGATGAAGAGTTAGAAGCTCATATAACAGAGAATACTCACCTA
TTTGTGATGGCCACTGGTGGTGAACCTCAAACCCTCGAAGAAGCGCTCAATGGTGACGAATCTGATGCTTGGT
CAGCGGCTATCAAAGCTGAACTTGATCAAATCAAAAAACTCGAGACCTGGGAGATCGTCGAGCAGCCCGACGA
TGCCAACGTTATTGGATGTCGCTACATTTTCAAACACAAACGCAACCAAAATGGGAATATCATCAAACATAAG
GCACGTCTCGTAGCAAAAGGTTACTCACAGGTTTACGGTGTTGATTATTTTGACACTCGCGTTTGGATTGTTC
GTTGGGAGACTCTTCGCAACCTCCTAAGCGCTGCTGCCGCGAAATCTGCTGTCATTCACCAAGCCGACGTCAA
GAACTTAAACTCTCTTGTCAAAGAGAAAATTCTCGTCCACCTACCCCCTGGCTACCTCCTCTTTCGTAAATTA
CCTCGTAATAAAAGATGGAAGCGCCCAGTTTGCCGACTCAAAAAGGGACTCTATGGCACCAAACAAGCAGGCC
GCGGATGGTACTTGAAGTTCAAGGAAGTTTTCGAAGCACTAGGTTATAAAGTCTCGCACTCTGACTTAGCTGT
TTTCTACAAATTCTCTGGGAGTGATTACACCATCGTTGCTGCAGCCACCAACGATTTTACCATCATTGGGAAC
TCTCTCGCATCCGTCGATGTTAAGGCGGATAGCCTTTACCAAGGTTCAAGCGTCGAGTATCGTCGAAGACATC
AAGTCAGAGTCTTGGGTCAATACCAACAAGACTATAGTCACTCCAAGTCAGGCTTTGACTTGGGTATCAAAGG
TTCAGTGATCAAGGTTCAGAGGTAAAGGGTGTAGTATTGCCTAGGTCAAGATGAAGAATAATGACAATGTATC
TAGGGTTTATATACCAGGGACCTTGAGGCGCGAGAACATAGTACAATGGGAAGTAAAGACAATGTAAGGTAAA
AGAGCTGTTGAAGCTCAGTCAGCTATGACATCATAGATGAGTCAGGTATCTAAGAATAGAGGCATGTGTTGGA
CATGCAATAGAACAATGAAGAATCAGAGAACAATGTAAGTATAG
>Cligib1_rnd-4_family-3420#TIR/MITE
GATATTTCCTTTTTGAGCTTTCCCAATTGGGCGCGATGGAAGAATGTGCACGTCCAGTACGCGTCGTGCGCTG
CGCATTCCACCATCGGGGATCTCAAAATAGCTTAGGATCGCAGATTTCACTAATCGGGAAAGCTCAAAATGG
>Cligib1_Cluster2#LTR/Gypsy
NtNaggacNNgtNNNNNtcNtttctttttccaactctttttcatcttatctaatctcttatctataggttatc
Ncatcttatctctgtagttatcttatcaacatcacgtgactgcttatgtaagccaaaccgcggttttgcatca

tgtagtacacatgtcgtttgtacatattatattcacgggctcttagNattagtatatagtttcattgtttgta
tgttcgtctcttaacttgattctctttggtttccgctNtaacaagtgggacttaagNcttcNtcttcatNNNt
tcNNttNNNcttgagtattcttgagAgaccgcacaNNcNNtNggNtgtNttAgcgtgtccttacagaagaatc
aagttcNtNNttgtgccctctctcaacgctttcNctNNtccacNacttcgaagcgcgtgaaNcaacatcaccg
cNcNtagtNNtNNgtNNNNgNNNNNAaNNNaNccNcccccNNtNNNtNNtaaaNNcNtNNNNNNccacNaNNa
NcNatcNNaNNNNcaNttgNNNtttNNNcgctNNtNNtNctcgNtNNgNtNttggcaacaactcttgNttagt
ggNtNNNaNtttNtNcNccNNNtNgtattgagtcagcaagtcccNNNNNNNNNNtNNNNNNgNNNNNNNNatt
NaccNagNcaacatNcgacacctctcactgcacgNtNggcccacAccttgcgcaggatcagcgtttaagggtt
agcNNcgtgTaacagtccgcctgGtcaggctctcgcggaacacactcgcatcattttcctAcaaNNactagaa
gNNNNgaNgNttNNNaNctNcNtcgctaNttNatNcctgttcgcgtttgaggttgcactcTcgcgtcctattN
gNNNcNNNNNtcgcccaatatttcgggcctattctcccgttccacNacccccttcaNTtgaagctggtgatNac
cgccggtctactcgattaagttcggacccgcggcccccTtcattttcttttcNcaNcttccttacgaacgcc
cgcNNagaatgtctacccactcctgccgaaatNctcNNNgcNgccggacaacaaNccggcccgtcccaacctc
NtNgNcccNagtacactaaNccctgggaagaacaNaccacgtacctgaatNcgaaaattcaggaactcacgaa
atcgatggagatgatgatgcaagtctccaagtNcaaaaNgagaagctcgaNNNtctcNcNggtcaactNgctN
cgagatcNgtccccctccNcccctccNNcNNcNcgNNNtcNtccccccttcNccggttNatNtgggtatNcNgN
cttNNaNaaNttcggagcNcatgttcgtatgccaaagatggctacaccccNgNtttNgatggNaaatggcaaa
NgcggaatctttcgAaaccgcatgtaaaatttataTggcaatgcgcaatcaggaattcccNGaNtatacgcat
caggttttNtgggtgctttcNttatgtcaAacgggcacagcggctttatggcgtgaccaaatggtcaaacatc
tNacggaacgtggcgtctccccagaNagcactatggaNggcttctgggaccttttaggagAacattcggaga
tcccgaTNtNcaaaaNNcaaaaatNtttaaaNtacggacaatNNaCaaggaactaaaacggcggaNgatcaNg
tccaNgaattNgaacgNatcgcgacggatctggctatgttgagGtagccNtNattcaagaatTaaaCggtcct
caacaaggcaNtacgNgAtaaaatatcacttggNgaataNNgaccccaaacaatcgaNgatggtatgaacgNg
ctaaaNtggttgatcgacaatggcgccaacatcaNagtgaacaaaaactNtaNtcNacggcaactacNNcaaN
NggcagattNaacAtcacaaccggcNagacagcaaactacgacaacgtcatcgtcaacagtccctgcaccacg
aaattactggccacctcgtcaacaaacccaaNctcctgcaaatacggtAcccgctacaacggctacacgcaac
ccggatgctatggatgttgatcgcggaaCtcgacgcccgNtcatttgttataaatgtcgtaaacccggtcacg
taatggcaaactgtccatcNtcgctAaacgttaggggaacNtacacNgaacttgaagatatttcaaacgcaag
ttcaatattaccgaatcacgcggcggtaatccctccacccttactcatgttgccaattcaacNtcgggtattg
tagaagtcccNaacaccccgtcttttccNaacggcgcacagtaaaatctccacctgtNgcgtcaaNNAatatg
ttttctgctctNtcNgtcgataattagagaatacgtcttccatgtaAccgtatctcagggggtctcccataag
gagcttggcggttcaacaccgtgctTaattcccttcaatNcatctatttcgacacaaagaacagAaaacggag
atAaaaatattNtaagtggtttNggtagtcggataggtgtccgtcctgctagtcagggtcccggcactacgat
tatNtcaacccaccaNgattccttaaatggaacaTcggtccgtcccggagctggtaactccgtgggtcccggc
cgtaNcgcctctgagcgtcttggtactattTatagagaacgNttatttatTcgcaccctgaatctNgaacgag
aaNtgaaaatgacggttaacatatccactctcgatacacatgagaaNgcgtcagTtcaggccttattagattc
aggagccactggttcattNatcgatcggaaataNgtcgaacaaatgggatggaaaacNcaagaacttaaatat
cccattaaagtttttaAtgtcgacggaaccctNaaNcaaggcggccaaatttcacgagaaatNtatctcatga
taacgcataaaggtcacaaagAgaaaatggtatttgaagtgaccgaccttggaaaaaccccgatGatcattgg
acacaCatggNtaaagaagcacaatccatcNatNgattgggctactggtgcgatagaattNtcacgatgtccc
ccggaatgtaatgtccgcattcgcgaaatgaaaacccaNaagaagcgcaaagcgatcgccgatcgNtggaaat
atAaagtctcgatcgaagaagttgatgatgaagatcatgatcgagtgatcggNggtTtaataaccgaacaagA
aggcgcgcttcttgatgaaataaNtttgacgatatacNcatgaAatcgTgaagaacattttgtaaacgcNaca
ttccaagaaccgaaNcttNcacagacgaaccaggtctgatggataaaattccccgacgNttccgacgatggtt
aaaggtgttcgaaaagaaagcttcNgaacgaatgcctgagaGaaaaccttgggatcaNgatatNgaactcaaa
ccagggttNgaacccAagaaagcNaaNatcattaatcTctcgcccatcgaacaaaagaagttgaNgatttcNT
aaccgatcaattgNcaaaggatatatccgcgaaagtaaatcacctcaaacttcagcggtcttctttgTcccaa
aaaggatggaaagaaacgNatggtacaggaNtatcgtcaaNtcaaNgctggactattcgaataattatccttt
accNatgatcgcNgacttaTcgataaagcgggaaatcaaaatggTtcacNaaactcgatctccgatggggata
caataatgttcgtatgaaggaaggcaaggaatggctcgcggcattttcaactcaccgcggctctttcgaacca
ctcgtcatgtatttcgggctcacaaattcacccgctacatTccaaaccatgatgaatgccttacttcgagaac
ttattgatgaaggacaNgttgtagtgtatAtNgacgacattctcatcttcacgatacccaagaagagaatgaa
cgcattaccgaacgNgtacttcaaatcctactggacaacgaccttttccttaaaccagaaaagtgtcttttca
gtcagaatcaaatcgagtttttgggccttattcttggagAtggagaagtNcgaatgatggaagagaaaaccga
cgcggtNaagaaatggcctgttccaaaaacagtccgtgatatccgaaaattcctcggatttgcaaatttctat
cgccggtttattgaaggtttNtcaaaacttgccAcgcctcttaacaattNattacgaaaagattcgaaattcg
aatggacggaaaaacaacaatccgcattcgatatgctcaaacatcgtttcactcgaatcctatattNgttacg
gcNgaNccttcaaaacctcttcgcgtggaatcggacgcttcggactttgctacgggaggNattttatctatgt

tatgtgatgacggtaaatggcgtccNtgttcttattattccaaatctctttcgatgttgaacgtaattaNgat
gtacaNgataaggaaatgttaggtatatacgatgtcttgaagcatggcgacattatttagaaggttccaAaca
tactatagacatttGgtcggaccacagaaaccttactattttatgactagtaaaaaaNtgaacCgtagacagg
cacgatgggctctttatttatctcgttTcgatttTactttaattcacaaggcaggatcctcgatgggaaaagc
ggatgcactgtccaggaggacagatcataaagagggagtggattcNgataaNgacgacgtNacgCttctcaaa
ccNgaatttttcgattaaagcattacaacaaggtcatgtcttgatcggaagcgaNgaagaatcNattctttc
caaNatcagNaaatctaaggagtacgatgaatcggtagtcaaggcaatcgaagaacTNaaacgatcgcccacc
aaacgtcttcgctctgaNgNatggNNtgaagaacaNaaccttgttctattccgtggaaaagtctaNgtaccac
agaataccgatcttcGacgagaaatcgttaaactccatcatgatactgGtgttgcaggacatcccggNcgttt
caaaaccctcgaactNattactcgtaattattggtggcccggaatgactaaNtttgtcaattcctatgtcgac
ggctgtgacaaatgcCaacgatataaaacataNcctgctcccccagttGgaaacttgtatctatgcctactcc
ggttgaaccgtggaaagctatctcttgcgaNttcattacaggacttccggaaagtaacggttatgatgctatt
gttgtagtcgttgatCgcgcttcaaaaatggctcattttattcctacaacttctcaaacttcgacgaccggtc
tcgcgaaactttatcatcaatatgTatggaagcttcatggtttaccagattcgataatctcggatcgtggccc
tcaGtttgcgtcatctatgatgcgtgaattgaattcgNtacttggtattaccactaatttatccacggcttac
catcctcaaacggacGgtcAaacggaacgagtcaatcaggaaattgaagcNtatctccgattatttatatcNc
atcttcaaagcgattggattgaatggattccttcagcNgaattcgcttacaacaacgccacgcattcctcgAc
aaaaatatccccttttattatgtcaatggacgtcacccacgcaTgggcgttgaaccNattcgtcaacccacaa
acgaNtcggcNgctgaattcgccagtcgaatgaagaagatacaagaagaagctcaggcaGctctaattaaatc
gcgcgacgaaatgaaacgtttcgctgatcaacaccgaggagaaacaatccagtttaaagtcggacaaaaggtt
tggttagaaaccaaggacctggatctNAaacgaccctcgaagaaactcacagaacgccgaattggtccatacg
AaattctcgAactagtaggaacaatgcggtaaaacttaaattacctgcctcgatNaaaAttcacccgtcatca
atgtctcgcgcattcgNccttatAcgccNNaNNNattccaaatcaacaacccgaaccacctgcgccagTacaa
atNGacggagaatttgaatatacggtagaagAaatcctcaattctcgtattaaNggaaacgacttgaatattg
ggtcaaatgggaaggctataccgccgaacataaNacNtgggaaccNatttcNaaNttgaagaacgcgatggat
cttgtcaacaatttccataaaaaaaaNccatccgcNccgcgaaaaataaaactcgccgttactcaagacgaat
tctttctatgttccaaccataNgagaaNttcaccgttccNgtcgaaaagaGcttattgttttcatagatagtg
atgcgcGcccttaggggagtgttNgcacctatctggNcgatgaagaAacagtgcatctNgttattttcctcgg
agagaaaaatcctaaagagggagttatgtcaggacttgtcttttctttctttttccaactctttttcatct
tatctaatctcttatctataggttAtctcatcttatctctgtagttatcttatcaacatcacgtgactgctta
tgtaagccaaaccgcggttttgcatcatgtagtacacatgtcgtttgtacatattatattcacgggctcttag
caatagtatatagtttcaNtgtttgtatgttcgNctcttaacttgattctctttggtttccgNtNtNaNNaNt
NNgaNNNagNNattNNNNNtNatNNNNNNNNNNNNNNNNNNtNNNNNgaNNNNgNaNNNNNNNNNNNNNN
>Cligib1_Cluster91#LTR/Copia
GTCGAATTCTCGTCCTCGCAGGTCCGGGCAACGTCAAAGCAAACGAGTGATTCaGATATATCTAGTATACCCC
GTTcGAACGCGCAAGAGAAAAAaCTAAACaAGCGACTAaGTAAATTATGTATGTATTATCTAATCGAGGAGTC
CGCCGGTCGCCTCACATATTTcGTCACaCCCCCTCGACCGCTGACTCCGAGAGGAATCGGAAACCGCGCGCTT
CGTGGGTTGcGCCCcCTTGAATCAATAAACCTCCCcGACCTCGCGATCTCCTAAGTCCcGTAAcGATGAGACG
TTGTAGTAGGCAGTATTAGCCTGTTCTTTCTTTCCTGGGGATTGCTCCTTCTTTTCCTGAGTGTCCGAcGAAT
CCTCaGTAGGTTTTTTCTTGCAGAACATCTTTATATGTCCCGTTTCGCCACATCGCCAGCAAgagcgcgacgc
gccatccttcgcgacGAGCGCCGTCTCCCTCCaATTcGGTTTGTCCACTGAGTCCTTATTCCCGCGACGCATT
TCTTCATTGAGTAAGCGATCCACAACATAcTTGAGAGTGAGGGTTTCGGGTGCGAGCGCCGCGTCGAGTGAAA
TAACAAAcGCTTCaTAAGAATCGTCCAGCCCaCCaGTGAGTACTAAAACTCGaTCCTcGTCaGTGACCACGAC
CCCGACATCTTCCAACTTAAATGAAAaACTCTTCACGCGGCCAACCCAGGTCaCCATTGTCTCGTCCGCaCCT
TTTACCAGGCGCATAAACTGCCGCCGCCACGCAAGCCGCGTTGCAAACCCTTGCGCaACaTGAACTTGGGCCA
GCGACTGCCAGATTTCCGCTGGGTCCCGACTCCGCATGTGTACCAACTGGGAATCCTCGACCTTCAAGATAAT
TTCaGCTCGcGCCTCGGCCATCTTCTTcGCCGTcCGCTTTTTCTGCCAGGCACCAAGTGCCTTCTCTGCTTCC
TCTGGTTCTTTCTTCACATCGACATCCATTTCGCACAcGACGATATCCCACAAACTGGTCTTGATGAGCTGGG
CCTCCATCCTCATAGCCCATTCCGGGTAATTGGATTCATTGAGGTGaGCAAACCGCaTTTTTTCGTCACTGGG
[truncated: 610,362 more chars]
